# Sex-Associated Effects of Thioredoxin Reductase Inhibition in Hyperoxia-Induced Lung Injury in Adult Mice

**DOI:** 10.3390/metabo16090623

**Published:** 2026-08-28

**Authors:** Katelyn Dunigan-Russell, Matthew Ryan Smith, Hua Zhong, ViLinh Tran, Dean P. Jones, Lynette K. Rogers, Trent Tipple

**Affiliations:** 1 Neonatology, Department of Pediatrics, University of Alabama at Birmingham, Birmingham, AL 35294, USA; kdunigan2@gmail.com; 2 Atlanta VA Healthcare System, Decatur, GA 30033, USA; matthew.ryan.smith@emory.edu; 3 Division of Pulmonary, Allergy and Critical Care Medicine, Emory University, Atlanta, GA 30322, USA; vilinh.tran@emory.edu (V.T.); dpjones@emory.edu (D.P.J.); 4 Department of Pediatrics, College of Medicine, University of Oklahoma Health Sciences Center, Oklahoma City, OK 73104, USA; hua-zhong@ou.edu (H.Z.); lynette-rogers@ou.edu (L.K.R.); 5 Oklahoma Children’s Hospital OU Health, Oklahoma City, OK 73104, USA

**Keywords:** lung, hyperoxia, aurothioglucose, sex differences, metabolomics

## Abstract

**Highlights:**

**What are the main findings?**

- In survival studies, aurothioglucose administration prior to hyperoxia exposure prolonged survival in both males and females, with females surviving longer than males.

**What are the implications of the main findings?**

- Improved survival of hyperoxia-exposed, ATG-treated, female mice may be driven by alterations in glutathione synthesis, energy production, and metabolism resulting in decreased lung injury.
- These findings highlight previously unrecognized effects of ATG in the lung and may shape further research designed to improve outcomes in settings of hyperoxia exposure.

**Abstract:**

Background: Supraphysiological levels of oxygen are often used as therapy for acute respiratory distress and severe pulmonary morbidities but can cause excessive generation of reactive oxygen species resulting in oxidative and inflammatory injury. Aurothioglucose (ATG), an FDA-approved, gold-containing pharmaceutical, potently and irreversibly inhibits thioredoxin reductase 1, and ATG treatment preserves reduced glutathione levels and attenuates hyperoxic lung injury. Methods: Adult C3H mice were treated with saline or ATG and exposed to room air (RA) or >95% O_2_. All mice had succumbed or were euthanized at 200 h of >95% O_2_. In a separate cohort euthanized at 72 h, prior to the appearance of oxygen toxicity, lung tissues were collected for metabolomic analyses. Comparisons were performed between RA and >95% O_2_ exposure, saline and ATG treatment, and male and female. Results: In <95% O_2_, differences in survival between the sexes with and without ATG treatment were observed. ATG-treated females survived longer than all other hyperoxia-exposed groups. Features in the glutathione and selenoprotein pathways were significantly different. Metabolomic analysis revealed keratan sulfate (KS) biosynthesis and glycosphingolipid (GSL) biosynthesis as the primary pathways affected in the saline O_2_ vs. ATG O_2_ comparison. Carnitine shuttle was identified as the primary pathway between the sexes both with ATG and O_2_. Conclusions: The current data suggest that the improved survival of hyperoxia-exposed, ATG-treated, female C3H mice may be driven by alterations in glutathione synthesis, energy production, and metabolism resulting in decreased lung injury. These findings may provide direction for further research to improve outcomes after hyperoxia exposure.

## 1. Introduction

Supraphysiological levels of oxygen (O_2_) are often used as therapy for acute respiratory distress and other severe pulmonary morbidities. Pulmonary O_2_ toxicity is mediated through excessive generation of reactive O_2_ species that cause oxidative and inflammatory injury. Our earlier work identified thioredoxin reductase (TrxR) inhibition as a protective intervention for hyperoxia-induced lung injury in C3H/HeN (C3H) adult mice [1]. Further mechanistic studies revealed that TrxR inhibition enhanced endogenous nuclear factor E2-related factor 2 (Nrf2)-dependent antioxidant responses [1,2,3,4,5,6,7]. Aurothioglucose (ATG), an FDA-approved, gold-containing pharmaceutical clinically indicated for treatment of rheumatoid arthritis, potently and irreversibly inhibits TrxR1 and is used experimentally to inhibit TrxR1 in animal models [8]. In adult and neonatal mice, ATG treatment was well tolerated, preserved GSH levels, and attenuated hyperoxic lung injury [1,5,9]. Further, administration of ATG attenuated hyperoxia-induced alveolarization deficits in a newborn model of hyperoxic lung injury [5,6].

Sex differences in susceptibility to lung pathologies are well established [10]. Females have more robust immune responses likely due to estrogen responses in inflammatory genes, which manifest as more severe symptoms [11]. For example, females are more likely to develop asthma or acute respiratory distress syndrome than males [12,13,14]. Our previous studies using metabolomic analyses indicated significant differences in hyperoxia tolerance between the sexes in C3H and C57Bl/6 (C57B) strains at baseline [4]. Given our consistent finding of ATG-mediated protection against hyperoxia-induced lung injury in C3H strains, the current studies focused on C3H adult mice exposed to >95% O_2_ to identify potentially unrecognized mechanisms responsible for observed differences in survival between the sexes with and without ATG treatment. Our findings revealed that ATG-treated females survived longer than all other hyperoxia-exposed groups. To understand these differences, we utilized untargeted metabolomic analyses of lungs for this exploratory analysis.

## 2. Materials and Methods

### 2.1. Animal Model

C3H/HeN (C3H) adult mice (6–8 weeks old) were purchased from Harlan (Indianapolis, IN, USA). After a 2–4-week acclimation period, mice received a single intraperitoneal injection at time 0 of either saline or 25 mg/kg of ATG in saline and were allowed to remain in room air (RA) (21% O_2_) or exposed to hyperoxia (>95% O_2_). Hyperoxia exposures were performed in a custom-made plexiglass chamber with a continuous flow of oxygen (10 L/min) controlled using a BioSpherix ProOx P110 controller (Parish, NY, USA). Oxygen levels were measured twice daily (Hudson RCI, Temecula, CA, USA). Soda lime (Fisher Scientific, Fair Lawn, NJ, USA) was placed in the hyperoxia exposure chamber to prevent accumulation of CO_2_. Mice were housed 3–4 per cage with wire tops and all cages were either placed into a single oxygen chamber at >95% O_2_ or a comparable chamber at 21% O_2_.

**Figure 1:**
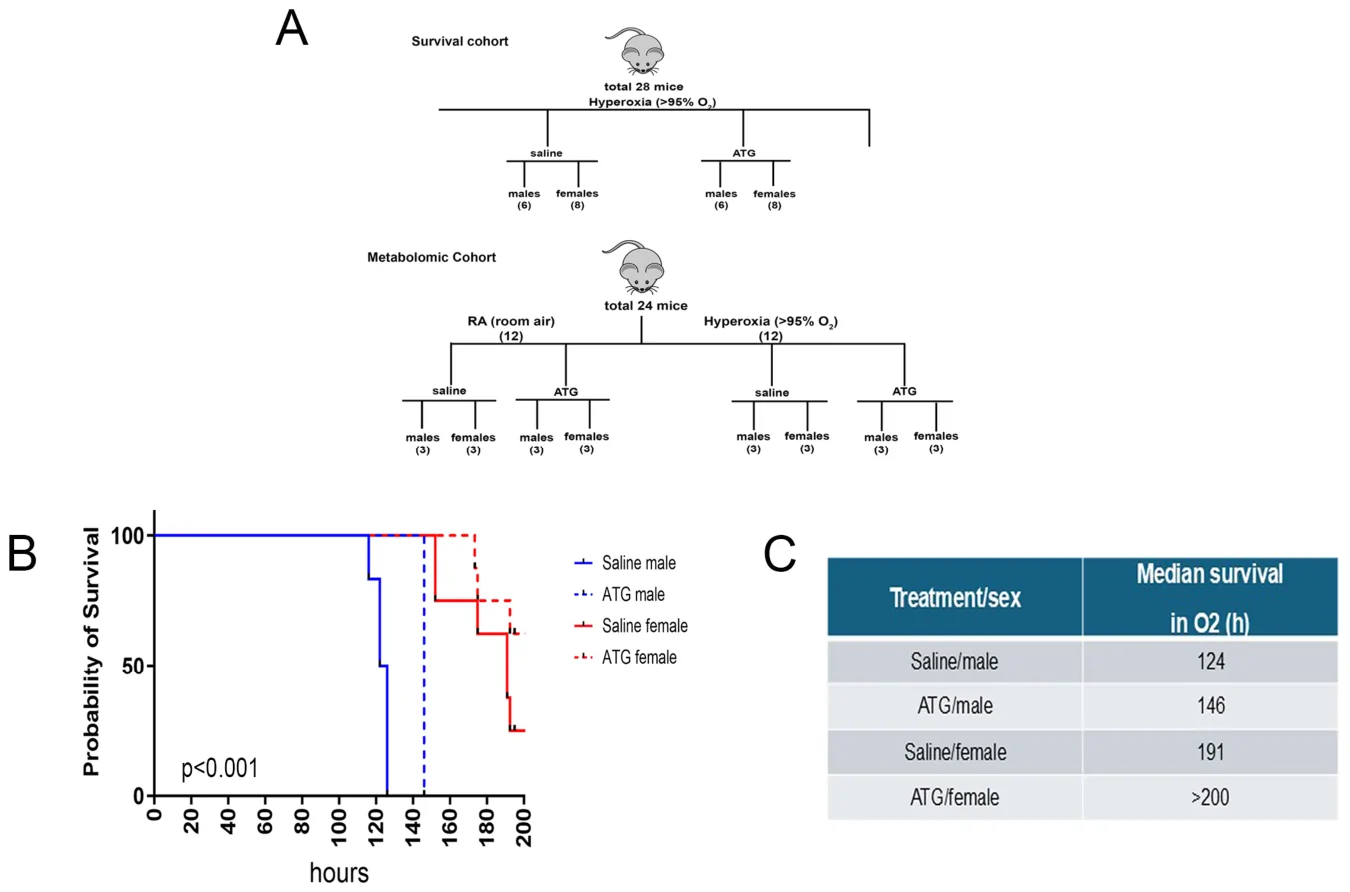
Survival in C3H/HeN male and female mice treated with saline or ATG and exposed to >95% O_2_. (**A**) Experimental design for survival cohort and metabolomics cohort. Parentheses indicate the number of mice in each group. (**B**) Survival was assessed up to a predetermined 200 h at which time the experiment was terminated. Survival curves were analyzed by the Kaplan–Meier and Logrank (Mantel–Cox) test post hoc for differences in survival curves, *p* < 0.0001. (**C**) The median survival time for each treatment group and sex is provided.

Two cohorts of mice were analyzed (Figure 1A). The first (survival) cohort were treated with saline or ATG as described above and all mice remained in >95% O_2_ until death or predetermined termination of the experiment at 200 h (total 28 mice: C3H male (M), saline-treated (6); C3H female (F), saline-treated (8); C3HM, ATG-treated (6); C3HF, ATG-treated (8)). Mice were monitored three times daily for signs of distress (end point criteria included: lethargy, ruffled fur, hunched posture, and agonal breathing).

The second (metabolomics) cohort was treated as described above (total 24 mice: C3HM, saline-treated; C3HF, saline-treated; C3HM, ATG-treated; C3HF, ATG-treated; RA or >95% O_2_, *n* = 3 in each group). The mice were euthanized with ketamine/xylazine (200/20 mg/kg, i.p.) at 72 h after ATG administration and hyperoxia exposure. Tissues were flash frozen in liquid nitrogen and stored at −80 °C for metabolomic analysis.

All treatments and exposures were performed in parallel for each cohort. Animal protocols were approved by the Institutional Animal Care and Use Committee at the University of Alabama at Birmingham (IACUC #20001, approved 17 May 2017). Studies were conducted in accordance with the ARRIVE guidelines.

### 2.2. High-Resolution Metabolomics

Samples were prepared as described in our previous report [4]. Metabolites were extracted from approximately 20–30 mg of lung tissue by homogenization in a mixture of acetonitrile:water (15 µL/mg of 2:1 ratio) which also contained our in-house mixture of stable isotope-labeled internal standards [15]. These extracts were incubated on ice for 30 min and then centrifuged (20,817 × rcf at 4 °C for 10 min) to remove precipitates and stored at 80 °C until metabolomics analyses were initiated. The samples were analyzed using an Orbitrap Fusion Tribrid Mass Spectrometer (Thermo Scientific, Waltham, MA, USA) coupled to a Thermo Dionex Ultimate 3000 liquid chromatography system. Samples were separated using a hydrophilic interaction liquid chromatography column (HILIC; ThermoFisher Scientific, Accucore, 50 × 2.1 mm, 2.6 μm). Electrospray ionization (ESI) was used, with positive ESI for analysis. In triplicate, samples were injected for analysis (10 µL). The flow rates were maintained at 0.35 mL/min until 1.5 min, increased to 0.4 mL/min at 4 min and held for 1 min, resulting in a total analytical run time of 5 min. Mobile phases A and B consisted of water and acetonitrile (LCMS grade). Mobile phase C was comprised of formic acid (2%; *v*/*v*) in water. Conditions for the mobile phases were held at 22.5% A, 75% B, and 2.5% C for 1.5 min, then a linear gradient was applied to 77.5% A, 20% B, and 2.5% C at 4 min, and held for 1 min. We then flushed the HILIC column for 5 min with a wash solution comprising 77.5% A, 20% B, and 2.5% C. Conditions for the fusion included spray voltage, 3500 (V); capillary temperature, 300 °C; sheath gas flow, 45 (arbitrary units); auxiliary gas flow, 25 (arbitrary units); spare gas flow, 1 (arbitrary units); max spray current, 100 (μA); and probe heater temperature, 200 °C. Data were acquired as accurate mass-to-charge ratio (*m*/*z*) in the scan range 85–1275.

### 2.3. Data Processing and Selection of Metabolic Features

Mass spectral files were generated in .raw format and were converted to .cdf files using XCalibur file converter software v. 3.6.1 (Thermo Fisher, Waltham, MA, USA). Mass spectral data were then extracted and collated into a feature table using the R software apLCMS [16] and xMSanalyzer [17], developed previously by the Jones group. Technical triplicates were median summarized, and Pearson correlation scores were calculated for the triplicate analysis. The average coefficient of variation for all metabolic feature intensities was evaluated, and the retention level was set to ≤30% CV. Total ion intensities were also evaluated for quality control of metabolic sample and batch qualities. Batch correction was conducted using Combat [18].

For post hoc bioinformatic analysis, the spectral intensities from the feature table were log2-transformed and quantile-normalized to reduce heteroscedasticity and minimize sample variability. Post hoc filtering criteria for the metabolic features included a 20% detection limit in the overall feature table and, in addition, each feature had to be present in 80% of the samples in either of the categorical groups (e.g., saline vs. ATG-exposed) to be retained for statistical comparison. For the HILIC+ feature table, after filtering, 16,120 features were retained for differential expression analysis using the linear models for microarray data (limma) package in R (v. 3.6.1) [19] using xmsPANDA (v. 1.3.2) (https://github.com/kuppal2/xmsPANDA, accessed on 13 August 2026) in R (v. 4.1.0). False discovery correction (Benjamini–Hochberg false discovery method, FDR) [20] was applied to account for multiple hypothesis testing correction (*q* < 0.05; *q* < 0.2). Two-way hierarchical clustering analysis (HCA; hclust()) plots were used to identify patterns of selected features in an unsupervised manner. Orthogonal partial least squares discriminant analysis (OPLS-DA) is a supervised statistical method which was used to determine the key metabolic features driving differences between comparisons and remove variance not related to the sample class by only focusing on the condition of interest (DOI: 10.1002/cem.695; https://www.sciencedirect.com/science/chapter/edited-volume/abs/pii/B978032385062900009X?, accessed on 13 August 2026).

### 2.4. Glutathione Metabolite Analysis 

Our previous experience in hyperoxic lung injury led us to focus on differential expression of antioxidant pathways, specifically glutathione (GSH) and selenium-dependent (selenoprotein) pathways. Consequently, specific metabolic features representative of these pathways were chosen for further analysis.

### 2.5. Metabolic Pathway Enrichment Analysis

Pathway enrichment analyses were performed on the differentially expressed features identified above using Mummichog v. 2.4.4 [19]. Selected thresholds for significance were set to a *p*-value < 0.05, detection of the primary metabolite ion (M + H), a 5 ppm mass tolerance, and 1000 permutation tests to protect from Type I error, and to increase accurate metabolic annotation. Using corrplot in R, bubble plots of the significant metabolic pathways were generated, using both the size and the color of the bubble (only sex analysis) to represent the pathway significance level based on the −log10 *p* value.

### 2.6. Statistics

The survival data was analyzed using Kaplan–Meier with the Logrank (Mantel–Cox) test post hoc to compare survival curves. Non-metabolomic data are expressed as means and 95% confidence intervals. Data were analyzed using Prism 6.0 (GraphPad, La Jolla, CA, USA). All data were tested for homogeneity of variances (Levene’s), logarithmically transformed when indicated, and analyzed by two-way ANOVA with Tukey’s post hoc test. Significance was accepted at *p* < 0.05.

## 3. Results

### 3.1. ATG Increases Survival in Females

Male and female mice were treated with saline or ATG and exposed to >95% O_2_ (Figure 1A). In saline-treated males, 100% death occurred at 124 h and in ATG-treated males at 146 h. Females were more resistant than males to hyperoxia exposure with saline-treated females surviving to 191 h and most ATG-treated females surviving for more than 200 h, the time point at which the experiment was terminated (Figure 1B,C).

### 3.2. Differentially Expressed Metabolites Between Treatment/Exposure and Sex

Metabolomic data was analyzed using MWAS, comparing treatment, exposure, and sex independently using LIMMA. A value of 0.05 was used for raw data (*p* < 0.05) and FDR-corrected data (*q* < 0.05). After FDR correction, no significant features were identified in the saline O_2_ vs. ATG O_2_ comparisons, so a more liberal threshold was investigated. Even with a threshold of *q* = 0.2, only one feature was identified. Table 1 provides a summary of the analyses with the number of significant features (FDR) and raw metabolite numbers.

#### 3.2.1. Analyses by Treatment/Exposure

**Figure 2:**
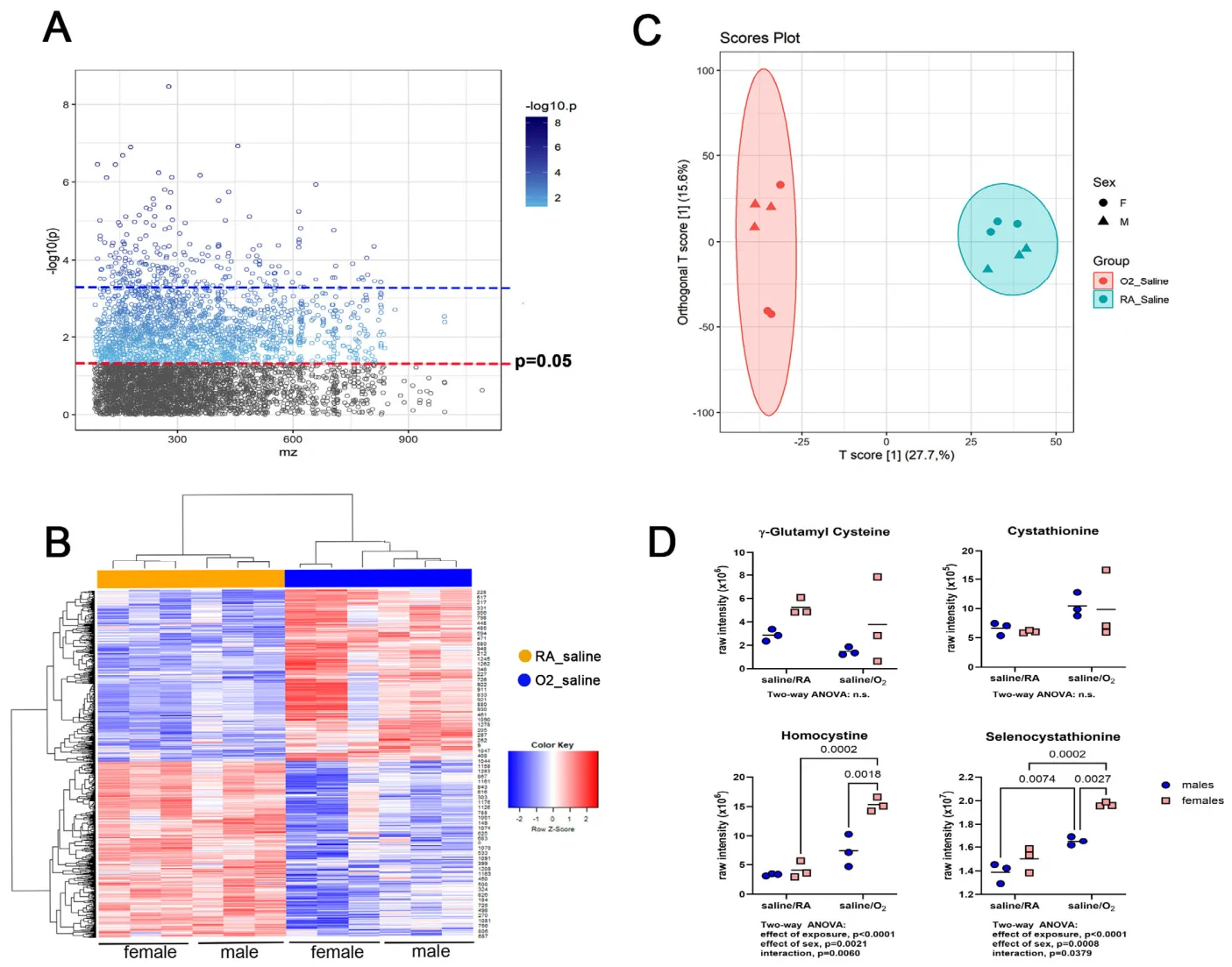
Metabolomic data was analyzed using *log2*-transformed spectral intensities. Differential expression analysis used linear models generated from the limma package in R using xmsPANDA. Differentially expressed metabolites specific to **room air (RA) or >95% O_2_ (O_2_) exposure** yielded changes in feature intensity levels. (**A**) Manhattan plot displaying the distribution of −log10(*p*) values against mass-to-charge ratio (mz), with color intensity corresponding to the −log10(*p*) value as indicated by the color scale on the right. The red dashed horizontal line and blue dashed horizontal line represent the significance threshold *p* = 0.05 and threshold *q* = 0.05, respectively. (**B**) Two-way hierarchal clustering heatmap of the significant metabolite features between saline-treated, RA- or O_2_-exposed conditions. The normalized z-score is represented by a color gradient from blue to red, indicating downregulated and upregulated values, respectively. (**C**) OPLS-DA score plot illustrates the separation between two experimental groups, saline-treated, RA- or O_2_-exposed, as well as within-group separation. Circles and triangles represent female and male mice, respectively. Inter-group separation is 27.7% on x-axis (predictive T-score, T1) and intra-group separation is 15.6% on y-axis (orthogonal T-score). (**D**) Differentially expressed GSH and selenoprotein metabolic features were plotted as individual graphs (each point represents one mouse). Data were analyzed by two-way ANOVA with Tukey’s post hoc and are expressed as mean with 95% confidence interval, *n* = 3 per group, n.s. = not significant.

Analyses of differentially expressed metabolites specific to oxygen exposure alone (saline-treated with RA or <95% O_2_) caused a change in the expression levels of 1876 metabolites with *p* < 0.05 and 1160 metabolites with *q* < 0.05 (Figure 2A, Supplemental Table S1). In total, 512 metabolites increased and 647 metabolites decreased in oxygen exposure as indicated by the heatmap in Figure 2B. An OPLS-DA plot demonstrates separation between exposure groups with more within-group variability in the saline/O_2_ group than the saline/RA group (Figure 2C). Metabolic features associated with redox pathways, specifically glutathione and selenoproteins, were identified and graphed using raw intensity levels. Two-way ANOVA with Tukey’s post hoc indicated increases in homocysteine and selenocystathionine with oxygen exposure (Figure 2D).

**Figure 3:**
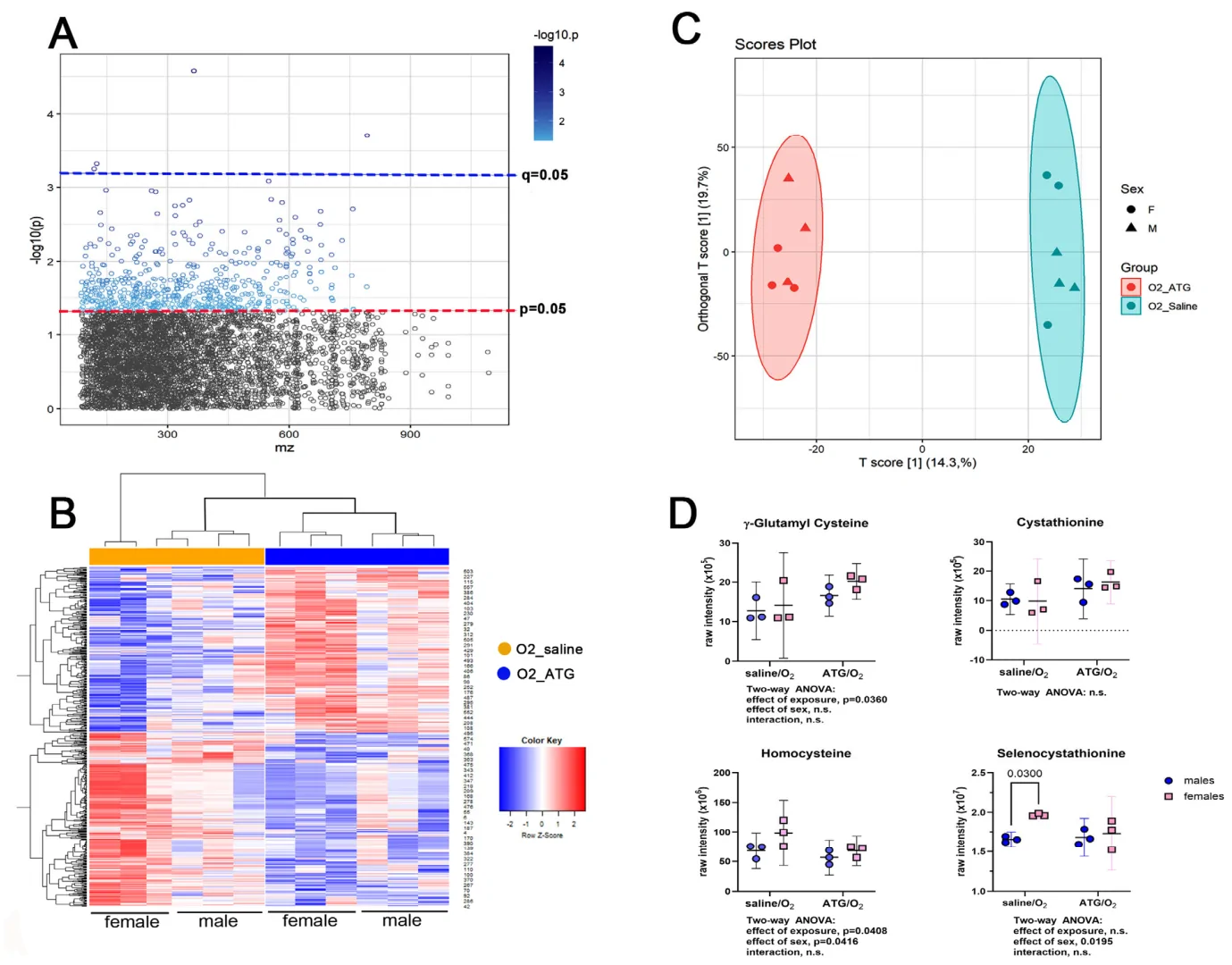
Metabolomic data was analyzed using *log2*-transformed spectral intensities. Differential expression analysis used linear models generated from the limma package in R using xmsPANDA. Differentially expressed metabolites due to **saline or ATG treatment** with O_2_ exposure caused changes in feature intensity levels. (**A**) Manhattan plot displaying the distribution of −log10(*p*) values against mass-to-charge ratio (mz), with color intensity corresponding to the −log10(*p*) value as indicated by the color scale on the right. The red dashed horizontal line and blue dashed horizontal line represent the significance threshold *p* = 0.05 and threshold *q* = 0.05, respectively. (**B**) Two-way hierarchal heatmap of the significant metabolite features between saline- or ATG-treated, O_2_-exposed conditions. The normalized z-score is represented by a color gradient from blue to red, indicating downregulated and upregulated values, respectively. (**C**) OPLS-DA score plot illustrating the separation between two experimental groups, saline- or ATG-treated, O_2_-exposed, as well as within-group separation. Circles and triangles represent female and male, respectively. Inter-group separation is 14.3% on x-axis and intra-group separation is19.7% on y-axis. (**D**) Differentially expressed glutathione and selenoprotein metabolic features were plotted as individual graphs (each point represents one mouse). Data were analyzed by two-way ANOVA with Tukey’s post hoc and are expressed as mean with 95% confidence interval, *n* = 3 per group, n.s.= not significant.

Analyses of differentially expressed metabolites specific to saline or ATG treatment in addition to oxygen exposure yielded 583 metabolites with *p* < 0.05 but only 1 metabolites with a value of *q* < 0.2 (Figure 3A, Supplemental Table S2). In total, 285 metabolites increased and 298 metabolites decreased with ATG treatment in oxygen as indicated by the heatmap in Figure 3B. The OPLS-DA plot indicates that the separation between groups is similar to other treatment/exposure comparisons but the within-group variability is less than in other comparisons (Figure 3C). Analyses of individual glutathione and selenoprotein metabolic features indicated only minor differences between groups (Figure 3D).

**Figure 4:**
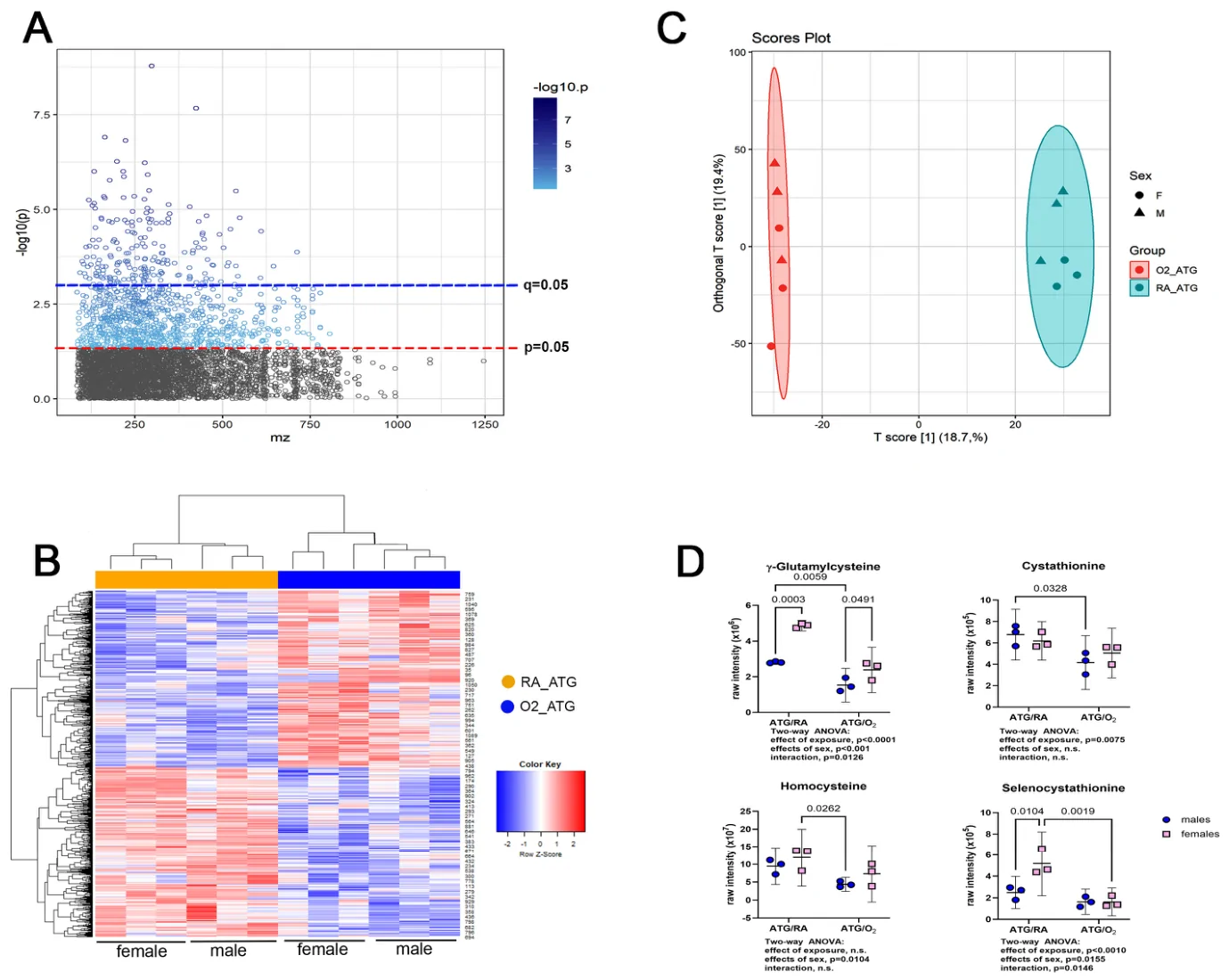
Metabolomic data was analyzed using *log2*-transformed spectral intensities. Differential expression analysis used linear models generated from the limma package in R using xmsPANDA. Differentially expressed metabolites specific to **ATG treatment with RA or O_2_ exposure** caused changes in feature intensity levels. (**A**) Manhattan plot displaying the distribution of −log10(*p*) values against mass-to-charge ratio (mz), with color intensity corresponding to the −log10(*p*) value as indicated by the color scale on the right. The red dashed horizontal line and blue dashed horizontal line represent the significance threshold *p* = 0.05 and threshold *q* = 0.05, respectively. (**B**) Two-way hierarchal heatmap of the significant metabolite features between ATG-treated, RA- or O_2_-exposed conditions. The normalized z-score is represented by a color gradient from blue to red, indicating downregulated and upregulated values, respectively. (**C**) OPLS-DA score plot illustrating the separation between two experimental groups, ATG-treated, RA- or O_2_-exposed, as well as within-group separation. Circle and triangle represent female and male, respectively. Inter-group separation is 18.7% on x-axis and intra-group separation is 19.4% on y-axis. (**D**) Differentially expressed GSH and selenoprotein metabolic features were plotted as individual graphs (each point represents one mouse). Data were analyzed by two-way ANOVA with Tukey’s post hoc and expressed as mean with 95% confidence interval, *n* = 3 per group, n.s.= not significant.

Analyses of differentially expressed metabolites specific to ATG treatment with and without exposure to oxygen yielded 1101 metabolites with *p* < 0.05 and 381 metabolites with *q* < 0.05 (Figure 4A, Supplemental Table S3). In total, 564 metabolites increased and 537 metabolites decreased as demonstrated in the heatmap (Figure 4B). The OPLS-DA plot indicated similar divergence between groups and variability within groups as observed with other treatment/exposure comparisons. This would imply that many of the differentially expressed metabolites are driven by oxygen exposure (Figure 4C). Analyses of individual redox metabolic features indicated that ATG/O_2_ exposures were associated with decreases in γ-glutamylcysteine, cystathionine, and selenocystathionine (Figure 4D).

#### 3.2.2. Analyses by Sex

**Figure 5:**
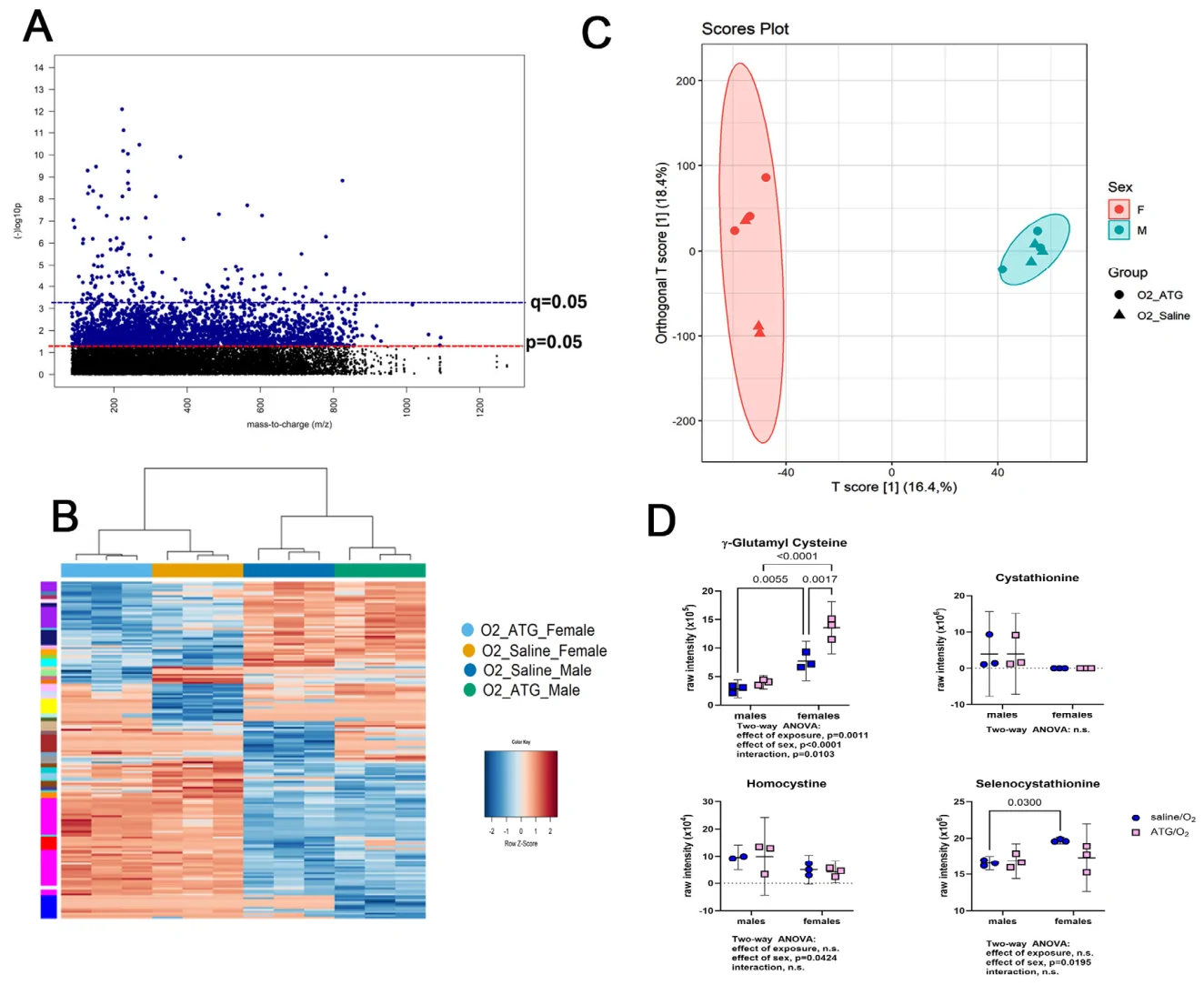
Metabolomic data was analyzed using log2-transformed spectral intensities. Differential expression analysis used linear models generated from the limma package in R using xmsPANDA. Differentially expressed metabolites due to **saline or ATG treatment with O_2_ exposure** caused changes in feature intensity levels. (**A**) Manhattan plot displaying the distribution of −log10(*p*) values against mass-to-charge ratio (mz). The red dashed horizontal line and blue dashed horizontal line represent the significance threshold *p* = 0.05 and threshold *q* = 0.05, respectively. (**B**) Two-way hierarchal heatmap of the significant metabolite features between saline- or ATG-treated, O_2_-exposed, males or females. The normalized z-score is represented by a color gradient from blue to red, indicating downregulated and upregulated values, respectively. (**C**) OPLS-DA score plot illustrating the separation between experimental groups, saline or ATG, RA- or O_2_-exposed, males and females. Circle and triangle represent female and male, respectively. Inter-group separation is 16.4% on x-axis and intra-group separation is 18.4% on y-axis. (**D**) Differentially expressed GSH and selenoprotein metabolic features were plotted as individual graphs (each point represents one mouse). Data were analyzed by two-way ANOVA with Tukey’s post hoc and are expressed as mean with 95% confidence interval, *n* = 3 per group, n.s. = not significant.

Analyses of differentially expressed metabolites due to sex with and without exposure to oxygen or ATG treatment yielded 2643 metabolites with *p* < 0.05 and 176 metabolites with *q* < 0.05 (Figure 5A, Supplemental Table S4). Of these significant metabolites, 1216 metabolites were increased either by sex or by treatment, and 1427 metabolites were decreased as a result of sex or treatment, as demonstrated in the heatmap (Figure 5B). Overall, the males had more features that were decreased than the females. The OPLS-DA plot indicated significantly greater divergence between groups and greater variability within females than observed in other treatment/exposure comparisons (Figure 5C). Analyses of individual redox metabolic features indicated that females had greater levels of homocysteine and selenocystathionine in response to oxygen exposure than males (Figure 5D).

### 3.3. Pathway Analyses

**Figure 6:**
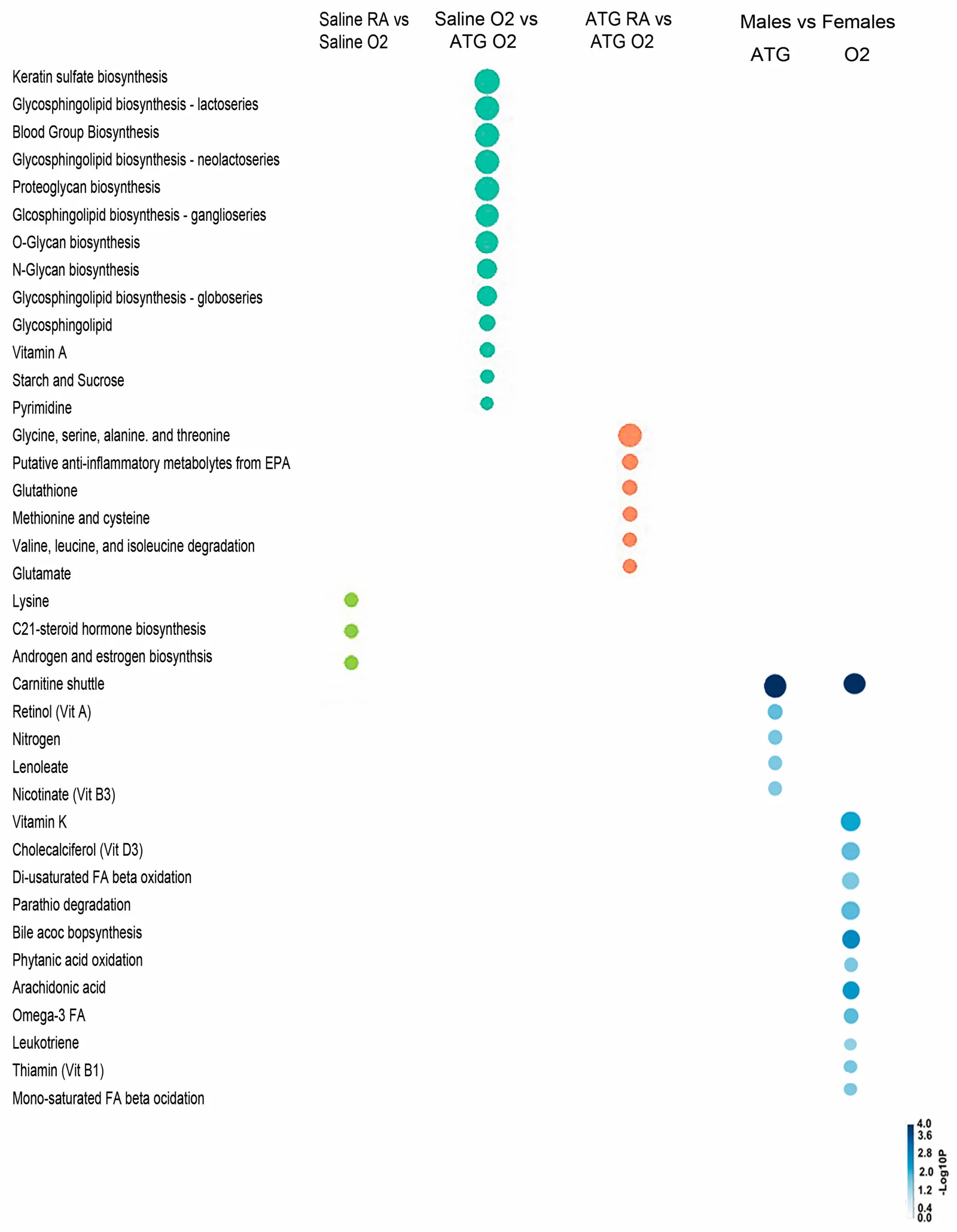
Pathway enrichment analyses were performed on the differentially expressed features identified above using mummichog 2.2. Selected thresholds for significance were set to a *p*-value < 0.05. Bubble plots of the significant metabolic pathways were generated, using size to represent the pathway significance level in the first 3 analyses and both color and size for the sex analyses. All data was based on the −log10 *p* value.

Mummichog (v. 2.2) pathway analysis (Figure 6) indicated metabolic pathways differentially expressed between exposure groups. Only three pathways were modestly different in the saline-treated, RA vs. O_2_ exposure comparison. Intriguingly, 13 pathways were dramatically different in the saline vs. ATG with O_2_ exposure comparison. This would imply that ATG causes disruption in several glycosphingolipid and carbohydrate pathways. Six pathways were identified as different in the comparison between RA and O_2_ in the presence of ATG. These included several amino acid pathways and several redox regulatory pathways such as glutathione and methionine and cysteine. We interpret these data to suggest that ATG treatment in the presence of O_2_ exposure may invoke redox pathways to provide protective effects.

Further analyses compared sexes with both O_2_ exposure and ATG treatment. ATG treatment and O_2_ exposure caused dramatic differences in the carnitine shuttle pathway. This suggests the presence of differences in energy metabolism as carnitine is responsible for shuttling lipids to the mitochondria. O_2_ exposure also caused modest differences in vitamin K, bile acid biosynthesis, and arachidonic acid pathways.

## 4. Discussion

Therapeutic oxygen administration is essential for treating pulmonary disease or injury, but exposure to hyperoxia can have deleterious effects. Our previous studies in murine models have provided a body of evidence that collectively supports the therapeutic value of ATG administration to lessen the injurious effects of hyperoxia [2,5,6]. Though the mechanisms by which ATG lessens lung injury are less clear, our data have implicated the glutathione system as a key contributor [1,5]. The present studies included evaluations of sex as a biological variable and were designed to further elucidate the mechanisms by which ATG attenuates hyperoxia-induced lung injury through focused metabolomic comparisons between males and females. Our new data indicate that metabolic differences are observed in response to O_2_ exposure or ATG treatment, and the effects differ by sex.

Decades of research have demonstrated that females have more robust immune responses than males, including faster clearance of pathogens and greater responses to vaccines, though females have an increased susceptibility to inflammatory and autoimmune diseases (reviewed by Klein and Flanagan) [11,14,21]. Females also have a greater likelihood of developing lung pathologies such as ARDS, asthma, and more robust responses to COVID-19 [12,13,22]. Recent studies in rats have identified enhanced antioxidant capacity and mitochondrial responses in females versus males as potential mechanisms underlying less severe responses to hyperoxia [23]. In metabolomic studies of selenium deficiency and high fat diet, males have demonstrated a more adaptive response to metabolic syndrome than females [24]. Further, Coarfa et al. investigated neonatal male versus female responses to hyperoxia [25]. Using RNA seq with Gene Set Enrichment Analyses, distinct transcriptomic responses were identified and genes encoding differentially expressed redox regulated transcription factors genes including *Nrf2, Ap1*, *NFkB* and *HIF-1α* [25].

The present studies revealed that hyperoxia exposure and ATG treatment caused differences in survival between the sexes (Figure 1). Hyperoxia-exposed females survived longer than hyperoxia-exposed males with the majority of ATG-treated females still alive at 200 h, the termination of the experiment. To better understand the influence of sex as a biological variable on our observed outcomes, we selected a 72 h timepoint for metabolomic analyses, a time that was consistently prior to the onset of observed morbidity in our collective body of work in hyperoxic lung injury. We chose to focus our metabolomic analyses on the glutathione and selenium antioxidant pathways based on our previously published hyperoxia-induced lung injury data [1,2,9,26,27].

For initial comparisons, we evaluated the impact of ATG treatment and/or hyperoxia exposure on lung tissue metabolic feature intensity. The metabolomic comparison between RA and hyperoxia exposure revealed the greatest number of differentially expressed metabolites (Table 1 and Figure 2A). Similar numbers of metabolites increased or decreased due to oxygen exposure (Figure 2B), and oxygen exposure caused a greater divergence in expression than observed in RA-exposed mice (Figure 2C). Homocysteine and selenocystathionine intensity were elevated in lungs from hyperoxia-exposed animals, and the findings suggested a potential upregulation of glutathione and/or selenoprotein synthesis (Figure 2D)—both of which may be protective against oxidant injury. The fewest differentially expressed metabolites were observed in the comparison between saline- or ATG-treated mice exposed to hyperoxia (Table 1 and Figure 3A). The heatmap in Figure 3B further demonstrated a less distinct pattern of differences and the OPLS plot in Figure 3C indicated less divergence between the groups. Further analyses of individual metabolic features indicated that of the four features chosen for further analysis, only selenocystathionine was statistically different between saline-treated males and females exposed to hyperoxia (Figure 3D). We interpret this observation to suggest that hyperoxia exposure may have a greater influence on glutathione and selenium metabolites than ATG treatment. Comparison of the effects of ATG treatment in the setting of either RA or hyperoxia exposure revealed a high number of differentially expressed metabolites compared to ATG or saline treatment in lungs from hyperoxia-exposed mice (Table 1 and Figure 4A) with equal numbers of metabolites being increased or decreased (Figure 4B). The OPLS plot indicated a greater divergence in the metabolites from ATG/hyperoxia-exposed lungs compared to ATG-treated mice in RA (Figure 4C). When compared to the more susceptible ATG/RA group, the present data revealed that the expression of the selected metabolic features, γ-glutamylcysteine, cystathionine, and selenocystathionine, were lower in ATG-treated mice exposed to hyperoxia, the longest surviving groups in our study (Figure 4D). Collectively, we conclude that the comparisons between each of these individual treatments or exposures may account for our observed differences in survival between males and females.

To determine whether an association existed between sex in lung metabolites in our groups, a final analysis was performed using metabolomic data from lung tissues of ATG- or saline-treated males or females exposed to room air or hyperoxia. While the number of significant features was less than observed in other comparisons (Table 1 and Figure 5A), the heatmap indicated differences in all groups, especially between male and female mice (Figure 5B). The OPLS plot indicated differences in the distribution of metabolites in females that were not observed in males. Given that ATG-treated female mice were the least susceptible to hyperoxia, we interpret these data to indicate that the differential response to ATG treatment between male and female mice is likely responsible for lower hyperoxia-induced mortality in females (Figure 5C). The expression of chosen metabolic features indicated substantially greater intensity of γ-glutamylcysteine and modestly greater intensity of selenocystathionine and very low intensity of cystathionine in females compared to males (Figure 5D). γ-glutamylcysteine is a precursor to glutathione synthesis and greater expression would imply that ATG-treated females are more capable of glutathione synthesis upregulation in response to hyperoxia than ATG-treated males. Importantly, these findings are consistent with our previous report that ATG-mediated pulmonary protection, which was lost with concomitant administration of the γ-glutamylcysteine inhibitor buthionine sufoximine, is glutathione-dependent [1]. Further, our findings of differential enhancement of glutathione synthesis in females vs. males in response to ATG are consistent with the findings of Coarfa et al. who demonstrated that redox transcriptomic responses were regulated in opposite directions for males vs. females in response to hyperoxia exposure [25].

Metabolomic pathway analysis for each of the comparisons revealed the greatest differences between lungs from hyperoxia-exposed animals treated with saline vs. ATG (Figure 6). These differences were in metabolites belonging to the keratan sulfate (KS) and glycosphingolipid (GSL) biosynthesis pathways. KS refers to several sulfated glycosaminoglycans that are large, highly hydrated molecules. Typically, they are found in the cornea, cartilage, and bone and can act as a cushion to absorb mechanical shock [28]. GSLs are a large group of bioactive lipids that play major roles in membrane structure, regulation of receptors and ion transporters, and cell–cell communication [29,30,31]. GSLs consist of a hydrophobic ceramide backbone and glucosidic carbohydrate. The subgroups are distinguished by the specific carbohydrate bound or the addition of other moieties. Both KSs and GSLs are formed in the endoplasmic reticulum and then trafficked to the Golgi for the addition of other functional groups. The species formed depends upon the substrates available and the specific enzyme acting upon the core molecule moiety. In the lungs, KSs are expressed in the interstitial, subepithelial tissues, bronchial walls, and airway secretions. Gao, et al. demonstrated that L4, a specific KS, is protective in a mouse model of COPD by decreasing the number of inflammatory cells, cytokines, and MMP expression [32]. Alternatively, sphingolipids are essential to the interaction of leukocytes with the blood vessel wall during inflammation and are involved in the production of reactive oxygen species and airway remodeling in both bronchopulmonary dysplasia and COPD [30]. Within the cell, GSLs can interact with several receptors including epidermal growth factor receptor, platelet-derived growth factor receptor, fibroblast growth factor receptor, and insulin receptor. Collectively, we proposed that ATG-associated differences in KS and GSL levels in hyperoxia may dampen inflammatory responses and enhance repair processes in our experimental model. In the setting of hyperoxia, modulation of KS and GSL levels may further contribute to our observation of improved survival of ATG-treated mice.

We focused our analyses of sex in the context of oxygen exposure and ATG treatment and noted a substantial difference between male and female mice in carnitine shuttle (Figure 6). Carnitine shuttle is associated with lipid metabolism and transport to the mitochondria for energy production. This observation would suggest that energy metabolism is substantially different between males and females in the setting of hyperoxia-mediated oxidative stress. Our finding is in line with a report by Teheri, et al. that identified enhanced antioxidant capacity and adaptive mitochondrial responses in female rats exposed to hyperoxia [23]. Other than carnitine shuttle, there were no other shared pathway discrepancies identified between ATG treatment and hyperoxia exposure when comparing male and female mice. While few metabolomic studies have been reported in hyperoxia-treated adult subjects, several have reported sex differences in metabolomic data in COPD and emphysema rodent models and human studies [33,34]. Common to both rodents and humans, sphingosine pathway and TCA cycle metabolites were noted as different between males and females. Our data indicate that hyperoxia exposure may alter pathways similar to those involved in other lung pathologies.

## 5. Conclusions

The present data add to our cumulative body of work demonstrating the protective effects of ATG pre-treatment on hyperoxia tolerance in mice and support the contribution of enhanced glutathione-dependent responses. Collectively, our work suggests that the improved survival of hyperoxia-exposed ATG-treated female C3H mice may be influenced by alterations in glutathione synthesis, energy production, and metabolism resulting in decreased lung injury through modulation of KS, GSL, and carnitine shuttle pathways. These findings may provide direction for further research to improve outcomes after hyperoxia exposure.

## Figures and Tables

**Table 1:** Results of treatment/exposure in C3H mice with ATG and/or O_2_.

Comparison	FDR (BH), *q* =	*p*-Value	Significant Features: Raw
Saline RA vs. saline O_2_	0.05	0.05	1159;1876
Saline O_2_ vs. ATG O_2_	0.20	0.05	1;583
ATG RA vs. ATG O_2_	0.05	0.05	381;1101
M vs. F vs. O_2_ vs. ATG	0.05	0.05	176;2643

RA, room air; ATG, aurothioglucose; O_2_ = >95% O_2_; M, male; F, female. See Supplemental Table S5.

## Data Availability

The metabolomic data presented in this study are available in Metabolomics Workbench [http://dev.metabolomicsworkbench.org:22222/data/DRCCMetadata.php?Mode=Study&StudyID=ST002264&Access=MzxA3369] (accessed on 17 June 2022) at [http://dx.doi.org/10.21228/M8T70R] (accessed on 30 August 2024).

## Supplementary Materials

The following supporting information can be downloaded at: https://www.mdpi.com/article/10.3390/metabo16090623/s1, Supplemental Table S1. Annotated data for saline treated, RA or O_2_ exposed; Supplemental Table S2. Annotated data from Saline or ATG treatment with oxygen exposure; Supplemental Table S3. Annotated data from ATG-treated, RA or >95% O_2_ exposed; Supplemental Table S4. Annotation of metabolites associated with sex by O_2_ and ATG; Supplemental Table S5. Annotation data from HILIC xMSAnnotator. Supplemental Materials contain the annotation tables from which Figure 2, Figure 3, Figure 4 and Figure 5 were generated as well as the total annotation table for HILICxMSAnnotator.

## Abbreviations

The following abbreviations are used in this manuscript:

ATG	Aurothioglucose
RA	Room air
TrxR1	Thioredoxin reductase 1
Nrf2	Nuclear factor E2-related factor 2
GSH	Reduced glutathione
OPLS-DA	Orthogonal least partial squares discriminant analyses
FDR	False discovery rate
MWAS	Metabolomic-wide association study
HILIC	Hydrophobic interaction liquid chromatography
C18	18-carbon chain used as stationary phase for chromatography
LC-MS	Liquid chromatography–mass spectrometry
KS	Keratan sulfate
GSL	Glycosphingolipid
O2	Hyperoxia (greater oxygen than room air (21%))
