# Supplementary material for: Sex-Associated Effects of Thioredoxin Reductase Inhibition in Hyperoxia-Induced Lung Injury in Adult Mice
Source: Metabolites. 2026 Aug 28;16(9):623. doi: 10.3390/metabo16090623 (PMC13609309; doi:10.3390/metabo16090623)
Supplement: Supplementary file 1 [file metabolites-16-00623-s001.zip › metabolites-4466093-supplementary.pdf]

Supplemental Table S1. Annotated data for saline treated, RA or O2 exposed.

feature	V1	V2	t.stat	p.value	X.log10.p.	FDR	mz	all_HMDBID	all_Name	all_Adduct	all_Formula
277.13944	1.851047	0.125477	19.042	3.47E-09	8.4602	1.73E-05	277.1394	HMDB00279 ; HMDB00535 ; HMDB00689 ; HMDB28780 ; HMDB28948 ; HMDB29762 ; HMDB30027 ; HMDB30056 ; HMDB30059 ; HMDB31207 ; HMDB31221 ; HMDB31246 ; HMDB31248 ; HMDB31325 ; HMDB31511 ; HMDB31580 ; HMDB33774 ; HMDB33889 ; HMDB34079 ; HMDB34163 ; HMDB37266 ; HMDB38574 ; HMDB38759 ; HMDB39150 ; HMDB39443 ; HMDB40279 ; HMDB40327 ; HMDB40398	Saccharopine ; Caproic acid ; Isocaproic acid ; Cysteinyl-Lysine ; Lysyl-Cysteine ; Methyl (S)-2-Methylbutanoate ; Methyl 3-methylbutanoate ; Isopropyl propionate ; Propyl propionate ; Methyl pentanoate ; 2-Ethylbutanoic acid ; 2-Methylpropyl acetate ; Ethyl 2-methylpropanoate ; n-Butyl acetate ; Diacetone alcohol ; ()-2-Methylpentanoic acid ; 3-Methylpentanoic acid ; Ethyl butyrate ; Homodolichosterone ; 3-Methylbutyl formate ; 224-Trimethyl-13-dioxolane ; Lansiumamide A ; Licoricesaponin C2 ; Flavidulol C ; (2alpha3alpha5alpha22R23R)-232223-Tetrahydroxy-25-methylergost-24(28)en-6-one ; 245-Trimethyl-13-dioxolane ; Pentyl formate ; 1alpha3beta22R-Trihydroxyergosta-524E-dien-26-oic acid 3-O-b-D-glucoside 26-O-b-D-glucosyl ester	M+H ; 2M+2Na-H ; 2M+2Na-H ; 2M+3H2O+2H ; 2M+3H2O+2H ; 2M+2Na-H ; 2M+2Na-H ; 2M+2Na-H ; 2M+2Na-H ; 2M+2Na-H ; 2M+2Na-H ; 2M+2Na-H ; 2M+2Na-H ; 2M+2Na-H ; 2M+2Na-H ; 2M+2Na-H ; 2M+2Na-H ; M+2K ; 2M+2Na-H ; 2M+2Na-H ; 2M+3H2O+2H ; M+2H+Na ; M+H+K ; M+2K ; 2M+2Na-H ; 2M+2Na-H ; M+H+2Na	C11H20N2O6 ; C6H12O2 ; C6H12O2 ; C9H19N3O3S ; C9H19N3O3S ; C6H12O2 ; C6H12O2 ; C6H12O2 ; C6H12O2 ; C6H12O2 ; C6H12O2 ; C6H12O2 ; C6H12O2 ; C6H12O2 ; C6H12O2 ; C6H12O2 ; C6H12O2 ; C6H12O2 ; C29H48O5 ; C6H12O2 ; C6H12O2 ; C17H15NO ; C42H62O15 ; C34H42O4 ; C29H48O5 ; C6H12O2 ; C6H12O2 ; C40H64O15
457.02009	1.823889	0.148604	-13.191	1.19E-07	6.9228	0.000215	457.0201	HMDB15541 ; HMDB29735 ; HMDB34590	Meticillin ; 5-Ethynyl-5-(1-propynyl)-22-bithiophene ; Shoyuflavone C	M+2K-H ; 2M+H ; M+K	C17H20N2O6S ; C13H8S2 ; C19H14O11
178.07099	1.815749	0.31919	-13.084	1.29E-07	6.8892	0.000215	178.071	HMDB00225 ; HMDB00398 ; HMDB14607 ; HMDB28808 ; HMDB29081 ; HMDB29097 ; HMDB29160 ; HMDB29379 ; HMDB33374 ; HMDB39447 ; HMDB59737 ; HMDB32418 ; HMDB32422	Oxoadipic acid ; 3-Oxoadipic acid ; Sodium Tetradecyl Sulfate ; Glutaminyl-Tryptophan ; Tryptophyl-Glutamine ; Tryptophyl-Gamma-glutamate ; Gamma-glutamyl-Tryptophan ; Sinapine ; Benzyl glycinate 4-methylbenzenesulfonate salt ; 2-Methyl-4-oxopentanedioic acid ; 3-Methyl-3-hydroxypentanedioate ; 2-(4-Methyl-5-thiazolyl)ethyl butanoate ; 2-(4-Methyl-5-thiazolyl)ethyl isobutyrate	M+NH4 ; M+NH4 ; M+Na+K ; M+H+Na ; M+H+Na ; M+H+Na ; M+H+Na ; M+2Na ; M+H+NH4 ; M+NH4 ; M+NH4 ; M+H-2H2O ; M+H-2H2O	C6H8O5 ; C6H8O5 ; C14H30O4S ; C16H20N4O4 ; C16H20N4O4 ; C16H20N4O4 ; C16H20N4O4 ; C16H24NO5 ; C16H19NO5S ; C6H8O5 ; C6H8O5 ; C10H15NO2S ; C10H15NO2S
157.55485	1.807791	0.427584	12.414	2.12E-07	6.6733	0.000265	157.5549	HMDB14683 ; HMDB29283 ; HMDB38944 ; HMDB02670 ; HMDB05897 ; HMDB13130 ; HMDB29631 ; HMDB30873 ; HMDB31824 ; HMDB33308 ; HMDB33928 ; HMDB34002 ; HMDB34105 ; HMDB34126 ; HMDB37254 ; HMDB38549 ; HMDB41656 ; HMDB41698 ; HMDB41704	Amoxapine ; Avenanthramide 1f ; Avenanthramide E ; Naringenin ; Dihydrogenistein ; Glutarylcarnitine ; Chalconaringenin ; 1-Hydroxy-37-dimethoxyxanthone ; 458-Trihydroxyflavanone ; Moracin J ; ()-2-Hydroxydihydrodaidzein ; 349-Trihydroxypterocarpan ; Glycinol ; Gyrocyanin ; (2R3R)-347-Trihydroxyflavanone ; Toralactone ; 3-Hydroxydihydrodaidzein ; 6-Hydroxydihydrodaidzein ; 8-Hydroxydihydrodaidzein	M+2H ; M+2H ; M+2H ; M+ACN+2H ; M+ACN+2H ; M+H+K ; M+ACN+2H ; M+ACN+2H ; M+ACN+2H ; M+ACN+2H ; M+ACN+2H ; M+ACN+2H ; M+ACN+2H ; M+H+NH4 ; M+ACN+2H ; M+ACN+2H ; M+ACN+2H ; M+ACN+2H ; M+ACN+2H	C17H16ClN3O ; C17H15NO5 ; C17H15NO5 ; C15H12O5 ; C15H12O5 ; C12H21NO6 ; C15H12O5 ; C15H12O5 ; C15H12O5 ; C15H12O5 ; C15H12O5 ; C15H12O5 ; C15H12O5 ; C17H12O5 ; C15H12O5 ; C15H12O5 ; C15H12O5 ; C15H12O5 ; C15H12O5
92.5758686	1.801224	0.136803	11.747	3.57E-07	6.4474	0.000299	92.57587	HMDB29742 ; HMDB31860 ; HMDB32933	3-Isopropyl-2-methoxy-5-methylpyrazine ; 2-Methoxy-3-(2-methylpropyl)pyrazine ; 2-Methoxy-3-(1-methylpropyl)pyrazine	M+H+NH4 ; M+H+NH4 ; M+H+NH4	C9H14N2O ; C9H14N2O ; C9H14N2O
138.58135	1.798556	0.15211	11.739	3.59E-07	6.4447	0.000299	138.5814	HMDB03869 ; HMDB04207 ; HMDB00508 ; HMDB00568 ; HMDB01851 ; HMDB02917 ; HMDB32341 ; HMDB33730 ; HMDB38433 ; HMDB38614 ; HMDB38676 ; HMDB38973	Epsilon-(gamma-Glutamyl)-lysine ; L-a-glutamyl-L-Lysine ; Ribitol ; D-Arabitol ; L-Arabitol ; D-Xylitol ; Isoamyl isothiocyanate ; L-2-(Hydroxymethyl)-1234-butanetetrol ; 1-Isothiocyanatopentane ; gamma-L-Glutamyl-L-pipecolic acid ; (2S2S)-Pyrosaccharopine ; 25-Dihydro-245-trimethylthiazole	M+2H ; M+2H ; M+3ACN+2H ; M+3ACN+2H ; M+3ACN+2H ; M+3ACN+2H ; 2M+H+NH4 ; M+3ACN+2H ; 2M+H+NH4 ; M+H+NH4 ; M+H+NH4 ; 2M+H+NH4	C11H21N3O5 ; C11H21N3O5 ; C5H12O5 ; C5H12O5 ; C5H12O5 ; C5H12O5 ; C6H11NS ; C5H12O5 ; C6H11NS ; C11H18N2O5 ; C11H18N2O5 ; C6H11NS
251.14295	1.838742	0.072518	11.134	5.89E-07	6.2299	0.00039	251.1427	HMDB01959 ; HMDB12560 ; HMDB31017 ; HMDB31028 ; HMDB31741 ; HMDB31896 ; HMDB32792 ; HMDB34286 ; HMDB35730 ; HMDB37163 ; HMDB37187 ; HMDB37226 ; HMDB37626 ; HMDB37907 ; HMDB38078 ; HMDB39805 ; HMDB40451 ; HMDB41365 ; HMDB00313 ; HMDB00335 ; HMDB00343 ; HMDB00406 ; HMDB05895 ; HMDB15182 ; HMDB60084 ; HMDB60085	Gamma-Aminobutyryl-lysine ; 13-Hydroxy-alpha-tocotrienol ; 10-Undecenyl acetate ; Methyl (E)-2-dodecenoate ; Coniferan ; Erinacine D ; ()-(E)-3-Methyl-4-decen-1-yl acetate ; Ethyl 10-undecenoate ; Camelledionol ; 12-Bis(1-ethoxyethoxy)propane ; Rhodinyl propionate ; Citronellyl propionate ; 1-Ethylhexyl tiglate ; 261010-Tetramethyl-1-oxaspiro45decan-6-ol ; 11-Diethoxy-26-nonadiene ; 4-(3-Hydroxybutyl)-335-trimethylcyclohexanone ; Oxacyclotetradecan-2-one ; 25-Acetylvulgaroside ; 16b-Hydroxyestrone ; 16a-Hydroxyestrone ; 2-Hydroxyestrone ; 16-Ketoestradiol ; 4-Hydroxyestrone ; Abacavir ; 17beta-Estradiol-23-quinone ; 17beta-Estradiol-34-quinone	2M+H+K ; M+Na+K ; M+K ; M+K ; M+K ; M+H+Na ; M+K ; M+K ; M+Na+K ; 2M+Na+K ; M+K ; M+K ; M+K ; M+K ; M+K ; M+K ; M+K ; M+H+Na ; M+H-2H2O ; M+H-2H2O ; M+H-2H2O ; M+H-2H2O ; M+H-2H2O ; M+H-2H2O ; M+H-2H2O ; M+H-2H2O	C10H21N3O3 ; C29H44O3 ; C13H24O2 ; C13H24O2 ; C13H24O2 ; C27H42O7 ; C13H24O2 ; C13H24O2 ; C29H44O3 ; C11H24O4 ; C13H24O2 ; C13H24O2 ; C13H24O2 ; C13H24O2 ; C13H24O2 ; C13H24O2 ; C13H24O2 ; C27H42O7 ; C18H22O3 ; C18H22O3 ; C18H22O3 ; C18H22O3 ; C18H22O3 ; C14H18N6O ; C18H22O3 ; C18H22O3
358.36770	1.823088	0.573652	10.959	6.83E-07	6.1658	0.00039	358.3677	HMDB00944 ; HMDB01018 ; HMDB11624 ; HMDB12301 ; HMDB12303 ; HMDB30941 ; HMDB30943 ; HMDB31016 ; HMDB31064 ; HMDB31065 ; HMDB31408 ; HMDB32371 ; HMDB32379 ; HMDB33713 ; HMDB34856 ; HMDB35246 ; HMDB35411 ; HMDB35651 ; HMDB35655 ; HMDB35660 ; HMDB35667 ; HMDB40290	Behenic acid ; UDP-D-Xylose ; Decyl alcohol ; UDP-D-apiose ; UDP-L-arabinose ; Undecanal ; 6-Undecanone ; 10-Undecen-1-ol ; (Z)-3-Methyl-3-decen-1-ol ; ()-(E)-3-Methyl-4-decen-1-ol ; ()-3-Decanol ; 1-Menthyl methyl ether ; trans- and cis-1-Methoxy-1-decene ; 2-Undecanone ; 2-Undecen-1-ol ; 37-Dimethyl-3-octanol ; (R)-Dihydrocitronellol ; 6-Hydroxy-8-docosanone ; 8-Hydroxy-6-docosanone ; 5-Hydroxy-7-docosanone ; 4-Hydroxy-6-docosanone ; Butyl octadecanoate	M+NH4 ; 2M+3H ; 2M+ACN+H ; 2M+3H ; 2M+3H ; 2M+NH4 ; 2M+NH4 ; 2M+NH4 ; 2M+NH4 ; 2M+NH4 ; 2M+ACN+H ; 2M+NH4 ; 2M+NH4 ; 2M+NH4 ; 2M+NH4 ; 2M+ACN+H ; 2M+ACN+H ; M+NH4 ; M+NH4 ; M+NH4 ; M+NH4 ; M+NH4	C22H44O2 ; C14H22N2O16P2 ; C10H22O ; C14H22N2O16P2 ; C14H22N2O16P2 ; C11H22O ; C11H22O ; C11H22O ; C11H22O ; C11H22O ; C10H22O ; C11H22O ; C11H22O ; C11H22O ; C11H22O ; C10H22O ; C10H22O ; C22H44O2 ; C22H44O2 ; C22H44O2 ; C22H44O2 ; C22H44O2
285.12073	1.841137	0.301578	-10.819	7.69E-07	6.1142	0.00039	285.1207	HMDB13129 ; HMDB15344 ; HMDB33428 ; HMDB34679 ; HMDB35475 ; HMDB61017	Glutaconylcarnitine ; Fomepizole ; ()-Setoclavine ; Cinncassiol D2 glucoside ; Simmondsin 2-ferulate ; 1-Pyrimidinylpiperazine	2M+H+Na ; 3M+K ; 2M+Na+K ; M+H+K ; M+H+NH4 ; 3M+2K	C12H19NO6 ; C4H6N2 ; C16H18N2O ; C26H42O11 ; C26H33NO12 ; C8H12N4

115.57866	1.786817	0.071596	10.803	7.80E-07	6.1081	0.00039	115.5787	HMDB00206 ; HMDB00446 ; HMDB00759 ; HMDB11180 ; HMDB14382 ; HMDB15442 ; HMDB28700 ; HMDB28844 ; HMDB28907 ; HMDB28929 ; HMDB29120 ; HMDB29449 ; HMDB33453 ; HMDB34778 ; HMDB60611 ; HMDB61157	N6-Acetyl-L-lysine ; N-Alpha-acetyllysine ; Glycyl-L-leucine ; L-prolyl-L-proline ; Butabarbital ; Butethal ; Alanyl-Valine ; Glycyl-Isoleucine ; Isoleucyl-Glycine ; Leucyl-Glycine ; Valyl-Alanine ; (2R3R4R)-2-Amino-4-hydroxy-3-methylpentanoic acid ; Fagomine ; (2R3R)-123-Butanetriol ; N-Desmethyl tapentadol ; N-(2-Hydroxyethyl)-morpholine N-oxide	M+ACN+2H ; M+ACN+2H ; M+ACN+2H ; M+H+NH4 ; M+H+NH4 ; M+H+NH4 ; M+ACN+2H ; M+ACN+2H ; M+ACN+2H ; M+ACN+2H ; M+ACN+2H ; M+2ACN+2H ; M+2ACN+2H ; M+3ACN+2H ; M+H+Na ; M+2ACN+2H	C8H16N2O3 ; C8H16N2O3 ; C8H16N2O3 ; C10H16N2O3 ; C10H16N2O3 ; C10H16N2O3 ; C8H16N2O3 ; C8H16N2O3 ; C8H16N2O3 ; C8H16N2O3 ; C8H16N2O3 ; C6H13NO3 ; C6H13NO3 ; C4H10O3 ; C13H21NO ; C6H13NO3
658.96406	1.80978	0.514095	-10.351	1.16E-06	5.9366	0.000526	NA	NA	NA	NA	NA
267.06688	1.840702	0.191554	10.008	1.58E-06	5.8019	0.000658	267.0669	HMDB01534 ; HMDB02062 ; HMDB05973 ; HMDB28727 ; HMDB28748 ; HMDB32926 ; HMDB32973 ; HMDB33961 ; HMDB37463 ; HMDB39741 ; HMDB14718 ; HMDB14967	5-Formiminotetrahydrofolic acid ; Norepinephrine sulfate ; Dimethyltryptamine ; Asparaginyl-Aspartate ; Aspartyl-Asparagine ; Polycartine B ; 25-Dimethyl-1H-pyrrole ; 2-(3-Methylbutyl)-1H-pyrrolo23-bpyridine ; Spinosin C ; alpha-L-Rhamnopyranosyl-(1-3)-alpha-D-galactopyranosyl-(1-3)-L-fucose ; Mazindol ; Diazepam	M+Na+K ; M+NH4 ; M+2K+H ; 2M+H+K ; 2M+H+K ; M+2K+H ; 2M+2K-H ; M+2K+H ; M+H+2Na ; M+Na+K ; M+H-H2O ; M+H-H2O	C20H24N8O6 ; C8H11NO6S ; C12H16N2 ; C8H13N3O6 ; C8H13N3O6 ; C12H16N2 ; C6H9N ; C12H16N2 ; C37H38O17 ; C18H32O14 ; C16H13ClN2O ; C16H13ClN2O
433.20262	1.810154	0.287139	9.8633	1.80E-06	5.744	0.000664	433.2026	HMDB36131 ; HMDB05020 ; HMDB28888 ; HMDB28889 ; HMDB28909 ; HMDB28931 ; HMDB30741 ; HMDB31361 ; HMDB31964 ; HMDB32005 ; HMDB34159 ; HMDB34637 ; HMDB35297 ; HMDB35298 ; HMDB36833 ; HMDB38622 ; HMDB39290 ; HMDB39294 ; HMDB39477 ; HMDB40569 ; HMDB40666 ; HMDB41941 ; HMDB61051 ; HMDB42013	S-Furanopetasitin ; Risperidone ; Histidinyl-Isoleucine ; Histidinyl-Leucine ; Isoleucyl-Histidine ; Leucyl-Histidine ; Heliocide H3 ; (R)-Kanzonol Y ; (3b6b8b12a)-812-Epoxy-7(11)-eremophilene-6-angeloyloxy-812-dimethoxy-3-ol ; 2-Geranyl-2344-tetrahydroxydihydrochalcone ; Acetyl tributyl citrate ; Phytolaccoside E ; Heliocide H1 ; Heliocide H4 ; Heliocide H2 ; Medicoside G ; Medicagenic acid beta-maltoside ; Medicoside L ; Acetylsoyasaponin A2 ; 3-Methoxy-6-Gingerdiol 35-diacetate ; Capsicoside B ; NN-dinitrosopiperazine ; N-desmethyltoremifene ; Sofalcone	M+H ; M+Na ; 3M+Na+K ; 3M+Na+K ; 3M+Na+K ; 3M+Na+K ; M+Na ; M+Na ; M+K ; M+Na ; 2M+Na+K ; M+H+K ; M+Na ; M+Na ; M+Na ; M+H+K ; M+H+K ; M+3H ; M+2H+Na ; M+K ; M+2H+Na ; 3M+H ; M+ACN+H ; M+H-H2O	C24H32O5S ; C23H27FN4O2 ; C12H20N4O3 ; C12H20N4O3 ; C12H20N4O3 ; C12H20N4O3 ; C25H30O5 ; C25H30O5 ; C22H34O6 ; C25H30O5 ; C20H34O8 ; C42H66O16 ; C25H30O5 ; C25H30O5 ; C25H30O5 ; C42H66O16 ; C42H66O16 ; C60H96O30 ; C61H94O28 ; C22H34O6 ; C58H98O30 ; C4H8N4O2 ; C25H26ClNO ; C27H30O6
282.11839	1.800499	0.635348	-9.8309	1.86E-06	5.731	0.000664	282.1184	HMDB01366 ; HMDB06592 ; HMDB12231 ; HMDB28913 ; HMDB28935 ; HMDB28976 ; HMDB28977 ; HMDB29954 ; HMDB31208 ; HMDB31664 ; HMDB31856 ; HMDB32025 ; HMDB32415 ; HMDB33562 ; HMDB36183 ; HMDB39750 ; HMDB39803 ; HMDB40245 ; HMDB41493 ; HMDB41622 ; HMDB60469 ; HMDB60956	Purine ; Lacto-N-triaose ; Geranylfarnesyl diphosphate ; Isoleucyl-Methionine ; Leucyl-Methionine ; Methionyl-Isoleucine ; Methionyl-Leucine ; D-glycero-L-galacto-Octulose ; 1-Phenyl-1-pentanone ; Alnustone ; ()-2-Methyl-3-(4-methylphenyl)propanal ; 4-Isopropylacetophenone ; 2-Methyl-4-phenylbutyraldehyde ; 3-Methyl-2-phenylbutanal ; 4-(4-Methylphenyl)-2-butanone ; Lacto-N-triose I ; 44alpha56-Tetrahydro-7-methyl-2(3H)-naphthalenone ; ()-2-Methyl-3-(2-methylphenyl)propanal ; 4-Isopropylphenylacetaldehyde ; N-Acetylgalactosaminyl lactose ; Dehydrodolichol diphosphate ; N-desmethylmirtazapine	2M+ACN+H ; M+H+NH4 ; M+2Na ; 2M+H+K ; 2M+H+K ; 2M+H+K ; 2M+H+K ; M+ACN+H ; 3M+2K ; 2M+H+K ; 3M+2K ; 3M+2K ; 3M+2K ; 3M+2K ; 3M+2K ; M+H+NH4 ; 3M+2K ; 3M+2K ; 3M+2K ; M+H+NH4 ; M+2Na ; 2M+Na+K	C5H4N4 ; C20H35NO16 ; C25H44O7P2 ; C11H22N2O3S ; C11H22N2O3S ; C11H22N2O3S ; C11H22N2O3S ; C8H16O8 ; C11H14O ; C19H18O ; C11H14O ; C11H14O ; C11H14O ; C11H14O ; C11H14O ; C20H35NO16 ; C11H14O ; C11H14O ; C11H14O ; C20H35NO16 ; C25H44O7P2 ; C16H17N3
237.01757	1.813654	0.592098	-9.4348	2.70E-06	5.5683	0.000901	237.0176	HMDB00379 ; HMDB01451 ; HMDB03518 ; HMDB06471 ; HMDB06492 ; HMDB14312 ; HMDB29715 ; HMDB33995 ; HMDB34858 ; HMDB59932	2-Methylcitric acid ; (R)-lipoic acid ; Homocitric acid ; Methylisocitric acid ; 4-Nitrophenyl sulfate ; (S)-lipoic acid ; 2-(2-Thienyl)furan ; Methyl (Z)-5-(5-methyl-2-thienyl)-2-penten-4-ynoate ; Methyl (Z)-5-(1-propynyl)-2-thienylacrylate ; 3-(Carboxymethyl)-3-hydroxypentanedioic acid	2M+Na+K ; 2M+Na+K ; 2M+Na+K ; 2M+Na+K ; M+NH4 ; 2M+Na+K ; 3M+H+Na ; 2M+Na+K ; 2M+Na+K ; 2M+Na+K	C7H10O7 ; C8H14O2S2 ; C7H10O7 ; C7H10O7 ; C6H5NO6S ; C8H14O2S2 ; C8H6OS ; C11H10O2S ; C11H10O2S ; C7H10O7
407.13935	1.775078	0.26001	-9.3206	3.02E-06	5.5204	0.000943	407.1394	HMDB00016 ; HMDB00374 ; HMDB00920 ; HMDB12622 ; HMDB14495 ; HMDB14828 ; HMDB15193 ; HMDB15281 ; HMDB28819 ; HMDB28840 ; HMDB29048 ; HMDB29721 ; HMDB29728 ; HMDB30500 ; HMDB31541 ; HMDB33126 ; HMDB34632 ; HMDB38391 ; HMDB40277 ; HMDB40278 ; HMDB40928 ; HMDB41585 ; HMDB60080 ; HMDB60906 ; HMDB60908 ; HMDB60909 ; HMDB60910 ; HMDB60911 ; HMDB60912 ; HMDB60973 ; HMDB61004	Deoxycorticosterone ; 17-Hydroxyprogesterone ; 11a-Hydroxyprogesterone ; 2-Hydroxyestrone sulfate ; Megestrol ; Flurazepam ; Amoxicillin ; Cefprozil ; Glutamyl-Glycine ; Glycyl-Glutamate ; Serinyl-Serine ; 235-Trimethylfuran ; 2-Ethyl-5-methylfuran ; Amoxycillin ; 3-Methyl-2-cyclohexen-1-one ; 10-Dehydroshogaol ; (4E9a)-9-(3-Methyl-2E-pentenoyloxy)-410(14)-oplopadien-3-one ; 12-Methoxy-81113-abietatrien-2011-olide ; 2-Propylfuran ; 2-Isopropylfuran ; 2-2-(4-Hydroxy-3-methoxyphenyl)ethyl-5-octylfuran ; Tussilagonone ; Thyrotropin releasing hormone ; 11-Hydroxy-delta-9-THC ; 7-beta-Hydroxy-delta-9-THC ; 7-alpha-Hydroxy-delta-9-THC ; 8-Hydroxy-delta-9-THC ; 8-beta-Hydroxy-delta-9-THC ; 9-alpha10-alpha-epoxyhexahydrocannabinol ; 6(beta)-hydroxyprogesterone ; 6-beta-hydroxyprogesterone	M+2K-H ; M+2K-H ; M+2K-H ; M+ACN+H ; M+2K+H ; 2M+H+K ; M+ACN+H ; M+NH4 ; 2M+H ; 2M+H ; 2M+Na ; 3M+2K-H ; 3M+2K-H ; M+ACN+H ; 3M+2K-H ; M+2K-H ; M+2K-H ; M+2K+H ; 3M+2K-H ; 3M+2K-H ; M+2K-H ; M+2K-H ; M+2Na-H ; M+2K-H ; M+2K-H ; M+2K-H ; M+2K-H ; M+2K-H ; M+2K-H ; M+2K-H ; M+2K-H	C21H30O3 ; C21H30O3 ; C21H30O3 ; C18H21O6S ; C21H28O3 ; C21H23ClFN3O ; C16H19N3O5S ; C18H19N3O5S ; C7H11N2O5 ; C7H11N2O5 ; C6H12N2O5 ; C7H10O ; C7H10O ; C16H19N3O5S ; C7H10O ; C21H30O3 ; C21H30O3 ; C21H28O3 ; C7H10O ; C7H10O ; C21H30O3 ; C21H30O3 ; C16H22N6O4 ; C21H30O3 ; C21H30O3 ; C21H30O3 ; C21H30O3 ; C21H30O3 ; C21H30O3 ; C21H30O3 ; C21H30O3
238.34456	1.804881	0.209421	-9.0772	3.83E-06	5.4166	0.001127	NA	NA	NA	NA	NA
276.15529	1.739825	0.261134	8.9258	4.46E-06	5.3509	0.001157	276.1553	HMDB12358 ; HMDB12368 ; HMDB12388 ; HMDB12398 ; HMDB15340 ; HMDB30993 ; HMDB36143 ; HMDB28711 ; HMDB28879	PS(16:018:2(9Z12Z)) ; PS(16:1(9Z)18:1(9Z)) ; PS(18:1(9Z)16:1(9Z)) ; PS(18:2(9Z12Z)16:0) ; Dezocine ; 5-Nonyltetrahydro-2-oxo-3-furancarboxylic acid ; Monomenthyl succinate ; Arginyl-Histidine ; Histidinyl-Arginine	M+3Na ; M+3Na ; M+3Na ; M+3Na ; 2M+Na+K ; 2M+H+K ; 2M+H+K ; M+H-2H2O ; M+H-2H2O	C40H74NO10P ; C40H74NO10P ; C40H74NO10P ; C40H74NO10P ; C16H23NO ; C14H24O4 ; C14H24O4 ; C12H21N7O3 ; C12H21N7O3

277.15857	1.733285	0.204381	8.9215	4.48E-06	5.349	0.001157	277.1586	HMDB14514 ; HMDB15689 ; HMDB29350 ; HMDB30428 ; HMDB31383 ; HMDB31384 ; HMDB32054 ; HMDB32209 ; HMDB32291 ; HMDB32343 ; HMDB33228 ; HMDB34673 ; HMDB35287 ; HMDB35395 ; HMDB35449 ; HMDB35887 ; HMDB36032 ; HMDB36730 ; HMDB36880 ; HMDB37195 ; HMDB37279 ; HMDB37284 ; HMDB38136 ; HMDB38138 ; HMDB38196 ; HMDB38197 ; HMDB38244 ; HMDB38247 ; HMDB38248 ; HMDB38250 ; HMDB39130 ; HMDB39545 ; HMDB39546 ; HMDB39745 ; HMDB40123 ; HMDB40273 ; HMDB40281 ; HMDB41589 ; HMDB59959 ; HMDB60745 ; HMDB31976 ; HMDB33294 ; HMDB40761 ; HMDB61027	Mirtazapine ; Antazoline ; Geranyl 3-methylbutanoate ; Linalyl isovalerate ; (3S4S6R7S)-110-Bisaboladiene-34-diol ; beta-Kessyl alcohol ; alpha-Terpinyl pentanoate ; Citronellyl trans-2-methyl-2-butenoate ; Geranyl 2-methylbutyrate ; Isobornyl 2-methylbutyrate ; Guaidiol ; Calamendiol ; Daucol ; Ginsenoside C ; 1beta2alpha3beta19alpha-Tetrahydroxy-12-ursen-28-oic acid 28-O-b-D-Glucopyranosyl-(1-2)-b-D-glucopyranosyl ester ; Germacrenone ; 3beta-7-Drimene-311-diol ; Tricyclohumuladiol ; 169-Farnesatriene-311-diol ; 2-Propenyl cyclohexanehexanoate ; 210-Bisaboladiene-14-diol ; 5(1-10)-Abeo-112-patchoulanediol ; Curcumadiol ; alpha-Bisabolol oxide C ; alpha-Bisabolol oxide A ; alpha-Bisabolol oxide B ; Panaxatriol ; Bornyl valerate ; Bornyl isovalerate ; Isobornyl isovalerate ; Methyl (ZZ)-58-tetradecadienoate ; Ginsenoside F2 ; Ginsenoside Rg3 ; (2E6E9xi)-Farnesol ; Centellasaponin B ; Terpenyl isovalerate ; Geranyl valerate ; alpha-Kessyl alcohol ; 2-(2-Dimethyl(2-phosphanylethyl)ammonioethyldimethylammonio)ethyl phosphinite ; 3-keto Fusidic acid ; Methyl (9Z)-10-oxo-610-diapo-6-carotenoate ; 5-Methoxy-7-(4-hydroxyphenyl)-1-phenyl-3-heptanone ; 4-Hydroxy-55-diisopropyl-22-dimethyl-34-biphenylquinone ; 2-hydroxyethinylestradiol	2M+H+Na ; 2M+H+Na ; M+K ; M+K ; M+K ; M+K ; M+K ; M+K ; M+K ; M+K ; M+K ; M+K ; M+K ; M+H+2Na ; M+3H ; M+K ; M+K ; M+K ; M+K ; M+K ; M+K ; M+K ; M+K ; M+K ; M+K ; M+K ; M+2K ; M+K ; M+K ; M+K ; M+K ; M+H+2Na ; M+H+2Na ; M+K ; M+3H ; M+K ; M+K ; M+K ; M+Na ; M+H+Na ; M+H-2H2O ; M+H-2H2O ; M+H-2H2O ; M+H-2H2O	C17H19N3 ; C17H19N3 ; C15H26O2 ; C15H26O2 ; C15H26O2 ; C15H26O2 ; C15H26O2 ; C15H26O2 ; C15H26O2 ; C15H26O2 ; C15H26O2 ; C15H26O2 ; C15H26O2 ; C42H72O13 ; C42H68O16 ; C15H26O2 ; C15H26O2 ; C15H26O2 ; C15H26O2 ; C15H26O2 ; C15H26O2 ; C15H26O2 ; C15H26O2 ; C15H26O2 ; C15H26O2 ; C15H26O2 ; C30H52O4 ; C15H26O2 ; C15H26O2 ; C15H26O2 ; C15H26O2 ; C42H72O13 ; C42H72O13 ; C15H26O2 ; C42H68O16 ; C15H26O2 ; C15H26O2 ; C15H26O2 ; C10H28N2OP2 ; C31H46O7 ; C20H24O3 ; C20H24O3 ; C20H24O3 ; C20H24O3
219.00100	1.791877	0.13851	8.8887	4.63E-06	5.3347	0.001157	219.001	HMDB01527 ; HMDB30577 ; HMDB30773 ; HMDB35646 ; HMDB61173	3-Methylthiopropionic acid ; 1-Oxo-1H-2-benzopyran-3-carboxaldehyde ; Juglone ; Methyl 2-(methylthio)acetate ; p-Chlorobenzenesulfonamide	3M+2K ; M+2Na-H ; M+2Na-H ; 3M+2K ; 2M+3H2O+2H	C4H8O2S ; C10H6O3 ; C10H6O3 ; C4H8O2S ; C6H6ClNO2S
228.05741	1.752951	0.128735	-8.8063	5.03E-06	5.2984	0.001198	228.0574	HMDB12127 ; HMDB15051 ; HMDB29185 ; HMDB33280 ; HMDB33542 ; HMDB34315 ; HMDB36055 ; HMDB37308 ; HMDB38510 ; HMDB40545 ; HMDB40892 ; HMDB41461 ; HMDB41692 ; HMDB14584	(R)-2-Benzylsuccinate ; Amantadine ; 5-(34-Dihydroxyphenyl)-gamma-valerolactone ; 8-Acetoxypinoresinol ; Gomphidic acid ; 3-(34-Dimethoxyphenyl)-2-propenoic acid ; Furapiole ; 1-(2-Methoxy-34-methylenedioxyphenyl)-1-propanone ; 6-Methoxymellein ; 4-Methylliquiritigenin 7-rhamnoside ; 3-(3-Methoxy-45-methylenedioxyphenyl)-2-propen-1-ol ; Anthriscinol ; 5-(35-Dihydroxyphenyl)-gamma-valerolactone ; Gemcitabine	2M+H+K ; M+2K-H ; 2M+H+K ; M+H+K ; M+2ACN+2H ; 2M+H+K ; 2M+H+K ; 2M+H+K ; 2M+H+K ; M+H+K ; 2M+H+K ; 2M+H+K ; 2M+H+K ; M+H-2H2O	C11H12O4 ; C10H17N ; C11H12O4 ; C22H24O8 ; C18H12O9 ; C11H12O4 ; C11H12O4 ; C11H12O4 ; C11H12O4 ; C22H24O8 ; C11H12O4 ; C11H12O4 ; C11H12O4 ; C9H11F2N3O4
615.12399	1.789331	0.262497	-8.6723	5.77E-06	5.2389	0.001311	615.124	HMDB01202 ; HMDB30133 ; HMDB33489 ; HMDB33496 ; HMDB33498 ; HMDB33929 ; HMDB34449 ; HMDB36637 ; HMDB36783 ; HMDB39230 ; HMDB40716	dCMP ; Asticolorin A ; Isoaurasperone A ; Nigerone ; Isonigerone ; Aurasperone A ; Hydroxyanigorufone ; Mulberrofuran Q ; Diosbulbinoside F ; Citrusin A ; Irenolone	2M+H ; M+2K-H ; M+2Na-H ; M+2Na-H ; M+2Na-H ; M+2Na-H ; 2M+K ; M+Na ; M+2K-H ; M+2K-H ; 2M+K	C9H14N3O7P ; C33H30O7 ; C32H26O10 ; C32H26O10 ; C32H26O10 ; C32H26O10 ; C19H12O3 ; C34H24O10 ; C26H34O12 ; C26H34O12 ; C19H12O3
200.48656	1.81438	0.095709	8.4725	7.10E-06	5.1488	0.001501	200.4866	HMDB30100	(8R)-Neochrome	M+3H	C41H58O3
241.04891	1.773228	0.497468	-8.4051	7.62E-06	5.1181	0.001501	241.0489	HMDB29752 ; HMDB36006 ; HMDB60873 ; HMDB01934 ; HMDB03219 ; HMDB04350 ; HMDB13846 ; HMDB14330 ; HMDB14847 ; HMDB29901 ; HMDB29935 ; HMDB29952 ; HMDB31735 ; HMDB32306 ; HMDB33092 ; HMDB34246 ; HMDB35175 ; HMDB37205 ; HMDB37470 ; HMDB37483 ; HMDB37484 ; HMDB37579 ; HMDB38235 ; HMDB39982 ; HMDB40789 ; HMDB59862 ; HMDB59880	Dantron ; Xanthopurpurin ; Sorafenib N-oxide ; L(-)-Nicotine pestanal ; Sedoheptulose ; Anabasine ; E-3179 ; Nicotine ; Lamivudine ; L-Galacto-2-heptulose ; D-Manno-2-heptulose ; D-altro-D-manno-Heptose ; Ethyl maltol ; 4-Heptenal diethyl acetal ; 3-(1-Hydroxymethyl-1-propenyl)pentanedioic acid ; Furfuryl acetate ; 2-Amino-16-dimethylfuro32-eimidazo45-bpyridine ; (2R)-2-Hydroxy-2-phenylethyl glucosinolate ; Spinacetin 3-rutinoside ; 246-Trihydroxydihydrochalcone 2-glucoside ; 246-Trihydroxydihydrochalcone 4-glucoside ; Limocitrin 3-rutinoside ; Rhamnazin 3-sophoroside ; Trimethyl-2-propenylpyrazine ; Bikoeniquinone A ; Ethyl furoate ; Ethyl 3-furoate	M+H ; M+H ; M+2H ; M+2K+H ; 2M+Na+K ; M+2K+H ; M+Na+K ; M+2K+H ; 2M+H+Na ; 2M+Na+K ; 2M+Na+K ; 2M+Na+K ; 3M+Na+K ; M+K ; M+K ; 3M+Na+K ; M+K ; M+ACN+2H ; M+3Na ; M+Na+K ; M+Na+K ; M+3Na ; M+3Na ; M+2K+H ; M+Na+K ; 3M+Na+K ; 3M+Na+K	C14H8O4 ; C14H8O4 ; C21H16ClF3N4O4 ; C10H14N2 ; C7H14O7 ; C10H14N2 ; C22H21ClN6O ; C10H14N2 ; C8H11N3O3S ; C7H14O7 ; C7H14O7 ; C7H14O7 ; C7H8O3 ; C9H14O5 ; C9H14O5 ; C7H8O3 ; C10H10N4O ; C15H21NO10S2 ; C29H34O17 ; C21H24O9 ; C21H24O9 ; C29H34O17 ; C29H34O17 ; C10H14N2 ; C27H20N2O3 ; C7H8O3 ; C7H8O3
487.21319	1.797451	0.544365	-8.3905	7.74E-06	5.1113	0.001501	487.2132	HMDB35590 ; HMDB01396 ; HMDB34947 ; HMDB36273 ; HMDB39295 ; HMDB39476	15-Acetoxyscirpene-34-diol 4-O-a-D-glucopyranoside ; 5-Methyltetrahydrofolic acid ; Rebaudioside C ; Quillaic acid 3-galactosyl-(1-2)-glucuronide 28-xylosyl-(1-4)-rhamnosyl-(1-2)-rhamnosyl-(1-3)-4-acetyl-fucosyl ester ; Medicagenic acid 28-O-b-D-xylosyl-(1-4)-a-L-rhamnosyl-(1-2)-a-L-arabinosyl ester ; Acetylsoyasaponin A1	M+H ; 2M+3H2O+2H ; M+H+Na ; M+2H+Na ; M+Na+K ; M+2H+Na	C23H34O11 ; C20H25N7O6 ; C44H70O22 ; C67H104O33 ; C46H72O18 ; C67H104O33
166.05328	1.806218	0.003119	-8.3823	7.81E-06	5.1076	0.001501	166.0533	HMDB02005 ; HMDB37057 ; HMDB40621 ; HMDB00999 ; HMDB02305 ; HMDB29776 ; HMDB31245 ; HMDB31322 ; HMDB31666 ; HMDB40576	Methionine sulfoxide ; Ethiin ; 3-Glucosyl-246-trihydroxyacetophenone ; Phosphoribosylformylglycineamidine ; 5-phosphoribosyl-a-N-formylglycineamidine ; Dhurrin 6-glucoside ; 2-Methyl-1-propanethiol ; 1-Butanethiol ; Diethyl sulfide ; 2-(Methylthio)propane	M+H ; M+H ; M+2H ; M+H+NH4 ; M+H+NH4 ; M+2H+Na ; 3M+Na+K ; 3M+Na+K ; 3M+Na+K ; 3M+Na+K	C5H11NO3S ; C5H11NO3S ; C14H18O9 ; C8H16N3O8P ; C8H16N3O8P ; C20H27NO12 ; C4H10S ; C4H10S ; C4H10S ; C4H10S

287.09062	1.806068	0.364371	8.2052	9.43E-06	5.0257	0.001671	287.0906	HMDB29527 ; HMDB29870 ; HMDB30090 ; HMDB30622 ; HMDB30793 ; HMDB30807 ; HMDB30852 ; HMDB31420 ; HMDB31620 ; HMDB33291 ; HMDB33309 ; HMDB34011 ; HMDB34012 ; HMDB34114 ; HMDB34115 ; HMDB34438 ; HMDB37320 ; HMDB37322 ; HMDB37489 ; HMDB38448 ; HMDB41726 ; HMDB01086 ; HMDB02211 ; HMDB15190 ; HMDB31584 ; HMDB32493 ; HMDB32985 ; HMDB34882 ; HMDB36544 ; HMDB38409 ; HMDB38439 ; HMDB41084 ; HMDB01067 ; HMDB29175 ; HMDB29179 ; HMDB29228 ; HMDB29231 ; HMDB30662 ; HMDB30745 ; HMDB33866 ; HMDB38519 ; HMDB41294 ; HMDB41507	Melilotocarpan B ; 37-Dihydroxy-4-methoxyisoflavanone ; Sakuranetin ; (R)-Oxypeucedanin ; Moracin B ; 34-Dihydro-8-hydroxy-3-(3-hydroxy-4-methoxyphenyl)-1H-2-benzopyran-1-one ; Asperxanthone ; Heliannone C ; Vestitone ; 38-Dihydroxy-9-methoxypterocarpan ; Moracin F ; 2-(24-Dihydroxyphenyl)-56-dimethoxybenzofuran ; 34-Dihydroxy-9-methoxypterocarpan ; 6alpha-Hydroxyisomedicarpin ; 6alpha-Hydroxymedicarpin ; (R)-Pabulenol ; Licochalcone B ; Dihydrooroxylin ; Dihydrowogonin ; (-)-57-Dihydroxy-3-(4-hydroxybenzyl)-4-chromanone ; Dihydroglycitein ; Uroporphyrinogen III ; Uroporphyrinogen I ; Echothiophate ; Myricatomentoside II ; 2-Propionyl-2-thiazoline ; 5-(2-Hydroxyethyl)-4-methylthiazole ; 5-Acetyl-23-dihydro-14-thiazine ; 1-Acetoxyeugenol acetate ; 6-(Methylthio)hexyl glucosinolate ; (-)-5-(2-Propenyl)-2-oxazolidinethione ; Raphanusamide ; N-Acetylaspartylglutamic acid ; 3-O-methyl-(-)-epicatechin ; 4-O-methyl-(-)-epicatechin ; 4-Methyl-epicatechin ; 3-Methyl-epicatechin ; 4-O-Methylcatechin ; (R)-Heraclenol ; Fonsecin B ; Arachidoside ; 6-Methoxy-alpha-pyrufuran ; 3-O-Methylcatechin	M+H ; M+2H+Na ; M+2H+Na ; 2M+Na+K ; M+H+K ; 2M+H ; 2M+H ; 2M+H ; M+Na ; M+3ACN+2H ; 2M+H ; 2M+H ; M+H-H2O ; M+H-H2O ; M+H-H2O ; M+H-H2O ; M+H-H2O ; M+H-H2O ; M+H-H2O ; M+H-H2O ; M+H-H2O ; M+H-H2O ; M+H-H2O	C16H14O5 ; C40H44N4O16 ; C40H44N4O16 ; C9H23NO3PS ; C27H34O11 ; C6H9NOS ; C6H9NOS ; C6H9NOS ; C14H16O5 ; C14H27NO9S3 ; C6H9NOS ; C6H9NOS ; C11H16N2O8 ; C16H16O6 ; C16H16O6 ; C16H16O6 ; C16H16O6 ; C16H16O6 ; C16H16O6 ; C16H16O6 ; C16H16O6 ; C16H16O6 ; C16H16O6
424.10208	1.79121	0.685247	8.1836	9.65E-06	5.0156	0.001671	424.1021	HMDB03572 ; HMDB15573 ; HMDB30812 ; HMDB30882 ; HMDB32838 ; HMDB33819 ; HMDB37334 ; HMDB37335 ; HMDB38356 ; HMDB39027 ; HMDB40344 ; HMDB40472 ; HMDB40794 ; HMDB40877 ; HMDB60868	Rosmarinic acid ; Niflumic Acid ; Acerosin ; (S)-Rutaretin ; Dorsteniol ; Jaceidin ; Majoranin ; Sudachitin ; Sideritiflavone ; Celereoin ; 356-Trihydroxy-347-trimethoxyflavone ; Agamanone ; 5-Hydroxy-4-methoxy-6-canthinone 3-N-oxide ; 358-Trihydroxy-347-trimethoxyflavone ; Phenytoin quinone	M+ACN+Na ; 3M+2H ; M+ACN+Na ; 3M+Na+K ; 3M+Na+K ; M+ACN+Na ; M+ACN+Na ; M+ACN+Na ; M+ACN+Na ; 3M+Na+K ; M+ACN+Na ; M+ACN+Na ; 3M+2H ; M+ACN+Na ; 3M+2H	C18H16O8 ; C13H9F3N2O2 ; C18H16O8 ; C14H14O5 ; C14H14O5 ; C18H16O8 ; C18H16O8 ; C18H16O8 ; C18H16O8 ; C14H14O5 ; C18H16O8 ; C18H16O8 ; C15H10N2O4 ; C18H16O8 ; C15H10N2O4
98.512055	1.763338	0.703585	8.1795	9.69E-06	5.0137	0.001671	NA	NA	NA	NA	NA
189.04099	1.743644	0.258431	8.0898	1.07E-05	4.9716	0.001717	189.041	HMDB32289 ; HMDB37157 ; HMDB36933 ; HMDB36934 ; HMDB36739 ; HMDB41133 ; HMDB60457 ; HMDB60515	Furfuryl propyl disulfide ; 2-Methyl-3-(propyldithio)furan ; 1-Methyl 2-galloylgalactarate ; 6-Methyl 2-galloylgalactarate ; Yuccaol C ; N-Methoxyspirobrassinol ; cis-3-Chloroacrylic acid ; trans-3-Chloroacrylic acid	M+H ; M+H ; M+2H ; M+2H ; M+2H+Na ; 2M+3H ; M+2ACN+H ; M+2ACN+H	C8H12OS2 ; C8H12OS2 ; C14H16O12 ; C14H16O12 ; C30H22O10 ; C12H14N2O2S2 ; C3H3ClO2 ; C3H3ClO2
218.92651	1.800993	0.21405	8.0727	1.09E-05	4.9635	0.001717	NA	NA	NA	NA	NA
147.03042	1.749689	0.470257	8.0628	1.10E-05	4.9588	0.001717	147.0304	HMDB33966 ; HMDB41390 ; HMDB41393 ; HMDB01199 ; HMDB03288 ; HMDB04185 ; HMDB12161 ; HMDB14350 ; HMDB28766 ; HMDB28873 ; HMDB29062 ; HMDB29123 ; HMDB29393 ; HMDB29445 ; HMDB29504 ; HMDB29730 ; HMDB29731 ; HMDB29732 ; HMDB30539 ; HMDB31337 ; HMDB33155 ; HMDB33270 ; HMDB34278 ; HMDB34300 ; HMDB36189 ; HMDB36605 ; HMDB36633 ; HMDB37456 ; HMDB37508 ; HMDB37599 ; HMDB37750 ; HMDB39775 ; HMDB40062 ; HMDB40096 ; HMDB40323 ; HMDB59759 ; HMDB60321 ; HMDB60537 ; HMDB60669 ; HMDB60670 ; HMDB60826	Di-2-propenyl disulfide 9CI ; (EE)-Di-1-propenyl disulfide ; (E)-1-Propenyl 2-propenyl disulfide ; N2-Succinyl-L-ornithine ; Selenocysteine ; 5-Hydroxyindoleacetylglycine ; 4-(Glutamylamino) butanoate ; Pyrimethamine ; Aspartyl-Valine ; Hydroxyprolyl-Threonine ; Threoninyl-Hydroxyproline ; Valyl-Aspartate ; L-N-(3-Carboxypropyl)glutamine ; L-Agaridoxin ; Marindinin ; 2-Ethyl-4-methylthiazole ; 4-Ethyl-2-methylthiazole ; 5-Ethyl-4-methylthiazole ; Tangeritin ; Thiiranebutanenitrile ; Trimethylthiazole ; Sesamolinol ; Auranetin ; 5-Isothiocyanato-1-pentene ; Tetrahydrofurfuryl cinnamate ; Pterosin E ; Sinensetin ; 34568-Pentamethoxyflavone ; Oxidihydroartocarpesin ; Isosinensetin ; Cyclokievitone hydrate ; Homoeriodictyol 4-isobutyrate ; 4-Ethyl-5-methylthiazole ; 5-Ethyl-2-methylthiazole ; 34578-Pentamethoxyflavone ; 1-Hydroxypyrene glucuronide ; 1-Hydroxy-6-methoxypyrene ; p-Hydroxyphenobarbital ; p-Hydroxyfelbamate ; 2-Hydroxyfelbamate ; Hydroxynalidixic acid	M+H ; M+H ; M+H ; M+Na+K ; M+3ACN+2H ; M+2Na ; M+Na+K ; M+2Na ; M+Na+K ; M+Na+K ; M+Na+K ; M+Na+K ; M+Na+K ; M+H+K ; M+Na+K ; 2M+H+K ; 2M+H+K ; 2M+H+K ; M+3Na ; 2M+H+K ; 2M+H+K ; M+3Na ; M+3Na ; 2M+H+K ; M+Na+K ; M+Na+K ; M+3Na ; M+3Na ; M+3Na ; M+3Na ; M+3Na ; M+3Na ; 2M+H+K ; 2M+H+K ; M+3Na ; M+H+2Na ; M+2Na ; M+2Na ; M+H+K ; M+H+K ; M+2Na	C6H10S2 ; C6H10S2 ; C6H10S2 ; C9H16N2O5 ; C3H7NO2Se ; C12H12N2O4 ; C9H16N2O5 ; C12H13ClN4 ; C9H16N2O5 ; C9H16N2O5 ; C9H16N2O5 ; C9H16N2O5 ; C9H16N2O5 ; C11H14N2O5 ; C14H16O3 ; C6H9NS ; C6H9NS ; C6H9NS ; C20H20O7 ; C6H9NS ; C6H9NS ; C20H20O7 ; C20H20O7 ; C6H9NS ; C14H16O3 ; C14H16O3 ; C20H20O7 ; C20H20O7 ; C20H20O7 ; C20H20O7 ; C20H20O7 ; C20H20O7 ; C6H9NS ; C6H9NS ; C20H20O7 ; C22H18O7 ; C17H12O2 ; C12H12N2O4 ; C11H14N2O5 ; C11H14N2O5 ; C12H12N2O4
320.10222	1.712707	0.176768	8.0353	1.13E-05	4.9458	0.001717	320.1022	HMDB14848 ; HMDB37462 ; HMDB37467 ; HMDB01935 ; HMDB02114 ; HMDB04400 ; HMDB11669 ; HMDB15585 ; HMDB29386 ; HMDB29789 ; HMDB30068 ; HMDB30312 ; HMDB33312 ; HMDB33313 ; HMDB33314 ; HMDB33488 ; HMDB33497 ; HMDB34100 ; HMDB37109 ; HMDB41200 ; HMDB41303 ; HMDB59771 ; HMDB60699	Ibandronate ; Tricin 7-neohesperidoside ; Rhamnazin 3-rutinoside ; Warfarin ; Bisdemethoxycurcumin ; 5-Acetylamino-6-amino-3-methyluracil ; 6-(N-Acetyl-alpha-D-glucosaminyl)-1-phosphatidyl-1D-myo-inositol ; Chlophedianol ; Indicaxanthin ; Kanzonol U ; Dimoracin ; Floribundine ; Moracin D ; Moracin E ; Moracin G ; Aurasperone D ; 6-O-Demethylnigerone ; Dianhydroaurasperone C ; (1S2R3S)-23-Dihydro-4-(4-hydroxyphenyl)-1H-phenalene-123-triol ; Dehydroxymethylflazine ; Glyinflanin H ; 6-amino-5N-methylformylamino-1-methyluracil ; (E)-2-Hydroxy-N-desmethyldoxepin	M+H ; M+2H ; M+2H ; 2M+H+Na ; 2M+H+Na ; 3M+2Na ; M+H+NH4 ; 2M+Na+K ; 2M+H+Na ; 2M+H+Na ; M+H+Na ; M+K ; 2M+H+Na ; 2M+H+Na ; 2M+H+Na ; M+2ACN+2H ; M+2ACN+2H ; M+2ACN+2H ; 2M+H+Na ; M+ACN+H ; 2M+H+Na ; 3M+2Na ; M+K	C9H23NO7P2 ; C29H34O16 ; C29H34O16 ; C19H16O4 ; C19H16O4 ; C7H10N4O3 ; C21H36NO18P ; C17H20ClNO ; C14H16N2O6 ; C19H16O4 ; C38H32O8 ; C18H19NO2 ; C19H16O4 ; C19H16O4 ; C19H16O4 ; C31H24O10 ; C31H24O10 ; C31H24O10 ; C19H16O4 ; C16H10N2O3 ; C19H16O4 ; C7H10N4O3 ; C18H19NO2

150.54742	1.806668	0.056658	-7.9186	1.29E-05	4.8902	0.001855	150.5474	HMDB14618 ; HMDB29284 ; HMDB38577 ; HMDB41141 ; HMDB41516 ; HMDB15471 ; HMDB29584 ; HMDB30831 ; HMDB30871 ; HMDB31335 ; HMDB31760 ; HMDB31898 ; HMDB33088 ; HMDB33588 ; HMDB33911 ; HMDB34445 ; HMDB36616 ; HMDB38677 ; HMDB39082	Chlordiazepoxide ; Avenanthramide 1c ; (Z)-N-Coumaroyl-5-hydroxyanthranilic acid ; Marshdine ; Avenanthramide G ; Salsalate ; 3-O-Methylcoumestrol ; Alternariol ; Isogentisin ; 2-Chloro-1-propanol ; Gentisin ; Porric acid C ; ()-2-(34-Dihydroxyphenyl)-13-benzodioxole-5-carboxaldehyde ; 9-O-Methylcoumestrol ; 568-Trihydroxy-2-methylbenzogchromen-4-one ; Cochliophilin A ; Pseudobaptigenin ; Alfafuran ; Camelinin	M+2H ; M+2H ; M+2H ; M+2H ; M+2H ; M+ACN+2H ; M+H+NH4 ; M+ACN+2H ; M+ACN+2H ; 3M+H+NH4 ; M+ACN+2H ; M+ACN+2H ; M+ACN+2H ; M+H+NH4 ; M+ACN+2H ; M+H+NH4 ; M+H+NH4 ; M+ACN+2H ; M+H+K	C16H14ClN3O ; C16H13NO5 ; C16H13NO5 ; C16H13NO5 ; C16H13NO5 ; C14H10O5 ; C16H10O5 ; C14H10O5 ; C14H10O5 ; C3H7ClO ; C14H10O5 ; C14H10O5 ; C14H10O5 ; C16H10O5 ; C14H10O5 ; C16H10O5 ; C16H10O5 ; C14H10O5 ; C12H23NOS2
238.03778	1.789133	0.049788	7.9113	1.30E-05	4.8867	0.001855	238.0378	HMDB15424 ; HMDB15563 ; HMDB29708 ; HMDB31255 ; HMDB32686 ; HMDB41147 ; HMDB41258 ; HMDB41418	Cefoperazone ; Pargyline ; 5678-Tetrahydro-24-dimethylquinoline ; Fluorocitric acid ; beta-D-3-5-Deoxy-5-(dimethylarsinyl)ribofuranosyloxy-2-hydroxy-1-propanesulfonic acid ; Isochinomin ; Homomangiferin ; Taxifolin 3-arabinoside	M+3Na ; M+2K+H ; M+2K-H ; 2M+3H2O+2H ; M+2ACN+2H ; M+H+K ; M+H+K ; M+H+K	C25H27N9O8S2 ; C11H13N ; C11H15N ; C6H7FO7 ; C10H21AsO9S ; C20H20O11 ; C20H20O11 ; C20H20O11
278.99574	1.764893	0.505377	-7.7872	1.49E-05	4.8268	0.00207	NA	NA	NA	NA	NA
619.56711	1.766867	0.652189	-7.7537	1.55E-05	4.8106	0.00209	619.5671	HMDB00772 ; HMDB00795 ; HMDB00937 ; HMDB01286 ; HMDB02374 ; HMDB04085 ; HMDB06845 ; HMDB06846 ; HMDB06851 ; HMDB30013 ; HMDB30021 ; HMDB30078 ; HMDB30125 ; HMDB30889 ; HMDB32146 ; HMDB33194 ; HMDB33495 ; HMDB33869 ; HMDB33873 ; HMDB33885 ; HMDB33934 ; HMDB34154 ; HMDB34197 ; HMDB34407 ; HMDB35474 ; HMDB35505 ; HMDB35674 ; HMDB35675 ; HMDB37397 ; HMDB38070 ; HMDB40586 ; HMDB60147	Nonadecanoic acid ; Pristanic acid ; Stigmasterol ; 44-Dimethyl-5a-cholesta-824-dien-3-b-ol ; Avenasterol ; Tuberculostearic acid ; 4a-Methylfecosterol ; 24-Methylenelophenol ; Delta7-Avenasterol ; (3beta5alpha24S)-Stigmasta-725-dien-3-ol ; Fucosterol ; 25-Methyl-24-methylenecholesterol ; 24-Methylenepollinasterol ; 2223-Dihydro-alpha-spinasterone ; (3beta5alpha)-14-Methyl-919-cycloergost-25-en-3-ol ; Triticusterol ; (3beta4beta5alpha)-4-Methylergosta-724(28)-dien-3-ol ; (3beta4alpha5alpha23E)-4-Methylergosta-723-dien-3-ol ; alpha-Spinasterol ; Chondrillasterol ; beta-Sitostenone ; Methyl stearate ; Clerosterol ; Peposterol ; Isopropyl hexadecanoate ; (5alpha24(28)Z)-Stigmast-24(28)-en-3-one ; 4-Hydroxy-6-nonadecanone ; 6-Hydroxy-4-nonadecanone ; xi-17-Methyloctadecanoic acid ; xi-16-Methyloctadecanoic acid ; Vitamin D5 ; 44-Dimethylcholesta-8(9)14-dien-3beta-ol	2M+Na ; 2M+Na ; 3M+2H ; 3M+2H ; 3M+2H ; 2M+Na ; 3M+2H ; 3M+2H ; 3M+2H ; 3M+2H ; 3M+2H ; 3M+2H ; 3M+2H ; 3M+2H ; 3M+2H ; 3M+2H ; 3M+2H ; 3M+2H ; 3M+2H ; 3M+2H ; 3M+2H ; 2M+Na ; 3M+2H ; 3M+2H ; 2M+Na ; 3M+2H ; 2M+Na ; 2M+Na ; 2M+Na ; 2M+Na ; 3M+2H ; 3M+2H	C19H38O2 ; C19H38O2 ; C29H48O ; C29H48O ; C29H48O ; C19H38O2 ; C29H48O ; C29H48O ; C29H48O ; C29H48O ; C29H48O ; C29H48O ; C29H48O ; C29H48O ; C29H48O ; C29H48O ; C29H48O ; C29H48O ; C29H48O ; C29H48O ; C29H48O ; C19H38O2 ; C29H48O ; C29H48O ; C19H38O2 ; C29H48O ; C19H38O2 ; C19H38O2 ; C19H38O2 ; C19H38O2 ; C29H48O ; C29H48O
267.56854	1.803097	0.218002	7.7109	1.62E-05	4.7897	0.002136	267.5685	HMDB14404 ; HMDB60032	Sulfanilamide ; (beta-1-O-N-(2-formyl-3-chlorophenyl)anthraniloyl-D-glucupyranuronic acid)	3M+H+NH4 ; M+2ACN+2H	C6H8N2O2S ; C20H18ClNO9
706.22382	1.80526	0.512396	-7.6575	1.72E-05	4.7635	0.00221	706.2238	HMDB06832 ; HMDB13204 ; HMDB34756	S-Glutaryldihydrolipoamide ; 6-Succinoaminopurine ; Methyl 345-trimethoxycinnamate arabinosyl-(1-3)-glucosyl-(1-6)-glucosyl ester	2M+ACN+Na ; 3M+H ; 2M+H+Na	C13H23NO4S2 ; C9H9N5O3 ; C29H42O19
306.12965	1.746625	0.945801	-7.4808	2.11E-05	4.6759	0.002636	306.1297	HMDB59949 ; HMDB41488 ; HMDB60989 ; HMDB14535 ; HMDB15135	Rhodin G7 ; 45-Dihydropiperlonguminine ; 4-hydroxypropanolol ; Sulpiride ; Cyclacillin	M+2H ; 2M+Na+K ; 2M+Na+K ; M+H-2H2O ; M+H-2H2O	C34H34N4O7 ; C16H21NO3 ; C16H21NO3 ; C15H23N3O4S ; C15H23N3O4S
168.50816	1.778359	0.675301	-7.4339	2.23E-05	4.6525	0.002715	168.5082	HMDB28820 ; HMDB28862	Glutamyl-Hydroxyproline ; Hydroxyprolyl-Glutamate	M+2K ; M+2K	C10H15N2O6 ; C10H15N2O6
218.06731	1.74701	0.89565	-7.3674	2.40E-05	4.619	0.002835	218.0673	HMDB30417 ; HMDB31347 ; HMDB33739 ; HMDB37480 ; HMDB38775 ; HMDB39935 ; HMDB40536 ; HMDB40832 ; HMDB41150 ; HMDB60772 ; HMDB00026 ; HMDB00168 ; HMDB11733 ; HMDB12265 ; HMDB30900 ; HMDB33780 ; HMDB37260 ; HMDB38347 ; HMDB39953 ; HMDB41623 ; HMDB60582	Lycoperdic acid ; 2-O-Caffeoylarbutin ; Floribundoside ; Eriodictin ; 578-Trihydroxyflavanone 7-glucoside ; Dihydrogenistin ; (2R3R)-3347-Tetrahydroxyflavanone 7-O-alpha-L-Rhamnopyranoside ; 5-Hydroxyaloin A ; 7-Hydroxyaloin B ; 4-Hydroxyfenoprofen glucuronide ; Ureidopropionic acid ; L-Asparagine ; Glycyl-glycine ; N-Carbamoylsarcosine ; Eremopetasitenin A2 ; D-Asparagine ; Todatriol glucoside ; Methylpicraquassioside A ; 1-(3-Methylbutanoyl)-6-apiosylglucose ; N6-Carbamoyl-L-threonyladenosine ; Benazeprilat	M+H ; M+2H ; M+2H ; M+2H ; M+2H ; M+2H ; M+2H ; M+2H ; M+2H ; M+2H ; 3M+H+K ; 3M+H+K ; 3M+H+K ; 3M+H+K ; M+H+K ; 3M+H+K ; M+2Na ; M+H+Na ; M+H+K ; M+H+Na ; M+H+K	C8H11NO6 ; C21H22O10 ; C21H22O10 ; C21H22O10 ; C21H22O10 ; C21H22O10 ; C21H22O10 ; C21H22O10 ; C21H22O10 ; C21H22O10 ; C4H8N2O3 ; C4H8N2O3 ; C4H8N2O3 ; C4H8N2O3 ; C20H28O6S ; C4H8N2O3 ; C17H26O10 ; C19H24O10 ; C16H28O11 ; C15H20N6O8 ; C22H24N2O5
98.961365	1.727393	0.394454	7.3554	2.44E-05	4.6129	0.002835	NA	NA	NA	NA	NA
202.98941	1.789146	0.098761	7.2078	2.90E-05	4.5377	0.003279	202.9894	HMDB38471 ; HMDB38472	Luteolin 4-sulfate ; Luteolin 7-sulfate	M+H+K ; M+H+K	C15H10O9S ; C15H10O9S
98.975493	1.716154	0.717622	7.1928	2.95E-05	4.53	0.003279	NA	NA	NA	NA	NA
615.72470	1.742161	0.027372	-7.1627	3.06E-05	4.5145	0.003324	615.7247	HMDB14663 ; HMDB34873	Bentiromide ; Ethyl 2-hydroxy-3-(3-indolyl)propanoate glucoside	3M+H+NH4 ; 3M+2Na	C23H20N2O5 ; C19H25NO8
159.02815	1.720358	0.518363	7.0643	3.44E-05	4.4636	0.003564	159.0282	HMDB31210 ; HMDB37759 ; HMDB60348 ; HMDB00294 ; HMDB11648 ; HMDB14026 ; HMDB29418 ; HMDB33118 ; HMDB33119 ; HMDB38789	Zymonic acid ; 2-Methylene-4-oxopentanedioic acid ; 2-Maleylacetate ; Urea ; 1-(beta-D-Ribofuranosyl)-14-dihydronicotinamide ; p-Chlorobenzene sulfonyl urea ; S-Cysteinosuccinic acid ; 2-Methylthiophene ; 3-Methylthiophene ; Torvanol A	M+H ; M+H ; M+H ; 2M+K ; M+Na+K ; M+2ACN+2H ; 2M+3H ; 3M+H+Na ; 3M+H+Na ; M+2H+Na	C6H6O5 ; C6H6O5 ; C6H6O5 ; CH4N2O ; C11H16N2O5 ; C7H7ClN2O3S ; C7H11NO6S ; C5H6S ; C5H6S ; C20H20O10S
615.52482	1.708823	0.38285	-7.044	3.52E-05	4.453	0.003564	615.5248	HMDB01419 ; HMDB01496 ; HMDB02103 ; HMDB02902 ; HMDB04035 ; HMDB05943 ; HMDB06119 ; HMDB06247 ; HMDB06283 ; HMDB06892 ; HMDB11643 ; HMDB13643 ; HMDB29320 ; HMDB34254 ; HMDB34872 ; HMDB35681 ; HMDB38959 ; HMDB39868 ; HMDB39869 ; HMDB39870 ; HMDB40180 ; HMDB59592 ; HMDB12084	24-Hydroxycholesterol ; 7a-Hydroxycholesterol ; 27-Hydroxycholesterol ; Delta-Tocopherol ; 22b-Hydroxycholesterol ; 5b-Pregnanediol ; 7b-Hydroxycholesterol ; 25-Hydroxycholesterol ; 20alpha-Hydroxycholesterol ; 7a-Hydroxy-5b-cholestan-3-one ; (24R)-Cholest-5-ene-3-beta24-diol ; 4b-Hydroxycholesterol ; (3beta5alpha6alpha)-Cholest-8-ene-36-diol ; eta-Tocopherol ; Cheritamine ; 3-Hydroxy-1-phenyl-1-heneicosanone ; 2-Methyl-6-phytylhydroquinone ; 5-(12-Heneicosenyl)-13-benzenediol ; 5-(14-Heneicosenyl)-13-benzenediol ; 5-(16-Heneicosenyl)-13-benzenediol ; Pentadecylbenzene ; Cholest-5-ene-3-beta7-alpha-diol ; SM(d18:012:0)	3M+H+Na ; 3M+H+Na ; 3M+H+Na ; 3M+H+Na ; 3M+H+Na ; 2M+K ; 3M+H+Na ; 3M+H+Na ; 3M+H+Na ; 3M+H+Na ; 3M+H+Na ; 3M+H+Na ; 3M+H+Na ; 3M+H+Na ; M+Na ; 3M+H+Na ; 3M+H+Na ; 3M+H+Na ; 3M+H+Na ; 3M+H+Na ; 2M+K ; 3M+H+Na ; M+H-2H2O	C27H46O2 ; C27H46O2 ; C27H46O2 ; C27H46O2 ; C27H46O2 ; C21H36 ; C27H46O2 ; C27H46O2 ; C27H46O2 ; C27H46O2 ; C27H46O2 ; C27H46O2 ; C27H46O2 ; C27H46O2 ; C40H68N2O ; C27H46O2 ; C27H46O2 ; C27H46O2 ; C27H46O2 ; C27H46O2 ; C21H36 ; C27H46O2 ; C35H75N2O6P

111.00361	1.786601	0.403298	7.0329	3.57E-05	4.4472	0.003564	111.0036	HMDB60689 ; HMDB60691 ; HMDB01090 ; HMDB01981	Phosphoramide mustard ; Isophosphamide mustard ; (S)-5-Diphosphomevalonic acid ; (R)-Mevalonic acid-5-pyrophosphate	M+2H ; M+2H ; M+2H+Na ; M+2H+Na	C4H11Cl2N2O2P ; C4H11Cl2N2O2P ; C6H14O10P2 ; C6H14O10P2
132.52222	1.744332	0.327899	7.0181	3.64E-05	4.4395	0.003564	132.5222	HMDB01120 ; HMDB01347 ; HMDB02096 ; HMDB15611 ; HMDB28682 ; HMDB28724 ; HMDB28797 ; HMDB28839 ; HMDB28855 ; HMDB29149 ; HMDB39656 ; HMDB41526	Dimethylallylpyrophosphate ; Isopentenyl pyrophosphate ; 3-Indolebutyric acid ; Methsuximide ; Alanyl-Asparagine ; Asparaginyl-Alanine ; Glutaminyl-Glycine ; Glycyl-Glutamine ; Glycyl-Gamma-glutamate ; Gamma-glutamyl-Glycine ; 5-(2-Furanyl)-123456-hexahydro-7H-cyclopentabpyridin-7-one ; Isosalsolidine	M+H+NH4 ; M+H+NH4 ; M+Na+K ; M+Na+K ; M+Na+K ; M+Na+K ; M+Na+K ; M+Na+K ; M+Na+K ; M+Na+K ; M+Na+K ; M+Na+K	C5H12O7P2 ; C5H12O7P2 ; C12H13NO2 ; C12H13NO2 ; C7H13N3O4 ; C7H13N3O4 ; C7H13N3O4 ; C7H13N3O4 ; C7H13N3O4 ; C7H13N3O4 ; C12H13NO2 ; C12H13NO2
161.98597	1.753165	0.061435	6.9775	3.82E-05	4.4182	0.003564	161.986	HMDB15419	Polythiazide	M+H+2Na	C11H13ClF3N3O4S3
332.09515	1.726491	0.20733	-6.96	3.90E-05	4.409	0.003564	332.0952	HMDB00195 ; HMDB00230 ; HMDB00425 ; HMDB00481 ; HMDB00773 ; HMDB03040 ; HMDB14930 ; HMDB28831 ; HMDB28847 ; HMDB28973 ; HMDB29104 ; HMDB40435	Inosine ; N-Acetylneuraminic acid ; 3-Deoxy-D-glycero-D-galacto-2-nonulosonic acid ; Allopurinol riboside ; N-Acetyl-a-neuraminic acid ; Arabinosylhypoxanthine ; Tripelennamine ; Glutamyl-Tyrosine ; Glycyl-Methionine ; Methionyl-Glycine ; Tyrosyl-Glutamate ; Dictyoquinazol A	M+ACN+Na ; M+Na ; M+ACN+Na ; M+ACN+Na ; M+Na ; M+ACN+Na ; M+2K-H ; M+Na ; 3M+2Na ; 3M+2Na ; M+Na ; 2M+H+K	C10H12N4O5 ; C11H19NO9 ; C9H16O9 ; C10H12N4O5 ; C11H19NO9 ; C10H12N4O5 ; C16H21N3 ; C14H17N2O6 ; C7H14N2O3S ; C7H14N2O3S ; C14H17N2O6 ; C17H16N2O4
407.31522	1.735891	0.228376	6.9498	3.95E-05	4.4036	0.003564	407.3152	HMDB07895 ; HMDB08917 ; HMDB10376 ; HMDB11203 ; HMDB11370 ; HMDB11543 ; HMDB11573 ; HMDB13402 ; HMDB13403 ; HMDB31680 ; HMDB36344 ; HMDB39288 ; HMDB59931	PC(14:0P-16:0) ; PE(15:0P-18:0) ; CE(24:0) ; PC(P-16:014:0) ; PE(P-18:015:0) ; MG(0:020:1(11Z)0:0) ; MG(20:1(11Z)0:00:0) ; PC(o-14:016:1(9Z)) ; PC(o-16:014:1(9Z)) ; 1-O-beta-D-Glucopyranosyl-23-di-O-palmitoylglycerol ; Tetrahydropersin ; beta-Sitosterol behenate ; 23-Diacetoxypropyl stearate	M+3ACN+2H ; M+3ACN+2H ; M+2K ; M+3ACN+2H ; M+3ACN+2H ; M+Na ; M+Na ; M+3ACN+2H ; M+3ACN+2H ; M+2ACN+2H ; M+Na ; M+2K ; M+H-2H2O	C38H76NO7P ; C38H76NO7P ; C51H92O2 ; C38H76NO7P ; C38H76NO7P ; C23H44O4 ; C23H44O4 ; C38H76NO7P ; C38H76NO7P ; C41H78O10 ; C23H44O4 ; C51H92O2 ; C25H46O6
242.11515	1.733802	0.736469	-6.948	3.96E-05	4.4026	0.003564	242.1152	HMDB02224 ; HMDB14922 ; HMDB29366 ; HMDB37696 ; HMDB40380 ; HMDB60750 ; HMDB36601 ; HMDB00732 ; HMDB11142 ; HMDB11554 ; HMDB11584 ; HMDB11631 ; HMDB12819 ; HMDB14787 ; HMDB29397 ; HMDB32584 ; HMDB33429 ; HMDB33430 ; HMDB37694 ; HMDB37695 ; HMDB40006 ; HMDB41278 ; HMDB41448 ; HMDB42008	5-Methyldeoxycytidine ; Mefenamic acid ; N-2-(4-Hydroxyphenyl)ethylbenzamide ; 2-Phenylethyl 2-aminobenzoate ; Schinifoline ; 3-Amino-3-deoxythimidine ; 3-Hydroxy-T2 Toxin ; Hydroxykynurenine ; DHAP(18:0e) ; MG(0:022:4(7Z10Z13Z16Z)0:0) ; MG(22:4(7Z10Z13Z16Z)0:00:0) ; L-3-Hydroxykynurenine ; 5-Hydroxykynurenine ; Stavudine ; L-Nicotianine ; 4-Hydroxychalcone ; Tanakamine ; Tanakine ; cis-3-Hexenyl 2-aminobenzoate ; Cyclohexyl 2-aminobenzoate ; 1-(23-Dihydro-6-methyl-1H-pyrrolizin-5-yl)-14-pentanedione ; 3b-Hydroxy-6b-(3-chloro-2-hydroxy-2-methylbutanoyloxy)-7(11)-eremophilen-128b-olide ; Dicyclohexyl disulfide ; Ritalinic acid	M+H ; M+H ; M+H ; M+H ; M+H ; M+H ; M+2H ; M+NH4 ; M+Na+K ; M+2K ; M+2K ; M+NH4 ; M+NH4 ; M+NH4 ; M+NH4 ; M+NH4 ; M+Na ; M+Na ; M+Na ; M+Na ; M+Na ; M+2ACN+2H ; 2M+H+Na ; M+Na	C10H15N3O4 ; C15H15NO2 ; C15H15NO2 ; C15H15NO2 ; C15H15NO2 ; C10H15N3O4 ; C24H34O10 ; C10H12N2O4 ; C21H43O6P ; C25H42O4 ; C25H42O4 ; C10H12N2O4 ; C10H12N2O4 ; C10H12N2O4 ; C10H12N2O4 ; C15H12O2 ; C13H17NO2 ; C13H17NO2 ; C13H17NO2 ; C13H17NO2 ; C13H17NO2 ; C20H29ClO6 ; C12H22S2 ; C13H17NO2
162.11244	1.714747	0.820443	-6.9418	3.99E-05	4.3994	0.003564	162.1124	HMDB00062 ; HMDB00596 ; HMDB00648 ; HMDB11531 ; HMDB11562 ; HMDB31841 ; HMDB32946 ; HMDB36132 ; HMDB36174 ; HMDB39160 ; HMDB39995 ; HMDB39996 ; HMDB41345	L-Carnitine ; Glucosylsphingosine ; Galactosylsphingosine ; MG(0:014:1(9Z)0:0) ; MG(14:1(9Z)0:00:0) ; 25-Dimethyl-3-propylpyrazine ; 35-Diethyl-2-methylpyrazine ; 25-Diethyl-3-methylpyrazine ; 2-Methyl-3-(2-methylpropyl)pyrazine ; L-Gizzerosine ; 35-Dimethyl-2-propylpyrazine ; 23-Dimethyl-5-propylpyrazine ; 23-Diethyl-5-methylpyrazine	M+H ; M+2H+Na ; M+2H+Na ; M+H+Na ; M+H+Na ; 2M+H+Na ; 2M+H+Na ; 2M+H+Na ; 2M+H+Na ; M+2ACN+2H ; 2M+H+Na ; 2M+H+Na ; 2M+H+Na	C7H15NO3 ; C24H47NO7 ; C24H47NO7 ; C17H32O4 ; C17H32O4 ; C9H14N2 ; C9H14N2 ; C9H14N2 ; C9H14N2 ; C11H20N4O2 ; C9H14N2 ; C9H14N2 ; C9H14N2
512.77066	1.726593	0.044611	-6.8982	4.20E-05	4.3764	0.003564	512.7707	HMDB41065	Dimethyl tetrasulfide	3M+K	C2H6S4
562.32696	1.703823	0.384321	-6.8904	4.24E-05	4.3723	0.003564	562.327	HMDB10384 ; HMDB11128 ; HMDB28736 ; HMDB28946 ; HMDB30361	LysoPC(18:0) ; LysoPC(0:018:0) ; Asparaginyl-Lysine ; Lysyl-Asparagine ; (-)-Aspidospermine	M+K ; M+K ; 2M+ACN+H ; 2M+ACN+H ; 3M+Na+K	C26H54NO7P ; C26H54NO7P ; C10H20N4O4 ; C10H20N4O4 ; C22H30N2O2
191.48134	1.777495	0.013968	6.8841	4.28E-05	4.3689	0.003564	NA	NA	NA	NA	NA
130.52580	1.750397	0.227741	6.8834	4.28E-05	4.3685	0.003564	130.5258	HMDB00592 ; HMDB01481 ; HMDB15239	Glucosamine 6-sulfate ; N-Sulfo-D-glucosamine ; Methyprylon	M+2H ; M+2H ; M+2K	C6H13NO8S ; C6H13NO8S ; C10H17NO2
408.09727	1.713291	0.368115	-6.8694	4.35E-05	4.3611	0.003564	408.0973	HMDB14038 ; HMDB15521 ; HMDB34754 ; HMDB36397 ; HMDB38858 ; HMDB39766 ; HMDB41158	N-Deisopropyl-fluvastatin ; Sulfamerazine ; 4-Methoxybenzyl O-(2-sulfoglucoside) ; Labadoside ; Oolongtheanin ; Pandamarilactone 31 ; Cyanidin 3-(6-feruloyl-2-sinapoylsophoroside) 5-glucoside	M+K ; 3M+H+Na ; 2M+3H2O+2H ; M+Na+K ; M+2ACN+2H ; M+2K-H ; M+3Na	C21H20FNO4 ; C11H12N4O2S ; C14H20O10S ; C38H42O16 ; C36H28O17 ; C19H25NO4 ; C54H59O28
278.03083	1.710197	0.05101	6.8619	4.39E-05	4.3571	0.003564	278.0308	HMDB02028 ; HMDB15343 ; HMDB31868 ; HMDB38866 ; HMDB41706 ; HMDB41708 ; HMDB60805	DOPA sulfate ; Ceftriaxone ; Monomethyl phenylphosphonate ; Quercetagetin 3-methylether 7-glucoside ; Caffeic acid 3-sulfate ; Caffeic acid 4-sulfate ; 9-Carboxymethoxymethylguanine	M+H ; M+2H ; 3M+H+K ; M+Na+K ; M+NH4 ; M+NH4 ; M+K	C9H11NO7S ; C18H18N8O7S3 ; C7H9O3P ; C22H22O13 ; C9H8O7S ; C9H8O7S ; C8H9N5O4

810.60404	1.745466	0.532695	6.8488	4.47E-05	4.3502	0.003564	810.604	HMDB07988 ; HMDB08048 ; HMDB08049 ; HMDB08079 ; HMDB08080 ; HMDB08112 ; HMDB08113 ; HMDB08145 ; HMDB08176 ; HMDB08209 ; HMDB08241 ; HMDB08273 ; HMDB08304 ; HMDB08305 ; HMDB08336 ; HMDB08367 ; HMDB08368 ; HMDB08400 ; HMDB08401 ; HMDB08431 ; HMDB08464 ; HMDB08626 ; HMDB06744 ; HMDB09050 ; HMDB09051 ; HMDB09083 ; HMDB09084 ; HMDB09115 ; HMDB09312 ; HMDB11349 ; HMDB11376 ; HMDB11407 ; HMDB11408 ; HMDB11440 ; HMDB11441	PC(16:022:4(7Z10Z13Z16Z)) ; PC(18:020:4(5Z8Z11Z14Z)) ; PC(18:020:4(8Z11Z14Z17Z)) ; PC(18:1(11Z)20:3(5Z8Z11Z)) ; PC(18:1(11Z)20:3(8Z11Z14Z)) ; PC(18:1(9Z)20:3(5Z8Z11Z)) ; PC(18:1(9Z)20:3(8Z11Z14Z)) ; PC(18:2(9Z12Z)20:2(11Z14Z)) ; PC(18:3(6Z9Z12Z)20:1(11Z)) ; PC(18:3(9Z12Z15Z)20:1(11Z)) ; PC(18:4(6Z9Z12Z15Z)20:0) ; PC(20:018:4(6Z9Z12Z15Z)) ; PC(20:1(11Z)18:3(6Z9Z12Z)) ; PC(20:1(11Z)18:3(9Z12Z15Z)) ; PC(20:2(11Z14Z)18:2(9Z12Z)) ; PC(20:3(5Z8Z11Z)18:1(11Z)) ; PC(20:3(5Z8Z11Z)18:1(9Z)) ; PC(20:3(8Z11Z14Z)18:1(11Z)) ; PC(20:3(8Z11Z14Z)18:1(9Z)) ; PC(20:4(5Z8Z11Z14Z)18:0) ; PC(20:4(8Z11Z14Z17Z)18:0) ; PC(22:4(7Z10Z13Z16Z)16:0) ; 3-Carboxy-1-hydroxypropylthiamine diphosphate ; PE(18:1(11Z)P-18:1(11Z)) ; PE(18:1(11Z)P-18:1(9Z)) ; PE(18:1(9Z)P-18:1(11Z)) ; PE(18:1(9Z)P-18:1(9Z)) ; PE(18:2(9Z12Z)P-18:0) ; PE(20:2(11Z14Z)P-16:0) ; PE(P-16:020:2(11Z14Z)) ; PE(P-18:018:2(9Z12Z)) ; PE(P-18:1(11Z)18:1(11Z)) ; PE(P-18:1(11Z)18:1(9Z)) ; PE(P-18:1(9Z)18:1(11Z)) ; PE(P-18:1(9Z)18:1(9Z))	M+H ; 3M+H+K ; M+2ACN+H ; M+2ACN+H ; M+2ACN+H ; M+2ACN+H ; M+2ACN+H ; M+2ACN+H ; M+2ACN+H ; M+2ACN+H ; M+2ACN+H ; M+2ACN+H ; M+2ACN+H ; M+2ACN+H	C46H84NO8P ; C16H25N4O10P2S ; C41H78NO7P ; C41H78NO7P ; C41H78NO7P ; C41H78NO7P ; C41H78NO7P ; C41H78NO7P ; C41H78NO7P ; C41H78NO7P ; C41H78NO7P ; C41H78NO7P ; C41H78NO7P ; C41H78NO7P
405.29932	1.741384	0.438281	6.844	4.49E-05	4.3476	0.003564	405.2993	HMDB11555 ; HMDB11556 ; HMDB11585 ; HMDB11586 ; HMDB12517 ; HMDB01186 ; HMDB11544 ; HMDB11574 ; HMDB13213 ; HMDB15498 ; HMDB31133 ; HMDB35959 ; HMDB36865	MG(0:022:5(4Z7Z10Z13Z16Z)0:0) ; MG(0:022:5(7Z10Z13Z16Z19Z)0:0) ; MG(22:5(4Z7Z10Z13Z16Z)0:00:0) ; MG(22:5(7Z10Z13Z16Z19Z)0:00:0) ; 11-Carboxy-gamma-chromanol ; N1-Acetylspermine ; MG(0:020:2(11Z14Z)0:0) ; MG(20:2(11Z14Z)0:00:0) ; Alpha-Linoleoylcholine ; Aprindine ; 1-O-beta-D-Glucopyranosyl-23-di-O-(8-hexadecenoyl)glycerol ; Persenone B ; Lepidiumterpenyl ester	M+H ; M+H ; M+H ; M+H ; M+H ; 3M+2K ; M+Na ; M+Na ; M+K ; M+2ACN+H ; M+2ACN+2H ; M+Na ; M+Na	C25H40O4 ; C25H40O4 ; C25H40O4 ; C25H40O4 ; C25H40O4 ; C12H28N4O ; C23H42O4 ; C23H42O4 ; C23H44NO2 ; C22H30N2 ; C41H74O10 ; C23H42O4 ; C23H42O4
615.32629	1.739631	0.329016	-6.8109	4.68E-05	4.33	0.003611	615.3263	HMDB35292 ; HMDB35294 ; HMDB35605 ; HMDB60583	Ganodermic acid P2 ; Ganoderic acid Mk ; 3alpha15alpha-Diacetoxy-(22R)-hydroxylanosta-79(11)24-trien-26-oic acid ; Trandolaprilat	M+2Na-H ; M+2Na-H ; M+2Na-H ; 3M+H+Na	C34H50O7 ; C34H50O7 ; C34H50O7 ; C22H30N2O5
380.28709	1.740633	0.256811	6.7975	4.76E-05	4.3229	0.003611	380.2871	HMDB06246 ; HMDB07941 ; HMDB07942 ; HMDB08165 ; HMDB08198 ; HMDB08876 ; HMDB08935 ; HMDB08936 ; HMDB08967 ; HMDB08995 ; HMDB08996 ; HMDB09027 ; HMDB09060 ; HMDB09091 ; HMDB09092 ; HMDB09123 ; HMDB09156 ; HMDB09287 ; HMDB09319 ; HMDB09352 ; HMDB09548 ; HMDB11539 ; HMDB11540 ; HMDB11569 ; HMDB11570 ; HMDB31678 ; HMDB35679 ; HMDB35680	Tetracosatetraenoic acid (24:4n-6) ; PC(15:018:3(6Z9Z12Z)) ; PC(15:018:3(9Z12Z15Z)) ; PC(18:3(6Z9Z12Z)15:0) ; PC(18:3(9Z12Z15Z)15:0) ; PE(14:1(9Z)22:2(13Z16Z)) ; PE(16:020:3(5Z8Z11Z)) ; PE(16:020:3(8Z11Z14Z)) ; PE(16:1(9Z)20:2(11Z14Z)) ; PE(18:018:3(6Z9Z12Z)) ; PE(18:018:3(9Z12Z15Z)) ; PE(18:1(11Z)18:2(9Z12Z)) ; PE(18:1(9Z)18:2(9Z12Z)) ; PE(18:2(9Z12Z)18:1(11Z)) ; PE(18:2(9Z12Z)18:1(9Z)) ; PE(18:3(6Z9Z12Z)18:0) ; PE(18:3(9Z12Z15Z)18:0) ; PE(20:2(11Z14Z)16:1(9Z)) ; PE(20:3(5Z8Z11Z)16:0) ; PE(20:3(8Z11Z14Z)16:0) ; PE(22:2(13Z16Z)14:1(9Z)) ; MG(0:018:3(6Z9Z12Z)0:0) ; MG(0:018:3(9Z12Z15Z)0:0) ; MG(18:3(6Z9Z12Z)0:00:0) ; MG(18:3(9Z12Z15Z)0:00:0) ; Pipericine ; 1-Hydroxy-1-phenyl-3-octadecanone ; 3-Hydroxy-1-phenyl-1-octadecanone	2M+H+K ; M+H+NH4 ; 2M+3H2O+2H ; 2M+3H2O+2H ; 2M+3H2O+2H ; 2M+3H2O+2H ; M+2Na-H ; 2M+H+K ; 2M+H+K	C24H40O2 ; C41H76NO8P ; C21H36O4 ; C21H36O4 ; C21H36O4 ; C21H36O4 ; C22H41NO ; C24H40O2 ; C24H40O2
265.02294	1.732124	0.409267	6.7956	4.77E-05	4.3218	0.003611	265.0229	HMDB15199 ; HMDB38888 ; HMDB39311 ; HMDB41942	Cefditoren ; Castamollissin ; Neocretanin ; N-Acetyl-S-(N-methylcarbamoyl)cysteine	M+H+Na ; M+Na+K ; M+Na+K ; M+2Na-H	C19H18N6O5S3 ; C20H20O13 ; C20H20O13 ; C7H12N2O4S
249.06326	1.738217	0.538552	-6.7788	4.87E-05	4.3128	0.003632	249.0633	HMDB36714 ; HMDB34251 ; HMDB38942 ; HMDB32437	Yuccaol A ; N-Benzoylaspartic acid ; Methyl 23-dihydro-35-dihydroxy-2-oxo-3-indoleacetic acid ; Monoglyceride citrate	M+2H ; 2M+H+Na ; 2M+H+Na ; M+H-H2O	C29H20O8 ; C11H11NO5 ; C11H11NO5 ; C9H14O9
154.99029	1.7364	0.930383	6.7309	5.16E-05	4.2871	0.003796	154.9903	HMDB06343 ; HMDB29726 ; HMDB31070 ; HMDB33746	Selenocystathionine ; 2-Vinylthiophene ; (2E11Z)-5-5-(Methylthio)-4-penten-2-ynyl-2-furanacrolein ; Benzenethiol	M+H+K ; M+2Na-H ; M+2K ; M+2Na-H	C7H14N2O4Se ; C6H6S ; C13H12O2S ; C6H6S

495.92679	1.717703	0.303528	6.7018	5.35E-05	4.2714	0.003853	495.9268	HMDB04610 ; HMDB45891 ; HMDB45943 ; HMDB46216 ; HMDB46218 ; HMDB50596 ; HMDB50601 ; HMDB50634 ; HMDB50639 ; HMDB50722 ; HMDB50724 ; HMDB50817 ; HMDB50819 ; HMDB51676 ; HMDB51710 ; HMDB51852 ; HMDB51854 ; HMDB52469 ; HMDB52568 ; HMDB52573 ; HMDB52602 ; HMDB52686 ; HMDB52700 ; HMDB52706 ; HMDB52776 ; HMDB53327 ; HMDB53412 ; HMDB53417 ; HMDB53442 ; HMDB53514 ; HMDB53526 ; HMDB53532 ; HMDB53592 ; HMDB54178 ; HMDB54200 ; HMDB54496 ; HMDB54516	Phytosphingosine ; TG(20:018:2(9Z12Z)22:6(4Z7Z10Z13Z16Z19Z)) ; TG(20:020:2n622:6(4Z7Z10Z13Z16Z19Z)) ; TG(20:022:6(4Z7Z10Z13Z16Z19Z)18:2(9Z12Z)) ; TG(20:022:6(4Z7Z10Z13Z16Z19Z)20:2n6) ; TG(20:1(11Z)18:2(9Z12Z)22:5(4Z7Z10Z13Z16Z)) ; TG(20:1(11Z)18:2(9Z12Z)22:5(7Z10Z13Z16Z19Z)) ; TG(20:1(11Z)20:2n622:5(4Z7Z10Z13Z16Z)) ; TG(20:1(11Z)20:2n622:5(7Z10Z13Z16Z19Z)) ; TG(20:1(11Z)22:5(4Z7Z10Z13Z16Z)18:2(9Z12Z)) ; TG(20:1(11Z)22:5(4Z7Z10Z13Z16Z)20:2n6) ; TG(20:1(11Z)22:5(7Z10Z13Z16Z19Z)18:2(9Z12Z)) ; TG(20:1(11Z)22:5(7Z10Z13Z16Z19Z)20:2n6) ; TG(22:1(13Z)18:2(9Z12Z)20:5(5Z8Z11Z14Z17Z)) ; TG(22:1(13Z)20:2n620:5(5Z8Z11Z14Z17Z)) ; TG(22:1(13Z)20:5(5Z8Z11Z14Z17Z)18:2(9Z12Z)) ; TG(22:1(13Z)20:5(5Z8Z11Z14Z17Z)20:2n6) ; TG(18:2(9Z12Z)20:022:6(4Z7Z10Z13Z16Z19Z)) ; TG(18:2(9Z12Z)20:1(11Z)22:5(4Z7Z10Z13Z16Z)) ; TG(18:2(9Z12Z)20:1(11Z)22:5(7Z10Z13Z16Z19Z)) ; TG(18:2(9Z12Z)22:1(13Z)20:5(5Z8Z11Z14Z17Z)) ; TG(18:2(9Z12Z)20:4(5Z8Z11Z14Z)22:2(13Z16Z)) ; TG(18:2(9Z12Z)22:2(13Z16Z)20:4(5Z8Z11Z14Z)) ; TG(18:2(9Z12Z)22:2(13Z16Z)20:4(8Z11Z14Z17Z)) ; TG(18:2(9Z12Z)20:4(8Z11Z14Z17Z)22:2(13Z16Z)) ; TG(20:2n620:022:6(4Z7Z10Z13Z16Z19Z)) ; TG(20:2n620:1(11Z)22:5(4Z7Z10Z13Z16Z)) ; TG(20:2n620:1(11Z)22:5(7Z10Z13Z16Z19Z)) ; TG(20:2n622:1(13Z)20:5(5Z8Z11Z14Z17Z)) ; TG(20:2n620:4(5Z8Z11Z14Z)22:2(13Z16Z)) ; TG(20:2n622:2(13Z16Z)20:4(5Z8Z11Z14Z)) ; TG(20:2n622:2(13Z16Z)20:4(8Z11Z14Z17Z)) ; TG(20:2n620:4(8Z11Z14Z17Z)22:2(13Z16Z)) ; TG(20:4(5Z8Z11Z14Z)18:2(9Z12Z)22:2(13Z16Z)) ; TG(20:4(5Z8Z11Z14Z)20:2n622:2(13Z16Z)) ; TG(22:2(13Z16Z)18:2(9Z12Z)20:4(8Z11Z14Z17Z)) ; TG(22:2(13Z16Z)20:2n620:4(8Z11Z14Z17Z))	3M+H+K ; M+H+NH4	C18H39NO3 ; C64H108O6
193.03575	1.726076	0.778747	6.6957	5.39E-05	4.2681	0.003853	193.0358	HMDB29841 ; HMDB30591 ; HMDB30640 ; HMDB38777 ; HMDB38817 ; HMDB39246 ; HMDB39323 ; HMDB60614 ; HMDB60780 ; HMDB00639 ; HMDB00663 ; HMDB29881	Vulgaxanthin II ; Dihydro-O-methylsterigmatocystin ; Biochanin A 7-(6-malonylglucoside) ; 26-Diacetylorientin ; 26-Di-O-acetylisoorientin ; 6-Malonyltrifolirhizin ; 6-O-Malonylglycitin ; Sulindac sulfide ; 5-Hydroxymethyl meloxicam ; Galactaric acid ; Glucaric acid ; D-Glucaric acid	M+2Na ; M+2Na ; M+H+2Na ; M+H+2Na ; M+H+2Na ; M+H+2Na ; M+H+2Na ; M+2Na ; M+H+NH4 ; M+H-H2O ; M+H-H2O ; M+H-H2O	C14H16N2O8 ; C19H16O6 ; C25H24O13 ; C25H24O13 ; C25H24O13 ; C25H24O13 ; C25H24O13 ; C20H17FO2S ; C14H13N3O5S2 ; C6H10O8 ; C6H10O8 ; C6H10O8
163.58553	1.711844	0.560706	-6.5942	6.12E-05	4.2131	0.004251	163.5855	HMDB01458	Biotin amide	M+2ACN+2H	C10H17N3O2S
214.03421	1.697335	0.89407	-6.5687	6.32E-05	4.1991	0.004251	214.0342	HMDB04220 ; HMDB12779 ; HMDB36377	Biotinyl-5-AMP ; 4-Hydroxyestrone sulfate ; 7-Hydroxy-2-methyl-4-oxo-4H-1-benzopyran-5-carboxylic acid 7-glucoside	M+3Na ; M+Na+K ; M+2Na	C20H28N7O9PS ; C18H22O6S ; C17H18O10
247.05781	1.718373	0.996858	-6.5665	6.34E-05	4.1979	0.004251	247.0578	HMDB05008 ; HMDB29968 ; HMDB30604 ; HMDB32657 ; HMDB33942 ; HMDB40894	Lansoprazole ; Ethyl beta-D-glucopyranoside ; epsilon-Viniferin ; trans-delta-Viniferin ; Dambonitol ; Ampelopsin D	2M+3H ; M+K ; M+H+K ; M+H+K ; M+K ; M+H+K	C16H14F3N3O2S ; C8H16O6 ; C28H22O6 ; C28H22O6 ; C8H16O6 ; C28H22O6
315.89707	1.766347	0.013193	6.5572	6.41E-05	4.1928	0.004251	315.8971	HMDB31381 ; HMDB36882	Methyl (7Z9Z9Z)-6-apo-y-caroten-6-oate ; Reticulataxanthin	2M+3H ; 2M+3H	C33H44O2 ; C33H44O2
140.53321	1.708604	0.22134	6.554	6.44E-05	4.1911	0.004251	140.5332	HMDB12204 ; HMDB14567 ; HMDB40288 ; HMDB41060	Cis-zeatin ; Methocarbamol ; 2-(Ethylsulfonylmethyl)phenyl methylcarbamate ; Dehydro-4-methoxycyclobrassinin	M+Na+K ; M+H+K ; M+H+Na ; M+H+NH4	C10H13N5O ; C11H15NO5 ; C11H15NO4S ; C12H10N2OS2
177.09499	1.717956	0.265561	6.5514	6.46E-05	4.1897	0.004251	177.095	HMDB32378 ; HMDB38300 ; HMDB38920 ; HMDB30389 ; HMDB30390 ; HMDB00369 ; HMDB00383 ; HMDB00412 ; HMDB00458 ; HMDB00493 ; HMDB00495 ; HMDB00551 ; HMDB00554 ; HMDB00895 ; HMDB01161 ; HMDB13811 ; HMDB13939 ; HMDB14447 ; HMDB15029 ; HMDB15593 ; HMDB29378 ; HMDB29428 ; HMDB29858 ; HMDB30344 ; HMDB33433 ; HMDB34371 ; HMDB34592 ; HMDB38539 ; HMDB38540 ; HMDB39086 ; HMDB39632 ; HMDB41314 ; HMDB60437	Methionyl butyrate ; Methylthiomethyl hexanoate ; (S)-3-Mercaptohexyl acetate ; Quebrachidine ; ()-Quebrachidine ; 3b17b-Dihydroxyetiocholane ; 3a17a-Dihydroxy-5b-androstane ; 3b17a-Dihydroxy-5a-androstane ; 5a-Androstane-3a17a-diol ; 5a-Androstane-3b17b-diol ; Androstanediol ; Etiocholanediol ; Dihydroandrosterone ; Acetylcholine ; 4-Trimethylammoniobutanoic acid ; 1-Nitroheptane ; N-Desmethylpromazine ; Tranexamic Acid ; Oxybuprocaine ; Ixabepilone ; (-)-Thebaine ; Hypoglycin B ; Lansimide 3 ; (R)-Pronuciferine ; (S)-Homostachydrine ; Piperettine ; Lansamide 4 ; Goshonoside F1 ; Goshonoside F2 ; Hydroxysintaxanthin 56-epoxide ; N-2-Methoxy-2-(4-methoxyphenyl)ethylcinnamide ; 4-tert-Butylphenyl salicylate ; Androstan-3alpha17beta-diol	M+H ; M+H ; M+H ; M+2H ; M+2H ; M+Na+K ; M+Na+K ; M+Na+K ; M+Na+K ; M+Na+K ; M+Na+K ; M+Na+K ; M+Na+K ; 2M+Na+K ; 2M+Na+K ; 2M+Na+K ; M+2ACN+2H ; 2M+H+K ; M+2Na ; M+2H+Na ; M+ACN+2H ; M+2ACN+2H ; M+ACN+2H ; M+ACN+2H ; 2M+H+K ; M+ACN+2H ; M+ACN+2H ; M+H+2Na ; M+H+2Na ; M+3Na ; M+ACN+2H ; M+2ACN+2H ; M+Na+K	C8H16O2S ; C8H16O2S ; C8H16O2S ; C21H24N2O3 ; C21H24N2O3 ; C19H32O2 ; C19H32O2 ; C19H32O2 ; C19H32O2 ; C19H32O2 ; C19H32O2 ; C19H32O2 ; C19H32O2 ; C7H16NO2 ; C7H16NO2 ; C7H16NO2 ; C16H18N2S ; C8H15NO2 ; C17H28N2O3 ; C27H42N2O5S ; C19H21NO3 ; C12H18N2O5 ; C19H21NO3 ; C19H21NO3 ; C8H15NO2 ; C19H21NO3 ; C19H21NO3 ; C26H44O8 ; C26H44O8 ; C31H42O3 ; C19H21NO3 ; C17H18O3 ; C19H32O2

								HMDB09243 ; HMDB09274 ; HMDB09275 ; HMDB09306 ; HMDB09404 ; HMDB09437 ; HMDB09469 ; HMDB09533 ; HMDB09564 ; HMDB09565 ; HMDB09594 ; HMDB09603 ; HMDB09604 ; HMDB09626 ; HMDB09636 ; HMDB09659 ; HMDB09669 ; HMDB09691 ; HMDB12372 ; HMDB12403 ; HMDB12413 ; HMDB12432	PE(20:022:6(4Z7Z10Z13Z16Z19Z)) ; PE(20:1(11Z)22:5(4Z7Z10Z13Z16Z)) ; PE(20:1(11Z)22:5(7Z10Z13Z16Z19Z)) ; PE(20:2(11Z14Z)22:4(7Z10Z13Z16Z)) ; PE(20:4(5Z8Z11Z14Z)22:2(13Z16Z)) ; PE(20:4(8Z11Z14Z17Z)22:2(13Z16Z)) ; PE(20:5(5Z8Z11Z14Z17Z)22:1(13Z)) ; PE(22:1(13Z)20:5(5Z8Z11Z14Z17Z)) ; PE(22:2(13Z16Z)20:4(5Z8Z11Z14Z)) ; PE(22:2(13Z16Z)20:4(8Z11Z14Z17Z)) ; PE(22:4(7Z10Z13Z16Z)20:2(11Z14Z)) ; PE(22:4(7Z10Z13Z16Z)22:5(4Z7Z10Z13Z16Z)) ; PE(22:4(7Z10Z13Z16Z)22:5(7Z10Z13Z16Z19Z)) ; PE(22:5(4Z7Z10Z13Z16Z)20:1(11Z)) ; PE(22:5(4Z7Z10Z13Z16Z)22:4(7Z10Z13Z16Z)) ; PE(22:5(7Z10Z13Z16Z19Z)20:1(11Z)) ; PE(22:5(7Z10Z13Z16Z19Z)22:4(7Z10Z13Z16Z)) ; PE(22:6(4Z7Z10Z13Z16Z19Z)20:0) ; PS(16:1(9Z)20:4(5Z8Z11Z14Z)) ; PS(18:2(9Z12Z)18:3(9Z12Z15Z)) ; PS(18:3(9Z12Z15Z)18:2(9Z12Z)) ; PS(20:4(5Z8Z11Z14Z)16:1(9Z))	M+2Na ; M+2Na ; M+2Na ; M+2Na ; M+2Na ; M+2Na ; M+2Na ; M+2Na ; M+2Na ; M+2Na ; M+2Na ; M+H+Na ; M+H+Na ; M+2Na ; M+H+Na ; M+2Na ; M+H+Na ; M+2Na ; M+2ACN+2H ; M+2ACN+2H ; M+2ACN+2H ; M+2ACN+2H	C47H82NO8P ; C47H82NO8P ; C47H82NO8P ; C47H82NO8P ; C47H82NO8P ; C47H82NO8P ; C47H82NO8P ; C47H82NO8P ; C47H82NO8P ; C47H82NO8P ; C47H82NO8P ; C49H80NO8P ; C49H80NO8P ; C47H82NO8P ; C49H80NO8P ; C47H82NO8P ; C49H80NO8P ; C47H82NO8P ; C42H72NO10P ; C42H72NO10P ; C42H72NO10P ; C42H72NO10P
432.77516	1.733421	0.699807	6.5315	6.63E-05	4.1787	0.004303	432.7752				
364.59643	1.695054	0.017947	-6.4877	7.00E-05	4.1547	0.00449	364.5964	HMDB41793	1-Aminopyrene	3M+2K	C16H11N
292.96762	1.725232	0.823907	-6.4671	7.19E-05	4.1433	0.004497	292.9676	HMDB41788	Vanillic acid 4-sulfate	M+2Na-H	C8H8O7S
254.01908	1.689353	1.005349	-6.4665	7.20E-05	4.143	0.004497	254.0191	HMDB01125 ; HMDB15138 ; HMDB32877 ; HMDB32982 ; HMDB41737 ; HMDB41738 ; HMDB41739 ; HMDB41832	Inositol cyclic phosphate ; Cromoglicic acid ; Glucorhein ; 22-Dithenyl sulfide ; Genistein 4-O-glucuronide ; Genistein 5-O-glucuronide ; Genistein 7-O-glucuronide ; Baicalin	2M+H+Na ; M+H+K ; M+Na+K ; 2M+3H2O+2H ; M+Na+K ; M+Na+K ; M+Na+K ; M+Na+K	C6H11O8P ; C23H16O11 ; C21H18O11 ; C10H10S3 ; C21H18O11 ; C21H18O11 ; C21H18O11 ; C21H18O11
318.97659	1.728546	0.672191	6.4304	7.53E-05	4.123	0.004643	318.9766	HMDB32040 ; HMDB32866 ; HMDB33307 ; HMDB60686 ; HMDB60690	Benzoyl peroxide ; 2456-Phenanthrenetetrol ; Moracin M ; 4-Ketocyclophosphamide ; 4-Ketoifosfamide	M+2K-H ; M+2K-H ; M+2K-H ; M+2Na-H ; M+2Na-H	C14H10O4 ; C14H10O4 ; C14H10O4 ; C7H13Cl2N2O3P ; C7H13Cl2N2O3P
125.10728	1.706202	1.068854	-6.4176	7.66E-05	4.1159	0.004643	125.1073	HMDB32181 ; HMDB29734 ; HMDB31288 ; HMDB32244 ; HMDB32972 ; HMDB33871 ; HMDB35584 ; HMDB39867 ; HMDB41077 ; HMDB41965 ; HMDB02038 ; HMDB06009	alpha-Butyl-omega-hydroxypoly(oxyethylene) poly(oxypropylene) ; 3-Ethylpyridine ; 121-Heneicosanediol ; 24-Dimethylpyridine ; 26-Dimethylpyridine ; Benzylamine ; 1-Phenyl-13-nonadecanedione ; 5-(1013-Nonadecadienyl)-13-benzenediol ; erythro-68-Heneicosanediol ; O-Toluidine ; N(6)-Methyllysine ; Isoputreanine	M+2H ; M+NH4 ; M+H+2Na ; M+NH4 ; M+NH4 ; M+NH4 ; M+3H ; M+3H ; M+H+2Na ; M+NH4 ; M+H-2H2O ; M+H-2H2O	C13H28O4 ; C7H9N ; C21H44O2 ; C7H9N ; C7H9N ; C7H9N ; C25H40O2 ; C25H40O2 ; C21H44O2 ; C7H9N ; C7H16N2O2 ; C7H16N2O2
379.28366	1.723283	0.190833	6.4115	7.72E-05	4.1125	0.004643	379.2837	HMDB06740 ; HMDB13208	CE(20:0) ; 9-Hexadecenoylcholine	M+2K ; M+K	C47H84O2 ; C21H42NO2
505.83220	1.699737	0.988077	-6.3979	7.85E-05	4.1049	0.004643	505.8322	HMDB05772	Postin	2M+H+NH4	C22H40N8O5
484.09662	1.729265	0.740518	-6.3878	7.96E-05	4.0993	0.004643	484.0966	HMDB29238	Cyanidin 3-O-(6-acetyl-arabinoside)	M+Na	C22H21O11
431.11911	1.699123	1.057372	-6.3785	8.05E-05	4.0941	0.004643	431.1191	HMDB36355 ; HMDB38420 ; HMDB38424	3-Methylpentyl glucosinolate ; 4-Methylpentyl glucosinolate ; Hexyl glucosinolate	2M+3H2O+2H ; 2M+3H2O+2H ; 2M+3H2O+2H	C13H25NO9S2 ; C13H25NO9S2 ; C13H25NO9S2
272.00291	1.688349	0.931563	-6.3759	8.08E-05	4.0927	0.004643	272.0029	HMDB60713	2-Chloro-2-deoxyadenosine-5-triphosphate	M+H+NH4	C10H15ClN5O12P3
404.28736	1.715071	0.231079	6.3415	8.44E-05	4.0735	0.004763	404.2874	HMDB07442 ; HMDB07470 ; HMDB07499 ; HMDB07639 ; HMDB07667 ; HMDB07806 ; HMDB07807 ; HMDB07834 ; HMDB07951 ; HMDB08494 ; HMDB08944 ; HMDB08945 ; HMDB08976 ; HMDB09005 ; HMDB09036 ; HMDB09037 ; HMDB09045 ; HMDB09069 ; HMDB09070 ; HMDB09078 ; HMDB09100 ; HMDB09101 ; HMDB09109 ; HMDB09110 ; HMDB09132 ; HMDB09141 ; HMDB09165 ; HMDB09174 ; HMDB09197 ; HMDB09261 ; HMDB09292 ; HMDB09293 ; HMDB09302 ; HMDB09324 ; HMDB09333 ; HMDB09334 ; HMDB09357 ; HMDB09366 ; HMDB09367 ; HMDB09388 ; HMDB09389 ; HMDB09397 ; HMDB09398 ; HMDB09421 ; HMDB09422 ; HMDB09430 ; HMDB09431 ; HMDB09453 ; HMDB09462 ; HMDB09584 ; HMDB09589 ; HMDB09590 ; HMDB09616 ; HMDB09621 ; HMDB09649 ; HMDB09654 ;	DG(20:2(11Z14Z)24:1(15Z)0:0) ; DG(20:3(5Z8Z11Z)24:00:0) ; DG(20:3(8Z11Z14Z)24:00:0) ; DG(22:1(13Z)22:2(13Z16Z)0:0) ; DG(22:2(13Z16Z)22:1(13Z)0:0) ; DG(24:020:3(5Z8Z11Z)0:0) ; DG(24:020:3(8Z11Z14Z)0:0) ; DG(24:1(15Z)20:2(11Z14Z)0:0) ; PC(15:020:5(5Z8Z11Z14Z17Z)) ; PC(20:5(5Z8Z11Z14Z17Z)15:0) ; PE(16:022:5(4Z7Z10Z13Z16Z)) ; PE(16:022:5(7Z10Z13Z16Z19Z)) ; PE(16:1(9Z)22:4(7Z10Z13Z16Z)) ; PE(18:020:5(5Z8Z11Z14Z17Z)) ; PE(18:1(11Z)20:4(5Z8Z11Z14Z)) ; PE(18:1(11Z)20:4(8Z11Z14Z17Z)) ; PE(18:1(11Z)22:6(4Z7Z10Z13Z16Z19Z)) ; PE(18:1(9Z)20:4(5Z8Z11Z14Z)) ; PE(18:1(9Z)20:4(8Z11Z14Z17Z)) ; PE(18:1(9Z)22:6(4Z7Z10Z13Z16Z19Z)) ; PE(18:2(9Z12Z)20:3(5Z8Z11Z)) ; PE(18:2(9Z12Z)20:3(8Z11Z14Z)) ; PE(18:2(9Z12Z)22:5(4Z7Z10Z13Z16Z)) ; PE(18:2(9Z12Z)22:5(7Z10Z13Z16Z19Z)) ; PE(18:3(6Z9Z12Z)20:2(11Z14Z)) ; PE(18:3(6Z9Z12Z)22:4(7Z10Z13Z16Z)) ; PE(18:3(9Z12Z15Z)20:2(11Z14Z)) ; PE(18:3(9Z12Z15Z)22:4(7Z10Z13Z16Z)) ; PE(18:4(6Z9Z12Z15Z)20:1(11Z)) ; PE(20:1(11Z)18:4(6Z9Z12Z15Z)) ; PE(20:2(11Z14Z)18:3(6Z9Z12Z)) ; PE(20:2(11Z14Z)18:3(9Z12Z15Z)) ; PE(20:2(11Z14Z)20:5(5Z8Z11Z14Z17Z)) ; PE(20:3(5Z8Z11Z)18:2(9Z12Z)) ; PE(20:3(5Z8Z11Z)20:4(5Z8Z11Z14Z)) ; PE(20:3(5Z8Z11Z)20:4(8Z11Z14Z17Z)) ; PE(20:3(8Z11Z14Z)18:2(9Z12Z)) ; PE(20:3(8Z11Z14Z)20:4(5Z8Z11Z14Z)) ; PE(20:3(8Z11Z14Z)20:4(8Z11Z14Z17Z)) ; PE(20:4(5Z8Z11Z14Z)18:1(11Z)) ; PE(20:4(5Z8Z11Z14Z)18:1(9Z)) ; PE(20:4(5Z8Z11Z14Z)20:3(5Z8Z11Z)) ; PE(20:4(5Z8Z11Z14Z)20:3(8Z11Z14Z)) ; PE(20:4(8Z11Z14Z17Z)18:1(11Z)) ; PE(20:4(8Z11Z14Z17Z)18:1(9Z)) ; PE(20:4(8Z11Z14Z17Z)20:3(5Z8Z11Z)) ; PE(20:4(8Z11Z14Z17Z)20:3(8Z11Z14Z)) ; PE(20:5(5Z8Z11Z14Z17Z)18:0) ; PE(20:5(5Z8Z11Z14Z17Z)20:2(11Z14Z)) ; PE(22:4(7Z10Z13Z16Z)16:1(9Z)) ; PE(22:4(7Z10Z13Z16Z)18:3(6Z9Z12Z)) ; PE(22:4(7Z10Z13Z16Z)18:3(9Z12Z15Z)) ; PE(22:5(4Z7Z10Z13Z16Z)16:0) ; PE(22:5(4Z7Z10Z13Z16Z)18:2(9Z12Z)) ; PE(22:5(7Z10Z13Z16Z19Z)16:0) ; PE(22:5(7Z10Z13Z16Z19Z)18:2(9Z12Z)) ; PE(22:6(4Z7Z10Z13Z16Z19Z)18:1(11Z)) ; PE(22:6(4Z7Z10Z13Z16Z19Z)18:1(9Z)) ; MG(0:020:5(5Z8Z11Z14Z17Z)0:0) ; MG(20:5(5Z8Z11Z14Z17Z)0:00:0) ; 9-Carboxy-gamma-chromanol ; 5-Decanoyl-2-nonylpyridine ; Homodihydrocapsaicin ; 12-Gingerdione ; Ipomeatetrahydrofuran ;	M+2K ; M+2K ; M+2K ; M+2K ; M+2K ; M+2K ; M+2K ; M+2K ; M+ACN+2H ; M+ACN+2H ; M+ACN+2H ; M+ACN+2H ; M+ACN+2H ; M+ACN+2H ; M+ACN+2H ; M+ACN+2H ; M+H+NH4 ; M+ACN+2H ; M+ACN+2H ; M+H+NH4 ; M+ACN+2H ; M+ACN+2H ; M+H+NH4 ; M+H+NH4 ; M+ACN+2H ; M+H+NH4 ; M+ACN+2H ; M+H+NH4 ; M+ACN+2H ; M+ACN+2H ; M+ACN+2H ; M+ACN+2H ; M+H+NH4 ; M+ACN+2H ; M+H+NH4 ; M+H+NH4 ; M+ACN+2H ; M+H+NH4 ; M+H+NH4 ; M+ACN+2H ; M+ACN+2H ; M+H+NH4 ; M+H+NH4 ; M+ACN+2H ; M+ACN+2H ; M+H+NH4 ; M+H+NH4 ; M+ACN+2H ; M+H+NH4 ; M+ACN+2H ; M+H+NH4 ; M+H+NH4 ; M+ACN+2H ; M+H+NH4 ; M+ACN+2H ; M+H+NH4 ; M+H+NH4 ; M+H+NH4 ; 2M+3H2O+2H ; 2M+3H2O+2H ; 2M+3H2O+2H ; M+2Na-H ; M+2ACN+H ; 2M+3H2O+2H ; 3M+H+K ; M+2K ; M+2K ; M+2K	C47H86O5 ; C47H86O5 ; C47H86O5 ; C47H86O5 ; C47H86O5 ; C47H86O5 ; C47H86O5 ; C47H86O5 ; C43H76NO8P ; C43H76NO8P ; C43H76NO8P ; C43H76NO8P ; C43H76NO8P ; C43H76NO8P ; C43H76NO8P ; C43H76NO8P ; C45H76NO8P ; C43H76NO8P ; C43H76NO8P ; C45H76NO8P ; C43H76NO8P ; C43H76NO8P ; C45H76NO8P ; C45H76NO8P ; C43H76NO8P ; C45H76NO8P ; C43H76NO8P ; C45H76NO8P ; C43H76NO8P ; C43H76NO8P ; C43H76NO8P ; C45H76NO8P ; C43H76NO8P ; C45H76NO8P ; C45H76NO8P ; C43H76NO8P ; C45H76NO8P ; C45H76NO8P ; C43H76NO8P ; C43H76NO8P ; C45H76NO8P ; C45H76NO8P ; C43H76NO8P ; C43H76NO8P ; C45H76NO8P ; C45H76NO8P ; C43H76NO8P ; C45H76NO8P ; C43H76NO8P ; C45H76NO8P ; C43H76NO8P ; C45H76NO8P ; C45H76NO8P ; C43H76NO8P ; C45H76NO8P ; C45H76NO8P ; C45H76NO8P ; C23H36O4 ; C23H36O4 ; C23H36O4 ; C24H41NO ; C19H31NO3 ; C23H36O4 ; C15H28O3 ; C47H86O5 ; C47H86O5 ; C47H86O5

137.07178	1.702882	0.996839	6.3332	8.53E-05	4.0688	0.004763	137.0718	HMDB03152 ; HMDB30001 ; HMDB33947 ; HMDB37819 ; HMDB59711 ; HMDB00696 ; HMDB04058 ; HMDB06803 ; HMDB12235 ; HMDB13751 ; HMDB14426 ; HMDB14997 ; HMDB29532 ; HMDB30032 ; HMDB30033 ; HMDB30034 ; HMDB30036 ; HMDB31173 ; HMDB31235 ; HMDB31236 ; HMDB31922 ; HMDB33567 ; HMDB33951 ; HMDB34017 ; HMDB35255 ; HMDB36057 ; HMDB39671 ; HMDB40317 ; HMDB41275 ; HMDB60318 ; HMDB60946 ; HMDB61022	N-Methylnicotinamide ; 2-Acetyl-3-methylpyrazine ; 2-Aminobenzamide ; 4-Acetyl-2-methylpyrimidine ; 2-Methylnicotinamide ; L-Methionine ; 56-Dihydroxyindole ; 2-Propyn-1-al ; Hydroxybupropion ; 2-Hydroxypyridine ; Lidocaine ; Penicillamine ; 3-Hydroxyglabrol ; Euglobal Ia1 ; Euglobal Ib ; Euglobal IIb ; Euglobal IIc ; 6-Hydroxy-1H-indole-3-acetamide ; (E)-2446-Tetrahydroxy-35-diprenylchalcone ; 3-Geranyl-2446-tetrahydroxychalcone ; 1-(24-Dihydroxyphenyl)-3-8-hydroxy-2-methyl-2-(4-methyl-3-pentenyl)-2H-1-benzopyran-5-yl-1-propanone ; Cyclotetradecane ; Racemethionine ; Kuwanol D ; 3-Geranyl-2344-tetrahydroxychalcone ; 1H-Pyrrole-2-carboxaldehyde ; 5-Methyl-25-di-1-pyrrolidinyl-2-cyclopenten-1-one ; (E)-2-Geranyl-347-trihydroxyflavanone ; 1-Hydroxy-35-dimethoxy-24-diprenylxanthone ; (S)-4-Hydroxymandelonitrile ; NAPQI ; 6-beta-hydroxydexamethasone	M+H ; M+H ; M+H ; M+H ; M+H ; M+3ACN+2H ; M+3ACN+2H ; M+2ACN+H ; M+H+NH4 ; M+ACN+H ; M+H+K ; M+3ACN+2H ; M+3H ; M+2H+Na ; M+2H+Na ; M+2H+Na ; M+2H+Na ; M+2ACN+2H ; M+3H ; M+3H ; M+3H ; M+2K ; M+3ACN+2H ; M+3H ; M+3H ; M+ACN+H ; M+H+K ; M+3H ; M+3H ; M+3ACN+2H ; M+3ACN+2H ; M+3H	C7H8N2O ; C7H8N2O ; C7H8N2O ; C7H8N2O ; C7H8N2O ; C5H11NO2S ; C8H7NO2 ; C3H2O ; C13H18ClNO2 ; C5H5NO ; C14H22N2O ; C5H11NO2S ; C25H28O5 ; C23H30O5 ; C23H30O5 ; C23H30O5 ; C23H30O5 ; C10H10N2O2 ; C25H28O5 ; C25H28O5 ; C25H28O5 ; C14H28 ; C5H11NO2S ; C25H28O5 ; C25H28O5 ; C5H5NO ; C14H22N2O ; C25H28O5 ; C25H28O5 ; C8H7NO2 ; C8H7NO2 ; C22H29FO6
220.99966	1.738819	0.33796	6.3254	8.62E-05	4.0645	0.004763	NA	NA	NA	NA	NA
271.23274	1.715045	0.344064	6.3088	8.81E-05	4.0551	0.004763	271.2327	HMDB30896 ; HMDB15222 ; HMDB42076 ; HMDB42078 ; HMDB42117 ; HMDB42511 ; HMDB42571 ; HMDB42842 ; HMDB42985 ; HMDB43171 ; HMDB43172 ; HMDB43181 ; HMDB43187 ; HMDB43199 ; HMDB43215 ; HMDB43227 ; HMDB43256 ; HMDB43517 ; HMDB43663 ; HMDB43691 ; HMDB47749 ; HMDB47750 ; HMDB47759 ; HMDB47765 ; HMDB48435 ; HMDB48451	Momordicilin ; Pentolinium ; TG(14:014:018:2(9Z12Z)) ; TG(14:014:020:2n6) ; TG(14:015:020:5(5Z8Z11Z14Z17Z)) ; TG(14:018:2(9Z12Z)14:0) ; TG(14:020:2n614:0) ; TG(14:020:5(5Z8Z11Z14Z17Z)15:0) ; TG(15:014:020:5(5Z8Z11Z14Z17Z)) ; TG(15:014:1(9Z)18:1(11Z)) ; TG(15:014:1(9Z)18:1(9Z)) ; TG(15:014:1(9Z)20:4(5Z8Z11Z14Z)) ; TG(15:014:1(9Z)20:4(8Z11Z14Z17Z)) ; TG(15:016:1(9Z)16:1(9Z)) ; TG(15:016:1(9Z)18:4(6Z9Z12Z15Z)) ; TG(15:018:1(11Z)14:1(9Z)) ; TG(15:018:1(9Z)14:1(9Z)) ; TG(15:020:4(5Z8Z11Z14Z)14:1(9Z)) ; TG(15:018:4(6Z9Z12Z15Z)16:1(9Z)) ; TG(15:020:4(8Z11Z14Z17Z)14:1(9Z)) ; TG(14:1(9Z)15:018:1(11Z)) ; TG(14:1(9Z)15:018:1(9Z)) ; TG(14:1(9Z)15:020:4(5Z8Z11Z14Z)) ; TG(14:1(9Z)15:020:4(8Z11Z14Z17Z)) ; TG(16:1(9Z)15:016:1(9Z)) ; TG(16:1(9Z)15:018:4(6Z9Z12Z15Z))	M+2H ; 2M+Na+K ; M+2H+Na ; M+2H+Na ; M+3H ; M+2H+Na ; M+2H+Na ; M+3H ; M+3H ; M+2H+Na ; M+2H+Na ; M+3H ; M+3H ; M+2H+Na ; M+3H ; M+2H+Na ; M+2H+Na ; M+3H ; M+3H ; M+3H ; M+2H+Na ; M+2H+Na ; M+3H ; M+3H ; M+2H+Na ; M+3H	C36H60O3 ; C15H32N2 ; C50H92O6 ; C50H92O6 ; C52H90O6 ; C50H92O6 ; C50H92O6 ; C52H90O6 ; C52H90O6 ; C50H92O6 ; C50H92O6 ; C52H90O6 ; C52H90O6 ; C50H92O6 ; C52H90O6 ; C50H92O6 ; C50H92O6 ; C52H90O6 ; C52H90O6 ; C52H90O6 ; C50H92O6 ; C50H92O6 ; C52H90O6 ; C52H90O6 ; C50H92O6 ; C52H90O6
791.57858	1.721395	0.241861	6.3041	8.86E-05	4.0525	0.004763	791.5786	HMDB08260 ; HMDB08261 ; HMDB08521 ; HMDB11222 ; HMDB11280 ; HMDB11313 ; HMDB30437 ; HMDB35990 ; HMDB39205	PC(18:4(6Z9Z12Z15Z)P-18:1(11Z)) ; PC(18:4(6Z9Z12Z15Z)P-18:1(9Z)) ; PC(20:5(5Z8Z11Z14Z17Z)P-16:0) ; PC(P-16:020:5(5Z8Z11Z14Z17Z)) ; PC(P-18:1(11Z)18:4(6Z9Z12Z15Z)) ; PC(P-18:1(9Z)18:4(6Z9Z12Z15Z)) ; Emblicanin A ; Cerebroside B ; Punicalin	2M+3H2O+2H ; 2M+3H2O+2H ; 2M+3H2O+2H ; 2M+3H2O+2H ; 2M+3H2O+2H ; 2M+3H2O+2H ; 2M+H+NH4 ; M+ACN+Na ; M+H+NH4	C44H78NO7P ; C44H78NO7P ; C44H78NO7P ; C44H78NO7P ; C44H78NO7P ; C44H78NO7P ; C34H22O22 ; C41H77NO9 ; C68H44O44
148.02289	1.669513	0.629142	6.2961	8.95E-05	4.048	0.004763	148.0229	HMDB37835 ; HMDB05760 ; HMDB15685 ; HMDB29462 ; HMDB29519 ; HMDB30808 ; HMDB32577 ; HMDB32670 ; HMDB33904 ; HMDB36457 ; HMDB37316 ; HMDB41647 ; HMDB60643	4-(24-Dihydroxyphenyl)azobenzenesulfonic acid ; Dihydrodaidzein ; Azidocillin ; (E)-244-Trihydroxychalcone ; (2S)-Liquiritigenin ; (S)-Pinocembrin ; 4-Methoxybenzophenone-2-carboxylic acid ; 2-(5-Methylsulfinyl)-4-penten-2-ynylidene-16-dioxaspiro44non-3-ene ; 7E-Mycosinyl acetate ; Emodinanthranol ; Isoliquiritigenin ; 2-Dehydro-O-desmethylangolensin ; Tizoxanide glucuronide	M+2H ; M+H+K ; M+3Na ; M+H+K ; M+H+K ; M+H+K ; M+H+K ; M+2Na ; M+H+K ; M+H+K ; M+H+K ; M+H+K ; M+3H	C12H10N2O5S ; C15H12O4 ; C16H17N5O4S ; C15H12O4 ; C15H12O4 ; C15H12O4 ; C15H12O4 ; C13H14O3S ; C15H12O4 ; C15H12O4 ; C15H12O4 ; C16H15N3O10S
165.04095	1.644838	0.152091	6.2962	8.95E-05	4.048	0.004763	165.041	HMDB32741 ; HMDB32748 ; HMDB32749 ; HMDB39965 ; HMDB01476 ; HMDB01972 ; HMDB12267 ; HMDB14378 ; HMDB14389 ; HMDB29481 ; HMDB30694 ; HMDB31586 ; HMDB31870 ; HMDB33691 ; HMDB33741 ; HMDB34032 ; HMDB34312 ; HMDB37134 ; HMDB37282 ; HMDB37485 ; HMDB37509 ; HMDB38778 ; HMDB38935 ; HMDB39989 ; HMDB40124 ; HMDB40412 ; HMDB40561 ; HMDB41525 ; HMDB41536 ; HMDB59749 ; HMDB59811	S-2-Propenyl 1-propanesulfinothioate ; R-1-Propenyl 1-propanesulfinothioate ; R-Propyl 1-propenesulfinothioate ; S-Propyl 2-propene-1-sulfinothioate ; 3-Hydroxyanthranilic acid ; 3-Aminosalicylic acid ; N-Succinyl-LL-26-diaminopimelate ; Aminosalicylic Acid ; Mesalazine ; Isosakuranin ; (S)-57-Dihydroxy-68-dimethylflavanone ; 7-Hydroxy-8-O-methylaloin B ; gamma-Glutamyl-S-(1-propenyl)cysteine sulfoxide ; 2-Hydroxy-46-dimethoxychalcone ; Puddumin A ; 6-(2-Carboxyethyl)-7-hydroxy-22-dimethyl-4-chromanone glucoside ; Isopimpinellin ; (1-Methylethenyl)pyrazine ; 2-Methyl-5-vinylpyrazine ; Stercurensin ; Chalconosakuranetin ; Batatasin I ; Kamahine C ; 2-Methyl-3-vinylpyrazine ; 1-Propenylpyrazine ; DL-Propylene glycol dibenzoate ; Aromadendrin 4-methyl ether 7-rhamnoside ; Coriandrone C ; Piperenol C ; 6-Methoxy-pyridine-3-carboxylic acid ; 2-Nitro-p-cresol	M+H ; M+H ; M+H ; M+H ; 2M+H+Na ; 2M+H+Na ; M+H+K ; 2M+H+Na ; 2M+H+Na ; M+H+2Na ; M+2Na ; M+H+2Na ; M+H+Na ; M+2Na ; M+H+2Na ; M+3Na ; 2M+3H ; M+2Na-H ; M+2Na-H ; M+2Na ; M+H+2Na ; M+2Na ; M+Na+K ; M+2Na-H ; M+2Na-H ; M+2Na ; M+H+2Na ; 2M+3H ; M+H+2Na ; 2M+H+Na ; 2M+H+Na	C6H12OS2 ; C6H12OS2 ; C6H12OS2 ; C6H12OS2 ; C7H7NO3 ; C7H7NO3 ; C11H18N2O7 ; C7H7NO3 ; C7H7NO3 ; C22H24O10 ; C17H16O4 ; C22H24O10 ; C11H18N2O6S ; C17H16O4 ; C22H24O10 ; C20H26O10 ; C13H10O5 ; C7H8N2 ; C7H8N2 ; C17H16O4 ; C22H24O10 ; C17H16O4 ; C14H20O5 ; C7H8N2 ; C7H8N2 ; C17H16O4 ; C22H24O10 ; C13H10O5 ; C22H24O10 ; C7H7NO3 ; C7H7NO3
92.522315	1.734019	0.313224	6.2748	9.20E-05	4.036	0.004844	NA	NA	NA	NA	NA
336.01002	1.714332	0.623834	-6.2325	9.72E-05	4.0122	0.005064	NA	NA	NA	NA	NA
174.07587	1.713699	0.908336	6.2157	9.94E-05	4.0026	0.005123	174.0759	HMDB06488 ; HMDB10329 ; HMDB15590 ; HMDB29362 ; HMDB29610 ; HMDB33504 ; HMDB34576 ; HMDB34681 ; HMDB35357 ; HMDB35847 ; HMDB35848 ; HMDB59731	N-Acetyl-L-glutamate 5-semialdehyde ; Dopamine glucuronide ; Fluspirilene ; (R)-1-O-b-D-glucopyranosyl-13-octanediol ; Ascladiol ; AF Toxin II ; 3-Acetoxyscirpene-415-diol ; 3-Hydroxy-4-butanolide ; Blumealactone C ; 4-Acetoxyscirpene-315-diol ; Monoacetoxyscirpenol ; 23-Methyleneglutaric acid	M+H ; M+H+NH4 ; M+H+2Na ; M+H+K ; M+NH4 ; M+H+Na ; M+H+Na ; M+2ACN+2H ; M+H+Na ; M+H+Na ; M+H+Na ; M+NH4	C7H11NO4 ; C14H19NO8 ; C29H31F2N3O ; C14H28O7 ; C7H8O4 ; C17H24O6 ; C17H24O6 ; C10H16O8 ; C17H24O6 ; C17H24O6 ; C17H24O6 ; C7H8O4

349.11754	1.661656	0.525726	6.2021	0.000101	3.9949	0.005162	349.1174	HMDB29299 ; HMDB00125 ; HMDB01081 ; HMDB02242 ; HMDB06581 ; HMDB06584 ; HMDB14918 ; HMDB15644 ; HMDB29727 ; HMDB29826 ; HMDB30311 ; HMDB30813 ; HMDB31182 ; HMDB32066 ; HMDB32276 ; HMDB33247 ; HMDB33330 ; HMDB33351 ; HMDB34052 ; HMDB35435 ; HMDB35918 ; HMDB36584 ; HMDB36808 ; HMDB36809 ; HMDB37151 ; HMDB38009 ; HMDB38033 ; HMDB38434 ; HMDB38911 ; HMDB40000 ; HMDB40035 ; HMDB40063 ; HMDB40064 ; HMDB40071 ; HMDB40072 ; HMDB40073 ; HMDB40074 ; HMDB40075 ; HMDB40102 ; HMDB40103 ; HMDB40104 ; HMDB40105 ; HMDB40299 ; HMDB40459 ; HMDB40742 ; HMDB40790 ; HMDB41252 ; HMDB41253 ; HMDB41325 ; HMDB59795	Sesamolinol 4-O-b-D-glucosyl (1-6)-O-b-D-glucoside ; Glutathione ; (N-acetylneuraminosyl(a2-6)lactosamine) ; Dehydroisocoproporphyrinogen ; 3-Sialyl-N-acetyllactosamine ; 6-Sialyl-N-acetyllactosamine ; Phenelzine ; Betahistine ; 5-Ethyl-23-dimethylpyrazine ; Hallacridone ; Harmine ; Cassiachromone ; 1H-Indol-3-ylacetyl-myo-inositol ; Carbanilide ; 2-Ethyl-3(5 or 6)-dimethylpyrazine ; Aristolodione ; Dihydrocoriandrin ; Methoxybrassinin ; Safflomin C ; Cinchonain Id 7-glucoside ; 7(14)-Bisabolene-231011-tetrol ; Tetramethylpyrazine ; 25-Diethylpyrazine ; 26-Diethylpyrazine ; 2-Isopropyl-5-methylpyrazine ; Malvin ; Malvidin 3-laminaribioside ; 7-Isothiocyanato-1-heptene ; (2S3S4S)-57911-Tridecatetrayne-1234-tetrol ; 2-Methyl-5-propylpyrazine ; 2-Pentylthiazole ; 4-Butyl-5-methylthiazole ; 4-Ethyl-5-propylthiazole ; 24-Dimethyl-5-propylthiazole ; 25-Dimethyl-4-propylthiazole ; 45-Dimethyl-2-propylthiazole ; 45-Dimethyl-2-(1-methylethyl)thiazole ; 25-Diethyl-4-methylthiazole ; 2-Butyl-4-methylthiazole ; 4-Butyl-2-methylthiazole ; 2-Ethyl-4-propylthiazole ; 4-Ethyl-2-propylthiazole ; 2-Ethyl-35-dimethylpyrazine ; Ethylene brassylate ; 23-Dehydrosalvipisone ; 4-Methoxybrassinin ; 2-Methyl-3-propylpyrazine ; 23-Diethylpyrazine ; Salfredin B11 ; Gluten exorphin B5	M+2H ; M+ACN+H ; M+H+Na ; M+2Na ; M+H+Na ; M+H+Na ; 2M+2K-H ; 2M+2K-H ; 2M+2K-H ; M+ACN+H ; 3M+Na+K ; 3M+2H ; 2M+H+Na ; 3M+Na+K ; 2M+2K-H ; M+ACN+H ; 3M+2H ; M+2ACN+H ; M+2ACN+2H ; M+2ACN+2H ; M+2K-H ; 2M+2K-H ; 2M+2K-H ; 2M+2K-H ; 2M+2K-H ; M+ACN+2H ; M+ACN+2H ; 2M+K ; 3M+2H ; 2M+2K-H ; 2M+K ; 2M+K ; 2M+K ; 2M+K ; 2M+K ; 2M+K ; 2M+K ; 2M+K ; 2M+K ; 2M+K ; 2M+K ; 2M+K ; 2M+2K-H ; M+2K+H ; M+K ; M+2ACN+H ; 2M+2K-H ; 2M+2K-H ; 3M+2H ; M+H+K	C32H40O17 ; C10H17N3O6S ; C25H42N2O19 ; C36H36N4O8 ; C25H42N2O19 ; C25H42N2O19 ; C8H12N2 ; C8H12N2 ; C8H12N2 ; C18H13NO4 ; C13H12N2O ; C13H12O4 ; C16H19NO7 ; C13H12N2O ; C8H12N2 ; C18H13NO4 ; C13H12O4 ; C12H14N2OS2 ; C30H30O14 ; C30H30O14 ; C15H28O4 ; C8H12N2 ; C8H12N2 ; C8H12N2 ; C8H12N2 ; C29H35O17 ; C29H35O17 ; C8H13NS ; C13H12O4 ; C8H12N2 ; C8H13NS ; C8H13NS ; C8H13NS ; C8H13NS ; C8H13NS ; C8H13NS ; C8H13NS ; C8H13NS ; C8H13NS ; C8H13NS ; C8H13NS ; C8H13NS ; C8H12N2 ; C15H26O4 ; C20H22O3 ; C12H14N2OS2 ; C8H12N2 ; C8H12N2 ; C13H12O4 ; C30H38N6O11
513.10502	1.698413	0.942693	-6.1835	0.000104	3.9844	0.005236	513.105	HMDB28776 ; HMDB28860 ; HMDB30415 ; HMDB30849 ; HMDB31585 ; HMDB32580 ; HMDB33694 ; HMDB33806 ; HMDB40358 ; HMDB40842 ; HMDB41212	Cysteinyl-Hydroxyproline ; Hydroxyprolyl-Cysteine ; Quercetin 3-(6-feruloylglucosyl)-(1-2)-galactosyl-(1-2)-glucoside ; Artocarpin ; Patuletin 3-(2-apiosyl-2-feruloylgentiobioside) ; 4-Hydroxybenzyl isothiocyanate ; trans-Caffeic acid apiosyl-(1-6)-glucosyl ester ; 2-O-Methylcajanone ; (R)-Bitalin A ; Quercetin 3-(2-feruloylsophoroside) 7-glucoside ; Kanzonol K	2M+2Na-H ; 2M+2Na-H ; M+Na+K ; M+2K-H ; M+Na+K ; 3M+NH4 ; M+K ; M+2K-H ; 2M+2K-H ; M+Na+K ; M+2K-H	C8H14N2O4S ; C8H14N2O4S ; C43H48O25 ; C26H28O6 ; C43H48O25 ; C8H7NOS ; C20H26O13 ; C26H28O6 ; C13H14O3 ; C43H48O25 ; C26H28O6
364.86043	1.688105	0.315842	6.168	0.000106	3.9755	0.005266	364.8604	HMDB34485	(23R)-Acetoxytomatine	M+3H	C52H85NO23
164.11779	1.702086	0.686928	-6.1639	0.000106	3.9732	0.005266	164.1178	HMDB00207 ; HMDB00573 ; HMDB01043 ; HMDB02177 ; HMDB03231 ; HMDB03450 ; HMDB03634 ; HMDB03667 ; HMDB03701 ; HMDB06036 ; HMDB13324 ; HMDB14573 ; HMDB14996 ; HMDB15334 ; HMDB15345 ; HMDB29740 ; HMDB30386 ; HMDB30892 ; HMDB30893 ; HMDB30906 ; HMDB30907 ; HMDB30908 ; HMDB31854 ; HMDB32120 ; HMDB32242 ; HMDB32536 ; HMDB33113 ; HMDB33114 ; HMDB34715 ; HMDB34916 ; HMDB34973 ; HMDB34974 ; HMDB34975 ; HMDB34985 ; HMDB35054 ; HMDB35077 ; HMDB35078 ; HMDB35092 ; HMDB35100 ; HMDB35120 ; HMDB35125 ; HMDB35158 ; HMDB35203 ; HMDB35241 ; HMDB35277 ; HMDB35280 ; HMDB35600 ; HMDB35604 ; HMDB35622 ; HMDB35656 ; HMDB35706 ; HMDB35737 ; HMDB35738 ; HMDB35741 ; HMDB35743 ; HMDB35744 ;	Oleic acid ; Elaidic acid ; Arachidonic acid ; Cis-8111417-Eicosatetraenoic acid ; Vaccenic acid ; (-)-trans-Carveol ; Perillyl alcohol ; Alpha-Pinene-oxide ; Dimethylbenzimidazole ; Mesterolone ; 2-Octenoylcarnitine ; Carboprost Tromethamine ; Drostanolone ; Nadolol ; Metipranolol ; 1H-Indole-3-methanamine ; Myosmine ; p-Menth-3-en-9-al ; Isocitral ; 14-Dimethylpyrrolo12-apyrazine ; 34-Dimethylpyrrolo12-apyrazine ; 13-Dimethylpyrrolo12-apyrazine ; 3-Methyl-5-propyl-2-cyclohexen-1-one ; 26-Dimethyl-17-octadien-3-one ; (E)-37-Dimethyl-157-octatrien-3-ol ; 2-(trans-2-Pentenyl)cyclopentanone ; 1-Methylpyrrole ; 2-Methylpyrrole ; p-Mentha-1(6)8-dien-3-ol ; cis-2-Thujen-4-ol ; (R)-Campholenic aldehyde ; (R)-Carvotanacetone ; Piperitone ; ()-Fenchone ; (1S4R)-p-Mentha-28-dien-1-ol ; (1R4R)-p-Mentha-28-dien-1-ol ; Geranial ; cis-Citral ; 2-Pinen-10-ol ; Dehydrolinalool ; Darwinol ; 12-Epoxy-p-menth-8-ene ; (S)-910-Cyclo-p-menth-1-en-4-ol ; (Z)-2-Methyl-6-methylene-27-octadien-1-ol ; (2S4R)-p-Mentha-1(7)5-dien-2-ol ; Teresantalol ; (S)-Phellandral ; (R)-p-Menth-4(8)-en-3-one ; ()-cis-Carveol ; 3-Pinanone ; alpha-Cyclocitral ; (R)-Piperitone ; (S)-Piperitone ; (-)-trans-Isopulegone ; Pinocarveol ; (-)-Isopinocamphone ; Marmelo oxide A ; Dihydrocarvone ; (1S4S)-Dihydrocarvone ; (-)-cis-Carveol ; Carveol ; Dehydro-18-cineole ; (R)-p-Mentha-18-dien-7-ol ; (S)-p-Mentha-18-dien-7-ol ; ()-3-Thujone ; (-)-3-Thujone ; (-)-3-Isothujone ; (-)-Pinocamphone ; (-)-trans-Pinocarveol ; Verbenol ; (EE)-24-Decadienal ; Sideridiol ; ent-17-Hydroxy-16beta-kauran-19-al ; Yucalexin P21 ; Copalic acid ; Junicedral ; 68-Epoxy-p-menth-2-ene ; Terpinolene oxide ; (2S4R)-p-Mentha-1(7)8-dien-2-ol ; p-Mentha-18-dien-4-ol ; (R)-p-Mentha-18-dien-10-ol ; 4-Acetyl-14-dimethyl-1-cyclohexene ; Hop ether ; Tetrahydro-5-isopropenyl-2-methyl-2-vinylfuran ; Junionone ; xi-p-Mentha-38-dien-1-ol ; xi-p-Mentha-1(7)2-dien-4-ol ; 713-Eperudien-15-oic acid ; xi-Pinol ; Photocitral A ; xi-36-Dihydro-4-methyl-2-(2-methyl-1-propenyl)-2H-pyran ; 34-Epoxy-p-menth-1(7)-ene ; Oryzalexin S ; Oryzalexin E ; 2-Hexylfuran ; Anethofuran ; beta-Cyclocitral ; Isocyclocitral ; (Z)-13-Octadecenoic acid ; 3-Thujanone ; ()-trans-Carveol ; 4-(1-Methylethyl)-1-cyclohexene-4-carboxaldehyde ; 3-Cyclohexene-1-carboxaldehyde 134-trimethyl- ; Camphor ; Ethyl 9-hexadecenoate ; Carvotanacetone ; p-Menth-1-en-9-al	M+2Na ; M+2Na ; M+H+Na ; M+H+Na ; M+2Na ; 2M+H+Na ; 2M+H+Na ; 2M+H+Na ; M+NH4 ; M+H+Na ; M+ACN+2H ; M+3H ; M+H+Na ; M+H+NH4 ; M+H+NH4 ; M+NH4 ; M+NH4 ; 2M+H+Na ; 2M+H+Na ; M+NH4 ; M+NH4 ; M+NH4 ; 2M+H+Na ; 2M+H+Na ; 2M+H+Na ; 2M+H+Na ; M+2ACN+H ; M+2ACN+H ; 2M+H+Na ; M+H+Na ; M+H+Na ; M+H+Na ; M+H+Na ; M+H+Na ; 2M+H+Na ; 2M+H+Na ; 2M+H+Na ; 2M+H+Na ; 2M+H+Na ; 2M+H+Na ; 2M+H+Na ; 2M+H+Na ; 2M+H+Na ; 2M+H+Na ; 2M+H+Na ; M+H+Na ; 2M+H+Na ; 2M+H+Na ; 2M+H+Na ; 2M+H+Na ; M+H+Na ; M+H+Na ; 2M+H+Na ; 2M+H+Na ; 2M+H+Na ; 2M+H+Na ; M+2Na ; 2M+H+Na ; 2M+H+Na ; 2M+H+Na ; 2M+H+Na ; 2M+H+Na ; M+2Na ;	C18H34O2 ; C18H34O2 ; C20H32O2 ; C20H32O2 ; C18H34O2 ; C10H16O ; C10H16O ; C10H16O ; C9H10N2 ; C20H32O2 ; C15H27NO4 ; C25H47NO8 ; C20H32O2 ; C17H27NO4 ; C17H27NO4 ; C9H10N2 ; C9H10N2 ; C10H16O ; C10H16O ; C9H10N2 ; C9H10N2 ; C9H10N2 ; C10H16O ; C10H16O ; C10H16O ; C10H16O ; C5H7N ; C5H7N ; C10H16O ; C20H32O2 ; C20H32O2 ; C20H32O2 ; C20H32O2 ; C20H32O2 ; C10H16O ; C10H16O ; C10H16O ; C10H16O ; C10H16O ; C10H16O ; C10H16O ; C10H16O ; C10H16O ; C10H16O ; C10H16O ; C20H32O2 ; C10H16O ; C10H16O ; C10H16O ; C10H16O ; C20H32O2 ; C20H32O2 ; C10H16O ; C10H16O ; C10H16O ; C10H16O ; C18H34O2 ; C10H16O ; C10H16O ; C10H16O ; C10H16O ; C10H16O ; C18H34O2 ; C10H16O ; C10H16O

439.10105	1.725484	0.751787	-6.145	0.000109	3.9624	0.005345	439.1011	HMDB13995 ; HMDB14823 ; HMDB15561 ; HMDB29329 ; HMDB30009 ; HMDB30671 ; HMDB31783 ; HMDB33091 ; HMDB33162 ; HMDB33651 ; HMDB33991 ; HMDB34759 ; HMDB36408 ; HMDB36618 ; HMDB39098 ; HMDB61068 ; HMDB01520 ; HMDB29528 ; HMDB37341 ; HMDB37342 ; HMDB37945 ; HMDB39328 ; HMDB39746 ; HMDB39747 ; HMDB39748 ; HMDB41215	Hydroxycelecoxib ; Trovafloxacin ; Zuclopenthixol ; Gravacridonolchlorine ; 36-Dihydro-4-(4-methyl-3-pentenyl)-12-dithiin ; Pulmatin ; Etrimfos ; (Z)-3-(1-Formyl-1-propenyl)pentanedioic acid ; (Z)-46-Dihydroxyaurone 6-glucoside ; Genistein 4-rhamnoside ; Daidzin ; Methyl 5-(1-Propynyl)-2-thiophenepropanoate ; Franguloside ; Toringin ; Chrysophanein ; S-methylazathioprine ; Flavin Mononucleotide ; 6-O-Acetylgenistin ; Apigenin 7-O-(2-O-acetylglucoside) ; Apigenin 7-O-(6-O-acetylglucoside) ; Epicatechin 3-O-(3-O-methylgallate) ; Epicatechin 3-O-(4-methylgallate) ; 4-O-Acetylafzelin ; 3-O-Acetylafzelin ; Kaempferol 3-(2-acetylrhamnoside) ; Betavulgarin glucoside	M+ACN+H ; M+Na ; M+K ; M+ACN+Na ; 2M+K ; M+Na ; 3M+2H ; 2M+K ; M+Na ; M+Na ; M+Na ; 2M+Na ; M+Na ; M+Na ; M+Na ; 3M+2H ; M+H-H2O ; M+H-2H2O ; M+H-2H2O ; M+H-2H2O ; M+H-H2O ; M+H-H2O ; M+H-2H2O ; M+H-2H2O ; M+H-2H2O ; M+H-2H2O	C17H14F3N3O3S ; C20H15F3N4O3 ; C22H25ClN2OS ; C19H18ClNO5 ; C10H16S2 ; C21H20O9 ; C10H17N2O4PS ; C9H12O5 ; C21H20O9 ; C21H20O9 ; C21H20O9 ; C11H12O2S ; C21H20O9 ; C21H20O9 ; C21H20O9 ; C10H10N7O2S ; C17H21N4O9P ; C23H22O11 ; C23H22O11 ; C23H22O11 ; C23H20O10 ; C23H20O10 ; C23H22O11 ; C23H22O11 ; C23H22O11 ; C23H22O11
503.22780	1.737428	0.471194	-6.1315	0.000111	3.9547	0.00537	503.2278	HMDB35222 ; HMDB35863 ; HMDB02522 ; HMDB02586 ; HMDB02642 ; HMDB11144 ; HMDB12562 ; HMDB15543 ; HMDB36304 ; HMDB36968 ; HMDB40696 ; HMDB41241 ; HMDB36525 ; HMDB38143	Isocyclocalamin ; Cyclocalamin ; Chenodeoxycholic acid sulfate ; Chenodeoxycholic acid 3-sulfate ; Ursodeoxycholic acid 3-sulfate ; LPA(18:0e0:0) ; 13-Hydroxy-gamma-tocotrienol ; Pivmecillinam ; (3beta5alpha6alpha7alpha22E24R)-56-Epoxyergosta-81422-triene-37-diol ; Araliasaponin II ; Lyciumin C ; Kudzusaponin SA2 ; Myricanol 5-glucoside ; Calamin	M+H ; M+H ; 2M+Na+K ; 2M+Na+K ; 2M+Na+K ; M+2K+H ; M+2K-H ; M+ACN+Na ; M+2K-H ; M+Na+K ; M+ACN+2H ; M+Na+K ; M+H-H2O ; M+H-H2O	C27H34O9 ; C27H34O9 ; C24H40O7S ; C24H40O7S ; C24H40O7S ; C21H45O6P ; C28H42O3 ; C21H33N3O5S ; C28H42O3 ; C47H76O19 ; C49H57N9O12 ; C47H76O19 ; C27H36O10 ; C27H36O10
206.02600	1.682401	0.540366	6.1266	0.000112	3.9519	0.00537	206.026	HMDB15485 ; HMDB00812 ; HMDB03470 ; HMDB29289 ; HMDB29290 ; HMDB31577 ; HMDB39046 ; HMDB39163 ; HMDB39936 ; HMDB60273	Ceftibuten ; N-Acetyl-L-aspartic acid ; N-Formyl-L-glutamic acid ; 3-Feruloyl-15-quinolactone ; 4-Feruloyl-15-quinolactone ; Berteroin ; Daphnoretin methyl ether ; D-N-(Carboxyacetyl)alanine ; Cyanidin 3-(6-dioxalylglucoside) ; 2-Amino-3-oxoadipate	M+2H ; 2M+Na+K ; 2M+Na+K ; M+Na+K ; M+Na+K ; 2M+Na+K ; M+2Na ; 2M+Na+K ; M+2H+Na ; 2M+Na+K	C15H14N4O6S2 ; C6H9NO5 ; C6H9NO5 ; C17H18O8 ; C17H18O8 ; C7H13NS2 ; C20H14O7 ; C6H9NO5 ; C25H21O17 ; C6H9NO5
179.97341	1.737151	0.43128	6.0843	0.000118	3.9277	0.005584	179.9734	HMDB60688	Nornitrogen mustard	M+K	C4H9Cl2N
201.16366	1.707653	0.252593	6.0799	0.000119	3.9252	0.005584	201.1637	HMDB36451 ; HMDB36453 ; HMDB38204 ; HMDB59829 ; HMDB59858 ; HMDB01449 ; HMDB01455 ; HMDB01471 ; HMDB06012 ; HMDB06759 ; HMDB07074 ; HMDB07242 ; HMDB30983 ; HMDB36740 ; HMDB55967 ; HMDB55969 ; HMDB60408	(S)-gamma-Calacorene ; 34-Dihydrocadalene ; alpha-Corocalene ; Isolongifolene 45910-dehydro- ; beta-Calacorene ; Allopregnanolone ; Alloepipregnanolone ; Epipregnanolone ; Epimetendiol ; 3a-Hydroxy-5b-pregnane-20-one ; DG(15:018:2(9Z12Z)0:0) ; DG(18:2(9Z12Z)15:00:0) ; 5-(8-Pentadecenyl)-13-benzenediol ; (2E5E12Z15Z)-1-Hydroxy-251215-heneicosatetraen-4-one ; DG(14:00:018:2n6) ; DG(14:00:020:2n6) ; 5alpha-Pregnan-20alpha-ol-3-one	M+H ; M+H ; M+H ; M+H ; M+H ; M+2ACN+2H ; M+2ACN+2H ; M+2ACN+2H ; M+2ACN+2H ; M+2ACN+2H ; M+2H+Na ; M+2H+Na ; M+2ACN+2H ; M+2ACN+2H ; M+2H+Na ; M+2H+Na ; M+2ACN+2H	C15H20 ; C15H20 ; C15H20 ; C15H20 ; C15H20 ; C21H34O2 ; C21H34O2 ; C21H34O2 ; C21H34O2 ; C21H34O2 ; C36H66O5 ; C36H66O5 ; C21H34O2 ; C21H34O2 ; C36H66O5 ; C36H66O5 ; C21H34O2
139.53104	1.707133	0.086266	6.0755	0.00012	3.9226	0.005584	139.531	HMDB00832 ; HMDB13116 ; HMDB15505 ; HMDB33519 ; HMDB41540 ; HMDB59745 ; HMDB60961	Capryloylglycine ; Valproylglycine ; Glutethimide ; N-Ornithyl-L-taurine ; N-(5-Methyl-3-oxohexyl)alanine ; N-Acetylaminooctanoic acid ; N-desisopropylpropranolol	M+2K ; M+2K ; M+Na+K ; M+H+K ; M+2K ; M+2K ; M+Na+K	C10H19NO3 ; C10H19NO3 ; C13H15NO2 ; C7H17N3O4S ; C10H19NO3 ; C10H19NO3 ; C13H15NO2
513.27237	1.707553	0.019853	-6.0417	0.000125	3.9032	0.005745	513.2724	HMDB35342 ; HMDB00344 ; HMDB05079 ; HMDB05832 ; HMDB06254 ; HMDB12451 ; HMDB12452 ; HMDB15211 ; HMDB31773 ; HMDB34630 ; HMDB34631 ; HMDB34691 ; HMDB35367 ; HMDB35710 ; HMDB36147 ; HMDB36725 ; HMDB36747 ; HMDB36750 ; HMDB36762 ; HMDB36812 ; HMDB37233 ; HMDB38885 ; HMDB39291 ; HMDB39442 ; HMDB39484 ; HMDB39560 ; HMDB39563 ; HMDB40810 ; HMDB41056 ; HMDB41057 ; HMDB59791 ; HMDB60093 ; HMDB61095	TR-Saponin B ; 2-Methoxyestradiol-3-methylether ; 15-Deoxy-d-1214-PGJ2 ; 6b-Hydroxymethandienone ; 4-Hydroxyretinoic acid ; all-trans-56-Epoxyretinoic acid ; all-trans-18-Hydroxyretinoic acid ; Deslanoside ; Phytocassane E ; (4Z9a)-9-(3-Methyl-2-butenoyloxy)-410(14)-oplopadien-3-one ; (4Z9a)-9-Angeloyloxy-410(14)-oplopadien-3-one ; Yucalexin B9 ; Cytochalasin Npho ; Cafestol ; Furanojaponin ; ent-17-Oxo-15-kauren-19-oic acid ; ent-7-Oxo-8(14)15-pimaradien-19-oic acid ; Momilactone C ; ent-15-Oxo-16-kauren-19-oic acid ; 7-Oxo-815-isopimaradien-18-oic acid ; Rosmaridiphenol ; Trilobinone ; Medicoside H ; ent-1516-Epoxy-1(10)13(16)14-halimatrien-19-oic acid ; Gibberellin A12 7-aldehyde ; Cynarasaponin J ; Cynarasaponin D ; Phytolaccoside I ; Phytocassane A ; Phytocassane D ; Adrenorphin ; rac-56-Epoxy-retinoate ; 18-Hydroxyretinoic acid	M+2H ; 3M+2K ; 3M+2K ; 3M+2K ; 3M+2K ; 3M+2K ; 3M+2K ; M+2ACN+2H ; 3M+2K ; 3M+2K ; 3M+2K ; 3M+2K ; 2M+H+K ; 3M+2K ; 3M+2K ; 3M+2K ; 3M+2K ; 3M+2K ; 3M+2K ; 3M+2K ; 3M+2K ; 3M+2K ; M+2ACN+2H ; 3M+2K ; 3M+2K ; M+2ACN+2H ; M+2ACN+2H ; M+2ACN+2H ; 3M+2K ; 3M+2K ; M+ACN+2H ; 3M+2K ; 3M+2K	C52H80O20 ; C20H28O3 ; C20H28O3 ; C20H28O3 ; C20H28O3 ; C20H28O3 ; C20H28O3 ; C47H74O19 ; C20H28O3 ; C20H28O3 ; C20H28O3 ; C20H28O3 ; C30H39NO5 ; C20H28O3 ; C20H28O3 ; C20H28O3 ; C20H28O3 ; C20H28O3 ; C20H28O3 ; C20H28O3 ; C20H28O3 ; C20H28O3 ; C47H74O19 ; C20H28O3 ; C20H28O3 ; C47H74O19 ; C47H74O19 ; C47H74O19 ; C20H28O3 ; C20H28O3 ; C44H69N15O9S ; C20H28O3 ; C20H28O3
754.54260	1.647678	0.245674	6.0402	0.000125	3.9023	0.005745	754.5426	HMDB01219 ; HMDB02685 ; HMDB02689 ; HMDB03180 ; HMDB04239 ; HMDB05821 ; HMDB07872 ; HMDB07873 ; HMDB07904 ; HMDB07969 ; HMDB08001 ; HMDB08032 ; HMDB08064 ; HMDB08097 ; HMDB08885 ; HMDB08886 ; HMDB08900 ; HMDB09252 ; HMDB10569 ; HMDB11402 ; HMDB11435 ; HMDB13649 ; HMDB29216 ; HMDB37961 ; HMDB59599	Chlordecone alcohol ; Prostaglandin F1a ; 1314-Dihydro PGE1 ; Cortol ; 1314-Dihydro PGF2a ; Beta-Cortol ; PC(14:018:1(11Z)) ; PC(14:018:1(9Z)) ; PC(14:1(9Z)18:0) ; PC(16:016:1(9Z)) ; PC(16:1(9Z)16:0) ; PC(18:014:1(9Z)) ; PC(18:1(11Z)14:0) ; PC(18:1(9Z)14:0) ; PE(14:1(9Z)P-18:1(11Z)) ; PE(14:1(9Z)P-18:1(9Z)) ; PE(15:020:1(11Z)) ; PE(20:1(11Z)15:0) ; PE-NMe(16:018:1(9Z)) ; PE(P-18:1(11Z)14:1(9Z)) ; PE(P-18:1(9Z)14:1(9Z)) ; Eicosapentaenoyl Ethanolamide ; SM C16:1 ; gamma-Eudesmol rhamnoside ; CDP-glycerol	3M+2Na ; 2M+ACN+H ; 2M+ACN+H ; 2M+NH4 ; 2M+ACN+H ; 2M+NH4 ; M+Na ; M+Na ; M+Na ; M+Na ; M+Na ; M+Na ; M+Na ; M+Na ; M+2ACN+H ; M+2ACN+H ; M+Na ; M+Na ; M+Na ; M+2ACN+H ; M+2ACN+H ; 2M+ACN+Na ; M+K ; 2M+NH4 ; 3M+2K	C10H2Cl10O ; C20H36O5 ; C20H36O5 ; C21H36O5 ; C20H36O5 ; C21H36O5 ; C40H78NO8P ; C40H78NO8P ; C40H78NO8P ; C40H78NO8P ; C40H78NO8P ; C40H78NO8P ; C40H78NO8P ; C40H78NO8P ; C40H78NO8P ; C37H70NO7P ; C37H70NO7P ; C40H78NO8P ; C40H78NO8P ; C40H78NO8P ; C37H70NO7P ; C37H70NO7P ; C22H35NO2 ; C40H80N2O6P ; C21H36O5 ; C12H21N3O13P2

217.06663	1.683976	1.142059	-6.0318	0.000127	3.8975	0.005756	217.0666	HMDB00715 ; HMDB03134 ; HMDB05032 ; HMDB15600 ; HMDB29915 ; HMDB29916 ; HMDB29965 ; HMDB30988 ; HMDB30992 ; HMDB31437 ; HMDB31449 ; HMDB32055 ; HMDB33284 ; HMDB33816 ; HMDB34145 ; HMDB34219 ; HMDB34221 ; HMDB34222 ; HMDB35045 ; HMDB35046 ; HMDB35110 ; HMDB35828 ; HMDB36557 ; HMDB38922 ; HMDB39048 ; HMDB39059 ; HMDB41148 ; HMDB41197 ; HMDB41608 ; HMDB60331 ; HMDB60332 ; HMDB32660	Kynurenic acid ; Biocytin ; Cetirizine ; Bepotastine ; D-4-O-Methyl-myo-inositol ; 4-O-Methyl-myo-inositol ; Methyl beta-D-glucopyranoside ; 5-(2-Methylpropyl)tetrahydro-2-oxo-3-furancarboxylic acid ; 5-Butyltetrahydro-2-oxo-3-furancarboxylic acid ; (-)-Bornesitol ; Sequoyitol ; N-Acetylhistidine ; 8-Hydroxy-4-methoxypinoresinol ; Mytilitol ; (-)-cis-Rotenolone ; D-Pinitol ; L-Quebrachitol ; L-Pinitol ; Gibberellin A39 ; Gibberellin A43 ; 8-Epidiosbulbin E acetate ; Lactupicrin ; MS 3 ; (7R)-()-Lyoniresinol 9-glucoside ; (5a6a8a11a)-8-Hydroxy-2-oxo-1(10)3-guaiadien-126-olide-15-al 8-(4-hydroxyphenylacetate) ; (R)-Heraclenol 2-(3-methylbutanoate) ; ()-Lyoniresinol 9-glucoside ; Edulisin III ; Methional diethyl acetal ; 1-Nitronaphthalene-56-oxide ; 1-Nitronaphthalene-78-oxide ; (2Z4Z)-2-(5-Methylthio-4-penten-2-ynylidene)-16-dioxaspiro44non-3-ene	2M+3H2O+2H ; M+Na+K ; M+2Na ; M+2Na ; M+Na ; M+Na ; M+Na ; 2M+Na+K ; 2M+Na+K ; M+Na ; M+Na ; 2M+H+K ; M+2Na ; M+Na ; M+H+Na ; M+Na ; M+Na ; M+Na ; M+H+K ; M+H+K ; M+2Na ; M+H+Na ; M+2Na ; M+3Na ; M+H+Na ; M+2Na ; M+3Na ; M+2Na ; M+K ; 2M+3H2O+2H ; 2M+3H2O+2H ; M+H-H2O	C10H7NO3 ; C16H28N4O4S ; C21H25ClN2O3 ; C21H25ClN2O3 ; C7H14O6 ; C7H14O6 ; C7H14O6 ; C9H14O4 ; C9H14O4 ; C7H14O6 ; C7H14O6 ; C8H11N3O3 ; C21H24O7 ; C7H14O6 ; C23H22O7 ; C7H14O6 ; C7H14O6 ; C7H14O6 ; C20H26O8 ; C20H26O8 ; C21H24O7 ; C23H22O7 ; C21H24O7 ; C28H38O13 ; C23H22O7 ; C21H24O7 ; C28H38O13 ; C21H24O7 ; C8H18O2S ; C10H7NO3 ; C10H7NO3 ; C13H14O2S
213.16361	1.708949	0.16563	6.024	0.000128	3.8929	0.005763	213.1636	HMDB59902 ; HMDB00067 ; HMDB00871 ; HMDB01170 ; HMDB05805 ; HMDB06841 ; HMDB07022 ; HMDB07032 ; HMDB07033 ; HMDB07050 ; HMDB07060 ; HMDB07103 ; HMDB07114 ; HMDB07130 ; HMDB07131 ; HMDB07141 ; HMDB07142 ; HMDB07186 ; HMDB07193 ; HMDB07215 ; HMDB07222 ; HMDB07243 ; HMDB07249 ; HMDB07250 ; HMDB07277 ; HMDB07288 ; HMDB07306 ; HMDB07317 ; HMDB07333 ; HMDB07334 ; HMDB07344 ; HMDB07345 ; HMDB07386 ; HMDB07414 ; HMDB07505 ; HMDB07512 ; HMDB07534 ; HMDB07541 ; HMDB07562 ; HMDB07568 ; HMDB07569 ; HMDB07676 ; HMDB07704 ; HMDB07733 ; HMDB11182 ; HMDB29815 ; HMDB31461 ; HMDB32627 ; HMDB32714 ; HMDB34302 ; HMDB34560 ; HMDB34924 ; HMDB37013 ; HMDB37050 ; HMDB37051 ; HMDB55974 ;	26-Diisopropylnaphthalene ; Cholesterol ; 5alpha-Cholestanone ; Lathosterol ; p-Cymene ; 5a-Cholest-8-en-3b-ol ; DG(14:020:2(11Z14Z)0:0) ; DG(14:022:5(4Z7Z10Z13Z16Z)0:0) ; DG(14:022:5(7Z10Z13Z16Z19Z)0:0) ; DG(14:1(9Z)20:1(11Z)0:0) ; DG(14:1(9Z)22:4(7Z10Z13Z16Z)0:0) ; DG(16:018:2(9Z12Z)0:0) ; DG(16:020:5(5Z8Z11Z14Z17Z)0:0) ; DG(16:1(9Z)18:1(11Z)0:0) ; DG(16:1(9Z)18:1(9Z)0:0) ; DG(16:1(9Z)20:4(5Z8Z11Z14Z)0:0) ; DG(16:1(9Z)20:4(8Z11Z14Z17Z)0:0) ; DG(18:1(11Z)16:1(9Z)0:0) ; DG(18:1(11Z)18:4(6Z9Z12Z15Z)0:0) ; DG(18:1(9Z)16:1(9Z)0:0) ; DG(18:1(9Z)18:4(6Z9Z12Z15Z)0:0) ; DG(18:2(9Z12Z)16:00:0) ; DG(18:2(9Z12Z)18:3(6Z9Z12Z)0:0) ; DG(18:2(9Z12Z)18:3(9Z12Z15Z)0:0) ; DG(18:3(6Z9Z12Z)18:2(9Z12Z)0:0) ; DG(18:3(6Z9Z12Z)20:5(5Z8Z11Z14Z17Z)0:0) ; DG(18:3(9Z12Z15Z)18:2(9Z12Z)0:0) ; DG(18:3(9Z12Z15Z)20:5(5Z8Z11Z14Z17Z)0:0) ; DG(18:4(6Z9Z12Z15Z)18:1(11Z)0:0) ; DG(18:4(6Z9Z12Z15Z)18:1(9Z)0:0) ; DG(18:4(6Z9Z12Z15Z)20:4(5Z8Z11Z14Z)0:0) ; DG(18:4(6Z9Z12Z15Z)20:4(8Z11Z14Z17Z)0:0) ; DG(20:1(11Z)14:1(9Z)0:0) ; DG(20:2(11Z14Z)14:00:0) ; DG(20:4(5Z8Z11Z14Z)16:1(9Z)0:0) ; DG(20:4(5Z8Z11Z14Z)18:4(6Z9Z12Z15Z)0:0) ; DG(20:4(8Z11Z14Z17Z)16:1(9Z)0:0) ; DG(20:4(8Z11Z14Z17Z)18:4(6Z9Z12Z15Z)0:0) ; DG(20:5(5Z8Z11Z14Z17Z)16:00:0) ; DG(20:5(5Z8Z11Z14Z17Z)18:3(6Z9Z12Z)0:0) ; DG(20:5(5Z8Z11Z14Z17Z)18:3(9Z12Z15Z)0:0) ; DG(22:4(7Z10Z13Z16Z)14:1(9Z)0:0) ; DG(22:5(4Z7Z10Z13Z16Z)14:00:0) ; DG(22:5(7Z10Z13Z16Z19Z)14:00:0) ; 5beta-Cholestanone ; Doristerol ; Xylene ; 1-Methyl-2-propylbenzene ; cis-Uvariamicin IB ; Reticulatain 2 ; cis-Uvariamicin IV ; Neoreticulatacin A ; p-Mentha-138-triene ; 1-Isopropyl-2-methylbenzene ; 1-Isopropyl-3-methylbenzene ; DG(14:00:022:5n6) ; DG(14:00:022:5n3) ; DG(15:00:018:2n6) ; DG(15:00:020:2n6) ; DG(16:00:020:5n3) ; DG(14:1n50:020:1n9) ; DG(14:1n50:022:4n6) ; DG(16:1n70:018:1n7) ; DG(16:1n70:018:1n9) ; DG(16:1n70:020:4n6) ; DG(16:1n70:020:4n3) ; DG(18:1n70:018:4n3) ; DG(18:1n90:018:4n3) ; DG(18:3n60:020:5n3) ; DG(20:4n60:018:4n3) ; DG(18:3n30:020:5n3) ; DG(18:4n30:020:4n3) ; 5-beta-Cholestan-3-one ; Butylbenzene ; m-Propyltoluene ; Prehnitene ; 4-Ethyl-o-xylene ; 2-Ethyl-p-xylene	M+H ; M+H+K ; M+H+K ; M+H+K ; 3M+H+Na ; M+H+K ; M+H+2Na ; M+2H+Na ; M+2H+Na ; M+H+2Na ; M+2H+Na ; M+H+2Na ; M+2H+Na ; M+H+2Na ; M+H+2Na ; M+2H+Na ; M+2H+Na ; M+H+2Na ; M+2H+Na ; M+H+2Na ; M+2H+Na ; M+H+2Na ; M+2H+Na ; M+2H+Na ; M+2H+Na ; M+3H ; M+2H+Na ; M+3H ; M+2H+Na ; M+2H+Na ; M+3H ; M+3H ; M+H+2Na ; M+H+2Na ; M+2H+Na ; M+3H ; M+2H+Na ; M+3H ; M+2H+Na ; M+3H ; M+3H ; M+2H+Na ; M+2H+Na ; M+2H+Na ; M+H+K ; M+H+K ; 2M+3H ; 3M+H+Na ; M+H+2Na ; M+H+2Na ; M+H+2Na ; M+H+2Na ; 3M+H+Na ; 3M+H+Na ; 3M+H+Na ; M+2H+Na ; M+2H+Na ; M+H+2Na ; M+H+2Na ; M+2H+Na ; M+H+2Na ; M+2H+Na ; M+H+2Na ; M+H+2Na ; M+2H+Na ; M+2H+Na ; M+2H+Na ; M+2H+Na ; M+3H ; M+3H ; M+3H ; M+3H ; M+H+K ; 3M+H+Na ; 3M+H+Na ; 3M+H+Na ; 3M+H+Na ; 3M+H+Na	C16H20 ; C27H46O ; C27H46O ; C27H46O ; C10H14 ; C27H46O ; C37H68O5 ; C39H66O5 ; C39H66O5 ; C37H68O5 ; C39H66O5 ; C37H68O5 ; C39H66O5 ; C37H68O5 ; C37H68O5 ; C39H66O5 ; C39H66O5 ; C37H68O5 ; C39H66O5 ; C37H68O5 ; C39H66O5 ; C37H68O5 ; C39H66O5 ; C39H66O5 ; C39H66O5 ; C41H64O5 ; C39H66O5 ; C41H64O5 ; C39H66O5 ; C39H66O5 ; C41H64O5 ; C41H64O5 ; C37H68O5 ; C37H68O5 ; C39H66O5 ; C41H64O5 ; C39H66O5 ; C41H64O5 ; C39H66O5 ; C41H64O5 ; C41H64O5 ; C39H66O5 ; C39H66O5 ; C39H66O5 ; C27H46O ; C27H46O ; C24H30 ; C10H14 ; C37H68O5 ; C37H68O5 ; C37H68O5 ; C37H68O5 ; C10H14 ; C10H14 ; C10H14 ; C39H66O5 ; C39H66O5 ; C37H68O5 ; C37H68O5 ; C39H66O5 ; C37H68O5 ; C39H66O5 ; C37H68O5 ; C37H68O5 ; C39H66O5 ; C39H66O5 ; C39H66O5 ; C39H66O5 ; C41H64O5 ; C41H64O5 ; C41H64O5 ; C41H64O5 ; C27H46O ; C10H14 ; C10H14 ; C10H14 ; C10H14 ; C10H14
311.23649	1.695952	0.000692	6.0108	0.00013	3.8853	0.005763	311.2365	HMDB14449 ; HMDB06482 ; HMDB10398 ; HMDB11618 ; HMDB32317 ; HMDB34736 ; HMDB36727 ; HMDB40983 ; HMDB31465 ; HMDB33897 ; HMDB34944 ; HMDB36722 ; HMDB41001 ; HMDB41302	Desogestrel ; LysoSM(d18:1) ; LysoPC(22:0) ; All-trans-1314-dihydroretinol ; Hexanal hexyl isoamyl acetal ; Abietinol ; ent-16-Kauren-19-ol ; 117-Heptadecanediol ; Methyl-10-shogaol ; Ginkgoic acid ; Siderol ; ent-17-Acetoxy-16b-kauran-19-al ; Volemolide ; (R)-34-Dihydro-8-hydroxy-3-tridecyl-1H-2-benzopyran-1-one	M+H ; 2M+3H ; M+ACN+2H ; M+Na ; M+K ; M+Na ; M+Na ; M+K ; M+H-2H2O ; M+H-2H2O ; M+H-2H2O ; M+H-2H2O ; M+H-2H2O ; M+H-2H2O	C22H30O ; C23H50N2O5P ; C30H62NO7P ; C20H32O ; C17H36O2 ; C20H32O ; C20H32O ; C17H36O2 ; C22H34O3 ; C22H34O3 ; C22H34O3 ; C22H34O3 ; C22H34O3 ; C22H34O3
219.01764	1.700729	0.35399	6.0062	0.000131	3.8826	0.005763	219.0176	HMDB00496 ; HMDB14394 ; HMDB32311 ; HMDB35476 ; HMDB36176 ; HMDB37167 ; HMDB37196 ; HMDB37625 ; HMDB37691 ; HMDB39651 ; HMDB39652 ; HMDB39673 ; HMDB39680 ; HMDB39681 ; HMDB40463 ; HMDB40526 ; HMDB60682	3-Methoxy-4-hydroxyphenylglycol glucuronide ; Idoxuridine ; 24-Hexadienyl acetate ; 6-Methoxypolygoacetophenoside ; 3-Ethyl-2-hydroxy-4-methyl-2-cyclopenten-1-one ; 3-Ethyl-5-methyl-12-cyclopentanedione ; Ethyl 2-methyl-34-pentadienoate ; Allyl tiglate ; Limocitrol ; 3-Propyl-12-cyclopentanedione ; 2-Hydroxy-345-trimethyl-2-cyclopenten-1-one ; 4-Ethyl-2-hydroxy-3-methyl-2-cyclopenten-1-one ; 355-Trimethyl-12-cyclopentanedione ; 3-Isopropyl-12-cyclopentanedione ; Ethyl sorbate ; 344-Trimethyl-12-cyclopentanedione ; 2-Propyl-24-pentadienoic acid	M+2K ; M+2ACN+2H ; M+2K+H ; M+2K ; M+2K+H ; M+2K+H ; M+2K+H ; M+2K+H ; M+Na+K ; M+2K+H ; M+2K+H ; M+2K+H ; M+2K+H ; M+2K+H ; M+2K+H ; M+2K+H ; M+2K+H	C15H20O10 ; C9H11IN2O5 ; C8H12O2 ; C15H20O10 ; C8H12O2 ; C8H12O2 ; C8H12O2 ; C8H12O2 ; C18H16O9 ; C8H12O2 ; C8H12O2 ; C8H12O2 ; C8H12O2 ; C8H12O2 ; C8H12O2 ; C8H12O2 ; C8H12O2
512.60364	1.682073	0.835117	-6.004	0.000131	3.8814	0.005763	512.6036	HMDB03937 ; HMDB11120 ; HMDB11876 ; HMDB60372	3-Oxododecanoyl-CoA ; trans-2-Enoyl-OPC4-CoA ; Ganglioside GM1 (d18:014:0) ; 3-Hydroxy-3-(4-methylpent-3-en-1-yl)glutaryl-CoA	M+Na+K ; M+H+K ; M+H+2Na ; M+2Na	C33H56N7O18P3S ; C35H54N7O18P3S ; C70H126N2O31 ; C32H52N7O20P3S
236.01695	1.695945	0.94152	-5.9836	0.000135	3.8696	0.005871	236.017	HMDB03850 ; HMDB33338	1-Phosphatidyl-1D-myo-inositol 3-phosphate ; Mollicellin F	M+2H ; M+H+K	C11H20O16P2 ; C21H17ClO8
163.11569	1.686927	0.764197	-5.9494	0.000141	3.8497	0.00597	163.1157	HMDB32380 ; HMDB30200 ; HMDB33526	(S1)-Methoxy-3-heptanethiol ; Hovenine A ; Chalciporone	M+H ; M+3H ; 2M+3H	C8H18OS ; C27H42N4O4 ; C16H21NO
403.28357	1.695688	0.35616	5.9491	0.000141	3.8495	0.00597	403.2836	HMDB14633	Buspirone	M+NH4	C21H31N5O2
710.35721	1.720006	0.466999	-5.9401	0.000143	3.8443	0.00597	710.3572	HMDB35350 ; HMDB08822 ; HMDB08854 ; HMDB08855 ; HMDB14775	(3b6a12b17a20S)-Dammar-24-ene-36121720-pentol 20-glucosyl-(1-2)-rhamnosyl-(1-6)-glucoside 3-glucosyl-(1-6)-glucoside 6-xyloside ; PE(14:014:1(9Z)) ; PE(14:1(9Z)14:0) ; PE(14:1(9Z)14:1(9Z)) ; Astemizole	M+2H ; M+2K-H ; M+2K-H ; M+2K+H ; 3M+2Na	C65H110O33 ; C33H64NO8P ; C33H64NO8P ; C33H62NO8P ; C28H31FN4O

248.14875	1.664024	0.275727	5.9395	0.000143	3.8439	0.00597	248.1488	HMDB13127 ; HMDB07867 ; HMDB07899 ; HMDB08891 ; HMDB08955 ; HMDB14859 ; HMDB30157 ; HMDB37530	Hydroxybutyrylcarnitine ; PC(14:014:1(9Z)) ; PC(14:1(9Z)14:0) ; PE(15:016:1(9Z)) ; PE(16:1(9Z)15:0) ; Procaine ; Austalide K ; 1-O-Acetylpaxilline	M+H ; M+3Na ; M+3Na ; M+3Na ; M+3Na ; 2M+H+Na ; M+2ACN+2H ; M+H+NH4	C11H21NO5 ; C36H70NO8P ; C36H70NO8P ; C36H70NO8P ; C36H70NO8P ; C13H20N2O2 ; C25H32O5 ; C29H35NO5
545.47828	1.703139	0.614647	-5.9322	0.000145	3.8397	0.00597	545.4783	HMDB10734 ; HMDB12094 ; HMDB31057 ; HMDB31445 ; HMDB37267 ; HMDB37268 ; HMDB39000 ; HMDB47302 ; HMDB47304 ; HMDB47371 ; HMDB47377 ; HMDB47419 ; HMDB47425 ; HMDB47446 ; HMDB47448 ; HMDB50980 ; HMDB50982 ; HMDB51670 ; HMDB51704 ; HMDB51750 ; HMDB51752 ; HMDB52489 ; HMDB52596 ; HMDB53343 ; HMDB53436	(R)-3-Hydroxy-hexadecanoic acid ; SM(d18:024:0) ; (R)-2-Hydroxyhexadecanoic acid ; Undecane ; xi-3-Methyldecane ; xi-4-Methyldecane ; 2-Methyldecane ; TG(24:020:3(5Z8Z11Z)18:2(9Z12Z)) ; TG(24:020:3(5Z8Z11Z)20:2n6) ; TG(24:018:2(9Z12Z)20:3(5Z8Z11Z)) ; TG(24:018:2(9Z12Z)20:3n6) ; TG(24:020:2n620:3(5Z8Z11Z)) ; TG(24:020:2n620:3n6) ; TG(24:020:3n618:2(9Z12Z)) ; TG(24:020:3n620:2n6) ; TG(20:3(5Z8Z11Z)24:018:2(9Z12Z)) ; TG(20:3(5Z8Z11Z)24:020:2n6) ; TG(22:1(13Z)18:2(9Z12Z)22:2(13Z16Z)) ; TG(22:1(13Z)20:2n622:2(13Z16Z)) ; TG(22:1(13Z)22:2(13Z16Z)18:2(9Z12Z)) ; TG(22:1(13Z)22:2(13Z16Z)20:2n6) ; TG(18:2(9Z12Z)24:020:3n6) ; TG(18:2(9Z12Z)22:1(13Z)22:2(13Z16Z)) ; TG(20:2n624:020:3n6) ; TG(20:2n622:1(13Z)22:2(13Z16Z))	2M+H ; 2M+3H ; 2M+H ; 3M+2K-H ; 3M+2K-H ; 3M+2K-H ; 3M+2K-H ; M+2ACN+2H ; M+2ACN+2H ; M+2ACN+2H ; M+2ACN+2H ; M+2ACN+2H ; M+2ACN+2H ; M+2ACN+2H ; M+2ACN+2H ; M+2ACN+2H ; M+2ACN+2H ; M+2ACN+2H ; M+2ACN+2H ; M+2ACN+2H ; M+2ACN+2H ; M+2ACN+2H ; M+2ACN+2H ; M+2ACN+2H	C16H32O3 ; C47H97N2O6P ; C16H32O3 ; C11H24 ; C11H24 ; C11H24 ; C11H24 ; C66H118O6 ; C66H118O6 ; C66H118O6 ; C66H118O6 ; C66H118O6 ; C66H118O6 ; C66H118O6 ; C66H118O6 ; C66H118O6 ; C66H118O6 ; C66H118O6 ; C66H118O6 ; C66H118O6 ; C66H118O6 ; C66H118O6 ; C66H118O6 ; C66H118O6
563.33035	1.646647	0.703774	-5.9289	0.000145	3.8378	0.00597	563.3304	HMDB00705 ; HMDB00756 ; HMDB04890 ; HMDB34441	Hexanoylcarnitine ; L-Hexanoylcarnitine ; Ganglioside GA2 (d18:116:0) ; Americine	2M+2Na-H ; 2M+2Na-H ; M+Na+K ; M+NH4	C13H25NO4 ; C13H25NO4 ; C54H100N2O18 ; C31H39N5O4
128.02311	1.69439	0.287126	5.927	0.000146	3.8366	0.00597	128.0231	HMDB12250 ; HMDB14407 ; HMDB31517 ; HMDB31984 ; HMDB32575 ; HMDB32590 ; HMDB34206 ; HMDB34633 ; HMDB34767 ; HMDB34990 ; HMDB36432 ; HMDB37269 ; HMDB37271 ; HMDB39815 ; HMDB40730 ; HMDB41194 ; HMDB41505 ; HMDB41506 ; HMDB41666 ; HMDB59898 ; HMDB59940 ; HMDB59941 ; HMDB59942 ; HMDB59943 ; HMDB59944 ; HMDB59945	L-Aspartyl-4-phosphate ; Carmustine ; (R)-3-Hydroxy-5-phenylpentanoic acid ; 25-Dimethoxy-4-(2-propenyl)phenol ; Butylparaben ; Zingerone ; Ethyl 4-methylphenoxyacetate ; 3-Chloro-1-(4-hydroxy-3-methoxyphenyl)-12-propanediol ; (1R3R3S)-1234-Tetrahydro-1-(2-thio-3-pyrrolidinyl)-beta-carboline-3-carboxylic acid ; 4-Methoxybenzyl propanoate ; Isobutyl salicylate ; Desacetycefapirin ; 26-Dimethoxy-4-(1-propenyl)phenol ; Flusilazole ; Butyl salicylate ; Methoxyeugenol ; 5-Hydroxy-2-benzyl-13-dioxane ; 2-Benzyl-5-hydroxymethyl-13-dioxolane ; 3-Hydroxyphenyl-valeric acid ; Ethyl 4-ethoxybenzoate ; 2-(2-Methylphenyl)-13-dioxolan-4-ylmethanol ; 2-(3-Methylphenyl)-13-dioxolan-4-ylmethanol ; 2-(4-Methylphenyl)-13-dioxolan-4-ylmethanol ; 2-(2-Methylphenyl)-13-dioxan-5-ol ; 2-(3-Methylphenyl)-13-dioxan-5-ol ; 2-(4-Methylphenyl)-13-dioxan-5-ol	M+ACN+2H ; M+ACN+2H ; M+Na+K ; M+Na+K ; M+Na+K ; M+Na+K ; M+Na+K ; M+H+Na ; M+3Na ; M+Na+K ; M+Na+K ; M+3H ; M+Na+K ; M+3Na ; M+Na+K ; M+Na+K ; M+Na+K ; M+Na+K ; M+Na+K ; M+Na+K ; M+Na+K ; M+Na+K ; M+Na+K ; M+Na+K ; M+Na+K ; M+Na+K	C4H8NO7P ; C5H9Cl2N3O2 ; C11H14O3 ; C11H14O3 ; C11H14O3 ; C11H14O3 ; C11H14O3 ; C10H13ClO4 ; C16H17N3O2S ; C11H14O3 ; C11H14O3 ; C15H15N3O5S2 ; C11H14O3 ; C16H15F2N3Si ; C11H14O3 ; C11H14O3 ; C11H14O3 ; C11H14O3 ; C11H14O3 ; C11H14O3 ; C11H14O3 ; C11H14O3 ; C11H14O3 ; C11H14O3 ; C11H14O3 ; C11H14O3
121.52052	1.681057	0.588927	5.9078	0.000149	3.8254	0.006077	121.5205	HMDB01943 ; HMDB04366 ; HMDB15451 ; HMDB35652 ; HMDB38597 ; HMDB39665 ; HMDB39666 ; HMDB39678 ; HMDB39679 ; HMDB39682 ; HMDB39836 ; HMDB40049 ; HMDB60767	Pseudoephedrine ; Hordenine ; Ephedrine ; Perilla sugar ; Nepetalactam ; 5-Ethyl-123456-hexahydro-7H-cyclopentabpyridin-7-one ; 6-Ethyl-123456-hexahydro-7H-cyclopentabpyridin-7-one ; 3-Methyl-2-(1-pyrrolidinyl)-2-cyclopenten-1-one ; 5-Methyl-2-(1-pyrrolidinyl)-2-cyclopenten-1-one ; 234567-Hexahydro-7-methylcyclopentbazepin-8(1H)-one ; 2-(5-Methyl-2-furanyl)piperidine ; 1-(5-Methyl-2-furanyl)methylpyrrolidine ; 4-Hydroxymethamphetamine	M+2K ; M+2K ; M+2K ; M+2K ; M+2K ; M+2K ; M+2K ; M+2K ; M+2K ; M+2K ; M+2K ; M+2K ; M+2K	C10H15NO ; C10H15NO ; C10H15NO ; C10H15NO ; C10H15NO ; C10H15NO ; C10H15NO ; C10H15NO ; C10H15NO ; C10H15NO ; C10H15NO ; C10H15NO ; C10H15NO
267.96682	1.715148	0.369927	-5.9008	0.000151	3.8213	0.006084	NA	NA	NA	NA	NA
260.04421	1.681127	0.208874	5.8849	0.000154	3.812	0.006167	260.0442	HMDB33868 ; HMDB35145 ; HMDB35195 ; HMDB36090 ; HMDB39784 ; HMDB39838 ; HMDB40038 ; HMDB41806 ; HMDB60748	beta-O-Methylynephrine ; Chrycolide ; cis-6-Nitro-p-mentha-1(7)2-diene ; (R)-2-Nitro-p-mentha-15-diene ; 25-Dimethyl-4-(1-pyrrolidinyl)-3(2H)-furanone ; 2-(5-Methyl-2-furanyl)-3-piperidinol ; 5-(1-Pyrrolidinylmethyl)-2-furanmethanol ; 34-Dimethoxyphenylethylamine ; 3-O-Methyl-a-methyldopamine	M+2K+H ; 2M+3H2O+2H ; M+2K+H ; M+2K+H ; M+2K+H ; M+2K+H ; M+2K+H ; M+2K+H ; M+2K+H	C10H15NO2 ; C12H8O3S ; C10H15NO2 ; C10H15NO2 ; C10H15NO2 ; C10H15NO2 ; C10H15NO2 ; C10H15NO2 ; C10H15NO2
271.06777	1.711283	0.187563	5.8695	0.000157	3.803	0.00617	271.0678	HMDB00978 ; HMDB01128 ; HMDB06766 ; HMDB10333 ; HMDB10335 ; HMDB10353 ; HMDB10354 ; HMDB29262 ; HMDB30414 ; HMDB37220 ; HMDB37542 ; HMDB37937 ; HMDB38680 ; HMDB38776 ; HMDB39026 ; HMDB39069 ; HMDB39070 ; HMDB40487 ; HMDB60328 ; HMDB60491 ; HMDB60634 ; HMDB60901	4-(2-Aminophenyl)-24-dioxobutanoic acid ; 5-Phosphoribosylamine ; Estriol-16-Glucuronide ; Estriol-17-glucuronide ; Estriol-3-glucuronide ; 15-Hydroxynorandrostene-317-dione glucuronide ; 16-alpha17-beta-estriol 17-beta-D-glucuronide ; Quercetin 374-O-triglucoside ; Quercetin 3-glucosyl-(1-2)-galactosyl-(1-2)-glucoside ; Quercetin 3-(2-glucosylgalactoside) 7-glucoside ; Patuletin 3-(2-apiosylgentiobioside) ; Quercetin 3-(2G-glucosylgentiobioside) ; Musabalbisiane A ; Medicarpin 3-O-(6-malonylglucoside) ; (R)-Isobyakangelicin 3-glucoside ; (R)-Byakangelicin 3-glucoside ; (R)-Byakangelicin 2-glucoside ; Moracetin ; 1-Nitro-56-dihydroxy-dihydronaphthalene ; Mycophenolic acid O-acyl-glucuronide ; Mycophenolic acid glucuronide ; Clozapine glucuronide	M+ACN+Na ; M+ACN+H ; M+2K ; M+2K ; M+2K ; M+2K ; M+2K ; M+2H+Na ; M+2H+Na ; M+2H+Na ; M+2H+Na ; M+2H+Na ; M+2Na ; M+H+Na ; M+2Na ; M+2Na ; M+2Na ; M+2H+Na ; M+ACN+Na ; M+2Na ; M+2Na ; M+H+K	C10H9NO4 ; C5H12NO7P ; C24H32O9 ; C24H32O9 ; C24H32O9 ; C24H32O9 ; C24H32O9 ; C33H40O22 ; C33H40O22 ; C33H40O22 ; C33H40O22 ; C33H40O22 ; C23H28O12 ; C25H26O12 ; C23H28O12 ; C23H28O12 ; C23H28O12 ; C33H40O22 ; C10H9NO4 ; C23H28O12 ; C23H28O12 ; C24H27ClN4O6
412.55920	1.714466	0.702876	-5.864	0.000159	3.7998	0.00617	NA	NA	NA	NA	NA
286.10502	1.664483	0.231039	5.8639	0.000159	3.7997	0.00617	286.105	HMDB34611 ; HMDB34613 ; HMDB35445 ; HMDB02034 ; HMDB05808 ; HMDB11679 ; HMDB29747 ; HMDB30720 ; HMDB32259 ; HMDB33036 ; HMDB33994 ; HMDB34617 ; HMDB36586 ; HMDB61055 ; HMDB31787	Rutaretin 9-rutinoside ; Torachrysone 8-beta-gentiobioside ; 2-Methoxy-(S)-oleuropein ; Homolanthionine ; Formononetin ; DHAP(6:0) ; 2-Amino-5-phenylpyridine ; Trigraecum ; Ethyl 4-(acetylthio)butyrate ; 8-Hydroxy-1-methoxy-3-methylanthraquinone ; Isoformononetin ; (3b4b11b14b)-11-Ethoxy-34-epoxy-14-hydroxy-12-cyathen-15-al 14-xyloside ; 13-Dimethoxyanthraquinone ; desethylzaleplon ; Fenamiphos	M+2H ; M+2H ; M+2H ; M+NH4 ; M+NH4 ; M+NH4 ; 3M+Na+K ; M+NH4 ; 3M+2H ; M+NH4 ; M+NH4 ; M+2K ; M+NH4 ; M+Na ; M+H-H2O	C26H34O14 ; C26H34O14 ; C26H34O14 ; C8H16N2O6S ; C16H12O4 ; C9H17O7P ; C11H10N2 ; C16H12O4 ; C8H14O3S ; C16H12O4 ; C16H12O4 ; C27H42O8 ; C16H12O4 ; C15H13N5 ; C13H22NO3PS

158.04542	1.68011	0.058754	-5.8612	0.000159	3.7981	0.00617	158.0454	HMDB29307 ; HMDB30544 ; HMDB30545 ; HMDB30546 ; HMDB33146 ; HMDB33255 ; HMDB33732 ; HMDB33989 ; HMDB35208 ; HMDB37243 ; HMDB37917 ; HMDB37918 ; HMDB41644 ; HMDB05767 ; HMDB06804 ; HMDB14780 ; HMDB15292 ; HMDB28898 ; HMDB29129 ; HMDB29900 ; HMDB30120 ; HMDB36334 ; HMDB37180 ; HMDB39030 ; HMDB59808 ; HMDB60459 ; HMDB60518	47-Dihydroxy-25-dimethoxyisoflavone ; Gnaphaliin ; 5-Hydroxy-78-dimethoxyflavonol ; 45-Dihydroxy-78-dimethoxyflavone ; 34-Dihydroxycinnamoyl-(Z)-2-(34-dihydroxyphenyl)ethenol ; Muscomosin ; Pisatin ; 45-Dihydroxy-37-dimethoxyisoflavone ; Aflatoxin B2 ; Sylpin ; 25-Dihydroxy-47-dimethoxyisoflavone ; 37-Dihydroxy-48-dimethoxyisoflavone ; 27-Dihydroxy-45-dimethoxyisoflavone ; Homoanserine ; Propynoic acid ; Pemetrexed ; Chloroprocaine ; Histidinyl-Valine ; Valyl-Histidine ; Lucuminoside ; (S)-Angelicain ; (S)-2-Hydroxy-2-phenylacetonitrile O-b-D-apiosyl-(1-2)-b-D-glucoside ; (S)-Argpyrimidine ; trans-Grandmarin ; Acetyl citrate ; cis-4-Carboxymethylenebut-2-en-4-olide ; trans-4-Carboxymethylenebut-2-en-4-olide	M+2H ; M+2H ; M+2H ; M+2H ; M+2H ; M+2H ; M+2H ; M+2H ; M+2H ; M+2H ; M+2H ; M+2H ; M+2H ; M+Na+K ; 2M+NH4 ; M+H+2Na ; M+2Na ; M+Na+K ; M+Na+K ; M+H+2Na ; M+H+Na ; M+H+2Na ; M+Na+K ; M+H+Na ; M+2ACN+2H ; M+NH4 ; M+NH4	C17H14O6 ; C17H14O6 ; C17H14O6 ; C17H14O6 ; C17H14O6 ; C17H14O6 ; C17H14O6 ; C17H14O6 ; C17H14O6 ; C17H14O6 ; C17H14O6 ; C17H14O6 ; C17H14O6 ; C11H18N4O3 ; C3H2O2 ; C20H21N5O6 ; C13H19ClN2O2 ; C11H18N4O3 ; C11H18N4O3 ; C19H25NO10 ; C15H16O6 ; C19H25NO10 ; C11H18N4O3 ; C15H16O6 ; C8H8O8 ; C6H4O4 ; C6H4O4
250.48312	1.695371	0.808399	5.8438	0.000163	3.7879	0.006269	NA	NA	NA	NA	NA
271.10384	1.686351	0.955762	-5.8287	0.000166	3.779	0.006349	271.1038	HMDB28755 ; HMDB28881 ; HMDB29955 ; HMDB03459 ; HMDB04081 ; HMDB13075 ; HMDB13134 ; HMDB14433 ; HMDB14457 ; HMDB15349 ; HMDB15440 ; HMDB28680 ; HMDB28917 ; HMDB28939 ; HMDB29064 ; HMDB29065 ; HMDB31411 ; HMDB32769 ; HMDB32809 ; HMDB32839 ; HMDB35626 ; HMDB38028 ; HMDB38085 ; HMDB40672 ; HMDB60069 ; HMDB60499 ; HMDB60721 ; HMDB61071	Aspartyl-Histidine ; Histidinyl-Aspartate ; D-erythro-L-galacto-Nonulose ; D-Alanyl-D-alanine ; 6-Hydroxymelatonin ; Spermic acid 2 ; Fumarycarnitine ; Amcinonide ; Pentobarbital ; Halofantrine ; Amobarbital ; Alanyl-Alanine ; Isoleucyl-Threonine ; Leucyl-Threonine ; Threoninyl-Isoleucine ; Threoninyl-Leucine ; 4-Acetamido-2-aminobutanoic acid ; Osmaronin ; 4-Hydroxyphenylacetonitrile triacetylrhamnoside ; Citronellyl beta-sophoroside ; Glaucarubin ; Eriojaposide A ; Pelargonidin 3-rhamnoside ; 3-Oxo-alpha-ionol 9-apiosyl-(1-6)-glucoside ; cyclic 3-Hydroxymelatonin ; NPC ; 2-Oxomelatonin ; N-desalkyludenafil	M+H ; M+H ; M+H ; 3M+Na+K ; M+Na ; M+K ; 2M+H+Na ; M+H+K ; M+2Na-H ; M+ACN+2H ; M+2Na-H ; 3M+Na+K ; M+K ; M+K ; M+K ; M+K ; 3M+Na+K ; 2M+H+Na ; 2M+3H ; M+Na+K ; M+2Na ; M+H+K ; M+3ACN+2H ; M+H+K ; M+Na ; M+H+Na ; M+Na ; 2M+3H	C10H14N4O5 ; C10H14N4O5 ; C9H18O9 ; C6H12N2O3 ; C13H16N2O3 ; C10H20N2O4 ; C11H17NO6 ; C28H35FO7 ; C11H18N2O3 ; C26H30Cl2F3NO ; C11H18N2O3 ; C6H12N2O3 ; C10H20N2O4 ; C10H20N2O4 ; C10H20N2O4 ; C10H20N2O4 ; C6H12N2O3 ; C11H17NO6 ; C20H23NO8 ; C22H40O11 ; C25H36O10 ; C24H38O11 ; C21H21O9 ; C24H38O11 ; C13H16N2O3 ; C28H30N4O6 ; C13H16N2O3 ; C18H23N5O4S
432.22187	1.729684	0.743196	-5.8147	0.00017	3.7707	0.006402	432.2219	HMDB60754 ; HMDB14875 ; HMDB33295 ; HMDB33613 ; HMDB61178	31-Hydroxy rifabutin ; Meclizine ; 7-(4-Hydroxyphenyl)-1-phenyl-4-hepten-3-one ; Glabrin D ; Trandolapril-d5 Diketopiperazine	M+2H ; M+ACN+H ; 3M+H+Na ; M+3ACN+2H ; 2M+H+K	C46H62N4O12 ; C25H27ClN2 ; C19H20O2 ; C36H49N7O10 ; C24H32N2O4
103.03905	1.685979	0.360486	-5.8062	0.000172	3.7657	0.006402	103.0391	HMDB00005 ; HMDB00060 ; HMDB01172 ; HMDB01259 ; HMDB02217 ; HMDB03381 ; HMDB31646 ; HMDB41031 ; HMDB00303 ; HMDB02284 ; HMDB04476 ; HMDB11177 ; HMDB11179 ; HMDB12135 ; HMDB14555 ; HMDB14617 ; HMDB14935 ; HMDB15407 ; HMDB29891 ; HMDB38456 ; HMDB60064	2-Ketobutyric acid ; Acetoacetic acid ; 2-Methyl-3-oxopropanoic acid ; Succinic acid semialdehyde ; (S)-Methylmalonic acid semialdehyde ; 4-Hydroxycrotonic acid ; Acetic anhydride ; 356-Trihydroxy-5-(hydroxymethyl)-2-methoxy-2-cyclohexen-1-one ; Tryptamine ; N-Acetylcadaverine ; Anatabine ; L-phenylalanyl-L-proline ; L-prolyl-L-phenylalanine ; 1-(3-Aminopropyl)-4-aminobutanal ; Carbachol ; Methohexital ; Tolazoline ; Pirbuterol ; Starch acetate ; N-Norgramine ; Tryptaminium	M+H ; M+H ; M+H ; M+H ; M+H ; M+H ; M+H ; M+2H ; M+2Na ; M+Na+K ; M+2Na ; M+H+2Na ; M+H+2Na ; M+Na+K ; M+H+Na ; M+H+2Na ; M+2Na ; M+3Na ; M+3H ; M+2Na ; M+2Na	C4H6O3 ; C4H6O3 ; C4H6O3 ; C4H6O3 ; C4H6O3 ; C4H6O3 ; C4H6O3 ; C8H12O6 ; C10H12N2 ; C7H16N2O ; C10H12N2 ; C14H18N2O3 ; C14H18N2O3 ; C7H16N2O ; C6H15ClN2O2 ; C14H18N2O3 ; C10H12N2 ; C12H20N2O3 ; C12H18O9 ; C10H12N2 ; C10H12N2
163.05350	1.632248	0.845169	5.8049	0.000172	3.7649	0.006402	163.0535	HMDB31804 ; HMDB35400 ; HMDB00574 ; HMDB03417 ; HMDB31674 ; HMDB31873 ; HMDB32228 ; HMDB35620 ; HMDB36051 ; HMDB36094 ; HMDB36095 ; HMDB36221 ; HMDB36640 ; HMDB36695 ; HMDB37713 ; HMDB37719 ; HMDB38545 ; HMDB40608 ; HMDB40893 ; HMDB41003 ; HMDB41144 ; HMDB60116	Methomyl ; 1-Tridecene-357911-pentayne ; L-Cysteine ; D-Cysteine ; Armillarinin ; 17-Diphenyl-4-hepten-3-one ; Dimethylbenzyl carbinyl hexanoate ; Artabsinolide A ; Dihydromarasmone ; Nigellic acid ; 13-Hydroxyabscisic acid ; 2-(Dimethoxymethyl)-1-heptenylbenzene ; Furanofukinin ; Crispolide ; Octyl phenylacetate ; 2-Phenylethyl octanoate ; (1beta4alpha5alpha6beta8alpha10b)-110:45-Diepoxy-6-hydroxy-7(11)-germacren-128-olide ; Kanzonol F ; (EE)-17-Diphenyl-46-heptadien-3-ol ; Vulgarolide ; 8-Deoxy-1113-dihydroxygrosheimin ; Dibenzoalpyrene	M+H ; M+H ; M+ACN+H ; M+ACN+H ; M+2H+Na ; M+Na+K ; M+2K ; M+2Na ; M+2Na ; M+2Na ; M+2Na ; M+2K ; M+2K ; M+2Na ; M+2K ; M+2K ; M+2Na ; M+3Na ; M+Na+K ; M+2Na ; M+2Na ; M+H+Na	C5H10N2O2S ; C13H6 ; C3H7NO2S ; C3H7NO2S ; C24H29ClO7 ; C19H20O ; C16H24O2 ; C15H20O5 ; C15H20O5 ; C15H20O5 ; C15H20O5 ; C16H24O2 ; C16H24O2 ; C15H20O5 ; C16H24O2 ; C16H24O2 ; C15H20O5 ; C26H28O5 ; C19H20O ; C15H20O5 ; C15H20O5 ; C24H14
298.06395	1.697712	1.023841	-5.7978	0.000173	3.7607	0.006402	298.064	HMDB11716 ; HMDB12179 ; HMDB12277 ; HMDB12278 ; HMDB14480 ; HMDB14626 ; HMDB38773 ; HMDB41725 ; HMDB60399	N-Acetylvanilalanine ; 6-Hydroxycyclohex-1-ene-1-carboxyl-CoA ; PhosphoribosylformiminoAICAR-phosphate ; Phosphoribulosylformimino-AICAR-P ; Nitrofurazone ; Gallamine Triethiodide ; Formononetin 7-(2-p-hydroxybenzoylglucoside) ; Dihydroferuloylglycine ; 5-Methyl-3-oxo-4-hexenoyl-CoA	M+2Na-H ; M+3H ; M+H+NH4 ; M+H+NH4 ; 3M+2H ; M+3H ; M+2Na ; M+2Na-H ; M+3H	C12H15NO5 ; C28H44N7O18P3S ; C15H25N5O15P2 ; C15H25N5O15P2 ; C6H6N4O4 ; C30H60I3N3O3 ; C29H26O11 ; C12H15NO5 ; C28H44N7O18P3S
479.65047	1.709602	0.018464	5.795	0.000174	3.7591	0.006402	NA	NA	NA	NA	NA
228.96188	1.734983	0.173728	5.7887	0.000176	3.7554	0.006411	228.9619	HMDB37985 ; HMDB40585 ; HMDB41565	Cyananin ; ()-Sulfobutanedioic acid ; 57-Dihydro-2-methylthieno34-dpyrimidine	M+2K ; 2M+Na+K ; M+2K-H	C16H10Cl2N2O5 ; C4H6O7S ; C7H8N2S
102.09140	1.685718	0.568173	-5.7816	0.000177	3.7512	0.006426	102.0914	HMDB12815 ; HMDB00820 ; HMDB00863 ; HMDB01184 ; HMDB02117 ; HMDB12157 ; HMDB31407 ; HMDB31512 ; HMDB31530 ; HMDB31601 ; HMDB31606 ; HMDB31607 ; HMDB32458 ; HMDB37788 ; HMDB40584 ; HMDB41081	5-Aminopentanal ; Propyl alcohol ; Isopropyl alcohol ; Methyl propenyl ketone ; Oleamide ; 3-Methyl-2-butenal ; Cyclopentanone ; Tiglic aldehyde ; 3-Methyl-3-buten-2-one ; 2-Pentenal ; 4-Penten-2-one ; 1-Penten-3-one ; 4-Pentenal ; xi-23-Dihydro-3-methylfuran ; 23-Dihydro-4-methylfuran ; (Z)-15-Tridecadiene	M+H ; M+ACN+H ; M+ACN+H ; M+NH4 ; M+2H+Na ; M+NH4 ; M+NH4 ; M+NH4 ; M+NH4 ; M+NH4 ; M+NH4 ; M+NH4 ; M+NH4 ; M+NH4 ; M+NH4 ; M+H+Na	C5H11NO ; C3H8O ; C3H8O ; C5H8O ; C18H35NO ; C5H8O ; C5H8O ; C5H8O ; C5H8O ; C5H8O ; C5H8O ; C5H8O ; C5H8O ; C5H8O ; C5H8O ; C13H24

325.16008	1.661646	0.737303	-5.7708	0.00018	3.7448	0.006474	325.1601	HMDB05783 ; HMDB10342 ; HMDB10360 ; HMDB29741 ; HMDB30251 ; HMDB30258 ; HMDB31146 ; HMDB33426 ; HMDB33435 ; HMDB34591 ; HMDB34779 ; HMDB35722 ; HMDB35754 ; HMDB36476 ; HMDB38173 ; HMDB38649 ; HMDB39073 ; HMDB40551 ; HMDB40646 ; HMDB40951 ; HMDB40952	Gingerol ; 25-Hydroxyvitamin D2-25-glucuronide ; 25-Hydroxyvitamin D2 25-(beta-glucuronide) ; 2-Isopropyl-35-dimethoxy-6-methylpyrazine ; Oripavine ; ()-Erythraline ; Chlorophyll b ; Betavulgaroside VI ; ()-Aegeline ; Lansamide 3 ; Nordihydrocapsiate ; Tanacetol A ; Phytuberin ; Araliasaponin I ; Halocins ; ()-Clausenamide ; Hoduloside IV ; L-alpha-Amino-1H-pyrrole-1-hexanoic acid ; Myrsinone ; Medicoside F ; Medicoside E	2M+Na+K ; M+Na+K ; M+Na+K ; 3M+Na+K ; 2M+3H2O+2H ; 2M+3H2O+2H ; M+3Na ; M+3H ; 2M+3H2O+2H ; 2M+3H2O+2H ; 2M+Na+K ; 2M+Na+K ; 2M+Na+K ; M+H+2Na ; 2M+H+K ; 2M+3H2O+2H ; M+H+2Na ; 3M+Na+K ; 2M+Na+K ; M+H+2Na ; M+H+2Na	C17H26O4 ; C34H52O8 ; C34H52O8 ; C10H16N2O2 ; C18H19NO3 ; C18H19NO3 ; C55H70MgN4O6 ; C47H72O21 ; C18H19NO3 ; C18H19NO3 ; C17H26O4 ; C17H26O4 ; C17H26O4 ; C47H76O18 ; C21H23NO ; C18H19NO3 ; C47H76O18 ; C10H16N2O2 ; C17H26O4 ; C47H76O18 ; C47H76O18
198.07157	1.649808	0.152023	-5.765	0.000181	3.7413	0.006479	198.0716	HMDB30817 ; HMDB35734 ; HMDB01337 ; HMDB05081 ; HMDB06027 ; HMDB10202 ; HMDB10205 ; HMDB10209 ; HMDB10210 ; HMDB10212 ; HMDB10217 ; HMDB12534 ; HMDB12611 ; HMDB13633 ; HMDB29569 ; HMDB30101 ; HMDB35116 ; HMDB35311 ; HMDB36698 ; HMDB36706 ; HMDB36712 ; HMDB36713 ; HMDB36724 ; HMDB36752 ; HMDB36776 ; HMDB36806 ; HMDB36839 ; HMDB36930 ; HMDB37523 ; HMDB38521 ; HMDB39012 ; HMDB39716 ; HMDB40057 ; HMDB40348 ; HMDB41058 ; HMDB60053 ; HMDB61114	6-Hydroxymusizin 8-O-b-D-glucopyranoside ; 2-Oxoaloesol 7-glucoside ; Leukotriene A4 ; 5-HEPE ; Oxymesterone ; 12-HEPE ; 1415-EpETE ; 15-HEPE ; 15-KETE ; 1718-EpETE ; 5-KETE ; 11R-HEPE ; 18R-HEPE ; 12-KETE ; Dibutyl disulfide ; Methyl 8-Shogaol ; (ent-7alpha)-7-Hydroxy-8(14)15-pimaradien-19-oic acid ; Galanal A ; Ineketone ; Steviol ; Yucalexin B20 ; Yucalexin B22 ; ent-17-Hydroxy-15-kauren-19-oic acid ; Yucalexin P17 ; Yucalexin A19 ; Aframodial ; (ent-15beta)-15-Hydroxy-19-trachylobanoic acid ; Grandiflorolic acid ; Galanolactone ; 2-Hydroxy-6-(8-tridecenyl)benzoic acid ; (ent-15beta)-1519-Dihydroxy-7-trachylobanone ; 12alpha-12-Hydroxy-713-abietadien-18-oic acid ; ()-2-Pentylthiazolidine ; 18-Octanedithiol ; Phytocassane C ; 9-HEPE ; Atractyligenin	M+2H ; M+2H ; M+2K ; M+2K ; M+2K ; M+2K ; M+2K ; M+2K ; M+2K ; M+2K ; M+2K ; M+2K ; M+2K ; M+2K ; 2M+H+K ; M+2K ; M+2K ; M+2K ; M+2K ; M+2K ; M+2K ; M+2K ; M+2K ; M+2K ; M+2K ; M+2K ; M+2K ; M+2K ; M+2K ; M+2K ; M+2K ; M+2K ; M+K ; 2M+H+K ; M+2K ; M+2K ; M+2K	C19H22O9 ; C19H22O9 ; C20H30O3 ; C20H30O3 ; C20H30O3 ; C20H30O3 ; C20H30O3 ; C20H30O3 ; C20H30O3 ; C20H30O3 ; C20H30O3 ; C20H30O3 ; C20H30O3 ; C20H30O3 ; C8H18S2 ; C20H30O3 ; C20H30O3 ; C20H30O3 ; C20H30O3 ; C20H30O3 ; C20H30O3 ; C20H30O3 ; C20H30O3 ; C20H30O3 ; C20H30O3 ; C20H30O3 ; C20H30O3 ; C20H30O3 ; C20H30O3 ; C20H30O3 ; C20H30O3 ; C20H30O3 ; C8H17NS ; C8H18S2 ; C20H30O3 ; C20H30O3 ; C20H30O3
250.15327	1.654934	0.223281	5.7191	0.000193	3.714	0.006821	250.1533	HMDB01389 ; HMDB02006 ; HMDB14501 ; HMDB15434 ; HMDB32879 ; HMDB33553 ; HMDB34726 ; HMDB35728 ; HMDB36787 ; HMDB36840 ; HMDB39111	Melatonin ; 23-Diaminopropionic acid ; Aminoglutethimide ; Forasartan ; all-trans-Carophyll yellow ; 3-(3-Methylbutyl)nitrosoamino-2-butanone ; Momordicinin ; Ganoderol A ; alphagamma-Onoceradienedione ; Thujyl 19-trachylobanoate ; L-Acetopine	M+NH4 ; 2M+ACN+H ; M+NH4 ; M+2ACN+2H ; M+H+K ; M+ACN+Na ; M+Na+K ; M+Na+K ; M+Na+K ; M+Na+K ; M+NH4	C13H16N2O2 ; C3H8N2O2 ; C13H16N2O2 ; C23H28N8 ; C32H44O2 ; C9H18N2O2 ; C30H46O2 ; C30H46O2 ; C30H46O2 ; C30H46O2 ; C8H16N4O4
176.00952	1.657137	0.921251	-5.716	0.000194	3.7122	0.006821	176.0095	HMDB13680 ; HMDB29276 ; HMDB31242 ; HMDB31253 ; HMDB31779 ; HMDB32160 ; HMDB32334 ; HMDB35088 ; HMDB39877 ; HMDB59651 ; HMDB59978 ; HMDB59979 ; HMDB61134 ; HMDB61160	Caftaric acid ; Cis-Caffeoyl tartaric acid ; Dihydro-3(2H)-thiophenone ; Dihydro-2(3H)-thiophenone ; Isoprothiolane ; 3-(Acetylthio)-2-methylfuran ; 1-(3-Hydroxy-5-methyl-2-thienyl)ethanone ; Furfuryl thioacetate ; Charine ; 2-(34-Dihydroxybenzoyloxy)-46-dihydroxybenzoate ; 4-Hydroxy-5-(dihydroxyphenyl)-valeric acid-O-sulphate ; 4-Hydroxy-5-(dihydroxyphenyl)-valeric acid-O-sulphate III ; Dedimethylchlorpromazine ; N-Dedimethylchlorpromazine	M+H+K ; M+H+K ; 3M+2Na ; 3M+2Na ; M+Na+K ; 2M+H+K ; 2M+H+K ; 2M+H+K ; M+2K ; M+2Na ; M+2Na ; M+2Na ; M+Na+K ; M+Na+K	C13H12O9 ; C13H12O9 ; C4H6OS ; C4H6OS ; C12H18O4S2 ; C7H8O2S ; C7H8O2S ; C7H8O2S ; C9H14N4O6 ; C14H10O8 ; C11H14O8S ; C11H14O8S ; C15H15ClN2S ; C15H15ClN2S
262.12841	1.653556	0.06968	-5.7119	0.000195	3.7098	0.006821	262.1284	HMDB13133 ; HMDB33865 ; HMDB34777 ; HMDB34346 ; HMDB15443 ; HMDB29876 ; HMDB31237 ; HMDB33462 ; HMDB60742	Methylmalonylcarnitine ; Lotaustralin ; Epidermin ; 4-Deoxyphysalolactone ; Heptabarbital ; Paucine ; threo-Syringoylglycerol ; 4-Coumaroyl-2-hydroxyputrescine ; 3-Hydroxymelatonin	M+H ; M+H ; M+H ; M+2H ; 2M+H+Na ; 2M+H+Na ; M+NH4 ; 2M+H+Na ; 2M+H+Na	C11H19NO6 ; C11H19NO6 ; C11H19NO6 ; C28H39ClO7 ; C13H18N2O3 ; C13H18N2O3 ; C11H16O6 ; C13H18N2O3 ; C13H18N2O3
132.98721	1.649485	1.056397	5.6894	0.000201	3.6963	0.00696	132.9872	HMDB35291 ; HMDB15320 ; HMDB60648	Isoplumbagin ; Desflurane ; Hexafluoroisopropanol	M+2K ; M+H-2H2O ; M+H-2H2O	C11H8O3 ; C3H2F6O ; C3H2F6O
286.06971	1.709464	0.710578	-5.6796	0.000204	3.6904	0.00696	286.0697	HMDB14387 ; HMDB01265 ; HMDB02326 ; HMDB29218 ; HMDB29463	Cladribine ; Fucose 1-phosphate ; Coumesterol ; Urolithin C ; Gentisein	M+H ; M+ACN+H ; M+NH4 ; M+ACN+H ; M+ACN+H	C10H12ClN5O3 ; C6H13O8P ; C15H8O5 ; C13H8O5 ; C13H8O5
182.02179	1.709962	0.42453	5.6683	0.000207	3.6837	0.00696	182.0218	HMDB00894 ; HMDB01494 ; HMDB04068 ; HMDB04110 ; HMDB12134 ; HMDB14491 ; HMDB30152 ; HMDB30521 ; HMDB32161 ; HMDB34024 ; HMDB34690 ; HMDB36204 ; HMDB40590 ; HMDB41168 ; HMDB41169 ; HMDB41462 ; HMDB41671 ; HMDB41680 ; HMDB61082	Vinylacetylglycine ; Acetylphosphate ; Melanin ; Phosphonoacetate ; 12-Dihydroxy-3-keto-5-methylthiopentene ; Trimethadione ; 4-Hydroxy-57-dimethoxyflavan ; Sativan ; Acrylamide-acrylic acid resin ; Isosativan ; 9-(4-Hydroxyphenyl)-2-methoxy-1H-phenalen-1-one ; 1-(Methylsulfanyl)-1-oxopropan-2-yl acetate ; Benzyl methyl disulfide ; Myrigalone G ; Myrigalone H ; 2-Hydroxy-4-(4-methoxyphenyl)-1H-phenalen-1-one ; 4-Hydroxy-345-trimethoxystilbene ; 4-Hydroxy-354-trimethoxystilbene ; 5-ethyl-5-methyl-24-oxazolidinedione	M+K ; M+ACN+H ; M+2Na ; M+ACN+H ; 2M+H+K ; M+K ; M+2K ; M+2K ; M+K ; M+2K ; M+Na+K ; 2M+H+K ; 2M+H+Na ; M+2K ; M+2K ; M+Na+K ; M+2K ; M+2K ; M+K	C6H9NO3 ; C2H5O5P ; C18H10N2O4 ; C2H5O5P ; C6H10O3S ; C6H9NO3 ; C17H18O4 ; C17H18O4 ; C6H9NO3 ; C17H18O4 ; C20H14O3 ; C6H10O3S ; C8H10S2 ; C17H18O4 ; C17H18O4 ; C20H14O3 ; C17H18O4 ; C17H18O4 ; C6H9NO3
341.03711	1.670941	0.702674	-5.6631	0.000209	3.6806	0.00696	341.0371	HMDB06336 ; HMDB33832 ; HMDB41059	ADP-ribose 2-phosphate ; Cinnamyl cinnamate ; N-Methoxyspirobrassinol methyl ether	M+ACN+2H ; M+2K-H ; M+2Na-H	C15H24N5O17P3 ; C18H16O2 ; C13H16N2O2S2

202.04066	1.669415	0.312152	5.6626	0.000209	3.6803	0.00696	202.0407	HMDB00072 ; HMDB00958 ; HMDB01264 ; HMDB13682 ; HMDB14332 ; HMDB30107 ; HMDB31332 ; HMDB32767 ; HMDB34218 ; HMDB35070 ; HMDB37252 ; HMDB37552 ; HMDB37598 ; HMDB38805 ; HMDB38845 ; HMDB39334 ; HMDB40200 ; HMDB40322 ; HMDB40663 ; HMDB40724 ; HMDB41273	cis-Aconitic acid ; trans-Aconitic acid ; Dehydroascorbic acid ; Coniferin ; Lorazepam ; 3-Hydroxy-3-(34-dihydroxy-4-methylpentanoyl)-5-(3-methylbutyl)-124-cyclopentanetrione ; Chloromethyl methyl ether ; Sphalleroside A ; Chrysoobtusin ; 12alpha-Hydroxymunduserone ; 58-Dihydroxy-3-(4-hydroxybenzyl)-7-methoxy-4-chromanone 8-acetate ; 4-Hydroxy-3-(2-hydroxyethyl)acetophenone 4-glucoside ; 5-Hydroxy-3478-tetramethoxyflavone ; Aflatoxin ExB2 ; 7-Hydroxy-3456-tetramethoxyflavone ; Citrusin D ; 25-Dimethyl-3-(methyldithio)furan ; 3-Hydroxy-4578-tetramethoxyflavone ; Veranisatin A ; 4-Hydroxy-5678-tetramethoxyflavone ; Myzodendrone	2M+3H2O+2H ; 2M+3H2O+2H ; 2M+3H2O+2H ; M+Na+K ; M+2ACN+2H ; M+Na+K ; 2M+ACN+H ; M+Na+K ; M+2Na ; M+2Na ; M+2Na ; M+Na+K ; M+2Na ; M+2Na ; M+2Na ; M+Na+K ; 2M+3H2O+2H ; M+2Na ; M+Na+K ; M+2Na ; M+Na+K	C6H6O6 ; C6H6O6 ; C6H6O6 ; C16H22O8 ; C15H10Cl2N2O2 ; C16H22O8 ; C2H5ClO ; C16H22O8 ; C19H18O7 ; C19H18O7 ; C19H18O7 ; C16H22O8 ; C19H18O7 ; C19H18O7 ; C19H18O7 ; C16H22O8 ; C7H10OS2 ; C19H18O7 ; C16H22O8 ; C19H18O7 ; C16H22O8
152.97987	1.720094	0.57515	5.6621	0.000209	3.68	0.00696	152.9799	HMDB02127 ; HMDB31853 ; HMDB32939 ; HMDB39787 ; HMDB39788	3-Mercaptolactic acid ; 2-Thiophenemethanethiol ; 3-(Methylthio)thiophene ; 2-Methyl-3-thiophenethiol ; 5-Methyl-3-thiophenethiol	2M+Na+K ; M+Na ; M+Na ; M+Na ; M+Na	C3H6O3S ; C5H6S2 ; C5H6S2 ; C5H6S2 ; C5H6S2
582.98688	1.613887	0.770363	5.6571	0.00021	3.677	0.00696	582.9869	HMDB01059 ; HMDB01187 ; HMDB03848 ; HMDB04527 ; HMDB39738 ; HMDB46798 ; HMDB46803 ; HMDB46822 ; HMDB46947 ; HMDB47315 ; HMDB47459 ; HMDB47477 ; HMDB47482 ; HMDB47529 ; HMDB47546 ; HMDB47552 ; HMDB47621 ; HMDB47626 ; HMDB47644 ; HMDB47666 ; HMDB47672 ; HMDB47683 ; HMDB47689 ; HMDB50993 ; HMDB51131 ; HMDB51136 ; HMDB51267 ; HMDB51357 ; HMDB51763 ; HMDB51773 ; HMDB51891 ; HMDB52117 ; HMDB52122 ; HMDB52213 ; HMDB52218 ; HMDB52228 ; HMDB52258 ; HMDB52264 ; HMDB52273 ; HMDB52324 ; HMDB52353 ; HMDB53735 ; HMDB53825 ; HMDB53830 ; HMDB54081 ; HMDB54086 ; HMDB54168 ; HMDB54479 ; HMDB54543 ; HMDB54548 ; HMDB54561 ; HMDB54611 ; HMDB54686 ; HMDB54691 ; HMDB54699 ; HMDB54757 ;	Inositol 1345-tetraphosphate ; 1D-Myo-inositol 1346-tetrakisphosphate ; D-Myo-inositol 3456-tetrakisphosphate ; 1D-Myo-inositol 1456-tetrakisphosphate ; Pipereicosalidine ; TG(22:022:4(7Z10Z13Z16Z)22:5(4Z7Z10Z13Z16Z)) ; TG(22:022:4(7Z10Z13Z16Z)22:5(7Z10Z13Z16Z19Z)) ; TG(22:022:5(4Z7Z10Z13Z16Z)22:4(7Z10Z13Z16Z)) ; TG(22:022:5(7Z10Z13Z16Z19Z)22:4(7Z10Z13Z16Z)) ; TG(24:020:3(5Z8Z11Z)22:6(4Z7Z10Z13Z16Z19Z)) ; TG(24:020:3n622:6(4Z7Z10Z13Z16Z19Z)) ; TG(24:020:4(5Z8Z11Z14Z)22:5(4Z7Z10Z13Z16Z)) ; TG(24:020:4(5Z8Z11Z14Z)22:5(7Z10Z13Z16Z19Z)) ; TG(24:022:4(7Z10Z13Z16Z)20:5(5Z8Z11Z14Z17Z)) ; TG(24:022:5(4Z7Z10Z13Z16Z)20:4(5Z8Z11Z14Z)) ; TG(24:022:5(4Z7Z10Z13Z16Z)20:4(8Z11Z14Z17Z)) ; TG(24:020:4(8Z11Z14Z17Z)22:5(4Z7Z10Z13Z16Z)) ; TG(24:020:4(8Z11Z14Z17Z)22:5(7Z10Z13Z16Z19Z)) ; TG(24:020:5(5Z8Z11Z14Z17Z)22:4(7Z10Z13Z16Z)) ; TG(24:022:5(7Z10Z13Z16Z19Z)20:4(5Z8Z11Z14Z)) ; TG(24:022:5(7Z10Z13Z16Z19Z)20:4(8Z11Z14Z17Z)) ; TG(24:022:6(4Z7Z10Z13Z16Z19Z)20:3(5Z8Z11Z)) ; TG(24:022:6(4Z7Z10Z13Z16Z19Z)20:3n6) ; TG(20:3(5Z8Z11Z)24:022:6(4Z7Z10Z13Z16Z19Z)) ; TG(20:3(5Z8Z11Z)24:1(15Z)22:5(4Z7Z10Z13Z16Z)) ; TG(20:3(5Z8Z11Z)24:1(15Z)22:5(7Z10Z13Z16Z19Z)) ; TG(20:3(5Z8Z11Z)22:5(4Z7Z10Z13Z16Z)24:1(15Z)) ; TG(20:3(5Z8Z11Z)22:5(7Z10Z13Z16Z19Z)24:1(15Z)) ; TG(22:1(13Z)22:2(13Z16Z)22:6(4Z7Z10Z13Z16Z19Z)) ; TG(22:1(13Z)22:4(7Z10Z13Z16Z)22:4(7Z10Z13Z16Z)) ; TG(22:1(13Z)22:6(4Z7Z10Z13Z16Z19Z)22:2(13Z16Z)) ; TG(24:1(15Z)20:3(5Z8Z11Z)22:5(4Z7Z10Z13Z16Z)) ; TG(24:1(15Z)20:3(5Z8Z11Z)22:5(7Z10Z13Z16Z19Z)) ; TG(24:1(15Z)20:3n622:5(4Z7Z10Z13Z16Z)) ; TG(24:1(15Z)20:3n622:5(7Z10Z13Z16Z19Z)) ; TG(24:1(15Z)20:4(5Z8Z11Z14Z)22:4(7Z10Z13Z16Z)) ; TG(24:1(15Z)22:4(7Z10Z13Z16Z)20:4(5Z8Z11Z14Z)) ; TG(24:1(15Z)22:4(7Z10Z13Z16Z)20:4(8Z11Z14Z17Z)) ; TG(24:1(15Z)22:5(4Z7Z10Z13Z16Z)20:3n6) ;	M+2ACN+H ; M+2ACN+H ; M+2ACN+H ; M+2ACN+H ; 3M+2Na ; M+3ACN+2H	C6H16O18P4 ; C6H16O18P4 ; C6H16O18P4 ; C6H16O18P4 ; C25H43NO ; C69H116O6
552.95031	1.713299	0.500596	-5.6413	0.000215	3.6675	0.00696	552.9503	HMDB00189 ; HMDB13303 ; HMDB47699 ; HMDB52283 ; HMDB52363 ; HMDB52373 ; HMDB52378 ; HMDB55027 ; HMDB55847 ; HMDB55905	Inosine triphosphate ; Pristanoylglycine ; TG(24:022:6(4Z7Z10Z13Z16Z19Z)22:6(4Z7Z10Z13Z16Z19Z)) ; TG(24:1(15Z)22:5(4Z7Z10Z13Z16Z)22:6(4Z7Z10Z13Z16Z19Z)) ; TG(24:1(15Z)22:5(7Z10Z13Z16Z19Z)22:6(4Z7Z10Z13Z16Z19Z)) ; TG(24:1(15Z)22:6(4Z7Z10Z13Z16Z19Z)22:5(4Z7Z10Z13Z16Z)) ; TG(24:1(15Z)22:6(4Z7Z10Z13Z16Z19Z)22:5(7Z10Z13Z16Z19Z)) ; TG(22:5(4Z7Z10Z13Z16Z)24:1(15Z)22:6(4Z7Z10Z13Z16Z19Z)) ; TG(22:5(7Z10Z13Z16Z19Z)24:1(15Z)22:6(4Z7Z10Z13Z16Z19Z)) ; TG(22:6(4Z7Z10Z13Z16Z19Z)24:022:6(4Z7Z10Z13Z16Z19Z))	M+2Na-H ; 3M+H+K ; M+ACN+2H ; M+ACN+2H ; M+ACN+2H ; M+ACN+2H ; M+ACN+2H ; M+ACN+2H ; M+ACN+2H ; M+ACN+2H	C10H15N4O14P3 ; C21H41NO3 ; C71H114O6 ; C71H114O6 ; C71H114O6 ; C71H114O6 ; C71H114O6 ; C71H114O6 ; C71H114O6 ; C71H114O6
290.14569	1.685037	0.915285	-5.6385	0.000216	3.6658	0.00696	290.1457	HMDB28705 ; HMDB28747 ; HMDB00145 ; HMDB15219 ; HMDB32673 ; HMDB37778	Arginyl-Aspartate ; Aspartyl-Arginine ; Estrone ; Primaquine ; 15-Octadecene-91113-triynoic acid ; Ganoderiol I	M+H ; M+H ; 2M+H+K ; 2M+Na+K ; 2M+H+K ; M+2K	C10H19N5O5 ; C10H19N5O5 ; C18H22O2 ; C15H21N3O ; C18H22O2 ; C31H50O5
258.09707	1.655987	0.670595	5.6377	0.000216	3.6654	0.00696	258.0971	HMDB04999 ; HMDB13713 ; HMDB14987 ; HMDB28726 ; HMDB29925 ; HMDB30269 ; HMDB32763 ; HMDB32906 ; HMDB33370 ; HMDB60810	Loperamide ; N-acetyltryptophan ; Methylphenobarbital ; Asparaginyl-Asparagine ; Methyl cellulose ; Nigellicine ; N2-Oxalylarginine ; Isolariciresinol 9-O-alpha-L-arabinofuranoside ; Ethyl cellulose ; cyclic 6-Hydroxymelatonin	M+H+K ; 2M+H+Na ; 2M+H+Na ; 2M+H+Na ; M+Na+K ; 2M+H+Na ; 2M+H+Na ; M+H+Na ; M+Na+K ; 2M+H+Na	C29H33ClN2O2 ; C13H14N2O3 ; C13H14N2O3 ; C8H14N4O5 ; C20H38O11 ; C13H14N2O3 ; C8H14N4O5 ; C25H32O10 ; C20H38O11 ; C13H14N2O3
534.12875	1.629961	0.506301	5.6266	0.000219	3.6587	0.00696	534.1288	HMDB12253 ; HMDB29271 ; HMDB30658 ; HMDB33894 ; HMDB37366 ; HMDB37459 ; HMDB38465 ; HMDB38772 ; HMDB39904 ; HMDB39923 ; HMDB40633 ; HMDB41757	Maltohexaose ; Quercetin 3-O-(6-acetyl-glucoside) ; Carmine red ; Tricin 7-glucuronoside ; Quercetin 3-(6-acetylglucoside) ; 6-Methoxyluteolin 7-glucuronide ; Glyphoside ; 8-Hydroxyluteolin 4-methyl ether 8-glucuronide ; 3-Methoxytricetin 7-glucuronide ; 4-O-Acetylmyricitrin ; Isobiflorin 6-gallate ; Isorhamnetin 4-O-glucuronide	M+2K ; 2M+3H2O+2H ; M+ACN+H ; 2M+3H2O+2H ; 2M+3H2O+2H ; M+ACN+H ; 2M+3H2O+2H ; M+ACN+H ; M+ACN+H ; 2M+3H2O+2H ; 2M+3H2O+2H ; M+ACN+H	C36H62O31 ; C23H22O13 ; C22H20O13 ; C23H22O13 ; C23H22O13 ; C22H20O13 ; C23H22O13 ; C22H20O13 ; C22H20O13 ; C23H22O13 ; C23H22O13 ; C22H20O13
552.83865	1.717714	0.490803	-5.6264	0.00022	3.6585	0.00696	552.8387	HMDB00063 ; HMDB00319 ; HMDB06758 ; HMDB14879 ; HMDB30026 ; HMDB30138 ; HMDB32095 ; HMDB34090	Cortisol ; 18-Hydroxycorticosterone ; 17a21-Dihydroxy-5b-pregnane-31120-trione ; Hydrocortisone ; Isohumulone A ; Adhumulone ; 5-Hydroxy-4-methoxy-5-(1-oxo-91215-hexadecatrienyl)-2(5H)-furanone ; (R)-Humulone	3M+H+NH4 ; 3M+H+NH4 ; 3M+H+NH4 ; 3M+H+NH4 ; 3M+H+NH4 ; 3M+H+NH4 ; 3M+H+NH4 ; 3M+H+NH4	C21H30O5 ; C21H30O5 ; C21H30O5 ; C21H30O5 ; C21H30O5 ; C21H30O5 ; C21H30O5 ; C21H30O5
614.92657	1.658774	0.614486	-5.6239	0.00022	3.657	0.00696	NA	NA	NA	NA	NA
550.61485	1.647873	0.811392	-5.6226	0.000221	3.6563	0.00696	550.6149	HMDB32229	Dimethyl dialkyl ammonium chloride	M+ACN+H	C35H74N

169.09714	1.708775	0.330698	5.6209	0.000221	3.6552	0.00696	169.0971	HMDB01431 ; HMDB00549 ; HMDB01859 ; HMDB02210 ; HMDB02523 ; HMDB03407 ; HMDB10720 ; HMDB12219 ; HMDB12992 ; HMDB15506 ; HMDB29339 ; HMDB29618 ; HMDB29703 ; HMDB29864 ; HMDB30263 ; HMDB31209 ; HMDB31415 ; HMDB32344 ; HMDB32609 ; HMDB33903 ; HMDB33977 ; HMDB34393 ; HMDB34439 ; HMDB37777 ; HMDB59846 ; HMDB60680	Pyridoxamine ; Gamma-Butyrolactone ; Acetaminophen ; 2-Phenylglycine ; Oxolan-3-one ; Diacetyl ; But-2-enoic acid ; Dopamine quinone ; Leukoaminochrome ; Phenazopyridine ; Ceanothine B ; Macrophorin D ; Methyl 2-aminobenzoate ; 2-Amino-38-dimethyl-3H-imidazo45-fquinoxaline ; Rhazidigenine Nb-oxide ; Ethenyl acetate ; 26-Dimethyl-24-heptadiene ; Isobutylene-isoprene copolymer ; 2-(Methylamino)benzoic acid ; 3alpha477alpha-Tetrahydro-1H-isoindole-13(2H)-dione ; Methyl acrylate ; Ixocarpalactone A ; Isocrotonic acid ; 2-Isopropyl-14-hexadiene ; Ethyl nicotinate ; 2-Amino-3-methylbenzoate	M+H ; M+2ACN+H ; M+NH4 ; M+NH4 ; M+2ACN+H ; M+2ACN+H ; M+2ACN+H ; M+NH4 ; M+NH4 ; M+3ACN+2H ; M+3H ; M+3H ; M+NH4 ; M+3ACN+2H ; M+H+Na ; M+2ACN+H ; M+2Na-H ; M+2Na-H ; M+NH4 ; M+NH4 ; M+2ACN+H ; M+3H ; M+2ACN+H ; M+2Na-H ; M+NH4 ; M+NH4	C8H12N2O2 ; C4H6O2 ; C8H9NO2 ; C8H9NO2 ; C4H6O2 ; C4H6O2 ; C4H6O2 ; C8H9NO2 ; C8H9NO2 ; C11H11N5 ; C29H36N4O4 ; C28H40O8 ; C8H9NO2 ; C11H11N5 ; C19H26N2O2 ; C4H6O2 ; C9H16 ; C9H16 ; C8H9NO2 ; C8H9NO2 ; C4H6O2 ; C28H40O8 ; C4H6O2 ; C9H16 ; C8H9NO2 ; C8H9NO2
494.54825	1.691056	0.124512	-5.6164	0.000223	3.6525	0.00696	NA	NA	NA	NA	NA
176.12805	1.660433	1.05291	-5.616	0.000223	3.6523	0.00696	176.1281	HMDB01252 ; HMDB11534 ; HMDB11565 ; HMDB31043 ; HMDB31044 ; HMDB31045 ; HMDB32478 ; HMDB39979 ; HMDB39980 ; HMDB39981 ; HMDB40001	Betaine aldehyde ; MG(0:016:1(9Z)0:0) ; MG(16:1(9Z)0:00:0) ; Avocadene 1-acetate ; Avocadene 2-acetate ; Avocadene 4-acetate ; Polypropylene glycol (m w 1200-3000) ; 25-Diethyl-36-dimethylpyrazine ; 23-Diethyl-56-dimethylpyrazine ; 26-Diethyl-35-dimethylpyrazine ; Trimethylpropylpyrazine	3M+2Na ; M+H+Na ; M+H+Na ; M+H+Na ; M+H+Na ; M+H+Na ; M+ACN+H ; 2M+H+Na ; 2M+H+Na ; 2M+H+Na ; 2M+H+Na	C5H12NO ; C19H36O4 ; C19H36O4 ; C19H36O4 ; C19H36O4 ; C19H36O4 ; C6H14O3 ; C10H16N2 ; C10H16N2 ; C10H16N2 ; C10H16N2
146.02709	1.628945	0.594355	5.6091	0.000225	3.6482	0.006982	146.0271	HMDB01068 ; HMDB60509 ; HMDB00575 ; HMDB00676 ; HMDB15492 ; HMDB01890	D-Sedoheptulose 7-phosphate ; Sedoheptulose 1-phosphate ; DL-Homocystine ; L-Homocystine ; Stepronin ; Acetylcysteine	M+2H ; M+2H ; M+H+Na ; M+H+Na ; M+H+NH4 ; M+H-H2O	C7H15O10P ; C7H15O10P ; C8H16N2O4S2 ; C8H16N2O4S2 ; C10H11NO4S2 ; C5H9NO3S
151.03899	1.670816	0.313419	5.5967	0.000229	3.6407	0.007039	151.039	HMDB01587 ; HMDB31515 ; HMDB32598 ; HMDB32612 ; HMDB40528 ; HMDB29492 ; HMDB29676 ; HMDB30617 ; HMDB30659 ; HMDB30667 ; HMDB32695 ; HMDB32696 ; HMDB33321 ; HMDB33720 ; HMDB33760 ; HMDB34443 ; HMDB37339 ; HMDB37441 ; HMDB42024 ; HMDB15522	Phenylglyoxylic acid ; (E)-8-Hydroxy-2-octene-46-diynoic acid ; 4-Hydroxyphthalide ; 34-Methylenedioxybenzaldehyde ; 14-Benzodioxin-2(3H)-one ; 337-Trihydroxy-4-methoxyflavone ; Diosmetin ; Pratensein ; Takakin ; Chrysoeriol ; Barpisoflavone A ; Cajanin ; 35-Dihydroxy-67-methylenedioxyflavanone ; 6alpha-Hydroxymaackiain ; Santal ; Questinol ; Luteolin 7-methyl ether ; Kaempferide ; Tectorigenin ; Sulfamethazine	M+H ; M+H ; M+H ; M+H ; M+H ; M+2H ; M+2H ; M+2H ; M+2H ; M+2H ; M+2H ; M+2H ; M+2H ; M+2H ; M+2H ; M+2H ; M+2H ; M+2H ; M+2H ; M+H+Na	C8H6O3 ; C8H6O3 ; C8H6O3 ; C8H6O3 ; C8H6O3 ; C16H12O6 ; C16H12O6 ; C16H12O6 ; C16H12O6 ; C16H12O6 ; C16H12O6 ; C16H12O6 ; C16H12O6 ; C16H12O6 ; C16H12O6 ; C16H12O6 ; C16H12O6 ; C16H12O6 ; C16H12O6 ; C12H14N4O2S
196.93114	1.683443	0.38562	5.5916	0.00023	3.6376	0.007039	NA	NA	NA	NA	NA
197.11864	1.670352	0.582394	5.59	0.000231	3.6366	0.007039	197.1186	HMDB36226 ; HMDB37725 ; HMDB37734 ; HMDB14354 ; HMDB34662 ; HMDB32423 ; HMDB30984 ; HMDB30985 ; HMDB30986	26-Dimethoxy-4-propylphenol ; Hexyl 2-furoate ; Isobutyl 2-furanpropionate ; Trospium ; Dihydrofukinolide ; 2-(4-Methyl-5-thiazolyl)ethyl octanoate ; 5-Hexyltetrahydro-2-oxo-3-furancarboxylic acid ; alpha-Carboxy-delta-decalactone ; 2-Carboxy-57-dimethyl-4-octanolide	M+H ; M+H ; M+H ; M+2H ; M+2H ; M+3ACN+2H ; M+H-H2O ; M+H-H2O ; M+H-H2O	C11H16O3 ; C11H16O3 ; C11H16O3 ; C25H30NO3 ; C22H32O6 ; C14H23NO2S ; C11H18O4 ; C11H18O4 ; C11H18O4
285.06926	1.716798	0.330791	-5.5666	0.000239	3.6225	0.007213	285.0693	HMDB35996 ; HMDB38846	2-(Methylthio)-3H-phenoxazin-3-one ; (-)-Epiafzelechin 3-gallate	M+ACN+H ; 2M+3H	C13H9NO2S ; C22H18O9
793.59689	1.665378	0.319485	5.5638	0.000239	3.6208	0.007213	793.5969	HMDB07038 ; HMDB09578 ; HMDB09579 ; HMDB11423 ; HMDB11456 ; HMDB39199 ; HMDB39245 ; HMDB39256 ; HMDB39257 ; HMDB39271 ; HMDB41119 ; HMDB56134	DG(14:1(9Z)14:1(9Z)0:0) ; PE(22:2(13Z16Z)P-18:1(11Z)) ; PE(22:2(13Z16Z)P-18:1(9Z)) ; PE(P-18:1(11Z)22:2(13Z16Z)) ; PE(P-18:1(9Z)22:2(13Z16Z)) ; Camelliin A ; Castanin ; Sanguiin H10 ; Sanguiin H3 ; Granatin A ; Camelliatannin H ; DG(14:1n50:014:1n5)	3M+Na+K ; 2M+H+Na ; 2M+H+Na ; 2M+H+Na ; 2M+H+Na ; M+H+NH4 ; 2M+H+NH4 ; M+H+NH4 ; M+H+NH4 ; 2M+H+NH4 ; M+H+NH4 ; 3M+Na+K	C31H56O5 ; C45H84NO7P ; C45H84NO7P ; C45H84NO7P ; C45H84NO7P ; C68H48O44 ; C34H24O22 ; C68H48O44 ; C68H48O44 ; C34H24O22 ; C68H48O44 ; C31H56O5
240.02444	1.617061	0.307457	-5.5194	0.000255	3.5939	0.00753	240.0244	HMDB11750 ; HMDB60006	Dihydroxyacetone Phosphate Acyl Ester ; N-(2-formyl-3-chlorophenyl)anthranilic acid	M+ACN+H ; M+H-2H2O	C4H7O7P ; C14H10ClNO3

302.59317	1.679396	0.937017	-5.5179	0.000255	3.593	0.00753	302.5932	HMDB05370 ; HMDB05411 ; HMDB05445 ; HMDB05455 ; HMDB05462 ; HMDB10455 ; HMDB10456 ; HMDB10458 ; HMDB10464 ; HMDB10469 ; HMDB10474 ; HMDB10480 ; HMDB10492 ; HMDB10521 ; HMDB29213 ; HMDB29554 ; HMDB30624 ; HMDB31113 ; HMDB31114 ; HMDB34358 ; HMDB34359 ; HMDB37756 ; HMDB40387 ; HMDB42172 ; HMDB42200 ; HMDB42206 ; HMDB42235 ; HMDB42403 ; HMDB42409 ; HMDB42419 ; HMDB42432 ; HMDB42442 ; HMDB42467 ; HMDB42474 ; HMDB42554 ; HMDB42612 ; HMDB42622 ; HMDB42635 ; HMDB42687 ; HMDB42694 ; HMDB42703 ; HMDB42709 ; HMDB42764 ; HMDB42786 ; HMDB42815 ; HMDB42861 ; HMDB42912 ; HMDB43416 ; HMDB43421 ; HMDB43474 ; HMDB43479 ; HMDB43612 ; HMDB43614 ; HMDB43757 ; HMDB43759 ; HMDB43904 ;	TG(16:018:020:4(5Z8Z11Z14Z))iso6 ; TG(18:018:2(9Z12Z)18:2(9Z12Z))iso3 ; TG(16:1(9Z)18:2(9Z12Z)20:1(11Z))iso6 ; TG(18:1(9Z)18:1(9Z)18:2(9Z12Z))iso3 ; TG(18:1(9Z)18:2(9Z12Z)20:4(5Z8Z11Z14Z))iso6 ; TG(18:1(9Z)16:022:6(4Z7Z10Z13Z16Z19Z))iso6 ; TG(18:1(9Z)18:018:3(9Z12Z15Z))iso6 ; TG(18:1(9Z)18:1(11Z)18:2(9Z12Z))iso6 ; TG(18:1(9Z)18:1(9Z)20:5(5Z8Z11Z14Z17Z))iso3 ; TG(18:2(9Z12Z)16:022:5(7Z10Z13Z16Z19Z))iso6 ; TG(18:2(9Z12Z)16:020:2(11Z14Z))iso6 ; TG(18:2(9Z12Z)18:1(11Z)20:4(5Z8Z11Z14Z))iso6 ; TG(18:2(9Z12Z)18:2(9Z12Z)20:3(8Z11Z14Z))iso3 ; TG(20:4(5Z8Z11Z14Z)18:018:3(9Z12Z15Z))iso6 ; Quercetin-3-glucuronide ; Glycerol 12-di-(9Z12Z-octadecadienoate) 3-octadecanoate ; Quercetagitrin ; Glycerol 13-di-(9Z-octadecenoate) 2-(9Z12Z-octadecadienoate) ; Glycerol 13-di-(9Z12Z-octadecadienoate) 2-octadecanoate ; Myricetin 3-galactoside ; Myricetin 3-glucoside ; Tagetiin ; Acrimarine I ; TG(14:018:022:4(7Z10Z13Z16Z)) ; TG(14:020:020:4(5Z8Z11Z14Z)) ; TG(14:020:020:4(8Z11Z14Z17Z)) ; TG(14:022:018:4(6Z9Z12Z15Z)) ; TG(14:020:1(11Z)20:3(5Z8Z11Z)) ; TG(14:020:1(11Z)20:3n6) ; TG(14:020:1(11Z)22:6(4Z7Z10Z13Z16Z19Z)) ; TG(14:020:3(5Z8Z11Z)20:1(11Z)) ; TG(14:020:3(5Z8Z11Z)22:4(7Z10Z13Z16Z)) ; TG(14:022:1(13Z)18:3(6Z9Z12Z)) ; TG(14:022:1(13Z)18:3(9Z12Z15Z)) ; TG(14:018:3(6Z9Z12Z)22:1(13Z)) ; TG(14:020:3n620:1(11Z)) ; TG(14:020:3n622:4(7Z10Z13Z16Z)) ; TG(14:020:4(5Z8Z11Z14Z)20:0) ; TG(14:022:2(13Z16Z)20:5(5Z8Z11Z14Z17Z)) ; TG(14:022:4(7Z10Z13Z16Z)18:0) ; TG(14:022:4(7Z10Z13Z16Z)20:3(5Z8Z11Z)) ; TG(14:022:4(7Z10Z13Z16Z)20:3n6) ; TG(14:018:3(9Z12Z15Z)22:1(13Z)) ; TG(14:018:4(6Z9Z12Z15Z)22:0) ; TG(14:020:4(8Z11Z14Z17Z)20:0) ; TG(14:020:5(5Z8Z11Z14Z17Z)22:2(13Z16Z)) ; TG(14:022:6(4Z7Z10Z13Z16Z19Z)20:1(11Z)) ; TG(15:018:2(9Z12Z)22:5(4Z7Z10Z13Z16Z)) ; TG(15:018:2(9Z12Z)22:5(7Z10Z13Z16Z19Z)) ; TG(15:020:2n622:5(4Z7Z10Z13Z16Z)) ; TG(15:020:2n622:5(7Z10Z13Z16Z19Z)) ; TG(15:022:5(4Z7Z10Z13Z16Z)18:2(9Z12Z)) ; TG(15:022:5(4Z7Z10Z13Z16Z)20:2n6) ; TG(15:022:5(7Z10Z13Z16Z19Z)18:2(9Z12Z)) ; TG(15:022:5(7Z10Z13Z16Z19Z)20:2n6) ; TG(16:016:022:4(7Z10Z13Z16Z)) ; TG(16:018:020:4(8Z11Z14Z17Z)) ; TG(16:020:018:4(6Z9Z12Z15Z)) ; TG(16:018:1(11Z)20:3(5Z8Z11Z)) ; TG(16:018:1(11Z)20:3n6) ; TG(16:018:1(11Z)22:6(4Z7Z10Z13Z16Z19Z)) ; TG(16:018:1(9Z)20:3(5Z8Z11Z)) ;	M+2H+Na ; M+2H+Na ; M+2H+Na ; M+2H+Na ; M+3H ; M+3H ; M+2H+Na ; M+2H+Na ; M+3H ; M+3H ; M+2H+Na ; M+3H ; M+3H ; M+3H ; M+3ACN+2H ; M+2H+Na ; M+3ACN+2H ; M+2H+Na ; M+2H+Na ; M+3ACN+2H ; M+3ACN+2H ; M+3ACN+2H ; M+H+K ; M+2H+Na ; M+2H+Na ; M+2H+Na ; M+2H+Na ; M+2H+Na ; M+2H+Na ; M+3H ; M+2H+Na ; M+3H ; M+2H+Na ; M+2H+Na ; M+2H+Na ; M+2H+Na ; M+3H ; M+2H+Na ; M+3H ; M+2H+Na ; M+3H ; M+3H ; M+2H+Na ; M+2H+Na ; M+2H+Na ; M+3H ; M+3H ; M+3H ; M+3H ; M+3H ; M+3H ; M+3H ; M+3H ; M+3H ; M+3H ; M+2H+Na ; M+2H+Na ; M+2H+Na ; M+2H+Na ; M+2H+Na ; M+3H ; M+2H+Na ; M+2H+Na ; M+3H ; M+2H+Na ; M+2H+Na ; M+2H+Na ; M+3H ; M+3H ; M+2H+Na ; M+2H+Na ; M+3H ; M+2H+Na ; M+2H+Na ; M+2H+Na ; M+2H+Na ; M+3H ; M+3H ; M+2H+Na ; M+3H ; M+3H ; M+2H+Na ; M+3H ; M+3H ; M+2H+Na ; M+3H ; M+2H+Na ; M+2H+Na ; M+3H ; M+3H ; M+3H ; M+3H ; M+2H+Na ; M+2H+Na ; M+2H+Na ; M+2H+Na ; M+2H+Na ; M+2H+Na ; M+3H ; M+2H+Na ; M+2H+Na ; M+2H+Na ; M+2H+Na ; M+2H+Na ; M+3H ; M+2H+Na ; M+2H+Na ; M+3H ; M+3H ; M+2H+Na ; M+3H ; M+3H ; M+3H ; M+2H+Na ; M+2H+Na ; M+3H ; M+3H ; M+2H+Na ; M+3H	C57H102O6 ; C57H102O6 ; C57H102O6 ; C57H102O6 ; C59H100O6 ; C59H100O6 ; C57H102O6 ; C57H102O6 ; C59H100O6 ; C59H100O6 ; C57H102O6 ; C59H100O6 ; C59H100O6 ; C59H100O6 ; C21H20O13 ; C57H102O6 ; C21H20O13 ; C57H102O6 ; C57H102O6 ; C21H20O13 ; C21H20O13 ; C21H20O13 ; C34H31NO7 ; C57H102O6 ; C57H102O6 ; C57H102O6 ; C57H102O6 ; C57H102O6 ; C57H102O6 ; C59H100O6 ; C57H102O6 ; C59H100O6 ; C57H102O6 ; C57H102O6 ; C57H102O6 ; C59H100O6 ; C57H102O6 ; C59H100O6 ; C57H102O6 ; C59H100O6 ; C57H102O6 ; C59H100O6 ; C57H102O6 ; C57H102O6 ; C57H102O6 ; C59H100O6 ; C59H100O6 ; C59H100O6 ; C59H100O6 ; C59H100O6 ; C59H100O6 ; C59H100O6 ; C59H100O6 ; C59H100O6 ; C59H100O6 ; C57H102O6 ; C57H102O6 ; C57H102O6 ; C57H102O6 ; C57H102O6 ; C59H100O6 ; C57H102O6 ; C57H102O6 ; C59H100O6 ; C57H102O6 ; C57H102O6 ; C57H102O6 ; C57H102O6 ; C59H100O6 ; C59H100O6 ; C57H102O6 ; C57H102O6 ; C59H100O6 ; C57H102O6 ; C57H102O6 ; C57H102O6 ; C57H102O6 ; C59H100O6 ; C59H100O6 ; C57H102O6 ; C59H100O6 ; C59H100O6 ; C57H102O6 ; C59H100O6 ; C59H100O6 ; C57H102O6 ; C59H100O6 ; C57H102O6 ; C57H102O6 ; C59H100O6 ; C59H100O6 ; C59H100O6 ; C57H102O6 ; C57H102O6 ; C57H102O6 ; C57H102O6 ; C57H102O6
186.07580	1.695201	0.727688	5.5123	0.000257	3.5896	0.00753	186.0758	HMDB00393 ; HMDB00522 ; HMDB01167 ; HMDB02266 ; HMDB06112 ; HMDB06240 ; HMDB13311 ; HMDB28962 ; HMDB29088 ; HMDB29171 ; HMDB29739 ; HMDB29784 ; HMDB31257 ; HMDB31647 ; HMDB33019 ; HMDB33110 ; HMDB33215 ; HMDB33220 ; HMDB33221 ; HMDB33644 ; HMDB34772 ; HMDB34988 ; HMDB35025 ; HMDB35043 ; HMDB35044 ; HMDB35048 ; HMDB35049 ; HMDB35502 ; HMDB35634 ; HMDB36232 ; HMDB36331 ; HMDB37004 ; HMDB37773 ; HMDB38080 ; HMDB38747 ; HMDB38980 ; HMDB38981 ; HMDB39055 ; HMDB39062 ; HMDB39622 ; HMDB40207 ; HMDB40944 ; HMDB60012	3-Hexenedioic acid ; 3-Methylglutaconic acid ; Pyruvaldehyde ; (E)-2-Methylglutaconic acid ; Malondialdehyde ; Demethylated antipyrine ; trans-2-Hexenedioic acid ; Lysyl-Tryptophan ; Tryptophyl-Lysine ; 3-Hydroxyadipic acid 36-lactone ; 1H-Indole-3-acetamide ; Unshuoside A ; Dimethyl fumarate ; Acrylic acid ; 7-Hydroxyterpineol 8-glucoside ; (1S2S4R)-18-Epoxy-p-menthan-2-ol glucoside ; 18-Epoxy-p-menthan-4-ol glucoside ; (1R2R4S6R)-26-Fenchanediol 2-O-b-D-glucoside ; (1R2S4R5S)-25-Fenchanediol 2-O-b-D-glucoside ; (1S2S4S5S)-24-Thujanediol 4-O-beta-D-Glucopyranoside ; (1R3S4S)-18-Epoxy-p-menthan-3-ol glucoside ; Gibberellin A60 ; 6Z-8-Hydroxygeraniol 8-O-glucoside ; Gibberellin A58 ; Gibberellin A63 ; Gibberellin A54 ; Gibberellin A67 ; Sambucus nigra Degraded cyanogenic glycosides (2-Epimer) ; Betulalbuside A ; Maleic acid homopolymer ; 6-O-Acetylholocalin ; Gibberellin A77 ; 8-Angeloylegelolide ; Gibberellin A81 ; (2E4E7R)-27-Dimethyl-24-octadiene-18-diol 8-O-b-D-glucopyranoside ; (4R6S)-p-Menth-1-ene-46-diol 4-glucoside ; (S)-p-Menth-1-ene-47-diol 4-glucoside ; (1S2R4R)-p-Menth-8-ene-210-diol 2-glucoside ; Triphasiol ; Gibberellin A90 ; Ethyl hydrogen fumarate ; 3-Epigibberellin A1 ; Neomenthol-glucuronide	M+ACN+H ; M+ACN+H ; 2M+ACN+H ; M+ACN+H ; 2M+ACN+H ; 2M+H+Na ; M+ACN+H ; M+H+K ; M+H+K ; M+ACN+H ; 2M+H+Na ; M+H+K ; M+ACN+H ; 2M+ACN+H ; M+H+K ; M+H+K ; M+H+K ; M+H+K ; M+H+K ; M+H+K ; M+H+K ; M+H+Na ; M+H+K ; M+H+Na ; M+H+Na ; M+H+Na ; M+H+Na ; M+H+NH4 ; M+H+K ; M+ACN+H ; M+H+NH4 ; M+H+Na ; M+H+Na ; M+H+Na ; M+H+K ; M+H+K ; M+H+K ; M+H+K ; M+H+Na ; M+H+Na ; M+ACN+H ; M+H+Na ; M+H+K	C6H8O4 ; C6H8O4 ; C3H4O2 ; C6H8O4 ; C3H4O2 ; C10H10N2O ; C6H8O4 ; C17H24N4O3 ; C17H24N4O3 ; C6H8O4 ; C10H10N2O ; C16H28O7 ; C6H8O4 ; C3H4O2 ; C16H28O7 ; C16H28O7 ; C16H28O7 ; C16H28O7 ; C16H28O7 ; C16H28O7 ; C16H28O7 ; C19H24O6 ; C16H28O7 ; C19H24O6 ; C19H24O6 ; C19H24O6 ; C19H24O6 ; C16H19NO8 ; C16H28O7 ; C6H8O4 ; C16H19NO8 ; C19H24O6 ; C19H24O6 ; C19H24O6 ; C16H28O7 ; C16H28O7 ; C16H28O7 ; C16H28O7 ; C19H24O6 ; C19H24O6 ; C6H8O4 ; C19H24O6 ; C16H28O7
249.15400	1.637117	0.367135	5.5078	0.000259	3.5868	0.00753	249.154	HMDB00520 ; HMDB00524 ; HMDB00655 ; HMDB01231 ; HMDB01968 ; HMDB13267 ; HMDB14474 ; HMDB28744 ; HMDB29122 ; HMDB29851 ; HMDB32669 ; HMDB35142 ; HMDB35888 ; HMDB35995 ; HMDB37065 ; HMDB38569	5a-Cholestane-3a7a12a25-tetrol ; 5b-Cholestane-3a7a12a25-tetrol ; Cholestane-371225-tetrol ; 27-Deoxy-5b-cyprinol ; 5b-Cholestane-3a7a12a23-Tetrol ; N-Decanoylglycine ; Ethambutol ; Asparaginyl-Valine ; Valyl-Asparagine ; 27-Deoxy-5alpha-cyprinol ; (3beta5alpha9alpha22E24R)-359-Trihydroxy-23-methylergosta-722-dien-6-one ; (3beta17alpha23S)-1723-Epoxy-329-dihydroxy-27-norlanost-8-en-24-one ; Tyromycic acid ; 2-(Methoxycarbonyl)-5-methyl-24-bis(3-methyl-2-butenyl)-6-(2-methyl-1-oxopropyl)-5-(4-methyl-3-pentenyl)cyclohexanone ; 3-Oxo-1218-ursadien-28-oic acid ; PC-M5	M+Na+K ; M+Na+K ; M+Na+K ; M+Na+K ; M+Na+K ; 2M+H+K ; M+2Na-H ; M+NH4 ; M+NH4 ; M+Na+K ; M+H+K ; M+H+K ; M+2Na ; M+H+K ; M+2Na ; M+H+NH4	C27H48O4 ; C27H48O4 ; C27H48O4 ; C27H48O4 ; C27H48O4 ; C12H23NO3 ; C10H24N2O2 ; C9H17N3O4 ; C9H17N3O4 ; C27H48O4 ; C29H46O4 ; C29H46O4 ; C30H44O3 ; C29H46O4 ; C30H44O3 ; C29H37NO5
297.06925	1.693583	0.79988	-5.5052	0.00026	3.5852	0.00753	297.0693	HMDB14576 ; HMDB34711 ; HMDB12710 ; HMDB13852 ; HMDB28818 ; HMDB28825 ; HMDB28972 ; HMDB33980 ; HMDB37815 ; HMDB38370 ; HMDB38592 ; HMDB38850 ; HMDB41447 ; HMDB59927 ; HMDB60529 ; HMDB60985	Trifluridine ; Protoleucomelone ; 3-Dehydroquinate ; Sulfamethoxazole N4-hydroxylamine ; Glutamyl-Glutamate ; Glutamyl-Methionine ; Methionyl-Glutamate ; 7-Methoxy-2-methylisoflavone ; 5-(Methylthio)-2-(methylthio)methyl-2-pentenal ; Procyanidin C1 ; Brassicanal B ; Cycasin ; (E)-4-5-(4-Hydroxyphenoxy)-3-penten-1-ynylphenol ; 2-Hydroxy-2-(2-oxopropyl)butanedioic acid ; 5-Hydroxysulfamethoxazole ; sulfamethoxazole hydroxylamine	M+H ; M+2H ; 3M+H+Na ; 2M+3H2O+2H ; M+Na ; 2M+H+K ; 2M+H+K ; 2M+Na+K ; 3M+H+Na ; M+2H+Na ; M+ACN+Na ; M+2Na-H ; 2M+Na+K ; 3M+H+Na ; 2M+3H2O+2H ; 2M+3H2O+2H	C10H11F3N2O5 ; C30H24O13 ; C7H10O6 ; C10H11N3O4S ; C10H14N2O7 ; C10H17N2O5S ; C10H17N2O5S ; C17H14O3 ; C8H14OS2 ; C45H38O18 ; C12H11NO2S ; C8H16N2O7 ; C17H14O3 ; C7H10O6 ; C10H11N3O4S ; C10H11N3O4S
131.02749	1.680312	0.281694	5.5016	0.000261	3.583	0.00753	131.0275	HMDB00630 ; HMDB11625 ; HMDB13989 ; HMDB14558 ; HMDB30647 ; HMDB32035 ; HMDB32656 ; HMDB37723 ; HMDB38017	Cytosine ; Dimethylarsinate ; O-Desmethylnaproxen ; Acetohexamide ; Artemidinol ; 6-(34-Methylenedioxyphenyl)-35-hexadien-2-one ; (Z)-6-(2-Methoxyvinyl)-7-methyl-2H-1-benzopyran-2-one ; 2-Phenylethyl 2-furancarboxylate ; Ethyl 2-phenyl-3-furancarboxylate	2M+H+K ; M+3ACN+2H ; M+2Na ; M+3Na ; M+2Na ; M+2Na ; M+2Na ; M+2Na ; M+2Na	C4H5N3O ; C2H6AsO2 ; C13H12O3 ; C15H20N2O4S ; C13H12O3 ; C13H12O3 ; C13H12O3 ; C13H12O3 ; C13H12O3

322.565429	1.666224	0.409654	-5.4996	0.000262	3.5818	0.00753	NA	NA	NA	NA	NA
178.965533	1.69784	0.427114	5.4993	0.000262	3.5816	0.00753	NA	NA	NA	NA	NA
434.772194	1.688865	0.75656	5.4899	0.000266	3.5759	0.007584	434.7722	HMDB11470 ; HMDB11500 ; HMDB32255 ; HMDB34601 ; HMDB35981	LysoPE(0:014:0) ; LysoPE(14:00:0) ; N-(Ethoxycarbonyl)methyl)-p-menthane-3-carboxamide ; Hebevinoside XI ; Hebevinoside V	2M+H+NH4 ; 2M+H+NH4 ; 3M+Na+K ; M+H+NH4 ; M+H+NH4	C19H40NO7P ; C19H40NO7P ; C15H27NO3 ; C46H74O14 ; C46H74O14
424.743415	1.680045	0.924022	5.4861	0.000267	3.5736	0.007584	424.7434	HMDB12380 ; HMDB12390 ; HMDB12394 ; HMDB12400 ; HMDB12404 ; HMDB12424 ; HMDB12434 ; HMDB14652 ; HMDB15116	PS(18:018:2(9Z12Z)) ; PS(18:1(9Z)18:1(9Z)) ; PS(18:1(9Z)20:4(5Z8Z11Z14Z)) ; PS(18:2(9Z12Z)18:0) ; PS(18:2(9Z12Z)20:3(8Z11Z14Z)) ; PS(20:3(8Z11Z14Z)18:2(9Z12Z)) ; PS(20:4(5Z8Z11Z14Z)18:1(9Z)) ; Acetyldigitoxin ; Physostigmine	M+Na+K ; M+Na+K ; M+H+K ; M+Na+K ; M+H+K ; M+H+K ; M+H+K ; M+ACN+2H ; 3M+H+Na	C42H78NO10P ; C42H78NO10P ; C44H76NO10P ; C42H78NO10P ; C44H76NO10P ; C44H76NO10P ; C44H76NO10P ; C43H66O14 ; C15H21N3O2
708.077592	1.59986	0.085919	-5.4386	0.000285	3.5445	0.007985	708.0776	HMDB02135 ; HMDB32937 ; HMDB33342 ; HMDB39421	S-(3-oxo-3-carboxy-n-propyl)cysteine ; Methyl 4-chloro-1H-indole-3-acetate ; Mollicellin E ; Fenugreekine	3M+2Na-H ; 3M+K ; 3M+2K ; M+2Na-H	C7H11NO5S ; C11H10ClNO2 ; C22H19ClO8 ; C21H27N7O14P2
424.608542	1.694146	0.571598	-5.4382	0.000286	3.5443	0.007985	424.6085	HMDB04896	Ganglioside GA2 (d18:126:0)	M+3Na	C64H120N2O18
778.540788	1.645204	0.559122	5.4375	0.000286	3.5439	0.007985	778.5408	HMDB07892 ; HMDB07923 ; HMDB07924 ; HMDB08017 ; HMDB08142 ; HMDB08172 ; HMDB08173 ; HMDB08205 ; HMDB08206 ; HMDB08237 ; HMDB08496 ; HMDB08657 ; HMDB08690 ; HMDB08722 ; HMDB00564 ; HMDB07871 ; HMDB07881 ; HMDB07882 ; HMDB07913 ; HMDB07974 ; HMDB07975 ; HMDB08006 ; HMDB08031 ; HMDB08134 ; HMDB08166 ; HMDB08199 ; HMDB08329 ; HMDB08361 ; HMDB08394 ; HMDB08899 ; HMDB09219 ; HMDB12382 ; HMDB12422	PC(14:022:6(4Z7Z10Z13Z16Z19Z)) ; PC(14:1(9Z)22:5(4Z7Z10Z13Z16Z)) ; PC(14:1(9Z)22:5(7Z10Z13Z16Z19Z)) ; PC(16:1(9Z)20:5(5Z8Z11Z14Z17Z)) ; PC(18:2(9Z12Z)18:4(6Z9Z12Z15Z)) ; PC(18:3(6Z9Z12Z)18:3(6Z9Z12Z)) ; PC(18:3(6Z9Z12Z)18:3(9Z12Z15Z)) ; PC(18:3(9Z12Z15Z)18:3(6Z9Z12Z)) ; PC(18:3(9Z12Z15Z)18:3(9Z12Z15Z)) ; PC(18:4(6Z9Z12Z15Z)18:2(9Z12Z)) ; PC(20:5(5Z8Z11Z14Z17Z)16:1(9Z)) ; PC(22:5(4Z7Z10Z13Z16Z)14:1(9Z)) ; PC(22:5(7Z10Z13Z16Z19Z)14:1(9Z)) ; PC(22:6(4Z7Z10Z13Z16Z19Z)14:0) ; PC(16:016:0) ; PC(14:018:0) ; PC(14:020:3(5Z8Z11Z)) ; PC(14:020:3(8Z11Z14Z)) ; PC(14:1(9Z)20:2(11Z14Z)) ; PC(16:018:3(6Z9Z12Z)) ; PC(16:018:3(9Z12Z15Z)) ; PC(16:1(9Z)18:2(9Z12Z)) ; PC(18:014:0) ; PC(18:2(9Z12Z)16:1(9Z)) ; PC(18:3(6Z9Z12Z)16:0) ; PC(18:3(9Z12Z15Z)16:0) ; PC(20:2(11Z14Z)14:1(9Z)) ; PC(20:3(5Z8Z11Z)14:0) ; PC(20:3(8Z11Z14Z)14:0) ; PE(15:020:0) ; PE(20:015:0) ; PS(18:020:3(8Z11Z14Z)) ; PS(20:3(8Z11Z14Z)18:0)	M+H ; M+H ; M+H ; M+H ; M+H ; M+H ; M+H ; M+H ; M+H ; M+H ; M+H ; M+H ; M+H ; M+H ; M+2Na-H ; M+2Na-H ; M+Na ; M+Na ; M+Na ; M+Na ; M+Na ; M+Na ; M+2Na-H ; M+Na ; M+Na ; M+Na ; M+Na ; M+Na ; M+Na ; M+2Na-H ; M+2Na-H ; M+H-2H2O ; M+H-2H2O	C44H76NO8P ; C44H76NO8P ; C44H76NO8P ; C44H76NO8P ; C44H76NO8P ; C44H76NO8P ; C44H76NO8P ; C44H76NO8P ; C44H76NO8P ; C44H76NO8P ; C44H76NO8P ; C44H76NO8P ; C44H76NO8P ; C44H76NO8P ; C40H80NO8P ; C40H80NO8P ; C42H78NO8P ; C42H78NO8P ; C42H78NO8P ; C42H78NO8P ; C42H78NO8P ; C42H78NO8P ; C40H80NO8P ; C42H78NO8P ; C42H78NO8P ; C42H78NO8P ; C42H78NO8P ; C42H78NO8P ; C42H78NO8P ; C40H80NO8P ; C40H80NO8P ; C44H80NO10P ; C44H80NO10P
702.987562	1.628636	1.128286	-5.4333	0.000288	3.5413	0.007988	702.9876	HMDB11925 ; HMDB30492 ; HMDB31105 ; HMDB45857 ; HMDB46007 ; HMDB46413 ; HMDB46419 ; HMDB46557 ; HMDB46596 ; HMDB46707 ; HMDB46764 ; HMDB47131 ; HMDB47137 ; HMDB47159 ; HMDB47166 ; HMDB47283 ; HMDB47389 ; HMDB47490 ; HMDB47557 ; HMDB49371 ; HMDB49985 ; HMDB50423 ; HMDB50550 ; HMDB50568 ; HMDB51500 ; HMDB51596 ; HMDB51629 ; HMDB51987 ; HMDB52061 ; HMDB52077	Ganglioside GM3 (d18:025:0) ; Spirolide B ; Trierucin ; TG(20:024:1(15Z)22:2(13Z16Z)) ; TG(20:022:2(13Z16Z)24:1(15Z)) ; TG(22:024:020:3(5Z8Z11Z)) ; TG(22:024:020:3n6) ; TG(22:020:3(5Z8Z11Z)24:0) ; TG(22:022:1(13Z)22:2(13Z16Z)) ; TG(22:020:3n624:0) ; TG(22:022:2(13Z16Z)22:1(13Z)) ; TG(24:022:020:3(5Z8Z11Z)) ; TG(24:022:020:3n6) ; TG(24:024:018:3(6Z9Z12Z)) ; TG(24:024:018:3(9Z12Z15Z)) ; TG(24:020:1(11Z)22:2(13Z16Z)) ; TG(24:018:3(6Z9Z12Z)24:0) ; TG(24:022:2(13Z16Z)20:1(11Z)) ; TG(24:018:3(9Z12Z15Z)24:0) ; TG(18:1(11Z)24:1(15Z)24:1(15Z)) ; TG(18:1(9Z)24:1(15Z)24:1(15Z)) ; TG(20:1(11Z)24:022:2(13Z16Z)) ; TG(20:1(11Z)22:1(13Z)24:1(15Z)) ; TG(20:1(11Z)24:1(15Z)22:1(13Z)) ; TG(22:1(13Z)22:022:2(13Z16Z)) ; TG(22:1(13Z)20:1(11Z)24:1(15Z)) ; TG(22:1(13Z)22:1(13Z)22:1(13Z)) ; TG(24:1(15Z)20:022:2(13Z16Z)) ; TG(24:1(15Z)18:1(11Z)24:1(15Z)) ; TG(24:1(15Z)18:1(9Z)24:1(15Z))	M+3ACN+2H ; 2M+H+NH4 ; 2M+3H	C66H124N2O21 ; C42H63NO7 ; C69H128O6
160.999824	1.685754	0.81958	5.4118	0.000296	3.5281	0.008188	160.9998	HMDB01870 ; HMDB04215 ; HMDB11718 ; HMDB13856 ; HMDB13861 ; HMDB30595 ; HMDB30946 ; HMDB32918 ; HMDB33735 ; HMDB34170 ; HMDB39430 ; HMDB40876 ; HMDB41016 ; HMDB60253 ; HMDB60433 ; HMDB60588	Benzoic acid ; 3345-Tetrahydroxystilbene ; 4-Hydroxybenzaldehyde ; 4-Hydroxycyclophosphamide ; 4-Hydroxyifosfamide ; Suberenone ; (2E11Z)-Wyerone acid ; 3-(2-Furanyl)-2-propenal ; Graveolone ; 2-Hydroxybenzaldehyde ; 910-Dihydro-2357-Phenanthrenetetrol ; (R)-Apiumetin ; Eriobofuran ; 1-Methylpyrrolinium ; Aldophosphamide ; Aldoifosfamide	M+K ; M+2K ; M+K ; M+2Na ; M+2Na ; M+2K ; M+2K ; M+K ; M+2K ; M+K ; M+2K ; M+2K ; M+2K ; M+2K-H ; M+2Na ; M+2Na	C7H6O2 ; C14H12O4 ; C7H6O2 ; C7H15Cl2N2O3P ; C7H15Cl2N2O3P ; C14H12O4 ; C14H12O4 ; C7H6O2 ; C14H12O4 ; C7H6O2 ; C14H12O4 ; C14H12O4 ; C14H12O4 ; C5H10N ; C7H15Cl2N2O3P ; C7H15Cl2N2O3P
263.087348	1.678818	0.298507	-5.404	0.0003	3.5233	0.008234	263.0873	HMDB11164 ; HMDB30413 ; HMDB30419 ; HMDB00585 ; HMDB00728 ; HMDB00737 ; HMDB00815 ; HMDB28728 ; HMDB28770 ; HMDB29394 ; HMDB29989 ; HMDB29990 ; HMDB31367 ; HMDB33316 ; HMDB33614 ; HMDB33653 ; HMDB34494 ; HMDB35489 ; HMDB36571 ; HMDB38416 ; HMDB39424 ; HMDB28979 ; HMDB60842	L-beta-aspartyl-L-glutamic acid ; Na-L-Glutamyl-L-aspartic acid ; Ng-L-Glutamyl-L-aspartic acid ; Glucosylgalactosyl hydroxylysine ; Hydroxypropionic porphyrin III ; Heptacarboxylporphyrin I ; Pseudouroporphyrin ; Asparaginyl-Cysteine ; Cysteinyl-Asparagine ; gamma-L-Glutamyl-S-(2-carboxy-1-propyl)cysteinylglycine ; Dulciol B ; Dulciol A ; Linalool oxide D 3-apiosyl-(1-6)-glucoside ; Garcinone E ; Licoagrone ; 1-alpha-Acevaltrate ; Acevaltrate ; Linalool 36-oxide primeveroside ; Linalool 37-oxide beta-primeveroside ; 5-Hexenyl glucosinolate ; (gamma-Glutamyl-gamma-glutamyl)-S-methylcysteine ; Methionyl-Methionine ; N-Despyridinyl rosiglitazone	M+H ; M+H ; M+H ; M+H+K ; M+3H ; M+3H ; M+3H ; 2M+3H2O+2H ; 2M+3H2O+2H ; 2M+3H ; M+Na+K ; M+Na+K ; M+Na+K ; M+Na+K ; M+H+2Na ; M+2Na ; M+2Na ; M+Na+K ; M+Na+K ; M+3ACN+2H ; 2M+3H ; M+H-H2O ; M+H-H2O	C9H14N2O7 ; C9H14N2O7 ; C9H14N2O7 ; C18H34N2O13 ; C39H38N4O14 ; C39H38N4O14 ; C39H38N4O14 ; C7H13N3O4S ; C7H13N3O4S ; C14H23N3O8S ; C28H32O6 ; C28H32O6 ; C21H36O11 ; C28H32O6 ; C45H42O10 ; C24H32O10 ; C24H32O10 ; C21H36O11 ; C21H36O11 ; C13H23NO9S2 ; C14H23N3O8S ; C10H20N2O3S2 ; C13H16N2O3S
512.9381_2	1.655271	0.55277	-5.394	0.000304	3.5171	0.008306	NA	NA	NA	NA	NA

164.11102	1.625997	1.044564	-5.3852	0.000308	3.5117	0.00832	164.111	HMDB14410 ; HMDB15639 ; HMDB28846 ; HMDB28951 ; HMDB29667 ; HMDB31312 ; HMDB31446 ; HMDB31627 ; HMDB31628 ; HMDB32029 ; HMDB32149 ; HMDB32441 ; HMDB32544 ; HMDB32621 ; HMDB32624 ; HMDB32625 ; HMDB33962 ; HMDB34378 ; HMDB37621 ; HMDB38984 ; HMDB38985 ; HMDB38986 ; HMDB38987 ; HMDB38988 ; HMDB39465 ; HMDB39468 ; HMDB39782 ; HMDB40186 ; HMDB59896	Crotamiton ; Mepyramine ; Glycyl-Lysine ; Lysyl-Glycine ; 236-Trimethylphenol ; Benzyl ethyl ether ; p-Isopropylphenol ; 1-Phenyl-1-propanol ; 2-Phenyl-1-propanol ; 2-Isopropylphenol ; 1-Methoxy-24-dimethylbenzene ; Nona-246-trienal ; 246-Trimethylphenol ; (2-Methoxyethyl)benzene ; 2-Propylphenol ; 4-Propylphenol ; 3-Phenyl-1-propanol ; ()-1-(4-Methylphenyl)ethanol ; 4-Methyl-4-(methylthio)-2-pentanone ; 4-Ethyl-2-methylphenol ; 3-Ethyl-5-methylphenol ; 3-Ethyl-4-methylphenol ; 2-Ethyl-5-methylphenol ; 2-Ethyl-4-methylphenol ; S-Methyl hexanethioate ; S-Methyl 4-methylpentanethioate ; 2-(1-Pentenyl)furan ; 3-(Methylthio)hexanal ; 6-Ethyl-o-cresol	M+3ACN+2H ; M+ACN+2H ; M+3ACN+2H ; M+3ACN+2H ; 2M+3H2O+2H ; 2M+3H2O+2H ; 2M+3H2O+2H ; 2M+3H2O+2H ; 2M+3H2O+2H ; 2M+3H2O+2H ; 2M+3H2O+2H ; 2M+3H2O+2H ; 2M+3H2O+2H ; 2M+3H2O+2H ; 2M+3H2O+2H ; 2M+3H2O+2H ; 2M+3H2O+2H ; 2M+3H2O+2H ; M+NH4 ; 2M+3H2O+2H ; 2M+3H2O+2H ; 2M+3H2O+2H ; 2M+3H2O+2H ; 2M+3H2O+2H ; M+NH4 ; M+NH4 ; 2M+3H2O+2H ; M+NH4 ; 2M+3H2O+2H	C13H17NO ; C17H23N3O ; C8H17N3O3 ; C8H17N3O3 ; C9H12O ; C9H12O ; C9H12O ; C9H12O ; C9H12O ; C9H12O ; C9H12O ; C9H12O ; C9H12O ; C9H12O ; C9H12O ; C9H12O ; C9H12O ; C9H12O ; C7H14OS ; C9H12O ; C9H12O ; C9H12O ; C9H12O ; C9H12O ; C7H14OS ; C7H14OS ; C9H12O ; C7H14OS ; C9H12O
99.495605	1.693797	0.489664	5.3851	0.000308	3.5117	0.00832	NA	NA	NA	NA	NA
110.01250	1.655785	0.107603	5.3797	0.00031	3.5083	0.008339	110.0125	HMDB00392 ; HMDB01568 ; HMDB03269 ; HMDB13897 ; HMDB13902 ; HMDB13903 ; HMDB13904 ; HMDB28752 ; HMDB28815 ; HMDB29761 ; HMDB31293 ; HMDB31343 ; HMDB31352 ; HMDB31403 ; HMDB32164 ; HMDB32207 ; HMDB32266 ; HMDB32267 ; HMDB32268 ; HMDB32278 ; HMDB32533 ; HMDB32861 ; HMDB35422 ; HMDB36212 ; HMDB36236 ; HMDB37622 ; HMDB37624 ; HMDB37630 ; HMDB38036 ; HMDB38310 ; HMDB39793 ; HMDB39872 ; HMDB40212 ; HMDB40215 ; HMDB41912 ; HMDB59766 ; HMDB59908 ; HMDB60303 ; HMDB60322	2-Octenoic acid ; trans-2-Octenoic acid ; Nicotinuric acid ; 4-ene-Valproic acid ; 2-ene-Valproic acid ; (3Z)-2-Propylpent-3-enoic acid ; (3E)-2-Propylpent-3-enoic acid ; Aspartyl-Glutamate ; Glutamyl-Aspartate ; Ethyl ()-2-methyl-4-pentenoate ; 23-Octanedione ; Methyl cyclohexanecarboxylate ; Cyclohexyl acetate ; Cyclohexaneacetic acid ; Allyl valerate ; cis-5-Octenoic acid ; Ethyl (E)-3-hexenoate ; Ethyl 5-hexenoate ; Ethyl 2E-hexenoate ; Ethyl trans-2-methyl-2-pentenoate ; trans-3-Hexenyl acetate ; 2-Methoxynaphthalene ; 4-Butyl-gamma-butyrolactone ; 2-Methylpropyl (2E)-butenoate ; 2-Methylallyl butyrate ; Ethyl 2-methyl-3-pentenoate ; Isopropyl tiglate ; xi-Tetrahydro-3-propyl-2H-pyran-2-one ; 2-Propenyl 3-methylbutanoate ; xi-Tetrahydro-6-propyl-2H-pyran-2-one ; (E)-4-Octenoic acid ; Methyl 2-heptenoate ; 2-Hexenyl acetate ; cis-3-Hexenyl acetate ; Isonicotinylglycine ; Picolinoylglycine ; Ethyl-3-hexanoate ; (2-Naphthyl)methanol ; 1-Hydroxymethylnaphthalene	M+2K ; M+2K ; M+H+K ; M+2K ; M+2K ; M+2K ; M+2K ; M+3Na ; M+3Na ; M+2K ; M+2K ; M+2K ; M+2K ; M+2K ; M+2K ; M+2K ; M+2K ; M+2K ; M+2K ; M+2K ; M+2K ; M+2K ; M+Na+K ; M+2K ; M+2K ; M+2K ; M+2K ; M+2K ; M+2K ; M+2K ; M+2K ; M+2K ; M+2K ; M+H+K ; M+H+K ; M+2K ; M+Na+K ; M+Na+K	C8H14O2 ; C8H14O2 ; C8H8N2O3 ; C8H14O2 ; C8H14O2 ; C8H14O2 ; C8H14O2 ; C9H13N2O7 ; C9H13N2O7 ; C8H14O2 ; C8H14O2 ; C8H14O2 ; C8H14O2 ; C8H14O2 ; C8H14O2 ; C8H14O2 ; C8H14O2 ; C8H14O2 ; C8H14O2 ; C8H14O2 ; C8H14O2 ; C11H10O ; C8H14O2 ; C8H14O2 ; C8H14O2 ; C8H14O2 ; C8H14O2 ; C8H14O2 ; C8H14O2 ; C8H14O2 ; C8H14O2 ; C8H14O2 ; C8H14O2 ; C8H8N2O3 ; C8H8N2O3 ; C8H14O2 ; C11H10O ; C11H10O
248.05067	1.663824	0.816651	-5.3662	0.000316	3.5	0.00843	248.0507	HMDB15017 ; HMDB01866 ; HMDB13198 ; HMDB14446 ; HMDB29650 ; HMDB37264 ; HMDB60026 ; HMDB60715	Emtricitabine ; 34-Dihydroxymandelic acid ; 4-O-Methylgallic acid ; Flucloxacillin ; Methyl 246-trihydroxybenzoate ; 2-Hydroxy-47-dimethoxy-2H-14-benzoxazin-3(4H)-one ; Vanilloylglycine ; 2-Hydroxy-4-trifluoromethyl benzoic acid	M+H ; M+ACN+Na ; M+ACN+Na ; M+ACN+2H ; M+ACN+Na ; M+Na ; M+Na ; M+ACN+H	C8H10FN3O3S ; C8H8O5 ; C8H8O5 ; C19H17ClFN3O5S ; C8H8O5 ; C10H11NO5 ; C10H11NO5 ; C8H5F3O3
659.46263	1.640616	0.841883	-5.3593	0.000319	3.4957	0.00843	659.4626	HMDB31060	(R)-2-Hydroxysterculic acid	2M+K	C19H34O3
293.20887	1.634472	1.023767	5.3574	0.00032	3.4945	0.00843	293.2089	HMDB13131 ; HMDB40820	Hydroxyhexanoycarnitine ; Nb-Pentacosanoyltryptamine	M+NH4 ; M+Na+K	C13H25NO5 ; C35H60N2O
159.00087	1.66632	0.05088	5.3571	0.00032	3.4944	0.00843	159.0009	HMDB32559	()-2-(1-Methylpropyl)-46-dinitrophenol	M+2K	C10H12N2O5
770.60460	1.639471	0.288702	5.3518	0.000323	3.4911	0.008433	770.6046	HMDB08095 ; HMDB08096 ; HMDB08128 ; HMDB08129 ; HMDB08160 ; HMDB08357 ; HMDB11217 ; HMDB11244 ; HMDB11275 ; HMDB11276 ; HMDB11308 ; HMDB11309 ; HMDB13425 ; HMDB13429 ; HMDB39527 ; HMDB07790 ; HMDB07846 ; HMDB13405 ; HMDB56281	PC(18:1(11Z)P-18:1(11Z)) ; PC(18:1(11Z)P-18:1(9Z)) ; PC(18:1(9Z)P-18:1(11Z)) ; PC(18:1(9Z)P-18:1(9Z)) ; PC(18:2(9Z12Z)P-18:0) ; PC(20:2(11Z14Z)P-16:0) ; PC(P-16:020:2(11Z14Z)) ; PC(P-18:018:2(9Z12Z)) ; PC(P-18:1(11Z)18:1(11Z)) ; PC(P-18:1(11Z)18:1(9Z)) ; PC(P-18:1(9Z)18:1(11Z)) ; PC(P-18:1(9Z)18:1(9Z)) ; PC(o-18:1(11Z)18:2(9Z12Z)) ; PC(o-18:1(9Z)18:2(9Z12Z)) ; 1-O-Hexadecyl-2-O-dihomogammalinolenoylglycero-3-phosphocholine ; DG(22:6(4Z7Z10Z13Z16Z19Z)24:1(15Z)0:0) ; DG(24:1(15Z)22:6(4Z7Z10Z13Z16Z19Z)0:0) ; PC(o-16:018:0) ; DG(24:1n90:022:6n3)	M+H ; M+H ; M+H ; M+H ; M+H ; M+H ; M+H ; M+H ; M+H ; M+H ; M+H ; M+H ; M+H ; M+H ; M+H ; 2M+H+K ; 2M+H+K ; M+Na ; 2M+H+K	C44H84NO7P ; C44H84NO7P ; C44H84NO7P ; C44H84NO7P ; C44H84NO7P ; C44H84NO7P ; C44H84NO7P ; C44H84NO7P ; C44H84NO7P ; C44H84NO7P ; C44H84NO7P ; C44H84NO7P ; C44H84NO7P ; C44H84NO7P ; C44H84NO7P ; C49H82O5 ; C49H82O5 ; C42H86NO7P ; C49H82O5
418.74781	1.649883	0.480497	5.3495	0.000324	3.4897	0.008433	418.7478	HMDB40793	Acidissiminol epoxide	2M+H+NH4	C25H31NO4
782.57520	1.666119	0.107653	5.3316	0.000332	3.4786	0.008606	782.5752	HMDB07889 ; HMDB07982 ; HMDB07983 ; HMDB08013 ; HMDB08014 ; HMDB08042 ; HMDB08073 ; HMDB08074 ; HMDB08106 ; HMDB08107 ; HMDB08138 ; HMDB08169 ; HMDB08170 ; HMDB08202 ; HMDB08203 ; HMDB08234 ; HMDB08365 ; HMDB08398 ; HMDB08429 ; HMDB08462 ; HMDB08623 ; HMDB07699 ; HMDB07700 ; HMDB07727 ; HMDB07756 ; HMDB08984 ; HMDB08985 ; HMDB09114 ; HMDB11343 ; HMDB11405 ; HMDB11438 ; HMDB15064 ; HMDB56352 ; HMDB56357	PC(14:022:4(7Z10Z13Z16Z)) ; PC(16:020:4(5Z8Z11Z14Z)) ; PC(16:020:4(8Z11Z14Z17Z)) ; PC(16:1(9Z)20:3(5Z8Z11Z)) ; PC(16:1(9Z)20:3(8Z11Z14Z)) ; PC(18:018:4(6Z9Z12Z15Z)) ; PC(18:1(11Z)18:3(6Z9Z12Z)) ; PC(18:1(11Z)18:3(9Z12Z15Z)) ; PC(18:1(9Z)18:3(6Z9Z12Z)) ; PC(18:1(9Z)18:3(9Z12Z15Z)) ; PC(18:2(9Z12Z)18:2(9Z12Z)) ; PC(18:3(6Z9Z12Z)18:1(11Z)) ; PC(18:3(6Z9Z12Z)18:1(9Z)) ; PC(18:3(9Z12Z15Z)18:1(11Z)) ; PC(18:3(9Z12Z15Z)18:1(9Z)) ; PC(18:4(6Z9Z12Z15Z)18:0) ; PC(20:3(5Z8Z11Z)16:1(9Z)) ; PC(20:3(8Z11Z14Z)16:1(9Z)) ; PC(20:4(5Z8Z11Z14Z)16:0) ; PC(20:4(8Z11Z14Z17Z)16:0) ; PC(22:4(7Z10Z13Z16Z)14:0) ; DG(22:4(7Z10Z13Z16Z)22:5(4Z7Z10Z13Z16Z)0:0) ; DG(22:4(7Z10Z13Z16Z)22:5(7Z10Z13Z16Z19Z)0:0) ; DG(22:5(4Z7Z10Z13Z16Z)22:4(7Z10Z13Z16Z)0:0) ; DG(22:5(7Z10Z13Z16Z19Z)22:4(7Z10Z13Z16Z)0:0) ; PE(16:1(9Z)P-18:1(11Z)) ; PE(16:1(9Z)P-18:1(9Z)) ; PE(18:2(9Z12Z)P-16:0) ; PE(P-16:018:2(9Z12Z)) ; PE(P-18:1(11Z)16:1(9Z)) ; PE(P-18:1(9Z)16:1(9Z)) ; Misoprostol ; DG(22:4n60:022:5n6) ; DG(22:4n60:022:5n3)	M+H ; M+ACN+Na ; M+ACN+Na ; M+ACN+Na ; M+ACN+Na ; M+2ACN+H ; M+2ACN+H ; M+2ACN+H ; M+2ACN+H ; M+2ACN+H ; M+2ACN+H ; 2M+NH4 ; M+ACN+Na ; M+ACN+Na	C44H80NO8P ; C47H74O5 ; C47H74O5 ; C47H74O5 ; C47H74O5 ; C39H74NO7P ; C39H74NO7P ; C39H74NO7P ; C39H74NO7P ; C39H74NO7P ; C39H74NO7P ; C22H38O5 ; C47H74O5 ; C47H74O5

156.07677	1.62783	1.003933	5.3235	0.000336	3.4736	0.008607	156.0768	HMDB00177 ; HMDB34267 ; HMDB00076 ; HMDB00301 ; HMDB01988 ; HMDB02730 ; HMDB03646 ; HMDB15391 ; HMDB28704 ; HMDB28725 ; HMDB28733 ; HMDB28826 ; HMDB28880 ; HMDB28994 ; HMDB29353 ; HMDB29354 ; HMDB31176 ; HMDB31338 ; HMDB33249 ; HMDB34085 ; HMDB34174 ; HMDB36594 ; HMDB37114 ; HMDB40433 ; HMDB60245 ; HMDB60493 ; HMDB04225 ; HMDB29384	L-Histidine ; L-2-Amino-3-(1-pyrazolyl)propanoic acid ; Dihydrouracil ; Urocanic acid ; 4-Hydroxycyclohexylcarboxylic acid ; Nicotinamide N-oxide ; N-Methylhydantoin ; Decitabine ; Arginyl-Asparagine ; Asparaginyl-Arginine ; Asparaginyl-Histidine ; Glutamyl-Phenylalanine ; Histidinyl-Asparagine ; Phenylalanyl-Glutamate ; Galactosyl 4-hydroxyproline ; 4-Hydroxyproline galactoside ; Tetrahydrofurfuryl acetate ; 5-Oxo-2(5H)-isoxazolepropanenitrile ; 6-Chloro-N-(1-methylethyl)-135-triazine-24-diamine ; (3R)-34-Dihydroxy-3-(hydroxymethyl)butanenitrile 4-glucoside ; (E)-Urocanic acid ; Botryodiplodin ; Methyl (R)-3-methyl-2-oxopentanoate ; Ethyl levulinate ; L-3-Cyanoalanine ; N-Acetylmuramate ; 2-Oxoarginine ; 4-Hydroxycitrulline	M+H ; M+H ; M+ACN+H ; M+NH4 ; 2M+H+Na ; M+NH4 ; M+ACN+H ; M+2ACN+2H ; M+H+Na ; M+H+Na ; M+ACN+2H ; M+H+NH4 ; M+ACN+2H ; M+H+NH4 ; M+H+NH4 ; M+H+NH4 ; 2M+H+Na ; M+NH4 ; M+3ACN+2H ; M+H+NH4 ; M+NH4 ; 2M+H+Na ; 2M+H+Na ; 2M+H+Na ; M+ACN+H ; M+H+NH4 ; M+H-H2O ; M+H-2H2O	C6H9N3O2 ; C6H9N3O2 ; C4H6N2O2 ; C6H6N2O2 ; C7H12O3 ; C6H6N2O2 ; C4H6N2O2 ; C8H12N4O4 ; C10H20N6O4 ; C10H20N6O4 ; C10H15N5O4 ; C14H17N2O5 ; C10H15N5O4 ; C14H17N2O5 ; C11H19NO8 ; C11H19NO8 ; C7H12O3 ; C6H6N2O2 ; C6H10ClN5 ; C11H19NO8 ; C6H6N2O2 ; C7H12O3 ; C7H12O3 ; C7H12O3 ; C4H6N2O2 ; C11H19NO8 ; C6H11N3O3 ; C6H13N3O4
552.72702	1.690083	0.725665	-5.3208	0.000337	3.4719	0.008607	552.727	HMDB06991 ; HMDB06992 ; HMDB06999 ; HMDB07000	CDP-DG(18:1(11Z)20:4(5Z8Z11Z14Z)) ; CDP-DG(18:1(11Z)20:4(8Z11Z14Z17Z)) ; CDP-DG(18:1(9Z)20:4(5Z8Z11Z14Z)) ; CDP-DG(18:1(9Z)20:4(8Z11Z14Z17Z))	M+2K ; M+2K ; M+2K ; M+2K	C50H83N3O15P2 ; C50H83N3O15P2 ; C50H83N3O15P2 ; C50H83N3O15P2
200.54592	1.638068	0.135937	5.3207	0.000337	3.4719	0.008607	200.5459	HMDB15565 ; HMDB37200 ; HMDB37203	Etoricoxib ; 2-Galloyl-14-galactarolactone methyl ester ; 5-Methoxy-galloyl-14-galactarolactone	M+ACN+2H ; M+ACN+2H ; M+ACN+2H	C18H15ClN2O2S ; C14H14O11 ; C14H14O11
317.99778	1.67435	0.011148	5.313	0.000341	3.467	0.008659	317.9978	HMDB37333 ; HMDB40289	8-Hydroxyluteolin 4-methyl ether 8-glucoside-3-sulfate ; 2-(Ethylsulfinylmethyl)phenyl methylcarbamate	M+2K ; M+2K-H	C22H22O15S ; C11H15NO3S
269.22607	1.666166	0.521561	5.3066	0.000344	3.4631	0.008694	269.2261	HMDB00561 ; HMDB02297 ; HMDB02935 ; HMDB03000 ; HMDB03993 ; HMDB35776 ; HMDB36925 ; HMDB07645 ; HMDB07673 ; HMDB07813 ; HMDB07841 ; HMDB34555 ; HMDB47907 ; HMDB48391 ; HMDB56125 ; HMDB56252	B-Carotene ; cis-yy-Carotene ; cis-bb-Carotene ; Lycopene ; Alpha-Carotene ; Prolycopene ; delta-Carotene ; DG(22:1(13Z)24:1(15Z)0:0) ; DG(22:2(13Z16Z)24:00:0) ; DG(24:022:2(13Z16Z)0:0) ; DG(24:1(15Z)22:1(13Z)0:0) ; (25S)-26-Hydroxy-24-methylenecycloartan-3-one ; TG(14:1(9Z)14:1(9Z)o-18:0) ; TG(14:1(9Z)o-18:014:1(9Z)) ; DG(24:00:022:2n6) ; DG(22:1n90:024:1n9)	M+2H ; M+2H ; M+2H ; M+2H ; M+2H ; M+2H ; M+2H ; M+H+2Na ; M+H+2Na ; M+H+2Na ; M+H+2Na ; M+2ACN+2H ; M+H+2Na ; M+H+2Na ; M+H+2Na ; M+H+2Na	C40H56 ; C40H56 ; C40H56 ; C40H56 ; C40H56 ; C40H56 ; C40H56 ; C49H92O5 ; C49H92O5 ; C49H92O5 ; C49H92O5 ; C31H50O2 ; C49H92O5 ; C49H92O5 ; C49H92O5 ; C49H92O5
141.52753	1.655644	0.175621	5.2967	0.000349	3.4569	0.008773	141.5275	HMDB00089 ; HMDB00223 ; HMDB04231 ; HMDB14798 ; HMDB15122 ; HMDB31770 ; HMDB33701 ; HMDB38142 ; HMDB39791 ; HMDB39792 ; HMDB39796 ; HMDB60478	Cytidine ; Oxalacetic acid ; Pantothenol ; Metaxalone ; Cytarabine ; Carbofuran ; Hydrocotarnine ; 58-Dihydro-6-(4-methyl-3-pentenyl)-1234-tetrathiocin ; 23-Dihydro-5-methyl-3-thiophenethiol ; 45-Dihydro-2-methyl-3-thiophenethiol ; 45-Dihydro-5-methyl-3-thiophenethiol ; gamma-Glutamyl-beta-cyanoalanine	M+H+K ; 2M+H+NH4 ; M+2K ; M+Na+K ; M+H+K ; M+Na+K ; M+Na+K ; M+H+NH4 ; 2M+H+NH4 ; 2M+H+NH4 ; 2M+H+NH4 ; M+H+K	C9H13N3O5 ; C4H4O5 ; C9H19NO4 ; C12H15NO3 ; C9H13N3O5 ; C12H15NO3 ; C12H15NO3 ; C10H16S4 ; C5H8S2 ; C5H8S2 ; C5H8S2 ; C9H13N3O5
291.52740	1.588903	0.221051	-5.2864	0.000354	3.4505	0.008818	NA	NA	NA	NA	NA
369.85619	1.635205	0.945521	-5.2815	0.000357	3.4475	0.008818	369.8562	HMDB33599 ; HMDB34308 ; HMDB36290 ; HMDB38607	Vignatic acid A ; Neomacrostemonoside D ; Campesteryl brassidate ; Soyasaponin A2	2M+3H ; M+3H ; M+H+NH4 ; M+3H	C30H39N3O7 ; C53H86O24 ; C50H88O2 ; C53H86O24
198.05262	1.626048	1.193551	-5.2812	0.000357	3.4473	0.008818	198.0526	HMDB39047 ; HMDB00197 ; HMDB00226 ; HMDB00339 ; HMDB00678 ; HMDB00927 ; HMDB04073 ; HMDB11757 ; HMDB12154 ; HMDB12175 ; HMDB29230 ; HMDB29409 ; HMDB29412 ; HMDB31345 ; HMDB31756 ; HMDB32469 ; HMDB33431 ; HMDB33558 ; HMDB35321 ; HMDB36384 ; HMDB37499 ; HMDB38593 ; HMDB38625 ; HMDB38628 ; HMDB39475 ; HMDB40972 ; HMDB41026 ; HMDB41370 ; HMDB41414 ; HMDB41487 ; HMDB41723	9-Hydroxy-4-methoxypsoralen 9-glucoside ; Indoleacetic acid ; Orotic acid ; 2-Methylbutyrylglycine ; Isovalerylglycine ; Valerylglycine ; 5-Hydroxyindoleacetaldehyde ; N-Acetylvaline ; 3-Dehydrocarnitine ; 5-Acetamidovalerate ; 4-Hydroxystachydrine ; Turicine ; Betonicine ; Calystegine A6 ; Veranisatin C ; Phthalide ; Moschamindole ; 4-O-Methylgalactinol ; Galactopinitol B ; Calystegine A7 ; 3-N-Acetylfusarochromanone ; Calystegin A3 ; Medicanine ; Hydroxymethyl indol-3-yl ketone ; Galactopinitol A ; 1-Methoxy-1H-indole-3-carboxaldehyde ; Erinapyrone C ; Na-p-Hydroxycoumaroyltryptophan ; Chloromarmin ; D-Pinitol 2-O-alpha-D-Galactopyranosyl ; Dihydroferulic acid 4-O-glucuronide	M+2H ; M+Na ; M+ACN+H ; M+K ; M+K ; M+K ; M+Na ; M+K ; M+K ; M+K ; M+K ; M+K ; M+K ; M+K ; M+H+Na ; M+ACN+Na ; M+2Na ; M+H+K ; M+H+K ; M+K ; M+Na+K ; M+K ; M+K ; M+Na ; M+H+K ; M+Na ; 2M+H+Na ; M+2Na ; M+2Na ; M+H+K ; M+H+Na	C18H18O10 ; C10H9NO2 ; C5H4N2O4 ; C7H13NO3 ; C7H13NO3 ; C7H13NO3 ; C10H9NO2 ; C7H13NO3 ; C7H13NO3 ; C7H13NO3 ; C7H13NO3 ; C7H13NO3 ; C7H13NO3 ; C7H13NO3 ; C16H20O10 ; C8H6O2 ; C20H18N2O4 ; C13H24O11 ; C13H24O11 ; C7H13NO3 ; C17H22N2O5 ; C7H13NO3 ; C7H13NO3 ; C10H9NO2 ; C13H24O11 ; C10H9NO2 ; C8H10O5 ; C20H18N2O4 ; C19H23ClO4 ; C13H24O11 ; C16H20O10
811.60800	1.614383	0.59599	5.2792	0.000358	3.4461	0.008818	811.608	HMDB06728 ; HMDB39202 ; HMDB39261 ; HMDB40714	CE(24:1(15Z)) ; Punigluconin ; Sanguiin H7 ; Vitilagin	M+2K-H ; 2M+H+NH4 ; 2M+H+NH4 ; 2M+H+NH4	C51H90O2 ; C34H26O23 ; C34H26O23 ; C34H26O23
524.80123	1.612296	1.209742	5.2694	0.000363	3.4399	0.008891	524.8012	HMDB41467	Melilotoside D	M+H+NH4	C52H86O20
184.05597	1.601015	0.969718	-5.2614	0.000367	3.4349	0.008891	184.056	HMDB29601 ; HMDB00073 ; HMDB01513 ; HMDB03551 ; HMDB04825 ; HMDB12309 ; HMDB29007 ; HMDB29112 ; HMDB31098 ; HMDB33050 ; HMDB33051 ; HMDB38340 ; HMDB39783 ; HMDB40116 ; HMDB40117 ; HMDB40441 ; HMDB41914 ; HMDB60857 ; HMDB60862	Furocoumarinic acid glucoside ; Dopamine ; Dolichyl diphosphate ; Carbamic acid ; p-Octopamine ; Vanillylamine ; Phenylalanyl-Tyrosine ; Tyrosyl-Phenylalanine ; (9Z11E13E15Z)-4-Oxo-9111315-octadecatetraenoic acid ; Butyl isopropyl disulfide ; Butyl propyl disulfide ; N6-cis-p-Coumaroylserotonin ; 1-(Methylthio)-1-hexanethiol ; ()-2-Butylthiazolidine ; ()-2-(2-Methylpropyl)thiazolidine ; Dictyoquinazol B ; Ketophenylbutazone ; o-O-glucuronide rosiglitazone ; p-O-glucuronide rosiglitazone	M+2H ; 2M+Na+K ; M+H+Na ; 3M+H ; 2M+Na+K ; 2M+Na+K ; M+H+K ; M+H+K ; M+2K ; 2M+H+K ; 2M+H+K ; M+2Na ; 2M+H+K ; M+K ; M+K ; M+H+K ; M+2Na ; M+3H ; M+3H	C17H18O9 ; C8H11NO2 ; C12H26O7P2 ; CH3NO2 ; C8H11NO2 ; C8H11NO2 ; C18H20N2O4 ; C18H20N2O4 ; C18H26O3 ; C7H16S2 ; C7H16S2 ; C19H18N2O3 ; C7H16S2 ; C7H15NS ; C7H15NS ; C18H20N2O4 ; C19H18N2O3 ; C24H27N3O10S ; C24H27N3O10S
354.89454	1.614545	1.014387	5.259	0.000369	3.4334	0.008891	NA	NA	NA	NA	NA

324.15301	1.618013	0.463621	5.2575	0.000369	3.4326	0.008891	324.153	HMDB11170 ; HMDB11171 ; HMDB13888 ; HMDB28958 ; HMDB29000 ; HMDB41368 ; HMDB60996 ; HMDB14509 ; HMDB29861	L-gamma-glutamyl-L-isoleucine ; L-gamma-glutamyl-L-leucine ; E-10-Hydroxyamitriptyline ; Lysyl-Phenylalanine ; Phenylalanyl-Lysine ; 24-Acetyl- 25-cinnamoylvulgaroside ; N-Acetyl-3-hydroxyprocainamide ; Grepafloxacin ; Enrofloxacin	M+ACN+Na ; M+ACN+Na ; 2M+Na+K ; 2M+Na+K ; 2M+Na+K ; M+H+K ; 2M+Na+K ; M+H-2H2O ; M+H-2H2O	C11H20N2O5 ; C11H20N2O5 ; C20H23NO ; C15H23N3O3 ; C15H23N3O3 ; C36H48O8 ; C15H23N3O3 ; C19H22FN3O3 ; C19H22FN3O3
238.84158	1.672848	0.566596	5.2566	0.00037	3.4319	0.008891	238.8416	HMDB07868 ; HMDB07932 ; HMDB08826 ; HMDB08836 ; HMDB08837 ; HMDB08868 ; HMDB08923 ; HMDB08929 ; HMDB08930 ; HMDB08961 ; HMDB08986 ; HMDB09089 ; HMDB09121 ; HMDB09154 ; HMDB09284 ; HMDB09316 ; HMDB09349 ; HMDB29174	PC(14:015:0) ; PC(15:014:0) ; PE(14:018:0) ; PE(14:020:3(5Z8Z11Z)) ; PE(14:020:3(8Z11Z14Z)) ; PE(14:1(9Z)20:2(11Z14Z)) ; PE(16:016:0) ; PE(16:018:3(6Z9Z12Z)) ; PE(16:018:3(9Z12Z15Z)) ; PE(16:1(9Z)18:2(9Z12Z)) ; PE(18:014:0) ; PE(18:2(9Z12Z)16:1(9Z)) ; PE(18:3(6Z9Z12Z)16:0) ; PE(18:3(9Z12Z15Z)16:0) ; PE(20:2(11Z14Z)14:1(9Z)) ; PE(20:3(5Z8Z11Z)14:0) ; PE(20:3(8Z11Z14Z)14:0) ; 3-Sn-phosphatidylethanolamine	M+2H+Na ; M+2H+Na ; M+2H+Na ; M+3H ; M+3H ; M+3H ; M+2H+Na ; M+3H ; M+3H ; M+3H ; M+2H+Na ; M+3H ; M+3H ; M+3H ; M+3H ; M+3H ; M+3H ; M+2H+Na	C37H74NO8P ; C37H74NO8P ; C37H74NO8P ; C39H72NO8P ; C39H72NO8P ; C39H72NO8P ; C37H74NO8P ; C39H72NO8P ; C39H72NO8P ; C39H72NO8P ; C37H74NO8P ; C39H72NO8P ; C39H72NO8P ; C39H72NO8P ; C39H72NO8P ; C39H72NO8P ; C39H72NO8P ; C37H74NO8P
200.04423	1.629103	0.049991	5.2494	0.000374	3.4275	0.008927	200.036	HMDB01015 ; HMDB02336 ; HMDB03164 ; HMDB29278 ; HMDB29417 ; HMDB29441 ; HMDB30652 ; HMDB30653 ; HMDB32849 ; HMDB32964 ; HMDB33592 ; HMDB40632 ; HMDB40690	N-Formyl-L-methionine ; Biflorin ; Chlorogenic acid ; Cis-5-Caffeoylquinic acid ; Cycloalliin ; Isoalliin ; 1-O-Caffeoylquinic acid ; Cryptochlorogenic acid ; trans-Chlorogenic acid ; 2-Acetylthiazole ; (R)C(S)S-Alliin ; Isobiflorin ; 5Z-Caffeoylquinic acid	M+Na ; M+2Na ; M+2Na ; M+2Na ; M+Na ; M+Na ; M+2Na ; M+2Na ; M+2Na ; 3M+H+NH4 ; M+Na ; M+2Na ; M+2Na	C6H11NO3S ; C16H18O9 ; C16H18O9 ; C16H18O9 ; C6H11NO3S ; C6H11NO3S ; C16H18O9 ; C16H18O9 ; C16H18O9 ; C5H5NOS ; C6H11NO3S ; C16H18O9 ; C16H18O9
199.05573	1.59497	0.010222	-5.2472	0.000375	3.4261	0.008927	199.0557	HMDB40164 ; HMDB37058 ; HMDB00402 ; HMDB02025 ; HMDB04448 ; HMDB12156 ; HMDB12281 ; HMDB29169 ; HMDB30234 ; HMDB30487 ; HMDB30573 ; HMDB30709 ; HMDB31572 ; HMDB31712 ; HMDB31993 ; HMDB32420 ; HMDB32426 ; HMDB34341 ; HMDB34837 ; HMDB35129 ; HMDB35467 ; HMDB35698 ; HMDB35789 ; HMDB36837 ; HMDB38113 ; HMDB38480 ; HMDB38481 ; HMDB38903 ; HMDB38999 ; HMDB39081 ; HMDB41113 ; HMDB41172 ; HMDB41276 ; HMDB60402 ; HMDB04821 ; HMDB60618	4-Methyldibenzothiophene ; Thiazopyr ; 2-Isopropylmalic acid ; 23-Dimethyl-3-hydroxyglutaric acid ; Estradiol-17beta 3-sulfate ; 3-Isopropylmalate ; Portulacaxanthin II ; 3-Hydroxy-2-methylglutarate ; Xanthohumol C ; Artonol A ; Sanshodiol ; Dihydrocubebin ; 2-Methyl-4-propyl-13-oxathiane ; ()-Glycerol 12-diacetate ; Glyurallin A ; 2-(4-Methyl-5-thiazolyl)ethyl formate ; (-)-3-(Methylthio)heptanal ; Physalin L ; Lansiumarin A ; Citronobin ; Glicophenone ; (-)-Matairesinol ; Matairesinol ; Isocolumbin ; 5-Deoxykievitone hydrate ; Gancaonin G ; Gancaonin A ; Gancaonin M ; 6-Gingesulfonic acid ; Physalin O ; Artonin U ; 1-Methoxyphaseollin ; (7x8x)-47-Epoxy-38-bilign-7-ene-35-dimethoxy-499-triol ; 56-Dihydro-5-fluorouracil ; Bisnorbiotin ; 6-Aminopenicillanic acid	M+H ; M+2H ; M+Na ; M+Na ; M+2Na ; M+Na ; M+H+Na ; M+Na ; M+2Na ; M+2Na ; M+H+K ; M+H+K ; M+K ; M+Na ; M+2Na ; 2M+3H2O+2H ; M+K ; M+3Na ; M+2Na ; M+2Na ; M+H+K ; M+H+K ; M+H+K ; M+H+K ; M+H+K ; M+2Na ; M+2Na ; M+2Na ; M+H+K ; M+3Na ; M+2Na ; M+2Na ; M+H+K ; 3M+2H ; M+H-H2O ; M+H-H2O	C13H10S ; C16H17F5N2O2S ; C7H12O5 ; C7H12O5 ; C18H24O5S ; C7H12O5 ; C18H18N2O7 ; C7H12O5 ; C21H20O5 ; C21H20O5 ; C20H22O6 ; C20H22O6 ; C8H16OS ; C7H12O5 ; C21H20O5 ; C7H9NO2S ; C8H16OS ; C28H32O10 ; C21H20O5 ; C21H20O5 ; C20H22O6 ; C20H22O6 ; C20H22O6 ; C20H22O6 ; C20H22O6 ; C21H20O5 ; C21H20O5 ; C21H20O5 ; C17H26O6S ; C28H32O10 ; C21H20O5 ; C21H20O5 ; C20H22O6 ; C4H5FN2O2 ; C8H12N2O3S ; C8H12N2O3S
270.22944	1.660249	0.452525	5.233	0.000383	3.4172	0.009031	270.2294	HMDB39535	3-Tritriacontanone	M+Na+K	C33H66O
316.99454	1.645874	0.08732	5.2308	0.000384	3.4158	0.009031	316.9945	HMDB32129 ; HMDB32130 ; HMDB36738 ; HMDB39612	25-Dihydroxychalcone ; 34-Dihydroxychalcone ; Lettucenin A ; 24-Dihydroxychalcone	M+2K-H ; M+2K-H ; M+2K-H ; M+2K-H	C15H12O3 ; C15H12O3 ; C15H12O3 ; C15H12O3
827.91924	1.685837	0.843637	-5.2288	0.000385	3.4146	0.009031	827.9192	HMDB40687	(3x5x10x)-910-Didehydroisohumbertiol O-rhamnosyl-(1-4)-rhamnosyl-(1-2)-rhamnosyl-(1-6)-glucoside	2M+H+NH4	C39H62O18
167.05714	1.642582	0.55246	-5.2233	0.000388	3.4111	0.009031	167.0571	HMDB01886 ; HMDB01991 ; HMDB10738 ; HMDB13029 ; HMDB34394 ; HMDB59716 ; HMDB11741 ; HMDB11747 ; HMDB14999 ; HMDB30019 ; HMDB30555 ; HMDB31744 ; HMDB33178 ; HMDB33310 ; HMDB34175 ; HMDB35664 ; HMDB36635 ; HMDB38576 ; HMDB39044 ; HMDB39858 ; HMDB41298 ; HMDB60768 ; HMDB60897	3-Methylxanthine ; 7-Methylxanthine ; 1-Methylxanthine ; Noradrenochrome o-semiquinone ; S-Propyl 1-propanesulfinothioate ; 9-Methylxanthine ; Gamma-Glutamyltyrosine ; Ciliatine ; Diflunisal ; 4-Methoxymucidin ; Tocopheronic acid ; Artocarbene ; 5-Methylquinoxaline ; Moracin C ; 2-Aminoquinoline ; Dukunolide B ; Hydroxytanshinone ; Avenanthramide C ; Ethylsuberenol ; Angeloylsenkyunolide F ; Moracin N ; 4-Hydroxymethylpyrazole ; Diphenhydramine N-glucuronide	M+H ; M+H ; M+H ; M+H ; M+H ; M+H ; M+H+Na ; M+ACN+H ; M+2ACN+2H ; M+2Na ; M+H+K ; M+H+Na ; M+Na ; M+H+Na ; M+Na ; M+3H ; M+H+Na ; M+H+NH4 ; M+2Na ; M+2Na ; M+H+Na ; 3M+H+K ; M+3Na	C6H6N4O2 ; C6H6N4O2 ; C6H6N4O2 ; C8H8NO3 ; C6H14OS2 ; C6H6N4O2 ; C14H18N2O6 ; C2H8NO3P ; C13H8F2O3 ; C17H20O4 ; C16H22O5 ; C19H18O4 ; C9H8N2 ; C19H18O4 ; C9H8N2 ; C26H26O10 ; C19H18O4 ; C16H13NO6 ; C17H20O4 ; C17H20O4 ; C19H18O4 ; C4H6N2O ; C23H30NO7
491.08791	1.677907	0.459479	5.2217	0.000389	3.4101	0.009031	491.0879	HMDB00580 ; HMDB06552 ; HMDB30561 ; HMDB30563 ; HMDB33658 ; HMDB33915 ; HMDB33993 ; HMDB34785 ; HMDB35496 ; HMDB36984 ; HMDB37101 ; HMDB41097	Chondroitin sulfate ; Aflatoxin B1 ; 3-Hydroxy-89-dimethoxycoumestan ; 344-Trihydroxypulvinone ; De-O-methyldihydrosterigmatocystin ; Aloe emodin w-acetate ; Wairol ; Vitisifuran A ; Vitisifuran B ; Garcimangosone C ; 8-Hydroxy-3-methoxy-1-methylanthraquinone-2-carboxylic acid ; (E)-Antibiotic BE 23372M	2M+3H2O+2H ; 3M+2Na ; 3M+2Na ; 3M+2Na ; 3M+2Na ; 3M+2Na ; 3M+2Na ; M+2K ; M+2K ; M+2K+H ; 3M+2Na ; 3M+2Na	C13H21NO15S ; C17H12O6 ; C17H12O6 ; C17H12O6 ; C17H12O6 ; C17H12O6 ; C17H12O6 ; C56H40O12 ; C56H40O12 ; C23H24O7 ; C17H12O6 ; C17H12O6
598.61423	1.658845	1.032602	5.2195	0.00039	3.4088	0.009031	NA	NA	NA	NA	NA

149.02634	1.620018	0.401527	5.2105	0.000395	3.4031	0.009108	149.0263	HMDB01553 ; HMDB00267 ; HMDB00805 ; HMDB00884 ; HMDB01369 ; HMDB01843 ; HMDB02234 ; HMDB02331 ; HMDB04086 ; HMDB04813 ; HMDB06239 ; HMDB06801 ; HMDB13870 ; HMDB13871 ; HMDB13872 ; HMDB15457 ; HMDB15619 ; HMDB35993 ; HMDB41441 ; HMDB60262 ; HMDB60587 ; HMDB61093	2-Oxo-4-methylthiobutanoic acid ; Pyroglutamic acid ; Pyrrolidonecarboxylic acid ; Ribothymidine ; Pyrroline hydroxycarboxylic acid ; N-Acryloylglycine ; 1-Pyrroline-4-hydroxy-2-carboxylate ; Imidazoleacetic acid riboside ; 5-Hydroxy-N-formylkynurenine ; 3-Methyluridine ; S-aminomethyldihydrolipoamide ; 2-Oxo-3-hydroxy-4-phosphobutanoic acid ; cistrans-5-Hydroxythalidomide ; 5-Hydroxythalidomide ; Thalidomide arene oxide ; Dihydroxyaluminium ; Sulfathiazole ; N5-(34-Dioxo-15-cyclohexadien-1-yl)-L-glutamine ; 11-Methylgerberinol ; 5-Oxoprolinate ; Aminoethoxyacetic acid ; dimethadione	M+H ; 2M+H+K ; 2M+H+K ; M+H+K ; 2M+H+K ; 2M+H+K ; 2M+H+K ; M+H+K ; M+2Na ; M+H+K ; M+Na+K ; M+2ACN+2H ; M+H+Na ; M+H+Na ; M+H+Na ; 2M+H+Na ; M+ACN+2H ; M+2Na ; M+3Na ; 2M+H+K ; 2M+Na+K ; 2M+H+K	C5H8O3S ; C5H7NO3 ; C5H7NO3 ; C10H14N2O6 ; C5H7NO3 ; C5H7NO3 ; C5H7NO3 ; C10H14N2O6 ; C11H12N2O5 ; C10H14N2O6 ; C9H20N2OS2 ; C4H7O8P ; C13H10N2O5 ; C13H10N2O5 ; C13H10N2O5 ; C2H8AlNO4 ; C9H9N3O2S2 ; C11H12N2O5 ; C22H18O6 ; C5H7NO3 ; C4H8NO3 ; C5H7NO3
457.97097	1.579296	1.132579	5.1887	0.000408	3.3894	0.009356	457.971	HMDB41797	24-Dichlorophenoxyacetic acid	2M+NH4	C8H6Cl2O3
246.18124	1.63316	0.321663	-5.1802	0.000413	3.3841	0.009428	246.1812	HMDB28964 ; HMDB29132 ; HMDB39090 ; HMDB07930 ; HMDB07931 ; HMDB11270 ; HMDB11303 ; HMDB13331 ; HMDB13341 ; HMDB30951 ; HMDB32677	Lysyl-Valine ; Valyl-Lysine ; 78-Dihydro-8-hydroxyreticulataxanthin ; PC(14:1(9Z)P-18:1(11Z)) ; PC(14:1(9Z)P-18:1(9Z)) ; PC(P-18:1(11Z)14:1(9Z)) ; PC(P-18:1(9Z)14:1(9Z)) ; 3 5-Tetradecadiencarnitine ; PC(o-14:016:0) ; (EE)-24-Decadienoic isobutylamide ; Soyacerebroside I	M+H ; M+H ; M+2H ; M+2H+Na ; M+2H+Na ; M+2H+Na ; M+2H+Na ; M+3ACN+2H ; M+H+2Na ; M+Na ; M+2H+Na	C11H23N3O3 ; C11H23N3O3 ; C33H46O3 ; C40H76NO7P ; C40H76NO7P ; C40H76NO7P ; C40H76NO7P ; C21H37NO4 ; C38H78NO7P ; C14H25NO ; C40H75NO9
178.03265	1.656092	0.578472	-5.1709	0.000419	3.3782	0.009484	178.0327	HMDB38591 ; HMDB13742 ; HMDB14831 ; HMDB14944 ; HMDB29609 ; HMDB30111 ; HMDB33298 ; HMDB33683 ; HMDB33692 ; HMDB33797 ; HMDB33924 ; HMDB37251 ; HMDB37477 ; HMDB37481 ; HMDB37749 ; HMDB39825 ; HMDB40145 ; HMDB40357 ; HMDB41234 ; HMDB41790	Brassicanal A ; 2-Furanmethanol ; Fluorescein ; Pentoxifylline ; 5-Methyl-2(3H)-furanone ; Homoferreirin ; 3-(34-Dihydroxybenzyl)-7-hydroxy-5-methoxy-4-chromanone ; Melilotocarpan E ; Melilotocarpan D ; Olivin ; Cajanol ; 58-Dihydroxy-3-(4-hydroxybenzyl)-7-methoxy-4-chromanone ; 57-Dihydroxy-3-(3-hydroxy-4-methoxybenzyl)-4-chromanone ; 35-Dihydroxy-47-dimethoxyflavanone ; 47-Dihydroxy-25-dimethoxyisoflavanone ; Cyclocalopin F ; 5-Hydroxy-4-pentenoic acid d-lactone ; 611-Dihydroxy-22-dimethylpyrano32-cxanthen-7(2H)-one ; Artocarpanone A ; Violanone	M+H ; 3M+Na+K ; M+H+Na ; M+2K ; 3M+Na+K ; M+H+K ; M+H+K ; M+H+K ; M+H+K ; M+H+K ; M+H+K ; M+H+K ; M+H+K ; M+H+K ; M+H+K ; M+Na+K ; 3M+Na+K ; M+2Na ; M+H+K ; M+H+K	C9H7NOS ; C5H6O2 ; C20H12O5 ; C13H18N4O3 ; C5H6O2 ; C17H16O6 ; C17H16O6 ; C17H16O6 ; C17H16O6 ; C17H16O6 ; C17H16O6 ; C17H16O6 ; C17H16O6 ; C17H16O6 ; C17H16O6 ; C15H18O6 ; C5H6O2 ; C18H14O5 ; C17H16O6 ; C17H16O6
167.45756	1.650782	0.621008	5.1698	0.000419	3.3776	0.009484	NA	NA	NA	NA	NA
131.51688	1.660662	0.244478	5.161	0.000425	3.372	0.009563	131.5169	HMDB06348 ; HMDB06548 ; HMDB31805	Pseudoecgonine ; Ecgonine ; (E)-Monocrotophos	M+2K ; M+2K ; M+H+K	C9H15NO3 ; C9H15NO3 ; C7H14NO5P
269.09496	1.67914	0.769755	-5.1545	0.000429	3.3679	0.00961	269.095	HMDB28782 ; HMDB28992 ; HMDB60090 ; HMDB60091 ; HMDB60439 ; HMDB03141 ; HMDB10332 ; HMDB28981 ; HMDB29023 ; HMDB29308 ; HMDB29540 ; HMDB30153 ; HMDB30214 ; HMDB32085 ; HMDB32175 ; HMDB32761 ; HMDB35415 ; HMDB37755 ; HMDB39379 ; HMDB41542 ; HMDB41938 ; HMDB60169 ; HMDB60773	Cysteinyl-Phenylalanine ; Phenylalanyl-Cysteine ; Benzoapyrene-910-oxide ; Benzoapyrene-45-oxide ; Benzoapyrene-78-oxide ; Retinoyl b-glucuronide ; Bilirubin glucuronide ; Methionyl-Proline ; Prolyl-Methionine ; Hexamethylquercetagetin ; Nobiletin ; Austalide H ; Mukonidine ; Koeniginequinone A ; N-Benzoylanthranilic acid ; Nb-trans-Feruloylserotonin glucoside ; 334568-Hexamethoxyflavone ; 334578-Hexamethoxyflavone ; Chrysaloin ; N2-Maltulosylarginine ; Moxonidine ; Mono(glucosyluronic acid)bilirubin ; 4-Hydroxyphenyl Carvedilol	M+H ; M+H ; M+H ; M+H ; M+H ; M+Na+K ; M+H+2Na ; M+Na ; M+Na ; 2M+3H ; 2M+3H ; M+Na+K ; 2M+3H2O+2H ; 2M+3H2O+2H ; 2M+3H2O+2H ; M+H+Na ; 2M+3H ; 2M+3H ; 2M+3H ; M+H+K ; 2M+3H2O+2H ; M+H+2Na ; M+H+K	C12H16N2O3S ; C12H16N2O3S ; C20H12O ; C20H12O ; C20H12O ; C26H36O8 ; C39H44N4O12 ; C10H18N2O3S ; C10H18N2O3S ; C21H22O8 ; C21H22O8 ; C26H36O8 ; C14H11NO3 ; C14H11NO3 ; C14H11NO3 ; C26H30N2O9 ; C21H22O8 ; C21H22O8 ; C21H22O8 ; C18H34N4O12 ; C9H12ClN5O ; C39H44N4O12 ; C30H30N2O5
376.05105	1.599588	0.996344	-5.1492	0.000432	3.3646	0.009642	376.0511	HMDB29368 ; HMDB29629 ; HMDB38632	Saxitoxin ; 26-Digalloyliriflophenone 3-C-glucoside ; 6-Methoxy-3-(2-thiazolyl)-1H-indole	M+2K-H ; M+H+K ; 3M+Na+K	C10H17N7O4 ; C33H28O18 ; C12H10N2OS
349.07298	1.602182	0.133053	-5.1387	0.000439	3.3579	0.009747	349.073	HMDB34067 ; HMDB34068 ; HMDB11720 ; HMDB32943 ; HMDB34073 ; HMDB35462 ; HMDB37457 ; HMDB37461 ; HMDB38262 ; HMDB38263 ; HMDB39374 ; HMDB39723 ; HMDB39929 ; HMDB40474 ; HMDB40737	Maclurin 3-C-(2-galloyl-6-p-hydroxybenzoyl-glucoside) ; Maclurin 3-C-(2-p-hydroxybenzoyl-6-galloyl-glucoside) ; UDP-N-acetylmuraminate ; Diazinon ; Vicine ; Myricetin 3-(6-acetylgalactoside) ; Spinatoside ; Tricin 7-rhamnosyl-(1-2)-galacturonide ; 3456-Tetrahydroxy-37-dimethoxyflavone 3-glucuronide ; 3356-Tetrahydroxy-47-dimethoxyflavone 3-glucuronide ; 6-Acetylhyperin 7-rhamnoside ; 3-O-alpha-D-Glucopyranuronosyl-D-xylose ; 2-O-Acetylrutin ; 2-(6-Acetylglucosyl)astragalin ; Aldobiouronic acid D3	M+2H ; M+2H ; M+H+NH4 ; M+2Na-H ; M+2Na-H ; 2M+3H ; 2M+3H ; M+2Na ; 2M+3H ; 2M+3H ; M+2Na ; M+Na ; M+2Na ; M+2Na ; M+Na	C33H28O17 ; C33H28O17 ; C20H31N3O19P2 ; C12H21N2O3PS ; C10H16N4O7 ; C23H22O14 ; C23H22O14 ; C29H32O17 ; C23H22O14 ; C23H22O14 ; C29H32O17 ; C11H18O11 ; C29H32O17 ; C29H32O17 ; C11H18O11

183.07817	1.603509	0.825598	5.1276	0.000446	3.3509	0.009862	183.0782	HMDB00246 ; HMDB00309 ; HMDB00322 ; HMDB00324 ; HMDB00352 ; HMDB00388 ; HMDB00474 ; HMDB00482 ; HMDB01280 ; HMDB01877 ; HMDB03039 ; HMDB03454 ; HMDB03543 ; HMDB03956 ; HMDB04089 ; HMDB04291 ; HMDB04611 ; HMDB04624 ; HMDB06031 ; HMDB06259 ; HMDB06769 ; HMDB12533 ; HMDB12654 ; HMDB13030 ; HMDB14602 ; HMDB29980 ; HMDB30368 ; HMDB30698 ; HMDB30719 ; HMDB31230 ; HMDB31243 ; HMDB31290 ; HMDB31324 ; HMDB31464 ; HMDB31478 ; HMDB31567 ; HMDB31579 ; HMDB31587 ; HMDB31588 ; HMDB31596 ; HMDB31638 ; HMDB31703 ; HMDB32046 ; HMDB32047 ; HMDB32203 ; HMDB32225 ; HMDB32272 ; HMDB32335 ; HMDB32551 ; HMDB32560 ; HMDB33358 ; HMDB33853 ; HMDB33858 ; HMDB34161 ; HMDB34164 ; HMDB34234 ;	Tetrahydrofuran ; 3a16b-Dihydroxyandrostenone ; 16-Oxoandrostenediol ; 3a16a-Dihydroxyandrostenone ; 16a-Hydroxydehydroisoandrosterone ; 3a16-Dihydroxyandrostenone ; Butanone ; Caprylic acid ; 2-Aminomuconic acid semialdehyde ; Valproic acid ; Isoacitretin ; 4-Pyridoxolactone ; Butanal ; 7a-Hydroxytestosterone ; Formylanthranilic acid ; 5-Pyridoxolactone ; 7a-Hydroxydehydroepiandrosterone ; 7b-Hydroxydehydroepiandrosterone ; 11-Ketoetiocholanolone ; 6beta-Hydroxytestosterone ; 19-Hydroxytestosterone ; 11beta-Hydroxytestosterone ; 2beta-Hydroxytestosterone ; Noradrenochrome ; Acitretin ; Hexyl acetate ; (3R5Z)-5-Octene-13-diol ; 57-Dimethoxyisoflavone ; 56-Dimethoxyflavone ; 2-Ethylhexanoic acid ; 2-Methylpropanal ; 1-Hydroxy-3-octanone ; 3-Buten-1-ol ; cis-8-Shogaol ; Methyl heptanoate ; 3-Methyl-4-phenyl-3-buten-2-one ; Ethyl ()-2-methylpentanoate ; 2-Methylheptanoic acid ; 6-Methylheptanoic acid ; Methyl 5-methylhexanoate ; Pentyl propanoate ; 2-Ethylbutyl acetate ; Isopropyl 3-methylbutanoate ; Propyl 3-methylbutanoate ; cis- and trans-2-Isobutyl-4-methyl-13-dioxolane ; 11-Dimethoxy-trans-2-hexene ; Ethyl 4-methylpentanoate ; 3-Hydroxy-2-octanone ; Valeraldehyde propyleneglycol acetal ; Dinoseb acetate ; (S)-Neolitsine ; Rutacridone epoxide ; 2-Buten-1-ol ; 2-Methylpropyl butanoate ; 3-Methylbutyl propanoate ; Ethyl ()-3-methylpentanoate ; 22-Dimethyloxirane ; Heptyl formate ; 2-Phenyl-4-pentenal ; ()-5-Hydroxy-4-octanone ; Mascaroside ; 3-(Hydroxymethyl)-2-heptanone ; 6-Methoxy-2(3H)-benzoxazolone ; 57-Dimethoxyflavone ; Gibberellin A38 glucosyl ester ; (8R8R)-Secoisolariciresinol 9-glucoside ; 5-Hydroxy-7-methoxy-6-methylflavone ; 24-Dihydroxy-67-dimethoxy-2H-14-benzoxazin-3(4H)-one ; 24-Dihydroxy-78-dimethoxy-2H-14-benzoxazin-3(4H)-one ; (S)-1016-Dihydroxyhexadecanoic acid ; (R)-2-Hydroxy-2H-14-benzoxazin-3(4H)-one ; 3-Phenyl-4-pentenal ; Isopropyl 2-methylbutanoate ; Ginsenoyne G ; Butyl butyrate ; 10-Acetoxy-8-heptadecene-46-diyn-3-ol ; Gravacridonol ; Ethyl hexanoate ; Butyl isobutyrate ; Isobutyl isobutyrate ; Propyl pentanoate ; Ethyl 2-ethylbutanoate ; 12-Dimethoxy-13-methyl-13benzodioxolo56-cphenanthridine ; N-Methoxycarbonylanonaine ; w Hydroxy testosterone ; 11beta17beta-Dihydroxy-4-androsten-3-one	2M+K ; M+Na+K ; M+Na+K ; M+Na+K ; M+Na+K ; M+Na+K ; 2M+K ; M+K ; M+ACN+H ; M+K ; M+H+K ; M+NH4 ; 2M+K ; M+Na+K ; M+NH4 ; M+NH4 ; M+Na+K ; M+Na+K ; M+Na+K ; M+Na+K ; M+Na+K ; M+Na+K ; M+Na+K ; M+NH4 ; M+H+K ; M+K ; M+K ; M+2ACN+2H ; M+2ACN+2H ; M+K ; 2M+K ; M+K ; 2M+K ; M+Na+K ; M+K ; M+Na ; M+K ; M+K ; M+K ; M+K ; M+K ; M+K ; M+K ; M+K ; M+K ; M+K ; M+K ; M+K ; M+2ACN+2H ; M+ACN+2H ; M+ACN+2H ; 2M+K ; M+K ; M+K ; M+K ; 2M+K ; M+K ; M+Na ; M+K ; M+2H+Na ; M+K ; M+NH4 ; M+2ACN+2H ; M+2H+Na ; M+2H+Na ; M+2ACN+2H ; M+3ACN+2H ; M+3ACN+2H ; M+2K ; M+NH4 ; M+Na ; M+K ; M+Na+K ; M+K ; M+Na+K ; M+ACN+2H ; M+K ; M+K ; M+K ; M+K ; M+K ; M+H+NH4 ; M+ACN+2H ; M+Na+K ; M+Na+K	C4H8O ; C19H28O3 ; C19H28O3 ; C19H28O3 ; C19H28O3 ; C19H28O3 ; C4H8O ; C8H16O2 ; C6H7NO3 ; C8H16O2 ; C21H26O3 ; C8H7NO3 ; C4H8O ; C19H28O3 ; C8H7NO3 ; C8H7NO3 ; C19H28O3 ; C19H28O3 ; C19H28O3 ; C19H28O3 ; C19H28O3 ; C19H28O3 ; C19H28O3 ; C8H7NO3 ; C21H26O3 ; C8H16O2 ; C8H16O2 ; C17H14O4 ; C17H14O4 ; C8H16O2 ; C4H8O ; C8H16O2 ; C4H8O ; C19H28O3 ; C8H16O2 ; C11H12O ; C8H16O2 ; C8H16O2 ; C8H16O2 ; C8H16O2 ; C8H16O2 ; C8H16O2 ; C8H16O2 ; C8H16O2 ; C8H16O2 ; C8H16O2 ; C8H16O2 ; C8H16O2 ; C8H16O2 ; C12H14N2O6 ; C19H17NO4 ; C19H17NO4 ; C4H8O ; C8H16O2 ; C8H16O2 ; C8H16O2 ; C4H8O ; C8H16O2 ; C11H12O ; C8H16O2 ; C26H36O11 ; C8H16O2 ; C8H7NO3 ; C17H14O4 ; C26H36O11 ; C26H36O11 ; C17H14O4 ; C10H11NO6 ; C10H11NO6 ; C16H32O4 ; C8H7NO3 ; C11H12O ; C8H16O2 ; C19H28O3 ; C8H16O2 ; C19H28O3 ; C19H17NO4 ; C8H16O2 ; C8H16O2 ; C8H16O2 ; C8H16O2 ; C8H16O2 ; C21H17NO4 ; C19H17NO4 ; C19H28O3 ; C19H28O3
226.94032	1.64235	0.394004	-5.1205	0.00045	3.3464	0.009863	226.9403	HMDB14706	Succimer	M+2Na-H	C4H6O4S2
280.09194	1.687292	0.649486	-5.119	0.000451	3.3455	0.009863	280.0919	HMDB34347 ; HMDB14965 ; HMDB30134 ; HMDB32730 ; HMDB33281 ; HMDB33282 ; HMDB33610 ; HMDB34787 ; HMDB37998	Physalin K ; Cinoxacin ; Asticolorin B ; Divanillyltetrahydrofuran ferulate ; 8-Hydroxypinoresinol 8-glucoside ; 8-Hydroxypinoresinol 4-glucoside ; Pandamarilactam 3x ; Neoacrimarine J ; Delphinidin 3-arabinoside	M+2H ; M+NH4 ; M+H+Na ; M+H+K ; M+H+Na ; M+H+Na ; M+2Na-H ; M+ACN+2H ; M+3ACN+2H	C28H30O12 ; C12H10N2O5 ; C33H28O7 ; C30H32O8 ; C26H32O12 ; C26H32O12 ; C13H17NO3 ; C28H23NO9 ; C20H19O11
320.22780	1.637897	0.664113	5.1184	0.000452	3.3451	0.009863	320.2278	HMDB37129 ; HMDB39554 ; HMDB39555 ; HMDB15500 ; HMDB29816 ; HMDB60931	Sanchinoside B1 ; Ginsenoside Rh1 ; Ginsenoside F1 ; Allylestrenol ; Cardanoldiene ; 2-ethyl-15-dimethyl-33-diphenylpyrrolinium (EDDP)	M+2H ; M+2H ; M+2H ; 2M+H+K ; 2M+H+K ; M+ACN+H	C36H62O9 ; C36H62O9 ; C36H62O9 ; C21H32O ; C21H32O ; C20H24N
555.58890	1.551588	0.840316	5.1112	0.000457	3.3406	0.009924	555.5889	HMDB01399 ; HMDB60081	46-Dideoxy-4-oxo-dTDP-D-glucose ; dTDP-4-dehydro-6-deoxy-alpha-D-glucose	2M+H+NH4 ; 2M+H+NH4	C16H24N2O15P2 ; C16H24N2O15P2
531.96626	1.623623	0.903596	-5.0983	0.000465	3.3324	0.010026	531.9663	HMDB05401 ; HMDB05470 ; HMDB35705 ; HMDB36566 ; HMDB42232 ; HMDB42260 ; HMDB42266 ; HMDB42435 ; HMDB42493 ; HMDB42499 ; HMDB42615 ; HMDB42637 ; HMDB42681 ; HMDB42696 ; HMDB42817 ; HMDB43960 ; HMDB43986 ; HMDB43992 ; HMDB44019 ; HMDB44176 ; HMDB44203 ; HMDB44209 ; HMDB44235 ; HMDB44242 ; HMDB44289 ; HMDB44344 ; HMDB44364 ; HMDB44419 ; HMDB44485 ; HMDB44505 ; HMDB44532 ; HMDB44767 ; HMDB44798 ; HMDB44824 ; HMDB44974 ; HMDB44980 ; HMDB45000 ; HMDB45032 ; HMDB45039 ; HMDB45110 ; HMDB45162 ; HMDB45182 ; HMDB45235 ; HMDB45299 ; HMDB45318 ; HMDB45344 ; HMDB45546 ; HMDB45570 ; HMDB45576 ; HMDB45601 ; HMDB45719 ; HMDB45725 ; HMDB45745 ; HMDB45751 ; HMDB45775 ; HMDB45782 ;	TG(18:020:020:4(5Z8Z11Z14Z))iso6 ; TG(18:2(9Z12Z)20:1(11Z)20:1(11Z))iso3 ; Zeaxanthin dipalmitate ; Xantofyl palmitate ; TG(14:022:022:4(7Z10Z13Z16Z)) ; TG(14:024:020:4(5Z8Z11Z14Z)) ; TG(14:024:020:4(8Z11Z14Z17Z)) ; TG(14:020:3(5Z8Z11Z)24:1(15Z)) ; TG(14:024:1(15Z)20:3(5Z8Z11Z)) ; TG(14:024:1(15Z)20:3n6) ; TG(14:020:3n624:1(15Z)) ; TG(14:020:4(5Z8Z11Z14Z)24:0) ; TG(14:022:2(13Z16Z)22:2(13Z16Z)) ; TG(14:022:4(7Z10Z13Z16Z)22:0) ; TG(14:020:4(8Z11Z14Z17Z)24:0) ; TG(16:020:022:4(7Z10Z13Z16Z)) ; TG(16:022:020:4(5Z8Z11Z14Z)) ; TG(16:022:020:4(8Z11Z14Z17Z)) ; TG(16:024:018:4(6Z9Z12Z15Z)) ; TG(16:020:3(5Z8Z11Z)22:1(13Z)) ; TG(16:022:1(13Z)20:3(5Z8Z11Z)) ; TG(16:022:1(13Z)20:3n6) ; TG(16:024:1(15Z)18:3(6Z9Z12Z)) ; TG(16:024:1(15Z)18:3(9Z12Z15Z)) ; TG(16:018:3(6Z9Z12Z)24:1(15Z)) ; TG(16:020:3n622:1(13Z)) ; TG(16:020:4(5Z8Z11Z14Z)22:0) ; TG(16:022:4(7Z10Z13Z16Z)20:0) ; TG(16:018:3(9Z12Z15Z)24:1(15Z)) ; TG(16:018:4(6Z9Z12Z15Z)24:0) ; TG(16:020:4(8Z11Z14Z17Z)22:0) ; TG(18:018:022:4(7Z10Z13Z16Z)) ; TG(18:020:020:4(8Z11Z14Z17Z)) ; TG(18:022:018:4(6Z9Z12Z15Z)) ; TG(18:020:1(11Z)20:3(5Z8Z11Z)) ; TG(18:020:1(11Z)20:3n6) ; TG(18:020:3(5Z8Z11Z)20:1(11Z)) ; TG(18:022:1(13Z)18:3(6Z9Z12Z)) ; TG(18:022:1(13Z)18:3(9Z12Z15Z)) ; TG(18:018:3(6Z9Z12Z)22:1(13Z)) ; TG(18:020:3n620:1(11Z)) ; TG(18:020:4(5Z8Z11Z14Z)20:0) ; TG(18:022:4(7Z10Z13Z16Z)18:0) ; TG(18:018:3(9Z12Z15Z)22:1(13Z)) ; TG(18:018:4(6Z9Z12Z15Z)22:0) ; TG(18:020:4(8Z11Z14Z17Z)20:0) ; TG(20:016:022:4(7Z10Z13Z16Z)) ; TG(20:018:020:4(5Z8Z11Z14Z)) ; TG(20:018:020:4(8Z11Z14Z17Z)) ; TG(20:020:018:4(6Z9Z12Z15Z)) ; TG(20:018:1(11Z)20:3(5Z8Z11Z)) ; TG(20:018:1(11Z)20:3n6) ; TG(20:018:1(9Z)20:3(5Z8Z11Z)) ; TG(20:018:1(9Z)20:3n6) ; TG(20:020:1(11Z)18:3(6Z9Z12Z)) ; TG(20:020:1(11Z)18:3(9Z12Z15Z)) ; TG(20:020:3(5Z8Z11Z)18:1(11Z)) ; TG(20:020:3(5Z8Z11Z)18:1(9Z)) ; TG(20:018:2(9Z12Z)18:2(9Z12Z)) ; TG(20:018:2(9Z12Z)20:2n6) ; TG(20:018:3(6Z9Z12Z)20:1(11Z)) ; TG(20:020:2n618:2(9Z12Z)) ; TG(20:020:2n620:2n6) ; TG(20:020:3n618:1(11Z)) ; TG(20:020:3n618:1(9Z)) ; TG(20:018:3(9Z12Z15Z)20:1(11Z)) ; TG(20:018:4(6Z9Z12Z15Z)20:0) ; TG(22:014:022:4(7Z10Z13Z16Z)) ; TG(22:016:020:4(5Z8Z11Z14Z)) ; TG(22:016:020:4(8Z11Z14Z17Z)) ; TG(22:018:018:4(6Z9Z12Z15Z)) ; TG(22:016:1(9Z)20:3(5Z8Z11Z)) ; TG(22:016:1(9Z)20:3n6) ; TG(22:018:1(11Z)18:3(6Z9Z12Z)) ; TG(22:018:1(11Z)18:3(9Z12Z15Z)) ;	M+3ACN+2H ; M+3ACN+2H ; M+H+NH4 ; M+H+NH4 ; M+3ACN+2H ;	C61H110O6 ; C61H110O6 ; C72H116O4 ; C72H116O4 ; C61H110O6 ;
151.54069	1.644056	0.148635	5.0982	0.000465	3.3323	0.010026	151.5407	HMDB00086 ; HMDB00982 ; HMDB14643 ; HMDB39229 ; HMDB40093 ; HMDB40094 ; HMDB40115	Glycerophosphocholine ; 5-Methylcytidine ; Tolmetin ; Pyro-L-glutaminyl-L-glutamine ; 45-Dimethyl-2-octylthiazole ; 4-Ethyl-2-heptyl-5-methylthiazole ; 4-Ethyl-2-octylthiazole	M+2Na ; M+2Na ; M+2Na ; M+2Na ; M+2K ; M+2K ; M+2K	C8H20NO6P ; C10H15N3O5 ; C15H15NO3 ; C10H15N3O5 ; C13H23NS ; C13H23NS ; C13H23NS
391.94418	1.584226	0.634419	-5.0827	0.000476	3.3225	0.010192	391.9442	HMDB14763	Efavirenz	M+2K-H	C14H9ClF3NO2

413.06082	1.660767	0.272033	-5.0812	0.000477	3.3215	0.010192	413.0608	HMDB34141 ; HMDB39355 ; HMDB15260 ; HMDB60067	12alpha-Hydroxyerosone ; Methyl 6-O-digalloyl-beta-D-glucopyranoside ; Bicalutamide ; CMP-2-aminoethylphosphonate	M+2Na-H ; M+Na ; M+H-H2O ; M+H-H2O	C20H16O7 ; C17H19BrN4O2 ; C18H14F4N2O4S ; C11H20N4O10P2
263.23662	1.639172	0.300237	5.0638	0.000489	3.3104	0.01041	263.2366	HMDB34495 ; HMDB37519 ; HMDB03648 ; HMDB60338 ; HMDB01551 ; HMDB11558 ; HMDB11588 ; HMDB31052 ; HMDB10736 ; HMDB30979 ; HMDB30980 ; HMDB30981 ; HMDB31127 ; HMDB34074 ; HMDB34297	61014-Trimethyl-5913-pentadecatrien-2-one ; 2-(58-Tetradecadienyl)cyclobutanone ; Retinyl palmitate ; 11-cis-Retinyl palmitate ; Palmitaldehyde ; MG(0:024:00:0) ; MG(24:00:00:0) ; 2-Hexadecanone ; 3-Oxooctadecanoic acid ; 9-Oxooctadecanoic acid ; 10-Oxooctadecanoic acid ; 11-Oxooctadecanoic acid ; 5-Hexyltetrahydro-2-furanoctanoic acid ; 5-Oxooctadecanoic acid ; Ricinoleic acid	M+H ; M+H ; M+2H ; M+2H ; M+Na ; M+2ACN+2H ; M+2ACN+2H ; M+Na ; M+H-2H2O ; M+H-2H2O ; M+H-2H2O ; M+H-2H2O ; M+H-2H2O ; M+H-2H2O ; M+H-2H2O	C18H30O ; C18H30O ; C36H60O2 ; C36H60O2 ; C16H32O ; C27H54O4 ; C27H54O4 ; C16H32O ; C18H34O3 ; C18H34O3 ; C18H34O3 ; C18H34O3 ; C18H34O3 ; C18H34O3 ; C18H34O3
537.19409	1.663974	0.882938	-5.0542	0.000496	3.3044	0.010485	537.1941	HMDB06602 ; HMDB06610 ; HMDB06628 ; HMDB38329 ; HMDB41413	Lacto-N-hexaose ; Lacto-N-neohexaose ; p-Lacto-N-hexaose ; Dide-O-methyl-4-O-alpha-D-glucopyranosylsimmondsin ; Marmesin rutinoside	M+2H ; M+2H ; M+2H ; 2M+3H2O+2H ; M+H-H2O	C40H68N2O31 ; C40H68N2O31 ; C40H68N2O31 ; C20H31NO14 ; C26H34O13
377.14571	1.591214	1.045727	5.0531	0.000497	3.3037	0.010485	377.1457	HMDB33065 ; HMDB33385 ; HMDB39504 ; HMDB40845 ; HMDB40846 ; HMDB41093 ; HMDB41339 ; HMDB41434 ; HMDB41436 ; HMDB60405	(x)-12-Propanediol 1-O-b-D-glucopyranoside ; CI Acid Violet 49 ; gamma-Glutamyl-S-methylcysteinyl-beta-alanine ; (S)-Nerolidol 3-O-a-L-Rhamnopyranosyl-(1-4)-a-L-rhamnopyranosyl-(1-2)-b-D-glucopyranoside ; (S)-Nerolidol 3-O-a-L-rhamnopyranosyl-(1-4)-a-L-rhamnopyranosyl-(1-6)-b-D-glucopyranoside ; Gingerglycolipid A ; 5-Chloro-2-(35-di-tert-butyl-2-hydroxyphenyl)-2H-benzotriazole ; Citbismine B ; Citbismine E ; 5-Deoxy-5-fluorocytidine	3M+H+K ; M+ACN+2H ; M+ACN+H ; M+2K ; M+2K ; M+2K ; 2M+H+K ; M+2ACN+2H ; M+2ACN+2H ; 3M+H+NH4	C9H18O7 ; C39H41N3O6S2 ; C12H21N3O6S ; C33H56O14 ; C33H56O14 ; C33H56O14 ; C20H24ClN3O ; C36H34N2O11 ; C36H34N2O11 ; C9H12FN3O4
209.11845	1.627877	0.010735	5.0439	0.000504	3.2978	0.010582	209.1185	HMDB29867 ; HMDB29872 ; HMDB30150 ; HMDB30197 ; HMDB30681 ; HMDB31469 ; HMDB33778 ; HMDB34991 ; HMDB35224 ; HMDB38604 ; HMDB40218 ; HMDB40225 ; HMDB41411 ; HMDB15389 ; HMDB30522 ; HMDB30749 ; HMDB35849 ; HMDB39145 ; HMDB02249 ; HMDB14536 ; HMDB14685 ; HMDB30210 ; HMDB33172 ; HMDB33737 ; HMDB36173 ; HMDB40671	Isoelemicin ; gamma-Asarone ; Jasmine ketolactone ; (Z)-3-Oxo-2-(2-pentenyl)-1-cyclopenteneacetic acid ; 5-Deoxydiplosporin ; trans-Isoasarone ; Elemicin ; 4-Methoxybenzyl butanoate ; 34-Dihydro-6-methoxy-37-dimethyl-1H-2-benzopyran-8-ol ; Amyl salicylate ; 2-Phenoxyethyl isobutyrate ; Isoamyl salicylate ; 34-Dihydro-6-methoxy-22-dimethyl-2H-1-benzopyran-4-ol ; Desonide ; Citreoviridin D ; Heterotropan ; Armillyl everninate ; Diasarone 2 ; 6-Methyltetrahydropterin ; Ethopropazine ; Pyridostigmine ; Koenimbine ; 3-Methylpyrrolo12-apyrazine ; Dehydronuciferine ; 2-(3-Phenylpropyl)pyridine ; Physapruin B	M+H ; M+H ; M+H ; M+H ; M+H ; M+H ; M+H ; M+H ; M+H ; M+H ; M+H ; M+H ; M+H ; M+2H ; M+2H ; M+2H ; M+2H ; M+2H ; 2M+3H2O+2H ; 2M+3H ; 2M+3H2O+2H ; M+3ACN+2H ; 3M+H+NH4 ; M+3ACN+2H ; 2M+H+Na ; M+2H+Na	C12H16O3 ; C12H16O3 ; C12H16O3 ; C12H16O3 ; C12H16O3 ; C12H16O3 ; C12H16O3 ; C12H16O3 ; C12H16O3 ; C12H16O3 ; C12H16O3 ; C12H16O3 ; C12H16O3 ; C24H32O6 ; C24H32O6 ; C24H32O6 ; C24H32O6 ; C24H32O6 ; C7H11N5O ; C19H24N2S ; C9H13N2O2 ; C19H19NO2 ; C7H7N3 ; C19H19NO2 ; C14H15N ; C34H50O9
222.02917	1.612775	1.201623	-5.0377	0.000508	3.2938	0.010582	222.0292	HMDB33339 ; HMDB33340 ; HMDB34752 ; HMDB35717 ; HMDB40673 ; HMDB60504	Mollicellin A ; Mollicellin B ; 7-beta-D-Glucopyranosyloxybutylidenephthalide ; S-Acetyl dihydroasparagusic acid ; Artonin K ; S-(12-Dichlorovinyl)glutathione	M+Na+K ; M+Na+K ; M+2K ; 2M+3H2O+2H ; M+Na+K ; M+ACN+2H	C21H18O7 ; C21H18O7 ; C18H22O8 ; C6H10O3S2 ; C21H18O7 ; C12H17Cl2N3O6S
619.44484	1.643522	0.646086	-5.0373	0.000509	3.2936	0.010582	619.4448	HMDB10399 ; HMDB33862	LysoPC(22:1(13Z)) ; 3-(81114-Pentadecatrienyl)phenol	M+ACN+H ; 2M+Na	C30H60NO7P ; C21H30O
192.10193	1.595324	0.183874	5.0354	0.00051	3.2924	0.010582	192.1019	HMDB01896 ; HMDB33560 ; HMDB15699 ; HMDB01939 ; HMDB05045 ; HMDB10213 ; HMDB11482 ; HMDB11493 ; HMDB11512 ; HMDB11523 ; HMDB13620 ; HMDB13621 ; HMDB14398 ; HMDB14842 ; HMDB15489 ; HMDB30184 ; HMDB30755 ; HMDB30922 ; HMDB31754 ; HMDB31845 ; HMDB31998 ; HMDB32502 ; HMDB32727 ; HMDB33896 ; HMDB36802 ; HMDB37210 ; HMDB37245 ; HMDB37246 ; HMDB38325 ; HMDB39885 ; HMDB41430 ; HMDB41858 ; HMDB60037 ; HMDB60040 ; HMDB60047 ; HMDB60048 ; HMDB60049 ; HMDB60050 ; HMDB60051 ; HMDB60052 ; HMDB60112 ; HMDB60947	5-Methoxytryptophol ; (E)-5-(3456-Tetrahydro-3-pyridylidenemethyl)-2-furanmethanol ; Tofisopam ; Medroxyprogesterone ; 15(S)-Hydroxyeicosatrienoic acid ; 17-HDoHE ; LysoPE(0:020:1(11Z)) ; LysoPE(0:022:4(7Z10Z13Z16Z)) ; LysoPE(20:1(11Z)0:0) ; LysoPE(22:4(7Z10Z13Z16Z)0:0) ; 19(20)-EpDPE ; 16(17)-EpDPE ; Medrysone ; Naltrexone ; Testosterone Propionate ; (S)-Isocorydine ; Myrigalone E ; 13-Diacetoxy-4612-tetradecatriene-810-diyne ; Nequinate ; 3-Propylidene-1(3H)-isobenzofuranone ; Peroxysimulenoline ; Santalyl phenylacetate ; 4-Methoxy-1-naphthol ; Anacardic acid ; Austroinulin ; alpha-Santalyl phenylacetate ; Myrigalone A ; Myrigalon B ; Verimol C ; 3-Hydroxyneogrifolin ; Gymnodimine ; Clobenzorex ; 10-HDoHE ; 11-HDoHE ; 16-HDoHE ; 20-HDoHE ; 4-HDoHE ; 7-HDoHE ; 8-HDoHE ; 8-HETrE ; 4-Hydroxy-all-trans-retinyl acetate ; desmethylclomipramine	M+H ; M+H ; M+2H ; M+H+K ; M+Na+K ; M+H+K ; M+3Na ; M+H+2Na ; M+3Na ; M+H+2Na ; M+H+K ; M+H+K ; M+H+K ; M+ACN+2H ; M+H+K ; M+ACN+2H ; M+2ACN+2H ; M+2ACN+2H ; M+H+NH4 ; M+NH4 ; M+ACN+2H ; M+2Na ; M+NH4 ; M+H+K ; M+Na+K ; M+2Na ; M+2ACN+2H ; M+2ACN+2H ; M+2ACN+2H ; M+H+K ; M+3Na ; M+3ACN+2H ; M+H+K ; M+H+K ; M+H+K ; M+H+K ; M+H+K ; M+H+K ; M+H+K ; M+Na+K ; M+H+K ; M+2ACN+2H	C11H13NO2 ; C11H13NO2 ; C22H26N2O4 ; C22H32O3 ; C20H34O3 ; C22H32O3 ; C25H50NO7P ; C27H48NO7P ; C25H50NO7P ; C27H48NO7P ; C22H32O3 ; C22H32O3 ; C22H32O3 ; C20H23NO4 ; C22H32O3 ; C20H23NO4 ; C18H20O4 ; C18H20O4 ; C22H23NO4 ; C11H10O2 ; C20H23NO4 ; C23H30O2 ; C11H10O2 ; C22H32O3 ; C20H34O3 ; C23H30O2 ; C18H20O4 ; C18H20O4 ; C18H20O4 ; C22H32O3 ; C32H45NO4 ; C16H18ClN ; C22H32O3 ; C22H32O3 ; C22H32O3 ; C22H32O3 ; C22H32O3 ; C22H32O3 ; C22H32O3 ; C20H34O3 ; C22H32O3 ; C18H21ClN2
628.62646	1.629352	0.596914	-5.0245	0.000518	3.2854	0.010709	NA	NA	NA	NA	NA

552.50387	1.676053	0.608803	-5.0046	0.000534	3.2727	0.010974	552.5039	HMDB47171 ; HMDB47357 ; HMDB47362 ; HMDB47541 ; HMDB47661 ; HMDB47677 ; HMDB52021 ; HMDB52026 ; HMDB52148 ; HMDB52253 ; HMDB32494 ; HMDB46621 ; HMDB46765 ; HMDB47155 ; HMDB47161 ; HMDB47293 ; HMDB47331 ; HMDB47437 ; HMDB47492 ; HMDB50569 ; HMDB51517 ; HMDB51630 ; HMDB51646 ; HMDB52003 ; HMDB52093	TG(24:024:022:6(4Z7Z10Z13Z16Z19Z)) ; TG(24:024:1(15Z)22:5(4Z7Z10Z13Z16Z)) ; TG(24:024:1(15Z)22:5(7Z10Z13Z16Z19Z)) ; TG(24:022:5(4Z7Z10Z13Z16Z)24:1(15Z)) ; TG(24:022:5(7Z10Z13Z16Z19Z)24:1(15Z)) ; TG(24:022:6(4Z7Z10Z13Z16Z19Z)24:0) ; TG(24:1(15Z)24:022:5(4Z7Z10Z13Z16Z)) ; TG(24:1(15Z)24:022:5(7Z10Z13Z16Z19Z)) ; TG(24:1(15Z)24:1(15Z)22:4(7Z10Z13Z16Z)) ; TG(24:1(15Z)22:4(7Z10Z13Z16Z)24:1(15Z)) ; Propylene glycol mono- and diesters of fats and fatty acids ; TG(22:024:1(15Z)22:2(13Z16Z)) ; TG(22:022:2(13Z16Z)24:1(15Z)) ; TG(24:024:020:3(5Z8Z11Z)) ; TG(24:024:020:3n6) ; TG(24:020:3(5Z8Z11Z)24:0) ; TG(24:022:1(13Z)22:2(13Z16Z)) ; TG(24:020:3n624:0) ; TG(24:022:2(13Z16Z)22:1(13Z)) ; TG(20:1(11Z)24:1(15Z)24:1(15Z)) ; TG(22:1(13Z)24:022:2(13Z16Z)) ; TG(22:1(13Z)22:1(13Z)24:1(15Z)) ; TG(22:1(13Z)24:1(15Z)22:1(13Z)) ; TG(24:1(15Z)22:022:2(13Z16Z)) ; TG(24:1(15Z)20:1(11Z)24:1(15Z))	M+2H ; M+2H ; M+2H ; M+2H ; M+2H ; M+2H ; M+2H ; M+2H ; M+2H ; M+2H ; M+ACN+H ; M+H+Na ; M+H+Na ; M+H+Na ; M+H+Na ; M+H+Na ; M+H+Na ; M+H+Na ; M+H+Na ; M+H+Na ; M+H+Na ; M+H+Na ; M+H+Na ; M+H+Na ; M+H+Na	C73H130O6 ; C73H130O6 ; C73H130O6 ; C73H130O6 ; C73H130O6 ; C73H130O6 ; C73H130O6 ; C73H130O6 ; C73H130O6 ; C73H130O6 ; C32H62O4 ; C71H132O6 ; C71H132O6 ; C71H132O6 ; C71H132O6 ; C71H132O6 ; C71H132O6 ; C71H132O6 ; C71H132O6 ; C71H132O6 ; C71H132O6 ; C71H132O6 ; C71H132O6 ; C71H132O6 ; C71H132O6
231.17015	1.59534	0.993313	-5.0023	0.000536	3.2712	0.010974	231.1702	HMDB01492 ; HMDB04666 ; HMDB06335 ; HMDB06463 ; HMDB11549 ; HMDB11578 ; HMDB11579 ; HMDB11626 ; HMDB12112 ; HMDB12173 ; HMDB12932 ; HMDB34253 ; HMDB34401 ; HMDB34840 ; HMDB35273 ; HMDB36356 ; HMDB36568 ; HMDB41288	Gamma-Tocopherol ; 2-Arachidonylglycerol ; Beta-tocopherol ; Stearidonyl carnitine ; MG(0:020:4(8Z11Z14Z17Z)0:0) ; MG(20:4(5Z8Z11Z14Z)0:00:0) ; MG(20:4(8Z11Z14Z17Z)0:00:0) ; Dodecanol ; (22Alpha)-hydroxy-5alpha-campestan-3-one ; 4Alpha-hydroxymethyl-5alpha-cholesta-8-en-3beta-ol ; Dynorphin A (6-8) ; z2-Tocopherol ; Cyclostenol ; Faradiol laurate ; 1-Acetoxy-2-hydroxy-51215-heneicosatrien-4-one ; 12-Gingerol ; Persenone A ; 2-Butyl-1-octanol	M+2Na ; M+2ACN+2H ; M+2Na ; M+ACN+2H ; M+2ACN+2H ; M+2ACN+2H ; M+2ACN+2H ; M+2Na-H ; M+2Na ; M+2Na ; M+H+NH4 ; M+2Na ; M+2Na ; M+3Na ; M+2ACN+2H ; M+2ACN+2H ; M+2ACN+2H ; M+2Na-H	C28H48O2 ; C23H38O4 ; C28H48O2 ; C25H41NO4 ; C23H38O4 ; C23H38O4 ; C23H38O4 ; C12H26O ; C28H48O2 ; C28H48O2 ; C18H37N9O4 ; C28H48O2 ; C28H48O2 ; C42H72O3 ; C23H38O4 ; C23H38O4 ; C23H38O4 ; C12H26O
290.99054	1.598781	0.172408	-4.9926	0.000543	3.265	0.010981	290.9905	HMDB00362 ; HMDB00807 ; HMDB00963 ; HMDB01299 ; HMDB03391 ; HMDB06039 ; HMDB30483 ; HMDB60180	2-Phosphoglyceric acid ; 3-Phosphoglyceric acid ; 5-Methylthioribose 1-phosphate ; 5-Methylthioribulose 1-phosphate ; 2-Phospho-D-glyceric acid ; 8-Chloroxanthine ; Questiomycin A ; (2R)-2-Hydroxy-3-(phosphonatooxy)propanoate	3M+H+Na ; 3M+H+Na ; 2M+Na+K ; 2M+Na+K ; 3M+H+Na ; 3M+H+Na ; M+2K+H ; 3M+H+Na	C3H7O7P ; C3H7O7P ; C6H13O7PS ; C6H13O7PS ; C3H7O7P ; C5H3ClN4O2 ; C12H8N2O2 ; C3H7O7P
448.86562	1.575591	1.143677	-4.9913	0.000544	3.2642	0.010981	448.8656	HMDB02274 ; HMDB09743 ; HMDB09744 ; HMDB09775 ; HMDB11396 ; HMDB11428 ; HMDB11461	Methylcobalamin ; PE(24:0P-18:1(11Z)) ; PE(24:0P-18:1(9Z)) ; PE(24:1(15Z)P-18:0) ; PE(P-18:024:1(15Z)) ; PE(P-18:1(11Z)24:0) ; PE(P-18:1(9Z)24:0)	M+3H ; M+2ACN+2H ; M+2ACN+2H ; M+2ACN+2H ; M+2ACN+2H ; M+2ACN+2H ; M+2ACN+2H	C63H91CoN13O14P ; C47H92NO7P ; C47H92NO7P ; C47H92NO7P ; C47H92NO7P ; C47H92NO7P ; C47H92NO7P
721.46966	1.580488	1.064435	-4.9901	0.000545	3.2634	0.010981	721.4697	HMDB08840 ; HMDB08871 ; HMDB08872 ; HMDB08964 ; HMDB09188 ; HMDB09383 ; HMDB09416 ; HMDB09448 ; HMDB12330 ; HMDB12344 ; HMDB12364 ; HMDB13326 ; HMDB30180	PE(14:020:5(5Z8Z11Z14Z17Z)) ; PE(14:1(9Z)20:4(5Z8Z11Z14Z)) ; PE(14:1(9Z)20:4(8Z11Z14Z17Z)) ; PE(16:1(9Z)18:4(6Z9Z12Z15Z)) ; PE(18:4(6Z9Z12Z15Z)16:1(9Z)) ; PE(20:4(5Z8Z11Z14Z)14:1(9Z)) ; PE(20:4(8Z11Z14Z17Z)14:1(9Z)) ; PE(20:5(5Z8Z11Z14Z17Z)14:0) ; PS(14:014:0) ; PS(14:1(9Z)16:1(9Z)) ; PS(16:1(9Z)14:1(9Z)) ; trans-2-Dodecenoylcarnitine ; Crustecdysone	2M+H+Na ; 2M+H+Na ; 2M+H+Na ; 2M+H+Na ; 2M+H+Na ; 2M+H+Na ; 2M+H+Na ; 2M+H+Na ; M+ACN+H ; M+NH4 ; M+NH4 ; 2M+K ; 3M+2H	C39H68NO8P ; C39H68NO8P ; C39H68NO8P ; C39H68NO8P ; C39H68NO8P ; C39H68NO8P ; C39H68NO8P ; C39H68NO8P ; C34H66NO10P ; C36H66NO10P ; C36H66NO10P ; C19H35NO4 ; C27H44O7
243.01551	1.611702	1.017556	4.988	0.000547	3.262	0.010981	243.0155	HMDB00998 ; HMDB01564 ; HMDB12236 ; HMDB31334 ; HMDB38589 ; HMDB39567	dCTP ; CDP-Ethanolamine ; Imidazole acetol-phosphate ; Glycerol alpha-monochlorohydrin ; Brassicanal C ; BL V	M+H+NH4 ; M+H+K ; M+Na ; 2M+Na ; 2M+H+K ; M+Na+K	C9H16N3O13P3 ; C11H20N4O11P2 ; C6H9N2O5P ; C3H7ClO2 ; C10H9NO3S ; C22H16O9
216.06320	1.599283	1.305912	-4.9868	0.000548	3.2613	0.010981	216.0632	HMDB00114 ; HMDB59660 ; HMDB14805 ; HMDB15068 ; HMDB33319 ; HMDB38442 ; HMDB40776 ; HMDB41099 ; HMDB61008	Glycerylphosphorylethanolamine ; sn-glycero-3-Phosphoethanolamine ; Histamine Phosphate ; Mesoridazine ; Licarin C ; 1-Isothiocyanato-2-(methylthio)ethane ; 11-Bis(2-hydroxy-3-methylcarbazole) ; Kanzonol R ; m-chlorophenylpiperazine (m-CPP)	M+H ; M+H ; M+3ACN+2H ; M+2Na ; M+Na+K ; M+2ACN+H ; M+H+K ; M+Na+K ; 2M+H+K	C5H14NO6P ; C5H14NO6P ; C5H15N3O8P2 ; C21H26N2OS2 ; C22H26O5 ; C4H7NS2 ; C26H20N2O2 ; C22H26O5 ; C10H13ClN2
155.01122	1.578015	0.840574	-4.9831	0.000551	3.2589	0.010981	155.0112	HMDB00019 ; HMDB00310 ; HMDB00720 ; HMDB01865 ; HMDB02124 ; HMDB03217 ; HMDB03771 ; HMDB12233 ; HMDB29372 ; HMDB29521 ; HMDB29971 ; HMDB30829 ; HMDB30967 ; HMDB31640 ; HMDB31643 ; HMDB31715 ; HMDB33790 ; HMDB33860 ; HMDB34004 ; HMDB34409 ; HMDB34466 ; HMDB35214 ; HMDB39449 ; HMDB41655	Alpha-ketoisovaleric acid ; Methylacetoacetic acid ; Levulinic acid ; 2-Oxovaleric acid ; Apigenin ; Genistein ; 2-Methylacetoacetic acid ; Glutarate semialdehyde ; 2-Hydroxydaidzein ; Norizalpinin ; Coriandrone E ; Aloeemodin ; 246-Octatriynoic acid ; Glycerol 1-propanoate diacetate ; Ethyl pyruvate ; 6-Hydroxydaidzein ; 128-Trihydroxy-3-methylanthraquinone ; 8-Hydroxydaidzein ; 347-Trihydroxyflavone ; Rhababerone ; Acetoxyacetone ; Emodin ; Rheinanthrone ; 3-Hydroxydaidzein	M+K ; M+K ; M+K ; M+K ; M+H+K ; M+H+K ; M+K ; M+K ; M+H+K ; M+H+K ; M+Na+K ; M+H+K ; M+Na ; M+2K ; M+K ; M+H+K ; M+H+K ; M+H+K ; M+H+K ; M+H+K ; M+K ; M+H+K ; M+H+K ; M+H+K	C5H8O3 ; C5H8O3 ; C5H8O3 ; C5H8O3 ; C15H10O5 ; C15H10O5 ; C5H8O3 ; C5H8O3 ; C15H10O5 ; C15H10O5 ; C13H12O5 ; C15H10O5 ; C8H4O2 ; C10H16O6 ; C5H8O3 ; C15H10O5 ; C15H10O5 ; C15H10O5 ; C15H10O5 ; C15H10O5 ; C5H8O3 ; C15H10O5 ; C15H10O5 ; C15H10O5
110.02013	1.632126	1.063436	4.9824	0.000551	3.2585	0.010981	110.0201	HMDB41785	Tyrosol 4-sulfate	M+2H	C8H10O5S
376.05534	1.61351	1.200716	-4.9793	0.000554	3.2565	0.010981	376.0553	HMDB39464	(E)-2-Propenyl 3-(2-propenylthio)-2-propenyl sulfate	3M+2H	C9H14O4S2

140.03298	1.612546	0.067447	4.9773	0.000556	3.2552	0.010981	140.033	HMDB01232 ; HMDB02658 ; HMDB13188 ; HMDB59710 ; HMDB00115 ; HMDB00350 ; HMDB00424 ; HMDB11169 ; HMDB14937 ; HMDB15625 ; HMDB28763 ; HMDB29057 ; HMDB30486 ; HMDB30648 ; HMDB31608 ; HMDB33109 ; HMDB35712 ; HMDB40906 ; HMDB41005 ; HMDB41006 ; HMDB60981 ; HMDB61105	4-Nitrophenol ; 6-Hydroxynicotinic acid ; 3-Hydroxypicolinic acid ; 2-Hydroxynicotinic acid ; Glycolic acid ; 3-Hydroxysebacic acid ; 2-Hydroxydecanedioic acid ; L-beta-aspartyl-L-threonine ; Tazarotene ; Prasugrel ; Aspartyl-Threonine ; Threoninyl-Aspartate ; (S)-23-Dihydro-6-hydroxy-5-(hydroxyacetyl)-2-isopropenylbenzofuran ; Artemidiol ; Peracetic acid ; Methyl (2R3S)-23-dihydro-3-hydroxy-2-isopropenyl-5-benzofurancarboxylate ; Aloesol ; Coixinden B ; 1-Acetoxychavicol acetate ; 1-Acetoxychavicol ; para-hydroxyrosiglitazone ; ortho-hydroxyrosiglitazone	M+H ; M+H ; M+H ; M+H ; M+ACN+Na ; M+Na+K ; M+Na+K ; M+2Na ; M+3Na ; M+H+2Na ; M+2Na ; M+2Na ; M+2Na ; M+2Na ; M+ACN+Na ; M+2Na ; M+2Na ; M+2Na ; M+2Na ; M+2Na ; M+H+2Na ; M+H+2Na	C6H5NO3 ; C6H5NO3 ; C6H5NO3 ; C6H5NO3 ; C2H4O3 ; C10H18O5 ; C10H18O5 ; C8H14N2O6 ; C21H21NO2S ; C20H20FNO3S ; C8H14N2O6 ; C8H14N2O6 ; C13H14O4 ; C13H14O4 ; C2H4O3 ; C13H14O4 ; C13H14O4 ; C13H14O4 ; C13H14O4 ; C13H14O4 ; C18H19N3O4S ; C18H19N3O4S
346.57002	1.573849	0.68902	4.9701	0.000562	3.2506	0.01103	346.57	HMDB60465 ; HMDB60775	cyclic GMP-AMP ; 5-Carboxy-lumiracoxib	M+H+NH4 ; 2M+H+NH4	C20H24N10O13P2 ; C16H13ClFNO4
148.02997	1.588812	0.818007	4.9689	0.000563	3.2498	0.01103	148.0276	HMDB02339 ; HMDB06809 ; HMDB13894 ; HMDB28778 ; HMDB28779 ; HMDB28904 ; HMDB28926 ; HMDB30227 ; HMDB30759 ; HMDB31473 ; HMDB31474 ; HMDB32227 ; HMDB33052 ; HMDB35227 ; HMDB36179 ; HMDB37704 ; HMDB37738 ; HMDB38281 ; HMDB38637 ; HMDB40250 ; HMDB41064 ; HMDB41318 ; HMDB41389 ; HMDB41391 ; HMDB60968	5-Methoxytryptophan ; Nicotinate D-ribonucleoside ; S-4-Hydroxymephenytoin ; Cysteinyl-Isoleucine ; Cysteinyl-Leucine ; Isoleucyl-Cysteine ; Leucyl-Cysteine ; 5-Methoxycanthin-6-one ; (R)-Pterosin B ; 13-Dithiane ; 14-Dithiane ; Dimethylbenzyl carbinyl crotonate ; Ethyl vinyl disulfide ; Hydroxypropanedioic acid ; 2-Methyl-13-dithiolane ; Isoamyl cinnamate ; 2-(3-Hydroxy-4-methylphenyl)-5-methyl-4-hexen-3-one ; cis-3-Hexenyl phenylacetate ; Methoxybrassitin ; xi-Tetrahydro-2-thiophenethiol ; 2-Methoxycanthin-6-one ; Cinnamyl isovalerate ; Methyl 2-propenyl disulfide ; (E)-Methyl 1-propenyl disulfide ; 4-hydroxymephenytoin	M+Na+K ; M+H+K ; M+Na+K ; M+Na+K ; M+Na+K ; M+Na+K ; M+Na+K ; M+2Na ; M+2K ; 2M+3H2O+2H ; 2M+3H2O+2H ; M+2K ; 2M+3H2O+2H ; 2M+3H2O+2H ; 2M+3H2O+2H ; M+2K ; M+2K ; M+2K ; M+2Na ; 2M+3H2O+2H ; M+2Na ; M+2K ; 2M+3H2O+2H ; 2M+3H2O+2H ; M+Na+K	C12H14N2O3 ; C11H14NO6 ; C12H14N2O3 ; C9H18N2O3S ; C9H18N2O3S ; C9H18N2O3S ; C9H18N2O3S ; C15H10N2O2 ; C14H18O2 ; C4H8S2 ; C4H8S2 ; C14H18O2 ; C4H8S2 ; C3H4O5 ; C4H8S2 ; C14H18O2 ; C14H18O2 ; C14H18O2 ; C12H14N2O2S ; C4H8S2 ; C15H10N2O2 ; C14H18O2 ; C4H8S2 ; C4H8S2 ; C12H14N2O3
370.03991	1.560433	0.853976	-4.9629	0.000568	3.246	0.011086	370.0399	HMDB11640 ; HMDB14678 ; HMDB15143 ; HMDB61052 ; HMDB61053	Uridine 23-cyclic phosphate ; Gadoversetamide ; Busulfan ; 4-hydroxytriazolam ; 1-hydroxytriazolam	M+ACN+Na ; M+2K ; 3M+2H ; 2M+H+Na ; 2M+H+Na	C9H11N2O8P ; C20H34GdN5O10 ; C6H14O6S2 ; C17H12Cl2N4O ; C17H12Cl2N4O
100.02161	1.576157	0.695297	4.9534	0.000576	3.2399	0.011111	100.0216	HMDB05843 ; HMDB29755 ; HMDB33116 ; HMDB33117 ; HMDB38624 ; HMDB00555 ; HMDB00709 ; HMDB00727 ; HMDB00845 ; HMDB00857 ; HMDB00877 ; HMDB02109 ; HMDB02441 ; HMDB05784 ; HMDB29167 ; HMDB29573 ; HMDB29720 ; HMDB29758 ; HMDB29895 ; HMDB30570 ; HMDB31054 ; HMDB32012 ; HMDB32883 ; HMDB32990 ; HMDB33813 ; HMDB34158 ; HMDB37724 ; HMDB37728 ; HMDB41605 ; HMDB59622 ; HMDB59722 ; HMDB59738 ; HMDB59893 ; HMDB59966	Allyl isothiocyanate ; 3-Thiocyanato-1-propene ; 4-Methylthiazole ; 5-Methylthiazole ; Thiiraneacetonitrile ; 3-Methyladipic acid ; L-Cysteinylglycine disulfide ; L-Threoneopterin ; Neopterin ; Pimelic acid ; Umanopterin ; Hydroxysepiapterin ; 33-Dimethylglutaric acid ; Hydroxytyrosol ; 2-Methyladipic acid ; Diethyl malonate ; 2-Thiophenethiol ; Herniarin ; D-erythro-Eritadenine ; Sylvopinol ; 10-Hydroxy-28-decadiene-46-diynoic acid ; 4-Hydroxy-3-methoxybenzenemethanol ; 145-Naphthalenetriol ; 7-Hydroxy-6-methyl-2H-1-benzopyran-2-one ; 3-(34-Methylenedioxyphenyl)propenal ; 26-Dimethoxyphenol ; Propyl 2-furoate ; 2-Furanylmethyl propanoate ; Propyleneglycol diacetate ; 4-Methylumbelliferone ; Mono-methyl-adipate ; 2-Ethylglutaric acid ; Ethyl methylsuccinate ; 35-Dimethoxyphenol	M+H ; M+H ; M+H ; M+H ; M+H ; M+H+K ; M+3H ; M+H+2Na ; M+H+2Na ; M+H+K ; M+H+2Na ; M+H+2Na ; M+H+K ; M+2Na ; M+H+K ; M+H+K ; M+2ACN+2H ; M+H+Na ; M+H+2Na ; M+2Na ; M+H+Na ; M+2Na ; M+H+Na ; M+H+Na ; M+H+Na ; M+2Na ; M+2Na ; M+2Na ; M+H+K ; M+H+Na ; M+H+K ; M+H+K ; M+H+K ; M+2Na	C4H5NS ; C4H5NS ; C4H5NS ; C4H5NS ; C4H5NS ; C7H12O4 ; C8H15N3O5S2 ; C9H11N5O4 ; C9H11N5O4 ; C7H12O4 ; C9H11N5O4 ; C9H11N5O4 ; C7H12O4 ; C8H10O3 ; C7H12O4 ; C7H12O4 ; C4H4S2 ; C10H8O3 ; C9H11N5O4 ; C8H10O3 ; C10H8O3 ; C8H10O3 ; C10H8O3 ; C10H8O3 ; C10H8O3 ; C8H10O3 ; C8H10O3 ; C8H10O3 ; C7H12O4 ; C10H8O3 ; C7H12O4 ; C7H12O4 ; C7H12O4 ; C8H10O3
439.11639	1.626886	0.921612	4.9531	0.000576	3.2397	0.011111	439.1164	HMDB32503 ; HMDB29460 ; HMDB31791 ; HMDB33246 ; HMDB33250 ; HMDB36862 ; HMDB38600 ; HMDB39126 ; HMDB39387 ; HMDB41508 ; HMDB60426	Silica aerogel ; Xerocomic acid ; Fenvalerate ; Grevilline D ; Grevilline C ; Ginkgolide A ; Torachrysone 8-glucoside ; Wybutoxine ; Methyl 26-dihydroxy-4-quinolinecarboxylate ; Marmesin galactoside ; 8-Methoxykynurenate	M+H ; M+2ACN+H ; 2M+H+K ; M+2ACN+H ; M+2ACN+H ; 2M+Na+K ; 2M+Na+K ; 2M+Na+K ; 2M+H ; 2M+Na+K ; 2M+H	C23H22N2O3S2 ; C18H12O8 ; C25H22ClNO3 ; C18H12O8 ; C18H12O8 ; C20H24O9 ; C20H24O9 ; C16H20N6O7 ; C11H9NO4 ; C20H24O9 ; C11H9NO4
179.51415	1.634158	0.050955	4.9512	0.000578	3.2384	0.011111	179.514	HMDB33954 ; HMDB40693	Juzirine ; N2-(gamma-Glutamyl)-4-carboxyphenylhydrazine	M+2K ; M+2K	C17H15NO3 ; C12H15N3O5
549.73287	1.64632	0.758591	-4.9509	0.000578	3.2382	0.011111	549.7329	HMDB34626 ; HMDB34627 ; HMDB37028 ; HMDB38381 ; HMDB39473 ; HMDB40283 ; HMDB60595	1-(34-Dimethoxyphenyl)-12-ethanediol 1-O-b-D-glucoside ; 1-(34-Dimethoxyphenyl)-12-ethanediol 2-O-b-D-glucoside ; Deoxyloganic acid ; 2-(4-Hydroxy-35-dimethoxyphenyl)ethanol 4-glucoside ; 2-Methoxy-3-(24-dihydroxyphenyl)-12-propanediol 4-glucoside ; (8R8R)-Secoisolariciresinol 99-bis-4-carboxy-3-hydroxy-3-methylbutanoyl-(-6)-glucoside ; Olopatadine n-oxide	3M+H+NH4 ; 3M+H+NH4 ; 3M+H+NH4 ; 3M+H+NH4 ; 3M+H+NH4 ; M+3ACN+2H ; 3M+H+K	C16H24O9 ; C16H24O9 ; C16H24O9 ; C16H24O9 ; C16H24O9 ; C44H62O24 ; C21H23NO4
120.00891	1.6468	0.862009	4.946	0.000582	3.2351	0.011148	120.0089	HMDB33532 ; HMDB37192 ; HMDB41503 ; HMDB59918 ; HMDB61002 ; HMDB02916 ; HMDB06200	Dimethicone ; Potassium 2-(1-ethoxy) ethoxypropanoate ; Bis(2-furanylmethyl) sulfide ; 2-(1-Ethoxyethoxy)propanoic acid ; norsertraline ; 4-Nitrocatechol ; 24-Dihydroxy-nitrophenol	M+2K ; M+H+K ; M+2Na ; M+2K ; M+3Na ; M+H-2H2O ; M+H-2H2O	C6H18OSi2 ; C7H13KO4 ; C10H10O2S ; C7H14O4 ; C16H15Cl2N ; C6H5NO4 ; C6H5NO4
215.04202	1.587159	0.462396	-4.9348	0.000592	3.2279	0.011231	215.042	HMDB14815 ; HMDB29549 ; HMDB33900 ; HMDB34223 ; HMDB35608 ; HMDB41693 ; HMDB59989	Isoflurophate ; Eleutheroside B1 ; Tomenin ; 23456-Penta-O-acetyl-D-glucose ; Monotropein ; 5-(35-Dihydroxyphenyl)-gamma-valerolactone 3-O-glucuronide ; 5-(34-Dihydroxyphenyl)-gamma-valerolactone-4-O-glucuronide	2M+Na+K ; M+2Na ; M+2Na ; M+H+K ; M+H+K ; M+2Na ; M+2Na	C6H14FO3P ; C17H20O10 ; C17H20O10 ; C16H22O11 ; C16H22O11 ; C17H20O10 ; C17H20O10
207.44065	1.668779	0.381754	4.9332	0.000593	3.2268	0.011231	NA	NA	NA	NA	NA
246.07945	1.556279	0.972005	4.932	0.000594	3.2261	0.011231	246.0795	HMDB40512 ; HMDB00445 ; HMDB15559 ; HMDB39253 ; HMDB60072 ; HMDB61037 ; HMDB42056	456-Trimethylscutellarein 7-glucoside ; Alpha-Tetrasaccharide ; Thioproperazine ; Crosatoside B ; CMP-N-trimethyl-2-aminoethylphosphonate ; 44-methanol-bisbenzonitrile ; Tulobuterol	M+2H ; M+H+2Na ; M+2Na ; M+2Na ; M+H+NH4 ; 2M+H+Na ; M+H-H2O	C24H26O11 ; C26H45NO20 ; C22H30N4O2S2 ; C20H30O11 ; C14H27N4O10P2 ; C15H10N2O ; C12H19Cl2NO

197.05906	1.64278	1.008582	4.9308	0.000595	3.2253	0.011231	197.0591	HMDB00439 ; HMDB12247 ; HMDB12271 ; HMDB15025 ; HMDB15412 ; HMDB29319 ; HMDB29415 ; HMDB30716 ; HMDB30735 ; HMDB32907 ; HMDB33657 ; HMDB34025 ; HMDB34048 ; HMDB34264 ; HMDB34330 ; HMDB34353 ; HMDB34414 ; HMDB34749 ; HMDB34838 ; HMDB35002 ; HMDB35230 ; HMDB35371 ; HMDB35428 ; HMDB37155 ; HMDB37479 ; HMDB38183 ; HMDB38711 ; HMDB38756 ; HMDB38927 ; HMDB39067 ; HMDB40471 ; HMDB41500	2-Furoylglycine ; L-23-Dihydrodipicolinate ; O-Ureidohomoserine ; Mechlorethamine ; Sulfacytine ; 1-Methoxyphaseollidin ; S-Carboxymethyl-L-cysteine ; 23-Dihydro-7-methoxy-2-(3-methoxy-45-methylenedioxyphenyl)-3-methyl-5-(1-propenyl)benzofuran ; Aurantiumal ; Isolariciresinol 9-O-beta-D-glucoside ; De-O-methylsterigmatocystin ; Cristacarpin ; Isoxanthohumol ; Glyceollin IV ; Physalin E ; Physalin D ; 3-Hydroxy-4-methoxyglabridin ; Icariside E5 ; Lansiumarin C ; Xanthogalenol ; Aurantricholide B ; Licoagrochalcone D ; Licoagrochalcone C ; 44-Thiobis-2-butanone ; Xanthohumol ; 3-(4-Methyl-3-pentenyl)thiophene ; (7R8R)-47-Epoxy-35-dimethoxy-499-lignanetriol 9-glucoside ; Gancaonin I ; Isolariciresinol 9-O-beta-D-glucoside ; Epoxybergamottin ; Isolariciresinol 4-O-beta-D-glucoside ; 1-Deoxy-D-glucitol	2M+3H2O+2H ; 2M+3H2O+2H ; 2M+H+K ; M+ACN+H ; 2M+3H ; M+H+K ; M+NH4 ; M+H+K ; M+H+K ; M+3Na ; M+2ACN+2H ; M+H+K ; M+H+K ; M+H+K ; M+H+2Na ; M+H+2Na ; M+H+K ; M+3Na ; M+H+K ; M+H+K ; M+2ACN+2H ; M+H+K ; M+H+K ; M+Na ; M+H+K ; 2M+Na+K ; M+3Na ; M+H+K ; M+3Na ; M+H+K ; M+3Na ; 2M+Na+K	C7H7NO4 ; C7H7NO4 ; C5H11N3O4 ; C5H11Cl2N ; C12H14N4O3S ; C21H22O5 ; C5H9NO4S ; C21H22O5 ; C21H22O5 ; C26H34O11 ; C17H10O6 ; C21H22O5 ; C21H22O5 ; C21H22O5 ; C28H32O11 ; C28H32O11 ; C21H22O5 ; C26H34O11 ; C21H22O5 ; C21H22O5 ; C17H10O6 ; C21H22O5 ; C21H22O5 ; C8H14O2S ; C21H22O5 ; C10H14S ; C26H34O11 ; C21H22O5 ; C26H34O11 ; C21H22O5 ; C26H34O11 ; C6H14O5
250.86775	1.639885	0.368519	4.9198	0.000605	3.2182	0.011374	250.8678	HMDB32079 ; HMDB32080 ; HMDB04972 ; HMDB10709	24-Dibromophenol ; 26-Dibromophenol ; Glucosylceramide (d18:118:0) ; Galactosylceramide (d18:118:0)	M+H ; M+H ; M+2H+Na ; M+2H+Na	C6H4Br2O ; C6H4Br2O ; C42H81NO8 ; C42H81NO8
285.24229	1.623706	0.522377	4.9146	0.00061	3.2149	0.011392	285.2423	HMDB35473 ; HMDB38685 ; HMDB12167 ; HMDB13224 ; HMDB30015 ; HMDB31080 ; HMDB31931 ; HMDB32084 ; HMDB37382 ; HMDB38063 ; HMDB39425 ; HMDB11530 ; HMDB11561	Avocadyne ; Muricatacin ; 4alpha-Formyl-4beta-methyl-5alpha-cholesta-824-dien-3beta-ol ; Caproylcholine ; 7-Oxostigmasterol ; Momordenol ; Stigmast-22-ene-36-dione ; 29-Norcycloartane-324-dione ; (3beta23E)-3-Hydroxy-27-norcycloart-23-en-25-one ; Stigmast-4-ene-36-dione ; (6beta22E)-6-Hydroxystigmasta-422-dien-3-one ; MG(0:014:00:0) ; MG(14:00:00:0)	M+H ; M+H ; 2M+3H ; M+2ACN+H ; 2M+3H ; 2M+3H ; 2M+3H ; 2M+3H ; 2M+3H ; 2M+3H ; 2M+3H ; M+H-H2O ; M+H-H2O	C17H32O3 ; C17H32O3 ; C29H46O2 ; C11H24NO2 ; C29H46O2 ; C29H46O2 ; C29H46O2 ; C29H46O2 ; C29H46O2 ; C29H46O2 ; C29H46O2 ; C17H34O4 ; C17H34O4
489.59784	1.597544	0.745137	-4.911	0.000613	3.2125	0.011392	489.5978	HMDB30835 ; HMDB40453	trans-334557-Hexahydroxyflavanone ; Theaflavonin	3M+H+NH4 ; M+ACN+2H	C15H12O8 ; C43H36O24
348.50455	1.559073	0.771909	-4.9081	0.000616	3.2107	0.011392	NA	NA	NA	NA	NA
108.50090	1.641448	0.605699	4.9064	0.000617	3.2095	0.011392	108.5009	HMDB12234	Histidinal	M+2K	C6H9N3O
374.19783	1.638982	0.267936	-4.9061	0.000617	3.2094	0.011392	374.1978	HMDB30206 ; HMDB30333 ; HMDB30666 ; HMDB30798 ; HMDB30914 ; HMDB33461 ; HMDB33879 ; HMDB33880 ; HMDB35403 ; HMDB35440 ; HMDB41401	Jubanine B ; Fragransol C ; Chalepin acetate ; Myricanone ; Alliospiroside C ; 4-Coumaroylputrescine ; Mammea BAD cyclo D ; Mammea BAC cyclo D ; Gingerenone A ; alphabeta-Dihydroxanthohumol ; 1234-Tetrahydro-1-1-hydroxy-3-(4-hydroxyphenyl)-2-propenyl-67-dimethoxy-2-naphthalenol	M+H+NH4 ; M+NH4 ; M+NH4 ; M+NH4 ; M+H+Na ; 3M+2Na ; M+NH4 ; M+NH4 ; M+NH4 ; M+NH4 ; M+NH4	C43H47N5O6 ; C21H24O5 ; C21H24O5 ; C21H24O5 ; C38H60O13 ; C13H18N2O2 ; C21H24O5 ; C21H24O5 ; C21H24O5 ; C21H24O5 ; C21H24O5
198.09759	1.601894	0.261505	4.8948	0.000628	3.202	0.011544	198.0976	HMDB14758 ; HMDB31690 ; HMDB37298 ; HMDB00122 ; HMDB00143 ; HMDB00169 ; HMDB00190 ; HMDB00211 ; HMDB00346 ; HMDB00516 ; HMDB00660 ; HMDB00700 ; HMDB01051 ; HMDB01151 ; HMDB01240 ; HMDB01266 ; HMDB01311 ; HMDB01329 ; HMDB01497 ; HMDB01860 ; HMDB01882 ; HMDB01889 ; HMDB02825 ; HMDB03345 ; HMDB03418 ; HMDB03449 ; HMDB06088 ; HMDB12224 ; HMDB12326 ; HMDB29580 ; HMDB31232 ; HMDB32222 ; HMDB33704 ; HMDB34220 ; HMDB41911 ; HMDB41929 ; HMDB60679 ; HMDB60903	Triamcinolone ; cis-3-Hexenyl b-primeveroside ; 138-Trihydroxy-4-methyl-27-diprenylxanthone ; D-Glucose ; D-Galactose ; D-Mannose ; L-Lactic acid ; Myoinositol ; 3-Deoxyarabinohexonic acid ; Beta-D-Glucose ; D-Fructose ; Hydroxypropionic acid ; Glyceraldehyde ; Allose ; Pseudooxynicotine ; L-Sorbose ; D-Lactic acid ; 2-Hydroxynicotine ; Nicotine-1-N-oxide ; Paraxanthine ; Dihydroxyacetone ; Theophylline ; Theobromine ; Alpha-D-Glucose ; D-Tagatose ; Beta-D-Galactose ; Scyllitol ; Emopamil ; L-Gulose ; Dimethyl carbonate ; Monoethyl carbonate ; Dihydroxyacetone (dimer) ; L-Galactose ; Levoinositol ; Isepamicin ; Methoxyacetic acid ; Glycinexylidide ; Reduced haloperidol	M+2H ; M+2H ; M+2H ; M+NH4 ; M+NH4 ; M+NH4 ; 2M+NH4 ; M+NH4 ; M+NH4 ; M+NH4 ; M+NH4 ; 2M+NH4 ; 2M+NH4 ; M+NH4 ; 2M+H+K ; M+NH4 ; 2M+NH4 ; 2M+H+K ; 2M+H+K ; M+NH4 ; 2M+NH4 ; M+NH4 ; M+NH4 ; M+NH4 ; M+NH4 ; M+NH4 ; M+NH4 ; M+Na+K ; M+NH4 ; 2M+NH4 ; 2M+NH4 ; M+NH4 ; M+NH4 ; M+NH4 ; M+2H+Na ; 2M+NH4 ; 2M+H+K ; M+H+NH4	C21H27FO6 ; C17H30O10 ; C24H26O5 ; C6H12O6 ; C6H12O6 ; C6H12O6 ; C3H6O3 ; C6H12O6 ; C6H12O6 ; C6H12O6 ; C6H12O6 ; C3H6O3 ; C3H6O3 ; C6H12O6 ; C10H14N2O ; C6H12O6 ; C3H6O3 ; C10H14N2O ; C10H14N2O ; C7H8N4O2 ; C3H6O3 ; C7H8N4O2 ; C7H8N4O2 ; C6H12O6 ; C6H12O6 ; C6H12O6 ; C6H12O6 ; C23H30N2 ; C6H12O6 ; C3H6O3 ; C3H6O3 ; C6H12O6 ; C6H12O6 ; C6H12O6 ; C22H43N5O12 ; C3H6O3 ; C10H14N2O ; C21H25ClFNO2
284.08030	1.611522	0.52151	-4.892	0.000631	3.2002	0.011544	284.0803	HMDB36254 ; HMDB36255 ; HMDB10356 ; HMDB15072 ; HMDB15702 ; HMDB29237 ; HMDB29241 ; HMDB30630 ; HMDB33935 ; HMDB60843 ; HMDB60861	Gladiatoside C2 ; Gladiatoside C1 ; Estriol 3-sulfate 16-glucuronide ; Diethylpropion ; Ticagrelor ; Cyanidin 3-(6-succinyl-glucoside) ; Peonidin 3-(6-malonyl-glucoside) ; Rubraflavone C ; Anigorufone ; N-Desthienylethyl-rotigotine ; OR-1896	M+2H ; M+2H ; M+H+Na ; M+2K+H ; M+2Na ; M+H+NH4 ; M+H+NH4 ; M+2K ; 2M+H+Na ; M+2K+H ; M+K	C29H26O12 ; C29H26O12 ; C24H32O12S ; C13H19NO ; C23H28F2N6O4S ; C25H25O14 ; C25H25O14 ; C30H34O6 ; C19H12O2 ; C13H19NO ; C13H15N3O2
198.06118	1.617117	1.13201	4.8877	0.000635	3.1975	0.011544	198.0612	HMDB01267 ; HMDB04145 ; HMDB04146 ; HMDB04634 ; HMDB13253 ; HMDB15253 ; HMDB30025 ; HMDB31189 ; HMDB31636 ; HMDB31718 ; HMDB32234 ; HMDB33556 ; HMDB37732 ; HMDB38896 ; HMDB40346 ; HMDB61003	4-Oxo-4-(3-pyridyl)-butanamide ; BOX B ; BOX A ; 4-Chloromethandienone ; N-Acetylhistamine ; Phenacemide ; 1-Mercapto-2-propanone ; S-Methyl thioacetate ; (E)-1-Propene-1-sulfenic acid ; (Methylthio)acetaldehyde ; 25-Dimethyl-3-furanthiol acetate ; 25-Dimethyl-14-dithiane-25-diol ; S-(2-Furanylmethyl) propanethioate ; Ethyl 2-(methyldithio)propionate ; Thial-1-Propene-1-thiol S-oxide ; lansoprazole sulfide	2M+H+K ; 2M+H+K ; 2M+H+K ; M+Na+K ; M+2Na-H ; 2M+H+K ; 2M+NH4 ; 2M+NH4 ; 2M+NH4 ; 2M+NH4 ; 2M+3H2O+2H ; M+NH4 ; 2M+3H2O+2H ; M+NH4 ; 2M+NH4 ; M+ACN+2H	C9H10N2O2 ; C9H10N2O2 ; C9H10N2O2 ; C20H27ClO2 ; C7H11N3O ; C9H10N2O2 ; C3H6OS ; C3H6OS ; C3H6OS ; C3H6OS ; C8H10O2S ; C6H12O2S2 ; C8H10O2S ; C6H12O2S2 ; C3H6OS ; C16H14F3N3OS
197.98391	1.648091	0.497928	4.8874	0.000635	3.1973	0.011544	197.9839	HMDB02755 ; HMDB33206 ; HMDB37357	Myricetin ; Arsenobetaine ; 334558-Hexahydroxyflavone	M+2K ; 2M+H+K ; M+2K	C15H10O8 ; C5H11AsO2 ; C15H10O8

190.01259	1.569397	0.214067	4.8765	0.000645	3.1902	0.011691	190.0126	HMDB00232 ; HMDB00870 ; HMDB01941 ; HMDB02937 ; HMDB03903 ; HMDB06462 ; HMDB14417 ; HMDB14549 ; HMDB31233 ; HMDB33161 ; HMDB33240 ; HMDB33892 ; HMDB37501 ; HMDB41773 ; HMDB60263 ; HMDB61113	Quinolinic acid ; Histamine ; Brompheniramine ; Tamarixetin ; 2-Hydroxyethanesulfonate ; Homocysteinesulfinic acid ; Betazole ; Dexbrompheniramine ; Ethyl hydrogen sulfate ; 26-Pyridinedicarboxylic acid ; Grevilline B ; Mycochromone ; Dihydroisorhamnetin ; Salvianolic acid D ; Histamium ; Sulforaphane-N-acetylcysteine	M+Na ; M+2K+H ; M+Na+K ; M+Na+K ; M+ACN+Na ; M+Na ; M+2K+H ; M+Na+K ; M+ACN+Na ; M+Na ; M+H+K ; M+Na+K ; M+Na+K ; M+H+K ; M+2K+H ; M+H+K	C7H5NO4 ; C5H9N3 ; C16H19BrN2 ; C16H14O7 ; C2H6O4S ; C4H9NO4S ; C5H9N3 ; C16H19BrN2 ; C2H6O4S ; C7H5NO4 ; C18H12O7 ; C16H14O7 ; C16H14O7 ; C18H12O7 ; C5H9N3 ; C11H20N2O4S3
296.06581	1.625822	1.212457	-4.8715	0.00065	3.187	0.011692	296.0658	HMDB01235 ; HMDB33261 ; HMDB02338 ; HMDB05781 ; HMDB14572 ; HMDB14659 ; HMDB29522 ; HMDB30768 ; HMDB31428 ; HMDB31720 ; HMDB34127 ; HMDB36629 ; HMDB39166 ; HMDB39578 ; HMDB41758 ; HMDB41776 ; HMDB60624	5-Aminoimidazole ribonucleotide ; Mulberrofuran M ; Biochanin A ; Glycitein ; Streptozocin ; Albendazole ; 57-Dihydroxy-3-methoxyflavone ; 2-(6-Hydroxy-2-methoxy-34-methylenedioxyphenyl)benzofuran ; Farnisin ; Xenognosin B ; Prunetin ; (-)-Maackiain ; Cajaquinone ; 15-Dihydroxy-2-methoxy-6-methylanthraquinone ; Melanettin ; Stevenin ; Ethionamide sulphoxide	M+H ; M+2H ; 2M+H+Na ; 2M+H+Na ; 2M+Na+K ; 2M+Na+K ; 2M+H+Na ; 2M+H+Na ; 2M+H+Na ; 2M+H+Na ; 2M+H+Na ; 2M+H+Na ; 2M+H+Na ; 2M+H+Na ; 2M+H+Na ; 2M+H+Na ; 3M+2Na	C8H14N3O7P ; C34H22O10 ; C16H12O5 ; C16H12O5 ; C8H15N3O7 ; C12H15N3O2S ; C16H12O5 ; C16H12O5 ; C16H12O5 ; C16H12O5 ; C16H12O5 ; C16H12O5 ; C16H12O5 ; C16H12O5 ; C16H12O5 ; C16H12O5 ; C8H10N2OS
383.03893	1.585422	0.786587	-4.8693	0.000652	3.1856	0.011692	383.0389	HMDB14939 ; HMDB39287	Halazepam ; Theogallin	2M+Na+K ; M+K	C17H12ClF3N2O ; C14H16O10
553.17195	1.649534	0.919574	-4.8692	0.000652	3.1855	0.011692	553.172	HMDB14789 ; HMDB30629	Dyphylline ; Rubraflavone B	2M+2Na-H ; M+2K+H	C10H14N4O4 ; C30H34O5
180.52197	1.637393	0.512404	4.8667	0.000655	3.1839	0.011693	180.522	HMDB01919 ; HMDB02238 ; HMDB02372 ; HMDB03563 ; HMDB15536 ; HMDB30191 ; HMDB31812 ; HMDB32845 ; HMDB34669 ; HMDB39521 ; HMDB40940	Famotidine ; L-Homocysteine sulfonic acid ; N-Phenylacetylphenylalanine ; Morphinone ; Imipenem ; N-2-Hydroxy-2-(4-hydroxyphenyl)ethylcinnamide ; Oxfendazole ; 2-Methyl(3-phenylpropanoyl)aminobenzoic acid ; Anaxagoreine ; N-(p-Hydroxyphenyl)ethyl p-hydroxycinnamide ; Capsiamide	M+H+Na ; 2M+H+NH4 ; M+2K ; M+2K ; M+Na+K ; M+2K ; M+2Na ; M+2K ; M+2K ; M+2K ; 2M+3H	C8H15N7O2S3 ; C3H9NO3S2 ; C17H17NO3 ; C17H17NO3 ; C12H17N3O4S ; C17H17NO3 ; C15H13N3O3S ; C17H17NO3 ; C17H17NO3 ; C17H17NO3 ; C17H35NO
380.11885	1.556559	0.207204	-4.8619	0.000659	3.1808	0.011735	380.1189	HMDB00629 ; HMDB60071 ; HMDB34569 ; HMDB01204 ; HMDB06045 ; HMDB15187 ; HMDB34019 ; HMDB35184 ; HMDB37424 ; HMDB37425 ; HMDB38127 ; HMDB38904 ; HMDB40304 ; HMDB60431 ; HMDB60905	Chondroitin ; Hyaluronan biosynthesis precursor 1 ; Rubrofusarin 6-glucosyl-(1-3)-glucosyl-(1-6)-glucoside ; trans-3-Hydroxycotinine glucuronide ; Dityrosine ; Nitrendipine ; Parvisoflavone A ; Semilicoisoflavone B ; Citrusinol ; Cycloartocarpesin ; Lupinisoflavone A ; 3-(24-Dihydroxyphenyl)-89-dihydro-5-hydroxy-8-(1-methylethenyl)-4H-furo23-h-1-benzopyran-5-one ; Cyclocommunol ; Aflatoxin B1exo-89-epoxide-GSH ; Haloperidol 1236-tetrahydropyridine	M+H ; M+H ; M+2H ; 2M+H+Na ; 2M+H+K ; 2M+H+K ; 2M+3H2O+2H ; 2M+3H2O+2H ; 2M+3H2O+2H ; 2M+3H2O+2H ; 2M+3H2O+2H ; 2M+3H2O+2H ; 2M+3H2O+2H ; M+3ACN+2H ; M+Na	C14H21NO11 ; C14H21NO11 ; C33H42O20 ; C16H20N2O8 ; C18H20N2O6 ; C18H20N2O6 ; C20H16O6 ; C20H16O6 ; C20H16O6 ; C20H16O6 ; C20H16O6 ; C20H16O6 ; C20H16O6 ; C27H29N3O13S ; C21H21ClFNO
222.01182	1.591711	0.452273	-4.8504	0.000671	3.1733	0.011887	222.0118	HMDB03484 ; HMDB11185 ; HMDB13679 ; HMDB29457 ; HMDB30774 ; HMDB37176 ; HMDB38432 ; HMDB38435 ; HMDB60483	O-Phosphohomoserine ; O-Phosphothreonine ; Bergaptol ; Xanthotoxol ; ()-12a-Hydroxypachyrrhizone ; 2-Ethyl-25-dihydro-45-dimethylthiazole ; 1-Isothiocyanatohexane ; 1-Isothiocyanato-4-methylpentane ; Iminoerythrose 4-phosphate	M+Na ; M+Na ; 2M+H+K ; 2M+H+K ; M+Na+K ; M+2K+H ; M+2K+H ; M+2K+H ; M+Na	C4H10NO6P ; C4H10NO6P ; C11H6O4 ; C11H6O4 ; C20H14O8 ; C7H13NS ; C7H13NS ; C7H13NS ; C4H10NO6P
108.01141	1.540629	0.489862	-4.8481	0.000673	3.1718	0.011887	108.0114	HMDB00094 ; HMDB00193 ; HMDB01209 ; HMDB01874 ; HMDB05971 ; HMDB06511 ; HMDB33717 ; HMDB33845 ; HMDB41862 ; HMDB60008 ; HMDB60009 ; HMDB60010	Citric acid ; Isocitric acid ; Allantoic acid ; D-threo-Isocitric acid ; Diketogulonic acid ; 23-Diketo-L-gulonate ; (1R2R)-Isocitric acid ; (Z)-5-(5-Methyl-2-thienyl)methylene-2(5H)-furanone ; D-Glucaro-14-lactone ; N-(2-hydroxymethyl-3-chlorophenyl)anthranilic acid ; N-(2-methyl-3-chloro-4-hydroxyphenyl)anthranilic acid ; N-(2-methyl-3-chloro-5-hydroxyphenyl)anthranilic acid	M+H+Na ; M+H+Na ; M+H+K ; M+H+Na ; M+H+Na ; M+H+Na ; M+H+Na ; M+H+Na ; M+H+Na ; M+H+2Na ; M+H+2Na ; M+H+2Na	C6H8O7 ; C6H8O7 ; C4H8N4O4 ; C6H8O7 ; C6H8O7 ; C6H8O7 ; C6H8O7 ; C10H8O2S ; C6H8O7 ; C14H12ClNO3 ; C14H12ClNO3 ; C14H12ClNO3
190.08942	1.53806	0.933838	4.8462	0.000675	3.1706	0.011887	190.0894	HMDB12286 ; HMDB30657 ; HMDB34474 ; HMDB35053 ; HMDB36694 ; HMDB36897 ; HMDB36900 ; HMDB36945 ; HMDB41002 ; HMDB41231 ; HMDB41349 ; HMDB02128 ; HMDB02640 ; HMDB02644 ; HMDB03192 ; HMDB15333 ; HMDB29714 ; HMDB32611 ; HMDB33218 ; HMDB33230 ; HMDB33231 ; HMDB33591 ; HMDB33830 ; HMDB33833 ; HMDB33926 ; HMDB34771 ; HMDB38228 ; HMDB38893 ; HMDB40577 ; HMDB40592 ; HMDB41208 ; HMDB41986 ; HMDB60335 ; HMDB61100	S-Prenyl-L-cysteine ; Carinol ; Niveusin C ; Gibberellin A52 ; 15-Hydroxyleptocarpin ; Gibberellin A17 ; Gibberellin A66 ; Gibberellin A125 ; (1E4Z6a8b10a)-8-(2-Methylbutanoyloxy)-1015-dihydroxy-3-oxo-1411(13)-germacratrien-126-olide ; Gibberellin A99 ; Gibberellin A102 ; 24-Diamino-6-hydroxypyrimidine ; N-(3a5b7a12a)-312-dihydroxy-24-oxo-7-(sulfooxy)cholan-24-yl-Glycine ; N-(3a5b7a12a)-37-dihydroxy-24-oxo-12-(sulfooxy)cholan-24-yl-Glycine ; 5-Aminoimidazole-4-carboxamide ; Levetiracetam ; 45-Dihydro-1-benzoxepin-3(2H)-one ; 3-(4-Methoxyphenyl)-2-propenal ; 26-Dimethyl-7-octene-16-diol 8-O-glucoside ; trans-p-Menthane-78-diol 7-glucoside ; trans-p-Menthane-78-diol 8-glucoside ; Safrole ; Cassiastearoptene ; Methyl cinnamate ; (3xi6xi)-Cyclo(alanylvalyl) ; 37-Dimethyl-5-octene-17-diol 1-glucoside ; 4-O-alpha-D-Galactopyranosylcalystegine B2 ; 36-Dimethyl-2(3H)-benzofuranone ; Cinnamyl formate ; Allyl benzoate ; De-O-methylsimmondsin ; Pibutidine ; 12-Dihydronaphthalene-12-diol ; 6-oxo-famciclovir	M+H ; M+2H ; M+2H ; M+2H ; M+2H ; M+2H ; M+2H ; M+2H ; M+2H ; M+2H ; M+2H ; 3M+2H ; M+2H+Na ; M+2H+Na ; 3M+2H ; 2M+H+K ; 2M+3H2O+2H ; 2M+3H2O+2H ; M+2Na ; M+2Na ; M+2Na ; 2M+3H2O+2H ; 2M+3H2O+2H ; 2M+3H2O+2H ; 2M+H+K ; M+2Na ; M+ACN+2H ; 2M+3H2O+2H ; 2M+3H2O+2H ; 2M+3H2O+2H ; M+H+NH4 ; M+H+Na ; 2M+3H2O+2H ; M+ACN+2H	C8H15NO2S ; C20H26O7 ; C20H26O7 ; C20H26O7 ; C20H26O7 ; C20H26O7 ; C20H26O7 ; C20H26O7 ; C20H26O7 ; C20H26O7 ; C20H26O7 ; C4H6N4O ; C26H43NO9S ; C26H43NO9S ; C4H6N4O ; C8H14N2O2 ; C10H10O2 ; C10H10O2 ; C16H30O7 ; C16H30O7 ; C16H30O7 ; C10H10O2 ; C10H10O2 ; C10H10O2 ; C8H14N2O2 ; C16H30O7 ; C13H23NO9 ; C10H10O2 ; C10H10O2 ; C10H10O2 ; C15H23NO9 ; C19H24N4O3 ; C10H10O2 ; C14H19N5O5
414.97166	1.613625	0.104433	4.8401	0.000681	3.1666	0.011955	NA	NA	NA	NA	NA
221.01825	1.635072	0.959622	-4.8362	0.000685	3.1641	0.011978	221.0183	HMDB00793 ; HMDB29940	Monoiodothyronine ; Alginic acid	M+ACN+2H ; M+H+Na	C15H14INO4 ; C12H20O12P2
191.54061	1.556431	0.800784	4.833	0.000689	3.162	0.011978	191.5406	HMDB01554 ; HMDB28715 ; HMDB28967 ; HMDB30934 ; HMDB31942 ; HMDB39161	Xanthylic acid ; Arginyl-Methionine ; Methionyl-Arginine ; Triglochinin ; Niazinin A ; Syndesine	M+H+NH4 ; M+2K ; M+2K ; M+H+Na ; M+H+K ; M+2K	C10H13N4O9P ; C11H23N5O3S ; C11H23N5O3S ; C14H17NO10 ; C15H21NO6S ; C12H23N3O6

278.58215	1.639146	0.483176	-4.8314	0.00069	3.1609	0.011978	278.5822	HMDB02235 ; HMDB02276 ; HMDB13862 ; HMDB30203 ; HMDB32986 ; HMDB38508 ; HMDB60621	O-6-deoxy-a-L-galactopyranosyl-(1-2)-O-b-D-galactopyranosyl-(1-3)-2-(acetylamino)-15-anhydro-2-deoxy-D-arabino-Hex-1-enitol ; O-6-deoxy-a-L-galactopyranosyl-(1-2)-O-b-D-galactopyranosyl-(1-4)-2-(acetylamino)-15-anhydro-2-deoxy-D-arabino-Hex-1-enitol ; N-desmethylimatinib ; Fumitremorgin B ; 5-(2-Hydroxyethyl)-4-methylthiazole acetate ; Rheidin C ; n-Demethylated piperazine	M+2Na ; M+2Na ; M+2K ; M+2K ; 3M+2H ; M+H+NH4 ; M+2K	C20H33NO14 ; C20H33NO14 ; C28H29N7O ; C27H33N3O5 ; C8H11NO2S ; C31H22O9 ; C28H29N7O
121.06891	1.615538	0.710978	4.8234	0.000699	3.1557	0.011978	121.0689	HMDB32373 ; HMDB36149 ; HMDB39781 ; HMDB41476 ; HMDB41562 ; HMDB00194 ; HMDB00745 ; HMDB05769 ; HMDB00532 ; HMDB02004 ; HMDB06454 ; HMDB12249 ; HMDB30358 ; HMDB31670 ; HMDB60477 ; HMDB60625	3-Mercapto-2-methyl-1-butanol ; 3-Mercapto-3-methyl-1-butanol ; xi-2-Mercapto-3-methyl-1-butanol ; 4-(Methylthio)-2-butanol ; 4-(Methylthio)-1-butanol ; Anserine ; Homocarnosine ; Balenine ; Acetylglycine ; 5-Methoxydimethyltryptamine ; L-2-Amino-3-oxobutanoic acid ; L-Aspartate-semialdehyde ; Aspidospermatine ; 1-(Hydroxymethyl)-55-dimethyl-24-imidazolidinedione ; gamma-Glutamyl-beta-aminopropiononitrile ; N-despropyl ropinirole	M+H ; M+H ; M+H ; M+H ; M+H ; M+2H ; M+2H ; M+2H ; M+3ACN+2H ; M+H+Na ; M+3ACN+2H ; M+3ACN+2H ; M+2H+Na ; M+2ACN+2H ; M+ACN+2H ; M+H+Na	C5H12OS ; C5H12OS ; C5H12OS ; C5H12OS ; C5H12OS ; C10H16N4O3 ; C10H16N4O3 ; C10H16N4O3 ; C4H7NO3 ; C13H18N2O ; C4H7NO3 ; C4H7NO3 ; C21H26N2O2 ; C6H10N2O3 ; C8H13N3O3 ; C13H18N2O
422.74629	1.628405	0.932784	4.8178	0.000705	3.1521	0.011978	422.7463	HMDB38232 ; HMDB38233 ; HMDB08248 ; HMDB08503	Azaspiracid 4 ; Azaspiracid 5 ; PC(18:4(6Z9Z12Z15Z)20:5(5Z8Z11Z14Z17Z)) ; PC(20:5(5Z8Z11Z14Z17Z)18:4(6Z9Z12Z15Z))	M+2H ; M+2H ; M+2Na ; M+2Na	C46H69NO13 ; C46H69NO13 ; C46H74NO8P ; C46H74NO8P
199.47882	1.668213	0.351018	4.8171	0.000705	3.1516	0.011978	199.4788	HMDB42052	Trimethylselenonium	3M+H+Na	C3H9Se
405.63512	1.610463	1.224432	-4.8165	0.000706	3.1512	0.011978	405.6351	HMDB00855 ; HMDB37994	Nicotinamide riboside ; Cyanidin 3-(2G-xylosylrutinoside)	3M+2Na ; M+2ACN+2H	C11H15N2O5 ; C32H39O19
294.96463	1.592811	1.18899	-4.8161	0.000706	3.151	0.011978	NA	NA	NA	NA	NA
326.16390	1.580061	1.158977	-4.816	0.000706	3.1509	0.011978	326.1639	HMDB15078 ; HMDB34229	Zalcitabine ; Hygromycin B	3M+H+NH4 ; M+3ACN+2H	C9H13N3O3 ; C20H37N3O13
238.04492	1.574379	1.266665	-4.8158	0.000707	3.1508	0.011978	238.0378	HMDB15424 ; HMDB15563 ; HMDB29708 ; HMDB31255 ; HMDB32686 ; HMDB41147 ; HMDB41258 ; HMDB41418	Cefoperazone ; Pargyline ; 5678-Tetrahydro-24-dimethylquinoline ; Fluorocitric acid ; beta-D-3-5-Deoxy-5-(dimethylarsinyl)ribofuranosyloxy-2-hydroxy-1-propanesulfonic acid ; Isochinomin ; Homomangiferin ; Taxifolin 3-arabinoside	M+3Na ; M+2K+H ; M+2K-H ; 2M+3H2O+2H ; M+2ACN+2H ; M+H+K ; M+H+K ; M+H+K	C25H27N9O8S2 ; C11H13N ; C11H15N ; C6H7FO7 ; C10H21AsO9S ; C20H20O11 ; C20H20O11 ; C20H20O11
169.97739	1.578626	1.192826	4.8024	0.000721	3.142	0.01218	NA	NA	NA	NA	NA
302.90576	1.530312	0.771459	-4.795	0.000729	3.1372	0.012273	NA	NA	NA	NA	NA
828.00843	1.591638	0.861501	-4.7879	0.000737	3.1326	0.012356	828.0084	HMDB11868 ; HMDB30442 ; HMDB37597 ; HMDB41770	Ganglioside GD3 (d18:025:0) ; Dinophysistoxin 1 ; 8-Hydroxyluteolin 8-sulfate ; Quercetin 3-sulfate	M+2ACN+2H ; 2M+H+NH4 ; 2M+ACN+Na ; 2M+ACN+Na	C77H141N3O29 ; C45H70O13 ; C15H10O10S ; C15H10O10S
677.44062	1.542122	0.641916	-4.7802	0.000746	3.1275	0.012356	677.4406	HMDB29962 ; HMDB29963 ; HMDB30440 ; HMDB30445 ; HMDB31136 ; HMDB31391 ; HMDB32113 ; HMDB32588 ; HMDB33423 ; HMDB34622 ; HMDB34628 ; HMDB34739 ; HMDB34740 ; HMDB34819 ; HMDB36547 ; HMDB40839	Annoglaucin ; Muricatin C ; Rollidecin A ; Bullatetrocin ; Rollitacin ; Mucocin ; Glabracin A ; Purpurenin ; 1215-cis-Squamostatin A ; Rollimusin ; 2023-cis-Bullatalicinone ; Bullatanocin ; Annonin XIV ; 27-Hydroxybullatacin ; 9-Hydroxyasimicinone ; Purpureacin 2	M+K ; M+K ; M+K ; M+K ; M+K ; M+K ; M+K ; M+K ; M+K ; M+K ; M+K ; M+K ; M+K ; M+K ; M+K ; M+K	C37H66O8 ; C37H66O8 ; C37H66O8 ; C37H66O8 ; C37H66O8 ; C37H66O8 ; C37H66O8 ; C37H66O8 ; C37H66O8 ; C37H66O8 ; C37H66O8 ; C37H66O8 ; C37H66O8 ; C37H66O8 ; C37H66O8 ; C37H66O8
217.07046	1.601088	1.091333	4.7786	0.000747	3.1265	0.012356	217.0666	HMDB00715 ; HMDB03134 ; HMDB05032 ; HMDB15600 ; HMDB29915 ; HMDB29916 ; HMDB29965 ; HMDB30988 ; HMDB30992 ; HMDB31437 ; HMDB31449 ; HMDB32055 ; HMDB33284 ; HMDB33816 ; HMDB34145 ; HMDB34219 ; HMDB34221 ; HMDB34222 ; HMDB35045 ; HMDB35046 ; HMDB35110 ; HMDB35828 ; HMDB36557 ; HMDB38922 ; HMDB39048 ; HMDB39059 ; HMDB41148 ; HMDB41197 ; HMDB41608 ; HMDB60331 ; HMDB60332 ; HMDB32660	Kynurenic acid ; Biocytin ; Cetirizine ; Bepotastine ; D-4-O-Methyl-myo-inositol ; 4-O-Methyl-myo-inositol ; Methyl beta-D-glucopyranoside ; 5-(2-Methylpropyl)tetrahydro-2-oxo-3-furancarboxylic acid ; 5-Butyltetrahydro-2-oxo-3-furancarboxylic acid ; (-)-Bornesitol ; Sequoyitol ; N-Acetylhistidine ; 8-Hydroxy-4-methoxypinoresinol ; Mytilitol ; (-)-cis-Rotenolone ; D-Pinitol ; L-Quebrachitol ; L-Pinitol ; Gibberellin A39 ; Gibberellin A43 ; 8-Epidiosbulbin E acetate ; Lactupicrin ; MS 3 ; (7R)-()-Lyoniresinol 9-glucoside ; (5a6a8a11a)-8-Hydroxy-2-oxo-1(10)3-guaiadien-126-olide-15-al 8-(4-hydroxyphenylacetate) ; (R)-Heraclenol 2-(3-methylbutanoate) ; ()-Lyoniresinol 9-glucoside ; Edulisin III ; Methional diethyl acetal ; 1-Nitronaphthalene-56-oxide ; 1-Nitronaphthalene-78-oxide ; (2Z4Z)-2-(5-Methylthio-4-penten-2-ynylidene)-16-dioxaspiro44non-3-ene	2M+3H2O+2H ; M+Na+K ; M+2Na ; M+2Na ; M+Na ; M+Na ; M+Na ; 2M+Na+K ; 2M+Na+K ; M+Na ; M+Na ; 2M+H+K ; M+2Na ; M+Na ; M+H+Na ; M+Na ; M+Na ; M+Na ; M+H+K ; M+H+K ; M+2Na ; M+H+Na ; M+2Na ; M+3Na ; M+H+Na ; M+2Na ; M+3Na ; M+2Na ; M+K ; 2M+3H2O+2H ; 2M+3H2O+2H ; M+H-H2O	C10H7NO3 ; C16H28N4O4S ; C21H25ClN2O3 ; C21H25ClN2O3 ; C7H14O6 ; C7H14O6 ; C7H14O6 ; C9H14O4 ; C9H14O4 ; C7H14O6 ; C7H14O6 ; C8H11N3O3 ; C21H24O7 ; C7H14O6 ; C23H22O7 ; C7H14O6 ; C7H14O6 ; C7H14O6 ; C20H26O8 ; C20H26O8 ; C21H24O7 ; C23H22O7 ; C21H24O7 ; C28H38O13 ; C23H22O7 ; C21H24O7 ; C28H38O13 ; C21H24O7 ; C8H18O2S ; C10H7NO3 ; C10H7NO3 ; C13H14O2S
345.10560	1.579049	0.159777	-4.7774	0.000749	3.1257	0.012356	345.1056	HMDB06061 ; HMDB34888 ; HMDB39337	4-Hydroxyphenylacetylglutamine ; 4-Acetylimidazo45-cpyridine ; Tetramethylquercetin 3-rutinoside	M+ACN+Na ; 2M+Na ; M+H+Na	C13H15NO6 ; C8H7N3O ; C31H38O16
501.06615	1.563121	1.1191	-4.7774	0.000749	3.1257	0.012356	501.0662	HMDB41988 ; HMDB38030	Pinazepam ; 3355-Tetrahydroxy-67-methyleneoxy-4-methoxyflavone 3-glucuronide	3M+2K ; M+H-2H2O	C18H13ClN2O ; C23H20O15
303.95266	1.550365	0.10847	-4.7757	0.000751	3.1246	0.012356	303.9527	HMDB05874 ; HMDB40414	3-Bromotyrosine ; (3beta24(241)Z)-24-Ethylidenelanost-9(11)-en-3-ol	M+2Na-H ; 2M+3H	C9H10BrNO3 ; C32H54O
217.57228	1.61084	1.118707	4.7751	0.000751	3.1242	0.012356	217.5723	HMDB14867 ; HMDB28784 ; HMDB29036 ; HMDB39503	Diphemanil Methylsulfate ; Cysteinyl-Serine ; Serinyl-Cysteine ; N-Jasmonoyltyrosine	M+2Na ; 2M+H+NH4 ; 2M+H+NH4 ; M+Na+K	C21H27NO4S ; C6H12N2O4S ; C6H12N2O4S ; C21H27NO5
248.86880	1.630391	0.206782	4.769	0.000758	3.1202	0.01243	248.8688	HMDB07996 ; HMDB07997 ; HMDB08028 ; HMDB08093 ; HMDB08126 ; HMDB11151 ; HMDB11209 ; HMDB11210 ; HMDB11240 ; HMDB11272 ; HMDB11305	PC(16:0P-18:1(11Z)) ; PC(16:0P-18:1(9Z)) ; PC(16:1(9Z)P-18:0) ; PC(18:1(11Z)P-16:0) ; PC(18:1(9Z)P-16:0) ; PC(O-16:018:2(9Z12Z)) ; PC(P-16:018:1(11Z)) ; PC(P-16:018:1(9Z)) ; PC(P-18:016:1(9Z)) ; PC(P-18:1(11Z)16:0) ; PC(P-18:1(9Z)16:0)	M+3H ; M+3H ; M+3H ; M+3H ; M+3H ; M+3H ; M+3H ; M+3H ; M+3H ; M+3H ; M+3H	C42H82NO7P ; C42H82NO7P ; C42H82NO7P ; C42H82NO7P ; C42H82NO7P ; C42H82NO7P ; C42H82NO7P ; C42H82NO7P ; C42H82NO7P ; C42H82NO7P ; C42H82NO7P

183.06118	1.548703	1.055123	-4.7625	0.000766	3.116	0.01251	183.0612	HMDB15024 ; HMDB00901 ; HMDB10347 ; HMDB14883 ; HMDB15052 ; HMDB29471 ; HMDB30685 ; HMDB31534 ; HMDB32683 ; HMDB33710 ; HMDB34665 ; HMDB34667 ; HMDB34720 ; HMDB35187 ; HMDB36409 ; HMDB40762 ; HMDB41484 ; HMDB41820 ; HMDB60900	Bumetanide ; Valproic acid glucuronide ; Octanoylglucuronide ; Modafinil ; Metronidazole ; Isocycloheterophyllin ; Cycloheterophyllin ; ()-Galeon ; Arsenocholine ; 1-(4-Hydroxy-3-methoxyphenyl)-7-phenyl-35-heptanedione ; Artonin A ; Artonin B ; Gingerenone C ; Artonin F ; 12-Dihydrodehydroguaiaretic acid ; 55-Diisopropyl-22-dimethylbiphenyl-3344-tetrone ; 5-Acetyl-24-dimethylthiazole ; 8-Oxoguanine ; Clozapine N-oxide	M+2H ; M+2Na ; M+2Na ; 2M+3H ; 2M+H+Na ; M+H+2Na ; M+H+2Na ; M+H+K ; M+NH4 ; M+H+K ; M+H+2Na ; M+H+2Na ; M+H+K ; M+H+2Na ; M+H+K ; M+H+K ; 2M+3H2O+2H ; M+NH4 ; M+H+Na	C17H20N2O5S ; C14H24O8 ; C14H24O8 ; C15H15NO2S ; C6H9N3O3 ; C30H30O7 ; C30H30O7 ; C20H22O4 ; C5H14AsO ; C20H22O4 ; C30H30O7 ; C30H30O7 ; C20H22O4 ; C30H30O7 ; C20H22O4 ; C20H22O4 ; C7H9NOS ; C5H3N5O2 ; C18H19ClN4O
168.04955	1.631432	0.370701	-4.7587	0.00077	3.1135	0.012542	168.0496	HMDB00052 ; HMDB00956 ; HMDB11677 ; HMDB14886 ; HMDB29878 ; HMDB29883 ; HMDB29897 ; HMDB30679 ; HMDB30842 ; HMDB31385 ; HMDB32765 ; HMDB33639 ; HMDB35030 ; HMDB36411 ; HMDB37330 ; HMDB37331 ; HMDB38475 ; HMDB38854 ; HMDB38863 ; HMDB38864 ; HMDB38889 ; HMDB39408 ; HMDB40354 ; HMDB59916	Argininosuccinic acid ; Tartaric acid ; Galactose-beta-14-xylose ; Carbinoxamine ; D-Tartaric acid ; Sambubiose ; Serotinose ; Cabreuvin ; 457-Trimethoxyflavone ; ()-Dulciol E ; N2-(3-Hydroxysuccinoyl)arginine ; 345-Trimethoxyflavone ; Amygdalin ; 5-Hydroxy-47-dimethoxy-6-methylflavone ; 356-Trimethoxyflavone ; 256-Trimethoxyflavone ; Mandelonitrile sophoroside ; 3-O-beta-D-Galactopyranosyl-L-arabinose ; 2-O-b-D-Galactopyranosyl-D-xylose ; 4-O-beta-D-Galactopyranosyl-D-xylose ; 2-Methyl-2-(methyldithio)propanal ; N2-(3-Carboxy-2-hydroxy-1-oxopropyl)arginine ; 145-Trihydroxy-3-prenylxanthone ; 23-Dihydroxybutanedioic acid	M+2Na ; M+NH4 ; M+H+Na ; M+2Na ; M+NH4 ; M+H+Na ; M+H+Na ; M+H+Na ; M+H+Na ; M+H+Na ; M+2Na ; M+H+Na ; M+H+2Na ; M+H+Na ; M+H+Na ; M+H+Na ; M+H+2Na ; M+H+Na ; M+H+Na ; M+H+Na ; M+NH4 ; M+2Na ; M+H+Na ; M+NH4	C10H18N4O6 ; C4H6O6 ; C11H20O10 ; C16H19ClN2O ; C4H6O6 ; C11H20O10 ; C11H20O10 ; C18H16O5 ; C18H16O5 ; C18H16O5 ; C10H18N4O6 ; C18H16O5 ; C20H27NO11 ; C18H16O5 ; C18H16O5 ; C18H16O5 ; C20H27NO11 ; C11H20O10 ; C11H20O10 ; C11H20O10 ; C5H10OS2 ; C10H18N4O6 ; C18H16O5 ; C4H6O6
684.01124	1.543004	0.941764	-4.7525	0.000777	3.1094	0.012618	684.0112	HMDB01163 ; HMDB03351 ; HMDB10039 ; HMDB10062 ; HMDB10063 ; HMDB10074 ; HMDB10087 ; HMDB10115	Guanosine diphosphate mannose ; GDP-glucose ; PIP2(16:020:1(11Z)) ; PIP2(18:018:1(11Z)) ; PIP2(18:018:1(9Z)) ; PIP2(18:1(11Z)18:0) ; PIP2(18:1(9Z)18:0) ; PIP2(20:1(11Z)16:0)	M+2K+H ; M+2K+H ; 2M+3H ; 2M+3H ; 2M+3H ; 2M+3H ; 2M+3H ; 2M+3H	C16H25N5O16P2 ; C16H25N5O16P2 ; C45H87O19P3 ; C45H87O19P3 ; C45H87O19P3 ; C45H87O19P3 ; C45H87O19P3 ; C45H87O19P3
249.03265	1.581746	0.313908	4.7445	0.000787	3.1042	0.0127	249.0327	HMDB37204 ; HMDB00217 ; HMDB14732 ; HMDB15130	35-Di-O-galloyl-14-galactarolactone ; NADP ; Amiloride ; Auranofin	M+2H ; M+3H ; 2M+H+K ; M+3Na	C20H16O15 ; C21H29N7O17P3 ; C6H8ClN7O ; C20H34AuO9PS
406.13685	1.602624	1.203717	-4.744	0.000787	3.1038	0.0127	406.1369	HMDB13202 ; HMDB14502 ; HMDB14546 ; HMDB14898 ; HMDB15178 ; HMDB15329 ; HMDB30447 ; HMDB38092 ; HMDB40442	6-Keto-decanoylcarnitine ; Mefloquine ; Eszopiclone ; Meropenem ; Gatifloxacin ; Zopiclone ; Phomopsin A ; Peonidin 3-(6-p-coumaroyl-glucoside) 5-glucoside ; Dictyoquinazol C	M+2K-H ; 2M+3H2O+2H ; M+NH4 ; M+Na ; 2M+Na+K ; M+NH4 ; M+H+Na ; M+ACN+2H ; M+ACN+Na	C17H31NO5 ; C17H16F6N2O ; C17H17ClN6O3 ; C17H25N3O5S ; C19H22FN3O4 ; C17H17ClN6O3 ; C36H45ClN6O12 ; C38H41O17 ; C18H18N2O5
205.11924	1.582305	1.213313	-4.7322	0.000802	3.0961	0.012886	205.1192	HMDB29052 ; HMDB29136 ; HMDB33891 ; HMDB39948 ; HMDB00385 ; HMDB12176 ; HMDB12867 ; HMDB30006 ; HMDB30424 ; HMDB31315 ; HMDB32050 ; HMDB32463 ; HMDB34708 ; HMDB34730 ; HMDB35156 ; HMDB36191 ; HMDB36215 ; HMDB36803 ; HMDB36804 ; HMDB37107 ; HMDB37388 ; HMDB37634 ; HMDB37829 ; HMDB38005 ; HMDB38068 ; HMDB38079 ; HMDB38245 ; HMDB38279 ; HMDB38387 ; HMDB38393 ; HMDB40432 ; HMDB40436 ; HMDB40595 ; HMDB59816	Serinyl-Valine ; Valyl-Serine ; N6-Acetyl-5S-hydroxy-L-lysine ; 34-Dihydroxy-2-hydroxymethyl-1-pyrrolidinepropanamide ; 12a-Hydroxy-3-oxocholadienic acid ; 5-Aminopentanamide ; 9-Carboxy-alpha-tocotrienol ; 2277-Tetramethyl-16-dioxaspiro44non-3-ene ; Linalyl formate ; 1-(beta-D-Glucopyranosyloxy)-3-octanone ; alpha-Terpineol formate ; 5-Pentyl-3h-furan-2-one ; Methyl 48-decadienoate ; Methyl (2E4Z)-decadienoate ; Neryl formate ; 4-Hydroxy-4-methyl-7-decenoic acid gamma-lactone ; Ethyl octynecarboxylate ; 6-O-Acetylaustroinulin ; 7-O-Acetylaustroinulin ; N1-trans-Feruloylagmatine ; Tsibulin 2 ; xi-1-Ethoxy-1-pentyloxyethane ; 6-(3-Hexenyl)tetrahydro-2H-pyran-2-one ; xi-2-Hexyl-5-methyl-3(2H)-furanone ; (3E6Z)-Nonadien-1-yl acetate ; (2E6Z)-26-Nonadien-1-Yl acetate ; Bornyl formate ; cis-3-Hexenyl tiglate ; 33-Deoxy-33-hydroperoxyfurohyperforin ; 11-Diethoxypentane ; 11-Dimethoxyheptane ; endo-14-beta-Xylanase ; Allyl cyclohexylacetate ; Methyl geranate	M+H ; M+H ; M+H ; M+H ; M+H+Na ; 3M+Na+K ; M+H+Na ; M+Na ; M+Na ; 2M+3H ; M+Na ; M+Na ; M+Na ; M+Na ; M+Na ; M+Na ; M+Na ; M+2Na ; M+2Na ; 2M+3H ; M+Na ; M+2Na-H ; M+Na ; M+Na ; M+Na ; M+Na ; M+Na ; M+Na ; M+H+2Na ; M+2Na-H ; M+2Na-H ; M+3Na ; M+Na ; M+Na	C8H16N2O4 ; C8H16N2O4 ; C8H16N2O4 ; C8H16N2O4 ; C24H34O4 ; C5H12N2O ; C24H34O4 ; C11H18O2 ; C11H18O2 ; C14H26O7 ; C11H18O2 ; C11H18O2 ; C11H18O2 ; C11H18O2 ; C11H18O2 ; C11H18O2 ; C11H18O2 ; C22H36O4 ; C22H36O4 ; C15H22N4O3 ; C11H18O2 ; C9H20O2 ; C11H18O2 ; C11H18O2 ; C11H18O2 ; C11H18O2 ; C11H18O2 ; C11H18O2 ; C35H52O6 ; C9H20O2 ; C9H20O2 ; C34H50N4O2 ; C11H18O2 ; C11H18O2
828.08872	1.630771	1.052703	-4.7283	0.000806	3.0936	0.01292	NA	NA	NA	NA	NA
199.09470	1.597293	0.93259	4.7257	0.000809	3.0918	0.01293	199.0947	HMDB04998 ; HMDB30991 ; HMDB33093 ; HMDB34099 ; HMDB59768 ; HMDB59779 ; HMDB32844 ; HMDB36608 ; HMDB38717 ; HMDB61092 ; HMDB14751 ; HMDB29782	Guaifenesin ; 5-(3E-Pentenyl)tetrahydro-2-oxo-3-furancarboxylic acid ; 3-(4-Hydroxy-3-methoxyphenyl)-12-propanediol ; (2E4E)-27-Dimethyl-24-octadienedioic acid ; 34-Methylene suberic acid ; 23-Methylene suberic acid ; 45-Dihydroniveusin A ; Pteroside P ; (10S11R)-Pterosin C 4-glucoside ; fluticasone 17beta-carboxylic acid ; Amodiaquine ; 6-Epi-7-isocucurbic acid glucoside	M+H ; M+H ; M+H ; M+H ; M+H ; M+H ; M+2H ; M+2H ; M+2H ; M+2H ; M+ACN+2H ; M+H+Na	C10H14O4 ; C10H14O4 ; C10H14O4 ; C10H14O4 ; C10H14O4 ; C10H14O4 ; C20H28O8 ; C20H28O8 ; C20H28O8 ; C21H26F2O5 ; C20H22ClN3O ; C18H30O8
828.41821	1.632728	0.848337	-4.7213	0.000815	3.0889	0.012976	828.4182	HMDB12553 ; HMDB34871 ; HMDB35164 ; HMDB35396 ; HMDB36859	12-Oxo-20-trihydroxy-leukotriene B4 ; Spinacoside D ; Cinncassiol A ; T2 Triol ; Cinncassiol C3	2M+ACN+Na ; M+ACN+Na ; 2M+ACN+Na ; 2M+ACN+Na ; 2M+ACN+Na	C20H30O7 ; C40H60O14 ; C20H30O7 ; C20H30O7 ; C20H30O7
542.65806	1.591354	1.257204	4.7134	0.000825	3.0837	0.013069	542.6581	HMDB11882 ; HMDB33612	Ganglioside GM1 (d18:022:0) ; Glabrin C	M+2H+Na ; 2M+3H	C78H142N2O31 ; C41H64N8O9
482.70191	1.591483	1.164294	4.7124	0.000826	3.083	0.013069	482.7019	HMDB33754	Albanin H	M+3ACN+2H	C50H48O12

349.11744	1.563255	1.249371	4.7083	0.000831	3.0804	0.013072	349.1174	HMDB29299 ; HMDB00125 ; HMDB01081 ; HMDB02242 ; HMDB06581 ; HMDB06584 ; HMDB14918 ; HMDB15644 ; HMDB29727 ; HMDB29826 ; HMDB30311 ; HMDB30813 ; HMDB31182 ; HMDB32066 ; HMDB32276 ; HMDB33247 ; HMDB33330 ; HMDB33351 ; HMDB34052 ; HMDB35435 ; HMDB35918 ; HMDB36584 ; HMDB36808 ; HMDB36809 ; HMDB37151 ; HMDB38009 ; HMDB38033 ; HMDB38434 ; HMDB38911 ; HMDB40000 ; HMDB40035 ; HMDB40063 ; HMDB40064 ; HMDB40071 ; HMDB40072 ; HMDB40073 ; HMDB40074 ; HMDB40075 ; HMDB40102 ; HMDB40103 ; HMDB40104 ; HMDB40105 ; HMDB40299 ; HMDB40459 ; HMDB40742 ; HMDB40790 ; HMDB41252 ; HMDB41253 ; HMDB41325 ; HMDB59795	Sesamolinol 4-O-b-D-glucosyl (1-6)-O-b-D-glucoside ; Glutathione ; (N-acetylneuraminosyl(a2-6)lactosamine) ; Dehydroisocoproporphyrinogen ; 3-Sialyl-N-acetyllactosamine ; 6-Sialyl-N-acetyllactosamine ; Phenelzine ; Betahistine ; 5-Ethyl-23-dimethylpyrazine ; Hallacridone ; Harmine ; Cassiachromone ; 1H-Indol-3-ylacetyl-myo-inositol ; Carbanilide ; 2-Ethyl-3(5 or 6)-dimethylpyrazine ; Aristolodione ; Dihydrocoriandrin ; Methoxybrassinin ; Safflomin C ; Cinchonain Id 7-glucoside ; 7(14)-Bisabolene-231011-tetrol ; Tetramethylpyrazine ; 25-Diethylpyrazine ; 26-Diethylpyrazine ; 2-Isopropyl-5-methylpyrazine ; Malvin ; Malvidin 3-laminaribioside ; 7-Isothiocyanato-1-heptene ; (2S3S4S)-57911-Tridecatetrayne-1234-tetrol ; 2-Methyl-5-propylpyrazine ; 2-Pentylthiazole ; 4-Butyl-5-methylthiazole ; 4-Ethyl-5-propylthiazole ; 24-Dimethyl-5-propylthiazole ; 25-Dimethyl-4-propylthiazole ; 45-Dimethyl-2-propylthiazole ; 45-Dimethyl-2-(1-methylethyl)thiazole ; 25-Diethyl-4-methylthiazole ; 2-Butyl-4-methylthiazole ; 4-Butyl-2-methylthiazole ; 2-Ethyl-4-propylthiazole ; 4-Ethyl-2-propylthiazole ; 2-Ethyl-35-dimethylpyrazine ; Ethylene brassylate ; 23-Dehydrosalvipisone ; 4-Methoxybrassinin ; 2-Methyl-3-propylpyrazine ; 23-Diethylpyrazine ; Salfredin B11 ; Gluten exorphin B5	M+2H ; M+ACN+H ; M+H+Na ; M+2Na ; M+H+Na ; M+H+Na ; 2M+2K-H ; 2M+2K-H ; 2M+2K-H ; M+ACN+H ; 3M+Na+K ; 3M+2H ; 2M+H+Na ; 3M+Na+K ; 2M+2K-H ; M+ACN+H ; 3M+2H ; M+2ACN+H ; M+2ACN+2H ; M+2ACN+2H ; M+2K-H ; 2M+2K-H ; 2M+2K-H ; 2M+2K-H ; 2M+2K-H ; M+ACN+2H ; M+ACN+2H ; 2M+K ; 3M+2H ; 2M+2K-H ; 2M+K ; 2M+K ; 2M+K ; 2M+K ; 2M+K ; 2M+K ; 2M+K ; 2M+K ; 2M+K ; 2M+K ; 2M+K ; 2M+K ; 2M+2K-H ; M+2K+H ; M+K ; M+2ACN+H ; 2M+2K-H ; 2M+2K-H ; 3M+2H ; M+H+K	C32H40O17 ; C10H17N3O6S ; C25H42N2O19 ; C36H36N4O8 ; C25H42N2O19 ; C25H42N2O19 ; C8H12N2 ; C8H12N2 ; C8H12N2 ; C18H13NO4 ; C13H12N2O ; C13H12O4 ; C16H19NO7 ; C13H12N2O ; C8H12N2 ; C18H13NO4 ; C13H12O4 ; C12H14N2OS2 ; C30H30O14 ; C30H30O14 ; C15H28O4 ; C8H12N2 ; C8H12N2 ; C8H12N2 ; C8H12N2 ; C29H35O17 ; C29H35O17 ; C8H13NS ; C13H12O4 ; C8H12N2 ; C8H13NS ; C8H13NS ; C8H13NS ; C8H13NS ; C8H13NS ; C8H13NS ; C8H13NS ; C8H13NS ; C8H13NS ; C8H13NS ; C8H13NS ; C8H13NS ; C8H12N2 ; C15H26O4 ; C20H22O3 ; C12H14N2OS2 ; C8H12N2 ; C8H12N2 ; C13H12O4 ; C30H38N6O11
101.00729	1.577223	0.783979	4.7073	0.000832	3.0797	0.013072	101.0073	HMDB31414 ; HMDB31582 ; HMDB31686 ; HMDB32446 ; HMDB32447 ; HMDB32927 ; HMDB32948 ; HMDB33209 ; HMDB33238 ; HMDB34345 ; HMDB39844 ; HMDB40246 ; HMDB40272 ; HMDB40408 ; HMDB42032	23-Dimethyl-2-cyclohexen-1-one ; 6-Methyl-35-heptadien-2-one ; (EE)-24-Octadienal ; trans trans-35-Octadien-2-one ; 15-Octadien-3-one ; Quindoxin ; 5-Phenyl-13-pentadiyne ; Fenthion ; 2-Isopropyl-5-methylfuran ; 7-Acetoxy-6-methoxycoumarin ; (EE)-26-Octadienal ; 355-Trimethyl-2-cyclopenten-1-one ; 2-Butylfuran ; 7-Hydroxy-2-methyl-4-oxo-4H-1-benzopyran-5-acetic acid ; Thiodiacetic acid	M+2K ; M+2K ; M+2K ; M+2K ; M+2K ; M+H+K ; M+Na+K ; M+2H+Na ; M+2K ; M+3Na ; M+2K ; M+2K ; M+2K ; M+3Na ; 2M+3H	C8H12O ; C8H12O ; C8H12O ; C8H12O ; C8H12O ; C8H6N2O2 ; C11H8 ; C10H15O3PS2 ; C8H12O ; C12H10O5 ; C8H12O ; C8H12O ; C8H12O ; C12H10O5 ; C4H6O4S
789.05209	1.568032	1.284042	-4.706	0.000834	3.0788	0.013072	789.0521	HMDB10238 ; HMDB10239 ; HMDB10242 ; HMDB10243 ; HMDB11933 ; HMDB56406 ; HMDB56424 ; HMDB56425 ; HMDB56434 ; HMDB56435 ; HMDB56460 ; HMDB56461 ; HMDB56470 ; HMDB56471 ; HMDB56478 ; HMDB56495 ; HMDB56531 ; HMDB56584 ; HMDB56585 ; HMDB56601 ; HMDB56611 ; HMDB56620 ; HMDB56621 ; HMDB56623 ; HMDB56628 ; HMDB56640 ; HMDB56641 ; HMDB56657 ; HMDB56667 ; HMDB56676 ; HMDB56677 ; HMDB56679 ; HMDB56684 ; HMDB56747 ; HMDB56748 ; HMDB56751 ; HMDB56752 ; HMDB56789 ; HMDB56794 ; HMDB56841 ; HMDB56846 ; HMDB56884 ; HMDB56957 ; HMDB57586 ; HMDB57634 ; HMDB57661 ; HMDB57665 ; HMDB57669 ; HMDB57670 ; HMDB57854 ; HMDB57864 ; HMDB57873 ; HMDB57874 ; HMDB57876 ; HMDB57881 ; HMDB57952 ;	CL(18:2(9Z12Z)18:2(9Z12Z)18:1(11Z)18:1(11Z)) ; CL(18:2(9Z12Z)18:2(9Z12Z)18:1(11Z)18:1(9Z)) ; CL(18:2(9Z12Z)18:2(9Z12Z)18:1(9Z)18:1(11Z)) ; CL(18:2(9Z12Z)18:2(9Z12Z)18:1(9Z)18:1(9Z)) ; Ganglioside GQ1c (d18:014:0) ; CL(16:016:018:022:6(4Z7Z10Z13Z16Z19Z)) ; CL(16:016:018:1(11Z)22:5(4Z7Z10Z13Z16Z)) ; CL(16:016:018:1(11Z)22:5(7Z10Z13Z16Z19Z)) ; CL(16:016:018:1(9Z)22:5(4Z7Z10Z13Z16Z)) ; CL(16:016:018:1(9Z)22:5(7Z10Z13Z16Z19Z)) ; CL(16:016:022:5(4Z7Z10Z13Z16Z)18:1(11Z)) ; CL(16:016:022:5(4Z7Z10Z13Z16Z)18:1(9Z)) ; CL(16:016:022:5(7Z10Z13Z16Z19Z)18:1(11Z)) ; CL(16:016:022:5(7Z10Z13Z16Z19Z)18:1(9Z)) ; CL(16:016:022:6(4Z7Z10Z13Z16Z19Z)18:0) ; CL(16:018:016:022:6(4Z7Z10Z13Z16Z19Z)) ; CL(16:018:022:6(4Z7Z10Z13Z16Z19Z)16:0) ; CL(16:018:1(11Z)16:022:5(4Z7Z10Z13Z16Z)) ; CL(16:018:1(11Z)16:022:5(7Z10Z13Z16Z19Z)) ; CL(16:018:1(11Z)18:1(11Z)20:4(5Z8Z11Z14Z)) ; CL(16:018:1(11Z)18:1(9Z)20:4(5Z8Z11Z14Z)) ; CL(16:018:1(11Z)20:4(5Z8Z11Z14Z)18:1(11Z)) ; CL(16:018:1(11Z)20:4(5Z8Z11Z14Z)18:1(9Z)) ; CL(16:018:1(11Z)22:5(4Z7Z10Z13Z16Z)16:0) ; CL(16:018:1(11Z)22:5(7Z10Z13Z16Z19Z)16:0) ; CL(16:018:1(9Z)16:022:5(4Z7Z10Z13Z16Z)) ; CL(16:018:1(9Z)16:022:5(7Z10Z13Z16Z19Z)) ; CL(16:018:1(9Z)18:1(11Z)20:4(5Z8Z11Z14Z)) ; CL(16:018:1(9Z)18:1(9Z)20:4(5Z8Z11Z14Z)) ; CL(16:018:1(9Z)20:4(5Z8Z11Z14Z)18:1(11Z)) ; CL(16:018:1(9Z)20:4(5Z8Z11Z14Z)18:1(9Z)) ; CL(16:018:1(9Z)22:5(4Z7Z10Z13Z16Z)16:0) ; CL(16:018:1(9Z)22:5(7Z10Z13Z16Z19Z)16:0) ; CL(16:020:4(5Z8Z11Z14Z)18:1(11Z)18:1(11Z)) ; CL(16:020:4(5Z8Z11Z14Z)18:1(11Z)18:1(9Z)) ; CL(16:020:4(5Z8Z11Z14Z)18:1(9Z)18:1(11Z)) ; CL(16:020:4(5Z8Z11Z14Z)18:1(9Z)18:1(9Z)) ; CL(16:022:5(4Z7Z10Z13Z16Z)18:1(11Z)16:0) ; CL(16:022:5(4Z7Z10Z13Z16Z)18:1(9Z)16:0) ; CL(16:022:5(7Z10Z13Z16Z19Z)18:1(11Z)16:0) ; CL(16:022:5(7Z10Z13Z16Z19Z)18:1(9Z)16:0) ; CL(16:022:6(4Z7Z10Z13Z16Z19Z)18:016:0) ; CL(18:016:022:6(4Z7Z10Z13Z16Z19Z)16:0) ; CL(16:1(9Z)18:1(11Z)18:2(9Z12Z)18:2(9Z12Z)) ; CL(16:1(9Z)18:1(9Z)18:2(9Z12Z)18:2(9Z12Z)) ; CL(16:1(9Z)18:2(9Z12Z)18:1(11Z)18:2(9Z12Z)) ; CL(16:1(9Z)18:2(9Z12Z)18:1(9Z)18:2(9Z12Z)) ; CL(16:1(9Z)18:2(9Z12Z)18:2(9Z12Z)18:1(11Z)) ; CL(16:1(9Z)18:2(9Z12Z)18:2(9Z12Z)18:1(9Z)) ; CL(18:1(11Z)16:018:1(11Z)20:4(5Z8Z11Z14Z)) ; CL(18:1(11Z)16:018:1(9Z)20:4(5Z8Z11Z14Z)) ; CL(18:1(11Z)16:020:4(5Z8Z11Z14Z)18:1(11Z)) ; CL(18:1(11Z)16:020:4(5Z8Z11Z14Z)18:1(9Z)) ; CL(18:1(11Z)16:022:5(4Z7Z10Z13Z16Z)16:0) ; CL(18:1(11Z)16:022:5(7Z10Z13Z16Z19Z)16:0) ; CL(18:1(11Z)16:1(9Z)18:2(9Z12Z)18:2(9Z12Z)) ;	M+3ACN+2H ; M+3ACN+2H ; M+3ACN+2H ; M+3ACN+2H ; M+3H ; M+3ACN+2H	C81H146O17P2 ; C81H146O17P2 ; C81H146O17P2 ; C81H146O17P2 ; C103H177N5O55 ; C81H146O17P2
414.71449	1.599664	1.252808	4.7003	0.000841	3.0751	0.013104	414.7145	HMDB15556 ; HMDB32305 ; HMDB36338 ; HMDB41907 ; HMDB60706 ; HMDB60948 ; HMDB61049	Phenindamine ; N-(Heptan-4-yl)benzod13dioxole-5-carboxamide ; 25-Acetyl-67-didehydrofevicordin F 3-glucoside ; Imidapril ; 13-Demethyl tacrolimus ; desmethylcyclobenzaprine ; 31-O-Demethyltacrolimus	3M+2Na ; 3M+H+K ; M+3ACN+2H ; 2M+H+NH4 ; M+H+K ; 3M+2Na ; M+H+K	C19H19N ; C15H21NO3 ; C37H52O13 ; C20H27N3O6 ; C43H67NO12 ; C19H19N ; C43H67NO12
206.53795	1.531211	0.962147	-4.7002	0.000841	3.0751	0.013104	206.538	HMDB14686	Adinazolam	M+Na+K	C19H18ClN5

316.11554	1.570533	1.372212	-4.6915	0.000853	3.0693	0.013238	316.1155	HMDB29324 ; HMDB14440 ; HMDB14472 ; HMDB29377 ; HMDB30256 ; HMDB32961 ; HMDB35988 ; HMDB36360 ; HMDB39456 ; HMDB40522 ; HMDB40987 ; HMDB60472 ; HMDB60657 ; HMDB60929 ; HMDB61070	Citpressine II ; Morphine ; Hydromorphone ; Piperine ; Erysopine ; Secodemethylclausenamide ; Ganoderic acid F ; Pipercyclobutanamide A ; Dipiperamide A ; Dipiperamide E ; (-)-Morphine ; Didemethylcitalopram ; Norcodeine ; Dihydroisomorphine ; norhydrocodone	M+H ; 2M+Na+K ; 2M+Na+K ; 2M+Na+K ; 2M+Na+K ; 2M+Na+K ; M+Na+K ; M+Na+K ; M+Na+K ; M+Na+K ; 2M+Na+K ; 2M+H+K ; 2M+Na+K ; 2M+Na+K ; 2M+Na+K	C17H17NO5 ; C17H19NO3 ; C17H19NO3 ; C17H19NO3 ; C17H19NO3 ; C17H19NO3 ; C32H42O9 ; C34H38N2O6 ; C34H38N2O6 ; C34H38N2O6 ; C17H19NO3 ; C18H17FN2O ; C17H19NO3 ; C17H19NO3 ; C17H19NO3
274.01854	1.537533	0.449766	-4.6856	0.00086	3.0654	0.013287	274.0185	HMDB14023	para-Trifluoromethylphenol	3M+Na+K	C7H5F3O
146.16541	1.617556	0.119874	4.685	0.000861	3.065	0.013287	146.1654	HMDB01257 ; HMDB39492	Spermidine ; 117-Diamino-4913-triazaheptadecane	M+H ; M+H+NH4	C7H19N3 ; C14H35N5
216.08635	1.587838	1.206117	4.6819	0.000865	3.063	0.013308	216.0864	HMDB12245 ; HMDB01557 ; HMDB03070 ; HMDB12149 ; HMDB14586 ; HMDB15364 ; HMDB15462 ; HMDB29531 ; HMDB32688 ; HMDB33656 ; HMDB34003 ; HMDB38102 ; HMDB38872 ; HMDB41373 ; HMDB41382 ; HMDB61388 ; HMDB35923	Kinetin ; Riboflavin reduced ; Shikimic acid ; 2-Isopropyl-3-oxosuccinate ; Betamethasone ; Dexamethasone ; Paramethasone ; (S)-47-Dihydroxy-38-diprenylflavanone ; (Z)-1-(Methylthio)-5-phenyl-1-penten-3-yne ; Mulberrofuran A ; Erythrabyssin II ; (R)-Hispaglabridin A ; Erybraedin A ; Ustiloxin B ; (S)-8-Prenylphaseollinisoflavan ; Dimethyl 2-oxoglutarate ; Hirsutin	M+H ; M+2ACN+2H ; M+ACN+H ; M+ACN+H ; M+H+K ; M+H+K ; M+H+K ; M+H+K ; 2M+3H2O+2H ; M+H+K ; M+H+K ; M+H+K ; M+H+K ; M+3H ; M+H+K ; M+ACN+H ; M+H-H2O	C10H9N5O ; C15H16N4O6 ; C7H10O5 ; C7H10O5 ; C22H29FO5 ; C22H29FO5 ; C22H29FO5 ; C25H28O4 ; C12H12S ; C25H28O4 ; C25H28O4 ; C25H28O4 ; C25H28O4 ; C26H39N5O12S ; C25H28O4 ; C7H10O5 ; C10H19NOS2
187.97078	1.633336	0.147443	4.6762	0.000873	3.0592	0.013371	187.9708	HMDB31719	1246-Tetrathiepane	M+NH4	C3H6S4
365.06752	1.611158	0.185242	-4.6748	0.000874	3.0583	0.013371	365.0675	HMDB14901 ; HMDB14975 ; HMDB31950 ; HMDB39864 ; HMDB33287	Methimazole ; Progabide ; L-2-Aminoethyl seryl phosphate ; 3-O-beta-D-Galactopyranosylcinnamtannin B1 ; Theaflagallin	3M+Na ; 2M+Na+K ; 3M+2Na ; M+3Na ; M+H-2H2O	C4H6N2S ; C17H16ClFN2O2 ; C5H13N2O6P ; C51H46O23 ; C20H16O9
219.14205	1.576643	0.490823	-4.6681	0.000883	3.0539	0.013432	219.1421	HMDB32798 ; HMDB01365 ; HMDB30898 ; HMDB30956 ; HMDB31394 ; HMDB31926 ; HMDB33603 ; HMDB35821 ; HMDB36966 ; HMDB37531 ; HMDB38587	(R)-1-O-b-D-Apiofuranosyl-(1-2)-b-D-glucopyranoside-13-octanediol ; Cotinine methonium ion ; (22E 24x)-Ergosta-46822-tetraen-3-one ; 5-Nonadecyl-13-benzenediol ; Annonisin ; Trilobalicin ; Annoglaxin ; Momordicoside G ; 28-Glucopyranosyl-3-methyloleanolic acid ; Dehydroxypaxilline ; Hericerin	M+2H ; 2M+3H2O+2H ; M+2Na ; M+Na+K ; M+H+2Na ; M+H+2Na ; M+H+2Na ; M+2H+Na ; M+2H+Na ; M+H+NH4 ; M+H+NH4	C21H40O9 ; C11H15N2O ; C28H40O ; C25H44O2 ; C35H62O8 ; C35H62O8 ; C35H62O8 ; C37H60O8 ; C37H60O8 ; C27H33NO3 ; C27H33NO3
138.04896	1.579687	0.059626	4.6662	0.000886	3.0526	0.013432	138.049	HMDB03306 ; HMDB30377 ; HMDB30541 ; HMDB30634 ; HMDB30635 ; HMDB30744 ; HMDB30754 ; HMDB30822 ; HMDB30823 ; HMDB30949 ; HMDB33017 ; HMDB33333 ; HMDB33590 ; HMDB37232 ; HMDB37760 ; HMDB40828 ; HMDB41654 ; HMDB41685 ; HMDB41695 ; HMDB00351 ; HMDB00354 ; HMDB00396 ; HMDB00407 ; HMDB00410 ; HMDB00531 ; HMDB00642 ; HMDB00754 ; HMDB01863 ; HMDB01987 ; HMDB02011 ; HMDB14877 ; HMDB29733 ; HMDB30668 ; HMDB33160 ; HMDB38551 ; HMDB40735 ; HMDB41603 ; HMDB59844	Phloretin ; (Z)-4-Methoxy-3355-tetrahydroxystilbene ; 3347-Tetrahydroxyflavan ; beta-Pyrufuran ; alpha-Pyrufuran ; Hemigossypolone ; 11-Methoxynoryangonin ; (-)-Epiafzelechin ; Afzelechin ; Wyerone epoxide ; 11-Hydroxyyangonin ; 23-Epoxysesamone ; (R)-Methysticin ; (2xi4xi)-4457-Tetrahydroxyflavan ; Hydroxysesamone ; (2S3S4R)-3447-Tetrahydroxyflavan ; 3-Hydroxy-O-desmethylangolensin ; 5-Hydroxy-O-desmethylangolensin ; 6-Hydroxy-O-desmethylangolensin ; 3-Hydroxy-2-methyl-R-(RS)-butanoic acid ; 2-Methyl-3-hydroxybutyric acid ; 2-Ethylhydracrylic acid ; 2-Hydroxy-3-methylbutyric acid ; 3-Hydroxy-2-methyl-S-(RR)-butanoic acid ; 3-Hydroxyvaleric acid ; Erythronilic acid ; 3-Hydroxyisovaleric acid ; 2-Hydroxyvaleric acid ; 2-Hydroxy-2-methylbutyric acid ; 4-Hydroxyisovaleric acid ; Hetacillin ; 2-Ethyl-5-methylthiophene ; 1-(246-Trimethoxyphenyl)-13-butanedione ; 2-Propylthiophene ; Methyl 345-trimethoxycinnamate ; Ethyl lactate ; Methyl 3-hydroxybutyrate ; Diethyl carbonate	M+2H ; M+2H ; M+2H ; M+2H ; M+2H ; M+2H ; M+2H ; M+2H ; M+2H ; M+2H ; M+2H ; M+2H ; M+2H ; M+2H ; M+2H ; M+2H ; M+2H ; M+2H ; M+2H ; 2M+H+K ; 2M+H+K ; 2M+H+K ; 2M+H+K ; 2M+H+K ; 2M+H+K ; 2M+H+K ; 2M+H+K ; 2M+H+K ; 2M+H+K ; 2M+H+K ; M+2H+Na ; 2M+H+Na ; M+H+Na ; 2M+H+Na ; M+H+Na ; 2M+H+K ; 2M+H+K ; 2M+H+K	C15H14O5 ; C15H14O5 ; C15H14O5 ; C15H14O5 ; C15H14O5 ; C15H14O5 ; C15H14O5 ; C15H14O5 ; C15H14O5 ; C15H14O5 ; C15H14O5 ; C15H14O5 ; C15H14O5 ; C15H14O5 ; C15H14O5 ; C15H14O5 ; C15H14O5 ; C15H14O5 ; C15H14O5 ; C5H10O3 ; C5H10O3 ; C5H10O3 ; C5H10O3 ; C5H10O3 ; C5H10O3 ; C5H10O3 ; C5H10O3 ; C5H10O3 ; C5H10O3 ; C5H10O3 ; C19H23N3O4S ; C7H10S ; C13H16O5 ; C7H10S ; C13H16O5 ; C5H10O3 ; C5H10O3 ; C5H10O3
210.51487	1.548774	0.543607	-4.664	0.000889	3.0511	0.013432	210.5149	HMDB29285 ; HMDB29295 ; HMDB29296 ; HMDB60043	Avenanthramide 1s ; Caffeoyl tyrosine ; p-Coumaroyl 3-hydroxytyrosine ; 13-HDoHE	M+2K ; M+2K ; M+2K ; 2M+3H	C18H17NO6 ; C18H17NO6 ; C18H17NO6 ; C22H34O
377.05146	1.582867	1.266557	-4.6636	0.000889	3.0509	0.013432	377.0515	HMDB01107 ; HMDB04808 ; HMDB06101 ; HMDB13938 ; HMDB15206 ; HMDB15533 ; HMDB29543 ; HMDB30695 ; HMDB37376 ; HMDB38453 ; HMDB40570	7-Methylguanosine ; 7C-aglycone ; Enterolactone ; Promazine 5-sulfoxide ; Fludarabine ; Cinolazepam ; ()-Ligballinol ; 7-Hydroxy-5-methoxy-68-dimethylflavanone ; 1-(24-Dihydroxy-6-methoxy-35-dimethylphenyl)-3-phenyl-2-propen-1-one ; 57-Dimethoxy-6-methylflavanone ; 2-O-p-Coumaroylhydroxycitric acid	M+2K+H ; M+2K+H ; M+2K+H ; M+2K-H ; 2M+H+Na ; 2M+H+K ; M+2K+H ; M+2K+H ; M+2K+H ; M+2K+H ; M+Na	C11H16N5O5 ; C18H18O4 ; C18H18O4 ; C17H20N2OS ; C10H13FN5O7P ; C18H13ClFN3O2 ; C18H18O4 ; C18H18O4 ; C18H18O4 ; C18H18O4 ; C15H14O10
472.67347	1.585867	1.3184	4.6618	0.000892	3.0497	0.013432	472.6735	HMDB57062 ; HMDB57076 ; HMDB57203 ; HMDB57448 ; HMDB57462 ; HMDB57507	CL(18:016:1(9Z)16:1(9Z)18:2(9Z12Z)) ; CL(18:016:1(9Z)18:2(9Z12Z)16:1(9Z)) ; CL(18:018:2(9Z12Z)16:1(9Z)16:1(9Z)) ; CL(16:1(9Z)18:016:1(9Z)18:2(9Z12Z)) ; CL(16:1(9Z)18:018:2(9Z12Z)16:1(9Z)) ; CL(16:1(9Z)16:1(9Z)18:2(9Z12Z)18:0)	M+3H ; M+3H ; M+3H ; M+3H ; M+3H ; M+3H	C78H144O17P2 ; C78H144O17P2 ; C78H144O17P2 ; C78H144O17P2 ; C78H144O17P2 ; C78H144O17P2
329.13977	1.587741	0.363411	4.6591	0.000896	3.0479	0.013447	329.1398	HMDB40670 ; HMDB60958 ; HMDB60987 ; HMDB06825 ; HMDB14484 ; HMDB14857 ; HMDB15311 ; HMDB32967 ; HMDB34816 ; HMDB37841 ; HMDB38315 ; HMDB32715 ; HMDB32716 ; HMDB33094 ; HMDB33095	Acutilobin ; 7-hydroxyolanzapine ; 2-hydroxymethylolanzapine ; Tetrahydrofolyl-Glu(2) ; Metixene ; Azatadine ; Rescinnamine ; 24-Dimethyl-1H-indole ; Betavulgaroside VIII ; N-(1-Deoxy-1-fructosyl)methionine ; 28-Galloylglucosylpomolate 3-arabinoside ; Lepidine B ; Lepidine D ; Lepidine F ; Lepidine E	M+H ; M+H ; M+H ; M+2ACN+2H ; 2M+H+K ; M+K ; M+H+Na ; 2M+K ; M+H+2Na ; M+NH4 ; M+3Na ; M+H-H2O ; M+H-H2O ; M+H-H2O ; M+H-H2O	C19H20O5 ; C17H20N4OS ; C17H20N4OS ; C24H30N8O9 ; C20H23NS ; C20H22N2 ; C35H42N2O9 ; C10H11N ; C46H68O20 ; C11H21NO7S ; C48H70O17 ; C20H18N4O2 ; C20H18N4O2 ; C20H18N4O2 ; C20H18N4O2
600.61713	1.586924	1.347344	4.6553	0.000901	3.0454	0.013484	600.6171	HMDB40695	Lyciumin D	2M+3H	C45H57N9O11

660.55687	1.603437	0.22976	4.6488	0.00091	3.0411	0.013562	660.5569	HMDB01372 ; HMDB07119 ; HMDB07120 ; HMDB07147 ; HMDB07172 ; HMDB07199 ; HMDB07200 ; HMDB07228 ; HMDB07229 ; HMDB07255 ; HMDB07256 ; HMDB07283 ; HMDB07312 ; HMDB07340 ; HMDB07396 ; HMDB07423 ; HMDB07424 ; HMDB07451 ; HMDB07480 ; HMDB07507 ; HMDB07508 ; HMDB07536 ; HMDB07537 ; HMDB07564 ; HMDB07679 ; HMDB07707 ; HMDB07736 ; HMDB15426 ; HMDB56029 ; HMDB56034 ; HMDB56059 ; HMDB56169 ; HMDB56183 ; HMDB56185 ; HMDB56187 ; HMDB56193 ; HMDB56202 ; HMDB56204 ; HMDB56206 ; HMDB56212 ; HMDB56229	Thiamine pyrophosphate ; DG(16:022:5(4Z7Z10Z13Z16Z)0:0) ; DG(16:022:5(7Z10Z13Z16Z19Z)0:0) ; DG(16:1(9Z)22:4(7Z10Z13Z16Z)0:0) ; DG(18:020:5(5Z8Z11Z14Z17Z)0:0) ; DG(18:1(11Z)20:4(5Z8Z11Z14Z)0:0) ; DG(18:1(11Z)20:4(8Z11Z14Z17Z)0:0) ; DG(18:1(9Z)20:4(5Z8Z11Z14Z)0:0) ; DG(18:1(9Z)20:4(8Z11Z14Z17Z)0:0) ; DG(18:2(9Z12Z)20:3(5Z8Z11Z)0:0) ; DG(18:2(9Z12Z)20:3(8Z11Z14Z)0:0) ; DG(18:3(6Z9Z12Z)20:2(11Z14Z)0:0) ; DG(18:3(9Z12Z15Z)20:2(11Z14Z)0:0) ; DG(18:4(6Z9Z12Z15Z)20:1(11Z)0:0) ; DG(20:1(11Z)18:4(6Z9Z12Z15Z)0:0) ; DG(20:2(11Z14Z)18:3(6Z9Z12Z)0:0) ; DG(20:2(11Z14Z)18:3(9Z12Z15Z)0:0) ; DG(20:3(5Z8Z11Z)18:2(9Z12Z)0:0) ; DG(20:3(8Z11Z14Z)18:2(9Z12Z)0:0) ; DG(20:4(5Z8Z11Z14Z)18:1(11Z)0:0) ; DG(20:4(5Z8Z11Z14Z)18:1(9Z)0:0) ; DG(20:4(8Z11Z14Z17Z)18:1(11Z)0:0) ; DG(20:4(8Z11Z14Z17Z)18:1(9Z)0:0) ; DG(20:5(5Z8Z11Z14Z17Z)18:00:0) ; DG(22:4(7Z10Z13Z16Z)16:1(9Z)0:0) ; DG(22:5(4Z7Z10Z13Z16Z)16:00:0) ; DG(22:5(7Z10Z13Z16Z19Z)16:00:0) ; Cefoxitin ; DG(16:00:022:5n6) ; DG(16:00:022:5n3) ; DG(18:00:020:5n3) ; DG(16:1n70:022:4n6) ; DG(18:1n70:018:2n6) ; DG(18:1n70:020:2n6) ; DG(18:1n70:020:4n6) ; DG(18:1n70:020:4n3) ; DG(18:1n90:018:2n6) ; DG(18:1n90:020:2n6) ; DG(18:1n90:020:4n6) ; DG(18:1n90:020:4n3) ; DG(20:1n90:018:4n3)	3M+2Na ; M+NH4 ; 3M+H+K ; M+NH4 ; M+NH4 ; M+NH4 ; M+NH4 ; 2M+3H2O+2H ; 2M+3H2O+2H ; M+NH4 ; M+NH4 ; 2M+3H2O+2H ; 2M+3H2O+2H ; M+NH4 ; M+NH4 ; M+NH4	C12H19N4O7P2S ; C41H70O5 ; C16H17N3O7S2 ; C41H70O5 ; C41H70O5 ; C41H70O5 ; C41H70O5 ; C40H72O5 ; C40H72O5 ; C41H70O5 ; C41H70O5 ; C40H72O5 ; C40H72O5 ; C41H70O5 ; C41H70O5 ; C41H70O5
242.15786	1.618513	0.140398	-4.6452	0.000915	3.0387	0.013562	242.1579	HMDB02126 ; HMDB32690 ; HMDB35188 ; HMDB35189 ; HMDB38498 ; HMDB38500 ; HMDB38501	27-Nor-5b-cholestane-3a7a12a2425-pentol ; (3beta5alpha9alpha14alpha22E24R)-35914-Tetrahydroxyergosta-722-dien-6-one ; Fasciculol F ; Fasciculol E ; Polyporusterone E ; Polyporusterone D ; Polyporusterone G	M+2Na ; M+H+Na ; M+3H ; M+3H ; M+H+Na ; M+H+Na ; M+H+Na	C26H46O5 ; C28H44O5 ; C39H65NO11 ; C39H65NO11 ; C28H44O5 ; C28H44O5 ; C28H44O5
138.07551	1.555501	1.12132	4.6443	0.000916	3.0381	0.013562	138.0755	HMDB00042 ; HMDB00337 ; HMDB00360 ; HMDB00498 ; HMDB00942 ; HMDB02281 ; HMDB02453 ; HMDB02601 ; HMDB02649 ; HMDB03344 ; HMDB06293	Acetic acid ; (S)-34-Dihydroxybutyric acid ; 24-Dihydroxybutanoic acid ; 4-Deoxyerythronic acid ; Tetrahydroneopterin ; 4a-Hydroxytetrahydrobiopterin ; 4-Deoxythreonic acid ; Ab-Dihydroxyisobutyric acid ; Erythrose ; Glycolaldehyde ; L-Erythrulose	2M+NH4 ; M+NH4 ; M+NH4 ; M+NH4 ; M+H+NH4 ; M+H+NH4 ; M+NH4 ; M+NH4 ; M+NH4 ; 2M+NH4 ; M+NH4	C2H4O2 ; C4H8O4 ; C4H8O4 ; C4H8O4 ; C9H15N5O4 ; C9H15N5O4 ; C4H8O4 ; C4H8O4 ; C4H8O4 ; C2H4O2 ; C4H8O4
298.09662	1.521166	0.799952	4.6437	0.000917	3.0377	0.013562	298.0966	HMDB06408 ; HMDB30188 ; HMDB30840 ; HMDB32240 ; HMDB33758 ; HMDB38840 ; HMDB59806 ; HMDB60014 ; HMDB60438 ; HMDB60966 ; HMDB61009	4-Hydroxy tolbutamide ; N-Isobutyl-248-decatrienamide ; 2-Methoxy-9-phenyl-1H-phenalen-1-one ; N-37-Dimethyl-26-octadienylcyclopropylcarboxamide ; L-Agaritine ; Sarmentine ; Phenylglucuronide ; Phenol glucuronide ; Benzoapyrene-78-diol ; norzolmitripan ; 4-amino-MX	2M+H+Na ; M+2K-H ; 2M+H+Na ; M+2K-H ; 2M+Na+K ; M+2K-H ; 2M+3H2O+2H ; 2M+3H2O+2H ; 2M+H+Na ; M+K ; 2M+Na+K	C12H18N2O4S ; C14H23NO ; C20H14O2 ; C14H23NO ; C12H17N3O4 ; C14H23NO ; C12H14O7 ; C12H14O7 ; C20H14O2 ; C14H17N3O2 ; C12H17N3O4
196.55742	1.616608	1.07243	4.6373	0.000926	3.0335	0.013594	196.5574	HMDB29360 ; HMDB29401	Isorheagenine ; Ochratoxin B	M+H+Na ; M+H+Na	C20H19NO6 ; C20H19NO6
710.64950	1.627565	1.069504	-4.6365	0.000927	3.033	0.013594	710.6495	HMDB34373	N-(14-Methylhexadecanoyl)pyrrolidine	2M+ACN+Na	C21H41NO
168.00620	1.579425	0.052395	4.6364	0.000927	3.0329	0.013594	168.0062	HMDB32721 ; HMDB04629 ; HMDB14499 ; HMDB30616 ; HMDB30947 ; HMDB31004 ; HMDB31842 ; HMDB32725 ; HMDB33931 ; HMDB33986 ; HMDB34144 ; HMDB34255 ; HMDB40679 ; HMDB40939 ; HMDB41120 ; HMDB41652 ; HMDB41657 ; HMDB41710	2-Methyl-14-naphthalenediol bis(dihydrogen phosphate) ; O-Desmethylangolensin ; Aztreonam ; Archangin ; Wyerone ; Diatretin 2 ; Rhapontigenin ; Acetomenaphthone ; Xanthoxyletin ; Demethylvestitol ; Yangonin ; 5-Methoxyseselin ; (2S3R4R)-344-Trihydroxyflavan ; Methyl phenyl disulfide ; 4-Phenyl-1H3H-naphtho18-cdpyran-13-dione ; 347-Trihydroxyisoflavan ; 3-Hydroxyequol ; cis-4-Hydroxyequol	M+2H ; M+2K ; M+3Na ; M+2K ; M+2K ; M+Na ; M+2K ; M+2K ; M+2K ; M+2K ; M+2K ; M+2K ; M+2K ; 2M+H+Na ; M+Na+K ; M+2K ; M+2K ; M+2K	C11H12O8P2 ; C15H14O4 ; C13H17N5O8S2 ; C15H14O4 ; C15H14O4 ; C8H3NO2 ; C15H14O4 ; C15H14O4 ; C15H14O4 ; C15H14O4 ; C15H14O4 ; C15H14O4 ; C15H14O4 ; C7H8S2 ; C18H10O3 ; C15H14O4 ; C15H14O4 ; C15H14O4
105.10405	1.545576	1.069303	4.6244	0.000944	3.0249	0.013759	105.1041	HMDB14795 ; HMDB31270 ; HMDB38191	Mecamylamine ; 1-Nonene ; Isopropylcyclohexane	M+ACN+2H ; M+2ACN+2H ; M+2ACN+2H	C11H21N ; C9H18 ; C9H18
251.14274	1.577976	1.241257	4.6241	0.000945	3.0247	0.013759	251.1427	HMDB01959 ; HMDB12560 ; HMDB31017 ; HMDB31028 ; HMDB31741 ; HMDB31896 ; HMDB32792 ; HMDB34286 ; HMDB35730 ; HMDB37163 ; HMDB37187 ; HMDB37226 ; HMDB37626 ; HMDB37907 ; HMDB38078 ; HMDB39805 ; HMDB40451 ; HMDB41365 ; HMDB00313 ; HMDB00335 ; HMDB00343 ; HMDB00406 ; HMDB05895 ; HMDB15182 ; HMDB60084 ; HMDB60085	Gamma-Aminobutyryl-lysine ; 13-Hydroxy-alpha-tocotrienol ; 10-Undecenyl acetate ; Methyl (E)-2-dodecenoate ; Coniferan ; Erinacine D ; ()-(E)-3-Methyl-4-decen-1-yl acetate ; Ethyl 10-undecenoate ; Camelledionol ; 12-Bis(1-ethoxyethoxy)propane ; Rhodinyl propionate ; Citronellyl propionate ; 1-Ethylhexyl tiglate ; 261010-Tetramethyl-1-oxaspiro45decan-6-ol ; 11-Diethoxy-26-nonadiene ; 4-(3-Hydroxybutyl)-335-trimethylcyclohexanone ; Oxacyclotetradecan-2-one ; 25-Acetylvulgaroside ; 16b-Hydroxyestrone ; 16a-Hydroxyestrone ; 2-Hydroxyestrone ; 16-Ketoestradiol ; 4-Hydroxyestrone ; Abacavir ; 17beta-Estradiol-23-quinone ; 17beta-Estradiol-34-quinone	2M+H+K ; M+Na+K ; M+K ; M+K ; M+K ; M+H+Na ; M+K ; M+K ; M+Na+K ; 2M+Na+K ; M+K ; M+K ; M+K ; M+K ; M+K ; M+K ; M+K ; M+H+Na ; M+H-2H2O ; M+H-2H2O ; M+H-2H2O ; M+H-2H2O ; M+H-2H2O ; M+H-2H2O ; M+H-2H2O ; M+H-2H2O	C10H21N3O3 ; C29H44O3 ; C13H24O2 ; C13H24O2 ; C13H24O2 ; C27H42O7 ; C13H24O2 ; C13H24O2 ; C29H44O3 ; C11H24O4 ; C13H24O2 ; C13H24O2 ; C13H24O2 ; C13H24O2 ; C13H24O2 ; C13H24O2 ; C13H24O2 ; C27H42O7 ; C18H22O3 ; C18H22O3 ; C18H22O3 ; C18H22O3 ; C18H22O3 ; C14H18N6O ; C18H22O3 ; C18H22O3
164.93508	1.61871	0.534427	4.6227	0.000947	3.0238	0.013759	NA	NA	NA	NA	NA

256.06054[illegible]	1.594838	1.138326	-4.6156	0.000957	3.0191	0.013833	256.0605	HMDB01031 ; HMDB01213 ; HMDB01245 ; HMDB01343 ; HMDB01351 ; HMDB14362 ; HMDB29905 ; HMDB31782 ; HMDB34020 ; HMDB34899 ; HMDB35884 ; HMDB36561 ; HMDB37043 ; HMDB38877 ; HMDB40481 ; HMDB40835 ; HMDB41311 ; HMDB59654 ; HMDB60034 ; HMDB60393	Deoxyribose 5-phosphate ; 1-Deoxy-D-xylulose 5-phosphate ; dCDP ; Mevalonic acid-5P ; Deoxyribose 1-phosphate ; Bethanidine ; Artonin T ; Ethiofencarb ; Cajaisoflavone ; 11-Ethylidenebistryptophan ; Melledonol ; Agnuside ; Melledonal B ; Dihydrocycloartomunin ; Dihydroisocycloartomunin ; Cycloaltilisin ; Myristicanol A ; Deoxyribose 5-monophosphate ; (beta-1-O-N-(2-hydroxymethyl-3-chlorophenyl)anthraniloyl-D-glucupyranuronic acid) ; 5-Deoxyribose-1-phosphate	M+ACN+H ; M+ACN+H ; M+3ACN+2H ; 2M+3H2O+2H ; M+ACN+H ; M+2K+H ; M+Na+K ; 2M+Na+K ; M+Na+K ; M+2K ; M+2K ; M+2Na ; M+2Na ; M+Na+K ; M+Na+K ; M+Na+K ; M+2K ; M+ACN+H ; M+ACN+2H ; M+ACN+H	C5H11O7P ; C5H11O7P ; C9H15N3O10P2 ; C6H13O7P ; C5H11O7P ; C10H15N3 ; C26H26O7 ; C11H15NO2S ; C26H26O7 ; C24H26N4O4 ; C23H30O8 ; C22H26O11 ; C23H27ClO8 ; C26H26O7 ; C26H26O7 ; C26H26O7 ; C23H30O8 ; C5H11O7P ; C20H20ClNO10 ; C5H11O7P
247.47624[illegible]	1.628274	0.172616	4.6144	0.000959	3.0183	0.013833	247.4762	HMDB07900 ; HMDB37737	PC(14:1(9Z)14:1(9Z)) ; CI Pigment Blue 63	M+3Na ; M+2Na	C36H68NO8P ; C16H10AlN2O8S2
225.96999[illegible]	1.575826	0.335258	-4.6135	0.00096	3.0177	0.013833	NA	NA	NA	NA	NA
406.63787[illegible]	1.572442	1.167031	-4.6117	0.000963	3.0165	0.013833	406.6379	HMDB07338 ; HMDB28737 ; HMDB28968 ; HMDB30350 ; HMDB37748 ; HMDB39541 ; HMDB39850 ; HMDB39873 ; HMDB56372	DG(18:4(6Z9Z12Z15Z)18:4(6Z9Z12Z15Z)0:0) ; Asparaginyl-Methionine ; Methionyl-Asparagine ; Dehydroanonaine ; Isorhamnetin 3-rutinoside 4-rhamnoside ; Typhaneoside ; Isorhamnetin 3-O-a-L-rhamnopyranosyl-(1-3)-a-L-rhamnopyranosyl-(1-6)-b-D-glucopyranoside ; 2-Naphthalenol 2-aminobenzoate ; DG(18:4n30:018:4n3)	2M+3H ; 3M+H+Na ; 3M+H+Na ; 3M+H+Na ; M+ACN+2H ; M+ACN+2H ; M+ACN+2H ; 3M+H+Na ; 2M+3H	C39H60O5 ; C9H17N3O4S ; C9H17N3O4S ; C17H13NO2 ; C34H42O20 ; C34H42O20 ; C34H42O20 ; C17H13NO2 ; C39H60O5
576.80501[illegible]	1.625075	0.054831	-4.6075	0.000969	3.0137	0.013846	576.805	HMDB38316 ; HMDB39392 ; HMDB39394	Pisumsaponin I ; 54-Deoxy-50-hydroxyciguatoxin ; 52-Epiciguatoxin	M+3ACN+2H ; M+ACN+2H ; M+ACN+2H	C51H80O21 ; C60H86O19 ; C60H86O19
790.57396[illegible]	1.589333	0.006864	4.6041	0.000974	3.0114	0.013846	790.574	HMDB08523 ; HMDB08524 ; HMDB08751 ; HMDB11229 ; HMDB11288 ; HMDB11321 ; HMDB05085 ; HMDB07995 ; HMDB08060 ; HMDB08161 ; HMDB08162 ; HMDB08193 ; HMDB08226 ; HMDB08390 ; HMDB08422 ; HMDB10602 ; HMDB11208 ; HMDB11218 ; HMDB11219 ; HMDB11239 ; HMDB11245 ; HMDB11246 ; HMDB11277 ; HMDB11310 ; HMDB13407 ; HMDB13412 ; HMDB13424 ; HMDB13426 ; HMDB13435 ; HMDB13630 ; HMDB13652 ; HMDB13659 ; HMDB13892 ; HMDB14339 ; HMDB15646 ; HMDB29567 ; HMDB31125 ; HMDB36015 ; HMDB38018 ; HMDB60532 ; HMDB07989 ; HMDB07990 ; HMDB08021 ; HMDB08050 ; HMDB08081 ; HMDB08082 ; HMDB08114 ; HMDB08115 ; HMDB08139 ; HMDB08146 ; HMDB08177 ; HMDB08210 ; HMDB08242 ; HMDB08306 ; HMDB08337 ; HMDB08338 ; [illegible]	PC(20:5(5Z8Z11Z14Z17Z)P-18:1(11Z)) ; PC(20:5(5Z8Z11Z14Z17Z)P-18:1(9Z)) ; PC(22:6(4Z7Z10Z13Z16Z19Z)P-16:0) ; PC(P-16:022:6(4Z7Z10Z13Z16Z19Z)) ; PC(P-18:1(11Z)20:5(5Z8Z11Z14Z17Z)) ; PC(P-18:1(9Z)20:5(5Z8Z11Z14Z17Z)) ; Leukotriene B4 dimethylamide ; PC(16:0P-18:0) ; PC(18:0P-16:0) ; PC(18:2(9Z12Z)P-18:1(11Z)) ; PC(18:2(9Z12Z)P-18:1(9Z)) ; PC(18:3(6Z9Z12Z)P-18:0) ; PC(18:3(9Z12Z15Z)P-18:0) ; PC(20:3(5Z8Z11Z)P-16:0) ; PC(20:3(8Z11Z14Z)P-16:0) ; PG(18:018:0) ; PC(P-16:018:0) ; PC(P-16:020:3(5Z8Z11Z)) ; PC(P-16:020:3(8Z11Z14Z)) ; PC(P-18:016:0) ; PC(P-18:018:3(6Z9Z12Z)) ; PC(P-18:018:3(9Z12Z15Z)) ; PC(P-18:1(11Z)18:2(9Z12Z)) ; PC(P-18:1(9Z)18:2(9Z12Z)) ; PC(o-16:020:4(8Z11Z14Z17Z)) ; PC(o-16:1(9Z)18:0) ; PC(o-18:1(11Z)16:0) ; PC(o-18:1(9Z)16:0) ; PC(o-18:2(9Z12Z)18:2(9Z12Z)) ; 20-HETE ethanolamide ; ()11(12)-EET Ethanolamide[illegible] ()8(9)-EET Ethanolamide ; N-Desmethylvenlafaxine ; Tramadol ; Desvenlafaxine ; Hydroxy-alpha-sanshool ; Glycerol trihexanoate ; Mangalkanyl glucoside ; Cryptomeridiol 11-rhamnoside ; O-Desmethylvenlafaxine ; PC(16:022:5(4Z7Z10Z13Z16Z)) ; PC(16:022:5(7Z10Z13Z16Z19Z)) ; PC(16:1(9Z)22:4(7Z10Z13Z16Z)) ; PC(18:020:5(5Z8Z11Z14Z17Z)) ; PC(18:1(11Z)20:4(5Z8Z11Z14Z)) ; PC(18:1(11Z)20:4(8Z11Z14Z17Z)) ; PC(18:1(9Z)20:4(5Z8Z11Z14Z)) ; PC(18:1(9Z)20:4(8Z11Z14Z17Z)) ; PC(18:2(9Z12Z)20:3(5Z8Z11Z)) ; PC(18:2(9Z12Z)20:3(8Z11Z14Z)) ; PC(18:3(6Z9Z12Z)20:2(11Z14Z)) ; PC(18:3(9Z12Z15Z)20:2(11Z14Z)) ; PC(18:4(6Z9Z12Z15Z)20:1(11Z)) ; PC(20:1(11Z)18:4(6Z9Z12Z15Z)) ; PC(20:2(11Z14Z)18:3(6Z9Z12Z)) ; PC(20:2(11Z14Z)18:3(9Z12Z15Z)) ; PC(20:3(5Z8Z11Z)18:2(9Z12Z)) ; PC(20:3(8Z11Z14Z)18:2(9Z12Z)) ; PC(20:4(5Z8Z11Z14Z)18:1(11Z)) ; PC(20:4(5Z8Z11Z14Z)18:1(9Z)) ; PC(20:4(8Z11Z14Z17Z)18:1(11Z)) ; PC(20:4(8Z11Z14Z17Z)18:1(9Z)) ; PC(20:5(5Z8Z11Z14Z17Z)18:0) ; PC(22:4(7Z10Z13Z16Z)16:1(9Z)) ; PC(22:5(4Z7Z10Z13Z16Z)16:0) ; PC(22:5(7Z10Z13Z16Z19Z)16:0)	M+H ; M+H ; M+H ; M+H ; M+H ; M+H ; 2M+ACN+Na ; M+2Na-H ; M+2Na-H ; M+Na ; M+Na ; M+Na ; M+Na ; M+Na ; M+Na ; 2M+H+Na ; M+2Na-H ; M+Na ; M+Na ; M+2Na-H ; M+Na ; M+Na ; M+Na ; M+Na ; M+Na ; M+2Na-H ; M+2Na-H ; M+2Na-H ; M+Na ; 2M+ACN+Na ; 2M+ACN+Na ; 2M+ACN+Na ; 3M+H ; 3M+H ; 3M+H ; 3M+H ; 2M+NH4 ; 2M+NH4 ; 2M+NH4 ; 3M+H ; M+H-H2O	C46H80NO7P ; C46H80NO7P ; C46H80NO7P ; C46H80NO7P ; C46H80NO7P ; C46H80NO7P ; C22H37NO3 ; C42H84NO7P ; C42H84NO7P ; C44H82NO7P ; C44H82NO7P ; C44H82NO7P ; C44H82NO7P ; C44H82NO7P ; C44H82NO7P ; C42H83O10P ; C42H84NO7P ; C44H82NO7P ; C44H82NO7P ; C42H84NO7P ; C44H82NO7P ; C44H82NO7P ; C44H82NO7P ; C44H82NO7P ; C44H82NO7P ; C42H84NO7P ; C42H84NO7P ; C42H84NO7P ; C44H82NO7P ; C22H37NO3 ; C22H37NO3 ; C22H37NO3 ; C16H25NO2 ; C16H25NO2 ; C16H25NO2 ; C16H25NO2 ; C21H38O6 ; C21H38O6 ; C21H38O6 ; C16H25NO2 ; C46H82NO8P

506.65676	1.548882	0.935023	-4.603	0.000976	3.0107	0.013846	506.6568	HMDB10246 ; HMDB10247 ; HMDB10269 ; HMDB10276 ; HMDB10277 ; HMDB10278 ; HMDB10283 ; HMDB10286 ; HMDB10291 ; HMDB10294 ; HMDB10693 ; HMDB10694 ; HMDB10695 ; HMDB10696 ; HMDB11908 ; HMDB56426 ; HMDB56436 ; HMDB56456 ; HMDB56480 ; HMDB56481 ; HMDB56483 ; HMDB56579 ; HMDB56586 ; HMDB56633 ; HMDB56642 ; HMDB56689 ; HMDB56739 ; HMDB56775 ; HMDB56889 ; HMDB56890 ; HMDB56894 ; HMDB56901 ; HMDB56914 ; HMDB57064 ; HMDB57065 ; HMDB57082 ; HMDB57086 ; HMDB57280 ; HMDB57328 ; HMDB57443 ; HMDB57450 ; HMDB57451 ; HMDB57468 ; HMDB57472 ; HMDB57527 ; HMDB57535 ; HMDB57537 ; HMDB57545 ; HMDB57553 ; HMDB57554 ; HMDB57569 ; HMDB57579 ; HMDB57588 ; HMDB57589 ; HMDB57590 ; HMDB57617 ;	CL(18:2(9Z12Z)18:2(9Z12Z)18:2(9Z12Z)18:1(11Z)) ; CL(18:2(9Z12Z)18:2(9Z12Z)18:2(9Z12Z)18:1(9Z)) ; CL(20:4(5Z8Z11Z14Z)18:2(9Z12Z)18:2(9Z12Z)18:2(9Z12Z)) ; CL(20:4(5Z8Z11Z14Z)20:4(5Z8Z11Z14Z)18:2(9Z12Z)16:0) ; CL(20:4(5Z8Z11Z14Z)20:4(8Z11Z14Z17Z)18:2(9Z12Z)16:0) ; CL(20:4(8Z11Z14Z17Z)18:2(9Z12Z)18:2(9Z12Z)18:2(9Z12Z)) ; CL(20:4(8Z11Z14Z17Z)20:4(5Z8Z11Z14Z)18:2(9Z12Z)16:0) ; CL(20:4(8Z11Z14Z17Z)20:4(8Z11Z14Z17Z)18:2(9Z12Z)16:0) ; CL(22:6(4Z7Z10Z13Z16Z19Z)18:2(9Z12Z)18:1(11Z)16:1(9Z)) ; CL(22:6(4Z7Z10Z13Z16Z19Z)18:2(9Z12Z)18:1(9Z)16:1(9Z)) ; CL(20:4(5Z8Z11Z14Z)20:3(5Z8Z11Z)18:2(9Z12Z)16:1(9Z)) ; CL(20:4(5Z8Z11Z14Z)20:3(8Z11Z14Z)18:2(9Z12Z)16:1(9Z)) ; CL(20:4(8Z11Z14Z17Z)20:3(5Z8Z11Z)18:2(9Z12Z)16:1(9Z)) ; CL(20:4(8Z11Z14Z17Z)20:3(8Z11Z14Z)18:2(9Z12Z)16:1(9Z)) ; Ganglioside GM2 (d18:026:1(17Z)) ; CL(16:016:018:1(11Z)22:6(4Z7Z10Z13Z16Z19Z)) ; CL(16:016:018:1(9Z)22:6(4Z7Z10Z13Z16Z19Z)) ; CL(16:016:020:4(5Z8Z11Z14Z)22:6(4Z7Z10Z13Z16Z19Z)) ; CL(16:016:022:6(4Z7Z10Z13Z16Z19Z)18:1(11Z)) ; CL(16:016:022:6(4Z7Z10Z13Z16Z19Z)18:1(9Z)) ; CL(16:016:022:6(4Z7Z10Z13Z16Z19Z)20:4(5Z8Z11Z14Z)) ; CL(16:016:1(9Z)22:6(4Z7Z10Z13Z16Z19Z)22:6(4Z7Z10Z13Z16Z19Z)) ; CL(16:018:1(11Z)16:022:6(4Z7Z10Z13Z16Z19Z)) ; CL(16:018:1(11Z)22:6(4Z7Z10Z13Z16Z19Z)16:0) ; CL(16:018:1(9Z)16:022:6(4Z7Z10Z13Z16Z19Z)) ; CL(16:018:1(9Z)22:6(4Z7Z10Z13Z16Z19Z)16:0) ; CL(16:020:4(5Z8Z11Z14Z)16:022:6(4Z7Z10Z13Z16Z19Z)) ; CL(16:020:4(5Z8Z11Z14Z)22:6(4Z7Z10Z13Z16Z19Z)16:0) ; CL(16:022:6(4Z7Z10Z13Z16Z19Z)16:1(9Z)22:6(4Z7Z10Z13Z16Z19Z)) ; CL(16:022:6(4Z7Z10Z13Z16Z19Z)18:1(11Z)16:0) ; CL(16:022:6(4Z7Z10Z13Z16Z19Z)18:1(9Z)16:0) ; CL(16:022:6(4Z7Z10Z13Z16Z19Z)20:4(5Z8Z11Z14Z)16:0) ; CL(16:022:6(4Z7Z10Z13Z16Z19Z)22:6(4Z7Z10Z13Z16Z19Z)16:1(9Z)) ; CL(18:016:1(9Z)16:1(9Z)22:5(4Z7Z10Z13Z16Z)) ; CL(18:016:1(9Z)16:1(9Z)22:5(7Z10Z13Z16Z19Z)) ; CL(18:016:1(9Z)22:5(4Z7Z10Z13Z16Z)16:1(9Z)) ; CL(18:016:1(9Z)22:5(7Z10Z13Z16Z19Z)16:1(9Z)) ;	M+3Na ; M+3Na ; M+H+2Na ; M+H+2Na ; M+H+2Na ; M+H+2Na ; M+H+2Na ; M+H+2Na ; M+H+2Na ; M+H+2Na ; M+H+2Na ; M+H+2Na ; M+H+2Na ; M+H+2Na ; M+2H+Na ; M+3Na ; M+3Na ; M+H+2Na ; M+3Na ; M+3Na ; M+H+2Na ; M+2H+Na ; M+3Na ; M+3Na ; M+3Na ; M+3Na ; M+H+2Na ; M+H+2Na ; M+2H+Na ; M+3Na ; M+3Na ; M+H+2Na ; M+2H+Na ; M+3Na ; M+3Na ; M+3Na ; M+3Na ; M+3Na ; M+3Na ; M+2H+Na ; M+3Na ; M+3Na ; M+3Na ; M+3Na ; M+3Na ; M+2H+Na ; M+3Na ; M+2H+Na ; M+2H+Na ; M+2H+Na ; M+3Na ; M+3Na ; M+3Na ; M+3Na ; M+H+2Na ; M+3Na ; M+3Na ; M+3Na ; M+3Na ; M+H+2Na ; M+3Na ; M+3Na ; M+H+2Na ; M+3Na ; M+3Na ; M+H+2Na ; M+H+2Na ; M+2H+Na ; M+2H+Na ; M+2H+Na ; M+2H+Na ; M+2H+Na ; M+2H+Na ; M+2H+Na ; M+2H+Na ; M+3Na ; M+3Na ; M+3Na ; M+3Na ; M+3Na ; M+H+2Na ; M+3Na ; M+3Na ; M+3Na ; M+H+2Na ; M+3Na ; M+3Na ; M+3Na ; M+3Na ; M+H+2Na ; M+3Na ; M+3Na ; M+H+2Na ; M+H+2Na ; M+H+2Na ; M+H+2Na ; M+2H+Na ; M+2H+Na ; M+2H+Na ; M+2H+Na	C81H144O17P2 ; C81H144O17P2 ; C83H142O17P2 ; C83H142O17P2 ; C83H142O17P2 ; C83H142O17P2 ; C83H142O17P2 ; C83H142O17P2 ; C83H142O17P2 ; C83H142O17P2 ; C83H142O17P2 ; C83H142O17P2 ; C83H142O17P2 ; C83H142O17P2 ; C76H138N2O26 ; C81H144O17P2 ; C81H144O17P2 ; C83H142O17P2 ; C81H144O17P2 ; C81H144O17P2 ; C83H142O17P2 ; C85H140O17P2 ; C81H144O17P2 ; C81H144O17P2 ; C81H144O17P2 ; C81H144O17P2 ; C83H142O17P2 ; C83H142O17P2 ; C85H140O17P2 ; C81H144O17P2 ; C81H144O17P2 ; C83H142O17P2 ; C85H140O17P2 ; C81H144O17P2 ; C81H144O17P2 ; C81H144O17P2 ; C81H144O17P2 ; C81H144O17P2 ; C81H144O17P2 ; C85H140O17P2 ; C81H144O17P2 ; C81H144O17P2 ; C81H144O17P2 ; C81H144O17P2 ; C81H144O17P2 ; C85H140O17P2 ; C81H144O17P2 ; C85H140O17P2 ; C85H140O17P2 ; C85H140O17P2 ; C81H144O17P2 ; C81H144O17P2 ; C81H144O17P2 ; C81H144O17P2 ; C83H142O17P2 ; C81H144O17P2 ; C81H144O17P2 ; C81H144O17P2 ; C81H144O17P2 ; C83H142O17P2 ; C81H144O17P2 ; C81H144O17P2 ; C83H142O17P2 ; C81H144O17P2 ; C81H144O17P2 ; C83H142O17P2 ; C83H142O17P2 ; C83H142O17P2 ; C85H140O17P2 ; C85H140O17P2 ; C85H140O17P2 ; C85H140O17P2 ; C85H140O17P2 ; C85H140O17P2 ; C85H140O17P2 ; C85H140O17P2 ; C81H144O17P2 ; C81H144O17P2 ; C81H144O17P2 ; C81H144O17P2 ; C81H144O17P2 ; C83H142O17P2 ; C81H144O17P2 ; C81H144O17P2 ;
275.92509	1.594625	1.125957	4.6018	0.000977	3.0099	0.013846	NA	NA	NA	NA	NA
237.14827	1.582162	0.823444	4.6017	0.000978	3.0099	0.013846	237.1483	HMDB33899 ; HMDB37379 ; HMDB38704 ; HMDB38733 ; HMDB38734 ; HMDB41586 ; HMDB14494 ; HMDB29225 ; HMDB29886 ; HMDB30183 ; HMDB30226 ; HMDB32298 ; HMDB41176 ; HMDB41578	Ixocarpanolide ; Vamonolide ; Macrocarpal B ; Macrocarpal D ; Macrocarpal E ; Macrocarpal H ; Minoxidil ; Coutaric acid ; Sorbitan oleate ; Murrayazolinine ; Mahanimbinine ; Glyceryl lactooleate ; (3S5R6S7E9x)-7-Megastigmene-369-triol 9-glucoside ; 78-Dihydro-3b6a-dihydroxy-alpha-ionol 9-glucoside	M+2H ; M+2H ; M+2H ; M+2H ; M+2H ; M+2H ; 2M+3H2O+2H ; M+3ACN+2H ; M+2Na ; M+3ACN+2H ; M+3ACN+2H ; M+2Na ; M+2ACN+2H ; M+2ACN+2H	C28H40O6 ; C28H40O6 ; C28H40O6 ; C28H40O6 ; C28H40O6 ; C28H40O6 ; C9H15N5O ; C18H27N3O4 ; C24H44O6 ; C23H27NO2 ; C23H27NO2 ; C24H44O6 ; C19H34O8 ; C19H34O8
827.00448	1.553654	1.26077	-4.5976	0.000984	3.0071	0.013894	827.0045	HMDB04921 ; HMDB10395 ; HMDB10396	Ganglioside GD3 (d18:125:0) ; LysoPC(20:4(5Z8Z11Z14Z)) ; LysoPC(20:4(8Z11Z14Z17Z))	M+2ACN+2H ; 3M+H+Na ; 3M+H+Na	C77H139N3O29 ; C28H50NO7P ; C28H50NO7P
250.49829	1.552095	0.86017	-4.5935	0.00099	3.0044	0.013943	250.4983	HMDB37168 ; HMDB38423	2-Propanoylthiazole ; Gluconasturtiin	3M+2K ; M+2K	C6H7NOS ; C15H21NO9S2
201.50973	1.544392	0.702455	-4.584	0.001004	2.9981	0.014106	201.5097	HMDB33193	Avenanthramide L	M+2K	C18H15NO5
234.89367	1.573132	1.365676	4.581	0.001009	2.9961	0.014133	234.8937	HMDB11771	Cer(d18:026:0)	M+2H+Na	C44H89NO3
406.88892	1.505029	1.050692	4.5729	0.001022	2.9907	0.014269	NA	NA	NA	NA	NA
405.45945	1.551865	0.417021	4.5711	0.001025	2.9895	0.014269	NA	NA	NA	NA	NA
359.54716	1.55898	1.102037	-4.5656	0.001033	2.9858	0.014339	359.5472	HMDB30806 ; HMDB38096 ; HMDB39164 ; HMDB60586	Petunin ; Petunidin 3-gentiobioside ; L-Oxalylalbizziine ; N-Trifluoroacetyladriamycinol	M+2K ; M+2K ; 3M+Na+K ; M+2K	C28H33O17 ; C28H33O17 ; C6H9N3O6 ; C29H30F3NO12
325.02166	1.575866	0.421229	-4.5618	0.001039	2.9833	0.014339	325.0217	HMDB02022	Glycineamideribotide	M+K	C7H15N2O8P
133.54399	1.569992	0.778636	-4.5618	0.001039	2.9833	0.014339	133.544	HMDB32337 ; HMDB33060 ; HMDB34319 ; HMDB02205 ; HMDB32928 ; HMDB40300 ; HMDB60804	Hydroxypropyl cellulose ; Piperolactam A ; Furofoline ; L-Homocysteic acid ; 2-Amino-348-trimethyl-3H-imidazo45-fquinoxaline ; 2-Amino-179-trimethylimidazo45-gquinoxaline ; 8-Hydroxythioguanine	M+2H ; M+2H ; M+2H ; M+2ACN+2H ; M+H+K ; M+H+K ; M+2ACN+2H	C16H11NO3 ; C16H11NO3 ; C16H11NO3 ; C4H9NO5S ; C12H13N5 ; C12H13N5 ; C5H5N5OS
121.02082	1.589183	0.771496	-4.5599	0.001042	2.982	0.014339	121.0208	HMDB41813	6-Demethylgriseofulvin	M+2H+Na	C16H15ClO6
281.57237	1.532689	0.403139	4.5589	0.001044	2.9813	0.014339	281.5724	HMDB14464 ; HMDB15574 ; HMDB29496 ; HMDB40680 ; HMDB61040	Piperacillin ; Latamoxef ; Fukiic acid ; Melitric acid B ; hydroxynefazodone	M+2Na ; M+ACN+2H ; 2M+H+NH4 ; M+ACN+2H ; M+2K	C23H27N5O7S ; C20H20N6O9S ; C11H12O8 ; C27H20O11 ; C25H32ClN5O3
253.03217	1.525366	0.646328	-4.5501	0.001058	2.9754	0.014495	253.0322	HMDB01155 ; HMDB03357 ; HMDB28854 ; HMDB29127 ; HMDB30237 ; HMDB31190 ; HMDB31192 ; HMDB31857 ; HMDB31982 ; HMDB34365 ; HMDB34709 ; HMDB39670 ; HMDB40170 ; HMDB41015 ; HMDB60769 ; HMDB60829	Diadenosine triphosphate ; N-Acetylornithine ; Glycyl-Valine ; Valyl-Glycine ; Syzyginin B ; S-Ethyl thioacetate ; S-Methyl propanethioate ; 3-(Methylthio)propanal ; 3-Mercapto-2-butanone ; L-Theanine ; BL IV ; 33-Dithiobis45-dihydro-2-methylfuran ; (Methylthio)acetone ; 4-Mercapto-2-butanone ; 4-Hydroxyphenytoin glucuronide ; Lacosamide-glucuronide	M+3H ; M+2K+H ; M+2K+H ; M+2K+H ; M+3H ; 2M+2Na-H ; 2M+2Na-H ; 2M+2Na-H ; 2M+2Na-H ; M+2K+H ; M+H+K ; M+Na ; 2M+2Na-H ; 2M+2Na-H ; M+Na+K ; M+2K	C20H27N10O16P3 ; C7H14N2O3 ; C7H14N2O3 ; C7H14N2O3 ; C33H24O21 ; C4H8OS ; C4H8OS ; C4H8OS ; C4H8OS ; C7H14N2O3 ; C24H18O10 ; C10H14O2S2 ; C4H8OS ; C4H8OS ; C21H20N2O9 ; C18H24N2O10
408.88596	1.515666	1.097223	4.5464	0.001064	2.973	0.014537	NA	NA	NA	NA	NA

290.07628	1.541447	0.063479	4.5415	0.001072	2.9697	0.014601	290.0763	HMDB13690 ; HMDB29298 ; HMDB29754 ; HMDB32900 ; HMDB33973 ; HMDB33974 ; HMDB37347 ; HMDB37471 ; HMDB37968 ; HMDB38366 ; HMDB38367 ; HMDB38368 ; HMDB38369 ; HMDB38741 ; HMDB41239 ; HMDB14636 ; HMDB29678 ; HMDB31612 ; HMDB33305 ; HMDB33702 ; HMDB33759 ; HMDB33940 ; HMDB34376 ; HMDB34612 ; HMDB35352 ; HMDB35872 ; HMDB37100 ; HMDB37132 ; HMDB37319 ; HMDB40868 ; HMDB59782	Procyanidin ; 4-O-85-5-Dehydrotriferulic acid ; Procyanidin B1 ; Epicatechin-(6-8)-epicatechin ; Procyanidin B2 ; Procyanidin B3 ; Terniflorin ; 2-O-p-Coumaroylvitexin ; ent-Epicatechin(4alpha-8)catechin ; Procyanidin B5 ; Procyanidin B6 ; Procyanidin B7 ; Procyanidin B8 ; Genistein 7-O-(2-p-coumaroylglucoside) ; ent-Epicatechin-(4alpha-6)-ent-epicatechin ; Cefotaxime ; Hibiscitrin ; 35-Dimethoxy-27-phenanthrenediol ; Isomedicarpin ; Pinostrobin chalcone ; Dihydroformononetin ; Vignafuran ; Imperatorin ; Torachrysone 8-(2-apiosylglucoside) ; Oleuroside ; Oleuropein ; Orientaloside ; (2R)-2-Hydroxy-2-(4-hydroxyphenyl)ethyl glucosinolate ; 3-(4-Hydroxyphenyl)-1-(4-hydroxy-2-methoxyphenyl)-2-propen-1-one ; 4-O-Methylpinosylvic acid ; N-Phenylacetyl pyroglutamic acid	M+2H ; M+2H ; M+2H ; M+2H ; M+2H ; M+2H ; M+2H ; M+2H ; M+2H ; M+2H ; M+2H ; M+2H ; M+2H ; M+2H ; M+2H ; M+3ACN+2H ; M+2ACN+2H ; 2M+H+K ; 2M+H+K ; 2M+H+K ; 2M+H+K ; 2M+H+K ; 2M+H+K ; M+H+K ; M+H+K ; M+H+K ; M+H+K ; M+3ACN+2H ; 2M+H+K ; 2M+H+K ; M+2Na-H	C30H26O12 ; C30H26O12 ; C30H26O12 ; C30H26O12 ; C30H26O12 ; C30H26O12 ; C30H26O12 ; C30H26O12 ; C30H26O12 ; C30H26O12 ; C30H26O12 ; C30H26O12 ; C30H26O12 ; C30H26O12 ; C30H26O12 ; C16H17N5O7S2 ; C21H20O14 ; C16H14O4 ; C16H14O4 ; C16H14O4 ; C16H14O4 ; C16H14O4 ; C16H14O4 ; C25H32O13 ; C25H32O13 ; C25H32O13 ; C25H32O13 ; C15H21NO11S2 ; C16H14O4 ; C16H14O4 ; C14H15NO3
402.01210	1.538172	0.724144	-4.5401	0.001075	2.9688	0.014601	402.0121	HMDB52141	TG(24:1(15Z)24:1(15Z)24:1(15Z))	M+3Na	C75H140O6
223.11130	1.570841	0.912892	4.5381	0.001078	2.9675	0.014605	223.1113	HMDB14037 ; HMDB14039 ; HMDB14557 ; HMDB29625 ; HMDB31062 ; HMDB31063 ; HMDB31087 ; HMDB31272 ; HMDB31406 ; HMDB31691 ; HMDB31843 ; HMDB32308 ; HMDB32320 ; HMDB32396 ; HMDB32404 ; HMDB32453 ; HMDB32461 ; HMDB32489 ; HMDB32534 ; HMDB33371 ; HMDB33724 ; HMDB34428 ; HMDB34429 ; HMDB35076 ; HMDB36140 ; HMDB36784 ; HMDB37106 ; HMDB37185 ; HMDB37225 ; HMDB37309 ; HMDB38073 ; HMDB38273 ; HMDB38274 ; HMDB38277 ; HMDB38278 ; HMDB38311 ; HMDB38906 ; HMDB40593	6-Hydroxyfluvastatin ; 5-Hydroxyfluvastatin ; Pramipexole ; 3-O-Protocatechuoylceanothic acid ; (Z)-3-Methyl-3-decenoic acid ; (Z)-3-Methyl-4-decenoic acid ; trans-2-Hexyl-1-cyclopropaneacetic acid ; Ethyl (E)-2-nonenoate ; Ethyl 3-cyclohexylpropionate ; cis-3-Hexenyl pentanoate ; 23-Undecanedione ; (E)-3-Heptenyl 2-methylpropanoate ; 3-Hexenyl 2-methylbutyrate ; Methyl 2-decenoate ; 6-Methyl-5-hepten-2-one propyleneglycol acetal ; cis-3-Octenyl propionate ; Pent-2-enyl hexanoate ; Prenyl caproate ; trans-2-Hexenyl 2-methylbutyrate ; Citronellyl formate ; Undecylenic acid ; Cyclohexyl 3-methylbutanoate ; Cyclohexyl pentanoate ; 2-(2-Methylpropoxy)naphthalene ; 5-Hexyldihydro-5-methyl-2(3H)-furanone ; 6-Hexyltetrahydro-2H-pyran-2-one ; (x)-1-Nonen-3-yl acetate ; Rhodinyl formate ; Methyl citronellate ; 5-Hexyldihydro-4-methyl-2(3H)-furanone ; Octadecyl fumarate ; 2-Hexenyl valerate ; 2-Hexenyl isovalerate ; cis-3-Hexenyl 2-methylbutanoate ; cis-3-Hexenyl 3-methylbutanoate ; xi-gamma-Undecalactone ; 5-Octenyl propanoate ; 2-Propenyl octanoate	M+H+NH4 ; M+H+NH4 ; 2M+H+Na ; M+H+2Na ; M+K ; M+K ; M+K ; M+K ; M+K ; M+K ; M+K ; M+K ; M+K ; M+K ; M+K ; M+K ; M+K ; M+K ; M+K ; M+K ; M+K ; M+K ; M+K ; M+Na ; M+K ; M+K ; M+K ; M+K ; M+K ; M+K ; M+2K ; M+K ; M+K ; M+K ; M+K ; M+K ; M+K ; M+K	C24H26FNO5 ; C24H26FNO5 ; C10H17N3S ; C37H50O8 ; C11H20O2 ; C11H20O2 ; C11H20O2 ; C11H20O2 ; C11H20O2 ; C11H20O2 ; C11H20O2 ; C11H20O2 ; C11H20O2 ; C11H20O2 ; C11H20O2 ; C11H20O2 ; C11H20O2 ; C11H20O2 ; C11H20O2 ; C11H20O2 ; C11H20O2 ; C11H20O2 ; C11H20O2 ; C14H16O ; C11H20O2 ; C11H20O2 ; C11H20O2 ; C11H20O2 ; C11H20O2 ; C11H20O2 ; C22H40O4 ; C11H20O2 ; C11H20O2 ; C11H20O2 ; C11H20O2 ; C11H20O2 ; C11H20O2 ; C11H20O2
160.06028	1.588408	1.164273	4.5347	0.001084	2.9652	0.014614	160.0603	HMDB29433 ; HMDB34442 ; HMDB00699 ; HMDB02349 ; HMDB02432 ; HMDB06331 ; HMDB14871 ; HMDB28824 ; HMDB28950 ; HMDB30342 ; HMDB32923 ; HMDB32988 ; HMDB36156 ; HMDB36867 ; HMDB39810 ; HMDB39812 ; HMDB39814 ; HMDB40825 ; HMDB41617 ; HMDB59762	L-2-Amino-4-methylenepentanedioic acid ; L-trans-alpha-Amino-2-carboxycyclopropaneacetic acid ; 1-Methylnicotinamide ; trans-trans-Muconic acid ; Sumikis acid ; ciscis-Muconic acid ; Pralidoxime ; Glutamyl-Lysine ; Lysyl-Glutamate ; Platydesminium ; Kojic acid ; 5-Hydroxymaltol ; Deoxynivalenol ; Marasmal ; Cyclocalopin A ; Cyclocalopin C1 ; Cyclocalopin E ; Tryptophol xylosyl-(1-6)-glucoside ; Chavicol O-beta-glucopyranoside ; 23-Methylenesuccinic acid	M+H ; M+H ; M+Na ; M+NH4 ; M+NH4 ; M+NH4 ; M+Na ; M+2Na ; M+2Na ; M+2Na ; M+NH4 ; M+NH4 ; M+H+Na ; M+H+Na ; M+H+Na ; M+H+Na ; M+H+Na ; M+2H+Na ; M+H+Na ; M+NH4	C6H9NO4 ; C6H9NO4 ; C7H9N2O ; C6H6O4 ; C6H6O4 ; C6H6O4 ; C7H9N2O ; C11H20N3O5 ; C11H20N3O5 ; C16H20NO3 ; C6H6O4 ; C6H6O4 ; C15H20O6 ; C15H20O6 ; C15H20O6 ; C15H20O6 ; C15H20O6 ; C21H29NO10 ; C15H20O6 ; C6H6O4

305.24713	1.594597	0.063669	4.5341	0.001085	2.9647	0.014614	305.2471	HMDB02364 ; HMDB02388 ; HMDB02395 ; HMDB30094 ; HMDB31882 ; HMDB32256 ; HMDB33020 ; HMDB34652 ; HMDB34654 ; HMDB34655 ; HMDB34961 ; HMDB34962 ; HMDB34964 ; HMDB35119 ; HMDB35263 ; HMDB35315 ; HMDB35353 ; HMDB35511 ; HMDB35775 ; HMDB36675 ; HMDB36757 ; HMDB36962 ; HMDB38177 ; HMDB38703 ; HMDB38713 ; HMDB39690 ; HMDB40455 ; HMDB40456 ; HMDB40652 ; HMDB40976 ; HMDB42104 ; HMDB42452 ; HMDB42533 ; HMDB42538 ; HMDB42593 ; HMDB42598 ; HMDB42736 ; HMDB42738 ; HMDB42886 ; HMDB42888 ; HMDB42972 ; HMDB43028 ; HMDB43038 ; HMDB43055 ; HMDB43056 ; HMDB43065 ; HMDB43071 ; HMDB43083 ; HMDB43099 ; HMDB43111 ; HMDB43167 ; HMDB43195 ; HMDB43223 ; HMDB43232 ; HMDB43238 ; HMDB43248 ;	Oleanolic acid ; Boswellic acid ; Ursolic acid ; Betulinic acid ; Sandosapogenol ; (-)-Ethyl 3-hydroxy-2-methylbutyrate ; Ganodermanondiol ; Soyasapogenol E ; Katonic acid ; 3-Epikatonic acid ; beta-Elemolic acid ; 3alpha-3-Hydroxytirucalla-724-dien-21-oic acid ; alpha-Elemolic acid ; Isomangiferolic acid ; Oxyallobetulin ; Ganodermatriol ; Epoxyganoderiol C ; Trametenolic acid B ; 11-Deoxoglycyrrhetinic acid ; 3beta-Hydroxy-2813-ursanolide ; Bryonolic acid ; 3-Epioleanolic acid ; 3-Hydroxycycloart-24-en-21-oic acid ; Carissic acid ; Erythrinasinate A ; Glycyrrhetol ; Lucidadiol ; Ganoderic acid Z ; 3-Hydroxy-2813-lupanolide ; 3-Epimasticadienolic acid ; TG(14:015:022:1(13Z)) ; TG(14:022:1(13Z)15:0) ; TG(14:018:2(9Z12Z)22:5(4Z7Z10Z13Z16Z)) ; TG(14:018:2(9Z12Z)22:5(7Z10Z13Z16Z19Z)) ; TG(14:020:2n622:5(4Z7Z10Z13Z16Z)) ; TG(14:020:2n622:5(7Z10Z13Z16Z19Z)) ; TG(14:022:5(4Z7Z10Z13Z16Z)18:2(9Z12Z)) ; TG(14:022:5(4Z7Z10Z13Z16Z)20:2n6) ; TG(14:022:5(7Z10Z13Z16Z19Z)18:2(9Z12Z)) ; TG(14:022:5(7Z10Z13Z16Z19Z)20:2n6) ; TG(15:014:022:1(13Z)) ; TG(15:016:020:1(11Z)) ; TG(15:016:022:4(7Z10Z13Z16Z)) ; TG(15:018:018:1(11Z)) ; TG(15:018:018:1(9Z)) ; TG(15:018:020:4(5Z8Z11Z14Z)) ; TG(15:018:020:4(8Z11Z14Z17Z)) ; TG(15:020:016:1(9Z)) ; TG(15:020:018:4(6Z9Z12Z15Z)) ; TG(15:022:014:1(9Z)) ; TG(15:014:1(9Z)22:0) ; TG(15:016:1(9Z)20:0) ; TG(15:018:1(11Z)18:0) ; TG(15:018:1(11Z)20:3(5Z8Z11Z)) ; TG(15:018:1(11Z)20:3n6) ; TG(15:018:1(11Z)22:6(4Z7Z10Z13Z16Z19Z)) ; TG(15:018:1(9Z)18:0) ; TG(15:018:1(9Z)20:3(5Z8Z11Z)) ; TG(15:018:1(9Z)20:3n6) ; TG(15:018:1(9Z)22:6(4Z7Z10Z13Z16Z19Z)) ; TG(15:020:1(11Z)16:0) ; TG(15:020:1(11Z)18:3(6Z9Z12Z)) ; TG(15:020:1(11Z)18:3(9Z12Z15Z)) ; TG(15:020:3(5Z8Z11Z)18:1(11Z)) ; TG(15:020:3(5Z8Z11Z)18:1(9Z)) ; TG(15:020:3(5Z8Z11Z)20:4(5Z8Z11Z14Z)) ; TG(15:020:3(5Z8Z11Z)20:4(8Z11Z14Z17Z)) ; TG(15:018:2(9Z12Z)20:2n6) ; TG(15:018:3(6Z9Z12Z)20:1(11Z)) ; TG(15:018:3(6Z9Z12Z)22:4(7Z10Z13Z16Z)) ; TG(15:020:2n618:2(9Z12Z)) ; TG(15:020:2n620:2n6) ; TG(15:020:3n618:1(11Z)) ; TG(15:020:3n618:1(9Z)) ; TG(15:020:3n620:4(5Z8Z11Z14Z)) ; TG(15:020:3n620:4(8Z11Z14Z17Z)) ; TG(15:020:4(5Z8Z11Z14Z)18:0) ; TG(15:020:4(5Z8Z11Z14Z)20:3(5Z8Z11Z)) ; TG(15:020:4(5Z8Z11Z14Z)20:3n6) ; TG(15:020:4(5Z8Z11Z14Z)22:6(4Z7Z10Z13Z16Z19Z)) ; TG(15:022:4(7Z10Z13Z16Z)16:0) ; TG(15:022:4(7Z10Z13Z16Z)18:3(6Z9Z12Z)) ; TG(15:022:4(7Z10Z13Z16Z)18:3(9Z12Z15Z)) ;	2M+3H ; M+H+Na ; 2M+3H ; 2M+3H ; 2M+3H ; 2M+3H ; 2M+3H ; M+3Na ; M+3Na ; M+2H+Na ; M+2H+Na ; M+2H+Na ; M+2H+Na ; M+2H+Na ; M+2H+Na ; M+2H+Na ; M+2H+Na ; M+3Na ; M+3Na ; M+H+2Na ; M+3Na ; M+3Na ; M+H+2Na ; M+H+2Na ; M+3Na ; M+H+2Na ; M+3Na ; M+3Na ; M+3Na ; M+3Na ; M+H+2Na ; M+H+2Na ; M+2H+Na ; M+3Na ; M+H+2Na ; M+H+2Na ; M+2H+Na ; M+3Na ; M+H+2Na ; M+H+2Na ; M+H+2Na ; M+H+2Na ; M+2H+Na ; M+2H+Na ; M+H+2Na ; M+H+2Na ; M+2H+Na ; M+H+2Na ; M+H+2Na ; M+H+2Na ; M+H+2Na ; M+2H+Na ; M+2H+Na ; M+H+2Na ; M+2H+Na ; M+2H+Na ; M+3H ; M+H+2Na ; M+2H+Na ; M+2H+Na ; M+3H ; M+H+2Na ; M+2H+Na ; M+H+2Na ; M+H+2Na ; M+2H+Na ; M+2H+Na ; M+3H ; M+3H ; M+3H ; M+3H ; M+2H+Na ; M+2H+Na ; M+3H ; M+3H ; M+3Na ; M+H+2Na ; M+2H+Na ; M+2H+Na ; M+2H+Na ; M+2H+Na ; M+3Na ; M+3Na ; M+H+2Na ; M+H+2Na ; M+3Na ; M+H+2Na ; M+3Na ; M+H+2Na ; M+H+2Na ; M+H+2Na ; M+2H+Na ; M+H+2Na ; M+2H+Na ; M+2H+Na	C30H48O3 ; C38H66O4 ; C30H48O3 ; C30H48O3 ; C30H48O3 ; C30H48O3 ; C30H48O3 ; C54H102O6 ; C54H102O6 ; C58H98O6 ; C58H98O6 ; C58H98O6 ; C58H98O6 ; C58H98O6 ; C58H98O6 ; C58H98O6 ; C58H98O6 ; C54H102O6 ; C54H102O6 ; C56H100O6 ; C54H102O6 ; C54H102O6 ; C56H100O6 ; C56H100O6 ; C54H102O6 ; C56H100O6 ; C54H102O6 ; C54H102O6 ; C54H102O6 ; C54H102O6 ; C56H100O6 ; C56H100O6 ; C58H98O6 ; C54H102O6 ; C56H100O6 ; C56H100O6 ; C58H98O6 ; C54H102O6 ; C56H100O6 ; C56H100O6 ; C56H100O6 ; C56H100O6 ; C58H98O6 ; C58H98O6 ; C56H100O6 ; C56H100O6 ; C58H98O6 ; C56H100O6 ; C56H100O6 ; C56H100O6 ; C56H100O6 ; C58H98O6 ; C58H98O6 ; C56H100O6 ; C58H98O6 ; C58H98O6 ; C60H96O6 ; C56H100O6 ; C58H98O6 ; C58H98O6 ; C60H96O6 ; C56H100O6 ; C58H98O6 ; C56H100O6 ; C56H100O6 ; C58H98O6 ; C58H98O6 ; C60H96O6 ; C60H96O6 ; C60H96O6 ; C60H96O6 ; C58H98O6 ; C58H98O6 ; C60H96O6 ; C60H96O6 ; C54H102O6 ; C56H100O6 ; C58H98O6 ; C58H98O6 ; C58H98O6 ; C58H98O6 ; C54H102O6 ; C54H102O6 ; C56H100O6 ; C56H100O6 ; C54H102O6 ; C56H100O6
315.17397	1.57668	0.990038	4.5323	0.001088	2.9635	0.014614	315.174	HMDB02697 ; HMDB03156 ; HMDB04590 ; HMDB05886 ; HMDB06547 ; HMDB13824 ; HMDB14812 ; HMDB14887 ; HMDB15005 ; HMDB30061 ; HMDB31084 ; HMDB31152 ; HMDB31195 ; HMDB31213 ; HMDB31261 ; HMDB31320 ; HMDB31685 ; HMDB32464 ; HMDB32466 ; HMDB32672 ; HMDB33007 ; HMDB33434 ; HMDB33857 ; HMDB33874 ; HMDB34382 ; HMDB34603 ; HMDB35229 ; HMDB35929 ; HMDB36069 ; HMDB36091 ; HMDB36092 ; HMDB37956 ; HMDB40338 ; HMDB40699 ; HMDB41956 ; HMDB60548 ; HMDB60796 ; HMDB60798 ; HMDB60854	19-Norandrosterone ; 23-Butanediol ; 19-Nor-5-androstenediol ; 19-Noretiocholanolone ; Stearidonic acid ; 2-Pentylfuran ; Galantamine ; Etodolac ; Ritodrine ; 11-Dimethoxyethane ; 2-Pentylfuran n ; (3Z6Z)-36-Nonadienal ; 355-Trimethyl-2-cyclohexen-1-one ; 2-Ethoxyethanol ; (2E6E)-26-Nonadienal ; 13-Butanediol ; 24-Nonadienal ; Phenethyl decanoate ; Phenol-formaldehyde cross-linked triethylenetetramine activated ; 481215-Octadecatetraenoic acid ; (2R3R)-23-Butanediol ; 4-Acetyl-1-methylcyclohexene ; Violet-leaf aldehyde ; Piperanine ; (EE)-1113-Octadecadien-9-ynoic acid ; Hebevinoside XIII ; 5-Isopropylbicyclo310hexan-2-one ; (R)-Cryptone ; Pulegenone ; exo-56-Dimethylbicyclo221hept-5-en-2-ol ; exo-2-Methyl-3-methylenebicyclo221heptan-2-ol ; (24R)-5b8b-Epidioxyergosta-622E-dien-3b-ol 3-glucoside ; 355-Trimethyl-3-cyclohexen-1-one ; Feruperine ; Nordihydrocodeine ; Dihydromorphine ; 6alpha-Hydroxy-hydromorphone ; 6beta-Hydroxy-hydromorphone ; Nordihydroisomorphine	M+K ; 3M+2Na-H ; M+K ; M+K ; M+K ; 2M+K ; 2M+3H2O+2H ; 2M+3H2O+2H ; 2M+3H2O+2H ; 3M+2Na-H ; 2M+K ; 2M+K ; 2M+K ; 3M+2Na-H ; 2M+K ; 3M+2Na-H ; 2M+K ; M+K ; M+2Na-H ; M+K ; 3M+2Na-H ; 2M+K ; 2M+K ; 2M+3H2O+2H ; M+K ; M+2H+Na ; 2M+K ; 2M+K ; 2M+K ; 2M+K ; 2M+K ; M+H+K ; 2M+K ; 2M+3H2O+2H ; 2M+3H2O+2H ; 2M+3H2O+2H ; 2M+3H2O+2H ; 2M+3H2O+2H ; 2M+3H2O+2H	C18H28O2 ; C4H10O2 ; C18H28O2 ; C18H28O2 ; C18H28O2 ; C9H14O ; C17H21NO3 ; C17H21NO3 ; C17H21NO3 ; C4H10O2 ; C9H14O ; C9H14O ; C9H14O ; C4H10O2 ; C9H14O ; C4H10O2 ; C9H14O ; C18H28O2 ; C13H26N4O2 ; C18H28O2 ; C4H10O2 ; C9H14O ; C9H14O ; C17H21NO3 ; C18H28O2 ; C49H76O16 ; C9H14O ; C9H14O ; C9H14O ; C9H14O ; C9H14O ; C34H54O8 ; C9H14O ; C17H21NO3 ; C17H21NO3 ; C17H21NO3 ; C17H21NO3 ; C17H21NO3 ; C17H21NO3
262.10318	1.564184	0.123501	-4.5296	0.001092	2.9617	0.014614	262.1032	HMDB28751 ; HMDB28767 ; HMDB28793 ; HMDB29145 ; HMDB00296 ; HMDB00767 ; HMDB13970 ; HMDB15230 ; HMDB15303 ; HMDB30043 ; HMDB34463 ; HMDB38238 ; HMDB40977	Aspartyl-Glutamine ; Aspartyl-Gamma-glutamate ; Glutaminyl-Aspartate ; Gamma-glutamyl-Aspartate ; Uridine ; Pseudouridine ; Dehydroxyzyleuton ; Rosuvastatin ; Kanamycin ; Isoaustin ; Austin ; Plantaricin BN ; Brassitin	M+H ; M+H ; M+H ; M+H ; M+NH4 ; M+NH4 ; M+ACN+H ; M+ACN+2H ; M+H+K ; M+H+Na ; M+H+Na ; M+H+K ; M+ACN+H	C9H15N3O6 ; C9H15N3O6 ; C9H15N3O6 ; C9H15N3O6 ; C9H12N2O6 ; C9H12N2O6 ; C11H12N2OS ; C22H28FN3O6S ; C18H36N4O11 ; C27H32O9 ; C27H32O9 ; C24H36O10 ; C11H12N2OS
409.72235	1.503449	0.924695	4.5289	0.001093	2.9613	0.014614	409.7224	HMDB12338 ; HMDB12340 ; HMDB12359 ; HMDB12369 ; HMDB12399 ; HMDB12409 ; HMDB12414 ; HMDB12418 ; HMDB12440 ; HMDB31826	PS(14:020:3(8Z11Z14Z)) ; PS(14:022:6(4Z7Z10Z13Z16Z19Z)) ; PS(16:018:3(9Z12Z15Z)) ; PS(16:1(9Z)18:2(9Z12Z)) ; PS(18:2(9Z12Z)16:1(9Z)) ; PS(18:3(9Z12Z15Z)16:0) ; PS(18:3(9Z12Z15Z)18:3(9Z12Z15Z)) ; PS(20:3(8Z11Z14Z)14:0) ; PS(22:6(4Z7Z10Z13Z16Z19Z)14:0) ; Fumonisin FP1	M+Na+K ; M+H+K ; M+Na+K ; M+Na+K ; M+Na+K ; M+Na+K ; M+H+K ; M+Na+K ; M+H+K ; M+H+NH4	C40H72NO10P ; C42H70NO10P ; C40H72NO10P ; C40H72NO10P ; C40H72NO10P ; C40H72NO10P ; C42H70NO10P ; C40H72NO10P ; C42H70NO10P ; C39H62NO16
348.93908	1.53822	1.115208	-4.5272	0.001096	2.9602	0.014614	348.9391	HMDB41280 ; HMDB41805	Raphanusamic acid ; 2-Thiothiazolidine-4-carboxylic acid	2M+Na ; 2M+Na	C4H5NO2S2 ; C4H5NO2S2

213.12339	1.524384	0.796311	4.5206	0.001107	2.9557	0.014725	213.1234	HMDB15631 ; HMDB12296 ; HMDB13657 ; HMDB13804 ; HMDB14903 ; HMDB29217 ; HMDB29525 ; HMDB30083 ; HMDB30407 ; HMDB31128 ; HMDB32093 ; HMDB32238 ; HMDB33577 ; HMDB35417 ; HMDB36178 ; HMDB36572 ; HMDB36685 ; HMDB37639 ; HMDB37763 ; HMDB37807 ; HMDB40247 ; HMDB40267 ; HMDB41943 ; HMDB59904 ; HMDB59906 ; HMDB60065 ; HMDB60351 ; HMDB28876 ; HMDB29128	Alvimopan ; Trimethylaminoacetone ; 2-Arachidonyl Glycerol ether ; beta-Damascenone ; Metyrosine ; Tyrosine methylester ; 7-(4-Hydroxy-3-methoxyphenyl)-5-methoxy-1-phenyl-3-heptanone ; ()-Hydroxycitronellol ; Merodesmosine ; 10-Undecenal ; 4-Hydroxy-3-(16-methylheptadecyl)-2H-pyran-2-one ; (-)-trans- and cis-48-Dimethyl-37-nonadien-2-ol ; 10-Undecen-2-one ; (1R2R)-1277-Tetramethylbicyclo221heptan-2-ol ; 2-(3-Phenylpropyl)tetrahydrofuran ; 358-Megastigmatrien-7-one ; 2267-Tetramethylbicyclo430nona-1(9)4-dien-8-one ; xi-1-Ethoxy-1-hexyloxyethane ; xi-3-(4-Isopropylphenyl)-2-methylpropanal ; 9-Undecenal ; 2-Undecenal ; 11-Dimethoxyoctane ; N-Acetyldopamine ; dehydro-beta-Ionone ; Megastigmatrienone ; N-methyl-467-trihydroxy-1234-tetrahydroisoquinoline ; 2-Phenyl-13-propanediol monocarbamate ; Hydroxyprolyl-Valine ; Valyl-Hydroxyproline	M+2H ; 3M+2K ; M+Na+K ; M+Na ; M+NH4 ; M+NH4 ; M+2ACN+2H ; M+K ; M+H+Na ; M+2Na-H ; M+Na+K ; M+2Na-H ; M+2Na-H ; M+2Na-H ; M+Na ; M+Na ; M+Na ; M+K ; M+Na ; M+2Na-H ; M+2Na-H ; M+K ; M+NH4 ; M+Na ; M+Na ; M+NH4 ; M+NH4 ; M+H-H2O ; M+H-H2O	C25H32N2O4 ; C6H14NO ; C23H40O3 ; C13H18O ; C10H13NO3 ; C10H13NO3 ; C21H26O4 ; C10H22O2 ; C18H34N4O6 ; C11H20O ; C23H40O3 ; C11H20O ; C11H20O ; C11H20O ; C13H18O ; C13H18O ; C13H18O ; C10H22O2 ; C13H18O ; C11H20O ; C11H20O ; C10H22O2 ; C10H13NO3 ; C13H18O ; C13H18O ; C10H13NO3 ; C10H13NO3 ; C10H18N2O4 ; C10H18N2O4
552.39235	1.626544	0.716651	-4.5179	0.001112	2.9539	0.014747	552.3924	HMDB14866 ; HMDB52171 ; HMDB52203 ; HMDB52366 ; HMDB52368 ; HMDB52619 ; HMDB53457	Rocuronium ; TG(24:1(15Z)18:2(9Z12Z)22:6(4Z7Z10Z13Z16Z19Z)) ; TG(24:1(15Z)20:2n622:6(4Z7Z10Z13Z16Z19Z)) ; TG(24:1(15Z)22:6(4Z7Z10Z13Z16Z19Z)18:2(9Z12Z)) ; TG(24:1(15Z)22:6(4Z7Z10Z13Z16Z19Z)20:2n6) ; TG(18:2(9Z12Z)24:1(15Z)22:6(4Z7Z10Z13Z16Z19Z)) ; TG(20:2n624:1(15Z)22:6(4Z7Z10Z13Z16Z19Z))	M+Na ; M+2K ; M+2K ; M+2K ; M+2K ; M+2K ; M+2K	C32H53N2O4 ; C68H114O6 ; C68H114O6 ; C68H114O6 ; C68H114O6 ; C68H114O6 ; C68H114O6
311.17899	1.572972	0.866983	4.5111	0.001124	2.9494	0.014863	311.179	HMDB01036 ; HMDB06046 ; HMDB09793 ; HMDB09794 ; HMDB09815 ; HMDB09816 ; HMDB09830 ; HMDB09831 ; HMDB09842 ; HMDB09843 ; HMDB09853 ; HMDB09877 ; HMDB09881 ; HMDB09882 ; HMDB09888 ; HMDB09889 ; HMDB09894 ; HMDB09900 ; HMDB09912 ; HMDB09914 ; HMDB15189 ; HMDB34406 ; HMDB37386 ; HMDB39579 ; HMDB40659	Angiotensin III ; 5a-Androst-3-en-17-one ; PI(16:022:4(10Z13Z16Z19Z)) ; PI(16:022:4(7Z10Z13Z16Z)) ; PI(18:020:4(5Z8Z11Z14Z)) ; PI(18:020:4(8Z11Z14Z17Z)) ; PI(18:1(11Z)20:3(5Z8Z11Z)) ; PI(18:1(11Z)20:3(8Z11Z14Z)) ; PI(18:1(9Z)20:3(5Z8Z11Z)) ; PI(18:1(9Z)20:3(8Z11Z14Z)) ; PI(18:2(9Z12Z)20:2(11Z14Z)) ; PI(20:2(11Z14Z)18:2(9Z12Z)) ; PI(20:3(5Z8Z11Z)18:1(11Z)) ; PI(20:3(5Z8Z11Z)18:1(9Z)) ; PI(20:3(8Z11Z14Z)18:1(11Z)) ; PI(20:3(8Z11Z14Z)18:1(9Z)) ; PI(20:4(5Z8Z11Z14Z)18:0) ; PI(20:4(8Z11Z14Z17Z)18:0) ; PI(22:4(10Z13Z16Z19Z)16:0) ; PI(22:4(7Z10Z13Z16Z)16:0) ; Tocainide ; 5alpha-Androst-16-en-3-one ; (S)-Menthone 8-thioacetate ; Sucrose monopalmitate ; Hoduloside VII	M+3H ; M+K ; M+H+2Na ; M+H+2Na ; M+H+2Na ; M+H+2Na ; M+H+2Na ; M+H+2Na ; M+H+2Na ; M+H+2Na ; M+H+2Na ; M+H+2Na ; M+H+2Na ; M+H+2Na ; M+H+2Na ; M+H+2Na ; M+H+2Na ; M+H+2Na ; M+H+2Na ; M+H+2Na ; 3M+2Na ; M+K ; M+2ACN+H ; M+H+Na ; M+3H	C46H66N12O9 ; C19H28O ; C47H83O13P ; C47H83O13P ; C47H83O13P ; C47H83O13P ; C47H83O13P ; C47H83O13P ; C47H83O13P ; C47H83O13P ; C47H83O13P ; C47H83O13P ; C47H83O13P ; C47H83O13P ; C47H83O13P ; C47H83O13P ; C47H83O13P ; C47H83O13P ; C47H83O13P ; C47H83O13P ; C11H16N2O ; C19H28O ; C12H20O2S ; C28H54O13 ; C47H78O18
512.91846	1.528421	0.660915	4.5069	0.001131	2.9466	0.01492	512.9185	HMDB00816 ; HMDB15284 ; HMDB45260 ; HMDB45281 ; HMDB45286 ; HMDB45416 ; HMDB45421 ; HMDB45442 ; HMDB45995 ; HMDB46071 ; HMDB46151 ; HMDB46171 ; HMDB46176 ; HMDB46201 ; HMDB46220 ; HMDB46226 ; HMDB46229 ; HMDB46879 ; HMDB46927 ; HMDB46975 ; HMDB49526 ; HMDB49531 ; HMDB49546 ; HMDB49651 ; HMDB50133 ; HMDB50138 ; HMDB50152 ; HMDB50252 ; HMDB50545 ; HMDB50659 ; HMDB50672 ; HMDB50677 ; HMDB50714 ; HMDB50726 ; HMDB50732 ; HMDB50735 ; HMDB50786 ; HMDB50791 ; HMDB50804 ; HMDB50821 ; HMDB50827 ; HMDB50830 ; HMDB50833 ; HMDB50839 ; HMDB50843 ; HMDB50848 ; HMDB51083 ; HMDB51094 ; HMDB51202 ; HMDB51243 ; HMDB51247 ; HMDB51253 ; HMDB51257 ; HMDB51262 ; HMDB51274 ; HMDB51345 ;	Phosphoglycolic acid ; Sertaconazole ; TG(18:022:4(7Z10Z13Z16Z)22:6(4Z7Z10Z13Z16Z19Z)) ; TG(18:022:5(4Z7Z10Z13Z16Z)22:5(4Z7Z10Z13Z16Z)) ; TG(18:022:5(4Z7Z10Z13Z16Z)22:5(7Z10Z13Z16Z19Z)) ; TG(18:022:5(7Z10Z13Z16Z19Z)22:5(4Z7Z10Z13Z16Z)) ; TG(18:022:5(7Z10Z13Z16Z19Z)22:5(7Z10Z13Z16Z19Z)) ; TG(18:022:6(4Z7Z10Z13Z16Z19Z)22:4(7Z10Z13Z16Z)) ; TG(20:020:4(5Z8Z11Z14Z)22:6(4Z7Z10Z13Z16Z19Z)) ; TG(20:022:5(4Z7Z10Z13Z16Z)20:5(5Z8Z11Z14Z17Z)) ; TG(20:020:4(8Z11Z14Z17Z)22:6(4Z7Z10Z13Z16Z19Z)) ; TG(20:020:5(5Z8Z11Z14Z17Z)22:5(4Z7Z10Z13Z16Z)) ; TG(20:020:5(5Z8Z11Z14Z17Z)22:5(7Z10Z13Z16Z19Z)) ; TG(20:022:5(7Z10Z13Z16Z19Z)20:5(5Z8Z11Z14Z17Z)) ; TG(20:022:6(4Z7Z10Z13Z16Z19Z)20:4(5Z8Z11Z14Z)) ; TG(20:022:6(4Z7Z10Z13Z16Z19Z)20:4(8Z11Z14Z17Z)) ; TG(20:022:6(4Z7Z10Z13Z16Z19Z)22:6(4Z7Z10Z13Z16Z19Z)) ; TG(22:018:4(6Z9Z12Z15Z)22:6(4Z7Z10Z13Z16Z19Z)) ; TG(22:020:5(5Z8Z11Z14Z17Z)20:5(5Z8Z11Z14Z17Z)) ; TG(22:022:6(4Z7Z10Z13Z16Z19Z)18:4(6Z9Z12Z15Z)) ; TG(18:1(11Z)22:4(7Z10Z13Z16Z)22:5(4Z7Z10Z13Z16Z)) ; TG(18:1(11Z)22:4(7Z10Z13Z16Z)22:5(7Z10Z13Z16Z19Z)) ; TG(18:1(11Z)22:5(4Z7Z10Z13Z16Z)22:4(7Z10Z13Z16Z)) ; TG(18:1(11Z)22:5(7Z10Z13Z16Z19Z)22:4(7Z10Z13Z16Z)) ; TG(18:1(9Z)22:4(7Z10Z13Z16Z)22:5(4Z7Z10Z13Z16Z)) ; TG(18:1(9Z)22:4(7Z10Z13Z16Z)22:5(7Z10Z13Z16Z19Z)) ; TG(18:1(9Z)22:5(4Z7Z10Z13Z16Z)22:4(7Z10Z13Z16Z)) ; TG(18:1(9Z)22:5(7Z10Z13Z16Z19Z)22:4(7Z10Z13Z16Z)) ; TG(20:1(11Z)20:3(5Z8Z11Z)22:6(4Z7Z10Z13Z16Z19Z)) ; TG(20:1(11Z)20:3n622:6(4Z7Z10Z13Z16Z19Z)) ;	3M+2Na-H ; M+2K-H ; M+ACN+2H ; M+ACN+2H ; M+ACN+2H ; M+ACN+2H ; M+ACN+2H ; M+ACN+2H ; M+ACN+2H ; M+ACN+2H ; M+ACN+2H ; M+ACN+2H ; M+ACN+2H ; M+ACN+2H ; M+ACN+2H ; M+ACN+2H ; M+H+NH4 ; M+ACN+2H ; M+ACN+2H ; M+ACN+2H ; M+ACN+2H ; M+ACN+2H ; M+ACN+2H ; M+ACN+2H ; M+ACN+2H ; M+ACN+2H ; M+ACN+2H ; M+ACN+2H ; M+ACN+2H ; M+ACN+2H ; M+ACN+2H ; M+ACN+2H ; M+H+NH4 ; M+ACN+2H ; M+ACN+2H ; M+ACN+2H ; M+ACN+2H ; M+ACN+2H ; M+H+NH4 ; M+ACN+2H ; M+ACN+2H ; M+H+NH4 ; M+H+NH4 ; M+ACN+2H ; M+ACN+2H ; M+ACN+2H ; M+ACN+2H ; M+ACN+2H ; M+ACN+2H ; M+H+NH4 ; M+H+NH4 ; M+H+NH4 ; M+ACN+2H ; M+H+NH4 ; M+ACN+2H ; M+ACN+2H ; M+ACN+2H ; M+ACN+2H ; M+ACN+2H ; M+ACN+2H ; M+ACN+2H ; M+ACN+2H ; M+ACN+2H ; M+H+NH4 ; M+ACN+2H ; M+ACN+2H ; M+ACN+2H ; M+H+NH4 ; M+ACN+2H ; M+ACN+2H ; M+ACN+2H ; M+ACN+2H ; M+ACN+2H ; M+ACN+2H ; M+ACN+2H ; M+ACN+2H ; M+ACN+2H ; M+ACN+2H ; M+ACN+2H ; M+ACN+2H ; M+H+NH4 ;	C2H5O6P ; C20H15Cl3N2OS ; C65H106O6 ; C65H106O6 ; C65H106O6 ; C65H106O6 ; C65H106O6 ; C65H106O6 ; C65H106O6 ; C65H106O6 ; C65H106O6 ; C65H106O6 ; C65H106O6 ; C65H106O6 ; C65H106O6 ; C65H106O6 ; C67H106O6 ; C65H106O6 ; C65H106O6 ; C65H106O6 ; C65H106O6 ; C65H106O6 ; C65H106O6 ; C65H106O6 ; C65H106O6 ; C65H106O6 ; C65H106O6 ; C65H106O6 ; C65H106O6 ; C65H106O6 ; C65H106O6 ; C65H106O6 ; C65H106O6 ; C67H106O6 ; C65H106O6 ; C65H106O6 ; C65H106O6 ; C65H106O6 ; C65H106O6 ; C67H106O6 ; C65H106O6 ; C65H106O6 ; C67H106O6 ; C67H106O6 ; C65H106O6 ; C65H106O6 ; C65H106O6 ; C65H106O6 ; C65H106O6 ; C65H106O6 ; C67H106O6 ; C67H106O6 ; C67H106O6 ; C65H106O6 ; C67H106O6 ; C65H106O6 ; C65H106O6 ; C65H106O6 ; C65H106O6 ; C65H106O6 ; C65H106O6 ; C65H106O6 ; C65H106O6 ; C65H106O6 ; C65H106O6 ; C67H106O6 ; C65H106O6 ; C65H106O6 ; C65H106O6 ; C65H106O6 ; C65H106O6 ; C65H106O6 ; C65H106O6 ; C67H106O6 ; C67H106O6 ; C67H106O6 ; C65H106O6 ; C67H106O6 ; C65H106O6 ; C65H106O6 ; C65H106O6 ; C65H106O6 ; C65H106O6 ; C65H106O6 ; C65H106O6 ; C65H106O6 ; C67H106O6 ; C67H106O6 ; C65H106O6
422.88198	1.594148	0.62752	4.5041	0.001136	2.9447	0.014946	NA	NA	NA	NA	NA
158.06797	1.546676	1.22624	4.5009	0.001142	2.9425	0.014949	158.068	HMDB00641 ; HMDB02031 ; HMDB03423 ; HMDB06899 ; HMDB11687 ; HMDB15155 ; HMDB28687 ; HMDB29616 ; HMDB33514 ; HMDB41900	L-Glutamine ; Ureidoisobutyric acid ; D-Glutamine ; Alanylglycine ; Phenylbutyrylglutamine ; Isosorbide Mononitrate ; Alanyl-Glycine ; Diazenedicarboxamide ; Fusarochromanone ; gamma-Carboxyglutamic acid	2M+H+Na ; 2M+H+Na ; 2M+H+Na ; 2M+H+Na ; M+H+Na ; M+3ACN+2H ; 2M+H+Na ; M+ACN+H ; M+H+Na ; M+3ACN+2H	C5H10N2O3 ; C5H10N2O3 ; C5H10N2O3 ; C5H10N2O3 ; C15H20N2O4 ; C6H9NO6 ; C5H10N2O3 ; C2H4N4O2 ; C15H20N2O4 ; C6H9NO6
791.04830	1.542759	1.266826	-4.5006	0.001142	2.9423	0.014949	NA	NA	NA	NA	NA

484.69910	1.56872	1.223397	4.4979	0.001147	2.9405	0.014968	484.6991	HMDB09116 ; HMDB09117 ; HMDB09148 ; HMDB09181 ; HMDB09345 ; HMDB09378 ; HMDB11350 ; HMDB11351 ; HMDB11377 ; HMDB11378 ; HMDB11409 ; HMDB11442 ; HMDB40669	PE(18:2(9Z12Z)P-18:1(11Z)) ; PE(18:2(9Z12Z)P-18:1(9Z)) ; PE(18:3(6Z9Z12Z)P-18:0) ; PE(18:3(9Z12Z15Z)P-18:0) ; PE(20:3(5Z8Z11Z)P-16:0) ; PE(20:3(8Z11Z14Z)P-16:0) ; PE(P-16:020:3(5Z8Z11Z)) ; PE(P-16:020:3(8Z11Z14Z)) ; PE(P-18:018:3(6Z9Z12Z)) ; PE(P-18:018:3(9Z12Z15Z)) ; PE(P-18:1(11Z)18:2(9Z12Z)) ; PE(P-18:1(9Z)18:2(9Z12Z)) ; ()-Pinoresinol 4-O-beta-D-Glucopyranosyl-(1-2)-beta-D-glucopyranosyl-(1-6)-beta-D-glucopyranoside	2M+3H ; 2M+3H ; 2M+3H ; 2M+3H ; 2M+3H ; 2M+3H ; 2M+3H ; 2M+3H ; 2M+3H ; 2M+3H ; 2M+3H ; 2M+3H ; M+3ACN+2H	C41H76NO7P ; C41H76NO7P ; C41H76NO7P ; C41H76NO7P ; C41H76NO7P ; C41H76NO7P ; C41H76NO7P ; C41H76NO7P ; C41H76NO7P ; C41H76NO7P ; C41H76NO7P ; C41H76NO7P ; C38H52O21
223.07470	1.513748	1.034371	4.4963	0.00115	2.9395	0.014968	223.0747	HMDB00099 ; HMDB00455 ; HMDB03075 ; HMDB14641 ; HMDB28785 ; HMDB29058 ; HMDB00840 ; HMDB01229 ; HMDB01908 ; HMDB01979 ; HMDB02016 ; HMDB02404 ; HMDB03235 ; HMDB03247 ; HMDB04067 ; HMDB04241 ; HMDB04242 ; HMDB05011 ; HMDB05099 ; HMDB06116 ; HMDB12110 ; HMDB12633 ; HMDB12635 ; HMDB13678 ; HMDB14334 ; HMDB14380 ; HMDB14708 ; HMDB14797 ; HMDB14920 ; HMDB30628 ; HMDB30726 ; HMDB30778 ; HMDB30779 ; HMDB30780 ; HMDB30975 ; HMDB31380 ; HMDB31710 ; HMDB33099 ; HMDB33536 ; HMDB33611 ; HMDB34269 ; HMDB34678 ; HMDB34917 ; HMDB36854 ; HMDB37444 ; HMDB37550 ; HMDB38055 ; HMDB38875 ; HMDB38876 ; HMDB38917 ; HMDB39119 ; HMDB60602 ; HMDB60704 ; HMDB60778 ; HMDB60800 ; HMDB60844 ;	L-Cystathionine ; Allocystathionine ; Flavone ; Phenindione ; Cysteinyl-Threonine ; Threoninyl-Cysteine ; Salicyluric acid ; Dopaquinone ; 19-Hydroxy-PGE2 ; 615-Diketo1314-dihydro-PGF1a ; 4-Carboxyphenylglycine ; Alpha-Hydroxyhippuric acid ; Prostaglandin G2 ; 20-Hydroxy-PGE2 ; Leucodopachrome ; 6-Ketoprostaglandin E1 ; 11-Dehydro-thromboxane B2 ; Clopidogrel ; Thromboxane B3 ; 3-Hydroxyhippuric acid ; 5(6)-Epoxy Prostaglandin E1 ; 20-COOH-1011-dihydro-LTB4 ; 20-dihydroxyleukotriene B4 ; 4-Hydroxyhippuric acid ; Bortezomib ; Milrinone ; Cinnarizine ; Acamprosate ; Propantheline ; Rubraflavone A ; 2-Hydroxy-67-dimethoxybenzoxazole ; Mammea AAB ; Mammea ABA ; Mammea ABB ; Corchoionoside B ; (6beta7alpha12beta13beta)-7-Hydroxy-1116-dioxo-814-apianadien-226-olide ; 19-Nonanedithiol ; Kanzonol Z ; Honyucitrin ; Licocoumarin A ; Mammeisin ; Cinncassiol D2 ; Xanthohumol E ; Cinncassiol D3 ; (S)-Cajaflavanone ; 24-Dihydroxy-78-dimethoxy-2H-14-benzoxazin-3(4H)-one 2-glucoside ; 2-Hydroxy-7-methoxy-2H-14-benzoxazin-3(4H)-one ; Gancaonin Q ; Albanin D ; Armillarivin ; L-Dopaquinone ; N-acetyl-5-aminosalicylic acid ; 1-Hydroxylorcaserin ; 5-Hydroxylorcaserin ; 7-Hydroxylorcaserin ; N-Hydroxylorcaserin ; 4-(4-chlorophenyl)-4-hydroxypiperidine	M+H ; M+H ; M+H ; M+H ; M+H ; M+H ; 2M+3H2O+2H ; 2M+3H2O+2H ; M+2K ; M+2K ; 2M+3H2O+2H ; 2M+3H2O+2H ; M+2K ; M+2K ; 2M+3H2O+2H ; M+2K ; M+2K ; M+3ACN+2H ; M+2K ; 2M+3H2O+2H ; M+2K ; M+2K ; M+2K ; 2M+3H2O+2H ; M+Na+K ; 2M+H+Na ; M+2K ; M+ACN+H ; M+2K ; M+H+K ; 2M+3H2O+2H ; M+H+K ; M+Na+K ; M+H+K ; M+H+K ; M+2Na ; M+Na+K ; 2M+Na+K ; M+H+K ; M+H+K ; M+H+K ; M+H+K ; M+2K ; M+H+K ; M+2K ; M+H+K ; M+ACN+2H ; 2M+3H2O+2H ; M+H+K ; M+H+K ; M+Na+K ; 2M+3H2O+2H ; 2M+3H2O+2H ; 2M+H+Na ; 2M+H+Na ; 2M+H+Na ; 2M+H+Na ; 2M+H+Na	C7H14N2O4S ; C7H14N2O4S ; C15H10O2 ; C15H10O2 ; C7H14N2O4S ; C7H14N2O4S ; C9H9NO4 ; C9H9NO4 ; C20H32O6 ; C20H32O6 ; C9H9NO4 ; C9H9NO4 ; C20H32O6 ; C20H32O6 ; C9H9NO4 ; C20H32O6 ; C20H32O6 ; C16H16ClNO2S ; C20H32O6 ; C9H9NO4 ; C20H32O6 ; C20H32O6 ; C20H32O6 ; C9H9NO4 ; C19H25BN4O4 ; C12H9N3O ; C26H28N2 ; C5H11NO4S ; C23H30NO3 ; C25H26O5 ; C9H9NO4 ; C25H26O5 ; C25H26O5 ; C25H26O5 ; C19H28O9 ; C23H28O5 ; C9H20S2 ; C25H26O5 ; C25H26O5 ; C25H26O5 ; C25H26O5 ; C20H32O6 ; C25H26O5 ; C20H32O6 ; C25H26O5 ; C16H21NO11 ; C9H9NO4 ; C25H26O5 ; C25H26O5 ; C23H28O5 ; C9H9NO4 ; C9H9NO4 ; C11H14ClNO ; C11H14ClNO ; C11H14ClNO ; C11H14ClNO ; C11H14ClNO
783.57347	1.599548	0.098009	4.494	0.001154	2.9379	0.014968	783.5735	HMDB06737 ; HMDB07181 ; HMDB07209 ; HMDB07238 ; HMDB07377 ; HMDB07405 ; HMDB07601 ; HMDB07629 ; HMDB07797 ; HMDB07798 ; HMDB07825 ; HMDB07946 ; HMDB08330 ; HMDB08882 ; HMDB08942 ; HMDB08974 ; HMDB09000 ; HMDB09032 ; HMDB09065 ; HMDB09097 ; HMDB09225 ; HMDB09256 ; HMDB09257 ; HMDB09288 ; HMDB09518 ; HMDB09550 ; HMDB09746 ; HMDB10564 ; HMDB30926 ; HMDB30927 ; HMDB30933 ; HMDB40373 ; HMDB56047 ; HMDB56071 ; HMDB56093 ; HMDB56114 ; HMDB56115	CE(22:2(13Z16Z)) ; DG(18:024:1(15Z)0:0) ; DG(18:1(11Z)24:00:0) ; DG(18:1(9Z)24:00:0) ; DG(20:022:1(13Z)0:0) ; DG(20:1(11Z)22:00:0) ; DG(22:020:1(11Z)0:0) ; DG(22:1(13Z)20:00:0) ; DG(24:018:1(11Z)0:0) ; DG(24:018:1(9Z)0:0) ; DG(24:1(15Z)18:00:0) ; PC(15:020:2(11Z14Z)) ; PC(20:2(11Z14Z)15:0) ; PE(14:1(9Z)24:1(15Z)) ; PE(16:022:2(13Z16Z)) ; PE(16:1(9Z)22:1(13Z)) ; PE(18:020:2(11Z14Z)) ; PE(18:1(11Z)20:1(11Z)) ; PE(18:1(9Z)20:1(11Z)) ; PE(18:2(9Z12Z)20:0) ; PE(20:018:2(9Z12Z)) ; PE(20:1(11Z)18:1(11Z)) ; PE(20:1(11Z)18:1(9Z)) ; PE(20:2(11Z14Z)18:0) ; PE(22:1(13Z)16:1(9Z)) ; PE(22:2(13Z16Z)16:0) ; PE(24:1(15Z)14:1(9Z)) ; PE-NMe2(18:1(9Z)18:1(9Z)) ; Avocadienofuran ; Isoavocadienofuran ; Avocadynofuran ; Ginsenoyne J ; DG(18:00:024:1n9) ; DG(20:00:022:1n9) ; DG(22:00:020:1n9) ; DG(24:00:018:1n7) ; DG(24:00:018:1n9)	M+2K+H ; M+2K-H ; M+2K-H ; M+2K-H ; M+2K-H ; M+2K-H ; M+2K-H ; M+2K-H ; M+2K-H ; M+2K-H ; M+2K-H ; 2M+H+Na ; 2M+H+Na ; 2M+H+Na ; 2M+H+Na ; 2M+H+Na ; 2M+H+Na ; 2M+H+Na ; 2M+H+Na ; 2M+H+Na ; 2M+H+Na ; 2M+H+Na ; 2M+H+Na ; 2M+H+Na ; 2M+H+Na ; 2M+H+Na ; 2M+H+Na ; 2M+H+Na ; 3M+2Na-H ; 3M+2Na-H ; 3M+2Na-H ; 3M+2Na-H ; M+2K-H ; M+2K-H ; M+2K-H ; M+2K-H ; M+2K-H	C49H84O2 ; C45H86O5 ; C45H86O5 ; C45H86O5 ; C45H86O5 ; C45H86O5 ; C45H86O5 ; C45H86O5 ; C45H86O5 ; C45H86O5 ; C45H86O5 ; C43H82NO8P ; C43H82NO8P ; C43H82NO8P ; C43H82NO8P ; C43H82NO8P ; C43H82NO8P ; C43H82NO8P ; C43H82NO8P ; C43H82NO8P ; C43H82NO8P ; C43H82NO8P ; C43H82NO8P ; C43H82NO8P ; C43H82NO8P ; C43H82NO8P ; C43H82NO8P ; C17H26O ; C17H26O ; C17H26O ; C17H26O ; C45H86O5 ; C45H86O5 ; C45H86O5 ; C45H86O5 ; C45H86O5
231.38244	1.513131	0.739018	4.4915	0.001158	2.9362	0.014968	231.3824	HMDB32199 ; HMDB38098 ; HMDB38099 ; HMDB41677 ; HMDB41766	Chymosin preparation escherichia coli k-12 ; Petunidin 3-rutinoside ; Petunidin 3-rhamnoside 5-glucoside ; 4-O-Methyldelphinidin 3-O-rutinoside ; Peonidin 3-sophoroside	M+2H+Na ; M+3Na ; M+3Na ; M+3Na ; M+3Na	C30H27N3O15 ; C28H33O16 ; C28H33O16 ; C28H33O16 ; C28H33O16
296.55872	1.569893	0.596224	-4.4913	0.001159	2.9361	0.014968	NA	NA	NA	NA	NA
651.13356	1.572767	1.094765	-4.487	0.001166	2.9332	0.014999	651.1336	HMDB35464 ; HMDB41235	6-Acetylapiin ; 6-O-Acetylvicenin 1	M+2Na-H ; M+2Na-H	C28H30O15 ; C28H30O15
284.03365	1.603817	1.194822	4.4867	0.001167	2.933	0.014999	284.0337	HMDB00286 ; HMDB00302 ; HMDB60079 ; HMDB59620 ; HMDB60266	Uridine diphosphate glucose ; Uridine diphosphategalactose ; (2R3S4R5R)-5-(24-dioxo-1234-tetrahydropyrimidin-1-yl)-34-dihydroxyoxolan-2-ylmethyl (3R4S5S6R)-345-trihydroxy-6-(hydroxymethyl)oxan-2-yl phosphonatooxyphosphonate ; 5-(methylthio)-23-Dioxopentyl phosphate ; 23-Diketo-5-methylthio-1-phosphopentane	M+2H ; M+2H ; M+2H ; M+ACN+H ; M+ACN+H	C15H24N2O17P2 ; C15H24N2O17P2 ; C15H24N2O17P2 ; C6H11O6PS ; C6H11O6PS

								HMDB14904 ; HMDB29414 ; HMDB33192 ; HMDB35209 ; HMDB36939 ; HMDB38003 ; HMDB43451 ; HMDB43536 ; HMDB43621 ; HMDB43654 ; HMDB43677 ; HMDB43682 ; HMDB43710 ; HMDB43732 ; HMDB43738 ; HMDB43766 ; HMDB43787 ; HMDB43794 ; HMDB48090 ; HMDB48136 ; HMDB48376 ; HMDB48378 ; HMDB52514 ; HMDB52645 ; HMDB52750 ; HMDB52757 ; HMDB52764 ; HMDB52871 ; HMDB53063 ; HMDB53091 ; HMDB53182 ; HMDB53366 ; HMDB53479 ; HMDB53570 ; HMDB53582 ; HMDB54030 ; HMDB54919 ; HMDB55160 ; HMDB55254 ; HMDB55268 ; HMDB55366 ; HMDB55542	Clavulanate ; (S)-alpha-Amino-4-carboxy-3-furanpropanoic acid ; Neoacrimarine K ; Cartormin ; Glucosinalbin 4-(4-acetylrhamnoside) ; Delphinidin 3-sambubioside ; TG(15:018:3(6Z9Z12Z)22:6(4Z7Z10Z13Z16Z19Z)) ; TG(15:020:4(5Z8Z11Z14Z)20:5(5Z8Z11Z14Z17Z)) ; TG(15:022:5(4Z7Z10Z13Z16Z)18:4(6Z9Z12Z15Z)) ; TG(15:018:3(9Z12Z15Z)22:6(4Z7Z10Z13Z16Z19Z)) ; TG(15:018:4(6Z9Z12Z15Z)22:5(4Z7Z10Z13Z16Z)) ; TG(15:018:4(6Z9Z12Z15Z)22:5(7Z10Z13Z16Z19Z)) ; TG(15:020:4(8Z11Z14Z17Z)20:5(5Z8Z11Z14Z17Z)) ; TG(15:020:5(5Z8Z11Z14Z17Z)20:4(5Z8Z11Z14Z)) ; TG(15:020:5(5Z8Z11Z14Z17Z)20:4(8Z11Z14Z17Z)) ; TG(15:022:5(7Z10Z13Z16Z19Z)18:4(6Z9Z12Z15Z)) ; TG(15:022:6(4Z7Z10Z13Z16Z19Z)18:3(6Z9Z12Z)) ; TG(15:022:6(4Z7Z10Z13Z16Z19Z)18:3(9Z12Z15Z)) ; TG(14:1(9Z)18:2(9Z12Z)22:6(4Z7Z10Z13Z16Z19Z)) ; TG(14:1(9Z)20:2n622:6(4Z7Z10Z13Z16Z19Z)) ; TG(14:1(9Z)22:6(4Z7Z10Z13Z16Z19Z)18:2(9Z12Z)) ; TG(14:1(9Z)22:6(4Z7Z10Z13Z16Z19Z)20:2n6) ; TG(18:2(9Z12Z)14:1(9Z)22:6(4Z7Z10Z13Z16Z19Z)) ; TG(18:2(9Z12Z)18:3(6Z9Z12Z)18:4(6Z9Z12Z15Z)) ; TG(18:2(9Z12Z)18:3(9Z12Z15Z)18:4(6Z9Z12Z15Z)) ; TG(18:2(9Z12Z)18:4(6Z9Z12Z15Z)18:3(6Z9Z12Z)) ; TG(18:2(9Z12Z)18:4(6Z9Z12Z15Z)18:3(9Z12Z15Z)) ; TG(18:3(6Z9Z12Z)15:022:6(4Z7Z10Z13Z16Z19Z)) ; TG(18:3(6Z9Z12Z)18:2(9Z12Z)18:4(6Z9Z12Z15Z)) ; TG(18:3(6Z9Z12Z)20:2n618:4(6Z9Z12Z15Z)) ; TG(18:3(6Z9Z12Z)18:4(6Z9Z12Z15Z)20:2n6) ; TG(20:2n614:1(9Z)22:6(4Z7Z10Z13Z16Z19Z)) ; TG(20:2n618:3(6Z9Z12Z)18:4(6Z9Z12Z15Z)) ; TG(20:2n618:3(9Z12Z15Z)18:4(6Z9Z12Z15Z)) ; TG(20:2n618:4(6Z9Z12Z15Z)18:3(9Z12Z15Z)) ; TG(20:4(5Z8Z11Z14Z)15:020:5(5Z8Z11Z14Z17Z)) ; TG(22:5(4Z7Z10Z13Z16Z)15:018:4(6Z9Z12Z15Z)) ; TG(18:3(9Z12Z15Z)15:022:6(4Z7Z10Z13Z16Z19Z)) ; TG(18:3(9Z12Z15Z)18:2(9Z12Z)18:4(6Z9Z12Z15Z)) ; TG(18:3(9Z12Z15Z)20:2n618:4(6Z9Z12Z15Z)) ; TG(18:4(6Z9Z12Z15Z)15:022:5(7Z10Z13Z16Z19Z)) ; TG(20:4(8Z11Z14Z17Z)15:020:5(5Z8Z11Z14Z17Z))	3M+H+K ; 3M+H+K ; M+2K ; M+Na+K ; M+H+Na ; M+H+K ; M+3Na	C8H9NO5 ; C8H9NO5 ; C31H29NO9 ; C27H29NO13 ; C22H31NO15S2 ; C26H29O16 ; C58H94O6
318.55574(	1.613301	0.103211	-4.4848	0.00117	2.9317	0.015004	318.5557				
286.77153:	1.578708	1.289713	4.4821	0.001175	2.9299	0.015014	286.7715	HMDB35519 ; HMDB38337	11-(14-Dihydro-4-nonyl-35-pyridinediyl)bis1-dodecanone ; Helianyl octanoate	M+2H ; M+H+NH4	C38H69NO2 ; C38H66O2
405.21112:	1.569216	0.229316	-4.4811	0.001177	2.9292	0.015014	405.2111	HMDB15204 ; HMDB15313 ; HMDB30339 ; HMDB33450 ; HMDB41530 ; HMDB32960	Mequitazine ; Propafenone ; Pipernonaline ; Retrofractamide D ; Torvoside E ; 1-Octen-3-yl primeveroside	M+2ACN+H ; M+ACN+Na ; M+ACN+Na ; M+ACN+Na ; M+H+K ; M+H-H2O	C20H22N2S ; C21H27NO3 ; C21H27NO3 ; C21H27NO3 ; C40H66O14 ; C19H34O10
489.43090:	1.561274	0.222539	-4.477	0.001185	2.9265	0.01507	489.4309	HMDB43161 ; HMDB43387 ; HMDB43392 ; HMDB43611 ; HMDB43756 ; HMDB43777 ; HMDB45884 ; HMDB45936 ; HMDB46034 ; HMDB46036 ; HMDB46645 ; HMDB46651 ; HMDB46695 ; HMDB46701 ; HMDB46741 ; HMDB46743 ; HMDB46891 ; HMDB46893 ; HMDB47051 ; HMDB47383 ; HMDB47431 ; HMDB47590 ; HMDB47592 ; HMDB51106 ; HMDB51108 ; HMDB51140 ; HMDB51176 ; HMDB51614 ; HMDB51616 ; HMDB51668 ; HMDB51702 ; HMDB51716 ; HMDB51718 ; HMDB51941 ; HMDB51946 ; HMDB52159 ; HMDB52166 ; HMDB52174 ; HMDB52176 ; HMDB52191 ; HMDB52198 ; HMDB52286 ; HMDB52288 ; HMDB52462 ; HMDB52475 ; HMDB52481 ; HMDB52495 ; HMDB52594 ; HMDB52607 ; HMDB52614 ; HMDB53042 ; HMDB53320 ; HMDB53331 ; HMDB53337 ; HMDB53349 ; HMDB53434 ;	TG(15:024:022:6(4Z7Z10Z13Z16Z19Z)) ; TG(15:024:1(15Z)22:5(4Z7Z10Z13Z16Z)) ; TG(15:024:1(15Z)22:5(7Z10Z13Z16Z19Z)) ; TG(15:022:5(4Z7Z10Z13Z16Z)24:1(15Z)) ; TG(15:022:5(7Z10Z13Z16Z19Z)24:1(15Z)) ; TG(15:022:6(4Z7Z10Z13Z16Z19Z)24:0) ; TG(20:018:2(9Z12Z)22:4(7Z10Z13Z16Z)) ; TG(20:020:2n622:4(7Z10Z13Z16Z)) ; TG(20:022:4(7Z10Z13Z16Z)18:2(9Z12Z)) ; TG(20:022:4(7Z10Z13Z16Z)20:2n6) ; TG(22:018:2(9Z12Z)20:4(5Z8Z11Z14Z)) ; TG(22:018:2(9Z12Z)20:4(8Z11Z14Z17Z)) ; TG(22:020:2n620:4(5Z8Z11Z14Z)) ; TG(22:020:2n620:4(8Z11Z14Z17Z)) ; TG(22:020:4(5Z8Z11Z14Z)18:2(9Z12Z)) ; TG(22:020:4(5Z8Z11Z14Z)20:2n6) ; TG(22:020:4(8Z11Z14Z17Z)18:2(9Z12Z)) ; TG(22:020:4(8Z11Z14Z17Z)20:2n6) ; TG(24:015:022:6(4Z7Z10Z13Z16Z19Z)) ; TG(24:018:2(9Z12Z)18:4(6Z9Z12Z15Z)) ; TG(24:020:2n618:4(6Z9Z12Z15Z)) ; TG(24:018:4(6Z9Z12Z15Z)18:2(9Z12Z)) ; TG(24:018:4(6Z9Z12Z15Z)20:2n6) ; TG(20:3(5Z8Z11Z)22:1(13Z)18:2(9Z12Z)) ; TG(20:3(5Z8Z11Z)22:1(13Z)20:2n6) ; TG(20:3(5Z8Z11Z)18:2(9Z12Z)22:1(13Z)) ; TG(20:3(5Z8Z11Z)20:2n622:1(13Z)) ; TG(22:1(13Z)20:3(5Z8Z11Z)18:2(9Z12Z)) ; TG(22:1(13Z)20:3(5Z8Z11Z)20:2n6) ; TG(22:1(13Z)18:2(9Z12Z)20:3n6) ; TG(22:1(13Z)20:2n620:3n6) ; TG(22:1(13Z)20:3n618:2(9Z12Z)) ; TG(22:1(13Z)20:3n620:2n6) ; TG(24:1(15Z)15:022:5(4Z7Z10Z13Z16Z)) ; TG(24:1(15Z)15:022:5(7Z10Z13Z16Z19Z)) ; TG(24:1(15Z)18:2(9Z12Z)18:3(6Z9Z12Z)) ; TG(24:1(15Z)18:2(9Z12Z)18:3(9Z12Z15Z)) ; TG(24:1(15Z)18:3(6Z9Z12Z)18:2(9Z12Z)) ; TG(24:1(15Z)18:3(6Z9Z12Z)20:2n6) ; TG(24:1(15Z)20:2n618:3(6Z9Z12Z)) ; TG(24:1(15Z)20:2n618:3(9Z12Z15Z)) ; TG(24:1(15Z)18:3(9Z12Z15Z)18:2(9Z12Z)) ; TG(24:1(15Z)18:3(9Z12Z15Z)20:2n6) ; TG(18:2(9Z12Z)20:022:4(7Z10Z13Z16Z)) ; TG(18:2(9Z12Z)22:020:4(5Z8Z11Z14Z)) ; TG(18:2(9Z12Z)22:020:4(8Z11Z14Z17Z)) ; TG(18:2(9Z12Z)24:018:4(6Z9Z12Z15Z)) ; TG(18:2(9Z12Z)22:1(13Z)20:3n6) ; TG(18:2(9Z12Z)24:1(15Z)18:3(6Z9Z12Z)) ; TG(18:2(9Z12Z)24:1(15Z)18:3(9Z12Z15Z)) ; TG(18:3(6Z9Z12Z)24:1(15Z)20:2n6) ; TG(20:2n620:022:4(7Z10Z13Z16Z)) ; TG(20:2n622:020:4(5Z8Z11Z14Z)) ; TG(20:2n622:020:4(8Z11Z14Z17Z)) ; TG(20:2n624:018:4(6Z9Z12Z15Z)) ; TG(20:2n622:1(13Z)20:3n6) ; TG(20:2n624:1(15Z)18:3(9Z12Z15Z)) ; Glycerol trinonadecanoate ; TG(15:018:024:0) ;	M+2H ; M+2Na ; M+2Na ; M+2Na ; M+2Na ; M+2Na ; M+H+Na ; M+H+Na ; M+H+Na ; M+H+Na ; M+H+Na ; M+H+Na ; M+H+Na ; M+H+Na ; M+H+Na ; M+H+Na ; M+2Na ; M+H+Na ; M+H+Na ; M+H+Na ; M+H+Na ; M+2Na ; M+H+Na ; M+H-2H2O ; M+H-2H2O ; M+H-2H2O	C64H112O6 ; C60H116O6 ; C60H116O6 ; C60H116O6 ; C60H116O6 ; C60H116O6 ; C62H114O6 ; C62H114O6 ; C62H114O6 ; C62H114O6 ; C62H114O6 ; C62H114O6 ; C62H114O6 ; C62H114O6 ; C62H114O6 ; C62H114O6 ; C60H116O6 ; C62H114O6 ; C62H114O6 ; C62H114O6 ; C62H114O6 ; C60H116O6 ; C62H114O6 ; C62H114O6 ; C62H114O6 ; C62H114O6 ; C62H114O6 ; C62H114O6 ; C62H114O6 ; C62H114O6 ; C62H114O6 ; C62H114O6 ; C62H114O6 ; C62H114O6 ; C62H114O6 ; C62H114O6 ; C62H114O6 ; C62H114O6 ; C62H114O6 ; C62H114O6 ;
621.94513:	1.561383	0.326615	-4.4752	0.001188	2.9252	0.015073	NA	NA	NA	NA	NA
577.97914:	1.560744	0.994008	-4.472	0.001194	2.9231	0.015073	NA	NA	NA	NA	NA
206.0372_:	1.506302	0.990416	-4.4704	0.001197	2.922	0.015073	206.0372	HMDB30560 ; HMDB01425 ; HMDB41787 ; HMDB41826	71112-Triacetoxycoumestan ; Estrone sulfate ; Urolithin B 3-O-glucuronide ; Amifloxacin	M+2H ; M+Na+K ; M+H+Na ; M+2K	C21H14O9 ; C18H22O5S ; C19H16O9 ; C16H19FN4O3

148.02763	1.540028	0.780941	4.4666	0.001204	2.9195	0.015073	148.0276	HMDB02339 ; HMDB06809 ; HMDB13894 ; HMDB28778 ; HMDB28779 ; HMDB28904 ; HMDB28926 ; HMDB30227 ; HMDB30759 ; HMDB31473 ; HMDB31474 ; HMDB32227 ; HMDB33052 ; HMDB35227 ; HMDB36179 ; HMDB37704 ; HMDB37738 ; HMDB38281 ; HMDB38637 ; HMDB40250 ; HMDB41064 ; HMDB41318 ; HMDB41389 ; HMDB41391 ; HMDB60968	5-Methoxytryptophan ; Nicotinate D-ribonucleoside ; S-4-Hydroxymephenytoin ; Cysteinyl-Isoleucine ; Cysteinyl-Leucine ; Isoleucyl-Cysteine ; Leucyl-Cysteine ; 5-Methoxycanthin-6-one ; (R)-Pterosin B ; 13-Dithiane ; 14-Dithiane ; Dimethylbenzyl carbinyl crotonate ; Ethyl vinyl disulfide ; Hydroxypropanedioic acid ; 2-Methyl-13-dithiolane ; Isoamyl cinnamate ; 2-(3-Hydroxy-4-methylphenyl)-5-methyl-4-hexen-3-one ; cis-3-Hexenyl phenylacetate ; Methoxybrassitin ; xi-Tetrahydro-2-thiophenethiol ; 2-Methoxycanthin-6-one ; Cinnamyl isovalerate ; Methyl 2-propenyl disulfide ; (E)-Methyl 1-propenyl disulfide ; 4-hydroxymephenytoin	M+Na+K ; M+H+K ; M+Na+K ; M+Na+K ; M+Na+K ; M+Na+K ; M+Na+K ; M+2Na ; M+2K ; 2M+3H2O+2H ; 2M+3H2O+2H ; M+2K ; 2M+3H2O+2H ; 2M+3H2O+2H ; 2M+3H2O+2H ; M+2K ; M+2K ; M+2K ; M+2Na ; 2M+3H2O+2H ; M+2Na ; M+2K ; 2M+3H2O+2H ; 2M+3H2O+2H ; M+Na+K	C12H14N2O3 ; C11H14NO6 ; C12H14N2O3 ; C9H18N2O3S ; C9H18N2O3S ; C9H18N2O3S ; C9H18N2O3S ; C15H10N2O2 ; C14H18O2 ; C4H8S2 ; C4H8S2 ; C14H18O2 ; C4H8S2 ; C3H4O5 ; C4H8S2 ; C14H18O2 ; C14H18O2 ; C14H18O2 ; C12H14N2O2S ; C4H8S2 ; C15H10N2O2 ; C14H18O2 ; C4H8S2 ; C4H8S2 ; C12H14N2O3
124.09594	1.517908	1.101592	-4.4661	0.001205	2.9191	0.015073	124.0959	HMDB01256 ; HMDB04080 ; HMDB13655 ; HMDB32182	Spermine ; Anandamide ; O-Arachidonoyl Ethanolamine ; Cadinene	M+2Na ; M+2H+Na ; M+2H+Na ; M+H+K	C10H26N4 ; C22H37NO2 ; C22H37NO2 ; C15H28
314.07493	1.569645	0.633726	-4.4659	0.001205	2.919	0.015073	314.0749	HMDB01168 ; HMDB05782 ; HMDB06530 ; HMDB06540 ; HMDB14911 ; HMDB14980 ; HMDB14991 ; HMDB29491 ; HMDB30746 ; HMDB31657 ; HMDB31858 ; HMDB31912 ; HMDB32245 ; HMDB35616 ; HMDB37507 ; HMDB39377 ; HMDB40316 ; HMDB40560 ; HMDB41346 ; HMDB41673 ; HMDB41867 ; HMDB60970 ; HMDB60975 ; HMDB61121 ; HMDB61175	24-Decadienoyl-CoA ; Hesperetin ; trans-2-Methyl-5-isopropylhexa-25-dienoyl-CoA ; cis-2-Methyl-5-isopropylhexa-25-dienoyl-CoA ; Etoposide ; Oxazepam ; Temozolomide ; Isoferreirin ; 573-Trihydroxy-4-methoxyflavanone ; Ferreirin ; 4-Methyl-5-vinylthiazole ; Porric acid A ; Divanillin ; 3344-Tetrahydroxy-2-methoxychalcone ; Folerogenin ; 236-Trihydroxy-4-methoxybenzylcoumaranone ; Blumeatin ; 457-Trihydroxy-3-methoxyflavanone ; 2-Pyridinemethanethiol ; 4-Methoxy-237-trihydroxyisoflavanone ; Demoxepam ; norclobazam ; nortemazepam ; 3-Hydroxynordiazepam ; p-Hydroxynordiazepam	M+2H+Na ; 2M+H+Na ; M+2H+Na ; M+2H+Na ; M+H+K ; 2M+3H2O+2H ; 3M+2Na ; 2M+H+Na ; 2M+H+Na ; 2M+H+Na ; 2M+ACN+Na ; 2M+H+Na ; 2M+H+Na ; 2M+H+Na ; 2M+H+Na ; 2M+H+Na ; 2M+H+Na ; 2M+H+Na ; 2M+ACN+Na ; 2M+H+Na ; 2M+3H2O+2H ; 2M+3H2O+2H ; 2M+3H2O+2H ; 2M+3H2O+2H ; 2M+3H2O+2H	C31H50N7O17P3S ; C16H14O6 ; C31H50N7O17P3S ; C31H50N7O17P3S ; C29H32O13 ; C15H11ClN2O2 ; C6H6N6O2 ; C16H14O6 ; C16H14O6 ; C16H14O6 ; C6H7NS ; C16H14O6 ; C16H14O6 ; C16H14O6 ; C16H14O6 ; C16H14O6 ; C16H14O6 ; C16H14O6 ; C6H7NS ; C16H14O6 ; C15H11ClN2O2 ; C15H11ClN2O2 ; C15H11ClN2O2 ; C15H11ClN2O2 ; C15H11ClN2O2
264.04297	1.619725	0.212163	-4.4655	0.001206	2.9187	0.015073	264.043	HMDB37080 ; HMDB37081 ; HMDB37082 ; HMDB38069 ; HMDB38808 ; HMDB39177 ; HMDB41384 ; HMDB59596	3-Methylellagic acid 8-(2-acetylrhamnoside) ; 3-Methylellagic acid 8-(3-acetylrhamnoside) ; 3-Methylellagic acid 8-(4-acetylrhamnoside) ; Gomphrenol 3-methylether 4-glucuronide ; Luteolin 3-(3-acetylglucuronide) ; 1-O-Galloylglycerol ; Luteolin 3-(4-acetylglucuronide) ; ETF	M+H+Na ; M+H+Na ; M+H+Na ; M+H+Na ; M+H+Na ; 2M+H+K ; M+H+Na ; 2M+ACN+Na	C23H20O13 ; C23H20O13 ; C23H20O13 ; C23H20O13 ; C23H20O13 ; C10H12O7 ; C23H20O13 ; C2H3F3O
281.99363	1.503923	1.220955	4.4639	0.001209	2.9176	0.015073	281.9936	HMDB60484	Indolepyruvate	M+2K+H	C11H9NO3
384.61176	1.582154	0.494624	-4.454	0.001228	2.911	0.015194	384.6118	HMDB29406	Amaranthin	M+ACN+2H	C30H34N2O19
363.01676	1.580863	1.068375	4.4534	0.001229	2.9106	0.015194	363.0168	HMDB13895 ; HMDB28787 ; HMDB29102 ; HMDB35190 ; HMDB60865 ; HMDB60866	34-Dihydrodiol ; Cysteinyl-Tyrosine ; Tyrosyl-Cysteine ; Chlorohydrin ; Phenytoin catechol ; Phenytoin dihydrodiol	M+2K-H ; M+2K+H ; M+2K+H ; 3M+2K ; M+2K+H ; M+2K-H	C15H14N2O4 ; C12H16N2O4S ; C12H16N2O4S ; C13H9ClO ; C15H12N2O4 ; C15H14N2O4
549.39832	1.614309	0.370758	-4.4528	0.00123	2.9102	0.015194	549.3983	HMDB13122 ; HMDB46829 ; HMDB46954 ; HMDB46973 ; HMDB46978 ; HMDB47651 ; HMDB47697 ; HMDB51780 ; HMDB51791 ; HMDB51796 ; HMDB51876 ; HMDB51881 ; HMDB51892 ; HMDB52235 ; HMDB52281 ; HMDB52331 ; HMDB52341 ; HMDB52346 ; HMDB52361 ; HMDB52370 ; HMDB52376 ; HMDB54175 ; HMDB54553 ; HMDB54558 ; HMDB54562 ; HMDB54612 ; HMDB54764 ; HMDB54821 ; HMDB54826 ; HMDB54955 ; HMDB55013 ; HMDB55018 ; HMDB55025 ; HMDB55609 ; HMDB55711 ; HMDB55742 ; HMDB55820 ; HMDB55843	LysoPC(P-18:0) ; TG(22:022:5(4Z7Z10Z13Z16Z)22:6(4Z7Z10Z13Z16Z19Z)) ; TG(22:022:5(7Z10Z13Z16Z19Z)22:6(4Z7Z10Z13Z16Z19Z)) ; TG(22:022:6(4Z7Z10Z13Z16Z19Z)22:5(4Z7Z10Z13Z16Z)) ; TG(22:022:6(4Z7Z10Z13Z16Z19Z)22:5(7Z10Z13Z16Z19Z)) ; TG(24:020:5(5Z8Z11Z14Z17Z)22:6(4Z7Z10Z13Z16Z19Z)) ; TG(24:022:6(4Z7Z10Z13Z16Z19Z)20:5(5Z8Z11Z14Z17Z)) ; TG(22:1(13Z)22:4(7Z10Z13Z16Z)22:6(4Z7Z10Z13Z16Z19Z)) ; TG(22:1(13Z)22:5(4Z7Z10Z13Z16Z)22:5(4Z7Z10Z13Z16Z)) ; TG(22:1(13Z)22:5(4Z7Z10Z13Z16Z)22:5(7Z10Z13Z16Z19Z)) ; TG(22:1(13Z)22:5(7Z10Z13Z16Z19Z)22:5(4Z7Z10Z13Z16Z)) ; TG(22:1(13Z)22:5(7Z10Z13Z16Z19Z)22:5(7Z10Z13Z16Z19Z)) ; TG(22:1(13Z)22:6(4Z7Z10Z13Z16Z19Z)22:4(7Z10Z13Z16Z)) ; TG(24:1(15Z)20:4(5Z8Z11Z14Z)22:6(4Z7Z10Z13Z16Z19Z)) ; TG(24:1(15Z)22:5(4Z7Z10Z13Z16Z)20:5(5Z8Z11Z14Z17Z)) ; TG(24:1(15Z)20:4(8Z11Z14Z17Z)22:6(4Z7Z10Z13Z16Z19Z)) ; TG(24:1(15Z)20:5(5Z8Z11Z14Z17Z)22:5(4Z7Z10Z13Z16Z)) ; TG(24:1(15Z)20:5(5Z8Z11Z14Z17Z)22:5(7Z10Z13Z16Z19Z)) ; TG(24:1(15Z)22:5(7Z10Z13Z16Z19Z)20:5(5Z8Z11Z14Z17Z)) ; TG(24:1(15Z)22:6(4Z7Z10Z13Z16Z19Z)20:4(5Z8Z11Z14Z)) ; TG(24:1(15Z)22:6(4Z7Z10Z13Z16Z19Z)20:4(8Z11Z14Z17Z)) ; TG(20:4(5Z8Z11Z14Z)24:1(15Z)22:6(4Z7Z10Z13Z16Z19Z)) ; TG(22:2(13Z16Z)22:4(7Z10Z13Z16Z)22:5(4Z7Z10Z13Z16Z)) ; TG(22:2(13Z16Z)22:4(7Z10Z13Z16Z)22:5(7Z10Z13Z16Z19Z)) ; TG(22:2(13Z16Z)22:5(4Z7Z10Z13Z16Z)22:4(7Z10Z13Z16Z)) ; TG(22:2(13Z16Z)22:5(7Z10Z13Z16Z19Z)22:4(7Z10Z13Z16Z)) ; TG(22:4(7Z10Z13Z16Z)22:1(13Z)22:6(4Z7Z10Z13Z16Z19Z)) ; TG(22:4(7Z10Z13Z16Z)22:2(13Z16Z)22:5(4Z7Z10Z13Z16Z)) ; TG(22:4(7Z10Z13Z16Z)22:2(13Z16Z)22:5(7Z10Z13Z16Z19Z)) ;	M+ACN+H ; M+Na+K	C26H54NO6P ; C69H112O6
234.51205	1.492969	0.964834	-4.4523	0.001231	2.9098	0.015194	NA	NA	NA	NA	NA

241.15461	1.59082	0.204931	-4.4371	0.00126	2.8996	0.015519	241.1546	HMDB01009 ; HMDB01457 ; HMDB03990 ; HMDB04649 ; HMDB11472 ; HMDB11502 ; HMDB12455 ; HMDB13687 ; HMDB13693 ; HMDB15168 ; HMDB30395 ; HMDB32153 ; HMDB32247 ; HMDB35081 ; HMDB35082 ; HMDB35643 ; HMDB35657 ; HMDB35747 ; HMDB35786 ; HMDB35835 ; HMDB35983 ; HMDB36071 ; HMDB36109 ; HMDB36172 ; HMDB36201 ; HMDB36443 ; HMDB36667 ; HMDB36718 ; HMDB36733 ; HMDB36797 ; HMDB36813 ; HMDB37000 ; HMDB37061 ; HMDB37068 ; HMDB37275 ; HMDB37310 ; HMDB38164 ; HMDB38187 ; HMDB38195 ; HMDB38200 ; HMDB38201 ; HMDB38215 ; HMDB38511 ; HMDB39463 ; HMDB39637 ; HMDB40686 ; HMDB41404 ; HMDB41490 ; HMDB60138	N-Methylnicotinium ; 5-b-Cholestane-3a 7a 12a-triol ; 3b5a6b-Cholestanetriol ; Demethylphylloquinone ; LysoPE(0:015:0) ; LysoPE(15:00:0) ; 3 alpha7 alpha26-Trihydroxy-5beta-cholestane ; Nookatone ; Sinensal ; Cerulenin ; Manglupenone ; Acetaldehyde diisoamyl acetal ; (Z)-4-Dodecenal ; (5alpha10alpha)-37(11)-Eudesmadien-2-one ; (5beta7beta10beta)-311-Eudesmadien-2-one ; beta-Sinensal ; Solavetivone ; Patchoulenone ; Valerenal ; Furanoeremophilane ; Ganoderal A ; Dendrolasin ; 135-Bisabolatrien-10-one ; 4-(266-Trimethyl-1-cyclohexenyl)-2-butanol ; (E)-1011-Dihydro-alpha-atlantone ; Rotundone ; Zerumbone ; beta-Santalal ; Vulgarone A ; Mustakone ; alpha-Santalal ; alpha-Turmerone ; ()-411-Eudesmadien-3-one ; Curlone ; 2-Tridecenal ; 11-Dimethoxydecane ; Vulgarone B ; beta-Atlantone ; Xanthorrhizol ; Calamusenone ; 47(11)-Guaiadien-8-one ; alpha-Sinensal ; Bisacumol ; (3alpha5alpha22R23R)-Cholestane-32223-triol ; beta-Elemenone ; Isohumbertiol ; 2-Methylcyclododecanone ; Dihydrogeranylacetone ; 5beta-Cholestane-3alpha7alpha27-triol	M+ACN+Na ; M+Na+K ; M+Na+K ; M+2Na ; M+ACN+2H ; M+ACN+2H ; M+Na+K ; M+Na ; M+Na ; M+NH4 ; M+2Na ; M+K ; M+2Na-H ; M+Na ; M+Na ; M+Na ; M+Na ; M+Na ; M+Na ; M+Na ; M+2Na ; M+Na ; M+Na ; M+2Na-H ; M+Na ; M+Na ; M+Na ; M+Na ; M+Na ; M+Na ; M+Na ; M+Na ; M+Na ; M+Na ; M+2Na-H ; M+K ; M+Na ; M+Na ; M+Na ; M+Na ; M+Na ; M+Na ; M+Na ; M+Na+K ; M+Na ; M+Na ; M+2Na-H ; M+2Na-H ; M+Na+K	C11H17N2 ; C27H48O3 ; C27H48O3 ; C30H44O2 ; C20H42NO7P ; C20H42NO7P ; C27H48O3 ; C15H22O ; C15H22O ; C12H17NO3 ; C30H44O2 ; C12H26O2 ; C13H24O ; C15H22O ; C15H22O ; C15H22O ; C15H22O ; C15H22O ; C15H22O ; C15H22O ; C30H44O2 ; C15H22O ; C15H22O ; C13H24O ; C15H22O ; C15H22O ; C15H22O ; C15H22O ; C15H22O ; C15H22O ; C15H22O ; C15H22O ; C15H22O ; C15H22O ; C13H24O ; C12H26O2 ; C15H22O ; C15H22O ; C15H22O ; C15H22O ; C15H22O ; C15H22O ; C15H22O ; C27H48O3 ; C15H22O ; C15H22O ; C13H24O ; C13H24O ; C27H48O3
287.98043	1.531577	0.925517	-4.4277	0.001279	2.8933	0.015708	NA	NA	NA	NA	NA
287.23662	1.565825	0.174762	4.4207	0.001293	2.8885	0.015842	287.2366	HMDB00305 ; HMDB06216 ; HMDB06217 ; HMDB06221 ; HMDB30403 ; HMDB34735 ; HMDB36728 ; HMDB36841 ; HMDB38709 ; HMDB03071 ; HMDB15430 ; HMDB36916 ; HMDB41023	Vitamin A ; 11-cis-Retinol ; 9-cis-Retinol ; 13-cis Retinol ; (x)-p-Menth-1-en-4-yl 5-isopropyl-2-methylphenyl ether ; Abietinal ; ent-16-Kauren-19-al ; ent-19-Trachylobanal ; 813-Abietadien-18-al ; 56-Dihydro-56-dihydroxy-yy-carotene ; Pancuronium ; 78-Dihydroparasiloxanthin ; Reticulatamol	M+H ; M+H ; M+H ; M+H ; M+H ; M+H ; M+H ; M+H ; M+H ; M+2H ; M+2H ; M+2H ; M+H+K	C20H30O ; C20H30O ; C20H30O ; C20H30O ; C20H30O ; C20H30O ; C20H30O ; C20H30O ; C20H30O ; C40H60O2 ; C35H60N2O4 ; C40H60O2 ; C35H66O3
156.07212	1.554999	1.102216	4.4185	0.001297	2.887	0.015857	156.0721	HMDB00012 ; HMDB00898 ; HMDB01861 ; HMDB01924 ; HMDB03323 ; HMDB03324 ; HMDB15023 ; HMDB15126 ; HMDB15411 ; HMDB30146 ; HMDB31700 ; HMDB34913 ; HMDB35293 ; HMDB37554 ; HMDB37631 ; HMDB37712	Deoxyuridine ; 1-Methylhistamine ; 3-Methylhistamine ; Atenolol ; Biotripyrrin-a ; Biotripyrrin-b ; Pemirolast ; Oxaprozin ; Practolol ; (3xi6E)-17-Diphenyl-6-hepten-3-ol ; 1-(Malonylamino)cyclopropanecarboxylic acid ; Imazamethabenz-methyl ; Norambreinolide ; ()-Rollipyrrole ; 3-Methyl-alpha-ionyl acetate ; 22-Bis(2-methylpropoxy)ethylbenzene	M+2ACN+2H ; 2M+Na+K ; 2M+Na+K ; M+2Na ; M+3H ; M+3H ; M+2ACN+2H ; M+H+NH4 ; M+2Na ; M+2Na ; M+3ACN+2H ; M+H+Na ; M+Na+K ; M+H+Na ; M+Na+K ; M+Na+K	C9H12N2O5 ; C6H11N3 ; C6H11N3 ; C14H22N2O3 ; C25H27N3O6 ; C25H27N3O6 ; C10H8N6O ; C18H15NO3 ; C14H22N2O3 ; C19H22O ; C7H9NO5 ; C16H20N2O3 ; C16H26O2 ; C16H20N2O3 ; C16H26O2 ; C16H26O2
752.56071	1.601206	0.050838	4.4047	0.001325	2.8777	0.016163	752.5607	HMDB05779 ; HMDB09347 ; HMDB09348 ; HMDB09380 ; HMDB09381 ; HMDB09412 ; HMDB09445 ; HMDB09609 ; HMDB11358 ; HMDB11386 ; HMDB11416 ; HMDB11417 ; HMDB11449 ; HMDB07963 ; HMDB07964 ; HMDB09017 ; HMDB09018 ; HMDB09049 ; HMDB09082 ; HMDB09279 ; HMDB11271 ; HMDB11304 ; HMDB11348 ; HMDB11374 ; HMDB11375 ; HMDB11406 ; HMDB11439 ; HMDB13025 ; HMDB35582	PE(O-18:1(1Z)20:4(5Z8Z11Z14Z)) ; PE(20:3(5Z8Z11Z)P-18:1(11Z)) ; PE(20:3(5Z8Z11Z)P-18:1(9Z)) ; PE(20:3(8Z11Z14Z)P-18:1(11Z)) ; PE(20:3(8Z11Z14Z)P-18:1(9Z)) ; PE(20:4(5Z8Z11Z14Z)P-18:0) ; PE(20:4(8Z11Z14Z17Z)P-18:0) ; PE(22:4(7Z10Z13Z16Z)P-16:0) ; PE(P-16:022:4(7Z10Z13Z16Z)) ; PE(P-18:020:4(8Z11Z14Z17Z)) ; PE(P-18:1(11Z)20:3(5Z8Z11Z)) ; PE(P-18:1(11Z)20:3(8Z11Z14Z)) ; PE(P-18:1(9Z)20:3(5Z8Z11Z)) ; PC(15:0P-18:1(11Z)) ; PC(15:0P-18:1(9Z)) ; PE(18:0P-18:1(11Z)) ; PE(18:0P-18:1(9Z)) ; PE(18:1(11Z)P-18:0) ; PE(18:1(9Z)P-18:0) ; PE(20:1(11Z)P-16:0) ; PC(P-18:1(11Z)15:0) ; PC(P-18:1(9Z)15:0) ; PE(P-16:020:1(11Z)) ; PE(dm18:018:1(11Z)) ; PE(P-18:018:1(9Z)) ; PE(P-18:1(11Z)18:0) ; PE(P-18:1(9Z)18:0) ; 6912151821-Tetracosahexaenoic acid ; 1-Phenyl-13-heptadecanedione	M+H ; M+H ; M+H ; M+H ; M+H ; M+H ; M+H ; M+H ; M+H ; M+H ; M+H ; M+H ; M+H ; M+Na ; M+Na ; M+Na ; M+Na ; M+Na ; M+Na ; M+Na ; M+Na ; M+Na ; M+Na ; M+Na ; M+Na ; M+Na ; M+Na ; 2M+ACN+H ; 2M+ACN+Na	C43H78NO7P ; C43H78NO7P ; C43H78NO7P ; C43H78NO7P ; C43H78NO7P ; C43H78NO7P ; C43H78NO7P ; C43H78NO7P ; C43H78NO7P ; C43H78NO7P ; C43H78NO7P ; C43H78NO7P ; C43H78NO7P ; C41H80NO7P ; C41H80NO7P ; C41H80NO7P ; C41H80NO7P ; C41H80NO7P ; C41H80NO7P ; C41H80NO7P ; C41H80NO7P ; C41H80NO7P ; C41H80NO7P ; C41H80NO7P ; C41H80NO7P ; C41H80NO7P ; C41H80NO7P ; C24H35O2 ; C23H36O2
619.18947	1.574154	0.284642	-4.402	0.001331	2.8759	0.016191	619.1895	HMDB10357 ; HMDB38722	Tetrahydroaldosterone-3-glucuronide ; 56-Dihydro-11-methoxyyangonin	M+2K+H ; 2M+K	C27H40O11 ; C16H18O5

269.11352	1.613382	0.433718	4.3996	0.001336	2.8742	0.016212	269.1135	HMDB29051 ; HMDB29114 ; HMDB32198 ; HMDB00623 ; HMDB14889 ; HMDB29355 ; HMDB30143 ; HMDB30911 ; HMDB31900 ; HMDB31916 ; HMDB33666 ; HMDB35599 ; HMDB35902 ; HMDB35908 ; HMDB36455 ; HMDB36473 ; HMDB36488 ; HMDB36767 ; HMDB37558 ; HMDB37609 ; HMDB37801 ; HMDB38188 ; HMDB38202 ; HMDB38547 ; HMDB39102 ; HMDB40334 ; HMDB40335 ; HMDB40336 ; HMDB40337 ; HMDB40903 ; HMDB41613 ; HMDB41614 ; HMDB41920 ; HMDB59747 ; HMDB33909 ; HMDB34894 ; HMDB38696	Serinyl-Tyrosine ; Tyrosyl-Serine ; Cellulose triacetate ; Dodecanedioic acid ; Epinastine ; N-Phenylacetylaspartic acid ; Talaromycin A ; 2-Oxo-511(13)-eudesmadien-128-olide ; Methyl (2E4Z6E8E10E)-48-dimethyl-12-oxo-246810-dodecapentaenoate ; 5-Hydroxy-2-(1-hydroxy-5-methyl-4-hexenyl)benzofuran ; KB 2 ; Lucidenic acid G ; Enokipodin B ; Lucidenic acid H ; Lacinilene C ; 8-Epixanthatin ; Annuolide C ; Zederone ; 3-Oxo-1411(13)-eudesmatrien-12-oic acid ; Lucidenic acid C ; Annuolide A ; Curcolone ; Zedoarol ; Glandulone A ; N-Carboxyacetyl-D-phenylalanine ; Dihydro-24-dimethyl-6-(2-methylpropyl)-4H-135-dithiazine ; Dihydro-46-dimethyl-2-(2-methylpropyl)-4H-135-dithiazine ; Dihydro-24-dimethyl-6-(1-methylpropyl)-4H-135-dithiazine ; Dihydro-46-dimethyl-2-(1-methylpropyl)-4H-135-dithiazine ; Ipomeabisfuran ; Bis(1-methylethyl) hexanedioate ; Dipropyl hexanedioate ; Loxoprofen ; 4-Hydroxy-3-methoxy-cinnamoylglycine ; 2-Deoxymugineic acid ; 2-(1234-Tetrahydroxybutyl)-6-(234-trihydroxybutyl)pyrazine ; Deoxyfructosazine	M+H ; M+H ; M+2H ; M+K ; 2M+H+K ; M+NH4 ; M+K ; M+Na ; M+Na ; M+Na ; M+2ACN+2H ; M+Na+K ; M+Na ; M+Na+K ; M+Na ; M+Na ; M+Na ; M+Na ; M+Na ; M+Na+K ; M+Na ; M+Na ; M+Na ; M+Na ; M+NH4 ; M+ACN+Na ; M+ACN+Na ; M+ACN+Na ; M+ACN+Na ; M+Na ; M+K ; M+K ; M+Na ; M+NH4 ; M+H-2H2O ; M+H-2H2O ; M+H-2H2O	C12H16N2O5 ; C12H16N2O5 ; C30H34Cl2N4O ; C12H22O4 ; C16H15N3 ; C12H13NO5 ; C12H22O4 ; C15H18O3 ; C15H18O3 ; C15H18O3 ; C25H26O8 ; C27H40O7 ; C15H18O3 ; C27H40O7 ; C15H18O3 ; C15H18O3 ; C15H18O3 ; C15H18O3 ; C15H18O3 ; C27H40O7 ; C15H18O3 ; C15H18O3 ; C15H18O3 ; C15H18O3 ; C12H13NO5 ; C9H19NS2 ; C9H19NS2 ; C9H19NS2 ; C9H19NS2 ; C15H18O3 ; C12H22O4 ; C12H22O4 ; C15H18O3 ; C12H13NO5 ; C12H20N2O7 ; C12H20N2O7 ; C12H20N2O7
306.25041	1.588757	0.075516	4.3965	0.001342	2.8721	0.016251	NA	NA	NA	NA	NA
621.82129	1.598784	1.073244	-4.3939	0.001348	2.8704	0.016278	621.8213	HMDB34868 ; HMDB40858	Calendulaglycoside A ; Elatoside D	M+3ACN+2H ; M+3ACN+2H	C54H86O24 ; C54H86O24
565.60366	1.542829	0.851258	-4.3846	0.001368	2.864	0.016477	565.6037	HMDB60001	Indole-3-acetic-acid-O-glucuronide	3M+2K	C16H17NO8
256.00810	1.496577	1.071816	-4.3725	0.001394	2.8559	0.016749	256.0081	HMDB01511 ; HMDB29499 ; HMDB30860 ; HMDB33795 ; HMDB37595 ; HMDB37927	Phosphocreatine ; Fukinolic acid ; Avicularin ; Quercetin 3-arabinoside ; 6-Hydroxyluteolin 6-xyloside ; Quercetin 7-xyloside	M+2Na-H ; M+2K ; M+2K ; M+2K ; M+2K ; M+2K	C4H10N3O5P ; C20H18O11 ; C20H18O11 ; C20H18O11 ; C20H18O11 ; C20H18O11
337.02757	1.600053	0.054345	-4.3694	0.0014	2.8537	0.016792	337.0276	HMDB02377 ; HMDB29871 ; HMDB31457 ; HMDB34310 ; HMDB39200 ; HMDB39201 ; HMDB39743	Urothion ; Sanguiin H4 ; Corilagin ; Cordeauxione ; Punicacortein A ; Punicacortein B ; Pterocaryanin B	2M+H+Na ; M+H+K ; M+H+K ; M+2Na-H ; M+H+K ; M+H+K ; M+H+K	C11H11N5O3S2 ; C27H22O18 ; C27H22O18 ; C14H12O7 ; C27H22O18 ; C27H22O18 ; C27H22O18
119.01708	1.58791	0.192384	4.3663	0.001407	2.8516	0.016794	119.0171	HMDB39786 ; HMDB28684 ; HMDB28696 ; HMDB28768 ; HMDB28851 ; HMDB29032 ; HMDB29061 ; HMDB29783 ; HMDB35589 ; HMDB38341 ; HMDB40464 ; HMDB60272 ; HMDB60764	Dihydro-4-mercapto-3(2H)-furanone ; Alanyl-Cysteine ; Alanyl-Serine ; Cysteinyl-Alanine ; Glycyl-Threonine ; Serinyl-Alanine ; Threoninyl-Glycine ; Mono-trans-p-coumaroylmesotartaric acid ; Phlorin ; Glucosylisomaltol ; Dowicide A ; 4-Nitrophenyl phosphate ; 4-Hydroxy-estazolam	M+H ; M+2Na ; M+Na+K ; M+2Na ; M+Na+K ; M+Na+K ; M+Na+K ; M+H+2Na ; M+3Na ; M+3Na ; M+2Na ; M+H+NH4 ; M+H+2Na	C4H6O2S ; C6H12N2O3S ; C6H12N2O4 ; C6H12N2O3S ; C6H12N2O4 ; C6H12N2O4 ; C6H12N2O4 ; C14H14O8 ; C12H16O8 ; C12H16O8 ; C12H9NaO ; C6H6NO6P ; C16H11ClN4O
432.12336	1.540763	0.663797	-4.3663	0.001407	2.8516	0.016794	432.1234	HMDB30488 ; HMDB31254 ; HMDB33343 ; HMDB34207 ; HMDB60913 ; HMDB60918 ; HMDB60983 ; HMDB61072	Artonol B ; Margrapine A ; Mollicellin D ; 6-Apiosyllotaustralin ; Zopiclone N-oxide ; Flurbiprofen glucuronide ; zopiclone-N-oxide ; nopiclone N-oxide	2M+H+Na ; 2M+Na+K ; 2M+3H2O+2H ; M+K ; 2M+3H2O+2H ; 2M+H+Na ; 2M+3H2O+2H ; 2M+3H2O+2H	C24H20O7 ; C21H23NO7 ; C21H21ClO6 ; C16H27NO10 ; C17H17ClN6O4 ; C21H21FO8 ; C17H17ClN6O4 ; C17H17ClN6O4
318.70267	1.526039	0.326056	-4.3608	0.001419	2.8479	0.016897	318.7027	HMDB38189 ; HMDB38745	Carindone ; Glyuranolide	M+3ACN+2H ; M+3ACN+2H	C31H44O6 ; C31H44O6
500.68129	1.526755	1.273145	4.3575	0.001427	2.8457	0.016926	NA	NA	NA	NA	NA
158.01745	1.60553	0.122527	-4.3567	0.001429	2.8451	0.016926	158.0175	HMDB00500 ; HMDB01895 ; HMDB02466 ; HMDB04062 ; HMDB15486 ; HMDB32576 ; HMDB32920 ; HMDB33649 ; HMDB33812 ; HMDB59965 ; HMDB60538	4-Hydroxybenzoic acid ; Salicylic acid ; 3-Hydroxybenzoic acid ; Gentisate aldehyde ; Cefpodoxime ; 2-Methoxy-14-benzoquinone ; alpha-Furyl methyl diketone ; O-Demethylfonsecin ; Sesamol ; 34-Dihydroxybenzaldehyde ; Nordiazepam	2M+H+K ; 2M+H+K ; 2M+H+K ; 2M+H+K ; M+H+2Na ; 2M+H+K ; 2M+H+K ; M+H+K ; 2M+H+K ; 2M+H+K ; M+2Na	C7H6O3 ; C7H6O3 ; C7H6O3 ; C7H6O3 ; C15H17N5O6S2 ; C7H6O3 ; C7H6O3 ; C14H12O6 ; C7H6O3 ; C7H6O3 ; C15H11ClN2O
621.06662	1.62156	0.737554	-4.3549	0.001433	2.8439	0.016933	621.0666	HMDB05790	Tannin	M+H	C26H20O18
290.76572	1.563987	1.287266	4.3515	0.00144	2.8416	0.016948	290.7657	HMDB02080 ; HMDB40821	Petroselinic acid ; Nb-Hexacosanoyltryptamine	2M+H+NH4 ; M+ACN+2H	C18H33O2 ; C36H62N2O
181.01781	1.580445	0.307932	4.3502	0.001443	2.8407	0.016948	181.0178	HMDB31671 ; HMDB32115 ; HMDB02273 ; HMDB02467 ; HMDB02655 ; HMDB29497 ; HMDB30547 ; HMDB30804 ; HMDB33764 ; HMDB38446 ; HMDB38515 ; HMDB38572 ; HMDB39210 ; HMDB41271 ; HMDB60495	246-Trimethyl-135-trithiane ; 1-(Methylthio)ethyl 2-propenyl disulfide ; 4-Hydroxy-L-glutamic acid ; A-Ketoglutaric acid oxime ; Isorhamnetin ; Dimethyl fukiic acid ; 3457-Tetrahydroxy-6-methoxyflavone ; 2-(34-Dihydroxyphenyl)-56-dihydroxy-7-methoxy-4H-1-benzopyran-4-one ; Erucin ; 1-Thiocyanato-4-(methylthio)butane ; L-gamma-Glutamyl-S-allylthio-L-cysteine ; Pollenitin ; 1358-Tetrahydroxy-6-methoxy-2-methylanthraquinone ; Salicylic acid beta-D-glucoside ; N-Formyl-L-aspartate	M+H ; M+H ; 2M+H+K ; 2M+H+K ; M+2Na ; M+Na+K ; M+2Na ; M+2Na ; 2M+H+K ; 2M+H+K ; M+H+K ; M+2Na ; M+2Na ; M+Na+K ; 2M+H+K	C6H12S3 ; C6H12S3 ; C5H7NO5 ; C5H7NO5 ; C16H12O7 ; C13H16O8 ; C16H12O7 ; C16H12O7 ; C6H11NS2 ; C6H11NS2 ; C11H18N2O5S2 ; C16H12O7 ; C16H12O7 ; C13H16O8 ; C5H7NO5
147.12096	1.535958	0.977399	-4.3485	0.001447	2.8396	0.016948	147.121	HMDB31660 ; HMDB41477 ; HMDB30285 ; HMDB35215 ; HMDB35549 ; HMDB35550 ; HMDB35551 ; HMDB35552 ; HMDB35553 ; HMDB35554	Dibutyl sulfide ; 2-Ethyl-1-hexanethiol ; (S)-2-Propylpiperidine ; Ximenic acid ; 1113-Hexacosanedione ; 1012-Hexacosanedione ; 79-Hexacosanedione ; 68-Hexacosanedione ; 57-Hexacosanedione ; 46-Hexacosanedione	M+H ; M+H ; 2M+H+K ; M+H+2Na ; M+H+2Na ; M+H+2Na ; M+H+2Na ; M+H+2Na ; M+H+2Na ; M+H+2Na	C8H18S ; C8H18S ; C8H17N ; C26H50O2 ; C26H50O2 ; C26H50O2 ; C26H50O2 ; C26H50O2 ; C26H50O2 ; C26H50O2
359.16676	1.566273	0.709682	4.3483	0.001447	2.8394	0.016948	359.1668	HMDB15535 ; HMDB01514 ; HMDB02030 ; HMDB06591 ; HMDB11668 ; HMDB11690 ; HMDB30091 ; HMDB33572 ; HMDB34693 ; HMDB39348 ; HMDB39351	Cilastatin ; Glucosamine ; Fructosamine ; Lactosamine ; 6-(alpha-D-Glucosaminyl)-1D-myo-inositol ; 7-Aminomethyl-7-carbaguanine ; beta-D-Glucosamine ; 1-Acetyl-31420-trihydroxywitha-524-dienolide 3-glucoside ; Medicagenic acid 3-O-b-D-glucuronide ; Myricanene B 5-arabinosyl-(1-6)-glucoside ; Myricanene A 5-arabinosyl-(1-6)-glucoside	M+H ; 2M+H ; 2M+H ; M+NH4 ; M+NH4 ; 2M+H ; 2M+H ; M+H+K ; M+H+K ; M+2ACN+2H ; M+2ACN+2H	C16H26N2O5S ; C6H13NO5 ; C6H13NO5 ; C12H23NO10 ; C12H23NO10 ; C7H9N5O ; C6H13NO5 ; C36H54O12 ; C36H54O12 ; C32H42O13 ; C32H42O13

345.04552	1.510422	1.130778	-4.3446	0.001456	2.8369	0.017007	345.0455	HMDB30558 ; HMDB40125 ; HMDB40559 ; HMDB40677 ; HMDB36932	Safflomin A ; Carthamidin 67-diglucoside ; Aromadendrin 37-diglucoside ; Hydroxysafflor yellow A ; 2-O-Galloylgalactaric acid	M+2K ; M+2K ; M+2K ; M+2K ; M+H-H2O	C27H32O16 ; C27H32O16 ; C27H32O16 ; C27H32O16 ; C13H14O12
739.94897	1.511116	1.349543	-4.3392	0.001468	2.8332	0.017112	NA	NA	NA	NA	NA
200.09141	1.545515	1.01651	4.3363	0.001475	2.8312	0.017151	200.0914	HMDB60898 ; HMDB00118 ; HMDB00332 ; HMDB00333 ; HMDB00423 ; HMDB00755 ; HMDB02643 ; HMDB04061 ; HMDB06078 ; HMDB15154 ; HMDB15503 ; HMDB29232 ; HMDB29273 ; HMDB29646 ; HMDB31932 ; HMDB32013 ; HMDB33624 ; HMDB37274 ; HMDB39557 ; HMDB41024 ; HMDB41270 ; HMDB59763 ; HMDB61016	Eletriptan N-oxide ; Homovanillic acid ; 18-Oxocortisol ; Isohomovanillic acid ; 34-Dihydroxyhydrocinnamic acid ; Hydroxyphenyllactic acid ; 3-(3-Hydroxyphenyl)-3-hydroxypropanoic acid ; 3-Methoxy-4-hydroxyphenylglycolaldehyde ; Putreanine ; Bethanechol ; Antipyrine ; 3-Hydroxyphenyllactate ; 26-Dimethoxybenzoic acid ; 26-Dihydroxy-4-methoxyacetophenone ; 111214-Trihydroxy-7-methoxy-81113-abietatrien-206-olide ; Vanilloloside ; ()-2-Hydroxy-3-(2-hydroxyphenyl)propanoic acid ; Maltol propionate ; 12alpha-Hydroxy-1318-dehydroparain ; Hydroxytyrosol 1-O-glucoside ; 24-Dihydroxy-6-methoxyacetophenone ; 34-Dimethoxybenzoic acid ; 16alpha;-hydroxyprednisolone	M+2H ; M+NH4 ; M+H+Na ; M+NH4 ; M+NH4 ; M+NH4 ; M+NH4 ; M+NH4 ; M+K ; M+K ; 2M+H+Na ; M+NH4 ; M+NH4 ; M+NH4 ; M+H+Na ; M+2ACN+2H ; M+NH4 ; M+NH4 ; M+H+Na ; M+2ACN+2H ; M+NH4 ; M+NH4 ; M+H+Na	C22H26N2O3S ; C9H10O4 ; C21H28O6 ; C9H10O4 ; C9H10O4 ; C9H10O4 ; C9H10O4 ; C9H10O4 ; C7H17N2O2 ; C7H17N2O2 ; C11H12N2O ; C9H10O4 ; C9H10O4 ; C9H10O4 ; C21H28O6 ; C14H20O8 ; C9H10O4 ; C9H10O4 ; C21H28O6 ; C14H20O8 ; C9H10O4 ; C9H10O4 ; C21H28O6
701.99400	1.51738	1.375666	-4.3337	0.001481	2.8295	0.017157	701.994	HMDB57481 ; HMDB57508	CL(16:1(9Z)16:1(9Z)16:1(9Z)18:2(9Z12Z)) ; CL(16:1(9Z)16:1(9Z)18:2(9Z12Z)16:1(9Z))	M+H+NH4 ; M+H+NH4	C76H138O17P2 ; C76H138O17P2
87.004073	1.518344	1.255574	4.3331	0.001482	2.829	0.017157	NA	NA	NA	NA	NA
703.49748	1.526533	1.360637	-4.3311	0.001487	2.8277	0.017159	703.4975	HMDB01201 ; HMDB07028 ; HMDB07107 ; HMDB07158 ; HMDB07359 ; HMDB07588 ; HMDB08825 ; HMDB08830 ; HMDB08831 ; HMDB08857 ; HMDB08862 ; HMDB08921 ; HMDB08953 ; HMDB09086 ; HMDB09118 ; HMDB09151 ; HMDB30016 ; HMDB31012 ; HMDB31349 ; HMDB32081 ; HMDB32837 ; HMDB33252 ; HMDB33253 ; HMDB33960 ; HMDB34515 ; HMDB34562 ; HMDB34742 ; HMDB34956 ; HMDB35202 ; HMDB35407 ; HMDB35408 ; HMDB35413 ; HMDB35434 ; HMDB35639 ; HMDB35794 ; HMDB35869 ; HMDB35886 ; HMDB35889 ; HMDB35935 ; HMDB36035 ; HMDB36390 ; HMDB36392 ; HMDB36427 ; HMDB36449 ; HMDB36466 ; HMDB36647 ; HMDB37038 ; HMDB37391 ; HMDB37640 ; HMDB37710 ; HMDB37739 ; HMDB38190 ; HMDB38198 ; HMDB38698 ; HMDB38795 ; HMDB38796 ;	Guanosine diphosphate ; DG(14:022:00:0) ; DG(16:020:00:0) ; DG(18:018:00:0) ; DG(20:016:00:0) ; DG(22:014:00:0) ; PE(14:016:1(9Z)) ; PE(14:018:3(6Z9Z12Z)) ; PE(14:018:3(9Z12Z15Z)) ; PE(14:1(9Z)16:0) ; PE(14:1(9Z)18:2(9Z12Z)) ; PE(16:014:1(9Z)) ; PE(16:1(9Z)14:0) ; PE(18:2(9Z12Z)14:1(9Z)) ; PE(18:3(6Z9Z12Z)14:0) ; PE(18:3(9Z12Z15Z)14:0) ; Valerenic acid ; Glycerol 12-dioctadecanoate ; Epiacoronene ; 410-Longipinanedione ; Ganoderic acid DM ; Curcumanolide A ; Curcumenone ; Curcumenol ; Glabrolide ; 9-Pentadecene-1214-diyne-111-diol ; Saussurea lactone ; Oxysolavetivone ; 7-Hydroxycostal ; Dehydrocurdione ; 110-Epoxygermacrone ; (4alpha5alpha)-11-Eremophilene-29-dione ; Isomasticadienonalic acid ; Petasalbin ; beta-Costic acid ; alpha-Rotunol ; Isoglabrolide ; Germacrone 45-epoxide ; Dihydroisoalantolactone ; Marasmene ; 3-Phenylpropyl hexanoate ; (Z)-alpha-Bergamotenoic acid ; Procurcumenol ; (1alpha4beta5beta)-4-Hydroxy-7(11)10(14)-guaiadien-8-one ; Zerumbone oxide ; Isocurcumenol ; Cyclodehydroisolubimin ; p-Hydroxynonanophenone ; 2-Methyl-4-phenyl-2-butyl 2-methylpropanoate ; 4-Methylphenyl octanoate ; 7-(13-Cyclohexadienyl)-5-hydroxy-26-dimethyl-2-hepten-4-one ; Rishitinol ; 37-Bisaboladiene-28-dione ; xi-2-Hydroxy-135-bisabolatrien-9-one ; 79-Illudadiene-314-diol ; 27(14)-Illudadiene-1015-diol ; Uralenolide ; 46-Decadiyn-1-ol isovalerate ; Colupone ; beta-Santalic acid ; 16beta-16-Hydroxy-3-oxo-112-oleanadien-28-oic acid ; Sugeonol ; DG(14:00:022:0) ; DG(16:00:020:0) ; DG(18:00:018:0) ; 8-oxo-dGDP ; PG(16:016:1(9Z)) ; PG(16:1(9Z)16:0)	3M+2K ; M+2K+H ; M+2K+H ; M+2K+H ; M+2K+H ; M+2K+H ; M+ACN+H ; M+NH4 ; M+NH4 ; M+ACN+H ; M+NH4 ; M+ACN+H ; M+ACN+H ; M+NH4 ; M+NH4 ; M+NH4 ; 3M+H ; M+2K+H ; 3M+H ; 3M+H ; 3M+2H ; 3M+H ; 3M+H ; 3M+H ; 3M+2H ; 3M+H ; 3M+H ; 3M+H ; 3M+H ; 3M+H ; 3M+H ; 3M+H ; 3M+2H ; 3M+H ; 3M+H ; 3M+H ; 3M+2H ; 3M+H ; 3M+H ; 3M+H ; 3M+H ; 3M+H ; 3M+H ; 3M+H ; 3M+H ; 3M+H ; 3M+H ; 3M+H ; 3M+H ; 3M+H ; 3M+H ; 3M+H ; 3M+H ; 3M+H ; 3M+2H ; 3M+H ; 3M+2H ; 3M+H ; 3M+2H ; 3M+H ; M+2K+H ; M+2K+H ; M+2K+H ; 3M+2K ; M+H-H2O ; M+H-H2O	C10H15N5O11P2 ; C39H76O5 ; C39H76O5 ; C39H76O5 ; C39H76O5 ; C39H76O5 ; C35H68NO8P ; C37H68NO8P ; C37H68NO8P ; C35H68NO8P ; C37H68NO8P ; C35H68NO8P ; C35H68NO8P ; C37H68NO8P ; C37H68NO8P ; C37H68NO8P ; C15H22O2 ; C39H76O5 ; C15H22O2 ; C15H22O2 ; C30H44O4 ; C15H22O2 ; C15H22O2 ; C15H22O2 ; C30H44O4 ; C15H22O2 ; C15H22O2 ; C15H22O2 ; C15H22O2 ; C15H22O2 ; C15H22O2 ; C15H22O2 ; C30H44O4 ; C15H22O2 ; C15H22O2 ; C15H22O2 ; C30H44O4 ; C15H22O2 ; C15H22O2 ; C15H22O2 ; C15H22O2 ; C15H22O2 ; C15H22O2 ; C15H22O2 ; C15H22O2 ; C15H22O2 ; C15H22O2 ; C15H22O2 ; C15H22O2 ; C15H22O2 ; C15H22O2 ; C15H22O2 ; C15H22O2 ; C15H22O2 ; C15H22O2 ; C15H22O2 ; C30H44O4 ; C15H22O2 ; C30H44O4 ; C15H22O2 ; C30H44O4 ; C15H22O2 ; C39H76O5 ; C39H76O5 ; C39H76O5 ; C10H15N5O11P2 ; C38H73O10P ; C38H73O10P
634.87955	1.534599	1.152138	4.3301	0.001489	2.827	0.017159	NA	NA	NA	NA	NA
827.75624	1.58325	1.195017	-4.3234	0.001505	2.8225	0.017284	827.7562	HMDB00534 ; HMDB36744 ; HMDB11700 ; HMDB11703 ; HMDB11707 ; HMDB42111 ; HMDB42166 ; HMDB42168 ; HMDB42514 ; HMDB42574 ; HMDB42662 ; HMDB42979 ; HMDB43175 ; HMDB43202 ; HMDB43229 ; HMDB43230 ; HMDB43258 ; HMDB43286 ; HMDB43343 ; HMDB43900 ; HMDB44249 ; HMDB44305 ; HMDB44682 ; HMDB47753 ; HMDB48438 ; HMDB49093 ; HMDB49094 ; HMDB49721	Campestanol ; alpha-Amyrin cerotate ; TG(15:016:020:2(11Z14Z))iso6 ; TG(15:018:018:2(9Z12Z))iso6 ; TG(15:018:1(9Z)18:1(9Z))iso3 ; TG(14:015:022:2(13Z16Z)) ; TG(14:018:018:2(9Z12Z)) ; TG(14:018:020:2n6) ; TG(14:018:2(9Z12Z)18:0) ; TG(14:020:2n618:0) ; TG(14:022:2(13Z16Z)15:0) ; TG(15:014:022:2(13Z16Z)) ; TG(15:014:1(9Z)22:1(13Z)) ; TG(15:016:1(9Z)20:1(11Z)) ; TG(15:018:1(11Z)18:1(11Z)) ; TG(15:018:1(11Z)18:1(9Z)) ; TG(15:018:1(9Z)18:1(11Z)) ; TG(15:020:1(11Z)16:1(9Z)) ; TG(15:022:1(13Z)14:1(9Z)) ; TG(16:016:020:2n6) ; TG(16:018:2(9Z12Z)16:0) ; TG(16:020:2n616:0) ; TG(18:014:020:2n6) ; TG(14:1(9Z)15:022:1(13Z)) ; TG(16:1(9Z)15:020:1(11Z)) ; TG(18:1(11Z)15:018:1(11Z)) ; TG(18:1(11Z)15:018:1(9Z)) ; TG(18:1(9Z)15:018:1(9Z))	2M+Na ; M+Na ; M+H-H2O	C28H50O ; C56H100O2 ; C54H100O6
256.56332	1.537288	0.972842	-4.3192	0.001515	2.8196	0.017284	256.5633	HMDB37073 ; HMDB37074 ; HMDB37075 ; HMDB37076 ; HMDB37077 ; HMDB37078 ; HMDB60942	(E)-Resveratrol 3-glucoside 4-sulfate ; (Z)-Resveratrol 3-glucoside 5-sulfate ; (Z)-Resveratrol 3-(2-sulfoglucoside) ; (Z)-Resveratrol 3-(4-sulfoglucoside) ; (Z)-Resveratrol 3-(6-sulfoglucopyranoside) ; (Z)-Resveratrol 3-(3-sulfoglucoside) ; N-Desmethylrosuvastatin	M+ACN+2H ; M+ACN+2H ; M+ACN+2H ; M+ACN+2H ; M+ACN+2H ; M+ACN+2H ; M+2Na	C20H22O11S ; C20H22O11S ; C20H22O11S ; C20H22O11S ; C20H22O11S ; C20H22O11S ; C21H26FN3O6S
304.01912	1.520928	0.972734	-4.3174	0.001519	2.8184	0.017284	304.0191	HMDB00293 ; HMDB01109 ; HMDB01254 ; HMDB34890 ; HMDB60436	Xanthosine 5-triphosphate ; Glucosamine-1P ; Glucosamine 6-phosphate ; Bis(4-isothiocyanatobutyl) disulfide ; Aminofructose 6-phosphate	M+2ACN+2H ; M+2Na-H ; M+2Na-H ; 2M+H+Na ; M+2Na-H	C10H15N4O15P3 ; C6H14NO8P ; C6H14NO8P ; C10H16N2S4 ; C6H14NO8P
164.48779	1.598259	0.646601	4.317	0.00152	2.8181	0.017284	NA	NA	NA	NA	NA
662.73949	1.557281	1.230387	-4.317	0.00152	2.8181	0.017284	662.7395	HMDB15331	Bromocriptine	2M+H+NH4	C32H40BrN5O5

278.08441	1.538512	1.358807	-4.3167	0.001521	2.8179	0.017284	278.0844	HMDB01049 ; HMDB02095 ; HMDB02275 ; HMDB15008 ; HMDB15139 ; HMDB15181 ; HMDB15538 ; HMDB30128 ; HMDB33412 ; HMDB33699 ; HMDB34094 ; HMDB37084 ; HMDB38610 ; HMDB38826 ; HMDB39696 ; HMDB39697 ; HMDB38583	Gamma-Glutamylcysteine ; Malonylcarnitine ; 78-Dihydroneopterin ; Suprofen ; Ganciclovir ; Fluocinonide ; Tiaprofenic acid ; Physalin B ; Gibberellin A20 13-glucoside ; Linamarin ; Physalin C ; (8S8S)-Secoisolariciresinol 9-xyloside ; Gibberellin A4 glucosyl ester ; Alatanin 2 ; 2527-Dihydro-47-didehydro-7-deoxyphysalin A ; Isophysalin B ; Piperolactam D	2M+3H2O+2H ; 2M+Na+K ; M+Na ; M+NH4 ; M+Na ; M+Na+K ; M+NH4 ; M+2Na ; M+Na+K ; 2M+Na+K ; M+2Na ; M+Na+K ; M+Na+K ; M+3H ; M+2Na ; M+2Na ; M+H-H2O	C8H14N2O5S ; C10H17NO6 ; C9H13N5O4 ; C14H12O3S ; C9H13N5O4 ; C26H32F2O7 ; C14H12O3S ; C28H30O9 ; C25H34O10 ; C10H17NO6 ; C28H30O9 ; C25H34O10 ; C25H34O10 ; C39H43O20 ; C28H30O9 ; C28H30O9 ; C17H13NO4
569.16035	1.600607	0.573442	-4.3054	0.001548	2.8102	0.017512	569.1604	HMDB00299 ; HMDB11166 ; HMDB11172 ; HMDB14367 ; HMDB28756 ; HMDB28757 ; HMDB28903 ; HMDB28925 ; HMDB30279 ; HMDB41124 ; HMDB41380 ; HMDB59717 ; HMDB60123 ; HMDB12947	Xanthosine ; L-beta-aspartyl-L-leucine ; L-gamma-glutamyl-L-valine ; Glimepiride ; Aspartyl-Isoleucine ; Aspartyl-Leucine ; Isoleucyl-Aspartate ; Leucyl-Aspartate ; Garciduol C ; Artonin Q ; Garciduol A ; Glu-Val ; rac-56-Epoxy-retinoyl-beta-D-glucuronide ; Ferrocytochrome	2M+H ; 2M+2K-H ; 2M+2K-H ; M+2K+H ; 2M+2K-H ; 2M+2K-H ; 2M+2K-H ; 2M+2K-H ; M+2ACN+H ; M+K ; M+2ACN+H ; 2M+2K-H ; M+2K-H ; M+H-2H2O	C10H12N4O6 ; C10H18N2O5 ; C10H18N2O5 ; C24H34N4O5S ; C10H18N2O5 ; C10H18N2O5 ; C10H18N2O5 ; C10H18N2O5 ; C27H18O9 ; C31H30O8 ; C27H18O9 ; C10H18N2O5 ; C26H36O9 ; C33H32FeN4O4
404.95753	1.471098	0.120654	4.3046	0.00155	2.8096	0.017512	404.9575	HMDB29723 ; HMDB41910	Saccharin ; Iopromide	2M+K ; M+H+NH4	C7H5NO3S ; C18H24I3N3O8
191.03911	1.530346	0.901721	4.3041	0.001552	2.8092	0.017512	191.0391	HMDB12289 ; HMDB14610 ; HMDB15159 ; HMDB29461 ; HMDB29533 ; HMDB29787 ; HMDB29791 ; HMDB29877 ; HMDB30141 ; HMDB30450 ; HMDB30575 ; HMDB30742 ; HMDB31289 ; HMDB31992 ; HMDB32581 ; HMDB32736 ; HMDB34027 ; HMDB34050 ; HMDB34182 ; HMDB35486 ; HMDB37321 ; HMDB37455 ; HMDB37922 ; HMDB41452 ; HMDB59746	Tetrahydrodipicolinate ; Enoxacin ; Mycophenolic acid ; Zapotin ; Glabrone ; Kanzonol W ; Dulciol D ; Myricetin 3-(34-diacetylrhamnoside) ; Isosojagol ; Atalantoflavone ; 4567-Tetramethoxyflavone ; Helipyrone ; 146-Trihydroxy-5-methoxy-7-prenylxanthone ; Dulxanthone A ; 1-(Isothiocyanatomethyl)-4-methoxybenzene ; Dulxanthone D ; Sojagol ; Psoralidin ; 158-Trihydroxy-3-methyl-2-prenylxanthone ; 6-Caffeoylsucrose ; Oleacein ; Chrysoeriol 7-O-(6-malonyl-glucoside) ; Phaseol ; Musanolone D ; (1R2S5R6S)-6-(34-Dihydroxyphenyl)-2-(34-methylenedioxyphenyl)-37-dioxabicyclo-330octane	2M+H+K ; M+Na+K ; M+Na+K ; M+H+K ; M+2Na ; M+2Na ; M+2Na ; M+2H+Na ; M+2Na ; M+2Na ; M+H+K ; M+Na+K ; M+H+K ; M+H+K ; 2M+H+Na ; M+H+K ; M+2Na ; M+2Na ; M+H+K ; M+3Na ; M+Na+K ; M+2H+Na ; M+2Na ; M+2Na ; M+H+K	C7H9NO4 ; C15H17FN4O3 ; C17H20O6 ; C19H18O6 ; C20H16O5 ; C20H16O5 ; C20H16O5 ; C25H24O14 ; C20H16O5 ; C20H16O5 ; C19H18O6 ; C17H20O6 ; C19H18O6 ; C19H18O6 ; C9H9NOS ; C19H18O6 ; C20H16O5 ; C20H16O5 ; C19H18O6 ; C21H28O14 ; C17H20O6 ; C25H24O14 ; C20H16O5 ; C20H16O5 ; C19H18O6
358.02087	1.536074	0.643568	4.3025	0.001555	2.8082	0.017514	358.0209	HMDB39266 ; HMDB39267 ; HMDB40827 ; HMDB60297	Isochesnatin ; Chesnatin ; Scutellarein 7-glucuronosyl-(1-2)-glucuronide ; Luteolin 7-O-beta-D-glucuronosyl-(1-2)-beta-D-glucuronide	M+2K ; M+2K ; M+2K ; M+2K	C27H26O18 ; C27H26O18 ; C27H26O18 ; C27H26O18
222.52471	1.522615	0.902468	-4.2999	0.001562	2.8064	0.017548	222.5247	HMDB34045 ; HMDB38403 ; HMDB38413 ; HMDB41596	Glucocleomin ; Glucoerucin ; 2-Hydroxypentyl glucosinolate ; Vitisidin A	M+H+K ; M+H+Na ; M+H+K ; M+2Na	C12H23NO10S2 ; C12H23NO9S3 ; C12H23NO10S2 ; C20H15O9
318.05405	1.599062	0.084448	-4.2974	0.001568	2.8047	0.017576	318.0541	HMDB10324 ; HMDB13854 ; HMDB30244 ; HMDB32026 ; HMDB33480 ; HMDB37355 ; HMDB37364 ; HMDB37536 ; HMDB38955 ; HMDB40488 ; HMDB60603	Benzoyl glucuronide (Benzoic acid) ; N4-Acetylsulfamethoxazole ; Graveolinine ; Sinalexin ; Graveoline ; Quercetin 3-arabinoside 7-glucoside ; Quercetin 3-glucoside 7-xyloside ; Quercetin 3-lathyroside ; Avenalumin II ; Quercetin 3-xylosyl-(1-6)-glucoside ; N-acetyl zonisamide	2M+H+K ; M+Na ; M+K ; 3M+H+Na ; M+K ; M+H+K ; M+H+K ; M+H+K ; M+K ; M+H+K ; M+ACN+Na	C13H14O8 ; C12H13N3O4S ; C17H13NO3 ; C10H8N2OS ; C17H13NO3 ; C26H28O16 ; C26H28O16 ; C26H28O16 ; C17H13NO3 ; C26H28O16 ; C10H10N2O4S
254.13286	1.564969	0.621323	4.2942	0.001576	2.8025	0.017617	254.1329	HMDB10364 ; HMDB05021 ; HMDB14682 ; HMDB36606	11-Hydroxyprogesterone 11-glucuronide ; Quetiapine ; Benazepril ; Pterosin F	M+2H ; M+3ACN+2H ; M+2ACN+2H ; M+NH4	C27H38O9 ; C21H25N3O2S ; C24H28N2O5 ; C14H17ClO
198.48770	1.60736	0.094045	4.2931	0.001578	2.8018	0.017617	198.4877	HMDB31168 ; HMDB31169	Cohibin A ; Cohibin B	M+H+2Na ; M+H+2Na	C35H64O4 ; C35H64O4
292.10211	1.553695	0.576754	-4.2891	0.001588	2.7991	0.017688	292.1021	HMDB35217 ; HMDB00706 ; HMDB02381 ; HMDB11167 ; HMDB14632 ; HMDB15378 ; HMDB28759 ; HMDB28760 ; HMDB28969 ; HMDB28991 ; HMDB31985 ; HMDB35191 ; HMDB39926 ; HMDB42023 ; HMDB61039 ; HMDB14552 ; HMDB60977	10-Acetoxyligustroside ; L-Aspartyl-L-phenylalanine ; N-Acetylcystathionine ; L-beta-aspartyl-L-phenylalanine ; Sotalol ; Docetaxel ; Aspartyl-Methionine ; Aspartyl-Phenylalanine ; Methionyl-Aspartate ; Phenylalanyl-Aspartate ; gamma-Glutamyl-S-methylcysteine ; (2S4R)-4-(9H-Pyrido34-bindol-1-yl)-124-butanetriol ; Lyoniresinol 9-sulfate ; Taxotere ; modafinil acid ; Loxapine ; desethylamodiaquine	M+2H ; 2M+H+Na ; 2M+3H2O+2H ; 2M+H+Na ; 2M+H+K ; M+3Na ; 2M+3H2O+2H ; 2M+H+Na ; 2M+3H2O+2H ; 2M+H+Na ; 2M+3H2O+2H ; 2M+H+K ; M+2ACN+2H ; M+3Na ; M+NH4 ; M+H-2H2O ; M+H-2H2O	C27H34O14 ; C13H16N2O5 ; C9H16N2O5S ; C13H16N2O5 ; C12H20N2O3S ; C43H53NO14 ; C9H16N2O5S ; C13H16N2O5 ; C9H16N2O5S ; C13H16N2O5 ; C9H16N2O5S ; C15H16N2O3 ; C22H28O11S ; C43H53NO14 ; C15H14O3S ; C18H18ClN3O ; C18H18ClN3O
434.89908	1.522744	1.35909	-4.2783	0.001616	2.7916	0.017922	434.8891	HMDB43684 ; HMDB43824 ; HMDB55368	TG(15:018:4(6Z9Z12Z15Z)o-18:0) ; TG(15:0o-18:018:4(6Z9Z12Z15Z)) ; TG(18:4(6Z9Z12Z15Z)15:0o-18:0)	M+ACN+2H ; M+ACN+2H ; M+ACN+2H	C54H98O5 ; C54H98O5 ; C54H98O5
237.11686	1.546172	0.870333	4.2756	0.001623	2.7898	0.017922	237.1169	HMDB60717 ; HMDB15255 ; HMDB15548 ; HMDB33337 ; HMDB34064 ; HMDB36946 ; HMDB60613 ; HMDB60827 ; HMDB60933 ; HMDB61056	2-Hydroxy-imipramine glucuronide ; Proflavine ; Valganciclovir ; Fusarin C ; 2-Angeloyl-9-(3-methyl-2E-pentenoyl)-2b9a-dihydroxy-4Z10(14)-oplopadien-3-one ; Scillipheosidin 3-glucosyl-(1-2)-rhamnoside ; Pefloxacin N-oxide ; ID11614 ; Propofol glucuronide ; benzisothiazole piperazine	M+2H ; 2M+3H2O+2H ; 2M+3H ; M+ACN+2H ; M+2Na ; M+3H ; M+3ACN+2H ; M+NH4 ; 2M+3H ; M+NH4	C25H32N2O7 ; C13H11N3 ; C14H22N6O5 ; C23H29NO7 ; C26H36O5 ; C36H52O14 ; C17H20FN3O4 ; C11H13N3S ; C18H26O7 ; C11H13N3S
532.62869	1.546653	1.384314	4.2726	0.00163	2.7877	0.017922	532.6287	HMDB40189	11-Dibromo-3-iodo-2-propanone	3M+2Na	C3H3Br2IO

302.19594	1.519394	0.574189	4.2724	0.001631	2.7876	0.017922	302.1959	HMDB35999 ; HMDB07010 ; HMDB07066 ; HMDB08054 ; HMDB08152 ; HMDB08184 ; HMDB08217 ; HMDB08249 ; HMDB08279 ; HMDB08280 ; HMDB08310 ; HMDB08311 ; HMDB08321 ; HMDB08342 ; HMDB08352 ; HMDB08353 ; HMDB08374 ; HMDB08384 ; HMDB08416 ; HMDB08438 ; HMDB08471 ; HMDB08514 ; HMDB08536 ; HMDB08566 ; HMDB08567 ; HMDB08598 ; HMDB08609 ; HMDB08628 ; HMDB08638 ; HMDB08639 ; HMDB08670 ; HMDB08703 ; HMDB08735 ; HMDB11201 ; HMDB13962 ; HMDB30260 ; HMDB30360 ; HMDB30528 ; HMDB31537 ; HMDB33483 ; HMDB39143 ; HMDB55953 ; HMDB60593 ; HMDB60962 ; HMDB61088	Ganoderic acid Mg ; DG(14:015:00:0) ; DG(15:014:00:0) ; PC(18:022:4(7Z10Z13Z16Z)) ; PC(18:2(9Z12Z)22:2(13Z16Z)) ; PC(18:3(6Z9Z12Z)22:1(13Z)) ; PC(18:3(9Z12Z15Z)22:1(13Z)) ; PC(18:4(6Z9Z12Z15Z)22:0) ; PC(20:020:4(5Z8Z11Z14Z)) ; PC(20:020:4(8Z11Z14Z17Z)) ; PC(20:1(11Z)20:3(5Z8Z11Z)) ; PC(20:1(11Z)20:3(8Z11Z14Z)) ; PC(20:1(11Z)22:6(4Z7Z10Z13Z16Z19Z)) ; PC(20:2(11Z14Z)20:2(11Z14Z)) ; PC(20:2(11Z14Z)22:5(4Z7Z10Z13Z16Z)) ; PC(20:2(11Z14Z)22:5(7Z10Z13Z16Z19Z)) ; PC(20:3(5Z8Z11Z)20:1(11Z)) ; PC(20:3(5Z8Z11Z)22:4(7Z10Z13Z16Z)) ; PC(20:3(8Z11Z14Z)22:4(7Z10Z13Z16Z)) ; PC(20:4(5Z8Z11Z14Z)20:0) ; PC(20:4(8Z11Z14Z17Z)20:0) ; PC(20:5(5Z8Z11Z14Z17Z)22:2(13Z16Z)) ; PC(22:018:4(6Z9Z12Z15Z)) ; PC(22:1(13Z)18:3(6Z9Z12Z)) ; PC(22:1(13Z)18:3(9Z12Z15Z)) ; PC(22:2(13Z16Z)18:2(9Z12Z)) ; PC(22:2(13Z16Z)20:5(5Z8Z11Z14Z17Z)) ; PC(22:4(7Z10Z13Z16Z)18:0) ; PC(22:4(7Z10Z13Z16Z)20:3(5Z8Z11Z)) ; PC(22:4(7Z10Z13Z16Z)20:3(8Z11Z14Z)) ; PC(22:5(4Z7Z10Z13Z16Z)20:2(11Z14Z)) ; PC(22:5(7Z10Z13Z16Z19Z)20:2(11Z14Z)) ; PC(22:6(4Z7Z10Z13Z16Z19Z)20:1(11Z)) ; PC(20:3(8Z11Z14Z)20:1(11Z)) ; Verapamil metabolite D-617 ; (-)-Quebrachamine ; ()-Aspidospermidine ; Janthitrem B ; 3-Methylcyclohexanethiol ; 67-Dihydro-4-(hydroxymethyl)-2-(p-hydroxyphenethyl)-7-methyl-5H-2-pyrindinium ; beta-Doradecin ; DG(14:00:015:0) ; Alpha-dihydroartemisinin ; 3-hydroxyropivacaine ; Dihydroartemisinin (DHA)	M+2H ; M+2K ; M+2K ; M+3Na ; M+3Na ; M+3Na ; M+3Na ; M+3Na ; M+3Na ; M+3Na ; M+3Na ; M+3Na ; M+H+2Na ; M+3Na ; M+H+2Na ; M+H+2Na ; M+3Na ; M+H+2Na ; M+H+2Na ; M+3Na ; M+3Na ; M+H+2Na ; M+3Na ; M+3Na ; M+3Na ; M+3Na ; M+H+2Na ; M+3Na ; M+H+2Na ; M+H+2Na ; M+H+2Na ; M+H+2Na ; M+H+2Na ; M+3Na ; 2M+H+Na ; 2M+H+K ; 2M+H+K ; M+H+NH4 ; 2M+ACN+H ; M+NH4 ; M+H+Na ; M+2K ; M+NH4 ; 2M+H+Na ; M+NH4	C35H54O8 ; C32H62O5 ; C32H62O5 ; C48H88NO8P ; C48H88NO8P ; C48H88NO8P ; C48H88NO8P ; C48H88NO8P ; C48H88NO8P ; C48H88NO8P ; C48H88NO8P ; C48H88NO8P ; C50H86NO8P ; C48H88NO8P ; C50H86NO8P ; C50H86NO8P ; C48H88NO8P ; C50H86NO8P ; C50H86NO8P ; C48H88NO8P ; C48H88NO8P ; C50H86NO8P ; C48H88NO8P ; C48H88NO8P ; C48H88NO8P ; C48H88NO8P ; C50H86NO8P ; C48H88NO8P ; C50H86NO8P ; C50H86NO8P ; C50H86NO8P ; C50H86NO8P ; C50H86NO8P ; C48H88NO8P ; C17H26N2O2 ; C19H26N2 ; C19H26N2 ; C37H47NO5 ; C7H14S ; C18H22NO2 ; C40H52O3 ; C32H62O5 ; C15H24O5 ; C17H26N2O2 ; C15H24O5
773.31995	1.554802	1.218329	-4.2709	0.001635	2.7866	0.017922	773.32	HMDB30462	Hordatine A glucoside	M+2Na-H	C34H48N8O10
443.03870	1.5052	0.218534	-4.2703	0.001636	2.7862	0.017922	443.0387	HMDB15270 ; HMDB30451 ; HMDB34034 ; HMDB40869	Cephapirin ; Cefapirin ; Bikojic acid ; 2-O-Caffeoyltartronic acid	2M+H+K ; 2M+H+K ; 3M+H+K ; 3M+H+K	C17H17N3O6S2 ; C17H17N3O6S2 ; C12H10O8 ; C12H10O8
220.10003	1.520034	0.683794	4.2695	0.001638	2.7856	0.017922	220.1	HMDB38562 ; HMDB14565 ; HMDB36340	8-Propanoylneosolaniol ; Spironolactone ; Ethyl 7-epi-12-hydroxyjasmonate glucoside	M+2H ; M+H+Na ; M+H+Na	C22H30O9 ; C24H32O4S ; C20H32O9
269.03962	1.53469	1.403319	-4.269	0.00164	2.7852	0.017922	269.0396	HMDB38634 ; HMDB02399 ; HMDB14733 ; HMDB31816 ; HMDB31888 ; HMDB33138 ; HMDB33652 ; HMDB39717 ; HMDB39730	(-)-Dioxibrassinin ; Cystathionine sulfoxide ; Oxytetracycline ; 3-Hydroxyflavone ; Theacitrin A ; Methyl salicylate O-rhamnosyl-(1-6)-glucoside ; Prunin 46-di-O-gallate ; Samarangenin A ; Prodelphinidin A2 3-gallate	M+H ; 2M+Na+K ; M+2K ; 2M+Na+K ; M+H+2Na ; M+2K ; M+3Na ; M+H+2Na ; M+H+2Na	C11H12N2O2S2 ; C7H14N2O5S ; C22H24N2O9 ; C15H10O3 ; C37H28O18 ; C20H28O12 ; C35H30O18 ; C37H28O18 ; C37H28O18
358.03777	1.470323	0.206022	4.2682	0.001642	2.7847	0.017922	358.0378	HMDB06032 ; HMDB13636 ; HMDB60413	Imidazoleacetic acid ribotide ; Pyrroloquinoline quinone ; 6-Methylthioguanosine monophosphate	2M+H+K ; 2M+3H2O+2H ; M+H-2H2O	C10H15N2O9P ; C14H6N2O8 ; C11H16N5O7PS
316.17666	1.527158	1.338264	4.2658	0.001648	2.7831	0.017951	316.1767	HMDB15494 ; HMDB14357 ; HMDB14521 ; HMDB30283 ; HMDB36159 ; HMDB37560 ; HMDB60952	Alizapride ; Remikiren ; Palonosetron ; Hydrocinchonine ; Toxin T2 tetrol ; 37815-Scirpenetetrol ; 2-hydroxyimipramine	M+H ; M+2H ; 2M+H+K ; 2M+H+K ; M+NH4 ; M+NH4 ; 2M+H+K	C16H21N5O2 ; C33H50N4O6S ; C19H24N2O ; C19H24N2O ; C15H22O6 ; C15H22O6 ; C19H24N2O
229.92863	1.475183	0.505993	-4.2613	0.00166	2.78	0.01804	229.9286	HMDB00996 ; HMDB39431 ; HMDB60179	3-Sulfinoalanine ; 12457-Pentathiocane ; 3-Sulfinato-L-alaninate	M+2K-H ; 2M+3H2O+2H ; M+2K-H	C3H7NO4S ; C3H6S5 ; C3H7NO4S
150.03782	1.564258	1.26836	-4.2585	0.001667	2.7781	0.018081	150.0378	HMDB33001 ; HMDB33969 ; HMDB34273 ; HMDB60834 ; HMDB04818 ; HMDB06790 ; HMDB11118 ; HMDB15144 ; HMDB15477 ; HMDB29687 ; HMDB30110 ; HMDB30578 ; HMDB30690 ; HMDB30788 ; HMDB30853 ; HMDB30854 ; HMDB30948 ; HMDB32890 ; HMDB32953 ; HMDB33015 ; HMDB33016 ; HMDB33191 ; HMDB33771 ; HMDB34046 ; HMDB34133 ; HMDB35254 ; HMDB36050 ; HMDB38664 ; HMDB39142 ; HMDB39592 ; HMDB41192 ; HMDB41873	2-Methylbenzothiazole ; Benzyl isothiocyanate ; Benzyl thiocyanate ; N-Acetylserotonin sulfate ; Biotin sulfone ; Galactosylglycerol ; Se-Adenosylselenomethionine ; Ketoprofen ; Clenbuterol ; 36-Dimethoxy-4-phenanthrenol ; Celerin ; Apigravin ; (E)-Suberenol ; Citrusal ; (R)-Meranzin ; (S)-Auraptenol ; Dihydrowyerone ; 26-Dimethoxy-4-phenanthrenol ; 7-Methoxy-5-prenyloxycoumarin ; 56-Dihydroyangonin ; 78-Dihydroyangonin ; 2244-Tetramethyl-6-(1-oxopropyl)-135-cyclohexanetrione ; Cyperine ; 1234-Tetramethoxy-5-(2-propenyl)benzene ; 3-(11-Dimethylallyl)scopoletin ; 7-Hydroxy-5-isopropyl-2-methoxy-3-methyl-14-naphthoquinone ; Anhydromarasmone ; (2R)-1-O-beta-D-Galactopyranosylglycerol ; Wyerol ; Pergillin ; Kanzonol Q ; Dexketoprofen	M+H ; M+H ; M+H ; M+2H ; M+H+Na ; M+2Na ; M+3H ; M+2Na ; M+H+Na ; M+2Na ; M+H+K ; M+H+K ; M+H+K ; M+H+K ; M+H+K ; M+H+K ; M+H+K ; M+2Na ; M+H+K ; M+H+K ; M+H+K ; M+Na+K ; M+H+K ; M+Na+K ; M+H+K ; M+H+K ; M+H+K ; M+2Na ; M+H+K ; M+H+K ; M+H+K ; M+2Na	C8H7NS ; C8H7NS ; C8H7NS ; C12H14N2O5S ; C10H16N2O5S ; C9H18O8 ; C15H23N6O5Se ; C16H14O3 ; C12H18Cl2N2O ; C16H14O3 ; C15H16O4 ; C15H16O4 ; C15H16O4 ; C15H16O4 ; C15H16O4 ; C15H16O4 ; C15H16O4 ; C16H14O3 ; C15H16O4 ; C15H16O4 ; C15H16O4 ; C13H18O4 ; C15H16O4 ; C13H18O4 ; C15H16O4 ; C15H16O4 ; C15H16O4 ; C9H18O8 ; C15H16O4 ; C15H16O4 ; C15H16O4 ; C16H14O3

138.05497	1.54761	0.157169	-4.2552	0.001676	2.7758	0.018136	138.049	HMDB03306 ; HMDB30377 ; HMDB30541 ; HMDB30634 ; HMDB30635 ; HMDB30744 ; HMDB30754 ; HMDB30822 ; HMDB30823 ; HMDB30949 ; HMDB33017 ; HMDB33333 ; HMDB33590 ; HMDB37232 ; HMDB37760 ; HMDB40828 ; HMDB41654 ; HMDB41685 ; HMDB41695 ; HMDB00351 ; HMDB00354 ; HMDB00396 ; HMDB00407 ; HMDB00410 ; HMDB00531 ; HMDB00642 ; HMDB00754 ; HMDB01863 ; HMDB01987 ; HMDB02011 ; HMDB14877 ; HMDB29733 ; HMDB30668 ; HMDB33160 ; HMDB38551 ; HMDB40735 ; HMDB41603 ; HMDB59844	Phloretin ; (Z)-4-Methoxy-3355-tetrahydroxystilbene ; 3347-Tetrahydroxyflavan ; beta-Pyrufuran ; alpha-Pyrufuran ; Hemigossypolone ; 11-Methoxynoryangonin ; (-)-Epiafzelechin ; Afzelechin ; Wyerone epoxide ; 11-Hydroxyyangonin ; 23-Epoxysesamone ; (R)-Methysticin ; (2xi4xi)-4457-Tetrahydroxyflavan ; Hydroxysesamone ; (2S3S4R)-3447-Tetrahydroxyflavan ; 3-Hydroxy-O-desmethylangolensin ; 5-Hydroxy-O-desmethylangolensin ; 6-Hydroxy-O-desmethylangolensin ; 3-Hydroxy-2-methyl-R-(RS)-butanoic acid ; 2-Methyl-3-hydroxybutyric acid ; 2-Ethylhydracrylic acid ; 2-Hydroxy-3-methylbutyric acid ; 3-Hydroxy-2-methyl-S-(RR)-butanoic acid ; 3-Hydroxyvaleric acid ; Erythronilic acid ; 3-Hydroxyisovaleric acid ; 2-Hydroxyvaleric acid ; 2-Hydroxy-2-methylbutyric acid ; 4-Hydroxyisovaleric acid ; Hetacillin ; 2-Ethyl-5-methylthiophene ; 1-(246-Trimethoxyphenyl)-13-butanedione ; 2-Propylthiophene ; Methyl 345-trimethoxycinnamate ; Ethyl lactate ; Methyl 3-hydroxybutyrate ; Diethyl carbonate	M+2H ; M+2H ; M+2H ; M+2H ; M+2H ; M+2H ; M+2H ; M+2H ; M+2H ; M+2H ; M+2H ; M+2H ; M+2H ; M+2H ; M+2H ; M+2H ; M+2H ; M+2H ; M+2H ; 2M+H+K ; 2M+H+K ; 2M+H+K ; 2M+H+K ; 2M+H+K ; 2M+H+K ; 2M+H+K ; 2M+H+K ; 2M+H+K ; 2M+H+K ; 2M+H+K ; M+2H+Na ; 2M+H+Na ; M+H+Na ; 2M+H+Na ; M+H+Na ; 2M+H+K ; 2M+H+K ; 2M+H+K	C15H14O5 ; C15H14O5 ; C15H14O5 ; C15H14O5 ; C15H14O5 ; C15H14O5 ; C15H14O5 ; C15H14O5 ; C15H14O5 ; C15H14O5 ; C15H14O5 ; C15H14O5 ; C15H14O5 ; C15H14O5 ; C15H14O5 ; C15H14O5 ; C15H14O5 ; C15H14O5 ; C15H14O5 ; C5H10O3 ; C5H10O3 ; C5H10O3 ; C5H10O3 ; C5H10O3 ; C5H10O3 ; C5H10O3 ; C5H10O3 ; C5H10O3 ; C5H10O3 ; C5H10O3 ; C19H23N3O4S ; C7H10S ; C13H16O5 ; C7H10S ; C13H16O5 ; C5H10O3 ; C5H10O3 ; C5H10O3
288.76841	1.550527	1.326064	4.2478	0.001695	2.7707	0.018309	288.7684	HMDB29982 ; HMDB29983	Epomusenin A ; Epomusenin B	M+H+NH4 ; M+H+NH4	C37H66O3 ; C37H66O3
380.83498	1.527495	1.182798	4.2446	0.001704	2.7685	0.018343	380.835	HMDB07087 ; HMDB07619 ; HMDB55993	DG(15:022:1(13Z)0:0) ; DG(22:1(13Z)15:00:0) ; DG(15:00:022:1n9)	M+3ACN+2H ; M+3ACN+2H ; M+3ACN+2H	C40H76O5 ; C40H76O5 ; C40H76O5
164.09150	1.493753	0.370691	4.2391	0.001719	2.7648	0.018343	164.0915	HMDB00473 ; HMDB11727 ; HMDB32862 ; HMDB35359 ; HMDB00422 ; HMDB00448 ; HMDB00752 ; HMDB00858 ; HMDB02074 ; HMDB02173 ; HMDB06900 ; HMDB12936 ; HMDB33837 ; HMDB14331 ; HMDB61079 ; HMDB61080 ; HMDB61081	6-Dimethylaminopurine ; Bicine ; 2-Amino-4-ethoxy-3-hydroxybutanoic acid ; 1-Deoxynojirimycin ; 2-Methylglutaric acid ; Adipic acid ; Methylglutaric acid ; Monomethyl glutaric acid ; 22-Dimethylsuccinic acid ; Solerol ; (S)-2-Aceto-2-hydroxybutanoic acid ; Dynorphin B (10-13) ; Dimethyl succinate ; Cevimeline ; cevimeline trans-sulfoxide ; cevimeline cis-sulfoxide ; cevimeline N-oxide	M+H ; M+H ; M+H ; M+H ; M+NH4 ; M+NH4 ; M+NH4 ; M+NH4 ; M+NH4 ; M+NH4 ; M+H+2Na ; M+NH4 ; M+H-2H2O ; M+H-2H2O ; M+H-2H2O ; M+H-2H2O	C7H9N5 ; C6H13NO4 ; C6H13NO4 ; C6H13NO4 ; C6H10O4 ; C6H10O4 ; C6H10O4 ; C6H10O4 ; C6H10O4 ; C6H10O4 ; C6H10O4 ; C20H39N5O6 ; C6H10O4 ; C10H17NOS ; C10H17NOS ; C10H17NOS ; C10H17NOS
621.44069	1.59651	0.804364	-4.239	0.001719	2.7647	0.018343	621.4407	HMDB36439 ; HMDB37034 ; HMDB39520 ; HMDB41534 ; HMDB07075 ; HMDB07076 ; HMDB07271 ; HMDB07300 ; HMDB13322 ; HMDB13623 ; HMDB31906 ; HMDB31907 ; HMDB31909 ; HMDB32996 ; HMDB33505 ; HMDB35337 ; HMDB38484 ; HMDB55996 ; HMDB56003 ; HMDB56142 ; HMDB56144	Fasciculic acid A ; (3b7b22x)-Cucurbita-524-diene-3723-triol 7-glucoside ; Soyasapogenol B 24-O-b-D-glucoside ; Ginsenoside Rh4 ; DG(15:018:3(6Z9Z12Z)0:0) ; DG(15:018:3(9Z12Z15Z)0:0) ; DG(18:3(6Z9Z12Z)15:00:0) ; DG(18:3(9Z12Z15Z)15:00:0) ; Undecanoylcholine ; 12(13)Ep-9-KODE ; Muridienin 4 ; Muridienin 3 ; Muridienin 2 ; 2-Undecyl-4(1H)-quinolinone ; (9Z11R12S13S15Z)-1213-Epoxy-11-hydroxy-915-octadecadienoic acid ; Sterebin A ; Auxin b ; DG(15:00:018:3n6) ; DG(15:00:018:3n3) ; DG(14:1n50:018:2n6) ; DG(14:1n50:020:2n6)	M+H ; M+H ; M+H ; M+H ; M+2Na-H ; M+2Na-H ; M+2Na-H ; M+2Na-H ; 2M+2K-H ; 2M+H ; M+2K+H ; M+2K+H ; M+2K+H ; 2M+Na ; 2M+H ; 2M+H ; 2M+H ; M+2Na-H ; M+2Na-H ; M+2Na-H ; M+2Na-H	C36H60O8 ; C36H60O8 ; C36H60O8 ; C36H60O8 ; C36H64O5 ; C36H64O5 ; C36H64O5 ; C36H64O5 ; C16H34NO2 ; C18H30O4 ; C37H66O2 ; C37H66O2 ; C37H66O2 ; C20H29NO ; C18H30O4 ; C18H30O4 ; C18H30O4 ; C36H64O5 ; C36H64O5 ; C36H64O5 ; C36H64O5
828.92234	1.595511	0.602748	-4.2387	0.00172	2.7645	0.018343	828.9223	HMDB11852 ; HMDB36317	Ganglioside GD2 (d18:114:0) ; Canavalioside	M+H+K ; 3M+H+NH4	C75H131N3O34 ; C26H42O12
249.53438	1.536098	0.208836	4.2387	0.00172	2.7645	0.018343	NA	NA	NA	NA	NA
246.15612	1.504257	0.118277	-4.2385	0.001721	2.7643	0.018343	246.1561	HMDB28681 ; HMDB28702 ; HMDB60441 ; HMDB41587 ; HMDB00649 ; HMDB00852 ; HMDB06840 ; HMDB30240 ; HMDB31041 ; HMDB33214 ; HMDB37524 ; HMDB39444 ; HMDB39699 ; HMDB41999 ; HMDB59641	Alanyl-Arginine ; Arginyl-Alanine ; beta-Alanyl-L-arginine ; Macrocarpal I ; Clionasterol ; Beta-Sitosterol ; 44-Dimethyl-5a-cholesta-8-en-3b-ol ; Piperundecalidine ; (8E15E)-1815-Heptadecatriene-1113-diyne ; Schottenol ; (3beta5alpha24R)-Stigmast-8(14)-en-3-ol ; 24alpha-Methyllophenol ; (5alpha14alpha24R)-14-Methylergostan-3-one ; Propiverine ; 44-Dimethyl-5-alpha-cholest-7-en-3-beta-ol	M+H ; M+H ; M+H ; M+2H ; M+2K ; M+2K ; M+2K ; M+3ACN+2H ; 2M+H+K ; M+2K ; M+2K ; M+2K ; M+2K ; M+3ACN+2H ; M+2K	C9H19N5O3 ; C9H19N5O3 ; C9H19N5O3 ; C28H42O7 ; C29H50O ; C29H50O ; C29H50O ; C23H29NO3 ; C17H22 ; C29H50O ; C29H50O ; C29H50O ; C29H50O ; C23H29NO3 ; C29H50O
206.50707	1.538684	0.379482	-4.2329	0.001736	2.7605	0.018467	206.5071	HMDB07021 ; HMDB07049 ; HMDB07101 ; HMDB07102 ; HMDB07129 ; HMDB07157 ; HMDB07185 ; HMDB07214 ; HMDB07357 ; HMDB07385 ; HMDB14971 ; HMDB55963 ; HMDB56016 ; HMDB56017 ; HMDB56041 ; HMDB56065	DG(14:020:1(11Z)0:0) ; DG(14:1(9Z)20:00:0) ; DG(16:018:1(11Z)0:0) ; DG(16:018:1(9Z)0:0) ; DG(16:1(9Z)18:00:0) ; DG(18:016:1(9Z)0:0) ; DG(18:1(11Z)16:00:0) ; DG(18:1(9Z)16:00:0) ; DG(20:014:1(9Z)0:0) ; DG(20:1(11Z)14:00:0) ; Cefaclor ; DG(14:00:020:1n9) ; DG(16:00:018:1n7) ; DG(16:00:018:1n9) ; DG(18:00:016:1n7) ; DG(20:00:014:1n5)	M+2H+Na ; M+2H+Na ; M+2H+Na ; M+2H+Na ; M+2H+Na ; M+2H+Na ; M+2H+Na ; M+2H+Na ; M+2H+Na ; M+2H+Na ; M+2Na ; M+2H+Na ; M+2H+Na ; M+2H+Na ; M+2H+Na ; M+2H+Na	C37H70O5 ; C37H70O5 ; C37H70O5 ; C37H70O5 ; C37H70O5 ; C37H70O5 ; C37H70O5 ; C37H70O5 ; C37H70O5 ; C37H70O5 ; C15H14ClN3O4S ; C37H70O5 ; C37H70O5 ; C37H70O5 ; C37H70O5 ; C37H70O5
200.03596	1.495178	1.43707	4.2308	0.001742	2.759	0.018489	200.036	HMDB01015 ; HMDB02336 ; HMDB03164 ; HMDB29278 ; HMDB29417 ; HMDB29441 ; HMDB30652 ; HMDB30653 ; HMDB32849 ; HMDB32964 ; HMDB33592 ; HMDB40632 ; HMDB40690	N-Formyl-L-methionine ; Biflorin ; Chlorogenic acid ; Cis-5-Caffeoylquinic acid ; Cycloalliin ; Isoalliin ; 1-O-Caffeoylquinic acid ; Cryptochlorogenic acid ; trans-Chlorogenic acid ; 2-Acetylthiazole ; (R)C(S)S-Alliin ; Isobiflorin ; 5Z-Caffeoylquinic acid	M+Na ; M+2Na ; M+2Na ; M+2Na ; M+Na ; M+Na ; M+2Na ; M+2Na ; M+2Na ; 3M+H+NH4 ; M+Na ; M+2Na ; M+2Na	C6H11NO3S ; C16H18O9 ; C16H18O9 ; C16H18O9 ; C6H11NO3S ; C6H11NO3S ; C16H18O9 ; C16H18O9 ; C16H18O9 ; C5H5NOS ; C6H11NO3S ; C16H18O9 ; C16H18O9

227.69393	1.544085	1.000383	-4.2255	0.001756	2.7554	0.018604	227.6939	HMDB15662 ; HMDB31274 ; HMDB31745 ; HMDB38525 ; HMDB39182 ; HMDB39185 ; HMDB39368 ; HMDB39369 ; HMDB39370	Fosaprepitant ; 134-Trigalloyl-beta-D-glucopyranose ; 124-Trigalloyl-beta-D-glucopyranose ; 3-(10-Heptadecenyl)phenol ; 126-Trigalloyl-beta-D-glucopyranose ; 146-Trigalloyl-beta-D-glucopyranose ; 125-Tri-O-galloylhamamelofuranose ; Kurigalin ; 3-O-Galloylhamamelitannin	M+3Na ; M+H+2Na ; M+H+2Na ; M+3ACN+2H ; M+H+2Na ; M+H+2Na ; M+H+2Na ; M+H+2Na ; M+H+2Na	C23H22F7N4O6P ; C27H24O18 ; C27H24O18 ; C23H38O ; C27H24O18 ; C27H24O18 ; C27H24O18 ; C27H24O18 ; C27H24O18
232.07638	1.557528	0.600525	-4.2196	0.001773	2.7514	0.018738	232.0764	HMDB01833 ; HMDB12503 ; HMDB14537 ; HMDB14772 ; HMDB14934 ; HMDB15321 ; HMDB15459 ; HMDB28843 ; HMDB28885 ; HMDB30523 ; HMDB30868 ; HMDB31996 ; HMDB32935 ; HMDB33750 ; HMDB35027 ; HMDB35774 ; HMDB36462 ; HMDB39860 ; HMDB40231 ; HMDB40589 ; HMDB60951 ; HMDB28773 ; HMDB28789 ; HMDB28794 ; HMDB29146	Aminopterin ; 1011-dihydro-20-trihydroxy-leukotriene B4 ; Nimodipine ; Sulfacetamide ; Candesartan ; Clindamycin ; Cortisone acetate ; Glycyl-Histidine ; Histidinyl-Glycine ; Citreoviridin C ; Citreoviridin A ; Licorice glycoside E ; 1-(2-Thienyl)-1-butanone ; D-Glycero-D-galacto-heptitol ; Melleolide F ; Armillyl orsellinate ; Virolongin B ; Propylene glycol alginate ; 1-(5-Methyl-2-thienyl)-1-propanone ; 3-Acetyl-25-dimethylthiophene ; fluorobenzoylpropionic acid ; Cysteinyl-Glutamine ; Cysteinyl-Gamma-glutamate ; Glutaminyl-Cysteine ; Gamma-glutamyl-Cysteine	M+H+Na ; M+2K ; M+2Na ; M+NH4 ; M+H+Na ; M+H+K ; M+Na+K ; 2M+H+K ; 2M+H+K ; M+Na+K ; M+Na+K ; M+3H ; 3M+2H ; 2M+H+K ; M+Na+K ; M+Na+K ; M+Na+K ; M+H+Na ; 3M+2H ; 3M+2H ; M+NH4 ; M+H-H2O ; M+H-H2O ; M+H-H2O ; M+H-H2O	C19H20N8O5 ; C20H34O7 ; C21H26N2O7 ; C8H10N2O3S ; C24H20N6O3 ; C18H33ClN2O5S ; C23H30O6 ; C8H12N4O3 ; C8H12N4O3 ; C23H30O6 ; C23H30O6 ; C35H35NO14 ; C8H10OS ; C7H16O7 ; C23H30O6 ; C23H30O6 ; C23H30O6 ; C17H28O13 ; C8H10OS ; C8H10OS ; C10H8F2O3 ; C8H15N3O4S ; C8H15N3O4S ; C8H15N3O4S ; C8H15N3O4S
290.93448	1.478146	1.068884	-4.2166	0.001781	2.7493	0.01875	NA	NA	NA	NA	NA
678.44135	1.491265	1.384648	-4.2157	0.001784	2.7487	0.01875	678.4414	HMDB14341 ; HMDB15121 ; HMDB29850 ; HMDB32368 ; HMDB38457 ; HMDB60777 ; HMDB60785	Betaxolol ; Glycopyrrolate ; L-Citronellol glucoside ; Menthyl pyrrolidone carboxylate ; Dihydrocapsaicin ; 5-Hydroxyemedastine ; 6-Hydroxyemedastine	2M+ACN+Na ; 2M+ACN+H ; 2M+ACN+H ; 2M+ACN+H ; 2M+ACN+Na ; 2M+ACN+H ; 2M+ACN+H	C18H29NO3 ; C19H28NO3 ; C16H30O6 ; C16H30O6 ; C18H29NO3 ; C17H26N4O2 ; C17H26N4O2
210.52835	1.538852	1.233021	-4.2153	0.001785	2.7484	0.01875	210.5284	HMDB11610 ; HMDB29286 ; HMDB33211 ; HMDB37845 ; HMDB39157	5-Taurinomethyl-2-thiouridine ; Avenanthramide 2s ; Avenanthramide 2 ; N-(1-Deoxy-1-fructosyl)tyrosine ; Clovamide	M+H+Na ; M+Na+K ; M+Na+K ; M+2K ; M+Na+K	C12H19N3O8S2 ; C18H17NO7 ; C18H17NO7 ; C15H21NO8 ; C18H17NO7
549.50912	1.604617	0.71725	-4.2124	0.001793	2.7464	0.018763	549.5091	HMDB00790 ; HMDB04949 ; HMDB14098 ; HMDB43147 ; HMDB43371 ; HMDB47037	N-Palmitoylsphingosine ; Ceramide (d18:116:0) ; 5-Hydroxytenoxicam ; TG(15:024:024:1(15Z)) ; TG(15:024:1(15Z)24:0) ; TG(24:015:024:1(15Z))	2M+H+Na ; 2M+H+Na ; 3M+H+K ; M+2ACN+2H ; M+2ACN+2H ; M+2ACN+2H	C34H67NO3 ; C34H67NO3 ; C13H11N3O5S2 ; C66H126O6 ; C66H126O6 ; C66H126O6
624.18114	1.588372	0.442549	-4.2122	0.001794	2.7462	0.018763	624.1811	HMDB39922	Eriodictyol 7-(6-trans-p-coumaroylglucoside)	2M+3H2O+2H	C30H28O13
124.92268	1.461395	0.585487	-4.2107	0.001798	2.7452	0.018767	124.9227	HMDB03227 ; HMDB31258	Methanethiol ; Lenthionine	M+2K-H ; M+Na+K	CH4S ; C2H4S5
188.09150	1.543028	0.997048	4.2074	0.001807	2.743	0.018826	188.0915	HMDB12150 ; HMDB15587 ; HMDB00259 ; HMDB01046 ; HMDB15316 ; HMDB39132 ; HMDB36394	2-Keto-6-acetamidocaproate ; Cyproterone ; Serotonin ; Cotinine ; Fluoxymesterone ; 4-Gingerdiol 35-diacetate ; N-Methylcalystegine C1	M+H ; M+2H ; 2M+H+Na ; 2M+H+Na ; M+H+K ; M+H+Na ; M+H-H2O	C8H13NO4 ; C22H27ClO3 ; C10H12N2O ; C10H12N2O ; C20H29FO3 ; C19H28O6 ; C8H15NO5
476.14477	1.546976	1.342727	-4.2056	0.001812	2.7417	0.01884	476.1448	HMDB14448 ; HMDB01846 ; HMDB32801 ; HMDB59970	Ertapenem ; Tetrahydrofolic acid ; 4-Hydroxybenzyl isothiocyanate rhamnoside ; 4-Hydroxy-5-(345-trihydroxyphenyl)-valeric acid-O-methyl-O-glucuronide	M+H ; 2M+Na+K ; 3M+H+NH4 ; 2M+3H2O+2H	C22H25N3O7S ; C19H23N7O6 ; C14H17NO5S ; C18H24O13
269.99339	1.494944	1.479314	4.2024	0.001822	2.7395	0.018879	269.9934	HMDB00763 ; HMDB11745 ; HMDB31832 ; HMDB35514 ; HMDB60400	5-Hydroxyindoleacetic acid ; N-Acetyl-L-methionine ; Methyl methanethiosulfonate ; xi-23-Dihydro-2-oxo-1H-indole-3-acetic acid ; 5-Phenyl-13-oxazinane-24-dione	M+2K+H ; M+2K+H ; 2M+NH4 ; M+2K+H ; M+2K+H	C10H9NO3 ; C7H13NO3S ; C2H6O2S2 ; C10H9NO3 ; C10H9NO3
386.13255	1.544735	0.266969	-4.2017	0.001824	2.7391	0.018879	386.1326	HMDB00988 ; HMDB02067 ; HMDB02209 ; HMDB06834 ; HMDB14713 ; HMDB29302 ; HMDB29530 ; HMDB30598 ; HMDB32869 ; HMDB33279 ; HMDB34173 ; HMDB35019 ; HMDB36347 ; HMDB37679 ; HMDB38111 ; HMDB38112 ; HMDB39146 ; HMDB40912 ; HMDB41167 ; HMDB41399	S-Adenosylmethioninamine ; O-b-D-Gal-(1-3)-O-O-b-D-Gal-(1-4)-2-(acetylamino)-2-deoxy-b-D-Glc-(1-6)-2-(acetylamino)-2-deoxy- D-Galactose ; Equol ; D-Pantothenoyl-L-cysteine ; Fenoprofen ; 1-Phenyl-67-dihydroxy-isochroman ; Glabrene ; (-)-Wikstromol ; 34578-Pentamethoxyflavanone ; 8-Hydroxypinoresinol ; Phaseollin ; Phenethyl salicylate ; 23-Dihydroxy-13-diphenyl-1-propanone ; Guaicyl phenylacetate ; Kievitone hydrate ; Kievitol ; Citrubuntin ; N-(5-Hydroxy-2-pyridinyl)methyladenosine ; (-)-Shinpterocarpin ; 23-Dihydro-4-(4-methoxyphenyl)-1H-phenalene-123-triol	2M+Na+K ; M+H+Na ; 3M+2Na ; M+ACN+Na ; 3M+2Na ; 3M+2Na ; M+ACN+Na ; 2M+H+Na ; 2M+H+Na ; 2M+H+Na ; M+ACN+Na ; 3M+2Na ; 3M+2Na ; 3M+2Na ; 2M+H+Na ; 2M+H+Na ; 3M+2Na ; 2M+H+Na ; M+ACN+Na ; M+ACN+Na	C14H23N6O3S ; C28H48N2O21 ; C15H14O3 ; C12H22N2O6S ; C15H14O3 ; C15H14O3 ; C20H18O4 ; C20H22O7 ; C20H22O7 ; C20H22O7 ; C20H18O4 ; C15H14O3 ; C15H14O3 ; C15H14O3 ; C20H22O7 ; C20H22O7 ; C15H14O3 ; C16H18N6O5 ; C20H18O4 ; C20H18O4
152.05663	1.490773	1.052466	-4.2002	0.001828	2.738	0.018887	152.0566	HMDB00132 ; HMDB00403 ; HMDB00542 ; HMDB00156 ; HMDB00744 ; HMDB02144 ; HMDB02271 ; HMDB02820 ; HMDB04988 ; HMDB29633 ; HMDB29634 ; HMDB31518 ; HMDB32872 ; HMDB34448 ; HMDB38974 ; HMDB39789 ; HMDB39790	Guanine ; 2-Hydroxyadenine ; 8-Hydroxyadenine ; L-Malic acid ; Malic acid ; 13-Dimethyluracil ; Imidazolepropionic acid ; Methylimidazoleacetic acid ; Pi-Methylimidazoleacetic acid ; Phenylmethanethiol ; 2-Methylbenzenethiol ; D-Malic acid ; Velcorin ; Methyl phenyl sulfide ; 33-Dimethyl-12-dithiolane ; Tetrahydro-2-methyl-2-thiophenethiol ; Tetrahydro-2-methyl-3-thiophenethiol	M+H ; M+H ; M+H ; M+NH4 ; M+NH4 ; 2M+H+Na ; 2M+H+Na ; 2M+H+Na ; 2M+H+Na ; 2M+3H2O+2H ; 2M+3H2O+2H ; M+NH4 ; M+NH4 ; 2M+3H2O+2H ; M+NH4 ; M+NH4 ; M+NH4	C5H5N5O ; C5H5N5O ; C5H5N5O ; C4H6O5 ; C4H6O5 ; C6H8N2O2 ; C6H8N2O2 ; C6H8N2O2 ; C6H8N2O2 ; C7H8S ; C7H8S ; C4H6O5 ; C4H6O5 ; C7H8S ; C5H10S2 ; C5H10S2 ; C5H10S2
163.10759	1.524187	0.986146	-4.1864	0.001869	2.7285	0.019198	163.1076	HMDB00450 ; HMDB00730 ; HMDB00808 ; HMDB01263 ; HMDB03681 ; HMDB12131 ; HMDB12151 ; HMDB15127 ; HMDB29425 ; HMDB29426 ; HMDB59595 ; HMDB60265 ; HMDB61156	5-Hydroxylysine ; Isobutyrylglycine ; N-Butyrylglycine ; Allysine ; 4-Acetamidobutanoic acid ; (S)-5-Amino-3-oxohexanoate ; 2-Keto-6-aminocaproate ; Methyl aminolevulinate ; L-cis-4-(Hydroxymethyl)-2-pyrrolidinecarboxylic acid ; L-trans-5-Hydroxy-2-piperidinecarboxylic acid ; (S)-2-amino-6-oxohexanoate ; 4-Acetamidobutanoate ; N-(2-Carboxymethyl)-morpholine	M+H ; M+NH4 ; M+NH4 ; M+NH4 ; M+NH4 ; M+NH4 ; M+NH4 ; M+NH4 ; M+NH4 ; M+NH4 ; M+NH4 ; M+NH4 ; M+NH4	C6H14N2O3 ; C6H11NO3 ; C6H11NO3 ; C6H11NO3 ; C6H11NO3 ; C6H11NO3 ; C6H11NO3 ; C6H11NO3 ; C6H11NO3 ; C6H11NO3 ; C6H11NO3 ; C6H11NO3 ; C6H11NO3
412.71752	1.534319	1.391571	4.1857	0.001871	2.728	0.019198	NA	NA	NA	NA	NA

112.51521	1.56202	0.604369	4.1846	0.001874	2.7272	0.019198	112.5152	HMDB14868 ; HMDB29709 ; HMDB30346 ; HMDB32124 ; HMDB59752 ; HMDB60016 ; HMDB60018	Thiabendazole ; 5678-Tetrahydro-4-methylquinoline ; (S)-Actinidine ; (Z)-Methyl 3-(methylsulfinyl)-1-propenyl disulfide ; 5-Sulfoxymethylfurfural ; Pyrogallol-1-O-sulphate ; Pyrogallol-2-O-sulphate	M+H+Na ; M+2K ; M+2K ; M+ACN+2H ; M+H+NH4 ; M+H+NH4 ; M+H+NH4	C10H7N3S ; C10H13N ; C10H13N ; C5H10OS3 ; C6H6O6S ; C6H6O6S ; C6H6O6S
282.82328	1.51336	1.481908	4.1842	0.001875	2.7269	0.019198	282.8233	HMDB29373 ; HMDB30930	Solacauline ; 1-Tridecene	M+2H+Na ; 3M+H+NH4	C43H69NO14 ; C13H26
282.25060	1.550123	0.555608	4.1825	0.00188	2.7258	0.019198	282.2506	HMDB11698 ; HMDB13297	SM(d18:126:0) ; Pentacosanoylglycine	M+3H ; M+3ACN+2H	C49H100N2O6P ; C27H53NO3
335.53064	1.548083	0.896465	4.1811	0.001884	2.7248	0.019198	NA	NA	NA	NA	NA
333.99375	1.529021	1.148249	-4.1808	0.001885	2.7246	0.019198	NA	NA	NA	NA	NA
162.13990	1.524451	0.617154	-4.1775	0.001895	2.7223	0.019242	162.1399	HMDB30944 ; HMDB32791 ; HMDB35792	Cystophorene ; Methyl 15-cyanopentadecanoate ; (E)-48-Dimethyl-137-nonatriene	2M+H+Na ; M+ACN+2H ; 2M+H+Na	C11H18 ; C17H31NO2 ; C11H18
576.63781	1.585857	0.034457	-4.1768	0.001897	2.7219	0.019242	576.6378	HMDB59612 ; HMDB59787	7-Methylguanosine 5-phosphate ; Casomorphin	3M+H+NH4 ; 2M+3H	C11H17N5O8P ; C44H61N7O11
473.59704	1.559622	0.53722	-4.1709	0.001915	2.7178	0.019332	NA	NA	NA	NA	NA
214.91736	1.560939	1.278625	4.1701	0.001918	2.7173	0.019332	NA	NA	NA	NA	NA
452.91834	1.553862	0.156965	4.17	0.001918	2.7172	0.019332	452.9183	HMDB05383 ; HMDB05388 ; HMDB05397 ; HMDB05403 ; HMDB05424 ; HMDB05437 ; HMDB10445 ; HMDB10446 ; HMDB31142 ; HMDB31144 ; HMDB42171 ; HMDB42315 ; HMDB42344 ; HMDB42374 ; HMDB42402 ; HMDB42460 ; HMDB42461 ; HMDB42489 ; HMDB42664 ; HMDB43090 ; HMDB43092 ; HMDB43398 ; HMDB43456 ; HMDB43903 ; HMDB44037 ; HMDB44064 ; HMDB44090 ; HMDB44144 ; HMDB44145 ; HMDB44199 ; HMDB44226 ; HMDB44389 ; HMDB44685 ; HMDB44867 ; HMDB44892 ; HMDB44917 ; HMDB44918 ; HMDB44944 ; HMDB44970 ; HMDB45023 ; HMDB45514 ; HMDB45516 ; HMDB45666 ; HMDB45690 ; HMDB45691 ; HMDB45715 ; HMDB45741 ; HMDB45766 ; HMDB46435 ; HMDB46436 ; HMDB46459 ; HMDB46483 ; HMDB46508 ; HMDB47175 ; HMDB47198 ; HMDB47777 ;	TG(16:018:1(9Z)20:1(11Z))iso6 ; TG(16:018:2(9Z12Z)20:0)iso6 ; TG(18:018:018:2(9Z12Z))iso3 ; TG(18:018:1(9Z)18:1(9Z))iso3 ; TG(16:1(9Z)18:020:1(11Z))iso6 ; TG(16:1(9Z)18:1(9Z)20:0)iso6 ; TG(18:1(11Z)18:018:1(11Z))iso3 ; TG(18:1(11Z)18:018:1(9Z))iso6 ; Glycerol 13-di-(9Z-octadecenoate) 2-octadecanoate ; Glycerol 12-di-(9Z-octadecenoate) 3-octadecanoate ; TG(14:018:022:2(13Z16Z)) ; TG(14:016:1(9Z)24:1(15Z)) ; TG(14:018:1(11Z)22:1(13Z)) ; TG(14:018:1(9Z)22:1(13Z)) ; TG(14:020:1(11Z)20:1(11Z)) ; TG(14:022:1(13Z)18:1(11Z)) ; TG(14:022:1(13Z)18:1(9Z)) ; TG(14:024:1(15Z)16:1(9Z)) ; TG(14:022:2(13Z16Z)18:0) ; TG(15:020:018:2(9Z12Z)) ; TG(15:020:020:2n6) ; TG(15:018:2(9Z12Z)20:0) ; TG(15:020:2n620:0) ; TG(16:016:022:2(13Z16Z)) ; TG(16:014:1(9Z)24:1(15Z)) ; TG(16:016:1(9Z)22:1(13Z)) ; TG(16:018:1(11Z)20:1(11Z)) ; TG(16:020:1(11Z)18:1(11Z)) ; TG(16:020:1(11Z)18:1(9Z)) ; TG(16:022:1(13Z)16:1(9Z)) ; TG(16:024:1(15Z)14:1(9Z)) ; TG(16:022:2(13Z16Z)16:0) ; TG(18:014:022:2(13Z16Z)) ; TG(18:014:1(9Z)22:1(13Z)) ; TG(18:016:1(9Z)20:1(11Z)) ; TG(18:018:1(11Z)18:1(11Z)) ; TG(18:018:1(11Z)18:1(9Z)) ; TG(18:018:1(9Z)18:1(11Z)) ; TG(18:020:1(11Z)16:1(9Z)) ; TG(18:022:1(13Z)14:1(9Z)) ; TG(20:015:018:2(9Z12Z)) ; TG(20:015:020:2n6) ; TG(20:014:1(9Z)20:1(11Z)) ; TG(20:016:1(9Z)18:1(11Z)) ; TG(20:016:1(9Z)18:1(9Z)) ; TG(20:018:1(11Z)16:1(9Z)) ; TG(20:018:1(9Z)16:1(9Z)) ; TG(20:020:1(11Z)14:1(9Z)) ; TG(22:014:1(9Z)18:1(11Z)) ; TG(22:014:1(9Z)18:1(9Z)) ; TG(22:016:1(9Z)16:1(9Z)) ; TG(22:018:1(11Z)14:1(9Z)) ; TG(22:018:1(9Z)14:1(9Z)) ; TG(24:014:1(9Z)16:1(9Z)) ; TG(24:016:1(9Z)14:1(9Z)) ; TG(14:1(9Z)16:024:1(15Z)) ; TG(14:1(9Z)18:022:1(13Z)) ; TG(14:1(9Z)20:020:1(11Z)) ; TG(14:1(9Z)22:018:1(11Z)) ; TG(14:1(9Z)22:018:1(9Z)) ; TG(14:1(9Z)24:016:1(9Z)) ; TG(16:1(9Z)14:024:1(15Z)) ; TG(16:1(9Z)16:022:1(13Z)) ; TG(16:1(9Z)20:018:1(11Z)) ; TG(16:1(9Z)20:018:1(9Z)) ; TG(16:1(9Z)22:016:1(9Z)) ; TG(18:1(11Z)14:022:1(13Z)) ; TG(18:1(11Z)16:020:1(11Z)) ; TG(18:1(9Z)14:022:1(13Z)) ; TG(18:1(9Z)16:020:1(11Z)) ; TG(18:1(9Z)18:018:1(9Z)) ; TG(20:1(11Z)14:020:1(11Z))	M+H+NH4 ; M+H+NH4	C57H106O6 ; C57H106O6
534.18840	1.538589	1.193256	-4.1688	0.001922	2.7163	0.019332	534.1884	HMDB32776	3-Oxo-carbofuran	2M+ACN+Na	C12H13NO4
166.06276	1.541866	1.285666	-4.164	0.001937	2.713	0.019413	166.0628	HMDB11672 ; HMDB32655 ; HMDB33299 ; HMDB33697 ; HMDB33817 ; HMDB37253 ; HMDB37315 ; HMDB37955 ; HMDB38494 ; HMDB39381 ; HMDB39740 ; HMDB00957 ; HMDB01555 ; HMDB01936 ; HMDB02434 ; HMDB15510 ; HMDB31180 ; HMDB31667 ; HMDB32037 ; HMDB33002 ; HMDB33115 ; HMDB33127 ; HMDB33662 ; HMDB40030 ; HMDB41802 ; HMDB42004 ; HMDB59734	Aflatoxin B1 dialcohol ; (R)-2-Feruloyl-1-(4-Hydroxyphenyl)-12-ethanediol ; 7-Hydroxy-3-(3-hydroxy-4-methoxybenzyl)-5-methoxy-4-chromanone ; Melilotocarpan C ; 4-Hydroxy-239-trimethoxypterocarpan ; 5-Hydroxy-3-(4-hydroxybenzyl)-78-dimethoxy-4-chromanone ; Cerasinone ; ()-34-Methylenedioxy-57-dimethylepicatechin ; Americanol ; Americanol A ; Isoamericanol A ; Pyrocatechol ; Pyridoxamine 5-phosphate ; Doxylamine ; Hydroquinone ; Flunitrazepam ; (EE)-24-Hexadienedial ; Nor-psi-tropine ; 13-Benzenediol ; 5-Methyl-2-furancarboxaldehyde ; 6-Methylquinoline ; 2-Acetylfuran ; (S)-5-Deoxy-5-(methylsulfinyl)adenosine ; 1-(1-Pyrrolidinyl)-2-propanone ; 2-Aminonaphthalene ; Quinaldine ; Pyrazin-2-carboxylic acid	M+2H ; M+2H ; M+2H ; M+2H ; M+2H ; M+2H ; M+2H ; M+2H ; M+2H ; M+2H ; M+2H ; 3M+2H ; M+2ACN+2H ; M+Na+K ; 3M+2H ; M+H+NH4 ; 3M+2H ; M+K ; 3M+2H ; 3M+2H ; M+Na ; 3M+2H ; M+H+NH4 ; M+K ; M+Na ; M+Na ; M+ACN+H	C18H18O6 ; C18H18O6 ; C18H18O6 ; C18H18O6 ; C18H18O6 ; C18H18O6 ; C18H18O6 ; C18H18O6 ; C18H18O6 ; C18H18O6 ; C18H18O6 ; C6H6O2 ; C8H13N2O5P ; C17H22N2O ; C6H6O2 ; C16H12FN3O3 ; C6H6O2 ; C7H13NO ; C6H6O2 ; C6H6O2 ; C10H9N ; C6H6O2 ; C11H15N5O4S ; C7H13NO ; C10H9N ; C10H9N ; C5H4N2O2
189.13465	1.446817	0.583273	-4.1637	0.001937	2.7128	0.019413	189.1347	HMDB00670 ; HMDB29416 ; HMDB31675	Homo-L-arginine ; L-Targinine ; 3beta-Acetoxy-11alpha-methoxy-12-ursen-28-oic acid	M+H ; M+H ; M+2H+Na	C7H16N4O2 ; C7H16N4O2 ; C34H54O5
488.87267	1.533245	1.192836	4.1617	0.001944	2.7114	0.019436	488.8727	HMDB15170	Tolterodine	3M+2H	C22H31NO
604.69537	1.533671	1.39138	-4.1584	0.001954	2.7092	0.019498	NA	NA	NA	NA	NA
319.12489	1.473965	0.582251	-4.1517	0.001975	2.7045	0.01963	319.1249	HMDB60950 ; HMDB00668 ; HMDB14882 ; HMDB15038 ; HMDB15363 ; HMDB32697 ; HMDB33090 ; HMDB38939 ; HMDB40515 ; HMDB40926 ; HMDB60354 ; HMDB60388	fluvoxamino acid ; Hematoporphyrin IX ; Zileuton ; Methdilazine ; Metoclopramide ; 1b-Furanoeudesm-4(15)-en-1-ol acetate ; 6-Dehydroshogaol ; Panaquinquecol 4 ; gamma-Glutamyl-beta-(isoxazolin-5-on-2-yl)alanine ; 2-Butyl-5-2-(4-hydroxy-3-methoxyphenyl)ethylfuran ; 2-Polyprenyl-6-methoxy-14-benzoquinone ; 4-Hydroxy-3-polyprenylbenzoate	M+H ; M+H+K ; M+2ACN+H ; M+Na ; 2M+H+K ; M+2Na-H ; M+2Na-H ; M+2Na-H ; M+NH4 ; M+2Na-H ; M+2Na-H ; M+2Na-H	C14H17F3N2O3 ; C34H38N4O6 ; C11H12N2O2S ; C18H20N2S ; C14H22ClN3O2 ; C17H22O3 ; C17H22O3 ; C17H22O3 ; C11H15N3O7 ; C17H22O3 ; C17H22O3 ; C17H22O3
449.26614	1.479669	0.130731	-4.151	0.001977	2.704	0.01963	449.2661	HMDB05764 ; HMDB10331 ; HMDB29559 ; HMDB37123 ; HMDB59616	Melanostatin ; Palmitoyl glucuronide ; (4-Hydroxybenzoyl)choline ; Goyaglycoside h ; all-trans-Decaprenyl diphosphate	3M+2Na ; 2M+Na+K ; 2M+H ; M+2ACN+2H ; M+H+K	C13H24N4O3 ; C22H42O7 ; C12H18NO3 ; C42H70O15 ; C50H84O7P2

516.21669	1.537531	1.383831	-4.1498	0.001981	2.7032	0.01963	516.2167	HMDB14729 ; HMDB15352 ; HMDB33251 ; HMDB33405 ; HMDB33946 ; HMDB34660 ; HMDB37158 ; HMDB38560 ; HMDB38561 ; HMDB38983 ; HMDB41352 ; HMDB41564	Fluocinolone Acetonide ; Ketamine ; Phenylmethyl benzeneacetate ; Quillaic acid 3-rhamnosyl-(1-3)-galactosyl-(1-2)-glucuronide ; 2-Phenylethyl benzoate ; Heterophyllin ; 3-(2-Methyl-3-furanyl)thio-4-heptanone ; 8-Butanoylneosolaniol ; 8-Isobutanoylneosolaniol ; 28-Glucosyl-3b-hydroxy-12-oleanene-30-methoxy-28-oic acid 3-arabinosyl-(1-3)-glucuronide ; Amaranthussaponin II ; p-Tolyl phenylacetate	M+ACN+Na ; 2M+ACN+H ; 2M+ACN+Na ; M+Na+K ; 2M+ACN+Na ; 2M+H+Na ; 2M+ACN+Na ; M+ACN+Na ; M+ACN+Na ; M+Na+K ; M+Na+K ; 2M+ACN+Na	C24H30F2O6 ; C13H16ClNO ; C15H14O2 ; C48H74O20 ; C15H14O2 ; C30H32O7 ; C12H18O2S ; C23H32O9 ; C23H32O9 ; C48H74O20 ; C48H74O20 ; C15H14O2
489.85730	1.501041	1.301296	-4.1492	0.001983	2.7028	0.01963	NA	NA	NA	NA	NA
543.01385	1.49224	1.393701	-4.1467	0.001991	2.701	0.019653	543.0139	HMDB30385 ; HMDB32032 ; HMDB47350 ; HMDB47352 ; HMDB47373 ; HMDB47421 ; HMDB52014 ; HMDB52016	248-Eicosatrienoic acid isobutylamide ; 2414-Eicosatrienoic acid isobutylamide ; TG(24:024:1(15Z)18:2(9Z12Z)) ; TG(24:024:1(15Z)20:2n6) ; TG(24:018:2(9Z12Z)24:1(15Z)) ; TG(24:020:2n624:1(15Z)) ; TG(24:1(15Z)24:018:2(9Z12Z)) ; TG(24:1(15Z)24:020:2n6)	3M+2H ; 3M+2H ; M+H+NH4 ; M+H+NH4 ; M+H+NH4 ; M+H+NH4 ; M+H+NH4 ; M+H+NH4	C24H43NO ; C24H43NO ; C70H130O6 ; C70H130O6 ; C70H130O6 ; C70H130O6 ; C70H130O6 ; C70H130O6
864.96093	1.488857	1.290163	-4.1445	0.001998	2.6995	0.019653	NA	NA	NA	NA	NA
318.87922	1.421868	0.485133	-4.1442	0.001999	2.6993	0.019653	318.8792	HMDB39460	Di-2-propenyl hexasulfide	M+2Na-H	C6H10S6
197.09436	1.515193	1.168539	4.1431	0.002002	2.6985	0.019653	197.0944	HMDB59631 ; HMDB00315 ; HMDB00363 ; HMDB00798 ; HMDB00847 ; HMDB03851 ; HMDB04026 ; HMDB11532 ; HMDB11563 ; HMDB11653 ; HMDB29442 ; HMDB30331 ; HMDB31008 ; HMDB31291 ; HMDB31340 ; HMDB31462 ; HMDB31480 ; HMDB31557 ; HMDB31622 ; HMDB31693 ; HMDB32042 ; HMDB32309 ; HMDB32406 ; HMDB32454 ; HMDB33918 ; HMDB34162 ; HMDB34165 ; HMDB34606 ; HMDB36234 ; HMDB37143 ; HMDB37164 ; HMDB37281 ; HMDB37288 ; HMDB37555 ; HMDB38040 ; HMDB38603 ; HMDB39153 ; HMDB39216 ; HMDB39219 ; HMDB40221 ; HMDB40295 ; HMDB40430 ; HMDB40823 ; HMDB59633 ; HMDB59822 ; HMDB60407 ; HMDB60424 ; HMDB61034	Cis-stilbene oxide ; 16-a-Hydroxypregnenolone ; 17a-Hydroxypregnenolone ; Oenanthic ether ; Pelargonic acid ; 3a7a-Dihydroxy-5b-cholestanate ; 21-Hydroxypregnenolone ; MG(0:015:00:0) ; MG(15:00:00:0) ; 17-alpha20-alpha-Dihydroxypregn-4-en-3-one ; (R)C(R)S-S-Propylcysteine sulfoxide ; xi-2-Ethylheptanoic acid ; ()-1516-Dihydroxyoctadecanoic acid ; Methyl caprylate ; Cyclamic acid ; 10-Shogaol ; Heptyl acetate ; 4-Methyloctanoic acid ; 6-Phenyl-3-hexen-2-one ; Ethyl (S)-3-hydroxybutyrate glucoside ; Butyl 3-methylbutanoate ; 3-Heptyl acetate ; 5-Methylhexyl acetate ; 3-Octyl formate ; (15alpha20R)-1520-Dihydroxypregn-4-en-3-one ; Pentyl butanoate ; Propyl hexanoate ; Hexyl propionate ; 3-Methylbutyl 2-methylpropanoate ; 1-Hydroxy-3-nonanone ; 3-(Hydroxymethyl)-2-octanone ; 4-Methyl-2-phenyl-2-pentenal ; 4-Methyl-2-pentyl-13-dioxolane ; Sandoricin ; 2-Methylpropyl 3-methylbutanoate ; Octyl formate ; (R)-111213-Trinor-1(5)69-guaiatrien-8-one ; Butyl 2-methylbutanoate ; 1-Methylbutyl butanoate ; Isoamyl butyrate ; Butyl pentanoate ; Isopropyl hexanoate ; (S)-111213-Trinor-7-calamenone ; (9S10S)-910-dihydroxyoctadecanoate ; Ethyl 5-methylhexanoate ; 5alpha-Dihydrodeoxycorticosterone ; 7alpha-Hydroxypregnenolone ; Metabolite M6	M+H ; M+Na+K ; M+Na+K ; M+K ; M+K ; M+Na+K ; M+Na+K ; M+2K ; M+2K ; M+Na+K ; M+NH4 ; M+K ; M+2K ; M+K ; M+NH4 ; M+Na+K ; M+K ; M+K ; M+Na ; 2M+3H ; M+K ; M+K ; M+K ; M+K ; M+Na+K ; M+K ; M+K ; M+K ; M+K ; M+K ; M+K ; M+Na ; M+K ; M+3H ; M+K ; M+K ; M+Na ; M+K ; M+K ; M+K ; M+K ; M+K ; M+Na ; M+2K ; M+K ; M+Na+K ; M+Na+K ; M+3Na	C14H12O ; C21H32O3 ; C21H32O3 ; C9H18O2 ; C9H18O2 ; C21H32O3 ; C21H32O3 ; C18H36O4 ; C18H36O4 ; C21H32O3 ; C6H13NO3S ; C9H18O2 ; C18H36O4 ; C9H18O2 ; C6H13NO3S ; C21H32O3 ; C9H18O2 ; C9H18O2 ; C12H14O ; C12H22O8 ; C9H18O2 ; C9H18O2 ; C9H18O2 ; C9H18O2 ; C21H32O3 ; C9H18O2 ; C9H18O2 ; C9H18O2 ; C9H18O2 ; C9H18O2 ; C9H18O2 ; C12H14O ; C9H18O2 ; C31H40O11 ; C9H18O2 ; C9H18O2 ; C12H14O ; C9H18O2 ; C9H18O2 ; C9H18O2 ; C9H18O2 ; C9H18O2 ; C12H14O ; C18H36O4 ; C9H18O2 ; C21H32O3 ; C21H32O3 ; C30H42N4O4
490.08462	1.50201	0.831961	4.1414	0.002008	2.6973	0.019653	490.0846	HMDB38043 ; HMDB39941 ; HMDB40476 ; HMDB41456	Isovitexin 2-O-4-hydroxy-(E)-cinnamoyl-(-6)-beta-D-glucopyranosyl 4-O-beta-D-glucopyranoside ; Kaempferol 3-2-(6-coumaroylglucosyl)-rhamnoside 7-glucoside ; Kaempferol 3-(6-4-glucosyl-p-coumarylglucosyl)(1-2)-rhamnoside ; Kaempferol 3-neohesperidoside-7-(2-p-coumarylglucoside)	M+2K ; M+2K ; M+2K ; M+2K	C42H46O22 ; C42H46O22 ; C42H46O22 ; C42H46O22
153.06003	1.490541	0.951561	-4.14	0.002012	2.6964	0.019653	153.06	HMDB38665 ; HMDB00228 ; HMDB00416 ; HMDB00569 ; HMDB02327 ; HMDB03747 ; HMDB14445 ; HMDB15497 ; HMDB15575 ; HMDB29073 ; HMDB29115 ; HMDB29818 ; HMDB30104 ; HMDB30137 ; HMDB30373 ; HMDB31658 ; HMDB31844 ; HMDB31862 ; HMDB31902 ; HMDB32217 ; HMDB32356 ; HMDB33229 ; HMDB33660 ; HMDB34118 ; HMDB34257 ; HMDB34270 ; HMDB34696 ; HMDB35210 ; HMDB35644 ; HMDB35647 ; HMDB35740 ; HMDB36038 ; HMDB36039 ; HMDB36141 ; HMDB36151 ; HMDB36152 ; HMDB36456 ; HMDB37005 ; HMDB37059 ; HMDB37070 ; HMDB37140 ; HMDB37156 ; HMDB37184 ; HMDB37278 ; HMDB37667 ; HMDB38148 ; HMDB38436 ; HMDB38476 ; HMDB39499 ; HMDB39516 ; HMDB40068 ; HMDB40069 ; HMDB40070 ; HMDB40097 ; HMDB40100 ; HMDB40101 ;	L-N-(1H-Indol-3-ylacetyl)glutamic acid ; Phenol ; 17-Hydroxypregnenolone sulfate ; Deoxypyridinoline ; 111-Undecanedicarboxylic acid ; Resveratrol ; Tenofovir ; Oxybenzone ; Trioxsalen ; Threoninyl-Tyrosine ; Tyrosyl-Threonine ; Benzyl salicylate ; Humulinic acid A ; Adhumulinic acid ; Citrusinine II ; L-2-Amino-5-hydroxypentanoic acid ; Trimethylpyrazine ; 2-(2-Methylpropyl)thiazole ; Blennin D ; 24-Difurfurylfuran ; N-Lactoyl ethanolamine ; Absindiol ; (R)-Athanagrandione ; (Z)-Resveratrol ; Seselin ; 56-Dihydro-5-hydroxy-6-methyl-2H-pyran-2-one ; Artemin ; 10-Hydroxymyoporone ; (6beta8betaOH)-68-Dihydroxy-7(11)-eremophilen-128-olide ; 314-Dihydroxy-1113-dihydrocostunolide ; Arlatin ; 1alpha15-Dihydroxymarasmene ; 3alpha15-Dihydroxymarasmene ; Menthyl ethylene glycol carbonate ; Arabsin ; Alkhanol ; 10-Hydroxy-3-methoxy-1357-cadinatetraen-9-one ; (1alpha4alpha5beta6alpha11betaH)-14-Epoxy-5-hydroxy-10(14)-germacren-126-olide ; 1alpha-Hydroxyarbusculin A ; Sporol ; 2-(1-Methylpropyl)thiazole ; 4-Methyl-2-(1-methylethyl)thiazole ; ()-14-Nonanediol diacetate ; Isoeugenol phenylacetate ; o-Tolyl salicylate ; Ipomeamaronol ; 6-Isothiocyanato-1-hexene ; 7-Hydroxytrichodermol ; 14-Dideoxy-14-imino-D-ribitol ; 4-Dihydroabscisic acid ; 2-Ethyl-45-dimethylthiazole ; 4-Ethyl-25-dimethylthiazole ; 5-Ethyl-24-dimethylthiazole ; 25-Diethylthiazole ; 2-Methyl-4-(1-methylethyl)thiazole ; 24-Diethylthiazole ; Grandisine III ; Ipomeamaronolide ; Cycloartocarpin ; 2-Ethyl-3-methylpyrazine 9CI ; Isoleptospermone ; Isopentyl gentiobioside ; 2-Ethyl-5-methylpyrazine 9CI 8CI ; 2-Ethyl-6-methylpyrazine ; Isopropylpyrazine ; Propylpyrazine ; 24-Toluenediamine ; 26-Toluenediamine ; Isothiocyanatocyclohexane ; Deoxyartemsinin ; Hydroxy-lacosamide	M+2H ; 3M+H+Na ; M+H+2Na ; M+H+2Na ; M+Na+K ; 2M+3H ; M+H+NH4 ; 2M+3H ; 2M+3H ; M+H+Na ; M+H+Na ; 2M+3H ; M+H+K ; M+H+K ; M+H+NH4 ; 2M+H+K ; 2M+Na+K ; 2M+H+Na ; M+H+K ; 2M+3H ; 2M+H+K ; M+H+K ; M+H+K ; 2M+3H ; 2M+3H ; 2M+3H ; M+H+K ; M+H+K ; M+H+K ; M+H+K ; M+H+K ; M+H+K ; M+H+K ; M+Na+K ; M+H+K ; M+H+K ; M+2Na ; M+H+K ; M+H+K ; M+H+K ; 2M+H+Na ; 2M+H+Na ; M+Na+K ; M+H+Na ; 2M+3H ; M+H+K ; 2M+H+Na ; M+H+K ; 2M+H+K ; M+H+K ; 2M+H+Na ; 2M+H+Na ; 2M+H+Na ; 2M+H+Na ; 2M+H+Na ; 2M+H+Na ; M+H+NH4 ; M+H+K ; M+2H+Na ; 2M+Na+K ; M+H+K ; M+H+2Na ; 2M+Na+K ; 2M+Na+K ; 2M+Na+K ; 2M+Na+K ; 2M+Na+K ; 2M+Na+K ; 2M+H+Na ; M+H+K ; M+H+Na	C15H16N2O5 ; C6H6O ; C21H32O6S ; C18H28N4O7 ; C13H24O4 ; C14H12O3 ; C9H14N5O4P ; C14H12O3 ; C14H12O3 ; C13H18N2O5 ; C13H18N2O5 ; C14H12O3 ; C15H22O4 ; C15H22O4 ; C15H13NO5 ; C5H11NO3 ; C7H10N2 ; C7H11NS ; C15H22O4 ; C14H12O3 ; C5H11NO3 ; C15H22O4 ; C15H22O4 ; C14H12O3 ; C14H12O3 ; C14H12O3 ; C15H22O4 ; C15H22O4 ; C15H22O4 ; C15H22O4 ; C15H22O4 ; C15H22O4 ; C15H22O4 ; C13H24O4 ; C15H22O4 ; C15H22O4 ; C16H20O3 ; C15H22O4 ; C15H22O4 ; C15H22O4 ; C7H11NS ; C7H11NS ; C13H24O4 ; C18H18O3 ; C14H12O3 ; C15H22O4 ; C7H11NS ; C15H22O4 ; C5H11NO3 ; C15H22O4 ; C7H11NS ; C7H11NS ; C7H11NS ; C7H11NS ; C7H11NS ; C7H11NS ; C15H13NO5 ; C15H22O4 ; C26H26O6 ; C7H10N2 ; C15H22O4 ; C17H32O11 ; C7H10N2 ; C7H10N2 ; C7H10N2 ; C7H10N2 ; C7H10N2 ; C7H10N2 ; C7H11NS ; C15H22O4 ; C13H18N2O5

416.80120	1.51504	1.468786	4.1398	0.002012	2.6963	0.019653	416.8012	HMDB06895 ; HMDB09413 ; HMDB09414 ; HMDB09446 ; HMDB09447 ; HMDB09478 ; HMDB09642 ; HMDB09675 ; HMDB11359 ; HMDB11360 ; HMDB11387 ; HMDB11418 ; HMDB11419 ; HMDB11451 ; HMDB11452	3a7a-Dihydroxy-5b-cholest-24-enoyl-CoA ; PE(20:4(5Z8Z11Z14Z)P-18:1(11Z)) ; PE(20:4(5Z8Z11Z14Z)P-18:1(9Z)) ; PE(20:4(8Z11Z14Z17Z)P-18:1(11Z)) ; PE(20:4(8Z11Z14Z17Z)P-18:1(9Z)) ; PE(20:5(5Z8Z11Z14Z17Z)P-18:0) ; PE(22:5(4Z7Z10Z13Z16Z)P-16:0) ; PE(22:5(7Z10Z13Z16Z19Z)P-16:0) ; PE(P-16:022:5(4Z7Z10Z13Z16Z)) ; PE(P-16:022:5(7Z10Z13Z16Z19Z)) ; PE(P-18:020:5(5Z8Z11Z14Z17Z)) ; PE(P-18:1(11Z)20:4(5Z8Z11Z14Z)) ; PE(P-18:1(11Z)20:4(8Z11Z14Z17Z)) ; PE(P-18:1(9Z)20:4(5Z8Z11Z14Z)) ; PE(P-18:1(9Z)20:4(8Z11Z14Z17Z))	M+3Na ; M+2ACN+2H ; M+2ACN+2H ; M+2ACN+2H ; M+2ACN+2H ; M+2ACN+2H ; M+2ACN+2H ; M+2ACN+2H ; M+2ACN+2H ; M+2ACN+2H ; M+2ACN+2H ; M+2ACN+2H ; M+2ACN+2H ; M+2ACN+2H ; M+2ACN+2H	C48H78N7O19P3S ; C43H76NO7P ; C43H76NO7P ; C43H76NO7P ; C43H76NO7P ; C43H76NO7P ; C43H76NO7P ; C43H76NO7P ; C43H76NO7P ; C43H76NO7P ; C43H76NO7P ; C43H76NO7P ; C43H76NO7P ; C43H76NO7P ; C43H76NO7P
709.79095	1.563631	1.092902	-4.1365	0.002023	2.694	0.019691	709.791	HMDB01957	Pentacarboxyl porphyrinogen III	2M+H+NH4	C37H40N4O10
516.94616	1.503537	1.196686	4.1361	0.002025	2.6937	0.019691	516.9462	HMDB44023 ; HMDB44241 ; HMDB44246 ; HMDB44457 ; HMDB44597 ; HMDB44617 ; HMDB44828 ; HMDB45038 ; HMDB45043 ; HMDB45069 ; HMDB45226 ; HMDB45252 ; HMDB45272 ; HMDB45381 ; HMDB45407 ; HMDB45426 ; HMDB45605 ; HMDB45781 ; HMDB45786 ; HMDB45837 ; HMDB45987 ; HMDB46012 ; HMDB46018 ; HMDB46021 ; HMDB46040 ; HMDB46056 ; HMDB46143 ; HMDB46162 ; HMDB46186 ; HMDB46205 ; HMDB46221 ; HMDB46354 ; HMDB46498 ; HMDB46503 ; HMDB46523 ; HMDB46528 ; HMDB46552 ; HMDB46563 ; HMDB46569 ; HMDB46573 ; HMDB46578 ; HMDB46713 ; HMDB46719 ; HMDB46723 ; HMDB46728 ; HMDB46747 ; HMDB46775 ; HMDB46795 ; HMDB46801 ; HMDB46810 ; HMDB46811 ; HMDB46813 ; HMDB46819 ; HMDB46871 ; HMDB46897 ; HMDB46912 ;	TG(16:024:022:6(4Z7Z10Z13Z16Z19Z)) ; TG(16:024:1(15Z)22:5(4Z7Z10Z13Z16Z)) ; TG(16:024:1(15Z)22:5(7Z10Z13Z16Z19Z)) ; TG(16:022:5(4Z7Z10Z13Z16Z)24:1(15Z)) ; TG(16:022:5(7Z10Z13Z16Z19Z)24:1(15Z)) ; TG(16:022:6(4Z7Z10Z13Z16Z19Z)24:0) ; TG(18:022:022:6(4Z7Z10Z13Z16Z19Z)) ; TG(18:022:1(13Z)22:5(4Z7Z10Z13Z16Z)) ; TG(18:022:1(13Z)22:5(7Z10Z13Z16Z19Z)) ; TG(18:024:1(15Z)20:5(5Z8Z11Z14Z17Z)) ; TG(18:022:2(13Z16Z)22:4(7Z10Z13Z16Z)) ; TG(18:022:4(7Z10Z13Z16Z)22:2(13Z16Z)) ; TG(18:022:5(4Z7Z10Z13Z16Z)22:1(13Z)) ; TG(18:020:5(5Z8Z11Z14Z17Z)24:1(15Z)) ; TG(18:022:5(7Z10Z13Z16Z19Z)22:1(13Z)) ; TG(18:022:6(4Z7Z10Z13Z16Z19Z)22:0) ; TG(20:020:022:6(4Z7Z10Z13Z16Z19Z)) ; TG(20:020:1(11Z)22:5(4Z7Z10Z13Z16Z)) ; TG(20:020:1(11Z)22:5(7Z10Z13Z16Z19Z)) ; TG(20:022:1(13Z)20:5(5Z8Z11Z14Z17Z)) ; TG(20:020:4(5Z8Z11Z14Z)22:2(13Z16Z)) ; TG(20:022:2(13Z16Z)20:4(5Z8Z11Z14Z)) ; TG(20:022:2(13Z16Z)20:4(8Z11Z14Z17Z)) ; TG(20:022:2(13Z16Z)22:6(4Z7Z10Z13Z16Z19Z)) ; TG(20:022:4(7Z10Z13Z16Z)22:4(7Z10Z13Z16Z)) ; TG(20:022:5(4Z7Z10Z13Z16Z)20:1(11Z)) ; TG(20:020:4(8Z11Z14Z17Z)22:2(13Z16Z)) ; TG(20:020:5(5Z8Z11Z14Z17Z)22:1(13Z)) ; TG(20:022:5(7Z10Z13Z16Z19Z)20:1(11Z)) ; TG(20:022:6(4Z7Z10Z13Z16Z19Z)20:0) ; TG(20:022:6(4Z7Z10Z13Z16Z19Z)22:2(13Z16Z)) ; TG(22:018:022:6(4Z7Z10Z13Z16Z19Z)) ; TG(22:018:1(11Z)22:5(4Z7Z10Z13Z16Z)) ; TG(22:018:1(11Z)22:5(7Z10Z13Z16Z19Z)) ; TG(22:018:1(9Z)22:5(4Z7Z10Z13Z16Z)) ; TG(22:018:1(9Z)22:5(7Z10Z13Z16Z19Z)) ; TG(22:020:1(11Z)20:5(5Z8Z11Z14Z17Z)) ; TG(22:020:3(5Z8Z11Z)20:3(5Z8Z11Z)) ; TG(22:020:3(5Z8Z11Z)20:3n6) ; TG(22:020:3(5Z8Z11Z)22:5(4Z7Z10Z13Z16Z)) ; TG(22:020:3(5Z8Z11Z)22:5(7Z10Z13Z16Z19Z)) ; TG(22:020:3n620:3(5Z8Z11Z)) ; TG(22:020:3n620:3n6) ; TG(22:020:3n622:5(4Z7Z10Z13Z16Z)) ; TG(22:020:3n622:5(7Z10Z13Z16Z19Z)) ; TG(22:020:4(5Z8Z11Z14Z)22:4(7Z10Z13Z16Z)) ; TG(22:022:2(13Z16Z)18:4(6Z9Z12Z15Z)) ; TG(22:022:4(7Z10Z13Z16Z)20:4(5Z8Z11Z14Z)) ; TG(22:022:4(7Z10Z13Z16Z)20:4(8Z11Z14Z17Z)) ; TG(22:022:5(4Z7Z10Z13Z16Z)18:1(11Z)) ; TG(22:022:5(4Z7Z10Z13Z16Z)18:1(9Z)) ; TG(22:022:5(4Z7Z10Z13Z16Z)20:3(5Z8Z11Z)) ; TG(22:022:5(4Z7Z10Z13Z16Z)20:3n6) ; TG(22:018:4(6Z9Z12Z15Z)22:2(13Z16Z)) ; TG(22:020:4(8Z11Z14Z17Z)22:4(7Z10Z13Z16Z)) ; TG(22:020:5(5Z8Z11Z14Z17Z)20:1(11Z)) ;	M+ACN+2H ; M+H+NH4 ; M+H+NH4 ; M+ACN+2H ; M+ACN+2H ; M+ACN+2H ; M+ACN+2H ; M+ACN+2H ; M+H+NH4 ; M+ACN+2H ; M+ACN+2H ; M+ACN+2H ; M+ACN+2H ; M+ACN+2H ; M+ACN+2H ; M+ACN+2H ; M+ACN+2H ; M+H+NH4 ; M+H+NH4 ; M+ACN+2H ; M+ACN+2H ; M+H+NH4 ; M+H+NH4 ; M+H+NH4 ; M+ACN+2H ; M+H+NH4 ; M+H+NH4 ; M+ACN+2H ; M+ACN+2H ; M+H+NH4 ; M+H+NH4 ; M+ACN+2H ; M+H+NH4 ; M+ACN+2H ; M+ACN+2H ; M+ACN+2H ; M+ACN+2H ; M+H+NH4 ; M+H+NH4 ; M+ACN+2H ; M+ACN+2H ; M+ACN+2H ; M+ACN+2H ; M+ACN+2H ; M+ACN+2H ; M+ACN+2H ; M+H+NH4 ; M+ACN+2H ; M+ACN+2H ; M+H+NH4 ; M+H+NH4 ; M+ACN+2H ; M+ACN+2H ; M+H+NH4 ; M+H+NH4 ; M+H+NH4 ; M+H+NH4 ; M+ACN+2H ; M+H+NH4 ; M+H+NH4 ; M+ACN+2H ; M+ACN+2H ; M+H+NH4 ;	C65H114O6 ; C67H114O6 ; C67H114O6 ; C65H114O6 ; C65H114O6 ; C65H114O6 ; C65H114O6 ; C65H114O6 ; C67H114O6 ; C65H114O6 ; C65H114O6 ; C65H114O6 ; C65H114O6 ; C65H114O6 ; C65H114O6 ; C65H114O6 ; C65H114O6 ; C65H114O6 ; C67H114O6 ; C67H114O6 ; C65H114O6 ; C65H114O6 ; C67H114O6 ; C67H114O6 ; C67H114O6 ; C65H114O6 ; C67H114O6 ; C67H114O6 ; C65H114O6 ; C65H114O6 ; C67H114O6 ; C67H114O6 ; C65H114O6 ; C67H114O6 ; C65H114O6 ; C65H114O6 ; C65H114O6 ; C67H114O6 ; C67H114O6 ; C65H114O6 ; C65H114O6 ; C65H114O6 ; C65H114O6 ; C65H114O6 ; C65H114O6 ; C67H114O6 ; C65H114O6 ; C65H114O6 ; C67H114O6 ; C67H114O6 ; C65H114O6 ; C65H114O6 ; C67H114O6 ; C67H114O6 ; C67H114O6 ; C67H114O6 ; C65H114O6 ; C67H114O6 ; C67H114O6 ; C65H114O6 ; C65H114O6 ; C67H114O6 ; C67H114O6 ; C67H114O6 ; C67H114O6 ; C67H114O6 ; C65H114O6 ; C65H114O6 ; C67H114O6 ; C67H114O6 ; C65H114O6 ; C67H114O6 ; C67H114O6 ; C65H114O6 ; C65H114O6 ; C65H114O6 ;
474.67051	1.52055	1.407271	4.1342	0.002031	2.6924	0.019691	474.6705	HMDB13240	Indoleacetyl glutamine	3M+H+K	C15H17N3O4
373.02034	1.527201	0.028756	4.1337	0.002032	2.692	0.019691	373.0203	HMDB15034 ; HMDB36168	Triazolam ; Bis(2-methyl-3-furanyl)tetrasulfide	2M+Na+K ; M+2ACN+H	C17H12Cl2N4 ; C10H10O2S4
207.03673	1.448834	0.939875	-4.1294	0.002046	2.6891	0.019745	207.0367	HMDB01933 ; HMDB14650 ; HMDB14675 ; HMDB15526 ; HMDB28850 ; HMDB29039 ; HMDB29235 ; HMDB29424 ; HMDB29546 ; HMDB30611 ; HMDB30660 ; HMDB30870 ; HMDB30872 ; HMDB33254 ; HMDB33276 ; HMDB33306 ; HMDB33770 ; HMDB33841 ; HMDB35446 ; HMDB40506 ; HMDB41519 ; HMDB41667	Furosemide ; Triflupromazine ; Cefdinir ; Ketazolam ; Glycyl-Serine ; Serinyl-Glycine ; Mulberrofuran P ; L-4-Hydroxyglutamine ; Pebrellin ; 58-Dihydroxy-3347-tetramethoxyflavone ; Casticin ; 1-Hydroxy-3-methoxy-7-primeverosyloxyxanthone ; Gentioside ; Comosin ; 45-Dihydroxy-3578-tetramethoxyflavone ; Hymenoxin ; 7-Hydroxy-3-methoxy-1-primeverosyloxyxanthone ; Menadiol disuccinate ; Methyl rosmarinate ; 356-Trihydroxy-3478-tetramethoxyflavone 3-glucoside ; Nb-Feruloyltryptamine ; 3-O-Methylrosmarinic acid	M+2ACN+2H ; M+Na+K ; M+H+NH4 ; M+2Na ; M+2Na-H ; M+2Na-H ; M+H+2Na ; M+2Na-H ; M+H+K ; M+H+K ; M+H+K ; M+3Na ; M+3Na ; M+H+K ; M+H+K ; M+H+K ; M+3Na ; M+H+K ; M+H+K ; M+3Na ; M+2K ; M+H+K	C12H11ClN2O5S ; C18H19F3N2S ; C14H13N5O5S2 ; C20H17ClN2O3 ; C5H10N2O4 ; C5H10N2O4 ; C34H22O9 ; C5H10N2O4 ; C19H18O8 ; C19H18O8 ; C19H18O8 ; C25H28O14 ; C25H28O14 ; C19H18O8 ; C19H18O8 ; C19H18O8 ; C25H28O14 ; C19H18O8 ; C19H18O8 ; C25H28O14 ; C20H20N2O3 ; C19H18O8
304.04477	1.549575	0.035581	-4.1286	0.002049	2.6885	0.019745	304.0448	HMDB01157 ; HMDB01316 ; HMDB06467 ; HMDB40924 ; HMDB41953 ; HMDB60140	2-Methylacetoacetyl-CoA ; 6-Phosphogluconic acid ; 4-hydroxybenzoyl-CoA ; 2-Carboxyarabinitol 5-phosphate ; Nnal-N-oxide ; 4-Hydroxybenoyl-CoA	M+H+2Na ; 2M+3H2O+2H ; M+2H+Na ; 2M+3H2O+2H ; M+2K+H ; M+2H+Na	C26H42N7O18P3S ; C6H13O10P ; C28H40N7O18P3S ; C6H13O10P ; C10H15N3O3 ; C28H40N7O18P3S
594.94093	1.500566	0.457772	-4.127	0.002054	2.6874	0.019745	594.9409	HMDB00328 ; HMDB00460 ; HMDB00467 ; HMDB00503 ; HMDB12866 ; HMDB41454	12-Ketodeoxycholic acid ; 7-Hydroxy-3-oxocholanoic acid ; Nutriacholic acid ; 7a-Hydroxy-3-oxo-5b-cholanoic acid ; 9-Carboxy-alpha-chromanol ; D8-Merulinic acid A	3M+H+NH4 ; 3M+H+NH4 ; 3M+H+NH4 ; 3M+H+NH4 ; 3M+H+NH4 ; 3M+H+NH4	C24H38O4 ; C24H38O4 ; C24H38O4 ; C24H38O4 ; C24H38O4 ; C24H38O4

526.68409	1.514876	1.461894	4.1263	0.002056	2.6869	0.019745	526.6841	HMDB15294 ; HMDB37520 ; HMDB37547 ; HMDB56966 ; HMDB56967 ; HMDB57020 ; HMDB57028 ; HMDB57030 ; HMDB57038 ; HMDB57046 ; HMDB57047 ; HMDB57134 ; HMDB57135 ; HMDB57139 ; HMDB57140 ; HMDB57186 ; HMDB57187 ; HMDB57191 ; HMDB57192 ; HMDB57267 ; HMDB57268 ; HMDB57271 ; HMDB57272 ; HMDB57278 ; HMDB57286 ; HMDB57287 ; HMDB57291 ; HMDB57292 ; HMDB57299 ; HMDB57300 ; HMDB57304 ; HMDB57305 ; HMDB57307 ; HMDB57314 ; HMDB57315 ; HMDB57317 ; HMDB57321 ; HMDB57326 ; HMDB57334 ; HMDB57335 ; HMDB57339 ; HMDB57340 ; HMDB57347 ; HMDB57348 ; HMDB57352 ; HMDB57353 ; HMDB57355 ; HMDB57362 ; HMDB57363 ; HMDB57365 ; HMDB57369 ; HMDB57391 ; HMDB57395 ; HMDB57920 ; HMDB57921 ; HMDB57925 ;	Amdinocillin ; Tetrabromodiphenyl ethers ; 11-Oxybis24-dibromobenzene ; CL(18:018:018:022:5(4Z7Z10Z13Z16Z)) ; CL(18:018:018:022:5(7Z10Z13Z16Z19Z)) ; CL(18:018:022:5(4Z7Z10Z13Z16Z)18:0) ; CL(18:018:022:5(4Z7Z10Z13Z16Z)22:6(4Z7Z10Z13Z16Z19Z)) ; CL(18:018:022:5(7Z10Z13Z16Z19Z)18:0) ; CL(18:018:022:5(7Z10Z13Z16Z19Z)22:6(4Z7Z10Z13Z16Z19Z)) ; CL(18:018:022:6(4Z7Z10Z13Z16Z19Z)22:5(4Z7Z10Z13Z16Z)) ; CL(18:018:022:6(4Z7Z10Z13Z16Z19Z)22:5(7Z10Z13Z16Z19Z)) ; CL(18:018:1(11Z)22:5(4Z7Z10Z13Z16Z)22:5(4Z7Z10Z13Z16Z)) ; CL(18:018:1(11Z)22:5(4Z7Z10Z13Z16Z)22:5(7Z10Z13Z16Z19Z)) ; CL(18:018:1(11Z)22:5(7Z10Z13Z16Z19Z)22:5(4Z7Z10Z13Z16Z)) ; CL(18:018:1(11Z)22:5(7Z10Z13Z16Z19Z)22:5(7Z10Z13Z16Z19Z)) ; CL(18:018:1(9Z)22:5(4Z7Z10Z13Z16Z)22:5(4Z7Z10Z13Z16Z)) ; CL(18:018:1(9Z)22:5(4Z7Z10Z13Z16Z)22:5(7Z10Z13Z16Z19Z)) ; CL(18:018:1(9Z)22:5(7Z10Z13Z16Z19Z)22:5(4Z7Z10Z13Z16Z)) ; CL(18:018:1(9Z)22:5(7Z10Z13Z16Z19Z)22:5(7Z10Z13Z16Z19Z)) ; CL(18:020:4(5Z8Z11Z14Z)22:5(4Z7Z10Z13Z16Z)22:5(4Z7Z10Z13Z16Z)) ; CL(18:020:4(5Z8Z11Z14Z)22:5(4Z7Z10Z13Z16Z)22:5(7Z10Z13Z16Z19Z)) ; CL(18:020:4(5Z8Z11Z14Z)22:5(7Z10Z13Z16Z19Z)22:5(4Z7Z10Z13Z16Z)) ; CL(18:020:4(5Z8Z11Z14Z)22:5(7Z10Z13Z16Z19Z)22:5(7Z10Z13Z16Z19Z)) ; CL(18:022:5(4Z7Z10Z13Z16Z)18:022:6(4Z7Z10Z13Z16Z19Z)) ; CL(18:022:5(4Z7Z10Z13Z16Z)18:1(11Z)22:5(4Z7Z10Z13Z16Z)) ; CL(18:022:5(4Z7Z10Z13Z16Z)18:1(11Z)22:5(7Z10Z13Z16Z19Z)) ; CL(18:022:5(4Z7Z10Z13Z16Z)18:1(9Z)22:5(4Z7Z10Z13Z16Z)) ; CL(18:022:5(4Z7Z10Z13Z16Z)18:1(9Z)22:5(7Z10Z13Z16Z19Z)) ; CL(18:022:5(4Z7Z10Z13Z16Z)20:4(5Z8Z11Z14Z)22:5(4Z7Z10Z13Z16Z)) ; CL(18:022:5(4Z7Z10Z13Z16Z)20:4(5Z8Z11Z14Z)22:5(7Z10Z13Z16Z19Z)) ; CL(18:022:5(4Z7Z10Z13Z16Z)22:5(4Z7Z10Z13Z16Z)18:1(11Z)) ;	3M+2K ; M+2Na-H ; M+2Na-H ; M+3Na ; M+3Na ; M+3Na ; M+2H+Na ; M+3Na ; M+2H+Na ; M+2H+Na ; M+2H+Na ; M+2H+Na ; M+2H+Na ; M+2H+Na ; M+2H+Na ; M+2H+Na ; M+2H+Na ; M+2H+Na ; M+2H+Na ; M+3H ; M+3H ; M+3H ; M+3H ; M+2H+Na ; M+2H+Na ; M+2H+Na ; M+2H+Na ; M+2H+Na ; M+3H ; M+3H ; M+2H+Na ; M+2H+Na ; M+3H ; M+2H+Na ; M+2H+Na ; M+3H ; M+2H+Na ; M+2H+Na ; M+2H+Na ; M+2H+Na ; M+2H+Na ; M+2H+Na ; M+3H ; M+3H ; M+2H+Na ; M+2H+Na ; M+3H ; M+2H+Na ; M+2H+Na ; M+3H ; M+2H+Na ; M+2H+Na ; M+2H+Na ; M+2H+Na ; M+2H+Na ; M+2H+Na ; M+2H+Na ; M+2H+Na ; M+2H+Na ; M+2H+Na ; M+2H+Na ; M+2H+Na ; M+2H+Na ; M+2H+Na ; M+2H+Na ; M+2H+Na ; M+2H+Na ; M+3H ; M+3H ; M+3H ; M+3H ; M+3H ; M+3H ; M+3H ; M+3H ; M+3H ; M+3H ; M+3H ; M+3H ; M+3H ; M+3H ; M+3H ; M+3H ; M+3H ; M+3H ; M+3H ; M+2H+Na ; M+2H+Na ; M+3H ; M+2H+Na ; M+2H+Na ; M+3H ; M+2H+Na ; M+2H+Na ; M+2H+Na ; M+3H ; M+3H ; M+3H ; M+2H+Na ; M+2H+Na ; M+3H ; M+2H+Na ; M+3H	C15H23N3O3S ; C12H6Br4O ; C12H6Br4O ; C85H156O17P2 ; C85H156O17P2 ; C85H156O17P2 ; C89H152O17P2 ; C85H156O17P2 ; C89H152O17P2 ; C89H152O17P2 ; C89H152O17P2 ; C89H152O17P2 ; C89H152O17P2 ; C89H152O17P2 ; C89H152O17P2 ; C89H152O17P2 ; C89H152O17P2 ; C89H152O17P2 ; C89H152O17P2 ; C91H150O17P2 ; C91H150O17P2 ; C91H150O17P2 ; C91H150O17P2 ; C89H152O17P2 ; C89H152O17P2 ; C89H152O17P2 ; C89H152O17P2 ; C89H152O17P2 ; C91H150O17P2 ; C91H150O17P2 ; C89H152O17P2 ; C89H152O17P2 ; C91H150O17P2 ; C89H152O17P2 ; C89H152O17P2 ; C91H150O17P2 ; C89H152O17P2 ; C89H152O17P2 ; C89H152O17P2 ; C89H152O17P2 ; C89H152O17P2 ; C89H152O17P2 ; C91H150O17P2 ; C91H150O17P2 ; C89H152O17P2 ; C89H152O17P2 ; C91H150O17P2 ; C89H152O17P2 ; C89H152O17P2 ; C91H150O17P2 ; C89H152O17P2 ; C89H152O17P2 ; C89H152O17P2 ; C89H152O17P2 ; C89H152O17P2 ; C89H152O17P2 ; C89H152O17P2 ; C89H152O17P2 ; C89H152O17P2 ; C89H152O17P2 ; C89H152O17P2 ; C89H152O17P2 ; C89H152O17P2 ; C89H152O17P2 ; C89H152O17P2 ; C89H152O17P2 ; C89H152O17P2 ; C91H150O17P2 ; C91H150O17P2 ; C91H150O17P2 ; C91H150O17P2 ; C91H150O17P2 ; C91H150O17P2 ; C91H150O17P2 ; C91H150O17P2 ; C91H150O17P2 ; C91H150O17P2 ; C91H150O17P2 ; C91H150O17P2 ; C91H150O17P2 ; C91H150O17P2 ; C91H150O17P2 ;
252.04821	1.531534	0.621791	-4.126	0.002057	2.6867	0.019745	252.0482	HMDB01142 ; HMDB29689 ; HMDB29868 ; HMDB30294 ; HMDB30593 ; HMDB30689 ; HMDB30845 ; HMDB34291 ; HMDB36982 ; HMDB39912 ; HMDB59607	FMNH2 ; 2-Naphthalenethiol ; Mangostanol ; 1-O-E-Cinnamoyl-(6-arabinosylglucose) ; Edulisin II ; 6-O-Acetyldaidzin ; Archangelicin ; Sampangine ; Atrovirisidone ; Mangostenol ; apo-3-methylcrotonoyl-CoA:carbon-dioxide ligase (ADP-forming)	M+2Na ; 3M+H+Na ; M+2K ; M+Na+K ; M+2K ; M+2Na ; M+2K ; 2M+H+K ; M+2K ; M+2K ; M+2K+H	C17H23N4O9P ; C10H8S ; C24H26O7 ; C20H26O11 ; C24H26O7 ; C23H22O10 ; C24H26O7 ; C15H8N2O ; C24H26O7 ; C24H26O7 ; C7H15N3O2
281.07059	1.475302	0.383235	4.1241	0.002064	2.6854	0.019765	281.0706	HMDB37687 ; HMDB40676 ; HMDB05036 ; HMDB14726 ; HMDB15059 ; HMDB28777 ; HMDB28882 ; HMDB30489 ; HMDB38384 ; HMDB39316	Prunus inhibitor b ; (2S2R3S3R4S)-3457-Tetrahydroxyflavan(2-74-8)-33557-pentahydroxyflavan ; Fluticasone ; Fluticasone Propionate ; Ceforanide ; Cysteinyl-Histidine ; Histidinyl-Cysteine ; Artonol C ; Neoglucobrassicin ; Luteone 7-glucoside	M+2H ; M+2H ; M+Na+K ; M+Na+K ; M+ACN+2H ; M+Na ; M+Na ; M+Na+K ; M+2ACN+2H ; M+2Na	C30H24O11 ; C30H24O11 ; C25H31F3O5S ; C25H31F3O5S ; C20H21N7O6S2 ; C9H14N4O3S ; C9H14N4O3S ; C30H28O7 ; C17H22N2O10S2 ; C26H28O11
367.53590	1.512264	1.082419	-4.1214	0.002073	2.6835	0.019813	NA	NA	NA	NA	NA
232.89645	1.522441	1.434261	4.1181	0.002083	2.6812	0.01988	NA	NA	NA	NA	NA
792.55268	1.589285	0.10494	4.1144	0.002096	2.6786	0.01996	792.5527	HMDB07958 ; HMDB08724 ; HMDB09012 ; HMDB09043 ; HMDB09044 ; HMDB09076 ; HMDB09077 ; HMDB09108 ; HMDB09206 ; HMDB09269 ; HMDB09300 ; HMDB09301 ; HMDB09331 ; HMDB09332 ; HMDB09364 ; HMDB09365 ; HMDB09396 ; HMDB09429 ; HMDB09461 ; HMDB09558 ; HMDB09588 ; HMDB09619 ; HMDB09620 ; HMDB09652 ; HMDB09653 ; HMDB09684 ; HMDB07947 ; HMDB07948 ; HMDB08363 ; HMDB08396 ; HMDB08975 ; HMDB09001 ; HMDB09002 ; HMDB09033 ; HMDB09066 ; HMDB09098 ; HMDB09130 ; HMDB09163 ; HMDB09226 ; HMDB09227 ; HMDB09258 ; HMDB09289 ; HMDB09290 ; HMDB09321 ; HMDB09354 ; HMDB09551	PC(15:022:6(4Z7Z10Z13Z16Z19Z)) ; PC(22:6(4Z7Z10Z13Z16Z19Z)15:0) ; PE(18:022:6(4Z7Z10Z13Z16Z19Z)) ; PE(18:1(11Z)22:5(4Z7Z10Z13Z16Z)) ; PE(18:1(11Z)22:5(7Z10Z13Z16Z19Z)) ; PE(18:1(9Z)22:5(4Z7Z10Z13Z16Z)) ; PE(18:1(9Z)22:5(7Z10Z13Z16Z19Z)) ; PE(18:2(9Z12Z)22:4(7Z10Z13Z16Z)) ; PE(18:4(6Z9Z12Z15Z)22:2(13Z16Z)) ; PE(20:1(11Z)20:5(5Z8Z11Z14Z17Z)) ; PE(20:2(11Z14Z)20:4(5Z8Z11Z14Z)) ; PE(20:2(11Z14Z)20:4(8Z11Z14Z17Z)) ; PE(20:3(5Z8Z11Z)20:3(5Z8Z11Z)) ; PE(20:3(5Z8Z11Z)20:3(8Z11Z14Z)) ; PE(20:3(8Z11Z14Z)20:3(5Z8Z11Z)) ; PE(20:3(8Z11Z14Z)20:3(8Z11Z14Z)) ; PE(20:4(5Z8Z11Z14Z)20:2(11Z14Z)) ; PE(20:4(8Z11Z14Z17Z)20:2(11Z14Z)) ; PE(20:5(5Z8Z11Z14Z17Z)20:1(11Z)) ; PE(22:2(13Z16Z)18:4(6Z9Z12Z15Z)) ; PE(22:4(7Z10Z13Z16Z)18:2(9Z12Z)) ; PE(22:5(4Z7Z10Z13Z16Z)18:1(11Z)) ; PE(22:5(4Z7Z10Z13Z16Z)18:1(9Z)) ; PE(22:5(7Z10Z13Z16Z19Z)18:1(11Z)) ; PE(22:5(7Z10Z13Z16Z19Z)18:1(9Z)) ; PE(22:6(4Z7Z10Z13Z16Z19Z)18:0) ; PC(15:020:3(5Z8Z11Z)) ; PC(15:020:3(8Z11Z14Z)) ; PC(20:3(5Z8Z11Z)15:0) ; PC(20:3(8Z11Z14Z)15:0) ; PE(16:1(9Z)22:2(13Z16Z)) ; PE(18:020:3(5Z8Z11Z)) ; PE(18:020:3(8Z11Z14Z)) ; PE(18:1(11Z)20:2(11Z14Z)) ; PE(18:1(9Z)20:2(11Z14Z)) ; PE(18:2(9Z12Z)20:1(11Z)) ; PE(18:3(6Z9Z12Z)20:0) ; PE(18:3(9Z12Z15Z)20:0) ; PE(20:018:3(6Z9Z12Z)) ; PE(20:018:3(9Z12Z15Z)) ; PE(20:1(11Z)18:2(9Z12Z)) ; PE(20:2(11Z14Z)18:1(11Z)) ; PE(20:2(11Z14Z)18:1(9Z)) ; PE(20:3(5Z8Z11Z)18:0) ; PE(20:3(8Z11Z14Z)18:0) ; PE(22:2(13Z16Z)16:1(9Z))	M+H ; M+Na ; M+Na ; M+Na ; M+Na ; M+Na ; M+Na ; M+Na ; M+Na ; M+Na ; M+Na ; M+Na ; M+Na ; M+Na ; M+Na ; M+Na ; M+Na ; M+Na ; M+Na ; M+Na ; M+Na	C45H78NO8P ; C43H80NO8P ; C43H80NO8P ; C43H80NO8P ; C43H80NO8P ; C43H80NO8P ; C43H80NO8P ; C43H80NO8P ; C43H80NO8P ; C43H80NO8P ; C43H80NO8P ; C43H80NO8P ; C43H80NO8P ; C43H80NO8P ; C43H80NO8P ; C43H80NO8P ; C43H80NO8P ; C43H80NO8P ; C43H80NO8P ; C43H80NO8P ; C43H80NO8P
257.00877	1.443456	0.227859	-4.1088	0.002115	2.6748	0.020092	257.0088	HMDB15153 ; HMDB30559 ; HMDB32658 ; HMDB33061 ; HMDB36374 ; HMDB60555	Guanfacine ; m-Trigallic acid ; 7-Chloro-334568-hexamethoxyflavone ; 1-(Methylsulfinyl)propyl propyl disulfide ; Methyl 1-(propylsulfinyl)propyl disulfide ; 4-Hydroxyclonidine	2M+H+Na ; M+H+K ; M+2K ; M+2Na-H ; M+2Na-H ; 2M+H+Na	C9H9Cl2N3O ; C21H14O13 ; C21H21ClO8 ; C7H16OS3 ; C7H16OS3 ; C9H9Cl2N3O
462.64455	1.515664	1.468879	4.1079	0.002118	2.6741	0.020092	NA	NA	NA	NA	NA
552.16953	1.572393	0.723535	-4.1062	0.002123	2.673	0.020107	552.1695	HMDB14408 ; HMDB15595 ; HMDB15688 ; HMDB32880 ; HMDB34038 ; HMDB36593 ; HMDB59794	Sulfisoxazole ; Nilotinib ; Sulfamoxole ; trans-Zeatin-O-glucoside riboside ; Palmidin A ; Validamycin B ; Gluten exorphin B4	2M+NH4 ; M+Na ; 2M+NH4 ; M+K ; M+ACN+H ; M+K ; M+Na	C11H13N3O3S ; C28H22F3N7O ; C11H13N3O3S ; C21H31N5O10 ; C30H22O8 ; C20H35NO14 ; C24H27N5O9

710.50302	1.515084	1.097653	-4.105	0.002128	2.6721	0.02011	710.503	HMDB07469 ; HMDB07498 ; HMDB07525 ; HMDB07526 ; HMDB07554 ; HMDB07555 ; HMDB07582 ; HMDB07694 ; HMDB07721 ; HMDB07722 ; HMDB07750 ; HMDB07751 ; HMDB07777 ; HMDB07778 ; HMDB12321 ; HMDB29885 ; HMDB37834 ; HMDB56250 ; HMDB56331 ; HMDB56335 ; HMDB56340 ; HMDB56356 ; HMDB56362 ; HMDB56379 ; HMDB30501	DG(20:3(5Z8Z11Z)22:6(4Z7Z10Z13Z16Z19Z)0:0) ; DG(20:3(8Z11Z14Z)22:6(4Z7Z10Z13Z16Z19Z)0:0) ; DG(20:4(5Z8Z11Z14Z)22:5(4Z7Z10Z13Z16Z)0:0) ; DG(20:4(5Z8Z11Z14Z)22:5(7Z10Z13Z16Z19Z)0:0) ; DG(20:4(8Z11Z14Z17Z)22:5(4Z7Z10Z13Z16Z)0:0) ; DG(20:4(8Z11Z14Z17Z)22:5(7Z10Z13Z16Z19Z)0:0) ; DG(20:5(5Z8Z11Z14Z17Z)22:4(7Z10Z13Z16Z)0:0) ; DG(22:4(7Z10Z13Z16Z)20:5(5Z8Z11Z14Z17Z)0:0) ; DG(22:5(4Z7Z10Z13Z16Z)20:4(5Z8Z11Z14Z)0:0) ; DG(22:5(4Z7Z10Z13Z16Z)20:4(8Z11Z14Z17Z)0:0) ; DG(22:5(7Z10Z13Z16Z19Z)20:4(5Z8Z11Z14Z)0:0) ; DG(22:5(7Z10Z13Z16Z19Z)20:4(8Z11Z14Z17Z)0:0) ; DG(22:6(4Z7Z10Z13Z16Z19Z)20:3(5Z8Z11Z)0:0) ; DG(22:6(4Z7Z10Z13Z16Z19Z)20:3(8Z11Z14Z)0:0) ; Galactosylceramide (d18:114:0) ; Sorbitan laurate ; Ethyl menthane carboxamide ; DG(20:3n90:022:6n3) ; DG(20:3n60:022:6n3) ; DG(20:4n60:022:5n6) ; DG(20:4n60:022:5n3) ; DG(22:4n60:020:5n3) ; DG(22:5n60:020:4n3) ; DG(20:4n30:022:5n3) ; Spirolide F	2M+H+K ; 2M+H+K ; 2M+H+K ; 2M+H+K ; 2M+H+K ; 2M+H+K ; 2M+H+K ; 2M+H+K ; 2M+H+K ; 2M+H+K ; 2M+H+K ; 2M+H+K ; 2M+H+K ; 2M+H+K ; M+K ; 2M+NH4 ; 3M+2K-H ; 2M+H+K ; 2M+H+K ; 2M+H+K ; 2M+H+K ; 2M+H+K ; 2M+H+K ; 2M+H+K ; M+H-H2O	C45H70O5 ; C45H70O5 ; C45H70O5 ; C45H70O5 ; C45H70O5 ; C45H70O5 ; C45H70O5 ; C45H70O5 ; C45H70O5 ; C45H70O5 ; C45H70O5 ; C45H70O5 ; C45H70O5 ; C45H70O5 ; C38H73NO8 ; C18H34O6 ; C13H25NO ; C45H70O5 ; C45H70O5 ; C45H70O5 ; C45H70O5 ; C45H70O5 ; C45H70O5 ; C45H70O5 ; C43H69NO8
152.01114	1.508158	1.169367	-4.1004	0.002143	2.6689	0.020216	152.0111	HMDB02899 ; HMDB60626 ; HMDB00245 ; HMDB01301 ; HMDB06875 ; HMDB14774 ; HMDB36354 ; HMDB38417 ; HMDB38419 ; HMDB39035 ; HMDB59667	Ellagic acid ; Malathion monocarboxylic acid ; Porphobilinogen ; 1-Pyrroline-5-carboxylic acid ; 1-Pyrroline-2-carboxylic acid ; Clofibrate ; 1-Pentenyl glucosinolate ; Glucobrassicanapin ; Glucotropaeolin ; Necatorine ; 3-Methyl sulfolene	M+2H ; M+2H ; M+2K ; M+K ; M+K ; M+Na+K ; M+3Na ; M+3Na ; M+H+2Na ; M+H+K ; 2M+H+K	C14H6O8 ; C8H15O6PS2 ; C10H14N2O4 ; C5H7NO2 ; C5H7NO2 ; C12H15ClO3 ; C12H21NO9S2 ; C12H21NO9S2 ; C14H19NO9S2 ; C15H8N2O3 ; C5H8O2S
697.41770	1.494203	1.031658	-4.0993	0.002147	2.6682	0.020216	697.4177	HMDB35697 ; HMDB38141 ; HMDB60707 ; HMDB60719 ; HMDB60755 ; HMDB60756 ; HMDB36300	Momordicoside E ; Glucosyl passiflorate ; 15beta-Hydroxydesogestrel ; 2-Hydroxydesogestrel ; 3a-Hydroxydesogestrel ; 3b-Hydroxydesogestrel ; Cyclopassifloside VII	M+H ; M+H ; 2M+2Na-H ; 2M+2Na-H ; 2M+2Na-H ; 2M+2Na-H ; M+H-H2O	C37H60O12 ; C37H60O12 ; C22H30O2 ; C22H30O2 ; C22H30O2 ; C22H30O2 ; C37H62O13
319.67033	1.503377	0.476966	4.0968	0.002156	2.6664	0.020229	319.6703	HMDB01045 ; HMDB10699 ; HMDB14021 ; HMDB15171 ; HMDB15583 ; HMDB60576	Enkephalin L ; CerP(d18:112:0) ; N-Desmethylcitalopram ; Selegiline ; Bifonazole ; Desmethylcitalopram	M+2ACN+2H ; M+2K ; 2M+H+NH4 ; 3M+2K ; 2M+H+NH4 ; 2M+H+NH4	C28H37N5O7 ; C30H60NO6P ; C19H19FN2O ; C13H17N ; C22H18N2 ; C19H19FN2O
332.95632	1.493992	1.323244	4.0956	0.00216	2.6656	0.020229	NA	NA	NA	NA	NA
269.59620	1.582493	0.19418	-4.0954	0.00216	2.6655	0.020229	269.5962	HMDB15499	Almitrine	M+Na+K	C26H29F2N7
709.64887	1.563147	1.205162	-4.092	0.002172	2.6631	0.020295	709.6489	HMDB31851 ; HMDB36831 ; HMDB37997	3-(566-Trimethylbicyclo221hept-1-yl)cyclohexanol ; Ambronide ; Delphinidin 3-glucoside	3M+H ; 3M+H ; 3M+H+Na	C16H28O ; C16H28O ; C21H21O12
724.52708	1.538934	0.021934	4.091	0.002176	2.6624	0.020295	724.5271	HMDB09149 ; HMDB09150 ; HMDB09182 ; HMDB09183 ; HMDB09214 ; HMDB09411 ; HMDB09444 ; HMDB11352 ; HMDB11353 ; HMDB11379 ; HMDB11410 ; HMDB11411 ; HMDB11443 ; HMDB11444 ; HMDB56501 ; HMDB56518 ; HMDB56699 ; HMDB56927 ; HMDB56944 ; HMDB56999 ; HMDB08951 ; HMDB08952 ; HMDB08983 ; HMDB09048 ; HMDB09081 ; HMDB11341 ; HMDB11342 ; HMDB11372 ; HMDB11404 ; HMDB11437 ; HMDB34430 ; HMDB39713	PE(18:3(6Z9Z12Z)P-18:1(11Z)) ; PE(18:3(6Z9Z12Z)P-18:1(9Z)) ; PE(18:3(9Z12Z15Z)P-18:1(11Z)) ; PE(18:3(9Z12Z15Z)P-18:1(9Z)) ; PE(18:4(6Z9Z12Z15Z)P-18:0) ; PE(20:4(5Z8Z11Z14Z)P-16:0) ; PE(20:4(8Z11Z14Z17Z)P-16:0) ; PE(P-16:020:4(5Z8Z11Z14Z)) ; PE(P-16:020:4(8Z11Z14Z17Z)) ; PE(P-18:018:4(6Z9Z12Z15Z)) ; PE(P-18:1(11Z)18:3(6Z9Z12Z)) ; PE(P-18:1(11Z)18:3(9Z12Z15Z)) ; PE(P-18:1(9Z)18:3(6Z9Z12Z)) ; PE(P-18:1(9Z)18:3(9Z12Z15Z)) ; CL(16:018:018:018:2(9Z12Z)) ; CL(16:018:018:2(9Z12Z)18:0) ; CL(16:018:2(9Z12Z)18:018:0) ; CL(18:016:018:018:2(9Z12Z)) ; CL(18:016:018:2(9Z12Z)18:0) ; CL(18:018:018:2(9Z12Z)16:0) ; PE(16:0P-18:1(11Z)) ; PE(16:0P-18:1(9Z)) ; PE(16:1(9Z)P-18:0) ; PE(18:1(11Z)P-16:0) ; PE(18:1(9Z)P-16:0) ; PEP-16:018:1(11Z)) ; PE(P-16:018:1(9Z)) ; PE(P-18:016:1(9Z)) ; PE(P-18:1(11Z)16:0) ; PE(P-18:1(9Z)16:0) ; 6-Deoxohomodolichosterone ; (3beta22R23R24S)-32223-Trihydroxystigmastan-6-one	M+H ; M+H ; M+H ; M+H ; M+H ; M+H ; M+H ; M+H ; M+H ; M+H ; M+H ; M+H ; M+H ; M+H ; M+2H ; M+2H ; M+2H ; M+2H ; M+2H ; M+2H ; M+Na ; M+Na ; M+Na ; M+Na ; M+Na ; M+Na ; M+Na ; M+Na ; M+Na ; M+Na ; 3M+Na+K ; 3M+Na+K	C41H74NO7P ; C41H74NO7P ; C41H74NO7P ; C41H74NO7P ; C41H74NO7P ; C41H74NO7P ; C41H74NO7P ; C41H74NO7P ; C41H74NO7P ; C41H74NO7P ; C41H74NO7P ; C41H74NO7P ; C41H74NO7P ; C41H74NO7P ; C80H152O17P2 ; C80H152O17P2 ; C80H152O17P2 ; C80H152O17P2 ; C80H152O17P2 ; C80H152O17P2 ; C39H76NO7P ; C39H76NO7P ; C39H76NO7P ; C39H76NO7P ; C39H76NO7P ; C39H76NO7P ; C39H76NO7P ; C39H76NO7P ; C39H76NO7P ; C39H76NO7P ; C29H50O4 ; C29H50O4

155.06909	1.508383	1.100301	4.0898	0.00218	2.6616	0.020296	155.0691	HMDB37772 ; HMDB00039 ; HMDB00050 ; HMDB00085 ; HMDB00317 ; HMDB00409 ; HMDB00525 ; HMDB00624 ; HMDB00665 ; HMDB00746 ; HMDB00830 ; HMDB00990 ; HMDB01624 ; HMDB01873 ; HMDB01975 ; HMDB03243 ; HMDB10718 ; HMDB11105 ; HMDB12843 ; HMDB14340 ; HMDB14638 ; HMDB15620 ; HMDB29166 ; HMDB30062 ; HMDB31217 ; HMDB31305 ; HMDB31507 ; HMDB31688 ; HMDB32158 ; HMDB32456 ; HMDB33851 ; HMDB35690 ; HMDB35864 ; HMDB36162 ; HMDB37842 ; HMDB38159 ; HMDB40253 ; HMDB40409 ; HMDB40579 ; HMDB41844 ; HMDB59770	8-Acetylegelolide ; Butyric acid ; Adenosine ; Deoxyguanosine ; 2-Hydroxy-3-methylpentanoic acid ; (5R)-5-Hydroxyhexanoic acid ; 5-Hydroxyhexanoic acid ; D-Leucic acid ; Leucinic acid ; Hydroxyisocaproic acid ; Neuraminic acid ; Acetaldehyde ; 2-Hydroxycaproic acid ; Isobutyric acid ; 2-Ethyl-2-Hydroxybutyric acid ; Acetoin ; (R)-3-Hydroxyhexanoic acid ; 5-Acetylamino-6-formylamino-3-methyluracil ; 6-Hydroxyhexanoic acid ; Vidarabine ; Zidovudine ; Mianserin ; 2-Methyl-3-hydroxyvaleric acid ; Methyl propionate ; Ethyl acetate ; Oxirane ; 1-Hydroxy-2-butanone ; Hexyl glucoside ; (-)-1-Acetoxy-1-ethoxyethane ; Paraldehyde ; 44-Dimethoxy-2-butanone ; 6-O-Methylarmillaridin ; 13-Hydroxy-5-O-methylmelledonal ; 3-Hydroxy-HT2 toxin ; N-(1-Deoxy-1-fructosyl)serine ; Retrocalamin ; Propyl formate ; Ethyl ()-3-hydroxybutyrate ; Isopropyl formate ; Butoxyacetic acid ; Threo-3-Hydroxy-2-methylbutyric acid	M+2H ; 3M+2Na ; M+ACN+2H ; M+ACN+2H ; M+Na ; M+Na ; M+Na ; M+Na ; M+Na ; M+Na ; M+ACN+2H ; 3M+Na ; M+Na ; 3M+2Na ; M+Na ; 3M+2Na ; M+Na ; M+2ACN+2H ; M+Na ; M+ACN+2H ; M+ACN+2H ; M+2Na ; M+Na ; 3M+2Na ; 3M+2Na ; 3M+Na ; 3M+2Na ; M+2Na ; M+Na ; M+Na ; M+Na ; M+3H ; M+3H ; M+2H+Na ; M+ACN+2H ; M+3H ; 3M+2Na ; M+Na ; 3M+2Na ; M+Na ; M+Na	C16H20O6 ; C4H8O2 ; C10H13N5O4 ; C10H13N5O4 ; C6H12O3 ; C6H12O3 ; C6H12O3 ; C6H12O3 ; C6H12O3 ; C6H12O3 ; C9H17NO8 ; C2H4O ; C6H12O3 ; C4H8O2 ; C6H12O3 ; C4H8O2 ; C6H12O3 ; C8H10N4O4 ; C6H12O3 ; C10H13N5O4 ; C10H13N5O4 ; C18H20N2 ; C6H12O3 ; C4H8O2 ; C4H8O2 ; C2H4O ; C4H8O2 ; C12H24O6 ; C6H12O3 ; C6H12O3 ; C6H12O3 ; C25H31ClO6 ; C24H30O9 ; C22H32O9 ; C9H17NO8 ; C24H30O9 ; C4H8O2 ; C6H12O3 ; C4H8O2 ; C6H12O3 ; C6H12O3
344.08900	1.483592	1.14075	-4.0878	0.002187	2.6602	0.020324	344.089	HMDB10328 ; HMDB13109 ; HMDB15097 ; HMDB15380 ; HMDB15527 ; HMDB29367 ; HMDB31929 ; HMDB37503 ; HMDB37504 ; HMDB38635 ; HMDB40320 ; HMDB40482 ; HMDB40985	Tyramine glucuronide ; Tridecanoyl-CoA ; Zaleplon ; Olsalazine ; Prazepam ; Sanguinarine ; Zanthodioline ; 345-Trihydroxy-37-dimethoxyflavanone ; 335-Trihydroxy-47-dimethoxyflavanone ; Methoxybrassenin A ; 357-Trihydroxy-46-dimethoxyflavanone ; 235-Trihydroxy-57-dimethoxyflavanone ; Polixetonium chloride	2M+Na+K ; M+3Na ; M+K ; M+ACN+H ; 2M+H+K ; 2M+H+Na ; M+K ; 2M+H+Na ; 2M+H+Na ; M+ACN+Na ; 2M+H+Na ; 2M+H+Na ; 2M+H+K	C14H19NO7 ; C34H60N7O17P3S ; C17H15N5O ; C14H10N2O6 ; C19H17ClN2O ; C20H14NO4 ; C16H19NO5 ; C17H16O7 ; C17H16O7 ; C13H16N2OS2 ; C17H16O7 ; C17H16O7 ; C10H28Cl2N2OP2
326.54527	1.541164	0.089159	4.0849	0.002197	2.6582	0.020352	326.5453	HMDB15437	Saprisartan	M+ACN+2H	C25H22BrF3N4O4S
584.63944	1.506581	1.446982	4.0843	0.002199	2.6577	0.020352	NA	NA	NA	NA	NA
170.05930	1.497503	1.367643	-4.0824	0.002206	2.6564	0.020352	170.0593	HMDB60244 ; HMDB00469 ; HMDB05785 ; HMDB30204 ; HMDB31716 ; HMDB32501 ; HMDB33957 ; HMDB37209 ; HMDB37832 ; HMDB38024 ; HMDB38544 ; HMDB38782 ; HMDB39134 ; HMDB39358 ; HMDB40009 ; HMDB40011 ; HMDB40185 ; HMDB40374 ; HMDB41061 ; HMDB41475 ; HMDB60561	Phosphodimethylethanolamine ; 5-Hydroxymethyluracil ; Indole-3-carbinol ; 1-(2-Furanylmethyl)-1H-pyrrole ; 3-(Methylthio)-1-propanol ; Santalyl acetate ; Crithmumdiol ; alpha-Santalyl acetate ; Vetiveryl acetate ; beta-Santalyl acetate ; Longistylin C ; 1-Heptadecene-46-diyne-39-diol ; Ginsenoyne B ; Ginsenoyne D ; 4-(2-Furanylmethylene)-34-dihydro-2H-pyrrole ; 3-(2-Furanylmethyl)-1H-pyrrole ; 3-Mercapto-2-butanol ; Ginsenoyne I ; Nigellidine ; 4-Mercapto-2-butanol ; Albendazole sulfone	M+H ; 2M+3H2O+2H ; M+Na ; M+Na ; M+ACN+Na ; M+2K ; M+2K ; M+2K ; M+2K ; M+2K ; M+Na+K ; M+2K ; M+2Na ; M+2K ; M+Na ; M+Na ; M+ACN+Na ; M+2K ; M+2Na ; M+ACN+Na ; M+ACN+2H	C4H12NO4P ; C5H6N2O3 ; C9H9NO ; C9H9NO ; C4H10OS ; C17H26O2 ; C17H26O2 ; C17H26O2 ; C17H26O2 ; C17H26O2 ; C20H22O ; C17H26O2 ; C17H23ClO2 ; C17H26O2 ; C9H9NO ; C9H9NO ; C4H10OS ; C17H26O2 ; C18H18N2O2 ; C4H10OS ; C12H15N3O4S
338.78488	1.506995	1.494449	4.0823	0.002206	2.6564	0.020352	338.7849	HMDB07070 ; HMDB07126 ; HMDB55988	DG(15:016:1(9Z)0:0) ; DG(16:1(9Z)15:00:0) ; DG(15:00:016:1n7)	M+3ACN+2H ; M+3ACN+2H ; M+3ACN+2H	C34H64O5 ; C34H64O5 ; C34H64O5
279.08012	1.511198	1.386881	-4.0774	0.002224	2.6529	0.020438	279.0801	HMDB04072 ; HMDB04135 ; HMDB06236 ; HMDB10338 ; HMDB13815 ; HMDB14623 ; HMDB15083 ; HMDB29636 ; HMDB29637 ; HMDB29638 ; HMDB30672 ; HMDB30722 ; HMDB30881 ; HMDB32006 ; HMDB32777 ; HMDB33654 ; HMDB33910 ; HMDB35009 ; HMDB36033 ; HMDB36161 ; HMDB38559 ; HMDB38997 ; HMDB39144 ; HMDB40723	4-Hydroxystyrene ; Poly-g-D-glutamate ; Phenylacetaldehyde ; 11-Oxo-androsterone glucuronide ; 23-Dihydrobenzofuran ; Lenalidomide ; Mezlocillin ; 2-Methylbenzaldehyde ; 3-Methylbenzaldehyde ; 4-Methylbenzaldehyde ; Obtustyrene ; (E)-35-Dimethoxystilbene ; 1-Acetoxy-46-tetradecadiene-81012-triyne ; Epifisetinidol-(4beta-8)-epicatechin-(6-4beta)-epifisetinidol ; Ciceritol ; 11-Deacetylvaltrate 11-(3-hydroxy-3-methylbutanoate) ; Acetophenone ; Phenethyl phenylacetate ; 7alpha-1(10-19)-Abeo-7-acetoxyobacun-9(11)-ene ; 4-Propanoyl-HT2 toxin ; 8-Hexanoylneosolaniol ; Lentialexin ; 48:48-Terflavan-3334444555777-tridecol ; (-)-Gossypol	2M+K ; M+H+K ; 2M+K ; M+2K ; 2M+K ; 2M+H+K ; M+H+NH4 ; 2M+K ; 2M+K ; 2M+K ; M+K ; M+K ; M+K ; M+3H ; M+H+K ; M+2K ; 2M+K ; M+K ; M+Na+K ; M+2K ; M+2K ; 2M+K ; M+3H ; M+H+K	C8H8O ; C20H30N4O12 ; C8H8O ; C25H36O9 ; C8H8O ; C13H13N3O3 ; C21H25N5O8S2 ; C8H8O ; C8H8O ; C8H8O ; C16H16O2 ; C16H16O2 ; C16H16O2 ; C45H38O16 ; C19H34O16 ; C25H36O9 ; C8H8O ; C16H16O2 ; C28H32O8 ; C25H36O9 ; C25H36O9 ; C8H8O ; C45H38O16 ; C30H30O8
211.55225	1.495445	0.829703	4.0774	0.002224	2.6529	0.020438	211.5523	HMDB32878 ; HMDB38554 ; HMDB59663	13-Oxocryptopine ; Gravacridonediolacetate ; 5-hydroxy-2-oxo-4-ureido-25-dihydro-1H-imidazole-5-carboxylate	M+H+K ; M+H+K ; 2M+H+NH4	C21H21NO6 ; C21H21NO6 ; C5H6N4O5
251.03657	1.469357	1.075677	-4.0758	0.002229	2.6518	0.020452	251.0366	HMDB04370 ; HMDB15014 ; HMDB29212 ; HMDB29214 ; HMDB33668 ; HMDB33755 ; HMDB35762 ; HMDB41769 ; HMDB59775 ; HMDB05800 ; HMDB05801 ; HMDB29711 ; HMDB30543 ; HMDB34014 ; HMDB34150 ; HMDB34444 ; HMDB41658 ; HMDB41688 ; HMDB41702	N-Methyltryptamine ; Eprosartan ; Quercetin 3-O-glucuronide ; Quercetin-4-glucuronide ; Quercetin 4-glucuronide ; 2-Butyl-1H-benzimidazole ; 3-(Dimethylaminomethyl)indole ; Quercetin 3-O-glucuronide ; 3-Hydroxy-3-carboxymethyl-adipic acid ; Luteolin ; Kaempferol ; Maritimetin ; Norartocarpetin ; 2-Hydroxygenistein ; Cernuine ; Citreorosein ; 3-Hydroxygenistein ; 5674-Tetrahydroxyisoflavone ; 7834-Tetrahydroxyisoflavone	M+2K-H ; M+2K ; M+H+Na ; M+H+Na ; M+H+Na ; M+2K-H ; M+2K-H ; M+H+Na ; 2M+Na+K ; M+H-2H2O ; M+H-2H2O ; M+H-2H2O ; M+H-2H2O ; M+H-2H2O ; M+H-2H2O ; M+H-2H2O ; M+H-2H2O ; M+H-2H2O ; M+H-2H2O	C11H14N2 ; C23H24N2O4S ; C21H18O13 ; C21H18O13 ; C21H18O13 ; C11H14N2 ; C11H14N2 ; C21H18O13 ; C8H12O7 ; C15H10O6 ; C15H10O6 ; C15H10O6 ; C15H10O6 ; C15H10O6 ; C15H10O6 ; C15H10O6 ; C15H10O6 ; C15H10O6 ; C15H10O6
642.51256	1.499399	1.238324	4.0719	0.002243	2.6491	0.020543	642.5126	HMDB07085 ; HMDB07561 ; HMDB31784 ; HMDB56006	DG(15:020:5(5Z8Z11Z14Z17Z)0:0) ; DG(20:5(5Z8Z11Z14Z17Z)15:00:0) ; Halofuginone ; DG(15:00:020:5n3)	M+ACN+H ; M+ACN+H ; 3M+2Na ; M+ACN+H	C38H64O5 ; C38H64O5 ; C16H17BrClN3O3 ; C38H64O5

149.06352	1.469914	0.301939	4.0674	0.00226	2.646	0.020594	149.0635	HMDB31717 ; HMDB32271 ; HMDB32410 ; HMDB32495 ; HMDB36193 ; HMDB37619 ; HMDB38299 ; HMDB39653 ; HMDB40413 ; HMDB41306 ; HMDB00070 ; HMDB00716 ; HMDB02352 ; HMDB04226 ; HMDB05960 ; HMDB13946 ; HMDB13947 ; HMDB13948 ; HMDB13950 ; HMDB14451 ; HMDB15212 ; HMDB28795 ; HMDB28811 ; HMDB29147 ; HMDB29163 ; HMDB29435 ; HMDB29444 ; HMDB30891 ; HMDB30916 ; HMDB30920 ; HMDB31348 ; HMDB31978 ; HMDB32048 ; HMDB32292 ; HMDB32681 ; HMDB32764 ; HMDB33083 ; HMDB33104 ; HMDB34714 ; HMDB34722 ; HMDB34885 ; HMDB34965 ; HMDB35032 ; HMDB35040 ; HMDB35127 ; HMDB35147 ; HMDB35301 ; HMDB35648 ; HMDB35827 ; HMDB36446 ; HMDB36549 ; HMDB36668 ; HMDB36680 ; HMDB36687 ; HMDB37393 ; HMDB37906 ;	3-(Methylthio)propyl acetate ; Ethyl 3-mercaptobutyrate ; Methyl 3-(methylthio)butanoate ; Propyl 2-mercaptopropionate ; 3-Mercapto-3-methylbutyl formate ; Methyl 4-(methylthio)butyrate ; Methylthiomethyl butyrate ; 246-Trimethyl-135-dioxathiane ; Ethyl 3-(methylthio)propanoate ; Methyl 2-(methylthio)butyrate ; Pipecolic acid ; L-Pipecolic acid ; Capsidiol ; N4-Acetylaminobutanal ; D-Pipecolic acid ; 8-Hydroxycarvedilol ; 4-Hydroxycarvedilol ; 5-Hydroxycarvedilol ; 1-Hydroxycarvedilol ; Talbutal ; Vigabatrin ; Glutaminyl-Glutamine ; Glutaminyl-Gamma-glutamate ; Gamma-glutamyl-Glutamine ; Gamma-glutamyl-Gamma-glutamate ; L-trans-4-Methyl-2-pyrrolidinecarboxylic acid ; 2-Pyrrolidineacetic acid ; (2E6E)-1-Hydroxy-2610-farnesatrien-9-one ; 2-Hydroxyacorenone ; Acorusnol ; Epiacorone ; Methyl (9Z)-6-oxo-66-diapo-6-carotenoate ; 26-Di-tert-butyl-4-hydroxymethylphenol ; Geranyl tiglate ; 10alpha-45-Seco-11-eudesmene-45-dione ; N2-Succinoylarginine ; (4S)-Dihydrocurcumenone ; Calamensesquiterpinenol ; Curdione ; 3-Hydroxy-110-bisaboladien-9-one ; Imazamethabenz ; 1-Methyl-3-(2-thiazolyl)-1H-indole ; Epilubimin ; 7-Hydroxycostol ; (-)-Myrtenyl isovalerate ; Rishitinone ; 4alpha5alpha-Epoxy-11-eudesmen-3a-ol ; Isolubimin ; Aubergenone ; beta-Kessyl ketone ; Apotrichothecene ; Humulene-8-hydroperoxide ; Cyperolone ; Tanavulgarol ; (3xi7(11)Z)-7(11)-Copaene-412-diol ; Hernandulcin ; beta-Ionyl acetate ; Curcumol ; Petasitolone ; Davanone ; Dihydro-alpha-santalic acid ; Tuberonone ; Humulene diepoxide A ; Trimethylammonioacetate ; 3-Acetamidobutanal	M+H ; M+H ; M+H ; M+H ; M+H ; M+H ; M+H ; M+H ; M+H ; M+H ; 2M+H+K ; 2M+H+K ; M+Na+K ; 2M+H+K ; 2M+H+K ; M+2H+Na ; M+2H+Na ; M+2H+Na ; M+2H+Na ; M+2Na ; 2M+H+K ; M+H+Na ; M+H+Na ; M+H+Na ; M+H+Na ; 2M+H+K ; 2M+H+K ; M+Na+K ; M+Na+K ; M+Na+K ; M+Na+K ; M+3Na ; M+Na+K ; M+Na+K ; M+Na+K ; M+H+Na ; M+Na+K ; M+Na+K ; M+Na+K ; M+Na+K ; M+H+Na ; M+2ACN+2H ; M+Na+K ; 2M+Na+K ; 2M+H+K	C6H12O2S ; C6H12O2S ; C6H12O2S ; C6H12O2S ; C6H12O2S ; C6H12O2S ; C6H12O2S ; C6H12O2S ; C6H12O2S ; C6H12O2S ; C6H11NO2 ; C6H11NO2 ; C15H24O2 ; C6H11NO2 ; C6H11NO2 ; C24H26N2O5 ; C24H26N2O5 ; C24H26N2O5 ; C24H26N2O5 ; C13H20N2O3 ; C6H11NO2 ; C10H18N4O5 ; C10H18N4O5 ; C10H18N4O5 ; C10H18N4O5 ; C6H11NO2 ; C6H11NO2 ; C15H24O2 ; C15H24O2 ; C15H24O2 ; C15H24O2 ; C25H30O3 ; C15H24O2 ; C15H24O2 ; C15H24O2 ; C10H18N4O5 ; C15H24O2 ; C15H24O2 ; C15H24O2 ; C15H24O2 ; C15H18N2O3 ; C12H10N2S ; C15H24O2 ; C5H12NO2 ; C6H11NO2
170.09249	1.505097	0.087817	4.067	0.002261	2.6457	0.020594	170.0925	HMDB00001 ; HMDB00479 ; HMDB13032 ; HMDB00079 ; HMDB00451 ; HMDB00909 ; HMDB03609 ; HMDB10721 ; HMDB11655 ; HMDB13211 ; HMDB14570 ; HMDB14864 ; HMDB28895 ; HMDB29063 ; HMDB29346 ; HMDB29856 ; HMDB29874 ; HMDB30303 ; HMDB30418 ; HMDB31177 ; HMDB31307 ; HMDB31547 ; HMDB36230 ; HMDB36395 ; HMDB37147 ; HMDB38305 ; HMDB39426 ; HMDB40447 ; HMDB40563 ; HMDB41421 ; HMDB41616 ; HMDB41830 ; HMDB59810 ; HMDB59851 ; HMDB59905 ; HMDB59924 ; HMDB59938 ; HMDB59939 ; HMDB60004 ; HMDB60683 ; HMDB60685	1-Methylhistidine ; 3-Methylhistidine ; Omega-Carboxy-trinor-leukotriene B4 ; Dihydrothymine ; cis-4-Hydroxycyclohexylacetic acid ; trans-4-Hydroxycyclohexylacetic acid ; 2-Aminoacrylic acid ; 3-Oxooctanoic acid ; 2-(3-Carboxy-3-aminopropyl)-L-histidine ; Alpha-Ketooctanoic acid ; Famciclovir ; Trimipramine ; Histidinyl-Threonine ; Threoninyl-Histidine ; Neryl glucoside ; (S)-alpha-Terpinyl glucoside ; Squamolone ; 6-Ethyl-1-methyl-278-trioxabicyclo321octane ; D-Linalool 3-glucoside ; Tetrahydrofurfuryl propionate ; Ethyl 3-oxohexanoate ; L-Cyclo(alanylglycyl) ; 1-Methyl-2-oxopropyl butyrate ; 2-Methylpropyl 3-oxobutanoate ; 5(6)-Butyl-14-dioxan-2-one ; 3-Methylbutyl 2-oxopropanoate ; 3-Isoxazolidinone ; Butyl acetoacetate ; Perilloside C ; Sarcodon scabrosus Depsipeptide ; Propyl levulinate ; Atrazine ; m-Xylene ; o-Xylene ; Ethylbenzene ; p-Xylene ; 5-Butyl-14-dioxan-2-one ; 6-Butyl-14-dioxan-2-one ; Menthol-glucoronide ; 2-n-Propyl-4-oxopentanoic acid ; 3-Oxovalproic acid	M+H ; M+H ; M+2H ; M+ACN+H ; 2M+H+Na ; 2M+H+Na ; M+2ACN+H ; 2M+H+Na ; M+2ACN+2H ; 2M+H+Na ; M+H+NH4 ; M+2Na ; M+2ACN+2H ; M+2ACN+2H ; M+H+Na ; M+H+Na ; M+ACN+H ; 2M+H+Na ; M+H+Na ; 2M+H+Na ; 2M+H+Na ; M+ACN+H ; 2M+H+Na ; 2M+H+Na ; M+H+Na ; 2M+H+Na ; M+2ACN+H ; 2M+H+Na ; M+H+Na ; M+2H+Na ; 2M+H+Na ; M+3ACN+2H ; M+ACN+Na ; M+ACN+Na ; M+ACN+Na ; M+ACN+Na ; 2M+H+Na ; 2M+H+Na ; M+H+Na ; 2M+H+Na ; 2M+H+Na	C7H11N3O2 ; C7H11N3O2 ; C18H26O6 ; C5H8N2O2 ; C8H14O3 ; C8H14O3 ; C3H5NO2 ; C8H14O3 ; C10H16N4O4 ; C8H14O3 ; C14H19N5O4 ; C20H26N2 ; C10H16N4O4 ; C10H16N4O4 ; C16H28O6 ; C16H28O6 ; C5H8N2O2 ; C8H14O3 ; C16H28O6 ; C8H14O3 ; C8H14O3 ; C5H8N2O2 ; C8H14O3 ; C8H14O3 ; C16H28O6 ; C8H14O3 ; C3H5NO2 ; C8H14O3 ; C16H28O6 ; C23H39N3O8 ; C8H14O3 ; C8H14ClN5 ; C8H10 ; C8H10 ; C8H10 ; C8H10 ; C8H14O3 ; C8H14O3 ; C16H28O6 ; C8H14O3 ; C8H14O3
134.06379	1.5533	0.974022	4.0669	0.002261	2.6456	0.020594	134.0638	HMDB40891 ; HMDB41582 ; HMDB00323 ; HMDB01526 ; HMDB01881 ; HMDB06115 ; HMDB06213 ; HMDB06878 ; HMDB14925 ; HMDB15466 ; HMDB30170 ; HMDB30299 ; HMDB32425 ; HMDB32472 ; HMDB38556 ; HMDB39848 ; HMDB40150 ; HMDB40258 ; HMDB40580 ; HMDB61162	345-Trimethoxycinnamyl alcohol acetate ; Sapidolide A ; 3-Amino-2-piperidone ; S-Acetyldihydrolipoamide ; Propylene glycol ; Benzaldehyde ; (S)-Propane-12-diol ; S-Acetyldihydrolipoamide-E ; Aciclovir ; Colchicine ; Glaudine ; Cibulins ; 2-(Methylthio)ethanol ; Polyethylene oxidized ; xl-Dihydro-2-methyl-3(2H)-thiophenone ; 3-Oxotetradecanoic acid glycerides ; Dihydro-5-methyl-2(3H)-thiophenone ; xi-2-Acetylthietane ; Dihydro-2-methyl-3(2H)-furanthione ; N-Mononitrosopiperazine	M+2H ; M+2H ; 2M+H+K ; M+H+NH4 ; 3M+H+K ; 2M+3H2O+2H ; 3M+H+K ; M+H+NH4 ; M+ACN+2H ; M+3H ; M+3H ; M+ACN+H ; M+ACN+H ; M+H+Na ; M+NH4 ; M+3Na ; M+NH4 ; M+NH4 ; M+NH4 ; 2M+H+K	C14H18O5 ; C14H18O5 ; C5H10N2O ; C10H19NO2S2 ; C3H8O2 ; C7H6O ; C3H8O2 ; C10H19NO2S2 ; C8H11N5O3 ; C22H25NO6 ; C22H25NO6 ; C3H8OS ; C3H8OS ; C12H20O5 ; C5H8OS ; C17H33O6 ; C5H8OS ; C5H8OS ; C5H8OS ; C5H10N2O
184.08155	1.510278	1.270397	4.065	0.002268	2.6443	0.020594	184.0816	HMDB11657 ; HMDB39610 ; HMDB00539 ; HMDB00721 ; HMDB00867 ; HMDB11178 ; HMDB12246 ; HMDB33438 ; HMDB34579 ; HMDB34900 ; HMDB36181 ; HMDB59750 ; HMDB60255 ; HMDB60256 ; HMDB60325 ; HMDB60349 ; HMDB61062	26-Diamino-4-hydroxy-5-N-methylformamidopyrimidine ; Curcumin II ; Arabinonic acid ; Glycylproline ; Ribonic acid ; L-prolyl-L-glycine ; Kynuramine ; NaNa-Dimethylhistamine ; 8-O-Methyloblongine ; 2-(12-Diamino-1-propenyl)phenol ; 2-(Isopropylthio)methylfuran ; 2345-Tetrahydroxypentanoic acid ; L-Lyxonate ; L-Xylonate ; 1-Naphthaldehyde ; 2-Naphthaldehyde ; 7-hydroxygranisetron	M+H ; M+2H ; M+NH4 ; 2M+H+Na ; M+NH4 ; 2M+H+Na ; 2M+H+K ; M+2Na-H ; M+H+K ; 2M+H+K ; 2M+3H2O+2H ; M+NH4 ; M+NH4 ; M+NH4 ; 2M+3H2O+2H ; 2M+3H2O+2H ; M+H+K	C6H9N5O2 ; C22H22O5 ; C5H10O6 ; C7H12N2O3 ; C5H10O6 ; C7H12N2O3 ; C9H12N2O ; C7H13N3 ; C20H26NO3 ; C9H12N2O ; C8H12OS ; C5H10O6 ; C5H10O6 ; C5H10O6 ; C11H8O ; C11H8O ; C18H24N4O2

153.09095	1.527663	0.614466	4.0627	0.002277	2.6427	0.020594	153.091	HMDB32132 ; HMDB32599 ; HMDB36169 ; HMDB36227 ; HMDB39801 ; HMDB39956 ; HMDB40175 ; HMDB40216 ; HMDB40449 ; HMDB41326 ; HMDB41804 ; HMDB59853 ; HMDB60352 ; HMDB60379 ; HMDB00907 ; HMDB30897 ; HMDB32593 ; HMDB33030 ; HMDB33630 ; HMDB36436 ; HMDB37511 ; HMDB40389 ; HMDB60605 ; HMDB30471 ; HMDB38276	2-Isopropyl-14-benzenediol ; 3-(4-Hydroxyphenyl)-1-propanol ; 3-(5-Methyl-2-furanyl)butanal ; 2-Propenyl 24-hexadienoate ; 1-(2-Furanyl)-1-pentanone ; 4-(Ethoxymethyl)phenol ; 4-Ethyl-2-methoxyphenol ; 266-Trimethyl-2-cyclohexene-14-dione ; 2-(Ethoxymethyl)phenol ; (Dimethoxymethyl)benzene ; 2-Isopropoxyphenol ; 35-Dimethoxytoluene ; 2-Polyprenyl-3-methyl-5-hydroxy-6-methoxy-14-benzoquinone ; 3-Polyprenyl-4-hydroxy-5-methoxybenzoate ; Sulfolithocholic acid ; Lucidenolactone ; 4-(Hydroxymethyl)benzenediazonium(1) ; (12S15S)-15-O-Demethyl-1029-dideoxy-1112-dihydro-striatin C ; Lactapiperanol C ; Lucidenic acid F ; Butyl oleate sulfate ; Erinacine C ; Metyrapol ; 14-Ipomeadiol ; cis-3-Hexenyl pyruvate	M+H ; M+H ; M+H ; M+H ; M+H ; M+H ; M+H ; M+H ; M+H ; M+H ; M+H ; M+H ; M+2H ; M+2H ; M+3H ; M+3H ; M+NH4 ; M+2H+Na ; M+H+Na ; M+3H ; M+2H+Na ; M+2H+Na ; 2M+3H ; M+H-H2O ; M+H-H2O	C9H12O2 ; C9H12O2 ; C9H12O2 ; C9H12O2 ; C9H12O2 ; C9H12O2 ; C9H12O2 ; C9H12O2 ; C9H12O2 ; C9H12O2 ; C9H12O2 ; C9H12O2 ; C18H24O4 ; C18H24O4 ; C24H40O6S ; C27H36O6 ; C7H7N2O ; C25H38O6 ; C16H26O4 ; C27H36O6 ; C22H42O6S ; C25H38O6 ; C14H16N2O ; C9H14O3 ; C9H14O3
468.01665	1.436471	0.569266	-4.0626	0.002277	2.6427	0.020594	NA	NA	NA	NA	NA
416.71182	1.525094	1.327187	4.0621	0.002279	2.6423	0.020594	NA	NA	NA	NA	NA
162.05023	1.497241	1.126022	4.06	0.002287	2.6408	0.020594	162.0502	HMDB37110 ; HMDB00043 ; HMDB00098 ; HMDB00283 ; HMDB00366 ; HMDB00466 ; HMDB00621 ; HMDB00646 ; HMDB00751 ; HMDB00883 ; HMDB01382 ; HMDB01644 ; HMDB02141 ; HMDB03355 ; HMDB03371 ; HMDB12194 ; HMDB12325 ; HMDB13716 ; HMDB15096 ; HMDB15327 ; HMDB15550 ; HMDB15602 ; HMDB29941 ; HMDB29942 ; HMDB32846 ; HMDB33564 ; HMDB33565 ; HMDB34236 ; HMDB59753 ; HMDB60254	4-(34-Dihydroxyphenyl)-23-dihydro-23-dihydroxy-1H-phenalen-1-one ; Betaine ; D-Xylose ; D-Ribose ; 2-Deoxyribonic acid ; 3-Methylindole ; D-Ribulose ; L-Arabinose ; L-Threo-2-pentulose ; L-Valine ; Vaporole ; D-Xylulose ; N-Methyl-a-aminoisobutyric acid ; 5-Aminopentanoic acid ; L-Ribulose ; Beta-D-ribopyranose ; Arabinofuranose ; Norvaline ; Mepivacaine ; Estramustine ; Amyl Nitrite ; Milnacipran ; D-Apiose ; Arabinose ; 5-Chloro-6-methoxy-2(3H)-benzoxazolone ; 23-Dihydro-2-methylthiophene ; 23-Dihydro-5-methylthiophene ; Benzenepropanenitrile ; 2-Deoxypentonic acid ; Aldehydo-D-xylose	M+2H ; M+2Na-H ; 2M+H+Na ; 2M+H+Na ; 2M+H+Na ; 2M+Na+K ; 2M+H+Na ; 2M+H+Na ; 2M+H+Na ; M+2Na-H ; M+2Na-H ; 2M+H+Na ; M+2Na-H ; M+2Na-H ; 2M+H+Na ; 2M+H+Na ; 2M+H+Na ; M+2Na-H ; M+2K ; M+H+2Na ; M+2Na-H ; M+2K ; 2M+H+Na ; 2M+H+Na ; M+3ACN+2H ; 3M+H+Na ; 3M+H+Na ; 2M+Na+K ; 2M+H+Na ; 2M+H+Na	C19H14O5 ; C5H11NO2 ; C5H10O5 ; C5H10O5 ; C5H10O5 ; C9H9N ; C5H10O5 ; C5H10O5 ; C5H10O5 ; C5H11NO2 ; C5H11NO2 ; C5H10O5 ; C5H11NO2 ; C5H11NO2 ; C5H10O5 ; C5H10O5 ; C5H10O5 ; C5H11NO2 ; C15H22N2O ; C23H31Cl2NO3 ; C5H11NO2 ; C15H22N2O ; C5H10O5 ; C5H10O5 ; C8H6ClNO3 ; C5H8S ; C5H8S ; C9H9N ; C5H10O5 ; C5H10O5
153.07719	1.536929	0.96331	4.0595	0.002288	2.6405	0.020594	153.0772	HMDB02056 ; HMDB02089 ; HMDB02261 ; HMDB03752 ; HMDB06037 ; HMDB10731 ; HMDB12486 ; HMDB13247 ; HMDB14539 ; HMDB14731 ; HMDB29226 ; HMDB29427 ; HMDB29470 ; HMDB30352 ; HMDB30725 ; HMDB31238 ; HMDB31634 ; HMDB31668 ; HMDB32417 ; HMDB33112 ; HMDB33486 ; HMDB33776 ; HMDB33941 ; HMDB34000 ; HMDB34056 ; HMDB34238 ; HMDB37130 ; HMDB37681 ; HMDB38563 ; HMDB38781 ; HMDB41379 ; HMDB59850 ; HMDB60355 ; HMDB61073	Monoisobutyl phthalic acid ; N-Ribosylhistidine ; 2-Hydroxymyristic acid ; LysoPC(10:0) ; 8-Hydroxy-7-methylguanine ; (R)-3-Hydroxy-tetradecanoic acid ; (RS)-Norlaudanosoline ; Monobutylphthalate ; Carisoprodol ; Ethosuximide ; 5-(3-Methoxy-4-hydroxyphenyl)-gamma-valerolactone ; L-Hypoglycin A ; Isodillapiole ; Arecaidine ; Dillapiol ; Ethyl methyl sulfide ; 2-Propanethiol ; Dihydrodeoxy-8-epiaustdiol ; 3-(L-Menthoxy)-2-methylpropane-12-diol ; Methylpyrazine ; Pipermethystine ; Apiole ; Falcarindiol ; Panaxydol ; 5-(34-Methylenedioxyphenyl)pentanoic acid ; 1-Propanethiol ; Piperonyl isobutyrate ; Vanillin isobutyrate ; (Z)-3-(245-Trimethoxyphenyl)-2-propenal ; 18-Heptadecadiene-46-diyne-310-diol ; 23-Dihydro-3-hydroxy-6-methoxy-22-dimethyl-4H-1-benzopyran-4-one ; Ethyl 3-hydroxydodecanoate ; 2-Polyprenyl-6-methoxyphenol ; noroxymorphone	M+2ACN+2H ; M+H+NH4 ; M+Na+K ; M+H+2Na ; M+3ACN+2H ; M+Na+K ; M+H+NH4 ; M+2ACN+2H ; M+2Na ; 2M+H+Na ; M+2ACN+2H ; 2M+H+Na ; M+2ACN+2H ; 2M+H+Na ; M+2ACN+2H ; 2M+H ; 2M+H ; M+2ACN+2H ; M+Na+K ; 3M+H+Na ; M+H+NH4 ; M+2ACN+2H ; M+2Na ; M+2Na ; M+2ACN+2H ; 2M+H ; M+2ACN+2H ; M+2ACN+2H ; M+2ACN+2H ; M+2Na ; M+2ACN+2H ; M+Na+K ; M+2Na ; M+H+NH4	C12H14O4 ; C11H17N3O6 ; C14H28O3 ; C18H39NO7P ; C6H7N5O2 ; C14H28O3 ; C16H17NO4 ; C12H14O4 ; C12H24N2O4 ; C7H11NO2 ; C12H14O4 ; C7H11NO2 ; C12H14O4 ; C7H11NO2 ; C12H14O4 ; C3H8S ; C3H8S ; C12H14O4 ; C14H28O3 ; C5H6N2 ; C16H17NO4 ; C12H14O4 ; C17H24O2 ; C17H24O2 ; C12H14O4 ; C3H8S ; C12H14O4 ; C12H14O4 ; C12H14O4 ; C17H24O2 ; C12H14O4 ; C14H28O3 ; C17H24O2 ; C16H17NO4
211.10751	1.523094	0.874734	4.0584	0.002292	2.6397	0.020594	211.1075	HMDB15441 ; HMDB15354 ; HMDB00821 ; HMDB00859 ; HMDB10323 ; HMDB11723 ; HMDB13245 ; HMDB13292 ; HMDB14619 ; HMDB31303 ; HMDB31306 ; HMDB31847 ; HMDB31855 ; HMDB31923 ; HMDB32388 ; HMDB33135 ; HMDB33285 ; HMDB33517 ; HMDB34250 ; HMDB37292 ; HMDB38072 ; HMDB40594 ; HMDB40732 ; HMDB41933 ; HMDB41984 ; HMDB59677 ; HMDB59683 ; HMDB59697 ; HMDB60366 ; HMDB60389 ; HMDB60758 ; HMDB60869	Aprobarbital ; Aminophylline ; Phenylacetylglycine ; Methylhippuric acid ; Phenethylamine glucuronide ; 2-Methylhippuric acid ; m-Methylhippuric acid ; p-Methylhippuric acid ; Duloxetine ; Osmundalactone ; 3-Hydroxy-45-dimethyl-2(5H)-furanone ; 2-Ethyl-34-dihydroxyfuran ; 5-Methyl-2-phenyl-2-hexenal ; 1020-Dihydroxyeicosanoic acid ; Methyl n-acetylanthranilate ; Pyrimethanil ; L-erythro-5-(1-Hydroxyethyl)-2(5H)-furanone ; 56-Dihydro-4-methoxy-2H-pyran-2-one ; Betamipron ; 2-Butyl-3-phenyl 2-propen-1-al ; xi-5-Acetyltetrahydro-2(3H)-furanone ; ()-Furaneol ; 27-Oxepanedione ; Mitragynine ; Pholcodine ; 3-Buten-2-one 1-(236-trimethyl phenyl) ; 1-H-Inden-1-one23-dihydro-3356-tetramethyl ; 1 (236-trimethyl phenyl)-3-Buten-2-one ; 3-Carbamoyl-2-phenylpropionaldehyde ; 4-Hydroxy-5-phenyltetrahydro-13-oxazin-2-one ; 4-Anilino-4-oxobutanoic acid ; Ramipril Diketopiperazine	M+H ; M+2H ; M+NH4 ; M+NH4 ; M+3ACN+2H ; M+NH4 ; M+NH4 ; M+NH4 ; M+3ACN+2H ; M+2ACN+H ; M+2ACN+H ; M+2ACN+H ; M+Na ; M+2K ; M+NH4 ; 2M+H+Na ; M+2ACN+H ; M+2ACN+H ; M+NH4 ; M+Na ; M+2ACN+H ; M+2ACN+H ; M+2ACN+H ; M+H+Na ; M+H+Na ; M+Na ; M+Na ; M+Na ; M+NH4 ; M+NH4 ; M+NH4 ; M+H+Na	C10H14N2O3 ; C16H24N10O4 ; C10H11NO3 ; C10H11NO3 ; C14H19NO6 ; C10H11NO3 ; C10H11NO3 ; C10H11NO3 ; C18H19NOS ; C6H8O3 ; C6H8O3 ; C6H8O3 ; C13H16O ; C20H40O4 ; C10H11NO3 ; C12H13N3 ; C6H8O3 ; C6H8O3 ; C10H11NO3 ; C13H16O ; C6H8O3 ; C6H8O3 ; C6H8O3 ; C23H30N2O4 ; C23H30N2O4 ; C13H16O ; C13H16O ; C13H16O ; C10H11NO3 ; C10H11NO3 ; C10H11NO3 ; C23H30N2O4
617.69418	1.579219	0.417644	-4.0569	0.002298	2.6387	0.020594	617.6942	HMDB29266 ; HMDB29548 ; HMDB32788 ; HMDB36009 ; HMDB37413 ; HMDB37414 ; HMDB37416 ; HMDB37448 ; HMDB37453 ; HMDB38810 ; HMDB38819 ; HMDB39856 ; HMDB39909 ; HMDB40376 ; HMDB59907	Kaempferol 3-O-feruloyl-caffeoyl-sophoroside 7-O-glucoside ; Diosmin ; Kaempferol 3-(2-feruloylsophoroside) 7-cellobioside ; Physcion 8-gentiobioside ; 36-Diglucopyranosyl-57-dihydroxy-4-methoxyflavone ; 8-Galactopyranosyl-6-glucopyranosyl-45-dihydroxy-7-methoxyflavone ; Isoswertisin 4-glucoside ; Diosmetin 8-C-(2-rhamnosylglucoside) ; Chrysoeriol 7-rutinoside ; Rhamnetin 3-(4-rhamnosylrhamnoside) ; Calendoflaside ; Diosmetin 7-neohesperidoside ; Isotectorigenin 4-glucoside 7-rhamnoside ; 24-Dimethyldodecane ; Tetradecane	M+3ACN+2H ; 2M+H+NH4 ; M+3ACN+2H ; 2M+H+NH4 ; 2M+H+NH4 ; 2M+H+NH4 ; 2M+H+NH4 ; 2M+H+NH4 ; 2M+H+NH4 ; 2M+H+NH4 ; 2M+H+NH4 ; 2M+H+NH4 ; 2M+H+NH4 ; 3M+Na ; 3M+Na	C52H54O27 ; C28H32O15 ; C49H58O29 ; C28H32O15 ; C28H32O15 ; C28H32O15 ; C28H32O15 ; C28H32O15 ; C28H32O15 ; C28H32O15 ; C28H32O15 ; C28H32O15 ; C28H32O15 ; C14H30 ; C14H30

500.87927	1.504236	1.50417	-4.0552	0.002304	2.6375	0.020594	NA	NA	NA	NA	NA
195.13795	1.533135	0.486187	4.0551	0.002305	2.6374	0.020594	195.138	HMDB30366 ; HMDB14995	Cavipetin C ; Terbinafine	M+2H ; 2M+3H	C24H36O4 ; C21H25N
474.75997	1.497439	1.483568	4.0539	0.002309	2.6366	0.020594	474.76	HMDB33404 ; HMDB36485 ; HMDB36943 ; HMDB39448 ; HMDB39512	Quillaic acid 3-galactosyl-(1-2)-glucuronide ; Trigoneoside XIb ; Oleragenoside ; Licoricesaponin J2 ; Periandrin III	M+3ACN+2H ; M+ACN+2H ; M+3ACN+2H ; M+3ACN+2H ; M+3ACN+2H	C42H64O16 ; C44H74O19 ; C42H64O16 ; C42H64O16 ; C42H64O16
149.54868	1.530967	0.796821	-4.0535	0.002311	2.6363	0.020594	149.5487	HMDB37297 ; HMDB01786 ; HMDB14444 ; HMDB28822 ; HMDB28823 ; HMDB28906 ; HMDB28928 ; HMDB30697 ; HMDB30809 ; HMDB32571 ; HMDB32922 ; HMDB33121 ; HMDB33130 ; HMDB33159 ; HMDB40269 ; HMDB41281 ; HMDB41502	Hexahydro-67-dihydroxy-5-(hydroxymethyl)-3-(2-hydroxyphenyl)-2H-pyrano23-doxazol-2-one ; Ethenodeoxyadenosine ; Penciclovir ; Glutamyl-Isoleucine ; Glutamyl-Leucine ; Isoleucyl-Glutamate ; Leucyl-Glutamate ; Sarmentosin ; (2R3S)-Piscidic acid ; 2-Methoxybenzenethiol ; Kahweofuran ; Paraquat dichloride ; 2-Acetyl-5-methylthiophene ; 2-Propanoylthiophene ; Choline bitartrate ; 2-(Methylthio)phenol ; 2-Acetyl-3-methylthiophene	M+2H ; M+H+Na ; M+2Na ; M+H+K ; M+H+K ; M+H+K ; M+H+K ; M+H+Na ; M+ACN+2H ; 2M+H+NH4 ; 2M+H+NH4 ; M+ACN+2H ; 2M+H+NH4 ; 2M+H+NH4 ; M+2Na ; 2M+H+NH4 ; 2M+H+NH4	C13H15NO7 ; C12H13N5O3 ; C10H15N5O3 ; C11H19N2O5 ; C11H19N2O5 ; C11H19N2O5 ; C11H19N2O5 ; C11H17NO7 ; C11H12O7 ; C7H8OS ; C7H8OS ; C12H14Cl2N2 ; C7H8OS ; C7H8OS ; C9H19NO7 ; C7H8OS ; C7H8OS
636.14305	1.514739	1.257437	4.0476	0.002332	2.6322	0.020751	636.1431	HMDB32389 ; HMDB33005 ; HMDB36336 ; HMDB40791 ; HMDB60927	Methyl acrylate-divinylbenzene completely hydrolyzed copolymer ; 4-Methyl-6-(34-dihydroxy-E-cinnamoyl)isoorientin ; Prodelphinidin A1 ; Acrimarine N ; Hydroxylumiracoxib	3M+2K ; 2M+H+Na ; 2M+3H2O+2H ; M+2K+H ; 2M+NH4	C18H18N6O3S ; C31H28O14 ; C30H24O14 ; C32H31NO8 ; C15H13ClFNO3
828.25825	1.540371	1.354668	-4.0436	0.002347	2.6294	0.020848	828.2583	HMDB11705 ; HMDB11712 ; HMDB11713 ; HMDB42102 ; HMDB42392 ; HMDB42970 ; HMDB43026 ; HMDB43027 ; HMDB43054 ; HMDB43166 ; HMDB43194 ; HMDB43222 ; HMDB43864 ; HMDB43865 ; HMDB44700 ; HMDB45506	TG(15:018:1(9Z)16:0)iso6 ; TG(15:020:014:1(9Z))iso6 ; TG(15:020:1(11Z)14:0)iso6 ; TG(14:015:020:1(11Z)) ; TG(14:020:1(11Z)15:0) ; TG(15:014:020:1(11Z)) ; TG(15:016:018:1(11Z)) ; TG(15:016:018:1(9Z)) ; TG(15:018:016:1(9Z)) ; TG(15:014:1(9Z)20:0) ; TG(15:016:1(9Z)18:0) ; TG(15:018:1(11Z)16:0) ; TG(16:015:018:1(11Z)) ; TG(16:015:018:1(9Z)) ; TG(18:015:016:1(9Z)) ; TG(20:015:014:1(9Z))	2M+H+NH4 ; 2M+H+NH4 ; 2M+H+NH4 ; 2M+H+NH4 ; 2M+H+NH4 ; 2M+H+NH4 ; 2M+H+NH4 ; 2M+H+NH4 ; 2M+H+NH4 ; 2M+H+NH4 ; 2M+H+NH4 ; 2M+H+NH4 ; 2M+H+NH4 ; 2M+H+NH4 ; 2M+H+NH4 ; 2M+H+NH4	C52H98O6 ; C52H98O6 ; C52H98O6 ; C52H98O6 ; C52H98O6 ; C52H98O6 ; C52H98O6 ; C52H98O6 ; C52H98O6 ; C52H98O6 ; C52H98O6 ; C52H98O6 ; C52H98O6 ; C52H98O6 ; C52H98O6 ; C52H98O6
322.06385	1.512667	1.237934	-4.0388	0.002366	2.626	0.020948	322.0639	HMDB01016 ; HMDB15167 ; HMDB29745 ; HMDB38956 ; HMDB59656 ; HMDB59773 ; HMDB60421 ; HMDB14473	D-4-Phosphopantothenate ; Mercaptopurine ; Fenbendazole ; Avenalumin III ; O-acetyl-ADP-ribose ; S-3-oxodecanoyl cysteamine ; 7-Methyl-3-oxo-6-octenoyl-CoA ; Indomethacin	M+Na ; 2M+NH4 ; M+Na ; 2M+Na+K ; M+ACN+2H ; M+2K-H ; M+H+2Na ; M+H-2H2O	C9H18NO8P ; C5H4N4S ; C15H13N3O2S ; C18H13NO3 ; C17H25N5O15P2 ; C12H23NO2S ; C30H48N7O18P3S ; C19H16ClNO4
534.68865	1.520704	1.143355	-4.0379	0.002369	2.6254	0.020948	534.6887	HMDB32602 ; HMDB57026 ; HMDB57027 ; HMDB57036 ; HMDB57037 ; HMDB57276 ; HMDB57277 ; HMDB57302 ; HMDB57310 ; HMDB57312 ; HMDB57320 ; HMDB57322 ; HMDB57323 ; HMDB57325 ; HMDB57350 ; HMDB57358 ; HMDB57360 ; HMDB57368 ; HMDB57370 ; HMDB57371 ; HMDB57392 ; HMDB57393 ; HMDB57396 ; HMDB57397 ; HMDB58217 ; HMDB58218 ; HMDB58227 ; HMDB58228 ; HMDB58264 ; HMDB58265 ; HMDB58274 ; HMDB58275 ; HMDB58610 ; HMDB58611 ; HMDB58620 ; HMDB58621 ; HMDB58647 ; HMDB58648 ; HMDB58657 ; HMDB58658 ; HMDB59209 ; HMDB59217 ; HMDB59219 ; HMDB59227 ; HMDB59229 ; HMDB59230 ; HMDB59258 ; HMDB59259 ; HMDB59268 ; HMDB59269 ; HMDB59279 ; HMDB59280 ; HMDB59289 ; HMDB59290 ; HMDB59340 ; HMDB59341 ;	4-O-Methylkanzonol W ; CL(18:018:022:5(4Z7Z10Z13Z16Z)22:5(4Z7Z10Z13Z16Z)) ; CL(18:018:022:5(4Z7Z10Z13Z16Z)22:5(7Z10Z13Z16Z19Z)) ; CL(18:018:022:5(7Z10Z13Z16Z19Z)22:5(4Z7Z10Z13Z16Z)) ; CL(18:018:022:5(7Z10Z13Z16Z19Z)22:5(7Z10Z13Z16Z19Z)) ; CL(18:022:5(4Z7Z10Z13Z16Z)18:022:5(4Z7Z10Z13Z16Z)) ; CL(18:022:5(4Z7Z10Z13Z16Z)18:022:5(7Z10Z13Z16Z19Z)) ; CL(18:022:5(4Z7Z10Z13Z16Z)22:5(4Z7Z10Z13Z16Z)18:0) ; CL(18:022:5(4Z7Z10Z13Z16Z)22:5(4Z7Z10Z13Z16Z)22:6(4Z7Z10Z13Z16Z19Z)) ; CL(18:022:5(4Z7Z10Z13Z16Z)22:5(7Z10Z13Z16Z19Z)18:0) ; CL(18:022:5(4Z7Z10Z13Z16Z)22:5(7Z10Z13Z16Z19Z)22:6(4Z7Z10Z13Z16Z19Z)) ; CL(18:022:5(4Z7Z10Z13Z16Z)22:6(4Z7Z10Z13Z16Z19Z)22:5(4Z7Z10Z13Z16Z)) ; CL(18:022:5(4Z7Z10Z13Z16Z)22:6(4Z7Z10Z13Z16Z19Z)22:5(7Z10Z13Z16Z19Z)) ; CL(18:022:5(7Z10Z13Z16Z19Z)18:022:5(7Z10Z13Z16Z19Z)) ; CL(18:022:5(7Z10Z13Z16Z19Z)22:5(4Z7Z10Z13Z16Z)18:0) ; CL(18:022:5(7Z10Z13Z16Z19Z)22:5(4Z7Z10Z13Z16Z)22:6(4Z7Z10Z13Z16Z19Z)) ; CL(18:022:5(7Z10Z13Z16Z19Z)22:5(7Z10Z13Z16Z19Z)18:0) ; CL(18:022:5(7Z10Z13Z16Z19Z)22:5(7Z10Z13Z16Z19Z)22:6(4Z7Z10Z13Z16Z19Z)) ; CL(18:022:5(7Z10Z13Z16Z19Z)22:6(4Z7Z10Z13Z16Z19Z)22:5(4Z7Z10Z13Z16Z)) ; CL(18:022:5(7Z10Z13Z16Z19Z)22:6(4Z7Z10Z13Z16Z19Z)22:5(7Z10Z13Z16Z19Z)) ; CL(18:022:6(4Z7Z10Z13Z16Z19Z)22:5(4Z7Z10Z13Z16Z)22:5(4Z7Z10Z13Z16Z)) ; CL(18:022:6(4Z7Z10Z13Z16Z19Z)22:5(4Z7Z10Z13Z16Z)22:5(7Z10Z13Z16Z19Z)) ; CL(18:022:6(4Z7Z10Z13Z16Z19Z)22:5(7Z10Z13Z16Z19Z)22:5(4Z7Z10Z13Z16Z)) ; CL(18:022:6(4Z7Z10Z13Z16Z19Z)22:5(7Z10Z13Z16Z19Z)22:5(7Z10Z13Z16Z19Z)) ; CL(18:1(11Z)22:5(4Z7Z10Z13Z16Z)22:5(4Z7Z10Z13Z16Z)22:5(4Z7Z10Z13Z16Z)) ; CL(18:1(11Z)22:5(4Z7Z10Z13Z16Z)22:5(4Z7Z10Z13Z16Z)22:5(7Z10Z13Z16Z19Z)) ; CL(18:1(11Z)22:5(4Z7Z10Z13Z16Z)22:5(7Z10Z13Z16Z19Z)22:5(4Z7Z10Z13Z16Z)) ; CL(18:1(11Z)22:5(4Z7Z10Z13Z16Z)22:5(7Z10Z13Z16Z19Z)22:5(7Z10Z13Z16Z19Z)) ; CL(18:1(11Z)22:5(7Z10Z13Z16Z19Z)22:5(4Z7Z10Z13Z16Z)22:5(4Z7Z10Z13Z16Z)) ;	3M+H+NH4 ; M+H+2Na ; M+H+2Na ; M+H+2Na ; M+H+2Na ; M+H+2Na ; M+H+2Na ; M+H+2Na ; M+3H ; M+H+2Na ; M+3H ; M+3H ; M+3H ; M+H+2Na ; M+H+2Na ; M+3H ; M+H+2Na ; M+3H ; M+H+2Na ; M+3H ; M+H+2Na ; M+3H ; M+3H ; M+3H ; M+3H ; M+3H ; M+3H ; M+3H ; M+3H ; M+3H ; M+3H ; M+3H ; M+3H ; M+3H ; M+3H ; M+3H ; M+3H ; M+3H ; M+3H ; M+3H ; M+H+2Na ; M+3H ; M+3H ; M+3H ; M+3H ; M+3H ; M+3H ; M+3H ; M+3H ; M+3H	C21H18O5 ; C89H154O17P2 ; C89H154O17P2 ; C89H154O17P2 ; C89H154O17P2 ; C89H154O17P2 ; C89H154O17P2 ; C89H154O17P2 ; C93H150O17P2 ; C89H154O17P2 ; C93H150O17P2 ; C93H150O17P2 ; C93H150O17P2 ; C89H154O17P2 ; C89H154O17P2 ; C93H150O17P2 ; C89H154O17P2 ; C93H150O17P2 ; C89H154O17P2 ; C93H150O17P2 ; C89H154O17P2 ; C93H150O17P2 ; C93H150O17P2 ; C93H150O17P2 ; C93H150O17P2 ; C93H150O17P2 ; C93H150O17P2 ; C93H150O17P2 ; C93H150O17P2 ; C93H150O17P2 ; C93H150O17P2 ; C93H150O17P2 ; C93H150O17P2 ; C93H150O17P2 ; C93H150O17P2 ; C93H150O17P2 ; C93H150O17P2 ; C89H154O17P2 ; C93H150O17P2 ; C93H150O17P2 ; C93H150O17P2 ; C93H150O17P2 ; C93H150O17P2 ; C93H150O17P2 ; C93H150O17P2 ; C93H150O17P2
758.92591	1.458805	0.765691	-4.0361	0.002376	2.6241	0.020948	NA	NA	NA	NA	NA
371.35787	1.53898	0.900448	4.036	0.002377	2.6241	0.020948	371.3579	HMDB10697 ; HMDB37105 ; HMDB46615 ; HMDB47163 ; HMDB47325 ; HMDB47348 ; HMDB47485 ; HMDB51511 ; HMDB51997	Cer(t18:016:0) ; Armillaramide ; TG(22:024:1(15Z)24:1(15Z)) ; TG(24:024:022:2(13Z16Z)) ; TG(24:022:1(13Z)24:1(15Z)) ; TG(24:024:1(15Z)22:1(13Z)) ; TG(24:022:2(13Z16Z)24:0) ; TG(22:1(13Z)24:024:1(15Z)) ; TG(24:1(15Z)22:024:1(15Z))	2M+3H ; 2M+3H ; M+3H ; M+3H ; M+3H ; M+3H ; M+3H ; M+3H ; M+3H	C34H69NO4 ; C34H69NO4 ; C73H138O6 ; C73H138O6 ; C73H138O6 ; C73H138O6 ; C73H138O6 ; C73H138O6 ; C73H138O6
232.03694	1.454959	0.577745	-4.035	0.00238	2.6234	0.020948	232.0369	HMDB30789 ; HMDB32645 ; HMDB33145 ; HMDB35086 ; HMDB36346 ; HMDB39820 ; HMDB61120 ; HMDB01319	Dracunculin ; 3-Glucosyl-23446-pentahydroxybenzophenone ; Azimsulfuron ; Buntansin A ; 7-Hydroxy-2-methyl-4-oxo-4H-1-benzopyran-5-carboxylic acid ; 8-Methoxy-67-methylenedioxycoumarin ; 3-Hydroxy-4-aminopyridine sulfate ; Pyridoxine 5-phosphate	2M+H+Na ; M+H+K ; M+H+K ; 2M+H+Na ; 2M+H+Na ; 2M+H+Na ; M+ACN+H ; M+H-H2O	C11H8O5 ; C19H20O11 ; C13H16N10O5S ; C11H8O5 ; C11H8O5 ; C11H8O5 ; C5H6N2O4S ; C8H12NO6P

412.34173	1.480099	0.025188	4.0334	0.002386	2.6223	0.020948	412.3417	HMDB10497 ; HMDB42089 ; HMDB42293 ; HMDB42298 ; HMDB42327 ; HMDB42557 ; HMDB42564 ; HMDB42728 ; HMDB42767 ; HMDB42774 ; HMDB42849 ; HMDB42878 ; HMDB42901 ; HMDB44049 ; HMDB44562 ; HMDB47739 ; HMDB47744 ; HMDB47789 ; HMDB47899 ; HMDB47920 ; HMDB47926 ; HMDB47948 ; HMDB47971 ; HMDB48162 ; HMDB48207 ; HMDB48278 ; HMDB48279 ; HMDB48300 ; HMDB48431 ; HMDB48578 ; HMDB48584 ; HMDB48605 ; HMDB48941 ; HMDB49234 ; HMDB49855 ; HMDB52845 ; HMDB52852 ; HMDB05432 ; HMDB10411 ; HMDB10422 ; HMDB40270 ; HMDB41619 ; HMDB42064 ; HMDB42073 ; HMDB42079 ; HMDB42123 ; HMDB42137 ; HMDB42144 ; HMDB42151 ; HMDB42421 ; HMDB42543 ; HMDB42601 ; HMDB42753 ; HMDB42990 ; HMDB43004 ; HMDB43011 ; [illegible]	TG(18:3(9Z12Z15Z)14:018:3(9Z12Z15Z))iso3 ; TG(14:014:022:6(4Z7Z10Z13Z16Z19Z)) ; TG(14:014:1(9Z)22:5(4Z7Z10Z13Z16Z)) ; TG(14:014:1(9Z)22:5(7Z10Z13Z16Z19Z)) ; TG(14:016:1(9Z)20:5(5Z8Z11Z14Z17Z)) ; TG(14:018:3(6Z9Z12Z)18:3(6Z9Z12Z)) ; TG(14:018:3(6Z9Z12Z)18:3(9Z12Z15Z)) ; TG(14:022:5(4Z7Z10Z13Z16Z)14:1(9Z)) ; TG(14:018:3(9Z12Z15Z)18:3(6Z9Z12Z)) ; TG(14:018:3(9Z12Z15Z)18:3(9Z12Z15Z)) ; TG(14:020:5(5Z8Z11Z14Z17Z)16:1(9Z)) ; TG(14:022:5(7Z10Z13Z16Z19Z)14:1(9Z)) ; TG(14:022:6(4Z7Z10Z13Z16Z19Z)14:0) ; TG(16:014:1(9Z)20:5(5Z8Z11Z14Z17Z)) ; TG(16:020:5(5Z8Z11Z14Z17Z)14:1(9Z)) ; TG(14:1(9Z)14:022:5(4Z7Z10Z13Z16Z)) ; TG(14:1(9Z)14:022:5(7Z10Z13Z16Z19Z)) ; TG(14:1(9Z)16:020:5(5Z8Z11Z14Z17Z)) ; TG(14:1(9Z)14:1(9Z)22:4(7Z10Z13Z16Z)) ; TG(14:1(9Z)16:1(9Z)20:4(5Z8Z11Z14Z)) ; TG(14:1(9Z)16:1(9Z)20:4(8Z11Z14Z17Z)) ; TG(14:1(9Z)18:1(11Z)18:4(6Z9Z12Z15Z)) ; TG(14:1(9Z)18:1(9Z)18:4(6Z9Z12Z15Z)) ; TG(14:1(9Z)20:4(5Z8Z11Z14Z)16:1(9Z)) ; TG(14:1(9Z)22:4(7Z10Z13Z16Z)14:1(9Z)) ; TG(14:1(9Z)18:4(6Z9Z12Z15Z)18:1(11Z)) ; TG(14:1(9Z)18:4(6Z9Z12Z15Z)18:1(9Z)) ; TG(14:1(9Z)20:4(8Z11Z14Z17Z)16:1(9Z)) ; TG(16:1(9Z)14:020:5(5Z8Z11Z14Z17Z)) ; TG(16:1(9Z)14:1(9Z)20:4(5Z8Z11Z14Z)) ; TG(16:1(9Z)14:1(9Z)20:4(8Z11Z14Z17Z)) ; TG(16:1(9Z)16:1(9Z)18:4(6Z9Z12Z15Z)) ; TG(16:1(9Z)18:4(6Z9Z12Z15Z)16:1(9Z)) ; TG(18:1(11Z)14:1(9Z)18:4(6Z9Z12Z15Z)) ; TG(18:1(9Z)14:1(9Z)18:4(6Z9Z12Z15Z)) ; TG(18:3(6Z9Z12Z)14:018:3(6Z9Z12Z)) ; TG(18:3(6Z9Z12Z)14:018:3(9Z12Z15Z)) ; TG(16:1(9Z)16:1(9Z)16:1(9Z))iso ; TG(16:014:016:0)iso3 ; TG(16:1(9Z)14:018:2(9Z12Z))iso6 ; Diethylhexyl adipate ; Dioctyl hexanedioate ; TG(14:014:018:0) ; TG(14:014:020:3(5Z8Z11Z)) ; TG(14:014:020:3n6) ; TG(14:016:016:0) ; TG(14:016:018:3(6Z9Z12Z)) ; TG(14:016:018:3(9Z12Z15Z)) ; TG(14:018:014:0) ; TG(14:020:3(5Z8Z11Z)14:0) ; TG(14:018:3(6Z9Z12Z)16:0) ; TG(14:020:3n614:0) ; TG(14:018:3(9Z12Z15Z)16:0) ; TG(15:015:016:0) ; TG(15:015:018:3(6Z9Z12Z)) ; TG(15:015:018:3(9Z12Z15Z)) ; TG(15:016:015:0) ; TG(15:014:1(9Z)18:2(9Z12Z)) ; TG(15:014:1(9Z)20:2n6) ; TG(15:018:2(9Z12Z)14:1(9Z)) ; TG(15:018:3(6Z9Z12Z)15:0) ; TG(15:020:2n614:1(9Z)) ; TG(15:018:3(9Z12Z15Z)15:0) ; TG(16:014:018:3(6Z9Z12Z)) ; TG(16:014:018:3(9Z12Z15Z)) ; TG(14:1(9Z)15:018:2(9Z12Z)) ; TG(14:1(9Z)15:020:2n6) ; TG(14:1(9Z)14:1(9Z)20:1(11Z)) ; TG(14:1(9Z)16:1(9Z)18:1(11Z)) ; [illegible]	M+2H ; M+H+Na ; M+2Na ; M+H+Na ; M+ACN+H ; M+ACN+H ; M+2Na ; M+H+Na ; M+H+Na ; M+2Na ; M+H+Na ; M+H+Na ; M+2Na ; M+H+Na ; M+H+Na ; M+H+Na ; M+H+Na ; M+2Na ; M+H+Na ; M+H+Na ; M+2Na ; M+H+Na ; M+H+Na ; M+H+Na ; M+H+Na ; M+H+Na ; M+H+Na ; M+H+Na ; M+H+Na ; M+H+Na ; M+H+Na ; M+H+Na ; M+H+Na ; M+H+Na ; M+H+Na ; M+H+Na ; M+H+Na ; M+H+Na ; M+H+Na	C53H90O6 ; C51H92O6 ; C49H94O6 ; C51H92O6 ; C22H42O4 ; C22H42O4 ; C49H94O6 ; C51H92O6 ; C51H92O6 ; C49H94O6 ; C51H92O6 ; C51H92O6 ; C49H94O6 ; C51H92O6 ; C51H92O6 ; C51H92O6 ; C51H92O6 ; C49H94O6 ; C51H92O6 ; C51H92O6 ; C49H94O6 ; C51H92O6 ; C51H92O6 ; C51H92O6 ; C51H92O6 ; C51H92O6 ; C51H92O6 ; C51H92O6 ; C51H92O6 ; C51H92O6 ; C51H92O6 ; C51H92O6 ; C51H92O6 ; C51H92O6 ; C51H92O6 ; C51H92O6 ; C51H92O6 ; C51H92O6 ; C51H92O6
569.86154	1.460212	0.193319	4.033	0.002388	2.622	0.020948	569.8615	HMDB00582 ; HMDB05869	35-Diiodothyronine ; 33-Diiodothyronine	M+2Na-H ; M+2Na-H	C15H13I2NO4 ; C15H13I2NO4
285.04487	1.471527	0.816868	-4.031	0.002396	2.6206	0.020978	285.0449	HMDB31821 ; HMDB32563 ; HMDB34240 ; HMDB34318 ; HMDB38507	12-Diphenylcyclobutane ; 24-Diphenyl-1-butene ; Styrene ; Sennidin C ; Rheidin A	M+2K-H ; M+2K-H ; 2M+2K-H ; M+2Na ; M+2Na	C16H16 ; C16H16 ; C8H8 ; C30H20O9 ; C30H20O9
266.07463	1.424522	0.858913	4.0243	0.002422	2.6159	0.021167	266.0746	HMDB60663 ; HMDB29438 ; HMDB30400 ; HMDB33073 ; HMDB33074 ; HMDB33557 ; HMDB35921 ; HMDB35984 ; HMDB36370 ; HMDB36375 ; HMDB37973 ; HMDB37995 ; HMDB38487 ; HMDB41716 ; HMDB42006	Isoniazid alpha-ketoglutaric acid ; L-Phosphoarginine ; L-4-Chlorotryptophan ; 1-Propenyl 1-(propylsulfinyl)propyl disulfide ; 1-(1-Propenylsulfinyl)propyl propyl disulfide ; ()-6-Chlorotryptophan ; Limonin ; Zapoterin ; 1-(2-Propenylsulfinyl)propyl propyl disulfide ; 2-Propenyl 1-(propylsulfinyl)propyl disulfide ; Cyanidin 335-triglucoside ; Cyanidin 3-sophorotrioside ; Cyanidin 3-sophoroside 5-glucoside ; Cyanidin 3-diglucoside 5-glucoside ; Ricinine	M+H ; 2M+H+Na ; 2M+3H2O+2H ; 2M+3H2O+2H ; 2M+3H2O+2H ; 2M+3H2O+2H ; M+Na+K ; M+Na+K ; 2M+3H2O+2H ; 2M+3H2O+2H ; M+2H+Na ; M+2H+Na ; M+2H+Na ; M+2H+Na ; 3M+H+K	C11H11N3O5 ; C6H15N4O5P ; C11H11ClN2O2 ; C9H18OS3 ; C9H18OS3 ; C11H11ClN2O2 ; C26H30O8 ; C26H30O8 ; C9H18OS3 ; C9H18OS3 ; C33H41O21 ; C33H41O21 ; C33H41O21 ; C33H41O21 ; C8H8N2O2
171.09579	1.487403	0.130376	4.0219	0.002431	2.6142	0.021214	171.0958	HMDB01091 ; HMDB40799 ; HMDB00673 ; HMDB03797 ; HMDB04995 ; HMDB05047 ; HMDB05048 ; HMDB06007 ; HMDB06227 ; HMDB06270 ; HMDB13036 ; HMDB13865 ; HMDB15091 ; HMDB29800 ; HMDB30208 ; HMDB30212 ; HMDB30248 ; HMDB30255 ; HMDB30420 ; HMDB30430 ; HMDB30957 ; HMDB30958 ; HMDB31051 ; HMDB31097 ; HMDB31262 ; HMDB31269 ; HMDB31527 ; HMDB31599 ; HMDB31834 ; HMDB32535 ; HMDB32667 ; HMDB32691 ; HMDB32962 ; HMDB33554 ; HMDB33647 ; HMDB33772 ; HMDB33777 ; HMDB33794 ; HMDB34758 ; HMDB34761 ; HMDB34764 ; HMDB34845 ; HMDB35916 ; HMDB35926 ; HMDB35927 ; HMDB37001 ; HMDB37150 ; HMDB37170 ; HMDB38067 ; HMDB38400 ; HMDB38447 ; HMDB38647 ; HMDB38725 ; HMDB38977 ; [illegible]	3-Hydroxyquinine ; 11-Hydroxytubotaiwine ; Linoleic acid ; Bovinic acid ; Codeine ; 9E11E-Octadecadienoic acid ; 10E12Z-Octadecadienoic acid ; Isopentanol ; 1-a24R25-Trihydroxyvitamin D2 ; Linoelaidic acid ; 1-Pentanol ; CGP72383 ; Hydrocodone ; Mangiferic acid ; Koenigicine ; Mukonicine ; Neopine ; Erysodine ; Strobilurin A ; Linalyl caprylate ; (3Z6Z)-36-Nonadien-1-ol ; (Z)-6-Nonenal ; Ethyl 2E4Z-hexadecadienoate ; 5-Octadecynoic acid ; 26-Nonadien-1-ol ; 2-Nonenal ; (S)-2-Methyl-1-butanol ; 2-Pentanol ; ()-26-Dimethyl-5-heptenal ; trans-4-Nonenal ; 59-Epidioxy-3-hydroxyergost-7-en-6-one ; (3beta5alpha6alpha7beta14alpha22E24R)-56-Epoxyergosta-822-diene-3714-triol ; Secoclausenamide ; 2-Methyl-2-octenal ; 56:89-Diepoxyergost-22-ene-37beta-diol ; 2-Methyl-2-butanol ; 3-Methyl-2-butanol ; ()-226-Trimethylcyclohexanone ; 8-Nonen-3-one ; 5-Nonen-2-one ; 3-Nonen-2-one ; 24-Nonadien-1-ol ; (E)-2-Nonen-4-one ; 8-Nonen-2-one ; 1-Nonen-4-one ; Santene hydrate ; 2-Ethyl-2-heptenal ; 3-Methylene-2-octanone ; (3E6Z)-36-Nonadien-1-ol ; (2xi4xi)-24-Nonadien-1-ol ; 1-Isothiocyanato-8-(methylthio)octane ; (2E6E)-Piperamide-C7:2 ; (R)-Juziphine ; (3beta5alpha6beta9alpha22E24R)-59-Epidioxyergosta-722-diene-36-diol ; 7-Methyl-3-octen-2-one ; Pandamarilactone 32 ; Physagulin A ; 3-Nonen-5-one ; (S)-N-Methylcoclaurine ; N-depropylpropafenone ; N-Monodemethylolopatadine ; N-desalkylpropafenone	M+2H ; M+2H ; M+Na+K ; M+Na+K ; M+ACN+2H ; M+Na+K ; M+Na+K ; 3M+2K ; M+3Na ; M+Na+K ; 3M+2K ; M+3H ; M+ACN+2H ; M+Na+K ; M+H+NH4 ; M+H+NH4 ; M+ACN+2H ; M+ACN+2H ; M+2ACN+2H ; M+Na+K ; 2M+Na+K ; 2M+Na+K ; M+Na+K ; M+Na+K ; 2M+Na+K ; 2M+Na+K ; 3M+2K ; 3M+2K ; 2M+Na+K ; 2M+Na+K ; M+3Na ; M+3Na ; M+ACN+2H ; 2M+Na+K ; M+3Na ; 3M+2K ; 3M+2K ; 2M+Na+K ; 2M+Na+K ; 2M+Na+K ; 2M+Na+K ; 2M+Na+K ; 2M+Na+K ; 2M+Na+K ; 2M+Na+K ; 2M+Na+K ; 2M+Na+K ; 2M+Na+K ; 2M+Na+K ; 2M+Na+K ; M+3ACN+2H ; M+ACN+2H ; M+ACN+2H ; M+3Na ; 2M+Na+K ; M+ACN+2H ; M+3H ; 2M+Na+K ; M+ACN+2H ; M+ACN+2H ; M+H+NH4 ; M+ACN+2H	C20H24N2O3 ; C20H24N2O3 ; C18H32O2 ; C18H32O2 ; C18H21NO3 ; C18H32O2 ; C18H32O2 ; C5H12O ; C28H44O4 ; C18H32O2 ; C5H12O ; C29H32N7O2 ; C18H21NO3 ; C18H32O2 ; C20H21NO3 ; C20H21NO3 ; C18H21NO3 ; C18H21NO3 ; C16H18O3 ; C18H32O2 ; C9H16O ; C9H16O ; C18H32O2 ; C18H32O2 ; C9H16O ; C9H16O ; C5H12O ; C5H12O ; C9H16O ; C9H16O ; C28H44O4 ; C28H44O4 ; C18H21NO3 ; C9H16O ; C28H44O4 ; C5H12O ; C5H12O ; C9H16O ; C9H16O ; C9H16O ; C9H16O ; C9H16O ; C9H16O ; C9H16O ; C9H16O ; C9H16O ; C9H16O ; C9H16O ; C9H16O ; C9H16O ; C10H19NS2 ; C18H21NO3 ; C18H21NO3 ; C28H44O4 ; C9H16O ; C18H21NO3 ; C30H38O7 ; C9H16O ; C18H21NO3 ; C18H21NO3 ; C20H21NO3 ; C18H21NO3

145.10530	1.462914	0.802014	-4.0174	0.002449	2.6111	0.021331	145.1053	HMDB31885 ; HMDB02368 ; HMDB29140 ; HMDB29799 ; HMDB35559 ; HMDB35560 ; HMDB35561 ; HMDB35562 ; HMDB35563 ; HMDB36371 ; HMDB41445 ; HMDB60656	6-Hydroxypentadecanedioic acid ; Nervonic acid ; Valyl-Valine ; (E)-2-Tetracosenoic acid ; 1012-Tetracosanedione ; 79-Tetracosanedione ; 68-Tetracosanedione ; 57-Tetracosanedione ; 46-Tetracosanedione ; 3-Hydroxy-1-phenyl-1-eicosanone ; Agrocybenine ; Monoethylglycinexylidide	M+2H ; M+3Na ; 2M+3H ; M+3Na ; M+3Na ; M+3Na ; M+3Na ; M+3Na ; M+3Na ; M+H+2Na ; M+2ACN+2H ; M+2ACN+2H	C15H28O5 ; C24H46O2 ; C10H20N2O3 ; C24H46O2 ; C24H46O2 ; C24H46O2 ; C24H46O2 ; C24H46O2 ; C24H46O2 ; C26H44O2 ; C12H18N2O ; C12H18N2O
517.21804	1.523961	1.36593	-4.0156	0.002456	2.6098	0.021356	517.218	HMDB05028 ; HMDB05038 ; HMDB10038 ; HMDB10054 ; HMDB10055 ; HMDB10057 ; HMDB10061 ; HMDB10073 ; HMDB10086 ; HMDB10098 ; HMDB14839 ; HMDB33012 ; HMDB33580 ; HMDB35269 ; HMDB35890 ; HMDB39009 ; HMDB60464	Escitalopram ; Citalopram ; PIP2(16:018:2(9Z12Z)) ; PIP2(16:1(9Z)18:1(11Z)) ; PIP2(16:1(9Z)18:1(9Z)) ; PIP2(16:2(9Z12Z)18:0) ; PIP2(18:016:2(9Z12Z)) ; PIP2(18:1(11Z)16:1(9Z)) ; PIP2(18:1(9Z)16:1(9Z)) ; PIP2(18:2(9Z12Z)16:0) ; Amprenavir ; Vestitone 7-glucoside ; 28-Glucosylarjunolate 3-rhamnosyl-(1-3)-glucuronide ; Azukisaponin IV ; Lucidenic acid K ; Calendasaponin B ; Codeine-6-glucuronide	3M+Na+K ; 3M+Na+K ; M+H+K ; M+H+K ; M+H+K ; M+H+K ; M+H+K ; M+H+K ; M+H+K ; M+H+K ; 2M+H+Na ; M+2ACN+H ; M+Na+K ; M+Na+K ; M+2Na-H ; M+Na+K ; M+ACN+H	C20H21FN2O ; C20H21FN2O ; C43H81O19P3 ; C43H81O19P3 ; C43H81O19P3 ; C43H81O19P3 ; C43H81O19P3 ; C43H81O19P3 ; C43H81O19P3 ; C43H81O19P3 ; C25H35N3O6S ; C22H26O9 ; C48H76O20 ; C48H76O20 ; C27H36O7 ; C48H76O20 ; C24H29NO9
198.98443	1.514546	0.715985	-4.0141	0.002462	2.6087	0.02137	198.9844	HMDB01928 ; HMDB13593 ; HMDB33042 ; HMDB33046 ; HMDB33056 ; HMDB33057 ; HMDB35901	Hydrochlorothiazide ; 14-Dithiothreitol ; Methyl 1-(methylthio)propyl disulfide ; Ethyl 1-(methylthio)ethyl disulfide ; Ethyl (ethylthio)methyl disulfide ; 1-(Ethylthio)ethyl methyl disulfide ; Thiacremonone	2M+3H ; M+2Na-H ; 2M+Na+K ; 2M+Na+K ; 2M+Na+K ; 2M+Na+K ; M+K	C7H8ClN3O4S2 ; C4H10O2S2 ; C5H12S3 ; C5H12S3 ; C5H12S3 ; C5H12S3 ; C6H8O3S
255.14514	1.472809	0.542426	-4.004	0.002502	2.6017	0.021659	255.1451	HMDB02366 ; HMDB12561 ; HMDB15693 ; HMDB29623 ; HMDB31092 ; HMDB31835 ; HMDB33493 ; HMDB33998 ; HMDB35194 ; HMDB35490 ; HMDB36011 ; HMDB36248 ; HMDB40960 ; HMDB60143 ; HMDB60725 ; HMDB61006	Tiglylcarnitine ; 13-Hydroxy-gamma-tocopherol ; Tymazoline ; Torvoside C ; 12-Di-(9Z12Z-octadecadienoyl)-sn-glycero-3-phosphate ; Asparagoside C ; Desglucodesrhamnoparillin ; gamma-Tocopheryl quinone ; beta-Tocopheryl quinone ; 25-Epiruizgenin 3-4-rhamnosylglucoside ; Cinnzeylanine ; Schidigerasaponin D5 ; Capsicoside B2 ; 8alpha-Hydroxy-gama-tocopherone ; 26-Pipecoloxylidide ; norfentanyl	2M+H+Na ; M+2K ; M+Na ; M+2H+Na ; M+3Na ; M+2H+Na ; M+2H+Na ; M+2K ; M+2K ; M+2H+Na ; M+2ACN+2H ; M+2H+Na ; M+2H+Na ; M+2K ; M+Na ; M+Na	C12H21NO4 ; C28H48O3 ; C14H20N2O ; C39H64O13 ; C39H69O8P ; C39H64O13 ; C39H64O13 ; C28H48O3 ; C28H48O3 ; C39H64O13 ; C22H34O8 ; C39H64O13 ; C39H64O13 ; C28H48O3 ; C14H20N2O ; C14H20N2O
200.06835	1.476967	0.067599	-4.0028	0.002507	2.6008	0.021659	200.0684	HMDB30376 ; HMDB29190 ; HMDB30055 ; HMDB41285 ; HMDB59988 ; HMDB59990 ; HMDB60030 ; HMDB00209 ; HMDB00244 ; HMDB00528 ; HMDB01855 ; HMDB02340 ; HMDB03349 ; HMDB03767 ; HMDB06853 ; HMDB12490 ; HMDB13785 ; HMDB15149 ; HMDB29635 ; HMDB29686 ; HMDB31459 ; HMDB32400 ; HMDB32568 ; HMDB32603 ; HMDB33128 ; HMDB33300 ; HMDB33301 ; HMDB33482 ; HMDB33766 ; HMDB33968 ; HMDB37693 ; HMDB38176 ; HMDB40045 ; HMDB40065 ; HMDB40066 ; HMDB40076 ; HMDB40077 ; HMDB40078 ; HMDB40079 ; HMDB40080 ; HMDB40081 ; HMDB40082 ; HMDB40098 ; HMDB40099 ; HMDB40106 ; HMDB40107 ; HMDB40108 ; HMDB40600 ; HMDB40638 ; HMDB40733 ; HMDB40768 ; HMDB41485 ; HMDB59767 ; HMDB60063	(S)-Isowillardiine ; 5-(34-dihydroxyphenyl)-gamma-valerolactone-3-O-glucuronide ; Taraxacoside ; Picraquassioside A ; 5-(34-Dihydroxyphenyl)-gamma-valerolactone-3-O-methyl-4-O-glucuronide ; 5-(34-Dihydroxyphenyl)-gamma-valerolactone-4-O-methyl-3-O-glucuronide ; 5-(35-Dihydroxyphenyl)-gamma-valerolactone-O-glucuronide-O-methyl ; Phenylacetic acid ; Riboflavin ; 45-Dihydroorotic acid ; 5-Hydroxytryptophol ; 2-Methylbenzoic acid ; L-Dihydroorotic acid ; 4-Hydroxyphenylacetaldehyde ; 3-Butyn-1-al ; 12-Dehydrosalsolinol ; Furan ; Balsalazide ; 4-Methylbenzoic acid ; 4-Methoxybenzaldehyde ; 3-Methoxybenzaldehyde ; 3-(5-Methyl-2-furyl)prop-2-enal ; 2-Hydroxyacetophenone ; 2-Hydroxy-4-methylbenzaldehyde ; 4-(2-Furanyl)-3-buten-2-one ; (1RS2RS)-Guaiacylglycerol 1-glucoside ; (1x2x)-Guaiacylglycerol 2-glucoside ; Plantagonine ; 2-Methoxybenzaldehyde ; Methyl benzoate ; 2-Propenyl 2-aminobenzoate ; alpha-Methyl-2-furanacrolein ; 5-2H-Pyrrol-4-(3H)-ylidenemethyl-2-furanmethanol ; 4-Butyl-5-ethylthiazole ; 5-Butyl-4-ethylthiazole ; 4-Butyl-25-dimethylthiazole ; 4-Ethyl-2-methyl-5-propylthiazole ; 5-Ethyl-2-methyl-4-propylthiazole ; 4-Ethyl-5-methyl-2-propylthiazole ; 5-Ethyl-4-methyl-2-propylthiazole ; 4-Ethyl-5-methyl-2-(1-methylethyl)thiazole ; 5-Ethyl-4-methyl-2-(1-methylethyl)thiazole ; 25-Dipropylthiazole ; 5-Methyl-2-pentylthiazole ; 4-Methyl-2-pentylthiazole ; 24-Dipropylthiazole ; 2-(1-Methylethyl)-4-propylthiazole ; (1x2x)-Guaiacylglycerol 3-glucoside ; 3-(3-Furanyl)-2-methyl-2-propenal ; Phenyl acetate ; N-(25-Dihydroxyphenyl)pyridinium(1) ; Benzyl formate ; Tetrahydro-25-furan-diacetic acid ; NN-dimethylindoliumolate	M+H ; M+2H ; M+2H ; M+2H ; M+2H ; M+2H ; M+2H ; M+ACN+Na ; M+H+Na ; M+ACN+H ; M+Na ; M+ACN+Na ; M+ACN+H ; M+ACN+Na ; 2M+ACN+Na ; M+Na ; 2M+ACN+Na ; M+ACN+2H ; M+ACN+Na ; M+ACN+Na ; M+ACN+Na ; M+ACN+Na ; M+ACN+Na ; M+ACN+Na ; M+ACN+Na ; M+H+Na ; M+H+Na ; M+Na ; M+ACN+Na ; M+ACN+Na ; M+Na ; M+ACN+Na ; M+Na ; 2M+Na+K ; 2M+Na+K ; 2M+Na+K ; 2M+Na+K ; 2M+Na+K ; 2M+Na+K ; 2M+Na+K ; 2M+Na+K ; 2M+Na+K ; 2M+Na+K ; 2M+Na+K ; 2M+Na+K ; 2M+Na+K ; 2M+Na+K ; 2M+Na+K ; 2M+Na+K ; M+H+Na ; M+ACN+Na ; M+ACN+Na ; 2M+H+Na ; M+ACN+Na ; 2M+H+Na ; M+Na	C7H9N3O4 ; C18H22O10 ; C18H22O10 ; C18H22O10 ; C18H22O10 ; C18H22O10 ; C18H22O10 ; C8H8O2 ; C17H20N4O6 ; C5H6N2O4 ; C10H11NO2 ; C8H8O2 ; C5H6N2O4 ; C8H8O2 ; C4H4O ; C10H11NO2 ; C4H4O ; C17H15N3O6 ; C8H8O2 ; C8H8O2 ; C8H8O2 ; C8H8O2 ; C8H8O2 ; C8H8O2 ; C8H8O2 ; C16H24O10 ; C16H24O10 ; C10H11NO2 ; C8H8O2 ; C8H8O2 ; C10H11NO2 ; C8H8O2 ; C10H11NO2 ; C9H15NS ; C9H15NS ; C9H15NS ; C9H15NS ; C9H15NS ; C9H15NS ; C9H15NS ; C9H15NS ; C9H15NS ; C9H15NS ; C9H15NS ; C9H15NS ; C9H15NS ; C9H15NS ; C9H15NS ; C9H15NS ; C16H24O10 ; C8H8O2 ; C8H8O2 ; C11H10NO2 ; C8H8O2 ; C8H12O5 ; C10H11NO2
626.31308	1.506918	0.454986	-4.0014	0.002513	2.5999	0.021659	626.3131	HMDB01198 ; HMDB05095 ; HMDB02160 ; HMDB11779 ; HMDB11809 ; HMDB14351 ; HMDB15129 ; HMDB15172 ; HMDB15196 ; HMDB15224 ; HMDB31448	Leukotriene C4 ; 11-trans-Leukotriene C4 ; Harderoporphyrinogen ; Ganglioside GD1a (d18:016:0) ; Ganglioside GD1b (d18:016:0) ; Reserpine ; Neomycin ; Carphenazine ; Acetophenazine ; Ouabain ; Glycosides	M+H ; M+H ; 2M+H+Na ; M+3Na ; M+3Na ; M+NH4 ; 2M+H+Na ; 3M+H+NH4 ; 3M+H+NH4 ; M+ACN+H ; M+ACN+H	C30H47N3O9S ; C30H47N3O9S ; C35H42N4O6 ; C83H147N3O39 ; C83H147N3O39 ; C33H40N2O9 ; C23H46N6O13 ; C23H29N3O2S ; C23H29N3O2S ; C29H44O12 ; C29H44O12
230.05132	1.492315	1.291034	4.0004	0.002517	2.5992	0.021659	230.0513	HMDB03153 ; HMDB41598 ; HMDB29356 ; HMDB31520 ; HMDB31813 ; HMDB41604	Epigallocatechin gallate ; 3-Galloylgallocatechin ; N-Feruloylglycyl-L-phenylalanine ; 2-Mercaptopropanoic acid ; N-Carbamoyl-2-amino-2-(4-hydroxyphenyl)acetic acid ; 3-Mercaptopropanoic acid	M+2H ; M+2H ; M+Na+K ; 2M+NH4 ; 2M+H+K ; 2M+NH4	C22H18O11 ; C22H18O11 ; C21H22N2O6 ; C3H6O2S ; C9H10N2O4 ; C3H6O2S
111.51363	1.481091	1.232997	-3.9962	0.002534	2.5962	0.021659	111.5136	HMDB33843	Polyvidone	M+2Na	C6H13NOP2
499.98906	1.457678	0.566769	-3.996	0.002535	2.5961	0.021659	NA	NA	NA	NA	NA
138.01299	1.497696	1.217425	3.9959	0.002535	2.596	0.021659	138.013	HMDB00202 ; HMDB00254 ; HMDB00349 ; HMDB00940 ; HMDB14606 ; HMDB31204 ; HMDB32431 ; HMDB33133 ; HMDB33134 ; HMDB38486 ; HMDB39324	Methylmalonic acid ; Succinic acid ; Erythrono-14-lactone ; Threonolactone ; Metharbital ; 4-Hydroxy-2-oxobutanoic acid ; 5-Methyl-2-thiophenecarboxaldehyde ; 2-Acetylthiophene ; 3-Acetylthiophene ; Tetraphyllin B sulfate ; xi-3-Hydroxy-2-oxobutanoic acid	2M+H+K ; 2M+H+K ; 2M+H+K ; 2M+H+K ; M+2K ; 2M+H+K ; 2M+H+Na ; 2M+H+Na ; 2M+H+Na ; M+H+2Na ; 2M+H+K	C4H6O4 ; C4H6O4 ; C4H6O4 ; C4H6O4 ; C9H14N2O3 ; C4H6O4 ; C6H6OS ; C6H6OS ; C6H6OS ; C12H17NO10S ; C4H6O4

178.53350	1.528507	0.365262	-3.9956	0.002536	2.5958	0.021659	178.5335	HMDB00370 ; HMDB15367 ; HMDB61168	2-Amino-3-phosphonopropionic acid ; Bromodiphenhydramine ; N2-Monodes-methylnizatidine	2M+H+NH4 ; M+H+Na ; M+H+K	C3H8NO5P ; C17H20BrNO ; C11H19N5O2S2
313.02934	1.539919	0.501745	3.9953	0.002538	2.5956	0.021659	313.0293	HMDB10716 ; HMDB29405 ; HMDB38820 ; HMDB40610 ; HMDB60007 ; HMDB40599	Gamma-Glutamyl-Se-methylselenocysteine ; Neobetanin ; Isorhamnetin 3-(6-malonylglucoside) ; Epitheaflavic acid 3-gallate ; N-(2-hydroxymethyl-3-chloro-4-hydroxyphenyl)anthranilic acid ; ()-Fenarimol	M+H ; M+2K ; M+Na+K ; M+2Na ; 2M+H+K ; M+H-H2O	C9H16N2O5Se ; C24H24N2O13 ; C25H24O15 ; C28H20O14 ; C14H12ClNO4 ; C17H12Cl2N2O
328.13632	1.502026	0.891227	3.9951	0.002538	2.5954	0.021659	328.1363	HMDB37846 ; HMDB03546 ; HMDB05773 ; HMDB14396 ; HMDB14950 ; HMDB15415 ; HMDB28890 ; HMDB28953 ; HMDB30293 ; HMDB31519 ; HMDB32237 ; HMDB32430 ; HMDB34023 ; HMDB35176 ; HMDB35880 ; HMDB38331 ; HMDB38964 ; HMDB39843 ; HMDB39958 ; HMDB40767 ; HMDB41845	N-(1-Deoxy-1-fructosyl)phenylalanine ; Salicin ; Endomorphin-1 ; Terconazole ; Phenylbutazone ; Oxtriphylline ; Histidinyl-Lysine ; Lysyl-Histidine ; (E)-1-O-Cinnamoyl-beta-D-glucose ; 4-Mercapto-4-methyl-2-pentanone ; 25-Dimethyl-3-mercaptotetrahydrofuran ; 4-(Methylthio)-2-pentanone ; Isosalicin ; 3-Mercapto-2-methylpentanal ; (E)-2-O-Cinnamoyl-beta-D-glucopyranose ; 3-Hydroxybenzyl alcohol glucoside ; 3-Hydroxy-46-heptadiyne-1-yl 1-glucoside ; S-Methyl 3-methylthiobutyrate ; 1-Pentadecanecarboxylic acid ; Neoacrimarine E ; Cadralazine	M+H ; M+ACN+H ; M+2Na ; M+3ACN+2H ; 2M+H+K ; M+2Na-H ; M+2Na-H ; M+2Na-H ; M+NH4 ; 2M+ACN+Na ; 2M+ACN+Na ; 2M+ACN+Na ; M+ACN+H ; 2M+ACN+Na ; M+NH4 ; M+ACN+H ; M+ACN+H ; 2M+ACN+Na ; M+NH4 ; M+ACN+2H ; M+2Na-H	C15H21NO7 ; C13H18O7 ; C34H38N6O5 ; C26H31Cl2N5O3 ; C19H20N2O2 ; C12H21N5O3 ; C12H21N5O3 ; C12H21N5O3 ; C15H18O7 ; C6H12OS ; C6H12OS ; C6H12OS ; C13H18O7 ; C6H12OS ; C15H18O7 ; C13H18O7 ; C13H18O7 ; C6H12OS ; C15H18O7 ; C35H35NO9 ; C12H21N5O3
159.01259	1.544201	0.43039	3.9938	0.002544	2.5945	0.021667	159.0126	HMDB30254 ; HMDB32884 ; HMDB38627 ; HMDB41756 ; HMDB61112	3-(345-Trimethoxyphenyl)propanoic acid ; Allura red AC ; Ethyl 345-trimethoxybenzoate ; Isopropyl 3-(34-dihydroxyphenyl)-2-hydroxypropanoate ; 3-Carboxy-4-methyl-5-propyl-2-furanpropionic acid	M+2K ; M+2H+Na ; M+2K ; M+2K ; M+2K	C12H16O5 ; C18H16N2O8S2 ; C12H16O5 ; C12H16O5 ; C12H16O5
703.98988	1.468977	1.425204	-3.9897	0.00256	2.5917	0.021754	NA	NA	NA	NA	NA
144.10194	1.460545	1.008713	-3.9892	0.002563	2.5913	0.021754	144.1019	HMDB04827 ; HMDB30410 ; HMDB38949 ; HMDB00378 ; HMDB00688 ; HMDB00826 ; HMDB13128 ; HMDB13812 ; HMDB15088 ; HMDB29582 ; HMDB30469 ; HMDB31222 ; HMDB31223 ; HMDB32067 ; HMDB32250 ; HMDB32328 ; HMDB32333 ; HMDB33629 ; HMDB34080 ; HMDB34835 ; HMDB35910 ; HMDB36153 ; HMDB36218 ; HMDB36220 ; HMDB37026 ; HMDB37027 ; HMDB37218 ; HMDB37224 ; HMDB37273 ; HMDB37306 ; HMDB38907 ; HMDB39630 ; HMDB40445 ; HMDB40998 ; HMDB41474 ; HMDB41588 ; HMDB41993 ; HMDB59820 ; HMDB59833	Proline betaine ; L-2-Amino-3-methylenehexanoic acid ; 3beta6beta-Dihydroxynortropane ; 2-Methylbutyroylcarnitine ; Isovalerylcarnitine ; Pentadecanoic acid ; Valerylcarnitine ; 1-Nitrohexane ; Rizatriptan ; Methyl sorbate ; Methyl tetradecanoate ; 3-Ethyl-12-cyclopentanedione ; Enol-3-Ethyl-12-cyclopentanedione ; Allyl crotonate ; Dodecyl propionate ; Hexyl nonanoate ; 4-Hydroxy-4-methyl-5-hexenoic acid gamma lactone ; xi-4-Hydroxy-4-methyl-2-cyclohexen-1-one ; 3-Methyl-12-cyclohexanedione ; 3-(3-Ethyloxiranyl)-2-propenal ; Cedryl acetate ; Shyobunyl acetate ; Heptyl octanoate ; Octyl heptanoate ; 34-Dimethyl-12-cyclopentanedione ; 35-Dimethyl-12-cyclopentanedione ; Guaiol acetate ; alpha-Caryophyllene alcohol acetate ; beta-Caryophyllene alcohol acetate ; Pentyl decanoate ; 3-Methylbutyl decanoate ; Nerolidyl acetate ; 2-(Ethoxymethyl)furan ; 7(14)-Farnesene-912-diol ; xi-5-Isopropyl-2(5H)-furanone ; 2610-Trimethyldodecanoic acid ; Pivaloylcarnitine ; Isopropyllaurate ; Ethyl tridecanoate	M+H ; M+H ; M+H ; M+ACN+2H ; M+ACN+2H ; M+2Na ; M+ACN+2H ; 2M+H+Na ; M+H+NH4 ; M+NH4 ; M+2Na ; M+NH4 ; M+NH4 ; M+NH4 ; M+2Na ; M+2Na ; M+NH4 ; M+NH4 ; M+NH4 ; M+NH4 ; M+H+Na ; M+H+Na ; M+2Na ; M+2Na ; M+NH4 ; M+NH4 ; M+H+Na ; M+H+Na ; M+H+Na ; M+2Na ; M+2Na ; M+H+Na ; M+NH4 ; M+2Na ; M+NH4 ; M+2Na ; M+ACN+2H ; M+2Na ; M+2Na	C7H13NO2 ; C7H13NO2 ; C7H13NO2 ; C12H23NO4 ; C12H23NO4 ; C15H30O2 ; C12H23NO4 ; C6H14NO2 ; C15H19N5 ; C7H10O2 ; C15H30O2 ; C7H10O2 ; C7H10O2 ; C7H10O2 ; C15H30O2 ; C15H30O2 ; C7H10O2 ; C7H10O2 ; C7H10O2 ; C7H10O2 ; C17H28O2 ; C17H28O2 ; C15H30O2 ; C15H30O2 ; C7H10O2 ; C7H10O2 ; C17H28O2 ; C17H28O2 ; C17H28O2 ; C15H30O2 ; C15H30O2 ; C17H28O2 ; C7H10O2 ; C15H30O2 ; C7H10O2 ; C15H30O2 ; C12H23NO4 ; C15H30O2 ; C15H30O2
596.95459	1.446343	1.254148	-3.9878	0.002569	2.5903	0.021768	NA	NA	NA	NA	NA
179.02953	1.524258	0.390721	-3.9866	0.002573	2.5895	0.021772	179.0295	HMDB30772 ; HMDB38518 ; HMDB40236 ; HMDB40873 ; HMDB40931 ; HMDB41451 ; HMDB60885 ; HMDB60886 ; HMDB60887 ; HMDB60888	Demethoxyegonol ; Desmosflavone ; 34-Diethylthiophene ; Dimethylstrobochrysin ; 1-(4-Hydroxy-3-methoxyphenyl)-5-(4-hydroxyphenyl)-14-pentadien-3-one ; Musanolone F ; 4-Hydroxy-R-phenprocoumon ; 6-Hydroxy-R-phenprocoumon ; 8-Hydroxy-R-phenprocoumon ; 7-Hydroxy-R-phenprocoumon	M+Na+K ; M+Na+K ; M+K ; M+Na+K ; M+Na+K ; M+H+K ; M+Na+K ; M+Na+K ; M+Na+K ; M+Na+K	C18H16O4 ; C18H16O4 ; C8H12S ; C18H16O4 ; C18H16O4 ; C20H14O4 ; C18H16O4 ; C18H16O4 ; C18H16O4 ; C18H16O4
488.21652	1.453274	0.09291	-3.983	0.002588	2.587	0.02186	488.2165	HMDB13335 ; HMDB15192	3-Hydroxyhexadecadienoylcarnitine ; Norfloxacin	M+2K-H ; 3M+H+NH4	C23H41NO5 ; C16H18FN3O3
273.12006	1.459969	1.471266	-3.9788	0.002606	2.584	0.021951	273.1201	HMDB14385 ; HMDB14844 ; HMDB38327 ; HMDB39233 ; HMDB41580	Alclometasone ; Tamsulosin ; (3b9R)-5-Megastigmene-39-diol 9-apiosyl-(1-6)-glucoside ; Verbasoside ; 4-Megastigmene-6a9R-diol 9-apiosyl-(1-6)-glucoside	2M+3H ; 2M+3H ; M+H+K ; M+2ACN+2H ; M+H+K	C22H29ClO5 ; C20H28N2O5S ; C24H42O11 ; C20H30O12 ; C24H42O11
560.48592	1.449337	1.258072	-3.9782	0.002609	2.5836	0.021951	560.4859	HMDB14621 ; HMDB15177	Rimantadine ; Memantine	3M+Na ; 3M+Na	C12H21N ; C12H21N
149.01856	1.546887	0.282356	-3.9773	0.002612	2.583	0.021951	149.0185	HMDB00008 ; HMDB00011 ; HMDB00023 ; HMDB00336 ; HMDB00357 ; HMDB00442 ; HMDB00710 ; HMDB00729 ; HMDB13968 ; HMDB13969 ; HMDB14406 ; HMDB14717 ; HMDB15175 ; HMDB15231 ; HMDB28781 ; HMDB28970 ; HMDB28982 ; HMDB29045 ; HMDB30968 ; HMDB31212 ; HMDB32929 ; HMDB38998 ; HMDB40549 ; HMDB41173 ; HMDB59748	2-Hydroxybutyric acid ; (R)-3-Hydroxybutyric acid ; (S)-3-Hydroxyisobutyric acid ; (R)-3-Hydroxyisobutyric acid ; 3-Hydroxybutyric acid ; (S)-3-Hydroxybutyric acid ; 4-Hydroxybutyric acid ; Alpha-Hydroxyisobutyric acid ; Hydroxyzileuton ; Zileuton sulfoxide ; Anagrelide ; Carbenicillin ; Thalidomide ; Flucytosine ; Cysteinyl-Methionine ; Methionyl-Cysteine ; Methionyl-Serine ; Serinyl-Methionine ; 246-Octatriyn-1-ol ; Ethoxyacetic acid ; Benzofuran ; 357-Octatriyn-1-ol ; xi-23-Octadiene-57-diyn-1-ol ; 1-O-Caffeoyl-(b-D-glucose 6-O-sulfate) ; 2-Methyl-3-hydroxypropanoate	M+2Na-H ; M+2Na-H ; M+2Na-H ; M+2Na-H ; M+2Na-H ; M+2Na-H ; M+2Na-H ; M+2Na-H ; M+2Na ; M+2Na ; M+ACN+2H ; M+3Na ; M+H+K ; 2M+H+K ; M+2Na ; M+2Na ; M+Na+K ; M+Na+K ; 2M+Na+K ; M+2Na-H ; 2M+Na+K ; 2M+Na+K ; 2M+Na+K ; M+2H+Na ; M+2Na-H	C4H8O3 ; C4H8O3 ; C4H8O3 ; C4H8O3 ; C4H8O3 ; C4H8O3 ; C4H8O3 ; C4H8O3 ; C11H12N2O3S ; C11H12N2O3S ; C10H7Cl2N3O ; C17H18N2O6S ; C13H10N2O4 ; C4H4FN3O ; C8H16N2O3S2 ; C8H16N2O3S2 ; C8H16N2O4S ; C8H16N2O4S ; C8H6O ; C4H8O3 ; C8H6O ; C8H6O ; C8H6O ; C15H18O12S ; C4H8O3

497.05827	1.449608	0.145622	3.9752	0.002621	2.5815	0.021992	497.0583	HMDB29520 ; HMDB30160 ; HMDB30161 ; HMDB32785 ; HMDB35219 ; HMDB37317 ; HMDB37318 ; HMDB37486 ; HMDB37490 ; HMDB37581 ; HMDB39902 ; HMDB40620 ; HMDB41098 ; HMDB41359 ; HMDB41731 ; HMDB41732 ; HMDB61142	Liquiritin ; Natsudaidain ; 5-Hydroxy-334678-hexamethoxyflavone ; 7-Hydroxy-334568-hexamethoxyflavone ; Aloin ; Neoisoliquiritin ; Isoliquiritin ; Isosakuranetin 7-xyloside ; Neoliquiritin ; Naringenin 5-rhamnoside ; Dihydrodaidzin ; Gaylussacin ; 2-Hydroxy-3-methyl-4H-pyran-4-one O-(6E-cinnamoyl-b-D-glucoside) ; 6-Hydroxy-334578-hexamethoxyflavone ; Equol 4-O-glucuronide ; Equol 7-O-glucuronide ; Fenoprofen glucuronide	M+2K+H ; M+2K+H ; M+2K+H ; M+2K+H ; M+2K+H ; M+2K+H ; M+2K+H ; M+2K+H ; M+2K+H ; M+2K+H ; M+2K+H ; M+2K+H ; M+2K+H ; M+2K+H ; M+2K+H ; M+2K+H ; M+2K+H	C21H22O9 ; C21H22O9 ; C21H22O9 ; C21H22O9 ; C21H22O9 ; C21H22O9 ; C21H22O9 ; C21H22O9 ; C21H22O9 ; C21H22O9 ; C21H22O9 ; C21H22O9 ; C21H22O9 ; C21H22O9 ; C21H22O9 ; C21H22O9 ; C21H22O9
179.98562	1.489482	1.054381	3.9741	0.002626	2.5807	0.021992	179.9856	HMDB32930 ; HMDB36807 ; HMDB40199	Benzothiazole ; (2-Furanylmethyl) methyl disulfide ; 2-Methyl-3-(methyldithio)furan	M+2Na-H ; 2M+H+K ; 2M+H+K	C7H5NS ; C6H8OS2 ; C6H8OS2
380.11232	1.515508	0.698513	-3.9728	0.002631	2.5798	0.022001	380.1123	HMDB01066 ; HMDB15596 ; HMDB28896 ; HMDB29085 ; HMDB30589 ; HMDB30619 ; HMDB31944 ; HMDB32864 ; HMDB38822	S-Lactoylglutathione ; Vildagliptin ; Histidinyl-Tryptophan ; Tryptophyl-Histidine ; O-Methylsterigmatocystin ; Ovalitenone ; Niazimicin A ; Mycotoxin T 2 ; Malvidin 3-(6-malonylglucoside) 5-glucoside	M+H ; M+2K-H ; M+K ; M+K ; M+ACN+H ; M+ACN+H ; M+Na ; M+K ; M+H+NH4	C13H21N3O8S ; C17H25N3O2 ; C17H19N5O3 ; C17H19N5O3 ; C19H14O6 ; C19H14O6 ; C16H23NO6S ; C16H23NO7 ; C32H37O20
283.03663	1.524494	1.379401	3.9699	0.002644	2.5778	0.022049	283.0366	HMDB14353 ; HMDB31829 ; HMDB33533 ; HMDB38779 ; HMDB39187 ; HMDB41394	Ticlopidine ; Bisosthenon B ; Ceftiofur ; 4-Methylisoscutellarein 8-(2-sulfoglucoside) ; Methyl 2346-tetra-O-galloyl-beta-D-glucopyranoside ; Khelmarin D	2M+H+K ; M+2K ; M+ACN+2H ; M+H+Na ; M+H+2Na ; M+2K	C14H14ClNS ; C28H24O8 ; C19H17N5O7S3 ; C22H22O14S ; C35H30O22 ; C28H24O8
194.99038	1.525844	0.858675	-3.9684	0.00265	2.5767	0.022049	194.9904	HMDB02151 ; HMDB29855 ; HMDB32885 ; HMDB37851 ; HMDB60496 ; HMDB61163	Dimethyl sulfoxide ; Cochineal Red A ; Amaranth ; Apigenin 7-sulfate ; NN-Diacetylhydrazine ; N-Nitroso-3-hydroxypyrrolidine	2M+K ; M+H+2Na ; M+H+2Na ; M+H+K ; M+2K+H ; M+2K+H	C2H6OS ; C20H14N2O10S3 ; C20H14N2O10S3 ; C15H10O8S ; C4H8N2O2 ; C4H8N2O2
489.76550	1.468848	0.372505	-3.9684	0.00265	2.5767	0.022049	489.7655	HMDB06972 ; HMDB32698 ; HMDB41824 ; HMDB41838	CDP-DG(16:018:2(9Z12Z)) ; Bis(2-methylpropanoyloxy)-910-epoxy-p-mentha-135-triene ; alpha-Zearalenol ; beta-Zearalenol	M+2H ; 3M+H+NH4 ; 3M+H+NH4 ; 3M+H+NH4	C46H81N3O15P2 ; C18H24O5 ; C18H24O5 ; C18H24O5
374.13746	1.521757	1.113705	3.9674	0.002655	2.576	0.022049	374.1375	HMDB02354 ; HMDB03601 ; HMDB15549 ; HMDB31366 ; HMDB33905 ; HMDB35050 ; HMDB36778 ; HMDB38724 ; HMDB39239 ; HMDB60609 ; HMDB15108	Hawkinsin ; (S)-Reticuline ; Hydroxychloroquine ; Gibberellin A93 ; 28-Dihydroxy-3457-tetramethoxyflavan ; Gibberellin A21 ; Diosbulbin C ; xi-Anomuricine ; Gibberellin A87 ; Orciprenaline-3-O-sulfate ; Ezetimibe	M+2ACN+H ; M+2Na-H ; M+K ; 2M+H+Na ; 2M+H+Na ; 2M+H+Na ; 2M+H+Na ; M+2Na-H ; 2M+H+Na ; M+2ACN+H ; M+H-2H2O	C11H17NO6S ; C19H23NO4 ; C18H26ClN3O ; C19H22O7 ; C19H22O7 ; C19H22O7 ; C19H22O7 ; C19H23NO4 ; C19H22O7 ; C11H17NO6S ; C24H21F2NO3
756.88522	1.454022	0.656986	3.9623	0.002676	2.5725	0.022172	NA	NA	NA	NA	NA
367.02956	1.485397	1.397488	3.9598	0.002688	2.5707	0.022172	367.0296	HMDB33622 ; HMDB59977	Cabbage identification factor 2 ; 4-Hydroxy-5-(dihydroxyphenyl)-valeric acid-O-methyl-O-sulphate	M+K ; 2M+Na+K	C15H12N4O3S ; C12H16O9S
406.77227	1.492975	1.529023	3.9592	0.00269	2.5702	0.022172	406.7723	HMDB10165 ; HMDB12383 ; HMDB12393 ; HMDB12423 ; HMDB12433 ; HMDB10163 ; HMDB10580 ; HMDB10584 ; HMDB10593 ; HMDB10594 ; HMDB10597 ; HMDB10598 ; HMDB10621 ; HMDB10622 ; HMDB10636 ; HMDB10637 ; HMDB10650 ; HMDB10655 ; HMDB10663 ; HMDB10664 ; HMDB10668 ; HMDB10669 ; HMDB10678 ; HMDB10679 ; HMDB10683 ; HMDB10684 ; HMDB12389	PS(18:020:4(8Z11Z14Z17Z)) ; PS(18:020:4(5Z8Z11Z14Z)) ; PS(18:1(9Z)20:3(8Z11Z14Z)) ; PS(20:3(8Z11Z14Z)18:1(9Z)) ; PS(20:4(5Z8Z11Z14Z)18:0) ; PS(18:018:1(9Z)) ; PG(16:020:4(5Z8Z11Z14Z)) ; PG(16:022:6(4Z7Z10Z13Z16Z19Z)) ; PG(16:1(9Z)20:3(5Z8Z11Z)) ; PG(16:1(9Z)20:3(8Z11Z14Z)) ; PG(16:1(9Z)22:5(4Z7Z10Z13Z16Z)) ; PG(16:1(9Z)22:5(7Z10Z13Z16Z19Z)) ; PG(18:1(11Z)18:3(6Z9Z12Z)) ; PG(18:1(11Z)18:3(9Z12Z15Z)) ; PG(18:1(9Z)18:3(6Z9Z12Z)) ; PG(18:1(9Z)18:3(9Z12Z15Z)) ; PG(18:2(9Z12Z)18:2(9Z12Z)) ; PG(18:2(9Z12Z)20:4(5Z8Z11Z14Z)) ; PG(18:3(6Z9Z12Z)18:1(11Z)) ; PG(18:3(6Z9Z12Z)18:1(9Z)) ; PG(18:3(6Z9Z12Z)20:3(5Z8Z11Z)) ; PG(18:3(6Z9Z12Z)20:3(8Z11Z14Z)) ; PG(18:3(9Z12Z15Z)18:1(11Z)) ; PG(18:3(9Z12Z15Z)18:1(9Z)) ; PG(18:3(9Z12Z15Z)20:3(5Z8Z11Z)) ; PG(18:3(9Z12Z15Z)20:3(8Z11Z14Z)) ; PS(18:1(9Z)18:0)	M+2H ; M+2H ; M+2H ; M+2H ; M+2H ; M+H+Na ; M+ACN+2H ; M+H+NH4 ; M+ACN+2H ; M+ACN+2H ; M+H+NH4 ; M+H+NH4 ; M+ACN+2H ; M+ACN+2H ; M+ACN+2H ; M+ACN+2H ; M+ACN+2H ; M+H+NH4 ; M+ACN+2H ; M+ACN+2H ; M+H+NH4 ; M+H+NH4 ; M+ACN+2H ; M+ACN+2H ; M+H+NH4 ; M+H+NH4 ; M+H+Na	C44H78NO10P ; C44H78NO10P ; C44H78NO10P ; C44H78NO10P ; C44H78NO10P ; C42H80NO10P ; C42H75O10P ; C44H75O10P ; C42H75O10P ; C42H75O10P ; C44H75O10P ; C44H75O10P ; C42H75O10P ; C42H75O10P ; C42H75O10P ; C42H75O10P ; C42H75O10P ; C44H75O10P ; C42H75O10P ; C42H75O10P ; C44H75O10P ; C44H75O10P ; C42H75O10P ; C42H75O10P ; C44H75O10P ; C44H75O10P ; C42H80NO10P
300.01795	1.456405	1.120045	-3.9591	0.002691	2.5702	0.022172	300.018	HMDB14841 ; HMDB37131 ; HMDB42043	Methazolamide ; ()-Furilazole ; Tolfenamic acid	M+ACN+Na ; M+Na ; M+K	C5H8N4O3S2 ; C11H13Cl2NO3 ; C14H12ClNO2
362.09702	1.516489	0.247835	3.9584	0.002693	2.5697	0.022172	362.097	HMDB15252 ; HMDB29840 ; HMDB30341 ; HMDB33446 ; HMDB33520 ; HMDB33521 ; HMDB33665 ; HMDB37839 ; HMDB38464 ; HMDB40318	Gliclazide ; Vulgaxanthin I ; Piplartine ; Rutagravine ; 1-Hydroxyrutacridone epoxide ; 20-Hydroxyrutacridone epoxide ; N-Hydroxyneosaxitoxin ; N-(1-Deoxy-1-fructosyl)histidine ; Nelumboside ; 5588-Tetrahydroxy-334477-hexamethoxy-55-biflavan	M+K ; M+Na ; M+2Na-H ; M+Na ; M+Na ; M+Na ; 2M+Na+K ; M+2Na-H ; M+2ACN+2H ; M+Na+K	C15H21N3O3S ; C14H17N3O7 ; C17H19NO5 ; C19H17NO5 ; C19H17NO5 ; C19H17NO5 ; C10H17N7O6 ; C12H19N3O7 ; C27H28O18 ; C36H38O12
157.51865	1.532478	0.809293	-3.9578	0.002696	2.5693	0.022172	157.5187	HMDB14543	Zoledronate	M+ACN+2H	C5H10N2O7P2
288.98387	1.480349	0.025734	-3.9477	0.002741	2.5622	0.022501	288.9839	HMDB11714 ; HMDB35484 ; HMDB60347	Vanilpyruvic acid ; 3-(34-Dihydroxy-5-methoxy)-2-propenoic acid ; 2-Chloromaleylacetate	M+2K+H ; M+2K+H ; 3M+2H	C10H10O5 ; C10H10O5 ; C6H5ClO5
665.04047	1.451823	1.306862	-3.9465	0.002746	2.5614	0.022505	665.0405	HMDB33044 ; HMDB33045	1-(Methylthio)propyl propyl disulfide ; Ethyl 1-(ethylthio)propyl disulfide	3M+2K-H ; 3M+2K-H	C7H16S3 ; C7H16S3
323.01423	1.542869	0.254661	3.9443	0.002756	2.5598	0.022551	323.0142	HMDB14949 ; HMDB15063	Ribavirin ; Azacitidine	M+2K+H ; M+2K+H	C8H12N4O5 ; C8H12N4O5
462.9706_	1.517019	0.547929	-3.9409	0.002771	2.5574	0.022637	462.9706	HMDB41624	Phosphocreatinine	2M+2K-H	C4H8N3O4P
330.06092	1.45154	1.043177	-3.9391	0.002779	2.5561	0.022666	330.0609	HMDB00058 ; HMDB11616 ; HMDB14492 ; HMDB31800 ; HMDB02044 ; HMDB15413 ; HMDB15621 ; HMDB29191 ; HMDB32956 ; HMDB60156 ; HMDB00045 ; HMDB01044 ; HMDB03540 ; HMDB11617 ; HMDB59594	Cyclic AMP ; Adenosine 23-cyclic phosphate ; Nitisinone ; Mecarbam ; 8-Hydroxyguanosine ; Sulfadoxine ; Sulfadimethoxine ; 5-(34-Dihydroxyphenyl)-gamma-valerolactone sulfate ; 2-O-p-Coumaroyltartronic acid ; 3-oxo-(2S)-Methylisocapryloyl-CoA ; Adenosine monophosphate ; 2-Deoxyguanosine 5-monophosphate ; 3-AMP ; Adenosine 2-phosphate ; 2-hydroxy-dAMP	M+H ; M+H ; M+H ; M+H ; 2M+Na+K ; 2M+H+K ; 2M+H+K ; M+ACN+H ; M+ACN+Na ; M+3Na ; M+H-H2O ; M+H-H2O ; M+H-H2O ; M+H-H2O ; M+H-H2O	C10H12N5O6P ; C10H12N5O6P ; C14H10F3NO5 ; C10H20NO5PS2 ; C10H13N5O6 ; C12H14N4O4S ; C12H14N4O4S ; C11H12O7S ; C12H10O7 ; C30H50N7O18P3S ; C10H14N5O7P ; C10H14N5O7P ; C10H14N5O7P ; C10H14N5O7P ; C10H14N5O7P

262.03998	1.541039	0.045799	3.9374	0.002787	2.5549	0.022692	262.04	HMDB14462 ; HMDB15289 ; HMDB32945 ; HMDB38748 ; HMDB40067 ; HMDB40083 ; HMDB40084 ; HMDB40085 ; HMDB40086 ; HMDB40087 ; HMDB40088 ; HMDB40089 ; HMDB40109 ; HMDB40110 ; HMDB40533 ; HMDB60549	Gefitinib ; Bretylium ; 1-Phenyl-24-pentadiyn-1-one ; Osmanthuside A ; 4-Butyl-5-propylthiazole ; 24-Dimethyl-5-pentylthiazole ; 45-Dimethyl-2-pentylthiazole ; 25-Dipropyl-4-methylthiazole ; 2-Butyl-5-ethyl-4-methylthiazole ; 4-Butyl-2-ethyl-5-methylthiazole ; 24-Diethyl-5-propylthiazole ; 25-Diethyl-4-propylthiazole ; 4-Ethyl-2-pentylthiazole ; 2-Butyl-4-propylthiazole ; Artomunoxanthentrione epoxide ; Norketamine	M+2K ; 2M+H+K ; 3M+Na+K ; M+2K ; M+2K+H ; M+2K+H ; M+2K+H ; M+2K+H ; M+2K+H ; M+2K+H ; M+2K+H ; M+2K+H ; M+2K+H ; M+2K+H ; M+Na+K ; M+K	C22H24ClFN4O3 ; C11H17BrN ; C11H6O ; C23H26O9 ; C10H17NS ; C10H17NS ; C10H17NS ; C10H17NS ; C10H17NS ; C10H17NS ; C10H17NS ; C10H17NS ; C10H17NS ; C10H17NS ; C26H22O8 ; C12H14ClNO
131.52788	1.531657	0.616836	3.9336	0.002804	2.5523	0.022795	131.5279	HMDB06049 ; HMDB00866 ; HMDB13068 ; HMDB15328 ; HMDB33757 ; HMDB36573 ; HMDB40452 ; HMDB42011	O-Phosphotyrosine ; N-Acetyl-L-tyrosine ; Salsolinol 1-carboxylate ; Captopril ; Marcanine A ; 135-Trithiane ; 123-Trithiane ; S-Phenylmercapturic acid	M+2H ; M+H+K ; M+H+K ; M+2Na ; M+H+Na ; M+3ACN+2H ; M+3ACN+2H ; M+H+Na	C9H12NO6P ; C11H13NO4 ; C11H13NO4 ; C9H15NO3S ; C14H9NO3 ; C3H6S3 ; C3H6S3 ; C11H13NO3S
588.59041	1.49125	1.496127	3.9302	0.002819	2.5499	0.022884	588.5904	HMDB41075	erythro-68-Pentacosanediol	3M+H+Na	C25H52O2
490.54802	1.425599	1.333417	-3.9258	0.002839	2.5468	0.02296	NA	NA	NA	NA	NA
404.68587	1.494479	1.509685	3.9255	0.002841	2.5466	0.02296	404.6859	HMDB31766 ; HMDB34713	Alachlor ; Fumonisin A1	3M+2H ; M+2Na	C14H20ClNO2 ; C36H61NO16
277.58526	1.494616	1.45497	-3.9252	0.002843	2.5463	0.02296	277.5853	HMDB29361	Alkaloid RC	M+H+Na	C26H29NO11
224.86485	1.4892	1.529052	3.915	0.00289	2.5392	0.023286	NA	NA	NA	NA	NA
322.81081	1.468746	1.544258	3.9127	0.002901	2.5375	0.023286	NA	NA	NA	NA	NA
358.60097	1.465472	0.536711	3.9118	0.002905	2.5369	0.023286	358.601	HMDB14370 ; HMDB14590 ; HMDB30463	Gadodiamide ; Loracarbef ; Hyperforin	M+3ACN+2H ; 2M+H+NH4 ; 2M+3H	C16H28GdN5O9 ; C16H16ClN3O4 ; C35H52O4
370.35454	1.552078	0.640822	3.9102	0.002912	2.5358	0.023286	NA	NA	NA	NA	NA
357.01564	1.473559	0.901287	-3.9096	0.002915	2.5354	0.023286	357.0156	HMDB14767 ; HMDB33884 ; HMDB41919	Guanabenz ; Gravolenic acid ; Lormetazepam	3M+H+Na ; M+2K-H ; M+Na	C8H8Cl2N4 ; C14H16O6 ; C16H12Cl2N2O2
266.88900	1.523533	0.945239	3.9095	0.002916	2.5353	0.023286	266.889	HMDB29627	1235-Tetrachloro-4-methoxybenzene	M+Na	C7H4Cl4O
109.06347	1.478183	1.381375	3.9092	0.002917	2.5351	0.023286	109.0635	HMDB29027 ; HMDB29069 ; HMDB38239 ; HMDB00380 ; HMDB00405 ; HMDB00421 ; HMDB00511 ; HMDB01292 ; HMDB01441 ; HMDB02201 ; HMDB03224 ; HMDB03955 ; HMDB03959 ; HMDB06478 ; HMDB06771 ; HMDB06773 ; HMDB06774 ; HMDB12141 ; HMDB12485 ; HMDB12535 ; HMDB12782 ; HMDB14018 ; HMDB29576 ; HMDB29760 ; HMDB30029 ; HMDB30124 ; HMDB30126 ; HMDB31038 ; HMDB31061 ; HMDB31206 ; HMDB31264 ; HMDB31525 ; HMDB31526 ; HMDB31604 ; HMDB31605 ; HMDB31739 ; HMDB31840 ; HMDB32045 ; HMDB32070 ; HMDB32155 ; HMDB32264 ; HMDB32302 ; HMDB32315 ; HMDB32567 ; HMDB32675 ; HMDB33125 ; HMDB33620 ; HMDB33854 ; HMDB34028 ; HMDB34167 ; HMDB34235 ; HMDB34252 ; HMDB34450 ; HMDB34457 ; HMDB34465 ; HMDB34849 ;	Prolyl-Threonine ; Threoninyl-Proline ; Sakacin P ; 2-Hydroxyestradiol-3-methyl ether ; 2-Methoxyestradiol ; 23-Dihydroxyvaleric acid ; Capric acid ; 1-Deoxy-D-xylulose ; Dihydropteridine ; N-Carboxyethyl-g-aminobutyric acid ; Deoxyribose ; 19-Hydroxyandrost-4-ene-317-dione ; 19-Oxotestosterone ; Iso-Valeraldehyde ; 7a-Hydroxyandrost-4-ene-317-dione ; 11b-Hydroxyandrost-4-ene-317-dione ; 16a-Hydroxyandrost-4-ene-317-dione ; (R)-23-Dihydroxy-isovalerate ; (R)-glycerol 1-acetate ; 12S-HHT ; 4-Methoxy-17beta-estradiol ; 4-Hydroxypropofol ; 23-Dihydroxy-2-methylbutanoic acid ; 11-Diethoxy-2-hexene ; Isopentyl isopentanoate ; Prenol ; 3-Methyl-3-buten-1-ol ; (2Z8S9Z)-29-Heptadecadiene-8-hydroxy-46-diyne-1-yl acetate ; 10-Hydroxymyristic acid methyl ester ; Pentanal ; Methyl nonanoate ; (S)-2-Methylbutanal ; ()-2-Methylbutanal ; (E)-2-Penten-1-ol ; 1-Penten-3-ol ; 7-Hydroxy-37-dimethyloctanal ; (SZ)-Lyratol acetate ; Pentyl 3-methylbutanoate ; Isooctyl acetate ; Acetaldehyde 13-octanediol acetal ; Ethyl 2-ethylhexanoate ; Heptanal propyleneglycol acetal ; Hexanal butane-23-diol acetal ; Hexylresorcinol ; Dihydropanaxacol ; 8-Dehydroshogaol ; Hexyl butyrate ; Isoprenyl alcohol ; 2-(37-Dimethyl-26-octadienyl)-4-hydroxy-6-methoxyacetophenone ; Falcarindiol 3-acetate ; 2-Pentanone ; 2-Aminoheptanedioic acid ; Neocnidilide ; Propyl heptanoate ; 2-Methyl-2-buten-1-ol ; 4-Methylnonanoic acid ; ()-Myrtenyl acetate ; 2-Acetyl-1567-tetrahydro-6-hydroxy-7-(hydroxymethyl)-4H-azepine-4-one ; Ethyl (2E4E7Z)-Decatrienoate ; Ethyl (2E4Z7Z)-Decatrienoate ; trans-p-Menthane-18-diol ; (1R2S3S4R)-p-Menthane-23-diol ; (2S4R)-p-Mentha-1(7)5-dien-2-ol acetate ; 4-Ethyloctanoic acid ; p-Menthane-38-diol ; 2-Methylpropyl hexanoate ; Calystegine B5 ; Fauronyl acetate ; Yucalexin B11 ; (S)-p-Mentha-18-dien-10-yl acetate ; Perillyl acetate ; (1S3R4R)-p-Menthane-13-diol ; (R)-2-Acetoxy-p-mentha-18-diene ; Hexyl 2-methylpropanoate ; 2-Methylbutyl 3-methylbutanoate ; Carvyl acetate ; Menthadienyl acetate ; Calystegine B2 ; Octyl acetate ; (Z)-1-(1-Ethoxyethoxy)-3-hexene ; 2-Methylbutyl 2-methylbutanoate ; Ginsenoyne H ; 8-Ocimenyl acetate ; xi-3-Methyl-3-buten-2-ol ; 3-Methylbutyl 2-methylbutanoate ; ()-3-Octyl acetate ; Ethyl octanoate ; Butyl hexanoate ; 1-(2-Furanyl)-1-octanone ; 3-Methyl-3H-imidazo45-fquinoxalin-2-amine ; 3-Methylbutyl pentanoate ; 2-Hexyl-5-2-(4-hydroxy-3-methoxyphenyl)ethylfuran ; Acetylpanaxydol ; 26-Dimethyl-7-octene-26-diol ; Valeracetate ; (S)-Neolyratyl acetate ; (22-Diethoxyethyl)benzene ; Ethyl 3-hydroxytridecanoate	M+2H ; M+2H ; M+2H ; M+2H+Na ; M+2H+Na ; M+2ACN+2H ; M+2Na ; M+2ACN+2H ; M+2ACN+2H ; M+ACN+2H ; M+2ACN+2H ; M+2H+Na ; M+2H+Na ; M+Na ; M+2H+Na ; M+2H+Na ; M+2H+Na ; M+2ACN+2H ; M+2ACN+2H ; M+H+2Na ; M+2H+Na ; M+H+Na ; M+2ACN+2H ; M+2Na ; M+2Na ; M+Na ; M+Na ; M+2H+Na ; M+3Na ; M+Na ; M+2Na ; M+Na ; M+Na ; M+Na ; M+Na ; M+2Na ; M+H+Na ; M+2Na ; M+2Na ; M+2Na ; M+2Na ; M+2Na ; M+2Na ; M+H+Na ; M+H+2Na ; M+2H+Na ; M+2Na ; M+Na ; M+2H+Na ; M+2H+Na ; M+Na ; M+ACN+2H ; M+H+Na ; M+2Na ; M+Na ; M+2Na ; M+H+Na ; M+H+NH4 ; M+H+Na ; M+H+Na ; M+2Na ; M+2Na ; M+H+Na ; M+2Na ; M+2Na ; M+2Na ; M+ACN+2H ; M+H+2Na ; M+2H+Na ; M+H+Na ; M+H+Na ; M+2Na ; M+H+Na ; M+2Na ; M+2Na ; M+H+Na ; M+H+Na ; M+ACN+2H ; M+2Na ; M+2Na ; M+2Na ; M+2H+Na ; M+H+Na ; M+Na ; M+2Na ; M+2Na ; M+2Na ; M+2Na ; M+H+Na ; M+H+NH4 ; M+2Na ; M+2H+Na ; M+2H+Na ; M+2Na ; M+H+2Na ; M+H+Na ; M+H+Na ; M+3Na	C9H16N2O4 ; C9H16N2O4 ; C9H16N2O4 ; C19H26O3 ; C19H26O3 ; C5H10O4 ; C10H20O2 ; C5H10O4 ; C6H6N4 ; C7H13NO4 ; C5H10O4 ; C19H26O3 ; C19H26O3 ; C5H10O ; C19H26O3 ; C19H26O3 ; C19H26O3 ; C5H10O4 ; C5H10O4 ; C17H28O3 ; C19H26O3 ; C12H18O2 ; C5H10O4 ; C10H20O2 ; C10H20O2 ; C5H10O ; C5H10O ; C19H26O3 ; C15H30O3 ; C5H10O ; C10H20O2 ; C5H10O ; C5H10O ; C5H10O ; C5H10O ; C10H20O2 ; C12H18O2 ; C10H20O2 ; C10H20O2 ; C10H20O2 ; C10H20O2 ; C10H20O2 ; C10H20O2 ; C12H18O2 ; C17H28O3 ; C19H26O3 ; C10H20O2 ; C5H10O ; C19H26O3 ; C19H26O3 ; C5H10O ; C7H13NO4 ; C12H18O2 ; C10H20O2 ; C5H10O ; C10H20O2 ; C12H18O2 ; C9H13NO4 ; C12H18O2 ; C12H18O2 ; C10H20O2 ; C10H20O2 ; C12H18O2 ; C10H20O2 ; C10H20O2 ; C10H20O2 ; C7H13NO4 ; C17H28O3 ; C19H26O3 ; C12H18O2 ; C12H18O2 ; C10H20O2 ; C12H18O2 ; C10H20O2 ; C10H20O2 ; C12H18O2 ; C12H18O2 ; C7H13NO4 ; C10H20O2 ; C10H20O2 ; C10H20O2 ; C19H26O3 ; C12H18O2 ; C5H10O ; C10H20O2 ; C10H20O2 ; C10H20O2 ; C10H20O2 ; C12H18O2 ; C10H9N5 ; C10H20O2 ; C19H26O3 ; C19H26O3 ; C10H20O2 ; C17H28O3 ; C12H18O2 ; C12H18O2 ; C15H30O3
223.06335	1.421922	0.550518	3.9086	0.00292	2.5346	0.023286	223.0634	HMDB06806 ; HMDB29399 ; HMDB29834 ; HMDB29869 ; HMDB30216 ; HMDB33258 ; HMDB38440 ; HMDB41307 ; HMDB41835 ; HMDB61023 ; HMDB33071	Propinol adenylate ; Ochratoxin A ; Harmalan ; Hemiariensin ; Mukonal ; Isoyatein ; 1-Isothiocyanato-7-(methylthio)heptane ; Italipyrone ; Benzidine ; N-Desmethyldiltiazem ; Propyl 1-(propylsulfinyl)propyl disulfide	M+ACN+2H ; M+ACN+2H ; M+K ; M+2Na ; 2M+H+Na ; M+2Na ; 2M+H+K ; M+2Na ; M+K ; M+2Na ; M+H-H2O	C13H18N5O8P ; C20H18ClNO6 ; C12H12N2 ; C22H24O7 ; C13H9NO2 ; C22H24O7 ; C9H17NS2 ; C22H24O7 ; C12H12N2 ; C21H24N2O4S ; C9H20OS3

234.14472	1.480822	0.222826	-3.9065	0.00293	2.5331	0.023327	234.1447	HMDB28960 ; HMDB29044 ; HMDB29447 ; HMDB01432 ; HMDB04980 ; HMDB10726 ; HMDB12183 ; HMDB12331 ; HMDB12341 ; HMDB29220 ; HMDB29358 ; HMDB29859 ; HMDB30031 ; HMDB31002 ; HMDB31003 ; HMDB31297 ; HMDB31362 ; HMDB31440 ; HMDB31441 ; HMDB32119 ; HMDB32275 ; HMDB32393 ; HMDB32452 ; HMDB33203 ; HMDB33377 ; HMDB34427 ; HMDB34581 ; HMDB34880 ; HMDB35122 ; HMDB35365 ; HMDB35837 ; HMDB35839 ; HMDB35907 ; HMDB36099 ; HMDB36211 ; HMDB36990 ; HMDB36992 ; HMDB37021 ; HMDB37116 ; HMDB37217 ; HMDB37497 ; HMDB37632 ; HMDB38744 ; HMDB39149 ; HMDB39768 ; HMDB39795 ; HMDB40194 ; HMDB40208 ; HMDB40213 ; HMDB40330 ; HMDB40530 ; HMDB40726 ; HMDB40727 ; HMDB41012 ; HMDB41630 ; HMDB59610 ;	Lysyl-Serine ; Serinyl-Lysine ; Lysinoalanine ; Agmatine ; cis-4-Decenoic acid ; trans-Dec-2-enoic acid ; 8-Methylnonenoate ; PS(14:014:1(9Z)) ; PS(14:1(9Z)14:0) ; lysoPC(26:1(5Z)) ; 5Z-Octenyl acetate ; Methyl (xi)-3-nonenoate ; ()-6-Methyl-5-hepten-2-yl acetate ; 3-Decenoic acid ; 9-Decenoic acid ; 2-Octenyl acetate ; Cyclohexylethyl acetate ; Linalool oxide (trans-pyranoid) ; Linalool oxide III ; 4-Hydroxy-26-dimethyl-7-octen-3-one ; Ethyl 3-octenoate ; 2-Methylbutyl 3-methyl-2-butenoate ; 1-Octen-3-yl acetate ; 6-Decanolide ; cis-3-Hexenyl butyrate ; Cyclohexyl butanoate ; 2-Hexenyl butanoate ; 3-Methyl-24-nonanedione ; (SE)-26-Dimethyl-57-octadiene-23-diol ; Cnidiol C ; Citronellic acid ; (E)-3-(Tetrahydro-55-dimethyl-2-furanyl)-2-buten-1-ol ; Linalyl oxide ; Lilac alcohol ; Hexyl crotonate ; 26-Dimethyl-37-octadiene-26-diol ; 1-Cyclopropyl-4-methyl-13-cyclohexanediol ; trans-p-Menth-2-ene-14-diol ; delta-Decalactone ; xi-5-Hexyldihydro-2(3H)-furanone ; Ethyl (E)-2-octenoate ; xi-Tetrahydro-3-pentyl-2H-pyran-2-one ; 9-Hydroxygeraniol ; ()-6-Hydroxy-26-dimethyl-7-octen-4-one ; 5-Decenoic acid ; Ethyl 4Z-octenoate ; Methyl 2-nonenoate ; 2-Propenyl heptanoate ; cis-3-Hexenyl isobutyrate ; Menthone lactone ; Isopentyl 3-methyl-2-butenoate ; ()-cis-Linalyl oxide ; ()-trans-Linalyl oxide ; 6-Decenoic acid ; ()-cis-Linalool 37-oxide ; 2-Exo-hydroxy-18-cineole ; 2-Hydroxycineol ; Agmatinium ; N-Didesmethyl-tamoxifen	M+H ; M+H ; M+H ; 3M+2K ; M+ACN+Na ; M+ACN+Na ; M+ACN+Na ; M+2H+Na ; M+2H+Na ; M+3Na ; M+ACN+Na ; 3M+2K ; M+3ACN+2H	C9H19N3O4 ; C9H19N3O4 ; C9H19N3O4 ; C5H14N4 ; C10H18O2 ; C10H18O2 ; C10H18O2 ; C34H64NO10P ; C34H64NO10P ; C34H68NO7P ; C10H18O2 ; C5H14N4 ; C24H25NO
205.08194	1.447882	0.100265	-3.9003	0.00296	2.5287	0.023514	205.0819	HMDB11162 ; HMDB11667 ; HMDB28683 ; HMDB28746 ; HMDB60830 ; HMDB12550 ; HMDB14989 ; HMDB29456 ; HMDB30899 ; HMDB31751 ; HMDB32607 ; HMDB33394 ; HMDB35168 ; HMDB36522 ; HMDB36665 ; HMDB36666 ; HMDB36944 ; HMDB37641 ; HMDB37727 ; HMDB38181 ; HMDB38354 ; HMDB38444 ; HMDB38746 ; HMDB39031 ; HMDB39874 ; HMDB40757 ; HMDB40758 ; HMDB41226 ; HMDB41279 ; HMDB41348 ; HMDB41496 ; HMDB41817 ; HMDB41892 ; HMDB59891 ; HMDB59992 ; HMDB60788	L-beta-aspartyl-L-alanine ; 5-L-Glutamylglycine ; Alanyl-Aspartate ; Aspartyl-Alanine ; Melatonin glucuronide ; 12-Oxo-20-carboxy-leukotriene B4 ; Dacarbazine ; Amyl 2-furoate ; Eremopetasitenin A1 ; y-Morphine ; ()-threo-Anethole glycol ; 4-Ethyl-26-dimethoxyphenol ; Cinncassiol C2 ; Verimol J ; Blumealactone A ; Blumealactone B ; Gibberellin A123 ; 4-(Ethoxymethyl)-2-methoxyphenol ; Furfuryl pentanoate ; Peperinic acid ; 2-Ethoxy-4-(methoxymethyl)phenol ; 1-Isothiocyanato-3-phenylpropane ; Gibberellin A74 ; trans-O-Methylgrandmarin ; Furfuryl isovalerate ; 3b8a-Dihydroxy-6b-angeloyloxy-7(11)-eremophilen-128-olide ; 3b8b-Dihydroxy-6b-angeloyloxy-7(11)-eremophilen-128-olide ; 3b9b-Dihydroxy-6b-angeloyloxy-7(11)-eremophilen-128b-olide ; 3b-Hydroxy-6b-(23-epoxy-2-methylbutanoyloxy)-7(11)-eremophilen-128b-olide ; Gibberellin A100 ; Gibberellin A97 ; 7-Aminoclonazepam ; Faropenem ; Isoamyl 2-furoate ; 5-(4-Hydroxyphenyl)-gamma-valerolactone-4-O-glucuronide ; 6-O-Desmethyl-mycophenolic acid	M+H ; M+H ; M+H ; M+H ; M+2H ; M+2Na ; M+Na ; M+Na ; M+2Na ; M+H+2Na ; M+Na ; M+Na ; M+2Na ; M+Na ; M+2Na ; M+2Na ; M+2Na ; M+Na ; M+Na ; M+Na ; M+Na ; 2M+3H2O+2H ; M+2Na ; 2M+3H ; M+Na ; M+2Na ; M+2Na ; M+2Na ; M+2Na ; M+2Na ; M+2Na ; M+3ACN+2H ; M+3ACN+2H ; M+Na ; M+2Na ; 2M+3H	C7H12N2O5 ; C7H12N2O5 ; C7H12N2O5 ; C7H12N2O5 ; C19H24N2O8 ; C20H28O6 ; C6H10N6O ; C10H14O3 ; C20H28O6 ; C34H36N2O6 ; C10H14O3 ; C10H14O3 ; C20H28O6 ; C10H14O3 ; C20H28O6 ; C20H28O6 ; C20H28O6 ; C10H14O3 ; C10H14O3 ; C10H14O3 ; C10H14O3 ; C10H11NS ; C20H28O6 ; C16H18O6 ; C10H14O3 ; C20H28O6 ; C20H28O6 ; C20H28O6 ; C20H28O6 ; C20H28O6 ; C20H28O6 ; C15H12ClN3O ; C12H15NO5S ; C10H14O3 ; C20H28O6 ; C16H18O6
715.63918	1.546029	0.780567	-3.8985	0.002968	2.5275	0.023514	NA	NA	NA	NA	NA
146.11757	1.465787	1.112174	-3.8971	0.002975	2.5265	0.023514	146.1176	HMDB06831 ; HMDB14347 ; HMDB06210 ; HMDB13238 ; HMDB29165 ; HMDB29314 ; HMDB30330 ; HMDB30997 ; HMDB31046 ; HMDB31174 ; HMDB31342 ; HMDB31351 ; HMDB31476 ; HMDB31477 ; HMDB31484 ; HMDB31500 ; HMDB31504 ; HMDB31593 ; HMDB31603 ; HMDB31681 ; HMDB32391 ; HMDB32460 ; HMDB33793 ; HMDB38271 ; HMDB38962 ; HMDB39819 ; HMDB40214 ; HMDB40591 ; HMDB40888 ; HMDB60038	3-Dehydroxycarnitine ; Succinylcholine ; Heptadecanoyl carnitine ; Heptanoylcarnitine ; 13-Diacetylpropane ; Isopentenyl acetate ; 1-Methoxy-3-methylene-2-pentanone ; Cyclohexaneundecanoic acid ; (Z)-9-Heptadecenoic acid ; 4-Methyl-5-hexanolide ; Cyclohexanecarboxylic acid ; Cyclohexyl formate ; 23-Heptanedione ; 34-Heptanedione ; 2-Heptenoic acid ; Methyl 2E-hexenoate ; Methyl (Z)-3-hexenoate ; 5-Methyl-23-hexanedione ; Ethyl 4-pentenoate ; Dihydro-5-propyl-2(3H)-furanone ; 3-Methyl-3-butenyl acetate ; 4-Pentenyl acetate ; 4-Heptenoic acid ; 2-Hexenyl formate ; Ethyl tiglate ; 24-Dimethyl-2-pentenoic acid ; cis-3-Hexenyl formate ; Allyl butyrate ; 24-Heptadecanedione ; 10Z-Heptadecenoic acid	M+H ; M+2H ; M+2H+Na ; M+H+NH4 ; M+NH4 ; M+NH4 ; M+NH4 ; M+H+Na ; M+H+Na ; M+NH4 ; M+NH4 ; M+NH4 ; M+NH4 ; M+NH4 ; M+NH4 ; M+NH4 ; M+NH4 ; M+NH4 ; M+NH4 ; M+NH4 ; M+NH4 ; M+NH4 ; M+NH4 ; M+NH4 ; M+NH4 ; M+NH4 ; M+NH4 ; M+NH4 ; M+H+Na ; M+H+Na	C7H15NO2 ; C14H30N2O4 ; C24H47NO4 ; C14H27NO4 ; C7H12O2 ; C7H12O2 ; C7H12O2 ; C17H32O2 ; C17H32O2 ; C7H12O2 ; C7H12O2 ; C7H12O2 ; C7H12O2 ; C7H12O2 ; C7H12O2 ; C7H12O2 ; C7H12O2 ; C7H12O2 ; C7H12O2 ; C7H12O2 ; C7H12O2 ; C7H12O2 ; C7H12O2 ; C7H12O2 ; C7H12O2 ; C7H12O2 ; C7H12O2 ; C7H12O2 ; C17H32O2 ; C17H32O2
284.14698	1.439652	0.990103	3.8966	0.002977	2.5262	0.023514	284.147	HMDB34525 ; HMDB34532 ; HMDB37783	Barringtogenol C ; Camelliagenin C ; Ganoderiol H	M+2K ; M+2K ; M+2K	C30H50O5 ; C30H50O5 ; C30H50O5
152.01646	1.440351	0.611307	3.8954	0.002983	2.5253	0.023514	152.0165	HMDB34413 ; HMDB40578 ; HMDB00033 ; HMDB12482 ; HMDB28689 ; HMDB28878 ; HMDB34303 ; HMDB36650 ; HMDB38126 ; HMDB39136 ; HMDB59741	12-Benzisothiazol-3(2H)-one ; 4-Thiocyanatophenol ; Carnosine ; (6R)-6-(L-Erythro-12-Dihydroxypropyl)-5678-tetrahydro-4a-hydroxypterin ; Alanyl-Histidine ; Histidinyl-Alanine ; 1-Methoxyspirobrassinin ; Sinalbin A ; Genipinic acid ; Elenaic acid ; xanthurenic acid 8-O-sulfate	M+H ; M+H ; M+2K ; M+2K ; M+2K ; M+2K ; M+H+Na ; M+H+Na ; M+Na+K ; M+Na+K ; M+H+NH4	C7H5NOS ; C7H5NOS ; C9H14N4O3 ; C9H14N4O3 ; C9H14N4O3 ; C9H14N4O3 ; C12H12N2O2S2 ; C12H12N2O2S2 ; C11H14O6 ; C11H14O6 ; C10H7NO7S

400.82712	1.470034	1.547509	3.8947	0.002987	2.5248	0.023514	400.8271	HMDB08293 ; HMDB08294 ; HMDB08325 ; HMDB08586 ; HMDB11224 ; HMDB11249 ; HMDB11281 ; HMDB11314 ; HMDB13431 ; HMDB13436	PC(20:0P-18:1(11Z)) ; PC(20:0P-18:1(9Z)) ; PC(20:1(11Z)P-18:0) ; PC(22:1(13Z)P-16:0) ; PC(P-16:022:1(13Z)) ; PC(P-18:020:1(11Z)) ; PC(P-18:1(11Z)20:0) ; PC(P-18:1(9Z)20:0) ; PC(o-18:1(9Z)20:1(11Z)) ; PC(o-18:2(9Z12Z)20:0)	M+2H ; M+2H ; M+2H ; M+2H ; M+2H ; M+2H ; M+2H ; M+2H ; M+2H ; M+2H	C46H90NO7P ; C46H90NO7P ; C46H90NO7P ; C46H90NO7P ; C46H90NO7P ; C46H90NO7P ; C46H90NO7P ; C46H90NO7P ; C46H90NO7P ; C46H90NO7P
363.16597	1.512546	0.029072	-3.8944	0.002988	2.5246	0.023514	363.166	HMDB14808 ; HMDB15058 ; HMDB30211 ; HMDB30270 ; HMDB31849 ; HMDB32971 ; HMDB33266 ; HMDB34552 ; HMDB34593 ; HMDB34636 ; HMDB35248 ; HMDB35289 ; HMDB38021 ; HMDB38351 ; HMDB40496 ; HMDB41347 ; HMDB60784 ; HMDB15613 ; HMDB31954 ; HMDB31956 ; HMDB41360	Pirenzepine ; Levosimendan ; (R)-Laudanidine ; Anomurine ; 2-Ethylpyrazine ; 23-Dimethylpyrazine ; Gossyrubilone ; Medicagenic acid 3-O-beta-D-glucoside ; (S)-Codamine ; Phytolaccasaponin G ; 26-Dimethylpyrazine ; 25-Dimethylpyrazine ; 3alpha-O-trans-Feruloyl-2alpha-hydroxy-12-ursen-28-oic acid ; 12-Ketoporrigenin 3-4-(2-glucosyl-3-xylosyl)-glucosyl)-galactoside ; trans-3-Feruloylcorosolic acid ; Elatoside G ; 6-beta-Naltrexol ; Ecabet ; 3-Methyl-3-butenyl apiosyl-(1-6)-glucoside ; Prenyl apiosyl-(1-6)-glucoside ; Prenyl arabinosyl-(1-6)-glucoside	2M+H+Na ; M+2ACN+H ; 2M+H+K ; 2M+H+K ; 3M+K ; 3M+K ; 2M+H+K ; M+Na+K ; 2M+H+K ; M+Na+K ; 3M+K ; 3M+K ; M+2K ; M+2H+Na ; M+2K ; M+Na+K ; 2M+H+K ; M+H-H2O ; M+H-H2O ; M+H-H2O ; M+H-H2O	C19H21N5O2 ; C14H12N6O ; C20H25NO4 ; C20H25NO4 ; C6H8N2 ; C6H8N2 ; C20H25NO4 ; C36H56O11 ; C20H25NO4 ; C36H56O11 ; C6H8N2 ; C6H8N2 ; C40H56O7 ; C50H80O24 ; C40H56O7 ; C36H56O11 ; C20H25NO4 ; C20H28O5S ; C16H28O10 ; C16H28O10 ; C16H28O10
993.29864	1.569078	0.298734	-3.8938	0.002991	2.5242	0.023514	993.2986	HMDB03936 ; HMDB30663 ; HMDB34464 ; HMDB60161 ; HMDB60596	(S)-3-Hydroxydodecanoyl-CoA ; 47-Di-O-methylcatechin ; Austinol ; 3(S)-Hydroxy-4(R)8-dimethyl-nonanoyl-CoA ; Ketotifen-N-glucuronide	2M+3H2O+2H ; 3M+K ; 2M+2K-H ; M+ACN+H ; 2M+Na	C33H58N7O18P3S ; C17H18O6 ; C25H30O8 ; C32H56N7O18P3S ; C25H27NO7S
705.79577	1.482273	0.492085	-3.8915	0.003002	2.5226	0.023565	705.7958	HMDB30901 ; HMDB41532	Eremopetasitenin B2 ; Eremosulphoxinolide A	3M+H+NH4 ; 3M+H+NH4	C24H32O7S ; C24H32O7S
191.06634	1.524666	0.040097	-3.8897	0.003011	2.5213	0.023599	191.0663	HMDB11165 ; HMDB15673 ; HMDB28753 ; HMDB28837 ; HMDB14414 ; HMDB01069 ; HMDB01164 ; HMDB02207 ; HMDB15271 ; HMDB29581 ; HMDB29680 ; HMDB30472 ; HMDB30473 ; HMDB31317 ; HMDB31344 ; HMDB31543 ; HMDB31631 ; HMDB31632 ; HMDB31759 ; HMDB32233 ; HMDB32252 ; HMDB32269 ; HMDB32912 ; HMDB32921 ; HMDB33569 ; HMDB34247 ; HMDB36231 ; HMDB37729 ; HMDB38445 ; HMDB38925 ; HMDB39040 ; HMDB39122 ; HMDB39407 ; HMDB39778 ; HMDB39817 ; HMDB40034 ; HMDB40254 ; HMDB40260 ; HMDB41821 ; HMDB41822 ; HMDB59842 ; HMDB59963	L-beta-aspartyl-L-glycine ; Carglumic acid ; Aspartyl-Glycine ; Glycyl-Aspartate ; Chlorotrianisene ; 2-Phenylaminoadenosine ; trans-12-Dihydrobenzene-12-diol ; 3-Hydroxyisoheptanoic acid ; Cefadroxil ; (2E4E)-24-Hexadienoic acid ; 26-Dimethoxy-4-methylphenol ; 4-Ipomeanol ; 1-Ipomeanol ; Bis(methylthio)methane ; 12-Cyclohexanedione ; 3-Methyl-12-cyclopentanedione ; 12-Propanedithiol ; 13-Propanedithiol ; (E)-4-Oxo-2-hexen-1-al ; 25-Dimethyl-3(2H)-furanone ; Epoxyoxophorone ; (-)-Ethyl 2-hydroxy-2-methylbutyrate ; Ethyl methyl disulfide ; Ethyl 2-furanpropionate ; Syoyualdehyde ; 2-(Methoxymethyl)furan ; Methyl DL-Leucate ; 2-Furanylmethyl butanoate ; 1-Isothiocyanato-2-phenylethane ; (4-Hydroxy-3-methoxyphenyl)ethanol ; Apiumoside ; Crocin 4 ; Methyl ()-3-hydroxyhexanoate ; xi-35-Dimethyl-2(5H)-furanone ; Dihydro-46-dimethyl-4H-135-dithiazine ; 2-Ethylbenzothiazole ; Butyl lactate ; 55-Dimethyl-2(5H)-furanone ; Acetylisoniazid ; Acrolein ; Ethyl 2-hydroxyisovalerate ; 135-Trimethoxybenzene	M+H ; M+H ; M+H ; M+H ; M+2H ; M+H+Na ; 3M+2Na ; M+2Na-H ; M+H+NH4 ; 3M+2Na ; M+Na ; M+Na ; M+Na ; M+2ACN+H ; 3M+2Na ; 3M+2Na ; M+2ACN+H ; M+2ACN+H ; 3M+2Na ; 3M+2Na ; M+Na ; M+2Na-H ; M+2ACN+H ; M+Na ; 3M+2Na ; 3M+2Na ; M+2Na-H ; M+Na ; 2M+3H2O+2H ; M+Na ; M+3H ; M+3Na ; M+2Na-H ; 3M+2Na ; M+ACN+H ; 2M+3H2O+2H ; M+2Na-H ; 3M+2Na ; 2M+H+Na ; 3M+Na ; M+2Na-H ; M+Na	C6H10N2O5 ; C6H10N2O5 ; C6H10N2O5 ; C6H10N2O5 ; C23H21ClO3 ; C16H18N6O4 ; C6H8O2 ; C7H14O3 ; C16H17N3O5S ; C6H8O2 ; C9H12O3 ; C9H12O3 ; C9H12O3 ; C3H8S2 ; C6H8O2 ; C6H8O2 ; C3H8S2 ; C3H8S2 ; C6H8O2 ; C6H8O2 ; C9H12O3 ; C7H14O3 ; C3H8S2 ; C9H12O3 ; C6H8O2 ; C6H8O2 ; C7H14O3 ; C9H12O3 ; C9H9NS ; C9H12O3 ; C29H30O12 ; C27H36O9 ; C7H14O3 ; C6H8O2 ; C5H11NS2 ; C9H9NS ; C7H14O3 ; C6H8O2 ; C8H9N3O2 ; C3H4O ; C7H14O3 ; C9H12O3
245.54578	1.526147	1.027365	3.8826	0.003046	2.5163	0.023833	NA	NA	NA	NA	NA
288.99235	1.444095	1.380464	-3.8803	0.003057	2.5147	0.023885	288.9924	HMDB01127 ; HMDB01376 ; HMDB14456 ; HMDB33048 ; HMDB33049	6-Phosphonoglucono-D-lactone ; 2-Keto-3-deoxy-6-phosphogluconic acid ; Ethoxzolamide ; Ethyl 1-(propylthio)propyl disulfide ; Butyl 1-(methylthio)propyl disulfide	2M+Na+K ; 2M+Na+K ; 2M+Na+K ; M+2K+H ; M+2K+H	C6H11O9P ; C6H11O9P ; C9H10N2O3S2 ; C8H18S3 ; C8H18S3
562.08476	1.463638	0.602141	-3.8764	0.003077	2.5119	0.023962	562.0848	HMDB13997 ; HMDB31943 ; HMDB31946 ; HMDB37596 ; HMDB60926	6-Hydroxymethyletoricoxib ; (Z)-N-(4-hydroxyphenyl)methylethoxycarbothioamide 4-(tri-acetylrhamnoside) ; N-(4-hydroxyphenyl)methylethoxycarbothioamide 4-(tri-acetylrhamnoside) ; 8-Hydroxyluteolin 8-glucoside 3-sulfate ; Etoricoxib 1-N-oxide	3M+2H ; M+2K+H ; M+2K+H ; M+NH4 ; 3M+2H	C18H15ClN2O3S ; C22H29NO9S ; C22H29NO9S ; C21H20O15S ; C18H15ClN2O3S
524.68692	1.483789	1.485208	3.8762	0.003078	2.5118	0.023962	524.6869	HMDB12360 ; HMDB12381 ; HMDB12391 ; HMDB12401 ; HMDB12411 ; HMDB12420 ; HMDB14853 ; HMDB61176	PS(16:020:3(8Z11Z14Z)) ; PS(18:018:3(9Z12Z15Z)) ; PS(18:1(9Z)18:2(9Z12Z)) ; PS(18:2(9Z12Z)18:1(9Z)) ; PS(18:3(9Z12Z15Z)18:0) ; PS(20:3(8Z11Z14Z)16:0) ; Paroxetine ; Phenylacetic acid-N-isopropylnortropine-ester methobromide	2M+3H ; 2M+3H ; 2M+3H ; 2M+3H ; 2M+3H ; 2M+3H ; 3M+Na+K ; 2M+H+NH4	C42H76NO10P ; C42H76NO10P ; C42H76NO10P ; C42H76NO10P ; C42H76NO10P ; C42H76NO10P ; C19H20FNO3 ; C27H34BrNO4
125.02088	1.521035	0.293018	-3.8751	0.003083	2.511	0.023962	125.0209	HMDB12199 ; HMDB12283 ; HMDB29612 ; HMDB33842 ; HMDB33893 ; HMDB39221	Chorismate ; Prephenate ; Asparagusic acid syn-S-oxide ; 34-Dimethoxy-12-benzenedicarboxylic acid ; 45-Dimethoxy-12-benzenedicarboxylic acid ; Methylcarbamodithioic acid K salt	M+H+Na ; M+H+Na ; M+2ACN+2H ; M+H+Na ; M+H+Na ; M+NH4	C10H10O6 ; C10H10O6 ; C4H6O3S2 ; C10H10O6 ; C10H10O6 ; C2H5NS2
340.08070	1.466933	0.759895	3.8745	0.003086	2.5106	0.023962	340.0807	HMDB30562 ; HMDB30830 ; HMDB33722 ; HMDB33820 ; HMDB33859 ; HMDB34139 ; HMDB35914 ; HMDB36305 ; HMDB37541	38-Dihydroxy-9-methoxycoumestan ; 38-Dihydroxy-1-methylanthraquinone-2-carboxylic acid ; Sativol ; Irilone ; Glyzaglabrin ; Trifoliol ; Aflatoxin P1 ; Isotrifoliol ; Patuletin 3-gentiobioside	M+ACN+H ; M+ACN+H ; M+ACN+H ; M+ACN+H ; M+ACN+H ; M+ACN+H ; M+ACN+H ; M+ACN+H ; M+H+Na	C16H10O6 ; C16H10O6 ; C16H10O6 ; C16H10O6 ; C16H10O6 ; C16H10O6 ; C16H10O6 ; C16H10O6 ; C28H32O18
198.04167	1.437637	0.842429	-3.8705	0.003107	2.5077	0.024082	198.0417	HMDB06955 ; HMDB14322 ; HMDB14450 ; HMDB29674 ; HMDB33163 ; HMDB36938 ; HMDB38428 ; HMDB40664 ; HMDB40866 ; HMDB60620 ; HMDB60793	3-Hydroxy-2-methylpyridine-45-dicarboxylate ; Fluvoxamine ; Mitomycin ; 2-Chloro-13-dimethoxy-5-methylbenzene ; Pyrocoll ; 1-O-Feruloylglucose ; 2-Hydroxypropyl glucosinolate ; Veranisatin B ; 1-O-2-Hydroxy-4-methoxycinnamoyl-b-D-glucose ; Sulindac sulfone ; 67-Dichloro-3-hydroxy-15 dihydro-imidazo21-bquinazolin-2-one	M+H ; M+2K ; M+Na+K ; 2M+H+Na ; 2M+H+Na ; M+H+K ; M+H+NH4 ; M+H+K ; M+H+K ; M+H+Na ; M+3ACN+2H	C8H7NO5 ; C15H21F3N2O2 ; C15H18N4O5 ; C9H11ClO2 ; C10H6N2O2 ; C16H20O9 ; C10H19NO10S2 ; C16H20O9 ; C16H20O9 ; C20H17FO4S ; C10H7Cl2N3O2

546.65426	1.509694	1.246275	-3.8641	0.003139	2.5032	0.024291	546.6543	HMDB01322 ; HMDB06521 ; HMDB06523 ; HMDB06529 ; HMDB11893 ; HMDB14559 ; HMDB15428 ; HMDB56830 ; HMDB56882 ; HMDB56907 ; HMDB56911 ; HMDB56919 ; HMDB56920 ; HMDB57756 ; HMDB57766 ; HMDB57768 ; HMDB57769 ; HMDB57800 ; HMDB57810 ; HMDB57812 ; HMDB57813 ; HMDB57831 ; HMDB57832 ; HMDB57835 ; HMDB57836 ; HMDB58186 ; HMDB58300 ; HMDB58317 ; HMDB58589 ; HMDB58673 ; HMDB58690 ; HMDB59042 ; HMDB59062 ; HMDB59080 ; HMDB59081 ; HMDB59089 ; HMDB59099 ; HMDB59112 ; HMDB59166 ; HMDB59178 ; HMDB59179 ; HMDB59181 ; HMDB59208 ; HMDB59239 ; HMDB59249 ; HMDB59251 ; HMDB59252 ; HMDB59349 ; HMDB59371 ; HMDB59382 ; HMDB59390 ; HMDB59413 ; HMDB59434 ; HMDB59436 ; HMDB59437 ; HMDB59484 ;	Oleoyl-CoA ; cis-Vaccenoyl CoA ; Arachidonyl-CoA ; trans-Octadec-2-enoyl-CoA ; Ganglioside GM1 (d18:123:0) ; Ampicillin ; Cefradine ; CL(16:022:5(4Z7Z10Z13Z16Z)22:6(4Z7Z10Z13Z16Z19Z)22:6(4Z7Z10Z13Z16Z19Z)) ; CL(16:022:5(7Z10Z13Z16Z19Z)22:6(4Z7Z10Z13Z16Z19Z)22:6(4Z7Z10Z13Z16Z19Z)) ; CL(16:022:6(4Z7Z10Z13Z16Z19Z)22:5(4Z7Z10Z13Z16Z)22:6(4Z7Z10Z13Z16Z19Z)) ; CL(16:022:6(4Z7Z10Z13Z16Z19Z)22:5(7Z10Z13Z16Z19Z)22:6(4Z7Z10Z13Z16Z19Z)) ; CL(16:022:6(4Z7Z10Z13Z16Z19Z)22:6(4Z7Z10Z13Z16Z19Z)22:5(4Z7Z10Z13Z16Z)) ; CL(16:022:6(4Z7Z10Z13Z16Z19Z)22:6(4Z7Z10Z13Z16Z19Z)22:5(7Z10Z13Z16Z19Z)) ; CL(16:1(9Z)22:5(4Z7Z10Z13Z16Z)22:5(4Z7Z10Z13Z16Z)22:6(4Z7Z10Z13Z16Z19Z)) ; CL(16:1(9Z)22:5(4Z7Z10Z13Z16Z)22:5(7Z10Z13Z16Z19Z)22:6(4Z7Z10Z13Z16Z19Z)) ; CL(16:1(9Z)22:5(4Z7Z10Z13Z16Z)22:6(4Z7Z10Z13Z16Z19Z)22:5(4Z7Z10Z13Z16Z)) ; CL(16:1(9Z)22:5(4Z7Z10Z13Z16Z)22:6(4Z7Z10Z13Z16Z19Z)22:5(7Z10Z13Z16Z19Z)) ; CL(16:1(9Z)22:5(7Z10Z13Z16Z19Z)22:5(4Z7Z10Z13Z16Z)22:6(4Z7Z10Z13Z16Z19Z)) ; CL(16:1(9Z)22:5(7Z10Z13Z16Z19Z)22:5(7Z10Z13Z16Z19Z)22:6(4Z7Z10Z13Z16Z19Z)) ; CL(16:1(9Z)22:5(7Z10Z13Z16Z19Z)22:6(4Z7Z10Z13Z16Z19Z)22:5(4Z7Z10Z13Z16Z)) ; CL(16:1(9Z)22:5(7Z10Z13Z16Z19Z)22:6(4Z7Z10Z13Z16Z19Z)22:5(7Z10Z13Z16Z19Z)) ; CL(16:1(9Z)22:6(4Z7Z10Z13Z16Z19Z)22:5(4Z7Z10Z13Z16Z)22:5(4Z7Z10Z13Z16Z)) ; CL(16:1(9Z)22:6(4Z7Z10Z13Z16Z19Z)22:5(4Z7Z10Z13Z16Z)22:5(7Z10Z13Z16Z19Z)) ; CL(16:1(9Z)22:6(4Z7Z10Z13Z16Z19Z)22:5(7Z10Z13Z16Z19Z)22:5(4Z7Z10Z13Z16Z)) ; CL(16:1(9Z)22:6(4Z7Z10Z13Z16Z19Z)22:5(7Z10Z13Z16Z19Z)22:5(7Z10Z13Z16Z19Z)) ; CL(18:1(11Z)20:4(5Z8Z11Z14Z)22:6(4Z7Z10Z13Z16Z19Z)22:6(4Z7Z10Z13Z16Z19Z)) ; CL(18:1(11Z)22:6(4Z7Z10Z13Z16Z19Z)20:4(5Z8Z11Z14Z)22:6(4Z7Z10Z13Z16Z19Z)) ; CL(18:1(11Z)22:6(4Z7Z10Z13Z16Z19Z)22:6(4Z7Z10Z13Z16Z19Z)20:4(5Z8Z11Z14Z)) ; CL(18:1(9Z)20:4(5Z8Z11Z14Z)22:6(4Z7Z10Z13Z16Z19Z)22:6(4Z7Z10Z13Z16Z19Z)) ; CL(18:1(9Z)22:6(4Z7Z10Z13Z16Z19Z)20:4(5Z8Z11Z14Z)22:6(4Z7Z10Z13Z16Z19Z)) ; CL(18:1(9Z)22:6(4Z7Z10Z13Z16Z19Z)22:6(4Z7Z10Z13Z16Z19Z)20:4(5Z8Z11Z14Z)) ; CL(20:4(5Z8Z11Z14Z)18:1(11Z)22:6(4Z7Z10Z13Z16Z19Z)22:6(4Z7Z10Z13Z16Z19Z)) ; CL(20:4(5Z8Z11Z14Z)18:1(9Z)22:6(4Z7Z10Z13Z16Z19Z)22:6(4Z7Z10Z13Z16Z19Z)) ;	M+Na+K ; M+Na+K ; M+H+K ; M+Na+K ; M+2H+Na ; 3M+2Na ; 3M+2Na ; M+3Na ; M+H+2Na ; M+H+2Na ; M+3Na ; M+3Na ; M+H+2Na ; M+3Na ; M+H+2Na ; M+H+K	C39H68N7O17P3S ; C39H68N7O17P3S ; C41H66N7O17P3S ; C39H68N7O17P3S ; C79H142N2O31 ; C16H19N3O4S ; C16H19N3O4S ; C91H144O17P2 ; C93H142O17P2 ; C93H142O17P2 ; C91H144O17P2 ; C91H144O17P2 ; C93H142O17P2 ; C91H144O17P2 ; C91H144O17P2 ; C91H144O17P2 ; C91H144O17P2 ; C91H144O17P2 ; C91H144O17P2 ; C91H144O17P2 ; C91H144O17P2 ; C91H144O17P2 ; C91H144O17P2 ; C91H144O17P2 ; C91H144O17P2 ; C91H144O17P2 ; C91H144O17P2 ; C91H144O17P2 ; C91H144O17P2 ; C91H144O17P2 ; C91H144O17P2 ; C91H144O17P2 ; C93H142O17P2 ; C41H66N7O17P3S
166.54277	1.491459	1.503667	-3.8633	0.003143	2.5026	0.024291	166.5428	HMDB00905 ; HMDB01915 ; HMDB01927 ; HMDB02039 ; HMDB14434 ; HMDB14766 ; HMDB15697 ; HMDB29508 ; HMDB42010 ; HMDB59975 ; HMDB59976 ; HMDB60427 ; HMDB60632	Deoxyadenosine monophosphate ; Alendronic acid ; Diphenhydramine ; 2-Pyrrolidinone ; Atomoxetine ; Clorazepate ; Bupranolol ; Laccaic acid D ; Rutaecarpine ; 4-Hydroxy-5-(3-hydroxyphenyl)-valeric acid-3-O-sulphate ; 4-Hydroxy-5-(4-hydroxyphenyl)-valeric acid-4-O-sulphate ; Acetone cyanohydrin ; N-Demethyl orphenadrine	M+2H ; M+2ACN+2H ; M+2K ; 3M+2K ; M+2K ; M+H+NH4 ; M+Na+K ; M+H+NH4 ; M+2Na ; M+ACN+2H ; M+ACN+2H ; 3M+2K ; M+2K	C10H14N5O6P ; C4H13NO7P2 ; C17H21NO ; C4H7NO ; C17H21NO ; C16H11ClN2O3 ; C14H22ClNO2 ; C16H10O7 ; C18H13N3O ; C11H14O7S ; C11H14O7S ; C4H7NO ; C17H21NO
751.54645	1.53469	0.289498	3.862	0.00315	2.5017	0.024306	751.5465	HMDB10572 ; HMDB10600 ; HMDB07672 ; HMDB07698 ; HMDB07784 ; HMDB08192 ; HMDB08225 ; HMDB11212 ; HMDB11213 ; HMDB33621 ; HMDB56350 ; HMDB56351 ; HMDB56402 ; HMDB56438 ; HMDB56491 ; HMDB56517 ; HMDB56698 ; HMDB56943	PG(16:018:0) ; PG(18:016:0) ; DG(22:2(13Z16Z)22:6(4Z7Z10Z13Z16Z19Z)0:0) ; DG(22:4(7Z10Z13Z16Z)22:4(7Z10Z13Z16Z)0:0) ; DG(22:6(4Z7Z10Z13Z16Z19Z)22:2(13Z16Z)0:0) ; PC(18:3(6Z9Z12Z)P-16:0) ; PC(18:3(9Z12Z15Z)P-16:0) ; PC(P-16:018:3(6Z9Z12Z)) ; PC(P-16:018:3(9Z12Z15Z)) ; Araliacerebroside ; DG(22:2n60:022:6n3) ; DG(22:4n60:022:4n6) ; CL(16:016:018:018:2(9Z12Z)) ; CL(16:016:018:2(9Z12Z)18:0) ; CL(16:018:016:018:2(9Z12Z)) ; CL(16:018:018:2(9Z12Z)16:0) ; CL(16:018:2(9Z12Z)18:016:0) ; CL(18:016:018:2(9Z12Z)16:0)	M+H ; M+H ; 2M+Na+K ; 2M+Na+K ; 2M+Na+K ; 2M+H+Na ; 2M+H+Na ; 2M+H+Na ; 2M+H+Na ; 2M+H+K ; 2M+Na+K ; 2M+Na+K ; M+2ACN+2H ; M+2ACN+2H ; M+2ACN+2H ; M+2ACN+2H ; M+2ACN+2H ; M+2ACN+2H	C40H79O10P ; C40H79O10P ; C47H76O5 ; C47H76O5 ; C47H76O5 ; C42H78NO7P ; C42H78NO7P ; C42H78NO7P ; C42H78NO7P ; C40H77NO10 ; C47H76O5 ; C47H76O5 ; C78H148O17P2 ; C78H148O17P2 ; C78H148O17P2 ; C78H148O17P2 ; C78H148O17P2 ; C78H148O17P2
622.06897	1.525696	0.939639	-3.8607	0.003157	2.5008	0.024319	622.069	HMDB00935 ; HMDB12302 ; HMDB12304 ; HMDB39232	Uridine diphosphate glucuronic acid ; UDP-D-galacturonate ; UDP-L-iduronate ; Randilongin	M+ACN+H ; M+ACN+H ; M+ACN+H ; 2M+H+NH4	C15H22N2O18P2 ; C15H22N2O18P2 ; C15H22N2O18P2 ; C41H72O3
221.18590	1.424758	0.648912	-3.8584	0.003169	2.4991	0.024371	221.1859	HMDB00222 ; HMDB02356 ; HMDB06461 ; HMDB06469 ; HMDB11131 ; HMDB11188 ; HMDB11535 ; HMDB30928 ; HMDB31075 ; HMDB34148 ; HMDB35628 ; HMDB60125 ; HMDB60145	L-Palmitoylcarnitine ; Hexacosanoic acid ; Linoelaidyl carnitine ; Linoleyl carnitine ; MG(18:00:00:0) ; TG(12:012:012:0) ; MG(0:018:00:0) ; Tridecanal ; Glycerol 1-octadecanoate ; 2-Tridecanone ; 6-Hydroxy-8-hexacosanone ; Cerotate ; alpha-Tocotrienoxyl radical	M+ACN+2H ; M+2Na ; M+H+NH4 ; M+H+NH4 ; M+2ACN+2H ; M+2H+Na ; M+2ACN+2H ; M+Na ; M+2ACN+2H ; M+Na ; M+2Na ; M+2Na ; M+H+NH4	C23H45NO4 ; C26H52O2 ; C25H45NO4 ; C25H45NO4 ; C21H42O4 ; C39H74O6 ; C21H42O4 ; C13H26O ; C21H42O4 ; C13H26O ; C26H52O2 ; C26H52O2 ; C29H43O2
369.35121	1.539195	0.734896	3.8575	0.003173	2.4985	0.024371	369.3512	HMDB07383 ; HMDB07608 ; HMDB07803 ; HMDB56064 ; HMDB56087 ; HMDB13078 ; HMDB13645 ; HMDB32316	DG(20:024:00:0) ; DG(22:022:00:0) ; DG(24:020:00:0) ; DG(20:00:024:0) ; DG(22:00:022:0) ; Stearoylethanolamide ; NN-Dimethylsphingosine ; Hexanal dihexyl acetal	M+2H ; M+2H ; M+2H ; M+2H ; M+2H ; M+ACN+H ; M+ACN+H ; M+2ACN+H	C47H92O5 ; C47H92O5 ; C47H92O5 ; C47H92O5 ; C47H92O5 ; C20H41NO2 ; C20H41NO2 ; C18H38O2
577.91218	1.472643	1.119686	3.8549	0.003187	2.4966	0.024439	NA	NA	NA	NA	NA
476.75698	1.435171	1.273981	3.8528	0.003198	2.4952	0.024451	476.757	HMDB31364 ; HMDB34321 ; HMDB61024	Ampeloside Bs1 ; Digitoxigenin 3-glucosyl-(1-6)-glucosyl-(1-4)-26-dideoxyribohexoside ; mono-isopropyl-disopyramide	M+H+NH4 ; M+3ACN+2H ; 3M+Na+K	C45H74O20 ; C41H64O17 ; C18H23N3O
246.09701	1.512494	1.05292	3.8527	0.003198	2.4951	0.024451	246.097	HMDB15146 ; HMDB28783 ; HMDB29014 ; HMDB31859 ; HMDB60461	Metyrapone ; Cysteinyl-Proline ; Prolyl-Cysteine ; Norfuraneol ; cis-Acetylacrylate	2M+H+K ; 2M+3H2O+2H ; 2M+3H2O+2H ; 2M+NH4 ; 2M+NH4	C14H14N2O ; C8H14N2O3S ; C8H14N2O3S ; C5H6O3 ; C5H6O3
300.51867	1.431302	0.811834	-3.8494	0.003215	2.4928	0.024529	300.5187	HMDB33887 ; HMDB41466	Chondrillasterol 3-glucosyl-(1-2)-glucosyl-(1-2)-glucoside ; Melilotoside C	M+3H ; M+3H	C47H78O16 ; C47H78O16
420.08575	1.487812	0.157543	-3.8473	0.003226	2.4913	0.024529	420.0858	HMDB06805 ; HMDB02307 ; HMDB14428 ; HMDB33522	Beta-Alanyl-CoA ; Acrylyl-CoA ; Clemastine ; Oxopurpureine	M+2H ; M+H+NH4 ; M+2K-H ; M+K	C24H41N8O17P3S ; C24H38N7O17P3S ; C21H26ClNO ; C21H19NO6

196.07278	1.451102	1.30747	-3.8472	0.003227	2.4912	0.024529	196.0728	HMDB30564 ; HMDB30565 ; HMDB31422 ; HMDB36294 ; HMDB01387 ; HMDB01490 ; HMDB01565 ; HMDB01942 ; HMDB03441 ; HMDB03633 ; HMDB12209 ; HMDB15416 ; HMDB15547 ; HMDB29710 ; HMDB29748 ; HMDB32072 ; HMDB32887 ; HMDB33459 ; HMDB33627 ; HMDB35056 ; HMDB36072 ; HMDB37113 ; HMDB39650 ; HMDB39658 ; HMDB39659 ; HMDB39663 ; HMDB39674 ; HMDB39835 ; HMDB39891 ; HMDB40037 ; HMDB40039 ; HMDB40044 ; HMDB40603 ; HMDB40895 ; HMDB59602 ; HMDB59730 ; HMDB59847 ; HMDB60283 ; HMDB60284 ; HMDB60671 ; HMDB60765	trans-Piceid ; (Z)-Resveratrol 4-glucoside ; cis-Piceid ; (E)-2-Glucosyl-345-trihydroxystilbene ; N-Methylphenylethanolamine ; Vanylglycol ; Phosphorylcholine ; Phenylpropanolamine ; Cinnamaldehyde ; N-Methyltyramine ; Diethylphosphate ; Fosamprenavir ; Deferasirox ; DIMBOA-Glc ; 2-Aminodipyrido12-a:32-dimidazole ; (Z)-3-Phenyl-2-propenal ; 4-Amino-2-methyl-1-naphthol ; Flazine ; D-Erythroascorbic acid 1-a-D-glucoside ; 1-(3-Hydroxy-4-methoxyphenyl)-12-ethanediol ; Genipic acid ; 1256-Tetrahydro-4H-pyrrolo321-ijquinolin-4-one ; 4-Formyl-3-(formylmethyl)-4-hexenoic acid ; 123456-Hexahydro-5-methyl-7H-cyclopentabpyridin-7-one ; 123456-Hexahydro-6-methyl-7H-cyclopentabpyridin-7-one ; 234567-Hexahydrocyclopentbazepin-8(1H)-one ; 2-(1-Pyrrolidinyl)-2-cyclopenten-1-one ; 2-(2-Furanyl)piperidine ; 4-Hydroxy-3-prenylbenzoic acid glucoside ; 1-Furfurylpyrrolidine ; 2-Acetyl-3-ethylidene-3456-tetrahydropyridine ; 2-(5-Methyl-2-furanyl)pyrrolidine ; ()-threo-1-(4-Hydroxyphenyl)-123-propanetriol ; Methyl (R)-9-hydroxy-10-undecene-57-diynoate glucoside ; Indanone ; 34-Methylenepimelic acid ; 2-Methylcumarone ; N-Methyltyraminium ; Tyraminium ; Atropaldehyde ; 4-Hydroxyamphetamine	M+2H ; M+2H ; M+2H ; M+2H ; M+2Na-H ; 2M+H+Na ; 2M+H+Na ; M+2Na-H ; M+ACN+Na ; M+2Na-H ; M+ACN+H ; M+3H ; M+H+NH4 ; M+H+NH4 ; 2M+H+Na ; M+ACN+Na ; M+Na ; M+2ACN+2H ; M+2ACN+2H ; 2M+H+Na ; 2M+H+Na ; M+Na ; 2M+H+Na ; M+2Na-H ; M+2Na-H ; M+2Na-H ; M+2Na-H ; M+2Na-H ; M+H+Na ; M+2Na-H ; M+2Na-H ; M+2Na-H ; 2M+H+Na ; M+H+Na ; M+ACN+Na ; 2M+H+Na ; M+ACN+Na ; M+2Na-H ; M+2Na-H ; M+ACN+Na ; M+2Na-H	C20H22O8 ; C20H22O8 ; C20H22O8 ; C20H22O8 ; C9H13NO ; C9H12O4 ; C5H15NO4P ; C9H13NO ; C9H8O ; C9H13NO ; C4H11O4P ; C25H36N3O9PS ; C21H15N3O4 ; C15H19NO10 ; C10H8N4 ; C9H8O ; C11H11NO ; C17H12N2O4 ; C11H16O10 ; C9H12O4 ; C9H12O4 ; C11H11NO ; C9H12O4 ; C9H13NO ; C9H13NO ; C9H13NO ; C9H13NO ; C9H13NO ; C18H24O8 ; C9H13NO ; C9H13NO ; C9H13NO ; C9H12O4 ; C18H24O8 ; C9H8O ; C9H12O4 ; C9H8O ; C9H13NO ; C9H13NO ; C9H8O ; C9H13NO
408.76942	1.476827	1.534176	3.847	0.003228	2.4911	0.024529	408.7694	HMDB08880 ; HMDB09129 ; HMDB09162 ; HMDB09193 ; HMDB09194 ; HMDB09680 ; HMDB31093	PE(14:1(9Z)22:6(4Z7Z10Z13Z16Z19Z)) ; PE(18:3(6Z9Z12Z)18:4(6Z9Z12Z15Z)) ; PE(18:3(9Z12Z15Z)18:4(6Z9Z12Z15Z)) ; PE(18:4(6Z9Z12Z15Z)18:3(6Z9Z12Z)) ; PE(18:4(6Z9Z12Z15Z)18:3(9Z12Z15Z)) ; PE(22:6(4Z7Z10Z13Z16Z19Z)14:1(9Z)) ; 91215-Octadecatrienoic acid 1-(phosphonoxy)methyl-12-ethanediyl ester	M+2ACN+2H ; M+2ACN+2H ; M+2ACN+2H ; M+2ACN+2H ; M+2ACN+2H ; M+2ACN+2H ; M+3ACN+2H	C41H68NO8P ; C41H68NO8P ; C41H68NO8P ; C41H68NO8P ; C41H68NO8P ; C41H68NO8P ; C39H65O8P
768.55326	1.512482	0.214439	3.841	0.00326	2.4868	0.024687	768.5533	HMDB07949 ; HMDB07950 ; HMDB08428 ; HMDB08461 ; HMDB08943 ; HMDB08969 ; HMDB09003 ; HMDB09004 ; HMDB09034 ; HMDB09035 ; HMDB09067 ; HMDB09068 ; HMDB09099 ; HMDB09131 ; HMDB09164 ; HMDB09196 ; HMDB09228 ; HMDB09259 ; HMDB09260 ; HMDB09291 ; HMDB09322 ; HMDB09323 ; HMDB09355 ; HMDB09356 ; HMDB09387 ; HMDB09420 ; HMDB09583 ; HMDB07938 ; HMDB07939 ; HMDB08066 ; HMDB08099 ; HMDB08842 ; HMDB08874 ; HMDB08933 ; HMDB08965 ; HMDB08992 ; HMDB08993 ; HMDB09024 ; HMDB09057 ; HMDB09221 ; HMDB09253 ; HMDB09482 ; HMDB09514 ; HMDB10568	PC(15:020:4(5Z8Z11Z14Z)) ; PC(15:020:4(8Z11Z14Z17Z)) ; PC(20:4(5Z8Z11Z14Z)15:0) ; PC(20:4(8Z11Z14Z17Z)15:0) ; PE(16:022:4(7Z10Z13Z16Z)) ; PE(16:1(9Z)20:3(8Z11Z14Z)) ; PE(18:020:4(5Z8Z11Z14Z)) ; PE(18:020:4(8Z11Z14Z17Z)) ; PE(18:1(11Z)20:3(5Z8Z11Z)) ; PE(18:1(11Z)20:3(8Z11Z14Z)) ; PE(18:1(9Z)20:3(5Z8Z11Z)) ; PE(18:1(9Z)20:3(8Z11Z14Z)) ; PE(18:2(9Z12Z)20:2(11Z14Z)) ; PE(18:3(6Z9Z12Z)20:1(11Z)) ; PE(18:3(9Z12Z15Z)20:1(11Z)) ; PE(18:4(6Z9Z12Z15Z)20:0) ; PE(20:018:4(6Z9Z12Z15Z)) ; PE(20:1(11Z)18:3(6Z9Z12Z)) ; PE(20:1(11Z)18:3(9Z12Z15Z)) ; PE(20:2(11Z14Z)18:2(9Z12Z)) ; PE(20:3(5Z8Z11Z)18:1(11Z)) ; PE(20:3(5Z8Z11Z)18:1(9Z)) ; PE(20:3(8Z11Z14Z)18:1(11Z)) ; PE(20:3(8Z11Z14Z)18:1(9Z)) ; PE(20:4(5Z8Z11Z14Z)18:0) ; PE(20:4(8Z11Z14Z17Z)18:0) ; PE(22:4(7Z10Z13Z16Z)16:0) ; PC(15:018:1(11Z)) ; PC(15:018:1(9Z)) ; PC(18:1(11Z)15:0) ; PC(18:1(9Z)15:0) ; PE(14:022:1(13Z)) ; PE(14:1(9Z)22:0) ; PE(16:020:1(11Z)) ; PE(16:1(9Z)20:0) ; PE(18:018:1(11Z)) ; PE(18:018:1(9Z)) ; PE(18:1(11Z)18:0) ; PE(18:1(9Z)18:0) ; PE(20:016:1(9Z)) ; PE(20:1(11Z)16:0) ; PE(22:014:1(9Z)) ; PE(22:1(13Z)14:0) ; PE-NMe2(16:018:1(9Z))	M+H ; M+Na ; M+Na ; M+Na ; M+Na ; M+Na ; M+Na ; M+Na ; M+Na ; M+Na ; M+Na ; M+Na ; M+Na ; M+Na ; M+Na ; M+Na ; M+Na ; M+Na	C43H78NO8P ; C41H80NO8P ; C41H80NO8P ; C41H80NO8P ; C41H80NO8P ; C41H80NO8P ; C41H80NO8P ; C41H80NO8P ; C41H80NO8P ; C41H80NO8P ; C41H80NO8P ; C41H80NO8P ; C41H80NO8P ; C41H80NO8P ; C41H80NO8P ; C41H80NO8P ; C41H80NO8P ; C41H80NO8P
402.73477	1.475494	1.526216	3.8399	0.003265	2.4861	0.024687	402.7348	HMDB09137 ; HMDB09170 ; HMDB09201 ; HMDB09202 ; HMDB09393 ; HMDB09426 ; HMDB09457 ; HMDB09458	PE(18:3(6Z9Z12Z)20:5(5Z8Z11Z14Z17Z)) ; PE(18:3(9Z12Z15Z)20:5(5Z8Z11Z14Z17Z)) ; PE(18:4(6Z9Z12Z15Z)20:4(5Z8Z11Z14Z)) ; PE(18:4(6Z9Z12Z15Z)20:4(8Z11Z14Z17Z)) ; PE(20:4(5Z8Z11Z14Z)18:4(6Z9Z12Z15Z)) ; PE(20:4(8Z11Z14Z17Z)18:4(6Z9Z12Z15Z)) ; PE(20:5(5Z8Z11Z14Z17Z)18:3(6Z9Z12Z)) ; PE(20:5(5Z8Z11Z14Z17Z)18:3(9Z12Z15Z))	M+2Na ; M+2Na ; M+2Na ; M+2Na ; M+2Na ; M+2Na ; M+2Na ; M+2Na	C43H70NO8P ; C43H70NO8P ; C43H70NO8P ; C43H70NO8P ; C43H70NO8P ; C43H70NO8P ; C43H70NO8P ; C43H70NO8P
296.03011	1.412252	0.232852	-3.8385	0.003273	2.485	0.024687	296.0301	HMDB00210 ; HMDB32825 ; HMDB32876 ; HMDB33055 ; HMDB33288 ; HMDB34042 ; HMDB34138 ; HMDB37921 ; HMDB39654 ; HMDB40032 ; HMDB40235 ; HMDB60924 ; HMDB60925	Pantothenic acid ; Dipropyl trisulfide ; Rhein ; Ethyl 1-(ethylthio)ethyl disulfide ; Epitheaflagallin 3-O-gallate ; Lucernol ; Repensol ; Aureol ; Ethyl 1-(methylthio)propyl disulfide ; 2-Hexylbenzothiazole ; 11-Thiobis-1-propanethiol ; Suprofen S-oxide ; Thiophene-45-epoxide	M+2K-H ; 3M+2Na ; 2M+H+Na ; 3M+2Na ; M+H+K ; 2M+H+Na ; 2M+H+Na ; 2M+H+Na ; 3M+2Na ; M+2K-H ; 3M+2Na ; 2M+H+K ; 2M+H+K	C9H17NO5 ; C6H14S3 ; C15H8O6 ; C6H14S3 ; C27H20O13 ; C15H8O6 ; C15H8O6 ; C15H8O6 ; C6H14S3 ; C13H17NS ; C6H14S3 ; C14H12O4S ; C14H12O4S

445.37135	1.477609	0.023833	3.8384	0.003274	2.485	0.024687	445.3714	HMDB11662 ; HMDB12165 ; HMDB33633 ; HMDB35804 ; HMDB42539 ; HMDB42599 ; HMDB42916 ; HMDB42918 ; HMDB43333 ; HMDB43445 ; HMDB43450 ; HMDB43507 ; HMDB43529 ; HMDB43535 ; HMDB43592 ; HMDB43613 ; HMDB43620 ; HMDB43648 ; HMDB43653 ; HMDB43676 ; HMDB43703 ; HMDB43709 ; HMDB43725 ; HMDB43731 ; HMDB43758 ; HMDB43765 ; HMDB48084 ; HMDB48089 ; HMDB48130 ; HMDB48135 ; HMDB48238 ; HMDB48240 ; HMDB48353 ; HMDB48355 ; HMDB48761 ; HMDB48805 ; HMDB48992 ; HMDB48994 ; HMDB50901 ; HMDB52409 ; HMDB52508 ; HMDB52513 ; HMDB52527 ; HMDB52637 ; HMDB52644 ; HMDB52742 ; HMDB52749 ; HMDB52865 ; HMDB52870 ; HMDB53055 ; HMDB53062 ; HMDB53070 ; HMDB53083 ; HMDB53090 ; HMDB53168 ; HMDB53275 ;	3-beta-Hydroxy-4-beta-methyl-5-alpha-cholest-7-ene-4-alpha-carboxylate ; 4alpha-Carboxy-4beta-methyl-5alpha-cholesta-8-en-3beta-ol ; (3beta5alpha6beta22E24R)-23-Methylergosta-722-diene-356-triol ; Schleicherastatin 5 ; TG(14:018:2(9Z12Z)22:6(4Z7Z10Z13Z16Z19Z)) ; TG(14:020:2n622:6(4Z7Z10Z13Z16Z19Z)) ; TG(14:022:6(4Z7Z10Z13Z16Z19Z)18:2(9Z12Z)) ; TG(14:022:6(4Z7Z10Z13Z16Z19Z)20:2n6) ; TG(15:020:3(5Z8Z11Z)20:5(5Z8Z11Z14Z17Z)) ; TG(15:018:3(6Z9Z12Z)22:5(4Z7Z10Z13Z16Z)) ; TG(15:018:3(6Z9Z12Z)22:5(7Z10Z13Z16Z19Z)) ; TG(15:020:3n620:5(5Z8Z11Z14Z17Z)) ; TG(15:020:4(5Z8Z11Z14Z)20:4(5Z8Z11Z14Z)) ; TG(15:020:4(5Z8Z11Z14Z)20:4(8Z11Z14Z17Z)) ; TG(15:022:4(7Z10Z13Z16Z)18:4(6Z9Z12Z15Z)) ; TG(15:022:5(4Z7Z10Z13Z16Z)18:3(6Z9Z12Z)) ; TG(15:022:5(4Z7Z10Z13Z16Z)18:3(9Z12Z15Z)) ; TG(15:018:3(9Z12Z15Z)22:5(4Z7Z10Z13Z16Z)) ; TG(15:018:3(9Z12Z15Z)22:5(7Z10Z13Z16Z19Z)) ; TG(15:018:4(6Z9Z12Z15Z)22:4(7Z10Z13Z16Z)) ; TG(15:020:4(8Z11Z14Z17Z)20:4(5Z8Z11Z14Z)) ; TG(15:020:4(8Z11Z14Z17Z)20:4(8Z11Z14Z17Z)) ; TG(15:020:5(5Z8Z11Z14Z17Z)20:3(5Z8Z11Z)) ; TG(15:020:5(5Z8Z11Z14Z17Z)20:3n6) ; TG(15:022:5(7Z10Z13Z16Z19Z)18:3(6Z9Z12Z)) ; TG(15:022:5(7Z10Z13Z16Z19Z)18:3(9Z12Z15Z)) ; TG(14:1(9Z)18:2(9Z12Z)22:5(4Z7Z10Z13Z16Z)) ; TG(14:1(9Z)18:2(9Z12Z)22:5(7Z10Z13Z16Z19Z)) ; TG(14:1(9Z)20:2n622:5(4Z7Z10Z13Z16Z)) ; TG(14:1(9Z)20:2n622:5(7Z10Z13Z16Z19Z)) ; TG(14:1(9Z)22:5(4Z7Z10Z13Z16Z)18:2(9Z12Z)) ; TG(14:1(9Z)22:5(4Z7Z10Z13Z16Z)20:2n6) ; TG(14:1(9Z)22:5(7Z10Z13Z16Z19Z)18:2(9Z12Z)) ; TG(14:1(9Z)22:5(7Z10Z13Z16Z19Z)20:2n6) ; TG(16:1(9Z)18:2(9Z12Z)20:5(5Z8Z11Z14Z17Z)) ; TG(16:1(9Z)20:2n620:5(5Z8Z11Z14Z17Z)) ; TG(16:1(9Z)20:5(5Z8Z11Z14Z17Z)18:2(9Z12Z)) ; TG(16:1(9Z)20:5(5Z8Z11Z14Z17Z)20:2n6) ; TG(20:3(5Z8Z11Z)15:020:5(5Z8Z11Z14Z17Z)) ; TG(18:2(9Z12Z)14:022:6(4Z7Z10Z13Z16Z19Z)) ; TG(18:2(9Z12Z)14:1(9Z)22:5(4Z7Z10Z13Z16Z)) ; TG(18:2(9Z12Z)14:1(9Z)22:5(7Z10Z13Z16Z19Z)) ; TG(18:2(9Z12Z)16:1(9Z)20:5(5Z8Z11Z14Z17Z)) ; TG(18:2(9Z12Z)18:3(6Z9Z12Z)18:3(6Z9Z12Z)) ; TG(18:2(9Z12Z)18:3(6Z9Z12Z)18:3(9Z12Z15Z)) ; TG(18:2(9Z12Z)18:3(9Z12Z15Z)18:3(6Z9Z12Z)) ; TG(18:2(9Z12Z)18:3(9Z12Z15Z)18:3(9Z12Z15Z)) ; TG(18:3(6Z9Z12Z)15:022:5(4Z7Z10Z13Z16Z)) ; TG(18:3(6Z9Z12Z)15:022:5(7Z10Z13Z16Z19Z)) ; TG(18:3(6Z9Z12Z)18:2(9Z12Z)18:3(6Z9Z12Z)) ; TG(18:3(6Z9Z12Z)18:2(9Z12Z)18:3(9Z12Z15Z)) ; TG(18:3(6Z9Z12Z)18:3(6Z9Z12Z)20:2n6) ; TG(18:3(6Z9Z12Z)20:2n618:3(6Z9Z12Z)) ; TG(18:3(6Z9Z12Z)20:2n618:3(9Z12Z15Z)) ; TG(18:3(6Z9Z12Z)18:3(9Z12Z15Z)20:2n6) ;	M+H ; M+H ; M+H ; M+H ; M+2H ; M+H+NH4 ; M+H+NH4 ; M+H+NH4 ; M+H+NH4 ; M+H+NH4 ; M+H+NH4 ; M+H+NH4 ; M+H+NH4 ; M+H+NH4 ; M+H+NH4 ; 2M+H+K	C29H48O3 ; C29H48O3 ; C29H48O3 ; C29H48O3 ; C58H96O6 ; C50H98NO8P ; C50H98NO8P ; C50H98NO8P ; C50H98NO8P ; C50H98NO8P ; C50H98NO8P ; C50H98NO8P ; C50H98NO8P ; C50H98NO8P ; C50H98NO8P ; C26H51NO3
548.79250	1.531882	1.017676	-3.8381	0.003275	2.4848	0.024687	548.7925	HMDB40505	Soyasaponin aa	M+ACN+2H	C53H82O21
273.14452	1.476616	1.202523	-3.8371	0.003281	2.484	0.024687	273.1445	HMDB00325 ; HMDB05770 ; HMDB05776 ; HMDB15371 ; HMDB28718 ; HMDB29033 ; HMDB30917 ; HMDB31901 ; HMDB32674 ; HMDB33234 ; HMDB33693 ; HMDB34200 ; HMDB34721 ; HMDB35117 ; HMDB35137 ; HMDB35148 ; HMDB35270 ; HMDB35299 ; HMDB35302 ; HMDB35358 ; HMDB35760 ; HMDB35798 ; HMDB35881 ; HMDB35972 ; HMDB36036 ; HMDB36037 ; HMDB36139 ; HMDB36550 ; HMDB36563 ; HMDB36664 ; HMDB37064 ; HMDB37212 ; HMDB37529 ; HMDB37559 ; HMDB39015 ; HMDB39156 ; HMDB39635 ; HMDB39644 ; HMDB40004 ; HMDB40220 ; HMDB40754 ; HMDB61150 ; HMDB34879	3-Hydroxysuberic acid ; Tuftsin ; Kentsin ; Gemfibrozil ; Arginyl-Serine ; Serinyl-Arginine ; 1-Hydroxyacorenone ; Blennin A ; (ZZ)-2916-Heptadecatriene-46-diyn-8-ol ; Ganoderic acid beta ; Falcarinone ; Physalolactone B ; Procurcumadiol ; (3beta6beta)-Furanoeremophilane-36-diol ; Heliannuol D ; (6beta8alpha)-6-Hydroxy-7(11)-eremophilen-128-olide ; Methyl 3-oxo-12-oleanen-28-oate ; Ganolucidic acid D ; Ganolucidic acid A ; Ketosantalic acid ; 3-Hydroxytrichothecene ; Piperdial ; 11-Hydroxy-9-tridecenoic acid ; Ambonic acid ; 3beta-Dihydroxymarasmene ; 13-Hydroxymarasmene ; Menthone 12-glyceryl ketal ; 3-Ketoapotrichothecene ; Valerenolic acid ; Ketopelenolide a ; FS4 toxin ; Menthyl lactate ; Lactaronecatorin A ; Cadabicilone ; Tangeraxanthin ; 3beta-Hydroxycinnamolide ; Abscisic alcohol ; Heliannuol A ; Menthone 13-glyceryl ketal ; Diethyl L-malate ; (3beta8beta)-3-Hydroxy-7(11)-eremophilen-128-olide ; Hydroxyl frovatriptan ; 1-(5-Amino-5-carboxypentyl)amino-1-deoxyfructose	M+2ACN+H ; M+2Na ; M+2Na ; M+Na ; 2M+H+Na ; 2M+H+Na ; M+Na ; M+Na ; 2M+Na+K ; M+2Na ; 2M+Na+K ; M+2Na ; M+Na ; M+Na ; M+Na ; M+Na ; M+2K ; M+2Na ; M+2Na ; M+Na ; M+Na ; M+Na ; M+2Na-H ; M+2K ; M+Na ; M+Na ; M+2Na-H ; M+Na ; M+Na ; M+Na ; M+Na ; M+2Na-H ; M+Na ; M+Na ; M+Na+K ; M+Na ; M+Na ; M+Na ; M+2Na-H ; M+2ACN+H ; M+Na ; 2M+H+Na ; M+H-2H2O	C8H14O5 ; C21H40N8O6 ; C21H40N8O6 ; C15H22O3 ; C9H19N5O4 ; C9H19N5O4 ; C15H22O3 ; C15H22O3 ; C17H22O ; C30H44O6 ; C17H22O ; C30H44O6 ; C15H22O3 ; C15H22O3 ; C15H22O3 ; C15H22O3 ; C31H48O3 ; C30H44O6 ; C30H44O6 ; C15H22O3 ; C15H22O3 ; C15H22O3 ; C13H24O3 ; C31H48O3 ; C15H22O3 ; C15H22O3 ; C13H24O3 ; C15H22O3 ; C15H22O3 ; C15H22O3 ; C15H22O3 ; C13H24O3 ; C15H22O3 ; C15H22O3 ; C34H44O2 ; C15H22O3 ; C15H22O3 ; C15H22O3 ; C13H24O3 ; C8H14O5 ; C15H22O3 ; C14H19N3O2 ; C12H24N2O7
222.86779	1.484476	1.54016	3.8366	0.003283	2.4837	0.024687	NA	NA	NA	NA	NA
511.11196	1.498776	0.719704	-3.8331	0.003302	2.4812	0.024758	511.112	HMDB30503 ; HMDB35865	(2S4S6S)-2-2-(4-Hydroxy-3-meyhoxyphenyl)ethyltetrahydro-6-(45-dihydroxy-3-methoxyphenyl)-2H-pyran-4-yl 4-acetate ; Melledonal A	M+2K+H ; M+2K+H	C23H28O8 ; C23H28O8
619.31532	1.510645	0.278221	-3.833	0.003303	2.4811	0.024758	619.3153	HMDB14355 ; HMDB36842	Adapalene ; Hydratopyrrhoxanthinol	3M+2H ; 2M+Na+K	C28H28O3 ; C37H48O6
431.90677	1.474472	1.497607	3.8308	0.003314	2.4796	0.024809	431.9068	HMDB04902 ; HMDB04960	Ganglioside GA1 (d18:116:0) ; Tetrahexosylceramide (d18:116:0)	M+3Na ; M+3Na	C60H110N2O23 ; C60H110N2O23
355.87920	1.503303	0.695541	-3.8295	0.003322	2.4786	0.024825	355.8792	HMDB06888 ; HMDB35310 ; HMDB35985 ; HMDB36309	5b-Cyprinol sulfate ; Ganoderic acid I ; Ganoderic acid G ; Ganoderic acid eta	2M+3H ; 2M+3H ; 2M+3H ; 2M+3H	C27H48O8S ; C30H44O8 ; C30H44O8 ; C30H44O8
652.88994	1.433531	1.420496	-3.8249	0.003347	2.4754	0.024974	652.8899	HMDB33202	Di-2-propenyl tetrasulfide	3M+Na	C6H10S4
161.51880	1.470273	0.682242	-3.8223	0.003361	2.4735	0.02502	161.5188	HMDB28832 ; HMDB29126 ; HMDB30277 ; HMDB39127	Glutamyl-Valine ; Valyl-Glutamate ; Dihydromaleimide beta-D-glucoside ; Pisatoside	M+2K ; M+2K ; M+Na+K ; M+Na+K	C10H17N2O5 ; C10H17N2O5 ; C10H15NO7 ; C10H15NO7
592.81133	1.447981	0.442321	-3.822	0.003363	2.4733	0.02502	592.8113	HMDB15092	Norgestimate	3M+2K	C23H31NO3

227.04854	1.465797	1.057002	-3.8202	0.003373	2.472	0.025057	227.0485	HMDB00107 ; HMDB00247 ; HMDB00434 ; HMDB00765 ; HMDB11632 ; HMDB14745 ; HMDB29495 ; HMDB29645 ; HMDB29648 ; HMDB29788 ; HMDB30571 ; HMDB32028 ; HMDB32061 ; HMDB32365 ; HMDB35721 ; HMDB37177 ; HMDB39427 ; HMDB39564 ; HMDB40256 ; HMDB40637 ; HMDB41219 ; HMDB60398 ; HMDB60664 ; HMDB60733 ; HMDB60735 ; HMDB61058	Galactitol ; Sorbitol ; Homoveratric acid ; Mannitol ; L-Iditol ; Nafcillin ; Trigoforin ; Xanthoxylin ; 245-Trimethoxybenzaldehyde ; Kanzonol V ; ()-3-(34-Methylenedioxyphenyl)-12-propanediol ; (E)-3-(2-Methylpropylidene)-1(3H)-isobenzofuranone ; 3-Butylidene-1(3H)-isobenzofuranone ; Maltyl isobutyrate ; (S)-Batatic acid ; 1-(245-Trihydroxyphenyl)-1-butanone ; 2-Hydroxy-3-(4-methoxyphenyl)propanoic acid ; Marmesin rhamnoside ; (S)-2-(4-Methoxyphenoxy)propanoic acid ; 4-Hydroxy-6-methyl-3-(1-oxobutyl)-2H-pyran-2-one ; Allyl cinnamate ; 5-Hydroxyconiferyl alcohol ; Isoniazid pyruvate ; 3-(3-Hydroxyphenyl)-2-methyllactic acid ; 3-(34-Dihydroxyphenyl)-2-methylpropionic acid ; triazolopropionic acid	M+2Na-H ; M+2Na-H ; 2M+Na+K ; M+2Na-H ; M+2Na-H ; M+H+K ; M+K ; 2M+Na+K ; 2M+Na+K ; M+2K ; 2M+Na+K ; M+K ; M+K ; 2M+Na+K ; 2M+Na+K ; 2M+Na+K ; 2M+Na+K ; M+Na+K ; 2M+Na+K ; 2M+Na+K ; M+K ; 2M+Na+K ; 2M+H+K ; 2M+Na+K ; 2M+Na+K ; 2M+H+K	C6H14O6 ; C6H14O6 ; C10H12O4 ; C6H14O6 ; C6H14O6 ; C21H22N2O5S ; C12H12O2 ; C10H12O4 ; C10H12O4 ; C24H24O4 ; C10H12O4 ; C12H12O2 ; C12H12O2 ; C10H12O4 ; C10H12O4 ; C10H12O4 ; C10H12O4 ; C20H24O8 ; C10H12O4 ; C10H12O4 ; C12H12O2 ; C10H12O4 ; C9H9N3O3 ; C10H12O4 ; C10H12O4 ; C9H9N3O3
327.59638	1.441112	1.165485	3.8183	0.003383	2.4707	0.025096	327.5964	HMDB60139	4-Hydroxy-17beta-estradiol-2-S-glutathione	M+Na+K	C28H39N3O9S
418.79818	1.467545	1.552378	3.812	0.003418	2.4662	0.025309	418.7982	HMDB12315 ; HMDB07788 ; HMDB07883 ; HMDB07884 ; HMDB07914 ; HMDB07915 ; HMDB07976 ; HMDB08007 ; HMDB08008 ; HMDB08167 ; HMDB08200 ; HMDB08232 ; HMDB08326 ; HMDB08327 ; HMDB08358 ; HMDB08362 ; HMDB08395 ; HMDB08426 ; HMDB08459 ; HMDB08619 ; HMDB08910 ; HMDB09582 ; HMDB11225 ; HMDB11250 ; HMDB11282 ; HMDB11315 ; HMDB13406 ; HMDB13417 ; HMDB13439 ; HMDB13440 ; HMDB56386	3-O-Sulfogalactosylceramide (d18:120:0) ; DG(22:6(4Z7Z10Z13Z16Z19Z)22:6(4Z7Z10Z13Z16Z19Z)0:0) ; PC(14:020:4(5Z8Z11Z14Z)) ; PC(14:020:4(8Z11Z14Z17Z)) ; PC(14:1(9Z)20:3(5Z8Z11Z)) ; PC(14:1(9Z)20:3(8Z11Z14Z)) ; PC(16:018:4(6Z9Z12Z15Z)) ; PC(16:1(9Z)18:3(6Z9Z12Z)) ; PC(16:1(9Z)18:3(9Z12Z15Z)) ; PC(18:3(6Z9Z12Z)16:1(9Z)) ; PC(18:3(9Z12Z15Z)16:1(9Z)) ; PC(18:4(6Z9Z12Z15Z)16:0) ; PC(20:1(11Z)P-18:1(11Z)) ; PC(20:1(11Z)P-18:1(9Z)) ; PC(20:2(11Z14Z)P-18:0) ; PC(20:3(5Z8Z11Z)14:1(9Z)) ; PC(20:3(8Z11Z14Z)14:1(9Z)) ; PC(20:4(5Z8Z11Z14Z)14:0) ; PC(20:4(8Z11Z14Z17Z)14:0) ; PC(22:2(13Z16Z)P-16:0) ; PE(15:022:4(7Z10Z13Z16Z)) ; PE(22:4(7Z10Z13Z16Z)15:0) ; PC(P-16:022:2(13Z16Z)) ; PC(P-18:020:2(11Z14Z)) ; PC(P-18:1(11Z)20:1(11Z)) ; PC(P-18:1(9Z)20:1(11Z)) ; PC(o-16:020:0) ; PC(o-18:018:0) ; PC(o-20:018:3(6Z9Z12Z)) ; PC(o-20:018:3(9Z12Z15Z)) ; DG(22:6n30:022:6n3)	M+2H ; M+3ACN+2H ; M+2ACN+2H ; M+2ACN+2H ; M+2ACN+2H ; M+2ACN+2H ; M+2ACN+2H ; M+2ACN+2H ; M+2ACN+2H ; M+2ACN+2H ; M+2ACN+2H ; M+2ACN+2H ; M+H+K ; M+H+K ; M+H+K ; M+2ACN+2H ; M+2ACN+2H ; M+2ACN+2H ; M+2ACN+2H ; M+H+K ; M+2ACN+2H ; M+2ACN+2H ; M+H+K ; M+H+K ; M+H+K ; M+H+K ; M+Na+K ; M+Na+K ; M+H+K ; M+H+K ; M+3ACN+2H	C44H85NO11S ; C47H68O5 ; C42H76NO8P ; C42H76NO8P ; C42H76NO8P ; C42H76NO8P ; C42H76NO8P ; C42H76NO8P ; C42H76NO8P ; C42H76NO8P ; C42H76NO8P ; C42H76NO8P ; C46H88NO7P ; C46H88NO7P ; C46H88NO7P ; C42H76NO8P ; C42H76NO8P ; C42H76NO8P ; C42H76NO8P ; C46H88NO7P ; C42H76NO8P ; C42H76NO8P ; C42H76NO8P ; C46H88NO7P ; C46H88NO7P ; C46H88NO7P ; C46H88NO7P ; C44H90NO7P ; C44H90NO7P ; C46H88NO7P ; C46H88NO7P ; C47H68O5
523.04835	1.399628	0.123735	3.8113	0.003422	2.4658	0.025309	523.0484	HMDB01512 ; HMDB14412 ; HMDB33785 ; HMDB39178 ; HMDB39179 ; HMDB39181 ; HMDB39325 ; HMDB39326 ; HMDB39367	Thiamine triphosphate ; Cefmenoxime ; Hamamelitannin ; 12-Digalloyl-beta-D-glucopyranose ; 16-Digalloyl-beta-D-glucopyranose ; 26-Digalloylglucose ; Gallic acid 3-O-(6-galloylglucoside) ; Gallic acid 4-O-(6-galloylglucoside) ; 12-Di-O-galloylhamamelofuranose	M+NH4 ; 2M+H+Na ; M+K ; M+K ; M+K ; M+K ; M+K ; M+K ; M+K	C12H20N4O10P3S ; C16H17N9O5S3 ; C20H20O14 ; C20H20O14 ; C20H20O14 ; C20H20O14 ; C20H20O14 ; C20H20O14 ; C20H20O14
277.05956	1.44515	1.432741	3.809	0.003435	2.4641	0.02537	277.0568	HMDB14917 ; HMDB15084 ; HMDB15305 ; HMDB28853 ; HMDB29105 ; HMDB29451 ; HMDB32357 ; HMDB35635 ; HMDB39249 ; HMDB39925 ; HMDB40988 ; HMDB41949 ; HMDB60316	Nalidixic Acid ; Felbamate ; Phenobarbital ; Glycyl-Tyrosine ; Tyrosyl-Glycine ; gamma-Glutaminyl-4-hydroxybenzene ; N-Lactoyl ethanolamine phosphate ; Kanokoside A ; 1-(2H-13-Benzodioxol-5-yl)-2-26 dimethoxy-4-(prop-2-en-1-yl)phenoxypropyl benzoate ; 26-Di-O-acetylononin ; Falimint ; Nitecapone ; (7R)-7-(5-Carboxy-5-oxopentanoyl)aminocephalosporinate	M+2Na-H ; M+K ; M+2Na-H ; M+K ; M+K ; M+K ; M+ACN+Na ; M+2K ; M+2K ; M+H+K ; M+K ; 2M+H+Na ; 2M+3H	C12H12N2O3 ; C11H14N2O4 ; C12H12N2O3 ; C11H14N2O4 ; C11H14N2O4 ; C11H14N2O4 ; C5H12NO6P ; C21H32O12 ; C28H28O7 ; C26H26O11 ; C11H14N2O4 ; C12H11NO6 ; C16H18N2O9S
254.95013	1.464325	1.235004	-3.8081	0.00344	2.4634	0.02537	254.9501	HMDB31162 ; HMDB34897	33-Thiobispropanoic acid ; 3-Chloro-4-(dichloromethylene)-25-pyrrolidinedione	M+2K-H ; M+ACN+H	C6H10O4S ; C5H2Cl3NO2
426.11121	1.48642	0.951356	-3.8061	0.003451	2.462	0.025373	426.1112	HMDB30824 ; HMDB31186 ; HMDB31423 ; HMDB38330 ; HMDB39039 ; HMDB39172 ; HMDB40555 ; HMDB40863 ; HMDB41265 ; HMDB60923 ; HMDB61059 ; HMDB61060	Afzelechin 7-apioside ; N-Nitrosothialdine ; omega-Salicoyisalicin ; Benzyl 26-dihydroxybenzoate 2-glucoside ; (R)-Apiumetin glucoside ; Flacourtin ; (E)-Oxyresveratrol 3-O-b-D-glucoside ; Piceatannol 4-glucoside ; Edulisin VI ; Naproxen O-glucuronide ; triazolopyridinone epoxide ; 4-hydroxytrazodone	2M+H+K ; 2M+ACN+H ; 2M+H+K ; 2M+H+K ; 2M+H+K ; 2M+H+K ; 2M+H+K ; 2M+H+K ; 2M+H+K ; 2M+H+K ; M+K ; M+K	C20H22O9 ; C6H12N2OS2 ; C20H22O9 ; C20H22O9 ; C20H22O9 ; C20H22O9 ; C20H22O9 ; C20H22O9 ; C20H22O9 ; C20H22O9 ; C19H22ClN5O2 ; C19H22ClN5O2
203.97333	1.460467	1.46526	-3.8057	0.003453	2.4617	0.025373	NA	NA	NA	NA	NA
195.06958	1.465564	1.019518	-3.8053	0.003456	2.4615	0.025373	195.0696	HMDB60943 ; HMDB14854 ; HMDB29992 ; HMDB30005 ; HMDB31753 ; HMDB31889 ; HMDB32955 ; HMDB33108 ; HMDB33296 ; HMDB34179 ; HMDB36691 ; HMDB38219 ; HMDB38690 ; HMDB40388 ; HMDB40421 ; HMDB41855	Sulfinpyrazone sulfide ; Nedocromil ; Tetrahydropentoxyline ; Austalide A ; 2-(4-Allyl-2-methoxyphenoxy)-1-(4-hydroxy-3-methoxyphenyl)-1-propanol ; (1(10)E4E6a8b)-8-Angeloyloxy-14-oxo-1(10)411(13)-germacratrien-126-olide ; 5-(6-Hydroxy-37-dimethyl-27-octadienyloxy)-7-methoxycoumarin ; Semilepidinoside B ; Kuwanon Y ; Malabaricano ; 3-Dehydronobilin ; Rosmadial ; Isoachifolidiene ; Columbaridione ; Safficinolide ; Chlorobenzene	M+2H ; M+H+NH4 ; M+H+Na ; M+3Na ; M+2Na ; M+2Na ; M+2Na ; M+H+Na ; M+3H ; M+2Na ; M+2Na ; M+2Na ; M+2Na ; M+2Na ; M+2Na ; M+2ACN+H	C23H20N2O2S ; C19H17NO7 ; C17H22N2O7 ; C28H36O9 ; C20H24O5 ; C20H24O5 ; C20H24O5 ; C17H22N2O7 ; C34H30O9 ; C20H24O5 ; C20H24O5 ; C20H24O5 ; C20H24O5 ; C20H24O5 ; C20H24O5 ; C6H5Cl
538.08272	1.447204	1.263565	3.8039	0.003464	2.4604	0.025395	538.0827	HMDB41377 ; HMDB41378 ; HMDB14672 ; HMDB15312 ; HMDB41936 ; HMDB41937 ; HMDB60824 ; HMDB60930	Camelliatannin E ; Camelliatannin C ; Cyclophosphamide ; Ifosfamide ; Morphine-3-glucuronide ; Morphine-6-glucuronide ; Hydromorphone-3-glucuronide ; Hydromorphone 3-beta-O-glucuronide	M+2H ; M+2H ; 2M+NH4 ; 2M+NH4 ; M+2K-H ; M+2K-H ; M+2K-H ; M+2K-H	C49H38O28 ; C49H38O28 ; C7H15Cl2N2O2P ; C7H15Cl2N2O2P ; C23H27NO9 ; C23H27NO9 ; C23H27NO9 ; C23H27NO9
166.99459	1.522687	0.483665	3.8007	0.003482	2.4582	0.025489	166.9946	HMDB41885 ; HMDB59993	Ebrotidine ; 5-(Hydroxyphenyl)-gamma-valerolactone-O-sulphate	M+2H+Na ; M+Na+K	C14H17BrN6O2S3 ; C11H12O6S

131.05305	1.39308	1.0701	3.7969	0.003503	2.4555	0.025582	131.0531	HMDB31370 ; HMDB37493 ; HMDB40238 ; HMDB32924 ; HMDB13076 ; HMDB13919 ; HMDB29743 ; HMDB29837 ; HMDB31163 ; HMDB31308 ; HMDB33631 ; HMDB34292 ; HMDB34910 ; HMDB37837 ; HMDB37838 ; HMDB37858 ; HMDB37859 ; HMDB37863 ; HMDB38580 ; HMDB39584 ; HMDB40148 ; HMDB41901 ; HMDB60678	2-(Methylthio)methyl-2-butenal ; S-2-Propenyl propanethioate ; 2-Ethyldihydro-3(2H)-thiophenone ; 3-Furanmethanol glucoside ; Spermine dialdehyde ; N-Desmethyleletriptan ; 9H-Carbazole-3-carboxaldehyde ; Nb-Acetyl-Nb-methyltryptamine ; N-Acetyl-23-dihydro-1H-pyrrole ; 5-Acetyl-34-dihydro-2H-pyrrole ; Lactapiperanol D ; Nb-Methyltetrahydroharmol ; (R)-2-Amino-N-(2244-tetramethyl-3-thietanyl)propanamide ; 2-Ethyl-4-methyloxazole ; 4-Ethyl-2-methyloxazole ; 2-Ethyl-5-methyloxazole ; 5-Ethyl-2-methyloxazole ; 5-Ethyl-4-methyloxazole ; Cibaric acid ; 5-Imino-2-methyl-1-cyclopenten-1-ol ; Trimethyloxazole ; Hexamethylene bisacetamide ; 3-Hydroxymonoethylglycinexylidide	M+H ; M+H ; M+H ; M+2H ; M+Na+K ; M+2H+Na ; 2M+3H ; M+2Na ; 2M+H+K ; 2M+H+K ; M+3Na ; M+2Na ; M+2Na ; 2M+H+K ; 2M+H+K ; 2M+H+K ; 2M+H+K ; 2M+H+K ; M+3Na ; 2M+H+K ; 2M+H+K ; M+Na+K ; M+H+K	C6H10OS ; C6H10OS ; C6H10OS ; C11H16O7 ; C10H20N2O2 ; C21H24N2O2S ; C13H9NO ; C13H16N2O ; C6H9NO ; C6H9NO ; C18H28O5 ; C13H16N2O ; C10H20N2OS ; C6H9NO ; C6H9NO ; C6H9NO ; C6H9NO ; C6H9NO ; C18H28O5 ; C6H9NO ; C6H9NO ; C10H20N2O2 ; C12H18N2O2
618.32260	1.544469	0.650076	-3.7967	0.003505	2.4554	0.025582	618.3226	HMDB33158 ; HMDB29838 ; HMDB39558 ; HMDB39886	Protoprimulagenin A 3-rhamnosyl-(1-4)-rhamnosyl-(1-4)-rhamnosyl-(1-2)-glucosyl-(1-)-glucuronide ; Harmalol ; 1alpha-O-Methylquassin ; 16-Methyl-epi-nigakihemiacetal B	M+2H ; 3M+NH4 ; 3M+H+Na ; 3M+H+Na	C60H98O26 ; C12H12N2O ; C23H32O6 ; C23H32O6
679.21226	1.482638	1.048261	-3.7944	0.003518	2.4537	0.025636	679.2123	HMDB29893 ; HMDB30069 ; HMDB33555 ; HMDB35284	Sucrose octaacetate ; Chalcomoracin ; Isomugineic acid ; D-Fructosazine	M+H ; 2M+Na+K ; 2M+K ; 2M+K	C28H38O19 ; C39H36O9 ; C12H20N2O8 ; C12H20N2O8
133.04359	1.473119	0.746777	-3.7928	0.003527	2.4526	0.025636	133.0436	HMDB00273 ; HMDB00291 ; HMDB00562 ; HMDB02085 ; HMDB03503 ; HMDB04083 ; HMDB15394 ; HMDB29170 ; HMDB32589 ; HMDB32759 ; HMDB33217 ; HMDB33642 ; HMDB33836 ; HMDB35133 ; HMDB37492 ; HMDB38941 ; HMDB39053 ; HMDB39495 ; HMDB39974 ; HMDB40424 ; HMDB41662 ; HMDB32867	Thymidine ; Vanillylmandelic acid ; Creatinine ; Syringic acid ; 3-(34-Dihydroxyphenyl)lactic acid ; 4-(2-Amino-3-hydroxyphenyl)-24-dioxobutanoic acid ; Telbivudine ; 3-Hydroxy-4-methoxymandelate ; 1-(5-Acetyl-2-hydroxyphenyl)-3-methyl-1-butanone ; Moschamine ; (2xi6xi)-7-Methyl-3-methylene-1267-octanetetrol ; (2xi3xi6E)-37-Dimethyl-6-octene-1238-tetrol ; Ethyl gallate ; 37-Dimethyl-3-octene-1267-tetrol ; Ethyl methyl-p-tolylglycidate ; (R)-23-Dihydro-35-dihydroxy-2-oxo-3-indoleacetic acid ; (1alpha2alpha4betaH6alpha8R)-p-Menthane-2689-tetrol ; 2-(Malonylamino)benzoic acid ; (1S2S4R8R)-p-Menthane-1289-tetrol ; Ethyl 2-benzylacetoacetate ; 34-O-Dimethylgallic acid ; Capillin	M+H+Na ; 2M+3H ; 2M+H+K ; 2M+3H ; 2M+3H ; M+ACN+2H ; M+H+Na ; 2M+3H ; M+2Na ; M+H+2Na ; M+Na+K ; M+Na+K ; 2M+3H ; M+Na+K ; M+2Na ; M+ACN+2H ; M+Na+K ; M+ACN+2H ; M+Na+K ; M+2Na ; 2M+3H ; M+H-2H2O	C10H14N2O5 ; C9H10O5 ; C4H7N3O ; C9H10O5 ; C9H10O5 ; C10H9NO5 ; C10H14N2O5 ; C9H10O5 ; C13H16O3 ; C20H20N2O4 ; C10H20O4 ; C10H20O4 ; C9H10O5 ; C10H20O4 ; C13H16O3 ; C10H9NO5 ; C10H20O4 ; C10H9NO5 ; C10H20O4 ; C13H16O3 ; C9H10O5 ; C12H8O
552.61905	1.501107	1.301525	-3.7927	0.003528	2.4525	0.025636	NA	NA	NA	NA	NA
677.49427	1.503499	0.518509	-3.7912	0.003537	2.4514	0.025648	677.4943	HMDB56387 ; HMDB08823 ; HMDB08887	CL(16:016:016:016:0) ; PE(14:015:0) ; PE(15:014:0)	M+2H ; 2M+3H2O+2H ; 2M+3H2O+2H	C73H142O17P2 ; C34H68NO8P ; C34H68NO8P
317.49616	1.489476	0.13231	3.7907	0.003539	2.4511	0.025648	NA	NA	NA	NA	NA
295.93186	1.462511	1.45505	3.7862	0.003566	2.4479	0.025801	295.9319	HMDB05358 ; HMDB05365 ; HMDB42067 ; HMDB42126 ; HMDB42155 ; HMDB42184 ; HMDB42213 ; HMDB42241 ; HMDB42993 ; HMDB43105 ; HMDB43832 ; HMDB43941 ; HMDB44669 ; HMDB44722	TG(16:016:020:0)iso3 ; TG(16:018:018:0)iso3 ; TG(14:014:024:0) ; TG(14:016:022:0) ; TG(14:018:020:0) ; TG(14:020:018:0) ; TG(14:022:016:0) ; TG(14:024:014:0) ; TG(15:015:022:0) ; TG(15:022:015:0) ; TG(16:014:022:0) ; TG(16:020:016:0) ; TG(18:014:020:0) ; TG(18:016:018:0)	M+2H+Na ; M+2H+Na ; M+2H+Na ; M+2H+Na ; M+2H+Na ; M+2H+Na ; M+2H+Na ; M+2H+Na ; M+2H+Na ; M+2H+Na ; M+2H+Na ; M+2H+Na ; M+2H+Na ; M+2H+Na	C55H106O6 ; C55H106O6 ; C55H106O6 ; C55H106O6 ; C55H106O6 ; C55H106O6 ; C55H106O6 ; C55H106O6 ; C55H106O6 ; C55H106O6 ; C55H106O6 ; C55H106O6 ; C55H106O6 ; C55H106O6
187.01396	1.473926	0.055753	3.7832	0.003583	2.4458	0.025869	187.014	HMDB00229 ; HMDB11685 ; HMDB28765 ; HMDB29101 ; HMDB32886 ; HMDB32902 ; HMDB37836 ; HMDB59913	Nicotinamide ribotide ; DHAP(8:0) ; Aspartyl-Tyrosine ; Tyrosyl-Aspartate ; Orange I ; Sodium 6-hydroxy-5-(phenylazo)-2-naphthalenesulfoniate ; 4-(2-Hydroxy-1-naphthalenyl)azobenzenesulfonic acid ; (5E)-6-Oxo-5-(phenylhydrazono)-56-dihydro-2-naphthalenesulfonic acid	M+H+K ; M+2K ; M+2K ; M+2K ; M+2Na ; M+H+Na ; M+2Na ; M+2Na	C11H15N2O8P ; C11H21O7P ; C13H16N2O6 ; C13H16N2O6 ; C16H12N2O4S ; C16H11N2NaO4S ; C16H12N2O4S ; C16H12N2O4S
280.65460	1.413059	0.116271	-3.7828	0.003586	2.4454	0.025869	280.6546	HMDB10320 ; HMDB31935	Cortolone-3-glucuronide ; Blumenol C O-rhamnosyl-(1-6)-glucoside	M+H+NH4 ; M+ACN+2H	C27H42O11 ; C25H42O11
251.32700	1.483511	0.02262	3.7805	0.003599	2.4438	0.025914	251.327	HMDB60581	Ceftaroline	M+3Na	C22H22N8O8PS4

157.08010	1.438692	1.463656	3.78	0.003602	2.4435	0.025914	157.0801	HMDB13302 ; HMDB00477 ; HMDB00541 ; HMDB01923 ; HMDB02335 ; HMDB11557 ; HMDB11587 ; HMDB11664 ; HMDB13809 ; HMDB14004 ; HMDB15453 ; HMDB28743 ; HMDB28761 ; HMDB29013 ; HMDB29100 ; HMDB29351 ; HMDB29374 ; HMDB29756 ; HMDB30429 ; HMDB30639 ; HMDB30961 ; HMDB30966 ; HMDB31086 ; HMDB31197 ; HMDB31301 ; HMDB31309 ; HMDB31328 ; HMDB31590 ; HMDB31591 ; HMDB31701 ; HMDB32340 ; HMDB32345 ; HMDB32403 ; HMDB32451 ; HMDB33103 ; HMDB33184 ; HMDB33547 ; HMDB33731 ; HMDB34130 ; HMDB34212 ; HMDB34244 ; HMDB35242 ; HMDB35390 ; HMDB35915 ; HMDB37152 ; HMDB37285 ; HMDB37805 ; HMDB38255 ; HMDB39097 ; HMDB39110 ; HMDB39131 ; HMDB39769 ; HMDB39770 ; HMDB40146 ; HMDB40149 ; HMDB40151 ;	Phenylalanylphenylalanine ; 7Z10Z-Hexadecadienoic acid ; 7b-Hydroxy-3-oxo-5b-cholanoic acid ; Naproxen ; Aspartyl-L-proline ; MG(0:022:6(4Z7Z10Z13Z16Z19Z)0:0) ; MG(22:6(4Z7Z10Z13Z16Z19Z)0:00:0) ; 3-Methylene-indolenine ; (E)-2-octenal ; Norhydromorphone ; Procaterol ; Asparaginyl-Tyrosine ; Aspartyl-Proline ; Prolyl-Aspartate ; Tyrosyl-Asparagine ; Geranyl hexanoate ; (EE)-Trichostachine ; (1R2S3R)-2-Acetyl-4(5)-(1234-tetrahydroxybutyl)imidazole ; Linalyl hexanoate ; ()-Tembamide ; 2-Octenal ; (3xi5Z)-15-Octadien-3-ol ; (Z)-7-Hexadecen-116-olide ; 244-Trimethylcyclopentanone ; 2-Octen-4-one ; 1-Octen-3-one ; 1-Isothiocyanatobutane ; 3-Methyl-3-hepten-2-one ; 5-Methyl-5-hepten-2-one ; 1-(gamma-Glutamylamino)cyclopropanecarboxylic acid ; Isoambrettolide ; Isobutyl isothiocyanate ; 6-Methyl-3-hepten-2-one trans- ; 4-Octen-3-one ; (R)-Higenamine ; Pondaplin ; 3-Octen-2-one ; Quinoline ; Osthenol ; (R)-Kawain ; Isoquinoline ; (SE)-Filbertone ; 5-Octen-2-one ; Sulcatone ; 2-Ethylidenehexanal ; 24-Dimethyl-5-vinylthiazole ; D6-Ambrettolide ; Geranyl 2-ethylbutyrate ; Demethylbatatasin IV ; (2S3S)-alpha-Amino-2-carboxy-5-oxo-1-pyrrolidinebutanoic acid ; Ethyl (ZZ)-58-tetradecadienoate ; 6-Octenal ; (Z)-5-Octenal ; 25-Octadien-1-ol ; (3E5Z)-35-Octadien-1-ol ; (2E4E)-24-Octadien-1-ol ; xi-25-Dihydro-24-dimethylthiazole ; 7-Hydroxyheptaphylline ; Normorphine ; Norfluoxetine	M+2H ; M+Na+K ; M+3Na ; M+2ACN+2H ; M+2ACN+2H ; M+3Na ; M+3Na ; 2M+3H2O+2H ; 2M+Na+K ; M+ACN+2H ; M+H+Na ; M+H+NH4 ; M+2ACN+2H ; M+2ACN+2H ; M+H+NH4 ; M+Na+K ; M+ACN+2H ; M+2ACN+2H ; M+Na+K ; M+ACN+2H ; 2M+Na+K ; 2M+Na+K ; M+Na+K ; 2M+Na+K ; 2M+Na+K ; 2M+Na+K ; M+ACN+H ; 2M+Na+K ; 2M+Na+K ; M+2ACN+2H ; M+Na+K ; M+ACN+H ; 2M+Na+K ; 2M+Na+K ; M+ACN+2H ; M+2ACN+2H ; 2M+Na+K ; 2M+3H2O+2H ; M+2ACN+2H ; M+2ACN+2H ; 2M+3H2O+2H ; 2M+Na+K ; 2M+Na+K ; 2M+Na+K ; 2M+Na+K ; M+NH4 ; M+Na+K ; M+Na+K ; M+2ACN+2H ; M+2ACN+2H ; M+Na+K ; 2M+Na+K ; 2M+Na+K ; 2M+Na+K ; 2M+Na+K ; 2M+Na+K ; M+ACN+H ; M+H+NH4 ; M+ACN+2H ; M+H+NH4	C18H20N2O3 ; C16H28O2 ; C25H38O4 ; C14H14O3 ; C9H14N2O5 ; C25H38O4 ; C25H38O4 ; C9H7N ; C8H14O ; C16H17NO3 ; C16H22N2O3 ; C13H17N3O5 ; C9H14N2O5 ; C9H14N2O5 ; C13H17N3O5 ; C16H28O2 ; C16H17NO3 ; C9H14N2O5 ; C16H28O2 ; C16H17NO3 ; C8H14O ; C8H14O ; C16H28O2 ; C8H14O ; C8H14O ; C8H14O ; C5H9NS ; C8H14O ; C8H14O ; C9H14N2O5 ; C16H28O2 ; C5H9NS ; C8H14O ; C8H14O ; C16H17NO3 ; C14H14O3 ; C8H14O ; C9H7N ; C14H14O3 ; C14H14O3 ; C9H7N ; C8H14O ; C8H14O ; C8H14O ; C8H14O ; C7H9NS ; C16H28O2 ; C16H28O2 ; C14H14O3 ; C9H14N2O5 ; C16H28O2 ; C8H14O ; C8H14O ; C8H14O ; C8H14O ; C8H14O ; C5H9NS ; C18H17NO3 ; C16H17NO3 ; C16H16F3NO
420.65275	1.411777	1.387714	-3.7772	0.003618	2.4415	0.025957	NA	NA	NA	NA	NA
111.07469	1.422955	1.424309	3.7764	0.003623	2.4409	0.025957	111.0747	HMDB02172 ; HMDB37162	N1N12-Diacetylspermine ; 123-Tris(1-ethoxyethoxy)propane	M+H+2Na ; M+2H+Na	C14H30N4O2 ; C15H32O6
622.31890	1.522107	0.44392	-3.7759	0.003626	2.4406	0.025957	622.3189	HMDB13020 ; HMDB14838 ; HMDB30404 ; HMDB37039 ; HMDB39380 ; HMDB41407	Neuromedin C ; Eplerenone ; Armillaripin ; 4-O-Methylmelleolide ; Armillarin ; Clausarinol	M+3ACN+2H ; 3M+2H ; 3M+2H ; 3M+2H ; 3M+2H ; 3M+2H	C50H73N17O11S ; C24H30O6 ; C24H30O6 ; C24H30O6 ; C24H30O6 ; C24H30O6
93.04477	1.422332	1.368256	3.7755	0.003629	2.4402	0.025957	93.04477	HMDB30761 ; HMDB30790 ; HMDB31000 ; HMDB35412 ; HMDB35906 ; HMDB35934 ; HMDB36110 ; HMDB36119 ; HMDB36148 ; HMDB36206 ; HMDB36688 ; HMDB36766 ; HMDB36881 ; HMDB37563 ; HMDB37608 ; HMDB37766 ; HMDB39011 ; HMDB40902 ; HMDB40981 ; HMDB41038 ; HMDB41062	Pterosin O ; 6-(34-Methylenedioxy)phenyl-33-dimethyl-1-hexene ; (Z)-8-Decene-46-diyn-1-yl 3-methylbutanoate ; 1(10)11-Eremophiladiene-29-dione ; Alantolactone ; Isoalantolactone ; (S)-Bilobanone ; Alloalantolactone ; Furoeremophilone 1 ; alpha-Amylcinnamyl formate ; Costunolide ; Furanogermenone ; Germacrone-13-al ; Turmeronol B ; Turmeronol A ; 3-Mercaptohexyl hexanoate ; 1311(13)-Eudesmatrien-12-oic acid ; (E)-9-(3-Furanyl)-26-dimethyl-26-nonadien-4-one ; (7b10a)-3-Hydroxy-135-cadinatrien-9-one ; Collybial ; 1011-Epidioxycalamene	M+H+2Na ; M+H+2Na	C15H20O2 ; C15H20O2 ; C15H20O2 ; C15H20O2 ; C15H20O2 ; C15H20O2 ; C15H20O2 ; C15H20O2 ; C15H20O2 ; C15H20O2 ; C15H20O2 ; C15H20O2 ; C15H20O2 ; C15H20O2 ; C15H20O2 ; C12H24O2S ; C15H20O2 ; C15H20O2 ; C15H20O2 ; C15H20O2 ; C15H20O2
822.43037	1.454912	1.275811	-3.7727	0.003645	2.4383	0.026005	822.4304	HMDB12351 ; HMDB12441 ; HMDB28800 ; HMDB28801 ; HMDB28868 ; HMDB28905 ; HMDB28921 ; HMDB28927 ; HMDB28943 ; HMDB28952 ; HMDB29152 ; HMDB29153 ; HMDB30466 ; HMDB31025 ; HMDB31774 ; HMDB34327 ; HMDB34540 ; HMDB34641 ; HMDB39352 ; HMDB39562 ; HMDB40840 ; HMDB40879 ; HMDB41356 ; HMDB60732	PS(14:1(9Z)22:6(4Z7Z10Z13Z16Z19Z)) ; PS(22:6(4Z7Z10Z13Z16Z19Z)14:1(9Z)) ; Glutaminyl-Isoleucine ; Glutaminyl-Leucine ; Hydroxyprolyl-Lysine ; Isoleucyl-Glutamine ; Isoleucyl-Gamma-glutamate ; Leucyl-Glutamine ; Leucyl-Gamma-glutamate ; Lysyl-Hydroxyproline ; Gamma-glutamyl-Isoleucine ; Gamma-glutamyl-Leucine ; Goldinodox ; Betavulgaroside IV ; Lucyoside J ; Glucoconvallasaponin B ; Spinasaponin B ; Phytolaccoside D ; Azukisaponin III ; Cynarasaponin E ; 15-Oxo-21-hydroxymabiogenin 3-rhamnosyl-(1-6)-glucoside ; Phytolaccoside D2 ; Elatoside H ; 25-O-Desacetyl rifabutin	M+2Na-H ; M+2Na-H ; 3M+2Na-H ; 3M+2Na-H ; 3M+2Na-H ; 3M+2Na-H ; 3M+2Na-H ; 3M+2Na-H ; 3M+2Na-H ; 3M+2Na-H ; 3M+2Na-H ; 3M+2Na-H ; 2M+H+Na ; 2M+3H2O+2H ; 2M+H+Na ; M+ACN+Na ; 2M+H+Na ; 2M+H+Na ; 2M+H+Na ; 2M+H+Na ; 2M+H+Na ; 2M+H+Na ; 2M+H+Na ; M+ACN+H	C42H68NO10P ; C42H68NO10P ; C11H21N3O4 ; C11H21N3O4 ; C11H21N3O4 ; C11H21N3O4 ; C11H21N3O4 ; C11H21N3O4 ; C11H21N3O4 ; C11H21N3O4 ; C11H21N3O4 ; C11H21N3O4 ; C44H62N2O12 ; C41H62O15 ; C42H66O15 ; C38H62O15 ; C42H66O15 ; C42H66O15 ; C42H66O15 ; C42H66O15 ; C42H66O15 ; C42H66O15 ; C42H66O15 ; C41H56N4O11
342.68286	1.399488	1.339235	-3.7726	0.003646	2.4382	0.026005	342.6829	HMDB36707	Steviobioside	M+ACN+2H	C32H50O13
340.78192	1.466223	1.546383	3.7638	0.003699	2.4319	0.026311	340.7819	HMDB02148 ; HMDB06402 ; HMDB11145 ; HMDB32126 ; HMDB40188 ; HMDB40190 ; HMDB41791	5-Methyltetrahydropteroylpentaglutamate ; 3-Oxohexadecanoyl-CoA ; PA(16:0e18:0) ; zeta-Carotene epoxide ; 11-Dibromo-2-propanone ; 1-Bromo-3-iodoacetone ; 111-Trichloroethane	M+H+2Na ; M+3H ; M+H+NH4 ; M+3ACN+2H ; 3M+H+K ; M+2K+H ; 2M+2K-H	C40H53N11O18 ; C37H64N7O18P3S ; C37H75O7P ; C40H60O ; C3H4Br2O ; C3H4BrIO ; C2H3Cl3
397.64608	1.524046	0.591577	-3.7638	0.003699	2.4319	0.026311	397.6461	HMDB35904 ; HMDB60787	Phyllanthusol B ; 6-Methoxy-2-naphthylacetic acid	M+H+Na ; M+2ACN+2H	C35H49NO18 ; C39H37NO12
452.96665	1.433886	1.501285	-3.7599	0.003723	2.4291	0.026434	452.9667	HMDB59912	Indigo Carmine	2M+Na+K	C16H10N2O8S2
269.92093	1.429441	1.532386	-3.7585	0.003731	2.4282	0.026434	269.9209	HMDB05356 ; HMDB10413 ; HMDB42065 ; HMDB42124 ; HMDB42153 ; HMDB42181 ; HMDB42991 ; HMDB43047	TG(16:016:016:0)iso ; TG(16:014:018:0)iso6 ; TG(14:014:020:0) ; TG(14:016:018:0) ; TG(14:018:016:0) ; TG(14:020:014:0) ; TG(15:015:018:0) ; TG(15:018:015:0)	M+3H ; M+3H ; M+3H ; M+3H ; M+3H ; M+3H ; M+3H ; M+3H	C51H98O6 ; C51H98O6 ; C51H98O6 ; C51H98O6 ; C51H98O6 ; C51H98O6 ; C51H98O6 ; C51H98O6
247.97810	1.438708	0.051472	3.7577	0.003737	2.4275	0.026434	247.9781	HMDB33670 ; HMDB35716 ; HMDB59664 ; HMDB60590	14-Dithiane-25-diol ; Dihydroasparagusic acid ; Oxidized dithiothreitol ; Roflumilast N-oxide	3M+H+K ; 3M+H+K ; 3M+H+K ; M+2K	C4H8O2S2 ; C4H8O2S2 ; C4H8O2S2 ; C17H14Cl2F2N2O4
648.76389	1.396284	0.400449	3.7575	0.003738	2.4274	0.026434	648.7639	HMDB59637	3-(35-Diiodo-4-hydroxyphenyl)pyruvate	3M+2H	C9H6I2O4

400.73764	1.460144	1.589852	3.7521	0.003771	2.4236	0.026609	400.7376	HMDB08979 ; HMDB09104 ; HMDB09135 ; HMDB09136 ; HMDB09168 ; HMDB09169 ; HMDB09199 ; HMDB09200 ; HMDB09327 ; HMDB09360 ; HMDB09391 ; HMDB09392 ; HMDB09424 ; HMDB09425 ; HMDB09456 ; HMDB09683	PE(16:1(9Z)22:6(4Z7Z10Z13Z16Z19Z)) ; PE(18:2(9Z12Z)20:5(5Z8Z11Z14Z17Z)) ; PE(18:3(6Z9Z12Z)20:4(5Z8Z11Z14Z)) ; PE(18:3(6Z9Z12Z)20:4(8Z11Z14Z17Z)) ; PE(18:3(9Z12Z15Z)20:4(5Z8Z11Z14Z)) ; PE(18:3(9Z12Z15Z)20:4(8Z11Z14Z17Z)) ; PE(18:4(6Z9Z12Z15Z)20:3(5Z8Z11Z)) ; PE(18:4(6Z9Z12Z15Z)20:3(8Z11Z14Z)) ; PE(20:3(5Z8Z11Z)18:4(6Z9Z12Z15Z)) ; PE(20:3(8Z11Z14Z)18:4(6Z9Z12Z15Z)) ; PE(20:4(5Z8Z11Z14Z)18:3(6Z9Z12Z)) ; PE(20:4(5Z8Z11Z14Z)18:3(9Z12Z15Z)) ; PE(20:4(8Z11Z14Z17Z)18:3(6Z9Z12Z)) ; PE(20:4(8Z11Z14Z17Z)18:3(9Z12Z15Z)) ; PE(20:5(5Z8Z11Z14Z17Z)18:2(9Z12Z)) ; PE(22:6(4Z7Z10Z13Z16Z19Z)16:1(9Z))	M+H+K ; M+H+K ; M+H+K ; M+H+K ; M+H+K ; M+H+K ; M+H+K ; M+H+K ; M+H+K ; M+H+K ; M+H+K ; M+H+K ; M+H+K ; M+H+K ; M+H+K ; M+H+K	C43H72NO8P ; C43H72NO8P ; C43H72NO8P ; C43H72NO8P ; C43H72NO8P ; C43H72NO8P ; C43H72NO8P ; C43H72NO8P ; C43H72NO8P ; C43H72NO8P ; C43H72NO8P ; C43H72NO8P ; C43H72NO8P ; C43H72NO8P ; C43H72NO8P ; C43H72NO8P
296.05716	1.447518	1.121786	-3.7515	0.003774	2.4232	0.026609	296.0572	HMDB03944 ; HMDB06503 ; HMDB06522 ; HMDB11636 ; HMDB13839 ; HMDB14642 ; HMDB38182 ; HMDB60791	trans-2-Hexenoyl-CoA ; Phenylacetyl-CoA ; trans-3-Hexenoyl-CoA ; Salicyl CoA ; N-Desmethylaminopyrine ; Flutamide ; 47-Dihydro-5-(4-methyl-3-pentenyl)-123-trithiepin ; 6-Thioinosinic acid	M+2H+Na ; M+3H ; M+2H+Na ; M+3H ; M+2K+H ; 2M+H+K ; M+ACN+Na ; 2M+H+Na	C27H44N7O17P3S ; C29H42N7O17P3S ; C27H44N7O17P3S ; C29H42N7O17P3S ; C12H15N3O ; C11H11F3N2O3 ; C10H16S3 ; C10H12N4O4S
621.56959	1.499817	1.26543	-3.7509	0.003779	2.4227	0.026609	621.5696	HMDB35950	Nervonoylacetone	3M+H+Na	C27H50O2
406.68299	1.468784	1.494937	3.7494	0.003788	2.4216	0.026635	NA	NA	NA	NA	NA
97.514393	1.442285	1.191992	3.7482	0.003795	2.4208	0.026651	NA	NA	NA	NA	NA
300.97422	1.45747	1.409157	3.7422	0.003833	2.4165	0.026878	NA	NA	NA	NA	NA
450.06034	1.457265	1.482622	-3.7379	0.00386	2.4134	0.027032	450.0603	HMDB14577 ; HMDB38669	Prochlorperazine ; S-(Allylthio)-L-cysteine	M+2K-H ; 2M+ACN+Na	C20H24ClN3S ; C6H11NO2S2
720.96656	1.465184	0.034291	-3.7326	0.003894	2.4096	0.027228	720.9666	HMDB30085	Pubescenol	3M+H+NH4	C28H42O6
473.95596	1.419825	1.552548	3.7249	0.003943	2.4041	0.027521	473.956	HMDB04910 ; HMDB04968	Ganglioside GA1 (d18:125:0) ; Tetrahexosylceramide (d18:125:0)	M+3Na ; M+3Na	C69H128N2O23 ; C69H128N2O23
382.01060	1.495156	1.130142	3.7225	0.003959	2.4024	0.027521	382.0106	HMDB00843 ; HMDB14436 ; HMDB29839 ; HMDB60176	N-Acetylgalactosamine 46-disulfate ; Chlorambucil ; Ascorbigen ; (S)-3-Sulfonatolactate	M+H ; M+2K+H ; M+2K-H ; 2M+ACN+H	C8H15NO12S2 ; C14H19Cl2NO2 ; C15H15NO6 ; C3H6O6S
226.95136	1.455863	1.141168	3.7223	0.00396	2.4023	0.027521	226.9514	HMDB60363 ; HMDB60345 ; HMDB60368	25-Dichloro-4-oxohex-2-enedioate ; 2-Chloro-5-methyl-cis-dienelactone ; 3-Chloro-2-methyldienelactone	M+H ; M+K ; M+K	C6H4Cl2O5 ; C7H5ClO4 ; C7H5ClO4
395.15626	1.455449	1.557792	3.7211	0.003968	2.4014	0.027521	395.1563	HMDB03920 ; HMDB12623 ; HMDB12624 ; HMDB12780 ; HMDB14600 ; HMDB30265 ; HMDB30322 ; HMDB30375 ; HMDB33445 ; HMDB38217 ; HMDB38630	Protopine ; 2-Hydroxyestrone-1-S-glutathione ; 2-Hydroxyestrone-4-S-glutathione ; 4-Hydroxyestrone-2-S-glutathione ; Prazosin ; 22-Dimethyl(pyrano-56:3:4)-15-dihydroxy-6-methoxy-10-methylacridone ; Papaveraldine ; Citracridone I ; Honyumine ; Pterosin H ; Niazimin A	M+ACN+H ; 2M+3H ; 2M+3H ; 2M+3H ; 2M+H+Na ; M+ACN+H ; M+ACN+H ; M+ACN+H ; M+ACN+H ; 3M+H+K ; 2M+H+Na	C20H19NO5 ; C28H37N3O9S ; C28H37N3O9S ; C28H37N3O9S ; C19H21N5O4 ; C20H19NO5 ; C20H19NO5 ; C20H19NO5 ; C20H19NO5 ; C15H19ClO ; C18H25NO8
385.12877	1.489995	0.077511	-3.7204	0.003972	2.401	0.027521	385.1288	HMDB14915 ; HMDB15607 ; HMDB29833 ; HMDB31991 ; HMDB33222 ; HMDB33223 ; HMDB35822 ; HMDB37125 ; HMDB37263 ; HMDB38149 ; HMDB60654	Propiomazine ; Lucanthone ; Cyclodopa glucoside ; Negletein 6-rhamnosyl-(1-2)-fucoside ; (1R3S4S6R)-69-Dihydroxyfenchone 6-O-b-D-glucoside ; (1S3R4R)-810-Dihydroxyfenchone 10-O-b-D-glucoside ; Glucosyl 6-hydroxy-26-dimethyl-2E7-octadienoate ; Pisumoside B ; HMBOA-Glc ; Nepetaside ; Citalopram N-oxide	M+2Na-H ; M+2Na-H ; 2M+3H2O+2H ; 2M+3H ; M+K ; M+K ; M+K ; M+2K ; 2M+3H2O+2H ; M+K ; M+2Na-H	C20H24N2OS ; C20H24N2OS ; C15H19NO9 ; C28H32O13 ; C16H26O8 ; C16H26O8 ; C16H26O8 ; C32H52O16 ; C15H19NO9 ; C16H26O8 ; C20H21FN2O2
464.64178	1.461172	1.565937	3.7203	0.003973	2.4009	0.027521	NA	NA	NA	NA	NA
340.17648	1.450796	0.307583	-3.7194	0.00398	2.4002	0.027521	340.1765	HMDB14736 ; HMDB31850 ; HMDB33460 ; HMDB36361 ; HMDB40054 ; HMDB40142 ; HMDB40144 ; HMDB40521 ; HMDB40911	Labetalol ; 2-Ethoxy-3-methylpyrazine ; 4-Hydroxycinnamoylagmatine ; Pipercyclobutanamide B ; 3-Ethyl-2-methoxypyrazine ; 2-Ethoxy-5-methylpyrazine ; 2-Ethoxy-6-methylpyrazine ; Dipiperamide D ; 2-Methoxy-35-dimethylpyrimidine	2M+H+Na ; 2M+ACN+Na ; M+ACN+Na ; M+2ACN+2H ; 2M+ACN+Na ; 2M+ACN+Na ; 2M+ACN+Na ; M+2ACN+2H ; 2M+ACN+Na	C19H24N2O3 ; C7H10N2O ; C14H20N4O2 ; C36H40N2O6 ; C7H10N2O ; C7H10N2O ; C7H10N2O ; C36H40N2O6 ; C7H10N2O
145.09721	1.420074	0.691227	-3.7193	0.00398	2.4002	0.027521	145.0972	HMDB60385 ; HMDB34554 ; HMDB00092 ; HMDB00112 ; HMDB00452 ; HMDB00650 ; HMDB01084 ; HMDB01906 ; HMDB02166 ; HMDB02299 ; HMDB03911 ; HMDB12130 ; HMDB28961 ; HMDB29066 ; HMDB29434 ; HMDB31329 ; HMDB31430 ; HMDB31447 ; HMDB31654 ; HMDB32262 ; HMDB38394 ; HMDB41945 ; HMDB59657 ; HMDB61050	4-Amino-1-piperidinecarboxylic acid ; Sakacin A ; Dimethylglycine ; Gamma-Aminobutyric acid ; L-Alpha-aminobutyric acid ; D-Alpha-aminobutyric acid ; D-1-Piperideine-2-carboxylic acid ; 2-Aminoisobutyric acid ; (S)-b-aminoisobutyric acid ; (R)-b-aminoisobutyric acid ; 3-Aminoisobutanoic acid ; (S)-2345-Tetrahydropiperidine-2-carboxylate ; Lysyl-Threonine ; Threoninyl-Lysine ; ()-4-Methylene-2-pyrrolidinecarboxylic acid ; Butyl nitrite ; Dimethyl peroxide ; N-Heptane ; 3-Aminobutanoic acid ; Ethylene oxidepropylene oxide copolymer (avg m w 9760 - 13200) ; O-Acetylethanolamine ; N-Ethylglycine ; (S)-2345-tetrahydropyridine-2-carboxylate ; Unknown	M+H ; M+2H ; M+ACN+H ; M+ACN+H ; M+ACN+H ; M+ACN+H ; M+NH4 ; M+ACN+H ; M+ACN+H ; M+ACN+H ; M+ACN+H ; M+NH4 ; M+ACN+2H ; M+ACN+2H ; M+NH4 ; M+ACN+H ; M+2ACN+H ; M+2Na-H ; M+ACN+H ; M+2ACN+H ; M+ACN+H ; M+ACN+H ; M+NH4 ; M+ACN+H	C6H12N2O2 ; C12H24N4O4 ; C4H9NO2 ; C4H9NO2 ; C4H9NO2 ; C4H9NO2 ; C6H9NO2 ; C4H9NO2 ; C4H9NO2 ; C4H9NO2 ; C4H9NO2 ; C6H9NO2 ; C10H21N3O4 ; C10H21N3O4 ; C6H9NO2 ; C4H9NO2 ; C2H6O2 ; C7H16 ; C4H9NO2 ; C2H6O2 ; C4H9NO2 ; C4H9NO2 ; C6H9NO2 ; C4H9NO2
460.14967	1.478617	1.29796	-3.7176	0.003991	2.3989	0.027556	460.1497	HMDB29999 ; HMDB30490 ; HMDB36185 ; HMDB38878	Cycloartomunoxanthone ; Artonol E ; 3-(2-Furanyl)-2-phenyl-2-propenal ; Cycloartomunin	2M+H+Na ; 2M+H+Na ; 2M+ACN+Na ; 2M+H+Na	C26H24O7 ; C26H24O7 ; C13H10O2 ; C26H24O7
524.59769	1.459399	1.529592	3.7169	0.003996	2.3984	0.027556	NA	NA	NA	NA	NA
295.09559	1.457479	0.314673	-3.716	0.004001	2.3978	0.027558	295.0956	HMDB29364 ; HMDB29534 ; HMDB31975 ; HMDB38613 ; HMDB00565 ; HMDB00625 ; HMDB01857 ; HMDB01982 ; HMDB02026 ; HMDB03290 ; HMDB04308 ; HMDB04810 ; HMDB11103 ; HMDB29722 ; HMDB30526 ; HMDB31772 ; HMDB32847 ; HMDB33800 ; HMDB34435 ; HMDB34675 ; HMDB35035 ; HMDB40352 ; HMDB41186 ; HMDB41187 ; HMDB41660 ; HMDB41669 ; HMDB41670 ; HMDB41701 ; HMDB59704	7-Acetoxy-2-methylisoflavone ; Glyzarin ; 3-Methoxy-2-(4-methylbenzoyl)-4H-1-benzopyran-4-one ; gamma-L-Glutamyl-L-methionine sulfoxide ; Galactonic acid ; Gluconic acid ; 13-Dimethyluric acid ; 37-Dimethyluric acid ; 19-Dimethyluric acid ; Gulonic acid ; 79-Dimethyluric acid ; 5C-aglycone ; 17-Dimethyluric acid ; 125-Trimethyl-1H-pyrrole ; Isovestitol ; 1-Methoxy-1-(245-trimethoxyphenyl)-2-propanol ; 12-Bis(4-hydroxy-3-methoxyphenyl)ethylene ; 246-Trimethoxybenzophenone ; 1-(26-Dihydroxy-4-methoxyphenyl)-3-phenyl-1-propanone ; (S)-23-Dihydro-35-dihydroxy-2-oxo-3-indoleacetic acid 5-glucosyl-(1-4)-b-D-glucoside ; Marminal ; 2-4-(3-Hydroxypropyl)-2-methoxyphenoxy-13-propanediol ; Ilicifolinoside A ; (Z)-2-Methyl-2-butene-14-diol 4-O-beta-D-Glucopyranoside ; 3-O-Methylequol ; 47-Dihydroxy-3-methoxyisoflavan ; 47-Dihydroxy-6-methoxyisoflavan ; 6-O-Methylequol ; 39-Dimethyluric acid	M+H ; M+H ; M+H ; M+H ; 3M+2H ; 3M+2H ; 3M+2H ; 3M+2H ; 3M+2H ; 3M+2H ; 3M+2H ; 3M+2H ; M+Na ; 3M+2H ; 2M+2K-H ; M+Na ; M+K ; M+Na ; M+Na ; M+Na ; M+ACN+2H ; M+Na ; M+K ; 2M+Na+K ; 2M+Na+K ; M+Na ; M+Na ; M+Na ; M+Na ; 3M+2H	C18H14O4 ; C18H14O4 ; C18H14O4 ; C10H18N2O6S ; C6H12O7 ; C6H12O7 ; C7H8N4O3 ; C7H8N4O3 ; C7H8N4O3 ; C6H12O7 ; C7H8N4O3 ; C16H16O4 ; C7H8N4O3 ; C7H11N ; C16H16O4 ; C13H20O5 ; C16H16O4 ; C16H16O4 ; C16H16O4 ; C22H29NO15 ; C16H16O4 ; C13H20O5 ; C11H20O7 ; C11H20O7 ; C16H16O4 ; C16H16O4 ; C16H16O4 ; C16H16O4 ; C7H8N4O3

679.06189[illegible]	1.470621	1.180616	-3.7129	0.004022	2.3956	0.027638	NA	NA	NA	NA	NA
621.19334[illegible]	1.513031	1.126276	-3.7117	0.00403	2.3947	0.027638	621.1933	HMDB30336 ; HMDB41517	2-Feruloyl-12-disinapoylgentiobiose ; beta-D-Glucopyranosyl anthranilate	2M+3H ; 2M+Na	C44H50O22 ; C13H17NO7
215.06967[illegible]	1.425411	0.243238	-3.7104	0.004039	2.3938	0.027638	215.0697	HMDB32018 ; HMDB32583 ; HMDB41807 ; HMDB00386 ; HMDB11691 ; HMDB15049 ; HMDB15359 ; HMDB29094 ; HMDB29516 ; HMDB30476 ; HMDB30478 ; HMDB30502 ; HMDB30580 ; HMDB30587 ; HMDB30752 ; HMDB31610 ; HMDB31623 ; HMDB32082 ; HMDB32591 ; HMDB33063 ; HMDB35275 ; HMDB35427 ; HMDB35873 ; HMDB35894 ; HMDB36746 ; HMDB37277 ; HMDB37877 ; HMDB38466 ; HMDB40268 ; HMDB40446 ; HMDB40809 ; HMDB40834 ; HMDB41007 ; HMDB41100 ; HMDB41417 ; HMDB41481 ; HMDB60430	Phenyl salicylate ; 4-Hydroxy-2-biphenylcarboxylic acid ; 3-Phenoxybenzoic acid ; 3b16a-Dihydroxyandrostenone sulfate ; Cytidine 23-cyclic phosphate ; Anileridine ; Encainide ; Tryptophyl-Tryptophan ; Limocitrin ; Aflatoxin G2a ; Aflatoxin GM2 ; (2S4S6S)-2-2-(4-Hydroxy-3-meyhoxyphenyl)ethyltetrahydro-6-(45-dihydroxy-3-methoxyphenyl)-2H-pyran-4-ol ; (R)-Shinanolone ; Spinacetin ; Methyl trans-p-methoxycinnamate ; Allyl phenoxyacetate ; Ethyl phenylglycidate ; Rosmic acid ; Dehydrozingerone ; 23-Butanediol apiosylglucoside ; Isomyristicin ; Glicoisoflavanone ; Myristicin ; (Z)-4-Methoxy-6-(1-propenyl)-13-benzodioxole ; Matsutakeside I ; Isoeugenol formate ; Eugenyl formate ; 5678-Tetrahydroxy-34-dimethoxyflavone ; Ethyl 3-oxo-3-phenylpropanoate ; Benzyl acetoacetate ; 57-Dihydroxy-34-dimethoxy-5-prenylflavanone ; O-Ethylcubebin ; 1-Hydroxychavicol acetate ; Kanzonol N ; 12-Dehydroporson ; 4-(34-Methylenedioxyphenyl)-2-butanone ; Aflatoxin B1 diol	M+H ; M+H ; M+H ; M+2Na ; M+3ACN+2H ; M+2K ; M+2K ; M+H+K ; M+2ACN+2H ; M+2ACN+2H ; M+2ACN+2H ; M+H+K ; M+Na ; M+2ACN+2H ; M+Na ; M+Na ; M+Na ; M+H+K ; M+Na ; M+2Na ; M+Na ; M+2Na ; M+Na ; M+Na ; M+2H+Na ; M+Na ; M+Na ; M+2ACN+2H ; M+Na ; M+Na ; M+2Na ; M+2Na ; M+Na ; M+2Na ; M+2Na ; M+Na ; M+2ACN+2H	C13H10O3 ; C13H10O3 ; C13H10O3 ; C19H28O6S ; C9H12N3O7P ; C22H28N2O2 ; C22H28N2O2 ; C22H22N4O3 ; C17H14O8 ; C17H14O8 ; C17H14O8 ; C21H26O7 ; C11H12O3 ; C17H14O8 ; C11H12O3 ; C11H12O3 ; C11H12O3 ; C21H26O7 ; C11H12O3 ; C15H28O11 ; C11H12O3 ; C22H24O6 ; C11H12O3 ; C11H12O3 ; C30H36O14 ; C11H12O3 ; C11H12O3 ; C17H14O8 ; C11H12O3 ; C11H12O3 ; C22H24O6 ; C22H24O6 ; C11H12O3 ; C22H24O6 ; C22H24O6 ; C11H12O3 ; C17H14O8
304.17560[illegible]	1.497891	0.34368	3.7103	0.004039	2.3937	0.027638	304.1756	HMDB13328 ; HMDB10361 ; HMDB14662 ; HMDB15557 ; HMDB29835 ; HMDB32756 ; HMDB28716 ; HMDB28989	Pimelylcarnitine ; (23S)-2325-dihdroxy-24-oxovitamine D3 23-(beta-glucuronide) ; Carteolol ; Pheniramine ; Tetrahydroharmol ; N-Methyl-1H-indole-3-propanamide ; Arginyl-Phenylalanine ; Phenylalanyl-Arginine	M+H ; M+2H ; 2M+H+Na ; M+ACN+Na ; 3M+2H ; 3M+2H ; M+H-H2O ; M+H-H2O	C14H25NO6 ; C33H50O10 ; C16H24N2O3 ; C16H20N2 ; C12H14N2O ; C12H14N2O ; C15H23N5O3 ; C15H23N5O3
617.82254[illegible]	1.526796	0.712026	-3.7101	0.004041	2.3935	0.027638	NA	NA	NA	NA	NA
359.05531[illegible]	1.393554	0.880791	-3.7077	0.004057	2.3918	0.027711	359.0553	HMDB14411 ; HMDB14587 ; HMDB15667 ; HMDB29292 ; HMDB29293 ; HMDB29413 ; HMDB34039 ; HMDB34049 ; HMDB34129 ; HMDB41295	Dicumarol ; Teniposide ; Sulfaphenazole ; 3-p-Coumaroyl-15-quinolactone ; 4-p-Coumaroyl-15-quinolactone ; Bis-gamma-glutamylcysteinylbis-beta-alanine ; Dolineone ; Pachyrrhizin ; Dehydroneotenone ; 7-Hydroxy-6-methoxy-alpha-pyrufuran	M+Na ; M+Na+K ; M+2Na-H ; M+K ; M+K ; M+2K ; M+Na ; M+Na ; M+Na ; M+K	C19H12O6 ; C32H32O13S ; C15H14N4O2S ; C16H16O7 ; C16H16O7 ; C22H36N6O12S2 ; C19H12O6 ; C19H12O6 ; C19H12O6 ; C16H16O7
247.54690[illegible]	1.45281	1.461849	-3.7052	0.004074	2.39	0.027787	247.5469	HMDB42044	Tolmetin glucuronide	M+Na+K	C21H23NO9
231.05467[illegible]	1.445703	1.230269	3.7035	0.004085	2.3888	0.027819	231.0547	HMDB01004 ; HMDB01309 ; HMDB01324 ; HMDB01390 ; HMDB01411 ; HMDB01427 ; HMDB04043 ; HMDB04472 ; HMDB05812 ; HMDB14612 ; HMDB30999 ; HMDB31410 ; HMDB32118 ; HMDB32530 ; HMDB32762 ; HMDB34848 ; HMDB34932 ; HMDB34969 ; HMDB34972 ; HMDB34976 ; HMDB35155 ; HMDB35162 ; HMDB35272 ; HMDB35783 ; HMDB35815 ; HMDB35818 ; HMDB35819 ; HMDB35820 ; HMDB35823 ; HMDB35825 ; HMDB35833 ; HMDB35838 ; HMDB35841 ; HMDB35842 ; HMDB35861 ; HMDB35954 ; HMDB35955 ; HMDB36041 ; HMDB36068 ; HMDB36077 ; HMDB36078 ; HMDB36086 ; HMDB36096 ; HMDB36097 ; HMDB36100 ; HMDB36101 ; HMDB36102 ; HMDB36107 ; HMDB36112 ; HMDB36126 ; HMDB36196 ; HMDB36991 ; HMDB36993 ; HMDB36996 ; HMDB37006 ; HMDB37007 ; [illegible]	Oxoamide ; m-Chlorohippuric acid ; N-Hydroxymethylnorcotinine ; Hydroxycotinine ; Cotinine N-oxide ; 5-Hydroxycotinine ; Alpha-Terpineol ; Eucalyptol ; Geraniol ; Tenoxicam ; 2-Decenal ; 3-Decen-2-one ; 26-Dimethyl-7-octen-3-one ; Thujyl alcohol ; Leucodelphinidin 3-galactosyl-(1-4)-glucoside ; 9-Decenal ; Fenchol ; (-)-Carvomenthone ; ()-Isomenthone ; (-)-Borneol ; beta-Geraniol ; (-)-Menthone ; p-Menthan-2-one ; ()-Menthone ; ()-Isoborneol ; ()-Borneol ; (-)-Isoborneol ; (R)-Citronellal ; (S)-p-Menth-1-en-4-ol ; (-)-Dihydrocarveol ; p-Menth-1-en-4-ol ; Piperitol ; Citronellal ; (S)-Citronellal ; trans-(-)-p-Menth-1-en-3-ol ; (1R3R6S)-3-Caranol ; (1R2S3S6S)-2-Caranol ; (R)-Lavandulol ; Campholenic alcohol ; p-Menth-8-en-3-ol ; (-)-Isopulegol ; (S)-alpha-Terpineol ; 14-Cineole ; Rose oxide ; 37-Dimethyl-16-octadien-3-ol ; (R)-37-Dimethyl-16-octadien-3-ol ; D-Linalool ; Myrcenol ; (1R4S5R)-4-Thujanol ; (-)-Pinocampheol ; (2E4E)-24-Decadien-1-ol ; delta-Terpineol ; gamma-Terpineol ; beta-Terpineol ; cis-Isopulegone ; p-Menth-1-en-9-ol ; p-Menth-1-en-5-ol ; 68-Epoxy-p-menthane ; (-)-trans-m-Menth-8-en-1-ol ; m-Menth-1-en-8-ol ; (R)-m-Menth-1(6)-en-8-ol ; Linaloyl oxide ; 2-Isopropyl-5-methyl-2-hexenal ; cis-beta-Terpineol ; trans-beta-Terpineol ; Nitrosylhaem ; xi-p-Menth-3-en-1-ol ; Ocimen quintoxide ; 2-(1-Methylpropyl)cyclohexanone ; 4-Decenal ; (4Z7Z)-47-Decadien-1-ol ; Isomenthone ; p-tert-Butylcyclohexanone ; Ocimenol ; 6-Carboxy-5678-tetrahydropterin ; 6-Methylthiopurine 5-monophosphate ribonucleotide	M+K ; M+NH4 ; M+K ; M+K ; M+K ; M+K ; M+2K-H ; M+2K-H ; M+2K-H ; M+3ACN+2H ; M+2K-H ; M+2K-H ; M+2K-H ; M+2K-H ; M+H+2Na ; M+2K-H ; M+H+2Na ; M+2K-H ; M+2K-H ; M+2K-H ; M+2K-H ; M+2K-H ; M+2K-H ; M+2K-H ; M+2K-H ; 2M+H+K ; M+2ACN+2H	C10H12N2O2 ; C9H8ClNO3 ; C10H12N2O2 ; C10H12N2O2 ; C10H12N2O2 ; C10H12N2O2 ; C10H18O ; C10H18O ; C10H18O ; C13H11N3O4S2 ; C10H18O ; C10H18O ; C10H18O ; C10H18O ; C27H34O18 ; C10H18O ; C34H32FeN5O5 ; C10H18O ; C10H18O ; C10H18O ; C10H18O ; C10H18O ; C10H18O ; C10H18O ; C10H18O ; C7H9N5O3 ; C11H15N4O7PS

149.01848	1.444147	0.845271	3.7021	0.004095	2.3878	0.027819	149.0185	HMDB00008 ; HMDB00011 ; HMDB00023 ; HMDB00336 ; HMDB00357 ; HMDB00442 ; HMDB00710 ; HMDB00729 ; HMDB13968 ; HMDB13969 ; HMDB14406 ; HMDB14717 ; HMDB15175 ; HMDB15231 ; HMDB28781 ; HMDB28970 ; HMDB28982 ; HMDB29045 ; HMDB30968 ; HMDB31212 ; HMDB32929 ; HMDB38998 ; HMDB40549 ; HMDB41173 ; HMDB59748	2-Hydroxybutyric acid ; (R)-3-Hydroxybutyric acid ; (S)-3-Hydroxyisobutyric acid ; (R)-3-Hydroxyisobutyric acid ; 3-Hydroxybutyric acid ; (S)-3-Hydroxybutyric acid ; 4-Hydroxybutyric acid ; Alpha-Hydroxyisobutyric acid ; Hydroxyzileuton ; Zileuton sulfoxide ; Anagrelide ; Carbenicillin ; Thalidomide ; Flucytosine ; Cysteinyl-Methionine ; Methionyl-Cysteine ; Methionyl-Serine ; Serinyl-Methionine ; 246-Octatriyn-1-ol ; Ethoxyacetic acid ; Benzofuran ; 357-Octatriyn-1-ol ; xi-23-Octadiene-57-diyn-1-ol ; 1-O-Caffeoyl-(b-D-glucose 6-O-sulfate) ; 2-Methyl-3-hydroxypropanoate	M+2Na-H ; M+2Na-H ; M+2Na-H ; M+2Na-H ; M+2Na-H ; M+2Na-H ; M+2Na-H ; M+2Na-H ; M+2Na ; M+2Na ; M+ACN+2H ; M+3Na ; M+H+K ; 2M+H+K ; M+2Na ; M+2Na ; M+Na+K ; M+Na+K ; 2M+Na+K ; M+2Na-H ; 2M+Na+K ; 2M+Na+K ; 2M+Na+K ; M+2H+Na ; M+2Na-H	C4H8O3 ; C4H8O3 ; C4H8O3 ; C4H8O3 ; C4H8O3 ; C4H8O3 ; C4H8O3 ; C4H8O3 ; C11H12N2O3S ; C11H12N2O3S ; C10H7Cl2N3O ; C17H18N2O6S ; C13H10N2O4 ; C4H4FN3O ; C8H16N2O3S2 ; C8H16N2O3S2 ; C8H16N2O4S ; C8H16N2O4S ; C8H6O ; C4H8O3 ; C8H6O ; C8H6O ; C8H6O ; C15H18O12S ; C4H8O3
580.55801	1.453397	1.577495	3.702	0.004095	2.3878	0.027819	580.558	HMDB40907 ; HMDB60753	7-Pentacosanone ; 3-p-Hydroxypaclitaxel	3M+Na+K ; 2M+3H	C25H50O ; C47H51NO15
119.08960	1.432198	0.446798	-3.6996	0.004111	2.3861	0.027883	119.0896	HMDB29579 ; HMDB31431 ; HMDB39806 ; HMDB31199 ; HMDB31404 ; HMDB31702 ; HMDB32416 ; HMDB37293	Diisopropyl sulfide ; Dipropyl sulfide ; 1-Hexanethiol ; 25-Dihydro-245-trimethyloxazole ; Cyclohexylamine ; 1-Piperidinecarboxaldehyde ; 2-Methylpiperidine ; 2-Acetylpyrrolidine	M+H ; M+H ; M+H ; M+3ACN+2H ; 2M+H+K ; M+3ACN+2H ; 2M+H+K ; M+3ACN+2H	C6H14S ; C6H14S ; C6H14S ; C6H11NO ; C6H13N ; C6H11NO ; C6H13N ; C6H11NO
123.11681	1.4977	0.892286	3.699	0.004116	2.3856	0.027883	123.1168	HMDB38140	Santene	M+H	C9H14
622.19657	1.479261	1.279387	-3.6959	0.004136	2.3834	0.027961	622.1966	HMDB01871 ; HMDB02780 ; HMDB15067 ; HMDB29253 ; HMDB30131 ; HMDB30144 ; HMDB32322 ; HMDB33783 ; HMDB34124 ; HMDB35399 ; HMDB37583 ; HMDB37953 ; HMDB37954 ; HMDB38357 ; HMDB41293 ; HMDB41310	Epicatechin ; Catechin ; Tipranavir ; 3-Hydroxyphloretin ; gamma-Pyrufuran ; alpha-Cotonefuran ; cis-3 and trans-2-Hexenyl propionate ; Marshrin ; Fonsecin ; Cartorimine ; 6-p-Coumaroylprunin ; (-)-Catechin ; ()-Epicatechin ; Gambiriin A1 ; 6-Hydroxy-alpha-pyrufuran ; Luteoforol	2M+ACN+H ; 2M+ACN+H ; 2M+H+K ; 2M+ACN+H ; 2M+ACN+H ; 2M+ACN+H ; 2M+ACN+H ; 2M+ACN+H ; 2M+ACN+H ; 2M+ACN+H ; M+ACN+H ; 2M+ACN+H ; 2M+ACN+H ; M+ACN+H ; 2M+ACN+H ; 2M+ACN+H	C15H14O6 ; C15H14O6 ; C31H33F3N2O5S ; C15H14O6 ; C15H14O6 ; C15H14O6 ; C15H14O6 ; C15H14O6 ; C15H14O6 ; C15H14O6 ; C30H28O12 ; C15H14O6 ; C15H14O6 ; C30H28O12 ; C15H14O6 ; C15H14O6
993.70609	1.532247	0.484195	-3.6956	0.004138	2.3832	0.027961	993.7061	HMDB05049 ; HMDB14443 ; HMDB14750 ; HMDB33503 ; HMDB41248 ; HMDB53250 ; HMDB53263 ; HMDB54308 ; HMDB54338 ; HMDB55108 ; HMDB55143 ; HMDB55350 ; HMDB55356 ; HMDB55494 ; HMDB55524 ; HMDB55534 ; HMDB55670 ; HMDB55682 ; HMDB55764 ; HMDB55788 ; HMDB55878 ; HMDB55926 ; HMDB55940	10-Nitrolinoleic acid ; Dapiprazole ; Bisoprolol ; Small bacteriocin ; N-3-Hydroxy-2-(2-pentenyl)cyclopentylacetylisoleucine ; TG(18:3(6Z9Z12Z)22:6(4Z7Z10Z13Z16Z19Z)o-18:0) ; TG(18:3(6Z9Z12Z)o-18:022:6(4Z7Z10Z13Z16Z19Z)) ; TG(20:4(5Z8Z11Z14Z)20:5(5Z8Z11Z14Z17Z)o-18:0) ; TG(20:4(5Z8Z11Z14Z)o-18:020:5(5Z8Z11Z14Z17Z)) ; TG(22:5(4Z7Z10Z13Z16Z)18:4(6Z9Z12Z15Z)o-18:0) ; TG(22:5(4Z7Z10Z13Z16Z)o-18:018:4(6Z9Z12Z15Z)) ; TG(18:3(9Z12Z15Z)22:6(4Z7Z10Z13Z16Z19Z)o-18:0) ; TG(18:3(9Z12Z15Z)o-18:022:6(4Z7Z10Z13Z16Z19Z)) ; TG(18:4(6Z9Z12Z15Z)22:5(4Z7Z10Z13Z16Z)o-18:0) ; TG(18:4(6Z9Z12Z15Z)22:5(7Z10Z13Z16Z19Z)o-18:0) ; TG(18:4(6Z9Z12Z15Z)o-18:022:5(7Z10Z13Z16Z19Z)) ; TG(20:4(8Z11Z14Z17Z)20:5(5Z8Z11Z14Z17Z)o-18:0) ; TG(20:4(8Z11Z14Z17Z)o-18:020:5(5Z8Z11Z14Z17Z)) ; TG(20:5(5Z8Z11Z14Z17Z)20:4(5Z8Z11Z14Z)o-18:0) ; TG(20:5(5Z8Z11Z14Z17Z)20:4(8Z11Z14Z17Z)o-18:0) ; TG(22:5(7Z10Z13Z16Z19Z)18:4(6Z9Z12Z15Z)o-18:0) ; TG(22:6(4Z7Z10Z13Z16Z19Z)18:3(6Z9Z12Z)o-18:0) ; TG(22:6(4Z7Z10Z13Z16Z19Z)18:3(9Z12Z15Z)o-18:0)	3M+NH4 ; 3M+NH4 ; 3M+NH4 ; 3M+NH4 ; 3M+NH4 ; M+2K+H ; M+2K+H ; M+2K+H ; M+2K+H ; M+2K+H ; M+2K+H ; M+2K+H ; M+2K+H ; M+2K+H ; M+2K+H ; M+2K+H ; M+2K+H ; M+2K+H ; M+2K+H ; M+2K+H ; M+2K+H ; M+2K+H ; M+2K+H	C18H31NO4 ; C19H27N5 ; C18H31NO4 ; C18H31NO4 ; C18H31NO4 ; C61H102O5 ; C61H102O5 ; C61H102O5 ; C61H102O5 ; C61H102O5 ; C61H102O5 ; C61H102O5 ; C61H102O5 ; C61H102O5 ; C61H102O5 ; C61H102O5 ; C61H102O5 ; C61H102O5 ; C61H102O5 ; C61H102O5 ; C61H102O5 ; C61H102O5 ; C61H102O5
101.06012	1.442719	0.510901	3.6943	0.004147	2.3823	0.027982	101.0601	HMDB00509 ; HMDB01470 ; HMDB01862 ; HMDB02167 ; HMDB29599 ; HMDB29608 ; HMDB31178 ; HMDB31598 ; HMDB31602 ; HMDB31648 ; HMDB32351 ; HMDB32385 ; HMDB32459 ; HMDB33840 ; HMDB33978 ; HMDB35143 ; HMDB00603 ; HMDB13227 ; HMDB30989 ; HMDB30990 ; HMDB34491 ; HMDB34971 ; HMDB36715 ; HMDB60606 ; HMDB13704 ; HMDB14571 ; HMDB29410 ; HMDB33141 ; HMDB33576 ; HMDB38513 ; HMDB39998 ; HMDB39999 ; HMDB40040	Senecioic acid ; Tiglic acid ; 2-Ethylacrylic acid ; 3-Methylbutyrolactone ; Glutaral ; Angelic acid ; Dihydro-2-methyl-3(2H)-furanone ; 23-Pentanedione ; 4-Pentenoic acid ; 24-Pentanedione ; Isopropenyl acetate ; Methyl methacrylate ; 2-Pentenoic acid ; Dihydro-5-methyl-2(3H)-furanone ; Ethyl acrylate ; Dihydro-3-methyl-2(3H)-furanone ; cis-4-Decenedioic acid ; cis-5-Decenedioic acid ; 5-Pentyltetrahydro-2-oxo-3-furancarboxylic acid ; alpha-Carboxy-delta-nonalactone ; ()-Camphoric acid ; (1R2R3S1R)-Nepetalinic acid ; Matsutakic acid A ; Hydralazine acetone hydrazone ; 6-Methylnicotinamide ; Triprolidine ; 2-Acetyl-3-ethylpyrazine ; 2-Amino-a-carboline ; 7-Azaindolizine ; 78-Dihydro-3-methylpyrrolo12-apyrimidin-2(6H)-one ; 2-Acetyl-36-dimethylpyrazine ; 2-Acetyl-35-dimethylpyrazine ; 5H-Cyclopentapyrazine	M+H ; M+H ; M+H ; M+H ; M+H ; M+H ; M+H ; M+H ; M+H ; M+H ; M+H ; M+H ; M+H ; M+H ; M+H ; M+H ; M+2H ; M+2H ; M+2H ; M+2H ; M+2H ; M+2H ; M+2H ; M+2H ; 2M+3H ; M+2H+Na ; 2M+3H ; M+H+NH4 ; M+2ACN+2H ; 2M+3H ; 2M+3H ; 2M+3H ; M+2ACN+2H	C5H8O2 ; C5H8O2 ; C5H8O2 ; C5H8O2 ; C5H8O2 ; C5H8O2 ; C5H8O2 ; C5H8O2 ; C5H8O2 ; C5H8O2 ; C5H8O2 ; C5H8O2 ; C5H8O2 ; C5H8O2 ; C5H8O2 ; C5H8O2 ; C10H16O4 ; C10H16O4 ; C10H16O4 ; C10H16O4 ; C10H16O4 ; C10H16O4 ; C10H16O4 ; C11H12N4 ; C8H10N2O ; C19H22N2 ; C8H10N2O ; C11H9N3 ; C7H6N2 ; C8H10N2O ; C8H10N2O ; C8H10N2O ; C7H6N2
246.14472	1.423378	1.434031	-3.6932	0.004155	2.3814	0.027999	246.1447	HMDB28734 ; HMDB28735 ; HMDB28810 ; HMDB28902 ; HMDB28924 ; HMDB29125 ; HMDB29141 ; HMDB29162 ; HMDB60118 ; HMDB60119 ; HMDB60120 ; HMDB60121 ; HMDB15508 ; HMDB29828 ; HMDB29875 ; HMDB30268 ; HMDB38343 ; HMDB39434	Asparaginyl-Isoleucine ; Asparaginyl-Leucine ; Glutaminyl-Valine ; Isoleucyl-Asparagine ; Leucyl-Asparagine ; Valyl-Glutamine ; Valyl-Gamma-glutamate ; Gamma-glutamyl-Valine ; 12-Hydroxy-13-O-D-glucuronoside-octadec-9Z-enoate ; 12-O-D-Glucuronoside-13-hydroxyoctadec-9Z-enoate ; 10-Hydroxy-octadec-12Z-enoate-9-beta-D-glucuronide ; 9-Hydroxy-10-O-D-glucuronoside-12Z-octadecenoate ; Fencamfamine ; Dihydrodioscorine ; Tigloidine ; Dumetorine ; 13-Demethylspirolide C ; Spirolide A	M+H ; M+H ; M+H ; M+H ; M+H ; M+H ; M+H ; M+H ; M+H ; M+2H ; M+2H ; M+2H ; M+2H ; 2M+Na+K ; M+Na ; M+Na ; M+Na ; M+H+2Na ; M+H+2Na	C10H19N3O4 ; C10H19N3O4 ; C10H19N3O4 ; C10H19N3O4 ; C10H19N3O4 ; C10H19N3O4 ; C10H19N3O4 ; C10H19N3O4 ; C24H42O10 ; C24H42O10 ; C24H42O10 ; C24H42O10 ; C15H21N ; C13H21NO2 ; C13H21NO2 ; C13H21NO2 ; C42H61NO7 ; C42H61NO7

239.70580	1.382199	0.314189	-3.6895	0.00418	2.3788	0.028131	239.7058	HMDB33274 ; HMDB37601 ; HMDB39312 ; HMDB41947	Guavin B ; 6-Malonylapiin ; 345-Trimethoxyphenyl 26-digalloylglucoside ; N1N8-Diacetylspermidine	M+2H+Na ; M+3Na ; M+3Na ; 2M+H+NH4	C33H26O17 ; C29H30O17 ; C29H30O17 ; C11H24N3O2
312.54575	1.405315	0.787435	-3.6824	0.004229	2.3737	0.028424	312.5458	HMDB01253 ; HMDB01430 ; HMDB11622 ; HMDB15057	56-Dihydroxyindole-2-carboxylic acid ; L-Dopachrome ; D-Dopachrome ; Buprenorphine	3M+2Na ; 3M+2Na ; 3M+2Na ; 2M+3H	C9H7NO4 ; C9H7NO4 ; C9H7NO4 ; C29H41NO4
468.07088	1.465939	1.188562	-3.6743	0.004286	2.3679	0.028767	468.0709	HMDB01570 ; HMDB07297 ; HMDB07326 ; HMDB07354 ; HMDB07379 ; HMDB07436 ; HMDB07464 ; HMDB07493 ; HMDB07521 ; HMDB07550 ; HMDB07605 ; HMDB07606 ; HMDB07632 ; HMDB07633 ; HMDB07660 ; HMDB07687 ; HMDB07802 ; HMDB07829 ; HMDB07830 ; HMDB14958 ; HMDB29498 ; HMDB31899 ; HMDB39718 ; HMDB41272 ; HMDB56079 ; HMDB56101 ; HMDB56107 ; HMDB56129 ; HMDB56235 ; HMDB56256 ; HMDB56269 ; HMDB56276 ; HMDB59998	Thymidine 35-cyclic monophosphate ; DG(18:3(6Z9Z12Z)24:1(15Z)0:0) ; DG(18:3(9Z12Z15Z)24:1(15Z)0:0) ; DG(18:4(6Z9Z12Z15Z)24:00:0) ; DG(20:022:4(7Z10Z13Z16Z)0:0) ; DG(20:2(11Z14Z)22:2(13Z16Z)0:0) ; DG(20:3(5Z8Z11Z)22:1(13Z)0:0) ; DG(20:3(8Z11Z14Z)22:1(13Z)0:0) ; DG(20:4(5Z8Z11Z14Z)22:00:0) ; DG(20:4(8Z11Z14Z17Z)22:00:0) ; DG(22:020:4(5Z8Z11Z14Z)0:0) ; DG(22:020:4(8Z11Z14Z17Z)0:0) ; DG(22:1(13Z)20:3(5Z8Z11Z)0:0) ; DG(22:1(13Z)20:3(8Z11Z14Z)0:0) ; DG(22:2(13Z16Z)20:2(11Z14Z)0:0) ; DG(22:4(7Z10Z13Z16Z)20:00:0) ; DG(24:018:4(6Z9Z12Z15Z)0:0) ; DG(24:1(15Z)18:3(6Z9Z12Z)0:0) ; DG(24:1(15Z)18:3(9Z12Z15Z)0:0) ; Tadalafil ; 3-Methoxyfukiic acid ; Theacitrin C ; Samarangenin B ; Uralenneoside ; DG(20:00:022:4n6) ; DG(22:00:020:4n6) ; DG(22:00:020:4n3) ; DG(24:00:018:4n3) ; DG(20:3n90:022:1n9) ; DG(22:1n90:020:3n6) ; DG(24:1n90:018:3n6) ; DG(24:1n90:018:3n3) ; Diphenol glucuronide	3M+H+Na ; 2M+3H ; 2M+3H ; 2M+3H ; 2M+3H ; 2M+3H ; 2M+3H ; 2M+3H ; 2M+3H ; 2M+3H ; 2M+3H ; 2M+3H ; 2M+3H ; 2M+3H ; 2M+3H ; 2M+3H ; 2M+3H ; 2M+3H ; 2M+3H ; M+2K+H ; 3M+2K ; M+H+Na ; M+H+Na ; 3M+2K ; 2M+3H ; 2M+3H ; 2M+3H ; 2M+3H ; 2M+3H ; 2M+3H ; 2M+3H ; 2M+3H ; 3M+2K	C10H13N2O7P ; C45H80O5 ; C45H80O5 ; C45H80O5 ; C45H80O5 ; C45H80O5 ; C45H80O5 ; C45H80O5 ; C45H80O5 ; C45H80O5 ; C45H80O5 ; C45H80O5 ; C45H80O5 ; C45H80O5 ; C45H80O5 ; C45H80O5 ; C45H80O5 ; C45H80O5 ; C45H80O5 ; C22H19N3O4 ; C12H14O8 ; C44H32O22 ; C44H32O22 ; C12H14O8 ; C45H80O5 ; C45H80O5 ; C45H80O5 ; C45H80O5 ; C45H80O5 ; C45H80O5 ; C45H80O5 ; C45H80O5 ; C12H14O8
217.19492	1.499795	1.250403	3.6731	0.004295	2.3671	0.028785	217.1949	HMDB36578 ; HMDB00256 ; HMDB07065 ; HMDB07117 ; HMDB07145 ; HMDB07167 ; HMDB07195 ; HMDB07224 ; HMDB07252 ; HMDB07364 ; HMDB07391 ; HMDB07392 ; HMDB07419 ; HMDB07621 ; HMDB07649 ; HMDB07821 ; HMDB34731 ; HMDB56027 ; HMDB56141 ; HMDB56161 ; HMDB56179 ; HMDB56198 ; HMDB40179	Cyperotundone ; Squalene ; DG(14:1(9Z)24:1(15Z)0:0) ; DG(16:022:2(13Z16Z)0:0) ; DG(16:1(9Z)22:1(13Z)0:0) ; DG(18:020:2(11Z14Z)0:0) ; DG(18:1(11Z)20:1(11Z)0:0) ; DG(18:1(9Z)20:1(11Z)0:0) ; DG(18:2(9Z12Z)20:00:0) ; DG(20:018:2(9Z12Z)0:0) ; DG(20:1(11Z)18:1(11Z)0:0) ; DG(20:1(11Z)18:1(9Z)0:0) ; DG(20:2(11Z14Z)18:00:0) ; DG(22:1(13Z)16:1(9Z)0:0) ; DG(22:2(13Z16Z)16:00:0) ; DG(24:1(15Z)14:1(9Z)0:0) ; Tomatidine ; DG(16:00:022:2n6) ; DG(14:1n50:024:1n9) ; DG(16:1n70:022:1n9) ; DG(18:1n70:020:1n9) ; DG(18:1n90:020:1n9) ; 26-Di-tert-butyl-4-ethylphenol	M+H ; M+H+Na ; M+3H ; M+3H ; M+3H ; M+3H ; M+3H ; M+3H ; M+3H ; M+3H ; M+3H ; M+3H ; M+3H ; M+3H ; M+3H ; M+3H ; M+H+NH4 ; M+3H ; M+3H ; M+3H ; M+3H ; M+3H ; M+H-H2O	C16H24 ; C30H50 ; C41H76O5 ; C41H76O5 ; C41H76O5 ; C41H76O5 ; C41H76O5 ; C41H76O5 ; C41H76O5 ; C41H76O5 ; C41H76O5 ; C41H76O5 ; C41H76O5 ; C41H76O5 ; C41H76O5 ; C41H76O5 ; C27H45NO2 ; C41H76O5 ; C41H76O5 ; C41H76O5 ; C41H76O5 ; C41H76O5 ; C16H26O
288.91811	1.455034	0.526554	-3.6722	0.004302	2.3664	0.028793	NA	NA	NA	NA	NA
167.04438	1.478863	1.43197	-3.6691	0.004324	2.3642	0.028901	167.0444	HMDB15661 ; HMDB34051 ; HMDB38728 ; HMDB38912 ; HMDB39286 ; HMDB39322 ; HMDB39366 ; HMDB39625 ; HMDB41593 ; HMDB00594 ; HMDB01894 ; HMDB15028 ; HMDB28774 ; HMDB28816 ; HMDB28875 ; HMDB29106 ; HMDB29291 ; HMDB29306 ; HMDB29562 ; HMDB30540 ; HMDB31865 ; HMDB32074 ; HMDB32076 ; HMDB32148 ; HMDB32150 ; HMDB32151 ; HMDB32619 ; HMDB33944 ; HMDB34881 ; HMDB34954 ; HMDB35974 ; HMDB37450 ; HMDB37568 ; HMDB38458 ; HMDB38514 ; HMDB38570 ; HMDB38686 ; HMDB41515 ; HMDB41609 ; HMDB59873 ; HMDB60708	Fospropofol ; 6-Galloylglucose ; beta-Glucogallin ; 4-Glucosyl gallate ; 1-O-Galloylfructose ; 3-Glucogallic acid ; 5-O-Galloylhamamelofuranose ; 2-Galloylglucose ; 4-Glucogallic acid ; Glutamylphenylalanine ; Aspartame ; Sulfapyridine ; Cysteinyl-Glutamate ; Glutamyl-Cysteine ; Hydroxyprolyl-Tyrosine ; Tyrosyl-Hydroxyproline ; 34-Dicaffeoyl-15-quinolactone ; 4-Ethylphenol ; N-gamma-L-Glutamyl-L-phenylalanine ; 25-Dimethylphenol ; 4-tert-Butyl-26-dimethyl-35-dinitroacetophenone ; 1-Methoxy-2-methylbenzene ; 1-Methoxy-4-methylbenzene ; 23-Dimethylphenol ; 26-Dimethylphenol ; 34-Dimethylphenol ; 1-Phenylethanol ; 2-Phenylethanol ; 3-Carboxy-2349-tetrahydro-1H-pyrido34-bindole-1-propanoic acid ; Zizybeoside I ; (S)-Spirobrassinin ; Orientin 73-dimethyl ether ; 6-beta-D-Glucopyranosyl-45-dihydroxy-37-dimethoxyflavone ; NN-Bis(gamma-glutamyl)cystine ; L-gamma-Glutamyl-beta-phenyl-beta-L-alanine ; Cyclobrassinin sulfoxide ; Armillaricin ; Benzyl gentiobioside ; 4-Methylbenzyl alcohol ; 3-Ethylphenol ; 15beta-hydroxycyproterone acetate	M+2H ; M+2H ; M+2H ; M+2H ; M+2H ; M+2H ; M+2H ; M+2H ; M+2H ; M+H+K ; M+H+K ; 2M+3H ; 2M+3H ; 2M+3H ; M+H+K ; M+H+K ; M+3H ; M+2Na-H ; M+H+K ; M+2Na-H ; M+H+K ; M+2Na-H ; M+2Na-H ; M+2Na-H ; M+2Na-H ; M+2Na-H ; M+2Na-H ; M+2Na-H ; M+2Na ; M+3Na ; M+2ACN+2H ; M+2H+Na ; M+2H+Na ; M+3H ; M+H+K ; M+2ACN+2H ; M+3Na ; M+3Na ; M+2Na-H ; M+2Na-H ; M+3Na	C13H19Na2O5P ; C13H16O10 ; C13H16O10 ; C13H16O10 ; C13H16O10 ; C13H16O10 ; C13H16O10 ; C13H16O10 ; C13H16O10 ; C14H18N2O5 ; C14H18N2O5 ; C11H11N3O2S ; C8H13N2O5S ; C8H13N2O5S ; C14H18N2O5 ; C14H18N2O5 ; C25H22O11 ; C8H10O ; C14H18N2O5 ; C8H10O ; C14H18N2O5 ; C8H10O ; C8H10O ; C8H10O ; C8H10O ; C8H10O ; C8H10O ; C8H10O ; C15H16N2O4 ; C19H28O11 ; C11H10N2OS2 ; C23H24O11 ; C23H24O11 ; C16H26N4O10S2 ; C14H18N2O5 ; C11H10N2OS2 ; C24H29ClO5 ; C19H28O11 ; C8H10O ; C8H10O ; C24H29ClO5
140.00196	1.41553	0.622758	3.6656	0.004349	2.3616	0.029032	140.002	HMDB14952 ; HMDB34007 ; HMDB38463 ; HMDB42002	Meloxicam ; (EE)-1-Chloro-311-tridecadiene-579-triyn-2-ol ; 3-(Isothiocyanatomethyl)-1-methoxy-1H-indole ; Pyrene	M+3Na ; M+Na+K ; M+Na+K ; M+2K	C14H13N3O4S2 ; C13H11ClO ; C11H10N2OS ; C16H10
218.91962	1.463954	1.503436	3.6646	0.004356	2.3609	0.029041	NA	NA	NA	NA	NA
560.31116	1.383697	0.208917	-3.6617	0.004377	2.3588	0.029137	560.3112	HMDB02815 ; HMDB10385 ; HMDB11654 ; HMDB31919	LysoPC(18:1(9Z)) ; LysoPC(18:1(11Z)) ; 2-(3-Carboxy-3-(methylammonio)propyl)-L-histidine ; Chaetoglobosin N	M+K ; M+K ; 2M+NH4 ; M+NH4	C26H52NO7P ; C26H52NO7P ; C11H19N4O4 ; C33H38N2O5
169.02682	1.427283	1.307754	3.661	0.004382	2.3583	0.029137	169.0268	HMDB05797 ; HMDB01218 ; HMDB02359 ; HMDB32938 ; HMDB33183 ; HMDB39404 ; HMDB39405 ; HMDB41238 ; HMDB60627 ; HMDB60835	Peonidin ; Coumarin ; Phenylpropiolic acid ; Chromone ; 2-Benzofurancarboxaldehyde ; 3-Hydroxy-9-(4 hydroxyphenyl)-1H3H-naphtho18-cdpyran-1-one ; 3-Hydroxy-4-(4-hydroxyphenyl)-1H3H-naphtho18-cdpyran-1-one ; Loquatoside ; Malaoxon ; N-Desmethyl vandetanib	M+2H ; M+Na ; M+Na ; M+Na ; M+Na ; M+2Na ; M+2Na ; M+3Na ; M+H+Na ; M+H+2Na	C16H13ClO6 ; C9H6O2 ; C9H6O2 ; C9H6O2 ; C9H6O2 ; C18H12O4 ; C18H12O4 ; C20H22O11 ; C10H19O7PS ; C21H22BrFN4O2
408.62590	1.395897	1.489738	-3.6584	0.004401	2.3564	0.029223	408.6259	HMDB60566	Carbamazepine-O-quinone	3M+H+NH4	C15H10N2O3
106.10739	1.424943	1.376243	3.6538	0.004434	2.3532	0.029389	106.1074	HMDB32525 ; HMDB32526	Tetradec-2-enal ; (Z)-8-Tetradecenal	M+2H ; M+2H	C14H26O ; C14H26O
209.07278	1.462176	1.433792	3.6533	0.004438	2.3528	0.029389	209.0728	HMDB34157 ; HMDB05006 ; HMDB30359 ; HMDB39952 ; HMDB06005 ; HMDB40734	2-Hydroxybenzaldehyde O-xylosyl-(1-6)-glucoside ; Atorvastatin ; 1419-Dihydroaspidospermatine ; 1-(3-Methyl-2-butenoyl)-6-apiosylglucose ; Indolylacryloylglycine ; Haematopodin	M+2H ; M+3Na ; M+2K ; M+H+Na ; M+H-2H2O ; M+H-2H2O	C18H24O11 ; C33H35FN2O5 ; C21H28N2O2 ; C16H26O11 ; C13H12N2O3 ; C13H12N2O3
544.10714	1.405862	1.169985	-3.6525	0.004444	2.3523	0.029389	544.1071	HMDB15417 ; HMDB41088	Fosphenytoin ; (E)-Squamosamide	3M+2H ; M+2K+H	C16H15N2O6P ; C26H27NO7

679.35075	1.467563	1.059154	-3.6471	0.004483	2.3484	0.029598	679.3508	HMDB03020 ; HMDB03101 ; HMDB29656 ; HMDB33260 ; HMDB34977 ; HMDB34978 ; HMDB35320 ; HMDB35891 ; HMDB36588 ; HMDB36873 ; HMDB36876 ; HMDB36912 ; HMDB36913 ; HMDB36924 ; HMDB38106 ; HMDB38565 ; HMDB39023 ; HMDB39553 ; HMDB39597 ; HMDB40291 ; HMDB40390 ; HMDB41581 ; HMDB42025	Neoxanthin ; Violaxanthin ; Erinacine E ; Capsanthin 36-epoxide ; Karpoxanthin ; 6-Epikarpoxanthin ; Cucurbitaxanthin B ; Oryzanol A ; Capsanthin 56-epoxide ; Heteroxanthin ; Auroxanthin ; Salmoxanthin ; Capsochrome ; Capsorubin ; Cycloviolaxanthin ; 3-N-Acetyl-4-O-(14-methylheptadecanoyl)fusarochromanone ; beta-Carotenone ; Lyciumoside III ; Colupdox a ; Erinacine A ; Erinacine B ; Cucurbitachrome 1 ; Terodiline hydrochloride	M+2K+H ; M+2K+H ; 3M+Na+K ; M+2K+H ; M+2K-H ; M+2K-H ; M+2K+H ; M+2K-H ; M+2K+H ; M+2K+H ; M+2K+H ; M+2K+H ; M+2K+H ; M+2K+H ; M+2K+H ; M+2K+H ; M+2K+H ; 2M+Na+K ; 3M+Na+K ; 3M+Na+K ; 3M+Na+K ; M+2K+H ; 2M+2Na-H	C40H56O4 ; C40H56O4 ; C25H36O6 ; C40H56O4 ; C40H58O4 ; C40H58O4 ; C40H56O4 ; C40H58O4 ; C40H56O4 ; C40H56O4 ; C40H56O4 ; C40H56O4 ; C40H56O4 ; C40H56O4 ; C40H56O4 ; C35H56N2O6 ; C40H56O4 ; C32H56O13 ; C25H36O6 ; C25H36O6 ; C25H36O6 ; C40H56O4 ; C20H28ClN
334.89915	1.442739	0.135249	3.6467	0.004487	2.348	0.029598	334.8892	HMDB15275	Dichlorphenamide	2M+Na+K	C6H6Cl2N2O4S2
168.10191	1.432086	1.159602	3.6408	0.004531	2.3438	0.029846	168.1019	HMDB00022 ; HMDB02182 ; HMDB04826 ; HMDB12162 ; HMDB14748 ; HMDB15165 ; HMDB32020 ; HMDB38869 ; HMDB39837 ; HMDB41888 ; HMDB60592 ; HMDB60807 ; HMDB00031 ; HMDB00365 ; HMDB00490 ; HMDB00546 ; HMDB00764 ; HMDB00995 ; HMDB02097 ; HMDB02222 ; HMDB02237 ; HMDB02961 ; HMDB03654 ; HMDB03818 ; HMDB04626 ; HMDB04667 ; HMDB04670 ; HMDB04701 ; HMDB04702 ; HMDB05030 ; HMDB05849 ; HMDB06770 ; HMDB10223 ; HMDB11475 ; HMDB11476 ; HMDB11487 ; HMDB11488 ; HMDB11505 ; HMDB11506 ; HMDB11517 ; HMDB11518 ; HMDB11743 ; HMDB13744 ; HMDB14343 ; HMDB14511 ; HMDB14522 ; HMDB15234 ; HMDB15348 ; HMDB15398 ; HMDB29746 ; HMDB29796 ; HMDB29978 ; HMDB29998 ; HMDB31310 ; HMDB32073 ; HMDB32075 ; [illegible]	3-Methoxytyramine ; Phenylephrine ; p-Synephrine ; 4-Methoxytyramine ; Metaraminol ; Ethinamate ; 4-(beta-Methylaminoethyl)catechol ; Norpandamarilactonine A ; 2-(2-Furanyl)-3-piperidinol ; Epinine ; 4-Hydroxynorephedrine ; a-Methyldopamine ; Androsterone ; Epiandrosterone ; Etiocholanolone ; Epietiocholanolone ; Hydrocinnamic acid ; 16-Dehydroprogesterone ; 4-Ethylbenzoic acid ; 3-Methylphenylacetic acid ; 34-Dimethylbenzoic acid ; Dihydrotestosterone ; 4-Coumaryl alcohol ; 5-Androstenediol ; Tetrahydrogestrinone ; 13S-hydroxyoctadecadienoic acid ; Alpha-dimorphecolic acid ; 910-Epoxyoctadecenoic acid ; 1213-EpOME ; Fexofenadine ; 4-Androstenediol ; 5b-Dihydrotestosterone ; 9-HODE ; LysoPE(0:018:1(11Z)) ; LysoPE(0:018:1(9Z)) ; LysoPE(0:020:4(5Z8Z11Z14Z)) ; LysoPE(0:020:4(8Z11Z14Z17Z)) ; LysoPE(18:1(11Z)0:0) ; LysoPE(18:1(9Z)0:0) ; LysoPE(20:4(5Z8Z11Z14Z)0:0) ; LysoPE(20:4(8Z11Z14Z17Z)0:0) ; 2-Phenylpropionate ; 2-Methoxy-4-vinylphenol ; Oseltamivir ; Levonorgestrel ; Dydrogesterone ; Arbutamine ; Anastrozole ; Varenicline ; 3-Amino-14-dimethyl-5H-pyrido43-bindole ; (Z)-13-Oxo-9-octadecenoic acid ; Avenoleic acid ; 12-Hydroxy-810-octadecadienoic acid ; Benzyl acetate ; o-Tolyl acetate ; p-Tolyl acetate ; 2-Methoxyacetophenone ; 4-Methoxyacetophenone ; 2-Hydroxy-5-methylacetophenone ; Methyl phenylacetate ; Ethyl benzoate ; 4-Ethoxybenzaldehyde ; Pandamarilactonine A ; Pandanamine ; 4-Methylphenyl dodecanoate ; 1-Phenylethyl formate ; 2-Ethyl-4-(2-furanyl)-2-propenal ; Pandamarilactone 1 ; 2-Phenylethyl formate ; 2-(2-Furanyl)-3-methyl-2-butenal ; Cocaethylene ; 4-Hydroxy-3-methylacetophenone ; Ethisterone ; alpha-oxycodol ; beta-oxycodol ; L-Acetylcarnitine	M+H ; M+H ; M+H ; M+H ; M+H ; M+H ; M+H ; M+H ; M+H ; M+H ; M+H ; M+H ; M+2Na ; M+2Na ; M+2Na ; M+2Na ; M+NH4 ; M+H+Na ; M+NH4 ; M+NH4 ; M+NH4 ; M+2Na ; M+NH4 ; M+2Na ; M+H+Na ; M+H+K ; M+H+K ; M+H+K ; M+H+K ; M+3H ; M+2Na ; M+2Na ; M+H+K ; M+2H+Na ; M+2H+Na ; M+3H ; M+3H ; M+2H+Na ; M+2H+Na ; M+3H ; M+3H ; M+NH4 ; M+NH4 ; M+H+Na ; M+H+Na ; M+H+Na ; M+H+NH4 ; M+ACN+2H ; M+3ACN+2H ; M+3ACN+2H ; M+H+K ; M+H+K ; M+H+K ; M+NH4 ; M+NH4 ; M+NH4 ; M+NH4 ; M+NH4 ; M+NH4 ; M+NH4 ; M+NH4 ; M+NH4 ; M+H+NH4 ; M+H+NH4 ; M+2Na ; M+NH4 ; M+NH4 ; M+H+NH4 ; M+NH4 ; M+NH4 ; M+H+NH4 ; M+NH4 ; M+H+Na ; M+H+NH4 ; M+H+NH4 ; M+H-2H2O	C9H13NO2 ; C9H13NO2 ; C9H13NO2 ; C9H13NO2 ; C9H13NO2 ; C9H13NO2 ; C9H13NO2 ; C9H13NO2 ; C9H13NO2 ; C9H13NO2 ; C9H13NO2 ; C9H13NO2 ; C19H30O2 ; C19H30O2 ; C19H30O2 ; C19H30O2 ; C9H10O2 ; C21H28O2 ; C9H10O2 ; C9H10O2 ; C9H10O2 ; C19H30O2 ; C9H10O2 ; C19H30O2 ; C21H28O2 ; C18H32O3 ; C18H32O3 ; C18H32O3 ; C18H32O3 ; C32H39NO4 ; C19H30O2 ; C19H30O2 ; C18H32O3 ; C23H46NO7P ; C23H46NO7P ; C25H44NO7P ; C25H44NO7P ; C23H46NO7P ; C23H46NO7P ; C25H44NO7P ; C25H44NO7P ; C9H10O2 ; C9H10O2 ; C16H28N2O4 ; C21H28O2 ; C21H28O2 ; C18H23NO4 ; C17H19N5 ; C13H13N3 ; C13H13N3 ; C18H32O3 ; C18H32O3 ; C18H32O3 ; C9H10O2 ; C9H10O2 ; C9H10O2 ; C9H10O2 ; C9H10O2 ; C9H10O2 ; C9H10O2 ; C9H10O2 ; C9H10O2 ; C18H23NO4 ; C18H23NO4 ; C19H30O2 ; C9H10O2 ; C9H10O2 ; C18H23NO4 ; C9H10O2 ; C9H10O2 ; C18H23NO4 ; C9H10O2 ; C21H28O2 ; C18H23NO4 ; C18H23NO4 ; C9H17NO4
103.05838	1.396269	0.024349	3.6394	0.004541	2.3428	0.029878	103.0584	HMDB31529 ; HMDB38892 ; HMDB39771 ; HMDB40347 ; HMDB01406 ; HMDB15496 ; HMDB29765 ; HMDB31861 ; HMDB32347 ; HMDB33529 ; HMDB34455 ; HMDB36426 ; HMDB37190 ; HMDB37211 ; HMDB37229 ; HMDB38954 ; HMDB39715 ; HMDB40173 ; HMDB40997	3-Methyl-2-butene-1-thiol ; 1-(Methylthio)-1-butene ; Cyclopentanethiol ; 2-Methyl-1-propenethiol ; Niacinamide ; Amrinone ; 9-Pentadecenoic acid ; 2-Acetylpyrazine ; Isobutyl 10-undecenoate ; ()-23-Dihydro-3-methyl-1H-pyrrole ; Exaltolide ; Butyl undecylenate ; Rhodinyl isovalerate ; Menthyl isovalerate ; Citronellyl pentanoate ; Citronellyl isovalerate ; Auberganol ; Pentanenitrile ; 155-Farnesanolide	M+H ; M+H ; M+H ; M+H ; M+2ACN+2H ; M+H+NH4 ; M+3Na ; M+2ACN+2H ; M+3Na ; 2M+H+K ; M+3Na ; M+3Na ; M+3Na ; M+3Na ; M+3Na ; M+3Na ; M+3Na ; 2M+H+K ; M+3Na	C5H10S ; C5H10S ; C5H10S ; C5H10S ; C6H6N2O ; C10H9N3O ; C15H28O2 ; C6H6N2O ; C15H28O2 ; C5H9N ; C15H28O2 ; C15H28O2 ; C15H28O2 ; C15H28O2 ; C15H28O2 ; C15H28O2 ; C15H28O2 ; C5H9N ; C15H28O2
163.08391	1.427185	1.297722	-3.6363	0.004565	2.3406	0.02999	163.0839	HMDB00600 ; HMDB14716 ; HMDB11749 ; HMDB28707 ; HMDB28723 ; HMDB28791 ; HMDB28799 ; HMDB28883 ; HMDB28894 ; HMDB28899 ; HMDB29041 ; HMDB29143 ; HMDB29151 ; HMDB29953 ; HMDB30155 ; HMDB31094 ; HMDB34587 ; HMDB40248 ; HMDB40249 ; HMDB40518 ; HMDB59780 ; HMDB60309	Galactosylhydroxylysine ; Valaciclovir ; 2-Piperidinone ; Arginyl-Glutamine ; Arginyl-Gamma-glutamate ; Glutaminyl-Arginine ; Glutaminyl-Histidine ; Histidinyl-Glutamine ; Histidinyl-Serine ; Histidinyl-Gamma-glutamate ; Serinyl-Histidine ; Gamma-glutamyl-Arginine ; Gamma-glutamyl-Histidine ; D-erythro-D-galacto-octitol ; Austalide I ; Glycerol tributanoate ; 1-Pyrrolidinecarboxaldehyde ; (SS)-Nt-Histidinylalanine ; (SS)-Np-Histidinylalanine ; 25-Dihydro-24-dimethyloxazole ; 1N2-propanodeoxyguanosine ; (2R)-2-Hydroxy-2-methylbutanenitrile	M+2H ; M+2H ; M+ACN+Na ; M+H+Na ; M+H+Na ; M+H+Na ; M+ACN+2H ; M+ACN+2H ; M+2ACN+2H ; M+ACN+2H ; M+2ACN+2H ; M+H+Na ; M+ACN+2H ; M+2ACN+2H ; M+3H ; M+H+Na ; M+ACN+Na ; M+2ACN+2H ; M+2ACN+2H ; M+ACN+Na ; M+H+NH4 ; M+ACN+Na	C12H24N2O8 ; C13H20N6O4 ; C5H9NO ; C11H22N6O4 ; C11H22N6O4 ; C11H22N6O4 ; C11H17N5O4 ; C11H17N5O4 ; C9H14N4O4 ; C11H17N5O4 ; C9H14N4O4 ; C11H22N6O4 ; C11H17N5O4 ; C8H18O8 ; C27H34O8 ; C15H26O6 ; C5H9NO ; C9H14N4O4 ; C9H14N4O4 ; C5H9NO ; C13H17N5O4 ; C5H9NO
257.26669	1.49149	0.92813	3.6347	0.004577	2.3394	0.030033	NA	NA	NA	NA	NA
106.00387	1.402775	0.277456	3.6333	0.004587	2.3385	0.03006	106.0039	HMDB03162 ; HMDB13141 ; HMDB29578 ; HMDB31161 ; HMDB31472 ; HMDB31709 ; HMDB32180 ; HMDB32408 ; HMDB33875 ; HMDB33922 ; HMDB40056 ; HMDB60279 ; HMDB60412	7-Methylhypoxanthine ; 1-Methylhypoxanthine ; Diisopropyl disulfide ; Methyl pentyl disulfide ; Dipropyl disulfide ; 16-Hexanedithiol ; Butyl ethyl disulfide ; Methyl isopentyl disulfide ; 23-Dihydrothiophene ; Divinyl sulfide ; xi-1-(Propylthio)-1-propanethiol ; Aminochrome o-semiquinone ; 6-Methylmercaptopurine	M+Na+K ; M+Na+K ; M+Na+K ; M+Na+K ; M+Na+K ; M+Na+K ; M+Na+K ; M+Na+K ; 2M+H+K ; 2M+H+K ; M+Na+K ; M+Na+K ; M+2Na	C6H6N4O ; C6H6N4O ; C6H14S2 ; C6H14S2 ; C6H14S2 ; C6H14S2 ; C6H14S2 ; C6H14S2 ; C4H6S ; C4H6S ; C6H14S2 ; C8H8NO2 ; C6H6N4S
185.95972	1.406561	0.771639	3.6316	0.0046	2.3372	0.030107	NA	NA	NA	NA	NA
482.55861	1.384364	1.420475	-3.6212	0.00468	2.3297	0.03059	NA	NA	NA	NA	NA

100.03930	1.401901	0.04428	3.6165	0.004717	2.3263	0.030789	100.0393	HMDB30276 ; HMDB15188 ; HMDB00108 ; HMDB00134 ; HMDB00176 ; HMDB00486 ; HMDB00711 ; HMDB01241 ; HMDB01954 ; HMDB02264 ; HMDB05766 ; HMDB06954 ; HMDB10722 ; HMDB11738 ; HMDB13898 ; HMDB13899 ; HMDB13900 ; HMDB29699 ; HMDB31302 ; HMDB31509 ; HMDB32270 ; HMDB32314 ; HMDB32367 ; HMDB32442 ; HMDB32827 ; HMDB33345 ; HMDB33733 ; HMDB33834 ; HMDB35135 ; HMDB36177 ; HMDB36239 ; HMDB36905 ; HMDB37142 ; HMDB37145 ; HMDB37276 ; HMDB38054 ; HMDB38268 ; HMDB38697 ; HMDB40285 ; HMDB41329 ; HMDB59825 ; HMDB59867 ; HMDB60285	(R)-Dihydromaleimide ; Mimosine ; Ethanol ; Fumaric acid ; Maleic acid ; 7-Hydroxyoctanoic acid ; Hydroxyoctanoic acid ; 2-Aminomuconic acid ; 3-Hydroxyoctanoic acid ; (R)-2-Hydroxycaprylic acid ; Norophthalmic acid ; 2-Methyl-3-hydroxy-5-formylpyridine-4-carboxylate ; (R)-3-Hydroxyoctanoic acid ; Gamma-Glutamyl Glutamine ; 5-Hydroxyvalproic acid ; 3-Hydroxyvalproic acid ; 4-Hydroxyvalproic acid ; Cinnamyl acetate ; Methyl 2-octynoate ; Ethyl ()-3-hydroxyhexanoate ; (-)-Ethyl 2-hydroxy-3-methylvalerate ; 24-Hexadienyl propionate ; L-Menthone 12-glycerol ketal ; 2-Nonenoic acid gamma-lactone ; Peroxyoctanoic acid ; ()-Ribaline ; 24-Dihydroxy-2H-14-benzoxazin-3(4H)-one ; Ethyl cinnamate ; (E)-26-Dimethyl-25-heptadienoic acid ; 355-Trimethyl-12-cyclohexanedione ; Enol-355-Trimethyl-12-cyclohexanedione ; 2-Propenyl phenylacetate ; 2-(Phenylethenyl)-13-dioxolane ; 3-(4-Methoxyphenyl)-2-methyl-2-propenal ; Octahydro-2H-1-benzopyran-2-one ; (R)-27-Dihydroxy-2H-14-benzoxazin-3(4H)-one ; Allyl hexenoate ; xi-226-Trimethyl-14-cyclohexanedione ; Anapear ; 5-Amino-6-(4-hydroxy-2-butenoyl)-22-dimethyl-4-chromanone ; Isoamyl lactate ; Ethyl 2-hydroxy-4-methylpentanoate ; 4-Oxo-2-nonenal	M+H ; M+2H ; 3M+Na+K ; M+2ACN+2H ; M+2ACN+2H ; M+H+K ; M+H+K ; M+ACN+2H ; M+H+K ; M+H+K ; M+2H+Na ; M+H+NH4 ; M+H+K ; M+2H+Na ; M+H+K ; M+H+K ; M+H+K ; M+H+Na ; M+2Na ; M+H+K ; M+H+K ; M+2Na ; M+3H ; M+2Na ; M+H+K ; M+2H+Na ; M+H+NH4 ; M+H+Na ; M+2Na ; M+2Na ; M+2Na ; M+H+Na ; M+H+Na ; M+H+Na ; M+2Na ; M+H+NH4 ; M+2Na ; M+2Na ; M+2Na ; M+2H+Na ; M+H+K ; M+H+K ; M+2Na	C4H5NO2 ; C8H10N2O4 ; C2H6O ; C4H4O4 ; C4H4O4 ; C8H16O3 ; C8H16O3 ; C6H7NO4 ; C8H16O3 ; C8H16O3 ; C10H17N3O6 ; C8H7NO4 ; C8H16O3 ; C10H17N3O6 ; C8H16O3 ; C8H16O3 ; C8H16O3 ; C11H12O2 ; C9H14O2 ; C8H16O3 ; C8H16O3 ; C9H14O2 ; C16H15N3OS ; C9H14O2 ; C8H16O3 ; C15H17NO4 ; C8H7NO4 ; C11H12O2 ; C9H14O2 ; C9H14O2 ; C9H14O2 ; C11H12O2 ; C11H12O2 ; C11H12O2 ; C9H14O2 ; C8H7NO4 ; C9H14O2 ; C9H14O2 ; C9H14O2 ; C15H17NO4 ; C8H16O3 ; C8H16O3 ; C9H14O2
222.89907	1.464591	1.470796	3.6154	0.004725	2.3256	0.030804	NA	NA	NA	NA	NA
120.10191	1.471083	0.289704	3.6135	0.004741	2.3242	0.030863	120.1019	HMDB00718 ; HMDB00892 ; HMDB02176 ; HMDB30058 ; HMDB31175 ; HMDB31241 ; HMDB31247 ; HMDB31249 ; HMDB31516 ; HMDB32154 ; HMDB32230 ; HMDB32263 ; HMDB32550 ; HMDB33742 ; HMDB33890 ; HMDB34237 ; HMDB39799 ; HMDB40575 ; HMDB41992 ; HMDB59678	Isovaleric acid ; Valeric acid ; Ethylmethylacetic acid ; Ethyl propionate ; Tetrahydro-2-furanmethanol ; Isopropyl acetate ; 2-Methylpropyl formate ; Methyl isobutyrate ; 3-Hydroxy-2-pentanone ; Acetaldehyde hexyl isoamyl acetal ; 24-Dimethyl-13-dioxolane ; Ethylene oxidepropylene oxide copolymer ; Valeraldehyde dibutyl acetal ; (S)-2-Methylbutanoic acid ; Methyl butyrate ; Propyl acetate ; Tetrahydro-2-methyl-3-furanol ; Butyl formate ; Pivalic acid ; 1-Hydroxy-2-pentanone	M+NH4 ; M+NH4 ; M+NH4 ; M+NH4 ; M+NH4 ; M+NH4 ; M+NH4 ; M+NH4 ; M+NH4 ; M+H+Na ; M+NH4 ; M+NH4 ; M+H+Na ; M+NH4 ; M+NH4 ; M+NH4 ; M+NH4 ; M+NH4 ; M+NH4 ; M+NH4	C5H10O2 ; C5H10O2 ; C5H10O2 ; C5H10O2 ; C5H10O2 ; C5H10O2 ; C5H10O2 ; C5H10O2 ; C5H10O2 ; C13H28O2 ; C5H10O2 ; C5H10O2 ; C13H28O2 ; C5H10O2 ; C5H10O2 ; C5H10O2 ; C5H10O2 ; C5H10O2 ; C5H10O2 ; C5H10O2
368.61659	1.458018	1.285108	-3.6111	0.00476	2.3224	0.030921	368.6166	HMDB30189 ; HMDB30384 ; HMDB32855 ; HMDB36989 ; HMDB60836 ; HMDB60838	Isogravacridonechlorine ; Gravacridonechlorine ; N-Chloroacetyl-26-diethylaniline ; Romucosine B ; N-Desmethyl-O-hydroxy rosiglitazone ; N-Desmethyl-p-hydroxyrosiglitazone	2M+H+NH4 ; 2M+H+NH4 ; 3M+Na+K ; 2M+H+NH4 ; 2M+H+NH4 ; 2M+H+NH4	C19H18ClNO4 ; C19H18ClNO4 ; C12H16ClNO ; C19H18ClNO4 ; C17H17N3O4S ; C17H17N3O4S
550.50499	1.477836	1.170131	-3.6108	0.004762	2.3222	0.030921	NA	NA	NA	NA	NA
626.08075	1.403131	1.405184	-3.606	0.0048	2.3188	0.03106	626.0808	HMDB34589 ; HMDB59985 ; HMDB59987 ; HMDB60020	Shoyuflavone B ; 5-(345-Trihydroxyphenyl)-gamma-valerolactone-3-O-sulphate ; 5-(345-Trihydroxyphenyl)-gamma-valerolactone-4-O-sulphate ; Sinapinic acid-O-sulphate	3M+2Na ; 2M+NH4 ; 2M+NH4 ; 2M+NH4	C19H14O10 ; C11H12O8S ; C11H12O8S ; C11H12O8S
405.00880	1.456581	1.088577	-3.606	0.0048	2.3188	0.03106	405.0088	HMDB12468 ; HMDB13991 ; HMDB15099	(-)-Epigallocatechin sulfate ; 5-Hydroxylornoxicam ; Apraclonidine	2M+H+K ; M+NH4 ; 3M+2K	C15H13O10S ; C13H10ClN3O5S2 ; C9H10Cl2N4
240.06523	1.463816	1.413798	-3.6057	0.004802	2.3186	0.03106	240.0652	HMDB29479 ; HMDB30548 ; HMDB30805 ; HMDB33650 ; HMDB36120 ; HMDB37332 ; HMDB37360 ; HMDB39190 ; HMDB39211 ; HMDB39338 ; HMDB40509 ; HMDB40510 ; HMDB41743 ; HMDB41745 ; HMDB35001 ; HMDB60086	Isorhamnetin 7-glucoside ; Nepitrin ; Pedaliin ; Estragonoside ; Alliumoside A ; 8-Hydroxyluteolin 4-methyl ether 8-glucoside ; Azaleatin 3-glucoside ; 2-O-(4-Hydroxycinnamoyl)-1-O-galloyl-beta-D-glucopyranoside ; 1358-Tetrahydroxy-6-methoxy-2-methylanthraquinone 8-O-b-D-glucoside ; Pollenin B ; 6-Methoxyluteolin 3-glucoside ; Eupafolin 4-glucoside ; Hesperetin 3-O-glucuronide ; Hesperetin 7-O-glucuronide ; Melleolide K ; 5-S-Glutathionyl-aminochrome reduced	M+2H ; M+2H ; M+2H ; M+2H ; M+2H ; M+2H ; M+2H ; M+2H ; M+2H ; M+2H ; M+2H ; M+2H ; M+2H ; M+2H ; M+2Na ; M+H+Na	C22H22O12 ; C22H22O12 ; C22H22O12 ; C22H22O12 ; C22H22O12 ; C22H22O12 ; C22H22O12 ; C22H22O12 ; C22H22O12 ; C22H22O12 ; C22H22O12 ; C22H22O12 ; C22H22O12 ; C22H22O12 ; C23H27ClO6 ; C18H24N4O8S
402.82429	1.422672	1.600224	3.6018	0.004834	2.3157	0.031225	402.8243	HMDB07953 ; HMDB08527 ; HMDB08947 ; HMDB09006 ; HMDB09229 ; HMDB09486 ; HMDB09715 ; HMDB14506 ; HMDB15581 ; HMDB30129	PC(15:022:0) ; PC(22:015:0) ; PE(16:024:0) ; PE(18:022:0) ; PE(20:020:0) ; PE(22:018:0) ; PE(24:016:0) ; Anidulafungin ; Iodipamide ; Plastoquinone 8	M+2H ; M+2H ; M+2H ; M+2H ; M+2H ; M+2H ; M+2H ; M+3Na ; M+3Na ; M+3ACN+2H	C45H90NO8P ; C45H90NO8P ; C45H90NO8P ; C45H90NO8P ; C45H90NO8P ; C45H90NO8P ; C45H90NO8P ; C58H73N7O17 ; C20H14I6N2O6 ; C48H72O2
179.14303	1.4145	0.398224	3.6	0.004848	2.3145	0.031269	179.143	HMDB14956 ; HMDB31194 ; HMDB31568 ; HMDB31731 ; HMDB32579 ; HMDB35055 ; HMDB36681 ; HMDB37272 ; HMDB38107 ; HMDB38194 ; HMDB02007 ; HMDB60117 ; HMDB01010 ; HMDB02823 ; HMDB31099 ; HMDB34553	Propofol ; Tricycloekasantal ; 4-Methyl-1-phenyl-2-pentanol ; 3-Methyl-1-phenyl-3-pentanol ; 25-Diisopropylphenol ; 11-Methyl-7-oxatetracyclo6310160411dodecane ; 7-Isopropyl-5-methylbicyclo222oct-5-en-2-one ; 2-Ethoxy-1-methyl-4-(1-methylethyl)benzene ; Quinceoxepine ; Geijerone ; Tetracosahexaenoic acid ; Tetracosahexaenoic acid n-3 ; Nicotine imine ; Docosatrienoic acid ; (all-Z)-71013-Docosatrienoic acid ; (3b24R25x)-26-Benzoyloxystigmast-5-ene-3-ol	M+H ; M+H ; M+H ; M+H ; M+H ; M+H ; M+H ; M+H ; M+H ; M+H ; M+2H ; M+2H ; M+NH4 ; M+H+Na ; M+H+Na ; M+3H	C12H18O ; C12H18O ; C12H18O ; C12H18O ; C12H18O ; C12H18O ; C12H18O ; C12H18O ; C12H18O ; C12H18O ; C24H36O2 ; C24H36O2 ; C10H13N2 ; C22H38O2 ; C22H38O2 ; C36H54O3
512.66125	1.44558	1.548986	3.5994	0.004853	2.314	0.031269	512.6613	HMDB32808	Niazirinin	3M+Na+K	C16H19NO6
454.70227	1.423039	1.600424	3.5948	0.00489	2.3107	0.031465	454.7023	HMDB05037 ; HMDB31806 ; HMDB56289 ; HMDB56294 ; HMDB56314 ; HMDB56319	Sumatriptan ; ()-Naled ; DG(18:2n60:022:5n6) ; DG(18:2n60:022:5n3) ; DG(20:2n60:022:5n6) ; DG(20:2n60:022:5n3)	3M+H+Na ; M+2K-H ; 2M+3H ; 2M+3H ; 2M+3H ; 2M+3H	C14H21N3O2S ; C4H7Br2Cl2O4P ; C44H72O5 ; C44H72O5 ; C44H72O5 ; C44H72O5
222.02279	1.381934	0.253352	-3.5921	0.004912	2.3087	0.031496	222.0228	HMDB02329	Oxalic acid	2M+ACN+H	C2H2O4
403.00986	1.423949	0.031848	-3.592	0.004913	2.3087	0.031496	403.0099	HMDB03974 ; HMDB41777 ; HMDB41778 ; HMDB60418 ; HMDB60792	Oxalosuccinic acid ; Tectorigenin 4-sulfate ; Tectorigenin 7-sulfate ; 6-Thioxanthine 5-monophosphate ; 6-Thioxanthylic acid	2M+Na ; M+Na ; M+Na ; M+Na ; M+Na	C6H6O7 ; C16H12O9S ; C16H12O9S ; C10H13N4O8PS ; C10H13N4O8PS

292.07326	1.36101	0.756125	3.5912	0.004919	2.3081	0.031496	292.0733	HMDB00127 ; HMDB01940 ; HMDB02545 ; HMDB02704 ; HMDB03363 ; HMDB03402 ; HMDB06334 ; HMDB11731 ; HMDB11732 ; HMDB13279 ; HMDB15263 ; HMDB29943 ; HMDB32758 ; HMDB34265 ; HMDB37421 ; HMDB38170 ; HMDB38491 ; HMDB41319	D-Glucuronic acid ; Triamterene ; Galacturonic acid ; Iduronic acid ; Pectic acid ; Pectin ; 3-Dehydro-L-gulonate ; 5-Keto-D-gluconate ; 2-Keto-L-gluconate ; N-Nonanoylglycine ; Proguanil ; Arbutin ; Ethyl 3-methyl-9H-carbazole-9-carboxylate ; Lippioside I ; Apimaysin ; Isolimonic acid ; L-Altruronic acid ; Cinnamyl anthranilate	3M+2H ; M+K ; 3M+2H ; 3M+2H ; 3M+2H ; 3M+2H ; 3M+2H ; 3M+2H ; 3M+2H ; M+2K-H ; M+K ; 2M+H+K ; M+K ; M+2Na ; M+H+Na ; M+2K ; 3M+2H ; M+K	C6H10O7 ; C12H11N7 ; C6H10O7 ; C6H10O7 ; C6H10O7 ; C6H10O7 ; C6H10O7 ; C6H10O7 ; C6H10O7 ; C11H21NO3 ; C11H16ClN5 ; C12H16O7 ; C16H15NO2 ; C25H30O13 ; C27H28O13 ; C26H34O10 ; C6H10O7 ; C16H15NO2
776.55827	1.498126	0.484751	3.5912	0.00492	2.3081	0.031496	776.5583	HMDB34583 ; HMDB07956 ; HMDB07957 ; HMDB08658 ; HMDB08691 ; HMDB09010 ; HMDB09011 ; HMDB09042 ; HMDB09075 ; HMDB09140 ; HMDB09173 ; HMDB09205 ; HMDB09236 ; HMDB09267 ; HMDB09268 ; HMDB09298 ; HMDB09299 ; HMDB09330 ; HMDB09363 ; HMDB09395 ; HMDB09428 ; HMDB09460 ; HMDB09525 ; HMDB09556 ; HMDB09557 ; HMDB09586 ; HMDB09587 ; HMDB09618 ; HMDB09651	Secaloside C ; PC(15:022:5(4Z7Z10Z13Z16Z)) ; PC(15:022:5(7Z10Z13Z16Z19Z)) ; PC(22:5(4Z7Z10Z13Z16Z)15:0) ; PC(22:5(7Z10Z13Z16Z19Z)15:0) ; PE(18:022:5(4Z7Z10Z13Z16Z)) ; PE(18:022:5(7Z10Z13Z16Z19Z)) ; PE(18:1(11Z)22:4(7Z10Z13Z16Z)) ; PE(18:1(9Z)22:4(7Z10Z13Z16Z)) ; PE(18:3(6Z9Z12Z)22:2(13Z16Z)) ; PE(18:3(9Z12Z15Z)22:2(13Z16Z)) ; PE(18:4(6Z9Z12Z15Z)22:1(13Z)) ; PE(20:020:5(5Z8Z11Z14Z17Z)) ; PE(20:1(11Z)20:4(5Z8Z11Z14Z)) ; PE(20:1(11Z)20:4(8Z11Z14Z17Z)) ; PE(20:2(11Z14Z)20:3(5Z8Z11Z)) ; PE(20:2(11Z14Z)20:3(8Z11Z14Z)) ; PE(20:3(5Z8Z11Z)20:2(11Z14Z)) ; PE(20:3(8Z11Z14Z)20:2(11Z14Z)) ; PE(20:4(5Z8Z11Z14Z)20:1(11Z)) ; PE(20:4(8Z11Z14Z17Z)20:1(11Z)) ; PE(20:5(5Z8Z11Z14Z17Z)20:0) ; PE(22:1(13Z)18:4(6Z9Z12Z15Z)) ; PE(22:2(13Z16Z)18:3(6Z9Z12Z)) ; PE(22:2(13Z16Z)18:3(9Z12Z15Z)) ; PE(22:4(7Z10Z13Z16Z)18:1(11Z)) ; PE(22:4(7Z10Z13Z16Z)18:1(9Z)) ; PE(22:5(4Z7Z10Z13Z16Z)18:0) ; PE(22:5(7Z10Z13Z16Z19Z)18:0)	2M+3H ; M+H-H2O	C52H61NO29 ; C45H80NO8P
283.16269	1.385529	1.165271	-3.5882	0.004944	2.306	0.031609	283.1627	HMDB14563 ; HMDB32216	Miglustat ; Diethylenetriamine crosslinked with epichlorohydrin	M+ACN+Na ; 2M+3H2O+2H	C10H21NO4 ; C14H22ClNO
388.71898	1.399092	0.579384	-3.5866	0.004957	2.3048	0.031654	388.719	HMDB15429 ; HMDB36296	Metocurine ; Uzarigenin 3-xylosyl-(1-2)-rhamnoside	M+3ACN+2H ; M+3ACN+2H	C40H48N2O6 ; C34H52O12
414.61799	1.374433	1.373454	-3.5854	0.004967	2.3039	0.031676	414.618	HMDB14827 ; HMDB32911	Cephaloglycin ; Isoorientin 2-feruloyl-(-6)-glucoside	2M+H+NH4 ; M+ACN+2H	C18H19N3O6S ; C37H38O19
288.64084	1.35742	0.210018	-3.5845	0.004974	2.3033	0.031684	288.6408	HMDB28738 ; HMDB28990 ; HMDB40973 ; HMDB41323	Asparaginyl-Phenylalanine ; Phenylalanyl-Asparagine ; 1-Methoxy-1H-indole-3-acetonitrile ; Heteroartonin A	2M+H+NH4 ; 2M+H+NH4 ; 3M+H+NH4 ; M+3ACN+2H	C13H17N3O4 ; C13H17N3O4 ; C11H10N2O ; C26H28O7
677.63715	1.457018	0.930508	-3.5813	0.005001	2.3009	0.031813	677.6372	HMDB31203 ; HMDB35234 ; HMDB35536 ; HMDB35537 ; HMDB37327 ; HMDB37328	N-Heptanoylsolamine ; 1416-Nonacosanedione ; 1012-Nonacosanedione ; 68-Nonacosanedione ; 810-Nonacosanedione ; 1214-Nonacosanedione	2M+Na ; 3M+2Na ; 3M+2Na ; 3M+2Na ; 3M+2Na ; 3M+2Na	C19H41N3O ; C29H56O2 ; C29H56O2 ; C29H56O2 ; C29H56O2 ; C29H56O2
262.16475	1.420938	1.464165	-3.5805	0.005007	2.3004	0.031813	262.1648	HMDB07936 ; HMDB07943 ; HMDB08000 ; HMDB08231 ; HMDB08834 ; HMDB08844 ; HMDB08866 ; HMDB08926 ; HMDB08927 ; HMDB08937 ; HMDB08938 ; HMDB08958 ; HMDB08968 ; HMDB08990 ; HMDB08997 ; HMDB09022 ; HMDB09028 ; HMDB09029 ; HMDB09055 ; HMDB09061 ; HMDB09062 ; HMDB09093 ; HMDB09124 ; HMDB09125 ; HMDB09157 ; HMDB09158 ; HMDB09189 ; HMDB09218 ; HMDB09250 ; HMDB09320 ; HMDB09353 ; HMDB09385 ; HMDB09418 ; HMDB09580 ; HMDB15124 ; HMDB30199 ; HMDB33409 ; HMDB34216 ; HMDB34516 ; HMDB35257 ; HMDB60655 ; HMDB32419 ; HMDB41889	PC(15:016:1(9Z)) ; PC(15:018:4(6Z9Z12Z15Z)) ; PC(16:1(9Z)15:0) ; PC(18:4(6Z9Z12Z15Z)15:0) ; PE(14:020:1(11Z)) ; PE(14:022:4(7Z10Z13Z16Z)) ; PE(14:1(9Z)20:0) ; PE(16:018:1(11Z)) ; PE(16:018:1(9Z)) ; PE(16:020:4(5Z8Z11Z14Z)) ; PE(16:020:4(8Z11Z14Z17Z)) ; PE(16:1(9Z)18:0) ; PE(16:1(9Z)20:3(5Z8Z11Z)) ; PE(18:016:1(9Z)) ; PE(18:018:4(6Z9Z12Z15Z)) ; PE(18:1(11Z)16:0) ; PE(18:1(11Z)18:3(6Z9Z12Z)) ; PE(18:1(11Z)18:3(9Z12Z15Z)) ; PE(18:1(9Z)16:0) ; PE(18:1(9Z)18:3(6Z9Z12Z)) ; PE(18:1(9Z)18:3(9Z12Z15Z)) ; PE(18:2(9Z12Z)18:2(9Z12Z)) ; PE(18:3(6Z9Z12Z)18:1(11Z)) ; PE(18:3(6Z9Z12Z)18:1(9Z)) ; PE(18:3(9Z12Z15Z)18:1(11Z)) ; PE(18:3(9Z12Z15Z)18:1(9Z)) ; PE(18:4(6Z9Z12Z15Z)18:0) ; PE(20:014:1(9Z)) ; PE(20:1(11Z)14:0) ; PE(20:3(5Z8Z11Z)16:1(9Z)) ; PE(20:3(8Z11Z14Z)16:1(9Z)) ; PE(20:4(5Z8Z11Z14Z)16:0) ; PE(20:4(8Z11Z14Z17Z)16:0) ; PE(22:4(7Z10Z13Z16Z)14:0) ; Rivastigmine ; Frangulanine ; 26-Methyl nigranoate ; Adouetine X ; 3b18b-3-Methoxy-11-oxo-12-oleanen-30-oic acid ; Methyl 3b24-dihydroxy-1113(18)-oleanadien-30-oate ; 3-Hydroxylidocaine ; 2-(4-Methyl-5-thiazolyl)ethyl decanoate ; Etamiphylline	M+3Na ; M+H+2Na ; M+3Na ; M+H+2Na ; M+3Na ; M+H+2Na ; M+3Na ; M+3Na ; M+3Na ; M+H+2Na ; M+H+2Na ; M+3Na ; M+H+2Na ; M+3Na ; M+H+2Na ; M+3Na ; M+H+2Na ; M+H+2Na ; M+3Na ; M+H+2Na ; M+H+2Na ; M+H+2Na ; M+H+2Na ; M+H+2Na ; M+H+2Na ; M+H+2Na ; M+H+2Na ; M+3Na ; M+3Na ; M+H+2Na ; M+H+2Na ; M+H+2Na ; M+H+2Na ; M+H+2Na ; 2M+H+Na ; M+H+Na ; M+H+K ; M+H+Na ; M+H+K ; M+H+K ; 2M+H+Na ; M+H-2H2O ; M+H-H2O	C39H76NO8P ; C41H74NO8P ; C39H76NO8P ; C41H74NO8P ; C39H76NO8P ; C41H74NO8P ; C39H76NO8P ; C39H76NO8P ; C39H76NO8P ; C41H74NO8P ; C41H74NO8P ; C39H76NO8P ; C41H74NO8P ; C39H76NO8P ; C41H74NO8P ; C39H76NO8P ; C41H74NO8P ; C41H74NO8P ; C39H76NO8P ; C41H74NO8P ; C41H74NO8P ; C41H74NO8P ; C41H74NO8P ; C41H74NO8P ; C41H74NO8P ; C41H74NO8P ; C41H74NO8P ; C39H76NO8P ; C39H76NO8P ; C41H74NO8P ; C41H74NO8P ; C41H74NO8P ; C41H74NO8P ; C41H74NO8P ; C14H22N2O2 ; C28H44N4O4 ; C31H48O4 ; C28H44N4O4 ; C31H48O4 ; C31H48O4 ; C14H22N2O2 ; C16H27NO2S ; C13H21N5O2
376.02332	1.44578	1.494071	-3.5784	0.005025	2.2988	0.031886	376.0233	HMDB01314 ; HMDB11629 ; HMDB60416 ; HMDB01508	Cyclic GMP ; Guanosine 23-cyclic phosphate ; 6-Thioinosine-5-monophosphate ; dADP	2M+Na+K ; 2M+Na+K ; 2M+H+Na ; M+H-2H2O	C10H12N5O7P ; C10H12N5O7P ; C10H13N4O7PS ; C10H15N5O9P2
211.03297	1.505028	0.106825	-3.5776	0.005032	2.2983	0.031888	211.033	HMDB00375 ; HMDB00563 ; HMDB00748 ; HMDB00779 ; HMDB02072 ; HMDB02199 ; HMDB02229 ; HMDB05175 ; HMDB05788 ; HMDB14933 ; HMDB15201 ; HMDB29256 ; HMDB29483 ; HMDB29665 ; HMDB29817 ; HMDB31132 ; HMDB32030 ; HMDB32138 ; HMDB32573 ; HMDB32605 ; HMDB32639 ; HMDB33752 ; HMDB34243 ; HMDB34370 ; HMDB34993 ; HMDB40645 ; HMDB41611 ; HMDB41683 ; HMDB59969	3-(3-Hydroxyphenyl)propanoic acid ; D-Phenyllactic acid ; L-3-Phenyllactic acid ; Phenyllactic acid ; 4-Methoxyphenylacetic acid ; Desaminotyrosine ; 3-Phenoxypropionic acid ; Homovanillin ; Theaflavin ; Sulfasalazine ; Clonazepam ; Isoneotheaflavin ; Isotheaflavin ; Ethyl vanillin ; Ethyl salicylate ; 34-Dihydroxyphenylacetone ; Guaicyl acetate ; 34-Dimethoxybenzaldehyde ; Ethylparaben ; Methyl 2-methoxybenzoate ; Methyl 4-methoxybenzoate ; 3-(2-Hydroxyphenyl)propanoic acid ; Ipomeanine ; Ibervirin ; 4-Methoxybenzyl formate ; 3-Hydroxy-1-(4-hydroxyphenyl)-1-propanone ; Ethyl 2-furanacrylate ; 4-Hydroxyphenyl-2-propionic acid ; 3-Methoxyphenylacetic acid	M+2Na-H ; M+2Na-H ; M+2Na-H ; M+2Na-H ; M+2Na-H ; M+2Na-H ; M+2Na-H ; M+2Na-H ; M+3Na ; M+H+Na ; 2M+3H ; M+3Na ; M+3Na ; M+2Na-H ; M+2Na-H ; M+2Na-H ; M+2Na-H ; M+2Na-H ; M+2Na-H ; M+2Na-H ; M+2Na-H ; M+2Na-H ; M+2Na-H ; M+ACN+Na ; M+2Na-H ; M+2Na-H ; M+2Na-H ; M+2Na-H ; M+2Na-H	C9H10O3 ; C9H10O3 ; C9H10O3 ; C9H10O3 ; C9H10O3 ; C9H10O3 ; C9H10O3 ; C9H10O3 ; C29H24O12 ; C18H14N4O5S ; C15H10ClN3O3 ; C29H24O12 ; C29H24O12 ; C9H10O3 ; C9H10O3 ; C9H10O3 ; C9H10O3 ; C9H10O3 ; C9H10O3 ; C9H10O3 ; C9H10O3 ; C9H10O3 ; C9H10O3 ; C5H9NS2 ; C9H10O3 ; C9H10O3 ; C9H10O3 ; C9H10O3 ; C9H10O3

309.12936	1.388402	1.390977	-3.5757	0.005048	2.2969	0.031912	309.1294	HMDB01518 ; HMDB13248 ; HMDB13835 ; HMDB33244 ; HMDB40814 ; HMDB61151	Alpha-CEHC ; Monoethylhexyl phthalic acid ; Diisobutyl phthalate ; Dibutyl phthalate ; Isosyringinoside ; Hydroxylated N-acetyl desmethyl frovatriptan	2M+Na+K ; 2M+Na+K ; 2M+Na+K ; 2M+Na+K ; M+2ACN+2H ; 2M+H+K	C16H22O4 ; C16H22O4 ; C16H22O4 ; C16H22O4 ; C23H34O14 ; C15H19N3O3
219.13399	1.469405	1.138268	-3.5751	0.005053	2.2965	0.031912	219.134	HMDB00348 ; HMDB00361 ; HMDB00384 ; HMDB00411 ; HMDB00438 ; HMDB00478 ; HMDB00514 ; HMDB00518 ; HMDB00626 ; HMDB00664 ; HMDB00686 ; HMDB00733 ; HMDB00811 ; HMDB00946 ; HMDB02451 ; HMDB02488 ; HMDB02536 ; HMDB02585 ; HMDB12187 ; HMDB30354 ; HMDB39892 ; HMDB40851 ; HMDB41353	3b12a-Dihydroxy-5a-cholanoic acid ; 3b7a-Dihydroxy-5b-cholanoic acid ; 3a7a-Dihydroxycholanoic acid ; 3a12b-Dihydroxy-5b-cholanoic acid ; 3b12a-Dihydroxy-5b-cholanoic acid ; Allodeoxycholic acid ; Allochenodeoxycholic acid ; Chenodeoxycholic acid ; Deoxycholic acid ; Isohyodeoxycholic acid ; Isoursodeoxycholic acid ; Hyodeoxycholic acid ; Murocholic acid ; Ursodeoxycholic acid ; 7b12a-Dihydroxycholanoic acid ; 7a12b-dihydroxy-5b-Cholan-24-oic acid ; Isodeoxycholic acid ; 3b12b-Dihydroxy-5b-cholanoic acid ; All-trans-heptaprenyl diphosphate ; Arenaine ; Cloversaponin I ; Calenduloside E ; Lucyoside K	M+2Na ; M+2Na ; M+2Na ; M+2Na ; M+2Na ; M+2Na ; M+2Na ; M+2Na ; M+2Na ; M+2Na ; M+2Na ; M+2Na ; M+2Na ; M+2Na ; M+2Na ; M+2Na ; M+2Na ; M+2Na ; M+3H ; 2M+H+Na ; M+2H+Na ; M+2H+Na ; M+2H+Na	C24H40O4 ; C24H40O4 ; C24H40O4 ; C24H40O4 ; C24H40O4 ; C24H40O4 ; C24H40O4 ; C24H40O4 ; C24H40O4 ; C24H40O4 ; C24H40O4 ; C24H40O4 ; C24H40O4 ; C24H40O4 ; C24H40O4 ; C24H40O4 ; C24H40O4 ; C24H40O4 ; C35H60O7P2 ; C11H17N3O ; C36H56O9 ; C36H56O9 ; C36H56O9
565.94047	1.421889	1.470351	-3.5748	0.005055	2.2963	0.031912	NA	NA	NA	NA	NA
360.03670	1.46696	0.881752	3.5731	0.00507	2.295	0.031967	360.0367	HMDB37850 ; HMDB38286	Myricetin 33-digalactoside ; Eujambin	M+2K ; M+Na+K	C27H30O18 ; C30H26O17
156.51837	1.48354	0.259396	-3.5692	0.005103	2.2922	0.032124	156.5184	HMDB01410 ; HMDB14468 ; HMDB61025	2-Amino-4-oxo-6-(12-dioxoprolyl)-78-dihydroxypteridine ; Tolcapone ; 5-Chloro-13-dihydro-1-(4-piperidinyl)-2H-benzimidazol-2-one	M+2Na ; M+H+K ; M+Na+K	C9H9N5O5 ; C14H11NO5 ; C12H14ClN3O
216.09770	1.42032	0.936935	3.5686	0.005108	2.2918	0.032124	216.0977	HMDB32622 ; HMDB00191 ; HMDB00854 ; HMDB03626 ; HMDB03892 ; HMDB05005 ; HMDB05015 ; HMDB06028 ; HMDB06483 ; HMDB11753 ; HMDB12469 ; HMDB12484 ; HMDB12575 ; HMDB12993 ; HMDB14359 ; HMDB28786 ; HMDB29080 ; HMDB29450 ; HMDB31797 ; HMDB32766 ; HMDB35072 ; HMDB36906 ; HMDB37698 ; HMDB28741 ; HMDB28806 ; HMDB29037 ; HMDB29053 ; HMDB29056 ; HMDB29158	Phenethyl rutinoside ; L-Aspartic acid ; Formiminoglutamic acid ; (R)-N-Methylsalsolinol ; (S)-N-Methylsalsolinol ; Tolnaftate ; Gabapentin ; N-Acetylasparagine ; D-Aspartic acid ; Iminodiacetic acid ; (-)-Salsoline ; (R)-12-dimethyl-56-dihydroxy-tetrahydroisoquinoline ; 13E-Tetranor-16-carboxy-LTE4 ; Leukotriene C5 ; Torasemide ; Cysteinyl-Tryptophan ; Tryptophyl-Cysteine ; Aspergillomarasmine A ; 2-Isopropylphenyl methylcarbamate ; N2-(2-Carboxymethyl-2-hydroxysuccinoyl)arginine ; 2-Methylpropyl 2-aminobenzoate ; Butyl 2-aminobenzoate ; Ethyl N-ethylanthranilate ; Asparaginyl-Threonine ; Glutaminyl-Serine ; Serinyl-Glutamine ; Serinyl-Gamma-glutamate ; Threoninyl-Asparagine ; Gamma-glutamyl-Serine	M+2H ; M+2ACN+H ; M+ACN+H ; M+Na ; M+Na ; M+3ACN+2H ; M+2Na-H ; M+ACN+H ; M+2ACN+H ; M+2ACN+H ; M+Na ; M+Na ; M+H+NH4 ; M+2H+Na ; M+2ACN+2H ; M+3ACN+2H ; M+3ACN+2H ; M+3ACN+2H ; M+Na ; M+2ACN+2H ; M+Na ; M+Na ; M+Na ; M+H-H2O ; M+H-H2O ; M+H-H2O ; M+H-H2O ; M+H-H2O ; M+H-H2O	C20H30O10 ; C4H7NO4 ; C6H10N2O4 ; C11H15NO2 ; C11H15NO2 ; C19H17NOS ; C9H17NO2 ; C6H10N2O4 ; C4H7NO4 ; C4H7NO4 ; C11H15NO2 ; C11H15NO2 ; C19H27NO7S ; C30H45N3O9S ; C16H20N4O3S ; C14H17N3O3S ; C14H17N3O3S ; C10H17N3O8 ; C11H15NO2 ; C12H20N4O8 ; C11H15NO2 ; C11H15NO2 ; C8H15N3O5 ; C8H15N3O5 ; C8H15N3O5 ; C8H15N3O5 ; C8H15N3O5 ; C8H15N3O5
398.74057	1.428718	1.619032	3.5611	0.005172	2.2863	0.032487	NA	NA	NA	NA	NA
207.02889	1.444137	0.88518	-3.5595	0.005186	2.2852	0.032494	207.0289	HMDB02064 ; HMDB04363 ; HMDB33107 ; HMDB39306 ; HMDB60268 ; HMDB60541 ; HMDB60617 ; HMDB36314	N-Acetylputrescine ; Imidazolone ; Semilepidinoside A ; Mangiferin 6-gallate ; N-Acetylputrescinium ; N-Desmethylzopiclone ; Penicilloic acid ; Dehydrochorismic acid	M+2K-H ; 2M+K ; M+2K ; M+H+2Na ; M+2K-H ; M+H+K ; M+Na+K ; M+H-H2O	C6H14N2O ; C3H4N2O ; C16H20N2O6 ; C26H22O15 ; C6H14N2O ; C16H15ClN6O3 ; C16H20N2O5S ; C10H8O6
558.48632	1.352907	0.919913	-3.5594	0.005186	2.2852	0.032494	558.4863	HMDB01394 ; HMDB35480 ; HMDB47380 ; HMDB47428 ; HMDB47518 ; HMDB47520 ; HMDB52492 ; HMDB53346	Heparin ; N-(4E8E)-13-dihydroxyoctadeca-48-dien-2-ylhexadecanamide ; TG(24:018:2(9Z12Z)22:4(7Z10Z13Z16Z)) ; TG(24:020:2n622:4(7Z10Z13Z16Z)) ; TG(24:022:4(7Z10Z13Z16Z)18:2(9Z12Z)) ; TG(24:022:4(7Z10Z13Z16Z)20:2n6) ; TG(18:2(9Z12Z)24:022:4(7Z10Z13Z16Z)) ; TG(20:2n624:022:4(7Z10Z13Z16Z))	M+2K ; M+Na ; M+2ACN+2H ; M+2ACN+2H ; M+2ACN+2H ; M+2ACN+2H ; M+2ACN+2H ; M+2ACN+2H	C26H41NO34S4 ; C34H65NO3 ; C68H120O6 ; C68H120O6 ; C68H120O6 ; C68H120O6 ; C68H120O6 ; C68H120O6
337.98105	1.398815	0.229827	3.5581	0.005198	2.2842	0.032494	337.9811	HMDB01504 ; HMDB12285 ; HMDB15679 ; HMDB43110 ; HMDB43138 ; HMDB45852 ; HMDB45854 ; HMDB45877 ; HMDB45929 ; HMDB46282 ; HMDB46591 ; HMDB46593 ; HMDB46639 ; HMDB46689 ; HMDB47278 ; HMDB47280 ; HMDB47370 ; HMDB47418 ; HMDB50418 ; HMDB50420 ; HMDB51495 ; HMDB51497 ; HMDB51982 ; HMDB51984 ; HMDB61101	Aminoparathion ; Ribose-1-arsenate ; Niclosamide ; TG(15:022:024:0) ; TG(15:024:022:0) ; TG(20:024:1(15Z)18:2(9Z12Z)) ; TG(20:024:1(15Z)20:2n6) ; TG(20:018:2(9Z12Z)24:1(15Z)) ; TG(20:020:2n624:1(15Z)) ; TG(22:015:024:0) ; TG(22:022:1(13Z)18:2(9Z12Z)) ; TG(22:022:1(13Z)20:2n6) ; TG(22:018:2(9Z12Z)22:1(13Z)) ; TG(22:020:2n622:1(13Z)) ; TG(24:020:1(11Z)18:2(9Z12Z)) ; TG(24:020:1(11Z)20:2n6) ; TG(24:018:2(9Z12Z)20:1(11Z)) ; TG(24:020:2n620:1(11Z)) ; TG(20:1(11Z)24:018:2(9Z12Z)) ; TG(20:1(11Z)24:020:2n6) ; TG(22:1(13Z)22:018:2(9Z12Z)) ; TG(22:1(13Z)22:020:2n6) ; TG(24:1(15Z)20:018:2(9Z12Z)) ; TG(24:1(15Z)20:020:2n6) ; 2-oxobrimonidine	M+2K-H ; M+ACN+Na ; 2M+H+Na ; M+2H+Na ; M+2H+Na ; M+3H ; M+3H ; M+3H ; M+3H ; M+2H+Na ; M+3H ; M+3H ; M+3H ; M+3H ; M+3H ; M+3H ; M+3H ; M+3H ; M+3H ; M+3H ; M+3H ; M+3H ; M+3H ; M+3H ; 2M+Na+K	C10H16NO3PS ; C5H11AsO8 ; C13H8Cl2N2O4 ; C64H124O6 ; C64H124O6 ; C66H122O6 ; C66H122O6 ; C66H122O6 ; C66H122O6 ; C64H124O6 ; C66H122O6 ; C66H122O6 ; C66H122O6 ; C66H122O6 ; C66H122O6 ; C66H122O6 ; C66H122O6 ; C66H122O6 ; C66H122O6 ; C66H122O6 ; C66H122O6 ; C66H122O6 ; C66H122O6 ; C66H122O6 ; C11H10BrN5O
438.72833	1.420623	1.620381	3.5575	0.005203	2.2838	0.032494	438.7283	HMDB13889 ; HMDB15273	E-10-Hydroxynortriptyline ; Doxepin	3M+H+K ; 3M+H+K	C19H21NO ; C19H21NO
339.28906	1.459577	0.40686	3.5569	0.005208	2.2834	0.032494	339.2891	HMDB05181 ; HMDB13206 ; HMDB32112	Dolichol-14 ; 9-Decenoylcholine ; 124-Nonadecanetriol	M+H+2Na ; M+2ACN+H ; M+Na	C70H114O ; C15H30NO2 ; C19H40O3
332.03368	1.455705	1.218373	3.5558	0.005218	2.2825	0.032494	332.0337	HMDB01062 ; HMDB01121 ; HMDB01367 ; HMDB02817 ; HMDB06480 ; HMDB11671 ; HMDB15250 ; HMDB59626 ; HMDB60875 ; HMDB29177	N-Acetyl-D-Glucosamine 6-Phosphate ; N-Acetyl-D-mannosamine 6-phosphate ; N-Acetyl-glucosamine 1-phosphate ; N-Acetylglucosamine 6-phosphate ; N-Acetyl-D-galactosamine 1-phosphate ; ADP-ribose 1-2 cyclic phosphate ; Amiodarone ; N-acetyl-alpha-D-galactosamine 1-phosphate ; Thioguanosine 5-triphosphate ; 3-O-Methyl-(-)-epicatechin-7-O-sulphate	2M+Na+K ; 2M+Na+K ; 2M+Na+K ; 2M+Na+K ; 2M+Na+K ; M+ACN+2H ; M+H+NH4 ; 2M+Na+K ; M+3ACN+2H ; M+H-2H2O	C8H16NO9P ; C8H16NO9P ; C8H16NO9P ; C8H16NO9P ; C8H16NO9P ; C15H22N5O16P3 ; C25H29I2NO3 ; C8H16NO9P ; C10H16N5O13P3S ; C16H15O8S
232.14034	1.397224	0.827736	-3.5557	0.005219	2.2825	0.032494	232.1403	HMDB28709 ; HMDB28835 ; HMDB10340 ; HMDB01276 ; HMDB02189 ; HMDB04224 ; HMDB15696 ; HMDB32327 ; HMDB34326 ; HMDB37765 ; HMDB38948 ; HMDB40811 ; HMDB60288	Arginyl-Glycine ; Glycyl-Arginine ; Retinyl beta-glucuronide ; N1-Acetylspermidine ; N8-Acetylspermidine ; N-(o)-Hydroxyarginine ; Bopindolol ; Hexyl 3-mercaptobutanoate ; L-erythro-4-Hydroxyarginine ; (S)-3-Mercaptohexyl butyrate ; 3-Mercaptohexyl butyrate ; Dehydropipernonaline ; N(8)-Acetylspermidinium	M+H ; M+H ; M+2H ; M+2Na-H ; M+2Na-H ; M+ACN+H ; M+2ACN+2H ; 2M+3H2O+2H ; M+ACN+H ; 2M+3H2O+2H ; 2M+3H2O+2H ; M+3ACN+2H ; M+2Na-H	C8H17N5O3 ; C8H17N5O3 ; C26H38O7 ; C9H21N3O ; C9H21N3O ; C6H14N4O3 ; C23H28N2O3 ; C10H20O2S ; C6H14N4O3 ; C10H20O2S ; C10H20O2S ; C21H25NO3 ; C9H21N3O

362.16798	1.397087	1.050561	-3.5513	0.005257	2.2793	0.032652	362.168	HMDB39694 ; HMDB02928 ; HMDB06791 ; HMDB14869 ; HMDB15376 ; HMDB15592 ; HMDB29911 ; HMDB30221 ; HMDB30222 ; HMDB30223 ; HMDB30225 ; HMDB30262 ; HMDB30289 ; HMDB30318 ; HMDB32807 ; HMDB34915 ; HMDB35233 ; HMDB36479 ; HMDB36689 ; HMDB38970 ; HMDB40384 ; HMDB40937 ; HMDB61099 ; HMDB14593	Physagulin G ; Maltitol ; Melibiitol ; Nateglinide ; Trimeprazine ; Tetrabenazine ; 1-O-alpha-D-Glucopyranosyl-D-mannitol ; Bicyclomahanimbicine ; Bicyclomahanimbine ; ()-Currayangine ; ()-Mahanimbicine ; Isomurrayazoline ; Curryanine ; ()-Mahanimbine ; Niazirin ; Calonectrin ; Olomoucine ; Cyclolinopeptide F ; Millefin ; Formylfusarochromanone ; Neoacrimarine A ; Lactitol ; 5-hydroxy saxagliptin ; Droperidol	M+2H ; M+NH4 ; M+NH4 ; M+2Na-H ; M+ACN+Na ; M+2Na-H ; M+NH4 ; 2M+Na+K ; 2M+Na+K ; 2M+Na+K ; 2M+Na+K ; 2M+Na+K ; 2M+Na+K ; 2M+Na+K ; M+2ACN+H ; 2M+H+Na ; M+ACN+Na ; M+3H ; 2M+H+Na ; M+ACN+H ; M+ACN+2H ; M+NH4 ; 2M+Na+K ; M+H-H2O	C36H50O15 ; C12H24O11 ; C12H24O11 ; C19H27NO3 ; C18H22N2S ; C19H27NO3 ; C12H24O11 ; C23H25NO ; C23H25NO ; C23H25NO ; C23H25NO ; C23H25NO ; C23H25NO ; C23H25NO ; C14H17NO5 ; C19H26O6 ; C15H18N6O ; C55H73N9O10S2 ; C19H26O6 ; C16H20N2O5 ; C40H43NO9 ; C12H24O11 ; C18H25N3O3 ; C22H22FN3O2
186.96303	1.39545	0.48748	3.5513	0.005257	2.2793	0.032652	186.963	HMDB00119 ; HMDB02050 ; HMDB39797 ; HMDB40230	Glyoxylic acid ; Dihydroxyfumaric acid ; Dihydro-4-mercapto-5-methyl-3(2H)-thiophenone ; 3-Acetyl-12-dithiolane	2M+K ; M+K ; M+K ; M+K	C2H2O3 ; C4H4O6 ; C5H8OS2 ; C5H8OS2
214.00627	1.374307	1.453007	3.5486	0.00528	2.2774	0.03273	214.0063	HMDB01439 ; HMDB15276 ; HMDB29202	Phosphoribosyl formamidocarboxamide ; Sulfoxone ; hesperetin 3-O-sulfate	M+Na+K ; M+H+Na ; M+2Na	C10H15N4O9P ; C14H16N2O6S3 ; C16H14O9S
353.88220	1.395353	1.095	-3.5484	0.005283	2.2771	0.03273	NA	NA	NA	NA	NA
151.94956	1.46592	0.288237	3.5471	0.005294	2.2762	0.032746	NA	NA	NA	NA	NA
204.88858	1.447892	1.536855	3.5466	0.005298	2.2759	0.032746	NA	NA	NA	NA	NA
294.93913	1.42806	1.356876	3.545	0.005312	2.2747	0.03279	294.9391	HMDB31823	Diphenyl disulfide	M+2K-H	C12H10S2
264.85226	1.421133	1.638095	3.5425	0.005335	2.2729	0.032885	264.8523	HMDB41974 ; HMDB07937 ; HMDB08033 ; HMDB08841 ; HMDB08932 ; HMDB08991 ; HMDB09220 ; HMDB09481 ; HMDB60501	Pentachlorophenol ; PC(15:018:0) ; PC(18:015:0) ; PE(14:022:0) ; PE(16:020:0) ; PE(18:018:0) ; PE(20:016:0) ; PE(22:014:0) ; Phosphatidylethanolamine	M+H ; M+H+2Na ; M+H+2Na ; M+H+2Na ; M+H+2Na ; M+H+2Na ; M+H+2Na ; M+H+2Na ; M+H+2Na	C6HCl5O ; C41H82NO8P ; C41H82NO8P ; C41H82NO8P ; C41H82NO8P ; C41H82NO8P ; C41H82NO8P ; C41H82NO8P ; C41H82NO8P
251.20022	1.426061	0.668658	3.5417	0.005341	2.2724	0.032885	251.2002	HMDB00806 ; HMDB02221 ; HMDB03671 ; HMDB04814 ; HMDB05841 ; HMDB31072 ; HMDB31405 ; HMDB31429 ; HMDB31475 ; HMDB31482 ; HMDB31538 ; HMDB31549 ; HMDB32318 ; HMDB32405 ; HMDB32548 ; HMDB33788 ; HMDB34461 ; HMDB36216 ; HMDB37312 ; HMDB37495 ; HMDB38034 ; HMDB38052 ; HMDB38969 ; HMDB40191 ; HMDB41320 ; HMDB59693 ; HMDB59865 ; HMDB00672 ; HMDB41287 ; HMDB59777	Myristic acid ; 2610-Trimethylundecanoic acid ; 2-Heptanone ; 4-Heptanone ; Ethyl isobutyl ketone ; 12-Methyltridecanoic acid ; (Z)-4-Hepten-1-ol ; 24-Dimethyl-3-pentanone ; Heptanal ; 3-Heptanone ; 3-Methylcyclohexanol ; 5-Methyl-2-hexanone ; Hexanal octane-13-diol acetal ; 3-Methylhexanal ; Undecanal propyleneglycol acetal ; Ethyl dodecanoate ; Heptyl heptanoate ; Hexyl octanoate ; Decyl butanoate ; 3-Methylbutyl nonanoate ; Nonyl isovalerate ; 1-Ethoxy-3-methyl-2-butene ; Dodecyl acetate ; ()-2-Methylhexanal ; (2R4Z)-4-Hepten-2-ol ; 1-Methylcyclohexanol ; Isobutyl decanoate ; Hexadecanedioic acid ; 16-Hydroxy-10-oxohexadecanoic acid ; 224-Trimethyl-13-pentadienol diisobutyrate	M+Na ; M+Na ; 2M+Na ; 2M+Na ; 2M+Na ; M+Na ; 2M+Na ; 2M+Na ; 2M+Na ; 2M+Na ; 2M+Na ; 2M+Na ; M+Na ; 2M+Na ; M+Na ; M+Na ; M+Na ; M+Na ; M+Na ; M+Na ; M+Na ; 2M+Na ; M+Na ; 2M+Na ; 2M+Na ; 2M+Na ; M+Na ; M+H-2H2O ; M+H-2H2O ; M+H-2H2O	C14H28O2 ; C14H28O2 ; C7H14O ; C7H14O ; C7H14O ; C14H28O2 ; C7H14O ; C7H14O ; C7H14O ; C7H14O ; C7H14O ; C7H14O ; C14H28O2 ; C7H14O ; C14H28O2 ; C14H28O2 ; C14H28O2 ; C14H28O2 ; C14H28O2 ; C14H28O2 ; C14H28O2 ; C7H14O ; C14H28O2 ; C7H14O ; C7H14O ; C7H14O ; C14H28O2 ; C16H30O4 ; C16H30O4 ; C16H30O4
312.78212	1.404036	1.628961	3.5411	0.005347	2.2719	0.032885	312.7821	HMDB07071 ; HMDB07080 ; HMDB07155 ; HMDB07416 ; HMDB55983 ; HMDB56022 ; HMDB56024	DG(15:018:00:0) ; DG(15:020:2(11Z14Z)0:0) ; DG(18:015:00:0) ; DG(20:2(11Z14Z)15:00:0) ; DG(15:00:018:0) ; DG(16:00:018:2n6) ; DG(16:00:020:2n6)	M+ACN+2H ; M+H+NH4 ; M+ACN+2H ; M+H+NH4 ; M+ACN+2H ; M+H+NH4 ; M+H+NH4	C36H70O5 ; C38H70O5 ; C36H70O5 ; C38H70O5 ; C36H70O5 ; C38H70O5 ; C38H70O5
493.91279	1.379983	1.472303	-3.5398	0.005359	2.2709	0.032915	493.9128	HMDB36404 ; HMDB43567 ; HMDB43589 ; HMDB43791 ; HMDB45098 ; HMDB45152 ; HMDB45436 ; HMDB45438 ; HMDB49400 ; HMDB49405 ; HMDB49442 ; HMDB49447 ; HMDB49540 ; HMDB49542 ; HMDB49645 ; HMDB49647 ; HMDB50013 ; HMDB50018 ; HMDB50053 ; HMDB50058 ; HMDB50146 ; HMDB50148 ; HMDB50246 ; HMDB50248 ; HMDB50600 ; HMDB50638 ; HMDB50798 ; HMDB50800 ; HMDB51088 ; HMDB51090 ; HMDB51139 ; HMDB51145 ; HMDB51175 ; HMDB51181 ; HMDB51196 ; HMDB51198 ; HMDB52538 ; HMDB52543 ; HMDB52553 ; HMDB52572 ; HMDB52579 ; HMDB52669 ; HMDB52705 ; HMDB52761 ; HMDB53314 ; HMDB53386 ; HMDB53391 ; HMDB53399 ; HMDB53404 ; HMDB53416 ; HMDB53421 ; HMDB53499 ; HMDB53531 ; HMDB53579 ; HMDB53833 ; HMDB53857 ;	3beta-Acetoxy-19alpha-hydroxy-12-ursene ; TG(15:022:2(13Z16Z)22:6(4Z7Z10Z13Z16Z19Z)) ; TG(15:022:4(7Z10Z13Z16Z)22:4(7Z10Z13Z16Z)) ; TG(15:022:6(4Z7Z10Z13Z16Z19Z)22:2(13Z16Z)) ; TG(18:018:2(9Z12Z)22:6(4Z7Z10Z13Z16Z19Z)) ; TG(18:020:2n622:6(4Z7Z10Z13Z16Z19Z)) ; TG(18:022:6(4Z7Z10Z13Z16Z19Z)18:2(9Z12Z)) ; TG(18:022:6(4Z7Z10Z13Z16Z19Z)20:2n6) ; TG(18:1(11Z)18:2(9Z12Z)22:5(4Z7Z10Z13Z16Z)) ; TG(18:1(11Z)18:2(9Z12Z)22:5(7Z10Z13Z16Z19Z)) ; TG(18:1(11Z)20:2n622:5(4Z7Z10Z13Z16Z)) ; TG(18:1(11Z)20:2n622:5(7Z10Z13Z16Z19Z)) ; TG(18:1(11Z)22:5(4Z7Z10Z13Z16Z)18:2(9Z12Z)) ; TG(18:1(11Z)22:5(4Z7Z10Z13Z16Z)20:2n6) ; TG(18:1(11Z)22:5(7Z10Z13Z16Z19Z)18:2(9Z12Z)) ; TG(18:1(11Z)22:5(7Z10Z13Z16Z19Z)20:2n6) ; TG(18:1(9Z)18:2(9Z12Z)22:5(4Z7Z10Z13Z16Z)) ; TG(18:1(9Z)18:2(9Z12Z)22:5(7Z10Z13Z16Z19Z)) ; TG(18:1(9Z)20:2n622:5(4Z7Z10Z13Z16Z)) ; TG(18:1(9Z)20:2n622:5(7Z10Z13Z16Z19Z)) ; TG(18:1(9Z)22:5(4Z7Z10Z13Z16Z)18:2(9Z12Z)) ; TG(18:1(9Z)22:5(4Z7Z10Z13Z16Z)20:2n6) ; TG(18:1(9Z)22:5(7Z10Z13Z16Z19Z)18:2(9Z12Z)) ; TG(18:1(9Z)22:5(7Z10Z13Z16Z19Z)20:2n6) ; TG(20:1(11Z)18:2(9Z12Z)20:5(5Z8Z11Z14Z17Z)) ; TG(20:1(11Z)20:2n620:5(5Z8Z11Z14Z17Z)) ; TG(20:1(11Z)20:5(5Z8Z11Z14Z17Z)18:2(9Z12Z)) ; TG(20:1(11Z)20:5(5Z8Z11Z14Z17Z)20:2n6) ; TG(20:3(5Z8Z11Z)20:3(5Z8Z11Z)18:2(9Z12Z)) ; TG(20:3(5Z8Z11Z)20:3(5Z8Z11Z)20:2n6) ; TG(20:3(5Z8Z11Z)18:2(9Z12Z)20:3(5Z8Z11Z)) ; TG(20:3(5Z8Z11Z)18:2(9Z12Z)20:3n6) ; TG(20:3(5Z8Z11Z)20:2n620:3(5Z8Z11Z)) ; TG(20:3(5Z8Z11Z)20:2n620:3n6) ; TG(20:3(5Z8Z11Z)20:3n618:2(9Z12Z)) ; TG(20:3(5Z8Z11Z)20:3n620:2n6) ; TG(18:2(9Z12Z)18:1(11Z)22:5(4Z7Z10Z13Z16Z)) ; TG(18:2(9Z12Z)18:1(11Z)22:5(7Z10Z13Z16Z19Z)) ; TG(18:2(9Z12Z)18:1(9Z)22:5(4Z7Z10Z13Z16Z)) ; TG(18:2(9Z12Z)20:1(11Z)20:5(5Z8Z11Z14Z17Z)) ; TG(18:2(9Z12Z)20:3(5Z8Z11Z)20:3n6) ; TG(18:2(9Z12Z)20:3n620:3n6) ; TG(18:2(9Z12Z)22:2(13Z16Z)18:4(6Z9Z12Z15Z)) ; TG(18:2(9Z12Z)18:4(6Z9Z12Z15Z)22:2(13Z16Z)) ; TG(20:2n618:022:6(4Z7Z10Z13Z16Z19Z)) ; TG(20:2n618:1(11Z)22:5(4Z7Z10Z13Z16Z)) ; TG(20:2n618:1(11Z)22:5(7Z10Z13Z16Z19Z)) ; TG(20:2n618:1(9Z)22:5(4Z7Z10Z13Z16Z)) ; TG(20:2n618:1(9Z)22:5(7Z10Z13Z16Z19Z)) ; TG(20:2n620:1(11Z)20:5(5Z8Z11Z14Z17Z)) ; TG(20:2n620:3(5Z8Z11Z)20:3n6) ; TG(20:2n620:3n620:3n6) ; TG(20:2n622:2(13Z16Z)18:4(6Z9Z12Z15Z)) ; TG(20:2n618:4(6Z9Z12Z15Z)22:2(13Z16Z)) ; TG(20:3n618:2(9Z12Z)20:3n6) ; TG(20:3n620:2n620:3n6) ;	2M+H+NH4 ; M+ACN+2H	C32H52O3 ; C62H104O6

263.16826	1.405793	1.352203	-3.5337	0.005413	2.2665	0.033211	263.1683	HMDB13286 ; HMDB30448 ; HMDB32601 ; HMDB34065 ; HMDB39362 ; HMDB40418 ; HMDB40781 ; HMDB61107 ; HMDB61108 ; HMDB61109 ; HMDB61110 ; HMDB14943 ; HMDB61061	N-Undecanoylglycine ; Narasin ; Muzanzagenin ; 29-Bis(3-methyl-2E-pentenoyl)-2b9a-dihydroxy-4Z10(14)-oplopadien-3-one ; 37-Dihydroxy-25-methoxycucurbita-523-dien-19-al ; Majonoside R2 ; Vinaginsenoside R11 ; Buspirone N-oxide ; 3-Hydroxybuspirone ; 5-Hydroxybuspirone ; 6-Hydroxybuspirone ; Minaprine ; 9-desmethylgranisetron	2M+H+K ; M+2H+Na ; M+2ACN+2H ; M+2ACN+2H ; M+H+K ; M+3H ; M+3H ; M+3ACN+2H ; M+3ACN+2H ; M+3ACN+2H ; M+3ACN+2H ; M+H-2H2O ; M+H-2H2O	C13H25NO3 ; C43H72O11 ; C27H38O5 ; C27H38O5 ; C31H50O4 ; C41H70O14 ; C41H70O14 ; C21H31N5O3 ; C21H31N5O3 ; C21H31N5O3 ; C21H31N5O3 ; C17H22N4O ; C17H22N4O
288.11894	1.431784	0.60972	-3.5327	0.005423	2.2658	0.033226	288.1189	HMDB00962 ; HMDB15254 ; HMDB30680 ; HMDB30703 ; HMDB32552 ; HMDB32663 ; HMDB36203 ; HMDB38461 ; HMDB38735 ; HMDB39125 ; HMDB40629 ; HMDB40890 ; HMDB60299 ; HMDB60300 ; HMDB60302	Lipoamide ; Ambenonium ; Diplosporin ; Dhelwangin ; Vanillin 12-butylene glycol acetal ; Acoramone ; 2-Ethoxy-4-(4-methyl-13-dioxolan-2-yl)phenol ; 1-Isothiocyanato-6-(methylsulfinyl)hexane ; 5-O-Feruloylnigrumin ; Coumaperine ; Isoacoramone ; 345-Trimethoxycinnamyl alcohol ; (1R)-Glutathionyl-(2R)-hydroxy-12-dihydronaphthalene ; (1R)-Hydroxy-(2R)-glutathionyl-12-dihydronaphthalene ; (1S)-Hydroxy-(2S)-glutathionyl-12-dihydronaphthalene	M+2ACN+H ; M+H+K ; M+ACN+Na ; M+ACN+Na ; M+ACN+Na ; M+ACN+Na ; M+ACN+Na ; M+2ACN+H ; M+3ACN+2H ; 2M+Na+K ; M+ACN+Na ; M+ACN+Na ; M+3ACN+2H ; M+3ACN+2H ; M+3ACN+2H	C8H15NOS2 ; C28H42Cl2N4O2 ; C12H16O4 ; C12H16O4 ; C12H16O4 ; C12H16O4 ; C12H16O4 ; C8H15NOS2 ; C21H25NO10 ; C16H19NO2 ; C12H16O4 ; C12H16O4 ; C20H25N3O7S ; C20H25N3O7S ; C20H25N3O7S
390.79830	1.401461	1.575847	3.5313	0.005435	2.2648	0.033262	390.7983	HMDB09610 ; HMDB11391 ; HMDB09248 ; HMDB09249 ; HMDB09280 ; HMDB09543 ; HMDB11356 ; HMDB11381 ; HMDB11413 ; HMDB11446 ; HMDB60215 ; HMDB60234	PE(22:4(7Z10Z13Z16Z)P-18:0) ; PE(P-18:022:4(7Z10Z13Z16Z)) ; PE(20:0P-18:1(11Z)) ; PE(20:0P-18:1(9Z)) ; PE(20:1(11Z)P-18:0) ; PE(22:1(13Z)P-16:0) ; PE(P-16:022:1(13Z)) ; PE(P-18:020:1(11Z)) ; PE(P-18:1(11Z)20:0) ; PE(P-18:1(9Z)20:0) ; 3-Oxodocosanoyl-CoA ; 3(S)-Hydroxy-tetracosa-12151821-all-cis-tetraenoyl-CoA	M+2H ; M+2H ; M+H+Na ; M+H+Na ; M+H+Na ; M+H+Na ; M+H+Na ; M+H+Na ; M+H+Na ; M+H+Na ; M+3Na ; M+H+2Na	C45H82NO7P ; C45H82NO7P ; C43H84NO7P ; C43H84NO7P ; C43H84NO7P ; C43H84NO7P ; C43H84NO7P ; C43H84NO7P ; C43H84NO7P ; C43H84NO7P ; C43H76N7O18P3S ; C45H74N7O18P3S
541.01797	1.400574	1.556968	-3.5249	0.005493	2.2602	0.033551	541.018	HMDB29500 ; HMDB30775 ; HMDB33132 ; HMDB34360 ; HMDB34725 ; HMDB35883 ; HMDB37079 ; HMDB37362 ; HMDB37932 ; HMDB40478 ; HMDB41969	Kaempferol 3-glucuronide ; Quercetin 3-galactoside ; Myricetin 7-rhamnoside ; Myricitrin ; 6-Hydroxykaempferol 7-glucoside ; 2-Hydroxyisoorientin ; 3-Methylellagic acid 8-rhamnoside ; Isoquercitrin ; Quercetin 4-glucoside ; 6-Hydroxykaempferol 3-glucoside ; Oxazepam glucuronide	M+2K+H ; M+2K-H ; M+2K-H ; M+2K-H ; M+2K-H ; M+2K-H ; M+2K+H ; M+2K-H ; M+2K-H ; M+2K-H ; M+2K+H	C21H18O12 ; C21H20O12 ; C21H20O12 ; C21H20O12 ; C21H20O12 ; C21H20O12 ; C21H18O12 ; C21H20O12 ; C21H20O12 ; C21H20O12 ; C21H19ClN2O8
278.58777	1.441333	0.952038	-3.5244	0.005498	2.2598	0.033551	278.5878	HMDB37690 ; HMDB38757	Limocitrol 3-glucoside ; Isolimocitrol 3-glucoside	M+H+NH4 ; M+H+NH4	C24H26O14 ; C24H26O14
594.68483	1.419877	1.110322	-3.5239	0.005502	2.2595	0.033551	594.6848	HMDB00768 ; HMDB12221 ; HMDB40450	N-Acetyl-9-O-lactoylneuraminic acid ; Dopaxanthin ; 23456-Pentachlorobenzyl alcohol	3M+2Na ; 3M+H+NH4 ; 2M+K	C14H23NO11 ; C18H18N2O8 ; C7H3Cl5O
746.51610	1.400536	0.637959	-3.5222	0.005518	2.2582	0.033608	746.5161	HMDB57992 ; HMDB58001 ; HMDB58058 ; HMDB58067 ; HMDB58103 ; HMDB58113 ; HMDB58127 ; HMDB58128 ; HMDB58154 ; HMDB58159 ; HMDB58160 ; HMDB58427 ; HMDB58436 ; HMDB58481 ; HMDB58490 ; HMDB58526 ; HMDB58540 ; HMDB58541 ; HMDB58562 ; HMDB58563 ; HMDB58770 ; HMDB58771 ; HMDB58794 ; HMDB58795 ; HMDB15579 ; HMDB57981 ; HMDB57989 ; HMDB57990 ; HMDB58037 ; HMDB58047 ; HMDB58055 ; HMDB58056 ; HMDB58110 ; HMDB58111 ; HMDB58416 ; HMDB58424 ; HMDB58425 ; HMDB58478 ; HMDB58479	CL(18:1(11Z)18:1(11Z)18:2(9Z12Z)20:4(5Z8Z11Z14Z)) ; CL(18:1(11Z)18:1(11Z)20:4(5Z8Z11Z14Z)18:2(9Z12Z)) ; CL(18:1(11Z)18:1(9Z)18:2(9Z12Z)20:4(5Z8Z11Z14Z)) ; CL(18:1(11Z)18:1(9Z)20:4(5Z8Z11Z14Z)18:2(9Z12Z)) ; CL(18:1(11Z)18:2(9Z12Z)18:1(11Z)20:4(5Z8Z11Z14Z)) ; CL(18:1(11Z)18:2(9Z12Z)18:1(9Z)20:4(5Z8Z11Z14Z)) ; CL(18:1(11Z)18:2(9Z12Z)20:4(5Z8Z11Z14Z)18:1(11Z)) ; CL(18:1(11Z)18:2(9Z12Z)20:4(5Z8Z11Z14Z)18:1(9Z)) ; CL(18:1(11Z)20:4(5Z8Z11Z14Z)18:1(9Z)18:2(9Z12Z)) ; CL(18:1(11Z)20:4(5Z8Z11Z14Z)18:2(9Z12Z)18:1(11Z)) ; CL(18:1(11Z)20:4(5Z8Z11Z14Z)18:2(9Z12Z)18:1(9Z)) ; CL(18:1(9Z)18:1(11Z)18:2(9Z12Z)20:4(5Z8Z11Z14Z)) ; CL(18:1(9Z)18:1(11Z)20:4(5Z8Z11Z14Z)18:2(9Z12Z)) ; CL(18:1(9Z)18:1(9Z)18:2(9Z12Z)20:4(5Z8Z11Z14Z)) ; CL(18:1(9Z)18:1(9Z)20:4(5Z8Z11Z14Z)18:2(9Z12Z)) ; CL(18:1(9Z)18:2(9Z12Z)18:1(9Z)20:4(5Z8Z11Z14Z)) ; CL(18:1(9Z)18:2(9Z12Z)20:4(5Z8Z11Z14Z)18:1(11Z)) ; CL(18:1(9Z)18:2(9Z12Z)20:4(5Z8Z11Z14Z)18:1(9Z)) ; CL(18:1(9Z)20:4(5Z8Z11Z14Z)18:2(9Z12Z)18:1(11Z)) ; CL(18:1(9Z)20:4(5Z8Z11Z14Z)18:2(9Z12Z)18:1(9Z)) ; CL(18:2(9Z12Z)18:1(11Z)20:4(5Z8Z11Z14Z)18:1(11Z)) ; CL(18:2(9Z12Z)18:1(11Z)20:4(5Z8Z11Z14Z)18:1(9Z)) ; CL(18:2(9Z12Z)18:1(9Z)20:4(5Z8Z11Z14Z)18:1(11Z)) ; CL(18:2(9Z12Z)18:1(9Z)20:4(5Z8Z11Z14Z)18:1(9Z)) ; Quinestrol ; CL(18:1(11Z)18:1(11Z)18:1(9Z)18:2(9Z12Z)) ; CL(18:1(11Z)18:1(11Z)18:2(9Z12Z)18:1(11Z)) ; CL(18:1(11Z)18:1(11Z)18:2(9Z12Z)18:1(9Z)) ; CL(18:1(11Z)18:1(9Z)18:1(11Z)18:2(9Z12Z)) ; CL(18:1(11Z)18:1(9Z)18:1(9Z)18:2(9Z12Z)) ; CL(18:1(11Z)18:1(9Z)18:2(9Z12Z)18:1(11Z)) ; CL(18:1(11Z)18:1(9Z)18:2(9Z12Z)18:1(9Z)) ; CL(18:1(11Z)18:2(9Z12Z)18:1(9Z)18:1(11Z)) ;	M+2H ; 2M+NH4 ; M+H+Na ; M+H+Na ; M+H+Na ; M+H+Na ; M+H+Na ; M+H+Na ; M+H+Na ; M+H+Na ; M+H+Na ; M+H+Na ; M+H+Na ; M+H+Na ; M+H+Na ; M+H+Na	C84H148O17P2 ; C25H32O2 ; C82H150O17P2 ; C82H150O17P2 ; C82H150O17P2 ; C82H150O17P2 ; C82H150O17P2 ; C82H150O17P2 ; C82H150O17P2 ; C82H150O17P2 ; C82H150O17P2 ; C82H150O17P2 ; C82H150O17P2 ; C82H150O17P2 ; C82H150O17P2 ; C82H150O17P2
431.91697	1.416597	1.395292	3.5205	0.005534	2.257	0.033646	431.917	HMDB36350 ; HMDB42180 ; HMDB42934 ; HMDB43423 ; HMDB43481 ; HMDB43815 ; HMDB43817 ; HMDB43912 ; HMDB44641 ; HMDB44694 ; HMDB47999 ; HMDB48395 ; HMDB48632 ; HMDB48654 ; HMDB49052 ; HMDB49053 ; HMDB49260 ; HMDB49880 ; HMDB50451 ; HMDB52425 ; HMDB53289	Methyl 2-propenyl tetrasulfide ; TG(14:018:0o-18:0) ; TG(14:0o-18:018:0) ; TG(15:018:2(9Z12Z)o-18:0) ; TG(15:020:2n6o-18:0) ; TG(15:0o-18:018:2(9Z12Z)) ; TG(15:0o-18:020:2n6) ; TG(16:016:0o-18:0) ; TG(16:0o-18:016:0) ; TG(18:014:0o-18:0) ; TG(14:1(9Z)20:1(11Z)o-18:0) ; TG(14:1(9Z)o-18:020:1(11Z)) ; TG(16:1(9Z)18:1(11Z)o-18:0) ; TG(16:1(9Z)18:1(9Z)o-18:0) ; TG(16:1(9Z)o-18:018:1(11Z)) ; TG(16:1(9Z)o-18:018:1(9Z)) ; TG(18:1(11Z)16:1(9Z)o-18:0) ; TG(18:1(9Z)16:1(9Z)o-18:0) ; TG(20:1(11Z)14:1(9Z)o-18:0) ; TG(18:2(9Z12Z)15:0o-18:0) ; TG(20:2n615:0o-18:0)	2M+ACN+Na ; M+ACN+2H ; M+ACN+2H ; M+H+NH4 ; M+H+NH4 ; M+H+NH4 ; M+H+NH4 ; M+ACN+2H ; M+ACN+2H ; M+ACN+2H ; M+H+NH4 ; M+H+NH4 ; M+H+NH4 ; M+H+NH4 ; M+H+NH4 ; M+H+NH4 ; M+H+NH4 ; M+H+NH4 ; M+H+NH4 ; M+H+NH4 ; M+H+NH4	C4H8S4 ; C53H104O5 ; C53H104O5 ; C55H104O5 ; C55H104O5 ; C55H104O5 ; C55H104O5 ; C53H104O5 ; C53H104O5 ; C53H104O5 ; C55H104O5 ; C55H104O5 ; C55H104O5 ; C55H104O5 ; C55H104O5 ; C55H104O5 ; C55H104O5 ; C55H104O5 ; C55H104O5 ; C55H104O5 ; C55H104O5
240.56681	1.442827	1.417686	-3.52	0.005538	2.2566	0.033646	240.5668	HMDB38093 ; HMDB38097 ; HMDB41684 ; HMDB29737 ; HMDB36617 ; HMDB59614	Petunidin 3-galactoside ; Petunidin 3-glucoside ; 4-O-Methyldelphinidin 3-O-beta-D-glucoside ; 1H-Indole-3-carboxaldehyde ; ()-Chebulic acid ; Riboflavin cyclic-45-phosphate	M+2H ; M+2H ; M+2H ; 3M+2Na ; M+3ACN+2H ; M+ACN+2H	C22H23O12 ; C22H23O12 ; C22H23O12 ; C9H7NO ; C14H12O11 ; C17H19N4O8P

259.03849	1.45105	1.049814	-3.5186	0.005551	2.2556	0.033677	259.0385	HMDB28750 ; HMDB28771 ; HMDB29467 ; HMDB29473 ; HMDB40055 ; HMDB41442 ; HMDB60002	Aspartyl-Cysteine ; Cysteinyl-Aspartate ; Eugenitin ; Isoeugenitin ; Ethyl 3-(2-furanylmethyl)thiopropanoate ; Hispolone ; Indole-3-carboxilic acid-O-sulphate	M+Na ; M+Na ; M+K ; M+K ; M+2Na-H ; M+K ; M+NH4	C7H12N2O5S ; C7H12N2O5S ; C12H12O4 ; C12H12O4 ; C10H14O3S ; C12H12O4 ; C9H7NO5S
218.35004	1.408603	0.424979	-3.5164	0.005572	2.254	0.033677	218.35	HMDB37512	CI Food Brown 3	M+H+2Na	C27H20N4O9S2
201.08685	1.411263	0.682632	3.5163	0.005573	2.2539	0.033677	201.0869	HMDB00017 ; HMDB02043 ; HMDB05026 ; HMDB15221 ; HMDB31250 ; HMDB31864 ; HMDB33376 ; HMDB33386 ; HMDB33597 ; HMDB33625 ; HMDB34061 ; HMDB35013 ; HMDB35099 ; HMDB36235 ; HMDB36385 ; HMDB37680 ; HMDB37708 ; HMDB38179 ; HMDB40287 ; HMDB40356 ; HMDB40411 ; HMDB40425 ; HMDB40426 ; HMDB40583 ; HMDB40634 ; HMDB40800 ; HMDB41553 ; HMDB59701 ; HMDB59733	4-Pyridoxic acid ; 5-Phenylvaleric acid ; Rabeprazole ; Deserpidine ; Phenylmethyl 2-methylpropanoate ; Methyleugenol ; Phenylmethyl butanoate ; 3-Phenylpropyl acetate ; 4-(4-Methoxyphenyl)-2-butanone ; (3R7R)-137-Octanetriol ; Withaperuvin H ; 2-Phenylethyl propanoate ; gamma-Crocetin ; alphaalpha-Dimethylphenethyl formate ; Methyl 4-phenylbutanoate ; 2-Methylphenyl 2-methylpropanoate ; p-Tolyl isobutyrate ; 3-(2-Hydroxy-4-methylphenyl)-2-butanone ; 1-Phenylethyl propanoate ; Osmorhizole ; Ethyl 3-phenylpropanoate ; Propyl phenylacetate ; Isopropyl phenylacetate ; 2-Methylpropyl benzoate ; Piperochromanoic acid ; 2-Ethoxy-5-(1-propenyl)phenol ; Methylisoeugenol ; 167-Trimethylnaphthalene ; 145-Trimethyl-naphtalene	M+NH4 ; M+Na ; M+ACN+2H ; M+2H+Na ; M+Na ; M+Na ; M+Na ; M+Na ; M+Na ; M+K ; M+2H+Na ; M+Na ; M+2Na ; M+Na ; M+Na ; M+Na ; M+Na ; M+Na ; M+Na ; M+Na ; M+Na ; M+Na ; M+Na ; M+Na ; M+2Na ; M+Na ; M+Na ; 2M+Na+K ; 2M+Na+K	C8H9NO4 ; C11H14O2 ; C18H21N3O3S ; C32H38N2O8 ; C11H14O2 ; C11H14O2 ; C11H14O2 ; C11H14O2 ; C11H14O2 ; C8H18O3 ; C30H42O9S ; C11H14O2 ; C22H28O4 ; C11H14O2 ; C11H14O2 ; C11H14O2 ; C11H14O2 ; C11H14O2 ; C11H14O2 ; C11H14O2 ; C11H14O2 ; C11H14O2 ; C11H14O2 ; C11H14O2 ; C22H28O4 ; C11H14O2 ; C11H14O2 ; C13H14 ; C13H14
364.06508	1.384687	1.060031	-3.5159	0.005577	2.2536	0.033677	364.0651	HMDB01397 ; HMDB11670 ; HMDB59639 ; HMDB00833 ; HMDB02171 ; HMDB15544 ; HMDB15692 ; HMDB34682 ; HMDB37337 ; HMDB39087 ; HMDB39088 ; HMDB39776 ; HMDB41563 ; HMDB61089 ; HMDB61090	Guanosine monophosphate ; 8-Oxo-dGMP ; Cyclic pyranopterin monophosphate ; N-Glycolylneuraminic acid ; Glycylprolylhydroxyproline ; Tazobactam ; Isothipendyl ; cis-Ferulic acid arabinosyl-(1-3)-glucosyl-(1-6)-glucosyl ester ; 7-(4-Carboxy-3-hydroxy-3-methylbutanoyl)sudachitin 4-glucoside ; Sudachiin B ; Sudachiin C ; 6-O-Malonylnaringin ; Cerdlan ; Alpha-hydroxymidazolam ; 4-hydroxymidazolam	M+H ; M+H ; M+H ; M+K ; M+2K+H ; M+ACN+Na ; M+2K+H ; M+2K ; M+Na+K ; M+Na+K ; M+Na+K ; M+Na+K ; M+2Na ; M+Na ; M+Na	C10H14N5O8P ; C10H14N5O8P ; C10H14N5O8P ; C11H19NO10 ; C12H19N3O5 ; C10H12N4O5S ; C16H19N3S ; C27H38O18 ; C30H34O17 ; C30H34O17 ; C30H34O17 ; C30H34O17 ; C27H44I2N2O2 ; C18H13ClFN3O ; C18H13ClFN3O
342.60172	1.467791	0.075772	-3.5159	0.005577	2.2536	0.033677	342.6017	HMDB35267	Methyl 3b-hydroxy-13(18)-oleanen-28-oate	2M+3H	C33H52O4
290.66821	1.424576	0.589386	-3.5124	0.00561	2.2511	0.033833	290.6682	HMDB05771 ; HMDB11116 ; HMDB11491 ; HMDB11521	Rigin ; Phytyl diphosphate ; LysoPE(0:022:1(13Z)) ; LysoPE(22:1(13Z)0:0)	M+3ACN+2H ; M+3ACN+2H ; M+2Na ; M+2Na	C18H32N8O6 ; C20H42O7P2 ; C27H54NO7P ; C27H54NO7P
456.69946	1.414136	1.62528	3.5106	0.005626	2.2498	0.033889	NA	NA	NA	NA	NA
178.05069	1.445871	1.500922	-3.5076	0.005654	2.2476	0.034019	178.0507	HMDB40612 ; HMDB31981 ; HMDB00839 ; HMDB14742 ; HMDB15102 ; HMDB15104 ; HMDB35028 ; HMDB60386 ; HMDB60395	4-Acetyl-2(3H)-benzoxazolone ; Niazidin ; Pentaporphyrin I ; Cisapride ; Desloratadine ; Alosetron ; (x)-2-Heptanol glucoside ; 4-Fluoromuconolactone ; 5-Fluoromuconolactone	M+H ; M+2H ; M+2Na ; M+3Na ; M+2Na ; M+Na+K ; M+2K ; M+NH4 ; M+NH4	C9H7NO3 ; C15H18N2O6S ; C20H14N4 ; C23H29ClFN3O4 ; C19H19ClN2 ; C17H18N4O ; C13H26O6 ; C6H5FO4 ; C6H5FO4
742.53860	1.413785	0.508644	3.5046	0.005682	2.2455	0.034148	742.5386	HMDB10254 ; HMDB10255 ; HMDB10256 ; HMDB10257 ; HMDB56503 ; HMDB56504 ; HMDB56524 ; HMDB56528 ; HMDB56782 ; HMDB56834 ; HMDB56929 ; HMDB56930 ; HMDB56950 ; HMDB56954 ; HMDB56985 ; HMDB56995 ; HMDB57012 ; HMDB57013 ; HMDB57019 ; HMDB57029 ; HMDB57095 ; HMDB57126 ; HMDB57127 ; HMDB57147 ; HMDB57178 ; HMDB57179 ; HMDB57208 ; HMDB57212 ; HMDB57216 ; HMDB57217 ; HMDB57244 ; HMDB57248 ; HMDB57912 ; HMDB57913 ; HMDB58118 ; HMDB58365 ; HMDB58366 ; HMDB58531 ; HMDB58720 ; HMDB58721 ; HMDB07730 ; HMDB07759 ; HMDB07786 ; HMDB07787 ; HMDB07861 ; HMDB07862 ; HMDB07863 ; HMDB07864 ; HMDB07865 ; HMDB07935 ; HMDB07967 ; HMDB08833 ; HMDB08925 ; HMDB08989 ; HMDB09217 ; HMDB10566 ;	CL(20:1(11Z)18:2(9Z12Z)18:1(11Z)18:1(11Z)) ; CL(20:1(11Z)18:2(9Z12Z)18:1(11Z)18:1(9Z)) ; CL(20:1(11Z)18:2(9Z12Z)18:1(9Z)18:1(11Z)) ; CL(20:1(11Z)18:2(9Z12Z)18:1(9Z)18:1(9Z)) ; CL(16:018:018:022:5(4Z7Z10Z13Z16Z)) ; CL(16:018:018:022:5(7Z10Z13Z16Z19Z)) ; CL(16:018:022:5(4Z7Z10Z13Z16Z)18:0) ; CL(16:018:022:5(7Z10Z13Z16Z19Z)18:0) ; CL(16:022:5(4Z7Z10Z13Z16Z)18:018:0) ; CL(16:022:5(7Z10Z13Z16Z19Z)18:018:0) ; CL(18:016:018:022:5(4Z7Z10Z13Z16Z)) ; CL(18:016:018:022:5(7Z10Z13Z16Z19Z)) ; CL(18:016:022:5(4Z7Z10Z13Z16Z)18:0) ; CL(18:016:022:5(7Z10Z13Z16Z19Z)18:0) ; CL(18:018:018:1(11Z)20:4(5Z8Z11Z14Z)) ; CL(18:018:018:1(9Z)20:4(5Z8Z11Z14Z)) ; CL(18:018:020:4(5Z8Z11Z14Z)18:1(11Z)) ; CL(18:018:020:4(5Z8Z11Z14Z)18:1(9Z)) ; CL(18:018:022:5(4Z7Z10Z13Z16Z)16:0) ; CL(18:018:022:5(7Z10Z13Z16Z19Z)16:0) ; CL(18:018:1(11Z)18:020:4(5Z8Z11Z14Z)) ; CL(18:018:1(11Z)18:2(9Z12Z)18:2(9Z12Z)) ; CL(18:018:1(11Z)20:4(5Z8Z11Z14Z)18:0) ; CL(18:018:1(9Z)18:020:4(5Z8Z11Z14Z)) ; CL(18:018:1(9Z)18:2(9Z12Z)18:2(9Z12Z)) ; CL(18:018:1(9Z)20:4(5Z8Z11Z14Z)18:0) ; CL(18:018:2(9Z12Z)18:1(11Z)18:2(9Z12Z)) ; CL(18:018:2(9Z12Z)18:1(9Z)18:2(9Z12Z)) ; CL(18:018:2(9Z12Z)18:2(9Z12Z)18:1(11Z)) ; CL(18:018:2(9Z12Z)18:2(9Z12Z)18:1(9Z)) ; CL(18:020:4(5Z8Z11Z14Z)18:1(11Z)18:0) ; CL(18:020:4(5Z8Z11Z14Z)18:1(9Z)18:0) ; CL(18:1(11Z)18:018:2(9Z12Z)18:2(9Z12Z)) ; CL(18:1(11Z)18:020:4(5Z8Z11Z14Z)18:0) ; CL(18:1(11Z)18:2(9Z12Z)18:2(9Z12Z)18:0) ; CL(18:1(9Z)18:018:2(9Z12Z)18:2(9Z12Z)) ; CL(18:1(9Z)18:020:4(5Z8Z11Z14Z)18:0) ; CL(18:1(9Z)18:2(9Z12Z)18:2(9Z12Z)18:0) ; CL(18:2(9Z12Z)18:018:2(9Z12Z)18:1(11Z)) ; CL(18:2(9Z12Z)18:018:2(9Z12Z)18:1(9Z)) ; DG(22:5(4Z7Z10Z13Z16Z)22:6(4Z7Z10Z13Z16Z19Z)0:0) ; DG(22:5(7Z10Z13Z16Z19Z)22:6(4Z7Z10Z13Z16Z19Z)0:0) ; DG(22:6(4Z7Z10Z13Z16Z19Z)22:5(4Z7Z10Z13Z16Z)0:0) ; DG(22:6(4Z7Z10Z13Z16Z19Z)22:5(7Z10Z13Z16Z19Z)0:0) ; PA(18:018:2(9Z12Z)) ; PA(18:1(11Z)18:1(11Z)) ; PA(18:1(11Z)18:1(9Z)) ; PA(18:1(9Z)18:1(11Z)) ; PA(18:1(9Z)18:1(9Z)) ; PC(15:016:0) ; PC(16:015:0) ; PE(14:020:0) ; PE(16:018:0) ; PE(18:016:0) ; PE(20:014:0) ; PE-NMe2(16:016:0) ; 9-Octadecenoic acid 1-(phosphonoxy)methyl-12-ethanediyl ester ; Soyasapogenol A ; Camelliagenin A ; Priverogenin B ; (3alphaOH20S24S)-319:2024-Dienoxydammarane-325-diol ;	M+2H ; 2M+3H2O+2H ; 2M+3H2O+2H ; 2M+3H2O+2H ; 2M+3H2O+2H ; M+ACN+H ; M+ACN+H ; M+ACN+H ; M+ACN+H ; M+ACN+H ; M+Na ; M+Na ; M+Na ; M+Na ; M+Na ; M+Na ; M+Na ; M+ACN+H ; 3M+Na+K ; 3M+Na+K ; 3M+Na+K ; 3M+Na+K ; 3M+Na+K ; 3M+Na+K ; M+ACN+Na ; M+ACN+Na ; 2M+3H2O+2H ; 2M+3H2O+2H ; M+H+Na ; M+H+Na ; M+H+Na ; M+H+Na ; M+H+Na ; M+H+Na ; M+H+Na ; M+H+Na ; M+H+Na ; M+H+Na ; M+H+Na ; M+H+Na ; M+H+Na ; M+H+Na	C83H152O17P2 ; C47H70O5 ; C47H70O5 ; C47H70O5 ; C47H70O5 ; C39H73O8P ; C39H73O8P ; C39H73O8P ; C39H73O8P ; C39H73O8P ; C39H78NO8P ; C39H78NO8P ; C39H78NO8P ; C39H78NO8P ; C39H78NO8P ; C39H78NO8P ; C39H78NO8P ; C39H73O8P ; C30H50O4 ; C30H50O4 ; C30H50O4 ; C30H50O4 ; C30H50O4 ; C30H50O4 ; C44H70O5 ; C44H70O5 ; C47H70O5 ; C47H70O5 ; C81H154O17P2 ; C81H154O17P2 ; C81H154O17P2 ; C81H154O17P2 ; C81H154O17P2 ; C81H154O17P2 ; C81H154O17P2 ; C81H154O17P2 ; C81H154O17P2 ; C81H154O17P2 ; C81H154O17P2 ; C81H154O17P2 ; C81H154O17P2 ; C81H154O17P2 ; C81H154O17P2
621.32012	1.462825	1.273081	-3.5018	0.00571	2.2434	0.034272	621.3201	HMDB00153 ; HMDB00338 ; HMDB00347 ; HMDB00356 ; HMDB00431 ; HMDB04843 ; HMDB05896 ; HMDB12075 ; HMDB15564 ; HMDB32522 ; HMDB40864 ; HMDB60144 ; HMDB60353 ; HMDB60999	Estriol ; 2-Hydroxyestradiol ; 16b-Hydroxyestradiol ; 17-Epiestriol ; 1617-Epiestriol ; Ganglioside GM3 (d18:19Z-18:1) ; 4-Hydroxyestradiol ; Ganglioside GT3 (d18:122:1(13Z)) ; Lincomycin ; Stearyl monoglyceridyl citrate ; O-Geranylvanillin ; Neocasomorphin (1-5) ; 2-Polyprenyl-3-methyl-6-methoxy-14-benzoquinone ; 4-hydroxystradiol	2M+2Na-H ; 2M+2Na-H ; 2M+2Na-H ; 2M+2Na-H ; 2M+2Na-H ; M+2K ; 2M+2Na-H ; M+H+2Na ; 3M+H+Na ; M+K ; 2M+2Na-H ; M+NH4 ; 2M+2Na-H ; 2M+2Na-H	C18H24O3 ; C18H24O3 ; C18H24O3 ; C18H24O3 ; C18H24O3 ; C58H104N2O21 ; C18H24O3 ; C85H148N4O37 ; C18H34N2O6S ; C28H54O12 ; C18H24O3 ; C29H41N5O9 ; C18H24O3 ; C18H24O3

173.02781	1.376423	0.873077	-3.4992	0.005734	2.2415	0.034339	173.0278	HMDB59933 ; HMDB11683 ; HMDB37199 ; HMDB37201 ; HMDB37202 ; HMDB01227 ; HMDB15606 ; HMDB29544 ; HMDB31555 ; HMDB33850 ; HMDB37476 ; HMDB38363 ; HMDB39360 ; HMDB40293 ; HMDB41768 ; HMDB41849	p-Toluenesulfonic acid ; Molybdopterin precursor Z ; 2-O-Galloyl-14-galactarolactone ; 3-O-Galloyl-14-galactarolactone ; 5-O-Galloyl-14-galactarolactone ; 5-Thymidylic acid ; Lofexidine ; Miscanthoside ; Maesopsin 6-glucoside ; Astilbin ; Neocarthamin ; (-)-Epigallocatechin 3-(3-methyl-gallate) ; ()-Flufenprox ; (-)-Epigallocatechin 3-(4-methyl-gallate) ; Phloretin 2-O-glucuronide ; Carboxytolbutamide	M+H ; M+2H ; M+2H ; M+2H ; M+2H ; M+H+Na ; 2M+3H ; M+3Na ; M+3Na ; M+3Na ; M+3Na ; M+H+2Na ; M+3Na ; M+H+2Na ; M+3Na ; M+2Na	C7H8O3S ; C10H11N5O7P ; C13H12O11 ; C13H12O11 ; C13H12O11 ; C10H15N2O8P ; C11H12Cl2N2O ; C21H22O11 ; C21H22O11 ; C21H22O11 ; C21H22O11 ; C23H20O11 ; C24H22ClF3O3 ; C23H20O11 ; C21H22O11 ; C12H16N2O5S
402.68879	1.427566	1.610389	3.4992	0.005735	2.2415	0.034339	402.6888	HMDB14671 ; HMDB38449 ; HMDB38643	Erlotinib ; Vomifoliol 9-glucosyl-(1-4)-xylosyl-(1-6)-glucoside ; Epoxyfumitremorgin C	2M+H+NH4 ; M+3ACN+2H ; 2M+H+NH4	C22H23N3O4 ; C30H48O17 ; C22H23N3O4
500.26072	1.440641	1.083338	-3.4975	0.005751	2.2403	0.034396	500.2607	HMDB06709 ; HMDB29991 ; HMDB30064 ; HMDB30324 ; HMDB30325 ; HMDB30338 ; HMDB31373 ; HMDB32383 ; HMDB32421 ; HMDB33364 ; HMDB34760 ; HMDB35218 ; HMDB35344 ; HMDB35500 ; HMDB38732 ; HMDB39277 ; HMDB39839 ; HMDB39840	Ubiquinone-2 ; Dulciol C ; Cohulupone ; (R)-Pelletierine ; ()-Pelletierine ; Physoperuvine ; (1(10)E4E6a9b)-9-(2-Methylpropanoyloxy)-1(10)411(13)-germacratrien-126-olide ; N1-(2-Methoxy-4-methylbenzyl)-n2-(2-(pyridin-2-yl) ethyl)oxalamide ; 2-(4-Methyl-5-thiazolyl)ethyl hexanoate ; Conhydrinone ; Hydroxystrobilurin D ; Cinereain ; (3b16a20R)-316202225-Pentahydroxy-5-cucurbiten-11-one 3-rhamnosyl-(1-4)-glucosyl-(1-6)-glucoside ; Panaquinquecol 6 ; alpha-Ionol O-arabinosyl-(1-6)-glucoside ; 8-Dehydrogingerdione ; 1-(1-Pyrrolidinyl)-2-butanone ; 3-(1-Pyrrolidinyl)-2-butanone	3M+2Na ; M+NH4 ; 3M+2Na ; 3M+2K-H ; 3M+2K-H ; 3M+2K-H ; 3M+2Na ; 3M+H+NH4 ; 2M+NH4 ; 3M+2K-H ; M+ACN+H ; 3M+H+NH4 ; M+H+Na ; 3M+2Na ; 2M+H+Na ; 3M+2Na ; 3M+2K-H ; 3M+2K-H	C19H26O4 ; C28H34O7 ; C19H26O4 ; C8H15NO ; C8H15NO ; C8H15NO ; C19H26O4 ; C18H21N3O3 ; C12H19NO2S ; C8H15NO ; C26H34O7 ; C18H21N3O3 ; C48H80O20 ; C19H26O4 ; C24H40O10 ; C19H26O4 ; C8H15NO ; C8H15NO
460.69364	1.4144	1.614337	3.4959	0.005766	2.2391	0.034406	460.6936	HMDB13019 ; HMDB14830 ; HMDB59786 ; HMDB60955 ; HMDB60957	Neuromedin C 1-8 ; Phentolamine ; Gliadorphin ; mirtazapine-N-oxide ; 8-hydroxymirtazapine	M+2Na ; 3M+2K ; M+2Na ; 3M+2K ; 3M+2K	C39H53N15O9 ; C17H19N3O ; C43H57N9O11 ; C17H19N3O ; C17H19N3O
344.87502	1.460426	1.220046	3.4958	0.005766	2.2391	0.034406	NA	NA	NA	NA	NA
516.85414	1.38104	1.491491	-3.4937	0.005787	2.2376	0.034464	NA	NA	NA	NA	NA
624.03705	1.406788	0.649365	-3.4934	0.00579	2.2373	0.034464	NA	NA	NA	NA	NA
274.79171	1.412709	1.667606	3.4897	0.005826	2.2346	0.034638	NA	NA	NA	NA	NA
144.55158	1.413104	0.866736	-3.4882	0.00584	2.2336	0.034675	144.5516	HMDB33718 ; HMDB00881 ; HMDB15229 ; HMDB15636 ; HMDB28805 ; HMDB29015 ; HMDB29031 ; HMDB29157 ; HMDB32931 ; HMDB32963 ; HMDB33528 ; HMDB39845 ; HMDB40694 ; HMDB60945	Clitocine ; Xanthurenic acid ; Leflunomide ; Agomelatine ; Glutaminyl-Proline ; Prolyl-Glutamine ; Prolyl-Gamma-glutamate ; Gamma-glutamyl-Proline ; 2-Benzoxazolol ; Zeanic acid ; 46-Dihydroxy-2-quinolinecarboxylic acid ; 3-Oxooctanoic acid glycerides ; Agaritinal ; A771726	M+2H ; M+2ACN+2H ; M+H+NH4 ; M+2Na ; M+2Na ; M+2Na ; M+2Na ; M+2Na ; 2M+H+NH4 ; M+2ACN+2H ; M+2ACN+2H ; M+H+K ; M+H+Na ; M+H+NH4	C9H13N5O6 ; C10H7NO4 ; C12H9F3N2O2 ; C15H17NO2 ; C10H17N3O4 ; C10H17N3O4 ; C10H17N3O4 ; C10H17N3O4 ; C7H5NO2 ; C10H7NO4 ; C10H7NO4 ; C11H21O6 ; C12H15N3O4 ; C12H9F3N2O2
228.04551	1.384364	1.409628	3.487	0.005852	2.2327	0.034675	228.0455	HMDB00797 ; HMDB00181 ; HMDB00609 ; HMDB12210 ; HMDB15318 ; HMDB29510 ; HMDB34436 ; HMDB38336 ; HMDB38750 ; HMDB40932 ; HMDB41620 ; HMDB41868 ; HMDB60589 ; HMDB60833 ; HMDB60599	SAICAR ; L-Dopa ; DL-Dopa ; Dihydrolipoate ; Iophendylate ; Garcinone B ; Rotenone ; 2-Hydroxy-3-(34-dihydroxyphenyl)propanamide ; N-Hydroxy-L-tyrosine ; 4-Chloro-35-dimethoxybenzaldehyde ; 17-Beta-Estradiol-317-beta-sulfate ; Desacetylcefotaxime ; Aminofurantoin ; N-Acetylserotonin glucuronide ; Lamivudine sulfoxide	M+2H ; 2M+Na+K ; 2M+Na+K ; 2M+H+K ; M+H+K ; M+Na+K ; M+Na+K ; 2M+Na+K ; 2M+Na+K ; 2M+3H2O+2H ; M+H+Na ; M+ACN+2H ; 2M+H+K ; M+Na+K ; M+H-H2O	C13H19N4O12P ; C9H11NO4 ; C9H11NO4 ; C8H16O2S2 ; C19H29IO2 ; C23H22O6 ; C23H22O6 ; C9H11NO4 ; C9H11NO4 ; C9H9ClO3 ; C18H24O8S2 ; C14H15N5O6S2 ; C8H8N4O3 ; C18H22N2O8 ; C8H11N3O4S
386.76077	1.389989	1.427876	3.486	0.005862	2.2319	0.034675	386.7608	HMDB34994 ; HMDB36430 ; HMDB38348	Chikusetsusaponin Ia ; Momordicoside K ; Goyaglycoside a	M+H+NH4 ; M+3ACN+2H ; M+3ACN+2H	C41H70O12 ; C37H60O9 ; C37H60O9
357.05553	1.422969	0.085873	3.4854	0.005868	2.2315	0.034675	357.0555	HMDB30590 ; HMDB31795 ; HMDB33100 ; HMDB36343 ; HMDB37570	Dihydrosterigmatocystin ; Iprodione ; Atroviridin ; O-Demethylforbexanthone ; Demethoxykanugin	2M+Na+K ; 2M+3H2O+2H ; 2M+Na+K ; 2M+Na+K ; 2M+Na+K	C18H14O6 ; C13H13Cl2N3O3 ; C18H14O6 ; C18H14O6 ; C18H14O6
328.94602	1.382593	1.543924	-3.4853	0.005869	2.2314	0.034675	328.946	HMDB01474 ; HMDB34395 ; HMDB60000	34-Dihydroxyphenylglycol O-sulfate ; 2-Propenyl 3-(2-propenylsulfonyl)-1-propenyl disulfide ; Hydroxymethoxyphenylcarboxylic acid-O-sulphate	M+2K+H ; M+2K+H ; M+2K+H	C8H10O7S ; C9H14O2S3 ; C8H10O7S
277.08369	1.418111	1.607662	-3.4848	0.005874	2.2311	0.034675	277.0837	HMDB13162 ; HMDB38710	2-Hexenoylcholine ; (7R8R)-47-Epoxy-3-methoxy-4599-lignanetetrol 9-glucoside	M+2K-H ; M+2Na	C11H22NO2 ; C25H32O11
315.17413	1.443082	0.417589	3.4838	0.005884	2.2303	0.034694	315.174	HMDB02697 ; HMDB03156 ; HMDB04590 ; HMDB05886 ; HMDB06547 ; HMDB13824 ; HMDB14812 ; HMDB14887 ; HMDB15005 ; HMDB30061 ; HMDB31084 ; HMDB31152 ; HMDB31195 ; HMDB31213 ; HMDB31261 ; HMDB31320 ; HMDB31685 ; HMDB32464 ; HMDB32466 ; HMDB32672 ; HMDB33007 ; HMDB33434 ; HMDB33857 ; HMDB33874 ; HMDB34382 ; HMDB34603 ; HMDB35229 ; HMDB35929 ; HMDB36069 ; HMDB36091 ; HMDB36092 ; HMDB37956 ; HMDB40338 ; HMDB40699 ; HMDB41956 ; HMDB60548 ; HMDB60796 ; HMDB60798 ; HMDB60854	19-Norandrosterone ; 23-Butanediol ; 19-Nor-5-androstenediol ; 19-Noretiocholanolone ; Stearidonic acid ; 2-Pentylfuran ; Galantamine ; Etodolac ; Ritodrine ; 11-Dimethoxyethane ; 2-Pentylfuran n ; (3Z6Z)-36-Nonadienal ; 355-Trimethyl-2-cyclohexen-1-one ; 2-Ethoxyethanol ; (2E6E)-26-Nonadienal ; 13-Butanediol ; 24-Nonadienal ; Phenethyl decanoate ; Phenol-formaldehyde cross-linked triethylenetetramine activated ; 481215-Octadecatetraenoic acid ; (2R3R)-23-Butanediol ; 4-Acetyl-1-methylcyclohexene ; Violet-leaf aldehyde ; Piperanine ; (EE)-1113-Octadecadien-9-ynoic acid ; Hebevinoside XIII ; 5-Isopropylbicyclo310hexan-2-one ; (R)-Cryptone ; Pulegenone ; exo-56-Dimethylbicyclo221hept-5-en-2-ol ; exo-2-Methyl-3-methylenebicyclo221heptan-2-ol ; (24R)-5b8b-Epidioxyergosta-622E-dien-3b-ol 3-glucoside ; 355-Trimethyl-3-cyclohexen-1-one ; Feruperine ; Nordihydrocodeine ; Dihydromorphine ; 6alpha-Hydroxy-hydromorphone ; 6beta-Hydroxy-hydromorphone ; Nordihydroisomorphine	M+K ; 3M+2Na-H ; M+K ; M+K ; M+K ; 2M+K ; 2M+3H2O+2H ; 2M+3H2O+2H ; 2M+3H2O+2H ; 3M+2Na-H ; 2M+K ; 2M+K ; 2M+K ; 3M+2Na-H ; 2M+K ; 3M+2Na-H ; 2M+K ; M+K ; M+2Na-H ; M+K ; 3M+2Na-H ; 2M+K ; 2M+K ; 2M+3H2O+2H ; M+K ; M+2H+Na ; 2M+K ; 2M+K ; 2M+K ; 2M+K ; 2M+K ; M+H+K ; 2M+K ; 2M+3H2O+2H ; 2M+3H2O+2H ; 2M+3H2O+2H ; 2M+3H2O+2H ; 2M+3H2O+2H ; 2M+3H2O+2H	C18H28O2 ; C4H10O2 ; C18H28O2 ; C18H28O2 ; C18H28O2 ; C9H14O ; C17H21NO3 ; C17H21NO3 ; C17H21NO3 ; C4H10O2 ; C9H14O ; C9H14O ; C9H14O ; C4H10O2 ; C9H14O ; C4H10O2 ; C9H14O ; C18H28O2 ; C13H26N4O2 ; C18H28O2 ; C4H10O2 ; C9H14O ; C9H14O ; C17H21NO3 ; C18H28O2 ; C49H76O16 ; C9H14O ; C9H14O ; C9H14O ; C9H14O ; C9H14O ; C34H54O8 ; C9H14O ; C17H21NO3 ; C17H21NO3 ; C17H21NO3 ; C17H21NO3 ; C17H21NO3 ; C17H21NO3

218.13865	1.414524	0.706429	-3.4826	0.005896	2.2295	0.034714	218.1387	HMDB00824 ; HMDB00666 ; HMDB30028 ; HMDB30052 ; HMDB31528 ; HMDB31594 ; HMDB31595 ; HMDB31600 ; HMDB32204 ; HMDB32874 ; HMDB33745 ; HMDB34166 ; HMDB34239 ; HMDB35238 ; HMDB36209 ; HMDB36237 ; HMDB36238 ; HMDB38227 ; HMDB39095 ; HMDB39618 ; HMDB39619 ; HMDB40297	Propionylcarnitine ; Heptanoic acid ; Ethyl isovalerate ; Butyl propionate ; Isopentyl acetate ; 2-Methylhexanoic acid ; 5-Methylhexanoic acid ; ()-2-Pentanol acetate ; cis- and trans-2-Isopropyl-4-methyl-13-dioxolane ; Hexyl formate ; Ethyl 2-methylbutyrate ; 2-Methylbutyl acetate ; 2-Methylpropyl propanoate ; Methyl hexanoate ; Propyl 2-methylpropanoate ; Methyl ()-2-methylpentanoate ; Methyl 4-methylpentanoate ; Isopropyl 2-methylpropanoate ; Pentyl acetate ; Propyl butyrate ; Isopropyl butyrate ; Ethyl pentanoate	M+H ; 3M+2Na	C10H19NO4 ; C7H14O2
458.69635	1.417013	1.612933	3.482	0.005901	2.229	0.034714	458.6964	HMDB15406	Glisoxepide	2M+H+NH4	C20H27N5O5S
470.57886	1.348207	0.851347	-3.4796	0.005925	2.2273	0.034779	470.5789	HMDB11660 ; HMDB12969 ; HMDB41160	2-Methylhexanoyl-CoA ; Heptanoyl-CoA ; Cyanidin 3-O-b-D-Xylopyranosyl-(1-2)-(4-hydroxybenzoyl)-(-6)-b-D-glucopyranosyl-(1-6)-b-D-galactopyranoside	M+Na+K ; M+Na+K ; M+2K	C28H48N7O17P3S ; C28H48N7O17P3S ; C39H43O22
225.49927	1.427739	1.566613	-3.4795	0.005926	2.2272	0.034779	225.4993	HMDB34071	Progoitrin	M+Na+K	C11H19NO10S2
260.19684	1.369524	0.989916	3.478	0.005941	2.2262	0.034782	260.1968	HMDB28912 ; HMDB28934 ; HMDB28954 ; HMDB28955 ; HMDB09281 ; HMDB09282 ; HMDB09313 ; HMDB09576 ; HMDB09611 ; HMDB09612 ; HMDB09643 ; HMDB09676 ; HMDB11357 ; HMDB11382 ; HMDB11392 ; HMDB11393 ; HMDB11414 ; HMDB11424 ; HMDB11447 ; HMDB11457 ; HMDB13334	Isoleucyl-Lysine ; Leucyl-Lysine ; Lysyl-Isoleucine ; Lysyl-Leucine ; PE(20:1(11Z)P-18:1(11Z)) ; PE(20:1(11Z)P-18:1(9Z)) ; PE(20:2(11Z14Z)P-18:0) ; PE(22:2(13Z16Z)P-16:0) ; PE(22:4(7Z10Z13Z16Z)P-18:1(11Z)) ; PE(22:4(7Z10Z13Z16Z)P-18:1(9Z)) ; PE(22:5(4Z7Z10Z13Z16Z)dm18:0) ; PE(22:5(7Z10Z13Z16Z19Z)P-18:0) ; PE(P-16:022:2(13Z16Z)) ; PE(P-18:020:2(11Z14Z)) ; PE(P-18:022:5(4Z7Z10Z13Z16Z)) ; PE(P-18:022:5(7Z10Z13Z16Z19Z)) ; PE(P-18:1(11Z)20:1(11Z)) ; PE(P-18:1(11Z)22:4(7Z10Z13Z16Z)) ; PE(P-18:1(9Z)20:1(11Z)) ; PE(P-18:1(9Z)22:4(7Z10Z13Z16Z)) ; 912-Hexadecadienoylcarnitine	M+H ; M+H ; M+H ; M+H ; M+2H+Na ; M+2H+Na ; M+2H+Na ; M+2H+Na ; M+3H ; M+3H ; M+3H ; M+3H ; M+2H+Na ; M+2H+Na ; M+3H ; M+3H ; M+2H+Na ; M+3H ; M+2H+Na ; M+3H ; M+3ACN+2H	C12H25N3O3 ; C12H25N3O3 ; C12H25N3O3 ; C12H25N3O3 ; C43H82NO7P ; C43H82NO7P ; C43H82NO7P ; C43H82NO7P ; C45H80NO7P ; C45H80NO7P ; C45H80NO7P ; C45H80NO7P ; C43H82NO7P ; C43H82NO7P ; C45H80NO7P ; C45H80NO7P ; C43H82NO7P ; C45H80NO7P ; C43H82NO7P ; C45H80NO7P ; C23H41NO4
266.75294	1.369908	1.534682	-3.4778	0.005943	2.226	0.034782	NA	NA	NA	NA	NA
337.90855	1.395169	1.525128	-3.4769	0.005952	2.2254	0.034782	337.9086	HMDB10408	LysoPC(P-18:1(9Z))	2M+3H	C26H52NO6P
310.55007	1.449302	1.083112	-3.4766	0.005955	2.2251	0.034782	310.5501	HMDB01072 ; HMDB02139 ; HMDB14588 ; HMDB15132	Coenzyme Q10 ; Ubisemiquinone ; Epirubicin ; Doxorubicin	M+3Na ; M+3Na ; M+2K ; M+2K	C59H90O4 ; C59H90O4 ; C27H29NO11 ; C27H29NO11
455.95652	1.370143	1.475181	-3.4751	0.00597	2.224	0.034812	455.9565	HMDB60415 ; HMDB60789	6-Thioguanosine monophosphate ; 6-Thioguanylic acid	M+2K-H ; M+2K-H	C10H14N5O7PS ; C10H14N5O7PS
331.59839	1.432231	1.40087	-3.4747	0.005974	2.2238	0.034812	NA	NA	NA	NA	NA
243.10875	1.388657	0.546631	-3.4728	0.005993	2.2223	0.034846	243.1088	HMDB00312 ; HMDB00320 ; HMDB00326 ; HMDB00330 ; HMDB00364 ; HMDB00371 ; HMDB00376 ; HMDB00390 ; HMDB00395 ; HMDB00404 ; HMDB00414 ; HMDB00415 ; HMDB00419 ; HMDB00427 ; HMDB00432 ; HMDB00505 ; HMDB00506 ; HMDB00527 ; HMDB00619 ; HMDB00726 ; HMDB00760 ; HMDB00865 ; HMDB00917 ; HMDB00953 ; HMDB01189 ; HMDB05199 ; HMDB10337 ; HMDB13845 ; HMDB29223 ; HMDB29224 ; HMDB30372 ; HMDB30412 ; HMDB31811 ; HMDB31949 ; HMDB33763 ; HMDB39655 ; HMDB39887 ; HMDB40021 ; HMDB40155 ; HMDB40546 ; HMDB41190 ; HMDB41514 ; HMDB41931 ; HMDB42012 ; HMDB42037 ; HMDB61123	3a7a12b-Trihydroxy-5b-cholanoic acid ; 3a4b7a-Trihydroxy-5b-cholanoic acid ; 1b3a12a-Trihydroxy-5b-cholanoic acid ; 3a4b12a-Trihydroxy-5b-cholanoic acid ; 3a6a7b-Trihydroxy-5b-cholanoic acid ; 1312-Trihydroxycholan-24-oic acid ; 3b7a12a-Trihydroxy-5a-Cholanoic acid ; 3a7b12b-Trihydroxy-5b-cholanoic acid ; 3b7b12a-Trihydroxy-5b-cholanoic acid ; 2b3a7a-Trihydroxy-5b-cholanoic acid ; 1b3a7a-Trihydroxy-5b-cholanoic acid ; 3a6b7b-Trihydroxy-5b-cholanoic acid ; 3b7a12a-Trihydroxy-5b-cholanoic acid ; 3b7b12a-Trihydroxy-5a-Cholanoic acid ; 3a7b12a-Trihydroxy-5a-Cholanoic acid ; Allocholic acid ; Alpha-Muricholic acid ; 6a12a-Dihydroxylithocholic acid ; Cholic acid ; Isovalerylglutamic acid ; Hyocholic acid ; Muricholic acid ; Ursocholic acid ; Suberylglycine ; 2(N)-Methyl-norsalsolinol ; (R)-Salsolinol ; 6-Dehydrotestosterone glucuronide ; SR 49498 ; alpha-Methylphenylalanine ; N-methylphenylalanine ; Maltoxazine ; Tricholomic acid ; 35-Dimethylphenyl methylcarbamate ; Bis-N-butyl phthalate ; Ethyl N-methylanthranilate ; 123456-Hexahydro-5-(1-hydroxyethylidene)-7H-cyclopentabpyridin-7-one ; Methyl nomilinate 17-glucoside ; 23-Dihydro-5-(3-hydroxypropanoyl)-1H-pyrrolizine ; Paradisin C ; Pyriminobac-methyl ; Benzyl beta-primeveroside ; Benzyl O-arabinofuranosyl-(1-6)-glucoside ; 34-Methylenedioxyamphetamine ; Salsolinol ; Thymine glycol ; 4-Hydroxytamoxifen sulfate	M+2K ; M+2K ; M+2K ; M+2K ; M+2K ; M+2K ; M+2K ; M+2K ; M+2K ; M+2K ; M+2K ; M+2K ; M+2K ; M+2K ; M+2K ; M+2K ; M+2K ; M+2K ; M+2K ; 2M+H+Na ; M+2K ; M+2K ; M+2K ; 2M+H+Na ; M+ACN+Na ; M+ACN+Na ; M+H+Na ; M+H+K ; M+ACN+Na ; M+ACN+Na ; M+ACN+Na ; M+2ACN+H ; M+ACN+Na ; M+H+NH4 ; M+ACN+Na ; M+ACN+Na ; M+3H ; M+ACN+Na ; M+3H ; M+3ACN+2H ; M+2ACN+2H ; M+2ACN+2H ; M+ACN+Na ; M+ACN+Na ; M+2ACN+H ; M+H+NH4	C24H40O5 ; C24H40O5 ; C24H40O5 ; C24H40O5 ; C24H40O5 ; C24H40O5 ; C24H40O5 ; C24H40O5 ; C24H40O5 ; C24H40O5 ; C24H40O5 ; C24H40O5 ; C24H40O5 ; C24H40O5 ; C24H40O5 ; C24H40O5 ; C24H40O5 ; C24H40O5 ; C24H40O5 ; C10H17NO5 ; C24H40O5 ; C24H40O5 ; C24H40O5 ; C10H17NO5 ; C10H13NO2 ; C10H13NO2 ; C25H34O8 ; C25H30N6O2 ; C10H13NO2 ; C10H13NO2 ; C10H13NO2 ; C5H8N2O4 ; C10H13NO2 ; C22H29NO10 ; C10H13NO2 ; C10H13NO2 ; C35H50O16 ; C10H13NO2 ; C42H46O11 ; C17H19N3O6 ; C18H26O10 ; C18H26O10 ; C10H13NO2 ; C10H13NO2 ; C5H8N2O4 ; C26H29NO5S
166.08414	1.353248	0.671399	-3.4728	0.005993	2.2223	0.034846	166.0841	HMDB05765 ; HMDB11474 ; HMDB11504 ; HMDB60990	Ophthalmic acid ; LysoPE(0:016:1(9Z)) ; LysoPE(16:1(9Z)0:0) ; 8-Hydroxycarteolol	M+ACN+2H ; M+H+2Na ; M+H+2Na ; M+H+Na	C11H19N3O6 ; C21H42NO7P ; C21H42NO7P ; C16H24N2O4
470.00482	1.357577	0.178653	-3.4689	0.006032	2.2195	0.03502	470.0048	HMDB01192 ; HMDB38407	Diadenosine pentaphosphate ; Glucocheirolin	M+H+Na ; 2M+Na+K	C20H29N10O22P5 ; C11H21NO11S3
710.08034	1.451143	1.470432	-3.4684	0.006038	2.2191	0.03502	710.0803	HMDB00221	NADPH	M+H-2H2O	C21H30N7O17P3
342.49449	1.415371	1.211334	-3.4677	0.006044	2.2186	0.03502	342.4945	HMDB31376 ; HMDB36656 ; HMDB38608 ; HMDB38886 ; HMDB39075 ; HMDB39076 ; HMDB39517 ; HMDB39946 ; HMDB40120 ; HMDB40942	Congmuyenoside A ; Asiaticoside ; Soyasaponin A3 ; Mabioside A ; Hoduloside I ; Hoduloside II ; Soyasaponin V ; Centellasaponin C ; Centellasaponin A ; Mabiogenin 3-rhamnosyl-(1-6)-glucosyl-(1-2)-glucoside	M+3Na ; M+3Na ; M+3Na ; M+3Na ; M+3Na ; M+3Na ; M+3Na ; M+3Na ; M+3Na ; M+3Na	C48H78O19 ; C48H78O19 ; C48H78O19 ; C48H78O19 ; C48H78O19 ; C48H78O19 ; C48H78O19 ; C48H78O19 ; C48H78O19 ; C48H78O19

277.05675	1.382677	1.023663	-3.466	0.006062	2.2174	0.03508	277.0568	HMDB14917 ; HMDB15084 ; HMDB15305 ; HMDB28853 ; HMDB29105 ; HMDB29451 ; HMDB32357 ; HMDB35635 ; HMDB39249 ; HMDB39925 ; HMDB40988 ; HMDB41949 ; HMDB60316	Nalidixic Acid ; Felbamate ; Phenobarbital ; Glycyl-Tyrosine ; Tyrosyl-Glycine ; gamma-Glutaminyl-4-hydroxybenzene ; N-Lactoyl ethanolamine phosphate ; Kanokoside A ; 1-(2H-13-Benzodioxol-5-yl)-2-26-dimethoxy-4-(prop-2-en-1-yl)phenoxypropyl benzoate ; 26-Di-O-acetylononin ; Falimint ; Nitecapone ; (7R)-7-(5-Carboxy-5-oxopentanoyl)aminocephalosporinate	M+2Na-H ; M+K ; M+2Na-H ; M+K ; M+K ; M+K ; M+ACN+Na ; M+2K ; M+2K ; M+H+K ; M+K ; 2M+H+Na ; 2M+3H	C12H12N2O3 ; C11H14N2O4 ; C12H12N2O3 ; C11H14N2O4 ; C11H14N2O4 ; C11H14N2O4 ; C5H12NO6P ; C21H32O12 ; C28H28O7 ; C26H26O11 ; C11H14N2O4 ; C12H11NO6 ; C16H18N2O9S
509.92269	1.439685	0.917573	3.464	0.006082	2.2159	0.035157	509.9227	HMDB13631	Oleoyl glycine	3M+2H	C20H37NO3
193.56985	1.436145	0.216803	-3.4629	0.006093	2.2152	0.035179	193.5699	HMDB15657 ; HMDB39526 ; HMDB41143 ; HMDB61041	Nilvadipine ; Sarafloxacin ; 8-Methyldihydrochelerythrine ; dehydronifedipine	M+2H ; M+2H ; M+H+Na ; M+ACN+2H	C19H19N3O6 ; C20H17F2N3O3 ; C22H21NO4 ; C17H16N2O6
708.35344	1.356403	1.43507	-3.4615	0.006108	2.2141	0.035184	708.3534	HMDB30736 ; HMDB38376	Capsianoside IV ; Goshonoside F3	M+ACN+Na ; M+ACN+Na	C32H52O13 ; C32H52O13
255.94387	1.449999	1.325701	3.4605	0.006117	2.2134	0.035184	NA	NA	NA	NA	NA
170.98370	1.436527	0.328917	3.4605	0.006118	2.2134	0.035184	170.9837	HMDB15551 ; HMDB36649	Erythrityl Tetranitrate ; Sinalbin B	M+H+K ; M+2K	C4H6N4O12 ; C12H12N2OS2
221.10342	1.380102	1.166511	3.4596	0.006127	2.2127	0.035184	221.1034	HMDB60724 ; HMDB33468 ; HMDB02082 ; HMDB03905 ; HMDB04159 ; HMDB14526 ; HMDB15686 ; HMDB31076 ; HMDB31095 ; HMDB32156 ; HMDB33156 ; HMDB33538 ; HMDB34989 ; HMDB35844 ; HMDB35879 ; HMDB36175 ; HMDB38015 ; HMDB39240 ; HMDB41350 ; HMDB41808 ; HMDB59632 ; HMDB60741	24-Diamino-67-dimethoxyquinazoline ; Diferuloylputrescine ; Bisnorcholic acid ; Imidazole-4-acetaldehyde ; L-Urobilin ; Tacrine ; Pipazethate ; Gibberellin A105 ; Gibberellin A108 ; alpha-Acetolactate decarboxylase (enzyme preparation from bacillus subtilis recombinant) ; Methoxypyrazine ; Fragransol B ; Gibberellin A62 ; Lucidone A ; Hericenone A ; (Cyclohexylmethyl)pyrazine ; Gibberellin A121 ; Gibberellin A88 ; Gibberellin A95 ; 44-Methylenedianiline ; (9S10S)-10-hydroxy-9-(phosphonooxy)octadecanoate ; 3-Hydroxy-4-aminopyridine	M+H ; M+2H ; M+Na+K ; 2M+H ; M+3Na ; M+Na ; M+ACN+2H ; 2M+3H ; 2M+3H ; M+ACN+2H ; 2M+H ; 2M+3H ; 2M+3H ; M+H+K ; 2M+3H ; M+2Na-H ; 2M+3H ; 2M+3H ; 2M+3H ; M+Na ; M+2Na ; 2M+H	C10H12N4O2 ; C24H28N2O6 ; C22H36O5 ; C5H6N2O ; C33H46N4O6 ; C13H14N2 ; C21H25N3O3S ; C19H22O5 ; C19H22O5 ; C21H25N3O3S ; C5H6N2O ; C19H22O5 ; C19H22O5 ; C24H34O5 ; C19H22O5 ; C11H16N2 ; C19H22O5 ; C19H22O5 ; C19H22O5 ; C13H14N2 ; C18H37O7P ; C5H6N2O
270.79741	1.40734	1.640871	3.4591	0.006132	2.2124	0.035184	NA	NA	NA	NA	NA
162.94673	1.427569	1.398427	-3.4587	0.006136	2.2121	0.035184	162.9467	HMDB39734	23579-Pentathiadecane 22-dioxide	M+Na+K	C5H12O2S5
518.65242	1.398892	1.614467	3.4561	0.006163	2.2102	0.035299	518.6524	HMDB41267 ; HMDB56724 ; HMDB56756 ; HMDB56762 ; HMDB58706 ; HMDB58859 ; HMDB58967 ; HMDB60165	Quercetin 3-(6-sinapoylsophorotrioside) ; CL(16:018:2(9Z12Z)20:4(5Z8Z11Z14Z)20:4(5Z8Z11Z14Z)) ; CL(16:020:4(5Z8Z11Z14Z)18:2(9Z12Z)20:4(5Z8Z11Z14Z)) ; CL(16:020:4(5Z8Z11Z14Z)20:4(5Z8Z11Z14Z)18:2(9Z12Z)) ; CL(18:2(9Z12Z)16:020:4(5Z8Z11Z14Z)20:4(5Z8Z11Z14Z)) ; CL(18:2(9Z12Z)20:4(5Z8Z11Z14Z)20:4(5Z8Z11Z14Z)16:0) ; CL(20:4(5Z8Z11Z14Z)16:020:4(5Z8Z11Z14Z)18:2(9Z12Z)) ; Pentadecanoyl Coenzyme A	M+ACN+2H ; M+3Na ; M+3Na ; M+3Na ; M+3Na ; M+3Na ; M+3Na ; M+2Na	C44H50O26 ; C84H144O17P2 ; C84H144O17P2 ; C84H144O17P2 ; C84H144O17P2 ; C84H144O17P2 ; C84H144O17P2 ; C36H64N7O17P3S
94.502250	1.436569	0.706797	3.4548	0.006177	2.2092	0.035336	NA	NA	NA	NA	NA
135.06666	1.427222	0.86868	3.4537	0.006188	2.2084	0.03536	135.0667	HMDB12881 ; HMDB14524 ; HMDB14692 ; HMDB28865 ; HMDB28886 ; HMDB00014 ; HMDB01085 ; HMDB01193 ; HMDB01552 ; HMDB02236 ; HMDB02656 ; HMDB02982 ; HMDB04243 ; HMDB04244 ; HMDB04688 ; HMDB04690 ; HMDB04692 ; HMDB04693 ; HMDB04696 ; HMDB04699 ; HMDB05050 ; HMDB05087 ; HMDB05088 ; HMDB05089 ; HMDB10204 ; HMDB10211 ; HMDB10216 ; HMDB10219 ; HMDB11135 ; HMDB12498 ; HMDB12839 ; HMDB14825 ; HMDB28920 ; HMDB28942 ; HMDB29130 ; HMDB29131 ; HMDB29300 ; HMDB29317 ; HMDB30023 ; HMDB32215 ; HMDB32492 ; HMDB32578 ; HMDB32708 ; HMDB33443 ; HMDB33452 ; HMDB34225 ; HMDB34686 ; HMDB35832 ; HMDB35856 ; HMDB35862 ; HMDB36058 ; HMDB36621 ; HMDB37587 ; HMDB39033 ; HMDB39043 ; HMDB39171 ;	Acetylcarnosine ; Dexrazoxane ; Pentostatin ; Hydroxyprolyl-Histidine ; Histidinyl-Hydroxyproline ; Deoxycytidine ; Leukotriene B4 ; 5(S)-Hydroperoxyeicosatetraenoic acid ; 2-Keto-glutaramic acid ; 8-iso-PGA1 ; Prostaglandin A1 ; Prostaglandin B1 ; 12(S)-HPETE ; 15(S)-HPETE ; Hepoxilin A3 ; Hepoxilin B3 ; 12(R)-HPETE ; 11H-1415-EETA ; 11(R)-HPETE ; 8(S)-HPETE ; 15H-1112-EETA ; 6-trans-Leukotriene B4 ; 6-trans-12-epi-Leukotriene B4 ; 12(S)-Leukotriene B4 ; 1415-DiHETE ; 1718-DiHETE ; 515-DiHETE ; 815-DiHETE ; 5-HPETE ; 1011-dihydro-12-oxo-LTB4 ; 67-dihydro-5-oxo-12-epi-LTB4 ; Fludrocortisone ; Isoleucyl-Valine ; Leucyl-Valine ; Valyl-Isoleucine ; Valyl-Leucine ; p-Anisidine ; Angelicolide ; Angeolide ; 2-Decylfuran ; 2-Propionylpyrrole ; 4-Hydroxybenzylamine ; 1-Naphthaleneacetic acid ; Sorgoleone 358 ; Koenoline ; Levistolide A ; (14S)-1415-Dihydroxy-8(17)13(16)-labdadien-19-oic acid ; Norbixin ; 1112-Dimethoxy-81113-abietatrien-207-olide ; (E)-610-Dimethyl-9-methylene-5-undecen-2-one ; 1-Ethyl-1H-pyrrole-2-carboxaldehyde ; Conferone ; Gancaonin U ; Kinocoumarin ; (3x5a9x10b)-O-(6-Oxo-7-drimen-11-yl)umbelliferone ; 9alpha-(3-Methylbutanoyloxy)-4S-hydroxy-10(14)-oplopen-3-one ; ent-1(10)-Halimene-1519-dioic acid ; 2-Cyclotetradecen-1-one ; 2-Acetyl-1-methylpyrrole ; Piperoic acid ; Tokinolide A ; Mukoline ; 9-Deoxy-delta12-PGD2 ; Prostaglandin C1 ; 1220-DiHETE ; cycloguanil ; Lenticin	M+2H ; M+2H ; M+2H ; M+2H ; M+2H ; M+ACN+2H ; M+3Na ; M+3Na ; M+3ACN+2H ; M+3Na ; M+2H+Na ; M+H+K ; M+H+K ; M+H+K ; M+H+K ; 2M+H+Na ; M+2H+Na ; M+2H+Na ; M+Na+K ; 2M+H+Na ; 2M+H+Na ; M+2ACN+2H ; M+H+2Na ; M+ACN+2H ; M+2H+Na ; M+3Na ; M+2H+Na ; M+H+2Na ; M+Na+K ; 2M+H+Na ; M+2H+Na ; M+2H+Na ; M+2H+Na ; M+2H+Na ; M+3Na ; M+3Na ; M+Na+K ; 2M+H+Na ; M+H+2Na ; M+2H+Na ; M+ACN+2H ; M+3Na ; M+3Na ; M+3Na ; M+H+NH4 ; M+H+Na	C11H16N4O4 ; C11H16N4O4 ; C11H16N4O4 ; C11H16N4O4 ; C11H16N4O4 ; C9H13N3O4 ; C20H32O4 ; C20H32O4 ; C5H7NO4 ; C20H32O4 ; C21H29FO5 ; C11H22N2O3 ; C11H22N2O3 ; C11H22N2O3 ; C11H22N2O3 ; C7H9NO ; C24H28O4 ; C24H28O4 ; C14H24O ; C7H9NO ; C7H9NO ; C12H10O2 ; C22H30O4 ; C14H13NO2 ; C24H28O4 ; C20H32O4 ; C24H28O4 ; C22H30O4 ; C14H24O ; C7H9NO ; C24H28O4 ; C24H28O4 ; C24H28O4 ; C24H28O4 ; C20H32O4 ; C20H32O4 ; C14H24O ; C7H9NO ; C22H30O4 ; C24H28O4 ; C14H13NO2 ; C20H32O4 ; C20H32O4 ; C20H32O4 ; C11H14ClN5 ; C14H18N2O2
430.74789	1.409476	0.853724	3.4527	0.006199	2.2077	0.03538	430.7479	HMDB15290	Halothane	2M+K	C2HBrClF3
378.52069	1.393826	0.941866	-3.4513	0.006212	2.2067	0.035418	378.5207	HMDB29313	Canescein	2M+3H	C29H42O11
167.01265	1.469096	1.068049	3.4501	0.006225	2.2059	0.03545	167.0127	HMDB11117 ; HMDB31548 ; HMDB31778 ; HMDB42033 ; HMDB61161	Se-Adenosylselenohomocysteine ; Dichloromethane ; Diflubenzuron ; Thiodiglycol ; N-Desalkyl flurazepam	M+3Na ; M+2ACN+H ; M+H+Na ; M+2Na-H ; M+2Na	C14H20N6O5Se ; CH2Cl2 ; C14H9ClF2N2O2 ; C4H10O2S ; C15H10ClFN2O
784.58315	1.422281	0.118004	3.4482	0.006245	2.2045	0.035523	784.5832	HMDB10164	PS(18:020:0)	M+H-2H2O	C44H86NO10P
115.02843	1.477052	0.924088	3.4461	0.006267	2.2029	0.035607	115.0284	HMDB13695 ; HMDB30723 ; HMDB30724 ; HMDB34263 ; HMDB41037	Urolithin A ; 15-Dihydroxyxanthone ; Euxanthone ; Triethyl citrate ; 8-Deoxy-11-hydroxy-13-chlorogrosheimin	M+2H ; M+2H ; M+2H ; M+3Na ; M+H+2Na	C13H8O4 ; C13H8O4 ; C13H8O4 ; C12H20O7 ; C15H19ClO4

451.06360	1.450148	0.258968	-3.4447	0.006281	2.202	0.035607	451.0636	HMDB32738 ; HMDB04078 ; HMDB29503 ; HMDB32414 ; HMDB36187 ; HMDB40609	CI Food Red 6 ; Cinnavalininate ; Kaempferol 3-(36-diacetyl-24-di-p-coumaroylrhamnoside) ; 2-Methyl-3 or 5 or 6-(furfurylthio)pyrazine (mixture of isomers) ; 2-(2-Furanylmethyl)thio-6-methylpyrazine ; Theaflavic acid	M+H ; 3M+2H ; M+2K ; 2M+K ; 2M+K ; M+Na	C19H18N2O7S2 ; C14H8N2O6 ; C43H36O17 ; C10H10N2OS ; C10H10N2OS ; C21H16O10
178.55235	1.440714	1.188075	-3.4428	0.006302	2.2005	0.035607	178.5524	HMDB01517 ; HMDB01961 ; HMDB04824 ; HMDB15237 ; HMDB30398 ; HMDB34621	AICAR ; 17-Dimethylguanosine ; N2N2-Dimethylguanosine ; Sibutramine ; N-(Carbomethoxyacetyl)-4-S-chlorotryptophan ; 2-O-Acetyl-trans-coutaric acid	M+H+NH4 ; M+2Na ; M+2Na ; M+2K ; M+H+NH4 ; M+H+NH4	C9H15N4O8P ; C12H17N5O5 ; C12H17N5O5 ; C17H26ClN ; C15H15ClN2O5 ; C15H14O9
110.07135	1.397443	1.551302	3.4424	0.006306	2.2003	0.035607	110.0714	HMDB14515 ; HMDB28916 ; HMDB28938 ; HMDB29042 ; HMDB29043 ; HMDB29074 ; HMDB29137 ; HMDB33675 ; HMDB41313 ; HMDB01216 ; HMDB01525 ; HMDB02084 ; HMDB03315 ; HMDB30332 ; HMDB30867 ; HMDB30945 ; HMDB31030 ; HMDB31196 ; HMDB31496 ; HMDB31497 ; HMDB31498 ; HMDB31563 ; HMDB31564 ; HMDB31692 ; HMDB32051 ; HMDB32193 ; HMDB32331 ; HMDB32901 ; HMDB33549 ; HMDB33714 ; HMDB34903 ; HMDB35157 ; HMDB35170 ; HMDB35239 ; HMDB35360 ; HMDB36048 ; HMDB36599 ; HMDB37301 ; HMDB38270 ; HMDB38280 ; HMDB39522 ; HMDB40206 ; HMDB40227 ; HMDB40264 ; HMDB40271 ; HMDB40397 ; HMDB40725 ; HMDB41469 ; HMDB41494 ; HMDB60292 ; HMDB03464	Meprobamate ; Isoleucyl-Serine ; Leucyl-Serine ; Serinyl-Isoleucine ; Serinyl-Leucine ; Threoninyl-Valine ; Valyl-Threonine ; L-Lysopine ; Pantothenamide ; Tetrahydropteridine ; Imidazole ; Cyanide ; Cyclohexanone ; 2-Methyl-4-pentenal ; Dihydro-5-(2-octenyl)-2(3H)-furanone ; Methyl 2-undecynoate ; 6-Heptyl-56-dihydro-2H-pyran-2-one ; 4-Methyl-2-pentenal ; 2-Hexenal ; 3-Hexenal ; (Z)-3-Hexenal ; 4-Methyl-3-penten-2-one 9CI ; 4-Methyl-4-penten-2-one ; cis-3-Hexenyl trans-4-hexenoate ; alpha-Terpineol acetate ; alpha-Campholene acetate ; (Z)-4-Hydroxy-6-dodecenoic acid lactone ; Yuzu lactone ; (E)-4-Hexenal ; 2-Methyl-2-pentenal ; (3R3aR7aS)-3-Butylhexahydro-1(3H)-isobenzofuranone ; Geranyl acetate ; 12-Dihydro-11-dimethyl-2-oxo-44-bipyridinium(1) ; 4-Hexen-3-one ; N-Methyl-1-deoxynojirimycin ; Artemisia alcohol acetate ; Ethyl 2Z4E-decadienoic acid ; p-Menth-1-en-9-ol acetate ; cis-3-Hexenyl cis-3-hexenoate ; cis-3-Hexenyl trans-2-hexenoate ; Linalyl acetate ; Allyl 3-cyclohexylpropionate ; Isopulegol acetate ; (Z)-2-Hexenal ; (Z)-4-Hexenal ; (-)-Bornyl acetate ; Fenchyl acetate ; Dihydrocarveol acetate ; (S)-Santolina acetate ; Hydrogen cyanide ; 4-Guanidinobutanoic acid	M+2H ; M+2H ; M+2H ; M+2H ; M+2H ; M+2H ; M+2H ; M+2H ; M+2H ; M+2ACN+2H ; M+ACN+H ; M+2ACN+H ; 2M+H+Na ; 2M+H+Na ; M+H+Na ; M+H+Na ; M+H+Na ; 2M+H+Na ; 2M+H+Na ; 2M+H+Na ; 2M+H+Na ; 2M+H+Na ; 2M+H+Na ; M+H+Na ; M+H+Na ; M+H+Na ; M+H+Na ; M+H+Na ; 2M+H+Na ; 2M+H+Na ; M+H+Na ; M+H+Na ; M+H+NH4 ; 2M+H+Na ; M+ACN+2H ; M+H+Na ; M+H+Na ; M+H+Na ; M+H+Na ; M+H+Na ; M+H+Na ; M+H+Na ; M+H+Na ; 2M+H+Na ; 2M+H+Na ; M+H+Na ; M+H+Na ; M+H+Na ; M+H+Na ; M+2ACN+H ; M+H-2H2O	C9H18N2O4 ; C9H18N2O4 ; C9H18N2O4 ; C9H18N2O4 ; C9H18N2O4 ; C9H18N2O4 ; C9H18N2O4 ; C9H18N2O4 ; C9H18N2O4 ; C6H8N4 ; C3H4N2 ; CHN ; C6H10O ; C6H10O ; C12H20O2 ; C12H20O2 ; C12H20O2 ; C6H10O ; C6H10O ; C6H10O ; C6H10O ; C6H10O ; C6H10O ; C12H20O2 ; C12H20O2 ; C12H20O2 ; C12H20O2 ; C12H20O2 ; C6H10O ; C6H10O ; C12H20O2 ; C12H20O2 ; C12H13N2O ; C6H10O ; C7H15NO4 ; C12H20O2 ; C12H20O2 ; C12H20O2 ; C12H20O2 ; C12H20O2 ; C12H20O2 ; C12H20O2 ; C12H20O2 ; C6H10O ; C6H10O ; C12H20O2 ; C12H20O2 ; C12H20O2 ; C12H20O2 ; CHN ; C5H11N3O2
127.05025	1.456934	0.437984	3.4416	0.006315	2.1996	0.035607	127.0503	HMDB00262 ; HMDB02024 ; HMDB29736 ; HMDB60261 ; HMDB00071 ; HMDB29956 ; HMDB00590 ; HMDB01138 ; HMDB03581 ; HMDB15191 ; HMDB15217 ; HMDB29030 ; HMDB29135 ; HMDB29304 ; HMDB31544 ; HMDB31574 ; HMDB31695 ; HMDB32499 ; HMDB35037 ; HMDB37942 ; HMDB39137 ; HMDB40461 ; HMDB40558 ; HMDB60811 ; HMDB60813	Thymine ; Imidazoleacetic acid ; 1H-Imidazole-1-acetic acid ; Imidazol-4-ylacetate ; Deoxyinosine ; Nebularine ; Glutarylglycine ; N-Acetylglutamic acid ; Dethiobiotin ; Praziquantel ; Pilocarpine ; Prolyl-Valine ; Valyl-Proline ; 34-DHPEA-EA ; 3-Methylcyclopentene ; 1-Isothiocyanato-6-(methylthio)hexane ; xi-3-Hydroxy-5-phenylpentanoic acid O-beta-D-Glucopyranoside ; Pyrrolidino-12E-4H-24-dimethyl-135-dithiazine ; Gibberellin A32 ; (1xi3xi)-1234-Tetrahydro-1-methyl-beta-carboline-3-carboxylic acid ; 2-(34-Dihydroxyphenylethyl)-6-epi-elenaiate ; 15-Dibutyl methyl hydroxycitrate ; Zingerone glucoside ; cyclic Melatonin ; Descarbonyl-lacosamide	M+H ; M+H ; M+H ; M+H ; M+2H ; M+2H ; 2M+3H ; 2M+3H ; M+H+K ; M+3Na ; M+2Na ; M+H+K ; M+H+K ; M+3H ; M+2Na-H ; 2M+3H ; M+2H+Na ; 2M+3H ; M+3H ; M+H+Na ; M+3H ; M+H+2Na ; M+2H+Na ; M+H+Na ; M+2Na	C5H6N2O2 ; C5H6N2O2 ; C5H6N2O2 ; C5H6N2O2 ; C10H12N4O4 ; C10H12N4O4 ; C7H11NO5 ; C7H11NO5 ; C10H18N2O3 ; C19H24N2O2 ; C11H16N2O2 ; C10H18N2O3 ; C10H18N2O3 ; C19H22O8 ; C6H10 ; C8H15NS2 ; C17H24O8 ; C8H15NS2 ; C19H22O8 ; C13H14N2O2 ; C19H22O8 ; C15H26O8 ; C17H24O8 ; C13H14N2O2 ; C11H16N2O2
566.88837	1.395931	1.311605	3.4404	0.006328	2.1988	0.035607	566.8884	HMDB12261 ; HMDB42210 ; HMDB42935 ; HMDB43940 ; HMDB44642 ; HMDB44748 ; HMDB45502 ; HMDB60455	Molybdenum cofactor (sulfide) ; TG(14:020:0o-18:0) ; TG(14:0o-18:020:0) ; TG(16:018:0o-18:0) ; TG(16:0o-18:018:0) ; TG(18:016:0o-18:0) ; TG(20:014:0o-18:0) ; cis-2-Chloro-4-carboxymethylenebut-2-en-14-olide	2M+3H2O+2H ; 2M+3H ; 2M+3H ; 2M+3H ; 2M+3H ; 2M+3H ; 2M+3H ; 3M+2Na-H	C10H12MoN5O7PS3 ; C55H108O5 ; C55H108O5 ; C55H108O5 ; C55H108O5 ; C55H108O5 ; C55H108O5 ; C6H3ClO4
416.00347	1.385363	1.558765	-3.4403	0.006328	2.1987	0.035607	416.0035	HMDB31554	Sucralose	2M+H+K	C12H19Cl3O8
169.03560	1.352734	1.0465	-3.4401	0.00633	2.1986	0.035607	169.0356	HMDB00289 ; HMDB05033 ; HMDB15109 ; HMDB15373 ; HMDB34271 ; HMDB40622 ; HMDB40623 ; HMDB41638 ; HMDB41639 ; HMDB41640	Uric acid ; Valdecoxib ; Edetic Acid ; Chloroxine ; Hypericin ; 3-(2-Galloylglucosyl)-phloroacetophenone ; 3-(6-Galloylglucosyl)-phloroacetophenone ; (-)-Epigallocatechin 3-glucuronide ; (-)-Epigallocatechin 3-glucuronide ; (-)-Epigallocatechin 7-glucuronide	M+H ; M+H+Na ; M+2Na ; M+3ACN+2H ; M+3H ; M+2H+Na ; M+2H+Na ; M+2H+Na ; M+2H+Na ; M+2H+Na	C5H4N4O3 ; C16H14N2O3S ; C10H16N2O8 ; C9H5Cl2NO ; C30H16O8 ; C21H22O13 ; C21H22O13 ; C21H22O13 ; C21H22O13 ; C21H22O13
362.92633	1.402727	1.397926	3.4397	0.006335	2.1983	0.035607	362.9263	HMDB14786	Mitotane	M+2Na-H	C14H10Cl4
261.88961	1.398379	0.294422	3.4395	0.006336	2.1982	0.035607	261.8896	HMDB43170 ; HMDB43186 ; HMDB43198 ; HMDB43662 ; HMDB47748 ; HMDB47764	TG(15:014:1(9Z)16:1(9Z)) ; TG(15:014:1(9Z)18:4(6Z9Z12Z15Z)) ; TG(15:016:1(9Z)14:1(9Z)) ; TG(15:018:4(6Z9Z12Z15Z)14:1(9Z)) ; TG(14:1(9Z)15:016:1(9Z)) ; TG(14:1(9Z)15:018:4(6Z9Z12Z15Z))	M+2H+Na ; M+3H ; M+2H+Na ; M+3H ; M+2H+Na ; M+3H	C48H88O6 ; C50H86O6 ; C48H88O6 ; C50H86O6 ; C48H88O6 ; C50H86O6
444.67347	1.399379	1.654962	3.4394	0.006338	2.198	0.035607	444.6735	HMDB31304 ; HMDB32992	Osmundalin ; Furaneol 4-glucoside	3M+H+NH4 ; 3M+H+NH4	C12H18O8 ; C12H18O8
520.60318	1.371969	1.50607	3.4366	0.006368	2.196	0.035707	520.6032	HMDB60375	3-Oxo-OPC4-CoA	M+H+K	C35H54N7O19P3S
268.00666	1.452257	0.124615	3.4362	0.006372	2.1958	0.035707	268.0067	HMDB28731 ; HMDB28836 ; HMDB29419 ; HMDB41917	Asparaginyl-Glycine ; Glycyl-Asparagine ; Glycylglycylglycine ; Lorazepam glucuronide	M+2K+H ; M+2K+H ; M+2K+H ; M+H+K	C6H11N3O4 ; C6H11N3O4 ; C6H11N3O4 ; C21H18Cl2N2O8
96.921823	1.408471	1.63868	3.4357	0.006377	2.1954	0.035707	NA	NA	NA	NA	NA
138.52966	1.411331	1.602455	-3.4345	0.00639	2.1945	0.035737	138.5297	HMDB00238 ; HMDB00468 ; HMDB00633 ; HMDB00817 ; HMDB01195 ; HMDB02263 ; HMDB29928 ; HMDB33566 ; HMDB34453 ; HMDB60711	Sepiapterin ; Biopterin ; D-Biopterin ; Orinapterin ; Dyspropterin ; Primapterin ; Deoxyeritadenine ; Ajoene ; 4-22-Bithiophen-5-yl-3-butyn-1-ol ; 2-Amino-5-benzoylbenzimidazole	M+H+K ; M+H+K ; M+H+K ; M+H+K ; M+H+K ; M+H+K ; M+H+K ; M+ACN+2H ; M+ACN+2H ; M+H+K	C9H11N5O3 ; C9H11N5O3 ; C9H11N5O3 ; C9H11N5O3 ; C9H11N5O3 ; C9H11N5O3 ; C9H11N5O3 ; C9H14OS3 ; C12H10OS2 ; C14H11N3O
508.71316	1.382067	1.603388	3.432	0.006417	2.1927	0.035743	508.7132	HMDB14379 ; HMDB15509 ; HMDB29381 ; HMDB30259	Reboxetine ; Ethylmorphine ; 6-O-Methylcodeine ; ()-Erysotrine	3M+2K ; 3M+2K ; 3M+2K ; 3M+2K	C19H23NO3 ; C19H23NO3 ; C19H23NO3 ; C19H23NO3

95.085522	1.425357	1.035332	3.432	0.006417	2.1927	0.035743	95.08552	HMDB31532 ; HMDB32395	1-Methyl-13-cyclohexadiene ; 2-Methyl-13-cyclohexadiene	M+H ; M+H	C7H10 ; C7H10
230.11354	1.362451	0.856405	-3.4319	0.006418	2.1926	0.035743	230.1135	HMDB28739 ; HMDB29012 ; HMDB00489 ; HMDB03929 ; HMDB12607 ; HMDB14984 ; HMDB15319 ; HMDB29370 ; HMDB29862 ; HMDB30274 ; HMDB37697 ; HMDB38971 ; HMDB39939 ; HMDB40010	Asparaginyl-Proline ; Prolyl-Asparagine ; Aspartylglycosamine ; 5-Aminoimidazole ; 18-Carboxy-dinor-LTE4 ; Flurandrenolide ; Ciclopirox ; O-Methylcorypalline ; Cyromazine ; (E)-Casimiroedine ; Isobutyl N-methylanthranilate ; Diacetylfusarochromanone ; Synephrine acetonide ; 1-(23-Dihydro-67-dimethyl-1H-pyrrolizin-5-yl)-2-hydroxy-1-propanone	M+H ; M+H ; M+3ACN+2H ; 2M+ACN+Na ; M+H+NH4 ; M+H+Na ; M+Na ; M+Na ; M+ACN+Na ; M+ACN+2H ; M+Na ; M+2ACN+2H ; M+Na ; M+Na	C9H15N3O4 ; C9H15N3O4 ; C12H21N3O8 ; C3H5N3 ; C21H31NO7S ; C24H33FO6 ; C12H17NO2 ; C12H17NO2 ; C6H10N6 ; C21H27N3O6 ; C12H17NO2 ; C19H24N2O6 ; C12H17NO2 ; C12H17NO2
336.07693	1.403309	0.287548	3.4317	0.00642	2.1925	0.035743	336.0769	HMDB01849 ; HMDB12226 ; HMDB13943 ; HMDB15115 ; HMDB29829 ; HMDB30121 ; HMDB31148 ; HMDB41954 ; HMDB60554 ; HMDB41837	Propranolol ; Entacapone ; Alpha-hydroxyalprazolam ; Ramelteon ; Norchalciporyl propionate ; Kuwanon L ; Protochlorophyllide ; Norbutorphanol ; 4-hydroxyalprazolam ; Berberine chloride	M+2K-H ; 2M+Na+K ; 2M+H+Na ; M+2K-H ; M+2K-H ; M+2Na ; M+Na+K ; M+2K-H ; 2M+H+Na ; M+H-2H2O	C16H21NO2 ; C14H15N3O5 ; C17H13ClN4O ; C16H21NO2 ; C16H21NO2 ; C35H30O11 ; C35H30MgN4O5 ; C16H21NO2 ; C17H13ClN4O ; C20H18ClNO4
186.98022	1.360091	0.822522	3.4311	0.006427	2.192	0.035743	186.9802	HMDB04812 ; HMDB29719 ; HMDB33831 ; HMDB37731 ; HMDB37762	25-Furandicarboxylic acid ; Methyl thiophene-2-carboxylate ; Medicagol ; S-(2-Furanylmethyl) methanethioate ; Methyl 2-thiofuroate	2M+Na+K ; M+2Na-H ; M+2K ; M+2Na-H ; M+2Na-H	C6H4O5 ; C6H6O2S ; C16H8O6 ; C6H6O2S ; C6H6O2S
384.76353	1.392962	1.66784	3.428	0.00646	2.1897	0.035891	NA	NA	NA	NA	NA
388.71171	1.399145	1.621149	3.426	0.006482	2.1883	0.035922	388.7117	HMDB09195 ; HMDB31227 ; HMDB32529 ; HMDB38642	PE(18:4(6Z9Z12Z15Z)18:4(6Z9Z12Z15Z)) ; Marshdimerin ; Thaumatin b recombinant ; Fumitremorgin C	M+2Na ; M+H+NH4 ; 2M+H+NH4 ; 2M+H+NH4	C41H66NO8P ; C48H54O8 ; C19H29N3O3S ; C22H25N3O3
550.39194	1.489693	0.216417	-3.4259	0.006483	2.1882	0.035922	550.3919	HMDB10391 ; HMDB11148 ; HMDB15282 ; HMDB15307 ; HMDB30356	LysoPC(20:1(11Z)) ; PC(18:1(9Z)e2:0) ; Desipramine ; Cyclizine ; 1419-Didehydrocondyfolan	M+H ; M+H ; 2M+NH4 ; 2M+NH4 ; 2M+NH4	C28H56NO7P ; C28H56NO7P ; C18H22N2 ; C18H22N2 ; C18H22N2
181.12226	1.485968	0.308558	3.4255	0.006487	2.1879	0.035922	181.1223	HMDB30007 ; HMDB30039 ; HMDB32468 ; HMDB35240 ; HMDB36669 ; HMDB36817 ; HMDB39159 ; HMDB39957 ; HMDB59925 ; HMDB59926 ; HMDB03689 ; HMDB04038 ; HMDB30281 ; HMDB30454 ; HMDB31848 ; HMDB61063 ; HMDB61064 ; HMDB61065 ; HMDB02265 ; HMDB02311 ; HMDB02314 ; HMDB02343 ; HMDB02995 ; HMDB12504 ; HMDB12838 ; HMDB35379 ; HMDB60106	2277-Tetramethyl-16-dioxaspiro44nona-38-diene ; Jasmolone ; 2-Phenylpropionaldehyde dimethyl acetal ; Dihydroactinidiolide ; ()-Myrtenyl formate ; Norecasantalic acid ; Bovolide ; 4-(Butoxymethyl)phenol ; 3-tert-Butyl-4-hydroxyanisole ; 2-tert-Butyl-4-hydroxyanisole ; Neuroprotectin D1 ; Resolvin D5 ; Calycanthidine ; Macrophorin A ; Butylated hydroxyanisole ; 6-beta-hydroxymedroxyprogesterone ; 2-beta-hydroxymedroxyprogesterone ; 1-beta-hydroxymedroxyprogesterone ; 1415-DiHETrE ; 89-DiHETrE ; 1112-DiHETrE ; 56-DHET ; 12-Keto-tetrahydro-leukotriene B4 ; 1011-dihydro-leukotriene B4 ; 67-dihydro-12-epi-LTB4 ; Sterebin E ; 15-Hydroperoxyeicosa-8Z11Z13E-trienoate	M+H ; M+H ; M+H ; M+H ; M+H ; M+H ; M+H ; M+H ; M+H ; M+H ; M+2H ; M+2H ; M+2H ; M+2H ; M+2H ; M+2H ; M+2H ; M+2H ; M+H+Na ; M+H+Na ; M+H+Na ; M+H+Na ; M+H+Na ; M+H+Na ; M+H+Na ; M+H+Na ; M+H+Na	C11H16O2 ; C11H16O2 ; C11H16O2 ; C11H16O2 ; C11H16O2 ; C11H16O2 ; C11H16O2 ; C11H16O2 ; C11H16O2 ; C11H16O2 ; C22H32O4 ; C22H32O4 ; C23H28N4 ; C22H32O4 ; C22H32O4 ; C22H32O4 ; C22H32O4 ; C22H32O4 ; C20H34O4 ; C20H34O4 ; C20H34O4 ; C20H34O4 ; C20H34O4 ; C20H34O4 ; C20H34O4 ; C20H34O4 ; C20H34O4
706.65376	1.444936	1.074605	-3.4226	0.006519	2.1858	0.036054	706.6538	HMDB05193 ; HMDB30508 ; HMDB36288 ; HMDB36660	CE(20:1(11Z)) ; Cyclolaudenyl palmitate ; Campesteryl elaidate ; alpha-Amyrin palmitate	2M+3H2O+2H ; 2M+3H2O+2H ; M+ACN+H ; M+ACN+H	C47H82O2 ; C47H82O2 ; C46H80O2 ; C46H80O2
134.01879	1.389218	1.376102	3.4215	0.00653	2.1851	0.03608	134.0188	HMDB04095 ; HMDB04369 ; HMDB14373 ; HMDB14891 ; HMDB30533 ; HMDB31635 ; HMDB33041 ; HMDB33072 ; HMDB40243 ; HMDB40262 ; HMDB40345 ; HMDB59983	5-Methoxytryptamine ; N-Methylserotonin ; Enflurane ; Isoflurane ; Alline ; 2-Propene-1-thiol ; 1-(1-Propenylthio)propyl propyl disulfide ; (E)-1-Propenyl 1-(propylthio)propyl disulfide ; 224466-Hexamethyl-135-trithiane ; 246-Triethyl-135-trithiane ; 1-Propene-1-thiol ; 4-phenylbutanic acid-O-sulphate	M+2K ; M+2K ; M+2ACN+2H ; M+2ACN+2H ; M+2K ; 3M+2Na ; M+2Na ; M+2Na ; M+2Na ; M+2Na ; 3M+2Na ; M+H+Na	C11H14N2O ; C11H14N2O ; C3H2ClF5O ; C3H2ClF5O ; C11H14N2O ; C3H6S ; C9H18S3 ; C9H18S3 ; C9H18S3 ; C9H18S3 ; C3H6S ; C10H12O5S
216.92257	1.412268	1.608055	3.418	0.006569	2.1825	0.036255	216.9226	HMDB32059 ; HMDB60446 ; HMDB60448	2-Bromophenol ; Bromobenzene-23-oxide ; Bromobenzene-34-oxide	M+2Na-H ; M+2Na-H ; M+2Na-H	C6H5BrO ; C6H5BrO ; C6H5BrO
230.09584	1.470446	0.711535	3.4172	0.006579	2.1819	0.036266	230.0958	HMDB03045 ; HMDB41887 ; HMDB29909 ; HMDB37108 ; HMDB37603 ; HMDB39852 ; HMDB06406 ; HMDB15238 ; HMDB15519 ; HMDB31820 ; HMDB32201 ; HMDB32202 ; HMDB32635 ; HMDB32854 ; HMDB37764 ; HMDB38437	Ergothioneine ; Endalin ; Gein ; 3-Hydroxychavicol 1-rhamnosyl-(1-6)-glucoside ; Eugenol O-a-L-Arabinofuranosyl-(1-6)-b-D-glucopyranoside ; Deoxynivalenol 3-glucoside ; Ecgonine methyl ester ; Levocabastine ; Phendimetrazine ; Neotussilagine ; cis- and trans-5-Ethyl-4-methyl-2-(2-butyl)-thiazoline ; cis and trans-5-Ethyl-4-methyl-2-(2-methylpropyl)-thiazoline ; NN-Diethylbenzeneacetamide ; N-Acetyl-26-diethylaniline ; S-1-2-(Acetyloxy)ethylbutyl ethanethioate ; 9-(Methylthio)nonanenitrile	M+H ; M+H ; M+2H ; M+2H ; M+2H ; M+2H ; 2M+Na+K ; M+H+K ; M+K ; 2M+Na+K ; M+2Na-H ; M+2Na-H ; M+K ; M+K ; 2M+H+Na ; M+2Na-H	C9H15N3O2S ; C9H12FN3O3 ; C21H30O11 ; C21H30O11 ; C21H30O11 ; C21H30O11 ; C10H17NO3 ; C26H29FN2O2 ; C12H17NO ; C10H17NO3 ; C10H19NS ; C10H19NS ; C12H17NO ; C12H17NO ; C10H18O3S ; C10H19NS
164.98299	1.391571	1.388813	-3.4153	0.006599	2.1805	0.03634	164.983	HMDB29198	Dimethylurea	M+2K-H	C3H8N2O
158.51627	1.462783	0.203392	-3.4127	0.006628	2.1786	0.036422	158.5163	HMDB01510	Bupropion	M+2K	C13H18ClNO
495.11851	1.44403	0.476763	-3.4113	0.006644	2.1776	0.036422	495.1185	HMDB14560 ; HMDB14837 ; HMDB29464 ; HMDB30828 ; HMDB31368 ; HMDB34607 ; HMDB35067 ; HMDB36781 ; HMDB36929 ; HMDB37040 ; HMDB38785 ; HMDB38786	Metocurine Iodide ; Nicergoline ; Asteltoxin ; 2-(4-Allyl-26-dimethoxyphenoxy)-1-(345-trimethoxyphenyl)-1-propanol ; (1a5b6a)-7-Protoilludene-15614-tetrol 14-(24-dihydroxy-6-methylbenzoic acid) ; Judeol ; Melleolide L ; Diosbulbin H ; 13-Hydroxydihydromelleolide ; Armillaric acid ; Melleolide E ; 10-Hydroxymelleolide	M+2ACN+2H ; 2M+H+Na ; M+2K-H ; M+2K-H ; M+2K-H ; M+2K-H ; M+2Na-H ; M+2K-H ; M+2K-H ; M+2K+H ; M+2K-H ; M+2K+H	C40H48I2N2O6 ; C24H26BrN3O3 ; C23H30O7 ; C23H30O7 ; C23H30O7 ; C23H30O7 ; C23H27ClO7 ; C23H30O7 ; C23H30O7 ; C23H28O7 ; C23H30O7 ; C23H28O7
481.01778	1.399807	0.089966	-3.4107	0.006651	2.1771	0.036422	481.0178	HMDB01117 ; HMDB03665 ; HMDB33392 ; HMDB34577 ; HMDB34850 ; HMDB36795	4-Phosphopantothenoylcysteine ; Phosphoribosyl-ATP ; CI Acid Red 13 ; 4-Chloro-67-dimethoxy-2-benzoxazolinone ; Metosulam ; Azorubine	M+2K+H ; 2M+3H ; M+Na ; 2M+Na ; M+ACN+Na ; M+Na	C12H23N2O9PS ; C15H26N5O20P4 ; C20H14N2O7S2 ; C9H8ClNO4 ; C14H13Cl2N5O4S ; C20H14N2O7S2
328.59432	1.356447	1.14737	3.4093	0.006666	2.1761	0.036422	328.5943	HMDB01176	Cytidine monophosphate N-acetylneuraminic acid	M+ACN+2H	C20H31N4O16P
169.98576	1.394263	1.694505	3.4087	0.006673	2.1757	0.036422	169.9858	HMDB01131 ; HMDB01530 ; HMDB05831 ; HMDB13924 ; HMDB32644 ; HMDB60011 ; HMDB60350	Iminoaspartic acid ; Mannitol 1-phosphate ; Sorbitol-6-phosphate ; Thienodihydropyridinium ; Maclurin ; N-acetyl-S-(N-allylthiocarbamoyl)-L-cysteine ; 2-Oxosuccinamate	M+K ; M+2K ; M+2K ; M+2K ; M+2K ; M+2K ; M+K	C4H5NO4 ; C6H15O9P ; C6H15O9P ; C14H13ClNS ; C13H10O6 ; C9H14N2O3S2 ; C4H5NO4

594.43396	1.418365	1.109348	-3.4079	0.006681	2.1751	0.036422	594.434	HMDB02020 ; HMDB03448 ; HMDB37122 ; HMDB40657 ; HMDB40743	3-Hydroxy-ee-caroten-3-one ; 3-Hydroxy-be-caroten-3-one ; Nigroxanthin ; Cryptocapsone ; 2-Dehydroplectaniaxanthin	2M+3H2O+2H ; 2M+3H2O+2H ; 2M+3H2O+2H ; 2M+3H2O+2H ; 2M+3H2O+2H	C40H54O2 ; C40H54O2 ; C40H54O2 ; C40H54O2 ; C40H54O2
392.70598	1.399507	1.642892	3.4078	0.006682	2.1751	0.036422	392.706	HMDB02248 ; HMDB04985 ; HMDB04987 ; HMDB12311 ; HMDB28758 ; HMDB28947	Gamma glutamyl ornithine ; Aspartylysine ; Alpha-Aspartyl-lysine ; 3-O-Sulfogalactosylceramide (d18:112:0) ; Aspartyl-Lysine ; Lysyl-Aspartate	3M+2H ; 3M+2H ; 3M+2H ; M+Na+K ; 3M+2H ; 3M+2H	C10H19N3O5 ; C10H19N3O5 ; C10H19N3O5 ; C36H69NO11S ; C10H19N3O5 ; C10H19N3O5
396.74356	1.400643	1.652378	3.4075	0.006686	2.1748	0.036422	396.7436	HMDB13868 ; HMDB60513 ; HMDB60530 ; HMDB60585	3-Hydroxytamoxifen (Droloxifene) ; Tamoxifen N-oxide ; 4-Hydroxytamoxifen ; alpha-Hydroxytamoxifen	2M+H+NH4 ; 2M+H+NH4 ; 2M+H+NH4 ; 2M+H+NH4	C26H29NO2 ; C26H29NO2 ; C26H29NO2 ; C26H29NO2
392.79538	1.382378	1.668113	3.4071	0.00669	2.1745	0.036422	392.7954	HMDB07921 ; HMDB07980 ; HMDB07981 ; HMDB08012 ; HMDB08040 ; HMDB08041 ; HMDB08072 ; HMDB08105 ; HMDB08136 ; HMDB08137 ; HMDB08168 ; HMDB08201 ; HMDB08332 ; HMDB08364 ; HMDB08397 ; HMDB08591 ; HMDB06516 ; HMDB07878 ; HMDB07970 ; HMDB08034 ; HMDB08262 ; HMDB08907 ; HMDB09483 ; HMDB60233	PC(14:1(9Z)22:2(13Z16Z)) ; PC(16:020:3(5Z8Z11Z)) ; PC(16:020:3(8Z11Z14Z)) ; PC(16:1(9Z)20:2(11Z14Z)) ; PC(18:018:3(6Z9Z12Z)) ; PC(18:018:3(9Z12Z15Z)) ; PC(18:1(11Z)18:2(9Z12Z)) ; PC(18:1(9Z)18:2(9Z12Z)) ; PC(18:2(9Z12Z)18:1(11Z)) ; PC(18:2(9Z12Z)18:1(9Z)) ; PC(18:3(6Z9Z12Z)18:0) ; PC(18:3(9Z12Z15Z)18:0) ; PC(20:2(11Z14Z)16:1(9Z)) ; PC(20:3(5Z8Z11Z)16:0) ; PC(20:3(8Z11Z14Z)16:0) ; PC(22:2(13Z16Z)14:1(9Z)) ; Tetracosatetraenoyl CoA ; PC(14:020:0) ; PC(16:018:0) ; PC(18:016:0) ; PC(20:014:0) ; PE(15:022:0) ; PE(22:015:0) ; all-cis-12151821-Tetracosatetraenoyl-CoA	M+2H ; M+2H ; M+2H ; M+2H ; M+2H ; M+2H ; M+2H ; M+2H ; M+2H ; M+2H ; M+2H ; M+2H ; M+2H ; M+2H ; M+2H ; M+2H ; M+3Na ; M+H+Na ; M+H+Na ; M+H+Na ; M+H+Na ; M+H+Na ; M+H+Na ; M+3Na	C44H82NO8P ; C44H82NO8P ; C44H82NO8P ; C44H82NO8P ; C44H82NO8P ; C44H82NO8P ; C44H82NO8P ; C44H82NO8P ; C44H82NO8P ; C44H82NO8P ; C44H82NO8P ; C44H82NO8P ; C44H82NO8P ; C44H82NO8P ; C44H82NO8P ; C44H82NO8P ; C45H74N7O17P3S ; C42H84NO8P ; C42H84NO8P ; C42H84NO8P ; C42H84NO8P ; C42H84NO8P ; C42H84NO8P ; C45H74N7O17P3S
569.08457	1.367381	0.574961	-3.4069	0.006694	2.1743	0.036422	569.0846	HMDB14582 ; HMDB32295	Ceftazidime ; Glucose isomerase from streptomyces rubiginosus	M+Na ; 3M+2Na	C22H22N6O7S2 ; C17H17ClN2O3S
289.98499	1.377767	0.728409	-3.4068	0.006695	2.1743	0.036422	289.985	HMDB14929 ; HMDB15627 ; HMDB41967	Uracil mustard ; Droxidopa ; Oltipraz	M+K ; M+2K-H ; M+ACN+Na	C8H11Cl2N3O2 ; C9H11NO5 ; C8H6N2S3
347.22131	1.438924	0.098917	3.4048	0.006716	2.1729	0.036485	347.2213	HMDB00015 ; HMDB01547 ; HMDB04030 ; HMDB06756 ; HMDB12612 ; HMDB12849 ; HMDB29476 ; HMDB29561 ; HMDB30280 ; HMDB35130 ; HMDB36624 ; HMDB37378 ; HMDB38031 ; HMDB10402 ; HMDB10403 ; HMDB31007 ; HMDB40684 ; HMDB60098 ; HMDB60099	Cortexolone ; Corticosterone ; 21-Deoxycortisol ; 21-Hydroxy-5b-pregnane-31120-trione ; 19-Hydroxydeoxycorticosterone ; 7-Carboxy-alpha-tocotrienol ; 10-Dehydrogingerdione ; ()-Calycanthine ; (-)-Chimonanthine ; Pinusolide ; 4-Deoxyhumulone ; 4-Deoxyadhumulone ; 12-Hydroxy-11-methoxy-81113-abietatrien-20-oic acid ; LysoPC(22:5(4Z7Z10Z13Z16Z)) ; LysoPC(22:5(7Z10Z13Z16Z19Z)) ; 1-Acetoxy-2-hydroxy-16-heptadecyn-4-one ; Janthitrem C ; 9-peroxy-5Z7E11Z14Z-eicosatetraenoate ; 11-peroxy-5Z8Z12E14Z-eicosatetraenoate	M+H ; M+H ; M+H ; M+H ; M+H ; M+H ; M+H ; M+H ; M+H ; M+H ; M+H ; M+H ; M+H ; M+3ACN+2H ; M+3ACN+2H ; M+Na ; M+3ACN+2H ; 2M+H+Na ; 2M+H+Na	C21H30O4 ; C21H30O4 ; C21H30O4 ; C21H30O4 ; C21H30O4 ; C21H30O4 ; C21H30O4 ; C22H26N4 ; C22H26N4 ; C21H30O4 ; C21H30O4 ; C21H30O4 ; C21H30O4 ; C30H52NO7P ; C30H52NO7P ; C19H32O4 ; C37H47NO4 ; C20H31O4 ; C20H31O4
131.97430	1.354322	1.065007	3.4045	0.006721	2.1726	0.036485	NA	NA	NA	NA	NA
197.70713	1.368306	1.251827	-3.4032	0.006735	2.1717	0.0365	197.7071	HMDB30641 ; HMDB33006 ; HMDB37087 ; HMDB39083	Biochanin A 7-(6-methylmalonylglucoside) ; Neobignonoside ; Chrysophanol 8-(6-galloylglucoside) ; 6-O-Malonylwistin	M+H+2Na ; M+2H+Na ; M+2H+Na ; M+H+2Na	C26H26O13 ; C28H24O13 ; C28H24O13 ; C26H26O13
549.17682	1.400271	1.093356	-3.4027	0.00674	2.1713	0.0365	549.1768	HMDB12897 ; HMDB15016 ; HMDB29203 ; HMDB33773 ; HMDB34092 ; HMDB34340 ; HMDB38609 ; HMDB39080 ; HMDB39092 ; HMDB39639 ; HMDB39698 ; HMDB41415 ; HMDB41597 ; HMDB41728 ; HMDB41729	Beta-Carboline ; Chlorhexidine ; hesperetin-7-O-glucuronide ; Physalin A ; Physalin F ; Physalin G ; Gibberellin A1 glucosyl ester ; Physalin N ; Stachyoside A ; Physalin P ; Isophysalin G ; Aeglin ; Glucodistylin ; (-)-Epicatechin 3-O-glucuronide ; (-)-Epicatechin 7-O-glucuronide	3M+2Na-H ; M+2Na-H ; M+2ACN+H ; M+Na ; M+Na ; M+Na ; M+K ; M+Na ; M+Na ; M+Na ; M+Na ; M+K ; M+2ACN+H ; M+2ACN+H ; M+2ACN+H	C11H8N2 ; C22H30Cl2N10 ; C21H22O12 ; C28H30O10 ; C28H30O10 ; C28H30O10 ; C25H34O11 ; C28H30O10 ; C21H34O15 ; C28H30O10 ; C28H30O10 ; C25H34O11 ; C21H22O12 ; C21H22O12 ; C21H22O12
430.69663	1.383838	1.640453	3.4023	0.006745	2.171	0.0365	430.6966	HMDB07118 ; HMDB07170 ; HMDB07171 ; HMDB07198 ; HMDB07226 ; HMDB07227 ; HMDB07254 ; HMDB07282 ; HMDB07311 ; HMDB07339 ; HMDB07367 ; HMDB07394 ; HMDB07395 ; HMDB07422 ; HMDB07449 ; HMDB07450 ; HMDB07478 ; HMDB07479 ; HMDB07506 ; HMDB07535 ; HMDB07678 ; HMDB37844 ; HMDB56028 ; HMDB56052 ; HMDB56058 ; HMDB56082 ; HMDB56180 ; HMDB56186 ; HMDB56199 ; HMDB56205 ; HMDB56221 ; HMDB56228 ; HMDB56282 ; HMDB56284 ; HMDB56309	DG(16:022:4(7Z10Z13Z16Z)0:0) ; DG(18:020:4(5Z8Z11Z14Z)0:0) ; DG(18:020:4(8Z11Z14Z17Z)0:0) ; DG(18:1(11Z)20:3(8Z11Z14Z)0:0) ; DG(18:1(9Z)20:3(5Z8Z11Z)0:0) ; DG(18:1(9Z)20:3(8Z11Z14Z)0:0) ; DG(18:2(9Z12Z)20:2(11Z14Z)0:0) ; DG(18:3(6Z9Z12Z)20:1(11Z)0:0) ; DG(18:3(9Z12Z15Z)20:1(11Z)0:0) ; DG(18:4(6Z9Z12Z15Z)20:00:0) ; DG(20:018:4(6Z9Z12Z15Z)0:0) ; DG(20:1(11Z)18:3(6Z9Z12Z)0:0) ; DG(20:1(11Z)18:3(9Z12Z15Z)0:0) ; DG(20:2(11Z14Z)18:2(9Z12Z)0:0) ; DG(20:3(5Z8Z11Z)18:1(11Z)0:0) ; DG(20:3(5Z8Z11Z)18:1(9Z)0:0) ; DG(20:3(8Z11Z14Z)18:1(11Z)0:0) ; DG(20:3(8Z11Z14Z)18:1(9Z)0:0) ; DG(20:4(5Z8Z11Z14Z)18:00:0) ; DG(20:4(8Z11Z14Z17Z)18:00:0) ; DG(22:4(7Z10Z13Z16Z)16:00:0) ; N-(1-Deoxy-1-fructosyl)valine ; DG(16:00:022:4n6) ; DG(18:00:020:4n6) ; DG(18:00:020:4n3) ; DG(20:00:018:4n3) ; DG(18:1n70:020:3n9) ; DG(18:1n70:020:3n6) ; DG(18:1n90:020:3n9) ; DG(18:1n90:020:3n6) ; DG(20:1n90:018:3n6) ; DG(20:1n90:018:3n3) ; DG(18:2n60:018:2n6) ; DG(18:2n60:020:2n6) ; DG(20:2n60:020:2n6)	2M+3H ; 3M+H+Na ; 2M+3H ; 2M+3H ; 2M+3H ; 2M+3H ; 2M+3H ; 2M+3H ; 2M+3H ; 2M+3H ; 2M+3H ; 2M+3H ; 2M+3H ; 2M+3H ; 2M+3H	C41H72O5 ; C11H21NO7 ; C41H72O5 ; C41H72O5 ; C41H72O5 ; C41H72O5 ; C41H72O5 ; C41H72O5 ; C41H72O5 ; C41H72O5 ; C41H72O5 ; C41H72O5 ; C41H72O5 ; C41H72O5 ; C41H72O5
504.62956	1.392098	1.643021	3.4002	0.006768	2.1695	0.036585	504.6296	HMDB29263 ; HMDB30475 ; HMDB30480 ; HMDB30696 ; HMDB30811 ; HMDB31706 ; HMDB33268 ; HMDB34009 ; HMDB35598 ; HMDB37096 ; HMDB37351 ; HMDB37354 ; HMDB38659 ; HMDB41690 ; HMDB60619 ; HMDB60969	37-Dimethylquercetin ; Aflatoxin G2 ; Aflatoxin M2 ; Demethoxysudachitin ; Prudomestin ; 34-Dihydroxyphenacyl caffeate ; 135-Trihydroxy-67-dimethoxy-2-methylanthraquinone ; Trifolian ; Aflatoxin B2a ; Pilosin ; Caryatin ; 34-Di-O-methylquercetin ; Cicerin ; 57-Dihydroxy-84-dimethoxyisoflavone ; Tazarotenic acid ; desmethyladinazolam	3M+H+NH4 ; 3M+H+NH4 ; 3M+H+NH4 ; 3M+H+NH4 ; 3M+H+NH4 ; 3M+H+NH4 ; 3M+H+NH4 ; 3M+H+NH4 ; 3M+H+NH4 ; 3M+H+NH4 ; 3M+H+NH4 ; 3M+H+NH4 ; 3M+H+NH4 ; 3M+H+NH4 ; 3M+H+K ; 3M+H+K	C17H14O7 ; C17H14O7 ; C17H14O7 ; C17H14O7 ; C17H14O7 ; C17H14O7 ; C17H14O7 ; C17H14O7 ; C17H14O7 ; C17H14O7 ; C17H14O7 ; C17H14O7 ; C17H14O7 ; C17H14O7 ; C19H17NO2S ; C17H14ClN5

378.05646	1.451474	0.57252	-3.3993	0.006779	2.1688	0.036594	378.0565	HMDB13646 ; HMDB05786 ; HMDB10362 ; HMDB10363 ; HMDB29254 ; HMDB29272 ; HMDB30603 ; HMDB32904 ; HMDB32905	Adenosine thiamine triphosphate ; Theaflavin-3-gallate ; 6-Hydroxy-5-methoxyindole glucuronide ; 5-Hydroxy-6-methoxyindole glucuronide ; Isoneotheaflavin 3-O-gallate ; 54-Dihydroxy-33-dimethoxy-6:7-methylenedioxyflavone ; alpha-Viniferin ; Isotheaflavin 3-gallate ; Neotheaflavin 3-gallate	M+2H ; M+H+K ; M+K ; M+K ; M+H+K ; 2M+H+K ; M+2K ; M+H+K ; M+H+K	C22H31N9O13P3S ; C36H28O16 ; C15H17NO8 ; C15H17NO8 ; C36H28O16 ; C18H14O8 ; C42H30O9 ; C36H28O16 ; C36H28O16
538.19887	1.419731	0.875068	-3.3988	0.006784	2.1685	0.036594	538.1989	HMDB29651 ; HMDB34938 ; HMDB35039 ; HMDB40649 ; HMDB40848 ; HMDB60917	Phlorisobutyrophenone 2-glucoside ; Cappariloside B ; Phaseolus e ; 1617-Dihydro-16a17-dihydroxygibberellin A7 17-glucoside ; (S)-Nerolidol 3-O-a-L-Rhamnopyranosyl-(1-4)-a-L-rhamnopyranosyl-(1-2)-4-(4-hydroxy-3-methoxycinnamoyl)-(E)-a-L-rhamnopyranosyl-(1-6)-b-D-glucopyranoside ; 4-Hydroxyflurbiprofen	3M+2H ; M+ACN+H ; 2M+H+Na ; 2M+H+Na ; M+2K ; 2M+NH4	C16H22O9 ; C22H28N2O11 ; C25H34O12 ; C25H34O12 ; C49H74O21 ; C15H13FO3
450.75424	1.381038	1.664152	3.3973	0.006802	2.1674	0.036649	450.7542	HMDB28919 ; HMDB28941 ; HMDB29108 ; HMDB29109 ; HMDB33464 ; HMDB33499 ; HMDB59571	Isoleucyl-Tyrosine ; Leucyl-Tyrosine ; Tyrosyl-Isoleucine ; Tyrosyl-Leucine ; Sinapoylputrescine ; Lyciumoside IV ; Oxychlordane	3M+H+NH4 ; 3M+H+NH4 ; 3M+H+NH4 ; 3M+H+NH4 ; 3M+H+NH4 ; M+3ACN+2H ; 2M+Na+K	C15H22N2O4 ; C15H22N2O4 ; C15H22N2O4 ; C15H22N2O4 ; C15H22N2O4 ; C38H64O16 ; C10H4Cl8O
227.95482	1.385677	1.432045	3.3952	0.006826	2.1658	0.03674	227.9548	HMDB31252	3-Iodopropanoic acid	2M+3H2O+2H	C3H5IO2
189.05754	1.396418	0.526027	3.393	0.006851	2.1642	0.036834	189.0575	HMDB29778 ; HMDB00216 ; HMDB00239 ; HMDB01272 ; HMDB01537 ; HMDB04817 ; HMDB14698 ; HMDB15010 ; HMDB29260 ; HMDB30803 ; HMDB30843 ; HMDB31642 ; HMDB31758 ; HMDB33571 ; HMDB33952 ; HMDB34765 ; HMDB34768 ; HMDB35178 ; HMDB37569 ; HMDB37575 ; HMDB37685 ; HMDB38636 ; HMDB39319 ; HMDB39321 ; HMDB60831	Furaneol 4-(6-malonylglucoside) ; Norepinephrine ; Pyridoxine ; Nicotine glucuronide ; 6-Hydroxydopamine ; 5-Hydroxydopamine ; Zanamivir ; Conivaptan ; Apigenin 6-C-glucoside 8-C-arabinoside ; Corymboside ; Apiin ; N-Nitroso-pyrrolidine ; Allithiamine ; 6-beta-D-Glucopyranosyl-8-beta-D-ribopyranosylapigenin ; Gyromitrin ; Wasalexin A ; Wasalexin B ; 6-Acetyl-23-dihydro-2-(hydroxymethyl)-4(1H)-pyridinone ; Isovitexin 2-O-arabinoside ; Kaempferol 3-arabinofuranoside 7-rhamnofuranoside ; xi-Norepinephrine ; Methoxybrassenin B ; Kaempferol 3-rhamnoside 7-xyloside ; Kaempferol 3-rhamnoside 4-xyloside ; meta-O-Dealkylated flecainide	M+2H ; 2M+H+K ; 2M+H+K ; M+H+K ; 2M+H+K ; 2M+H+K ; M+2Na ; M+3Na ; M+3H ; M+3H ; M+3H ; 3M+2K ; M+H+Na ; M+3H ; 3M+2K ; M+2ACN+2H ; M+2ACN+2H ; 2M+H+K ; M+3H ; M+3H ; 2M+H+K ; M+2ACN+2H ; M+3H ; M+3H ; M+2Na	C15H20O11 ; C8H11NO3 ; C8H11NO3 ; C16H22N2O6 ; C8H11NO3 ; C8H11NO3 ; C12H20N4O7 ; C32H26N4O2 ; C26H28O14 ; C26H28O14 ; C26H28O14 ; C4H8N2O ; C15H22N4O2S2 ; C26H28O14 ; C4H8N2O ; C13H14N2O2S2 ; C13H14N2O2S2 ; C8H11NO3 ; C26H28O14 ; C26H28O14 ; C8H11NO3 ; C13H14N2O2S2 ; C26H28O14 ; C26H28O14 ; C15H19F3N2O3
201.99114	1.344773	0.316938	-3.3868	0.006923	2.1597	0.037058	201.9911	HMDB59638 ; HMDB60397	Guanosine 35-bis(diphosphate) ; 5-Fluorouridine monophosphate	M+3H ; M+Na+K	C10H17N5O17P4 ; C9H12FN2O9P
345.01856	1.32711	0.076681	-3.3864	0.006928	2.1594	0.037058	345.0186	HMDB15302 ; HMDB33267 ; HMDB35179 ; HMDB38429 ; HMDB60876	Moclobemide ; Bisnorbadioquinone A ; Mumefural ; Methyl glucosinolate ; Thioxanthine monophosphate	M+2K-H ; M+H+K ; M+2Na-H ; 2M+H+Na ; M+2ACN+H	C13H17ClN2O2 ; C34H18O14 ; C12H12O9 ; C8H15NO9S2 ; C5H3N4O5PS
785.58755	1.466288	0.22149	3.3863	0.006929	2.1593	0.037058	785.5876	HMDB07180 ; HMDB07376 ; HMDB07600 ; HMDB07796 ; HMDB07880 ; HMDB07912 ; HMDB07940 ; HMDB07945 ; HMDB07973 ; HMDB08004 ; HMDB08005 ; HMDB08068 ; HMDB08101 ; HMDB08132 ; HMDB08133 ; HMDB08296 ; HMDB08297 ; HMDB08328 ; HMDB08843 ; HMDB08849 ; HMDB08875 ; HMDB08881 ; HMDB08909 ; HMDB08934 ; HMDB08941 ; HMDB08966 ; HMDB08973 ; HMDB08994 ; HMDB08999 ; HMDB09025 ; HMDB09026 ; HMDB09031 ; HMDB09058 ; HMDB09059 ; HMDB09064 ; HMDB09090 ; HMDB09223 ; HMDB09224 ; HMDB09254 ; HMDB09255 ; HMDB09286 ; HMDB09485 ; HMDB09515 ; HMDB09517 ; HMDB09547 ; HMDB09549 ; HMDB09713 ; HMDB09745 ; HMDB10565 ; HMDB56039 ; HMDB56063	DG(18:024:00:0) ; DG(20:022:00:0) ; DG(22:020:00:0) ; DG(24:018:00:0) ; PC(14:020:2(11Z14Z)) ; PC(14:1(9Z)20:1(11Z)) ; PC(15:018:2(9Z12Z)) ; PC(15:020:1(11Z)) ; PC(16:018:2(9Z12Z)) ; PC(16:1(9Z)18:1(11Z)) ; PC(16:1(9Z)18:1(9Z)) ; PC(18:1(11Z)16:1(9Z)) ; PC(18:1(9Z)16:1(9Z)) ; PC(18:2(9Z12Z)15:0) ; PC(18:2(9Z12Z)16:0) ; PC(20:1(11Z)14:1(9Z)) ; PC(20:1(11Z)15:0) ; PC(20:2(11Z14Z)14:0) ; PE(14:022:2(13Z16Z)) ; PE(14:024:1(15Z)) ; PE(14:1(9Z)22:1(13Z)) ; PE(14:1(9Z)24:0) ; PE(15:022:2(13Z16Z)) ; PE(16:020:2(11Z14Z)) ; PE(16:022:1(13Z)) ; PE(16:1(9Z)20:1(11Z)) ; PE(16:1(9Z)22:0) ; PE(18:018:2(9Z12Z)) ; PE(18:020:1(11Z)) ; PE(18:1(11Z)18:1(11Z)) ; PE(18:1(11Z)18:1(9Z)) ; PE(18:1(11Z)20:0) ; PE(18:1(9Z)18:1(11Z)) ; PE(18:1(9Z)18:1(9Z)) ; PE(18:1(9Z)20:0) ; PE(18:2(9Z12Z)18:0) ; PE(20:018:1(11Z)) ; PE(20:018:1(9Z)) ; PE(20:1(11Z)16:1(9Z)) ; PE(20:1(11Z)18:0) ; PE(20:2(11Z14Z)16:0) ; PE(22:016:1(9Z)) ; PE(22:1(13Z)14:1(9Z)) ; PE(22:1(13Z)16:0) ; PE(22:2(13Z16Z)14:0) ; PE(22:2(13Z16Z)15:0) ; PE(24:014:1(9Z)) ; PE(24:1(15Z)14:0) ; PE-NMe(18:1(9Z)18:1(9Z)) ; DG(18:00:024:0) ; DG(20:00:022:0)	M+2K-H ; M+2K-H ; M+2K-H ; M+2K-H ; 2M+3H2O+2H ; 2M+3H2O+2H ; M+ACN+H ; 2M+H+Na ; 2M+3H2O+2H ; 2M+3H2O+2H ; 2M+3H2O+2H ; 2M+3H2O+2H ; 2M+3H2O+2H ; M+ACN+H ; 2M+3H2O+2H ; 2M+3H2O+2H ; 2M+H+Na ; 2M+3H2O+2H ; M+ACN+H ; 2M+H+Na ; M+ACN+H ; 2M+H+Na ; 2M+3H2O+2H ; M+ACN+H ; 2M+H+Na ; M+ACN+H ; 2M+H+Na ; M+ACN+H ; 2M+H+Na ; M+ACN+H ; M+ACN+H ; 2M+H+Na ; M+ACN+H ; M+ACN+H ; 2M+H+Na ; M+ACN+H ; 2M+H+Na ; 2M+H+Na ; M+ACN+H ; 2M+H+Na ; M+ACN+H ; 2M+H+Na ; M+ACN+H ; 2M+H+Na ; M+ACN+H ; 2M+3H2O+2H ; 2M+H+Na ; 2M+H+Na ; 2M+3H2O+2H ; M+2K-H ; M+2K-H	C45H88O5 ; C45H88O5 ; C45H88O5 ; C45H88O5 ; C42H80NO8P ; C42H80NO8P ; C41H78NO8P ; C43H84NO8P ; C42H80NO8P ; C42H80NO8P ; C42H80NO8P ; C42H80NO8P ; C42H80NO8P ; C41H78NO8P ; C42H80NO8P ; C42H80NO8P ; C43H84NO8P ; C42H80NO8P ; C41H78NO8P ; C43H84NO8P ; C41H78NO8P ; C43H84NO8P ; C42H80NO8P ; C41H78NO8P ; C43H84NO8P ; C41H78NO8P ; C43H84NO8P ; C41H78NO8P ; C43H84NO8P ; C41H78NO8P ; C41H78NO8P ; C43H84NO8P ; C41H78NO8P ; C41H78NO8P ; C43H84NO8P ; C41H78NO8P ; C43H84NO8P ; C43H84NO8P ; C41H78NO8P ; C43H84NO8P ; C41H78NO8P ; C43H84NO8P ; C41H78NO8P ; C43H84NO8P ; C41H78NO8P ; C42H80NO8P ; C43H84NO8P ; C43H84NO8P ; C42H80NO8P ; C45H88O5 ; C45H88O5
261.11699	1.426271	1.462435	-3.3862	0.00693	2.1593	0.037058	261.117	HMDB28729 ; HMDB28745 ; HMDB28792 ; HMDB29144 ; HMDB14538 ; HMDB30142 ; HMDB15337 ; HMDB15614 ; HMDB28740 ; HMDB29034 ; HMDB29904 ; HMDB30620 ; HMDB30797 ; HMDB30909 ; HMDB32521 ; HMDB33756 ; HMDB38352 ; HMDB39225 ; HMDB39913 ; HMDB41114	Asparaginyl-Glutamine ; Asparaginyl-Gamma-glutamate ; Glutaminyl-Asparagine ; Gamma-glutamyl-Asparagine ; Beclometasone dipropionate ; Physalolactone C ; Lomustine ; Ibudilast ; Asparaginyl-Serine ; Serinyl-Asparagine ; Kanzonol T ; Morusinol ; Mulberranol ; 23-Secoporrigenin ; Stearyl citrate ; Albanin C ; Lucidenic acid N ; 2-Stearyl citrate ; 16-Dihydroxy-37-dimethoxy-2-(3-methyl-2-butenyl)-8-(2-oxo-3-methyl-3-butenyl)-xanthone ; Artonin V	M+H ; M+H ; M+H ; M+H ; M+2H ; M+2H ; 2M+3H2O+2H ; 2M+Na+K ; M+ACN+H ; M+ACN+H ; M+2ACN+2H ; M+2ACN+2H ; M+2ACN+2H ; M+Na+K ; M+2K ; M+2ACN+2H ; M+Na+K ; M+2K ; M+2ACN+2H ; M+2ACN+2H	C9H16N4O5 ; C9H16N4O5 ; C9H16N4O5 ; C9H16N4O5 ; C28H37ClO7 ; C28H37ClO7 ; C9H16ClN3O2 ; C14H18N2O ; C7H13N3O5 ; C7H13N3O5 ; C25H26O7 ; C25H26O7 ; C25H26O7 ; C27H40O6 ; C24H44O7 ; C25H26O7 ; C27H40O6 ; C24H44O7 ; C25H26O7 ; C25H26O7
413.87413	1.47298	0.478353	3.386	0.006932	2.1592	0.037058	NA	NA	NA	NA	NA
490.92259	1.354573	1.493703	-3.3853	0.00694	2.1586	0.037058	NA	NA	NA	NA	NA
176.46287	1.421695	0.163973	3.3849	0.006945	2.1584	0.037058	176.4629	HMDB36673 ; HMDB37780	3alpha-Acetomethoxy-11alpha-oxo-12-ursen-24-oic acid ; Ganoderiol G	M+3H ; M+2H+Na	C33H50O5 ; C31H52O5

186.828226	1.341226	1.531332	-3.3835	0.006961	2.1573	0.037108	NA	NA	NA	NA	NA
710.222578	1.440943	1.449691	-3.3794	0.00701	2.1543	0.037326	710.2226	HMDB33456	2-Methoxy-3-methyl-9H-carbazole	3M+2K-H	C14H13NO
266.849224	1.385757	1.672089	3.3767	0.007042	2.1523	0.037424	266.8492	HMDB07962 ; HMDB09016 ; HMDB09246 ; HMDB09314 ; HMDB09315 ; HMDB09346 ; HMDB09379 ; HMDB09644 ; HMDB09645 ; HMDB09677 ; HMDB09678 ; HMDB09709 ; HMDB11238 ; HMDB11347 ; HMDB11373 ; HMDB11383 ; HMDB11384 ; HMDB11394 ; HMDB11415 ; HMDB11425 ; HMDB11426 ; HMDB11448 ; HMDB11458 ; HMDB11459	PC(15:0P-18:0) ; PE(18:0P-18:0) ; PE(20:0P-16:0) ; PE(20:2(11Z14Z)P-18:1(11Z)) ; PE(20:2(11Z14Z)P-18:1(9Z)) ; PE(20:3(5Z8Z11Z)P-18:0) ; PE(20:3(8Z11Z14Z)P-18:0) ; PE(22:5(4Z7Z10Z13Z16Z)P-18:1(11Z)) ; PE(22:5(4Z7Z10Z13Z16Z)P-18:1(9Z)) ; PE(22:5(7Z10Z13Z16Z19Z)P-18:1(11Z)) ; PE(22:5(7Z10Z13Z16Z19Z)dm18:1(9Z)) ; PE(22:6(4Z7Z10Z13Z16Z19Z)P-18:0) ; PC(P-18:015:0) ; PE(P-16:020:0) ; PE(dm18:018:0) ; PE(P-18:020:3(5Z8Z11Z)) ; PE(P-18:020:3(8Z11Z14Z)) ; PE(P-18:022:6(4Z7Z10Z13Z16Z19Z)) ; PE(P-18:1(11Z)20:2(11Z14Z)) ; PE(P-18:1(11Z)22:5(4Z7Z10Z13Z16Z)) ; PE(P-18:1(11Z)22:5(7Z10Z13Z16Z19Z)) ; PE(P-18:1(9Z)20:2(11Z14Z)) ; PE(P-18:1(9Z)22:5(4Z7Z10Z13Z16Z)) ; PE(P-18:1(9Z)22:5(7Z10Z13Z16Z19Z))	M+3Na ; M+3Na ; M+3Na ; M+H+2Na ; M+H+2Na ; M+H+2Na ; M+H+2Na ; M+2H+Na ; M+2H+Na ; M+2H+Na ; M+2H+Na ; M+2H+Na ; M+3Na ; M+3Na ; M+3Na ; M+H+2Na ; M+H+2Na ; M+2H+Na ; M+H+2Na ; M+2H+Na ; M+2H+Na ; M+H+2Na ; M+2H+Na ; M+2H+Na	C41H82NO7P ; C41H82NO7P ; C41H82NO7P ; C43H80NO7P ; C43H80NO7P ; C43H80NO7P ; C43H80NO7P ; C45H78NO7P ; C45H78NO7P ; C45H78NO7P ; C45H78NO7P ; C45H78NO7P ; C41H82NO7P ; C41H82NO7P ; C41H82NO7P ; C43H80NO7P ; C43H80NO7P ; C45H78NO7P ; C43H80NO7P ; C45H78NO7P ; C45H78NO7P ; C43H80NO7P ; C45H78NO7P ; C45H78NO7P
227.042078	1.394091	1.644946	3.3766	0.007043	2.1522	0.037424	227.0421	HMDB03337 ; HMDB13163 ; HMDB15484 ; HMDB30411 ; HMDB31200 ; HMDB32049 ; HMDB33587 ; HMDB34249 ; HMDB34757 ; HMDB39103 ; HMDB41340 ; HMDB41717 ; HMDB41718 ; HMDB59802 ; HMDB59803 ; HMDB60027 ; HMDB60028	Oxidized glutathione ; 2-Hydroxyfluorene ; Cefacetrile ; (2R2S)-Isobuteine ; 2-Methyl-2-(1-oxo-2-propenyl)amino-1-propanesulfonic acid ; Benzophenone ; Coumestrin ; Caryoptosidic acid ; 4-Methoxybenzenepropanol 1-(2-sulfoglucoside) ; Gossypetin 8-glucuronide 3-glucoside ; Benzofenap ; Daidzein 4-O-glucuronide ; Daidzein 7-O-glucuronide ; 3-Hydroxyfluorene ; 9-Hydroxyfluorene ; 5-(345-trihydroxyphenyl)-gamma-valerolactone-O-methyl-4-O-glucuronide ; 5-(345-trihydroxyphenyl)-gamma-valerolactone-O-methyl-5-O-glucuronide	M+3Na ; M+2Na-H ; 2M+3H ; 2M+H+K ; 2M+H+K ; M+2Na-H ; M+H+Na ; M+Na+K ; M+2Na ; M+2H+Na ; M+H+Na ; M+H+Na ; M+H+Na ; M+2Na-H ; M+2Na-H ; M+H+K ; M+H+K	C20H32N6O12S2 ; C13H10O ; C13H13N3O6S ; C7H13NO4S ; C7H13NO4S ; C13H10O ; C21H18O10 ; C16H24O11 ; C16H24O10S ; C27H28O19 ; C22H20Cl2N2O3 ; C21H18O10 ; C21H18O10 ; C13H10O ; C13H10O ; C18H22O11 ; C18H22O11
163.990629	1.370103	1.034243	3.3758	0.007052	2.1517	0.037431	163.9906	HMDB14503 ; HMDB31803 ; HMDB32429 ; HMDB34901 ; HMDB40259 ; HMDB60639	Sulfadiazine ; ()-Methamidophos ; 3-(Methylthio)methylthiophene ; 2-Vinyl-4H-13-dithiine ; 2-Methyl-5-(methylthio)thiophene ; Gemcitabine diphosphate	M+2K ; M+Na ; 2M+H+K ; 2M+H+K ; 2M+H+K ; M+3Na	C10H10N4O2S ; C2H8NO2PS ; C6H8S2 ; C6H8S2 ; C6H8S2 ; C9H13F2N3O10P2
348.716952	1.319575	1.241157	-3.3751	0.00706	2.1512	0.037434	348.717	HMDB14369 ; HMDB14497	Indinavir ; Methylergonovine	M+2ACN+2H ; 2M+H+NH4	C36H47N5O4 ; C20H25N3O2
212.053713	1.362123	1.144942	3.3741	0.007072	2.1505	0.037458	212.0537	HMDB11639 ; HMDB29842 ; HMDB34864 ; HMDB59999 ; HMDB00426 ; HMDB00428 ; HMDB00606 ; HMDB00694 ; HMDB01900 ; HMDB05807 ; HMDB11676 ; HMDB29649 ; HMDB31468 ; HMDB32819 ; HMDB32896 ; HMDB33031 ; HMDB33912 ; HMDB34605 ; HMDB35047 ; HMDB35812 ; HMDB36474 ; HMDB36661 ; HMDB36690 ; HMDB36894 ; HMDB36901 ; HMDB37986 ; HMDB37991 ; HMDB37996 ; HMDB38001 ; HMDB38002 ; HMDB38890 ; HMDB40795 ; HMDB41392 ; HMDB41637 ; HMDB41795 ; HMDB59655 ; HMDB59964	Topaquinone ; Betalamic acid ; 24-Dihydroxy-7-methoxy-2H-14-benzoxazin-3(4H)-one ; Gentisuric acid ; Citramalic acid ; 3-Hydroxyglutaric acid ; D-2-Hydroxyglutaric acid ; L-2-Hydroxyglutaric acid ; Ribonolactone ; Gallic acid ; D-Xylono-15-lactone ; 246-Trihydroxybenzoic acid ; Cyanidin 3-glucogalactoside ; Gibberellin A113 ; 3-Oxo-6b-angeloyloxy-7(11)-eremophilen-128a-olide ; Neoacrimarine H ; 2-Propenyl propyl disulfide ; Annuolide G ; Gibberellin A37 ; Epirosmanol ; Verimol G ; Isorosmanol ; 3-Epinobilin ; Gibberellin A64 ; Gibberellin A44 ; Cyanidin 33-diglucoside ; Cyanidin 34-diglucoside ; Cyanidin 3-laminaribioside ; Delphinidin 3-rutinoside ; Delphinidin 3-rhamnoside 5-glucoside ; Methyl 3-methyl-1-butenyl disulfide ; 7-O-Methylmarmin ; 1-Propenyl propyl disulfide ; Cyanidin 3-gentiobioside ; 19-Noraldosterone ; 2-Hydroxyglutarate ; 234-Trihydroxybenzoic acid	M+H ; M+H ; M+H ; M+H ; M+ACN+Na ; M+ACN+Na ; M+ACN+Na ; M+ACN+Na ; M+ACN+Na ; M+ACN+H ; M+ACN+Na ; M+ACN+H ; M+2H+Na ; M+2K ; M+2K ; M+3Na ; M+ACN+Na ; M+2K ; M+2K ; M+2K ; M+2K ; M+2K ; M+2K ; M+2K ; M+2K ; M+2H+Na ; M+2H+Na ; M+2H+Na ; M+2H+Na ; M+2H+Na ; M+ACN+Na ; M+2K ; M+ACN+Na ; M+2H+Na ; M+2K ; M+ACN+Na ; M+ACN+H	C9H9NO5 ; C9H9NO5 ; C9H9NO5 ; C9H9NO5 ; C5H8O5 ; C5H8O5 ; C5H8O5 ; C5H8O5 ; C5H8O5 ; C7H6O5 ; C5H8O5 ; C7H6O5 ; C27H31O16 ; C20H26O5 ; C20H26O5 ; C33H29NO8 ; C6H12S2 ; C20H26O5 ; C20H26O5 ; C20H26O5 ; C20H26O5 ; C20H26O5 ; C20H26O5 ; C20H26O5 ; C20H26O5 ; C27H31O16 ; C27H31O16 ; C27H31O16 ; C27H31O16 ; C27H31O16 ; C6H12S2 ; C20H26O5 ; C6H12S2 ; C27H31O16 ; C20H26O5 ; C5H8O5 ; C7H6O5
468.368028	1.426233	0.567181	3.3735	0.00708	2.15	0.03746	468.368	HMDB00601 ; HMDB03873 ; HMDB08389 ; HMDB08421 ; HMDB08453 ; HMDB08486 ; HMDB08548 ; HMDB08612 ; HMDB08643 ; HMDB08684 ; HMDB08717 ; HMDB08749 ; HMDB08772 ; HMDB08773 ; HMDB08781 ; HMDB08803 ; HMDB08804 ; HMDB08812 ; HMDB08813 ; HMDB09776 ; HMDB09777 ; HMDB11429 ; HMDB11462 ; HMDB44276 ; HMDB44332 ; HMDB44654 ; HMDB44656 ; HMDB52440 ; HMDB53302 ; HMDB60137	Coprocholic acid ; 3a7a12a-Trihydroxy-5b-cholestanoic acid ; PC(20:3(5Z8Z11Z)24:1(15Z)) ; PC(20:3(8Z11Z14Z)24:1(15Z)) ; PC(20:4(5Z8Z11Z14Z)24:0) ; PC(20:4(8Z11Z14Z17Z)24:0) ; PC(22:022:4(7Z10Z13Z16Z)) ; PC(22:2(13Z16Z)22:2(13Z16Z)) ; PC(22:4(7Z10Z13Z16Z)22:0) ; PC(22:5(4Z7Z10Z13Z16Z)24:1(15Z)) ; PC(22:5(7Z10Z13Z16Z19Z)24:1(15Z)) ; PC(22:6(4Z7Z10Z13Z16Z19Z)24:0) ; PC(24:020:4(5Z8Z11Z14Z)) ; PC(24:020:4(8Z11Z14Z17Z)) ; PC(24:022:6(4Z7Z10Z13Z16Z19Z)) ; PC(24:1(15Z)20:3(5Z8Z11Z)) ; PC(24:1(15Z)20:3(8Z11Z14Z)) ; PC(24:1(15Z)22:5(4Z7Z10Z13Z16Z)) ; PC(24:1(15Z)22:5(7Z10Z13Z16Z19Z)) ; PE(24:1(15Z)P-18:1(11Z)) ; PE(24:1(15Z)P-18:1(9Z)) ; PE(P-18:1(11Z)24:1(15Z)) ; PE(P-18:1(9Z)24:1(15Z)) ; TG(16:018:2(9Z12Z)o-18:0) ; TG(16:020:2n6o-18:0) ; TG(16:0o-18:018:2(9Z12Z)) ; TG(16:0o-18:020:2n6) ; TG(18:2(9Z12Z)16:0o-18:0) ; TG(20:2n616:0o-18:0) ; 3alpha7alpha12alpha25-Tetrahydroxy-5beta-cholestane-24-one	M+NH4 ; M+NH4 ; M+ACN+2H ; M+ACN+2H ; M+ACN+2H ; M+ACN+2H ; M+ACN+2H ; M+ACN+2H ; M+ACN+2H ; M+H+NH4 ; M+H+NH4 ; M+H+NH4 ; M+ACN+2H ; M+ACN+2H ; M+H+NH4 ; M+ACN+2H ; M+ACN+2H ; M+H+NH4 ; M+H+NH4 ; M+3ACN+2H ; M+3ACN+2H ; M+3ACN+2H ; M+3ACN+2H ; M+2K ; M+2K ; M+2K ; M+2K ; M+2K ; M+2K ; M+NH4	C27H46O5 ; C27H46O5 ; C52H96NO8P ; C52H96NO8P ; C52H96NO8P ; C52H96NO8P ; C52H96NO8P ; C52H96NO8P ; C52H96NO8P ; C54H96NO8P ; C54H96NO8P ; C54H96NO8P ; C52H96NO8P ; C52H96NO8P ; C54H96NO8P ; C52H96NO8P ; C52H96NO8P ; C54H96NO8P ; C54H96NO8P ; C47H90NO7P ; C47H90NO7P ; C47H90NO7P ; C47H90NO7P ; C56H106O5 ; C56H106O5 ; C56H106O5 ; C56H106O5 ; C56H106O5 ; C56H106O5 ; C27H46O5

365.983408	1.334485	0.242224	3.3726	0.00709	2.1494	0.037473	365.9834	HMDB45650 ; HMDB46025 ; HMDB46397 ; HMDB46420 ; HMDB46426 ; HMDB46565 ; HMDB46613 ; HMDB46619 ; HMDB46629 ; HMDB46715 ; HMDB46732 ; HMDB46771 ; HMDB46781 ; HMDB46882 ; HMDB46965 ; HMDB47116 ; HMDB47138 ; HMDB47144 ; HMDB47167 ; HMDB47300 ; HMDB47323 ; HMDB47329 ; HMDB47339 ; HMDB47351 ; HMDB47358 ; HMDB47397 ; HMDB47444 ; HMDB47501 ; HMDB47506 ; HMDB47547 ; HMDB47565 ; HMDB47581 ; HMDB47667 ; HMDB47684 ; HMDB50575 ; HMDB50683 ; HMDB50961 ; HMDB50978 ; HMDB51515 ; HMDB51525 ; HMDB51636 ; HMDB51655 ; HMDB51660 ; HMDB51748 ; HMDB51783 ; HMDB51868 ; HMDB52001 ; HMDB52011 ; HMDB52015 ; HMDB52022 ; HMDB52099 ; HMDB52133 ; HMDB52138 ; HMDB52153 ; HMDB52244 ; HMDB52259 ;	TG(20:024:022:4(7Z10Z13Z16Z)) ; TG(20:022:4(7Z10Z13Z16Z)24:0) ; TG(22:022:022:4(7Z10Z13Z16Z)) ; TG(22:024:020:4(5Z8Z11Z14Z)) ; TG(22:024:020:4(8Z11Z14Z17Z)) ; TG(22:020:3(5Z8Z11Z)24:1(15Z)) ; TG(22:024:1(15Z)20:3(5Z8Z11Z)) ; TG(22:024:1(15Z)20:3n6) ; TG(22:024:1(15Z)22:6(4Z7Z10Z13Z16Z19Z)) ; TG(22:020:3n624:1(15Z)) ; TG(22:020:4(5Z8Z11Z14Z)24:0) ; TG(22:022:2(13Z16Z)22:2(13Z16Z)) ; TG(22:022:4(7Z10Z13Z16Z)22:0) ; TG(22:020:4(8Z11Z14Z17Z)24:0) ; TG(22:022:6(4Z7Z10Z13Z16Z19Z)24:1(15Z)) ; TG(24:020:022:4(7Z10Z13Z16Z)) ; TG(24:022:020:4(5Z8Z11Z14Z)) ; TG(24:022:020:4(8Z11Z14Z17Z)) ; TG(24:024:018:4(6Z9Z12Z15Z)) ; TG(24:020:3(5Z8Z11Z)22:1(13Z)) ; TG(24:022:1(13Z)20:3(5Z8Z11Z)) ; TG(24:022:1(13Z)20:3n6) ; TG(24:022:1(13Z)22:6(4Z7Z10Z13Z16Z19Z)) ; TG(24:024:1(15Z)18:3(6Z9Z12Z)) ; TG(24:024:1(15Z)18:3(9Z12Z15Z)) ; TG(24:018:3(6Z9Z12Z)24:1(15Z)) ; TG(24:020:3n622:1(13Z)) ; TG(24:022:2(13Z16Z)22:5(4Z7Z10Z13Z16Z)) ; TG(24:022:2(13Z16Z)22:5(7Z10Z13Z16Z19Z)) ; TG(24:022:5(4Z7Z10Z13Z16Z)22:2(13Z16Z)) ; TG(24:018:3(9Z12Z15Z)24:1(15Z)) ; TG(24:018:4(6Z9Z12Z15Z)24:0) ; TG(24:022:5(7Z10Z13Z16Z19Z)22:2(13Z16Z)) ; TG(24:022:6(4Z7Z10Z13Z16Z19Z)22:1(13Z)) ; TG(20:1(11Z)24:1(15Z)22:2(13Z16Z)) ; TG(20:1(11Z)22:2(13Z16Z)24:1(15Z)) ; TG(20:3(5Z8Z11Z)22:024:1(15Z)) ; TG(20:3(5Z8Z11Z)24:022:1(13Z)) ; TG(22:1(13Z)24:020:3n6) ; TG(22:1(13Z)24:022:6(4Z7Z10Z13Z16Z19Z)) ; TG(22:1(13Z)22:1(13Z)22:2(13Z16Z)) ; TG(22:1(13Z)24:1(15Z)22:5(4Z7Z10Z13Z16Z)) ; TG(22:1(13Z)24:1(15Z)22:5(7Z10Z13Z16Z19Z)) ; TG(22:1(13Z)22:2(13Z16Z)22:1(13Z)) ; TG(22:1(13Z)22:5(4Z7Z10Z13Z16Z)24:1(15Z)) ; TG(22:1(13Z)22:5(7Z10Z13Z16Z19Z)24:1(15Z)) ; TG(24:1(15Z)22:020:3n6) ; TG(24:1(15Z)22:022:6(4Z7Z10Z13Z16Z19Z)) ; TG(24:1(15Z)24:018:3(6Z9Z12Z)) ; TG(24:1(15Z)24:018:3(9Z12Z15Z)) ; TG(24:1(15Z)20:1(11Z)22:2(13Z16Z)) ; TG(24:1(15Z)22:1(13Z)22:5(4Z7Z10Z13Z16Z)) ; TG(24:1(15Z)22:1(13Z)22:5(7Z10Z13Z16Z19Z)) ; TG(24:1(15Z)24:1(15Z)20:5(5Z8Z11Z14Z17Z)) ; TG(24:1(15Z)22:2(13Z16Z)22:4(7Z10Z13Z16Z)) ; TG(24:1(15Z)22:4(7Z10Z13Z16Z)22:2(13Z16Z)) ; TG(24:1(15Z)20:5(5Z8Z11Z14Z17Z)24:1(15Z)) ; TG(22:2(13Z16Z)22:022:2(13Z16Z)) ; TG(22:2(13Z16Z)24:022:5(4Z7Z10Z13Z16Z)) ; TG(22:2(13Z16Z)24:022:5(7Z10Z13Z16Z19Z)) ; TG(22:2(13Z16Z)24:1(15Z)22:4(7Z10Z13Z16Z))	M+H+2Na ; M+H+2Na ; M+H+2Na ; M+H+2Na ; M+H+2Na ; M+H+2Na ; M+H+2Na ; M+H+2Na ; M+2H+Na ; M+H+2Na ; M+H+2Na ; M+H+2Na ; M+H+2Na ; M+H+2Na ; M+2H+Na ; M+H+2Na ; M+H+2Na ; M+H+2Na ; M+H+2Na ; M+H+2Na ; M+2H+Na ; M+H+2Na ; M+H+2Na ; M+H+2Na ; M+H+2Na ; M+2H+Na ; M+2H+Na ; M+2H+Na ; M+H+2Na ; M+H+2Na ; M+2H+Na ; M+2H+Na ; M+H+2Na ; M+H+2Na ; M+H+2Na ; M+H+2Na ; M+H+2Na ; M+H+2Na ; M+2H+Na ; M+H+2Na ; M+2H+Na ; M+2H+Na ; M+H+2Na ; M+2H+Na ; M+2H+Na ; M+H+2Na ; M+2H+Na ; M+H+2Na ; M+H+2Na ; M+H+2Na ; M+2H+Na ; M+2H+Na ; M+2H+Na ; M+2H+Na ; M+2H+Na ; M+2H+Na ; M+H+2Na ; M+2H+Na ; M+2H+Na ; M+2H+Na	C69H126O6 ; C69H126O6 ; C69H126O6 ; C69H126O6 ; C69H126O6 ; C69H126O6 ; C69H126O6 ; C69H126O6 ; C71H124O6 ; C69H126O6 ; C69H126O6 ; C69H126O6 ; C69H126O6 ; C69H126O6 ; C71H124O6 ; C69H126O6 ; C69H126O6 ; C69H126O6 ; C69H126O6 ; C69H126O6 ; C71H124O6 ; C69H126O6 ; C69H126O6 ; C69H126O6 ; C69H126O6 ; C71H124O6 ; C71H124O6 ; C71H124O6 ; C69H126O6 ; C69H126O6 ; C71H124O6 ; C71H124O6 ; C69H126O6 ; C69H126O6 ; C69H126O6 ; C69H126O6 ; C69H126O6 ; C71H124O6 ; C69H126O6 ; C71H124O6 ; C71H124O6 ; C69H126O6 ; C71H124O6 ; C71H124O6 ; C69H126O6 ; C71H124O6 ; C69H126O6 ; C69H126O6 ; C69H126O6 ; C71H124O6 ; C71H124O6 ; C71H124O6 ; C71H124O6 ; C71H124O6 ; C71H124O6 ; C69H126O6 ; C71H124O6 ; C71H124O6 ; C71H124O6
386.714697	1.378869	1.688625	3.3715	0.007103	2.1485	0.037475	386.7147	HMDB08838 ; HMDB08839 ; HMDB08869 ; HMDB08870 ; HMDB08931 ; HMDB08962 ; HMDB08963 ; HMDB09122 ; HMDB09155 ; HMDB09187 ; HMDB09317 ; HMDB09350 ; HMDB09382 ; HMDB09415 ; HMDB14344 ; HMDB61020	PE(14:020:4(5Z8Z11Z14Z)) ; PE(14:020:4(8Z11Z14Z17Z)) ; PE(14:1(9Z)20:3(5Z8Z11Z)) ; PE(14:1(9Z)20:3(8Z11Z14Z)) ; PE(16:018:4(6Z9Z12Z15Z)) ; PE(16:1(9Z)18:3(6Z9Z12Z)) ; PE(16:1(9Z)18:3(9Z12Z15Z)) ; PE(18:3(6Z9Z12Z)16:1(9Z)) ; PE(18:3(9Z12Z15Z)16:1(9Z)) ; PE(18:4(6Z9Z12Z15Z)16:0) ; PE(20:3(5Z8Z11Z)14:1(9Z)) ; PE(20:3(8Z11Z14Z)14:1(9Z)) ; PE(20:4(5Z8Z11Z14Z)14:0) ; PE(20:4(8Z11Z14Z17Z)14:0) ; Erythromycin ; N-desmethylclarithromycin	M+Na+K ; M+Na+K ; M+Na+K ; M+Na+K ; M+Na+K ; M+Na+K ; M+Na+K ; M+Na+K ; M+Na+K ; M+Na+K ; M+Na+K ; M+Na+K ; M+Na+K ; M+Na+K ; M+H+K ; M+H+K	C39H70NO8P ; C39H70NO8P ; C39H70NO8P ; C39H70NO8P ; C39H70NO8P ; C39H70NO8P ; C39H70NO8P ; C39H70NO8P ; C39H70NO8P ; C39H70NO8P ; C39H70NO8P ; C39H70NO8P ; C39H70NO8P ; C39H70NO8P ; C37H67NO13 ; C37H67NO13
221.076956	1.367703	0.85045	-3.3709	0.007111	2.1481	0.037475	221.077	HMDB11168 ; HMDB28762 ; HMDB29035 ; HMDB00903 ; HMDB03259 ; HMDB06753 ; HMDB06754 ; HMDB06760 ; HMDB13157 ; HMDB14803 ; HMDB30905 ; HMDB32027 ; HMDB32064 ; HMDB32177 ; HMDB32346 ; HMDB32372 ; HMDB32427 ; HMDB32488 ; HMDB32553 ; HMDB33033 ; HMDB34277 ; HMDB37627 ; HMDB37700 ; HMDB37701 ; HMDB41315 ; HMDB41720	L-beta-aspartyl-L-serine ; Aspartyl-Serine ; Serinyl-Aspartate ; Tetrahydrocortisone ; Dihydrocortisol ; 3a11b21-Trihydroxy-20-oxo-5b-pregnan-18-al ; 11b21-Dihydroxy-320-oxo-5b-pregnan-18-al ; 11b17a21-Trihydroxypreg-nenolone ; 18-Hydroxy-11-dehydrotetrahydrocorticosterone ; Nilutamide ; 1-(4-Methoxyphenyl)-1-penten-3-one ; 3-(2-Methylpropylidene)-3alpha4-dihydro-1(3H)-isobenzofuranone ; (S)-3-Butyl-1(3H)-isobenzofuranone ; Benzyl trans-2-methyl-2-butenoate ; (-)-Isobutyl 3-methylthiobutyrate ; 3-Mercaptoheptyl acetate ; (S)-3-Methylthiohexyl acetate ; Prenyl benzoate ; Vanillin 3-(L-menthoxy)propane-12-diol acetal ; (R)-37-Dimethyl-5-indanecarboxylic acid ; Ligustilide ; Benzyl tiglate ; Propyl cinnamate ; Isopropyl cinnamate ; Cinnamyl propionate ; Dihydrocaffeic acid 3-O-glucuronide	M+H ; M+H ; M+H ; M+2K ; M+2K ; M+2K ; M+2K ; M+2K ; M+2K ; M+3ACN+2H ; 2M+Na+K ; 2M+Na+K ; 2M+Na+K ; 2M+Na+K ; 2M+Na+K ; 2M+Na+K ; 2M+Na+K ; 2M+Na+K ; M+2K ; 2M+Na+K ; 2M+Na+K ; 2M+Na+K ; 2M+Na+K ; 2M+Na+K ; 2M+Na+K ; M+2ACN+2H	C7H12N2O6 ; C7H12N2O6 ; C7H12N2O6 ; C21H32O5 ; C21H32O5 ; C21H32O5 ; C21H32O5 ; C21H32O5 ; C21H32O5 ; C12H10F3N3O4 ; C12H14O2 ; C12H14O2 ; C12H14O2 ; C12H14O2 ; C9H18O2S ; C9H18O2S ; C9H18O2S ; C12H14O2 ; C21H32O5 ; C12H14O2 ; C12H14O2 ; C12H14O2 ; C12H14O2 ; C12H14O2 ; C12H14O2 ; C15H18O10
370.831852	1.441193	0.788612	3.3707	0.007113	2.148	0.037475	NA	NA	NA	NA	NA

176.11814	1.430238	0.519317	3.3657	0.007173	2.1443	0.037752	176.1181	HMDB06543 ; HMDB00926 ; HMDB02183 ; HMDB02925 ; HMDB10378 ; HMDB13825 ; HMDB29653 ; HMDB30053 ; HMDB30347 ; HMDB30446 ; HMDB31058 ; HMDB31311 ; HMDB31323 ; HMDB31454 ; HMDB31624 ; HMDB31625 ; HMDB31866 ; HMDB32060 ; HMDB33154 ; HMDB34891 ; HMDB35185 ; HMDB35601 ; HMDB35924 ; HMDB35989 ; HMDB36150 ; HMDB36719 ; HMDB36814 ; HMDB36816 ; HMDB36835 ; HMDB37148 ; HMDB37149 ; HMDB37294 ; HMDB38153 ; HMDB38154 ; HMDB38165 ; HMDB38503 ; HMDB38908 ; HMDB39990 ; HMDB39991 ; HMDB39992 ; HMDB39993 ; HMDB39994 ; HMDB39997 ; HMDB60039 ; HMDB60570	Debrisoquine ; Pyridine ; Docosahexaenoic acid ; 81114-Eicosatrienoic acid ; 5811-Eicosatrienoic acid ; 4-(1133-Tetramethylbutyl)-phenol ; 1-(1-Methoxy-1-methylethyl)-4-methylbenzene ; Neogrifolin ; N-(p-Hydroxyphenethyl)actinidine ; Grifolin ; Sciadonic acid ; Benzyl butyl ether ; 3-Butenenitrile ; trans-Jasmone ; 5-Phenyl-1-pentanol ; 1-Phenyl-2-pentanol ; 2-Methyl-4-phenyl-2-butanol ; 2-Butyl-4-methylphenol ; 5678-Tetrahydroquinoxaline ; 35-Dimethyl-2-vinylpyrazine ; Retinol acetate ; (Z)-3-Methyl-2-(2-pentenyl)-2-cyclopenten-1-one ; Pyrrole ; Thymol methyl ether ; 4111315-Tetrahydroridentin B ; Norbicycloekasantalal ; Santalone ; Norecasantalal ; Sagittariol ; 67-Dihydro-2-methyl-5H-cyclopentabpyrazine ; 67-Dihydro-5-methyl-5H-cyclopentabpyrazine ; 2-Methyl-3-(2-pentenyl)-2-cyclopenten-1-one ; 1-(3-Furanyl)-67-dihydroxy-48-dimethyl-1-nonanone ; 31112-Trihydroxy-1(10)-spirovetiven-2-one ; Acoric acid ; Bisacurone epoxide ; (ZZ)-2-Methyl-5-(81114-pentadecatrienyl)-13-benzenediol ; 2-Methyl-6-(1-propenyl)pyrazine ; 2-Methyl-3-(1-propenyl)pyrazine ; 2-Methyl-6-(2-propenyl)pyrazine ; 2-Methyl-5-(2-propenyl)pyrazine ; 2-Methyl-3-(2-propenyl)pyrazine ; 2-Methyl-5-(1-propenyl)pyrazine ; 111417-Eicosatrienoic acid ; Deoxydihydro-artemisinin	M+H ; 2M+NH4 ; M+H+Na ; M+2Na ; M+2Na ; 2M+H+Na ; 2M+H+Na ; M+H+Na ; M+2ACN+2H ; M+H+Na ; M+2Na ; 2M+H+Na ; 2M+ACN+H ; 2M+H+Na ; 2M+H+Na ; 2M+H+Na ; 2M+H+Na ; 2M+H+Na ; M+ACN+H ; M+ACN+H ; M+H+Na ; 2M+H+Na ; 2M+ACN+H ; 2M+H+Na ; M+2ACN+2H ; 2M+H+Na ; 2M+H+Na ; 2M+H+Na ; M+2Na ; M+ACN+H ; M+ACN+H ; 2M+H+Na ; M+2ACN+2H ; M+2ACN+2H ; M+2ACN+2H ; M+2ACN+2H ; M+H+Na ; M+ACN+H ; M+ACN+H ; M+ACN+H ; M+ACN+H ; M+ACN+H ; M+ACN+H ; M+2Na ; M+2ACN+2H	C10H13N3 ; C5H5N ; C22H32O2 ; C20H34O2 ; C20H34O2 ; C11H16O ; C11H16O ; C22H32O2 ; C18H22NO ; C22H32O2 ; C20H34O2 ; C11H16O ; C4H5N ; C11H16O ; C11H16O ; C11H16O ; C11H16O ; C11H16O ; C8H10N2 ; C8H10N2 ; C22H32O2 ; C11H16O ; C4H5N ; C11H16O ; C15H24O4 ; C11H16O ; C11H16O ; C11H16O ; C20H34O2 ; C8H10N2 ; C8H10N2 ; C11H16O ; C15H24O4 ; C15H24O4 ; C15H24O4 ; C15H24O4 ; C22H32O2 ; C8H10N2 ; C8H10N2 ; C8H10N2 ; C8H10N2 ; C8H10N2 ; C8H10N2 ; C20H34O2 ; C15H24O4
98.918883	1.390883	1.684004	3.365	0.007181	2.1438	0.037756	NA	NA	NA	NA	NA
206.89377	1.38609	1.667791	3.3632	0.007204	2.1424	0.037835	NA	NA	NA	NA	NA
540.02532	1.368002	1.61027	-3.3599	0.007244	2.14	0.037997	540.0253	HMDB29251 ; HMDB60015 ; HMDB60260	Pinotin A aglycone ; Phenol sulphate ; Urate radical	M+2K-H ; 3M+NH4 ; 3M+K	C25H19O9 ; C6H6O4S ; C5H3N4O3
231.02503	1.304393	0.470194	3.3591	0.007254	2.1394	0.037997	231.025	HMDB00618 ; HMDB00868 ; HMDB01489 ; HMDB01548 ; HMDB06534 ; HMDB11734 ; HMDB12195 ; HMDB00939 ; HMDB01124 ; HMDB03072 ; HMDB06789 ; HMDB29541 ; HMDB30621 ; HMDB34914 ; HMDB35479 ; HMDB36281 ; HMDB37166 ; HMDB38396 ; HMDB38874 ; HMDB38950 ; HMDB39617 ; HMDB39634 ; HMDB40134 ; HMDB40813 ; HMDB40837 ; HMDB40934 ; HMDB59984 ; HMDB59986 ; HMDB60019 ; HMDB60257	D-Ribulose 5-phosphate ; Xylulose 5-phosphate ; Ribose 1-phosphate ; D-Ribose 5-phosphate ; D-Xylulose 1-phosphate ; D-Arabinose 5-phosphate ; Beta-L-arabinose 1-phosphate ; S-Adenosylhomocysteine ; Trehalose 6-phosphate ; Quinic acid ; Lactose 6-phosphate ; ()-Zeylenol ; Oxyisocyclointegrin ; Dulxanthone F ; Isolicopyranocoumarin ; Licofuranocoumarin ; 2-(Methylthiomethyl)-3-phenyl-2-propenal ; Gancaonin D ; Licopyranocoumarin ; 5-Methoxyhinokinin ; Piperenol B ; Piperenol A ; Calebin A ; 3-O-Methylgancaonin P ; 3-O-Methyluralenol ; Uralene ; 5-(345-Trihydroxyphenyl)-gamma-valerolactone-3-O-glucuronide ; 5-(345-Trihydroxyphenyl)-gamma-valerolactone-4-O-glucuronide ; Sinapinic acid-O-glucuronide isomer ; Selenomethionine se-oxide	M+H ; M+H ; M+H ; M+H ; M+H ; M+H ; M+H ; M+2K ; M+H+K ; M+K ; M+H+K ; M+2K ; M+2K ; M+2K ; M+2K ; M+2K ; M+K ; M+2K ; M+2K ; M+2K ; M+2K ; M+2K ; M+2K ; M+2K ; M+2K ; M+2K ; M+Na+K ; M+Na+K ; M+Na+K ; M+NH4	C5H11O8P ; C5H11O8P ; C5H11O8P ; C5H11O8P ; C5H11O8P ; C5H11O8P ; C5H11O8P ; C14H20N6O5S ; C12H23O14P ; C7H12O6 ; C12H23O14P ; C21H20O7 ; C21H20O7 ; C21H20O7 ; C21H20O7 ; C21H20O7 ; C11H12OS ; C21H20O7 ; C21H20O7 ; C21H20O7 ; C21H20O7 ; C21H20O7 ; C21H20O7 ; C21H20O7 ; C21H20O7 ; C21H20O7 ; C17H20O11 ; C17H20O11 ; C17H20O11 ; C5H11NO3Se
694.18004	1.436637	0.572007	-3.3588	0.007257	2.1392	0.037997	694.18	HMDB15066 ; HMDB15369 ; HMDB30569 ; HMDB37449 ; HMDB37451 ; HMDB37473 ; HMDB37567 ; HMDB38049 ; HMDB38455 ; HMDB39189 ; HMDB39195 ; HMDB39314 ; HMDB39758	Methacycline ; Chlorprothixene ; Swertiajaponin ; Isoswertiajaponin ; Diosmetin 7-O-beta-D-glucopyranoside ; Leptosin ; 6-beta-D-Glucopyranosyldiosmetin ; Pratensein 3-glucoside ; Kaempferide 7-glucoside ; 2-Cinnamoyl-1-galloyl-beta-D-glucopyranose ; 6-Cinnamoyl-1-galloylglucose ; 4-(4-Hydroxyphenyl)-2-butanone O-26-digalloylglucoside ; Tricin arabinoside	3M+Na+K ; 2M+ACN+Na ; 3M+2H ; 3M+2H ; 3M+2H ; 3M+2H ; 3M+2H ; 3M+2H ; 3M+2H ; 3M+2H ; 3M+2H ; M+ACN+Na ; 3M+2H	C22H22N2O8 ; C18H18ClNS ; C22H22O11 ; C22H22O11 ; C22H22O11 ; C22H22O11 ; C22H22O11 ; C22H22O11 ; C22H22O11 ; C22H22O11 ; C22H22O11 ; C30H30O15 ; C22H22O11
349.13551	1.404468	0.455011	-3.3577	0.00727	2.1385	0.038024	349.1355	HMDB13930 ; HMDB14011 ; HMDB15552 ; HMDB15553	R-95913 ; 5-O-Desmethyl omeprazole ; Acepromazine ; Acepromethazine	M+NH4 ; M+NH4 ; M+Na ; M+Na	C18H18FNO2S ; C16H17N3O3S ; C19H22N2OS ; C19H22N2OS

331.26276	1.417996	0.055503	3.3565	0.007285	2.1376	0.038029	331.2628	HMDB01976 ; HMDB06528 ; HMDB32257 ; HMDB35024 ; HMDB35581 ; HMDB38909 ; HMDB39133 ; HMDB39530 ; HMDB60113 ; HMDB07029 ; HMDB07057 ; HMDB07108 ; HMDB07136 ; HMDB07159 ; HMDB07160 ; HMDB07187 ; HMDB07216 ; HMDB07360 ; HMDB07388 ; HMDB07589 ; HMDB07617 ; HMDB29957 ; HMDB29960 ; HMDB31887 ; HMDB32754 ; HMDB38037 ; HMDB43096 ; HMDB43123 ; HMDB43129 ; HMDB43157 ; HMDB43320 ; HMDB43348 ; HMDB43354 ; HMDB43364 ; HMDB43381 ; HMDB43388 ; HMDB43437 ; HMDB43494 ; HMDB43515 ; HMDB43561 ; HMDB43566 ; HMDB43572 ; HMDB43617 ; HMDB43640 ; HMDB43661 ; HMDB43689 ; HMDB43762 ; HMDB43784 ; HMDB44267 ; HMDB44323 ; HMDB44402 ; HMDB44404 ; HMDB45092 ; HMDB45097 ; HMDB45146 ; HMDB45151 ;	Docosapentaenoic acid (22n-6) ; Docosapentaenoic acid ; Ethyl abietate ; ent-16-Kauren-19-ol acetate ; 1-Phenyl-13-hexadecanedione ; 2-Methyl-5-(811-pentadecadienyl)-13-benzenediol ; 48121519-Docosapentaenoic acid ; Ethyl icosapentate ; Docosa-47101316-pentaenoic acid ; DG(14:022:1(13Z)0:0) ; DG(14:1(9Z)22:00:0) ; DG(16:020:1(11Z)0:0) ; DG(16:1(9Z)20:00:0) ; DG(18:018:1(11Z)0:0) ; DG(18:018:1(9Z)0:0) ; DG(18:1(11Z)18:00:0) ; DG(18:1(9Z)18:00:0) ; DG(20:016:1(9Z)0:0) ; DG(20:1(11Z)16:00:0) ; DG(22:014:1(9Z)0:0) ; DG(22:1(13Z)14:00:0) ; Squamocin K ; Asimilobin ; Rollidecin C ; Isomurisolenin ; Corossolone ; TG(15:020:022:4(7Z10Z13Z16Z)) ; TG(15:022:020:4(5Z8Z11Z14Z)) ; TG(15:022:020:4(8Z11Z14Z17Z)) ; TG(15:024:018:4(6Z9Z12Z15Z)) ; TG(15:020:3(5Z8Z11Z)22:1(13Z)) ; TG(15:022:1(13Z)20:3(5Z8Z11Z)) ; TG(15:022:1(13Z)20:3n6) ; TG(15:022:1(13Z)22:6(4Z7Z10Z13Z16Z19Z)) ; TG(15:024:1(15Z)18:3(6Z9Z12Z)) ; TG(15:024:1(15Z)18:3(9Z12Z15Z)) ; TG(15:018:3(6Z9Z12Z)24:1(15Z)) ; TG(15:020:3n622:1(13Z)) ; TG(15:020:4(5Z8Z11Z14Z)22:0) ; TG(15:022:2(13Z16Z)22:5(4Z7Z10Z13Z16Z)) ; TG(15:022:2(13Z16Z)22:5(7Z10Z13Z16Z19Z)) ; TG(15:022:4(7Z10Z13Z16Z)20:0) ; TG(15:022:5(4Z7Z10Z13Z16Z)22:2(13Z16Z)) ; TG(15:018:3(9Z12Z15Z)24:1(15Z)) ; TG(15:018:4(6Z9Z12Z15Z)24:0) ; TG(15:020:4(8Z11Z14Z17Z)22:0) ; TG(15:022:5(7Z10Z13Z16Z19Z)22:2(13Z16Z)) ; TG(15:022:6(4Z7Z10Z13Z16Z19Z)22:1(13Z)) ; TG(16:018:2(9Z12Z)22:2(13Z16Z)) ; TG(16:020:2n622:2(13Z16Z)) ; TG(16:022:2(13Z16Z)18:2(9Z12Z)) ; TG(16:022:2(13Z16Z)20:2n6) ; TG(18:018:2(9Z12Z)22:5(4Z7Z10Z13Z16Z)) ; TG(18:018:2(9Z12Z)22:5(7Z10Z13Z16Z19Z)) ; TG(18:020:2n622:5(4Z7Z10Z13Z16Z)) ; TG(18:020:2n622:5(7Z10Z13Z16Z19Z)) ; TG(18:022:5(4Z7Z10Z13Z16Z)18:2(9Z12Z)) ; TG(18:022:5(4Z7Z10Z13Z16Z)20:2n6) ; TG(18:022:5(7Z10Z13Z16Z19Z)18:2(9Z12Z)) ; TG(18:022:5(7Z10Z13Z16Z19Z)20:2n6) ; TG(20:015:022:4(7Z10Z13Z16Z)) ; TG(20:018:2(9Z12Z)20:5(5Z8Z11Z14Z17Z)) ; TG(20:020:2n620:5(5Z8Z11Z14Z17Z)) ; TG(20:020:5(5Z8Z11Z14Z17Z)18:2(9Z12Z)) ; TG(20:020:5(5Z8Z11Z14Z17Z)20:2n6) ; TG(22:015:020:4(5Z8Z11Z14Z)) ; TG(22:015:020:4(8Z11Z14Z17Z)) ; TG(24:015:018:4(6Z9Z12Z15Z)) ; TG(14:1(9Z)24:1(15Z)18:2(9Z12Z)) ; TG(14:1(9Z)24:1(15Z)20:2n6) ; TG(14:1(9Z)18:2(9Z12Z)24:1(15Z)) ; TG(14:1(9Z)20:2n624:1(15Z)) ;	M+H ; M+H ; M+H ; M+H ; M+H ; M+H ; M+H ; M+H ; M+H ; M+H+K ; M+H+K ; M+H+K ; M+H+K ; M+H+K ; M+H+K ; M+H+K ; M+H+K ; M+H+K ; M+H+K ; M+H+K ; M+H+K ; M+2ACN+2H ; M+2ACN+2H ; M+2ACN+2H ; M+2ACN+2H ; M+2ACN+2H ; M+3Na ; M+3Na ; M+3Na ; M+3Na ; M+3Na ; M+3Na ; M+3Na ; M+H+2Na ; M+3Na ; M+3Na ; M+3Na ; M+3Na ; M+3Na ; M+H+2Na ; M+H+2Na ; M+3Na ; M+H+2Na ; M+3Na ; M+3Na ; M+3Na ; M+H+2Na ; M+H+2Na ; M+3Na ; M+3Na ; M+3Na ; M+3Na ; M+H+2Na ; M+H+2Na ; M+H+2Na ; M+H+2Na ; M+H+2Na ; M+H+2Na ; M+H+2Na ; M+H+2Na ; M+3Na ; M+H+2Na ; M+H+2Na ; M+H+2Na ; M+H+2Na ; M+3Na ; M+3Na ; M+3Na ; M+3Na ; M+3Na ; M+3Na ; M+3Na ; M+3Na ; M+3Na ; M+3Na ; M+3Na ; M+3Na ; M+3Na ; M+3Na ; M+H+2Na ; M+3Na ; M+H+2Na ; M+H+2Na ; M+H+2Na ; M+3Na ; M+3Na ; M+H+2Na ; M+3Na ; M+H+2Na ; M+H+2Na ; M+H+2Na ; M+3Na ; M+3Na ; M+3Na ; M+3Na ; M+H+2Na ; M+H+2Na ; M+H+2Na ; M+H+2Na ; M+H+2Na ; M+H+2Na ; M+H+2Na ; M+H+2Na ; M+3Na ; M+2H+Na ; M+2H+Na ; M+2H+Na ; M+2H+Na ; M+2H+Na ; M+2H+Na ; M+2H+Na ; M+2H+Na ; M+3Na ; M+H+2Na ; M+3Na ; M+3Na ; M+H+2Na ; M+H+2Na ; M+H+2Na ; M+H+2Na ; M+3Na ;	C22H34O2 ; C22H34O2 ; C22H34O2 ; C22H34O2 ; C22H34O2 ; C22H34O2 ; C22H34O2 ; C22H34O2 ; C22H34O2 ; C39H74O5 ; C39H74O5 ; C39H74O5 ; C39H74O5 ; C39H74O5 ; C39H74O5 ; C39H74O5 ; C39H74O5 ; C39H74O5 ; C39H74O5 ; C39H74O5 ; C39H74O5 ; C35H62O6 ; C35H62O6 ; C35H62O6 ; C35H62O6 ; C35H62O6 ; C60H108O6 ; C60H108O6 ; C60H108O6 ; C60H108O6 ; C60H108O6 ; C60H108O6 ; C60H108O6 ; C62H106O6 ; C60H108O6 ; C60H108O6 ; C60H108O6 ; C60H108O6 ; C60H108O6 ; C62H106O6 ; C62H106O6 ; C60H108O6 ; C62H106O6 ; C60H108O6 ; C60H108O6 ; C60H108O6 ; C62H106O6 ; C62H106O6 ; C60H108O6 ; C60H108O6 ; C60H108O6 ; C60H108O6 ; C62H106O6 ; C62H106O6 ; C62H106O6 ; C62H106O6 ; C62H106O6 ; C62H106O6 ; C62H106O6 ; C62H106O6 ; C60H108O6 ; C62H106O6 ; C62H106O6 ; C62H106O6 ; C62H106O6 ; C60H108O6 ; C60H108O6 ; C60H108O6 ; C60H108O6 ; C60H108O6 ; C60H108O6 ; C60H108O6 ; C60H108O6 ; C60H108O6 ; C60H108O6 ; C60H108O6 ; C60H108O6 ; C60H108O6 ; C62H106O6 ; C60H108O6 ; C62H106O6 ; C62H106O6 ; C62H106O6 ; C60H108O6 ; C60H108O6 ; C62H106O6 ; C60H108O6 ; C62H106O6 ; C62H106O6 ; C62H106O6 ; C60H108O6 ; C60H108O6 ; C60H108O6 ; C60H108O6 ; C62H106O6 ; C62H106O6 ; C62H106O6 ; C62H106O6 ; C62H106O6 ; C62H106O6 ; C62H106O6
523.67372	1.490003	0.233787	-3.3564	0.007287	2.1375	0.038029	523.6737	HMDB06498 ; HMDB12715 ; HMDB41557	3-Oxooctadecanoyl-CoA ; 3-hydroxyoctadecanoyl-CoA ; Foeniculoside III	M+2H ; M+2H ; M+ACN+2H	C39H66N7O18P3S ; C39H66N7O18P3S ; C54H52O19
416.91760	1.381784	1.656403	-3.3555	0.007297	2.1369	0.038029	416.9176	HMDB00968 ; HMDB01047 ; HMDB01058 ; HMDB03514 ; HMDB06234 ; HMDB06235 ; HMDB06872 ; HMDB60269 ; HMDB60444	1D-Myo-inositol 14-bisphosphate ; D-Fructose 26-bisphosphate ; Fructose 16-bisphosphate ; Alpha-D-Glucose 16-bisphosphate ; 1D-Myo-inositol 13-bisphosphate ; 1D-Myo-inositol 34-bisphosphate ; D-Tagatose 16-bisphosphate ; D-Mannose 16-bisphosphate ; beta-D-Fructose 16-bisphosphate	M+2K-H ; M+2K-H ; M+2K-H ; M+2K-H ; M+2K-H ; M+2K-H ; M+2K-H ; M+2K-H ; M+2K-H	C6H14O12P2 ; C6H14O12P2 ; C6H14O12P2 ; C6H14O12P2 ; C6H14O12P2 ; C6H14O12P2 ; C6H14O12P2 ; C6H14O12P2 ; C6H14O12P2
157.04552	1.357375	1.579632	-3.3547	0.007307	2.1363	0.038029	157.0455	HMDB01944 ; HMDB15086 ; HMDB29542 ; HMDB31874 ; HMDB32056 ; HMDB33197 ; HMDB33561 ; HMDB33921 ; HMDB34016 ; HMDB34132 ; HMDB34688 ; HMDB36047 ; HMDB37178 ; HMDB38430 ; HMDB38553 ; HMDB39660 ; HMDB40557 ; HMDB40929 ; HMDB41282 ; HMDB59661 ; HMDB60786	Chlorpheniramine ; Isoniazid ; Clusin ; N-gamma-Glutamyl-S-allylcysteine ; Eugenyl benzoate ; 2244-Tetramethyl-6-(1-oxobutyl)-135-cyclohexanetrione ; 2-Acetyl-45-dihydrothiazole ; 3-(11-Dimethyl-2-propenyl)-78-dimethoxy-2H-1-benzopyran-2-one ; 6-Hydroxy-99-dimethyl-5-(3-methyl-1-oxobutyl)-1-propyl-3H9H-12-dioxolo34:45furo23-f1benzopyran-3-one ; Rutacultin ; 23-Dihydro-23-dihydroxy-9-phenyl-1H-phenalen-1-one ; 3alpha-Hydroxyoreadone ; 5-Methoxy-2-methylthiazole ; 5-(1-Propynyl)-5-vinyl-22-bithiophene ; N-gamma-Glutamyl-S-(1-propenyl)cysteine ; Gossyvertin ; N-gamma-Glutamyl-S-trans-(1-propenyl)cysteine ; Batatasin II ; 2-Ethoxythiazole ; QH(2) ; 6-Hydroxymelatonin glucuronide	M+H+K ; 2M+H+K ; M+3Na ; M+H+Na ; M+2Na ; M+Na+K ; 2M+3H2O+2H ; M+H+K ; M+3Na ; M+H+K ; M+H+Na ; M+Na+K ; 2M+3H2O+2H ; M+2ACN+2H ; M+H+Na ; M+H+K ; M+H+Na ; M+H+K ; 2M+3H2O+2H ; M+Na+K ; M+H+2Na	C16H19ClN2 ; C6H7N3O ; C22H26O7 ; C11H18N2O5S ; C17H16O3 ; C14H20O4 ; C5H7NOS ; C16H18O4 ; C22H26O7 ; C16H18O4 ; C19H14O3 ; C14H20O4 ; C5H7NOS ; C13H10S2 ; C11H18N2O5S ; C16H18O4 ; C11H18N2O5S ; C16H18O4 ; C5H7NOS ; C14H20O4 ; C19H24N2O9
245.91509	1.43216	1.279362	3.3545	0.007309	2.1361	0.038029	NA	NA	NA	NA	NA
188.82524	1.317925	1.381341	-3.3537	0.00732	2.1355	0.038046	NA	NA	NA	NA	NA
99.013696	1.398273	0.755937	3.353	0.007328	2.135	0.038049	NA	NA	NA	NA	NA
690.84193	1.351731	1.43113	-3.3513	0.007349	2.1338	0.038117	690.8419	HMDB33396	(3b16a)-Dihydroxy-12-oleanen-28-oic acid 3-glucoside 28-glucosyl-(1-3)-xylosyl-(1-3)-xylosyl-(1-4)-rhamnosyl-(1-2)-arabinosyl ester	M+ACN+2H	C63H102O30
256.26339	1.385713	0.030075	3.35	0.007365	2.1328	0.038162	256.2634	HMDB12273 ; HMDB11638 ; HMDB34181 ; HMDB37544 ; HMDB60482	Palmitic amide ; Tetradecanol ; 3-Methylcyclopentadecanone ; ()-2-Dodecylcyclobutanone ; Hexadecenal	M+H ; M+ACN+H ; M+NH4 ; M+NH4 ; M+NH4	C16H33NO ; C14H30O ; C16H30O ; C16H30O ; C16H30O
529.87038	1.364128	1.534168	-3.3483	0.007386	2.1316	0.038228	529.8704	HMDB31224	Nabam	2M+NH4	C4H6N2Na2S4
576.47047	1.45982	0.035623	-3.3472	0.0074	2.1308	0.038228	576.4705	HMDB07016 ; HMDB07043 ; HMDB07044 ; HMDB07128 ; HMDB07183 ; HMDB07212 ; HMDB07240 ; HMDB31390 ; HMDB31910 ; HMDB32094 ; HMDB32647 ; HMDB32732 ; HMDB56136 ; HMDB56137 ; HMDB56156	DG(14:018:2(9Z12Z)0:0) ; DG(14:1(9Z)18:1(11Z)0:0) ; DG(14:1(9Z)18:1(9Z)0:0) ; DG(16:1(9Z)16:1(9Z)0:0) ; DG(18:1(11Z)14:1(9Z)0:0) ; DG(18:1(9Z)14:1(9Z)0:0) ; DG(18:2(9Z12Z)14:00:0) ; Annotemoyin 1 ; Chatenaytrienin 1 ; Panatellin ; Chatenaytrienin 2 ; cis-Solamin ; DG(14:1n50:018:1n7) ; DG(14:1n50:018:1n9) ; DG(16:1n70:016:1n7)	2M+H+Na ; 2M+H+Na ; 2M+H+Na ; 2M+H+Na ; 2M+H+Na ; 2M+H+Na ; 2M+H+Na ; 2M+H+Na ; M+ACN+Na ; 2M+H+Na ; M+ACN+Na ; 2M+H+Na ; 2M+H+Na ; 2M+H+Na ; 2M+H+Na	C35H64O5 ; C35H64O5 ; C35H64O5 ; C35H64O5 ; C35H64O5 ; C35H64O5 ; C35H64O5 ; C35H64O5 ; C35H60O2 ; C35H64O5 ; C35H60O2 ; C35H64O5 ; C35H64O5 ; C35H64O5 ; C35H64O5
828.59423	1.400906	1.614681	-3.3468	0.007405	2.1305	0.038228	828.5942	HMDB07886 ; HMDB07977 ; HMDB08036 ; HMDB08265 ; HMDB08525 ; HMDB08914 ; HMDB09714 ; HMDB36368 ; HMDB37522 ; HMDB09540 ; HMDB09571 ; HMDB09572 ; HMDB09635 ; HMDB09668 ; HMDB09700	PC(14:022:0) ; PC(16:020:0) ; PC(18:018:0) ; PC(20:016:0) ; PC(22:014:0) ; PE(15:024:0) ; PE(24:015:0) ; (R)-34-Dihydro-2-methyl-2-(4812-trimethyl-3711-tridecatrienyl)-2H-1-benzopyran-6-ol ; Orange B ; PE(22:1(13Z)22:6(4Z7Z10Z13Z16Z19Z)) ; PE(22:2(13Z16Z)22:5(4Z7Z10Z13Z16Z)) ; PE(22:2(13Z16Z)22:5(7Z10Z13Z16Z19Z)) ; PE(22:5(4Z7Z10Z13Z16Z)22:2(13Z16Z)) ; PE(22:5(7Z10Z13Z16Z19Z)22:2(13Z16Z)) ; PE(22:6(4Z7Z10Z13Z16Z19Z)22:1(13Z))	M+K ; M+K ; M+K ; M+K ; M+K ; M+K ; M+K ; 2M+ACN+Na ; 3M+H+NH4 ; M+H-H2O ; M+H-H2O ; M+H-H2O ; M+H-H2O ; M+H-H2O ; M+H-H2O	C44H88NO8P ; C44H88NO8P ; C44H88NO8P ; C44H88NO8P ; C44H88NO8P ; C44H88NO8P ; C44H88NO8P ; C26H38O2 ; C22H18N4O9S2 ; C49H84NO8P ; C49H84NO8P ; C49H84NO8P ; C49H84NO8P ; C49H84NO8P ; C49H84NO8P

88.057878	1.399329	1.148482	3.3465	0.007409	2.1303	0.038228	88.05788	HMDB00131 ; HMDB00459 ; HMDB00959 ; HMDB01183 ; HMDB13818 ; HMDB14755 ; HMDB15003 ; HMDB30070 ; HMDB31231 ; HMDB31589 ; HMDB34261 ; HMDB36166 ; HMDB37791 ; HMDB40095 ; HMDB40114 ; HMDB59859	Glycerol ; 3-Methylcrotonylglycine ; Tiglylglycine ; Octanol ; 2-Ethyl-4-methyl-1-pentanol ; Paramethadione ; Benzphetamine ; (S)-3-Octanol ; xi-2-Ethyl-1-hexanol ; 4-Methyl-2-heptanol ; ()-2-Octanol ; 234-Trimethyl-3-pentanol ; (2xi4xi)-24-Dimethyl-1-hexanol ; 5-Ethyl-4-methyl-2-octylthiazole ; 2-Octyl-4-propylthiazole ; (S)-3-Ethyl-4-methylpentanol	M+2ACN+2H ; M+H+NH4 ; M+H+NH4 ; M+2Na ; M+2Na ; M+H+NH4 ; M+2H+Na ; M+2Na ; M+2Na ; M+2Na ; M+2Na ; M+2Na ; M+2Na ; M+2H+Na ; M+2H+Na ; M+2Na	C3H8O3 ; C7H11NO3 ; C7H11NO3 ; C8H18O ; C8H18O ; C7H11NO3 ; C17H21N ; C8H18O ; C8H18O ; C8H18O ; C8H18O ; C8H18O ; C8H18O ; C14H25NS ; C14H25NS ; C8H18O
221.12275	1.388566	0.874759	3.3459	0.007417	2.1298	0.038231	221.1228	HMDB14913 ; HMDB05798 ; HMDB07005 ; HMDB07006 ; HMDB11545 ; HMDB11546 ; HMDB11547 ; HMDB11575 ; HMDB11576 ; HMDB11577 ; HMDB14773 ; HMDB15235 ; HMDB30784 ; HMDB30792 ; HMDB32735 ; HMDB34096 ; HMDB34275 ; HMDB39403 ; HMDB40405 ; HMDB41103	Tirofiban ; Malabaricone C ; CPA(18:1(11Z)0:0) ; CPA(18:1(9Z)0:0) ; MG(0:020:3(11Z14Z17Z)0:0) ; MG(0:020:3(5Z8Z11Z)0:0) ; MG(0:020:3(8Z11Z14Z)0:0) ; MG(20:3(11Z14Z17Z)0:00:0) ; MG(20:3(5Z8Z11Z)0:00:0) ; MG(20:3(8Z11Z14Z)0:00:0) ; Prednisone ; Quinacrine ; Normammein ; Monascoflavin ; Isopersin ; Mammea BBD ; Mammea BAC ; 2-Hydroxy-4-oxo-512-heneicosadien-1-yl acetate ; 26-Glucosyl-131122-tetrahydroxyergosta-524-dien-26-oate ; Persin	M+2H ; M+2ACN+2H ; M+H+Na ; M+H+Na ; M+Na+K ; M+Na+K ; M+Na+K ; M+Na+K ; M+Na+K ; M+Na+K ; M+2ACN+2H ; M+ACN+2H ; M+2ACN+2H ; M+2ACN+2H ; M+Na+K ; M+2ACN+2H ; M+2ACN+2H ; M+Na+K ; M+2H+Na ; M+Na+K	C22H36N2O5S ; C21H26O5 ; C21H39O6P ; C21H39O6P ; C23H40O4 ; C23H40O4 ; C23H40O4 ; C23H40O4 ; C23H40O4 ; C23H40O4 ; C21H26O5 ; C23H30ClN3O ; C21H26O5 ; C21H26O5 ; C23H40O4 ; C21H26O5 ; C21H26O5 ; C23H40O4 ; C34H54O11 ; C23H40O4
442.72252	1.377921	1.678984	3.3439	0.007442	2.1283	0.038321	NA	NA	NA	NA	NA
621.69853	1.439653	1.394922	-3.3404	0.007486	2.1258	0.038413	NA	NA	NA	NA	NA
141.53000	1.387054	0.552844	-3.3402	0.007488	2.1256	0.038413	141.5275	HMDB00089 ; HMDB00223 ; HMDB04231 ; HMDB14798 ; HMDB15122 ; HMDB31770 ; HMDB33701 ; HMDB38142 ; HMDB39791 ; HMDB39792 ; HMDB39796 ; HMDB60478	Cytidine ; Oxalacetic acid ; Pantothenol ; Metaxalone ; Cytarabine ; Carbofuran ; Hydrocotarnine ; 58-Dihydro-6-(4-methyl-3-pentenyl)-1234-tetrathiocin ; 23-Dihydro-5-methyl-3-thiophenethiol ; 45-Dihydro-2-methyl-3-thiophenethiol ; 45-Dihydro-5-methyl-3-thiophenethiol ; gamma-Glutamyl-beta-cyanoalanine	M+H+K ; 2M+H+NH4 ; M+2K ; M+Na+K ; M+H+K ; M+Na+K ; M+Na+K ; M+H+NH4 ; 2M+H+NH4 ; 2M+H+NH4 ; 2M+H+NH4 ; M+H+K	C9H13N3O5 ; C4H4O5 ; C9H19NO4 ; C12H15NO3 ; C9H13N3O5 ; C12H15NO3 ; C12H15NO3 ; C10H16S4 ; C5H8S2 ; C5H8S2 ; C5H8S2 ; C9H13N3O5
303.00759	1.388068	1.471369	-3.3396	0.007496	2.1252	0.038413	303.0076	HMDB14627 ; HMDB33040 ; HMDB33956 ; HMDB03074	Brimonidine ; Propyl 1-(propylthio)propyl disulfide ; Dichlorvos ; Delphinidin	2M+H+Na ; M+2K+H ; M+2ACN+H ; M+H-2H2O	C11H10BrN5 ; C9H20S3 ; C4H7Cl2O4P ; C15H11ClO7
468.12184	1.319844	1.140538	3.3394	0.007499	2.125	0.038413	468.1218	HMDB29268 ; HMDB37938 ; HMDB00656 ; HMDB12248 ; HMDB30381 ; HMDB32299 ; HMDB33913 ; HMDB39307 ; HMDB41711 ; HMDB41713 ; HMDB41782 ; HMDB41783 ; HMDB61135	Kaempferol 3-O-caffeoyl-sophoroside 7-O-glucoside ; Quercetin 3-p-coumaroyl-(-6)-glucosyl-(1-2)-glucosyl-(1-2)-glucoside ; Cysteineglutathione disulfide ; L-3-Aminobutyryl-CoA ; Roquefortine ; Glyceryl lactopalmitate ; Cassiaside ; 35-Dihydroxyphenyl 1-O-(6-O-galloyl-beta-D-glucopyranoside) ; cis-Resveratrol 3-O-glucuronide ; cis-Resveratrol 4-O-glucuronide ; trans-Resveratrol 3-O-glucuronide ; trans-Resveratrol 4-O-glucuronide ; Demonomethylchlorpromazine	M+2H ; M+2H ; M+ACN+H ; M+2ACN+2H ; M+2K+H ; M+ACN+Na ; M+ACN+Na ; 2M+3H2O+2H ; M+ACN+Na ; M+ACN+Na ; M+ACN+Na ; M+ACN+Na ; 3M+H+Na	C42H46O24 ; C42H46O24 ; C13H22N4O8S2 ; C25H43N8O17P3S ; C22H23N5O2 ; C20H16N6O2S ; C20H20O9 ; C19H20O12 ; C20H20O9 ; C20H20O9 ; C20H20O9 ; C20H20O9 ; C16H17ClN2S
199.10092	1.385268	1.082169	3.339	0.007503	2.1248	0.038413	199.1009	HMDB01220 ; HMDB01320 ; HMDB01335 ; HMDB01381 ; HMDB01403 ; HMDB01452 ; HMDB01509 ; HMDB02122 ; HMDB02132 ; HMDB02363 ; HMDB02400 ; HMDB02776 ; HMDB04240 ; HMDB04385 ; HMDB05077 ; HMDB05082 ; HMDB05844 ; HMDB11651 ; HMDB12481 ; HMDB12564 ; HMDB12587 ; HMDB12983 ; HMDB15094 ; HMDB30456 ; HMDB34676 ; HMDB35064 ; HMDB35338 ; HMDB35339 ; HMDB35987 ; HMDB36756 ; HMDB39505 ; HMDB40525 ; HMDB60041 ; HMDB60042	Prostaglandin E2 ; (13E)-11a-Hydroxy-915-dioxoprost-13-enoic acid ; Prostaglandin I2 ; Prostaglandin H2 ; Prostaglandin D2 ; Thromboxane A2 ; 20-Hydroxy-leukotriene B4 ; Prostaglandin F3a ; 8-iso-PGF3a ; Levuglandin E2 ; Levuglandin D2 ; 1314-Dihydro-15-keto-PGE2 ; 15-Keto-prostaglandin F2a ; Lipoxin A4 ; 8-iso-15-keto-PGF2a ; Lipoxin B4 ; 8-isoprostaglandin E2 ; 11beta20-Dihydroxy-3-oxopregn-4-en-21-oic acid ; (5Z)-(15S)-11alpha-Hydroxy-915-dioxoprostanoate ; 1314-Dihydro-15-oxo-lipoxin A4 ; 15-Epi-lipoxin A4 ; Kinetensin 1-3 ; Methylprednisolone ; Macrophorin B ; Cinncassiol D1 ; Cinncassiol D4 ; Sterebin B ; Sterebin C ; Ganoderic acid H ; (ent-6alpha7alpha16alphaH)-6717-Trihydroxy-19-kauranoic acid ; 2-(Arabinosylamino)-3-(glucosylamino)propanenitrile ; 1112-Dimethylrosmanol ; 11b-PGE2 ; 1314-Dihydro-15-keto-PGD2	M+2Na ; M+2Na ; M+2Na ; M+2Na ; M+2Na ; M+2Na ; M+2Na ; M+2Na ; M+2Na ; M+2Na ; M+2Na ; M+2Na ; M+2Na ; M+2Na ; M+2Na ; M+2Na ; M+2Na ; M+H+Na ; M+2Na ; M+2Na ; M+2Na ; M+H+K ; M+H+Na ; M+H+Na ; M+2Na ; M+2Na ; M+2Na ; M+2Na ; M+2H+Na ; M+2Na ; M+H+NH4 ; M+H+Na ; M+2Na ; M+2Na	C20H32O5 ; C20H32O5 ; C20H32O5 ; C20H32O5 ; C20H32O5 ; C20H32O5 ; C20H32O5 ; C20H32O5 ; C20H32O5 ; C20H32O5 ; C20H32O5 ; C20H32O5 ; C20H32O5 ; C20H32O5 ; C20H32O5 ; C20H32O5 ; C20H32O5 ; C22H30O5 ; C20H32O5 ; C20H32O5 ; C20H32O5 ; C15H30N6O4 ; C22H30O5 ; C22H30O5 ; C20H32O5 ; C20H32O5 ; C20H32O5 ; C20H32O5 ; C32H44O9 ; C20H32O5 ; C14H25N3O9 ; C22H30O5 ; C20H32O5 ; C20H32O5

483.93027	1.422325	0.158428	-3.3377	0.00752	2.1238	0.038413	483.9303	HMDB11898 ; HMDB11899 ; HMDB42450 ; HMDB42630 ; HMDB42943 ; HMDB42949 ; HMDB43132 ; HMDB43358 ; HMDB43363 ; HMDB43391 ; HMDB43560 ; HMDB43588 ; HMDB43610 ; HMDB43727 ; HMDB43755 ; HMDB43776 ; HMDB44304 ; HMDB44500 ; HMDB44655 ; HMDB44662 ; HMDB45091 ; HMDB45145 ; HMDB45247 ; HMDB45249 ; HMDB45882 ; HMDB45888 ; HMDB45934 ; HMDB45940 ; HMDB45982 ; HMDB45984 ; HMDB46138 ; HMDB46140 ; HMDB46304 ; HMDB46650 ; HMDB46700 ; HMDB46866 ; HMDB46868 ; HMDB50532 ; HMDB50534 ; HMDB50586 ; HMDB50592 ; HMDB50624 ; HMDB50630 ; HMDB50646 ; HMDB50648 ; HMDB50886 ; HMDB51070 ; HMDB51072 ; HMDB51434 ; HMDB51439 ; HMDB51666 ; HMDB51673 ; HMDB51682 ; HMDB51684 ; HMDB51700 ; HMDB51707 ;	Ganglioside GM2 (d18:018:1(11Z)) ; Ganglioside GM2 (d18:018:1(9Z)) ; TG(14:020:3(5Z8Z11Z)o-18:0) ; TG(14:020:3n6o-18:0) ; TG(14:0o-18:020:3(5Z8Z11Z)) ; TG(14:0o-18:020:3n6) ; TG(15:022:022:6(4Z7Z10Z13Z16Z19Z)) ; TG(15:022:1(13Z)22:5(4Z7Z10Z13Z16Z)) ; TG(15:022:1(13Z)22:5(7Z10Z13Z16Z19Z)) ; TG(15:024:1(15Z)20:5(5Z8Z11Z14Z17Z)) ; TG(15:022:2(13Z16Z)22:4(7Z10Z13Z16Z)) ; TG(15:022:4(7Z10Z13Z16Z)22:2(13Z16Z)) ; TG(15:022:5(4Z7Z10Z13Z16Z)22:1(13Z)) ; TG(15:020:5(5Z8Z11Z14Z17Z)24:1(15Z)) ; TG(15:022:5(7Z10Z13Z16Z19Z)22:1(13Z)) ; TG(15:022:6(4Z7Z10Z13Z16Z19Z)22:0) ; TG(16:018:3(6Z9Z12Z)o-18:0) ; TG(16:018:3(9Z12Z15Z)o-18:0) ; TG(16:0o-18:018:3(6Z9Z12Z)) ; TG(16:0o-18:018:3(9Z12Z15Z)) ; TG(18:018:2(9Z12Z)22:4(7Z10Z13Z16Z)) ; TG(18:020:2n622:4(7Z10Z13Z16Z)) ; TG(18:022:4(7Z10Z13Z16Z)18:2(9Z12Z)) ; TG(18:022:4(7Z10Z13Z16Z)20:2n6) ; TG(20:018:2(9Z12Z)20:4(5Z8Z11Z14Z)) ; TG(20:018:2(9Z12Z)20:4(8Z11Z14Z17Z)) ; TG(20:020:2n620:4(5Z8Z11Z14Z)) ; TG(20:020:2n620:4(8Z11Z14Z17Z)) ; TG(20:020:4(5Z8Z11Z14Z)18:2(9Z12Z)) ; TG(20:020:4(5Z8Z11Z14Z)20:2n6) ; TG(20:020:4(8Z11Z14Z17Z)18:2(9Z12Z)) ; TG(20:020:4(8Z11Z14Z17Z)20:2n6) ; TG(22:015:022:6(4Z7Z10Z13Z16Z19Z)) ; TG(22:018:2(9Z12Z)18:4(6Z9Z12Z15Z)) ; TG(22:020:2n618:4(6Z9Z12Z15Z)) ; TG(22:018:4(6Z9Z12Z15Z)18:2(9Z12Z)) ; TG(22:018:4(6Z9Z12Z15Z)20:2n6) ; TG(20:1(11Z)20:3(5Z8Z11Z)18:2(9Z12Z)) ; TG(20:1(11Z)20:3(5Z8Z11Z)20:2n6) ; TG(20:1(11Z)18:2(9Z12Z)20:3(5Z8Z11Z)) ; TG(20:1(11Z)18:2(9Z12Z)20:3n6) ; TG(20:1(11Z)20:2n620:3(5Z8Z11Z)) ; TG(20:1(11Z)20:2n620:3n6) ; TG(20:1(11Z)20:3n618:2(9Z12Z)) ; TG(20:1(11Z)20:3n620:2n6) ; TG(20:3(5Z8Z11Z)14:0o-18:0) ; TG(20:3(5Z8Z11Z)20:1(11Z)18:2(9Z12Z)) ; TG(20:3(5Z8Z11Z)20:1(11Z)20:2n6) ; TG(22:1(13Z)15:022:5(4Z7Z10Z13Z16Z)) ; TG(22:1(13Z)15:022:5(7Z10Z13Z16Z19Z)) ; TG(22:1(13Z)18:2(9Z12Z)18:3(6Z9Z12Z)) ; TG(22:1(13Z)18:2(9Z12Z)18:3(9Z12Z15Z)) ; TG(22:1(13Z)18:3(6Z9Z12Z)18:2(9Z12Z)) ; TG(22:1(13Z)18:3(6Z9Z12Z)20:2n6) ; TG(22:1(13Z)20:2n618:3(6Z9Z12Z)) ; TG(22:1(13Z)20:2n618:3(9Z12Z15Z)) ; TG(22:1(13Z)18:3(9Z12Z15Z)18:2(9Z12Z)) ; TG(22:1(13Z)18:3(9Z12Z15Z)20:2n6) ; TG(24:1(15Z)15:020:5(5Z8Z11Z14Z17Z)) ; TG(18:2(9Z12Z)18:022:4(7Z10Z13Z16Z)) ; TG(18:2(9Z12Z)20:020:4(8Z11Z14Z17Z)) ;	M+3Na ; M+3Na ; M+3ACN+2H ; M+3ACN+2H ; M+3ACN+2H ; M+3ACN+2H ; M+H+NH4 ; M+H+NH4 ; M+H+NH4 ; M+H+NH4 ; M+H+NH4 ; M+H+NH4 ; M+H+NH4 ; M+H+NH4 ; M+H+NH4 ; M+H+NH4 ; M+3ACN+2H ; M+3ACN+2H ; M+3ACN+2H ; M+3ACN+2H ; M+H+NH4 ; M+3ACN+2H ; M+H+NH4 ; M+H+NH4 ; M+H+NH4 ; M+H+NH4 ; M+H+NH4 ; M+H+NH4 ; M+H+NH4 ; M+H+NH4 ; M+H+NH4 ; M+H+NH4 ; M+H+NH4 ; M+H+NH4 ; M+H+NH4 ; M+H+NH4 ; M+H+NH4 ; M+H+NH4 ; M+3ACN+2H ; M+H+NH4 ; M+H+NH4 ; M+H+NH4 ; M+H+NH4 ; M+H+NH4 ; M+H+NH4 ; M+H+NH4 ; M+3ACN+2H ; M+H+NH4 ; M+3ACN+2H	C68H122N2O26 ; C68H122N2O26 ; C55H102O5 ; C55H102O5 ; C55H102O5 ; C55H102O5 ; C62H108O6 ; C62H108O6 ; C62H108O6 ; C62H108O6 ; C62H108O6 ; C62H108O6 ; C62H108O6 ; C62H108O6 ; C62H108O6 ; C62H108O6 ; C55H102O5 ; C55H102O5 ; C55H102O5 ; C55H102O5 ; C62H108O6 ; C55H102O5 ; C62H108O6 ; C62H108O6 ; C62H108O6 ; C62H108O6 ; C62H108O6 ; C62H108O6 ; C62H108O6 ; C62H108O6 ; C62H108O6 ; C62H108O6 ; C62H108O6 ; C62H108O6 ; C62H108O6 ; C62H108O6 ; C55H102O5 ; C62H108O6 ; C62H108O6 ; C62H108O6 ; C62H108O6 ; C62H108O6 ; C62H108O6 ; C62H108O6 ; C55H102O5 ; C62H108O6 ; C55H102O5
499.90466	1.348638	1.301647	3.3372	0.007526	2.1234	0.038413	NA	NA	NA	NA	NA
380.76960	1.37731	1.687273	3.3371	0.007527	2.1234	0.038413	380.7696	HMDB08258 ; HMDB11214 ; HMDB32843 ; HMDB33208 ; HMDB36928	PC(18:4(6Z9Z12Z15Z)P-16:0) ; PC(P-16:018:4(6Z9Z12Z15Z)) ; AS 1-5 ; Dimethyl diselenide ; Mactraxanthin	M+H+Na ; M+H+Na ; M+2Na ; 2M+H ; M+3ACN+2H	C42H76NO7P ; C42H76NO7P ; C40H77NO9 ; C2H6Se2 ; C40H60O6
594.18121	1.426128	1.04676	-3.3364	0.007536	2.1229	0.038413	594.1812	HMDB06865 ; HMDB12310 ; HMDB29813 ; HMDB30439 ; HMDB31552 ; HMDB34836 ; HMDB34867 ; HMDB35003 ; HMDB35499 ; HMDB37419 ; HMDB38114 ; HMDB38236 ; HMDB38477 ; HMDB40126 ; HMDB41199 ; HMDB41459	3-Methyl-1-hydroxybutyl-ThPP ; 2-Methyl-1-hydroxybutyl-ThPP ; 5-Hydroxycastavinol ; Fragransol D ; Dihydrocurcumin ; Lansiumarin B ; Xanthohumol B ; Xanthohumol D ; Isoxanthohumol B ; Maysin ; 4-O-Methylkievitone ; ()-Fargesin ; Cassiaside B ; Mulberroside F ; Vitexin 6-(3-hydroxy-3-methylglutarate) ; Chamaemeloside	M+2ACN+H ; M+2ACN+H ; 2M+3H2O+2H ; 3M+2K ; 3M+2K ; 3M+2K ; 3M+2K ; 3M+2K ; 3M+2K ; M+NH4 ; 3M+2K ; 3M+2K ; 2M+3H2O+2H ; 2M+3H2O+2H ; M+NH4 ; M+NH4	C17H29N4O8P2S ; C17H29N4O8P2S ; C26H30O14 ; C21H22O6 ; C21H22O6 ; C21H22O6 ; C21H22O6 ; C21H22O6 ; C21H22O6 ; C27H28O14 ; C21H22O6 ; C21H22O6 ; C26H30O14 ; C26H30O14 ; C27H28O14 ; C27H28O14
137.04581	1.364069	0.021945	3.3364	0.007537	2.1228	0.038413	137.0458	HMDB00157 ; HMDB00613 ; HMDB00943 ; HMDB14581 ; HMDB31160 ; HMDB33053 ; HMDB33054 ; HMDB61177 ; HMDB06695 ; HMDB14604 ; HMDB28871 ; HMDB29018 ; HMDB29388 ; HMDB30601 ; HMDB32133 ; HMDB32870 ; HMDB32944 ; HMDB33420 ; HMDB33601 ; HMDB33878 ; HMDB34629 ; HMDB35038 ; HMDB35052 ; HMDB35085 ; HMDB36890 ; HMDB36898 ; HMDB37326 ; HMDB39045 ; HMDB39058 ; HMDB39147 ; HMDB39623 ; HMDB40292 ; HMDB40324 ; HMDB40355 ; HMDB40573 ; HMDB40651 ; HMDB41572 ; HMDB60494	Hypoxanthine ; Erythronic acid ; Threonic acid ; Allopurinol ; 1-Pentanesulfenothioic acid ; Ethyl propyl disulfide ; Ethyl isopropyl disulfide ; Pyridine N-oxide glucuronide ; Prolylhydroxyproline ; Nabumetone ; Hydroxyprolyl-Proline ; Prolyl-Hydroxyproline ; Cucurbic acid ; Edultin ; Bisphenol A ; Jasmolone glucoside ; Coumarin-4-carboxylic acid ; Gibberellin A55 ; Dihydrojasmonic acid ; (S)-Curzeone ; (7R8S)-Methyl 47-epoxy-38-bilign-7-ene-49-dihydroxy-35-dimethoxy-9-oate ; Gibberellin A50 ; Gibberellin A49 ; Vulgarole ; Gibberellin A72 ; Gibberellin A76 ; Traumatin ; 8H-13-Dioxolo45-h1benzopyran-8-one ; (R)-Heraclenol 2-(3-methyl-2-butenoate) ; 6-Formylumbelliferone ; Gibberellin A79 ; Imidacloprid ; (7R8R)-3-Methoxy-34799-pentahydroxy-84-oxyneolignan ; 7-Hydroxy-17-bis(4-hydroxy-3-methoxyphenyl)-1-heptene-35-dione ; Tetraacetylethylenediamine ; Gibberellin A91 ; 3-Ethenyl-4-hydroxy-25-dimethylhex-5-en-2-yl acetate ; N-Acetylmuramoyl-Ala	M+H ; M+H ; M+H ; M+H ; M+H ; M+H ; M+H ; M+2H ; M+2Na ; M+2Na ; M+2Na ; M+2Na ; M+Na+K ; M+2H+Na ; M+2Na ; M+3Na ; M+2ACN+2H ; M+H+2Na ; M+Na+K ; M+2Na ; M+2H+Na ; M+H+2Na ; M+H+2Na ; M+Na+K ; M+H+2Na ; M+H+2Na ; M+Na+K ; M+2ACN+2H ; M+2H+Na ; M+2ACN+2H ; M+H+2Na ; M+H+NH4 ; M+H+2Na ; M+2H+Na ; M+2Na ; M+H+2Na ; M+Na+K ; M+H+2Na	C5H4N4O ; C4H8O5 ; C4H8O5 ; C5H4N4O ; C5H12S2 ; C5H12S2 ; C5H12S2 ; C11H14NO7 ; C10H16N2O4 ; C15H16O2 ; C10H16N2O4 ; C10H16N2O4 ; C12H20O3 ; C21H22O7 ; C15H16O2 ; C17H26O7 ; C10H6O4 ; C19H24O7 ; C12H20O3 ; C15H16O2 ; C21H22O7 ; C19H24O7 ; C19H24O7 ; C12H20O3 ; C19H24O7 ; C19H24O7 ; C12H20O3 ; C10H6O4 ; C21H22O7 ; C10H6O4 ; C19H24O7 ; C9H10ClN5O2 ; C19H24O7 ; C21H22O7 ; C10H16N2O4 ; C19H24O7 ; C12H20O3 ; C14H24N2O9
226.55291	1.329237	1.052167	3.3357	0.007545	2.1223	0.038419	226.5529	HMDB02068 ; HMDB02884 ; HMDB02894 ; HMDB04339 ; HMDB11601 ; HMDB31013 ; HMDB32470 ; HMDB35566 ; HMDB35567 ; HMDB35568 ; HMDB35569	Erucic acid ; Cetoleic acid ; 5-Methylcytosine ; 2-O-Methylcytosine ; 3-Methylcytosine ; Catelaidic acid ; Phytyl acetate ; 79-Docosanedione ; 68-Docosanedione ; 57-Docosanedione ; 46-Docosanedione	2M+3H ; 2M+3H ; 3M+2K ; 3M+2K ; 3M+2K ; 2M+3H ; 2M+3H ; 2M+3H ; 2M+3H ; 2M+3H ; 2M+3H	C22H42O2 ; C22H42O2 ; C5H7N3O ; C5H7N3O ; C5H7N3O ; C22H42O2 ; C22H42O2 ; C22H42O2 ; C22H42O2 ; C22H42O2 ; C22H42O2

154.05871	1.391573	1.167628	3.3336	0.007572	2.1208	0.038515	154.0587	HMDB14342 ; HMDB00064 ; HMDB01536 ; HMDB13222 ; HMDB14400 ; HMDB14761 ; HMDB29639 ; HMDB29697 ; HMDB29698 ; HMDB30839 ; HMDB31626 ; HMDB31897 ; HMDB32024 ; HMDB32142 ; HMDB32608 ; HMDB32623 ; HMDB33716 ; HMDB34107 ; HMDB34563 ; HMDB36133 ; HMDB36207 ; HMDB36441 ; HMDB37705 ; HMDB38598 ; HMDB38947 ; HMDB41250 ; HMDB59601 ; HMDB59895	Fluconazole ; Creatine ; Formamide ; Beta-Guanidinopropionic acid ; Diethylstilbestrol ; Fluphenazine ; (4-Methylphenyl)acetaldehyde ; Cinnamyl alcohol ; trans-Cinnamyl alcohol ; (Z)-4-(1-Propenyl)phenol ; 2-Phenylpropanal ; Phaseolic acid ; 4-Ethylbenzaldehyde ; 24-Dimethylbenzaldehyde ; 4-Methylacetophenone ; 1-Phenyl-1-propanone ; 3-Phenylpropanal ; Chavicol ; 7-Nonene-35-diyn-1-ol ; 3- 5-Methyl-2-(1-methylethyl)cyclohexyloxy-12-propanediol ; alpha-Amylcinnamyl acetate ; o-Vinylanisole ; Heptyl cinnamate ; Narceinone ; (S)-3-Methylthiohexyl hexanoate ; Demethoxyshogaol ; Indan-1-ol ; m-Methylacetophenone	M+2H ; M+Na ; 2M+ACN+Na ; M+Na ; M+H+K ; M+2H+Na ; 2M+H+K ; 2M+H+K ; 2M+H+K ; 2M+H+K ; 2M+H+K ; M+2Na ; 2M+H+K ; 2M+H+K ; 2M+H+K ; 2M+H+K ; 2M+H+K ; 2M+H+K ; 2M+H+K ; M+2K ; M+Na+K ; 2M+H+K ; M+Na+K ; M+3H ; M+Na+K ; M+Na+K ; 2M+H+K ; 2M+H+K	C13H12F2N6O ; C4H9N3O2 ; CH3NO ; C4H9N3O2 ; C18H20O2 ; C22H26F3N3OS ; C9H10O ; C9H10O ; C9H10O ; C9H10O ; C9H10O ; C12H22O6 ; C9H10O ; C9H10O ; C9H10O ; C9H10O ; C9H10O ; C9H10O ; C9H10O ; C13H26O3 ; C16H22O2 ; C9H10O ; C16H22O2 ; C23H25NO9 ; C13H26O2S ; C16H22O2 ; C9H10O ; C9H10O
508.62380	1.377962	1.661437	3.3325	0.007586	2.12	0.038545	508.6238	HMDB12470 ; HMDB38298 ; HMDB38301 ; HMDB38302	(2S6R10R)-Trimethyl-2E-hendecenoyl-CoA ; Apigenin 4-feruloyl-(-2)-glucuronyl-(1-2)-glucuronide 7-glucuronide ; Apigenin 7-feruloyl-(-2)-glucuronyl-(1-2)-glucuronide 4-glucuronide ; Apigenin 7-feruloyl-(-2)-glucuronyl-(1-3)-glucuronyl-(1-2)-glucuronide	M+2Na ; M+ACN+2H ; M+ACN+2H ; M+ACN+2H	C35H56N7O17P3S ; C43H42O26 ; C43H42O26 ; C43H42O26
317.85075	1.330992	1.544967	3.3302	0.007615	2.1183	0.038614	317.8508	HMDB11478 ; HMDB11479 ; HMDB11508 ; HMDB11509	LysoPE(0:018:3(6Z9Z12Z)) ; LysoPE(0:018:3(9Z12Z15Z)) ; LysoPE(18:3(6Z9Z12Z)0:0) ; LysoPE(18:3(9Z12Z15Z)0:0)	2M+3H ; 2M+3H ; 2M+3H ; 2M+3H	C23H42NO7P ; C23H42NO7P ; C23H42NO7P ; C23H42NO7P
592.93325	1.421865	0.681203	-3.33	0.007618	2.1182	0.038614	NA	NA	NA	NA	NA
232.17272	1.364269	0.454705	-3.329	0.007631	2.1174	0.038614	232.1727	HMDB34336 ; HMDB38499 ; HMDB29804 ; HMDB30507 ; HMDB32145 ; HMDB34919 ; HMDB35124 ; HMDB35265 ; HMDB35274 ; HMDB35817 ; HMDB36016 ; HMDB36659 ; HMDB39243 ; HMDB01932	Dolichosterone ; Polyporusterone F ; Momordol ; Cycloeucalenone ; 31-Norcyclolaudenone ; Taraxasterone ; Moretenone ; Anhydrosophoradiol ; Momodicaursenol ; gamma-Taraxasterone ; Mangiferoleanone ; alpha-Amyrone ; Butyrospermone ; Metoprolol	M+2H ; M+2H ; M+H+Na ; M+H+K ; M+H+K ; M+H+K ; M+H+K ; M+H+K ; M+H+K ; M+H+K ; M+H+K ; M+H+K ; M+H+K ; M+H-2H2O	C28H46O5 ; C28H46O5 ; C26H48O5 ; C30H48O ; C30H48O ; C30H48O ; C30H48O ; C30H48O ; C30H48O ; C30H48O ; C30H48O ; C30H48O ; C30H48O ; C15H25NO3
246.22968	1.354043	1.168263	3.3289	0.007632	2.1173	0.038614	NA	NA	NA	NA	NA
182.05779	1.348319	1.529646	-3.3285	0.007638	2.117	0.038614	182.0578	HMDB00318 ; HMDB01190 ; HMDB14895 ; HMDB30678 ; HMDB32966 ; HMDB35441 ; HMDB37620 ; HMDB38084 ; HMDB38760 ; HMDB38761 ; HMDB39227 ; HMDB59756 ; HMDB60021	34-Dihydroxyphenylglycol ; Indoleacetaldehyde ; Dolasetron ; Linocinnamarin ; Furaneol acetate ; Oenanthoside A ; Ethyl 4-(methylthio)butyrate ; Ethyl 3-(methylthio)butanoate ; Herierin III ; Herierin IV ; 3-Mercapto-3-methyl-1-butyl acetate ; 34-Methyleneadipic acid ; trans-isoeugenol-O-glucuronide	2M+H+Na ; M+Na ; M+H+K ; M+H+Na ; 2M+H+Na ; M+H+Na ; 2M+H+K ; 2M+H+K ; 2M+H+Na ; 2M+H+Na ; 2M+H+K ; 2M+H+Na ; M+H+Na	C8H10O4 ; C10H9NO ; C19H20N2O3 ; C16H20O8 ; C8H10O4 ; C16H20O8 ; C7H14O2S ; C7H14O2S ; C8H10O4 ; C8H10O4 ; C7H14O2S ; C8H10O4 ; C16H20O8
285.22098	1.426389	0.040497	3.3227	0.007712	2.1128	0.03894	285.221	HMDB01358 ; HMDB02152 ; HMDB06218 ; HMDB06220 ; HMDB13117 ; HMDB35151 ; HMDB02198 ; HMDB02789 ; HMDB02950 ; HMDB03112 ; HMDB03146 ; HMDB03233 ; HMDB03240 ; HMDB03415 ; HMDB03451 ; HMDB14284 ; HMDB30538 ; HMDB35316 ; HMDB35928 ; HMDB36870 ; HMDB36877 ; HMDB36919 ; HMDB36922 ; HMDB36923 ; HMDB36971 ; HMDB39017 ; HMDB40162 ; HMDB13272 ; HMDB34033 ; HMDB39536 ; HMDB40920 ; HMDB40922 ; HMDB40923 ; HMDB01999 ; HMDB03598 ; HMDB15655 ; HMDB35692 ; HMDB36703 ; HMDB36709 ; HMDB36775 ; HMDB36805 ; HMDB36811 ; HMDB36826 ; HMDB38172 ; HMDB38687 ; HMDB39486 ; HMDB41928	Retinal ; 11-cis-Retinaldehyde ; 9-cis-Retinal ; 13-cis-Retinal ; Vitamin A2 ; delta6-Dehydroferruginol ; (3R3R6R9-cis)-Carotene-33-diol ; Zeaxanthin ; (3R3R6R9-cis)-be-Carotene-33-diol ; (3R3R13-cis)-bb-Carotene-33-diol ; (3R3R9-cis)-bb-Carotene-33-diol ; Lutein ; (3R3R15-cis)-bb-Carotene-33-diol ; (3R3S6R)-be-Carotene-33-diol ; (3R3R6R13-cis)-be-Carotene-33-diol ; Xanthophyll ; Cryptoxanthin epoxide ; (5R5R6S8R)-Luteochrome ; betabeta-Carotene-33-diol ; Cryptoflavin ; Aurochrome ; Lycophyll ; Isozeaxanthin ; Cryptocapsin ; 56-Epoxy-56-dihydro-betabeta-caroten-3-ol ; Semi-beta-carotenone ; (3S3Sall-E)-Zeaxanthin ; N-Lauroylglycine ; Sabadelin ; 4-Hydroxy-1618-tritriacontanedione ; Epoxymurin A ; Epoxymurin B ; Epomuricenin B ; Eicosapentaenoic acid ; Retinyl ester ; Methyltestosterone ; 815-Isopimaradien-18-oic acid ; Siderone ; Yucalexin B14 ; Yucalexin A16 ; 8(17)12-Labdadiene-1516-dial ; Isopimaric acid ; Cascarillone ; 813-Abietadien-18-oic acid ; (ent-15beta16beta)-1516-Epoxy-3-kauranone ; ent-8(17)13(16)14-Labdatrien-18-oic acid ; Methenolone	M+H ; M+H ; M+H ; M+H ; M+H ; M+H ; M+2H ; 2M+3H2O+2H ; M+H+K ; M+Na+K ; M+H+K ; M+H+K ; M+H+K ; M+H-H2O ; M+H-H2O ; M+H-H2O ; M+H-H2O ; M+H-H2O ; M+H-H2O ; M+H-H2O ; M+H-H2O ; M+H-H2O ; M+H-H2O ; M+H-H2O ; M+H-H2O ; M+H-H2O ; M+H-H2O	C20H28O ; C20H28O ; C20H28O ; C20H28O ; C20H28O ; C20H28O ; C40H56O2 ; C14H27NO3 ; C35H62O3 ; C33H64O3 ; C35H62O3 ; C35H62O3 ; C35H62O3 ; C20H30O2 ; C20H30O2 ; C20H30O2 ; C20H30O2 ; C20H30O2 ; C20H30O2 ; C20H30O2 ; C20H30O2 ; C20H30O2 ; C20H30O2 ; C20H30O2 ; C20H30O2 ; C20H30O2 ; C20H30O2
148.00399	1.312648	1.388626	3.3223	0.007718	2.1125	0.03894	148.004	HMDB00251 ; HMDB29592	Taurine ; Triacetin	M+Na ; M+2K	C2H7NO3S ; C9H14O6
130.96651	1.387959	1.211383	3.3214	0.007729	2.1119	0.038955	130.9665	HMDB33075 ; HMDB34232 ; HMDB41792 ; HMDB60417 ; HMDB60456 ; HMDB60514 ; HMDB60601	Methyl 1-(methylsulfinyl)propyl disulfide ; (Chloromethyl)oxirane ; 13-Butadiene ; 6-Thiourate ; cis-3-Chloro-2-propene-1-ol ; trans-3-Chloro-2-propene-1-ol ; 6-thiouric acid	M+2K ; M+K ; M+2K-H ; M+2K ; M+K ; M+K ; M+2K	C5H12OS3 ; C3H5ClO ; C4H6 ; C5H4N4O2S ; C3H5ClO ; C3H5ClO ; C5H4N4O2S
319.84795	1.341353	1.589874	3.3204	0.007742	2.1112	0.038982	NA	NA	NA	NA	NA
208.89081	1.375028	1.675405	3.3188	0.007763	2.11	0.039048	208.8908	HMDB60451	Chloral hydrate	M+2Na-H	C2H3Cl3O2

272.12530	1.377167	1.533188	-3.3134	0.007835	2.106	0.039371	272.1253	HMDB35167 ; HMDB29771 ; HMDB30929 ; HMDB32610 ; HMDB33527 ; HMDB34699 ; HMDB38754 ; HMDB41577 ; HMDB60535 ; HMDB61165	Cinncassiol C1 19-glucoside ; 78-Dihydrovomifoliol 9-apiosyl-(1-6)-glucoside ; 2468-Tridecatetrayne ; 4-Methylbiphenyl ; Dehydroisochalciporone ; Fumonisin A2 ; (EEE)-13511-Tridecatetraene-79-diyne ; 3b6a-Dihydroxy-alpha-ionol 9-apiosyl-(1-6)-glucoside ; N-Desmethyldiphenhydramine ; NN-Didemethyl orphenadrine	M+2H ; M+H+Na ; 3M+H+K ; 3M+H+K ; 2M+Na+K ; M+3Na ; 3M+H+K ; M+H+Na ; 2M+Na+K ; 2M+Na+K	C26H38O12 ; C24H40O12 ; C13H12 ; C13H12 ; C16H19NO ; C36H61NO15 ; C13H12 ; C24H40O12 ; C16H19NO ; C16H19NO
394.79242	1.379084	1.666148	3.3114	0.007861	2.1045	0.039454	NA	NA	NA	NA	NA
283.22555	1.472085	0.257176	3.3103	0.007875	2.1038	0.039454	283.2256	HMDB06294 ; HMDB12093	16-Hydroxy hexadecanoic acid ; SM(d18:023:0)	2M+H+Na ; M+H+2Na	C16H31O3 ; C46H95N2O6P
272.7946_	1.379986	1.697763	3.3103	0.007875	2.1037	0.039454	272.7946	HMDB37822	Tridodecylamine	M+H+Na	C36H75N
534.71581	1.387019	1.643736	3.3096	0.007884	2.1033	0.03946	534.7158	HMDB10608 ; HMDB10609 ; HMDB32224	PG(18:020:3(5Z8Z11Z)) ; PG(18:020:3(8Z11Z14Z)) ; N1-(24-Dimethoxybenzyl)-n2-(2-(pyridin-2-yl) ethyl)oxalamide	2M+3H ; 2M+3H ; 3M+H+K	C44H81O10P ; C44H81O10P ; C18H21N3O4
498.58461	1.357871	1.133039	-3.3062	0.00793	2.1007	0.039634	498.5846	HMDB01291 ; HMDB02137 ; HMDB02145 ; HMDB03948 ; HMDB12145 ; HMDB29246 ; HMDB32294 ; HMDB35458 ; HMDB41162	trans-D-Decenoyl-CoA ; trans-3-Decenoyl-CoA ; 4-cis-Decenoyl-CoA ; (2E)-Decenoyl-CoA ; 2-Carboxymethyl-3-hydroxyphenylpropionyl-CoA ; Cyanidin 3-O-(2-xylosyl-6-(6-feruloyl-glucosyl)-galactoside) ; N-Gluconyl ethanolamine phosphate ; Cyanidin 3-O-4-Hydroxy-E-cinnamoyl-(-6)-b-D-glucopyranosyl-(1-2)-b-D-glucopyranoside 5-glucoside ; Cyanidin 3-O-b-D-Xylopyranosyl-(1-2)-(4-hydroxy-3-methoxycinnamoyl)-(-6)-b-D-glucopyranosyl-(1-6)-b-D-galactopyranoside	M+2K ; M+2K ; M+2K ; M+2K ; M+H+Na ; M+2K ; 3M+H+K ; M+2K ; M+2K	C31H52N7O17P3S ; C31H52N7O17P3S ; C31H52N7O17P3S ; C31H52N7O17P3S ; C32H46N7O20P3S ; C42H47O23 ; C8H18NO10P ; C42H47O23 ; C42H47O23
232.92789	1.427613	1.449466	3.3058	0.007935	2.1005	0.039634	232.9279	HMDB29643 ; HMDB31871 ; HMDB32547 ; HMDB33047 ; HMDB39524 ; HMDB40239	135-Trichloro-2-methoxybenzene ; Methyl propyl trisulfide ; 246-Trithiaheptane ; Ethyl (methylthio)methyl disulfide ; Diethyl trisulfide ; 11-Thiobisethanethiol	M+Na ; M+2K+H ; M+2K+H ; M+2K+H ; M+2K+H ; M+2K+H	C7H5Cl3O ; C4H10S3 ; C4H10S3 ; C4H10S3 ; C4H10S3 ; C4H10S3
568.86906	1.402534	0.173455	-3.3049	0.007948	2.0998	0.039659	NA	NA	NA	NA	NA
214.91764	1.413005	0.988061	3.3009	0.008002	2.0968	0.039888	NA	NA	NA	NA	NA
772.31675	1.389358	1.610911	-3.2986	0.008033	2.0951	0.040003	NA	NA	NA	NA	NA
353.00622	1.341506	0.795137	-3.2926	0.008115	2.0907	0.040373	353.0062	HMDB41712 ; HMDB41714 ; HMDB41772 ; HMDB41784 ; HMDB41831 ; HMDB60274	cis-Resveratrol 3-sulfate ; cis-Resveratrol 4-sulfate ; Resveratrol 3-sulfate ; trans-Resveratrol 4-sulfate ; Azosemide ; Sedoheptulose 17-bisphosphate	M+2Na-H ; M+2Na-H ; M+2Na-H ; M+2Na-H ; M+H-H2O ; M+H-H2O	C14H12O6S ; C14H12O6S ; C14H12O6S ; C14H12O6S ; C12H11ClN6O2S2 ; C7H16O13P2
120.00241	1.379146	1.316849	3.2911	0.008135	2.0896	0.040414	120.0024	HMDB00123 ; HMDB01268 ; HMDB01297 ; HMDB02052 ; HMDB12916 ; HMDB14691 ; HMDB31239 ; HMDB32523 ; HMDB33043 ; HMDB34440	Glycine ; 4-Fumarylacetoacetic acid ; Norcotinine ; Maleylacetoacetic acid ; Cyanosulfurous acid anion ; Acetohydroxamic Acid ; Ethyl nitrite ; Succinic anhydride ; Methyl 1-(1-propenylthio)propyl disulfide ; (-)-1-(Methylthio)propyl 1-propenyl disulfide	M+2Na-H ; M+H+K ; M+2K ; M+H+K ; 2M+3H2O+2H ; M+2Na-H ; M+2Na-H ; 2M+H+K ; M+2Na ; M+2Na	C2H5NO2 ; C8H8O6 ; C9H10N2O ; C8H8O6 ; CH2NO2S ; C2H5NO2 ; C2H5NO2 ; C4H4O3 ; C7H14S3 ; C7H14S3
531.13798	1.369853	1.361384	-3.2908	0.008139	2.0894	0.040414	531.138	HMDB35419 ; HMDB37682 ; HMDB38334 ; HMDB39354 ; HMDB39777	Pyranodelphinin B ; Ethylvanillin glucoside ; Dihydromelilotoside ; Methyl 6-O-galloyl-beta-D-glucopyranoside ; Orcein	2M+3H2O+2H ; 3M+2K ; 3M+2K ; 3M+H+Na ; 2M+Na+K	C24H23O12 ; C15H20O8 ; C15H20O8 ; C14H18O10 ; C28H24N2O7
434.52876	1.383707	0.003439	-3.2901	0.008149	2.0889	0.040423	NA	NA	NA	NA	NA
394.11823	1.383968	0.44751	-3.2875	0.008185	2.087	0.040518	394.1182	HMDB14421 ; HMDB38254 ; HMDB00828 ; HMDB29402 ; HMDB30399 ; HMDB30574 ; HMDB38668	Amsacrine ; Isorhamnetin 3-O-b-D-glucopyranosyl-(1-2)-a-L-rhamnopyranosyl-(1-6)-b-D-glucopyranoside ; Ureidosuccinic acid ; L-2-Amino-3-(oxalylamino)propanoic acid ; N-(Carbethoxyacetyl)-4-chloro-L-tryptophan ; Occidentoside ; L-3-Amino-2-(oxalylamino)propanoic acid	M+H ; M+2H ; 2M+ACN+H ; 2M+ACN+H ; M+ACN+H ; M+2ACN+2H ; 2M+ACN+H	C21H19N3O3S ; C34H42O21 ; C5H8N2O5 ; C5H8N2O5 ; C16H17ClN2O5 ; C36H32O15 ; C5H8N2O5
241.06776	1.412672	0.27741	-3.2869	0.008193	2.0866	0.040518	241.0678	HMDB15483 ; HMDB13070 ; HMDB15589 ; HMDB29187 ; HMDB29233 ; HMDB30230 ; HMDB33373 ; HMDB33798 ; HMDB34047 ; HMDB34876 ; HMDB35369 ; HMDB36199 ; HMDB37088 ; HMDB39428 ; HMDB39947 ; HMDB40787 ; HMDB41225 ; HMDB41336 ; HMDB41406 ; HMDB60705 ; HMDB60720 ; HMDB60737 ; HMDB60743 ; HMDB61130	Cefepime ; Sinapyl alcohol ; Flunarizine ; 5-(35)-Dihydroxyphenyl-gamma-valerolactone ; 34-Dihydroxyphenylvaleric acid ; 2-(E)-Feruloyl-3-(arabinosylxylose) ; 2-Ethyl-1-hexanol sulfate ; 3-Methyl-1-(246-trihydroxyphenyl)-1-butanone ; 2-Hydroxy-46-dimethoxy-3-methylacetophenone ; Castaneiolide ; Citreoviridinol A1 ; 2-Methoxy-4-(4-methyl-13-dioxolan-2-yl)phenol ; cis-p-Coumaric acid 4-apiosyl-(1-2)-glucoside ; 2-Methoxy-3-(4-methoxyphenyl)propanoic acid ; Pisumionoside ; Murrastifoline F ; b-D-Xylopyranosyl-(1-4)-a-L-rhamnopyranosyl-(1-2)-D-fucose ; 7-Hydroxy-4-methylphthalide O-arabinosyl-(1-6)-glucoside ; Bancroftinone ; 12-Hydroxynevirapine glucuronide ; 2-Hydroxynevirapine glucuronide ; 3-(4-Hydroxy-3-methoxyphenyl)-2-methylpropionic acid ; 3-Hydroxynevirapine glucuronide ; 8-Hydroxynevirapine glucuronide	M+2H ; 2M+Na+K ; M+2K ; 2M+Na+K ; 2M+Na+K ; M+H+Na ; 2M+Na+K ; 2M+Na+K ; 2M+Na+K ; M+H+Na+K ; M+Na+K ; 2M+Na+K ; M+H+Na ; 2M+Na+K ; M+2K ; M+Na+K ; M+H+K ; M+H+Na ; 2M+Na+K ; M+H+Na ; M+H+Na ; 2M+Na+K ; M+H+Na ; M+H+Na	C19H24N6O5S2 ; C11H14O4 ; C26H26F2N2 ; C11H14O4 ; C11H14O4 ; C20H26O12 ; C8H18O4S ; C11H14O4 ; C11H14O4 ; C22H28O8 ; C22H28O8 ; C11H14O4 ; C20H26O12 ; C11H14O4 ; C19H32O9 ; C28H24N2O2 ; C17H30O13 ; C20H26O12 ; C11H14O4 ; C21H22N4O8 ; C21H22N4O8 ; C11H14O4 ; C21H22N4O8 ; C21H22N4O8
489.61526	1.307044	0.52165	3.2869	0.008193	2.0866	0.040518	489.6153	HMDB04047 ; HMDB33021	Adipoyl-CoA ; Pelargonidin 3-O-4-Hydroxy-3-methoxy-E-cinnamoyl-(-4)-a-L-rhamnopyranosyl-(1-6)-b-D-glucopyranoside 5-O-b-D-glucopyranoside	M+2ACN+2H ; M+Na+K	C27H44N7O19P3S ; C43H49O22
170.06543	1.386578	0.209816	-3.285	0.008219	2.0852	0.04061	170.0654	HMDB30618 ; HMDB30771 ; HMDB33315 ; HMDB33801 ; HMDB33901 ; HMDB34041 ; HMDB34087 ; HMDB35373 ; HMDB36636 ; HMDB37920 ; HMDB38902 ; HMDB01173 ; HMDB01885 ; HMDB12143 ; HMDB38323 ; HMDB38324 ; HMDB38780 ; HMDB39510 ; HMDB39607 ; HMDB40534 ; HMDB60803	Otobanone ; 6alpha-Hydroxyphaseollin ; Moracin H ; Demethoxycurcumin ; Glyceollin I ; Glyceollin III ; Glyceollin II ; Licoagroaurone ; Methuyl tanshinonate ; 3-(gammagamma-Dimethylallyl)genistein ; Lupiwighteone ; 5-Methylthioadenosine ; 3-Chlorotyrosine ; 2-Amino-4-oxo-4-alpha-hydroxy-6-(erythro-123-trihydroxypropyl)-5678-tetrahydroxypterin ; Verimol B ; Verimol A ; 7-Hydroxy-245-trimethoxyisoflavan ; 7-Hydroxy-234-trimethoxyisoflavan ; Sorgolactone ; 5-Hydroxy-347-trimethoxyflavan ; 8-Hydroxydesmethylclomipramine	M+2H ; M+2H ; M+2H ; M+2H ; M+2H ; M+2H ; M+2H ; M+2H ; M+2H ; M+2H ; M+2H ; M+ACN+2H ; M+3ACN+2H ; M+H+NH4 ; M+H+Na ; M+H+Na ; M+H+Na ; M+H+Na ; M+H+Na ; M+H+Na ; M+H+Na	C20H18O5 ; C20H18O5 ; C20H18O5 ; C20H18O5 ; C20H18O5 ; C20H18O5 ; C20H18O5 ; C20H18O5 ; C20H18O5 ; C20H18O5 ; C20H18O5 ; C11H15N5O3S ; C9H10ClNO3 ; C9H15N5O8 ; C18H20O5 ; C18H20O5 ; C18H20O5 ; C18H20O5 ; C18H20O5 ; C18H20O5 ; C18H21ClN2O
94.502312	1.436288	0.899279	3.2839	0.008235	2.0844	0.040623	NA	NA	NA	NA	NA

470.38366	1.406033	0.650715	3.2835	0.00824	2.0841	0.040623	470.3837	HMDB00483 ; HMDB00513 ; HMDB00556 ; HMDB00558 ; HMDB02180 ; HMDB02208 ; HMDB08323 ; HMDB08355 ; HMDB08547 ; HMDB08578 ; HMDB08610 ; HMDB08650 ; HMDB08769 ; HMDB08778 ; HMDB08801 ; HMDB09742 ; HMDB10737 ; HMDB11395 ; HMDB37396 ; HMDB43104 ; HMDB43804 ; HMDB45528 ; HMDB59928	5b-Cholestane-3a7a12a23S25-pentol ; 5b-Cholestane-3a7a12a23R25-pentol ; 5b-Cholestane-3a7a12a2425-pentol ; 5a-Cholestane-3a7a12a2325-pentol ; 5b-Cholestane-3a7a12a2526-pentol ; Cholestane-37122425-pentol ; PC(20:1(11Z)24:1(15Z)) ; PC(20:2(11Z14Z)24:0) ; PC(22:022:2(13Z16Z)) ; PC(22:1(13Z)22:1(13Z)) ; PC(22:2(13Z16Z)22:0) ; PC(22:4(7Z10Z13Z16Z)24:0) ; PC(24:020:2(11Z14Z)) ; PC(24:022:4(7Z10Z13Z16Z)) ; PC(24:1(15Z)20:1(11Z)) ; PE(24:0P-18:0) ; (R)-3-Hydroxy-Octadecanoic acid ; PE(P-18:024:0) ; xi-10-Hydroxyoctadecanoic acid ; TG(15:020:0o-18:0) ; TG(15:0o-18:020:0) ; TG(20:015:0o-18:0) ; 3-(Acetyloxy)-2-hydroxypropyl icosanoate	M+NH4 ; M+NH4 ; M+NH4 ; M+NH4 ; M+NH4 ; M+NH4 ; M+ACN+2H ; M+ACN+2H ; M+ACN+2H ; M+ACN+2H ; M+ACN+2H ; M+H+NH4 ; M+ACN+2H ; M+H+NH4 ; M+ACN+2H ; M+3ACN+2H ; 3M+H+K ; M+3ACN+2H ; 3M+H+K ; M+2K ; M+2K ; M+2K ; M+ACN+H	C27H48O5 ; C27H48O5 ; C27H48O5 ; C27H48O5 ; C27H48O5 ; C27H48O5 ; C52H100NO8P ; C52H100NO8P ; C52H100NO8P ; C52H100NO8P ; C52H100NO8P ; C54H100NO8P ; C52H100NO8P ; C54H100NO8P ; C52H100NO8P ; C47H94NO7P ; C18H36O3 ; C47H94NO7P ; C18H36O3 ; C56H110O5 ; C56H110O5 ; C56H110O5 ; C25H48O5
165.97583	1.352481	1.603265	-3.283	0.008247	2.0837	0.040623	165.9758	HMDB04113 ; HMDB04119 ; HMDB31466	Se-Methylselenocysteine ; Selenohomocysteine ; Selenomethyl selenocysteine	M+H-H2O ; M+H-H2O ; M+H-H2O	C4H9NO2Se ; C4H9NO2Se ; C4H9NO2Se
704.52184	1.348062	1.065568	-3.2819	0.008263	2.0829	0.040662	704.5218	HMDB07870 ; HMDB07902 ; HMDB07966 ; HMDB07998 ; HMDB08893 ; HMDB08894 ; HMDB09021 ; HMDB09054 ; HMDB56400 ; HMDB56401 ; HMDB56418 ; HMDB56428 ; HMDB56489 ; HMDB56490 ; HMDB56498 ; HMDB56507 ; HMDB56509 ; HMDB56513 ; HMDB56543 ; HMDB56587 ; HMDB56643 ; HMDB56924 ; HMDB56933 ; HMDB56935 ; HMDB56939 ; HMDB56969 ; HMDB07121 ; HMDB07148 ; HMDB07149 ; HMDB07201 ; HMDB07230 ; HMDB07257 ; HMDB07258 ; HMDB07284 ; HMDB07285 ; HMDB07295 ; HMDB07313 ; HMDB07314 ; HMDB07324 ; HMDB07341 ; HMDB07351 ; HMDB07352 ; HMDB07425 ; HMDB07452 ; HMDB07453 ; HMDB07481 ; HMDB07482 ; HMDB07509 ; HMDB07520 ; HMDB07538 ; HMDB07549 ; HMDB07565 ; HMDB07566 ; HMDB07576 ; HMDB07577 ; HMDB07585 ;	PC(14:016:1(9Z)) ; PC(14:1(9Z)16:0) ; PC(16:014:1(9Z)) ; PC(16:1(9Z)14:0) ; PE(15:018:1(11Z)) ; PE(15:018:1(9Z)) ; PE(18:1(11Z)15:0) ; PE(18:1(9Z)15:0) ; CL(16:016:018:018:1(11Z)) ; CL(16:016:018:018:1(9Z)) ; CL(16:016:018:1(11Z)18:0) ; CL(16:016:018:1(9Z)18:0) ; CL(16:018:016:018:1(11Z)) ; CL(16:018:016:018:1(9Z)) ; CL(16:018:018:016:1(9Z)) ; CL(16:018:016:1(9Z)18:0) ; CL(16:018:018:1(11Z)16:0) ; CL(16:018:018:1(9Z)16:0) ; CL(16:016:1(9Z)18:018:0) ; CL(16:018:1(11Z)18:016:0) ; CL(16:018:1(9Z)18:016:0) ; CL(18:016:018:016:1(9Z)) ; CL(18:016:016:1(9Z)18:0) ; CL(18:016:018:1(11Z)16:0) ; CL(18:016:018:1(9Z)16:0) ; CL(18:018:016:1(9Z)16:0) ; DG(16:022:6(4Z7Z10Z13Z16Z19Z)0:0) ; DG(16:1(9Z)22:5(4Z7Z10Z13Z16Z)0:0) ; DG(16:1(9Z)22:5(7Z10Z13Z16Z19Z)0:0) ; DG(18:1(11Z)20:5(5Z8Z11Z14Z17Z)0:0) ; DG(18:1(9Z)20:5(5Z8Z11Z14Z17Z)0:0) ; DG(18:2(9Z12Z)20:4(5Z8Z11Z14Z)0:0) ; DG(18:2(9Z12Z)20:4(8Z11Z14Z17Z)0:0) ; DG(18:3(6Z9Z12Z)20:3(5Z8Z11Z)0:0) ; DG(18:3(6Z9Z12Z)20:3(8Z11Z14Z)0:0) ; DG(18:3(6Z9Z12Z)22:6(4Z7Z10Z13Z16Z19Z)0:0) ; DG(18:3(9Z12Z15Z)20:3(5Z8Z11Z)0:0) ; DG(18:3(9Z12Z15Z)20:3(8Z11Z14Z)0:0) ; DG(18:3(9Z12Z15Z)22:6(4Z7Z10Z13Z16Z19Z)0:0) ; DG(18:4(6Z9Z12Z15Z)20:2(11Z14Z)0:0) ; DG(18:4(6Z9Z12Z15Z)22:5(4Z7Z10Z13Z16Z)0:0) ; DG(18:4(6Z9Z12Z15Z)22:5(7Z10Z13Z16Z19Z)0:0) ; DG(20:2(11Z14Z)18:4(6Z9Z12Z15Z)0:0) ; DG(20:3(5Z8Z11Z)18:3(6Z9Z12Z)0:0) ; DG(20:3(5Z8Z11Z)18:3(9Z12Z15Z)0:0) ; DG(20:3(8Z11Z14Z)18:3(6Z9Z12Z)0:0) ; DG(20:3(8Z11Z14Z)18:3(9Z12Z15Z)0:0) ; DG(20:4(5Z8Z11Z14Z)18:2(9Z12Z)0:0) ; DG(20:4(5Z8Z11Z14Z)20:5(5Z8Z11Z14Z17Z)0:0) ; DG(20:4(8Z11Z14Z17Z)18:2(9Z12Z)0:0) ; DG(20:4(8Z11Z14Z17Z)20:5(5Z8Z11Z14Z17Z)0:0) ; DG(20:5(5Z8Z11Z14Z17Z)18:1(11Z)0:0) ; DG(20:5(5Z8Z11Z14Z17Z)18:1(9Z)0:0) ; DG(20:5(5Z8Z11Z14Z17Z)20:4(5Z8Z11Z14Z)0:0) ; DG(20:5(5Z8Z11Z14Z17Z)20:4(8Z11Z14Z17Z)0:0) ; DG(20:5(5Z8Z11Z14Z17Z)22:6(4Z7Z10Z13Z16Z19Z)0:0) ; DG(22:5(4Z7Z10Z13Z16Z)16:1(9Z)0:0) ; DG(22:5(4Z7Z10Z13Z16Z)18:4(6Z9Z12Z15Z)0:0) ; DG(22:5(7Z10Z13Z16Z19Z)16:1(9Z)0:0) ; DG(22:5(7Z10Z13Z16Z19Z)18:4(6Z9Z12Z15Z)0:0) ; DG(22:6(4Z7Z10Z13Z16Z19Z)16:00:0) ; DG(22:6(4Z7Z10Z13Z16Z19Z)18:3(6Z9Z12Z)0:0) ; DG(22:6(4Z7Z10Z13Z16Z19Z)18:3(9Z12Z15Z)0:0) ; DG(22:6(4Z7Z10Z13Z16Z19Z)20:5(5Z8Z11Z14Z17Z)0:0) ; CerP(d18:120:0) ; YGM 1A ; DG(16:00:022:6n3) ; DG(16:1n70:022:5n6) ; DG(16:1n70:022:5n3) ; DG(18:1n70:020:5n3) ;	M+H ; M+H ; M+H ; M+H ; M+H ; M+H ; M+H ; M+H ; M+2H ; M+2H ; M+2H ; M+2H ; M+2H ; M+2H ; M+2H ; M+2H ; M+2H ; M+2H ; M+2H ; M+2H ; M+2H ; M+2H ; M+2H ; M+2H ; M+2H ; M+2H ; M+ACN+Na ; M+ACN+Na ; M+ACN+Na ; M+ACN+Na ; M+ACN+Na ; M+ACN+Na ; M+ACN+Na ; M+ACN+Na ; M+ACN+Na ; M+ACN+H ; M+ACN+Na ; M+ACN+Na ; M+ACN+H ; M+ACN+Na ; M+ACN+H ; M+ACN+H ; M+ACN+Na ; M+ACN+Na ; M+ACN+Na ; M+ACN+Na ; M+ACN+Na ; M+ACN+Na ; M+ACN+H ; M+ACN+Na ; M+ACN+H ; M+ACN+Na ; M+ACN+Na ; M+ACN+H ; M+ACN+H ; M+NH4 ; M+ACN+Na ; M+ACN+H ; M+ACN+Na ; M+ACN+H ; M+ACN+Na ; M+ACN+H ; M+ACN+H ; M+NH4 ; 2M+Na+K ; 2M+3H ; M+ACN+Na ; M+ACN+Na ; M+ACN+Na ; M+ACN+Na ; M+ACN+Na ; M+ACN+Na ; M+ACN+Na ; M+ACN+Na ; M+ACN+H ; M+ACN+Na ; M+ACN+H ; M+ACN+H ; M+ACN+H ; M+ACN+H ; M+ACN+H ; M+NH4	C38H74NO8P ; C38H74NO8P ; C38H74NO8P ; C38H74NO8P ; C38H74NO8P ; C38H74NO8P ; C38H74NO8P ; C38H74NO8P ; C77H148O17P2 ; C77H148O17P2 ; C77H148O17P2 ; C77H148O17P2 ; C77H148O17P2 ; C77H148O17P2 ; C77H148O17P2 ; C77H148O17P2 ; C77H148O17P2 ; C77H148O17P2 ; C77H148O17P2 ; C77H148O17P2 ; C77H148O17P2 ; C77H148O17P2 ; C77H148O17P2 ; C77H148O17P2 ; C77H148O17P2 ; C41H68O5 ; C41H68O5 ; C41H68O5 ; C41H68O5 ; C41H68O5 ; C41H68O5 ; C41H68O5 ; C41H68O5 ; C41H68O5 ; C43H66O5 ; C41H68O5 ; C41H68O5 ; C43H66O5 ; C41H68O5 ; C43H66O5 ; C43H66O5 ; C41H68O5 ; C41H68O5 ; C41H68O5 ; C41H68O5 ; C41H68O5 ; C41H68O5 ; C43H66O5 ; C41H68O5 ; C43H66O5 ; C41H68O5 ; C41H68O5 ; C43H66O5 ; C43H66O5 ; C45H66O5 ; C41H68O5 ; C43H66O5 ; C41H68O5 ; C43H66O5 ; C41H68O5 ; C43H66O5 ; C43H66O5 ; C45H66O5 ; C38H76NO6P ; C49H51O26 ; C41H68O5 ; C41H68O5 ; C41H68O5 ; C41H68O5 ; C41H68O5 ; C41H68O5 ; C41H68O5 ; C41H68O5 ; C43H66O5 ; C41H68O5 ; C43H66O5 ; C43H66O5 ; C43H66O5 ; C43H66O5 ; C43H66O5 ; C45H66O5
166.08625	1.329124	1.275491	3.2796	0.008294	2.0812	0.04074	166.0863	HMDB00159 ; HMDB01007 ; HMDB04992 ; HMDB06044 ; HMDB33485 ; HMDB33589 ; HMDB34169 ; HMDB59934 ; HMDB00053 ; HMDB00567 ; HMDB00930 ; HMDB02720 ; HMDB10733 ; HMDB11480 ; HMDB11510 ; HMDB15690 ; HMDB29365 ; HMDB29575 ; HMDB29774 ; HMDB30288 ; HMDB30972 ; HMDB30973 ; HMDB30978 ; HMDB30996 ; HMDB31460 ; HMDB31725 ; HMDB32947 ; HMDB33357 ; HMDB33515 ; HMDB35243 ; HMDB35366 ; HMDB36381 ; HMDB36626 ; HMDB38035 ; HMDB38957 ; HMDB40806 ; HMDB40974 ; HMDB40986 ; HMDB41140 ; HMDB41836 ; HMDB41955	L-Phenylalanine ; 3-Pyridinebutanoic acid ; Benzocaine ; Norsalsolinol ; Gentiatibetine ; Ethyl 2-aminobenzoate ; Methyl N-methylanthranilate ; Benzyl glycinate ; Androstenedione ; Cinnamic acid ; trans-Cinnamic acid ; Gestrinone ; 3-Oxohexadecanoic acid ; LysoPE(0:018:4(6Z9Z12Z15Z)) ; LysoPE(18:4(6Z9Z12Z15Z)0:0) ; Chloropyramine ; Moupinamide ; ()-cis-56-Dihydro-5-hydroxy-4-methoxy-6-(2-phenylethyl)-2H-pyran-2-one ; 7-Hydroxy-6-(methoxyacetyl)-22-dimethyl-2H-1-benzopyran ; Muricinine ; 8-Oxohexadecanoic acid ; 9-Oxohexadecanoic acid ; 11-Oxohexadecanoic acid ; (2E4Z7Z8E)-Colnelenic acid ; Anthocyanidins ; (E)-3-(2-Hydroxyphenyl)-2-propenal ; Di-2-furanylmethane ; Laurelliptine ; Citreovirenone ; 1-Phenyl-12-propanedione ; Cytochalasin Opho ; N-cis-Feruloyltyramine ; 34-Dihydro-2H-1-benzopyran-2-one ; Panaquinquecol 1 ; Citronellyl cinnamate ; 7-Paradol ; Laurolitsine ; (E)-3-(4-Hydroxyphenyl)-2-propenal ; Prenyl cis-caffeate ; Benzoyl ecgonine ; Norcocaine	M+H ; M+H ; M+H ; M+H ; M+H ; M+H ; M+H ; M+H ; M+2Na ; M+NH4 ; M+NH4 ; M+H+Na ; M+Na+K ; M+2H+Na ; M+2H+Na ; M+ACN+2H ; M+H+NH4 ; M+2ACN+2H ; M+2ACN+2H ; M+H+NH4 ; M+Na+K ; M+Na+K ; M+Na+K ; M+H+K ; M+3ACN+2H ; M+NH4 ; M+NH4 ; M+H+NH4 ; M+2ACN+2H ; M+NH4 ; M+H+2Na ; M+H+NH4 ; M+NH4 ; M+H+K ; M+2Na ; M+H+K ; M+H+NH4 ; M+NH4 ; M+2ACN+2H ; M+ACN+2H ; M+ACN+2H	C9H11NO2 ; C9H11NO2 ; C9H11NO2 ; C9H11NO2 ; C9H11NO2 ; C9H11NO2 ; C9H11NO2 ; C9H11NO2 ; C19H26O2 ; C9H8O2 ; C9H8O2 ; C21H24O2 ; C16H30O3 ; C23H40NO7P ; C23H40NO7P ; C16H20ClN3 ; C18H19NO4 ; C14H16O4 ; C14H16O4 ; C18H19NO4 ; C16H30O3 ; C16H30O3 ; C16H30O3 ; C18H28O3 ; C15H11O ; C9H8O2 ; C9H8O2 ; C18H19NO4 ; C14H16O4 ; C9H8O2 ; C28H37NO4 ; C18H19NO4 ; C9H8O2 ; C18H28O3 ; C19H26O2 ; C18H28O3 ; C18H19NO4 ; C9H8O2 ; C14H16O4 ; C16H19NO4 ; C16H19NO4
230.10190	1.384558	0.156884	3.2795	0.008296	2.0811	0.04074	230.0958	HMDB03045 ; HMDB41887 ; HMDB29909 ; HMDB37108 ; HMDB37603 ; HMDB39852 ; HMDB06406 ; HMDB15238 ; HMDB15519 ; HMDB31820 ; HMDB32201 ; HMDB32202 ; HMDB32635 ; HMDB32854 ; HMDB37764 ; HMDB38437	Ergothioneine ; Endalin ; Gein ; 3-Hydroxychavicol 1-rhamnosyl-(1-6)-glucoside ; Eugenol O-a-L-Arabinofuranosyl-(1-6)-b-D-glucopyranoside ; Deoxynivalenol 3-glucoside ; Ecgonine methyl ester ; Levocabastine ; Phendimetrazine ; Neotussilagine ; cis- and trans-5-Ethyl-4-methyl-2-(2-butyl)-thiazoline ; cis and trans-5-Ethyl-4-methyl-2-(2-methylpropyl)-thiazoline ; NN-Diethylbenzeneacetamide ; N-Acetyl-26-diethylaniline ; S-1-2-(Acetyloxy)ethylbutyl ethanethioate ; 9-(Methylthio)nonanenitrile	M+H ; M+H ; M+2H ; M+2H ; M+2H ; M+2H ; 2M+Na+K ; M+H+K ; M+K ; 2M+Na+K ; M+2Na-H ; M+2Na-H ; M+K ; M+K ; 2M+H+Na ; M+2Na-H	C9H15N3O2S ; C9H12FN3O3 ; C21H30O11 ; C21H30O11 ; C21H30O11 ; C21H30O11 ; C10H17NO3 ; C26H29FN2O2 ; C12H17NO ; C10H17NO3 ; C10H19NS ; C10H19NS ; C12H17NO ; C12H17NO ; C10H18O3S ; C10H19NS
277.95005	1.423443	1.206816	3.2789	0.008304	2.0807	0.04074	NA	NA	NA	NA	NA

244.09461	1.416201	0.506983	-3.2784	0.008311	2.0804	0.04074	244.0946	HMDB01904 ; HMDB15269 ; HMDB29436 ; HMDB29556 ; HMDB32706 ; HMDB32713 ; HMDB32860 ; HMDB32974 ; HMDB32975 ; HMDB32976 ; HMDB32998 ; HMDB33867 ; HMDB33925 ; HMDB34663 ; HMDB38115 ; HMDB38460 ; HMDB40036	3-Nitrotyrosine ; Sulfinpyrazone ; Penmacric acid ; Glycerol tribenzoate ; (2-Naphthalenyloxy)acetic acid ; 345-Biphenyltriol ; 1-Methylnaphthalene ; 24-Dimethylthiazole ; 25-Dimethylthiazole ; 45-Dimethylthiazole ; 2-Methoxyxanthone ; 4-Isothiocyanato-1-butene ; Oroselone ; 5-Hydroxy-23-dimethyl-14-naphthoquinone ; Setarin ; Thiiranepropanenitrile ; 2-Ethylthiazole	M+NH4 ; M+2ACN+2H ; M+ACN+H ; M+2ACN+2H ; M+ACN+H ; M+ACN+H ; 3M+Na+K ; 2M+NH4 ; 2M+NH4 ; 2M+NH4 ; M+NH4 ; 2M+NH4 ; M+NH4 ; M+ACN+H ; M+ACN+H ; 2M+NH4 ; 2M+NH4	C9H10N2O5 ; C23H20N2O3S ; C7H10N2O5 ; C24H20O6 ; C12H10O3 ; C12H10O3 ; C11H10 ; C5H7NS ; C5H7NS ; C5H7NS ; C14H10O3 ; C5H7NS ; C14H10O3 ; C12H10O3 ; C12H10O3 ; C5H7NS ; C5H7NS
179.95369	1.477582	0.196479	3.2777	0.008321	2.0798	0.040748	179.9537	HMDB04122	Selenocystine	M+H+Na	C6H12N2O4Se2
136.04867	1.377132	0.957982	3.2757	0.008349	2.0784	0.040847	136.0487	HMDB01542 ; HMDB06357 ; HMDB06535 ; HMDB06575 ; HMDB06583 ; HMDB14658 ; HMDB39506 ; HMDB40705 ; HMDB59729 ; HMDB60320	N-Acetyllactosamine ; cis-2-Methylaconitate ; Beta-14-mannose-N-acetylglucosamine ; Lacto-N-biose I ; Poly-N-acetyllactosamine ; Anisotropine Methylbromide ; N-Salicyloylaspartic acid ; Allixin ; 34-Methylenesebacic acid ; (Z)-But-1-ene-124-tricarboxylate	M+2H+Na ; M+2ACN+2H ; M+2H+Na ; M+2H+Na ; M+2H+Na ; M+H+2Na ; M+H+NH4 ; M+2Na ; M+2Na ; M+2ACN+2H	C14H25NO11 ; C7H8O6 ; C14H25NO11 ; C14H25NO11 ; C14H25NO11 ; C17H32BrNO2 ; C11H11NO6 ; C12H18O4 ; C12H18O4 ; C7H8O6
206.52739	1.399913	1.367709	-3.2728	0.00839	2.0762	0.041008	206.5274	HMDB28984 ; HMDB29089	Methionyl-Tryptophan ; Tryptophyl-Methionine	M+2K ; M+2K	C16H21N3O3S ; C16H21N3O3S
169.50753	1.363059	1.531693	-3.2715	0.008409	2.0753	0.041058	169.5075	HMDB31780	Dithianon	M+ACN+2H	C14H4N2O2S2
382.76668	1.359005	1.700145	3.2696	0.008437	2.0738	0.04115	382.7667	HMDB08946 ; HMDB08977 ; HMDB08978 ; HMDB09038 ; HMDB09071 ; HMDB09102 ; HMDB09103 ; HMDB09133 ; HMDB09134 ; HMDB09166 ; HMDB09167 ; HMDB09198 ; HMDB09294 ; HMDB09325 ; HMDB09326 ; HMDB09358 ; HMDB09359 ; HMDB09390 ; HMDB09423 ; HMDB09454 ; HMDB09455 ; HMDB09617 ; HMDB09650 ; HMDB09682 ; HMDB10169 ; HMDB13327 ; HMDB34616	PE(16:022:6(4Z7Z10Z13Z16Z19Z)) ; PE(16:1(9Z)22:5(4Z7Z10Z13Z16Z)) ; PE(16:1(9Z)22:5(7Z10Z13Z16Z19Z)) ; PE(18:1(11Z)20:5(5Z8Z11Z14Z17Z)) ; PE(18:1(9Z)20:5(5Z8Z11Z14Z17Z)) ; PE(18:2(9Z12Z)20:4(5Z8Z11Z14Z)) ; PE(18:2(9Z12Z)20:4(8Z11Z14Z17Z)) ; PE(18:3(6Z9Z12Z)20:3(5Z8Z11Z)) ; PE(18:3(6Z9Z12Z)20:3(8Z11Z14Z)) ; PE(18:3(9Z12Z15Z)20:3(5Z8Z11Z)) ; PE(18:3(9Z12Z15Z)20:3(8Z11Z14Z)) ; PE(18:4(6Z9Z12Z15Z)20:2(11Z14Z)) ; PE(20:2(11Z14Z)18:4(6Z9Z12Z15Z)) ; PE(20:3(5Z8Z11Z)18:3(6Z9Z12Z)) ; PE(20:3(5Z8Z11Z)18:3(9Z12Z15Z)) ; PE(20:3(8Z11Z14Z)18:3(6Z9Z12Z)) ; PE(20:3(8Z11Z14Z)18:3(9Z12Z15Z)) ; PE(20:4(5Z8Z11Z14Z)18:2(9Z12Z)) ; PE(20:4(8Z11Z14Z17Z)18:2(9Z12Z)) ; PE(20:5(5Z8Z11Z14Z17Z)18:1(11Z)) ; PE(20:5(5Z8Z11Z14Z17Z)18:1(9Z)) ; PE(22:5(4Z7Z10Z13Z16Z)16:1(9Z)) ; PE(22:5(7Z10Z13Z16Z19Z)16:1(9Z)) ; PE(22:6(4Z7Z10Z13Z16Z19Z)16:0) ; SM(d18:116:0) ; Dodecanedioylcarnitine ; 22-Dibromo-2-cyanoacetamide	M+2H ; M+Na+K ; 2M+H+NH4 ; 3M+2Na	C43H74NO8P ; C39H80N2O6P ; C19H35NO6 ; C3H2Br2N2O
427.04396	1.334912	0.759129	-3.269	0.008444	2.0735	0.04115	NA	NA	NA	NA	NA
244.04852	1.337601	0.903489	3.2637	0.008521	2.0695	0.041462	244.0485	HMDB14756 ; HMDB14976 ; HMDB15148 ; HMDB30371 ; HMDB36030 ; HMDB37864 ; HMDB37883 ; HMDB37884 ; HMDB37885 ; HMDB37886 ; HMDB37887 ; HMDB37888 ; HMDB38379 ; HMDB14513	Demeclocycline ; Clocortolone ; Clobetasol ; Myrtine ; Buccoxime ; 4-Ethyl-5-pentyloxazole ; 24-Diethyl-5-propyloxazole ; 2-Butyl-5-ethyl-4-methyloxazole ; 5-Butyl-4-ethyl-2-methyloxazole ; 2-Butyl-4-ethyl-5-methyloxazole ; 45-Dimethyl-2-pentyloxazole ; 4-Butyl-2-ethyl-5-methyloxazole ; 1-Piperazinecarbodithioic acid ; Cidofovir	M+H+Na ; M+2K ; M+2K ; M+2K-H ; M+2K-H ; M+2K-H ; M+2K-H ; M+2K-H ; M+2K-H ; M+2K-H ; M+2K-H ; M+2K-H ; 3M+2H ; M+H-2H2O	C21H21ClN2O8 ; C22H28ClFO4 ; C22H28ClFO4 ; C10H17NO ; C10H17NO ; C10H17NO ; C10H17NO ; C10H17NO ; C10H17NO ; C10H17NO ; C10H17NO ; C10H17NO ; C5H10N2S2 ; C8H14N3O6P
197.07678	1.322294	1.244936	-3.2634	0.008525	2.0693	0.041462	197.0768	HMDB60510 ; HMDB00704 ; HMDB00893 ; HMDB02023 ; HMDB02358 ; HMDB04031 ; HMDB04586 ; HMDB12851 ; HMDB14817 ; HMDB29337 ; HMDB29691 ; HMDB30103 ; HMDB30136 ; HMDB30408 ; HMDB30794 ; HMDB31085 ; HMDB31374 ; HMDB31375 ; HMDB31877 ; HMDB32062 ; HMDB32135 ; HMDB32462 ; HMDB32615 ; HMDB33009 ; HMDB33011 ; HMDB33219 ; HMDB33236 ; HMDB33645 ; HMDB33838 ; HMDB34786 ; HMDB34874 ; HMDB34912 ; HMDB34922 ; HMDB35063 ; HMDB35235 ; HMDB35328 ; HMDB35637 ; HMDB36042 ; HMDB36084 ; HMDB36146 ; HMDB36623 ; HMDB36700 ; HMDB36726 ; HMDB36761 ; HMDB36878 ; HMDB36879 ; HMDB37011 ; HMDB37012 ; HMDB37044 ; HMDB38752 ; HMDB38753 ; HMDB38966 ; HMDB39014 ; HMDB39382 ; HMDB39469 ; HMDB40176 ;	SN-38 ; Isoxanthopterin ; Suberic acid ; Ethyladipic acid ; Carnosic acid ; 11b-Hydroxyprogesterone ; Perillic acid ; 7-Carboxy-gamma-tocotrienol ; Thioridazine ; Oxoglaucine ; Wine lactone ; Hulupone ; Adhulupone ; 2-Amino-34-dihydroxypentanedioic acid ; Morellin ; 6-Pentyl-2H-pyran-2-one ; (1(10)E4E6a9b)-9-(2-Methylbutanoyloxy)-1(10)411(13)-germacratrien-126-olide ; (1(10)E4E6a9b)-9-(3-Methylbutanoyloxy)-1(10)411(13)-germacratrien-126-olide ; Acetylsalvipisone ; 2-tert-Butyl-14-benzenediol ; 2-Methoxy-4-propylphenol ; Pentyl 2-furyl ketone ; 11-Dimethoxy-2-phenylethane ; Foeniculoside VIII ; Foeniculoside IX ; (1S2S4R5S7S)-257-Fenchanetriol 2-O-b-D-glucoside ; cis-10-Hydroxylinalyl oxide 7-glucoside ; (1S2S4S5S)-247-Thujanetriol 4-glucoside ; Diethyl succinate ; (1S2S4S)-18-Epoxy-p-menthane-27-diol 2-O-b-D-glucoside ; Foeniculoside V ; (S)-N-(45-Dihydro-1-methyl-4-oxo-1H-imidazol-2-yl)alanine ; alpha-Teresantalic acid ; Epinepetalactone ; 247-Decatrienoic acid ; Perilla ketone ; Petasitin ; Marmelolactone A ; Isomintlactone ; 6-Angeloylfuranofukinol ; 4-Deoxycohumulone ; ent-7alpha12beta-Dihydroxy-16-kauren-196beta-olide ; ent-15-Kaurene-1719-dioic acid ; 718-Dihydroxykaurenolide ; 14-Epidioxy-p-mentha-28-diene ; 12:34-Diepoxy-p-menth-8-ene ; 5-Hydroxy-p-mentha-68-dien-2-one ; 2-Hydroxy-p-mentha-18-dien-6-one ; Rotundifolone ; Distichonic acid A ; Distichonic acid B ; (9Z11S16S)-1-Acetoxy-917-octadecadiene-1214-diyne-1116-diol ; Nepetariaside ; (1xi4xi6xi)-Carvone oxide ; (1S2S4R)-p-Menth-8-ene-1210-triol 2-glucoside ; 4-Ethyl-12-dimethoxybenzene ; Karahana lactone ; Foeniculoside VII ; 78-Dihydro-3b6a-dihydroxy-alpha-ionol 9-apiosyl-(1-6)-glucoside ; 3-Hydroxy-p-mentha-18-dien-7-al ; Dimethyl adipate ; N-nitrosonornicotine ; 24-Dimethyladipic acid ; 3-Methylpimelic acid ; 2-Propylglutaric acid ; Methyl bisnorbiotinyl ketone	M+2H ; M+NH4 ; M+Na ; M+Na ; M+Na+K ; M+Na+K ; 2M+Na+K ; M+Na+K ; M+H+Na ; M+ACN+2H ; 2M+Na+K ; M+Na+K ; M+Na+K ; M+NH4 ; M+H+2Na ; 2M+Na+K ; M+Na+K ; M+Na+K ; M+H+K ; 2M+Na+K ; 2M+Na+K ; 2M+Na+K ; 2M+Na+K ; M+2Na ; M+2Na ; M+2Na ; M+2Na ; M+2Na ; M+Na ; M+2Na ; M+2Na ; 2M+H+Na ; 2M+Na+K ; 2M+Na+K ; 2M+Na+K ; 2M+Na+K ; M+Na+K ; 2M+Na+K ; 2M+Na+K ; M+Na+K ; M+Na+K ; M+Na+K ; M+Na+K ; M+Na+K ; 2M+Na+K ; 2M+Na+K ; 2M+Na+K ; 2M+Na+K ; 2M+Na+K ; 2M+3H ; 2M+3H ; M+Na+K ; M+2Na ; 2M+Na+K ; M+2Na ; 2M+Na+K ; 2M+Na+K ; M+2Na ; M+3Na ; 2M+Na+K ; M+Na ; 2M+H+K ; M+Na ; M+Na ; M+Na ; M+H-H2O	C22H20N2O5 ; C6H5N5O2 ; C8H14O4 ; C8H14O4 ; C20H28O4 ; C20H28O4 ; C10H14O2 ; C20H28O4 ; C21H26N2S2 ; C20H17NO5 ; C10H14O2 ; C20H28O4 ; C20H28O4 ; C5H9NO6 ; C33H36O7 ; C10H14O2 ; C20H28O4 ; C20H28O4 ; C22H26O4 ; C10H14O2 ; C10H14O2 ; C10H14O2 ; C10H14O2 ; C16H28O8 ; C16H28O8 ; C16H28O8 ; C16H28O8 ; C16H28O8 ; C8H14O4 ; C16H28O8 ; C16H28O8 ; C7H11N3O3 ; C10H14O2 ; C10H14O2 ; C10H14O2 ; C10H14O2 ; C20H28O4 ; C10H14O2 ; C10H14O2 ; C20H28O4 ; C20H28O4 ; C20H28O4 ; C20H28O4 ; C20H28O4 ; C10H14O2 ; C10H14O2 ; C10H14O2 ; C10H14O2 ; C10H14O2 ; C10H18N2O8 ; C10H18N2O8 ; C20H28O4 ; C16H28O8 ; C10H14O2 ; C16H28O8 ; C10H14O2 ; C10H14O2 ; C16H28O8 ; C24H42O12 ; C10H14O2 ; C8H14O4 ; C9H11N3O ; C8H14O4 ; C8H14O4 ; C8H14O4 ; C9H14N2O2S
230.92200	1.357463	1.375844	-3.2604	0.008568	2.0671	0.041632	230.922	HMDB60364	25-Dichloro-carboxymethylenebut-2-en-4-olide	M+Na	C6H2Cl2O4
336.50023	1.397241	0.053791	-3.2594	0.008583	2.0664	0.041656	336.5002	HMDB11725 ; HMDB59968 ; HMDB59982	5-Sulfosalicylic acid ; 3-hydroxybenzoic acid-3-O-sulphate ; 4-hydroxybenzoic acid-4-O-sulphate	3M+H+NH4 ; 3M+H+NH4 ; 3M+H+NH4	C7H6O6S ; C7H6O6S ; C7H6O6S
292.50517	1.381256	0.781651	-3.259	0.008589	2.066	0.041656	NA	NA	NA	NA	NA

834.57658(	1.458742	0.030608	-3.2569	0.008619	2.0646	0.041733	834.5766	HMDB07885 ; HMDB07916 ; HMDB07917 ; HMDB08009 ; HMDB08233 ; HMDB08359 ; HMDB08360 ; HMDB08391 ; HMDB08423 ; HMDB08427 ; HMDB08460 ; HMDB08492 ; HMDB08911 ; HMDB08912 ; HMDB09615 ; HMDB09648 ; HMDB11251 ; HMDB11252 ; HMDB11283 ; HMDB11316 ; HMDB13420 ; HMDB15385 ; HMDB33479	PC(14:020:5(5Z8Z11Z14Z17Z)) ; PC(14:1(9Z)20:4(5Z8Z11Z14Z)) ; PC(14:1(9Z)20:4(8Z11Z14Z17Z)) ; PC(16:1(9Z)18:4(6Z9Z12Z15Z)) ; PC(18:4(6Z9Z12Z15Z)16:1(9Z)) ; PC(20:2(11Z14Z)P-18:1(11Z)) ; PC(20:2(11Z14Z)P-18:1(9Z)) ; PC(20:3(5Z8Z11Z)P-18:0) ; PC(20:3(8Z11Z14Z)P-18:0) ; PC(20:4(5Z8Z11Z14Z)14:1(9Z)) ; PC(20:4(8Z11Z14Z17Z)14:1(9Z)) ; PC(20:5(5Z8Z11Z14Z17Z)14:0) ; PE(15:022:5(4Z7Z10Z13Z16Z)) ; PE(15:022:5(7Z10Z13Z16Z19Z)) ; PE(22:5(4Z7Z10Z13Z16Z)15:0) ; PE(22:5(7Z10Z13Z16Z19Z)15:0) ; PC(P-18:020:3(5Z8Z11Z)) ; PC(P-18:020:3(8Z11Z14Z)) ; PC(P-18:1(11Z)20:2(11Z14Z)) ; PC(P-18:1(9Z)20:2(11Z14Z)) ; PC(o-18:020:4(8Z11Z14Z17Z)) ; Lisdexamfetamine ; Sinapoylspermine	M+2ACN+H ; M+2ACN+H ; M+2ACN+H ; M+2ACN+H ; M+2ACN+H ; M+K ; M+K ; M+K ; M+K ; M+2ACN+H ; M+2ACN+H ; M+2ACN+H ; M+2ACN+H ; M+2ACN+H ; M+2ACN+H ; M+2ACN+H ; M+K ; M+K ; M+K ; M+K ; M+K ; 3M+2Na-H ; 2M+NH4	C42H74NO8P ; C42H74NO8P ; C42H74NO8P ; C42H74NO8P ; C42H74NO8P ; C46H86NO7P ; C46H86NO7P ; C46H86NO7P ; C46H86NO7P ; C42H74NO8P ; C42H74NO8P ; C42H74NO8P ; C42H74NO8P ; C42H74NO8P ; C42H74NO8P ; C42H74NO8P ; C46H86NO7P ; C46H86NO7P ; C46H86NO7P ; C46H86NO7P ; C46H86NO7P ; C15H25N3O ; C21H36N4O4
161.55751	1.401261	0.471853	-3.2567	0.008622	2.0644	0.041733	161.5575	HMDB29395 ; HMDB30391 ; HMDB15176 ; HMDB29614 ; HMDB31863 ; HMDB31969 ; HMDB32804 ; HMDB32909 ; HMDB33026 ; HMDB33743 ; HMDB38322 ; HMDB39879 ; HMDB41450	L-L-Homoglutathione ; Cepharadione B ; Melphalan ; ()-Conen ; gamma-Glutamyl-S-methylcysteine sulfoxide ; 3-Hydroxy-2-(4-methylbenzoyl)-4H-1-benzopyran-4-one ; N-trans-p-Coumaroyloctopamine ; 2-(4-Methoxyphenyl)naphthalic anhydride ; N-cis-Caffeoyltyramine ; Neodunol ; 4-(34-Dihydroxyphenyl)-2-hydroxy-1H-phenalen-1-one ; N-Acetoxymethylflindersine ; Musanolone E	M+2H ; M+2H ; M+H+NH4 ; M+H+NH4 ; M+ACN+2H ; M+ACN+2H ; M+H+Na ; M+H+NH4 ; M+H+Na ; M+ACN+2H ; M+H+NH4 ; M+H+Na ; M+H+NH4	C11H19N3O6S ; C19H15NO4 ; C13H18Cl2N2O2 ; C13H21O2PS2 ; C9H16N2O6S ; C17H12O4 ; C17H17NO4 ; C19H12O4 ; C17H17NO4 ; C17H12O4 ; C19H12O4 ; C17H17NO4 ; C19H12O4
170.56709	1.389436	0.262877	-3.2547	0.008651	2.0629	0.041796	170.5671	HMDB02285 ; HMDB03320 ; HMDB04077 ; HMDB14693 ; HMDB15160 ; HMDB28874 ; HMDB29084 ; HMDB30242 ; HMDB30637 ; HMDB31172 ; HMDB33457 ; HMDB36326 ; HMDB41096 ; HMDB60289 ; HMDB60594 ; HMDB60630	2-Indolecarboxylic acid ; Indole-3-carboxylic acid ; 46-Dihydroxyquinoline ; Methoxsalen ; Amlexanox ; Hydroxyprolyl-Tryptophan ; Tryptophyl-Hydroxyproline ; (E)-Herclavine ; Bergapten ; 3-Formyl-6-hydroxyindole ; Flazine methyl ether ; N-(4-Hydroxy-3-methoxyphenyl)methyloctanamide ; Lepidimoic acid ; Quinoline-48-diol ; Doxepin N-oxide ; (E)-2-hydroxydoxepin	2M+H+NH4 ; 2M+H+NH4 ; 2M+H+NH4 ; M+3ACN+2H ; M+ACN+2H ; M+H+Na ; M+H+Na ; M+2Na ; M+3ACN+2H ; 2M+H+NH4 ; M+H+NH4 ; M+Na+K ; M+H+NH4 ; 2M+H+NH4 ; M+2Na ; M+2Na	C9H7NO2 ; C9H7NO2 ; C9H7NO2 ; C12H8O4 ; C16H14N2O4 ; C16H19N3O4 ; C16H19N3O4 ; C19H21NO2 ; C12H8O4 ; C9H7NO2 ; C18H14N2O4 ; C16H25NO3 ; C12H18O10 ; C9H7NO2 ; C19H21NO2 ; C19H21NO2
319.16130	1.347546	1.107014	-3.2545	0.008655	2.0627	0.041796	319.1613	HMDB02725 ; HMDB29310 ; HMDB35579 ; HMDB35963 ; HMDB37191 ; HMDB37230 ; HMDB41541	Nandrolone ; Collettiside I ; 1-Phenyl-13-dodecanedione ; (10Z14E16E)-101416-Octadecatrien-12-ynoic acid ; Rhodinyl phenylacetate ; Citronellyl alpha-toluate ; N2-Fructopyranosylarginine	M+2Na-H ; M+Na+K ; M+2Na-H ; M+2Na-H ; M+2Na-H ; M+2Na-H ; M+H-H2O	C18H26O2 ; C33H52O8 ; C18H26O2 ; C18H26O2 ; C18H26O2 ; C18H26O2 ; C12H24N4O7
405.89525	1.348655	1.610972	-3.2537	0.008666	2.0622	0.041796	405.8953	HMDB07818 ; HMDB31211 ; HMDB41188 ; HMDB42150 ; HMDB42933 ; HMDB43017 ; HMDB43801 ; HMDB43856 ; HMDB56111	DG(24:024:00:0) ; Endrin ; Bis(methylsulfonylmethyl) disulfide ; TG(14:016:0o-18:0) ; TG(14:0o-18:016:0) ; TG(15:015:0o-18:0) ; TG(15:0o-18:015:0) ; TG(16:014:0o-18:0) ; DG(24:00:024:0)	M+H+NH4 ; 2M+3H2O+2H ; 3M+Na+K ; M+H+NH4 ; M+H+NH4 ; M+H+NH4 ; M+H+NH4 ; M+H+NH4 ; M+H+NH4	C51H100O5 ; C12H8Cl6O ; C4H10O4S4 ; C51H100O5 ; C51H100O5 ; C51H100O5 ; C51H100O5 ; C51H100O5 ; C51H100O5
254.13864	1.419905	0.417337	3.2535	0.008669	2.0621	0.041796	254.1386	HMDB00736 ; HMDB00906 ; HMDB02013 ; HMDB12211 ; HMDB12213 ; HMDB12214 ; HMDB34006 ; HMDB35973 ; HMDB37683 ; HMDB60564 ; HMDB00552	Isobutyryl-L-carnitine ; Trimethylamine ; Butyrylcarnitine ; Dihydrozeatin-7-N-dihydrozeatin ; Dihydrozeatin-9-N-glucoside ; Dihydrozeatin-O-glucoside ; 1-Propylamine ; Lucidenic acid M ; Ethyl vanillin isobutyrate ; Carboxy-ibuprofen ; 3-Methylglutarylcarnitine	M+Na ; 3M+2K-H ; M+Na ; M+3ACN+2H ; M+3ACN+2H ; M+3ACN+2H ; 3M+2K-H ; M+2Na ; M+NH4 ; M+NH4 ; M+H-2H2O	C11H21NO4 ; C3H9N ; C11H21NO4 ; C16H25N5O6 ; C16H25N5O6 ; C16H25N5O6 ; C3H9N ; C27H42O6 ; C13H16O4 ; C13H16O4 ; C13H23NO6
440.72548	1.365827	1.659112	3.2525	0.008684	2.0613	0.041828	NA	NA	NA	NA	NA
448.75190	1.410902	1.331374	3.2502	0.008718	2.0596	0.041847	448.7519	HMDB41548	Hovenidulcioside B1	M+ACN+2H	C44H70O16
594.30666	1.38884	1.220175	-3.2498	0.008724	2.0593	0.041847	594.3067	HMDB02379 ; HMDB11793 ; HMDB11823 ; HMDB14257 ; HMDB30363 ; HMDB30764 ; HMDB32932 ; HMDB33023 ; HMDB33796 ; HMDB34925 ; HMDB35325 ; HMDB36310 ; HMDB38838 ; HMDB38976 ; HMDB40785 ; HMDB60840	Mesoporphyrin IX ; Ganglioside GD1a (d18:114:0) ; Ganglioside GD1b (d18:114:0) ; E-10-Hydroxydesmethylnortriptyline ; Cavipetin A ; Acetylpterosin C ; Tuberoside C (Ullucus tuberosus) ; 712-Dihydroxy-3111523-tetraoxolanost-8-en-26-oic acid ; Physapubescin ; Camellidin II ; Ganoderic acid N ; Ganoderic acid theta ; Lansiumamide C ; Camellioside A ; Mukoenine A ; N-Desmethyldoxepin	2M+3H2O+2H ; M+3H ; M+3H ; 2M+ACN+Na ; M+ACN+Na ; 2M+ACN+H ; M+2ACN+2H ; M+ACN+Na ; M+ACN+Na ; M+2ACN+2H ; M+ACN+Na ; M+ACN+Na ; 2M+ACN+Na ; M+2ACN+2H ; 2M+ACN+Na ; 2M+ACN+Na	C34H38N4O4 ; C81H141N3O39 ; C81H141N3O39 ; C18H19NO ; C30H42O8 ; C16H20O4 ; C53H84O24 ; C30H42O8 ; C30H42O8 ; C53H84O24 ; C30H42O8 ; C30H42O8 ; C18H19NO ; C53H84O24 ; C18H19NO ; C18H19NO
620.94125	1.331914	0.505883	3.2498	0.008724	2.0593	0.041847	NA	NA	NA	NA	NA
793.55775	1.367824	0.520283	3.2496	0.008726	2.0592	0.041847	793.5578	HMDB13330 ; HMDB13465 ; HMDB56268 ; HMDB56270	3-Hydroxy-cis-5-tetradecenoylcarnitine ; SM(d18:020:2(11Z14Z)) ; DG(24:1n90:018:2n6) ; DG(24:1n90:020:2n6)	2M+Na ; M+K ; M+2K-H ; M+2K-H	C21H39NO5 ; C43H83N2O6P ; C46H84O5 ; C46H84O5
434.69067	1.353531	1.703489	3.2494	0.008729	2.059	0.041847	NA	NA	NA	NA	NA
410.62314	1.311546	1.548936	-3.2487	0.00874	2.0585	0.041857	410.6231	HMDB14851	Oxacillin	2M+H+NH4	C19H19N3O5S
258.81754	1.352377	1.699853	3.2465	0.008773	2.0569	0.041975	258.8175	HMDB29596	Chloroform	2M+Na	CHCl3
506.62648	1.353323	1.66527	3.2424	0.008833	2.0539	0.042189	506.6265	HMDB02288 ; HMDB06866 ; HMDB11658 ; HMDB12471 ; HMDB13189 ; HMDB35452	5-cis-8-cis-Tetradecadienoyl-CoA ; 2-Methyl-1-hydroxypropyl-ThPP ; 28-Dihydroxyquinoline-beta-D-glucuronide ; (2S6R10R)-Trimethyl-hendecanoyl-CoA ; 3-Indole carboxylic acid glucuronide ; Pelargonidin 3-O-b-D-Glucopyranosyl-(1-2)-4-hydroxycinnamoyl-(-6)-b-D-glucopyranoside(E-) 5-O-(6-O-malonyl-b-D-glucopyranoside)	M+H+K ; 2M+H+NH4 ; 3M+2H ; M+H+K ; 3M+2H ; M+H+Na	C35H58N7O17P3S ; C16H27N4O8P2S ; C15H15NO8 ; C35H58N7O17P3S ; C15H15NO8 ; C45H49O25
138.94691	1.358487	0.722787	-3.2419	0.008842	2.0535	0.042189	NA	NA	NA	NA	NA
444.96040	1.308135	0.878547	-3.2415	0.008846	2.0532	0.042189	444.9604	HMDB33695	Poppy acid	2M+2Na-H	C7H4O7

703.50206	1.338461	0.425522	3.2412	0.008851	2.053	0.042189	703.4975	HMDB01201 ; HMDB07028 ; HMDB07107 ; HMDB07158 ; HMDB07359 ; HMDB07588 ; HMDB08825 ; HMDB08830 ; HMDB08831 ; HMDB08857 ; HMDB08862 ; HMDB08921 ; HMDB08953 ; HMDB09086 ; HMDB09118 ; HMDB09151 ; HMDB30016 ; HMDB31012 ; HMDB31349 ; HMDB32081 ; HMDB32837 ; HMDB33252 ; HMDB33253 ; HMDB33960 ; HMDB34515 ; HMDB34562 ; HMDB34742 ; HMDB34956 ; HMDB35202 ; HMDB35407 ; HMDB35408 ; HMDB35413 ; HMDB35434 ; HMDB35639 ; HMDB35794 ; HMDB35869 ; HMDB35886 ; HMDB35889 ; HMDB35935 ; HMDB36035 ; HMDB36390 ; HMDB36392 ; HMDB36427 ; HMDB36449 ; HMDB36466 ; HMDB36647 ; HMDB37038 ; HMDB37391 ; HMDB37640 ; HMDB37710 ; HMDB37739 ; HMDB38190 ; HMDB38198 ; HMDB38698 ; HMDB38795 ; HMDB38796 ;	Guanosine diphosphate ; DG(14:022:00:0) ; DG(16:020:00:0) ; DG(18:018:00:0) ; DG(20:016:00:0) ; DG(22:014:00:0) ; PE(14:016:1(9Z)) ; PE(14:018:3(6Z9Z12Z)) ; PE(14:018:3(9Z12Z15Z)) ; PE(14:1(9Z)16:0) ; PE(14:1(9Z)18:2(9Z12Z)) ; PE(16:014:1(9Z)) ; PE(16:1(9Z)14:0) ; PE(18:2(9Z12Z)14:1(9Z)) ; PE(18:3(6Z9Z12Z)14:0) ; PE(18:3(9Z12Z15Z)14:0) ; Valerenic acid ; Glycerol 12-dioctadecanoate ; Epiacoronene ; 410-Longipinanedione ; Ganoderic acid DM ; Curcumanolide A ; Curcumenone ; Curcumenol ; Glabrolide ; 9-Pentadecene-1214-diyne-111-diol ; Saussurea lactone ; Oxysolavetivone ; 7-Hydroxycostal ; Dehydrocurdione ; 110-Epoxygermacrone ; (4alpha5alpha)-11-Eremophilene-29-dione ; Isomasticadienonalic acid ; Petasalbin ; beta-Costic acid ; alpha-Rotunol ; Isoglabrolide ; Germacrone 45-epoxide ; Dihydroisoalantolactone ; Marasmene ; 3-Phenylpropyl hexanoate ; (Z)-alpha-Bergamotenoic acid ; Procurcumenol ; (1alpha4beta5beta)-4-Hydroxy-7(11)10(14)-guaiadien-8-one ; Zerumbone oxide ; Isocurcumenol ; Cyclodehydroisolubimin ; p-Hydroxynonanophenone ; 2-Methyl-4-phenyl-2-butyl 2-methylpropanoate ; 4-Methylphenyl octanoate ; 7-(13-Cyclohexadienyl)-5-hydroxy-26-dimethyl-2-hepten-4-one ; Rishitinol ; 37-Bisaboladiene-28-dione ; xi-2-Hydroxy-135-bisabolatrien-9-one ; 79-Illudadiene-314-diol ; 27(14)-Illudadiene-1015-diol ; Uralenolide ; 46-Decadiyn-1-ol isovalerate ; Colupone ; beta-Santalic acid ; 16beta-16-Hydroxy-3-oxo-112-oleanadien-28-oic acid ; Sugeonol ; DG(14:00:022:0) ; DG(16:00:020:0) ; DG(18:00:018:0) ; 8-oxo-dGDP ; PG(16:016:1(9Z)) ; PG(16:1(9Z)16:0)	3M+2K ; M+2K+H ; M+2K+H ; M+2K+H ; M+2K+H ; M+2K+H ; M+ACN+H ; M+NH4 ; M+NH4 ; M+ACN+H ; M+NH4 ; M+ACN+H ; M+ACN+H ; M+NH4 ; M+NH4 ; M+NH4 ; 3M+H ; M+2K+H ; 3M+H ; 3M+H ; 3M+2H ; 3M+H ; 3M+H ; 3M+H ; 3M+2H ; 3M+H ; 3M+H ; 3M+H ; 3M+H ; 3M+H ; 3M+H ; 3M+H ; 3M+2H ; 3M+H ; 3M+H ; 3M+H ; 3M+2H ; 3M+H ; 3M+H ; 3M+H ; 3M+H ; 3M+H ; 3M+H ; 3M+H ; 3M+H ; 3M+H ; 3M+H ; 3M+H ; 3M+H ; 3M+H ; 3M+H ; 3M+H ; 3M+H ; 3M+H ; 3M+H ; 3M+2H ; 3M+H ; 3M+2H ; 3M+H ; 3M+2H ; 3M+H ; M+2K+H ; M+2K+H ; M+2K+H ; 3M+2K ; M+H-H2O ; M+H-H2O	C10H15N5O11P2 ; C39H76O5 ; C39H76O5 ; C39H76O5 ; C39H76O5 ; C39H76O5 ; C35H68NO8P ; C37H68NO8P ; C37H68NO8P ; C35H68NO8P ; C37H68NO8P ; C35H68NO8P ; C35H68NO8P ; C37H68NO8P ; C37H68NO8P ; C37H68NO8P ; C15H22O2 ; C39H76O5 ; C15H22O2 ; C15H22O2 ; C30H44O4 ; C15H22O2 ; C15H22O2 ; C15H22O2 ; C30H44O4 ; C15H22O2 ; C15H22O2 ; C15H22O2 ; C15H22O2 ; C15H22O2 ; C15H22O2 ; C15H22O2 ; C30H44O4 ; C15H22O2 ; C15H22O2 ; C15H22O2 ; C30H44O4 ; C15H22O2 ; C15H22O2 ; C15H22O2 ; C15H22O2 ; C15H22O2 ; C15H22O2 ; C15H22O2 ; C15H22O2 ; C15H22O2 ; C15H22O2 ; C15H22O2 ; C15H22O2 ; C15H22O2 ; C15H22O2 ; C15H22O2 ; C15H22O2 ; C15H22O2 ; C15H22O2 ; C15H22O2 ; C30H44O4 ; C15H22O2 ; C30H44O4 ; C15H22O2 ; C30H44O4 ; C15H22O2 ; C39H76O5 ; C39H76O5 ; C39H76O5 ; C10H15N5O11P2 ; C38H73O10P ; C38H73O10P
203.04626	1.379699	1.429019	-3.2406	0.00886	2.0526	0.042191	203.0463	HMDB14994 ; HMDB40933 ; HMDB29222 ; HMDB60022 ; HMDB00329 ; HMDB00543 ; HMDB01955 ; HMDB02708 ; HMDB05802 ; HMDB05809 ; HMDB13749 ; HMDB15173 ; HMDB15338 ; HMDB29117 ; HMDB29430 ; HMDB29524 ; HMDB29830 ; HMDB30060 ; HMDB30063 ; HMDB30645 ; HMDB30728 ; HMDB30841 ; HMDB31597 ; HMDB31761 ; HMDB32007 ; HMDB32038 ; HMDB32336 ; HMDB32354 ; HMDB32586 ; HMDB32618 ; HMDB32620 ; HMDB32891 ; HMDB32954 ; HMDB33723 ; HMDB33945 ; HMDB34178 ; HMDB34471 ; HMDB34615 ; HMDB34732 ; HMDB35098 ; HMDB35213 ; HMDB35268 ; HMDB35414 ; HMDB36098 ; HMDB36459 ; HMDB36720 ; HMDB37160 ; HMDB37174 ; HMDB37612 ; HMDB37662 ; HMDB38184 ; HMDB38358 ; HMDB40359 ; HMDB40460 ; HMDB40760 ; HMDB41136 ;	Chlorphenesin ; 4-Chloro-35-dimethoxybenzyl alcohol ; Urolithin A-3-O-glucuronide ; Urolithin A-8-O-glucuronide ; 2-Phenylbutyric acid ; Benzenebutanoic acid ; 3-Phenylbutyric acid ; Cyanidin ; Isoeugenol ; Eugenol ; 2-Methylfuran ; Fenofibrate ; Ridogrel ; Tyrosyl-Tyrosine ; Methionine sulfoximine ; 5-Hydroxy-7-(4-hydroxy-3-methoxyphenyl)-1-phenyl-3-heptanone ; N-Caffeoyltryptophan ; Methyl 3-phenylpropanoate ; Benzyl propionate ; Sclareapinone ; 8-Methoxygravelliferone ; Sagequinone methide A ; 24-Pentadienal ; Propyl benzoate ; Epifisetinidol-(4beta-8)-catechin ; Isopropyl benzoate ; 3-Hydroxy-4-phenylbutan-2-one ; S-Isopropyl 3-methylbut-2-enethioate ; 3-(4-Methoxyphenyl)-2-propen-1-ol ; Ethyl phenylacetate ; 1-Phenylethyl acetate ; 1-(4-Methoxyphenyl)-2-propanone ; 5-Geranyloxy-7-methoxycoumarin ; 4-(4-Hydroxyphenyl)-2-butanone ; 2-Phenylethyl acetate ; Guaiacin ; 3-Phenylpropyl formate ; Epoxyanethole ; Thymoquinone ; (all-E)-Crocetin ; beta-Thujaplicin ; 4-Isopropylbenzoic acid ; Lepalone ; Isoegomaketone ; Macelignan ; Verimol H ; 4-Methyl-2-phenyl-13-dioxolane ; p-Mentha-24(8)-dien-93-olide ; CI Acid Blue 3 ; 33457-Pentahydroxyflavan(4-8)-3457-tetrahydroxyflavan ; Egomaketone ; Gambiriin B3 ; 12-Dimethoxy-4-vinylbenzene ; 4-Methylbenzyl alcohol acetate ; 44-Dihydroxy-55-diisopropyl-22-dimethyl-36-biphenyldione ; 3-Chloro-3-deoxytriphasiol ; 2-Methylbenzyl alcohol acetate ; (3-Methylphenyl)methyl acetate ; Phenyl butyrate ; Cyanuric acid	M+H ; M+H ; M+2H ; M+2H ; M+K ; M+K ; M+K ; M+2ACN+2H ; M+K ; M+K ; 2M+K ; M+2Na ; M+H+K ; M+Na+K ; M+Na ; M+2K ; M+H+K ; M+K ; M+K ; M+2K ; M+2K ; M+2K ; 2M+K ; M+K ; M+H+2Na ; M+K ; M+K ; M+2Na-H ; M+K ; M+K ; M+K ; M+K ; M+2K ; M+K ; M+K ; M+2K ; M+K ; M+K ; M+K ; M+2K ; M+K ; M+K ; M+K ; M+K ; M+2K ; M+2K ; M+K ; M+K ; M+2H+Na ; M+H+2Na ; M+K ; M+H+2Na ; M+K ; M+K ; M+2K ; M+H+K ; M+K ; M+K ; M+K ; 3M+H+NH4	C9H11ClO3 ; C9H11ClO3 ; C19H16O10 ; C19H16O10 ; C10H12O2 ; C10H12O2 ; C10H12O2 ; C15H11ClO6 ; C10H12O2 ; C10H12O2 ; C5H6O ; C20H21ClO4 ; C18H17F3N2O3 ; C18H20N2O5 ; C5H12N2O3S ; C20H24O4 ; C20H18N2O5 ; C10H12O2 ; C10H12O2 ; C20H24O4 ; C20H24O4 ; C20H24O4 ; C5H6O ; C10H12O2 ; C30H26O11 ; C10H12O2 ; C10H12O2 ; C8H14OS ; C10H12O2 ; C10H12O2 ; C10H12O2 ; C10H12O2 ; C20H24O4 ; C10H12O2 ; C10H12O2 ; C20H24O4 ; C10H12O2 ; C10H12O2 ; C10H12O2 ; C20H24O4 ; C10H12O2 ; C10H12O2 ; C10H12O2 ; C10H12O2 ; C20H24O4 ; C20H24O4 ; C10H12O2 ; C10H12O2 ; C27H32CaN2O6S2 ; C30H26O11 ; C10H12O2 ; C30H26O11 ; C10H12O2 ; C10H12O2 ; C20H24O4 ; C19H23ClO5 ; C10H12O2 ; C10H12O2 ; C10H12O2 ; C3H3N3O3
161.96275	1.409274	1.226514	3.2391	0.008883	2.0514	0.04226	NA	NA	NA	NA	NA
407.02645	1.394847	0.326671	3.2363	0.008925	2.0494	0.042419	407.0265	HMDB29677 ; HMDB30596 ; HMDB30727 ; HMDB30834 ; HMDB31894 ; HMDB33169 ; HMDB33318 ; HMDB34109 ; HMDB34186 ; HMDB37458 ; HMDB39601 ; HMDB40303 ; HMDB40719 ; HMDB40935 ; HMDB41544	Zapotinin ; Ugaxanthone ; Betagarin ; Americanin A ; Castacrenin F ; Garbogiol ; Isoamericanin A ; Frenolicin B ; Morusignin B ; 5-Hydroxy-478-trimethoxyflavone ; Americanin D ; 5-Hydroxy-446-trimethoxyaurone ; 8-Hydroxy-457-trimethoxyflavone ; Methyl 2-(2-methoxy-4-hydroxyphenyl)-6-methoxy-3-benzofurancarboxylate ; 7-Hydroxy-348-trimethoxyflavone	M+2K+H ; M+2K+H ; M+2K+H ; M+2K+H ; M+3H ; M+2K+H ; M+2K+H ; M+2K+H ; M+2K+H ; M+2K+H ; M+2K+H ; M+2K+H ; M+2K+H ; M+2K+H ; M+2K+H	C18H16O6 ; C18H16O6 ; C18H16O6 ; C18H16O6 ; C55H30O33 ; C18H16O6 ; C18H16O6 ; C18H16O6 ; C18H16O6 ; C18H16O6 ; C18H16O6 ; C18H16O6 ; C18H16O6 ; C18H16O6 ; C18H16O6
528.38412	1.359803	1.120061	-3.235	0.008945	2.0484	0.042474	528.3841	HMDB29704 ; HMDB34645 ; HMDB11498 ; HMDB11528	1-(266-Trimethyl-2-cyclohexen-1-yl)-16-heptadien-3-one ; 22-Acetylpriverogenin B ; LysoPE(0:024:1(15Z)) ; LysoPE(24:1(15Z)0:0)	2M+ACN+Na ; 2M+H+Na ; M+H-2H2O ; M+H-2H2O	C16H24O ; C32H52O5 ; C29H58NO7P ; C29H58NO7P
552.28086	1.454458	0.280808	-3.2343	0.008955	2.0479	0.042482	552.2809	HMDB32892 ; HMDB33157 ; HMDB06321 ; HMDB06496 ; HMDB12985 ; HMDB14852 ; HMDB15280 ; HMDB15454 ; HMDB29431 ; HMDB31402 ; HMDB33789 ; HMDB40381	Lablaboside A ; Protoprimulagenin A 3-glucosyl-(1-3)-galactosyl-(1-4)-rhamnosyl-(1-2)-glucuronide ; Docosa-47101316-pentaenoyl carnitine ; Clupanodonyl carnitine ; Kinetensin 1-8 ; Apomorphine ; Nefazodone ; Rasagiline ; (3b5a22x25S)-Furostane-22-methoxy-326-diol 3-rhamnosyl-(1-2)-glucosyl-(1-3)-glucoside 26-glucoside ; 23-Acetoxysoladulcidine ; 2-O-Methyl-L-fucose ; N-Methylschinifoline	M+2H ; M+2H ; M+2K+H ; M+2K+H ; M+2Na ; 2M+NH4 ; M+2ACN+H ; 3M+K ; M+H+Na ; M+2K+H ; 3M+NH4 ; 2M+ACN+H	C54H86O23 ; C54H86O23 ; C29H47NO4 ; C29H47NO4 ; C50H74N16O10 ; C17H17NO2 ; C25H32ClN5O2 ; C12H13N ; C52H88O23 ; C29H47NO4 ; C7H14O5 ; C16H17NO2

578.97728	1.333314	1.397215	-3.2328	0.008978	2.0468	0.042535	NA	NA	NA	NA	NA
117.06987	1.358057	0.926103	3.2318	0.008994	2.0461	0.042535	117.0699	HMDB00056 ; HMDB00161 ; HMDB00271 ; HMDB01310 ; HMDB31219 ; HMDB31846 ; HMDB31963 ; HMDB32355 ; HMDB32913 ; HMDB36026 ; HMDB36184 ; HMDB36820 ; HMDB36823 ; HMDB38026 ; HMDB38216 ; HMDB38731 ; HMDB38897 ; HMDB41629	Beta-Alanine ; L-Alanine ; Sarcosine ; D-Alanine ; Ethyl carbamate ; Geranylacetone ; (3b6b8a12a)-812-Epoxy-7(11)-eremophilene-6812-trimethoxy-3-ol ; (-)-R-(E)-5-Isopropyl-8-methylnona-68-dien-2-one ; 4-(266-Trimethyl-1-cyclohexen-1-yl)-2-butanone ; Octahydro-6-isopropyl-2(1H)-naphthalenone ; 78-Dehydro-34-dihydro-beta-ionol ; beta-Ionol ; Theaspirane ; Dihydro-alpha-ionone ; 5-Isopropyl-2-(2-methylpropyl)-2-cyclohexen-1-one ; 47-Megastigmadien-9-ol ; (E)-2-(2-Octenyl)cyclopentanone ; (3S6E)-Nerolidol	2M+3H2O+2H ; 2M+3H2O+2H ; 2M+3H2O+2H ; 2M+3H2O+2H ; 2M+3H2O+2H ; M+H+K ; M+2H+Na ; M+H+K ; M+H+K ; M+H+K ; M+H+K ; M+H+K ; M+H+K ; M+H+K ; M+H+K ; M+H+K ; M+H+K ; M+H+K	C3H7NO2 ; C3H7NO2 ; C3H7NO2 ; C3H7NO2 ; C3H7NO2 ; C13H22O ; C18H30O5 ; C13H22O ; C13H22O ; C13H22O ; C13H22O ; C13H22O ; C13H22O ; C13H22O ; C13H22O ; C13H22O ; C13H22O ; C13H22O
666.03352	1.336259	1.514718	-3.2317	0.008994	2.046	0.042535	666.0335	HMDB12028 ; HMDB32936	Ganglioside GT2 (d18:020:0) ; 4-Chloro-1H-indole-3-acetic acid	M+3H ; 3M+K	C92H162N4O42 ; C10H8ClNO2
231.60080	1.337399	1.372162	-3.2307	0.00901	2.0453	0.042535	231.6008	HMDB15433 ; HMDB29505 ; HMDB29506 ; HMDB30688 ; HMDB36631 ; HMDB40480	Cilazapril ; Kuwanon A ; Kuwanon B ; Cyclomulberrin ; Morusin ; Cyclocommunin	M+2Na ; M+ACN+2H ; M+ACN+2H ; M+ACN+2H ; M+ACN+2H ; M+ACN+2H	C22H31N3O5 ; C25H24O6 ; C25H24O6 ; C25H24O6 ; C25H24O6 ; C25H24O6
823.59488	1.35618	0.269898	-3.2302	0.009017	2.0449	0.042535	823.5949	HMDB04866 ; HMDB07879 ; HMDB07911 ; HMDB07955 ; HMDB07971 ; HMDB07972 ; HMDB07991 ; HMDB08003 ; HMDB08022 ; HMDB08024 ; HMDB08035 ; HMDB08067 ; HMDB08083 ; HMDB08100 ; HMDB08116 ; HMDB08147 ; HMDB08148 ; HMDB08178 ; HMDB08179 ; HMDB08211 ; HMDB08212 ; HMDB08243 ; HMDB08263 ; HMDB08295 ; HMDB08339 ; HMDB08370 ; HMDB08371 ; HMDB08403 ; HMDB08404 ; HMDB08434 ; HMDB08467 ; HMDB08498 ; HMDB08499 ; HMDB08625 ; HMDB08660 ; HMDB08693 ; HMDB08725 ; HMDB08908 ; HMDB09009 ; HMDB09107 ; HMDB09139 ; HMDB09172 ; HMDB09204 ; HMDB09234 ; HMDB09235 ; HMDB09265 ; HMDB09266 ; HMDB09297 ; HMDB09329 ; HMDB09362 ; HMDB09394 ; HMDB09427 ; HMDB09492 ; HMDB09516 ; HMDB09523 ; HMDB09524 ;	Lactosylceramide (d18:112:0) ; PC(14:020:1(11Z)) ; PC(14:1(9Z)20:0) ; PC(15:022:4(7Z10Z13Z16Z)) ; PC(16:018:1(11Z)) ; PC(16:018:1(9Z)) ; PC(16:022:6(4Z7Z10Z13Z16Z19Z)) ; PC(16:1(9Z)18:0) ; PC(16:1(9Z)22:5(4Z7Z10Z13Z16Z)) ; PC(16:1(9Z)22:5(7Z10Z13Z16Z19Z)) ; PC(18:016:1(9Z)) ; PC(18:1(11Z)16:0) ; PC(18:1(11Z)20:5(5Z8Z11Z14Z17Z)) ; PC(18:1(9Z)16:0) ; PC(18:1(9Z)20:5(5Z8Z11Z14Z17Z)) ; PC(18:2(9Z12Z)20:4(5Z8Z11Z14Z)) ; PC(18:2(9Z12Z)20:4(8Z11Z14Z17Z)) ; PC(18:3(6Z9Z12Z)20:3(5Z8Z11Z)) ; PC(18:3(6Z9Z12Z)20:3(8Z11Z14Z)) ; PC(18:3(9Z12Z15Z)20:3(5Z8Z11Z)) ; PC(18:3(9Z12Z15Z)20:3(8Z11Z14Z)) ; PC(18:4(6Z9Z12Z15Z)20:2(11Z14Z)) ; PC(20:014:1(9Z)) ; PC(20:1(11Z)14:0) ; PC(20:2(11Z14Z)18:4(6Z9Z12Z15Z)) ; PC(20:3(5Z8Z11Z)18:3(6Z9Z12Z)) ; PC(20:3(5Z8Z11Z)18:3(9Z12Z15Z)) ; PC(20:3(8Z11Z14Z)18:3(6Z9Z12Z)) ; PC(20:3(8Z11Z14Z)18:3(9Z12Z15Z)) ; PC(20:4(5Z8Z11Z14Z)18:2(9Z12Z)) ; PC(20:4(8Z11Z14Z17Z)18:2(9Z12Z)) ; PC(20:5(5Z8Z11Z14Z17Z)18:1(11Z)) ; PC(20:5(5Z8Z11Z14Z17Z)18:1(9Z)) ; PC(22:4(7Z10Z13Z16Z)15:0) ; PC(22:5(4Z7Z10Z13Z16Z)16:1(9Z)) ; PC(22:5(7Z10Z13Z16Z19Z)16:1(9Z)) ; PC(22:6(4Z7Z10Z13Z16Z19Z)16:0) ; PE(15:022:1(13Z)) ; PE(18:022:4(7Z10Z13Z16Z)) ; PE(18:2(9Z12Z)22:2(13Z16Z)) ; PE(18:3(6Z9Z12Z)22:1(13Z)) ; PE(18:3(9Z12Z15Z)22:1(13Z)) ; PE(18:4(6Z9Z12Z15Z)22:0) ; PE(20:020:4(5Z8Z11Z14Z)) ; PE(20:020:4(8Z11Z14Z17Z)) ; PE(20:1(11Z)20:3(5Z8Z11Z)) ; PE(20:1(11Z)20:3(8Z11Z14Z)) ; PE(20:2(11Z14Z)20:2(11Z14Z)) ; PE(20:3(5Z8Z11Z)20:1(11Z)) ; PE(20:3(8Z11Z14Z)20:1(11Z)) ; PE(20:4(5Z8Z11Z14Z)20:0) ; PE(20:4(8Z11Z14Z17Z)20:0) ; PE(22:018:4(6Z9Z12Z15Z)) ; PE(22:1(13Z)15:0) ; PE(22:1(13Z)18:3(6Z9Z12Z)) ; PE(22:1(13Z)18:3(9Z12Z15Z)) ; PE(22:2(13Z16Z)18:2(9Z12Z)) ; PE(22:4(7Z10Z13Z16Z)18:0) ; TG(14:1(9Z)14:1(9Z)20:5(5Z8Z11Z14Z17Z)) ; TG(14:1(9Z)20:5(5Z8Z11Z14Z17Z)14:1(9Z))	M+NH4 ; M+ACN+Na ; M+ACN+Na ; 2M+3H2O+2H ; M+ACN+Na ; M+ACN+Na ; M+NH4 ; M+ACN+Na ; M+NH4 ; M+NH4 ; M+ACN+Na ; M+ACN+Na ; M+NH4 ; M+ACN+Na ; M+NH4 ; M+NH4 ; M+NH4 ; M+NH4 ; M+NH4 ; M+NH4 ; M+NH4 ; M+NH4 ; M+ACN+Na ; M+ACN+Na ; M+NH4 ; M+NH4 ; M+NH4 ; M+NH4 ; M+NH4 ; M+NH4 ; M+NH4 ; M+NH4 ; M+NH4 ; 2M+3H2O+2H ; M+NH4 ; M+NH4 ; M+NH4 ; M+ACN+Na ; 2M+3H2O+2H ; 2M+3H2O+2H ; 2M+3H2O+2H ; 2M+3H2O+2H ; 2M+3H2O+2H ; 2M+3H2O+2H ; 2M+3H2O+2H ; 2M+3H2O+2H ; 2M+3H2O+2H ; 2M+3H2O+2H ; 2M+3H2O+2H ; 2M+3H2O+2H ; 2M+3H2O+2H ; M+ACN+Na ; 2M+3H2O+2H ; 2M+3H2O+2H ; 2M+3H2O+2H ; 2M+3H2O+2H ; 2M+Na+K ; 2M+Na+K	C42H79NO13 ; C42H82NO8P ; C42H82NO8P ; C45H82NO8P ; C42H82NO8P ; C42H82NO8P ; C46H80NO8P ; C42H82NO8P ; C46H80NO8P ; C46H80NO8P ; C42H82NO8P ; C42H82NO8P ; C46H80NO8P ; C42H82NO8P ; C46H80NO8P ; C46H80NO8P ; C46H80NO8P ; C46H80NO8P ; C46H80NO8P ; C46H80NO8P ; C46H80NO8P ; C46H80NO8P ; C42H82NO8P ; C42H82NO8P ; C46H80NO8P ; C46H80NO8P ; C46H80NO8P ; C46H80NO8P ; C46H80NO8P ; C46H80NO8P ; C46H80NO8P ; C46H80NO8P ; C46H80NO8P ; C45H82NO8P ; C46H80NO8P ; C46H80NO8P ; C46H80NO8P ; C42H82NO8P ; C45H82NO8P ; C45H82NO8P ; C45H82NO8P ; C45H82NO8P ; C45H82NO8P ; C45H82NO8P ; C45H82NO8P ; C45H82NO8P ; C45H82NO8P ; C45H82NO8P ; C45H82NO8P ; C45H82NO8P ; C45H82NO8P ; C45H82NO8P ; C45H82NO8P ; C42H82NO8P ; C45H82NO8P ; C45H82NO8P ; C45H82NO8P ; C45H82NO8P ; C51H84O6 ; C51H84O6
246.04743	1.414625	1.134692	3.23	0.009021	2.0447	0.042535	246.0474	HMDB14471 ; HMDB02219 ; HMDB29511 ; HMDB29617 ; HMDB30827 ; HMDB33646 ; HMDB33990 ; HMDB34149 ; HMDB35931 ; HMDB36630 ; HMDB38821 ; HMDB40511 ; HMDB40803 ; HMDB41256 ; HMDB41455	Calcium Gluceptate ; Glycitin ; Garcinone C ; Glucoobtusifolin ; 1-(2H-13-Benzodioxol-5-yl)-2-26-dimethoxy-4-(prop-2-en-1-yl)phenoxypropyl acetate ; Viniferifuran ; Sissotrin ; Prunitrin ; Rheochrysin ; Trifolirhizin ; Calycosin 7-galactoside ; Physcionin ; Kaempferide 3-rhamnoside ; Heteroflavanone C ; 367-Trihydroxy-4-methoxyflavone 7-rhamnoside	M+2H ; M+2Na ; M+2K ; M+2Na ; M+2K ; M+H+K ; M+2Na ; M+2Na ; M+2Na ; M+2Na ; M+2Na ; M+2Na ; M+2Na ; M+2K ; M+2Na	C14H26CaO16 ; C22H22O10 ; C23H26O7 ; C22H22O10 ; C23H26O7 ; C28H20O6 ; C22H22O10 ; C22H22O10 ; C22H22O10 ; C22H22O10 ; C22H22O10 ; C22H22O10 ; C22H22O10 ; C23H26O7 ; C22H22O10
463.17647	1.351714	0.564992	3.2297	0.009026	2.0445	0.042535	463.1765	HMDB33523	(-)-Pyrifolidine	M+2K+H	C23H32N2O3
326.30515	1.369032	0.782812	3.2274	0.009061	2.0428	0.042662	326.3052	HMDB02088 ; HMDB00918 ; HMDB05189 ; HMDB34420 ; HMDB00827 ; HMDB05060 ; HMDB13821 ; HMDB29557 ; HMDB29811 ; HMDB30959 ; HMDB31066 ; HMDB31266 ; HMDB31267 ; HMDB31268 ; HMDB31417 ; HMDB31684 ; HMDB31730 ; HMDB34152 ; HMDB36827 ; HMDB37304 ; HMDB40581 ; HMDB41498 ; HMDB41499 ; HMDB41601 ; HMDB59835 ; HMDB59887 ; HMDB15638	N-Oleoylethanolamine ; CE(18:1(9Z)) ; CE(18:1(11Z)) ; Palmitoylstigmasterol ; Stearic acid ; Eicosadienoic acid ; 2-Methyl-4-heptanone ; Dihydroisophorol ; Ethyl hexadecanoate ; (Z)-3-Nonen-1-ol ; 16-Methylheptadecanoic acid ; 2-Nonanone ; 3-Nonanone ; Amyl propyl ketone ; 26-Dimethyl-4-heptanone ; 2-Methyloctanal ; 355-Trimethylhexanal ; 1-Nonen-3-ol ; Sclareol ; ()-26-Dimethyl-6-hepten-1-ol ; (Z)-6-Nonen-1-ol ; 2-Nonen-1-ol ; (Z)-2-Nonen-1-ol ; (E)-3-Nonen-1-ol ; Nonanal ; (E)-6-Nonenol ; Ethanolamine Oleate	M+H ; M+2H ; M+2H ; M+2H ; M+ACN+H ; M+NH4 ; 2M+ACN+H ; 2M+ACN+H ; M+ACN+H ; 2M+ACN+H ; M+ACN+H ; 2M+ACN+H ; 2M+ACN+H ; 2M+ACN+H ; 2M+ACN+H ; 2M+ACN+H ; 2M+ACN+H ; 2M+ACN+H ; M+NH4 ; 2M+ACN+H ; 2M+ACN+H ; 2M+ACN+H ; 2M+ACN+H ; 2M+ACN+H ; 2M+ACN+H ; 2M+ACN+H ; M+H-H2O	C20H39NO2 ; C45H78O2 ; C45H78O2 ; C45H78O2 ; C18H36O2 ; C20H36O2 ; C9H18O ; C9H18O ; C18H36O2 ; C9H18O ; C18H36O2 ; C9H18O ; C9H18O ; C9H18O ; C9H18O ; C9H18O ; C9H18O ; C9H18O ; C20H36O2 ; C9H18O ; C9H18O ; C9H18O ; C9H18O ; C9H18O ; C9H18O ; C9H18O ; C20H41NO3

385.25561	1.322543	0.046907	3.225	0.009098	2.0411	0.042748	385.2556	HMDB14453 ; HMDB10595 ; HMDB10651 ; HMDB10652 ; HMDB10665 ; HMDB10680 ; HMDB10575 ; HMDB10588 ; HMDB10589 ; HMDB10616 ; HMDB10631 ; HMDB10645 ; HMDB11538 ; HMDB11568 ; HMDB15195 ; HMDB32718	Ibutilide ; PG(16:1(9Z)20:4(5Z8Z11Z14Z)) ; PG(18:2(9Z12Z)18:3(6Z9Z12Z)) ; PG(18:2(9Z12Z)18:3(9Z12Z15Z)) ; PG(18:3(6Z9Z12Z)18:2(9Z12Z)) ; PG(18:3(9Z12Z15Z)18:2(9Z12Z)) ; PG(16:018:2(9Z12Z)) ; PG(16:1(9Z)18:1(11Z)) ; PG(16:1(9Z)18:1(9Z)) ; PG(18:1(11Z)16:1(9Z)) ; PG(18:1(9Z)16:1(9Z)) ; PG(18:2(9Z12Z)16:0) ; MG(0:018:2(9Z12Z)0:0) ; MG(18:2(9Z12Z)0:00:0) ; Oxybutynin ; Dihydroretrofractamide B	M+H ; M+2H ; M+2H ; M+2H ; M+2H ; M+2H ; M+H+Na ; M+H+Na ; M+H+Na ; M+H+Na ; M+H+Na ; M+H+Na ; 2M+Na+K ; 2M+Na+K ; 2M+3H2O+2H ; 2M+3H2O+2H	C20H36N2O3S ; C42H73O10P ; C42H73O10P ; C42H73O10P ; C42H73O10P ; C42H73O10P ; C40H75O10P ; C40H75O10P ; C40H75O10P ; C40H75O10P ; C40H75O10P ; C40H75O10P ; C21H38O4 ; C21H38O4 ; C22H31NO3 ; C22H31NO3
277.54841	1.40871	0.93674	-3.2243	0.009108	2.0406	0.042748	277.5484	HMDB11694 ; HMDB15151	SM(d16:124:1(15Z)) ; Glyburide	M+H+2Na ; M+Na+K	C45H90N2O6P ; C23H28ClN3O5S
193.51234	1.307944	0.866316	-3.2241	0.009112	2.0404	0.042748	193.5123	HMDB14096 ; HMDB33359 ; HMDB38584 ; HMDB39757	5-Hydroxypiroxicam ; Cryptodorine ; Piperolactam C ; 7-Chloro-6-demethylcepharadione B	M+H+K ; M+2K ; M+2K ; M+2Na	C15H13N3O5S ; C18H15NO4 ; C18H15NO4 ; C18H12ClNO4
186.07995	1.35711	0.871068	-3.224	0.009114	2.0403	0.042748	186.0758	HMDB00393 ; HMDB00522 ; HMDB01167 ; HMDB02266 ; HMDB06112 ; HMDB06240 ; HMDB13311 ; HMDB28962 ; HMDB29088 ; HMDB29171 ; HMDB29739 ; HMDB29784 ; HMDB31257 ; HMDB31647 ; HMDB33019 ; HMDB33110 ; HMDB33215 ; HMDB33220 ; HMDB33221 ; HMDB33644 ; HMDB34772 ; HMDB34988 ; HMDB35025 ; HMDB35043 ; HMDB35044 ; HMDB35048 ; HMDB35049 ; HMDB35502 ; HMDB35634 ; HMDB36232 ; HMDB36331 ; HMDB37004 ; HMDB37773 ; HMDB38080 ; HMDB38747 ; HMDB38980 ; HMDB38981 ; HMDB39055 ; HMDB39062 ; HMDB39622 ; HMDB40207 ; HMDB40944 ; HMDB60012	3-Hexenedioic acid ; 3-Methylglutaconic acid ; Pyruvaldehyde ; (E)-2-Methylglutaconic acid ; Malondialdehyde ; Demethylated antipyrine ; trans-2-Hexenedioic acid ; Lysyl-Tryptophan ; Tryptophyl-Lysine ; 3-Hydroxyadipic acid 36-lactone ; 1H-Indole-3-acetamide ; Unshuoside A ; Dimethyl fumarate ; Acrylic acid ; 7-Hydroxyterpineol 8-glucoside ; (1S2S4R)-18-Epoxy-p-menthan-2-ol glucoside ; 18-Epoxy-p-menthan-4-ol glucoside ; (1R2R4S6R)-26-Fenchanediol 2-O-b-D-glucoside ; (1R2S4R5S)-25-Fenchanediol 2-O-b-D-glucoside ; (1S2S4S5S)-24-Thujanediol 4-O-beta-D-Glucopyranoside ; (1R3S4S)-18-Epoxy-p-menthan-3-ol glucoside ; Gibberellin A60 ; 6Z-8-Hydroxygeraniol 8-O-glucoside ; Gibberellin A58 ; Gibberellin A63 ; Gibberellin A54 ; Gibberellin A67 ; Sambucus nigra Degraded cyanogenic glycosides (2-Epimer) ; Betulalbuside A ; Maleic acid homopolymer ; 6-O-Acetylholocalin ; Gibberellin A77 ; 8-Angeloylegelolide ; Gibberellin A81 ; (2E4E7R)-27-Dimethyl-24-octadiene-18-diol 8-O-b-D-glucopyranoside ; (4R6S)-p-Menth-1-ene-46-diol 4-glucoside ; (S)-p-Menth-1-ene-47-diol 4-glucoside ; (1S2R4R)-p-Menth-8-ene-210-diol 2-glucoside ; Triphasiol ; Gibberellin A90 ; Ethyl hydrogen fumarate ; 3-Epigibberellin A1 ; Neomenthol-glucuronide	M+ACN+H ; M+ACN+H ; 2M+ACN+H ; M+ACN+H ; 2M+ACN+H ; 2M+H+Na ; M+ACN+H ; M+H+K ; M+H+K ; M+ACN+H ; 2M+H+Na ; M+H+K ; M+ACN+H ; 2M+ACN+H ; M+H+K ; M+H+K ; M+H+K ; M+H+K ; M+H+K ; M+H+K ; M+H+K ; M+H+Na ; M+H+K ; M+H+Na ; M+H+Na ; M+H+Na ; M+H+Na ; M+H+NH4 ; M+H+K ; M+ACN+H ; M+H+NH4 ; M+H+Na ; M+H+Na ; M+H+Na ; M+H+K ; M+H+K ; M+H+K ; M+H+K ; M+H+Na ; M+H+Na ; M+ACN+H ; M+H+Na ; M+H+K	C6H8O4 ; C6H8O4 ; C3H4O2 ; C6H8O4 ; C3H4O2 ; C10H10N2O ; C6H8O4 ; C17H24N4O3 ; C17H24N4O3 ; C6H8O4 ; C10H10N2O ; C16H28O7 ; C6H8O4 ; C3H4O2 ; C16H28O7 ; C16H28O7 ; C16H28O7 ; C16H28O7 ; C16H28O7 ; C16H28O7 ; C16H28O7 ; C19H24O6 ; C16H28O7 ; C19H24O6 ; C19H24O6 ; C19H24O6 ; C19H24O6 ; C16H19NO8 ; C16H28O7 ; C6H8O4 ; C16H19NO8 ; C19H24O6 ; C19H24O6 ; C19H24O6 ; C16H28O7 ; C16H28O7 ; C16H28O7 ; C16H28O7 ; C19H24O6 ; C19H24O6 ; C6H8O4 ; C19H24O6 ; C16H28O7
548.95875	1.421181	0.477612	-3.2231	0.009127	2.0397	0.04277	548.9588	HMDB60636	Malathion dicarboxylic acid	2M+H	C6H11O6PS2
236.06417	1.30599	1.164778	3.2219	0.009145	2.0388	0.042779	236.0642	HMDB01212 ; HMDB03556 ; HMDB13855 ; HMDB29432 ; HMDB29981 ; HMDB32587 ; HMDB32729 ; HMDB32772 ; HMDB34322 ; HMDB34425 ; HMDB35796 ; HMDB40242 ; HMDB41363 ; HMDB41521 ; HMDB41946 ; HMDB42040 ; HMDB60033 ; HMDB60035 ; HMDB60036	Hydantoin-5-propionic acid ; Chitobiose ; Sulfamethoxazole N1-glucuronide ; (S)C(S)S-S-Methylcysteine sulfoxide ; 1-Isomangostin ; Pteroside Z ; Dulxanthone B ; Eremopetasitenin C3 ; L-alpha-Amino-5-oxo-2(5H)-isoxazolepropanoic acid ; ()-1-Methylpropyl 3-(methylthio)-2-propenyl disulfide ; alpha-Mangostin ; 3-Methyl-5-pentyl-124-trithiolane ; Secoeremopetasitolide B ; 3-Isomangostin ; N-Nitrosoproline ; Tipredane ; (beta-1-O-N-(2-hydroxymethyl-3-chlorophenyl)-anthraniloyl-D-glucupyranuronic acid) ; (beta-1-O-N-(2-methyl-3-chlorophenyl-4-hydroxy)anthraniloyl-D-glucupyranuronic acid) ; (beta-1-O-N-(2-methyl-3-chlorophenyl-5-hydroxy) anthraniloyl-D-glucupyranuronic acid	M+ACN+Na ; M+2Na ; M+ACN+2H ; 3M+H+NH4 ; M+Na+K ; M+2K ; M+Na+K ; M+Na+K ; M+ACN+Na ; 2M+3H2O+2H ; M+Na+K ; 2M+3H2O+2H ; M+2K ; M+Na+K ; 3M+H+K ; M+Na+K ; M+H+NH4 ; M+H+NH4 ; M+H+NH4	C6H8N2O4 ; C15H26N2O12 ; C16H19N3O9S ; C4H9NO3S ; C24H26O6 ; C21H30O7 ; C24H26O6 ; C21H30O6S ; C6H8N2O4 ; C8H16S3 ; C24H26O6 ; C8H16S3 ; C21H30O7 ; C24H26O6 ; C5H8N2O3 ; C22H31FO2S2 ; C20H20ClNO9 ; C20H20ClNO9 ; C20H20ClNO9
399.12930	1.393209	1.54432	-3.2219	0.009146	2.0388	0.042779	399.1293	HMDB29948 ; HMDB31401 ; HMDB61044 ; HMDB59972 ; HMDB59974	Scleroglucan ; Dihydroxycitracridone I ; Hydroxy Ritonavir ; 4-Hydroxy-5-(34-dihydroxyphenyl)-valeric acid-O-methyl-O-glucuronide ; 4-Hydroxy-5-(35-dihydroxyphenyl)-valeric acid-O-methyl-O-glucuronide	M+2ACN+2H ; 2M+H+Na ; M+Na+K ; M+H-2H2O ; M+H-2H2O	C24H44O20P2 ; C20H21NO7 ; C37H48N6O6S2 ; C18H26O12 ; C18H26O12

286.58922	1.39777	0.773787	3.2201	0.009174	2.0375	0.042867	286.5892	HMDB30457 ; HMDB30458 ; HMDB05357 ; HMDB05384 ; HMDB05425 ; HMDB05434 ; HMDB05438 ; HMDB10427 ; HMDB10432 ; HMDB10439 ; HMDB10442 ; HMDB10443 ; HMDB29494 ; HMDB31940 ; HMDB33008 ; HMDB37092 ; HMDB41642 ; HMDB41643 ; HMDB42066 ; HMDB42125 ; HMDB42154 ; HMDB42163 ; HMDB42169 ; HMDB42183 ; HMDB42197 ; HMDB42204 ; HMDB42211 ; HMDB42321 ; HMDB42424 ; HMDB42545 ; HMDB42604 ; HMDB42669 ; HMDB42755 ; HMDB42992 ; HMDB43076 ; HMDB43235 ; HMDB43237 ; HMDB43266 ; HMDB43403 ; HMDB43404 ; HMDB43461 ; HMDB43462 ; HMDB43831 ; HMDB43895 ; HMDB43901 ; HMDB43913 ; HMDB43927 ; HMDB43934 ; HMDB44043 ; HMDB44165 ; HMDB44278 ; HMDB44333 ; HMDB44394 ; HMDB44474 ; HMDB44677 ; HMDB44683 ;	Cyclochlorotine ; Islanditoxin ; TG(16:016:018:0)iso3 ; TG(16:018:1(9Z)18:2(9Z12Z))iso6 ; TG(16:1(9Z)18:018:2(9Z12Z))iso6 ; TG(16:1(9Z)16:1(9Z)20:1(11Z))iso3 ; TG(16:1(9Z)18:1(9Z)18:1(9Z))iso3 ; TG(18:014:018:0)iso3 ; TG(18:016:018:3(9Z12Z15Z))iso6 ; TG(18:1(11Z)16:018:2(9Z12Z))iso3 ; TG(18:1(11Z)16:1(9Z)18:1(11Z))iso3 ; TG(18:1(11Z)16:1(9Z)18:1(9Z))iso6 ; Formononetin 7-(6-methylmalonylglucoside) ; Arachisprenol 12 ; 4-O-Caffeoyl-3-O-feruloylquinic acid ; 3-Caffeoyl-4-feruloylquinic acid ; 1-Caffeoyl-5-feruloylquinic acid ; 1-Feruloyl-5-caffeoylquinic acid ; TG(14:014:022:0) ; TG(14:016:020:0) ; TG(14:018:018:0) ; TG(14:018:020:3(5Z8Z11Z)) ; TG(14:018:020:3n6) ; TG(14:020:016:0) ; TG(14:020:018:3(6Z9Z12Z)) ; TG(14:020:018:3(9Z12Z15Z)) ; TG(14:022:014:0) ; TG(14:016:1(9Z)22:2(13Z16Z)) ; TG(14:020:3(5Z8Z11Z)18:0) ; TG(14:018:3(6Z9Z12Z)20:0) ; TG(14:020:3n618:0) ; TG(14:022:2(13Z16Z)16:1(9Z)) ; TG(14:018:3(9Z12Z15Z)20:0) ; TG(15:015:020:0) ; TG(15:020:015:0) ; TG(15:018:1(11Z)18:2(9Z12Z)) ; TG(15:018:1(11Z)20:2n6) ; TG(15:018:1(9Z)20:2n6) ; TG(15:018:2(9Z12Z)18:1(11Z)) ; TG(15:018:2(9Z12Z)18:1(9Z)) ; TG(15:020:2n618:1(11Z)) ; TG(15:020:2n618:1(9Z)) ; TG(16:014:020:0) ; TG(16:016:020:3(5Z8Z11Z)) ; TG(16:016:020:3n6) ; TG(16:018:016:0) ; TG(16:018:018:3(6Z9Z12Z)) ; TG(16:018:018:3(9Z12Z15Z)) ; TG(16:014:1(9Z)22:2(13Z16Z)) ; TG(16:020:3(5Z8Z11Z)16:0) ; TG(16:018:3(6Z9Z12Z)18:0) ; TG(16:020:3n616:0) ; TG(16:022:2(13Z16Z)14:1(9Z)) ; TG(16:018:3(9Z12Z15Z)18:0) ; TG(18:014:020:3(5Z8Z11Z)) ; TG(18:014:020:3n6) ; TG(18:016:018:3(6Z9Z12Z)) ; TG(20:014:018:3(6Z9Z12Z)) ; TG(20:014:018:3(9Z12Z15Z)) ; TG(14:1(9Z)16:022:2(13Z16Z)) ; TG(14:1(9Z)14:1(9Z)24:1(15Z)) ; TG(14:1(9Z)16:1(9Z)22:1(13Z)) ; TG(14:1(9Z)18:1(11Z)20:1(11Z)) ; TG(14:1(9Z)18:1(9Z)20:1(11Z)) ; TG(14:1(9Z)20:1(11Z)18:1(11Z)) ; TG(14:1(9Z)20:1(11Z)18:1(9Z)) ; TG(14:1(9Z)22:1(13Z)16:1(9Z)) ; TG(14:1(9Z)24:1(15Z)14:1(9Z)) ; TG(16:1(9Z)14:022:2(13Z16Z)) ; TG(16:1(9Z)14:1(9Z)22:1(13Z)) ; TG(16:1(9Z)18:1(11Z)18:1(11Z)) ; TG(16:1(9Z)18:1(11Z)18:1(9Z)) ; TG(16:1(9Z)18:1(9Z)18:1(11Z)) ; TG(16:1(9Z)20:1(11Z)16:1(9Z)) ; TG(18:1(11Z)15:018:2(9Z12Z)) ; TG(18:1(11Z)15:020:2n6) ; TG(18:1(11Z)14:1(9Z)20:1(11Z)) ; TG(18:1(9Z)15:018:2(9Z12Z)) ; TG(18:1(9Z)15:020:2n6) ; TG(18:1(9Z)14:1(9Z)20:1(11Z)) ; TG(18:1(9Z)16:1(9Z)18:1(9Z)) ; Chondroitin 6-sulfate	M+2H ; M+2H ; M+2H+Na ; M+3H ; M+3H ; M+3H ; M+3H ; M+2H+Na ; M+3H ; M+3H ; M+3H ; M+3H ; M+ACN+2H ; M+2H+Na ; M+ACN+2H ; M+ACN+2H ; M+ACN+2H ; M+ACN+2H ; M+2H+Na ; M+2H+Na ; M+2H+Na ; M+3H ; M+3H ; M+2H+Na ; M+3H ; M+3H ; M+2H+Na ; M+3H ; M+3H ; M+3H ; M+3H ; M+3H ; M+3H ; M+2H+Na ; M+2H+Na ; M+3H ; M+3H ; M+3H ; M+3H ; M+3H ; M+3H ; M+3H ; M+2H+Na ; M+3H ; M+3H ; M+2H+Na ; M+3H ; M+2ACN+2H	C24H31Cl2N5O7 ; C24H31Cl2N5O7 ; C53H102O6 ; C55H100O6 ; C55H100O6 ; C55H100O6 ; C55H100O6 ; C53H102O6 ; C55H100O6 ; C55H100O6 ; C55H100O6 ; C55H100O6 ; C26H26O12 ; C60H98O ; C26H26O12 ; C26H26O12 ; C26H26O12 ; C26H26O12 ; C53H102O6 ; C53H102O6 ; C53H102O6 ; C55H100O6 ; C55H100O6 ; C53H102O6 ; C55H100O6 ; C55H100O6 ; C53H102O6 ; C55H100O6 ; C55H100O6 ; C55H100O6 ; C55H100O6 ; C55H100O6 ; C55H100O6 ; C53H102O6 ; C53H102O6 ; C55H100O6 ; C55H100O6 ; C55H100O6 ; C55H100O6 ; C55H100O6 ; C55H100O6 ; C55H100O6 ; C53H102O6 ; C55H100O6 ; C55H100O6 ; C53H102O6 ; C55H100O6 ; C16H27NO14S
99.531153	1.42307	0.218673	3.2194	0.009184	2.037	0.042876	NA	NA	NA	NA	NA
225.12328	1.386518	0.475719	-3.2141	0.009267	2.0331	0.043223	225.1233	HMDB14386 ; HMDB29347 ; HMDB29348 ; HMDB30421 ; HMDB39573 ; HMDB39749 ; HMDB00068 ; HMDB00512 ; HMDB00819 ; HMDB00860 ; HMDB00947 ; HMDB02042 ; HMDB02373 ; HMDB02832 ; HMDB15652 ; HMDB29455 ; HMDB29564 ; HMDB30572 ; HMDB30838 ; HMDB31181 ; HMDB31313 ; HMDB32044 ; HMDB32325 ; HMDB32448 ; HMDB32531 ; HMDB33551 ; HMDB33618 ; HMDB33736 ; HMDB33848 ; HMDB34134 ; HMDB34230 ; HMDB34456 ; HMDB34458 ; HMDB34460 ; HMDB34635 ; HMDB35802 ; HMDB36034 ; HMDB36229 ; HMDB38108 ; HMDB38237 ; HMDB38267 ; HMDB38523 ; HMDB39215 ; HMDB39274 ; HMDB39339 ; HMDB39356 ; HMDB39590 ; HMDB40171 ; HMDB40193 ; HMDB40266 ; HMDB41426 ; HMDB41427 ; HMDB41559 ; HMDB59831 ; HMDB59860 ; HMDB60608 ;	Butalbital ; Acuminoside ; Neryl arabinofuranosyl-glucoside ; L-Linalool 3-xylosyl-(1-6)-glucoside ; Geranyl arabinopyranosyl-glucoside ; Kenposide B ; Epinephrine ; N-Acetyl-L-phenylalanine ; Normetanephrine ; Phenylpropionylglycine ; Undecanoic acid ; 3-Phenylpropionylglycine ; 46-Dimethylnonanoic acid ; Methylnoradrenaline ; Levonordefrin ; Ginkgotoxin ; Bufotenine O-glucoside ; ()-Anisoxide ; Feniculin ; Homodihydrojasmone ; 2-Pentyl-3-phenyl-2-propenal ; Hexyl 3-methylbutanoate ; 2-Hexyl-45-dimethyl-13-dioxolane ; Octanal propyleneglycol acetal ; 2-trans-6-cis-Dodecadienal ; 5-Ethyl-3-hydroxy-4-methyl-2(5H)-furanone ; 3-Methylbutyl hexanoate ; Homofureanol ; Methyl decanoate ; Octyl propanoate ; Mesifurane ; 9-Methyldecanoic acid ; Isobutyl enanthate ; Heptyl butanoate ; Esculentoside E ; S-Japonin ; 66-Dimethoxy-255-trimethyl-2-hexene ; Pentyl hexanoate ; cis-Quinceoxepane ; xi-8-Methyldecanoic acid ; 77-Diethoxy-3-heptene ; Methyl 2-(10-heptadecenyl)-6-hydroxybenzoate ; Hexyl 2-methylbutanoate ; Dictagymnin ; Tricycloekasantalol ; Panaquinquecol 5 ; (2E4E)-24-Dodecadienal ; Heptyl 2-methylpropanoate ; Ethyl nonanoate ; Nonyl acetate ; Erinapyrone A ; Erinapyrone B ; Butyl heptanoate ; Propyl octanoate ; 2-Methylbutyl hexanoate ; N-isopropylterephthalamic acid ; Digoxigenin bisdigitoxoside	M+H ; M+2H ; M+2H ; M+2H ; M+2H ; M+2H ; M+ACN+H ; M+NH4 ; M+ACN+H ; M+NH4 ; M+K ; M+NH4 ; M+K ; M+ACN+H ; M+ACN+H ; M+ACN+H ; M+2ACN+2H ; M+Na ; M+Na ; M+2Na-H ; M+Na ; M+K ; M+K ; M+K ; M+2Na-H ; M+2ACN+H ; M+K ; M+2ACN+H ; M+K ; M+K ; M+2ACN+H ; M+K ; M+K ; M+K ; M+2H+Na ; 2M+3H ; M+K ; M+K ; M+2Na-H ; M+K ; M+K ; M+Na+K ; M+K ; M+Na ; M+2Na-H ; M+Na ; M+2Na-H ; M+K ; M+K ; M+K ; M+2ACN+H ; M+2ACN+H ; M+K ; M+K ; M+K ; M+NH4 ; M+2H+Na	C11H16N2O3 ; C21H36O10 ; C21H36O10 ; C21H36O10 ; C21H36O10 ; C21H36O10 ; C9H13NO3 ; C11H13NO3 ; C9H13NO3 ; C11H13NO3 ; C11H22O2 ; C11H13NO3 ; C11H22O2 ; C9H13NO3 ; C9H13NO3 ; C9H13NO3 ; C18H26N2O6 ; C14H18O ; C14H18O ; C12H20O ; C14H18O ; C11H22O2 ; C11H22O2 ; C11H22O2 ; C12H20O ; C7H10O3 ; C11H22O2 ; C7H10O3 ; C11H22O2 ; C11H22O2 ; C7H10O3 ; C11H22O2 ; C11H22O2 ; C11H22O2 ; C35H54O11 ; C19H28O3S ; C11H22O2 ; C11H22O2 ; C12H20O ; C11H22O2 ; C11H22O2 ; C25H40O3 ; C11H22O2 ; C14H18O ; C12H20O ; C14H18O ; C12H20O ; C11H22O2 ; C11H22O2 ; C11H22O2 ; C7H10O3 ; C7H10O3 ; C11H22O2 ; C11H22O2 ; C11H22O2 ; C11H13NO3 ; C35H54O11
566.27156	1.377767	0.463299	-3.2136	0.009276	2.0326	0.043224	566.2716	HMDB29437 ; HMDB30362 ; HMDB30549 ; HMDB30691 ; HMDB31355 ; HMDB32781 ; HMDB33943 ; HMDB34059 ; HMDB34339 ; HMDB34400 ; HMDB35596 ; HMDB38399 ; HMDB39497 ; HMDB40662 ; HMDB14465	Nopalinic acid ; Cavipetin B ; 56-Dihydro-4-methoxy-6-2-(4-methoxyphenyl)ethyl-2H-pyran-2-one ; Dihydrosuberenol ; 28-Hydroxywithanolide E ; Acetyljujuboside B ; Withaperuvin G ; 4beta-Hydroxywithanolide E ; Ixocarpalactone B ; Withaperuvin F ; Armexifolin ; Enokipodin D ; Dihydrowyerol ; Hoduloside X ; Dihydroergotamine	2M+ACN+H ; M+ACN+Na ; 2M+ACN+H ; 2M+ACN+H ; M+ACN+Na ; M+2Na ; M+ACN+Na ; M+ACN+Na ; M+ACN+Na ; M+ACN+Na ; 2M+ACN+H ; 2M+ACN+H ; 2M+ACN+H ; M+H+K ; M+H-H2O	C10H18N2O6 ; C28H38O8 ; C15H18O4 ; C15H18O4 ; C28H38O8 ; C54H86O22 ; C28H38O8 ; C28H38O8 ; C28H38O8 ; C28H38O8 ; C15H18O4 ; C15H18O4 ; C15H18O4 ; C53H88O23 ; C33H37N5O5
400.14511	1.372454	0.076994	-3.2101	0.00933	2.0301	0.043429	400.1451	HMDB31945 ; HMDB41970 ; HMDB06889 ; HMDB06896 ; HMDB15633 ; HMDB29207 ; HMDB29509 ; HMDB30701 ; HMDB37847 ; HMDB38552 ; HMDB38884 ; HMDB40351 ; HMDB41292 ; HMDB41908 ; HMDB60633	Niaziminin A ; Oxmetidine ; 3a7a12a-Trihydroxy-5b-cholest-24-enoyl-CoA ; 3a7a-Dihydroxy-5b-24-oxocholestanoyl-CoA ; Amisulpride ; lysoPC(6:0) ; Garcinone A ; 8-Desoxygartanin ; N-(1-Deoxy-1-fructosyl)tryptophan ; 6-Deoxy-gamma-mangostin ; Phomopsin B ; (E)-4-(37-Dimethyl-26-octadienyl)-135-trihydroxyxanthone ; Mangostinone ; Imidaprilat ; N-Acetyl gemifloxacin	M+H ; M+H ; M+3H ; M+3H ; 2M+Na+K ; M+2Na-H ; 2M+H+K ; 2M+H+K ; 2M+H+K ; 2M+H+K ; M+2Na ; 2M+H+K ; 2M+H+K ; M+Na ; M+H-H2O	C18H25NO7S ; C19H21N5O3S ; C48H78N7O20P3S ; C48H78N7O20P3S ; C17H27N3O4S ; C14H30NO7P ; C23H24O5 ; C23H24O5 ; C18H24N2O7 ; C23H24O5 ; C36H46N6O12 ; C23H24O5 ; C23H24O5 ; C18H23N3O6 ; C19H20FN5O5

274.09185	1.402708	0.962755	-3.2093	0.009343	2.0295	0.043429	274.0919	HMDB00101 ; HMDB01983 ; HMDB02123 ; HMDB03254 ; HMDB15285 ; HMDB15678 ; HMDB31360 ; HMDB32916 ; HMDB34751 ; HMDB36431 ; HMDB36610 ; HMDB37808 ; HMDB37809 ; HMDB38117 ; HMDB38662 ; HMDB39586 ; HMDB39800 ; HMDB40197 ; HMDB41566 ; HMDB60676 ; HMDB60701 ; HMDB60702	Deoxyadenosine ; 5-Deoxyadenosine ; 137-Trimethyluric acid ; Muramic acid ; Thiamylal ; Nepafenac ; L-cis-Cyclo(aspartylphenylalanyl) ; 2-(Methylthiomethyl)furan ; Ligustroside ; 139-Trimethyluric acid ; 2-Methyl-3-(methylthio)furan ; Hexahydro-24-dimethylspiro13-dithiolo45-cfuran-23(2H)-furan ; Hexahydro-23alpha-dimethylspiro13-dithiolo45-bfuran-23(2H)-furan ; 6-O-Oleuropeoylsucrose ; N-(1-Deoxy-1-fructosyl)alanine ; 2-(1-Mercaptoethyl)furan ; 5-Methyl-2-furanmethanethiol ; 25-Dimethyl-3-furanthiol ; 2-Methyl-5-(methylthio)furan ; 10-Hydroxycarbazepine ; (R)-MHD ; (S)-MHD	M+Na ; M+Na ; M+ACN+Na ; M+Na ; 2M+H+K ; 2M+H+K ; 2M+H+Na ; 2M+NH4 ; M+H+Na ; M+ACN+Na ; 2M+NH4 ; M+ACN+H ; M+ACN+H ; M+H+K ; M+Na ; 2M+NH4 ; 2M+NH4 ; 2M+NH4 ; 2M+NH4 ; 2M+H+K ; 2M+H+K ; 2M+H+K	C10H13N5O3 ; C10H13N5O3 ; C8H10N4O3 ; C9H17NO7 ; C12H18N2O2S ; C15H14N2O2 ; C13H14N2O4 ; C6H8OS ; C25H32O12 ; C8H10N4O3 ; C6H8OS ; C10H16O2S2 ; C10H16O2S2 ; C22H36O13 ; C9H17NO7 ; C6H8OS ; C6H8OS ; C6H8OS ; C6H8OS ; C15H14N2O2 ; C15H14N2O2 ; C15H14N2O2
205.50688	1.400489	0.114659	-3.2091	0.009346	2.0294	0.043429	205.5069	HMDB15093	Carboplatin	M+H+K	C6H12N2O4Pt
616.06535	1.451254	0.002508	-3.2083	0.009359	2.0288	0.043449	616.0654	HMDB06893 ; HMDB06953 ; HMDB31035 ; HMDB34454 ; HMDB36844 ; HMDB37752	3a7a-Dihydroxy-5b-cholestane ; 1-Phosphatidyl-D-myo-inositol ; 5-Heneicosyl-13-benzenediol ; 5-(4-Acetoxy-1-butynyl)-22-bithiophene ; Lepidiumterpenoid ; Gossypetin 8-glucuronide 3-sulfate	3M+H+NH4 ; 3M+Na+K ; 3M+H+NH4 ; 2M+ACN+Na ; 3M+H+NH4 ; M+ACN+H	C27H48O2 ; C11H19O13P ; C27H48O2 ; C14H12O2S2 ; C27H48O2 ; C21H18O17S
167.08680	1.308074	0.137458	-3.2069	0.009382	2.0277	0.043486	167.0868	HMDB34294 ; HMDB39104 ; HMDB15055 ; HMDB30787 ; HMDB34987 ; HMDB35041 ; HMDB35042 ; HMDB38014 ; HMDB06867 ; HMDB06869 ; HMDB12153 ; HMDB14829 ; HMDB30020 ; HMDB30035 ; HMDB30037 ; HMDB30038 ; HMDB30166 ; HMDB32353 ; HMDB38174 ; HMDB38705 ; HMDB40756 ; HMDB40980 ; HMDB59856 ; HMDB60384 ; HMDB30816 ; HMDB31663	1311-Tridecatriene-579-triyne ; (3E5Z)-135-Tridecatriene-7911-triyne ; Spectinomycin ; (R)-Marmin ; Gibberellin A61 ; Gibberellin A51 ; Gibberellin A45 ; Gibberellin A70 ; S-(3-Methylbutanoyl)-dihydrolipoamide-E ; S-(2-Methylbutanoyl)-dihydrolipoamide ; 34-Dihydroxybenzylamine ; Moexipril ; Withanolide B ; Euglobal IVa ; Euglobal VII ; Euglobal V ; Euglobal III ; Isopropyl citrate ; 5-Acetyl-24-dimethyloxazole ; Macrocarpal C ; 3-Hydroxy-68-dimethoxy-7(11)-eremophilen-128-olide ; Valdiate ; Ethyl 2-pyrrolecarboxylate ; 4-Acetamido-2-amino-6-nitrotoluene ; 6-Acetyl-22-dimethyl-2H-1-benzopyran ; 3-(3-Methylbutylidene)-1(3H)-isobenzofuranone	M+H ; M+H ; M+2H ; M+2H ; M+2H ; M+2H ; M+2H ; M+2H ; M+ACN+2H ; M+ACN+2H ; 2M+3H2O+2H ; M+3H ; M+H+2Na ; M+H+2Na ; M+H+2Na ; M+H+2Na ; M+H+2Na ; M+2ACN+2H ; 2M+3H2O+2H ; M+H+2Na ; M+H+Na ; M+H+Na ; 2M+3H2O+2H ; M+3ACN+2H ; M+H-2H2O ; M+H-2H2O	C13H10 ; C13H10 ; C14H24N2O7 ; C19H24O5 ; C19H24O5 ; C19H24O5 ; C19H24O5 ; C19H24O5 ; C13H25NO2S2 ; C13H25NO2S2 ; C7H9NO2 ; C27H34N2O7 ; C28H38O5 ; C28H38O5 ; C28H38O5 ; C28H38O5 ; C28H38O5 ; C10H18O7 ; C7H9NO2 ; C28H38O5 ; C17H26O5 ; C17H26O5 ; C7H9NO2 ; C9H11N3O3 ; C13H14O2 ; C13H14O2
158.04021	1.331432	1.63671	-3.2067	0.009384	2.0276	0.043486	158.0402	HMDB01285 ; HMDB10313 ; HMDB10314 ; HMDB15350 ; HMDB11106 ; HMDB12182 ; HMDB14356 ; HMDB14684 ; HMDB15256 ; HMDB29390 ; HMDB29443 ; HMDB32058 ; HMDB32349 ; HMDB33143 ; HMDB37817 ; HMDB38256 ; HMDB60568	Geranyl-PP ; 1-Salicylate glucuronide ; Beta-D-Glucopyranuronic acid ; Dantrolene ; 5-Amino-6-ribitylamino uracil ; 8-Hydroxypurine ; Midodrine ; Fluorouracil ; Tolbutamide ; L-Furosine ; L-Pyridosine ; Isoeugenol benzyl ether ; Isoeugenyl benzyl ether ; Pyrraline ; Aldosine ; Geranyl acetoacetate ; 1011-Dihydroxycarbamazepine	M+2H ; M+2H ; M+2H ; M+2H ; M+H+K ; 2M+H+K ; M+Na+K ; 2M+3H2O+2H ; M+2Na ; M+Na+K ; M+Na+K ; M+Na+K ; M+Na+K ; M+Na+K ; M+Na+K ; M+2K ; M+2Na	C10H20O7P2 ; C13H14O9 ; C13H14O9 ; C14H10N4O5 ; C9H16N4O6 ; C5H6N4O ; C12H18N2O4 ; C4H3FN2O2 ; C12H18N2O3S ; C12H18N2O4 ; C12H18N2O4 ; C17H18O2 ; C17H18O2 ; C12H18N2O4 ; C12H18N2O4 ; C14H22O3 ; C15H14N2O3
126.98957	1.391889	0.377234	3.206	0.009395	2.0271	0.043494	126.9896	HMDB32723	Menadiol disulfate	M+H+2Na	C11H10O8S2
515.21318	1.3925	1.309037	-3.2052	0.009409	2.0265	0.043519	515.2132	HMDB02865 ; HMDB29332 ; HMDB29777 ; HMDB29827 ; HMDB29949 ; HMDB33524 ; HMDB38451 ; HMDB38670 ; HMDB39385 ; HMDB40465 ; HMDB60577 ; HMDB60631 ; HMDB60718 ; HMDB60802 ; HMDB61047	Oxytocin ; 5-Methoxynoracronycine ; Cymorcin monoglucoside ; Junosidine ; Pangamic acid ; Lambertine ; Licoricesaponin A3 ; S-Methylmethionine ; 3-Hydroxy-4-isopropylbenzyl alcohol 3-glucoside ; Perilloside B ; Clomipramine N-oxide ; Hydroxyclomipramine ; 2-Hydroxyclomipramine ; 8-Hydroxyclomipramine ; simvastatin hydroxy acid	M+H+Na ; 3M+H+NH4 ; 3M+2Na ; 3M+H+NH4 ; M+2K+H ; 3M+H+NH4 ; M+2Na ; 3M+Na ; 3M+2Na ; 3M+2Na ; 3M+H+K ; 3M+H+K ; 3M+H+K ; 3M+H+K ; M+2K+H	C43H66N12O12S2 ; C20H19NO4 ; C16H24O7 ; C20H19NO4 ; C20H40N2O8 ; C20H19NO4 ; C48H72O21 ; C6H14NO2S ; C16H24O7 ; C16H24O7 ; C19H23ClN2O ; C19H23ClN2O ; C19H23ClN2O ; C19H23ClN2O ; C25H40O6
514.65830	1.354684	1.706621	3.2033	0.009438	2.0251	0.043615	514.6583	HMDB03952 ; HMDB10308 ; HMDB10309 ; HMDB10310 ; HMDB10311 ; HMDB11123 ; HMDB31839 ; HMDB35362 ; HMDB39556 ; HMDB40491 ; HMDB57714 ; HMDB57724 ; HMDB57828 ; HMDB59010 ; HMDB59020 ; HMDB59105	ciscis-36-Dodecadienoyl-CoA ; CL(22:6(4Z7Z10Z13Z16Z19Z)20:4(5Z8Z11Z14Z)18:3(6Z9Z12Z)18:2(9Z12Z)) ; CL(22:6(4Z7Z10Z13Z16Z19Z)20:4(5Z8Z11Z14Z)18:3(9Z12Z15Z)18:2(9Z12Z)) ; CL(22:6(4Z7Z10Z13Z16Z19Z)20:4(8Z11Z14Z17Z)18:3(6Z9Z12Z)18:2(9Z12Z)) ; CL(22:6(4Z7Z10Z13Z16Z19Z)20:4(8Z11Z14Z17Z)18:3(9Z12Z15Z)18:2(9Z12Z)) ; transcis-Lauro-26-dienoyl-CoA ; Ginsenoside F5 ; Notoginsenoside R2 ; Ginsenoside F3 ; Pseudoginsenoside RT3 ; CL(16:1(9Z)20:4(5Z8Z11Z14Z)20:4(5Z8Z11Z14Z)22:6(4Z7Z10Z13Z16Z19Z)) ; CL(16:1(9Z)20:4(5Z8Z11Z14Z)22:6(4Z7Z10Z13Z16Z19Z)20:4(5Z8Z11Z14Z)) ; CL(16:1(9Z)22:6(4Z7Z10Z13Z16Z19Z)20:4(5Z8Z11Z14Z)20:4(5Z8Z11Z14Z)) ; CL(20:4(5Z8Z11Z14Z)16:1(9Z)20:4(5Z8Z11Z14Z)22:6(4Z7Z10Z13Z16Z19Z)) ; CL(20:4(5Z8Z11Z14Z)16:1(9Z)22:6(4Z7Z10Z13Z16Z19Z)20:4(5Z8Z11Z14Z)) ; CL(20:4(5Z8Z11Z14Z)20:4(5Z8Z11Z14Z)22:6(4Z7Z10Z13Z16Z19Z)16:1(9Z))	M+2ACN+2H ; M+2H+Na ; M+2H+Na ; M+2H+Na ; M+2H+Na ; M+2ACN+2H ; 2M+3H ; 2M+3H ; 2M+3H ; 2M+3H ; M+2H+Na ; M+2H+Na ; M+2H+Na ; M+2H+Na ; M+2H+Na ; M+2H+Na	C33H54N7O17P3S ; C87H140O17P2 ; C87H140O17P2 ; C87H140O17P2 ; C87H140O17P2 ; C33H54N7O17P3S ; C41H70O13 ; C41H70O13 ; C41H70O13 ; C41H70O13 ; C87H140O17P2 ; C87H140O17P2 ; C87H140O17P2 ; C87H140O17P2 ; C87H140O17P2 ; C87H140O17P2
308.02704	1.362645	1.302665	-3.2023	0.009455	2.0243	0.043653	308.027	HMDB06211	2-(Formamido)-N1-(5-phospho-D-ribosyl)acetamidine	M+Na	C6H12N3O8P
270.96520	1.353966	0.355403	-3.1991	0.009507	2.022	0.043851	270.9652	HMDB02206 ; HMDB12931 ; HMDB33245 ; HMDB41646 ; HMDB41789	Molybdopterin ; Dopachrome o-semiquinone ; 6-(Hydroxymethyl)-24(1H3H)-pteridinedione ; 24-Dihydroxyacetophenone 5-sulfate ; Vanillin 4-sulfate	M+H+NH4 ; M+2K-H ; M+2K-H ; M+K ; M+K	C10H12MoN5O8PS2 ; C9H8NO4 ; C7H6N4O3 ; C8H8O6S ; C8H8O6S
153.03544	1.362803	0.388926	3.1969	0.009541	2.0204	0.043969	153.0354	HMDB29694 ; HMDB40631 ; HMDB02994 ; HMDB04136 ; HMDB11174 ; HMDB11175 ; HMDB14437 ; HMDB28915 ; HMDB28937	S-Methyl benzenecarbothioate ; Pratenol B ; Erythritol ; D-Threitol ; L-isoleucyl-L-proline ; L-leucyl-L-proline ; Etomidate ; Isoleucyl-Proline ; Leucyl-Proline	M+H ; M+2H ; 2M+Na+K ; 2M+Na+K ; M+2K ; M+2K ; M+Na+K ; M+2K ; M+2K	C8H8OS ; C15H12O7 ; C4H10O4 ; C4H10O4 ; C11H20N2O3 ; C11H20N2O3 ; C14H16N2O2 ; C11H20N2O3 ; C11H20N2O3
145.55037	1.361746	0.884707	-3.196	0.009556	2.0197	0.043996	145.5504	HMDB14395 ; HMDB15599 ; HMDB33531 ; HMDB37770 ; HMDB39106 ; HMDB60816	Dapsone ; Enoximone ; 2468-Decatetraenoic acid dehydropiperidide ; Neopellitorine A ; 24-Undecadiene-810-diynoic acid isobutylamide ; Didesmethyl doxepin	M+ACN+2H ; M+ACN+2H ; M+Na+K ; M+Na+K ; M+Na+K ; M+H+K	C12H12N2O2S ; C12H12N2O2S ; C15H19NO ; C15H19NO ; C15H19NO ; C17H17NO

214.83599	1.360532	1.72463	3.1954	0.009565	2.0193	0.043999	NA	NA	NA	NA	NA
483.99444	1.322114	0.788205	-3.1941	0.009587	2.0183	0.044058	483.9944	HMDB00082 ; HMDB10229 ; HMDB10231 ; HMDB10233 ; HMDB10235 ; HMDB10236 ; HMDB10240 ; HMDB10692 ; HMDB15039 ; HMDB56394 ; HMDB56395 ; HMDB56420 ; HMDB56421 ; HMDB56423 ; HMDB56430 ; HMDB56431 ; HMDB56433 ; HMDB56450 ; HMDB56451 ; HMDB56453 ; HMDB56457 ; HMDB56467 ; HMDB56565 ; HMDB56580 ; HMDB56581 ; HMDB56583 ; HMDB56595 ; HMDB56605 ; HMDB56619 ; HMDB56637 ; HMDB56639 ; HMDB56651 ; HMDB56661 ; HMDB56675 ; HMDB56703 ; HMDB56714 ; HMDB56736 ; HMDB56746 ; HMDB56750 ; HMDB56757 ; HMDB56971 ; HMDB57049 ; HMDB57058 ; HMDB57066 ; HMDB57090 ; HMDB57375 ; HMDB57429 ; HMDB57444 ; HMDB57452 ; HMDB57476 ; HMDB57493 ; HMDB57494 ; HMDB57503 ; HMDB57504 ; HMDB57529 ; HMDB57530 ;	Cytidine triphosphate ; CL(18:2(9Z12Z)18:1(11Z)18:1(11Z)16:1(9Z)) ; CL(18:2(9Z12Z)18:1(11Z)18:1(9Z)16:1(9Z)) ; CL(18:2(9Z12Z)18:1(9Z)18:1(11Z)16:1(9Z)) ; CL(18:2(9Z12Z)18:1(9Z)18:1(9Z)16:1(9Z)) ; CL(18:2(9Z12Z)18:2(9Z12Z)18:1(11Z)16:0) ; CL(18:2(9Z12Z)18:2(9Z12Z)18:1(9Z)16:0) ; CL(18:2(9Z12Z)18:2(9Z12Z)18:2(9Z12Z)18:2(9Z12Z)) ; Ethacrynic acid ; CL(16:016:016:022:5(4Z7Z10Z13Z16Z)) ; CL(16:016:016:022:5(7Z10Z13Z16Z19Z)) ; CL(16:016:018:1(11Z)18:1(11Z)) ; CL(16:016:018:1(11Z)18:1(9Z)) ; CL(16:016:018:1(11Z)20:4(5Z8Z11Z14Z)) ; CL(16:016:018:1(9Z)18:1(11Z)) ; CL(16:016:018:1(9Z)18:1(9Z)) ; CL(16:016:018:1(9Z)20:4(5Z8Z11Z14Z)) ; CL(16:016:020:4(5Z8Z11Z14Z)18:1(11Z)) ; CL(16:016:020:4(5Z8Z11Z14Z)18:1(9Z)) ; CL(16:016:020:4(5Z8Z11Z14Z)20:4(5Z8Z11Z14Z)) ; CL(16:016:022:5(4Z7Z10Z13Z16Z)16:0) ; CL(16:016:022:5(7Z10Z13Z16Z19Z)16:0) ; CL(16:016:1(9Z)18:2(9Z12Z)18:2(9Z12Z)) ; CL(16:018:1(11Z)16:018:1(11Z)) ; CL(16:018:1(11Z)16:018:1(9Z)) ; CL(16:018:1(11Z)16:020:4(5Z8Z11Z14Z)) ; CL(16:018:1(11Z)18:1(11Z)16:0) ; CL(16:018:1(11Z)18:1(9Z)16:0) ; CL(16:018:1(11Z)20:4(5Z8Z11Z14Z)16:0) ; CL(16:018:1(9Z)16:018:1(9Z)) ; CL(16:018:1(9Z)16:020:4(5Z8Z11Z14Z)) ; CL(16:018:1(9Z)18:1(11Z)16:0) ; CL(16:018:1(9Z)18:1(9Z)16:0) ; CL(16:018:1(9Z)20:4(5Z8Z11Z14Z)16:0) ; CL(16:018:2(9Z12Z)16:1(9Z)18:2(9Z12Z)) ; CL(16:018:2(9Z12Z)18:2(9Z12Z)16:1(9Z)) ; CL(16:020:4(5Z8Z11Z14Z)16:020:4(5Z8Z11Z14Z)) ; CL(16:020:4(5Z8Z11Z14Z)18:1(11Z)16:0) ; CL(16:020:4(5Z8Z11Z14Z)18:1(9Z)16:0) ; CL(16:020:4(5Z8Z11Z14Z)20:4(5Z8Z11Z14Z)16:0) ; CL(18:018:016:1(9Z)16:1(9Z)) ; CL(18:016:1(9Z)18:016:1(9Z)) ; CL(18:016:1(9Z)16:1(9Z)18:0) ; CL(18:016:1(9Z)16:1(9Z)22:6(4Z7Z10Z13Z16Z19Z)) ; CL(18:016:1(9Z)22:6(4Z7Z10Z13Z16Z19Z)16:1(9Z)) ; CL(18:022:6(4Z7Z10Z13Z16Z19Z)16:1(9Z)16:1(9Z)) ; CL(16:1(9Z)16:018:2(9Z12Z)18:2(9Z12Z)) ; CL(16:1(9Z)18:016:1(9Z)18:0) ; CL(16:1(9Z)18:016:1(9Z)22:6(4Z7Z10Z13Z16Z19Z)) ; CL(16:1(9Z)18:022:6(4Z7Z10Z13Z16Z19Z)16:1(9Z)) ; CL(16:1(9Z)16:1(9Z)18:1(11Z)22:5(4Z7Z10Z13Z16Z)) ; CL(16:1(9Z)16:1(9Z)18:1(11Z)22:5(7Z10Z13Z16Z19Z)) ;	M+H ; M+2H+Na ; M+2H+Na ; M+2H+Na ; M+2H+Na ; M+2H+Na ; M+2H+Na ; M+3H ; 3M+Na+K ; M+2H+Na ; M+2H+Na ; M+H+2Na ; M+H+2Na ; M+2H+Na ; M+H+2Na ; M+H+2Na ; M+2H+Na ; M+2H+Na ; M+2H+Na ; M+3H ; M+2H+Na ; M+2H+Na ; M+2H+Na ; M+H+2Na ; M+H+2Na ; M+2H+Na ; M+H+2Na ; M+H+2Na ; M+2H+Na ; M+H+2Na ; M+2H+Na ; M+H+2Na ; M+H+2Na ; M+2H+Na ; M+2H+Na ; M+2H+Na ; M+3H ; M+2H+Na ; M+2H+Na ; M+3H ; M+H+2Na ; M+H+2Na ; M+H+2Na ; M+3H ; M+3H ; M+3H ; M+2H+Na ; M+H+2Na ; M+3H ; M+3H ; M+3H ; M+3H ; M+3H ; M+3H ; M+3H ; M+3H ; M+3H ; M+3H ; M+3H ; M+3H ; M+3H ; M+3H ; M+3H ; M+3H ; M+3H ; M+3H ; M+3H ; M+2H+Na ; M+3H ; M+3H ; M+3H ; M+3H ; M+H+2Na ; M+H+2Na ; M+2H+Na ; M+3H ; M+3H ; M+H+2Na ; M+2H+Na ; M+3H ; M+3H ; M+2H+Na ; M+3H	C9H16N3O14P3 ; C79H144O17P2 ; C79H144O17P2 ; C79H144O17P2 ; C79H144O17P2 ; C79H144O17P2 ; C79H144O17P2 ; C81H142O17P2 ; C13H12Cl2O4 ; C79H144O17P2 ; C79H144O17P2 ; C77H146O17P2 ; C77H146O17P2 ; C79H144O17P2 ; C77H146O17P2 ; C77H146O17P2 ; C79H144O17P2 ; C79H144O17P2 ; C79H144O17P2 ; C81H142O17P2 ; C79H144O17P2 ; C79H144O17P2 ; C79H144O17P2 ; C77H146O17P2 ; C77H146O17P2 ; C79H144O17P2 ; C77H146O17P2 ; C77H146O17P2 ; C79H144O17P2 ; C77H146O17P2 ; C79H144O17P2 ; C77H146O17P2 ; C77H146O17P2 ; C79H144O17P2 ; C79H144O17P2 ; C79H144O17P2 ; C81H142O17P2 ; C79H144O17P2 ; C79H144O17P2 ; C81H142O17P2 ; C77H146O17P2 ; C77H146O17P2 ; C77H146O17P2 ; C81H142O17P2 ; C81H142O17P2 ; C81H142O17P2 ; C79H144O17P2 ; C77H146O17P2 ; C81H142O17P2 ; C81H142O17P2 ; C81H142O17P2 ; C81H142O17P2 ; C81H142O17P2 ; C81H142O17P2 ; C81H142O17P2 ; C81H142O17P2 ; C81H142O17P2 ; C81H142O17P2 ; C81H142O17P2 ; C81H142O17P2 ; C81H142O17P2 ; C81H142O17P2 ; C81H142O17P2 ; C81H142O17P2 ; C79H144O17P2 ; C81H142O17P2 ; C81H142O17P2 ; C81H142O17P2 ; C81H142O17P2 ; C77H146O17P2 ; C77H146O17P2 ; C79H144O17P2 ; C81H142O17P2 ; C81H142O17P2 ; C77H146O17P2 ; C79H144O17P2 ; C81H142O17P2 ; C81H142O17P2 ; C79H144O17P2 ; C81H142O17P2
490.65233	1.340449	1.534661	3.1909	0.009639	2.016	0.044242	490.6523	HMDB06487 ; HMDB10237 ; HMDB10241 ; HMDB10244 ; HMDB10248 ; HMDB10249 ; HMDB14927 ; HMDB34538 ; HMDB35006 ; HMDB37206 ; HMDB56396 ; HMDB56414 ; HMDB56415 ; HMDB56459 ; HMDB56469 ; HMDB56477 ; HMDB56539 ; HMDB56540 ; HMDB56557 ; HMDB56558 ; HMDB56561 ; HMDB56562 ; HMDB56568 ; HMDB56569 ; HMDB56573 ; HMDB56593 ; HMDB56594 ; HMDB56597 ; HMDB56607 ; HMDB56649 ; HMDB56650 ; HMDB56653 ; HMDB56663 ; HMDB56745 ; HMDB56759 ; HMDB56785 ; HMDB56837 ; HMDB57060 ; HMDB57061 ; HMDB57063 ; HMDB57068 ; HMDB57072 ; HMDB57079 ; HMDB57100 ; HMDB57152 ; HMDB57242 ; HMDB57421 ; HMDB57422 ; HMDB57425 ; HMDB57426 ; HMDB57432 ; HMDB57433 ; HMDB57437 ; HMDB57446 ; HMDB57447 ; HMDB57449 ;	Pentaglutamyl folate ; CL(18:2(9Z12Z)18:2(9Z12Z)18:1(11Z)16:1(9Z)) ; CL(18:2(9Z12Z)18:2(9Z12Z)18:1(9Z)16:1(9Z)) ; CL(18:2(9Z12Z)18:2(9Z12Z)18:2(9Z12Z)16:0) ; CL(18:2(9Z12Z)18:2(9Z12Z)18:2(9Z12Z)18:3(6Z9Z12Z)) ; CL(18:2(9Z12Z)18:2(9Z12Z)18:2(9Z12Z)18:3(9Z12Z15Z)) ; Gadopentetate dimeglumine ; Mubenin B ; Isovitexin 7-(6-sinapoylglucoside) 4-glucoside ; Isoscoparin 2-(6-(E)-feruloylglucoside) 4-glucoside ; CL(16:016:016:022:6(4Z7Z10Z13Z16Z19Z)) ; CL(16:016:016:1(9Z)22:5(4Z7Z10Z13Z16Z)) ; CL(16:016:016:1(9Z)22:5(7Z10Z13Z16Z19Z)) ; CL(16:016:022:5(4Z7Z10Z13Z16Z)16:1(9Z)) ; CL(16:016:022:5(7Z10Z13Z16Z19Z)16:1(9Z)) ; CL(16:016:022:6(4Z7Z10Z13Z16Z19Z)16:0) ; CL(16:016:1(9Z)16:022:5(4Z7Z10Z13Z16Z)) ; CL(16:016:1(9Z)16:022:5(7Z10Z13Z16Z19Z)) ; CL(16:016:1(9Z)18:1(11Z)18:1(11Z)) ; CL(16:016:1(9Z)18:1(11Z)18:1(9Z)) ; CL(16:016:1(9Z)18:1(9Z)18:1(11Z)) ; CL(16:016:1(9Z)18:1(9Z)18:1(9Z)) ; CL(16:016:1(9Z)20:4(5Z8Z11Z14Z)20:4(5Z8Z11Z14Z)) ; CL(16:016:1(9Z)22:5(4Z7Z10Z13Z16Z)16:0) ; CL(16:016:1(9Z)22:5(7Z10Z13Z16Z19Z)16:0) ; CL(16:018:1(11Z)16:1(9Z)18:1(11Z)) ; CL(16:018:1(11Z)16:1(9Z)18:1(9Z)) ; CL(16:018:1(11Z)18:1(11Z)16:1(9Z)) ; CL(16:018:1(11Z)18:1(9Z)16:1(9Z)) ; CL(16:018:1(9Z)16:1(9Z)18:1(11Z)) ; CL(16:018:1(9Z)16:1(9Z)18:1(9Z)) ; CL(16:018:1(9Z)18:1(11Z)16:1(9Z)) ; CL(16:018:1(9Z)18:1(9Z)16:1(9Z)) ; CL(16:020:4(5Z8Z11Z14Z)16:1(9Z)20:4(5Z8Z11Z14Z)) ; CL(16:020:4(5Z8Z11Z14Z)20:4(5Z8Z11Z14Z)16:1(9Z)) ; CL(16:022:5(4Z7Z10Z13Z16Z)16:1(9Z)16:0) ; CL(16:022:5(7Z10Z13Z16Z19Z)16:1(9Z)16:0) ; CL(18:016:1(9Z)16:1(9Z)18:1(11Z)) ; CL(18:016:1(9Z)16:1(9Z)18:1(9Z)) ; CL(18:016:1(9Z)16:1(9Z)20:4(5Z8Z11Z14Z)) ; CL(18:016:1(9Z)18:1(11Z)16:1(9Z)) ; CL(18:016:1(9Z)18:1(9Z)16:1(9Z)) ; CL(18:016:1(9Z)20:4(5Z8Z11Z14Z)16:1(9Z)) ; CL(18:018:1(11Z)16:1(9Z)16:1(9Z)) ; CL(18:018:1(9Z)16:1(9Z)16:1(9Z)) ; CL(18:020:4(5Z8Z11Z14Z)16:1(9Z)16:1(9Z)) ; CL(16:1(9Z)16:018:1(11Z)18:1(11Z)) ; CL(16:1(9Z)16:018:1(11Z)18:1(9Z)) ; CL(16:1(9Z)16:018:1(9Z)18:1(11Z)) ; CL(16:1(9Z)16:018:1(9Z)18:1(9Z)) ; CL(16:1(9Z)16:020:4(5Z8Z11Z14Z)20:4(5Z8Z11Z14Z)) ; CL(16:1(9Z)16:022:5(4Z7Z10Z13Z16Z)16:0) ; CL(16:1(9Z)16:022:5(7Z10Z13Z16Z19Z)16:0) ; CL(16:1(9Z)18:016:1(9Z)18:1(11Z)) ; CL(16:1(9Z)18:016:1(9Z)18:1(9Z)) ; CL(16:1(9Z)18:016:1(9Z)20:4(5Z8Z11Z14Z)) ;	M+H+Na ; M+H+2Na ; M+H+2Na ; M+H+2Na ; M+2H+Na ; M+2H+Na ; M+ACN+2H ; 2M+3H ; M+H+NH4 ; M+H+NH4 ; M+H+2Na ; M+H+2Na ; M+H+2Na ; M+H+2Na ; M+H+2Na ; M+H+2Na ; M+H+2Na ; M+H+2Na ; M+3Na ; M+3Na ; M+3Na ; M+3Na ; M+2H+Na ; M+H+2Na ; M+H+2Na ; M+3Na ; M+3Na ; M+3Na ; M+3Na ; M+3Na ; M+3Na ; M+3Na ; M+3Na ; M+2H+Na ; M+2H+Na ; M+H+2Na ; M+H+2Na ; M+3Na ; M+3Na ; M+H+2Na ; M+3Na ; M+3Na ; M+H+2Na ; M+3Na ; M+3Na ; M+H+2Na ; M+3Na ; M+3Na ; M+3Na ; M+3Na ; M+2H+Na ; M+H+2Na ; M+H+2Na ; M+3Na ; M+3Na ; M+H+2Na ; M+3Na ; M+3Na ; M+H+2Na ; M+3Na ; M+2H+Na ; M+3Na ; M+2H+Na ; M+H+2Na ; M+H+2Na ; M+3H ; M+2H+Na ; M+2H+Na ; M+3H ; M+2H+Na ; M+3Na ; M+3Na ; M+2H+Na ; M+2H+Na ; M+3Na ; M+3Na ; M+2H+Na ; M+H+2Na ; M+H+2Na ; M+3H ; M+2H+Na ; M+3H ; M+2H+Na ; M+2H+Na ; M+3H ; M+3Na ; M+3Na ; M+3Na ; M+2H+Na ; M+3Na ; M+2H+Na ; M+H+2Na ; M+2H+Na ; M+3H	C39H47N11O18 ; C79H142O17P2 ; C79H142O17P2 ; C79H142O17P2 ; C81H140O17P2 ; C81H140O17P2 ; C28H54GdN5O20 ; C41H66O11 ; C44H50O24 ; C44H50O24 ; C79H142O17P2 ; C79H142O17P2 ; C79H142O17P2 ; C79H142O17P2 ; C79H142O17P2 ; C79H142O17P2 ; C79H142O17P2 ; C79H142O17P2 ; C77H144O17P2 ; C77H144O17P2 ; C77H144O17P2 ; C77H144O17P2 ; C81H140O17P2 ; C79H142O17P2 ; C79H142O17P2 ; C77H144O17P2 ; C77H144O17P2 ; C77H144O17P2 ; C77H144O17P2 ; C77H144O17P2 ; C77H144O17P2 ; C77H144O17P2 ; C77H144O17P2 ; C81H140O17P2 ; C81H140O17P2 ; C79H142O17P2 ; C79H142O17P2 ; C77H144O17P2 ; C77H144O17P2 ; C79H142O17P2 ; C77H144O17P2 ; C77H144O17P2 ; C79H142O17P2 ; C77H144O17P2 ; C77H144O17P2 ; C79H142O17P2 ; C77H144O17P2 ; C77H144O17P2 ; C77H144O17P2 ; C77H144O17P2 ; C81H140O17P2 ; C79H142O17P2 ; C79H142O17P2 ; C77H144O17P2 ; C77H144O17P2 ; C79H142O17P2 ; C77H144O17P2 ; C77H144O17P2 ; C79H142O17P2 ; C77H144O17P2 ; C81H140O17P2 ; C77H144O17P2 ; C81H140O17P2 ; C79H142O17P2 ; C79H142O17P2 ; C83H138O17P2 ; C81H140O17P2 ; C81H140O17P2 ; C83H138O17P2 ; C81H140O17P2 ; C77H144O17P2 ; C77H144O17P2 ; C81H140O17P2 ; C81H140O17P2 ; C77H144O17P2 ; C77H144O17P2 ; C81H140O17P2 ; C79H142O17P2 ; C79H142O17P2 ; C83H138O17P2 ; C81H140O17P2 ; C83H138O17P2 ; C81H140O17P2 ; C81H140O17P2 ;
160.01758	1.347013	1.663712	-3.1906	0.009645	2.0157	0.044242	160.0176	HMDB14737 ; HMDB14802 ; HMDB33506 ; HMDB33508 ; HMDB40229	Thiopental ; Sulfametopyrazine ; Gonyautoxin I ; Gonyautoxin IV ; 1H-Pyrrolo21-c14thiazine	M+2K ; M+H+K ; M+3Na ; M+3Na ; M+Na	C11H18N2O2S ; C11H12N4O3S ; C10H17N7O9S ; C10H17N7O9S ; C7H7NS
790.53836	1.419129	0.373339	3.1897	0.009659	2.015	0.044248	790.5384	HMDB01916 ; HMDB12332 ; HMDB12352 ; HMDB14352 ; HMDB30493 ; HMDB38065	Azythromycin ; PS(14:016:0) ; PS(16:014:0) ; Azithromycin ; Spirolide D ; 128-Octacosanediol diferulate	M+ACN+H ; M+2ACN+H ; M+2ACN+H ; M+ACN+H ; M+2ACN+H ; 2M+H+Na	C38H72N2O12 ; C36H70NO10P ; C36H70NO10P ; C38H72N2O12 ; C43H65NO7 ; C48H74O8
263.00237	1.338436	0.634253	-3.189	0.009671	2.0145	0.044248	263.0024	HMDB37323 ; HMDB40192	23-Dihydro-5577-tetrahydroxy-2-(4-hydroxyphenyl)38-bi-4H-1-benzopyran-44-dione ; N-Nitrosothiazolidine-4-carboxylic acid	M+2K ; 3M+H+K	C24H16O9 ; C4H6N2O3S
367.96372	1.37309	1.085117	3.1881	0.009686	2.0139	0.044248	367.9637	HMDB15420 ; HMDB52163 ; HMDB52195 ; HMDB52238 ; HMDB52240 ; HMDB52611 ; HMDB53449	Quinethazone ; TG(24:1(15Z)18:2(9Z12Z)22:2(13Z16Z)) ; TG(24:1(15Z)20:2n622:2(13Z16Z)) ; TG(24:1(15Z)22:2(13Z16Z)18:2(9Z12Z)) ; TG(24:1(15Z)22:2(13Z16Z)20:2n6) ; TG(18:2(9Z12Z)24:1(15Z)22:2(13Z16Z)) ; TG(20:2n624:1(15Z)22:2(13Z16Z))	M+2K+H ; M+3Na ; M+3Na ; M+3Na ; M+3Na ; M+3Na ; M+3Na	C10H12ClN3O3S ; C68H122O6 ; C68H122O6 ; C68H122O6 ; C68H122O6 ; C68H122O6 ; C68H122O6
258.00567	1.339378	0.444715	-3.1878	0.00969	2.0137	0.044248	NA	NA	NA	NA	NA
88.509291	1.374409	1.040415	3.1878	0.00969	2.0137	0.044248	NA	NA	NA	NA	NA

369.11830	1.364239	1.195711	-3.1837	0.009759	2.0106	0.04452	369.1183	HMDB30669 ; HMDB39959 ; HMDB39960 ; HMDB01261 ; HMDB02158 ; HMDB29330 ; HMDB32401 ; HMDB32742 ; HMDB35057 ; HMDB36562 ; HMDB40601 ; HMDB40602 ; HMDB41189 ; HMDB41537 ; HMDB60343 ; HMDB41175	3-O-Feruloylquinic acid ; 3-O-Caffeoyl-1-O-methylquinic acid ; 3-O-Caffeoyl-4-O-methylquinic acid ; Coproporphyrinogen III ; Coproporphyrinogen I ; Gravacridonetriol ; (-)-3-(2-methyl-3-furyl)thio-2-butanone ; Di-O-methylcrenatin ; 1-(3-Hydroxy-4-Methoxyphenyl)-12-ethanediol 3-O-b-D-glucoside ; Aucubin ; (1xi2xi)-1-(4-Hydroxyphenyl)-123-propanetriol 2-O-beta-D-glucopyranoside ; (1xi2xi)-1-(4-Hydroxyphenyl)-123-propanetriol 3-O-beta-D-Glucopyranoside ; 345-Trimethoxyphenyl glucoside ; N-Acetyldehydroanonaine ; 2-(S-Glutathionyl)acetyl glutathione ; 1-O-Sinapoylglucose	M+H ; M+H ; M+H ; M+2K ; M+2K ; 2M+H+Na ; 2M+H ; M+Na ; M+Na ; M+Na ; M+Na ; M+Na ; M+Na ; M+ACN+Na ; M+2ACN+2H ; M+H-H2O	C17H20O9 ; C17H20O9 ; C17H20O9 ; C36H44N4O8 ; C36H44N4O8 ; C19H19NO6 ; C9H12O2S ; C15H22O9 ; C15H22O9 ; C15H22O9 ; C15H22O9 ; C15H22O9 ; C15H22O9 ; C19H15NO3 ; C22H34N6O13S2 ; C17H22O10
454.16194	1.357611	1.146064	-3.1818	0.009789	2.0093	0.044575	454.1619	HMDB13842 ; HMDB14902 ; HMDB30329 ; HMDB35876 ; HMDB41891 ; HMDB60795	O-Deethylated candesartan ; Mometasone ; Neopetasitenine ; Mesuagin ; Famprofazone ; 6a 3-p-Dihydroxypaclitaxel	M+ACN+H ; 2M+3H2O+2H ; 2M+Na+K ; M+ACN+Na ; M+2K-H ; M+H+NH4	C22H16N6O3 ; C22H28Cl2O4 ; C21H29NO8 ; C24H22O5 ; C24H31N3O ; C45H47NO18
453.96011	1.327935	1.611449	-3.1792	0.009832	2.0074	0.044732	NA	NA	NA	NA	NA
450.66474	1.352699	1.681096	3.1784	0.009845	2.0068	0.04474	450.6647	HMDB07858 ; HMDB07859 ; HMDB10366 ; HMDB31130	PA(16:018:1(11Z)) ; PA(16:018:1(9Z)) ; Hyaluronan ; 9-Octadecenoic acid 1-(1-oxohexadecyl)oxymethyl-2-(phosphonoxy)ethyl ester	2M+3H ; 2M+3H ; M+3ACN+2H ; 2M+3H	C37H71O8P ; C37H71O8P ; C28H44N2O23 ; C37H71O8P
314.77913	1.344826	1.715596	3.1781	0.009852	2.0065	0.04474	314.7791	HMDB33753 ; HMDB38058	Glycerol 13-didodecanoate 2-decanoate ; Glycerol 1-dodecanoate 2-tetradecanoate 3-octanoate	M+H+NH4 ; M+H+NH4	C37H70O6 ; C37H70O6
618.07615	1.423185	0.751616	-3.1742	0.009917	2.0036	0.044953	618.0762	HMDB60056	CE(15:1)	2M+H+NH4	C42H72O2
106.06845	1.358683	1.296963	3.1721	0.009952	2.0021	0.045071	106.0685	HMDB01505 ; HMDB02203 ; HMDB10725 ; HMDB15382 ; HMDB29846 ; HMDB30185 ; HMDB31653 ; HMDB33201 ; HMDB33574 ; HMDB33640 ; HMDB34783 ; HMDB36328 ; HMDB38186 ; HMDB39470 ; HMDB39668 ; HMDB39669 ; HMDB39807 ; HMDB39893 ; HMDB40329 ; HMDB41600 ; HMDB59843	Benzene ; 3-Hydroxycapric acid ; (R)-3-Hydroxydecanoic acid ; Mitiglinide ; Nonivamide ; (E)-Piperolein A ; 3-(Methylthio)-1-propene ; 9-Hydroxydecanoic acid ; (1R2R4R8R)-p-Menthane-289-triol ; 7-Methyl-3-methylene-167-octanetriol ; cis-p-Menthane-178-triol ; Nordihydrocapsaicin ; 26-Dimethyl-7-octene-236-triol ; (1S2S4R8R)-p-Menthane-129-triol ; 2-Hexyl-13-dioxan-5-ol ; 2-Hexyl-13-dioxolane-4-methanol ; cis-23-Dimethylthiirane ; (1R2R4S)-p-Menthane-128-triol ; xi-5-Hydroxydecanoic acid ; Ethyl ()-3-hydroxyoctanoate ; (1E)-1-(methylthio)prop-1-ene	2M+3H2O+2H ; M+H+Na ; M+H+Na ; M+3H ; M+2H+Na ; M+3H ; M+NH4 ; M+H+Na ; M+H+Na ; M+H+Na ; M+H+Na ; M+2H+Na ; M+H+Na ; M+H+Na ; M+H+Na ; M+H+Na ; M+NH4 ; M+H+Na ; M+H+Na ; M+H+Na ; M+NH4	C6H6 ; C10H20O3 ; C10H20O3 ; C19H25NO3 ; C17H27NO3 ; C19H25NO3 ; C4H8S ; C10H20O3 ; C10H20O3 ; C10H20O3 ; C10H20O3 ; C17H27NO3 ; C10H20O3 ; C10H20O3 ; C10H20O3 ; C10H20O3 ; C4H8S ; C10H20O3 ; C10H20O3 ; C10H20O3 ; C4H8S
468.58181	1.308068	1.127332	-3.1693	0.01	2	0.045238	NA	NA	NA	NA	NA
225.03546	1.3542	1.090487	3.1684	0.010014	1.9994	0.045238	225.0355	HMDB28772 ; HMDB00954 ; HMDB00955 ; HMDB02120 ; HMDB03745 ; HMDB12883 ; HMDB29227 ; HMDB29545 ; HMDB29547 ; HMDB29663 ; HMDB29979 ; HMDB30095 ; HMDB30765 ; HMDB31955 ; HMDB32614 ; HMDB32652 ; HMDB33275 ; HMDB36335 ; HMDB37571 ; HMDB37644 ; HMDB39234 ; HMDB39327 ; HMDB39606 ; HMDB40275 ; HMDB40722 ; HMDB59721	Cysteinyl-Cysteine ; trans-Ferulic acid ; Isoferulic acid ; Monoethyl phthalate ; Mesna ; Adrenochrome o-semiquinone ; 5-Hydroxy-33478-pentamethoxyflavone ; 3-Hydroxy-45678-pentamethoxyflavone ; 4-Hydroxy-35678-pentamethoxyflavone ; Vanillin acetate ; 2-Hydroxy-34578-pentamethoxyflavone ; Artemetin ; (-)-trans-34-Dihydro-48-dihydroxy-3-methyl-1H-2-benzopyran-1-one ; Dimethyl (1R2S3S)-2-carboxy-3-(34-dihydroxyphenyl)-23-dihydro-56-dihydroxy-1H-indene-1-acetate ; Piperonyl acetate ; Meconine ; 5-Hydroxyauranetin ; 246-Phenanthrenetriol 2-O-b-D-glucoside ; Demethylnobiletin ; 3-(2-Hydroxy-3-methoxyphenyl)-2-propenoic acid ; Citrusin E ; 3-(4-Hydroxybenzoyl)epicatechin ; Repandiol ; Ethyl aconitate ; 7-Hydroxy-34568-pentamethoxyflavone ; mono-Benzyl malonate	M+H ; 2M+Na+K ; 2M+Na+K ; 2M+Na+K ; M+2ACN+H ; M+2Na-H ; M+Na+K ; M+Na+K ; M+Na+K ; 2M+Na+K ; M+Na+K ; M+Na+K ; 2M+Na+K ; M+Na+K ; 2M+Na+K ; 2M+Na+K ; M+Na+K ; M+Na+K ; M+Na+K ; 2M+Na+K ; M+2K ; M+H+K ; 2M+Na+K ; M+Na ; M+Na+K ; 2M+Na+K	C6H12N2O3S2 ; C10H10O4 ; C10H10O4 ; C10H10O4 ; C2H6O3S2 ; C9H10NO3 ; C20H20O8 ; C20H20O8 ; C20H20O8 ; C10H10O4 ; C20H20O8 ; C20H20O8 ; C10H10O4 ; C20H20O8 ; C10H10O4 ; C10H10O4 ; C20H20O8 ; C20H20O8 ; C20H20O8 ; C10H10O4 ; C17H24O9 ; C22H18O8 ; C10H10O4 ; C8H10O6 ; C20H20O8 ; C10H10O4
284.26634	1.390588	0.502066	3.1683	0.010016	1.9993	0.045238	NA	NA	NA	NA	NA
173.02124	1.356864	1.144956	-3.1678	0.010025	1.9989	0.045239	173.0212	HMDB00126 ; HMDB02520 ; HMDB11686 ; HMDB14376 ; HMDB14493 ; HMDB33559 ; HMDB34618 ; HMDB38361 ; HMDB38365 ; HMDB38492 ; HMDB39875 ; HMDB40198	Glycerol 3-phosphate ; Beta-Glycerophosphoric acid ; p-Cresol glucuronide ; Temazepam ; Clobazam ; 77-Dihydroxy-68-bicoumarin ; 2-O-Benzoyl-D-glucose ; (-)-Epigallocatechin ; ()-Gallocatechin ; D-Vacciniin ; 2-(Ethylthio)methylfuran ; 25-Dimethyl-3-(methylthio)furan	M+H ; M+H ; M+Na+K ; M+2Na ; M+2Na ; M+H+Na ; M+Na+K ; M+H+K ; M+H+K ; M+Na+K ; 2M+Na+K ; 2M+Na+K	C3H9O6P ; C3H9O6P ; C13H16O7 ; C16H13ClN2O2 ; C16H13ClN2O2 ; C18H10O6 ; C13H16O7 ; C15H14O7 ; C15H14O7 ; C13H16O7 ; C7H10OS ; C7H10OS
549.12706	1.411219	0.923532	-3.1669	0.010041	1.9982	0.045269	549.1271	HMDB13159 ; HMDB15257 ; HMDB33979 ; HMDB35684 ; HMDB36259 ; HMDB36260 ; HMDB36261 ; HMDB59778	23-Diaminosalicylic acid ; Anisindione ; 7-Hydroxy-2-methylisoflavone ; Deacetylnomilin ; Gladiatoside A3 ; Gladiatoside A2 ; Gladiatoside A1 ; 2-Pyrroloylglycine	3M+2Na-H ; 2M+2Na-H ; 2M+2Na-H ; M+2K-H ; M+Na+K ; M+Na+K ; M+Na+K ; 3M+2Na-H	C7H8N2O3 ; C16H12O3 ; C16H12O3 ; C26H32O8 ; C47H56O26 ; C47H56O26 ; C47H56O26 ; C7H8N2O3
202.93310	1.31694	1.610294	-3.1622	0.01012	1.9948	0.045587	202.9331	HMDB14894	Hexachlorophene	M+2H	C13H6Cl6O2
390.70904	1.352889	1.725628	3.1609	0.010143	1.9938	0.045647	NA	NA	NA	NA	NA

106.99906	1.358021	1.203787	3.1593	0.010171	1.9926	0.045734	106.9991	HMDB00020 ; HMDB00440 ; HMDB00669 ; HMDB00703 ; HMDB01101 ; HMDB02390 ; HMDB03791 ; HMDB03976 ; HMDB04815 ; HMDB11659 ; HMDB12308 ; HMDB29657 ; HMDB29658 ; HMDB29659 ; HMDB29660 ; HMDB29661 ; HMDB29662 ; HMDB31201 ; HMDB31609 ; HMDB32399 ; HMDB32572 ; HMDB32594 ; HMDB32604 ; HMDB32606 ; HMDB32629 ; HMDB32919 ; HMDB33003 ; HMDB33919 ; HMDB34172 ; HMDB36907 ; HMDB41283 ; HMDB41486 ; HMDB60390 ; HMDB15677	p-Hydroxyphenylacetic acid ; 3-Hydroxyphenylacetic acid ; Ortho-Hydroxyphenylacetic acid ; Mandelic acid ; p-Anisic acid ; 3-Cresotinic acid ; 34-Dihydroxyphenylacetaldehyde ; D-Glucuronic acid 1-phosphate ; 4-Hydroxy-3-methylbenzoic acid ; 2-Methylerythritol ; Vanillin ; 24-Dihydroxyacetophenone ; 23-Dihydroxyacetophenone ; 24-Dihydroxyacetophenone ; 26-Dihydroxyacetophenone ; 34-Dihydroxyacetophenone ; 35-Dihydroxyacetophenone ; 3-Deoxy-D-arabinitol ; Phenoxyacetic acid ; Methyl furfuracrylate ; Methylparaben ; 2-(Hydroxymethyl)benzoic acid ; 2-Methoxybenzoic acid ; 3-Methoxybenzoic acid ; 25-Dihydroxyacetophenone ; Ethyl 2-furanyl diketone ; 1-(5-Methyl-2-furanyl)-12-propanedione ; 2-Deoxy-D-ribitol ; Methyl 2-hydroxybenzoate ; 2-Propenyl 2-furancarboxylate ; 1-(2-Furanyl)-13-butanedione ; 1-Deoxy-D-ribitol ; 4-Hydroxyphenyl acetate ; Dimercaprol	M+Na+K ; M+Na+K ; M+Na+K ; M+Na+K ; M+Na+K ; M+Na+K ; M+Na+K ; M+H+2Na ; M+Na+K ; M+2K ; M+Na+K ; M+Na+K ; M+Na+K ; M+Na+K ; M+Na+K ; M+Na+K ; M+Na+K ; M+2K ; M+Na+K ; M+Na+K ; M+Na+K ; M+Na+K ; M+Na+K ; M+Na+K ; M+Na+K ; M+Na+K ; M+Na+K ; M+2K ; M+Na+K ; M+Na+K ; M+Na+K ; M+2K ; M+Na+K ; M+H-H2O	C8H8O3 ; C8H8O3 ; C8H8O3 ; C8H8O3 ; C8H8O3 ; C8H8O3 ; C8H8O3 ; C6H11O10P ; C8H8O3 ; C5H12O4 ; C8H8O3 ; C8H8O3 ; C8H8O3 ; C8H8O3 ; C8H8O3 ; C8H8O3 ; C8H8O3 ; C5H12O4 ; C8H8O3 ; C8H8O3 ; C8H8O3 ; C8H8O3 ; C8H8O3 ; C8H8O3 ; C8H8O3 ; C8H8O3 ; C8H8O3 ; C5H12O4 ; C8H8O3 ; C8H8O3 ; C8H8O3 ; C5H12O4 ; C8H8O3 ; C3H8OS2
357.29958	1.368105	0.619016	3.1568	0.010214	1.9908	0.04574	357.2996	HMDB11536 ; HMDB11537 ; HMDB11566 ; HMDB11567 ; HMDB33372 ; HMDB34031 ; HMDB32034 ; HMDB39954	MG(0:018:1(11Z)0:0) ; MG(0:018:1(9Z)0:0) ; MG(18:1(11Z)0:00:0) ; MG(18:1(9Z)0:00:0) ; Stearoyllactic acid ; 3-(2-Heptenyloxy)-2-hydroxypropyl undecanoate ; 2412-Octadecatrienoic acid piperidide ; Macamide B	M+H ; M+H ; M+H ; M+H ; M+H ; M+H ; 2M+H+Na ; 2M+H+Na	C21H40O4 ; C21H40O4 ; C21H40O4 ; C21H40O4 ; C21H40O4 ; C21H40O4 ; C23H39NO ; C23H39NO
90.976696	1.349099	1.447104	3.1567	0.010215	1.9907	0.04574	90.9767	HMDB00142	Formic acid	M+2Na-H	CH2O2
186.06422	1.34375	1.573704	-3.1566	0.010218	1.9906	0.04574	186.0642	HMDB00788 ; HMDB05810 ; HMDB15487 ; HMDB30847 ; HMDB31911 ; HMDB32707 ; HMDB37314 ; HMDB37786 ; HMDB38575 ; HMDB40314 ; HMDB41651	Orotidine ; Eriodictyol ; Acenocoumarol ; Aromadendrin ; Porric acid B ; Dalbergioidin ; Norartocarpanone ; S-(45-Dihydro-2-methyl-3-furanyl) ethanethioate ; (Z)-N-Feruloyl-5-hydroxyanthranilic acid ; 2457-Tetrahydroxyflavanone ; 3457-Tetrahydroxyisoflavanone	M+2ACN+2H ; M+2ACN+2H ; M+H+NH4 ; M+2ACN+2H ; M+2ACN+2H ; M+2ACN+2H ; M+2ACN+2H ; 2M+3H2O+2H ; M+ACN+2H ; M+2ACN+2H ; M+2ACN+2H	C10H12N2O8 ; C15H12O6 ; C19H15NO6 ; C15H12O6 ; C15H12O6 ; C15H12O6 ; C15H12O6 ; C7H10O2S ; C17H15NO6 ; C15H12O6 ; C15H12O6
549.28860	1.359742	1.359584	-3.1551	0.010244	1.9896	0.045812	549.2886	HMDB01054 ; HMDB05777 ; HMDB15110 ; HMDB30434 ; HMDB40847 ; HMDB41876 ; HMDB30461	Dolichyl b-D-glucosyl phosphate ; Morphiceptin ; Dipyridamole ; Loquatifolin A ; (S)-Nerolidol 3-O-a-L-Rhamnopyranosyl-(1-4)-a-L-rhamnopyranosyl-(1-2)-a-L-rhamnopyranosyl-(1-6)-b-D-glucopyranoside ; Diacetylspermine ; Hordatine A	M+2ACN+H ; 2M+3H2O+2H ; M+2Na-H ; 2M+3H ; 2M+3H ; 3M+H+Na ; M+H-H2O	C21H39O9P ; C28H35N5O5 ; C24H40N8O4 ; C39H66O18 ; C39H66O18 ; C14H32Cl2N4O2 ; C28H38N8O5
320.02982	1.324825	0.66841	-3.1535	0.010272	1.9883	0.045881	NA	NA	NA	NA	NA
405.96787	1.302527	1.01023	3.153	0.01028	1.988	0.045881	405.9679	HMDB15142 ; HMDB34852 ; HMDB37112	Tioconazole ; Sulfentrazone ; Acifluorfen	2M+H+K ; 2M+H+K ; M+2Na-H	C16H13Cl3N2OS ; C11H10Cl2F2N4O3S ; C14H7ClF3NO5
205.09712	1.338224	1.557206	3.1521	0.010295	1.9874	0.045881	205.0971	HMDB00929 ; HMDB13609 ; HMDB13840 ; HMDB14892 ; HMDB30396 ; HMDB60533 ; HMDB60878 ; HMDB60967 ; HMDB39084 ; HMDB00734 ; HMDB00810 ; HMDB01097 ; HMDB01424 ; HMDB02226 ; HMDB03826 ; HMDB04186 ; HMDB05923 ; HMDB14507 ; HMDB15141 ; HMDB15166 ; HMDB29095 ; HMDB29116 ; HMDB29342 ; HMDB29772 ; HMDB30002 ; HMDB30177 ; HMDB30252 ; HMDB30370 ; HMDB30795 ; HMDB31359 ; HMDB31565 ; HMDB31728 ; HMDB32540 ; HMDB33546 ; HMDB34101 ; HMDB35212 ; HMDB35676 ; HMDB35677 ; HMDB36190 ; HMDB36815 ; HMDB38910 ; HMDB60610	L-Tryptophan ; D-Tryptophan ; 3-Hydroxymethylantipyrine ; Ethotoin ; ()-Tryptophan ; Nirvanol ; 4-Hydroxyantipyrine ; S-nirvanol ; 3-O-Methylniveusin A ; Indoleacrylic acid ; Dimethylprotoporphyrin IX dimethyl ester ; Protoporphyrinogen IX ; 4-(3-Pyridyl)-3-butenoic acid ; Adrenic acid ; 67-Dimethyl-8-(1-D-ribityl)lumazine ; 3-Methyldioxyindole ; N4-Acetylcytidine ; Clozapine ; Letrozole ; Probenecid ; Tryptophyl-Tyrosine ; Tyrosyl-Tryptophan ; Ceanothine E ; Corchoionol C 9-glucoside ; (-)-Nopol ; Arborinine ; Limonene aldehyde ; Citroside A ; Moreollin ; 24-Undecadienal ; Dihydrojasmone ; 2-Hexylidenecyclopentanone ; 266-Trimethyl-1-cyclohexen-1-acetaldehyde ; 25-Undecadienal ; Adouetine Y ; Sonchuionoside C ; 1-Hydroxy-1-phenyl-3-hexadecanone ; 3-Hydroxy-1-phenyl-1-hexadecanone ; 2-Heptylfuran ; Norecasantalol ; 2-Methyl-5-(8-pentadecenyl)-13-benzenediol ; Hydroxyhexamide	M+H ; M+H ; M+H ; M+H ; M+H ; M+H ; M+H ; M+H ; M+2H ; M+NH4 ; M+2H+Na ; M+H+2Na ; M+ACN+H ; M+2K ; M+2ACN+2H ; M+ACN+H ; M+3ACN+2H ; M+2ACN+2H ; M+3ACN+2H ; M+3ACN+2H ; M+ACN+2H ; M+ACN+2H ; M+H+2Na ; M+H+Na ; M+K ; M+3ACN+2H ; M+K ; M+H+Na ; M+2H+Na ; M+K ; M+K ; M+K ; M+K ; M+K ; M+H+2Na ; M+H+Na ; M+2K ; M+2K ; M+K ; M+K ; M+2K ; M+2ACN+2H	C11H12N2O2 ; C11H12N2O2 ; C11H12N2O2 ; C11H12N2O2 ; C11H12N2O2 ; C11H12N2O2 ; C11H12N2O2 ; C11H12N2O2 ; C21H28O8 ; C11H9NO2 ; C36H38N4O4 ; C34H40N4O4 ; C9H9NO2 ; C22H36O2 ; C13H18N4O6 ; C9H9NO2 ; C11H15N3O6 ; C18H19ClN4 ; C17H11N5 ; C13H19NO4S ; C20H21N3O4 ; C20H21N3O4 ; C34H40N4O4 ; C19H30O8 ; C11H18O ; C16H15NO4 ; C11H18O ; C19H30O8 ; C35H42O8 ; C11H18O ; C11H18O ; C11H18O ; C11H18O ; C11H18O ; C34H40N4O4 ; C19H30O8 ; C22H36O2 ; C22H36O2 ; C11H18O ; C11H18O ; C22H36O2 ; C15H22N2O4S
318.86053	1.424915	0.995681	3.1519	0.0103	1.9872	0.045881	318.8605	HMDB37833 ; HMDB39459	Ficin ; Di-2-propenyl pentasulfide	M+NH4 ; M+2K-H	CH2FI2N ; C6H10S5
164.97713	1.315218	1.66962	-3.1516	0.010305	1.987	0.045881	164.983	HMDB29198	Dimethylurea	M+2K-H	C3H8N2O

274.87325	1.32447	1.462723	-3.1484	0.010362	1.9846	0.046094	274.8733	HMDB07954 ; HMDB08592 ; HMDB08981 ; HMDB09008 ; HMDB09040 ; HMDB09073 ; HMDB09105 ; HMDB09212 ; HMDB09231 ; HMDB09241 ; HMDB09242 ; HMDB09263 ; HMDB09273 ; HMDB09295 ; HMDB09338 ; HMDB09371 ; HMDB09403 ; HMDB09436 ; HMDB09468 ; HMDB09489 ; HMDB09500 ; HMDB09520 ; HMDB09521 ; HMDB09531 ; HMDB09532 ; HMDB09552 ; HMDB09562 ; HMDB09563 ; HMDB09593 ; HMDB09625 ; HMDB09658 ; HMDB09749 ; HMDB09756	PC(15:022:2(13Z16Z)) ; PC(22:2(13Z16Z)15:0) ; PE(16:1(9Z)24:1(15Z)) ; PE(18:022:2(13Z16Z)) ; PE(18:1(11Z)22:1(13Z)) ; PE(18:1(9Z)22:1(13Z)) ; PE(18:2(9Z12Z)22:0) ; PE(18:4(6Z9Z12Z15Z)24:1(15Z)) ; PE(20:020:2(11Z14Z)) ; PE(20:022:5(4Z7Z10Z13Z16Z)) ; PE(20:022:5(7Z10Z13Z16Z19Z)) ; PE(20:1(11Z)20:1(11Z)) ; PE(20:1(11Z)22:4(7Z10Z13Z16Z)) ; PE(20:2(11Z14Z)20:0) ; PE(20:3(5Z8Z11Z)22:2(13Z16Z)) ; PE(20:3(8Z11Z14Z)22:2(13Z16Z)) ; PE(20:4(5Z8Z11Z14Z)22:1(13Z)) ; PE(20:4(8Z11Z14Z17Z)22:1(13Z)) ; PE(20:5(5Z8Z11Z14Z17Z)22:0) ; PE(22:018:2(9Z12Z)) ; PE(22:020:5(5Z8Z11Z14Z17Z)) ; PE(22:1(13Z)18:1(11Z)) ; PE(22:1(13Z)18:1(9Z)) ; PE(22:1(13Z)20:4(5Z8Z11Z14Z)) ; PE(22:1(13Z)20:4(8Z11Z14Z17Z)) ; PE(22:2(13Z16Z)18:0) ; PE(22:2(13Z16Z)20:3(5Z8Z11Z)) ; PE(22:2(13Z16Z)20:3(8Z11Z14Z)) ; PE(22:4(7Z10Z13Z16Z)20:1(11Z)) ; PE(22:5(4Z7Z10Z13Z16Z)20:0) ; PE(22:5(7Z10Z13Z16Z19Z)20:0) ; PE(24:1(15Z)16:1(9Z)) ; PE(24:1(15Z)18:4(6Z9Z12Z15Z))	M+2H+Na ; M+2H+Na ; M+2H+Na ; M+2H+Na ; M+2H+Na ; M+2H+Na ; M+2H+Na ; M+3H ; M+2H+Na ; M+3H ; M+3H ; M+2H+Na ; M+3H ; M+2H+Na ; M+3H ; M+3H ; M+3H ; M+3H ; M+3H ; M+2H+Na ; M+3H ; M+2H+Na ; M+2H+Na ; M+3H ; M+3H ; M+2H+Na ; M+3H ; M+3H ; M+3H ; M+3H ; M+3H ; M+2H+Na ; M+3H	C45H86NO8P ; C45H86NO8P ; C45H86NO8P ; C45H86NO8P ; C45H86NO8P ; C45H86NO8P ; C45H86NO8P ; C47H84NO8P ; C45H86NO8P ; C47H84NO8P ; C47H84NO8P ; C45H86NO8P ; C47H84NO8P ; C45H86NO8P ; C47H84NO8P ; C47H84NO8P ; C47H84NO8P ; C47H84NO8P ; C47H84NO8P ; C45H86NO8P ; C47H84NO8P ; C45H86NO8P ; C45H86NO8P ; C47H84NO8P ; C47H84NO8P ; C45H86NO8P ; C47H84NO8P ; C47H84NO8P ; C47H84NO8P ; C47H84NO8P ; C47H84NO8P ; C45H86NO8P ; C47H84NO8P
196.01921	1.348441	0.089174	3.1456	0.01041	1.9825	0.046267	196.0192	HMDB00044 ; HMDB01412 ; HMDB06355 ; HMDB14544 ; HMDB30567 ; HMDB31193 ; HMDB32806 ; HMDB33548 ; HMDB33586 ; HMDB33787 ; HMDB37086 ; HMDB39720 ; HMDB40936 ; HMDB41645 ; HMDB41650 ; HMDB41672 ; HMDB41682 ; HMDB41694 ; HMDB41697 ; HMDB41700 ; HMDB41703	Ascorbic acid ; 78-Dihydropteroic acid ; D-Glucurono-63-lactone ; Griseofulvin ; (-)-349-Trimethoxypterocarpan ; 123-Propanetricarboxylic acid ; p-Hydroxyphenethyl trans-ferulate ; 4-Hydroxy-5-methyl-3(2H)-thiophenone ; 2-Hydroxy-446-trimethoxychalcone ; 239-Trimethoxypterocarpan ; 58-Dimethoxychalepensin ; a-L-threo-4-Hex-4-enopyranuronosyl-D-galacturonic acid ; beta2-Dihydroxy-46-dimethoxy-3-methylchalcone ; 2-Hydroxyenterolactone ; 2-Hydroxyenterolactone ; 4-Hydroxyenterolactone ; 4-Hydroxyenterolactone ; 5-Hydroxyenterolactone ; 6-Hydroxyenterolactone ; 6-Hydroxyenterolactone ; 7-Hydroxyenterolactone	2M+H+K ; M+2K ; 2M+H+K ; M+H+K ; M+2K ; 2M+H+K ; M+2K ; 3M+2H ; M+2K ; M+2K ; M+2K ; M+H+K ; M+2K ; M+2K ; M+2K ; M+2K ; M+2K ; M+2K ; M+2K ; M+2K ; M+2K	C6H8O6 ; C14H14N6O3 ; C6H8O6 ; C17H17ClO6 ; C18H18O5 ; C6H8O6 ; C18H18O5 ; C5H6O2S ; C18H18O5 ; C18H18O5 ; C18H18O5 ; C12H16O12 ; C18H18O5 ; C18H18O5 ; C18H18O5 ; C18H18O5 ; C18H18O5 ; C18H18O5 ; C18H18O5 ; C18H18O5 ; C18H18O5
549.62044	1.419446	0.916179	-3.1448	0.010425	1.9819	0.046294	549.6204	HMDB32802	4-Hydroxybenzyl isothiocyanate 4-acetylrhamnoside	3M+H+K	C16H19NO6S
715.20878	1.391089	0.927743	-3.1436	0.010445	1.9811	0.046341	715.2088	HMDB30074 ; HMDB30122 ; HMDB30196 ; HMDB40319 ; HMDB41435	Albanin F ; Kuwanon K ; Fagopyrine ; 588-Trihydroxy-3344577-heptamethoxy-55-biflavan ; Citbismine C	M+Na ; M+Na ; M+2Na-H ; M+K ; 2M+Na+K	C40H36O11 ; C40H36O11 ; C40H34N2O8 ; C37H40O12 ; C37H36N2O11
257.99337	1.326792	0.039908	-3.1431	0.010455	1.9807	0.046344	257.9934	HMDB00158 ; HMDB00714 ; HMDB00992 ; HMDB01119 ; HMDB02184 ; HMDB03831 ; HMDB06050 ; HMDB12884 ; HMDB15422 ; HMDB29274 ; HMDB32398 ; HMDB32595 ; HMDB59720 ; HMDB60280	L-Tyrosine ; Hippuric acid ; 3-Succinoylpyridine ; 4-Hydroxy-4-(3-pyridyl)-butanoic acid ; L-Threo-3-Phenylserine ; Beta-Tyrosine ; o-Tyrosine ; Adrenochrome ; Cefazolin ; Sanguisorbic acid dilactone ; Methyl n-formylanthranilate ; 1-(4-Methoxyphenyl)-2-nitroethylene ; Meta-Tyrosine ; 467-Trihydroxy-1234-tetrahydroisoquinoline	M+2K-H ; M+2K+H ; M+2K+H ; M+2K-H ; M+2K-H ; M+2K-H ; M+2K-H ; M+2K+H ; M+Na+K ; M+2Na ; M+2K+H ; M+2K+H ; M+2K-H ; M+2K-H	C9H11NO3 ; C9H9NO3 ; C9H9NO3 ; C9H11NO3 ; C9H11NO3 ; C9H11NO3 ; C9H11NO3 ; C9H9NO3 ; C14H14N8O4S3 ; C21H10O13 ; C9H9NO3 ; C9H9NO3 ; C9H11NO3 ; C9H11NO3
576.97598	1.319289	1.664972	-3.1381	0.010544	1.977	0.046695	NA	NA	NA	NA	NA
421.86978	1.303351	1.634758	-3.1337	0.010623	1.9738	0.046938	421.8698	HMDB31798 ; HMDB31959 ; HMDB56120 ; HMDB56122	()-Leptophos ; Lablaboside D ; DG(24:00:018:2n6) ; DG(24:00:020:2n6)	2M+H+Na ; M+3H ; M+3ACN+2H ; M+3ACN+2H	C13H10BrCl2O2PS ; C60H94O28 ; C46H86O5 ; C46H86O5
129.95137	1.332524	1.274488	3.1335	0.010627	1.9736	0.046938	NA	NA	NA	NA	NA
446.67048	1.34003	1.700122	3.1327	0.010642	1.973	0.046948	446.6705	HMDB01563 ; HMDB05862 ; HMDB06038 ; HMDB15401 ; HMDB41328	1-Methylguanosine ; 2-Methylguanosine ; 3-O-Methylguanosine ; Nelarabine ; 3-Deaminofusarochromanone	3M+2H ; 3M+2H ; 3M+2H ; 3M+2H ; 3M+Na+K	C11H15N5O5 ; C11H15N5O5 ; C11H15N5O5 ; C11H15N5O5 ; C15H19NO4
120.09048	1.361757	1.107768	-3.1324	0.010648	1.9727	0.046948	120.0905	HMDB04362 ; HMDB30305 ; HMDB31263 ; HMDB31271 ; HMDB31514 ; HMDB31531 ; HMDB32265 ; HMDB32307 ; HMDB32413 ; HMDB32491 ; HMDB33167 ; HMDB34716 ; HMDB35307 ; HMDB36208 ; HMDB36222 ; HMDB37023 ; HMDB37496 ; HMDB37618 ; HMDB37629 ; HMDB38272 ; HMDB38275 ; HMDB39736 ; HMDB39794 ; HMDB40210 ; HMDB59728 ; HMDB59817 ; HMDB59840 ; HMDB59894 ; HMDB60286 ; HMDB60850	4-Hydroxynonenal ; Dioscorine ; 24-Nonanedione ; 2-Nonenoic acid ; 6-Butyltetrahydro-2H-pyran-2-one ; Dihydro-5-pentyl-2(3H)-furanone ; cis-Ethyl 4-heptenoate ; Hept-trans-2-en-1-yl acetate ; Methyl 2-octenoate ; Prenyl isobutyrate ; Ethyl cyclohexanecarboxylate ; 4-Oxononanal ; Quercuslactone a ; 2-Propenyl 2-ethylbutanoate ; Cyclohexyl propionate ; 2-Hydroxy-266-trimethylcyclohexanone ; Methyl (E)-2-octenoate ; (E)-3-Heptenyl acetate ; Isobutyl angelate ; 2-Hexenyl propionate ; cis-3-Hexenyl propanoate ; 6-Hydroxy-46-dimethyl-3-hepten-2-one ; Methyl 4Z-octenoate ; 2-Propenyl hexanoate ; Methyl 3-Octenoate ; (Z)-Oak lactone ; (E)-2-Ethyl heptenoate ; (E)-Oak lactone ; 34-Epoxynonanal ; NNO-Tridesmethyl-tramadol	M+2ACN+2H ; M+H+NH4 ; M+2ACN+2H ; M+H+NH4	C9H16O2 ; C13H19NO2 ; C9H16O2 ; C13H19NO2
709.93819	1.379316	1.584428	-3.1219	0.01084	1.965	0.04772	NA	NA	NA	NA	NA
316.77620	1.326196	1.761914	3.1217	0.010842	1.9649	0.04772	NA	NA	NA	NA	NA
129.58174	1.313479	1.354804	-3.1185	0.010902	1.9625	0.047944	129.5817	HMDB00904 ; HMDB01522 ; HMDB03148 ; HMDB04620 ; HMDB12251 ; HMDB15169	Citrulline ; Methylguanidine ; Argininic acid ; N-a-Acetyl-L-arginine ; L-Canaline ; Procainamide	M+2ACN+2H ; 3M+H+K ; M+2ACN+2H ; M+ACN+2H ; M+3ACN+2H ; M+H+Na	C6H13N3O3 ; C2H7N3 ; C6H13N3O3 ; C8H16N4O3 ; C4H10N2O3 ; C13H21N3O
179.03169	1.33711	1.474748	-3.1172	0.010926	1.9615	0.047952	179.0295	HMDB30772 ; HMDB38518 ; HMDB40236 ; HMDB40873 ; HMDB40931 ; HMDB41451 ; HMDB60885 ; HMDB60886 ; HMDB60887 ; HMDB60888	Demethoxyegonol ; Desmosflavone ; 34-Diethylthiophene ; Dimethylstrobochrysin ; 1-(4-Hydroxy-3-methoxyphenyl)-5-(4-hydroxyphenyl)-14-pentadien-3-one ; Musanolone F ; 4-Hydroxy-R-phenprocoumon ; 6-Hydroxy-R-phenprocoumon ; 8-Hydroxy-R-phenprocoumon ; 7-Hydroxy-R-phenprocoumon	M+Na+K ; M+Na+K ; M+K ; M+Na+K ; M+Na+K ; M+H+K ; M+Na+K ; M+Na+K ; M+Na+K ; M+Na+K	C18H16O4 ; C18H16O4 ; C8H12S ; C18H16O4 ; C18H16O4 ; C20H14O4 ; C18H16O4 ; C18H16O4 ; C18H16O4 ; C18H16O4

432.76404	1.320989	0.725841	3.1168	0.010933	1.9613	0.047952	432.764	HMDB07887 ; HMDB07919 ; HMDB07978 ; HMDB08010 ; HMDB08037 ; HMDB08038 ; HMDB08069 ; HMDB08102 ; HMDB08266 ; HMDB08298 ; HMDB08526 ; HMDB08558 ; HMDB08915 ; HMDB09747 ; HMDB32208 ; HMDB35080 ; HMDB41090	PC(14:022:1(13Z)) ; PC(14:1(9Z)22:0) ; PC(16:020:1(11Z)) ; PC(16:1(9Z)20:0) ; PC(18:018:1(11Z)) ; PC(18:018:1(9Z)) ; PC(18:1(11Z)18:0) ; PC(18:1(9Z)18:0) ; PC(20:016:1(9Z)) ; PC(20:1(11Z)16:0) ; PC(22:014:1(9Z)) ; PC(22:1(13Z)14:0) ; PE(15:024:1(15Z)) ; PE(24:1(15Z)15:0) ; Citronellyl anthranilate ; Miltirone ; (-)-(E)-1-(4-Hydroxyphenyl)-7-phenyl-6-hepten-3-ol	M+2K ; M+2K ; M+2K ; M+2K ; M+2K ; M+2K ; M+2K ; M+2K ; M+2K ; M+2K ; M+2K ; M+2K ; M+2K ; M+2K ; M+2K ; M+2K ; M+2K ; M+2K ; 3M+H+K ; 3M+H+NH4 ; 3M+H+NH4	C44H86NO8P ; C44H86NO8P ; C44H86NO8P ; C44H86NO8P ; C44H86NO8P ; C44H86NO8P ; C44H86NO8P ; C44H86NO8P ; C44H86NO8P ; C44H86NO8P ; C44H86NO8P ; C44H86NO8P ; C44H86NO8P ; C44H86NO8P ; C17H25NO2 ; C19H22O2 ; C19H22O2
618.20060	1.369458	0.790436	-3.1127	0.01101	1.9582	0.048248	618.2006	HMDB01445 ; HMDB31819 ; HMDB38008 ; HMDB38443 ; HMDB39570 ; HMDB40031 ; HMDB41550 ; HMDB60498	N-Acetyl-D-glucosaminyldiphosphodolichol ; 45-Hydroxyyessotoxin ; Malvidin 3-(6-acetylglucoside) ; 1-Isothiocyanato-4-phenylbutane ; Methylsyringin ; 2-Butylbenzothiazole ; Oleoside 11-methyl ester ; NNAL-N-glucuronide	2M+Na+K ; M+2K ; M+2ACN+H ; 3M+2Na-H ; 3M+2K ; 3M+2Na-H ; 3M+H+Na ; 3M+2K	C23H43NO12P2 ; C55H82O22S2 ; C25H27O13 ; C11H13NS ; C18H26O9 ; C11H13NS ; C17H24O11 ; C16H24N3O8
589.04369	1.34194	0.901356	-3.11	0.011061	1.9562	0.048352	589.0437	HMDB30497 ; HMDB39198 ; HMDB39372 ; HMDB41182 ; HMDB61153	Eugeniflorin D1 ; Camelliin B ; Degalloylrugosin F ; Camellioferin A ; Lorcaserin sulfamate	M+H+2Na ; M+H+2Na ; M+H+2Na ; M+H+2Na ; 2M+K	C75H52O48 ; C75H52O48 ; C75H52O48 ; C75H52O48 ; C11H14ClNO3S
140.46402	1.380258	1.154868	3.1097	0.011066	1.956	0.048352	NA	NA	NA	NA	NA
420.01544	1.307884	1.708463	3.1097	0.011066	1.956	0.048352	420.0154	HMDB07090 ; HMDB07091 ; HMDB07706 ; HMDB07735 ; HMDB41247 ; HMDB56002 ; HMDB56007 ; HMDB56283 ; HMDB56290 ; HMDB56297 ; HMDB56315 ; HMDB60980 ; HMDB61029	DG(15:022:5(4Z7Z10Z13Z16Z)0:0) ; DG(15:022:5(7Z10Z13Z16Z19Z)0:0) ; DG(22:5(4Z7Z10Z13Z16Z)15:00:0) ; DG(22:5(7Z10Z13Z16Z19Z)15:00:0) ; Alkaloid A6 ; DG(15:00:022:5n6) ; DG(15:00:022:5n3) ; DG(18:2n60:018:3n6) ; DG(18:2n60:018:3n3) ; DG(18:3n60:020:2n6) ; DG(20:2n60:018:3n3) ; N-desmethylrosiglitazone ; dehydrofelodipine	2M+3H ; 2M+3H ; 2M+3H ; 2M+3H ; M+2K+H ; 2M+3H ; 2M+3H ; 2M+3H ; 2M+3H ; 2M+3H ; 2M+3H ; M+2K-H ; M+K	C40H68O5 ; C40H68O5 ; C40H68O5 ; C40H68O5 ; C19H16ClNO3 ; C40H68O5 ; C40H68O5 ; C40H68O5 ; C40H68O5 ; C40H68O5 ; C40H68O5 ; C17H17N3O3S ; C18H17Cl2NO4
234.90811	1.41145	0.030962	3.1068	0.011121	1.9539	0.048521	NA	NA	NA	NA	NA
345.54721	1.306156	0.091876	-3.1054	0.011147	1.9528	0.048591	345.5472	HMDB06409	Tyramine-O-sulfate	3M+H+K	C8H11NO4S
207.99826	1.345365	1.320153	-3.1038	0.011177	1.9517	0.048664	207.9983	HMDB15242 ; HMDB00272 ; HMDB01721 ; HMDB11635 ; HMDB33384 ; HMDB34782 ; HMDB59996	Miconazole ; Phosphoserine ; DL-O-Phosphoserine ; p-Cresol sulfate ; CI Direct Red 45 ; Benzyl sulfate ; Dihydronaringenin-O-sulphate	M+2H ; M+Na ; M+Na ; 2M+H+K ; M+3Na ; 2M+H+K ; M+Na+K	C18H14Cl4N2O ; C3H8NO6P ; C3H8NO6P ; C7H8O4S ; C24H17N3O7S3 ; C7H8O4S ; C15H14O8S
200.07606	1.300139	1.374948	-3.102	0.011212	1.9503	0.04871	200.0761	HMDB34030 ; HMDB38921 ; HMDB00462 ; HMDB01892 ; HMDB01970 ; HMDB01973 ; HMDB03099 ; HMDB05031 ; HMDB06029 ; HMDB11107 ; HMDB11173 ; HMDB13035 ; HMDB28842 ; HMDB30081 ; HMDB32105 ; HMDB32731 ; HMDB33765 ; HMDB33906 ; HMDB35108 ; HMDB36780 ; HMDB39032 ; HMDB39575 ; HMDB60659	Dulxanthone E ; 5-Methoxycurcumin ; Allantoin ; Menadione ; 3-Methyluric acid ; 9-Methyluric acid ; 1-Methyluric acid ; Rosiglitazone ; N-Acetylglutamine ; 7-Methyluric acid ; L-glycyl-L-hydroxyproline ; Paraoxon ; Glycyl-Hydroxyproline ; Carissanol ; 12-Anhydridoniveusin ; (1E4Z6a8b10a)-8-Angeloyloxy-1015-dihydroxy-3-oxo-1411(13)-germacratrien-126-olide ; Methyl (Z)-2-decene-468-triynoate ; 2-Hydroxy-34578-pentamethoxyflavan ; Diosbulbin A ; Diosbulbin F ; trans-Grandmarin isovalerate ; alpha-Peroxyachifolide ; 1-Methyl-4-nitroimidazole	M+2H ; M+2H ; M+ACN+H ; 2M+3H2O+2H ; M+NH4 ; M+NH4 ; M+NH4 ; M+ACN+2H ; 2M+H+Na ; M+NH4 ; 2M+H+Na ; M+3ACN+2H ; 2M+H+Na ; M+H+Na ; M+H+Na ; M+H+Na ; 2M+3H2O+2H ; M+H+Na ; M+H+Na ; M+H+Na ; M+H+Na ; M+H+Na ; 3M+H+NH4	C22H22O7 ; C22H22O7 ; C4H6N4O3 ; C11H8O2 ; C6H6N4O3 ; C6H6N4O3 ; C6H6N4O3 ; C18H19N3O3S ; C7H12N2O4 ; C6H6N4O3 ; C7H12N2O4 ; C10H14NO6P ; C7H12N2O4 ; C20H24O7 ; C20H24O7 ; C20H24O7 ; C11H8O2 ; C20H24O7 ; C20H24O7 ; C20H24O7 ; C20H24O7 ; C20H24O7 ; C4H5N3O2
131.03444	1.301477	1.002964	3.102	0.011213	1.9503	0.04871	131.0275	HMDB00630 ; HMDB11625 ; HMDB13989 ; HMDB14558 ; HMDB30647 ; HMDB32035 ; HMDB32656 ; HMDB37723 ; HMDB38017	Cytosine ; Dimethylarsinate ; O-Desmethylnaproxen ; Acetohexamide ; Artemidinol ; 6-(34-Methylenedioxyphenyl)-35-hexadien-2-one ; (Z)-6-(2-Methoxyvinyl)-7-methyl-2H-1-benzopyran-2-one ; 2-Phenylethyl 2-furancarboxylate ; Ethyl 2-phenyl-3-furancarboxylate	2M+H+K ; M+3ACN+2H ; M+2Na ; M+3Na ; M+2Na ; M+2Na ; M+2Na ; M+2Na ; M+2Na	C4H5N3O ; C2H6AsO2 ; C13H12O3 ; C15H20N2O4S ; C13H12O3 ; C13H12O3 ; C13H12O3 ; C13H12O3 ; C13H12O3
414.00699	1.313057	1.612053	-3.1002	0.011248	1.9489	0.048818	414.007	HMDB10698	CerP(d18:016:0)	2M+3H	C34H70NO6P
150.01870	1.388215	0.10777	-3.0982	0.011284	1.9475	0.048934	150.0187	HMDB00237 ; HMDB02511 ; HMDB03052 ; HMDB03312 ; HMDB03453 ; HMDB04195 ; HMDB06458 ; HMDB06961 ; HMDB11721 ; HMDB30670 ; HMDB30699 ; HMDB30874 ; HMDB31229 ; HMDB31523 ; HMDB31771 ; HMDB32705 ; HMDB33153 ; HMDB33265 ; HMDB36619 ; HMDB39616 ; HMDB40630	Propionic acid ; 345-Trimethoxycinnamic acid ; Lactaldehyde ; Daidzein ; 3-Hydroxypropanal ; 5-L-Glutamyl-taurine ; D-Lactaldehyde ; Hydroxyacetone ; Trans-2 3 4-Trimethoxycinnamate ; Chrysophanol ; 57-Dihydroxyisoflavone ; Phomarin ; Ethyl formate ; Methyl acetate ; 1-(245-Trimethoxyphenyl)-12-propanedione ; Isopropyl beta-D-glucoside ; (Z)-46-Dihydroxyaurone ; 2-Methoxystypandrone ; 57-Dihydroxyflavone ; Orientalone ; Pratenol A	3M+2K ; M+Na+K ; 3M+2K ; M+2Na ; 3M+2K ; M+2Na ; 3M+2K ; 3M+2K ; M+Na+K ; M+2Na ; M+2Na ; M+2Na ; 3M+2K ; 3M+2K ; M+Na+K ; M+2K ; M+2Na ; M+H+K ; M+2Na ; M+H+K ; M+H+K	C3H6O2 ; C12H14O5 ; C3H6O2 ; C15H10O4 ; C3H6O2 ; C7H14N2O6S ; C3H6O2 ; C3H6O2 ; C12H14O5 ; C15H10O4 ; C15H10O4 ; C15H10O4 ; C3H6O2 ; C3H6O2 ; C12H14O5 ; C9H18O6 ; C15H10O4 ; C14H12O5 ; C15H10O4 ; C14H12O5 ; C14H12O5
195.95868	1.356733	1.569991	3.0977	0.011295	1.9471	0.04894	195.9587	HMDB02287	Homocysteine thiolactone	M+2K+H	C4H7NOS
114.96704	1.321468	1.10897	3.0971	0.011306	1.9467	0.048944	114.967	HMDB00263 ; HMDB60445 ; HMDB60447	Phosphoenolpyruvic acid ; Bromobenzene-23-dihydrodiol ; Bromobenzene-34-dihydrodiol	M+Na+K ; M+H+K ; M+H+K	C3H5O6P ; C6H7BrO2 ; C6H7BrO2

452.37311	1.349449	0.233059	3.0963	0.011322	1.9461	0.048972	452.3731	HMDB05392 ; HMDB05475 ; HMDB10476 ; HMDB10483 ; HMDB10523 ; HMDB42443 ; HMDB42448 ; HMDB42623 ; HMDB42628 ; HMDB42652 ; HMDB42710 ; HMDB42716 ; HMDB42733 ; HMDB42739 ; HMDB42832 ; HMDB42883 ; HMDB42889 ; HMDB43422 ; HMDB43480 ; HMDB43786 ; HMDB43788 ; HMDB44189 ; HMDB44297 ; HMDB44302 ; HMDB44357 ; HMDB44384 ; HMDB44439 ; HMDB44459 ; HMDB44466 ; HMDB44493 ; HMDB44498 ; HMDB44520 ; HMDB44546 ; HMDB44552 ; HMDB44567 ; HMDB44573 ; HMDB44599 ; HMDB44606 ; HMDB45123 ; HMDB45202 ; HMDB45312 ; HMDB45332 ; HMDB45338 ; HMDB45364 ; HMDB45383 ; HMDB45390 ; HMDB46121 ; HMDB47998 ; HMDB48014 ; HMDB48152 ; HMDB48203 ; HMDB48212 ; HMDB48218 ; HMDB48335 ; HMDB48372 ; HMDB48631 ;	TG(16:020:4(5Z8Z11Z14Z)20:4(5Z8Z11Z14Z))iso3 ; TG(18:2(9Z12Z)18:2(9Z12Z)20:4(5Z8Z11Z14Z))iso3 ; TG(18:2(9Z12Z)16:022:6(4Z7Z10Z13Z16Z19Z))iso6 ; TG(18:2(9Z12Z)18:1(9Z)20:5(5Z8Z11Z14Z17Z))iso6 ; TG(20:4(5Z8Z11Z14Z)18:1(9Z)18:3(9Z12Z15Z))iso6 ; TG(14:020:3(5Z8Z11Z)22:5(4Z7Z10Z13Z16Z)) ; TG(14:020:3(5Z8Z11Z)22:5(7Z10Z13Z16Z19Z)) ; TG(14:020:3n622:5(4Z7Z10Z13Z16Z)) ; TG(14:020:3n622:5(7Z10Z13Z16Z19Z)) ; TG(14:020:4(5Z8Z11Z14Z)22:4(7Z10Z13Z16Z)) ; TG(14:022:4(7Z10Z13Z16Z)20:4(5Z8Z11Z14Z)) ; TG(14:022:4(7Z10Z13Z16Z)20:4(8Z11Z14Z17Z)) ; TG(14:022:5(4Z7Z10Z13Z16Z)20:3(5Z8Z11Z)) ; TG(14:022:5(4Z7Z10Z13Z16Z)20:3n6) ; TG(14:020:4(8Z11Z14Z17Z)22:4(7Z10Z13Z16Z)) ; TG(14:022:5(7Z10Z13Z16Z19Z)20:3(5Z8Z11Z)) ; TG(14:022:5(7Z10Z13Z16Z19Z)20:3n6) ; TG(15:018:2(9Z12Z)22:6(4Z7Z10Z13Z16Z19Z)) ; TG(15:020:2n622:6(4Z7Z10Z13Z16Z19Z)) ; TG(15:022:6(4Z7Z10Z13Z16Z19Z)18:2(9Z12Z)) ; TG(15:022:6(4Z7Z10Z13Z16Z19Z)20:2n6) ; TG(16:020:3(5Z8Z11Z)20:5(5Z8Z11Z14Z17Z)) ; TG(16:018:3(6Z9Z12Z)22:5(4Z7Z10Z13Z16Z)) ; TG(16:018:3(6Z9Z12Z)22:5(7Z10Z13Z16Z19Z)) ; TG(16:020:3n620:5(5Z8Z11Z14Z17Z)) ; TG(16:020:4(5Z8Z11Z14Z)20:4(8Z11Z14Z17Z)) ; TG(16:022:4(7Z10Z13Z16Z)18:4(6Z9Z12Z15Z)) ; TG(16:022:5(4Z7Z10Z13Z16Z)18:3(6Z9Z12Z)) ; TG(16:022:5(4Z7Z10Z13Z16Z)18:3(9Z12Z15Z)) ; TG(16:018:3(9Z12Z15Z)22:5(4Z7Z10Z13Z16Z)) ; TG(16:018:3(9Z12Z15Z)22:5(7Z10Z13Z16Z19Z)) ; TG(16:018:4(6Z9Z12Z15Z)22:4(7Z10Z13Z16Z)) ; TG(16:020:4(8Z11Z14Z17Z)20:4(5Z8Z11Z14Z)) ; TG(16:020:4(8Z11Z14Z17Z)20:4(8Z11Z14Z17Z)) ; TG(16:020:5(5Z8Z11Z14Z17Z)20:3(5Z8Z11Z)) ; TG(16:020:5(5Z8Z11Z14Z17Z)20:3n6) ; TG(16:022:5(7Z10Z13Z16Z19Z)18:3(6Z9Z12Z)) ; TG(16:022:5(7Z10Z13Z16Z19Z)18:3(9Z12Z15Z)) ; TG(18:018:3(6Z9Z12Z)20:5(5Z8Z11Z14Z17Z)) ; TG(18:020:4(5Z8Z11Z14Z)18:4(6Z9Z12Z15Z)) ; TG(18:018:3(9Z12Z15Z)20:5(5Z8Z11Z14Z17Z)) ; TG(18:018:4(6Z9Z12Z15Z)20:4(5Z8Z11Z14Z)) ; TG(18:018:4(6Z9Z12Z15Z)20:4(8Z11Z14Z17Z)) ; TG(18:020:4(8Z11Z14Z17Z)18:4(6Z9Z12Z15Z)) ; TG(18:020:5(5Z8Z11Z14Z17Z)18:3(6Z9Z12Z)) ; TG(18:020:5(5Z8Z11Z14Z17Z)18:3(9Z12Z15Z)) ; TG(20:018:4(6Z9Z12Z15Z)18:4(6Z9Z12Z15Z)) ; TG(14:1(9Z)20:1(11Z)22:6(4Z7Z10Z13Z16Z19Z)) ; TG(14:1(9Z)20:3(5Z8Z11Z)22:4(7Z10Z13Z16Z)) ; TG(14:1(9Z)20:3n622:4(7Z10Z13Z16Z)) ; TG(14:1(9Z)22:2(13Z16Z)20:5(5Z8Z11Z14Z17Z)) ; TG(14:1(9Z)22:4(7Z10Z13Z16Z)20:3(5Z8Z11Z)) ;	M+2H ; M+NH4 ; M+NH4 ; M+2Na ; M+2Na ; M+2Na ; M+H+Na ; M+2Na ; M+H+Na ; M+2Na ; M+H+Na ; M+2K ;	C59H98O6 ; C59H98O6 ;
762.60199	1.349237	1.292498	3.0943	0.01136	1.9446	0.049056	762.602	HMDB07355 ; HMDB07380 ; HMDB07381 ; HMDB07408 ; HMDB07465 ; HMDB07494 ; HMDB07522 ; HMDB07551 ; HMDB07579 ; HMDB07607 ; HMDB07634 ; HMDB07635 ; HMDB07661 ; HMDB07662 ; HMDB07688 ; HMDB07716 ; HMDB07745 ; HMDB07831 ; HMDB32195 ; HMDB38961 ; HMDB56080 ; HMDB56085 ; HMDB56108 ; HMDB56226 ; HMDB56242 ; HMDB56257 ; HMDB56263 ; HMDB56277 ; HMDB56323	DG(18:4(6Z9Z12Z15Z)24:1(15Z)0:0) ; DG(20:022:5(4Z7Z10Z13Z16Z)0:0) ; DG(20:022:5(7Z10Z13Z16Z19Z)0:0) ; DG(20:1(11Z)22:4(7Z10Z13Z16Z)0:0) ; DG(20:3(5Z8Z11Z)22:2(13Z16Z)0:0) ; DG(20:3(8Z11Z14Z)22:2(13Z16Z)0:0) ; DG(20:4(5Z8Z11Z14Z)22:1(13Z)0:0) ; DG(20:4(8Z11Z14Z17Z)22:1(13Z)0:0) ; DG(20:5(5Z8Z11Z14Z17Z)22:00:0) ; DG(22:020:5(5Z8Z11Z14Z17Z)0:0) ; DG(22:1(13Z)20:4(5Z8Z11Z14Z)0:0) ; DG(22:1(13Z)20:4(8Z11Z14Z17Z)0:0) ; DG(22:2(13Z16Z)20:3(5Z8Z11Z)0:0) ; DG(22:2(13Z16Z)20:3(8Z11Z14Z)0:0) ; DG(22:4(7Z10Z13Z16Z)20:1(11Z)0:0) ; DG(22:5(4Z7Z10Z13Z16Z)20:00:0) ; DG(22:5(7Z10Z13Z16Z19Z)20:00:0) ; DG(24:1(15Z)18:4(6Z9Z12Z15Z)0:0) ; Carrageenan and salts of carrageenan ; N-2-(1H-Indol-3-yl)ethyldocosanamide ; DG(20:00:022:5n6) ; DG(20:00:022:5n3) ; DG(22:00:020:5n3) ; DG(20:1n90:022:4n6) ; DG(20:3n90:022:2n6) ; DG(22:1n90:020:4n6) ; DG(22:1n90:020:4n3) ; DG(24:1n90:018:4n3) ; DG(20:3n60:022:2n6)	M+ACN+Na ; M+ACN+Na ; M+ACN+Na ; M+ACN+Na ; M+ACN+Na ; M+ACN+Na ; M+ACN+Na ; M+ACN+Na ; M+ACN+Na ; M+ACN+Na ; M+ACN+Na ; M+ACN+Na ; M+ACN+Na ; M+ACN+Na ; M+ACN+Na ; M+ACN+Na ; M+ACN+Na ; M+ACN+Na ; M+H+NH4 ; 3M+2K ; M+ACN+Na ; M+ACN+Na ; M+ACN+Na ; M+ACN+Na ; M+ACN+Na ; M+ACN+Na ; M+ACN+Na ; M+ACN+Na ; M+ACN+Na	C45H78O5 ; C45H78O5 ; C45H78O5 ; C45H78O5 ; C45H78O5 ; C45H78O5 ; C45H78O5 ; C45H78O5 ; C45H78O5 ; C45H78O5 ; C45H78O5 ; C45H78O5 ; C45H78O5 ; C45H78O5 ; C45H78O5 ; C45H78O5 ; C45H78O5 ; C45H78O5 ; C42H74O46S6 ; C32H54N2O ; C45H78O5 ; C45H78O5 ; C45H78O5 ; C45H78O5 ; C45H78O5 ; C45H78O5 ; C45H78O5 ; C45H78O5 ; C45H78O5
480.82138	1.303346	0.162772	3.0943	0.011361	1.9446	0.049056	480.8214	HMDB08750 ; HMDB08814 ; HMDB09782 ; HMDB09783 ; HMDB09799 ; HMDB09806 ; HMDB09822 ; HMDB09834	PC(22:6(4Z7Z10Z13Z16Z19Z)24:1(15Z)) ; PC(24:1(15Z)22:6(4Z7Z10Z13Z16Z19Z)) ; PI(16:018:1(11Z)) ; PI(16:018:1(9Z)) ; PI(16:1(9Z)18:0) ; PI(18:016:1(9Z)) ; PI(18:1(11Z)16:0) ; PI(18:1(9Z)16:0)	M+2Na ; M+2Na ; M+3ACN+2H ; M+3ACN+2H ; M+3ACN+2H ; M+3ACN+2H ; M+3ACN+2H ; M+3ACN+2H	C54H94NO8P ; C54H94NO8P ; C43H81O13P ; C43H81O13P ; C43H81O13P ; C43H81O13P ; C43H81O13P ; C43H81O13P
615.93217	1.31561	1.660442	-3.0925	0.011394	1.9433	0.049156	NA	NA	NA	NA	NA
539.02543	1.304926	1.648909	-3.0909	0.011426	1.9421	0.04925	539.0254	HMDB01491 ; HMDB44833 ; HMDB45609 ; HMDB45634 ; HMDB46357 ; HMDB46381 ; HMDB47077 ; HMDB60728	Pyridoxal 5-phosphate ; TG(18:024:024:0) ; TG(20:022:024:0) ; TG(20:024:022:0) ; TG(22:020:024:0) ; TG(22:022:022:0) ; TG(24:018:024:0) ; 23-Dideoxyadenosine-5-triphosphate	2M+2Na-H ; M+H+NH4 ; M+H+NH4 ; M+H+NH4 ; M+H+NH4 ; M+H+NH4 ; M+H+NH4 ; M+ACN+Na	C8H10NO6P ; C69H134O6 ; C69H134O6 ; C69H134O6 ; C69H134O6 ; C69H134O6 ; C69H134O6 ; C10H16N5O11P3
196.86509	1.325583	1.738633	3.089	0.011464	1.9407	0.049361	NA	NA	NA	NA	NA
463.06982	1.323327	0.672033	-3.0881	0.011482	1.94	0.049361	463.0698	HMDB01409 ; HMDB14580 ; HMDB35504 ; HMDB37672 ; HMDB37673 ; HMDB37674 ; HMDB37810 ; HMDB38857 ; HMDB39572	dUMP ; Bendroflumethiazide ; Epicatechin-(4beta-6)-epicatechin-(2beta-74beta-8)-epicatechin ; Epicatechin-(2beta-74beta-8)-epicatechin-(4beta-8)-epicatechin ; Cinnamtannin D1 ; Pavetannin B6 ; 411-Dichloro-512-dihydroquino23-bacridine-714-dione ; Epicatechin-(4beta-8)-epicatechin-(2beta-74beta-8)-catechin ; Pavetannin B2	3M+2H ; M+ACN+H ; M+Na+K ; M+Na+K ; M+Na+K ; M+Na+K ; M+2ACN+H ; M+Na+K ; M+Na+K	C9H13N2O8P ; C15H14F3N3O4S2 ; C45H36O18 ; C45H36O18 ; C45H36O18 ; C45H36O18 ; C20H10Cl2N2O2 ; C45H36O18 ; C45H36O18
303.01465	1.316943	1.467014	-3.0877	0.011489	1.9397	0.049361	303.0076	HMDB14627 ; HMDB33040 ; HMDB33956 ; HMDB03074	Brimonidine ; Propyl 1-(propylthio)propyl disulfide ; Dichlorvos ; Delphinidin	2M+H+Na ; M+2K+H ; M+2ACN+H ; M+H-2H2O	C11H10BrN5 ; C9H20S3 ; C4H7Cl2O4P ; C15H11ClO7
494.69151	1.394875	0.769029	-3.0876	0.011491	1.9396	0.049361	NA	NA	NA	NA	NA
95.501661	1.368783	0.41376	3.0857	0.011527	1.9383	0.049473	NA	NA	NA	NA	NA
179.96193	1.436346	0.366269	3.0839	0.011563	1.9369	0.049585	179.9619	HMDB34895 ; HMDB29555	3-(Dichloromethylene)-25-pyrrolidinedione ; 45-Dihydro-2-methylthiazole	M+H ; M+2K+H	C5H3Cl2NO2 ; C4H7NS
412.11317	1.357662	1.451018	-3.0828	0.011586	1.9361	0.049639	412.1132	HMDB36321 ; HMDB36322 ; HMDB36570 ; HMDB39896 ; HMDB40556 ; HMDB60534	(7E7R8R)-e-Viniferin 35-diglucoside ; (7Z7R8R)-e-Viniferin 35-diglucoside ; Pallidol 33-diglucoside ; xi-8-Acetonyldihydrosanguinarine ; 5-Hydroxyflavone ; Desmethylazelastine	M+2Na ; M+2Na ; M+2Na ; M+Na ; 2M+H+Na ; M+2Na-H	C40H42O16 ; C40H42O16 ; C40H42O16 ; C23H19NO5 ; C21H20O8 ; C21H22ClN3O

663.45539	1.37025	0.260762	3.0795	0.011651	1.9337	0.049874	663.4554	HMDB02190 ; HMDB02232 ; HMDB02283 ; HMDB03876 ; HMDB04264 ; HMDB04673 ; HMDB04679 ; HMDB04680 ; HMDB04682 ; HMDB05998 ; HMDB06111 ; HMDB06245 ; HMDB10222 ; HMDB10409 ; HMDB11134 ; HMDB11136 ; HMDB12508 ; HMDB12567 ; HMDB12598 ; HMDB14384 ; HMDB30438 ; HMDB31749 ; HMDB31893 ; HMDB33186 ; HMDB35389 ; HMDB35436 ; HMDB36262 ; HMDB36705 ; HMDB36810 ; HMDB38520 ; HMDB38688 ; HMDB38689 ; HMDB38694 ; HMDB60101 ; HMDB60102	56-Epoxy-81114-eicosatrienoic acid ; 89-Epoxyeicosatrienoic acid ; 14R15S-EpETrE ; 15(S)-HETE ; 1415-Epoxy-5811-eicosatrienoic acid ; 1112-Epoxyeicosatrienoic acid ; 8-HETE ; 16(R)-HETE ; 11(R)-HETE ; 20-Hydroxyeicosatetraenoic acid ; 12-HETE ; 18-Hydroxyarachidonic acid ; 9-HETE ; 1112-EpETrE ; 5-HETE ; 19(S)-HETE ; 10-HETE ; 13-HETE ; 17-HETE ; Oxiconazole ; Rollinecin A ; Annomutacin ; Squamoxinone ; Annoglacin A ; Asitrilobin D ; Asitrilobin C ; Annoglabasin E ; Ucriol ; 8alpha13R-Epoxy-14-labden-19-oic acid ; 2-Hydroxy-6-tridecylbenzoic acid ; (ent-2alpha3beta15beta16beta)-1516-Epoxy-23-kauranediol ; (ent-2alpha3beta15beta)-16-Kaurene-2315-triol ; Crispane ; 12 Hydroxy arachidonic acid ; Arachidonate	2M+Na ; 2M+Na ; 2M+Na ; 2M+Na ; 2M+Na ; 2M+Na ; 2M+Na ; 2M+Na ; 2M+Na ; 2M+Na ; 2M+Na ; 2M+Na ; 2M+Na ; 2M+Na ; 2M+Na ; 2M+Na ; 2M+Na ; 2M+Na ; 2M+Na ; 3M+2Na ; M+K ; M+K ; M+K ; M+K ; M+K ; M+K ; 2M+Na ; 2M+Na ; 2M+Na ; 2M+Na ; 2M+Na ; 2M+Na ; 2M+Na ; 2M+Na ; 2M+Na	C20H32O3 ; C20H32O3 ; C20H32O3 ; C20H32O3 ; C20H32O3 ; C20H32O3 ; C20H32O3 ; C20H32O3 ; C20H32O3 ; C20H32O3 ; C20H32O3 ; C20H32O3 ; C20H32O3 ; C20H32O3 ; C20H32O3 ; C20H32O3 ; C20H32O3 ; C20H32O3 ; C20H32O3 ; C18H13Cl4N3O ; C37H68O7 ; C37H68O7 ; C37H68O7 ; C37H68O7 ; C37H68O7 ; C37H68O7 ; C20H32O3 ; C20H32O3 ; C20H32O3 ; C20H32O3 ; C20H32O3 ; C20H32O3 ; C20H32O3 ; C20H32O3 ; C20H32O3
628.29568	1.309943	0.946166	-3.0735	0.011771	1.9292	0.050252	628.2957	HMDB12212 ; HMDB15397 ; HMDB30163 ; HMDB31672 ; HMDB33881 ; HMDB33972 ; HMDB34142 ; HMDB35431 ; HMDB39036	Dihydrozeatin-9-N-glucoside-O-glucoside ; Sunitinib ; Galbanic acid ; Armillaribin ; Mammea CAB cyclo D ; Asacoumarin A ; 5-Hydroxy-88-dimethyl-6-(3-methyl-1-oxobutyl)-4-pentyl-2H8H-benzo12-b:34-bdipyran-2-one ; Assamsaponin D ; Kamolonol	M+2ACN+H ; 3M+Na+K ; 3M+Na+K ; 3M+Na+K ; 3M+Na+K ; 3M+Na+K ; 3M+Na+K ; M+H+Na ; 3M+Na+K	C22H35N5O11 ; C22H27FN4O2 ; C24H30O5 ; C24H30O5 ; C24H30O5 ; C24H30O5 ; C24H30O5 ; C59H92O27 ; C24H30O5
460.77991	1.313896	1.560123	3.0734	0.011773	1.9291	0.050252	460.7799	HMDB08649 ; HMDB08680 ; HMDB08681 ; HMDB08713 ; HMDB08714 ; HMDB08745 ; HMDB34651 ; HMDB35958 ; HMDB40943 ; HMDB40946	PC(22:4(7Z10Z13Z16Z)22:6(4Z7Z10Z13Z16Z19Z)) ; PC(22:5(4Z7Z10Z13Z16Z)22:5(4Z7Z10Z13Z16Z)) ; PC(22:5(4Z7Z10Z13Z16Z)22:5(7Z10Z13Z16Z19Z)) ; PC(22:5(7Z10Z13Z16Z19Z)22:5(4Z7Z10Z13Z16Z)) ; PC(22:5(7Z10Z13Z16Z19Z)22:5(7Z10Z13Z16Z19Z)) ; PC(22:6(4Z7Z10Z13Z16Z19Z)22:4(7Z10Z13Z16Z)) ; Soyasaponin III ; Saponin E ; Mabiogenin 3-rhamnosyl-(1-6)-glucoside ; Jujubasaponin VI	M+H+K ; M+H+K ; M+H+K ; M+H+K ; M+H+K ; M+H+K ; M+3ACN+2H ; M+3ACN+2H ; M+3ACN+2H ; M+3ACN+2H	C52H84NO8P ; C52H84NO8P ; C52H84NO8P ; C52H84NO8P ; C52H84NO8P ; C52H84NO8P ; C42H68O14 ; C42H68O14 ; C42H68O14 ; C42H68O14
192.88866	1.364352	0.873756	3.073	0.011781	1.9288	0.050252	NA	NA	NA	NA	NA
500.42840	1.360522	0.568528	-3.0728	0.011785	1.9287	0.050252	500.4284	HMDB08782 ; HMDB51354 ; HMDB51405 ; HMDB53166 ; HMDB53236 ; HMDB53257 ; HMDB53262 ; HMDB53976 ; HMDB54009 ; HMDB54231 ; HMDB54297 ; HMDB54331 ; HMDB54337 ; HMDB54864 ; HMDB54904 ; HMDB55044 ; HMDB55100 ; HMDB55142 ; HMDB55308 ; HMDB55343 ; HMDB55355 ; HMDB55488 ; HMDB55635 ; HMDB55665 ; HMDB55681 ; HMDB55736 ; HMDB55760 ; HMDB55854 ; HMDB55875	PC(24:024:0) ; TG(20:3(5Z8Z11Z)20:5(5Z8Z11Z14Z17Z)o-18:0) ; TG(20:3(5Z8Z11Z)o-18:020:5(5Z8Z11Z14Z17Z)) ; TG(18:3(6Z9Z12Z)22:5(4Z7Z10Z13Z16Z)o-18:0) ; TG(18:3(6Z9Z12Z)22:5(7Z10Z13Z16Z19Z)o-18:0) ; TG(18:3(6Z9Z12Z)o-18:022:5(4Z7Z10Z13Z16Z)) ; TG(18:3(6Z9Z12Z)o-18:022:5(7Z10Z13Z16Z19Z)) ; TG(20:3n620:5(5Z8Z11Z14Z17Z)o-18:0) ; TG(20:3n6o-18:020:5(5Z8Z11Z14Z17Z)) ; TG(20:4(5Z8Z11Z14Z)20:4(5Z8Z11Z14Z)o-18:0) ; TG(20:4(5Z8Z11Z14Z)20:4(8Z11Z14Z17Z)o-18:0) ; TG(20:4(5Z8Z11Z14Z)o-18:020:4(5Z8Z11Z14Z)) ; TG(20:4(5Z8Z11Z14Z)o-18:020:4(8Z11Z14Z17Z)) ; TG(22:4(7Z10Z13Z16Z)18:4(6Z9Z12Z15Z)o-18:0) ; TG(22:4(7Z10Z13Z16Z)o-18:018:4(6Z9Z12Z15Z)) ; TG(22:5(4Z7Z10Z13Z16Z)18:3(6Z9Z12Z)o-18:0) ; TG(22:5(4Z7Z10Z13Z16Z)18:3(9Z12Z15Z)o-18:0) ; TG(22:5(4Z7Z10Z13Z16Z)o-18:018:3(9Z12Z15Z)) ; TG(18:3(9Z12Z15Z)22:5(4Z7Z10Z13Z16Z)o-18:0) ; TG(18:3(9Z12Z15Z)22:5(7Z10Z13Z16Z19Z)o-18:0) ; TG(18:3(9Z12Z15Z)o-18:022:5(7Z10Z13Z16Z19Z)) ; TG(18:4(6Z9Z12Z15Z)22:4(7Z10Z13Z16Z)o-18:0) ; TG(20:4(8Z11Z14Z17Z)20:4(5Z8Z11Z14Z)o-18:0) ; TG(20:4(8Z11Z14Z17Z)20:4(8Z11Z14Z17Z)o-18:0) ; TG(20:4(8Z11Z14Z17Z)o-18:020:4(8Z11Z14Z17Z)) ; TG(20:5(5Z8Z11Z14Z17Z)20:3(5Z8Z11Z)o-18:0) ; TG(20:5(5Z8Z11Z14Z17Z)20:3n6o-18:0) ; TG(22:5(7Z10Z13Z16Z19Z)18:3(6Z9Z12Z)o-18:0) ; TG(22:5(7Z10Z13Z16Z19Z)18:3(9Z12Z15Z)o-18:0)	M+ACN+2H ; M+2ACN+2H	C56H112NO8P ; C61H104O5
162.00734	1.39179	1.316666	3.0631	0.011981	1.9215	0.050895	162.0073	HMDB00497 ; HMDB11160 ; HMDB14483 ; HMDB28754 ; HMDB30786 ; HMDB33914 ; HMDB34301 ; HMDB34384 ; HMDB38473 ; HMDB39493 ; HMDB60396 ; HMDB60432 ; HMDB60693 ; HMDB03705	56-Dihydrouridine ; L-alpha-Aspartyl-L-hydroxyproline ; Pyrazinamide ; Aspartyl-Hydroxyproline ; Marmesin ; Aegelinol ; Piperidine ; Corticrocin ; Torachrysone ; Dihydrowyerone acid ; 5-Fluorouridine ; Alcophosphamide ; Alcoifosfamide ; Phosphoguanidinoacetate	M+2K ; M+2K ; M+K ; M+2K ; M+2K ; M+2K ; M+2K-H ; M+2K ; M+2K ; M+2K ; M+Na+K ; M+2Na ; M+2Na ; M+H-2H2O	C9H14N2O6 ; C9H14N2O6 ; C5H5N3O ; C9H14N2O6 ; C14H14O4 ; C14H14O4 ; C5H11N ; C14H14O4 ; C14H14O4 ; C14H14O4 ; C9H11FN2O6 ; C7H17Cl2N2O3P ; C7H17Cl2N2O3P ; C3H8N3O5P
185.51412	1.352693	1.108945	-3.0618	0.012008	1.9205	0.050936	185.5141	HMDB15461	Glycodiazine	M+Na+K	C13H15N3O4S
468.81097	1.355481	1.055942	3.0616	0.012011	1.9204	0.050936	468.811	HMDB00024 ; HMDB08452 ; HMDB08485 ; HMDB08516 ; HMDB08517 ; HMDB08675 ; HMDB08708 ; HMDB08739 ; HMDB08740 ; HMDB09244 ; HMDB09501 ; HMDB09724 ; HMDB13450 ; HMDB13457	3-O-Sulfogalactosylceramide (d18:124:0) ; PC(20:4(5Z8Z11Z14Z)22:6(4Z7Z10Z13Z16Z19Z)) ; PC(20:4(8Z11Z14Z17Z)22:6(4Z7Z10Z13Z16Z19Z)) ; PC(20:5(5Z8Z11Z14Z17Z)22:5(4Z7Z10Z13Z16Z)) ; PC(20:5(5Z8Z11Z14Z17Z)22:5(7Z10Z13Z16Z19Z)) ; PC(22:5(4Z7Z10Z13Z16Z)20:5(5Z8Z11Z14Z17Z)) ; PC(22:5(7Z10Z13Z16Z19Z)20:5(5Z8Z11Z14Z17Z)) ; PC(22:6(4Z7Z10Z13Z16Z19Z)20:4(5Z8Z11Z14Z)) ; PC(22:6(4Z7Z10Z13Z16Z19Z)20:4(8Z11Z14Z17Z)) ; PE(20:024:0) ; PE(22:022:0) ; PE(24:020:0) ; PC(o-22:022:6(4Z7Z10Z13Z16Z19Z)) ; PC(o-22:3(10Z13Z16Z)22:3(10Z13Z16Z))	M+2Na ; M+2ACN+2H ; M+2ACN+2H ; M+2ACN+2H ; M+2ACN+2H ; M+2ACN+2H ; M+2ACN+2H ; M+2ACN+2H ; M+2ACN+2H ; M+2K ; M+2K ; M+2K ; M+Na+K ; M+Na+K	C48H93NO11S ; C50H80NO8P ; C50H80NO8P ; C50H80NO8P ; C50H80NO8P ; C50H80NO8P ; C50H80NO8P ; C50H80NO8P ; C50H80NO8P ; C49H98NO8P ; C49H98NO8P ; C49H98NO8P ; C52H94NO7P ; C52H94NO7P

358.59298	1.418362	0.211472	-3.0603	0.012038	1.9194	0.05101	358.593	HMDB01260 ; HMDB54845 ; HMDB54890 ; HMDB54893 ; HMDB54898 ; HMDB55083 ; HMDB55085 ; HMDB55090 ; HMDB55125 ; HMDB55130 ; HMDB55868 ; HMDB55870 ; HMDB55885	ADP-Ribosyl-L-arginine ; TG(22:4(7Z10Z13Z16Z)22:5(4Z7Z10Z13Z16Z)22:6(4Z7Z10Z13Z16Z19Z)) ; TG(22:4(7Z10Z13Z16Z)22:5(7Z10Z13Z16Z19Z)22:6(4Z7Z10Z13Z16Z19Z)) ; TG(22:4(7Z10Z13Z16Z)22:6(4Z7Z10Z13Z16Z19Z)22:5(4Z7Z10Z13Z16Z)) ; TG(22:4(7Z10Z13Z16Z)22:6(4Z7Z10Z13Z16Z19Z)22:5(7Z10Z13Z16Z19Z)) ; TG(22:5(4Z7Z10Z13Z16Z)22:4(7Z10Z13Z16Z)22:6(4Z7Z10Z13Z16Z19Z)) ; TG(22:5(4Z7Z10Z13Z16Z)22:5(4Z7Z10Z13Z16Z)22:5(4Z7Z10Z13Z16Z)) ; TG(22:5(4Z7Z10Z13Z16Z)22:5(4Z7Z10Z13Z16Z)22:5(7Z10Z13Z16Z19Z)) ; TG(22:5(4Z7Z10Z13Z16Z)22:5(7Z10Z13Z16Z19Z)22:5(4Z7Z10Z13Z16Z)) ; TG(22:5(4Z7Z10Z13Z16Z)22:5(7Z10Z13Z16Z19Z)22:5(7Z10Z13Z16Z19Z)) ; TG(22:5(7Z10Z13Z16Z19Z)22:4(7Z10Z13Z16Z)22:6(4Z7Z10Z13Z16Z19Z)) ; TG(22:5(7Z10Z13Z16Z19Z)22:5(4Z7Z10Z13Z16Z)22:5(7Z10Z13Z16Z19Z)) ; TG(22:5(7Z10Z13Z16Z19Z)22:5(7Z10Z13Z16Z19Z)22:5(7Z10Z13Z16Z19Z))	M+2H ; M+H+2Na ; M+H+2Na ; M+H+2Na ; M+H+2Na ; M+H+2Na ; M+H+2Na ; M+H+2Na ; M+H+2Na ; M+H+2Na ; M+H+2Na ; M+H+2Na ; M+H+2Na	C21H35N9O15P2 ; C69H104O6 ; C69H104O6 ; C69H104O6 ; C69H104O6 ; C69H104O6 ; C69H104O6 ; C69H104O6 ; C69H104O6 ; C69H104O6 ; C69H104O6 ; C69H104O6 ; C69H104O6
374.73468	1.311796	1.747819	3.0582	0.012081	1.9179	0.051146	374.7347	HMDB07928 ; HMDB08918 ; HMDB08919 ; HMDB11204 ; HMDB11403 ; HMDB11436 ; HMDB13410 ; HMDB60531	PC(14:1(9Z)P-16:0) ; PE(15:0P-18:1(11Z)) ; PE(15:0P-18:1(9Z)) ; PC(P-16:014:1(9Z)) ; PE(P-18:1(11Z)15:0) ; PE(P-18:1(9Z)15:0) ; PC(o-16:1(9Z)14:1(9Z)) ; Desmethylnortriptyline	M+Na+K ; M+Na+K ; M+Na+K ; M+Na+K ; M+Na+K ; M+Na+K ; M+Na+K ; 3M+2H	C38H74NO7P ; C38H74NO7P ; C38H74NO7P ; C38H74NO7P ; C38H74NO7P ; C38H74NO7P ; C38H74NO7P ; C18H19N
432.69367	1.32229	1.746428	3.0562	0.012123	1.9164	0.05128	432.6937	HMDB30187 ; HMDB38482 ; HMDB38834	(EE)-Piperlonguminine ; Neolinustatin ; (2E)-Piperamide-C5:1	3M+2Na ; 2M+H+NH4 ; 3M+2Na	C16H19NO3 ; C17H29NO11 ; C16H19NO3
172.09674	1.329944	0.794124	3.054	0.012169	1.9147	0.051432	172.0967	HMDB12798 ; HMDB34363 ; HMDB37591 ; HMDB37592 ; HMDB39276 ; HMDB39821 ; HMDB36604	5-Carboxy-alpha-chromanol ; Monocrotaline ; Oryzalide A ; Oryzalide B ; 8-Gingerdione ; 10-Acetylpanaxytriol ; N-Methylcalystegine B2	M+H+Na ; M+H+NH4 ; M+H+Na ; M+H+Na ; M+H+Na ; M+H+Na ; M+H-H2O	C19H28O4 ; C16H23NO6 ; C19H28O4 ; C19H28O4 ; C19H28O4 ; C19H28O4 ; C8H15NO4
593.05849	1.337862	1.225286	-3.0531	0.012187	1.9141	0.051443	593.0585	HMDB14744 ; HMDB31807	Cyclothiazide ; m-Hydroxydiphenyldimercury(1)	3M+H+NH4 ; M+NH4	C14H16ClN3O4S2 ; C12H11Hg2O
198.86213	1.310695	1.754949	3.0529	0.012192	1.9139	0.051443	NA	NA	NA	NA	NA
614.06438	1.426367	0.066377	-3.0507	0.012237	1.9123	0.051524	NA	NA	NA	NA	NA
296.98946	1.363224	1.302153	-3.0502	0.012247	1.912	0.051524	296.9895	HMDB12228 ; HMDB30568	Ethylphosphate ; Trisjuglone	2M+2Na-H ; M+2K	C2H7O4P ; C30H12O9
256.82059	1.319253	1.753076	3.05	0.012252	1.9118	0.051524	256.8206	HMDB07903 ; HMDB07999 ; HMDB08895 ; HMDB09087	PC(14:1(9Z)16:1(9Z)) ; PC(16:1(9Z)14:1(9Z)) ; PE(15:018:2(9Z12Z)) ; PE(18:2(9Z12Z)15:0)	M+3Na ; M+3Na ; M+3Na ; M+3Na	C38H72NO8P ; C38H72NO8P ; C38H72NO8P ; C38H72NO8P
211.05293	1.380544	1.304189	-3.0491	0.012271	1.9111	0.051559	211.0529	HMDB11751 ; HMDB12206 ; HMDB12915 ; HMDB13922 ; HMDB29685 ; HMDB29688 ; HMDB31240 ; HMDB31621 ; HMDB31769 ; HMDB32128 ; HMDB32174 ; HMDB32574 ; HMDB32640 ; HMDB33761 ; HMDB33932 ; HMDB37235 ; HMDB37602 ; HMDB37733 ; HMDB39034 ; HMDB39100 ; HMDB40311 ; HMDB40796 ; HMDB59611 ; HMDB60734 ; HMDB14467 ; HMDB60406	3-Methoxybenzenepropanoic acid ; CMP-N-glycoloylneuraminate ; Coniferyl alcohol ; 2-Chloroticlopidine ; 4-Ethoxy-3-methoxybenzaldehyde ; 2-Ethoxynaphthalene ; 2-Phenyl-13-dioxolane-4-methanol ; Cinnamylideneacetone ; Carbendazim ; 1-(24-Dihydroxyphenyl)-1-butanone ; Benzaldehyde glyceryl acetal ; Propylparaben ; Ethyl p-anisate ; 4-Methoxybenzyl acetate ; Cinnatriacetin A ; Quassinol ; 4-Hydroxy-266-trimethyl-3-oxo-14-cyclohexadiene-1-carboxaldehyde ; Propyl 2-furanacrylate ; Casegravol isovalerate ; 4-Acetylzearalenone ; 34578-Pentamethoxyflavan ; 6-O-Formylmarmin ; Thiomorpholine 3-carboxylate ; 3-(3-Hydroxyphenyl)-2-methylpropionic acid ; Floxuridine ; 5-Deoxy-5-fluorouridine	2M+Na+K ; M+3H ; 2M+Na+K ; M+3ACN+2H ; 2M+Na+K ; M+K ; 2M+Na+K ; M+K ; 2M+H+K ; 2M+Na+K ; 2M+Na+K ; 2M+Na+K ; 2M+Na+K ; 2M+Na+K ; M+2Na ; M+Na+K ; 2M+Na+K ; 2M+Na+K ; M+Na+K ; M+Na+K ; M+Na+K ; M+Na+K ; M+ACN+Na ; 2M+Na+K ; M+H-2H2O ; M+H-2H2O	C10H12O3 ; C20H31N4O17P ; C10H12O3 ; C14H13Cl2NS ; C10H12O3 ; C12H12O ; C10H12O3 ; C12H12O ; C9H9N3O2 ; C10H12O3 ; C10H12O3 ; C10H12O3 ; C10H12O3 ; C10H12O3 ; C23H20O5 ; C20H24O6 ; C10H12O3 ; C10H12O3 ; C20H24O6 ; C20H24O6 ; C20H24O6 ; C20H24O6 ; C5H9NO2S ; C10H12O3 ; C9H11FN2O5 ; C9H11FN2O5
254.82355	1.319104	1.746053	3.0407	0.012447	1.9049	0.052075	NA	NA	NA	NA	NA
337.52904	1.391077	0.325124	-3.0403	0.012457	1.9046	0.052075	NA	NA	NA	NA	NA
102.97033	1.351188	0.706235	3.0399	0.012465	1.9043	0.052075	NA	NA	NA	NA	NA
337.51960	1.378359	0.216216	-3.0395	0.012473	1.904	0.052075	NA	NA	NA	NA	NA
294.57979	1.330186	1.369216	-3.0394	0.012476	1.9039	0.052075	294.5798	HMDB15381 ; HMDB15491	Gliquidone ; Nitroxoline	M+Na+K ; 3M+H+NH4	C27H33N3O6S ; C9H6N2O3
419.31509	1.378213	0.741816	3.0393	0.012477	1.9039	0.052075	419.3151	HMDB12515 ; HMDB39317 ; HMDB00529 ; HMDB10729 ; HMDB29585 ; HMDB29763 ; HMDB31683 ; HMDB32214 ; HMDB32235 ; HMDB32248 ; HMDB33378 ; HMDB34160 ; HMDB34844 ; HMDB36182 ; HMDB37186 ; HMDB37305 ; HMDB37329 ; HMDB37498 ; HMDB37742 ; HMDB37813 ; HMDB38081 ; HMDB38269 ; HMDB38895 ; HMDB38924 ; HMDB39220 ; HMDB40158 ; HMDB40163 ; HMDB40361 ; HMDB41264 ; HMDB41449 ; HMDB02195	11-Carboxy-alpha-chromanol ; Hexadecyl ferulate ; 5-Dodecenoic acid ; trans-Dodec-2-enoic acid ; Methyl 10-undecenoate ; Allyl nonanoate ; xi-Dihydro-5-octyl-2(3H)-furanone ; cis-4-Decenyl acetate ; 26-Dimethyl-5-heptenal propyleneglycol acetal ; 11-Dodecenoic acid ; cis-3-Hexenyl hexanoate ; ()-Citronellyl acetate ; Citronellyl acetate ; 55-Dibutyl-45-dihydro-2(3H)furanone ; Rhodinyl acetate ; Methyl 9-undecenoate ; Ethyl 2-decenoate ; 1-Ethenylhexyl butanoate ; xi-5-Dodecanolide ; 3-Heptyldihydro-5-methyl-2(3H)-furanone ; 2-Octenyl butyrate ; Hexyl 2E-hexenoate ; 7-Hexyl-2-oxepanone ; 2-Hexenyl hexanoate ; Ethyl 4-decenoate ; Hexyl 2-methyl-3-pentenoate ; Hexyl 2-methyl-4-pentenoate ; 11-Dimethoxy-37-dimethyl-26-octadiene ; ()-Menthyl acetate ; (37-Dimethyl-6-octenyl)oxyacetaldehyde ; Varanic acid	M+H ; M+H ; 2M+Na ; M+H-H2O	C26H42O4 ; C26H42O4 ; C12H22O2 ; C26H44O5

225.07730	1.34135	1.187746	3.0378	0.012509	1.9028	0.052159	225.0773	HMDB30018 ; HMDB32616 ; HMDB34069 ; HMDB41691 ; HMDB12869 ; HMDB14800 ; HMDB29400 ; HMDB29836 ; HMDB30012 ; HMDB30044 ; HMDB32784 ; HMDB32977 ; HMDB32978 ; HMDB32979 ; HMDB32980 ; HMDB35384 ; HMDB35385 ; HMDB37144 ; HMDB41872 ; HMDB42005 ; HMDB60579 ; HMDB28730 ; HMDB28814	Elenolide ; Sinapic acid ; cis-Sinapic acid ; 5-(345-Trihydroxyphenyl)-gamma-valerolactone ; 9-Carboxy-gamma-tocotrienol ; Trimethobenzamide ; Ochratoxin C ; 1234-Tetrahydro-2-methyl-b-carboline ; Scutigeral ; Neriantogenin ; 5-Ethoxysorgoleone 358 ; 23-Dimethylthiophene ; 24-Dimethylthiophene ; 25-Dimethylthiophene ; 34-Dimethylthiophene ; 2-Ethylthiophene ; 3-Ethylthiophene ; 1-Phenyl-5-propyl-1H-pyrazole ; Detomidine ; Quinaprilat ; Ramiprilat ; Asparaginyl-Glutamate ; Glutamyl-Asparagine	M+H ; M+H ; M+H ; M+H ; M+2K ; M+Na+K ; M+H+NH4 ; M+K ; M+2K ; M+2K ; M+2K ; 2M+H ; 2M+H ; 2M+H ; 2M+H ; 2M+H ; 2M+H ; M+K ; M+K ; M+H+K ; M+Na+K ; M+H-2H2O ; M+H-2H2O	C11H12O5 ; C11H12O5 ; C11H12O5 ; C11H12O5 ; C23H32O4 ; C21H28N2O5 ; C22H22ClNO6 ; C12H14N2 ; C23H32O4 ; C23H32O4 ; C23H32O4 ; C6H8S ; C6H8S ; C6H8S ; C6H8S ; C6H8S ; C6H8S ; C12H14N2 ; C12H14N2 ; C23H26N2O5 ; C21H28N2O5 ; C9H14N3O6 ; C9H14N3O6
347.59310	1.331061	1.70801	-3.0362	0.012545	1.9015	0.052183	NA	NA	NA	NA	NA
594.61481	1.321258	0.615933	-3.036	0.012548	1.9014	0.052183	594.6148	HMDB12631 ; HMDB35455 ; HMDB35461 ; HMDB41156	20-CoA-20-oxo-18R-hydroxyleucotriene B4 ; YGM 4B ; YGM 3 ; Cyanidin 3-6-(4-glucosylferuloyl)sophoroside 5-glucoside	M+Na+K ; M+2K ; M+2K ; M+2K	C41H60N7O22P3S ; C52H55O27 ; C52H55O27 ; C49H59O29
164.05608	1.345719	0.729411	3.0351	0.012568	1.9007	0.052192	164.0561	HMDB00802 ; HMDB30393 ; HMDB31187 ; HMDB33581 ; HMDB36735 ; HMDB36936 ; HMDB39167 ; HMDB39169 ; HMDB39509 ; HMDB60077 ; HMDB00208 ; HMDB04193 ; HMDB04194 ; HMDB13701 ; HMDB15200 ; HMDB31825 ; HMDB60763	Pterin ; L-N-Carboxymethylserine ; Thialdine ; trans-o-Coumaric acid 2-glucoside ; Bilobalide A ; 1-O-p-Coumaroyl-beta-D-glucose ; 2-O-p-Coumaroyl-D-glucose ; 6-O-p-Coumaroyl-D-glucose ; trans-p-Coumaric acid 4-glucoside ; cis-beta-D-Glucosyl-2-hydroxycinnamate ; Oxoglutaric acid ; N1-Methyl-2-pyridone-5-carboxamide ; N1-Methyl-4-pyridone-3-carboxamide ; 3-Oxoglutaric acid ; Glipizide ; Phenyl vinyl sulfide ; 4-Hydroxy duloxetine glucuronide	M+H ; M+H ; M+H ; M+2H ; M+2H ; M+2H ; M+2H ; M+2H ; M+2H ; M+2H ; M+NH4 ; 2M+H+Na ; 2M+H+Na ; M+NH4 ; M+H+2Na ; 2M+3H2O+2H ; M+3H	C6H5N5O ; C5H9NO5 ; C6H13NS2 ; C15H18O8 ; C15H18O8 ; C15H18O8 ; C15H18O8 ; C15H18O8 ; C15H18O8 ; C15H18O8 ; C5H6O5 ; C7H8N2O2 ; C7H8N2O2 ; C5H6O5 ; C21H27N5O4S ; C8H8S ; C24H27NO8S
186.10603	1.385984	0.653863	3.0344	0.012583	1.9002	0.052212	186.106	HMDB32841 ; HMDB32842 ; HMDB36822 ; HMDB28807 ; HMDB29059 ; HMDB29075 ; HMDB29159 ; HMDB30326 ; HMDB31179 ; HMDB39466 ; HMDB39833 ; HMDB39834	5-Megastigmen-7-yne-39-diol 9-glucoside ; 5-Megastigmen-7-yne-39-diol 3-glucoside ; (3S7E9S)-9-Hydroxy-47-megastigmadien-3-one 9-glucoside ; Glutaminyl-Threonine ; Threoninyl-Glutamine ; Threoninyl-Gamma-glutamate ; Gamma-glutamyl-Threonine ; Methylisopelletierine ; 2266-Tetramethyl-4-piperidinone ; Hexanethioic acid S-propyl ester ; 3-(1-Pyrrolidinyl)-2-pentanone ; 2-(1-Pyrrolidinyl)-3-pentanone	M+2H ; M+2H ; M+2H ; M+3ACN+2H ; M+3ACN+2H ; M+3ACN+2H ; M+3ACN+2H ; 2M+Na+K ; 2M+Na+K ; 2M+H+Na ; 2M+Na+K ; 2M+Na+K	C19H30O7 ; C19H30O7 ; C19H30O7 ; C9H17N3O5 ; C9H17N3O5 ; C9H17N3O5 ; C9H17N3O5 ; C9H17NO ; C9H17NO ; C9H18OS ; C9H17NO ; C9H17NO
193.05418	1.313717	1.695559	-3.0332	0.012607	1.8994	0.052233	193.0542	HMDB03559 ; HMDB05794 ; HMDB29468 ; HMDB29620 ; HMDB29866 ; HMDB30610 ; HMDB30796 ; HMDB31365 ; HMDB33264 ; HMDB33290 ; HMDB33985 ; HMDB34026 ; HMDB34214 ; HMDB34268 ; HMDB35609 ; HMDB36423 ; HMDB36632 ; HMDB36677 ; HMDB36779 ; HMDB36782 ; HMDB36891 ; HMDB36892 ; HMDB37247 ; HMDB37585 ; HMDB37916 ; HMDB38314 ; HMDB38755 ; HMDB40306 ; HMDB40309 ; HMDB40310 ; HMDB40648 ; HMDB41251 ; HMDB41372 ; HMDB41687 ; HMDB41689	Gibberellin A3 ; Quercetin ; Dolichin B ; Tricetin ; (S)-457-Trihydroxy-3-prenylflavanone ; Desmethylxanthohumol ; Morin ; Gibberellin A94 ; 24578-Pentahydroxyisoflavone ; Junosmarin ; Mulberrofuran S ; Glyceollidin I ; 5-Deoxykievitone ; Lippioside II ; Gibberellin A68 ; Cynaropicrin ; 6-Hydroxyluteolin ; Strigol ; Diosbulbin E ; Diosbulbin G ; Gibberellin A22 ; 11beta-Hydroxygibberellin A7 ; (S)-457-Trihydroxy-6-prenylflavanone ; Selinone ; Glyceollidin II ; Gibberellin GA126 ; Licocoumarone ; Morachalcone A ; 8-Hydroxy-3457-tetramethoxyflavan ; 5-Hydroxy-3478-tetramethoxyflavan ; Gibberellin A92 ; 1-Hydroxy-35-dimethoxy-2-prenylxanthone ; Alectrol ; 56734-Pentahydroxyisoflavone ; 57834-Pentahydroxyisoflavone	M+H+K ; M+2ACN+2H ; M+2Na ; M+2ACN+2H ; M+2Na ; M+2Na ; M+2ACN+2H ; M+H+K ; M+2ACN+2H ; M+H+K ; M+3H ; M+2Na ; M+2Na ; M+2H+Na ; M+H+K ; M+H+K ; M+2ACN+2H ; M+H+K ; M+H+K ; M+H+K ; M+H+K ; M+H+K ; M+2Na ; M+2Na ; M+2Na ; M+H+K ; M+2Na ; M+2Na ; M+H+K ; M+H+K ; M+H+K ; M+2Na ; M+H+K ; M+2ACN+2H ; M+2ACN+2H	C19H22O6 ; C15H10O7 ; C20H20O5 ; C15H10O7 ; C20H20O5 ; C20H20O5 ; C15H10O7 ; C19H22O6 ; C15H10O7 ; C19H22O6 ; C34H24O9 ; C20H20O5 ; C20H20O5 ; C25H30O14 ; C19H22O6 ; C19H22O6 ; C15H10O7 ; C19H22O6 ; C19H22O6 ; C19H22O6 ; C19H22O6 ; C19H22O6 ; C20H20O5 ; C20H20O5 ; C20H20O5 ; C19H22O6 ; C20H20O5 ; C20H20O5 ; C19H22O6 ; C19H22O6 ; C19H22O6 ; C20H20O5 ; C19H22O6 ; C15H10O7 ; C15H10O7
766.57419	1.322132	0.106675	3.0332	0.012609	1.8993	0.052233	766.5742	HMDB08194 ; HMDB08195 ; HMDB08227 ; HMDB08228 ; HMDB08259 ; HMDB08455 ; HMDB08488 ; HMDB11220 ; HMDB11221 ; HMDB11247 ; HMDB11278 ; HMDB11279 ; HMDB11311 ; HMDB11312 ; HMDB13415 ; HMDB39528	PC(18:3(6Z9Z12Z)P-18:1(11Z)) ; PC(18:3(6Z9Z12Z)P-18:1(9Z)) ; PC(18:3(9Z12Z15Z)P-18:1(11Z)) ; PC(18:3(9Z12Z15Z)P-18:1(9Z)) ; PC(18:4(6Z9Z12Z15Z)dm18:0) ; PC(20:4(5Z8Z11Z14Z)P-16:0) ; PC(20:4(8Z11Z14Z17Z)P-16:0) ; PC(P-16:020:4(5Z8Z11Z14Z)) ; PC(P-16:020:4(8Z11Z14Z17Z)) ; PC(P-18:018:4(6Z9Z12Z15Z)) ; PC(P-18:1(11Z)18:3(6Z9Z12Z)) ; PC(P-18:1(11Z)18:3(9Z12Z15Z)) ; PC(P-18:1(9Z)18:3(6Z9Z12Z)) ; PC(P-18:1(9Z)18:3(9Z12Z15Z)) ; PC(o-16:1(9Z)20:4(8Z11Z14Z17Z)) ; 2-O-(58111417-Eicosapentaenoyl)-1-O-hexadecylglycero-3-phosphocholine	M+H ; M+H ; M+H ; M+H ; M+H ; M+H ; M+H ; M+H ; M+H ; M+H ; M+H ; M+H ; M+H ; M+H ; M+H ; M+H	C44H80NO7P ; C44H80NO7P ; C44H80NO7P ; C44H80NO7P ; C44H80NO7P ; C44H80NO7P ; C44H80NO7P ; C44H80NO7P ; C44H80NO7P ; C44H80NO7P ; C44H80NO7P ; C44H80NO7P ; C44H80NO7P ; C44H80NO7P ; C44H80NO7P ; C44H80NO7P

211.10771	1.387587	0.230853	3.0288	0.012704	1.8961	0.052583	211.1075	HMDB15441 ; HMDB15354 ; HMDB00821 ; HMDB00859 ; HMDB10323 ; HMDB11723 ; HMDB13245 ; HMDB13292 ; HMDB14619 ; HMDB31303 ; HMDB31306 ; HMDB31847 ; HMDB31855 ; HMDB31923 ; HMDB32388 ; HMDB33135 ; HMDB33285 ; HMDB33517 ; HMDB34250 ; HMDB37292 ; HMDB38072 ; HMDB40594 ; HMDB40732 ; HMDB41933 ; HMDB41984 ; HMDB59677 ; HMDB59683 ; HMDB59697 ; HMDB60366 ; HMDB60389 ; HMDB60758 ; HMDB60869	Aprobarbital ; Aminophylline ; Phenylacetylglycine ; Methylhippuric acid ; Phenethylamine glucuronide ; 2-Methylhippuric acid ; m-Methylhippuric acid ; p-Methylhippuric acid ; Duloxetine ; Osmundalactone ; 3-Hydroxy-45-dimethyl-2(5H)-furanone ; 2-Ethyl-34-dihydroxyfuran ; 5-Methyl-2-phenyl-2-hexenal ; 1020-Dihydroxyeicosanoic acid ; Methyl n-acetylanthranilate ; Pyrimethanil ; L-erythro-5-(1-Hydroxyethyl)-2(5H)-furanone ; 56-Dihydro-4-methoxy-2H-pyran-2-one ; Betamipron ; 2-Butyl-3-phenyl-2-propen-1-al ; xi-5-Acetyltetrahydro-2(3H)-furanone ; ()-Furaneol ; 27-Oxepanedione ; Mitragynine ; Pholcodine ; 3-Buten-2-one 1-(236-trimethyl phenyl) ; 1-H-Inden-1-one23-dihydro-3356-tetramethyl ; 1-(236-trimethyl phenyl)-3-Buten-2-one ; 3-Carbamoyl-2-phenylpropionaldehyde ; 4-Hydroxy-5-phenyltetrahydro-13-oxazin-2-one ; 4-Anilino-4-oxobutanoic acid ; Ramipril Diketopiperazine	M+H ; M+2H ; M+NH4 ; M+NH4 ; M+3ACN+2H ; M+NH4 ; M+NH4 ; M+NH4 ; M+3ACN+2H ; M+2ACN+H ; M+2ACN+H ; M+2ACN+H ; M+Na ; M+2K ; M+NH4 ; 2M+H+Na ; M+2ACN+H ; M+2ACN+H ; M+NH4 ; M+Na ; M+2ACN+H ; M+2ACN+H ; M+2ACN+H ; M+H+Na ; M+H+Na ; M+Na ; M+Na ; M+Na ; M+NH4 ; M+NH4 ; M+NH4 ; M+H+Na	C10H14N2O3 ; C16H24N10O4 ; C10H11NO3 ; C10H11NO3 ; C14H19NO6 ; C10H11NO3 ; C10H11NO3 ; C10H11NO3 ; C18H19NOS ; C6H8O3 ; C6H8O3 ; C6H8O3 ; C13H16O ; C20H40O4 ; C10H11NO3 ; C12H13N3 ; C6H8O3 ; C6H8O3 ; C10H11NO3 ; C13H16O ; C6H8O3 ; C6H8O3 ; C6H8O3 ; C23H30N2O4 ; C23H30N2O4 ; C13H16O ; C13H16O ; C13H16O ; C10H11NO3 ; C10H11NO3 ; C10H11NO3 ; C23H30N2O4
567.04420	1.336834	0.091913	-3.0277	0.012727	1.8953	0.052616	567.0442	HMDB01413 ; HMDB06941 ; HMDB13926 ; HMDB39638	Citicoline ; L-2-Aminoadipate adenylate ; Dehydrogenated ticlopidine ; Brassilexin	M+2K+H ; M+2K-H ; 2M+2Na-H ; 3M+2Na-H	C14H26N4O11P2 ; C16H23N6O10P ; C14H12ClNS ; C9H6N2S
442.35232	1.315552	0.255097	3.0252	0.012781	1.8934	0.052727	442.3523	HMDB13339 ; HMDB13340 ; HMDB08026 ; HMDB08053 ; HMDB08085 ; HMDB08118 ; HMDB08150 ; HMDB08191 ; HMDB08224 ; HMDB08256 ; HMDB08276 ; HMDB08285 ; HMDB08308 ; HMDB08340 ; HMDB08350 ; HMDB08382 ; HMDB08414 ; HMDB08446 ; HMDB08479 ; HMDB08533 ; HMDB08542 ; HMDB08543 ; HMDB08564 ; HMDB08572 ; HMDB08573 ; HMDB08595 ; HMDB08604 ; HMDB08635 ; HMDB08766 ; HMDB08792 ; HMDB08797 ; HMDB08798 ; HMDB09247 ; HMDB09510 ; HMDB11202 ; HMDB11355 ; HMDB11380 ; HMDB13466 ; HMDB43046 ; HMDB43802 ; HMDB43884 ; HMDB59929	3-Hydroxy-11Z-octadecenoylcarnitine ; 3-Hydroxy-9Z-octadecenoylcarnitine ; PC(16:1(9Z)24:1(15Z)) ; PC(18:022:2(13Z16Z)) ; PC(18:1(11Z)22:1(13Z)) ; PC(18:1(9Z)22:1(13Z)) ; PC(18:2(9Z12Z)22:0) ; PC(18:3(6Z9Z12Z)24:1(15Z)) ; PC(18:3(9Z12Z15Z)24:1(15Z)) ; PC(18:4(6Z9Z12Z15Z)24:0) ; PC(20:020:2(11Z14Z)) ; PC(20:022:4(7Z10Z13Z16Z)) ; PC(20:1(11Z)20:1(11Z)) ; PC(20:2(11Z14Z)20:0) ; PC(20:2(11Z14Z)22:2(13Z16Z)) ; PC(20:3(5Z8Z11Z)22:1(13Z)) ; PC(20:3(8Z11Z14Z)22:1(13Z)) ; PC(20:4(5Z8Z11Z14Z)22:0) ; PC(20:4(8Z11Z14Z17Z)22:0) ; PC(22:018:2(9Z12Z)) ; PC(22:020:4(5Z8Z11Z14Z)) ; PC(22:020:4(8Z11Z14Z17Z)) ; PC(22:1(13Z)18:1(9Z)) ; PC(22:1(13Z)20:3(5Z8Z11Z)) ; PC(22:1(13Z)20:3(8Z11Z14Z)) ; PC(22:2(13Z16Z)18:0) ; PC(22:2(13Z16Z)20:2(11Z14Z)) ; PC(22:4(7Z10Z13Z16Z)20:0) ; PC(24:018:4(6Z9Z12Z15Z)) ; PC(24:1(15Z)16:1(9Z)) ; PC(24:1(15Z)18:3(6Z9Z12Z)) ; PC(24:1(15Z)18:3(9Z12Z15Z)) ; PE(20:0dm18:0) ; PE(22:0P-16:0) ; PC(22:1(13Z)18:1(11Z)) ; PE(P-16:022:0) ; PE(P-18:020:0) ; SM(d18:022:1(13Z)(OH)) ; TG(15:016:0o-18:0) ; TG(15:0o-18:016:0) ; TG(16:015:0o-18:0) ; 3-(Acetyloxy)-2-hydroxypropyl octadecanoate	M+H ; M+H ; M+ACN+2H ; M+ACN+2H ; M+ACN+2H ; M+ACN+2H ; M+ACN+2H ; M+H+NH4 ; M+H+NH4 ; M+H+NH4 ; M+ACN+2H ; M+H+NH4 ; M+ACN+2H ; M+ACN+2H ; M+H+NH4 ; M+H+NH4 ; M+H+NH4 ; M+H+NH4 ; M+H+NH4 ; M+ACN+2H ; M+H+NH4 ; M+H+NH4 ; M+ACN+2H ; M+H+NH4 ; M+H+NH4 ; M+ACN+2H ; M+H+NH4 ; M+H+NH4 ; M+H+NH4 ; M+ACN+2H ; M+H+NH4 ; M+H+NH4 ; M+3ACN+2H ; M+3ACN+2H ; M+ACN+2H ; M+3ACN+2H ; M+3ACN+2H ; M+2ACN+2H ; M+2K ; M+2K ; M+2K ; M+ACN+H	C25H47NO5 ; C25H47NO5 ; C48H92NO8P ; C48H92NO8P ; C48H92NO8P ; C48H92NO8P ; C48H92NO8P ; C50H92NO8P ; C50H92NO8P ; C50H92NO8P ; C48H92NO8P ; C50H92NO8P ; C48H92NO8P ; C48H92NO8P ; C50H92NO8P ; C50H92NO8P ; C50H92NO8P ; C50H92NO8P ; C50H92NO8P ; C48H92NO8P ; C50H92NO8P ; C50H92NO8P ; C48H92NO8P ; C50H92NO8P ; C50H92NO8P ; C48H92NO8P ; C50H92NO8P ; C50H92NO8P ; C50H92NO8P ; C48H92NO8P ; C50H92NO8P ; C50H92NO8P ; C43H86NO7P ; C43H86NO7P ; C48H92NO8P ; C43H86NO7P ; C43H86NO7P ; C45H89N2O7P ; C52H102O5 ; C52H102O5 ; C52H102O5 ; C23H44O5
574.60943	1.31865	1.733828	3.0231	0.012827	1.8919	0.052829	574.6094	HMDB32828 ; HMDB60387	Corchorusoside E ; 4-Glutathionyl cyclophosphamide	2M+3H ; 2M+H+NH4	C41H64O19 ; C17H30Cl2N5O8PS
407.63162	1.32113	1.321049	-3.0194	0.012908	1.8891	0.05312	407.6316	HMDB32109 ; HMDB40382 ; HMDB41819 ; HMDB60647	Koeniginequinone B ; Pummeline ; 7-Aminonitrazepam ; 4-hydroxy ketorolac	3M+2H ; 3M+2H ; 3M+Na+K ; 3M+2H	C15H13NO4 ; C15H13NO4 ; C15H13N3O ; C15H13NO4
626.18457	1.418944	0.539436	-3.017	0.012962	1.8873	0.053297	626.1846	HMDB37418 ; HMDB37950 ; HMDB37951 ; HMDB37952 ; HMDB39934 ; HMDB59609 ; HMDB60560	Molludistin 2-rhamnoside ; Catechin 34-diglucoside ; Catechin 35-diglucoside ; Catechin 37-diglucoside ; Chrysin 7-rhamnosyl-(1-4)-glucoside ; albendazole S-oxide ; Albendazole sulfoxide	M+ACN+Na ; 2M+H+Na ; 2M+H+Na ; 2M+H+Na ; M+ACN+Na ; 2M+ACN+Na ; 2M+ACN+Na	C27H30O13 ; C27H34O16 ; C27H34O16 ; C27H34O16 ; C27H30O13 ; C12H15N3O3S ; C12H15N3O3S
594.81225	1.349502	0.787086	-3.0154	0.012996	1.8862	0.053395	594.8123	HMDB35483 ; HMDB38312 ; HMDB38615 ; HMDB39397 ; HMDB39482	Tuberoside C (Allium tuberosum) ; Balanitoside ; Trigofoenoside F ; Tuberoside L ; Trigofoenoside D	M+3ACN+2H ; M+3ACN+2H ; M+3ACN+2H ; M+3ACN+2H ; M+3ACN+2H	C51H84O23 ; C51H84O23 ; C51H84O23 ; C51H84O23 ; C51H84O23
234.98531	1.309778	1.243661	-3.0127	0.013057	1.8842	0.05351	234.9853	HMDB00544 ; HMDB01014 ; HMDB02320 ; HMDB60013 ; HMDB60712	5-Hydroxymethyl-4-methyluracil ; 4-Imidazolone-5-propionic acid ; Imidazolelactic acid ; O-methoxycatechol-O-sulphate ; 2-Amino-56-dichloro-34-dihydroquinazoline	M+2K+H ; M+2K+H ; M+2K+H ; 2M+Na+K ; 2M+H+K	C6H8N2O3 ; C6H8N2O3 ; C6H8N2O3 ; C7H8O5S ; C8H7Cl2N3
594.55652	1.348265	0.89708	-3.0123	0.013067	1.8838	0.05351	594.5565	HMDB00577 ; HMDB00908 ; HMDB01569 ; HMDB11146 ; HMDB11759	5beta-Coprostanol ; 5alpha-Cholestanol ; Epi-coprostanol ; DG(16:0e18:00:0) ; Cer(d18:014:0)	3M+H+Na ; 3M+H+Na ; 3M+H+Na ; 2M+H+Na ; M+2ACN+H	C27H48O ; C27H48O ; C27H48O ; C37H74O4 ; C32H65NO3
626.43715	1.352793	0.039519	-3.0104	0.013109	1.8824	0.053532	626.4372	HMDB15648 ; HMDB29887 ; HMDB40627 ; HMDB59736	Fesoterodine ; Sorbitan palmitate ; 3-(2346-Tetrakisgalloylglucosyl)-phloroacetophenone ; 2-Hydroxyundecanoate	3M+H+NH4 ; 3M+2Na ; 2M+3H ; 3M+Na	C26H37NO3 ; C22H42O6 ; C42H34O25 ; C11H21O3
122.06006	1.376757	0.749317	3.0103	0.013112	1.8823	0.053532	122.0601	HMDB04461 ; HMDB33131 ; HMDB35281 ; HMDB13816 ; HMDB29744 ; HMDB31020 ; HMDB31102 ; HMDB31341 ; HMDB31776 ; HMDB32279 ; HMDB32382 ; HMDB32394 ; HMDB32542 ; HMDB32983 ; HMDB36073 ; HMDB36170 ; HMDB36461 ; HMDB36648 ; HMDB38638 ; HMDB38849 ; HMDB38972 ; HMDB41009 ; HMDB41443 ; HMDB41482 ; HMDB59698 ; HMDB60497	Benzamide ; 3-Acetylpyridine ; 2-Acetylpyridine ; 24-Di-tert-butylphenol ; Methyl 3-carbazolecarboxylate ; 2-Dodecenal ; (ZZ)-36-Dodecadien-1-ol ; Cyclododecanone ; (6R7S)-67-Epoxy-13-tetradecadiyne ; 2-Ethyl-133-trimethyl-2-norbornanol ; N1-(2-Methoxy-4-methylbenzyl)-n2-(2-(5-methylpyridin-2-yl)ethyl)oxalamide ; 6-Methylcoumarin ; trans- and cis-248-Trimethyl-37-nona-dien-2-ol ; 1-Hydroxy-10-methylacridone ; Viburtinal ; 2-Benzylidene-1-heptanol ; Geosmin ; 7-Isopropyl-14-dimethylazulene ; 6-Methoxy-9H-carbazole-3-carboxaldehyde ; Murrayanine ; (E)-2-Butyl-2-octenal ; 7-Methyl-2-benzofurancarboxaldehyde ; Mukolidine ; 3-Benzyl-4-heptanone ; Cadalene ; Naphthalene-12-diol	M+H ; M+H ; M+H ; M+H+K ; M+H+NH4 ; M+Na+K ; M+Na+K ; M+Na+K ; M+H+K ; M+Na+K ; M+2H+Na ; M+2ACN+2H ; M+Na+K ; M+H+NH4 ; M+2ACN+2H ; M+H+K ; M+Na+K ; M+2Na ; M+H+NH4 ; M+H+NH4 ; M+Na+K ; M+2ACN+2H ; M+H+NH4 ; M+H+K ; M+2Na ; M+2ACN+2H	C7H7NO ; C7H7NO ; C7H7NO ; C14H20O ; C14H11NO2 ; C12H22O ; C12H22O ; C12H22O ; C14H20O ; C12H22O ; C19H23N3O3 ; C10H8O2 ; C12H22O ; C14H11NO2 ; C10H8O2 ; C14H20O ; C12H22O ; C15H18 ; C14H11NO2 ; C14H11NO2 ; C12H22O ; C10H8O2 ; C14H11NO2 ; C14H20O ; C15H18 ; C10H8O2

420.04117	1.385594	0.095216	-3.0095	0.01313	1.8817	0.053532	420.0412	HMDB60506 ; HMDB05014 ; HMDB30000 ; HMDB34368 ; HMDB37553 ; HMDB39343 ; HMDB39344 ; HMDB39345 ; HMDB39346 ; HMDB39940	S-(22-Dichloro-1-hydroxy)ethyl glutathione ; Celecoxib ; Bissulfine ; Hydroxyminaline ; SS-Ethylidene dithioacetate ; 1345-Tetra-O-galloylquinic acid ; 3-Digalloyl-45-di-O-galloylquinic acid ; 5-O-Digalloyl-34-di-O-galloylquinic acid ; 4-O-Digalloyl-35-di-O-galloylquinic acid ; S-2-Propenyl 2-propene-1-sulfonothioate	M+H ; M+K ; 2M+ACN+Na ; 3M+K ; 2M+ACN+Na ; M+H+K ; M+H+K ; M+H+K ; M+H+K ; 2M+ACN+Na	C12H19Cl2N3O7S ; C17H14F3N3O2S ; C6H10O2S2 ; C5H5NO3 ; C6H10O2S2 ; C35H28O22 ; C35H28O22 ; C35H28O22 ; C35H28O22 ; C6H10O2S2
383.16650	1.303097	0.49467	3.0093	0.013133	1.8817	0.053532	383.1665	HMDB10343 ; HMDB35201 ; HMDB38916 ; HMDB39811 ; HMDB60919 ; HMDB02278 ; HMDB02282 ; HMDB12492 ; HMDB14909 ; HMDB29327 ; HMDB29769 ; HMDB31346 ; HMDB35438 ; HMDB36764 ; HMDB38590 ; HMDB38992 ; HMDB40332 ; HMDB40333 ; HMDB41046	1-(alpha-Methyl-4-(2-methylpropyl)benzeneacetate)-beta-D-Glucopyranuronic acid ; 48-Diacetyl-T2-tetrol ; 7alpha8alpha-Dihydroxycalonectrin ; Cyclocalopin B ; Ibuprofen glucuronide ; 2-(acetylamino)-15-anhydro-2-deoxy-3-O-b-D-galactopyranosyl-D-arabino-Hex-1-enitol ; 2-(acetylamino)-15-anhydro-2-deoxy-4-O-b-D-galactopyranosyl-D-arabino-Hex-1-enitol ; 1-(12345-Pentahydroxypent-1-yl)-1234-tetrahydro-beta-carboline-3-carboxylate ; Clidinium ; Gravacridonediol methyl ether ; Isoeruboside B ; Calystegine C1 ; Promucosine ; Romucosine C ; Buntanine ; Cistocardin ; Dihydro-6-isopropyl-24-dimethyl-4H-135-dithiazine ; Dihydro-46-dimethyl-2-(1-methylethyl)-4H-135-dithiazine ; Marshmine	M+H ; M+H ; M+H ; M+H ; M+H ; M+NH4 ; M+NH4 ; M+NH4 ; 2M+Na+K ; 2M+3H2O+2H ; M+3Na ; 2M+H ; 2M+3H2O+2H ; 2M+3H2O+2H ; 2M+3H2O+2H ; M+3Na ; 2M+H ; 2M+H ; 2M+3H2O+2H	C19H26O8 ; C19H26O8 ; C19H26O8 ; C19H26O8 ; C19H26O8 ; C14H23NO10 ; C14H23NO10 ; C17H21N2O7 ; C22H26NO3 ; C20H21NO5 ; C51H84O24 ; C7H13NO5 ; C20H21NO5 ; C20H21NO5 ; C20H21NO5 ; C51H84O24 ; C8H17NS2 ; C8H17NS2 ; C20H21NO5
199.51369	1.328038	0.909403	-3.0091	0.013137	1.8815	0.053532	199.5137	HMDB59724 ; HMDB60770	Pyrocatechol sulfate ; 4-Hydroxy-5-carboxy-lumiracoxib	2M+H+NH4 ; M+2Na	C6H6O5S ; C16H13ClFNO5
298.53970	1.404289	0.226494	3.0033	0.013268	1.8772	0.053936	298.5397	HMDB06821 ; HMDB06823 ; HMDB29247	25-Diaminopyrimidine nucleoside triphosphate ; 25-Diamino-6-(5-triphosphoryl-34-trihydroxy-2-oxopentyl)-amino-4-oxopyrimidine ; Delphinidin 3-(6-malonyl-glucoside)	M+2ACN+2H ; M+2ACN+2H ; M+2Na	C9H18N5O14P3 ; C9H18N5O14P3 ; C24H23O15
500.59521	1.402459	0.010556	-3.0017	0.013306	1.876	0.054002	NA	NA	NA	NA	NA
195.10153	1.38404	1.240985	3.0007	0.013327	1.8753	0.054044	195.1015	HMDB30711 ; HMDB30712 ; HMDB30714 ; HMDB30715 ; HMDB30825 ; HMDB33539 ; HMDB33540 ; HMDB33688 ; HMDB36587 ; HMDB39029 ; HMDB00235 ; HMDB03116 ; HMDB06059 ; HMDB12549 ; HMDB12551 ; HMDB30308 ; HMDB30348 ; HMDB36863 ; HMDB37062 ; HMDB37594 ; HMDB39105 ; HMDB40597 ; HMDB41518 ; HMDB41543	Cyclomammein ; Furomammea G ; Cycloneomammein ; MAB 4 ; 2-(4-Allyl-26-dimethoxyphenoxy)-1-(34-dimethoxyphenyl)-1-propanol ; Fragransin D1 ; Fragransin D3 ; Pyranomammea C ; Quassin ; Pyranomammea B ; Thiamine ; Stanozolol ; 20-Carboxy-leukotriene B4 ; 12-oxo-1011-dihydro-20-COOH-LTB4 ; 12-oxo-20-dihydroxy-leukotriene B4 ; O-Isopentenylhalfordinol ; Anonaine ; Anhydrocinnzeylanol ; Sporotrichiol ; Eduleine ; 3-Oxo-1415-dehydrorhazinilam ; Eduline ; Nb-p-Coumaroyltryptamine ; 25-Dibenzyl-3-hydroxy-6-methoxypyrazine	M+2H ; M+2H ; M+2H ; M+2H ; M+2H ; M+2H ; M+2H ; M+2H ; M+2H ; M+2H ; M+3ACN+2H ; M+Na+K ; M+H+Na ; M+H+Na ; M+H+Na ; M+2ACN+2H ; M+3ACN+2H ; M+H+Na ; M+H+Na ; M+3ACN+2H ; M+2ACN+2H ; M+3ACN+2H ; M+2ACN+2H ; M+2ACN+2H	C22H28O6 ; C22H28O6 ; C22H28O6 ; C22H28O6 ; C22H28O6 ; C22H28O6 ; C22H28O6 ; C22H28O6 ; C22H28O6 ; C22H28O6 ; C12H17N4OS ; C21H32N2O ; C20H30O6 ; C20H30O6 ; C20H30O6 ; C19H18N2O2 ; C17H15NO2 ; C20H30O6 ; C20H30O6 ; C17H15NO2 ; C19H18N2O2 ; C17H15NO2 ; C19H18N2O2 ; C19H18N2O2
100.02438	1.371712	0.166162	2.9997	0.013351	1.8745	0.054096	100.0216	HMDB05843 ; HMDB29755 ; HMDB33116 ; HMDB33117 ; HMDB38624 ; HMDB00555 ; HMDB00709 ; HMDB00727 ; HMDB00845 ; HMDB00857 ; HMDB00877 ; HMDB02109 ; HMDB02441 ; HMDB05784 ; HMDB29167 ; HMDB29573 ; HMDB29720 ; HMDB29758 ; HMDB29895 ; HMDB30570 ; HMDB31054 ; HMDB32012 ; HMDB32883 ; HMDB32990 ; HMDB33813 ; HMDB34158 ; HMDB37724 ; HMDB37728 ; HMDB41605 ; HMDB59622 ; HMDB59722 ; HMDB59738 ; HMDB59893 ; HMDB59966	Allyl isothiocyanate ; 3-Thiocyanato-1-propene ; 4-Methylthiazole ; 5-Methylthiazole ; Thiiraneacetonitrile ; 3-Methyladipic acid ; L-Cysteinylglycine disulfide ; L-Threoneopterin ; Neopterin ; Pimelic acid ; Umanopterin ; Hydroxysepiapterin ; 33-Dimethylglutaric acid ; Hydroxytyrosol ; 2-Methyladipic acid ; Diethyl malonate ; 2-Thiophenethiol ; Herniarin ; D-erythro-Eritadenine ; Sylvopinol ; 10-Hydroxy-28-decadiene-46-diynoic acid ; 4-Hydroxy-3-methoxybenzenemethanol ; 145-Naphthalenetriol ; 7-Hydroxy-6-methyl-2H-1-benzopyran-2-one ; 3-(34-Methylenedioxyphenyl)propenal ; 26-Dimethoxyphenol ; Propyl 2-furoate ; 2-Furanylmethyl propanoate ; Propyleneglycol diacetate ; 4-Methylumbelliferone ; Mono-methyl-adipate ; 2-Ethylglutaric acid ; Ethyl methylsuccinate ; 35-Dimethoxyphenol	M+H ; M+H ; M+H ; M+H ; M+H ; M+H+K ; M+3H ; M+H+2Na ; M+H+2Na ; M+H+K ; M+H+2Na ; M+H+2Na ; M+H+K ; M+2Na ; M+H+K ; M+H+K ; M+2ACN+2H ; M+H+Na ; M+H+2Na ; M+2Na ; M+H+Na ; M+2Na ; M+H+Na ; M+H+Na ; M+H+Na ; M+2Na ; M+2Na ; M+2Na ; M+H+K ; M+H+Na ; M+H+K ; M+H+K ; M+H+K ; M+2Na	C4H5NS ; C4H5NS ; C4H5NS ; C4H5NS ; C4H5NS ; C7H12O4 ; C8H15N3O5S2 ; C9H11N5O4 ; C9H11N5O4 ; C7H12O4 ; C9H11N5O4 ; C9H11N5O4 ; C7H12O4 ; C8H10O3 ; C7H12O4 ; C7H12O4 ; C4H4S2 ; C10H8O3 ; C9H11N5O4 ; C8H10O3 ; C10H8O3 ; C8H10O3 ; C10H8O3 ; C10H8O3 ; C10H8O3 ; C8H10O3 ; C8H10O3 ; C8H10O3 ; C7H12O4 ; C10H8O3 ; C7H12O4 ; C7H12O4 ; C7H12O4 ; C8H10O3

329.24712	1.33169	0.184421	2.9964	0.013426	1.8721	0.05434	329.2471	HMDB11155 ; HMDB06320 ; HMDB07020 ; HMDB07059 ; HMDB07100 ; HMDB07110 ; HMDB07111 ; HMDB07138 ; HMDB07156 ; HMDB07162 ; HMDB07163 ; HMDB07190 ; HMDB07219 ; HMDB07246 ; HMDB07247 ; HMDB07274 ; HMDB07303 ; HMDB07356 ; HMDB07418 ; HMDB07446 ; HMDB07475 ; HMDB07647 ; HMDB13288 ; HMDB29768 ; HMDB33775 ; HMDB34204 ; HMDB34354 ; HMDB34419 ; HMDB43306 ; HMDB43328 ; HMDB43502 ; HMDB43565 ; HMDB43580 ; HMDB43586 ; HMDB43596 ; HMDB43619 ; HMDB43624 ; HMDB43733 ; HMDB43764 ; HMDB43769 ; HMDB43782 ; HMDB43792 ; HMDB44269 ; HMDB44274 ; HMDB44325 ; HMDB44330 ; HMDB44458 ; HMDB44460 ; HMDB44598 ; HMDB44600 ; HMDB45096 ; HMDB45150 ; HMDB45382 ; HMDB45384 ; HMDB48756 ; HMDB48800 ;	PA(P-16:0e18:2(9Z12Z)) ; 26 Dimethylheptanoyl carnitine ; DG(14:020:00:0) ; DG(14:1(9Z)22:2(13Z16Z)0:0) ; DG(16:018:00:0) ; DG(16:020:3(5Z8Z11Z)0:0) ; DG(16:020:3(8Z11Z14Z)0:0) ; DG(16:1(9Z)20:2(11Z14Z)0:0) ; DG(18:016:00:0) ; DG(18:018:3(6Z9Z12Z)0:0) ; DG(18:018:3(9Z12Z15Z)0:0) ; DG(18:1(11Z)18:2(9Z12Z)0:0) ; DG(18:1(9Z)18:2(9Z12Z)0:0) ; DG(18:2(9Z12Z)18:1(11Z)0:0) ; DG(18:2(9Z12Z)18:1(9Z)0:0) ; DG(18:3(6Z9Z12Z)18:00:0) ; DG(18:3(9Z12Z15Z)18:00:0) ; DG(20:014:00:0) ; DG(20:2(11Z14Z)16:1(9Z)0:0) ; DG(20:3(5Z8Z11Z)16:00:0) ; DG(20:3(8Z11Z14Z)16:00:0) ; DG(22:2(13Z16Z)14:1(9Z)0:0) ; Nonanoylcarnitine ; Isofucosterol glucoside ; alpha-Spinasterol 3-glucoside ; Clerosterol 3-glucoside ; Tocophersolan ; Stigmasteryl glucoside ; TG(15:020:1(11Z)22:6(4Z7Z10Z13Z16Z19Z)) ; TG(15:020:3(5Z8Z11Z)22:4(7Z10Z13Z16Z)) ; TG(15:020:3n622:4(7Z10Z13Z16Z)) ; TG(15:022:2(13Z16Z)20:5(5Z8Z11Z14Z17Z)) ; TG(15:022:4(7Z10Z13Z16Z)20:3(5Z8Z11Z)) ; TG(15:022:4(7Z10Z13Z16Z)20:3n6) ; TG(15:022:4(7Z10Z13Z16Z)22:6(4Z7Z10Z13Z16Z19Z)) ; TG(15:022:5(4Z7Z10Z13Z16Z)22:5(4Z7Z10Z13Z16Z)) ; TG(15:022:5(4Z7Z10Z13Z16Z)22:5(7Z10Z13Z16Z19Z)) ; TG(15:020:5(5Z8Z11Z14Z17Z)22:2(13Z16Z)) ; TG(15:022:5(7Z10Z13Z16Z19Z)22:5(4Z7Z10Z13Z16Z)) ; TG(15:022:5(7Z10Z13Z16Z19Z)22:5(7Z10Z13Z16Z19Z)) ; TG(15:022:6(4Z7Z10Z13Z16Z19Z)20:1(11Z)) ; TG(15:022:6(4Z7Z10Z13Z16Z19Z)22:4(7Z10Z13Z16Z)) ; TG(16:018:2(9Z12Z)22:5(4Z7Z10Z13Z16Z)) ; TG(16:018:2(9Z12Z)22:5(7Z10Z13Z16Z19Z)) ; TG(16:020:2n622:5(4Z7Z10Z13Z16Z)) ; TG(16:020:2n622:5(7Z10Z13Z16Z19Z)) ; TG(16:022:5(4Z7Z10Z13Z16Z)18:2(9Z12Z)) ; TG(16:022:5(4Z7Z10Z13Z16Z)20:2n6) ; TG(16:022:5(7Z10Z13Z16Z19Z)18:2(9Z12Z)) ; TG(16:022:5(7Z10Z13Z16Z19Z)20:2n6) ; TG(18:018:2(9Z12Z)20:5(5Z8Z11Z14Z17Z)) ; TG(18:020:2n620:5(5Z8Z11Z14Z17Z)) ; TG(18:020:5(5Z8Z11Z14Z17Z)18:2(9Z12Z)) ; TG(18:020:5(5Z8Z11Z14Z17Z)20:2n6) ; TG(16:1(9Z)18:2(9Z12Z)22:4(7Z10Z13Z16Z)) ; TG(16:1(9Z)20:2n622:4(7Z10Z13Z16Z)) ; TG(16:1(9Z)22:4(7Z10Z13Z16Z)18:2(9Z12Z)) ; TG(16:1(9Z)22:4(7Z10Z13Z16Z)20:2n6) ; TG(18:1(11Z)18:2(9Z12Z)20:4(5Z8Z11Z14Z)) ; TG(18:1(11Z)18:2(9Z12Z)20:4(8Z11Z14Z17Z)) ; TG(18:1(11Z)20:2n620:4(5Z8Z11Z14Z)) ; TG(18:1(11Z)20:2n620:4(8Z11Z14Z17Z)) ; TG(18:1(11Z)20:4(5Z8Z11Z14Z)18:2(9Z12Z)) ;	M+2H ; 2M+3H2O+2H ; M+Na+K ; M+H+K ; M+Na+K ; M+H+K ; M+H+K ; M+H+K ; M+Na+K ; M+H+K ; M+H+K ; M+H+K ; M+H+K ; M+H+K ; M+H+K ; M+H+K ; M+H+K ; M+Na+K ; M+H+K ; M+H+K ; M+H+K ; M+H+K ; 2M+3H2O+2H ; M+2ACN+2H ; M+2ACN+2H ; M+2ACN+2H ; M+2ACN+2H ; M+2ACN+2H ; M+3Na ; M+3Na ; M+3Na ; M+3Na ; M+3Na ; M+3Na ; M+H+2Na ; M+H+2Na ; M+H+2Na ; M+3Na ; M+H+2Na ; M+H+2Na ; M+3Na ; M+H+2Na ; M+3Na ; M+H+2Na ; M+H+2Na ; M+H+2Na ; M+H+2Na ; M+3Na ; M+3Na ; M+3Na ; M+3Na ; M+3Na ; M+3Na ; M+3Na ; M+H+2Na ; M+H+2Na ; M+H+2Na ; M+H+2Na ; M+H+2Na ; M+H+2Na ; M+H+2Na ; M+H+2Na ; M+H+2Na ; M+H+2Na ; M+H+2Na ; M+H+2Na ; M+H+2Na ; M+H+2Na ; M+2H+Na ; M+H+2Na ; M+H+2Na ; M+2H+Na ; M+H+2Na ; M+H+2Na ; M+H+2Na ; M+H+2Na ; M+H+2Na ; M+H+2Na ; M+3Na ; M+3Na ; M+3Na ; M+3Na ; M+3Na ; M+3Na ;	C37H69O7P ; C16H31NO4 ; C37H72O5 ; C39H70O5 ; C37H72O5 ; C39H70O5 ; C39H70O5 ; C39H70O5 ; C37H72O5 ; C39H70O5 ; C39H70O5 ; C39H70O5 ; C39H70O5 ; C39H70O5 ; C39H70O5 ; C39H70O5 ; C39H70O5 ; C37H72O5 ; C39H70O5 ; C39H70O5 ; C39H70O5 ; C39H70O5 ; C16H31NO4 ; C35H58O6 ; C35H58O6 ; C35H58O6 ; C35H58O6 ; C35H58O6 ; C60H102O6 ; C60H102O6 ; C60H102O6 ; C60H102O6 ; C60H102O6 ; C60H102O6 ; C62H100O6 ; C62H100O6 ; C62H100O6 ; C60H102O6 ; C62H100O6 ; C62H100O6 ; C60H102O6 ; C62H100O6 ; C60H102O6 ; C62H100O6 ; C62H100O6 ; C62H100O6 ; C62H100O6 ; C60H102O6 ; C60H102O6 ; C60H102O6 ; C60H102O6 ; C60H102O6 ; C60H102O6 ; C60H102O6 ; C62H100O6 ; C62H100O6 ; C62H100O6 ; C62H100O6 ; C62H100O6 ; C62H100O6 ; C62H100O6 ; C62H100O6 ; C62H100O6 ; C62H100O6 ; C62H100O6 ; C62H100O6 ; C62H100O6 ;
534.62690	1.36235	1.149284	2.9961	0.013433	1.8718	0.05434	534.6269	HMDB14374	Cefotiam	2M+H+NH4	C18H23N9O4S3
290.57791	1.404357	0.085962	-2.9935	0.013492	1.8699	0.054536	290.5779	HMDB39523 ; HMDB40681	Isomelitric acid A ; Melitric acid A	M+ACN+2H ; M+ACN+2H	C27H22O12 ; C27H22O12
425.47490	1.35303	0.111011	2.9911	0.013547	1.8682	0.054714	425.4749	HMDB33180 ; HMDB34287 ; HMDB41951	15-Nonacosanol ; (S)-10-Nonacosanol ; Nitrofen	M+H ; M+H ; 3M+2H	C29H60O ; C29H60O ; C12H7Cl2NO3
444.36798	1.327486	0.336	2.9862	0.013662	1.8645	0.055002	444.368	HMDB13154 ; HMDB07992 ; HMDB08051 ; HMDB08092 ; HMDB08125 ; HMDB08157 ; HMDB08274 ; HMDB08284 ; HMDB08316 ; HMDB08348 ; HMDB08530 ; HMDB08539 ; HMDB08570 ; HMDB08602 ; HMDB08757 ; HMDB08758 ; HMDB08763 ; HMDB08794 ; HMDB08795	12-Hydroxy-12-octadecanoylcarnitine ; PC(16:024:0) ; PC(18:022:0) ; PC(18:1(11Z)24:1(15Z)) ; PC(18:1(9Z)24:1(15Z)) ; PC(18:2(9Z12Z)24:0) ; PC(20:020:0) ; PC(20:022:2(13Z16Z)) ; PC(20:1(11Z)22:1(13Z)) ; PC(20:2(11Z14Z)22:0) ; PC(22:018:0) ; PC(22:020:2(11Z14Z)) ; PC(22:1(13Z)20:1(11Z)) ; PC(22:2(13Z16Z)20:0) ; PC(24:015:0) ; PC(24:016:0) ; PC(24:018:2(9Z12Z)) ; PC(24:1(15Z)18:1(11Z)) ; PC(24:1(15Z)18:1(9Z))	M+H ; M+ACN+2H ; M+ACN+2H ; M+H+NH4 ; M+H+NH4 ; M+H+NH4 ; M+ACN+2H ; M+H+NH4 ; M+H+NH4 ; M+H+NH4 ; M+ACN+2H ; M+H+NH4 ; M+H+NH4 ; M+H+NH4 ; M+ACN+2H ; M+ACN+2H ; M+H+NH4 ; M+H+NH4 ; M+H+NH4	C25H49NO5 ; C48H96NO8P ; C48H96NO8P ; C50H96NO8P ; C50H96NO8P ; C50H96NO8P ; C48H96NO8P ; C50H96NO8P ; C50H96NO8P ; C50H96NO8P ; C48H96NO8P ; C50H96NO8P ; C50H96NO8P ; C50H96NO8P ; C48H96NO8P ; C48H96NO8P ; C50H96NO8P ; C50H96NO8P ; C50H96NO8P
613.93881	1.382061	0.300373	-2.9853	0.013683	1.8638	0.055035	NA	NA	NA	NA	NA
473.88340	1.301722	1.685235	-2.9849	0.013693	1.8635	0.055035	473.8834	HMDB36351	Methyl 2-propenyl pentasulfide	2M+ACN+H	C4H8S5
151.14411	1.363439	0.520447	-2.9812	0.01378	1.8608	0.055191	151.1441	HMDB00925 ; HMDB12136	Trimethylamine N-oxide ; 1-Amino-propan-2-ol	2M+H ; 2M+H	C3H9NO ; C3H9NO
372.18862	1.3089	1.487942	2.9809	0.013785	1.8606	0.055191	372.1886	HMDB30460 ; HMDB00037 ; HMDB02802 ; HMDB12613 ; HMDB14749 ; HMDB14998 ; HMDB15658 ; HMDB28720 ; HMDB29077 ; HMDB30165 ; HMDB31979 ; HMDB35813 ; HMDB38389 ; HMDB41513	Hordatine B glucoside ; Aldosterone ; Cortisone ; 19-Oic-deoxycorticosterone ; Butorphanol ; Prednisolone ; Norelgestromin ; Arginyl-Tryptophan ; Tryptophyl-Arginine ; Tricyclodehydroisohumulone ; Methyl (9Z)-8-oxo-68-diapo-6-carotenoate ; 7-Methylrosmanol ; Cyclopassifloside II ; Isopropyl apiosylglucoside	M+2H ; 2M+H+Na ; 2M+H+Na ; 2M+H+Na ; M+2Na-H ; 2M+H+Na ; M+2Na-H ; 2M+H+Na ; 2M+H+Na ; 2M+H+Na ; 2M+H+K ; 2M+H+Na ; M+Na+K ; M+NH4	C35H50N8O10 ; C21H28O5 ; C21H28O5 ; C21H28O5 ; C21H29NO2 ; C21H28O5 ; C21H29NO2 ; C17H24N6O3 ; C17H24N6O3 ; C21H28O5 ; C23H28O3 ; C21H28O5 ; C37H62O11 ; C14H26O10
706.08407	1.318472	1.275395	-2.9788	0.013837	1.859	0.055327	706.0841	HMDB06460 ; HMDB07382 ; HMDB07409 ; HMDB07410 ; HMDB07437 ; HMDB07523 ; HMDB07552 ; HMDB07580 ; HMDB07636 ; HMDB07663 ; HMDB07664 ; HMDB07689 ; HMDB07717 ; HMDB07746 ; HMDB07774 ; HMDB30693 ; HMDB34350 ; HMDB38006 ; HMDB38007 ; HMDB41594 ; HMDB56086 ; HMDB56227 ; HMDB56232 ; HMDB56264 ; HMDB56333 ; HMDB56347	Arachidyl carnitine ; DG(20:022:6(4Z7Z10Z13Z16Z19Z)0:0) ; DG(20:1(11Z)22:5(4Z7Z10Z13Z16Z)0:0) ; DG(20:1(11Z)22:5(7Z10Z13Z16Z19Z)0:0) ; DG(20:2(11Z14Z)22:4(7Z10Z13Z16Z)0:0) ; DG(20:4(5Z8Z11Z14Z)22:2(13Z16Z)0:0) ; DG(20:4(8Z11Z14Z17Z)22:2(13Z16Z)0:0) ; DG(20:5(5Z8Z11Z14Z17Z)22:1(13Z)0:0) ; DG(22:1(13Z)20:5(5Z8Z11Z14Z17Z)0:0) ; DG(22:2(13Z16Z)20:4(5Z8Z11Z14Z)0:0) ; DG(22:2(13Z16Z)20:4(8Z11Z14Z17Z)0:0) ; DG(22:4(7Z10Z13Z16Z)20:2(11Z14Z)0:0) ; DG(22:5(4Z7Z10Z13Z16Z)20:1(11Z)0:0) ; DG(22:5(7Z10Z13Z16Z19Z)20:1(11Z)0:0) ; DG(22:6(4Z7Z10Z13Z16Z19Z)20:00:0) ; Delphinidin 35-diglucoside ; Norbadione A ; Delphinidin 3-gentiobioside ; Delphinidin 3-sophoroside ; Delphinidin 3-glucosylglucoside ; DG(20:00:022:6n3) ; DG(20:1n90:022:5n6) ; DG(20:1n90:022:5n3) ; DG(22:1n90:020:5n3) ; DG(20:4n60:022:2n6) ; DG(22:2n60:020:4n3)	3M+2Na ; 2M+H+NH4 ; 2M+H+NH4 ; 2M+H+NH4 ; 2M+H+NH4 ; 2M+H+NH4 ; 2M+H+NH4 ; 2M+H+NH4 ; 2M+H+NH4 ; 2M+H+NH4 ; 2M+H+NH4 ; 2M+H+NH4 ; 2M+H+NH4 ; 2M+H+NH4 ; 2M+H+NH4 ; M+2K+H ; 2M+3H2O+2H ; M+2K+H ; M+2K+H ; M+2K+H ; 2M+H+NH4 ; 2M+H+NH4 ; 2M+H+NH4 ; 2M+H+NH4 ; 2M+H+NH4 ; 2M+H+NH4	C27H53NO4 ; C45H76O5 ; C45H76O5 ; C45H76O5 ; C45H76O5 ; C45H76O5 ; C45H76O5 ; C45H76O5 ; C45H76O5 ; C45H76O5 ; C45H76O5 ; C45H76O5 ; C45H76O5 ; C45H76O5 ; C45H76O5 ; C27H31O17 ; C35H18O15 ; C27H31O17 ; C27H31O17 ; C27H31O17 ; C45H76O5 ; C45H76O5 ; C45H76O5 ; C45H76O5 ; C45H76O5 ; C45H76O5

123.03044	1.379875	0.012493	2.9785	0.013843	1.8588	0.055327	123.0304	HMDB29572 ; HMDB31157 ; HMDB31708 ; HMDB31872 ; HMDB33552 ; HMDB41492 ; HMDB01228 ; HMDB04230 ; HMDB14738 ; HMDB29049 ; HMDB29070 ; HMDB29469 ; HMDB30266 ; HMDB30646 ; HMDB31682 ; HMDB34013 ; HMDB36117 ; HMDB36460 ; HMDB36998 ; HMDB37600 ; HMDB37692 ; HMDB38175 ; HMDB38332 ; HMDB38807 ; HMDB39052 ; HMDB39710 ; HMDB40321 ; HMDB40883 ; HMDB41405	Diethyl disulfide ; 23-Butanedithiol ; 12-Bis(methylthio)ethane ; Methyl propyl disulfide ; ()-13-Butanedithiol ; 12-Butanedithiol ; L-Glutamic acid 5-phosphate ; Pyrrole-2-carboxylic acid ; Monobenzone ; Serinyl-Threonine ; Threoninyl-Serine ; Eupatilin ; Berberrubine ; (Z)-3-(1-Butenyl)-1H-2-benzopyran-1-one ; ()-2-Methylthiazolidine ; Lathycarpin ; (1R)-Nepetalic acid ; Safynol ; (S)-Oleuropeic acid ; Xanthomicrol ; 57-Dihydroxy-345-trimethoxyflavone ; 2-Acetyloxazole ; 2-(3-Hydroxyphenyl)ethanol 1-glucoside ; Aflatoxin Ex2B1 ; (4S8R)-89-Dihydroxy-p-menth-1(6)-en-2-one ; 1-Acetylcyclohexyl acetate ; 38-Dihydroxy-457-trimethoxyflavone ; (E)-10-Oxo-8-decenoic acid ; 4-Methoxybenzyl glucoside	M+H ; M+H ; M+H ; M+H ; M+H ; M+H ; M+H+NH4 ; 2M+H+Na ; M+2Na ; M+H+K ; M+H+K ; M+2H+Na ; M+H+2Na ; M+2Na ; 2M+H+K ; M+2H+Na ; M+Na+K ; M+2Na ; M+Na+K ; M+2H+Na ; M+2H+Na ; 2M+H+Na ; M+3Na ; M+2H+Na ; M+Na+K ; M+Na+K ; M+2H+Na ; M+Na+K ; M+3Na	C4H10S2 ; C4H10S2 ; C4H10S2 ; C4H10S2 ; C4H10S2 ; C4H10S2 ; C5H10NO7P ; C5H5NO2 ; C13H12O2 ; C7H14N2O5 ; C7H14N2O5 ; C18H16O7 ; C19H16NO4 ; C13H12O2 ; C4H9NS ; C18H16O7 ; C10H16O3 ; C13H12O2 ; C10H16O3 ; C18H16O7 ; C18H16O7 ; C5H5NO2 ; C14H20O7 ; C18H16O7 ; C10H16O3 ; C10H16O3 ; C18H16O7 ; C10H16O3 ; C14H20O7
523.27309	1.348502	0.000939	-2.9747	0.013934	1.8559	0.055602	523.2731	HMDB01937 ; HMDB14366 ; HMDB29341 ; HMDB30516 ; HMDB33454 ; HMDB34102 ; HMDB34750 ; HMDB41935 ; HMDB37035	Salbutamol ; Isoetharine ; Ceanothine D ; Petasinine ; 3alpha-Tigloyloxytropane N-oxide ; N-Nitrosotomatidine ; Isopentyl beta-D-glucoside ; Moprolol ; Glycinoeclepin B	2M+2Na-H ; 2M+2Na-H ; M+K ; 2M+2Na-H ; 2M+2Na-H ; M+2K+H ; 2M+Na ; 2M+2Na-H ; M+H-2H2O	C13H21NO3 ; C13H21NO3 ; C27H40N4O4 ; C13H21NO3 ; C13H21NO3 ; C27H44N2O3 ; C11H22O6 ; C13H21NO3 ; C31H42O9
152.07381	1.338034	1.202944	2.971	0.014022	1.8532	0.055839	152.0738	HMDB00214 ; HMDB00873 ; HMDB01398 ; HMDB03374 ; HMDB11724 ; HMDB29696 ; HMDB31521 ; HMDB32409 ; HMDB32455 ; HMDB32917 ; HMDB34377 ; HMDB34877 ; HMDB35226 ; HMDB38963 ; HMDB40263 ; HMDB40280 ; HMDB40410 ; HMDB59709 ; HMDB59712	Ornithine ; 4-Methylcatechol ; Guaiacol ; D-Ornithine ; 4-Hydroxybenzyl alcohol ; Mequinol ; Ethyl 2-mercaptopropionate ; Methyl 3-mercaptobutanoate ; L-Ornithine monochlorohydrateornithine ; 1-(2-Furanyl)-2-propanone ; Methyl 3-(methylthio)propanoate ; 2-Acetyl-3-methylfuran ; 2-Acetyl-5-methylfuran ; 46-Heptadiyne-13-diol ; Ethyl 2-(methylthio)acetate ; 1-(2-Furanyl)-1-propanone ; Ethyl 3-mercaptopropanoic acid ; 2-Hydroxybenzyl alcohol ; 3-Hydroxybenzyl alcohol	2M+H+K ; 2M+3H2O+2H ; 2M+3H2O+2H ; 2M+H+K ; 2M+3H2O+2H ; 2M+3H2O+2H ; M+NH4 ; M+NH4 ; 2M+H+K ; 2M+3H2O+2H ; M+NH4 ; 2M+3H2O+2H ; 2M+3H2O+2H ; 2M+3H2O+2H ; M+NH4 ; 2M+3H2O+2H ; M+NH4 ; 2M+3H2O+2H ; 2M+3H2O+2H	C5H12N2O2 ; C7H8O2 ; C7H8O2 ; C5H12N2O2 ; C7H8O2 ; C7H8O2 ; C5H10O2S ; C5H10O2S ; C5H12N2O2 ; C7H8O2 ; C5H10O2S ; C7H8O2 ; C7H8O2 ; C7H8O2 ; C5H10O2S ; C7H8O2 ; C5H10O2S ; C7H8O2 ; C7H8O2
617.95048	1.311998	1.147184	-2.9684	0.014085	1.8512	0.055991	NA	NA	NA	NA	NA
594.05823	1.335717	0.735104	-2.9621	0.014236	1.8466	0.056448	594.0582	HMDB01330 ; HMDB29513 ; HMDB36825	2-Amino-3-carboxymuconic acid semialdehyde ; Myricatin ; Vitisin B	3M+K ; M+ACN+H ; M+2K-H	C7H7NO5 ; C22H16O15S ; C25H25O12
149.46924	1.321436	0.870898	2.9609	0.014267	1.8457	0.056461	NA	NA	NA	NA	NA
138.02977	1.307978	0.280204	2.9568	0.014366	1.8427	0.056649	138.0298	HMDB33326 ; HMDB38715 ; HMDB41243 ; HMDB03012 ; HMDB13674 ; HMDB13675 ; HMDB15037 ; HMDB29172 ; HMDB29750 ; HMDB30776 ; HMDB31191 ; HMDB31256 ; HMDB31316 ; HMDB32330 ; HMDB32376 ; HMDB32651 ; HMDB33856 ; HMDB34355 ; HMDB34896 ; HMDB35171 ; HMDB35244 ; HMDB36138 ; HMDB37193 ; HMDB37474 ; HMDB38412 ; HMDB38414 ; HMDB39826 ; HMDB41195 ; HMDB41491 ; HMDB41558 ; HMDB59735 ; HMDB61143	BR-Xanthone B ; Alloathyriol ; 136-Trihydroxy-5-methoxyxanthone ; Aniline ; 123-Trihydroxybenzene ; 135-Trihydroxybenzene ; Didanosine ; 3-keto-2-Methylbutyrate ; Methyl 2-furoate ; Maltol ; S-Methyl butanethioate ; 4-Hydroxy-2H-pyran-3-carboxaldehyde ; 4-(Methylthio)-2-butanone ; 4-Hydroxy-2-butenoic acid gamma-lactone ; 4-Mercapto-2-pentanone ; (Z)-Tamarindienal ; S-Propyl thioacetate ; 5-Hydroxymethyl-2-furancarboxaldehyde ; 3-Mercapto-2-pentanone ; 2-Mercapto-3-pentanone ; Tetrahydro-2-methylthiophen-3-ol ; Tetrahydro-2-methyl-3-furanthiol ; 1-(Methylthio)-2-butanone ; 4-(Methylthio)butanal ; 3-Methylbutyl glucosinolate ; Pentyl glucosinolate ; xi-2-Methyl-13-oxathiane ; 2-O-(Z-p-Hydroxycinnamoyl)-(x)-glyceric acid ; 3-(Methylthio)butanal ; 3-(Methylthio)-2-butanone ; 5-Methylfuran-2-carboxylic acid ; Hydralazine pyruvate hydrazone	M+2H ; M+2H ; M+2H ; M+2Na-H ; 2M+H+Na ; 2M+H+Na ; M+H+K ; M+Na ; 2M+H+Na ; 2M+H+Na ; 2M+H+K ; 2M+H+Na ; 2M+H+K ; 3M+H+Na ; 2M+H+K ; 2M+H+Na ; 2M+H+K ; 2M+H+Na ; 2M+H+K ; 2M+H+K ; 2M+H+K ; 2M+H+K ; 2M+H+K ; 2M+H+K ; M+2H+Na ; M+2H+Na ; 2M+H+K ; M+H+Na ; 2M+H+K ; 2M+H+K ; 2M+H+Na ; M+2Na	C14H10O6 ; C14H10O6 ; C14H10O6 ; C6H7N ; C6H6O3 ; C6H6O3 ; C10H12N4O3 ; C5H7O3 ; C6H6O3 ; C6H6O3 ; C5H10OS ; C6H6O3 ; C5H10OS ; C4H4O2 ; C5H10OS ; C6H6O3 ; C5H10OS ; C6H6O3 ; C5H10OS ; C5H10OS ; C5H10OS ; C5H10OS ; C5H10OS ; C5H10OS ; C12H23NO9S2 ; C12H23NO9S2 ; C5H10OS ; C12H12O6 ; C5H10OS ; C5H10OS ; C6H6O3 ; C11H10N4O2
139.05832	1.302231	0.107114	-2.9524	0.014476	1.8394	0.056991	139.0583	HMDB31314 ; HMDB32019 ; HMDB32467 ; HMDB32637 ; HMDB41361 ; HMDB30145 ; HMDB30287 ; HMDB30791 ; HMDB35814 ; HMDB41520 ; HMDB00985 ; HMDB13949 ; HMDB14452 ; HMDB15695 ; HMDB29939 ; HMDB32600	Benzyl methyl sulfide ; 26-Dimethylbenzenethiol ; (-)-1-Phenylethylmercaptan ; 2-Phenylethanethiol ; 2-Ethylbenzenethiol ; Ascochitine ; 10-Acetoxytoxol ; Dihydromethysticin ; Lactucin ; 5-De-O-methyltoddanol ; Dihydrolipoamide ; O-Desmethylcarvedilol ; Bexarotene ; Roxatidine acetate ; 6-Amino-9H-purine-9-propanoic acid ; Ptelatoside A	M+H ; M+H ; M+H ; M+H ; M+H ; M+2H ; M+2H ; M+2H ; M+2H ; M+2H ; 2M+3H ; M+2H+Na ; M+3Na ; M+3Na ; 2M+3H ; M+3H	C8H10S ; C8H10S ; C8H10S ; C8H10S ; C8H10S ; C15H16O5 ; C15H16O5 ; C15H16O5 ; C15H16O5 ; C15H16O5 ; C8H17NOS2 ; C23H24N2O4 ; C24H28O2 ; C19H28N2O4 ; C8H9N5O2 ; C19H26O10
374.03750	1.318609	0.193097	2.947	0.014609	1.8354	0.057379	374.0375	HMDB01507 ; HMDB31781 ; HMDB33062 ; HMDB34934 ; HMDB34981 ; HMDB35022 ; HMDB36372 ; HMDB36373 ; HMDB38968 ; HMDB39961 ; HMDB60462 ; HMDB60545 ; HMDB60646	Diguanosine diphosphate ; O-Ethyl SS-diphenyl phosphorodithioate ; 2-Propenyl 1-(2-propenylsulfinyl)propyl disulfide ; Prunasin ; Sambunigrin ; Prulaurasin ; 1-Propenyl 1-(2-propenylsulfinyl)propyl disulfide ; 2-Propenyl 1-(1-propenylsulfinyl)propyl disulfide ; 1-Propenyl 1-(1-propenylsulfinyl)propyl disulfide ; (S)-2-Hydroxy-2-phenylacetonitrile O-b-D-allopyranoside ; Citalopram aldehyde ; O-Desmethylindomethacin ; Nor-ketotifen	M+H+K ; M+ACN+Na ; 3M+H+K ; M+2K+H ; M+2K+H ; M+2K+H ; 3M+H+K ; 3M+H+K ; 3M+H+K ; M+2K+H ; M+2K+H ; 2M+Na+K ; M+2K+H	C20H26N10O15P2 ; C14H15O2PS2 ; C9H16OS3 ; C14H17NO6 ; C14H17NO6 ; C14H17NO6 ; C9H16OS3 ; C9H16OS3 ; C9H16OS3 ; C14H17NO6 ; C18H14FNO2 ; C18H14ClNO4 ; C18H17NOS

150.04935	1.356608	0.334542	-2.9459	0.014636	1.8346	0.057441	150.0494	HMDB29512 ; HMDB30718 ; HMDB38811 ; HMDB00408 ; HMDB00491 ; HMDB00695 ; HMDB01864 ; HMDB04437 ; HMDB06024 ; HMDB10717 ; HMDB12882 ; HMDB30328 ; HMDB31214 ; HMDB31216 ; HMDB31218 ; HMDB32857 ; HMDB33286 ; HMDB33302 ; HMDB33698 ; HMDB38091 ; HMDB41602 ; HMDB42041 ; HMDB59876	45-Dihydroxy-7-methoxy-6-methylflavone ; Sayanedin ; Alfalone ; 2-Methyl-3-ketovaleric acid ; 3-Methyl-2-oxovaleric acid ; Ketoleucine ; 2-Ketohexanoic acid ; Diethanolamine ; Mevalonolactone ; 3-Oxohexanoic acid ; Adipate semialdehyde ; Petasitenine ; 5-Ethoxy-45-dihydro-2(3H)furanone ; Ethyl acetoacetate ; Ethyl 3-oxobutanoate ; Glycerol tripropanoate ; Sherry lactone ; (4S6S)-3456-Tetrahydro-4-hydroxy-6-methyl-2H-pyran-2-one ; Acetoin acetate ; Peonidin 3-rhamnoside ; Methyl levulinate ; Tiracizine ; Pantolactone	M+2H ; M+2H ; M+2H ; 2M+H+K ; 2M+H+K ; 2M+H+K ; 2M+H+K ; M+2Na-H ; 2M+H+K ; 2M+H+K ; 2M+H+K ; M+3Na ; 2M+H+K ; 2M+H+K ; 2M+H+K ; M+H+K ; 2M+H+K ; 2M+H+K ; 2M+H+K ; M+3H ; 2M+H+K ; M+H+2Na ; 2M+H+K	C17H14O5 ; C17H14O5 ; C17H14O5 ; C6H10O3 ; C6H10O3 ; C6H10O3 ; C6H10O3 ; C4H11NO2 ; C6H10O3 ; C6H10O3 ; C6H10O3 ; C19H27NO7 ; C6H10O3 ; C6H10O3 ; C6H10O3 ; C12H20O6 ; C6H10O3 ; C6H10O3 ; C6H10O3 ; C22H23O10 ; C6H10O3 ; C21H26ClN3O3 ; C6H10O3
828.55115	1.317811	0.493235	2.9449	0.014663	1.8338	0.057457	828.5512	HMDB08189 ; HMDB08222 ; HMDB08253 ; HMDB08254 ; HMDB08445 ; HMDB08478 ; HMDB08509 ; HMDB08510 ; HMDB08667 ; HMDB08700 ; HMDB08731 ; HMDB08732 ; HMDB33726	PC(18:3(6Z9Z12Z)22:6(4Z7Z10Z13Z16Z19Z)) ; PC(18:3(9Z12Z15Z)22:6(4Z7Z10Z13Z16Z19Z)) ; PC(18:4(6Z9Z12Z15Z)22:5(4Z7Z10Z13Z16Z)) ; PC(18:4(6Z9Z12Z15Z)22:5(7Z10Z13Z16Z19Z)) ; PC(20:4(5Z8Z11Z14Z)20:5(5Z8Z11Z14Z17Z)) ; PC(20:4(8Z11Z14Z17Z)20:5(5Z8Z11Z14Z17Z)) ; PC(20:5(5Z8Z11Z14Z17Z)20:4(5Z8Z11Z14Z)) ; PC(20:5(5Z8Z11Z14Z17Z)20:4(8Z11Z14Z17Z)) ; PC(22:5(4Z7Z10Z13Z16Z)18:4(6Z9Z12Z15Z)) ; PC(22:5(7Z10Z13Z16Z19Z)18:4(6Z9Z12Z15Z)) ; PC(22:6(4Z7Z10Z13Z16Z19Z)18:3(6Z9Z12Z)) ; PC(22:6(4Z7Z10Z13Z16Z19Z)18:3(9Z12Z15Z)) ; Cyanidin 3-O-4-Hydroxy-35-dimethoxycinnamoyl-(-2)-b-D-glucopyranosyl-(1-2)-4-hydroxy-3-methoxycinnamoyl-(-6)-b-D-glucopyranoside 5-O-(6-O-malonyl-b-D-glucopyranoside)	M+H ; M+H ; M+H ; M+H ; M+H ; M+H ; M+H ; M+H ; M+H ; M+H ; M+H ; M+H ; 2M+3H	C48H78NO8P ; C48H78NO8P ; C48H78NO8P ; C48H78NO8P ; C48H78NO8P ; C48H78NO8P ; C48H78NO8P ; C48H78NO8P ; C48H78NO8P ; C48H78NO8P ; C48H78NO8P ; C48H78NO8P ; C57H61O31
337.27355	1.317998	0.482408	2.9422	0.014731	1.8318	0.057587	337.2736	HMDB06070 ; HMDB31005 ; HMDB38105 ; HMDB00560 ; HMDB05407 ; HMDB05450 ; HMDB05467 ; HMDB30426 ; HMDB30427 ; HMDB31015 ; HMDB31483 ; HMDB31486 ; HMDB31487 ; HMDB31488 ; HMDB31539 ; HMDB31540 ; HMDB31550 ; HMDB31551 ; HMDB32053 ; HMDB32301 ; HMDB32342 ; HMDB32524 ; HMDB33667 ; HMDB33827 ; HMDB34372 ; HMDB36424 ; HMDB37814 ; HMDB38246 ; HMDB38259 ; HMDB38260 ; HMDB39129 ; HMDB40339 ; HMDB42231 ; HMDB42405 ; HMDB42464 ; HMDB42492 ; HMDB42666 ; HMDB43148 ; HMDB43150 ; HMDB43400 ; HMDB43458 ; HMDB43959 ; HMDB44093 ; HMDB44121 ; HMDB44148 ; HMDB44202 ; HMDB44228 ; HMDB44229 ; HMDB44391 ; HMDB44766 ; HMDB44895 ; HMDB44921 ; HMDB44948 ; HMDB45025 ; HMDB45026 ; HMDB45051 ;	Pregnanetriol ; 3-Methyl-5-pentyl-2-furanundecanoic acid ; Isolinderanolide ; 58-Tetradecadienoic acid ; TG(18:020:1(11Z)20:1(11Z))iso3 ; TG(18:1(9Z)20:020:1(11Z))iso6 ; TG(18:2(9Z12Z)20:020:0)iso3 ; Linalyl isobutyrate ; Linalyl butyrate ; Ethyl (2E6Z)-dodecadienoate ; (Z)-4-Heptenal ; 3-Hepten-2-one ; (E)-4-Hepten-2-one ; 2-Hepten-4-one ; 2-Methylcyclohexanone ; 4-Methylcyclohexanone ; 5-Methyl-3-hexen-2-one ; 5-Methyl-5-hexen-2-one ; alpha-Terpineol butanoate ; trans-2-trans-4-Heptadien-1-ol ; Isobornyl isobutyrate ; Terpinyl isobutyrate ; (R)-3-Methylcyclohexanone ; 2-Heptenal ; xi-3-Methyl-3-cyclohexen-1-ol ; Allyl undecylenate ; 2-Propenyl cyclohexanepentanoate ; Bornyl butyrate ; Neryl butyrate ; Geranyl 2-methylpropanoate ; Goshuyic acid ; 25-Heptadien-1-ol ; TG(14:022:022:2(13Z16Z)) ; TG(14:020:1(11Z)24:1(15Z)) ; TG(14:022:1(13Z)22:1(13Z)) ; TG(14:024:1(15Z)20:1(11Z)) ; TG(14:022:2(13Z16Z)22:0) ; TG(15:024:018:2(9Z12Z)) ; TG(15:024:020:2n6) ; TG(15:018:2(9Z12Z)24:0) ; TG(15:020:2n624:0) ; TG(16:020:022:2(13Z16Z)) ; TG(16:018:1(11Z)24:1(15Z)) ; TG(16:018:1(9Z)24:1(15Z)) ; TG(16:020:1(11Z)22:1(13Z)) ; TG(16:022:1(13Z)20:1(11Z)) ; TG(16:024:1(15Z)18:1(11Z)) ; TG(16:024:1(15Z)18:1(9Z)) ; TG(16:022:2(13Z16Z)20:0) ; TG(18:018:022:2(13Z16Z)) ; TG(18:016:1(9Z)24:1(15Z)) ; TG(18:018:1(11Z)22:1(13Z)) ; TG(18:018:1(9Z)22:1(13Z)) ; TG(18:022:1(13Z)18:1(11Z)) ; TG(18:022:1(13Z)18:1(9Z)) ; TG(18:024:1(15Z)16:1(9Z)) ; TG(18:022:2(13Z16Z)18:0) ; TG(20:016:022:2(13Z16Z)) ; TG(20:014:1(9Z)24:1(15Z)) ; TG(20:016:1(9Z)22:1(13Z)) ; TG(20:018:1(11Z)20:1(11Z)) ; TG(20:018:1(9Z)20:1(11Z)) ; TG(20:020:1(11Z)18:1(11Z)) ; TG(20:020:1(11Z)18:1(9Z)) ; TG(20:022:1(13Z)16:1(9Z)) ; TG(20:024:1(15Z)14:1(9Z)) ; TG(22:014:022:2(13Z16Z)) ; TG(22:014:1(9Z)22:1(13Z)) ; TG(22:016:1(9Z)20:1(11Z)) ; TG(22:018:1(11Z)18:1(11Z)) ; TG(22:018:1(11Z)18:1(9Z)) ; TG(22:018:1(9Z)18:1(11Z)) ; TG(22:018:1(9Z)18:1(9Z)) ; TG(22:020:1(11Z)16:1(9Z)) ; TG(22:022:1(13Z)14:1(9Z)) ; TG(24:015:018:2(9Z12Z)) ; TG(24:015:020:2n6) ; TG(24:014:1(9Z)20:1(11Z)) ; TG(24:016:1(9Z)18:1(11Z)) ; TG(24:016:1(9Z)18:1(9Z)) ; TG(24:018:1(11Z)16:1(9Z)) ; TG(24:018:1(9Z)16:1(9Z)) ; TG(24:020:1(11Z)14:1(9Z)) ; TG(14:1(9Z)20:024:1(15Z)) ; TG(14:1(9Z)22:022:1(13Z)) ; TG(14:1(9Z)24:020:1(11Z)) ; TG(16:1(9Z)18:024:1(15Z)) ; TG(16:1(9Z)20:022:1(13Z)) ; TG(16:1(9Z)22:020:1(11Z)) ; TG(16:1(9Z)24:018:1(11Z)) ; TG(16:1(9Z)24:018:1(9Z)) ; TG(18:1(11Z)16:024:1(15Z)) ; TG(18:1(11Z)18:022:1(13Z)) ;	M+H ; M+H ; M+H ; 3M+2H ; M+3Na ; M+3Na ; M+3Na ; 3M+2H ; 3M+2H ; 3M+2H ; 3M+H ; 3M+H ; 3M+H ; 3M+H ; 3M+H ; 3M+H ; 3M+H ; 3M+H ; 3M+2H ; 3M+H ; 3M+2H ; 3M+2H ; 3M+H ; 3M+H ; 3M+H ; 3M+2H ; 3M+2H ; 3M+2H ; 3M+2H ; 3M+2H ; 3M+2H ; 3M+H ; M+3Na	C21H36O3 ; C21H36O3 ; C21H36O3 ; C14H24O2 ; C61H114O6 ; C61H114O6 ; C61H114O6 ; C14H24O2 ; C14H24O2 ; C14H24O2 ; C7H12O ; C7H12O ; C7H12O ; C7H12O ; C7H12O ; C7H12O ; C7H12O ; C7H12O ; C14H24O2 ; C7H12O ; C14H24O2 ; C14H24O2 ; C7H12O ; C7H12O ; C7H12O ; C14H24O2 ; C14H24O2 ; C14H24O2 ; C14H24O2 ; C14H24O2 ; C14H24O2 ; C7H12O ; C61H114O6
210.89930	1.300633	1.350577	2.9392	0.014807	1.8295	0.057838	NA	NA	NA	NA	NA
158.96405	1.331767	1.018649	2.9307	0.015023	1.8233	0.058499	158.9641	HMDB14118	Trifluoroacetic acid	M+2Na-H	C2HF3O2
131.54227	1.303317	1.46533	-2.9271	0.015115	1.8206	0.058723	131.5423	HMDB00856 ; HMDB28685 ; HMDB28701 ; HMDB28790 ; HMDB29142 ; HMDB29189 ; HMDB38967	N-a-Acetylcitrulline ; Alanyl-Glutamine ; Alanyl-Gamma-glutamate ; Glutaminyl-Alanine ; Gamma-glutamyl-Alanine ; 5-Hydroxysebacate ; 1-Propenyl 1-(1-propenylthio)propyl disulfide	M+2Na ; M+2Na ; M+2Na ; M+2Na ; M+2Na ; M+2Na ; M+ACN+2H	C8H15N3O4 ; C8H15N3O4 ; C8H15N3O4 ; C8H15N3O4 ; C8H15N3O4 ; C10H17O5 ; C9H16S3
279.58161	1.313418	1.4338	-2.9262	0.015139	1.8199	0.058768	279.5816	HMDB00707 ; HMDB01879 ; HMDB01964 ; HMDB02130 ; HMDB03501 ; HMDB06915 ; HMDB11663 ; HMDB29279 ; HMDB29280 ; HMDB29493 ; HMDB30093 ; HMDB30705 ; HMDB30706 ; HMDB30707 ; HMDB32057 ; HMDB32131 ; HMDB34766 ; HMDB36592 ; HMDB37343 ; HMDB37344 ; HMDB40138 ; HMDB40139	4-Hydroxyphenylpyruvic acid ; Aspirin ; Caffeic acid ; Monomethyl phthalate ; 34-Dihydroxy-trans-cinnamate ; 2-Hydroxy-3-(4-hydroxyphenyl)propenoic acid ; 3-Hydroxyphenylpyruvic acid ; 13-Dicaffeoylquinic acid ; Dicaffeoylquinic acid ; Formononetin 7-(6-malonylglucoside) ; 15-Dicaffeoylquinic acid ; 34-Di-O-caffeoylquinic acid ; 35-Di-O-caffeoylquinic acid ; 45-Di-O-caffeoylquinic acid ; (E)-3-(23-Dihydroxyphenyl)-2-propenoic acid ; 3-(35-Dihydroxyphenyl)-2-propenoic acid ; 14-Di-O-caffeoylquinic acid ; Validamycin A ; 23-Diacetylcosmosiin ; 34-Diacetylcosmosiin ; 24-Diacetylafzelin ; 34-Diacetylafzelin	3M+H+NH4 ; 3M+H+NH4 ; 3M+H+NH4 ; 3M+H+NH4 ; 3M+H+NH4 ; 3M+H+NH4 ; 3M+H+NH4 ; M+ACN+2H ; M+ACN+2H ; M+ACN+2H ; M+ACN+2H ; M+ACN+2H ; M+ACN+2H ; M+ACN+2H ; M+ACN+2H ; 3M+H+NH4 ; 3M+H+NH4 ; M+ACN+2H ; M+Na+K ; M+ACN+2H ; M+ACN+2H ; M+ACN+2H ; M+ACN+2H	C9H8O4 ; C9H8O4 ; C9H8O4 ; C9H8O4 ; C9H8O4 ; C9H8O4 ; C9H8O4 ; C25H24O12 ; C25H24O12 ; C25H24O12 ; C25H24O12 ; C25H24O12 ; C25H24O12 ; C25H24O12 ; C9H8O4 ; C9H8O4 ; C25H24O12 ; C20H35NO13 ; C25H24O12 ; C25H24O12 ; C25H24O12 ; C25H24O12

238.89410	1.324201	1.195592	2.9174	0.01537	1.8133	0.059526	NA	NA	NA	NA	NA
480.34464	1.306047	0.363176	2.9112	0.015532	1.8088	0.059896	480.3446	HMDB10407 ; HMDB00430 ; HMDB01420 ; HMDB01903 ; HMDB02197 ; HMDB03871 ; HMDB04706 ; HMDB06226 ; HMDB06720 ; HMDB06940 ; HMDB10201 ; HMDB10208 ; HMDB10221 ; HMDB12453 ; HMDB12457 ; HMDB12459 ; HMDB13628 ; HMDB15046 ; HMDB30024 ; HMDB30047 ; HMDB30048 ; HMDB34381 ; HMDB35300 ; HMDB40900 ; HMDB60133 ; HMDB60425 ; HMDB60508	LysoPC(P-16:0) ; 2425-Dihydroxyvitamin D ; 2526-dihydroxyvitamin D ; Calcitriol ; 7a12a-Dihydroxy-cholestene-3-one ; 13-L-Hydroperoxylinoleic acid ; 8(R)-Hydroperoxylinoleic acid ; 24R25-Dihydroxyvitamin D3 ; 23S25-dihydroxyvitamin D3 ; 9(S)-HPODE ; 1213-DiHODE ; 1516-DiHODE ; 910-DiHODE ; 3 beta-Hydroxy-5-cholestenoate ; 7 alpha24-Dihydroxy-4-cholesten-3-one ; 7 alpha26-Dihydroxy-4-cholesten-3-one ; PGF2a ethanolamide ; Paricalcitol ; Sarsasapogenin ; Neotigogenin ; Smilagenin ; Methyl linoleate ; Octadecyl cis-p-coumarate ; ()-(E)-13-Hydroxy-10-oxo-11-octadecenoic acid ; (24R)-2425-Dihydroxycalciol ; 7alpha25-Dihydroxy-4-cholesten-3-one ; Secalciferol	M+H ; M+ACN+Na ; M+ACN+Na ; M+ACN+Na ; M+ACN+Na ; 3M+H+Na ; 3M+H+Na ; M+ACN+Na ; M+ACN+Na ; 3M+H+Na ; 3M+H+Na ; 3M+H+Na ; 3M+H+Na ; M+ACN+Na ; M+ACN+Na ; M+ACN+Na ; M+2ACN+H ; M+ACN+Na ; M+ACN+Na ; M+ACN+Na ; M+ACN+Na ; 3M+2K ; M+ACN+Na ; 3M+H+Na ; M+ACN+Na ; M+ACN+Na ; M+ACN+Na	C24H50NO6P ; C27H44O3 ; C27H44O3 ; C27H44O3 ; C27H44O3 ; C18H32O4 ; C18H32O4 ; C27H44O3 ; C27H44O3 ; C18H32O4 ; C18H32O4 ; C18H32O4 ; C18H32O4 ; C27H44O3 ; C27H44O3 ; C27H44O3 ; C22H39NO5 ; C27H44O3 ; C27H44O3 ; C27H44O3 ; C27H44O3 ; C19H34O2 ; C27H44O3 ; C18H32O4 ; C27H44O3 ; C27H44O3 ; C27H44O3
179.51402	1.342344	0.272862	2.9107	0.015546	1.8084	0.059896	179.514	HMDB33954 ; HMDB40693	Juzirine ; N2-(gamma-Glutamyl)-4-carboxyphenylhydrazine	M+2K ; M+2K	C17H15NO3 ; C12H15N3O5
494.40505	1.345646	0.317589	-2.9023	0.015771	1.8021	0.060518	494.4051	HMDB45814 ; HMDB45970 ; HMDB46239 ; HMDB46245 ; HMDB46680 ; HMDB46855 ; HMDB46992 ; HMDB46999 ; HMDB49512 ; HMDB49692 ; HMDB50120 ; HMDB50291 ; HMDB50958 ; HMDB52928 ; HMDB53712 ; HMDB54440 ; HMDB54450 ; HMDB55189	TG(20:020:3(5Z8Z11Z)o-18:0) ; TG(20:020:3n6o-18:0) ; TG(20:0o-18:020:3(5Z8Z11Z)) ; TG(20:0o-18:020:3n6) ; TG(22:018:3(6Z9Z12Z)o-18:0) ; TG(22:018:3(9Z12Z15Z)o-18:0) ; TG(22:0o-18:018:3(6Z9Z12Z)) ; TG(22:0o-18:018:3(9Z12Z15Z)) ; TG(18:1(11Z)22:2(13Z16Z)o-18:0) ; TG(18:1(11Z)o-18:022:2(13Z16Z)) ; TG(18:1(9Z)22:2(13Z16Z)o-18:0) ; TG(18:1(9Z)o-18:022:2(13Z16Z)) ; TG(20:3(5Z8Z11Z)20:0o-18:0) ; TG(18:3(6Z9Z12Z)22:0o-18:0) ; TG(20:3n620:0o-18:0) ; TG(22:2(13Z16Z)18:1(11Z)o-18:0) ; TG(22:2(13Z16Z)18:1(9Z)o-18:0) ; TG(18:3(9Z12Z15Z)22:0o-18:0)	M+Na+K ; M+Na+K ; M+Na+K ; M+Na+K ; M+Na+K ; M+Na+K ; M+Na+K ; M+Na+K ; M+Na+K ; M+Na+K ; M+Na+K ; M+Na+K ; M+Na+K ; M+Na+K ; M+Na+K ; M+Na+K ; M+Na+K ; M+Na+K	C61H114O5 ; C61H114O5 ; C61H114O5 ; C61H114O5 ; C61H114O5 ; C61H114O5 ; C61H114O5 ; C61H114O5 ; C61H114O5 ; C61H114O5 ; C61H114O5 ; C61H114O5 ; C61H114O5 ; C61H114O5 ; C61H114O5 ; C61H114O5 ; C61H114O5 ; C61H114O5
522.59634	1.314657	1.725922	2.9012	0.015801	1.8013	0.060585	522.5963	HMDB30298 ; HMDB32236 ; HMDB36345 ; HMDB59830 ; HMDB59874	3610-Trimethyltetradecane ; (-)-NN-Dimethyl menthyl succinamide ; 3-Cyclohexyldodecane ; Heptadecane ; 1-Dodecene	2M+ACN+H ; 3M+NH4 ; 2M+NH4 ; 2M+ACN+H ; 3M+NH4	C17H36 ; C12H24 ; C18H36 ; C17H36 ; C12H24
311.56652	1.357039	0.066043	2.8958	0.015949	1.7973	0.060951	311.5665	HMDB33136 ; HMDB60031	2-Amino-4-hydroxy-6-pteridinecarboxylic acid ; 5-(35-Dihydroxyphenyl)-gamma-valerolactone-O-sulphate-O-methyl	3M+2H ; 2M+H+NH4	C7H5N5O3 ; C12H14O7S
155.09114	1.340255	0.386673	2.8928	0.01603	1.7951	0.061137	155.0911	HMDB12128 ; HMDB12295 ; HMDB13961 ; HMDB14246 ; HMDB14328 ; HMDB15322 ; HMDB15516 ; HMDB30008 ; HMDB32934 ; HMDB34192 ; HMDB34421 ; HMDB37287 ; HMDB37291 ; HMDB38504 ; HMDB38505 ; HMDB38839 ; HMDB39228 ; HMDB60540	(R)-Amphetamine ; Trimethyl sulfonium ; O-Desmethylverapamil (D-702) ; O-Desmethylverapamil (D-703) ; Amphetamine ; Dexfenfluramine ; Dextroamphetamine ; d-Tocotrienol ; 3-Butylpyridine ; delta35-Deoxytigogenin ; delta35-Deoxyneotigogenin ; 3-(2-Methylpropyl)pyridine ; 2-(2-Methylpropyl)pyridine ; alpha-Micropteroxanthin B ; beta-Micropteroxanthin ; 1-(m-Methoxycinnamoyl)pyrrolidine ; (3-Aminopropoxy)guanidine ; Norverapamil	2M+H+K ; 2M+H ; M+2H+Na ; M+2H+Na ; 2M+H+K ; 2M+3H ; 2M+H+K ; M+3Na ; 2M+H+K ; M+3Na ; M+3Na ; 2M+H+K ; 2M+H+K ; M+3Na ; M+3Na ; 2M+3H ; M+Na ; M+2H+Na	C9H13N ; C3H9S ; C26H36N2O4 ; C26H36N2O4 ; C9H13N ; C12H16F3N ; C9H13N ; C27H40O2 ; C9H13N ; C27H40O2 ; C27H40O2 ; C9H13N ; C9H13N ; C27H40O2 ; C27H40O2 ; C14H17NO2 ; C4H12N4O ; C26H36N2O4
180.95748	1.334745	1.177116	-2.879	0.016415	1.7848	0.062037	180.9575	HMDB29611	Asparagusic acid	2M+Na+K	C4H6O2S2
617.56781	1.365888	0.361076	-2.8777	0.016449	1.7839	0.062069	617.5678	HMDB38418 ; HMDB34841	Glucoconringiin ; Faradiol myristate	3M+Na+K ; M+H-2H2O	C11H21NO10S2 ; C44H76O3
828.76232	1.318168	1.562189	-2.8769	0.016473	1.7832	0.062069	828.7623	HMDB34773 ; HMDB34774 ; HMDB47953 ; HMDB47976 ; HMDB48393 ; HMDB48394 ; HMDB48610 ; HMDB49051 ; HMDB49239 ; HMDB49860	4-Demethylsimmondsin 2-(E)-ferulate ; 3-Demethylsimmondsin 2-(Z)-ferulate ; TG(14:1(9Z)18:1(11Z)o-18:0) ; TG(14:1(9Z)18:1(9Z)o-18:0) ; TG(14:1(9Z)o-18:018:1(11Z)) ; TG(14:1(9Z)o-18:018:1(9Z)) ; TG(16:1(9Z)16:1(9Z)o-18:0) ; TG(16:1(9Z)o-18:016:1(9Z)) ; TG(18:1(11Z)14:1(9Z)o-18:0) ; TG(18:1(9Z)14:1(9Z)o-18:0)	3M+2Na ; 3M+2Na ; 2M+H+Na ; 2M+H+Na ; 2M+H+Na ; 2M+H+Na ; 2M+H+Na ; 2M+H+Na ; 2M+H+Na ; 2M+H+Na	C25H31NO12 ; C25H31NO12 ; C53H100O5 ; C53H100O5 ; C53H100O5 ; C53H100O5 ; C53H100O5 ; C53H100O5 ; C53H100O5 ; C53H100O5
297.24191	1.336663	0.595563	2.8758	0.016505	1.7824	0.062104	297.2419	HMDB00782 ; HMDB04704 ; HMDB04705 ; HMDB30935 ; HMDB31679 ; HMDB34295 ; HMDB41220	Octadecanedioic acid ; 910-DHOME ; 1213-DHOME ; 91013-Trihydroxystearic acid ; (9xi10xi12xi)-910-Dihydroxy-12-octadecenoic acid ; Floionolic acid ; Dibutyl decanedioate	M+H-H2O ; M+H-H2O ; M+H-H2O ; M+H-2H2O ; M+H-H2O ; M+H-2H2O ; M+H-H2O	C18H34O4 ; C18H34O4 ; C18H34O4 ; C18H36O5 ; C18H34O4 ; C18H36O5 ; C18H34O4
239.16381	1.341924	0.701569	2.8663	0.016774	1.7754	0.062684	239.1638	HMDB38496 ; HMDB38497 ; HMDB00387 ; HMDB02059 ; HMDB02157 ; HMDB10728 ; HMDB12215 ; HMDB15358 ; HMDB15502 ; HMDB40092 ; HMDB40113 ; HMDB40169 ; HMDB00394	Polyporusterone B ; Polyporusterone C ; 3-Hydroxydodecanoic acid ; 12-Hydroxydodecanoic acid ; 27-Norcholestanehexol ; (R)-3-Hydroxydodecanoic acid ; Dihydrozeatin ; Levomethadyl Acetate ; Methadyl Acetate ; 2-Heptyl-45-dimethylthiazole ; 4-Ethyl-2-heptylthiazole ; xi-5-Hydroxydodecanoic acid ; 3-Hydroxytetradecanedioic acid	M+2H ; M+2H ; M+Na ; M+Na ; M+H+Na ; M+Na ; M+NH4 ; M+3ACN+2H ; M+3ACN+2H ; 2M+3H2O+2H ; 2M+3H2O+2H ; M+Na ; M+H-2H2O	C28H44O6 ; C28H44O6 ; C12H24O3 ; C12H24O3 ; C26H46O6 ; C12H24O3 ; C10H15N5O ; C23H31NO2 ; C23H31NO2 ; C12H21NS ; C12H21NS ; C12H24O3 ; C14H26O5
120.06558	1.307277	0.379934	2.8626	0.016882	1.7726	0.062993	120.0656	HMDB00167 ; HMDB00719 ; HMDB04041 ; HMDB61148 ; HMDB00212 ; HMDB00215 ; HMDB00389 ; HMDB00803 ; HMDB00853 ; HMDB00888 ; HMDB01129 ; HMDB37137 ; HMDB38751	L-Threonine ; L-Homoserine ; L-Allothreonine ; Hydroxyethyl glycine ; N-Acetylgalactosamine ; N-Acetyl-D-glucosamine ; 2-Deoxysepiapterin ; Beta-N-Acetylglucosamine ; N-Acetyl-b-D-galactosamine ; Undecanedioic acid ; N-Acetylmannosamine ; Butyl butyryllactate ; Avenic acid B	M+H ; M+H ; M+H ; M+H ; M+H+NH4 ; M+H+NH4 ; M+H+NH4 ; M+H+NH4 ; M+H+NH4 ; M+H+Na ; M+H+NH4 ; M+H+Na ; M+H+NH4	C4H9NO3 ; C4H9NO3 ; C4H9NO3 ; C4H9NO3 ; C8H15NO6 ; C8H15NO6 ; C9H11N5O2 ; C8H15NO6 ; C8H15NO6 ; C11H20O4 ; C8H15NO6 ; C11H20O4 ; C8H15NO6

125.07080	1.303266	1.172376	2.86	0.016956	1.7707	0.063094	125.0708	HMDB04181 ; HMDB31852 ; HMDB40141 ; HMDB40143 ; HMDB02053 ; HMDB31876 ; HMDB33064 ; HMDB02341 ; HMDB02664 ; HMDB03034 ; HMDB03175 ; HMDB10410 ; HMDB11603 ; HMDB12552 ; HMDB12588 ; HMDB12590 ; HMDB12641 ; HMDB13040 ; HMDB15377 ; HMDB30781 ; HMDB30782 ; HMDB30783 ; HMDB35919 ; HMDB36701 ; HMDB37593 ; HMDB38483 ; HMDB38693 ; HMDB38742 ; HMDB39060 ; HMDB40611 ; HMDB41330 ; HMDB60096	Methylimidazole acetaldehyde ; 2-Methoxy-3-methylpyrazine ; 2-Methoxy-5-methylpyrazine ; 2-Methoxy-6-methylpyrazine ; Histidylproline diketopiperazine ; Prenyl glucoside ; (E)-2-Methyl-2-buten-1-ol O-beta-D-Glucopyranoside ; 8-iso-15-keto-PGE2 ; Prostaglandin E3 ; Prostaglandin D3 ; 15-Keto-prostaglandin E2 ; Resolvin E1 ; 4-(Methylnitrosamino)-1-(3-pyridyl)-1-butanone ; 12-Oxo-20-hydroxy-leukotriene B4 ; 15-Epi-lipoxin B5 ; 15-Oxo-lipoxin A4 ; 20-oxo-leukotriene B4 ; PGH3 ; Isocarboxazid ; Isomammein ; Mammea BAB ; Mammea BBA ; Corchorifatty acid F ; (ent-6alpha7alpha12alpha)-6712-Trihydroxy-16-kauren-19-oic acid ; Oryzalic acid A ; Auxin a ; Oryzalic acid B ; Armillasin ; (S)-Neomammein ; (9R10S12Z)-910-Dihydroxy-8-oxo-12-octadecenoic acid ; Tanabalin ; 51218R-TriHEPE	M+H ; M+H ; M+H ; M+H ; M+2H ; M+2H ; M+2H ; M+2H+Na ; M+2H+Na ; M+2H+Na ; M+2H+Na ; M+2H+Na ; M+ACN+2H ; M+2H+Na ; M+2H+Na ; M+2H+Na ; M+2H+Na ; M+2H+Na ; M+H+NH4 ; M+3H ; M+3H ; M+3H ; M+H+2Na ; M+2H+Na ; M+2H+Na ; M+H+2Na ; M+2H+Na ; M+3H ; M+3H ; M+H+2Na ; M+3H ; M+2H+Na	C6H8N2O ; C6H8N2O ; C6H8N2O ; C6H8N2O ; C12H16N4O2 ; C11H20O6 ; C11H20O6 ; C20H30O5 ; C20H30O5 ; C20H30O5 ; C20H30O5 ; C20H30O5 ; C10H13N3O2 ; C20H30O5 ; C20H30O5 ; C20H30O5 ; C20H30O5 ; C20H30O5 ; C12H13N3O2 ; C22H28O5 ; C22H28O5 ; C22H28O5 ; C18H32O5 ; C20H30O5 ; C20H30O5 ; C18H32O5 ; C20H30O5 ; C22H28O5 ; C22H28O5 ; C18H32O5 ; C22H28O5 ; C20H30O5
446.99660	1.316931	0.406726	2.8505	0.017236	1.7636	0.063744	446.9966	HMDB01274 ; HMDB39721	dTDP ; 4-O-alpha-D-Galactopyranuronosyl-D-galacturonic acid	M+2Na-H ; M+2K-H	C10H16N2O11P2 ; C12H18O13
538.52996	1.334366	0.448602	-2.8479	0.017314	1.7616	0.063888	NA	NA	NA	NA	NA
113.03460	1.310184	0.561117	2.8445	0.017412	1.7592	0.064087	113.0346	HMDB00300 ; HMDB60760 ; HMDB00130 ; HMDB00484 ; HMDB00750 ; HMDB00822 ; HMDB01336 ; HMDB01868 ; HMDB02818 ; HMDB04076 ; HMDB06242 ; HMDB13319 ; HMDB28695 ; HMDB29010 ; HMDB29287 ; HMDB29288 ; HMDB29644 ; HMDB29664 ; HMDB29673 ; HMDB30397 ; HMDB30654 ; HMDB32565 ; HMDB33129 ; HMDB33137 ; HMDB33563 ; HMDB33997 ; HMDB33999 ; HMDB34060 ; HMDB34335 ; HMDB38333 ; HMDB38335 ; HMDB40720 ; HMDB41109 ; HMDB41269 ; HMDB59713 ; HMDB60003 ; HMDB60281 ; HMDB60562 ; HMDB32332	Uracil ; 4-Carboxypyrazole ; Homogentisic acid ; Vanillic acid ; 3-Hydroxymandelic acid ; p-Hydroxymandelic acid ; 34-Dihydroxybenzeneacetic acid ; 5-Methoxysalicylic acid ; Alloxan ; 5-Hydroxykynurenamine ; 34-Dihydroxymandelaldehyde ; Tyrosinamide ; Alanyl-Proline ; Prolyl-Alanine ; 3-Caffeoyl-15-quinolactone ; 4-Caffeoyl-15-quinolactone ; 246-Trihydroxyacetophenone ; Vanilloside ; 26-Dimethoxy-14-benzoquinone ; L-2-Amino-3-(4-aminophenyl)propanoic acid ; 3-O-Caffeoylshikimic acid ; (4-Ethoxyphenyl)urea ; 3-Acetyl-4-hydroxy-6-methyl-2H-pyran-2-one ; 2-Methoxycarbonylphenyl beta-D-glucopyranoside ; Juglone glucoside ; 4-O-Caffeoylshikimic acid ; Dattelic acid ; 6-O-Acetylarbutin ; Thermophillin ; 2-Hydroxyphenylacetic acid O-b-D-glucoside ; (S)-Mandelic acid O-beta-D-Glucopyranoside ; 2-O-Acetylarbutin ; (S)-3-Ethylidenehexahydropyrrolo12-apyrazine-14-dione ; Pantoyllactone glucoside ; 3-Methoxysalicylic acid ; Isovanillic acid ; 3-Hydroxykynurenamine ; Captopril-cysteine disulfide ; Hydroxylated lecithin	M+H ; M+H ; 2M+3H ; 2M+3H ; 2M+3H ; 2M+3H ; 2M+3H ; 2M+3H ; M+2ACN+2H ; M+2Na ; 2M+3H ; M+2Na ; M+H+K ; M+H+K ; M+3H ; M+3H ; 2M+3H ; M+2H+Na ; 2M+3H ; M+2Na ; M+3H ; M+2Na ; 2M+3H ; M+2H+Na ; M+3H ; M+3H ; M+3H ; M+2H+Na ; 2M+3H ; M+2H+Na ; M+2H+Na ; M+2H+Na ; M+2Na ; M+H+2Na ; 2M+3H ; 2M+3H ; M+2Na ; M+3H ; M+H-2H2O	C4H4N2O2 ; C4H4N2O2 ; C8H8O4 ; C8H8O4 ; C8H8O4 ; C8H8O4 ; C8H8O4 ; C8H8O4 ; C4H2N2O4 ; C9H12N2O2 ; C8H8O4 ; C9H12N2O2 ; C8H14N2O3 ; C8H14N2O3 ; C16H16O8 ; C16H16O8 ; C8H8O4 ; C14H18O8 ; C8H8O4 ; C9H12N2O2 ; C16H16O8 ; C9H12N2O2 ; C8H8O4 ; C14H18O8 ; C16H16O8 ; C16H16O8 ; C16H16O8 ; C14H18O8 ; C8H8O4 ; C14H18O8 ; C14H18O8 ; C14H18O8 ; C9H12N2O2 ; C12H20O8 ; C8H8O4 ; C8H8O4 ; C9H12N2O2 ; C12H20N2O5S2 ; C4H8N2O4
420.54301	1.35595	0.501071	-2.8407	0.017529	1.7563	0.064254	420.543	HMDB03419 ; HMDB29200 ; HMDB32780 ; HMDB35492 ; HMDB41748	Formyl-CoA ; Ferulic acid 4-sulfate ; Jujuboside C ; (3b16a)-Dihydroxy-12-oleanen-28-oic acid 3-glucosyl-(1-2)-arabinoside 28-rhamnosyl-(1-4)-glucosyl-(1-4)-glucosyl ester ; Isoferulic acid 3-sulfate	M+2Na ; 3M+H+NH4 ; M+2H+Na ; M+2H+Na ; 3M+H+NH4	C22H36N7O17P3S ; C10H10O7S ; C59H96O27 ; C59H96O27 ; C10H10O7S
445.59701	1.30085	0.4902	-2.8303	0.017843	1.7485	0.064788	445.597	HMDB02289	2-trans4-trans-Octadienoyl-CoA	M+2H	C29H46N7O17P3S
357.08230	1.314404	0.972158	-2.8297	0.01786	1.7481	0.064805	357.0823	HMDB03841 ; HMDB41705 ; HMDB41707 ; HMDB41709 ; HMDB29270 ; HMDB04662 ; HMDB06273 ; HMDB12268 ; HMDB28985 ; HMDB29111 ; HMDB29411 ; HMDB33329 ; HMDB39141 ; HMDB39209 ; HMDB39733 ; HMDB60536 ; HMDB60573 ; HMDB60991	5-Amino-6-(5-phosphoribitylamino)uracil ; Caffeic acid 3-O-glucuronide ; Caffeic acid 4-O-glucuronide ; Caffeoyl C1-glucuronide ; Quercetin 3-O-(6-malonyl-glucoside) 7-O-glucoside ; S-(Hydroxymethyl)glutathione ; 5-amino-1-(5-phospho-D-ribosyl)imidazole-4-carboxylate ; N5-Carboxyaminoimidazole ribonucleotide ; Methionyl-Tyrosine ; Tyrosyl-Methionine ; Prebetanin ; Coriandrin ; Wyeronic acid ; 24-Dihydroxy-2-biphenylcarboxylic acid ; gamma-Glutamylcysteinylserine ; Norclozapine ; Chlorpromazine-N-oxide ; hydroxychlorpromazine	M+H ; M+H ; M+H ; M+H ; M+2H ; 2M+H+K ; M+NH4 ; M+NH4 ; M+2Na-H ; M+2Na-H ; M+2ACN+2H ; 3M+H+Na ; 3M+H+Na ; 3M+H+Na ; 2M+H+K ; M+2Na-H ; M+Na ; M+Na	C9H17N4O9P ; C15H16O10 ; C15H16O10 ; C15H16O10 ; C30H32O20 ; C11H19N3O7S ; C9H14N3O9P ; C9H14N3O9P ; C14H20N2O4S ; C14H20N2O4S ; C24H26N2O16S ; C13H10O4 ; C13H10O4 ; C13H10O4 ; C11H19N3O7S ; C17H17ClN4 ; C17H19ClN2OS ; C17H19ClN2OS
306.15969	1.309698	0.7181	-2.8266	0.017956	1.7458	0.064863	306.1597	HMDB33749 ; HMDB33920 ; HMDB35443 ; HMDB38543 ; HMDB38546	Isoliensinine ; Liensinine ; (EE)-16-bis(4-methoxyphenyl)-15-hexadiene ; Longistylin A ; ent-6R16bOH17-Trihydroxy-7-oxo-67-seco-196-kauranolide 6-O-glucoside	M+2H ; M+2H ; 2M+H+Na ; 2M+H+Na ; M+2ACN+2H	C37H42N2O6 ; C37H42N2O6 ; C20H22O2 ; C20H22O2 ; C26H40O11
149.95269	1.343302	0.22724	2.8263	0.017967	1.7455	0.064863	NA	NA	NA	NA	NA
548.62552	1.338603	1.1572	-2.825	0.018004	1.7446	0.06495	NA	NA	NA	NA	NA
232.09155	1.351219	0.619391	2.8144	0.018336	1.7367	0.065908	232.0916	HMDB13010 ; HMDB15698 ; HMDB29707 ; HMDB30182 ; HMDB32277 ; HMDB32374 ; HMDB32375 ; HMDB34492 ; HMDB34878 ; HMDB36194 ; HMDB37790 ; HMDB38835 ; HMDB39013 ; HMDB39914 ; HMDB39915 ; HMDB39916 ; HMDB39917 ; HMDB40007 ; HMDB40152 ; HMDB59744	N-Heptanoylglycine ; Cinitapride ; 2-Amino-34-dimethylimidazo45-fquinoline ; Eupolauridine ; (-)-3-(Ethylthio)butanol ; 2-Mercapto-2-methyl-1-pentanol ; (-)-4-Mercapto-4-methyl-2-pentanol ; Dihydrovaltrate ; 3-Mercapto-2-methylpentanol ; 4-Methoxy-2-methyl-2-butanethiol ; Polyethylene glycol ; (E)-1-Cinnamoylpyrrolidine ; 8beta-Angeloyloxy-15-hydroxy-1alpha10R-dimethoxy-3-oxo-11(13)-germacren-126alpha-olide ; 16-Dihydroxy-37-dimethoxy-2-(3-methyl-2-butenyl)-8-(3-hydroxy-3-methyl-1E-butenyl)-xanthone ; 16-Dihydroxy-37-dimethoxy-2-(2-hydroxy-3-methyl-3-butenyl)-8-(3-methyl-2-butenyl)-xanthone ; 16-Dihydroxy-37-dimethoxy-2-(3-methyl-2-butenyl)-8-(2-hydroxy-3-methyl-3-butenyl)-xanthone ; 13-Dihydroxy-67-dimethoxy-2-(2-hydroxy-3-methyl-3-butenyl)-8-(3-methyl-2-butenyl)-xanthone ; 23-Dihydro-6-methyl-5-(5-methyl-2-furanyl)-1H-pyrrolizine ; 3-Mercapto-1-hexanol ; 34-Methyleneazelaic acid	M+2Na-H ; M+Na+K ; 2M+H+K ; 2M+3H2O+2H ; 3M+Na+K ; 3M+Na+K ; 3M+Na+K ; M+H+K ; 3M+Na+K ; 3M+Na+K ; M+2Na-H ; 2M+Na+K ; M+H+K ; M+H+Na ; M+H+Na ; M+H+Na ; M+H+Na ; 2M+Na+K ; 3M+Na+K ; 2M+H+K	C9H17NO3 ; C21H30N4O4 ; C12H12N4 ; C14H8N2 ; C6H14OS ; C6H14OS ; C6H14OS ; C22H32O8 ; C6H14OS ; C6H14OS ; C9H17NO3 ; C13H15NO ; C22H32O8 ; C25H28O7 ; C25H28O7 ; C25H28O7 ; C25H28O7 ; C13H15NO ; C6H14OS ; C11H16O4

219.64801	1.330886	1.48257	2.8121	0.018407	1.735	0.06607	219.648	HMDB32099 ; HMDB32797 ; HMDB32871 ; HMDB33102 ; HMDB33458 ; HMDB37104 ; HMDB37135 ; HMDB37642 ; HMDB37735 ; HMDB37816 ; HMDB41573	Dimethyl 3-methoxy-4-oxo-5-(81114-pentadecatrienyl)-2-hexenedioate ; Jasmonic acid ; Sedanonic acid ; Dihydro-3-(2-octenyl)-25-furandione ; N-Carbamoylputrescine ; (R)-8-Acetoxycarvotanacetone ; 1-Benzyloxy-1-(2-methoxyethoxy)ethane ; 4-(Butoxymethyl)-2-methoxyphenol ; Isoamyl 2-furonpropionate ; Dihydro-3-(1-octenyl)-25-furandione ; 3-Ethenyl-25-dimethyl-4-oxohex-5-en-2-yl acetate	M+H+NH4 ; 2M+H+NH4 ; 2M+H+NH4 ; 2M+H+NH4 ; 3M+2Na ; 2M+H+NH4 ; 2M+H+NH4 ; 2M+H+NH4 ; 2M+H+NH4 ; 2M+H+NH4 ; 2M+H+NH4	C24H36O6 ; C12H18O3 ; C12H18O3 ; C12H18O3 ; C5H13N3O ; C12H18O3 ; C12H18O3 ; C12H18O3 ; C12H18O3 ; C12H18O3 ; C12H18O3
106.04995	1.303097	0.779599	2.8069	0.018574	1.7311	0.066525	106.05	HMDB00187 ; HMDB03406 ; HMDB00243 ; HMDB00784 ; HMDB11111 ; HMDB11717 ; HMDB15145 ; HMDB28717 ; HMDB29011 ; HMDB29369 ; HMDB32200 ; HMDB32258 ; HMDB32387 ; HMDB33664 ; HMDB36233 ; HMDB37840 ; HMDB39780 ; HMDB40261 ; HMDB59760 ; HMDB59783 ; HMDB59814 ; HMDB59879	L-Serine ; D-Serine ; Pyruvic acid ; Azelaic acid ; Malonic semialdehyde ; Nonate ; Edrophonium ; Arginyl-Proline ; Prolyl-Arginine ; Neosaxitoxin ; cis- and trans-Ethyl 24-dimethyl-13-dioxolane-2-acetate ; (-)-Ethyl 3-acetoxy-2-methylbutyrate ; (-)-Methyl 5-acetoxyhexanoate ; N-Hydroxysaxitoxin ; Butyl ethyl malonate ; N-(1-Deoxy-1-fructosyl)leucine ; N-(1-Deoxy-1-fructosyl)isoleucine ; Glucosereductone ; 24-Dimethylpimelic acid ; 3-Methylsuberic acid ; Diethyl methylsuccinate ; Diethyl glutarate	M+H ; M+H ; M+NH4 ; M+H+Na ; M+NH4 ; M+H+Na ; M+2Na ; M+H+2Na ; M+H+2Na ; M+3H ; M+H+Na ; M+H+Na ; M+H+Na ; M+3H ; M+H+Na ; M+2H+Na ; M+2H+Na ; M+NH4 ; M+H+Na ; M+H+Na ; M+H+Na ; M+H+Na	C3H7NO3 ; C3H7NO3 ; C3H4O3 ; C9H16O4 ; C3H4O3 ; C9H16O4 ; C10H16NO ; C11H21N5O3 ; C11H21N5O3 ; C10H17N7O5 ; C9H16O4 ; C9H16O4 ; C9H16O4 ; C10H17N7O5 ; C9H16O4 ; C12H23NO7 ; C12H23NO7 ; C3H4O3 ; C9H16O4 ; C9H16O4 ; C9H16O4 ; C9H16O4
150.07597	1.318572	0.510322	2.8047	0.018642	1.7295	0.066641	150.076	HMDB02099 ; HMDB11599 ; HMDB11600 ; HMDB11614 ; HMDB00576 ; HMDB00622 ; HMDB00661 ; HMDB01383 ; HMDB01844 ; HMDB02001 ; HMDB03157 ; HMDB03331 ; HMDB04044 ; HMDB04326 ; HMDB06023 ; HMDB06833 ; HMDB06855 ; HMDB29884 ; HMDB33958 ; HMDB34260 ; HMDB37843 ; HMDB40531	6-Methyladenine ; 1-Methyladenine ; 3-Methyladenine ; 7-Methyladenine ; Monoethyl malonic acid ; Ethylmalonic acid ; Glutaric acid ; Sphinganine 1-phosphate ; Methylsuccinic acid ; Dimethylmalonic acid ; Guanidinosuccinic acid ; 1-Methyladenosine ; N6-Methyladenosine ; 2-O-Methyladenosine ; 3-O-Methyladenosine ; 2-Acetolactate ; (S)-2-Acetolactate ; 2-C-Methyl-14-erythrono-D-lactone ; 2-Deoxy-L-ribono-14-lactone ; 22-Dihydroperoxypropane ; N-(1-Deoxy-1-fructosyl)threonine ; 2-Hydroxy-4-oxopentanoic acid	M+H ; M+H ; M+H ; M+H ; M+NH4 ; M+NH4 ; M+NH4 ; M+3Na ; M+NH4 ; M+NH4 ; M+3ACN+2H ; M+H+NH4 ; M+H+NH4 ; M+H+NH4 ; M+H+NH4 ; M+NH4 ; M+NH4 ; M+NH4 ; M+NH4 ; M+ACN+H ; M+H+NH4 ; M+NH4	C6H7N5 ; C6H7N5 ; C6H7N5 ; C6H7N5 ; C5H8O4 ; C5H8O4 ; C5H8O4 ; C18H40NO5P ; C5H8O4 ; C5H8O4 ; C5H9N3O4 ; C11H15N5O4 ; C11H15N5O4 ; C11H15N5O4 ; C11H15N5O4 ; C5H8O4 ; C5H8O4 ; C5H8O4 ; C5H8O4 ; C3H8O4 ; C10H19NO8 ; C5H8O4
116.00189	1.34023	1.314373	2.8039	0.018669	1.7289	0.066675	116.0019	HMDB14690 ; HMDB29571 ; HMDB32653 ; HMDB32684 ; HMDB34437 ; HMDB34968	Propylthiouracil ; 12-Dichloroethane ; (Z)-3-(Methylsulfinyl)-1-propenyl 2-propenyl disulfide ; Lactyltrimethylammonium betaine ; Biphenyl ; Capillene	M+Na+K ; M+NH4 ; M+H+Na ; M+H+Na ; M+2K ; M+2K	C7H10N2OS ; C2H4Cl2 ; C7H12OS3 ; C6H13AsO3 ; C12H10 ; C12H10
322.55577	1.329519	0.436374	-2.8012	0.018755	1.7269	0.06684	322.5558	HMDB10508 ; HMDB10513 ; HMDB10539 ; HMDB30096 ; HMDB42659 ; HMDB42747 ; HMDB42839 ; HMDB42863 ; HMDB42868 ; HMDB42897 ; HMDB42920 ; HMDB42926 ; HMDB44527 ; HMDB44581 ; HMDB44635 ; HMDB48021 ; HMDB48159 ; HMDB48176 ; HMDB48181 ; HMDB48226 ; HMDB48242 ; HMDB48248 ; HMDB48314 ; HMDB48319 ; HMDB48336 ; HMDB48357 ; HMDB48363 ; HMDB48373 ; HMDB48379 ; HMDB48389 ; HMDB48785 ; HMDB48849 ; HMDB48913 ; HMDB48939 ; HMDB48955 ; HMDB48960 ; HMDB48981 ; HMDB48996 ; HMDB49002 ; HMDB49023 ; HMDB49037 ; HMDB49044 ; HMDB49593 ; HMDB49633 ; HMDB50197 ; HMDB50235 ; HMDB51011 ; HMDB51169 ; HMDB51295 ; HMDB51305 ; HMDB51312 ; HMDB52969 ; HMDB53021 ; HMDB53072 ; HMDB53078 ; HMDB53105 ;	TG(18:3(9Z12Z15Z)18:2(9Z12Z)20:5(5Z8Z11Z14Z17Z))iso6 ; TG(18:3(9Z12Z15Z)20:4(5Z8Z11Z14Z)18:3(9Z12Z15Z))iso3 ; TG(20:5(5Z8Z11Z14Z17Z)18:3(9Z12Z15Z)20:5(5Z8Z11Z14Z17Z))iso3 ; Cyanidin 3-(36-dimalonylglucoside) ; TG(14:020:4(5Z8Z11Z14Z)22:6(4Z7Z10Z13Z16Z19Z)) ; TG(14:022:5(4Z7Z10Z13Z16Z)20:5(5Z8Z11Z14Z17Z)) ; TG(14:020:4(8Z11Z14Z17Z)22:6(4Z7Z10Z13Z16Z19Z)) ; TG(14:020:5(5Z8Z11Z14Z17Z)22:5(4Z7Z10Z13Z16Z)) ; TG(14:020:5(5Z8Z11Z14Z17Z)22:5(7Z10Z13Z16Z19Z)) ; TG(14:022:5(7Z10Z13Z16Z19Z)20:5(5Z8Z11Z14Z17Z)) ; TG(14:022:6(4Z7Z10Z13Z16Z19Z)20:4(5Z8Z11Z14Z)) ; TG(14:022:6(4Z7Z10Z13Z16Z19Z)20:4(8Z11Z14Z17Z)) ; TG(16:018:4(6Z9Z12Z15Z)22:6(4Z7Z10Z13Z16Z19Z)) ; TG(16:020:5(5Z8Z11Z14Z17Z)20:5(5Z8Z11Z14Z17Z)) ; TG(16:022:6(4Z7Z10Z13Z16Z19Z)18:4(6Z9Z12Z15Z)) ; TG(14:1(9Z)20:3(5Z8Z11Z)22:6(4Z7Z10Z13Z16Z19Z)) ; TG(14:1(9Z)20:3n622:6(4Z7Z10Z13Z16Z19Z)) ; TG(14:1(9Z)20:4(5Z8Z11Z14Z)22:5(4Z7Z10Z13Z16Z)) ; TG(14:1(9Z)20:4(5Z8Z11Z14Z)22:5(7Z10Z13Z16Z19Z)) ; TG(14:1(9Z)22:4(7Z10Z13Z16Z)20:5(5Z8Z11Z14Z17Z)) ; TG(14:1(9Z)22:5(4Z7Z10Z13Z16Z)20:4(5Z8Z11Z14Z)) ; TG(14:1(9Z)22:5(4Z7Z10Z13Z16Z)20:4(8Z11Z14Z17Z)) ; TG(14:1(9Z)20:4(8Z11Z14Z17Z)22:5(4Z7Z10Z13Z16Z)) ; TG(14:1(9Z)20:4(8Z11Z14Z17Z)22:5(7Z10Z13Z16Z19Z)) ; TG(14:1(9Z)20:5(5Z8Z11Z14Z17Z)22:4(7Z10Z13Z16Z)) ; TG(14:1(9Z)22:5(7Z10Z13Z16Z19Z)20:4(5Z8Z11Z14Z)) ; TG(14:1(9Z)22:5(7Z10Z13Z16Z19Z)20:4(8Z11Z14Z17Z)) ; TG(14:1(9Z)22:6(4Z7Z10Z13Z16Z19Z)20:3(5Z8Z11Z)) ; TG(14:1(9Z)22:6(4Z7Z10Z13Z16Z19Z)20:3n6) ; TG(14:1(9Z)22:6(4Z7Z10Z13Z16Z19Z)22:6(4Z7Z10Z13Z16Z19Z)) ; TG(16:1(9Z)18:3(6Z9Z12Z)22:6(4Z7Z10Z13Z16Z19Z)) ;	M+3Na ; M+3Na ; M+H+2Na ; M+H+Na ; M+3Na ; M+H+2Na ; M+3Na ; M+H+2Na ; M+3Na ; M+3Na ; M+H+2Na ; M+H+2Na ; M+3Na ; M+3Na ; M+3Na ; M+3Na ; M+3Na ; M+3Na ; M+3Na ; M+3Na ; M+3Na ; M+3Na ; M+H+2Na ; M+H+2Na ; M+3Na ; M+3Na ; M+3Na ; M+3Na ; M+H+2Na ; M+3Na ; M+3Na ; M+3Na ; M+3Na ; M+3Na ; M+H+2Na ; M+3Na ; M+H+2Na ; M+H+2Na ; M+3Na ; M+3Na ; M+3Na ; M+3Na ; M+H+2Na ; M+H+2Na ; M+H+2Na ; M+H+2Na ; M+H+2Na ; M+H+2Na ; M+H+2Na ; M+H+2Na ; M+3Na ; M+3Na ; M+3Na ; M+H+2Na ; M+3Na ; M+3Na ; M+H+2Na ; M+H+2Na	C59H94O6 ; C59H94O6 ; C61H92O6 ; C27H25O17 ; C59H94O6 ; C61H92O6 ; C59H94O6 ; C61H92O6 ; C59H94O6 ; C59H94O6 ; C61H92O6 ; C61H92O6 ; C59H94O6 ; C59H94O6 ; C59H94O6 ; C59H94O6 ; C59H94O6 ; C59H94O6 ; C59H94O6 ; C59H94O6 ; C59H94O6 ; C59H94O6 ; C61H92O6 ; C61H92O6 ; C59H94O6 ; C59H94O6 ; C59H94O6 ; C59H94O6 ; C61H92O6 ; C59H94O6 ; C59H94O6 ; C59H94O6 ; C59H94O6 ; C59H94O6 ; C61H92O6 ; C59H94O6 ; C61H92O6 ; C61H92O6 ; C59H94O6 ; C59H94O6 ; C59H94O6 ; C59H94O6 ; C61H92O6 ; C61H92O6 ; C61H92O6 ; C61H92O6 ; C61H92O6 ; C61H92O6 ; C61H92O6 ; C61H92O6 ; C59H94O6 ; C59H94O6 ; C59H94O6 ; C61H92O6 ; C59H94O6 ; C59H94O6 ; C61H92O6 ; C61H92O6
454.63112	1.3088	0.531696	-2.7981	0.018855	1.7246	0.066966	454.6311	HMDB28803 ; HMDB28971 ; HMDB28987 ; HMDB29155 ; HMDB33355 ; HMDB34110	Glutaminyl-Methionine ; Methionyl-Glutamine ; Methionyl-Gamma-glutamate ; Gamma-glutamyl-Methionine ; Dehydroaporheine ; Murrayacine	3M+2K ; 3M+2K ; 3M+2K ; 3M+2K ; 3M+2K ; 3M+2K	C10H19N3O4S ; C10H19N3O4S ; C10H19N3O4S ; C10H19N3O4S ; C18H15NO2 ; C18H15NO2
211.52287	1.328989	0.349401	-2.7938	0.018995	1.7214	0.067358	211.5229	HMDB00295 ; HMDB05017 ; HMDB31796 ; HMDB60440	Uridine 5-diphosphate ; Pantoprazole ; Isofenphos ; Benzoyl phosphate	M+H+NH4 ; M+H+K ; M+2K ; 2M+H+NH4	C9H14N2O12P2 ; C16H15F2N3O4S ; C15H24NO4PS ; C7H7O5P
285.53737	1.316381	1.215405	-2.7897	0.019128	1.7183	0.067783	285.5374	HMDB09511 ; HMDB09741 ; HMDB11362 ; HMDB11388	PE(22:0P-18:0) ; PE(24:0P-16:0) ; PE(P-16:024:0) ; PE(P-18:022:0)	M+3Na ; M+3Na ; M+3Na ; M+3Na	C45H90NO7P ; C45H90NO7P ; C45H90NO7P ; C45H90NO7P
465.24962	1.317671	0.08911	-2.7657	0.019933	1.7004	0.069951	465.2496	HMDB03193 ; HMDB10327 ; HMDB10348 ; HMDB29655 ; HMDB32925 ; HMDB33469 ; HMDB10351	Testosterone glucuronide ; Dehydroisoandrosterone 3-glucuronide ; Dehydroepiandrosterone 3-glucuronide ; Erinacine G ; 1-Phenyl-13-eicosanedione ; N1N10-Dicoumaroylspermidine ; 11-beta-Hydroxyandrosterone-3-glucuronide	M+H ; M+H ; M+H ; M+H ; M+2K+H ; 2M+3H2O+2H ; M+H-H2O	C25H36O8 ; C25H36O8 ; C25H36O8 ; C25H36O8 ; C26H42O2 ; C25H31N3O4 ; C25H38O9

577.13850	1.348992	0.613402	-2.7656	0.019936	1.7004	0.069951	577.1385	HMDB34707 ; HMDB37656 ; HMDB37657 ; HMDB38716 ; HMDB40678 ; HMDB37668 ; HMDB37669 ; HMDB37670 ; HMDB30351 ; HMDB30592 ; HMDB37974 ; HMDB41409 ; HMDB36303 ; HMDB37345 ; HMDB37348 ; HMDB37651 ; HMDB37653 ; HMDB38766 ; HMDB40473 ; HMDB40477 ; HMDB40689	Epicatechin-(2beta-74beta-6)-catechin ; Proanthocyanidin A1 ; Proanthocyanidin A5 ; Pavetannin A2 ; Epicatechin-(2beta-54beta-6)-ent-epicatechin ; Cinnamtannin B2 ; Cinnamtannin D2 ; Pavetannin C1 ; Arborine ; 56-Dimethoxysterigmatocystin ; Cyanidin 3-(6-malonylglucoside) ; Cyanidin 3-(3-malonyl-glucoside) ; Epicatechin-(4beta-8)-gallocatechin ; 3-O-Caffeoylcosmosiin ; Piperitoside ; Epigallocatechin-(4beta-8)-catechin ; Gallocatechin-(4alpha-8)-epicatechin ; 2-O-trans-p-Coumaroylastragalin ; 7-O-(4-Hydroxycinnamoyl) astragalin ; Buddlenoid A ; 6-O-p-Coumaroyltrifolin	M+H ; M+H ; M+H ; M+H ; M+H ; M+2H ; M+2H ; M+2H ; 2M+2K-H ; 3M+2H ; M+ACN+H ; M+ACN+H ; M+H-H2O ; M+H-H2O ; M+H-H2O ; M+H-H2O ; M+H-H2O ; M+H-H2O ; M+H-H2O ; M+H-H2O ; M+H-H2O	C30H24O12 ; C30H24O12 ; C30H24O12 ; C30H24O12 ; C30H24O12 ; C60H48O24 ; C60H48O24 ; C60H48O24 ; C16H14N2O ; C20H16O8 ; C24H23O14 ; C24H23O14 ; C30H26O13 ; C30H26O13 ; C30H26O13 ; C30H26O13 ; C30H26O13 ; C30H26O13 ; C30H26O13 ; C30H26O13 ; C30H26O13
266.13277	1.323127	1.08273	2.7642	0.019984	1.6993	0.070064	266.1328	HMDB00413 ; HMDB31696 ; HMDB37141 ; HMDB37154 ; HMDB60851	3-Hydroxydodecanedioic acid ; Dibutyl malate ; 4-(2-Methyl-3-furanyl)thio-5-nonanone ; 26-Dimethyl-3-(2-methyl-3-furanyl)thio-4-heptanone ; NO-Didesmethyltramadol	2M+H+K ; 2M+H+K ; 2M+H+Na ; 2M+H+Na ; 2M+Na+K	C12H22O5 ; C12H22O5 ; C14H22O2S ; C14H22O2S ; C14H21NO2
496.18400	1.307745	0.214979	2.7618	0.020066	1.6975	0.070161	496.184	HMDB14734 ; HMDB29181 ; HMDB29183 ; HMDB30470 ; HMDB30550 ; HMDB32760 ; HMDB37482 ; HMDB38383 ; HMDB39378 ; HMDB40721 ; HMDB41398	Halobetasol Propionate ; 4-O-methyl-(-)-epicatechin-5-O-beta-glucuronide ; 4-O-methyl-(-)-epicatechin-7-O-beta-glucuronide ; Lindleyin ; Thamnosin ; Nb-trans-p-Coumaroylserotonin glucoside ; Persiconin ; 3b-Pregnadienolone 3-rhamnosyl-(1-4)-rhamnosyl-(1-4)-rhamnosyl-(1-4)-glucoside ; Isolindleyin ; Eugenol O-345-Trihydroxybenzoyl-(-6)-b-D-glucopyranoside ; Persicogenin 3-glucoside	2M+H+Na ; M+NH4 ; M+NH4 ; M+NH4 ; 2M+H+Na ; 2M+H+Na ; M+NH4 ; M+2K ; M+NH4 ; M+NH4 ; M+NH4	C25H31ClF2O5 ; C23H26O11 ; C23H26O11 ; C23H26O11 ; C30H28O6 ; C25H28N2O8 ; C23H26O11 ; C45H70O19 ; C23H26O11 ; C23H26O11 ; C23H26O11
265.25230	1.318202	0.412413	2.7574	0.02022	1.6942	0.070452	265.2523	HMDB37543 ; HMDB03424	()-(Z)-2-(5-Tetradecenyl)cyclobutanone ; 1-Hexadecanol	M+H ; M+Na	C18H32O ; C16H34O
203.54869	1.310136	0.487879	-2.7516	0.020424	1.6899	0.070817	203.5487	HMDB29297	Avenanthramide A2	M+2Na	C18H19NO7
456.91622	1.306778	1.437685	2.7465	0.0206	1.6861	0.071134	NA	NA	NA	NA	NA
121.95789	1.332694	0.056077	2.7261	0.021336	1.6709	0.073067	121.9579	HMDB40457	3-Bromosulfolane	M+2Na	C4H7BrO2S
286.98394	1.315006	0.711398	-2.7073	0.022034	1.6569	0.074692	286.9839	HMDB11104 ; HMDB60005	3-Phosphoadenylylselenate ; Methylgallic acid-O-sulphate	M+H+NH4 ; M+Na	C10H15N5O13P2Se ; C8H8O8S
150.53523	1.315035	1.018075	-2.7063	0.022073	1.6561	0.074773	150.5352	HMDB04983 ; HMDB14608 ; HMDB15472 ; HMDB29322 ; HMDB30215 ; HMDB30320 ; HMDB38639 ; HMDB38640 ; HMDB41996	Dimethyl sulfone ; Ketorolac ; Neostigmine ; 1-Hydroxy-3-methoxy-10-methylacridone ; Clausine L ; Mukonine ; Methyl 6-methoxy-9H-carbazole-3-carboxylate ; 16-Dimethoxy-9H-carbazole-3-carboxaldehyde ; Pranoprofen	3M+H+NH4 ; M+2Na ; M+2K ; M+2Na ; M+2Na ; M+2Na ; M+2Na ; M+2Na ; M+2Na	C2H6O2S ; C15H13NO3 ; C12H19N2O2 ; C15H13NO3 ; C15H13NO3 ; C15H13NO3 ; C15H13NO3 ; C15H13NO3 ; C15H13NO3
748.53087	1.304097	0.183122	2.6967	0.022439	1.649	0.075554	748.5309	HMDB05780 ; HMDB09479 ; HMDB09480 ; HMDB09708 ; HMDB11420 ; HMDB11453 ; HMDB57005 ; HMDB57014 ; HMDB57198 ; HMDB57218 ; HMDB57223 ; HMDB57252 ; HMDB58722 ; HMDB58727 ; HMDB15004 ; HMDB56984 ; HMDB56994 ; HMDB57002 ; HMDB57003 ; HMDB57094 ; HMDB57123 ; HMDB57146 ; HMDB57175 ; HMDB57205 ; HMDB57209 ; HMDB57909 ; HMDB58362 ; HMDB60852 ; HMDB60997 ; HMDB61007	PE(O-16:1(1Z)22:6(4Z7Z10Z13Z16Z19Z)) ; PE(20:5(5Z8Z11Z14Z17Z)P-18:1(11Z)) ; PE(20:5(5Z8Z11Z14Z17Z)P-18:1(9Z)) ; PE(22:6(4Z7Z10Z13Z16Z19Z)P-16:0) ; PE(P-18:1(11Z)20:5(5Z8Z11Z14Z17Z)) ; PE(P-18:1(9Z)20:5(5Z8Z11Z14Z17Z)) ; CL(18:018:018:2(9Z12Z)20:4(5Z8Z11Z14Z)) ; CL(18:018:020:4(5Z8Z11Z14Z)18:2(9Z12Z)) ; CL(18:018:2(9Z12Z)18:020:4(5Z8Z11Z14Z)) ; CL(18:018:2(9Z12Z)18:2(9Z12Z)18:2(9Z12Z)) ; CL(18:018:2(9Z12Z)20:4(5Z8Z11Z14Z)18:0) ; CL(18:020:4(5Z8Z11Z14Z)18:2(9Z12Z)18:0) ; CL(18:2(9Z12Z)18:018:2(9Z12Z)18:2(9Z12Z)) ; CL(18:2(9Z12Z)18:020:4(5Z8Z11Z14Z)18:0) ; Alprenolol ; CL(18:018:018:1(11Z)18:2(9Z12Z)) ; CL(18:018:018:1(9Z)18:2(9Z12Z)) ; CL(18:018:018:2(9Z12Z)18:1(11Z)) ; CL(18:018:018:2(9Z12Z)18:1(9Z)) ; CL(18:018:1(11Z)18:018:2(9Z12Z)) ; CL(18:018:1(11Z)18:2(9Z12Z)18:0) ; CL(18:018:1(9Z)18:018:2(9Z12Z)) ; CL(18:018:1(9Z)18:2(9Z12Z)18:0) ; CL(18:018:2(9Z12Z)18:1(11Z)18:0) ; CL(18:018:2(9Z12Z)18:1(9Z)18:0) ; CL(18:1(11Z)18:018:2(9Z12Z)18:0) ; CL(18:1(9Z)18:018:2(9Z12Z)18:0) ; NO-Didesmethylvenlafaxine ; O-Desmethyltramadol ; N-Desmethyltramadol	M+H ; M+H ; M+H ; M+H ; M+H ; M+H ; M+2H ; M+2H ; M+2H ; M+2H ; M+2H ; M+2H ; M+2H ; M+2H ; 3M+H ; M+H+Na ; M+H+Na ; M+H+Na ; M+H+Na ; M+H+Na ; M+H+Na ; M+H+Na ; M+H+Na ; M+H+Na ; M+H+Na ; M+H+Na ; M+H+Na ; 3M+H ; 3M+H ; 3M+H	C43H74NO7P ; C43H74NO7P ; C43H74NO7P ; C43H74NO7P ; C43H74NO7P ; C43H74NO7P ; C84H152O17P2 ; C84H152O17P2 ; C84H152O17P2 ; C84H152O17P2 ; C84H152O17P2 ; C84H152O17P2 ; C84H152O17P2 ; C84H152O17P2 ; C15H23NO2 ; C82H154O17P2 ; C82H154O17P2 ; C82H154O17P2 ; C82H154O17P2 ; C82H154O17P2 ; C82H154O17P2 ; C82H154O17P2 ; C82H154O17P2 ; C82H154O17P2 ; C82H154O17P2 ; C82H154O17P2 ; C82H154O17P2 ; C15H23NO2 ; C15H23NO2 ; C15H23NO2
151.07932	1.310011	1.035571	2.6955	0.022485	1.6481	0.075604	151.0793	HMDB00172 ; HMDB00557 ; HMDB00687 ; HMDB01645 ; HMDB01901 ; HMDB03640 ; HMDB03764 ; HMDB06248 ; HMDB13243 ; HMDB13773 ; HMDB15119 ; HMDB15163 ; HMDB28798 ; HMDB28861 ; HMDB28872 ; HMDB28877 ; HMDB28914 ; HMDB28998 ; HMDB29040 ; HMDB29150 ; HMDB29168 ; HMDB33923 ; HMDB34952 ; HMDB36301 ; HMDB36382 ; HMDB36425 ; HMDB38516 ; HMDB60650	L-Isoleucine ; L-Alloisoleucine ; L-Leucine ; L-Norleucine ; Aminocaproic acid ; Beta-Leucine ; Glutamylalanine ; 5-L-Glutamyl-L-alanine ; Leucyl-phenylalanine ; D-Leucine ; Nandrolone phenpropionate ; Irbesartan ; Glutaminyl-Hydroxyproline ; Hydroxyprolyl-Glutamine ; Hydroxyprolyl-Serine ; Hydroxyprolyl-Gamma-glutamate ; Isoleucyl-Phenylalanine ; Phenylalanyl-Isoleucine ; Serinyl-Hydroxyproline ; Gamma-glutamyl-Hydroxyproline ; 3-Aminocaproic acid ; ()-erythro-Isoleucine ; Persicaxanthin ; N-gamma-L-Glutamyl-D-alanine ; 6-Deoxyfagomine ; Persicachrome ; Linatine ; N-(2-Hydroxyethyl)-morpholine	2M+H+K ; 2M+H+K ; 2M+H+K ; 2M+H+K ; 2M+H+K ; 2M+H+K ; M+2ACN+2H ; M+2ACN+2H ; M+H+Na ; 2M+H+K ; M+H+2Na ; M+2H+Na ; M+ACN+2H ; M+ACN+2H ; M+2ACN+2H ; M+ACN+2H ; M+H+Na ; M+H+Na ; M+2ACN+2H ; M+ACN+2H ; 2M+H+K ; 2M+H+K ; M+3Na ; M+2ACN+2H ; 2M+H+K ; M+3Na ; M+ACN+2H ; 2M+H+K	C6H13NO2 ; C6H13NO2 ; C6H13NO2 ; C6H13NO2 ; C6H13NO2 ; C6H13NO2 ; C8H14N2O5 ; C8H14N2O5 ; C15H22N2O3 ; C6H13NO2 ; C27H34O3 ; C25H28N6O ; C10H17N3O5 ; C10H17N3O5 ; C8H14N2O5 ; C10H17N3O5 ; C15H22N2O3 ; C15H22N2O3 ; C8H14N2O5 ; C10H17N3O5 ; C6H13NO2 ; C6H13NO2 ; C25H36O3 ; C8H14N2O5 ; C6H13NO2 ; C25H36O3 ; C10H17N3O5 ; C6H13NO2
94.045096	1.30252	0.477888	2.6951	0.022502	1.6478	0.075613	NA	NA	NA	NA	NA
476.75786	1.30141	1.106326	-2.6797	0.023103	1.6363	0.077112	476.757	HMDB31364 ; HMDB34321 ; HMDB61024	Ampeloside Bs1 ; Digitoxigenin 3-glucosyl-(1-6)-glucosyl-(1-4)-26-dideoxyribohexoside ; mono-isopropyl-disopyramide	M+H+NH4 ; M+3ACN+2H ; 3M+Na+K	C45H74O20 ; C41H64O17 ; C18H23N3O

127.03255	1.324323	0.48143	2.6788	0.023142	1.6356	0.077192	127.0326	HMDB33152 ; HMDB36186 ; HMDB00684 ; HMDB01112 ; HMDB01473 ; HMDB03066 ; HMDB12948 ; HMDB29713 ; HMDB29938 ; HMDB31545 ; HMDB31546 ; HMDB31571 ; HMDB31614 ; HMDB31618 ; HMDB31809 ; HMDB32043 ; HMDB32965 ; HMDB33182 ; HMDB33380 ; HMDB34729 ; HMDB35010 ; HMDB35014 ; HMDB35015 ; HMDB35447 ; HMDB36388 ; HMDB36734 ; HMDB37165 ; HMDB37709 ; HMDB37716 ; HMDB37717 ; HMDB37792 ; HMDB38180 ; HMDB39496 ; HMDB39643 ; HMDB39722 ; HMDB39724 ; HMDB39726 ; HMDB39841 ; HMDB40427 ; HMDB40587 ; HMDB40588 ; HMDB60323 ; HMDB60757 ; HMDB14335	2-(Methylthio)pyrazine ; Pyrazinemethanethiol ; L-Kynurenine ; D-Glyceraldehyde 3-phosphate ; Dihydroxyacetone phosphate ; Chalcone ; Formyl-5-hydroxykynurenamine ; Thiazole ; Acaciabiuronic acid ; 2-Methyl-2-cyclopenten-1-one ; 3-Methyl-2-cyclopenten-1-one ; 2-Methyl-1-phenyl-2-propanyl acetate ; 4-Phenyl-2-butyl acetate ; Ethyl 4-phenylbutanoate ; Tricyclazole ; Benzyl 3-methylbutanoate ; 24-Dimethylfuran ; 25-Dimethylfuran ; 3-Methylbutyl benzoate ; (EE)-24-Hexadienal ; 2-Methylpropyl phenylacetate ; 2-Phenylethyl butanoate ; 2-Phenylethyl 2-methylpropanoate ; 5-Isopropyl-2-methylphenol acetate ; 3-Phenylpropyl propanoate ; 111213-Trinor-1(10)-spirovetivene-27-dione ; 4-Methyl-2-(1-phenylethyl)-13-dioxolane 9CI ; 4-Methylphenyl 3-methylbutanoate ; 1-Phenylethyl isobutyrate ; 1-Phenylethyl butyrate ; 1-Ethoxy-2-methoxy-4-(1-propenyl)benzene ; 2-(2-Hydroxy-4-methylphenyl)-3-pentanone ; 2-Ethoxy-1-methoxy-4-(1-propenyl)benzene ; 111213-Trinor-135-bisabolatrien-10-oic acid ; 2-O-beta-D-Glucopyranuronosyl-D-mannose ; 4-O-alpha-D-Glucopyranuronosyl-D-galactose ; 3-O-beta-D-Galactopyranuronosyl-D-galactose ; 2-Benzyl-45-dimethyl-13-dioxolane ; Butyl phenylacetate ; 2-Ethylfuran ; 3-Ethylfuran ; 1-Methoxyphenanthrene ; 4-Aminobenzoyl-(beta)-alanine ; Ethchlorvynol	M+H ; M+H ; M+2Na ; M+2ACN+2H ; M+2ACN+2H ; M+2Na ; M+2Na ; M+ACN+H ; M+2H+Na ; 2M+Na+K ; 2M+Na+K ; M+Na+K ; M+Na+K ; M+Na+K ; 2M+3H ; M+Na+K ; 2M+Na+K ; 2M+Na+K ; M+Na+K ; 2M+Na+K ; M+Na+K ; M+Na+K ; M+Na+K ; M+Na+K ; M+Na+K ; M+Na+K ; M+Na+K ; M+Na+K ; M+Na+K ; M+Na+K ; M+Na+K ; M+Na+K ; M+Na+K ; M+Na+K ; M+2H+Na ; M+2H+Na ; M+2H+Na ; M+Na+K ; M+Na+K ; 2M+Na+K ; 2M+Na+K ; M+2Na ; M+2Na ; M+H-H2O	C5H6N2S ; C5H6N2S ; C10H12N2O3 ; C3H7O6P ; C3H7O6P ; C15H12O ; C10H12N2O3 ; C3H3NS ; C12H20O12 ; C6H8O ; C6H8O ; C12H16O2 ; C12H16O2 ; C12H16O2 ; C9H7N3S ; C12H16O2 ; C6H8O ; C6H8O ; C12H16O2 ; C6H8O ; C12H16O2 ; C12H16O2 ; C12H16O2 ; C12H16O2 ; C12H16O2 ; C12H16O2 ; C12H16O2 ; C12H16O2 ; C12H16O2 ; C12H16O2 ; C12H16O2 ; C12H16O2 ; C12H16O2 ; C12H16O2 ; C12H20O12 ; C12H20O12 ; C12H20O12 ; C12H16O2 ; C12H16O2 ; C6H8O ; C6H8O ; C15H12O ; C10H12N2O3 ; C7H9ClO
242.17490	1.307205	0.471098	2.6756	0.023267	1.6333	0.0774	242.1749	HMDB38588 ; HMDB32111 ; HMDB00138 ; HMDB00331 ; HMDB12555 ; HMDB29670 ; HMDB32596 ; HMDB32662 ; HMDB32679 ; HMDB32689 ; HMDB33634 ; HMDB34564 ; HMDB36029 ; HMDB36583 ; HMDB37179 ; HMDB37244 ; HMDB37726 ; HMDB37730 ; HMDB37736 ; HMDB59834	Dioscoretine ; Adlupone ; Glycocholic acid ; 3a7b12a-Trihydroxyoxocholanyl-Glycine ; 13-Carboxy-alpha-tocopherol ; Epoxyeremopetasinorol ; Sodium glycocholate ; (S)-9-Hydroxy-10-undecenoic acid ; Annuionone A ; Annuionone C ; (3beta5alpha6beta9alpha22E24R)-23-Methylergosta-722-diene-3569-tetrol ; 13-Oxo-911-tridecadienoic acid ; Clarycet ; Methyl jasmonate ; 3-Nonanon-1-yl acetate ; (8alpha10beta11beta)-3-Hydroxy-415-dinor-1(5)-xanthen-128-olide ; Octyl 2-furoate ; Furfuryl octanoate ; 3-Methylbutyl 2-furanbutanoate ; Pentylbenzene	M+H ; M+2H ; M+H+NH4 ; M+H+NH4 ; M+H+Na ; M+NH4 ; M+H+NH4 ; M+ACN+H ; M+NH4 ; M+NH4 ; M+H+Na ; M+NH4 ; M+ACN+H ; M+NH4 ; M+ACN+H ; M+NH4 ; M+NH4 ; M+NH4 ; M+NH4 ; 3M+H+K	C13H23NO3 ; C31H46O4 ; C26H43NO6 ; C26H43NO6 ; C29H48O4 ; C13H20O3 ; C26H43NO6 ; C11H20O3 ; C13H20O3 ; C13H20O3 ; C29H48O4 ; C13H20O3 ; C11H20O3 ; C13H20O3 ; C11H20O3 ; C13H20O3 ; C13H20O3 ; C13H20O3 ; C13H20O3 ; C11H16
587.45231	1.304346	0.078813	-2.6263	0.025325	1.5965	0.082383	587.4523	HMDB01325 ; HMDB02272 ; HMDB02871 ; HMDB02936 ; HMDB09606 ; HMDB09735 ; HMDB30954 ; HMDB31281 ; HMDB35278 ; HMDB39094 ; HMDB41073 ; HMDB36295	N6N6N6-Trimethyl-L-lysine ; 778811111212-Hexahydro-yy-Carotene ; Phytofluene ; cis-77881112-Hexahydro-Carotene ; PE(22:4(7Z10Z13Z16Z)24:0) ; PE(24:022:4(7Z10Z13Z16Z)) ; Grewinol ; erythro-79-Tetratriacontanediol ; 28-Hydroxy-6-methyl-5-tritriacontanone ; (15Z9Z)-77881112-Hexahydrolycopene ; (6R8S)-68-Tetratriacontanediol ; Cycloartanyl ferulate	3M+Na ; M+2Na-H ; M+2Na-H ; M+2Na-H ; 2M+3H ; 2M+3H ; M+2K+H ; M+2K-H ; M+2K+H ; M+2Na-H ; M+2K-H ; M+H-H2O	C9H20N2O2 ; C40H62 ; C40H62 ; C40H62 ; C51H94NO8P ; C51H94NO8P ; C34H68O2 ; C34H70O2 ; C34H68O2 ; C40H62 ; C34H70O2 ; C40H60O4
190.47346	1.300607	0.41544	2.5809	0.027374	1.5627	0.086616	190.4735	HMDB15681	Plerixafor	M+3Na	C28H54N8

Supplemental Table S2. Annotated data from Saline or ATG treatment with oxygen exposure.

feature	V1	V2	t.stat	p.value	X.log10.p.	FDR	mz	all_HMDBID	all_Name	all_Adduct	all_Formula
4321_45.1	2.423802	0.300801	-7.28	2.66E-05	4.5746	0.13315	364.8604	HMDB34485	(23R)-Acetoxytomatine	M+3H	C52H85NO23
5651_290.	2.279529	0.308904	5.7034	0.000197	3.7047	0.42361	792.7426	HMDB07615 ; HMDB07811 ; HMDB42090 ; HMDB42931 ; HMDB56088	DG(22:024:00:0) ; DG(24:022:00:0) ; TG(14:014:0o-18:0) ; TG(14:0o-18:014:0) ; DG(22:00:024:0)	2M+3H2O+2H ; 2M+3H2O+2H ; 2M+3H2O+2H ; 2M+3H2O+2H ; 2M+3H2O+2H	C49H96O5 ; C49H96O5 ; C49H96O5 ; C49H96O5 ; C49H96O5
6879_46.5	2.214987	0.493183	5.0717	0.000484	3.3155	0.42361	124.9227	HMDB03227 ; HMDB31258	Methanethiol ; Lenthionine	M+2K-H ; M+Na+K	CH4S ; C2H4S5
2981_284.	2.209597	0.394974	4.7111	0.000828	3.0822	0.42361	549.9683	HMDB04925	Ganglioside GD2 (d18:116:0)	M+3H	C76H134N4O34
1941_56.2	2.205414	0.717584	4.9659	0.000565	3.2479	0.42361	118.0612	HMDB00128 ; HMDB15140 ; HMDB28686 ; HMDB28812 ; HMDB29006 ; HMDB29090 ; HMDB31321 ; HMDB32179 ; HMDB34198 ; HMDB34778 ; HMDB41878	Guanidoacetic acid ; Hydroxyurea ; Alanyl-Glutamate ; Glutamyl-Alanine ; Phenylalanyl-Tryptophan ; Tryptophyl-Phenylalanine ; 1-Butylamine ; sec-Butylamine ; 2-Methyl-1-propylamine ; (2R3R)-123-Butanetriol ; Diethylamine	M+H ; M+ACN+H ; M+H+NH4 ; M+H+NH4 ; M+3H ; M+3H ; M+2Na-H ; M+2Na-H ; M+2Na-H ; 2M+H+Na ; M+2Na-H	C3H7N3O2 ; CH4N2O2 ; C8H13N2O5 ; C8H13N2O5 ; C20H21N3O3 ; C20H21N3O3 ; C4H11N ; C4H11N ; C4H11N ; C4H10O3 ; C4H11N
7385_58.6	2.176484	0.382541	-4.516	0.001115	2.9526	0.42361	NA	NA	NA	NA	NA
2318_263.	2.14293	0.707042	-4.4941	0.001154	2.938	0.42361	270.2142	HMDB02364 ; HMDB02388 ; HMDB02395 ; HMDB13336 ; HMDB13337 ; HMDB29801 ; HMDB30094 ; HMDB31259 ; HMDB31336 ; HMDB31882 ; HMDB32256 ; HMDB33020 ; HMDB34652 ; HMDB34654 ; HMDB34655 ; HMDB34961 ; HMDB34962 ; HMDB34964 ; HMDB35119 ; HMDB35263 ; HMDB35315 ; HMDB35353 ; HMDB35511 ; HMDB35530 ; HMDB35576 ; HMDB35577 ; HMDB35775 ; HMDB36675 ; HMDB36757 ; HMDB36962 ; HMDB38177 ; HMDB38703 ; HMDB39690 ; HMDB40455 ; HMDB40456 ; HMDB40652 ; HMDB40976 ; HMDB41334	Oleanolic acid ; Boswellic acid ; Ursolic acid ; 3-Hydroxyhexadecanoylcarnitine ; 2-Hydroxyhexadecanoylcarnitine ; 8-Dotriacontenoic acid ; Betulinic acid ; Neurine ; (Z)-9-Cycloheptadecen-1-one ; Sandosapogenol ; (-)-Ethyl 3-hydroxy-2-methylbutyrate ; Ganodermanondiol ; Soyasapogenol E ; Katonic acid ; 3-Epikatonic acid ; beta-Elemolic acid ; 3alpha-3-Hydroxytirucalla-724-dien-21-oic acid ; alpha-Elemolic acid ; Isomangiferolic acid ; Oxyallobetulin ; Ganodermatriol ; Epoxyganoderiol C ; Trametenolic acid B ; 1012-Dotriacontanedione ; 1113-Dotriacontanedione ; 1214-Dotriacontanedione ; 11-Deoxoglycyrrhetinic acid ; 3beta-Hydroxy-2813-ursanolide ; Bryonolic acid ; 3-Epioleanolic acid ; 3-Hydroxycycloart-24-en-21-oic acid ; Carissic acid ; Glycyrrhetol ; Lucidadiol ; Ganoderic acid Z ; 3-Hydroxy-2813-lupanolide ; 3-Epimasticadienolic acid ; 811-Heptadecadienal	M+2ACN+2H ; M+2ACN+2H ; M+2ACN+2H ; M+3ACN+2H ; M+3ACN+2H ; M+Na+K ; M+2ACN+2H ; 2M+ACN+Na ; 2M+H+K ; M+2ACN+2H ; M+2ACN+2H ; M+2ACN+2H ; M+2ACN+2H ; M+2ACN+2H ; M+2ACN+2H ; M+2ACN+2H ; M+2ACN+2H ; M+2ACN+2H ; M+2ACN+2H ; M+2ACN+2H ; M+2ACN+2H ; M+2ACN+2H ; M+2ACN+2H ; M+Na+K ; M+Na+K ; M+Na+K ; M+2ACN+2H ; M+2ACN+2H ; M+2ACN+2H ; M+2ACN+2H ; M+2ACN+2H ; M+2ACN+2H ; M+2ACN+2H ; M+2ACN+2H ; M+2ACN+2H ; M+2ACN+2H ; M+2ACN+2H ; 2M+H+K	C30H48O3 ; C30H48O3 ; C30H48O3 ; C23H45NO5 ; C23H45NO5 ; C32H62O2 ; C30H48O3 ; C5H13NO ; C17H30O ; C30H48O3 ; C30H48O3 ; C30H48O3 ; C30H48O3 ; C30H48O3 ; C30H48O3 ; C30H48O3 ; C30H48O3 ; C30H48O3 ; C30H48O3 ; C30H48O3 ; C30H48O3 ; C30H48O3 ; C30H48O3 ; C32H62O2 ; C32H62O2 ; C32H62O2 ; C30H48O3 ; C30H48O3 ; C30H48O3 ; C30H48O3 ; C30H48O3 ; C30H48O3 ; C30H48O3 ; C30H48O3 ; C30H48O3 ; C30H48O3 ; C30H48O3 ; C17H30O
3995_55.61	2.132574	0.846214	-4.5246	0.001101	2.9584	0.42361	148.004	HMDB00251 ; HMDB29592	Taurine ; Triacetin	M+Na ; M+2K	C2H7NO3S ; C9H14O6
2516_278.	2.131197	0.707451	4.1555	0.001963	2.7072	0.42361	NA	NA	NA	NA	NA

5762_260.3	2.115184	0.553827	4.2289	0.001747	2.7578	0.42361	337.0276	HMDB02377 ; HMDB15492 ; HMDB29871 ; HMDB31457 ; HMDB33042 ; HMDB33046 ; HMDB33056 ; HMDB33057 ; HMDB34310 ; HMDB39200 ; HMDB39201 ; HMDB39743 ; HMDB60834	Urothion ; Stepronin ; Sanguiin H4 ; Corilagin ; Methyl 1-(methylthio)propyl disulfide ; Ethyl 1-(methylthio)ethyl disulfide ; Ethyl (ethylthio)methyl disulfide ; 1-(Ethylthio)ethyl methyl disulfide ; Cordeauxione ; Punicacortein A ; Punicacortein B ; Pterocaryanin B ; N-Acetylserotonin sulfate	2M+H+Na ; M+ACN+Na ; M+H+K ; M+H+K ; 2M+H ; 2M+H ; 2M+H ; 2M+H ; M+2Na-H ; M+H+K ; M+H+K ; M+H+K ; M+K	C11H11N5O3S2 ; C10H11NO4S2 ; C27H22O18 ; C27H22O18 ; C5H12S3 ; C5H12S3 ; C5H12S3 ; C5H12S3 ; C14H12O7 ; C27H22O18 ; C27H22O18 ; C27H22O18 ; C12H14N2O5S
9015_191.1	2.111221	0.874338	-4.3454	0.001454	2.8374	0.42361	555.5889	HMDB01399 ; HMDB15533 ; HMDB60081	46-Dideoxy-4-oxo-dTDP-D-glucose ; Cinolazepam ; dTDP-4-dehydro-6-deoxy-alpha-D-glucose	2M+H+NH4 ; 3M+H+K ; 2M+H+NH4	C16H24N2O15P2 ; C18H13ClFN3O2 ; C16H24N2O15P2
6687_21.97	2.102537	0.635595	-3.9112	0.002907	2.5365	0.42361	379.2837	HMDB04666 ; HMDB11549 ; HMDB11578 ; HMDB11579 ; HMDB35273 ; HMDB36356 ; HMDB36568 ; HMDB06740 ; HMDB11536 ; HMDB11537 ; HMDB11566 ; HMDB11567 ; HMDB13208 ; HMDB29220 ; HMDB33372 ; HMDB34031	2-Arachidonylglycerol ; MG(0:020:4(8Z11Z14Z17Z)0:0) ; MG(20:4(5Z8Z11Z14Z)0:00:0) ; MG(20:4(8Z11Z14Z17Z)0:00:0) ; 1-Acetoxy-2-hydroxy-51215-heneicosatrien-4-one ; 12-Gingerol ; Persenone A ; CE(20:0) ; MG(0:018:1(11Z)0:0) ; MG(0:018:1(9Z)0:0) ; MG(18:1(11Z)0:00:0) ; MG(18:1(9Z)0:00:0) ; 9-Hexadecenoylcholine ; lysoPC(26:1(5Z)) ; Stearoyllactic acid ; 3-(2-Heptenyloxy)-2-hydroxypropyl undecanoate	M+H ; M+H ; M+H ; M+H ; M+H ; M+H ; M+H ; M+2K ; M+Na ; M+Na ; M+Na ; M+Na ; M+K ; M+3ACN+2H ; M+Na ; M+Na	C23H38O4 ; C23H38O4 ; C23H38O4 ; C23H38O4 ; C23H38O4 ; C23H38O4 ; C23H38O4 ; C47H84O2 ; C21H40O4 ; C21H40O4 ; C21H40O4 ; C21H40O4 ; C21H42NO2 ; C34H68NO7P ; C21H40O4 ; C21H40O4
4052_262.5	2.100391	0.491077	4.2171	0.00178	2.7496	0.42361	318.0541	HMDB10324 ; HMDB13854 ; HMDB13856 ; HMDB13861 ; HMDB14445 ; HMDB30244 ; HMDB30373 ; HMDB33480 ; HMDB37355 ; HMDB37364 ; HMDB37536 ; HMDB38955 ; HMDB40488 ; HMDB40683 ; HMDB60433 ; HMDB60588 ; HMDB60603	Benzoyl glucuronide (Benzoic acid) ; N4-Acetylsulfamethoxazole ; 4-Hydroxycyclophosphamide ; 4-Hydroxyifosfamide ; Tenofovir ; Graveolinine ; Citrusinine II ; Graveoline ; Quercetin 3-arabinoside 7-glucoside ; Quercetin 3-glucoside 7-xyloside ; Quercetin 3-lathyroside ; Avenalumin II ; Quercetin 3-xylosyl-(1-6)-glucoside ; Grandisine III ; Aldophosphamide ; Aldoifosfamide ; N-acetyl zonisamide	2M+H+K ; M+Na ; M+ACN+H ; M+ACN+H ; 2M+Na+K ; M+K ; 2M+Na+K ; M+K ; M+H+K ; M+H+K ; M+H+K ; M+K ; M+H+K ; 2M+Na+K ; M+ACN+H ; M+ACN+H ; M+ACN+Na	C13H14O8 ; C12H13N3O4S ; C7H15Cl2N2O3P ; C7H15Cl2N2O3P ; C9H14N5O4P ; C17H13NO3 ; C15H13NO5 ; C17H13NO3 ; C26H28O16 ; C26H28O16 ; C26H28O16 ; C17H13NO3 ; C26H28O16 ; C15H13NO5 ; C7H15Cl2N2O3P ; C7H15Cl2N2O3P ; C10H10N2O4S

39939_286.	2.09075	0.575534	3.9976	0.002528	2.5972	0.42361	615.124	HMDB15662 ; HMDB01117 ; HMDB01202 ; HMDB29247 ; HMDB29549 ; HMDB30133 ; HMDB33281 ; HMDB33282 ; HMDB33489 ; HMDB33496 ; HMDB33498 ; HMDB33900 ; HMDB33929 ; HMDB34126 ; HMDB34449 ; HMDB36637 ; HMDB36783 ; HMDB39230 ; HMDB40464 ; HMDB40716 ; HMDB41693 ; HMDB59989	Fosaprepitant ; 4-Phosphopantothenoylcysteine ; dCMP ; Delphinidin 3-(6-malonyl-glucoside) ; Eleutheroside B1 ; Asticolorin A ; 8-Hydroxypinoresinol 8-glucoside ; 8-Hydroxypinoresinol 4-glucoside ; Isoaurasperone A ; Nigerone ; Isonigerone ; Tomenin ; Aurasperone A ; Gyrocyanin ; Hydroxyanigorufone ; Mulberrofuran Q ; Diosbulbinoside F ; Citrusin A ; Dowicide A ; Irenolone ; 5-(35-Dihydroxyphenyl)-gamma-valerolactone 3-O-glucuronide ; 5-(34-Dihydroxyphenyl)-gamma-valerolactone-4-O-glucuronide	M+H ; 3M+H+Na ; 2M+H ; M+ACN+Na ; 3M+2K ; M+2K-H ; M+2K+H ; M+2K+H ; M+2Na-H ; M+2Na-H ; M+2Na-H ; 3M+2K ; M+2Na-H ; 2M+Na ; 2M+K ; M+Na ; M+2K-H ; M+2K-H ; 3M+K ; 2M+K ; 3M+2K ; 3M+2K	C23H22F7N4O6P ; C12H23N2O9PS ; C9H14N3O7P ; C24H23O15 ; C17H20O10 ; C33H30O7 ; C26H32O12 ; C26H32O12 ; C32H26O10 ; C32H26O10 ; C32H26O10 ; C17H20O10 ; C32H26O10 ; C17H12O5 ; C19H12O3 ; C34H24O10 ; C26H34O12 ; C26H34O12 ; C12H9NaO ; C19H12O3 ; C17H20O10 ; C17H20O10
5792_27.20	2.088952	0.549884	4.3299	0.00149	2.8269	0.42361	353.9358	HMDB30877 ; HMDB30880 ; HMDB41893	Methyl (2-propenylthio) selenide ; Methyl 1-propene-1-sulfenoselenoate ; Fenitrothion	2M+NH4 ; 2M+NH4 ; M+2K-H	C4H8SSe ; C4H8SSe ; C9H12NO5PS
10405_54.74	2.0848	0.570728	4.0877	0.002187	2.6601	0.42361	133.074	HMDB30102 ; HMDB31972 ; HMDB33227 ; HMDB33661 ; HMDB34719 ; HMDB35140 ; HMDB35715 ; HMDB35805 ; HMDB35874 ; HMDB36043 ; HMDB36045 ; HMDB36093 ; HMDB36125 ; HMDB36130 ; HMDB36135 ; HMDB36202 ; HMDB36773 ; HMDB36931 ; HMDB37055 ; HMDB37528 ; HMDB38156 ; HMDB40119 ; HMDB41227 ; HMDB41284 ; HMDB00107 ; HMDB00247 ; HMDB00765 ; HMDB00774 ; HMDB01976 ; HMDB02095 ; HMDB06528 ; HMDB11632 ; HMDB12497 ; HMDB14555 ; HMDB14606 ; HMDB29172 ; HMDB30987 ; HMDB31689 ; HMDB32257 ; HMDB33699 ; HMDB35024 ; HMDB35581 ; HMDB37163 ; HMDB38736 ; HMDB38909 ; HMDB39133 ; HMDB39530 ; HMDB60113 ; HMDB60246 ; HMDB60382	Hulupinic acid ; Heliespirone A ; Curcolonol ; 4-Hydroxydehydromyoporone ; Isoamberboin ; (S)-Abscisic acid ; Tanacetin ; Thellungianin G ; (10R11R)-Pterosin L ; 15-Hydroxymarasmen-3-one ; O-Formyloreadone ; Abscisic acid ; (1beta8beta)-18-Dihydroxy-37(11)-eudesmadien-128-olide ; Vulgarin ; 3-Epiarmefolin ; Alkhanin ; Tavulin ; Tatridin B ; Istanbulin A ; Blennin B ; Umbellifolide ; Enokipodin C ; (8betaOH10beta)-8-Hydroxy-3-oxo-7(11)-eremophilen-128-olide ; 2-Methyl-1-246-trihydroxy-3-(3-methyl-2-butenyl)phenyl-1-propanone ; Galactitol ; Sorbitol ; Mannitol ; Pregnenolone sulfate ; Docosapentaenoic acid (22n-6) ; Malonylcarnitine ; Docosapentaenoic acid ; L-Iditol ; 1-Pyrroline ; Carbachol ; Metharbital ; 3-keto-2-Methylbutyrate ; 2-Carboxy-4-dodecanolide ; 1-Hexanol arabinosylglucoside ; Ethyl abietate ; Linamarin ; ent-16-Kauren-19-ol acetate ; 1-Phenyl-13-hexadecanedione ; 12-Bis(1-ethoxyethoxy)propane ; (3S5R6R7E)-356-Trihydroxy-7-megastigmen-9-one ; 2-Methyl-5-(811-pentadecadienyl)-13-benzenediol ; 48121519-Docosapentaenoic acid ; Ethyl icosapentate ; Docosa-47101316-pentaenoic acid ; 1-Pyrrolinium ; 3beta-Hydroxypregn-5-en-20-one sulfate	M+2H ; M+2ACN+2H ; M+2ACN+2H ; M+2ACN+2H ; M+3H ; M+3Na ; M+H+NH4 ; M+3Na ; M+2ACN+2H ; M+ACN+Na ; M+2ACN+2H ; 2M+3H ; M+NH4 ; M+H+Na ; M+3H ; M+3Na ; M+H+NH4 ; M+3Na ; M+3Na ; M+2Na ; M+H+Na ; M+3Na ; M+3Na ; M+3Na ; M+3Na ; M+ACN+Na ; M+3H	C15H20O4 ; C6H14O6 ; C6H14O6 ; C6H14O6 ; C21H32O5S ; C22H34O2 ; C10H17NO6 ; C22H34O2 ; C6H14O6 ; C4H7N ; C6H15ClN2O2 ; C9H14N2O3 ; C5H7O3 ; C13H22O4 ; C17H32O10 ; C22H34O2 ; C10H17NO6 ; C22H34O2 ; C22H34O2 ; C11H24O4 ; C13H22O4 ; C22H34O2 ; C22H34O2 ; C22H34O2 ; C22H34O2 ; C4H7N ; C21H32O5S
71504_288.7	2.082841	0.7336	4.1104	0.002109	2.6759	0.42361	677.6372	HMDB31203	N-Heptanoylsolamine	2M+Na	C19H41N3O

7469_260.7	2.08027	0.556957	4.027	0.002411	2.6177	0.42361	318.5557	HMDB14904 ; HMDB29414 ; HMDB33192 ; HMDB35209 ; HMDB36939 ; HMDB38003 ; HMDB43451 ; HMDB43536 ; HMDB43621 ; HMDB43654 ; HMDB43677 ; HMDB43682 ; HMDB43710 ; HMDB43732 ; HMDB43738 ; HMDB43766 ; HMDB43787 ; HMDB43794 ; HMDB48090 ; HMDB48136 ; HMDB48376 ; HMDB48378 ; HMDB52514 ; HMDB52645 ; HMDB52750 ; HMDB52757 ; HMDB52764 ; HMDB52871 ; HMDB53063 ; HMDB53091 ; HMDB53182 ; HMDB53366 ; HMDB53479 ; HMDB53570 ; HMDB53582 ; HMDB54030 ; HMDB54919 ; HMDB55160 ; HMDB55254 ; HMDB55268 ; HMDB55366 ; HMDB55542	Clavulanate ; (S)-alpha-Amino-4-carboxy-3-furanpropanoic acid ; Neoacrimarine K ; Cartormin ; Glucosinalbin 4-(4-acetylrhamnoside) ; Delphinidin 3-sambubioside ; TG(15:018:3(6Z9Z12Z)22:6(4Z7Z10Z13Z16Z19Z)) ; TG(15:020:4(5Z8Z11Z14Z)20:5(5Z8Z11Z14Z17Z)) ; TG(15:022:5(4Z7Z10Z13Z16Z)18:4(6Z9Z12Z15Z)) ; TG(15:018:3(9Z12Z15Z)22:6(4Z7Z10Z13Z16Z19Z)) ; TG(15:018:4(6Z9Z12Z15Z)22:5(4Z7Z10Z13Z16Z)) ; TG(15:018:4(6Z9Z12Z15Z)22:5(7Z10Z13Z16Z19Z)) ; TG(15:020:4(8Z11Z14Z17Z)20:5(5Z8Z11Z14Z17Z)) ; TG(15:020:5(5Z8Z11Z14Z17Z)20:4(5Z8Z11Z14Z)) ; TG(15:020:5(5Z8Z11Z14Z17Z)20:4(8Z11Z14Z17Z)) ; TG(15:022:5(7Z10Z13Z16Z19Z)18:4(6Z9Z12Z15Z)) ; TG(15:022:6(4Z7Z10Z13Z16Z19Z)18:3(6Z9Z12Z)) ; TG(15:022:6(4Z7Z10Z13Z16Z19Z)18:3(9Z12Z15Z)) ; TG(14:1(9Z)18:2(9Z12Z)22:6(4Z7Z10Z13Z16Z19Z)) ; TG(14:1(9Z)20:2n622:6(4Z7Z10Z13Z16Z19Z)) ; TG(14:1(9Z)22:6(4Z7Z10Z13Z16Z19Z)18:2(9Z12Z)) ; TG(14:1(9Z)22:6(4Z7Z10Z13Z16Z19Z)20:2n6) ; TG(18:2(9Z12Z)14:1(9Z)22:6(4Z7Z10Z13Z16Z19Z)) ; TG(18:2(9Z12Z)18:3(6Z9Z12Z)18:4(6Z9Z12Z15Z)) ; TG(18:2(9Z12Z)18:3(9Z12Z15Z)18:4(6Z9Z12Z15Z)) ; TG(18:2(9Z12Z)18:4(6Z9Z12Z15Z)18:3(6Z9Z12Z)) ; TG(18:2(9Z12Z)18:4(6Z9Z12Z15Z)18:3(9Z12Z15Z)) ; TG(18:3(6Z9Z12Z)15:022:6(4Z7Z10Z13Z16Z19Z)) ; TG(18:3(6Z9Z12Z)18:2(9Z12Z)18:4(6Z9Z12Z15Z)) ; TG(18:3(6Z9Z12Z)20:2n618:4(6Z9Z12Z15Z)) ; TG(18:3(6Z9Z12Z)18:4(6Z9Z12Z15Z)20:2n6) ;	3M+H+K ; 3M+H+K ; M+2K ; M+Na+K ; M+H+Na ; M+H+K ; M+3Na	C8H9NO5 ; C8H9NO5 ; C31H29NO9 ; C27H29NO13 ; C22H31NO15S2 ; C26H29O16 ; C58H94O6
2529_47.8	2.076023	0.780999	4.067	0.002261	2.6457	0.42361	274.8733	HMDB07954 ; HMDB08592 ; HMDB08981 ; HMDB09008 ; HMDB09040 ; HMDB09073 ; HMDB09105 ; HMDB09212 ; HMDB09231 ; HMDB09241 ; HMDB09242 ; HMDB09263 ; HMDB09273 ; HMDB09295 ; HMDB09338 ; HMDB09371 ; HMDB09403 ; HMDB09436 ; HMDB09468 ; HMDB09489 ; HMDB09500 ; HMDB09520 ; HMDB09521 ; HMDB09531 ; HMDB09532 ; HMDB09552 ; HMDB09562 ; HMDB09563 ; HMDB09593 ; HMDB09625 ; HMDB09658 ; HMDB09749 ; HMDB09756	PC(15:022:2(13Z16Z)) ; PC(22:2(13Z16Z)15:0) ; PE(16:1(9Z)24:1(15Z)) ; PE(18:022:2(13Z16Z)) ; PE(18:1(11Z)22:1(13Z)) ; PE(18:1(9Z)22:1(13Z)) ; PE(18:2(9Z12Z)22:0) ; PE(18:4(6Z9Z12Z15Z)24:1(15Z)) ; PE(20:020:2(11Z14Z)) ; PE(20:022:5(4Z7Z10Z13Z16Z)) ; PE(20:022:5(7Z10Z13Z16Z19Z)) ; PE(20:1(11Z)20:1(11Z)) ; PE(20:1(11Z)22:4(7Z10Z13Z16Z)) ; PE(20:2(11Z14Z)20:0) ; PE(20:3(5Z8Z11Z)22:2(13Z16Z)) ; PE(20:3(8Z11Z14Z)22:2(13Z16Z)) ; PE(20:4(5Z8Z11Z14Z)22:1(13Z)) ; PE(20:4(8Z11Z14Z17Z)22:1(13Z)) ; PE(20:5(5Z8Z11Z14Z17Z)22:0) ; PE(22:018:2(9Z12Z)) ; PE(22:020:5(5Z8Z11Z14Z17Z)) ; PE(22:1(13Z)18:1(11Z)) ; PE(22:1(13Z)18:1(9Z)) ; PE(22:1(13Z)20:4(5Z8Z11Z14Z)) ; PE(22:1(13Z)20:4(8Z11Z14Z17Z)) ; PE(22:2(13Z16Z)18:0) ; PE(22:2(13Z16Z)20:3(5Z8Z11Z)) ; PE(22:2(13Z16Z)20:3(8Z11Z14Z)) ; PE(22:4(7Z10Z13Z16Z)20:1(11Z)) ; PE(22:5(4Z7Z10Z13Z16Z)20:0) ; PE(22:5(7Z10Z13Z16Z19Z)20:0) ; PE(24:1(15Z)16:1(9Z)) ; PE(24:1(15Z)18:4(6Z9Z12Z15Z))	M+2H+Na ; M+2H+Na ; M+2H+Na ; M+2H+Na ; M+2H+Na ; M+2H+Na ; M+2H+Na ; M+3H ; M+2H+Na ; M+3H ; M+3H ; M+2H+Na ; M+3H ; M+2H+Na ; M+3H ; M+3H ; M+3H ; M+3H ; M+3H ; M+2H+Na ; M+3H ; M+2H+Na ; M+2H+Na ; M+3H ; M+3H ; M+2H+Na ; M+3H ; M+3H ; M+3H ; M+3H ; M+3H ; M+2H+Na ; M+3H	C45H86NO8P ; C45H86NO8P ; C45H86NO8P ; C45H86NO8P ; C45H86NO8P ; C45H86NO8P ; C45H86NO8P ; C47H84NO8P ; C45H86NO8P ; C47H84NO8P ; C47H84NO8P ; C45H86NO8P ; C47H84NO8P ; C45H86NO8P ; C47H84NO8P ; C47H84NO8P ; C47H84NO8P ; C47H84NO8P ; C47H84NO8P ; C45H86NO8P ; C47H84NO8P ; C45H86NO8P ; C45H86NO8P ; C47H84NO8P ; C47H84NO8P ; C45H86NO8P ; C47H84NO8P ; C47H84NO8P ; C47H84NO8P ; C47H84NO8P ; C47H84NO8P ; C45H86NO8P ; C47H84NO8P
78179_279.	2.065109	0.190954	3.9395	0.002777	2.5564	0.42361	576.6378	HMDB14854 ; HMDB59612 ; HMDB59787	Nedocromil ; 7-Methylguanosine 5-phosphate ; Casomorphin	3M+H+K ; 3M+H+NH4 ; 2M+3H	C19H17NO7 ; C11H17N5O8P ; C44H61N7O11

8855_53.9	2.061904	0.714815	-4.284	0.001601	2.7956	0.42361	582.9869	HMDB39738 ; HMDB46798 ;<br>HMDB46803 ; HMDB46822 ;<br>HMDB46947 ; HMDB47315 ;<br>HMDB47459 ; HMDB47477 ;<br>HMDB47482 ; HMDB47529 ;<br>HMDB47546 ; HMDB47552 ;<br>HMDB47621 ; HMDB47626 ;<br>HMDB47644 ; HMDB47666 ;<br>HMDB47672 ; HMDB47683 ;<br>HMDB47689 ; HMDB50993 ;<br>HMDB51131 ; HMDB51136 ;<br>HMDB51267 ; HMDB51357 ;<br>HMDB51763 ; HMDB51773 ;<br>HMDB51891 ; HMDB52117 ;<br>HMDB52122 ; HMDB52213 ;<br>HMDB52218 ; HMDB52228 ;<br>HMDB52258 ; HMDB52264 ;<br>HMDB52273 ; HMDB52324 ;<br>HMDB52353 ; HMDB53735 ;<br>HMDB53825 ; HMDB53830 ;<br>HMDB54081 ; HMDB54086 ;<br>HMDB54168 ; HMDB54479 ;<br>HMDB54543 ; HMDB54548 ;<br>HMDB54561 ; HMDB54611 ;<br>HMDB54686 ; HMDB54691 ;<br>HMDB54699 ; HMDB54757 ;<br>HMDB54770 ; HMDB54960 ;<br>HMDB55568	Pipereicosalidine ; TG(22:022:4(7Z10Z13Z16Z)22:5(4Z7Z10Z13Z16Z)) ;<br>TG(22:022:4(7Z10Z13Z16Z)22:5(7Z10Z13Z16Z19Z)) ;<br>TG(22:022:5(4Z7Z10Z13Z16Z)22:4(7Z10Z13Z16Z)) ;<br>TG(22:022:5(7Z10Z13Z16Z19Z)22:4(7Z10Z13Z16Z)) ;<br>TG(24:020:3(5Z8Z11Z)22:6(4Z7Z10Z13Z16Z19Z)) ;<br>TG(24:020:3n622:6(4Z7Z10Z13Z16Z19Z)) ;<br>TG(24:020:4(5Z8Z11Z14Z)22:5(4Z7Z10Z13Z16Z)) ;<br>TG(24:020:4(5Z8Z11Z14Z)22:5(7Z10Z13Z16Z19Z)) ;<br>TG(24:022:4(7Z10Z13Z16Z)20:5(5Z8Z11Z14Z17Z)) ;<br>TG(24:022:5(4Z7Z10Z13Z16Z)20:4(5Z8Z11Z14Z)) ;<br>TG(24:022:5(4Z7Z10Z13Z16Z)20:4(8Z11Z14Z17Z)) ;<br>TG(24:020:4(8Z11Z14Z17Z)22:5(4Z7Z10Z13Z16Z)) ;<br>TG(24:020:4(8Z11Z14Z17Z)22:5(7Z10Z13Z16Z19Z)) ;<br>TG(24:020:5(5Z8Z11Z14Z17Z)22:4(7Z10Z13Z16Z)) ;<br>TG(24:022:5(7Z10Z13Z16Z19Z)20:4(5Z8Z11Z14Z)) ;<br>TG(24:022:5(7Z10Z13Z16Z19Z)20:4(8Z11Z14Z17Z)) ;<br>TG(24:022:6(4Z7Z10Z13Z16Z19Z)20:3(5Z8Z11Z)) ;<br>TG(24:022:6(4Z7Z10Z13Z16Z19Z)20:3n6) ;<br>TG(20:3(5Z8Z11Z)24:022:6(4Z7Z10Z13Z16Z19Z)) ;<br>TG(20:3(5Z8Z11Z)24:1(15Z)22:5(4Z7Z10Z13Z16Z)) ;<br>TG(20:3(5Z8Z11Z)24:1(15Z)22:5(7Z10Z13Z16Z19Z)) ;<br>TG(20:3(5Z8Z11Z)22:5(4Z7Z10Z13Z16Z)24:1(15Z)) ;<br>TG(20:3(5Z8Z11Z)22:5(7Z10Z13Z16Z19Z)24:1(15Z)) ;<br>TG(22:1(13Z)22:2(13Z16Z)22:6(4Z7Z10Z13Z16Z19Z)) ;<br>TG(22:1(13Z)22:4(7Z10Z13Z16Z)22:4(7Z10Z13Z16Z)) ;<br>TG(22:1(13Z)22:6(4Z7Z10Z13Z16Z19Z)22:2(13Z16Z)) ;<br>TG(24:1(15Z)20:3(5Z8Z11Z)22:5(4Z7Z10Z13Z16Z)) ;<br>TG(24:1(15Z)20:3(5Z8Z11Z)22:5(7Z10Z13Z16Z19Z)) ;	3M+2Na ; M+3ACN+2H ; M+3ACN+2H ;<br>M+3ACN+2H ; M+3ACN+2H	C25H43NO ; C69H116O6 ; C69H116O6 ;<br>C69H116O6 ; C69H116O6 ; C69H116O6<br>; C69H116O6 ; C69H116O6 ;<br>C69H116O6 ; C69H116O6 ; C69H116O6<br>; C69H116O6 ; C69H116O6 ;<br>C69H116O6 ; C69H116O6 ; C69H116O6<br>; C69H116O6 ; C69H116O6 ;<br>C69H116O6 ; C69H116O6 ; C69H116O6<br>; C69H116O6 ; C69H116O6 ;<br>C69H116O6 ; C69H116O6 ; C69H116O6<br>; C69H116O6 ; C69H116O6 ;<br>C69H116O6 ; C69H116O6 ; C69H116O6<br>; C69H116O6 ; C69H116O6 ;<br>C69H116O6 ; C69H116O6 ; C69H116O6<br>; C69H116O6 ; C69H116O6 ;<br>C69H116O6 ; C69H116O6 ; C69H116O6<br>; C69H116O6 ; C69H116O6 ;<br>C69H116O6 ; C69H116O6 ; C69H116O6<br>; C69H116O6 ; C69H116O6 ;<br>C69H116O6 ; C69H116O6 ; C69H116O6<br>; C69H116O6 ; C69H116O6 ;<br>C69H116O6 ; C69H116O6
4547_188.8	2.057823	0.495055	-3.8483	0.003221	2.492	0.42361	135.0805	HMDB29639 ; HMDB29697 ;<br>HMDB29698 ; HMDB30839 ;<br>HMDB31626 ; HMDB32024 ;<br>HMDB32142 ; HMDB32608 ;<br>HMDB32623 ; HMDB33716 ;<br>HMDB34107 ; HMDB34563 ;<br>HMDB36441 ; HMDB59601 ;<br>HMDB59895 ; HMDB14400 ;<br>HMDB15671 ; HMDB36133 ;<br>HMDB36207 ; HMDB37705 ;<br>HMDB38947 ; HMDB41250	(4-Methylphenyl)acetaldehyde ; Cinnamyl alcohol ; trans-Cinnamyl alcohol ; (Z)-4-(1-Propenyl)phenol ; 2-Phenylpropanal ; 4-Ethylbenzaldehyde ; 24-Dimethylbenzaldehyde ; 4-Methylacetophenone ; 1-Phenyl-1-propanone ; 3-Phenylpropanal ; Chavicol ; 7-Nonene-35-diyn-1-ol ; o-Vinylanisole ; Indan-1-ol ; m-Methylacetophenone ; Diethylstilbestrol ; Ammonium lactate ; 3-5-Methyl-2-(1-methylethyl)cyclohexyloxy-12-propanediol ; alpha-Amylcinnamyl acetate ; Heptyl cinnamate ; (S)-3-Methylthiohexyl hexanoate ; Demethoxyshogaol	M+H ; M+H ; M+H ; M+H ; M+H ; M+H ;<br>M+H ; M+H ; M+H ; M+H ; M+H ; M+H ;<br>M+H ; M+H ; M+H ; M+2H ; 2M+3H2O+2H<br>; M+H+K ; M+H+Na ; M+H+Na ; M+H+Na ;<br>M+H+Na	C9H10O ; C9H10O ; C9H10O ; C9H10O ;<br>C9H10O ; C9H10O ; C9H10O ; C9H10O ;<br>C9H10O ; C9H10O ; C9H10O ; C9H10O ;<br>C9H10O ; C9H10O ; C9H10O ;<br>C18H20O2 ; C3H9NO3 ; C13H26O3 ;<br>C16H22O2 ; C16H22O2 ; C13H26O2S ;<br>C16H22O2

…2769_288.6	2.055666	0.685873	3.8552	0.003185	2.4969	0.42361	677.4943	HMDB56387 ; HMDB02265 ; HMDB02311 ; HMDB02314 ; HMDB02343 ; HMDB02995 ; HMDB04649 ; HMDB08823 ; HMDB08887 ; HMDB11532 ; HMDB11563 ; HMDB12504 ; HMDB12838 ; HMDB13687 ; HMDB13693 ; HMDB30395 ; HMDB31008 ; HMDB35081 ; HMDB35082 ; HMDB35379 ; HMDB35643 ; HMDB35657 ; HMDB35747 ; HMDB35786 ; HMDB35835 ; HMDB35983 ; HMDB36071 ; HMDB36109 ; HMDB36201 ; HMDB36443 ; HMDB36667 ; HMDB36718 ; HMDB36733 ; HMDB36797 ; HMDB36813 ; HMDB37000 ; HMDB37061 ; HMDB37068 ; HMDB38164 ; HMDB38187 ; HMDB38195 ; HMDB38200 ; HMDB38201 ; HMDB38215 ; HMDB38511 ; HMDB39637 ; HMDB40686 ; HMDB59633 ; HMDB60106	CL(16:016:016:016:0) ; 1415-DiHETrE ; 89-DiHETrE ; 1112-DiHETrE ; 56-DHET ; 12-Keto-tetrahydro-leukotriene B4 ; Demethylphylloquinone ; PE(14:015:0) ; PE(15:014:0) ; MG(0:015:00:0) ; MG(15:00:00:0) ; 1011-dihydro-leukotriene B4 ; 67-dihydro-12-epi-LTB4 ; Nookatone ; Sinensal ; Manglupenone ; ()-1516-Dihydroxyoctadecanoic acid ; (5alpha10alpha)-37(11)-Eudesmadien-2-one ; (5beta7beta10beta)-311-Eudesmadien-2-one ; Sterebin E ; beta-Sinensal ; Solavetivone ; Patchoulenone ; Valerenal ; Furanoeremophilane ; Ganoderal A ; Dendrolasin ; 135-Bisabolatrien-10-one ; (E)-1011-Dihydro-alpha-atlantone ; Rotundone ; Zerumbone ; beta-Santalal ; Vulgarone A ; Mustakone ; alpha-Santalal ; alpha-Turmerone ; ()-411-Eudesmadien-3-one ; Curlone ; Vulgarone B ; beta-Atlantone ; Xanthorrhizol ; Calamusenone ; 47(11)-Guaiadien-8-one ; alpha-Sinensal ; Bisacumol ; beta-Elemenone ; Isohumbertiol ; (9S10S)-910-dihydroxyoctadecanoate ; 15-Hydroperoxyeicosa-8Z11Z13E-trienoate	M+2H ; 2M+H ; 2M+H ; 2M+H ; 2M+H ; 2M+H ; 3M+2Na ; 2M+3H2O+2H ; 2M+3H2O+2H ; 2M+2Na-H ; 2M+2Na-H ; 2M+H ; 2M+H ; 3M+Na ; 3M+Na ; 3M+2Na ; 2M+2Na-H ; 3M+Na ; 3M+Na ; 2M+H ; 3M+Na ; 3M+Na ; 3M+Na ; 3M+Na ; 3M+Na ; 3M+2Na ; 3M+Na ; 2M+2Na-H ; 2M+H	C73H142O17P2 ; C20H34O4 ; C20H34O4 ; C20H34O4 ; C20H34O4 ; C20H34O4 ; C30H44O2 ; C34H68NO8P ; C34H68NO8P ; C18H36O4 ; C18H36O4 ; C20H34O4 ; C20H34O4 ; C15H22O ; C15H22O ; C30H44O2 ; C18H36O4 ; C15H22O ; C15H22O ; C20H34O4 ; C15H22O ; C15H22O ; C15H22O ; C15H22O ; C15H22O ; C30H44O2 ; C15H22O ; C18H36O4 ; C20H34O4
…0928_21.78	2.038603	0.65668	-3.6453	0.004497	2.3471	0.42361	380.2871	HMDB06246 ; HMDB07936 ; HMDB07941 ; HMDB07942 ; HMDB08000 ; HMDB08165 ; HMDB08198 ; HMDB08834 ; HMDB08866 ; HMDB08876 ; HMDB08926 ; HMDB08927 ; HMDB08935 ; HMDB08936 ; HMDB08958 ; HMDB08967 ; HMDB08990 ; HMDB08995 ; HMDB08996 ; HMDB09022 ; HMDB09027 ; HMDB09055 ; HMDB09060 ; HMDB09091 ; HMDB09092 ; HMDB09123 ; HMDB09156 ; HMDB09218 ; HMDB09250 ; HMDB09287 ; HMDB09319 ; HMDB09352 ; HMDB09548 ; HMDB11539 ; HMDB11540 ; HMDB11569 ; HMDB11570 ; HMDB31678 ; HMDB35679 ; HMDB35680	Tetracosatetraenoic acid (24:4n-6) ; PC(15:016:1(9Z)) ; PC(15:018:3(6Z9Z12Z)) ; PC(15:018:3(9Z12Z15Z)) ; PC(16:1(9Z)15:0) ; PC(18:3(6Z9Z12Z)15:0) ; PC(18:3(9Z12Z15Z)15:0) ; PE(14:020:1(11Z)) ; PE(14:1(9Z)20:0) ; PE(14:1(9Z)22:2(13Z16Z)) ; PE(16:018:1(11Z)) ; PE(16:018:1(9Z)) ; PE(16:020:3(5Z8Z11Z)) ; PE(16:020:3(8Z11Z14Z)) ; PE(16:1(9Z)18:0) ; PE(16:1(9Z)20:2(11Z14Z)) ; PE(18:016:1(9Z)) ; PE(18:018:3(6Z9Z12Z)) ; PE(18:018:3(9Z12Z15Z)) ; PE(18:1(11Z)16:0) ; PE(18:1(11Z)18:2(9Z12Z)) ; PE(18:1(9Z)16:0) ; PE(18:1(9Z)18:2(9Z12Z)) ; PE(18:2(9Z12Z)18:1(11Z)) ; PE(18:2(9Z12Z)18:1(9Z)) ; PE(18:3(6Z9Z12Z)18:0) ; PE(18:3(9Z12Z15Z)18:0) ; PE(20:014:1(9Z)) ; PE(20:1(11Z)14:0) ; PE(20:2(11Z14Z)16:1(9Z)) ; PE(20:3(5Z8Z11Z)16:0) ; PE(20:3(8Z11Z14Z)16:0) ; PE(22:2(13Z16Z)14:1(9Z)) ; MG(0:018:3(6Z9Z12Z)0:0) ; MG(0:018:3(9Z12Z15Z)0:0) ; MG(18:3(6Z9Z12Z)0:00:0) ; MG(18:3(9Z12Z15Z)0:00:0) ; Pipericine ; 1-Hydroxy-1-phenyl-3-octadecanone ; 3-Hydroxy-1-phenyl-1-octadecanone	2M+H+K ; M+ACN+2H ; M+H+NH4 ; M+H+NH4 ; M+ACN+2H ; M+H+NH4 ; M+H+NH4 ; M+ACN+2H ; M+ACN+2H ; M+H+NH4 ; M+ACN+2H ; M+ACN+2H ; M+H+NH4 ; M+H+NH4 ; M+ACN+2H ; M+H+NH4 ; M+ACN+2H ; M+H+NH4 ; M+H+NH4 ; M+ACN+2H ; M+H+NH4 ; M+ACN+2H ; M+H+NH4 ; M+H+NH4 ; M+H+NH4 ; M+H+NH4 ; M+H+NH4 ; M+ACN+2H ; M+ACN+2H ; M+H+NH4 ; M+H+NH4 ; M+H+NH4 ; M+H+NH4 ; 2M+3H2O+2H ; 2M+3H2O+2H ; 2M+3H2O+2H ; 2M+3H2O+2H ; M+2Na-H ; 2M+H+K ; 2M+H+K	C24H40O2 ; C39H76NO8P ; C41H76NO8P ; C41H76NO8P ; C39H76NO8P ; C41H76NO8P ; C41H76NO8P ; C39H76NO8P ; C39H76NO8P ; C41H76NO8P ; C39H76NO8P ; C39H76NO8P ; C41H76NO8P ; C41H76NO8P ; C39H76NO8P ; C41H76NO8P ; C39H76NO8P ; C41H76NO8P ; C41H76NO8P ; C39H76NO8P ; C41H76NO8P ; C39H76NO8P ; C41H76NO8P ; C41H76NO8P ; C41H76NO8P ; C41H76NO8P ; C41H76NO8P ; C39H76NO8P ; C39H76NO8P ; C41H76NO8P ; C41H76NO8P ; C41H76NO8P ; C41H76NO8P ; C21H36O4 ; C21H36O4 ; C21H36O4 ; C21H36O4 ; C22H41NO ; C24H40O2 ; C24H40O2
…3931_297.9	2.037369	0.711333	3.5676	0.005116	2.2911	0.42361	353.0034	HMDB02708 ; HMDB13636 ; HMDB29508 ; HMDB38429	Cyanidin ; Pyrroloquinoline quinone ; Laccaic acid D ; Methyl glucosinolate	2M+Na+K ; M+Na ; M+K ; 2M+H+K	C15H11ClO6 ; C14H6N2O8 ; C16H10O7 ; C8H15NO9S2

1921_54.1	2.027515	0.871619	3.8499	0.003213	2.4931	0.42361	369.8562	HMDB33599 ; HMDB34308 ; HMDB36290 ; HMDB38607	Vignatic acid A ; Neomacrostemonoside D ; Campesteryl brassidate ; Soyasaponin A2	2M+3H ; M+3H ; M+H+NH4 ; M+3H	C30H39N3O7 ; C53H86O24 ; C50H88O2 ; C53H86O24
49395_231.	2.006499	0.213728	-3.776	0.003626	2.4406	0.42361	162.0502	HMDB37110 ; HMDB00098 ; HMDB00283 ; HMDB00366 ; HMDB00621 ; HMDB00646 ; HMDB00751 ; HMDB01644 ; HMDB03371 ; HMDB12194 ; HMDB12325 ; HMDB15327 ; HMDB29941 ; HMDB29942 ; HMDB32846 ; HMDB33564 ; HMDB33565 ; HMDB59753 ; HMDB60254	4-(34-Dihydroxyphenyl)-23-dihydro-23-dihydroxy-1H-phenalen-1-one ; D-Xylose ; D-Ribose ; 2-Deoxyribonic acid ; D-Ribulose ; L-Arabinose ; L-Threo-2-pentulose ; D-Xylulose ; L-Ribulose ; Beta-D-ribopyranose ; Arabinofuranose ; Estramustine ; D-Apiose ; Arabinose ; 5-Chloro-6-methoxy-2(3H)-benzoxazolone ; 23-Dihydro-2-methylthiophene ; 23-Dihydro-5-methylthiophene ; 2-Deoxypentonic acid ; Aldehydo-D-xylose	M+2H ; 2M+H+Na ; 2M+H+Na ; 2M+H+Na ; 2M+H+Na ; 2M+H+Na ; 2M+H+Na ; 2M+H+Na ; 2M+H+Na ; 2M+H+Na ; 2M+H+Na ; M+H+2Na ; 2M+H+Na ; 2M+H+Na ; M+3ACN+2H ; 3M+H+Na ; 3M+H+Na ; 2M+H+Na ; 2M+H+Na	C19H14O5 ; C5H10O5 ; C5H10O5 ; C5H10O5 ; C5H10O5 ; C5H10O5 ; C5H10O5 ; C5H10O5 ; C5H10O5 ; C5H10O5 ; C5H10O5 ; C23H31Cl2NO3 ; C5H10O5 ; C5H10O5 ; C8H6ClNO3 ; C5H8S ; C5H8S ; C5H10O5 ; C5H10O5
8483_218.	2.004574	0.253869	-3.4928	0.005796	2.2369	0.42361	109.0648	HMDB01858 ; HMDB02048 ; HMDB02055 ; HMDB03119 ; HMDB33895 ; HMDB40833 ; HMDB00380 ; HMDB00405 ; HMDB03955 ; HMDB03959 ; HMDB06771 ; HMDB06773 ; HMDB06774 ; HMDB12782 ; HMDB31038 ; HMDB33125 ; HMDB34028 ; HMDB34167 ; HMDB36696 ; HMDB39568 ; HMDB40927 ; HMDB41204 ; HMDB60359	p-Cresol ; m-Cresol ; o-Cresol ; Benzyl alcohol ; Anisole ; 3-4-Hydroxy-3-(3-methyl-2-butenyl)phenyl-2-propenal ; 2-Hydroxyestradiol-3-methyl ether ; 2-Methoxyestradiol ; 19-Hydroxyandrost-4-ene-317-dione ; 19-Oxotestosterone ; 7a-Hydroxyandrost-4-ene-317-dione ; 11b-Hydroxyandrost-4-ene-317-dione ; 16a-Hydroxyandrost-4-ene-317-dione ; 4-Methoxy-17beta-estradiol ; (2Z8S9Z)-29-Heptadecadiene-8-hydroxy-46-diyne-1-yl acetate ; 8-Dehydroshogaol ; 2-(37-Dimethyl-26-octadienyl)-4-hydroxy-6-methoxyacetophenone ; Falcarindiol 3-acetate ; Yucalexin B11 ; Ginsenoyne H ; 2-Hexyl-5-2-(4-hydroxy-3-methoxyphenyl)ethylfuran ; Acetylpanaxydol ; 23-Epoxymenaquinone	M+H ; M+H ; M+H ; M+H ; M+H ; M+2H ; M+2H+Na ; M+2H+Na ; M+2H+Na ; M+2H+Na ; M+2H+Na ; M+2H+Na ; M+2H+Na ; M+2H+Na ; M+2H+Na ; M+2H+Na ; M+2H+Na ; M+2H+Na ; M+2H+Na ; M+2H+Na ; M+2H+Na ; M+2H+Na ; M+3H	C7H8O ; C7H8O ; C7H8O ; C7H8O ; C7H8O ; C14H16O2 ; C19H26O3 ; C19H26O3 ; C19H26O3 ; C19H26O3 ; C19H26O3 ; C19H26O3 ; C19H26O3 ; C19H26O3 ; C19H26O3 ; C19H26O3 ; C19H26O3 ; C19H26O3 ; C19H26O3 ; C19H26O3 ; C19H26O3 ; C19H26O3 ; C21H24O3
8354_286.	2.000653	0.914382	3.5763	0.005043	2.2973	0.42361	609.0898	HMDB03705 ; HMDB12222 ; HMDB14387 ; HMDB15573	Phosphoguanidinoacetate ; dTDP-4-acetamido-46-dideoxy-D-galactose ; Cladribine ; Niflumic Acid	3M+NH4 ; 2M+H+K ; 2M+K ; 2M+2Na-H	C3H8N3O5P ; C18H29N3O15P2 ; C10H12ClN5O3 ; C13H9F3N2O2
3307_288.	1.993492	0.624737	3.674	0.004289	2.3677	0.42361	592.8113	HMDB15092	Norgestimate	3M+2K	C23H31NO3

5124_263.4	1.989893	0.47731	-3.6917	0.004165	2.3804	0.42361	315.1545	HMDB35528 ; HMDB00459 ; HMDB00959 ; HMDB10323 ; HMDB13129 ; HMDB13484 ; HMDB13485 ; HMDB13498 ; HMDB13527 ; HMDB13542 ; HMDB13555 ; HMDB13556 ; HMDB13561 ; HMDB13574 ; HMDB13575 ; HMDB13589 ; HMDB13590 ; HMDB14619 ; HMDB14755 ; HMDB15082 ; HMDB31640 ; HMDB33632 ; HMDB34658 ; HMDB34738 ; HMDB34930 ; HMDB35237 ; HMDB35374 ; HMDB35687 ; HMDB36693 ; HMDB39275 ; HMDB40133 ; HMDB40459 ; HMDB41048 ; HMDB41249 ; HMDB60841 ; HMDB15055	Capsicum annuum Fluorescent chlorophyll catabolite ; 3-Methylcrotonylglycine ; Tiglylglycine ; Phenethylamine glucuronide ; Glutaconylcarnitine ; PGP(16:022:5(4Z7Z10Z13Z16Z)) ; PGP(16:022:5(7Z10Z13Z16Z19Z)) ; PGP(16:1(9Z)22:4(7Z10Z13Z16Z)) ; PGP(18:1(11Z)20:4(5Z8Z11Z14Z)) ; PGP(18:1(9Z)20:4(5Z8Z11Z14Z)) ; PGP(18:2(9Z12Z)20:3(5Z8Z11Z)) ; PGP(18:2(9Z12Z)20:3(8Z11Z14Z)) ; PGP(18:2(9Z12Z)22:6(4Z7Z10Z13Z16Z19Z)) ; PGP(18:3(6Z9Z12Z)22:5(4Z7Z10Z13Z16Z)) ; PGP(18:3(6Z9Z12Z)22:5(7Z10Z13Z16Z19Z)) ; PGP(18:3(9Z12Z15Z)22:5(4Z7Z10Z13Z16Z)) ; PGP(18:3(9Z12Z15Z)22:5(7Z10Z13Z16Z19Z)) ; Duloxetine ; Paramethadione ; Fulvestrant ; Glycerol 1-propanoate diacetate ; Furohyperforin ; 9-Acetoxyfukinanolide ; Isotrichodermin ; (3beta6beta)-Furanoeremophilane-36-diol 6-acetate ; 8-Hydroxyhyperforin 81-hemiacetal ; Oxepahyperforin ; Acetylvalerenolic acid ; Acetylbalchanolide ; 6-Gingerdione ; Pectenotoxin 2 secoacid ; Ethylene brassylate ; Physagulin B ; 6-Hydroxyshogaol ; N-Despropyl-rotigotine ; Spectinomycin	M+2H ; 2M+H ; 2M+H ; M+NH4 ; M+ACN+H ; M+3Na ; M+3Na ; M+3Na ; M+3Na ; M+3Na ; M+3Na ; M+3Na ; M+H+2Na ; M+H+2Na ; M+H+2Na ; M+H+2Na ; M+H+2Na ; M+NH4 ; 2M+H ; M+H+Na ; M+2ACN+H ; M+2K ; M+Na ; M+Na ; M+Na ; M+2K ; M+2K ; M+Na ; M+Na ; M+Na ; M+3Na ; M+2Na-H ; M+2ACN+2H ; M+Na ; M+ACN+H ; M+H-H2O	C35H40N4O7 ; C7H11NO3 ; C7H11NO3 ; C14H19NO6 ; C12H19NO6 ; C44H78O13P2 ; C44H78O13P2 ; C44H78O13P2 ; C44H78O13P2 ; C44H78O13P2 ; C44H78O13P2 ; C44H78O13P2 ; C46H76O13P2 ; C46H76O13P2 ; C46H76O13P2 ; C46H76O13P2 ; C46H76O13P2 ; C18H19NOS ; C7H11NO3 ; C32H47F5O3S ; C10H16O6 ; C35H52O5 ; C17H24O4 ; C17H24O4 ; C17H24O4 ; C35H52O5 ; C35H52O5 ; C17H24O4 ; C17H24O4 ; C17H24O4 ; C47H72O15 ; C15H26O4 ; C30H39ClO7 ; C17H24O4 ; C16H19NOS ; C14H24N2O7
2393_196.1	1.98638	0.751233	-3.7049	0.004075	2.3899	0.42361	338.0472	HMDB00639 ; HMDB00663 ; HMDB01176 ; HMDB02044 ; HMDB04068 ; HMDB14332 ; HMDB14618 ; HMDB15512 ; HMDB28798 ; HMDB28861 ; HMDB28877 ; HMDB29150 ; HMDB29881 ; HMDB31781 ; HMDB37785 ; HMDB38516 ; HMDB39168 ; HMDB39170 ; HMDB39193	Galactaric acid ; Glucaric acid ; Cytidine monophosphate N-acetylneuraminic acid ; 8-Hydroxyguanosine ; Melanin ; Lorazepam ; Chlordiazepoxide ; Clotiazepam ; Glutaminyl-Hydroxyproline ; Hydroxyprolyl-Glutamine ; Hydroxyprolyl-Gamma-glutamate ; Gamma-glutamyl-Hydroxyproline ; D-Glucaric acid ; O-Ethyl SS-diphenyl phosphorodithioate ; 2-C-Methylmyricetin 3-rhamnoside 5-gallate ; Linatine ; 2-O-p-Coumaroyl-16-digalloyl-beta-D-glucopyranoside ; 6-O-p-Coumaroyl-12-digalloylglucose ; 2-O-(4-Hydroxycinnamoyl)-16-di-O-galloyl-beta-D-glucopyranose	3M+2Na ; 3M+2Na ; M+Na+K ; M+K ; 2M+H+K ; M+NH4 ; M+K ; 2M+H+K ; M+2K+H ; M+2K+H ; M+2K+H ; M+2K+H ; 3M+2Na ; 2M+3H2O+2H ; M+2Na ; M+2K+H ; M+2Na ; M+2Na ; M+2Na	C6H10O8 ; C6H10O8 ; C20H31N4O16P ; C10H13N5O6 ; C18H10N2O4 ; C15H10Cl2N2O2 ; C16H14ClN3O ; C16H15ClN2OS ; C10H17N3O5 ; C10H17N3O5 ; C10H17N3O5 ; C10H17N3O5 ; C10H17N3O5 ; C6H10O8 ; C14H15O2PS2 ; C29H26O16 ; C10H17N3O5 ; C29H26O16 ; C29H26O16 ; C29H26O16

4552_22.2	1.985463	0.939878	-3.6381	0.004551	2.3419	0.42361	209.1185	HMDB29867 ; HMDB29872 ; HMDB30150 ; HMDB30197 ; HMDB30681 ; HMDB31469 ; HMDB33778 ; HMDB34991 ; HMDB35224 ; HMDB38604 ; HMDB40218 ; HMDB40225 ; HMDB41411 ; HMDB15389 ; HMDB30522 ; HMDB30749 ; HMDB35849 ; HMDB39145 ; HMDB04634 ; HMDB14536 ; HMDB30210 ; HMDB33172 ; HMDB33737 ; HMDB36173 ; HMDB40671 ; HMDB32472	Isoelemicin ; gamma-Asarone ; Jasmine ketolactone ; (Z)-3-Oxo-2-(2-pentenyl)-1-cyclopenteneacetic acid ; 5-Deoxydiplosporin ; trans-Isoasarone ; Elemicin ; 4-Methoxybenzyl butanoate ; 34-Dihydro-6-methoxy-37-dimethyl-1H-2-benzopyran-8-ol ; Amyl salicylate ; 2-Phenoxyethyl isobutyrate ; Isoamyl salicylate ; 34-Dihydro-6-methoxy-22-dimethyl-2H-1-benzopyran-4-ol ; Desonide ; Citreoviridin D ; Heterotropan ; Armillyl everninate ; Diasarone 2 ; 4-Chloromethandienone ; Ethopropazine ; Koenimbine ; 3-Methylpyrrolo12-apyrazine ; Dehydronuciferine ; 2-(3-Phenylpropyl)pyridine ; Physapruin B ; Polyethylene oxidized	M+H ; M+H ; M+H ; M+H ; M+H ; M+H ; M+H ; M+H ; M+H ; M+H ; M+H ; M+H ; M+H ; M+2H ; M+2H ; M+2H ; M+2H ; M+2H ; M+2ACN+2H ; 2M+3H ; M+3ACN+2H ; 3M+H+NH4 ; M+3ACN+2H ; 2M+H+Na ; M+2H+Na ; M+H-2H2O	C12H16O3 ; C12H16O3 ; C12H16O3 ; C12H16O3 ; C12H16O3 ; C12H16O3 ; C12H16O3 ; C12H16O3 ; C12H16O3 ; C12H16O3 ; C12H16O3 ; C12H16O3 ; C12H16O3 ; C24H32O6 ; C24H32O6 ; C24H32O6 ; C24H32O6 ; C24H32O6 ; C20H27ClO2 ; C19H24N2S ; C19H19NO2 ; C7H7N3 ; C19H19NO2 ; C14H15N ; C34H50O9 ; C12H20O5
55265_289.	1.980787	1.034826	3.5337	0.005413	2.2666	0.42361	NA	NA	NA	NA	NA
9084_54.7	1.980574	0.953966	3.726	0.003936	2.405	0.42361	434.8891	HMDB43684 ; HMDB43824 ; HMDB55368	TG(15:018:4(6Z9Z12Z15Z)o-18:0) ; TG(15:0o-18:018:4(6Z9Z12Z15Z)) ; TG(18:4(6Z9Z12Z15Z)15:0o-18:0)	M+ACN+2H ; M+ACN+2H ; M+ACN+2H	C54H98O5 ; C54H98O5 ; C54H98O5
0121_277.	1.980497	0.750199	3.5554	0.005221	2.2822	0.42361	730.897	HMDB38569	PC-M5	3M+H+Na	C29H37NO5
5093_293.	1.977719	0.266017	3.6126	0.004748	2.3235	0.42361	NA	NA	NA	NA	NA
3075_55.3	1.976784	0.946376	3.7102	0.00404	2.3936	0.42361	NA	NA	NA	NA	NA
5465_263.	1.976122	0.25535	-3.2968	0.008057	2.0938	0.42361	394.0185	HMDB15369	Chlorprothixene	M+2K+H	C18H18ClNS
8585_54.6	1.975224	0.753813	-3.4447	0.006282	2.2019	0.42361	119.9929	HMDB14627 ; HMDB31185 ; HMDB35717	Brimonidine ; 1245-Tetrathiane ; S-Acetyl dihydroasparagusic acid	M+3Na ; M+2ACN+2H ; M+2Na	C11H10BrN5 ; C2H4S4 ; C6H10O3S2
9104_264.	1.972604	0.242228	3.4977	0.005749	2.2404	0.42361	610.0629	HMDB15309 ; HMDB36242 ; HMDB37522 ; HMDB41567 ; HMDB60387	Chlormezanone ; 4-O-alpha-L-Rhamnopyranosylglucosinalbin ; Orange B ; Lithol Rubine ; 4-Glutathionyl cyclophosphamide	2M+ACN+Na ; M+K ; M+ACN+Na ; 3M+Na+K ; M+2Na-H	C11H12ClNO3S ; C20H29NO14S2 ; C22H18N4O9S2 ; C18H14N2O6S ; C17H30Cl2N5O8PS
5404_239.	1.971143	1.014492	3.5471	0.005293	2.2763	0.42361	243.5125	HMDB07869 ; HMDB07875 ; HMDB07876 ; HMDB07907 ; HMDB07934 ; HMDB07965 ; HMDB08131 ; HMDB08163 ; HMDB08196 ; HMDB08892 ; HMDB08902 ; HMDB08903 ; HMDB08988 ; HMDB09318 ; HMDB09351 ; HMDB10567 ; HMDB60643	PC(14:016:0) ; PC(14:018:3(6Z9Z12Z)) ; PC(14:018:3(9Z12Z15Z)) ; PC(14:1(9Z)18:2(9Z12Z)) ; PC(15:015:0) ; PC(16:014:0) ; PC(18:2(9Z12Z)14:1(9Z)) ; PC(18:3(6Z9Z12Z)14:0) ; PC(18:3(9Z12Z15Z)14:0) ; PE(15:018:0) ; PE(15:020:3(5Z8Z11Z)) ; PE(15:020:3(8Z11Z14Z)) ; PE(18:015:0) ; PE(20:3(5Z8Z11Z)15:0) ; PE(20:3(8Z11Z14Z)15:0) ; PE-NMe(16:016:0) ; Tizoxanide glucuronide	M+2H+Na ; M+3H ; M+3H ; M+3H ; M+2H+Na ; M+2H+Na ; M+3H ; M+3H ; M+3H ; M+2H+Na ; M+3H ; M+3H ; M+2H+Na ; M+3H ; M+3H ; M+2H+Na ; M+2Na	C38H76NO8P ; C40H74NO8P ; C40H74NO8P ; C40H74NO8P ; C38H76NO8P ; C38H76NO8P ; C40H74NO8P ; C40H74NO8P ; C40H74NO8P ; C38H76NO8P ; C40H74NO8P ; C40H74NO8P ; C38H76NO8P ; C40H74NO8P ; C40H74NO8P ; C38H76NO8P ; C16H15N3O10S
64618_252.	1.970686	0.83374	3.7446	0.003817	2.4182	0.42361	NA	NA	NA	NA	NA

1518_27.5	1.970216	0.73088	-3.2676	0.008465	2.0724	0.42361	96.96102	HMDB14575 ; HMDB59785	Lindane ; beta-Hexachlorocyclohexane	M+3H ; M+3H	C6H6Cl6 ; C6H6Cl6
5258_60.8	1.96767	0.648533	3.6638	0.004361	2.3604	0.42361	223.5675	HMDB14969	Trifluoperazine	M+H+K	C21H24F3N3S
99694_290.	1.966869	0.776581	3.5817	0.004997	2.3013	0.42361	650.9	HMDB43393 ; HMDB43785 ; HMDB45885 ; HMDB45890 ; HMDB45937 ; HMDB45942 ; HMDB46060 ; HMDB46062 ; HMDB46190 ; HMDB46192 ; HMDB46652 ; HMDB46702 ; HMDB46916 ; HMDB46918 ; HMDB50595 ; HMDB50633 ; HMDB50703 ; HMDB50705 ; HMDB51147 ; HMDB51183 ; HMDB51232 ; HMDB51234 ; HMDB51669 ; HMDB51675 ; HMDB51703 ; HMDB51709 ; HMDB51733 ; HMDB51735 ; HMDB51835 ; HMDB51837 ; HMDB51947 ; HMDB52167 ; HMDB52199 ; HMDB52302 ; HMDB52304 ; HMDB52463 ; HMDB52468 ; HMDB52482 ; HMDB52567 ; HMDB52581 ; HMDB52595 ; HMDB52601 ; HMDB52615 ; HMDB52671 ; HMDB52699 ; HMDB53321 ; HMDB53326 ; HMDB53338 ; HMDB53411 ; HMDB53423 ; HMDB53435 ; HMDB53441 ; HMDB53453 ; HMDB53501 ; [illegible]	TG(15:024:1(15Z)22:6(4Z7Z10Z13Z16Z19Z)) ; TG(15:022:6(4Z7Z10Z13Z16Z19Z)24:1(15Z)) ; TG(20:018:2(9Z12Z)22:5(4Z7Z10Z13Z16Z)) ; TG(20:018:2(9Z12Z)22:5(7Z10Z13Z16Z19Z)) ; TG(20:020:2n622:5(4Z7Z10Z13Z16Z)) ; TG(20:020:2n622:5(7Z10Z13Z16Z19Z)) ; TG(20:022:5(4Z7Z10Z13Z16Z)18:2(9Z12Z)) ; TG(20:022:5(4Z7Z10Z13Z16Z)20:2n6) ; TG(20:022:5(7Z10Z13Z16Z19Z)18:2(9Z12Z)) ; TG(20:022:5(7Z10Z13Z16Z19Z)20:2n6) ; TG(22:018:2(9Z12Z)20:5(5Z8Z11Z14Z17Z)) ; TG(22:020:2n620:5(5Z8Z11Z14Z17Z)) ; TG(22:020:5(5Z8Z11Z14Z17Z)18:2(9Z12Z)) ; TG(22:020:5(5Z8Z11Z14Z17Z)20:2n6) ; TG(20:1(11Z)18:2(9Z12Z)22:4(7Z10Z13Z16Z)) ; TG(20:1(11Z)20:2n622:4(7Z10Z13Z16Z)) ; TG(20:1(11Z)22:4(7Z10Z13Z16Z)18:2(9Z12Z)) ; TG(20:1(11Z)22:4(7Z10Z13Z16Z)20:2n6) ; TG(20:3(5Z8Z11Z)18:2(9Z12Z)22:2(13Z16Z)) ; TG(20:3(5Z8Z11Z)20:2n622:2(13Z16Z)) ; TG(20:3(5Z8Z11Z)22:2(13Z16Z)18:2(9Z12Z)) ; TG(20:3(5Z8Z11Z)22:2(13Z16Z)20:2n6) ; TG(22:1(13Z)18:2(9Z12Z)20:4(5Z8Z11Z14Z)) ; TG(22:1(13Z)18:2(9Z12Z)20:4(8Z11Z14Z17Z)) ; TG(22:1(13Z)20:2n620:4(5Z8Z11Z14Z)) ; TG(22:1(13Z)20:2n620:4(8Z11Z14Z17Z)) ; TG(22:1(13Z)20:4(5Z8Z11Z14Z)18:2(9Z12Z)) ; TG(22:1(13Z)20:4(5Z8Z11Z14Z)20:2n6) ;	2M+3H ; 2M+3H	C64H110O6 ; C64H110O6
0114_279.8	1.964272	0.499428	3.7543	0.003757	2.4251	0.42361	576.805	HMDB38316 ; HMDB39392 ; HMDB39394	Pisumsaponin I ; 54-Deoxy-50-hydroxyciguatoxin ; 52-Epiciguatoxin	M+3ACN+2H ; M+ACN+2H ; M+ACN+2H	C51H80O21 ; C60H86O19 ; C60H86O19
6334_43.0	1.963065	0.665186	3.3062	0.00793	2.1007	0.42361	229.9286	HMDB00996 ; HMDB60179	3-Sulfinoalanine ; 3-Sulfinato-L-alaninate	M+2K-H ; M+2K-H	C3H7NO4S ; C3H7NO4S
44989_287.	1.96228	0.746556	3.6446	0.004503	2.3465	0.42361	342.4945	HMDB31376 ; HMDB36656 ; HMDB38608 ; HMDB38886 ; HMDB39075 ; HMDB39076 ; HMDB39517 ; HMDB39946 ; HMDB40120 ; HMDB40942	Congmuyenoside A ; Asiaticoside ; Soyasaponin A3 ; Mabioside A ; Hoduloside I ; Hoduloside II ; Soyasaponin V ; Centellasaponin C ; Centellasaponin A ; Mabiogenin 3-rhamnosyl-(1-6)-glucosyl-(1-2)-glucoside	M+3Na ; M+3Na ; M+3Na ; M+3Na ; M+3Na ; M+3Na ; M+3Na ; M+3Na ; M+3Na ; M+3Na	C48H78O19 ; C48H78O19 ; C48H78O19 ; C48H78O19 ; C48H78O19 ; C48H78O19 ; C48H78O19 ; C48H78O19 ; C48H78O19 ; C48H78O19

9023_284.2	1.959092	0.636871	3.3198	0.00775	2.1107	0.42361	489.4309	HMDB43161 ; HMDB43387 ; HMDB43392 ; HMDB43611 ; HMDB43756 ; HMDB43777 ; HMDB45884 ; HMDB45936 ; HMDB46034 ; HMDB46036 ; HMDB46645 ; HMDB46651 ; HMDB46695 ; HMDB46701 ; HMDB46741 ; HMDB46743 ; HMDB46891 ; HMDB46893 ; HMDB47051 ; HMDB47383 ; HMDB47431 ; HMDB47590 ; HMDB47592 ; HMDB51106 ; HMDB51108 ; HMDB51140 ; HMDB51176 ; HMDB51614 ; HMDB51616 ; HMDB51668 ; HMDB51702 ; HMDB51716 ; HMDB51718 ; HMDB51941 ; HMDB51946 ; HMDB52159 ; HMDB52166 ; HMDB52174 ; HMDB52176 ; HMDB52191 ; HMDB52198 ; HMDB52286 ; HMDB52288 ; HMDB52462 ; HMDB52475 ; HMDB52481 ; HMDB52495 ; HMDB52594 ; HMDB52607 ; HMDB52614 ; HMDB53042 ; HMDB53320 ; HMDB53331 ; HMDB53337 ; [illegible]	TG(15:024:022:6(4Z7Z10Z13Z16Z19Z)) ; TG(15:024:1(15Z)22:5(4Z7Z10Z13Z16Z)) ; TG(15:024:1(15Z)22:5(7Z10Z13Z16Z19Z)) ; TG(15:022:5(4Z7Z10Z13Z16Z)24:1(15Z)) ; TG(15:022:5(7Z10Z13Z16Z19Z)24:1(15Z)) ; TG(15:022:6(4Z7Z10Z13Z16Z19Z)24:0) ; TG(20:018:2(9Z12Z)22:4(7Z10Z13Z16Z)) ; TG(20:020:2n622:4(7Z10Z13Z16Z)) ; TG(20:022:4(7Z10Z13Z16Z)18:2(9Z12Z)) ; TG(20:022:4(7Z10Z13Z16Z)20:2n6) ; TG(22:018:2(9Z12Z)20:4(5Z8Z11Z14Z)) ; TG(22:018:2(9Z12Z)20:4(8Z11Z14Z17Z)) ; TG(22:020:2n620:4(5Z8Z11Z14Z)) ; TG(22:020:2n620:4(8Z11Z14Z17Z)) ; TG(22:020:4(5Z8Z11Z14Z)18:2(9Z12Z)) ; TG(22:020:4(5Z8Z11Z14Z)20:2n6) ; TG(22:020:4(8Z11Z14Z17Z)18:2(9Z12Z)) ; TG(22:020:4(8Z11Z14Z17Z)20:2n6) ; TG(24:015:022:6(4Z7Z10Z13Z16Z19Z)) ; TG(24:018:2(9Z12Z)18:4(6Z9Z12Z15Z)) ; TG(24:020:2n618:4(6Z9Z12Z15Z)) ; TG(24:018:4(6Z9Z12Z15Z)18:2(9Z12Z)) ; TG(24:018:4(6Z9Z12Z15Z)20:2n6) ; TG(20:3(5Z8Z11Z)22:1(13Z)18:2(9Z12Z)) ; TG(20:3(5Z8Z11Z)22:1(13Z)20:2n6) ; TG(20:3(5Z8Z11Z)18:2(9Z12Z)22:1(13Z)) ; TG(20:3(5Z8Z11Z)20:2n622:1(13Z)) ; TG(22:1(13Z)20:3(5Z8Z11Z)18:2(9Z12Z)) ; [illegible]	M+2H ; M+2Na ; M+2Na ; M+2Na ; M+2Na ; M+2Na ; M+H+Na ; M+H+Na ; M+H+Na ; M+H+Na ; M+H+Na ; M+H+Na ; M+H+Na ; M+H+Na ; M+H+Na ; M+H+Na ; M+2Na ; M+H+Na ; M+H+Na ; M+H+Na ; M+H+Na ; M+2Na ; M+H+Na ; M+H-2H2O ; M+H-2H2O ; M+H-2H2O	C64H112O6 ; C60H116O6 ; C60H116O6 ; C60H116O6 ; C60H116O6 ; C60H116O6 ; C62H114O6 ; C62H114O6 ; C62H114O6 ; C62H114O6 ; C62H114O6 ; C62H114O6 ; [illegible]
2116_238.	1.958343	0.60313	-3.496	0.005765	2.2392	0.42361	154.0862	HMDB00073 ; HMDB04825 ; HMDB12309 ; HMDB01164 ; HMDB03422 ; HMDB11657 ; HMDB15041 ; HMDB15073 ; HMDB29581 ; HMDB30432 ; HMDB30680 ; HMDB30703 ; HMDB31344 ; HMDB31543 ; HMDB31759 ; HMDB32233 ; HMDB32501 ; HMDB32552 ; HMDB32663 ; HMDB33141 ; HMDB33569 ; HMDB33957 ; HMDB34247 ; HMDB34557 ; HMDB36203 ; HMDB37209 ; HMDB37832 ; HMDB38024 ; HMDB38050 ; HMDB38782 ; HMDB39358 ; HMDB39778 ; HMDB40260 ; HMDB40374 ; HMDB40629 ; HMDB40890 ; HMDB41008 ; HMDB41822 ; HMDB41836 ; HMDB41922 ; HMDB41955 ; HMDB36604	Dopamine ; p-Octopamine ; Vanillylamine ; trans-12-Dihydrobenzene-12-diol ; Boldione ; 26-Diamino-4-hydroxy-5-N-methylformamidopyrimidine ; Bimatoprost ; Salmeterol ; (2E4E)-24-Hexadienoic acid ; Linalyl cinnamate ; Diplosporin ; Dhelwangin ; 12-Cyclohexanedione ; 3-Methyl-12-cyclopentanedione ; (E)-4-Oxo-2-hexen-1-al ; 25-Dimethyl-3(2H)-furanone ; Santalyl acetate ; Vanillin 12-butylene glycol acetal ; Acoramone ; 2-Amino-a-carboline ; Syoyualdehyde ; Crithmumdiol ; 2-(Methoxymethyl)furan ; 88-Diethoxy-26-dimethyl-2-octanol ; 2-Ethoxy-4-(4-methyl-13-dioxolan-2-yl)phenol ; alpha-Santalyl acetate ; Vetiveryl acetate ; beta-Santalyl acetate ; alpha-Terpinyl cinnamate ; 1-Heptadecene-46-diyne-39-diol ; Ginsenoyne D ; xi-35-Dimethyl-2(5H)-furanone ; 55-Dimethyl-2(5H)-furanone ; Ginsenoyne I ; Isoacoramone ; 345-Trimethoxycinnamyl alcohol ; 1-Methyl-6-phenyl-1H-imidazo45-bpyridin-2-amine ; Acrolein ; Benzoyl ecgonine ; Melamine ; Norcocaine ; N-Methylcalystegine B2	M+H ; M+H ; M+H ; M+ACN+H ; M+H+Na ; M+3ACN+2H ; M+H+2Na ; M+H+2Na ; M+ACN+H ; M+H+Na ; M+2ACN+2H ; M+2ACN+2H ; M+ACN+H ; M+ACN+H ; M+ACN+H ; M+ACN+H ; M+2Na ; M+2ACN+2H ; M+2ACN+2H ; M+3ACN+2H ; M+ACN+H ; M+2Na ; M+ACN+H ; M+Na+K ; M+2ACN+2H ; M+2Na ; M+2Na ; M+2Na ; M+H+Na ; M+2Na ; M+2Na ; M+ACN+H ; M+ACN+H ; M+2Na ; M+2ACN+2H ; M+2ACN+2H ; M+2ACN+2H ; 2M+ACN+H ; M+H+NH4 ; 2M+3H2O+2H ; M+H+NH4 ; M+H-2H2O	C8H11NO2 ; C8H11NO2 ; C8H11NO2 ; C6H8O2 ; C19H24O2 ; C6H9N5O2 ; C25H37NO4 ; C25H37NO4 ; C6H8O2 ; C19H24O2 ; C12H16O4 ; C12H16O4 ; C6H8O2 ; C6H8O2 ; C6H8O2 ; C6H8O2 ; C17H26O2 ; C12H16O4 ; C12H16O4 ; C11H9N3 ; C6H8O2 ; C17H26O2 ; C6H8O2 ; C14H30O3 ; C12H16O4 ; C17H26O2 ; C17H26O2 ; C17H26O2 ; C19H24O2 ; C17H26O2 ; C17H26O2 ; C6H8O2 ; C6H8O2 ; C17H26O2 ; C12H16O4 ; C12H16O4 ; C13H12N4 ; C3H4O ; C16H19NO4 ; C3H6N6 ; C16H19NO4 ; C8H15NO4
3521_52.9	1.954943	0.674299	-3.3659	0.007171	2.1444	0.42361	523.0484	HMDB01512 ; HMDB29212 ; HMDB29214 ; HMDB33668 ; HMDB41769 ; HMDB60769	Thiamine triphosphate ; Quercetin 3-O-glucuronide ; Quercetin-4-glucuronide ; Quercetin 4-glucuronide ; Quercetin 3-O-glucuronide ; 4-Hydroxyphenytoin glucuronide	M+NH4 ; M+2Na-H ; M+2Na-H ; M+2Na-H ; M+2Na-H ; M+2K+H	C12H20N4O10P3S ; C21H18O13 ; C21H18O13 ; C21H18O13 ; C21H18O13 ; C21H20N2O9

7712_58.53	1.953812	0.735358	3.3543	0.007313	2.1359	0.42361	271.1377	HMDB10357 ; HMDB02163 ; HMDB06029 ; HMDB11173 ; HMDB15557 ; HMDB28739 ; HMDB28842 ; HMDB29012 ; HMDB41135 ; HMDB60716	Tetrahydroaldosterone-3-glucuronide ; Trihydroxycoprostanoic acid ; N-Acetylglutamine ; L-glycyl-L-hydroxyproline ; Pheniramine ; Asparaginyl-Proline ; Glycyl-Hydroxyproline ; Prolyl-Asparagine ; 2-Deoxybrassinolide ; 2-Hydroxy-desipramine glucuronide	M+2H ; M+2K ; M+2ACN+H ; M+2ACN+H ; 2M+Na+K ; M+ACN+H ; M+2ACN+H ; M+ACN+H ; M+2K ; M+2ACN+2H	C27H40O11 ; C28H48O5 ; C7H12N2O4 ; C7H12N2O4 ; C16H20N2 ; C9H15N3O4 ; C7H12N2O4 ; C9H15N3O4 ; C28H48O5 ; C24H30N2O7
26921_262.6	1.94759	0.305501	-3.3959	0.006819	2.1663	0.42361	176.0827	HMDB15026 ; HMDB15302 ; HMDB29524 ; HMDB30645 ; HMDB30728 ; HMDB30841 ; HMDB32457 ; HMDB32954 ; HMDB33902 ; HMDB34178 ; HMDB35098 ; HMDB35418 ; HMDB36180 ; HMDB36459 ; HMDB36720 ; HMDB40760 ; HMDB41010	Granisetron ; Moclobemide ; 5-Hydroxy-7-(4-hydroxy-3-methoxyphenyl)-1-phenyl-3-heptanone ; Sclareapinone ; 8-Methoxygravelliferone ; Sagequinone methide A ; 1-Pentanethiol ; 5-Geranyloxy-7-methoxycoumarin ; Isopentyl mercaptan ; Guaiacin ; (all-E)-Crocetin ; 2-Methyl-1-butanethiol ; 3-Methyl-2-butanethiol ; Macelignan ; Verimol H ; 44-Dihydroxy-55-diisopropyl-22-dimethyl-36-biphenyldione ; ()-2-Pentanethiol	M+H+K ; M+2ACN+2H ; M+H+Na ; M+H+Na ; M+H+Na ; M+H+Na ; 3M+H+K ; M+H+Na ; 3M+H+K ; M+H+Na ; M+H+Na ; 3M+H+K ; 3M+H+K ; M+H+Na ; M+H+Na ; M+H+Na ; 3M+H+K	C18H24N4O ; C13H17ClN2O2 ; C20H24O4 ; C20H24O4 ; C20H24O4 ; C20H24O4 ; C5H12S ; C20H24O4 ; C5H12S ; C20H24O4 ; C20H24O4 ; C5H12S ; C5H12S ; C20H24O4 ; C20H24O4 ; C20H24O4 ; C5H12S
3964_286.2	1.947502	1.037511	3.3569	0.00728	2.1379	0.42361	646.0344	HMDB15485	Ceftibuten	3M+Na+K	C15H14N4O6S2
8283_294.3	1.945988	0.645232	3.263	0.008531	2.069	0.42361	605.0083	HMDB37752	Gossypetin 8-glucuronide 3-sulfate	2M+Na+K	C21H18O17S
5924_86.7	1.943525	0.749915	3.3499	0.007367	2.1327	0.42361	279.0646	HMDB05760 ; HMDB14408 ; HMDB15688 ; HMDB29462 ; HMDB29519 ; HMDB30254 ; HMDB30808 ; HMDB32577 ; HMDB33653 ; HMDB33904 ; HMDB34494 ; HMDB36376 ; HMDB36457 ; HMDB37316 ; HMDB38627 ; HMDB41647 ; HMDB41756 ; HMDB61112 ; HMDB29307 ; HMDB30544 ; HMDB30545 ; HMDB30546 ; HMDB33146 ; HMDB33255 ; HMDB33732 ; HMDB33989 ; HMDB35208 ; HMDB37243 ; HMDB37917 ; HMDB37918 ; HMDB41644	Dihydrodaidzein ; Sulfisoxazole ; Sulfamoxole ; (E)-244-Trihydroxychalcone ; (2S)-Liquiritigenin ; 3-(345-Trimethoxyphenyl)propanoic acid ; (S)-Pinocembrin ; 4-Methoxybenzophenone-2-carboxylic acid ; 1-alpha-Acevaltrate ; 7E-Mycosinyl acetate ; Acevaltrate ; 3-(4-Hydroxy-3-methoxyphenyl)-12-propanediol 2-O-(galloyl-glucoside) ; Emodinanthranol ; Isoliquiritigenin ; Ethyl 345-trimethoxybenzoate ; 2-Dehydro-O-desmethylangolensin ; Isopropyl 3-(34-dihydroxyphenyl)-2-hydroxypropanoate ; 3-Carboxy-4-methyl-5-propyl-2-furanpropionic acid ; 47-Dihydroxy-25-dimethoxyisoflavone ; Gnaphaliin ; 5-Hydroxy-78-dimethoxyflavonol ; 45-Dihydroxy-78-dimethoxyflavone ; 34-Dihydroxycinnamoyl-(Z)-2-(34-dihydroxyphenyl)ethenol ; Muscomosin ; Pisatin ; 45-Dihydroxy-37-dimethoxyisoflavone ; Aflatoxin B2 ; Sylpin ; 25-Dihydroxy-47-dimethoxyisoflavone ; 37-Dihydroxy-48-dimethoxyisoflavone ; 27-Dihydroxy-45-dimethoxyisoflavone	M+Na ; 2M+H+Na ; 2M+H+Na ; M+Na ; M+Na ; M+K ; M+Na ; M+Na ; M+2K ; M+Na ; M+2K ; M+2Na ; M+Na ; M+Na ; M+K ; M+Na ; M+K ; M+K ; M+H-2H2O ; M+H-2H2O ; M+H-2H2O ; M+H-2H2O ; M+H-2H2O ; M+H-2H2O ; M+H-2H2O ; M+H-2H2O ; M+H-2H2O ; M+H-2H2O ; M+H-2H2O ; M+H-2H2O ; M+H-2H2O	C15H12O4 ; C11H13N3O3S ; C11H13N3O3S ; C15H12O4 ; C15H12O4 ; C12H16O5 ; C15H12O4 ; C15H12O4 ; C24H32O10 ; C15H12O4 ; C24H32O10 ; C23H28O13 ; C15H12O4 ; C15H12O4 ; C12H16O5 ; C15H12O4 ; C12H16O5 ; C12H16O5 ; C17H14O6 ; C17H14O6 ; C17H14O6 ; C17H14O6 ; C17H14O6 ; C17H14O6 ; C17H14O6 ; C17H14O6 ; C17H14O6 ; C17H14O6 ; C17H14O6 ; C17H14O6 ; C17H14O6
5098_286.4	1.938944	1.010058	3.3061	0.007932	2.1006	0.42361	608.0885	HMDB29361 ; HMDB39355	Alkaloid RC ; Methyl 6-O-digalloyl-beta-D-glucopyranoside	M+2K-H ; 3M+2Na	C26H29NO11 ; C17H19BrN4O2
8655_88.66	1.937808	0.661827	-3.1434	0.010449	1.9809	0.42361	NA	NA	NA	NA	NA

6051_190.2	1.936693	0.835746	-3.5371	0.005383	2.269	0.42361	213.1486	HMDB29388 ; HMDB33601 ; HMDB35085 ; HMDB37326 ; HMDB41572 ; HMDB00307 ; HMDB00311 ; HMDB00316 ; HMDB00340 ; HMDB00367 ; HMDB00377 ; HMDB00399 ; HMDB00433 ; HMDB00437 ; HMDB13158 ; HMDB13192 ; HMDB29887 ; HMDB36438 ; HMDB39446 ; HMDB39943 ; HMDB59736 ; HMDB00623 ; HMDB30143 ; HMDB41613 ; HMDB41614	Cucurbic acid ; Dihydrojasmonic acid ; Vulgarole ; Traumatin ; 3-Ethenyl-4-hydroxy-25-dimethylhex-5-en-2-yl acetate ; 1b-Hydroxycholic acid ; 3b4b7a12a-Tetrahydroxy-5b-cholanoic acid ; 2b3a7a12a-Tetrahydroxy-5b-cholanoic acid ; 3a7a12a19-Tetrahydroxy-5b-cholanoic acid ; 3a6b7b12a-Tetrahydroxy-5b-cholanoic acid ; 3a6b7b12b-Tetrahydroxy-5b-cholanoic acid ; 3a6b7a12a-Tetrahydroxy-5b-cholanoic acid ; 13712-Tetrahydroxycholan-24-oic acid ; 3a4b7a12a-Tetrahydroxy-5b-cholanoic acid ; 1b3a7b-Trihydroxy-5b-cholanoic acid ; 3a7b21-Trihydroxy-5b-cholanoic acid ; Sorbitan palmitate ; Fasciculic acid B ; Ginsenoside Rh7 ; Ginsenoside Rh8 ; 2-Hydroxyundecanoate ; Dodecanedioic acid ; Talaromycin A ; Bis(1-methylethyl) hexanedioate ; Dipropyl hexanedioate	M+H ; M+H ; M+H ; M+H ; M+H ; M+2H ; M+2H ; M+2H ; M+2H ; M+2H ; M+2H ; M+2H ; M+2H ; M+2H ; M+2H ; M+2H ; M+H+Na ; M+3H ; M+3H ; M+3H ; 2M+H+Na ; M+H-H2O ; M+H-H2O ; M+H-H2O ; M+H-H2O	C12H20O3 ; C12H20O3 ; C12H20O3 ; C12H20O3 ; C12H20O3 ; C24H40O6 ; C24H40O6 ; C24H40O6 ; C24H40O6 ; C24H40O6 ; C24H40O6 ; C24H40O6 ; C24H40O6 ; C24H40O6 ; C24H40O6 ; C24H40O6 ; C22H42O6 ; C36H60O9 ; C36H60O9 ; C36H60O9 ; C11H21O3 ; C12H22O4 ; C12H22O4 ; C12H22O4 ; C12H22O4
8753_59.6	1.934772	0.39059	3.5749	0.005054	2.2964	0.42361	133.0799	HMDB15568 ; HMDB29008 ; HMDB29134 ; HMDB33463 ; HMDB12971 ; HMDB13688 ; HMDB14989 ; HMDB15168 ; HMDB28807 ; HMDB29059 ; HMDB29075 ; HMDB29159 ; HMDB29456 ; HMDB29622 ; HMDB30232 ; HMDB31326 ; HMDB31328 ; HMDB31493 ; HMDB32220 ; HMDB32345 ; HMDB32607 ; HMDB33394 ; HMDB33676 ; HMDB33826 ; HMDB34661 ; HMDB34718 ; HMDB34940 ; HMDB35020 ; HMDB35026 ; HMDB35097 ; HMDB35306 ; HMDB35391 ; HMDB35645 ; HMDB35704 ; HMDB35718 ; HMDB35720 ; HMDB35739 ; HMDB35793 ; HMDB36118 ; HMDB36192 ; HMDB36402 ; HMDB36420 ; HMDB36522 ; HMDB36716 ; HMDB36717 ; HMDB36788 ; HMDB36789 ; HMDB36793 ; HMDB36798 ; HMDB37069 ; HMDB37392 ; HMDB37395 ; HMDB37641 ; HMDB37727 ; [illegible]	Vorinostat ; Phenylalanyl-Valine ; Valyl-Phenylalanine ; Subaphylline ; 1-Hexanol ; Nootkatol ; Dacarbazine ; Cerulenin ; Glutaminyl-Threonine ; Threoninyl-Glutamine ; Threoninyl-Gamma-glutamate ; Gamma-glutamyl-Threonine ; Amyl 2-furoate ; Isopetasoside ; (2R6S7S10Z)-beta-Santala-3(15)10-dien-12-ol ; 2-Ethoxybutane ; 1-Isothiocyanatobutane ; 3-Hexanol ; Dihydronootkatone ; Isobutyl isothiocyanate ; ()-threo-Anethole glycol ; 4-Ethyl-26-dimethoxyphenol ; ()-3-Methyl-1-pentanol ; 26-Di-tert-butyl-4-methylphenol ; alpha-Valerenol ; Isospathulenol ; alpha-Santal-10-en-12-ol ; Acolamone ; alpha-Cyperol ; beta-Costol ; Epishyobunone ; Preisocalamendiol ; Epoxyguaiene ; Acorenone ; Isocyperol ; Isoacolamone ; (R)-27(14)9-Bisabolatrien-11-ol ; Fukinone ; (3S6E)-6-Caryophyllen-15-al ; Apritone ; alpha-Bergamotenol ; Spathulenol ; Verimol J ; beta-Santalol ; trans-beta-Santalol ; beta-Betulenol ; Caryophyllene alpha-oxide ; 315-Epoxy-6-caryophyllene ; 8alpha-3-Copaen-8-ol ; Acoragermacrone ; 4(15)-Copaen-11-ol ; 11-Copaen-4-ol ; 4-(Ethoxymethyl)-2-methoxyphenol ; Furfuryl pentanoate ; Vetiverol ; Eremofukinone ; 15-Epoxy-4(14)-salvialene ; Bisabolene oxide ; Calacone ; 7(14)-Isodaucen-10-one ; Peperinic acid ; Isoshyobunone ; Humuladienone ; Humulene epoxide I ; Humulene epoxide II ; Humulenol I ; Humulenol II ; 2-Ethoxy-4-(methoxymethyl)phenol ; Bisacurol ; 4-Nonylphenol ; (6Z9Z12Z)-6912-Pentadecatrien-2-one ; Aromadendrene epoxide ; Furfuryl isovalerate ; xi-25-Dihydro-24-dimethylthiazole ; Italicene ether ; Cabreuva oxide D ; Isohexanol ; Isoamyl 2-furoate ; 2-trans6-trans-Farnesal	M+2H ; M+2H ; M+2H ; M+2H ; 2M+Na+K ; M+2Na ; M+2ACN+2H ; M+ACN+2H ; M+H+NH4 ; M+H+NH4 ; M+H+NH4 ; M+H+NH4 ; M+2ACN+2H ; M+3H ; M+2Na ; 2M+Na+K ; M+NH4 ; 2M+Na+K ; M+2Na ; M+NH4 ; M+2ACN+2H ; M+2ACN+2H ; 2M+Na+K ; M+2Na ; M+2Na ; M+2Na ; M+2Na ; M+2Na ; M+2Na ; M+2Na ; M+2Na ; M+2Na ; M+2Na ; M+2Na ; M+2Na ; M+2Na ; M+2Na ; M+2Na ; M+2Na ; M+2Na ; M+2Na ; M+2Na ; M+2ACN+2H ; M+2Na ; M+2Na ; M+2Na ; M+2Na ; M+2Na ; M+2Na ; M+2Na ; M+2Na ; M+2Na ; M+2ACN+2H ; M+2ACN+2H ; M+2Na ; M+2Na ; M+2Na ; M+2Na ; M+2Na ; M+2Na ; M+2ACN+2H ; M+2Na ; M+2Na ; M+2Na ; M+2Na ; M+2Na ; M+2Na ; M+2ACN+2H ; M+2Na ; M+2Na ; M+2Na ; M+2Na ; M+2ACN+2H ; M+NH4 ; M+2Na ; M+2Na ; 2M+Na+K ; M+2ACN+2H ; M+2Na	C14H20N2O3 ; C14H20N2O3 ; C14H20N2O3 ; C14H20N2O3 ; C6H14O ; C15H24O ; C6H10N6O ; C12H17NO3 ; C9H17N3O5 ; C9H17N3O5 ; C9H17N3O5 ; C9H17N3O5 ; C10H14O3 ; C21H32O7 ; C15H24O ; C6H14O ; C5H9NS ; C6H14O ; C15H24O ; C5H9NS ; C10H14O3 ; C10H14O3 ; C6H14O ; C15H24O ; C15H24O ; C15H24O ; C15H24O ; C15H24O ; C15H24O ; C15H24O ; C15H24O ; C15H24O ; C15H24O ; C15H24O ; C15H24O ; C15H24O ; C15H24O ; C15H24O ; C15H24O ; C15H24O ; C15H24O ; C15H24O ; C10H14O3 ; C15H24O ; C15H24O ; C15H24O ; C15H24O ; C15H24O ; C15H24O ; C15H24O ; C15H24O ; C15H24O ; C10H14O3 ; C10H14O3 ; C15H24O ; C15H24O ; C15H24O ; C15H24O ; C15H24O ; C15H24O ; C10H14O3 ; C15H24O ; C15H24O ; C15H24O ; C15H24O ; C15H24O ; C15H24O ; C10H14O3 ; C15H24O ; C15H24O ; C15H24O ; C15H24O ; C10H14O3 ; C5H9NS ; C15H24O ; C15H24O ; C6H14O ; C10H14O3 ; C15H24O
3509_71.7	1.933105	0.794087	-3.4849	0.005873	2.2311	0.42361	318.9884	HMDB14774 ; HMDB29200 ; HMDB37751 ; HMDB41748 ; HMDB59600	Clofibrate ; Ferulic acid 4-sulfate ; Gossypetin 8-glucoside 3-sulfate ; Isoferulic acid 3-sulfate ; 5-phosphonooxy-L-lysine	M+2K-H ; M+2Na-H ; M+2K ; M+2Na-H ; M+2K-H	C12H15ClO3 ; C10H10O7S ; C21H20O16S ; C10H10O7S ; C6H15N2O6P

74104_263.6	1.931914	0.814379	3.0759	0.011723	1.931	0.42361	372.6074	HMDB37980 ; HMDB41159 ; HMDB15271 ; HMDB28828 ; HMDB29038 ; HMDB29406 ; HMDB30532	Cyanidin 3-sambubioside 5-glucoside ; Cyanidin 3-O-b-D-Xylopyranosyl-(1-2)-b-D-glucopyranosyl-(1-6)-b-D-galactopyranoside ; Cefadroxil ; Glutamyl-Serine ; Serinyl-Glutamate ; Amaranthin ; Agrocin 84	M+2H ; M+2H ; 2M+H+NH4 ; 3M+2Na ; 3M+2Na ; M+H+NH4 ; M+ACN+2H	C32H39O20 ; C32H39O20 ; C16H17N3O5S ; C8H13N2O6 ; C8H13N2O6 ; C30H34N2O19 ; C22H36N6O16P2
6969_68.98	1.928162	0.940192	3.4878	0.005845	2.2333	0.42361	208.647	HMDB39848 ; HMDB61030	3-Oxotetradecanoic acid glycerides ; omega-hydroxyfinasteride	M+2ACN+2H ; M+ACN+2H	C17H33O6 ; C22H34N2O3
9233_286.2	1.924091	1.044881	3.306	0.007933	2.1005	0.42361	442.9559	HMDB02205 ; HMDB39491	L-Homocysteic acid ; 118-Diamino-5914-triazaoctadecane	2M+2K-H ; 3M+H+Na	C4H9NO5S ; C15H37N5
23651_294.	1.921937	0.912329	3.3916	0.006868	2.1632	0.42361	406.0124	HMDB38575 ; HMDB60799 ; HMDB60801 ; HMDB61069	(Z)-N-Feruloyl-5-hydroxyanthranilic acid ; 7-Hydroxyamoxapine ; 8-Hydroxyamoxapine ; clofarabind-5-monophosphate	M+2K-H ; M+2K-H ; M+2K-H ; M+Na	C17H15NO6 ; C17H16ClN3O2 ; C17H16ClN3O2 ; C10H12ClFN5O6P
5847_197.1	1.921698	0.912504	-3.3344	0.007561	2.1214	0.42361	428.0986	HMDB01088 ; HMDB01243 ; HMDB33287 ; HMDB38626 ; HMDB61177	Butyryl-CoA ; Isobutyryl-CoA ; Theaflagallin ; Xanthotoxol glucoside ; Pyridine N-oxide glucuronide	M+H+NH4 ; M+H+NH4 ; 2M+3H2O+2H ; M+ACN+Na ; 3M+H+K	C25H42N7O17P3S ; C25H42N7O17P3S ; C20H16O9 ; C17H16O9 ; C11H14NO7
5041_44.87	1.920114	1.027261	-3.2877	0.008182	2.0872	0.42361	163.0535	HMDB31804 ; HMDB35400 ; HMDB00039 ; HMDB00317 ; HMDB00409 ; HMDB00525 ; HMDB00574 ; HMDB00624 ; HMDB00665 ; HMDB00746 ; HMDB01624 ; HMDB01873 ; HMDB01975 ; HMDB03243 ; HMDB03417 ; HMDB10718 ; HMDB12843 ; HMDB29166 ; HMDB30062 ; HMDB31217 ; HMDB31507 ; HMDB31674 ; HMDB31688 ; HMDB31873 ; HMDB32158 ; HMDB32228 ; HMDB32456 ; HMDB32931 ; HMDB33851 ; HMDB35620 ; HMDB36051 ; HMDB36094 ; HMDB36095 ; HMDB36221 ; HMDB36640 ; HMDB36695 ; HMDB37713 ; HMDB37719 ; HMDB38545 ; HMDB40253 ; HMDB40409 ; HMDB40579 ; HMDB40608 ; HMDB40893 ; HMDB41003 ; HMDB41144 ; HMDB41844 ; HMDB59770 ; HMDB60116 ; HMDB29430 ; HMDB38915	Methomyl ; 1-Tridecene-357911-pentayne ; Butyric acid ; 2-Hydroxy-3-methylpentanoic acid ; (5R)-5-Hydroxyhexanoic acid ; 5-Hydroxyhexanoic acid ; L-Cysteine ; D-Leucic acid ; Leucinic acid ; Hydroxyisocaproic acid ; 2-Hydroxycaproic acid ; Isobutyric acid ; 2-Ethyl-2-Hydroxybutyric acid ; Acetoin ; D-Cysteine ; (R)-3-Hydroxyhexanoic acid ; 6-Hydroxyhexanoic acid ; 2-Methyl-3-hydroxyvaleric acid ; Methyl propionate ; Ethyl acetate ; 1-Hydroxy-2-butanone ; Armillarinin ; Hexyl glucoside ; 17-Diphenyl-4-hepten-3-one ; (-)-1-Acetoxy-1-ethoxyethane ; Dimethylbenzyl carbinyl hexanoate ; Paraldehyde ; 2-Benzoxazolol ; 44-Dimethoxy-2-butanone ; Artabsinolide A ; Dihydromarasmone ; Nigellic acid ; 13-Hydroxyabscisic acid ; 2-(Dimethoxymethyl)-1-heptenylbenzene ; Furanofukinin ; Crispolide ; Octyl phenylacetate ; 2-Phenylethyl octanoate ; (1beta4alpha5alpha6beta8alpha10b)-110:45-Diepoxy-6-hydroxy-7(11)-germacren-128-olide ; Propyl formate ; Ethyl ()-3-hydroxybutyrate ; Isopropyl formate ; Kanzonol F ; (EE)-17-Diphenyl-46-heptadien-3-ol ; Vulgarolide ; 8-Deoxy-1113-dihydroxygrosheimin ; Butoxyacetic acid ; Threo-3-Hydroxy-2-methylbutyric acid ; Dibenzoalpyrene ; Methionine sulfoximine ; 12-Tridecene-46810-tetraynal	M+H ; M+H ; 3M+Na+K ; 2M+Na+K ; 2M+Na+K ; 2M+Na+K ; M+ACN+H ; 2M+Na+K ; 2M+Na+K ; 2M+Na+K ; 2M+Na+K ; 3M+Na+K ; 2M+Na+K ; 3M+Na+K ; M+ACN+H ; 2M+Na+K ; 2M+Na+K ; 2M+Na+K ; 3M+Na+K ; 3M+Na+K ; 3M+Na+K ; M+2H+Na ; M+Na+K ; M+Na+K ; 2M+Na+K ; M+2K ; 2M+Na+K ; 2M+3H2O+2H ; 2M+Na+K ; M+2Na ; M+2Na ; M+2Na ; M+2Na ; M+2K ; M+2K ; M+2Na ; M+2K ; M+2K ; M+2Na ; 3M+Na+K ; 2M+Na+K ; 3M+Na+K ; M+3Na ; M+Na+K ; M+2Na ; M+2Na ; 2M+Na+K ; 2M+Na+K ; M+H+Na ; M+H-H2O ; M+H-H2O	C5H10N2O2S ; C13H6 ; C4H8O2 ; C6H12O3 ; C6H12O3 ; C6H12O3 ; C3H7NO2S ; C6H12O3 ; C6H12O3 ; C6H12O3 ; C6H12O3 ; C4H8O2 ; C6H12O3 ; C4H8O2 ; C3H7NO2S ; C6H12O3 ; C6H12O3 ; C6H12O3 ; C4H8O2 ; C4H8O2 ; C4H8O2 ; C24H29ClO7 ; C12H24O6 ; C19H20O ; C6H12O3 ; C16H24O2 ; C6H12O3 ; C7H5NO2 ; C6H12O3 ; C15H20O5 ; C15H20O5 ; C15H20O5 ; C15H20O5 ; C16H24O2 ; C16H24O2 ; C15H20O5 ; C16H24O2 ; C16H24O2 ; C15H20O5 ; C4H8O2 ; C6H12O3 ; C4H8O2 ; C26H28O5 ; C19H20O ; C15H20O5 ; C15H20O5 ; C6H12O3 ; C6H12O3 ; C24H14 ; C5H12N2O3S ; C13H8O
7224_283.1	1.919132	0.762123	3.4084	0.006676	2.1755	0.42361	523.6737	HMDB06498 ; HMDB12715 ; HMDB41557	3-Oxooctadecanoyl-CoA ; 3-hydroxyoctadecanoyl-CoA ; Foeniculoside III	M+2H ; M+2H ; M+ACN+2H	C39H66N7O18P3S ; C39H66N7O18P3S ; C54H52O19

6031_19.5	1.916775	0.216104	-2.9102	0.01556	1.808	0.42361	754.5426	HMDB07883 ; HMDB07884 ; HMDB07914 ; HMDB07915 ; HMDB07976 ; HMDB08007 ; HMDB08008 ; HMDB08167 ; HMDB08200 ; HMDB08232 ; HMDB08362 ; HMDB08395 ; HMDB08426 ; HMDB08459 ; HMDB08910 ; HMDB09582 ; HMDB01219 ; HMDB02685 ; HMDB02689 ; HMDB03180 ; HMDB04239 ; HMDB05821 ; HMDB07469 ; HMDB07498 ; HMDB07525 ; HMDB07526 ; HMDB07554 ; HMDB07555 ; HMDB07582 ; HMDB07694 ; HMDB07721 ; HMDB07722 ; HMDB07750 ; HMDB07751 ; HMDB07777 ; HMDB07778 ; HMDB07788 ; HMDB07872 ; HMDB07873 ; HMDB07904 ; HMDB07969 ; HMDB08001 ; HMDB08032 ; HMDB08064 ; HMDB08097 ; HMDB08885 ; HMDB08886 ; HMDB08900 ; HMDB09252 ; HMDB10569 ; HMDB11402 ; HMDB11435 ; HMDB13649 ; HMDB29216 ; [illegible]	PC(14:020:4(5Z8Z11Z14Z)) ; PC(14:020:4(8Z11Z14Z17Z)) ; PC(14:1(9Z)20:3(5Z8Z11Z)) ; PC(14:1(9Z)20:3(8Z11Z14Z)) ; PC(16:018:4(6Z9Z12Z15Z)) ; PC(16:1(9Z)18:3(6Z9Z12Z)) ; PC(16:1(9Z)18:3(9Z12Z15Z)) ; PC(18:3(6Z9Z12Z)16:1(9Z)) ; PC(18:3(9Z12Z15Z)16:1(9Z)) ; PC(18:4(6Z9Z12Z15Z)16:0) ; PC(20:3(5Z8Z11Z)14:1(9Z)) ; PC(20:3(8Z11Z14Z)14:1(9Z)) ; PC(20:4(5Z8Z11Z14Z)14:0) ; PC(20:4(8Z11Z14Z17Z)14:0) ; PE(15:022:4(7Z10Z13Z16Z)) ; PE(22:4(7Z10Z13Z16Z)15:0) ; Chlordecone alcohol ; Prostaglandin F1a ; 1314-Dihydro PGE1 ; Cortol ; 1314-Dihydro PGF2a ; Beta-Cortol ; DG(20:3(5Z8Z11Z)22:6(4Z7Z10Z13Z16Z19Z)0:0) ; DG(20:3(8Z11Z14Z)22:6(4Z7Z10Z13Z16Z19Z)0:0) ; DG(20:4(5Z8Z11Z14Z)22:5(4Z7Z10Z13Z16Z)0:0) ; DG(20:4(5Z8Z11Z14Z)22:5(7Z10Z13Z16Z19Z)0:0) ; DG(20:4(8Z11Z14Z17Z)22:5(4Z7Z10Z13Z16Z)0:0) ; DG(20:4(8Z11Z14Z17Z)22:5(7Z10Z13Z16Z19Z)0:0) ; DG(20:5(5Z8Z11Z14Z17Z)22:4(7Z10Z13Z16Z)0:0) ; DG(22:4(7Z10Z13Z16Z)20:5(5Z8Z11Z14Z17Z)0:0) ; DG(22:5(4Z7Z10Z13Z16Z)20:4(5Z8Z11Z14Z)0:0) ; DG(22:5(4Z7Z10Z13Z16Z)20:4(8Z11Z14Z17Z)0:0) ; DG(22:5(7Z10Z13Z16Z19Z)20:4(5Z8Z11Z14Z)0:0) ; DG(22:5(7Z10Z13Z16Z19Z)20:4(8Z11Z14Z17Z)0:0) ; DG(22:6(4Z7Z10Z13Z16Z19Z)20:3(5Z8Z11Z)0:0) ; DG(22:6(4Z7Z10Z13Z16Z19Z)20:3(8Z11Z14Z)0:0) ; DG(22:6(4Z7Z10Z13Z16Z19Z)22:6(4Z7Z10Z13Z16Z19Z)0:0) ; PC(14:018:1(11Z)) ; PC(14:018:1(9Z)) ; PC(14:1(9Z)18:0) ; PC(16:016:1(9Z)) ; PC(16:1(9Z)16:0) ; PC(18:014:1(9Z)) ; PC(18:1(11Z)14:0) ; PC(18:1(9Z)14:0) ; PE(14:1(9Z)P-18:1(11Z)) ; [illegible]	M+H ; M+H ; M+H ; M+H ; M+H ; M+H ; M+H ; M+H ; M+H ; M+H ; M+H ; M+H ; M+H ; M+H ; M+H ; M+H ; 3M+2Na ; 2M+ACN+H ; 2M+ACN+H ; 2M+NH4 ; 2M+ACN+H ; 2M+NH4 ; M+ACN+Na ; M+ACN+Na ; M+ACN+Na ; M+ACN+Na ; M+ACN+Na ; M+ACN+Na ; M+ACN+Na ; M+ACN+Na ; M+ACN+Na ; M+ACN+Na ; M+ACN+Na ; M+ACN+Na ; M+ACN+Na ; M+ACN+Na ; M+ACN+H ; M+Na ; M+Na ; M+Na ; M+Na ; M+Na ; M+Na ; M+Na ; M+Na ; M+2ACN+H ; M+2ACN+H ; M+Na ; M+Na ; M+Na ; M+2ACN+H ; M+2ACN+H ; 2M+ACN+Na ; M+K ; 2M+NH4 ; M+ACN+Na ; M+ACN+Na ; M+ACN+Na ; M+ACN+Na ; M+ACN+Na ; M+ACN+Na ; M+ACN+Na ; M+ACN+H	C42H76NO8P ; C42H76NO8P ; C42H76NO8P ; C42H76NO8P ; C42H76NO8P ; C42H76NO8P ; C42H76NO8P ; C42H76NO8P ; C42H76NO8P ; C42H76NO8P ; C42H76NO8P ; C42H76NO8P ; C42H76NO8P ; C42H76NO8P ; C42H76NO8P ; C42H76NO8P ; C10H2Cl10O ; C20H36O5 ; C20H36O5 ; C21H36O5 ; C20H36O5 ; C21H36O5 ; C45H70O5 ; C45H70O5 ; C45H70O5 ; C45H70O5 ; C45H70O5 ; C45H70O5 ; C45H70O5 ; C45H70O5 ; C45H70O5 ; C45H70O5 ; C45H70O5 ; C45H70O5 ; C45H70O5 ; C45H70O5 ; C47H68O5 ; C40H78NO8P ; C40H78NO8P ; C40H78NO8P ; C40H78NO8P ; C40H78NO8P ; C40H78NO8P ; C40H78NO8P ; C40H78NO8P ; C37H70NO7P ; C37H70NO7P ; C40H78NO8P ; C40H78NO8P ; C40H78NO8P ; C37H70NO7P ; C37H70NO7P ; C22H35NO2 ; C40H80N2O6P ; C21H36O5 ; C45H70O5 ; C45H70O5 ; C45H70O5 ; C45H70O5 ; C45H70O5 ; C45H70O5 ; C45H70O5 ; C47H68O5
2267_289.0	1.916413	0.785181	3.4718	0.006003	2.2216	0.42361	679.2123	HMDB39922	Eriodictyol 7-(6-trans-p-coumaroylglucoside)	M+2ACN+H	C30H28O13
7041_45.6	1.909852	0.241591	3.2213	0.009155	2.0384	0.42361	219.0797	HMDB28783 ; HMDB29014 ; HMDB60553 ; HMDB00763 ; HMDB00784 ; HMDB03689 ; HMDB04038 ; HMDB11717 ; HMDB14469 ; HMDB14487 ; HMDB14687 ; HMDB30007 ; HMDB30039 ; HMDB30281 ; HMDB30454 ; HMDB31848 ; HMDB31981 ; HMDB32200 ; HMDB32258 ; HMDB32387 ; HMDB32468 ; HMDB34473 ; HMDB35240 ; HMDB35514 ; HMDB36233 ; HMDB36669 ; HMDB36817 ; HMDB39159 ; HMDB39502 ; HMDB39957 ; HMDB40241 ; HMDB41423 ; HMDB59631 ; HMDB59760 ; HMDB59783 ; HMDB59814 ; HMDB59879 ; HMDB59925 ; HMDB59926 ; HMDB60400 ; HMDB60477 ; HMDB61063 ; HMDB61064 ; HMDB61065 ; HMDB15144 ; HMDB28982 ; HMDB29045 ; HMDB29445 ; HMDB29687 ; HMDB32890 ; HMDB41873 ; HMDB60669 ; HMDB60670	Cysteinyl-Proline ; Prolyl-Cysteine ; E-3174 ; 5-Hydroxyindoleacetic acid ; Azelaic acid ; Neuroprotectin D1 ; Resolvin D5 ; Nonate ; Fluorometholone ; Diltiazem ; Desoximetasone ; 2277-Tetramethyl-16-dioxaspiro44nona-38-diene ; Jasmolone ; Calycanthidine ; Macrophorin A ; Butylated hydroxyanisole ; Niazidin ; cis- and trans-Ethyl 24-dimethyl-13-dioxolane-2-acetate ; (-)-Ethyl 3-acetoxy-2-methylbutyrate ; (-)-Methyl 5-acetoxyhexanoate ; 2-Phenylpropionaldehyde dimethyl acetal ; Citbismine F ; Dihydroactinidiolide ; xi-23-Dihydro-2-oxo-1H-indole-3-acetic acid ; Butyl ethyl malonate ; ()-Myrtenyl formate ; Norecasantalic acid ; Bovolide ; N-(4-Hydroxycinnamoyl)tyrosine ; 4-(Butoxymethyl)phenol ; 1-(2-Thienyl)-1-heptanone ; Mulberrofuran E ; Cis-stilbene oxide ; 24-Dimethylpimelic acid ; 3-Methylsuberic acid ; Diethyl methylsuccinate ; Diethyl glutarate ; 3-tert-Butyl-4-hydroxyanisole ; 2-tert-Butyl-4-hydroxyanisole ; 5-Phenyl-13-oxazinane-24-dione ; gamma-Glutamyl-beta-aminopropiononitrile ; 6-beta-hydroxymedroxyprogesterone ; 2-beta-hydroxymedroxyprogesterone ; 1-beta-hydroxymedroxyprogesterone ; Ketoprofen ; Methionyl-Serine ; Serinyl-Methionine ; L-Agaridoxin ; 36-Dimethoxy-4-phenanthrenol ; 26-Dimethoxy-4-phenanthrenol ; Dexketoprofen ; p-Hydroxyfelbamate ; 2-Hydroxyfelbamate	M+H ; M+H ; M+2H ; 2M+3H2O+2H ; 2M+Na+K ; M+2K ; M+2K ; 2M+Na+K ; M+Na+K ; M+H+Na ; M+Na+K ; M+K ; M+K ; M+2K ; M+2K ; M+2K ; M+2ACN+2H ; 2M+Na+K ; 2M+Na+K ; 2M+Na+K ; M+K ; M+3H ; M+K ; 2M+3H2O+2H ; 2M+Na+K ; M+K ; M+K ; M+K ; 2M+3H ; M+K ; M+Na ; M+2H+Na ; M+Na ; 2M+Na+K ; 2M+Na+K ; 2M+Na+K ; 2M+Na+K ; M+K ; M+K ; 2M+3H2O+2H ; 2M+H+K ; M+2K ; M+2K ; M+2K ; M+H-2H2O ; M+H-H2O ; M+H-H2O ; M+H-2H2O ; M+H-2H2O ; M+H-2H2O ; M+H-2H2O ; M+H-2H2O ; M+H-2H2O	C8H14N2O3S ; C8H14N2O3S ; C22H21ClN6O2 ; C10H9NO3 ; C9H16O4 ; C22H32O4 ; C22H32O4 ; C9H16O4 ; C22H29FO4 ; C22H26N2O4S ; C22H29FO4 ; C11H16O2 ; C11H16O2 ; C23H28N4 ; C22H32O4 ; C22H32O4 ; C15H18N2O6S ; C9H16O4 ; C9H16O4 ; C9H16O4 ; C11H16O2 ; C36H34N2O10 ; C11H16O2 ; C10H9NO3 ; C9H16O4 ; C11H16O2 ; C11H16O2 ; C11H16O2 ; C18H17NO5 ; C11H16O2 ; C11H16OS ; C39H36O8 ; C14H12O ; C9H16O4 ; C9H16O4 ; C9H16O4 ; C9H16O4 ; C11H16O2 ; C11H16O2 ; C10H9NO3 ; C8H13N3O3 ; C22H32O4 ; C22H32O4 ; C22H32O4 ; C16H14O3 ; C8H16N2O4S ; C8H16N2O4S ; C11H14N2O5 ; C16H14O3 ; C16H14O3 ; C16H14O3 ; C11H14N2O5 ; C11H14N2O5

0221_86.7	1.908901	0.512289	-3.4994	0.005733	2.2416	0.42361	362.097	HMDB00012 ; HMDB15023 ; HMDB15252 ; HMDB15415 ; HMDB28890 ; HMDB28953 ; HMDB29289 ; HMDB29290 ; HMDB29461 ; HMDB29512 ; HMDB29840 ; HMDB30341 ; HMDB30575 ; HMDB30718 ; HMDB31289 ; HMDB31992 ; HMDB32736 ; HMDB33446 ; HMDB33520 ; HMDB33521 ; HMDB33665 ; HMDB34182 ; HMDB37839 ; HMDB38464 ; HMDB38811 ; HMDB40318 ; HMDB41845 ; HMDB59746	Deoxyuridine ; Pemirolast ; Gliclazide ; Oxtriphylline ; Histidinyl-Lysine ; Lysyl-Histidine ; 3-Feruloyl-15-quinolactone ; 4-Feruloyl-15-quinolactone ; Zapotin ; 45-Dihydroxy-7-methoxy-6-methylflavone ; Vulgaxanthin I ; Piplartine ; 4567-Tetramethoxyflavone ; Sayanedin ; 146-Trihydroxy-5-methoxy-7-prenylxanthone ; Dulxanthone A ; Dulxanthone D ; Rutagravine ; 1-Hydroxyrutacridone epoxide ; 20-Hydroxyrutacridone epoxide ; N-Hydroxyneosaxitoxin ; 158-Trihydroxy-3-methyl-2-prenylxanthone ; N-(1-Deoxy-1-fructosyl)histidine ; Nelumboside ; Alfalone ; 5588-Tetrahydroxy-334477-hexamethoxy-55-biflavan ; Cadralazine ; (1R2S5R6S)-6-(34-Dihydroxyphenyl)-2-(34-methylenedioxyphenyl)-37-dioxabicyclo-330octane	3M+H+K ; 3M+H+K ; M+K ; M+2K+H ; M+2K+H ; M+2K+H ; 2M+H+Na ; 2M+H+Na ; 2M+H+K ; M+ACN+Na ; M+Na ; M+2Na-H ; 2M+H+K ; M+ACN+Na ; 2M+H+K ; 2M+H+K ; 2M+H+K ; M+Na ; M+Na ; M+Na ; 2M+Na+K ; 2M+H+K ; M+2Na-H ; M+2ACN+2H ; M+ACN+Na ; M+Na+K ; M+2K+H ; 2M+H+K	C9H12N2O5 ; C10H8N6O ; C15H21N3O3S ; C12H21N5O3 ; C12H21N5O3 ; C12H21N5O3 ; C17H18O8 ; C17H18O8 ; C19H18O6 ; C17H14O5 ; C14H17N3O7 ; C17H19NO5 ; C19H18O6 ; C17H14O5 ; C19H18O6 ; C19H18O6 ; C19H18O6 ; C19H17NO5 ; C19H17NO5 ; C19H17NO5 ; C10H17N7O6 ; C19H18O6 ; C12H19N3O7 ; C27H28O18 ; C17H14O5 ; C36H38O12 ; C12H21N5O3 ; C19H18O6
3963_289.	1.906766	0.800736	3.4067	0.006695	2.1742	0.42361	594.434	HMDB02020 ; HMDB03448 ; HMDB37122 ; HMDB40657 ; HMDB40743	3-Hydroxy-ee-caroten-3-one ; 3-Hydroxy-be-caroten-3-one ; Nigroxanthin ; Cryptocapsone ; 2-Dehydroplectaniaxanthin	2M+3H2O+2H ; 2M+3H2O+2H ; 2M+3H2O+2H ; 2M+3H2O+2H ; 2M+3H2O+2H	C40H54O2 ; C40H54O2 ; C40H54O2 ; C40H54O2 ; C40H54O2
03588_286.	1.90655	1.069229	3.1541	0.010262	1.9888	0.42361	569.1604	HMDB11166 ; HMDB11172 ; HMDB12779 ; HMDB14367 ; HMDB15338 ; HMDB28756 ; HMDB28757 ; HMDB28903 ; HMDB28925 ; HMDB30279 ; HMDB41124 ; HMDB41380 ; HMDB59717 ; HMDB60123 ; HMDB12947	L-beta-aspartyl-L-leucine ; L-gamma-glutamyl-L-valine ; 4-Hydroxyestrone sulfate ; Glimepiride ; Ridogrel ; Aspartyl-Isoleucine ; Aspartyl-Leucine ; Isoleucyl-Aspartate ; Leucyl-Aspartate ; Garciduol C ; Artonin Q ; Garciduol A ; Glu-Val ; rac-56-Epoxy-retinoyl-beta-D-glucuronide ; Ferrocytochrome	2M+2K-H ; 2M+2K-H ; 3M+H+K ; M+2K+H ; 3M+H+K ; 2M+2K-H ; 2M+2K-H ; 2M+2K-H ; 2M+2K-H ; M+2ACN+H ; M+K ; M+2ACN+H ; 2M+2K-H ; M+2K-H ; M+H-2H2O	C10H18N2O5 ; C10H18N2O5 ; C18H22O6S ; C24H34N4O5S ; C18H17F3N2O3 ; C10H18N2O5 ; C10H18N2O5 ; C10H18N2O5 ; C10H18N2O5 ; C27H18O9 ; C31H30O8 ; C27H18O9 ; C10H18N2O5 ; C26H36O9 ; C33H32FeN4O4
4951_288.	1.906178	0.975327	3.3168	0.007789	2.1085	0.42361	593.0585	HMDB01212 ; HMDB06211 ; HMDB14744 ; HMDB31807 ; HMDB34322 ; HMDB60008 ; HMDB60009 ; HMDB60010	Hydantoin-5-propionic acid ; 2-(Formamido)-N1-(5-phospho-D-ribosyl)acetamidine ; Cyclothiazide ; m-Hydroxydiphenyldimercury(1) ; L-alpha-Amino-5-oxo-2(5H)-isoxazolepropanoic acid ; N-(2-hydroxymethyl-3-chlorophenyl)anthranilic acid ; N-(2-methyl-3-chloro-4-hydroxyphenyl)anthranilic acid ; N-(2-methyl-3-chloro-5-hydroxyphenyl)anthranilic acid	3M+2K-H ; 2M+Na ; 3M+H+NH4 ; M+NH4 ; 3M+2K-H ; 2M+K ; 2M+K ; 2M+K	C6H8N2O4 ; C6H12N3O8P ; C14H16ClN3O4S2 ; C12H11Hg2O ; C6H8N2O4 ; C14H12ClNO3 ; C14H12ClNO3 ; C14H12ClNO3

2704_54.8	1.903229	1.050019	3.4191	0.006558	2.1833	0.42361	500.8793	HMDB08684 ; HMDB08717 ; HMDB08749 ; HMDB08781 ; HMDB08812 ; HMDB08813	PC(22:5(4Z7Z10Z13Z16Z)24:1(15Z)) ; PC(22:5(7Z10Z13Z16Z19Z)24:1(15Z)) ; PC(22:6(4Z7Z10Z13Z16Z19Z)24:0) ; PC(24:022:6(4Z7Z10Z13Z16Z19Z)) ; PC(24:1(15Z)22:5(4Z7Z10Z13Z16Z)) ; PC(24:1(15Z)22:5(7Z10Z13Z16Z19Z))	M+2ACN+2H ; M+2ACN+2H ; M+2ACN+2H ; M+2ACN+2H ; M+2ACN+2H ; M+2ACN+2H	C54H96NO8P ; C54H96NO8P ; C54H96NO8P ; C54H96NO8P ; C54H96NO8P ; C54H96NO8P
1225_205.	1.901632	0.870834	3.1746	0.009911	2.0039	0.42361	185.5141	HMDB15126 ; HMDB15461	Oxaprozin ; Glycodiazine	M+2K ; M+Na+K	C18H15NO3 ; C13H15N3O4S
6039_101.	1.900645	0.587897	-3.0006	0.013331	1.8751	0.42361	NA	NA	NA	NA	NA
9352_54.5	1.900641	1.090882	3.3519	0.007342	2.1342	0.42361	269.9209	HMDB05356 ; HMDB10413 ; HMDB42065 ; HMDB42124 ; HMDB42153 ; HMDB42181 ; HMDB42991 ; HMDB43047	TG(16:016:016:0)iso ; TG(16:014:018:0)iso6 ; TG(14:014:020:0) ; TG(14:016:018:0) ; TG(14:018:016:0) ; TG(14:020:014:0) ; TG(15:015:018:0) ; TG(15:018:015:0)	M+3H ; M+3H ; M+3H ; M+3H ; M+3H ; M+3H ; M+3H ; M+3H	C51H98O6 ; C51H98O6 ; C51H98O6 ; C51H98O6 ; C51H98O6 ; C51H98O6 ; C51H98O6 ; C51H98O6
3527_23.7	1.897619	0.552369	-3.2629	0.008533	2.0689	0.42361	223.0634	HMDB33041 ; HMDB33072 ; HMDB40243 ; HMDB40262 ; HMDB00426 ; HMDB00428 ; HMDB00606 ; HMDB00694 ; HMDB01900 ; HMDB06806 ; HMDB11676 ; HMDB29399 ; HMDB29869 ; HMDB30216 ; HMDB33258 ; HMDB33912 ; HMDB38890 ; HMDB41307 ; HMDB41392 ; HMDB59655 ; HMDB61023 ; HMDB33071	1-(1-Propenylthio)propyl propyl disulfide ; (E)-1-Propenyl 1-(propylthio)propyl disulfide ; 224466-Hexamethyl-135-trithiane ; 246-Triethyl-135-trithiane ; Citramalic acid ; 3-Hydroxyglutaric acid ; D-2-Hydroxyglutaric acid ; L-2-Hydroxyglutaric acid ; Ribonolactone ; Propinol adenylate ; D-Xylono-15-lactone ; Ochratoxin A ; Hemiariensin ; Mukonal ; Isoyatein ; 2-Propenyl propyl disulfide ; Methyl 3-methyl-1-butenyl disulfide ; Italipyrone ; 1-Propenyl propyl disulfide ; 2-Hydroxyglutarate ; N-Desmethyldiltiazem ; Propyl 1-(propylsulfinyl)propyl disulfide	M+H ; M+H ; M+H ; M+H ; 3M+2H ; 3M+2H ; 3M+2H ; 3M+2H ; 3M+2H ; 3M+2H ; M+ACN+2H ; 3M+2H ; M+ACN+2H ; M+2Na ; 2M+H+Na ; M+2Na ; 3M+2H ; 3M+2H ; M+2Na ; 3M+2H ; 3M+2H ; M+2Na ; M+H-H2O	C9H18S3 ; C9H18S3 ; C9H18S3 ; C9H18S3 ; C5H8O5 ; C5H8O5 ; C5H8O5 ; C5H8O5 ; C5H8O5 ; C13H18N5O8P ; C5H8O5 ; C20H18ClNO6 ; C22H24O7 ; C13H9NO2 ; C22H24O7 ; C6H12S2 ; C6H12S2 ; C22H24O7 ; C6H12S2 ; C5H8O5 ; C21H24N2O4S ; C9H20OS3
1686_50.66	1.894036	0.339898	-3.3437	0.007444	2.1282	0.42361	468.8417	HMDB08323 ; HMDB08355 ; HMDB08547 ; HMDB08578 ; HMDB08610 ; HMDB08769 ; HMDB08801	PC(20:1(11Z)24:1(15Z)) ; PC(20:2(11Z14Z)24:0) ; PC(22:022:2(13Z16Z)) ; PC(22:1(13Z)22:1(13Z)) ; PC(22:2(13Z16Z)22:0) ; PC(24:020:2(11Z14Z)) ; PC(24:1(15Z)20:1(11Z))	M+H+K ; M+H+K ; M+H+K ; M+H+K ; M+H+K ; M+H+K ; M+H+K	C52H100NO8P ; C52H100NO8P ; C52H100NO8P ; C52H100NO8P ; C52H100NO8P ; C52H100NO8P ; C52H100NO8P
1866_285.	1.892399	0.927277	3.2439	0.008812	2.0549	0.42361	523.4732	HMDB31288 ; HMDB41077 ; HMDB07011 ; HMDB07068 ; HMDB07095 ; HMDB55954 ; HMDB55981	121-Heneicosanediol ; erythro-68-Heneicosanediol ; DG(14:016:00:0) ; DG(15:015:00:0) ; DG(16:014:00:0) ; DG(14:00:016:0) ; DG(15:00:015:0)	3M+Na+K ; 3M+Na+K ; M+H-H2O ; M+H-H2O ; M+H-H2O ; M+H-H2O ; M+H-H2O	C21H44O2 ; C21H44O2 ; C33H64O5 ; C33H64O5 ; C33H64O5 ; C33H64O5 ; C33H64O5
4319_290.	1.891412	0.687161	3.2934	0.008103	2.0913	0.42361	705.2174	HMDB01081 ; HMDB06581 ; HMDB06584 ; HMDB32726 ; HMDB34095 ; HMDB38013 ; HMDB39063	(N-acetylneuraminosyl(a2-6)lactosamine) ; 3-Sialyl-N-acetyllactosamine ; 6-Sialyl-N-acetyllactosamine ; Menadiol dibutyrate ; Gambogic acid ; Gibberellin A120 ; 7-(6-Hydroxy-37-dimethyl-27-octadienyl)oxy-2H-1-benzopyran-2-one	2M+Na+K ; 2M+Na+K ; 2M+Na+K ; 2M+2K-H ; M+2K-H ; 2M+2K-H ; 2M+2K-H	C25H42N2O19 ; C25H42N2O19 ; C25H42N2O19 ; C19H22O4 ; C38H44O8 ; C19H22O4 ; C19H22O4

0623_88.77	1.888652	0.896436	-3.307	0.007919	2.1013	0.42361	287.0906	HMDB29527 ; HMDB29870 ; HMDB30090 ; HMDB30622 ; HMDB30793 ; HMDB30807 ; HMDB30852 ; HMDB31420 ; HMDB31620 ; HMDB33291 ; HMDB33309 ; HMDB34011 ; HMDB34012 ; HMDB34114 ; HMDB34115 ; HMDB34438 ; HMDB37320 ; HMDB37322 ; HMDB37489 ; HMDB38448 ; HMDB41726 ; HMDB00402 ; HMDB01086 ; HMDB02025 ; HMDB02053 ; HMDB02211 ; HMDB04181 ; HMDB11655 ; HMDB12156 ; HMDB15190 ; HMDB28895 ; HMDB29063 ; HMDB29169 ; HMDB31584 ; HMDB31653 ; HMDB31712 ; HMDB31852 ; HMDB31876 ; HMDB32493 ; HMDB32985 ; HMDB33064 ; HMDB34341 ; HMDB34882 ; HMDB35466 ; HMDB36544 ; HMDB38409 ; HMDB38439 ; HMDB38816 ; HMDB39081 ; HMDB39807 ; HMDB40141 ; HMDB40143 ; HMDB41084 ; HMDB41746 ; [illegible]	Melilotocarpan B ; 37-Dihydroxy-4-methoxyisoflavanone ; Sakuranetin ; (R)-Oxypeucedanin ; Moracin B ; 34-Dihydro-8-hydroxy-3-(3-hydroxy-4-methoxyphenyl)-1H-2-benzopyran-1-one ; Asperxanthone ; Heliannone C ; Vestitone ; 38-Dihydroxy-9-methoxypterocarpan ; Moracin F ; 2-(24-Dihydroxyphenyl)-56-dimethoxybenzofuran ; 34-Dihydroxy-9-methoxypterocarpan ; 6alpha-Hydroxyisomedicarpin ; 6alpha-Hydroxymedicarpin ; (R)-Pabulenol ; Licochalcone B ; Dihydrooroxylin ; Dihydrowogonin ; (-)-57-Dihydroxy-3-(4-hydroxybenzyl)-4-chromanone ; Dihydroglycitein ; 2-Isopropylmalic acid ; Uroporphyrinogen III ; 23-Dimethyl-3-hydroxyglutaric acid ; Histidylproline diketopiperazine ; Uroporphyrinogen I ; Methylimidazole acetaldehyde ; 2-(3-Carboxy-3-aminopropyl)-L-histidine ; 3-Isopropylmalate ; Echothiophate ; Histidinyl-Threonine ; Threoninyl-Histidine ; 3-Hydroxy-2-methylglutarate ; Myricatomentoside II ; 3-(Methylthio)-1-propene ; ()-Glycerol 12-diacetate ; 2-Methoxy-3-methylpyrazine ; Prenyl glucoside ; 2-Propionyl-2-thiazoline ; 5-(2-Hydroxyethyl)-4-methylthiazole ; (E)-2-Methyl-2-buten-1-ol O-beta-D-Glucopyranoside ; Physalin L ; 5-Acetyl-23-dihydro-14-thiazine ; Kaempferol 3-(6-acetylgalactoside) ; 1-Acetoxyeugenol acetate ; 6-(Methylthio)hexyl glucosinolate ; (-)-5-(2-Propenyl)-2-oxazolidinethione ; 2-O-Acetylisoorientin ; Physalin O ; cis-23-Dimethylthiirane ; 2-Methoxy-5-methylpyrazine ; 2-Methoxy-6-methylpyrazine ; Raphanusamide ; Irisolidone 7-O-glucuronide ; (1E)-1-(methylthio)prop-1-ene ; Norketamine	M+H ; 3M+2Na ; M+2H+Na ; 3M+2Na ; M+K ; M+2H+Na ; 2M+K ; 2M+Na+K ; 3M+2Na ; 2M+Na+K ; 2M+Na+K ; 2M+Na+K ; 3M+2Na ; M+H+K ; 3M+Na ; 3M+2Na ; 2M+K ; M+K ; 2M+H ; 2M+H ; M+K ; M+2Na ; 2M+H ; M+2ACN+2H ; M+Na ; M+3ACN+2H ; 2M+H ; M+2ACN+2H ; M+2Na ; 3M+Na ; 2M+K ; 2M+K ; 2M+H ; M+2ACN+2H ; 3M+Na ; M+ACN+Na	C16H14O5 ; C7H12O5 ; C40H44N4O16 ; C7H12O5 ; C12H16N4O2 ; C40H44N4O16 ; C6H8N2O ; C10H16N4O4 ; C7H12O5 ; C9H23NO3PS ; C10H16N4O4 ; C10H16N4O4 ; C7H12O5 ; C27H34O11 ; C4H8S ; C7H12O5 ; C6H8N2O ; C11H20O6 ; C6H9NOS ; C6H9NOS ; C11H20O6 ; C28H32O10 ; C6H9NOS ; C23H22O12 ; C14H16O5 ; C14H27NO9S3 ; C6H9NOS ; C23H22O12 ; C28H32O10 ; C4H8S ; C6H8N2O ; C6H8N2O ; C6H9NOS ; C23H22O12 ; C4H8S ; C12H14ClNO
7081_257.8	1.887992	0.399791	-3.4335	0.006401	2.1937	0.42361	NA	NA	NA	NA	NA
5526_34.47	1.887369	0.798949	-3.4216	0.00653	2.1851	0.42361	NA	NA	NA	NA	NA
0432_87.74	1.882345	0.393916	-2.9504	0.014526	1.8379	0.42361	161.023	HMDB60386 ; HMDB60395 ; HMDB29465 ; HMDB00192 ; HMDB30562 ; HMDB30830 ; HMDB33722 ; HMDB33820 ; HMDB33859 ; HMDB34139 ; HMDB35914 ; HMDB36305 ; HMDB38442 ; HMDB30800 ; HMDB30819 ; HMDB32950 ; HMDB32951 ; HMDB59809	4-Fluoromuconolactone ; 5-Fluoromuconolactone ; Erosnin ; L-Cystine ; 38-Dihydroxy-9-methoxycoumestan ; 38-Dihydroxy-1-methylanthraquinone-2-carboxylic acid ; Sativol ; Irilone ; Glyzaglabrin ; Trifoliol ; Aflatoxin P1 ; Isotrifoliol ; 1-Isothiocyanato-2-(methylthio)ethane ; 3-Methoxy-45-methylenedioxybenzoic acid ; Aesculetin ; 57-Dihydroxy-4H-1-benzopyran-4-one ; 47-Dihydroxy-2H-1-benzopyran-2-one ; 4-Hydroxy-benzenepropanedioate	M+H ; M+H ; M+2H ; 2M+3H ; M+H+Na ; M+H+Na ; M+H+Na ; M+H+Na ; M+H+Na ; M+H+Na ; M+H+Na ; M+H+Na ; 2M+3H2O+2H ; M+H-2H2O ; M+H-H2O ; M+H-H2O ; M+H-H2O ; M+H-2H2O	C6H5FO4 ; C6H5FO4 ; C18H8O6 ; C6H12N2O4S2 ; C16H10O6 ; C16H10O6 ; C16H10O6 ; C16H10O6 ; C16H10O6 ; C16H10O6 ; C16H10O6 ; C16H10O6 ; C4H7NS2 ; C9H8O5 ; C9H6O4 ; C9H6O4 ; C9H6O4 ; C9H8O5
3796_282.2	1.878695	0.869147	3.0205	0.012884	1.89	0.42361	254.0084	HMDB00226 ; HMDB01564 ; HMDB13593 ; HMDB33566 ; HMDB38589 ; HMDB59724 ; HMDB59911 ; HMDB59994	Orotic acid ; CDP-Ethanolamine ; 14-Dithiothreitol ; Ajoene ; Brassicanal C ; Pyrocatechol sulfate ; Paracetamol sulfate ; Benzeneacetamide-4-O-sulphate	3M+H+K ; M+Na+K ; 3M+2Na ; 2M+H+K ; 2M+Na+K ; M+ACN+Na ; M+Na ; M+Na	C5H4N2O4 ; C11H20N4O11P2 ; C4H10O2S2 ; C9H14OS3 ; C10H9NO3S ; C6H6O5S ; C8H9NO5S ; C8H9NO5S
2112_87.18	1.873624	0.987652	3.1361	0.010579	1.9755	0.42361	557.1222	HMDB03882 ; HMDB14743 ; HMDB33659 ; HMDB41720	N1-(5-Phospho-a-D-ribosyl)-56-dimethylbenzimidazole ; Sulindac ; Dihydrohydroxy-O-methylsterigmatocystin ; Dihydrocaffeic acid 3-O-glucuronide	3M+H+K ; 3M+2Na ; 3M+2Na ; 3M+H+K	C14H19N2O7P ; C20H17FO3S ; C19H16O7 ; C15H18O10
7564_125.0	1.872273	0.948586	-3.0982	0.011285	1.9475	0.42361	142.9868	HMDB39431	12457-Pentathiocane	M+2ACN+2H	C3H6S5

23556_79.3	1.872054	0.769397	-3.4915	0.005808	2.236	0.42361	409.7224	HMDB12338 ; HMDB12359 ; HMDB12369 ; HMDB12399 ; HMDB12409 ; HMDB12418 ; HMDB31826	PS(14:020:3(8Z11Z14Z)) ; PS(16:018:3(9Z12Z15Z)) ; PS(16:1(9Z)18:2(9Z12Z)) ; PS(18:2(9Z12Z)16:1(9Z)) ; PS(18:3(9Z12Z15Z)16:0) ; PS(20:3(8Z11Z14Z)14:0) ; Fumonisin FP1	M+Na+K ; M+Na+K ; M+Na+K ; M+Na+K ; M+Na+K ; M+Na+K ; M+H+NH4	C40H72NO10P ; C40H72NO10P ; C40H72NO10P ; C40H72NO10P ; C40H72NO10P ; C40H72NO10P ; C39H62NO16
43653_279.	1.871882	0.574216	3.0713	0.011815	1.9275	0.42361	435.8944	HMDB13023	Neurotensin 1-10	M+2H+Na	C57H90N18O16
5248_183.7	1.871124	0.411322	-3.1029	0.011195	1.951	0.42361	365.0675	HMDB01919 ; HMDB14975 ; HMDB31950 ; HMDB39864 ; HMDB60573 ; HMDB60991 ; HMDB30774	Famotidine ; Progabide ; L-2-Aminoethyl seryl phosphate ; 3-O-beta-D-Galactopyranosylcinnamtannin B1 ; Chlorpromazine-N-oxide ; hydroxychlorpromazine ; ()-12a-Hydroxypachyrrhizone	2M+3H2O+2H ; 2M+Na+K ; 3M+2Na ; M+3Na ; 2M+Na+K ; 2M+Na+K ; M+H-H2O	C8H15N7O2S3 ; C17H16ClFN2O2 ; C5H13N2O6P ; C51H46O23 ; C17H19ClN2OS ; C17H19ClN2OS ; C20H14O8
31396_82.0	1.86849	0.497364	3.1769	0.009871	2.0056	0.42361	306.0931	HMDB00788 ; HMDB03546 ; HMDB03950 ; HMDB14911 ; HMDB15598 ; HMDB28821 ; HMDB28884 ; HMDB28891 ; HMDB28975 ; HMDB29569 ; HMDB31150 ; HMDB33345 ; HMDB34023 ; HMDB38126 ; HMDB38331 ; HMDB38964 ; HMDB39136 ; HMDB40348 ; HMDB41200 ; HMDB41329 ; HMDB60984	Orotidine ; Salicin ; 7-Methylinosine ; Etoposide ; Voglibose ; Glutamyl-Histidine ; Histidinyl-Glutamate ; Histidinyl-Methionine ; Methionyl-Histidine ; Dibutyl disulfide ; Pyrophaeophorbide a ; ()-Ribaline ; Isosalicin ; Genipinic acid ; 3-Hydroxybenzyl alcohol glucoside ; 3-Hydroxy-46-heptadiyne-1-yl 1-glucoside ; Elenaic acid ; 18-Octanedithiol ; Dehydroxymethylflazine ; 5-Amino-6-(4-hydroxy-2-butenoyl)-22-dimethyl-4-chromanone ; dapsone hydroxylamine	M+NH4 ; 2M+H+K ; M+Na ; M+H+Na ; M+K ; M+Na ; M+Na ; 2M+H+K ; 2M+H+K ; 3M+2K ; M+2K ; 2M+Na+K ; 2M+H+K ; M+ACN+Na ; 2M+H+K ; 2M+H+K ; M+ACN+Na ; 3M+2K ; 2M+3H2O+2H ; 2M+Na+K ; M+ACN+H	C10H12N2O8 ; C13H18O7 ; C11H15N4O5 ; C29H32O13 ; C10H21NO7 ; C11H15N4O5 ; C11H15N4O5 ; C11H18N4O3S ; C11H18N4O3S ; C8H18S2 ; C33H34N4O3 ; C15H17NO4 ; C13H18O7 ; C11H14O6 ; C13H18O7 ; C13H18O7 ; C11H14O6 ; C8H18S2 ; C16H10N2O3 ; C15H17NO4 ; C12H12N2O3S
2645_139.4	1.868155	0.484201	-2.8696	0.01668	1.7778	0.42361	185.1193	HMDB14708 ; HMDB14920 ; HMDB35150 ; HMDB35853 ; HMDB59773 ; HMDB60995 ; HMDB00792 ; HMDB31510 ; HMDB40196 ; HMDB59708 ; HMDB59719 ; HMDB59754	Cinnarizine ; Propantheline ; 2-Apo-beta-carotenal ; Fasciculol C ; S-3-oxodecanoyl cysteamine ; 10-alpha-methoxy-910-dihydrolysergol ; Sebacic acid ; R-2-Hydroxy-3-methylbutanoic acid 3-Methylbutanoyl ; Oxalic acid dibutyl ester ; 2-Ethylsuberic acid ; Heptylmalonic acid ; 3-Methylazelaic acid	M+2H ; M+2H ; M+H+2Na ; M+H+2Na ; M+3ACN+2H ; M+2ACN+2H ; M+H-H2O ; M+H-H2O ; M+H-H2O ; M+H-H2O ; M+H-H2O ; M+H-H2O	C26H28N2 ; C23H30NO3 ; C37H48O ; C30H52O6 ; C12H23NO2S ; C17H22N2O2 ; C10H18O4 ; C10H18O4 ; C10H18O4 ; C10H18O4 ; C10H18O4 ; C10H18O4

8293_284.6	1.866869	0.856083	3.0662	0.011918	1.9238	0.42361	593.3038	HMDB04224 ; HMDB15696 ; HMDB28802 ; HMDB28949 ; HMDB28965 ; HMDB29154 ; HMDB29488 ; HMDB29489 ; HMDB29490 ; HMDB30381 ; HMDB32711 ; HMDB33271 ; HMDB33543 ; HMDB34326 ; HMDB34529 ; HMDB34645 ; HMDB34647 ; HMDB35510 ; HMDB36325 ; HMDB37588 ; HMDB39051 ; HMDB39065 ; HMDB60847 ; HMDB33749 ; HMDB33920	N-(o)-Hydroxyarginine ; Bopindolol ; Glutaminyl-Lysine ; Lysyl-Glutamine ; Lysyl-Gamma-glutamate ; Gamma-glutamyl-Lysine ; Farnesiferol A ; Farnesiferol B ; (-)-Farnesiferol C ; Roquefortine ; Pectenotoxin 7 ; Foetidin ; Assafoetidin ; L-erythro-4-Hydroxyarginine ; 16-Acetylpriverogenin A ; 22-Acetylpriverogenin B ; Oleanderolide 3-acetate ; Tsugaric acid C ; Dinorcapsaicin ; Gancaonin R ; Notoginsenoside R10 ; (3alpha5alpha9xi10beta)-O-(3-Hydroxy-7-drimen-11-yl)umbelliferone ; N-Monodesmethyl-rizatriptan ; Isoliensinine ; Liensinine	3M+Na ; 3M+2Na ; 2M+2Na-H ; 2M+2Na-H ; 2M+2Na-H ; 2M+2Na-H ; 3M+H+K ; 3M+H+K ; 3M+H+K ; 3M+H+NH4 ; 2M+3H ; 3M+H+K ; 3M+H+K ; 3M+Na ; M+2K+H ; M+2K-H ; M+2K+H ; M+2K+H ; 2M+K ; 3M+H+K ; M+K ; 3M+H+K ; 2M+Na ; M+H-H2O ; M+H-H2O	C6H14N4O3 ; C23H28N2O3 ; C11H22N4O4 ; C11H22N4O4 ; C11H22N4O4 ; C11H22N4O4 ; C24H30O4 ; C24H30O4 ; C24H30O4 ; C22H23N5O2 ; C47H68O16 ; C24H30O4 ; C24H30O4 ; C6H14N4O3 ; C32H50O5 ; C32H52O5 ; C32H50O5 ; C32H50O5 ; C16H23NO3 ; C24H30O4 ; C30H50O9 ; C24H30O4 ; C15H19N5O ; C37H42N2O6 ; C37H42N2O6
25909_270.5	1.861898	0.861071	3.3434	0.007448	2.128	0.42361	596.9826	HMDB06317 ; HMDB13207 ; HMDB29219	trans-Hexadec-2-enoyl carnitine ; 9-Hexadecenoylcarnitine ; Urolithin D	3M+2H ; 3M+2H ; 2M+2K-H	C23H43NO4 ; C23H43NO4 ; C13H8O6
.9452_72.12	1.858934	0.311955	2.9703	0.014037	1.8527	0.42361	205.0819	HMDB11162 ; HMDB11667 ; HMDB28683 ; HMDB28746 ; HMDB00315 ; HMDB00363 ; HMDB00486 ; HMDB00711 ; HMDB00802 ; HMDB01954 ; HMDB02264 ; HMDB03851 ; HMDB04026 ; HMDB10722 ; HMDB11653 ; HMDB13898 ; HMDB13899 ; HMDB13900 ; HMDB14452 ; HMDB30393 ; HMDB31187 ; HMDB31462 ; HMDB31509 ; HMDB31622 ; HMDB31700 ; HMDB31751 ; HMDB32270 ; HMDB32827 ; HMDB33918 ; HMDB37281 ; HMDB38444 ; HMDB39031 ; HMDB39153 ; HMDB40823 ; HMDB41817 ; HMDB41892 ; HMDB59825 ; HMDB59867 ; HMDB60407 ; HMDB60424 ; HMDB60788	L-beta-aspartyl-L-alanine ; 5-L-Glutamylglycine ; Alanyl-Aspartate ; Aspartyl-Alanine ; 16-a-Hydroxypregnenolone ; 17a-Hydroxypregnenolone ; 7-Hydroxyoctanoic acid ; Hydroxyoctanoic acid ; Pterin ; 3-Hydroxyoctanoic acid ; (R)-2-Hydroxycaprylic acid ; 3a7a-Dihydroxy-5b-cholestanate ; 21-Hydroxypregnenolone ; (R)-3-Hydroxyoctanoic acid ; 17-alpha20-alpha-Dihydroxypregn-4-en-3-one ; 5-Hydroxyvalproic acid ; 3-Hydroxyvalproic acid ; 4-Hydroxyvalproic acid ; Bexarotene ; L-N-Carboxymethylserine ; Thialdine ; 10-Shogaol ; Ethyl ()-3-hydroxyhexanoate ; 6-Phenyl-3-hexen-2-one ; 1-(Malonylamino)cyclopropanecarboxylic acid ; y-Morphine ; (-)-Ethyl 2-hydroxy-3-methylvalerate ; Peroxyoctanoic acid ; (15alpha20R)-1520-Dihydroxypregn-4-en-3-one ; 4-Methyl-2-phenyl-2-pentenal ; 1-Isothiocyanato-3-phenylpropane ; trans-O-Methylgrandmarin ; (R)-111213-Trinor-1(5)69-guaiatrien-8-one ; (S)-111213-Trinor-7-calamenone ; 7-Aminoclonazepam ; Faropenem ; Isoamyl lactate ; Ethyl 2-hydroxy-4-methylpentanoate ; 5alpha-Dihydrodeoxycorticosterone ; 7alpha-Hydroxypregnenolone ; 6-O-Desmethyl-mycophenolic acid	M+H ; M+H ; M+H ; M+H ; M+2K ; M+2K ; M+2Na-H ; M+2Na-H ; M+ACN+H ; M+2Na-H ; M+2Na-H ; M+2K ; M+2K ; M+2Na-H ; M+2K ; M+2Na-H ; M+2Na-H ; M+2Na-H ; M+Na+K ; M+ACN+H ; M+ACN+H ; M+2K ; M+2Na-H ; 2M+Na+K ; M+NH4 ; M+H+2Na ; M+2Na-H ; M+2Na-H ; M+2K ; 2M+Na+K ; 2M+3H2O+2H ; 2M+3H ; 2M+Na+K ; 2M+Na+K ; M+3ACN+2H ; M+3ACN+2H ; M+2Na-H ; M+2Na-H ; M+2K ; M+2K ; 2M+3H	C7H12N2O5 ; C7H12N2O5 ; C7H12N2O5 ; C7H12N2O5 ; C21H32O3 ; C21H32O3 ; C8H16O3 ; C8H16O3 ; C6H5N5O ; C8H16O3 ; C8H16O3 ; C21H32O3 ; C21H32O3 ; C8H16O3 ; C21H32O3 ; C8H16O3 ; C8H16O3 ; C8H16O3 ; C24H28O2 ; C5H9NO5 ; C6H13NS2 ; C21H32O3 ; C8H16O3 ; C12H14O ; C7H9NO5 ; C34H36N2O6 ; C8H16O3 ; C8H16O3 ; C21H32O3 ; C12H14O ; C10H11NS ; C16H18O6 ; C12H14O ; C12H14O ; C15H12ClN3O ; C12H15NO5S ; C8H16O3 ; C8H16O3 ; C21H32O3 ; C21H32O3 ; C16H18O6

4077_129.6	1.857962	0.152989	2.8232	0.018062	1.7432	0.42361	112.0064	HMDB03701 ; HMDB12258 ; HMDB15098 ; HMDB29740 ; HMDB30386 ; HMDB30906 ; HMDB30907 ; HMDB30908	Dimethylbenzimidazole ; Methylarsonate ; Bromfenac ; 1H-Indole-3-methanamine ; Myosmine ; 14-Dimethylpyrrolo12-apyrazine ; 34-Dimethylpyrrolo12-apyrazine ; 13-Dimethylpyrrolo12-apyrazine	M+2K ; M+2ACN+2H ; M+3H ; M+2K ; M+2K ; M+2K ; M+2K ; M+2K	C9H10N2 ; CH5AsO3 ; C15H12BrNO3 ; C9H10N2 ; C9H10N2 ; C9H10N2 ; C9H10N2 ; C9H10N2
0401_24.14	1.857	0.724383	-2.8963	0.015935	1.7977	0.42361	114.967	HMDB00263 ; HMDB60445 ; HMDB60447	Phosphoenolpyruvic acid ; Bromobenzene-23-dihydrodiol ; Bromobenzene-34-dihydrodiol	M+Na+K ; M+H+K ; M+H+K	C3H5O6P ; C6H7BrO2 ; C6H7BrO2
6096_284.5	1.855848	0.484797	3.0838	0.011566	1.9368	0.42361	NA	NA	NA	NA	NA
2721_293.2	1.855763	0.686949	3.0162	0.01298	1.8867	0.42361	419.9763	HMDB32086 ; HMDB39757 ; HMDB41362 ; HMDB60545 ; HMDB60779	Muricoreacin ; 7-Chloro-6-demethylcepharadione B ; Annohexocin ; O-Desmethylindomethacin ; 5-Carboxy meloxicam	2M+3H ; M+2K+H ; 2M+3H ; M+2K-H ; M+K	C35H64O9 ; C18H12ClNO4 ; C35H64O9 ; C18H14ClNO4 ; C14H11N3O6S2
4185_275.4	1.855511	0.688666	3.0776	0.011688	1.9322	0.42361	NA	NA	NA	NA	NA
.8172_44.80	1.851006	1.086537	3.1287	0.010713	1.9701	0.42361	468.5818	HMDB14626	Gallamine Triethiodide	M+2Na	C30H60I3N3O3
4376_261.9	1.849387	0.452116	3.0647	0.011947	1.9227	0.42361	NA	NA	NA	NA	NA
.8939_289.3	1.848671	0.965229	3.1966	0.009547	2.0201	0.42361	679.0619	HMDB33338	Mollicellin F	3M+Na+K	C21H17ClO8
3428_38.75	1.847461	0.431708	3.3994	0.006778	2.1689	0.42361	284.0643	HMDB01209 ; HMDB02381 ; HMDB02670 ; HMDB05897 ; HMDB14016 ; HMDB14017 ; HMDB15050 ; HMDB28732 ; HMDB28759 ; HMDB28858 ; HMDB28969 ; HMDB29631 ; HMDB30084 ; HMDB30873 ; HMDB31824 ; HMDB31985 ; HMDB33308 ; HMDB33422 ; HMDB33519 ; HMDB33928 ; HMDB34002 ; HMDB34105 ; HMDB37254 ; HMDB38549 ; HMDB39386 ; HMDB41656 ; HMDB41698 ; HMDB41704 ; HMDB60550 ; HMDB60774	Allantoic acid ; N-Acetylcystathionine ; Naringenin ; Dihydrogenistein ; 5-Hydroxyketamine ; 4-Hydroxyketamine ; Phenformin ; Asparaginyl-Hydroxyproline ; Aspartyl-Methionine ; Hydroxyprolyl-Asparagine ; Methionyl-Aspartate ; Chalconaringenin ; Diosbulbinoside D ; 1-Hydroxy-37-dimethoxyxanthone ; 458-Trihydroxyflavanone ; gamma-Glutamyl-S-methylcysteine ; Moracin J ; (2S2S3S3R4S)-3457-Tetrahydroxyflavan(2-74-8)-3457-tetrahydroxyflavan ; N-Ornithyl-L-taurine ; ()-2-Hydroxydihydrodaidzein ; 349-Trihydroxypterocarpan ; Glycinol ; (2R3R)-347-Trihydroxyflavanone ; Toralactone ; Cymorcin diglucoside ; 3-Hydroxydihydrodaidzein ; 6-Hydroxydihydrodaidzein ; 8-Hydroxydihydrodaidzein ; 6-Hydroxyketamine ; 5-Carboxy-2-deoxyuridine	3M+H+K ; 2M+H+K ; 2M+H+Na ; 2M+H+Na ; 2M+Na+K ; 2M+Na+K ; M+2K+H ; M+K ; 2M+H+K ; M+K ; 2M+H+K ; 2M+H+Na ; M+Na+K ; 2M+H+Na ; 2M+H+Na ; 2M+H+K ; 2M+H+Na ; M+H+Na ; M+2Na-H ; 2M+H+Na ; 2M+H+Na ; 2M+H+Na ; 2M+H+Na ; 2M+H+Na ; M+2K ; 2M+H+Na ; 2M+H+Na ; 2M+H+Na ; 2M+Na+K ; 2M+H+Na	C4H8N4O4 ; C9H16N2O5S ; C15H12O5 ; C15H12O5 ; C13H16ClNO2 ; C13H16ClNO2 ; C10H15N5 ; C9H15N3O5 ; C9H16N2O5S ; C9H15N3O5 ; C9H16N2O5S ; C15H12O5 ; C25H30O11 ; C15H12O5 ; C15H12O5 ; C9H16N2O5S ; C15H12O5 ; C30H24O10 ; C7H17N3O4S ; C15H12O5 ; C15H12O5 ; C15H12O5 ; C15H12O5 ; C15H12O5 ; C22H34O12 ; C15H12O5 ; C15H12O5 ; C15H12O5 ; C13H16ClNO2 ; C10H12N2O7
9874_279.3	1.846305	0.188905	3.1067	0.011122	1.9538	0.42361	275.489	HMDB12340 ; HMDB12414 ; HMDB12440	PS(14:022:6(4Z7Z10Z13Z16Z19Z)) ; PS(18:3(9Z12Z15Z)18:3(9Z12Z15Z)) ; PS(22:6(4Z7Z10Z13Z16Z19Z)14:0)	M+H+2Na ; M+H+2Na ; M+H+2Na	C42H70NO10P ; C42H70NO10P ; C42H70NO10P
6802_240.4	1.844953	1.101549	3.1176	0.010919	1.9618	0.42361	243.0107	HMDB59620 ; HMDB60266 ; HMDB14480 ; HMDB15244 ; HMDB33061 ; HMDB36374 ; HMDB40002 ; HMDB00963 ; HMDB01299	5-(methylthio)-23-Dioxopentyl phosphate ; 23-Diketo-5-methylthio-1-phosphopentane ; Nitrofurazone ; Cefuroxime ; 1-(Methylsulfinyl)propyl propyl disulfide ; Methyl 1-(propylsulfinyl)propyl disulfide ; 1-(2-Thienyl)-12-propanedione ; 5-Methylthioribose 1-phosphate ; 5-Methylthioribulose 1-phosphate	M+H ; M+H ; M+2Na-H ; M+Na+K ; 2M+Na+K ; 2M+Na+K ; 3M+H+Na ; M+H-H2O ; M+H-H2O	C6H11O6PS ; C6H11O6PS ; C6H6N4O4 ; C16H16N4O8S ; C7H16OS3 ; C7H16OS3 ; C7H6O2S ; C6H13O7PS ; C6H13O7PS

5331_266.:	1.844236	0.402956	3.1557	0.010234	1.99	0.42361	478.2355	HMDB01054 ; HMDB09924 ; HMDB09925 ; HMDB09947 ; HMDB09958 ; HMDB09970 ; HMDB11472 ; HMDB11502 ; HMDB14634 ; HMDB14775 ; HMDB36122 ; HMDB36123 ; HMDB40660	Dolichyl b-D-glucosyl phosphate ; PIP(16:018:1(11Z)) ; PIP3(16:018:1(9Z)) ; PIP(18:016:1(9Z)) ; PIP(18:1(11Z)16:0) ; PIP(18:1(9Z)16:0) ; LysoPE(0:015:0) ; LysoPE(15:00:0) ; Miglitol ; Astemizole ; 6-Gingerdiol 4-O-beta-D-glucopyranoside ; 6-Gingerdiol 5-O-beta-D-glucopyranoside ; Hoduloside VIII	2M+H+Na ; M+H+K ; M+H+K ; M+H+K ; M+H+K ; M+H+K ; M+K ; M+K ; 2M+ACN+Na ; 2M+H+K ; 2M+H+K ; 2M+H+K ; M+H+K	C21H39O9P ; C43H82O16P2 ; C43H82O16P2 ; C43H82O16P2 ; C43H82O16P2 ; C43H82O16P2 ; C20H42NO7P ; C20H42NO7P ; C8H17NO5 ; C28H31FN4O ; C23H38O9 ; C23H38O9 ; C46H76O18
8846_19.2	1.844027	0.473836	-2.7502	0.02047	1.6889	0.42361	97.96885	HMDB36577	Trifluoromethanesulfonic acid	M+2Na	CHF3O3S
7524_209.:	1.843924	0.435696	3.152	0.010298	1.9873	0.42361	224.0538	HMDB04083 ; HMDB38941 ; HMDB39495 ; HMDB00225 ; HMDB00398 ; HMDB01554 ; HMDB02759 ; HMDB06278 ; HMDB11690 ; HMDB13232 ; HMDB29308 ; HMDB29540 ; HMDB30182 ; HMDB32199 ; HMDB33319 ; HMDB35415 ; HMDB37755 ; HMDB38098 ; HMDB38099 ; HMDB39126 ; HMDB39379 ; HMDB39447 ; HMDB39570 ; HMDB41099 ; HMDB41677 ; HMDB41766 ; HMDB41918 ; HMDB59737 ; HMDB60498 ; HMDB60715 ; HMDB37548 ; HMDB37549	4-(2-Amino-3-hydroxyphenyl)-24-dioxobutanoic acid ; (R)-23-Dihydro-35-dihydroxy-2-oxo-3-indoleacetic acid ; 2-(Malonylamino)benzoic acid ; Oxoadipic acid ; 3-Oxoadipic acid ; Xanthylic acid ; Androsterone sulfate ; 5a-Dihydrotestosterone sulfate ; 7-Aminomethyl-7-carbaguanine ; Etiocholanolone sulfate ; Hexamethylquercetagetin ; Nobiletin ; Eupolauridine ; Chymosin preparation escherichia coli k-12 ; Licarin C ; 334568-Hexamethoxyflavone ; 334578-Hexamethoxyflavone ; Petunidin 3-rutinoside ; Petunidin 3-rhamnoside 5-glucoside ; Wybutoxine ; Chrysaloin ; 2-Methyl-4-oxopentanedioic acid ; Methylsyringin ; Kanzonol R ; 4-O-Methyldelphinidin 3-O-rutinoside ; Peonidin 3-sophoroside ; Lorcainide ; 3-Methyl-3-hydroxypentanedioate ; NNAL-N-glucuronide ; 2-Hydroxy-4-trifluoromethyl benzoic acid ; 24-Dihydroxy-67-dimethoxy-2H-14-benzoxazin-3(4H)-one ; 24-Dihydroxy-78-dimethoxy-2H-14-benzoxazin-3(4H)-one	M+H ; M+H ; M+H ; M+ACN+Na ; M+ACN+Na ; M+2ACN+2H ; M+2K ; M+2K ; M+2Na-H ; M+2K ; M+2Na ; M+2Na ; 2M+H+K ; M+3H ; M+2K ; M+2Na ; M+2Na ; M+H+2Na ; M+H+2Na ; M+H+K ; M+2Na ; M+ACN+Na ; M+Na+K ; M+2K ; M+H+2Na ; M+H+2Na ; M+2K ; M+ACN+Na ; M+Na+K ; M+NH4 ; M+H-H2O ; M+H-H2O	C10H9NO5 ; C10H9NO5 ; C10H9NO5 ; C6H8O5 ; C6H8O5 ; C10H13N4O9P ; C19H30O5S ; C19H30O5S ; C7H9N5O ; C19H30O5S ; C21H22O8 ; C21H22O8 ; C14H8N2 ; C30H27N3O15 ; C22H26O5 ; C21H22O8 ; C21H22O8 ; C28H33O16 ; C28H33O16 ; C16H20N6O7 ; C21H22O8 ; C6H8O5 ; C18H26O9 ; C22H26O5 ; C28H33O16 ; C28H33O16 ; C22H27ClN2O ; C6H8O5 ; C16H24N3O8 ; C8H5F3O3 ; C10H11NO6 ; C10H11NO6
7152_123.:	1.837735	1.13838	2.9494	0.014549	1.8372	0.42361	129.1387	HMDB31202 ; HMDB31659 ; HMDB32392 ; HMDB41075	Solamine ; 3-Methyl-1-butylamine ; 2-Methylbutylamine ; erythro-68-Pentacosanediol	M+ACN+2H ; M+ACN+H ; M+ACN+H ; M+3H	C12H29N3 ; C5H13N ; C5H13N ; C25H52O2
045438_48.(	1.835429	1.045717	-3.1218	0.01084	1.965	0.42361	NA	NA	NA	NA	NA

'1239_75.7(	1.832954	0.268903	-2.8051	0.018631	1.7298	0.42361	290.0871	HMDB12266 ; HMDB05799 ; HMDB30235 ; HMDB33992 ; HMDB35023 ; HMDB37349 ; HMDB37438 ; HMDB37580 ; HMDB38848 ; HMDB39488 ; HMDB39855 ; HMDB40504 ; HMDB00050 ; HMDB00085 ; HMDB00830 ; HMDB11105 ; HMDB12883 ; HMDB14340 ; HMDB14638 ; HMDB29496 ; HMDB37842 ; HMDB40814 ; HMDB41289 ; HMDB41858 ; HMDB59734 ; HMDB60499 ; HMDB60861 ; HMDB01177	N-Succinyl-2-amino-6-ketopimelate ; Scolymoside ; eq-4-Hydroxymaysin ; Daidzein 47-diglucoside ; Acacetin 7-apiosyl(1-6)-glucoside ; Isorhoifolin ; Kaempferitrin ; Galangin 3-galactosyl-(1-4)-rhamnoside ; Rhoifolin ; Chrysophanol 8-gentiobioside ; Vitexin 4-O-alpha-L-Rhamnopyranoside ; Camellianin B ; Adenosine ; Deoxyguanosine ; Neuraminic acid ; 5-Acetylamino-6-formylamino-3-methyluracil ; Adrenochrome o-semiquinone ; Vidarabine ; Zidovudine ; Fukiic acid ; N-(1-Deoxy-1-fructosyl)serine ; Isosyringinoside ; Cassitoroside ; Clobenzorex ; Pyrazin-2-carboxylic acid ; NPC ; OR-1896 ; (S)-Succinyldihydrolipoamide	M+H ; M+2H ; M+2H ; M+2H ; M+2H ; M+2H ; M+2H ; M+2H ; M+2H ; M+2H ; M+2H ; M+2H ; M+Na ; M+Na ; M+Na ; M+ACN+Na ; 3M+H+K ; M+Na ; M+Na ; M+NH4 ; M+Na ; M+2Na ; M+H+Na ; 2M+Na+K ; 2M+ACN+H ; M+Na+K ; M+2Na-H ; M+H-H2O	C11H15NO8 ; C27H30O14 ; C27H30O14 ; C27H30O14 ; C27H30O14 ; C27H30O14 ; C27H30O14 ; C27H30O14 ; C27H30O14 ; C27H30O14 ; C27H30O14 ; C27H30O14 ; C10H13N5O4 ; C10H13N5O4 ; C9H17NO8 ; C8H10N4O4 ; C9H10NO3 ; C10H13N5O4 ; C10H13N5O4 ; C11H12O8 ; C9H17NO8 ; C23H34O14 ; C25H32O14 ; C16H18ClN ; C5H4N2O2 ; C28H30N4O6 ; C13H15N3O2 ; C12H21NO4S2
)381_285.9:	1.832842	0.697819	3.1321	0.010652	1.9726	0.42361	NA	NA	NA	NA	NA
:2577_288.:	1.828355	0.872877	3.0871	0.011501	1.9393	0.42361	NA	NA	NA	NA	NA
.2134_289.2	1.82478	0.88801	3.1242	0.010797	1.9667	0.42361	594.1812	HMDB06865 ; HMDB12310 ; HMDB14642 ; HMDB29813 ; HMDB30439 ; HMDB30870 ; HMDB30872 ; HMDB31552 ; HMDB33770 ; HMDB33985 ; HMDB34836 ; HMDB34867 ; HMDB35003 ; HMDB35499 ; HMDB37419 ; HMDB38114 ; HMDB38236 ; HMDB38477 ; HMDB40126 ; HMDB40506 ; HMDB41199 ; HMDB41459 ; HMDB60943	3-Methyl-1-hydroxybutyl-ThPP ; 2-Methyl-1-hydroxybutyl-ThPP ; Flutamide ; 5-Hydroxycastavinol ; Fragransol D ; 1-Hydroxy-3-methoxy-7-primeverosyloxyxanthone ; Gentioside ; Dihydrocurcumin ; 7-Hydroxy-3-methoxy-1-primeverosyloxyxanthone ; Mulberrofuran S ; Lansiumarin B ; Xanthohumol B ; Xanthohumol D ; Isoxanthohumol B ; Maysin ; 4-O-Methylkievitone ; ()-Fargesin ; Cassiaside B ; Mulberroside F ; 356-Trihydroxy-3478-tetramethoxyflavone 3-glucoside ; Vitexin 6-(3-hydroxy-3-methylglutarate) ; Chamaemeloside ; Sulfinpyrazone sulfide	M+2ACN+H ; M+2ACN+H ; 2M+ACN+H ; 2M+3H2O+2H ; 3M+2K ; M+ACN+H ; M+ACN+H ; 3M+2K ; M+ACN+H ; M+NH4 ; 3M+2K ; 3M+2K ; 3M+2K ; 3M+2K ; M+NH4 ; 3M+2K ; 3M+2K ; 2M+3H2O+2H ; 2M+3H2O+2H ; M+ACN+H ; M+NH4 ; M+NH4 ; 3M+H+Na	C17H29N4O8P2S ; C17H29N4O8P2S ; C11H11F3N2O3 ; C26H30O14 ; C21H22O6 ; C25H28O14 ; C25H28O14 ; C21H22O6 ; C25H28O14 ; C34H24O9 ; C21H22O6 ; C21H22O6 ; C21H22O6 ; C21H22O6 ; C27H28O14 ; C21H22O6 ; C21H22O6 ; C26H30O14 ; C26H30O14 ; C25H28O14 ; C27H28O14 ; C27H28O14 ; C23H20N2O2S
3523_58.99	1.824257	0.867008	3.0787	0.011666	1.9331	0.42361	91.05835	HMDB12234 ; HMDB31641 ; HMDB60768	Histidinal ; Pyrrolidine ; 4-Hydroxymethylpyrazole	M+ACN+2H ; 2M+H+K ; M+2ACN+2H	C6H9N3O ; C4H9N ; C4H6N2O

[illegible]0876_290.[illegible]	1.82348	0.802019	3.0924	0.011397	1.9432	0.42361	578.3031	HMDB15254 ; HMDB34362 ; HMDB34927 ; HMDB35381 ; HMDB35627 ; HMDB35709 ; HMDB36308 ; HMDB36403 ; HMDB39125 ; HMDB39641 ; HMDB39649 ; HMDB40668 ; HMDB40701 ; HMDB60709	Ambenonium ; Desglucocheirotoxin ; Cucurbitacin B ; Ganoderenic acid A ; Ganoderic acid C1 ; Ganoderenic acid B ; Ganoderic acid xi ; Ganosporelactone B ; Coumaperine ; Ganoderic acid J ; Ganoderic acid V1 ; Blumenol C glucoside ; 9-Hydroxy-7-megastigmen-3-one glucoside ; 17-Desacetyl-rocuronium	M+ACN+H ; 2M+3H2O+2H ; 2M+H+K ; M+ACN+Na ; M+ACN+Na ; M+ACN+Na ; M+ACN+Na ; M+ACN+Na ; 2M+ACN+Na ; M+ACN+Na ; M+ACN+Na ; 3M+H+K ; 3M+H+K ; 2M+H+Na	C28H42Cl2N4O2 ; C29H42O10 ; C32H46O8 ; C30H42O7 ; C30H42O7 ; C30H42O7 ; C30H42O7 ; C30H42O7 ; C16H19NO2 ; C30H42O7 ; C30H42O7 ; C19H32O7 ; C19H32O7 ; C30H51BrN2O3
[illegible]2372_39.12	1.82141	0.646338	3.037	0.012527	1.9021	0.42361	443.0824	HMDB01022 ; HMDB01269 ; HMDB01372 ; HMDB02135 ; HMDB02255 ; HMDB02310 ; HMDB14802 ; HMDB15269 ; HMDB32571 ; HMDB32922 ; HMDB33130 ; HMDB33159 ; HMDB33420 ; HMDB35038 ; HMDB35052 ; HMDB36890 ; HMDB36898 ; HMDB39421 ; HMDB39623 ; HMDB40324 ; HMDB40651 ; HMDB40794 ; HMDB41281 ; HMDB41502 ; HMDB60494 ; HMDB60868	Succinyl-CoA ; Methylmalonyl-CoA ; Thiamine pyrophosphate ; S-(3-oxo-3-carboxy-n-propyl)cysteine ; R-Methylmalonyl-CoA ; S-Methylmalonyl-CoA ; Sulfametopyrazine ; Sulfinpyrazone ; 2-Methoxybenzenethiol ; Kahweofuran ; 2-Acetyl-5-methylthiophene ; 2-Propanoylthiophene ; Gibberellin A55 ; Gibberellin A50 ; Gibberellin A49 ; Gibberellin A72 ; Gibberellin A76 ; Fenugreekine ; Gibberellin A79 ; (7R8R)-3-Methoxy-34799-pentahydroxy-84-oxyneolignan ; Gibberellin A91 ; 5-Hydroxy-4-methoxy-6-canthinone 3-N-oxide ; 2-(Methylthio)phenol ; 2-Acetyl-3-methylthiophene ; N-Acetylmuramoyl-Ala ; Phenytoin quinone	M+H+NH4 ; M+H+NH4 ; M+NH4 ; 2M+H ; M+H+NH4 ; M+H+NH4 ; 3M+2Na ; M+K ; 3M+Na ; 3M+Na ; 3M+Na ; 3M+Na ; M+2K+H ; M+2K+H ; M+2K+H ; M+2K+H ; M+2K+H ; 2M+3H ; M+2K+H ; M+2K+H ; M+2K+H ; 3M+H+K ; 3M+Na ; 3M+Na ; M+2K+H ; 3M+H+K	C25H40N7O19P3S ; C25H40N7O19P3S ; C12H19N4O7P2S ; C7H11NO5S ; C25H40N7O19P3S ; C25H40N7O19P3S ; C11H12N4O3S ; C23H20N2O3S ; C7H8OS ; C7H8OS ; C7H8OS ; C7H8OS ; C19H24O7 ; C19H24O7 ; C19H24O7 ; C19H24O7 ; C19H24O7 ; C21H27N7O14P2 ; C19H24O7 ; C19H24O7 ; C19H24O7 ; C15H10N2O4 ; C7H8OS ; C7H8OS ; C14H24N2O9 ; C15H10N2O4
[illegible]8743_288.[illegible]	1.820341	0.796483	2.877	0.016471	1.7833	0.42361	593.1859	HMDB30238 ; HMDB35444 ; HMDB37415 ; HMDB37417 ; HMDB37540 ; HMDB00961 ; HMDB31388 ; HMDB34812 ; HMDB36434 ; HMDB36435 ; HMDB37036 ; HMDB59922 ; HMDB59923 ; HMDB60759 ; HMDB03265 ; HMDB30748 ; HMDB36333	ax-4-Hydroxy-3--methoxymaysin ; Liquiritigenin 4-3-acetylapiosyl-(1-2)-glucoside ; Isomargaritene ; Isowertin 2-rhamnoside ; Margaritene ; Farnesyl pyrophosphate ; 23-Hydroxyphysalolactone ; 57-Dihydroxy-34-dimethoxy-8-(3-hydroxy-3-methylbutyl)-isoflavone 7-glucoside ; Lucidenic acid E2 ; Lucidenic acid D2 ; Glycinoeclepin C ; Cavipetin E isomer 1 ; Cavipetin E isomer 2 ; 4-Carboxynevirapine ; Hesperidin ; Hesperetin 7-neohesperidoside ; 4-Hydroxyacetophenone 4-4-hydroxy-35-dimethoxybenzoyl-(-5)-apiosyl-(1-2)-glucoside	M+H ; M+H ; M+H ; M+H ; M+H ; 3M+H+K ; M+K ; 2M+Na+K ; M+2K-H ; M+2K+H ; M+2K+H ; M+2K-H ; M+2K-H ; 2M+H ; M+H-H2O ; M+H-H2O ; M+H-H2O	C28H32O14 ; C28H32O14 ; C28H32O14 ; C28H32O14 ; C28H32O14 ; C15H28O7P2 ; C28H39ClO9 ; C28H34O12 ; C29H40O8 ; C29H38O8 ; C29H38O8 ; C29H40O8 ; C29H40O8 ; C15H12N4O3 ; C28H34O15 ; C28H34O15 ; C28H34O15
[illegible]4416_198.[illegible]	1.81831	0.575616	2.8327	0.017771	1.7503	0.42361	328.9944	HMDB01049	Gamma-Glutamylcysteine	M+2K+H	C8H14N2O5S
[illegible]3105_93.2	1.818113	0.565785	-2.9887	0.013604	1.8663	0.42361	NA	NA	NA	NA	NA
[illegible]6007_258.[illegible]	1.817176	0.728126	2.8596	0.016968	1.7704	0.42361	NA	NA	NA	NA	NA

8032_240.4	1.814297	1.148384	3.0279	0.012722	1.8954	0.42361	124.0158	HMDB02039 ; HMDB12210 ; HMDB12467 ; HMDB14436 ; HMDB15333 ; HMDB31190 ; HMDB31192 ; HMDB31857 ; HMDB31982 ; HMDB33926 ; HMDB40170 ; HMDB40973 ; HMDB41015 ; HMDB60427 ; HMDB60457 ; HMDB60515 ; HMDB60589	2-Pyrrolidinone ; Dihydrolipoate ; (-)-Epicatechin sulfate ; Chlorambucil ; Levetiracetam ; S-Ethyl thioacetate ; S-Methyl propanethioate ; 3-(Methylthio)propanal ; 3-Mercapto-2-butanone ; (3xi6xi)-Cyclo(alanylvalyl) ; (Methylthio)acetone ; 1-Methoxy-1H-indole-3-acetonitrile ; 4-Mercapto-2-butanone ; Acetone cyanohydrin ; cis-3-Chloroacrylic acid ; trans-3-Chloroacrylic acid ; Aminofurantoin	M+K ; M+H+K ; M+3H ; M+3Na ; M+2K ; 2M+H+K ; 2M+H+K ; 2M+H+K ; 2M+H+K ; M+2K ; 2M+H+K ; M+Na+K ; 2M+H+K ; M+K ; M+NH4 ; M+NH4 ; M+H+K	C4H7NO ; C8H16O2S2 ; C15H13O9S ; C14H19Cl2NO2 ; C8H14N2O2 ; C4H8OS ; C4H8OS ; C4H8OS ; C4H8OS ; C8H14N2O2 ; C4H8OS ; C11H10N2O ; C4H8OS ; C4H7NO ; C3H3ClO2 ; C3H3ClO2 ; C8H8N4O3
5221_21.65	1.811612	0.573952	-2.7017	0.022247	1.6527	0.42361	407.3152	HMDB11554 ; HMDB11584 ; HMDB07895 ; HMDB08917 ; HMDB11203 ; HMDB11370 ; HMDB11543 ; HMDB11573 ; HMDB13402 ; HMDB13403 ; HMDB31680 ; HMDB36344 ; HMDB59931	MG(0:022:4(7Z10Z13Z16Z)0:0) ; MG(22:4(7Z10Z13Z16Z)0:00:0) ; PC(14:0P-16:0) ; PE(15:0P-18:0) ; PC(P-16:014:0) ; PE(P-18:015:0) ; MG(0:020:1(11Z)0:0) ; MG(20:1(11Z)0:00:0) ; PC(o-14:016:1(9Z)) ; PC(o-16:014:1(9Z)) ; 1-O-beta-D-Glucopyranosyl-23-di-O-palmitoylglycerol ; Tetrahydropersin ; 23-Diacetoxypropyl stearate	M+H ; M+H ; M+3ACN+2H ; M+3ACN+2H ; M+3ACN+2H ; M+3ACN+2H ; M+Na ; M+Na ; M+3ACN+2H ; M+3ACN+2H ; M+2ACN+2H ; M+Na ; M+H-2H2O	C25H42O4 ; C25H42O4 ; C38H76NO7P ; C38H76NO7P ; C38H76NO7P ; C38H76NO7P ; C23H44O4 ; C23H44O4 ; C38H76NO7P ; C38H76NO7P ; C41H78O10 ; C23H44O4 ; C25H46O6
9164_20.82	1.81097	0.698472	-2.7789	0.019486	1.7103	0.42361	NA	NA	NA	NA	NA
9602_99.44	1.810289	0.70919	-3.108	0.011099	1.9547	0.42361	140.002	HMDB14952 ; HMDB29571 ; HMDB34007 ; HMDB38463 ; HMDB42002	Meloxicam ; 12-Dichloroethane ; (EE)-1-Chloro-311-tridecadiene-579-triyn-2-ol ; 3-(Isothiocyanatomethyl)-1-methoxy-1H-indole ; Pyrene	M+3Na ; M+ACN+H ; M+Na+K ; M+Na+K ; M+2K	C14H13N3O4S2 ; C2H4Cl2 ; C13H11ClO ; C11H10N2OS ; C16H10
2206_292.2	1.809258	0.770274	2.9533	0.014453	1.84	0.42361	650.7492	HMDB30401	AK toxin I	3M+Na+K	C23H27NO6
4473_53.54	1.806122	1.168618	2.9484	0.014575	1.8364	0.42361	359.8274	HMDB06729 ; HMDB06734 ; HMDB11771 ; HMDB32260 ; HMDB34333	CE(22:4(7Z10Z13Z16Z) ; CE(20:2(6Z9Z)) ; Cer(d18:026:0) ; Ethylene glycol distearate ; Physalolactone	M+H+NH4 ; M+ACN+2H ; M+H+K ; M+3ACN+2H ; 2M+3H	C49H80O2 ; C47H80O2 ; C44H89NO3 ; C38H74O4 ; C28H39ClO8
90275_23.7	1.805725	0.765864	-2.7456	0.020632	1.6855	0.42361	138.959	HMDB31808 ; HMDB60455	Tecnazene ; cis-2-Chloro-4-carboxymethylenebut-2-en-14-olide	M+H+NH4 ; M+H-2H2O	C6HCl4NO2 ; C6H3ClO4
0664_87.99	1.804866	0.62086	-2.7678	0.019861	1.702	0.42361	149.5151	HMDB13922 ; HMDB14798 ; HMDB15150 ; HMDB31770 ; HMDB33701 ; HMDB60006 ; HMDB60649	2-Chloroticlopidine ; Metaxalone ; Sulfamethoxazole ; Carbofuran ; Hydrocotarnine ; N-(2-formyl-3-chlorophenyl)anthranilic acid ; Ascorbic acid-2-sulfate	M+2H ; M+2K ; M+2Na ; M+2K ; M+2K ; M+H+Na ; M+ACN+2H	C14H13Cl2NS ; C12H15NO3 ; C10H11N3O3S ; C12H15NO3 ; C12H15NO3 ; C14H10ClNO3 ; C6H8O9S

.5304_256.9	1.80454	1.011177	-2.8363	0.017662	1.753	0.42361	195.1015	HMDB30711 ; HMDB30712 ; HMDB30714 ; HMDB30715 ; HMDB30825 ; HMDB33539 ; HMDB33540 ; HMDB33688 ; HMDB36587 ; HMDB39029 ; HMDB00235 ; HMDB03116 ; HMDB06059 ; HMDB12549 ; HMDB12551 ; HMDB30308 ; HMDB30348 ; HMDB36863 ; HMDB37062 ; HMDB37594 ; HMDB39105 ; HMDB40597 ; HMDB40694 ; HMDB41518 ; HMDB41543 ; HMDB59744	Cyclomammein ; Furomammea G ; Cycloneomammein ; MAB 4 ; 2-(4-Allyl-26-dimethoxyphenoxy)-1-(34-dimethoxyphenyl)-1-propanol ; Fragransin D1 ; Fragransin D3 ; Pyranomammea C ; Quassin ; Pyranomammea B ; Thiamine ; Stanozolol ; 20-Carboxy-leukotriene B4 ; 12-oxo-1011-dihydro-20-COOH-LTB4 ; 12-oxo-20-dihydroxy-leukotriene B4 ; O-Isopentenylhalfordinol ; Anonaine ; Anhydrocinnzeylanol ; Sporotrichiol ; Eduleine ; 3-Oxo-1415-dehydrorhazinilam ; Eduline ; Agaritinal ; Nb-p-Coumaroyltryptamine ; 25-Dibenzyl-3-hydroxy-6-methoxypyrazine ; 34-Methyleneazelaic acid	M+2H ; M+2H ; M+2H ; M+2H ; M+2H ; M+2H ; M+2H ; M+2H ; M+2H ; M+2H ; M+3ACN+2H ; M+Na+K ; M+H+Na ; M+H+Na ; M+H+Na ; M+2ACN+2H ; M+3ACN+2H ; M+H+Na ; M+H+Na ; M+3ACN+2H ; M+2ACN+2H ; M+3ACN+2H ; M+3ACN+2H ; M+2ACN+2H ; M+2ACN+2H ; M+H-H2O	C22H28O6 ; C22H28O6 ; C22H28O6 ; C22H28O6 ; C22H28O6 ; C22H28O6 ; C22H28O6 ; C22H28O6 ; C22H28O6 ; C22H28O6 ; C12H17N4OS ; C21H32N2O ; C20H30O6 ; C20H30O6 ; C20H30O6 ; C19H18N2O2 ; C17H15NO2 ; C20H30O6 ; C20H30O6 ; C17H15NO2 ; C19H18N2O2 ; C17H15NO2 ; C12H15N3O4 ; C19H18N2O2 ; C19H18N2O2 ; C11H16O4
09703_53.74	1.804482	0.735105	-3.173	0.009937	2.0027	0.42361	457.971	HMDB41797 ; HMDB60415 ; HMDB60789	24-Dichlorophenoxyacetic acid ; 6-Thioguanosine monophosphate ; 6-Thioguanylic acid	2M+NH4 ; M+2K+H ; M+2K+H	C8H6Cl2O3 ; C10H14N5O7PS ; C10H14N5O7PS
4251_239.5	1.804313	0.72919	2.7947	0.018967	1.722	0.42361	485.0143	HMDB33069	7-Chloro-34568-pentamethoxyflavone	M+2K+H	C20H19ClO7
9337_58.43	1.804004	0.799416	2.8185	0.018209	1.7397	0.42361	327.9893	HMDB01090 ; HMDB01981 ; HMDB11725 ; HMDB15028 ; HMDB15420 ; HMDB28774 ; HMDB28816 ; HMDB35956 ; HMDB59968 ; HMDB59982	(S)-5-Diphosphomevalonic acid ; (R)-Mevalonic acid-5-pyrophosphate ; 5-Sulfosalicylic acid ; Sulfapyridine ; Quinethazone ; Cysteinyl-Glutamate ; Glutamyl-Cysteine ; Lumequoylacetone ; 3-hydroxybenzoic acid-3-O-sulphate ; 4-hydroxybenzoic acid-4-O-sulphate	2M+H+K ; 2M+H+K ; 3M+2H ; M+2K+H ; M+K ; M+2K+H ; M+2K+H ; 2M+3H ; 3M+2H ; 3M+2H	C6H14O10P2 ; C6H14O10P2 ; C7H6O6S ; C11H11N3O2S ; C10H12ClN3O3S ; C8H13N2O5S ; C8H13N2O5S ; C33H62O2 ; C7H6O6S ; C7H6O6S
8671_44.79	1.803927	1.081796	2.9013	0.015799	1.8014	0.42361	470.5789	HMDB11660 ; HMDB12969 ; HMDB41160	2-Methylhexanoyl-CoA ; Heptanoyl-CoA ; Cyanidin 3-O-b-D-Xylopyranosyl-(1-2)-(4-hydroxybenzoyl)-(-6)-b-D-glucopyranosyl-(1-6)-b-D-galactopyranoside	M+Na+K ; M+Na+K ; M+2K	C28H48N7O17P3S ; C28H48N7O17P3S ; C39H43O22
.4951_20.18	1.801102	0.919003	-2.7719	0.019722	1.705	0.42361	114.967	HMDB00263 ; HMDB60445 ; HMDB60447	Phosphoenolpyruvic acid ; Bromobenzene-23-dihydrodiol ; Bromobenzene-34-dihydrodiol	M+Na+K ; M+H+K ; M+H+K	C3H5O6P ; C6H7BrO2 ; C6H7BrO2
2219_277.4	1.801005	0.262809	2.9227	0.015229	1.8173	0.42361	NA	NA	NA	NA	NA
08634_87.69	1.796775	0.518821	-2.7323	0.021109	1.6755	0.42361	NA	NA	NA	NA	NA

3555_257.4	1.796022	0.328603	-2.7617	0.020072	1.6974	0.42361	227.1024	HMDB00245 ; HMDB00473 ; HMDB00735 ; HMDB01216 ; HMDB01301 ; HMDB01525 ; HMDB02203 ; HMDB06875 ; HMDB10329 ; HMDB10725 ; HMDB11727 ; HMDB29052 ; HMDB29136 ; HMDB30006 ; HMDB30424 ; HMDB30770 ; HMDB30904 ; HMDB31553 ; HMDB31661 ; HMDB31850 ; HMDB32021 ; HMDB32050 ; HMDB32463 ; HMDB32564 ; HMDB32862 ; HMDB33201 ; HMDB33379 ; HMDB33574 ; HMDB33640 ; HMDB33891 ; HMDB34433 ; HMDB34708 ; HMDB34730 ; HMDB34783 ; HMDB35156 ; HMDB35359 ; HMDB36191 ; HMDB36215 ; HMDB36224 ; HMDB36887 ; HMDB37388 ; HMDB37628 ; HMDB37702 ; HMDB37703 ; HMDB37721 ; HMDB37829 ; HMDB38005 ; HMDB38068 ; HMDB38079 ; HMDB38162 ; HMDB38163 ; HMDB38186 ; HMDB38245 ; HMDB38279 ; [illegible]	Porphobilinogen ; 6-Dimethylaminopurine ; Hydroxyphenylacetylglycine ; Tetrahydropteridine ; 1-Pyrroline-5-carboxylic acid ; Imidazole ; 3-Hydroxycapric acid ; 1-Pyrroline-2-carboxylic acid ; Dopamine glucuronide ; (R)-3-Hydroxydecanoic acid ; Bicine ; Serinyl-Valine ; Valyl-Serine ; 2277-Tetramethyl-16-dioxaspiro44non-3-ene ; Linalyl formate ; 4-Hydroxy-3-(3-methyl-2-butenyl)acetophenone ; alphaalpha-Dimethylanisalacetone ; 6-(1-Hydroxyethyl)-22-dimethyl-2H-1-benzopyran ; 3-Isovalidene-3alpha4-dihydrophthalide ; 2-Ethoxy-3-methylpyrazine ; cis-Methylbixin ; alpha-Terpineol formate ; 5-Pentyl-3h-furan-2-one ; 13-Diphenylpropane ; 2-Amino-4-ethoxy-3-hydroxybutanoic acid ; 9-Hydroxydecanoic acid ; cis-3-Hexenyl benzoate ; (1R2R4R8R)-p-Menthane-289-triol ; 7-Methyl-3-methylene-167-octanetriol ; N6-Acetyl-5S-hydroxy-L-lysine ; Riboflavine 2345-tetrabutanoate ; Methyl 48-decadienoate ; Methyl (2E4Z)-decadienoate ; cis-p-Menthane-178-triol ; Neryl formate ; 1-Deoxynojirimycin ; 4-Hydroxy-4-methyl-7-decenoic acid gamma-lactone ; Ethyl octynecarboxylate ; Benzyl 23-dimethyl-2-butenoate ; 10-Apo-beta-caroten-10-al ; Tsibulin 2 ; Phenethyl tiglate ; Butyl cinnamate ; Isobutyl cinnamate ; 2-Phenylethyl 3-methyl-2-butenoate ; 6-(3-Hexenyl)tetrahydro-2H-pyran-2-one ; xi-2-Hexyl-5-methyl-3(2H)-furanone ; (3E6Z)-Nonadien-1-yl acetate ; (2E6Z)-26-Nonadien-1-Yl acetate ; Lactarazulene ; Lactarofulvene ; 26-Dimethyl-7-octene-236-triol ; Bornyl formate ; cis-3-Hexenyl tiglate ; Hv-NCC-1 ; trans-Methylbixin ; (1S2S4R8R)-p-Menthane-129-triol ; 2-Hexyl-13-dioxan-5-ol ; 2-Hexyl-13-dioxolane-4-methanol ; (1R2R4S)-p-Menthane-128-triol ; 34-Dihydroxy-2-hydroxymethyl-1-pyrrolidinepropanamide ; 3-Ethyl-2-methoxypyrazine ; 2-Ethoxy-5-methylpyrazine ; 2-Ethoxy-6-methylpyrazine ; xi-5-Hydroxydecanoic acid ; Allyl cyclohexylacetate ; 2-Methoxy-35-dimethylpyrimidine ; Heterophylol ; Cinnamyl butyrate ; Cinnamyl isobutyrate ; Ethyl (-)-3-hydroxyoctanoate ; 10 [illegible]	M+H ; M+ACN+Na ; M+NH4 ; 3M+2Na ; 2M+H ; 3M+Na ; M+K ; 2M+H ; M+3ACN+2H ; M+K ; M+ACN+Na ; M+Na ; M+Na ; M+2Na-H ; M+2Na-H ; M+Na ; M+Na ; M+Na ; M+Na ; 3M+H+K ; M+2Na ; M+2Na-H ; M+2Na-H ; 2M+Na+K ; M+ACN+Na ; M+K ; M+Na ; M+K ; M+K ; M+Na ; M+2H+Na ; M+2Na-H ; M+2Na-H ; M+K ; M+2Na-H ; M+ACN+Na ; M+2Na-H ; M+2Na-H ; M+Na ; M+2K ; M+2Na-H ; M+Na ; M+Na ; M+Na ; M+Na ; M+2Na-H ; M+2Na-H ; M+2Na-H ; M+2Na-H ; 2M+Na+K ; 2M+Na+K ; M+K ; M+2Na-H ; M+2Na-H ; M+3H ; M+2Na ; M+K ; M+K ; M+K ; M+K ; M+Na ; 3M+H+K ; 3M+H+K ; 3M+H+K ; M+K ; M+2Na-H ; 3M+H+K ; M+2Na ; M+Na ; M+Na ; M+K ; M+2K ; M+2Na-H ; M+NH4 ; M+Na+K ; M+H-H2O ; M+H-H2O ; M+H-H2O	C10H14N2O4 ; C7H9N5 ; C10H11NO4 ; C6H8N4 ; C5H7NO2 ; C3H4N2 ; C10H20O3 ; C5H7NO2 ; C14H19NO8 ; C10H20O3 ; C6H13NO4 ; C8H16N2O4 ; C8H16N2O4 ; C11H18O2 ; C11H18O2 ; C13H16O2 ; C13H16O2 ; C13H16O2 ; C13H16O2 ; C7H10N2O ; C26H32O4 ; C11H18O2 ; C11H18O2 ; C15H16 ; C6H13NO4 ; C10H20O3 ; C13H16O2 ; C10H20O3 ; C10H20O3 ; C8H16N2O4 ; C33H44N4O10 ; C11H18O2 ; C11H18O2 ; C10H20O3 ; C11H18O2 ; C6H13NO4 ; C11H18O2 ; C11H18O2 ; C13H16O2 ; C27H36O ; C11H18O2 ; C13H16O2 ; C13H16O2 ; C13H16O2 ; C13H16O2 ; C11H18O2 ; C11H18O2 ; C11H18O2 ; C11H18O2 ; C15H16 ; C15H16 ; C10H20O3 ; C11H18O2 ; C11H18O2 ; C35H42N4O10 ; C26H32O4 ; C10H20O3 ; C10H20O3 ; C10H20O3 ; C10H20O3 ; C8H16N2O4 ; C7H10N2O ; C7H10N2O ; C7H10N2O ; C10H20O3 ; C11H18O2 ; C7H10N2O ; C26H32O4 ; C13H16O2 ; C13H16O2 ; C10H20O3 ; C27H36O ; C11H18O2 ; C10H11NO4 ; C23H36O5 ; C10H16N2O5 ; C10H16N2O5 ; C10H16N2O5
9284_251.9	1.794443	1.070758	-2.8547	0.01711	1.7667	0.42361	217.1949	HMDB36578 ; HMDB00256 ; HMDB02284 ; HMDB07065 ; HMDB07117 ; HMDB07145 ; HMDB07167 ; HMDB07195 ; HMDB07224 ; HMDB07252 ; HMDB07364 ; HMDB07391 ; HMDB07392 ; HMDB07419 ; HMDB07621 ; HMDB07649 ; HMDB07821 ; HMDB12135 ; HMDB34731 ; HMDB56027 ; HMDB56141 ; HMDB56161 ; HMDB56179 ; HMDB56198	Cyperotundone ; Squalene ; N-Acetylcadaverine ; DG(14:1(9Z)24:1(15Z)0:0) ; DG(16:022:2(13Z16Z)0:0) ; DG(16:1(9Z)22:1(13Z)0:0) ; DG(18:020:2(11Z14Z)0:0) ; DG(18:1(11Z)20:1(11Z)0:0) ; DG(18:1(9Z)20:1(11Z)0:0) ; DG(18:2(9Z12Z)20:00:0) ; DG(20:018:2(9Z12Z)0:0) ; DG(20:1(11Z)18:1(11Z)0:0) ; DG(20:1(11Z)18:1(9Z)0:0) ; DG(20:2(11Z14Z)18:00:0) ; DG(22:1(13Z)16:1(9Z)0:0) ; DG(22:2(13Z16Z)16:00:0) ; DG(24:1(15Z)14:1(9Z)0:0) ; 1-(3-Aminopropyl)-4-aminobutanal ; Tomatidine ; DG(16:00:022:2n6) ; DG(14:1n50:024:1n9) ; DG(16:1n70:022:1n9) ; DG(18:1n70:020:1n9) ; DG(18:1n90:020:1n9)	M+H ; M+H+Na ; 3M+2H ; M+3H ; M+3H ; M+3H ; M+3H ; M+3H ; M+3H ; M+3H ; M+3H ; M+3H ; M+3H ; M+3H ; M+3H ; M+3H ; M+3H ; 3M+2H ; M+H+NH4 ; M+3H ; M+3H ; M+3H ; M+3H ; M+3H	C16H24 ; C30H50 ; C7H16N2O ; C41H76O5 ; C41H76O5 ; C41H76O5 ; C41H76O5 ; C41H76O5 ; C41H76O5 ; C41H76O5 ; C41H76O5 ; C41H76O5 ; C41H76O5 ; C41H76O5 ; C41H76O5 ; C41H76O5 ; C41H76O5 ; C7H16N2O ; C27H45NO2 ; C41H76O5 ; C41H76O5 ; C41H76O5 ; C41H76O5 ; C41H76O5
1278_190.8	1.794101	0.928188	2.8873	0.016181	1.791	0.42361	370.0211	HMDB06273 ; HMDB12268 ; HMDB32902 ; HMDB38956 ; HMDB01245	5-amino-1-(5-phospho-D-ribosyl)imidazole-4-carboxylate ; N5-Carboxyaminoimidazole ribonucleotide ; Sodium 6-hydroxy-5-(phenylazo)-2-naphthalenesulfonate ; Avenalumin III ; dCDP	2M+Na+K ; 2M+Na+K ; 2M+H+K ; M+2K+H ; M+H-H2O	C9H14N3O9P ; C9H14N3O9P ; C16H11N2NaO4S ; C18H13NO3 ; C9H15N3O10P2

6203_21.49	1.794079	0.683513	-2.706	0.022085	1.6559	0.42361	287.2366	HMDB00305 ; HMDB06216 ; HMDB06217 ; HMDB06221 ; HMDB30403 ; HMDB34735 ; HMDB36728 ; HMDB36841 ; HMDB38709 ; HMDB03071 ; HMDB15430 ; HMDB36916 ; HMDB37543 ; HMDB41023 ; HMDB01043 ; HMDB02177 ; HMDB05045 ; HMDB06036 ; HMDB14996 ; HMDB36702 ; HMDB36721 ; HMDB36754 ; HMDB36802 ; HMDB36829 ; HMDB36830 ; HMDB37824 ; HMDB39687 ; HMDB39702 ; HMDB60052	Vitamin A ; 11-cis-Retinol ; 9-cis-Retinol ; 13-cis Retinol ; (x)-p-Menth-1-en-4-yl 5-isopropyl-2-methylphenyl ether ; Abietinal ; ent-16-Kauren-19-al ; ent-19-Trachylobanal ; 813-Abietadien-18-al ; 56-Dihydro-56-dihydroxy-yy-carotene ; Pancuronium ; 78-Dihydroparasiloxanthin ; ()-(Z)-2-(5-Tetradecenyl)cyclobutanone ; Reticulatamol ; Arachidonic acid ; Cis-8111417-Eicosatetraenoic acid ; 15(S)-Hydroxyeicosatrienoic acid ; Mesterolone ; Drostanolone ; Sideridiol ; ent-17-Hydroxy-16beta-kauran-19-al ; Yucalexin P21 ; Austroinulin ; Copalic acid ; Junicedral ; 713-Eperudien-15-oic acid ; Oryzalexin S ; Oryzalexin E ; 8-HETrE	M+H ; M+H ; M+H ; M+H ; M+H ; M+H ; M+H ; M+H ; M+H ; M+2H ; M+2H ; M+2H ; M+Na ; M+H+K ; M+H-H2O ; M+H-H2O ; M+H-2H2O ; M+H-H2O ; M+H-H2O ; M+H-H2O ; M+H-H2O ; M+H-H2O ; M+H-2H2O ; M+H-H2O ; M+H-H2O ; M+H-H2O ; M+H-H2O ; M+H-H2O ; M+H-2H2O	C20H30O ; C20H30O ; C20H30O ; C20H30O ; C20H30O ; C20H30O ; C20H30O ; C20H30O ; C20H30O ; C40H60O2 ; C35H60N2O4 ; C40H60O2 ; C18H32O ; C35H66O3 ; C20H32O2 ; C20H32O2 ; C20H34O3 ; C20H32O2 ; C20H32O2 ; C20H32O2 ; C20H32O2 ; C20H32O2 ; C20H34O3 ; C20H32O2 ; C20H32O2 ; C20H32O2 ; C20H32O2 ; C20H32O2 ; C20H34O3
2102_85.91	1.79311	0.512472	-2.7159	0.021712	1.6633	0.42361	139.5122	HMDB31853 ; HMDB32939 ; HMDB39787 ; HMDB39788	2-Thiophenemethanethiol ; 3-(Methylthio)thiophene ; 2-Methyl-3-thiophenethiol ; 5-Methyl-3-thiophenethiol	2M+H+NH4 ; 2M+H+NH4 ; 2M+H+NH4 ; 2M+H+NH4	C5H6S2 ; C5H6S2 ; C5H6S2 ; C5H6S2
4703_288.0	1.792506	0.83118	2.798	0.018858	1.7245	0.42361	NA	NA	NA	NA	NA
8219_286.5	1.791675	0.905847	2.9783	0.013848	1.8586	0.42361	615.5248	HMDB01419 ; HMDB01496 ; HMDB02103 ; HMDB02902 ; HMDB04035 ; HMDB05943 ; HMDB06119 ; HMDB06247 ; HMDB06283 ; HMDB06892 ; HMDB11643 ; HMDB13643 ; HMDB29320 ; HMDB34254 ; HMDB34872 ; HMDB35681 ; HMDB38959 ; HMDB39868 ; HMDB39869 ; HMDB39870 ; HMDB40180 ; HMDB59592 ; HMDB12084	24-Hydroxycholesterol ; 7a-Hydroxycholesterol ; 27-Hydroxycholesterol ; Delta-Tocopherol ; 22b-Hydroxycholesterol ; 5b-Pregnanediol ; 7b-Hydroxycholesterol ; 25-Hydroxycholesterol ; 20alpha-Hydroxycholesterol ; 7a-Hydroxy-5b-cholestan-3-one ; (24R)-Cholest-5-ene-3-beta24-diol ; 4b-Hydroxycholesterol ; (3beta5alpha6alpha)-Cholest-8-ene-36-diol ; eta-Tocopherol ; Cheritamine ; 3-Hydroxy-1-phenyl-1-heneicosanone ; 2-Methyl-6-phytylhydroquinone ; 5-(12-Heneicosenyl)-13-benzenediol ; 5-(14-Heneicosenyl)-13-benzenediol ; 5-(16-Heneicosenyl)-13-benzenediol ; Pentadecylbenzene ; Cholest-5-ene-3-beta7-alpha-diol ; SM(d18:012:0)	3M+H+Na ; 3M+H+Na ; 3M+H+Na ; 3M+H+Na ; 3M+H+Na ; 2M+K ; 3M+H+Na ; 3M+H+Na ; 3M+H+Na ; 3M+H+Na ; 3M+H+Na ; 3M+H+Na ; 3M+H+Na ; 3M+H+Na ; M+Na ; 3M+H+Na ; 3M+H+Na ; 3M+H+Na ; 3M+H+Na ; 3M+H+Na ; 2M+K ; 3M+H+Na ; M+H-2H2O	C27H46O2 ; C27H46O2 ; C27H46O2 ; C27H46O2 ; C27H46O2 ; C21H36 ; C27H46O2 ; C27H46O2 ; C27H46O2 ; C27H46O2 ; C27H46O2 ; C27H46O2 ; C27H46O2 ; C27H46O2 ; C40H68N2O ; C27H46O2 ; C27H46O2 ; C27H46O2 ; C27H46O2 ; C27H46O2 ; C21H36 ; C27H46O2 ; C35H75N2O6P
4643_68.5	1.791338	0.306622	3.133	0.010635	1.9733	0.42361	272.6765	HMDB07004	CPA(18:00:0)	M+3ACN+2H	C21H41O6P
8685_259.7	1.786571	1.130408	-2.8538	0.017137	1.7661	0.42361	195.9587	HMDB02287	Homocysteine thiolactone	M+2K+H	C4H7NOS

58963_290.	1.786414	0.644457	3.0598	0.012049	1.919	0.42361	651.1259	HMDB00656 ; HMDB29914 ; HMDB30558 ; HMDB35464 ; HMDB40125 ; HMDB40559 ; HMDB40677 ; HMDB41235 ; HMDB42010	Cysteineglutathione disulfide ; Tetraphyllin B ; Safflomin A ; 6-Acetylapiin ; Carthamidin 67-diglucoside ; Aromadendrin 37-diglucoside ; Hydroxysafflor yellow A ; 6-O-Acetylvicenin 1 ; Rutaecarpine	3M+H+Na ; 2M+2K-H ; M+K ; M+2Na-H ; M+K ; M+K ; M+K ; M+2Na-H ; 2M+2K-H	C13H22N4O8S2 ; C12H17NO7 ; C27H32O16 ; C28H30O15 ; C27H32O16 ; C27H32O16 ; C27H32O16 ; C28H30O15 ; C18H13N3O
8252_280.0	1.785302	0.109765	3.1384	0.01054	1.9772	0.42361	494.5483	HMDB36273 ; HMDB39476	Quillaic acid 3-galactosyl-(1-2)-glucuronide 28-xylosyl-(1-4)-rhamnosyl-(1-2)-rhamnosyl-(1-3)-4-acetyl-fucosyl ester ; Acetylsoyasaponin A1	M+H+2Na ; M+H+2Na	C67H104O33 ; C67H104O33
25354_189.3	1.783805	1.06162	2.8364	0.017657	1.7531	0.42361	177.5254	HMDB28706 ; HMDB28769	Arginyl-Cysteine ; Cysteinyl-Arginine	M+2K ; M+2K	C9H19N5O3S ; C9H19N5O3S
51334_288.1	1.783064	0.480452	3.131	0.010673	1.9717	0.42361	621.9451	HMDB36168	Bis(2-methyl-3-furanyl)tetrasulfide	2M+ACN+H	C10H10O2S4
07214_292.8	1.782396	0.85774	2.6762	0.023243	1.6337	0.42361	378.9497	HMDB32723	Menadiol disulfate	M+2Na-H	C11H10O8S2
0757_289.7	1.780036	0.896479	2.9767	0.013885	1.8574	0.42361	679.3508	HMDB03020 ; HMDB03101 ; HMDB14736 ; HMDB29656 ; HMDB33260 ; HMDB34977 ; HMDB34978 ; HMDB35320 ; HMDB35891 ; HMDB36588 ; HMDB36873 ; HMDB36876 ; HMDB36912 ; HMDB36913 ; HMDB36924 ; HMDB38106 ; HMDB38565 ; HMDB39023 ; HMDB39553 ; HMDB39597 ; HMDB40291 ; HMDB40390 ; HMDB41581 ; HMDB42025	Neoxanthin ; Violaxanthin ; Labetalol ; Erinacine E ; Capsanthin 36-epoxide ; Karpoxanthin ; 6-Epikarpoxanthin ; Cucurbitaxanthin B ; Oryzanol A ; Capsanthin 56-epoxide ; Heteroxanthin ; Auroxanthin ; Salmoxanthin ; Capsochrome ; Capsorubin ; Cycloviolaxanthin ; 3-N-Acetyl-4-O-(14-methylheptadecanoyl)fusarochromanone ; beta-Carotenone ; Lyciumoside III ; Colupdox a ; Erinacine A ; Erinacine B ; Cucurbitachrome 1 ; Terodiline hydrochloride	M+2K+H ; M+2K+H ; 2M+Na ; 3M+Na+K ; M+2K+H ; M+2K-H ; M+2K-H ; M+2K+H ; M+2K-H ; M+2K+H ; M+2K+H ; M+2K+H ; M+2K+H ; M+2K+H ; M+2K+H ; M+2K+H ; M+2K+H ; M+2K+H ; 2M+Na+K ; 3M+Na+K ; 3M+Na+K ; 3M+Na+K ; M+2K+H ; 2M+2Na-H	C40H56O4 ; C40H56O4 ; C19H24N2O3 ; C25H36O6 ; C40H56O4 ; C40H58O4 ; C40H58O4 ; C40H56O4 ; C40H58O4 ; C40H56O4 ; C40H56O4 ; C40H56O4 ; C40H56O4 ; C40H56O4 ; C40H56O4 ; C40H56O4 ; C35H56N2O6 ; C40H56O4 ; C32H56O13 ; C25H36O6 ; C25H36O6 ; C25H36O6 ; C40H56O4 ; C20H28ClN
4131_277.2	1.779679	0.908173	-2.6827	0.022985	1.6386	0.42361	125.9641	HMDB02306 ; HMDB59917	Hydrochloric acid ; 12-Ethanediyldicarbamodithioic acid	3M+NH4 ; M+H+K	ClH ; C4H8N2S4

8129_196.2	1.774853	0.949159	-2.9406	0.014769	1.8306	0.42361	431.0978	HMDB35174 ; HMDB37972 ; HMDB37984 ; HMDB37989 ; HMDB29203 ; HMDB29208 ; HMDB29209 ; HMDB30614 ; HMDB30864 ; HMDB32903 ; HMDB33751 ; HMDB35588 ; HMDB37359 ; HMDB37377 ; HMDB37429 ; HMDB37572 ; HMDB38467 ; HMDB38468 ; HMDB41597 ; HMDB41728 ; HMDB41729 ; HMDB41759 ; HMDB41760 ; HMDB41761	3-1-Formyl-2-(2-furanyl)ethenyl-2-(2-furanyl)-5-(2-furanylmethylene)-45-dihydro-a-methyl-4-oxo-1H-pyrrole-1-acetic acid 9CI ; Cyanidin 3-arabinoside ; Cyanidin 3-xyloside ; Cyanidin 7-arabinoside ; hesperetin-7-O-glucuronide ; naringenin-4-O-glucuronide ; naringenin-7-O-glucuronide ; Orientin ; Trifolin ; 1268-Tetrahydroxy-3-methylanthraquinone 2-O-b-D-glucoside ; Quercitrin ; Luteolin 7-glucoside ; Azaleatin 3-arabinoside ; Carthamone ; Astragalin ; Kaempferol 5-glucoside ; Luteolin 7-galactoside ; Luteolin 4-glucoside ; Glucodistylin ; (-)-Epicatechin 3-O-glucuronide ; (-)-Epicatechin 7-O-glucuronide ; Naringenin 4-O-glucuronide ; Naringenin 5-O-glucuronide ; Naringenin 7-O-glucuronide	2M+H+Na ; 2M+H+Na ; 2M+H+Na ; 2M+H+Na ; M+H-2H2O ; M+H-H2O ; M+H-H2O ; M+H-H2O ; M+H-H2O ; M+H-H2O ; M+H-H2O ; M+H-H2O ; M+H-H2O ; M+H-H2O ; M+H-H2O ; M+H-H2O ; M+H-H2O ; M+H-2H2O ; M+H-2H2O ; M+H-2H2O ; M+H-H2O ; M+H-H2O ; M+H-H2O	C23H17NO7 ; C20H19O10 ; C20H19O10 ; C20H19O10 ; C21H22O12 ; C21H20O11 ; C21H20O11 ; C21H20O11 ; C21H20O11 ; C21H20O11 ; C21H20O11 ; C21H20O11 ; C21H20O11 ; C21H20O11 ; C21H20O11 ; C21H20O11 ; C21H20O11 ; C21H20O11 ; C21H22O12 ; C21H22O12 ; C21H22O12 ; C21H20O11 ; C21H20O11 ; C21H20O11
2285_292.6	1.77458	0.896688	2.6674	0.023596	1.6272	0.42361	322.9922	HMDB04249 ; HMDB32876 ; HMDB34042 ; HMDB34138 ; HMDB37921 ; HMDB39177 ; HMDB41850 ; HMDB60440 ; HMDB60449 ; HMDB60687 ; HMDB60692	Phosphatidylinositol-345-trisphosphate ; Rhein ; Lucernol ; Repensol ; Aureol ; 1-O-Galloylglycerol ; Cefodizime ; Benzoyl phosphate ; Carboxyphosphamide ; Carboxycyclophosphamide ; Carboxyifosfamide	M+2H ; M+K ; M+K ; M+K ; M+K ; M+2K+H ; M+Na+K ; 3M+H+K ; 2M+Na+K ; 2M+Na+K ; 2M+Na+K	C12H24O22P4 ; C15H8O6 ; C15H8O6 ; C15H8O6 ; C15H8O6 ; C10H12O7 ; C20H20N6O7S4 ; C7H7O5P ; C7H15Cl2N2O4P ; C7H15Cl2N2O4P ; C7H15Cl2N2O4P
59625_46.6	1.774407	1.111156	-2.8889	0.016136	1.7922	0.42361	200.036	HMDB00126 ; HMDB01015 ; HMDB02336 ; HMDB02520 ; HMDB03164 ; HMDB29278 ; HMDB29417 ; HMDB29441 ; HMDB30652 ; HMDB30653 ; HMDB32849 ; HMDB33592 ; HMDB33843 ; HMDB40632 ; HMDB40690	Glycerol 3-phosphate ; N-Formyl-L-methionine ; Biflorin ; Beta-Glycerophosphoric acid ; Chlorogenic acid ; Cis-5-Caffeoylquinic acid ; Cycloalliin ; Isoalliin ; 1-O-Caffeoylquinic acid ; Cryptochlorogenic acid ; trans-Chlorogenic acid ; (R)C(S)S-Alliin ; Polyvidone ; Isobiflorin ; 5Z-Caffeoylquinic acid	2M+3H2O+2H ; M+Na ; M+2Na ; 2M+3H2O+2H ; M+2Na ; M+2Na ; M+Na ; M+Na ; M+2Na ; M+2Na ; M+2Na ; M+Na ; M+Na ; M+2Na ; M+2Na	C3H9O6P ; C6H11NO3S ; C16H18O9 ; C3H9O6P ; C16H18O9 ; C16H18O9 ; C6H11NO3S ; C6H11NO3S ; C16H18O9 ; C16H18O9 ; C16H18O9 ; C6H11NO3S ; C6H13NOP2 ; C16H18O9 ; C16H18O9
1103_293.9	1.77417	0.908898	2.7443	0.020678	1.6845	0.42361	444.0311	HMDB01201 ; HMDB59648 ; HMDB14580 ; HMDB37475 ; HMDB37810 ; HMDB60418 ; HMDB60792 ; HMDB60825	Guanosine diphosphate ; 8-oxo-dGDP ; Bendroflumethiazide ; 4beta-(2-Aminoethylthio)catechin ; 411-Dichloro-512-dihydroquino23-bacridine-714-dione ; 6-Thioxanthine 5-monophosphate ; 6-Thioxanthylic acid ; Hydromorphone-3-sulphate	M+H ; M+H ; M+Na ; M+2K+H ; M+ACN+Na ; M+ACN+Na ; M+ACN+Na ; M+2K+H	C10H15N5O11P2 ; C10H15N5O11P2 ; C15H14F3N3O4S2 ; C17H19NO6S ; C20H10Cl2N2O2 ; C10H13N4O8PS ; C10H13N4O8PS ; C17H19NO6S

)2314_53.3!	1.772762	1.150958	-2.8225	0.018082	1.7427	0.42361	162.0502	HMDB37110 ; HMDB00098 ; HMDB00283 ; HMDB00366 ; HMDB00621 ; HMDB00646 ; HMDB00751 ; HMDB01644 ; HMDB03371 ; HMDB12194 ; HMDB12325 ; HMDB15327 ; HMDB29941 ; HMDB29942 ; HMDB32846 ; HMDB33564 ; HMDB33565 ; HMDB59753 ; HMDB60254	4-(34-Dihydroxyphenyl)-23-dihydro-23-dihydroxy-1H-phenalen-1-one ; D-Xylose ; D-Ribose ; 2-Deoxyribonic acid ; D-Ribulose ; L-Arabinose ; L-Threo-2-pentulose ; D-Xylulose ; L-Ribulose ; Beta-D-ribopyranose ; Arabinofuranose ; Estramustine ; D-Apiose ; Arabinose ; 5-Chloro-6-methoxy-2(3H)-benzoxazolone ; 23-Dihydro-2-methylthiophene ; 23-Dihydro-5-methylthiophene ; 2-Deoxypentonic acid ; Aldehydo-D-xylose	M+2H ; 2M+H+Na ; 2M+H+Na ; 2M+H+Na ; 2M+H+Na ; 2M+H+Na ; 2M+H+Na ; 2M+H+Na ; 2M+H+Na ; 2M+H+Na ; 2M+H+Na ; M+H+2Na ; 2M+H+Na ; 2M+H+Na ; M+3ACN+2H ; 3M+H+Na ; 3M+H+Na ; 2M+H+Na ; 2M+H+Na	C19H14O5 ; C5H10O5 ; C5H10O5 ; C5H10O5 ; C5H10O5 ; C5H10O5 ; C5H10O5 ; C5H10O5 ; C5H10O5 ; C5H10O5 ; C5H10O5 ; C23H31Cl2NO3 ; C5H10O5 ; C5H10O5 ; C8H6ClNO3 ; C5H8S ; C5H8S ; C5H10O5 ; C5H10O5
)328_21.64	1.77195	0.351784	-2.5162	0.030588	1.5145	0.42361	405.2993	HMDB11555 ; HMDB11556 ; HMDB11585 ; HMDB11586 ; HMDB12517 ; HMDB01186 ; HMDB11544 ; HMDB11574 ; HMDB13213 ; HMDB15498 ; HMDB31133 ; HMDB35959 ; HMDB36865	MG(0:022:5(4Z7Z10Z13Z16Z)0:0) ; MG(0:022:5(7Z10Z13Z16Z19Z)0:0) ; MG(22:5(4Z7Z10Z13Z16Z)0:00:0) ; MG(22:5(7Z10Z13Z16Z19Z)0:00:0) ; 11-Carboxy-gamma-chromanol ; N1-Acetylspermine ; MG(0:020:2(11Z14Z)0:0) ; MG(20:2(11Z14Z)0:00:0) ; Alpha-Linoleoylcholine ; Aprindine ; 1-O-beta-D-Glucopyranosyl-23-di-O-(8-hexadecenoyl)glycerol ; Persenone B ; Lepidiumterpenyl ester	M+H ; M+H ; M+H ; M+H ; M+H ; 3M+2K ; M+Na ; M+Na ; M+K ; M+2ACN+H ; M+2ACN+2H ; M+Na ; M+Na	C25H40O4 ; C25H40O4 ; C25H40O4 ; C25H40O4 ; C25H40O4 ; C12H28N4O ; C23H42O4 ; C23H42O4 ; C23H44NO2 ; C22H30N2 ; C41H74O10 ; C23H42O4 ; C23H42O4
!7683_102.7	1.771486	0.956374	-2.7331	0.02108	1.6761	0.42361	167.0928	HMDB41500 ; HMDB00077 ; HMDB00234 ; HMDB00628 ; HMDB00899 ; HMDB02894 ; HMDB03769 ; HMDB04339 ; HMDB04363 ; HMDB05962 ; HMDB06035 ; HMDB06921 ; HMDB10734 ; HMDB11601 ; HMDB14640 ; HMDB14889 ; HMDB30178 ; HMDB30181 ; HMDB30353 ; HMDB31057 ; HMDB33028 ; HMDB33948 ; HMDB35599 ; HMDB35908 ; HMDB36205 ; HMDB37609	1-Deoxy-D-glucitol ; Dehydroepiandrosterone ; Testosterone ; Epitestosterone ; Androstanedione ; 5-Methylcytosine ; Etiocholanedione ; 2-O-Methylcytosine ; Imidazolone ; Dehydroandrosterone ; 4-Dihydroboldenone ; (S)-3-Hydroxy-N-methylcoclaurine ; (R)-3-Hydroxy-hexadecanoic acid ; 3-Methylcytosine ; Oxycodone ; Epinastine ; (S)-Edulinine ; Erythratine ; Arecoline ; (R)-2-Hydroxyhexadecanoic acid ; N-Dihydroferuloyltyramine ; Codeine N-oxide ; Lucidenic acid G ; Lucidenic acid H ; alpha-Amylcinnamyl isovalerate ; Lucidenic acid C	M+H ; M+2Na ; M+2Na ; M+2Na ; M+2Na ; M+ACN+H ; M+2Na ; M+ACN+H ; M+2ACN+H ; M+2Na ; M+2Na ; M+H+NH4 ; M+Na+K ; M+ACN+H ; M+H+NH4 ; 2M+3H ; M+ACN+2H ; M+H+NH4 ; 2M+H+Na ; M+Na+K ; M+H+NH4 ; M+H+NH4 ; M+2H+Na ; M+2H+Na ; M+2Na ; M+2H+Na	C6H14O5 ; C19H28O2 ; C19H28O2 ; C19H28O2 ; C19H28O2 ; C5H7N3O ; C19H28O2 ; C5H7N3O ; C3H4N2O ; C19H28O2 ; C19H28O2 ; C18H21NO4 ; C16H32O3 ; C5H7N3O ; C18H21NO4 ; C16H15N3 ; C16H21NO4 ; C18H21NO4 ; C8H13NO2 ; C16H32O3 ; C18H21NO4 ; C18H21NO4 ; C27H40O7 ; C27H40O7 ; C19H28O2 ; C27H40O7
'4513_272.!	1.770489	0.821838	2.6734	0.023354	1.6316	0.42361	251.5175	HMDB00580 ; HMDB60358 ; HMDB60381	Chondroitin sulfate ; 23-Dihydro-2-S-glutathionyl-3-hydroxy bromobenzene ; 34-Dihydro-3-hydroxy-4-S-glutathionyl bromobenzene	M+H+K ; M+H+Na ; M+H+Na	C13H21NO15S ; C16H22BrN3O7S ; C16H22BrN3O7S

75591_290.3	1.770304	0.422642	2.6859	0.02286	1.6409	0.42361	470.5376	HMDB01290 ; HMDB03377 ; HMDB34379 ; HMDB39481 ; HMDB60124	Glutaconyl-CoA ; Itaconyl-CoA ; Silenoside A ; Acetylsoyasaponin A4 ; Mesaconyl-CoA	M+Na+K ; M+Na+K ; M+H+2Na ; M+H+2Na ; M+Na+K	C26H40N7O19P3S ; C26H40N7O19P3S ; C64H100O31 ; C64H100O31 ; C26H40N7O19P3S
56399_158.	1.769853	0.267557	-2.6254	0.02536	1.5959	0.42361	NA	NA	NA	NA	NA
.2905_47.73	1.769061	1.116064	-2.8141	0.018347	1.7364	0.42361	NA	NA	NA	NA	NA
35533_55.32	1.768639	1.12255	2.9214	0.015264	1.8163	0.42361	337.9086	HMDB10408	LysoPC(P-18:1(9Z))	2M+3H	C26H52NO6P
35005_29.84	1.768481	0.896192	-2.6609	0.023863	1.6223	0.42361	NA	NA	NA	NA	NA
09501_54.75	1.76799	1.074172	-2.9929	0.013506	1.8695	0.42361	NA	NA	NA	NA	NA
2784_198.3	1.765458	0.910492	-2.8544	0.017119	1.7665	0.42361	214.5528	HMDB00742 ; HMDB02108 ; HMDB14958 ; HMDB15605 ; HMDB29095 ; HMDB29116 ; HMDB30192 ; HMDB34582	Homocysteine ; Methylcysteine ; Tadalafil ; Tamibarotene ; Tryptophyl-Tyrosine ; Tyrosyl-Tryptophan ; Marmeline ; Shoyuflavone A	3M+H+Na ; 3M+H+Na ; M+H+K ; M+2K ; M+Na+K ; M+Na+K ; M+2K ; M+ACN+2H	C4H9NO2S ; C4H9NO2S ; C22H19N3O4 ; C22H25NO3 ; C20H21N3O4 ; C20H21N3O4 ; C22H25NO3 ; C19H14O9
01115_274.4	1.764995	0.460994	-2.5491	0.028908	1.539	0.42361	NA	NA	NA	NA	NA
.4141_207.9	1.764962	0.445726	2.611	0.025996	1.5851	0.42361	218.0414	HMDB00152 ; HMDB00379 ; HMDB00397 ; HMDB00840 ; HMDB01229 ; HMDB01321 ; HMDB01451 ; HMDB01514 ; HMDB01856 ; HMDB02016 ; HMDB02030 ; HMDB02404 ; HMDB03518 ; HMDB04067 ; HMDB06116 ; HMDB06471 ; HMDB06488 ; HMDB12221 ; HMDB13676 ; HMDB13677 ; HMDB13678 ; HMDB14312 ; HMDB15366 ; HMDB15535 ; HMDB29666 ; HMDB30091 ; HMDB30726 ; HMDB34299 ; HMDB38055 ; HMDB39119 ; HMDB40912 ; HMDB59932 ; HMDB60602	Gentisic acid ; 2-Methylcitric acid ; 2-Pyrocatechuic acid ; Salicyluric acid ; Dopaquinone ; D-Erythrose 4-phosphate ; (R)-lipoic acid ; Glucosamine ; Protocatechuic acid ; 4-Carboxyphenylglycine ; Fructosamine ; Alpha-Hydroxyhippuric acid ; Homocitric acid ; Leucodopachrome ; 3-Hydroxyhippuric acid ; Methylisocitric acid ; N-Acetyl-L-glutamate 5-semialdehyde ; Dopaxanthin ; 26-Dihydroxybenzoic acid ; 35-Dihydroxybenzoic acid ; 4-Hydroxyhippuric acid ; (S)-lipoic acid ; Sevoflurane ; Cilastatin ; 24-Dihydroxybenzoic acid ; beta-D-Glucosamine ; 2-Hydroxy-67-dimethoxybenzoxazole ; Patulin ; 2-Hydroxy-7-methoxy-2H-14-benzoxazin-3(4H)-one ; L-Dopaquinone ; N-(5-Hydroxy-2-pyridinyl)methyladenosine ; 3-(Carboxymethyl)-3-hydroxypentanedioic acid ; N-acetyl-5-aminosalicylic acid	M+ACN+Na ; 2M+H+Na ; M+ACN+Na ; M+Na ; M+Na ; M+NH4 ; 2M+H+Na ; M+K ; M+ACN+Na ; M+Na ; M+K ; M+Na ; 2M+H+Na ; M+Na ; M+Na ; 2M+H+Na ; M+2Na-H ; M+2Na ; M+ACN+Na ; M+ACN+Na ; M+Na ; 2M+H+Na ; M+NH4 ; M+2K ; M+ACN+Na ; M+K ; M+Na ; M+ACN+Na ; M+Na ; M+Na ; M+Na+K ; 2M+H+Na ; M+Na	C7H6O4 ; C7H10O7 ; C7H6O4 ; C9H9NO4 ; C9H9NO4 ; C4H9O7P ; C8H14O2S2 ; C6H13NO5 ; C7H6O4 ; C9H9NO4 ; C6H13NO5 ; C9H9NO4 ; C7H10O7 ; C9H9NO4 ; C9H9NO4 ; C7H10O7 ; C7H11NO4 ; C18H18N2O8 ; C7H6O4 ; C7H6O4 ; C9H9NO4 ; C8H14O2S2 ; C4H3F7O ; C16H26N2O5S ; C7H6O4 ; C6H13NO5 ; C9H9NO4 ; C7H6O4 ; C9H9NO4 ; C9H9NO4 ; C16H18N6O5 ; C7H10O7 ; C9H9NO4

1324_179.9	1.763106	0.435347	-2.5848	0.02719	1.5656	0.42361	224.1281	HMDB13845 ; HMDB00312 ; HMDB00320 ; HMDB00326 ; HMDB00330 ; HMDB00364 ; HMDB00371 ; HMDB00376 ; HMDB00390 ; HMDB00395 ; HMDB00404 ; HMDB00414 ; HMDB00415 ; HMDB00419 ; HMDB00427 ; HMDB00432 ; HMDB00505 ; HMDB00506 ; HMDB00527 ; HMDB00619 ; HMDB00760 ; HMDB00865 ; HMDB00917 ; HMDB30762 ; HMDB31729 ; HMDB31973 ; HMDB32348 ; HMDB33594 ; HMDB34122 ; HMDB34135 ; HMDB35071 ; HMDB41410	SR 49498 ; 3a7a12b-Trihydroxy-5b-cholanoic acid ; 3a4b7a-Trihydroxy-5b-cholanoic acid ; 1b3a12a-Trihydroxy-5b-cholanoic acid ; 3a4b12a-Trihydroxy-5b-cholanoic acid ; 3a6a7b-Trihydroxy-5b-cholanoic acid ; 1312-Trihydroxycholan-24-oic acid ; 3b7a12a-Trihydroxy-5a-Cholanoic acid ; 3a7b12b-Trihydroxy-5b-cholanoic acid ; 3b7b12a-Trihydroxy-5b-cholanoic acid ; 2b3a7a-Trihydroxy-5b-cholanoic acid ; 1b3a7a-Trihydroxy-5b-cholanoic acid ; 3a6b7b-Trihydroxy-5b-cholanoic acid ; 3b7a12a-Trihydroxy-5b-cholanoic acid ; 3b7b12a-Trihydroxy-5a-Cholanoic acid ; 3a7b12a-Trihydroxy-5a-Cholanoic acid ; Allocholic acid ; Alpha-Muricholic acid ; 6a12a-Dihydroxylithocholic acid ; Cholic acid ; Hyocholic acid ; Muricholic acid ; Ursocholic acid ; Ethyl trans-p-methoxycinnamate ; Phenylmethylglycidic ester ; (2RS5RS)-(E)-2-(2-Phenylethenyl)-13-dioxan-5-ol ; Isoeugenyl acetate ; Cuelure ; Aceteugenol ; Isoeugenol acetate ; Verimol I ; 23-Dihydro-6-methoxy-22-dimethyl-4H-1-benzopyran-4-one	M+2H ; M+H+K ; M+H+K ; M+H+K ; M+H+K ; M+H+K ; M+H+K ; M+H+K ; M+H+K ; M+H+K ; M+H+K ; M+H+K ; M+H+K ; M+H+K ; M+H+K ; M+H+K ; M+H+K ; M+H+K ; M+H+K ; M+NH4 ; M+NH4 ; M+NH4 ; M+NH4 ; M+NH4 ; M+NH4 ; M+NH4 ; M+NH4 ; M+NH4	C25H30N6O2 ; C24H40O5 ; C12H14O3 ; C12H14O3 ; C12H14O3 ; C12H14O3 ; C12H14O3 ; C12H14O3 ; C12H14O3 ; C12H14O3 ; C12H14O3
0589_191.8	1.761547	0.746887	-2.6735	0.023351	1.6317	0.42361	221.0961	HMDB15618 ; HMDB00821 ; HMDB00859 ; HMDB11723 ; HMDB13245 ; HMDB13292 ; HMDB29442 ; HMDB29464 ; HMDB29651 ; HMDB30828 ; HMDB31340 ; HMDB31368 ; HMDB32388 ; HMDB33068 ; HMDB33082 ; HMDB34159 ; HMDB34250 ; HMDB34607 ; HMDB36781 ; HMDB36929 ; HMDB38304 ; HMDB38785 ; HMDB41438 ; HMDB60366 ; HMDB60389 ; HMDB60758 ; HMDB61168 ; HMDB29539 ; HMDB34992 ; HMDB37686 ; HMDB41678	Sertindole ; Phenylacetylglycine ; Methylhippuric acid ; 2-Methylhippuric acid ; m-Methylhippuric acid ; p-Methylhippuric acid ; (R)C(R)S-S-Propylcysteine sulfoxide ; Asteltoxin ; Phlorisobutyrophenone 2-glucoside ; 2-(4-Allyl-26-dimethoxyphenoxy)-1-(345-trimethoxyphenyl)-1-propanol ; Cyclamic acid ; (1a5b6a)-7-Protoilludene-15614-tetrol 14-(24-dihydroxy-6-methylbenzoic acid) ; Methyl n-acetylanthranilate ; ()-threo-1-(p-Hydroxyphenyl)propylene glycol 4-glucoside ; ()-3-(4-Hydroxyphenyl)-12-propanediol 4-O-glucoside ; Acetyl tributyl citrate ; Betamipron ; Judeol ; Diosbulbin H ; 13-Hydroxydihydromelleolide ; 3-(34-Dihydroxyphenyl)-1-propanol 3-glucoside ; Melleolide E ; Pseudomonine ; 3-Carbamoyl-2-phenylpropionaldehyde ; 4-Hydroxy-5-phenyltetrahydro-13-oxazin-2-one ; 4-Anilino-4-oxobutanoic acid ; N2-Monodes-methylnizatidine ; Xenognosin A ; 4-Methoxybenzyl phenylacetate ; 7-Hydroxy-5-methoxyflavan ; 4-O-Methylequol	M+2H ; 2M+3H2O+2H ; 2M+3H2O+2H ; 2M+3H2O+2H ; 2M+3H2O+2H ; 2M+3H2O+2H ; M+ACN+H ; M+H+Na ; M+2ACN+2H ; M+H+Na ; M+ACN+H ; M+H+Na ; 2M+3H2O+2H ; 2M+3H ; 2M+3H ; M+H+K ; 2M+3H2O+2H ; M+H+Na ; M+H+Na ; M+H+Na ; 2M+3H ; M+H+Na ; 2M+3H ; 2M+3H2O+2H ; 2M+3H2O+2H ; 2M+3H2O+2H ; M+3ACN+2H ; M+H-2H2O ; M+H-2H2O ; M+H-2H2O ; M+H-2H2O	C24H26ClFN4O ; C10H11NO3 ; C10H11NO3 ; C10H11NO3 ; C10H11NO3 ; C10H11NO3 ; C6H13NO3S ; C23H30O7 ; C16H22O9 ; C23H30O7 ; C6H13NO3S ; C23H30O7 ; C10H11NO3 ; C15H22O8 ; C15H22O8 ; C20H34O8 ; C10H11NO3 ; C23H30O7 ; C23H30O7 ; C23H30O7 ; C15H22O8 ; C23H30O7 ; C16H18N4O4 ; C10H11NO3 ; C10H11NO3 ; C10H11NO3 ; C11H19N5O2S2 ; C16H16O3 ; C16H16O3 ; C16H16O3 ; C16H16O3

8113_42.3[illegible]	1.760152	0.811755	2.8187	0.018201	1.7399	0.42361	307.0438	HMDB00162 ; HMDB00195 ; HMDB00425 ; HMDB00481 ; HMDB02349 ; HMDB02432 ; HMDB03040 ; HMDB03411 ; HMDB06331 ; HMDB06695 ; HMDB06944 ; HMDB12248 ; HMDB12880 ; HMDB28871 ; HMDB28876 ; HMDB29018 ; HMDB29128 ; HMDB30409 ; HMDB30865 ; HMDB31521 ; HMDB31762 ; HMDB32409 ; HMDB32923 ; HMDB32988 ; HMDB34043 ; HMDB34208 ; HMDB34377 ; HMDB40263 ; HMDB40410 ; HMDB40573 ; HMDB59762	L-Proline ; Inosine ; 3-Deoxy-D-glycero-D-galacto-2-nonulosonic acid ; Allopurinol riboside ; trans-trans-Muconic acid ; Sumikis acid ; Arabinosylhypoxanthine ; D-Proline ; ciscis-Muconic acid ; Prolylhydroxyproline ; 14-beta-D-Glucan ; L-3-Aminobutyryl-CoA ; Acetamidopropanal ; Hydroxyprolyl-Proline ; Hydroxyprolyl-Valine ; Prolyl-Hydroxyproline ; Valyl-Hydroxyproline ; 4-Amino-2-methylenebutanoic acid ; Rubroskyrin ; Ethyl 2-mercaptopropionate ; Carbadox ; Methyl 3-mercaptobutanoate ; Kojic acid ; 5-Hydroxymaltol ; Tricetanidin ; Pterolactam ; Methyl 3-(methylthio)propanoate ; Ethyl 2-(methylthio)acetate ; Ethyl 3-mercaptopropanoic acid ; Tetraacetylethylenediamine ; 23-Methylenesuccinic acid	2M+2K-H ; M+K ; M+K ; M+K ; 2M+Na ; 2M+Na ; M+K ; 2M+2K-H ; 2M+Na ; M+2K+H ; M+2K ; M+3Na ; 2M+2K-H ; M+2K+H ; M+2K-H ; M+2K+H ; M+2K-H ; 2M+2K-H ; M+H+K ; 2M+K ; M+2Na-H ; 2M+K ; 2M+Na ; 2M+Na ; 2M+H+K ; 2M+2K-H ; 2M+K ; 2M+K ; 2M+K ; M+2K+H ; 2M+Na	C5H9NO2 ; C10H12N4O5 ; C9H16O9 ; C10H12N4O5 ; C6H6O4 ; C6H6O4 ; C10H12N4O5 ; C5H9NO2 ; C6H6O4 ; C10H16N2O4 ; C18H32O18 ; C25H43N8O17P3S ; C5H9NO2 ; C10H16N2O4 ; C10H18N2O4 ; C10H16N2O4 ; C10H18N2O4 ; C5H9NO2 ; C30H22O12 ; C5H10O2S ; C11H10N4O4 ; C5H10O2S ; C6H6O4 ; C6H6O4 ; C15H11O6 ; C5H9NO2 ; C5H10O2S ; C5H10O2S ; C5H10O2S ; C10H16N2O4 ; C6H6O4
5192_262.[illegible]	1.758199	1.041774	-2.9373	0.014853	1.8282	0.42361	233.1185	HMDB29504 ; HMDB36189 ; HMDB36605 ; HMDB29989 ; HMDB29990 ; HMDB31367 ; HMDB33316 ; HMDB35489 ; HMDB36571 ; HMDB01539 ; HMDB03334 ; HMDB14361 ; HMDB14535 ; HMDB15135 ; HMDB32864 ; HMDB34361 ; HMDB36394 ; HMDB40334 ; HMDB40335 ; HMDB40336 ; HMDB40337 ; HMDB41886 ; HMDB38935	Marindinin ; Tetrahydrofurfuryl cinnamate ; Pterosin E ; Dulciol B ; Dulciol A ; Linalool oxide D 3-apiosyl-(1-6)-glucoside ; Garcinone E ; Linalool 36-oxide primeveroside ; Linalool 37-oxide beta-primeveroside ; Asymmetric dimethylarginine ; Symmetric dimethylarginine ; Eletriptan ; Sulpiride ; Cyclacillin ; Mycotoxin T 2 ; Olitorin ; N-Methylcalystegine C1 ; Dihydro-24-dimethyl-6-(2-methylpropyl)-4H-135-dithiazine ; Dihydro-46-dimethyl-2-(2-methylpropyl)-4H-135-dithiazine ; Dihydro-24-dimethyl-6-(1-methylpropyl)-4H-135-dithiazine ; Dihydro-46-dimethyl-2-(1-methylpropyl)-4H-135-dithiazine ; Enalaprilat ; Kamahine C	M+H ; M+H ; M+H ; M+2H ; M+2H ; M+2H ; M+2H ; M+2H ; M+2H ; 2M+Na+K ; 2M+Na+K ; M+2ACN+2H ; M+3ACN+2H ; M+3ACN+2H ; M+3ACN+2H ; M+3H ; 2M+3H2O+2H ; 2M+3H2O+2H ; 2M+3H2O+2H ; 2M+3H2O+2H ; 2M+3H2O+2H ; 2M+3H ; M+H-2H2O	C14H16O3 ; C14H16O3 ; C14H16O3 ; C28H32O6 ; C28H32O6 ; C21H36O11 ; C28H32O6 ; C21H36O11 ; C21H36O11 ; C8H18N4O2 ; C8H18N4O2 ; C22H26N2O2S ; C15H23N3O4S ; C15H23N3O4S ; C16H23NO7 ; C35H52O14 ; C8H15NO5 ; C9H19NS2 ; C9H19NS2 ; C9H19NS2 ; C9H19NS2 ; C18H24N2O5 ; C14H20O5
466_289.9[illegible]	1.758046	0.88132	2.9401	0.014782	1.8303	0.42361	650.9747	HMDB01364 ; HMDB31913 ; HMDB34022 ; HMDB35282	Adenosine tetraphosphate ; 5alpha-Tomatidan-3-one ; 6-Hydroxy-5-(4-sulfophenyl)azo-2-naphthalenesulfonic acid ; Solasodine	M+ACN+Na ; 3M+Na+K ; 3M+2K ; 3M+Na+K	C10H17N5O16P4 ; C27H43NO2 ; C16H12N2O7S2 ; C27H43NO2
6198_254.[illegible]	1.75712	1.131158	-2.7366	0.020954	1.6787	0.42361	NA	NA	NA	NA	NA
3563_82.5[illegible]	1.756721	1.058154	-2.6302	0.025154	1.5994	0.42361	NA	NA	NA	NA	NA

1309_25.31	1.755852	0.931874	-2.7097	0.021944	1.6587	0.42361	223.1113	HMDB14037 ; HMDB14039 ; HMDB14557 ; HMDB14903 ; HMDB15119 ; HMDB15398 ; HMDB28846 ; HMDB28951 ; HMDB29217 ; HMDB29625 ; HMDB29746 ; HMDB31062 ; HMDB31063 ; HMDB31087 ; HMDB31272 ; HMDB31406 ; HMDB31691 ; HMDB31843 ; HMDB32308 ; HMDB32320 ; HMDB32396 ; HMDB32404 ; HMDB32453 ; HMDB32461 ; HMDB32489 ; HMDB32534 ; HMDB33371 ; HMDB33724 ; HMDB34428 ; HMDB34429 ; HMDB34952 ; HMDB35076 ; HMDB36140 ; HMDB36425 ; HMDB36784 ; HMDB37106 ; HMDB37185 ; HMDB37225 ; HMDB37309 ; HMDB38073 ; HMDB38273 ; HMDB38274 ; HMDB38277 ; HMDB38278 ; HMDB38311 ; HMDB38906 ; HMDB40593 ; HMDB41943 ; HMDB60065 ; HMDB60351 ; HMDB30420 ; HMDB30672 ; HMDB30722 ; HMDB30881 ; [illegible]	6-Hydroxyfluvastatin ; 5-Hydroxyfluvastatin ; Pramipexole ; Metyrosine ; Nandrolone phenpropionate ; Varenicline ; Glycyl-Lysine ; Lysyl-Glycine ; Tyrosine methylester ; 3-O-Protocatechuoylceanothic acid ; 3-Amino-14-dimethyl-5H-pyrido43-bindole ; (Z)-3-Methyl-3-decenoic acid ; (Z)-3-Methyl-4-decenoic acid ; trans-2-Hexyl-1-cyclopropaneacetic acid ; Ethyl (E)-2-nonenoate ; Ethyl 3-cyclohexylpropionate ; cis-3-Hexenyl pentanoate ; 23-Undecanedione ; (E)-3-Heptenyl 2-methylpropanoate ; 3-Hexenyl 2-methylbutyrate ; Methyl 2-decenoate ; 6-Methyl-5-hepten-2-one propyleneglycol acetal ; cis-3-Octenyl propionate ; Pent-2-enyl hexanoate ; Prenyl caproate ; trans-2-Hexenyl 2-methylbutyrate ; Citronellyl formate ; Undecylenic acid ; Cyclohexyl 3-methylbutanoate ; Cyclohexyl pentanoate ; Persicaxanthin ; 2-(2-Methylpropoxy)naphthalene ; 5-Hexyldihydro-5-methyl-2(3H)-furanone ; Persicachrome ; 6-Hexyltetrahydro-2H-pyran-2-one ; (x)-1-Nonen-3-yl acetate ; Rhodinyl formate ; Methyl citronellate ; 5-Hexyldihydro-4-methyl-2(3H)-furanone ; Octadecyl fumarate ; 2-Hexenyl valerate ; 2-Hexenyl isovalerate ; cis-3-Hexenyl 2-methylbutanoate ; cis-3-Hexenyl 3-methylbutanoate ; xi-gamma-Undecalactone ; 5-Octenyl propanoate ; 2-Propenyl octanoate ; N-Acetyldopamine ; N-methyl-467-trihydroxy-1234-tetrahydroisoquinoline ; 2-Phenyl-13-propanediol monocarbamate ; Strobilurin A ; Obtustyrene ; (E)-35-Dimethoxystilbene ; 1-Acetoxy-46-tetradecadiene-81012-triyne ; Phenethyl phenylacetate	M+H+NH4 ; M+H+NH4 ; 2M+H+Na ; 2M+3H2O+2H ; M+H+K ; 2M+H+Na ; 2M+H+K ; 2M+H+K ; 2M+3H2O+2H ; M+H+2Na ; 2M+H+Na ; M+K ; M+K ; M+K ; M+K ; M+K ; M+K ; M+K ; M+K ; M+K ; M+K ; M+K ; M+K ; M+K ; M+K ; M+K ; M+K ; M+K ; M+Na+K ; M+Na ; M+K ; M+Na+K ; M+K ; M+K ; M+K ; M+K ; M+K ; M+2K ; M+K ; M+K ; M+K ; M+K ; M+K ; M+K ; M+K ; 2M+3H2O+2H ; 2M+3H2O+2H ; 2M+3H2O+2H ; M+H-2H2O ; M+H-H2O ; M+H-H2O ; M+H-H2O ; M+H-H2O	C24H26FNO5 ; C24H26FNO5 ; C10H17N3S ; C10H13NO3 ; C27H34O3 ; C13H13N3 ; C8H17N3O3 ; C8H17N3O3 ; C10H13NO3 ; C37H50O8 ; C13H13N3 ; C11H20O2 ; C11H20O2 ; C11H20O2 ; C11H20O2 ; C11H20O2 ; C11H20O2 ; C11H20O2 ; C11H20O2 ; C11H20O2 ; C11H20O2 ; C11H20O2 ; C11H20O2 ; C11H20O2 ; C11H20O2 ; C11H20O2 ; C11H20O2 ; C11H20O2 ; C11H20O2 ; C11H20O2 ; C25H36O3 ; C14H16O ; C11H20O2 ; C25H36O3 ; C11H20O2 ; C11H20O2 ; C11H20O2 ; C11H20O2 ; C11H20O2 ; C22H40O4 ; C11H20O2 ; C11H20O2 ; C11H20O2 ; C11H20O2 ; C11H20O2 ; C11H20O2 ; C11H20O2 ; C10H13NO3 ; C10H13NO3 ; C10H13NO3 ; C16H18O3 ; C16H16O2 ; C16H16O2 ; C16H16O2 ; C16H16O2
9894_270.5	1.753416	1.18461	-2.711	0.021895	1.6597	0.42361	NA	NA	NA	NA	NA
602_20.278	1.751976	0.394387	-2.6101	0.026038	1.5844	0.42361	112.956	HMDB60333 ; HMDB60357 ; HMDB60452 ; HMDB15162	11-Dichloroethylene epoxide ; 22-Dichloroacetaldehyde ; Chloroacetyl chloride ; Methoxyflurane	M+H ; M+H ; M+H ; M+Na+K	C2H2Cl2O ; C2H2Cl2O ; C2H2Cl2O ; C3H4Cl2F2O
9591_257.4	1.751852	0.863087	-2.6653	0.023683	1.6256	0.42361	153.091	HMDB32132 ; HMDB32599 ; HMDB36169 ; HMDB36227 ; HMDB39801 ; HMDB39956 ; HMDB40175 ; HMDB40216 ; HMDB40449 ; HMDB41326 ; HMDB41804 ; HMDB59853 ; HMDB60352 ; HMDB60379 ; HMDB00907 ; HMDB30897 ; HMDB32593 ; HMDB33030 ; HMDB33630 ; HMDB36436 ; HMDB37511 ; HMDB40389 ; HMDB60605	2-Isopropyl-14-benzenediol ; 3-(4-Hydroxyphenyl)-1-propanol ; 3-(5-Methyl-2-furanyl)butanal ; 2-Propenyl 24-hexadienoate ; 1-(2-Furanyl)-1-pentanone ; 4-(Ethoxymethyl)phenol ; 4-Ethyl-2-methoxyphenol ; 266-Trimethyl-2-cyclohexene-14-dione ; 2-(Ethoxymethyl)phenol ; (Dimethoxymethyl)benzene ; 2-Isopropoxyphenol ; 35-Dimethoxytoluene ; 2-Polyprenyl-3-methyl-5-hydroxy-6-methoxy-14-benzoquinone ; 3-Polyprenyl-4-hydroxy-5-methoxybenzoate ; Sulfolithocholic acid ; Lucidenolactone ; 4-(Hydroxymethyl)benzenediazonium(1) ; (12S15S)-15-O-Demethyl-1029-dideoxy-1112-dihydro-striatin C ; Lactapiperanol C ; Lucidenic acid F ; Butyl oleate sulfate ; Erinacine C ; Metyrapol	M+H ; M+H ; M+H ; M+H ; M+H ; M+H ; M+H ; M+H ; M+H ; M+H ; M+H ; M+H ; M+2H ; M+2H ; M+3H ; M+3H ; M+NH4 ; M+2H+Na ; M+H+Na ; M+3H ; M+2H+Na ; M+2H+Na ; 2M+3H	C9H12O2 ; C9H12O2 ; C9H12O2 ; C9H12O2 ; C9H12O2 ; C9H12O2 ; C9H12O2 ; C9H12O2 ; C9H12O2 ; C9H12O2 ; C9H12O2 ; C9H12O2 ; C18H24O4 ; C18H24O4 ; C24H40O6S ; C27H36O6 ; C7H7N2O ; C25H38O6 ; C16H26O4 ; C27H36O6 ; C22H42O6S ; C25H38O6 ; C14H16N2O

37545_289.5	1.751041	0.462889	2.8562	0.017069	1.7678	0.42361	578.4238	HMDB10399 ; HMDB01848 ; HMDB02304 ; HMDB30357 ; HMDB30463 ; HMDB40172	LysoPC(22:1(13Z)) ; Imipramine ; Leukotriene B4 ethanolamide ; N-Methylaspidospermatidine ; Hyperforin ; Didodecyl thiobispropanoate	M+H ; 2M+NH4 ; 3M+H+NH4 ; 2M+NH4 ; M+ACN+H ; M+ACN+Na	C30H60NO7P ; C19H24N2 ; C22H37NO4 ; C19H24N2 ; C35H52O4 ; C30H58O4S
9329_73.37	1.75082	0.91336	-2.7412	0.020789	1.6822	0.42361	NA	NA	NA	NA	NA
29531_188.1	1.74953	0.793221	-2.6143	0.02585	1.5875	0.42361	251.143	HMDB01959 ; HMDB12560 ; HMDB31896 ; HMDB35730 ; HMDB39086 ; HMDB41365 ; HMDB00313 ; HMDB00335 ; HMDB00343 ; HMDB00406 ; HMDB05895 ; HMDB15182 ; HMDB60084 ; HMDB60085	Gamma-Aminobutyryl-lysine ; 13-Hydroxy-alpha-tocotrienol ; Erinacine D ; Camelledionol ; Hydroxysintaxanthin 56-epoxide ; 25-Acetylvulgaroside ; 16b-Hydroxyestrone ; 16a-Hydroxyestrone ; 2-Hydroxyestrone ; 16-Ketoestradiol ; 4-Hydroxyestrone ; Abacavir ; 17beta-Estradiol-23-quinone ; 17beta-Estradiol-34-quinone	2M+H+K ; M+Na+K ; M+H+Na ; M+Na+K ; M+H+K ; M+H+Na ; M+H-2H2O ; M+H-2H2O ; M+H-2H2O ; M+H-2H2O ; M+H-2H2O ; M+H-2H2O ; M+H-2H2O ; M+H-2H2O	C10H21N3O3 ; C29H44O3 ; C27H42O7 ; C29H44O3 ; C31H42O3 ; C27H42O7 ; C18H22O3 ; C18H22O3 ; C18H22O3 ; C18H22O3 ; C18H22O3 ; C14H18N6O ; C18H22O3 ; C18H22O3
0384_205.0	1.748452	0.903484	2.7572	0.020226	1.6941	0.42361	191.0104	HMDB61120 ; HMDB00020 ; HMDB00440 ; HMDB00669 ; HMDB00703 ; HMDB01067 ; HMDB01101 ; HMDB02390 ; HMDB03791 ; HMDB04815 ; HMDB05797 ; HMDB12308 ; HMDB29175 ; HMDB29179 ; HMDB29228 ; HMDB29231 ; HMDB29657 ; HMDB29658 ; HMDB29659 ; HMDB29660 ; HMDB29661 ; HMDB29662 ; HMDB29689 ; HMDB30662 ; HMDB30745 ; HMDB31609 ; HMDB32399 ; HMDB32572 ; HMDB32594 ; HMDB32604 ; HMDB32606 ; HMDB32629 ; HMDB32919 ; HMDB33003 ; HMDB33866 ; HMDB34172 ; HMDB36907 ; HMDB38519 ; HMDB40237 ; HMDB41283 ; HMDB41294 ; HMDB41507 ; HMDB60390	3-Hydroxy-4-aminopyridine sulfate ; p-Hydroxyphenylacetic acid ; 3-Hydroxyphenylacetic acid ; Ortho-Hydroxyphenylacetic acid ; Mandelic acid ; N-Acetylaspartylglutamic acid ; p-Anisic acid ; 3-Cresotinic acid ; 34-Dihydroxyphenylacetaldehyde ; 4-Hydroxy-3-methylbenzoic acid ; Peonidin ; Vanillin ; 3-O-methyl-(-)-epicatechin ; 4-O-methyl-(-)-epicatechin ; 4-Methyl-epicatechin ; 3-Methyl-epicatechin ; 24-Dihydroxyacetophenone ; 23-Dihydroxyacetophenone ; 24-Dihydroxyacetophenone ; 26-Dihydroxyacetophenone ; 34-Dihydroxyacetophenone ; 35-Dihydroxyacetophenone ; 2-Naphthalenethiol ; 4-O-Methylcatechin ; (R)-Heraclenol ; Phenoxyacetic acid ; Methyl furfuracrylate ; Methylparaben ; 2-(Hydroxymethyl)benzoic acid ; 2-Methoxybenzoic acid ; 3-Methoxybenzoic acid ; 25-Dihydroxyacetophenone ; Ethyl 2-furanyl diketone ; 1-(5-Methyl-2-furanyl)-12-propanedione ; Fonsecin B ; Methyl 2-hydroxybenzoate ; 2-Propenyl 2-furancarboxylate ; Arachidoside ; Dihydro-2-methoxy-2-methyl-3(2H)-thiophenone ; 1-(2-Furanyl)-13-butanedione ; 6-Methoxy-alpha-pyrufuran ; 3-O-Methylcatechin ; 4-Hydroxyphenyl acetate	M+H ; M+K ; M+K ; M+K ; M+K ; M+2K ; M+K ; M+K ; M+K ; M+K ; M+2Na ; M+K ; M+2K ; M+2K ; M+2K ; M+2K ; M+K ; M+K ; M+K ; M+K ; M+K ; M+K ; 2M+Na+K ; M+2K ; M+2K ; M+K ; M+K ; M+K ; M+K ; M+K ; M+K ; M+K ; M+K ; M+K ; M+2K ; M+K ; M+K ; M+2K ; M+2Na-H ; M+K ; M+2K ; M+2K ; M+K	C5H6N2O4S ; C8H8O3 ; C8H8O3 ; C8H8O3 ; C8H8O3 ; C11H16N2O8 ; C8H8O3 ; C8H8O3 ; C8H8O3 ; C8H8O3 ; C16H13ClO6 ; C8H8O3 ; C16H16O6 ; C16H16O6 ; C16H16O6 ; C16H16O6 ; C8H8O3 ; C8H8O3 ; C8H8O3 ; C8H8O3 ; C8H8O3 ; C8H8O3 ; C10H8S ; C16H16O6 ; C16H16O6 ; C8H8O3 ; C8H8O3 ; C8H8O3 ; C8H8O3 ; C8H8O3 ; C8H8O3 ; C8H8O3 ; C8H8O3 ; C8H8O3 ; C16H16O6 ; C8H8O3 ; C8H8O3 ; C16H16O6 ; C6H10O2S ; C8H8O3 ; C16H16O6 ; C16H16O6 ; C8H8O3
36535_259.7	1.748155	0.598216	-2.7156	0.021725	1.663	0.42361	NA	NA	NA	NA	NA
06974_102.0	1.747946	0.60939	-2.9552	0.014406	1.8415	0.42361	107.0397	HMDB00325 ; HMDB14620 ; HMDB34885 ; HMDB39466 ; HMDB40220	3-Hydroxysuberic acid ; Chlorpromazine ; Imazamethabenz ; Hexanethioic acid S-propyl ester ; Diethyl L-malate	M+H+Na ; M+3H ; M+H+2Na ; M+H+K ; M+H+Na	C8H14O5 ; C17H19ClN2S ; C15H18N2O3 ; C9H18OS ; C8H14O5
3035_64.18	1.746876	0.420489	-2.8066	0.018584	1.7309	0.42361	186.963	HMDB39797 ; HMDB40230	Dihydro-4-mercapto-5-methyl-3(2H)-thiophenone ; 3-Acetyl-12-dithiolane	M+K ; M+K	C5H8OS2 ; C5H8OS2

.5333_56.65	1.746176	1.031433	2.8213	0.018119	1.7419	0.42361	462.8615	HMDB01999 ; HMDB03598 ; HMDB08026 ; HMDB08053 ; HMDB08085 ; HMDB08118 ; HMDB08150 ; HMDB08276 ; HMDB08308 ; HMDB08340 ; HMDB08533 ; HMDB08564 ; HMDB08595 ; HMDB08792 ; HMDB11202 ; HMDB15655 ; HMDB35692 ; HMDB36703 ; HMDB36709 ; HMDB36775 ; HMDB36805 ; HMDB36811 ; HMDB36826 ; HMDB38172 ; HMDB38687 ; HMDB39486 ; HMDB41928	Eicosapentaenoic acid ; Retinyl ester ; PC(16:1(9Z)24:1(15Z)) ; PC(18:022:2(13Z16Z)) ; PC(18:1(11Z)22:1(13Z)) ; PC(18:1(9Z)22:1(13Z)) ; PC(18:2(9Z12Z)22:0) ; PC(20:020:2(11Z14Z)) ; PC(20:1(11Z)20:1(11Z)) ; PC(20:2(11Z14Z)20:0) ; PC(22:018:2(9Z12Z)) ; PC(22:1(13Z)18:1(9Z)) ; PC(22:2(13Z16Z)18:0) ; PC(24:1(15Z)16:1(9Z)) ; PC(22:1(13Z)18:1(11Z)) ; Methyltestosterone ; 815-Isopimaradien-18-oic acid ; Siderone ; Yucalexin B14 ; Yucalexin A16 ; 8(17)12-Labdadiene-1516-dial ; Isopimaric acid ; Cascarillone ; 813-Abietadien-18-oic acid ; (ent-15beta16beta)-1516-Epoxy-3-kauranone ; ent-8(17)13(16)14-Labdatrien-18-oic acid ; Methenolone	3M+H+NH4 ; 3M+H+NH4 ; M+2ACN+2H ; M+2ACN+2H ; M+2ACN+2H ; M+2ACN+2H ; M+2ACN+2H ; M+2ACN+2H ; M+2ACN+2H ; M+2ACN+2H ; M+2ACN+2H ; M+2ACN+2H ; M+2ACN+2H ; M+2ACN+2H ; M+2ACN+2H ; 3M+H+NH4 ; 3M+H+NH4 ; 3M+H+NH4 ; 3M+H+NH4 ; 3M+H+NH4 ; 3M+H+NH4 ; 3M+H+NH4 ; 3M+H+NH4 ; 3M+H+NH4 ; 3M+H+NH4 ; 3M+H+NH4 ; 3M+H+NH4	C20H30O2 ; C20H30O2 ; C48H92NO8P ; C48H92NO8P ; C48H92NO8P ; C48H92NO8P ; C48H92NO8P ; C48H92NO8P ; C48H92NO8P ; C48H92NO8P ; C48H92NO8P ; C48H92NO8P ; C48H92NO8P ; C48H92NO8P ; C48H92NO8P ; C20H30O2 ; C20H30O2 ; C20H30O2 ; C20H30O2 ; C20H30O2 ; C20H30O2 ; C20H30O2 ; C20H30O2 ; C20H30O2 ; C20H30O2 ; C20H30O2 ; C20H30O2
;7741_56.47	1.744701	1.182917	-2.7241	0.021409	1.6694	0.42361	431.9068	HMDB04902 ; HMDB04960	Ganglioside GA1 (d18:116:0) ; Tetrahexosylceramide (d18:116:0)	M+3Na ; M+3Na	C60H110N2O23 ; C60H110N2O23
30407_286.8	1.744104	0.767642	2.7007	0.022287	1.6519	0.42361	527.163	HMDB00071 ; HMDB01262 ; HMDB03213 ; HMDB03539 ; HMDB06599 ; HMDB06857 ; HMDB11729 ; HMDB11730 ; HMDB14567 ; HMDB29347 ; HMDB29348 ; HMDB29910 ; HMDB29921 ; HMDB29922 ; HMDB29929 ; HMDB29930 ; HMDB29937 ; HMDB29956 ; HMDB30421 ; HMDB30618 ; HMDB30771 ; HMDB32867 ; HMDB33315 ; HMDB33673 ; HMDB33748 ; HMDB33801 ; HMDB33901 ; HMDB33927 ; HMDB34041 ; HMDB34072 ; HMDB34087 ; HMDB35322 ; HMDB35373 ; HMDB35860 ; HMDB36636 ; HMDB37920 ; HMDB38851 ; HMDB38852 ; HMDB38853 ; HMDB38902 ; HMDB39364 ; HMDB39573 ; HMDB39703 ; HMDB39704 ; HMDB39706 ; HMDB39707 ; HMDB39708 ; HMDB39749 ; HMDB40181 ; HMDB40984 ; HMDB41948 ; HMDB60468 ; HMDB10356	Deoxyinosine ; Maltotriose ; Raffinose ; Levan ; 3-Galactosyllactose ; Dextrin ; 1-Kestose ; Melezitose ; Methocarbamol ; Acuminoside ; Neryl arabinofuranosyl-glucoside ; Gentiotriose ; Neokestose ; Galactotriose ; 6-O-Glucosylmaltose ; beta-D-Fructofuranosyl alpha-D-glucopyranosyl-(1-4)-D-glucopyranoside ; Panose ; Nebularine ; L-Linalool 3-xylosyl-(1-6)-glucoside ; Otobanone ; 6alpha-Hydroxyphaseollin ; Capillin ; Moracin H ; 6-Kestose ; Umbelliferose ; Demethoxycurcumin ; Glyceollin I ; 3-Hydroxymugineic acid ; Glyceollin III ; Gentianose ; Glyceollin II ; Fagopyritol B2 ; Licoagroaurone ; Terretonin ; Methuyl tanshinonate ; 3-(gammagamma-Dimethylallyl)genistein ; beta-D-Galactopyranosyl-(1-2)-beta-D-galactopyranosyl-(1-4)-D-galactose ; beta-D-Galactopyranosyl-(1-4)-beta-D-galactopyranosyl-(1-4)-D-galactose ; beta-D-Galactopyranosyl-(1-3)-beta-D-galactopyranosyl-(1-6)-D-galactose ; Lupiwighteone ; Isolimonic acid 16-17-lactone ; Geranyl arabinopyranosyl-glucoside ; 3-beta-Gentiobiosylglucose ; alpha-D-Glucopyranosyl-(1-6)-alpha-D-glucopyranosyl-(1-2)-D-glucose ; 4-beta-Laminaribiosylglucose ; alpha-D-Glucopyranosyl-(1-4)-alpha-D-glucopyranosyl-(1-6)-D-glucose ; 3-beta-Cellobiosylglucose ; Kenposide B ; Fagopyritol A2 ; Sophorotriose ; Nephritogenoside ; D-Gal alpha 1-6D-Gal alpha 1-6D-Glucose ; Estriol 3-sulfate 16-glucuronide	2M+Na ; M+Na ; M+Na ; M+Na ; M+Na ; M+Na ; M+Na ; M+Na ; 2M+2Na-H ; M+2K+H ; M+2K+H ; M+Na ; M+Na ; M+Na ; M+Na ; M+Na ; M+Na ; 2M+Na ; M+2K+H ; 3M+H+K ; 3M+H+K ; 3M+Na ; 3M+H+K ; M+Na ; M+Na ; 3M+H+K ; 3M+H+K ; 3M+2Na ; 3M+H+K ; M+Na ; 3M+H+K ; M+Na ; 3M+H+K ; M+K ; 3M+H+K ; 3M+H+K ; M+Na ; M+Na ; M+Na ; 3M+H+K ; M+K ; M+2K+H ; M+Na ; M+Na ; M+Na ; M+Na ; M+Na ; M+2K+H ; M+Na ; M+Na ; M+Na ; M+Na ; M+H-H2O	C10H12N4O4 ; C18H32O16 ; C18H32O16 ; C18H32O16 ; C18H32O16 ; C18H32O16 ; C18H32O16 ; C18H32O16 ; C11H15NO5 ; C21H36O10 ; C21H36O10 ; C18H32O16 ; C18H32O16 ; C18H32O16 ; C18H32O16 ; C18H32O16 ; C18H32O16 ; C10H12N4O4 ; C21H36O10 ; C20H18O5 ; C20H18O5 ; C12H8O ; C20H18O5 ; C18H32O16 ; C18H32O16 ; C20H18O5 ; C20H18O5 ; C12H20N2O9 ; C20H18O5 ; C18H32O16 ; C20H18O5 ; C18H32O16 ; C20H18O5 ; C26H32O9 ; C20H18O5 ; C20H18O5 ; C18H32O16 ; C18H32O16 ; C18H32O16 ; C20H18O5 ; C26H32O9 ; C21H36O10 ; C18H32O16 ; C18H32O16 ; C18H32O16 ; C18H32O16 ; C18H32O16 ; C21H36O10 ; C18H32O16 ; C18H32O16 ; C18H32O16 ; C18H32O16 ; C24H32O12S

)6639_218.2	1.742206	1.193282	2.7047	0.022135	1.6549	0.42361	357.0697	HMDB29629 ; HMDB14944 ; HMDB29433 ; HMDB30679 ; HMDB30842 ; HMDB31385 ; HMDB33639 ; HMDB34442 ; HMDB36411 ; HMDB37330 ; HMDB37331 ; HMDB40354	26-Digalloyliriflophenone 3-C-glucoside ; Pentoxifylline ; L-2-Amino-4-methylenepentanedioic acid ; Cabreuvin ; 457-Trimethoxyflavone ; ()-Dulciol E ; 345-Trimethoxyflavone ; L-trans-alpha-Amino-2-carboxycyclopropaneacetic acid ; 5-Hydroxy-47-dimethoxy-6-methylflavone ; 356-Trimethoxyflavone ; 256-Trimethoxyflavone ; 145-Trihydroxy-3-prenylxanthone	M+2H ; M+2K+H ; 2M+K ; M+2Na-H ; M+2Na-H ; M+2Na-H ; M+2Na-H ; 2M+K ; M+2Na-H ; M+2Na-H ; M+2Na-H ; M+2Na-H	C33H28O18 ; C13H18N4O3 ; C6H9NO4 ; C18H16O5 ; C18H16O5 ; C18H16O5 ; C18H16O5 ; C6H9NO4 ; C18H16O5 ; C18H16O5 ; C18H16O5 ; C18H16O5
)8799_50.42	1.741267	0.457966	-2.8599	0.016959	1.7706	0.42361	538.7809	HMDB11728 ; HMDB29321 ; HMDB34536 ; HMDB39490 ; HMDB40992 ; HMDB41022	Bradykinin hydroxyproline ; Asparagoside F ; Araloside B ; Licoricesaponin F3 ; Mahanimbinol ; Hemsloside Ma 2	M+2H ; M+ACN+2H ; M+H+NH4 ; M+3ACN+2H ; 3M+2K ; M+H+NH4	C50H73N15O12 ; C50H82O22 ; C52H82O22 ; C48H72O19 ; C23H27NO ; C52H82O22
i6559_281.8	1.740716	1.080505	-2.7731	0.019683	1.7059	0.42361	172.0967	HMDB00303 ; HMDB00408 ; HMDB00491 ; HMDB00695 ; HMDB01864 ; HMDB04476 ; HMDB05784 ; HMDB06024 ; HMDB10717 ; HMDB12798 ; HMDB12882 ; HMDB14935 ; HMDB30570 ; HMDB31214 ; HMDB31216 ; HMDB31218 ; HMDB32012 ; HMDB32857 ; HMDB33286 ; HMDB33302 ; HMDB33698 ; HMDB34158 ; HMDB34363 ; HMDB37591 ; HMDB37592 ; HMDB37724 ; HMDB37728 ; HMDB38456 ; HMDB39276 ; HMDB39821 ; HMDB41602 ; HMDB59876 ; HMDB59966 ; HMDB60064	Tryptamine ; 2-Methyl-3-ketovaleric acid ; 3-Methyl-2-oxovaleric acid ; Ketoleucine ; 2-Ketohexanoic acid ; Anatabine ; Hydroxytyrosol ; Mevalonolactone ; 3-Oxohexanoic acid ; 5-Carboxy-alpha-chromanol ; Adipate semialdehyde ; Tolazoline ; Sylvopinol ; 5-Ethoxy-45-dihydro-2(3H)furanone ; Ethyl acetoacetate ; Ethyl 3-oxobutanoate ; 4-Hydroxy-3-methoxybenzenemethanol ; Glycerol tripropanoate ; Sherry lactone ; (4S6S)-3456-Tetrahydro-4-hydroxy-6-methyl-2H-pyran-2-one ; Acetoin acetate ; 26-Dimethoxyphenol ; Monocrotaline ; Oryzalide A ; Oryzalide B ; Propyl 2-furoate ; 2-Furanylmethyl propanoate ; N-Norgramine ; 8-Gingerdione ; 10-Acetylpanaxytriol ; Methyl levulinate ; Pantolactone ; 35-Dimethoxyphenol ; Tryptaminium	2M+H+Na ; M+ACN+H ; M+ACN+H ; M+ACN+H ; M+ACN+H ; 2M+H+Na ; M+NH4 ; M+ACN+H ; M+ACN+H ; M+H+Na ; M+ACN+H ; 2M+H+Na ; M+NH4 ; M+ACN+H ; M+ACN+H ; M+ACN+H ; M+NH4 ; M+2ACN+2H ; M+ACN+H ; M+ACN+H ; M+ACN+H ; M+NH4 ; M+H+NH4 ; M+H+Na ; M+H+Na ; M+NH4 ; M+NH4 ; 2M+H+Na ; M+H+Na ; M+H+Na ; M+ACN+H ; M+ACN+H ; M+NH4 ; 2M+H+Na	C10H12N2 ; C6H10O3 ; C6H10O3 ; C6H10O3 ; C6H10O3 ; C10H12N2 ; C8H10O3 ; C6H10O3 ; C6H10O3 ; C19H28O4 ; C6H10O3 ; C10H12N2 ; C8H10O3 ; C6H10O3 ; C6H10O3 ; C6H10O3 ; C8H10O3 ; C12H20O6 ; C6H10O3 ; C6H10O3 ; C6H10O3 ; C8H10O3 ; C16H23NO6 ; C19H28O4 ; C19H28O4 ; C8H10O3 ; C8H10O3 ; C10H12N2 ; C19H28O4 ; C19H28O4 ; C6H10O3 ; C6H10O3 ; C8H10O3 ; C10H12N2

)7158_284.2	1.739429	0.800706	2.8496	0.017261	1.7629	0.42361	205.0897	HMDB00353 ; HMDB00382 ; HMDB00517 ; HMDB03416 ; HMDB06348 ; HMDB06465 ; HMDB06548 ; HMDB06755 ; HMDB06757 ; HMDB06762 ; HMDB12848 ; HMDB29475 ; HMDB30605 ; HMDB30606 ; HMDB30730 ; HMDB30935 ; HMDB34015 ; HMDB34084 ; HMDB34279 ; HMDB34295 ; HMDB39123 ; HMDB39773 ; HMDB40313 ; HMDB40315 ; HMDB40641 ; HMDB40930 ; HMDB40954 ; HMDB41299	3b15b17a-Trihydroxy-pregnenone ; 3b17a21-Trihydroxypregnenone ; L-Arginine ; D-Arginine ; Pseudoecgonine ; Leukotriene F4 ; Ecgonine ; 3a21-Dihydroxy-5b-pregnane-1120-dione ; 11b21-Dihydroxy-5b-pregnane-320-dione ; 17a21-Dihydroxypreg-nenolone ; 7-Carboxy-alpha-chromanol ; 10-Gingerdione ; 15-Dihydroxy-3-methoxy-2-prenylxanthone ; 17-Dihydroxy-3-methoxy-2-prenylxanthone ; 3-(11-Dimethyl-2-propenyl)-8-(3-methyl-2-butenyl)xanthyletin ; 91013-Trihydroxystearic acid ; 16-Dihydroxy-3-methoxy-2-prenylxanthone ; Canesceol ; Egonol ; Floionolic acid ; Humilixanthin ; 11-alpha-O-beta-D-Glucopyranosyl-16alpha-O-methylneoquassin ; Moracin O ; Moracin P ; 8-Paradyl acetate ; 15-Bis(4-hydroxy-3-methoxyphenyl)-14-pentadien-3-one ; 9alpha-(3-Methyl-2E-pentenoyloxy)-4S-hydroxy-10(14)-oplopen-3-one ; omega-Hydroxymoracin N	M+Na+K ; M+Na+K ; 2M+Na+K ; 2M+Na+K ; 2M+H+K ; M+H+2Na ; 2M+H+K ; M+Na+K ; M+Na+K ; M+Na+K ; M+Na+K ; M+Na+K ; M+2ACN+2H ; M+2ACN+2H ; M+2Na ; M+2K ; M+2ACN+2H ; M+H+2Na ; M+2ACN+2H ; M+2K ; M+2ACN+2H ; M+H+2Na ; M+2ACN+2H ; M+2ACN+2H ; M+Na+K ; M+2ACN+2H ; M+Na+K ; M+2ACN+2H	C21H32O4 ; C21H32O4 ; C6H14N4O2 ; C6H14N4O2 ; C9H15NO3 ; C28H44N2O8S ; C9H15NO3 ; C21H32O4 ; C21H32O4 ; C21H32O4 ; C21H32O4 ; C21H32O4 ; C19H18O5 ; C19H18O5 ; C24H28O3 ; C18H36O5 ; C19H18O5 ; C29H44O11 ; C19H18O5 ; C18H36O5 ; C14H18N2O7 ; C29H44O11 ; C19H18O5 ; C19H18O5 ; C21H32O4 ; C19H18O5 ; C21H32O4 ; C19H18O5
09355_238.	1.739421	1.090049	2.7746	0.019631	1.7071	0.42361	346.0409	HMDB31812	Oxfendazole	2M+Na+K	C15H13N3O3S
0008_47.98	1.738627	1.021438	-2.7763	0.019574	1.7083	0.42361	510.71	HMDB14450 ; HMDB15294 ; HMDB15314 ; HMDB30220 ; HMDB30249 ; HMDB30286 ; HMDB31921 ; HMDB33363 ; HMDB39085 ; HMDB41812	Mitomycin ; Amdinocillin ; Naloxone ; Laurotetanine ; Norisocorydine ; Coreximine ; (R)-Norisocorydine ; Norcorydine ; (S)-Boldine ; 6-Acetylmorphine	3M+H+NH4 ; 3M+2Na ; 3M+H+K ; 3M+H+K ; 3M+H+K ; 3M+H+K ; 3M+H+K ; 3M+H+K ; 3M+H+K ; 3M+H+K	C15H18N4O5 ; C15H23N3O3S ; C19H21NO4 ; C19H21NO4 ; C19H21NO4 ; C19H21NO4 ; C19H21NO4 ; C19H21NO4 ; C19H21NO4 ; C19H21NO4
35049_92.9	1.737705	0.946789	-2.6644	0.023721	1.6249	0.42361	222.0335	HMDB01406 ; HMDB29992 ; HMDB31861 ; HMDB33108 ; HMDB38405 ; HMDB40275 ; HMDB60504 ; HMDB60913 ; HMDB60983 ; HMDB61072	Niacinamide ; Tetrahydropentoxyline ; 2-Acetylpyrazine ; Semilepidinoside B ; 6-Sinapoylglucoraphenin ; Ethyl aconitate ; S-(12-Dichlorovinyl)glutathione ; Zopiclone N-oxide ; zopiclone-N-oxide ; nopiclone N-oxide	3M+2K ; M+2K ; 3M+2K ; M+2K ; M+2H+Na ; 2M+H+K ; M+ACN+2H ; M+H+K ; M+H+K ; M+H+K	C6H6N2O ; C17H22N2O7 ; C6H6N2O ; C17H22N2O7 ; C23H31NO14S3 ; C8H10O6 ; C12H17Cl2N3O6S ; C17H17ClN6O4 ; C17H17ClN6O4 ; C17H17ClN6O4

27596_153.	1.736371	1.005886	-2.6395	0.024756	1.6063	0.42361	221.1228	HMDB14913 ; HMDB00214 ; HMDB00832 ; HMDB03374 ; HMDB05798 ; HMDB07005 ; HMDB07006 ; HMDB11545 ; HMDB11546 ; HMDB11547 ; HMDB11575 ; HMDB11576 ; HMDB11577 ; HMDB13116 ; HMDB14773 ; HMDB15235 ; HMDB30784 ; HMDB30792 ; HMDB32455 ; HMDB32735 ; HMDB34096 ; HMDB34275 ; HMDB39403 ; HMDB40405 ; HMDB41103 ; HMDB41540 ; HMDB59745	Tirofiban ; Ornithine ; Capryloylglycine ; D-Ornithine ; Malabaricone C ; CPA(18:1(11Z)0:0) ; CPA(18:1(9Z)0:0) ; MG(0:020:3(11Z14Z17Z)0:0) ; MG(0:020:3(5Z8Z11Z)0:0) ; MG(0:020:3(8Z11Z14Z)0:0) ; MG(20:3(11Z14Z17Z)0:00:0) ; MG(20:3(5Z8Z11Z)0:00:0) ; MG(20:3(8Z11Z14Z)0:00:0) ; Valproylglycine ; Prednisone ; Quinacrine ; Normammein ; Monascoflavin ; L-Ornithine monochlorohydrateornithine ; Isopersin ; Mammea BBD ; Mammea BAC ; 2-Hydroxy-4-oxo-512-heneicosadien-1-yl acetate ; 26-Glucosyl-131122-tetrahydroxyergosta-524-dien-26-oate ; Persin ; N-(5-Methyl-3-oxohexyl)alanine ; N-Acetylaminooctanoic acid	M+2H ; 3M+2Na ; 2M+H+K ; 3M+2Na ; M+2ACN+2H ; M+H+Na ; M+H+Na ; M+Na+K ; M+Na+K ; M+Na+K ; M+Na+K ; M+Na+K ; M+Na+K ; 2M+H+K ; M+2ACN+2H ; M+ACN+2H ; M+2ACN+2H ; M+2ACN+2H ; 3M+2Na ; M+Na+K ; M+2ACN+2H ; M+2ACN+2H ; M+Na+K ; M+2H+Na ; M+Na+K ; 2M+H+K ; 2M+H+K	C22H36N2O5S ; C5H12N2O2 ; C10H19NO3 ; C5H12N2O2 ; C21H26O5 ; C21H39O6P ; C21H39O6P ; C23H40O4 ; C23H40O4 ; C23H40O4 ; C23H40O4 ; C23H40O4 ; C23H40O4 ; C10H19NO3 ; C21H26O5 ; C23H30ClN3O ; C21H26O5 ; C21H26O5 ; C5H12N2O2 ; C23H40O4 ; C21H26O5 ; C21H26O5 ; C23H40O4 ; C34H54O11 ; C23H40O4 ; C10H19NO3 ; C10H19NO3
75255_286.	1.736111	0.770289	2.856	0.017072	1.7677	0.42361	363.0075	HMDB15408 ; HMDB14718 ; HMDB14878 ; HMDB14967	Bismuth Subsalicylate ; Mazindol ; Riluzole ; Diazepam	M+H ; M+2K+H ; 3M+H+Na ; M+2K+H	C7H5BiO4 ; C16H13ClN2O ; C8H5F3N2OS ; C16H13ClN2O
1817_282.3	1.735436	0.744166	2.9125	0.015497	1.8097	0.42361	611.4618	HMDB07063 ; HMDB07280 ; HMDB07309 ; HMDB07336 ; HMDB07337 ; HMDB07763 ; HMDB56155 ; HMDB56304 ; HMDB56367 ; HMDB07014 ; HMDB07015 ; HMDB07025 ; HMDB07026 ; HMDB07042 ; HMDB07052 ; HMDB07053 ; HMDB07099 ; HMDB07106 ; HMDB07127 ; HMDB07133 ; HMDB07134 ; HMDB07154 ; HMDB07182 ; HMDB07211 ; HMDB07273 ; HMDB07302 ; HMDB07330 ; HMDB07444 ; HMDB07473 ; HMDB07501 ; HMDB07530 ; HMDB31042 ; HMDB41092 ; HMDB55961 ; HMDB55962 ; HMDB55971 ; HMDB55977 ; HMDB56015 ; HMDB56031 ; HMDB56040 ; HMDB56139 ; HMDB56145 ; HMDB56164 ; HMDB56171	DG(14:1(9Z)22:6(4Z7Z10Z13Z16Z19Z)0:0) ; DG(18:3(6Z9Z12Z)18:4(6Z9Z12Z15Z)0:0) ; DG(18:3(9Z12Z15Z)18:4(6Z9Z12Z15Z)0:0) ; DG(18:4(6Z9Z12Z15Z)18:3(6Z9Z12Z)0:0) ; DG(18:4(6Z9Z12Z15Z)18:3(9Z12Z15Z)0:0) ; DG(22:6(4Z7Z10Z13Z16Z19Z)14:1(9Z)0:0) ; DG(14:1n50:022:6n3) ; DG(18:3n60:018:4n3) ; DG(18:3n30:018:4n3) ; DG(14:018:1(11Z)0:0) ; DG(14:018:1(9Z)0:0) ; DG(14:020:4(5Z8Z11Z14Z)0:0) ; DG(14:020:4(8Z11Z14Z17Z)0:0) ; DG(14:1(9Z)18:00:0) ; DG(14:1(9Z)20:3(5Z8Z11Z)0:0) ; DG(14:1(9Z)20:3(8Z11Z14Z)0:0) ; DG(16:016:1(9Z)0:0) ; DG(16:018:4(6Z9Z12Z15Z)0:0) ; DG(16:1(9Z)16:00:0) ; DG(16:1(9Z)18:3(6Z9Z12Z)0:0) ; DG(16:1(9Z)18:3(9Z12Z15Z)0:0) ; DG(18:014:1(9Z)0:0) ; DG(18:1(11Z)14:00:0) ; DG(18:1(9Z)14:00:0) ; DG(18:3(6Z9Z12Z)16:1(9Z)0:0) ; DG(18:3(9Z12Z15Z)16:1(9Z)0:0) ; DG(18:4(6Z9Z12Z15Z)16:00:0) ; DG(20:3(5Z8Z11Z)14:1(9Z)0:0) ; DG(20:3(8Z11Z14Z)14:1(9Z)0:0) ; DG(20:4(5Z8Z11Z14Z)14:00:0) ; DG(20:4(8Z11Z14Z17Z)14:00:0) ; Avocadene ; Tripoxyrollin ; DG(14:00:018:1n7) ; DG(14:00:018:1n9) ; DG(14:00:020:4n6) ; DG(14:00:020:4n3) ; DG(16:00:016:1n7) ; DG(16:00:018:4n3) ; DG(18:00:014:1n5) ; DG(14:1n50:020:3n9) ; DG(14:1n50:020:3n6) ; DG(16:1n70:018:3n6) ; DG(16:1n70:018:3n3)	M+H ; M+H ; M+H ; M+H ; M+H ; M+H ; M+H ; M+H ; M+H ; M+2Na-H ; M+2Na-H ; M+Na ; M+Na ; M+2Na-H ; M+Na ; M+Na ; M+2Na-H ; M+Na ; M+2Na-H ; M+Na ; M+Na ; M+2Na-H ; M+2Na-H ; M+2Na-H ; M+Na ; M+Na ; M+Na ; M+Na ; M+Na ; M+Na ; M+Na ; 2M+K ; M+Na ; M+2Na-H ; M+2Na-H ; M+Na ; M+Na ; M+2Na-H ; M+Na ; M+2Na-H ; M+Na ; M+Na ; M+Na ; M+Na	C39H62O5 ; C39H62O5 ; C39H62O5 ; C39H62O5 ; C39H62O5 ; C39H62O5 ; C39H62O5 ; C39H62O5 ; C39H62O5 ; C35H66O5 ; C35H66O5 ; C37H64O5 ; C37H64O5 ; C35H66O5 ; C37H64O5 ; C37H64O5 ; C35H66O5 ; C37H64O5 ; C35H66O5 ; C37H64O5 ; C37H64O5 ; C35H66O5 ; C35H66O5 ; C35H66O5 ; C37H64O5 ; C37H64O5 ; C37H64O5 ; C37H64O5 ; C37H64O5 ; C37H64O5 ; C37H64O5 ; C17H34O3 ; C37H64O5 ; C35H66O5 ; C35H66O5 ; C37H64O5 ; C37H64O5 ; C35H66O5 ; C37H64O5 ; C35H66O5 ; C37H64O5 ; C37H64O5 ; C37H64O5 ; C37H64O5

9296_114.2	1.734635	1.036197	2.6245	0.0254	1.5952	0.42361	464.0808	HMDB00536 ; HMDB59653 ; HMDB06779 ; HMDB14576 ; HMDB30481 ; HMDB38370 ; HMDB39907 ; HMDB41059 ; HMDB60383	Adenylsuccinic acid ; N(6)-(12-dicarboxyethyl)AMP ; Indole-56-quinone ; Trifluridine ; Agrocybin ; Procyanidin C1 ; Tricin 7-sinapoyl-(-2)-glucuronyl-(1-2)-glucuronide ; N-Methoxyspirobrassinol methyl ether ; 4-(Methylnitrosamino)-1-(3-pyridyl)-1-butanol glucuronide	M+H ; M+H ; 3M+Na ; 3M+H+K ; 3M+Na ; M+Na+K ; M+H+K ; 3M+H+K ; M+2K+H	C14H18N5O11P ; C14H18N5O11P ; C8H5NO2 ; C10H11F3N2O5 ; C8H5NO2 ; C45H38O18 ; C40H40O23 ; C13H16N2O2S2 ; C16H23N3O8
9482_280.8	1.734354	0.186231	3.0526	0.012197	1.9137	0.42361	713.3719	HMDB02835 ; HMDB10340 ; HMDB15409 ; HMDB33218 ; HMDB33230 ; HMDB33231 ; HMDB34771 ; HMDB38721 ; HMDB39342 ; HMDB39474 ; HMDB41446 ; HMDB41986 ; HMDB60264 ; HMDB60336	Danazol ; Retinyl beta-glucuronide ; Bevantolol ; 26-Dimethyl-7-octene-16-diol 8-O-glucoside ; trans-p-Menthane-78-diol 7-glucoside ; trans-p-Menthane-78-diol 8-glucoside ; 37-Dimethyl-5-octene-17-diol 1-glucoside ; Soyasaponin bg ; Capsianoside G ; Capsianoside H ; Rosmaricine ; Pibutidine ; Apelin (1-12) ; 14-Bipiperidine-1-carboxylic acid	2M+K ; 3M+H+K ; 2M+Na ; 2M+2Na-H ; 2M+2Na-H ; 2M+2Na-H ; 2M+2Na-H ; 2M+3H ; M+H+Na ; M+H+Na ; 2M+Na ; 2M+H ; M+H+Na ; 3M+2K-H	C22H27NO2 ; C26H38O7 ; C20H27NO4 ; C16H30O7 ; C16H30O7 ; C16H30O7 ; C16H30O7 ; C54H84O21 ; C70H114O28 ; C70H114O28 ; C20H27NO4 ; C19H24N4O3 ; C60H102N22O15S ; C11H20N2O2
2587_94.3	1.73373	0.601855	-2.7466	0.0206	1.6861	0.42361	230.5753	HMDB37551	1-(2-Hydroxyphenylamino)-1-deoxy-beta-D-gentiobioside 12-carbamate	M+2H	C19H25NO12
1664_239.5	1.732142	1.177423	2.8162	0.018281	1.738	0.42361	441.9722	HMDB15206 ; HMDB59912	Fludarabine ; Indigo Carmine	M+2K-H ; 2M+H+K	C10H13FN5O7P ; C16H10N2O8S2
4851_38.12	1.731907	0.641736	-2.5178	0.030504	1.5156	0.42361	137.0593	HMDB00209 ; HMDB02340 ; HMDB03767 ; HMDB29635 ; HMDB29686 ; HMDB31459 ; HMDB32400 ; HMDB32568 ; HMDB32603 ; HMDB33128 ; HMDB33766 ; HMDB33968 ; HMDB38176 ; HMDB40638 ; HMDB40733 ; HMDB41485 ; HMDB04810 ; HMDB30526 ; HMDB32847 ; HMDB33800 ; HMDB34435 ; HMDB35035 ; HMDB41660 ; HMDB41669 ; HMDB41670 ; HMDB41701 ; HMDB02012 ; HMDB06853 ; HMDB12550 ; HMDB13785 ; HMDB30810 ; HMDB30899 ; HMDB31920 ; HMDB32116 ; HMDB33303 ; HMDB35095 ; HMDB35168 ; HMDB35404 ; HMDB35405 ; HMDB36665 ; HMDB36666 ; HMDB36944 ; HMDB36970 ; HMDB37241 ; HMDB37242 ; HMDB37299 ; HMDB37815 ; HMDB38746 ; HMDB38804 ; HMDB40757 ; HMDB40758 ; HMDB41226 ; HMDB41279 ; HMDB41348 ; [illegible]	Phenylacetic acid ; 2-Methylbenzoic acid ; 4-Hydroxyphenylacetaldehyde ; 4-Methylbenzoic acid ; 4-Methoxybenzaldehyde ; 3-Methoxybenzaldehyde ; 3-(5-Methyl-2-furyl)prop-2-enal ; 2-Hydroxyacetophenone ; 2-Hydroxy-4-methylbenzaldehyde ; 4-(2-Furanyl)-3-buten-2-one ; 2-Methoxybenzaldehyde ; Methyl benzoate ; alpha-Methyl-2-furanacrolein ; 3-(3-Furanyl)-2-methyl-2-propenal ; Phenyl acetate ; Benzyl formate ; 5C-aglycone ; Isovestitol ; 12-Bis(4-hydroxy-3-methoxyphenyl)ethylene ; 246-Trimethoxybenzophenone ; 1-(26-Dihydroxy-4-methoxyphenyl)-3-phenyl-1-propanone ; Marminal ; 3-O-Methylequol ; 47-Dihydroxy-3-methoxyisoflavan ; 47-Dihydroxy-6-methoxyisoflavan ; 6-O-Methylequol ; Ubiquinone-1 ; 3-Butyn-1-al ; 12-Oxo-20-carboxy-leukotriene B4 ; Furan ; Porson ; Eremopetasitenin A1 ; 9-Hydroxycalabaxanthone ; Helinorbisabone ; Anofinic acid ; 7-Ethoxy-4-methyl-2H-1-benzopyran-2-one ; Cinncassiol C2 ; Isogingerenone B ; Gingerenone B ; Blumealactone A ; Blumealactone B ; Gibberellin A123 ; Garcimangosone B ; 89-Dihydro-5-hydroxy-8-(1-hydroxy-1-methylethyl)-6-(2-methyl-1-oxopropyl)-4-phenyl-2H-furo23-h-1-benzopyran-2-one ; Mammea AAC cyclo F ; 68-Dihydroxy-17-diprenylxanthone-2-carboxylic acid ; 5-(Methylthio)-2-(methylthio)methyl-2-pentenal ; Gibberellin A74 ; 34-Dihydro-6-hydroxy-2578-tetramethyl-2H-1-benzopyran-2-carboxylic acid ; 3b8a-Dihydroxy-6b-angeloyloxy-7(11)-eremophilen-128-olide ; 3b8b-Dihydroxy-6b-angeloyloxy-7(11)-eremophilen-128-olide ; 3b9b-Dihydroxy-6b-angeloyloxy-7(11)-eremophilen-128b-olide ; 3b-Hydroxy-6b-(23-epoxy-2-methylbutanoyloxy)-7(11)-eremophilen-128b-olide ; Gibberellin A100 ; Gibberellin A97 ; 5-(4-Hydroxyphenyl)-gamma-valerolactone-4-O-glucuronide ; Melatonin glucuronide	M+H ; M+H ; M+H ; M+H ; M+H ; M+H ; M+H ; M+H ; M+H ; M+H ; M+H ; M+H ; M+H ; M+H ; M+H ; M+H ; M+2H ; M+2H ; M+2H ; M+2H ; M+2H ; M+2H ; M+2H ; M+2H ; M+2H ; M+2H ; M+H+Na ; 2M+H ; M+H+2Na ; 2M+H ; M+2H+Na ; M+H+2Na ; M+3H ; M+H+Na ; 2M+3H ; 2M+3H ; M+H+2Na ; M+2H+Na ; M+2H+Na ; M+H+2Na ; M+H+2Na ; M+H+2Na ; M+3H ; M+3H ; M+3H ; M+3H ; M+2ACN+2H ; M+H+2Na ; M+H+Na ; M+H+2Na ; M+H+2Na ; M+H+2Na ; M+H+2Na ; M+H+2Na ; M+H+2Na ; M+H+2Na ; M+3H	C8H8O2 ; C8H8O2 ; C8H8O2 ; C8H8O2 ; C8H8O2 ; C8H8O2 ; C8H8O2 ; C8H8O2 ; C8H8O2 ; C8H8O2 ; C8H8O2 ; C8H8O2 ; C8H8O2 ; C8H8O2 ; C8H8O2 ; C8H8O2 ; C16H16O4 ; C16H16O4 ; C16H16O4 ; C16H16O4 ; C16H16O4 ; C16H16O4 ; C16H16O4 ; C16H16O4 ; C16H16O4 ; C16H16O4 ; C14H18O4 ; C4H4O ; C20H28O6 ; C4H4O ; C22H26O6 ; C20H28O6 ; C24H24O6 ; C14H18O4 ; C12H12O3 ; C12H12O3 ; C20H28O6 ; C22H26O6 ; C22H26O6 ; C20H28O6 ; C20H28O6 ; C20H28O6 ; C24H24O6 ; C24H24O6 ; C24H24O6 ; C24H24O6 ; C8H14OS2 ; C20H28O6 ; C14H18O4 ; C20H28O6 ; C20H28O6 ; C20H28O6 ; C20H28O6 ; C20H28O6 ; C20H28O6 ; C20H28O6 ; C19H24N2O8

5763_238.7(	1.731132	1.172395	2.814	0.018347	1.7364	0.42361	447.0676	HMDB15278 ; HMDB32197 ; HMDB33147 ; HMDB39567 ; HMDB41257 ; HMDB59975 ; HMDB59976 ; HMDB60342 ; HMDB60505	Cloxacillin ; Cellulose microcrystalline ; Cloransulam-methyl ; BL V ; 5-Hydroxy-3378-tetramethoxy-45-methylenedioxyflavone ; 4-Hydroxy-5-(3-hydroxyphenyl)-valeric acid-3-O-sulphate ; 4-Hydroxy-5-(4-hydroxyphenyl)-valeric acid-4-O-sulphate ; 2-(S-Glutathionyl)acetyl chloride ; S-(2-Chloroacetyl)glutathione	2M+H+Na ; M+2K-H ; M+NH4 ; M+Na ; M+2Na-H ; 3M+H+Na ; 3M+H+Na ; M+ACN+Na ; M+ACN+Na	C19H18ClN3O5S ; C14H26O11 ; C15H13ClFN5O5S ; C22H16O9 ; C20H18O9 ; C11H14O7S ; C11H14O7S ; C12H18ClN3O7S ; C12H18ClN3O7S
.7461_196.1	1.730988	0.960683	-2.7866	0.019231	1.716	0.42361	429.1017	HMDB02706 ; HMDB29190 ; HMDB29743 ; HMDB30055 ; HMDB30389 ; HMDB30390 ; HMDB31370 ; HMDB37493 ; HMDB38416 ; HMDB40238 ; HMDB41285 ; HMDB59988 ; HMDB59990 ; HMDB60030 ; HMDB60484	Canavanine ; 5-(34-dihydroxyphenyl)-gamma-valerolactone-3-O-glucuronide ; 9H-Carbazole-3-carboxaldehyde ; Taraxacoside ; Quebrachidine ; ()-Quebrachidine ; 2-(Methylthio)methyl-2-butenal ; S-2-Propenyl propanethioate ; 5-Hexenyl glucosinolate ; 2-Ethyldihydro-3(2H)-thiophenone ; Picraquassioside A ; 5-(34-Dihydroxyphenyl)-gamma-valerolactone-3-O-methyl-4-O-glucuronide ; 5-(34-Dihydroxyphenyl)-gamma-valerolactone-4-O-methyl-3-O-glucuronide ; 5-(35-Dihydroxyphenyl)-gamma-valerolactone-O-glucuronide-O-methyl ; Indolepyruvate	2M+2K-H ; 2M+Na+K ; 2M+K ; 2M+Na+K ; M+2K-H ; M+2K-H ; 3M+K ; 3M+K ; 2M+3H2O+2H ; 3M+K ; 2M+Na+K ; 2M+Na+K ; 2M+Na+K ; 2M+Na+K ; 2M+Na	C5H12N4O3 ; C18H22O10 ; C13H9NO ; C18H22O10 ; C21H24N2O3 ; C21H24N2O3 ; C6H10OS ; C6H10OS ; C13H23NO9S2 ; C6H10OS ; C18H22O10 ; C18H22O10 ; C18H22O10 ; C18H22O10 ; C11H9NO3
52289_249.4	1.730796	1.132337	-2.6432	0.024601	1.6091	0.42361	95.08552	HMDB31483 ; HMDB31486 ; HMDB31487 ; HMDB31488 ; HMDB31539 ; HMDB31540 ; HMDB31550 ; HMDB31551 ; HMDB32301 ; HMDB33667 ; HMDB33827 ; HMDB34372 ; HMDB40339	(Z)-4-Heptenal ; 3-Hepten-2-one ; (E)-4-Hepten-2-one ; 2-Hepten-4-one ; 2-Methylcyclohexanone ; 4-Methylcyclohexanone ; 5-Methyl-3-hexen-2-one ; 5-Methyl-5-hexen-2-one ; trans-2-trans-4-Heptadien-1-ol ; (R)-3-Methylcyclohexanone ; 2-Heptenal ; xi-3-Methyl-3-cyclohexen-1-ol ; 25-Heptadien-1-ol	M+H-H2O ; M+H-H2O ; M+H-H2O ; M+H-H2O ; M+H-H2O ; M+H-H2O ; M+H-H2O ; M+H-H2O ; M+H-H2O ; M+H-H2O ; M+H-H2O ; M+H-H2O ; M+H-H2O	C7H12O ; C7H12O ; C7H12O ; C7H12O ; C7H12O ; C7H12O ; C7H12O ; C7H12O ; C7H12O ; C7H12O ; C7H12O ; C7H12O ; C7H12O
52243_57.04	1.729068	1.096531	-2.6394	0.024761	1.6062	0.42361	300.9742	HMDB00300 ; HMDB60760	Uracil ; 4-Carboxypyrazole	2M+2K-H ; 2M+2K-H	C4H4N2O2 ; C4H4N2O2

3625_284.4	1.727073	0.920797	2.7601	0.020128	1.6962	0.42361	204.0864	HMDB38440 ; HMDB00079 ; HMDB00321 ; HMDB00345 ; HMDB00355 ; HMDB00368 ; HMDB00640 ; HMDB01004 ; HMDB01324 ; HMDB01390 ; HMDB01411 ; HMDB01427 ; HMDB02249 ; HMDB02278 ; HMDB02282 ; HMDB06730 ; HMDB12492 ; HMDB14685 ; HMDB15527 ; HMDB29834 ; HMDB29874 ; HMDB29934 ; HMDB31547 ; HMDB32873 ; HMDB34426 ; HMDB40985 ; HMDB41026 ; HMDB41561 ; HMDB41835 ; HMDB59758 ; HMDB32293	1-Isothiocyanato-7-(methylthio)heptane ; Dihydrothymine ; 2-Hydroxyadipic acid ; 3-Hydroxyadipic acid ; 3-Hydroxymethylglutaric acid ; 2(R)-Hydroxyadipic acid ; Levoglucosan ; Oxoamide ; N-Hydroxymethylnorcotinine ; Hydroxycotinine ; Cotinine N-oxide ; 5-Hydroxycotinine ; 6-Methyltetrahydropterin ; 2-(acetylamino)-15-anhydro-2-deoxy-3-O-b-D-galactopyranosyl-D-arabino-Hex-1-enitol ; 2-(acetylamino)-15-anhydro-2-deoxy-4-O-b-D-galactopyranosyl-D-arabino-Hex-1-enitol ; QYNAD ; 1-(12345-Pentahydroxypent-1-yl)-1234-tetrahydro-beta-carboline-3-carboxylate ; Pyridostigmine ; Prazepam ; Harmalan ; Squamolone ; D-1-Deoxy-erythro-hexo-23-diulose ; L-Cyclo(alanylglycyl) ; Diethyl dicarbonate ; (-)-1-Methylpropyl 1-propenyl disulfide ; Polixetonium chloride ; Erinapyrone C ; D-15-Anhydrofructose ; Benzidine ; 2-Hydroxy-2-ethylsuccinic acid ; N-Gluconyl ethanolamine	M+H ; 3M+H+Na ; M+ACN+H ; M+ACN+H ; M+ACN+H ; M+ACN+H ; M+ACN+H ; 2M+H+Na ; 2M+H+Na ; 2M+H+Na ; 2M+H+Na ; 2M+H+Na ; M+Na ; M+ACN+2H ; M+ACN+2H ; M+3H ; M+ACN+2H ; M+Na ; M+2ACN+2H ; 2M+H+K ; 3M+H+Na ; M+ACN+H ; 3M+H+Na ; M+ACN+H ; M+ACN+H ; M+2ACN+2H ; M+NH4 ; M+ACN+H ; 2M+H+K ; M+ACN+H ; M+H-2H2O	C9H17NS2 ; C5H8N2O2 ; C6H10O5 ; C6H10O5 ; C6H10O5 ; C6H10O5 ; C6H10O5 ; C10H12N2O2 ; C10H12N2O2 ; C10H12N2O2 ; C10H12N2O2 ; C10H12N2O2 ; C7H11N5O ; C14H23NO10 ; C14H23NO10 ; C25H35N7O11 ; C17H21N2O7 ; C9H13N2O2 ; C19H17ClN2O ; C12H12N2 ; C5H8N2O2 ; C6H10O5 ; C5H8N2O2 ; C6H10O5 ; C7H14S2 ; C10H28Cl2N2OP2 ; C8H10O5 ; C6H10O5 ; C12H12N2 ; C6H10O5 ; C8H17NO7
1947_240.2	1.726968	1.188781	2.7594	0.020151	1.6957	0.42361	252.0162	HMDB00904 ; HMDB01143 ; HMDB01498 ; HMDB03148 ; HMDB15470 ; HMDB32887 ; HMDB34209 ; HMDB37113	Citrulline ; Inositol 134-trisphosphate ; Inositol 145-trisphosphate ; Argininic acid ; Salicylate-sodium ; 4-Amino-2-methyl-1-naphthol ; Cyclobrassinone ; 1256-Tetrahydro-4H-pyrrolo321-ijquinolin-4-one	M+2K-H ; M+2ACN+2H ; M+2ACN+2H ; M+2K-H ; 3M+H+Na ; M+2K+H ; 2M+H+K ; M+2K+H	C6H13N3O3 ; C6H15O15P3 ; C6H15O15P3 ; C6H13N3O3 ; C7H5NaO3 ; C11H11NO ; C11H8N2O2S ; C11H11NO
74988_291.0	1.726907	0.216359	2.7283	0.021255	1.6725	0.42361	705.9275	HMDB35697 ; HMDB38141 ; HMDB60188	Momordicoside E ; Glucosyl passiflorate ; ciscis-1114-Eicosadienoyl-CoA	2M+H+NH4 ; 2M+H+NH4 ; 2M+3H	C37H60O12 ; C37H60O12 ; C41H70N7O17P3S
07899_55.22	1.726765	1.140209	2.8519	0.017195	1.7646	0.42361	421.8698	HMDB31798 ; HMDB31959 ; HMDB56120 ; HMDB56122	()-Leptophos ; Lablaboside D ; DG(24:00:018:2n6) ; DG(24:00:020:2n6)	2M+H+Na ; M+3H ; M+3ACN+2H ; M+3ACN+2H	C13H10BrCl2O2PS ; C60H94O28 ; C46H86O5 ; C46H86O5
08493_42.87	1.72273	0.941338	2.6144	0.025843	1.5877	0.42361	420.9298	HMDB00280 ; HMDB29716	Phosphoribosyl pyrophosphate ; Liquid thiophthene	2M+Na+K ; 3M+H	C5H13O14P3 ; C6H4S2

73673_21.8	1.722632	0.768234	-2.4752	0.032813	1.4839	0.42361	404.2874	HMDB00348 ; HMDB00361 ; HMDB00384 ; HMDB00411 ; HMDB00438 ; HMDB00478 ; HMDB00514 ; HMDB00518 ; HMDB00626 ; HMDB00664 ; HMDB00686 ; HMDB00733 ; HMDB00811 ; HMDB00946 ; HMDB02451 ; HMDB02488 ; HMDB02536 ; HMDB02585 ; HMDB07442 ; HMDB07470 ; HMDB07499 ; HMDB07639 ; HMDB07667 ; HMDB07806 ; HMDB07807 ; HMDB07834 ; HMDB07951 ; HMDB08494 ; HMDB08944 ; HMDB08945 ; HMDB08976 ; HMDB09005 ; HMDB09036 ; HMDB09037 ; HMDB09045 ; HMDB09069 ; HMDB09070 ; HMDB09078 ; HMDB09100 ; HMDB09101 ; HMDB09109 ; HMDB09110 ; HMDB09132 ; HMDB09141 ; HMDB09165 ; HMDB09174 ; HMDB09197 ; HMDB09261 ; HMDB09292 ; HMDB09293 ; HMDB09302 ; HMDB09324 ; HMDB09333 ; HMDB09334 ; HMDB09357 ; HMDB09366 ;	3b12a-Dihydroxy-5a-cholanoic acid ; 3b7a-Dihydroxy-5b-cholanoic acid ; 3a7a-Dihydroxycholanoic acid ; 3a12b-Dihydroxy-5b-cholanoic acid ; 3b12a-Dihydroxy-5b-cholanoic acid ; Allodeoxycholic acid ; Allochenodeoxycholic acid ; Chenodeoxycholic acid ; Deoxycholic acid ; Isohyodeoxycholic acid ; Isoursodeoxycholic acid ; Hyodeoxycholic acid ; Murocholic acid ; Ursodeoxycholic acid ; 7b12a-Dihydroxycholanoic acid ; 7a12b-dihydroxy-5b-Cholan-24-oic acid ; Isodeoxycholic acid ; 3b12b-Dihydroxy-5b-cholanoic acid ; DG(20:2(11Z14Z)24:1(15Z)0:0) ; DG(20:3(5Z8Z11Z)24:00:0) ; DG(20:3(8Z11Z14Z)24:00:0) ; DG(22:1(13Z)22:2(13Z16Z)0:0) ; DG(22:2(13Z16Z)22:1(13Z)0:0) ; DG(24:020:3(5Z8Z11Z)0:0) ; DG(24:020:3(8Z11Z14Z)0:0) ; DG(24:1(15Z)20:2(11Z14Z)0:0) ; PC(15:020:5(5Z8Z11Z14Z17Z)) ; PC(20:5(5Z8Z11Z14Z17Z)15:0) ; PE(16:022:5(4Z7Z10Z13Z16Z)) ; PE(16:022:5(7Z10Z13Z16Z19Z)) ; PE(16:1(9Z)22:4(7Z10Z13Z16Z)) ; PE(18:020:5(5Z8Z11Z14Z17Z)) ; PE(18:1(11Z)20:4(5Z8Z11Z14Z)) ; PE(18:1(11Z)20:4(8Z11Z14Z17Z)) ; PE(18:1(11Z)22:6(4Z7Z10Z13Z16Z19Z)) ; PE(18:1(9Z)20:4(5Z8Z11Z14Z)) ; PE(18:1(9Z)20:4(8Z11Z14Z17Z)) ; PE(18:1(9Z)22:6(4Z7Z10Z13Z16Z19Z)) ; PE(18:2(9Z12Z)20:3(5Z8Z11Z)) ; PE(18:2(9Z12Z)20:3(8Z11Z14Z)) ; PE(18:2(9Z12Z)22:5(4Z7Z10Z13Z16Z)) ; PE(18:2(9Z12Z)22:5(7Z10Z13Z16Z19Z)) ; PE(18:3(6Z9Z12Z)20:2(11Z14Z)) ; PE(18:3(6Z9Z12Z)22:4(7Z10Z13Z16Z)) ; PE(18:3(9Z12Z15Z)20:2(11Z14Z)) ; PE(18:3(9Z12Z15Z)22:4(7Z10Z13Z16Z)) ; PE(18:4(6Z9Z12Z15Z)20:1(11Z)) ; PE(20:1(11Z)18:4(6Z9Z12Z15Z)) ; PE(20:2(11Z14Z)18:3(6Z9Z12Z)) ; PE(20:2(11Z14Z)18:3(9Z12Z15Z)) ; PE(20:2(11Z14Z)20:5(5Z8Z11Z14Z17Z)) ; PE(20:3(5Z8Z11Z)18:2(9Z12Z)) ; PE(20:3(5Z8Z11Z)20:4(5Z8Z11Z14Z)) ; PE(20:3(5Z8Z11Z)20:4(8Z11Z14Z17Z)) ; PE(20:3(8Z11Z14Z)18:2(9Z12Z)) ; PE(20:3(8Z11Z14Z)20:4(5Z8Z11Z14Z)) ; PE(20:3(8Z11Z14Z)20:4(8Z11Z14Z17Z)) ; PE(20:4(5Z8Z11Z14Z)18:1(11Z)) ;	2M+H+Na ; 2M+H+Na ; 2M+H+Na ; 2M+H+Na ; 2M+H+Na ; 2M+H+Na ; 2M+H+Na ; 2M+H+Na ; 2M+H+Na ; 2M+H+Na ; 2M+H+Na ; 2M+H+Na ; 2M+H+Na ; 2M+H+Na ; 2M+H+Na ; 2M+H+Na ; 2M+H+Na ; 2M+H+Na ; M+2K ; M+2K ; M+2K ; M+2K ; M+2K ; M+2K ; M+2K ; M+2K ; M+ACN+2H ; M+ACN+2H ; M+ACN+2H ; M+ACN+2H ; M+ACN+2H ; M+ACN+2H ; M+ACN+2H ; M+ACN+2H ; M+H+NH4 ; M+ACN+2H ; M+ACN+2H ; M+H+NH4 ; M+ACN+2H ; M+ACN+2H ; M+H+NH4 ; M+H+NH4 ; M+ACN+2H ; M+H+NH4 ; M+ACN+2H ; M+H+NH4 ; M+ACN+2H ; M+ACN+2H ; M+ACN+2H ; M+ACN+2H ; M+H+NH4 ; M+ACN+2H ; M+H+NH4 ; M+H+NH4 ; M+ACN+2H ; M+H+NH4 ; M+H+NH4 ; M+ACN+2H ; M+ACN+2H ; M+H+NH4 ; M+H+NH4 ; M+ACN+2H ; M+ACN+2H ; M+H+NH4 ; M+H+NH4 ; M+H+NH4 ; M+ACN+2H ; M+H+NH4 ; M+ACN+2H ; M+H+NH4 ; M+H+NH4 ; M+ACN+2H ; M+H+NH4 ; M+ACN+2H ; M+H+NH4 ; M+H+NH4 ; M+H+NH4 ; 2M+3H2O+2H ; 2M+3H2O+2H ; 2M+3H2O+2H ; M+2Na-H ; M+2ACN+H ; 2M+3H2O+2H ; 3M+H+K ; M+2K ; M+2K ; M+2K	C24H40O4 ; C24H40O4 ; C24H40O4 ; C24H40O4 ; C24H40O4 ; C24H40O4 ; C24H40O4 ; C24H40O4 ; C24H40O4 ; C24H40O4 ; C24H40O4 ; C24H40O4 ; C24H40O4 ; C24H40O4 ; C24H40O4 ; C24H40O4 ; C24H40O4 ; C24H40O4 ; C47H86O5 ; C47H86O5 ; C47H86O5 ; C47H86O5 ; C47H86O5 ; C47H86O5 ; C47H86O5 ; C47H86O5 ; C43H76NO8P ; C43H76NO8P ; C43H76NO8P ; C43H76NO8P ; C43H76NO8P ; C43H76NO8P ; C43H76NO8P ; C43H76NO8P ; C45H76NO8P ; C43H76NO8P ; C43H76NO8P ; C45H76NO8P ; C43H76NO8P ; C43H76NO8P ; C45H76NO8P ; C45H76NO8P ; C43H76NO8P ; C45H76NO8P ; C43H76NO8P ; C45H76NO8P ; C43H76NO8P ; C43H76NO8P ; C43H76NO8P ; C43H76NO8P ; C45H76NO8P ; C43H76NO8P ; C45H76NO8P ; C45H76NO8P ; C43H76NO8P ; C45H76NO8P ; C45H76NO8P ; C43H76NO8P ; C43H76NO8P ; C45H76NO8P ; C45H76NO8P ; C43H76NO8P ; C43H76NO8P ; C45H76NO8P ; C43H76NO8P ; C45H76NO8P ; C45H76NO8P ;
50323_61.6	1.722314	0.735377	2.8358	0.017676	1.7526	0.42361	269.055	HMDB34035 ; HMDB00362 ; HMDB00539 ; HMDB00807 ; HMDB00867 ; HMDB00881 ; HMDB01488 ; HMDB02243 ; HMDB03391 ; HMDB06039 ; HMDB14467 ; HMDB14929 ; HMDB30553 ; HMDB30556 ; HMDB31713 ; HMDB31784 ; HMDB32584 ; HMDB32963 ; HMDB33528 ; HMDB35448 ; HMDB36983 ; HMDB37578 ; HMDB38825 ; HMDB39336 ; HMDB40479 ; HMDB41852 ; HMDB41950 ; HMDB59750 ; HMDB60180 ; HMDB60255 ; HMDB60256 ; HMDB60406 ; HMDB60665	Sagecoumarin ; 2-Phosphoglyceric acid ; Arabinonic acid ; 3-Phosphoglyceric acid ; Ribonic acid ; Xanthurenic acid ; Nicotinic acid ; Picolinic acid ; 2-Phospho-D-glyceric acid ; 8-Chloroxanthine ; Floxuridine ; Uracil mustard ; Tricin 7-glucoside ; Tovophyllin B ; 2-Hydroxy-4-imino-25-cyclohexadienone ; Halofuginone ; 4-Hydroxychalcone ; Zeanic acid ; 46-Dihydroxy-2-quinolinecarboxylic acid ; Licoagroside A ; Garcimangosone A ; Limocitrin 3-rhamnoside ; 38-Dimethoxyapigenin 7-glucoside ; 345-Trihydroxy-37-dimethoxyflavone 5-glucoside ; 37-Dimethoxy-458-trihydroxyflavone 8-glucoside ; Cefpirome ; Nitrobenzene ; 2345-Tetrahydroxypentanoic acid ; (2R)-2-Hydroxy-3-(phosphonatooxy)propanoate ; L-Lyxonate ; L-Xylonate ; 5-Deoxy-5-fluorouridine ; Isonicotinic acid	M+2H ; M+2ACN+H ; 3M+H+K ; M+2ACN+H ; 3M+H+K ; M+ACN+Na ; 2M+Na ; 2M+Na ; M+2ACN+H ; M+2ACN+H ; M+Na ; M+NH4 ; M+2Na ; M+2K ; 2M+Na ; M+3ACN+2H ; M+2Na-H ; M+ACN+Na ; M+ACN+Na ; M+2Na ; M+2K ; M+2Na ; M+2Na ; M+2Na ; M+2Na ; M+H+Na ; 2M+Na ; 3M+H+K ; M+2ACN+H ; 3M+H+K ; 3M+H+K ; M+Na ; 2M+Na	C27H20O12 ; C3H7O7P ; C5H10O6 ; C3H7O7P ; C5H10O6 ; C10H7NO4 ; C6H5NO2 ; C6H5NO2 ; C3H7O7P ; C5H3ClN4O2 ; C9H11FN2O5 ; C8H11Cl2N3O2 ; C23H24O12 ; C28H28O6 ; C6H5NO2 ; C16H17BrClN3O3 ; C15H12O2 ; C10H7NO4 ; C10H7NO4 ; C23H24O12 ; C28H28O6 ; C23H24O12 ; C23H24O12 ; C23H24O12 ; C23H24O12 ; C22H22N6O5S2 ; C6H5NO2 ; C5H10O6 ; C3H7O7P ; C5H10O6 ; C5H10O6 ; C9H11FN2O5 ; C6H5NO2
58328_290.3	1.72223	0.724227	2.6977	0.0224	1.6498	0.42361	792.2458	HMDB01196 ; HMDB06598 ; HMDB15609 ; HMDB36075 ; HMDB60635 ; HMDB60974	NN-Diacetylchitobiosyldiphosphodolichol ; Isoglobotriaose ; Trabectedin ; Limonexic acid ; Etoposide glucuronide ; cycloguanil	2M+H+Na ; 3M+2K ; 2M+Na+K ; 3M+2K ; 2M+3H2O+2H ; 3M+K	C28H50N2O19P2 ; C19H34O15 ; C39H43N3O11S ; C26H30O10 ; C35H40O19 ; C11H14ClN5

09916_286.4	1.722121	1.105012	2.7335	0.021065	1.6764	0.42361	323.521	HMDB15250	Amiodarone	M+2H	C25H29I2NO3
28751_287.4	1.722101	1.039445	2.5124	0.030785	1.5117	0.42361	549.7329	HMDB34626 ; HMDB34627 ; HMDB37028 ; HMDB38381 ; HMDB39473 ; HMDB40283 ; HMDB60595	1-(34-Dimethoxyphenyl)-12-ethanediol 1-O-b-D-glucoside ; 1-(34-Dimethoxyphenyl)-12-ethanediol 2-O-b-D-glucoside ; Deoxyloganic acid ; 2-(4-Hydroxy-35-dimethoxyphenyl)ethanol 4-glucoside ; 2-Methoxy-3-(24-dihydroxyphenyl)-12-propanediol 4-glucoside ; (8R8R)-Secoisolariciresinol 99-bis-4-carboxy-3-hydroxy-3-methylbutanoyl-(-6)-glucoside ; Olopatadine n-oxide	3M+H+NH4 ; 3M+H+NH4 ; 3M+H+NH4 ; 3M+H+NH4 ; 3M+H+NH4 ; M+3ACN+2H ; 3M+H+K	C16H24O9 ; C16H24O9 ; C16H24O9 ; C16H24O9 ; C16H24O9 ; C44H62O24 ; C21H23NO4
73531_75.48	1.721986	0.952191	-2.7602	0.020122	1.6963	0.42361	398.0274	HMDB01155 ; HMDB06832 ; HMDB14848 ; HMDB15528 ; HMDB29177 ; HMDB30237 ; HMDB35974 ; HMDB37985 ; HMDB38570 ; HMDB41831 ; HMDB60414	Diadenosine triphosphate ; S-Glutaryldihydrolipoamide ; Ibandronate ; Quazepam ; 3-O-Methyl-(-)-epicatechin-7-O-sulphate ; Syzyginin B ; (S)-Spirobrassinin ; Cyananin ; Cyclobrassinin sulfoxide ; Azosemide ; 6-Methylthiopurine 5-monophosphate ribonucleotide	M+H+K ; M+2K-H ; M+2K+H ; 2M+H+Na ; 2M+Na+K ; M+H+K ; 3M+2Na ; M+NH4 ; 3M+2Na ; 2M+3H2O+2H ; 2M+H+K	C20H27N10O16P3 ; C13H23NO4S2 ; C9H23NO7P2 ; C17H11ClF4N2S ; C16H15O8S ; C33H24O21 ; C11H10N2OS2 ; C16H10Cl2N2O5 ; C11H10N2OS2 ; C12H11ClN6O2S2 ; C11H15N4O7PS
57338_45.04	1.720845	1.169256	2.6558	0.024074	1.6184	0.42361	322.7137	HMDB35999 ; HMDB39580	Ganoderic acid Mg ; Sucrose monostearate	M+ACN+2H ; M+H+NH4	C35H54O8 ; C30H58O13
9683_289.5	1.719774	0.697873	2.8288	0.017888	1.7474	0.42361	NA	NA	NA	NA	NA
96774_54.7	1.718942	1.033833	-2.7971	0.018888	1.7238	0.42361	149.9997	HMDB00124 ; HMDB00213 ; HMDB00645 ; HMDB00994 ; HMDB01076 ; HMDB01078 ; HMDB01313 ; HMDB01401 ; HMDB01586 ; HMDB02985 ; HMDB03498 ; HMDB03971 ; HMDB06328 ; HMDB06330 ; HMDB06797 ; HMDB06800 ; HMDB06814 ; HMDB06873 ; HMDB10325 ; HMDB32964 ; HMDB39494 ; HMDB60467	Fructose 6-phosphate ; Myo-inositol 1-phosphate ; Galactose 1-phosphate ; Dolichyl phosphate D-mannose ; Fructose 1-phosphate ; Mannose 6-phosphate ; D-Myo-inositol 4-phosphate ; Glucose 6-phosphate ; Glucose 1-phosphate ; Inositol phosphate ; Beta-D-Glucose 6-phosphate ; Beta-D-Fructose 6-phosphate ; D-Tagatose 1-phosphate ; D-Mannose 1-phosphate ; Sorbose 1-phosphate ; Beta-D-Fructose 2-phosphate ; 1D-myo-Inositol 3-phosphate ; D-Tagatose 6-phosphate ; Ethyl glucuronide ; 2-Acetylthiazole ; Benzoylmalic acid ; D-fructose 1-phosphate	M+H+K ; M+H+K ; M+H+K ; M+H+K ; M+H+K ; M+H+K ; M+H+K ; M+H+K ; M+H+K ; M+H+K ; M+H+K ; M+H+K ; M+H+K ; M+H+K ; M+H+K ; M+H+K ; M+H+K ; M+H+K ; M+2K ; M+Na ; M+Na+K ; M+H+K	C6H13O9P ; C6H13O9P ; C6H13O9P ; C6H13O9P ; C6H13O9P ; C6H13O9P ; C6H13O9P ; C6H13O9P ; C6H13O9P ; C6H13O9P ; C6H13O9P ; C6H13O9P ; C6H13O9P ; C6H13O9P ; C6H13O9P ; C6H13O9P ; C6H13O9P ; C6H13O9P ; C8H14O7 ; C5H5NOS ; C11H10O6 ; C6H13O9P
06015_70.60	1.717434	0.990505	2.8	0.018793	1.726	0.42361	138.5996	HMDB00070 ; HMDB00716 ; HMDB04226 ; HMDB05960 ; HMDB15212 ; HMDB29435 ; HMDB29444 ; HMDB59649	Pipecolic acid ; L-Pipecolic acid ; N4-Acetylaminobutanal ; D-Pipecolic acid ; Vigabatrin ; L-trans-4-Methyl-2-pyrrolidinecarboxylic acid ; 2-Pyrrolidineacetic acid ; 3-Acetamidobutanal	2M+H+NH4 ; 2M+H+NH4 ; 2M+H+NH4 ; 2M+H+NH4 ; 2M+H+NH4 ; 2M+H+NH4 ; 2M+H+NH4 ; 2M+H+NH4	C6H11NO2 ; C6H11NO2 ; C6H11NO2 ; C6H11NO2 ; C6H11NO2 ; C6H11NO2 ; C6H11NO2 ; C6H11NO2

2836_89.14	1.716797	0.58382	-2.5336	0.029685	1.5275	0.42361	326.1103	HMDB00833 ; HMDB34682 ; HMDB00299 ; HMDB00469 ; HMDB01412 ; HMDB05037 ; HMDB12209 ; HMDB13240 ; HMDB30312 ; HMDB31149 ; HMDB33459 ; HMDB33627 ; HMDB35420 ; HMDB38090 ; HMDB41753 ; HMDB60699 ; HMDB60865	N-Glycolylneuraminic acid ; cis-Ferulic acid arabinosyl-(1-3)-glucosyl-(1-6)-glucosyl ester ; Xanthosine ; 5-Hydroxymethyluracil ; 78-Dihydropteroic acid ; Sumatriptan ; Diethylphosphate ; Indoleacetyl glutamine ; Floribundine ; Phaeophorbide b ; Flazine ; D-Erythroascorbic acid 1-a-D-glucoside ; Pyranocyanin A ; Peonidin 3-rhamnoside 5-glucoside ; Isopeonidin 3-rutinoside ; (E)-2-Hydroxy-N-desmethyldoxepin ; Phenytoin catechol	M+H ; M+2H ; M+ACN+H ; 2M+ACN+H ; 2M+H+Na ; 2M+Na+K ; 2M+NH4 ; M+Na ; M+2Na-H ; M+2Na ; M+NH4 ; M+NH4 ; M+H+NH4 ; M+ACN+2H ; M+ACN+2H ; M+2Na-H ; M+ACN+H	C11H19NO10 ; C27H38O18 ; C10H12N4O6 ; C5H6N2O3 ; C14H14N6O3 ; C14H21N3O2S ; C4H11O4P ; C15H17N3O4 ; C18H19NO2 ; C35H34N4O6 ; C17H12N2O4 ; C11H16O10 ; C30H33O15 ; C28H33O15 ; C28H33O15 ; C18H19NO2 ; C15H12N2O4
7622_56.15	1.716022	1.245907	-2.6767	0.023225	1.6341	0.42361	169.9858	HMDB01131 ; HMDB01530 ; HMDB05831 ; HMDB13924 ; HMDB32644 ; HMDB60011 ; HMDB60350	Iminoaspartic acid ; Mannitol 1-phosphate ; Sorbitol-6-phosphate ; Thienodihydropyridinium ; Maclurin ; N-acetyl-S-(N-allylthiocarbamoyl)-L-cysteine ; 2-Oxosuccinamate	M+K ; M+2K ; M+2K ; M+2K ; M+2K ; M+2K ; M+K	C4H5NO4 ; C6H15O9P ; C6H15O9P ; C14H13ClNS ; C13H10O6 ; C9H14N2O3S2 ; C4H5NO4
9144_59.60	1.714816	0.330079	2.7595	0.020147	1.6958	0.42361	329.0929	HMDB00230 ; HMDB00773 ; HMDB01107 ; HMDB28831 ; HMDB28847 ; HMDB28973 ; HMDB29104 ; HMDB30542 ; HMDB32337 ; HMDB33060 ; HMDB34319 ; HMDB35006 ; HMDB37206	N-Acetylneuraminic acid ; N-Acetyl-a-neuraminic acid ; 7-Methylguanosine ; Glutamyl-Tyrosine ; Glycyl-Methionine ; Methionyl-Glycine ; Tyrosyl-Glutamate ; Neohesperidin dihydrochalcone ; Hydroxypropyl cellulose ; Piperolactam A ; Furofoline ; Isovitexin 7-(6-sinapoylglucoside) 4-glucoside ; Isoscoparin 2-(6-(E)-feruloylglucoside) 4-glucoside	2M+H+K ; 2M+H+K ; 2M+Na+K ; 2M+H+K ; 3M+H+K ; 3M+H+K ; 2M+H+K ; M+2Na ; M+ACN+Na ; M+ACN+Na ; M+ACN+Na ; M+2H+Na ; M+2H+Na	C11H19NO9 ; C11H19NO9 ; C11H16N5O5 ; C14H17N2O6 ; C7H14N2O3S ; C7H14N2O3S ; C14H17N2O6 ; C28H36O15 ; C16H11NO3 ; C16H11NO3 ; C16H11NO3 ; C44H50O24 ; C44H50O24
2243_54.94	1.714153	1.018817	-2.8139	0.018353	1.7363	0.42361	149.0072	HMDB02050 ; HMDB13139 ; HMDB14882 ; HMDB28693 ; HMDB28788 ; HMDB28966 ; HMDB29124	Dihydroxyfumaric acid ; 1-Hydroxypyrene ; Zileuton ; Alanyl-Methionine ; Cysteinyl-Valine ; Methionyl-Alanine ; Valyl-Cysteine	M+H ; M+2K ; M+Na+K ; M+2K ; M+2K ; M+2K ; M+2K	C4H4O6 ; C16H12O ; C11H12N2O2S ; C8H16N2O3S ; C8H16N2O3S ; C8H16N2O3S ; C8H16N2O3S
3618_86.12	1.712991	0.664942	-2.5042	0.03122	1.5056	0.42361	160.0254	HMDB31576 ; HMDB41083 ; HMDB02755 ; HMDB37357 ; HMDB29318 ; HMDB31522 ; HMDB32384 ; HMDB32957 ; HMDB34620 ; HMDB37169 ; HMDB40060 ; HMDB40061 ; HMDB59599 ; HMDB05792	(E)-4-Isothiocyanato-1-(methylthio)-1-butene ; (E)-Raphanusanin ; Myricetin ; 334558-Hexahydroxyflavone ; Caffeoylmalic acid ; Mesoxalic acid ; 2-Methyl-(3 or 5 or 6)-(methylthio)pyrazine (mixture of isomers) ; 2-O-Feruloyltartronic acid ; cis-Coutaric acid ; Pyrazineethanethiol ; 2-Methyl-5-(methylthio)pyrazine ; 2-Methyl-6-(methylthio)pyrazine ; CDP-glycerol ; Sulforaphane	M+H ; M+H ; M+2H ; M+2H ; M+H+Na ; M+ACN+H ; 2M+H+K ; M+H+Na ; M+H+Na ; 2M+H+K ; 2M+H+K ; 2M+H+K ; M+3H ; M+H-H2O	C6H9NS2 ; C6H9NS2 ; C15H10O8 ; C15H10O8 ; C13H12O8 ; C3H2O5 ; C6H8N2S ; C13H12O8 ; C13H12O8 ; C6H8N2S ; C6H8N2S ; C6H8N2S ; C12H21N3O13P2 ; C6H11NOS2
2488_52.93	1.712362	0.923222	-2.743	0.020726	1.6835	0.42361	525.0432	HMDB02757 ; HMDB32195 ; HMDB59970	Cysteic acid ; Carrageenan and salts of carrageenan ; 4-Hydroxy-5-(345-trihydroxyphenyl)-valeric acid-O-methyl-O-glucuronide	3M+NH4 ; M+3Na ; M+2K-H	C3H7NO5S ; C42H74O46S6 ; C18H24O13

9841_256.9	1.710972	0.324737	-2.8491	0.017276	1.7626	0.42361	284.147	HMDB15620 ; HMDB34525 ; HMDB34532 ; HMDB37783 ; HMDB38238 ; HMDB40891 ; HMDB41582 ; HMDB61034	Mianserin ; Barringtogenol C ; Camelliagenin C ; Ganoderiol H ; Plantaricin BN ; 345-Trimethoxycinnamyl alcohol acetate ; Sapidolide A ; Metabolite M6	2M+H+K ; M+2K ; M+2K ; M+2K ; M+2ACN+2H ; M+NH4 ; M+NH4 ; M+2Na	C18H20N2 ; C30H50O5 ; C30H50O5 ; C30H50O5 ; C24H36O10 ; C14H18O5 ; C14H18O5 ; C30H42N4O4
5402_53.74	1.710915	1.06385	2.6399	0.024738	1.6066	0.42361	192.0145	HMDB60600 ; HMDB14342 ; HMDB14404 ; HMDB29611 ; HMDB31868	Olsalazine-O-sulfate ; Fluconazole ; Sulfanilamide ; Asparagusic acid ; Monomethyl phenylphosphonate	M+2H ; M+2K ; 2M+H+K ; M+ACN+H ; 2M+H+K	C14H10N2O9S ; C13H12F2N6O ; C6H8N2O2S ; C4H6O2S2 ; C7H9O3P
9487_76.85	1.710759	0.878016	-2.8526	0.017172	1.7652	0.42361	265.0229	HMDB38142 ; HMDB01904 ; HMDB15199 ; HMDB30988 ; HMDB30992 ; HMDB32974 ; HMDB32975 ; HMDB32976 ; HMDB33112 ; HMDB33867 ; HMDB38460 ; HMDB38888 ; HMDB39311 ; HMDB39791 ; HMDB39792 ; HMDB39796 ; HMDB40036 ; HMDB41942 ; HMDB04078	58-Dihydro-6-(4-methyl-3-pentenyl)-1234-tetrathiocin ; 3-Nitrotyrosine ; Cefditoren ; 5-(2-Methylpropyl)tetrahydro-2-oxo-3-furancarboxylic acid ; 5-Butyltetrahydro-2-oxo-3-furancarboxylic acid ; 24-Dimethylthiazole ; 25-Dimethylthiazole ; 45-Dimethylthiazole ; Methylpyrazine ; 4-Isothiocyanato-1-butene ; Thiiranepropanenitrile ; Castamollissin ; Neocretanin ; 23-Dihydro-5-methyl-3-thiophenethiol ; 45-Dihydro-2-methyl-3-thiophenethiol ; 45-Dihydro-5-methyl-3-thiophenethiol ; 2-Ethylthiazole ; N-Acetyl-S-(N-methylcarbamoyl)cysteine ; Cinnavalininate	M+H ; M+K ; M+H+Na ; M+2K+H ; M+2K+H ; 2M+K ; 2M+K ; 2M+K ; 2M+2K-H ; 2M+K ; 2M+K ; M+Na+K ; M+Na+K ; 2M+H ; 2M+H ; 2M+H ; 2M+K ; M+2Na-H ; M+H-2H2O	C10H16S4 ; C9H10N2O5 ; C19H18N6O5S3 ; C9H14O4 ; C9H14O4 ; C5H7NS ; C5H7NS ; C5H7NS ; C5H6N2 ; C5H7NS ; C5H7NS ; C20H20O13 ; C20H20O13 ; C5H8S2 ; C5H8S2 ; C5H8S2 ; C5H7NS ; C7H12N2O4S ; C14H8N2O6
0628_55.23	1.710492	0.642563	2.754	0.020337	1.6917	0.42361	NA	NA	NA	NA	NA
8353_239.9	1.709964	1.177	2.6845	0.022915	1.6399	0.42361	442.9758	HMDB01439 ; HMDB15276	Phosphoribosyl formamidocarboxamide ; Sulfoxone	M+2K-H ; M+K	C10H15N4O9P ; C14H16N2O6S3

64991_21.5	1.706661	0.629372	-2.378	0.038741	1.4118	0.42361	311.2365	HMDB14449 ; HMDB06482 ; HMDB10398 ; HMDB11618 ; HMDB31846 ; HMDB32317 ; HMDB32355 ; HMDB32913 ; HMDB34736 ; HMDB36026 ; HMDB36184 ; HMDB36727 ; HMDB36820 ; HMDB36823 ; HMDB38026 ; HMDB38216 ; HMDB38731 ; HMDB38897 ; HMDB40983 ; HMDB41629 ; HMDB02183 ; HMDB30053 ; HMDB30446 ; HMDB31465 ; HMDB33897 ; HMDB34944 ; HMDB35185 ; HMDB36722 ; HMDB38908 ; HMDB41001 ; HMDB41302	Desogestrel ; LysoSM(d18:1) ; LysoPC(22:0) ; All-trans-1314-dihydroretinol ; Geranylacetone ; Hexanal hexyl isoamyl acetal ; (-)-R-(E)-5-Isopropyl-8-methylnona-68-dien-2-one ; 4-(266-Trimethyl-1-cyclohexen-1-yl)-2-butanone ; Abietinol ; Octahydro-6-isopropyl-2(1H)-naphthalenone ; 78-Dehydro-34-dihydro-beta-ionol ; ent-16-Kauren-19-ol ; beta-Ionol ; Theaspirane ; Dihydro-alpha-ionone ; 5-Isopropyl-2-(2-methylpropyl)-2-cyclohexen-1-one ; 47-Megastigmadien-9-ol ; (E)-2-(2-Octenyl)cyclopentanone ; 117-Heptadecanediol ; (3S6E)-Nerolidol ; Docosahexaenoic acid ; Neogrifolin ; Grifolin ; Methyl-10-shogaol ; Ginkgoic acid ; Siderol ; Retinol acetate ; ent-17-Acetoxy-16b-kauran-19-al ; (ZZ)-2-Methyl-5-(81114-pentadecatrienyl)-13-benzenediol ; Volemolide ; (R)-34-Dihydro-8-hydroxy-3-tridecyl-1H-2-benzopyran-1-one	M+H ; 2M+3H ; M+ACN+2H ; M+Na ; 3M+H+K ; M+K ; 3M+H+K ; 3M+H+K ; M+Na ; 3M+H+K ; 3M+H+K ; M+Na ; 3M+H+K ; 3M+H+K ; 3M+H+K ; 3M+H+K ; 3M+H+K ; 3M+H+K ; M+K ; 3M+H+K ; M+H-H2O ; M+H-H2O ; M+H-H2O ; M+H-2H2O ; M+H-2H2O ; M+H-2H2O ; M+H-H2O ; M+H-2H2O ; M+H-H2O ; M+H-2H2O ; M+H-2H2O	C22H30O ; C23H50N2O5P ; C30H62NO7P ; C20H32O ; C13H22O ; C17H36O2 ; C13H22O ; C13H22O ; C20H32O ; C13H22O ; C13H22O ; C20H32O ; C13H22O ; C13H22O ; C13H22O ; C13H22O ; C13H22O ; C13H22O ; C17H36O2 ; C13H22O ; C22H32O2 ; C22H32O2 ; C22H32O2 ; C22H34O3 ; C22H34O3 ; C22H34O3 ; C22H32O2 ; C22H34O3 ; C22H32O2 ; C22H34O3 ; C22H34O3
10027_35.7	1.70657	0.87079	-2.8159	0.018289	1.7378	0.42361	132.081	HMDB00466 ; HMDB34236 ; HMDB02006 ; HMDB13246 ; HMDB00022 ; HMDB02182 ; HMDB04826 ; HMDB12162 ; HMDB14748 ; HMDB15165 ; HMDB30175 ; HMDB32020 ; HMDB38869 ; HMDB39662 ; HMDB39837 ; HMDB40016 ; HMDB40029 ; HMDB40043 ; HMDB40047 ; HMDB40050 ; HMDB40051 ; HMDB41888 ; HMDB60592 ; HMDB60807	3-Methylindole ; Benzenepropanenitrile ; 23-Diaminopropionic acid ; Margaroylglycine ; 3-Methoxytyramine ; Phenylephrine ; p-Synephrine ; 4-Methoxytyramine ; Metaraminol ; Ethinamate ; Venoterpine ; 4-(beta-Methylaminoethyl)catechol ; Norpandamarilactonine A ; 2367-Tetrahydrocyclopentbazepin-8(1H)-one ; 2-(2-Furanyl)-3-piperidinol ; 2-(2-Furanyl)-3456-tetrahydropyridine ; 23-Dihydro-5-methyl-1H-pyrrolizine-7-carboxaldehyde ; 3-(2-Furanylmethylene)pyrrolidine ; 23-Dihydro-6-methyl-1H-pyrrolizine-5-carboxaldehyde ; 34-Dihydro-5-(5-methyl-2-furanyl)-2H-pyrrole ; 5-Acetyl-23-dihydro-1H-pyrrolizine ; Epinine ; 4-Hydroxynorephedrine ; a-Methyldopamine	M+H ; M+H ; 2M+3H2O+2H ; M+3Na ; M+H-2H2O ; M+H-2H2O ; M+H-2H2O ; M+H-2H2O ; M+H-2H2O ; M+H-2H2O ; M+H-H2O ; M+H-2H2O ; M+H-2H2O ; M+H-H2O ; M+H-2H2O ; M+H-H2O ; M+H-H2O ; M+H-H2O ; M+H-H2O ; M+H-H2O ; M+H-H2O ; M+H-2H2O ; M+H-2H2O ; M+H-2H2O	C9H9N ; C9H9N ; C3H8N2O2 ; C19H37NO3 ; C9H13NO2 ; C9H13NO2 ; C9H13NO2 ; C9H13NO2 ; C9H13NO2 ; C9H13NO2 ; C9H11NO ; C9H13NO2 ; C9H13NO2 ; C9H11NO ; C9H13NO2 ; C9H11NO ; C9H11NO ; C9H11NO ; C9H11NO ; C9H11NO ; C9H11NO ; C9H13NO2 ; C9H13NO2 ; C9H13NO2
2846_269.7	1.705312	0.912097	2.6895	0.022717	1.6436	0.42361	463.6083	HMDB39187	Methyl 2346-tetra-O-galloyl-beta-D-glucopyranoside	M+3ACN+2H	C35H30O22

.8354_218.5	1.704436	0.612291	2.6504	0.024298	1.6144	0.42361	297.5618	HMDB00094 ; HMDB00193 ; HMDB01874 ; HMDB05971 ; HMDB06511 ; HMDB08520 ; HMDB08551 ; HMDB08581 ; HMDB08582 ; HMDB08613 ; HMDB08645 ; HMDB08677 ; HMDB08710 ; HMDB08742 ; HMDB08807 ; HMDB14412 ; HMDB32986 ; HMDB33717 ; HMDB41862 ; HMDB60857 ; HMDB60862	Citric acid ; Isocitric acid ; D-threo-Isocitric acid ; Diketogulonic acid ; 23-Diketo-L-gulonate ; PC(20:5(5Z8Z11Z14Z17Z)24:1(15Z)) ; PC(22:022:6(4Z7Z10Z13Z16Z19Z)) ; PC(22:1(13Z)22:5(4Z7Z10Z13Z16Z)) ; PC(22:1(13Z)22:5(7Z10Z13Z16Z19Z)) ; PC(22:2(13Z16Z)22:4(7Z10Z13Z16Z)) ; PC(22:4(7Z10Z13Z16Z)22:2(13Z16Z)) ; PC(22:5(4Z7Z10Z13Z16Z)22:1(13Z)) ; PC(22:5(7Z10Z13Z16Z19Z)22:1(13Z)) ; PC(22:6(4Z7Z10Z13Z16Z19Z)22:0) ; PC(24:1(15Z)20:5(5Z8Z11Z14Z17Z)) ; Cefmenoxime ; 5-(2-Hydroxyethyl)-4-methylthiazole acetate ; (1R2R)-Isocitric acid ; D-Glucaro-14-lactone ; o-O-glucuronide rosiglitazone ; p-O-glucuronide rosiglitazone	3M+H+NH4 ; 3M+H+NH4 ; 3M+H+NH4 ; 3M+H+NH4 ; 3M+H+NH4 ; M+3H ; M+3H ; M+3H ; M+3H ; M+3H ; M+3H ; M+3H ; M+3H ; M+3H ; M+3H ; M+3H ; M+2ACN+2H ; 3M+H+K ; 3M+H+NH4 ; 3M+H+NH4 ; M+2Na ; M+2Na	C6H8O7 ; C6H8O7 ; C6H8O7 ; C6H8O7 ; C6H8O7 ; C52H92NO8P ; C52H92NO8P ; C52H92NO8P ; C52H92NO8P ; C52H92NO8P ; C52H92NO8P ; C52H92NO8P ; C52H92NO8P ; C52H92NO8P ; C52H92NO8P ; C16H17N9O5S3 ; C8H11NO2S ; C6H8O7 ; C6H8O7 ; C24H27N3O10S ; C24H27N3O10S
1954_56.41	1.704297	1.090204	2.6446	0.024539	1.6101	0.42361	394.8742	HMDB14786	Mitotane	M+2K-H	C14H10Cl4
.9782_260.0	1.702574	0.570501	-2.6265	0.025312	1.5967	0.42361	412.992	HMDB00229 ; HMDB04645 ; HMDB14674 ; HMDB31790 ; HMDB61129	Nicotinamide ribotide ; S-Nitrosoglutathione ; Chlormerodrin ; Hydroxytriphenylstannane ; 6-Methyl-griseofulvin	M+2K+H ; M+2K-H ; M+2Na-H ; M+2Na-H ; M+2K-H	C11H15N2O8P ; C10H16N4O7S ; C5H11ClHgN2O2 ; C18H16OSn ; C17H17ClO5
8007_85.83	1.702358	0.738492	-2.7971	0.01889	1.7238	0.42361	130.5258	HMDB00592 ; HMDB01481 ; HMDB15239	Glucosamine 6-sulfate ; N-Sulfo-D-glucosamine ; Methyprylon	M+2H ; M+2H ; M+2K	C6H13NO8S ; C6H13NO8S ; C10H17NO2
9775_140.1	1.702287	1.046094	-2.6114	0.02598	1.5854	0.42361	254.0398	HMDB33785 ; HMDB39178 ; HMDB39179 ; HMDB39181 ; HMDB39325 ; HMDB39326 ; HMDB39367 ; HMDB33602	Hamamelitannin ; 12-Digalloyl-beta-D-glucopyranose ; 16-Digalloyl-beta-D-glucopyranose ; 26-Digalloylglucose ; Gallic acid 3-O-(6-galloylglucoside) ; Gallic acid 4-O-(6-galloylglucoside) ; 12-Di-O-galloylhamamelofuranose ; Brassica oleracea Alkaloid	M+H+Na ; M+H+Na ; M+H+Na ; M+H+Na ; M+H+Na ; M+H+Na ; M+H+Na ; M+H-H2O	C20H20O14 ; C20H20O14 ; C20H20O14 ; C20H20O14 ; C20H20O14 ; C20H20O14 ; C20H20O14 ; C13H9N3O2S
6037_276.5	1.701222	0.161131	2.6601	0.023894	1.6217	0.42361	665.9566	HMDB12932	Dynorphin A (6-8)	3M+2H	C18H37N9O4
3901_74.48	1.700044	1.022311	-2.5774	0.027537	1.5601	0.42361	87.05534	HMDB01536 ; HMDB31841 ; HMDB32946 ; HMDB36132 ; HMDB36174 ; HMDB36468 ; HMDB36469 ; HMDB38151 ; HMDB39995 ; HMDB39996 ; HMDB41345	Formamide ; 25-Dimethyl-3-propylpyrazine ; 35-Diethyl-2-methylpyrazine ; 25-Diethyl-3-methylpyrazine ; 2-Methyl-3-(2-methylpropyl)pyrazine ; 1558-Tetramethyl-12-thiabicyclo910dodeca-37-diene ; 37710-Tetramethyl-12-thiabicyclo910dodeca-37-diene ; Mintsulfide ; 35-Dimethyl-2-propylpyrazine ; 23-Dimethyl-5-propylpyrazine ; 23-Diethyl-5-methylpyrazine	M+ACN+H ; M+H+Na ; M+H+Na ; M+H+Na ; M+H+Na ; M+2H+Na ; M+2H+Na ; M+2H+Na ; M+H+Na ; M+H+Na ; M+H+Na	CH3NO ; C9H14N2 ; C9H14N2 ; C9H14N2 ; C9H14N2 ; C15H24S ; C15H24S ; C15H24S ; C9H14N2 ; C9H14N2 ; C9H14N2

2687_56.3	1.698436	1.245928	-2.6498	0.024323	1.614	0.42361	395.1563	HMDB39952 ; HMDB03920 ;<br>HMDB12623 ; HMDB12624 ;<br>HMDB12780 ; HMDB14600 ;<br>HMDB30265 ; HMDB30322 ;<br>HMDB30375 ; HMDB31461 ;<br>HMDB33445 ; HMDB38217 ;<br>HMDB38630 ; HMDB59810 ;<br>HMDB59851 ; HMDB59905 ;<br>HMDB59924	1-(3-Methyl-2-butenoyl)-6-apiosylglucose ; Protopine ; 2-Hydroxyestrone-1-S-glutathione ; 2-Hydroxyestrone-4-S-glutathione ; 4-Hydroxyestrone-2-S-glutathione ; Prazosin ; 22-Dimethyl(pyrano-56:3:4)-15-dihydroxy-6-methoxy-10-methylacridone ; Papaveraldine ; Citracridone I ; Xylene ; Honyumine ; Pterosin H ; Niazimin A ; m-Xylene ; o-Xylene ; Ethylbenzene ; p-Xylene	M+H ; M+ACN+H ; 2M+3H ; 2M+3H ;<br>2M+3H ; 2M+H+Na ; M+ACN+H ;<br>M+ACN+H ; M+ACN+H ; M+2K-H ;<br>M+ACN+H ; 3M+H+K ; 2M+H+Na ; 3M+2K-H ; 3M+2K-H ; 3M+2K-H ; 3M+2K-H	C16H26O11 ; C20H19NO5 ;<br>C28H37N3O9S ; C28H37N3O9S ;<br>C28H37N3O9S ; C19H21N5O4 ;<br>C20H19NO5 ; C20H19NO5 ;<br>C20H19NO5 ; C24H30 ; C20H19NO5 ;<br>C15H19ClO ; C18H25NO8 ; C8H10 ;<br>C8H10 ; C8H10 ; C8H10
41407_54.4	1.698118	1.168023	2.7042	0.022153	1.6546	0.42361	516.8541	HMDB11482 ; HMDB11512 ;<br>HMDB32502 ; HMDB37210 ;<br>HMDB41430	LysoPE(0:020:1(11Z)) ; LysoPE(20:1(11Z)0:0) ; Santalyl phenylacetate ; alpha-Santalyl phenylacetate ; Gymnodimine	2M+H+NH4 ; 2M+H+NH4 ; 3M+H+NH4 ;<br>3M+H+NH4 ; 2M+H+NH4	C25H50NO7P ; C25H50NO7P ;<br>C23H30O2 ; C23H30O2 ; C32H45NO4
1335_52.9	1.696987	1.115099	-2.5074	0.031051	1.5079	0.42361	NA	NA	NA	NA	NA
6802_26.2	1.696597	0.713743	-2.5629	0.028234	1.5492	0.42361	534.2957	HMDB10382 ; HMDB29688 ;<br>HMDB31621 ; HMDB38029 ;<br>HMDB39150 ; HMDB40978	LysoPC(16:0) ; 2-Ethoxynaphthalene ; Cinnamylideneacetone ; Eriojaposide B ; Flavidulol C ; 14alpha-Hydroxypaxilline	M+K ; 3M+NH4 ; 3M+NH4 ; M+NH4 ;<br>2M+H+K ; M+2ACN+H	C24H50NO7P ; C12H12O ; C12H12O ;<br>C25H40O11 ; C34H42O4 ; C27H33NO5
0912_47.7	1.696013	1.241231	-2.6599	0.023903	1.6215	0.42361	526.6841	HMDB37520 ; HMDB37547 ;<br>HMDB56966 ; HMDB56967 ;<br>HMDB57020 ; HMDB57028 ;<br>HMDB57030 ; HMDB57038 ;<br>HMDB57046 ; HMDB57047 ;<br>HMDB57134 ; HMDB57135 ;<br>HMDB57139 ; HMDB57140 ;<br>HMDB57186 ; HMDB57187 ;<br>HMDB57191 ; HMDB57192 ;<br>HMDB57267 ; HMDB57268 ;<br>HMDB57271 ; HMDB57272 ;<br>HMDB57278 ; HMDB57286 ;<br>HMDB57287 ; HMDB57291 ;<br>HMDB57292 ; HMDB57299 ;<br>HMDB57300 ; HMDB57304 ;<br>HMDB57305 ; HMDB57307 ;<br>HMDB57314 ; HMDB57315 ;<br>HMDB57317 ; HMDB57321 ;<br>HMDB57326 ; HMDB57334 ;<br>HMDB57335 ; HMDB57339 ;<br>HMDB57340 ; HMDB57347 ;<br>HMDB57348 ; HMDB57352 ;<br>HMDB57353 ; HMDB57355 ;<br>HMDB57362 ; HMDB57363 ;<br>HMDB57365 ; HMDB57369 ;<br>HMDB57391 ; HMDB57395 ;<br>HMDB57920 ; HMDB57921 ;<br>[illegible]	Tetrabromodiphenyl ethers ; 11-Oxybis24-dibromobenzene ;<br>CL(18:018:018:022:5(4Z7Z10Z13Z16Z)) ;<br>CL(18:018:018:022:5(7Z10Z13Z16Z19Z)) ;<br>CL(18:018:022:5(4Z7Z10Z13Z16Z)18:0) ;<br>CL(18:018:022:5(4Z7Z10Z13Z16Z)22:6(4Z7Z10Z13Z16Z19Z)) ;<br>CL(18:018:022:5(7Z10Z13Z16Z19Z)18:0) ;<br>CL(18:018:022:5(7Z10Z13Z16Z19Z)22:6(4Z7Z10Z13Z16Z19Z)) ;<br>CL(18:018:022:6(4Z7Z10Z13Z16Z19Z)22:5(4Z7Z10Z13Z16Z)) ;<br>CL(18:018:022:6(4Z7Z10Z13Z16Z19Z)22:5(7Z10Z13Z16Z19Z)) ;<br>CL(18:018:1(11Z)22:5(4Z7Z10Z13Z16Z)22:5(4Z7Z10Z13Z16Z)) ;<br>CL(18:018:1(11Z)22:5(4Z7Z10Z13Z16Z)22:5(7Z10Z13Z16Z19Z)) ;<br>CL(18:018:1(11Z)22:5(7Z10Z13Z16Z19Z)22:5(4Z7Z10Z13Z16Z)) ;<br>CL(18:018:1(11Z)22:5(7Z10Z13Z16Z19Z)22:5(7Z10Z13Z16Z19Z)) ;<br>CL(18:018:1(9Z)22:5(4Z7Z10Z13Z16Z)22:5(4Z7Z10Z13Z16Z)) ;<br>CL(18:018:1(9Z)22:5(4Z7Z10Z13Z16Z)22:5(7Z10Z13Z16Z19Z)) ;<br>CL(18:018:1(9Z)22:5(7Z10Z13Z16Z19Z)22:5(4Z7Z10Z13Z16Z)) ;<br>CL(18:018:1(9Z)22:5(7Z10Z13Z16Z19Z)22:5(7Z10Z13Z16Z19Z)) ;<br>CL(18:020:4(5Z8Z11Z14Z)22:5(4Z7Z10Z13Z16Z)22:5(4Z7Z10Z13Z16Z)) ;<br>CL(18:020:4(5Z8Z11Z14Z)22:5(4Z7Z10Z13Z16Z)22:5(7Z10Z13Z16Z19Z)) ;<br>CL(18:020:4(5Z8Z11Z14Z)22:5(7Z10Z13Z16Z19Z)22:5(4Z7Z10Z13Z16Z)) ;<br>CL(18:020:4(5Z8Z11Z14Z)22:5(7Z10Z13Z16Z19Z)22:5(7Z10Z13Z16Z19Z)) ; CL(18:022:5(4Z7Z10Z13Z16Z)18:022:6(4Z7Z10Z13Z16Z19Z)) ;<br>CL(18:022:5(4Z7Z10Z13Z16Z)18:1(11Z)22:5(4Z7Z10Z13Z16Z)) ;<br>CL(18:022:5(4Z7Z10Z13Z16Z)18:1(11Z)22:5(7Z10Z13Z16Z19Z)) ;<br>CL(18:022:5(4Z7Z10Z13Z16Z)18:1(9Z)22:5(4Z7Z10Z13Z16Z)) ;<br>CL(18:022:5(4Z7Z10Z13Z16Z)18:1(9Z)22:5(7Z10Z13Z16Z19Z)) ;<br>CL(18:022:5(4Z7Z10Z13Z16Z)20:4(5Z8Z11Z14Z)22:5(4Z7Z10Z13Z16Z)) ;<br>[illegible]	M+2Na-H ; M+2Na-H ; M+3Na ; M+3Na ;<br>M+3Na ; M+2H+Na ; M+3Na ; M+2H+Na ;<br>M+2H+Na ; M+2H+Na ; M+2H+Na ;<br>M+2H+Na ; M+2H+Na ; M+2H+Na ;<br>M+2H+Na ; M+2H+Na ; M+2H+Na ;<br>M+2H+Na ; M+3H ; M+3H ; M+3H ; M+3H ; M+2H+Na ; M+2H+Na ; M+2H+Na ;<br>M+2H+Na ; M+2H+Na ; M+3H ; M+3H ;<br>M+2H+Na ; M+2H+Na ; M+3H ;<br>M+2H+Na ; M+2H+Na ; M+3H ;<br>M+2H+Na ; M+2H+Na ; M+2H+Na ;<br>M+2H+Na ; M+2H+Na ; M+2H+Na ;<br>M+3H ; M+3H ; M+2H+Na ; M+2H+Na ;<br>M+3H ; M+2H+Na ; M+2H+Na ; M+3H ;<br>M+2H+Na ; M+2H+Na ; M+2H+Na ;<br>M+2H+Na ; M+2H+Na ; M+2H+Na ;<br>M+2H+Na ; M+2H+Na ; M+2H+Na ;<br>M+2H+Na ; M+2H+Na ; M+2H+Na ;<br>M+2H+Na ; M+2H+Na ; M+2H+Na ;<br>M+2H+Na ; M+2H+Na ; M+2H+Na ;<br>M+2H+Na ; M+3H ; M+3H ; M+3H ; M+3H ; M+3H ; M+3H ; M+3H ; M+3H ; M+3H ;<br>M+3H ; M+3H ; M+3H ; M+3H ; M+3H ;<br>M+3H ; M+3H ; M+3H ; M+3H ; M+3H ;<br>M+2H+Na ; M+2H+Na ; M+3H ;<br>M+2H+Na ; M+2H+Na ; M+3H ;<br>M+2H+Na ; M+2H+Na ; M+2H+Na ;<br>[illegible]	C12H6Br4O ; C12H6Br4O ;<br>C85H156O17P2 ; C85H156O17P2 ;<br>C85H156O17P2 ; C89H152O17P2 ;<br>C85H156O17P2 ; C89H152O17P2 ;<br>C89H152O17P2 ; C89H152O17P2 ;<br>C89H152O17P2 ; C89H152O17P2 ;<br>C89H152O17P2 ; C89H152O17P2 ;<br>C89H152O17P2 ; C89H152O17P2 ;<br>C89H152O17P2 ; C89H152O17P2 ;<br>C91H150O17P2 ; C91H150O17P2 ;<br>C91H150O17P2 ; C91H150O17P2 ;<br>C89H152O17P2 ; C89H152O17P2 ;<br>C89H152O17P2 ; C89H152O17P2 ;<br>C89H152O17P2 ; C91H150O17P2 ;<br>C91H150O17P2 ; C89H152O17P2 ;<br>C89H152O17P2 ; C91H150O17P2 ;<br>C89H152O17P2 ; C89H152O17P2 ;<br>C91H150O17P2 ; C89H152O17P2 ;<br>C89H152O17P2 ; C89H152O17P2 ;<br>C89H152O17P2 ; C89H152O17P2 ;<br>C89H152O17P2 ; C91H150O17P2 ;<br>C91H150O17P2 ; C89H152O17P2 ;<br>C89H152O17P2 ; C91H150O17P2 ;<br>C89H152O17P2 ; C89H152O17P2 ;<br>C91H150O17P2 ; C89H152O17P2 ;<br>C89H152O17P2 ; C89H152O17P2 ;<br>C89H152O17P2 ; C89H152O17P2 ;<br>[illegible]

76822_177.9	1.695523	1.129661	-2.6795	0.023112	1.6362	0.42361	243.0377	HMDB05018 ; HMDB14986 ; HMDB15421 ; HMDB15623 ; HMDB29500 ; HMDB36185 ; HMDB37079 ; HMDB39307 ; HMDB41969 ; HMDB61017	Amlodipine ; Levamisole ; Cefamandole ; Rimonabant ; Kaempferol 3-glucuronide ; 3-(2-Furanyl)-2-phenyl-2-propenal ; 3-Methylellagic acid 8-rhamnoside ; 35-Dihydroxyphenyl 1-O-(6-O-galloyl-beta-D-glucopyranoside) ; Oxazepam glucuronide ; 1-Pyrimidinylpiperazine	M+2K ; M+K ; M+H+Na ; M+H+Na ; M+H+Na ; M+2Na-H ; M+H+Na ; M+2Na ; M+H+Na ; M+2K+H	C20H25ClN2O5 ; C11H12N2S ; C18H18N6O5S2 ; C22H21Cl3N4O ; C21H18O12 ; C13H10O2 ; C21H18O12 ; C19H20O12 ; C21H19ClN2O8 ; C8H12N4
0549_293.78	1.692924	0.598557	2.4343	0.035193	1.4535	0.42361	323.9955	HMDB02206 ; HMDB31780	Molybdopterin ; Dithianon	M+3ACN+2H ; 2M+3H2O+2H	C10H12MoN5O8PS2 ; C14H4N2O2S2
72932_84.85	1.692748	0.809729	-2.787	0.019217	1.7163	0.42361	101.0073	HMDB31414 ; HMDB31582 ; HMDB31686 ; HMDB32446 ; HMDB32447 ; HMDB32927 ; HMDB32948 ; HMDB33209 ; HMDB33238 ; HMDB34345 ; HMDB39844 ; HMDB40246 ; HMDB40272 ; HMDB40408 ; HMDB42032	23-Dimethyl-2-cyclohexen-1-one ; 6-Methyl-35-heptadien-2-one ; (EE)-24-Octadienal ; trans trans-35-Octadien-2-one ; 15-Octadien-3-one ; Quindoxin ; 5-Phenyl-13-pentadiyne ; Fenthion ; 2-Isopropyl-5-methylfuran ; 7-Acetoxy-6-methoxycoumarin ; (EE)-26-Octadienal ; 355-Trimethyl-2-cyclopenten-1-one ; 2-Butylfuran ; 7-Hydroxy-2-methyl-4-oxo-4H-1-benzopyran-5-acetic acid ; Thiodiacetic acid	M+2K ; M+2K ; M+2K ; M+2K ; M+2K ; M+H+K ; M+Na+K ; M+2H+Na ; M+2K ; M+3Na ; M+2K ; M+2K ; M+2K ; M+3Na ; 2M+3H	C8H12O ; C8H12O ; C8H12O ; C8H12O ; C8H12O ; C8H6N2O2 ; C11H8 ; C10H15O3PS2 ; C8H12O ; C12H10O5 ; C8H12O ; C8H12O ; C8H12O ; C12H10O5 ; C4H6O4S
57861_87.62	1.692056	1.164969	2.6654	0.02368	1.6256	0.42361	558.1268	HMDB00652 ; HMDB15653 ; HMDB38866	Chondroitin 4-sulfate ; Lumefantrine ; Quercetagetin 3-methylether 7-glucoside	M+2ACN+H ; 2M+Na+K ; M+ACN+Na	C14H21NO15S ; C30H32Cl3NO ; C22H22O13
54152_290.5	1.691529	0.975707	2.6714	0.023436	1.6301	0.42361	705.3054	HMDB02318 ; HMDB05796 ; HMDB29972 ; HMDB31382 ; HMDB32589 ; HMDB33111 ; HMDB33224 ; HMDB33225 ; HMDB33226 ; HMDB34091 ; HMDB34558 ; HMDB37492 ; HMDB38979 ; HMDB39056 ; HMDB39903 ; HMDB40424 ; HMDB61013 ; HMDB39694	Capsianoside I ; Picrocrocin ; Coriandrone D ; Portuloside A ; 1-(5-Acetyl-2-hydroxyphenyl)-3-methyl-1-butanone ; Epijasminoside A ; (1R4R5S)-5-Hydroxyfenchone glucoside ; (1R4S6R)-6-Hydroxyfenchone glucoside ; (1S4R)-10-Hydroxyfenchone glucoside ; Ponasteroside A ; 6-Hydroxy-2-bornanone glucoside ; Ethyl methyl-p-tolylglycidate ; (3b4b5b)-45-Epoxy-p-menth-1-en-3-ol 3-glucoside ; (4S6R)-p-Mentha-18-diene-67-diol 7-glucoside ; Mangostenone B ; Ethyl 2-benzylacetoacetate ; desmethylastemizole ; Physagulin G	M+2Na-H ; 2M+2Na-H ; 2M+H ; 2M+2Na-H ; 3M+2Na-H ; 2M+2Na-H ; 2M+2Na-H ; 2M+2Na-H ; 2M+2Na-H ; M+2K+H ; 2M+2Na-H ; 3M+2Na-H ; 2M+2Na-H ; 2M+2Na-H ; 3M+H+Na ; 3M+2Na-H ; 3M+2K ; M+H-H2O	C32H52O14 ; C16H26O7 ; C18H24O7 ; C16H26O7 ; C13H16O3 ; C16H26O7 ; C16H26O7 ; C16H26O7 ; C16H26O7 ; C33H54O11 ; C16H26O7 ; C13H16O3 ; C16H26O7 ; C16H26O7 ; C28H30O6 ; C13H16O3 ; C27H29FN4O ; C36H50O15
02698_263.8	1.691373	0.846914	-2.4559	0.033912	1.4696	0.42361	344.9893	HMDB03263 ; HMDB33351 ; HMDB40790	Pelargonidin ; Methoxybrassinin ; 4-Methoxybrassinin	M+K ; M+2K+H ; M+2K+H	C15H11ClO5 ; C12H14N2OS2 ; C12H14N2OS2

)4533_251.9	1.690413	0.965814	2.6114	0.025976	1.5854	0.42361	388.1095	HMDB01204 ; HMDB02269 ; HMDB03572 ; HMDB12479 ; HMDB29486 ; HMDB30304 ; HMDB30467 ; HMDB30686 ; HMDB30812 ; HMDB33086 ; HMDB33344 ; HMDB33819 ; HMDB34112 ; HMDB35526 ; HMDB36324 ; HMDB37334 ; HMDB37335 ; HMDB38225 ; HMDB38356 ; HMDB38488 ; HMDB38528 ; HMDB38873 ; HMDB39721 ; HMDB39755 ; HMDB40344 ; HMDB40368 ; HMDB40472 ; HMDB40877 ; HMDB60204 ; HMDB60205 ; HMDB60206 ; HMDB60613 ; HMDB60905 ; HMDB34860	trans-3-Hydroxycotinine glucuronide ; Curcumin ; Rosmarinic acid ; (3S)-Hydroxy-tetracosa-6912151821-all-cis-hexaenoyl-CoA ; Integrin ; Atherosperminine ; Artocarpetin A ; Cyclointegrin ; Acerosin ; Cyclocurcumin ; Mollicellin H ; Jaceidin ; 9-O-Methylglyceofuran ; Glycyrrhizaisoflavone C ; Glicoricone ; Majoranin ; Sudachitin ; Glycycoumarin ; Sideritiflavone ; Gancaonin B ; Gancaonin N ; Isoglycycoumarin ; 4-O-alpha-D-Galactopyranuronosyl-D-galacturonic acid ; N-trans-Feruloyl-4-O-methyldopamine ; 356-Trihydroxy-347-trimethoxyflavone ; Zanthosimuline ; Agamanone ; 358-Trihydroxy-347-trimethoxyflavone ; 3-Oxo-docosa-10131619-all-cis-tetraenoyl-CoA ; 3(S)-Hydroxy-docosa-710131619-all-cis-pentaenoyl-CoA ; 3-Oxo-docosa-7101316-all-cis-tetraenoyl-CoA ; Pefloxacin N-oxide ; Haloperidol 1236-tetrahydropyridine ; Flumiclorac-pentyl	2M+H+K ; 2M+H+K ; 2M+3H2O+2H ; M+H+2Na ; 2M+H+K ; M+2K+H ; 2M+H+K ; 2M+H+K ; 2M+3H2O+2H ; 2M+H+K ; 2M+H+K ; 2M+3H2O+2H ; 2M+H+K ; 2M+H+K ; 2M+H+K ; 2M+3H2O+2H ; 2M+3H2O+2H ; 2M+H+K ; 2M+3H2O+2H ; 2M+H+K ; 2M+H+K ; 2M+H+K ; M+NH4 ; M+2Na-H ; 2M+3H2O+2H ; M+2K+H ; 2M+3H2O+2H ; 2M+3H2O+2H ; M+3Na ; M+3Na ; M+3Na ; M+K ; 2M+Na+K ; M+H-2H2O	C16H20N2O8 ; C21H20O6 ; C18H16O8 ; C45H66N7O18P3S ; C21H20O6 ; C20H23NO2 ; C21H20O6 ; C21H20O6 ; C18H16O8 ; C21H20O6 ; C21H20O6 ; C18H16O8 ; C21H20O6 ; C21H20O6 ; C21H20O6 ; C18H16O8 ; C18H16O8 ; C21H20O6 ; C18H16O8 ; C21H20O6 ; C21H20O6 ; C21H20O6 ; C12H18O13 ; C19H21NO5 ; C18H16O8 ; C20H23NO2 ; C18H16O8 ; C18H16O8 ; C43H68N7O18P3S ; C43H68N7O18P3S ; C43H68N7O18P3S ; C17H20FN3O4 ; C21H21ClFNO ; C21H23ClFNO5
!7949_195.7	1.689547	1.0184	-2.6793	0.023121	1.636	0.42361	121.0148	HMDB31473 ; HMDB31474 ; HMDB33052 ; HMDB36179 ; HMDB40250 ; HMDB41389 ; HMDB41391 ; HMDB02655 ; HMDB11751 ; HMDB12915 ; HMDB15412 ; HMDB29436 ; HMDB29555 ; HMDB29685 ; HMDB30547 ; HMDB30804 ; HMDB31240 ; HMDB31671 ; HMDB32115 ; HMDB32128 ; HMDB32174 ; HMDB32574 ; HMDB32640 ; HMDB33761 ; HMDB37602 ; HMDB37733 ; HMDB38572 ; HMDB39210 ; HMDB60734 ; HMDB60771	13-Dithiane ; 14-Dithiane ; Ethyl vinyl disulfide ; 2-Methyl-13-dithiolane ; xi-Tetrahydro-2-thiophenethiol ; Methyl 2-propenyl disulfide ; (E)-Methyl 1-propenyl disulfide ; Isorhamnetin ; 3-Methoxybenzenepropanoic acid ; Coniferyl alcohol ; Sulfacytine ; Penmacric acid ; 45-Dihydro-2-methylthiazole ; 4-Ethoxy-3-methoxybenzaldehyde ; 3457-Tetrahydroxy-6-methoxyflavone ; 2-(34-Dihydroxyphenyl)-56-dihydroxy-7-methoxy-4H-1-benzopyran-4-one ; 2-Phenyl-13-dioxolane-4-methanol ; 246-Trimethyl-135-trithiane ; 1-(Methylthio)ethyl 2-propenyl disulfide ; 1-(24-Dihydroxyphenyl)-1-butanone ; Benzaldehyde glyceryl acetal ; Propylparaben ; Ethyl p-anisate ; 4-Methoxybenzyl acetate ; 4-Hydroxy-266-trimethyl-3-oxo-14-cyclohexadiene-1-carboxaldehyde ; Propyl 2-furanacrylate ; Pollenitin ; 1358-Tetrahydroxy-6-methoxy-2-methylanthraquinone ; 3-(3-Hydroxyphenyl)-2-methylpropionic acid ; 4-Hydroxyclobazam	M+H ; M+H ; M+H ; M+H ; M+H ; M+H ; M+H ; M+H+2Na ; M+Na+K ; M+Na+K ; M+3Na ; M+H+K ; 2M+H+K ; M+Na+K ; M+H+2Na ; M+H+2Na ; M+Na+K ; 2M+3H ; 2M+3H ; M+Na+K ; M+Na+K ; M+Na+K ; M+Na+K ; M+Na+K ; M+Na+K ; M+Na+K ; M+H+2Na ; M+H+2Na ; M+Na+K ; M+H+2Na	C4H8S2 ; C4H8S2 ; C4H8S2 ; C4H8S2 ; C4H8S2 ; C4H8S2 ; C4H8S2 ; C16H12O7 ; C10H12O3 ; C10H12O3 ; C12H14N4O3S ; C7H10N2O5 ; C4H7NS ; C10H12O3 ; C16H12O7 ; C16H12O7 ; C10H12O3 ; C6H12S3 ; C6H12S3 ; C10H12O3 ; C10H12O3 ; C10H12O3 ; C10H12O3 ; C10H12O3 ; C10H12O3 ; C10H12O3 ; C16H12O7 ; C16H12O7 ; C10H12O3 ; C16H13ClN2O3

8751_32.9	1.687107	0.626985	2.8062	0.018595	1.7306	0.42361	321.1309	HMDB15159 ; HMDB30742 ; HMDB33555 ; HMDB35284 ; HMDB37321 ; HMDB00942 ; HMDB02099 ; HMDB02281 ; HMDB11599 ; HMDB11600 ; HMDB11614 ; HMDB14464 ; HMDB14615 ; HMDB28809 ; HMDB29028 ; HMDB29091 ; HMDB29103 ; HMDB29119 ; HMDB29161 ; HMDB29474 ; HMDB29913 ; HMDB30459 ; HMDB32223 ; HMDB33460 ; HMDB36159 ; HMDB36361 ; HMDB36771 ; HMDB37560 ; HMDB39949 ; HMDB40521 ; HMDB41053 ; HMDB60380 ; HMDB60661 ; HMDB33444 ; HMDB36137 ; HMDB39824	Mycophenolic acid ; Helipyrone ; Isomugineic acid ; D-Fructosazine ; Oleacein ; Tetrahydroneopterin ; 6-Methyladenine ; 4a-Hydroxytetrahydrobiopterin ; 1-Methyladenine ; 3-Methyladenine ; 7-Methyladenine ; Piperacillin ; Fluoxetine ; Glutaminyl-Tyrosine ; Prolyl-Tryptophan ; Tryptophyl-Proline ; Tyrosyl-Glutamine ; Tyrosyl-Gamma-glutamate ; gln-tyr ; 6-Dehydrogingerdione ; Gynocardin ; Hordatine B ; Diisopentyl thiomalate ; 4-Hydroxycinnamoylagmatine ; Toxin T2 tetrol ; Pipercyclobutanamide B ; Laurenobiolide ; 37815-Scirpenetetrol ; Oudemansin A ; Dipiperamide D ; Ustiloxin C ; 3-Polyprenyl-45-dihydroxybenzoate ; APC ; 15-Acetyl-4-deoxynivalenol ; Acetyldeoxynivalenol ; O-Acetylcyclocalopin A	M+H ; M+H ; M+H ; M+H ; M+H ; M+ACN+Na ; 2M+Na ; M+ACN+Na ; 2M+Na ; 2M+Na ; 2M+Na ; M+3ACN+2H ; 2M+H+Na ; 2M+H+Na ; 2M+H+K ; 2M+H+K ; 2M+H+Na ; 2M+H+Na ; 2M+H+Na ; 2M+Na+K ; M+NH4 ; M+Na+K ; 2M+Na+K ; M+2Na-H ; M+Na ; M+2Na ; 2M+Na+K ; M+Na ; 2M+Na+K ; M+2Na ; M+2ACN+2H ; 2M+Na+K ; M+H+Na ; M+H-H2O ; M+H-H2O ; M+H-H2O	C17H20O6 ; C17H20O6 ; C12H20N2O8 ; C12H20N2O8 ; C17H20O6 ; C9H15N5O4 ; C6H7N5 ; C9H15N5O4 ; C6H7N5 ; C6H7N5 ; C6H7N5 ; C23H27N5O7S ; C17H18F3NO ; C14H19N3O5 ; C16H19N3O3 ; C16H19N3O3 ; C14H19N3O5 ; C14H19N3O5 ; C14H19N3O5 ; C17H22O4 ; C12H17NO8 ; C29H40N8O5 ; C14H26O4S ; C14H20N4O2 ; C15H22O6 ; C36H40N2O6 ; C17H22O4 ; C15H22O6 ; C17H22O4 ; C36H40N2O6 ; C23H34N4O10S ; C17H22O4 ; C33H38N4O8 ; C17H22O7 ; C17H22O7 ; C17H22O7
9661_22.4	1.686857	0.790139	-2.6053	0.02625	1.5809	0.42361	235.169	HMDB32837 ; HMDB34515 ; HMDB35434 ; HMDB35886 ; HMDB38797 ; HMDB39615 ; HMDB40711 ; HMDB12557 ; HMDB14393 ; HMDB31125 ; HMDB32107 ; HMDB32123 ; HMDB32875 ; HMDB36015 ; HMDB38018 ; HMDB40999 ; HMDB41527	Ganoderic acid DM ; Glabrolide ; Isomasticadienonalic acid ; Isoglabrolide ; Uralenolide ; Colupone ; 16beta-16-Hydroxy-3-oxo-112-oleanadien-28-oic acid ; 13-Carboxy-gamma-tocopherol ; Cabergoline ; Glycerol trihexanoate ; (3beta5alpha6beta7alpha22E24R)-Ergosta-822-diene-3567-tetrol ; (3beta5alpha6alpha9alpha22E24R)-Ergosta-722-diene-3569-tetrol ; 359-Trihydroxyergost-7-en-6-one ; Mangalkanyl glucoside ; Cryptomeridiol 11-rhamnoside ; Secasterone ; 3-Dehydroteasterone	M+2H ; M+2H ; M+2H ; M+2H ; M+2H ; M+2H ; M+2H ; M+H+Na ; M+H+NH4 ; M+2ACN+2H ; M+H+Na ; M+H+Na ; M+H+Na ; M+2ACN+2H ; M+2ACN+2H ; M+H+Na ; M+H+Na	C30H44O4 ; C30H44O4 ; C30H44O4 ; C30H44O4 ; C30H44O4 ; C30H44O4 ; C30H44O4 ; C28H46O4 ; C26H37N5O2 ; C21H38O6 ; C28H46O4 ; C28H46O4 ; C28H46O4 ; C21H38O6 ; C21H38O6 ; C28H46O4 ; C28H46O4
9847_56.5	1.68556	1.091858	-2.5851	0.027178	1.5658	0.42361	422.882	HMDB02080	Petroselinic acid	3M+2H	C18H33O2
8799_277.	1.685112	0.271531	2.6234	0.025448	1.5943	0.42361	266.4839	HMDB15397 ; HMDB34596 ; HMDB41465	Sunitinib ; Hebevinoside VI ; Melilotoside B	2M+3H ; M+H+2Na ; M+H+2Na	C22H27FN4O2 ; C41H68O12 ; C41H68O12
3574_43.3	1.68497	0.928992	2.5689	0.027941	1.5538	0.42361	NA	NA	NA	NA	NA
8549_246.	1.682796	1.094664	-2.5038	0.031245	1.5052	0.42361	327.0079	HMDB00224 ; HMDB29188 ; HMDB33831 ; HMDB34034 ; HMDB40869 ; HMDB60151 ; HMDB60152 ; HMDB61161	O-Phosphoethanolamine ; 5-Hydroxymethyl-2-furanoate ; Medicagol ; Bikojic acid ; 2-O-Caffeoyltartronic acid ; O-Phosphonatoethanaminium ; Ethamp ; N-Desalkyl flurazepam	2M+2Na-H ; 2M+2Na-H ; 2M+Na+K ; M+2Na-H ; M+2Na-H ; 2M+2Na-H ; 2M+2Na-H ; M+K	C2H8NO4P ; C6H5O4 ; C16H8O6 ; C12H10O8 ; C12H10O8 ; C2H8NO4P ; C2H8NO4P ; C15H10ClFN2O
9413_52.4	1.682496	0.976551	2.7846	0.019297	1.7145	0.42361	652.8899	HMDB33202	Di-2-propenyl tetrasulfide	3M+Na	C6H10S4

22266_241.3	1.681518	1.231547	2.6544	0.024129	1.6175	0.42361	325.0622	HMDB00286 ; HMDB00290 ; HMDB00302 ; HMDB00304 ; HMDB01931 ; HMDB03249 ; HMDB05010 ; HMDB06408 ; HMDB13112 ; HMDB14649 ; HMDB28979 ; HMDB29258 ; HMDB29364 ; HMDB29478 ; HMDB29534 ; HMDB29812 ; HMDB30757 ; HMDB30840 ; HMDB30888 ; HMDB30910 ; HMDB30911 ; HMDB31378 ; HMDB31386 ; HMDB31387 ; HMDB31900 ; HMDB31916 ; HMDB31970 ; HMDB31975 ; HMDB32649 ; HMDB32908 ; HMDB33707 ; HMDB33824 ; HMDB34123 ; HMDB35131 ; HMDB35132 ; HMDB35223 ; HMDB35503 ; HMDB35708 ; HMDB35902 ; HMDB35903 ; HMDB36005 ; HMDB36040 ; HMDB36455 ; HMDB36473 ; HMDB36488 ; HMDB36641 ; HMDB36767 ; HMDB36770 ; HMDB36772 ; HMDB37022 ; HMDB37431 ; HMDB37433 ; HMDB37534 ; HMDB37538 ; [illegible]	Uridine diphosphate glucose ; Uridine diphosphate-N-acetylglucosamine ; Uridine diphosphategalactose ; Uridine diphosphate-N-acetylgalactosamine ; Gamma-CEHC ; Rutin ; Sertraline ; 4-Hydroxy tolbutamide ; UDP-N-acetyl-D-mannosamine ; Nitazoxanide ; Methionyl-Methionine ; Luteolin 6-C-glucoside 8-C-arabinoside ; 7-Acetoxy-2-methylisoflavone ; Meloside L ; Glyzarin ; Quercetin 8-C-(2-rhamnosylglucoside) ; (S)-Pterosin A ; 2-Methoxy-9-phenyl-1H-phenalen-1-one ; Illudin C2 ; 2alpha-Hydroxyalantolactone ; 2-Oxo-511(13)-eudesmadien-128-olide ; 4-Epiisoinuviscolide ; (1(10)E4beta5alpha8beta)-45-Epoxy-1(10)11(13)-germacradien-128-olide ; (1beta4E8beta10alpha)-110-Epoxy-411(13)-germacradien-128-olide ; Methyl (2E4Z6E8E10E)-48-dimethyl-12-oxo-246810-dodecapentaenoate ; 5-Hydroxy-2-(1-hydroxy-5-methyl-4-hexenyl)benzofuran ; 1beta-Hydroxyalantolactone ; 3-Methoxy-2-(4-methylbenzoyl)-4H-1-benzopyran-4-one ; 45-Epoxy-11(13)-guaien-128-olide ; Kaempferol 7-sophoroside ; Heliannuol E ; Isoorientin 7-glucoside ; Eugenyl isovalerate ; Heliannuol C ; Heliannuol B ; (S)-Pterosin D ; Pseudoisoeugenol 2-methylbutanoate ; 8-Epiisoivangustin ; Enokipodin B ; Enokipodin A ; Istanbulin B ; Marasmen-3-one ; Lacinilene C ; 8-Epixanthatin ; Annuolide C ; Artabsin ; Zederone ; Desacetyllaurenobiolide ; Glechomafuran ; Cichoralexin ; Allivicin ; Paeonoside ; Panasenoside ; Quercetin 3-galactoside 7-rhamnoside ; 6-Caffeoylhyperin ; 1alpha-1-Hydroxy-24(18)11(13)-eudesmatrien-12-oic acid ; 3-Oxo-1411(13)-eudesmatrien-12-oic acid ; Annuolide B ; Annuolide A ; Calendoflavobioside ; Quercetin 7-rutinoside ; Multinoside A ; Curcolone ; Zedoarol ; Annuolide E ; 1-Piperazinecarbodithioic acid ; Glandulone A ; 212-Epoxy-7(14)-illudadiene-38-diol ; Quercetin 7-(rhamnosylglucoside) ; Glandulone C ; Quercetin 3-(2-glucosylrhamnoside) ; Isoquercitrin 4-[illegible]	M+2ACN+2H ; M+ACN+2H ; M+2ACN+2H ; M+ACN+2H ; M+2K-H ; M+H+K ; 2M+H+K ; M+K ; M+ACN+2H ; M+NH4 ; M+2Na-H ; M+H+K ; 2M+Na+K ; M+H+K ; 2M+Na+K ; M+H+K ; M+2K-H ; M+K ; M+2K-H ; M+2K-H ; M+2K+H ; M+2K-H ; M+2K-H ; M+2K-H ; M+2K+H ; M+2K+H ; M+2K-H ; 2M+Na+K ; M+2K-H ; M+H+K ; M+2K-H ; M+H+K ; M+2K-H ; M+2K-H ; M+2K-H ; M+2K-H ; M+2K-H ; M+2K-H ; M+2K+H ; M+2K-H ; M+2K-H ; M+2K-H ; M+2K+H ; M+2K+H ; M+2K+H ; M+2K-H ; M+2K+H ; M+2K-H ; M+2K-H ; M+2K-H ; M+H+K ; M+H+K ; M+H+K ; M+H+K ; M+H+Na ; M+2K-H ; M+2K+H ; M+2K-H ; M+2K+H ; M+H+K ; M+H+K ; M+H+K ; M+2K+H ; M+2K+H ; M+2K-H ; 2M+H ; M+2K+H ; M+2K-H ; M+H+K ; M+2K-H ; M+H+K ; M+H+K ; M+H+K ; M+H+K ; M+2K+H ; M+2K+H ; M+2ACN+2H ; M+K ; M+ACN+2H ; M+2Na-H	C15H24N2O17P2 ; C17H27N3O17P2 ; C15H24N2O17P2 ; C17H27N3O17P2 ; C15H20O3 ; C27H30O16 ; C17H17Cl2N ; C12H18N2O4S ; C17H27N3O17P2 ; C12H9N3O5S ; C10H20N2O3S2 ; C27H30O16 ; C18H14O4 ; C27H30O16 ; C18H14O4 ; C27H30O16 ; C15H20O3 ; C20H14O2 ; C15H20O3 ; C15H20O3 ; C15H18O3 ; C15H20O3 ; C15H20O3 ; C15H20O3 ; C15H18O3 ; C15H18O3 ; C15H20O3 ; C18H14O4 ; C15H20O3 ; C27H30O16 ; C15H20O3 ; C27H30O16 ; C15H20O3 ; C15H20O3 ; C15H20O3 ; C15H20O3 ; C15H20O3 ; C15H20O3 ; C15H18O3 ; C15H20O3 ; C15H20O3 ; C15H20O3 ; C15H18O3 ; C15H18O3 ; C15H18O3 ; C15H20O3 ; C15H18O3 ; C15H20O3 ; C15H20O3 ; C15H20O3 ; C27H30O16 ; C27H30O16 ; C27H30O16 ; C27H30O16 ; C30H26O15 ; C15H20O3 ; C15H18O3 ; C15H20O3 ; C15H18O3 ; C27H30O16 ; C27H30O16 ; C27H30O16 ; C15H18O3 ; C15H18O3 ; C15H20O3 ; C5H10N2S2 ; C15H18O3 ; C15H20O3 ; C27H30O16 ; C15H20O3 ; C27H30O16 ; C27H30O16 ; C27H30O16 ; C27H30O16 ; C15H18O3 ; C15H18O3 ; [illegible]
30247_44.05	1.681406	1.060326	2.7133	0.021808	1.6614	0.42361	NA	NA	NA	NA	NA
36012_277.8	1.680682	0.829935	2.4588	0.033744	1.4718	0.42361	NA	NA	NA	NA	NA
32848_73.53	1.680078	0.26032	2.603	0.026355	1.5791	0.42361	166.0533	HMDB02005 ; HMDB37057 ; HMDB40621 ; HMDB00500 ; HMDB00641 ; HMDB00999 ; HMDB01895 ; HMDB02031 ; HMDB02305 ; HMDB02466 ; HMDB03423 ; HMDB04062 ; HMDB06899 ; HMDB28687 ; HMDB29776 ; HMDB31245 ; HMDB31322 ; HMDB31666 ; HMDB32576 ; HMDB32920 ; HMDB33812 ; HMDB40576 ; HMDB59965	Methionine sulfoxide ; Ethiin ; 3-Glucosyl-246-trihydroxyacetophenone ; 4-Hydroxybenzoic acid ; L-Glutamine ; Phosphoribosylformylglycineamidine ; Salicylic acid ; Ureidoisobutyric acid ; 5-phosphoribosyl-a-N-formylglycineamidine ; 3-Hydroxybenzoic acid ; D-Glutamine ; Gentisate aldehyde ; Alanylglycine ; Alanyl-Glycine ; Dhurrin 6-glucoside ; 2-Methyl-1-propanethiol ; 1-Butanethiol ; Diethyl sulfide ; 2-Methoxy-14-benzoquinone ; alpha-Furyl methyl diketone ; Sesamol ; 2-(Methylthio)propane ; 34-Dihydroxybenzaldehyde	M+H ; M+H ; M+2H ; 2M+3H2O+2H ; 2M+H+K ; M+H+NH4 ; 2M+3H2O+2H ; 2M+H+K ; M+H+NH4 ; 2M+3H2O+2H ; 2M+H+K ; 2M+3H2O+2H ; 2M+H+K ; 2M+H+K ; M+2H+Na ; 3M+Na+K ; 3M+Na+K ; 3M+Na+K ; 2M+3H2O+2H ; 2M+3H2O+2H ; 2M+3H2O+2H ; 3M+Na+K ; 2M+3H2O+2H	C5H11NO3S ; C5H11NO3S ; C14H18O9 ; C7H6O3 ; C5H10N2O3 ; C8H16N3O8P ; C7H6O3 ; C5H10N2O3 ; C8H16N3O8P ; C7H6O3 ; C5H10N2O3 ; C7H6O3 ; C5H10N2O3 ; C5H10N2O3 ; C20H27NO12 ; C4H10S ; C4H10S ; C4H10S ; C7H6O3 ; C7H6O3 ; C7H6O3 ; C4H10S ; C7H6O3

2994_101.6	1.679923	0.881572	-2.5559	0.028571	1.5441	0.42361	239.0353	HMDB00797 ; HMDB01934 ; HMDB04350 ; HMDB14330 ; HMDB15318 ; HMDB33113 ; HMDB33114 ; HMDB39164 ; HMDB39982 ; HMDB41620 ; HMDB41851 ; HMDB33326 ; HMDB38715 ; HMDB41243	SAICAR ; L(-)-Nicotine pestanal ; Anabasine ; Nicotine ; Iophendylate ; 1-Methylpyrrole ; 2-Methylpyrrole ; L-Oxalylalbizziine ; Trimethyl-2-propenylpyrazine ; 17-Beta-Estradiol-317-beta-sulfate ; Cefpimizole ; BR-Xanthone B ; Alloathyriol ; 136-Trihydroxy-5-methoxyxanthone	M+H+Na ; M+2K-H ; M+2K-H ; M+2K-H ; M+Na+K ; 2M+2K-H ; 2M+2K-H ; 2M+H+K ; M+2K-H ; M+2Na ; M+H+2Na ; M+H-2H2O ; M+H-2H2O ; M+H-2H2O	C13H19N4O12P ; C10H14N2 ; C10H14N2 ; C10H14N2 ; C19H29IO2 ; C5H7N ; C5H7N ; C6H9N3O6 ; C10H14N2 ; C18H24O8S2 ; C28H26N6O10S2 ; C14H10O6 ; C14H10O6 ; C14H10O6
1246_20.1	1.678201	0.485485	2.6313	0.025107	1.6002	0.42361	399.3291	HMDB01551 ; HMDB05066 ; HMDB10376 ; HMDB31052 ; HMDB31061 ; HMDB31667 ; HMDB39288 ; HMDB40030 ; HMDB59866 ; HMDB00359 ; HMDB00430 ; HMDB01420 ; HMDB01903 ; HMDB02197 ; HMDB03533 ; HMDB06226 ; HMDB06720 ; HMDB12453 ; HMDB12457 ; HMDB12459 ; HMDB15046 ; HMDB30024 ; HMDB30047 ; HMDB30048 ; HMDB35300 ; HMDB60133 ; HMDB60425 ; HMDB60508	Palmitaldehyde ; Tetradecanoylcarnitine ; CE(24:0) ; 2-Hexadecanone ; 10-Hydroxymyristic acid methyl ester ; Nor-psi-tropine ; beta-Sitosterol behenate ; 1-(1-Pyrrolidinyl)-2-propanone ; Ethyl 3-hydroxytridecanoate ; 3a7a-Dihydroxycoprostanic acid ; 2425-Dihydroxyvitamin D ; 2526-dihydroxyvitamin D ; Calcitriol ; 7a12a-Dihydroxy-cholestene-3-one ; 3a7a12a-Trihydroxy-5b-cholestan-26-al ; 24R25-Dihydroxyvitamin D3 ; 23S25-dihydroxyvitamin D3 ; 3 beta-Hydroxy-5-cholestenoate ; 7 alpha24-Dihydroxy-4-cholesten-3-one ; 7 alpha26-Dihydroxy-4-cholesten-3-one ; Paricalcitol ; Sarsasapogenin ; Neotigogenin ; Smilagenin ; Octadecyl cis-p-coumarate ; (24R)-2425-Dihydroxycalciol ; 7alpha25-Dihydroxy-4-cholesten-3-one ; Secalciferol	3M+2K ; 2M+3H2O+2H ; M+Na+K ; 3M+2K ; 3M+H+Na ; 3M+NH4 ; M+Na+K ; 3M+NH4 ; 3M+H+Na ; M+H-2H2O ; M+H-H2O ; M+H-H2O ; M+H-H2O ; M+H-H2O ; M+H-2H2O ; M+H-H2O ; M+H-H2O ; M+H-H2O ; M+H-H2O ; M+H-H2O ; M+H-H2O ; M+H-H2O ; M+H-H2O ; M+H-H2O ; M+H-H2O ; M+H-H2O ; M+H-H2O ; M+H-H2O	C16H32O ; C21H41NO4 ; C51H92O2 ; C16H32O ; C15H30O3 ; C7H13NO ; C51H92O2 ; C7H13NO ; C15H30O3 ; C27H46O4 ; C27H44O3 ; C27H44O3 ; C27H44O3 ; C27H44O3 ; C27H46O4 ; C27H44O3 ; C27H44O3 ; C27H44O3 ; C27H44O3 ; C27H44O3 ; C27H44O3 ; C27H44O3 ; C27H44O3 ; C27H44O3 ; C27H44O3 ; C27H44O3 ; C27H44O3 ; C27H44O3
06216_67.6	1.676253	0.944154	2.5133	0.030737	1.5123	0.42361	229.9806	HMDB29202	hesperetin 3-O-sulfate	M+2K	C16H14O9S
80477_42.3	1.676231	0.794951	2.4881	0.032096	1.4936	0.42361	346.998	HMDB41712 ; HMDB41714 ; HMDB41772 ; HMDB41784 ; HMDB41951	cis-Resveratrol 3-sulfate ; cis-Resveratrol 4-sulfate ; Resveratrol 3-sulfate ; trans-Resveratrol 4-sulfate ; Nitrofen	M+K ; M+K ; M+K ; M+K ; M+ACN+Na	C14H12O6S ; C14H12O6S ; C14H12O6S ; C14H12O6S ; C12H7Cl2NO3
24602_55.4	1.675538	1.199209	-2.588	0.027041	1.568	0.42361	316.9825	HMDB30400 ; HMDB33073 ; HMDB33074 ; HMDB33557 ; HMDB36370 ; HMDB36375	L-4-Chlorotryptophan ; 1-Propenyl 1-(propylsulfinyl)propyl disulfide ; 1-(1-Propenylsulfinyl)propyl propyl disulfide ; ()-6-Chlorotryptophan ; 1-(2-Propenylsulfinyl)propyl propyl disulfide ; 2-Propenyl 1-(propylsulfinyl)propyl disulfide	M+2K+H ; M+2K+H ; M+2K+H ; M+2K+H ; M+2K+H ; M+2K+H	C11H11ClN2O2 ; C9H18OS3 ; C9H18OS3 ; C11H11ClN2O2 ; C9H18OS3 ; C9H18OS3

4662_177.9	1.675071	1.165947	-2.5958	0.026682	1.5738	0.42361	242.0345	HMDB01943 ; HMDB04366 ; HMDB15451 ; HMDB35652 ; HMDB36398 ; HMDB36794 ; HMDB38597 ; HMDB39661 ; HMDB39665 ; HMDB39666 ; HMDB39678 ; HMDB39679 ; HMDB39682 ; HMDB39836 ; HMDB40015 ; HMDB40022 ; HMDB40023 ; HMDB40024 ; HMDB40025 ; HMDB40026 ; HMDB40049 ; HMDB41341 ; HMDB41927 ; HMDB59614 ; HMDB60767	Pseudoephedrine ; Hordenine ; Ephedrine ; Perilla sugar ; Rumexoside ; Azorhodine 2G ; Nepetalactam ; 2367-Tetrahydro-7-methylcyclopentbazepin-8(1H)-one ; 5-Ethyl-123456-hexahydro-7H-cyclopentabpyridin-7-one ; 6-Ethyl-123456-hexahydro-7H-cyclopentabpyridin-7-one ; 3-Methyl-2-(1-pyrrolidinyl)-2-cyclopenten-1-one ; 5-Methyl-2-(1-pyrrolidinyl)-2-cyclopenten-1-one ; 234567-Hexahydro-7-methylcyclopentbazepin-8(1H)-one ; 2-(5-Methyl-2-furanyl)piperidine ; 2345-Tetrahydro-6-(5-methyl-2-furanyl)pyridine ; 23-Dihydro-5-propanoyl-1H-pyrrolizine ; 5-Acetyl-23-dihydro-7-methyl-1H-pyrrolizine ; 5-Acetyl-23-dihydro-6-methyl-1H-pyrrolizine ; 1-(23-Dihydro-5-methyl-1H-pyrrolizin-7-yl)ethanone ; 23-Dihydro-56-dimethyl-1H-pyrrolizine-7-carboxaldehyde ; 1-(5-Methyl-2-furanyl)methylpyrrolidine ; Flupoxam ; Methcathinone ; Riboflavin cyclic-45-phosphate ; 4-Hydroxymethamphetamine	M+2K-H ; M+2K-H ; M+2K-H ; M+2K-H ; M+Na+K ; M+H+NH4 ; M+2K-H ; M+2K+H ; M+2K-H ; M+2K-H ; M+2K-H ; M+2K-H ; M+2K-H ; M+2K-H ; M+2K+H ; M+2K+H ; M+2K+H ; M+2K+H ; M+2K+H ; M+2K+H ; M+2K-H ; M+H+Na ; M+2K+H ; M+2Na ; M+2K-H	C10H15NO ; C10H15NO ; C10H15NO ; C10H15NO ; C20H22O10 ; C18H15N3O8S2 ; C10H15NO ; C10H13NO ; C10H15NO ; C10H15NO ; C10H15NO ; C10H15NO ; C10H15NO ; C10H15NO ; C10H13NO ; C10H13NO ; C10H13NO ; C10H13NO ; C10H13NO ; C10H13NO ; C10H15NO ; C19H14ClF5N4O2 ; C10H13NO ; C17H19N4O8P ; C10H15NO
4844_28.76	1.673662	0.637597	-2.4441	0.034604	1.4609	0.42361	NA	NA	NA	NA	NA
4857_47.58	1.672446	1.228514	-2.5699	0.027897	1.5544	0.42361	506.6265	HMDB02288 ; HMDB06866 ; HMDB11658 ; HMDB12471 ; HMDB13189 ; HMDB35452	5-cis-8-cis-Tetradecadienoyl-CoA ; 2-Methyl-1-hydroxypropyl-ThPP ; 28-Dihydroxyquinoline-beta-D-glucuronide ; (2S6R10R)-Trimethyl-hendecanoyl-CoA ; 3-Indole carboxylic acid glucuronide ; Pelargonidin 3-O-b-D-Glucopyranosyl-(1-2)-4-hydroxycinnamoyl-(-6)-b-D-glucopyranoside(E-) 5-O-(6-O-malonyl-b-D-glucopyranoside)	M+H+K ; 2M+H+NH4 ; 3M+2H ; M+H+K ; 3M+2H ; M+H+Na	C35H58N7O17P3S ; C16H27N4O8P2S ; C15H15NO8 ; C35H58N7O17P3S ; C15H15NO8 ; C45H49O25
1024_210.0	1.672153	1.091683	2.5334	0.029696	1.5273	0.42361	275.5671	HMDB29237 ; HMDB29241	Cyanidin 3-(6-succinyl-glucoside) ; Peonidin 3-(6-malonyl-glucoside)	M+2H ; M+2H	C25H25O14 ; C25H25O14
5135_247.3	1.670199	0.215806	-2.4628	0.033517	1.4747	0.42361	145.0495	HMDB00393 ; HMDB00522 ; HMDB02266 ; HMDB13311 ; HMDB29171 ; HMDB31257 ; HMDB36232 ; HMDB40207 ; HMDB35589 ; HMDB38341 ; HMDB01167 ; HMDB06112 ; HMDB11174 ; HMDB11175 ; HMDB14437 ; HMDB14545 ; HMDB28915 ; HMDB28937 ; HMDB29249 ; HMDB29564 ; HMDB31647 ; HMDB35318 ; HMDB36523	3-Hexenedioic acid ; 3-Methylglutaconic acid ; (E)-2-Methylglutaconic acid ; trans-2-Hexenedioic acid ; 3-Hydroxyadipic acid 36-lactone ; Dimethyl fumarate ; Maleic acid homopolymer ; Ethyl hydrogen fumarate ; Phlorin ; Glucosylisomaltol ; Pyruvaldehyde ; Malondialdehyde ; L-isoleucyl-L-proline ; L-leucyl-L-proline ; Etomidate ; Nisoldipine ; Isoleucyl-Proline ; Leucyl-Proline ; Luteolinidin ; Bufotenine O-glucoside ; Acrylic acid ; Apiosylglucosyl 4-hydroxybenzoate ; Licoagroside B	M+H ; M+H ; M+H ; M+H ; M+H ; M+H ; M+H ; M+H ; M+2H ; M+2H ; 2M+H ; 2M+H ; M+Na+K ; M+Na+K ; M+2Na ; M+H+2Na ; M+Na+K ; M+Na+K ; M+H+NH4 ; M+3Na ; 2M+H ; M+3H ; M+3H	C6H8O4 ; C6H8O4 ; C6H8O4 ; C6H8O4 ; C6H8O4 ; C6H8O4 ; C6H8O4 ; C6H8O4 ; C12H16O8 ; C12H16O8 ; C3H4O2 ; C3H4O2 ; C11H20N2O3 ; C11H20N2O3 ; C14H16N2O2 ; C20H24N2O6 ; C11H20N2O3 ; C11H20N2O3 ; C15H11O5 ; C18H26N2O6 ; C3H4O2 ; C18H24O12 ; C18H24O12

3305_197.8	1.668814	0.573709	-2.5026	0.031309	1.5043	0.42361	250.0573	HMDB39180 ; HMDB00134 ; HMDB00176 ; HMDB00691 ; HMDB01352 ; HMDB05015 ; HMDB06938 ; HMDB13946 ; HMDB13947 ; HMDB13948 ; HMDB13950 ; HMDB14733 ; HMDB15078 ; HMDB31159 ; HMDB33163 ; HMDB38411 ; HMDB39585 ; HMDB59808 ; HMDB60827 ; HMDB61056 ; HMDB01410	Methyl 46-di-O-galloyl-beta-D-glucopyranoside ; Fumaric acid ; Maleic acid ; Malonic acid ; Hydroxypyruvic acid ; Gabapentin ; Tartronate semialdehyde ; 8-Hydroxycarvedilol ; 4-Hydroxycarvedilol ; 5-Hydroxycarvedilol ; 1-Hydroxycarvedilol ; Oxytetracycline ; Zalcitabine ; Garcinia acid ; Pyrocoll ; 6-(Methylsulfonyl)hexyl glucosinolate ; S-(25-Dimethyl-3-furanyl) 2-furancarbothioate ; Acetyl citrate ; ID11614 ; benzisothiazole piperazine ; 2-Amino-4-oxo-6-(12-dioxoprolyl)-78-dihydroxypteridine	M+2H ; 2M+NH4 ; 2M+NH4 ; 2M+ACN+H ; 2M+ACN+H ; M+2K+H ; 2M+ACN+H ; M+2K ; M+2K ; M+2K ; M+2K ; M+H+K ; M+K ; M+ACN+H ; M+ACN+Na ; M+H+NH4 ; 2M+3H2O+2H ; M+NH4 ; 2M+Na+K ; 2M+Na+K ; M+H-H2O	C21H22O14 ; C4H4O4 ; C4H4O4 ; C3H4O4 ; C3H4O4 ; C9H17NO2 ; C3H4O4 ; C24H26N2O5 ; C24H26N2O5 ; C24H26N2O5 ; C24H26N2O5 ; C22H24N2O9 ; C9H13N3O3 ; C6H8O8 ; C10H6N2O2 ; C14H27NO11S3 ; C11H10O3S ; C8H8O8 ; C11H13N3S ; C11H13N3S ; C9H9N5O5
9478_276.1	1.668692	0.321757	2.525	0.030127	1.521	0.42361	314.9759	HMDB14656 ; HMDB29513 ; HMDB33350	Cisplatin ; Myricatin ; Brassinin	M+NH4 ; M+2K ; M+2K+H	Cl2H4N2Pt ; C22H16O15S ; C11H12N2S2
7128_47.82	1.667741	1.259445	-2.5972	0.026618	1.5748	0.42361	400.8271	HMDB08293 ; HMDB08294 ; HMDB08325 ; HMDB08586 ; HMDB11224 ; HMDB11249 ; HMDB11281 ; HMDB11314 ; HMDB13431 ; HMDB13436	PC(20:0P-18:1(11Z)) ; PC(20:0P-18:1(9Z)) ; PC(20:1(11Z)P-18:0) ; PC(22:1(13Z)P-16:0) ; PC(P-16:022:1(13Z)) ; PC(P-18:020:1(11Z)) ; PC(P-18:1(11Z)20:0) ; PC(P-18:1(9Z)20:0) ; PC(o-18:1(9Z)20:1(11Z)) ; PC(o-18:2(9Z12Z)20:0)	M+2H ; M+2H ; M+2H ; M+2H ; M+2H ; M+2H ; M+2H ; M+2H ; M+2H ; M+2H	C46H90NO7P ; C46H90NO7P ; C46H90NO7P ; C46H90NO7P ; C46H90NO7P ; C46H90NO7P ; C46H90NO7P ; C46H90NO7P ; C46H90NO7P ; C46H90NO7P
2134_53.88	1.667378	1.20993	2.5962	0.026663	1.5741	0.42361	258.8992	HMDB13592	13-Dichloropropene	2M+K	C3H4Cl2
3262_193.2	1.66613	0.4572	2.4616	0.033587	1.4738	0.42361	238.0193	HMDB15260 ; HMDB32389	Bicalutamide ; Methyl acrylate-divinylbenzene completely hydrolyzed copolymer	M+2Na ; M+2K	C18H14F4N2O4S ; C18H18N6O3S
5717_21.82	1.664886	0.747053	-2.3289	0.042126	1.3755	0.42361	403.2836	HMDB14633	Buspirone	M+NH4	C21H31N5O2

4114_252.2	1.66435	0.949225	2.5925	0.026834	1.5713	0.42361	379.1044	HMDB32910 ; HMDB37437 ; HMDB39332 ; HMDB00010 ; HMDB01260 ; HMDB06768 ; HMDB06772 ; HMDB10336 ; HMDB11195 ; HMDB12491 ; HMDB13674 ; HMDB13675 ; HMDB14359 ; HMDB14917 ; HMDB15031 ; HMDB15305 ; HMDB15576 ; HMDB29452 ; HMDB29750 ; HMDB30776 ; HMDB31256 ; HMDB32651 ; HMDB32766 ; HMDB34355 ; HMDB38449 ; HMDB38684 ; HMDB39569 ; HMDB41195 ; HMDB59735 ; HMDB60088 ; HMDB60027 ; HMDB60028	Isoorientin 2-p-coumaroyl-(-6)-glucoside ; 6-(4-Hydroxycinnamoyl)astragalin 4-glucoside ; 2-(6-p-Coumaroylglucosyl)quercitrin ; 2-Methoxyestrone ; ADP-Ribosyl-L-arginine ; 19-Oxoandrost-4-ene-317-dione ; Adrenosterone ; Epinephrine glucuronide ; 2-Hydroxy-3-methoxyestrone ; 14-Benzothiazine-O-quinonimine ; 123-Trihydroxybenzene ; 135-Trihydroxybenzene ; Torasemide ; Nalidixic Acid ; Testolactone ; Phenobarbital ; Thiotepa ; L-DOPA 3-glucoside ; Methyl 2-furoate ; Maltol ; 4-Hydroxy-2H-pyran-3-carboxaldehyde ; (Z)-Tamarindienal ; N2-(2-Carboxymethyl-2-hydroxysuccinoyl)arginine ; 5-Hydroxymethyl-2-furancarboxaldehyde ; Vomifoliol 9-glucosyl-(1-4)-xylosyl-(1-6)-glucoside ; Sageone ; Ginsenoyne F ; 2-O-(Z-p-Hydroxycinnamoyl)-(x)-glyceric acid ; 5-Methylfuran-2-carboxylic acid ; 4-Methoxyestrone ; 5-(345-trihydroxyphenyl)-gamma-valerolactone-O-methyl-4-O-glucuronide ; 5-(345-trihydroxyphenyl)-gamma-valerolactone-O-methyl-5-O-glucuronide	M+2H ; M+2H ; M+2H ; M+2K+H ; M+ACN+2H ; M+2K+H ; M+2K+H ; 2M+H+K ; M+2K+H ; M+2ACN+H ; 3M+H ; 3M+H ; 2M+Na+K ; 3M+Na+K ; M+2K+H ; 3M+Na+K ; 2M+H ; 2M+H+K ; 3M+H ; 3M+H ; 3M+H ; 3M+H ; 2M+Na+K ; 3M+H ; M+2K ; M+2K+H ; M+2K+H ; 3M+2H ; 3M+H ; M+2K+H ; M+H-2H2O ; M+H-2H2O	C36H36O18 ; C36H36O18 ; C36H36O18 ; C19H24O3 ; C21H35N9O15P2 ; C19H24O3 ; C19H24O3 ; C15H21NO9 ; C19H24O3 ; C12H12N2O5S ; C6H6O3 ; C6H6O3 ; C16H20N4O3S ; C12H12N2O3 ; C19H24O3 ; C12H12N2O3 ; C6H12N3PS ; C15H21NO9 ; C6H6O3 ; C6H6O3 ; C6H6O3 ; C6H6O3 ; C12H20N4O8 ; C6H6O3 ; C30H48O17 ; C19H24O3 ; C19H24O3 ; C12H12O6 ; C6H6O3 ; C19H24O3 ; C18H22O11 ; C18H22O11
9689_50.48	1.664203	0.507583	-2.7859	0.019254	1.7155	0.42361	NA	NA	NA	NA	NA
5261_197.3	1.66414	0.999515	-2.6484	0.024379	1.613	0.42361	301.0525	HMDB35179 ; HMDB01268 ; HMDB02052 ; HMDB06809 ; HMDB32523 ; HMDB33626 ; HMDB38667 ; HMDB40005 ; HMDB59806 ; HMDB60014 ; HMDB14411 ; HMDB34039 ; HMDB34049 ; HMDB34129	Mumefural ; 4-Fumarylacetoacetic acid ; Maleylacetoacetic acid ; Nicotinate D-ribonucleoside ; Succinic anhydride ; D-Erythroascorbic acid 1-a-D-xylopyranoside ; NN-Bis(g-glutamyl)-33-(12-propylenedithio)dialanine ; 235-Trimethyl-6-4-(methylthio)butylpyrazine ; Phenylglucuronide ; Phenol glucuronide ; Dicumarol ; Dolineone ; Pachyrrhizin ; Dehydroneotenone	M+H ; 3M+2H ; 3M+2H ; M+2Na-H ; 3M+H ; M+Na ; M+Na+K ; M+2K-H ; 2M+Na+K ; 2M+Na+K ; M+H-2H2O ; M+H-2H2O ; M+H-2H2O ; M+H-2H2O	C12H12O9 ; C8H8O6 ; C8H8O6 ; C11H14NO6 ; C4H4O3 ; C10H14O9 ; C19H32N4O10S2 ; C12H20N2S ; C12H14O7 ; C12H14O7 ; C19H12O6 ; C19H12O6 ; C19H12O6 ; C19H12O6
7586_62.16	1.663714	0.944371	2.7239	0.021415	1.6693	0.42361	268.0518	HMDB03045 ; HMDB06941 ; HMDB11163 ; HMDB15519 ; HMDB28749 ; HMDB29909 ; HMDB29971 ; HMDB31793 ; HMDB32026 ; HMDB32635 ; HMDB32854 ; HMDB32884 ; HMDB33121 ; HMDB34395 ; HMDB37108 ; HMDB37603 ; HMDB39852 ; HMDB41887	Ergothioneine ; L-2-Aminoadipate adenylate ; L-beta-aspartyl-L-aspartic acid ; Phendimetrazine ; Aspartyl-Aspartate ; Gein ; Coriandrone E ; Heptenophos ; Sinalexin ; NN-Diethylbenzeneacetamide ; N-Acetyl-26-diethylaniline ; Allura red AC ; Paraquat dichloride ; 2-Propenyl 3-(2-propenylsulfonyl)-1-propenyl disulfide ; 3-Hydroxychavicol 1-rhamnosyl-(1-6)-glucoside ; Eugenol O-a-L-Arabinofuranosyl-(1-6)-b-D-glucopyranoside ; Deoxynivalenol 3-glucoside ; Endalin	M+K ; M+2Na ; 2M+H+K ; M+2K-H ; 2M+H+K ; M+2K ; 2M+H+K ; M+NH4 ; M+ACN+Na ; M+2K-H ; M+2K-H ; M+2ACN+2H ; 2M+H+Na ; M+NH4 ; M+2K ; M+2K ; M+2K ; M+K	C9H15N3O2S ; C16H23N6O10P ; C8H12N2O7 ; C12H17NO ; C8H12N2O7 ; C21H30O11 ; C13H12O5 ; C9H12ClO4P ; C10H8N2OS ; C12H17NO ; C12H17NO ; C18H16N2O8S2 ; C12H14Cl2N2 ; C9H14O2S3 ; C21H30O11 ; C21H30O11 ; C21H30O11 ; C9H12FN3O3

4297_47.86	1.663082	1.207407	-2.5279	0.029978	1.5232	0.42361	402.8243	HMDB07953 ; HMDB08527 ; HMDB08947 ; HMDB09006 ; HMDB09229 ; HMDB09486 ; HMDB09715 ; HMDB14506 ; HMDB15581 ; HMDB30129	PC(15:022:0) ; PC(22:015:0) ; PE(16:024:0) ; PE(18:022:0) ; PE(20:020:0) ; PE(22:018:0) ; PE(24:016:0) ; Anidulafungin ; Iodipamide ; Plastoquinone 8	M+2H ; M+2H ; M+2H ; M+2H ; M+2H ; M+2H ; M+2H ; M+3Na ; M+3Na ; M+3ACN+2H	C45H90NO8P ; C45H90NO8P ; C45H90NO8P ; C45H90NO8P ; C45H90NO8P ; C45H90NO8P ; C45H90NO8P ; C58H73N7O17 ; C20H14I6N2O6 ; C48H72O2
57736_288.6	1.662824	0.805392	2.4812	0.032478	1.4884	0.42361	705.7958	HMDB30901 ; HMDB41532	Eremopetasitenin B2 ; Eremosulphoxinolide A	3M+H+NH4 ; 3M+H+NH4	C24H32O7S ; C24H32O7S
28308_114.1	1.662237	0.423211	2.3457	0.040937	1.3879	0.42361	153.0328	HMDB01343 ; HMDB02994 ; HMDB04136 ; HMDB14391 ; HMDB28730 ; HMDB28814 ; HMDB30564 ; HMDB30565 ; HMDB31422 ; HMDB32924 ; HMDB33341 ; HMDB33980 ; HMDB36294 ; HMDB41447	Mevalonic acid-5P ; Erythritol ; D-Threitol ; Ziprasidone ; Asparaginyl-Glutamate ; Glutamyl-Asparagine ; trans-Piceid ; (Z)-Resveratrol 4-glucoside ; cis-Piceid ; 3-Furanmethanol glucoside ; Mollicellin C ; 7-Methoxy-2-methylisoflavone ; (E)-2-Glucosyl-345-trihydroxystilbene ; (E)-4-5-(4-Hydroxyphenoxy)-3-penten-1-ynylphenol	2M+3H ; 2M+Na+K ; 2M+Na+K ; M+H+2Na ; M+2Na ; M+2Na ; M+3Na ; M+3Na ; M+3Na ; M+2Na ; M+H+2Na ; M+H+K ; M+3Na ; M+H+K	C6H13O7P ; C4H10O4 ; C4H10O4 ; C21H21ClN4OS ; C9H14N3O6 ; C9H14N3O6 ; C20H22O8 ; C20H22O8 ; C20H22O8 ; C11H16O7 ; C22H20O8 ; C17H14O3 ; C20H22O8 ; C17H14O3
24403_65.96	1.662014	0.860131	-2.5744	0.027682	1.5578	0.42361	NA	NA	NA	NA	NA
13162_212.	1.661925	0.344007	2.7184	0.02162	1.6651	0.42361	344.1013	HMDB01932 ; HMDB06049 ; HMDB13926 ; HMDB15520 ; HMDB29072 ; HMDB29093 ; HMDB30069 ; HMDB31930 ; HMDB60376 ; HMDB60434 ; HMDB61122 ; HMDB00629 ; HMDB60071	Metoprolol ; O-Phosphotyrosine ; Dehydrogenated ticlopidine ; Oxprenolol ; Threoninyl-Tryptophan ; Tryptophyl-Threonine ; Chalcomoracin ; Benzosimuline ; 3-Oxo-OPC6-CoA ; alpha-Fluoro-beta-alanine ; 4-Hydroxy-alprenolol ; Chondroitin ; Hyaluronan biosynthesis precursor 1	M+2K-H ; M+2ACN+H ; M+2ACN+H ; M+2K+H ; M+K ; M+K ; M+H+K ; M+K ; M+3H ; 3M+Na ; M+2K+H ; M+H-2H2O ; M+H-2H2O	C15H25NO3 ; C9H12NO6P ; C14H12ClNS ; C15H23NO3 ; C15H19N3O4 ; C15H19N3O4 ; C39H36O9 ; C20H19NO2 ; C37H58N7O19P3S ; C3H6FNO2 ; C15H23NO3 ; C14H21NO11 ; C14H21NO11
34913_260.9	1.661752	1.17338	-2.5674	0.028015	1.5526	0.42361	364.0035	HMDB00843	N-Acetylgalactosamine 46-disulfate	M+H-H2O	C8H15NO12S2

)4121_45.04	1.661408	1.051934	2.5308	0.029828	1.5254	0.42361	318.7194	HMDB01925 ; HMDB29669 ; HMDB31879 ; HMDB32176 ; HMDB33196 ; HMDB34469 ; HMDB34470 ; HMDB34472 ; HMDB34539 ; HMDB34556 ; HMDB34646 ; HMDB35008 ; HMDB35016 ; HMDB35017 ; HMDB35181 ; HMDB35313 ; HMDB35330 ; HMDB35332 ; HMDB36025 ; HMDB36240 ; HMDB36299 ; HMDB36386 ; HMDB36391 ; HMDB36672 ; HMDB37194 ; HMDB37604 ; HMDB37623 ; HMDB37720 ; HMDB38605 ; HMDB39101 ; HMDB39140 ; HMDB39595 ; HMDB40431 ; HMDB40495	Ibuprofen ; Eremopetasinorone A ; 11a12a-Epoxy-3b-hydroxy-2813-oleananolide 3-acetate ; Benzyl hexanoate ; (E)-6-Methyl-6-(5-methyl-2-furanyl)-3-hepten-2-one ; 2-Phenylpropyl butyrate ; 2-Phenylpropyl isobutyrate ; 3-Phenylpropyl 2-methylpropanoate ; 3-O-cis-Coumaroylmaslinic acid ; 3-Acetoxy-3-methyl-1-phenylbutane ; 3beta-Acetoxy-12-oxo-2813beta-oleananolide ; 3-Methylbutyl phenylacetate ; 2-Phenylethyl pentanoate ; 2-Phenylethyl 3-methylbutanoate ; xi-89-Dehydrotheaspirone ; Ganoderic acid S ; Ganoderic acid Mf ; Ganoderic acid X ; 246-Trimethyl-4-phenyl-13-dioxane ; Methyl 4-tert-butylphenylacetate ; 3-O-p-trans-Coumaroylalphitolic acid ; 1-Phenylpropyl butyrate ; alpha-Methylphenethyl butyrate ; 3alpha-Acetoxy-11-keto-beta-boswellic acid ; Ethyl ()-2-ethyl-3-phenylpropanoate ; 10beta-1213-Dinor-8-oxo-6-eremophilen-11-al ; 1-Methyl-1-phenylethyl isobutyrate ; 2-Phenylethyl 2-methylbutanoate ; Amyl phenylacetate ; Etrogol ; Hericenone E ; Hericenone H ; Hexyl benzoate ; cis-p-Coumaroylcorosolic acid	3M+H+NH4 ; 3M+H+NH4 ; M+3ACN+2H ; 3M+H+NH4 ; 3M+H+NH4 ; 3M+H+NH4 ; 3M+H+NH4 ; 3M+H+NH4 ; M+H+NH4 ; 3M+H+NH4 ; M+3ACN+2H ; 3M+H+NH4 ; 3M+H+NH4 ; 3M+H+NH4 ; 3M+H+NH4 ; M+3ACN+2H ; M+3ACN+2H ; M+3ACN+2H ; 3M+H+NH4 ; 3M+H+NH4 ; M+H+NH4 ; 3M+H+NH4 ; 3M+H+NH4 ; M+3ACN+2H ; 3M+H+NH4 ; 3M+H+NH4 ; 3M+H+NH4 ; 3M+H+NH4 ; 3M+H+NH4 ; 3M+H+NH4 ; M+ACN+2H ; M+ACN+2H ; 3M+H+NH4 ; M+H+NH4	C13H18O2 ; C13H18O2 ; C32H48O5 ; C13H18O2 ; C13H18O2 ; C13H18O2 ; C13H18O2 ; C13H18O2 ; C39H54O6 ; C13H18O2 ; C32H48O5 ; C13H18O2 ; C13H18O2 ; C13H18O2 ; C13H18O2 ; C32H48O5 ; C32H48O5 ; C32H48O5 ; C13H18O2 ; C13H18O2 ; C39H54O6 ; C13H18O2 ; C13H18O2 ; C32H48O5 ; C13H18O2 ; C13H18O2 ; C13H18O2 ; C13H18O2 ; C13H18O2 ; C13H18O2 ; C37H54O6 ; C37H54O6 ; C13H18O2 ; C39H54O6
57687_136.	1.661032	0.771418	-2.5469	0.029019	1.5373	0.42361	124.0758	HMDB29300 ; HMDB32492 ; HMDB32578 ; HMDB36058 ; HMDB40360 ; HMDB61115 ; HMDB00329 ; HMDB00543 ; HMDB01955 ; HMDB05802 ; HMDB05809 ; HMDB06115 ; HMDB11626 ; HMDB13749 ; HMDB15170 ; HMDB30060 ; HMDB30063 ; HMDB31597 ; HMDB31761 ; HMDB32038 ; HMDB32215 ; HMDB32336 ; HMDB32586 ; HMDB32618 ; HMDB32620 ; HMDB32891 ; HMDB33723 ; HMDB33945 ; HMDB34471 ; HMDB34615 ; HMDB34732 ; HMDB35213 ; HMDB35268 ; HMDB35414 ; HMDB35707 ; HMDB35862 ; HMDB36098 ; HMDB36416 ; HMDB36796 ; HMDB37160 ; HMDB37174 ; HMDB37270 ; HMDB38125 ; HMDB38184 ; HMDB39884 ; HMDB40046 ; HMDB40359 ; HMDB40460 ; HMDB41288 ; HMDB41472 ; HMDB41473 ; HMDB41612 ; HMDB59676 ; HMDB59857 ; [illegible]	p-Anisidine ; 2-Propionylpyrrole ; 4-Hydroxybenzylamine ; 1-Ethyl-1H-pyrrole-2-carboxaldehyde ; 2-Acetyl-1-methylpyrrole ; Lenticin ; 2-Phenylbutyric acid ; Benzenebutanoic acid ; 3-Phenylbutyric acid ; Isoeugenol ; Eugenol ; Benzaldehyde ; Dodecanol ; 2-Methylfuran ; Tolterodine ; Methyl 3-phenylpropanoate ; Benzyl propionate ; 24-Pentadienal ; Propyl benzoate ; Isopropyl benzoate ; 2-Decylfuran ; 3-Hydroxy-4-phenylbutan-2-one ; 3-(4-Methoxyphenyl)-2-propen-1-ol ; Ethyl phenylacetate ; 1-Phenylethyl acetate ; 1-(4-Methoxyphenyl)-2-propanone ; 4-(4-Hydroxyphenyl)-2-butanone ; 2-Phenylethyl acetate ; 3-Phenylpropyl formate ; Epoxyanethole ; Thymoquinone ; beta-Thujaplicin ; 4-Isopropylbenzoic acid ; Lepalone ; (6alpha7alpha10alpha)-1(5)3-Aromadendradiene ; (E)-610-Dimethyl-9-methylene-5-undecen-2-one ; Isoegomaketone ; beta-Spathulene ; (1alpha6alpha7alphaH)-24(15)-Copadiene ; 4-Methyl-2-phenyl-13-dioxolane ; p-Mentha-24(8)-dien-93-olide ; Fenpropimorph ; 149-Cadinatriene ; Egomaketone ; 2-Cyclotetradecen-1-one ; 1-(23-Dihydro-1H-pyrrolizin-5-yl)-14-pentanedione ; 12-Dimethoxy-4-vinylbenzene ; 4-Methylbenzyl alcohol acetate ; 2-Butyl-1-octanol ; 2-Methylbenzyl alcohol acetate ; (3-Methylphenyl)methyl acetate ; Phenyl butyrate ; beta-Vatirenene ; beta-Vetivenene ; alpha-Curcumene ; (E)-Calamene ; 2-Methylbutyrylglycine ; Isovalerylglycine ; Valerylglycine ; N-Acetylvaline ; 3-Dehydrocarnitine ; 5-Acetamidovalerate ; Ethosuximide ; 4-Hydroxystachydrine ; Turicine ; Betonicine ; L-Hypoglycin A ; Arecaidine ; Calystegine A6 ; Calystegine A7 ; Calystegin A3 ; Medicanine	M+H ; M+H ; M+H ; M+H ; M+H ; M+2H ; M+2ACN+2H ; M+2ACN+2H ; M+2ACN+2H ; M+2ACN+2H ; M+2ACN+2H ; M+NH4 ; M+Na+K ; M+ACN+H ; M+H+2Na ; M+2ACN+2H ; M+2ACN+2H ; M+ACN+H ; M+2ACN+2H ; M+2ACN+2H ; M+H+K ; M+2ACN+2H ; M+2ACN+2H ; M+2ACN+2H ; M+2ACN+2H ; M+2ACN+2H ; M+2ACN+2H ; M+2ACN+2H ; M+2ACN+2H ; M+2ACN+2H ; M+2ACN+2H ; M+2ACN+2H ; M+2ACN+2H ; M+2ACN+2H ; M+2Na ; M+H+K ; M+2ACN+2H ; M+2Na ; M+2Na ; M+2ACN+2H ; M+2ACN+2H ; M+3Na ; M+2Na ; M+2ACN+2H ; M+H+K ; M+ACN+2H ; M+2ACN+2H ; M+2ACN+2H ; M+Na+K ; M+2ACN+2H ; M+2ACN+2H ; M+2ACN+2H ; M+2Na ; M+2Na ; M+2Na ; M+2Na ; M+H-2H2O ; M+H-2H2O ; M+H-2H2O ; M+H-2H2O ; M+H-2H2O ; M+H-2H2O ; M+H-H2O ; M+H-2H2O ; M+H-2H2O ; M+H-2H2O ; M+H-H2O ; M+H-H2O ; M+H-2H2O ; M+H-2H2O ; M+H-2H2O ; M+H-2H2O	C7H9NO ; C7H9NO ; C7H9NO ; C7H9NO ; C7H9NO ; C14H18N2O2 ; C10H12O2 ; C10H12O2 ; C10H12O2 ; C10H12O2 ; C10H12O2 ; C7H6O ; C12H26O ; C5H6O ; C22H31NO ; C10H12O2 ; C10H12O2 ; C5H6O ; C10H12O2 ; C10H12O2 ; C14H24O ; C10H12O2 ; C10H12O2 ; C10H12O2 ; C10H12O2 ; C10H12O2 ; C10H12O2 ; C10H12O2 ; C10H12O2 ; C10H12O2 ; C10H12O2 ; C10H12O2 ; C10H12O2 ; C10H12O2 ; C15H22 ; C14H24O ; C10H12O2 ; C15H22 ; C15H22 ; C10H12O2 ; C10H12O2 ; C20H33NO ; C15H22 ; C10H12O2 ; C14H24O ; C12H15NO2 ; C10H12O2 ; C10H12O2 ; C12H26O ; C10H12O2 ; C10H12O2 ; C10H12O2 ; C15H22 ; C15H22 ; C15H22 ; C15H22 ; C7H13NO3 ; C7H13NO3 ; C7H13NO3 ; C7H13NO3 ; C7H13NO3 ; C7H13NO3 ; C7H11NO2 ; C7H13NO3 ; C7H13NO3 ; C7H13NO3 ; C7H11NO2 ; C7H11NO2 ; C7H13NO3 ; C7H13NO3 ; C7H13NO3 ; C7H13NO3

[illegible]7558_289.3	1.660397	0.860051	2.4301	0.035441	1.4505	0.42361	548.9588	HMDB03850 ; HMDB14406 ; HMDB45891 ; HMDB45943 ; HMDB46216 ; HMDB46218 ; HMDB50596 ; HMDB50601 ; HMDB50634 ; HMDB50639 ; HMDB50722 ; HMDB50724 ; HMDB50817 ; HMDB50819 ; HMDB51676 ; HMDB51710 ; HMDB51852 ; HMDB51854 ; HMDB52469 ; HMDB52568 ; HMDB52573 ; HMDB52602 ; HMDB52686 ; HMDB52700 ; HMDB52706 ; HMDB52776 ; HMDB53327 ; HMDB53412 ; HMDB53417 ; HMDB53442 ; HMDB53514 ; HMDB53526 ; HMDB53532 ; HMDB53592 ; HMDB54178 ; HMDB54200 ; HMDB54496 ; HMDB54516 ; HMDB60636	1-Phosphatidyl-1D-myo-inositol 3-phosphate ; Anagrelide ; TG(20:018:2(9Z12Z)22:6(4Z7Z10Z13Z16Z19Z)) ; TG(20:020:2n622:6(4Z7Z10Z13Z16Z19Z)) ; TG(20:022:6(4Z7Z10Z13Z16Z19Z)18:2(9Z12Z)) ; TG(20:022:6(4Z7Z10Z13Z16Z19Z)20:2n6) ; TG(20:1(11Z)18:2(9Z12Z)22:5(4Z7Z10Z13Z16Z)) ; TG(20:1(11Z)18:2(9Z12Z)22:5(7Z10Z13Z16Z19Z)) ; TG(20:1(11Z)20:2n622:5(4Z7Z10Z13Z16Z)) ; TG(20:1(11Z)20:2n622:5(7Z10Z13Z16Z19Z)) ; TG(20:1(11Z)22:5(4Z7Z10Z13Z16Z)18:2(9Z12Z)) ; TG(20:1(11Z)22:5(4Z7Z10Z13Z16Z)20:2n6) ; TG(20:1(11Z)22:5(7Z10Z13Z16Z19Z)18:2(9Z12Z)) ; TG(20:1(11Z)22:5(7Z10Z13Z16Z19Z)20:2n6) ; TG(22:1(13Z)18:2(9Z12Z)20:5(5Z8Z11Z14Z17Z)) ; TG(22:1(13Z)20:2n620:5(5Z8Z11Z14Z17Z)) ; TG(22:1(13Z)20:5(5Z8Z11Z14Z17Z)18:2(9Z12Z)) ; TG(22:1(13Z)20:5(5Z8Z11Z14Z17Z)20:2n6) ; TG(18:2(9Z12Z)20:022:6(4Z7Z10Z13Z16Z19Z)) ; TG(18:2(9Z12Z)20:1(11Z)22:5(4Z7Z10Z13Z16Z)) ; TG(18:2(9Z12Z)20:1(11Z)22:5(7Z10Z13Z16Z19Z)) ; TG(18:2(9Z12Z)22:1(13Z)20:5(5Z8Z11Z14Z17Z)) ; TG(18:2(9Z12Z)20:4(5Z8Z11Z14Z)22:2(13Z16Z)) ; TG(18:2(9Z12Z)22:2(13Z16Z)20:4(5Z8Z11Z14Z)) ; TG(18:2(9Z12Z)22:2(13Z16Z)20:4(8Z11Z14Z17Z)) ; TG(18:2(9Z12Z)20:4(8Z11Z14Z17Z)22:2(13Z16Z)) ; TG(20:2n620:022:6(4Z7Z10Z13Z16Z19Z)) ; TG(20:2n620:1(11Z)22:5(4Z7Z10Z13Z16Z)) ; TG(20:2n620:1(11Z)22:5(7Z10Z13Z16Z19Z)) ;	M+2K+H ; 2M+K ; M+3ACN+2H ; 2M+H	C11H20O16P2 ; C10H7Cl2N3O ; C64H108O6 ; C6H11O6PS2
[illegible]6088_54.8[illegible]	1.660157	1.151279	2.6826	0.022989	1.6385	0.42361	416.9176	HMDB00968 ; HMDB01047 ; HMDB01058 ; HMDB01112 ; HMDB01473 ; HMDB03514 ; HMDB06234 ; HMDB06235 ; HMDB06872 ; HMDB60269 ; HMDB60444	1D-Myo-inositol 14-bisphosphate ; D-Fructose 26-bisphosphate ; Fructose 16-bisphosphate ; D-Glyceraldehyde 3-phosphate ; Dihydroxyacetone phosphate ; Alpha-D-Glucose 16-bisphosphate ; 1D-Myo-inositol 13-bisphosphate ; 1D-Myo-inositol 34-bisphosphate ; D-Tagatose 16-bisphosphate ; D-Mannose 16-bisphosphate ; beta-D-Fructose 16-bisphosphate	M+2K-H ; M+2K-H ; M+2K-H ; 2M+2K-H ; 2M+2K-H ; M+2K-H ; M+2K-H ; M+2K-H ; M+2K-H ; M+2K-H ; M+2K-H	C6H14O12P2 ; C6H14O12P2 ; C6H14O12P2 ; C3H7O6P ; C3H7O6P ; C6H14O12P2 ; C6H14O12P2 ; C6H14O12P2 ; C6H14O12P2 ; C6H14O12P2 ; C6H14O12P2

[illegible]452_219.2	1.659955	1.081007	2.6195	0.025619	1.5914	0.42361	290.5585	HMDB05376 ; HMDB10415 ; HMDB10420 ; HMDB10421 ; HMDB10471 ; HMDB36341 ; HMDB41224 ; HMDB42083 ; HMDB42088 ; HMDB42106 ; HMDB42108 ; HMDB42147 ; HMDB42282 ; HMDB42292 ; HMDB42310 ; HMDB42311 ; HMDB42320 ; HMDB42326 ; HMDB42339 ; HMDB42355 ; HMDB42369 ; HMDB42385 ; HMDB42398 ; HMDB42512 ; HMDB42567 ; HMDB42572 ; HMDB42639 ; HMDB42655 ; HMDB42698 ; HMDB42721 ; HMDB42777 ; HMDB42790 ; HMDB42791 ; HMDB42800 ; HMDB42806 ; HMDB42819 ; HMDB42835 ; HMDB42843 ; HMDB42857 ; HMDB42864 ; HMDB42871 ; HMDB42974 ; HMDB42976 ; HMDB43014 ; HMDB43714 ; HMDB43853 ; HMDB44032 ; HMDB44033 ; HMDB44042 ; HMDB44048 ; HMDB44075 ; HMDB44086 ; HMDB44114 ; HMDB44366 ; HMDB44507 ; HMDB44523 ;	TG(16:016:1(9Z)16:1(9Z))iso3 ; TG(16:014:018:2(9Z12Z))iso6 ; TG(16:1(9Z)14:018:1(11Z))iso6 ; TG(16:1(9Z)14:018:1(9Z))iso6 ; TG(18:2(9Z12Z)14:018:3(9Z12Z15Z))iso6 ; 15-Palmitoylsolamin ; beta-D-Galactopyranosyl-(1-4)-2-amino-2-deoxy-beta-D-glucopyranosyl-(1-6)-D-mannose ; TG(14:014:022:5(4Z7Z10Z13Z16Z)) ; TG(14:014:022:5(7Z10Z13Z16Z19Z)) ; TG(14:015:018:2(9Z12Z)) ; TG(14:015:020:2n6) ; TG(14:016:020:5(5Z8Z11Z14Z17Z)) ; TG(14:014:1(9Z)20:1(11Z)) ; TG(14:014:1(9Z)22:4(7Z10Z13Z16Z)) ; TG(14:016:1(9Z)18:1(11Z)) ; TG(14:016:1(9Z)18:1(9Z)) ; TG(14:016:1(9Z)20:4(5Z8Z11Z14Z)) ; TG(14:016:1(9Z)20:4(8Z11Z14Z17Z)) ; TG(14:018:1(11Z)16:1(9Z)) ; TG(14:018:1(11Z)18:4(6Z9Z12Z15Z)) ; TG(14:018:1(9Z)16:1(9Z)) ; TG(14:018:1(9Z)18:4(6Z9Z12Z15Z)) ; TG(14:020:1(11Z)14:1(9Z)) ; TG(14:018:2(9Z12Z)15:0) ; TG(14:018:3(6Z9Z12Z)20:5(5Z8Z11Z14Z17Z)) ; TG(14:020:2n615:0) ; TG(14:020:4(5Z8Z11Z14Z)16:1(9Z)) ; TG(14:020:4(5Z8Z11Z14Z)18:4(6Z9Z12Z15Z)) ; TG(14:022:4(7Z10Z13Z16Z)14:1(9Z)) ; TG(14:022:5(4Z7Z10Z13Z16Z)14:0) ; TG(14:018:3(9Z12Z15Z)20:5(5Z8Z11Z14Z17Z)) ; TG(14:018:4(6Z9Z12Z15Z)18:1(11Z)) ; TG(14:018:4(6Z9Z12Z15Z)18:1(9Z)) ; TG(14:018:4(6Z9Z12Z15Z)20:4(5Z8Z11Z14Z)) ; TG(14:018:4(6Z9Z12Z15Z)20:4(8Z11Z14Z17Z)) ; TG(14:020:4(8Z11Z14Z17Z)16:1(9Z)) ; TG(14:020:4(8Z11Z14Z17Z)18:4(6Z9Z12Z15Z)) ; TG(14:020:5(5Z8Z11Z14Z17Z)16:0) ; TG(14:020:5(5Z8Z11Z14Z17Z)18:3(6Z9Z12Z)) ; TG(14:020:5(5Z8Z11Z14Z17Z)18:3(9Z12Z15Z)) ; TG(14:022:5(7Z10Z13Z16Z19Z)14:0) ; TG(15:014:018:2(9Z12Z)) ; TG(15:014:020:2n6) ; TG(15:015:020:5(5Z8Z11Z14Z17Z)) ;	M+3Na ; M+3Na ; M+3Na ; M+3Na ; M+H+2Na ; M+3Na ; M+2K ; M+H+2Na ; M+H+2Na ; M+3Na ; M+3Na ; M+H+2Na ; M+3Na ; M+H+2Na ; M+3Na ; M+3Na ; M+H+2Na ; M+H+2Na ; M+3Na ; M+H+2Na ; M+3Na ; M+H+2Na ; M+3Na ; M+3Na ; M+2H+Na ; M+3Na ; M+H+2Na ; M+2H+Na ; M+H+2Na ; M+H+2Na ; M+2H+Na ; M+H+2Na ; M+H+2Na ; M+2H+Na ; M+2H+Na ; M+H+2Na ; M+2H+Na ; M+H+2Na ; M+2H+Na ; M+2H+Na ; M+H+2Na ; M+3Na ; M+3Na ; M+H+2Na ; M+H+2Na ; M+H+2Na ; M+3Na ; M+3Na ; M+H+2Na ; M+H+2Na ; M+H+2Na ; M+3Na ; M+3Na ; M+H+2Na ; M+H+2Na ; M+2H+Na ; M+H+2Na ; M+3Na ; M+H+2Na ; M+3Na ; M+H+2Na ; M+3Na ; M+3Na ; M+H+2Na ; M+3Na ; M+3Na ; M+H+2Na ; M+H+2Na ; M+3Na ; M+H+2Na ; M+3Na ; M+H+2Na ; M+H+2Na ; M+2H+Na ; M+H+2Na ; M+H+2Na ; M+H+2Na ; M+H+2Na ; M+H+2Na ; M+2H+Na ; M+H+2Na ; M+H+2Na ; M+2H+Na ; M+2H+Na ; M+H+2Na ; M+2H+Na ; M+2H+Na ; M+2H+Na ; M+H+2Na ; M+H+2Na ; M+2H+Na ; M+2H+Na ; M+2H+Na ; M+2H+Na ; M+2H ; M+2H+Na ;	C51H94O6 ; C51H94O6 ; C51H94O6 ; C51H94O6 ; C53H92O6 ; C51H94O6 ; C18H33NO15 ; C53H92O6 ; C53H92O6 ; C51H94O6 ; C51H94O6 ; C53H92O6 ; C51H94O6 ; C53H92O6 ; C51H94O6 ; C51H94O6 ; C53H92O6 ; C53H92O6 ; C51H94O6 ; C53H92O6 ; C51H94O6 ; C53H92O6 ; C51H94O6 ; C51H94O6 ; C55H90O6 ; C51H94O6 ; C53H92O6 ; C55H90O6 ; C53H92O6 ; C53H92O6 ; C55H90O6 ; C53H92O6 ; C53H92O6 ; C55H90O6 ; C55H90O6 ; C53H92O6 ; C55H90O6 ; C53H92O6 ; C55H90O6 ; C55H90O6 ; C53H92O6 ; C51H94O6 ; C51H94O6 ; C53H92O6 ; C53H92O6 ; C53H92O6 ; C51H94O6 ; C51H94O6 ; C53H92O6 ; C53H92O6 ; C53H92O6 ; C51H94O6 ; C51H94O6 ; C53H92O6 ; C53H92O6 ; C55H90O6 ; C53H92O6 ; C51H94O6 ; C53H92O6 ; C51H94O6 ; C53H92O6 ; C51H94O6 ; C51H94O6 ; C53H92O6 ; C51H94O6 ; C51H94O6 ; C53H92O6 ; C53H92O6 ; C51H94O6 ; C53H92O6 ; C51H94O6 ; C53H92O6 ; C53H92O6 ; C55H90O6 ; C53H92O6 ; C53H92O6 ; C53H92O6 ; C53H92O6 ; C53H92O6 ; C55H90O6 ; C53H92O6 ; C53H92O6 ; C55H90O6 ; C55H90O6 ;
[illegible]0779_177.[illegible]	1.659823	1.144941	-2.5497	0.02888	1.5394	0.42361	241.0311	HMDB00565 ; HMDB00625 ; HMDB00682 ; HMDB00965 ; HMDB01128 ; HMDB01857 ; HMDB01982 ; HMDB02026 ; HMDB03290 ; HMDB04308 ; HMDB11103 ; HMDB11714 ; HMDB14663 ; HMDB29255 ; HMDB30661 ; HMDB33048 ; HMDB33049 ; HMDB35484 ; HMDB37944 ; HMDB37947 ; HMDB40774 ; HMDB41550 ; HMDB41883 ; HMDB59704 ; HMDB60944 ; HMDB60924 ; HMDB60925	Galactonic acid ; Gluconic acid ; Indoxyl sulfate ; Hypotaurine ; 5-Phosphoribosylamine ; 13-Dimethyluric acid ; 37-Dimethyluric acid ; 19-Dimethyluric acid ; Gulonic acid ; 79-Dimethyluric acid ; 17-Dimethyluric acid ; Vanilpyruvic acid ; Bentiromide ; (-)-Catechin 3-O-gallate ; 3-Galloylcatechin ; Ethyl 1-(propylthio)propyl disulfide ; Butyl 1-(methylthio)propyl disulfide ; 3-(34-Dihydroxy-5-methoxy)-2-propenoic acid ; (-)-Epicatechin 3-O-gallate ; 7-Galloylcatechin ; Bismurrayaquinone A ; Oleoside 11-methyl ester ; Doripenem ; 39-Dimethyluric acid ; Sulfinpyrazone sulfone ; Suprofen S-oxide ; Thiophene-45-epoxide	M+2Na-H ; M+2Na-H ; 2M+3H2O+2H ; 2M+Na ; 2M+H+Na ; M+2Na-H ; M+2Na-H ; M+2Na-H ; M+2Na-H ; M+2Na-H ; M+2Na-H ; 2M+Na+K ; M+2K ; M+H+K ; M+H+K ; 2M+Na+K ; 2M+Na+K ; 2M+Na+K ; M+H+K ; M+H+K ; M+Na+K ; M+2K ; M+Na+K ; M+2Na-H ; M+Na+K ; M+H-2H2O ; M+H-2H2O	C6H12O7 ; C6H12O7 ; C8H7NO4S ; C2H7NO2S ; C5H12NO7P ; C7H8N4O3 ; C7H8N4O3 ; C7H8N4O3 ; C6H12O7 ; C7H8N4O3 ; C7H8N4O3 ; C10H10O5 ; C23H20N2O5 ; C22H18O10 ; C22H18O10 ; C8H18S3 ; C8H18S3 ; C10H10O5 ; C22H18O10 ; C22H18O10 ; C26H16N2O4 ; C17H24O11 ; C15H24N4O6S2 ; C7H8N4O3 ; C23H20N2O4S ; C14H12O4S ; C14H12O4S

2896_125.	1.659695	1.120766	-2.5713	0.02783	1.5555	0.42361	284.0643	HMDB01209 ; HMDB02381 ; HMDB02670 ; HMDB05897 ; HMDB14016 ; HMDB14017 ; HMDB15050 ; HMDB28732 ; HMDB28759 ; HMDB28858 ; HMDB28969 ; HMDB29631 ; HMDB30084 ; HMDB30873 ; HMDB31824 ; HMDB31985 ; HMDB33308 ; HMDB33422 ; HMDB33519 ; HMDB33928 ; HMDB34002 ; HMDB34105 ; HMDB37254 ; HMDB38549 ; HMDB39386 ; HMDB41656 ; HMDB41698 ; HMDB41704 ; HMDB60550 ; HMDB60774	Allantoic acid ; N-Acetylcystathionine ; Naringenin ; Dihydrogenistein ; 5-Hydroxyketamine ; 4-Hydroxyketamine ; Phenformin ; Asparaginyl-Hydroxyproline ; Aspartyl-Methionine ; Hydroxyprolyl-Asparagine ; Methionyl-Aspartate ; Chalconaringenin ; Diosbulbinoside D ; 1-Hydroxy-37-dimethoxyxanthone ; 458-Trihydroxyflavanone ; gamma-Glutamyl-S-methylcysteine ; Moracin J ; (2S2S3S3R4S)-3457-Tetrahydroxyflavan(2-74-8)-3457-tetrahydroxyflavan ; N-Ornithyl-L-taurine ; ()-2-Hydroxydihydrodaidzein ; 349-Trihydroxypterocarpan ; Glycinol ; (2R3R)-347-Trihydroxyflavanone ; Toralactone ; Cymorcin diglucoside ; 3-Hydroxydihydrodaidzein ; 6-Hydroxydihydrodaidzein ; 8-Hydroxydihydrodaidzein ; 6-Hydroxyketamine ; 5-Carboxy-2-deoxyuridine	3M+H+K ; 2M+H+K ; 2M+H+Na ; 2M+H+Na ; 2M+Na+K ; 2M+Na+K ; M+2K+H ; M+K ; 2M+H+K ; M+K ; 2M+H+K ; 2M+H+Na ; M+Na+K ; 2M+H+Na ; 2M+H+Na ; 2M+H+K ; 2M+H+Na ; M+H+Na ; M+2Na-H ; 2M+H+Na ; 2M+H+Na ; 2M+H+Na ; 2M+H+Na ; 2M+H+Na ; M+2K ; 2M+H+Na ; 2M+H+Na ; 2M+H+Na ; 2M+Na+K ; 2M+H+Na	C4H8N4O4 ; C9H16N2O5S ; C15H12O5 ; C15H12O5 ; C13H16ClNO2 ; C13H16ClNO2 ; C10H15N5 ; C9H15N3O5 ; C9H16N2O5S ; C9H15N3O5 ; C9H16N2O5S ; C15H12O5 ; C25H30O11 ; C15H12O5 ; C15H12O5 ; C9H16N2O5S ; C15H12O5 ; C30H24O10 ; C7H17N3O4S ; C15H12O5 ; C15H12O5 ; C15H12O5 ; C15H12O5 ; C15H12O5 ; C22H34O12 ; C15H12O5 ; C15H12O5 ; C15H12O5 ; C13H16ClNO2 ; C10H12N2O7
3286_53.2	1.658069	0.833569	-2.7369	0.020942	1.679	0.42361	NA	NA	NA	NA	NA
5734_47.9	1.657453	1.262985	-2.5173	0.030526	1.5153	0.42361	216.9226	HMDB32059 ; HMDB60446 ; HMDB60448	2-Bromophenol ; Bromobenzene-23-oxide ; Bromobenzene-34-oxide	M+2Na-H ; M+2Na-H ; M+2Na-H	C6H5BrO ; C6H5BrO ; C6H5BrO
8138_243.	1.656816	1.175997	-2.4442	0.034598	1.4609	0.42361	123.1168	HMDB38140 ; HMDB00798 ; HMDB00847 ; HMDB30331 ; HMDB30957 ; HMDB30958 ; HMDB31262 ; HMDB31269 ; HMDB31291 ; HMDB31480 ; HMDB31557 ; HMDB31834 ; HMDB32042 ; HMDB32309 ; HMDB32406 ; HMDB32454 ; HMDB32535 ; HMDB33554 ; HMDB33794 ; HMDB34162 ; HMDB34165 ; HMDB34606 ; HMDB34758 ; HMDB34761 ; HMDB34764 ; HMDB34845 ; HMDB35916 ; HMDB35926 ; HMDB35927 ; HMDB36234 ; HMDB37001 ; HMDB37143 ; HMDB37150 ; HMDB37164 ; HMDB37170 ; HMDB37288 ; HMDB38040 ; HMDB38067 ; HMDB38400 ; HMDB38603 ; HMDB39216 ; HMDB39219 ; HMDB39461 ; HMDB40221 ; HMDB40295 ; HMDB40430 ; HMDB59822 ; HMDB59828	Santene ; Oenanthic ether ; Pelargonic acid ; xi-2-Ethylheptanoic acid ; (3Z6Z)-36-Nonadien-1-ol ; (Z)-6-Nonenal ; 26-Nonadien-1-ol ; 2-Nonenal ; Methyl caprylate ; Heptyl acetate ; 4-Methyloctanoic acid ; ()-26-Dimethyl-5-heptenal ; Butyl 3-methylbutanoate ; 3-Heptyl acetate ; 5-Methylhexyl acetate ; 3-Octyl formate ; trans-4-Nonenal ; 2-Methyl-2-octenal ; ()-226-Trimethylcyclohexanone ; Pentyl butanoate ; Propyl hexanoate ; Hexyl propionate ; 8-Nonen-3-one ; 5-Nonen-2-one ; 3-Nonen-2-one ; 24-Nonadien-1-ol ; (E)-2-Nonen-4-one ; 8-Nonen-2-one ; 1-Nonen-4-one ; 3-Methylbutyl 2-methylpropanoate ; Santene hydrate ; 1-Hydroxy-3-nonanone ; 2-Ethyl-2-heptenal ; 3-(Hydroxymethyl)-2-octanone ; 3-Methylene-2-octanone ; 4-Methyl-2-pentyl-13-dioxolane ; 2-Methylpropyl 3-methylbutanoate ; (3E6Z)-36-Nonadien-1-ol ; (2xi4xi)-24-Nonadien-1-ol ; Octyl formate ; Butyl 2-methylbutanoate ; 1-Methylbutyl butanoate ; 7-Methyl-3-octen-2-one ; Isoamyl butyrate ; Butyl pentanoate ; Isopropyl hexanoate ; Ethyl 5-methylhexanoate ; 3-Nonen-5-one	M+H ; M+H-2H2O ; M+H-2H2O ; M+H-2H2O ; M+H-H2O ; M+H-H2O ; M+H-H2O ; M+H-H2O ; M+H-2H2O ; M+H-2H2O ; M+H-2H2O ; M+H-H2O ; M+H-2H2O ; M+H-2H2O ; M+H-2H2O ; M+H-H2O ; M+H-H2O ; M+H-H2O ; M+H-2H2O ; M+H-2H2O ; M+H-2H2O ; M+H-H2O ; M+H-H2O ; M+H-H2O ; M+H-H2O ; M+H-H2O ; M+H-H2O ; M+H-H2O ; M+H-2H2O ; M+H-H2O ; M+H-2H2O ; M+H-H2O ; M+H-2H2O ; M+H-H2O ; M+H-2H2O ; M+H-2H2O ; M+H-2H2O ; M+H-H2O ; M+H-2H2O ; M+H-2H2O ; M+H-H2O ; M+H-2H2O ; M+H-H2O ; M+H-2H2O ; M+H-2H2O ; M+H-2H2O ; M+H-2H2O ; M+H-H2O	C9H14 ; C9H18O2 ; C9H18O2 ; C9H18O2 ; C9H16O ; C9H16O ; C9H16O ; C9H16O ; C9H18O2 ; C9H18O2 ; C9H18O2 ; C9H16O ; C9H18O2 ; C9H18O2 ; C9H18O2 ; C9H18O2 ; C9H16O ; C9H16O ; C9H16O ; C9H18O2 ; C9H18O2 ; C9H18O2 ; C9H16O ; C9H16O ; C9H16O ; C9H16O ; C9H16O ; C9H16O ; C9H16O ; C9H18O2 ; C9H16O ; C9H18O2 ; C9H16O ; C9H18O2 ; C9H16O ; C9H18O2 ; C9H18O2 ; C9H16O ; C9H16O ; C9H18O2 ; C9H18O2 ; C9H18O2 ; C9H16O ; C9H18O2 ; C9H18O2 ; C9H18O2 ; C9H18O2 ; C9H16O

36906_294.7	1.656115	0.962569	2.608	0.026131	1.5828	0.42361	589.0437	HMDB30497 ; HMDB33542 ; HMDB37333 ; HMDB39198 ; HMDB39372 ; HMDB41182 ; HMDB61153	Eugeniflorin D1 ; Gomphidic acid ; 8-Hydroxyluteolin 4-methyl ether 8-glucoside-3-sulfate ; Camelliin B ; Degalloylrugosin F ; Camellioferin A ; Lorcaserin sulfamate	M+H+2Na ; 3M+Na+K ; 2M+Na+K ; M+H+2Na ; M+H+2Na ; M+H+2Na ; 2M+K	C75H52O48 ; C18H12O9 ; C22H22O15S ; C75H52O48 ; C75H52O48 ; C75H52O48 ; C11H14ClNO3S
04461_21.39	1.656114	0.736793	-2.4121	0.036549	1.4371	0.42361	270.2294	HMDB01276 ; HMDB02189 ; HMDB39535 ; HMDB60288	N1-Acetylspermidine ; N8-Acetylspermidine ; 3-Tritriacontanone ; N(8)-Acetylspermidinium	M+2ACN+H ; M+2ACN+H ; M+Na+K ; M+2ACN+H	C9H21N3O ; C9H21N3O ; C33H66O ; C9H21N3O
52071_35.93	1.65608	1.019976	-2.633	0.025033	1.6015	0.42361	118.0652	HMDB00738 ; HMDB34171 ; HMDB13250 ; HMDB41793 ; HMDB01250 ; HMDB10715 ; HMDB32628 ; HMDB32630 ; HMDB37071 ; HMDB37072 ; HMDB40008 ; HMDB40013 ; HMDB40222 ; HMDB40223 ; HMDB40224	Indole ; Benzeneacetonitrile ; Myristoylglycine ; 1-Aminopyrene ; N-Acetylarylamine ; 2-Phenylacetamide ; 2-Aminoacetophenone ; 2-Aminoacetophenone ; 2-Acetyl-4-methylpyridine ; 2-Acetyl-6-methylpyridine ; 23-Dihydro-1H-pyrrolizine-5-carboxaldehyde ; 5-(2-Furanyl)-34-dihydro-2H-pyrrole ; 2-Acetyl-5-methylpyridine ; 4-Acetyl-2-methylpyridine ; 4-Acetyl-3-methylpyridine	M+H ; M+H ; M+3Na ; M+H+NH4 ; M+H-H2O ; M+H-H2O ; M+H-H2O ; M+H-H2O ; M+H-H2O ; M+H-H2O ; M+H-H2O ; M+H-H2O ; M+H-H2O ; M+H-H2O ; M+H-H2O	C8H7N ; C8H7N ; C16H31NO3 ; C16H11N ; C8H9NO ; C8H9NO ; C8H9NO ; C8H9NO ; C8H9NO ; C8H9NO ; C8H9NO ; C8H9NO ; C8H9NO ; C8H9NO ; C8H9NO
07339_30.18	1.655984	1.123957	-2.5263	0.030059	1.522	0.42361	87.00407	HMDB30692 ; HMDB34344	8-Hydroxy-7-methoxy-2H-1-benzopyran-2-one ; Scopoletin	M+3Na ; M+3Na	C10H8O4 ; C10H8O4
33379_285.8	1.654236	1.021579	2.5277	0.029989	1.523	0.42361	293.8983	HMDB39460 ; HMDB42810 ; HMDB42955 ; HMDB48114 ; HMDB48275 ; HMDB48400 ; HMDB48407 ; HMDB52956 ; HMDB55203 ; HMDB55362	Di-2-propenyl hexasulfide ; TG(14:018:4(6Z9Z12Z15Z)o-18:0) ; TG(14:0o-18:018:4(6Z9Z12Z15Z)) ; TG(14:1(9Z)18:3(6Z9Z12Z)o-18:0) ; TG(14:1(9Z)18:3(9Z12Z15Z)o-18:0) ; TG(14:1(9Z)o-18:018:3(6Z9Z12Z)) ; TG(14:1(9Z)o-18:018:3(9Z12Z15Z)) ; TG(18:3(6Z9Z12Z)14:1(9Z)o-18:0) ; TG(18:3(9Z12Z15Z)14:1(9Z)o-18:0) ; TG(18:4(6Z9Z12Z15Z)14:0o-18:0)	2M+H+K ; M+3Na ; M+3Na ; M+3Na ; M+3Na ; M+3Na ; M+3Na ; M+3Na ; M+3Na ; M+3Na	C6H10S6 ; C53H96O5 ; C53H96O5 ; C53H96O5 ; C53H96O5 ; C53H96O5 ; C53H96O5 ; C53H96O5 ; C53H96O5 ; C53H96O5

1643_188.	1.651966	0.957968	-2.5237	0.030195	1.5201	0.42361	255.1592	HMDB00535 ; HMDB00689 ; HMDB00926 ; HMDB12488 ; HMDB13239 ; HMDB29762 ; HMDB30027 ; HMDB30056 ; HMDB30059 ; HMDB30200 ; HMDB31207 ; HMDB31221 ; HMDB31246 ; HMDB31248 ; HMDB31325 ; HMDB31511 ; HMDB31580 ; HMDB32380 ; HMDB33526 ; HMDB33774 ; HMDB33889 ; HMDB34163 ; HMDB37266 ; HMDB38524 ; HMDB40279 ; HMDB40327 ; HMDB32959	Caproic acid ; Isocaproic acid ; Pyridine ; 1234-Tetrahydro-beta-carboline ; Heptanoylcholine ; Methyl (S)-2-Methylbutanoate ; Methyl 3-methylbutanoate ; Isopropyl propionate ; Propyl propionate ; Hovenine A ; Methyl pentanoate ; 2-Ethylbutanoic acid ; 2-Methylpropyl acetate ; Ethyl 2-methylpropanoate ; n-Butyl acetate ; Diacetone alcohol ; ()-2-Methylpentanoic acid ; (S1)-Methoxy-3-heptanethiol ; Chalciporone ; 3-Methylpentanoic acid ; Ethyl butyrate ; 3-Methylbutyl formate ; 224-Trimethyl-13-dioxolane ; 5-Tricosyl-13-benzenediol ; 245-Trimethyl-13-dioxolane ; Pentyl formate ; 1-Octen-3-yl glucoside	2M+Na ; 2M+Na ; 3M+NH4 ; M+2ACN+H ; M+K ; 2M+Na ; 2M+Na ; 2M+Na ; 2M+Na ; 2M+Na ; M+H+Na ; 2M+Na ; 2M+Na ; 2M+Na ; 2M+Na ; 2M+Na ; 2M+Na ; 2M+Na ; 3M+H+Na ; 2M+H+Na ; 2M+Na ; 2M+Na ; 2M+Na ; 2M+Na ; M+2K ; 2M+Na ; 2M+Na ; M+H-2H2O	C6H12O2 ; C6H12O2 ; C5H5N ; C11H12N2 ; C12H26NO2 ; C6H12O2 ; C6H12O2 ; C6H12O2 ; C6H12O2 ; C27H42N4O4 ; C6H12O2 ; C6H12O2 ; C6H12O2 ; C6H12O2 ; C6H12O2 ; C6H12O2 ; C6H12O2 ; C8H18OS ; C16H21NO ; C6H12O2 ; C6H12O2 ; C6H12O2 ; C6H12O2 ; C29H52O2 ; C6H12O2 ; C6H12O2 ; C14H26O6
98686_187.	1.651701	0.938423	2.5052	0.031168	1.5063	0.42361	253.1799	HMDB30918 ; HMDB30919 ; HMDB32122 ; HMDB33149 ; HMDB34574 ; HMDB35309 ; HMDB35597 ; HMDB35613 ; HMDB35729 ; HMDB35767 ; HMDB36448 ; HMDB36663 ; HMDB37063 ; HMDB37969 ; HMDB38502 ; HMDB40685 ; HMDB34500 ; HMDB34501 ; HMDB34518 ; HMDB35487 ; HMDB35875 ; HMDB35948 ; HMDB36670 ; HMDB40772 ; HMDB40784 ; HMDB01383 ; HMDB13105 ; HMDB31156 ; HMDB31273 ; HMDB32219 ; HMDB32312 ; HMDB32313 ; HMDB32319 ; HMDB32434 ; HMDB33700 ; HMDB34452 ; HMDB34670 ; HMDB35714 ; HMDB35766 ; HMDB35829 ; HMDB36067 ; HMDB36103 ; HMDB37014 ; HMDB37175 ; HMDB37821 ; HMDB40093 ; HMDB40094 ; HMDB40115 ; HMDB40639	1-Hydroxyepiacorone ; Acorusdiol ; 3(4-5)-Abeo-411:412-diepoxy-3-eudesmanol ; (4R5S7R11R)-1112-Dihydroxy-1(10)-spirovetiven-2-one ; Apotrichodiol ; 6alpha-Carissanol ; alpha-Carissanol ; Epioxylubimin ; Dihydromyoporone ; Piperalol ; Zedoarondiol ; Hydroxypelenolide ; Toxin FS2 ; Urodiolenone ; Bisacurone B ; 23-Dihydroabscisic alcohol ; Protobassic acid ; Tomentosic acid ; Theasapogenol E ; (1beta2alpha3alpha)-12324-Tetrahydroxy-12-oleanen-28-oic acid ; Myrianthic acid ; Cyclopassifloic acid D ; 6beta-Hydroxyasiatic acid ; Isothankunic acid ; (3beta19alpha)-3192324-Tetrahydroxy-12-oleanen-28-oic acid ; Sphinganine 1-phosphate ; trans-45-epoxy-2(E)-decenal ; Ethyl (4Z)-47-octadienoate ; Methyl octynecarboxylate ; (-)-Dihydromintlactone ; 24-Hexadienyl butyrate ; 24-Hexadienyl isobutyrate ; cis-3-Hexenyl crotonate ; (-)-2-(5-Methyl-5-vinyltetrahydrofuran-2-yl)propionaldehyde ; (-)-(Z)-Tetrahydro-6-(2-pentenyl)-2H-pyran-2-one ; 56-Dihydro-6-pentyl-2H-pyran-2-one ; 6-Hydroxy-26-dimethyl-27-octadien-4-one ; Epoxyartemisia ketone ; Ascaridole ; Dihydronepetalactone ; Epoxycampholenic aldehyde ; Geranic acid ; gamma-Diosphenol ; 4-Isopropyl-3-cyclohexene-1-carboxylic acid ; xi-188-Trimethyl-2-oxabicyclo321octan-3-one ; 45-Dimethyl-2-octylthiazole ; 4-Ethyl-2-heptyl-5-methylthiazole ; 4-Ethyl-2-octylthiazole ; (xi)-(Z)-5-(3-Hexenyl)dihydro-2(3H)-furanone	M+H ; M+H ; M+H ; M+H ; M+H ; M+H ; M+H ; M+H ; M+H ; M+H ; M+H ; M+H ; M+H ; M+H ; M+H ; M+H ; M+2H ; M+2H ; M+2H ; M+2H ; M+2H ; M+2H ; M+2H ; M+2H ; M+2H ; M+3ACN+2H ; 3M+2H ; 3M+2H ; 3M+2H ; 3M+2H ; 3M+2H ; 3M+2H ; 3M+2H ; 3M+2H ; 3M+2H ; 3M+2H ; 3M+2H ; 3M+2H ; 3M+2H ; 3M+2H ; 3M+2H ; 3M+2H ; 3M+2H ; 3M+2H ; 2M+3H2O+2H ; 2M+3H2O+2H ; 2M+3H2O+2H ; 3M+2H	C15H24O3 ; C15H24O3 ; C15H24O3 ; C15H24O3 ; C15H24O3 ; C15H24O3 ; C15H24O3 ; C15H24O3 ; C15H24O3 ; C15H24O3 ; C15H24O3 ; C15H24O3 ; C15H24O3 ; C15H24O3 ; C15H24O3 ; C15H24O3 ; C30H48O6 ; C30H48O6 ; C30H48O6 ; C30H48O6 ; C30H48O6 ; C30H48O6 ; C30H48O6 ; C30H48O6 ; C30H48O6 ; C18H40NO5P ; C10H16O2 ; C10H16O2 ; C10H16O2 ; C10H16O2 ; C10H16O2 ; C10H16O2 ; C10H16O2 ; C10H16O2 ; C10H16O2 ; C10H16O2 ; C10H16O2 ; C10H16O2 ; C10H16O2 ; C10H16O2 ; C10H16O2 ; C10H16O2 ; C10H16O2 ; C10H16O2 ; C10H16O2 ; C13H23NS ; C13H23NS ; C13H23NS ; C10H16O2
5566_46.2	1.651581	0.465503	2.5895	0.026972	1.5691	0.42361	122.9246	HMDB00142	Formic acid	M+2K-H	CH2O2

9063_137.	1.651236	0.736145	-2.5483	0.028946	1.5384	0.42361	223.1191	HMDB32705 ; HMDB00227 ; HMDB00237 ; HMDB00391 ; HMDB00400 ; HMDB00502 ; HMDB02144 ; HMDB02271 ; HMDB02820 ; HMDB03052 ; HMDB03453 ; HMDB04988 ; HMDB06458 ; HMDB06961 ; HMDB12140 ; HMDB12518 ; HMDB13692 ; HMDB14570 ; HMDB14771 ; HMDB14861 ; HMDB14954 ; HMDB15197 ; HMDB15563 ; HMDB30065 ; HMDB31229 ; HMDB31523 ; HMDB31639 ; HMDB32820 ; HMDB33399 ; HMDB34475 ; HMDB35288 ; HMDB36692 ; HMDB36896 ; HMDB36899 ; HMDB36902 ; HMDB37103 ; HMDB37826 ; HMDB40759 ; HMDB41408 ; HMDB41510 ; HMDB59629 ; HMDB59705 ; HMDB59991 ; HMDB60080 ; HMDB00194 ; HMDB00745 ; HMDB05769	Isopropyl beta-D-glucoside ; Mevalonic acid ; Propionic acid ; 7-Ketodeoxycholic acid ; 37-Dihydroxy-12-oxocholanoic acid ; 3-Oxocholic acid ; 13-Dimethyluracil ; Imidazolepropionic acid ; Methylimidazoleacetic acid ; Lactaldehyde ; 3-Hydroxypropanal ; Pi-Methylimidazoleacetic acid ; D-Lactaldehyde ; Hydroxyacetone ; (R) 23-Dihydroxy-3-methylvalerate ; 11-Carboxy-gamma-tocotrienol ; Secoisolariciresinol ; Famciclovir ; Dexmedetomidine ; Methoxamine ; Orciprenaline ; Isoproterenol ; Pargyline ; Colupulone ; Ethyl formate ; Methyl acetate ; Glycerol 1-propanoate ; Gibberellin Ga115 ; Annocherin A ; Hydroxyisonobilin ; (6b7b13R)-67-Diacetoxy-814-labdadiene-13-ol ; Heliangin ; Gibberellin A19 ; Gibberellin A65 ; Gibberellin A38 ; Gibberellin A124 ; (E)-2627-Dinorergosta-422-dien-3-one ; 3-Oxo-6b-angeloyloxy-8b-hydroxy-7(11)-eremophilen-128-olide ; Gibberellin A101 ; Gibberellin A98 ; (R)-mevalonate ; (2-Methoxyethoxy)propanoic acid ; 5-(3-Hydroxyphenyl)-gamma-valerolactone-3-O-glucuronide ; Thyrotropin releasing hormone ; Anserine ; Homocarnosine ; Balenine	M+H ; 3M+2H ; 3M+H ; M+H+K ; M+H+K ; M+H+K ; M+2ACN+H ; M+2ACN+H ; M+2ACN+H ; 3M+H ; 3M+H ; M+2ACN+H ; 3M+H ; 3M+H ; 3M+2H ; M+2Na ; M+2ACN+2H ; M+3ACN+2H ; M+Na ; 2M+H+Na ; 2M+H+Na ; 2M+H+Na ; M+ACN+Na ; M+2Na ; 3M+H ; 3M+H ; 3M+2H ; M+2ACN+2H ; M+H+K ; M+2ACN+2H ; M+H+K ; M+2ACN+2H ; M+2ACN+2H ; M+2ACN+2H ; M+2ACN+2H ; M+2ACN+2H ; M+2K ; M+2ACN+2H ; M+2ACN+2H ; M+2ACN+2H ; 3M+2H ; 3M+2H ; M+2ACN+2H ; M+2ACN+2H ; M+H-H2O ; M+H-H2O ; M+H-H2O	C9H18O6 ; C6H12O4 ; C3H6O2 ; C24H38O5 ; C24H38O5 ; C24H38O5 ; C6H8N2O2 ; C6H8N2O2 ; C6H8N2O2 ; C3H6O2 ; C3H6O2 ; C6H8N2O2 ; C3H6O2 ; C3H6O2 ; C6H12O4 ; C25H36O4 ; C20H26O6 ; C14H19N5O4 ; C13H16N2 ; C11H17NO3 ; C11H17NO3 ; C11H17NO3 ; C11H13N ; C25H36O4 ; C3H6O2 ; C3H6O2 ; C6H12O4 ; C20H26O6 ; C24H38O5 ; C20H26O6 ; C24H38O5 ; C20H26O6 ; C20H26O6 ; C20H26O6 ; C20H26O6 ; C20H26O6 ; C26H40O ; C20H26O6 ; C20H26O6 ; C20H26O6 ; C6H12O4 ; C6H12O4 ; C20H26O6 ; C16H22N6O4 ; C10H16N4O3 ; C10H16N4O3 ; C10H16N4O3
3961_265.	1.651143	0.324794	-2.5292	0.02991	1.5242	0.42361	NA	NA	NA	NA	NA
2504_57.0	1.650226	1.184547	2.6438	0.024572	1.6096	0.42361	405.8953	HMDB07818 ; HMDB31211 ; HMDB41188 ; HMDB42150 ; HMDB42933 ; HMDB43017 ; HMDB43801 ; HMDB43856 ; HMDB56111	DG(24:024:00:0) ; Endrin ; Bis(methylsulfonylmethyl) disulfide ; TG(14:016:0o-18:0) ; TG(14:0o-18:016:0) ; TG(15:015:0o-18:0) ; TG(15:0o-18:015:0) ; TG(16:014:0o-18:0) ; DG(24:00:024:0)	M+H+NH4 ; 2M+3H2O+2H ; 3M+Na+K ; M+H+NH4 ; M+H+NH4 ; M+H+NH4 ; M+H+NH4 ; M+H+NH4 ; M+H+NH4	C51H100O5 ; C12H8Cl6O ; C4H10O4S4 ; C51H100O5 ; C51H100O5 ; C51H100O5 ; C51H100O5 ; C51H100O5 ; C51H100O5

5059_287.	1.650168	1.064926	2.6089	0.026088	1.5836	0.42361	527.3855	HMDB06070 ; HMDB06227 ; HMDB08523 ; HMDB08524 ; HMDB08751 ; HMDB11229 ; HMDB11288 ; HMDB11321 ; HMDB11481 ; HMDB11511 ; HMDB12108 ; HMDB13892 ; HMDB14339 ; HMDB15646 ; HMDB29567 ; HMDB31005 ; HMDB32667 ; HMDB32691 ; HMDB33647 ; HMDB35967 ; HMDB38105 ; HMDB38977 ; HMDB55529 ; HMDB55791 ; HMDB55797 ; HMDB55941 ; HMDB60532 ; HMDB01063 ; HMDB36284 ; HMDB36293	Pregnanetriol ; 1-a24R25-Trihydroxyvitamin D2 ; PC(20:5(5Z8Z11Z14Z17Z)P-18:1(11Z)) ; PC(20:5(5Z8Z11Z14Z17Z)P-18:1(9Z)) ; PC(22:6(4Z7Z10Z13Z16Z19Z)P-16:0) ; PC(P-16:022:6(4Z7Z10Z13Z16Z19Z)) ; PC(P-18:1(11Z)20:5(5Z8Z11Z14Z17Z)) ; PC(P-18:1(9Z)20:5(5Z8Z11Z14Z17Z)) ; LysoPE(0:020:0) ; LysoPE(20:00:0) ; LysoPC(17:0) ; N-Desmethylvenlafaxine ; Tramadol ; Desvenlafaxine ; Hydroxy-alpha-sanshool ; 3-Methyl-5-pentyl-2-furanundecanoic acid ; 59-Epidioxy-3-hydroxyergost-7-en-6-one ; (3beta5alpha6alpha7beta14alpha22E24R)-56-Epoxyergosta-822-diene-3714-triol ; 56:89-Diepoxyergost-22-ene-37beta-diol ; 24-Methylcycloart-23-en-3beta-yl acetate ; Isolinderanolide ; (3beta5alpha6beta9alpha22E24R)-59-Epidioxyergosta-722-diene-36-diol ; TG(18:4(6Z9Z12Z15Z)22:6(4Z7Z10Z13Z16Z19Z)22:6(4Z7Z10Z13Z16Z19Z)) ; TG(20:5(5Z8Z11Z14Z17Z)20:5(5Z8Z11Z14Z17Z)22:6(4Z7Z10Z13Z16Z19Z)) ; TG(20:5(5Z8Z11Z14Z17Z)22:6(4Z7Z10Z13Z16Z19Z)20:5(5Z8Z11Z14Z17Z)) ; TG(22:6(4Z7Z10Z13Z16Z19Z)18:4(6Z9Z12Z15Z)22:6(4Z7Z10Z13Z16Z19Z)) ; O-Desmethylvenlafaxine ; 3-Hexaprenyl-45-Dihydroxybenzoic acid ; Campesteryl caffeate ; Cholesteryl ferulate	3M+2Na ; M+2ACN+H ; 2M+3H ; 2M+3H ; 2M+3H ; 2M+3H ; 2M+3H ; 2M+3H ; M+NH4 ; M+NH4 ; M+NH4 ; 2M+H ; 2M+H ; 2M+H ; 2M+H ; 3M+2Na ; M+2ACN+H ; M+2ACN+H ; M+2ACN+H ; M+2Na-H ; 3M+2Na ; M+2ACN+H ; M+2ACN+2H ; M+2ACN+2H ; M+2ACN+2H ; M+2ACN+2H ; 2M+H ; M+H-2H2O ; M+H-2H2O ; M+H-2H2O	C21H36O3 ; C28H44O4 ; C46H80NO7P ; C46H80NO7P ; C46H80NO7P ; C46H80NO7P ; C46H80NO7P ; C46H80NO7P ; C25H52NO7P ; C25H52NO7P ; C25H52NO7P ; C16H25NO2 ; C16H25NO2 ; C16H25NO2 ; C16H25NO2 ; C21H36O3 ; C28H44O4 ; C28H44O4 ; C28H44O4 ; C33H54O2 ; C21H36O3 ; C28H44O4 ; C65H94O6 ; C65H94O6 ; C65H94O6 ; C65H94O6 ; C16H25NO2 ; C37H54O4 ; C37H54O4 ; C37H54O4
4376_268.	1.650092	1.206391	-2.4422	0.03472	1.4594	0.42361	115.0284	HMDB13695 ; HMDB30723 ; HMDB30724 ; HMDB34263 ; HMDB41037	Urolithin A ; 15-Dihydroxyxanthone ; Euxanthone ; Triethyl citrate ; 8-Deoxy-11-hydroxy-13-chlorogrosheimin	M+2H ; M+2H ; M+2H ; M+3Na ; M+H+2Na	C13H8O4 ; C13H8O4 ; C13H8O4 ; C12H20O7 ; C15H19ClO4
9134_33.8	1.648042	1.261877	-2.4939	0.031779	1.4979	0.42361	460.7799	HMDB08321 ; HMDB08352 ; HMDB08353 ; HMDB08384 ; HMDB08416 ; HMDB08514 ; HMDB08609 ; HMDB08638 ; HMDB08639 ; HMDB08649 ; HMDB08670 ; HMDB08680 ; HMDB08681 ; HMDB08703 ; HMDB08713 ; HMDB08714 ; HMDB08735 ; HMDB08745 ; HMDB34651 ; HMDB35958 ; HMDB40943 ; HMDB40946	PC(20:1(11Z)22:6(4Z7Z10Z13Z16Z19Z)) ; PC(20:2(11Z14Z)22:5(4Z7Z10Z13Z16Z)) ; PC(20:2(11Z14Z)22:5(7Z10Z13Z16Z19Z)) ; PC(20:3(5Z8Z11Z)22:4(7Z10Z13Z16Z)) ; PC(20:3(8Z11Z14Z)22:4(7Z10Z13Z16Z)) ; PC(20:5(5Z8Z11Z14Z17Z)22:2(13Z16Z)) ; PC(22:2(13Z16Z)20:5(5Z8Z11Z14Z17Z)) ; PC(22:4(7Z10Z13Z16Z)20:3(5Z8Z11Z)) ; PC(22:4(7Z10Z13Z16Z)20:3(8Z11Z14Z)) ; PC(22:4(7Z10Z13Z16Z)22:6(4Z7Z10Z13Z16Z19Z)) ; PC(22:5(4Z7Z10Z13Z16Z)20:2(11Z14Z)) ; PC(22:5(4Z7Z10Z13Z16Z)22:5(4Z7Z10Z13Z16Z)) ; PC(22:5(4Z7Z10Z13Z16Z)22:5(7Z10Z13Z16Z19Z)) ; PC(22:5(7Z10Z13Z16Z19Z)20:2(11Z14Z)) ; PC(22:5(7Z10Z13Z16Z19Z)22:5(4Z7Z10Z13Z16Z)) ; PC(22:5(7Z10Z13Z16Z19Z)22:5(7Z10Z13Z16Z19Z)) ; PC(22:6(4Z7Z10Z13Z16Z19Z)20:1(11Z)) ; PC(22:6(4Z7Z10Z13Z16Z19Z)22:4(7Z10Z13Z16Z)) ; Soyasaponin III ; Saponin E ; Mabiogenin 3-rhamnosyl-(1-6)-glucoside ; Jujubasaponin VI	M+Na+K ; M+Na+K ; M+Na+K ; M+Na+K ; M+Na+K ; M+Na+K ; M+Na+K ; M+Na+K ; M+Na+K ; M+H+K ; M+Na+K ; M+H+K ; M+H+K ; M+Na+K ; M+H+K ; M+H+K ; M+Na+K ; M+H+K ; M+3ACN+2H ; M+3ACN+2H ; M+3ACN+2H ; M+3ACN+2H	C50H86NO8P ; C50H86NO8P ; C50H86NO8P ; C50H86NO8P ; C50H86NO8P ; C50H86NO8P ; C50H86NO8P ; C50H86NO8P ; C50H86NO8P ; C52H84NO8P ; C50H86NO8P ; C52H84NO8P ; C52H84NO8P ; C50H86NO8P ; C52H84NO8P ; C52H84NO8P ; C50H86NO8P ; C52H84NO8P ; C42H68O14 ; C42H68O14 ; C42H68O14 ; C42H68O14

8963_114.3	1.64798	0.694833	2.3238	0.042495	1.3717	0.42361	465.0844	HMDB01847 ; HMDB06467 ; HMDB14962 ; HMDB15068 ; HMDB28787 ; HMDB29102 ; HMDB32659 ; HMDB36628 ; HMDB60033 ; HMDB60035 ; HMDB60036 ; HMDB60140 ; HMDB60723	Caffeine ; 4-hydroxybenzoyl-CoA ; Enprofylline ; Mesoridazine ; Cysteinyl-Tyrosine ; Tyrosyl-Cysteine ; 4-Hydroxy-8-methoxy-2H-furo23-h-1-benzopyran-2-one ; 9-Hydroxy-4-methoxypsoralen ; (beta-1-O-N-(2-hydroxymethyl-3-chlorophenyl)-anthraniloyl-D-glucupyranuronic acid) ; (beta-1-O-N-(2-methyl-3-chlorophenyl-4-hydroxy)anthraniloyl-D-glucupyranuronic acid) ; (beta-1-O-N-(2-methyl-3-chlorophenyl-5-hydroxy) anthraniloyl-D-glucupyranuronic acid ; 4-Hydroxybenoyl-CoA ; 23-diene-Valproic acid-CoA	2M+2K-H ; M+ACN+2H ; 2M+2K-H ; M+2K+H ; 3M+2K ; 3M+2K ; 2M+H ; 2M+H ; 2M+H+Na ; 2M+H+Na ; 2M+H+Na ; M+ACN+2H ; M+H+K	C8H10N4O2 ; C28H40N7O18P3S ; C8H10N4O2 ; C21H26N2OS2 ; C12H16N2O4S ; C12H16N2O4S ; C12H8O5 ; C12H8O5 ; C20H20ClNO9 ; C20H20ClNO9 ; C20H20ClNO9 ; C28H40N7O18P3S ; C29H47N7O17P3S
5062_85.71	1.647751	0.411931	-2.5402	0.029353	1.5323	0.42361	110.0125	HMDB00392 ; HMDB01568 ; HMDB03269 ; HMDB13897 ; HMDB13902 ; HMDB13903 ; HMDB13904 ; HMDB28752 ; HMDB28815 ; HMDB29761 ; HMDB31293 ; HMDB31343 ; HMDB31352 ; HMDB31403 ; HMDB32164 ; HMDB32207 ; HMDB32266 ; HMDB32267 ; HMDB32268 ; HMDB32278 ; HMDB32533 ; HMDB32861 ; HMDB35422 ; HMDB36212 ; HMDB36236 ; HMDB37622 ; HMDB37624 ; HMDB37630 ; HMDB38036 ; HMDB38310 ; HMDB39793 ; HMDB39872 ; HMDB40212 ; HMDB40215 ; HMDB41912 ; HMDB59766 ; HMDB59908 ; HMDB60303 ; HMDB60322	2-Octenoic acid ; trans-2-Octenoic acid ; Nicotinuric acid ; 4-ene-Valproic acid ; 2-ene-Valproic acid ; (3Z)-2-Propylpent-3-enoic acid ; (3E)-2-Propylpent-3-enoic acid ; Aspartyl-Glutamate ; Glutamyl-Aspartate ; Ethyl ()-2-methyl-4-pentenoate ; 23-Octanedione ; Methyl cyclohexanecarboxylate ; Cyclohexyl acetate ; Cyclohexaneacetic acid ; Allyl valerate ; cis-5-Octenoic acid ; Ethyl (E)-3-hexenoate ; Ethyl 5-hexenoate ; Ethyl 2E-hexenoate ; Ethyl trans-2-methyl-2-pentenoate ; trans-3-Hexenyl acetate ; 2-Methoxynaphthalene ; 4-Butyl-gamma-butyrolactone ; 2-Methylpropyl (2E)-butenoate ; 2-Methylallyl butyrate ; Ethyl 2-methyl-3-pentenoate ; Isopropyl tiglate ; xi-Tetrahydro-3-propyl-2H-pyran-2-one ; 2-Propenyl 3-methylbutanoate ; xi-Tetrahydro-6-propyl-2H-pyran-2-one ; (E)-4-Octenoic acid ; Methyl 2-heptenoate ; 2-Hexenyl acetate ; cis-3-Hexenyl acetate ; Isonicotinylglycine ; Picolinoylglycine ; Ethyl-3-hexanoate ; (2-Naphthyl)methanol ; 1-Hydroxymethylnaphthalene	M+2K ; M+2K ; M+H+K ; M+2K ; M+2K ; M+2K ; M+2K ; M+3Na ; M+3Na ; M+2K ; M+2K ; M+2K ; M+2K ; M+2K ; M+2K ; M+2K ; M+2K ; M+2K ; M+2K ; M+2K ; M+2K ; M+Na+K ; M+2K ; M+2K ; M+2K ; M+2K ; M+2K ; M+2K ; M+2K ; M+2K ; M+2K ; M+2K ; M+2K ; M+2K ; M+H+K ; M+H+K ; M+2K ; M+Na+K ; M+Na+K	C8H14O2 ; C8H14O2 ; C8H8N2O3 ; C8H14O2 ; C8H14O2 ; C8H14O2 ; C8H14O2 ; C9H13N2O7 ; C9H13N2O7 ; C8H14O2 ; C8H14O2 ; C8H14O2 ; C8H14O2 ; C8H14O2 ; C8H14O2 ; C8H14O2 ; C8H14O2 ; C8H14O2 ; C8H14O2 ; C8H14O2 ; C8H14O2 ; C11H10O ; C8H14O2 ; C8H14O2 ; C8H14O2 ; C8H14O2 ; C8H14O2 ; C8H14O2 ; C8H14O2 ; C8H14O2 ; C8H14O2 ; C8H14O2 ; C8H14O2 ; C8H14O2 ; C8H8N2O3 ; C8H8N2O3 ; C8H14O2 ; C11H10O ; C11H10O
3489_18.83	1.647357	0.951885	-2.328	0.042191	1.3748	0.42361	NA	NA	NA	NA	NA
8092_294.2	1.646012	1.046254	2.5485	0.02894	1.5385	0.42361	405.0088	HMDB00295 ; HMDB12468 ; HMDB13991 ; HMDB15099 ; HMDB30590 ; HMDB33100 ; HMDB36343 ; HMDB37570 ; HMDB60804	Uridine 5-diphosphate ; (-)-Epigallocatechin sulfate ; 5-Hydroxylornoxicam ; Apraclonidine ; Dihydrosterigmatocystin ; Atroviridin ; O-Demethylforbexanthone ; Demethoxykanugin ; 8-Hydroxythioguanine	M+H ; 2M+H+K ; M+NH4 ; 3M+2K ; M+2K+H ; M+2K+H ; M+2K+H ; M+2K+H ; 2M+K	C9H14N2O12P2 ; C15H13O10S ; C13H10ClN3O5S2 ; C9H10Cl2N4 ; C18H14O6 ; C18H14O6 ; C18H14O6 ; C18H14O6 ; C5H5N5OS
3052_30.44	1.645959	0.98685	-2.5622	0.028267	1.5487	0.42361	NA	NA	NA	NA	NA

.1369_256.4	1.645786	0.872624	2.5424	0.029242	1.534	0.42361	314.1711	HMDB00726 ; HMDB00953 ; HMDB02890 ; HMDB05783 ; HMDB10342 ; HMDB10360 ; HMDB13972 ; HMDB14607 ; HMDB14647 ; HMDB15038 ; HMDB15114 ; HMDB15330 ; HMDB15341 ; HMDB15371 ; HMDB28707 ; HMDB28723 ; HMDB28791 ; HMDB29143 ; HMDB30917 ; HMDB31094 ; HMDB31901 ; HMDB32255 ; HMDB32522 ; HMDB34721 ; HMDB34779 ; HMDB35117 ; HMDB35137 ; HMDB35148 ; HMDB35358 ; HMDB35722 ; HMDB35754 ; HMDB35760 ; HMDB35798 ; HMDB36036 ; HMDB36037 ; HMDB36327 ; HMDB36550 ; HMDB36563 ; HMDB36664 ; HMDB37064 ; HMDB37256 ; HMDB37529 ; HMDB37559 ; HMDB39156 ; HMDB39635 ; HMDB39644 ; HMDB40646 ; HMDB40754 ; HMDB31033	Isovalerylglutamic acid ; Suberylglycine ; 3-cis-Hydroxy-be-Caroten-3-one ; Gingerol ; 25-Hydroxyvitamin D2-25-glucuronide ; 25-Hydroxyvitamin D2 25-(beta-glucuronide) ; N-Dealkylated tolterodine ; Sodium Tetradecyl Sulfate ; Levallorphan ; Methdilazine ; Cyclopentolate ; Tubocurarine ; Levobunolol ; Gemfibrozil ; Arginyl-Glutamine ; Arginyl-Gamma-glutamate ; Glutaminyl-Arginine ; Gamma-glutamyl-Arginine ; 1-Hydroxyacorenone ; Glycerol tributanoate ; Blennin A ; N-(Ethoxycarbonyl)methyl)-p-menthane-3-carboxamide ; Stearyl monoglyceridyl citrate ; Procurcumadiol ; Nordihydrocapsiate ; (3beta6beta)-Furanoeremophilane-36-diol ; Heliannuol D ; (6beta8alpha)-6-Hydroxy-7(11)-eremophilen-128-olide ; Ketosantalic acid ; Tanacetol A ; Phytuberin ; 3-Hydroxytrichothecene ; Piperdial ; 3beta-Dihydroxymarasmene ; 13-Hydroxymarasmene ; Norcapsaicin ; 3-Ketoapotrichothecene ; Valerenolic acid ; Ketopelenolide a ; FS4 toxin ; Chlorophyll d ; Lactaronecatorin A ; Cadabicilone ; 3beta-Hydroxycinnamolide ; Abscisic alcohol ; Heliannuol A ; Myrsinone ; (3beta8beta)-3-Hydroxy-7(11)-eremophilen-128-olide ; 4-Dodecylbenzenesulfonic acid Na salt	M+2ACN+H ; M+2ACN+H ; M+2K ; 2M+H+K ; M+H+K ; M+H+K ; 2M+Na+K ; 2M+H+K ; 2M+Na+K ; M+NH4 ; M+Na ; M+H+NH4 ; M+Na ; M+ACN+Na ; 2M+H+Na ; 2M+H+Na ; 2M+H+Na ; 2M+H+Na ; M+ACN+Na ; 2M+H+Na ; M+ACN+Na ; M+2Na-H ; M+2Na ; M+ACN+Na ; 2M+H+K ; M+ACN+Na ; M+ACN+Na ; M+ACN+Na ; M+ACN+Na ; 2M+H+K ; 2M+H+K ; M+ACN+Na ; M+ACN+Na ; M+ACN+Na ; M+ACN+Na ; M+Na ; M+ACN+Na ; M+ACN+Na ; M+ACN+Na ; M+ACN+Na ; M+H+2Na ; M+ACN+Na ; M+ACN+Na ; M+ACN+Na ; M+ACN+Na ; M+ACN+Na ; 2M+H+K ; M+ACN+Na ; M+H-2H2O	C10H17NO5 ; C10H17NO5 ; C40H54O ; C17H26O4 ; C34H52O8 ; C34H52O8 ; C19H25NO ; C14H30O4S ; C19H25NO ; C18H20N2S ; C17H25NO3 ; C37H41N2O6 ; C17H25NO3 ; C15H22O3 ; C11H22N6O4 ; C11H22N6O4 ; C11H22N6O4 ; C11H22N6O4 ; C15H22O3 ; C15H26O6 ; C15H22O3 ; C15H27NO3 ; C28H54O12 ; C15H22O3 ; C17H26O4 ; C15H22O3 ; C15H22O3 ; C15H22O3 ; C15H22O3 ; C17H26O4 ; C17H26O4 ; C15H22O3 ; C15H22O3 ; C15H22O3 ; C15H22O3 ; C17H25NO3 ; C15H22O3 ; C15H22O3 ; C15H22O3 ; C15H22O3 ; C54H71MgN4O6 ; C15H22O3 ; C15H22O3 ; C15H22O3 ; C15H22O3 ; C15H22O3 ; C17H26O4 ; C15H22O3 ; C18H30NaO3S
.0975_239.3	1.645406	1.237885	2.5559	0.028571	1.5441	0.42361	448.0711	HMDB06802 ; HMDB14038 ; HMDB14415 ; HMDB14794 ; HMDB36355 ; HMDB38420 ; HMDB38424	O-Phospho-4-hydroxy-L-threonine ; N-Deisopropyl-fluvastatin ; Isradipine ; Trazodone ; 3-Methylpentyl glucosinolate ; 4-Methylpentyl glucosinolate ; Hexyl glucosinolate	2M+NH4 ; M+2K+H ; M+2K-H ; M+2K-H ; M+2Na-H ; M+2Na-H ; M+2Na-H	C4H10NO7P ; C21H20FNO4 ; C19H21N3O5 ; C19H22ClN5O ; C13H25NO9S2 ; C13H25NO9S2 ; C13H25NO9S2
6519_49.12	1.64508	1.014999	-2.3804	0.038585	1.4136	0.42361	NA	NA	NA	NA	NA

[illegible]4173_171.6	1.64494	1.069322	-2.3086	0.043609	1.3604	0.42361	263.1224	HMDB29437 ; HMDB35603 ; HMDB36903 ; HMDB37083 ; HMDB00122 ; HMDB00143 ; HMDB00169 ; HMDB00211 ; HMDB00212 ; HMDB00215 ; HMDB00346 ; HMDB00516 ; HMDB00660 ; HMDB00725 ; HMDB00766 ; HMDB00783 ; HMDB00803 ; HMDB00853 ; HMDB01129 ; HMDB01149 ; HMDB01151 ; HMDB01266 ; HMDB02104 ; HMDB02113 ; HMDB03345 ; HMDB03418 ; HMDB03449 ; HMDB06055 ; HMDB06088 ; HMDB06272 ; HMDB11156 ; HMDB12326 ; HMDB14671 ; HMDB15133 ; HMDB30761 ; HMDB30790 ; HMDB31000 ; HMDB31254 ; HMDB31567 ; HMDB32222 ; HMDB32783 ; HMDB33704 ; HMDB34220 ; HMDB34293 ; HMDB35207 ; HMDB35412 ; HMDB35906 ; HMDB35934 ; HMDB36110 ; HMDB36119 ; HMDB36148 ; HMDB36206 ; HMDB36576 ; HMDB36688 ; HMDB36766 ; HMDB36881 ;	Nopalinic acid ; Mascaroside ; Gibberellin A38 glucosyl ester ; (8R8R)-Secoisolariciresinol 9-glucoside ; D-Glucose ; D-Galactose ; D-Mannose ; Myoinositol ; N-Acetylgalactosamine ; N-Acetyl-D-glucosamine ; 3-Deoxyarabinohexonic acid ; Beta-D-Glucose ; D-Fructose ; 4-Hydroxyproline ; N-Acetyl-L-alanine ; Propionylglycine ; Beta-N-Acetylglucosamine ; N-Acetyl-b-D-galactosamine ; N-Acetylmannosamine ; 5-Aminolevulinic acid ; Allose ; L-Sorbose ; L-Glutamic gamma-semialdehyde ; 3-Hydroxy-L-proline ; Alpha-D-Glucose ; D-Tagatose ; Beta-D-Galactose ; 4-Hydroxy-L-proline ; Scyllitol ; 5-Amino-2-oxopentanoic acid ; PA(20:4(5Z8Z11Z14Z)e2:0) ; L-Gulose ; Erlotinib ; Frovatriptan ; Pterosin O ; 6-(34-Methylenedioxy)phenyl-33-dimethyl-1-hexene ; (Z)-8-Decene-46-diyn-1-yl 3-methylbutanoate ; Margrapine A ; 3-Methyl-4-phenyl-3-buten-2-one ; Dihydroxyacetone (dimer) ; Porrigenin A ; L-Galactose ; Levoinositol ; Asperagenin ; 2-Phenyl-4-pentenal ; 1(10)11-Eremophiladiene-29-dione ; Alantolactone ; Isoalantolactone ; (S)-Bilobanone ; Alloalantolactone ; Furoeremophilone 1 ; alpha-Amylcinnamyl formate ; 4-Hydroxy-2-pyrrolidinecarboxylic acid ; Costunolide ; Furanogermenone ; Germacrone-13-al ; Turmeronol B ; Turmeronol A ; Epoxyfumitremorgin C ; Avenic acid B ; 3-Phenyl-4-pentenal ; 1311(13)-Eudesmatrien-12-oic acid ; (E)-9-(3-Furanyl)-26-dimethyl-26-nonadien-4-one ; (7b10a)-3-Hydroxy-135-cadinatrien-9-one ; Collybial ; 1011-Epidioxycalamene ; Trans-3-hydroxy-L-proline ; cis-4-Hydroxy-D-proline	M+H ; M+2H ; M+2H ; M+2H ; M+2ACN+H ; M+2ACN+H ; M+2ACN+H ; M+2ACN+H ; M+ACN+H ; M+ACN+H ; M+2ACN+H ; M+2ACN+H ; M+2ACN+H ; 2M+H ; 2M+H ; 2M+H ; M+ACN+H ; M+ACN+H ; M+ACN+H ; 2M+H ; M+2ACN+H ; M+2ACN+H ; 2M+H ; 2M+H ; M+2ACN+H ; M+2ACN+H ; M+2ACN+H ; 2M+H ; M+2ACN+H ; 2M+H ; M+H+K ; M+2ACN+H ; 2M+3H ; 2M+H+K ; 2M+Na+K ; 2M+Na+K ; 2M+Na+K ; M+3ACN+2H ; 3M+2Na ; M+2ACN+H ; M+2K ; M+2ACN+H ; M+2ACN+H ; M+2K ; 3M+2Na ; 2M+Na+K ; 2M+Na+K ; 2M+Na+K ; 2M+Na+K ; 2M+Na+K ; 2M+Na+K ; 2M+Na+K ; 2M+H ; 2M+Na+K ; 2M+Na+K ; 2M+Na+K ; 2M+Na+K ; 2M+Na+K ; 2M+3H ; M+ACN+H ; 3M+2Na ; 2M+Na+K ; 2M+Na+K ; 2M+Na+K ; 2M+Na+K ; 2M+Na+K ; 2M+H ; 2M+H	C10H18N2O6 ; C26H36O11 ; C26H36O11 ; C26H36O11 ; C6H12O6 ; C6H12O6 ; C6H12O6 ; C6H12O6 ; C8H15NO6 ; C8H15NO6 ; C6H12O6 ; C6H12O6 ; C6H12O6 ; C5H9NO3 ; C5H9NO3 ; C5H9NO3 ; C8H15NO6 ; C8H15NO6 ; C8H15NO6 ; C5H9NO3 ; C6H12O6 ; C6H12O6 ; C5H9NO3 ; C5H9NO3 ; C6H12O6 ; C6H12O6 ; C6H12O6 ; C5H9NO3 ; C6H12O6 ; C5H9NO3 ; C25H43O7P ; C6H12O6 ; C22H23N3O4 ; C14H17N3O ; C15H20O2 ; C15H20O2 ; C15H20O2 ; C21H23NO7 ; C11H12O ; C6H12O6 ; C27H44O5 ; C6H12O6 ; C6H12O6 ; C27H44O5 ; C11H12O ; C15H20O2 ; C15H20O2 ; C15H20O2 ; C15H20O2 ; C15H20O2 ; C15H20O2 ; C15H20O2 ; C5H9NO3 ; C15H20O2 ; C15H20O2 ; C15H20O2 ; C15H20O2 ; C15H20O2 ; C22H23N3O4 ; C8H15NO6 ; C11H12O ; C15H20O2 ; C15H20O2 ; C15H20O2 ; C15H20O2 ; C15H20O2 ; C5H9NO3 ; C5H9NO3
[illegible]8067_53.3[illegible]	1.644573	0.904005	-2.6614	0.023841	1.6227	0.42361	334.9528	HMDB00816 ; HMDB15606 ; HMDB60148	Phosphoglycolic acid ; Lofexidine ; 2-Phosphonatoglycolate	2M+Na ; M+2K-H ; 2M+Na	C2H5O6P ; C11H12Cl2N2O ; C2H5O6P
[illegible]7579_28.6[illegible]	1.643415	0.750938	-2.4237	0.035833	1.4457	0.42361	113.9638	HMDB29612 ; HMDB31792 ; HMDB60490	Asparagusic acid syn-S-oxide ; Folpet ; Methylselenopyruvate	M+Na+K ; M+H+2Na ; M+2Na	C4H6O3S2 ; C9H4Cl3NO2S ; C4H6O3Se
[illegible]9663_287.[illegible]	1.643149	0.69828	2.4431	0.034664	1.4601	0.42361	708.114	HMDB10374 ; HMDB10375 ; HMDB33559	CE(22:5(4Z7Z10Z13Z16Z)) ; CE(22:5(7Z10Z13Z16Z19Z)) ; 77-Dihydroxy-68-bicoumarin	2M+H+NH4 ; 2M+H+NH4 ; 2M+ACN+Na	C49H78O2 ; C49H78O2 ; C18H10O6

1046_88.5	1.642375	1.10445	-2.5971	0.026624	1.5747	0.42361	157.0801	HMDB13302 ; HMDB00477 ; HMDB00541 ; HMDB00590 ; HMDB01138 ; HMDB01923 ; HMDB02335 ; HMDB11557 ; HMDB11587 ; HMDB11664 ; HMDB13809 ; HMDB14004 ; HMDB15453 ; HMDB28743 ; HMDB28761 ; HMDB29013 ; HMDB29100 ; HMDB29351 ; HMDB29374 ; HMDB29756 ; HMDB30429 ; HMDB30639 ; HMDB30961 ; HMDB30966 ; HMDB31086 ; HMDB31197 ; HMDB31301 ; HMDB31309 ; HMDB31574 ; HMDB31590 ; HMDB31591 ; HMDB31635 ; HMDB31701 ; HMDB32340 ; HMDB32403 ; HMDB32451 ; HMDB32499 ; HMDB33103 ; HMDB33184 ; HMDB33547 ; HMDB33731 ; HMDB34130 ; HMDB34212 ; HMDB34244 ; HMDB35242 ; HMDB35390 ; HMDB35915 ; HMDB37152 ; HMDB37285 ; HMDB37805 ; HMDB38255 ; HMDB39097 ; HMDB39110 ; HMDB39131 ; [illegible]	Phenylalanylphenylalanine ; 7Z10Z-Hexadecadienoic acid ; 7b-Hydroxy-3-oxo-5b-cholanoic acid ; Glutarylglycine ; N-Acetylglutamic acid ; Naproxen ; Aspartyl-L-proline ; MG(0:022:6(4Z7Z10Z13Z16Z19Z)0:0) ; MG(22:6(4Z7Z10Z13Z16Z19Z)0:00:0) ; 3-Methylene-indolenine ; (E)-2-octenal ; Norhydromorphone ; Procaterol ; Asparaginyl-Tyrosine ; Aspartyl-Proline ; Prolyl-Aspartate ; Tyrosyl-Asparagine ; Geranyl hexanoate ; (EE)-Trichostachine ; (1R2S3R)-2-Acetyl-4(5)-(1234-tetrahydroxybutyl)imidazole ; Linalyl hexanoate ; ()-Tembamide ; 2-Octenal ; (3xi5Z)-15-Octadien-3-ol ; (Z)-7-Hexadecen-116-olide ; 244-Trimethylcyclopentanone ; 2-Octen-4-one ; 1-Octen-3-one ; 1-Isothiocyanato-6-(methylthio)hexane ; 3-Methyl-3-hepten-2-one ; 5-Methyl-5-hepten-2-one ; 2-Propene-1-thiol ; 1-(gamma-Glutamylamino)cyclopropanecarboxylic acid ; Isoambrettolide ; 6-Methyl-3-hepten-2-one trans- ; 4-Octen-3-one ; Pyrrolidino-12E-4H-24-dimethyl-135-dithiazine ; (R)-Higenamine ; Pondaplin ; 3-Octen-2-one ; Quinoline ; Osthenol ; (R)-Kawain ; Isoquinoline ; (SE)-Filbertone ; 5-Octen-2-one ; Sulcatone ; 2-Ethylidenehexanal ; 24-Dimethyl-5-vinylthiazole ; D6-Ambrettolide ; Geranyl 2-ethylbutyrate ; Demethylbatatasin IV ; (2S3S)-alpha-Amino-2-carboxy-5-oxo-1-pyrrolidinebutanoic acid ; Ethyl (ZZ)-58-tetradecadienoate ; 6-Octenal ; (Z)-5-Octenal ; 25-Octadien-1-ol ; (3E5Z)-35-Octadien-1-ol ; (2E4E)-24-Octadien-1-ol ; 1-Propene-1-thiol ; 7-Hydroxyheptaphylline ; Normorphine ; Norfluoxetine	M+2H ; M+Na+K ; M+3Na ; M+3ACN+2H ; M+3ACN+2H ; M+2ACN+2H ; M+2ACN+2H ; M+3Na ; M+3Na ; 2M+3H2O+2H ; 2M+Na+K ; M+ACN+2H ; M+H+Na ; M+H+NH4 ; M+2ACN+2H ; M+2ACN+2H ; M+H+NH4 ; M+Na+K ; M+ACN+2H ; M+2ACN+2H ; M+Na+K ; M+ACN+2H ; 2M+Na+K ; 2M+Na+K ; M+Na+K ; 2M+Na+K ; 2M+Na+K ; 2M+Na+K ; M+3ACN+2H ; 2M+Na+K ; 2M+Na+K ; M+2ACN+H ; M+2ACN+2H ; M+Na+K ; 2M+Na+K ; 2M+Na+K ; M+3ACN+2H ; M+ACN+2H ; M+2ACN+2H ; 2M+Na+K ; 2M+3H2O+2H ; M+2ACN+2H ; M+2ACN+2H ; 2M+3H2O+2H ; 2M+Na+K ; 2M+Na+K ; 2M+Na+K ; 2M+Na+K ; M+NH4 ; M+Na+K ; M+Na+K ; M+2ACN+2H ; M+2ACN+2H ; M+Na+K ; 2M+Na+K ; 2M+Na+K ; 2M+Na+K ; 2M+Na+K ; 2M+Na+K ; M+2ACN+H ; M+H+NH4 ; M+ACN+2H ; M+H+NH4	C18H20N2O3 ; C16H28O2 ; C25H38O4 ; C7H11NO5 ; C7H11NO5 ; C14H14O3 ; C9H14N2O5 ; C25H38O4 ; C25H38O4 ; C9H7N ; C8H14O ; C16H17NO3 ; C16H22N2O3 ; C13H17N3O5 ; C9H14N2O5 ; C9H14N2O5 ; C13H17N3O5 ; C16H28O2 ; C16H17NO3 ; C9H14N2O5 ; C16H28O2 ; C16H17NO3 ; C8H14O ; C8H14O ; C16H28O2 ; C8H14O ; C8H14O ; C8H14O ; C8H15NS2 ; C8H14O ; C8H14O ; C3H6S ; C9H14N2O5 ; C16H28O2 ; C8H14O ; C8H14O ; C8H15NS2 ; C16H17NO3 ; C14H14O3 ; C8H14O ; C9H7N ; C14H14O3 ; C14H14O3 ; C9H7N ; C8H14O ; C8H14O ; C8H14O ; C8H14O ; C7H9NS ; C16H28O2 ; C16H28O2 ; C14H14O3 ; C9H14N2O5 ; C16H28O2 ; C8H14O ; C8H14O ; C8H14O ; C8H14O ; C8H14O ; C3H6S ; C18H17NO3 ; C16H17NO3 ; C16H16F3NO
5213_53.4	1.641728	1.206846	2.5813	0.027355	1.563	0.42361	157.0455	HMDB01944 ; HMDB15086 ; HMDB29542 ; HMDB29579 ; HMDB29582 ; HMDB31222 ; HMDB31223 ; HMDB31431 ; HMDB31874 ; HMDB32056 ; HMDB32067 ; HMDB32333 ; HMDB33197 ; HMDB33561 ; HMDB33629 ; HMDB33921 ; HMDB34016 ; HMDB34080 ; HMDB34132 ; HMDB34688 ; HMDB34835 ; HMDB36047 ; HMDB37026 ; HMDB37027 ; HMDB37178 ; HMDB38430 ; HMDB38553 ; HMDB39660 ; HMDB39806 ; HMDB40445 ; HMDB40557 ; HMDB40929 ; HMDB41282 ; HMDB41474 ; HMDB59661 ; HMDB60786 ; HMDB28684 ; HMDB28768	Chlorpheniramine ; Isoniazid ; Clusin ; Diisopropyl sulfide ; Methyl sorbate ; 3-Ethyl-12-cyclopentanedione ; Enol-3-Ethyl-12-cyclopentanedione ; Dipropyl sulfide ; N-gamma-Glutamyl-S-allylcysteine ; Eugenyl benzoate ; Allyl crotonate ; 4-Hydroxy-4-methyl-5-hexenoic acid gamma lactone ; 2244-Tetramethyl-6-(1-oxobutyl)-135-cyclohexanetrione ; 2-Acetyl-45-dihydrothiazole ; xi-4-Hydroxy-4-methyl-2-cyclohexen-1-one ; 3-(11-Dimethyl-2-propenyl)-78-dimethoxy-2H-1-benzopyran-2-one ; 6-Hydroxy-99-dimethyl-5-(3-methyl-1-oxobutyl)-1-propyl-3H9H-12-dioxolo34:45furo23-f1benzopyran-3-one ; 3-Methyl-12-cyclohexanedione ; Rutacultin ; 23-Dihydro-23-dihydroxy-9-phenyl-1H-phenalen-1-one ; 3-(3-Ethyloxiranyl)-2-propenal ; 3alpha-Hydroxyoreadone ; 34-Dimethyl-12-cyclopentanedione ; 35-Dimethyl-12-cyclopentanedione ; 5-Methoxy-2-methylthiazole ; 5-(1-Propynyl)-5-vinyl-22-bithiophene ; N-gamma-Glutamyl-S-(1-propenyl)cysteine ; Gossyvertin ; 1-Hexanethiol ; 2-(Ethoxymethyl)furan ; N-gamma-Glutamyl-S-trans-(1-propenyl)cysteine ; Batatasin II ; 2-Ethoxythiazole ; xi-5-Isopropyl-2(5H)-furanone ; QH(2) ; 6-Hydroxymelatonin glucuronide ; Alanyl-Cysteine ; Cysteinyl-Alanine	M+H+K ; 2M+H+K ; M+3Na ; M+K ; 2M+Na+K ; 2M+Na+K ; 2M+Na+K ; M+K ; M+H+Na ; M+2Na ; 2M+Na+K ; 2M+Na+K ; M+Na+K ; 2M+3H2O+2H ; 2M+Na+K ; M+H+K ; M+3Na ; 2M+Na+K ; M+H+K ; M+H+Na ; 2M+Na+K ; M+Na+K ; 2M+Na+K ; 2M+Na+K ; 2M+3H2O+2H ; M+2ACN+2H ; M+H+Na ; M+H+K ; M+K ; 2M+Na+K ; M+H+Na ; M+H+K ; 2M+3H2O+2H ; 2M+Na+K ; M+Na+K ; M+H+2Na ; M+H-2H2O ; M+H-2H2O	C16H19ClN2 ; C6H7N3O ; C22H26O7 ; C6H14S ; C7H10O2 ; C7H10O2 ; C7H10O2 ; C6H14S ; C11H18N2O5S ; C17H16O3 ; C7H10O2 ; C7H10O2 ; C14H20O4 ; C5H7NOS ; C7H10O2 ; C16H18O4 ; C22H26O7 ; C7H10O2 ; C16H18O4 ; C19H14O3 ; C7H10O2 ; C14H20O4 ; C7H10O2 ; C7H10O2 ; C5H7NOS ; C13H10S2 ; C11H18N2O5S ; C16H18O4 ; C6H14S ; C7H10O2 ; C11H18N2O5S ; C16H18O4 ; C5H7NOS ; C7H10O2 ; C14H20O4 ; C19H24N2O9 ; C6H12N2O3S ; C6H12N2O3S
7529_157.	1.641545	0.965172	2.5088	0.030977	1.509	0.42361	312.5458	HMDB01253 ; HMDB01430 ; HMDB11622 ; HMDB15057	56-Dihydroxyindole-2-carboxylic acid ; L-Dopachrome ; D-Dopachrome ; Buprenorphine	3M+2Na ; 3M+2Na ; 3M+2Na ; 2M+3H	C9H7NO4 ; C9H7NO4 ; C9H7NO4 ; C29H41NO4

4404_284.6	1.641536	1.061923	2.4824	0.032408	1.4893	0.42361	465.0844	HMDB01847 ; HMDB06467 ; HMDB14962 ; HMDB15068 ; HMDB28787 ; HMDB29102 ; HMDB32659 ; HMDB36628 ; HMDB60033 ; HMDB60035 ; HMDB60036 ; HMDB60140 ; HMDB60723	Caffeine ; 4-hydroxybenzoyl-CoA ; Enprofylline ; Mesoridazine ; Cysteinyl-Tyrosine ; Tyrosyl-Cysteine ; 4-Hydroxy-8-methoxy-2H-furo23-h-1-benzopyran-2-one ; 9-Hydroxy-4-methoxypsoralen ; (beta-1-O-N-(2-hydroxymethyl-3-chlorophenyl)-anthraniloyl-D-glucupyranuronic acid) ; (beta-1-O-N-(2-methyl-3-chlorophenyl-4-hydroxy)anthraniloyl-D-glucupyranuronic acid) ; (beta-1-O-N-(2-methyl-3-chlorophenyl-5-hydroxy) anthraniloyl-D-glucupyranuronic acid ; 4-Hydroxybenoyl-CoA ; 23-diene-Valproic acid-CoA	2M+2K-H ; M+ACN+2H ; 2M+2K-H ; M+2K+H ; 3M+2K ; 3M+2K ; 2M+H ; 2M+H ; 2M+H+Na ; 2M+H+Na ; 2M+H+Na ; M+ACN+2H ; M+H+K	C8H10N4O2 ; C28H40N7O18P3S ; C8H10N4O2 ; C21H26N2OS2 ; C12H16N2O4S ; C12H16N2O4S ; C12H8O5 ; C12H8O5 ; C20H20ClNO9 ; C20H20ClNO9 ; C20H20ClNO9 ; C28H40N7O18P3S ; C29H47N7O17P3S
6574_44.1	1.641244	1.040297	2.5536	0.028686	1.5423	0.42361	484.5557	HMDB15201	Clonazepam	3M+H+Na	C15H10ClN3O3
2136_251.4	1.640631	1.026202	2.2932	0.044768	1.349	0.42361	371.6042	HMDB38822 ; HMDB15047	Malvidin 3-(6-malonylglucoside) 5-glucoside ; Tinidazole	M+2H ; 3M+2H	C32H37O20 ; C8H13N3O4S
7099_160.5	1.640413	1.119465	2.5108	0.030869	1.5105	0.42361	223.0747	HMDB00099 ; HMDB00455 ; HMDB03075 ; HMDB14641 ; HMDB28785 ; HMDB29058 ; HMDB01908 ; HMDB01979 ; HMDB03235 ; HMDB03247 ; HMDB04241 ; HMDB04242 ; HMDB05011 ; HMDB05099 ; HMDB12110 ; HMDB12633 ; HMDB12635 ; HMDB14334 ; HMDB14380 ; HMDB14797 ; HMDB30628 ; HMDB30778 ; HMDB30779 ; HMDB30780 ; HMDB30975 ; HMDB31380 ; HMDB31710 ; HMDB33099 ; HMDB33536 ; HMDB33611 ; HMDB34269 ; HMDB34678 ; HMDB34917 ; HMDB36854 ; HMDB37444 ; HMDB37550 ; HMDB38875 ; HMDB38876 ; HMDB38917 ; HMDB41608 ; HMDB60704 ; HMDB60778 ; HMDB60800 ; HMDB60844 ; HMDB60902 ; HMDB04629 ; HMDB30616 ; HMDB30947 ; HMDB31842 ; HMDB32129 ; HMDB32130 ; HMDB32725 ; HMDB33931 ; HMDB33986 ; [illegible]	L-Cystathionine ; Allocystathionine ; Flavone ; Phenindione ; Cysteinyl-Threonine ; Threoninyl-Cysteine ; 19-Hydroxy-PGE2 ; 615-Diketo1314-dihydro-PGF1a ; Prostaglandin G2 ; 20-Hydroxy-PGE2 ; 6-Ketoprostaglandin E1 ; 11-Dehydro-thromboxane B2 ; Clopidogrel ; Thromboxane B3 ; 5(6)-Epoxy Prostaglandin E1 ; 20-COOH-1011-dihydro-LTB4 ; 20-dihydroxyleukotriene B4 ; Bortezomib ; Milrinone ; Acamprosate ; Rubraflavone A ; Mammea AAB ; Mammea ABA ; Mammea ABB ; Corchoionoside B ; (6beta7alpha12beta13beta)-7-Hydroxy-1116-dioxo-814-apianadien-226-olide ; 19-Nonanedithiol ; Kanzonol Z ; Honyucitrin ; Licocoumarin A ; Mammeisin ; Cinncassiol D2 ; Xanthohumol E ; Cinncassiol D3 ; (S)-Cajaflavanone ; 24-Dihydroxy-78-dimethoxy-2H-14-benzoxazin-3(4H)-one 2-glucoside ; Gancaonin Q ; Albanin D ; Armillarivin ; Methional diethyl acetal ; 1-Hydroxylorcaserin ; 5-Hydroxylorcaserin ; 7-Hydroxylorcaserin ; N-Hydroxylorcaserin ; 4-(4-chlorophenyl)-4-hydroxypiperidine ; O-Desmethylangolensin ; Archangin ; Wyerone ; Rhapontigenin ; 25-Dihydroxychalcone ; 34-Dihydroxychalcone ; Acetomenaphthone ; Xanthoxyletin ; Demethylvestitol ; Yangonin ; 5-Methoxyseselin ; Lettucenin A ; 24-Dihydroxychalcone ; (2S3R4R)-344-Trihydroxyflavan ; 347-Trihydroxyisoflavan ; 3-Hydroxyequol ; cis-4-Hydroxyequol	M+H ; M+H ; M+H ; M+H ; M+H ; M+H ; M+2K ; M+2K ; M+2K ; M+2K ; M+2K ; M+2K ; M+3ACN+2H ; M+2K ; M+2K ; M+2K ; M+2K ; M+Na+K ; 2M+H+Na ; M+ACN+H ; M+H+K ; M+H+K ; M+H+K ; M+H+K ; M+2Na ; M+Na+K ; 2M+Na+K ; M+H+K ; M+H+K ; M+H+K ; M+H+K ; M+2K ; M+H+K ; M+2K ; M+H+K ; M+ACN+2H ; M+H+K ; M+H+K ; M+Na+K ; M+2Na-H ; 2M+H+Na ; 2M+H+Na ; 2M+H+Na ; 2M+H+Na ; 2M+H+Na ; M+H-2H2O ; M+H-2H2O ; M+H-2H2O ; M+H-2H2O ; M+H-H2O ; M+H-H2O ; M+H-2H2O ; M+H-2H2O ; M+H-2H2O ; M+H-2H2O ; M+H-2H2O ; M+H-H2O ; M+H-H2O ; M+H-2H2O ; M+H-2H2O ; M+H-2H2O ; M+H-2H2O	C7H14N2O4S ; C7H14N2O4S ; C15H10O2 ; C15H10O2 ; C7H14N2O4S ; C7H14N2O4S ; C20H32O6 ; C20H32O6 ; C20H32O6 ; C20H32O6 ; C20H32O6 ; C20H32O6 ; C16H16ClNO2S ; C20H32O6 ; C20H32O6 ; C20H32O6 ; C20H32O6 ; C19H25BN4O4 ; C12H9N3O ; C5H11NO4S ; C25H26O5 ; C25H26O5 ; C25H26O5 ; C25H26O5 ; C19H28O9 ; C23H28O5 ; C9H20S2 ; C25H26O5 ; C25H26O5 ; C25H26O5 ; C25H26O5 ; C20H32O6 ; C25H26O5 ; C20H32O6 ; C25H26O5 ; C16H21NO11 ; C25H26O5 ; C25H26O5 ; C23H28O5 ; C8H18O2S ; C11H14ClNO ; C11H14ClNO ; C11H14ClNO ; C11H14ClNO ; C11H14ClNO ; C15H14O4 ; C15H14O4 ; C15H14O4 ; C15H14O4 ; C15H12O3 ; C15H12O3 ; C15H14O4 ; C15H14O4 ; C15H14O4 ; C15H14O4 ; C15H14O4 ; C15H12O3 ; C15H12O3 ; C15H14O4 ; C15H14O4 ; C15H14O4 ; C15H14O4

9047_241.(	1.639566	1.18271	2.5429	0.029219	1.5343	0.42361	324.0589	HMDB00095 ; HMDB11692 ; HMDB01163 ; HMDB03351 ; HMDB06258 ; HMDB06552 ; HMDB11640 ; HMDB15176 ; HMDB29614 ; HMDB30561 ; HMDB30563 ; HMDB32909 ; HMDB33533 ; HMDB33658 ; HMDB33915 ; HMDB33993 ; HMDB34425 ; HMDB37101 ; HMDB38322 ; HMDB40242 ; HMDB41097 ; HMDB41450 ; HMDB60688	Cytidine monophosphate ; Cytidine 2-phosphate ; Guanosine diphosphate mannose ; GDP-glucose ; 26-Dimethylheptanoyl-CoA ; Aflatoxin B1 ; Uridine 23-cyclic phosphate ; Melphalan ; ()-Conen ; 3-Hydroxy-89-dimethoxycoumestan ; 344-Trihydroxypulvinone ; 2-(4-Methoxyphenyl)naphthalic anhydride ; Ceftiofur ; De-O-methyldihydrosterigmatocystin ; Aloe emodin w-acetate ; Wairol ; ()-1-Methylpropyl 3-(methylthio)-2-propenyl disulfide ; 8-Hydroxy-3-methoxy-1-methylanthraquinone-2-carboxylic acid ; 4-(34-Dihydroxyphenyl)-2-hydroxy-1H-phenalen-1-one ; 3-Methyl-5-pentyl-124-trithiolane ; (E)-Antibiotic BE 23372M ; Musanolone E ; Nornitrogen mustard	M+H ; M+H ; M+ACN+2H ; M+ACN+2H ; M+3Na ; 2M+H+Na ; M+NH4 ; 2M+H+K ; 2M+H+K ; 2M+H+Na ; 2M+H+Na ; 2M+H+K ; M+3ACN+2H ; 2M+H+Na ; 2M+H+Na ; 2M+H+Na ; 3M+H+Na ; 2M+H+Na ; 2M+H+K ; 3M+H+Na ; 2M+H+Na ; 2M+H+K ; 2M+ACN+H	C9H14N3O8P ; C9H14N3O8P ; C16H25N5O16P2 ; C16H25N5O16P2 ; C30H48N7O17P3S ; C17H12O6 ; C9H11N2O8P ; C13H18Cl2N2O2 ; C13H21O2PS2 ; C17H12O6 ; C17H12O6 ; C19H12O4 ; C19H17N5O7S3 ; C17H12O6 ; C17H12O6 ; C17H12O6 ; C8H16S3 ; C17H12O6 ; C19H12O4 ; C8H16S3 ; C17H12O6 ; C19H12O4 ; C4H9Cl2N
6254_47.6!	1.639469	1.259287	-2.4763	0.032752	1.4848	0.42361	218.9196	HMDB01494 ; HMDB02818 ; HMDB04110	Acetylphosphate ; Alloxan ; Phosphonoacetate	M+2K+H ; M+2K-H ; M+2K+H	C2H5O5P ; C4H2N2O4 ; C2H5O5P
27429_21.5	1.639222	0.618963	-2.349	0.040708	1.3903	0.42361	271.2327	HMDB30896 ; HMDB15222 ; HMDB31181 ; HMDB32531 ; HMDB38108 ; HMDB39339 ; HMDB39590 ; HMDB42076 ; HMDB42078 ; HMDB42117 ; HMDB42511 ; HMDB42571 ; HMDB42842 ; HMDB42985 ; HMDB43171 ; HMDB43172 ; HMDB43181 ; HMDB43187 ; HMDB43199 ; HMDB43215 ; HMDB43227 ; HMDB43256 ; HMDB43517 ; HMDB43663 ; HMDB43691 ; HMDB47749 ; HMDB47750 ; HMDB47759 ; HMDB47765 ; HMDB48435 ; HMDB48451	Momordicilin ; Pentolinium ; Homodihydrojasmone ; 2-trans-6-cis-Dodecadienal ; cis-Quinceoxepane ; Tricycloekasantalol ; (2E4E)-24-Dodecadienal ; TG(14:014:018:2(9Z12Z)) ; TG(14:014:020:2n6) ; TG(14:015:020:5(5Z8Z11Z14Z17Z)) ; TG(14:018:2(9Z12Z)14:0) ; TG(14:020:2n614:0) ; TG(14:020:5(5Z8Z11Z14Z17Z)15:0) ; TG(15:014:020:5(5Z8Z11Z14Z17Z)) ; TG(15:014:1(9Z)18:1(11Z)) ; TG(15:014:1(9Z)18:1(9Z)) ; TG(15:014:1(9Z)20:4(5Z8Z11Z14Z)) ; TG(15:014:1(9Z)20:4(8Z11Z14Z17Z)) ; TG(15:016:1(9Z)16:1(9Z)) ; TG(15:016:1(9Z)18:4(6Z9Z12Z15Z)) ; TG(15:018:1(11Z)14:1(9Z)) ; TG(15:018:1(9Z)14:1(9Z)) ; TG(15:020:4(5Z8Z11Z14Z)14:1(9Z)) ; TG(15:018:4(6Z9Z12Z15Z)16:1(9Z)) ; TG(15:020:4(8Z11Z14Z17Z)14:1(9Z)) ; TG(14:1(9Z)15:018:1(11Z)) ; TG(14:1(9Z)15:018:1(9Z)) ; TG(14:1(9Z)15:020:4(5Z8Z11Z14Z)) ; TG(14:1(9Z)15:020:4(8Z11Z14Z17Z)) ; TG(16:1(9Z)15:016:1(9Z)) ; TG(16:1(9Z)15:018:4(6Z9Z12Z15Z))	M+2H ; 2M+Na+K ; 3M+2H ; 3M+2H ; 3M+2H ; 3M+2H ; 3M+2H ; M+2H+Na ; M+2H+Na ; M+3H ; M+2H+Na ; M+2H+Na ; M+3H ; M+3H ; M+2H+Na ; M+2H+Na ; M+3H ; M+3H ; M+2H+Na ; M+3H ; M+2H+Na ; M+2H+Na ; M+3H ; M+3H ; M+3H ; M+2H+Na ; M+2H+Na ; M+3H ; M+3H ; M+2H+Na ; M+3H	C36H60O3 ; C15H32N2 ; C12H20O ; C12H20O ; C12H20O ; C12H20O ; C12H20O ; C50H92O6 ; C50H92O6 ; C52H90O6 ; C50H92O6 ; C50H92O6 ; C52H90O6 ; C52H90O6 ; C50H92O6 ; C50H92O6 ; C52H90O6 ; C52H90O6 ; C50H92O6 ; C52H90O6 ; C50H92O6 ; C50H92O6 ; C52H90O6 ; C52H90O6 ; C50H92O6 ; C50H92O6 ; C52H90O6 ; C52H90O6 ; C50H92O6 ; C52H90O6

'4373_53.6(	1.636972	0.766881	-2.6657	0.023666	1.6259	0.42361	459.9674	HMDB29985 ; HMDB31951 ; HMDB34432 ; HMDB35532 ; HMDB35533 ; HMDB35534 ; HMDB35535 ; HMDB38307	1315-Triacontanedione ; 28-Methyl-27-nonacosenoic acid ; Lumequic acid ; 1214-Triacontanedione ; 1113-Triacontanedione ; 1012-Triacontanedione ; 79-Triacontanedione ; Cetyl myristoleate	2M+H+NH4 ; 2M+H+NH4 ; 2M+H+NH4 ; 2M+H+NH4 ; 2M+H+NH4 ; 2M+H+NH4 ; 2M+H+NH4 ; 2M+H+NH4	C30H58O2 ; C30H58O2 ; C30H58O2 ; C30H58O2 ; C30H58O2 ; C30H58O2 ; C30H58O2 ; C30H58O2
;1054_272.(	1.636721	0.95127	2.3975	0.037475	1.4263	0.42361	146.1651	HMDB01257 ; HMDB39492	Spermidine ; 117-Diamino-4913-triazaheptadecane	M+H ; M+H+NH4	C7H19N3 ; C14H35N5
;5226_288.8	1.636412	0.844459	2.5842	0.027222	1.5651	0.42361	594.5565	HMDB00577 ; HMDB00908 ; HMDB01569 ; HMDB11146 ; HMDB11759	5beta-Coprostanol ; 5alpha-Cholestanol ; Epi-coprostanol ; DG(16:0e18:00:0) ; Cer(d18:014:0)	3M+H+Na ; 3M+H+Na ; 3M+H+Na ; 2M+H+Na ; M+2ACN+H	C27H48O ; C27H48O ; C27H48O ; C37H74O4 ; C32H65NO3
16701_86.6	1.636094	0.661066	-2.4983	0.031539	1.5011	0.42361	102.0217	HMDB00119 ; HMDB01087 ; HMDB13067 ; HMDB32775 ; HMDB38670 ; HMDB41934	Glyoxylic acid ; 5-Methylthioribose ; Salsoline-1-carboxylate ; 3-Hydroxy-carbofuran ; S-Methylmethionine ; Mizoribine	2M+3H2O+2H ; M+H+Na ; M+3Na ; M+3Na ; M+H+K ; M+H+2Na	C2H2O3 ; C6H12O4S ; C12H15NO4 ; C12H15NO4 ; C6H14NO2S ; C9H13N3O6
;2382_47.1	1.634854	1.268157	-2.4897	0.032009	1.4947	0.42361	NA	NA	NA	NA	NA
'8689_286.(	1.634281	0.671896	2.3224	0.0426	1.3706	0.42361	476.7579	HMDB31364 ; HMDB34321	Ampeloside Bs1 ; Digitoxigenin 3-glucosyl-(1-6)-glucosyl-(1-4)-26-dideoxyribohexoside	M+H+NH4 ; M+3ACN+2H	C45H74O20 ; C41H64O17
;6157_45.3	1.633153	1.08375	2.5568	0.028531	1.5447	0.42361	498.5846	HMDB01291 ; HMDB02137 ; HMDB02145 ; HMDB03948 ; HMDB12145 ; HMDB14803 ; HMDB29246 ; HMDB32294 ; HMDB35458 ; HMDB41162	trans-D-Decenoyl-CoA ; trans-3-Decenoyl-CoA ; 4-cis-Decenoyl-CoA ; (2E)-Decenoyl-CoA ; 2-Carboxymethyl-3-hydroxyphenylpropionyl-CoA ; Nilutamide ; Cyanidin 3-O-(2-xylosyl-6-(6-feruloyl-glucosyl)-galactoside) ; N-Gluconyl ethanolamine phosphate ; Cyanidin 3-O-4-Hydroxy-E-cinnamoyl-(-6)-b-D-glucopyranosyl-(1-2)-b-D-glucopyranoside 5-glucoside ; Cyanidin 3-O-b-D-Xylopyranosyl-(1-2)-(4-hydroxy-3-methoxycinnamoyl)-(-6)-b-D-glucopyranosyl-(1-6)-b-D-galactopyranoside	M+2K ; M+2K ; M+2K ; M+2K ; M+H+Na ; 3M+2Na ; M+2K ; 3M+H+K ; M+2K ; M+2K	C31H52N7O17P3S ; C31H52N7O17P3S ; C31H52N7O17P3S ; C31H52N7O17P3S ; C32H46N7O20P3S ; C12H10F3N3O4 ; C42H47O23 ; C8H18NO10P ; C42H47O23 ; C42H47O23
'5959_293.9	1.632854	1.092994	2.5281	0.02997	1.5233	0.42361	303.0076	HMDB15551 ; HMDB33040 ; HMDB33956 ; HMDB41861 ; HMDB03074	Erythrityl Tetranitrate ; Propyl 1-(propylthio)propyl disulfide ; Dichlorvos ; Cyanuric acid ; Delphinidin	M+H ; M+2K+H ; M+2ACN+H ; 2M+2Na-H ; M+H-2H2O	C4H6N4O12 ; C9H20S3 ; C4H7Cl2O4P ; C3H3N3O3 ; C15H11ClO7
;4405_55.0	1.632626	1.124796	-2.621	0.025553	1.5926	0.42361	420.0154	HMDB07090 ; HMDB07091 ; HMDB07706 ; HMDB07735 ; HMDB41247 ; HMDB56002 ; HMDB56007 ; HMDB56283 ; HMDB56290 ; HMDB56297 ; HMDB56315 ; HMDB60980 ; HMDB61029	DG(15:022:5(4Z7Z10Z13Z16Z)0:0) ; DG(15:022:5(7Z10Z13Z16Z19Z)0:0) ; DG(22:5(4Z7Z10Z13Z16Z)15:00:0) ; DG(22:5(7Z10Z13Z16Z19Z)15:00:0) ; Alkaloid A6 ; DG(15:00:022:5n6) ; DG(15:00:022:5n3) ; DG(18:2n60:018:3n6) ; DG(18:2n60:018:3n3) ; DG(18:3n60:020:2n6) ; DG(20:2n60:018:3n3) ; N-desmethylrosiglitazone ; dehydrofelodipine	2M+3H ; 2M+3H ; 2M+3H ; 2M+3H ; M+2K+H ; 2M+3H ; 2M+3H ; 2M+3H ; 2M+3H ; 2M+3H ; 2M+3H ; M+2K-H ; M+K	C40H68O5 ; C40H68O5 ; C40H68O5 ; C40H68O5 ; C19H16ClNO3 ; C40H68O5 ; C40H68O5 ; C40H68O5 ; C40H68O5 ; C40H68O5 ; C40H68O5 ; C17H17N3O3S ; C18H17Cl2NO4

20614_54.7	1.632453	1.030325	2.3931	0.037756	1.423	0.42361	170.0321	HMDB06032 ; HMDB00064 ; HMDB00528 ; HMDB03349 ; HMDB13222 ; HMDB28913 ; HMDB28935 ; HMDB28976 ; HMDB28977 ; HMDB29719 ; HMDB31188 ; HMDB31664 ; HMDB33670 ; HMDB35716 ; HMDB37731 ; HMDB37762 ; HMDB38613 ; HMDB59664	Imidazoleacetic acid ribotide ; Creatine ; 45-Dihydroorotic acid ; L-Dihydroorotic acid ; Beta-Guanidinopropionic acid ; Isoleucyl-Methionine ; Leucyl-Methionine ; Methionyl-Isoleucine ; Methionyl-Leucine ; Methyl thiophene-2-carboxylate ; Ethanethioic acid ; Alnustone ; 14-Dithiane-25-diol ; Dihydroasparagusic acid ; S-(2-Furanylmethyl) methanethioate ; Methyl 2-thiofuroate ; gamma-L-Glutamyl-L-methionine sulfoxide ; Oxidized dithiothreitol	M+2H ; M+K ; 2M+H+Na ; 2M+H+Na ; M+K ; M+2K ; M+2K ; M+2K ; M+2K ; 2M+3H2O+2H ; 2M+NH4 ; M+2K ; M+NH4 ; M+NH4 ; 2M+3H2O+2H ; 2M+3H2O+2H ; M+2Na ; M+NH4	C10H15N2O9P ; C4H9N3O2 ; C5H6N2O4 ; C5H6N2O4 ; C4H9N3O2 ; C11H22N2O3S ; C11H22N2O3S ; C11H22N2O3S ; C11H22N2O3S ; C6H6O2S ; C2H4OS ; C19H18O ; C4H8O2S2 ; C4H8O2S2 ; C6H6O2S ; C6H6O2S ; C10H18N2O6S ; C4H8O2S2
3536_88.47	1.631606	0.900182	-2.4089	0.036749	1.4348	0.42361	89.00835	HMDB00753 ; HMDB01125 ; HMDB01318 ; HMDB02939 ; HMDB05842 ; HMDB05846 ; HMDB05994 ; HMDB30003 ; HMDB30789 ; HMDB30952 ; HMDB31220 ; HMDB31502 ; HMDB31503 ; HMDB31578 ; HMDB32411 ; HMDB35086 ; HMDB36346 ; HMDB38967 ; HMDB39820 ; HMDB40244	3-Hexanone ; Inositol cyclic phosphate ; 4-Methylpentanal ; Methyl isobutyl ketone ; 2-Oxohexane ; Ethyl isopropyl ketone ; Hexanal ; (E)-3-Hexen-1-ol ; Dracunculin ; 2-Hexen-1-ol ; 2-Ethylbutanal ; (E)-4-Hexen-1-ol ; 1-Hexen-3-ol ; 2-Methylpentanal ; 2-Methyl-1-methylthio-2-butene ; Buntansin A ; 7-Hydroxy-2-methyl-4-oxo-4H-1-benzopyran-5-carboxylic acid ; 1-Propenyl 1-(1-propenylthio)propyl disulfide ; 8-Methoxy-67-methylenedioxycoumarin ; 1-Propenyl propyl sulfide	M+2K ; M+2H+Na ; M+2K ; M+2K ; M+2K ; M+2K ; M+2K ; M+2K ; M+H+2Na ; M+2K ; M+2K ; M+2K ; M+2K ; M+2K ; M+Na+K ; M+H+2Na ; M+H+2Na ; M+H+2Na ; M+H+2Na ; M+Na+K	C6H12O ; C6H11O8P ; C6H12O ; C6H12O ; C6H12O ; C6H12O ; C6H12O ; C6H12O ; C11H8O5 ; C6H12O ; C6H12O ; C6H12O ; C6H12O ; C6H12O ; C6H12S ; C11H8O5 ; C11H8O5 ; C9H16S3 ; C11H8O5 ; C6H12S
95585_290.	1.63142	0.747198	2.4924	0.03186	1.4968	0.42361	705.1396	HMDB34857 ; HMDB35149 ; HMDB37337 ; HMDB37913 ; HMDB39087 ; HMDB39088 ; HMDB39720 ; HMDB39776 ; HMDB41563 ; HMDB59775 ; HMDB61089 ; HMDB61090	2-(2-Thienylmethylene)-16-dioxaspiro44non-3-ene ; Chrycorin ; 7-(4-Carboxy-3-hydroxy-3-methylbutanoyl)sudachitin 4-glucoside ; Artomunoxanthentrione ; Sudachiin B ; Sudachiin C ; a-L-threo-4-Hex-4-enopyranuronosyl-D-galacturonic acid ; 6-O-Malonylnaringin ; Cerdlan ; 3-Hydroxy-3-carboxymethyl-adipic acid ; Alpha-hydroxymidazolam ; 4-hydroxymidazolam	3M+2Na-H ; 3M+2Na-H ; M+K ; 3M+2K ; M+K ; M+K ; 2M+H ; M+K ; M+Na ; 3M+2Na-H ; 2M+Na ; 2M+Na	C12H12O2S ; C12H12O2S ; C30H34O17 ; C26H20O7 ; C30H34O17 ; C30H34O17 ; C12H16O12 ; C30H34O17 ; C27H44I2N2O2 ; C8H12O7 ; C18H13ClFN3O ; C18H13ClFN3O
9688_66.3	1.630452	0.708697	-2.418	0.036187	1.4414	0.42361	170.961	HMDB00228 ; HMDB15265 ; HMDB32856 ; HMDB60005	Phenol ; Tiludronate ; 3344-Tetrachloroazobenzene ; Methylgallic acid-O-sulphate	M+2K-H ; M+H+Na ; M+H+Na ; M+2K	C6H6O ; C7H9ClO6P2S ; C12H6Cl4N2 ; C8H8O8S
0511_40.7	1.630023	1.154068	-2.4733	0.032919	1.4826	0.42361	105.1041	HMDB14795 ; HMDB31270 ; HMDB38191	Mecamylamine ; 1-Nonene ; Isopropylcyclohexane	M+ACN+2H ; M+2ACN+2H ; M+2ACN+2H	C11H21N ; C9H18 ; C9H18
.3741_16.7	1.629611	0.809284	-2.2835	0.045513	1.3419	0.42361	NA	NA	NA	NA	NA

47774_46.6	1.628266	1.254799	-2.4853	0.032247	1.4915	0.42361	402.7348	HMDB09137 ; HMDB09170 ; HMDB09201 ; HMDB09202 ; HMDB09393 ; HMDB09426 ; HMDB09457 ; HMDB09458 ; HMDB31897	PE(18:3(6Z9Z12Z)20:5(5Z8Z11Z14Z17Z)) ; PE(18:3(9Z12Z15Z)20:5(5Z8Z11Z14Z17Z)) ; PE(18:4(6Z9Z12Z15Z)20:4(5Z8Z11Z14Z)) ; PE(18:4(6Z9Z12Z15Z)20:4(8Z11Z14Z17Z)) ; PE(20:4(5Z8Z11Z14Z)18:4(6Z9Z12Z15Z)) ; PE(20:4(8Z11Z14Z17Z)18:4(6Z9Z12Z15Z)) ; PE(20:5(5Z8Z11Z14Z17Z)18:3(6Z9Z12Z)) ; PE(20:5(5Z8Z11Z14Z17Z)18:3(9Z12Z15Z)) ; Phaseolic acid	M+2Na ; M+2Na ; M+2Na ; M+2Na ; M+2Na ; M+2Na ; M+2Na ; M+2Na ; 3M+H+NH4	C43H70NO8P ; C43H70NO8P ; C43H70NO8P ; C43H70NO8P ; C43H70NO8P ; C43H70NO8P ; C43H70NO8P ; C43H70NO8P ; C12H22O6
3006_47.7	1.627068	1.258294	-2.482	0.032434	1.489	0.42361	390.7983	HMDB09610 ; HMDB11391 ; HMDB09248 ; HMDB09249 ; HMDB09280 ; HMDB09543 ; HMDB11356 ; HMDB11381 ; HMDB11413 ; HMDB11446 ; HMDB60215 ; HMDB60234	PE(22:4(7Z10Z13Z16Z)P-18:0) ; PE(P-18:022:4(7Z10Z13Z16Z)) ; PE(20:0P-18:1(11Z)) ; PE(20:0P-18:1(9Z)) ; PE(20:1(11Z)P-18:0) ; PE(22:1(13Z)P-16:0) ; PE(P-16:022:1(13Z)) ; PE(P-18:020:1(11Z)) ; PE(P-18:1(11Z)20:0) ; PE(P-18:1(9Z)20:0) ; 3-Oxodocosanoyl-CoA ; 3(S)-Hydroxy-tetracosa-12151821-all-cis-tetraenoyl-CoA	M+2H ; M+2H ; M+H+Na ; M+H+Na ; M+H+Na ; M+H+Na ; M+H+Na ; M+H+Na ; M+H+Na ; M+H+Na ; M+3Na ; M+H+2Na	C45H82NO7P ; C45H82NO7P ; C43H84NO7P ; C43H84NO7P ; C43H84NO7P ; C43H84NO7P ; C43H84NO7P ; C43H84NO7P ; C43H84NO7P ; C43H84NO7P ; C43H76N7O18P3S ; C45H74N7O18P3S
1545_208.	1.626949	1.091283	2.3654	0.039588	1.4024	0.42361	269.5672	HMDB15349 ; HMDB32581	Halofantrine ; 1-(Isothiocyanatomethyl)-4-methoxybenzene	M+H+K ; 3M+2H	C26H30Cl2F3NO ; C9H9NOS
1035_51.8	1.626256	1.121295	2.584	0.027229	1.565	0.42361	202.9331	HMDB14894 ; HMDB32431 ; HMDB33133 ; HMDB33134	Hexachlorophene ; 5-Methyl-2-thiophenecarboxaldehyde ; 2-Acetylthiophene ; 3-Acetylthiophene	M+2H ; M+2K-H ; M+2K-H ; M+2K-H	C13H6Cl6O2 ; C6H6OS ; C6H6OS ; C6H6OS
6114_16.7	1.625046	0.846957	-2.3774	0.038783	1.4114	0.42361	137.9876	HMDB01031 ; HMDB01213 ; HMDB01351 ; HMDB14841 ; HMDB40164 ; HMDB59654 ; HMDB60393	Deoxyribose 5-phosphate ; 1-Deoxy-D-xylulose 5-phosphate ; Deoxyribose 1-phosphate ; Methazolamide ; 4-Methyldibenzothiophene ; Deoxyribose 5-monophosphate ; 5-Deoxyribose-1-phosphate	M+Na+K ; M+Na+K ; M+Na+K ; M+H+K ; M+2K ; M+Na+K ; M+Na+K	C5H11O7P ; C5H11O7P ; C5H11O7P ; C5H8N4O3S2 ; C13H10S ; C5H11O7P ; C5H11O7P
9055_287.	1.624809	0.966072	2.4199	0.036068	1.4429	0.42361	526.9429	HMDB12307	Undecaprenyl N-acetyl-glucosaminyl-N-acetyl-mannosaminuronate-4-acetamido-46-dideoxy-D-galactose pyrophosphate	M+H+2Na	C79H129N3O22P2
1433_52.4	1.624643	0.768298	2.6205	0.025576	1.5922	0.42361	577.9791	HMDB60260	Urate radical	3M+2K-H	C5H3N4O3
42486_47.8	1.623985	1.236912	-2.4694	0.033137	1.4797	0.42361	450.7542	HMDB28919 ; HMDB28941 ; HMDB29108 ; HMDB29109 ; HMDB33464 ; HMDB33499 ; HMDB59571	Isoleucyl-Tyrosine ; Leucyl-Tyrosine ; Tyrosyl-Isoleucine ; Tyrosyl-Leucine ; Sinapoylputrescine ; Lyciumoside IV ; Oxychlordane	3M+H+NH4 ; 3M+H+NH4 ; 3M+H+NH4 ; 3M+H+NH4 ; 3M+H+NH4 ; M+3ACN+2H ; 2M+Na+K	C15H22N2O4 ; C15H22N2O4 ; C15H22N2O4 ; C15H22N2O4 ; C15H22N2O4 ; C38H64O16 ; C10H4Cl8O
56256_53.7	1.623677	1.150873	2.5607	0.028337	1.5476	0.42361	448.8656	HMDB02274 ; HMDB09743 ; HMDB09744 ; HMDB09775 ; HMDB11396 ; HMDB11428 ; HMDB11461	Methylcobalamin ; PE(24:0P-18:1(11Z)) ; PE(24:0P-18:1(9Z)) ; PE(24:1(15Z)P-18:0) ; PE(P-18:024:1(15Z)) ; PE(P-18:1(11Z)24:0) ; PE(P-18:1(9Z)24:0)	M+3H ; M+2ACN+2H ; M+2ACN+2H ; M+2ACN+2H ; M+2ACN+2H ; M+2ACN+2H ; M+2ACN+2H	C63H91CoN13O14P ; C47H92NO7P ; C47H92NO7P ; C47H92NO7P ; C47H92NO7P ; C47H92NO7P ; C47H92NO7P
4557_47.0	1.62245	1.300151	-2.4245	0.035787	1.4463	0.42361	NA	NA	NA	NA	NA
6675_196.	1.622351	1.040473	-2.5219	0.030287	1.5187	0.42361	215.5507	HMDB13855 ; HMDB30257 ; HMDB38952 ; HMDB39526 ; HMDB61128	Sulfamethoxazole N1-glucuronide ; Erysothiopine ; Niazicin A ; Sarafloxacin ; 5-Hydroxy 6-methoxy duloxetine sulfate	M+2H ; M+H+Na ; M+2Na ; M+2Na ; M+H+Na	C16H19N3O9S ; C19H21NO7S ; C17H23NO7S ; C20H17F2N3O3 ; C19H21NO7S
2154_199.	1.622348	1.038156	-2.5129	0.03076	1.512	0.42361	430.0942	HMDB37208	Kaempferol 3-2-glucosyl-6-acetyl-galactoside 7-glucoside	M+2Na	C35H42O22
3125_42.9	1.619857	1.072962	2.3992	0.037367	1.4275	0.42361	NA	NA	NA	NA	NA

02421_100.3	1.619138	1.114713	-2.3425	0.041163	1.3855	0.42361	NA	NA	NA	NA	NA
7717_17.12	1.619029	0.328519	-2.4739	0.032885	1.483	0.42361	420.3188	HMDB00501 ; HMDB01993 ; HMDB03550 ; HMDB06046 ; HMDB06721 ; HMDB06722 ; HMDB07616 ; HMDB07644 ; HMDB07812 ; HMDB07840 ; HMDB07897 ; HMDB07898 ; HMDB07929 ; HMDB08027 ; HMDB09041 ; HMDB09074 ; HMDB09106 ; HMDB09138 ; HMDB09171 ; HMDB09232 ; HMDB09233 ; HMDB09264 ; HMDB09296 ; HMDB09328 ; HMDB09361 ; HMDB09490 ; HMDB09491 ; HMDB09522 ; HMDB09553 ; HMDB09554 ; HMDB11207 ; HMDB11237 ; HMDB11269 ; HMDB11302 ; HMDB13411 ; HMDB15504 ; HMDB32001 ; HMDB32031 ; HMDB32478 ; HMDB34328 ; HMDB34406 ; HMDB35585 ; HMDB36012 ; HMDB39871 ; HMDB42300 ; HMDB42938 ; HMDB47746 ; HMDB56096 ; HMDB56118 ; HMDB60131 ; HMDB60132	7-Ketocholesterol ; 7a-Hydroxy-cholestene-3-one ; Calcidiol ; 5a-Androst-3-en-17-one ; 56-trans-25-Hydroxyvitamin D3 ; 25-Hydroxytachysterol3 ; DG(22:024:1(15Z)0:0) ; DG(22:1(13Z)24:00:0) ; DG(24:022:1(13Z)0:0) ; DG(24:1(15Z)22:00:0) ; PC(14:0P-18:1(11Z)) ; PC(14:0P-18:1(9Z)) ; PC(14:1(9Z)P-18:0) ; PC(16:1(9Z)P-16:0) ; PE(18:1(11Z)22:2(13Z16Z)) ; PE(18:1(9Z)22:2(13Z16Z)) ; PE(18:2(9Z12Z)22:1(13Z)) ; PE(18:3(6Z9Z12Z)22:0) ; PE(18:3(9Z12Z15Z)22:0) ; PE(20:020:3(5Z8Z11Z)) ; PE(20:020:3(8Z11Z14Z)) ; PE(20:1(11Z)20:2(11Z14Z)) ; PE(20:2(11Z14Z)20:1(11Z)) ; PE(20:3(5Z8Z11Z)20:0) ; PE(20:3(8Z11Z14Z)20:0) ; PE(22:018:3(6Z9Z12Z)) ; PE(22:018:3(9Z12Z15Z)) ; PE(22:1(13Z)18:2(9Z12Z)) ; PE(22:2(13Z16Z)18:1(11Z)) ; PE(22:2(13Z16Z)18:1(9Z)) ; PC(P-16:016:1(9Z)) ; PC(P-18:014:1(9Z)) ; PC(P-18:1(11Z)14:0) ; PC(P-18:1(9Z)14:0) ; PC(o-16:1(9Z)16:1(9Z)) ; Alfacalcidol ; N-(24-Eicosadienoyl)piperidine ; N-(214-Eicosadienoyl)piperidine ; Polypropylene glycol (m w 1200-3000) ; (3beta5alpha6a)-Cholesta-814-diene-36-diol ; 5alpha-Androst-16-en-3-one ; 1-Phenyl-13-heneicosanedione ; 5-Methyl-24-bis(3-methyl-2-butenyl)-6-(2-methyl-1-oxopropyl)-5-(4-methyl-3-pentenyl)cyclohexanone ; 5-(1215-Heneicosadienyl)-13-benzenediol ; TG(14:014:1(9Z)o-18:0) ; TG(14:0o-18:014:1(9Z)) ; TG(14:1(9Z)14:0o-18:0) ; DG(22:00:024:1n9) ; DG(24:00:022:1n9) ; 3beta-Hydroxy-5-cholestenal ; 27alpha-Hydroxy-8-dehydrocholesterol	2M+H+K ; 2M+H+K ; 2M+H+K ; 3M+H+Na ; 2M+H+K ; 2M+H+K ; M+2K ; M+2K ; M+2K ; M+2K ; M+3ACN+2H ; M+3ACN+2H ; M+3ACN+2H ; M+3ACN+2H ; M+ACN+2H ; M+ACN+2H ; M+ACN+2H ; M+ACN+2H ; M+ACN+2H ; M+ACN+2H ; M+ACN+2H ; M+ACN+2H ; M+ACN+2H ; M+ACN+2H ; M+ACN+2H ; M+ACN+2H ; M+ACN+2H ; M+ACN+2H ; M+ACN+2H ; M+ACN+2H ; M+3ACN+2H ; M+3ACN+2H ; M+3ACN+2H ; M+3ACN+2H ; M+3ACN+2H ; 2M+H+K ; M+2Na-H ; M+2Na-H ; 3M+NH4 ; 2M+H+K ; 3M+H+Na ; 2M+H+K ; 2M+H+K ; 2M+H+K ; M+2K ; M+2K ; M+2K ; M+2K ; M+2K ; 2M+H+K ; 2M+H+K	C27H44O2 ; C27H44O2 ; C27H44O2 ; C19H28O ; C27H44O2 ; C27H44O2 ; C49H94O5 ; C49H94O5 ; C49H94O5 ; C49H94O5 ; C40H78NO7P ; C40H78NO7P ; C40H78NO7P ; C40H78NO7P ; C45H84NO8P ; C45H84NO8P ; C45H84NO8P ; C45H84NO8P ; C45H84NO8P ; C45H84NO8P ; C45H84NO8P ; C45H84NO8P ; C45H84NO8P ; C45H84NO8P ; C45H84NO8P ; C45H84NO8P ; C45H84NO8P ; C45H84NO8P ; C45H84NO8P ; C45H84NO8P ; C40H78NO7P ; C40H78NO7P ; C40H78NO7P ; C40H78NO7P ; C40H78NO7P ; C27H44O2 ; C25H45NO ; C25H45NO ; C6H14O3 ; C27H44O2 ; C19H28O ; C27H44O2 ; C27H44O2 ; C27H44O2 ; C49H94O5 ; C49H94O5 ; C49H94O5 ; C49H94O5 ; C49H94O5 ; C27H44O2 ; C27H44O2
9216_197.6	1.618984	0.965432	-2.5127	0.030769	1.5119	0.42361	183.0369	HMDB01147 ; HMDB14901 ; HMDB30412 ; HMDB31803 ; HMDB33581 ; HMDB34259 ; HMDB34318 ; HMDB34913 ; HMDB36735 ; HMDB36936 ; HMDB37554 ; HMDB38507 ; HMDB39167 ; HMDB39169 ; HMDB39509 ; HMDB42037 ; HMDB60077	Aminomalonic acid ; Methimazole ; Tricholomic acid ; ()-Methamidophos ; trans-o-Coumaric acid 2-glucoside ; Bovinocidin ; Sennidin C ; Imazamethabenz-methyl ; Bilobalide A ; 1-O-p-Coumaroyl-beta-D-glucose ; ()-Rollipyrrole ; Rheidin A ; 2-O-p-Coumaroyl-D-glucose ; 6-O-p-Coumaroyl-D-glucose ; trans-p-Coumaric acid 4-glucoside ; Thymine glycol ; cis-beta-D-Glucosyl-2-hydroxycinnamate	M+ACN+Na ; 3M+H+Na ; M+Na ; M+ACN+H ; M+H+K ; M+ACN+Na ; M+2H+Na ; M+2K ; M+H+K ; M+H+K ; M+2K ; M+2H+Na ; M+H+K ; M+H+K ; M+H+K ; M+Na ; M+H+K	C3H5NO4 ; C4H6N2S ; C5H8N2O4 ; C2H8NO2PS ; C15H18O8 ; C3H5NO4 ; C30H20O9 ; C16H20N2O3 ; C15H18O8 ; C15H18O8 ; C16H20N2O3 ; C30H20O9 ; C15H18O8 ; C15H18O8 ; C15H18O8 ; C5H8N2O4 ; C15H18O8

[illegible]0298_39.5[illegible]	1.61847	1.110242	2.3973	0.037489	1.4261	0.42361	286.105	HMDB05923 ; HMDB30177 ; HMDB34611 ; HMDB34613 ; HMDB35445 ; HMDB00296 ; HMDB00767 ; HMDB01870 ; HMDB02034 ; HMDB02056 ; HMDB04215 ; HMDB05808 ; HMDB11679 ; HMDB11718 ; HMDB13127 ; HMDB13247 ; HMDB13865 ; HMDB29226 ; HMDB29470 ; HMDB29747 ; HMDB30595 ; HMDB30720 ; HMDB30725 ; HMDB30946 ; HMDB31668 ; HMDB31829 ; HMDB32259 ; HMDB32469 ; HMDB32918 ; HMDB33036 ; HMDB33735 ; HMDB33776 ; HMDB33994 ; HMDB34056 ; HMDB34170 ; HMDB34617 ; HMDB36586 ; HMDB37130 ; HMDB37681 ; HMDB38563 ; HMDB39430 ; HMDB40876 ; HMDB41016 ; HMDB41047 ; HMDB41379 ; HMDB41394 ; HMDB61055 ; HMDB31787	N4-Acetylcytidine ; Arborinine ; Rutaretin 9-rutinoside ; Torachrysone 8-beta-gentiobioside ; 2-Methoxy-(S)-oleuropein ; Uridine ; Pseudouridine ; Benzoic acid ; Homolanthionine ; Monoisobutyl phthalic acid ; 3345-Tetrahydroxystilbene ; Formononetin ; DHAP(6:0) ; 4-Hydroxybenzaldehyde ; Hydroxybutyrylcarnitine ; Monobutylphthalate ; CGP72383 ; 5-(3-Methoxy-4-hydroxyphenyl)-gamma-valerolactone ; Isodillapiole ; 2-Amino-5-phenylpyridine ; Suberenone ; Trigraecum ; Dillapiol ; (2E11Z)-Wyerone acid ; Dihydrodeoxy-8-epiaustdiol ; Bisosthenon B ; Ethyl 4-(acetylthio)butyrate ; Phthalide ; 3-(2-Furanyl)-2-propenal ; 8-Hydroxy-1-methoxy-3-methylanthraquinone ; Graveolone ; Apiole ; Isoformononetin ; 5-(34-Methylenedioxyphenyl)pentanoic acid ; 2-Hydroxybenzaldehyde ; (3b4b11b14b)-11-Ethoxy-34-epoxy-14-hydroxy-12-cyathen-15-al 14-xyloside ; 13-Dimethoxyanthraquinone ; Piperonyl isobutyrate ; Vanillin isobutyrate ; (Z)-3-(245-Trimethoxyphenyl)-2-propenal ; 910-Dihydro-2357-Phenanthrenetetrol ; (R)-Apiumetin ; Eriobofuran ; Physagulin A ; 23-Dihydro-3-hydroxy-6-methoxy-22-dimethyl-4H-1-benzopyran-4-one ; Khelmarin D ; desethylzaleplon ; Fenamiphos	M+H ; M+H ; M+2H ; M+2H ; M+2H ; M+ACN+H ; M+ACN+H ; 2M+ACN+H ; M+NH4 ; M+ACN+Na ; M+ACN+H ; M+NH4 ; M+NH4 ; 2M+ACN+H ; M+K ; M+ACN+Na ; M+Na+K ; M+ACN+Na ; M+ACN+Na ; 3M+Na+K ; M+ACN+H ; M+NH4 ; M+ACN+Na ; M+ACN+H ; M+ACN+Na ; M+2ACN+2H ; 3M+2H ; 2M+NH4 ; 2M+ACN+H ; M+NH4 ; M+ACN+H ; M+ACN+Na ; M+NH4 ; M+ACN+Na ; 2M+ACN+H ; M+2K ; M+NH4 ; M+ACN+Na ; M+ACN+Na ; M+ACN+Na ; M+ACN+H ; M+ACN+H ; M+ACN+H ; M+Na+K ; M+ACN+Na ; M+2ACN+2H ; M+Na ; M+H-H2O	C11H15N3O6 ; C16H15NO4 ; C26H34O14 ; C26H34O14 ; C26H34O14 ; C9H12N2O6 ; C9H12N2O6 ; C7H6O2 ; C8H16N2O6S ; C12H14O4 ; C14H12O4 ; C16H12O4 ; C9H17O7P ; C7H6O2 ; C11H21NO5 ; C12H14O4 ; C29H32N7O2 ; C12H14O4 ; C12H14O4 ; C11H10N2 ; C14H12O4 ; C16H12O4 ; C12H14O4 ; C14H12O4 ; C12H14O4 ; C28H24O8 ; C8H14O3S ; C8H6O2 ; C7H6O2 ; C16H12O4 ; C14H12O4 ; C12H14O4 ; C16H12O4 ; C12H14O4 ; C7H6O2 ; C27H42O8 ; C16H12O4 ; C12H14O4 ; C12H14O4 ; C12H14O4 ; C14H12O4 ; C14H12O4 ; C14H12O4 ; C30H38O7 ; C12H14O4 ; C28H24O8 ; C15H13N5 ; C13H22NO3PS
[illegible]0627_73.1[illegible]	1.618313	1.170449	2.5778	0.027519	1.5604	0.42361	273.1201	HMDB11165 ; HMDB13133 ; HMDB15673 ; HMDB28753 ; HMDB28837 ; HMDB33865 ; HMDB34346 ; HMDB34777 ; HMDB38327 ; HMDB39082 ; HMDB39233 ; HMDB41580 ; HMDB00052 ; HMDB32765 ; HMDB39408	L-beta-aspartyl-L-glycine ; Methylmalonylcarnitine ; Carglumic acid ; Aspartyl-Glycine ; Glycyl-Aspartate ; Lotaustralin ; 4-Deoxyphysalolactone ; Epidermin ; (3b9R)-5-Megastigmene-39-diol 9-apiosyl-(1-6)-glucoside ; Camelinin ; Verbasoside ; 4-Megastigmene-6a9R-diol 9-apiosyl-(1-6)-glucoside ; Argininosuccinic acid ; N2-(3-Hydroxysuccinoyl)arginine ; N2-(3-Carboxy-2-hydroxy-1-oxopropyl)arginine	M+2ACN+H ; 2M+H+Na ; M+2ACN+H ; M+2ACN+H ; M+2ACN+H ; 2M+H+Na ; M+H+Na ; 2M+H+Na ; M+H+K ; 2M+H+Na ; M+2ACN+2H ; M+H+K ; M+H-H2O ; M+H-H2O ; M+H-H2O	C6H10N2O5 ; C11H19NO6 ; C6H10N2O5 ; C6H10N2O5 ; C6H10N2O5 ; C11H19NO6 ; C28H39ClO7 ; C11H19NO6 ; C24H42O11 ; C12H23NOS2 ; C20H30O12 ; C24H42O11 ; C10H18N4O6 ; C10H18N4O6 ; C10H18N4O6
[illegible]8375_275.[illegible]	1.617621	0.435934	2.5024	0.031316	1.5042	0.42361	428.7258	HMDB12313 ; HMDB15111	3-O-Sulfogalactosylceramide (d18:116:0) ; Telithromycin	M+2K ; M+2Na	C40H77NO11S ; C43H65N5O10
[illegible]2209_54.80	1.617133	0.962392	2.5319	0.029774	1.5262	0.42361	353.8822	HMDB11493 ; HMDB11523	LysoPE(0:022:4(7Z10Z13Z16Z)) ; LysoPE(22:4(7Z10Z13Z16Z)0:0)	2M+3H ; 2M+3H	C27H48NO7P ; C27H48NO7P
[illegible]9372_139.[illegible]	1.616527	1.086484	-2.4415	0.034761	1.4589	0.42361	235.5679	HMDB02061 ; HMDB04058 ; HMDB12199 ; HMDB12283 ; HMDB15315 ; HMDB33842 ; HMDB33893 ; HMDB36354 ; HMDB38417 ; HMDB60318 ; HMDB60946	Hyaluronic acid ; 56-Dihydroxyindole ; Chorismate ; Prephenate ; Domperidone ; 34-Dimethoxy-12-benzenedicarboxylic acid ; 45-Dimethoxy-12-benzenedicarboxylic acid ; 1-Pentenyl glucosinolate ; Glucobrassicanapin ; (S)-4-Hydroxymandelonitrile ; NAPQI	M+2Na ; 3M+H+Na ; 2M+H+NH4 ; 2M+H+NH4 ; M+2Na ; 2M+H+NH4 ; 2M+H+NH4 ; M+2ACN+2H ; M+2ACN+2H ; 3M+H+Na ; 3M+H+Na	C16H27NO12 ; C8H7NO2 ; C10H10O6 ; C10H10O6 ; C22H24ClN5O2 ; C10H10O6 ; C10H10O6 ; C12H21NO9S2 ; C12H21NO9S2 ; C8H7NO2 ; C8H7NO2

40216_54.5[illegible]	1.615885	1.213266	2.5211	0.030329	1.5181	0.42361	158.0402	HMDB01285 ; HMDB10313 ; HMDB10314 ; HMDB15350 ; HMDB01084 ; HMDB11106 ; HMDB12130 ; HMDB12182 ; HMDB14356 ; HMDB14684 ; HMDB15256 ; HMDB29390 ; HMDB29434 ; HMDB29443 ; HMDB32058 ; HMDB32349 ; HMDB33143 ; HMDB37817 ; HMDB38256 ; HMDB59657 ; HMDB60568	Geranyl-PP ; 1-Salicylate glucuronide ; Beta-D-Glucopyranuronic acid ; Dantrolene ; D-1-Piperideine-2-carboxylic acid ; 5-Amino-6-ribitylamino uracil ; (S)-2345-Tetrahydropiperidine-2-carboxylate ; 8-Hydroxypurine ; Midodrine ; Fluorouracil ; Tolbutamide ; L-Furosine ; ()-4-Methylene-2-pyrrolidinecarboxylic acid ; L-Pyridosine ; Isoeugenol benzyl ether ; Isoeugenyl benzyl ether ; Pyrraline ; Aldosine ; Geranyl acetoacetate ; (S)-2345-tetrahydropyridine-2-carboxylate ; 1011-Dihydroxycarbamazepine	M+2H ; M+2H ; M+2H ; M+2H ; 2M+Na+K ; M+H+K ; 2M+Na+K ; 2M+H+K ; M+Na+K ; 2M+3H2O+2H ; M+2Na ; M+Na+K ; 2M+Na+K ; M+Na+K ; M+Na+K ; M+Na+K ; M+Na+K ; M+Na+K ; M+2K ; 2M+Na+K ; M+2Na	C10H20O7P2 ; C13H14O9 ; C13H14O9 ; C14H10N4O5 ; C6H9NO2 ; C9H16N4O6 ; C6H9NO2 ; C5H6N4O ; C12H18N2O4 ; C4H3FN2O2 ; C12H18N2O3S ; C12H18N2O4 ; C6H9NO2 ; C12H18N2O4 ; C17H18O2 ; C17H18O2 ; C12H18N2O4 ; C12H18N2O4 ; C14H22O3 ; C6H9NO2 ; C15H14N2O3
22547_290.[illegible]	1.615589	0.842251	2.4346	0.035171	1.4538	0.42361	594.8123	HMDB35483 ; HMDB38312 ; HMDB38615 ; HMDB39397 ; HMDB39482 ; HMDB40457	Tuberoside C (Allium tuberosum) ; Balanitoside ; Trigofoenoside F ; Tuberoside L ; Trigofoenoside D ; 3-Bromosulfolane	M+3ACN+2H ; M+3ACN+2H ; M+3ACN+2H ; M+3ACN+2H ; M+3ACN+2H ; 3M+H	C51H84O23 ; C51H84O23 ; C51H84O23 ; C51H84O23 ; C51H84O23 ; C4H7BrO2S
3687_113.0	1.614573	1.075067	-2.3496	0.040672	1.3907	0.42361	332.0337	HMDB01062 ; HMDB01121 ; HMDB01367 ; HMDB02817 ; HMDB06480 ; HMDB11671 ; HMDB59626 ; HMDB60875	N-Acetyl-D-Glucosamine 6-Phosphate ; N-Acetyl-D-mannosamine 6-phosphate ; N-Acetyl-glucosamine 1-phosphate ; N-Acetylglucosamine 6-phosphate ; N-Acetyl-D-galactosamine 1-phosphate ; ADP-ribose 1-2 cyclic phosphate ; N-acetyl-alpha-D-galactosamine 1-phosphate ; Thioguanosine 5-triphosphate	2M+Na+K ; 2M+Na+K ; 2M+Na+K ; 2M+Na+K ; 2M+Na+K ; M+ACN+2H ; 2M+Na+K ; M+3ACN+2H	C8H16NO9P ; C8H16NO9P ; C8H16NO9P ; C8H16NO9P ; C8H16NO9P ; C15H22N5O16P3 ; C8H16NO9P ; C10H16N5O13P3S
5685_20.3[illegible]	1.614568	0.983238	-2.3546	0.040322	1.3945	0.42361	126.9556	HMDB60364	25-Dichloro-carboxymethylenebut-2-en-4-olide	M+2Na	C6H2Cl2O4
5011_141.[illegible]	1.61447	0.394661	2.2865	0.045283	1.3441	0.42361	112.9785	HMDB04122	Selenocystine	M+3H	C6H12N2O4Se2

.1884_54.5	1.613291	1.000396	2.5994	0.02652	1.5764	0.42361	183.0612	HMDB15024 ; HMDB00555 ; HMDB00857 ; HMDB00901 ; HMDB02441 ; HMDB10347 ; HMDB14883 ; HMDB15052 ; HMDB29167 ; HMDB29471 ; HMDB29573 ; HMDB29584 ; HMDB30685 ; HMDB31534 ; HMDB31885 ; HMDB32683 ; HMDB33588 ; HMDB33710 ; HMDB34445 ; HMDB34665 ; HMDB34667 ; HMDB34720 ; HMDB35187 ; HMDB36409 ; HMDB36616 ; HMDB40762 ; HMDB41484 ; HMDB41605 ; HMDB41820 ; HMDB59722 ; HMDB59738 ; HMDB59893 ; HMDB60900	Bumetanide ; 3-Methyladipic acid ; Pimelic acid ; Valproic acid glucuronide ; 33-Dimethylglutaric acid ; Octanoylglucuronide ; Modafinil ; Metronidazole ; 2-Methyladipic acid ; Isocycloheterophyllin ; Diethyl malonate ; 3-O-Methylcoumestrol ; Cycloheterophyllin ; ()-Galeon ; 6-Hydroxypentadecanedioic acid ; Arsenocholine ; 9-O-Methylcoumestrol ; 1-(4-Hydroxy-3-methoxyphenyl)-7-phenyl-35-heptanedione ; Cochliophilin A ; Artonin A ; Artonin B ; Gingerenone C ; Artonin F ; 12-Dihydrodehydroguaiaretic acid ; Pseudobaptigenin ; 55-Diisopropyl-22-dimethylbiphenyl-3344-tetrone ; 5-Acetyl-24-dimethylthiazole ; Propyleneglycol diacetate ; 8-Oxoguanine ; Mono-methyl-adipate ; 2-Ethylglutaric acid ; Ethyl methylsuccinate ; Clozapine N-oxide	M+2H ; M+Na ; M+Na ; M+2Na ; M+Na ; M+2Na ; 2M+3H ; 2M+H+Na ; M+Na ; M+H+2Na ; M+Na ; M+2ACN+2H ; M+H+2Na ; M+H+K ; M+2K ; M+NH4 ; M+2ACN+2H ; M+H+K ; M+2ACN+2H ; M+H+2Na ; M+H+2Na ; M+H+K ; M+H+2Na ; M+H+K ; M+2ACN+2H ; M+H+K ; 2M+3H2O+2H ; M+Na ; M+NH4 ; M+Na ; M+Na ; M+Na ; M+H+Na	C17H20N2O5S ; C7H12O4 ; C7H12O4 ; C14H24O8 ; C7H12O4 ; C14H24O8 ; C15H15NO2S ; C6H9N3O3 ; C7H12O4 ; C30H30O7 ; C7H12O4 ; C16H10O5 ; C30H30O7 ; C20H22O4 ; C15H28O5 ; C5H14AsO ; C16H10O5 ; C20H22O4 ; C16H10O5 ; C30H30O7 ; C30H30O7 ; C20H22O4 ; C30H30O7 ; C20H22O4 ; C16H10O5 ; C20H22O4 ; C7H9NOS ; C7H12O4 ; C5H3N5O2 ; C7H12O4 ; C7H12O4 ; C7H12O4 ; C18H19ClN4O
5155_87.9	1.612884	0.844476	-2.2872	0.045232	1.3446	0.42361	194.4285	HMDB37035 ; HMDB40916 ; HMDB41137 ; HMDB60517 ; HMDB60519	Glycinoeclepin B ; 2-(4-Methyl-3-pentenyl)anthraquinone ; Corchorosol A ; trans-34-Dihydro-34-dihydroxy-712-dimethylbenzaanthracene ; trans-56-Dihydro-56-dihydroxy-712-dimethylbenzaanthracene	M+2H+Na ; 2M+3H ; M+H+2Na ; 2M+3H ; 2M+3H	C31H42O9 ; C20H18O2 ; C29H44O9 ; C20H18O2 ; C20H18O2
3953_57.9	1.612335	1.074554	-2.5666	0.028054	1.552	0.42361	269.9934	HMDB11745 ; HMDB31832	N-Acetyl-L-methionine ; Methyl methanethiosulfonate	M+2K+H ; 2M+NH4	C7H13NO3S ; C2H6O2S2
1253_286.2	1.612238	1.070274	2.3304	0.042023	1.3765	0.42361	549.5091	HMDB00790 ; HMDB04949 ; HMDB14098 ; HMDB43147 ; HMDB43371 ; HMDB47037	N-Palmitoylsphingosine ; Ceramide (d18:116:0) ; 5-Hydroxytenoxicam ; TG(15:024:024:1(15Z)) ; TG(15:024:1(15Z)24:0) ; TG(24:015:024:1(15Z))	2M+H+Na ; 2M+H+Na ; 3M+H+K ; M+2ACN+2H ; M+2ACN+2H ; M+2ACN+2H	C34H67NO3 ; C34H67NO3 ; C13H11N3O5S2 ; C66H126O6 ; C66H126O6 ; C66H126O6

7176_98.5	1.611767	1.185468	-2.3814	0.038517	1.4143	0.42361	377.1457	HMDB00244 ; HMDB33300 ; HMDB33301 ; HMDB40600 ; HMDB01855 ; HMDB11531 ; HMDB11562 ; HMDB12490 ; HMDB33065 ; HMDB33385 ; HMDB33482 ; HMDB33576 ; HMDB37683 ; HMDB37693 ; HMDB39504 ; HMDB40040 ; HMDB40045 ; HMDB40065 ; HMDB40066 ; HMDB40076 ; HMDB40077 ; HMDB40078 ; HMDB40079 ; HMDB40080 ; HMDB40081 ; HMDB40082 ; HMDB40098 ; HMDB40099 ; HMDB40106 ; HMDB40107 ; HMDB40108 ; HMDB40845 ; HMDB40846 ; HMDB41093 ; HMDB41339 ; HMDB41434 ; HMDB41436 ; HMDB59767 ; HMDB60063 ; HMDB60405 ; HMDB60564	Riboflavin ; (1RS2RS)-Guaiacylglycerol 1-glucoside ; (1x2x)-Guaiacylglycerol 2-glucoside ; (1x2x)-Guaiacylglycerol 3-glucoside ; 5-Hydroxytryptophol ; MG(0:014:1(9Z)0:0) ; MG(14:1(9Z)0:00:0) ; 12-Dehydrosalsolinol ; (x)-12-Propanediol 1-O-b-D-glucopyranoside ; CI Acid Violet 49 ; Plantagonine ; 7-Azaindolizine ; Ethyl vanillin isobutyrate ; 2-Propenyl 2-aminobenzoate ; gamma-Glutamyl-S-methylcysteinyl-beta-alanine ; 5H-Cyclopentapyrazine ; 5-2H-Pyrrol-4-(3H)-ylidenemethyl-2-furanmethanol ; 4-Butyl-5-ethylthiazole ; 5-Butyl-4-ethylthiazole ; 4-Butyl-25-dimethylthiazole ; 4-Ethyl-2-methyl-5-propylthiazole ; 5-Ethyl-2-methyl-4-propylthiazole ; 4-Ethyl-5-methyl-2-propylthiazole ; 5-Ethyl-4-methyl-2-propylthiazole ; 4-Ethyl-5-methyl-2-(1-methylethyl)thiazole ; 5-Ethyl-4-methyl-2-(1-methylethyl)thiazole ; 25-Dipropylthiazole ; 5-Methyl-2-pentylthiazole ; 4-Methyl-2-pentylthiazole ; 24-Dipropylthiazole ; 2-(1-Methylethyl)-4-propylthiazole ; (S)-Nerolidol 3-O-a-L-Rhamnopyranosyl-(1-4)-a-L-rhamnopyranosyl-(1-2)-b-D-glucopyranoside ; (S)-Nerolidol 3-O-a-L-rhamnopyranosyl-(1-4)-a-L-rhamnopyranosyl-(1-6)-b-D-glucopyranoside ; Gingerglycolipid A ; 5-Chloro-2-(35-di-tert-butyl-2-hydroxyphenyl)-2H-benzotriazole ; Citbismine B ; Citbismine E ; Tetrahydro-25-furan-diacetic acid ; NN-dimethylindoliumolate ; 5-Deoxy-5-fluorocytidine ; Carboxy-ibuprofen	M+H ; M+H ; M+H ; M+H ; 2M+Na ; M+2K-H ; M+2K-H ; 2M+Na ; 3M+H+K ; M+ACN+2H ; 2M+Na ; 3M+Na ; 3M+2Na ; 2M+Na ; M+ACN+H ; 3M+Na ; 2M+Na ; 2M+K ; 2M+K ; 2M+K ; 2M+K ; 2M+K ; 2M+K ; 2M+K ; 2M+K ; 2M+K ; 2M+K ; 2M+K ; 2M+K ; 2M+K ; 2M+K ; M+2K ; M+2K ; M+2K ; 2M+H+K ; M+2ACN+2H ; M+2ACN+2H ; 2M+H ; 2M+Na ; 3M+H+NH4 ; 3M+2Na	C17H20N4O6 ; C16H24O10 ; C16H24O10 ; C16H24O10 ; C10H11NO2 ; C17H32O4 ; C17H32O4 ; C10H11NO2 ; C9H18O7 ; C39H41N3O6S2 ; C10H11NO2 ; C7H6N2 ; C13H16O4 ; C10H11NO2 ; C12H21N3O6S ; C7H6N2 ; C10H11NO2 ; C9H15NS ; C9H15NS ; C9H15NS ; C9H15NS ; C9H15NS ; C9H15NS ; C9H15NS ; C9H15NS ; C9H15NS ; C9H15NS ; C9H15NS ; C9H15NS ; C9H15NS ; C9H15NS ; C33H56O14 ; C33H56O14 ; C33H56O14 ; C20H24ClN3O ; C36H34N2O11 ; C36H34N2O11 ; C8H12O5 ; C10H11NO2 ; C9H12FN3O4 ; C13H16O4
1544_293.	1.611593	0.656089	2.3426	0.041154	1.3856	0.42361	266.9662	HMDB12710 ; HMDB32289 ; HMDB34228 ; HMDB37157 ; HMDB59927	3-Dehydroquinate ; Furfuryl propyl disulfide ; Thiolutin ; 2-Methyl-3-(propyldithio)furan ; 2-Hydroxy-2-(2-oxopropyl)butanedioic acid	M+2K-H ; M+2K+H ; M+K ; M+2K+H ; M+2K-H	C7H10O6 ; C8H12OS2 ; C8H8N2O2S2 ; C8H12OS2 ; C7H10O6
4203_264.	1.610296	0.747761	2.4577	0.03381	1.471	0.42361	478.4864	HMDB32246 ; HMDB38678	Dodecanal dimethyl acetal ; 11-Dihexyloxyethane	2M+NH4 ; 2M+NH4	C14H30O2 ; C14H30O2
4439_47.3	1.610081	1.272862	-2.4273	0.035611	1.4484	0.42361	NA	NA	NA	NA	NA

4057_289.	1.608545	1.105624	2.3909	0.037898	1.4214	0.42361	548.4584	HMDB46041 ; HMDB46046 ;<br>HMDB46066 ; HMDB46196 ;<br>HMDB46579 ; HMDB46729 ;<br>HMDB46748 ; HMDB46753 ;<br>HMDB46802 ; HMDB46820 ;<br>HMDB46826 ; HMDB46898 ;<br>HMDB46903 ; HMDB46922 ;<br>HMDB46945 ; HMDB46951 ;<br>HMDB46963 ; HMDB46969 ;<br>HMDB47411 ; HMDB47481 ;<br>HMDB47551 ; HMDB47579 ;<br>HMDB47597 ; HMDB47602 ;<br>HMDB47625 ; HMDB47642 ;<br>HMDB47648 ; HMDB47671 ;<br>HMDB47687 ; HMDB47694 ;<br>HMDB50697 ; HMDB50709 ;<br>HMDB50841 ; HMDB50975 ;<br>HMDB51113 ; HMDB51118 ;<br>HMDB51135 ; HMDB51238 ;<br>HMDB51255 ; HMDB51266 ;<br>HMDB51339 ; HMDB51356 ;<br>HMDB51621 ; HMDB51626 ;<br>HMDB51723 ; HMDB51728 ;<br>HMDB51739 ; HMDB51771 ;<br>HMDB51777 ; HMDB51787 ;<br>HMDB51841 ; HMDB51872 ;<br>HMDB52121 ; HMDB52181 ;<br>HMDB52186 ; HMDB52217 ;	TG(20:022:4(7Z10Z13Z16Z)22:5(4Z7Z10Z13Z16Z)) ;<br>TG(20:022:4(7Z10Z13Z16Z)22:5(7Z10Z13Z16Z19Z)) ;<br>TG(20:022:5(4Z7Z10Z13Z16Z)22:4(7Z10Z13Z16Z)) ;<br>TG(20:022:5(7Z10Z13Z16Z19Z)22:4(7Z10Z13Z16Z)) ;<br>TG(22:020:3(5Z8Z11Z)22:6(4Z7Z10Z13Z16Z19Z)) ;<br>TG(22:020:3n622:6(4Z7Z10Z13Z16Z19Z)) ;<br>TG(22:020:4(5Z8Z11Z14Z)22:5(4Z7Z10Z13Z16Z)) ;<br>TG(22:020:4(5Z8Z11Z14Z)22:5(7Z10Z13Z16Z19Z)) ;<br>TG(22:022:4(7Z10Z13Z16Z)20:5(5Z8Z11Z14Z17Z)) ;<br>TG(22:022:5(4Z7Z10Z13Z16Z)20:4(5Z8Z11Z14Z)) ;<br>TG(22:022:5(4Z7Z10Z13Z16Z)20:4(8Z11Z14Z17Z)) ;<br>TG(22:020:4(8Z11Z14Z17Z)22:5(4Z7Z10Z13Z16Z)) ;<br>TG(22:020:4(8Z11Z14Z17Z)22:5(7Z10Z13Z16Z19Z)) ;<br>TG(22:020:5(5Z8Z11Z14Z17Z)22:4(7Z10Z13Z16Z)) ;<br>TG(22:022:5(7Z10Z13Z16Z19Z)20:4(5Z8Z11Z14Z)) ;<br>TG(22:022:5(7Z10Z13Z16Z19Z)20:4(8Z11Z14Z17Z)) ;<br>TG(22:022:6(4Z7Z10Z13Z16Z19Z)20:3(5Z8Z11Z)) ;<br>TG(22:022:6(4Z7Z10Z13Z16Z19Z)20:3n6) ;<br>TG(24:018:3(6Z9Z12Z)22:6(4Z7Z10Z13Z16Z19Z)) ;<br>TG(24:020:4(5Z8Z11Z14Z)20:5(5Z8Z11Z14Z17Z)) ;<br>TG(24:022:5(4Z7Z10Z13Z16Z)18:4(6Z9Z12Z15Z)) ;<br>TG(24:018:3(9Z12Z15Z)22:6(4Z7Z10Z13Z16Z19Z)) ;<br>TG(24:018:4(6Z9Z12Z15Z)22:5(4Z7Z10Z13Z16Z)) ;<br>TG(24:018:4(6Z9Z12Z15Z)22:5(7Z10Z13Z16Z19Z)) ;<br>TG(24:020:4(8Z11Z14Z17Z)20:5(5Z8Z11Z14Z17Z)) ;<br>TG(24:020:5(5Z8Z11Z14Z17Z)20:4(5Z8Z11Z14Z)) ;<br>TG(24:020:5(5Z8Z11Z14Z17Z)20:4(8Z11Z14Z17Z)) ;<br>TG(24:022:5(7Z10Z13Z16Z19Z)18:4(6Z9Z12Z15Z)) ;	M+2ACN+2H ; M+2ACN+2H ;<br>M+2ACN+2H ; M+2ACN+2H ;	C67H112O6 ; C67H112O6 ; C67H112O6<br>; C67H112O6 ; C67H112O6 ;<br>C67H112O6 ; C67H112O6 ; C67H112O6<br>; C67H112O6 ; C67H112O6 ;<br>C67H112O6 ; C67H112O6 ; C67H112O6<br>; C67H112O6 ; C67H112O6 ;<br>C67H112O6 ; C67H112O6 ; C67H112O6<br>; C67H112O6 ; C67H112O6 ;<br>C67H112O6 ; C67H112O6 ; C67H112O6<br>; C67H112O6 ; C67H112O6 ;<br>C67H112O6 ; C67H112O6 ; C67H112O6<br>; C67H112O6 ; C67H112O6 ;<br>C67H112O6 ; C67H112O6 ; C67H112O6<br>; C67H112O6 ; C67H112O6 ;<br>C67H112O6 ; C67H112O6 ; C67H112O6<br>; C67H112O6 ; C67H112O6 ;<br>C67H112O6 ; C67H112O6 ; C67H112O6<br>; C67H112O6 ; C67H112O6 ;<br>C67H112O6 ; C67H112O6 ; C67H112O6<br>; C67H112O6 ; C67H112O6 ;<br>C67H112O6 ; C67H112O6 ; C67H112O6<br>; C67H112O6 ; C67H112O6 ;<br>C67H112O6 ; C67H112O6 ; C67H112O6<br>; C67H112O6 ; C67H112O6 ;<br>C67H112O6 ; C67H112O6 ; C67H112O6<br>; C67H112O6 ; C67H112O6 ;<br>C67H112O6 ; C67H112O6 ; C67H112O6<br>; C67H112O6 ; C67H112O6 ;
8561_174.	1.608352	0.900782	-2.3944	0.037673	1.424	0.42361	245.0399	HMDB01265 ; HMDB03201 ;<br>HMDB15507 ; HMDB30009 ;<br>HMDB32018 ; HMDB32583 ;<br>HMDB33091 ; HMDB33584 ;<br>HMDB36609 ; HMDB36984 ;<br>HMDB37115 ; HMDB41214 ;<br>HMDB41735 ; HMDB41807	Fucose 1-phosphate ; Malvidin ; Gamma Hydroxybutyric Acid ; 36-Dihydro-4-(4-methyl-3-pentenyl)-12-dithiin ; Phenyl salicylate ; 4-Hydroxy-2-biphenylcarboxylic acid ; (Z)-3-(1-Formyl-1-propenyl)pentanedioic acid ; Diethyl tartrate ; Rothindin ; Garcimangosone C ; ()-2-Hydroxy-4-(methylthio)butanoic acid ; Betavulgarin xyloside ; Formononetin 7-O-glucuronide ; 3-Phenoxybenzoic acid	M+H ; 2M+3H ; 2M+K ; M+2Na-H ; 2M+Na+K ; 2M+Na+K ; M+2Na-H ; M+K ; M+2Na ; M+2K ; 3M+H+K ; M+2Na ; M+2Na ; 2M+Na+K	C6H13O8P ; C17H15ClO7 ; C4H7O3 ; C10H16S2 ; C13H10O3 ; C13H10O3 ; C9H12O5 ; C8H14O6 ; C22H20O10 ; C23H24O7 ; C5H10O3S ; C22H20O10 ; C22H20O10 ; C13H10O3
8457_55.9	1.606788	1.261349	-2.4161	0.036306	1.44	0.42361	463.9788	HMDB60660	6-Mercaptopurine ribonucleoside 5-diphosphate	2M+H+K	C10H14N4O10P2S
8178_23.7	1.605937	0.606227	-2.302	0.044104	1.3555	0.42361	418.7478	HMDB28717 ; HMDB29011 ; HMDB40793	Arginyl-Proline ; Prolyl-Arginine ; Acidissiminol epoxide	3M+H+Na ; 3M+H+Na ; 2M+H+NH4	C11H21N5O3 ; C11H21N5O3 ; C25H31NO4
2693_255.	1.605333	0.950488	-2.3967	0.037525	1.4257	0.42361	347.0263	HMDB00288 ; HMDB01271 ; HMDB11641 ; HMDB15380 ; HMDB29389 ; HMDB32750 ; HMDB32751 ; HMDB32752 ; HMDB32753 ; HMDB33963 ; HMDB35463 ; HMDB38283 ; HMDB39208 ; HMDB40718 ; HMDB60282	Uridine 5-monophosphate ; Pseudouridine 5-phosphate ; Uridine 2-phosphate ; Olsalazine ; ()-threo-2-Amino-34-dihydroxybutanoic acid ; S-2-Propenyl 1-propenesulfinothioate ; S-1-Propenyl 2-propenesulfinothioate ; R-2-Propenyl 1-propenesulfinothioate ; R-1-Propenyl 2-propenesulfinothioate ; Allicin ; Myricetin 7-(6-galloylglucoside) ; 6-Chlorocatechin ; 8-C-Ascorbylepigallocatechin 3-gallate ; Zwiebelane B ; 3-UMP	M+Na ; M+Na ; M+Na ; M+2Na-H ; 2M+2K-H ; 2M+Na ; 2M+Na ; 2M+Na ; 2M+Na ; 2M+Na ; M+Na+K ; M+Na ; M+Na+K ; 2M+Na ; M+Na	C9H13N2O9P ; C9H13N2O9P ; C9H13N2O9P ; C14H10N2O6 ; C4H9NO4 ; C6H10OS2 ; C6H10OS2 ; C6H10OS2 ; C6H10OS2 ; C6H10OS2 ; C28H24O17 ; C15H13ClO6 ; C28H24O17 ; C6H10OS2 ; C9H13N2O9P

8958_22.9	1.605108	0.952668	-2.2589	0.047455	1.3237	0.42361	90.00809	HMDB00351 ; HMDB00354 ; HMDB00396 ; HMDB00407 ; HMDB00410 ; HMDB00531 ; HMDB00642 ; HMDB00754 ; HMDB01863 ; HMDB01987 ; HMDB02011 ; HMDB31735 ; HMDB32937 ; HMDB34246 ; HMDB40735 ; HMDB41603 ; HMDB59844 ; HMDB59862 ; HMDB59880 ; HMDB60488	3-Hydroxy-2-methyl-R-(RS)-butanoic acid ; 2-Methyl-3-hydroxybutyric acid ; 2-Ethylhydracrylic acid ; 2-Hydroxy-3-methylbutyric acid ; 3-Hydroxy-2-methyl-S-(RR)-butanoic acid ; 3-Hydroxyvaleric acid ; Erythronilic acid ; 3-Hydroxyisovaleric acid ; 2-Hydroxyvaleric acid ; 2-Hydroxy-2-methylbutyric acid ; 4-Hydroxyisovaleric acid ; Ethyl maltol ; Methyl 4-chloro-1H-indole-3-acetate ; Furfuryl acetate ; Ethyl lactate ; Methyl 3-hydroxybutyrate ; Diethyl carbonate ; Ethyl furoate ; Ethyl 3-furoate ; Methaneselenol	M+Na+K ; M+Na+K ; M+Na+K ; M+Na+K ; M+Na+K ; M+Na+K ; M+Na+K ; M+Na+K ; M+Na+K ; M+Na+K ; M+Na+K ; M+H+K ; M+H+2Na ; M+H+K ; M+Na+K ; M+Na+K ; M+Na+K ; M+H+K ; M+H+K ; M+2ACN+2H	C5H10O3 ; C5H10O3 ; C5H10O3 ; C5H10O3 ; C5H10O3 ; C5H10O3 ; C5H10O3 ; C5H10O3 ; C5H10O3 ; C5H10O3 ; C5H10O3 ; C7H8O3 ; C11H10ClNO2 ; C7H8O3 ; C5H10O3 ; C5H10O3 ; C5H10O3 ; C7H8O3 ; C7H8O3 ; CH4Se
9119_49.7	1.605083	1.075363	2.5194	0.030419	1.5169	0.42361	190.9119	HMDB14118	Trifluoroacetic acid	M+2K-H	C2HF3O2
9246_221.	1.604684	1.094207	2.333	0.041837	1.3784	0.42361	276.5649	HMDB12289 ; HMDB36292 ; HMDB60244	Tetrahydrodipicolinate ; Campesterol 6-(9Z-octadecenoyl)-glucoside ; Phosphodimethylethanolamine	3M+H+K ; M+3H ; 3M+2Na	C7H9NO4 ; C52H90O7 ; C4H12NO4P
8348_47.1	1.604586	1.265428	-2.4171	0.036241	1.4408	0.42361	NA	NA	NA	NA	NA
4927_86.2	1.604297	0.842182	-2.5315	0.029794	1.5259	0.42361	131.0275	HMDB00509 ; HMDB00603 ; HMDB00630 ; HMDB01470 ; HMDB01862 ; HMDB02167 ; HMDB11625 ; HMDB13227 ; HMDB13989 ; HMDB14558 ; HMDB29599 ; HMDB29608 ; HMDB30647 ; HMDB30989 ; HMDB30990 ; HMDB31178 ; HMDB31598 ; HMDB31602 ; HMDB31648 ; HMDB32035 ; HMDB32351 ; HMDB32385 ; HMDB32459 ; HMDB32656 ; HMDB33840 ; HMDB33978 ; HMDB34491 ; HMDB34971 ; HMDB35143 ; HMDB36715 ; HMDB37723 ; HMDB38017	Senecioic acid ; cis-4-Decenedioic acid ; Cytosine ; Tiglic acid ; 2-Ethylacrylic acid ; 3-Methylbutyrolactone ; Dimethylarsinate ; cis-5-Decenedioic acid ; O-Desmethylnaproxen ; Acetohexamide ; Glutaral ; Angelic acid ; Artemidinol ; 5-Pentyltetrahydro-2-oxo-3-furancarboxylic acid ; alpha-Carboxy-delta-nonalactone ; Dihydro-2-methyl-3(2H)-furanone ; 23-Pentanedione ; 4-Pentenoic acid ; 24-Pentanedione ; 6-(34-Methylenedioxyphenyl)-35-hexadien-2-one ; Isopropenyl acetate ; Methyl methacrylate ; 2-Pentenoic acid ; (Z)-6-(2-Methoxyvinyl)-7-methyl-2H-1-benzopyran-2-one ; Dihydro-5-methyl-2(3H)-furanone ; Ethyl acrylate ; ()-Camphoric acid ; (1R2R3S1R)-Nepetalinic acid ; Dihydro-3-methyl-2(3H)-furanone ; Matsutakic acid A ; 2-Phenylethyl 2-furancarboxylate ; Ethyl 2-phenyl-3-furancarboxylate	2M+Na+K ; M+Na+K ; 2M+H+K ; 2M+Na+K ; 2M+Na+K ; 2M+Na+K ; M+3ACN+2H ; M+Na+K ; M+2Na ; M+3Na ; 2M+Na+K ; 2M+Na+K ; M+2Na ; M+Na+K ; M+Na+K ; 2M+Na+K ; 2M+Na+K ; 2M+Na+K ; 2M+Na+K ; M+2Na ; 2M+Na+K ; 2M+Na+K ; 2M+Na+K ; M+2Na ; 2M+Na+K ; 2M+Na+K ; M+Na+K ; M+Na+K ; 2M+Na+K ; M+Na+K ; M+2Na ; M+2Na	C5H8O2 ; C10H16O4 ; C4H5N3O ; C5H8O2 ; C5H8O2 ; C5H8O2 ; C2H6AsO2 ; C10H16O4 ; C13H12O3 ; C15H20N2O4S ; C5H8O2 ; C5H8O2 ; C13H12O3 ; C10H16O4 ; C10H16O4 ; C5H8O2 ; C5H8O2 ; C5H8O2 ; C5H8O2 ; C13H12O3 ; C5H8O2 ; C5H8O2 ; C5H8O2 ; C13H12O3 ; C5H8O2 ; C5H8O2 ; C10H16O4 ; C10H16O4 ; C5H8O2 ; C10H16O4 ; C13H12O3 ; C13H12O3
03823_52.1	1.603684	1.093996	2.5526	0.028734	1.5416	0.42361	529.8704	HMDB31224	Nabam	2M+NH4	C4H6N2Na2S4

0146_196.3	1.602994	1.002468	-2.4795	0.032573	1.4871	0.42361	191.5501	HMDB29286 ; HMDB33211 ; HMDB33240 ; HMDB37845 ; HMDB39157 ; HMDB41773 ; HMDB61011 ; HMDB61113	Avenanthramide 2s ; Avenanthramide 2 ; Grevilline B ; N-(1-Deoxy-1-fructosyl)tyrosine ; Clovamide ; Salvianolic acid D ; N-desmethylalmotriptan ; Sulforaphane-N-acetylcysteine	M+H+Na ; M+H+Na ; M+ACN+2H ; M+H+K ; M+H+Na ; M+ACN+2H ; M+Na+K ; M+ACN+2H	C18H17NO7 ; C18H17NO7 ; C18H12O7 ; C15H21NO8 ; C18H17NO7 ; C18H12O7 ; C16H23N3O2S ; C11H20N2O4S3
9242_47.3[illegible]	1.602468	1.281398	-2.4239	0.035821	1.4459	0.42361	340.7819	HMDB02148 ; HMDB06402 ; HMDB11145 ; HMDB32126 ; HMDB40188 ; HMDB40190 ; HMDB41791	5-Methyltetrahydropteroylpentaglutamate ; 3-Oxohexadecanoyl-CoA ; PA(16:0e18:0) ; zeta-Carotene epoxide ; 11-Dibromo-2-propanone ; 1-Bromo-3-iodoacetone ; 111-Trichloroethane	M+H+2Na ; M+3H ; M+H+NH4 ; M+3ACN+2H ; 3M+H+K ; M+2K+H ; 2M+2K-H	C40H53N11O18 ; C37H64N7O18P3S ; C37H75O7P ; C40H60O ; C3H4Br2O ; C3H4BrIO ; C2H3Cl3
0571_222.[illegible]	1.602345	1.185234	-2.4507	0.034218	1.4657	0.42361	636.1431	HMDB60431 ; HMDB30376 ; HMDB33005 ; HMDB36336 ; HMDB40791 ; HMDB60927	Aflatoxin B1exo-89-epoxide-GSH ; (S)-Isowillardiine ; 4-Methyl-6-(34-dihydroxy-E-cinnamoyl)isoorientin ; Prodelphinidin A1 ; Acrimarine N ; Hydroxylumiracoxib	M+H ; 3M+K ; 2M+H+Na ; 2M+3H2O+2H ; M+2K+H ; 2M+NH4	C27H29N3O13S ; C7H9N3O4 ; C31H28O14 ; C30H24O14 ; C32H31NO8 ; C15H13ClFNO3
0057_286.[illegible]	1.601813	0.666959	2.306	0.043802	1.3585	0.42361	372.013	HMDB14781	Mebendazole	M+2K-H	C16H13N3O3
2899999_1[illegible]	1.6014	1.23774	-2.3543	0.040343	1.3942	0.42361	282.556	HMDB00812 ; HMDB03470 ; HMDB05432 ; HMDB10411 ; HMDB10422 ; HMDB12105 ; HMDB31577 ; HMDB39163 ; HMDB42064 ; HMDB42073 ; HMDB42079 ; HMDB42123 ; HMDB42137 ; HMDB42144 ; HMDB42151 ; HMDB42421 ; HMDB42543 ; HMDB42601 ; HMDB42753 ; HMDB42990 ; HMDB43004 ; HMDB43011 ; HMDB43018 ; HMDB43177 ; HMDB43179 ; HMDB43401 ; HMDB43424 ; HMDB43459 ; HMDB43627 ; HMDB43843 ; HMDB43850 ; HMDB47755 ; HMDB47757 ; HMDB47889 ; HMDB47910 ; HMDB47911 ; HMDB47932 ; HMDB47955 ; HMDB47977 ; HMDB48568 ; HMDB48569 ; HMDB60273	N-Acetyl-L-aspartic acid ; N-Formyl-L-glutamic acid ; TG(16:1(9Z)16:1(9Z)16:1(9Z))iso ; TG(16:014:016:0)iso3 ; TG(16:1(9Z)14:018:2(9Z12Z))iso6 ; SM(d18:123:0) ; Berteroin ; D-N-(Carboxyacetyl)alanine ; TG(14:014:018:0) ; TG(14:014:020:3(5Z8Z11Z)) ; TG(14:014:020:3n6) ; TG(14:016:016:0) ; TG(14:016:018:3(6Z9Z12Z)) ; TG(14:016:018:3(9Z12Z15Z)) ; TG(14:018:014:0) ; TG(14:020:3(5Z8Z11Z)14:0) ; TG(14:018:3(6Z9Z12Z)16:0) ; TG(14:020:3n614:0) ; TG(14:018:3(9Z12Z15Z)16:0) ; TG(15:015:016:0) ; TG(15:015:018:3(6Z9Z12Z)) ; TG(15:015:018:3(9Z12Z15Z)) ; TG(15:016:015:0) ; TG(15:014:1(9Z)18:2(9Z12Z)) ; TG(15:014:1(9Z)20:2n6) ; TG(15:018:2(9Z12Z)14:1(9Z)) ; TG(15:018:3(6Z9Z12Z)15:0) ; TG(15:020:2n614:1(9Z)) ; TG(15:018:3(9Z12Z15Z)15:0) ; TG(16:014:018:3(6Z9Z12Z)) ; TG(16:014:018:3(9Z12Z15Z)) ; TG(14:1(9Z)15:018:2(9Z12Z)) ; TG(14:1(9Z)15:020:2n6) ; TG(14:1(9Z)14:1(9Z)20:1(11Z)) ; TG(14:1(9Z)16:1(9Z)18:1(11Z)) ; TG(14:1(9Z)16:1(9Z)18:1(9Z)) ; TG(14:1(9Z)18:1(11Z)16:1(9Z)) ; TG(14:1(9Z)18:1(9Z)16:1(9Z)) ; TG(14:1(9Z)20:1(11Z)14:1(9Z)) ; TG(16:1(9Z)14:1(9Z)18:1(11Z)) ; TG(16:1(9Z)14:1(9Z)18:1(9Z)) ; 2-Amino-3-oxoadipate	3M+H+K ; 3M+H+K ; M+H+2Na ; M+3Na ; M+H+2Na ; M+H+2Na ; 3M+H+K ; 3M+H+K ; M+3Na ; M+H+2Na ; M+H+2Na ; M+3Na ; M+H+2Na ; M+H+2Na ; M+3Na ; M+H+2Na ; M+H+2Na ; M+H+2Na ; M+H+2Na ; M+3Na ; M+H+2Na ; M+H+2Na ; M+3Na ; M+H+2Na ; M+H+2Na ; M+H+2Na ; M+H+2Na ; M+H+2Na ; M+H+2Na ; M+H+2Na ; M+H+2Na ; M+H+2Na ; M+H+2Na ; M+H+2Na ; M+H+2Na ; M+H+2Na ; M+H+2Na ; M+H+2Na ; M+H+2Na ; M+H+2Na ; M+H+2Na ; 3M+H+K	C6H9NO5 ; C6H9NO5 ; C51H92O6 ; C49H94O6 ; C51H92O6 ; C46H93N2O6P ; C7H13NS2 ; C6H9NO5 ; C49H94O6 ; C51H92O6 ; C51H92O6 ; C49H94O6 ; C51H92O6 ; C51H92O6 ; C49H94O6 ; C51H92O6 ; C51H92O6 ; C51H92O6 ; C51H92O6 ; C49H94O6 ; C51H92O6 ; C51H92O6 ; C49H94O6 ; C51H92O6 ; C51H92O6 ; C51H92O6 ; C51H92O6 ; C51H92O6 ; C51H92O6 ; C51H92O6 ; C51H92O6 ; C51H92O6 ; C51H92O6 ; C51H92O6 ; C51H92O6 ; C51H92O6 ; C51H92O6 ; C51H92O6 ; C51H92O6 ; C51H92O6 ; C51H92O6 ; C6H9NO5
2644_62.0[illegible]	1.600894	1.011413	2.3591	0.040015	1.3978	0.42361	207.9983	HMDB15242 ; HMDB00272 ; HMDB01721 ; HMDB11635 ; HMDB30398 ; HMDB33384 ; HMDB34782 ; HMDB59996	Miconazole ; Phosphoserine ; DL-O-Phosphoserine ; p-Cresol sulfate ; N-(Carbomethoxyacetyl)-4-S-chlorotryptophan ; CI Direct Red 45 ; Benzyl sulfate ; Dihydronaringenin-O-sulphate	M+2H ; M+Na ; M+Na ; 2M+H+K ; M+2K ; M+3Na ; 2M+H+K ; M+Na+K	C18H14Cl4N2O ; C3H8NO6P ; C3H8NO6P ; C7H8O4S ; C15H15ClN2O5 ; C24H17N3O7S3 ; C7H8O4S ; C15H14O8S

215_56.22	1.600844	1.088557	2.2861	0.04531	1.3438	0.42361	152.0222	HMDB00273 ; HMDB00562 ; HMDB02209 ; HMDB14713 ; HMDB14809 ; HMDB15175 ; HMDB15231 ; HMDB15394 ; HMDB29302 ; HMDB35019 ; HMDB36347 ; HMDB37679 ; HMDB39146 ; HMDB40705 ; HMDB59729	Thymidine ; Creatinine ; Equol ; Fenoprofen ; Cefixime ; Thalidomide ; Flucytosine ; Telbivudine ; 1-Phenyl-67-dihydroxy-isochroman ; Phenethyl salicylate ; 23-Dihydroxy-13-diphenyl-1-propanone ; Guaicyl phenylacetate ; Citrubuntin ; Allixin ; 34-Methylenesebacic acid	M+Na+K ; M+K ; M+Na+K ; M+Na+K ; M+3H ; M+2Na ; M+Na ; M+Na+K ; M+Na+K ; M+Na+K ; M+Na+K ; M+Na+K ; M+Na+K ; M+2K ; M+2K	C10H14N2O5 ; C4H7N3O ; C15H14O3 ; C15H14O3 ; C16H15N5O7S2 ; C13H10N2O4 ; C4H4FN3O ; C10H14N2O5 ; C15H14O3 ; C15H14O3 ; C15H14O3 ; C15H14O3 ; C15H14O3 ; C12H18O4 ; C12H18O4
7463_54.5	1.600698	1.102399	2.4253	0.035738	1.4469	0.42361	437.8437	HMDB02728	Thyroxine sulfate	M+H+NH4	C15H11I4NO7S
2516_197.6	1.600639	1.047906	-2.3969	0.037514	1.4258	0.42361	372.2825	HMDB06738 ; HMDB07355 ; HMDB07380 ; HMDB07381 ; HMDB07408 ; HMDB07465 ; HMDB07494 ; HMDB07522 ; HMDB07551 ; HMDB07579 ; HMDB07607 ; HMDB07634 ; HMDB07635 ; HMDB07661 ; HMDB07662 ; HMDB07672 ; HMDB07688 ; HMDB07698 ; HMDB07716 ; HMDB07745 ; HMDB07784 ; HMDB07831 ; HMDB13078 ; HMDB13645 ; HMDB36289 ; HMDB56080 ; HMDB56085 ; HMDB56108 ; HMDB56226 ; HMDB56242 ; HMDB56257 ; HMDB56263 ; HMDB56277 ; HMDB56323 ; HMDB56350 ; HMDB56351	CE(19:0) ; DG(18:4(6Z9Z12Z15Z)24:1(15Z)0:0) ; DG(20:022:5(4Z7Z10Z13Z16Z)0:0) ; DG(20:022:5(7Z10Z13Z16Z19Z)0:0) ; DG(20:1(11Z)22:4(7Z10Z13Z16Z)0:0) ; DG(20:3(5Z8Z11Z)22:2(13Z16Z)0:0) ; DG(20:3(8Z11Z14Z)22:2(13Z16Z)0:0) ; DG(20:4(5Z8Z11Z14Z)22:1(13Z)0:0) ; DG(20:4(8Z11Z14Z17Z)22:1(13Z)0:0) ; DG(20:5(5Z8Z11Z14Z17Z)22:00:0) ; DG(22:020:5(5Z8Z11Z14Z17Z)0:0) ; DG(22:1(13Z)20:4(5Z8Z11Z14Z)0:0) ; DG(22:1(13Z)20:4(8Z11Z14Z17Z)0:0) ; DG(22:2(13Z16Z)20:3(5Z8Z11Z)0:0) ; DG(22:2(13Z16Z)20:3(8Z11Z14Z)0:0) ; DG(22:2(13Z16Z)22:6(4Z7Z10Z13Z16Z19Z)0:0) ; DG(22:4(7Z10Z13Z16Z)20:1(11Z)0:0) ; DG(22:4(7Z10Z13Z16Z)22:4(7Z10Z13Z16Z)0:0) ; DG(22:5(4Z7Z10Z13Z16Z)20:00:0) ; DG(22:5(7Z10Z13Z16Z19Z)20:00:0) ; DG(22:6(4Z7Z10Z13Z16Z19Z)22:2(13Z16Z)0:0) ; DG(24:1(15Z)18:4(6Z9Z12Z15Z)0:0) ; Stearoylethanolamide ; NN-Dimethylsphingosine ; Campesteryl stearate ; DG(20:00:022:5n6) ; DG(20:00:022:5n3) ; DG(22:00:020:5n3) ; DG(20:1n90:022:4n6) ; DG(20:3n90:022:2n6) ; DG(22:1n90:020:4n6) ; DG(22:1n90:020:4n3) ; DG(24:1n90:018:4n3) ; DG(20:3n60:022:2n6) ; DG(22:2n60:022:6n3) ; DG(22:4n60:022:4n6)	M+2K ; M+2Na ; M+2Na ; M+2Na ; M+2Na ; M+2Na ; M+2Na ; M+2Na ; M+2Na ; M+2Na ; M+2Na ; M+2Na ; M+2Na ; M+2Na ; M+2Na ; M+H+Na ; M+2Na ; M+H+Na ; M+2Na ; M+2Na ; M+H+Na ; M+2Na ; M+2Na-H ; M+2Na-H ; M+2K ; M+2Na ; M+2Na ; M+2Na ; M+2Na ; M+2Na ; M+2Na ; M+2Na ; M+2Na ; M+2Na ; M+H+Na ; M+H+Na	C46H82O2 ; C45H78O5 ; C45H78O5 ; C45H78O5 ; C45H78O5 ; C45H78O5 ; C45H78O5 ; C45H78O5 ; C45H78O5 ; C45H78O5 ; C45H78O5 ; C45H78O5 ; C45H78O5 ; C45H78O5 ; C45H78O5 ; C47H76O5 ; C45H78O5 ; C47H76O5 ; C45H78O5 ; C45H78O5 ; C47H76O5 ; C45H78O5 ; C20H41NO2 ; C20H41NO2 ; C46H82O2 ; C45H78O5 ; C45H78O5 ; C45H78O5 ; C45H78O5 ; C45H78O5 ; C45H78O5 ; C45H78O5 ; C45H78O5 ; C45H78O5 ; C47H76O5 ; C47H76O5

4085_55.6	1.600216	1.250686	2.4712	0.033035	1.481	0.42361	473.8834	HMDB10497 ; HMDB36351 ;<br>HMDB42089 ; HMDB42293 ;<br>HMDB42298 ; HMDB42327 ;<br>HMDB42557 ; HMDB42564 ;<br>HMDB42728 ; HMDB42767 ;<br>HMDB42774 ; HMDB42849 ;<br>HMDB42878 ; HMDB42901 ;<br>HMDB44049 ; HMDB44562 ;<br>HMDB47739 ; HMDB47744 ;<br>HMDB47789 ; HMDB47899 ;<br>HMDB47920 ; HMDB47926 ;<br>HMDB47948 ; HMDB47971 ;<br>HMDB48162 ; HMDB48207 ;<br>HMDB48278 ; HMDB48279 ;<br>HMDB48300 ; HMDB48431 ;<br>HMDB48578 ; HMDB48584 ;<br>HMDB48605 ; HMDB48941 ;<br>HMDB49234 ; HMDB49855 ;<br>HMDB52845 ; HMDB52852	TG(18:3(9Z12Z15Z)14:018:3(9Z12Z15Z))iso3 ; Methyl 2-propenyl<br>pentasulfide ; TG(14:014:022:6(4Z7Z10Z13Z16Z19Z)) ;<br>TG(14:014:1(9Z)22:5(4Z7Z10Z13Z16Z)) ;<br>TG(14:014:1(9Z)22:5(7Z10Z13Z16Z19Z)) ;<br>TG(14:016:1(9Z)20:5(5Z8Z11Z14Z17Z)) ;<br>TG(14:018:3(6Z9Z12Z)18:3(6Z9Z12Z)) ;<br>TG(14:018:3(6Z9Z12Z)18:3(9Z12Z15Z)) ;<br>TG(14:022:5(4Z7Z10Z13Z16Z)14:1(9Z)) ;<br>TG(14:018:3(9Z12Z15Z)18:3(6Z9Z12Z)) ;<br>TG(14:018:3(9Z12Z15Z)18:3(9Z12Z15Z)) ;<br>TG(14:020:5(5Z8Z11Z14Z17Z)16:1(9Z)) ;<br>TG(14:022:5(7Z10Z13Z16Z19Z)14:1(9Z)) ;<br>TG(14:022:6(4Z7Z10Z13Z16Z19Z)14:0) ;<br>TG(16:014:1(9Z)20:5(5Z8Z11Z14Z17Z)) ;<br>TG(16:020:5(5Z8Z11Z14Z17Z)14:1(9Z)) ;<br>TG(14:1(9Z)14:022:5(4Z7Z10Z13Z16Z)) ;<br>TG(14:1(9Z)14:022:5(7Z10Z13Z16Z19Z)) ;<br>TG(14:1(9Z)16:020:5(5Z8Z11Z14Z17Z)) ;<br>TG(14:1(9Z)14:1(9Z)22:4(7Z10Z13Z16Z)) ;<br>TG(14:1(9Z)16:1(9Z)20:4(5Z8Z11Z14Z)) ;<br>TG(14:1(9Z)16:1(9Z)20:4(8Z11Z14Z17Z)) ;<br>TG(14:1(9Z)18:1(11Z)18:4(6Z9Z12Z15Z)) ;<br>TG(14:1(9Z)18:1(9Z)18:4(6Z9Z12Z15Z)) ;<br>TG(14:1(9Z)20:4(5Z8Z11Z14Z)16:1(9Z)) ;<br>TG(14:1(9Z)22:4(7Z10Z13Z16Z)14:1(9Z)) ;<br>TG(14:1(9Z)18:4(6Z9Z12Z15Z)18:1(11Z)) ;<br>TG(14:1(9Z)18:4(6Z9Z12Z15Z)18:1(9Z)) ;<br>TG(14:1(9Z)20:4(8Z11Z14Z17Z)16:1(9Z)) ;	M+3ACN+2H ; 2M+ACN+H ;<br>M+3ACN+2H ; M+3ACN+2H ;<br>M+3ACN+2H ; M+3ACN+2H ;<br>M+3ACN+2H ; M+3ACN+2H ;<br>M+3ACN+2H ; M+3ACN+2H ;<br>M+3ACN+2H ; M+3ACN+2H ;<br>M+3ACN+2H ; M+3ACN+2H ;<br>M+3ACN+2H ; M+3ACN+2H ;<br>M+3ACN+2H ; M+3ACN+2H ;<br>M+3ACN+2H ; M+3ACN+2H ;<br>M+3ACN+2H ; M+3ACN+2H ;<br>M+3ACN+2H ; M+3ACN+2H ;<br>M+3ACN+2H ; M+3ACN+2H ;<br>M+3ACN+2H ; M+3ACN+2H ;<br>M+3ACN+2H ; M+3ACN+2H ;<br>M+3ACN+2H ; M+3ACN+2H ;<br>M+3ACN+2H ; M+3ACN+2H ;<br>M+3ACN+2H ; M+3ACN+2H ;<br>M+3ACN+2H ; M+3ACN+2H	C53H90O6 ; C4H8S5 ; C53H90O6 ;<br>C53H90O6 ; C53H90O6 ; C53H90O6 ;<br>C53H90O6 ; C53H90O6 ; C53H90O6 ;<br>C53H90O6 ; C53H90O6 ; C53H90O6 ;<br>C53H90O6 ; C53H90O6 ; C53H90O6 ;<br>C53H90O6 ; C53H90O6 ; C53H90O6 ;<br>C53H90O6 ; C53H90O6 ; C53H90O6 ;<br>C53H90O6 ; C53H90O6 ; C53H90O6 ;<br>C53H90O6 ; C53H90O6 ; C53H90O6 ;<br>C53H90O6 ; C53H90O6 ; C53H90O6 ;<br>C53H90O6 ; C53H90O6 ; C53H90O6 ;<br>C53H90O6 ; C53H90O6 ; C53H90O6 ;<br>C53H90O6 ; C53H90O6
0966_256.6	1.600106	0.730824	2.2431	0.048746	1.3121	0.42643	524.0561	HMDB00232 ; HMDB00618 ;<br>HMDB00868 ; HMDB01489 ;<br>HMDB01548 ; HMDB06534 ;<br>HMDB11734 ; HMDB12195 ;<br>HMDB33161 ; HMDB59646 ;<br>HMDB01178 ; HMDB12276	Quinolinic acid ; D-Ribulose 5-phosphate ; Xylulose 5-phosphate ; Ribose 1-phosphate ; D-Ribose 5-phosphate ; D-Xylulose 1-phosphate ; D-Arabinose 5-phosphate ; Beta-L-arabinose 1-phosphate ; 26-Pyridinedicarboxylic acid ; nicotinate beta-D-ribonucleotide ; Adenosine diphosphate ribose ; Phosphoribosyl-AMP	3M+Na ; 2M+ACN+Na ; 2M+ACN+Na ;<br>2M+ACN+Na ; 2M+ACN+Na ;<br>2M+ACN+Na ; 2M+ACN+Na ;<br>2M+ACN+Na ; 3M+Na ; 3M+H+K ; M+H-2H2O ; M+H-2H2O	C7H5NO4 ; C5H11O8P ; C5H11O8P ;<br>C5H11O8P ; C5H11O8P ; C5H11O8P ;<br>C5H11O8P ; C5H11O8P ; C7H5NO4 ;<br>C11H15NO9P ; C15H23N5O14P2 ;<br>C15H23N5O14P2
8589_211.7	1.599484	1.215026	2.4042	0.037047	1.4313	0.42361	165.0009	HMDB03537 ; HMDB15257 ;<br>HMDB31004 ; HMDB33979	2-Deoxyinosine triphosphate ; Anisindione ; Diatretin 2 ; 7-Hydroxy-2-methylisoflavone	M+3H ; M+2K ; 2M+H+K ; M+2K	C10H15N4O13P3 ; C16H12O3 ;<br>C8H3NO2 ; C16H12O3

76439_47.44	1.599387	1.297229	-2.4027	0.037146	1.4301	0.42361	400.7376	HMDB01849 ; HMDB07943 ; HMDB08231 ; HMDB08844 ; HMDB08937 ; HMDB08938 ; HMDB08968 ; HMDB08979 ; HMDB08997 ; HMDB09028 ; HMDB09029 ; HMDB09061 ; HMDB09062 ; HMDB09093 ; HMDB09104 ; HMDB09124 ; HMDB09125 ; HMDB09135 ; HMDB09136 ; HMDB09157 ; HMDB09158 ; HMDB09168 ; HMDB09169 ; HMDB09189 ; HMDB09199 ; HMDB09200 ; HMDB09320 ; HMDB09327 ; HMDB09353 ; HMDB09360 ; HMDB09385 ; HMDB09391 ; HMDB09392 ; HMDB09418 ; HMDB09424 ; HMDB09425 ; HMDB09456 ; HMDB09580 ; HMDB09683 ; HMDB15115 ; HMDB29829 ; HMDB41954	Propranolol ; PC(15:018:4(6Z9Z12Z15Z)) ; PC(18:4(6Z9Z12Z15Z)15:0) ; PE(14:022:4(7Z10Z13Z16Z)) ; PE(16:020:4(5Z8Z11Z14Z)) ; PE(16:020:4(8Z11Z14Z17Z)) ; PE(16:1(9Z)20:3(5Z8Z11Z)) ; PE(16:1(9Z)22:6(4Z7Z10Z13Z16Z19Z)) ; PE(18:018:4(6Z9Z12Z15Z)) ; PE(18:1(11Z)18:3(6Z9Z12Z)) ; PE(18:1(11Z)18:3(9Z12Z15Z)) ; PE(18:1(9Z)18:3(6Z9Z12Z)) ; PE(18:1(9Z)18:3(9Z12Z15Z)) ; PE(18:2(9Z12Z)18:2(9Z12Z)) ; PE(18:2(9Z12Z)20:5(5Z8Z11Z14Z17Z)) ; PE(18:3(6Z9Z12Z)18:1(11Z)) ; PE(18:3(6Z9Z12Z)18:1(9Z)) ; PE(18:3(6Z9Z12Z)20:4(5Z8Z11Z14Z)) ; PE(18:3(6Z9Z12Z)20:4(8Z11Z14Z17Z)) ; PE(18:3(9Z12Z15Z)18:1(11Z)) ; PE(18:3(9Z12Z15Z)18:1(9Z)) ; PE(18:3(9Z12Z15Z)20:4(5Z8Z11Z14Z)) ; PE(18:3(9Z12Z15Z)20:4(8Z11Z14Z17Z)) ; PE(18:4(6Z9Z12Z15Z)18:0) ; PE(18:4(6Z9Z12Z15Z)20:3(5Z8Z11Z)) ; PE(18:4(6Z9Z12Z15Z)20:3(8Z11Z14Z)) ; PE(20:3(5Z8Z11Z)16:1(9Z)) ; PE(20:3(5Z8Z11Z)18:4(6Z9Z12Z15Z)) ; PE(20:3(8Z11Z14Z)16:1(9Z)) ; PE(20:3(8Z11Z14Z)18:4(6Z9Z12Z15Z)) ; PE(20:4(5Z8Z11Z14Z)16:0) ; PE(20:4(5Z8Z11Z14Z)18:3(6Z9Z12Z)) ; PE(20:4(5Z8Z11Z14Z)18:3(9Z12Z15Z)) ; PE(20:4(8Z11Z14Z17Z)16:0) ; PE(20:4(8Z11Z14Z17Z)18:3(6Z9Z12Z)) ; PE(20:4(8Z11Z14Z17Z)18:3(9Z12Z15Z)) ; PE(20:5(5Z8Z11Z14Z17Z)18:2(9Z12Z)) ; PE(22:4(7Z10Z13Z16Z)14:0) ; PE(22:6(4Z7Z10Z13Z16Z19Z)16:1(9Z)) ; Ramelteon ; Norchalciporyl propionate ; Norbutorphanol	3M+H+Na ; M+Na+K ; M+Na+K ; M+Na+K ; M+Na+K ; M+Na+K ; M+Na+K ; M+H+K ; M+Na+K ; M+Na+K ; M+Na+K ; M+Na+K ; M+Na+K ; M+Na+K ; M+H+K ; M+Na+K ; M+Na+K ; M+H+K ; M+H+K ; M+Na+K ; M+Na+K ; M+H+K ; M+H+K ; M+Na+K ; M+H+K ; M+H+K ; M+Na+K ; M+H+K ; M+Na+K ; M+H+K ; M+Na+K ; M+H+K ; M+H+K ; M+Na+K ; M+H+K ; M+H+K ; M+H+K ; M+Na+K ; M+H+K ; 3M+H+Na ; 3M+H+Na ; 3M+H+Na	C16H21NO2 ; C41H74NO8P ; C41H74NO8P ; C41H74NO8P ; C41H74NO8P ; C41H74NO8P ; C41H74NO8P ; C43H72NO8P ; C41H74NO8P ; C41H74NO8P ; C41H74NO8P ; C41H74NO8P ; C41H74NO8P ; C41H74NO8P ; C43H72NO8P ; C41H74NO8P ; C41H74NO8P ; C43H72NO8P ; C43H72NO8P ; C41H74NO8P ; C41H74NO8P ; C43H72NO8P ; C43H72NO8P ; C41H74NO8P ; C43H72NO8P ; C43H72NO8P ; C41H74NO8P ; C43H72NO8P ; C41H74NO8P ; C43H72NO8P ; C41H74NO8P ; C43H72NO8P ; C43H72NO8P ; C41H74NO8P ; C43H72NO8P ; C43H72NO8P ; C43H72NO8P ; C41H74NO8P ; C43H72NO8P ; C16H21NO2 ; C16H21NO2 ; C16H21NO2
2625_264.5	1.599199	0.905321	-2.4308	0.035405	1.4509	0.42361	NA	NA	NA	NA	NA
5922_39.74	1.599167	1.158196	2.3107	0.043458	1.3619	0.42361	161.1366	HMDB15651 ; HMDB35234 ; HMDB35536 ; HMDB35537 ; HMDB37327 ; HMDB37328	Isometheptene ; 1416-Nonacosanedione ; 1012-Nonacosanedione ; 68-Nonacosanedione ; 810-Nonacosanedione ; 1214-Nonacosanedione	2M+H+K ; M+H+2Na ; M+H+2Na ; M+H+2Na ; M+H+2Na ; M+H+2Na	C9H19N ; C29H56O2 ; C29H56O2 ; C29H56O2 ; C29H56O2 ; C29H56O2
465312_102	1.598938	0.980949	2.394	0.037699	1.4237	0.42361	241.9465	HMDB06095	3-Methylsulfinylpropyl isothiocyanate	M+2K+H	C5H9NOS2
9222_47.68	1.598878	1.253823	-2.4013	0.037234	1.4291	0.42361	524.6869	HMDB12360 ; HMDB12381 ; HMDB12391 ; HMDB12401 ; HMDB12411 ; HMDB12420 ; HMDB14853 ; HMDB61176	PS(16:020:3(8Z11Z14Z)) ; PS(18:018:3(9Z12Z15Z)) ; PS(18:1(9Z)18:2(9Z12Z)) ; PS(18:2(9Z12Z)18:1(9Z)) ; PS(18:3(9Z12Z15Z)18:0) ; PS(20:3(8Z11Z14Z)16:0) ; Paroxetine ; Phenylacetic acid-N-isopropylnortropine-ester methobromide	2M+3H ; 2M+3H ; 2M+3H ; 2M+3H ; 2M+3H ; 2M+3H ; 3M+Na+K ; 2M+H+NH4	C42H76NO10P ; C42H76NO10P ; C42H76NO10P ; C42H76NO10P ; C42H76NO10P ; C42H76NO10P ; C19H20FNO3 ; C27H34BrNO4
9498_68.26	1.598762	0.68885	-2.4312	0.035379	1.4513	0.42361	127.1059	HMDB15169	Procainamide	M+H+NH4	C13H21N3O
594_289.93	1.597469	0.968065	2.2473	0.048404	1.3151	0.42643	428.0359	HMDB00061 ; HMDB00960 ; HMDB01341 ; HMDB00785 ; HMDB00794 ; HMDB00796 ; HMDB04381 ; HMDB06268 ; HMDB06880 ; HMDB11610 ; HMDB15325 ; HMDB32930 ; HMDB59993 ; HMDB60416 ; HMDB60492	Adenosine 35-diphosphate ; dGDP ; ADP ; N-Acetyl-7-O-acetylneuraminic acid ; N-Acetyl-9-O-acetylneuraminic acid ; N-Acetyl-4-O-acetylneuraminic acid ; N-Acetylneuraminic acid 9-phosphate ; N-Acetylneuraminate 9-phosphate ; Acetyl adenylate ; 5-Taurinomethyl-2-thiouridine ; Brinzolamide ; Benzothiazole ; 5-(Hydroxyphenyl)-gamma-valerolactone-O-sulphate ; 6-Thioinosine-5-monophosphate ; N-acetyl-O-acetylneuraminate	M+H ; M+H ; M+H ; M+2K-H ; M+2K-H ; M+2K-H ; M+K ; M+K ; M+K ; 2M+Na+K ; M+2Na-H ; 3M+Na ; 3M+H+K ; M+ACN+Na ; M+2K-H	C10H15N5O10P2 ; C10H15N5O10P2 ; C10H15N5O10P2 ; C13H21NO10 ; C13H21NO10 ; C13H21NO10 ; C11H20NO12P ; C11H20NO12P ; C12H16N5O8P ; C12H19N3O8S2 ; C12H21N3O5S3 ; C7H5NS ; C11H12O6S ; C10H13N4O7PS ; C13H21NO10
7773_56.22	1.59669	1.139632	2.4781	0.03265	1.4861	0.42361	389.9218	HMDB29695	Hexacosanal	2M+H+NH4	C26H52O

6335_47.8	1.595711	1.203766	-2.4184	0.036163	1.4417	0.42361	430.6966	HMDB07118 ; HMDB07170 ; HMDB07171 ; HMDB07198 ; HMDB07226 ; HMDB07227 ; HMDB07254 ; HMDB07282 ; HMDB07311 ; HMDB07339 ; HMDB07367 ; HMDB07394 ; HMDB07395 ; HMDB07422 ; HMDB07449 ; HMDB07450 ; HMDB07478 ; HMDB07479 ; HMDB07506 ; HMDB07535 ; HMDB07678 ; HMDB37844 ; HMDB56028 ; HMDB56052 ; HMDB56058 ; HMDB56082 ; HMDB56180 ; HMDB56186 ; HMDB56199 ; HMDB56205 ; HMDB56221 ; HMDB56228 ; HMDB56282 ; HMDB56284 ; HMDB56309 ; HMDB61044	DG(16:022:4(7Z10Z13Z16Z)0:0) ; DG(18:020:4(5Z8Z11Z14Z)0:0) ; DG(18:020:4(8Z11Z14Z17Z)0:0) ; DG(18:1(11Z)20:3(8Z11Z14Z)0:0) ; DG(18:1(9Z)20:3(5Z8Z11Z)0:0) ; DG(18:1(9Z)20:3(8Z11Z14Z)0:0) ; DG(18:2(9Z12Z)20:2(11Z14Z)0:0) ; DG(18:3(6Z9Z12Z)20:1(11Z)0:0) ; DG(18:3(9Z12Z15Z)20:1(11Z)0:0) ; DG(18:4(6Z9Z12Z15Z)20:00:0) ; DG(20:018:4(6Z9Z12Z15Z)0:0) ; DG(20:1(11Z)18:3(6Z9Z12Z)0:0) ; DG(20:1(11Z)18:3(9Z12Z15Z)0:0) ; DG(20:2(11Z14Z)18:2(9Z12Z)0:0) ; DG(20:3(5Z8Z11Z)18:1(11Z)0:0) ; DG(20:3(5Z8Z11Z)18:1(9Z)0:0) ; DG(20:3(8Z11Z14Z)18:1(11Z)0:0) ; DG(20:3(8Z11Z14Z)18:1(9Z)0:0) ; DG(20:4(5Z8Z11Z14Z)18:00:0) ; DG(20:4(8Z11Z14Z17Z)18:00:0) ; DG(22:4(7Z10Z13Z16Z)16:00:0) ; N-(1-Deoxy-1-fructosyl)valine ; DG(16:00:022:4n6) ; DG(18:00:020:4n6) ; DG(18:00:020:4n3) ; DG(20:00:018:4n3) ; DG(18:1n70:020:3n9) ; DG(18:1n70:020:3n6) ; DG(18:1n90:020:3n9) ; DG(18:1n90:020:3n6) ; DG(20:1n90:018:3n6) ; DG(20:1n90:018:3n3) ; DG(18:2n60:018:2n6) ; DG(18:2n60:020:2n6) ; DG(20:2n60:020:2n6) ; Hydroxy Ritonavir	2M+3H ; 3M+H+Na ; 2M+3H ; 2M+3H ; 2M+3H ; 2M+3H ; 2M+3H ; 2M+3H ; 2M+3H ; 2M+3H ; 2M+3H ; 2M+3H ; 2M+3H ; 2M+3H ; 2M+3H ; M+3ACN+2H	C41H72O5 ; C11H21NO7 ; C41H72O5 ; C41H72O5 ; C41H72O5 ; C41H72O5 ; C41H72O5 ; C41H72O5 ; C41H72O5 ; C41H72O5 ; C41H72O5 ; C41H72O5 ; C41H72O5 ; C41H72O5 ; C41H72O5 ; C37H48N6O6S2
5633_47.4	1.595525	1.219645	-2.4134	0.036471	1.438	0.42361	504.6296	HMDB60619 ; HMDB60969	Tazarotenic acid ; desmethyladinazolam	3M+H+K ; 3M+H+K	C19H17NO2S ; C17H14ClN5
7046_51.3	1.594331	0.516745	-2.4504	0.034232	1.4656	0.42361	452.8677	HMDB00607 ; HMDB14520	Cyanocobalamin ; Trihexyphenidyl	M+3H ; 3M+2H	C63H89CoN14O14P ; C20H31NO
1183_66.5	1.594234	0.78519	-2.4396	0.034874	1.4575	0.42361	274.0921	HMDB00101 ; HMDB00118 ; HMDB00333 ; HMDB00423 ; HMDB00755 ; HMDB01983 ; HMDB02123 ; HMDB02643 ; HMDB03219 ; HMDB03254 ; HMDB04061 ; HMDB15285 ; HMDB15678 ; HMDB29232 ; HMDB29273 ; HMDB29646 ; HMDB29901 ; HMDB29935 ; HMDB29952 ; HMDB30809 ; HMDB31360 ; HMDB32916 ; HMDB33624 ; HMDB34751 ; HMDB36431 ; HMDB36610 ; HMDB37274 ; HMDB37808 ; HMDB37809 ; HMDB38117 ; HMDB38556 ; HMDB38662 ; HMDB39586 ; HMDB39800 ; HMDB40150 ; HMDB40197 ; HMDB40258 ; HMDB40580 ; HMDB41270 ; HMDB41566 ; HMDB59763 ; HMDB60676 ; HMDB60701 ; HMDB60702	Deoxyadenosine ; Homovanillic acid ; Isohomovanillic acid ; 34-Dihydroxyhydrocinnamic acid ; Hydroxyphenyllactic acid ; 5-Deoxyadenosine ; 137-Trimethyluric acid ; 3-(3-Hydroxyphenyl)-3-hydroxypropanoic acid ; Sedoheptulose ; Muramic acid ; 3-Methoxy-4-hydroxyphenylglycolaldehyde ; Thiamylal ; Nepafenac ; 3-Hydroxyphenyllactate ; 26-Dimethoxybenzoic acid ; 26-Dihydroxy-4-methoxyacetophenone ; L-Galacto-2-heptulose ; D-Manno-2-heptulose ; D-altro-D-manno-Heptose ; (2R3S)-Piscidic acid ; L-cis-Cyclo(aspartylphenylalanyl) ; 2-(Methylthiomethyl)furan ; ()-2-Hydroxy-3-(2-hydroxyphenyl)propanoic acid ; Ligustroside ; 139-Trimethyluric acid ; 2-Methyl-3-(methylthio)furan ; Maltol propionate ; Hexahydro-24-dimethylspiro13-dithiolo45-cfuran-23(2H)-furan ; Hexahydro-23alpha-dimethylspiro13-dithiolo45-bfuran-23(2H)-furan ; 6-O-Oleuropeoylsucrose ; xl-Dihydro-2-methyl-3(2H)-thiophenone ; N-(1-Deoxy-1-fructosyl)alanine ; 2-(1-Mercaptoethyl)furan ; 5-Methyl-2-furanmethanethiol ; Dihydro-5-methyl-2(3H)-thiophenone ; 25-Dimethyl-3-furanthiol ; xi-2-Acetylthietane ; Dihydro-2-methyl-3(2H)-furanthione ; 24-Dihydroxy-6-methoxyacetophenone ; 2-Methyl-5-(methylthio)furan ; 34-Dimethoxybenzoic acid ; 10-Hydroxycarbazepine ; (R)-MHD ; (S)-MHD	M+Na ; 3M+2H ; 3M+2H ; 3M+2H ; 3M+2H ; M+Na ; M+ACN+Na ; 3M+2H ; M+ACN+Na ; M+Na ; 3M+2H ; 2M+H+K ; 2M+H+K ; 3M+2H ; 3M+2H ; 3M+2H ; M+ACN+Na ; M+ACN+Na ; M+ACN+Na ; M+NH4 ; 2M+H+Na ; 2M+NH4 ; 3M+2H ; M+H+Na ; M+ACN+Na ; 2M+NH4 ; 3M+2H ; M+ACN+H ; M+ACN+H ; M+H+K ; 2M+ACN+H ; M+Na ; 2M+NH4 ; 2M+NH4 ; 2M+ACN+H ; 2M+NH4 ; 2M+ACN+H ; 2M+ACN+H ; 3M+2H ; 2M+NH4 ; 3M+2H ; 2M+H+K ; 2M+H+K ; 2M+H+K	C10H13N5O3 ; C9H10O4 ; C9H10O4 ; C9H10O4 ; C9H10O4 ; C10H13N5O3 ; C8H10N4O3 ; C9H10O4 ; C7H14O7 ; C9H17NO7 ; C9H10O4 ; C12H18N2O2S ; C15H14N2O2 ; C9H10O4 ; C9H10O4 ; C9H10O4 ; C7H14O7 ; C7H14O7 ; C7H14O7 ; C11H12O7 ; C13H14N2O4 ; C6H8OS ; C9H10O4 ; C25H32O12 ; C8H10N4O3 ; C6H8OS ; C9H10O4 ; C10H16O2S2 ; C10H16O2S2 ; C22H36O13 ; C5H8OS ; C9H17NO7 ; C6H8OS ; C6H8OS ; C5H8OS ; C6H8OS ; C5H8OS ; C5H8OS ; C9H10O4 ; C6H8OS ; C9H10O4 ; C15H14N2O2 ; C15H14N2O2 ; C15H14N2O2
2037_263.2	1.594016	1.092769	2.2829	0.045557	1.3414	0.42361	NA	NA	NA	NA	NA

23738_54.4!	1.592711	0.715309	2.3268	0.042282	1.3738	0.42361	263.0024	HMDB14815 ; HMDB35227 ; HMDB37323 ; HMDB40192	Isoflurophate ; Hydroxypropanedioic acid ; 23-Dihydro-5577-tetrahydroxy-2-(4-hydroxyphenyl)38-bi-4H-1-benzopyran-44-dione ; N-Nitrosothiazolidine-4-carboxylic acid	M+2K+H ; 2M+Na ; M+2K ; 3M+H+K	C6H14FO3P ; C3H4O5 ; C24H16O9 ; C4H6N2O3S
07219_104.!	1.592412	0.375221	-2.3111	0.043425	1.3623	0.42361	393.1197	HMDB38066 ; HMDB37464 ; HMDB00344 ; HMDB05079 ; HMDB05832 ; HMDB06254 ; HMDB12451 ; HMDB12452 ; HMDB14577 ; HMDB29329 ; HMDB31773 ; HMDB31858 ; HMDB33289 ; HMDB33522 ; HMDB33768 ; HMDB34630 ; HMDB34631 ; HMDB34691 ; HMDB35710 ; HMDB36147 ; HMDB36725 ; HMDB36747 ; HMDB36750 ; HMDB36762 ; HMDB36812 ; HMDB37233 ; HMDB38885 ; HMDB39442 ; HMDB39484 ; HMDB41056 ; HMDB41057 ; HMDB41346 ; HMDB60093 ; HMDB60764 ; HMDB61095	Garcimangosone D ; Spinosin B ; 2-Methoxyestradiol-3-methylether ; 15-Deoxy-d-1214-PGJ2 ; 6b-Hydroxymethandienone ; 4-Hydroxyretinoic acid ; all-trans-56-Epoxyretinoic acid ; all-trans-18-Hydroxyretinoic acid ; Prochlorperazine ; Gravacridonolchlorine ; Phytocassane E ; 4-Methyl-5-vinylthiazole ; Aurasperone E ; Oxopurpureine ; Fonsecinone B ; (4Z9a)-9-(3-Methyl-2-butenoyloxy)-410(14)-oplopadien-3-one ; (4Z9a)-9-Angeloyloxy-410(14)-oplopadien-3-one ; Yucalexin B9 ; Cafestol ; Furanojaponin ; ent-17-Oxo-15-kauren-19-oic acid ; ent-7-Oxo-8(14)15-pimaradien-19-oic acid ; Momilactone C ; ent-15-Oxo-16-kauren-19-oic acid ; 7-Oxo-815-isopimaradien-18-oic acid ; Rosmaridiphenol ; Trilobinone ; ent-1516-Epoxy-1(10)13(16)14-halimatrien-19-oic acid ; Gibberellin A12 7-aldehyde ; Phytocassane A ; Phytocassane D ; 2-Pyridinemethanethiol ; rac-56-Epoxy-retinoate ; 4-Hydroxy-estazolam ; 18-Hydroxyretinoic acid	M+H ; M+2H ; M+2K-H ; M+2K-H ; M+2K-H ; M+2K-H ; M+2K-H ; M+2K-H ; 2M+H+K ; M+NH4 ; M+2K-H ; 3M+NH4 ; 2M+3H ; 2M+H+Na ; 2M+3H ; M+2K-H ; M+2K-H ; M+2K-H ; M+2K-H ; M+2K-H ; M+2K-H ; M+2K-H ; M+2K-H ; M+2K-H ; M+2K-H ; M+2K-H ; M+2K-H ; M+2K-H ; M+2K-H ; M+2K-H ; 3M+NH4 ; M+2K-H ; M+2ACN+H ; M+2K-H	C19H20O9 ; C38H40O18 ; C20H28O3 ; C20H28O3 ; C20H28O3 ; C20H28O3 ; C20H28O3 ; C20H28O3 ; C20H24ClN3S ; C19H18ClNO5 ; C20H28O3 ; C6H7NS ; C32H28O11 ; C21H19NO6 ; C32H28O11 ; C20H28O3 ; C20H28O3 ; C20H28O3 ; C20H28O3 ; C20H28O3 ; C20H28O3 ; C20H28O3 ; C20H28O3 ; C20H28O3 ; C20H28O3 ; C20H28O3 ; C20H28O3 ; C20H28O3 ; C20H28O3 ; C20H28O3 ; C6H7NS ; C20H28O3 ; C16H11ClN4O ; C20H28O3
03327_256.!	1.592328	0.513238	-2.6333	0.025018	1.6017	0.42361	137.0593	HMDB00209 ; HMDB02340 ; HMDB03767 ; HMDB29635 ; HMDB29686 ; HMDB31459 ; HMDB32400 ; HMDB32568 ; HMDB32603 ; HMDB33128 ; HMDB33766 ; HMDB33968 ; HMDB38176 ; HMDB40638 ; HMDB40733 ; HMDB41485 ; HMDB04810 ; HMDB30526 ; HMDB32847 ; HMDB33800 ; HMDB34435 ; HMDB35035 ; HMDB41660 ; HMDB41669 ; HMDB41670 ; HMDB41701 ; HMDB02012 ; HMDB06853 ; HMDB12550 ; HMDB13785 ; HMDB30810 ; HMDB30899 ; HMDB31920 ; HMDB32116 ; HMDB33303 ; HMDB35095 ; HMDB35168 ; HMDB35404 ; HMDB35405 ; HMDB36665 ; HMDB36666 ; HMDB36944 ; HMDB36970 ; HMDB37241 ; HMDB37242 ; HMDB37299 ; HMDB37815 ; HMDB38746 ; HMDB38804 ; HMDB40757 ; HMDB40758 ; HMDB41226 ; HMDB41279 ; HMDB41348 ; HMDB41496 ; HMDB59992 ;	Phenylacetic acid ; 2-Methylbenzoic acid ; 4-Hydroxyphenylacetaldehyde ; 4-Methylbenzoic acid ; 4-Methoxybenzaldehyde ; 3-Methoxybenzaldehyde ; 3-(5-Methyl-2-furyl)prop-2-enal ; 2-Hydroxyacetophenone ; 2-Hydroxy-4-methylbenzaldehyde ; 4-(2-Furanyl)-3-buten-2-one ; 2-Methoxybenzaldehyde ; Methyl benzoate ; alpha-Methyl-2-furanacrolein ; 3-(3-Furanyl)-2-methyl-2-propenal ; Phenyl acetate ; Benzyl formate ; 5C-aglycone ; Isovestitol ; 12-Bis(4-hydroxy-3-methoxyphenyl)ethylene ; 246-Trimethoxybenzophenone ; 1-(26-Dihydroxy-4-methoxyphenyl)-3-phenyl-1-propanone ; Marminal ; 3-O-Methylequol ; 47-Dihydroxy-3-methoxyisoflavan ; 47-Dihydroxy-6-methoxyisoflavan ; 6-O-Methylequol ; Ubiquinone-1 ; 3-Butyn-1-al ; 12-Oxo-20-carboxy-leukotriene B4 ; Furan ; Porson ; Eremopetasitenin A1 ; 9-Hydroxycalabaxanthone ; Helinorbisabone ; Anofinic acid ; 7-Ethoxy-4-methyl-2H-1-benzopyran-2-one ; Cinncassiol C2 ; Isogingerenone B ; Gingerenone B ; Blumealactone A ; Blumealactone B ; Gibberellin A123 ; Garcimangosone B ; 89-Dihydro-5-hydroxy-8-(1-hydroxy-1-methylethyl)-6-(2-methyl-1-oxopropyl)-4-phenyl-2H-furo23-h-1-benzopyran-2-one ; Mammea AAC cyclo F ; 68-Dihydroxy-17-diprenylxanthone-2-carboxylic acid ; 5-(Methylthio)-2-(methylthio)methyl-2-pentenal ; Gibberellin A74 ; 34-Dihydro-6-hydroxy-2578-tetramethyl-2H-1-benzopyran-2-carboxylic acid ; 3b8a-Dihydroxy-6b-angeloyloxy-7(11)-eremophilen-128-olide ; 3b8b-Dihydroxy-6b-angeloyloxy-7(11)-eremophilen-128-olide ; 3b9b-Dihydroxy-6b-angeloyloxy-7(11)-eremophilen-128b-olide ; 3b-Hydroxy-6b-(23-epoxy-2-methylbutanoyloxy)-7(11)-eremophilen-128b-olide ; Gibberellin A100 ; Gibberellin A97 ; 5-(4-Hydroxyphenyl)-gamma-valerolactone-4-O-glucuronide ; Melatonin glucuronide	M+H ; M+H ; M+H ; M+H ; M+H ; M+H ; M+H ; M+H ; M+H ; M+H ; M+H ; M+H ; M+H ; M+H ; M+H ; M+H ; M+2H ; M+2H ; M+2H ; M+2H ; M+2H ; M+2H ; M+2H ; M+2H ; M+2H ; M+2H ; M+H+Na ; 2M+H ; M+H+2Na ; 2M+H ; M+2H+Na ; M+H+2Na ; M+3H ; M+H+Na ; 2M+3H ; 2M+3H ; M+H+2Na ; M+2H+Na ; M+2H+Na ; M+H+2Na ; M+H+2Na ; M+H+2Na ; M+3H ; M+3H ; M+3H ; M+3H ; M+2ACN+2H ; M+H+2Na ; M+H+Na ; M+H+2Na ; M+H+2Na ; M+H+2Na ; M+H+2Na ; M+H+2Na ; M+H+2Na ; M+H+2Na ; M+3H	C8H8O2 ; C8H8O2 ; C8H8O2 ; C8H8O2 ; C8H8O2 ; C8H8O2 ; C8H8O2 ; C8H8O2 ; C8H8O2 ; C8H8O2 ; C8H8O2 ; C8H8O2 ; C8H8O2 ; C8H8O2 ; C8H8O2 ; C8H8O2 ; C16H16O4 ; C16H16O4 ; C16H16O4 ; C16H16O4 ; C16H16O4 ; C16H16O4 ; C16H16O4 ; C16H16O4 ; C16H16O4 ; C16H16O4 ; C14H18O4 ; C4H4O ; C20H28O6 ; C4H4O ; C22H26O6 ; C20H28O6 ; C24H24O6 ; C14H18O4 ; C12H12O3 ; C12H12O3 ; C20H28O6 ; C22H26O6 ; C22H26O6 ; C20H28O6 ; C20H28O6 ; C20H28O6 ; C24H24O6 ; C24H24O6 ; C24H24O6 ; C24H24O6 ; C8H14OS2 ; C20H28O6 ; C14H18O4 ; C20H28O6 ; C20H28O6 ; C20H28O6 ; C20H28O6 ; C20H28O6 ; C20H28O6 ; C20H28O6 ; C19H24N2O8

48195_197.	1.591918	1.035952	-2.4774	0.032688	1.4856	0.42361	215.5507	HMDB13855 ; HMDB30257 ; HMDB38952 ; HMDB39526 ; HMDB61128	Sulfamethoxazole N1-glucuronide ; Erysothiopine ; Niazicin A ; Sarafloxacin ; 5-Hydroxy 6-methoxy duloxetine sulfate	M+2H ; M+H+Na ; M+2Na ; M+2Na ; M+H+Na	C16H19N3O9S ; C19H21NO7S ; C17H23NO7S ; C20H17F2N3O3 ; C19H21NO7S
2341_45.9	1.59167	1.265561	-2.3355	0.041658	1.3803	0.42361	598.6142	HMDB29710	DIMBOA-Glc	3M+2K	C15H19NO10
2879_75.3	1.59046	0.876348	2.3416	0.041228	1.3848	0.42361	438.1373	HMDB00291 ; HMDB02085 ; HMDB02376 ; HMDB03503 ; HMDB14383 ; HMDB14902 ; HMDB29170 ; HMDB33836 ; HMDB34207 ; HMDB34942 ; HMDB41662	Vanillylmandelic acid ; Syringic acid ; Tricrocin ; 3-(34-Dihydroxyphenyl)lactic acid ; Nevirapine ; Mometasone ; 3-Hydroxy-4-methoxymandelate ; Ethyl gallate ; 6-Apiosyllotaustralin ; Geniposidic acid ; 34-O-Dimethylgallic acid	2M+ACN+H ; 2M+ACN+H ; M+Na+K ; 2M+ACN+H ; 3M+2K ; 2M+H+Na ; 2M+ACN+H ; 2M+ACN+H ; M+2Na-H ; M+ACN+Na ; 2M+ACN+H	C9H10O5 ; C9H10O5 ; C38H54O19 ; C9H10O5 ; C15H14N4O ; C22H28Cl2O4 ; C9H10O5 ; C9H10O5 ; C16H27NO10 ; C16H22O10 ; C9H10O5
1383_47.7	1.58986	1.231803	-2.4048	0.037008	1.4317	0.42361	NA	NA	NA	NA	NA
8888_107.	1.589665	1.125533	-2.4692	0.033151	1.4795	0.42361	185.4959	HMDB15403	Lumiracoxib	M+2K	C15H13ClFNO2
1777_45.3	1.588343	0.628693	2.3616	0.039843	1.3996	0.42361	266.8418	HMDB07925 ; HMDB08174 ; HMDB08207 ; HMDB08238 ; HMDB08239 ; HMDB08723 ; HMDB33621	PC(14:1(9Z)22:6(4Z7Z10Z13Z16Z19Z)) ; PC(18:3(6Z9Z12Z)18:4(6Z9Z12Z15Z)) ; PC(18:3(9Z12Z15Z)18:4(6Z9Z12Z15Z)) ; PC(18:4(6Z9Z12Z15Z)18:3(6Z9Z12Z)) ; PC(18:4(6Z9Z12Z15Z)18:3(9Z12Z15Z)) ; PC(22:6(4Z7Z10Z13Z16Z19Z)14:1(9Z)) ; Araliacerebroside	M+2H+Na ; M+2H+Na ; M+2H+Na ; M+2H+Na ; M+2H+Na ; M+2H+Na ; M+3Na	C44H74NO8P ; C44H74NO8P ; C44H74NO8P ; C44H74NO8P ; C44H74NO8P ; C44H74NO8P ; C40H77NO10
3112_52.2	1.588257	0.790644	2.229	0.04993	1.3016	0.42721	263.1453	HMDB15468 ; HMDB28832 ; HMDB28959 ; HMDB29022 ; HMDB29126 ; HMDB30180 ; HMDB33610 ; HMDB36358 ; HMDB37643 ; HMDB38345 ; HMDB39160 ; HMDB30729 ; HMDB33295 ; HMDB33711 ; HMDB34054 ; HMDB41089	Digitoxin ; Glutamyl-Valine ; Lysyl-Proline ; Prolyl-Lysine ; Valyl-Glutamate ; Crustecdysone ; Pandamarilactam 3x ; Momordin I ; 28-Glucosyl-3b23-dihydroxy-1219(29)-ursadien-28-oate 3-arabinoside ; Alliosterol 1-rhamnoside 16-galactoside ; L-Gizzerosine ; Gravelliferone ; 7-(4-Hydroxyphenyl)-1-phenyl-4-hepten-3-one ; 5-Hydroxy-7-(4-hydroxyphenyl)-1-phenyl-3-heptanone ; Auraptene ; 4-(3-Hydroxy-7-phenyl-6-heptenyl)-12-benzenediol	M+2H+Na ; M+NH4 ; 2M+H+K ; 2M+H+K ; M+NH4 ; M+2Na ; 2M+3H2O+2H ; M+2H+Na ; M+2H+Na ; M+H+2Na ; M+Na ; M+H-2H2O ; M+H-H2O ; M+H-2H2O ; M+H-2H2O ; M+H-2H2O	C41H64O13 ; C10H17N2O5 ; C11H21N3O3 ; C11H21N3O3 ; C10H17N2O5 ; C27H44O7 ; C13H17NO3 ; C41H64O13 ; C41H64O13 ; C39H66O13 ; C11H20N4O2 ; C19H22O3 ; C19H20O2 ; C19H22O3 ; C19H22O3 ; C19H22O3
6396_191.	1.588196	0.822543	2.2928	0.044802	1.3487	0.42361	545.0666	HMDB32660 ; HMDB39638	(2Z4Z)-2-(5-Methylthio-4-penten-2-ynylidene)-16-dioxaspiro44non-3-ene ; Brassilexin	2M+2K-H ; 3M+Na	C13H14O2S ; C9H6N2S

[illegible]6623_34.73	1.587654	0.960183	-2.4304	0.035423	1.4507	0.42361	188.0707	HMDB00734 ; HMDB01218 ; HMDB02359 ; HMDB06285 ; HMDB12789 ; HMDB32759 ; HMDB32938 ; HMDB33183 ; HMDB34712 ; HMDB35200 ; HMDB35602 ; HMDB36748 ; HMDB39404 ; HMDB39405 ; HMDB39614 ; HMDB40746 ; HMDB41152 ; HMDB42003 ; HMDB00671 ; HMDB00866 ; HMDB04096 ; HMDB11621 ; HMDB13068 ; HMDB30179 ; HMDB32755 ; HMDB59765	Indoleacrylic acid ; Coumarin ; Phenylpropiolic acid ; 4-oxo-Retinoic acid ; 4-Oxo-13-cis-retinoate ; Moschamine ; Chromone ; 2-Benzofurancarboxaldehyde ; Prexanthoperol ; Yucalexin B5 ; Kahweol ; Momilactone A ; 3-Hydroxy-9-(4-hydroxyphenyl)-1H3H-naphtho18-cdpyran-1-one ; 3-Hydroxy-4-(4-hydroxyphenyl)-1H3H-naphtho18-cdpyran-1-one ; 344-Trihydroxy-55-diisopropyl-22-dimethylbiphenyl ; 12-Hydroxy-7-oxo-81113-abietatrien-18-al ; Methyl cis-p-coumarate 3-(37-dimethyl-26-octadienyl) ; Pyronaridine ; Indolelactic acid ; N-Acetyl-L-tyrosine ; 5-Methoxyindoleacetate ; Cinnamoylglycine ; Salsolinol 1-carboxylate ; Edulitine ; Methyl 1-methoxy-1H-indole-3-carboxylate ; 3-Indolehydracrylic acid	M+H ; M+ACN+H ; M+ACN+H ; M+Na+K ; M+Na+K ; M+H+Na ; M+ACN+H ; M+ACN+H ; M+Na+K ; M+Na+K ; M+Na+K ; M+Na+K ; M+2ACN+2H ; M+2ACN+2H ; M+Na+K ; M+Na+K ; M+Na+K ; M+H+2Na ; M+H-H2O ; M+H-2H2O ; M+H-H2O ; M+H-H2O ; M+H-2H2O ; M+H-H2O ; M+H-H2O ; M+H-H2O	C11H9NO2 ; C9H6O2 ; C9H6O2 ; C20H26O3 ; C20H26O3 ; C20H20N2O4 ; C9H6O2 ; C9H6O2 ; C20H26O3 ; C20H26O3 ; C20H26O3 ; C20H26O3 ; C18H12O4 ; C18H12O4 ; C20H26O3 ; C20H26O3 ; C20H26O3 ; C29H32ClN5O2 ; C11H11NO3 ; C11H13NO4 ; C11H11NO3 ; C11H11NO3 ; C11H13NO4 ; C11H11NO3 ; C11H11NO3 ; C11H11NO3
[illegible]6466_67.27	1.586824	0.942465	-2.4624	0.033537	1.4745	0.42361	292.1026	HMDB33296 ; HMDB35217 ; HMDB00706 ; HMDB11167 ; HMDB14632 ; HMDB15058 ; HMDB15378 ; HMDB15593 ; HMDB28760 ; HMDB28991 ; HMDB32670 ; HMDB39926 ; HMDB42023 ; HMDB60341 ; HMDB60419 ; HMDB60422 ; HMDB61039 ; HMDB14552 ; HMDB60977	Kuwanon Y ; 10-Acetoxyligustroside ; L-Aspartyl-L-phenylalanine ; L-beta-aspartyl-L-phenylalanine ; Sotalol ; Levosimendan ; Docetaxel ; Ixabepilone ; Aspartyl-Phenylalanine ; Phenylalanyl-Aspartate ; 2-(5-Methylsulfinyl)-4-penten-2-ynylidene-16-dioxaspiro44non-3-ene ; Lyoniresinol 9-sulfate ; Taxotere ; 1a11b-Dihydro-49-dimethylbenzaanthra34-boxirene ; 7-Hydroxymethyl-12-methylbenzaanthracene ; 712-Dimethylbenzaanthracene 56-oxide ; modafinil acid ; Loxapine ; desethylamodiaquine	M+2H ; M+2H ; 2M+H+Na ; 2M+H+Na ; 2M+H+K ; 2M+H+Na ; M+3Na ; M+2K ; 2M+H+Na ; 2M+H+Na ; M+ACN+H ; M+2ACN+2H ; M+3Na ; 2M+H+K ; 2M+H+K ; 2M+H+K ; M+NH4 ; M+H-2H2O ; M+H-2H2O	C34H30O9 ; C27H34O14 ; C13H16N2O5 ; C13H16N2O5 ; C12H20N2O3S ; C14H12N6O ; C43H53NO14 ; C27H42N2O5S ; C13H16N2O5 ; C13H16N2O5 ; C13H14O3S ; C22H28O11S ; C43H53NO14 ; C20H16O ; C20H16O ; C20H16O ; C15H14O3S ; C18H18ClN3O ; C18H18ClN3O

4206_264.6	1.586706	0.195377	-2.3621	0.039808	1.4	0.42361	221.1534	HMDB13817 ; HMDB36389 ; HMDB37606 ; HMDB37714 ; HMDB37718 ; HMDB38996 ; HMDB39539 ; HMDB40217 ; HMDB40226 ; HMDB12558 ; HMDB34349 ; HMDB40748 ; HMDB40764 ; HMDB00016 ; HMDB00374 ; HMDB00920 ; HMDB12515 ; HMDB15223 ; HMDB29721 ; HMDB29728 ; HMDB31541 ; HMDB33126 ; HMDB34632 ; HMDB39317 ; HMDB40277 ; HMDB40278 ; HMDB40928 ; HMDB41585 ; HMDB60906 ; HMDB60908 ; HMDB60909 ; HMDB60910 ; HMDB60911 ; HMDB60912 ; HMDB60973 ; HMDB61004 ; HMDB30993 ; HMDB36143	26-Di-tert-butylbenzoquinone ; 3-Phenylpropyl isovalerate ; 13-Nor-6-eremophilene-811-dione ; Hexyl phenylacetate ; 2-Phenylethyl hexanoate ; (Z)-6-Tetradecene-13-diyne-58-diol ; 13-Tetradecene-13-diyne-67-diol ; 34-Dihydro-22578-pentamethyl-2H-1-benzopyran-6-ol ; 2-Methyl-1-phenyl-2-propanyl butyrate ; 13-Carboxy-gamma-tocotrienol ; Suillin ; 2-Acetoxy-3-geranylgeranyl-14-dihydroxybenzene ; Bolegrevilol ; Deoxycorticosterone ; 17-Hydroxyprogesterone ; 11a-Hydroxyprogesterone ; 11-Carboxy-alpha-chromanol ; Butenafine ; 235-Trimethylfuran ; 2-Ethyl-5-methylfuran ; 3-Methyl-2-cyclohexen-1-one ; 10-Dehydroshogaol ; (4E9a)-9-(3-Methyl-2E-pentenoyloxy)-410(14)-oplopadien-3-one ; Hexadecyl ferulate ; 2-Propylfuran ; 2-Isopropylfuran ; 2-2-(4-Hydroxy-3-methoxyphenyl)ethyl-5-octylfuran ; Tussilagonone ; 11-Hydroxy-delta-9-THC ; 7-beta-Hydroxy-delta-9-THC ; 7-alpha-Hydroxy-delta-9-THC ; 8-Hydroxy-delta-9-THC ; 8-beta-Hydroxy-delta-9-THC ; 9-alpha10-alpha-epoxyhexahydrocannabinol ; 6(beta)-hydroxyprogesterone ; 6-beta-hydroxyprogesterone ; 5-Nonyltetrahydro-2-oxo-3-furancarboxylic acid ; Monomenthyl succinate	M+H ; M+H ; M+H ; M+H ; M+H ; M+H ; M+H ; M+H ; M+H ; M+H ; M+2H ; M+2H ; M+2H ; M+2H ; 2M+3H ; 2M+3H ; 2M+3H ; M+H+Na ; M+3ACN+2H ; 2M+H ; 2M+H ; 2M+H ; 2M+3H ; 2M+3H ; M+H+Na ; 2M+H ; 2M+H ; 2M+3H ; 2M+3H ; 2M+3H ; 2M+3H ; 2M+3H ; 2M+3H ; 2M+3H ; 2M+3H ; 2M+3H ; 2M+3H ; M+H-2H2O ; M+H-2H2O	C14H20O2 ; C14H20O2 ; C14H20O2 ; C14H20O2 ; C14H20O2 ; C14H20O2 ; C14H20O2 ; C14H20O2 ; C14H20O2 ; C28H40O4 ; C28H40O4 ; C28H40O4 ; C28H40O4 ; C21H30O3 ; C21H30O3 ; C21H30O3 ; C26H42O4 ; C23H27N ; C7H10O ; C7H10O ; C7H10O ; C21H30O3 ; C21H30O3 ; C26H42O4 ; C7H10O ; C7H10O ; C21H30O3 ; C21H30O3 ; C21H30O3 ; C21H30O3 ; C21H30O3 ; C21H30O3 ; C21H30O3 ; C21H30O3 ; C21H30O3 ; C21H30O3 ; C14H24O4 ; C14H24O4
2248_47.72	1.585428	1.255978	-2.3997	0.037336	1.4279	0.42361	266.8492	HMDB07962 ; HMDB09016 ; HMDB09246 ; HMDB09314 ; HMDB09315 ; HMDB09346 ; HMDB09379 ; HMDB09644 ; HMDB09645 ; HMDB09677 ; HMDB09678 ; HMDB09709 ; HMDB11238 ; HMDB11347 ; HMDB11373 ; HMDB11383 ; HMDB11384 ; HMDB11394 ; HMDB11415 ; HMDB11425 ; HMDB11426 ; HMDB11448 ; HMDB11458 ; HMDB11459	PC(15:0P-18:0) ; PE(18:0P-18:0) ; PE(20:0P-16:0) ; PE(20:2(11Z14Z)P-18:1(11Z)) ; PE(20:2(11Z14Z)P-18:1(9Z)) ; PE(20:3(5Z8Z11Z)P-18:0) ; PE(20:3(8Z11Z14Z)P-18:0) ; PE(22:5(4Z7Z10Z13Z16Z)P-18:1(11Z)) ; PE(22:5(4Z7Z10Z13Z16Z)P-18:1(9Z)) ; PE(22:5(7Z10Z13Z16Z19Z)P-18:1(11Z)) ; PE(22:5(7Z10Z13Z16Z19Z)dm18:1(9Z)) ; PE(22:6(4Z7Z10Z13Z16Z19Z)P-18:0) ; PC(P-18:015:0) ; PE(P-16:020:0) ; PE(dm18:018:0) ; PE(P-18:020:3(5Z8Z11Z)) ; PE(P-18:020:3(8Z11Z14Z)) ; PE(P-18:022:6(4Z7Z10Z13Z16Z19Z)) ; PE(P-18:1(11Z)20:2(11Z14Z)) ; PE(P-18:1(11Z)22:5(4Z7Z10Z13Z16Z)) ; PE(P-18:1(11Z)22:5(7Z10Z13Z16Z19Z)) ; PE(P-18:1(9Z)20:2(11Z14Z)) ; PE(P-18:1(9Z)22:5(4Z7Z10Z13Z16Z)) ; PE(P-18:1(9Z)22:5(7Z10Z13Z16Z19Z))	M+3Na ; M+3Na ; M+3Na ; M+H+2Na ; M+H+2Na ; M+H+2Na ; M+H+2Na ; M+2H+Na ; M+2H+Na ; M+2H+Na ; M+2H+Na ; M+2H+Na ; M+3Na ; M+3Na ; M+3Na ; M+H+2Na ; M+H+2Na ; M+2H+Na ; M+H+2Na ; M+2H+Na ; M+2H+Na ; M+H+2Na ; M+2H+Na ; M+2H+Na	C41H82NO7P ; C41H82NO7P ; C41H82NO7P ; C43H80NO7P ; C43H80NO7P ; C43H80NO7P ; C43H80NO7P ; C45H78NO7P ; C45H78NO7P ; C45H78NO7P ; C45H78NO7P ; C45H78NO7P ; C41H82NO7P ; C41H82NO7P ; C41H82NO7P ; C43H80NO7P ; C43H80NO7P ; C45H78NO7P ; C43H80NO7P ; C45H78NO7P ; C45H78NO7P ; C43H80NO7P ; C45H78NO7P ; C45H78NO7P
9146_139.5	1.584902	1.093324	-2.3347	0.041718	1.3797	0.42361	226.5529	HMDB02068 ; HMDB02884 ; HMDB31013 ; HMDB32470 ; HMDB35566 ; HMDB35567 ; HMDB35568 ; HMDB35569	Erucic acid ; Cetoleic acid ; Catelaidic acid ; Phytyl acetate ; 79-Docosanedione ; 68-Docosanedione ; 57-Docosanedione ; 46-Docosanedione	2M+3H ; 2M+3H ; 2M+3H ; 2M+3H ; 2M+3H ; 2M+3H ; 2M+3H ; 2M+3H	C22H42O2 ; C22H42O2 ; C22H42O2 ; C22H42O2 ; C22H42O2 ; C22H42O2 ; C22H42O2 ; C22H42O2

.0788_208.3	1.583057	1.201807	2.2895	0.045055	1.3463	0.42361	308.5811	HMDB29235 ; HMDB36379	Mulberrofuran P ; 2-Decarboxyphyllocactin	M+ACN+2H ; M+H+Na	C34H22O9 ; C26H29N2O14
8428_258.6	1.582677	1.028179	2.4579	0.033798	1.4711	0.42361	306.0484	HMDB11691 ; HMDB00223 ; HMDB01057 ; HMDB13126 ; HMDB29408 ; HMDB32429 ; HMDB32928 ; HMDB33762 ; HMDB33937 ; HMDB34765 ; HMDB34768 ; HMDB34901 ; HMDB38636 ; HMDB38773 ; HMDB40259 ; HMDB40300 ; HMDB41895 ; HMDB60392	Cytidine 23-cyclic phosphate ; Oxalacetic acid ; 3-Methylglutaconyl-CoA ; Butenylcarnitine ; Betanin ; 3-(Methylthio)methylthiophene ; 2-Amino-348-trimethyl-3H-imidazo45-fquinoxaline ; Ginkgetin ; Isoginkgetin ; Wasalexin A ; Wasalexin B ; 2-Vinyl-4H-13-dithiine ; Methoxybrassenin B ; Formononetin 7-(2-p-hydroxybenzoylglucoside) ; 2-Methyl-5-(methylthio)thiophene ; 2-Amino-179-trimethylimidazo45-gquinoxaline ; Flumequine ; 5-Carboxy-2-pentenoyl-CoA	M+H ; 2M+ACN+H ; M+2H+Na ; M+2K-H ; M+Na+K ; 2M+NH4 ; M+2K+H ; M+2Na ; M+2Na ; 2M+H+Na ; 2M+H+Na ; 2M+NH4 ; 2M+H+Na ; M+Na+K ; 2M+NH4 ; M+2K+H ; M+2Na-H ; M+2H+Na	C9H12N3O7P ; C4H4O5 ; C27H42N7O19P3S ; C11H19NO4 ; C24H26N2O13 ; C6H8S2 ; C12H13N5 ; C32H22O10 ; C32H22O10 ; C13H14N2O2S2 ; C13H14N2O2S2 ; C6H8S2 ; C13H14N2O2S2 ; C29H26O11 ; C6H8S2 ; C12H13N5 ; C14H12FNO3 ; C27H42N7O19P3S
.7141_47.37	1.582653	1.263572	-2.3907	0.037913	1.4212	0.42361	NA	NA	NA	NA	NA
35384_46.6	1.58248	1.277751	-2.3659	0.039552	1.4028	0.42361	384.7635	HMDB13286	N-Undecanoylglycine	3M+H+K	C13H25NO3
.1227_47.82	1.58197	1.262253	-2.364	0.039681	1.4014	0.42361	452.7511	HMDB09939 ; HMDB09943 ; HMDB31786 ; HMDB34343 ; HMDB36250 ; HMDB36297 ; HMDB41343	PIP(16:1(9Z)16:1(9Z)) ; PIP(16:2(9Z12Z)16:0) ; 9-(2-Carboxyphenyl)-36-bis(diethylamino)xanthylium(1) ; Furcogenin 3-2-glucosyl-6-arabinosylglucoside ; Schidigerasaponin C1 ; Schidigerasaponin E1 ; Chinenoside III	M+H+NH4 ; M+H+NH4 ; 2M+H+NH4 ; M+H+NH4 ; M+H+NH4 ; M+H+NH4 ; M+H+NH4	C41H76O16P2 ; C41H76O16P2 ; C28H31N2O3 ; C44H70O18 ; C44H70O18 ; C44H70O18 ; C44H70O18
57779_87.73	1.581422	1.085838	-2.4321	0.035326	1.4519	0.42361	156.0768	HMDB00177 ; HMDB34267 ; HMDB00076 ; HMDB00301 ; HMDB01988 ; HMDB02730 ; HMDB03646 ; HMDB15391 ; HMDB28704 ; HMDB28725 ; HMDB28733 ; HMDB28826 ; HMDB28880 ; HMDB28994 ; HMDB29353 ; HMDB29354 ; HMDB31176 ; HMDB31338 ; HMDB33249 ; HMDB34085 ; HMDB34174 ; HMDB36594 ; HMDB37114 ; HMDB40433 ; HMDB60245 ; HMDB60493 ; HMDB04225 ; HMDB29384	L-Histidine ; L-2-Amino-3-(1-pyrazolyl)propanoic acid ; Dihydrouracil ; Urocanic acid ; 4-Hydroxycyclohexylcarboxylic acid ; Nicotinamide N-oxide ; N-Methylhydantoin ; Decitabine ; Arginyl-Asparagine ; Asparaginyl-Arginine ; Asparaginyl-Histidine ; Glutamyl-Phenylalanine ; Histidinyl-Asparagine ; Phenylalanyl-Glutamate ; Galactosyl 4-hydroxyproline ; 4-Hydroxyproline galactoside ; Tetrahydrofurfuryl acetate ; 5-Oxo-2(5H)-isoxazolepropanenitrile ; 6-Chloro-N-(1-methylethyl)-135-triazine-24-diamine ; (3R)-34-Dihydroxy-3-(hydroxymethyl)butanenitrile 4-glucoside ; (E)-Urocanic acid ; Botryodiplodin ; Methyl (R)-3-methyl-2-oxopentanoate ; Ethyl levulinate ; L-3-Cyanoalanine ; N-Acetylmuramate ; 2-Oxoarginine ; 4-Hydroxycitrulline	M+H ; M+H ; M+ACN+H ; M+NH4 ; 2M+H+Na ; M+NH4 ; M+ACN+H ; M+2ACN+2H ; M+H+Na ; M+H+Na ; M+ACN+2H ; M+H+NH4 ; M+ACN+2H ; M+H+NH4 ; M+H+NH4 ; M+H+NH4 ; 2M+H+Na ; M+NH4 ; M+3ACN+2H ; M+H+NH4 ; M+NH4 ; 2M+H+Na ; 2M+H+Na ; 2M+H+Na ; M+ACN+H ; M+H+NH4 ; M+H-H2O ; M+H-2H2O	C6H9N3O2 ; C6H9N3O2 ; C4H6N2O2 ; C6H6N2O2 ; C7H12O3 ; C6H6N2O2 ; C4H6N2O2 ; C8H12N4O4 ; C10H20N6O4 ; C10H20N6O4 ; C10H15N5O4 ; C14H17N2O5 ; C10H15N5O4 ; C14H17N2O5 ; C11H19NO8 ; C11H19NO8 ; C7H12O3 ; C6H6N2O2 ; C6H10ClN5 ; C11H19NO8 ; C6H6N2O2 ; C7H12O3 ; C7H12O3 ; C7H12O3 ; C4H6N2O2 ; C11H19NO8 ; C6H11N3O3 ; C6H13N3O4
25052_289.5	1.580032	1.101979	2.4188	0.036134	1.4421	0.42361	548.7925	HMDB33580 ; HMDB35269 ; HMDB39009 ; HMDB40505	28-Glucosylarjunolate 3-rhamnosyl-(1-3)-glucuronide ; Azukisaponin IV ; Calendasaponin B ; Soyasaponin aa	M+3ACN+2H ; M+3ACN+2H ; M+3ACN+2H ; M+ACN+2H	C48H76O20 ; C48H76O20 ; C48H76O20 ; C53H82O21

2662_55.16	1.579659	1.178463	-2.4595	0.033705	1.4723	0.42361	295.0003	HMDB15022	Risedronate	2M+H+Na	C7H11NO7P2
2525_42.08	1.579216	1.045518	-2.4002	0.037302	1.4283	0.42361	166.0863	HMDB00159 ; HMDB01007 ; HMDB04992 ; HMDB06044 ; HMDB33485 ; HMDB33589 ; HMDB34169 ; HMDB59934 ; HMDB00053 ; HMDB00567 ; HMDB00873 ; HMDB00930 ; HMDB00985 ; HMDB01398 ; HMDB02720 ; HMDB10733 ; HMDB11474 ; HMDB11480 ; HMDB11504 ; HMDB11510 ; HMDB11724 ; HMDB15690 ; HMDB29365 ; HMDB29575 ; HMDB29696 ; HMDB29774 ; HMDB29939 ; HMDB30288 ; HMDB30972 ; HMDB30973 ; HMDB30978 ; HMDB30996 ; HMDB31460 ; HMDB31725 ; HMDB32917 ; HMDB32947 ; HMDB33357 ; HMDB33515 ; HMDB34877 ; HMDB35226 ; HMDB35243 ; HMDB35366 ; HMDB36381 ; HMDB36626 ; HMDB38035 ; HMDB38957 ; HMDB38963 ; HMDB40280 ; HMDB40806 ; HMDB40974 ; HMDB40986 ; HMDB41140 ; HMDB59709 ; HMDB59712 ; HMDB60990	L-Phenylalanine ; 3-Pyridinebutanoic acid ; Benzocaine ; Norsalsolinol ; Gentiatibetine ; Ethyl 2-aminobenzoate ; Methyl N-methylanthranilate ; Benzyl glycinate ; Androstenedione ; Cinnamic acid ; 4-Methylcatechol ; trans-Cinnamic acid ; Dihydrolipoamide ; Guaiacol ; Gestrinone ; 3-Oxohexadecanoic acid ; LysoPE(0:016:1(9Z)) ; LysoPE(0:018:4(6Z9Z12Z15Z)) ; LysoPE(16:1(9Z)0:0) ; LysoPE(18:4(6Z9Z12Z15Z)0:0) ; 4-Hydroxybenzyl alcohol ; Chloropyramine ; Moupinamide ; ()-cis-56-Dihydro-5-hydroxy-4-methoxy-6-(2-phenylethyl)-2H-pyran-2-one ; Mequinol ; 7-Hydroxy-6-(methoxyacetyl)-22-dimethyl-2H-1-benzopyran ; 6-Amino-9H-purine-9-propanoic acid ; Muricinine ; 8-Oxohexadecanoic acid ; 9-Oxohexadecanoic acid ; 11-Oxohexadecanoic acid ; (2E4Z7Z8E)-Colnelenic acid ; Anthocyanidins ; (E)-3-(2-Hydroxyphenyl)-2-propenal ; 1-(2-Furanyl)-2-propanone ; Di-2-furanylmethane ; Laurelliptine ; Citreovirenone ; 2-Acetyl-3-methylfuran ; 2-Acetyl-5-methylfuran ; 1-Phenyl-12-propanedione ; Cytochalasin Opho ; N-cis-Feruloyltyramine ; 34-Dihydro-2H-1-benzopyran-2-one ; Panaquinquecol 1 ; Citronellyl cinnamate ; 46-Heptadiyne-13-diol ; 1-(2-Furanyl)-1-propanone ; 7-Paradol ; Laurolitsine ; (E)-3-(4-Hydroxyphenyl)-2-propenal ; Prenyl cis-caffeate ; 2-Hydroxybenzyl alcohol ; 3-Hydroxybenzyl alcohol ; 8-Hydroxycarteolol	M+H ; M+H ; M+H ; M+H ; M+H ; M+H ; M+H ; M+H ; M+2Na ; M+NH4 ; M+ACN+H ; M+NH4 ; M+3ACN+2H ; M+ACN+H ; M+H+Na ; M+Na+K ; M+H+2Na ; M+2H+Na ; M+H+2Na ; M+2H+Na ; M+ACN+H ; M+ACN+2H ; M+H+NH4 ; M+2ACN+2H ; M+ACN+H ; M+2ACN+2H ; M+3ACN+2H ; M+H+NH4 ; M+Na+K ; M+Na+K ; M+Na+K ; M+H+K ; M+3ACN+2H ; M+NH4 ; M+ACN+H ; M+NH4 ; M+H+NH4 ; M+2ACN+2H ; M+ACN+H ; M+ACN+H ; M+NH4 ; M+H+2Na ; M+H+NH4 ; M+NH4 ; M+H+K ; M+2Na ; M+ACN+H ; M+ACN+H ; M+H+K ; M+H+NH4 ; M+NH4 ; M+2ACN+2H ; M+ACN+H ; M+ACN+H ; M+H+Na	C9H11NO2 ; C9H11NO2 ; C9H11NO2 ; C9H11NO2 ; C9H11NO2 ; C9H11NO2 ; C9H11NO2 ; C9H11NO2 ; C19H26O2 ; C9H8O2 ; C7H8O2 ; C9H8O2 ; C8H17NOS2 ; C7H8O2 ; C21H24O2 ; C16H30O3 ; C21H42NO7P ; C23H40NO7P ; C21H42NO7P ; C23H40NO7P ; C7H8O2 ; C16H20ClN3 ; C18H19NO4 ; C14H16O4 ; C7H8O2 ; C14H16O4 ; C8H9N5O2 ; C18H19NO4 ; C16H30O3 ; C16H30O3 ; C16H30O3 ; C18H28O3 ; C15H11O ; C9H8O2 ; C7H8O2 ; C9H8O2 ; C18H19NO4 ; C14H16O4 ; C7H8O2 ; C7H8O2 ; C9H8O2 ; C28H37NO4 ; C18H19NO4 ; C9H8O2 ; C18H28O3 ; C19H26O2 ; C7H8O2 ; C7H8O2 ; C18H28O3 ; C18H19NO4 ; C9H8O2 ; C14H16O4 ; C7H8O2 ; C7H8O2 ; C16H24N2O4
6972_47.28	1.578788	1.168235	-2.388	0.038086	1.4192	0.42361	NA	NA	NA	NA	NA
4788_110.5	1.578767	1.200407	2.4237	0.035837	1.4457	0.42361	NA	NA	NA	NA	NA
2569_42.48	1.578712	1.154983	-2.2784	0.045912	1.3381	0.42361	642.5126	HMDB07032 ; HMDB07033 ; HMDB07060 ; HMDB07074 ; HMDB07085 ; HMDB07114 ; HMDB07141 ; HMDB07142 ; HMDB07193 ; HMDB07222 ; HMDB07242 ; HMDB07249 ; HMDB07250 ; HMDB07277 ; HMDB07306 ; HMDB07333 ; HMDB07334 ; HMDB07505 ; HMDB07534 ; HMDB07561 ; HMDB07562 ; HMDB07676 ; HMDB07704 ; HMDB07733 ; HMDB55967 ; HMDB55969 ; HMDB55974 ; HMDB55979 ; HMDB56006 ; HMDB56033 ; HMDB56148 ; HMDB56167 ; HMDB56173 ; HMDB56192 ; HMDB56211	DG(14:022:5(4Z7Z10Z13Z16Z)0:0) ; DG(14:022:5(7Z10Z13Z16Z19Z)0:0) ; DG(14:1(9Z)22:4(7Z10Z13Z16Z)0:0) ; DG(15:018:2(9Z12Z)0:0) ; DG(15:020:5(5Z8Z11Z14Z17Z)0:0) ; DG(16:020:5(5Z8Z11Z14Z17Z)0:0) ; DG(16:1(9Z)20:4(5Z8Z11Z14Z)0:0) ; DG(16:1(9Z)20:4(8Z11Z14Z17Z)0:0) ; DG(18:1(11Z)18:4(6Z9Z12Z15Z)0:0) ; DG(18:1(9Z)18:4(6Z9Z12Z15Z)0:0) ; DG(18:2(9Z12Z)15:00:0) ; DG(18:2(9Z12Z)18:3(6Z9Z12Z)0:0) ; DG(18:2(9Z12Z)18:3(9Z12Z15Z)0:0) ; DG(18:3(6Z9Z12Z)18:2(9Z12Z)0:0) ; DG(18:3(9Z12Z15Z)18:2(9Z12Z)0:0) ; DG(18:4(6Z9Z12Z15Z)18:1(11Z)0:0) ; DG(18:4(6Z9Z12Z15Z)18:1(9Z)0:0) ; DG(20:4(5Z8Z11Z14Z)16:1(9Z)0:0) ; DG(20:4(8Z11Z14Z17Z)16:1(9Z)0:0) ; DG(20:5(5Z8Z11Z14Z17Z)15:00:0) ; DG(20:5(5Z8Z11Z14Z17Z)16:00:0) ; DG(22:4(7Z10Z13Z16Z)14:1(9Z)0:0) ; DG(22:5(4Z7Z10Z13Z16Z)14:00:0) ; DG(22:5(7Z10Z13Z16Z19Z)14:00:0) ; DG(14:00:018:2n6) ; DG(14:00:020:2n6) ; DG(14:00:022:5n6) ; DG(14:00:022:5n3) ; DG(15:00:020:5n3) ; DG(16:00:020:5n3) ; DG(14:1n50:022:4n6) ; DG(16:1n70:020:4n6) ; DG(16:1n70:020:4n3) ; DG(18:1n70:018:4n3) ; DG(18:1n90:018:4n3)	2M+3H2O+2H ; 2M+3H2O+2H ; 2M+3H2O+2H ; M+ACN+Na ; M+ACN+H ; 2M+3H2O+2H ; 2M+3H2O+2H ; 2M+3H2O+2H ; 2M+3H2O+2H ; 2M+3H2O+2H ; M+ACN+Na ; 2M+3H2O+2H ; 2M+3H2O+2H ; 2M+3H2O+2H ; 2M+3H2O+2H ; 2M+3H2O+2H ; 2M+3H2O+2H ; 2M+3H2O+2H ; 2M+3H2O+2H ; M+ACN+H ; 2M+3H2O+2H ; 2M+3H2O+2H ; 2M+3H2O+2H ; 2M+3H2O+2H ; M+ACN+Na ; M+ACN+Na ; 2M+3H2O+2H ; 2M+3H2O+2H ; M+ACN+H ; 2M+3H2O+2H ; 2M+3H2O+2H ; 2M+3H2O+2H ; 2M+3H2O+2H ; 2M+3H2O+2H ; 2M+3H2O+2H	C39H66O5 ; C39H66O5 ; C39H66O5 ; C36H66O5 ; C38H64O5 ; C39H66O5 ; C39H66O5 ; C39H66O5 ; C39H66O5 ; C39H66O5 ; C36H66O5 ; C39H66O5 ; C39H66O5 ; C39H66O5 ; C39H66O5 ; C39H66O5 ; C39H66O5 ; C39H66O5 ; C39H66O5 ; C38H64O5 ; C39H66O5 ; C39H66O5 ; C39H66O5 ; C39H66O5 ; C36H66O5 ; C36H66O5 ; C39H66O5 ; C39H66O5 ; C38H64O5 ; C39H66O5 ; C39H66O5 ; C39H66O5 ; C39H66O5 ; C39H66O5 ; C39H66O5

3083_196.7	1.577134	1.061559	-2.4469	0.03444	1.4629	0.42361	216.0523	HMDB33587 ; HMDB41717 ; HMDB41718 ; HMDB00072 ; HMDB00613 ; HMDB00943 ; HMDB00958 ; HMDB01264 ; HMDB06357 ; HMDB14817 ; HMDB31825 ; HMDB34249 ; HMDB34757 ; HMDB40200 ; HMDB41940 ; HMDB60320 ; HMDB60510 ; HMDB61101	Coumestrin ; Daidzein 4-O-glucuronide ; Daidzein 7-O-glucuronide ; cis-Aconitic acid ; Erythronic acid ; Threonic acid ; trans-Aconitic acid ; Dehydroascorbic acid ; cis-2-Methylaconitate ; Thioridazine ; Phenyl vinyl sulfide ; Caryoptosidic acid ; 4-Methoxybenzenepropanol 1-(2-sulfoglucoside) ; 25-Dimethyl-3-(methyldithio)furan ; N-nitrosonornicotine ; (Z)-But-1-ene-124-tricarboxylate ; SN-38 ; 2-oxobrimonidine	M+2H ; M+2H ; M+2H ; M+ACN+H ; 3M+H+Na ; 3M+H+Na ; M+ACN+H ; M+ACN+H ; 2M+3H2O+2H ; M+Na+K ; 3M+H+Na ; M+H+K ; M+H+Na ; M+ACN+H ; M+K ; 2M+3H2O+2H ; M+H+K ; M+3ACN+2H	C21H18O10 ; C21H18O10 ; C21H18O10 ; C6H6O6 ; C4H8O5 ; C4H8O5 ; C6H6O6 ; C6H6O6 ; C7H8O6 ; C21H26N2S2 ; C8H8S ; C16H24O11 ; C16H24O10S ; C7H10OS2 ; C9H11N3O ; C7H8O6 ; C22H20N2O5 ; C11H10BrN5O
1004_283.3	1.575771	0.966303	2.3086	0.043615	1.3604	0.42361	385.3461	HMDB00032 ; HMDB00876 ; HMDB00921 ; HMDB02027 ; HMDB02719 ; HMDB06271 ; HMDB06500 ; HMDB06505 ; HMDB06560 ; HMDB06719 ; HMDB06842 ; HMDB12178 ; HMDB14315 ; HMDB34399 ; HMDB59615 ; HMDB13206 ; HMDB15638 ; HMDB35397 ; HMDB35398 ; HMDB01457 ; HMDB03990 ; HMDB12455 ; HMDB39463 ; HMDB60138	7-Dehydrocholesterol ; Vitamin D3 ; Cholestenone ; 8-Dehydrocholesterol ; Desmosterol ; Zymosterol intermediate 2 ; Previtamin D3 ; Lumisterol 3 ; Tachysterol 3 ; 56-trans-Vitamin D3 ; 5a-Cholesta-724-dien-3b-ol ; 5Alpha-cholesta-8-en-3-one ; Cholecalciferol ; 5alpha-Cholest-7-en-3-one ; 5-alpha-Cholesta-724-dien-3-beta-ol ; 9-Decenoylcholine ; Ethanolamine Oleate ; Cohibin C ; Cohibin D ; 5-b-Cholestane-3a 7a 12a-triol ; 3b5a6b-Cholestanetriol ; 3 alpha7 alpha26-Trihydroxy-5beta-cholestane ; (3alpha5alpha22R23R)-Cholestane-32223-triol ; 5beta-Cholestane-3alpha7alpha27-triol	M+H ; M+H ; M+H ; M+H ; M+H ; M+H ; M+H ; M+H ; M+H ; M+H ; M+H ; M+H ; M+H ; M+H ; M+H ; 3M+2H ; M+ACN+H ; 2M+3H ; 2M+3H ; M+H-2H2O ; M+H-2H2O ; M+H-2H2O ; M+H-2H2O ; M+H-2H2O	C27H44O ; C27H44O ; C27H44O ; C27H44O ; C27H44O ; C27H44O ; C27H44O ; C27H44O ; C27H44O ; C27H44O ; C27H44O ; C27H44O ; C27H44O ; C27H44O ; C27H44O ; C15H30NO2 ; C20H41NO3 ; C37H68O4 ; C37H68O4 ; C27H48O3 ; C27H48O3 ; C27H48O3 ; C27H48O3 ; C27H48O3
2353_287.4	1.575283	0.55886	2.4703	0.033085	1.4804	0.42361	510.1032	HMDB14831 ; HMDB15138 ; HMDB32877 ; HMDB38458 ; HMDB41737 ; HMDB41738 ; HMDB41739 ; HMDB41832	Fluorescein ; Cromoglicic acid ; Glucorhein ; NN-Bis(gamma-glutamyl)cystine ; Genistein 4-O-glucuronide ; Genistein 5-O-glucuronide ; Genistein 7-O-glucuronide ; Baicalin	3M+H+Na ; M+ACN+H ; M+ACN+Na ; 2M+H+Na ; M+ACN+Na ; M+ACN+Na ; M+ACN+Na ; M+ACN+Na	C20H12O5 ; C23H16O11 ; C21H18O11 ; C16H26N4O10S2 ; C21H18O11 ; C21H18O11 ; C21H18O11 ; C21H18O11
6823_253.2	1.575198	0.929683	2.4959	0.03167	1.4994	0.42361	628.2957	HMDB12212 ; HMDB15255 ; HMDB30163 ; HMDB31672 ; HMDB33881 ; HMDB33972 ; HMDB34142 ; HMDB35431 ; HMDB39036	Dihydrozeatin-9-N-glucoside-O-glucoside ; Proflavine ; Galbanic acid ; Armillaribin ; Mammea CAB cyclo D ; Asacoumarin A ; 5-Hydroxy-88-dimethyl-6-(3-methyl-1-oxobutyl)-4-pentyl-2H8H-benzo12-b:34-bdipyran-2-one ; Assamsaponin D ; Kamolonol	M+2ACN+H ; 3M+H ; 3M+Na+K ; 3M+Na+K ; 3M+Na+K ; 3M+Na+K ; 3M+Na+K ; M+H+Na ; 3M+Na+K	C22H35N5O11 ; C13H11N3 ; C24H30O5 ; C24H30O5 ; C24H30O5 ; C24H30O5 ; C24H30O5 ; C59H92O27 ; C24H30O5

3229_275.	1.575017	0.149221	2.3676	0.039438	1.4041	0.42361	328.9733	HMDB03585 ; HMDB13968 ; HMDB13969 ; HMDB30228 ; HMDB38459	Thiocysteine ; Hydroxyzileuton ; Zileuton sulfoxide ; Nigakinone ; 5-(4-Acetoxy-3-oxo-1-butynyl)-22-bithiophene	2M+Na ; M+2K-H ; M+2K-H ; M+2K-H ; M+K	C3H7NO2S2 ; C11H12N2O3S ; C11H12N2O3S ; C14H8N2O3 ; C14H10O3S2
2173_275.	1.574707	0.703563	2.3756	0.038904	1.41	0.42361	NA	NA	NA	NA	NA
.4011_46.4	1.574553	1.115872	2.2593	0.047423	1.324	0.42361	102.0914	HMDB12815 ; HMDB00820 ; HMDB00863 ; HMDB01184 ; HMDB02117 ; HMDB12157 ; HMDB31407 ; HMDB31512 ; HMDB31530 ; HMDB31601 ; HMDB31606 ; HMDB31607 ; HMDB32458 ; HMDB37788 ; HMDB40584 ; HMDB41081	5-Aminopentanal ; Propyl alcohol ; Isopropyl alcohol ; Methyl propenyl ketone ; Oleamide ; 3-Methyl-2-butenal ; Cyclopentanone ; Tiglic aldehyde ; 3-Methyl-3-buten-2-one ; 2-Pentenal ; 4-Penten-2-one ; 1-Penten-3-one ; 4-Pentenal ; xi-23-Dihydro-3-methylfuran ; 23-Dihydro-4-methylfuran ; (Z)-15-Tridecadiene	M+H ; M+ACN+H ; M+ACN+H ; M+NH4 ; M+2H+Na ; M+NH4 ; M+NH4 ; M+NH4 ; M+NH4 ; M+NH4 ; M+NH4 ; M+NH4 ; M+NH4 ; M+NH4 ; M+NH4 ; M+H+Na	C5H11NO ; C3H8O ; C3H8O ; C5H8O ; C18H35NO ; C5H8O ; C5H8O ; C5H8O ; C5H8O ; C5H8O ; C5H8O ; C5H8O ; C5H8O ; C5H8O ; C5H8O ; C13H24
9577_146.	1.572429	1.231398	-2.233	0.049586	1.3046	0.42721	NA	NA	NA	NA	NA
2739_125.	1.572405	1.099743	2.3172	0.042976	1.3668	0.42361	428.1763	HMDB01913 ; HMDB05009 ; HMDB14679 ; HMDB14874 ; HMDB15020 ; HMDB29532 ; HMDB31235 ; HMDB31236 ; HMDB31796 ; HMDB31922 ; HMDB34017 ; HMDB35255 ; HMDB40317 ; HMDB41275 ; HMDB41314 ; HMDB41907 ; HMDB61022	Omeprazole ; (S)-Esomeprazole ; Toremifene ; Esomeprazole ; Clomifene ; 3-Hydroxyglabrol ; (E)-2446-Tetrahydroxy-35-diprenylchalcone ; 3-Geranyl-2446-tetrahydroxychalcone ; Isofenphos ; 1-(24-Dihydroxyphenyl)-3-8-hydroxy-2-methyl-2-(4-methyl-3-pentenyl)-2H-1-benzopyran-5-yl-1-propanone ; Kuwanol D ; 3-Geranyl-2344-tetrahydroxychalcone ; (E)-2-Geranyl-347-trihydroxyflavanone ; 1-Hydroxy-35-dimethoxy-24-diprenylxanthone ; 4-tert-Butylphenyl salicylate ; Imidapril ; 6-beta-hydroxydexamethasone	M+2ACN+H ; M+2ACN+H ; M+Na ; M+2ACN+H ; M+Na ; 2M+H+K ; 2M+H+K ; 2M+H+K ; M+2ACN+H ; 2M+H+K ; 2M+H+K ; 2M+H+K ; 2M+H+K ; 2M+H+K ; 3M+2Na ; M+Na ; 2M+H+K	C17H19N3O3S ; C17H19N3O3S ; C26H28ClNO ; C17H19N3O3S ; C26H28ClNO ; C25H28O5 ; C25H28O5 ; C25H28O5 ; C15H24NO4PS ; C25H28O5 ; C25H28O5 ; C25H28O5 ; C25H28O5 ; C25H28O5 ; C17H18O3 ; C20H27N3O6 ; C22H29FO6
8032_47.5	1.572197	1.163673	-2.382	0.038483	1.4147	0.42361	508.6238	HMDB12470 ; HMDB38298 ; HMDB38301 ; HMDB38302	(2S6R10R)-Trimethyl-2E-hendecenoyl-CoA ; Apigenin 4-feruloyl-(-2)-glucuronyl-(1-2)-glucuronide 7-glucuronide ; Apigenin 7-feruloyl-(-2)-glucuronyl-(1-2)-glucuronide 4-glucuronide ; Apigenin 7-feruloyl-(-2)-glucuronyl-(1-3)-glucuronyl-(1-2)-glucuronide	M+2Na ; M+ACN+2H ; M+ACN+2H ; M+ACN+2H	C35H56N7O17P3S ; C43H42O26 ; C43H42O26 ; C43H42O26
6987_88.9	1.570882	1.118572	-2.4398	0.034858	1.4577	0.42361	111.0747	HMDB02172 ; HMDB37162	N1N12-Diacetylspermine ; 123-Tris(1-ethoxyethoxy)propane	M+H+2Na ; M+2H+Na	C14H30N4O2 ; C15H32O6

5389_47.07	1.570254	1.294882	-2.3437	0.041082	1.3864	0.42361	392.7954	HMDB07921 ; HMDB07980 ; HMDB07981 ; HMDB08012 ; HMDB08040 ; HMDB08041 ; HMDB08072 ; HMDB08105 ; HMDB08136 ; HMDB08137 ; HMDB08168 ; HMDB08201 ; HMDB08332 ; HMDB08364 ; HMDB08397 ; HMDB08591 ; HMDB06516 ; HMDB07878 ; HMDB07970 ; HMDB08034 ; HMDB08262 ; HMDB08907 ; HMDB09483 ; HMDB60233	PC(14:1(9Z)22:2(13Z16Z)) ; PC(16:020:3(5Z8Z11Z)) ; PC(16:020:3(8Z11Z14Z)) ; PC(16:1(9Z)20:2(11Z14Z)) ; PC(18:018:3(6Z9Z12Z)) ; PC(18:018:3(9Z12Z15Z)) ; PC(18:1(11Z)18:2(9Z12Z)) ; PC(18:1(9Z)18:2(9Z12Z)) ; PC(18:2(9Z12Z)18:1(11Z)) ; PC(18:2(9Z12Z)18:1(9Z)) ; PC(18:3(6Z9Z12Z)18:0) ; PC(18:3(9Z12Z15Z)18:0) ; PC(20:2(11Z14Z)16:1(9Z)) ; PC(20:3(5Z8Z11Z)16:0) ; PC(20:3(8Z11Z14Z)16:0) ; PC(22:2(13Z16Z)14:1(9Z)) ; Tetracosatetraenoyl CoA ; PC(14:020:0) ; PC(16:018:0) ; PC(18:016:0) ; PC(20:014:0) ; PE(15:022:0) ; PE(22:015:0) ; all-cis-12151821-Tetracosatetraenoyl-CoA	M+2H ; M+2H ; M+2H ; M+2H ; M+2H ; M+2H ; M+2H ; M+2H ; M+2H ; M+2H ; M+2H ; M+2H ; M+2H ; M+2H ; M+2H ; M+2H ; M+3Na ; M+H+Na ; M+H+Na ; M+H+Na ; M+H+Na ; M+H+Na ; M+H+Na ; M+3Na	C44H82NO8P ; C44H82NO8P ; C44H82NO8P ; C44H82NO8P ; C44H82NO8P ; C44H82NO8P ; C44H82NO8P ; C44H82NO8P ; C44H82NO8P ; C44H82NO8P ; C44H82NO8P ; C44H82NO8P ; C44H82NO8P ; C44H82NO8P ; C44H82NO8P ; C44H82NO8P ; C45H74N7O17P3S ; C42H84NO8P ; C42H84NO8P ; C42H84NO8P ; C42H84NO8P ; C42H84NO8P ; C42H84NO8P ; C45H74N7O17P3S
7134_85.9	1.569873	1.170561	-2.4421	0.034724	1.4594	0.42361	93.04477	HMDB01490 ; HMDB01565 ; HMDB29748 ; HMDB35056 ; HMDB36072 ; HMDB39650 ; HMDB40603 ; HMDB59730 ; HMDB00895 ; HMDB01161 ; HMDB13811 ; HMDB37766 ; HMDB03905 ; HMDB33156 ; HMDB60741	Vanylglycol ; Phosphorylcholine ; 2-Aminodipyrido12-a:32-dimidazole ; 1-(3-Hydroxy-4-methoxyphenyl)-12-ethanediol ; Genipic acid ; 4-Formyl-3-(formylmethyl)-4-hexenoic acid ; ()-threo-1-(4-Hydroxyphenyl)-123-propanetriol ; 34-Methylenepimelic acid ; Acetylcholine ; 4-Trimethylammoniobutanoic acid ; 1-Nitroheptane ; 3-Mercaptohexyl hexanoate ; Imidazole-4-acetaldehyde ; Methoxypyrazine ; 3-Hydroxy-4-aminopyridine	M+2H ; M+2H ; M+2H ; M+2H ; M+2H ; M+2H ; M+2H ; M+2H ; M+H+K ; M+H+K ; M+H+K ; M+H+2Na ; M+H-H2O ; M+H-H2O ; M+H-H2O	C9H12O4 ; C5H15NO4P ; C10H8N4 ; C9H12O4 ; C9H12O4 ; C9H12O4 ; C9H12O4 ; C9H12O4 ; C7H16NO2 ; C7H16NO2 ; C7H16NO2 ; C12H24O2S ; C5H6N2O ; C5H6N2O ; C5H6N2O

2767_47.5	1.569327	1.332327	-2.3286	0.042148	1.3752	0.42361	406.7723	HMDB10165 ; HMDB12383 ; HMDB12393 ; HMDB12423 ; HMDB12433 ; HMDB10163 ; HMDB10580 ; HMDB10584 ; HMDB10593 ; HMDB10594 ; HMDB10597 ; HMDB10598 ; HMDB10621 ; HMDB10622 ; HMDB10636 ; HMDB10637 ; HMDB10650 ; HMDB10655 ; HMDB10663 ; HMDB10664 ; HMDB10668 ; HMDB10669 ; HMDB10678 ; HMDB10679 ; HMDB10683 ; HMDB10684 ; HMDB12389	PS(18:020:4(8Z11Z14Z17Z)) ; PS(18:020:4(5Z8Z11Z14Z)) ; PS(18:1(9Z)20:3(8Z11Z14Z)) ; PS(20:3(8Z11Z14Z)18:1(9Z)) ; PS(20:4(5Z8Z11Z14Z)18:0) ; PS(18:018:1(9Z)) ; PG(16:020:4(5Z8Z11Z14Z)) ; PG(16:022:6(4Z7Z10Z13Z16Z19Z)) ; PG(16:1(9Z)20:3(5Z8Z11Z)) ; PG(16:1(9Z)20:3(8Z11Z14Z)) ; PG(16:1(9Z)22:5(4Z7Z10Z13Z16Z)) ; PG(16:1(9Z)22:5(7Z10Z13Z16Z19Z)) ; PG(18:1(11Z)18:3(6Z9Z12Z)) ; PG(18:1(11Z)18:3(9Z12Z15Z)) ; PG(18:1(9Z)18:3(6Z9Z12Z)) ; PG(18:1(9Z)18:3(9Z12Z15Z)) ; PG(18:2(9Z12Z)18:2(9Z12Z)) ; PG(18:2(9Z12Z)20:4(5Z8Z11Z14Z)) ; PG(18:3(6Z9Z12Z)18:1(11Z)) ; PG(18:3(6Z9Z12Z)18:1(9Z)) ; PG(18:3(6Z9Z12Z)20:3(5Z8Z11Z)) ; PG(18:3(6Z9Z12Z)20:3(8Z11Z14Z)) ; PG(18:3(9Z12Z15Z)18:1(11Z)) ; PG(18:3(9Z12Z15Z)18:1(9Z)) ; PG(18:3(9Z12Z15Z)20:3(5Z8Z11Z)) ; PG(18:3(9Z12Z15Z)20:3(8Z11Z14Z)) ; PS(18:1(9Z)18:0)	M+2H ; M+2H ; M+2H ; M+2H ; M+2H ; M+H+Na ; M+ACN+2H ; M+H+NH4 ; M+ACN+2H ; M+ACN+2H ; M+H+NH4 ; M+H+NH4 ; M+ACN+2H ; M+ACN+2H ; M+ACN+2H ; M+ACN+2H ; M+ACN+2H ; M+H+NH4 ; M+ACN+2H ; M+ACN+2H ; M+H+NH4 ; M+H+NH4 ; M+ACN+2H ; M+ACN+2H ; M+H+NH4 ; M+H+NH4 ; M+H+Na	C44H78NO10P ; C44H78NO10P ; C44H78NO10P ; C44H78NO10P ; C44H78NO10P ; C42H80NO10P ; C42H75O10P ; C44H75O10P ; C42H75O10P ; C42H75O10P ; C44H75O10P ; C44H75O10P ; C42H75O10P ; C42H75O10P ; C42H75O10P ; C42H75O10P ; C42H75O10P ; C44H75O10P ; C42H75O10P ; C42H75O10P ; C44H75O10P ; C44H75O10P ; C42H75O10P ; C42H75O10P ; C44H75O10P ; C44H75O10P ; C42H80NO10P
9974_47.65	1.568814	1.323917	-2.3185	0.042879	1.3678	0.42361	474.76	HMDB33404 ; HMDB36485 ; HMDB36943 ; HMDB39448 ; HMDB39512 ; HMDB40742	Quillaic acid 3-galactosyl-(1-2)-glucuronide ; Trigoneoside XIb ; Oleragenoside ; Licoricesaponin J2 ; Periandrin III ; 23-Dehydrosalvipisone	M+3ACN+2H ; M+ACN+2H ; M+3ACN+2H ; M+3ACN+2H ; M+3ACN+2H ; 3M+H+NH4	C42H64O16 ; C44H74O19 ; C42H64O16 ; C42H64O16 ; C42H64O16 ; C20H22O3
13218_154	1.568814	0.146277	-2.4591	0.033728	1.472	0.42361	140.9913	HMDB32914 ; HMDB33205 ; HMDB60503	2-Furancarboxaldehyde ; Roxarsone ; Protoanemonin	M+2Na-H ; M+H+NH4 ; M+2Na-H	C5H4O2 ; C6H6AsNO6 ; C5H4O2
.0231_48.4	1.567636	0.71123	-2.359	0.040019	1.3977	0.42361	343.861	HMDB15357 ; HMDB30737 ; HMDB34677 ; HMDB35065 ; HMDB35096	Mivacurium ; Capsianoside V ; Cinncassiol D1 glucoside ; Cinncassiol D4 2-glucoside ; Cofaryloside	M+3H ; 2M+3H ; 2M+3H ; 2M+3H ; 2M+3H	C58H80N2O14 ; C26H42O10 ; C26H42O10 ; C26H42O10 ; C26H42O10

9501_196.6	1.566687	0.985812	-2.3898	0.037975	1.4205	0.42361	206.0395	HMDB30560 ; HMDB00127 ; HMDB00197 ; HMDB00510 ; HMDB02545 ; HMDB02704 ; HMDB03363 ; HMDB03402 ; HMDB03551 ; HMDB04073 ; HMDB06334 ; HMDB11731 ; HMDB11732 ; HMDB15249 ; HMDB29423 ; HMDB31756 ; HMDB33431 ; HMDB33747 ; HMDB37499 ; HMDB38491 ; HMDB38628 ; HMDB40972 ; HMDB41370 ; HMDB41414 ; HMDB41503 ; HMDB41723 ; HMDB41787	71112-Triacetoxycoumestan ; D-Glucuronic acid ; Indoleacetic acid ; Aminoadipic acid ; Galacturonic acid ; Iduronic acid ; Pectic acid ; Pectin ; Carbamic acid ; 5-Hydroxyindoleacetaldehyde ; 3-Dehydro-L-gulonate ; 5-Keto-D-gluconate ; 2-Keto-L-gluconate ; Atovaquone ; Acetylhomoserine ; Veranisatin C ; Moschamindole ; ()-22-Iminobispropanoic acid ; 3-N-Acetylfusarochromanone ; L-Altruronic acid ; Hydroxymethyl indol-3-yl ketone ; 1-Methoxy-1H-indole-3-carboxaldehyde ; Na-p-Hydroxycoumaroyltryptophan ; Chloromarmin ; Bis(2-furanylmethyl) sulfide ; Dihydroferulic acid 4-O-glucuronide ; Urolithin B 3-O-glucuronide	M+2H ; 2M+H+Na ; 2M+Na+K ; M+2Na-H ; 2M+H+Na ; 2M+H+Na ; 2M+H+Na ; 2M+H+Na ; 3M+Na ; 2M+Na+K ; 2M+H+Na ; 2M+H+Na ; 2M+H+Na ; M+2Na ; M+2Na-H ; M+H+K ; M+Na+K ; M+2Na-H ; M+2K ; 2M+H+Na ; 2M+Na+K ; 2M+Na+K ; M+Na+K ; M+Na+K ; 2M+H+Na ; M+H+K ; M+H+Na	C21H14O9 ; C6H10O7 ; C10H9NO2 ; C6H11NO4 ; C6H10O7 ; C6H10O7 ; C6H10O7 ; C6H10O7 ; CH3NO2 ; C10H9NO2 ; C6H10O7 ; C6H10O7 ; C6H10O7 ; C22H19ClO3 ; C6H11NO4 ; C16H20O10 ; C20H18N2O4 ; C6H11NO4 ; C17H22N2O5 ; C6H10O7 ; C10H9NO2 ; C10H9NO2 ; C20H18N2O4 ; C19H23ClO4 ; C10H10O2S ; C16H20O10 ; C19H16O9
20593_47.6	1.566189	1.255521	-2.3514	0.040545	1.3921	0.42361	256.8206	HMDB07903 ; HMDB07999 ; HMDB08895 ; HMDB09087	PC(14:1(9Z)16:1(9Z)) ; PC(16:1(9Z)14:1(9Z)) ; PE(15:018:2(9Z12Z)) ; PE(18:2(9Z12Z)15:0)	M+3Na ; M+3Na ; M+3Na ; M+3Na	C38H72NO8P ; C38H72NO8P ; C38H72NO8P ; C38H72NO8P
0929_47.75	1.565995	1.245378	-2.3441	0.041048	1.3867	0.42361	NA	NA	NA	NA	NA
88488_60.6	1.565943	0.552757	2.3675	0.039446	1.404	0.42361	334.9188	HMDB00265 ; HMDB60074	Liothyronine ; Reverse-triiodthyronine	M+H+NH4 ; M+H+NH4	C15H12I3NO4 ; C15H12I3NO4
2957_26.41	1.56586	1.081935	-2.229	0.049927	1.3017	0.42721	154.9903	HMDB06343 ; HMDB29726 ; HMDB31070 ; HMDB33746	Selenocystathionine ; 2-Vinylthiophene ; (2E11Z)-5-5-(Methylthio)-4-penten-2-ynyl-2-furanacrolein ; Benzenethiol	M+H+K ; M+2Na-H ; M+2K ; M+2Na-H	C7H14N2O4Se ; C6H6S ; C13H12O2S ; C6H6S
2803_287.8	1.564519	0.860852	2.2598	0.047386	1.3244	0.42361	618.5753	HMDB11764 ; HMDB15207	Cer(d18:020:0) ; Perhexiline	M+Na ; 2M+ACN+Na	C38H77NO3 ; C19H35N
6381_278.6	1.56398	1.016936	2.3368	0.041563	1.3813	0.42361	465.9826	HMDB01003 ; HMDB08898 ; HMDB09186 ; HMDB11688 ; HMDB56389 ; HMDB56407 ; HMDB57479 ; HMDB57480 ; HMDB57482 ; HMDB57488 ; HMDB57498 ; HMDB57518 ; HMDB00082	Adenosine phosphosulfate ; PE(15:018:4(6Z9Z12Z15Z)) ; PE(18:4(6Z9Z12Z15Z)15:0) ; Ribose 15-bisphosphate ; CL(16:016:016:016:1(9Z)) ; CL(16:016:016:1(9Z)16:0) ; CL(16:1(9Z)16:1(9Z)16:1(9Z)18:1(11Z)) ; CL(16:1(9Z)16:1(9Z)16:1(9Z)18:1(9Z)) ; CL(16:1(9Z)16:1(9Z)16:1(9Z)20:4(5Z8Z11Z14Z)) ; CL(16:1(9Z)16:1(9Z)18:1(11Z)16:1(9Z)) ; CL(16:1(9Z)16:1(9Z)18:1(9Z)16:1(9Z)) ; CL(16:1(9Z)16:1(9Z)20:4(5Z8Z11Z14Z)16:1(9Z)) ; Cytidine triphosphate	M+K ; 2M+3H ; 2M+3H ; 3M+2H ; M+H+2Na ; M+H+2Na ; M+2H+Na ; M+2H+Na ; M+3H ; M+2H+Na ; M+2H+Na ; M+3H ; M+H-H2O	C10H14N5O10PS ; C38H68NO8P ; C38H68NO8P ; C5H12O11P2 ; C73H140O17P2 ; C73H140O17P2 ; C75H138O17P2 ; C75H138O17P2 ; C77H136O17P2 ; C75H138O17P2 ; C75H138O17P2 ; C77H136O17P2 ; C9H16N3O14P3

5127_237.5	1.563969	0.188362	-2.3692	0.039328	1.4053	0.42361	135.0805	HMDB29639 ; HMDB29697 ; HMDB29698 ; HMDB30839 ; HMDB31626 ; HMDB32024 ; HMDB32142 ; HMDB32608 ; HMDB32623 ; HMDB33716 ; HMDB34107 ; HMDB34563 ; HMDB36441 ; HMDB59601 ; HMDB59895 ; HMDB14400 ; HMDB15671 ; HMDB36133 ; HMDB36207 ; HMDB37705 ; HMDB38947 ; HMDB41250	(4-Methylphenyl)acetaldehyde ; Cinnamyl alcohol ; trans-Cinnamyl alcohol ; (Z)-4-(1-Propenyl)phenol ; 2-Phenylpropanal ; 4-Ethylbenzaldehyde ; 24-Dimethylbenzaldehyde ; 4-Methylacetophenone ; 1-Phenyl-1-propanone ; 3-Phenylpropanal ; Chavicol ; 7-Nonene-35-diyn-1-ol ; o-Vinylanisole ; Indan-1-ol ; m-Methylacetophenone ; Diethylstilbestrol ; Ammonium lactate ; 3-5-Methyl-2-(1-methylethyl)cyclohexyloxy-12-propanediol ; alpha-Amylcinnamyl acetate ; Heptyl cinnamate ; (S)-3-Methylthiohexyl hexanoate ; Demethoxyshogaol	M+H ; M+H ; M+H ; M+H ; M+H ; M+H ; M+H ; M+H ; M+H ; M+H ; M+H ; M+H ; M+H ; M+H ; M+H ; M+2H ; 2M+3H2O+2H ; M+H+K ; M+H+Na ; M+H+Na ; M+H+Na ; M+H+Na	C9H10O ; C9H10O ; C9H10O ; C9H10O ; C9H10O ; C9H10O ; C9H10O ; C9H10O ; C9H10O ; C9H10O ; C9H10O ; C9H10O ; C9H10O ; C9H10O ; C9H10O ; C18H20O2 ; C3H9NO3 ; C13H26O3 ; C16H22O2 ; C16H22O2 ; C13H26O2S ; C16H22O2
7163_50.4	1.56341	1.172308	-2.3178	0.042934	1.3672	0.42361	388.7117	HMDB09195 ; HMDB31227 ; HMDB32529 ; HMDB38642 ; HMDB60956	PE(18:4(6Z9Z12Z15Z)18:4(6Z9Z12Z15Z)) ; Marshdimerin ; Thaumatin b recombinant ; Fumitremorgin C ; N-desmethylmirtazapine	M+2Na ; M+H+NH4 ; 2M+H+NH4 ; 2M+H+NH4 ; 3M+H+Na	C41H66NO8P ; C48H54O8 ; C19H29N3O3S ; C22H25N3O3 ; C16H17N3
7565_260.7	1.561123	1.054926	2.3939	0.037705	1.4236	0.42361	265.0508	HMDB05973 ; HMDB14868 ; HMDB29271 ; HMDB29418 ; HMDB29836 ; HMDB30967 ; HMDB32124 ; HMDB32438 ; HMDB32926 ; HMDB33894 ; HMDB33961 ; HMDB34180 ; HMDB37144 ; HMDB37366 ; HMDB38083 ; HMDB38465 ; HMDB38867 ; HMDB39923 ; HMDB40633 ; HMDB41872 ; HMDB29492 ; HMDB29676 ; HMDB30617 ; HMDB30659 ; HMDB30667 ; HMDB32695 ; HMDB32696 ; HMDB33321 ; HMDB33720 ; HMDB33760 ; HMDB34443 ; HMDB37339 ; HMDB37441 ; HMDB42024	Dimethyltryptamine ; Thiabendazole ; Quercetin 3-O-(6-acetyl-glucoside) ; S-Cysteinosuccinic acid ; 1234-Tetrahydro-2-methyl-b-carboline ; 246-Octatriynoic acid ; (Z)-Methyl 3-(methylsulfinyl)-1-propenyl disulfide ; 1-Isopropyl citrate ; Polycartine B ; Tricin 7-glucuronoside ; 2-(3-Methylbutyl)-1H-pyrrolo23-bpyridine ; Protohypericin ; 1-Phenyl-5-propyl-1H-pyrazole ; Quercetin 3-(6-acetylglucoside) ; 2-Isopropyl citrate ; Glyphoside ; Diospyrin ; 4-O-Acetylmyricitrin ; Isobiflorin 6-gallate ; Detomidine ; 337-Trihydroxy-4-methoxyflavone ; Diosmetin ; Pratensein ; Takakin ; Chrysoeriol ; Barpisoflavone A ; Cajanin ; 35-Dihydroxy-67-methylenedioxyflavanone ; 6alpha-Hydroxymaackiain ; Santal ; Questinol ; Luteolin 7-methyl ether ; Kaempferide ; Tectorigenin	M+2K-H ; M+ACN+Na ; M+H+Na ; 2M+3H2O+2H ; M+2K+H ; 2M+H ; M+2ACN+H ; 2M+Na+K ; M+2K-H ; M+H+Na ; M+2K-H ; M+H+Na ; M+2K+H ; M+H+Na ; 2M+Na+K ; M+H+Na ; M+2Na ; M+H+Na ; M+H+Na ; M+2K+H ; M+H-2H2O ; M+H-2H2O ; M+H-2H2O ; M+H-2H2O ; M+H-2H2O ; M+H-2H2O ; M+H-2H2O ; M+H-2H2O ; M+H-2H2O ; M+H-2H2O ; M+H-2H2O ; M+H-2H2O ; M+H-2H2O ; M+H-2H2O	C12H16N2 ; C10H7N3S ; C23H22O13 ; C7H11NO6S ; C12H14N2 ; C8H4O2 ; C5H10OS3 ; C9H14O7 ; C12H16N2 ; C23H22O13 ; C12H16N2 ; C30H18O8 ; C12H14N2 ; C23H22O13 ; C9H14O7 ; C23H22O13 ; C21H24O13 ; C23H22O13 ; C23H22O13 ; C12H14N2 ; C16H12O6 ; C16H12O6 ; C16H12O6 ; C16H12O6 ; C16H12O6 ; C16H12O6 ; C16H12O6 ; C16H12O6 ; C16H12O6 ; C16H12O6 ; C16H12O6 ; C16H12O6 ; C16H12O6 ; C16H12O6
1582_274.4	1.561049	0.094797	2.4051	0.036991	1.4319	0.42361	342.9816	HMDB32437 ; HMDB32653 ; HMDB59647	Monoglyceride citrate ; (Z)-3-(Methylsulfinyl)-1-propenyl 2-propenyl disulfide ; Adenosine 5-pentaphosphate	M+2K-H ; 3M+Na+K ; M+H+NH4	C9H14O9 ; C7H12OS3 ; C10H18N5O19P5

36409_285.9	1.561006	1.084275	2.2703	0.046546	1.3321	0.42361	512.6036	HMDB03937 ; HMDB11120 ; HMDB11876 ; HMDB60372	3-Oxododecanoyl-CoA ; trans-2-Enoyl-OPC4-CoA ; Ganglioside GM1 (d18:014:0) ; 3-Hydroxy-3-(4-methylpent-3-en-1-yl)glutaryl-CoA	M+Na+K ; M+H+K ; M+H+2Na ; M+2Na	C33H56N7O18P3S ; C35H54N7O18P3S ; C70H126N2O31 ; C32H52N7O20P3S
30756_234.5	1.560002	1.22019	2.3776	0.038774	1.4115	0.42361	290.0981	HMDB01267 ; HMDB02091 ; HMDB04145 ; HMDB04146 ; HMDB14395 ; HMDB15253 ; HMDB15292 ; HMDB15599 ; HMDB31722 ; HMDB33136 ; HMDB35830 ; HMDB38883 ; HMDB41560 ; HMDB41679 ; HMDB41727 ; HMDB60326 ; HMDB60327 ; HMDB60329 ; HMDB60330 ; HMDB60736 ; HMDB05005 ; HMDB28786 ; HMDB29080 ; HMDB29450	4-Oxo-4-(3-pyridyl)-butanamide ; Isovalerylglucuronide ; BOX B ; BOX A ; Dapsone ; Phenacemide ; Chloroprocaine ; Enoximone ; 345-Trimethoxyphenyl acetate ; 2-Amino-4-hydroxy-6-pteridinecarboxylic acid ; Genipin ; Sambacin ; 246-Trimethoxyphenyl acetate ; 4-Hydroxy-(34-dihydroxyphenyl)-valeric acid ; Dihydrosinapic acid ; 1-Nitro-5-glutathionyl-6-hydroxy-56-dihydronaphthalene ; 1-Nitro-5-hydroxy-6-glutathionyl-56-dihydronaphthalene ; 1-Nitro-7-glutathionyl-8-hydroxy-78-dihydronaphthalene ; 1-Nitro-7-hydroxy-8-glutathionyl-78-dihydronaphthalene ; 3-(4-Hydroxy-3-methoxyphenyl)-2-methyllactic acid ; Tolnaftate ; Cysteinyl-Tryptophan ; Tryptophyl-Cysteine ; Aspergillomarasmine A	3M+2Na ; 2M+H+Na ; 3M+2Na ; 3M+2Na ; M+ACN+H ; 3M+2Na ; 2M+H+K ; M+ACN+H ; M+ACN+Na ; M+2ACN+H ; M+ACN+Na ; M+H+K ; M+ACN+Na ; M+ACN+Na ; M+ACN+Na ; M+2ACN+2H ; M+2ACN+2H ; M+2ACN+2H ; M+2ACN+2H ; M+ACN+Na ; M+H-H2O ; M+H-H2O ; M+H-H2O ; M+H-H2O	C9H10N2O2 ; C11H18O8 ; C9H10N2O2 ; C9H10N2O2 ; C12H12N2O2S ; C9H10N2O2 ; C13H19ClN2O2 ; C12H12N2O2S ; C11H14O5 ; C7H5N5O3 ; C11H14O5 ; C26H36O12 ; C11H14O5 ; C11H14O5 ; C11H14O5 ; C20H24N4O9S ; C20H24N4O9S ; C20H24N4O9S ; C20H24N4O9S ; C11H14O5 ; C19H17NOS ; C14H17N3O3S ; C14H17N3O3S ; C10H17N3O8
00868_248.9	1.559883	0.933719	-2.3878	0.038099	1.4191	0.42361	161.1001	HMDB31572 ; HMDB32426 ; HMDB01345 ; HMDB02195 ; HMDB04063 ; HMDB14562 ; HMDB14862 ; HMDB15228 ; HMDB29096 ; HMDB29138 ; HMDB30324 ; HMDB30325 ; HMDB30338 ; HMDB33364 ; HMDB36074 ; HMDB39839 ; HMDB39840 ; HMDB60558	2-Methyl-4-propyl-13-oxathiane ; (-)-3-(Methylthio)heptanal ; 4-Trimethylammoniobutanal ; Varanic acid ; Metanephrine ; Secobarbital ; Imiquimod ; Oxamniquine ; Tryptophyl-Valine ; Valyl-Tryptophan ; (R)-Pelletierine ; ()-Pelletierine ; Physoperuvine ; Conhydrinone ; Tenuazonic acid ; 1-(1-Pyrrolidinyl)-2-butanone ; 3-(1-Pyrrolidinyl)-2-butanone ; Desglymidodrine	M+H ; M+H ; 2M+Na+K ; M+H+2Na ; M+3ACN+2H ; M+2ACN+2H ; 2M+3H ; M+ACN+2H ; M+H+NH4 ; M+H+NH4 ; 2M+H+K ; 2M+H+K ; 2M+H+K ; 2M+H+K ; M+3ACN+2H ; 2M+H+K ; 2M+H+K ; M+3ACN+2H	C8H16OS ; C8H16OS ; C7H16NO ; C26H44O5 ; C10H15NO3 ; C12H18N2O3 ; C14H16N4 ; C14H21N3O3 ; C16H21N3O3 ; C16H21N3O3 ; C8H15NO ; C8H15NO ; C8H15NO ; C8H15NO ; C10H15NO3 ; C8H15NO ; C8H15NO ; C10H15NO3
0787_288.7	1.55964	1.125796	2.2439	0.048679	1.3127	0.42643	464.0808	HMDB00536 ; HMDB59653 ; HMDB06779 ; HMDB14576 ; HMDB30481 ; HMDB38370 ; HMDB39907 ; HMDB41059 ; HMDB60383	Adenylsuccinic acid ; N(6)-(12-dicarboxyethyl)AMP ; Indole-56-quinone ; Trifluridine ; Agrocybin ; Procyanidin C1 ; Tricin 7-sinapoyl-(-2)-glucuronyl-(1-2)-glucuronide ; N-Methoxyspirobrassinol methyl ether ; 4-(Methylnitrosamino)-1-(3-pyridyl)-1-butanol glucuronide	M+H ; M+H ; 3M+Na ; 3M+H+K ; 3M+Na ; M+Na+K ; M+H+K ; 3M+H+K ; M+2K+H	C14H18N5O11P ; C14H18N5O11P ; C8H5NO2 ; C10H11F3N2O5 ; C8H5NO2 ; C45H38O18 ; C40H40O23 ; C13H16N2O2S2 ; C16H23N3O8
24244_47.8	1.559487	1.291901	-2.3255	0.042375	1.3729	0.42361	NA	NA	NA	NA	NA

24286_47.5	1.559421	1.25062	-2.3379	0.041489	1.3821	0.42361	518.6524	HMDB41267 ; HMDB56724 ; HMDB56756 ; HMDB56762 ; HMDB58706 ; HMDB58859 ; HMDB58967 ; HMDB60165	Quercetin 3-(6-sinapoylsophorotrioside) ; CL(16:018:2(9Z12Z)20:4(5Z8Z11Z14Z)20:4(5Z8Z11Z14Z)) ; CL(16:020:4(5Z8Z11Z14Z)18:2(9Z12Z)20:4(5Z8Z11Z14Z)) ; CL(16:020:4(5Z8Z11Z14Z)20:4(5Z8Z11Z14Z)18:2(9Z12Z)) ; CL(18:2(9Z12Z)16:020:4(5Z8Z11Z14Z)20:4(5Z8Z11Z14Z)) ; CL(18:2(9Z12Z)20:4(5Z8Z11Z14Z)20:4(5Z8Z11Z14Z)16:0) ; CL(20:4(5Z8Z11Z14Z)16:020:4(5Z8Z11Z14Z)18:2(9Z12Z)) ; Pentadecanoyl Coenzyme A	M+ACN+2H ; M+3Na ; M+3Na ; M+3Na ; M+3Na ; M+3Na ; M+3Na ; M+2Na	C44H50O26 ; C84H144O17P2 ; C84H144O17P2 ; C84H144O17P2 ; C84H144O17P2 ; C84H144O17P2 ; C84H144O17P2 ; C36H64N7O17P3S
22603_47.74	1.559096	1.275377	-2.3104	0.043478	1.3617	0.42361	264.8523	HMDB41974 ; HMDB07937 ; HMDB08033 ; HMDB08841 ; HMDB08932 ; HMDB08991 ; HMDB09220 ; HMDB09481 ; HMDB60501	Pentachlorophenol ; PC(15:018:0) ; PC(18:015:0) ; PE(14:022:0) ; PE(16:020:0) ; PE(18:018:0) ; PE(20:016:0) ; PE(22:014:0) ; Phosphatidylethanolamine	M+H ; M+H+2Na ; M+H+2Na ; M+H+2Na ; M+H+2Na ; M+H+2Na ; M+H+2Na ; M+H+2Na ; M+H+2Na	C6HCl5O ; C41H82NO8P ; C41H82NO8P ; C41H82NO8P ; C41H82NO8P ; C41H82NO8P ; C41H82NO8P ; C41H82NO8P ; C41H82NO8P
9527_56.99	1.559037	1.181812	2.2887	0.045113	1.3457	0.42361	176.0095	HMDB00875 ; HMDB01123 ; HMDB01392 ; HMDB01538 ; HMDB01891 ; HMDB13680 ; HMDB15687 ; HMDB29276 ; HMDB29806 ; HMDB31242 ; HMDB31253 ; HMDB31779 ; HMDB32160 ; HMDB32334 ; HMDB35088 ; HMDB35115 ; HMDB39877 ; HMDB59651 ; HMDB59978 ; HMDB59979 ; HMDB60722 ; HMDB61134 ; HMDB61160	Trigonelline ; 2-Aminobenzoic acid ; p-Aminobenzoic acid ; 3-Pyridylacetic acid ; m-Aminobenzoic acid ; Caftaric acid ; Salicylamide ; Cis-Caffeoyl tartaric acid ; Methyl nicotinate ; Dihydro-3(2H)-thiophenone ; Dihydro-2(3H)-thiophenone ; Isoprothiolane ; 3-(Acetylthio)-2-methylfuran ; 1-(3-Hydroxy-5-methyl-2-thienyl)ethanone ; Furfuryl thioacetate ; (1xi3S)-1234-Tetrahydro-1-methyl-beta-carboline-13-dicarboxylic acid ; Charine ; 2-(34-Dihydroxybenzoyloxy)-46-dihydroxybenzoate ; 4-Hydroxy-5-(dihydroxyphenyl)-valeric acid-O-sulphate ; 4-Hydroxy-5-(dihydroxyphenyl)-valeric acid-O-sulphate III ; 2-Pyridylacetic acid ; Dedimethylchlorpromazine ; N-Dedimethylchlorpromazine	M+K ; M+K ; M+K ; M+K ; M+K ; M+H+K ; M+K ; M+H+K ; M+K ; 3M+2Na ; 3M+2Na ; M+Na+K ; 2M+H+K ; 2M+H+K ; 2M+H+K ; M+2K ; M+2K ; M+2Na ; M+2Na ; M+2Na ; M+K ; M+Na+K ; M+Na+K	C7H7NO2 ; C7H7NO2 ; C7H7NO2 ; C7H7NO2 ; C7H7NO2 ; C13H12O9 ; C7H7NO2 ; C13H12O9 ; C7H7NO2 ; C4H6OS ; C4H6OS ; C12H18O4S2 ; C7H8O2S ; C7H8O2S ; C7H8O2S ; C14H14N2O4 ; C9H14N4O6 ; C14H10O8 ; C11H14O8S ; C11H14O8S ; C7H7NO2 ; C15H15ClN2S ; C15H15ClN2S
25231_47.66	1.557674	1.253374	-2.3212	0.042684	1.3697	0.42361	442.7225	HMDB14812 ; HMDB14887 ; HMDB15005 ; HMDB33874 ; HMDB40699 ; HMDB41956 ; HMDB60548 ; HMDB60796 ; HMDB60798 ; HMDB60854	Galantamine ; Etodolac ; Ritodrine ; Piperanine ; Feruperine ; Nordihydrocodeine ; Dihydromorphine ; 6alpha-Hydroxy-hydromorphone ; 6beta-Hydroxy-hydromorphone ; Nordihydroisomorphine	3M+H+Na ; 3M+H+Na ; 3M+H+Na ; 3M+H+Na ; 3M+H+Na ; 3M+H+Na ; 3M+H+Na ; 3M+H+Na ; 3M+H+Na ; 3M+H+Na	C17H21NO3 ; C17H21NO3 ; C17H21NO3 ; C17H21NO3 ; C17H21NO3 ; C17H21NO3 ; C17H21NO3 ; C17H21NO3 ; C17H21NO3 ; C17H21NO3

1314_45.9	1.557542	0.851157	2.4775	0.03268	1.4857	0.42361	351.0991	HMDB14561 ; HMDB00394 ; HMDB02326 ; HMDB03066 ; HMDB04816 ; HMDB05034 ; HMDB06524 ; HMDB14405 ; HMDB14723 ; HMDB29893 ; HMDB30600 ; HMDB33535 ; HMDB33718 ; HMDB35478 ; HMDB37586 ; HMDB40829 ; HMDB60323 ; HMDB60739	Penicillin V ; 3-Hydroxytetradecanedioic acid ; Coumesterol ; Chalcone ; FAPy-adenine ; Topiramate ; 3-Indoleacetonitrile ; Cycloserine ; Nizatidine ; Sucrose octaacetate ; 4-(3-Methyl-1-butenyl)-3345-tetrahydroxystilbene ; Honyudisin ; Clitocine ; 2345-Tetrahydroxy-4-prenylstilbene ; Gancaonin V ; N-(1-Deoxy-b-D-fructopyranosyl) (R)C(S)S-alliin ; 1-Methoxyphenanthrene ; 3-Amino-2-oxazolidone	M+H ; M+2K-H ; M+2ACN+H ; 3M+2K ; 2M+2Na-H ; 2M+H+Na ; 2M+K ; 3M+2Na-H ; 2M+H+K ; M+H+Na ; M+K ; M+K ; M+ACN+Na ; M+K ; M+K ; 2M+H+Na ; 3M+2K ; 3M+2Na-H	C16H18N2O5S ; C14H26O5 ; C15H8O5 ; C15H12O ; C5H7N5O ; C12H21NO8S ; C10H8N2 ; C3H6N2O2 ; C12H21N5O2S2 ; C28H38O19 ; C19H20O4 ; C19H20O4 ; C9H13N5O6 ; C19H20O4 ; C19H20O4 ; C12H21NO8S ; C15H12O ; C3H6N2O2
8361_289.	1.55734	0.974009	2.3843	0.038328	1.4165	0.42361	594.6848	HMDB00768 ; HMDB32878 ; HMDB38554 ; HMDB40450	N-Acetyl-9-O-lactoylneuraminic acid ; 13-Oxocryptopine ; Gravacridonediolacetate ; 23456-Pentachlorobenzyl alcohol	3M+2Na ; 3M+H+K ; 3M+H+K ; 2M+K	C14H23NO11 ; C21H21NO6 ; C21H21NO6 ; C7H3Cl5O
6798_47.6	1.557173	1.216162	-2.329	0.042119	1.3755	0.42361	434.6907	HMDB15585 ; HMDB32423	Chlophedianol ; 2-(4-Methyl-5-thiazolyl)ethyl octanoate	3M+2H ; 3M+Na+K	C17H20ClNO ; C14H23NO2S
4455_199.	1.556409	1.254429	-2.3378	0.041497	1.382	0.42361	450.0804	HMDB28818	Glutamyl-Glutamate	3M+2K	C10H14N2O7
6196_239.	1.555403	1.224204	2.3603	0.039933	1.3987	0.42361	291.9996	HMDB01059 ; HMDB01187 ; HMDB03848 ; HMDB04527 ; HMDB12245 ; HMDB12285 ; HMDB15627	Inositol 1345-tetraphosphate ; 1D-Myo-inositol 1346-tetrakisphosphate ; D-Myo-inositol 3456-tetrakisphosphate ; 1D-Myo-inositol 1456-tetrakisphosphate ; Kinetin ; Ribose-1-arsenate ; Droxidopa	M+2ACN+2H ; M+2ACN+2H ; M+2ACN+2H ; M+2ACN+2H ; M+2K-H ; M+NH4 ; M+2K+H	C6H16O18P4 ; C6H16O18P4 ; C6H16O18P4 ; C6H16O18P4 ; C10H9N5O ; C5H11AsO8 ; C9H11NO5
7946_47.4	1.555012	1.310315	-2.3075	0.043696	1.3596	0.42361	NA	NA	NA	NA	NA
6301_199.	1.553577	1.016291	-2.3744	0.038984	1.4091	0.42361	146.5246	HMDB01355 ; HMDB00727 ; HMDB00845 ; HMDB00877 ; HMDB02109 ; HMDB03976 ; HMDB29895	Parathion ; L-Threoneopterin ; Neopterin ; Umanopterin ; Hydroxysepiapterin ; D-Glucuronic acid 1-phosphate ; D-erythro-Eritadenine	M+2H ; M+H+K ; M+H+K ; M+H+K ; M+H+K ; M+H+NH4 ; M+H+K	C10H14NO5PS ; C9H11N5O4 ; C9H11N5O4 ; C9H11N5O4 ; C9H11N5O4 ; C6H11O10P ; C9H11N5O4
5987_239.	1.552694	1.17218	2.2699	0.04658	1.3318	0.42361	549.5896	HMDB39203	Camelliatannin A	M+ACN+2H	C49H36O27
3399_198.	1.552611	1.043642	-2.3723	0.039121	1.4076	0.42361	340.0433	HMDB01316 ; HMDB10716 ; HMDB14589 ; HMDB28852 ; HMDB29083 ; HMDB29294 ; HMDB34454 ; HMDB40924 ; HMDB41724 ; HMDB60872	6-Phosphogluconic acid ; Gamma-Glutamyl-Se-methylselenocysteine ; Chloramphenicol ; Glycyl-Tryptophan ; Tryptophyl-Glycine ; Caffeoyl aspartic acid ; 5-(4-Acetoxy-1-butynyl)-22-bithiophene ; 2-Carboxyarabinitol 5-phosphate ; Dihydroferulic acid 4-sulfate ; Sorafenib beta-D-Glucuronide	M+ACN+Na ; 2M+3H2O+2H ; M+NH4 ; M+2K+H ; M+2K+H ; M+2Na-H ; M+ACN+Na ; M+ACN+Na ; M+ACN+Na ; M+H+K	C6H13O10P ; C9H16N2O5Se ; C11H12Cl2N2O5 ; C13H15N3O3 ; C13H15N3O3 ; C13H13NO7 ; C14H12O2S2 ; C6H13O10P ; C10H12O7S ; C27H24ClF3N4O9

;6802_47.62	1.552168	1.265301	-2.3183	0.0429	1.3675	0.42361	382.7667	HMDB08946 ; HMDB08977 ; HMDB08978 ; HMDB09038 ; HMDB09071 ; HMDB09102 ; HMDB09103 ; HMDB09133 ; HMDB09134 ; HMDB09166 ; HMDB09167 ; HMDB09198 ; HMDB09294 ; HMDB09325 ; HMDB09326 ; HMDB09358 ; HMDB09359 ; HMDB09390 ; HMDB09423 ; HMDB09454 ; HMDB09455 ; HMDB09617 ; HMDB09650 ; HMDB09682 ; HMDB10169 ; HMDB13327 ; HMDB34616	PE(16:022:6(4Z7Z10Z13Z16Z19Z)) ; PE(16:1(9Z)22:5(4Z7Z10Z13Z16Z)) ; PE(16:1(9Z)22:5(7Z10Z13Z16Z19Z)) ; PE(18:1(11Z)20:5(5Z8Z11Z14Z17Z)) ; PE(18:1(9Z)20:5(5Z8Z11Z14Z17Z)) ; PE(18:2(9Z12Z)20:4(5Z8Z11Z14Z)) ; PE(18:2(9Z12Z)20:4(8Z11Z14Z17Z)) ; PE(18:3(6Z9Z12Z)20:3(5Z8Z11Z)) ; PE(18:3(6Z9Z12Z)20:3(8Z11Z14Z)) ; PE(18:3(9Z12Z15Z)20:3(5Z8Z11Z)) ; PE(18:3(9Z12Z15Z)20:3(8Z11Z14Z)) ; PE(18:4(6Z9Z12Z15Z)20:2(11Z14Z)) ; PE(20:2(11Z14Z)18:4(6Z9Z12Z15Z)) ; PE(20:3(5Z8Z11Z)18:3(6Z9Z12Z)) ; PE(20:3(5Z8Z11Z)18:3(9Z12Z15Z)) ; PE(20:3(8Z11Z14Z)18:3(6Z9Z12Z)) ; PE(20:3(8Z11Z14Z)18:3(9Z12Z15Z)) ; PE(20:4(5Z8Z11Z14Z)18:2(9Z12Z)) ; PE(20:4(8Z11Z14Z17Z)18:2(9Z12Z)) ; PE(20:5(5Z8Z11Z14Z17Z)18:1(11Z)) ; PE(20:5(5Z8Z11Z14Z17Z)18:1(9Z)) ; PE(22:5(4Z7Z10Z13Z16Z)16:1(9Z)) ; PE(22:5(7Z10Z13Z16Z19Z)16:1(9Z)) ; PE(22:6(4Z7Z10Z13Z16Z19Z)16:0) ; SM(d18:116:0) ; Dodecanedioylcarnitine ; 22-Dibromo-2-cyanoacetamide	M+2H ; M+Na+K ; 2M+H+NH4 ; 3M+2Na	C43H74NO8P ; C39H80N2O6P ; C19H35NO6 ; C3H2Br2N2O
'8515_58.69	1.551772	1.051765	-2.375	0.038947	1.4095	0.42361	255.9779	HMDB00714 ; HMDB00992 ; HMDB12884 ; HMDB32398 ; HMDB32595 ; HMDB40612	Hippuric acid ; 3-Succinoylpyridine ; Adrenochrome ; Methyl n-formylanthranilate ; 1-(4-Methoxyphenyl)-2-nitroethylene ; 4-Acetyl-2(3H)-benzoxazolone	M+2K-H ; M+2K-H ; M+2K-H ; M+2K-H ; M+2K-H ; M+2K+H	C9H9NO3 ; C9H9NO3 ; C9H9NO3 ; C9H9NO3 ; C9H9NO3 ; C9H7NO3
;2411_110.9	1.551564	0.716232	-2.2675	0.046772	1.33	0.42361	NA	NA	NA	NA	NA
;9259_24.09	1.551432	0.535557	-2.3135	0.043252	1.364	0.42361	352.3389	HMDB01028	25-Azacholesterol	M+H-2H2O	C26H45NO
;3543_47.45	1.551092	1.293477	-2.2951	0.044627	1.3504	0.42361	458.6964	HMDB15097 ; HMDB31929	Zaleplon ; Zanthodioline	3M+2H ; 3M+2H	C17H15N5O ; C16H19NO5

3597_196.9	1.549792	1.057917	-2.3926	0.037792	1.4226	0.42361	353.0664	HMDB14939 ; HMDB30756 ; HMDB29263 ; HMDB29664 ; HMDB30475 ; HMDB30480 ; HMDB30696 ; HMDB30811 ; HMDB31706 ; HMDB33137 ; HMDB33268 ; HMDB34009 ; HMDB34060 ; HMDB35598 ; HMDB37096 ; HMDB37351 ; HMDB37354 ; HMDB37460 ; HMDB38333 ; HMDB38335 ; HMDB38659 ; HMDB40720 ; HMDB41096 ; HMDB41690 ; HMDB37371	Halazepam ; 13alpha-Hydroxydolineone ; 37-Dimethylquercetin ; Vanilloside ; Aflatoxin G2 ; Aflatoxin M2 ; Demethoxysudachitin ; Prudomestin ; 34-Dihydroxyphenacyl caffeate ; 2-Methoxycarbonylphenyl beta-D-glucopyranoside ; 135-Trihydroxy-67-dimethoxy-2-methylanthraquinone ; Trifolian ; 6-O-Acetylarbutin ; Aflatoxin B2a ; Pilosin ; Caryatin ; 34-Di-O-methylquercetin ; Tricin 7-diglucuronoside ; 2-Hydroxyphenylacetic acid O-b-D-glucoside ; (S)-Mandelic acid O-beta-D-Glucopyranoside ; Cicerin ; 2-O-Acetylarbutin ; Lepidimoic acid ; 57-Dihydroxy-84-dimethoxyisoflavone ; Suillusin	M+H ; M+H ; M+Na ; M+K ; M+Na ; M+Na ; M+Na ; M+Na ; M+Na ; M+K ; M+Na ; M+Na ; M+K ; M+Na ; M+Na ; M+Na ; M+Na ; M+H+Na ; M+K ; M+K ; M+Na ; M+K ; 2M+Na+K ; M+Na ; M+H-H2O	C17H12ClF3N2O ; C19H12O7 ; C17H14O7 ; C14H18O8 ; C17H14O7 ; C17H14O7 ; C17H14O7 ; C17H14O7 ; C17H14O7 ; C14H18O8 ; C17H14O7 ; C17H14O7 ; C14H18O8 ; C17H14O7 ; C17H14O7 ; C17H14O7 ; C17H14O7 ; C29H30O19 ; C14H18O8 ; C14H18O8 ; C17H14O7 ; C14H18O8 ; C12H18O10 ; C17H14O7 ; C19H14O8
8464_284.2	1.549255	1.145924	2.2694	0.046615	1.3315	0.42361	489.5978	HMDB30835 ; HMDB40453 ; HMDB60873	trans-334557-Hexahydroxyflavanone ; Theaflavonin ; Sorafenib N-oxide	3M+H+NH4 ; M+ACN+2H ; 2M+H+NH4	C15H12O8 ; C43H36O24 ; C21H16ClF3N4O4
2086_47.72	1.548423	1.258796	-2.3129	0.04329	1.3636	0.42361	NA	NA	NA	NA	NA
.8943_276.7	1.547994	0.279411	2.5277	0.029988	1.5231	0.42361	NA	NA	NA	NA	NA
90504_53.3	1.546756	1.060955	-2.3635	0.039715	1.401	0.42361	400.0291	HMDB12143 ; HMDB29395	2-Amino-4-oxo-4-alpha-hydroxy-6-(erythro-123-trihydroxypropyl)-5678-tetrahydroxypterin ; L-L-Homoglutathione	M+2K+H ; M+2K+H	C9H15N5O8 ; C11H19N3O6S
5529_47.79	1.546675	1.239642	-2.311	0.043431	1.3622	0.42361	NA	NA	NA	NA	NA
84218_215.	1.546408	1.165906	2.316	0.043067	1.3659	0.42361	306.5784	HMDB30099 ; HMDB37089 ; HMDB35475	Delphinidin 3-(3-p-coumaroylglucoside) ; Delphinidin 3-(6-p-coumaroylgalactoside) ; Simmondsin 2-ferulate	M+2H ; M+2H ; M+Na+K	C30H27O14 ; C30H27O14 ; C26H33NO12
9689_40.04	1.546235	0.825125	-2.2842	0.045462	1.3424	0.42361	86.0965	HMDB34301 ; HMDB31630	Piperidine ; Propane	M+H ; M+ACN+H	C5H11N ; C3H8
4729_190.4	1.54614	1.171946	2.355	0.040294	1.3948	0.42361	213.9895	HMDB00034 ; HMDB00699 ; HMDB01000 ; HMDB14871 ; HMDB37597 ; HMDB41770	Adenine ; 1-Methylnicotinamide ; dUDP ; Pralidoxime ; 8-Hydroxyluteolin 8-sulfate ; Quercetin 3-sulfate	M+2K+H ; M+2K-H ; M+H+K ; M+2K-H ; M+2Na ; M+2Na	C5H5N5 ; C7H9N2O ; C9H14N2O11P2 ; C7H9N2O ; C15H10O10S ; C15H10O10S
6182_205.1	1.543785	1.208585	2.2985	0.044367	1.3529	0.42361	174.0062	HMDB01409 ; HMDB02022 ; HMDB31612 ; HMDB32973 ; HMDB33305 ; HMDB33702 ; HMDB33759 ; HMDB33940 ; HMDB34376 ; HMDB37319 ; HMDB39734 ; HMDB40868	dUMP ; Glycineamideribotide ; 35-Dimethoxy-27-phenanthrenediol ; 25-Dimethyl-1H-pyrrole ; Isomedicarpin ; Pinostrobin chalcone ; Dihydroformononetin ; Vignafuran ; Imperatorin ; 3-(4-Hydroxyphenyl)-1-(4-hydroxy-2-methoxyphenyl)-2-propen-1-one ; 23579-Pentathiadecane 22-dioxide ; 4-O-Methylpinosylvic acid	M+H+K ; M+Na+K ; M+2K ; M+2K+H ; M+2K ; M+2K ; M+2K ; M+2K ; M+2K ; M+2K ; M+2ACN+2H ; M+2K	C9H13N2O8P ; C7H15N2O8P ; C16H14O4 ; C6H9N ; C16H14O4 ; C16H14O4 ; C16H14O4 ; C16H14O4 ; C16H14O4 ; C16H14O4 ; C5H12O2S5 ; C16H14O4

3098_47.5	1.543684	1.260872	-2.2826	0.045586	1.3412	0.42361	514.6583	HMDB03952 ; HMDB10308 ; HMDB10309 ; HMDB10310 ; HMDB10311 ; HMDB11123 ; HMDB31839 ; HMDB35362 ; HMDB39556 ; HMDB40491 ; HMDB57714 ; HMDB57724 ; HMDB57828 ; HMDB59010 ; HMDB59020 ; HMDB59105	ciscis-36-Dodecadienoyl-CoA ; CL(22:6(4Z7Z10Z13Z16Z19Z)20:4(5Z8Z11Z14Z)18:3(6Z9Z12Z)18:2(9Z12Z)) ; CL(22:6(4Z7Z10Z13Z16Z19Z)20:4(5Z8Z11Z14Z)18:3(9Z12Z15Z)18:2(9Z12Z)) ; CL(22:6(4Z7Z10Z13Z16Z19Z)20:4(8Z11Z14Z17Z)18:3(6Z9Z12Z)18:2(9Z12Z)) ; CL(22:6(4Z7Z10Z13Z16Z19Z)20:4(8Z11Z14Z17Z)18:3(9Z12Z15Z)18:2(9Z12Z)) ; transcis-Lauro-26-dienoyl-CoA ; Ginsenoside F5 ; Notoginsenoside R2 ; Ginsenoside F3 ; Pseudoginsenoside RT3 ; CL(16:1(9Z)20:4(5Z8Z11Z14Z)20:4(5Z8Z11Z14Z)22:6(4Z7Z10Z13Z16Z19Z)) ; CL(16:1(9Z)20:4(5Z8Z11Z14Z)22:6(4Z7Z10Z13Z16Z19Z)20:4(5Z8Z11Z14Z)) ; CL(16:1(9Z)22:6(4Z7Z10Z13Z16Z19Z)20:4(5Z8Z11Z14Z)20:4(5Z8Z11Z14Z)) ; CL(20:4(5Z8Z11Z14Z)16:1(9Z)20:4(5Z8Z11Z14Z)22:6(4Z7Z10Z13Z16Z19Z)) ; CL(20:4(5Z8Z11Z14Z)16:1(9Z)22:6(4Z7Z10Z13Z16Z19Z)20:4(5Z8Z11Z14Z)) ; CL(20:4(5Z8Z11Z14Z)20:4(5Z8Z11Z14Z)22:6(4Z7Z10Z13Z16Z19Z)16:1(9Z))	M+2ACN+2H ; M+2H+Na ; M+2H+Na ; M+2H+Na ; M+2H+Na ; M+2ACN+2H ; 2M+3H ; 2M+3H ; 2M+3H ; 2M+3H ; M+2H+Na ; M+2H+Na ; M+2H+Na ; M+2H+Na ; M+2H+Na ; M+2H+Na	C33H54N7O17P3S ; C87H140O17P2 ; C87H140O17P2 ; C87H140O17P2 ; C87H140O17P2 ; C33H54N7O17P3S ; C41H70O13 ; C41H70O13 ; C41H70O13 ; C41H70O13 ; C87H140O17P2 ; C87H140O17P2 ; C87H140O17P2 ; C87H140O17P2 ; C87H140O17P2 ; C87H140O17P2
0254_273.5	1.542857	1.138975	-2.3355	0.041659	1.3803	0.42361	NA	NA	NA	NA	NA
.5814_45.9	1.542715	0.788874	2.3804	0.038585	1.4136	0.42361	NA	NA	NA	NA	NA
7718_47.6	1.541525	1.292321	-2.279	0.045863	1.3385	0.42361	NA	NA	NA	NA	NA
0888_49.8	1.541091	0.41069	-2.3285	0.042158	1.3751	0.42361	477.7501	HMDB38173	Halocins	3M+H+K	C21H23NO
4889_47.7	1.539468	1.265332	-2.2801	0.045777	1.3394	0.42361	440.7255	HMDB37840 ; HMDB39780	N-(1-Deoxy-1-fructosyl)leucine ; N-(1-Deoxy-1-fructosyl)isoleucine	3M+2H ; 3M+2H	C12H23NO7 ; C12H23NO7
5459_47.8	1.539359	1.243931	-2.2954	0.044601	1.3507	0.42361	258.8175	HMDB29596	Chloroform	2M+Na	CHCl3
8132_47.7	1.536143	1.29371	-2.2709	0.046496	1.3326	0.42361	208.8908	HMDB60451 ; HMDB29627	Chloral hydrate ; 1235-Tetrachloro-4-methoxybenzene	M+2Na-H ; M+H-2H2O	C2H3Cl3O2 ; C7H4Cl4O
0427_58.3	1.535845	0.402522	2.3735	0.039041	1.4085	0.42361	192.123	HMDB00893 ; HMDB02023 ; HMDB28800 ; HMDB28801 ; HMDB28868 ; HMDB28905 ; HMDB28921 ; HMDB28927 ; HMDB28943 ; HMDB28952 ; HMDB29152 ; HMDB29153 ; HMDB33838 ; HMDB41606 ; HMDB59727 ; HMDB59757 ; HMDB60684 ; HMDB14494	Suberic acid ; Ethyladipic acid ; Glutaminyl-Isoleucine ; Glutaminyl-Leucine ; Hydroxyprolyl-Lysine ; Isoleucyl-Glutamine ; Isoleucyl-Gamma-glutamate ; Leucyl-Glutamine ; Leucyl-Gamma-glutamate ; Lysyl-Hydroxyproline ; Gamma-glutamyl-Isoleucine ; Gamma-glutamyl-Leucine ; Diethyl succinate ; Dimethyl adipate ; 24-Dimethyladipic acid ; 3-Methylpimelic acid ; 2-Propylglutaric acid ; Minoxidil	M+NH4 ; M+NH4 ; M+3ACN+2H ; M+3ACN+2H ; M+3ACN+2H ; M+3ACN+2H ; M+3ACN+2H ; M+3ACN+2H ; M+3ACN+2H ; M+3ACN+2H ; M+3ACN+2H ; M+3ACN+2H ; M+NH4 ; M+NH4 ; M+NH4 ; M+NH4 ; M+NH4 ; M+H-H2O	C8H14O4 ; C8H14O4 ; C11H21N3O4 ; C11H21N3O4 ; C11H21N3O4 ; C11H21N3O4 ; C11H21N3O4 ; C11H21N3O4 ; C11H21N3O4 ; C11H21N3O4 ; C11H21N3O4 ; C11H21N3O4 ; C8H14O4 ; C8H14O4 ; C8H14O4 ; C8H14O4 ; C8H14O4 ; C9H15N5O
1991_260.	1.535283	1.160348	2.2818	0.045649	1.3406	0.42361	368.0452	HMDB14971 ; HMDB13647 ; HMDB37851 ; HMDB38182	Cefaclor ; Adenosine thiamine diphosphate ; Apigenin 7-sulfate ; 47-Dihydro-5-(4-methyl-3-pentenyl)-123-trithiepin	M+H ; M+Na+K ; M+NH4 ; 3M+H+K	C15H14ClN3O4S ; C22H30N9O10P2S ; C15H10O8S ; C10H16S3
3514_55.9	1.534941	0.960599	-2.2441	0.048663	1.3128	0.42643	223.9644	HMDB00731 ; HMDB60274	Cysteine-S-sulfate ; Sedoheptulose 17-bisphosphate	M+Na ; M+2K	C3H7NO5S2 ; C7H16O13P2

)4192_260.6	1.534826	1.08235	2.2498	0.048197	1.317	0.42643	509.0904	HMDB36986 ; HMDB06215 ; HMDB12134 ; HMDB12138 ; HMDB12322 ; HMDB28728 ; HMDB28770 ; HMDB29479 ; HMDB30548 ; HMDB30805 ; HMDB31001 ; HMDB33650 ; HMDB36120 ; HMDB36167 ; HMDB36204 ; HMDB37332 ; HMDB37360 ; HMDB38686 ; HMDB39190 ; HMDB39211 ; HMDB39338 ; HMDB39511 ; HMDB40509 ; HMDB40510 ; HMDB41300 ; HMDB41743 ; HMDB41745 ; HMDB60301 ; HMDB60708	14-Dimethoxyglucobrassicin ; Naphthalene epoxide ; 12-Dihydroxy-3-keto-5-methylthiopentene ; 1-Naphthol ; 2-Naphthol ; Asparaginyl-Cysteine ; Cysteinyl-Asparagine ; Isorhamnetin 7-glucoside ; Nepitrin ; Pedaliin ; (Z)-2-Decene-468-triyn-1-ol ; Estragonoside ; Alliumoside A ; Bis(25-dimethyl-3-furanyl) disulfide ; 1-(Methylsulfanyl)-1-oxopropan-2-yl acetate ; 8-Hydroxyluteolin 4-methyl ether 8-glucoside ; Azaleatin 3-glucoside ; Armillaricin ; 2-O-(4-Hydroxycinnamoyl)-1-O-galloyl-beta-D-glucopyranoside ; 1358-Tetrahydroxy-6-methoxy-2-methylanthraquinone 8-O-b-D-glucoside ; Pollenin B ; Benzoyl meso-tartaric acid ; 6-Methoxyluteolin 3-glucoside ; Eupafolin 4-glucoside ; PRE ; Hesperetin 3-O-glucuronide ; Hesperetin 7-O-glucuronide ; (1S2R)-Naphthalene 12-oxide ; 15beta-hydroxycyproterone acetate	M+H ; 3M+2K-H ; 3M+Na ; 3M+2K-H ; 3M+2K-H ; 2M+K ; 2M+K ; 2M+Na+K ; 2M+Na+K ; 2M+Na+K ; 3M+2K-H ; 2M+Na+K ; 2M+Na+K ; 2M+H ; 3M+Na ; 2M+Na+K ; 2M+Na+K ; M+2K-H ; 2M+Na+K ; 2M+Na+K ; 2M+Na+K ; 2M+H ; 2M+Na+K ; 2M+Na+K ; M+Na+K ; 2M+Na+K ; 2M+Na+K ; 3M+2K-H ; M+2K-H	C18H24N2O11S2 ; C10H8O ; C6H10O3S ; C10H8O ; C10H8O ; C7H13N3O4S ; C7H13N3O4S ; C22H22O12 ; C22H22O12 ; C22H22O12 ; C10H8O ; C22H22O12 ; C22H22O12 ; C12H14O2S2 ; C6H10O3S ; C22H22O12 ; C22H22O12 ; C24H29ClO5 ; C22H22O12 ; C22H22O12 ; C22H22O12 ; C11H10O7 ; C22H22O12 ; C22H22O12 ; C44H44O24 ; C22H22O12 ; C22H22O12 ; C10H8O ; C24H29ClO5
8552_73.81	1.534348	0.379467	2.3684	0.039382	1.4047	0.42361	234.1086	HMDB28741 ; HMDB28806 ; HMDB29037 ; HMDB29053 ; HMDB29056 ; HMDB29158 ; HMDB00132 ; HMDB00403 ; HMDB00542 ; HMDB14410 ; HMDB15406 ; HMDB29048 ; HMDB30471 ; HMDB38276	Asparaginyl-Threonine ; Glutaminyl-Serine ; Serinyl-Glutamine ; Serinyl-Gamma-glutamate ; Threoninyl-Asparagine ; Gamma-glutamyl-Serine ; Guanine ; 2-Hydroxyadenine ; 8-Hydroxyadenine ; Crotamiton ; Glisoxepide ; Serinyl-Serine ; 14-Ipomeadiol ; cis-3-Hexenyl pyruvate	M+H ; M+H ; M+H ; M+H ; M+H ; M+H ; M+2ACN+H ; M+2ACN+H ; M+2ACN+H ; 2M+Na+K ; M+H+NH4 ; M+ACN+H ; M+ACN+Na ; M+ACN+Na	C8H15N3O5 ; C8H15N3O5 ; C8H15N3O5 ; C8H15N3O5 ; C8H15N3O5 ; C8H15N3O5 ; C5H5N5O ; C5H5N5O ; C5H5N5O ; C13H17NO ; C20H27N5O5S ; C6H12N2O5 ; C9H14O3 ; C9H14O3
34758_47.4	1.533397	1.248764	-2.2888	0.045102	1.3458	0.42361	444.6735	HMDB28799 ; HMDB28883 ; HMDB28899 ; HMDB29151 ; HMDB31304 ; HMDB32992	Glutaminyl-Histidine ; Histidinyl-Glutamine ; Histidinyl-Gamma-glutamate ; Gamma-glutamyl-Histidine ; Osmundalin ; Furaneol 4-glucoside	3M+H+K ; 3M+H+K ; 3M+H+K ; 3M+H+K ; 3M+H+NH4 ; 3M+H+NH4	C11H17N5O4 ; C11H17N5O4 ; C11H17N5O4 ; C11H17N5O4 ; C12H18O8 ; C12H18O8

4401_55.8	1.533128	0.884186	-2.2719	0.046424	1.3333	0.42361	123.0304	HMDB29572 ; HMDB31157 ; HMDB31708 ; HMDB31872 ; HMDB33552 ; HMDB41492 ; HMDB01228 ; HMDB04230 ; HMDB14738 ; HMDB29049 ; HMDB29070 ; HMDB29469 ; HMDB30266 ; HMDB30646 ; HMDB31682 ; HMDB34013 ; HMDB36117 ; HMDB36460 ; HMDB36998 ; HMDB37600 ; HMDB37692 ; HMDB38175 ; HMDB38332 ; HMDB38807 ; HMDB39052 ; HMDB39710 ; HMDB40321 ; HMDB40883 ; HMDB41405 ; HMDB00462	Diethyl disulfide ; 23-Butanedithiol ; 12-Bis(methylthio)ethane ; Methyl propyl disulfide ; ()-13-Butanedithiol ; 12-Butanedithiol ; L-Glutamic acid 5-phosphate ; Pyrrole-2-carboxylic acid ; Monobenzone ; Serinyl-Threonine ; Threoninyl-Serine ; Eupatilin ; Berberrubine ; (Z)-3-(1-Butenyl)-1H-2-benzopyran-1-one ; ()-2-Methylthiazolidine ; Lathycarpin ; (1R)-Nepetalic acid ; Safynol ; (S)-Oleuropeic acid ; Xanthomicrol ; 57-Dihydroxy-345-trimethoxyflavone ; 2-Acetyloxazole ; 2-(3-Hydroxyphenyl)ethanol 1-glucoside ; Aflatoxin Ex2B1 ; (4S8R)-89-Dihydroxy-p-menth-1(6)-en-2-one ; 1-Acetylcyclohexyl acetate ; 38-Dihydroxy-457-trimethoxyflavone ; (E)-10-Oxo-8-decenoic acid ; 4-Methoxybenzyl glucoside ; Allantoin	M+H ; M+H ; M+H ; M+H ; M+H ; M+H ; M+H+NH4 ; 2M+H+Na ; M+2Na ; M+H+K ; M+H+K ; M+2H+Na ; M+H+2Na ; M+2Na ; 2M+H+K ; M+2H+Na ; M+Na+K ; M+2Na ; M+Na+K ; M+2H+Na ; M+2H+Na ; 2M+H+Na ; M+3Na ; M+2H+Na ; M+Na+K ; M+Na+K ; M+2H+Na ; M+Na+K ; M+3Na ; M+H-2H2O	C4H10S2 ; C4H10S2 ; C4H10S2 ; C4H10S2 ; C4H10S2 ; C4H10S2 ; C5H10NO7P ; C5H5NO2 ; C13H12O2 ; C7H14N2O5 ; C7H14N2O5 ; C18H16O7 ; C19H16NO4 ; C13H12O2 ; C4H9NS ; C18H16O7 ; C10H16O3 ; C13H12O2 ; C10H16O3 ; C18H16O7 ; C18H16O7 ; C5H5NO2 ; C14H20O7 ; C18H16O7 ; C10H16O3 ; C10H16O3 ; C18H16O7 ; C10H16O3 ; C14H20O7 ; C4H6N4O3
.5384_51.8	1.533114	1.130267	2.3743	0.038992	1.409	0.42361	442.8315	HMDB07992 ; HMDB08051 ; HMDB08274 ; HMDB08530 ; HMDB08757 ; HMDB08758 ; HMDB12216 ; HMDB14531 ; HMDB15077	PC(16:024:0) ; PC(18:022:0) ; PC(20:020:0) ; PC(22:018:0) ; PC(24:015:0) ; PC(24:016:0) ; Dimethylarsinous acid ; Procyclidine ; Cycrimine	M+H+K ; M+H+K ; M+H+K ; M+H+K ; M+H+K ; M+H+K ; 3M+2K-H ; 3M+H+Na ; 3M+H+Na	C48H96NO8P ; C48H96NO8P ; C48H96NO8P ; C48H96NO8P ; C48H96NO8P ; C48H96NO8P ; C2H7AsO ; C19H29NO ; C19H29NO

23323_47.7	1.532297	1.146849	-2.2643	0.047025	1.3277	0.42361	490.6523	HMDB06487 ; HMDB10237 ;<br>HMDB10241 ; HMDB10244 ;<br>HMDB10248 ; HMDB10249 ;<br>HMDB14927 ; HMDB34538 ;<br>HMDB56396 ; HMDB56414 ;<br>HMDB56415 ; HMDB56459 ;<br>HMDB56469 ; HMDB56477 ;<br>HMDB56539 ; HMDB56540 ;<br>HMDB56557 ; HMDB56558 ;<br>HMDB56561 ; HMDB56562 ;<br>HMDB56568 ; HMDB56569 ;<br>HMDB56573 ; HMDB56593 ;<br>HMDB56594 ; HMDB56597 ;<br>HMDB56607 ; HMDB56649 ;<br>HMDB56650 ; HMDB56653 ;<br>HMDB56663 ; HMDB56745 ;<br>HMDB56759 ; HMDB56785 ;<br>HMDB56837 ; HMDB57060 ;<br>HMDB57061 ; HMDB57063 ;<br>HMDB57068 ; HMDB57072 ;<br>HMDB57079 ; HMDB57100 ;<br>HMDB57152 ; HMDB57242 ;<br>HMDB57421 ; HMDB57422 ;<br>HMDB57425 ; HMDB57426 ;<br>HMDB57432 ; HMDB57433 ;<br>HMDB57437 ; HMDB57446 ;<br>HMDB57447 ; HMDB57449 ;<br>HMDB57454 ; HMDB57458 ;	Pentaglutamyl folate ; CL(18:2(9Z12Z)18:2(9Z12Z)18:1(11Z)16:1(9Z)) ;<br>CL(18:2(9Z12Z)18:2(9Z12Z)18:1(9Z)16:1(9Z)) ;<br>CL(18:2(9Z12Z)18:2(9Z12Z)18:2(9Z12Z)16:0) ;<br>CL(18:2(9Z12Z)18:2(9Z12Z)18:2(9Z12Z)18:3(6Z9Z12Z)) ;<br>CL(18:2(9Z12Z)18:2(9Z12Z)18:2(9Z12Z)18:3(9Z12Z15Z)) ; Gadopentetate<br>dimeglumine ; Mubenin B ; CL(16:016:016:022:6(4Z7Z10Z13Z16Z19Z)) ;<br>CL(16:016:016:1(9Z)22:5(4Z7Z10Z13Z16Z)) ;<br>CL(16:016:016:1(9Z)22:5(7Z10Z13Z16Z19Z)) ;<br>CL(16:016:022:5(4Z7Z10Z13Z16Z)16:1(9Z)) ;<br>CL(16:016:022:5(7Z10Z13Z16Z19Z)16:1(9Z)) ;<br>CL(16:016:022:6(4Z7Z10Z13Z16Z19Z)16:0) ;<br>CL(16:016:1(9Z)16:022:5(4Z7Z10Z13Z16Z)) ;<br>CL(16:016:1(9Z)16:022:5(7Z10Z13Z16Z19Z)) ;<br>CL(16:016:1(9Z)18:1(11Z)18:1(11Z)) ; CL(16:016:1(9Z)18:1(11Z)18:1(9Z)) ;<br>CL(16:016:1(9Z)18:1(9Z)18:1(11Z)) ; CL(16:016:1(9Z)18:1(9Z)18:1(9Z)) ;<br>CL(16:016:1(9Z)20:4(5Z8Z11Z14Z)20:4(5Z8Z11Z14Z)) ;<br>CL(16:016:1(9Z)22:5(4Z7Z10Z13Z16Z)16:0) ;<br>CL(16:016:1(9Z)22:5(7Z10Z13Z16Z19Z)16:0) ;<br>CL(16:018:1(11Z)16:1(9Z)18:1(11Z)) ; CL(16:018:1(11Z)16:1(9Z)18:1(9Z)) ;<br>CL(16:018:1(11Z)18:1(11Z)16:1(9Z)) ; CL(16:018:1(11Z)18:1(9Z)16:1(9Z)) ;<br>CL(16:018:1(9Z)16:1(9Z)18:1(11Z)) ; CL(16:018:1(9Z)16:1(9Z)18:1(9Z)) ;<br>CL(16:018:1(9Z)18:1(11Z)16:1(9Z)) ; CL(16:018:1(9Z)18:1(9Z)16:1(9Z)) ;<br>CL(16:020:4(5Z8Z11Z14Z)16:1(9Z)20:4(5Z8Z11Z14Z)) ;<br>CL(16:020:4(5Z8Z11Z14Z)20:4(5Z8Z11Z14Z)16:1(9Z)) ;<br>CL(16:022:5(4Z7Z10Z13Z16Z)16:1(9Z)16:0) ;<br>CL(16:022:5(7Z10Z13Z16Z19Z)16:1(9Z)16:0) ;<br>CL(18:016:1(9Z)16:1(9Z)18:1(11Z)) ; CL(18:016:1(9Z)16:1(9Z)18:1(9Z)) ;<br>CL(18:016:1(9Z)16:1(9Z)20:4(5Z8Z11Z14Z)) ;	M+H+Na ; M+H+2Na ; M+H+2Na ;<br>M+H+2Na ; M+2H+Na ; M+2H+Na ;<br>M+ACN+2H ; 2M+3H ; M+H+2Na ;<br>M+H+2Na ; M+H+2Na ; M+H+2Na ;<br>M+H+2Na ; M+H+2Na ; M+H+2Na ;<br>M+H+2Na ; M+3Na ; M+3Na ; M+3Na ;<br>M+3Na ; M+2H+Na ; M+H+2Na ;<br>M+H+2Na ; M+3Na ; M+3Na ; M+3Na ;<br>M+3Na ; M+3Na ; M+3Na ; M+3Na ;<br>M+3Na ; M+2H+Na ; M+2H+Na ;<br>M+H+2Na ; M+H+2Na ; M+3Na ; M+3Na ;<br>M+H+2Na ; M+3Na ; M+3Na ; M+H+2Na ;<br>M+3Na ; M+3Na ; M+H+2Na ; M+3Na ;<br>M+3Na ; M+3Na ; M+3Na ; M+2H+Na ;<br>M+H+2Na ; M+H+2Na ; M+3Na ; M+3Na ;<br>M+H+2Na ; M+3Na ; M+3Na ; M+H+2Na ;<br>M+3Na ; M+2H+Na ; M+3Na ; M+2H+Na ;<br>M+H+2Na ; M+H+2Na ; M+3H ;<br>M+2H+Na ; M+2H+Na ; M+3H ;<br>M+2H+Na ; M+3Na ; M+3Na ; M+2H+Na ;<br>M+2H+Na ; M+3Na ; M+3Na ; M+2H+Na ;<br>M+H+2Na ; M+H+2Na ; M+3H ;<br>M+2H+Na ; M+3H ; M+2H+Na ;<br>M+2H+Na ; M+3H ; M+3Na ; M+3Na ;<br>M+3Na ; M+2H+Na ; M+3Na ; M+2H+Na ;<br>M+H+2Na ; M+2H+Na ; M+3H	C39H47N11O18 ; C79H142O17P2 ;<br>C79H142O17P2 ; C79H142O17P2 ;<br>C81H140O17P2 ; C81H140O17P2 ;<br>C28H54GdN5O20 ; C41H66O11 ;<br>C79H142O17P2 ; C79H142O17P2 ;<br>C79H142O17P2 ; C79H142O17P2 ;<br>C79H142O17P2 ; C79H142O17P2 ;<br>C79H142O17P2 ; C79H142O17P2 ;<br>C77H144O17P2 ; C77H144O17P2 ;<br>C77H144O17P2 ; C77H144O17P2 ;<br>C81H140O17P2 ; C79H142O17P2 ;<br>C79H142O17P2 ; C77H144O17P2 ;<br>C77H144O17P2 ; C77H144O17P2 ;<br>C77H144O17P2 ; C77H144O17P2 ;<br>C77H144O17P2 ; C77H144O17P2 ;<br>C77H144O17P2 ; C81H140O17P2 ;<br>C81H140O17P2 ; C79H142O17P2 ;<br>C79H142O17P2 ; C77H144O17P2 ;<br>C77H144O17P2 ; C79H142O17P2 ;<br>C77H144O17P2 ; C77H144O17P2 ;<br>C79H142O17P2 ; C77H144O17P2 ;<br>C77H144O17P2 ; C79H142O17P2 ;<br>C77H144O17P2 ; C77H144O17P2 ;<br>C77H144O17P2 ; C77H144O17P2 ;<br>C81H140O17P2 ; C79H142O17P2 ;<br>C79H142O17P2 ; C77H144O17P2 ;<br>C77H144O17P2 ; C79H142O17P2 ;<br>C77H144O17P2 ; C77H144O17P2 ;
05141_47.1	1.531046	1.328978	-2.2392	0.049068	1.3092	0.42721	NA	NA	NA	NA	NA
74166_47.3	1.528861	1.266363	-2.272	0.04641	1.3334	0.42361	NA	NA	NA	NA	NA
04196_47.3	1.52789	1.231332	-2.2584	0.047496	1.3233	0.42361	NA	NA	NA	NA	NA
06071_47.6	1.527254	1.260118	-2.2713	0.046465	1.3329	0.42361	380.7696	HMDB08258 ; HMDB11214 ;<br>HMDB32843 ; HMDB33208 ;<br>HMDB36928	PC(18:4(6Z9Z12Z15Z)P-16:0) ; PC(P-16:018:4(6Z9Z12Z15Z)) ; AS 1-5 ;<br>Dimethyl diselenide ; Mactraxanthin	M+H+Na ; M+H+Na ; M+2Na ; 2M+H ;<br>M+3ACN+2H	C42H76NO7P ; C42H76NO7P ;<br>C40H77NO9 ; C2H6Se2 ; C40H60O6
59829_47.4	1.526603	1.256345	-2.2688	0.046667	1.331	0.42361	392.706	HMDB01261 ; HMDB02158 ;<br>HMDB02248 ; HMDB04985 ;<br>HMDB04987 ; HMDB12311 ;<br>HMDB28758 ; HMDB28947 ;<br>HMDB30460 ; HMDB35188 ;<br>HMDB35189	Coproporphyrinogen III ; Coproporphyrinogen I ; Gamma glutamyl ornithine<br>; Aspartylysine ; Alpha-Aspartyl-lysine ; 3-O-Sulfogalactosylceramide<br>(d18:112:0) ; Aspartyl-Lysine ; Lysyl-Aspartate ; Hordatine B glucoside ;<br>Fasciculol F ; Fasciculol E	M+3ACN+2H ; M+3ACN+2H ; 3M+2H ;<br>3M+2H ; 3M+2H ; M+Na+K ; 3M+2H ;<br>3M+2H ; M+ACN+2H ; M+Na+K ; M+Na+K	C36H44N4O8 ; C36H44N4O8 ;<br>C10H19N3O5 ; C10H19N3O5 ;<br>C10H19N3O5 ; C36H69NO11S ;<br>C10H19N3O5 ; C10H19N3O5 ;<br>C35H50N8O10 ; C39H65NO11 ;<br>C39H65NO11
04622_47.4	1.525824	1.294263	-2.2495	0.048221	1.3168	0.42643	456.6995	HMDB04808 ; HMDB06101 ;<br>HMDB28705 ; HMDB28747 ;<br>HMDB29543 ; HMDB30695 ;<br>HMDB37376 ; HMDB38453 ;<br>HMDB61151	7C-aglycone ; Enterolactone ; Arginyl-Aspartate ; Aspartyl-Arginine ; ()-<br>Ligballinol ; 7-Hydroxy-5-methoxy-68-dimethylflavanone ; 1-(24-Dihydroxy-<br>6-methoxy-35-dimethylphenyl)-3-phenyl-2-propen-1-one ; 57-Dimethoxy-6-<br>methylflavanone ; Hydroxylated N-acetyl desmethyl frovatriptan	3M+H+NH4 ; 3M+H+NH4 ; 3M+2Na ;<br>3M+2Na ; 3M+H+NH4 ; 3M+H+NH4 ;<br>3M+H+NH4 ; 3M+H+NH4 ; 3M+2Na	C18H18O4 ; C18H18O4 ; C10H19N5O5 ;<br>C10H19N5O5 ; C18H18O4 ; C18H18O4 ;<br>C18H18O4 ; C18H18O4 ; C15H19N3O3
67842_274.9	1.525699	0.291279	2.2892	0.045074	1.3461	0.42361	759.0788	HMDB12208 ; HMDB14836	D-Erythro-imidazole-glycerol-phosphate ; Nitrofurantoin	3M+2Na-H ; 3M+2Na-H	C6H11N2O6P ; C8H6N4O5
07215_52.64	1.525354	0.428329	-2.3254	0.042379	1.3729	0.42361	185.9597	HMDB29720	2-Thiophenethiol	3M+H+Na	C4H4S2

0666_196.4	1.523748	0.976204	-2.3383	0.04146	1.3824	0.42361	430.1031	HMDB39906 ; HMDB14808 ; HMDB15390 ; HMDB37465 ; HMDB29250	Tricin 7-feruloyl-(-2)-glucuronyl-(1-2)-glucuronide ; Pirenzepine ; Sitagliptin ; Spinosin A ; Pigment A aglycone	M+2H ; M+2K+H ; M+Na ; M+2Na ; M+H-H2O	C39H38O22 ; C19H21N5O2 ; C16H15F6N5O ; C39H42O19 ; C25H19O8
8833_56.16	1.523693	1.014609	2.2835	0.045515	1.3418	0.42361	153.9869	HMDB13780 ; HMDB15143	Dimethyl trisulfide ; Busulfan	2M+3H2O+2H ; M+Na+K	C2H6S3 ; C6H14O6S2
1142_141.5	1.523145	1.086962	-2.2474	0.048391	1.3152	0.42643	469.1251	HMDB12197 ; HMDB15608 ; HMDB15705 ; HMDB33259 ; HMDB33277 ; HMDB34698 ; HMDB38931 ; HMDB40502	Canavaninosuccinate ; Indacaterol ; Ivacaftor ; Cubebinone ; 8-Acetoxy-4-methoxypinoresinol ; 7-Hydroxy-5-(4-hydroxy-2-oxopentyl)-2-methylchromone 7-glucoside ; Sesartemin ; Cnidimol 7-glucoside	3M+Na+K ; M+2K-H ; M+2K-H ; M+K ; M+K ; 2M+Na+K ; M+K ; 2M+Na+K	C9H16N4O7 ; C24H28N2O3 ; C24H28N2O3 ; C23H26O8 ; C23H26O8 ; C21H26O10 ; C23H26O8 ; C21H26O10
21505_197.0	1.52097	1.063345	-2.3155	0.043105	1.3655	0.42361	411.0722	HMDB14096 ; HMDB38294 ; HMDB41596 ; HMDB60831 ; HMDB40609	5-Hydroxypiroxicam ; Apigenin 7-glucuronyl-(1-2)-glucuronide 4-glucuronide ; Vitisidin A ; meta-O-Dealkylated flecainide ; Theaflavic acid	M+ACN+Na ; M+H+Na ; 2M+H+Na ; M+2K+H ; M+H-H2O	C15H13N3O5S ; C33H34O23 ; C20H15O9 ; C15H19F3N2O3 ; C21H16O10
1992_196.0	1.520483	1.075327	-2.2815	0.045669	1.3404	0.42361	237.0362	HMDB15021 ; HMDB00902 ; HMDB03072 ; HMDB04400 ; HMDB14752 ; HMDB29466 ; HMDB29472 ; HMDB29775 ; HMDB30818 ; HMDB32558 ; HMDB32952 ; HMDB35303 ; HMDB36134 ; HMDB36627 ; HMDB37850 ; HMDB40565 ; HMDB40897 ; HMDB41079 ; HMDB41635 ; HMDB59771	Isosorbide Dinitrate ; NAD ; Quinic acid ; 5-Acetylamino-6-amino-3-methyluracil ; Furazolidone ; Eugenitol ; Isoeugenitol ; Vinyl caffeate ; Scoparone ; Dinitolmide ; Citropten ; 3-Methoxy-45-methylenedioxycinnamaldehyde ; 4-(2-Furanylmethyl)thio-2-pentanone ; Eugenin ; Myricetin 33-digalactoside ; Aloesol 7-glucoside ; Methyl (3x4E10R)-310-dihydroxy-411-dodecadiene-68-diynoate 10-glucoside ; (E)-S-1-Propenyl thiosulfate ; Myricetin 3-arabinoside ; 6-amino-5N-methylformylamino-1-methyluracil	M+H ; M+H+2Na ; M+2Na-H ; M+K ; 2M+H+Na ; 2M+Na+K ; 2M+Na+K ; 2M+Na+K ; 2M+Na+K ; 2M+H+Na ; 2M+Na+K ; 2M+Na+K ; M+K ; 2M+Na+K ; M+3Na ; M+2K ; M+2K ; M+2ACN+H ; M+H+Na ; M+K	C6H8N2O8 ; C21H28N7O14P2 ; C7H12O6 ; C7H10N4O3 ; C8H7N3O5 ; C11H10O4 ; C11H10O4 ; C11H10O4 ; C11H10O4 ; C8H7N3O5 ; C11H10O4 ; C11H10O4 ; C10H14O2S ; C11H10O4 ; C27H30O18 ; C19H24O9 ; C19H24O9 ; C3H6O3S2 ; C20H18O12 ; C7H10N4O3
21762_54.7	1.520413	1.23438	2.2998	0.044266	1.3539	0.42361	156.0422	HMDB60173 ; HMDB29783 ; HMDB04094 ; HMDB14966 ; HMDB15686 ; HMDB29757 ; HMDB32156 ; HMDB32390 ; HMDB34666 ; HMDB36602 ; HMDB37997 ; HMDB38730 ; HMDB59805 ; HMDB60007 ; HMDB60486	N-Methylethanolaminium phosphate ; Mono-trans-p-coumaroylmesotartaric acid ; Indoxyl ; Fosfomycin ; Pipazethate ; 4-Hydroxybenzeneacetonitrile ; alpha-Acetolactate decarboxylase (enzyme preparation from bacillus subtilis recombinant) ; 2-Methyl-45-benzoxazole ; ()-2-Hydroxy-2-phenylacetonitrile ; (S)-Pterosin K ; Delphinidin 3-glucoside ; 5-O-p-Coumaroylnigrumin ; 5-Hydroxyindole ; N-(2-hydroxymethyl-3-chloro-4-hydroxyphenyl)anthranilic acid ; Mandelonitrile	M+H ; M+2H ; M+Na ; M+NH4 ; M+3Na ; M+Na ; M+3Na ; M+Na ; M+Na ; M+2Na ; M+3H ; M+H+2Na ; M+Na ; M+H+NH4 ; M+Na	C3H10NO4P ; C14H14O8 ; C8H7NO ; C3H7O4P ; C21H25N3O3S ; C8H7NO ; C21H25N3O3S ; C8H7NO ; C8H7NO ; C15H19ClO2 ; C21H21O12 ; C20H23NO9 ; C8H7NO ; C14H12ClNO4 ; C8H7NO
9216_50.48	1.518766	0.583472	-2.4473	0.034419	1.4632	0.42361	NA	NA	NA	NA	NA
946_47.336	1.517886	1.272096	-2.2546	0.047805	1.3205	0.42456	272.7946	HMDB37822	Tridodecylamine	M+H+Na	C36H75N

2783_47.44	1.516407	1.290195	-2.2297	0.049869	1.3022	0.42721	454.7023	HMDB15200 ; HMDB31806 ; HMDB56289 ; HMDB56294 ; HMDB56314 ; HMDB56319	Glipizide ; ()-Naled ; DG(18:2n60:022:5n6) ; DG(18:2n60:022:5n3) ; DG(20:2n60:022:5n6) ; DG(20:2n60:022:5n3)	2M+H+NH4 ; M+2K-H ; 2M+3H ; 2M+3H ; 2M+3H ; 2M+3H	C21H27N5O4S ; C4H7Br2Cl2O4P ; C44H72O5 ; C44H72O5 ; C44H72O5 ; C44H72O5
2268_52.05	1.51523	1.144725	2.2645	0.047006	1.3278	0.42361	NA	NA	NA	NA	NA
6976_47.49	1.514254	1.276373	-2.2454	0.048554	1.3138	0.42643	386.7147	HMDB08838 ; HMDB08839 ; HMDB08869 ; HMDB08870 ; HMDB08931 ; HMDB08962 ; HMDB08963 ; HMDB09122 ; HMDB09155 ; HMDB09187 ; HMDB09317 ; HMDB09350 ; HMDB09382 ; HMDB09415 ; HMDB14344 ; HMDB61020	PE(14:020:4(5Z8Z11Z14Z)) ; PE(14:020:4(8Z11Z14Z17Z)) ; PE(14:1(9Z)20:3(5Z8Z11Z)) ; PE(14:1(9Z)20:3(8Z11Z14Z)) ; PE(16:018:4(6Z9Z12Z15Z)) ; PE(16:1(9Z)18:3(6Z9Z12Z)) ; PE(16:1(9Z)18:3(9Z12Z15Z)) ; PE(18:3(6Z9Z12Z)16:1(9Z)) ; PE(18:3(9Z12Z15Z)16:1(9Z)) ; PE(18:4(6Z9Z12Z15Z)16:0) ; PE(20:3(5Z8Z11Z)14:1(9Z)) ; PE(20:3(8Z11Z14Z)14:1(9Z)) ; PE(20:4(5Z8Z11Z14Z)14:0) ; PE(20:4(8Z11Z14Z17Z)14:0) ; Erythromycin ; N-desmethylclarithromycin	M+Na+K ; M+Na+K ; M+Na+K ; M+Na+K ; M+Na+K ; M+Na+K ; M+Na+K ; M+Na+K ; M+Na+K ; M+Na+K ; M+Na+K ; M+Na+K ; M+Na+K ; M+Na+K ; M+H+K ; M+H+K	C39H70NO8P ; C39H70NO8P ; C39H70NO8P ; C39H70NO8P ; C39H70NO8P ; C39H70NO8P ; C39H70NO8P ; C39H70NO8P ; C39H70NO8P ; C39H70NO8P ; C39H70NO8P ; C39H70NO8P ; C39H70NO8P ; C39H70NO8P ; C37H67NO13 ; C37H67NO13
211_280.24	1.514059	1.116698	2.273	0.046332	1.3341	0.42361	758.9721	HMDB03665 ; HMDB04943 ; HMDB12935	Phosphoribosyl-ATP ; Ganglioside GM2 (d18:126:1(17Z)) ; Dynorphin A	M+K ; M+H+Na ; M+ACN+2H	C15H26N5O20P4 ; C75H135N3O26 ; C69H116N23O13
8619_44.02	1.513954	1.165188	2.3117	0.04338	1.3627	0.42361	NA	NA	NA	NA	NA
2208_84.8	1.512901	0.854153	-2.3116	0.04339	1.3626	0.42361	132.5222	HMDB01120 ; HMDB01347 ; HMDB02096 ; HMDB15611 ; HMDB28682 ; HMDB28724 ; HMDB28797 ; HMDB28839 ; HMDB28855 ; HMDB29149 ; HMDB39656 ; HMDB41526	Dimethylallylpyrophosphate ; Isopentenyl pyrophosphate ; 3-Indolebutyric acid ; Methsuximide ; Alanyl-Asparagine ; Asparaginyl-Alanine ; Glutaminyl-Glycine ; Glycyl-Glutamine ; Glycyl-Gamma-glutamate ; Gamma-glutamyl-Glycine ; 5-(2-Furanyl)-123456-hexahydro-7H-cyclopentabpyridin-7-one ; Isosalsolidine	M+H+NH4 ; M+H+NH4 ; M+Na+K ; M+Na+K ; M+Na+K ; M+Na+K ; M+Na+K ; M+Na+K ; M+Na+K ; M+Na+K ; M+Na+K ; M+Na+K	C5H12O7P2 ; C5H12O7P2 ; C12H13NO2 ; C12H13NO2 ; C7H13N3O4 ; C7H13N3O4 ; C7H13N3O4 ; C7H13N3O4 ; C7H13N3O4 ; C7H13N3O4 ; C12H13NO2 ; C12H13NO2
32257_58.9	1.511185	0.488812	-2.3029	0.044035	1.3562	0.42361	222.9682	HMDB14335 ; HMDB33075 ; HMDB33206 ; HMDB34232 ; HMDB60417 ; HMDB60456 ; HMDB60514 ; HMDB60601	Ethchlorvynol ; Methyl 1-(methylsulfinyl)propyl disulfide ; Arsenobetaine ; (Chloromethyl)oxirane ; 6-Thiourate ; cis-3-Chloro-2-propene-1-ol ; trans-3-Chloro-2-propene-1-ol ; 6-thiouric acid	M+2K+H ; M+K ; M+2Na-H ; 2M+K ; M+K ; 2M+K ; 2M+K ; M+K	C7H9ClO ; C5H12OS3 ; C5H11AsO2 ; C3H5ClO ; C5H4N4O2S ; C3H5ClO ; C3H5ClO ; C5H4N4O2S
4449_52.03	1.510683	1.077658	2.3233	0.042535	1.3713	0.42361	203.9374	HMDB36350 ; HMDB42048 ; HMDB61387	Methyl 2-propenyl tetrasulfide ; Trichloroacetic acid ; Triclosan sulfate	2M+H+K ; M+ACN+H ; M+H+K	C4H8S4 ; C2HCl3O2 ; C12H7Cl3O5S
02043_51.2	1.505144	0.860988	-2.2558	0.047704	1.3214	0.42441	450.8702	HMDB08290 ; HMDB08322 ; HMDB08546 ; HMDB08577 ; HMDB08768 ; HMDB08800 ; HMDB13339 ; HMDB13340	PC(20:024:1(15Z)) ; PC(20:1(11Z)24:0) ; PC(22:022:1(13Z)) ; PC(22:1(13Z)22:0) ; PC(24:020:1(11Z)) ; PC(24:1(15Z)20:0) ; 3-Hydroxy-11Z-octadecenoylcarnitine ; 3-Hydroxy-9Z-octadecenoylcarnitine	M+2H ; M+2H ; M+2H ; M+2H ; M+2H ; M+2H ; 2M+H+NH4 ; 2M+H+NH4	C52H102NO8P ; C52H102NO8P ; C52H102NO8P ; C52H102NO8P ; C52H102NO8P ; C52H102NO8P ; C25H47NO5 ; C25H47NO5

[illegible]3421_54.0[illegible]	1.502463	1.129826	-2.2612	0.047275	1.3254	0.42361	149.0263	HMDB01553 ; HMDB00267 ; HMDB00805 ; HMDB00884 ; HMDB01369 ; HMDB01843 ; HMDB02234 ; HMDB02331 ; HMDB04086 ; HMDB04813 ; HMDB06239 ; HMDB06801 ; HMDB13870 ; HMDB13871 ; HMDB13872 ; HMDB15457 ; HMDB15619 ; HMDB35993 ; HMDB41441 ; HMDB60262 ; HMDB60587 ; HMDB61093	2-Oxo-4-methylthiobutanoic acid ; Pyroglutamic acid ; Pyrrolidonecarboxylic acid ; Ribothymidine ; Pyrroline hydroxycarboxylic acid ; N-Acryloylglycine ; 1-Pyrroline-4-hydroxy-2-carboxylate ; Imidazoleacetic acid riboside ; 5-Hydroxy-N-formylkynurenine ; 3-Methyluridine ; S-aminomethyldihydrolipoamide ; 2-Oxo-3-hydroxy-4-phosphobutanoic acid ; cistrans-5-Hydroxythalidomide ; 5-Hydroxythalidomide ; Thalidomide arene oxide ; Dihydroxyaluminium ; Sulfathiazole ; N5-(34-Dioxo-15-cyclohexadien-1-yl)-L-glutamine ; 11-Methylgerberinol ; 5-Oxoprolinate ; Aminoethoxyacetic acid ; dimethadione	M+H ; 2M+H+K ; 2M+H+K ; M+H+K ; 2M+H+K ; 2M+H+K ; 2M+H+K ; M+H+K ; M+2Na ; M+H+K ; M+Na+K ; M+2ACN+2H ; M+H+Na ; M+H+Na ; M+H+Na ; 2M+H+Na ; M+ACN+2H ; M+2Na ; M+3Na ; 2M+H+K ; 2M+Na+K ; 2M+H+K	C5H8O3S ; C5H7NO3 ; C5H7NO3 ; C10H14N2O6 ; C5H7NO3 ; C5H7NO3 ; C5H7NO3 ; C10H14N2O6 ; C11H12N2O5 ; C10H14N2O6 ; C9H20N2OS2 ; C4H7O8P ; C13H10N2O5 ; C13H10N2O5 ; C13H10N2O5 ; C2H8AlNO4 ; C9H9N3O2S2 ; C11H12N2O5 ; C22H18O6 ; C5H7NO3 ; C4H8NO3 ; C5H7NO3
[illegible]6976_33.2[illegible]	1.499491	0.943797	-2.2416	0.048875	1.3109	0.42648	148.0757	HMDB05785 ; HMDB30204 ; HMDB40009 ; HMDB40011 ; HMDB39134 ; HMDB41061 ; HMDB01940 ; HMDB14814 ; HMDB15263 ; HMDB28803 ; HMDB28971 ; HMDB28987 ; HMDB29155 ; HMDB32039 ; HMDB32758 ; HMDB33355 ; HMDB34110 ; HMDB40179 ; HMDB41319 ; HMDB00068 ; HMDB00819 ; HMDB02832 ; HMDB15652 ; HMDB29455	Indole-3-carbinol ; 1-(2-Furanylmethyl)-1H-pyrrole ; 4-(2-Furanylmethylene)-34-dihydro-2H-pyrrole ; 3-(2-Furanylmethyl)-1H-pyrrole ; Ginsenoyne B ; Nigellidine ; Triamterene ; Benzyl Benzoate ; Proguanil ; Glutaminyl-Methionine ; Methionyl-Glutamine ; Methionyl-Gamma-glutamate ; Gamma-glutamyl-Methionine ; Benzoin ; Ethyl 3-methyl-9H-carbazole-9-carboxylate ; Dehydroaporheine ; Murrayacine ; 26-Di-tert-butyl-4-ethylphenol ; Cinnamyl anthranilate ; Epinephrine ; Normetanephrine ; Methylnoradrenaline ; Levonordefrin ; Ginkgotoxin	M+H ; M+H ; M+H ; M+H ; M+2H ; M+2H ; M+ACN+2H ; M+2ACN+2H ; M+ACN+2H ; M+H+NH4 ; M+H+NH4 ; M+H+NH4 ; M+H+NH4 ; M+2ACN+2H ; M+ACN+2H ; M+H+NH4 ; M+H+NH4 ; M+Na+K ; M+ACN+2H ; M+H-2H2O ; M+H-2H2O ; M+H-2H2O ; M+H-2H2O ; M+H-2H2O	C9H9NO ; C9H9NO ; C9H9NO ; C9H9NO ; C17H23ClO2 ; C18H18N2O2 ; C12H11N7 ; C14H12O2 ; C11H16ClN5 ; C10H19N3O4S ; C10H19N3O4S ; C10H19N3O4S ; C10H19N3O4S ; C14H12O2 ; C16H15NO2 ; C18H15NO2 ; C18H15NO2 ; C16H26O ; C16H15NO2 ; C9H13NO3 ; C9H13NO3 ; C9H13NO3 ; C9H13NO3 ; C9H13NO3
[illegible]0333_278.[illegible]	1.498957	1.24743	2.259	0.04745	1.3238	0.42361	112.112	HMDB31455 ; HMDB31532 ; HMDB32395 ; HMDB35141 ; HMDB41402	p-Menthane ; 1-Methyl-13-cyclohexadiene ; 2-Methyl-13-cyclohexadiene ; 1-[illegible] Isopropyl-23-dimethylcyclopentane ; Butylcyclohexane	M+2ACN+2H ; M+NH4 ; M+NH4 ; M+2ACN+2H ; M+2ACN+2H	C10H20 ; C7H10 ; C7H10 ; C10H20 ; C10H20

2265_38.2	1.497806	1.231548	-2.2427	0.048783	1.3117	0.42643	131.0492	HMDB60917 ; HMDB02455 ; HMDB04620 ; HMDB11602 ; HMDB14611 ; HMDB15044 ; HMDB29700 ; HMDB31162 ; HMDB32121 ; HMDB32585 ; HMDB33222 ; HMDB33223 ; HMDB35822 ; HMDB36819 ; HMDB38149 ; HMDB38894 ; HMDB39891 ; HMDB40286 ; HMDB40895 ; HMDB60565 ; HMDB60920 ; HMDB60921 ; HMDB00375 ; HMDB00563 ; HMDB00748 ; HMDB00779 ; HMDB02072 ; HMDB02199 ; HMDB02229 ; HMDB05175 ; HMDB29665 ; HMDB29817 ; HMDB31132 ; HMDB32030 ; HMDB32138 ; HMDB32573 ; HMDB32605 ; HMDB32639 ; HMDB33752 ; HMDB34243 ; HMDB34993 ; HMDB40645 ; HMDB41611 ; HMDB41683 ; HMDB59969	4-Hydroxyflurbiprofen ; Pyruvatoxime ; N-a-Acetyl-L-arginine ; 3-Oxoalanine ; Quinine ; Quinidine ; Cinnamyl benzoate ; 33-Thiobispropanoic acid ; Annuionone B ; 4-Methoxychalcone ; (1R3S4S6R)-69-Dihydroxyfenchone 6-O-b-D-glucoside ; (1S3R4R)-810-Dihydroxyfenchone 10-O-b-D-glucoside ; Glucosyl 6-hydroxy-26-dimethyl-2E7-octadienoate ; Dehydrovomifoliol ; Nepetaside ; Isoamyl p-anisate ; 4-Hydroxy-3-prenylbenzoic acid glucoside ; Benzyl cinnamate ; Methyl (R)-9-hydroxy-10-undecene-57-diynoate glucoside ; 1-Hydroxyibuprofen ; 2-Hydroxyibuprofen ; 3-Hydroxyibuprofen ; 3-(3-Hydroxyphenyl)propanoic acid ; D-Phenyllactic acid ; L-3-Phenyllactic acid ; Phenyllactic acid ; 4-Methoxyphenylacetic acid ; Desaminotyrosine ; 3-Phenoxypropionic acid ; Homovanillin ; Ethyl vanillin ; Ethyl salicylate ; 34-Dihydroxyphenylacetone ; Guaicyl acetate ; 34-Dimethoxybenzaldehyde ; Ethylparaben ; Methyl 2-methoxybenzoate ; Methyl 4-methoxybenzoate ; 3-(2-Hydroxyphenyl)propanoic acid ; Ipomeanine ; 4-Methoxybenzyl formate ; 3-Hydroxy-1-(4-hydroxyphenyl)-1-propanone ; Ethyl 2-furanacrylate ; 4-Hydroxyphenyl-2-propionic acid ; 3-Methoxyphenylacetic acid	M+2H ; 2M+3H2O+2H ; M+2Na ; 2M+3H2O+2H ; M+3Na ; M+3Na ; M+H+Na ; M+2ACN+2H ; M+H+K ; M+H+Na ; M+H+2Na ; M+H+2Na ; M+H+2Na ; M+H+K ; M+H+2Na ; M+H+K ; M+2H+Na ; M+H+Na ; M+2H+Na ; M+H+K ; M+H+K ; M+H+K ; M+H-2H2O	C15H13FO3 ; C3H5NO3 ; C8H16N4O3 ; C3H5NO3 ; C20H24N2O2 ; C20H24N2O2 ; C16H14O2 ; C6H10O4S ; C13H18O3 ; C16H14O2 ; C16H26O8 ; C16H26O8 ; C16H26O8 ; C13H18O3 ; C16H26O8 ; C13H18O3 ; C18H24O8 ; C16H14O2 ; C18H24O8 ; C13H18O3 ; C13H18O3 ; C13H18O3 ; C9H10O3
9868_82.9	1.496542	0.605695	-2.3149	0.043148	1.365	0.42361	140.033	HMDB01232 ; HMDB02658 ; HMDB13188 ; HMDB59710 ; HMDB00115 ; HMDB00350 ; HMDB00424 ; HMDB00532 ; HMDB06454 ; HMDB11169 ; HMDB12249 ; HMDB14937 ; HMDB15625 ; HMDB28763 ; HMDB29057 ; HMDB30486 ; HMDB30648 ; HMDB31608 ; HMDB33109 ; HMDB35712 ; HMDB40906 ; HMDB41005 ; HMDB41006 ; HMDB60981 ; HMDB61105	4-Nitrophenol ; 6-Hydroxynicotinic acid ; 3-Hydroxypicolinic acid ; 2-Hydroxynicotinic acid ; Glycolic acid ; 3-Hydroxysebacic acid ; 2-Hydroxydecanedioic acid ; Acetylglycine ; L-2-Amino-3-oxobutanoic acid ; L-beta-aspartyl-L-threonine ; L-Aspartate-semialdehyde ; Tazarotene ; Prasugrel ; Aspartyl-Threonine ; Threoninyl-Aspartate ; (S)-23-Dihydro-6-hydroxy-5-(hydroxyacetyl)-2-isopropenylbenzofuran ; Artemidiol ; Peracetic acid ; Methyl (2R3S)-23-dihydro-3-hydroxy-2-isopropenyl-5-benzofurancarboxylate ; Aloesol ; Coixinden B ; 1-Acetoxychavicol acetate ; 1-Acetoxychavicol ; para-hydroxyrosiglitazone ; ortho-hydroxyrosiglitazone	M+H ; M+H ; M+H ; M+H ; M+ACN+Na ; M+Na+K ; M+Na+K ; M+Na ; M+Na ; M+2Na ; M+Na ; M+3Na ; M+H+2Na ; M+2Na ; M+2Na ; M+2Na ; M+2Na ; M+ACN+Na ; M+2Na ; M+2Na ; M+2Na ; M+2Na ; M+2Na ; M+H+2Na ; M+H+2Na	C6H5NO3 ; C6H5NO3 ; C6H5NO3 ; C6H5NO3 ; C2H4O3 ; C10H18O5 ; C10H18O5 ; C4H7NO3 ; C4H7NO3 ; C8H14N2O6 ; C4H7NO3 ; C21H21NO2S ; C20H20FNO3S ; C8H14N2O6 ; C8H14N2O6 ; C13H14O4 ; C13H14O4 ; C2H4O3 ; C13H14O4 ; C13H14O4 ; C13H14O4 ; C13H14O4 ; C13H14O4 ; C18H19N3O4S ; C18H19N3O4S
73912_38.8	1.492149	1.228058	-2.2335	0.049549	1.305	0.42721	106.1074	HMDB32525 ; HMDB32526	Tetradec-2-enal ; (Z)-8-Tetradecenal	M+2H ; M+2H	C14H26O ; C14H26O
0209_52.2	1.490247	1.103029	2.296	0.044559	1.3511	0.42361	328.946	HMDB01474 ; HMDB60000	34-Dihydroxyphenylglycol O-sulfate ; Hydroxymethoxyphenylcarboxylic acid-O-sulphate	M+2K+H ; M+2K+H	C8H10O7S ; C8H10O7S
5903_273.2	1.487598	1.071911	-2.2348	0.049435	1.306	0.42721	351.5626	HMDB15034 ; HMDB30806 ; HMDB38096 ; HMDB60586	Triazolam ; Petunin ; Petunidin 3-gentiobioside ; N-Trifluoroacetyladriamycinol	2M+H+NH4 ; M+Na+K ; M+Na+K ; M+Na+K	C17H12Cl2N4 ; C28H33O17 ; C28H33O17 ; C29H30F3NO12

8262_52.34	1.487197	0.109395	-2.3544	0.040337	1.3943	0.42361	NA	NA	NA	NA	NA
7895_275.3	1.486676	0.737531	2.279	0.045862	1.3386	0.42361	475.0058	HMDB31823 ; HMDB33383 ; HMDB38404	Diphenyl disulfide ; CI Food Red 1 ; Glucoraphanin	2M+K ; M+K ; M+K	C12H10S2 ; C18H16N2O7S2 ; C12H22NO10S3
1186_83.05	1.486325	0.784727	-2.2935	0.044748	1.3492	0.42361	128.0231	HMDB12250 ; HMDB14407 ; HMDB31517 ; HMDB31984 ; HMDB32575 ; HMDB32590 ; HMDB34206 ; HMDB34633 ; HMDB34767 ; HMDB34990 ; HMDB36432 ; HMDB37269 ; HMDB37271 ; HMDB39815 ; HMDB40730 ; HMDB41194 ; HMDB41505 ; HMDB41506 ; HMDB41666 ; HMDB59898 ; HMDB59940 ; HMDB59941 ; HMDB59942 ; HMDB59943 ; HMDB59944 ; HMDB59945	L-Aspartyl-4-phosphate ; Carmustine ; (R)-3-Hydroxy-5-phenylpentanoic acid ; 25-Dimethoxy-4-(2-propenyl)phenol ; Butylparaben ; Zingerone ; Ethyl 4-methylphenoxyacetate ; 3-Chloro-1-(4-hydroxy-3-methoxyphenyl)-12-propanediol ; (1R3R3S)-1234-Tetrahydro-1-(2-thio-3-pyrrolidinyl)-beta-carboline-3-carboxylic acid ; 4-Methoxybenzyl propanoate ; Isobutyl salicylate ; Desacetycefapirin ; 26-Dimethoxy-4-(1-propenyl)phenol ; Flusilazole ; Butyl salicylate ; Methoxyeugenol ; 5-Hydroxy-2-benzyl-13-dioxane ; 2-Benzyl-5-hydroxymethyl-13-dioxolane ; 3-Hydroxyphenyl-valeric acid ; Ethyl 4-ethoxybenzoate ; 2-(2-Methylphenyl)-13-dioxolan-4-ylmethanol ; 2-(3-Methylphenyl)-13-dioxolan-4-ylmethanol ; 2-(4-Methylphenyl)-13-dioxolan-4-ylmethanol ; 2-(2-Methylphenyl)-13-dioxan-5-ol ; 2-(3-Methylphenyl)-13-dioxan-5-ol ; 2-(4-Methylphenyl)-13-dioxan-5-ol	M+ACN+2H ; M+ACN+2H ; M+Na+K ; M+Na+K ; M+Na+K ; M+Na+K ; M+Na+K ; M+H+Na ; M+3Na ; M+Na+K ; M+Na+K ; M+3H ; M+Na+K ; M+3Na ; M+Na+K ; M+Na+K ; M+Na+K ; M+Na+K ; M+Na+K ; M+Na+K ; M+Na+K ; M+Na+K ; M+Na+K ; M+Na+K ; M+Na+K ; M+Na+K	C4H8NO7P ; C5H9Cl2N3O2 ; C11H14O3 ; C11H14O3 ; C11H14O3 ; C11H14O3 ; C11H14O3 ; C10H13ClO4 ; C16H17N3O2S ; C11H14O3 ; C11H14O3 ; C15H15N3O5S2 ; C11H14O3 ; C16H15F2N3Si ; C11H14O3 ; C11H14O3 ; C11H14O3 ; C11H14O3 ; C11H14O3 ; C11H14O3 ; C11H14O3 ; C11H14O3 ; C11H14O3 ; C11H14O3 ; C11H14O3 ; C11H14O3
6259_49.61	1.485864	0.902184	2.258	0.047529	1.323	0.42361	282.0486	HMDB00089 ; HMDB00201 ; HMDB02124 ; HMDB03217 ; HMDB04231 ; HMDB15122 ; HMDB29372 ; HMDB29521 ; HMDB30829 ; HMDB31715 ; HMDB33790 ; HMDB33860 ; HMDB34004 ; HMDB34409 ; HMDB35214 ; HMDB37999 ; HMDB39449 ; HMDB41655 ; HMDB60478	Cytidine ; L-Acetylcarnitine ; Apigenin ; Genistein ; Pantothenol ; Cytarabine ; 2-Hydroxydaidzein ; Norizalpinin ; Aloeemodin ; 6-Hydroxydaidzein ; 128-Trihydroxy-3-methylanthraquinone ; 8-Hydroxydaidzein ; 347-Trihydroxyflavone ; Rhababerone ; Emodin ; Delphinidin 35-di(6-O-malonylglucoside) ; Rheinanthrone ; 3-Hydroxydaidzein ; gamma-Glutamyl-beta-cyanoalanine	M+K ; M+2K+H ; 2M+H+Na ; 2M+H+Na ; M+2K-H ; M+K ; 2M+H+Na ; 2M+H+Na ; 2M+H+Na ; 2M+H+Na ; 2M+H+Na ; 2M+H+Na ; 2M+H+Na ; 2M+H+Na ; 2M+H+Na ; M+H+2Na ; 2M+H+Na ; 2M+H+Na ; M+K	C9H13N3O5 ; C9H17NO4 ; C15H10O5 ; C15H10O5 ; C9H19NO4 ; C9H13N3O5 ; C15H10O5 ; C15H10O5 ; C15H10O5 ; C15H10O5 ; C15H10O5 ; C15H10O5 ; C15H10O5 ; C15H10O5 ; C15H10O5 ; C33H35O23 ; C15H10O5 ; C15H10O5 ; C9H13N3O5
80763_55.4	1.484504	1.216221	-2.2282	0.049992	1.3011	0.42721	318.9781	HMDB32040 ; HMDB32866 ; HMDB33307 ; HMDB60686 ; HMDB60690	Benzoyl peroxide ; 2456-Phenanthrenetetrol ; Moracin M ; 4-Ketocyclophosphamide ; 4-Ketoifosfamide	M+2K-H ; M+2K-H ; M+2K-H ; M+2Na-H ; M+2Na-H	C14H10O4 ; C14H10O4 ; C14H10O4 ; C7H13Cl2N2O3P ; C7H13Cl2N2O3P
0601_55.28	1.481683	1.208018	2.2311	0.049746	1.3032	0.42721	NA	NA	NA	NA	NA

25968_52.4	1.476909	1.069819	2.29	0.045012	1.3467	0.42361	490.9226	HMDB05384 ; HMDB05425 ; HMDB05434 ; HMDB05438 ; HMDB10432 ; HMDB10439 ; HMDB10442 ; HMDB10443 ; HMDB42163 ; HMDB42169 ; HMDB42197 ; HMDB42204 ; HMDB42321 ; HMDB42424 ; HMDB42545 ; HMDB42604 ; HMDB42669 ; HMDB42755 ; HMDB43235 ; HMDB43237 ; HMDB43266 ; HMDB43403 ; HMDB43404 ; HMDB43461 ; HMDB43462 ; HMDB43895 ; HMDB43901 ; HMDB43927 ; HMDB43934 ; HMDB44043 ; HMDB44165 ; HMDB44278 ; HMDB44333 ; HMDB44394 ; HMDB44474 ; HMDB44677 ; HMDB44683 ; HMDB44735 ; HMDB45489 ; HMDB45496 ; HMDB47783 ; HMDB47892 ; HMDB47914 ; HMDB47935 ; HMDB47958 ; HMDB47979 ; HMDB47980 ; HMDB48024 ; HMDB48046 ; HMDB48425 ; HMDB48572 ; HMDB48612 ; HMDB48613 ; HMDB48634 ; HMDB48655 ; [illegible]	TG(16:018:1(9Z)18:2(9Z12Z))iso6 ; TG(16:1(9Z)18:018:2(9Z12Z))iso6 ; TG(16:1(9Z)16:1(9Z)20:1(11Z))iso3 ; TG(16:1(9Z)18:1(9Z)18:1(9Z))iso3 ; TG(18:016:018:3(9Z12Z15Z))iso6 ; TG(18:1(11Z)16:018:2(9Z12Z))iso3 ; TG(18:1(11Z)16:1(9Z)18:1(11Z))iso3 ; TG(18:1(11Z)16:1(9Z)18:1(9Z))iso6 ; TG(14:018:020:3(5Z8Z11Z)) ; TG(14:018:020:3n6) ; TG(14:020:018:3(6Z9Z12Z)) ; TG(14:020:018:3(9Z12Z15Z)) ; TG(14:016:1(9Z)22:2(13Z16Z)) ; TG(14:020:3(5Z8Z11Z)18:0) ; TG(14:018:3(6Z9Z12Z)20:0) ; TG(14:020:3n618:0) ; TG(14:022:2(13Z16Z)16:1(9Z)) ; TG(14:018:3(9Z12Z15Z)20:0) ; TG(15:018:1(11Z)18:2(9Z12Z)) ; TG(15:018:1(11Z)20:2n6) ; TG(15:018:1(9Z)20:2n6) ; TG(15:018:2(9Z12Z)18:1(11Z)) ; TG(15:018:2(9Z12Z)18:1(9Z)) ; TG(15:020:2n618:1(11Z)) ; TG(15:020:2n618:1(9Z)) ; TG(16:016:020:3(5Z8Z11Z)) ; TG(16:016:020:3n6) ; TG(16:018:018:3(6Z9Z12Z)) ; TG(16:018:018:3(9Z12Z15Z)) ; TG(16:014:1(9Z)22:2(13Z16Z)) ; TG(16:020:3(5Z8Z11Z)16:0) ; TG(16:018:3(6Z9Z12Z)18:0) ; TG(16:020:3n616:0) ; TG(16:022:2(13Z16Z)14:1(9Z)) ; TG(16:018:3(9Z12Z15Z)18:0) ; TG(18:014:020:3(5Z8Z11Z)) ; TG(18:014:020:3n6) ; TG(18:016:018:3(6Z9Z12Z)) ; TG(20:014:018:3(6Z9Z12Z)) ; TG(20:014:018:3(9Z12Z15Z)) ; TG(14:1(9Z)16:022:2(13Z16Z)) ; TG(14:1(9Z)14:1(9Z)24:1(15Z)) ; TG(14:1(9Z)16:1(9Z)22:1(13Z)) ; TG(14:1(9Z)18:1(11Z)20:1(11Z)) ; TG(14:1(9Z)18:1(9Z)20:1(11Z)) ; TG(14:1(9Z)20:1(11Z)18:1(11Z)) ; TG(14:1(9Z)20:1(11Z)18:1(9Z)) ; TG(14:1(9Z)22:1(13Z)16:1(9Z)) ; TG(14:1(9Z)24:1(15Z)14:1(9Z)) ; TG(16:1(9Z)14:022:2(13Z16Z)) ; TG(16:1(9Z)14:1(9Z)22:1(13Z)) ; TG(16:1(9Z)18:1(11Z)18:1(11Z)) ; TG(16:1(9Z)18:1(11Z)18:1(9Z)) ; TG(16:1(9Z)18:1(9Z)18:1(11Z)) ; TG(16:1(9Z)20:1(11Z)16:1(9Z)) ; TG(18:1(11Z)15:018:2(9Z12Z)) ;	M+3ACN+2H ; M+3ACN+2H ;	C55H100O6 ; C55H100O6
4726_281.	1.468959	0.317056	2.2621	0.047203	1.326	0.42361	576.4705	HMDB07016 ; HMDB07043 ; HMDB07044 ; HMDB07128 ; HMDB07183 ; HMDB07212 ; HMDB07240 ; HMDB14621 ; HMDB15177 ; HMDB31390 ; HMDB31910 ; HMDB32094 ; HMDB32647 ; HMDB32732 ; HMDB56136 ; HMDB56137 ; HMDB56156	DG(14:018:2(9Z12Z)0:0) ; DG(14:1(9Z)18:1(11Z)0:0) ; DG(14:1(9Z)18:1(9Z)0:0) ; DG(16:1(9Z)16:1(9Z)0:0) ; DG(18:1(11Z)14:1(9Z)0:0) ; DG(18:1(9Z)14:1(9Z)0:0) ; DG(18:2(9Z12Z)14:00:0) ; Rimantadine ; Memantine ; Annotemoyin 1 ; Chatenaytrienin 1 ; Panatellin ; Chatenaytrienin 2 ; cis-Solamin ; DG(14:1n50:018:1n7) ; DG(14:1n50:018:1n9) ; DG(16:1n70:016:1n7)	2M+H+Na ; 2M+H+Na ; 2M+H+Na ; 2M+H+Na ; 2M+H+Na ; 2M+H+Na ; 2M+H+Na ; 3M+K ; 3M+K ; 2M+H+Na ; M+ACN+Na ; 2M+H+Na ; M+ACN+Na ; 2M+H+Na ; 2M+H+Na ; 2M+H+Na ; 2M+H+Na	C35H64O5 ; C35H64O5 ; C35H64O5 ; C35H64O5 ; C35H64O5 ; C35H64O5 ; C35H64O5 ; C12H21N ; C12H21N ; C35H64O5 ; C35H60O2 ; C35H64O5 ; C35H60O2 ; C35H64O5 ; C35H64O5 ; C35H64O5 ; C35H64O5
8975_52.57	1.461554	1.006687	2.2636	0.047083	1.3271	0.42361	NA	NA	NA	NA	NA
9035_252.	1.461296	1.086824	2.2377	0.049197	1.3081	0.42721	113.0539	HMDB30379 ; HMDB12897 ; HMDB14662 ; HMDB15372 ; HMDB29835 ; HMDB31544 ; HMDB32756 ; HMDB33553 ; HMDB60675	N-Methoxy-1-vinyl-beta-carboline ; Beta-Carboline ; Carteolol ; Clomipramine ; Tetrahydroharmol ; 3-Methylcyclopentene ; N-Methyl-1H-indole-3-propanamide ; 3-(3-Methylbutyl)nitrosoamino-2-butanone ; Carbamazepine iminoquinone	M+2H ; 2M+3H ; M+H+2Na ; M+2H+Na ; M+H+Na ; 2M+Na+K ; M+H+Na ; M+H+K ; M+H+NH4	C14H12N2O ; C11H8N2 ; C16H24N2O3 ; C19H23ClN2 ; C12H14N2O ; C6H10 ; C12H14N2O ; C9H18N2O2 ; C14H9NO
1331_282.	1.451714	0.274406	2.3229	0.042563	1.371	0.42361	NA	NA	NA	NA	NA

'9615_152.7	1.426774	0.400409	-2.2604	0.04734	1.3248	0.42361	131.118	HMDB02064 ; HMDB60268 ; HMDB31199 ; HMDB31702 ; HMDB32231 ; HMDB37293	N-Acetylputrescine ; N-Acetylputrescinium ; 25-Dihydro-245-trimethyloxazole ; 1-Piperidinecarboxaldehyde ; Dimethylethanolamine ; 2-Acetylpyrrolidine	M+H ; M+H ; M+NH4 ; M+NH4 ; M+ACN+H ; M+NH4	C6H14N2O ; C6H14N2O ; C6H11NO ; C6H11NO ; C4H11NO ; C6H11NO

Supplemental Table S3. Annotated data from ATG-treated, RA or >95% O2 exposed.

feature	V1	V2	t.stat	p.value	X.log10.p.	FDR	mz	all_HMDBID	all_Name	all_Adduct	all_Formula
7833_102.[illegible]	2.276335	0.173368	-20.555	1.64E-09	8.7847	8.21E-06	297.0598	HMDB14628 ; HMDB39462 ; HMDB39890 ; HMDB15350	Dicloxacillin ; Epiafzelechin-(4beta-6)-epicatechin 33-digallate ; Epiafzelechin-(4b-8)-epicatechin 33-digallate ; Dantrolene	M+3ACN+2H ; M+2H+Na ; M+2H+Na ; M+H-H2O	C19H17Cl2N3O5S ; C44H34O19 ; C44H34O19 ; C14H10N4O5
0841_77.4[illegible]	2.260943	0.176074	15.774	2.15E-08	7.6667	5.39E-05	NA	NA	NA	NA	NA
7099_160.[illegible]	2.234254	0.289882	12.865	1.51E-07	6.8199	0.000189	223.0663	HMDB33041 ; HMDB33072 ; HMDB40243 ; HMDB40262 ; HMDB37913 ; HMDB60769 ; HMDB00056 ; HMDB00161 ; HMDB00271 ; HMDB01310 ; HMDB06806 ; HMDB15417 ; HMDB29869 ; HMDB31219 ; HMDB31635 ; HMDB33258 ; HMDB33912 ; HMDB37337 ; HMDB38890 ; HMDB39087 ; HMDB39088 ; HMDB39776 ; HMDB40345 ; HMDB41307 ; HMDB41392 ; HMDB33071	1-(1-Propenylthio)propyl propyl disulfide ; (E)-1-Propenyl 1-(propylthio)propyl disulfide ; 224466-Hexamethyl-135-trithiane ; 246-Triethyl-135-trithiane ; Artomunoxanthentrione ; 4-Hydroxyphenytoin glucuronide ; Beta-Alanine ; L-Alanine ; Sarcosine ; D-Alanine ; Propinol adenylate ; Fosphenytoin ; Hemiariensin ; Ethyl carbamate ; 2-Propene-1-thiol ; Isoyatein ; 2-Propenyl propyl disulfide ; 7-(4-Carboxy-3-hydroxy-3-methylbutanoyl)sudachitin 4-glucoside ; Methyl 3-methyl-1-butenyl disulfide ; Sudachiin B ; Sudachiin C ; 6-O-Malonylnaringin ; 1-Propene-1-thiol ; Italipyrone ; 1-Propenyl propyl disulfide ; Propyl 1-(propylsulfinyl)propyl disulfide	M+H ; M+H ; M+H ; M+H ; M+2H ; M+2H ; 2M+2Na-H ; 2M+2Na-H ; 2M+2Na-H ; 2M+2Na-H ; M+ACN+2H ; M+2ACN+2H ; M+2Na ; 2M+2Na-H ; 3M+H ; M+2Na ; 3M+2H ; M+3H ; 3M+2H ; M+3H ; M+3H ; M+3H ; 3M+H ; M+2Na ; 3M+2H ; M+H-H2O	C9H18S3 ; C9H18S3 ; C9H18S3 ; C9H18S3 ; C26H20O7 ; C21H20N2O9 ; C3H7NO2 ; C3H7NO2 ; C3H7NO2 ; C3H7NO2 ; C13H18N5O8P ; C16H15N2O6P ; C22H24O7 ; C3H7NO2 ; C3H6S ; C22H24O7 ; C6H12S2 ; C30H34O17 ; C6H12S2 ; C30H34O17 ; C30H34O17 ; C30H34O17 ; C3H6S ; C22H24O7 ; C6H12S2 ; C9H20OS3

50889_61.1(	2.233874	0.367474	13.141	1.24E-07	6.9074	0.000189	164.0561	HMDB00802 ; HMDB30393 ; HMDB31187 ; HMDB33581 ; HMDB36735 ; HMDB36936 ; HMDB39167 ; HMDB39169 ; HMDB39509 ; HMDB60077 ; HMDB00208 ; HMDB01924 ; HMDB04193 ; HMDB04194 ; HMDB13701 ; HMDB15200 ; HMDB15411 ; HMDB29572 ; HMDB31157 ; HMDB31708 ; HMDB31825 ; HMDB31872 ; HMDB33552 ; HMDB41492 ; HMDB60763	Pterin ; L-N-Carboxymethylserine ; Thialdine ; trans-o-Coumaric acid 2-glucoside ; Bilobalide A ; 1-O-p-Coumaroyl-beta-D-glucose ; 2-O-p-Coumaroyl-D-glucose ; 6-O-p-Coumaroyl-D-glucose ; trans-p-Coumaric acid 4-glucoside ; cis-beta-D-Glucosyl-2-hydroxycinnamate ; Oxoglutaric acid ; Atenolol ; N1-Methyl-2-pyridone-5-carboxamide ; N1-Methyl-4-pyridone-3-carboxamide ; 3-Oxoglutaric acid ; Glipizide ; Practolol ; Diethyl disulfide ; 23-Butanedithiol ; 12-Bis(methylthio)ethane ; Phenyl vinyl sulfide ; Methyl propyl disulfide ; ()-13-Butanedithiol ; 12-Butanedithiol ; 4-Hydroxy duloxetine glucuronide	M+H ; M+H ; M+H ; M+2H ; M+2H ; M+2H ; M+2H ; M+2H ; M+2H ; M+2H ; M+NH4 ; M+Na+K ; 2M+H+Na ; 2M+H+Na ; M+NH4 ; M+H+2Na ; M+Na+K ; M+ACN+H ; M+ACN+H ; M+ACN+H ; 2M+3H2O+2H ; M+ACN+H ; M+ACN+H ; M+ACN+H ; M+3H	C6H5N5O ; C5H9NO5 ; C6H13NS2 ; C15H18O8 ; C15H18O8 ; C15H18O8 ; C15H18O8 ; C15H18O8 ; C15H18O8 ; C15H18O8 ; C5H6O5 ; C14H22N2O3 ; C7H8N2O2 ; C7H8N2O2 ; C5H6O5 ; C21H27N5O4S ; C14H22N2O3 ; C4H10S2 ; C4H10S2 ; C4H10S2 ; C8H8S ; C4H10S2 ; C4H10S2 ; C4H10S2 ; C24H27NO8S
27923_139.2	2.228373	0.349351	11.136	5.88E-07	6.2306	0.00049	278.1428	HMDB11140 ; HMDB33337 ; HMDB60717	Hypusine ; Fusarin C ; 2-Hydroxy-imipramine glucuronide	M+2Na-H ; M+3ACN+2H ; M+2ACN+2H	C10H23N3O3 ; C23H29NO7 ; C25H32N2O7
26279_88.73	2.218618	0.032653	-11.227	5.45E-07	6.2636	0.00049	198.0526	HMDB39047 ; HMDB00197 ; HMDB00226 ; HMDB00339 ; HMDB00678 ; HMDB00927 ; HMDB04073 ; HMDB11757 ; HMDB12154 ; HMDB12175 ; HMDB29230 ; HMDB29409 ; HMDB29412 ; HMDB31345 ; HMDB31756 ; HMDB32469 ; HMDB33431 ; HMDB33558 ; HMDB35321 ; HMDB36384 ; HMDB37499 ; HMDB38593 ; HMDB38625 ; HMDB38628 ; HMDB39475 ; HMDB40972 ; HMDB41026 ; HMDB41370 ; HMDB41414 ; HMDB41487 ; HMDB41723	9-Hydroxy-4-methoxypsoralen 9-glucoside ; Indoleacetic acid ; Orotic acid ; 2-Methylbutyrylglycine ; Isovalerylglycine ; Valerylglycine ; 5-Hydroxyindoleacetaldehyde ; N-Acetylvaline ; 3-Dehydrocarnitine ; 5-Acetamidovalerate ; 4-Hydroxystachydrine ; Turicine ; Betonicine ; Calystegine A6 ; Veranisatin C ; Phthalide ; Moschamindole ; 4-O-Methylgalactinol ; Galactopinitol B ; Calystegine A7 ; 3-N-Acetylfusarochromanone ; Calystegin A3 ; Medicanine ; Hydroxymethyl indol-3-yl ketone ; Galactopinitol A ; 1-Methoxy-1H-indole-3-carboxaldehyde ; Erinapyrone C ; Na-p-Hydroxycoumaroyltryptophan ; Chloromarmin ; D-Pinitol 2-O-alpha-D-Galactopyranosyl ; Dihydroferulic acid 4-O-glucuronide	M+2H ; M+Na ; M+ACN+H ; M+K ; M+K ; M+K ; M+Na ; M+K ; M+K ; M+K ; M+K ; M+K ; M+K ; M+K ; M+H+Na ; M+ACN+Na ; M+2Na ; M+H+K ; M+H+K ; M+K ; M+Na+K ; M+K ; M+K ; M+Na ; M+H+K ; M+Na ; 2M+H+Na ; M+2Na ; M+2Na ; M+H+K ; M+H+Na	C18H18O10 ; C10H9NO2 ; C5H4N2O4 ; C7H13NO3 ; C7H13NO3 ; C7H13NO3 ; C10H9NO2 ; C7H13NO3 ; C7H13NO3 ; C7H13NO3 ; C7H13NO3 ; C7H13NO3 ; C7H13NO3 ; C7H13NO3 ; C16H20O10 ; C8H6O2 ; C20H18N2O4 ; C13H24O11 ; C13H24O11 ; C7H13NO3 ; C17H22N2O5 ; C7H13NO3 ; C7H13NO3 ; C10H9NO2 ; C13H24O11 ; C10H9NO2 ; C8H10O5 ; C20H18N2O4 ; C19H23ClO4 ; C13H24O11 ; C16H20O10

2019_88.7	2.221202	0.05492	-10.522	9.95E-07	6.0021	0.000622	216.0632	HMDB00114 ; HMDB59660 ; HMDB00821 ; HMDB00859 ; HMDB11723 ; HMDB13245 ; HMDB13292 ; HMDB14805 ; HMDB32388 ; HMDB33319 ; HMDB34250 ; HMDB35360 ; HMDB38412 ; HMDB38414 ; HMDB40776 ; HMDB41099 ; HMDB60366 ; HMDB60389 ; HMDB60758 ; HMDB61008	Glycerylphosphorylethanolamine ; sn-glycero-3-Phosphoethanolamine ; Phenylacetylglycine ; Methylhippuric acid ; 2-Methylhippuric acid ; m-Methylhippuric acid ; p-Methylhippuric acid ; Histamine Phosphate ; Methyl n-acetylanthranilate ; Licarin C ; Betamipron ; N-Methyl-1-deoxynojirimycin ; 3-Methylbutyl glucosinolate ; Pentyl glucosinolate ; 11-Bis(2-hydroxy-3-methylcarbazole) ; Kanzonol R ; 3-Carbamoyl-2-phenylpropionaldehyde ; 4-Hydroxy-5-phenyltetrahydro-13-oxazin-2-one ; 4-Anilino-4-oxobutanoic acid ; m-chlorophenylpiperazine (m-CPP)	M+H ; M+H ; M+Na ; M+Na ; M+Na ; M+Na ; M+Na ; M+3ACN+2H ; M+Na ; M+Na+K ; M+Na ; M+K ; M+ACN+2H ; M+ACN+2H ; M+H+K ; M+Na+K ; M+Na ; M+Na ; M+Na ; 2M+H+K	C5H14NO6P ; C5H14NO6P ; C10H11NO3 ; C10H11NO3 ; C10H11NO3 ; C10H11NO3 ; C10H11NO3 ; C5H15N3O8P2 ; C10H11NO3 ; C22H26O5 ; C10H11NO3 ; C7H15NO4 ; C12H23NO9S2 ; C12H23NO9S2 ; C26H20N2O2 ; C22H26O5 ; C10H11NO3 ; C10H11NO3 ; C10H11NO3 ; C10H13ClN2
0405_54.7	2.201369	0.052851	10.529	9.89E-07	6.0046	0.000622	133.074	HMDB30102 ; HMDB31972 ; HMDB33227 ; HMDB33661 ; HMDB34719 ; HMDB35140 ; HMDB35715 ; HMDB35805 ; HMDB35874 ; HMDB36043 ; HMDB36045 ; HMDB36093 ; HMDB36125 ; HMDB36130 ; HMDB36135 ; HMDB36202 ; HMDB36773 ; HMDB36931 ; HMDB37055 ; HMDB37528 ; HMDB38156 ; HMDB40119 ; HMDB41227 ; HMDB41284 ; HMDB00107 ; HMDB00247 ; HMDB00765 ; HMDB00774 ; HMDB01976 ; HMDB02095 ; HMDB06528 ; HMDB11632 ; HMDB12497 ; HMDB14606 ; HMDB29172 ; HMDB30987 ; HMDB31689 ; HMDB32257 ; HMDB33699 ; HMDB35024 ; HMDB35581 ; HMDB37163 ; HMDB38736 ; HMDB38909 ; HMDB39133 ; HMDB39530 ; HMDB60113 ; HMDB60246 ; HMDB60382	Hulupinic acid ; Heliespirone A ; Curcolonol ; 4-Hydroxydehydromyoporone ; Isoamberboin ; (S)-Abscisic acid ; Tanacetin ; Thellungianin G ; (10R11R)-Pterosin L ; 15-Hydroxymarasmen-3-one ; O-Formyloreadone ; Abscisic acid ; (1beta8beta)-18-Dihydroxy-37(11)-eudesmadien-128-olide ; Vulgarin ; 3-Epiarmefolin ; Alkhanin ; Tavulin ; Tatridin B ; Istanbulin A ; Blennin B ; Umbellifolide ; Enokipodin C ; (8betaOH10beta)-8-Hydroxy-3-oxo-7(11)-eremophilen-128-olide ; 2-Methyl-1-246-trihydroxy-3-(3-methyl-2-butenyl)phenyl-1-propanone ; Galactitol ; Sorbitol ; Mannitol ; Pregnenolone sulfate ; Docosapentaenoic acid (22n-6) ; Malonylcarnitine ; Docosapentaenoic acid ; L-Iditol ; 1-Pyrroline ; Metharbital ; 3-keto-2-Methylbutyrate ; 2-Carboxy-4-dodecanolide ; 1-Hexanol arabinosylglucoside ; Ethyl abietate ; Linamarin ; ent-16-Kauren-19-ol acetate ; 1-Phenyl-13-hexadecanedione ; 12-Bis(1-ethoxyethoxy)propane ; (3S5R6R7E)-	M+2H ; M+2ACN+2H ; M+2ACN+2H ; M+2ACN+2H ; M+3H ; M+3Na ; M+H+NH4 ; M+3Na ; M+2ACN+2H ; M+ACN+Na ; 2M+3H ; M+NH4 ; M+H+Na ; M+3H ; M+3Na ; M+H+NH4 ; M+3Na ; M+3Na ; M+2Na ; M+H+Na ; M+3Na ; M+3Na ; M+3Na ; M+3Na ; M+ACN+Na ; M+3H	C15H20O4 ; C6H14O6 ; C6H14O6 ; C6H14O6 ; C21H32O5S ; C22H34O2 ; C10H17NO6 ; C22H34O2 ; C6H14O6 ; C4H7N ; C9H14N2O3 ; C5H7O3 ; C13H22O4 ; C17H32O10 ; C22H34O2 ; C10H17NO6 ; C22H34O2 ; C22H34O2 ; C11H24O4 ; C13H22O4 ; C22H34O2 ; C22H34O2 ; C22H34O2 ; C22H34O2 ; C4H7N ; C21H32O5S

0298_39.5(	2.201994	0.462547	10.296	1.22E-06	5.9151	0.000676	286.105	HMDB05923 ; HMDB30177 ; HMDB34611 ; HMDB34613 ; HMDB35445 ; HMDB00296 ; HMDB00767 ; HMDB02034 ; HMDB02056 ; HMDB05808 ; HMDB11679 ; HMDB13247 ; HMDB29226 ; HMDB29470 ; HMDB29747 ; HMDB30720 ; HMDB30725 ; HMDB31668 ; HMDB31829 ; HMDB33036 ; HMDB33776 ; HMDB33994 ; HMDB34056 ; HMDB34617 ; HMDB36586 ; HMDB37130 ; HMDB37681 ; HMDB38563 ; HMDB41379 ; HMDB41394 ; HMDB61055 ; HMDB31787	N4-Acetylcytidine ; Arborinine ; Rutaretin 9-rutinoside ; Torachrysone 8-beta-gentiobioside ; 2-Methoxy-(S)-oleuropein ; Uridine ; Pseudouridine ; Homolanthionine ; Monoisobutyl phthalic acid ; Formononetin ; DHAP(6:0) ; Monobutylphthalate ; 5-(3-Methoxy-4-hydroxyphenyl)-gamma-valerolactone ; Isodillapiole ; 2-Amino-5-phenylpyridine ; Trigraecum ; Dillapiol ; Dihydrodeoxy-8-epiaustdiol ; Bisosthenon B ; 8-Hydroxy-1-methoxy-3-methylanthraquinone ; Apiole ; Isoformononetin ; 5-(34-Methylenedioxyphenyl)pentanoic acid ; (3b4b11b14b)-11-Ethoxy-34-epoxy-14-hydroxy-12-cyathen-15-al 14-xyloside ; 13-Dimethoxyanthraquinone ; Piperonyl isobutyrate ; Vanillin isobutyrate ; (Z)-3-(245-Trimethoxyphenyl)-2-propenal ; 23-Dihydro-3-hydroxy-6-methoxy-22-dimethyl-4H-1-benzopyran-4-one ; Khelmarin D ; desethylzaleplon ; Fenamiphos	M+H ; M+H ; M+2H ; M+2H ; M+2H ; M+ACN+H ; M+ACN+H ; M+NH4 ; M+ACN+Na ; M+NH4 ; M+NH4 ; M+ACN+Na ; M+ACN+Na ; M+ACN+Na ; 3M+Na+K ; M+NH4 ; M+ACN+Na ; M+ACN+Na ; M+2ACN+2H ; M+NH4 ; M+ACN+Na ; M+NH4 ; M+ACN+Na ; M+2K ; M+NH4 ; M+ACN+Na ; M+ACN+Na ; M+ACN+Na ; M+ACN+Na ; M+2ACN+2H ; M+Na ; M+H-H2O	C11H15N3O6 ; C16H15NO4 ; C26H34O14 ; C26H34O14 ; C26H34O14 ; C9H12N2O6 ; C9H12N2O6 ; C8H16N2O6S ; C12H14O4 ; C16H12O4 ; C9H17O7P ; C12H14O4 ; C12H14O4 ; C12H14O4 ; C11H10N2 ; C16H12O4 ; C12H14O4 ; C12H14O4 ; C28H24O8 ; C16H12O4 ; C12H14O4 ; C16H12O4 ; C12H14O4 ; C27H42O8 ; C16H12O4 ; C12H14O4 ; C12H14O4 ; C12H14O4 ; C12H14O4 ; C28H24O8 ; C15H13N5 ; C13H22NO3PS
73146_88.8	2.222932	0.073201	-10.158	1.38E-06	5.8613	0.000688	218.0673	HMDB30417 ; HMDB00026 ; HMDB00168 ; HMDB01268 ; HMDB02052 ; HMDB11733 ; HMDB12265 ; HMDB30900 ; HMDB33780 ; HMDB39953 ; HMDB60582 ; HMDB13204	Lycoperdic acid ; Ureidopropionic acid ; L-Asparagine ; 4-Fumarylacetoacetic acid ; Maleylacetoacetic acid ; Glycyl-glycine ; N-Carbamoylsarcosine ; Eremopetasitenin A2 ; D-Asparagine ; 1-(3-Methylbutanoyl)-6-apiosylglucose ; Benazeprilat ; 6-Succinoaminopurine	M+H ; 3M+H+K ; 3M+H+K ; M+NH4 ; M+NH4 ; 3M+H+K ; 3M+H+K ; M+H+K ; 3M+H+K ; M+H+K ; M+H+K ; M+H-H2O	C8H11NO6 ; C4H8N2O3 ; C4H8N2O3 ; C8H8O6 ; C8H8O6 ; C4H8N2O3 ; C4H8N2O3 ; C20H28O6S ; C4H8N2O3 ; C16H28O11 ; C22H24N2O5 ; C9H9N5O3

9969_71.47	2.184244	0.215432	-9.9137	1.72E-06	5.7642	0.000782	178.071	HMDB00157 ; HMDB00225 ; HMDB00398 ; HMDB00613 ; HMDB00943 ; HMDB14581 ; HMDB14607 ; HMDB28808 ; HMDB29081 ; HMDB29097 ; HMDB29160 ; HMDB29379 ; HMDB31160 ; HMDB33053 ; HMDB33054 ; HMDB33374 ; HMDB39447 ; HMDB59737 ; HMDB61177 ; HMDB32418 ; HMDB32422	Hypoxanthine ; Oxoadipic acid ; 3-Oxoadipic acid ; Erythronic acid ; Threonic acid ; Allopurinol ; Sodium Tetradecyl Sulfate ; Glutaminyl-Tryptophan ; Tryptophyl-Glutamine ; Tryptophyl-Gamma-glutamate ; Gamma-glutamyl-Tryptophan ; Sinapine ; 1-Pentanesulfenothioic acid ; Ethyl propyl disulfide ; Ethyl isopropyl disulfide ; Benzyl glycinate 4-methylbenzenesulfonate salt ; 2-Methyl-4-oxopentanedioic acid ; 3-Methyl-3-hydroxypentanedioate ; Pyridine N-oxide glucuronide ; 2-(4-Methyl-5-thiazolyl)ethyl butanoate ; 2-(4-Methyl-5-thiazolyl)ethyl isobutyrate	M+ACN+H ; M+NH4 ; M+NH4 ; M+ACN+H ; M+ACN+H ; M+ACN+H ; M+Na+K ; M+H+Na ; M+H+Na ; M+H+Na ; M+H+Na ; M+2Na ; M+ACN+H ; M+ACN+H ; M+ACN+H ; M+H+NH4 ; M+NH4 ; M+NH4 ; M+2ACN+2H ; M+H-2H2O ; M+H-2H2O	C5H4N4O ; C6H8O5 ; C6H8O5 ; C4H8O5 ; C4H8O5 ; C5H4N4O ; C14H30O4S ; C16H20N4O4 ; C16H20N4O4 ; C16H20N4O4 ; C16H20N4O4 ; C16H24NO5 ; C5H12S2 ; C5H12S2 ; C5H12S2 ; C16H19NO5S ; C6H8O5 ; C6H8O5 ; C11H14NO7 ; C10H15NO2S ; C10H15NO2S
1239_75.70	2.194884	0.496548	-9.2767	3.15E-06	5.5018	0.001255	290.0871	HMDB05799 ; HMDB30235 ; HMDB33992 ; HMDB35023 ; HMDB37349 ; HMDB37438 ; HMDB37580 ; HMDB38848 ; HMDB39488 ; HMDB39855 ; HMDB40504 ; HMDB11105 ; HMDB15256 ; HMDB40814 ; HMDB41289 ; HMDB41858 ; HMDB60568	Scolymoside ; eq-4-Hydroxymaysin ; Daidzein 47-diglucoside ; Acacetin 7-apiosyl(1-6)-glucoside ; Isorhoifolin ; Kaempferitrin ; Galangin 3-galactosyl-(1-4)-rhamnoside ; Rhoifolin ; Chrysophanol 8-gentiobioside ; Vitexin 4-O-alpha-L-Rhamnopyranoside ; Camellianin B ; 5-Acetylamino-6-formylamino-3-methyluracil ; Tolbutamide ; Isosyringinoside ; Cassitoroside ; Clobenzorex ; 1011-Dihydroxycarbamazepine	M+2H ; M+2H ; M+2H ; M+2H ; M+2H ; M+2H ; M+2H ; M+2H ; M+2H ; M+2H ; M+2H ; M+ACN+Na ; 2M+H+K ; M+2Na ; M+H+Na ; 2M+Na+K ; 2M+H+K	C27H30O14 ; C27H30O14 ; C27H30O14 ; C27H30O14 ; C27H30O14 ; C27H30O14 ; C27H30O14 ; C27H30O14 ; C27H30O14 ; C27H30O14 ; C27H30O14 ; C8H10N4O4 ; C12H18N2O3S ; C23H34O14 ; C25H32O14 ; C16H18ClN ; C15H14N2O3
7227_282.1	2.175183	0.48887	9.2402	3.26E-06	5.4863	0.001255	538.0827	HMDB41377 ; HMDB41378 ; HMDB14672 ; HMDB15312 ; HMDB37204 ; HMDB41936 ; HMDB41937 ; HMDB60824 ; HMDB60930	Camelliatannin E ; Camelliatannin C ; Cyclophosphamide ; Ifosfamide ; 35-Di-O-galloyl-14-galactarolactone ; Morphine-3-glucuronide ; Morphine-6-glucuronide ; Hydromorphone-3-glucuronide ; Hydromorphone 3-beta-O-glucuronide	M+2H ; M+2H ; 2M+NH4 ; 2M+NH4 ; M+ACN+H ; M+2K-H ; M+2K-H ; M+2K-H ; M+2K-H	C49H38O28 ; C49H38O28 ; C7H15Cl2N2O2P ; C7H15Cl2N2O2P ; C20H16O15 ; C23H27NO9 ; C23H27NO9 ; C23H27NO9 ; C23H27NO9

6302_88.52	2.187826	0.345824	-8.8378	4.87E-06	5.3123	0.001484	217.0666	HMDB00715 ; HMDB03134 ; HMDB05032 ; HMDB15600 ; HMDB29915 ; HMDB29916 ; HMDB29965 ; HMDB30988 ; HMDB30992 ; HMDB31437 ; HMDB31449 ; HMDB32055 ; HMDB33284 ; HMDB33816 ; HMDB34145 ; HMDB34219 ; HMDB34221 ; HMDB34222 ; HMDB35045 ; HMDB35046 ; HMDB35110 ; HMDB35828 ; HMDB36557 ; HMDB38922 ; HMDB39048 ; HMDB39059 ; HMDB41148 ; HMDB41197 ; HMDB60331 ; HMDB60332 ; HMDB32660	Kynurenic acid ; Biocytin ; Cetirizine ; Bepotastine ; D-4-O-Methyl-myo-inositol ; 4-O-Methyl-myo-inositol ; Methyl beta-D-glucopyranoside ; 5-(2-Methylpropyl)tetrahydro-2-oxo-3-furancarboxylic acid ; 5-Butyltetrahydro-2-oxo-3-furancarboxylic acid ; (-)-Bornesitol ; Sequoyitol ; N-Acetylhistidine ; 8-Hydroxy-4-methoxypinoresinol ; Mytilitol ; (-)-cis-Rotenolone ; D-Pinitol ; L-Quebrachitol ; L-Pinitol ; Gibberellin A39 ; Gibberellin A43 ; 8-Epidiosbulbin E acetate ; Lactupicrin ; MS 3 ; (7R)-()-Lyoniresinol 9-glucoside ; (5a6a8a11a)-8-Hydroxy-2-oxo-1(10)3-guaiadien-126-olide-15-al 8-(4-hydroxyphenylacetate) ; (R)-Heraclenol 2-(3-methylbutanoate) ; ()-Lyoniresinol 9-glucoside ; Edulisin III ; 1-Nitronaphthalene-56-oxide ; 1-Nitronaphthalene-78-oxide ; (2Z4Z)-2-(5-Methylthio-4-penten-2-ynylidene)-16-dioxaspiro44non-3-ene	2M+3H2O+2H ; M+Na+K ; M+2Na ; M+2Na ; M+Na ; M+Na ; M+Na ; 2M+Na+K ; 2M+Na+K ; M+Na ; M+Na ; 2M+H+K ; M+2Na ; M+Na ; M+H+Na ; M+Na ; M+Na ; M+Na ; M+H+K ; M+H+K ; M+2Na ; M+H+Na ; M+2Na ; M+3Na ; M+H+Na ; M+2Na ; M+3Na ; M+2Na ; 2M+3H2O+2H ; 2M+3H2O+2H ; M+H-H2O	C10H7NO3 ; C16H28N4O4S ; C21H25ClN2O3 ; C21H25ClN2O3 ; C7H14O6 ; C7H14O6 ; C7H14O6 ; C9H14O4 ; C9H14O4 ; C7H14O6 ; C7H14O6 ; C8H11N3O3 ; C21H24O7 ; C7H14O6 ; C23H22O7 ; C7H14O6 ; C7H14O6 ; C7H14O6 ; C20H26O8 ; C20H26O8 ; C21H24O7 ; C23H22O7 ; C21H24O7 ; C28H38O13 ; C23H22O7 ; C21H24O7 ; C28H38O13 ; C21H24O7 ; C10H7NO3 ; C10H7NO3 ; C13H14O2S
1941_56.23	2.180478	0.22887	8.6944	5.64E-06	5.2488	0.001484	118.0612	HMDB00128 ; HMDB15140 ; HMDB28686 ; HMDB28812 ; HMDB29006 ; HMDB29090 ; HMDB31321 ; HMDB32179 ; HMDB34198 ; HMDB41878	Guanidoacetic acid ; Hydroxyurea ; Alanyl-Glutamate ; Glutamyl-Alanine ; Phenylalanyl-Tryptophan ; Tryptophyl-Phenylalanine ; 1-Butylamine ; sec-Butylamine ; 2-Methyl-1-propylamine ; Diethylamine	M+H ; M+ACN+H ; M+H+NH4 ; M+H+NH4 ; M+3H ; M+3H ; M+2Na-H ; M+2Na-H ; M+2Na-H ; M+2Na-H	C3H7N3O2 ; CH4N2O2 ; C8H13N2O5 ; C8H13N2O5 ; C20H21N3O3 ; C20H21N3O3 ; C4H11N ; C4H11N ; C4H11N ; C4H11N
8244_99.63	2.16558	0.292897	8.8953	4.60E-06	5.3376	0.001484	160.9998	HMDB04983 ; HMDB13856 ; HMDB13861 ; HMDB60253 ; HMDB60433 ; HMDB60588	Dimethyl sulfone ; 4-Hydroxycyclophosphamide ; 4-Hydroxyifosfamide ; 1-Methylpyrrolinium ; Aldophosphamide ; Aldoifosfamide	3M+H+K ; M+2Na ; M+2Na ; M+2K-H ; M+2Na ; M+2Na	C2H6O2S ; C7H15Cl2N2O3P ; C7H15Cl2N2O3P ; C5H10N ; C7H15Cl2N2O3P ; C7H15Cl2N2O3P
2985_93.46	2.165092	0.345014	8.8025	5.05E-06	5.2967	0.001484	164.0083	HMDB32437 ; HMDB61161	Monoglyceride citrate ; N-Desalkyl flurazepam	M+Na+K ; M+H+K	C9H14O9 ; C15H10ClFN2O
7571_36.44	2.150914	0.13249	8.9208	4.48E-06	5.3487	0.001484	248.1488	HMDB13127 ; HMDB07867 ; HMDB07899 ; HMDB08891 ; HMDB08955 ; HMDB14859 ; HMDB37530	Hydroxybutyrylcarnitine ; PC(14:014:1(9Z)) ; PC(14:1(9Z)14:0) ; PE(15:016:1(9Z)) ; PE(16:1(9Z)15:0) ; Procaine ; 1-O-Acetylpaxilline	M+H ; M+3Na ; M+3Na ; M+3Na ; M+3Na ; 2M+H+Na ; M+H+NH4	C11H21NO5 ; C36H70NO8P ; C36H70NO8P ; C36H70NO8P ; C36H70NO8P ; C13H20N2O2 ; C29H35NO5

4482_139.9	2.140972	0.097404	8.7287	5.44E-06	5.264	0.001484	277.1394	HMDB00279 ; HMDB01847 ; HMDB14962 ; HMDB28780 ; HMDB28948 ; HMDB34079 ; HMDB37295 ; HMDB38574 ; HMDB38759 ; HMDB39150 ; HMDB39443 ; HMDB40398	Saccharopine ; Caffeine ; Enprofylline ; Cysteinyl-Lysine ; Lysyl-Cysteine ; Homodolichosterone ; 3-(2-Mercapto-1-methylpropyl)thio-2-butanol ; Lansiumamide A ; Licoricesaponin C2 ; Flavidulol C ; (2alpha3alpha5alpha22R23R)-232223-Tetrahydroxy-25-methylergost-24(28)en-6-one ; 1alpha3beta22R-Trihydroxyergosta-524E-dien-26-oic acid 3-O-b-D-glucoside 26-O-b-D-glucosyl ester	M+H ; M+2ACN+H ; M+2ACN+H ; 2M+3H2O+2H ; 2M+3H2O+2H ; M+2K ; M+2ACN+H ; 2M+3H2O+2H ; M+2H+Na ; M+H+K ; M+2K ; M+H+2Na	C11H20N2O6 ; C8H10N4O2 ; C8H10N4O2 ; C9H19N3O3S ; C9H19N3O3S ; C29H48O5 ; C8H18OS2 ; C17H15NO ; C42H62O15 ; C34H42O4 ; C29H48O5 ; C40H64O15
8753_59.66	2.172531	0.424175	8.4917	6.96E-06	5.1575	0.001739	133.0799	HMDB15568 ; HMDB29008 ; HMDB29134 ; HMDB33463 ; HMDB12971 ; HMDB13688 ; HMDB14989 ; HMDB15168 ; HMDB28807 ; HMDB29059 ; HMDB29075 ; HMDB29159 ; HMDB29456 ; HMDB29622 ; HMDB30232 ; HMDB31326 ; HMDB31328 ; HMDB31493 ; HMDB32220 ; HMDB32345 ; HMDB32607 ; HMDB33394 ; HMDB33676 ; HMDB33826 ; HMDB34661 ; HMDB34718 ; HMDB34940 ; HMDB35020 ; HMDB35026 ; HMDB35097 ; HMDB35306 ; HMDB35391 ; HMDB35645 ; HMDB35704 ; HMDB35718 ; HMDB35720 ; HMDB35739 ; HMDB35793 ; HMDB36118 ; HMDB36192 ; HMDB36402 ; HMDB36420 ; HMDB36522 ; HMDB36716 ; HMDB36717 ; HMDB36788 ; HMDB36789 ; HMDB36793 ; HMDB36798 ; HMDB37069 ; HMDB37392 ; HMDB37395 ; HMDB37641 ; HMDB37727 ; HMDB37811 ; HMDB38119 ;	Vorinostat ; Phenylalanyl-Valine ; Valyl-Phenylalanine ; Subaphylline ; 1-Hexanol ; Nootkatol ; Dacarbazine ; Cerulenin ; Glutaminyl-Threonine ; Threoninyl-Glutamine ; Threoninyl-Gamma-glutamate ; Gamma-glutamyl-Threonine ; Amyl 2-furoate ; Isopetasoside ; (2R6S7S10Z)-beta-Santala-3(15)10-dien-12-ol ; 2-Ethoxybutane ; 1-Isothiocyanatobutane ; 3-Hexanol ; Dihydronootkatone ; Isobutyl isothiocyanate ; ()-threo-Anethole glycol ; 4-Ethyl-26-dimethoxyphenol ; ()-3-Methyl-1-pentanol ; 26-Di-tert-butyl-4-methylphenol ; alpha-Valerenol ; Isospathulenol ; alpha-Santal-10-en-12-ol ; Acolamone ; alpha-Cyperol ; beta-Costol ; Epishyobunone ; Preisocalamendiol ; Epoxyguaiene ; Acorenone ; Isocyperol ; Isoacolamone ; (R)-27(14)9-Bisabolatrien-11-ol ; Fukinone ; (3S6E)-6-Caryophyllen-15-al ; Apritone ; alpha-Bergamotenol ; Spathulenol ; Verimol J ; beta-Santalol ; trans-beta-Santalol ; beta-Betulenol ; Caryophyllene alpha-oxide ; 315-Epoxy-6-caryophyllene ;	M+2H ; M+2H ; M+2H ; M+2H ; 2M+Na+K ; M+2Na ; M+2ACN+2H ; M+ACN+2H ; M+H+NH4 ; M+H+NH4 ; M+H+NH4 ; M+H+NH4 ; M+2ACN+2H ; M+3H ; M+2Na ; 2M+Na+K ; M+NH4 ; 2M+Na+K ; M+2Na ; M+NH4 ; M+2ACN+2H ; M+2ACN+2H ; 2M+Na+K ; M+2Na ; M+2Na ; M+2Na ; M+2Na ; M+2Na ; M+2Na ; M+2Na ; M+2Na ; M+2Na ; M+2Na ; M+2Na ; M+2Na ; M+2Na ; M+2Na ; M+2Na ; M+2Na ; M+2Na ; M+2ACN+2H ; M+2Na ; M+2Na ; M+2Na ; M+2Na ; M+2Na ; M+2Na ; M+2Na ; M+2Na ; M+2Na ; M+2ACN+2H ; M+2ACN+2H ; M+2Na ; M+2Na ; M+2Na ; M+2Na ; M+2Na ; M+2Na ; M+2ACN+2H ; M+2Na ; M+2Na ; M+2Na ; M+2Na ; M+2Na ; M+2Na ; M+2ACN+2H ; M+2Na ; M+2Na ; M+2Na ; M+2Na ;	C14H20N2O3 ; C14H20N2O3 ; C14H20N2O3 ; C14H20N2O3 ; C6H14O ; C15H24O ; C6H10N6O ; C12H17NO3 ; C9H17N3O5 ; C9H17N3O5 ; C9H17N3O5 ; C9H17N3O5 ; C10H14O3 ; C21H32O7 ; C15H24O ; C6H14O ; C5H9NS ; C6H14O ; C15H24O ; C5H9NS ; C10H14O3 ; C10H14O3 ; C6H14O ; C15H24O ; C15H24O ; C15H24O ; C15H24O ; C15H24O ; C15H24O ; C15H24O ; C15H24O ; C15H24O ; C15H24O ; C15H24O ; C15H24O ; C15H24O ; C15H24O ; C15H24O ; C15H24O ; C15H24O ; C10H14O3 ; C15H24O ; C15H24O ; C15H24O ; C15H24O ; C15H24O ; C15H24O ; C15H24O ; C15H24O ; C15H24O ; C10H14O3 ; C10H14O3 ; C15H24O ; C15H24O ;
9355_238.	2.163624	0.383504	8.423	7.48E-06	5.1263	0.00178	346.0409	HMDB38430	5-(1-Propynyl)-5-vinyl-22-bithiophene	3M+2H	C13H10S2

3922_73.92	2.168899	0.263043	-8.3621	7.97E-06	5.0984	0.001812	128.0764	HMDB05767 ; HMDB28898 ; HMDB29129 ; HMDB37180 ; HMDB00721 ; HMDB00725 ; HMDB00766 ; HMDB00783 ; HMDB01149 ; HMDB02104 ; HMDB02113 ; HMDB06055 ; HMDB06272 ; HMDB11178 ; HMDB15693 ; HMDB36576 ; HMDB59659 ; HMDB60460 ; HMDB60725 ; HMDB61006	Homoanserine ; Histidinyl-Valine ; Valyl-Histidine ; (S)-Argpyrimidine ; Glycylproline ; 4-Hydroxyproline ; N-Acetyl-L-alanine ; Propionylglycine ; 5-Aminolevulinic acid ; L-Glutamic gamma-semialdehyde ; 3-Hydroxy-L-proline ; 4-Hydroxy-L-proline ; 5-Amino-2-oxopentanoic acid ; L-prolyl-L-glycine ; Tymazoline ; 4-Hydroxy-2-pyrrolidinecarboxylic acid ; Trans-3-hydroxy-L-proline ; cis-4-Hydroxy-D-proline ; 26-Pipecoloxylidide ; norfentanyl	M+2H ; M+2H ; M+2H ; M+2H ; M+2ACN+2H ; M+3ACN+2H ; M+3ACN+2H ; M+3ACN+2H ; M+3ACN+2H ; M+3ACN+2H ; M+3ACN+2H ; M+3ACN+2H ; M+3ACN+2H ; M+2ACN+2H ; M+H+Na ; M+3ACN+2H ; M+3ACN+2H ; M+3ACN+2H ; M+H+Na ; M+H+Na	C11H18N4O3 ; C11H18N4O3 ; C11H18N4O3 ; C11H18N4O3 ; C7H12N2O3 ; C5H9NO3 ; C5H9NO3 ; C5H9NO3 ; C5H9NO3 ; C5H9NO3 ; C5H9NO3 ; C5H9NO3 ; C5H9NO3 ; C7H12N2O3 ; C14H20N2O ; C5H9NO3 ; C5H9NO3 ; C5H9NO3 ; C14H20N2O ; C14H20N2O
2644_89.03	2.169337	0.339724	-8.293	8.58E-06	5.0664	0.001866	406.7113	HMDB30206 ; HMDB60056	Jubanine B ; CE(15:1)	M+2ACN+2H ; 2M+3H	C43H47N5O6 ; C42H72O2
6751_55.52	2.17261	0.582977	8.2006	9.47E-06	5.0236	0.001894	136.0487	HMDB00562 ; HMDB01542 ; HMDB06357 ; HMDB06535 ; HMDB06575 ; HMDB06583 ; HMDB14658 ; HMDB39506 ; HMDB40705 ; HMDB59729 ; HMDB60320	Creatinine ; N-Acetyllactosamine ; cis-2-Methylaconitate ; Beta-14-mannose-N-acetylglucosamine ; Lacto-N-biose I ; Poly-N-acetyllactosamine ; Anisotropine Methylbromide ; N-Salicyloylaspartic acid ; Allixin ; 34-Methylenesebacic acid ; (Z)-But-1-ene-124-tricarboxylate	M+Na ; M+2H+Na ; M+2ACN+2H ; M+2H+Na ; M+2H+Na ; M+2H+Na ; M+H+2Na ; M+H+NH4 ; M+2Na ; M+2Na ; M+2ACN+2H	C4H7N3O ; C14H25NO11 ; C7H8O6 ; C14H25NO11 ; C14H25NO11 ; C14H25NO11 ; C17H32BrNO2 ; C11H11NO6 ; C12H18O4 ; C12H18O4 ; C7H8O6
0722_58.97	2.150496	0.257318	8.2107	9.37E-06	5.0282	0.001894	285.1281	HMDB61152 ; HMDB00050 ; HMDB00085 ; HMDB00830 ; HMDB06901 ; HMDB14340 ; HMDB14638 ; HMDB30363 ; HMDB30378 ; HMDB31697 ; HMDB33023 ; HMDB33796 ; HMDB34352 ; HMDB35325 ; HMDB36310 ; HMDB37842 ; HMDB41419 ; HMDB61124	Indacaterol-8-O-glucuronide ; Adenosine ; Deoxyguanosine ; Neuraminic acid ; Heme A ; Vidarabine ; Zidovudine ; Cavipetin A ; (Z)-Narceine imide ; Lucuminamide ; 712-Dihydroxy-3111523-tetraoxolanost-8-en-26-oic acid ; Physapubescin ; Morellinol ; Ganoderic acid N ; Ganoderic acid theta ; N-(1-Deoxy-1-fructosyl)serine ; Astin I ; 4-Hydroxytamoxifen-N-glucuronide	M+2H ; M+NH4 ; M+NH4 ; M+NH4 ; M+3H ; M+NH4 ; M+NH4 ; M+H+K ; 2M+3H ; M+3ACN+2H ; M+H+K ; M+H+K ; M+H+Na ; M+H+K ; M+H+K ; M+NH4 ; M+H+NH4 ; M+H+NH4	C30H36N2O9 ; C10H13N5O4 ; C10H13N5O4 ; C9H17NO8 ; C49H56FeN4O6 ; C10H13N5O4 ; C10H13N5O4 ; C30H42O8 ; C23H26N2O6 ; C19H27NO11 ; C30H42O8 ; C30H42O8 ; C33H38O7 ; C30H42O8 ; C30H42O8 ; C9H17NO8 ; C25H34ClN5O7 ; C30H33NO9
1689_100.5	2.155216	0.131018	-8.0518	1.11E-05	4.9536	0.002114	296.0572	HMDB01970 ; HMDB01973 ; HMDB03099 ; HMDB03944 ; HMDB06503 ; HMDB06522 ; HMDB11107 ; HMDB11636 ; HMDB13839 ; HMDB14642	3-Methyluric acid ; 9-Methyluric acid ; 1-Methyluric acid ; trans-2-Hexenoyl-CoA ; Phenylacetyl-CoA ; trans-3-Hexenoyl-CoA ; 7-Methyluric acid ; Salicyl CoA ; N-Desmethylaminopyrine ; Flutamide	3M+2Na ; 3M+2Na ; 3M+2Na ; M+2H+Na ; M+3H ; M+2H+Na ; 3M+2Na ; M+3H ; M+2K+H ; 2M+H+K	C6H6N4O3 ; C6H6N4O3 ; C6H6N4O3 ; C27H44N7O17P3S ; C29H42N7O17P3S ; C27H44N7O17P3S ; C6H6N4O3 ; C29H42N7O17P3S ; C12H15N3O ; C11H11F3N2O3

6802_240.4	2.154682	0.494225	8.0283	1.14E-05	4.9425	0.002114	243.0107	HMDB59620 ; HMDB60266 ; HMDB14480 ; HMDB15244 ; HMDB33061 ; HMDB36374 ; HMDB40002	5-(methylthio)-23-Dioxopentyl phosphate ; 23-Diketo-5-methylthio-1-phosphopentane ; Nitrofurazone ; Cefuroxime ; 1-(Methylsulfinyl)propyl propyl disulfide ; Methyl 1-(propylsulfinyl)propyl disulfide ; 1-(2-Thienyl)-12-propanedione	M+H ; M+H ; M+2Na-H ; M+Na+K ; 2M+Na+K ; 2M+Na+K ; 3M+H+Na	C6H11O6PS ; C6H11O6PS ; C6H6N4O4 ; C16H16N4O8S ; C7H16OS3 ; C7H16OS3 ; C7H6O2S
3965_114.9	2.145164	0.28358	-7.9458	1.25E-05	4.9032	0.002232	282.1184	HMDB00576 ; HMDB00622 ; HMDB00661 ; HMDB01844 ; HMDB02001 ; HMDB06592 ; HMDB06833 ; HMDB06855 ; HMDB12231 ; HMDB13939 ; HMDB28822 ; HMDB28823 ; HMDB28906 ; HMDB28928 ; HMDB29428 ; HMDB29884 ; HMDB33958 ; HMDB34681 ; HMDB39750 ; HMDB40531 ; HMDB41622 ; HMDB60469 ; HMDB60956	Monoethyl malonic acid ; Ethylmalonic acid ; Glutaric acid ; Methylsuccinic acid ; Dimethylmalonic acid ; Lacto-N-triaose ; 2-Acetolactate ; (S)-2-Acetolactate ; Geranylfarnesyl diphosphate ; N-Desmethylpromazine ; Glutamyl-Isoleucine ; Glutamyl-Leucine ; Isoleucyl-Glutamate ; Leucyl-Glutamate ; Hypoglycin B ; 2-C-Methyl-14-erythrono-D-lactone ; 2-Deoxy-L-ribono-14-lactone ; 3-Hydroxy-4-butanolide ; Lacto-N-triose I ; 2-Hydroxy-4-oxopentanoic acid ; N-Acetylgalactosaminyl lactose ; Dehydrodolichol diphosphate ; N-desmethylmirtazapine	2M+NH4 ; 2M+NH4 ; 2M+NH4 ; 2M+NH4 ; 2M+NH4 ; M+H+NH4 ; 2M+NH4 ; 2M+NH4 ; M+2Na ; 2M+H+Na ; M+Na ; M+Na ; M+Na ; M+Na ; 2M+H+Na ; 2M+NH4 ; 2M+NH4 ; M+NH4 ; M+H+NH4 ; 2M+NH4 ; M+H+NH4 ; M+2Na ; 2M+Na+K	C5H8O4 ; C5H8O4 ; C5H8O4 ; C5H8O4 ; C5H8O4 ; C20H35NO16 ; C5H8O4 ; C5H8O4 ; C25H44O7P2 ; C16H18N2S ; C11H19N2O5 ; C11H19N2O5 ; C11H19N2O5 ; C11H19N2O5 ; C12H18N2O5 ; C5H8O4 ; C5H8O4 ; C10H16O8 ; C20H35NO16 ; C5H8O4 ; C20H35NO16 ; C25H44O7P2 ; C16H17N3

4444_59.3[illegible]	2.156687	0.135938	7.9052	1.31E-05	4.8838	0.002253	349.1174	HMDB01557 ; HMDB29299 ; HMDB01081 ; HMDB02242 ; HMDB04220 ; HMDB06581 ; HMDB06584 ; HMDB14507 ; HMDB15141 ; HMDB15166 ; HMDB30311 ; HMDB31182 ; HMDB32066 ; HMDB38009 ; HMDB38033 ; HMDB38434 ; HMDB40035 ; HMDB40063 ; HMDB40064 ; HMDB40071 ; HMDB40072 ; HMDB40073 ; HMDB40074 ; HMDB40075 ; HMDB40102 ; HMDB40103 ; HMDB40104 ; HMDB40105 ; HMDB40459 ; HMDB59795 ; HMDB60610	Riboflavin reduced ; Sesamolinol 4-O-b-D-glucosyl (1-6)-O-b-D-glucoside ; (N-acetylneuraminosyl(a2-6)lactosamine) ; Dehydroisocoproporphyrinogen ; Biotinyl-5-AMP ; 3-Sialyl-N-acetyllactosamine ; 6-Sialyl-N-acetyllactosamine ; Clozapine ; Letrozole ; Probenecid ; Harmine ; 1H-Indol-3-ylacetyl-myo-inositol ; Carbanilide ; Malvin ; Malvidin 3-laminaribioside ; 7-Isothiocyanato-1-heptene ; 2-Pentylthiazole ; 4-Butyl-5-methylthiazole ; 4-Ethyl-5-propylthiazole ; 24-Dimethyl-5-propylthiazole ; 25-Dimethyl-4-propylthiazole ; 45-Dimethyl-2-propylthiazole ; 45-Dimethyl-2-(1-methylethyl)thiazole ; 25-Diethyl-4-methylthiazole ; 2-Butyl-4-methylthiazole ; 4-Butyl-2-methylthiazole ; 2-Ethyl-4-propylthiazole ; 4-Ethyl-2-propylthiazole ; Ethylene brassylate ; Gluten exorphin B5 ; Hydroxyhexamide	M+H ; M+2H ; M+H+Na ; M+2Na ; M+3ACN+2H ; M+H+Na ; M+H+Na ; M+Na ; M+ACN+Na ; M+ACN+Na ; 3M+Na+K ; 2M+H+Na ; 3M+Na+K ; M+ACN+2H ; M+ACN+2H ; 2M+K ; 2M+K ; 2M+K ; 2M+K ; 2M+K ; 2M+K ; 2M+K ; 2M+K ; 2M+K ; 2M+K ; 2M+K ; 2M+K ; 2M+K ; M+2K+H ; M+H+K ; M+Na	C15H16N4O6 ; C32H40O17 ; C25H42N2O19 ; C36H36N4O8 ; C20H28N7O9PS ; C25H42N2O19 ; C25H42N2O19 ; C18H19ClN4 ; C17H11N5 ; C13H19NO4S ; C13H12N2O ; C16H19NO7 ; C13H12N2O ; C29H35O17 ; C29H35O17 ; C8H13NS ; C8H13NS ; C8H13NS ; C8H13NS ; C8H13NS ; C8H13NS ; C8H13NS ; C8H13NS ; C8H13NS ; C8H13NS ; C8H13NS ; C8H13NS ; C8H13NS ; C15H26O4 ; C30H38N6O11 ; C15H22N2O4S
3057_46.46	2.151716	0.491335	-7.503	2.06E-05	4.687	0.002398	176.1281	HMDB01252 ; HMDB11534 ; HMDB11565 ; HMDB31043 ; HMDB31044 ; HMDB31045 ; HMDB32478 ; HMDB39979 ; HMDB39980 ; HMDB39981 ; HMDB40001	Betaine aldehyde ; MG(0:016:1(9Z)0:0) ; MG(16:1(9Z)0:00:0) ; Avocadene 1-acetate ; Avocadene 2-acetate ; Avocadene 4-acetate ; Polypropylene glycol (m w 1200-3000) ; 25-Diethyl-36-dimethylpyrazine ; 23-Diethyl-56-dimethylpyrazine ; 26-Diethyl-35-dimethylpyrazine ; Trimethylpropylpyrazine	3M+2Na ; M+H+Na ; M+H+Na ; M+H+Na ; M+H+Na ; M+H+Na ; M+ACN+H ; 2M+H+Na ; 2M+H+Na ; 2M+H+Na ; 2M+H+Na	C5H12NO ; C19H36O4 ; C19H36O4 ; C19H36O4 ; C19H36O4 ; C19H36O4 ; C6H14O3 ; C10H16N2 ; C10H16N2 ; C10H16N2 ; C10H16N2
9417_102.[illegible]	2.147219	0.061097	-7.7997	1.47E-05	4.8329	0.002398	454.1619	HMDB13842 ; HMDB14854 ; HMDB30329 ; HMDB35876 ; HMDB60795	O-Deethylated candesartan ; Nedocromil ; Neopetasitenine ; Mesuagin ; 6a 3-p-Dihydroxypaclitaxel	M+ACN+H ; M+2ACN+H ; 2M+Na+K ; M+ACN+Na ; M+H+NH4	C22H16N6O3 ; C19H17NO7 ; C21H29NO8 ; C24H22O5 ; C45H47NO18

[illegible]7146_58.4[illegible]	2.144319	0.515471	7.6819	1.68E-05	4.7755	0.002398	154.0587	HMDB14342 ; HMDB14400 ; HMDB14761 ; HMDB29639 ; HMDB29697 ; HMDB29698 ; HMDB30839 ; HMDB31626 ; HMDB31897 ; HMDB32024 ; HMDB32142 ; HMDB32337 ; HMDB32608 ; HMDB32623 ; HMDB33060 ; HMDB33716 ; HMDB34107 ; HMDB34319 ; HMDB34563 ; HMDB36133 ; HMDB36207 ; HMDB36441 ; HMDB37705 ; HMDB38598 ; HMDB38947 ; HMDB41250 ; HMDB59601 ; HMDB59895 ; HMDB60804	Fluconazole ; Diethylstilbestrol ; Fluphenazine ; (4-Methylphenyl)acetaldehyde ; Cinnamyl alcohol ; trans-Cinnamyl alcohol ; (Z)-4-(1-Propenyl)phenol ; 2-Phenylpropanal ; Phaseolic acid ; 4-Ethylbenzaldehyde ; 24-Dimethylbenzaldehyde ; Hydroxypropyl cellulose ; 4-Methylacetophenone ; 1-Phenyl-1-propanone ; Piperolactam A ; 3-Phenylpropanal ; Chavicol ; Furofoline ; 7-Nonene-35-diyn-1-ol ; 3-5-Methyl-2-(1-methylethyl)cyclohexyloxy-12-propanediol ; alpha-Amylcinnamyl acetate ; o-Vinylanisole ; Heptyl cinnamate ; Narceinone ; (S)-3-Methylthiohexyl hexanoate ; Demethoxyshogaol ; Indan-1-ol ; m-Methylacetophenone ; 8-Hydroxythioguanine	M+2H ; M+H+K ; M+2H+Na ; 2M+H+K ; 2M+H+K ; 2M+H+K ; 2M+H+K ; 2M+H+K ; M+2Na ; 2M+H+K ; 2M+H+K ; M+ACN+2H ; 2M+H+K ; 2M+H+K ; M+ACN+2H ; 2M+H+K ; 2M+H+K ; M+ACN+2H ; 2M+H+K ; M+2K ; M+Na+K ; 2M+H+K ; M+Na+K ; M+3H ; M+Na+K ; M+Na+K ; 2M+H+K ; 2M+H+K ; M+3ACN+2H	C13H12F2N6O ; C18H20O2 ; C22H26F3N3OS ; C9H10O ; C9H10O ; C9H10O ; C9H10O ; C9H10O ; C12H22O6 ; C9H10O ; C9H10O ; C16H11NO3 ; C9H10O ; C9H10O ; C16H11NO3 ; C9H10O ; C9H10O ; C16H11NO3 ; C9H10O ; C13H26O3 ; C16H22O2 ; C9H10O ; C16H22O2 ; C23H25NO9 ; C13H26O2S ; C16H22O2 ; C9H10O ; C9H10O ; C5H5N5OS

0435_58.5	2.142716	0.436231	7.6142	1.81E-05	4.7422	0.002398	155.062	HMDB01330 ; HMDB01904 ; HMDB15618 ; HMDB29464 ; HMDB30828 ; HMDB31245 ; HMDB31322 ; HMDB31368 ; HMDB31666 ; HMDB34607 ; HMDB36781 ; HMDB36929 ; HMDB38785 ; HMDB39914 ; HMDB39915 ; HMDB39916 ; HMDB39917 ; HMDB40576	2-Amino-3-carboxymuconic acid semialdehyde ; 3-Nitrotyrosine ; Sertindole ; Asteltoxin ; 2-(4-Allyl-26-dimethoxyphenoxy)-1-(345-trimethoxyphenyl)-1-propanol ; 2-Methyl-1-propanethiol ; 1-Butanethiol ; (1a5b6a)-7-Protoilludene-15614-tetrol 14-(24-dihydroxy-6-methylbenzoic acid) ; Diethyl sulfide ; Judeol ; Diosbulbin H ; 13-Hydroxydihydromelleolide ; Melleolide E ; 16-Dihydroxy-37-dimethoxy-2-(3-methyl-2-butenyl)-8-(3-hydroxy-3-methyl-1E-butenyl)-xanthone ; 16-Dihydroxy-37-dimethoxy-2-(2-hydroxy-3-methyl-3-butenyl)-8-(3-methyl-2-butenyl)-xanthone ; 16-Dihydroxy-37-dimethoxy-2-(3-methyl-2-butenyl)-8-(2-hydroxy-3-methyl-3-butenyl)-xanthone ; 13-Dihydroxy-67-dimethoxy-2-(2-hydroxy-3-methyl-3-butenyl)-8-(3-methyl-2-butenyl)-xanthone ; 2-(Methylthio)propane	M+3ACN+2H ; M+2ACN+2H ; M+2H+Na ; M+H+2Na ; M+H+2Na ; 3M+H+K ; 3M+H+K ; M+H+2Na ; 3M+H+K ; M+H+2Na ; M+H+2Na ; M+H+2Na ; M+H+2Na ; M+2H+Na ; M+2H+Na ; M+2H+Na ; M+2H+Na ; 3M+H+K	C7H7NO5 ; C9H10N2O5 ; C24H26ClFN4O ; C23H30O7 ; C23H30O7 ; C4H10S ; C4H10S ; C23H30O7 ; C4H10S ; C23H30O7 ; C23H30O7 ; C23H30O7 ; C23H30O7 ; C25H28O7 ; C25H28O7 ; C25H28O7 ; C25H28O7 ; C4H10S
2662_279.	2.141199	0.501664	7.6415	1.76E-05	4.7556	0.002398	288.5183	HMDB31807 ; HMDB61045	m-Hydroxydiphenyldimercury(1) ; alpha-hydroxysalmeterol	M+2H ; 2M+3H	C12H11Hg2O ; C25H37NO5
7235_101.	2.140045	0.21583	-7.6048	1.83E-05	4.7376	0.002398	NA	NA	NA	NA	NA
6651_89.0	2.133443	0.219011	7.5698	1.90E-05	4.7202	0.002398	220.9997	HMDB39791 ; HMDB39792 ; HMDB39796	23-Dihydro-5-methyl-3-thiophenethiol ; 45-Dihydro-2-methyl-3-thiophenethiol ; 45-Dihydro-5-methyl-3-thiophenethiol	3M+2Na ; 3M+2Na ; 3M+2Na	C5H8S2 ; C5H8S2 ; C5H8S2
2981_284.	2.129797	0.088421	7.6832	1.67E-05	4.7761	0.002398	549.9683	HMDB04925	Ganglioside GD2 (d18:116:0)	M+3H	C76H134N4O34
6716_101.	2.128118	0.328258	-7.5883	1.86E-05	4.7294	0.002398	198.0417	HMDB06955 ; HMDB04812 ; HMDB14322 ; HMDB14450 ; HMDB33163 ; HMDB36938 ; HMDB38428 ; HMDB40664 ; HMDB40866 ; HMDB40939 ; HMDB60620 ; HMDB60793	3-Hydroxy-2-methylpyridine-45-dicarboxylate ; 25-Furandicarboxylic acid ; Fluvoxamine ; Mitomycin ; Pyrocoll ; 1-O-Feruloylglucose ; 2-Hydroxypropyl glucosinolate ; Veranisatin B ; 1-O-2-Hydroxy-4-methoxycinnamoyl-b-D-glucose ; Methyl phenyl disulfide ; Sulindac sulfone ; 67-Dichloro-3-hydroxy-15 dihydro-imidazo21-bquinazolin-2-one	M+H ; M+ACN+H ; M+2K ; M+Na+K ; 2M+H+Na ; M+H+K ; M+H+NH4 ; M+H+K ; M+H+K ; M+ACN+H ; M+H+Na ; M+3ACN+2H	C8H7NO5 ; C6H4O5 ; C15H21F3N2O2 ; C15H18N4O5 ; C10H6N2O2 ; C16H20O9 ; C10H19NO10S2 ; C16H20O9 ; C16H20O9 ; C7H8S2 ; C20H17FO4S ; C10H7Cl2N3O2

2777_65.99	2.126209	0.443243	-7.4049	2.30E-05	4.6379	0.002398	310.1128	HMDB00230 ; HMDB00773 ; HMDB01107 ; HMDB28726 ; HMDB32763 ; HMDB34075 ; HMDB61014 ; HMDB61015	N-Acetylneuraminic acid ; N-Acetyl-a-neuraminic acid ; 7-Methylguanosine ; Asparaginyl-Asparagine ; N2-Oxalylarginine ; Austalide C ; iparai-hydroxyatorvastatin ; iorthoi-hydroxyatorvastatin	M+H ; M+H ; 2M+H+Na ; M+ACN+Na ; M+ACN+Na ; M+2Na ; M+2Na ; M+2Na	C11H19NO9 ; C11H19NO9 ; C11H16N5O5 ; C8H14N4O5 ; C8H14N4O5 ; C30H38O11 ; C33H35FN2O6 ; C33H35FN2O6
8721_96.3[illegible]	2.123767	0.528862	-7.4969	2.07E-05	4.684	0.002398	432.2219	HMDB60754 ; HMDB14875 ; HMDB33295 ; HMDB33613 ; HMDB61107 ; HMDB61108 ; HMDB61109 ; HMDB61110 ; HMDB61178	31-Hydroxy rifabutin ; Meclizine ; 7-(4-Hydroxyphenyl)-1-phenyl-4-hepten-3-one ; Glabrin D ; Buspirone N-oxide ; 3-Hydroxybuspirone ; 5-Hydroxybuspirone ; 6-Hydroxybuspirone ; Trandolapril-d5 Diketopiperazine	M+2H ; M+ACN+H ; 3M+H+Na ; M+3ACN+2H ; 2M+Na+K ; 2M+Na+K ; 2M+Na+K ; 2M+Na+K ; 2M+H+K	C46H62N4O12 ; C25H27ClN2 ; C19H20O2 ; C36H49N7O10 ; C21H31N5O3 ; C21H31N5O3 ; C21H31N5O3 ; C21H31N5O3 ; C24H32N2O4
5232_99.9[illegible]	2.122808	0.373133	-7.6401	1.76E-05	4.7549	0.002398	345.0455	HMDB37199 ; HMDB37201 ; HMDB37202 ; HMDB01227 ; HMDB01285 ; HMDB01476 ; HMDB01972 ; HMDB14378 ; HMDB14389 ; HMDB59749 ; HMDB59811 ; HMDB36932	2-O-Galloyl-14-galactarolactone ; 3-O-Galloyl-14-galactarolactone ; 5-O-Galloyl-14-galactarolactone ; 5-Thymidylic acid ; Geranyl-PP ; 3-Hydroxyanthranilic acid ; 3-Aminosalicylic acid ; Aminosalicylic Acid ; Mesalazine ; 6-Methoxy-pyridine-3-carboxylic acid ; 2-Nitro-p-cresol ; 2-O-Galloylgalactaric acid	M+H ; M+H ; M+H ; M+Na ; 2M+Na+K ; 2M+K ; 2M+K ; 2M+K ; 2M+K ; 2M+K ; 2M+K ; M+H-H2O	C13H12O11 ; C13H12O11 ; C13H12O11 ; C10H15N2O8P ; C10H20O7P2 ; C7H7NO3 ; C7H7NO3 ; C7H7NO3 ; C7H7NO3 ; C7H7NO3 ; C7H7NO3 ; C13H14O12
1179_256.[illegible]	2.122127	0.266656	-7.4344	2.22E-05	4.6527	0.002398	292.1021	HMDB33296 ; HMDB35217 ; HMDB00706 ; HMDB02381 ; HMDB11167 ; HMDB14632 ; HMDB15378 ; HMDB15593 ; HMDB28759 ; HMDB28760 ; HMDB28969 ; HMDB28991 ; HMDB31985 ; HMDB35191 ; HMDB38327 ; HMDB39926 ; HMDB41580 ; HMDB42023 ; HMDB61039	Kuwanon Y ; 10-Acetoxyligustroside ; L-Aspartyl-L-phenylalanine ; N-Acetylcystathionine ; L-beta-aspartyl-L-phenylalanine ; Sotalol ; Docetaxel ; Ixabepilone ; Aspartyl-Methionine ; Aspartyl-Phenylalanine ; Methionyl-Aspartate ; Phenylalanyl-Aspartate ; gamma-Glutamyl-S-methylcysteine ; (2S4R)-4-(9H-Pyrido34-bindol-1-yl)-124-butanetriol ; (3b9R)-5-Megastigmene-39-diol 9-apiosyl-(1-6)-glucoside ; Lyoniresinol 9-sulfate ; 4-Megastigmene-6a9R-diol 9-apiosyl-(1-6)-glucoside ; Taxotere ; modafinil acid	M+2H ; M+2H ; 2M+H+Na ; 2M+3H2O+2H ; 2M+H+Na ; 2M+H+K ; M+3Na ; M+2K ; 2M+3H2O+2H ; 2M+H+Na ; 2M+3H2O+2H ; 2M+H+Na ; 2M+3H2O+2H ; 2M+H+K ; M+2K ; M+2ACN+2H ; M+2K ; M+3Na ; M+NH4	C34H30O9 ; C27H34O14 ; C13H16N2O5 ; C9H16N2O5S ; C13H16N2O5 ; C12H20N2O3S ; C43H53NO14 ; C27H42N2O5S ; C9H16N2O5S ; C13H16N2O5 ; C9H16N2O5S ; C13H16N2O5 ; C9H16N2O5S ; C15H16N2O3 ; C24H42O11 ; C22H28O11S ; C24H42O11 ; C43H53NO14 ; C15H14O3S
7418_111.7	2.11911	0.395587	-7.4339	2.23E-05	4.6525	0.002398	228.0574	HMDB15051 ; HMDB33280 ; HMDB40545	Amantadine ; 8-Acetoxypinoresinol ; 4-Methylliquiritigenin 7-rhamnoside	M+2K-H ; M+H+K ; M+H+K	C10H17N ; C22H24O8 ; C22H24O8

[illegible]3112_52.2[illegible]	2.118778	0.236621	7.5684	1.91E-05	4.7195	0.002398	263.1453	HMDB15468 ; HMDB28959 ; HMDB29022 ; HMDB30180 ; HMDB36358 ; HMDB37643 ; HMDB38345 ; HMDB30729 ; HMDB33711 ; HMDB34054 ; HMDB41089	Digitoxin ; Lysyl-Proline ; Prolyl-Lysine ; Crustecdysone ; Momordin I ; 28-Glucosyl-3b23-dihydroxy-1219(29)-ursadien-28-oate 3-arabinoside ; Alliosterol 1-rhamnoside 16-galactoside ; Gravelliferone ; 5-Hydroxy-7-(4-hydroxyphenyl)-1-phenyl-3-heptanone ; Auraptene ; 4-(3-Hydroxy-7-phenyl-6-heptenyl)-12-benzenediol	M+2H+Na ; 2M+H+K ; 2M+H+K ; M+2Na ; M+2H+Na ; M+2H+Na ; M+H+2Na ; M+H-2H2O ; M+H-2H2O ; M+H-2H2O ; M+H-2H2O	C41H64O13 ; C11H21N3O3 ; C11H21N3O3 ; C27H44O7 ; C41H64O13 ; C41H64O13 ; C39H66O13 ; C19H22O3 ; C19H22O3 ; C19H22O3 ; C19H22O3
[illegible]2716_112.[illegible]	2.115642	0.173091	-7.4423	2.20E-05	4.6567	0.002398	184.0733	HMDB34752 ; HMDB03447 ; HMDB04072 ; HMDB06236 ; HMDB13815 ; HMDB14736 ; HMDB29636 ; HMDB29637 ; HMDB29638 ; HMDB30267 ; HMDB33066 ; HMDB33067 ; HMDB33910 ; HMDB38997 ; HMDB40028 ; HMDB40042 ; HMDB40048 ; HMDB41509 ; HMDB60865	7-beta-D-Glucopyranosyloxybutylidenephthalide ; Tryptophanol ; 4-Hydroxystyrene ; Phenylacetaldehyde ; 23-Dihydrobenzofuran ; Labetalol ; 2-Methylbenzaldehyde ; 3-Methylbenzaldehyde ; 4-Methylbenzaldehyde ; (R)-Boschniakine ; (-)-erythro-Anethole glycol 1-glucoside ; (-)-erythro-Anethole glycol 2-glucoside ; Acetophenone ; Lentialexin ; 1-(23-Dihydro-1H-pyrrolizin-5-yl)-2-propen-1-one ; 3-(5-Methyl-2-furanyl)methyl-1H-pyrrole ; 34-Dihydro-4-(5-methyl-2-furanyl)methylene-2H-pyrrole ; Dihydroconiferin ; Phenytoin catechol	M+2H ; M+Na ; M+ACN+Na ; M+ACN+Na ; M+ACN+Na ; M+H+K ; M+ACN+Na ; M+ACN+Na ; M+ACN+Na ; M+Na ; M+H+Na ; M+H+Na ; M+ACN+Na ; M+ACN+Na ; M+Na ; M+Na ; M+Na ; M+H+Na ; M+2ACN+2H	C18H22O8 ; C10H11NO ; C8H8O ; C8H8O ; C8H8O ; C19H24N2O3 ; C8H8O ; C8H8O ; C8H8O ; C10H11NO ; C16H24O8 ; C16H24O8 ; C8H8O ; C8H8O ; C10H11NO ; C10H11NO ; C10H11NO ; C16H24O8 ; C15H12N2O4

6139_49.1	2.110589	0.461544	-7.4848	2.10E-05	4.6779	0.002398	241.1546	HMDB01009 ; HMDB01457 ; HMDB03990 ; HMDB04649 ; HMDB11472 ; HMDB11502 ; HMDB12455 ; HMDB13687 ; HMDB13693 ; HMDB30395 ; HMDB32153 ; HMDB32247 ; HMDB35081 ; HMDB35082 ; HMDB35643 ; HMDB35657 ; HMDB35747 ; HMDB35786 ; HMDB35835 ; HMDB35983 ; HMDB36071 ; HMDB36109 ; HMDB36172 ; HMDB36201 ; HMDB36443 ; HMDB36667 ; HMDB36718 ; HMDB36733 ; HMDB36797 ; HMDB36813 ; HMDB37000 ; HMDB37061 ; HMDB37068 ; HMDB37275 ; HMDB37310 ; HMDB38164 ; HMDB38187 ; HMDB38195 ; HMDB38200 ; HMDB38201 ; HMDB38215 ; HMDB38511 ; HMDB39463 ; HMDB39637 ; HMDB40686 ; HMDB41404 ; HMDB41490 ; HMDB60138	N-Methylnicotinium ; 5-b-Cholestane-3a 7a 12a-triol ; 3b5a6b-Cholestanetriol ; Demethylphylloquinone ; LysoPE(0:015:0) ; LysoPE(15:00:0) ; 3 alpha7 alpha26-Trihydroxy-5beta-cholestane ; Nookatone ; Sinensal ; Manglupenone ; Acetaldehyde diisoamyl acetal ; (Z)-4-Dodecenal ; (5alpha10alpha)-37(11)-Eudesmadien-2-one ; (5beta7beta10beta)-311-Eudesmadien-2-one ; beta-Sinensal ; Solavetivone ; Patchoulenone ; Valerenal ; Furanoeremophilane ; Ganoderal A ; Dendrolasin ; 135-Bisabolatrien-10-one ; 4-(266-Trimethyl-1-cyclohexenyl)-2-butanol ; (E)-1011-Dihydro-alpha-atlantone ; Rotundone ; Zerumbone ; beta-Santalal ; Vulgarone A ; Mustakone ; alpha-Santalal ; alpha-Turmerone ; ()-411-Eudesmadien-3-one ; Curlone ; 2-Tridecenal ; 11-Dimethoxydecane ; Vulgarone B ; beta-Atlantone ; Xanthorrhizol ; Calamusenone ; 47(11)-Guaiadien-8-one ; alpha-Sinensal ; Bisacumol ; (3alpha5alpha22R23R)-Cholestane-32223-triol ; beta-	M+ACN+Na ; M+Na+K ; M+Na+K ; M+2Na ; M+ACN+2H ; M+ACN+2H ; M+Na+K ; M+Na ; M+Na ; M+2Na ; M+K ; M+2Na-H ; M+Na ; M+Na ; M+Na ; M+Na ; M+Na ; M+Na ; M+Na ; M+2Na ; M+Na ; M+Na ; M+2Na-H ; M+Na ; M+Na ; M+Na ; M+Na ; M+Na ; M+Na ; M+Na ; M+Na ; M+Na ; M+Na ; M+2Na-H ; M+K ; M+Na ; M+Na ; M+Na ; M+Na ; M+Na ; M+Na ; M+Na ; M+Na+K ; M+Na ; M+Na ; M+2Na-H ; M+2Na-H ; M+Na+K	C11H17N2 ; C27H48O3 ; C27H48O3 ; C30H44O2 ; C20H42NO7P ; C20H42NO7P ; C27H48O3 ; C15H22O ; C15H22O ; C30H44O2 ; C12H26O2 ; C13H24O ; C15H22O ; C15H22O ; C15H22O ; C15H22O ; C15H22O ; C15H22O ; C15H22O ; C30H44O2 ; C15H22O ; C15H22O ; C13H24O ; C15H22O ; C15H22O ; C15H22O ; C15H22O ; C15H22O ; C15H22O ; C15H22O ; C15H22O ; C15H22O ; C15H22O ; C13H24O ; C12H26O2 ; C15H22O ; C15H22O ; C15H22O ; C15H22O ; C15H22O ; C15H22O ; C15H22O ; C27H48O3 ; C15H22O ; C15H22O ; C13H24O ; C13H24O ; C27H48O3
2827_100.9	2.103939	0.230065	-7.4228	2.25E-05	4.6469	0.002398	NA	NA	NA	NA	NA
2104_100.4	2.10222	0.235364	-7.4115	2.28E-05	4.6412	0.002398	NA	NA	NA	NA	NA
8697_49.48	2.101309	0.394519	-7.2624	2.72E-05	4.5657	0.002774	242.1579	HMDB02126 ; HMDB32690 ; HMDB35188 ; HMDB35189 ; HMDB38498 ; HMDB38500 ; HMDB38501	27-Nor-5b-cholestane-3a7a12a2425-pentol ; (3beta5alpha9alpha14alpha22E24R)-35914-Tetrahydroxyergosta-722-dien-6-one ; Fasciculol F ; Fasciculol E ; Polyporusterone E ; Polyporusterone D ; Polyporusterone G	M+2Na ; M+H+Na ; M+3H ; M+3H ; M+H+Na ; M+H+Na ; M+H+Na	C26H46O5 ; C28H44O5 ; C39H65NO11 ; C39H65NO11 ; C28H44O5 ; C28H44O5 ; C28H44O5
4138_58.05	2.106342	0.281769	7.1701	3.03E-05	4.5183	0.003032	137.0514	HMDB29943 ; HMDB15068 ; HMDB15491 ; HMDB29525 ; HMDB32353 ; HMDB60336 ; HMDB60605	Arbutin ; Mesoridazine ; Nitroxoline ; 7-(4-Hydroxy-3-methoxyphenyl)-5-methoxy-1-phenyl-3-heptanone ; Isopropyl citrate ; 14-Bipiperidine-1-carboxylic acid ; Metyrapol	M+2H ; M+2H+Na ; M+2ACN+2H ; M+3Na ; M+H+Na ; M+Na+K ; M+2Na	C12H16O7 ; C21H26N2OS2 ; C9H6N2O3 ; C21H26O4 ; C10H18O7 ; C11H20N2O2 ; C14H16N2O

)4078_75.4	2.126569	0.689294	-7.0836	3.36E-05	4.4736	0.003232	291.0904	HMDB11166 ; HMDB11172 ; HMDB14959 ; HMDB28756 ; HMDB28757 ; HMDB28903 ; HMDB28925 ; HMDB29766 ; HMDB30244 ; HMDB31936 ; HMDB33480 ; HMDB36525 ; HMDB38143 ; HMDB38955 ; HMDB41308 ; HMDB59717	L-beta-aspartyl-L-leucine ; L-gamma-glutamyl-L-valine ; Carprofen ; Aspartyl-Isoleucine ; Aspartyl-Leucine ; Isoleucyl-Aspartate ; Leucyl-Aspartate ; (3S7E9R)-47-Megastigmadiene-39-diol 9-apiosyl-(1-6)-glucoside ; Graveolinine ; Blumenol C O-apiosyl-(1-6)-glucoside ; Graveoline ; Myricanol 5-glucoside ; Calamin ; Avenalumin II ; Italidipyrone ; Glu-Val	M+2Na-H ; M+2Na-H ; M+NH4 ; M+2Na-H ; M+2Na-H ; M+2Na-H ; M+2Na-H ; M+2K ; 2M+H+Na ; M+2K ; 2M+H+Na ; M+Na+K ; M+Na+K ; 2M+H+Na ; M+H+K ; M+2Na-H	C10H18N2O5 ; C10H18N2O5 ; C15H12ClNO2 ; C10H18N2O5 ; C10H18N2O5 ; C10H18N2O5 ; C10H18N2O5 ; C24H40O11 ; C17H13NO3 ; C24H40O11 ; C17H13NO3 ; C27H36O10 ; C27H36O10 ; C17H13NO3 ; C29H34O10 ; C10H18N2O5
3958_99.57	2.106331	0.186638	-7.0904	3.33E-05	4.4771	0.003232	522.5884	HMDB41117	Camelliatannin F	M+H+NH4	C48H34O26
8842_99.43	2.130539	0.444154	6.9741	3.83E-05	4.4164	0.00355	228.9619	HMDB40585 ; HMDB41565	()-Sulfobutanedioic acid ; 57-Dihydro-2-methylthieno34-dpyrimidine	2M+Na+K ; M+2K-H	C4H6O7S ; C7H8N2S
9104_264.5	2.07878	0.210806	6.9855	3.78E-05	4.4223	0.00355	610.0629	HMDB15309 ; HMDB36242 ; HMDB37522 ; HMDB60387	Chlormezanone ; 4-O-alpha-L-Rhamnopyranosylglucosinalbin ; Orange B ; 4-Glutathionyl cyclophosphamide	2M+ACN+Na ; M+K ; M+ACN+Na ; M+2Na-H	C11H12ClNO3S ; C20H29NO14S2 ; C22H18N4O9S2 ; C17H30Cl2N5O8PS
5404_239.3	2.09462	0.685067	6.9317	4.04E-05	4.3941	0.003669	243.5125	HMDB07869 ; HMDB07875 ; HMDB07876 ; HMDB07907 ; HMDB07934 ; HMDB07965 ; HMDB08131 ; HMDB08163 ; HMDB08196 ; HMDB08892 ; HMDB08902 ; HMDB08903 ; HMDB08988 ; HMDB09318 ; HMDB09351 ; HMDB10567	PC(14:016:0) ; PC(14:018:3(6Z9Z12Z)) ; PC(14:018:3(9Z12Z15Z)) ; PC(14:1(9Z)18:2(9Z12Z)) ; PC(15:015:0) ; PC(16:014:0) ; PC(18:2(9Z12Z)14:1(9Z)) ; PC(18:3(6Z9Z12Z)14:0) ; PC(18:3(9Z12Z15Z)14:0) ; PE(15:018:0) ; PE(15:020:3(5Z8Z11Z)) ; PE(15:020:3(8Z11Z14Z)) ; PE(18:015:0) ; PE(20:3(5Z8Z11Z)15:0) ; PE(20:3(8Z11Z14Z)15:0) ; PE-NMe(16:016:0)	M+2H+Na ; M+3H ; M+3H ; M+3H ; M+2H+Na ; M+2H+Na ; M+3H ; M+3H ; M+3H ; M+2H+Na ; M+3H ; M+3H ; M+2H+Na ; M+3H ; M+3H ; M+2H+Na	C38H76NO8P ; C40H74NO8P ; C40H74NO8P ; C40H74NO8P ; C38H76NO8P ; C38H76NO8P ; C40H74NO8P ; C40H74NO8P ; C40H74NO8P ; C38H76NO8P ; C40H74NO8P ; C40H74NO8P ; C38H76NO8P ; C40H74NO8P ; C40H74NO8P ; C38H76NO8P
8353_239.9	2.111441	0.631239	6.8922	4.23E-05	4.3732	0.003781	NA	NA	NA	NA	NA

0018_49.3	2.064025	0.155251	6.8773	4.31E-05	4.3653	0.003783	249.154	HMDB00520 ; HMDB00524 ; HMDB00655 ; HMDB01231 ; HMDB01968 ; HMDB13267 ; HMDB14474 ; HMDB28744 ; HMDB29122 ; HMDB29851 ; HMDB32669 ; HMDB35142 ; HMDB35888 ; HMDB35995 ; HMDB37065 ; HMDB38569	5a-Cholestane-3a7a12a25-tetrol ; 5b-Cholestane-3a7a12a25-tetrol ; Cholestane-371225-tetrol ; 27-Deoxy-5b-cyprinol ; 5b-Cholestane-3a7a12a23-Tetrol ; N-Decanoylglycine ; Ethambutol ; Asparaginyl-Valine ; Valyl-Asparagine ; 27-Deoxy-5alpha-cyprinol ; (3beta5alpha9alpha22E24R)-359-Trihydroxy-23-methylergosta-722-dien-6-one ; (3beta17alpha23S)-1723-Epoxy-329-dihydroxy-27-norlanost-8-en-24-one ; Tyromycic acid ; 2-(Methoxycarbonyl)-5-methyl-24-bis(3-methyl-2-butenyl)-6-(2-methyl-1-oxopropyl)-5-(4-methyl-3-pentenyl)cyclohexanone ; 3-Oxo-1218-ursadien-28-oic acid ; PC-M5	M+Na+K ; M+Na+K ; M+Na+K ; M+Na+K ; M+Na+K ; 2M+H+K ; M+2Na-H ; M+NH4 ; M+NH4 ; M+Na+K ; M+H+K ; M+H+K ; M+2Na ; M+H+K ; M+2Na ; M+H+NH4	C27H48O4 ; C27H48O4 ; C27H48O4 ; C27H48O4 ; C27H48O4 ; C12H23NO3 ; C10H24N2O2 ; C9H17N3O4 ; C9H17N3O4 ; C27H48O4 ; C29H46O4 ; C29H46O4 ; C30H44O3 ; C29H46O4 ; C30H44O3 ; C29H37NO5
1664_239.	2.09503	0.571247	6.812	4.67E-05	4.3306	0.003959	441.9722	HMDB15206	Fludarabine	M+2K-H	C10H13FN5O7P
8777_96.7	2.064601	0.585392	-6.8194	4.63E-05	4.3345	0.003959	492.0109	HMDB01532	Deoxyadenosine triphosphate	M+H	C10H16N5O12P3
0555_98.4	2.092838	0.247788	6.6742	5.54E-05	4.2565	0.004616	277.9501	HMDB34895	3-(Dichloromethylene)-25-pyrrolidinedione	3M+H+NH4	C5H3Cl2NO2
8799_277.	2.093793	0.372975	6.6432	5.76E-05	4.2397	0.00472	266.4839	HMDB15397 ; HMDB34596 ; HMDB41465	Sunitinib ; Hebevinoside VI ; Melilotoside B	2M+3H ; M+H+2Na ; M+H+2Na	C22H27FN4O2 ; C41H68O12 ; C41H68O12
3414_99.9	2.095048	0.269718	6.5723	6.29E-05	4.2011	0.004973	NA	NA	NA	NA	NA
763_238.7	2.080472	0.544484	6.5867	6.18E-05	4.209	0.004973	447.0676	HMDB15278 ; HMDB32197 ; HMDB33147 ; HMDB41257	Cloxacillin ; Cellulose microcrystalline ; Cloransulam-methyl ; 5-Hydroxy-3378-tetramethoxy-45-methylenedioxyflavone	2M+H+Na ; M+2K-H ; M+NH4 ; M+2Na-H	C19H18ClN3O5S ; C14H26O11 ; C15H13ClFN5O5S ; C20H18O9

4235_37.6	2.062058	0.306195	6.5633	6.37E-05	4.1962	0.004973	147.0304	HMDB33966 ; HMDB41390 ; HMDB41393 ; HMDB00888 ; HMDB01199 ; HMDB03288 ; HMDB04185 ; HMDB12161 ; HMDB14350 ; HMDB15229 ; HMDB28766 ; HMDB28873 ; HMDB29062 ; HMDB29123 ; HMDB29393 ; HMDB29445 ; HMDB29504 ; HMDB29730 ; HMDB29731 ; HMDB29732 ; HMDB30539 ; HMDB31337 ; HMDB32931 ; HMDB33155 ; HMDB33270 ; HMDB34278 ; HMDB34300 ; HMDB36189 ; HMDB36605 ; HMDB36633 ; HMDB37137 ; HMDB37456 ; HMDB37508 ; HMDB37599 ; HMDB37750 ; HMDB39775 ; HMDB40062 ; HMDB40096 ; HMDB40323 ; HMDB59759 ; HMDB60321 ; HMDB60537 ; HMDB60669 ; HMDB60670 ; HMDB60826 ; HMDB60945	Di-2-propenyl disulfide 9CI ; (EE)-Di-1-propenyl disulfide ; (E)-1-Propenyl 2-propenyl disulfide ; Undecanedioic acid ; N2-Succinyl-L-ornithine ; Selenocysteine ; 5-Hydroxyindoleacetylglycine ; 4-(Glutamylamino) butanoate ; Pyrimethamine ; Leflunomide ; Aspartyl-Valine ; Hydroxyprolyl-Threonine ; Threoninyl-Hydroxyproline ; Valyl-Aspartate ; L-N-(3-Carboxypropyl)glutamine ; L-Agaridoxin ; Marindinin ; 2-Ethyl-4-methylthiazole ; 4-Ethyl-2-methylthiazole ; 5-Ethyl-4-methylthiazole ; Tangeritin ; Thiiranebutanenitrile ; 2-Benzoxazolol ; Trimethylthiazole ; Sesamolinol ; Auranetin ; 5-Isothiocyanato-1-pentene ; Tetrahydrofurfuryl cinnamate ; Pterosin E ; Sinensetin ; Butyl butyryllactate ; 34568-Pentamethoxyflavone ; Oxidihydroartocarpesin ; Isosinensetin ; Cyclokievitone hydrate ; Homoeriodictyol 4-isobutyrate ; 4-Ethyl-5-methylthiazole ; 5-Ethyl-2-methylthiazole ; 34578-	M+H ; M+H ; M+H ; M+2K ; M+Na+K ; M+3ACN+2H ; M+2Na ; M+Na+K ; M+2Na ; M+H+Na ; M+Na+K ; M+Na+K ; M+Na+K ; M+Na+K ; M+Na+K ; M+H+K ; M+Na+K ; 2M+H+K ; 2M+H+K ; 2M+H+K ; M+3Na ; 2M+H+K ; 2M+H+Na ; 2M+H+K ; M+3Na ; M+3Na ; 2M+H+K ; M+Na+K ; M+Na+K ; M+3Na ; M+2K ; M+3Na ; M+3Na ; M+3Na ; M+3Na ; M+3Na ; 2M+H+K ; 2M+H+K ; M+3Na ; M+H+2Na ; M+2Na ; M+2Na ; M+H+K ; M+H+K ; M+2Na ; M+H+Na	C6H10S2 ; C6H10S2 ; C6H10S2 ; C11H20O4 ; C9H16N2O5 ; C3H7NO2Se ; C12H12N2O4 ; C9H16N2O5 ; C12H13ClN4 ; C12H9F3N2O2 ; C9H16N2O5 ; C9H16N2O5 ; C9H16N2O5 ; C9H16N2O5 ; C9H16N2O5 ; C11H14N2O5 ; C14H16O3 ; C6H9NS ; C6H9NS ; C6H9NS ; C20H20O7 ; C6H9NS ; C7H5NO2 ; C6H9NS ; C20H20O7 ; C20H20O7 ; C6H9NS ; C14H16O3 ; C14H16O3 ; C20H20O7 ; C11H20O4 ; C20H20O7 ; C20H20O7 ; C20H20O7 ; C20H20O7 ; C20H20O7 ; C6H9NS ; C6H9NS ; C20H20O7 ; C22H18O7 ; C17H12O2 ; C12H12N2O4 ; C11H14N2O5 ; C11H14N2O5 ; C12H12N2O4 ; C12H9F3N2O2
4307_110.	2.074265	0.493677	-6.5445	6.52E-05	4.1859	0.005014	129.5244	HMDB31332 ; HMDB33188	Chloromethyl methyl ether ; 3-Amino-1-methyl-5H-pyrido43-bindole	3M+H+NH4 ; M+Na+K	C2H5ClO ; C12H11N3
3722_99.9	2.095711	0.152138	-6.4939	6.95E-05	4.1581	0.005264	NA	NA	NA	NA	NA
1347_101.	2.074253	0.387738	-6.4267	7.57E-05	4.121	0.005566	197.7071	HMDB30641 ; HMDB33006 ; HMDB37087 ; HMDB39083	Biochanin A 7-(6-methylmalonylglucoside) ; Neobignonoside ; Chrysophanol 8-(6-galloylglucoside) ; 6-O-Malonylwistin	M+H+2Na ; M+2H+Na ; M+2H+Na ; M+H+2Na	C26H26O13 ; C28H24O13 ; C28H24O13 ; C26H26O13
3542_100.8	2.073596	0.176951	-6.4308	7.53E-05	4.1232	0.005566	424.6085	HMDB04896	Ganglioside GA2 (d18:126:0)	M+3Na	C64H120N2O18

38436_124.	2.0901	0.244052	-6.3897	7.94E-05	4.1004	0.005669	271.1038	HMDB28755 ; HMDB28881 ; HMDB29955 ; HMDB12883 ; HMDB14433 ; HMDB14457 ; HMDB15440 ; HMDB30230 ; HMDB32809 ; HMDB37088 ; HMDB38028 ; HMDB38085 ; HMDB40672 ; HMDB41336 ; HMDB60705 ; HMDB60720 ; HMDB60743 ; HMDB61071 ; HMDB61130	Aspartyl-Histidine ; Histidinyl-Aspartate ; D-erythro-L-galacto-Nonulose ; Adrenochrome o-semiquinone ; Amcinonide ; Pentobarbital ; Amobarbital ; 2-(E)-Feruloyl-3-(arabinosylxylose) ; 4-Hydroxyphenylacetonitrile triacetylrhamnoside ; cis-p-Coumaric acid 4-apiosyl-(1-2)-glucoside ; Eriojaposide A ; Pelargonidin 3-rhamnoside ; 3-Oxo-alpha-ionol 9-apiosyl-(1-6)-glucoside ; 7-Hydroxy-4-methylphthalide O-arabinosyl-(1-6)-glucoside ; 12-Hydroxynevirapine glucuronide ; 2-Hydroxynevirapine glucuronide ; 3-Hydroxynevirapine glucuronide ; N-desalkyludenafil ; 8-Hydroxynevirapine glucuronide	M+H ; M+H ; M+H ; 3M+2H ; M+H+K ; M+2Na-H ; M+2Na-H ; M+2ACN+2H ; 2M+3H ; M+2ACN+2H ; M+H+K ; M+3ACN+2H ; M+H+K ; M+2ACN+2H ; M+2ACN+2H ; M+2ACN+2H ; M+2ACN+2H ; 2M+3H ; M+2ACN+2H	C10H14N4O5 ; C10H14N4O5 ; C9H18O9 ; C9H10NO3 ; C28H35FO7 ; C11H18N2O3 ; C11H18N2O3 ; C20H26O12 ; C20H23NO8 ; C20H26O12 ; C24H38O11 ; C21H21O9 ; C24H38O11 ; C20H26O12 ; C21H22N4O8 ; C21H22N4O8 ; C21H22N4O8 ; C18H23N5O4S ; C21H22N4O8
)6531_78.2(	2.085191	0.209258	-6.3928	7.91E-05	4.1021	0.005669	306.1266	HMDB59949 ; HMDB14737 ; HMDB15384 ; HMDB29313 ; HMDB32852 ; HMDB33331 ; HMDB37555 ; HMDB37688 ; HMDB41305 ; HMDB41488 ; HMDB60989 ; HMDB15252	Rhodin G7 ; Thiopental ; Dasatinib ; Canescein ; 2-(Ethylamino)-45-dihydroxybenzamide ; (E)-1-4-Hydroxy-3-(3-methyl-13-butadienyl)phenyl-2-(35-dihydroxyphenyl)ethylene ; Sandoricin ; 5-Nitro-2-propoxyaniline ; 1-Methoxy-4-5-(4-methoxyphenoxy)-3-penten-1-ynylbenzene ; 45-Dihydropiperlonguminine ; 4-hydroxypropanolol ; Gliclazide	M+2H ; M+ACN+Na ; M+3ACN+2H ; M+2Na ; 3M+H+Na ; 2M+H+Na ; M+H+Na ; 3M+H+Na ; 2M+H+Na ; 2M+Na+K ; 2M+Na+K ; M+H-H2O	C34H34N4O7 ; C11H18N2O2S ; C22H26ClN7O2S ; C29H42O11 ; C9H12N2O3 ; C19H18O3 ; C31H40O11 ; C9H12N2O3 ; C19H18O3 ; C16H21NO3 ; C16H21NO3 ; C15H21N3O3S

[illegible]1435_70.2[illegible]	2.076229	0.219865	-6.2829	9.11E-05	4.0406	0.006372	255.1451	HMDB02366 ; HMDB12561 ; HMDB15519 ; HMDB29623 ; HMDB31092 ; HMDB31835 ; HMDB32635 ; HMDB32854 ; HMDB33493 ; HMDB33998 ; HMDB35194 ; HMDB35490 ; HMDB36248 ; HMDB40960 ; HMDB60143	Tiglylcarnitine ; 13-Hydroxy-gamma-tocopherol ; Phendimetrazine ; Torvoside C ; 12-Di-(9Z12Z-octadecadienoyl)-sn-glycero-3-phosphate ; Asparagoside C ; NN-Diethylbenzeneacetamide ; N-Acetyl-26-diethylaniline ; Desglucodesrhamnoparillin ; gamma-Tocopheryl quinone ; beta-Tocopheryl quinone ; 25-Epiruizgenin 3-4-rhamnosylglucoside ; Schidigerasaponin D5 ; Capsicoside B2 ; 8alpha-Hydroxy-gama-tocopherone	2M+H+Na ; M+2K ; M+ACN+Na ; M+2H+Na ; M+3Na ; M+2H+Na ; M+ACN+Na ; M+ACN+Na ; M+2H+Na ; M+2K ; M+2K ; M+2H+Na ; M+2H+Na ; M+2H+Na ; M+2K	C12H21NO4 ; C28H48O3 ; C12H17NO ; C39H64O13 ; C39H69O8P ; C39H64O13 ; C12H17NO ; C12H17NO ; C39H64O13 ; C28H48O3 ; C28H48O3 ; C39H64O13 ; C39H64O13 ; C39H64O13 ; C28H48O3
[illegible]6257_99.9[illegible]	2.059435	0.151645	6.2772	9.18E-05	4.0374	0.006372	120.5106	HMDB41821	Acetylisoniazid	M+Na+K	C8H9N3O2
[illegible]3229_275.[illegible]	2.037173	0.551612	6.2598	9.39E-05	4.0275	0.006428	328.9733	HMDB03585 ; HMDB30228 ; HMDB38459	Thiocysteine ; Nigakinone ; 5-(4-Acetoxy-3-oxo-1-butynyl)-22-bithiophene	2M+Na ; M+2K-H ; M+K	C3H7NO2S2 ; C14H8N2O3 ; C14H10O3S2
[illegible]2683_100.[illegible]	2.086201	0.375817	-6.2421	9.60E-05	4.0176	0.006489	277.5853	HMDB29361	Alkaloid RC	M+H+Na	C26H29NO11
[illegible]6623_107.[illegible]	2.073459	0.552748	-6.2254	9.81E-05	4.0081	0.006494	138.5297	HMDB00238 ; HMDB00468 ; HMDB00633 ; HMDB00817 ; HMDB01195 ; HMDB02263 ; HMDB29928 ; HMDB33566 ; HMDB34453 ; HMDB60711	Sepiapterin ; Biopterin ; D-Biopterin ; Orinapterin ; Dyspropterin ; Primapterin ; Deoxyeritadenine ; Ajoene ; 4-22-Bithiophen-5-yl-3-butyn-1-ol ; 2-Amino-5-benzoylbenzimidazole	M+H+K ; M+H+K ; M+H+K ; M+H+K ; M+H+K ; M+H+K ; M+H+K ; M+ACN+2H ; M+ACN+2H ; M+H+K	C9H11N5O3 ; C9H11N5O3 ; C9H11N5O3 ; C9H11N5O3 ; C9H11N5O3 ; C9H11N5O3 ; C9H11N5O3 ; C9H14OS3 ; C12H10OS2 ; C14H11N3O
[illegible]6148_101.[illegible]	2.073068	0.425804	6.2211	9.87E-05	4.0057	0.006494	111.0036	HMDB60689 ; HMDB60691 ; HMDB01090 ; HMDB01981	Phosphoramide mustard ; Isophosphamide mustard ; (S)-5-Diphosphomevalonic acid ; (R)-Mevalonic acid-5-pyrophosphate	M+2H ; M+2H ; M+2H+Na ; M+2H+Na	C4H11Cl2N2O2P ; C4H11Cl2N2O2P ; C6H14O10P2 ; C6H14O10P2

3838_100.5	2.084132	0.205421	-6.1936	0.000102	3.9901	0.006522	167.0444	HMDB15661 ; HMDB34051 ; HMDB38728 ; HMDB38912 ; HMDB39286 ; HMDB39322 ; HMDB39366 ; HMDB39625 ; HMDB41593 ; HMDB00594 ; HMDB01894 ; HMDB15028 ; HMDB28774 ; HMDB28816 ; HMDB28875 ; HMDB29106 ; HMDB29291 ; HMDB29306 ; HMDB29562 ; HMDB30540 ; HMDB31865 ; HMDB32074 ; HMDB32076 ; HMDB32148 ; HMDB32150 ; HMDB32151 ; HMDB32619 ; HMDB33944 ; HMDB34881 ; HMDB34954 ; HMDB35974 ; HMDB37450 ; HMDB37568 ; HMDB38458 ; HMDB38514 ; HMDB38570 ; HMDB38686 ; HMDB41515 ; HMDB41609 ; HMDB59873 ; HMDB60708	Fospropofol ; 6-Galloylglucose ; beta-Glucogallin ; 4-Glucosyl gallate ; 1-O-Galloylfructose ; 3-Glucogallic acid ; 5-O-Galloylhamamelofuranose ; 2-Galloylglucose ; 4-Glucogallic acid ; Glutamylphenylalanine ; Aspartame ; Sulfapyridine ; Cysteinyl-Glutamate ; Glutamyl-Cysteine ; Hydroxyprolyl-Tyrosine ; Tyrosyl-Hydroxyproline ; 34-Dicaffeoyl-15-quinolactone ; 4-Ethylphenol ; N-gamma-L-Glutamyl-L-phenylalanine ; 25-Dimethylphenol ; 4-tert-Butyl-26-dimethyl-35-dinitroacetophenone ; 1-Methoxy-2-methylbenzene ; 1-Methoxy-4-methylbenzene ; 23-Dimethylphenol ; 26-Dimethylphenol ; 34-Dimethylphenol ; 1-Phenylethanol ; 2-Phenylethanol ; 3-Carboxy-2349-tetrahydro-1H-pyrido34-bindole-1-propanoic acid ; Zizybeoside I ; (S)-Spirobrassinin ; Orientin 73-dimethyl ether ; 6-beta-D-Glucopyranosyl-45-dihydroxy-37-dimethoxyflavone ; NN-Bis(gamma-glutamyl)cystine ; L-gamma-Glutamyl-beta-phenyl-beta-L-alanine ; Cyclobrassinin sulfoxide ; Armillaricin ; Benzyl gentiobioside ; 4-	M+2H ; M+2H ; M+2H ; M+2H ; M+2H ; M+2H ; M+2H ; M+2H ; M+2H ; M+H+K ; M+H+K ; 2M+3H ; 2M+3H ; 2M+3H ; M+H+K ; M+H+K ; M+3H ; M+2Na-H ; M+H+K ; M+2Na-H ; M+H+K ; M+2Na-H ; M+2Na-H ; M+2Na-H ; M+2Na-H ; M+2Na-H ; M+2Na-H ; M+2Na-H ; M+2Na ; M+3Na ; M+2ACN+2H ; M+2H+Na ; M+2H+Na ; M+3H ; M+H+K ; M+2ACN+2H ; M+3Na ; M+3Na ; M+2Na-H ; M+2Na-H ; M+3Na	C13H19Na2O5P ; C13H16O10 ; C13H16O10 ; C13H16O10 ; C13H16O10 ; C13H16O10 ; C13H16O10 ; C13H16O10 ; C13H16O10 ; C14H18N2O5 ; C14H18N2O5 ; C11H11N3O2S ; C8H13N2O5S ; C8H13N2O5S ; C14H18N2O5 ; C14H18N2O5 ; C25H22O11 ; C8H10O ; C14H18N2O5 ; C8H10O ; C14H18N2O5 ; C8H10O ; C8H10O ; C8H10O ; C8H10O ; C8H10O ; C8H10O ; C8H10O ; C15H16N2O4 ; C19H28O11 ; C11H10N2OS2 ; C23H24O11 ; C23H24O11 ; C16H26N4O10S2 ; C14H18N2O5 ; C11H10N2OS2 ; C24H29ClO5 ; C19H28O11 ; C8H10O ; C8H10O ; C24H29ClO5
9768_279.7	2.05768	0.39587	-6.1873	0.000103	3.9865	0.006522	503.063	HMDB32195 ; HMDB33511 ; HMDB60837 ; HMDB60839	Carrageenan and salts of carrageenan ; Protogonyautoxin I ; N-Desmethyl-o-O-sulfate rosiglitazone ; N-Desmethyl-p-O-sulfate rosiglitazone	M+3H ; 2M+3H2O+2H ; M+ACN+Na ; M+ACN+Na	C42H74O46S6 ; C10H17N7O11S2 ; C17H17N3O7S2 ; C17H17N3O7S2
9167_126.6	2.055946	0.075846	-6.169	0.000106	3.9761	0.006522	241.0489	HMDB60873 ; HMDB01934 ; HMDB04350 ; HMDB13846 ; HMDB14330 ; HMDB14847 ; HMDB37205 ; HMDB37470 ; HMDB37579 ; HMDB38235 ; HMDB39982 ; HMDB40789 ; HMDB33649	Sorafenib N-oxide ; L(-)-Nicotine pestanal ; Anabasine ; E-3179 ; Nicotine ; Lamivudine ; (2R)-2-Hydroxy-2-phenylethyl glucosinolate ; Spinacetin 3-rutinoside ; Limocitrin 3-rutinoside ; Rhamnazin 3-sophoroside ; Trimethyl-2-propenylpyrazine ; Bikoeniquinone A ; O-Demethylfonsecin	M+2H ; M+2K+H ; M+2K+H ; M+Na+K ; M+2K+H ; 2M+H+Na ; M+ACN+2H ; M+3Na ; M+3Na ; M+3Na ; M+2K+H ; M+Na+K ; M+H-2H2O	C21H16ClF3N4O4 ; C10H14N2 ; C10H14N2 ; C22H21ClN6O ; C10H14N2 ; C8H11N3O3S ; C15H21NO10S2 ; C29H34O17 ; C29H34O17 ; C29H34O17 ; C10H14N2 ; C27H20N2O3 ; C14H12O6
1151_87.84	2.055706	0.406189	-6.2023	0.000101	3.995	0.006522	431.1191	HMDB36355 ; HMDB38420 ; HMDB38424 ; HMDB39132 ; HMDB39877 ; HMDB61023	3-Methylpentyl glucosinolate ; 4-Methylpentyl glucosinolate ; Hexyl glucosinolate ; 4-Gingerdiol 35-diacetate ; Charine ; N-Desmethyldiltiazem	2M+3H2O+2H ; 2M+3H2O+2H ; 2M+3H2O+2H ; M+2K+H ; 3M+H+K ; 2M+Na+K	C13H25NO9S2 ; C13H25NO9S2 ; C13H25NO9S2 ; C19H28O6 ; C9H14N4O6 ; C21H24N2O4S

0975_239.3	2.050678	0.693311	6.1775	0.000104	3.9809	0.006522	448.0711	HMDB14415	Isradipine	M+2K-H	C19H21N3O5
3625_284.4	2.056537	0.290913	6.133	0.000111	3.9556	0.006754	204.0864	HMDB38440 ; HMDB00321 ; HMDB00345 ; HMDB00355 ; HMDB00368 ; HMDB00640 ; HMDB01004 ; HMDB01324 ; HMDB01390 ; HMDB01411 ; HMDB01427 ; HMDB02249 ; HMDB02278 ; HMDB02282 ; HMDB06730 ; HMDB12492 ; HMDB14685 ; HMDB15527 ; HMDB29834 ; HMDB29934 ; HMDB32873 ; HMDB34426 ; HMDB40985 ; HMDB41561 ; HMDB41835 ; HMDB59758 ; HMDB32293	1-Isothiocyanato-7-(methylthio)heptane ; 2-Hydroxyadipic acid ; 3-Hydroxyadipic acid ; 3-Hydroxymethylglutaric acid ; 2(R)-Hydroxyadipic acid ; Levoglucosan ; Oxoamide ; N-Hydroxymethylnorcotinine ; Hydroxycotinine ; Cotinine N-oxide ; 5-Hydroxycotinine ; 6-Methyltetrahydropterin ; 2-(acetylamino)-15-anhydro-2-deoxy-3-O-b-D-galactopyranosyl-D-arabino-Hex-1-enitol ; 2-(acetylamino)-15-anhydro-2-deoxy-4-O-b-D-galactopyranosyl-D-arabino-Hex-1-enitol ; QYNAD ; 1-(12345-Pentahydroxypent-1-yl)-1234-tetrahydro-beta-carboline-3-carboxylate ; Pyridostigmine ; Prazepam ; Harmalan ; D-1-Deoxy-erythro-hexo-23-diulose ; Diethyl dicarbonate ; (-)-1-Methylpropyl 1-propenyl disulfide ; Polixetonium chloride ; D-15-Anhydrofructose ; Benzidine ; 2-Hydroxy-2-ethylsuccinic acid ; N-Gluconyl ethanolamine	M+H ; M+ACN+H ; M+ACN+H ; M+ACN+H ; M+ACN+H ; M+ACN+H ; 2M+H+Na ; 2M+H+Na ; 2M+H+Na ; 2M+H+Na ; 2M+H+Na ; M+Na ; M+ACN+2H ; M+ACN+2H ; M+3H ; M+ACN+2H ; M+Na ; M+2ACN+2H ; 2M+H+K ; M+ACN+H ; M+ACN+H ; M+ACN+H ; M+2ACN+2H ; M+ACN+H ; 2M+H+K ; M+ACN+H ; M+H-2H2O	C9H17NS2 ; C6H10O5 ; C6H10O5 ; C6H10O5 ; C6H10O5 ; C6H10O5 ; C10H12N2O2 ; C10H12N2O2 ; C10H12N2O2 ; C10H12N2O2 ; C10H12N2O2 ; C7H11N5O ; C14H23NO10 ; C14H23NO10 ; C25H35N7O11 ; C17H21N2O7 ; C9H13N2O2 ; C19H17ClN2O ; C12H12N2 ; C6H10O5 ; C6H10O5 ; C7H14S2 ; C10H28Cl2N2OP2 ; C6H10O5 ; C12H12N2 ; C6H10O5 ; C8H17NO7
5184_98.6	2.043005	0.305128	-6.0595	0.000122	3.9134	0.007353	416.0555	HMDB00658 ; HMDB01131 ; HMDB40626 ; HMDB60350	CE(16:1(9Z)) ; Iminoaspartic acid ; 236-Tris-O-(345-trihydroxybenzoyl)-3-Glucosyl-246-trihydroxyacetophenone ; 2-Oxosuccinamate	2M+3H ; 3M+Na ; M+2Na ; 3M+Na	C43H74O2 ; C4H5NO4 ; C35H30O21 ; C4H5NO4
9482_280.8	2.051805	0.096202	5.9925	0.000133	3.8747	0.007943	713.3719	HMDB02835 ; HMDB33218 ; HMDB33230 ; HMDB33231 ; HMDB34771 ; HMDB38721 ; HMDB39342 ; HMDB39474 ; HMDB41986 ; HMDB60264	Danazol ; 26-Dimethyl-7-octene-16-diol 8-O-glucoside ; trans-p-Menthane-78-diol 7-glucoside ; trans-p-Menthane-78-diol 8-glucoside ; 37-Dimethyl-5-octene-17-diol 1-glucoside ; Soyasaponin bg ; Capsianoside G ; Capsianoside H ; Pibutidine ; Apelin (1-12)	2M+K ; 2M+2Na-H ; 2M+2Na-H ; 2M+2Na-H ; 2M+2Na-H ; 2M+3H ; M+H+Na ; M+H+Na ; 2M+H ; M+H+Na	C22H27NO2 ; C16H30O7 ; C16H30O7 ; C16H30O7 ; C16H30O7 ; C54H84O21 ; C70H114O28 ; C70H114O28 ; C19H24N4O3 ; C60H102N22O15S
1832_50.1	2.025035	0.50612	-5.9771	0.000136	3.8658	0.008013	391.9442	HMDB14763	Efavirenz	M+2K-H	C14H9ClF3NO2

9144_59.6	2.066278	0.008781	5.9334	0.000144	3.8404	0.008024	329.0929	HMDB01184 ; HMDB12157 ; HMDB28831 ; HMDB28847 ; HMDB28973 ; HMDB29104 ; HMDB30542 ; HMDB30553 ; HMDB30918 ; HMDB30919 ; HMDB31407 ; HMDB31512 ; HMDB31530 ; HMDB31601 ; HMDB31606 ; HMDB31607 ; HMDB32122 ; HMDB32458 ; HMDB33149 ; HMDB34574 ; HMDB35006 ; HMDB35309 ; HMDB35448 ; HMDB35597 ; HMDB35613 ; HMDB35729 ; HMDB35767 ; HMDB36448 ; HMDB36663 ; HMDB37063 ; HMDB37206 ; HMDB37578 ; HMDB37788 ; HMDB37969 ; HMDB38502 ; HMDB38825 ; HMDB39336 ; HMDB40479 ; HMDB40584 ; HMDB40685 ; HMDB40916 ; HMDB60517 ; HMDB60519	Methyl propenyl ketone ; 3-Methyl-2-butenal ; Glutamyl-Tyrosine ; Glycyl-Methionine ; Methionyl-Glycine ; Tyrosyl-Glutamate ; Neohesperidin dihydrochalcone ; Tricin 7-glucoside ; 1-Hydroxyepiacorone ; Acorusdiol ; Cyclopentanone ; Tiglic aldehyde ; 3-Methyl-3-buten-2-one ; 2-Pentenal ; 4-Penten-2-one ; 1-Penten-3-one ; 3(4-5)-Abeo-411:412-diepoxy-3-eudesmanol ; 4-Pentenal ; (4R5S7R11R)-1112-Dihydroxy-1(10)-spirovetiven-2-one ; Apotrichodiol ; Isovitexin 7-(6-sinapoylglucoside) 4-glucoside ; 6alpha-Carissanol ; Licoagroside A ; alpha-Carissanol ; Epioxylubimin ; Dihydromyoporone ; Piperalol ; Zedoarondiol ; Hydroxypelenolide ; Toxin FS2 ; Isoscoparin 2-(6-(E)-feruloylglucoside) 4-glucoside ; Limocitrin 3-rhamnoside ; xi-23-Dihydro-3-methylfuran ; Urodiolenone ; Bisacurone B ; 38-Dimethoxyapigenin 7-glucoside ; 345-Trihydroxy-37-dimethoxyflavone 5-glucoside ; 37-Dimethoxy-458-trihydroxyflavone 8-glucoside ; 23-Dihydro-4-methylfuran ; 23-	3M+2K-H ; 3M+2K-H ; 2M+H+K ; 3M+H+K ; 3M+H+K ; 2M+H+K ; M+2Na ; 2M+3H ; M+2K-H ; M+2K-H ; 3M+2K-H ; 3M+2K-H ; 3M+2K-H ; 3M+2K-H ; 3M+2K-H ; 3M+2K-H ; M+2K-H ; 3M+2K-H ; M+2K-H ; M+2K-H ; M+2H+Na ; M+2K-H ; 2M+3H ; M+2K-H ; M+2K-H ; M+2K-H ; M+2K-H ; M+2K-H ; M+2K-H ; M+2K-H ; M+2H+Na ; 2M+3H ; 3M+2K-H ; M+2K-H ; M+2K-H ; 2M+3H ; 2M+3H ; 2M+3H ; 3M+2K-H ; M+2K-H ; M+K ; M+K ; M+K	C5H8O ; C5H8O ; C14H17N2O6 ; C7H14N2O3S ; C7H14N2O3S ; C14H17N2O6 ; C28H36O15 ; C23H24O12 ; C15H24O3 ; C15H24O3 ; C5H8O ; C5H8O ; C5H8O ; C5H8O ; C5H8O ; C5H8O ; C15H24O3 ; C5H8O ; C15H24O3 ; C15H24O3 ; C44H50O24 ; C15H24O3 ; C23H24O12 ; C15H24O3 ; C15H24O3 ; C15H24O3 ; C15H24O3 ; C15H24O3 ; C15H24O3 ; C15H24O3 ; C44H50O24 ; C23H24O12 ; C5H8O ; C15H24O3 ; C15H24O3 ; C23H24O12 ; C23H24O12 ; C23H24O12 ; C5H8O ; C15H24O3 ; C20H18O2 ; C20H18O2 ; C20H18O2
2739_100.	2.06315	0.324892	-5.9395	0.000143	3.8439	0.008024	225.4993	HMDB34071	Progoitrin	M+Na+K	C11H19NO10S2
4231_99.4	2.044878	0.09669	-5.9451	0.000142	3.8472	0.008024	415.5554	HMDB01379	Diguanosine triphosphate	M+ACN+2H	C20H27N10O18P3
1538_33.6	2.025931	0.035195	-5.9438	0.000142	3.8464	0.008024	231.1702	HMDB01492 ; HMDB04666 ; HMDB06335 ; HMDB06463 ; HMDB11549 ; HMDB11578 ; HMDB11579 ; HMDB12112 ; HMDB12173 ; HMDB12932 ; HMDB34253 ; HMDB34401 ; HMDB34840 ; HMDB35273 ; HMDB36356 ; HMDB36568	Gamma-Tocopherol ; 2-Arachidonylglycerol ; Beta-tocopherol ; Stearidonyl carnitine ; MG(0:020:4(8Z11Z14Z17Z)0:0) ; MG(20:4(5Z8Z11Z14Z)0:00:0) ; MG(20:4(8Z11Z14Z17Z)0:00:0) ; (22Alpha)-hydroxy-5alpha-campestan-3-one ; 4Alpha-hydroxymethyl-5alpha-cholesta-8-en-3beta-ol ; Dynorphin A (6-8) ; z2-Tocopherol ; Cyclostenol ; Faradiol laurate ; 1-Acetoxy-2-hydroxy-51215-heneicosatrien-4-one ; 12-Gingerol ; Persenone A	M+2Na ; M+2ACN+2H ; M+2Na ; M+ACN+2H ; M+2ACN+2H ; M+2ACN+2H ; M+2ACN+2H ; M+2Na ; M+2Na ; M+H+NH4 ; M+2Na ; M+2Na ; M+3Na ; M+2ACN+2H ; M+2ACN+2H ; M+2ACN+2H	C28H48O2 ; C23H38O4 ; C28H48O2 ; C25H41NO4 ; C23H38O4 ; C23H38O4 ; C23H38O4 ; C28H48O2 ; C28H48O2 ; C18H37N9O4 ; C28H48O2 ; C28H48O2 ; C42H72O3 ; C23H38O4 ; C23H38O4 ; C23H38O4

2836_89.1	2.014672	0.844988	-5.9429	0.000143	3.8459	0.008024	326.1103	HMDB34682 ; HMDB05037 ; HMDB12209 ; HMDB13240 ; HMDB31149 ; HMDB33459 ; HMDB33627 ; HMDB35420 ; HMDB38090 ; HMDB41753	cis-Ferulic acid arabinosyl-(1-3)-glucosyl-(1-6)-glucosyl ester ; Sumatriptan ; Diethylphosphate ; Indoleacetyl glutamine ; Phaeophorbide b ; Flazine ; D-Erythroascorbic acid 1-a-D-glucoside ; Pyranocyanin A ; Peonidin 3-rhamnoside 5-glucoside ; Isopeonidin 3-rutinoside	M+2H ; 2M+Na+K ; 2M+NH4 ; M+Na ; M+2Na ; M+NH4 ; M+NH4 ; M+H+NH4 ; M+ACN+2H ; M+ACN+2H	C27H38O18 ; C14H21N3O2S ; C4H11O4P ; C15H17N3O4 ; C35H34N4O6 ; C17H12N2O4 ; C11H16O10 ; C30H33O15 ; C28H33O15 ; C28H33O15
4188_97.8	2.051912	0.487548	5.8421	0.000163	3.7869	0.008975	179.9734	HMDB60688	Nornitrogen mustard	M+K	C4H9Cl2N
7994_32.0	2.060819	0.039394	5.7594	0.000183	3.738	0.009622	182.0218	HMDB00894 ; HMDB01494 ; HMDB04110 ; HMDB12134 ; HMDB14491 ; HMDB30152 ; HMDB30521 ; HMDB32161 ; HMDB34024 ; HMDB34690 ; HMDB36204 ; HMDB40590 ; HMDB41168 ; HMDB41169 ; HMDB41462 ; HMDB41671 ; HMDB41680 ; HMDB61082	Vinylacetylglycine ; Acetylphosphate ; Phosphonoacetate ; 12-Dihydroxy-3-keto-5-methylthiopentene ; Trimethadione ; 4-Hydroxy-57-dimethoxyflavan ; Sativan ; Acrylamide-acrylic acid resin ; Isosativan ; 9-(4-Hydroxyphenyl)-2-methoxy-1H-phenalen-1-one ; 1-(Methylsulfanyl)-1-oxopropan-2-yl acetate ; Benzyl methyl disulfide ; Myrigalone G ; Myrigalone H ; 2-Hydroxy-4-(4-methoxyphenyl)-1H-phenalen-1-one ; 4-Hydroxy-345-trimethoxystilbene ; 4-Hydroxy-354-trimethoxystilbene ; 5-ethyl-5-methyl-24-oxazolidinedione	M+K ; M+ACN+H ; M+ACN+H ; 2M+H+K ; M+K ; M+2K ; M+2K ; M+K ; M+2K ; M+Na+K ; 2M+H+K ; 2M+H+Na ; M+2K ; M+2K ; M+Na+K ; M+2K ; M+2K ; M+K	C6H9NO3 ; C2H5O5P ; C2H5O5P ; C6H10O3S ; C6H9NO3 ; C17H18O4 ; C17H18O4 ; C6H9NO3 ; C17H18O4 ; C20H14O3 ; C6H10O3S ; C8H10S2 ; C17H18O4 ; C17H18O4 ; C20H14O3 ; C17H18O4 ; C17H18O4 ; C6H9NO3
6964_68.4	2.050857	0.136925	-5.7679	0.000181	3.7431	0.009622	306.1597	HMDB28715 ; HMDB28967 ; HMDB33749 ; HMDB33920 ; HMDB38546	Arginyl-Methionine ; Methionyl-Arginine ; Isoliensinine ; Liensinine ; ent-6R16bOH17-Trihydroxy-7-oxo-67-seco-196-kauranolide 6-O-glucoside	M+H ; M+H ; M+2H ; M+2H ; M+2ACN+2H	C11H23N5O3S ; C11H23N5O3S ; C37H42N2O6 ; C37H42N2O6 ; C26H40O11
0576_99.6	2.033679	0.221768	-5.7615	0.000182	3.7392	0.009622	112.9911	HMDB31717 ; HMDB32271 ; HMDB32410 ; HMDB32495 ; HMDB36193 ; HMDB37619 ; HMDB38299 ; HMDB39653 ; HMDB40413 ; HMDB41306	3-(Methylthio)propyl acetate ; Ethyl 3-mercaptobutyrate ; Methyl 3-(methylthio)butanoate ; Propyl 2-mercaptopropionate ; 3-Mercapto-3-methylbutyl formate ; Methyl 4-(methylthio)butyrate ; Methylthiomethyl butyrate ; 246-Trimethyl-135-dioxathiane ; Ethyl 3-(methylthio)propanoate ; Methyl 2-(methylthio)butyrate	M+2K ; M+2K ; M+2K ; M+2K ; M+2K ; M+2K ; M+2K ; M+2K ; M+2K ; M+2K	C6H12O2S ; C6H12O2S ; C6H12O2S ; C6H12O2S ; C6H12O2S ; C6H12O2S ; C6H12O2S ; C6H12O2S ; C6H12O2S ; C6H12O2S
8876_99.1	2.002342	0.373685	5.7777	0.000178	3.7489	0.009622	131.5169	HMDB06348 ; HMDB06548 ; HMDB31805	Pseudoecgonine ; Ecgonine ; (E)-Monocrotophos	M+2K ; M+2K ; M+H+K	C9H15NO3 ; C9H15NO3 ; C7H14NO5P

3286_93.3	2.047293	0.436686	-5.714	0.000195	3.711	0.010131	256.5633	HMDB29776 ; HMDB37073 ; HMDB37074 ; HMDB37075 ; HMDB37076 ; HMDB37077 ; HMDB37078 ; HMDB60942	Dhurrin 6-glucoside ; (E)-Resveratrol 3-glucoside 4-sulfate ; (Z)-Resveratrol 3-glucoside 5-sulfate ; (Z)-Resveratrol 3-(2-sulfoglucoside) ; (Z)-Resveratrol 3-(4-sulfoglucoside) ; (Z)-Resveratrol 3-(6-sulfoglucopyranoside) ; (Z)-Resveratrol 3-(3-sulfoglucoside) ; N-Desmethylrosuvastatin	M+H+K ; M+ACN+2H ; M+ACN+2H ; M+ACN+2H ; M+ACN+2H ; M+ACN+2H ; M+ACN+2H ; M+2Na	C20H27NO12 ; C20H22O11S ; C20H22O11S ; C20H22O11S ; C20H22O11S ; C20H22O11S ; C20H22O11S ; C21H26FN3O6S
1274_100.	2.043467	0.523103	-5.6957	0.000199	3.7001	0.010283	279.0801	HMDB04135 ; HMDB10338 ; HMDB15083 ; HMDB28820 ; HMDB28862 ; HMDB29575 ; HMDB29774 ; HMDB30672 ; HMDB30722 ; HMDB30881 ; HMDB32006 ; HMDB32777 ; HMDB33515 ; HMDB33654 ; HMDB35009 ; HMDB36033 ; HMDB36161 ; HMDB38559 ; HMDB39144 ; HMDB40723 ; HMDB41140	Poly-g-D-glutamate ; 11-Oxo-androsterone glucuronide ; Mezlocillin ; Glutamyl-Hydroxyproline ; Hydroxyprolyl-Glutamate ; ()-cis-56-Dihydro-5-hydroxy-4-methoxy-6-(2-phenylethyl)-2H-pyran-2-one ; 7-Hydroxy-6-(methoxyacetyl)-22-dimethyl-2H-1-benzopyran ; Obtustyrene ; (E)-35-Dimethoxystilbene ; 1-Acetoxy-46-tetradecadiene-81012-triyne ; Epifisetinidol-(4beta-8)-epicatechin-(6-4beta)-epifisetinidol ; Ciceritol ; Citreovirenone ; 11-Deacetylvaltrate 11-(3-hydroxy-3-methylbutanoate) ; Phenethyl phenylacetate ; 7alpha-1(10-19)-Abeo-7-acetoxyobacun-9(11)-ene ; 4-Propanoyl-HT2 toxin ; 8-Hexanoylneosolaniol ; 48:48-Terflavan-3334444555777-tridecol ; (-)-Gossypol ; Prenyl cis-caffeate	M+H+K ; M+2K ; M+H+NH4 ; 2M+H+K ; 2M+H+K ; 2M+Na+K ; 2M+Na+K ; M+K ; M+K ; M+K ; M+3H ; M+H+K ; 2M+Na+K ; M+2K ; M+K ; M+Na+K ; M+2K ; M+2K ; M+3H ; M+H+K ; 2M+Na+K	C20H30N4O12 ; C25H36O9 ; C21H25N5O8S2 ; C10H15N2O6 ; C10H15N2O6 ; C14H16O4 ; C14H16O4 ; C16H16O2 ; C16H16O2 ; C16H16O2 ; C45H38O16 ; C19H34O16 ; C14H16O4 ; C25H36O9 ; C16H16O2 ; C28H32O8 ; C25H36O9 ; C25H36O9 ; C45H38O16 ; C30H30O8 ; C14H16O4
3999_68.9	2.037027	0.410159	-5.6574	0.00021	3.6772	0.010666	278.1534	HMDB28957 ; HMDB28978 ; HMDB06888 ; HMDB14481 ; HMDB30272 ; HMDB34578 ; HMDB59701 ; HMDB59733 ; HMDB14379 ; HMDB15509 ; HMDB29381 ; HMDB30259	Lysyl-Methionine ; Methionyl-Lysine ; 5b-Cyprinol sulfate ; Pimecrolimus ; Carpaine ; Deterrol stearate ; 167-Trimethylnaphthalene ; 145-Trimethyl-naphtalene ; Reboxetine ; Ethylmorphine ; 6-O-Methylcodeine ; ()-Erysotrine	M+H ; M+H ; M+H+Na ; M+2H+Na ; M+2K ; M+2K ; 3M+2Na ; 3M+2Na ; M+H-2H2O ; M+H-2H2O ; M+H-2H2O ; M+H-2H2O	C11H23N3O3S ; C11H23N3O3S ; C27H48O8S ; C43H68ClNO11 ; C28H50N2O4 ; C33H50O2 ; C13H14 ; C13H14 ; C19H23NO3 ; C19H23NO3 ; C19H23NO3 ; C19H23NO3
1341_65.7	2.009355	0.763149	-5.6543	0.000211	3.6753	0.010666	457.1671	HMDB01846 ; HMDB14701 ; HMDB35867 ; HMDB41793	Tetrahydrofolic acid ; Doxapram ; 1113-Dihydrotaraxinic acid glucosyl ester ; 1-Aminopyrene	2M+H+Na ; M+2K+H ; 2M+Na+K ; 2M+Na	C19H23N7O6 ; C24H30N2O2 ; C21H30O9 ; C16H11N
4424_139.	2.031357	0.8051	-5.6404	0.000215	3.667	0.010765	333.5174	HMDB12222	dTDP-4-acetamido-46-dideoxy-D-galactose	M+2K	C18H29N3O15P2

06713_66.1[illegible]	2.032998	0.554729	-5.6173	0.000222	3.6531	0.010791	300.1207	HMDB04629 ; HMDB30616 ; HMDB30698 ; HMDB30719 ; HMDB30947 ; HMDB31622 ; HMDB31842 ; HMDB32423 ; HMDB32725 ; HMDB33931 ; HMDB33986 ; HMDB34144 ; HMDB34255 ; HMDB36620 ; HMDB37255 ; HMDB37281 ; HMDB37683 ; HMDB39153 ; HMDB40679 ; HMDB40823 ; HMDB41328 ; HMDB41652 ; HMDB41657 ; HMDB41710 ; HMDB60564 ; HMDB61034 ; HMDB37839	O-Desmethylangolensin ; Archangin ; 57-Dimethoxyisoflavone ; 56-Dimethoxyflavone ; Wyerone ; 6-Phenyl-3-hexen-2-one ; Rhapontigenin ; 2-(4-Methyl-5-thiazolyl)ethyl octanoate ; Acetomenaphthone ; Xanthoxyletin ; Demethylvestitol ; Yangonin ; 5-Methoxyseselin ; 57-Dimethoxyflavone ; 5-Hydroxy-7-methoxy-6-methylflavone ; 4-Methyl-2-phenyl-2-pentenal ; Ethyl vanillin isobutyrate ; (R)-111213-Trinor-1(5)69-guaiatrien-8-one ; (2S3R4R)-344-Trihydroxyflavan ; (S)-111213-Trinor-7-calamenone ; 3-Deaminofusarochromanone ; 347-Trihydroxyisoflavan ; 3-Hydroxyequol ; cis-4-Hydroxyequol ; Carboxy-ibuprofen ; Metabolite M6 ; N-(1-Deoxy-1-fructosyl)histidine	M+ACN+H ; M+ACN+H ; M+NH4 ; M+NH4 ; M+ACN+H ; 3M+2K ; M+ACN+H ; 2M+Na+K ; M+ACN+H ; M+ACN+H ; M+ACN+H ; M+ACN+H ; M+ACN+H ; M+NH4 ; M+NH4 ; 3M+2K ; M+ACN+Na ; 3M+2K ; M+ACN+H ; 3M+2K ; M+Na ; M+ACN+H ; M+ACN+H ; M+ACN+H ; M+ACN+Na ; M+2K ; M+H-H2O	C15H14O4 ; C15H14O4 ; C17H14O4 ; C17H14O4 ; C15H14O4 ; C12H14O ; C15H14O4 ; C14H23NO2S ; C15H14O4 ; C15H14O4 ; C15H14O4 ; C15H14O4 ; C15H14O4 ; C17H14O4 ; C17H14O4 ; C12H14O ; C13H16O4 ; C12H14O ; C15H14O4 ; C12H14O ; C15H19NO4 ; C15H14O4 ; C15H14O4 ; C15H14O4 ; C13H16O4 ; C30H42N4O4 ; C12H19N3O7
[illegible]3995_55.61	2.008353	0.051212	-5.626	0.00022	3.6583	0.010791	148.004	HMDB29592 ; HMDB34345 ; HMDB40408	Triacetin ; 7-Acetoxy-6-methoxycoumarin ; 7-Hydroxy-2-methyl-4-oxo-4H-1-benzopyran-5-acetic acid	M+2K ; M+Na+K ; M+Na+K	C9H14O6 ; C12H10O5 ; C12H10O5
[illegible]5601_105.[illegible]	1.998184	0.263967	-5.6186	0.000222	3.6539	0.010791	342.0626	HMDB14257 ; HMDB15007 ; HMDB15336 ; HMDB30190 ; HMDB30241 ; HMDB38405 ; HMDB38573 ; HMDB38838 ; HMDB38967 ; HMDB40785 ; HMDB60840	E-10-Hydroxydesmethylnortriptyline ; Dorzolamide ; Flumazenil ; (EE)-Lansamide I ; Girinimbine ; 6-Sinapoylglucoraphenin ; Lansiumamide B ; Lansiumamide C ; 1-Propenyl 1-(1-propenylthio)propyl disulfide ; Mukoenine A ; N-Desmethyldoxepin	M+2K-H ; M+NH4 ; M+K ; M+2K+H ; M+2K+H ; M+ACN+2H ; M+2K+H ; M+2K-H ; 3M+H+Na ; M+2K-H ; M+2K-H	C18H19NO ; C10H16N2O4S3 ; C15H14FN3O3 ; C18H17NO ; C18H17NO ; C23H31NO14S3 ; C18H17NO ; C18H19NO ; C9H16S3 ; C18H19NO ; C18H19NO

3244_100.2	2.043787	0.021611	-5.5829	0.000233	3.6323	0.011103	223.0663	HMDB33041 ; HMDB33072 ; HMDB40243 ; HMDB40262 ; HMDB37913 ; HMDB60769 ; HMDB00056 ; HMDB00161 ; HMDB00271 ; HMDB01310 ; HMDB06806 ; HMDB15417 ; HMDB29869 ; HMDB31219 ; HMDB31635 ; HMDB33258 ; HMDB33912 ; HMDB37337 ; HMDB38890 ; HMDB39087 ; HMDB39088 ; HMDB39776 ; HMDB40345 ; HMDB41307 ; HMDB41392 ; HMDB33071	1-(1-Propenylthio)propyl propyl disulfide ; (E)-1-Propenyl 1-(propylthio)propyl disulfide ; 224466-Hexamethyl-135-trithiane ; 246-Triethyl-135-trithiane ; Artomunoxanthentrione ; 4-Hydroxyphenytoin glucuronide ; Beta-Alanine ; L-Alanine ; Sarcosine ; D-Alanine ; Propinol adenylate ; Fosphenytoin ; Hemiariensin ; Ethyl carbamate ; 2-Propene-1-thiol ; Isoyatein ; 2-Propenyl propyl disulfide ; 7-(4-Carboxy-3-hydroxy-3-methylbutanoyl)sudachitin 4-glucoside ; Methyl 3-methyl-1-butenyl disulfide ; Sudachiin B ; Sudachiin C ; 6-O-Malonylnaringin ; 1-Propene-1-thiol ; Italipyrone ; 1-Propenyl propyl disulfide ; Propyl 1-(propylsulfinyl)propyl disulfide	M+H ; M+H ; M+H ; M+H ; M+2H ; M+2H ; 2M+2Na-H ; 2M+2Na-H ; 2M+2Na-H ; 2M+2Na-H ; M+ACN+2H ; M+2ACN+2H ; M+2Na ; 2M+2Na-H ; 3M+H ; M+2Na ; 3M+2H ; M+3H ; 3M+2H ; M+3H ; M+3H ; M+3H ; 3M+H ; M+2Na ; 3M+2H ; M+H-H2O	C9H18S3 ; C9H18S3 ; C9H18S3 ; C9H18S3 ; C26H20O7 ; C21H20N2O9 ; C3H7NO2 ; C3H7NO2 ; C3H7NO2 ; C3H7NO2 ; C13H18N5O8P ; C16H15N2O6P ; C22H24O7 ; C3H7NO2 ; C3H6S ; C22H24O7 ; C6H12S2 ; C30H34O17 ; C6H12S2 ; C30H34O17 ; C30H34O17 ; C30H34O17 ; C3H6S ; C22H24O7 ; C6H12S2 ; C9H20OS3
33927_49.4	1.986778	0.280456	-5.587	0.000232	3.6348	0.011103	228.9734	HMDB02332 ; HMDB15259 ; HMDB31470 ; HMDB34416 ; HMDB37290 ; HMDB41777 ; HMDB41778 ; HMDB60418 ; HMDB60792	B-Sulfinyl pyruvate ; Econazole ; 35-Dimethyl-124-trithiolane ; Methyl 2-propenyl trisulfide ; 3-Methyl-124-trithiane ; Tectorigenin 4-sulfate ; Tectorigenin 7-sulfate ; 6-Thioxanthine 5-monophosphate ; 6-Thioxanthylic acid	3M+2H ; M+2K ; 3M+2H ; 3M+2H ; 3M+2H ; M+2K ; M+2K ; M+2K ; M+2K	C3H4O5S ; C18H15Cl3N2O ; C4H8S3 ; C4H8S3 ; C4H8S3 ; C16H12O9S ; C16H12O9S ; C10H13N4O8PS ; C10H13N4O8PS
0528_98.9	2.033282	0.461151	5.5743	0.000236	3.6272	0.01113	NA	NA	NA	NA	NA
9478_276.1	2.021566	0.392154	5.5626	0.00024	3.6201	0.011207	314.9759	HMDB14656 ; HMDB29513 ; HMDB33350	Cisplatin ; Myricatin ; Brassinin	M+NH4 ; M+2K ; M+2K+H	Cl2H4N2Pt ; C22H16O15S ; C11H12N2S2
4366_120.2	2.014792	0.693344	5.5405	0.000247	3.6067	0.011312	205.0224	HMDB11640 ; HMDB14582 ; HMDB32394 ; HMDB36073 ; HMDB39721 ; HMDB41009 ; HMDB60497	Uridine 23-cyclic phosphate ; Ceftazidime ; 6-Methylcoumarin ; Viburtinal ; 4-O-alpha-D-Galactopyranuronosyl-D-galacturonic acid ; 7-Methyl-2-benzofurancarboxaldehyde ; Naphthalene-12-diol	2M+3H ; M+3Na ; M+2Na-H ; M+2Na-H ; M+H+K ; M+2Na-H ; M+2Na-H	C9H11N2O8P ; C22H22N6O7S2 ; C10H8O2 ; C10H8O2 ; C12H18O13 ; C10H8O2 ; C10H8O2
0325_88.63	1.996377	0.369077	-5.5406	0.000247	3.6067	0.011312	226.9403	HMDB14706	Succimer	M+2Na-H	C4H6O4S2
5914_99.4	1.990139	0.105593	5.5362	0.000249	3.6041	0.011312	318.9766	HMDB15289 ; HMDB32040 ; HMDB32866 ; HMDB33307	Bretylium ; Benzoyl peroxide ; 2456-Phenanthrenetetrol ; Moracin M	M+2K-H ; M+2K-H ; M+2K-H ; M+2K-H	C11H17BrN ; C14H10O4 ; C14H10O4 ; C14H10O4

1677_99.07	1.989428	0.37358	5.5208	0.000254	3.5947	0.011454	516.9462	HMDB44023 ; HMDB44241 ;<br>HMDB44246 ; HMDB44457 ;<br>HMDB44597 ; HMDB44617 ;<br>HMDB44828 ; HMDB45038 ;<br>HMDB45043 ; HMDB45069 ;<br>HMDB45226 ; HMDB45252 ;<br>HMDB45272 ; HMDB45381 ;<br>HMDB45407 ; HMDB45426 ;<br>HMDB45605 ; HMDB45781 ;<br>HMDB45786 ; HMDB45837 ;<br>HMDB45987 ; HMDB46012 ;<br>HMDB46018 ; HMDB46021 ;<br>HMDB46040 ; HMDB46056 ;<br>HMDB46143 ; HMDB46162 ;<br>HMDB46186 ; HMDB46205 ;<br>HMDB46221 ; HMDB46354 ;<br>HMDB46498 ; HMDB46503 ;<br>HMDB46523 ; HMDB46528 ;<br>HMDB46552 ; HMDB46563 ;<br>HMDB46569 ; HMDB46573 ;<br>HMDB46578 ; HMDB46713 ;<br>HMDB46719 ; HMDB46723 ;<br>HMDB46728 ; HMDB46747 ;<br>HMDB46775 ; HMDB46795 ;<br>HMDB46801 ; HMDB46810 ;<br>HMDB46811 ; HMDB46813 ;<br>HMDB46819 ; HMDB46871 ;<br>HMDB46897 ; HMDB46912 ;	TG(16:024:022:6(4Z7Z10Z13Z16Z19Z)<br>) ;<br>TG(16:024:1(15Z)22:5(4Z7Z10Z13Z16<br>Z)) ;<br>TG(16:024:1(15Z)22:5(7Z10Z13Z16Z1<br>9Z)) ;<br>TG(16:022:5(4Z7Z10Z13Z16Z)24:1(15<br>Z)) ;<br>TG(16:022:5(7Z10Z13Z16Z19Z)24:1(1<br>5Z)) ;<br>TG(16:022:6(4Z7Z10Z13Z16Z19Z)24:0<br>) ;<br>TG(18:022:022:6(4Z7Z10Z13Z16Z19Z)<br>) ;<br>TG(18:022:1(13Z)22:5(4Z7Z10Z13Z16<br>Z)) ;<br>TG(18:022:1(13Z)22:5(7Z10Z13Z16Z1<br>9Z)) ;<br>TG(18:024:1(15Z)20:5(5Z8Z11Z14Z17<br>Z)) ;<br>TG(18:022:2(13Z16Z)22:4(7Z10Z13Z1<br>6Z)) ;<br>TG(18:022:4(7Z10Z13Z16Z)22:2(13Z1<br>6Z)) ;<br>TG(18:022:5(4Z7Z10Z13Z16Z)22:1(13<br>Z)) ;<br>TG(18:020:5(5Z8Z11Z14Z17Z)24:1(15<br>Z)) ;	M+ACN+2H ; M+ACN+2H ;<br>M+ACN+2H ; M+ACN+2H ;<br>M+ACN+2H ; M+ACN+2H ;<br>M+ACN+2H ; M+ACN+2H ;<br>M+ACN+2H ; M+ACN+2H ;<br>M+ACN+2H ; M+ACN+2H ;<br>M+ACN+2H ; M+ACN+2H ;<br>M+ACN+2H ; M+ACN+2H ;<br>M+ACN+2H ; M+ACN+2H ;<br>M+ACN+2H ; M+ACN+2H ;<br>M+ACN+2H ; M+ACN+2H ;<br>M+ACN+2H ; M+H+NH4 ;<br>M+H+NH4 ; M+ACN+2H ;<br>M+ACN+2H ; M+ACN+2H ;<br>M+ACN+2H ; M+ACN+2H ;<br>M+H+NH4 ; M+ACN+2H ;<br>M+ACN+2H ; M+ACN+2H ;<br>M+ACN+2H ; M+ACN+2H ;<br>M+ACN+2H ; M+ACN+2H ;<br>M+ACN+2H ; M+H+NH4 ;<br>M+H+NH4 ; M+ACN+2H ;<br>M+ACN+2H ; M+H+NH4 ;<br>M+H+NH4 ; M+H+NH4 ;<br>M+ACN+2H ; M+H+NH4 ;<br>M+H+NH4 ; M+ACN+2H ;<br>M+ACN+2H ; M+H+NH4 ;<br>M+H+NH4 ; M+ACN+2H ;<br>M+H+NH4 ; M+ACN+2H ;	C65H114O6 ; C65H114O6 ;<br>C65H114O6 ; C65H114O6 ;<br>C65H114O6 ; C65H114O6 ;<br>C65H114O6 ; C65H114O6 ;<br>C65H114O6 ; C65H114O6 ;<br>C65H114O6 ; C65H114O6 ;<br>C65H114O6 ; C65H114O6 ;<br>C65H114O6 ; C65H114O6 ;<br>C65H114O6 ; C65H114O6 ;<br>C65H114O6 ; C65H114O6 ;<br>C65H114O6 ; C65H114O6 ;<br>C65H114O6 ; C67H114O6 ;<br>C67H114O6 ; C65H114O6 ;<br>C65H114O6 ; C65H114O6 ;<br>C65H114O6 ; C65H114O6 ;<br>C67H114O6 ; C65H114O6 ;<br>C65H114O6 ; C65H114O6 ;<br>C65H114O6 ; C65H114O6 ;<br>C65H114O6 ; C65H114O6 ;<br>C65H114O6 ; C67H114O6 ;<br>C67H114O6 ; C65H114O6 ;<br>C65H114O6 ; C67H114O6 ;<br>C67H114O6 ; C67H114O6 ;<br>C65H114O6 ; C67H114O6 ;<br>C67H114O6 ; C65H114O6 ;<br>C65H114O6 ; C67H114O6 ;<br>C67H114O6 ; C65H114O6 ;<br>C67H114O6 ; C65H114O6 ;
5651_100.5	2.02554	0.460856	5.5076	0.000259	3.5867	0.011562	200.4866	HMDB30100	(8R)-Neochrome	M+3H	C41H58O3
75255_286.	2.010061	0.1895	5.498	0.000263	3.5809	0.011615	363.0075	HMDB15408 ; HMDB14878 ;<br>HMDB37810	Bismuth Subsalicylate ; Riluzole ; 411-<br>Dichloro-512-dihydroquino23-<br>bacridine-714-dione	M+H ; 3M+H+Na ; M+H-H2O	C7H5BiO4 ; C8H5F3N2OS ;<br>C20H10Cl2N2O2
071_100.06	1.990037	0.815682	5.4868	0.000267	3.574	0.011682	144.0307	HMDB05800 ; HMDB05801 ;<br>HMDB29711 ; HMDB30543 ;<br>HMDB34014 ; HMDB34150 ;<br>HMDB34444 ; HMDB41658 ;<br>HMDB41688 ; HMDB41702 ;<br>HMDB00033 ; HMDB12482 ;<br>HMDB15146 ; HMDB28689 ;<br>HMDB28878 ; HMDB34276 ;<br>HMDB60402	Luteolin ; Kaempferol ; Maritimetin ;<br>Norartocarpetin ; 2-Hydroxygenistein ;<br>Cernuine ; Citreorosein ; 3-<br>Hydroxygenistein ; 5674-<br>Tetrahydroxyisoflavone ; 7834-<br>Tetrahydroxyisoflavone ; Carnosine ;<br>(6R)-6-(L-Erythro-12-Dihydroxypropyl)-<br>5678-tetrahydro-4a-hydroxypterin ;<br>Metyrapone ; Alanyl-Histidine ;<br>Histidinyl-Alanine ; LL-<br>Cyclo(leucylprolyl) ; 56-Dihydro-5-<br>fluorouracil	M+2H ; M+2H ; M+2H ; M+2H ;<br>M+2H ; M+2H ; M+2H ; M+2H ;<br>M+2H ; M+2H ; M+Na+K ;<br>M+Na+K ; M+Na+K ; M+Na+K ;<br>M+Na+K ; M+2K ; 2M+H+Na	C15H10O6 ; C15H10O6 ;<br>C15H10O6 ; C15H10O6 ;<br>C15H10O6 ; C15H10O6 ;<br>C15H10O6 ; C15H10O6 ;<br>C15H10O6 ; C15H10O6 ;<br>C9H14N4O3 ; C9H14N4O3 ;<br>C14H14N2O ; C9H14N4O3 ;<br>C9H14N4O3 ; C11H18N2O2 ;<br>C4H5FN2O2

[illegible]7324_21.5[illegible]	1.981359	0.749851	-5.4814	0.000269	3.5707	0.011682	328.3207	HMDB13078 ; HMDB13645 ; HMDB02231 ; HMDB12925 ; HMDB32316 ; HMDB34296 ; HMDB34451 ; HMDB35159 ; HMDB35572 ; HMDB35573 ; HMDB35574 ; HMDB37127 ; HMDB60275	Stearoylethanolamide ; NN-Dimethylsphingosine ; Eicosenoic acid ; Dehydrospermidine ; Hexanal dihexyl acetal ; 11-Eicosenoic acid ; Ethyl oleate ; Paullinic acid ; 68-Icosanedione ; 57-Icosanedione ; 46-Icosanedione ; Oleyl acetate ; N-(E)-4-Ammoniobutylidenepropane-13-diaminium	M+H ; M+H ; M+NH4 ; 2M+ACN+H ; M+ACN+H ; M+NH4 ; M+NH4 ; M+NH4 ; M+NH4 ; M+NH4 ; M+NH4 ; M+NH4 ; 2M+ACN+H	C20H41NO2 ; C20H41NO2 ; C20H38O2 ; C7H17N3 ; C18H38O2 ; C20H38O2 ; C20H38O2 ; C20H38O2 ; C20H38O2 ; C20H38O2 ; C20H38O2 ; C20H38O2 ; C7H17N3
[illegible]8522_99.9[illegible]	2.002431	0.246717	-5.4509	0.000281	3.552	0.011988	343.0659	HMDB02377 ; HMDB13921 ; HMDB13923 ; HMDB13925 ; HMDB15201 ; HMDB32943 ; HMDB34058 ; HMDB34073 ; HMDB39315 ; HMDB60899 ; HMDB59612	Urothion ; 7-Hydroxyticlopidine ; 2-Oxoticlopidine ; Ticlopidine S-oxide ; Clonazepam ; Diazinon ; Quinacridone ; Vicine ; 4-(4-Hydroxyphenyl)-2-butanone O-2-galloyl-6-p-coumaroylglucoside ; Ticlopidine N-oxide ; 7-Methylguanosine 5-phosphate	M+NH4 ; M+ACN+Na ; M+ACN+Na ; M+ACN+Na ; 2M+3H2O+2H ; M+K ; 2M+Na+K ; M+K ; M+Na+K ; M+ACN+Na ; M+H-2H2O	C11H11N5O3S2 ; C14H14ClNOS ; C14H14ClNOS ; C14H14ClNOS ; C15H10ClN3O3 ; C12H21N2O3PS ; C20H12N2O2 ; C10H16N4O7 ; C32H32O13 ; C14H14ClNOS ; C11H17N5O8P
[illegible]3813_65.7[illegible]	1.975452	0.818207	5.4516	0.00028	3.5525	0.011988	103.0584	HMDB31529 ; HMDB38892 ; HMDB39771 ; HMDB40347 ; HMDB01406 ; HMDB15496 ; HMDB29765 ; HMDB31861 ; HMDB32347 ; HMDB33529 ; HMDB34455 ; HMDB36426 ; HMDB37190 ; HMDB37211 ; HMDB37229 ; HMDB38954 ; HMDB39715 ; HMDB40173 ; HMDB40997	3-Methyl-2-butene-1-thiol ; 1-(Methylthio)-1-butene ; Cyclopentanethiol ; 2-Methyl-1-propenethiol ; Niacinamide ; Amrinone ; 9-Pentadecenoic acid ; 2-Acetylpyrazine ; Isobutyl 10-undecenoate ; ()-23-Dihydro-3-methyl-1H-pyrrole ; Exaltolide ; Butyl undecylenate ; Rhodinyl isovalerate ; Menthyl isovalerate ; Citronellyl pentanoate ; Citronellyl isovalerate ; Auberganol ; Pentanenitrile ; 155-Farnesanolide	M+H ; M+H ; M+H ; M+H ; M+2ACN+2H ; M+H+NH4 ; M+3Na ; M+2ACN+2H ; M+3Na ; 2M+H+K ; M+3Na ; M+3Na ; M+3Na ; M+3Na ; M+3Na ; M+3Na ; M+3Na ; 2M+H+K ; M+3Na	C5H10S ; C5H10S ; C5H10S ; C5H10S ; C6H6N2O ; C10H9N3O ; C15H28O2 ; C6H6N2O ; C15H28O2 ; C5H9N ; C15H28O2 ; C15H28O2 ; C15H28O2 ; C15H28O2 ; C15H28O2 ; C15H28O2 ; C15H28O2 ; C5H9N ; C15H28O2
[illegible]5417_64.9[illegible]	2.014619	0.036994	-5.4417	0.000284	3.5464	0.012041	230.1135	HMDB28739 ; HMDB29012 ; HMDB12607 ; HMDB15319 ; HMDB29370 ; HMDB37697 ; HMDB38971 ; HMDB39939 ; HMDB40010	Asparaginyl-Proline ; Prolyl-Asparagine ; 18-Carboxy-dinor-LTE4 ; Ciclopirox ; O-Methylcorypalline ; Isobutyl N-methylanthranilate ; Diacetylfusarochromanone ; Synephrine acetonide ; 1-(23-Dihydro-67-dimethyl-1H-pyrrolizin-5-yl)-2-hydroxy-1-propanone	M+H ; M+H ; M+H+NH4 ; M+Na ; M+Na ; M+Na ; M+2ACN+2H ; M+Na ; M+Na	C9H15N3O4 ; C9H15N3O4 ; C21H31NO7S ; C12H17NO2 ; C12H17NO2 ; C12H17NO2 ; C19H24N2O6 ; C12H17NO2 ; C12H17NO2

8032_240.4	1.981218	0.575813	5.4317	0.000288	3.5403	0.012089	124.0158	HMDB02039 ; HMDB12210 ; HMDB12467 ; HMDB14436 ; HMDB15333 ; HMDB31190 ; HMDB31192 ; HMDB31857 ; HMDB31982 ; HMDB33926 ; HMDB40170 ; HMDB40973 ; HMDB41015 ; HMDB60427 ; HMDB60457 ; HMDB60515 ; HMDB60589	2-Pyrrolidinone ; Dihydrolipoate ; (-)-Epicatechin sulfate ; Chlorambucil ; Levetiracetam ; S-Ethyl thioacetate ; S-Methyl propanethioate ; 3-(Methylthio)propanal ; 3-Mercapto-2-butanone ; (3xi6xi)-Cyclo(alanylvalyl) ; (Methylthio)acetone ; 1-Methoxy-1H-indole-3-acetonitrile ; 4-Mercapto-2-butanone ; Acetone cyanohydrin ; cis-3-Chloroacrylic acid ; trans-3-Chloroacrylic acid ; Aminofurantoin	M+K ; M+H+K ; M+3H ; M+3Na ; M+2K ; 2M+H+K ; 2M+H+K ; 2M+H+K ; 2M+H+K ; M+2K ; 2M+H+K ; M+Na+K ; 2M+H+K ; M+K ; M+NH4 ; M+NH4 ; M+H+K	C4H7NO ; C8H16O2S2 ; C15H13O9S ; C14H19Cl2NO2 ; C8H14N2O2 ; C4H8OS ; C4H8OS ; C4H8OS ; C4H8OS ; C8H14N2O2 ; C4H8OS ; C11H10N2O ; C4H8OS ; C4H7NO ; C3H3ClO2 ; C3H3ClO2 ; C8H8N4O3
2742_227.8	1.972497	0.373623	5.4228	0.000292	3.5349	0.012089	471.0773	HMDB15011 ; HMDB34449 ; HMDB40716	Loteprednol ; Hydroxyanigorufone ; Irenolone	M+2K-H ; 3M+2K ; 3M+2K	C21H27ClO5 ; C19H12O3 ; C19H12O3
7069_98.79	1.972006	0.000206	-5.4211	0.000293	3.5338	0.012089	199.5171	HMDB06832 ; HMDB37985	S-Glutaryldihydrolipoamide ; Cyananin	M+2K ; M+H+NH4	C13H23NO4S2 ; C16H10Cl2N2O5
0476_100.8	2.02446	0.358159	-5.3926	0.000305	3.5163	0.012483	NA	NA	NA	NA	NA
8747_101.0	2.005357	0.174673	-5.3763	0.000312	3.5062	0.012672	538.1989	HMDB34938 ; HMDB35039 ; HMDB40649 ; HMDB40848	Cappariloside B ; Phaseolus e ; 1617-Dihydro-16a17-dihydroxygibberellin A7 17-glucoside ; (S)-Nerolidol 3-O-a-L-Rhamnopyranosyl-(1-4)-a-L-rhamnopyranosyl-(1-2)-4-(4-hydroxy-3-methoxycinnamoyl)-(E)-a-L-rhamnopyranosyl-(1-6)-b-D-glucopyranoside	M+ACN+H ; 2M+H+Na ; 2M+H+Na ; M+2K	C22H28N2O11 ; C25H34O12 ; C25H34O12 ; C49H74O21
8577_151.4	2.024708	0.165141	5.3491	0.000324	3.4894	0.01269	157.5549	HMDB14683 ; HMDB29283 ; HMDB38944 ; HMDB02670 ; HMDB05897 ; HMDB13130 ; HMDB29631 ; HMDB30873 ; HMDB31824 ; HMDB33308 ; HMDB33928 ; HMDB34002 ; HMDB34105 ; HMDB34126 ; HMDB37254 ; HMDB38549 ; HMDB41656 ; HMDB41698 ; HMDB41704	Amoxapine ; Avenanthramide 1f ; Avenanthramide E ; Naringenin ; Dihydrogenistein ; Glutarylcarnitine ; Chalconaringenin ; 1-Hydroxy-37-dimethoxyxanthone ; 458-Trihydroxyflavanone ; Moracin J ; ()-2-Hydroxydihydrodaidzein ; 349-Trihydroxypterocarpan ; Glycinol ; Gyrocyanin ; (2R3R)-347-Trihydroxyflavanone ; Toralactone ; 3-Hydroxydihydrodaidzein ; 6-Hydroxydihydrodaidzein ; 8-Hydroxydihydrodaidzein	M+2H ; M+2H ; M+2H ; M+ACN+2H ; M+ACN+2H ; M+H+K ; M+ACN+2H ; M+ACN+2H ; M+ACN+2H ; M+ACN+2H ; M+ACN+2H ; M+ACN+2H ; M+ACN+2H ; M+H+NH4 ; M+ACN+2H ; M+ACN+2H ; M+ACN+2H ; M+ACN+2H ; M+ACN+2H	C17H16ClN3O ; C17H15NO5 ; C17H15NO5 ; C15H12O5 ; C15H12O5 ; C12H21NO6 ; C15H12O5 ; C15H12O5 ; C15H12O5 ; C15H12O5 ; C15H12O5 ; C15H12O5 ; C15H12O5 ; C17H12O5 ; C15H12O5 ; C15H12O5 ; C15H12O5 ; C15H12O5 ; C15H12O5

27737_100.	2.018571	0.030117	-5.3574	0.00032	3.4945	0.01269	166.5428	HMDB00905 ; HMDB01915 ; HMDB01927 ; HMDB14434 ; HMDB14766 ; HMDB15697 ; HMDB29508 ; HMDB42010 ; HMDB59975 ; HMDB59976 ; HMDB60632	Deoxyadenosine monophosphate ; Alendronic acid ; Diphenhydramine ; Atomoxetine ; Clorazepate ; Bupranolol ; Laccaic acid D ; Rutaecarpine ; 4-Hydroxy-5-(3-hydroxyphenyl)-valeric acid-3-O-sulphate ; 4-Hydroxy-5-(4-hydroxyphenyl)-valeric acid-4-O-sulphate ; N-Demethyl orphenadrine	M+2H ; M+2ACN+2H ; M+2K ; M+2K ; M+H+NH4 ; M+Na+K ; M+H+NH4 ; M+2Na ; M+ACN+2H ; M+ACN+2H ; M+2K	C10H14N5O6P ; C4H13NO7P2 ; C17H21NO ; C17H21NO ; C16H11ClN2O3 ; C14H22ClNO2 ; C16H10O7 ; C18H13N3O ; C11H14O7S ; C11H14O7S ; C17H21NO
6919_100.3	2.015849	0.226724	-5.3418	0.000327	3.4849	0.01269	277.0837	HMDB30870 ; HMDB30872 ; HMDB33770 ; HMDB40506 ; HMDB03141 ; HMDB13162 ; HMDB30153 ; HMDB32761 ; HMDB33043 ; HMDB34440 ; HMDB38710 ; HMDB60027 ; HMDB60028	1-Hydroxy-3-methoxy-7-primeverosyloxyxanthone ; Gentioside ; 7-Hydroxy-3-methoxy-1-primeverosyloxyxanthone ; 356-Trihydroxy-3478-tetramethoxyflavone 3-glucoside ; Retinoyl b-glucuronide ; 2-Hexenoylcholine ; Austalide H ; Nb-trans-Feruloylserotonin glucoside ; Methyl 1-(1-propenylthio)propyl disulfide ; (-)-1-(Methylthio)propyl 1-propenyl disulfide ; (7R8R)-47-Epoxy-3-methoxy-4599-lignanetetrol 9-glucoside ; 5-(345-trihydroxyphenyl)-gamma-valerolactone-O-methyl-4-O-glucuronide ; 5-(345-trihydroxyphenyl)-gamma-valerolactone-O-methyl-5-O-glucuronide	M+2H ; M+2H ; M+2H ; M+2H ; M+2K ; M+2K-H ; M+2K ; M+H+K ; M+2ACN+H ; M+2ACN+H ; M+2Na ; 2M+3H ; 2M+3H	C25H28O14 ; C25H28O14 ; C25H28O14 ; C25H28O14 ; C26H36O8 ; C11H22NO2 ; C26H36O8 ; C26H30N2O9 ; C7H14S3 ; C7H14S3 ; C25H32O11 ; C18H22O11 ; C18H22O11
0381_285.9	1.979621	0.931409	-5.36	0.000319	3.4962	0.01269	NA	NA	NA	NA	NA
6622_151.5	1.97962	0.643887	5.3466	0.000325	3.4879	0.01269	115.5787	HMDB00206 ; HMDB00446 ; HMDB00759 ; HMDB11180 ; HMDB14382 ; HMDB15442 ; HMDB28700 ; HMDB28844 ; HMDB28907 ; HMDB28929 ; HMDB29120 ; HMDB29449 ; HMDB33453 ; HMDB34778 ; HMDB60611 ; HMDB61157	N6-Acetyl-L-lysine ; N-Alpha-acetyllysine ; Glycyl-L-leucine ; L-prolyl-L-proline ; Butabarbital ; Butethal ; Alanyl-Valine ; Glycyl-Isoleucine ; Isoleucyl-Glycine ; Leucyl-Glycine ; Valyl-Alanine ; (2R3R4R)-2-Amino-4-hydroxy-3-methylpentanoic acid ; Fagomine ; (2R3R)-123-Butanetriol ; N-Desmethyl tapentadol ; N-(2-Hydroxyethyl)-morpholine N-oxide	M+ACN+2H ; M+ACN+2H ; M+ACN+2H ; M+H+NH4 ; M+H+NH4 ; M+H+NH4 ; M+ACN+2H ; M+ACN+2H ; M+ACN+2H ; M+ACN+2H ; M+ACN+2H ; M+2ACN+2H ; M+2ACN+2H ; M+3ACN+2H ; M+H+Na ; M+2ACN+2H	C8H16N2O3 ; C8H16N2O3 ; C8H16N2O3 ; C10H16N2O3 ; C10H16N2O3 ; C10H16N2O3 ; C8H16N2O3 ; C8H16N2O3 ; C8H16N2O3 ; C8H16N2O3 ; C8H16N2O3 ; C6H13NO3 ; C6H13NO3 ; C4H10O3 ; C13H21NO ; C6H13NO3
3964_286.2	1.975373	0.713098	5.3654	0.000317	3.4995	0.01269	646.0344	HMDB14627 ; HMDB15485 ; HMDB15551	Brimonidine ; Ceftibuten ; Erythrityl Tetranitrate	2M+ACN+Na ; 3M+Na+K ; 2M+ACN+H	C11H10BrN5 ; C15H14N4O6S2 ; C4H6N4O12

1216_100.7	2.0167	0.519113	-5.302	0.000347	3.4602	0.013225	405.6351	HMDB37994	Cyanidin 3-(2G-xylosylrutinoside)	M+2ACN+2H	C32H39O19
0733_112.7	2.008638	0.073567	-5.3021	0.000347	3.4603	0.013225	141.5341	HMDB15352 ; HMDB28827 ; HMDB29016	Ketamine ; Glutamyl-Proline ; Prolyl-Glutamate	M+2Na ; M+H+K ; M+H+K	C13H16ClNO ; C10H15N2O5 ; C10H15N2O5
6675_283.5	1.993527	0.661057	5.2968	0.000349	3.457	0.013225	235.0387	HMDB13696 ; HMDB32259 ; HMDB32877 ; HMDB32937 ; HMDB32997 ; HMDB32999 ; HMDB33342 ; HMDB41737 ; HMDB41738 ; HMDB41739 ; HMDB41832	Urolithin B ; Ethyl 4-(acetylthio)butyrate ; Glucorhein ; Methyl 4-chloro-1H-indole-3-acetate ; 2-Hydroxyxanthone ; 4-Hydroxyxanthone ; Mollicellin E ; Genistein 4-O-glucuronide ; Genistein 5-O-glucuronide ; Genistein 7-O-glucuronide ; Baicalin	M+Na ; M+2Na-H ; M+H+Na ; 2M+H+Na ; M+Na ; M+Na ; M+H+Na ; M+H+Na ; M+H+Na ; M+H+Na ; M+H+Na	C13H8O3 ; C8H14O3S ; C21H18O11 ; C11H10ClNO2 ; C13H8O3 ; C13H8O3 ; C22H19ClO8 ; C21H18O11 ; C21H18O11 ; C21H18O11 ; C21H18O11
8225_89.1	1.972717	0.406608	-5.2787	0.000358	3.4458	0.01347	NA	NA	NA	NA	NA
7446_126.0	1.9791	0.00841	-5.2654	0.000365	3.4375	0.013626	278.9957	HMDB41917 ; HMDB42002	Lorazepam glucuronide ; Pyrene	M+Na+K ; M+2K-H	C21H18Cl2N2O8 ; C16H10
8523_122.4	1.995761	0.47692	-5.2542	0.000371	3.4305	0.013745	299.0559	HMDB06944 ; HMDB29259 ; HMDB30865 ; HMDB34043 ; HMDB41052 ; HMDB60771	14-beta-D-Glucan ; Luteolin 7-O-(2-apiosyl-4-glucosyl-6-malonyl)-glucoside ; Rubroskyrin ; Tricetanidin ; Ajocysteine ; 4-Hydroxyclobazam	M+Na+K ; M+3Na ; M+H+Na ; 2M+H+Na ; M+NH4 ; M+H-H2O	C18H32O18 ; C35H40O23 ; C30H22O12 ; C15H11O6 ; C9H15NO3S3 ; C16H13ClN2O3
32723_43.0	1.926759	0.163754	5.241	0.000378	3.4222	0.013907	250.1533	HMDB02157 ; HMDB32879 ; HMDB34726 ; HMDB35728 ; HMDB36787 ; HMDB36840 ; HMDB38256 ; HMDB38496 ; HMDB38497	27-Norcholestanehexol ; all-trans-Carophyll yellow ; Momordicinin ; Ganoderol A ; alphagamma-Onoceradienedione ; Thujyl 19-trachylobanoate ; Geranyl acetoacetate ; Polyporusterone B ; Polyporusterone C	M+2Na ; M+H+K ; M+Na+K ; M+Na+K ; M+Na+K ; M+Na+K ; 2M+H+Na ; M+H+Na ; M+H+Na	C26H46O6 ; C32H44O2 ; C30H46O2 ; C30H46O2 ; C30H46O2 ; C30H46O2 ; C14H22O3 ; C28H44O6 ; C28H44O6

1183_66.5	1.976353	0.453298	-5.2339	0.000382	3.4178	0.013946	274.0919	HMDB14883 ; HMDB00101 ; HMDB00118 ; HMDB00333 ; HMDB00423 ; HMDB00755 ; HMDB01983 ; HMDB02643 ; HMDB03254 ; HMDB04061 ; HMDB15285 ; HMDB29232 ; HMDB29273 ; HMDB29646 ; HMDB30809 ; HMDB31360 ; HMDB32916 ; HMDB33624 ; HMDB34751 ; HMDB36610 ; HMDB37274 ; HMDB38117 ; HMDB38662 ; HMDB39586 ; HMDB39800 ; HMDB40197 ; HMDB41270 ; HMDB41566 ; HMDB59763	Modafinil ; Deoxyadenosine ; Homovanillic acid ; Isohomovanillic acid ; 34-Dihydroxyhydrocinnamic acid ; Hydroxyphenyllactic acid ; 5-Deoxyadenosine ; 3-(3-Hydroxyphenyl)-3-hydroxypropanoic acid ; Muramic acid ; 3-Methoxy-4-hydroxyphenylglycolaldehyde ; Thiamylal ; 3-Hydroxyphenyllactate ; 26-Dimethoxybenzoic acid ; 26-Dihydroxy-4-methoxyacetophenone ; (2R3S)-Piscidic acid ; L-cis-Cyclo(aspartylphenylalanyl) ; 2-(Methylthiomethyl)furan ; ()-2-Hydroxy-3-(2-hydroxyphenyl)propanoic acid ; Ligustroside ; 2-Methyl-3-(methylthio)furan ; Maltol propionate ; 6-O-Oleuropeoylsucrose ; N-(1-Deoxy-1-fructosyl)alanine ; 2-(1-Mercaptoethyl)furan ; 5-Methyl-2-furanmethanethiol ; 25-Dimethyl-3-furanthiol ; 24-Dihydroxy-6-methoxyacetophenone ; 2-Methyl-5-(methylthio)furan ; 34-Dimethoxybenzoic acid	M+H ; M+Na ; 3M+2H ; 3M+2H ; 3M+2H ; 3M+2H ; M+Na ; 3M+2H ; M+Na ; 3M+2H ; 2M+H+K ; 3M+2H ; 3M+2H ; 3M+2H ; M+NH4 ; 2M+H+Na ; 2M+NH4 ; 3M+2H ; M+H+Na ; 2M+NH4 ; 3M+2H ; M+H+K ; M+Na ; 2M+NH4 ; 2M+NH4 ; 2M+NH4 ; 3M+2H ; 2M+NH4 ; 3M+2H	C15H15NO2S ; C10H13N5O3 ; C9H10O4 ; C9H10O4 ; C9H10O4 ; C9H10O4 ; C10H13N5O3 ; C9H10O4 ; C9H17NO7 ; C9H10O4 ; C12H18N2O2S ; C9H10O4 ; C9H10O4 ; C9H10O4 ; C11H12O7 ; C13H14N2O4 ; C6H8OS ; C9H10O4 ; C25H32O12 ; C6H8OS ; C9H10O4 ; C22H36O13 ; C9H17NO7 ; C6H8OS ; C6H8OS ; C6H8OS ; C9H10O4 ; C6H8OS ; C9H10O4
8627_99.8	1.96419	0.221609	-5.1925	0.000406	3.3918	0.014698	159.5339	HMDB00942 ; HMDB01316 ; HMDB02281 ; HMDB14427 ; HMDB29294 ; HMDB29715 ; HMDB34454 ; HMDB40924	Tetrahydroneopterin ; 6-Phosphogluconic acid ; 4a-Hydroxytetrahydrobiopterin ; Pamidronate ; Caffeoyl aspartic acid ; 2-(2-Thienyl)furan ; 5-(4-Acetoxy-1-butynyl)-22-bithiophene ; 2-Carboxyarabinitol 5-phosphate	M+Na+K ; M+ACN+2H ; M+Na+K ; M+2ACN+2H ; M+H+Na ; 2M+H+NH4 ; M+ACN+2H ; M+ACN+2H	C9H15N5O4 ; C6H13O10P ; C9H15N5O4 ; C3H11NO7P2 ; C13H13NO7 ; C8H6OS ; C14H12O2S2 ; C6H13O10P
9192_99.7	2.005103	0.420094	5.1669	0.000421	3.3757	0.015144	NA	NA	NA	NA	NA
9368_104.	1.975883	0.106132	5.1595	0.000426	3.3711	0.015197	144.9999	HMDB00115 ; HMDB00251 ; HMDB14901 ; HMDB31608 ; HMDB38442	Glycolic acid ; Taurine ; Methimazole ; Peracetic acid ; 1-Isothiocyanato-2-(methylthio)ethane	3M+Na+K ; 2M+H+K ; 2M+Na+K ; 3M+Na+K ; 2M+H+Na	C2H4O3 ; C2H7NO3S ; C4H6N2S ; C2H4O3 ; C4H7NS2

9047_241.(	1.986138	0.219148	5.1504	0.000431	3.3653	0.015291	324.0589	HMDB00095 ; HMDB11692 ; HMDB01163 ; HMDB03351 ; HMDB06258 ; HMDB06552 ; HMDB15176 ; HMDB30561 ; HMDB30563 ; HMDB32909 ; HMDB33533 ; HMDB33658 ; HMDB33915 ; HMDB33993 ; HMDB34425 ; HMDB37101 ; HMDB38322 ; HMDB40242 ; HMDB41097 ; HMDB41450	Cytidine monophosphate ; Cytidine 2-phosphate ; Guanosine diphosphate mannose ; GDP-glucose ; 26-Dimethylheptanoyl-CoA ; Aflatoxin B1 ; Melphalan ; 3-Hydroxy-89-dimethoxycoumestan ; 344-Trihydroxypulvinone ; 2-(4-Methoxyphenyl)naphthalic anhydride ; Ceftiofur ; De-O-methyldihydrosterigmatocystin ; Aloe emodin w-acetate ; Wairol ; ()-1-Methylpropyl 3-(methylthio)-2-propenyl disulfide ; 8-Hydroxy-3-methoxy-1-methylanthraquinone-2-carboxylic acid ; 4-(34-Dihydroxyphenyl)-2-hydroxy-1H-phenalen-1-one ; 3-Methyl-5-pentyl-124-trithiolane ; (E)-Antibiotic BE 23372M ; Musanolone E	M+H ; M+H ; M+ACN+2H ; M+ACN+2H ; M+3Na ; 2M+H+Na ; 2M+H+K ; 2M+H+Na ; 2M+H+Na ; 2M+H+K ; M+3ACN+2H ; 2M+H+Na ; 2M+H+Na ; 2M+H+Na ; 3M+H+Na ; 2M+H+Na ; 2M+H+K ; 3M+H+Na ; 2M+H+Na ; 2M+H+K	C9H14N3O8P ; C9H14N3O8P ; C16H25N5O16P2 ; C16H25N5O16P2 ; C30H48N7O17P3S ; C17H12O6 ; C13H18Cl2N2O2 ; C17H12O6 ; C17H12O6 ; C19H12O4 ; C19H17N5O7S3 ; C17H12O6 ; C17H12O6 ; C17H12O6 ; C8H16S3 ; C17H12O6 ; C19H12O4 ; C8H16S3 ; C17H12O6 ; C19H12O4
7195_102.1	1.994374	0.252898	-5.1437	0.000435	3.3611	0.015331	286.0697	HMDB14387 ; HMDB01265 ; HMDB02326 ; HMDB38634	Cladribine ; Fucose 1-phosphate ; Coumesterol ; (-)-Dioxibrassinin	M+H ; M+ACN+H ; M+NH4 ; M+NH4	C10H12ClN5O3 ; C6H13O8P ; C15H8O5 ; C11H12N2O2S2
4417_100.5	1.9994	0.499311	-5.1385	0.000439	3.3578	0.01534	278.0844	HMDB00202 ; HMDB00254 ; HMDB00349 ; HMDB00940 ; HMDB01049 ; HMDB02275 ; HMDB15008 ; HMDB15139 ; HMDB15538 ; HMDB30128 ; HMDB31204 ; HMDB34094 ; HMDB38826 ; HMDB39324 ; HMDB39696 ; HMDB39697	Methylmalonic acid ; Succinic acid ; Erythrono-14-lactone ; Threonolactone ; Gamma-Glutamylcysteine ; 78-Dihydroneopterin ; Suprofen ; Ganciclovir ; Tiaprofenic acid ; Physalin B ; 4-Hydroxy-2-oxobutanoic acid ; Physalin C ; Alatanin 2 ; xi-3-Hydroxy-2-oxobutanoic acid ; 2527-Dihydro-47-didehydro-7-deoxyphysalin A ; Isophysalin B	2M+ACN+H ; 2M+ACN+H ; 2M+ACN+H ; 2M+ACN+H ; 2M+3H2O+2H ; M+Na ; M+NH4 ; M+Na ; M+NH4 ; M+2Na ; 2M+ACN+H ; M+2Na ; M+3H ; 2M+ACN+H ; M+2Na ; M+2Na	C4H6O4 ; C4H6O4 ; C4H6O4 ; C4H6O4 ; C8H14N2O5S ; C9H13N5O4 ; C14H12O3S ; C9H13N5O4 ; C14H12O3S ; C28H30O9 ; C4H6O4 ; C28H30O9 ; C39H43O20 ; C4H6O4 ; C28H30O9 ; C28H30O9
6015_70.6(	2.000969	0.394514	5.0736	0.000482	3.3167	0.016129	NA	NA	NA	NA	NA
3489_99.9!	1.988875	0.588388	-5.0708	0.000484	3.3149	0.016129	450.0603	HMDB14577 ; HMDB41120	Prochlorperazine ; 4-Phenyl-1H3H-naphtho18-cdpyran-13-dione	M+2K-H ; 3M+2K	C20H24ClN3S ; C18H10O3

[illegible]1458_26.73	1.983233	0.328095	5.0846	0.000475	3.3237	0.016129	203.0463	HMDB14994 ; HMDB40933 ; HMDB29222 ; HMDB60022 ; HMDB02708 ; HMDB15173 ; HMDB15338 ; HMDB29117 ; HMDB29430 ; HMDB29830 ; HMDB32007 ; HMDB32354 ; HMDB37612 ; HMDB37662 ; HMDB38358 ; HMDB38915 ; HMDB41136 ; HMDB41861	Chlorphenesin ; 4-Chloro-35-dimethoxybenzyl alcohol ; Urolithin A-3-O-glucuronide ; Urolithin A-8-O-glucuronide ; Cyanidin ; Fenofibrate ; Ridogrel ; Tyrosyl-Tyrosine ; Methionine sulfoximine ; N-Caffeoyltryptophan ; Epifisetinidol-(4beta-8)-catechin ; S-Isopropyl 3-methylbut-2-enethioate ; CI Acid Blue 3 ; 33457-Pentahydroxyflavan(4-8)-3457-tetrahydroxyflavan ; Gambiriin B3 ; 12-Tridecene-46810-tetraynal ; 3-Chloro-3-deoxytriphasiol ; Cyanuric acid	M+H ; M+H ; M+2H ; M+2H ; M+2ACN+2H ; M+2Na ; M+H+K ; M+Na+K ; M+Na ; M+H+K ; M+H+2Na ; M+2Na-H ; M+2H+Na ; M+H+2Na ; M+H+2Na ; M+Na ; M+H+K ; 3M+H+NH4	C9H11ClO3 ; C9H11ClO3 ; C19H16O10 ; C19H16O10 ; C15H11ClO6 ; C20H21ClO4 ; C18H17F3N2O3 ; C18H20N2O5 ; C5H12N2O3S ; C20H18N2O5 ; C30H26O11 ; C8H14OS ; C27H32CaN2O6S2 ; C30H26O11 ; C30H26O11 ; C13H8O ; C19H23ClO5 ; C3H3N3O3
[illegible]8721_40.0[illegible]	1.975272	0.54407	5.0716	0.000484	3.3154	0.016129	106.0039	HMDB03162 ; HMDB13141 ; HMDB29578 ; HMDB31161 ; HMDB31472 ; HMDB31709 ; HMDB32180 ; HMDB32408 ; HMDB40056 ; HMDB60279 ; HMDB60412	7-Methylhypoxanthine ; 1-Methylhypoxanthine ; Diisopropyl disulfide ; Methyl pentyl disulfide ; Dipropyl disulfide ; 16-Hexanedithiol ; Butyl ethyl disulfide ; Methyl isopentyl disulfide ; xi-1-(Propylthio)-1-propanethiol ; Aminochrome o-semiquinone ; 6-Methylmercaptopurine	M+Na+K ; M+Na+K ; M+Na+K ; M+Na+K ; M+Na+K ; M+Na+K ; M+Na+K ; M+Na+K ; M+Na+K ; M+Na+K ; M+2Na	C6H6N4O ; C6H6N4O ; C6H14S2 ; C6H14S2 ; C6H14S2 ; C6H14S2 ; C6H14S2 ; C6H14S2 ; C6H14S2 ; C8H8NO2 ; C6H6N4S

2266_241.3	1.964279	0.790372	5.0668	0.000487	3.3124	0.016129	325.0622	HMDB00286 ; HMDB00290 ; HMDB00302 ; HMDB00304 ; HMDB00565 ; HMDB00625 ; HMDB01931 ; HMDB03249 ; HMDB03290 ; HMDB05010 ; HMDB13112 ; HMDB14649 ; HMDB28979 ; HMDB29258 ; HMDB29364 ; HMDB29478 ; HMDB29534 ; HMDB29812 ; HMDB30757 ; HMDB30888 ; HMDB30910 ; HMDB30911 ; HMDB31378 ; HMDB31386 ; HMDB31387 ; HMDB31900 ; HMDB31916 ; HMDB31970 ; HMDB31975 ; HMDB32649 ; HMDB32908 ; HMDB33707 ; HMDB33824 ; HMDB34123 ; HMDB35131 ; HMDB35132 ; HMDB35223 ; HMDB35503 ; HMDB35708 ; HMDB35902 ; HMDB35903 ; HMDB36005 ; HMDB36040 ; HMDB36455 ; HMDB36473 ; HMDB36488 ; HMDB36641 ; HMDB36767 ; HMDB36770 ; HMDB36772 ; HMDB37022 ; HMDB37431 ; HMDB37433 ; HMDB37534 ; HMDB37538 ; HMDB37539 ;	Uridine diphosphate glucose ; Uridine diphosphate-N-acetylglucosamine ; Uridine diphosphategalactose ; Uridine diphosphate-N-acetylgalactosamine ; Galactonic acid ; Gluconic acid ; Gamma-CEHC ; Rutin ; Gulonic acid ; Sertraline ; UDP-N-acetyl-D-mannosamine ; Nitazoxanide ; Methionyl-Methionine ; Luteolin 6-C-glucoside 8-C-arabinoside ; 7-Acetoxy-2-methylisoflavone ; Meloside L ; Glyzarin ; Quercetin 8-C-(2-rhamnosylglucoside) ; (S)-Pterosin A ; Illudin C2 ; 2alpha-Hydroxyalantolactone ; 2-Oxo-511(13)-eudesmadien-128-olide ; 4-Epiisoinuviscolide ; (1(10)E4beta5alpha8beta)-45-Epoxy-1(10)11(13)-germacradien-128-olide ; (1beta4E8beta10alpha)-110-Epoxy-411(13)-germacradien-128-olide ; Methyl (2E4Z6E8E10E)-48-dimethyl-12-oxo-246810-dodecapentaenoate ; 5-Hydroxy-2-(1-hydroxy-5-methyl-4-hexenyl)benzofuran ; 1beta-Hydroxyalantolactone ; 3-Methoxy-2-(4-methylbenzoyl)-4H-1-benzopyran-4-one ; 45-Epoxy-11(13)-guaien-128-	M+2ACN+2H ; M+ACN+2H ; M+2ACN+2H ; M+ACN+2H ; 3M+Na+K ; 3M+Na+K ; M+2K-H ; M+H+K ; 3M+Na+K ; 2M+H+K ; M+ACN+2H ; M+NH4 ; M+2Na-H ; M+H+K ; 2M+Na+K ; M+H+K ; 2M+Na+K ; M+H+K ; M+2K-H ; M+2K-H ; M+2K-H ; M+2K+H ; M+2K-H ; M+2K-H ; M+2K-H ; M+2K+H ; M+2K+H ; M+2K-H ; 2M+Na+K ; M+2K-H ; M+H+K ; M+2K-H ; M+H+K ; M+2K-H ; M+2K-H ; M+2K-H ; M+2K-H ; M+2K-H ; M+2K-H ; M+2K+H ; M+2K-H ; M+2K-H ; M+2K-H ; M+2K+H ; M+2K+H ; M+2K+H ; M+2K-H ; M+2K+H ; M+2K-H ; M+2K-H ; M+2K-H ; M+H+K ; M+H+K ; M+H+K ; M+H+K ; M+H+Na ; M+2K-H ; M+2K+H ; M+2K-H ; M+2K+H ; M+H+K ; M+H+K ; M+H+K ; M+2K+H ; M+2K+H ; M+2K-H ; M+2K+H ; M+2K-H ; M+H+K ; M+2K-H ; M+H+K ; M+H+K ; M+H+K ; M+H+K ; M+2K+H ; M+2K+H ; M+2ACN+2H ; M+ACN+2H ;	C15H24N2O17P2 ; C17H27N3O17P2 ; C15H24N2O17P2 ; C17H27N3O17P2 ; C6H12O7 ; C6H12O7 ; C15H20O3 ; C27H30O16 ; C6H12O7 ; C17H17Cl2N ; C17H27N3O17P2 ; C12H9N3O5S ; C10H20N2O3S2 ; C27H30O16 ; C18H14O4 ; C27H30O16 ; C18H14O4 ; C27H30O16 ; C15H20O3 ; C15H20O3 ; C15H20O3 ; C15H18O3 ; C15H20O3 ; C15H20O3 ; C15H20O3 ; C15H18O3 ; C15H18O3 ; C15H20O3 ; C18H14O4 ; C15H20O3 ; C27H30O16 ; C15H20O3 ; C27H30O16 ; C15H20O3 ; C15H20O3 ; C15H20O3 ; C15H20O3 ; C15H20O3 ; C15H20O3 ; C15H18O3 ; C15H20O3 ; C15H20O3 ; C15H20O3 ; C15H18O3 ; C15H18O3 ; C15H18O3 ; C15H20O3 ; C15H18O3 ; C15H20O3 ;
6891_150.3	1.959267	0.780664	5.0961	0.000467	3.331	0.016129	92.57587	HMDB12815 ; HMDB29742 ; HMDB31860 ; HMDB32933	5-Aminopentanal ; 3-Isopropyl-2-methoxy-5-methylpyrazine ; 2-Methoxy-3-(2-methylpropyl)pyrazine ; 2-Methoxy-3-(1-methylpropyl)pyrazine	M+2ACN+2H ; M+H+NH4 ; M+H+NH4 ; M+H+NH4	C5H11NO ; C9H14N2O ; C9H14N2O ; C9H14N2O
3891_158.4	1.941496	0.390244	5.0862	0.000473	3.3247	0.016129	327.5964	HMDB01941 ; HMDB02937 ; HMDB14549 ; HMDB33892 ; HMDB37501 ; HMDB60139	Brompheniramine ; Tamarixetin ; Dexbrompheniramine ; Mycochromone ; Dihydroisorhamnetin ; 4-Hydroxy-17beta-estradiol-2-S-glutathione	2M+H+NH4 ; 2M+H+NH4 ; 2M+H+NH4 ; 2M+H+NH4 ; 2M+H+NH4 ; M+Na+K	C16H19BrN2 ; C16H14O7 ; C16H19BrN2 ; C16H14O7 ; C16H14O7 ; C28H39N3O9S
1982_48.97	1.928713	0.123232	-5.0823	0.000476	3.3222	0.016129	NA	NA	NA	NA	NA
5206_100.0	1.969394	0.098339	-5.0595	0.000492	3.3077	0.016196	158.5355	HMDB15469 ; HMDB39955	Magnesium salicylate ; Carotamine	M+H+NH4 ; M+2Na	C14H10MgO6 ; C14H13N3O3
7776_99.95	1.988113	0.21797	-5.0113	0.000528	3.277	0.017271	278.5878	HMDB37690 ; HMDB38757	Limocitrol 3-glucoside ; Isolimocitrol 3-glucoside	M+H+NH4 ; M+H+NH4	C24H26O14 ; C24H26O14

6121_81.4[illegible]	1.966585	0.552649	-5.0052	0.000533	3.2731	0.017313	244.0927	HMDB00089 ; HMDB15122 ; HMDB60478 ; HMDB11751 ; HMDB12915 ; HMDB14639 ; HMDB14798 ; HMDB29436 ; HMDB29685 ; HMDB30901 ; HMDB31240 ; HMDB31640 ; HMDB31770 ; HMDB32128 ; HMDB32174 ; HMDB32574 ; HMDB32640 ; HMDB32860 ; HMDB32974 ; HMDB32975 ; HMDB32976 ; HMDB33701 ; HMDB33761 ; HMDB33867 ; HMDB37602 ; HMDB37733 ; HMDB38460 ; HMDB40036 ; HMDB41008 ; HMDB41532 ; HMDB60734 ; HMDB28751 ; HMDB28767 ; HMDB28793 ; HMDB29145	Cytidine ; Cytarabine ; gamma-Glutamyl-beta-cyanoalanine ; 3-Methoxybenzenepropanoic acid ; Coniferyl alcohol ; Darifenacin ; Metaxalone ; Penmacric acid ; 4-Ethoxy-3-methoxybenzaldehyde ; Eremopetasitenin B2 ; 2-Phenyl-13-dioxolane-4-methanol ; Glycerol 1-propanoate diacetate ; Carbofuran ; 1-(24-Dihydroxyphenyl)-1-butanone ; Benzaldehyde glyceryl acetal ; Propylparaben ; Ethyl p-anisate ; 1-Methylnaphthalene ; 24-Dimethylthiazole ; 25-Dimethylthiazole ; 45-Dimethylthiazole ; Hydrocotarnine ; 4-Methoxybenzyl acetate ; 4-Isothiocyanato-1-butene ; 4-Hydroxy-266-trimethyl-3-oxo-14-cyclohexadiene-1-carboxaldehyde ; Propyl 2-furanacrylate ; Thiiranepropanenitrile ; 2-Ethylthiazole ; 1-Methyl-6-phenyl-1H-imidazo45-bpyridin-2-amine ; Eremosulphoxinolide A ; 3-(3-Hydroxyphenyl)-2-methylpropionic acid ; Aspartyl-Glutamine ; Aspartyl-Gamma-glutamate ; Glutaminyl-[illegible]	M+H ; M+H ; M+H ; M+ACN+Na ; M+ACN+Na ; M+Na+K ; M+Na ; M+ACN+H ; M+ACN+Na ; M+H+Na ; M+ACN+Na ; 2M+H+Na ; M+Na ; M+ACN+Na ; M+ACN+Na ; M+ACN+Na ; M+ACN+Na ; 3M+Na+K ; 2M+NH4 ; 2M+NH4 ; 2M+NH4 ; M+Na ; M+ACN+Na ; 2M+NH4 ; M+ACN+Na ; M+ACN+Na ; 2M+NH4 ; 2M+NH4 ; 2M+H+K ; M+H+Na ; M+ACN+Na ; M+H-H2O ; M+H-H2O ; M+H-H2O ; M+H-H2O	C9H13N3O5 ; C9H13N3O5 ; C9H13N3O5 ; C10H12O3 ; C10H12O3 ; C28H30N2O2 ; C12H15NO3 ; C7H10N2O5 ; C10H12O3 ; C24H32O7S ; C10H12O3 ; C10H16O6 ; C12H15NO3 ; C10H12O3 ; C10H12O3 ; C10H12O3 ; C10H12O3 ; C11H10 ; C5H7NS ; C5H7NS ; C5H7NS ; C12H15NO3 ; C10H12O3 ; C5H7NS ; C10H12O3 ; C10H12O3 ; C5H7NS ; C5H7NS ; C13H12N4 ; C24H32O7S ; C10H12O3 ; C9H15N3O6 ; C9H15N3O6 ; C9H15N3O6 ; C9H15N3O6
30763_55.4	1.948206	0.000625	-4.9917	0.000544	3.2644	0.017323	318.9766	HMDB15289 ; HMDB32040 ; HMDB32866 ; HMDB33307	Bretylium ; Benzoyl peroxide ; 2456-Phenanthrenetetrol ; Moracin M	M+2K-H ; M+2K-H ; M+2K-H ; M+2K-H	C11H17BrN ; C14H10O4 ; C14H10O4 ; C14H10O4
7665_49.5[illegible]	1.928452	0.241028	-4.9921	0.000544	3.2647	0.017323	NA	NA	NA	NA	NA
1038_50.0[illegible]	1.91915	0.146542	-4.9991	0.000538	3.2692	0.017323	375.9701	HMDB42052	Trimethylselenonium	3M+H	C3H9Se
2467_60.5[illegible]	1.977766	0.027425	-4.9756	0.000557	3.2541	0.017408	269.1612	HMDB28888 ; HMDB28889 ; HMDB28909 ; HMDB28931 ; HMDB29560 ; HMDB15072 ; HMDB28695 ; HMDB29010 ; HMDB30290 ; HMDB30441 ; HMDB31651 ; HMDB34203 ; HMDB34521 ; HMDB35011 ; HMDB35777 ; HMDB35803 ; HMDB35998 ; HMDB38243 ; HMDB38517 ; HMDB39647 ; HMDB60843	Histidinyl-Isoleucine ; Histidinyl-Leucine ; Isoleucyl-Histidine ; Leucyl-Histidine ; Hesperaline ; Diethylpropion ; Alanyl-Proline ; Prolyl-Alanine ; Cuscohygrine ; Okadaic acid ; N-(3-Methylbutyl)acetamide ; N-(2-Methylpropyl)acetamide ; (3beta11alpha13beta)-31113-Oleananetriol ; Momordicoside D ; Betulafolienetriol ; Schleicherastatin 2 ; (3beta24xi)-Cycloartane-32425-triol ; Panaxadiol ; Dinophysistoxin 2 ; Ginsenoside La ; N-Desthienylethyl-rotigotine	M+H ; M+H ; M+H ; M+H ; M+H ; M+ACN+Na ; M+2ACN+H ; M+2ACN+H ; M+2Na-H ; M+3H ; 2M+K ; 2M+K ; M+2K ; M+2H+Na ; M+2K ; M+2K ; M+2K ; M+2K ; M+3H ; M+2H+Na ; M+ACN+Na	C12H20N4O3 ; C12H20N4O3 ; C12H20N4O3 ; C12H20N4O3 ; C14H22NO4 ; C13H19NO ; C8H14N2O3 ; C8H14N2O3 ; C13H24N2O ; C44H68O13 ; C6H13NO ; C6H13NO ; C30H52O3 ; C42H70O13 ; C30H52O3 ; C30H52O3 ; C30H52O3 ; C30H52O3 ; C44H68O13 ; C42H70O13 ; C13H19NO

3122_100.1	1.946781	0.100737	4.9784	0.000555	3.2559	0.017408	NA	NA	NA	NA	NA
0057_286.5	1.940465	0.138891	4.9779	0.000555	3.2556	0.017408	NA	NA	NA	NA	NA
13587_149.9	1.935697	0.658275	4.9682	0.000563	3.2494	0.01749	138.5814	HMDB03869 ; HMDB04207 ; HMDB00508 ; HMDB00568 ; HMDB01851 ; HMDB02917 ; HMDB32341 ; HMDB33730 ; HMDB38433 ; HMDB38614 ; HMDB38676 ; HMDB38973	Epsilon-(gamma-Glutamyl)-lysine ; L-a-glutamyl-L-Lysine ; Ribitol ; D-Arabitol ; L-Arabitol ; D-Xylitol ; Isoamyl isothiocyanate ; L-2-(Hydroxymethyl)-1234-butanetetrol ; 1-Isothiocyanatopentane ; gamma-L-Glutamyl-L-pipecolic acid ; (2S2S)-Pyrosaccharopine ; 25-Dihydro-245-trimethylthiazole	M+2H ; M+2H ; M+3ACN+2H ; M+3ACN+2H ; M+3ACN+2H ; M+3ACN+2H ; 2M+H+NH4 ; M+3ACN+2H ; 2M+H+NH4 ; M+H+NH4 ; M+H+NH4 ; 2M+H+NH4	C11H21N3O5 ; C11H21N3O5 ; C5H12O5 ; C5H12O5 ; C5H12O5 ; C5H12O5 ; C6H11NS ; C5H12O5 ; C6H11NS ; C11H18N2O5 ; C11H18N2O5 ; C6H11NS
78753_100.	1.981674	0.597682	-4.9507	0.000578	3.2381	0.017728	406.6379	HMDB07338 ; HMDB28737 ; HMDB28968 ; HMDB30350 ; HMDB39873 ; HMDB56372	DG(18:4(6Z9Z12Z15Z)18:4(6Z9Z12Z15Z)0:0) ; Asparaginyl-Methionine ; Methionyl-Asparagine ; Dehydroanonaine ; 2-Naphthalenol 2-aminobenzoate ; DG(18:4n30:018:4n3)	2M+3H ; 3M+H+Na ; 3M+H+Na ; 3M+H+Na ; 3M+H+Na ; 2M+3H	C39H60O5 ; C9H17N3O4S ; C9H17N3O4S ; C17H13NO2 ; C17H13NO2 ; C39H60O5
1492_100.1	1.969695	0.162405	-4.951	0.000578	3.2383	0.017728	544.1071	HMDB29213 ; HMDB30624 ; HMDB34358 ; HMDB34359 ; HMDB37756 ; HMDB41088	Quercetin-3-glucuronide ; Quercetagitrin ; Myricetin 3-galactoside ; Myricetin 3-glucoside ; Tagetiin ; (E)-Squamosamide	M+ACN+Na ; M+ACN+Na ; M+ACN+Na ; M+ACN+Na ; M+ACN+Na ; M+2K+H	C21H20O13 ; C21H20O13 ; C21H20O13 ; C21H20O13 ; C21H20O13 ; C26H27NO7
6376_98.79	1.926629	0.29414	4.9464	0.000582	3.2354	0.017732	142.0264	HMDB00224 ; HMDB29188 ; HMDB60151 ; HMDB60152 ; HMDB34034 ; HMDB40869 ; HMDB00092 ; HMDB00112 ; HMDB00452 ; HMDB00650 ; HMDB01906 ; HMDB02166 ; HMDB02299 ; HMDB03911 ; HMDB05031 ; HMDB06005 ; HMDB14949 ; HMDB15063 ; HMDB15270 ; HMDB30451 ; HMDB31329 ; HMDB31654 ; HMDB33513 ; HMDB38394 ; HMDB40734 ; HMDB41945 ; HMDB42033 ; HMDB61050	O-Phosphoethanolamine ; 5-Hydroxymethyl-2-furanoate ; O-Phosphonatoethanaminium ; Ethamp ; Bikojic acid ; 2-O-Caffeoyltartronic acid ; Dimethylglycine ; Gamma-Aminobutyric acid ; L-Alpha-aminobutyric acid ; D-Alpha-aminobutyric acid ; 2-Aminoisobutyric acid ; (S)-b-aminoisobutyric acid ; (R)-b-aminoisobutyric acid ; 3-Aminoisobutanoic acid ; Rosiglitazone ; Indolylacryloylglycine ; Ribavirin ; Azacitidine ; Cephapirin ; Cefapirin ; Butyl nitrite ; 3-Aminobutanoic acid ; Gonyautoxin V ; O-Acetylethanolamine ; Haematopodin ; N-Ethylglycine ; Thiodiglycol ; Unknown	M+H ; M+H ; M+H ; M+H ; M+2H ; M+2H ; M+K ; M+K ; M+K ; M+K ; M+K ; M+K ; M+K ; M+K ; M+3Na ; M+H+K ; M+H+K ; M+H+K ; M+3H ; M+3H ; M+K ; M+K ; M+H+2Na ; M+K ; M+H+K ; M+K ; 2M+H+K ; M+K	C2H8NO4P ; C6H5O4 ; C2H8NO4P ; C2H8NO4P ; C12H10O8 ; C12H10O8 ; C4H9NO2 ; C4H9NO2 ; C4H9NO2 ; C4H9NO2 ; C4H9NO2 ; C4H9NO2 ; C4H9NO2 ; C4H9NO2 ; C18H19N3O3S ; C13H12N2O3 ; C8H12N4O5 ; C8H12N4O5 ; C17H17N3O6S2 ; C17H17N3O6S2 ; C4H9NO2 ; C4H9NO2 ; C10H17N7O7S ; C4H9NO2 ; C13H12N2O3 ; C4H9NO2 ; C4H10O2S ; C4H9NO2

6363_58.58	1.980099	0.09305	4.9263	0.000599	3.2224	0.018012	281.9936	HMDB00671 ; HMDB04096 ; HMDB11621 ; HMDB30179 ; HMDB32755 ; HMDB59765 ; HMDB60484	Indolelactic acid ; 5-Methoxyindoleacetate ; Cinnamoylglycine ; Edulitine ; Methyl 1-methoxy-1H-indole-3-carboxylate ; 3-Indolehydracrylic acid ; Indolepyruvate	M+2K-H ; M+2K-H ; M+2K-H ; M+2K-H ; M+2K-H ; M+2K-H ; M+2K+H	C11H11NO3 ; C11H11NO3 ; C11H11NO3 ; C11H11NO3 ; C11H11NO3 ; C11H11NO3 ; C11H9NO3
7239_38.13	1.977357	0.028414	-4.9217	0.000603	3.2194	0.018012	153.0407	HMDB00292 ; HMDB00786 ; HMDB01182 ; HMDB01280 ; HMDB01520 ; HMDB01881 ; HMDB06213 ; HMDB11161 ; HMDB29403 ; HMDB31347 ; HMDB31849 ; HMDB31950 ; HMDB32472 ; HMDB32560 ; HMDB32971 ; HMDB33739 ; HMDB35248 ; HMDB35289 ; HMDB37260 ; HMDB37480 ; HMDB38347 ; HMDB38775 ; HMDB39935 ; HMDB40536 ; HMDB40832 ; HMDB41150 ; HMDB41623 ; HMDB60772	Xanthine ; Oxypurinol ; 68-Dihydroxypurine ; 2-Aminomuconic acid semialdehyde ; Flavin Mononucleotide ; Propylene glycol ; (S)-Propane-12-diol ; L-alpha-glutamyl-L-hydroxyproline ; Oxypinnatanine ; 2-O-Caffeoylarbutin ; 2-Ethylpyrazine ; L-2-Aminoethyl seryl phosphate ; Polyethylene oxidized ; Dinoseb acetate ; 23-Dimethylpyrazine ; Floribundoside ; 26-Dimethylpyrazine ; 25-Dimethylpyrazine ; Todatriol glucoside ; Eriodictin ; Methylpicraquassioside A ; 578-Trihydroxyflavanone 7-glucoside ; Dihydrogenistin ; (2R3R)-3347-Tetrahydroxyflavanone 7-O-alpha-L-Rhamnopyranoside ; 5-Hydroxyaloin A ; 7-Hydroxyaloin B ; N6-Carbamoyl-L-threonyladenosine ; 4-Hydroxyfenoprofen glucuronide	M+H ; M+H ; M+H ; 2M+H+Na ; M+3H ; 3M+2K ; 3M+2K ; M+2Na ; M+2Na ; M+2H+Na ; M+2Na-H ; 2M+3H ; M+Na+K ; M+H+Na ; M+2Na-H ; M+2H+Na ; M+2Na-H ; M+2Na-H ; M+3Na ; M+2H+Na ; M+H+2Na ; M+2H+Na ; M+2H+Na ; M+2H+Na ; M+2H+Na ; M+2H+Na ; M+H+2Na ; M+2H+Na	C5H4N4O2 ; C5H4N4O2 ; C5H4N4O2 ; C6H7NO3 ; C17H21N4O9P ; C3H8O2 ; C3H8O2 ; C10H16N2O6 ; C10H16N2O6 ; C21H22O10 ; C6H8N2 ; C5H13N2O6P ; C12H20O5 ; C12H14N2O6 ; C6H8N2 ; C21H22O10 ; C6H8N2 ; C6H8N2 ; C17H26O10 ; C21H22O10 ; C19H24O10 ; C21H22O10 ; C21H22O10 ; C21H22O10 ; C21H22O10 ; C21H22O10 ; C15H20N6O8 ; C21H22O10
7902_111.9	1.930108	0.36871	4.9196	0.000605	3.2181	0.018012	110.0088	HMDB00731 ; HMDB01087 ; HMDB01504 ; HMDB15684 ; HMDB42043	Cysteine-S-sulfate ; 5-Methylthioribose ; Aminoparathion ; Diloxanide ; Tolfenamic acid	M+H+NH4 ; M+H+K ; M+3Na ; M+3H ; M+3Na	C3H7NO5S2 ; C6H12O4S ; C10H16NO3PS ; C14H11Cl2NO4 ; C14H12ClNO2
0065_89.40	1.919157	0.108555	4.923	0.000602	3.2203	0.018012	177.547	HMDB61003 ; HMDB14011 ; HMDB15056 ; HMDB15507 ; HMDB30210 ; HMDB33737 ; HMDB36325 ; HMDB39846 ; HMDB59977	lansoprazole sulfide ; 5-O-Desmethyl omeprazole ; Ketotifen ; Gamma Hydroxybutyric Acid ; Koenimbine ; Dehydronuciferine ; Dinorcapsaicin ; 3-Oxodecanoic acid glycerides ; 4-Hydroxy-5-(dihydroxyphenyl)-valeric acid-O-methyl-O-sulphate	M+2H ; M+H+Na ; M+2Na ; 3M+2Na ; M+Na+K ; M+Na+K ; M+2K ; M+2K ; M+H+NH4	C16H14F3N3OS ; C16H17N3O3S ; C19H19NOS ; C4H7O3 ; C19H19NO2 ; C19H19NO2 ; C16H23NO3 ; C13H25O6 ; C12H16O9S

5752_150.4	1.938292	0.777206	4.9094	0.000614	3.2115	0.018179	277.1586	HMDB13825 ; HMDB14514 ; HMDB15689 ; HMDB29653 ; HMDB31311 ; HMDB31454 ; HMDB31624 ; HMDB31625 ; HMDB31866 ; HMDB32060 ; HMDB35395 ; HMDB35449 ; HMDB35601 ; HMDB35989 ; HMDB36719 ; HMDB36814 ; HMDB36816 ; HMDB37294 ; HMDB38244 ; HMDB39545 ; HMDB39546 ; HMDB39845 ; HMDB40123 ; HMDB60745 ; HMDB31976 ; HMDB33294 ; HMDB40761 ; HMDB61027	4-(1133-Tetramethylbutyl)-phenol ; Mirtazapine ; Antazoline ; 1-(1-Methoxy-1-methylethyl)-4-methylbenzene ; Benzyl butyl ether ; trans-Jasmone ; 5-Phenyl-1-pentanol ; 1-Phenyl-2-pentanol ; 2-Methyl-4-phenyl-2-butanol ; 2-Butyl-4-methylphenol ; Ginsenoside C ; 1beta2alpha3beta19alpha-Tetrahydroxy-12-ursen-28-oic acid 28-O-b-D-Glucopyranosyl-(1-2)-b-D-glucopyranosyl ester ; (Z)-3-Methyl-2-(2-pentenyl)-2-cyclopenten-1-one ; Thymol methyl ether ; Norbicycloekasantalal ; Santalone ; Norecasantalal ; 2-Methyl-3-(2-pentenyl)-2-cyclopenten-1-one ; Panaxatriol ; Ginsenoside F2 ; Ginsenoside Rg3 ; 3-Oxooctanoic acid glycerides ; Centellasaponin B ; 3-keto Fusidic acid ; Methyl (9Z)-10-oxo-610-diapo-6-carotenoate ; 5-Methoxy-7-(4-hydroxyphenyl)-1-phenyl-3-heptanone ; 4-Hydroxy-55-diisopropyl-22-dimethyl-34-biphenylquinone ; 2-hydroxyethinylestradiol	3M+Na+K ; 2M+H+Na ; 2M+H+Na ; 3M+Na+K ; 3M+Na+K ; 3M+Na+K ; 3M+Na+K ; 3M+Na+K ; 3M+Na+K ; 3M+Na+K ; M+H+2Na ; M+3H ; 3M+Na+K ; 3M+Na+K ; 3M+Na+K ; 3M+Na+K ; 3M+Na+K ; 3M+Na+K ; M+2K ; M+H+2Na ; M+H+2Na ; 2M+3H2O+2H ; M+3H ; M+H+Na ; M+H-2H2O ; M+H-2H2O ; M+H-2H2O ; M+H-2H2O	C11H16O ; C17H19N3 ; C17H19N3 ; C11H16O ; C11H16O ; C11H16O ; C11H16O ; C11H16O ; C11H16O ; C11H16O ; C42H72O13 ; C42H68O16 ; C11H16O ; C11H16O ; C11H16O ; C11H16O ; C11H16O ; C11H16O ; C30H52O4 ; C42H72O13 ; C42H72O13 ; C11H21O6 ; C42H68O16 ; C31H46O7 ; C20H24O3 ; C20H24O3 ; C20H24O3 ; C20H24O3
1076_99.96	1.951717	0.138886	-4.8938	0.000629	3.2014	0.018497	169.5471	HMDB11658 ; HMDB13189 ; HMDB30835 ; HMDB39815	28-Dihydroxyquinoline-beta-D-glucuronide ; 3-Indole carboxylic acid glucuronide ; trans-334557-Hexahydroxyflavanone ; Flusilazole	M+2H ; M+2H ; M+H+NH4 ; M+H+Na	C15H15NO8 ; C15H15NO8 ; C15H12O8 ; C16H15F2N3Si

2748_261.4	1.929903	0.873075	-4.8889	0.000634	3.1982	0.018524	408.0973	HMDB03601 ; HMDB14038 ; HMDB29227 ; HMDB29545 ; HMDB29547 ; HMDB29979 ; HMDB30095 ; HMDB31955 ; HMDB33275 ; HMDB34754 ; HMDB36335 ; HMDB36397 ; HMDB37571 ; HMDB38724 ; HMDB38858 ; HMDB39766 ; HMDB40722 ; HMDB41158	(S)-Reticuline ; N-Deisopropyl-fluvastatin ; 5-Hydroxy-33478-pentamethoxyflavone ; 3-Hydroxy-45678-pentamethoxyflavone ; 4-Hydroxy-35678-pentamethoxyflavone ; 2-Hydroxy-34578-pentamethoxyflavone ; Artemetin ; Dimethyl (1R2S3S)-2-carboxy-3-(34-dihydroxyphenyl)-23-dihydro-56-dihydroxy-1H-indene-1-acetate ; 5-Hydroxyauranetin ; 4-Methoxybenzyl O-(2-sulfoglucoside) ; 246-Phenanthrenetriol 2-O-b-D-glucoside ; Labadoside ; Demethylnobiletin ; xi-Anomuricine ; Oolongtheanin ; Pandamarilactone 31 ; 7-Hydroxy-34568-pentamethoxyflavone ; Cyanidin 3-(6-feruloyl-2-sinapoylsophoroside) 5-glucoside	M+2K+H ; M+K ; 2M+H+K ; 2M+H+K ; 2M+H+K ; 2M+H+K ; 2M+H+K ; 2M+H+K ; 2M+H+K ; 2M+3H2O+2H ; 2M+H+K ; M+Na+K ; 2M+H+K ; M+2K+H ; M+2ACN+2H ; M+2K-H ; 2M+H+K ; M+3Na	C19H23NO4 ; C21H20FNO4 ; C20H20O8 ; C20H20O8 ; C20H20O8 ; C20H20O8 ; C20H20O8 ; C20H20O8 ; C20H20O8 ; C14H20O10S ; C20H20O8 ; C38H42O16 ; C20H20O8 ; C19H23NO4 ; C36H28O17 ; C19H25NO4 ; C20H20O8 ; C54H59O28
5753_69.30	1.944794	0.38025	4.8809	0.000641	3.1931	0.018564	193.0358	HMDB02899 ; HMDB06954 ; HMDB29841 ; HMDB30591 ; HMDB30640 ; HMDB33733 ; HMDB38054 ; HMDB38777 ; HMDB38817 ; HMDB39246 ; HMDB39323 ; HMDB60614 ; HMDB60626 ; HMDB60780	Ellagic acid ; 2-Methyl-3-hydroxy-5-formylpyridine-4-carboxylate ; Vulgaxanthin II ; Dihydro-O-methylsterigmatocystin ; Biochanin A 7-(6-malonylglucoside) ; 24-Dihydroxy-2H-14-benzoxazin-3(4H)-one ; (R)-27-Dihydroxy-2H-14-benzoxazin-3(4H)-one ; 26-Diacetylorientin ; 26-Di-O-acetylisoorientin ; 6-Malonyltrifolirhizin ; 6-O-Malonylglycitin ; Sulindac sulfide ; Malathion monocarboxylic acid ; 5-Hydroxymethyl meloxicam	M+2ACN+2H ; 2M+H+Na ; M+2Na ; M+2Na ; M+H+2Na ; 2M+H+Na ; 2M+H+Na ; M+H+2Na ; M+H+2Na ; M+H+2Na ; M+H+2Na ; M+2Na ; M+2ACN+2H ; M+H+NH4	C14H6O8 ; C8H7NO4 ; C14H16N2O8 ; C19H16O6 ; C25H24O13 ; C8H7NO4 ; C8H7NO4 ; C25H24O13 ; C25H24O13 ; C25H24O13 ; C25H24O13 ; C20H17FO2S ; C8H15O6PS2 ; C14H13N3O5S2
5098_286.4	1.925188	1.020826	4.8796	0.000642	3.1923	0.018564	NA	NA	NA	NA	NA
3506_99.43	1.923443	0.242021	-4.8681	0.000653	3.1848	0.018778	178.5484	HMDB03012 ; HMDB30217 ; HMDB30341 ; HMDB33361 ; HMDB33362 ; HMDB34489 ; HMDB36369 ; HMDB41646 ; HMDB41789 ; HMDB41889 ; HMDB60627	Aniline ; Launobine ; Piplartine ; Norisodomesticine ; (S)-Nandigerine ; ()-Norushinsunine N-oxide ; Annocherine B ; 24-Dihydroxyacetophenone 5-sulfate ; Vanillin 4-sulfate ; Etamiphylline ; Malaoxon	3M+2K ; M+2Na ; M+H+K ; M+2Na ; M+2Na ; M+2Na ; M+2Na ; M+3ACN+2H ; M+3ACN+2H ; M+2K ; M+ACN+2H	C6H7N ; C18H17NO4 ; C17H19NO5 ; C18H17NO4 ; C18H17NO4 ; C18H17NO4 ; C18H17NO4 ; C8H8O6S ; C8H8O6S ; C13H21N5O2 ; C10H19O7PS

5604_101.[illegible]	1.92487	0.34231	-4.8616	0.00066	3.1806	0.018852	453.6606	HMDB12486 ; HMDB33486 ; HMDB40541 ; HMDB59903 ; HMDB61073	(RS)-Norlaudanosoline ; Pipermethystine ; Kaempferol 3-(2-rhamnosyl-6-acetylgalactoside) 7-rhamnoside ; Dibromochloromethane ; noroxymorphone	3M+2Na ; 3M+2Na ; M+3ACN+2H ; 2M+ACN+H ; 3M+2Na	C16H17NO4 ; C16H17NO4 ; C35H42O20 ; CHBr2Cl ; C16H17NO4
7518_107.[illegible]	1.95982	0.36235	-4.8534	0.000668	3.1753	0.018855	243.1088	HMDB28894 ; HMDB29041 ; HMDB29953 ; HMDB40248 ; HMDB40249 ; HMDB00312 ; HMDB00320 ; HMDB00326 ; HMDB00330 ; HMDB00364 ; HMDB00371 ; HMDB00376 ; HMDB00390 ; HMDB00395 ; HMDB00404 ; HMDB00414 ; HMDB00415 ; HMDB00419 ; HMDB00427 ; HMDB00432 ; HMDB00505 ; HMDB00506 ; HMDB00527 ; HMDB00619 ; HMDB00726 ; HMDB00760 ; HMDB00865 ; HMDB00917 ; HMDB00953 ; HMDB10337 ; HMDB11749 ; HMDB13845 ; HMDB14913 ; HMDB30412 ; HMDB31949 ; HMDB34587 ; HMDB39887 ; HMDB40155 ; HMDB40518 ; HMDB40546 ; HMDB41190 ; HMDB41514 ; HMDB42037 ; HMDB60309 ; HMDB61123	Histidinyl-Serine ; Serinyl-Histidine ; D-erythro-D-galacto-octitol ; (SS)-Nt-Histidinylalanine ; (SS)-Np-Histidinylalanine ; 3a7a12b-Trihydroxy-5b-cholanoic acid ; 3a4b7a-Trihydroxy-5b-cholanoic acid ; 1b3a12a-Trihydroxy-5b-cholanoic acid ; 3a4b12a-Trihydroxy-5b-cholanoic acid ; 3a6a7b-Trihydroxy-5b-cholanoic acid ; 1312-Trihydroxycholan-24-oic acid ; 3b7a12a-Trihydroxy-5a-Cholanoic acid ; 3a7b12b-Trihydroxy-5b-cholanoic acid ; 3b7b12a-Trihydroxy-5b-cholanoic acid ; 2b3a7a-Trihydroxy-5b-cholanoic acid ; 1b3a7a-Trihydroxy-5b-cholanoic acid ; 3a6b7b-Trihydroxy-5b-cholanoic acid ; 3b7a12a-Trihydroxy-5b-cholanoic acid ; 3b7b12a-Trihydroxy-5a-Cholanoic acid ; 3a7b12a-Trihydroxy-5a-Cholanoic acid ; Allocholic acid ; Alpha-Muricholic acid ; 6a12a-Dihydroxylithocholic acid ; Cholic acid ; Isovalerylglutamic acid ; Hyocholic acid ; Muricholic acid ; Ursocholic acid ; Suberylglycine ; 6-Dehydrotestosterone glucuronide ; 2-Piperidinone ; SR 49498 ; Tirofiban ; [illegible]	M+H ; M+H ; M+H ; M+H ; M+H ; M+2K ; M+2K ; M+2K ; M+2K ; M+2K ; M+2K ; M+2K ; M+2K ; M+2K ; M+2K ; M+2K ; M+2K ; M+2K ; M+2K ; M+2K ; M+2K ; M+2K ; M+2K ; M+2K ; 2M+H+Na ; M+2K ; M+2K ; M+2K ; 2M+H+Na ; M+H+Na ; 2M+2Na-H ; M+H+K ; M+2Na ; M+2ACN+H ; M+H+NH4 ; 2M+2Na-H ; M+3H ; M+3H ; 2M+2Na-H ; M+3ACN+2H ; M+2ACN+2H ; M+2ACN+2H ; M+2ACN+H ; 2M+2Na-H ; M+H+NH4	C9H14N4O4 ; C9H14N4O4 ; C8H18O8 ; C9H14N4O4 ; C9H14N4O4 ; C24H40O5 ; C24H40O5 ; C24H40O5 ; C24H40O5 ; C24H40O5 ; C24H40O5 ; C24H40O5 ; C24H40O5 ; C24H40O5 ; C24H40O5 ; C24H40O5 ; C24H40O5 ; C24H40O5 ; C24H40O5 ; C24H40O5 ; C24H40O5 ; C24H40O5 ; C24H40O5 ; C24H40O5 ; C10H17NO5 ; C24H40O5 ; C24H40O5 ; C24H40O5 ; C10H17NO5 ; C25H34O8 ; C5H9NO ; C25H30N6O2 ; C22H36N2O5S ; C5H8N2O4 ; C22H29NO10 ; C5H9NO ; C35H50O16 ; C42H46O11 ; C5H9NO ; C17H19N3O6 ; C18H26O10 ; C18H26O10 ; C5H8N2O4 ; C5H9NO ; C26H29NO5S
3913_100.3	1.946828	0.3441	4.8501	0.000671	3.1731	0.018855	120.0089	HMDB33532 ; HMDB37192 ; HMDB41503 ; HMDB59918 ; HMDB61002	Dimethicone ; Potassium 2-(1-ethoxy) ethoxypropanoate ; Bis(2-furanylmethyl) sulfide ; 2-(1-Ethoxyethoxy)propanoic acid ; norsertraline	M+2K ; M+H+K ; M+2Na ; M+2K ; M+3Na	C6H18OSi2 ; C7H13KO4 ; C10H10O2S ; C7H14O4 ; C16H15Cl2N
5257_100.[illegible]	1.94109	0.060547	-4.8532	0.000668	3.1751	0.018855	158.5355	HMDB15469 ; HMDB39955	Magnesium salicylate ; Carotamine	M+H+NH4 ; M+2Na	C14H10MgO6 ; C14H13N3O3
2516_99.3[illegible]	1.936558	0.426527	-4.8447	0.000677	3.1696	0.018902	244.5073	HMDB15486 ; HMDB40229	Cefpodoxime ; 1H-Pyrrolo21-c14thiazine	M+Na+K ; 3M+2K	C15H17N5O6S2 ; C7H7NS

[illegible]8565_100.[illegible]	1.968847	0.513215	-4.8322	0.000689	3.1615	0.019054	406.1369	HMDB13202 ; HMDB14502 ; HMDB14546 ; HMDB15178 ; HMDB15329 ; HMDB29510 ; HMDB30447 ; HMDB34436 ; HMDB38092 ; HMDB40442 ; HMDB60833	6-Keto-decanoylcarnitine ; Mefloquine ; Eszopiclone ; Gatifloxacin ; Zopiclone ; Garcinone B ; Phomopsin A ; Rotenone ; Peonidin 3-(6-p-coumaroyl-glucoside) 5-glucoside ; Dictyoquinazol C ; N-Acetylserotonin glucuronide	M+2K-H ; 2M+3H2O+2H ; M+NH4 ; 2M+Na+K ; M+NH4 ; 2M+H+Na ; M+H+Na ; 2M+H+Na ; M+ACN+2H ; M+ACN+Na ; 2M+H+Na	C17H31NO5 ; C17H16F6N2O ; C17H17ClN6O3 ; C19H22FN3O4 ; C17H17ClN6O3 ; C23H22O6 ; C36H45ClN6O12 ; C23H22O6 ; C38H41O17 ; C18H18N2O5 ; C18H22N2O8
[illegible]8838_96.9	1.954194	0.487496	-4.8319	0.00069	3.1613	0.019054	468.0709	HMDB07297 ; HMDB07326 ; HMDB07354 ; HMDB07379 ; HMDB07436 ; HMDB07464 ; HMDB07493 ; HMDB07521 ; HMDB07550 ; HMDB07605 ; HMDB07606 ; HMDB07632 ; HMDB07633 ; HMDB07660 ; HMDB07687 ; HMDB07802 ; HMDB07829 ; HMDB07830 ; HMDB14877 ; HMDB29498 ; HMDB31899 ; HMDB39718 ; HMDB41272 ; HMDB56079 ; HMDB56101 ; HMDB56107 ; HMDB56129 ; HMDB56235 ; HMDB56256 ; HMDB56269 ; HMDB56276 ; HMDB59998	DG(18:3(6Z9Z12Z)24:1(15Z)0:0) ; DG(18:3(9Z12Z15Z)24:1(15Z)0:0) ; DG(18:4(6Z9Z12Z15Z)24:00:0) ; DG(20:022:4(7Z10Z13Z16Z)0:0) ; DG(20:2(11Z14Z)22:2(13Z16Z)0:0) ; DG(20:3(5Z8Z11Z)22:1(13Z)0:0) ; DG(20:3(8Z11Z14Z)22:1(13Z)0:0) ; DG(20:4(5Z8Z11Z14Z)22:00:0) ; DG(20:4(8Z11Z14Z17Z)22:00:0) ; DG(22:020:4(5Z8Z11Z14Z)0:0) ; DG(22:020:4(8Z11Z14Z17Z)0:0) ; DG(22:1(13Z)20:3(5Z8Z11Z)0:0) ; DG(22:1(13Z)20:3(8Z11Z14Z)0:0) ; DG(22:2(13Z16Z)20:2(11Z14Z)0:0) ; DG(22:4(7Z10Z13Z16Z)20:00:0) ; DG(24:018:4(6Z9Z12Z15Z)0:0) ; DG(24:1(15Z)18:3(6Z9Z12Z)0:0) ; DG(24:1(15Z)18:3(9Z12Z15Z)0:0) ; Hetacillin ; 3-Methoxyfukiic acid ; Theacitrin C ; Samarangenin B ; Uralenneoside ; DG(20:00:022:4n6) ; DG(22:00:020:4n6) ; DG(22:00:020:4n3) ; DG(24:00:018:4n3) ; DG(20:3n90:022:1n9) ; DG(22:1n90:020:3n6) ; DG(24:1n90:018:3n6) ; DG(24:1n90:018:3n3) ; Diphenol [illegible]	2M+3H ; 2M+3H ; 2M+3H ; 2M+3H ; 2M+3H ; 2M+3H ; 2M+3H ; 2M+3H ; 2M+3H ; 2M+3H ; 2M+3H ; 2M+3H ; 2M+3H ; 2M+3H ; 2M+3H ; 2M+3H ; 2M+3H ; 2M+3H ; M+2K+H ; 3M+2K ; M+H+Na ; M+H+Na ; 3M+2K ; 2M+3H ; 2M+3H ; 2M+3H ; 2M+3H ; 2M+3H ; 2M+3H ; 2M+3H ; 2M+3H ; 3M+2K	C45H80O5 ; C45H80O5 ; C45H80O5 ; C45H80O5 ; C45H80O5 ; C45H80O5 ; C45H80O5 ; C45H80O5 ; C45H80O5 ; C45H80O5 ; C45H80O5 ; C45H80O5 ; C45H80O5 ; C45H80O5 ; C45H80O5 ; C45H80O5 ; C45H80O5 ; C45H80O5 ; C19H23N3O4S ; C12H14O8 ; C44H32O22 ; C44H32O22 ; C12H14O8 ; C45H80O5 ; C45H80O5 ; C45H80O5 ; C45H80O5 ; C45H80O5 ; C45H80O5 ; C45H80O5 ; C45H80O5 ; C12H14O8

6374_67.78	1.954963	0.099758	-4.8135	0.000709	3.1493	0.019319	218.1136	HMDB00856 ; HMDB28685 ; HMDB28701 ; HMDB28790 ; HMDB29142 ; HMDB29189 ; HMDB14326 ; HMDB61126 ; HMDB00034 ; HMDB12153 ; HMDB14977 ; HMDB29389 ; HMDB30274 ; HMDB31537 ; HMDB33939 ; HMDB38174 ; HMDB59856 ; HMDB60953 ; HMDB60954	N-a-Acetylcitrulline ; Alanyl-Glutamine ; Alanyl-Gamma-glutamate ; Glutaminyl-Alanine ; Gamma-glutamyl-Alanine ; 5-Hydroxysebacate ; Flunisolide ; 45-Dihydro-drospirenone-3-sulfate ; Adenine ; 34-Dihydroxybenzylamine ; Tolazamide ; ()-threo-2-Amino-34-dihydroxybutanoic acid ; (E)-Casimiroedine ; 3-Methylcyclohexanethiol ; Domoic acid ; 5-Acetyl-24-dimethyloxazole ; Ethyl 2-pyrrolecarboxylate ; 2-hydroxymexiletine ; p-hydroxymexiletine	M+H ; M+H ; M+H ; M+H ; M+H ; M+H ; M+2H ; M+2H ; M+2ACN+H ; 3M+H+NH4 ; M+3ACN+2H ; M+2ACN+H ; M+H+NH4 ; 3M+2Na ; M+3ACN+2H ; 3M+H+NH4 ; 3M+H+NH4 ; M+Na ; M+Na	C8H15N3O4 ; C8H15N3O4 ; C8H15N3O4 ; C8H15N3O4 ; C8H15N3O4 ; C10H17O5 ; C24H31FO6 ; C24H34O5S ; C5H5N5 ; C7H9NO2 ; C14H21N3O3S ; C4H9NO4 ; C21H27N3O6 ; C7H14S ; C15H21NO6 ; C7H9NO2 ; C7H9NO2 ; C11H17NO2 ; C11H17NO2
9404_143.5	1.939202	0.9291	-4.8118	0.000711	3.1482	0.019319	232.0369	HMDB30789 ; HMDB32645 ; HMDB33145 ; HMDB35086 ; HMDB36346 ; HMDB39820 ; HMDB01319	Dracunculin ; 3-Glucosyl-23446-pentahydroxybenzophenone ; Azimsulfuron ; Buntansin A ; 7-Hydroxy-2-methyl-4-oxo-4H-1-benzopyran-5-carboxylic acid ; 8-Methoxy-67-methylenedioxycoumarin ; Pyridoxine 5-phosphate	2M+H+Na ; M+H+K ; M+H+K ; 2M+H+Na ; 2M+H+Na ; 2M+H+Na ; M+H-H2O	C11H8O5 ; C19H20O11 ; C13H16N10O5S ; C11H8O5 ; C11H8O5 ; C11H8O5 ; C8H12NO6P
9735_96.69	1.918294	0.614273	4.8134	0.000709	3.1492	0.019319	180.522	HMDB01919 ; HMDB02238 ; HMDB02372 ; HMDB03563 ; HMDB15536 ; HMDB30191 ; HMDB31812 ; HMDB32845 ; HMDB34669 ; HMDB39521 ; HMDB40940	Famotidine ; L-Homocysteine sulfonic acid ; N-Phenylacetylphenylalanine ; Morphinone ; Imipenem ; N-2-Hydroxy-2-(4-hydroxyphenyl)ethylcinnamide ; Oxfendazole ; 2-Methyl(3-phenylpropanoyl)aminobenzoic acid ; Anaxagoreine ; N-(p-Hydroxyphenyl)ethyl p-hydroxycinnamide ; Capsiamide	M+H+Na ; 2M+H+NH4 ; M+2K ; M+2K ; M+Na+K ; M+2K ; M+2Na ; M+2K ; M+2K ; M+2K ; 2M+3H	C8H15N7O2S3 ; C3H9NO3S2 ; C17H17NO3 ; C17H17NO3 ; C12H17N3O4S ; C17H17NO3 ; C15H13N3O3S ; C17H17NO3 ; C17H17NO3 ; C17H17NO3 ; C17H35NO

8513_273.2	1.924262	0.964615	-4.8037	0.00072	3.1429	0.019449	274.0919	HMDB14883 ; HMDB00101 ; HMDB00118 ; HMDB00333 ; HMDB00423 ; HMDB00755 ; HMDB01983 ; HMDB02643 ; HMDB03254 ; HMDB04061 ; HMDB15285 ; HMDB29232 ; HMDB29273 ; HMDB29646 ; HMDB30809 ; HMDB31360 ; HMDB32916 ; HMDB33624 ; HMDB34751 ; HMDB36610 ; HMDB37274 ; HMDB38117 ; HMDB38662 ; HMDB39586 ; HMDB39800 ; HMDB40197 ; HMDB41270 ; HMDB41566 ; HMDB59763	Modafinil ; Deoxyadenosine ; Homovanillic acid ; Isohomovanillic acid ; 34-Dihydroxyhydrocinnamic acid ; Hydroxyphenyllactic acid ; 5-Deoxyadenosine ; 3-(3-Hydroxyphenyl)-3-hydroxypropanoic acid ; Muramic acid ; 3-Methoxy-4-hydroxyphenylglycolaldehyde ; Thiamylal ; 3-Hydroxyphenyllactate ; 26-Dimethoxybenzoic acid ; 26-Dihydroxy-4-methoxyacetophenone ; (2R3S)-Piscidic acid ; L-cis-Cyclo(aspartylphenylalanyl) ; 2-(Methylthiomethyl)furan ; ()-2-Hydroxy-3-(2-hydroxyphenyl)propanoic acid ; Ligustroside ; 2-Methyl-3-(methylthio)furan ; Maltol propionate ; 6-O-Oleuropeoylsucrose ; N-(1-Deoxy-1-fructosyl)alanine ; 2-(1-Mercaptoethyl)furan ; 5-Methyl-2-furanmethanethiol ; 25-Dimethyl-3-furanthiol ; 24-Dihydroxy-6-methoxyacetophenone ; 2-Methyl-5-(methylthio)furan ; 34-Dimethoxybenzoic acid	M+H ; M+Na ; 3M+2H ; 3M+2H ; 3M+2H ; 3M+2H ; M+Na ; 3M+2H ; M+Na ; 3M+2H ; 2M+H+K ; 3M+2H ; 3M+2H ; 3M+2H ; M+NH4 ; 2M+H+Na ; 2M+NH4 ; 3M+2H ; M+H+Na ; 2M+NH4 ; 3M+2H ; M+H+K ; M+Na ; 2M+NH4 ; 2M+NH4 ; 2M+NH4 ; 3M+2H ; 2M+NH4 ; 3M+2H	C15H15NO2S ; C10H13N5O3 ; C9H10O4 ; C9H10O4 ; C9H10O4 ; C9H10O4 ; C10H13N5O3 ; C9H10O4 ; C9H17NO7 ; C9H10O4 ; C12H18N2O2S ; C9H10O4 ; C9H10O4 ; C9H10O4 ; C11H12O7 ; C13H14N2O4 ; C6H8OS ; C9H10O4 ; C25H32O12 ; C6H8OS ; C9H10O4 ; C22H36O13 ; C9H17NO7 ; C6H8OS ; C6H8OS ; C6H8OS ; C9H10O4 ; C6H8OS ; C9H10O4
9916_286.4	1.916027	0.287184	4.7899	0.000735	3.1339	0.019749	323.521	HMDB15250	Amiodarone	M+2H	C25H29I2NO3
0969_48.94	1.889506	0.134431	-4.7638	0.000764	3.1168	0.020433	288.9291	HMDB01059 ; HMDB01187 ; HMDB03848 ; HMDB04527 ; HMDB59586	Inositol 1345-tetraphosphate ; 1D-Myo-inositol 1346-tetrakisphosphate ; D-Myo-inositol 3456-tetrakisphosphate ; 1D-Myo-inositol 1456-tetrakisphosphate ; Perfluorooctanesulfonic acid	M+2K ; M+2K ; M+2K ; M+2K ; M+2K	C6H16O18P4 ; C6H16O18P4 ; C6H16O18P4 ; C6H16O18P4 ; C8HF17O3S

5082_32.73	1.900052	0.001641	4.7475	0.000783	3.1061	0.020831	276.1805	HMDB13131 ; HMDB07879 ; HMDB07889 ; HMDB07911 ; HMDB07971 ; HMDB07972 ; HMDB07982 ; HMDB07983 ; HMDB08003 ; HMDB08013 ; HMDB08014 ; HMDB08035 ; HMDB08042 ; HMDB08067 ; HMDB08073 ; HMDB08074 ; HMDB08100 ; HMDB08106 ; HMDB08107 ; HMDB08138 ; HMDB08169 ; HMDB08170 ; HMDB08202 ; HMDB08203 ; HMDB08234 ; HMDB08263 ; HMDB08295 ; HMDB08365 ; HMDB08398 ; HMDB08429 ; HMDB08462 ; HMDB08623 ; HMDB08908 ; HMDB09516 ; HMDB35267	Hydroxyhexanoycarnitine ; PC(14:020:1(11Z)) ; PC(14:022:4(7Z10Z13Z16Z)) ; PC(14:1(9Z)20:0) ; PC(16:018:1(11Z)) ; PC(16:018:1(9Z)) ; PC(16:020:4(5Z8Z11Z14Z)) ; PC(16:020:4(8Z11Z14Z17Z)) ; PC(16:1(9Z)18:0) ; PC(16:1(9Z)20:3(5Z8Z11Z)) ; PC(16:1(9Z)20:3(8Z11Z14Z)) ; PC(18:016:1(9Z)) ; PC(18:018:4(6Z9Z12Z15Z)) ; PC(18:1(11Z)16:0) ; PC(18:1(11Z)18:3(6Z9Z12Z)) ; PC(18:1(11Z)18:3(9Z12Z15Z)) ; PC(18:1(9Z)16:0) ; PC(18:1(9Z)18:3(6Z9Z12Z)) ; PC(18:1(9Z)18:3(9Z12Z15Z)) ; PC(18:2(9Z12Z)18:2(9Z12Z)) ; PC(18:3(6Z9Z12Z)18:1(11Z)) ; PC(18:3(6Z9Z12Z)18:1(9Z)) ; PC(18:3(9Z12Z15Z)18:1(11Z)) ; PC(18:3(9Z12Z15Z)18:1(9Z)) ; PC(18:4(6Z9Z12Z15Z)18:0) ; PC(20:014:1(9Z)) ; PC(20:1(11Z)14:0) ; PC(20:3(5Z8Z11Z)16:1(9Z)) ; PC(20:3(8Z11Z14Z)16:1(9Z)) ; PC(20:4(5Z8Z11Z14Z)16:0) ;	M+H ; M+3Na ; M+H+2Na ; M+3Na ; M+3Na ; M+3Na ; M+H+2Na ; M+H+2Na ; M+3Na ; M+H+2Na ; M+H+2Na ; M+3Na ; M+H+2Na ; M+3Na ; M+H+2Na ; M+H+2Na ; M+3Na ; M+H+2Na ; M+H+2Na ; M+H+2Na ; M+H+2Na ; M+H+2Na ; M+H+2Na ; M+H+2Na ; M+H+2Na ; M+3Na ; M+3Na ; M+H+2Na ; M+H+2Na ; M+H+2Na ; M+H+2Na ; M+H+2Na ; M+3Na ; M+3Na ; M+H+K	C13H25NO5 ; C42H82NO8P ; C44H80NO8P ; C42H82NO8P ; C42H82NO8P ; C42H82NO8P ; C44H80NO8P ; C44H80NO8P ; C42H82NO8P ; C44H80NO8P ; C44H80NO8P ; C42H82NO8P ; C44H80NO8P ; C42H82NO8P ; C44H80NO8P ; C44H80NO8P ; C42H82NO8P ; C44H80NO8P ; C44H80NO8P ; C44H80NO8P ; C44H80NO8P ; C44H80NO8P ; C44H80NO8P ; C44H80NO8P ; C42H82NO8P ; C42H82NO8P ; C44H80NO8P ; C44H80NO8P ; C44H80NO8P ; C44H80NO8P ; C44H80NO8P ; C42H82NO8P ; C42H82NO8P ; C33H52O4
5006_101.1	1.918794	0.614655	-4.7353	0.000798	3.0981	0.021105	178.5335	HMDB00370 ; HMDB15367 ; HMDB28743 ; HMDB29100 ; HMDB61168	2-Amino-3-phosphonopropionic acid ; Bromodiphenhydramine ; Asparaginyl-Tyrosine ; Tyrosyl-Asparagine ; N2-Monodes-methylnizatidine	2M+H+NH4 ; M+H+Na ; M+Na+K ; M+Na+K ; M+H+K	C3H8NO5P ; C17H20BrNO ; C13H17N3O5 ; C13H17N3O5 ; C11H19N5O2S2
35194_100.	1.946545	0.36659	-4.7282	0.000806	3.0935	0.021109	495.1185	HMDB01157 ; HMDB14560 ; HMDB14837	2-Methylacetoacetyl-CoA ; Metocurine Iodide ; Nicergoline	M+3ACN+2H ; M+2ACN+2H ; 2M+H+Na	C26H42N7O18P3S ; C40H48I2N2O6 ; C24H26BrN3O3

7335_94.1[illegible]	1.895329	1.012021	-4.7306	0.000803	3.0951	0.021109	199.0557	HMDB40164 ; HMDB37058 ; HMDB00402 ; HMDB02025 ; HMDB04448 ; HMDB12156 ; HMDB12281 ; HMDB12295 ; HMDB29169 ; HMDB30234 ; HMDB30487 ; HMDB30573 ; HMDB30709 ; HMDB31572 ; HMDB31712 ; HMDB31993 ; HMDB32420 ; HMDB32426 ; HMDB34341 ; HMDB34837 ; HMDB35129 ; HMDB35467 ; HMDB35698 ; HMDB35789 ; HMDB36837 ; HMDB38113 ; HMDB38480 ; HMDB38481 ; HMDB38903 ; HMDB38999 ; HMDB39081 ; HMDB41113 ; HMDB41172 ; HMDB41276	4-Methyldibenzothiophene ; Thiazopyr ; 2-Isopropylmalic acid ; 23-Dimethyl-3-hydroxyglutaric acid ; Estradiol-17beta 3-sulfate ; 3-Isopropylmalate ; Portulacaxanthin II ; Trimethyl sulfonium ; 3-Hydroxy-2-methylglutarate ; Xanthohumol C ; Artonol A ; Sanshodiol ; Dihydrocubebin ; 2-Methyl-4-propyl-13-oxathiane ; ()-Glycerol 12-diacetate ; Glyurallin A ; 2-(4-Methyl-5-thiazolyl)ethyl formate ; (-)-3-(Methylthio)heptanal ; Physalin L ; Lansiumarin A ; Citrunobin ; Glicophenone ; (-)-Matairesinol ; Matairesinol ; Isocolumbin ; 5-Deoxykievitone hydrate ; Gancaonin G ; Gancaonin A ; Gancaonin M ; 6-Gingesulfonic acid ; Physalin O ; Artonin U ; 1-Methoxyphaseollin ; (7x8x)-47-Epoxy-38-bilign-7-ene-35-dimethoxy-499-triol	M+H ; M+2H ; M+Na ; M+Na ; M+2Na ; M+Na ; M+H+Na ; 2M+2Na-H ; M+Na ; M+2Na ; M+2Na ; M+H+K ; M+H+K ; M+K ; M+Na ; M+2Na ; 2M+3H2O+2H ; M+K ; M+3Na ; M+2Na ; M+2Na ; M+H+K ; M+H+K ; M+H+K ; M+H+K ; M+H+K ; M+2Na ; M+2Na ; M+2Na ; M+H+K ; M+3Na ; M+2Na ; M+2Na ; M+H+K	C13H10S ; C16H17F5N2O2S ; C7H12O5 ; C7H12O5 ; C18H24O5S ; C7H12O5 ; C18H18N2O7 ; C3H9S ; C7H12O5 ; C21H20O5 ; C21H20O5 ; C20H22O6 ; C20H22O6 ; C8H16OS ; C7H12O5 ; C21H20O5 ; C7H9NO2S ; C8H16OS ; C28H32O10 ; C21H20O5 ; C21H20O5 ; C20H22O6 ; C20H22O6 ; C20H22O6 ; C20H22O6 ; C20H22O6 ; C21H20O5 ; C21H20O5 ; C21H20O5 ; C17H26O6S ; C28H32O10 ; C21H20O5 ; C21H20O5 ; C20H22O6
9082_100.[illegible]	1.950759	0.575138	-4.7194	0.000817	3.0876	0.021282	NA	NA	NA	NA	NA
4161_80.5[illegible]	1.957762	0.713819	-4.7137	0.000824	3.084	0.021325	275.0344	HMDB14526 ; HMDB37080 ; HMDB37081 ; HMDB37082 ; HMDB38069 ; HMDB38808 ; HMDB39177 ; HMDB39343 ; HMDB39344 ; HMDB39345 ; HMDB39346 ; HMDB41384 ; HMDB41808	Tacrine ; 3-Methylellagic acid 8-(2-acetylrhamnoside) ; 3-Methylellagic acid 8-(3-acetylrhamnoside) ; 3-Methylellagic acid 8-(4-acetylrhamnoside) ; Gomphrenol 3-methylether 4-glucuronide ; Luteolin 3-(3-acetylglucuronide) ; 1-O-Galloylglycerol ; 1345-Tetra-O-galloylquinic acid ; 3-Digalloyl-45-di-O-galloylquinic acid ; 5-O-Digalloyl-34-di-O-galloylquinic acid ; 4-O-Digalloyl-35-di-O-galloylquinic acid ; Luteolin 3-(4-acetylglucuronide) ; 44-Methylenedianiline	M+2K-H ; M+2Na ; M+2Na ; M+2Na ; M+2Na ; M+2Na ; 2M+Na+K ; M+2H+Na ; M+2H+Na ; M+2H+Na ; M+2H+Na ; M+2Na ; M+2K-H	C13H14N2 ; C23H20O13 ; C23H20O13 ; C23H20O13 ; C23H20O13 ; C23H20O13 ; C10H12O7 ; C35H28O22 ; C35H28O22 ; C35H28O22 ; C35H28O22 ; C23H20O13 ; C13H14N2
3607_99.86	1.93185	0.493264	-4.711	0.000828	3.0822	0.021325	451.0636	HMDB32738 ; HMDB29503 ; HMDB40609 ; HMDB15138	CI Food Red 6 ; Kaempferol 3-(36-diacetyl-24-di-p-coumaroylrhamnoside) ; Theaflavic acid ; Cromoglicic acid	M+H ; M+2K ; M+Na ; M+H-H2O	C19H18N2O7S2 ; C43H36O17 ; C21H16O10 ; C23H16O11

8123_31.9	1.922114	0.246179	4.7078	0.000832	3.08	0.021325	181.0178	HMDB31671 ; HMDB32115 ; HMDB02273 ; HMDB02467 ; HMDB02655 ; HMDB29497 ; HMDB30547 ; HMDB30804 ; HMDB31473 ; HMDB31474 ; HMDB33052 ; HMDB33764 ; HMDB35227 ; HMDB36179 ; HMDB38446 ; HMDB38515 ; HMDB38572 ; HMDB39210 ; HMDB40250 ; HMDB41271 ; HMDB41389 ; HMDB41391 ; HMDB60495	246-Trimethyl-135-trithiane ; 1-(Methylthio)ethyl 2-propenyl disulfide ; 4-Hydroxy-L-glutamic acid ; A-Ketoglutaric acid oxime ; Isorhamnetin ; Dimethyl fukiic acid ; 3457-Tetrahydroxy-6-methoxyflavone ; 2-(34-Dihydroxyphenyl)-56-dihydroxy-7-methoxy-4H-1-benzopyran-4-one ; 13-Dithiane ; 14-Dithiane ; Ethyl vinyl disulfide ; Erucin ; Hydroxypropanedioic acid ; 2-Methyl-13-dithiolane ; 1-Thiocyanato-4-(methylthio)butane ; L-gamma-Glutamyl-S-allylthio-L-cysteine ; Pollenitin ; 1358-Tetrahydroxy-6-methoxy-2-methylanthraquinone ; xi-Tetrahydro-2-thiophenethiol ; Salicylic acid beta-D-glucoside ; Methyl 2-propenyl disulfide ; (E)-Methyl 1-propenyl disulfide ; N-Formyl-L-aspartate	M+H ; M+H ; 2M+H+K ; 2M+H+K ; M+2Na ; M+Na+K ; M+2Na ; M+2Na ; 3M+2H ; 3M+2H ; 3M+2H ; 2M+H+K ; 3M+2H ; 3M+2H ; 2M+H+K ; M+H+K ; M+2Na ; M+2Na ; 3M+2H ; M+Na+K ; 3M+2H ; 3M+2H ; 2M+H+K	C6H12S3 ; C6H12S3 ; C5H7NO5 ; C5H7NO5 ; C16H12O7 ; C13H16O8 ; C16H12O7 ; C16H12O7 ; C4H8S2 ; C4H8S2 ; C4H8S2 ; C6H11NS2 ; C3H4O5 ; C4H8S2 ; C6H11NS2 ; C11H18N2O5S2 ; C16H12O7 ; C16H12O7 ; C4H8S2 ; C13H16O8 ; C4H8S2 ; C4H8S2 ; C5H7NO5
6622_296.	1.914484	0.427986	4.6972	0.000845	3.0731	0.021559	425.0437	HMDB41868 ; HMDB41893	Desacetylcefotaxime ; Fenitrothion	2M+H+Na ; 3M+H+NH4	C14H15N5O6S2 ; C9H12NO5PS
9233_286.	1.912965	1.109668	4.6853	0.000861	3.0652	0.021842	442.9559	HMDB39491	118-Diamino-5914-triazaoctadecane	3M+H+Na	C15H37N5
4191_98.4	1.928928	0.205378	4.6795	0.000868	3.0614	0.021923	202.9894	HMDB00126 ; HMDB02520 ; HMDB38471 ; HMDB38472	Glycerol 3-phosphate ; Beta-Glycerophosphoric acid ; Luteolin 4-sulfate ; Luteolin 7-sulfate	2M+Na+K ; 2M+Na+K ; M+H+K ; M+H+K	C3H9O6P ; C3H9O6P ; C15H10O9S ; C15H10O9S
4457_100.	1.886697	0.647866	-4.6743	0.000875	3.0579	0.021988	198.0224	HMDB14374 ; HMDB33542 ; HMDB60950 ; HMDB04148 ; HMDB06275	Cefotiam ; Gomphidic acid ; fluvoxamino acid ; Dopamine 4-sulfate ; Dopamine 3-O-sulfate	M+3Na ; M+H+Na ; M+2K ; M+H-2H2O ; M+H-2H2O	C18H23N9O4S3 ; C18H12O9 ; C14H17F3N2O3 ; C8H11NO5S ; C8H11NO5S

;6536_100.9	1.933643	0.622599	-4.6211	0.000949	3.0228	0.023489	534.6887	HMDB06591 ; HMDB11668 ; HMDB32602 ; HMDB57026 ; HMDB57027 ; HMDB57036 ; HMDB57037 ; HMDB57276 ; HMDB57277 ; HMDB57302 ; HMDB57310 ; HMDB57312 ; HMDB57320 ; HMDB57322 ; HMDB57323 ; HMDB57325 ; HMDB57350 ; HMDB57358 ; HMDB57360 ; HMDB57368 ; HMDB57370 ; HMDB57371 ; HMDB57392 ; HMDB57393 ; HMDB57396 ; HMDB57397 ; HMDB58217 ; HMDB58218 ; HMDB58227 ; HMDB58228 ; HMDB58264 ; HMDB58265 ; HMDB58274 ; HMDB58275 ; HMDB58610 ; HMDB58611 ; HMDB58620 ; HMDB58621 ; HMDB58647 ; HMDB58648 ; HMDB58657 ; HMDB58658 ; HMDB59209 ; HMDB59217 ; HMDB59219 ; HMDB59227 ; HMDB59229 ; HMDB59230 ; HMDB59258 ; HMDB59259 ; HMDB59268 ; HMDB59269 ; HMDB59279 ; HMDB59280 ; HMDB59289 ; HMDB59290 ;	Lactosamine ; 6-(alpha-D-Glucosaminyl)-1D-myo-inositol ; 4-O-Methylkanzonol W ; CL(18:018:022:5(4Z7Z10Z13Z16Z)22:5(4Z7Z10Z13Z16Z)) ; CL(18:018:022:5(4Z7Z10Z13Z16Z)22:5(7Z10Z13Z16Z19Z)) ; CL(18:018:022:5(7Z10Z13Z16Z19Z)22:5(4Z7Z10Z13Z16Z)) ; CL(18:018:022:5(7Z10Z13Z16Z19Z)22:5(7Z10Z13Z16Z19Z)) ; CL(18:022:5(4Z7Z10Z13Z16Z)18:022:5(4Z7Z10Z13Z16Z)) ; CL(18:022:5(4Z7Z10Z13Z16Z)18:022:5(7Z10Z13Z16Z19Z)) ; CL(18:022:5(4Z7Z10Z13Z16Z)22:5(4Z7Z10Z13Z16Z)18:0) ; CL(18:022:5(4Z7Z10Z13Z16Z)22:5(4Z7Z10Z13Z16Z)22:6(4Z7Z10Z13Z16Z19Z)) ; CL(18:022:5(4Z7Z10Z13Z16Z)22:5(7Z10Z13Z16Z19Z)18:0) ; CL(18:022:5(4Z7Z10Z13Z16Z)22:5(7Z10Z13Z16Z19Z)22:6(4Z7Z10Z13Z16Z19Z)) ; CL(18:022:5(4Z7Z10Z13Z16Z)22:6(4Z7Z10Z13Z16Z19Z)22:5(4Z7Z10Z13Z16Z)) ;	3M+2Na ; 3M+2Na ; 3M+H+NH4 ; M+H+2Na ; M+H+2Na ; M+H+2Na ; M+H+2Na ; M+H+2Na ; M+H+2Na ; M+H+2Na ; M+3H ; M+H+2Na ; M+3H ; M+3H ; M+3H ; M+H+2Na ; M+H+2Na ; M+3H ; M+H+2Na ; M+3H ; M+H+2Na ; M+3H ; M+H+2Na ; M+3H ; M+3H ; M+3H ; M+3H ; M+3H ; M+3H ; M+3H ; M+3H ; M+3H ; M+3H ; M+3H ; M+3H ; M+3H ; M+3H ; M+3H ; M+H+2Na ; M+3H ; M+3H ; M+3H ; M+3H ; M+3H ; M+3H ; M+3H ; M+3H	C12H23NO10 ; C12H23NO10 ; C21H18O5 ; C89H154O17P2 ; C89H154O17P2 ; C89H154O17P2 ; C89H154O17P2 ; C89H154O17P2 ; C89H154O17P2 ; C89H154O17P2 ; C93H150O17P2 ; C89H154O17P2 ; C93H150O17P2 ; C93H150O17P2 ; C93H150O17P2 ; C89H154O17P2 ; C89H154O17P2 ; C93H150O17P2 ; C89H154O17P2 ; C93H150O17P2 ; C93H150O17P2 ; C93H150O17P2 ; C93H150O17P2 ; C93H150O17P2 ; C93H150O17P2 ; C93H150O17P2 ; C93H150O17P2 ; C93H150O17P2 ; C93H150O17P2 ; C93H150O17P2 ;

4429_51.5	1.915476	0.362469	4.6235	0.000946	3.0243	0.023489	131.0344	HMDB00620 ; HMDB00634 ; HMDB00749 ; HMDB02092 ; HMDB13233 ; HMDB33809 ; HMDB59743 ; HMDB60365 ; HMDB61185 ; HMDB11675 ; HMDB15247 ; HMDB15604 ; HMDB28848 ; HMDB28995 ; HMDB32671 ; HMDB33311 ; HMDB33669 ; HMDB34188 ; HMDB34411 ; HMDB34692 ; HMDB35665 ; HMDB36935 ; HMDB37445 ; HMDB60832 ; HMDB00539 ; HMDB00867 ; HMDB59750 ; HMDB60255 ; HMDB60256	Glutaconic acid ; Citraconic acid ; Mesaconic acid ; Itaconic acid ; Gamma-delta-Dioxovaleric acid ; Methyl hydrogen fumarate ; 2-Hydroxyglutaric acid lactone ; 25-Dioxopentanoate ; 2-Pentendioate ; DHAP(10:0) ; Nifedipine ; Permethrin ; Glycyl-Phenylalanine ; Phenylalanyl-Glycine ; (E)-244-Trihydroxy-3-prenylchalcone ; Moracin I ; Phaseollidin ; (R)-Glabridin ; Phaseollinisoflavan ; 9-(34-Dimethoxyphenyl)-2-methoxy-1H-phenalen-1-one ; L-1234-Tetrahydro-beta-carboline-3-carboxylic acid ; Dimethyl 2-galloylgalactarate ; (S)-57-Dihydroxy-6-prenylflavanone ; meta-O-Dealkylated flecainide lactam ; Arabinonic acid ; Ribonic acid ; 2345-Tetrahydroxypentanoic acid ; L-Lyxonate ; L-Xylonate	M+H ; M+H ; M+H ; M+H ; M+H ; M+H ; M+H ; M+H ; M+H ; M+H ; M+3Na ; M+H+2Na ; M+3H ; M+H+K ; M+H+K ; M+3Na ; M+3Na ; M+3Na ; M+3Na ; M+3Na ; M+H+2Na ; M+2Na ; M+3H ; M+3Na ; M+H+2Na ; M+H-2H2O ; M+H-2H2O ; M+H-2H2O ; M+H-2H2O ; M+H-2H2O	C5H6O4 ; C5H6O4 ; C5H6O4 ; C5H6O4 ; C5H6O4 ; C5H6O4 ; C5H6O4 ; C5H6O4 ; C5H6O4 ; C13H25O7P ; C17H18N2O6 ; C21H20Cl2O3 ; C11H14N2O3 ; C11H14N2O3 ; C20H20O4 ; C20H20O4 ; C20H20O4 ; C20H20O4 ; C20H20O4 ; C22H18O4 ; C12H12N2O2 ; C15H18O12 ; C20H20O4 ; C15H17F3N2O4 ; C5H10O6 ; C5H10O6 ; C5H10O6 ; C5H10O6 ; C5H10O6

5275_68.12	1.913598	1.015504	4.6265	0.000941	3.0263	0.023489	149.0587	HMDB00567 ; HMDB00930 ; HMDB31725 ; HMDB32947 ; HMDB35243 ; HMDB36626 ; HMDB40986 ; HMDB11685 ; HMDB28765 ; HMDB29101 ; HMDB30772 ; HMDB38518 ; HMDB40873 ; HMDB40931 ; HMDB60885 ; HMDB60886 ; HMDB60887 ; HMDB60888 ; HMDB00418 ; HMDB01944 ; HMDB14399 ; HMDB14897 ; HMDB29234 ; HMDB29507 ; HMDB30045 ; HMDB30105 ; HMDB30710 ; HMDB30713 ; HMDB30861 ; HMDB30863 ; HMDB31927 ; HMDB32162 ; HMDB32770 ; HMDB32773 ; HMDB33197 ; HMDB33804 ; HMDB33921 ; HMDB34098 ; HMDB34132 ; HMDB35689 ; HMDB36047 ; HMDB36889 ; HMDB38879 ; HMDB38905 ; HMDB38918 ; HMDB39660 ; HMDB40702 ; HMDB40929 ; HMDB59661 ; HMDB61033	Cinnamic acid ; trans-Cinnamic acid ; (E)-3-(2-Hydroxyphenyl)-2-propenal ; Di-2-furanylmethane ; 1-Phenyl-12-propanedione ; 34-Dihydro-2H-1-benzopyran-2-one ; (E)-3-(4-Hydroxyphenyl)-2-propenal ; DHAP(8:0) ; Aspartyl-Tyrosine ; Tyrosyl-Aspartate ; Demethoxyegonol ; Desmosflavone ; Dimethylstrobochrysin ; 1-(4-Hydroxy-3-methoxyphenyl)-5-(4-hydroxyphenyl)-14-pentadien-3-one ; 4-Hydroxy-R-phenprocoumon ; 6-Hydroxy-R-phenprocoumon ; 8-Hydroxy-R-phenprocoumon ; 7-Hydroxy-R-phenprocoumon ; 18-Hydroxycortisol ; Chlorpheniramine ; Doxycycline ; Tetracycline ; N-4-hydroxy-(E)-cinnamoyl-L-aspartic acid ; Mulberrin ; Isohumulinone A ; Humulinone ; Cyclomammeisin ; Mammea AAB cyclo F ; Kuwanon D ; Kuwanon F ; Glyurallin B ; Acrylic acid-2-acrylamido-2-methyl propane sulfonic acid copolymer ; Eremopetasitenin C1 ; Eremopetasitenin D1 ; 2244-Tetramethyl-6-(1-oxobutyl)-135-	M+H ; M+H ; M+H ; M+H ; M+H ; M+H ; M+H ; M+2H ; M+2H ; M+2H ; M+2H ; M+2H ; M+2H ; M+2H ; M+2H ; M+2H ; M+2H ; M+2H ; M+3Na ; M+H+Na ; M+3H ; M+3H ; M+H+NH4 ; M+2H+Na ; M+3Na ; M+3Na ; M+2H+Na ; M+2H+Na ; M+2H+Na ; M+2H+Na ; M+2H+Na ; M+H+NH4 ; M+3Na ; M+3Na ; M+2Na ; M+2H+Na ; M+H+Na ; M+2H+Na ; M+H+Na ; M+H+2Na ; M+2Na ; M+3Na ; M+2H+Na ; M+2H+Na ; M+H+2Na ; M+H+Na ; M+3Na ; M+H+Na ; M+2Na ; M+3Na	C9H8O2 ; C9H8O2 ; C9H8O2 ; C9H8O2 ; C9H8O2 ; C9H8O2 ; C9H8O2 ; C11H21O7P ; C13H16N2O6 ; C13H16N2O6 ; C18H16O4 ; C18H16O4 ; C18H16O4 ; C18H16O4 ; C18H16O4 ; C18H16O4 ; C18H16O4 ; C18H16O4 ; C21H30O6 ; C16H19ClN2 ; C22H24N2O8 ; C22H24N2O8 ; C13H13NO6 ; C25H26O6 ; C21H30O6 ; C21H30O6 ; C25H26O6 ; C25H26O6 ; C25H26O6 ; C25H26O6 ; C25H26O6 ; C10H17NO6S ; C21H30O6 ; C21H30O6 ; C14H20O4 ; C25H26O6 ; C16H18O4 ; C25H26O6 ; C16H18O4 ; C23H28O6 ; C14H20O4 ; C21H30O6 ; C25H26O6 ; C25H26O6 ; C23H28O6 ; C16H18O4 ; C21H30O6 ; C16H18O4 ; C14H20O4 ; C21H30O6
9493_287.6	1.880928	0.442134	4.5832	0.001006	2.9975	0.024771	469.0672	HMDB40628 ; HMDB00061 ; HMDB00960 ; HMDB01341 ; HMDB33352 ; HMDB38409 ; HMDB38830 ; HMDB39302 ; HMDB39304 ; HMDB39772 ; HMDB59614	3-(23-Digalloyl-46-hexahydroxydiphenoylglucosyl)-phloroacetophenone ; Adenosine 35-diphosphate ; dGDP ; ADP ; Cyclobrassinin ; 6-(Methylthio)hexyl glucosinolate ; Theasinensin F ; Epicatechin 3-O-gallate-(4beta-6)-epigallocatechin 3-O-gallate ; Epicatechin 3-O-gallate-(4beta-8)-epigallocatechin 3-O-gallate ; Epigallocatechin 3-O-gallate-(4beta-6)-epicatechin 3-O-gallate ; Riboflavin cyclic-45-phosphate	M+2H ; M+ACN+H ; M+ACN+H ; M+ACN+H ; 2M+H ; 2M+H+K ; M+H+K ; M+H+K ; M+H+K ; M+H+K ; 2M+Na+K	C42H32O25 ; C10H15N5O10P2 ; C10H15N5O10P2 ; C10H15N5O10P2 ; C11H10N2S2 ; C14H27NO9S3 ; C44H34O21 ; C44H34O21 ; C44H34O21 ; C44H34O21 ; C17H19N4O8P

5149_168.5	1.846469	0.098937	-4.573	0.001022	2.9908	0.025037	349.1355	HMDB05005 ; HMDB06046 ; HMDB14917 ; HMDB15305 ; HMDB15552 ; HMDB15553 ; HMDB28786 ; HMDB29080 ; HMDB29450 ; HMDB29707 ; HMDB34406 ; HMDB38228 ; HMDB59744 ; HMDB61100	Tolnaftate ; 5a-Androst-3-en-17-one ; Nalidixic Acid ; Phenobarbital ; Acepromazine ; Aceprometazine ; Cysteinyl-Tryptophan ; Tryptophyl-Cysteine ; Aspergillomarasmine A ; 2-Amino-34-dimethylimidazo45-fquinoline ; 5alpha-Androst-16-en-3-one ; 4-O-alpha-D-Galactopyranosylcalystegine B2 ; 34-Methyleneazelaic acid ; 6-oxo-famciclovir	M+ACN+H ; M+2K-H ; 3M+2H ; 3M+2H ; M+Na ; M+Na ; M+ACN+H ; M+ACN+H ; M+ACN+H ; 3M+Na+K ; M+2K-H ; 2M+H+Na ; 3M+Na+K ; 2M+H+Na	C19H17NOS ; C19H28O ; C12H12N2O3 ; C12H12N2O3 ; C19H22N2OS ; C19H22N2OS ; C14H17N3O3S ; C14H17N3O3S ; C10H17N3O8 ; C12H12N4 ; C19H28O ; C13H23NO9 ; C11H16O4 ; C14H19N5O5
1455_27.89	1.889549	0.551337	-4.555	0.00105	2.9787	0.025506	449.2661	HMDB06059 ; HMDB10331 ; HMDB12549 ; HMDB12551 ; HMDB14633 ; HMDB29559 ; HMDB36863 ; HMDB37062 ; HMDB37123 ; HMDB59616	20-Carboxy-leukotriene B4 ; Palmitoyl glucuronide ; 12-oxo-1011-dihydro-20-COOH-LTB4 ; 12-oxo-20-dihydroxy-leukotriene B4 ; Buspirone ; (4-Hydroxybenzoyl)choline ; Anhydrocinnzeylanol ; Sporotrichiol ; Goyaglycoside h ; all-trans-Decaprenyl diphosphate	M+2ACN+H ; 2M+Na+K ; M+2ACN+H ; M+2ACN+H ; M+ACN+Na ; 2M+H ; M+2ACN+H ; M+2ACN+H ; M+2ACN+2H ; M+H+K	C20H30O6 ; C22H42O7 ; C20H30O6 ; C20H30O6 ; C21H31N5O2 ; C12H18NO3 ; C20H30O6 ; C20H30O6 ; C42H70O15 ; C50H84O7P2
5765_99.31	1.876474	0.674018	4.5546	0.001051	2.9785	0.025506	418.9696	HMDB04122	Selenocystine	M+2ACN+H	C6H12N2O4Se2
1754_112.0	1.917571	0.286667	-4.5408	0.001073	2.9693	0.025927	225.0982	HMDB00833 ; HMDB12109 ; HMDB13627 ; HMDB14629 ; HMDB15506 ; HMDB29864 ; HMDB30247 ; HMDB31414 ; HMDB31582 ; HMDB31686 ; HMDB32446 ; HMDB32447 ; HMDB33238 ; HMDB34095 ; HMDB35501 ; HMDB39558 ; HMDB39609 ; HMDB39844 ; HMDB39886 ; HMDB40246 ; HMDB40272 ; HMDB41301	N-Glycolylneuraminic acid ; 56-Dihydroxyprostaglandin F1a ; Cervonoyl ethanolamide ; Nabilone ; Phenazopyridine ; 2-Amino-38-dimethyl-3H-imidazo45-fquinoxaline ; Oxynarcotine ; 23-Dimethyl-2-cyclohexen-1-one ; 6-Methyl-35-heptadien-2-one ; (EE)-24-Octadienal ; trans trans-35-Octadien-2-one ; 15-Octadien-3-one ; 2-Isopropyl-5-methylfuran ; Gambogic acid ; Licoagrochalcone B ; 1alpha-O-Methylquassin ; Curcumin III ; (EE)-26-Octadienal ; 16-Methyl-epi-nigakihemiacetal B ; 355-Trimethyl-2-cyclopenten-1-one ; 2-Butylfuran ; (3R 6Z)-34-Dihydro-8-hydroxy-3-(6-pentadecenyl)-1H-2-benzopyran-1-one	M+3ACN+2H ; M+Na+K ; M+2K ; M+2K ; 2M+H+Na ; 2M+H+Na ; M+H+NH4 ; 3M+2K ; 3M+2K ; 3M+2K ; 3M+2K ; 3M+2K ; 3M+2K ; M+H+2Na ; 2M+3H ; M+2Na ; 2M+3H ; 3M+2K ; M+2Na ; 3M+2K ; 3M+2K ; M+2K	C11H19NO10 ; C20H36O7 ; C24H36O3 ; C24H36O3 ; C11H11N5 ; C11H11N5 ; C22H25NO8 ; C8H12O ; C8H12O ; C8H12O ; C8H12O ; C8H12O ; C8H12O ; C38H44O8 ; C21H20O4 ; C23H32O6 ; C21H20O4 ; C8H12O ; C23H32O6 ; C8H12O ; C8H12O ; C24H36O3

)6964_100.3	1.929876	0.22485	-4.5223	0.001105	2.9568	0.02605	178.0507	HMDB40612 ; HMDB31981 ; HMDB00839 ; HMDB14742 ; HMDB15102 ; HMDB15104 ; HMDB30555 ; HMDB31744 ; HMDB33310 ; HMDB36635 ; HMDB41298 ; HMDB60386 ; HMDB60395	4-Acetyl-2(3H)-benzoxazolone ; Niazidin ; Pentaporphyrin I ; Cisapride ; Desloratadine ; Alosetron ; Tocopheronic acid ; Artocarbene ; Moracin C ; Hydroxytanshinone ; Moracin N ; 4-Fluoromuconolactone ; 5-Fluoromuconolactone	M+H ; M+2H ; M+2Na ; M+3Na ; M+2Na ; M+Na+K ; M+Na+K ; M+2Na ; M+2Na ; M+2Na ; M+2Na ; M+NH4 ; M+NH4	C9H7NO3 ; C15H18N2O6S ; C20H14N4 ; C23H29ClFN3O4 ; C19H19ClN2 ; C17H18N4O ; C16H22O5 ; C19H18O4 ; C19H18O4 ; C19H18O4 ; C19H18O4 ; C6H5FO4 ; C6H5FO4
4083_100.6	1.927781	0.620017	-4.5224	0.001104	2.9569	0.02605	534.1884	HMDB01892 ; HMDB33765 ; HMDB60577 ; HMDB60631 ; HMDB60718 ; HMDB60802	Menadione ; Methyl (Z)-2-decene-468-triynoate ; Clomipramine N-oxide ; Hydroxyclomipramine ; 2-Hydroxyclomipramine ; 8-Hydroxyclomipramine	3M+NH4 ; 3M+NH4 ; 3M+2K ; 3M+2K ; 3M+2K ; 3M+2K	C11H8O2 ; C11H8O2 ; C19H23ClN2O ; C19H23ClN2O ; C19H23ClN2O ; C19H23ClN2O
5603_114.(	1.903911	0.148791	-4.5257	0.001099	2.9591	0.02605	149.0186	HMDB00008 ; HMDB00011 ; HMDB00023 ; HMDB00336 ; HMDB00357 ; HMDB00442 ; HMDB00710 ; HMDB00729 ; HMDB13968 ; HMDB13969 ; HMDB14406 ; HMDB14717 ; HMDB15175 ; HMDB28781 ; HMDB28970 ; HMDB28982 ; HMDB29045 ; HMDB31212 ; HMDB41173 ; HMDB59748	2-Hydroxybutyric acid ; (R)-3-Hydroxybutyric acid ; (S)-3-Hydroxyisobutyric acid ; (R)-3-Hydroxyisobutyric acid ; 3-Hydroxybutyric acid ; (S)-3-Hydroxybutyric acid ; 4-Hydroxybutyric acid ; Alpha-Hydroxyisobutyric acid ; Hydroxyzileuton ; Zileuton sulfoxide ; Anagrelide ; Carbenicillin ; Thalidomide ; Cysteinyl-Methionine ; Methionyl-Cysteine ; Methionyl-Serine ; Serinyl-Methionine ; Ethoxyacetic acid ; 1-O-Caffeoyl-(b-D-glucose 6-O-sulfate) ; 2-Methyl-3-hydroxypropanoate	M+2Na-H ; M+2Na-H ; M+2Na-H ; M+2Na-H ; M+2Na-H ; M+2Na-H ; M+2Na-H ; M+2Na-H ; M+2Na ; M+2Na ; M+ACN+2H ; M+3Na ; M+H+K ; M+2Na ; M+2Na ; M+Na+K ; M+Na+K ; M+2Na-H ; M+2H+Na ; M+2Na-H	C4H8O3 ; C4H8O3 ; C4H8O3 ; C4H8O3 ; C4H8O3 ; C4H8O3 ; C4H8O3 ; C4H8O3 ; C11H12N2O3S ; C11H12N2O3S ; C10H7Cl2N3O ; C17H18N2O6S ; C13H10N2O4 ; C8H16N2O3S2 ; C8H16N2O3S2 ; C8H16N2O4S ; C8H16N2O4S ; C4H8O3 ; C15H18O12S ; C4H8O3
9874_279.3	1.899701	0.674409	4.5222	0.001105	2.9568	0.02605	275.489	HMDB12340 ; HMDB12414 ; HMDB12440	PS(14:022:6(4Z7Z10Z13Z16Z19Z)) ; PS(18:3(9Z12Z15Z)18:3(9Z12Z15Z)) ; PS(22:6(4Z7Z10Z13Z16Z19Z)14:0)	M+H+2Na ; M+H+2Na ; M+H+2Na	C42H70NO10P ; C42H70NO10P ; C42H70NO10P
2766_49.07	1.858712	0.299649	-4.5336	0.001085	2.9644	0.02605	250.9553	HMDB41188	Bis(methylsulfonylmethyl) disulfide	M+H	C4H10O4S4
6879_46.52	1.921582	0.138556	4.5068	0.001131	2.9465	0.026307	124.9227	HMDB03227 ; HMDB31258	Methanethiol ; Lenthionine	M+2K-H ; M+Na+K	CH4S ; C2H4S5

3812_111.7	1.881677	0.445571	-4.5106	0.001125	2.949	0.026307	141.0324	HMDB00639 ; HMDB00663 ; HMDB00718 ; HMDB00892 ; HMDB02176 ; HMDB02209 ; HMDB14713 ; HMDB15231 ; HMDB29302 ; HMDB29881 ; HMDB30058 ; HMDB30968 ; HMDB31175 ; HMDB31241 ; HMDB31247 ; HMDB31249 ; HMDB31516 ; HMDB32230 ; HMDB32263 ; HMDB32589 ; HMDB32929 ; HMDB33217 ; HMDB33642 ; HMDB33742 ; HMDB33890 ; HMDB34237 ; HMDB35019 ; HMDB35133 ; HMDB36347 ; HMDB37492 ; HMDB37679 ; HMDB38998 ; HMDB39053 ; HMDB39146 ; HMDB39799 ; HMDB39974 ; HMDB40424 ; HMDB40549 ; HMDB40575 ; HMDB40797 ; HMDB40898 ; HMDB41992 ; HMDB59678	Galactaric acid ; Glucaric acid ; Isovaleric acid ; Valeric acid ; Ethylmethylacetic acid ; Equol ; Fenoprofen ; Flucytosine ; 1-Phenyl-67-dihydroxy-isochroman ; D-Glucaric acid ; Ethyl propionate ; 246-Octatriyn-1-ol ; Tetrahydro-2-furanmethanol ; Isopropyl acetate ; 2-Methylpropyl formate ; Methyl isobutyrate ; 3-Hydroxy-2-pentanone ; 24-Dimethyl-13-dioxolane ; Ethylene oxidepropylene oxide copolymer ; 1-(5-Acetyl-2-hydroxyphenyl)-3-methyl-1-butanone ; Benzofuran ; (2xi6xi)-7-Methyl-3-methylene-1267-octanetetrol ; (2xi3xi6E)-37-Dimethyl-6-octene-1238-tetrol ; (S)-2-Methylbutanoic acid ; Methyl butyrate ; Propyl acetate ; Phenethyl salicylate ; 37-Dimethyl-3-octene-1267-tetrol ; 23-Dihydroxy-13-diphenyl-1-propanone ; Ethyl methyl-p-tolylglycidate ; Guaicyl phenylacetate ; 357-Octatriyn-1-ol ; (1alpha2alpha4betaH6alpha8R)-p-Menthane-2689-tetrol ; Citrubuntin ; Tetrahydro-2-methyl-3-furanol ; (1S2S4R8R)-p-Menthane-1289-tetrol ; Ethyl 2-benzylacetoacetate ; xi-23-	2M+3H ; 2M+3H ; M+K ; M+K ; M+K ; M+H+K ; M+H+K ; 2M+H+Na ; M+H+K ; 2M+3H ; M+K ; M+Na ; M+K ; M+K ; M+K ; M+K ; M+K ; M+K ; M+K ; M+Na+K ; M+Na ; M+2K ; M+2K ; M+K ; M+K ; M+K ; M+H+K ; M+2K ; M+H+K ; M+Na+K ; M+H+K ; M+Na ; M+2K ; M+H+K ; M+K ; M+2K ; M+Na+K ; M+Na ; M+K ; M+3Na ; M+3Na ; M+K ; M+K	C6H10O8 ; C6H10O8 ; C5H10O2 ; C5H10O2 ; C5H10O2 ; C15H14O3 ; C15H14O3 ; C4H4FN3O ; C15H14O3 ; C6H10O8 ; C5H10O2 ; C8H6O ; C5H10O2 ; C5H10O2 ; C5H10O2 ; C5H10O2 ; C5H10O2 ; C5H10O2 ; C5H10O2 ; C13H16O3 ; C8H6O ; C10H20O4 ; C10H20O4 ; C5H10O2 ; C5H10O2 ; C5H10O2 ; C15H14O3 ; C10H20O4 ; C15H14O3 ; C13H16O3 ; C15H14O3 ; C8H6O ; C10H20O4 ; C15H14O3 ; C5H10O2 ; C10H20O4 ; C13H16O3 ; C8H6O ; C5H10O2 ; C17H22O8 ; C17H22O8 ; C5H10O2 ; C5H10O2
6236_22.75	1.871469	0.822825	-4.5073	0.00113	2.9468	0.026307	705.5306	HMDB56398 ; HMDB56487 ; HMDB56496 ; HMDB56922 ; HMDB11533 ; HMDB11564 ; HMDB31074	CL(16:016:018:018:0) ; CL(16:018:016:018:0) ; CL(16:018:018:016:0) ; CL(18:016:018:016:0) ; MG(0:016:00:0) ; MG(16:00:00:0) ; Glycerol 1-hexadecanoate	M+2H ; M+2H ; M+2H ; M+2H ; 2M+2Na-H ; 2M+2Na-H ; 2M+2Na-H	C77H150O17P2 ; C77H150O17P2 ; C77H150O17P2 ; C77H150O17P2 ; C19H38O4 ; C19H38O4 ; C19H38O4

)6799_95.28	1.9305	0.159507	-4.5025	0.001139	2.9436	0.026357	248.0507	HMDB15017 ; HMDB00618 ; HMDB00868 ; HMDB01489 ; HMDB01548 ; HMDB01866 ; HMDB06534 ; HMDB11734 ; HMDB12195 ; HMDB13198 ; HMDB14446 ; HMDB29650 ; HMDB37264 ; HMDB38363 ; HMDB39360 ; HMDB40293 ; HMDB60026	Emtricitabine ; D-Ribulose 5-phosphate ; Xylulose 5-phosphate ; Ribose 1-phosphate ; D-Ribose 5-phosphate ; 34-Dihydroxymandelic acid ; D-Xylulose 1-phosphate ; D-Arabinose 5-phosphate ; Beta-L-arabinose 1-phosphate ; 4-O-Methylgallic acid ; Flucloxacillin ; Methyl 246-trihydroxybenzoate ; 2-Hydroxy-47-dimethoxy-2H-14-benzoxazin-3(4H)-one ; (-)-Epigallocatechin 3-(3-methyl-gallate) ; ()-Flufenprox ; (-)-Epigallocatechin 3-(4-methyl-gallate) ; Vanilloylglycine	M+H ; M+NH4 ; M+NH4 ; M+NH4 ; M+NH4 ; M+ACN+Na ; M+NH4 ; M+NH4 ; M+NH4 ; M+ACN+Na ; M+ACN+2H ; M+ACN+Na ; M+Na ; M+H+Na ; M+2Na ; M+H+Na ; M+Na	C8H10FN3O3S ; C5H11O8P ; C5H11O8P ; C5H11O8P ; C5H11O8P ; C8H8O5 ; C5H11O8P ; C5H11O8P ; C5H11O8P ; C8H8O5 ; C19H17ClFN3O5S ; C8H8O5 ; C10H11NO5 ; C23H20O11 ; C24H22ClF3O3 ; C23H20O11 ; C10H11NO5
)0853_43.19	1.931843	0.279792	4.4927	0.001156	2.937	0.026637	NA	NA	NA	NA	NA
'8729_54.83	1.936319	0.777342	4.4816	0.001176	2.9295	0.026874	244.0279	HMDB32982 ; HMDB39421	22-Dithenyl sulfide ; Fenugreekine	M+NH4 ; M+3Na	C10H10S3 ; C21H27N7O14P2
3421_54.02	1.872497	0.041526	4.4811	0.001177	2.9292	0.026874	149.0263	HMDB01553 ; HMDB00267 ; HMDB00805 ; HMDB00884 ; HMDB01369 ; HMDB01843 ; HMDB02234 ; HMDB02331 ; HMDB04086 ; HMDB04813 ; HMDB06239 ; HMDB06801 ; HMDB13870 ; HMDB13871 ; HMDB13872 ; HMDB15457 ; HMDB15619 ; HMDB35993 ; HMDB41441 ; HMDB60262 ; HMDB60587 ; HMDB61093	2-Oxo-4-methylthiobutanoic acid ; Pyroglutamic acid ; Pyrrolidonecarboxylic acid ; Ribothymidine ; Pyrroline hydroxycarboxylic acid ; N-Acryloylglycine ; 1-Pyrroline-4-hydroxy-2-carboxylate ; Imidazoleacetic acid riboside ; 5-Hydroxy-N-formylkynurenine ; 3-Methyluridine ; S-aminomethyldihydrolipoamide ; 2-Oxo-3-hydroxy-4-phosphobutanoic acid ; cistrans-5-Hydroxythalidomide ; 5-Hydroxythalidomide ; Thalidomide arene oxide ; Dihydroxyaluminium ; Sulfathiazole ; N5-(34-Dioxo-15-cyclohexadien-1-yl)-L-glutamine ; 11-Methylgerberinol ; 5-Oxoprolinate ; Aminoethoxyacetic acid ; dimethadione	M+H ; 2M+H+K ; 2M+H+K ; M+H+K ; 2M+H+K ; 2M+H+K ; 2M+H+K ; M+H+K ; M+2Na ; M+H+K ; M+Na+K ; M+2ACN+2H ; M+H+Na ; M+H+Na ; M+H+Na ; 2M+H+Na ; M+ACN+2H ; M+2Na ; M+3Na ; 2M+H+K ; 2M+Na+K ; 2M+H+K	C5H8O3S ; C5H7NO3 ; C5H7NO3 ; C10H14N2O6 ; C5H7NO3 ; C5H7NO3 ; C5H7NO3 ; C10H14N2O6 ; C11H12N2O5 ; C10H14N2O6 ; C9H20N2OS2 ; C4H7O8P ; C13H10N2O5 ; C13H10N2O5 ; C13H10N2O5 ; C2H8AlNO4 ; C9H9N3O2S2 ; C11H12N2O5 ; C22H18O6 ; C5H7NO3 ; C4H8NO3 ; C5H7NO3

[illegible]6019_51.78[illegible]	1.854064	0.357386	-4.4676	0.001202	2.9201	0.027317	169.0356	HMDB00289 ; HMDB01241 ; HMDB04181 ; HMDB05033 ; HMDB15109 ; HMDB15373 ; HMDB30120 ; HMDB31852 ; HMDB34271 ; HMDB39030 ; HMDB40141 ; HMDB40143 ; HMDB40622 ; HMDB40623 ; HMDB41638 ; HMDB41639 ; HMDB41640	Uric acid ; 2-Aminomuconic acid ; Methylimidazole acetaldehyde ; Valdecoxib ; Edetic Acid ; Chloroxine ; (S)-Angelicain ; 2-Methoxy-3-methylpyrazine ; Hypericin ; trans-Grandmarin ; 2-Methoxy-5-methylpyrazine ; 2-Methoxy-6-methylpyrazine ; 3-(2-Galloylglucosyl)-phloroacetophenone ; 3-(6-Galloylglucosyl)-phloroacetophenone ; (-)-Epigallocatechin 3-glucuronide ; (-)-Epigallocatechin 3-glucuronide ; (-)-Epigallocatechin 7-glucuronide	M+H ; 2M+H+Na ; M+2Na-H ; M+H+Na ; M+2Na ; M+3ACN+2H ; M+2Na ; M+2Na-H ; M+3H ; M+2Na ; M+2Na-H ; M+2Na-H ; M+2H+Na ; M+2H+Na ; M+2H+Na ; M+2H+Na ; M+2H+Na	C5H4N4O3 ; C6H7NO4 ; C6H8N2O ; C16H14N2O3S ; C10H16N2O8 ; C9H5Cl2NO ; C15H16O6 ; C6H8N2O ; C30H16O8 ; C15H16O6 ; C6H8N2O ; C6H8N2O ; C21H22O13 ; C21H22O13 ; C21H22O13 ; C21H22O13 ; C21H22O13
[illegible]8216_21.8[illegible]	1.858869	0.93187	-4.463	0.00121	2.9171	0.027385	434.2878	HMDB08255 ; HMDB08511 ; HMDB08733 ; HMDB13335 ; HMDB14940 ; HMDB28956 ; HMDB32564 ; HMDB38162 ; HMDB38163 ; HMDB39596 ; HMDB39598 ; HMDB60730 ; HMDB14393 ; HMDB60159	PC(18:4(6Z9Z12Z15Z)22:6(4Z7Z10Z13Z16Z19Z)) ; PC(20:5(5Z8Z11Z14Z17Z)20:5(5Z8Z11Z14Z17Z)) ; PC(22:6(4Z7Z10Z13Z16Z19Z)18:4(6Z9Z12Z15Z)) ; 3-Hydroxyhexadecadienoylcarnitine ; Alfentanil ; Lysyl-Lysine ; 13-Diphenylpropane ; Lactarazulene ; Lactarofulvene ; Colupox a ; Colupox b ; 20 22-Dihydrodigoxigenin ; Cabergoline ; Ebastine	M+ACN+2H ; M+ACN+2H ; M+ACN+2H ; M+Na ; M+NH4 ; 3M+2Na ; 2M+ACN+H ; 2M+ACN+H ; 2M+ACN+H ; M+NH4 ; M+NH4 ; M+ACN+H ; M+H-H2O ; M+H-2H2O	C48H76NO8P ; C48H76NO8P ; C48H76NO8P ; C23H41NO5 ; C21H32N6O3 ; C12H26N4O3 ; C15H16 ; C15H16 ; C15H16 ; C25H36O5 ; C25H36O5 ; C23H36O5 ; C26H37N5O2 ; C32H39NO2
[illegible]3187_38.4[illegible]	1.916436	0.179851	-4.4548	0.001226	2.9115	0.027443	136.0143	HMDB06343 ; HMDB02123 ; HMDB03219 ; HMDB15427 ; HMDB29901 ; HMDB29935 ; HMDB29952 ; HMDB31070 ; HMDB36431 ; HMDB37808 ; HMDB37809 ; HMDB38556 ; HMDB40150 ; HMDB40258 ; HMDB40580	Selenocystathionine ; 137-Trimethyluric acid ; Sedoheptulose ; Ceftizoxime ; L-Galacto-2-heptulose ; D-Manno-2-heptulose ; D-altro-D-manno-Heptose ; (2E11Z)-5-5-(Methylthio)-4-penten-2-ynyl-2-furanacrolein ; 139-Trimethyluric acid ; Hexahydro-24-dimethylspiro13-dithiolo45-cfuran-23(2H)-furan ; Hexahydro-23alpha-dimethylspiro13-dithiolo45-bfuran-23(2H)-furan ; xi-Dihydro-2-methyl-3(2H)-thiophenone ; Dihydro-5-methyl-2(3H)-thiophenone ; xi-2-Acetylthietane ; Dihydro-2-methyl-3(2H)-furanthione	M+2H ; M+Na+K ; M+Na+K ; M+2H+Na ; M+Na+K ; M+Na+K ; M+Na+K ; M+H+K ; M+Na+K ; M+H+K ; M+H+K ; 2M+H+K ; 2M+H+K ; 2M+H+K ; 2M+H+K	C7H14N2O4Se ; C8H10N4O3 ; C7H14O7 ; C13H13N5O5S2 ; C7H14O7 ; C7H14O7 ; C7H14O7 ; C13H12O2S ; C8H10N4O3 ; C10H16O2S2 ; C10H16O2S2 ; C5H8OS ; C5H8OS ; C5H8OS ; C5H8OS

6466_67.2	1.883243	0.505904	-4.4554	0.001225	2.9119	0.027443	292.1021	HMDB33296 ; HMDB35217 ; HMDB00706 ; HMDB02381 ; HMDB11167 ; HMDB14632 ; HMDB15378 ; HMDB15593 ; HMDB28759 ; HMDB28760 ; HMDB28969 ; HMDB28991 ; HMDB31985 ; HMDB35191 ; HMDB38327 ; HMDB39926 ; HMDB41580 ; HMDB42023 ; HMDB61039	Kuwanon Y ; 10-Acetoxyligustroside ; L-Aspartyl-L-phenylalanine ; N-Acetylcystathionine ; L-beta-aspartyl-L-phenylalanine ; Sotalol ; Docetaxel ; Ixabepilone ; Aspartyl-Methionine ; Aspartyl-Phenylalanine ; Methionyl-Aspartate ; Phenylalanyl-Aspartate ; gamma-Glutamyl-S-methylcysteine ; (2S4R)-4-(9H-Pyrido34-bindol-1-yl)-124-butanetriol ; (3b9R)-5-Megastigmene-39-diol 9-apiosyl-(1-6)-glucoside ; Lyoniresinol 9-sulfate ; 4-Megastigmene-6a9R-diol 9-apiosyl-(1-6)-glucoside ; Taxotere ; modafinil acid	M+2H ; M+2H ; 2M+H+Na ; 2M+3H2O+2H ; 2M+H+Na ; 2M+H+K ; M+3Na ; M+2K ; 2M+3H2O+2H ; 2M+H+Na ; 2M+3H2O+2H ; 2M+H+Na ; 2M+3H2O+2H ; 2M+H+K ; M+2K ; M+2ACN+2H ; M+2K ; M+3Na ; M+NH4	C34H30O9 ; C27H34O14 ; C13H16N2O5 ; C9H16N2O5S ; C13H16N2O5 ; C12H20N2O3S ; C43H53NO14 ; C27H42N2O5S ; C9H16N2O5S ; C13H16N2O5 ; C9H16N2O5S ; C13H16N2O5 ; C9H16N2O5S ; C15H16N2O3 ; C24H42O11 ; C22H28O11S ; C24H42O11 ; C43H53NO14 ; C15H14O3S
6891_51.0	1.843946	0.711365	4.453	0.00123	2.9103	0.027443	149.0587	HMDB00567 ; HMDB00930 ; HMDB31725 ; HMDB32947 ; HMDB35243 ; HMDB36626 ; HMDB40986 ; HMDB11685 ; HMDB28765 ; HMDB29101 ; HMDB30772 ; HMDB38518 ; HMDB40873 ; HMDB40931 ; HMDB60885 ; HMDB60886 ; HMDB60887 ; HMDB60888 ; HMDB00418 ; HMDB01944 ; HMDB14399 ; HMDB14897 ; HMDB29234 ; HMDB29507 ; HMDB30045 ; HMDB30105 ; HMDB30710 ; HMDB30713 ; HMDB30861 ; HMDB30863 ; HMDB31927 ; HMDB32162 ; HMDB32770 ; HMDB32773 ; HMDB33197 ; HMDB33804 ; HMDB33921 ; HMDB34098 ; HMDB34132 ; HMDB35689 ; HMDB36047 ; HMDB36889 ; HMDB38879 ; HMDB38905 ; HMDB38918 ; HMDB39660 ; HMDB40702 ; HMDB40929 ; HMDB59661 ; HMDB61033	Cinnamic acid ; trans-Cinnamic acid ; (E)-3-(2-Hydroxyphenyl)-2-propenal ; Di-2-furanylmethane ; 1-Phenyl-12-propanedione ; 34-Dihydro-2H-1-benzopyran-2-one ; (E)-3-(4-Hydroxyphenyl)-2-propenal ; DHAP(8:0) ; Aspartyl-Tyrosine ; Tyrosyl-Aspartate ; Demethoxyegonol ; Desmosflavone ; Dimethylstrobochrysin ; 1-(4-Hydroxy-3-methoxyphenyl)-5-(4-hydroxyphenyl)-14-pentadien-3-one ; 4-Hydroxy-R-phenprocoumon ; 6-Hydroxy-R-phenprocoumon ; 8-Hydroxy-R-phenprocoumon ; 7-Hydroxy-R-phenprocoumon ; 18-Hydroxycortisol ; Chlorpheniramine ; Doxycycline ; Tetracycline ; N-4-hydroxy-(E)-cinnamoyl-L-aspartic acid ; Mulberrin ; Isohumulinone A ; Humulinone ; Cyclomammeisin ; Mammea AAB cyclo F ; Kuwanon D ; Kuwanon F ; Glyurallin B ; Acrylic acid-2-acrylamido-2-methyl propane sulfonic acid copolymer ; Eremopetasitenin C1 ; Eremopetasitenin D1 ; 2244-Tetramethyl-6-(1-oxobutyl)-135- [illegible]	M+H ; M+H ; M+H ; M+H ; M+H ; M+H ; M+H ; M+2H ; M+2H ; M+2H ; M+2H ; M+2H ; M+2H ; M+2H ; M+2H ; M+2H ; M+2H ; M+2H ; M+3Na ; M+H+Na ; M+3H ; M+3H ; M+H+NH4 ; M+2H+Na ; M+3Na ; M+3Na ; M+2H+Na ; M+2H+Na ; M+2H+Na ; M+2H+Na ; M+2H+Na ; M+H+NH4 ; M+3Na ; M+3Na ; M+2Na ; M+2H+Na ; M+H+Na ; M+2H+Na ; M+H+Na ; M+H+2Na ; M+2Na ; M+3Na ; M+2H+Na ; M+2H+Na ; M+H+2Na ; M+H+Na ; M+3Na ; M+H+Na ; M+2Na ; M+3Na	C9H8O2 ; C9H8O2 ; C9H8O2 ; C9H8O2 ; C9H8O2 ; C9H8O2 ; C9H8O2 ; C11H21O7P ; C13H16N2O6 ; C13H16N2O6 ; C18H16O4 ; C18H16O4 ; C18H16O4 ; C18H16O4 ; C18H16O4 ; C18H16O4 ; C18H16O4 ; C18H16O4 ; C21H30O6 ; C16H19ClN2 ; C22H24N2O8 ; C22H24N2O8 ; C13H13NO6 ; C25H26O6 ; C21H30O6 ; C21H30O6 ; C25H26O6 ; C25H26O6 ; C25H26O6 ; C25H26O6 ; C25H26O6 ; C10H17NO6S ; C21H30O6 ; C21H30O6 ; C14H20O4 ; C25H26O6 ; C16H18O4 ; C25H26O6 ; C16H18O4 ; C23H28O6 ; C14H20O4 ; C21H30O6 ; C25H26O6 ; C25H26O6 ; C23H28O6 ; C16H18O4 ; C21H30O6 ; C16H18O4 ; C14H20O4 ; C21H30O6
99936_280.	1.880387	0.268147	4.4449	0.001245	2.9048	0.027546	649.05	HMDB11691	Cytidine 23-cyclic phosphate	2M+K	C9H12N3O7P
3222_165.	1.865298	0.555567	4.4466	0.001242	2.906	0.027546	328.5943	HMDB01176	Cytidine monophosphate N-acetylneuraminic acid	M+ACN+2H	C20H31N4O16P

9706_101.8	1.874235	0.48667	4.4391	0.001256	2.901	0.027608	321.997	HMDB15039	Ethacrynic acid	2M+H+K	C13H12Cl2O4
7842_274.9	1.851178	0.842739	4.4377	0.001259	2.9	0.027608	759.0788	HMDB12208 ; HMDB14836	D-Erythro-imidazole-glycerol-phosphate ; Nitrofurantoin	3M+2Na-H ; 3M+2Na-H	C6H11N2O6P ; C8H6N4O5
9598_104.6	1.888475	0.235371	-4.4345	0.001265	2.8978	0.027625	139.527	HMDB15128 ; HMDB00963 ; HMDB01299 ; HMDB28828 ; HMDB29038 ; HMDB31323 ; HMDB35924	Azathioprine ; 5-Methylthioribose 1-phosphate ; 5-Methylthioribulose 1-phosphate ; Glutamyl-Serine ; Serinyl-Glutamate ; 3-Butenenitrile ; Pyrrole	M+2H ; M+H+NH4 ; M+H+NH4 ; M+2Na ; M+2Na ; 3M+2K ; 3M+2K	C9H7N7O2S ; C6H13O7PS ; C6H13O7PS ; C8H13N2O6 ; C8H13N2O6 ; C4H5N ; C4H5N
55265_289.	1.919843	0.648932	4.4215	0.001291	2.889	0.027947	548.6255	HMDB06402	3-Oxohexadecanoyl-CoA	M+2K	C37H64N7O18P3S
0427_58.32	1.885801	0.751354	4.4223	0.001289	2.8896	0.027947	192.123	HMDB00893 ; HMDB02023 ; HMDB28800 ; HMDB28801 ; HMDB28868 ; HMDB28905 ; HMDB28921 ; HMDB28927 ; HMDB28943 ; HMDB28952 ; HMDB29152 ; HMDB29153 ; HMDB33838 ; HMDB41606 ; HMDB59727 ; HMDB59757 ; HMDB60684	Suberic acid ; Ethyladipic acid ; Glutaminyl-Isoleucine ; Glutaminyl-Leucine ; Hydroxyprolyl-Lysine ; Isoleucyl-Glutamine ; Isoleucyl-Gamma-glutamate ; Leucyl-Glutamine ; Leucyl-Gamma-glutamate ; Lysyl-Hydroxyproline ; Gamma-glutamyl-Isoleucine ; Gamma-glutamyl-Leucine ; Diethyl succinate ; Dimethyl adipate ; 24-Dimethyladipic acid ; 3-Methylpimelic acid ; 2-Propylglutaric acid	M+NH4 ; M+NH4 ; M+3ACN+2H ; M+3ACN+2H ; M+3ACN+2H ; M+3ACN+2H ; M+3ACN+2H ; M+3ACN+2H ; M+3ACN+2H ; M+3ACN+2H ; M+3ACN+2H ; M+3ACN+2H ; M+NH4 ; M+NH4 ; M+NH4 ; M+NH4 ; M+NH4	C8H14O4 ; C8H14O4 ; C11H21N3O4 ; C11H21N3O4 ; C11H21N3O4 ; C11H21N3O4 ; C11H21N3O4 ; C11H21N3O4 ; C11H21N3O4 ; C11H21N3O4 ; C11H21N3O4 ; C11H21N3O4 ; C8H14O4 ; C8H14O4 ; C8H14O4 ; C8H14O4 ; C8H14O4
8698_290.4	1.889107	1.092843	4.4122	0.00131	2.8827	0.028053	714.6449	HMDB29238 ; HMDB29251 ; HMDB29587 ; HMDB38530	Cyanidin 3-O-(6-acetyl-arabinoside) ; Pinotin A aglycone ; Lansiol ; 5-Heptadecyl-13-benzenediol	3M+2Na ; 3M+H+K ; 3M+H+Na ; 2M+NH4	C22H21O11 ; C25H19O9 ; C33H56O ; C23H40O2
5192_254.4	1.885008	0.548087	4.4107	0.001313	2.8818	0.028053	473.1195	HMDB06953 ; HMDB30294 ; HMDB31690 ; HMDB31814 ; HMDB33012 ; HMDB33013 ; HMDB36352 ; HMDB39861 ; HMDB41221 ; HMDB41291	1-Phosphatidyl-D-myo-inositol ; 1-O-E-Cinnamoyl-(6-arabinosylglucose) ; cis-3-Hexenyl b-primeveroside ; Vitisin C ; Vestitone 7-glucoside ; Isohopeaphenol ; Viniferol A ; 3-O-alpha-L-Arabinopyranosylproanthocyanidin A5 ; b-D-Xylopyranosyl-(1-4)-a-L-rhamnopyranosyl-(1-2)-L-arabinose ; r-Viniferin	M+2ACN+H ; 2M+Na+K ; M+2K+H ; M+H+K ; M+K ; M+H+K ; M+H+K ; 2M+3H ; M+2Na-H ; M+H+K	C11H19O13P ; C20H26O11 ; C17H30O10 ; C56H42O12 ; C22H26O9 ; C56H42O12 ; C56H42O12 ; C35H32O16 ; C16H28O13 ; C56H42O12
3073_33.64	1.860128	0.504461	4.4125	0.001309	2.883	0.028053	300.2003	HMDB00151 ; HMDB00429 ; HMDB03114 ; HMDB15090 ; HMDB35623 ; HMDB36909 ; HMDB37216 ; HMDB38257 ; HMDB39089 ; HMDB39754	Estradiol ; 17a-Estradiol ; Neurosporene ; Netilmicin ; beta-Zeacarotene ; alpha-Zeacarotene ; Linalyl phenylacetate ; Geranyl phenylacetate ; (9Z7Z9Z)-78-Dihydrolycopene ; Phygrine	2M+3H2O+2H ; 2M+3H2O+2H ; M+Na+K ; M+3ACN+2H ; M+Na+K ; M+Na+K ; 2M+3H2O+2H ; 2M+3H2O+2H ; M+Na+K ; 2M+H+K	C18H24O2 ; C18H24O2 ; C40H58 ; C21H41N5O7 ; C40H58 ; C40H58 ; C18H24O2 ; C18H24O2 ; C40H58 ; C16H28N2O2

0872_38.2	1.918421	0.18577	-4.3881	0.00136	2.8664	0.028103	154.0441	HMDB38361 ; HMDB38365 ; HMDB00421 ; HMDB01292 ; HMDB01441 ; HMDB03224 ; HMDB11686 ; HMDB12141 ; HMDB12485 ; HMDB12881 ; HMDB14524 ; HMDB14671 ; HMDB14692 ; HMDB28865 ; HMDB28886 ; HMDB28920 ; HMDB28942 ; HMDB29130 ; HMDB29131 ; HMDB29576 ; HMDB34207 ; HMDB34618 ; HMDB38492 ; HMDB38643 ; HMDB39875 ; HMDB40198	(-)-Epigallocatechin ; ()-Gallocatechin ; 23-Dihydroxyvaleric acid ; 1-Deoxy-D-xylulose ; Dihydropteridine ; Deoxyribose ; p-Cresol glucuronide ; (R)-23-Dihydroxy-isovalerate ; (R)-glycerol 1-acetate ; Acetylcarnosine ; Dexrazoxane ; Erlotinib ; Pentostatin ; Hydroxyprolyl-Histidine ; Histidinyl-Hydroxyproline ; Isoleucyl-Valine ; Leucyl-Valine ; Valyl-Isoleucine ; Valyl-Leucine ; 23-Dihydroxy-2-methylbutanoic acid ; 6-Apiosyllotaustralin ; 2-O-Benzoyl-D-glucose ; D-Vacciniin ; Epoxyfumitremorgin C ; 2-(Ethylthio)methylfuran ; 25-Dimethyl-3-(methylthio)furan	M+2H ; M+2H ; 2M+H+K ; 2M+H+K ; 2M+H+K ; 2M+H+K ; M+H+Na ; 2M+H+K ; 2M+H+K ; M+H+K ; M+H+K ; M+3Na ; M+H+K ; M+H+K ; M+H+K ; M+2K ; M+2K ; M+2K ; M+2K ; 2M+H+K ; M+3Na ; M+H+Na ; M+H+Na ; M+3Na ; 2M+H+Na ; 2M+H+Na	C15H14O7 ; C15H14O7 ; C5H10O4 ; C5H10O4 ; C6H6N4 ; C5H10O4 ; C13H16O7 ; C5H10O4 ; C5H10O4 ; C11H16N4O4 ; C11H16N4O4 ; C22H23N3O4 ; C11H16N4O4 ; C11H16N4O4 ; C11H16N4O4 ; C11H22N2O3 ; C11H22N2O3 ; C11H22N2O3 ; C11H22N2O3 ; C5H10O4 ; C16H27NO10 ; C13H16O7 ; C13H16O7 ; C22H23N3O4 ; C7H10OS ; C7H10OS
9337_58.43	1.871444	0.506806	4.4005	0.001334	2.8748	0.028103	327.9893	HMDB11725 ; HMDB13922 ; HMDB35956 ; HMDB59968 ; HMDB59982	5-Sulfosalicylic acid ; 2-Chloroticlopidine ; Lumequoylacetone ; 3-hydroxybenzoic acid-3-O-sulphate ; 4-hydroxybenzoic acid-4-O-sulphate	3M+2H ; 2M+Na+K ; 2M+3H ; 3M+2H ; 3M+2H	C7H6O6S ; C14H13Cl2NS ; C33H62O2 ; C7H6O6S ; C7H6O6S
0025_158.8	1.861714	0.340547	4.3946	0.001346	2.8709	0.028103	346.57	HMDB60465 ; HMDB60775	cyclic GMP-AMP ; 5-Carboxy-lumiracoxib	M+H+NH4 ; 2M+H+NH4	C20H24N10O13P2 ; C16H13ClFNO4
1947_240.	1.860462	0.657802	4.394	0.001348	2.8704	0.028103	252.0162	HMDB01143 ; HMDB01498 ; HMDB15470	Inositol 134-trisphosphate ; Inositol 145-trisphosphate ; Salicylate-sodium	M+2ACN+2H ; M+2ACN+2H ; 3M+H+Na	C6H15O15P3 ; C6H15O15P3 ; C7H5NaO3
2266_99.9	1.860117	0.076917	-4.39	0.001356	2.8677	0.028103	149.5342	HMDB14623 ; HMDB30305 ; HMDB60455 ; HMDB60712 ; HMDB60850	Lenalidomide ; Dioscorine ; cis-2-Chloro-4-carboxymethylenebut-2-en-14-olide ; 2-Amino-56-dichloro-34-dihydroquinazoline ; NNO-Tridesmethyl-tramadol	M+H+K ; M+2K ; M+3ACN+2H ; M+2ACN+2H ; M+2K	C13H13N3O3 ; C13H19NO2 ; C6H3ClO4 ; C8H7Cl2N3 ; C13H19NO2
2791_262.	1.859157	0.076203	-4.4022	0.00133	2.876	0.028103	222.0228	HMDB02329 ; HMDB14933 ; HMDB36377	Oxalic acid ; Sulfasalazine ; 7-Hydroxy-2-methyl-4-oxo-4H-1-benzopyran-5-carboxylic acid 7-glucoside	2M+ACN+H ; M+2Na ; M+Na+K	C2H2O4 ; C18H14N4O5S ; C17H18O10
7712_58.53	1.847937	0.633361	4.3945	0.001347	2.8708	0.028103	271.1377	HMDB10357 ; HMDB02163 ; HMDB15557 ; HMDB41135	Tetrahydroaldosterone-3-glucuronide ; Trihydroxycoprostanoic acid ; Pheniramine ; 2-Deoxybrassinolide	M+2H ; M+2K ; 2M+Na+K ; M+2K	C27H40O11 ; C28H48O5 ; C16H20N2 ; C28H48O5
0879_101.	1.845093	0.78981	4.3902	0.001356	2.8679	0.028103	320.9886	HMDB14966	Fosfomycin	2M+2Na-H	C3H7O4P
0808_48.4	1.835687	0.222909	-4.3796	0.001378	2.8607	0.02836	NA	NA	NA	NA	NA

5131_72.7(	1.858409	0.356722	4.3689	0.001402	2.8534	0.028721	212.0425	HMDB01511 ; HMDB28684 ; HMDB28768 ; HMDB29873 ; HMDB32930 ; HMDB35717 ; HMDB37269 ; HMDB60362	Phosphocreatine ; Alanyl-Cysteine ; Cysteinyl-Alanine ; (S)-Spinacine ; Benzothiazole ; S-Acetyl dihydroasparagusic acid ; Desacetycefapirin ; 24-Diamino-6-nitrotoluene	M+H ; 2M+H+K ; 2M+H+K ; M+2Na-H ; 3M+H+NH4 ; M+NH4 ; M+ACN+2H ; M+2Na-H	C4H10N3O5P ; C6H12N2O3S ; C6H12N2O3S ; C7H9N3O2 ; C7H5NS ; C6H10O3S2 ; C15H15N3O5S2 ; C7H9N3O2
5512_94.3	1.887822	0.205258	-4.352	0.001439	2.842	0.029366	228.9786	HMDB59752 ; HMDB60016 ; HMDB60018 ; HMDB60435	5-Sulfoxymethylfurfural ; Pyrogallol-1-O-sulphate ; Pyrogallol-2-O-sulphate ; alpha-Fluoro-beta-ureidopropionic acid	M+Na ; M+Na ; M+Na ; M+2K+H	C6H6O6S ; C6H6O6S ; C6H6O6S ; C4H7FN2O3
6958_101.8	1.874021	0.937353	-4.331	0.001487	2.8276	0.03023	199.5137	HMDB29395 ; HMDB30391 ; HMDB59724 ; HMDB60770	L-L-Homoglutathione ; Cepharadione B ; Pyrocatechol sulfate ; 4-Hydroxy-5-carboxy-lumiracoxib	M+2K ; M+2K ; 2M+H+NH4 ; M+2Na	C11H19N3O6S ; C19H15NO4 ; C6H6O5S ; C16H13ClFNO5
8961_35.2	1.830976	0.085052	4.3211	0.001511	2.8208	0.030579	148.0229	HMDB37835 ; HMDB05760 ; HMDB15685 ; HMDB29462 ; HMDB29519 ; HMDB30808 ; HMDB32577 ; HMDB32670 ; HMDB33904 ; HMDB36457 ; HMDB37316 ; HMDB41647 ; HMDB60643 ; HMDB32683	4-(24-Dihydroxyphenyl)azobenzenesulfonic acid ; Dihydrodaidzein ; Azidocillin ; (E)-244-Trihydroxychalcone ; (2S)-Liquiritigenin ; (S)-Pinocembrin ; 4-Methoxybenzophenone-2-carboxylic acid ; 2-(5-Methylsulfinyl)-4-penten-2-ynylidene-16-dioxaspiro44non-3-ene ; 7E-Mycosinyl acetate ; Emodinanthranol ; Isoliquiritigenin ; 2-Dehydro-O-desmethylangolensin ; Tizoxanide glucuronide ; Arsenocholine	M+2H ; M+H+K ; M+3Na ; M+H+K ; M+H+K ; M+H+K ; M+H+K ; M+2Na ; M+H+K ; M+H+K ; M+H+K ; M+H+K ; M+3H ; M+H-H2O	C12H10N2O5S ; C15H12O4 ; C16H17N5O4S ; C15H12O4 ; C15H12O4 ; C15H12O4 ; C15H12O4 ; C13H14O3S ; C15H12O4 ; C15H12O4 ; C15H12O4 ; C15H12O4 ; C16H15N3O10S ; C5H14AsO
3505_65.6	1.902829	0.454717	4.3091	0.001539	2.8127	0.031009	311.0984	HMDB06866 ; HMDB13847 ; HMDB15403 ; HMDB30336 ; HMDB39631 ; HMDB41517 ; HMDB04256	2-Methyl-1-hydroxypropyl-ThPP ; Losartan N2-glucuronide ; Lumiracoxib ; 2-Feruloyl-12-disinapoylgentiobiose ; Physagulin F ; beta-D-Glucopyranosyl anthranilate ; 7-Hydroxy-6-methyl-8-ribityl lumazine	M+3ACN+2H ; M+H+Na ; M+NH4 ; M+3H ; M+2K ; 2M+H+Na ; M+H-H2O	C16H27N4O8P2S ; C28H31ClN6O7 ; C15H13ClFNO2 ; C44H50O22 ; C30H40O9 ; C13H17NO7 ; C12H16N4O7
5236_40.7	1.855962	0.074866	-4.3023	0.001556	2.8081	0.031009	NA	NA	NA	NA	NA
0735_68.7	1.836354	0.192709	4.3026	0.001555	2.8082	0.031009	85.04781	HMDB40822	23-Butanediol glucoside	M+3H	C10H20O7
3397_107.	1.826669	0.622484	-4.302	0.001557	2.8078	0.031009	197.4863	HMDB15666 ; HMDB38429	Lornoxicam ; Methyl glucosinolate	M+H+Na ; M+Na+K	C13H10ClN3O4S2 ; C8H15NO9S2

8103_98.1	1.858049	0.337197	-4.2912	0.001583	2.8005	0.031289	247.0567	HMDB29481 ; HMDB29968 ; HMDB30018 ; HMDB30604 ; HMDB31586 ; HMDB32616 ; HMDB32657 ; HMDB33652 ; HMDB33741 ; HMDB33942 ; HMDB34069 ; HMDB37509 ; HMDB40561 ; HMDB40894 ; HMDB41536 ; HMDB41691 ; HMDB41946	Isosakuranin ; Ethyl beta-D-glucopyranoside ; Elenolide ; epsilon-Viniferin ; 7-Hydroxy-8-O-methylaloin B ; Sinapic acid ; trans-delta-Viniferin ; Prunin 46-di-O-gallate ; Puddumin A ; Dambonitol ; cis-Sinapic acid ; Chalconosakuranetin ; Aromadendrin 4-methyl ether 7-rhamnoside ; Ampelopsin D ; Piperenol C ; 5-(345-Trihydroxyphenyl)-gamma-valerolactone ; N-Nitrosoproline	M+2Na ; M+K ; M+Na ; M+H+K ; M+2Na ; M+Na ; M+H+K ; M+3H ; M+2Na ; M+K ; M+Na ; M+2Na ; M+2Na ; M+H+K ; M+2Na ; M+Na ; 3M+Na+K	C22H24O10 ; C8H16O6 ; C11H12O5 ; C28H22O6 ; C22H24O10 ; C11H12O5 ; C28H22O6 ; C35H30O18 ; C22H24O10 ; C8H16O6 ; C11H12O5 ; C22H24O10 ; C22H24O10 ; C28H22O6 ; C22H24O10 ; C11H12O5 ; C5H8N2O3
4466_159.1	1.844758	0.21969	4.2926	0.00158	2.8014	0.031289	NA	NA	NA	NA	NA
1575_88.1	1.866845	0.889079	-4.2863	0.001595	2.7971	0.031405	242.1152	HMDB02224 ; HMDB14922 ; HMDB29366 ; HMDB37696 ; HMDB40380 ; HMDB60750 ; HMDB36601 ; HMDB00732 ; HMDB02043 ; HMDB03323 ; HMDB03324 ; HMDB11142 ; HMDB11554 ; HMDB11584 ; HMDB11631 ; HMDB12819 ; HMDB14787 ; HMDB29397 ; HMDB29433 ; HMDB31250 ; HMDB31864 ; HMDB32584 ; HMDB33376 ; HMDB33386 ; HMDB33429 ; HMDB33430 ; HMDB33597 ; HMDB34442 ; HMDB35013 ; HMDB36235 ; HMDB36385 ; HMDB37680 ; HMDB37694 ; HMDB37695 ; HMDB37708 ; HMDB38179 ; HMDB40006 ; HMDB40092 ; HMDB40113 ; HMDB40287 ; HMDB40356 ; HMDB40411 ; HMDB40425 ; HMDB40426 ; HMDB40583 ; HMDB40800 ; HMDB41278 ; HMDB41448 ; HMDB41553 ; HMDB42008 ; HMDB28798 ; HMDB28861 ; HMDB28877 ; HMDB29150 ; HMDB38516	5-Methyldeoxycytidine ; Mefenamic acid ; N-2-(4-Hydroxyphenyl)ethylbenzamide ; 2-Phenylethyl 2-aminobenzoate ; Schinifoline ; 3-Amino-3-deoxythimidine ; 3-Hydroxy-T2 Toxin ; Hydroxykynurenine ; 5-Phenylvaleric acid ; Biotripyrrin-a ; Biotripyrrin-b ; DHAP(18:0e) ; MG(0:022:4(7Z10Z13Z16Z)0:0) ; MG(22:4(7Z10Z13Z16Z)0:00:0) ; L-3-Hydroxykynurenine ; 5-Hydroxykynurenine ; Stavudine ; L-Nicotianine ; L-2-Amino-4-methylenepentanedioic acid ; Phenylmethyl 2-methylpropanoate ; Methyleugenol ; 4-Hydroxychalcone ; Phenylmethyl butanoate ; 3-Phenylpropyl acetate ; Tanakamine ; Tanakine ; 4-(4-Methoxyphenyl)-2-butanone ; L-trans-alpha-Amino-2-carboxycyclopropaneacetic acid ; 2-Phenylethyl propanoate ; alphaalpha-Dimethylphenethyl formate ; Methyl 4-phenylbutanoate ; 2-Methylphenyl 2-methylpropanoate ; cis-3-Hexenyl 2-aminobenzoate ; Cyclohexyl 2-aminobenzoate ; p-Tolyl isobutyrate ; 3-	M+H ; M+H ; M+H ; M+H ; M+H ; M+H ; M+2H ; M+NH4 ; M+ACN+Na ; M+H+NH4 ; M+H+NH4 ; M+Na+K ; M+2K ; M+2K ; M+NH4 ; M+NH4 ; M+NH4 ; M+NH4 ; M+2ACN+H ; M+ACN+Na ; M+ACN+Na ; M+NH4 ; M+ACN+Na ; M+ACN+Na ; M+Na ; M+Na ; M+ACN+Na ; M+2ACN+H ; M+ACN+Na ; M+ACN+Na ; M+ACN+Na ; M+ACN+Na ; M+Na ; M+Na ; M+ACN+Na ; M+ACN+Na ; M+Na ; 2M+Na+K ; 2M+Na+K ; M+ACN+Na ; M+ACN+Na ; M+ACN+Na ; M+ACN+Na ; M+ACN+Na ; M+ACN+Na ; M+ACN+Na ; M+2ACN+2H ; 2M+H+Na ; M+ACN+Na ; M+Na ; M+H-H2O ; M+H-H2O ; M+H-H2O ; M+H-H2O ; M+H-H2O	C10H15N3O4 ; C15H15NO2 ; C15H15NO2 ; C15H15NO2 ; C15H15NO2 ; C10H15N3O4 ; C24H34O10 ; C10H12N2O4 ; C11H14O2 ; C25H27N3O6 ; C25H27N3O6 ; C21H43O6P ; C25H42O4 ; C25H42O4 ; C10H12N2O4 ; C10H12N2O4 ; C10H12N2O4 ; C10H12N2O4 ; C6H9NO4 ; C11H14O2 ; C11H14O2 ; C15H12O2 ; C11H14O2 ; C11H14O2 ; C13H17NO2 ; C13H17NO2 ; C11H14O2 ; C6H9NO4 ; C11H14O2 ; C11H14O2 ; C11H14O2 ; C11H14O2 ; C13H17NO2 ; C13H17NO2 ; C11H14O2 ; C11H14O2 ; C13H17NO2 ; C12H21NS ; C12H21NS ; C11H14O2 ; C11H14O2 ; C11H14O2 ; C11H14O2 ; C11H14O2 ; C11H14O2 ; C11H14O2 ; C20H29ClO6 ; C12H22S2 ; C11H14O2 ; C13H17NO2 ; C10H17N3O5 ; C10H17N3O5 ; C10H17N3O5 ; C10H17N3O5 ;
5363_113.0	1.841583	0.151361	-4.2827	0.001605	2.7946	0.031463	169.5075	HMDB31780	Dithianon	M+ACN+2H	C14H4N2O2S2
2219_277.4	1.846961	0.066598	4.2801	0.001611	2.7929	0.031468	NA	NA	NA	NA	NA

4224_291.[illegible]	1.889238	0.989546	4.277	0.001619	2.7907	0.031501	779.5154	HMDB12312 ; HMDB15584 ; HMDB57008 ; HMDB57044 ; HMDB57201 ; HMDB57234 ; HMDB57385 ; HMDB58738 ; HMDB10582 ; HMDB10583 ; HMDB10596 ; HMDB10625 ; HMDB10640 ; HMDB10653 ; HMDB10654	3-O-Sulfogalactosylceramide (d18:114:0) ; Maraviroc ; CL(18:018:018:2(9Z12Z)22:6(4Z7Z10Z13Z16Z19Z)) ; CL(18:018:022:6(4Z7Z10Z13Z16Z19Z)18:2(9Z12Z)) ; CL(18:018:2(9Z12Z)18:022:6(4Z7Z10Z13Z16Z19Z)) ; CL(18:018:2(9Z12Z)22:6(4Z7Z10Z13Z16Z19Z)18:0) ; CL(18:022:6(4Z7Z10Z13Z16Z19Z)18:2(9Z12Z)18:0) ; CL(18:2(9Z12Z)18:022:6(4Z7Z10Z13Z16Z19Z)18:0) ; PG(16:022:5(4Z7Z10Z13Z16Z)) ; PG(16:022:5(7Z10Z13Z16Z19Z)) ; PG(16:1(9Z)22:4(7Z10Z13Z16Z)) ; PG(18:1(11Z)20:4(5Z8Z11Z14Z)) ; PG(18:1(9Z)20:4(5Z8Z11Z14Z)) ; PG(18:2(9Z12Z)20:3(5Z8Z11Z)) ; PG(18:2(9Z12Z)20:3(8Z11Z14Z))	2M+3H2O+2H ; 3M+H+NH4 ; M+H+K ; M+H+K ; M+H+K ; M+H+K ; M+H+K ; M+H+K ; M+H-H2O ; M+H-H2O ; M+H-H2O ; M+H-H2O ; M+H-H2O ; M+H-H2O ; M+H-H2O	C38H73NO11S ; C29H41F2N5O ; C86H152O17P2 ; C86H152O17P2 ; C86H152O17P2 ; C86H152O17P2 ; C86H152O17P2 ; C86H152O17P2 ; C44H77O10P ; C44H77O10P ; C44H77O10P ; C44H77O10P ; C44H77O10P ; C44H77O10P ; C44H77O10P
0976_91.5[illegible]	1.871898	0.053063	-4.2686	0.001641	2.785	0.031704	292.1141	HMDB28818 ; HMDB30327 ; HMDB35165 ; HMDB37551 ; HMDB38637 ; HMDB39777 ; HMDB60623	Glutamyl-Glutamate ; Perlolyrine ; Cinncassiol A 19-glucoside ; 1-(2-Hydroxyphenylamino)-1-deoxy-beta-D-gentiobioside 12-carbamate ; Methoxybrassitin ; Orcein ; Perindopril Acyl-beta-D-glucuronide	M+NH4 ; 2M+3H2O+2H ; M+H+K ; M+3ACN+2H ; M+ACN+H ; M+2ACN+2H ; M+H+K	C10H14N2O7 ; C16H12N2O2 ; C26H40O12 ; C19H25NO12 ; C12H14N2O2S ; C28H24N2O7 ; C25H40N2O11
6716_254.[illegible]	1.865469	0.654228	4.268	0.001642	2.7846	0.031704	344.0657	HMDB60545 ; HMDB36014 ; HMDB40470 ; HMDB41537 ; HMDB60394	O-Desmethylindomethacin ; 7-Hydroxy-145-trimethoxyxanthone 7-O-b-D-glucosyl-(1-2)-b-D-galactoside ; 5-Methyleriodictyol 7-glucosyl-(1-4)-galactoside ; N-Acetyldehydroanonaine ; 5-Fluorodeoxyuridine monophosphate	M+H ; M+Na+K ; M+Na+K ; M+K ; M+NH4	C18H14ClNO4 ; C28H34O16 ; C28H34O16 ; C19H15NO3 ; C9H12FN2O8P
2739_125.[illegible]	1.847564	0.449658	4.2558	0.001674	2.7762	0.032133	428.1763	HMDB01913 ; HMDB05009 ; HMDB14679 ; HMDB14874 ; HMDB15020 ; HMDB41314 ; HMDB41907	Omeprazole ; (S)-Esomeprazole ; Toremifene ; Esomeprazole ; Clomifene ; 4-tert-Butylphenyl salicylate ; Imidapril	M+2ACN+H ; M+2ACN+H ; M+Na ; M+2ACN+H ; M+Na ; 3M+2Na ; M+Na	C17H19N3O3S ; C17H19N3O3S ; C26H28ClNO ; C17H19N3O3S ; C26H28ClNO ; C17H18O3 ; C20H27N3O6
3552_158.[illegible]	1.83234	0.46705	4.2546	0.001677	2.7754	0.032133	328.0984	HMDB10323 ; HMDB15690 ; HMDB37521 ; HMDB40367 ; HMDB41849 ; HMDB60764	Phenethylamine glucuronide ; Chloropyramine ; CI Solvent Red 80 ; Azacridone A ; Carboxytolbutamide ; 4-Hydroxy-estazolam	2M+Na+K ; M+K ; 2M+H+K ; 2M+H+K ; 2M+3H2O+2H ; M+NH4	C14H19NO6 ; C16H20ClN3 ; C18H16N2O3 ; C18H16N2O3 ; C12H16N2O5S ; C16H11ClN4O

6372_46.4	1.874922	0.563035	4.2484	0.001694	2.7711	0.032324	176.0386	HMDB00052 ; HMDB00544 ; HMDB01014 ; HMDB02320 ; HMDB32765 ; HMDB39408	Argininosuccinic acid ; 5-Hydroxymethyl-4-methyluracil ; 4-Imidazolone-5-propionic acid ; Imidazolelactic acid ; N2-(3-Hydroxysuccinoyl)arginine ; N2-(3-Carboxy-2-hydroxy-1-oxopropyl)arginine	M+Na+K ; 2M+H+K ; 2M+H+K ; 2M+H+K ; M+Na+K ; M+Na+K	C10H18N4O6 ; C6H8N2O3 ; C6H8N2O3 ; C6H8N2O3 ; C10H18N4O6 ; C10H18N4O6
8354_286.	1.818278	0.992274	4.2359	0.001728	2.7626	0.032842	NA	NA	NA	NA	NA
3817_87.5	1.860413	0.004601	-4.2316	0.001739	2.7596	0.032883	357.0434	HMDB36617 ; HMDB01132 ; HMDB32948	()-Chebulic acid ; Nicotinic acid mononucleotide ; 5-Phenyl-13-pentadiyne	M+H ; 2M+H+K ; 2M+2K-H	C14H12O11 ; C11H16NO9P ; C11H8
2394_238.	1.818946	0.806632	4.2304	0.001743	2.7588	0.032883	549.0882	HMDB06354 ; HMDB32234 ; HMDB37732	Deoxythymidine diphosphate-l-rhamnose ; 25-Dimethyl-3-furanthiol acetate ; S-(2-Furanylmethyl) propanethioate	M+H ; 3M+K ; 3M+K	C16H26N2O15P2 ; C8H10O2S ; C8H10O2S
5086_58.2	1.899616	0.742014	4.2179	0.001778	2.7502	0.033163	312.0155	HMDB14024 ; HMDB14025 ; HMDB41788	2-Hydroxychlorpropamide ; 3-Hydroxychlorpropamide ; Vanillic acid 4-sulfate	2M+H+K ; 2M+H+K ; M+ACN+Na	C10H13ClN2O4S ; C10H13ClN2O4S ; C8H8O7S
6939_109.	1.864967	0.752114	-4.2213	0.001768	2.7525	0.033163	179.0295	HMDB14772	Sulfacetamide	M+H-2H2O	C8H10N2O3S
0299_55.6	1.84412	0.398364	-4.2155	0.001784	2.7486	0.033163	257.147	HMDB35980 ; HMDB36059 ; HMDB36406 ; HMDB36414 ; HMDB00043 ; HMDB00883 ; HMDB01382 ; HMDB02141 ; HMDB03355 ; HMDB13716 ; HMDB15550 ; HMDB31017 ; HMDB31028 ; HMDB31741 ; HMDB32792 ; HMDB34286 ; HMDB37187 ; HMDB37226 ; HMDB37626 ; HMDB37907 ; HMDB38078 ; HMDB39805 ; HMDB40451	37111523-Pentaoxolanost-8-en-26-oic acid ; Ganoderenic acid D ; Ganosporelactone A ; Anabsin ; Betaine ; L-Valine ; Vaporole ; N-Methyl-a-aminoisobutyric acid ; 5-Aminopentanoic acid ; Norvaline ; Amyl Nitrite ; 10-Undecenyl acetate ; Methyl (E)-2-dodecenoate ; Coniferan ; ()-(E)-3-Methyl-4-decen-1-yl acetate ; Ethyl 10-undecenoate ; Rhodinyl propionate ; Citronellyl propionate ; 1-Ethylhexyl tiglate ; 261010-Tetramethyl-1-oxaspiro45decan-6-ol ; 11-Diethoxy-26-nonadiene ; 4-(3-Hydroxybutyl)-335-trimethylcyclohexanone ; Oxacyclotetradecan-2-one	M+2H ; M+2H ; M+2H ; M+2H ; 2M+Na ; 2M+Na ; 2M+Na ; 2M+Na ; 2M+Na ; 2M+Na ; 2M+Na ; M+2Na-H ; M+2Na-H ; M+2Na-H ; M+2Na-H ; M+2Na-H ; M+2Na-H ; M+2Na-H ; M+2Na-H ; M+2Na-H ; M+2Na-H ; M+2Na-H ; M+2Na-H	C30H40O7 ; C30H40O7 ; C30H40O7 ; C30H40O7 ; C5H11NO2 ; C5H11NO2 ; C5H11NO2 ; C5H11NO2 ; C5H11NO2 ; C5H11NO2 ; C5H11NO2 ; C13H24O2 ; C13H24O2 ; C13H24O2 ; C13H24O2 ; C13H24O2 ; C13H24O2 ; C13H24O2 ; C13H24O2 ; C13H24O2 ; C13H24O2 ; C13H24O2 ; C13H24O2

3191_89.5	1.83307	0.220275	4.219	0.001774	2.751	0.033163	371.1133	HMDB31941 ; HMDB34119 ; HMDB35527 ; HMDB36555 ; HMDB38398 ; HMDB38900 ; HMDB38932 ; HMDB39731 ; HMDB39732 ; HMDB11169 ; HMDB14686 ; HMDB14937 ; HMDB14945 ; HMDB28763 ; HMDB29057 ; HMDB33734 ; HMDB37261 ; HMDB30824 ; HMDB31423 ; HMDB38330 ; HMDB39039 ; HMDB39172 ; HMDB40555 ; HMDB40863 ; HMDB41265 ; HMDB60923	8-Episesaminone ; Sesamolin ; Glycyrrhizaflavonol A ; Glycyrrhizaisoflavone A ; Gancaonin P ; 23-Dehydrokievitol ; Justisolin ; Neouralenol ; Uralenol ; L-beta-aspartyl-L-threonine ; Adinazolam ; Tazarotene ; Proparacaine ; Aspartyl-Threonine ; Threoninyl-Aspartate ; DIBOA-Glc ; DHBOA-Glc ; Afzelechin 7-apioside ; omega-Salicoyisalicin ; Benzyl 26-dihydroxybenzoate 2-glucoside ; (R)-Apiumetin glucoside ; Flacourtin ; (E)-Oxyresveratrol 3-O-b-D-glucoside ; Piceatannol 4-glucoside ; Edulisin VI ; Naproxen O-glucuronide	M+H ; M+H ; M+H ; M+H ; M+H ; M+H ; M+H ; M+H ; M+H ; 3M+H+K ; 2M+H+K ; 2M+H+K ; M+2K-H ; 3M+H+K ; 3M+H+K ; 2M+3H2O+2H ; 2M+3H2O+2H ; M+H-2H2O ; M+H-2H2O ; M+H-2H2O ; M+H-2H2O ; M+H-2H2O ; M+H-2H2O ; M+H-2H2O ; M+H-2H2O ; M+H-2H2O	C20H18O7 ; C20H18O7 ; C20H18O7 ; C20H18O7 ; C20H18O7 ; C20H18O7 ; C20H18O7 ; C20H18O7 ; C20H18O7 ; C8H14N2O6 ; C19H18ClN5 ; C21H21NO2S ; C16H26N2O3 ; C8H14N2O6 ; C8H14N2O6 ; C14H17NO9 ; C14H17NO9 ; C20H22O9 ; C20H22O9 ; C20H22O9 ; C20H22O9 ; C20H22O9 ; C20H22O9 ; C20H22O9 ; C20H22O9 ; C20H22O9
75616_241.	1.88549	0.678533	4.1986	0.001833	2.7369	0.033941	250.9976	HMDB06240 ; HMDB29739 ; HMDB37296 ; HMDB38462	Demethylated antipyrine ; 1H-Indole-3-acetamide ; 3-Acetyl-27-naphthyridine ; 4-Hydroxy-1H-indole-3-acetonitrile	M+2K-H ; M+2K-H ; M+2K+H ; M+2K+H	C10H10N2O ; C10H10N2O ; C10H8N2O ; C10H8N2O
3879_210.9	1.866222	0.143066	4.1926	0.00185	2.7328	0.034055	318.0674	HMDB40624 ; HMDB40625 ; HMDB00956 ; HMDB29878 ; HMDB33527 ; HMDB34368 ; HMDB34402 ; HMDB59916 ; HMDB60535 ; HMDB61165	3-(23-Digalloylglucosyl)-phloroacetophenone ; 3-(26-Digalloylglucosyl)-phloroacetophenone ; Tartaric acid ; D-Tartaric acid ; Dehydroisochalciporone ; Hydroxyminaline ; Physalin I ; 23-Dihydroxybutanedioic acid ; N-Desmethyldiphenhydramine ; NN-Didemethyl orphenadrine	M+2H ; M+2H ; 2M+NH4 ; 2M+NH4 ; M+2K-H ; 2M+ACN+Na ; M+2K ; 2M+NH4 ; M+2K-H ; M+2K-H	C28H26O17 ; C28H26O17 ; C4H6O6 ; C4H6O6 ; C16H19NO ; C5H5NO3 ; C29H34O11 ; C4H6O6 ; C16H19NO ; C16H19NO
8214_57.4	1.861182	0.744655	4.1857	0.001871	2.728	0.034055	227.9548	HMDB31252	3-Iodopropanoic acid	2M+3H2O+2H	C3H5IO2
2516_278.4	1.856882	0.087684	4.1854	0.001872	2.7278	0.034055	NA	NA	NA	NA	NA

0162_65.39	1.833252	1.12993	4.1854	0.001872	2.7278	0.034055	102.055	HMDB29387 ; HMDB29615 ; HMDB36458 ; HMDB28688 ; HMDB28856 ; HMDB29026 ; HMDB29047 ; HMDB34266 ; HMDB00042 ; HMDB00337 ; HMDB00360 ; HMDB00498 ; HMDB00510 ; HMDB00705 ; HMDB00756 ; HMDB00798 ; HMDB00847 ; HMDB01366 ; HMDB01426 ; HMDB02453 ; HMDB02601 ; HMDB02649 ; HMDB03344 ; HMDB06293 ; HMDB29423 ; HMDB30007 ; HMDB30039 ; HMDB30331 ; HMDB31291 ; HMDB31480 ; HMDB31557 ; HMDB32042 ; HMDB32309 ; HMDB32330 ; HMDB32406 ; HMDB32454 ; HMDB32468 ; HMDB33747 ; HMDB34162 ; HMDB34165 ; HMDB34606 ; HMDB35240 ; HMDB36234 ; HMDB36669 ; HMDB36817 ; HMDB37143 ; HMDB37164 ; HMDB37288 ; HMDB38040 ; HMDB38603 ; HMDB39159 ; HMDB39216 ; HMDB39219 ; HMDB39423 ; HMDB39957 ; HMDB40221 ;	L-3-Aminodihydro-2(3H)-furanone ; (S)-2-Azetidinecarboxylic acid ; 1-Aminocyclopropanecarboxylic acid ; Alanyl-Hydroxyproline ; Hydroxyprolyl-Alanine ; Prolyl-Serine ; Serinyl-Proline ; L-Coprine ; Acetic acid ; (S)-34-Dihydroxybutyric acid ; 24-Dihydroxybutanoic acid ; 4-Deoxyerythronic acid ; Aminoadipic acid ; Hexanoylcarnitine ; L-Hexanoylcarnitine ; Oenanthic ether ; Pelargonic acid ; Purine ; Formaldehyde ; 4-Deoxythreonic acid ; Ab-Dihydroxyisobutyric acid ; Erythrose ; Glycolaldehyde ; L-Erythrulose ; Acetylhomoserine ; 2277-Tetramethyl-16-dioxaspiro44nona-38-diene ; Jasmolone ; xi-2-Ethylheptanoic acid ; Methyl caprylate ; Heptyl acetate ; 4-Methyloctanoic acid ; Butyl 3-methylbutanoate ; 3-Heptyl acetate ; 4-Hydroxy-2-butenoic acid gamma-lactone ; 5-Methylhexyl acetate ; 3-Octyl formate ; 2-Phenylpropionaldehyde dimethyl acetal ; ()-22-Iminobispropanoic acid ; Pentyl butanoate ; Propyl hexanoate ; Hexyl propionate ;	M+H ; M+H ; M+H ; M+2H ; M+2H ; M+2H ; M+2H ; M+2H ; M+ACN+H ; M+2ACN+2H ; M+2ACN+2H ; M+2ACN+2H ; M+ACN+2H ; M+H+2Na ; M+H+2Na ; M+2Na ; M+2Na ; M+2ACN+2H ; 2M+ACN+H ; M+2ACN+2H ; M+2ACN+2H ; M+2ACN+2H ; M+ACN+H ; M+2ACN+2H ; M+ACN+2H ; M+H+Na ; M+H+Na ; M+2Na ; M+2Na ; M+2Na ; M+2Na ; M+2Na ; M+2Na ; M+NH4 ; M+2Na ; M+2Na ; M+H+Na ; M+ACN+2H ; M+2Na ; M+2Na ; M+2Na ; M+H+Na ; M+2Na ; M+H+Na ; M+H+Na ; M+2Na ; M+2Na ; M+2Na ; M+2Na ; M+2Na ; M+H+Na ; M+2Na ; M+2Na ; M+3H ; M+H+Na ; M+2Na ; M+2Na ; M+2Na ; M+2Na ; M+H+Na ; M+H+Na ; M+H-H2O ; M+H-H2O ; M+H-H2O ; M+H-H2O	C4H7NO2 ; C4H7NO2 ; C4H7NO2 ; C8H14N2O4 ; C8H14N2O4 ; C8H14N2O4 ; C8H14N2O4 ; C8H14N2O4 ; C2H4O2 ; C4H8O4 ; C4H8O4 ; C4H8O4 ; C6H11NO4 ; C13H25NO4 ; C13H25NO4 ; C9H18O2 ; C9H18O2 ; C5H4N4 ; CH2O ; C4H8O4 ; C4H8O4 ; C4H8O4 ; C2H4O2 ; C4H8O4 ; C6H11NO4 ; C11H16O2 ; C11H16O2 ; C9H18O2 ; C9H18O2 ; C9H18O2 ; C9H18O2 ; C9H18O2 ; C9H18O2 ; C4H4O2 ; C9H18O2 ; C9H18O2 ; C11H16O2 ; C6H11NO4 ; C9H18O2 ; C9H18O2 ; C9H18O2 ; C11H16O2 ; C9H18O2 ; C11H16O2 ; C11H16O2 ; C9H18O2 ; C9H18O2 ; C9H18O2 ; C9H18O2 ; C9H18O2 ; C11H16O2 ; C9H18O2 ; C9H18O2 ; C12H21N3O6 ; C11H16O2 ; C9H18O2 ; C9H18O2 ; C9H18O2 ; C9H18O2 ;
0614_54.7	1.818993	0.94197	4.1849	0.001873	2.7275	0.034055	170.0321	HMDB06032 ; HMDB00528 ; HMDB03349 ; HMDB28913 ; HMDB28935 ; HMDB28976 ; HMDB28977 ; HMDB29719 ; HMDB31664 ; HMDB33670 ; HMDB35716 ; HMDB37731 ; HMDB37762 ; HMDB38613 ; HMDB59664	Imidazoleacetic acid ribotide ; 45-Dihydroorotic acid ; L-Dihydroorotic acid ; Isoleucyl-Methionine ; Leucyl-Methionine ; Methionyl-Isoleucine ; Methionyl-Leucine ; Methyl thiophene-2-carboxylate ; Alnustone ; 14-Dithiane-25-diol ; Dihydroasparagusic acid ; S-(2-Furanylmethyl) methanethioate ; Methyl 2-thiofuroate ; gamma-L-Glutamyl-L-methionine sulfoxide ; Oxidized dithiothreitol	M+2H ; 2M+H+Na ; 2M+H+Na ; M+2K ; M+2K ; M+2K ; M+2K ; 2M+3H2O+2H ; M+2K ; M+NH4 ; M+NH4 ; 2M+3H2O+2H ; 2M+3H2O+2H ; M+2Na ; M+NH4	C10H15N2O9P ; C5H6N2O4 ; C5H6N2O4 ; C11H22N2O3S ; C11H22N2O3S ; C11H22N2O3S ; C11H22N2O3S ; C6H6O2S ; C19H18O ; C4H8O2S2 ; C4H8O2S2 ; C6H6O2S ; C6H6O2S ; C10H18N2O6S ; C4H8O2S2

.4415_228.5	1.83299	0.938221	4.1824	0.001881	2.7257	0.034067	335.0514	HMDB00232 ; HMDB01871 ; HMDB02780 ; HMDB29253 ; HMDB30131 ; HMDB30144 ; HMDB30238 ; HMDB32322 ; HMDB33161 ; HMDB33783 ; HMDB34124 ; HMDB35193 ; HMDB35399 ; HMDB35444 ; HMDB37415 ; HMDB37417 ; HMDB37540 ; HMDB37953 ; HMDB37954 ; HMDB41293 ; HMDB41310 ; HMDB60260 ; HMDB60759	Quinolinic acid ; Epicatechin ; Catechin ; 3-Hydroxyphloretin ; gamma-Pyrufuran ; alpha-Cotonefuran ; ax-4-Hydroxy-3--methoxymaysin ; cis-3 and trans-2-Hexenyl propionate ; 26-Pyridinedicarboxylic acid ; Marshrin ; Fonsecin ; 1-(9H-Pyrido34-bindol-1-yl)-14-butanediol ; Cartorimine ; Liquiritigenin 4-3-acetylapiosyl-(1-2)-glucoside ; Isomargaritene ; Isowertin 2-rhamnoside ; Margaritene ; (-)-Catechin ; ()-Epicatechin ; 6-Hydroxy-alpha-pyrufuran ; Luteoforol ; Urate radical ; 4-Carboxynevirapine	2M+H ; M+2Na-H ; M+2Na-H ; M+2Na-H ; M+2Na-H ; M+2Na-H ; M+2K ; M+2Na-H ; 2M+H ; M+2Na-H ; M+2Na-H ; M+2K+H ; M+2Na-H ; M+2K ; M+2K ; M+2K ; M+2K ; M+2Na-H ; M+2Na-H ; M+2Na-H ; M+2Na-H ; 2M+H ; M+K	C7H5NO4 ; C15H14O6 ; C15H14O6 ; C15H14O6 ; C15H14O6 ; C15H14O6 ; C28H32O14 ; C15H14O6 ; C7H5NO4 ; C15H14O6 ; C15H14O6 ; C15H16N2O2 ; C15H14O6 ; C28H32O14 ; C28H32O14 ; C28H32O14 ; C28H32O14 ; C15H14O6 ; C15H14O6 ; C15H14O6 ; C15H14O6 ; C5H3N4O3 ; C15H12N4O3
70822_60.1	1.825307	0.457116	4.1794	0.001889	2.7237	0.034105	260.0771	HMDB38776 ; HMDB00149 ; HMDB00635 ; HMDB01857 ; HMDB01982 ; HMDB02026 ; HMDB04308 ; HMDB11103 ; HMDB12241 ; HMDB13639 ; HMDB36214 ; HMDB36380 ; HMDB38680 ; HMDB39026 ; HMDB39069 ; HMDB39070 ; HMDB59704 ; HMDB59815 ; HMDB60491 ; HMDB60634 ; HMDB60716	Medicarpin 3-O-(6-malonylglucoside) ; Ethanolamine ; Succinylacetone ; 13-Dimethyluric acid ; 37-Dimethyluric acid ; 19-Dimethyluric acid ; 79-Dimethyluric acid ; 17-Dimethyluric acid ; Isopropylmaleate ; 8-(Aminomethyl)sulfanyl-6-sulfanyloctanoic acid ; 6-Feruloylglucose 234-trihydroxy-3-methylbutylglycoside ; xi-23-Dihydro-35-dihydroxy-6-methyl-4H-pyran-4-one ; Musabalbisiane A ; (R)-Isobyakangelicin 3-glucoside ; (R)-Byakangelicin 3-glucoside ; (R)-Byakangelicin 2-glucoside ; 39-Dimethyluric acid ; Ethyl 5-oxotetrahydro-2-furancarboxylate ; Mycophenolic acid O-acyl-glucuronide ; Mycophenolic acid glucuronide ; 2-Hydroxy-desipramine glucuronide	M+2H ; 3M+2K-H ; 3M+2Na ; M+ACN+Na ; M+ACN+Na ; M+ACN+Na ; M+ACN+Na ; M+ACN+Na ; 3M+2Na ; M+Na ; M+2Na ; 3M+2Na ; M+H+Na ; M+H+Na ; M+H+Na ; M+H+Na ; M+ACN+Na ; 3M+2Na ; M+H+Na ; M+H+Na ; M+Na+K	C25H26O12 ; C2H7NO ; C7H10O4 ; C7H8N4O3 ; C7H8N4O3 ; C7H8N4O3 ; C7H8N4O3 ; C7H8N4O3 ; C7H10O4 ; C9H19NO2S2 ; C21H30O12 ; C7H10O4 ; C23H28O12 ; C23H28O12 ; C23H28O12 ; C23H28O12 ; C7H8N4O3 ; C7H10O4 ; C23H28O12 ; C23H28O12 ; C24H30N2O7

4783_196.7	1.831231	0.219768	-4.1718	0.001912	2.7184	0.034395	372.0805	HMDB03572 ; HMDB30609 ; HMDB30812 ; HMDB32580 ; HMDB33600 ; HMDB33819 ; HMDB34019 ; HMDB35184 ; HMDB37334 ; HMDB37335 ; HMDB37424 ; HMDB37425 ; HMDB37840 ; HMDB38127 ; HMDB38356 ; HMDB38904 ; HMDB39780 ; HMDB40304 ; HMDB40344 ; HMDB40472 ; HMDB40877 ; HMDB42026 ; HMDB60507	Rosmarinic acid ; Xanthochymuside ; Acerosin ; 4-Hydroxybenzyl isothiocyanate ; Sagerinic acid ; Jaceidin ; Parvisoflavone A ; Semilicoisoflavone B ; Majoranin ; Sudachitin ; Citrusinol ; Cycloartocarpesin ; N-(1-Deoxy-1-fructosyl)leucine ; Lupinisoflavone A ; Sideritiflavone ; 3-(24-Dihydroxyphenyl)-89-dihydro-5-hydroxy-8-(1-methylethenyl)-4H-furo23-h-1-benzopyran-5-one ; N-(1-Deoxy-1-fructosyl)isoleucine ; Cyclocommunol ; 356-Trihydroxy-347-trimethoxyflavone ; Agamanone ; 358-Trihydroxy-347-trimethoxyflavone ; Tertatolol ; S-(Formylmethyl)glutathione	2M+H+Na ; M+H+Na ; 2M+H+Na ; 2M+ACN+H ; M+H+Na ; 2M+H+Na ; 2M+H+K ; 2M+H+K ; 2M+H+Na ; 2M+H+Na ; 2M+H+K ; 2M+H+K ; M+2K+H ; 2M+H+K ; 2M+H+Na ; 2M+H+K ; M+2K+H ; 2M+H+K ; 2M+H+Na ; 2M+H+Na ; 2M+H+Na ; M+2K-H ; M+Na	C18H16O8 ; C36H32O16 ; C18H16O8 ; C8H7NOS ; C36H32O16 ; C18H16O8 ; C20H16O6 ; C20H16O6 ; C18H16O8 ; C18H16O8 ; C20H16O6 ; C20H16O6 ; C12H23NO7 ; C20H16O6 ; C18H16O8 ; C20H16O6 ; C12H23NO7 ; C20H16O6 ; C18H16O8 ; C18H16O8 ; C18H16O8 ; C16H25NO2S ; C12H19N3O7S
8542_102.9	1.845765	0.45276	-4.1686	0.001922	2.7162	0.034448	149.0263	HMDB01553 ; HMDB00267 ; HMDB00805 ; HMDB00884 ; HMDB01369 ; HMDB01843 ; HMDB02234 ; HMDB02331 ; HMDB04086 ; HMDB04813 ; HMDB06239 ; HMDB06801 ; HMDB13870 ; HMDB13871 ; HMDB13872 ; HMDB15457 ; HMDB15619 ; HMDB35993 ; HMDB41441 ; HMDB60262 ; HMDB60587 ; HMDB61093	2-Oxo-4-methylthiobutanoic acid ; Pyroglutamic acid ; Pyrrolidonecarboxylic acid ; Ribothymidine ; Pyrroline hydroxycarboxylic acid ; N-Acryloylglycine ; 1-Pyrroline-4-hydroxy-2-carboxylate ; Imidazoleacetic acid riboside ; 5-Hydroxy-N-formylkynurenine ; 3-Methyluridine ; S-aminomethyldihydrolipoamide ; 2-Oxo-3-hydroxy-4-phosphobutanoic acid ; cistrans-5-Hydroxythalidomide ; 5-Hydroxythalidomide ; Thalidomide arene oxide ; Dihydroxyaluminium ; Sulfathiazole ; N5-(34-Dioxo-15-cyclohexadien-1-yl)-L-glutamine ; 11-Methylgerberinol ; 5-Oxoprolinate ; Aminoethoxyacetic acid ; dimethadione	M+H ; 2M+H+K ; 2M+H+K ; M+H+K ; 2M+H+K ; 2M+H+K ; 2M+H+K ; M+H+K ; M+2Na ; M+H+K ; M+Na+K ; M+2ACN+2H ; M+H+Na ; M+H+Na ; M+H+Na ; 2M+H+Na ; M+ACN+2H ; M+2Na ; M+3Na ; 2M+H+K ; 2M+Na+K ; 2M+H+K	C5H8O3S ; C5H7NO3 ; C5H7NO3 ; C10H14N2O6 ; C5H7NO3 ; C5H7NO3 ; C5H7NO3 ; C10H14N2O6 ; C11H12N2O5 ; C10H14N2O6 ; C9H20N2OS2 ; C4H7O8P ; C13H10N2O5 ; C13H10N2O5 ; C13H10N2O5 ; C2H8AlNO4 ; C9H9N3O2S2 ; C11H12N2O5 ; C22H18O6 ; C5H7NO3 ; C4H8NO3 ; C5H7NO3

1977_248.6	1.826924	0.84928	-4.1582	0.001954	2.709	0.034899	487.2132	HMDB00585 ; HMDB35590 ; HMDB01396 ; HMDB01458 ; HMDB11116 ; HMDB29989 ; HMDB29990 ; HMDB31367 ; HMDB33316 ; HMDB34947 ; HMDB35489 ; HMDB36273 ; HMDB36571 ; HMDB39295 ; HMDB39476	Glucosylgalactosyl hydroxylysine ; 15-Acetoxyscirpene-34-diol 4-O-a-D-glucopyranoside ; 5-Methyltetrahydrofolic acid ; Biotin amide ; Phytyl diphosphate ; Dulciol B ; Dulciol A ; Linalool oxide D 3-apiosyl-(1-6)-glucoside ; Garcinone E ; Rebaudioside C ; Linalool 36-oxide primeveroside ; Quillaic acid 3-galactosyl-(1-2)-glucuronide 28-xylosyl-(1-4)-rhamnosyl-(1-2)-rhamnosyl-(1-3)-4-acetyl-fucosyl ester ; Linalool 37-oxide beta-primeveroside ; Medicagenic acid 28-O-b-D-xylosyl-(1-4)-a-L-rhamnosyl-(1-2)-a-L-arabinosyl ester ; Acetylsoyasaponin A1	M+H ; M+H ; 2M+3H2O+2H ; 2M+H ; 2M+Na+K ; M+Na ; M+Na ; M+Na ; M+Na ; M+H+Na ; M+Na ; M+2H+Na ; M+Na ; M+Na+K ; M+2H+Na	C18H34N2O13 ; C23H34O11 ; C20H25N7O6 ; C10H17N3O2S ; C20H42O7P2 ; C28H32O6 ; C28H32O6 ; C21H36O11 ; C28H32O6 ; C44H70O22 ; C21H36O11 ; C67H104O33 ; C21H36O11 ; C46H72O18 ; C67H104O33
9567_88.2	1.848485	0.656812	-4.1464	0.001991	2.7009	0.035307	221.077	HMDB11168 ; HMDB28762 ; HMDB29035 ; HMDB00903 ; HMDB03259 ; HMDB03441 ; HMDB04998 ; HMDB06753 ; HMDB06754 ; HMDB06760 ; HMDB13157 ; HMDB14825 ; HMDB29317 ; HMDB30023 ; HMDB30905 ; HMDB30991 ; HMDB32027 ; HMDB32064 ; HMDB32072 ; HMDB32177 ; HMDB32346 ; HMDB32372 ; HMDB32427 ; HMDB32488 ; HMDB32553 ; HMDB32844 ; HMDB33033 ; HMDB33093 ; HMDB34099 ; HMDB34225 ; HMDB34277 ; HMDB35832 ; HMDB36608 ; HMDB36621 ; HMDB37587 ; HMDB37627 ; HMDB37700 ; HMDB37701 ; HMDB38717 ; HMDB39033 ; HMDB39043 ; HMDB40707 ; HMDB41315 ; HMDB59602 ; HMDB59768 ; HMDB59779 ; HMDB59847 ; HMDB60384 ; HMDB60671 ; HMDB61092	L-beta-aspartyl-L-serine ; Aspartyl-Serine ; Serinyl-Aspartate ; Tetrahydrocortisone ; Dihydrocortisol ; Cinnamaldehyde ; Guaifenesin ; 3a11b21-Trihydroxy-20-oxo-5b-pregnan-18-al ; 11b21-Dihydroxy-320-oxo-5b-pregnan-18-al ; 11b17a21-Trihydroxypreg-nenolone ; 18-Hydroxy-11-dehydrotetrahydrocorticosterone ; Fludrocortisone ; Angelicolide ; Angeolide ; 1-(4-Methoxyphenyl)-1-penten-3-one ; 5-(3E-Pentenyl)tetrahydro-2-oxo-3-furancarboxylic acid ; 3-(2-Methylpropylidene)-3alpha4-dihydro-1(3H)-isobenzofuranone ; (S)-3-Butyl-1(3H)-isobenzofuranone ; (Z)-3-Phenyl-2-propenal ; Benzyl trans-2-methyl-2-butenoate ; (-)-Isobutyl 3-methylthiobutyrate ; 3-Mercaptoheptyl acetate ; (S)-3-Methylthiohexyl acetate ; Prenyl benzoate ; Vanillin 3-(L-menthoxy)propane-12-diol acetal ; 45-Dihydroniveusin A ; (R)-37-Dimethyl-5-indanecarboxylic acid ; 3-(4-Hydroxy-3-methoxyphenyl)-12-propanediol ; (2E4E)-27-Dimethyl-24-octadienedioic acid ; Levistolide A ;	M+H ; M+H ; M+H ; M+2K ; M+2K ; 3M+2Na ; M+Na ; M+2K ; M+2K ; M+2K ; M+2K ; M+Na+K ; M+Na+K ; M+Na+K ; 2M+Na+K ; M+Na ; 2M+Na+K ; 2M+Na+K ; 3M+2Na ; 2M+Na+K ; 2M+Na+K ; 2M+Na+K ; 2M+Na+K ; 2M+Na+K ; M+2K ; M+2Na ; 2M+Na+K ; M+Na ; M+Na ; M+Na+K ; 2M+Na+K ; M+Na+K ; M+2Na ; M+Na+K ; M+Na+K ; 2M+Na+K ; 2M+Na+K ; 2M+Na+K ; M+2Na ; M+Na+K ; M+Na+K ; M+Na+K ; 2M+Na+K ; 3M+2Na ; M+Na ; M+Na ; 3M+2Na ; 2M+H+Na ; 3M+2Na ; M+2Na	C7H12N2O6 ; C7H12N2O6 ; C7H12N2O6 ; C21H32O5 ; C21H32O5 ; C9H8O ; C10H14O4 ; C21H32O5 ; C21H32O5 ; C21H32O5 ; C21H32O5 ; C21H29FO5 ; C24H28O4 ; C24H28O4 ; C12H14O2 ; C10H14O4 ; C12H14O2 ; C12H14O2 ; C9H8O ; C12H14O2 ; C9H18O2S ; C9H18O2S ; C9H18O2S ; C12H14O2 ; C21H32O5 ; C20H28O8 ; C12H14O2 ; C10H14O4 ; C10H14O4 ; C24H28O4 ; C12H14O2 ; C24H28O4 ; C20H28O8 ; C24H28O4 ; C24H28O4 ; C12H14O2 ; C12H14O2 ; C12H14O2 ; C20H28O8 ; C24H28O4 ; C24H28O4 ; C24H28O4 ; C12H14O2 ; C9H8O ; C10H14O4 ; C10H14O4 ; C9H8O ; C9H11N3O3 ; C9H8O ; C21H26F2O5
5373_275.9	1.819825	0.548473	4.1486	0.001984	2.7024	0.035307	404.9575	HMDB29723 ; HMDB41910	Saccharin ; Iopromide	2M+K ; M+H+NH4	C7H5NO3S ; C18H24I3N3O8

5813_111.9	1.788682	0.399527	4.1415	0.002007	2.6975	0.035458	111.0096	HMDB01933	Furosemide	M+3H	C12H11ClN2O5S
6057_100.2	1.81318	0.062776	4.1294	0.002046	2.689	0.035962	446.9966	HMDB01274	dTDP	M+2Na-H	C10H16N2O11P2
6542_68.63	1.807155	0.16521	4.1283	0.00205	2.6883	0.035962	190.1187	HMDB00679 ; HMDB10393 ; HMDB10394 ; HMDB10404 ; HMDB13809 ; HMDB15671 ; HMDB30371 ; HMDB30961 ; HMDB30966 ; HMDB31197 ; HMDB31301 ; HMDB31309 ; HMDB31590 ; HMDB31591 ; HMDB32403 ; HMDB32451 ; HMDB33547 ; HMDB35242 ; HMDB35390 ; HMDB35915 ; HMDB36030 ; HMDB37152 ; HMDB37864 ; HMDB37883 ; HMDB37884 ; HMDB37885 ; HMDB37886 ; HMDB37887 ; HMDB37888 ; HMDB39769 ; HMDB39770 ; HMDB40146 ; HMDB40149 ; HMDB40151	Homocitrulline ; LysoPC(20:3(5Z8Z11Z)) ; LysoPC(20:3(8Z11Z14Z)) ; LysoPC(22:6(4Z7Z10Z13Z16Z19Z)) ; (E)-2-octenal ; Ammonium lactate ; Myrtine ; 2-Octenal ; (3xi5Z)-15-Octadien-3-ol ; 244-Trimethylcyclopentanone ; 2-Octen-4-one ; 1-Octen-3-one ; 3-Methyl-3-hepten-2-one ; 5-Methyl-5-hepten-2-one ; 6-Methyl-3-hepten-2-one trans- ; 4-Octen-3-one ; 3-Octen-2-one ; (SE)-Filbertone ; 5-Octen-2-one ; Sulcatone ; Buccoxime ; 2-Ethylidenehexanal ; 4-Ethyl-5-pentyloxazole ; 24-Diethyl-5-propyloxazole ; 2-Butyl-5-ethyl-4-methyloxazole ; 5-Butyl-4-ethyl-2-methyloxazole ; 2-Butyl-4-ethyl-5-methyloxazole ; 45-Dimethyl-2-pentyloxazole ; 4-Butyl-2-ethyl-5-methyloxazole ; 6-Octenal ; (Z)-5-Octenal ; 25-Octadien-1-ol ; (3E5Z)-35-Octadien-1-ol ; (2E4E)-24-Octadien-1-ol	M+H ; M+2H+Na ; M+2H+Na ; M+3H ; M+ACN+Na ; M+2ACN+H ; M+Na ; M+ACN+Na ; M+ACN+Na ; M+ACN+Na ; M+ACN+Na ; M+ACN+Na ; M+ACN+Na ; M+ACN+Na ; M+ACN+Na ; M+ACN+Na ; M+ACN+Na ; M+ACN+Na ; M+ACN+Na ; M+ACN+Na ; M+Na ; M+ACN+Na ; M+Na ; M+Na ; M+Na ; M+Na ; M+Na ; M+Na ; M+Na ; M+ACN+Na ; M+ACN+Na ; M+ACN+Na ; M+ACN+Na ; M+ACN+Na	C7H15N3O3 ; C28H52NO7P ; C28H52NO7P ; C30H50NO7P ; C8H14O ; C3H9NO3 ; C10H17NO ; C8H14O ; C8H14O ; C8H14O ; C8H14O ; C8H14O ; C8H14O ; C8H14O ; C8H14O ; C8H14O ; C8H14O ; C8H14O ; C8H14O ; C8H14O ; C10H17NO ; C8H14O ; C10H17NO ; C10H17NO ; C10H17NO ; C10H17NO ; C10H17NO ; C10H17NO ; C10H17NO ; C8H14O ; C8H14O ; C8H14O ; C8H14O ; C8H14O

8565_280.6	1.852707	0.126748	4.1243	0.002063	2.6856	0.036062	138.0549	HMDB00875 ; HMDB01123 ; HMDB01392 ; HMDB01538 ; HMDB01891 ; HMDB15687 ; HMDB29806 ; HMDB60722 ; HMDB35115 ; HMDB10727 ; HMDB13940 ; HMDB13941 ; HMDB14476 ; HMDB14644 ; HMDB15301 ; HMDB15409 ; HMDB28699 ; HMDB29004 ; HMDB29046 ; HMDB29098 ; HMDB30692 ; HMDB33235 ; HMDB33317 ; HMDB33349 ; HMDB34344 ; HMDB34462 ; HMDB35018 ; HMDB35425 ; HMDB36155 ; HMDB36768 ; HMDB37398 ; HMDB37706 ; HMDB37707 ; HMDB40778 ; HMDB41446 ; HMDB41468 ; HMDB06488	Trigonelline ; 2-Aminobenzoic acid ; p-Aminobenzoic acid ; 3-Pyridylacetic acid ; m-Aminobenzoic acid ; Salicylamide ; Methyl nicotinate ; 2-Pyridylacetic acid ; (1xi3S)-1234-Tetrahydro-1-methyl-beta-carboline-13-dicarboxylic acid ; 3-Oxododecanoic acid ; 3-Hydroxyhexobarbital ; Epoxy-hexobarbital ; Ipratropium bromide ; Cimetidine ; Guanethidine ; Bevantolol ; Alanyl-Tyrosine ; Phenylalanyl-Serine ; Serinyl-Phenylalanine ; Tyrosyl-Alanine ; 8-Hydroxy-7-methoxy-2H-1-benzopyran-2-one ; 812-Epoxy-4(15)711-eudesmatrien-1-one ; (1Z4Z)-15-bis(4-hydroxyphenyl)-14-pentadiene ; Casimiroin ; Scopoletin ; Heptyl 4-hydroxybenzoate ; 2-Phenylethyl 3-phenyl-2-propenoate ; (2E8Z)-Decadiene-46-diyn-1-yl 3-methylbutanoate ; Curzerenone ; Furanodienone ; 4-Acetoxy-2-hexyltetrahydrofuran ; Cyclohexyl cinnamate ; Cinnamyl phenylacetate ; Eremopetasidione ; Rosmaricine ; 135-Trihydroxy-10-methylacridone ; N-	M+H ; M+H ; M+H ; M+H ; M+H ; M+H ; M+H ; M+H ; M+H ; M+2H ; M+Na+K ; M+H+Na ; M+H+Na ; M+3H ; M+H+Na ; M+2K ; M+3Na ; M+H+Na ; M+H+Na ; M+H+Na ; M+H+Na ; M+2ACN+2H ; M+2Na ; M+H+Na ; M+ACN+2H ; M+2ACN+2H ; M+H+K ; M+H+Na ; M+2Na ; M+2Na ; M+2Na ; M+Na+K ; M+2Na ; M+H+Na ; M+H+K ; M+3Na ; M+H+NH4 ; M+H-2H2O	C7H7NO2 ; C7H7NO2 ; C7H7NO2 ; C7H7NO2 ; C7H7NO2 ; C7H7NO2 ; C7H7NO2 ; C7H7NO2 ; C14H14N2O4 ; C12H22O3 ; C12H16N2O4 ; C12H16N2O4 ; C20H30BrNO3 ; C10H16N6S ; C10H22N4 ; C20H27NO4 ; C12H16N2O4 ; C12H16N2O4 ; C12H16N2O4 ; C12H16N2O4 ; C10H8O4 ; C15H18O2 ; C17H16O2 ; C12H11NO4 ; C10H8O4 ; C14H20O3 ; C17H16O2 ; C15H18O2 ; C15H18O2 ; C15H18O2 ; C12H22O3 ; C15H18O2 ; C17H16O2 ; C14H20O3 ; C20H27NO4 ; C14H11NO4 ; C7H11NO4
7836_66.42	1.84071	0.812773	4.1155	0.002092	2.6794	0.03607	238.0378	HMDB29418 ; HMDB15424 ; HMDB15563 ; HMDB29356 ; HMDB29708 ; HMDB31255 ; HMDB32686 ; HMDB38463 ; HMDB38789 ; HMDB41147 ; HMDB41258 ; HMDB41418	S-Cysteinosuccinic acid ; Cefoperazone ; Pargyline ; N-Feruloylglycyl-L-phenylalanine ; 5678-Tetrahydro-24-dimethylquinoline ; Fluorocitric acid ; beta-D-3-5-Deoxy-5-(dimethylarsinyl)ribofuranosyloxy-2-hydroxy-1-propanesulfonic acid ; 3-(Isothiocyanatomethyl)-1-methoxy-1H-indole ; Torvanol A ; Isochinomin ; Homomangiferin ; Taxifolin 3-arabinoside	M+H ; M+3Na ; M+2K+H ; M+2K ; M+2K-H ; 2M+3H2O+2H ; M+2ACN+2H ; 2M+H+K ; M+H+Na ; M+H+K ; M+H+K ; M+H+K	C7H11NO6S ; C25H27N9O8S2 ; C11H13N ; C21H22N2O6 ; C11H15N ; C6H7FO7 ; C10H21AsO9S ; C11H10N2OS ; C20H20O10S ; C20H20O11 ; C20H20O11 ; C20H20O11
0689_129.1	1.838577	0.515431	4.1156	0.002092	2.6795	0.03607	248.5051	HMDB00980 ; HMDB12085	Dihydroneopterin triphosphate ; SM(d18:014:0)	M+2H ; M+3Na	C9H16N5O13P3 ; C37H77N2O6P
3622_127.9	1.818714	0.632455	4.1198	0.002078	2.6824	0.03607	293.0804	HMDB15534 ; HMDB32503 ; HMDB32796 ; HMDB37418 ; HMDB39934 ; HMDB59609 ; HMDB60560	Nitrazepam ; Silica aerogel ; Methyl 3-(23-dihydroxy-3-methylbutyl)-4-hydroxybenzoate ; Molludistin 2-rhamnoside ; Chrysin 7-rhamnosyl-(1-4)-glucoside ; albendazole S-oxide ; Albendazole sulfoxide	2M+H+Na ; 2M+3H ; M+K ; M+H+Na ; M+H+Na ; 2M+H+Na ; 2M+H+Na	C15H11N3O3 ; C23H22N2O3S2 ; C13H18O5 ; C27H30O13 ; C27H30O13 ; C12H15N3O3S ; C12H15N3O3S

8435_19.3	1.799707	0.970569	-4.1211	0.002074	2.6833	0.03607	704.5218	HMDB07870 ; HMDB07902 ; HMDB07966 ; HMDB07998 ; HMDB08893 ; HMDB08894 ; HMDB09021 ; HMDB09054 ; HMDB56400 ; HMDB56401 ; HMDB56418 ; HMDB56428 ; HMDB56489 ; HMDB56490 ; HMDB56498 ; HMDB56507 ; HMDB56509 ; HMDB56513 ; HMDB56543 ; HMDB56587 ; HMDB56643 ; HMDB56924 ; HMDB56933 ; HMDB56935 ; HMDB56939 ; HMDB56969 ; HMDB07121 ; HMDB07148 ; HMDB07149 ; HMDB07201 ; HMDB07230 ; HMDB07257 ; HMDB07258 ; HMDB07284 ; HMDB07285 ; HMDB07295 ; HMDB07313 ; HMDB07314 ; HMDB07324 ; HMDB07341 ; HMDB07351 ; HMDB07352 ; HMDB07425 ; HMDB07452 ; HMDB07453 ; HMDB07481 ; HMDB07482 ; HMDB07509 ; HMDB07520 ; HMDB07538 ; HMDB07549 ; HMDB07565 ; HMDB07566 ; HMDB07576 ; HMDB07577 ; HMDB07585 ;	PC(14:016:1(9Z)) ; PC(14:1(9Z)16:0) ; PC(16:014:1(9Z)) ; PC(16:1(9Z)14:0) ; PE(15:018:1(11Z)) ; PE(15:018:1(9Z)) ; PE(18:1(11Z)15:0) ; PE(18:1(9Z)15:0) ; CL(16:016:018:018:1(11Z)) ; CL(16:016:018:018:1(9Z)) ; CL(16:016:018:1(11Z)18:0) ; CL(16:016:018:1(9Z)18:0) ; CL(16:018:016:018:1(11Z)) ; CL(16:018:016:018:1(9Z)) ; CL(16:018:018:016:1(9Z)) ; CL(16:018:016:1(9Z)18:0) ; CL(16:018:018:1(11Z)16:0) ; CL(16:018:018:1(9Z)16:0) ; CL(16:016:1(9Z)18:018:0) ; CL(16:018:1(11Z)18:016:0) ; CL(16:018:1(9Z)18:016:0) ; CL(18:016:018:016:1(9Z)) ; CL(18:016:016:1(9Z)18:0) ; CL(18:016:018:1(11Z)16:0) ; CL(18:016:018:1(9Z)16:0) ; CL(18:018:016:1(9Z)16:0) ; DG(16:022:6(4Z7Z10Z13Z16Z19Z)0:0) ; DG(16:1(9Z)22:5(4Z7Z10Z13Z16Z)0:0) ; DG(16:1(9Z)22:5(7Z10Z13Z16Z19Z)0:0) ;	M+H ; M+H ; M+H ; M+H ; M+H ; M+H ; M+H ; M+H ; M+2H ; M+2H ; M+2H ; M+2H ; M+2H ; M+2H ; M+2H ; M+2H ; M+2H ; M+2H ; M+2H ; M+2H ; M+2H ; M+2H ; M+2H ; M+2H ; M+2H ; M+ACN+Na ; M+ACN+Na ; M+ACN+Na ; M+ACN+Na ; M+ACN+Na ; M+ACN+Na ; M+ACN+Na ; M+ACN+Na ; M+ACN+Na ; M+ACN+H ; M+ACN+Na ; M+ACN+Na ; M+ACN+H ; M+ACN+Na ; M+ACN+H ; M+ACN+H ; M+ACN+Na ; M+ACN+Na ; M+ACN+Na ; M+ACN+Na ; M+ACN+Na ; M+ACN+Na ; M+ACN+H ; M+ACN+Na ; M+ACN+H ; M+ACN+Na ; M+ACN+Na ; M+ACN+H ; M+ACN+H ; M+NH4 ; M+ACN+Na ; M+ACN+H ; M+ACN+Na ; M+ACN+H ; M+ACN+Na ; M+ACN+H ; M+ACN+H ; M+NH4 ; 2M+Na+K ; 2M+3H ; M+ACN+Na ; M+ACN+Na ; M+ACN+Na ; M+ACN+Na ;	C38H74NO8P ; C38H74NO8P ; C38H74NO8P ; C38H74NO8P ; C38H74NO8P ; C38H74NO8P ; C38H74NO8P ; C38H74NO8P ; C77H148O17P2 ; C77H148O17P2 ; C77H148O17P2 ; C77H148O17P2 ; C77H148O17P2 ; C77H148O17P2 ; C77H148O17P2 ; C77H148O17P2 ; C77H148O17P2 ; C77H148O17P2 ; C77H148O17P2 ; C77H148O17P2 ; C77H148O17P2 ; C77H148O17P2 ; C77H148O17P2 ; C77H148O17P2 ; C77H148O17P2 ; C77H148O17P2 ; C41H68O5 ; C41H68O5 ; C41H68O5 ; C41H68O5 ; C41H68O5 ; C41H68O5 ; C41H68O5 ; C41H68O5 ; C41H68O5 ; C43H66O5 ; C41H68O5 ;
0548_95.08	1.861561	1.034979	-4.0919	0.002172	2.6631	0.03693	256.0605	HMDB01031 ; HMDB01213 ; HMDB01245 ; HMDB01351 ; HMDB29905 ; HMDB31782 ; HMDB34020 ; HMDB34899 ; HMDB35884 ; HMDB36561 ; HMDB38877 ; HMDB40481 ; HMDB40835 ; HMDB41311 ; HMDB59654 ; HMDB60034 ; HMDB60393	Deoxyribose 5-phosphate ; 1-Deoxy-D-xylulose 5-phosphate ; dCDP ; Deoxyribose 1-phosphate ; Artonin T ; Ethiofencarb ; Cajaisoflavone ; 11-Ethylidenebistryptophan ; Melledonol ; Agnuside ; Dihydrocycloartomunin ; Dihydroisocycloartomunin ; Cycloaltilisin ; Myristicanol A ; Deoxyribose 5-monophosphate ; (beta-1-O-N-(2-hydroxymethyl-3-chlorophenyl)anthraniloyl-D-glucupyranuronic acid) ; 5-Deoxyribose-1-phosphate	M+ACN+H ; M+ACN+H ; M+3ACN+2H ; M+ACN+H ; M+Na+K ; 2M+Na+K ; M+Na+K ; M+2K ; M+2K ; M+2Na ; M+Na+K ; M+Na+K ; M+Na+K ; M+2K ; M+ACN+H ; M+ACN+2H ; M+ACN+H	C5H11O7P ; C5H11O7P ; C9H15N3O10P2 ; C5H11O7P ; C26H26O7 ; C11H15NO2S ; C26H26O7 ; C24H26N4O4 ; C23H30O8 ; C22H26O11 ; C26H26O7 ; C26H26O7 ; C26H26O7 ; C23H30O8 ; C5H11O7P ; C20H20ClNO10 ; C5H11O7P

4367_28.76	1.856971	0.302194	-4.0983	0.00215	2.6675	0.03693	237.1234	HMDB15567 ; HMDB29820 ; HMDB29821 ; HMDB29822 ; HMDB29823 ; HMDB29824 ; HMDB29825 ; HMDB31733 ; HMDB32141 ; HMDB32498 ; HMDB32541 ; HMDB33545 ; HMDB34552 ; HMDB34636 ; HMDB34671 ; HMDB34853 ; HMDB34959 ; HMDB34979 ; HMDB35682 ; HMDB35753 ; HMDB36022 ; HMDB36027 ; HMDB36144 ; HMDB36565 ; HMDB36684 ; HMDB36818 ; HMDB36848 ; HMDB36849 ; HMDB37139 ; HMDB38103 ; HMDB39022 ; HMDB40749 ; HMDB40765 ; HMDB41217 ; HMDB41347 ; HMDB41615 ; HMDB59883 ; HMDB14356 ; HMDB29390 ; HMDB29443 ; HMDB33143 ; HMDB37817	Calcipotriol ; 26-Diisopropyl-3-methylphenol ; 25-Diisopropyl-4-methylphenol ; 25-Diisopropyl-3-methylphenol ; 24-Diisopropyl-5-methylphenol ; 24-Diisopropyl-3-methylphenol ; 23-Diisopropyl-5-methylphenol ; 4-(4-Methyl-3-pentenyl)-3-cyclohexene-1-carboxaldehyde ; 4-(Dimethylamino)azobenzene ; Pseudoionone ; 4-(266-Trimethylcyclohex-1-enyl)but-2-en-4-one ; (2E4Z7Z)-247-Tridecatrienal ; Medicagenic acid 3-O-beta-D-glucoside ; Phytolaccasaponin G ; (E)-58-Megastigmadien-4-one ; Cyprodinil ; Edulan I ; gamma-Ionone ; delta-Damascone ; (R)-(E)-47-Megastigmadien-9-one ; Isospirene ; alpha-Damascone ; Cycloionone ; beta-Ionone ; 2457alpha-Tetrahydro-1447a-tetramethyl-1H-inden-2-ol ; Vitispirane ; (9E)-Valenciaxanthin ; Valenciachrome ; 4-(266-Trimethyl-13-cyclohexadien-1-yl)-2-butanone ; (R)-Hispaglabridin B ; Unknown 370 ; 4-Methoxy-3-geranylgeranyl-12-dihydroxybenzene ; 4-Methoxy-5-	M+Na+K ; M+2Na-H ; M+2Na-H ; M+2Na-H ; M+2Na-H ; M+2Na-H ; M+2Na-H ; M+2Na-H ; 2M+H+Na ; M+2Na-H ; M+2Na-H ; M+2Na-H ; M+H+2Na ; M+H+2Na ; M+2Na-H ; 2M+H+Na ; M+2Na-H ; M+2Na-H ; M+2Na-H ; M+2Na-H ; M+2Na-H ; M+2Na-H ; M+2Na-H ; M+2Na-H ; M+2Na-H ; M+2Na-H ; M+Na+K ; M+Na+K ; M+2Na-H ; M+2ACN+2H ; M+Na+K ; M+Na+K ; M+Na+K ; M+2ACN+2H ; M+H+2Na ; M+2Na-H ; M+2Na-H ; M+H-H2O ; M+H-H2O ; M+H-H2O ; M+H-H2O ; M+H-H2O	C27H40O3 ; C13H20O ; C13H20O ; C13H20O ; C13H20O ; C13H20O ; C13H20O ; C13H20O ; C14H15N3 ; C13H20O ; C13H20O ; C13H20O ; C36H56O11 ; C36H56O11 ; C13H20O ; C14H15N3 ; C13H20O ; C13H20O ; C13H20O ; C13H20O ; C13H20O ; C13H20O ; C13H20O ; C13H20O ; C13H20O ; C13H20O ; C27H40O3 ; C27H40O3 ; C13H20O ; C25H26O4 ; C27H40O3 ; C27H40O3 ; C27H40O3 ; C25H26O4 ; C36H56O11 ; C13H20O ; C13H20O ; C12H18N2O4 ; C12H18N2O4 ; C12H18N2O4 ; C12H18N2O4 ; C12H18N2O4
7821_100.3	1.853874	0.074819	-4.0947	0.002163	2.665	0.03693	NA	NA	NA	NA	NA

96085_261	1.846854	0.595186	4.0903	0.002178	2.6619	0.03693	381.1696	HMDB15613 ; HMDB29509 ; HMDB30701 ; HMDB38552 ; HMDB40351 ; HMDB41292 ; HMDB02277 ; HMDB02904 ; HMDB05798 ; HMDB14773 ; HMDB15454 ; HMDB15633 ; HMDB30784 ; HMDB30792 ; HMDB31023 ; HMDB33401 ; HMDB33502 ; HMDB34096 ; HMDB34275 ; HMDB34784 ; HMDB37939 ; HMDB38940 ; HMDB39894 ; HMDB39977 ; HMDB39978 ; HMDB61154	Ecabet ; Garcinone A ; 8-Desoxygartanin ; 6-Deoxy-gamma-mangostin ; (E)-4-(37-Dimethyl-26-octadienyl)-135-trihydroxyxanthone ; Mangostinone ; 23-Dinor-6-keto-prostaglandin F1 a ; 23-Dinor-TXB2 ; Malabaricone C ; Prednisone ; Rasagiline ; Amisulpride ; Normammein ; Monascoflavin ; Achyranthoside D ; Yayoisaponin C ; Lyciumoside IX ; Mammea BBD ; Mammea BAC ; trans-p-Menthane-178-triol 8-glucoside ; Tragopogonsaponin Q ; (-)-11-Hydroxy-91516-trioxooctadecanoic acid ; (1R2R4S)-p-Menthane-128-triol 8-glucoside ; (1S2R4R8S)-p-Menthane-289-triol 2-glucoside ; (1S2R4R8S)-p-Menthane-289-triol 9-glucoside ; Monic acid	M+H ; M+H ; M+H ; M+H ; M+H ; M+H ; M+K ; M+K ; M+Na ; M+Na ; 2M+K ; 2M+H+Na ; M+Na ; M+Na ; M+2H+Na ; M+H+2Na ; M+2Na ; M+Na ; M+Na ; 2M+Na+K ; M+3Na ; M+K ; 2M+Na+K ; 2M+Na+K ; 2M+Na+K ; M+K	C20H28O5S ; C23H24O5 ; C23H24O5 ; C23H24O5 ; C23H24O5 ; C23H24O5 ; C18H30O6 ; C18H30O6 ; C21H26O5 ; C21H26O5 ; C12H13N ; C17H27N3O4S ; C21H26O5 ; C21H26O5 ; C53H82O25 ; C51H84O25 ; C35H56O15 ; C21H26O5 ; C21H26O5 ; C16H30O8 ; C56H82O20 ; C18H30O6 ; C16H30O8 ; C16H30O8 ; C16H30O8 ; C18H30O6
2252_99.84	1.819205	0.249923	4.0901	0.002179	2.6618	0.03693	NA	NA	NA	NA	NA
1154_120.9	1.831073	0.495886	-4.0825	0.002205	2.6565	0.037124	341.0371	HMDB06336 ; HMDB33832 ; HMDB41059 ; HMDB61052 ; HMDB61053	ADP-ribose 2-phosphate ; Cinnamyl cinnamate ; N-Methoxyspirobrassinol methyl ether ; 4-hydroxytriazolam ; 1-hydroxytriazolam	M+ACN+2H ; M+2K-H ; M+2Na-H ; M+H-H2O ; M+H-H2O	C15H24N5O17P3 ; C18H16O2 ; C13H16N2O2S2 ; C17H12Cl2N4O ; C17H12Cl2N4O
7158_284.2	1.827348	0.452906	4.0805	0.002213	2.6551	0.037124	205.0858	HMDB33303 ; HMDB35095 ; HMDB31920 ; HMDB34998 ; HMDB36970 ; HMDB37241 ; HMDB37242 ; HMDB37299 ; HMDB60830 ; HMDB15145 ; HMDB15185 ; HMDB30810 ; HMDB32841 ; HMDB32842 ; HMDB35404 ; HMDB35405 ; HMDB36822	Anofinic acid ; 7-Ethoxy-4-methyl-2H-1-benzopyran-2-one ; 9-Hydroxycalabaxanthone ; Bakkenolide D ; Garcimangosone B ; 89-Dihydro-5-hydroxy-8-(1-hydroxy-1-methylethyl)-6-(2-methyl-1-oxopropyl)-4-phenyl-2H-furo23-h-1-benzopyran-2-one ; Mammea AAC cyclo F ; 68-Dihydroxy-17-diprenylxanthone-2-carboxylic acid ; Melatonin glucuronide ; Edrophonium ; Novobiocin ; Porson ; 5-Megastigmen-7-yne-39-diol 9-glucoside ; 5-Megastigmen-7-yne-39-diol 3-glucoside ; Isogingerenone B ; Gingerenone B ; (3S7E9S)-9-Hydroxy-47-megastigmadien-3-one 9-glucoside	M+H ; M+H ; M+2H ; M+2H ; M+2H ; M+2H ; M+2H ; M+2H ; M+2H ; M+K ; M+3H ; M+H+Na ; M+H+K ; M+H+K ; M+H+Na ; M+H+Na ; M+H+K	C12H12O3 ; C12H12O3 ; C24H24O6 ; C21H28O6S ; C24H24O6 ; C24H24O6 ; C24H24O6 ; C24H24O6 ; C19H24N2O8 ; C10H16NO ; C31H36N2O11 ; C22H26O6 ; C19H30O7 ; C19H30O7 ; C22H26O6 ; C22H26O6 ; C19H30O7

6196_239.4	1.822201	0.891138	4.0847	0.002198	2.658	0.037124	291.9996	HMDB00072 ; HMDB00958 ; HMDB01264 ; HMDB12245 ; HMDB12285 ; HMDB40200	cis-Aconitic acid ; trans-Aconitic acid ; Dehydroascorbic acid ; Kinetin ; Ribose-1-arsenate ; 25-Dimethyl-3-(methyldithio)furan	3M+Na+K ; 3M+Na+K ; 3M+Na+K ; M+2K-H ; M+NH4 ; 3M+Na+K	C6H6O6 ; C6H6O6 ; C6H6O6 ; C10H9N5O ; C5H11AsO8 ; C7H10OS2
9326_277.8	1.880999	0.013542	4.0754	0.002231	2.6515	0.037233	307.5009	HMDB29415	S-Carboxymethyl-L-cysteine	3M+2K	C5H9NO4S
9917_167.7	1.793672	0.24381	-4.0745	0.002234	2.6509	0.037233	289.985	HMDB15153 ; HMDB15627 ; HMDB60555	Guanfacine ; Droxidopa ; 4-Hydroxyclonidine	M+2Na-H ; M+2K-H ; M+2Na-H	C9H9Cl2N3O ; C9H11NO5 ; C9H9Cl2N3O
3521_52.97	1.861048	0.067307	-4.0662	0.002264	2.6451	0.037591	523.0484	HMDB29212 ; HMDB29214 ; HMDB33668 ; HMDB41769	Quercetin 3-O-glucuronide ; Quercetin-4-glucuronide ; Quercetin 4-glucuronide ; Quercetin 3-O-glucuronide	M+2Na-H ; M+2Na-H ; M+2Na-H ; M+2Na-H	C21H18O13 ; C21H18O13 ; C21H18O13 ; C21H18O13
9119_49.76	1.840098	0.423034	4.0644	0.002271	2.6439	0.037591	190.9119	HMDB14118 ; HMDB60333 ; HMDB60357 ; HMDB60452	Trifluoroacetic acid ; 11-Dichloroethylene epoxide ; 22-Dichloroacetaldehyde ; Chloroacetyl chloride	M+2K-H ; 3M+2Na ; 3M+2Na ; 3M+2Na	C2HF3O2 ; C2H2Cl2O ; C2H2Cl2O ; C2H2Cl2O
3676_67.37	1.852624	0.260059	-4.0585	0.002292	2.6398	0.037821	147.5944	HMDB28958 ; HMDB29000 ; HMDB60996 ; HMDB01920 ; HMDB14451 ; HMDB14861 ; HMDB14954 ; HMDB15197 ; HMDB30471 ; HMDB38276 ; HMDB38841 ; HMDB59570	Lysyl-Phenylalanine ; Phenylalanyl-Lysine ; N-Acetyl-3-hydroxyprocainamide ; Dextromethorphan ; Talbutal ; Methoxamine ; Orciprenaline ; Isoproterenol ; 14-Ipomeadiol ; cis-3-Hexenyl pyruvate ; 24-Dodecadienoic acid pyrrolidide ; trans-Nonachlor	M+2H ; M+2H ; M+2H ; M+H+Na ; M+ACN+2H ; M+2ACN+2H ; M+2ACN+2H ; M+2ACN+2H ; M+3ACN+2H ; M+3ACN+2H ; M+2Na ; M+3H	C15H23N3O3 ; C15H23N3O3 ; C15H23N3O3 ; C18H25NO ; C13H20N2O3 ; C11H17NO3 ; C11H17NO3 ; C11H17NO3 ; C9H14O3 ; C9H14O3 ; C16H27NO ; C10H5Cl9
7369_289.9	1.834949	1.120203	4.0522	0.002315	2.6354	0.038081	659.7447	HMDB30300 ; HMDB30627 ; HMDB34281 ; HMDB37825 ; HMDB41501 ; HMDB59886	3-Methyltetradecane ; 5-Hydroxy-734-trimethoxy-8-methylisoflavone 5-neohesperidoside ; Egonol gentiobioside ; xi-25-Dimethyltridecane ; Isopentadecane ; Pentadecane	3M+Na ; 2M+H+NH4 ; 2M+H+NH4 ; 3M+Na ; 3M+Na ; 3M+Na	C15H32 ; C31H38O15 ; C31H38O15 ; C15H32 ; C15H32 ; C15H32
9051_89.09	1.814904	0.226816	4.0437	0.002347	2.6294	0.038348	165.5339	HMDB00058 ; HMDB11616 ; HMDB14492 ; HMDB31800 ; HMDB31766	Cyclic AMP ; Adenosine 23-cyclic phosphate ; Nitisinone ; Mecarbam ; Alachlor	M+2H ; M+2H ; M+2H ; M+2H ; M+Na+K	C10H12N5O6P ; C10H12N5O6P ; C14H10F3NO5 ; C10H20NO5PS2 ; C14H20ClNO2
7072_168.3	1.809147	0.129182	-4.0418	0.002355	2.6281	0.038348	443.0387	HMDB34303 ; HMDB36650	1-Methoxyspirobrassinin ; Sinalbin A	3M+2Na ; 3M+2Na	C12H12N2O2S2 ; C12H12N2O2S2

2422_281.6	1.805889	0.153943	4.0422	0.002353	2.6284	0.038348	757.0522	HMDB57005 ; HMDB57014 ; HMDB57108 ; HMDB57118 ; HMDB57124 ; HMDB57125 ; HMDB57160 ; HMDB57170 ; HMDB57176 ; HMDB57177 ; HMDB57198 ; HMDB57206 ; HMDB57207 ; HMDB57210 ; HMDB57211 ; HMDB57218 ; HMDB57223 ; HMDB57252 ; HMDB57894 ; HMDB57904 ; HMDB57910 ; HMDB57911 ; HMDB57987 ; HMDB58053 ; HMDB58108 ; HMDB58357 ; HMDB58363 ; HMDB58364 ; HMDB58422 ; HMDB58476 ; HMDB58722 ; HMDB58727	CL(18:018:018:2(9Z12Z)20:4(5Z8Z11Z14Z)) ; CL(18:018:020:4(5Z8Z11Z14Z)18:2(9Z12Z)) ; CL(18:018:1(11Z)18:1(11Z)18:2(9Z12Z)) ; CL(18:018:1(11Z)18:1(9Z)18:2(9Z12Z)) ; CL(18:018:1(11Z)18:2(9Z12Z)18:1(11Z)) ; CL(18:018:1(11Z)18:2(9Z12Z)18:1(9Z)) ; CL(18:018:1(9Z)18:1(11Z)18:2(9Z12Z)) ; CL(18:018:1(9Z)18:1(9Z)18:2(9Z12Z)) ; CL(18:018:1(9Z)18:2(9Z12Z)18:1(11Z)) ; CL(18:018:1(9Z)18:2(9Z12Z)18:1(9Z)) ; CL(18:018:2(9Z12Z)18:020:4(5Z8Z11Z14Z)) ; CL(18:018:2(9Z12Z)18:1(11Z)18:1(11Z)) ; CL(18:018:2(9Z12Z)18:1(11Z)18:1(9Z)) ; CL(18:018:2(9Z12Z)18:1(9Z)18:1(11Z)) ;	M+H+NH4 ; M+H+NH4 ; M+ACN+2H ; M+ACN+2H ; M+ACN+2H ; M+ACN+2H ; M+ACN+2H ; M+ACN+2H ; M+ACN+2H ; M+ACN+2H ; M+H+NH4 ; M+ACN+2H ; M+ACN+2H ; M+ACN+2H ; M+ACN+2H ; M+H+NH4 ; M+H+NH4 ; M+H+NH4 ; M+ACN+2H ; M+ACN+2H ; M+ACN+2H ; M+ACN+2H ; M+ACN+2H ; M+ACN+2H ; M+ACN+2H ; M+ACN+2H ; M+ACN+2H ; M+ACN+2H ; M+ACN+2H ; M+ACN+2H ; M+H+NH4 ; M+H+NH4	C84H152O17P2 ; C84H152O17P2 ; C82H152O17P2 ; C82H152O17P2 ; C82H152O17P2 ; C82H152O17P2 ; C82H152O17P2 ; C82H152O17P2 ; C82H152O17P2 ; C82H152O17P2 ; C84H152O17P2 ; C82H152O17P2 ; C82H152O17P2 ; C82H152O17P2 ; C82H152O17P2 ; C84H152O17P2 ; C84H152O17P2 ; C84H152O17P2 ; C82H152O17P2 ; C82H152O17P2 ; C82H152O17P2 ; C82H152O17P2 ; C82H152O17P2 ; C82H152O17P2 ; C82H152O17P2 ; C82H152O17P2 ; C82H152O17P2 ; C82H152O17P2 ;

7041_45.62	1.860524	0.212168	4.0367	0.002374	2.6245	0.038413	219.0797	HMDB60553 ; HMDB00763 ; HMDB00784 ; HMDB00999 ; HMDB01015 ; HMDB02305 ; HMDB02336 ; HMDB03164 ; HMDB03689 ; HMDB04038 ; HMDB11717 ; HMDB12182 ; HMDB14469 ; HMDB14487 ; HMDB14687 ; HMDB29278 ; HMDB29417 ; HMDB29441 ; HMDB30281 ; HMDB30454 ; HMDB30652 ; HMDB30653 ; HMDB31460 ; HMDB31848 ; HMDB32200 ; HMDB32258 ; HMDB32387 ; HMDB32849 ; HMDB33592 ; HMDB34473 ; HMDB35514 ; HMDB36233 ; HMDB40241 ; HMDB40632 ; HMDB40690 ; HMDB41423 ; HMDB59631 ; HMDB59760 ; HMDB59783 ; HMDB59814 ; HMDB59879 ; HMDB60400 ; HMDB60477 ; HMDB61063 ; HMDB61064 ; HMDB61065	E-3174 ; 5-Hydroxyindoleacetic acid ; Azelaic acid ; Phosphoribosylformylglycineamidine ; N-Formyl-L-methionine ; 5-phosphoribosyl-a-N-formylglycineamidine ; Biflorin ; Chlorogenic acid ; Neuroprotectin D1 ; Resolvin D5 ; Nonate ; 8-Hydroxypurine ; Fluorometholone ; Diltiazem ; Desoximetasone ; Cis-5-Caffeoylquinic acid ; Cycloalliin ; Isoalliin ; Calycanthidine ; Macrophorin A ; 1-O-Caffeoylquinic acid ; Cryptochlorogenic acid ; Anthocyanidins ; Butylated hydroxyanisole ; cis- and trans-Ethyl 24-dimethyl-13-dioxolane-2-acetate ; (-)-Ethyl 3-acetoxy-2-methylbutyrate ; (-)-Methyl 5-acetoxyhexanoate ; trans-Chlorogenic acid ; (R)C(S)S-Alliin ; Citbismine F ; xi-23-Dihydro-2-oxo-1H-indole-3-acetic acid ; Butyl ethyl malonate ; 1-(2-Thienyl)-1-heptanone ; Isobiflorin ; 5Z-Caffeoylquinic acid ; Mulberrofuran E ; Cis-stilbene oxide ; 24-Dimethylpimelic acid ; 3-Methylsuberic acid ; Diethyl methylsuccinate ; Diethyl glutarate ; 5-	M+2H ; 2M+3H2O+2H ; 2M+Na+K ; M+3ACN+2H ; M+ACN+H ; M+3ACN+2H ; M+2ACN+2H ; M+2ACN+2H ; M+2K ; M+2K ; 2M+Na+K ; 3M+H+Na ; M+Na+K ; M+H+Na ; M+Na+K ; M+2ACN+2H ; M+ACN+H ; M+ACN+H ; M+2K ; M+2K ; M+2ACN+2H ; M+2ACN+2H ; 2M+H+Na ; M+2K ; 2M+Na+K ; 2M+Na+K ; 2M+Na+K ; M+2ACN+2H ; M+ACN+H ; M+3H ; 2M+3H2O+2H ; 2M+Na+K ; M+Na ; M+2ACN+2H ; M+2ACN+2H ; M+2H+Na ; M+Na ; 2M+Na+K ; 2M+Na+K ; 2M+Na+K ; 2M+Na+K ; 2M+3H2O+2H ; 2M+H+K ; M+2K ; M+2K ; M+2K	C22H21ClN6O2 ; C10H9NO3 ; C9H16O4 ; C8H16N3O8P ; C6H11NO3S ; C8H16N3O8P ; C16H18O9 ; C16H18O9 ; C22H32O4 ; C22H32O4 ; C9H16O4 ; C5H6N4O ; C22H29FO4 ; C22H26N2O4S ; C22H29FO4 ; C16H18O9 ; C6H11NO3S ; C6H11NO3S ; C23H28N4 ; C22H32O4 ; C16H18O9 ; C16H18O9 ; C15H11O ; C22H32O4 ; C9H16O4 ; C9H16O4 ; C9H16O4 ; C16H18O9 ; C6H11NO3S ; C36H34N2O10 ; C10H9NO3 ; C9H16O4 ; C11H16OS ; C16H18O9 ; C16H18O9 ; C39H36O8 ; C14H12O ; C9H16O4 ; C9H16O4 ; C9H16O4 ; C9H16O4 ; C10H9NO3 ; C8H13N3O3 ; C22H32O4 ; C22H32O4 ; C22H32O4
6139_276.2	1.816102	0.05239	4.038	0.002369	2.6255	0.038413	308.4596	HMDB60998	6-hydroxychlorzoxazone	3M+Na+K	C7H4ClNO3
3529_272.7	1.825915	0.612667	4.0323	0.002391	2.6215	0.03856	393.1614	HMDB12201 ; HMDB12202 ; HMDB12203 ; HMDB14853 ; HMDB15107 ; HMDB15464 ; HMDB32000 ; HMDB32010 ; HMDB32011 ; HMDB32895 ; HMDB33965 ; HMDB33981 ; HMDB34999 ; HMDB36895 ; HMDB40989 ; HMDB41369 ; HMDB60094	Cis-zeatin-7-N-glucoside ; Cis-zeatin-9-N-glucoside ; Cis-zeatin-O-glucoside ; Paroxetine ; Azelastine ; Yohimbine ; Raphanatin ; Gibberellin A110 ; Gibberellin A112 ; 3b-Hydroxy-6b-angeloyloxy-7(11)-eremophilen-128b-olide ; (R)-Pantothenic acid 4-O-b-D-glucoside ; Cohumulone ; Bakkenolide C ; Gibberellin A53 ; 3b-Hydroxy-6b-tigloyloxy-7(11)-eremophilen-128b-olide ; cis-Zeatin 9-glucoside ; 1220-Dioxo-leukotriene B4	2M+H+Na ; 2M+H+Na ; 2M+H+Na ; M+ACN+Na ; 2M+H+Na ; M+K ; 2M+H+Na ; M+2Na-H ; M+2Na-H ; M+2Na-H ; 2M+H+Na ; M+2Na-H ; M+2Na-H ; M+2Na-H ; M+2Na-H ; 2M+H+Na ; M+2Na-H	C16H23N5O6 ; C16H23N5O6 ; C16H23N5O6 ; C19H20FNO3 ; C22H24ClN3O ; C21H26N2O3 ; C16H23N5O6 ; C20H28O5 ; C20H28O5 ; C20H28O5 ; C15H27NO10 ; C20H28O5 ; C20H28O5 ; C20H28O5 ; C20H28O5 ; C16H23N5O6 ; C20H28O5
1192_127.0	1.845601	0.198864	-4.0276	0.002409	2.6182	0.038728	NA	NA	NA	NA	NA

[illegible]7585_101.[illegible]	1.799238	0.039683	4.0168	0.002451	2.6107	0.039152	534.1288	HMDB12253 ; HMDB29271 ; HMDB30658 ; HMDB33894 ; HMDB37366 ; HMDB37459 ; HMDB38465 ; HMDB38772 ; HMDB39904 ; HMDB39923 ; HMDB39925 ; HMDB40633 ; HMDB41757	Maltohexaose ; Quercetin 3-O-(6-acetyl-glucoside) ; Carmine red ; Tricin 7-glucuronoside ; Quercetin 3-(6-acetylglucoside) ; 6-Methoxyluteolin 7-glucuronide ; Glyphoside ; 8-Hydroxyluteolin 4-methyl ether 8-glucuronide ; 3-Methoxytricetin 7-glucuronide ; 4-O-Acetylmyricitrin ; 26-Di-O-acetylononin ; Isobiflorin 6-gallate ; Isorhamnetin 4-O-glucuronide	M+2K ; 2M+3H2O+2H ; M+ACN+H ; 2M+3H2O+2H ; 2M+3H2O+2H ; M+ACN+H ; 2M+3H2O+2H ; M+ACN+H ; M+ACN+H ; 2M+3H2O+2H ; 2M+H+K ; 2M+3H2O+2H ; M+ACN+H	C36H62O31 ; C23H22O13 ; C22H20O13 ; C23H22O13 ; C23H22O13 ; C22H20O13 ; C23H22O13 ; C22H20O13 ; C22H20O13 ; C23H22O13 ; C26H26O11 ; C23H22O13 ; C22H20O13
[illegible]5483_145.[illegible]	1.76941	0.003849	4.0183	0.002445	2.6117	0.039152	349.1174	HMDB01557 ; HMDB29299 ; HMDB01081 ; HMDB02242 ; HMDB04220 ; HMDB06581 ; HMDB06584 ; HMDB14507 ; HMDB15141 ; HMDB15166 ; HMDB30311 ; HMDB31182 ; HMDB32066 ; HMDB38009 ; HMDB38033 ; HMDB38434 ; HMDB40035 ; HMDB40063 ; HMDB40064 ; HMDB40071 ; HMDB40072 ; HMDB40073 ; HMDB40074 ; HMDB40075 ; HMDB40102 ; HMDB40103 ; HMDB40104 ; HMDB40105 ; HMDB40459 ; HMDB59795 ; HMDB60610	Riboflavin reduced ; Sesamolinol 4-O-b-D-glucosyl (1-6)-O-b-D-glucoside ; (N-acetylneuraminosyl(a2-6)lactosamine) ; Dehydroisocoproporphyrinogen ; Biotinyl-5-AMP ; 3-Sialyl-N-acetyllactosamine ; 6-Sialyl-N-acetyllactosamine ; Clozapine ; Letrozole ; Probenecid ; Harmine ; 1H-Indol-3-ylacetyl-myo-inositol ; Carbanilide ; Malvin ; Malvidin 3-laminaribioside ; 7-Isothiocyanato-1-heptene ; 2-Pentylthiazole ; 4-Butyl-5-methylthiazole ; 4-Ethyl-5-propylthiazole ; 24-Dimethyl-5-propylthiazole ; 25-Dimethyl-4-propylthiazole ; 45-Dimethyl-2-propylthiazole ; 45-Dimethyl-2-(1-methylethyl)thiazole ; 25-Diethyl-4-methylthiazole ; 2-Butyl-4-methylthiazole ; 4-Butyl-2-methylthiazole ; 2-Ethyl-4-propylthiazole ; 4-Ethyl-2-propylthiazole ; Ethylene brassylate ; Gluten exorphin B5 ; Hydroxyhexamide	M+H ; M+2H ; M+H+Na ; M+2Na ; M+3ACN+2H ; M+H+Na ; M+H+Na ; M+Na ; M+ACN+Na ; M+ACN+Na ; 3M+Na+K ; 2M+H+Na ; 3M+Na+K ; M+ACN+2H ; M+ACN+2H ; 2M+K ; 2M+K ; 2M+K ; 2M+K ; 2M+K ; 2M+K ; 2M+K ; 2M+K ; 2M+K ; 2M+K ; 2M+K ; 2M+K ; 2M+K ; M+2K+H ; M+H+K ; M+Na	C15H16N4O6 ; C32H40O17 ; C25H42N2O19 ; C36H36N4O8 ; C20H28N7O9PS ; C25H42N2O19 ; C25H42N2O19 ; C18H19ClN4 ; C17H11N5 ; C13H19NO4S ; C13H12N2O ; C16H19NO7 ; C13H12N2O ; C29H35O17 ; C29H35O17 ; C8H13NS ; C8H13NS ; C8H13NS ; C8H13NS ; C8H13NS ; C8H13NS ; C8H13NS ; C8H13NS ; C8H13NS ; C8H13NS ; C8H13NS ; C8H13NS ; C8H13NS ; C15H26O4 ; C30H38N6O11 ; C15H22N2O4S
[illegible]9296_114.[illegible]	1.813537	1.109735	4.0144	0.00246	2.609	0.039178	464.0808	HMDB00536 ; HMDB59653 ; HMDB06779 ; HMDB30481 ; HMDB39907	Adenylsuccinic acid ; N(6)-(12-dicarboxyethyl)AMP ; Indole-56-quinone ; Agrocybin ; Tricin 7-sinapoyl-(-2)-glucuronyl-(1-2)-glucuronide	M+H ; M+H ; 3M+Na ; 3M+Na ; M+H+K	C14H18N5O11P ; C14H18N5O11P ; C8H5NO2 ; C8H5NO2 ; C40H40O23

2039_91.04	1.830169	0.722614	-4.011	0.002474	2.6066	0.03924	244.1292	HMDB28805 ; HMDB29015 ; HMDB29031 ; HMDB29157 ; HMDB12634	Glutaminyl-Proline ; Prolyl-Glutamine ; Prolyl-Gamma-glutamate ; Gamma-glutamyl-Proline ; 20-COOH-leukotriene E4	M+H ; M+H ; M+H ; M+H ; M+H+NH4	C10H17N3O4 ; C10H17N3O4 ; C10H17N3O4 ; C10H17N3O4 ; C23H35NO7S
9416_291.1	1.821074	1.326046	4.0095	0.00248	2.6055	0.03924	714.5619	HMDB00950 ; HMDB11767 ; HMDB13464	Cer(d18:123:0) ; Cer(d18:023:0) ; SM(d18:016:1(9Z))	M+2K+H ; M+2K-H ; 2M+H+Na	C41H81NO3 ; C41H83NO3 ; C39H79N2O6P
78292_114.	1.831842	0.833999	-3.9974	0.002529	2.5971	0.039762	150.0378	HMDB33001 ; HMDB33969 ; HMDB34273 ; HMDB60834 ; HMDB04818 ; HMDB06790 ; HMDB11118 ; HMDB15144 ; HMDB15477 ; HMDB29687 ; HMDB30110 ; HMDB30578 ; HMDB30690 ; HMDB30788 ; HMDB30853 ; HMDB30854 ; HMDB30948 ; HMDB32890 ; HMDB32953 ; HMDB33015 ; HMDB33016 ; HMDB33191 ; HMDB33771 ; HMDB34046 ; HMDB34133 ; HMDB35254 ; HMDB36050 ; HMDB38664 ; HMDB39142 ; HMDB39592 ; HMDB41192 ; HMDB41873	2-Methylbenzothiazole ; Benzyl isothiocyanate ; Benzyl thiocyanate ; N-Acetylserotonin sulfate ; Biotin sulfone ; Galactosylglycerol ; Se-Adenosylselenomethionine ; Ketoprofen ; Clenbuterol ; 36-Dimethoxy-4-phenanthrenol ; Celerin ; Apigravin ; (E)-Suberenol ; Citrusal ; (R)-Meranzin ; (S)-Auraptenol ; Dihydrowyerone ; 26-Dimethoxy-4-phenanthrenol ; 7-Methoxy-5-prenyloxycoumarin ; 56-Dihydroyangonin ; 78-Dihydroyangonin ; 2244-Tetramethyl-6-(1-oxopropyl)-135-cyclohexanetrione ; Cyperine ; 1234-Tetramethoxy-5-(2-propenyl)benzene ; 3-(11-Dimethylallyl)scopoletin ; 7-Hydroxy-5-isopropyl-2-methoxy-3-methyl-14-naphthoquinone ; Anhydromarasmone ; (2R)-1-O-beta-D-Galactopyranosylglycerol ; Wyerol ; Pergillin ; Kanzonol Q ; Dexketoprofen	M+H ; M+H ; M+H ; M+2H ; M+H+Na ; M+2Na ; M+3H ; M+2Na ; M+H+Na ; M+2Na ; M+H+K ; M+H+K ; M+H+K ; M+H+K ; M+H+K ; M+H+K ; M+H+K ; M+2Na ; M+H+K ; M+H+K ; M+H+K ; M+Na+K ; M+H+K ; M+Na+K ; M+H+K ; M+H+K ; M+H+K ; M+2Na ; M+H+K ; M+H+K ; M+H+K ; M+2Na	C8H7NS ; C8H7NS ; C8H7NS ; C12H14N2O5S ; C10H16N2O5S ; C9H18O8 ; C15H23N6O5Se ; C16H14O3 ; C12H18Cl2N2O ; C16H14O3 ; C15H16O4 ; C15H16O4 ; C15H16O4 ; C15H16O4 ; C15H16O4 ; C15H16O4 ; C15H16O4 ; C16H14O3 ; C15H16O4 ; C15H16O4 ; C15H16O4 ; C13H18O4 ; C15H16O4 ; C13H18O4 ; C15H16O4 ; C15H16O4 ; C15H16O4 ; C9H18O8 ; C15H16O4 ; C15H16O4 ; C15H16O4 ; C16H14O3
824_160.35	1.787551	1.007556	-3.9988	0.002523	2.598	0.039762	195.9682	HMDB14373 ; HMDB14891	Enflurane ; Isoflurane	2M+H+Na ; 2M+H+Na	C3H2ClF5O ; C3H2ClF5O
7031_255.7	1.824889	0.344397	3.9952	0.002538	2.5955	0.039778	256.0367	HMDB13991 ; HMDB29218 ; HMDB29463 ; HMDB29544 ; HMDB30216 ; HMDB31555 ; HMDB33850 ; HMDB34709 ; HMDB37476 ; HMDB41768 ; HMDB59972 ; HMDB59974 ; HMDB59983	5-Hydroxylornoxicam ; Urolithin C ; Gentisein ; Miscanthoside ; Mukonal ; Maesopsin 6-glucoside ; Astilbin ; BL IV ; Neocarthamin ; Phloretin 2-O-glucuronide ; 4-Hydroxy-5-(34-dihydroxyphenyl)-valeric acid-O-methyl-O-glucuronide ; 4-Hydroxy-5-(35-dihydroxyphenyl)-valeric acid-O-methyl-O-glucuronide ; 4-phenylbutanic acid-O-sulphate	M+3ACN+2H ; 2M+H+Na ; 2M+H+Na ; M+Na+K ; M+2Na-H ; M+Na+K ; M+Na+K ; M+2Na ; M+Na+K ; M+Na+K ; M+2K ; M+2K ; 2M+H+Na	C13H10ClN3O5S2 ; C13H8O5 ; C13H8O5 ; C21H22O11 ; C13H9NO2 ; C21H22O11 ; C21H22O11 ; C24H18O10 ; C21H22O11 ; C21H22O11 ; C18H26O12 ; C18H26O12 ; C10H12O5S

2986_168.4	1.798807	0.298954	-3.9909	0.002556	2.5925	0.039929	250.4983	HMDB37168 ; HMDB38423	2-Propanoylthiazole ; Gluconasturtiin	3M+2K ; M+2K	C6H7NOS ; C15H21NO9S2
3595_224.2	1.77705	0.543781	3.9825	0.002591	2.5866	0.040351	261.5354	HMDB33042 ; HMDB33046 ; HMDB33056 ; HMDB33057	Methyl 1-(methylthio)propyl disulfide ; Ethyl 1-(methylthio)ethyl disulfide ; Ethyl (ethylthio)methyl disulfide ; 1-(Ethylthio)ethyl methyl disulfide	3M+H+NH4 ; 3M+H+NH4 ; 3M+H+NH4 ; 3M+H+NH4	C5H12S3 ; C5H12S3 ; C5H12S3 ; C5H12S3
6158_100.3	1.840453	0.342628	-3.9719	0.002635	2.5792	0.040795	279.5816	HMDB00707 ; HMDB01879 ; HMDB01964 ; HMDB02130 ; HMDB03501 ; HMDB06915 ; HMDB11663 ; HMDB32057 ; HMDB32131 ; HMDB36592	4-Hydroxyphenylpyruvic acid ; Aspirin ; Caffeic acid ; Monomethyl phthalate ; 34-Dihydroxy-trans-cinnamate ; 2-Hydroxy-3-(4-hydroxyphenyl)propenoic acid ; 3-Hydroxyphenylpyruvic acid ; (E)-3-(23-Dihydroxyphenyl)-2-propenoic acid ; 3-(35-Dihydroxyphenyl)-2-propenoic acid ; Validamycin A	3M+H+NH4 ; 3M+H+NH4 ; 3M+H+NH4 ; 3M+H+NH4 ; 3M+H+NH4 ; 3M+H+NH4 ; 3M+H+NH4 ; 3M+H+NH4 ; 3M+H+NH4 ; M+Na+K	C9H8O4 ; C9H8O4 ; C9H8O4 ; C9H8O4 ; C9H8O4 ; C9H8O4 ; C9H8O4 ; C9H8O4 ; C9H8O4 ; C20H35NO13
7334_242.1	1.812795	0.497767	-3.9736	0.002628	2.5803	0.040795	285.1195	HMDB13129 ; HMDB29868 ; HMDB30593 ; HMDB30845 ; HMDB33428 ; HMDB34679 ; HMDB35475 ; HMDB36982 ; HMDB39912 ; HMDB41048 ; HMDB60841	Glutaconylcarnitine ; Mangostanol ; Edulisin II ; Archangelicin ; ()-Setoclavine ; Cinncassiol D2 glucoside ; Simmondsin 2-ferulate ; Atrovirisidone ; Mangostenol ; Physagulin B ; N-Despropyl-rotigotine	2M+H+Na ; 2M+3H ; 2M+3H ; 2M+3H ; 2M+Na+K ; M+H+K ; M+H+NH4 ; 2M+3H ; 2M+3H ; M+H+Na ; 2M+H+Na	C12H19NO6 ; C24H26O7 ; C24H26O7 ; C24H26O7 ; C16H18N2O ; C26H42O11 ; C26H33NO12 ; C24H26O7 ; C24H26O7 ; C30H39ClO7 ; C16H19NOS
4811_57.06	1.848263	0.405794	3.9698	0.002644	2.5777	0.040803	244.9235	HMDB29718 ; HMDB36577	Thiophene ; Trifluoromethanesulfonic acid	2M+2K-H ; 3M+H+K	C4H4S ; CHF3O3S
92258_49.8	1.77429	0.603566	-3.9678	0.002653	2.5763	0.040813	318.8792	HMDB39460	Di-2-propenyl hexasulfide	M+2Na-H	C6H10S6

2549_107.5	1.833526	0.328899	-3.9636	0.002671	2.5734	0.040961	213.0983	HMDB28843 ; HMDB28885 ; HMDB33750 ; HMDB15321 ; HMDB01080 ; HMDB13287 ; HMDB13682 ; HMDB14600 ; HMDB14898 ; HMDB15052 ; HMDB15459 ; HMDB29683 ; HMDB30107 ; HMDB30175 ; HMDB30523 ; HMDB30868 ; HMDB31215 ; HMDB31581 ; HMDB32767 ; HMDB33870 ; HMDB35027 ; HMDB35477 ; HMDB35774 ; HMDB36462 ; HMDB36801 ; HMDB37552 ; HMDB38630 ; HMDB39334 ; HMDB39662 ; HMDB40016 ; HMDB40029 ; HMDB40043 ; HMDB40047 ; HMDB40050 ; HMDB40051 ; HMDB40663 ; HMDB41273 ; HMDB60247 ; HMDB60900	Glycyl-Histidine ; Histidinyl-Glycine ; D-Glycero-D-galacto-heptitol ; Clindamycin ; 4-Aminobutyraldehyde ; NeNe dimethyllysine ; Coniferin ; Prazosin ; Meropenem ; Metronidazole ; Cortisone acetate ; 2-Hydroxy-6-pentadecylbenzoic acid ; 3-Hydroxy-3-(34-dihydroxy-4-methylpentanoyl)-5-(3-methylbutyl)-124-cyclopentanetrione ; Venoterpine ; Citreoviridin C ; Citreoviridin A ; N-Ethylacetamide ; Morpholine ; Sphalleroside A ; Butyramide ; Melleolide F ; Tridecyl phloretate ; Armillyl orsellinate ; Virolongin B ; ent-16b19-Kauranediol 19-acetate ; 4-Hydroxy-3-(2-hydroxyethyl)acetophenone 4-glucoside ; Niazimin A ; Citrusin D ; 2367-Tetrahydrocyclopentbazepin-8(1H)-one ; 2-(2-Furanyl)-3456-tetrahydropyridine ; 23-Dihydro-5-methyl-1H-pyrrolizine-7-carboxaldehyde ; 3-(2-Furanylmethylene)pyrrolidine ; 23-Dihydro-6-methyl-1H-pyrrolizine-5-carboxaldehyde ; 34-Dihydro-5-(5-methyl-2-furanyl)-2H-pyrrole ; 5-	M+H ; M+H ; M+H ; M+2H ; 2M+K ; M+K ; M+2ACN+2H ; M+ACN+2H ; M+ACN+2H ; M+ACN+H ; M+H+Na ; M+2K ; M+2ACN+2H ; M+ACN+Na ; M+H+Na ; M+H+Na ; 2M+K ; 2M+K ; M+2ACN+2H ; 2M+K ; M+H+Na ; M+2K ; M+H+Na ; M+H+Na ; M+2K ; M+2ACN+2H ; M+ACN+2H ; M+2ACN+2H ; M+ACN+Na ; M+ACN+Na ; M+ACN+Na ; M+ACN+Na ; M+ACN+Na ; M+ACN+Na ; M+ACN+Na ; M+2ACN+2H ; M+2ACN+2H ; 2M+K ; M+2ACN+2H	C8H12N4O3 ; C8H12N4O3 ; C7H16O7 ; C18H33ClN2O5S ; C4H9NO ; C8H18N2O2 ; C16H22O8 ; C19H21N5O4 ; C17H25N3O5S ; C6H9N3O3 ; C23H30O6 ; C22H36O3 ; C16H22O8 ; C9H11NO ; C23H30O6 ; C23H30O6 ; C4H9NO ; C4H9NO ; C16H22O8 ; C4H9NO ; C23H30O6 ; C22H36O3 ; C23H30O6 ; C23H30O6 ; C22H36O3 ; C16H22O8 ; C18H25NO8 ; C16H22O8 ; C9H11NO ; C9H11NO ; C9H11NO ; C9H11NO ; C9H11NO ; C9H11NO ; C9H11NO ; C16H22O8 ; C16H22O8 ; C4H9NO ; C18H19ClN4O
6779_61.14	1.840586	0.151073	3.961	0.002682	2.5715	0.041009	132.0767	HMDB00064 ; HMDB13222 ; HMDB00076 ; HMDB01536 ; HMDB03646 ; HMDB14813 ; HMDB28832 ; HMDB29126 ; HMDB31659 ; HMDB32392 ; HMDB39160 ; HMDB60245	Creatine ; Beta-Guanidinopropionic acid ; Dihydrouracil ; Formamide ; N-Methylhydantoin ; Tamoxifen ; Glutamyl-Valine ; Valyl-Glutamate ; 3-Methyl-1-butylamine ; 2-Methylbutylamine ; L-Gizzerosine ; L-3-Cyanoalanine	M+H ; M+H ; M+NH4 ; 2M+ACN+H ; M+NH4 ; M+2H+Na ; M+H+NH4 ; M+H+NH4 ; M+2Na-H ; M+2Na-H ; M+H+Na ; M+NH4	C4H9N3O2 ; C4H9N3O2 ; C4H6N2O2 ; CH3NO ; C4H6N2O2 ; C26H29NO ; C10H17N2O5 ; C10H17N2O5 ; C5H13N ; C5H13N ; C11H20N4O2 ; C4H6N2O2
6875_92.02	1.792997	0.466466	-3.951	0.002726	2.5645	0.041553	NA	NA	NA	NA	NA
76362_101.7	1.809183	1.088844	-3.9487	0.002736	2.5629	0.04158	271.5576	HMDB09776 ; HMDB09777 ; HMDB11429 ; HMDB11462 ; HMDB15230	PE(24:1(15Z)P-18:1(11Z)) ; PE(24:1(15Z)P-18:1(9Z)) ; PE(P-18:1(11Z)24:1(15Z)) ; PE(P-18:1(9Z)24:1(15Z)) ; Rosuvastatin	M+3H ; M+3H ; M+3H ; M+3H ; M+Na+K	C47H90NO7P ; C47H90NO7P ; C47H90NO7P ; C47H90NO7P ; C22H28FN3O6S

3747_102.7	1.831007	0.35275	-3.939	0.002779	2.5561	0.04211	260.1137	HMDB13134 ; HMDB32769 ; HMDB60499 ; HMDB03459 ; HMDB04081 ; HMDB28680 ; HMDB31411 ; HMDB32839 ; HMDB35626 ; HMDB38126 ; HMDB39136 ; HMDB60069 ; HMDB60721 ; HMDB60768	Fumarycarnitine ; Osmaronin ; NPC ; D-Alanyl-D-alanine ; 6-Hydroxymelatonin ; Alanyl-Alanine ; 4-Acetamido-2-aminobutanoic acid ; Citronellyl beta-sophoroside ; Glaucarubin ; Genipinic acid ; Elenaic acid ; cyclic 3-Hydroxymelatonin ; 2-Oxomelatonin ; 4-Hydroxymethylpyrazole	M+H ; M+H ; M+2H ; 3M+H+K ; 2M+H+Na ; 3M+H+K ; 3M+H+K ; M+H+K ; M+H+Na ; M+NH4 ; M+NH4 ; 2M+H+Na ; 2M+H+Na ; 2M+ACN+Na	C11H17NO6 ; C11H17NO6 ; C28H30N4O6 ; C6H12N2O3 ; C13H16N2O3 ; C6H12N2O3 ; C6H12N2O3 ; C22H40O11 ; C25H36O10 ; C11H14O6 ; C11H14O6 ; C13H16N2O3 ; C13H16N2O3 ; C4H6N2O
2922_150.	1.783164	1.09887	3.9347	0.002799	2.5531	0.042158	276.1553	HMDB12358 ; HMDB12368 ; HMDB12388 ; HMDB12398 ; HMDB15340 ; HMDB29388 ; HMDB33601 ; HMDB35085 ; HMDB37326 ; HMDB41572 ; HMDB28711 ; HMDB28879	PS(16:018:2(9Z12Z)) ; PS(16:1(9Z)18:1(9Z)) ; PS(18:1(9Z)16:1(9Z)) ; PS(18:2(9Z12Z)16:0) ; Dezocine ; Cucurbic acid ; Dihydrojasmonic acid ; Vulgarole ; Traumatin ; 3-Ethenyl-4-hydroxy-25-dimethylhex-5-en-2-yl acetate ; Arginyl-Histidine ; Histidinyl-Arginine	M+3Na ; M+3Na ; M+3Na ; M+3Na ; 2M+Na+K ; M+ACN+Na ; M+ACN+Na ; M+ACN+Na ; M+ACN+Na ; M+ACN+Na ; M+H-2H2O ; M+H-2H2O	C40H74NO10P ; C40H74NO10P ; C40H74NO10P ; C40H74NO10P ; C16H23NO ; C12H20O3 ; C12H20O3 ; C12H20O3 ; C12H20O3 ; C12H20O3 ; C12H21N7O3 ; C12H21N7O3
7575_27.3	1.758673	0.567686	-3.9346	0.002799	2.5529	0.042158	262.1198	HMDB28852 ; HMDB29083 ; HMDB00030 ; HMDB00714 ; HMDB00992 ; HMDB01185 ; HMDB02012 ; HMDB12884 ; HMDB29442 ; HMDB30127 ; HMDB31340 ; HMDB32078 ; HMDB32116 ; HMDB32398 ; HMDB32595 ; HMDB33525 ; HMDB38804 ; HMDB39531 ; HMDB41779	Glycyl-Tryptophan ; Tryptophyl-Glycine ; Biotin ; Hippuric acid ; 3-Succinoylpyridine ; S-Adenosylmethionine ; Ubiquinone-1 ; Adrenochrome ; (R)C(R)S-S-Propylcysteine sulfoxide ; Withaperuvin E ; Cyclamic acid ; Dibenzyl ether ; Helinorbisabone ; Methyl n-formylanthranilate ; 1-(4-Methoxyphenyl)-2-nitroethylene ; 24-Undecadiene-810-diynoic acid 23-dehydropiperidide ; 34-Dihydro-6-hydroxy-2578-tetramethyl-2H-1-benzopyran-2-carboxylic acid ; Ethoxyquin ; Tigloylgomicin H	M+H ; M+H ; M+NH4 ; M+2ACN+H ; M+2ACN+H ; M+3ACN+2H ; 2M+H+Na ; M+2ACN+H ; M+2ACN+H ; M+H+Na ; M+2ACN+H ; M+ACN+Na ; 2M+H+Na ; M+2ACN+H ; M+2ACN+H ; M+Na ; 2M+H+Na ; M+2Na-H ; M+H+Na	C13H15N3O3 ; C13H15N3O3 ; C10H16N2O3S ; C9H9NO3 ; C9H9NO3 ; C15H23N6O5S ; C14H18O4 ; C9H9NO3 ; C6H13NO3S ; C28H36O8 ; C6H13NO3S ; C14H14O ; C14H18O4 ; C9H9NO3 ; C9H9NO3 ; C16H17NO ; C14H18O4 ; C14H19NO ; C28H36O8
1516_268.	1.80689	0.467453	-3.9262	0.002838	2.547	0.042608	673.4852	HMDB07860 ; HMDB29767 ; HMDB34599 ; HMDB41464	PA(16:018:2(9Z12Z)) ; Saringosterol 3-glucoside ; Hebevinoside IX ; Melilotoside A	M+H ; M+2ACN+H ; M+2ACN+H ; M+2ACN+H	C37H69O8P ; C35H58O7 ; C35H58O7 ; C35H58O7
5182_32.1	1.757323	0.385977	3.9218	0.002858	2.5439	0.042784	277.1835	HMDB13272 ; HMDB35113 ; HMDB35970 ; HMDB38683 ; HMDB39883 ; HMDB15026	N-Lauroylglycine ; 1723-Epoxy-29-hydroxy-27-norlanost-8-ene-31524-trione ; (3beta17alpha23S)-1723-Epoxy-329-dihydroxy-27-norlanosta-79(11)-diene-1524-dione ; 19alpha-19-Hydroxy-311-dioxo-12-ursen-28-oic acid ; Ascorbyl palmitate ; Granisetron	2M+H+K ; M+2ACN+2H ; M+2ACN+2H ; M+2ACN+2H ; 2M+3H ; M+H-2H2O	C14H27NO3 ; C29H42O5 ; C29H42O5 ; C29H42O5 ; C22H38O7 ; C18H24N4O

[illegible]1954_56.4[illegible]	1.822748	0.943167	3.9108	0.00291	2.5362	0.043296	394.8742	HMDB14786	Mitotane	M+2K-H	C14H10Cl4
[illegible]9424_101.[illegible]	1.820523	0.660796	-3.9113	0.002907	2.5365	0.043296	280.0919	HMDB34347 ; HMDB14643 ; HMDB30134 ; HMDB32730 ; HMDB33281 ; HMDB33282 ; HMDB34787 ; HMDB34767	Physalin K ; Tolmetin ; Asticolorin B ; Divanillyltetrahydrofuran ferulate ; 8-Hydroxypinoresinol 8-glucoside ; 8-Hydroxypinoresinol 4-glucoside ; Neoacrimarine J ; (1R3R3S)-1234-Tetrahydro-1-(2-thio-3-pyrrolidinyl)-beta-carboline-3-carboxylic acid	M+2H ; M+Na ; M+H+Na ; M+H+K ; M+H+Na ; M+H+Na ; M+ACN+2H ; M+H-2H2O	C28H30O12 ; C15H15NO3 ; C33H28O7 ; C30H32O8 ; C26H32O12 ; C26H32O12 ; C28H23NO9 ; C16H17N3O2S
[illegible]6775_216.[illegible]	1.774395	0.314954	3.9072	0.002927	2.5337	0.04342	247.0567	HMDB29481 ; HMDB29968 ; HMDB30018 ; HMDB30604 ; HMDB31586 ; HMDB32616 ; HMDB32657 ; HMDB33652 ; HMDB33741 ; HMDB33942 ; HMDB34069 ; HMDB37509 ; HMDB40561 ; HMDB40894 ; HMDB41536 ; HMDB41691 ; HMDB41946	Isosakuranin ; Ethyl beta-D-glucopyranoside ; Elenolide ; epsilon-Viniferin ; 7-Hydroxy-8-O-methylaloin B ; Sinapic acid ; trans-delta-Viniferin ; Prunin 46-di-O-gallate ; Puddumin A ; Dambonitol ; cis-Sinapic acid ; Chalconosakuranetin ; Aromadendrin 4-methyl ether 7-rhamnoside ; Ampelopsin D ; Piperenol C ; 5-(345-Trihydroxyphenyl)-gamma-valerolactone ; N-Nitrosoproline	M+2Na ; M+K ; M+Na ; M+H+K ; M+2Na ; M+Na ; M+H+K ; M+3H ; M+2Na ; M+K ; M+Na ; M+2Na ; M+2Na ; M+H+K ; M+2Na ; M+Na ; 3M+Na+K	C22H24O10 ; C8H16O6 ; C11H12O5 ; C28H22O6 ; C22H24O10 ; C11H12O5 ; C28H22O6 ; C35H30O18 ; C22H24O10 ; C8H16O6 ; C11H12O5 ; C22H24O10 ; C22H24O10 ; C28H22O6 ; C22H24O10 ; C11H12O5 ; C5H8N2O3

[illegible]7994_21.2[illegible]	1.837537	0.353941	3.8985	0.002968	2.5275	0.043552	443.3558	HMDB01181 ; HMDB06927 ; HMDB32668 ; HMDB35735 ; HMDB60146 ; HMDB43683 ; HMDB43739 ; HMDB43795 ; HMDB52765 ; HMDB53583 ; HMDB55367 ; HMDB55447 ; HMDB55459 ; HMDB55689 ; HMDB07993 ; HMDB08025 ; HMDB08052 ; HMDB08084 ; HMDB08117 ; HMDB08158 ; HMDB08190 ; HMDB08223 ; HMDB08275 ; HMDB08307 ; HMDB08317 ; HMDB08349 ; HMDB08381 ; HMDB08413 ; HMDB08531 ; HMDB08532 ; HMDB08540 ; HMDB08541 ; HMDB08563 ; HMDB08571 ; HMDB08603 ; HMDB08759 ; HMDB08764 ; HMDB08765 ; HMDB08791 ; HMDB08796 ; HMDB29206	4a-Carboxy-4b-methyl-5a-cholesta-824-dien-3b-ol ; 4a-Methylzymosterol-4-carboxylic acid ; (3beta22E24R)-58-Epidioxy-23-methylergosta-622-dien-3-ol ; Camellenodiol ; 4-Methylzymosterol intermediate 1 ; TG(15:018:4(6Z9Z12Z15Z)22:6(4Z7Z10Z13Z16Z19Z)) ; TG(15:020:5(5Z8Z11Z14Z17Z)20:5(5Z8Z11Z14Z17Z)) ; TG(15:022:6(4Z7Z10Z13Z16Z19Z)18:4(6Z9Z12Z15Z)) ; TG(18:2(9Z12Z)18:4(6Z9Z12Z15Z)18:4(6Z9Z12Z15Z)) ; TG(20:2n618:4(6Z9Z12Z15Z)18:4(6Z9Z12Z15Z)) ; TG(18:4(6Z9Z12Z15Z)15:022:6(4Z7Z10Z13Z16Z19Z)) ; TG(18:4(6Z9Z12Z15Z)18:2(9Z12Z)18:4(6Z9Z12Z15Z)) ; TG(18:4(6Z9Z12Z15Z)20:2n618:4(6Z9Z12Z15Z)) ; TG(20:5(5Z8Z11Z14Z17Z)15:020:5(5Z8Z11Z14Z17Z)) ; PC(16:024:1(15Z)) ; PC(16:1(9Z)24:0) ; PC(18:022:1(13Z)) ; PC(18:1(11Z)22:0) ; PC(18:1(9Z)22:0) ; PC(18:2(9Z12Z)24:1(15Z)) ;	M+H ; M+H ; M+H ; M+H ; M+H ; M+2H ; M+2H ; M+2H ; M+2H ; M+2H ; M+2H ; M+2H ; M+2H ; M+2H ; M+ACN+2H ; M+ACN+2H ; M+ACN+2H ; M+ACN+2H ; M+ACN+2H ; M+H+NH4 ; M+H+NH4 ; M+H+NH4 ; M+ACN+2H ; M+ACN+2H ; M+H+NH4 ; M+H+NH4 ; M+H+NH4 ; M+H+NH4 ; M+ACN+2H ; M+ACN+2H ; M+H+NH4 ; M+H+NH4 ; M+ACN+2H ; M+H+NH4 ; M+H+NH4 ; M+ACN+2H ; M+H+NH4 ; M+H+NH4 ; M+ACN+2H ; M+H+NH4 ; 2M+3H	C29H46O3 ; C29H46O3 ; C29H46O3 ; C29H46O3 ; C29H46O3 ; C58H92O6 ; C58H92O6 ; C58H92O6 ; C58H92O6 ; C58H92O6 ; C58H92O6 ; C58H92O6 ; C58H92O6 ; C58H92O6 ; C48H94NO8P ; C48H94NO8P ; C48H94NO8P ; C48H94NO8P ; C48H94NO8P ; C50H94NO8P ; C50H94NO8P ; C50H94NO8P ; C48H94NO8P ; C48H94NO8P ; C50H94NO8P ; C50H94NO8P ; C50H94NO8P ; C50H94NO8P ; C48H94NO8P ; C48H94NO8P ; C50H94NO8P ; C50H94NO8P ; C48H94NO8P ; C50H94NO8P ; C50H94NO8P ; C48H94NO8P ; C50H94NO8P ; C50H94NO8P ; C48H94NO8P ; C50H94NO8P ; C36H74NO7P

7282_103.4	1.813981	0.53376	-3.8996	0.002963	2.5283	0.043552	234.1447	HMDB28960 ; HMDB29044 ; HMDB29447 ; HMDB01432 ; HMDB04980 ; HMDB10726 ; HMDB12183 ; HMDB12331 ; HMDB12341 ; HMDB29027 ; HMDB29069 ; HMDB29220 ; HMDB29358 ; HMDB29859 ; HMDB30031 ; HMDB31002 ; HMDB31003 ; HMDB31297 ; HMDB31362 ; HMDB31440 ; HMDB31441 ; HMDB32119 ; HMDB32275 ; HMDB32393 ; HMDB32452 ; HMDB33203 ; HMDB33377 ; HMDB34427 ; HMDB34581 ; HMDB34880 ; HMDB35122 ; HMDB35365 ; HMDB35837 ; HMDB35839 ; HMDB35907 ; HMDB36099 ; HMDB36211 ; HMDB36990 ; HMDB36992 ; HMDB37021 ; HMDB37116 ; HMDB37217 ; HMDB37497 ; HMDB37632 ; HMDB38239 ; HMDB38744 ; HMDB39149 ; HMDB39768 ; HMDB39795 ; HMDB40194 ; HMDB40208 ; HMDB40213 ; HMDB40330 ; HMDB40530 ; HMDB40726 ; HMDB40727 ;	Lysyl-Serine ; Serinyl-Lysine ; Lysinoalanine ; Agmatine ; cis-4-Decenoic acid ; trans-Dec-2-enoic acid ; 8-Methylnonenoate ; PS(14:014:1(9Z)) ; PS(14:1(9Z)14:0) ; Prolyl-Threonine ; Threoninyl-Proline ; lysoPC(26:1(5Z)) ; 5Z-Octenyl acetate ; Methyl (xi)-3-nonenoate ; ()-6-Methyl-5-hepten-2-yl acetate ; 3-Decenoic acid ; 9-Decenoic acid ; 2-Octenyl acetate ; Cyclohexylethyl acetate ; Linalool oxide (trans-pyranoid) ; Linalool oxide III ; 4-Hydroxy-26-dimethyl-7-octen-3-one ; Ethyl 3-octenoate ; 2-Methylbutyl 3-methyl-2-butenoate ; 1-Octen-3-yl acetate ; 6-Decanolide ; cis-3-Hexenyl butyrate ; Cyclohexyl butanoate ; 2-Hexenyl butanoate ; 3-Methyl-24-nonanedione ; (SE)-26-Dimethyl-57-octadiene-23-diol ; Cnidiol C ; Citronellic acid ; (E)-3-(Tetrahydro-55-dimethyl-2-furanyl)-2-buten-1-ol ; Linalyl oxide ; Lilac alcohol ; Hexyl crotonate ; 26-Dimethyl-37-octadiene-26-diol ; 1-Cyclopropyl-4-methyl-13-cyclohexanediol ; trans-p-Menth-2-ene-14-diol ; delta-Decalactone ; xi-5-	M+H ; M+H ; M+H ; 3M+2K ; M+ACN+Na ; M+ACN+Na ; M+ACN+Na ; M+2H+Na ; M+2H+Na ; M+NH4 ; M+NH4 ; M+3Na ; M+ACN+Na ; M+NH4 ; M+ACN+Na ; M+ACN+Na ; M+ACN+Na ; M+ACN+Na ; M+ACN+Na ; M+ACN+Na ; M+ACN+Na ; M+ACN+Na ; M+ACN+Na ; M+ACN+Na ; M+ACN+Na ; M+ACN+Na ; M+ACN+Na ; M+ACN+Na ;	C9H19N3O4 ; C9H19N3O4 ; C9H19N3O4 ; C5H14N4 ; C10H18O2 ; C10H18O2 ; C10H18O2 ; C34H64NO10P ; C34H64NO10P ; C9H16N2O4 ; C9H16N2O4 ; C34H68NO7P ; C10H18O2 ; C9H16N2O4 ; C10H18O2 ; C10H18O2 ; C10H18O2 ; C10H18O2 ; C10H18O2 ; C10H18O2 ; C10H18O2 ; C10H18O2 ; C10H18O2 ; C10H18O2 ; C10H18O2 ; C10H18O2 ; C10H18O2 ;
4369_43.65	1.811047	0.21215	3.8963	0.002979	2.5259	0.043552	180.9664	HMDB40450	23456-Pentachlorobenzyl alcohol	M+2ACN+2H	C7H3Cl5O
7576_99.01	1.799522	0.06622	3.9022	0.00295	2.5301	0.043552	NA	NA	NA	NA	NA
1509_65.43	1.775347	0.800215	-3.8977	0.002972	2.5269	0.043552	332.0952	HMDB10313 ; HMDB10314 ; HMDB61134 ; HMDB61160	1-Salicylate glucuronide ; Beta-D-Glucopyranuronic acid ; Dedimethylchlorpromazine ; N-Dedimethylchlorpromazine	M+NH4 ; M+NH4 ; M+ACN+H ; M+ACN+H	C13H14O9 ; C13H14O9 ; C15H15ClN2S ; C15H15ClN2S

6381_48.7	1.776303	0.262613	3.8888	0.003015	2.5207	0.043828	266.0746	HMDB60663 ; HMDB29438 ; HMDB30400 ; HMDB30786 ; HMDB33073 ; HMDB33074 ; HMDB33557 ; HMDB33914 ; HMDB34384 ; HMDB35921 ; HMDB35984 ; HMDB36370 ; HMDB36375 ; HMDB37973 ; HMDB37995 ; HMDB38473 ; HMDB38487 ; HMDB39493 ; HMDB41716 ; HMDB42006	Isoniazid alpha-ketoglutaric acid ; L-Phosphoarginine ; L-4-Chlorotryptophan ; Marmesin ; 1-Propenyl 1-(propylsulfinyl)propyl disulfide ; 1-(1-Propenylsulfinyl)propyl propyl disulfide ; ()-6-Chlorotryptophan ; Aegelinol ; Corticrocin ; Limonin ; Zapoterin ; 1-(2-Propenylsulfinyl)propyl propyl disulfide ; 2-Propenyl 1-(propylsulfinyl)propyl disulfide ; Cyanidin 335-triglucoside ; Cyanidin 3-sophorotrioside ; Torachrysone ; Cyanidin 3-sophoroside 5-glucoside ; Dihydrowyerone acid ; Cyanidin 3-diglucoside 5-glucoside ; Ricinine	M+H ; 2M+H+Na ; 2M+3H2O+2H ; 2M+H+K ; 2M+3H2O+2H ; 2M+3H2O+2H ; 2M+3H2O+2H ; 2M+H+K ; 2M+H+K ; M+Na+K ; M+Na+K ; 2M+3H2O+2H ; 2M+3H2O+2H ; M+2H+Na ; M+2H+Na ; 2M+H+K ; M+2H+Na ; 2M+H+K ; M+2H+Na ; 3M+H+K	C11H11N3O5 ; C6H15N4O5P ; C11H11ClN2O2 ; C14H14O4 ; C9H18OS3 ; C9H18OS3 ; C11H11ClN2O2 ; C14H14O4 ; C14H14O4 ; C26H30O8 ; C26H30O8 ; C9H18OS3 ; C9H18OS3 ; C33H41O21 ; C33H41O21 ; C14H14O4 ; C33H41O21 ; C14H14O4 ; C33H41O21 ; C8H8N2O2
0571_211.8	1.760409	0.537435	3.8892	0.003014	2.5209	0.043828	355.0741	HMDB06607 ; HMDB13880 ; HMDB13881 ; HMDB13882 ; HMDB13883 ; HMDB13884 ; HMDB13885 ; HMDB13886 ; HMDB30293 ; HMDB35880 ; HMDB37469 ; HMDB39958 ; HMDB41296 ; HMDB41297 ; HMDB41621	3-Sialyllactosamine ; S-6-Hydroxywarfarin ; S-4-Hydroxywarfarin ; R-4-Hydroxywarfarin ; R-6-Hydroxywarfarin ; R-10-Hydroxywarfarin ; R-8-Hydroxywarfarin ; R-7-Hydroxywarfarin ; (E)-1-O-Cinnamoyl-beta-D-glucose ; (E)-2-O-Cinnamoyl-beta-D-glucopyranose ; Spinacetin 3-gentiobioside ; 1-Pentadecanecarboxylic acid ; Moracin K ; Moracin L ; 6-Sialyllactosamine	M+2K ; 2M+Na+K ; 2M+Na+K ; 2M+Na+K ; 2M+Na+K ; 2M+Na+K ; 2M+Na+K ; 2M+Na+K ; M+2Na-H ; M+2Na-H ; M+H+K ; M+2Na-H ; 2M+Na+K ; 2M+Na+K ; M+2K	C23H40N2O18 ; C19H16O5 ; C19H16O5 ; C19H16O5 ; C19H16O5 ; C19H16O5 ; C19H16O5 ; C19H16O5 ; C15H18O7 ; C15H18O7 ; C29H34O18 ; C15H18O7 ; C19H16O5 ; C19H16O5 ; C23H40N2O18
6148_21.4	1.812923	0.020978	-3.8808	0.003055	2.515	0.044276	408.2926	HMDB34557	88-Diethoxy-26-dimethyl-2-octanol	3M+2K	C14H30O3
7586_62.1	1.74276	0.392013	3.8631	0.003144	2.5025	0.045439	268.0518	HMDB06941 ; HMDB11163 ; HMDB28749 ; HMDB29971 ; HMDB31793 ; HMDB32026 ; HMDB34395	L-2-Aminoadipate adenylate ; L-beta-aspartyl-L-aspartic acid ; Aspartyl-Aspartate ; Coriandrone E ; Heptenophos ; Sinalexin ; 2-Propenyl 3-(2-propenylsulfonyl)-1-propenyl disulfide	M+2Na ; 2M+H+K ; 2M+H+K ; 2M+H+K ; M+NH4 ; M+ACN+Na ; M+NH4	C16H23N6O10P ; C8H12N2O7 ; C8H12N2O7 ; C13H12O5 ; C9H12ClO4P ; C10H8N2OS ; C9H14O2S3
9683_289.5	1.82928	0.32703	3.8587	0.003167	2.4994	0.04563	NA	NA	NA	NA	NA

6708_71.1	1.811826	0.133968	3.8524	0.0032	2.4949	0.045712	162.0217	HMDB00299 ; HMDB00469 ; HMDB01368 ; HMDB14718 ; HMDB14967 ; HMDB32523 ; HMDB33626 ; HMDB35179	Xanthosine ; 5-Hydroxymethyluracil ; 3-Mercaptopyruvic acid ; Mazindol ; Diazepam ; Succinic anhydride ; D-Erythroascorbic acid 1-a-D-xylopyranoside ; Mumefural	M+H+K ; 2M+H+K ; M+ACN+H ; M+H+K ; M+H+K ; 3M+H+Na ; M+2Na ; M+H+Na	C10H12N4O6 ; C5H6N2O3 ; C3H4O3S ; C16H13ClN2O ; C16H13ClN2O ; C4H4O3 ; C10H14O9 ; C12H12O9
94058_18.	1.763037	1.216753	-3.853	0.003197	2.4953	0.045712	300.2894	HMDB00252 ; HMDB01480 ; HMDB02100	Sphingosine ; 3-Dehydrosphinganine ; Palmitoylethanolamide	M+H ; M+H ; M+H	C18H37NO2 ; C18H37NO2 ; C18H37NO2
6112_43.3	1.755777	0.135506	3.8524	0.0032	2.4949	0.045712	100.0216	HMDB05843 ; HMDB29755 ; HMDB33116 ; HMDB33117 ; HMDB38624 ; HMDB00555 ; HMDB00709 ; HMDB00727 ; HMDB00845 ; HMDB00857 ; HMDB00877 ; HMDB02109 ; HMDB02441 ; HMDB05784 ; HMDB29167 ; HMDB29573 ; HMDB29720 ; HMDB29758 ; HMDB29895 ; HMDB30570 ; HMDB31054 ; HMDB32012 ; HMDB32883 ; HMDB32990 ; HMDB33813 ; HMDB34158 ; HMDB37724 ; HMDB37728 ; HMDB41605 ; HMDB59622 ; HMDB59722 ; HMDB59738 ; HMDB59893 ; HMDB59966	Allyl isothiocyanate ; 3-Thiocyanato-1-propene ; 4-Methylthiazole ; 5-Methylthiazole ; Thiiraneacetonitrile ; 3-Methyladipic acid ; L-Cysteinylglycine disulfide ; L-Threoneopterin ; Neopterin ; Pimelic acid ; Umanopterin ; Hydroxysepiapterin ; 33-Dimethylglutaric acid ; Hydroxytyrosol ; 2-Methyladipic acid ; Diethyl malonate ; 2-Thiophenethiol ; Herniarin ; D-erythro-Eritadenine ; Sylvopinol ; 10-Hydroxy-28-decadiene-46-diynoic acid ; 4-Hydroxy-3-methoxybenzenemethanol ; 145-Naphthalenetriol ; 7-Hydroxy-6-methyl-2H-1-benzopyran-2-one ; 3-(34-Methylenedioxyphenyl)propenal ; 26-Dimethoxyphenol ; Propyl 2-furoate ; 2-Furanylmethyl propanoate ; Propyleneglycol diacetate ; 4-Methylumbelliferone ; Mono-methyl-adipate ; 2-Ethylglutaric acid ; Ethyl methylsuccinate ; 35-Dimethoxyphenol	M+H ; M+H ; M+H ; M+H ; M+H ; M+H+K ; M+3H ; M+H+2Na ; M+H+2Na ; M+H+K ; M+H+2Na ; M+H+2Na ; M+H+K ; M+2Na ; M+H+K ; M+H+K ; M+2ACN+2H ; M+H+Na ; M+H+2Na ; M+2Na ; M+H+Na ; M+2Na ; M+H+Na ; M+H+Na ; M+H+Na ; M+2Na ; M+2Na ; M+2Na ; M+H+K ; M+H+Na ; M+H+K ; M+H+K ; M+H+K ; M+2Na	C4H5NS ; C4H5NS ; C4H5NS ; C4H5NS ; C4H5NS ; C7H12O4 ; C8H15N3O5S2 ; C9H11N5O4 ; C9H11N5O4 ; C7H12O4 ; C9H11N5O4 ; C9H11N5O4 ; C7H12O4 ; C8H10O3 ; C7H12O4 ; C7H12O4 ; C4H4S2 ; C10H8O3 ; C9H11N5O4 ; C8H10O3 ; C10H8O3 ; C8H10O3 ; C10H8O3 ; C10H8O3 ; C10H8O3 ; C8H10O3 ; C8H10O3 ; C8H10O3 ; C7H12O4 ; C10H8O3 ; C7H12O4 ; C7H12O4 ; C7H12O4 ; C8H10O3
6411_121.	1.760927	0.943516	3.8458	0.003234	2.4902	0.046072	261.9726	HMDB32986	5-(2-Hydroxyethyl)-4-methylthiazole acetate	M+2K-H	C8H11NO2S
9039_56.3	1.809742	0.326727	-3.8435	0.003246	2.4886	0.046107	599.9519	HMDB36062 ; HMDB41146	Ubiquinone 6 ; Feruloyl-beta-sitosterol	2M+H+NH4 ; 2M+H+NH4	C39H58O4 ; C39H58O4
0431_168.0	1.766159	0.398497	-3.8406	0.003262	2.4865	0.046107	NA	NA	NA	NA	NA
4933_99.8	1.742429	0.953778	3.8401	0.003264	2.4862	0.046107	283.0455	HMDB01328 ; HMDB06876 ; HMDB34280	dTDP-D-glucose ; dTDP-D-galactose ; Egonol glucoside	M+2H ; M+2H ; M+2K	C16H26N2O16P2 ; C16H26N2O16P2 ; C25H28O10

5183_276.2	1.786779	0.959298	-3.8381	0.003275	2.4847	0.046132	248.1125	HMDB15181 ; HMDB33412 ; HMDB37084 ; HMDB38610 ; HMDB00131 ; HMDB00907 ; HMDB30897 ; HMDB33584 ; HMDB36436 ; HMDB37511 ; HMDB38791 ; HMDB61143	Fluocinonide ; Gibberellin A20 13-glucoside ; (8S8S)-Secoisolariciresinol 9-xyloside ; Gibberellin A4 glucosyl ester ; Glycerol ; Sulfolithocholic acid ; Lucidenolactone ; Diethyl tartrate ; Lucidenic acid F ; Butyl oleate sulfate ; 246-Triethyl-135-oxadithiane ; Hydralazine pyruvate hydrazone	M+2H ; M+2H ; M+2H ; M+2H ; 2M+ACN+Na ; M+H+K ; M+H+K ; M+ACN+H ; M+H+K ; M+Na+K ; M+ACN+H ; M+NH4	C26H32F2O7 ; C25H34O10 ; C25H34O10 ; C25H34O10 ; C3H8O3 ; C24H40O6S ; C27H36O6 ; C8H14O6 ; C27H36O6 ; C22H42O6S ; C9H18OS2 ; C11H10N4O2
5979_101.4	1.808968	0.507958	3.8311	0.003313	2.4798	0.046402	159.0126	HMDB01232 ; HMDB02658 ; HMDB13188 ; HMDB30254 ; HMDB32884 ; HMDB38627 ; HMDB41756 ; HMDB59710 ; HMDB61112	4-Nitrophenol ; 6-Hydroxynicotinic acid ; 3-Hydroxypicolinic acid ; 3-(345-Trimethoxyphenyl)propanoic acid ; Allura red AC ; Ethyl 345-trimethoxybenzoate ; Isopropyl 3-(34-dihydroxyphenyl)-2-hydroxypropanoate ; 2-Hydroxynicotinic acid ; 3-Carboxy-4-methyl-5-propyl-2-furanpropionic acid	2M+H+K ; 2M+H+K ; 2M+H+K ; M+2K ; M+2H+Na ; M+2K ; M+2K ; 2M+H+K ; M+2K	C6H5NO3 ; C6H5NO3 ; C6H5NO3 ; C12H16O5 ; C18H16N2O8S2 ; C12H16O5 ; C12H16O5 ; C6H5NO3 ; C12H16O5
7532_86.46	1.763689	0.682038	-3.8321	0.003308	2.4805	0.046402	178.0588	HMDB34854 ; HMDB61000 ; HMDB12489 ; HMDB14890 ; HMDB29496 ; HMDB30801 ; HMDB30974 ; HMDB31112 ; HMDB31928 ; HMDB32861 ; HMDB34788 ; HMDB35443 ; HMDB37115 ; HMDB38329 ; HMDB38543 ; HMDB39002 ; HMDB39251 ; HMDB59911 ; HMDB59994 ; HMDB60303 ; HMDB60322 ; HMDB60931	Flumioxazin ; 4-(4-Chlorophenyl)-1-4-(4-fluorophenyl)-4-oxobutyl-pyridinium (HPP) ; 1234-Tetrahydroisoquinoline ; Tranylcypromine ; Fukiic acid ; 1-(4-Hydroxy-3-methoxyphenyl)-3-decanone ; 8-Acetoxy-4-acoren-3-one ; Neoacrimarine F ; Panaxytriol ; 2-Methoxynaphthalene ; Neoacrimarine I ; (EE)-16-bis(4-methoxyphenyl)-15-hexadiene ; ()-2-Hydroxy-4-(methylthio)butanoic acid ; Dide-O-methyl-4-O-alpha-D-glucopyranosylsimmondsin ; Longistylin A ; Mytilin A ; Panaxacol ; Paracetamol sulfate ; Benzeneacetamide-4-O-sulphate ; (2-Naphthyl)methanol ; 1-Hydroxymethylnaphthalene ; 2-ethyl-15-dimethyl-33-diphenylpyrrolinium (EDDP)	M+2H ; M+2H ; M+2Na-H ; M+2Na-H ; M+2ACN+2H ; M+2K ; M+2K ; M+3H ; M+2K ; 2M+H+K ; M+3H ; M+Na+K ; 2M+3H2O+2H ; M+2H+Na ; M+Na+K ; M+H+Na ; M+2K ; M+3ACN+2H ; M+3ACN+2H ; 2M+H+K ; 2M+H+K ; M+2K	C19H15FN2O4 ; C21H18ClFNO ; C9H11N ; C9H11N ; C11H12O8 ; C17H26O3 ; C17H26O3 ; C29H25NO9 ; C17H26O3 ; C11H10O ; C29H25NO9 ; C20H22O2 ; C5H10O3S ; C20H31NO14 ; C20H22O2 ; C13H20N2O8 ; C17H26O3 ; C8H9NO5S ; C8H9NO5S ; C11H10O ; C11H10O ; C20H24N

6292_58.0(	1.793669	0.579704	3.8293	0.003323	2.4785	0.046408	370.9686	HMDB00968 ; HMDB01047 ; HMDB01058 ; HMDB03514 ; HMDB06234 ; HMDB06235 ; HMDB06872 ; HMDB14026 ; HMDB34765 ; HMDB34768 ; HMDB38636 ; HMDB60269 ; HMDB60444 ; HMDB60500	1D-Myo-inositol 14-bisphosphate ; D-Fructose 26-bisphosphate ; Fructose 16-bisphosphate ; Alpha-D-Glucose 16-bisphosphate ; 1D-Myo-inositol 13-bisphosphate ; 1D-Myo-inositol 34-bisphosphate ; D-Tagatose 16-bisphosphate ; p-Chlorobenzene sulfonyl urea ; Wasalexin A ; Wasalexin B ; Methoxybrassenin B ; D-Mannose 16-bisphosphate ; beta-D-Fructose 16-bisphosphate ; NTP	2M+Na+K ; 2M+Na+K ; 2M+Na+K ; 2M+Na+K ; 2M+Na+K ; 2M+Na+K ; 2M+Na+K ; 3M+H+K ; M+2K-H ; M+2K-H ; M+2K-H ; 2M+Na+K ; 2M+Na+K ; M+H-H2O	C6H14O12P2 ; C6H14O12P2 ; C6H14O12P2 ; C6H14O12P2 ; C6H14O12P2 ; C6H14O12P2 ; C6H14O12P2 ; C7H7ClN2O3S ; C13H14N2O2S2 ; C13H14N2O2S2 ; C13H14N2O2S2 ; C6H14O12P2 ; C6H14O12P2 ; C6H15O13P3
9697_287.(	1.785186	0.540946	3.8235	0.003354	2.4744	0.046718	610.092	HMDB41174	1-O-p-Coumaroyl-(b-D-glucose 6-O-sulfate)	3M+2H	C15H18O11S
4049_60.0:	1.827031	0.307956	-3.8122	0.003417	2.4664	0.047456	263.074	HMDB06083 ; HMDB32911 ; HMDB32981 ; HMDB33738 ; HMDB37584 ; HMDB60635	Troxerutin ; Isoorientin 2-feruloyl-(-6)-glucoside ; 23-Diphenylpyrazine ; Naringin 4-glucoside ; Narirutin 4-glucoside ; Etoposide glucuronide	M+H+2Na ; M+3H ; 2M+Na+K ; M+H+2Na ; M+H+2Na ; M+2H+Na	C33H42O19 ; C37H38O19 ; C16H12N2 ; C33H42O19 ; C33H42O19 ; C35H40O19
7782_290.5	1.839458	0.451351	3.8057	0.003454	2.4617	0.047608	714.4778	HMDB08855 ; HMDB14951 ; HMDB32837 ; HMDB34515 ; HMDB35434 ; HMDB35886 ; HMDB38797 ; HMDB39615 ; HMDB40711 ; HMDB40782 ; HMDB56412 ; HMDB56439 ; HMDB56537 ; HMDB56563 ; HMDB56701 ; HMDB57427 ; HMDB12336 ; HMDB12346 ; HMDB12366 ; HMDB12386 ; HMDB12396	PE(14:1(9Z)14:1(9Z)) ; Fentanyl ; Ganoderic acid DM ; Glabrolide ; Isomasticadienonalic acid ; Isoglabrolide ; Uralenolide ; Colupone ; 16beta-16-Hydroxy-3-oxo-112-oleanadien-28-oic acid ; Vinaginsenoside R12 ; CL(16:016:016:1(9Z)18:2(9Z12Z)) ; CL(16:016:018:2(9Z12Z)16:1(9Z)) ; CL(16:016:1(9Z)16:018:2(9Z12Z)) ; CL(16:016:1(9Z)18:2(9Z12Z)16:0) ; CL(16:018:2(9Z12Z)16:1(9Z)16:0) ; CL(16:1(9Z)16:018:2(9Z12Z)16:0) ; PS(14:018:2(9Z12Z)) ; PS(14:1(9Z)18:1(9Z)) ; PS(16:1(9Z)16:1(9Z)) ; PS(18:1(9Z)14:1(9Z)) ; PS(18:2(9Z12Z)14:0)	M+2ACN+H ; 2M+ACN+H ; 3M+H+Na ; 3M+H+Na ; 3M+H+Na ; 3M+H+Na ; 3M+H+Na ; 3M+H+Na ; 3M+H+Na ; M+ACN+H ; M+H+K ; M+H+K ; M+H+K ; M+H+K ; M+H+K ; M+H+K ; M+H-H2O ; M+H-H2O ; M+H-H2O ; M+H-H2O ; M+H-H2O	C33H62NO8P ; C22H28N2O ; C30H44O4 ; C30H44O4 ; C30H44O4 ; C30H44O4 ; C30H44O4 ; C30H44O4 ; C30H44O4 ; C36H64O11 ; C76H142O17P2 ; C76H142O17P2 ; C76H142O17P2 ; C76H142O17P2 ; C76H142O17P2 ; C76H142O17P2 ; C38H70NO10P ; C38H70NO10P ; C38H70NO10P ; C38H70NO10P ; C38H70NO10P
0787_288.7	1.790785	0.779961	3.8052	0.003456	2.4614	0.047608	464.0808	HMDB00536 ; HMDB59653 ; HMDB06779 ; HMDB30481 ; HMDB39907	Adenylsuccinic acid ; N(6)-(12-dicarboxyethyl)AMP ; Indole-56-quinone ; Agrocybin ; Tricin 7-sinapoyl-(-2)-glucuronyl-(1-2)-glucuronide	M+H ; M+H ; 3M+Na ; 3M+Na ; M+H+K	C14H18N5O11P ; C14H18N5O11P ; C8H5NO2 ; C8H5NO2 ; C40H40O23
9066_168.0	1.748151	0.08923	-3.8059	0.003452	2.4619	0.047608	NA	NA	NA	NA	NA

[illegible]72316_91.[illegible]	1.824983	0.501129	-3.7838	0.00358	2.4462	0.047962	469.0672	HMDB40628 ; HMDB00061 ; HMDB00960 ; HMDB01341 ; HMDB33352 ; HMDB38409 ; HMDB38830 ; HMDB39302 ; HMDB39304 ; HMDB39772 ; HMDB59614	3-(23-Digalloyl-46-hexahydroxydiphenoylglucosyl)-phloroacetophenone ; Adenosine 35-diphosphate ; dGDP ; ADP ; Cyclobrassinin ; 6-(Methylthio)hexyl glucosinolate ; Theasinensin F ; Epicatechin 3-O-gallate-(4beta-6)-epigallocatechin 3-O-gallate ; Epicatechin 3-O-gallate-(4beta-8)-epigallocatechin 3-O-gallate ; Epigallocatechin 3-O-gallate-(4beta-6)-epicatechin 3-O-gallate ; Riboflavin cyclic-45-phosphate	M+2H ; M+ACN+H ; M+ACN+H ; M+ACN+H ; 2M+H ; 2M+H+K ; M+H+K ; M+H+K ; M+H+K ; M+H+K ; 2M+Na+K	C42H32O25 ; C10H15N5O10P2 ; C10H15N5O10P2 ; C10H15N5O10P2 ; C11H10N2S2 ; C14H27NO9S3 ; C44H34O21 ; C44H34O21 ; C44H34O21 ; C44H34O21 ; C17H19N4O8P
[illegible]5817_37.2[illegible]	1.812313	0.252078	-3.7927	0.003528	2.4525	0.047962	233.1496	HMDB13075 ; HMDB28917 ; HMDB28939 ; HMDB29064 ; HMDB29065 ; HMDB00577 ; HMDB00908 ; HMDB01569 ; HMDB13124 ; HMDB14631 ; HMDB15299 ; HMDB30425 ; HMDB31014 ; HMDB32052 ; HMDB32098 ; HMDB34151 ; HMDB37286 ; HMDB37307 ; HMDB37390 ; HMDB38249 ; HMDB38258 ; HMDB38291 ; HMDB40232 ; HMDB41554	Spermic acid 2 ; Isoleucyl-Threonine ; Leucyl-Threonine ; Threoninyl-Isoleucine ; Threoninyl-Leucine ; 5beta-Coprostanol ; 5alpha-Cholestanol ; Epi-coprostanol ; Propenoylcarnitine ; Altretamine ; Procarbazine ; Linalyl propionate ; Methyl (2E6Z)-dodecadienoate ; alpha-Terpineol propanoate ; Cepanone ; 11-Dimethoxynonane ; Citral propylene glycol acetal ; Propyl 24-decadienoate ; Tsibulin 1 ; Isobornyl propionate ; Neryl propionate ; 2-Propenyl cyclohexanebutanoate ; Octahydro-2558a-tetramethyl-7H-1-benzopyran-7-one ; Artemisyl propionate	M+H ; M+H ; M+H ; M+H ; M+H ; M+2K ; M+2K ; M+2K ; M+NH4 ; M+Na ; 2M+H+Na ; M+Na ; M+Na ; M+Na ; M+Na ; M+2Na-H ; M+Na ; M+Na ; M+Na ; M+Na ; M+Na ; M+Na ; M+Na ; M+Na	C10H20N2O4 ; C10H20N2O4 ; C10H20N2O4 ; C10H20N2O4 ; C10H20N2O4 ; C27H48O ; C27H48O ; C27H48O ; C10H17NO4 ; C9H18N6 ; C12H19N3O ; C13H22O2 ; C13H22O2 ; C13H22O2 ; C13H22O2 ; C11H24O2 ; C13H22O2 ; C13H22O2 ; C13H22O2 ; C13H22O2 ; C13H22O2 ; C13H22O2 ; C13H22O2 ; C13H22O2

3209_65.3[illegible]	1.808434	0.667699	3.7889	0.00355	2.4498	0.047962	181.0443	HMDB01216 ; HMDB01527 ; HMDB06834 ; HMDB11659 ; HMDB29530 ; HMDB30755 ; HMDB30922 ; HMDB31201 ; HMDB33209 ; HMDB33483 ; HMDB33919 ; HMDB34173 ; HMDB34888 ; HMDB35646 ; HMDB37245 ; HMDB37246 ; HMDB38325 ; HMDB41167 ; HMDB41399 ; HMDB41486 ; HMDB60538 ; HMDB60593 ; HMDB60947 ; HMDB61088	Tetrahydropteridine ; 3-Methylthiopropionic acid ; D-Pantothenoyl-L-cysteine ; 2-Methylerythritol ; Glabrene ; Myrigalone E ; 13-Diacetoxy-4612-tetradecatriene-810-diyne ; 3-Deoxy-D-arabinitol ; Fenthion ; 67-Dihydro-4-(hydroxymethyl)-2-(p-hydroxyphenethyl)-7-methyl-5H-2-pyrindinium ; 2-Deoxy-D-ribitol ; Phaseollin ; 4-Acetylimidazo45-cpyridine ; Methyl 2-(methylthio)acetate ; Myrigalone A ; Myrigalon B ; Verimol C ; (-)-Shinpterocarpin ; 23-Dihydro-4-(4-methoxyphenyl)-1H-phenalene-123-triol ; 1-Deoxy-D-ribitol ; Nordiazepam ; Alpha-dihydroartemisinin ; desmethylclomipramine ; Dihydroartemisinin (DHA)	M+2Na-H ; 3M+2H ; M+H+K ; M+2Na-H ; M+H+K ; M+Na+K ; M+Na+K ; M+2Na-H ; M+2ACN+2H ; M+2K ; M+2Na-H ; M+H+K ; 2M+H+K ; 3M+2H ; M+Na+K ; M+Na+K ; M+Na+K ; M+H+K ; M+H+K ; M+2Na-H ; 2M+3H ; M+2K ; M+Na+K ; M+2K	C6H8N4 ; C4H8O2S ; C12H22N2O6S ; C5H12O4 ; C20H18O4 ; C18H20O4 ; C18H20O4 ; C5H12O4 ; C10H15O3PS2 ; C18H22NO2 ; C5H12O4 ; C20H18O4 ; C8H7N3O ; C4H8O2S ; C18H20O4 ; C18H20O4 ; C18H20O4 ; C20H18O4 ; C20H18O4 ; C5H12O4 ; C15H11ClN2O ; C15H24O5 ; C18H21ClN2 ; C15H24O5
9021_98.8[illegible]	1.802502	0.729858	3.7812	0.003595	2.4443	0.047962	108.5009	HMDB12234	Histidinal	M+2K	C6H9N3O
1665_163.[illegible]	1.801373	0.190004	3.7967	0.003505	2.4553	0.047962	482.1982	HMDB09939 ; HMDB09943 ; HMDB13848 ; HMDB14703 ; HMDB30308 ; HMDB31786 ; HMDB31945 ; HMDB32692 ; HMDB33469 ; HMDB39105 ; HMDB41518 ; HMDB41543 ; HMDB41970	PIP(16:1(9Z)16:1(9Z)) ; PIP(16:2(9Z12Z)16:0) ; 4-Hydroxyvalsartan ; Methotrexate ; O-Isopentenylhalfordinol ; 9-(2-Carboxyphenyl)-36-bis(diethylamino)xanthylium(1) ; Niaziminin A ; Chinenoside VI ; N1N10-Dicoumaroylspermidine ; 3-Oxo-1415-dehydrorhazinilam ; Nb-p-Coumaroyltryptamine ; 25-Dibenzyl-3-hydroxy-6-methoxypyrazine ; Oxmetidine	M+2K ; M+2K ; 2M+Na+K ; 2M+3H2O+2H ; 3M+2Na ; M+K ; M+2ACN+H ; M+Na+K ; M+2Na-H ; 3M+2Na ; 3M+2Na ; 3M+2Na ; M+2ACN+H	C41H76O16P2 ; C41H76O16P2 ; C24H29N5O4 ; C20H22N8O5 ; C19H18N2O2 ; C28H31N2O3 ; C18H25NO7S ; C44H70O19 ; C25H31N3O4 ; C19H18N2O2 ; C19H18N2O2 ; C19H18N2O2 ; C19H21N5O3S
9591_32.3[illegible]	1.784479	0.642298	3.7946	0.003517	2.4539	0.047962	464.283	HMDB07851 ; HMDB07855 ; HMDB11133 ; HMDB35075 ; HMDB38798	LPA(0:018:1(9Z)) ; LPA(18:1(9Z)0:0) ; DHAP(18:0) ; Tanacetol B ; (1(10)E4a5E)-1(10)5-Germacradiene-12-acetoxy-411-diol	2M+3H2O+2H ; 2M+3H2O+2H ; 2M+3H2O+2H ; 3M+H+K ; 3M+H+K	C21H41O7P ; C21H41O7P ; C21H41O7P ; C17H28O4 ; C17H28O4
47182_168	1.776942	0.441491	-3.792	0.003532	2.452	0.047962	222.5247	HMDB02818 ; HMDB12250 ; HMDB34045 ; HMDB38403 ; HMDB38413 ; HMDB41596	Alloxan ; L-Aspartyl-4-phosphate ; Glucocleomin ; Glucoerucin ; 2-Hydroxypentyl glucosinolate ; Vitisidin A	3M+H+NH4 ; 2M+H+NH4 ; M+H+K ; M+H+Na ; M+H+K ; M+2Na	C4H2N2O4 ; C4H8NO7P ; C12H23NO10S2 ; C12H23NO9S3 ; C12H23NO10S2 ; C20H15O9

5994_99.28	1.770858	0.168908	-3.7898	0.003545	2.4504	0.047962	150.5286	HMDB00038 ; HMDB01127 ; HMDB01376 ; HMDB01510 ; HMDB02065 ; HMDB02215 ; HMDB13031 ; HMDB13642 ; HMDB14456 ; HMDB30277 ; HMDB39127	Dihydrobiopterin ; 6-Phosphonoglucono-D-lactone ; 2-Keto-3-deoxy-6-phosphogluconic acid ; Bupropion ; 6-Lactoyltetrahydropterin ; 4a-Carbinolamine tetrahydrobiopterin ; O2-4a-cyclic-tetrahydrobiopterin ; 1-hydroxy-2-Oxopropyl tetrahydropterin ; Ethoxzolamide ; Dihydromaleimide beta-D-glucoside ; Pisatoside	M+Na+K ; M+ACN+2H ; M+ACN+2H ; M+Na+K ; M+Na+K ; M+Na+K ; M+Na+K ; M+Na+K ; M+ACN+2H ; M+H+K ; M+H+K	C9H13N5O3 ; C6H11O9P ; C6H11O9P ; C13H18ClNO ; C9H13N5O3 ; C9H13N5O3 ; C9H13N5O3 ; C9H13N5O3 ; C9H10N2O3S2 ; C10H15NO7 ; C10H15NO7
6826_56.78	1.76888	0.793889	-3.7816	0.003593	2.4446	0.047962	384.9697	HMDB01112 ; HMDB01473	D-Glyceraldehyde 3-phosphate ; Dihydroxyacetone phosphate	2M+2Na-H ; 2M+2Na-H	C3H7O6P ; C3H7O6P
7958_129.(	1.766322	0.56528	3.7972	0.003502	2.4557	0.047962	274.4908	HMDB40655	Mabioside D	M+2H+Na	C42H70O14
6402_113.(	1.74696	0.897821	-3.7808	0.003597	2.444	0.047962	131.0016	HMDB00288 ; HMDB01271 ; HMDB11641 ; HMDB29748 ; HMDB38283 ; HMDB39585 ; HMDB60282	Uridine 5-monophosphate ; Pseudouridine 5-phosphate ; Uridine 2-phosphate ; 2-Aminodipyrido12-a:32-dimidazole ; 6-Chlorocatechin ; S-(25-Dimethyl-3-furanyl) 2-furancarbothioate ; 3-UMP	M+3Na ; M+3Na ; M+3Na ; M+2K ; M+3Na ; M+H+K ; M+3Na	C9H13N2O9P ; C9H13N2O9P ; C9H13N2O9P ; C10H8N4 ; C15H13ClO6 ; C11H10O3S ; C9H13N2O9P
0948_37.26	1.727569	0.091987	3.7843	0.003577	2.4465	0.047962	146.0271	HMDB01068 ; HMDB60509 ; HMDB00071 ; HMDB00262 ; HMDB00575 ; HMDB00676 ; HMDB02024 ; HMDB15492 ; HMDB29736 ; HMDB29956 ; HMDB60261 ; HMDB01890	D-Sedoheptulose 7-phosphate ; Sedoheptulose 1-phosphate ; Deoxyinosine ; Thymine ; DL-Homocystine ; L-Homocystine ; Imidazoleacetic acid ; Stepronin ; 1H-Imidazole-1-acetic acid ; Nebularine ; Imidazol-4-ylacetate ; Acetylcysteine	M+2H ; M+2H ; M+H+K ; 2M+H+K ; M+H+Na ; M+H+Na ; 2M+H+K ; M+H+NH4 ; 2M+H+K ; M+H+K ; 2M+H+K ; M+H-H2O	C7H15O10P ; C7H15O10P ; C10H12N4O4 ; C5H6N2O2 ; C8H16N2O4S2 ; C8H16N2O4S2 ; C5H6N2O2 ; C10H11NO4S2 ; C5H6N2O2 ; C10H12N4O4 ; C5H6N2O2 ; C5H9NO3S
4251_239.5	1.768902	0.948547	3.7575	0.003738	2.4274	0.049571	485.0143	HMDB33069 ; HMDB36794	7-Chloro-34568-pentamethoxyflavone ; Azorhodine 2G	M+2K+H ; 2M+H+K	C20H19ClO7 ; C18H15N3O8S2
448_94.00(	1.760465	0.848529	-3.7587	0.00373	2.4283	0.049571	290.9345	HMDB00263	Phosphoenolpyruvic acid	3M+2K	C3H5O6P

8465_64.02	1.808409	0.795872	3.7532	0.003764	2.4243	0.049588	180.0518	HMDB00704 ; HMDB30408 ; HMDB41720 ; HMDB02070 ; HMDB03431 ; HMDB13159 ; HMDB14803 ; HMDB14810 ; HMDB15376 ; HMDB29694 ; HMDB32750 ; HMDB32751 ; HMDB32752 ; HMDB32753 ; HMDB32977 ; HMDB32978 ; HMDB32979 ; HMDB32980 ; HMDB33963 ; HMDB35233 ; HMDB35384 ; HMDB35385 ; HMDB40718 ; HMDB59778 ; HMDB60466 ; HMDB60629 ; HMDB60700	Isoxanthopterin ; 2-Amino-34-dihydroxypentanedioic acid ; Dihydrocaffeic acid 3-O-glucuronide ; 4-Hydroxy-2-oxoglutaric acid ; L-Histidinol ; 23-Diaminosalicylic acid ; Nilutamide ; Chlorpropamide ; Trimeprazine ; S-Methyl benzenecarbothioate ; S-2-Propenyl 1-propenesulfinothioate ; S-1-Propenyl 2-propenesulfinothioate ; R-2-Propenyl 1-propenesulfinothioate ; R-1-Propenyl 2-propenesulfinothioate ; 23-Dimethylthiophene ; 24-Dimethylthiophene ; 25-Dimethylthiophene ; 34-Dimethylthiophene ; Allicin ; Olomoucine ; 2-Ethylthiophene ; 3-Ethylthiophene ; Zwiebelane B ; 2-Pyrroloylglycine ; D-4-Hydroxy-2-oxoglutarate ; Hydroxydesmthyl doxepin glucuronide ; (E)-2-Hydroxydoxepin glucuronide	M+H ; M+H ; M+2H ; M+NH4 ; M+K ; 2M+H+Na ; M+ACN+2H ; M+2ACN+2H ; M+Na+K ; 2M+3H2O+2H ; M+NH4 ; M+NH4 ; M+NH4 ; M+NH4 ; 3M+H+Na ; 3M+H+Na ; 3M+H+Na ; 3M+H+Na ; M+NH4 ; M+Na+K ; 3M+H+Na ; 3M+H+Na ; M+NH4 ; 2M+H+Na ; M+NH4 ; M+3Na ; M+3Na	C6H5N5O2 ; C5H9NO6 ; C15H18O10 ; C5H6O6 ; C6H11N3O ; C7H8N2O3 ; C12H10F3N3O4 ; C10H13ClN2O3S ; C18H22N2S ; C8H8OS ; C6H10OS2 ; C6H10OS2 ; C6H10OS2 ; C6H10OS2 ; C6H8S ; C6H8S ; C6H8S ; C6H8S ; C6H10OS2 ; C15H18N6O ; C6H8S ; C6H8S ; C6H10OS2 ; C7H8N2O3 ; C5H6O6 ; C25H29NO8 ; C25H29NO8

7213_60.2	1.807091	0.281354	-3.7536	0.003761	2.4247	0.049588	246.1447	HMDB28734 ; HMDB28735 ; HMDB28810 ; HMDB28902 ; HMDB28924 ; HMDB29125 ; HMDB29141 ; HMDB29162 ; HMDB60118 ; HMDB60119 ; HMDB60120 ; HMDB60121 ; HMDB00473 ; HMDB06695 ; HMDB11727 ; HMDB15508 ; HMDB28871 ; HMDB29018 ; HMDB32862 ; HMDB35359 ; HMDB38343 ; HMDB39434 ; HMDB40573	Asparaginyl-Isoleucine ; Asparaginyl-Leucine ; Glutaminyl-Valine ; Isoleucyl-Asparagine ; Leucyl-Asparagine ; Valyl-Glutamine ; Valyl-Gamma-glutamate ; Gamma-glutamyl-Valine ; 12-Hydroxy-13-O-D-glucuronoside-octadec-9Z-enoate ; 12-O-D-Glucuronoside-13-hydroxyoctadec-9Z-enoate ; 10-Hydroxy-octadec-12Z-enoate-9-beta-D-glucuronide ; 9-Hydroxy-10-O-D-glucuronoside-12Z-octadecenoate ; 6-Dimethylaminopurine ; Prolylhydroxyproline ; Bicine ; Fencamfamine ; Hydroxyprolyl-Proline ; Prolyl-Hydroxyproline ; 2-Amino-4-ethoxy-3-hydroxybutanoic acid ; 1-Deoxynojirimycin ; 13-Demethylspirolide C ; Spirolide A ; Tetraacetylethylenediamine	M+H ; M+H ; M+H ; M+H ; M+H ; M+H ; M+H ; M+H ; M+2H ; M+2H ; M+2H ; M+2H ; M+2ACN+H ; M+NH4 ; M+2ACN+H ; 2M+Na+K ; M+NH4 ; M+NH4 ; M+2ACN+H ; M+2ACN+H ; M+H+2Na ; M+H+2Na ; M+NH4	C10H19N3O4 ; C10H19N3O4 ; C10H19N3O4 ; C10H19N3O4 ; C10H19N3O4 ; C10H19N3O4 ; C10H19N3O4 ; C10H19N3O4 ; C24H42O10 ; C24H42O10 ; C24H42O10 ; C24H42O10 ; C7H9N5 ; C10H16N2O4 ; C6H13NO4 ; C15H21N ; C10H16N2O4 ; C10H16N2O4 ; C6H13NO4 ; C6H13NO4 ; C42H61NO7 ; C42H61NO7 ; C10H16N2O4
2647_36.1	1.793521	0.414829	3.7524	0.003769	2.4238	0.049588	652.3383	HMDB04902 ; HMDB04960 ; HMDB15505 ; HMDB34104 ; HMDB40965 ; HMDB60961	Ganglioside GA1 (d18:116:0) ; Tetrahexosylceramide (d18:116:0) ; Glutethimide ; Neferine ; Matesaponin 4 ; N-desisopropylpropranolol	M+2K ; M+2K ; 3M+H ; 2M+3H2O+2H ; M+2ACN+2H ; 3M+H	C60H110N2O23 ; C60H110N2O23 ; C13H15NO2 ; C38H44N2O6 ; C59H96O26 ; C13H15NO2

4529_225.7	1.736623	0.12982	-3.75	0.003784	2.422	0.04966	273.1445	HMDB05770 ; HMDB05776 ; HMDB15371 ; HMDB28718 ; HMDB29033 ; HMDB30917 ; HMDB31901 ; HMDB32674 ; HMDB33234 ; HMDB33693 ; HMDB34200 ; HMDB34721 ; HMDB35117 ; HMDB35137 ; HMDB35148 ; HMDB35270 ; HMDB35299 ; HMDB35302 ; HMDB35358 ; HMDB35760 ; HMDB35798 ; HMDB35972 ; HMDB36036 ; HMDB36037 ; HMDB36550 ; HMDB36563 ; HMDB36664 ; HMDB37064 ; HMDB37529 ; HMDB37559 ; HMDB39015 ; HMDB39156 ; HMDB39635 ; HMDB39644 ; HMDB40754 ; HMDB61150 ; HMDB34879	Tuftsin ; Kentsin ; Gemfibrozil ; Arginyl-Serine ; Serinyl-Arginine ; 1-Hydroxyacorenone ; Blennin A ; (ZZ)-2916-Heptadecatriene-46-diyn-8-ol ; Ganoderic acid beta ; Falcarinone ; Physalolactone B ; Procurcumadiol ; (3beta6beta)-Furanoeremophilane-36-diol ; Heliannuol D ; (6beta8alpha)-6-Hydroxy-7(11)-eremophilen-128-olide ; Methyl 3-oxo-12-oleanen-28-oate ; Ganolucidic acid D ; Ganolucidic acid A ; Ketosantalic acid ; 3-Hydroxytrichothecene ; Piperdial ; Ambonic acid ; 3beta-Dihydroxymarasmene ; 13-Hydroxymarasmene ; 3-Ketoapotrichothecene ; Valerenolic acid ; Ketopelenolide a ; FS4 toxin ; Lactaronecatorin A ; Cadabicilone ; Tangeraxanthin ; 3beta-Hydroxycinnamolide ; Abscisic alcohol ; Heliannuol A ; (3beta8beta)-3-Hydroxy-7(11)-eremophilen-128-olide ; Hydroxyl frovatriptan ; 1-(5-Amino-5-carboxypentyl)amino-1-deoxyfructose	M+2Na ; M+2Na ; M+Na ; 2M+H+Na ; 2M+H+Na ; M+Na ; M+Na ; 2M+Na+K ; M+2Na ; 2M+Na+K ; M+2Na ; M+Na ; M+Na ; M+Na ; M+Na ; M+2K ; M+2Na ; M+2Na ; M+Na ; M+Na ; M+Na ; M+2K ; M+Na ; M+Na ; M+Na ; M+Na ; M+Na ; M+Na ; M+Na ; M+Na ; M+Na+K ; M+Na ; M+Na ; M+Na ; M+Na ; 2M+H+Na ; M+H-2H2O	C21H40N8O6 ; C21H40N8O6 ; C15H22O3 ; C9H19N5O4 ; C9H19N5O4 ; C15H22O3 ; C15H22O3 ; C17H22O ; C30H44O6 ; C17H22O ; C30H44O6 ; C15H22O3 ; C15H22O3 ; C15H22O3 ; C15H22O3 ; C31H48O3 ; C30H44O6 ; C30H44O6 ; C15H22O3 ; C15H22O3 ; C15H22O3 ; C31H48O3 ; C15H22O3 ; C15H22O3 ; C15H22O3 ; C15H22O3 ; C15H22O3 ; C15H22O3 ; C15H22O3 ; C15H22O3 ; C34H44O2 ; C15H22O3 ; C15H22O3 ; C15H22O3 ; C15H22O3 ; C14H19N3O2 ; C12H24N2O7
30676_280.	1.739423	0.409717	-3.739	0.003853	2.4142	0.050303	314.1031	HMDB31147 ; HMDB31968 ; HMDB34362 ; HMDB38088 ; HMDB14258 ; HMDB14720 ; HMDB37797	Chlorophyll c ; Malvidin 3-glucoside-4-vinylphenol ; Desglucocheirotoxin ; 3-Ferulylpelargonidin 5-glucoside ; R-138727 ; Voriconazole ; Citbrasine	M+H+K ; M+H+NH4 ; M+2K ; M+H+NH4 ; M+H-2H2O ; M+H-2H2O ; M+H-H2O	C35H32N4O5 ; C31H29O13 ; C29H42O10 ; C31H29O13 ; C18H20FNO3S ; C16H14F3N5O ; C17H17NO6
1806_48.34	1.731093	0.383699	-3.7391	0.003852	2.4143	0.050303	234.9818	HMDB00816 ; HMDB34022 ; HMDB60148	Phosphoglycolic acid ; 6-Hydroxy-5-(4-sulfophenyl)azo-2-naphthalenesulfonic acid ; 2-Phosphonatoglycolate	3M+2H ; M+Na+K ; 3M+2H	C2H5O6P ; C16H12N2O7S2 ; C2H5O6P
3353_104.7	1.78596	0.714169	-3.7355	0.003875	2.4117	0.050314	NA	NA	NA	NA	NA
6757_289.9	1.760414	1.093753	3.7351	0.003878	2.4114	0.050314	659.8268	HMDB15543 ; HMDB33814	Pivmecillinam ; Agavasaponin D	3M+2H ; M+3ACN+2H	C21H33N3O5S ; C56H90O27
49732_101	1.740271	1.016501	-3.7341	0.003884	2.4107	0.050314	243.0107	HMDB59620 ; HMDB60266 ; HMDB14480 ; HMDB15244 ; HMDB33061 ; HMDB36374 ; HMDB40002	5-(methylthio)-23-Dioxopentyl phosphate ; 23-Diketo-5-methylthio-1-phosphopentane ; Nitrofurazone ; Cefuroxime ; 1-(Methylsulfinyl)propyl propyl disulfide ; Methyl 1-(propylsulfinyl)propyl disulfide ; 1-(2-Thienyl)-12-propanedione	M+H ; M+H ; M+2Na-H ; M+Na+K ; 2M+Na+K ; 2M+Na+K ; 3M+H+Na	C6H11O6PS ; C6H11O6PS ; C6H6N4O4 ; C16H16N4O8S ; C7H16OS3 ; C7H16OS3 ; C7H6O2S

6618_290.(	1.806996	0.775367	3.7273	0.003928	2.4059	0.050614	659.9767	HMDB00693 ; HMDB12023	Heparan sulfate ; Ganglioside GT2 (d18:014:0)	M+Na ; M+3Na	C14H23NO21S3 ; C86H150N4O42
2933_285.8	1.764226	0.848343	-3.7277	0.003925	2.4061	0.050614	615.3263	HMDB14453 ; HMDB29310 ; HMDB30463 ; HMDB33523 ; HMDB34554 ; HMDB35292 ; HMDB35294 ; HMDB35605 ; HMDB60583	Ibutilide ; Collettiside I ; Hyperforin ; (-)-Pyrifolidine ; Sakacin A ; Ganodermic acid P2 ; Ganoderic acid Mk ; 3alpha15alpha-Diacetoxy-(22R)-hydroxylanosta-79(11)24-trien-26-oic acid ; Trandolaprilat	3M+2K ; M+K ; M+2K+H ; 3M+2K ; 2M+K ; M+2Na-H ; M+2Na-H ; M+2Na-H ; 3M+H+Na	C20H36N2O3S ; C33H52O8 ; C35H52O4 ; C23H32N2O3 ; C12H24N4O4 ; C34H50O7 ; C34H50O7 ; C34H50O7 ; C22H30N2O5
444_17.121	1.720684	0.972967	-3.7252	0.003941	2.4044	0.050657	731.5444	HMDB56982 ; HMDB56983 ; HMDB56992 ; HMDB56993 ; HMDB57092 ; HMDB57093 ; HMDB57104 ; HMDB57114 ; HMDB57145 ; HMDB57156 ; HMDB57166 ; HMDB57890 ; HMDB57900 ; HMDB58353 ; HMDB07935 ; HMDB07967 ; HMDB08833 ; HMDB08925 ; HMDB08989 ; HMDB09217 ; HMDB10368 ; HMDB10566 ; HMDB11538 ; HMDB11568 ; HMDB34088 ; HMDB34505 ; HMDB34528 ; HMDB34644 ; HMDB34683 ; HMDB35326 ; HMDB35678 ; HMDB38527 ; HMDB38531 ; HMDB38532 ; HMDB39692	CL(18:018:018:1(11Z)18:1(11Z)) ; CL(18:018:018:1(11Z)18:1(9Z)) ; CL(18:018:018:1(9Z)18:1(11Z)) ; CL(18:018:018:1(9Z)18:1(9Z)) ; CL(18:018:1(11Z)18:018:1(11Z)) ; CL(18:018:1(11Z)18:018:1(9Z)) ; CL(18:018:1(11Z)18:1(11Z)18:0) ; CL(18:018:1(11Z)18:1(9Z)18:0) ; CL(18:018:1(9Z)18:018:1(9Z)) ; CL(18:018:1(9Z)18:1(11Z)18:0) ; CL(18:018:1(9Z)18:1(9Z)18:0) ; CL(18:1(11Z)18:018:1(11Z)18:0) ; CL(18:1(11Z)18:018:1(9Z)18:0) ; CL(18:1(9Z)18:018:1(9Z)18:0) ; PC(15:016:0) ; PC(16:015:0) ; PE(14:020:0) ; PE(16:018:0) ; PE(18:016:0) ; PE(20:014:0) ; CE(18:0) ; PE-NMe2(16:016:0) ; MG(0:018:2(9Z12Z)0:0) ; MG(18:2(9Z12Z)0:00:0) ; beta-Sitosterol palmitate ; Soyasapogenol A ; Camelliagenin A ; Priverogenin B ; (3alphaOH20S24S)-319:2024-Diepoxydammarane-325-diol ; Ganoderiol A ; 3-Hydroxy-1-phenyl-1-heptadecanone ; 5-(12-Heptadecenyl)-13-benzenediol ; 5-(2-Heptadecenyl)-13-benzenediol ; Irisresorcinol ; 2024-	M+2H ; M+2H ; M+2H ; M+2H ; M+2H ; M+2H ; M+2H ; M+2H ; M+2H ; M+2H ; M+2H ; M+2H ; M+2H ; M+2H ; 2M+H+Na ; 2M+H+Na ; 2M+H+Na ; 2M+H+Na ; 2M+H+Na ; 2M+H+Na ; M+2K+H ; 2M+H+Na ; 2M+Na ; 2M+Na ; M+2K+H ; 3M+H+K ; 3M+H+K ; 3M+H+K ; 3M+H+K ; 3M+H+K ; 2M+K ; 2M+K ; 2M+K ; 2M+K ; 3M+H+K	C81H154O17P2 ; C81H154O17P2 ; C81H154O17P2 ; C81H154O17P2 ; C81H154O17P2 ; C81H154O17P2 ; C81H154O17P2 ; C81H154O17P2 ; C81H154O17P2 ; C81H154O17P2 ; C81H154O17P2 ; C81H154O17P2 ; C81H154O17P2 ; C81H154O17P2 ; C39H78NO8P ; C39H78NO8P ; C39H78NO8P ; C39H78NO8P ; C39H78NO8P ; C39H78NO8P ; C45H80O2 ; C39H78NO8P ; C21H38O4 ; C21H38O4 ; C45H80O2 ; C30H50O4 ; C30H50O4 ; C30H50O4 ; C30H50O4 ; C30H50O4 ; C23H38O2 ; C23H38O2 ; C23H38O2 ; C23H38O2 ; C30H50O4
8889_75.92	1.771088	0.312202	3.7223	0.00396	2.4023	0.050774	437.1339	HMDB15625 ; HMDB36785 ; HMDB60633	Prasugrel ; Erioglaucine A ; N-Acetyl gemifloxacin	M+ACN+Na ; M+3ACN+2H ; 2M+H+K	C20H20FNO3S ; C37H37N2O9S3 ; C19H20FN5O5
5252_101.8	1.734449	1.105389	-3.7104	0.004039	2.3937	0.051648	326.6125	HMDB03265 ; HMDB30748 ; HMDB36333	Hesperidin ; Hesperetin 7-neohesperidoside ; 4-Hydroxyacetophenone 4-4-hydroxy-35-dimethoxybenzoyl-(-5)-apiosyl-(1-2)-glucoside	M+ACN+2H ; M+ACN+2H ; M+ACN+2H	C28H34O15 ; C28H34O15 ; C28H34O15

51924_126.	1.746721	1.013539	3.7078	0.004056	2.3919	0.051736	101.0252	HMDB01297 ; HMDB15544 ; HMDB31415 ; HMDB32344 ; HMDB37777 ; HMDB41200	Norcotinine ; Tazobactam ; 26-Dimethyl-24-heptadiene ; Isobutylene-isoprene copolymer ; 2-Isopropyl-14-hexadiene ; Dehydroxymethylflazine	M+H+K ; M+3H ; M+2K ; M+2K ; M+2K ; M+2H+Na	C9H10N2O ; C10H12N4O5S ; C9H16 ; C9H16 ; C9H16 ; C16H10N2O3
9723_65.5(	1.797688	0.86744	3.7059	0.004069	2.3905	0.051769	616.18	HMDB12947 ; HMDB31234 ; HMDB33185 ; HMDB33289 ; HMDB33679 ; HMDB33768	Ferrocytochrome ; 33-Bis(4-hydroxyanigorufone) ; Anigorootin ; Aurasperone E ; Pelargonidin 3-sophoroside ; Fonsecinone B	2M+H+Na ; M+ACN+H ; M+ACN+H ; 2M+3H2O+2H ; M+Na ; 2M+3H2O+2H	C33H32FeN4O4 ; C38H22O6 ; C38H22O6 ; C32H28O11 ; C28H33O14 ; C32H28O11
.7489_93.3	1.74739	0.027193	-3.7002	0.004107	2.3864	0.051992	129.5817	HMDB00194 ; HMDB00745 ; HMDB00904 ; HMDB01522 ; HMDB03148 ; HMDB04620 ; HMDB05769 ; HMDB12251 ; HMDB15169 ; HMDB32373 ; HMDB36149 ; HMDB39781 ; HMDB41476 ; HMDB41562	Anserine ; Homocarnosine ; Citrulline ; Methylguanidine ; Argininic acid ; N-a-Acetyl-L-arginine ; Balenine ; L-Canaline ; Procainamide ; 3-Mercapto-2-methyl-1-butanol ; 3-Mercapto-3-methyl-1-butanol ; xi-2-Mercapto-3-methyl-1-butanol ; 4-(Methylthio)-2-butanol ; 4-(Methylthio)-1-butanol	M+H+NH4 ; M+H+NH4 ; M+2ACN+2H ; 3M+H+K ; M+2ACN+2H ; M+ACN+2H ; M+H+NH4 ; M+3ACN+2H ; M+H+Na ; 2M+H+NH4 ; 2M+H+NH4 ; 2M+H+NH4 ; 2M+H+NH4 ; 2M+H+NH4	C10H16N4O3 ; C10H16N4O3 ; C6H13N3O3 ; C2H7N3 ; C6H13N3O3 ; C8H16N4O3 ; C10H16N4O3 ; C4H10N2O3 ; C13H21N3O ; C5H12OS ; C5H12OS ; C5H12OS ; C5H12OS ; C5H12OS
91118_101.	1.736885	1.016005	-3.7006	0.004104	2.3868	0.051992	NA	NA	NA	NA	NA
3265_241.	1.766909	1.091144	3.698	0.004122	2.3849	0.052044	327.9403	HMDB01928	Hydrochlorothiazide	2M+Na+K	C7H8ClN3O4S2
.0084_101.	1.766739	0.50581	3.6917	0.004165	2.3804	0.052324	496.184	HMDB14734 ; HMDB29181 ; HMDB29183 ; HMDB30470 ; HMDB30550 ; HMDB32760 ; HMDB37482 ; HMDB38383 ; HMDB39378 ; HMDB40721 ; HMDB41398	Halobetasol Propionate ; 4-O-methyl-(-)-epicatechin-5-O-beta-glucuronide ; 4-O-methyl-(-)-epicatechin-7-O-beta-glucuronide ; Lindleyin ; Thamnosin ; Nb-trans-p-Coumaroylserotonin glucoside ; Persiconin ; 3b-Pregnadienolone 3-rhamnosyl-(1-4)-rhamnosyl-(1-4)-rhamnosyl-(1-4)-glucoside ; Isolindleyin ; Eugenol O-345-Trihydroxybenzoyl-(-6)-b-D-glucopyranoside ; Persicogenin 3-glucoside	2M+H+Na ; M+NH4 ; M+NH4 ; M+NH4 ; 2M+H+Na ; 2M+H+Na ; M+NH4 ; M+2K ; M+NH4 ; M+NH4 ; M+NH4	C25H31ClF2O5 ; C23H26O11 ; C23H26O11 ; C23H26O11 ; C30H28O6 ; C25H28N2O8 ; C23H26O11 ; C45H70O19 ; C23H26O11 ; C23H26O11 ; C23H26O11

1781_45.46	1.750061	1.026122	3.692	0.004163	2.3806	0.052324	137.0718	HMDB03152 ; HMDB30001 ; HMDB33947 ; HMDB37819 ; HMDB59711 ; HMDB00696 ; HMDB04058 ; HMDB06803 ; HMDB12235 ; HMDB13751 ; HMDB14426 ; HMDB14997 ; HMDB15377 ; HMDB29532 ; HMDB30032 ; HMDB30033 ; HMDB30034 ; HMDB30036 ; HMDB31173 ; HMDB31235 ; HMDB31236 ; HMDB31922 ; HMDB33567 ; HMDB33951 ; HMDB34017 ; HMDB35255 ; HMDB36057 ; HMDB39671 ; HMDB40317 ; HMDB41275 ; HMDB60318 ; HMDB60946 ; HMDB61022	N-Methylnicotinamide ; 2-Acetyl-3-methylpyrazine ; 2-Aminobenzamide ; 4-Acetyl-2-methylpyrimidine ; 2-Methylnicotinamide ; L-Methionine ; 56-Dihydroxyindole ; 2-Propyn-1-al ; Hydroxybupropion ; 2-Hydroxypyridine ; Lidocaine ; Penicillamine ; Isocarboxazid ; 3-Hydroxyglabrol ; Euglobal Ia1 ; Euglobal Ib ; Euglobal IIb ; Euglobal IIc ; 6-Hydroxy-1H-indole-3-acetamide ; (E)-2446-Tetrahydroxy-35-diprenylchalcone ; 3-Geranyl-2446-tetrahydroxychalcone ; 1-(24-Dihydroxyphenyl)-3-8-hydroxy-2-methyl-2-(4-methyl-3-pentenyl)-2H-1-benzopyran-5-yl-1-propanone ; Cyclotetradecane ; Racemethionine ; Kuwanol D ; 3-Geranyl-2344-tetrahydroxychalcone ; 1H-Pyrrole-2-carboxaldehyde ; 5-Methyl-25-di-1-pyrrolidinyl-2-cyclopenten-1-one ; (E)-2-Geranyl-347-trihydroxyflavanone ; 1-Hydroxy-35-dimethoxy-24-diprenylxanthone ; (S)-4-Hydroxymandelonitrile ; NAPQI ; 6-beta-hydroxydexamethasone	M+H ; M+H ; M+H ; M+H ; M+H ; M+3ACN+2H ; M+3ACN+2H ; M+2ACN+H ; M+H+NH4 ; M+ACN+H ; M+H+K ; M+3ACN+2H ; M+ACN+2H ; M+3H ; M+2H+Na ; M+2H+Na ; M+2H+Na ; M+2H+Na ; M+2ACN+2H ; M+3H ; M+3H ; M+3H ; M+2K ; M+3ACN+2H ; M+3H ; M+3H ; M+ACN+H ; M+H+K ; M+3H ; M+3H ; M+3ACN+2H ; M+3ACN+2H ; M+3H	C7H8N2O ; C7H8N2O ; C7H8N2O ; C7H8N2O ; C7H8N2O ; C5H11NO2S ; C8H7NO2 ; C3H2O ; C13H18ClNO2 ; C5H5NO ; C14H22N2O ; C5H11NO2S ; C12H13N3O2 ; C25H28O5 ; C23H30O5 ; C23H30O5 ; C23H30O5 ; C23H30O5 ; C10H10N2O2 ; C25H28O5 ; C25H28O5 ; C25H28O5 ; C14H28 ; C5H11NO2S ; C25H28O5 ; C25H28O5 ; C5H5NO ; C14H22N2O ; C25H28O5 ; C25H28O5 ; C8H7NO2 ; C8H7NO2 ; C22H29FO6
4953_294.8	1.749887	0.441824	3.6895	0.00418	2.3788	0.052338	213.0255	HMDB38401 ; HMDB00854 ; HMDB06028 ; HMDB14359 ; HMDB32766 ; HMDB33304 ; HMDB34589 ; HMDB60624	Glucosinalbin ; Formiminoglutamic acid ; N-Acetylasparagine ; Torasemide ; N2-(2-Carboxymethyl-2-hydroxysuccinoyl)arginine ; Gerberinol ; Shoyuflavone B ; Ethionamide sulphoxide	M+2H ; M+K ; M+K ; M+2K ; M+2K ; M+Na+K ; M+H+Na ; 2M+Na+K	C14H18NO10S2 ; C6H10N2O4 ; C6H10N2O4 ; C16H20N4O3S ; C12H20N4O8 ; C21H16O6 ; C19H14O10 ; C8H10N2OS
6772_129.1	1.734368	0.360122	3.6885	0.004187	2.3781	0.052338	469.0007	HMDB39355 ; HMDB60449 ; HMDB60687 ; HMDB60692	Methyl 6-O-digalloyl-beta-D-glucopyranoside ; Carboxyphosphamide ; Carboxycyclophosphamide ; Carboxyifosfamide	M+2K+H ; 3M+Na+K ; 3M+Na+K ; 3M+Na+K	C17H19BrN4O2 ; C7H15Cl2N2O4P ; C7H15Cl2N2O4P ; C7H15Cl2N2O4P
0878_178.7	1.747589	0.015377	-3.6778	0.004262	2.3704	0.053139	364.0651	HMDB00574 ; HMDB03417	L-Cysteine ; D-Cysteine	3M+H ; 3M+H	C3H7NO2S ; C3H7NO2S

6625_50.08	1.736739	0.074937	3.6761	0.004274	2.3692	0.053161	244.0927	HMDB00089 ; HMDB15122 ; HMDB60478 ; HMDB11751 ; HMDB12915 ; HMDB14639 ; HMDB14798 ; HMDB29436 ; HMDB29685 ; HMDB30901 ; HMDB31240 ; HMDB31640 ; HMDB31770 ; HMDB32128 ; HMDB32174 ; HMDB32574 ; HMDB32640 ; HMDB32860 ; HMDB32974 ; HMDB32975 ; HMDB32976 ; HMDB33701 ; HMDB33761 ; HMDB33867 ; HMDB37602 ; HMDB37733 ; HMDB38460 ; HMDB40036 ; HMDB41008 ; HMDB41532 ; HMDB60734 ; HMDB28751 ; HMDB28767 ; HMDB28793 ; HMDB29145	Cytidine ; Cytarabine ; gamma-Glutamyl-beta-cyanoalanine ; 3-Methoxybenzenepropanoic acid ; Coniferyl alcohol ; Darifenacin ; Metaxalone ; Penmacric acid ; 4-Ethoxy-3-methoxybenzaldehyde ; Eremopetasitenin B2 ; 2-Phenyl-13-dioxolane-4-methanol ; Glycerol 1-propanoate diacetate ; Carbofuran ; 1-(24-Dihydroxyphenyl)-1-butanone ; Benzaldehyde glyceryl acetal ; Propylparaben ; Ethyl p-anisate ; 1-Methylnaphthalene ; 24-Dimethylthiazole ; 25-Dimethylthiazole ; 45-Dimethylthiazole ; Hydrocotarnine ; 4-Methoxybenzyl acetate ; 4-Isothiocyanato-1-butene ; 4-Hydroxy-266-trimethyl-3-oxo-14-cyclohexadiene-1-carboxaldehyde ; Propyl 2-furanacrylate ; Thiiranepropanenitrile ; 2-Ethylthiazole ; 1-Methyl-6-phenyl-1H-imidazo45-bpyridin-2-amine ; Eremosulphoxinolide A ; 3-(3-Hydroxyphenyl)-2-methylpropionic acid ; Aspartyl-Glutamine ; Aspartyl-	M+H ; M+H ; M+H ; M+ACN+Na ; M+ACN+Na ; M+Na+K ; M+Na ; M+ACN+H ; M+ACN+Na ; M+H+Na ; M+ACN+Na ; 2M+H+Na ; M+Na ; M+ACN+Na ; M+ACN+Na ; M+ACN+Na ; M+ACN+Na ; 3M+Na+K ; 2M+NH4 ; 2M+NH4 ; 2M+NH4 ; M+Na ; M+ACN+Na ; 2M+NH4 ; M+ACN+Na ; M+ACN+Na ; 2M+NH4 ; 2M+NH4 ; 2M+H+K ; M+H+Na ; M+ACN+Na ; M+H-H2O ; M+H-H2O ; M+H-H2O ; M+H-H2O	C9H13N3O5 ; C9H13N3O5 ; C9H13N3O5 ; C10H12O3 ; C10H12O3 ; C28H30N2O2 ; C12H15NO3 ; C7H10N2O5 ; C10H12O3 ; C24H32O7S ; C10H12O3 ; C10H16O6 ; C12H15NO3 ; C10H12O3 ; C10H12O3 ; C10H12O3 ; C10H12O3 ; C11H10 ; C5H7NS ; C5H7NS ; C5H7NS ; C12H15NO3 ; C10H12O3 ; C5H7NS ; C10H12O3 ; C10H12O3 ; C5H7NS ; C5H7NS ; C13H12N4 ; C24H32O7S ; C10H12O3 ; C9H15N3O6 ; C9H15N3O6 ; C9H15N3O6 ; C9H15N3O6
3796_282.2	1.789567	0.778383	3.6612	0.00438	2.3585	0.054344	254.0084	HMDB33587 ; HMDB41717 ; HMDB41718	Coumestrin ; Daidzein 4-O-glucuronide ; Daidzein 7-O-glucuronide	M+2K ; M+2K ; M+2K	C21H18O10 ; C21H18O10 ; C21H18O10
6259_49.61	1.737587	0.086251	3.651	0.004455	2.3512	0.055134	282.0486	HMDB02124 ; HMDB03217 ; HMDB29372 ; HMDB29521 ; HMDB30829 ; HMDB31715 ; HMDB33790 ; HMDB33860 ; HMDB34004 ; HMDB34409 ; HMDB35214 ; HMDB37999 ; HMDB39449 ; HMDB41655	Apigenin ; Genistein ; 2-Hydroxydaidzein ; Norizalpinin ; Aloeemodin ; 6-Hydroxydaidzein ; 128-Trihydroxy-3-methylanthraquinone ; 8-Hydroxydaidzein ; 347-Trihydroxyflavone ; Rhababerone ; Emodin ; Delphinidin 35-di(6-O-malonylglucoside) ; Rheinanthrone ; 3-Hydroxydaidzein	2M+H+Na ; 2M+H+Na ; 2M+H+Na ; 2M+H+Na ; 2M+H+Na ; 2M+H+Na ; 2M+H+Na ; 2M+H+Na ; 2M+H+Na ; 2M+H+Na ; 2M+H+Na ; M+H+2Na ; 2M+H+Na ; 2M+H+Na	C15H10O5 ; C15H10O5 ; C15H10O5 ; C15H10O5 ; C15H10O5 ; C15H10O5 ; C15H10O5 ; C15H10O5 ; C15H10O5 ; C15H10O5 ; C15H10O5 ; C33H35O23 ; C15H10O5 ; C15H10O5
6992_117.7	1.803548	0.587031	-3.6472	0.004483	2.3485	0.055303	203.5487	HMDB29297	Avenanthramide A2	M+2Na	C18H19NO7
5372_104.8	1.735017	0.633789	-3.6462	0.004491	2.3477	0.055303	179.0295	HMDB14772	Sulfacetamide	M+H-2H2O	C8H10N2O3S

)161_69.51	1.746574	0.002908	-3.6436	0.00451	2.3458	0.055404	319.1402	HMDB28742 ; HMDB28897 ; HMDB29078 ; HMDB29107 ; HMDB00306 ; HMDB01065 ; HMDB01200 ; HMDB01466 ; HMDB04989 ; HMDB29759 ; HMDB32711 ; HMDB37153 ; HMDB38582 ; HMDB39657 ; HMDB40059 ; HMDB60485 ; HMDB60681 ; HMDB60782 ; HMDB61131	Asparaginyl-Tryptophan ; Histidinyl-Tyrosine ; Tryptophyl-Asparagine ; Tyrosyl-Histidine ; Tyramine ; 2-Hydroxyphenethylamine ; N-Formylkynurenine ; Dimethylaniline-N-oxide ; m-Tyramine ; 2-Acetyl-1-ethylpyrrole ; Pectenotoxin 7 ; S-25-Dimethyl-3-furanyl 3-methylbutanethioate ; 5-Amino-23-dihydro-6-(3-hydroxy-4-methoxy-1-oxobutyl)-22-dimethyl-4H-1-benzopyran-4-one ; 123456-Hexahydro-7H-cyclopentabpyridin-7-one ; ()-2-(2-Furanyl)pyrrolidine ; L-Formylkynurenine ; 4-Hydroxy-26-dimethylaniline ; 6-allyl-8b-Carboxy-ergoline ; Carboxybupranolol 4-chloro-3-3-(11-dimethylethylamino)-2-hydroxy-propyloxybenzoic acid	M+H ; M+H ; M+H ; M+H ; 2M+2Na-H ; 2M+2Na-H ; M+2ACN+H ; 2M+2Na-H ; 2M+2Na-H ; 2M+2Na-H ; M+3Na ; 3M+2H ; 2M+H+Na ; 2M+2Na-H ; 2M+2Na-H ; M+2ACN+H ; 2M+2Na-H ; M+Na ; M+NH4	C15H18N4O4 ; C15H18N4O4 ; C15H18N4O4 ; C15H18N4O4 ; C8H11NO ; C8H11NO ; C11H12N2O4 ; C8H11NO ; C8H11NO ; C8H11NO ; C47H68O16 ; C11H16O2S ; C16H21NO5 ; C8H11NO ; C8H11NO ; C11H12N2O4 ; C8H11NO ; C18H20N2O2 ; C14H20ClNO4

39944_135.	1.785546	1.073013	-3.6405	0.004533	2.3436	0.055419	219.1339	HMDB14515 ; HMDB28916 ; HMDB28938 ; HMDB29042 ; HMDB29043 ; HMDB29074 ; HMDB29137 ; HMDB33675 ; HMDB41313 ; HMDB00348 ; HMDB00361 ; HMDB00384 ; HMDB00411 ; HMDB00438 ; HMDB00478 ; HMDB00514 ; HMDB00518 ; HMDB00626 ; HMDB00664 ; HMDB00686 ; HMDB00733 ; HMDB00811 ; HMDB00820 ; HMDB00863 ; HMDB00946 ; HMDB02451 ; HMDB02488 ; HMDB02536 ; HMDB02585 ; HMDB03315 ; HMDB12187 ; HMDB30332 ; HMDB30354 ; HMDB30867 ; HMDB30945 ; HMDB31030 ; HMDB31196 ; HMDB31496 ; HMDB31497 ; HMDB31498 ; HMDB31563 ; HMDB31564 ; HMDB31692 ; HMDB32051 ; HMDB32193 ; HMDB32331 ; HMDB32901 ; HMDB33549 ; HMDB33714 ; HMDB34903 ; HMDB35157 ; HMDB35239 ; HMDB36048 ; HMDB36599 ; HMDB37301 ; HMDB38270 ;	Meprobamate ; Isoleucyl-Serine ; Leucyl-Serine ; Serinyl-Isoleucine ; Serinyl-Leucine ; Threoninyl-Valine ; Valyl-Threonine ; L-Lysopine ; Pantothenamide ; 3b12a-Dihydroxy-5a-cholanoic acid ; 3b7a-Dihydroxy-5b-cholanoic acid ; 3a7a-Dihydroxycholanoic acid ; 3a12b-Dihydroxy-5b-cholanoic acid ; 3b12a-Dihydroxy-5b-cholanoic acid ; Allodeoxycholic acid ; Allochenodeoxycholic acid ; Chenodeoxycholic acid ; Deoxycholic acid ; Isohyodeoxycholic acid ; Isoursodeoxycholic acid ; Hyodeoxycholic acid ; Murocholic acid ; Propyl alcohol ; Isopropyl alcohol ; Ursodeoxycholic acid ; 7b12a-Dihydroxycholanoic acid ; 7a12b-dihydroxy-5b-Cholan-24-oic acid ; Isodeoxycholic acid ; 3b12b-Dihydroxy-5b-cholanoic acid ; Cyclohexanone ; All-trans-heptaprenyl diphosphate ; 2-Methyl-4-pentenal ; Arenaine ; Dihydro-5-(2-octenyl)-2(3H)-furanone ; Methyl 2-undecynoate ; 6-Heptyl-56-dihydro-2H-pyran-2-one ; 4-Methyl-2-pentenal ; 2-Hexenal ; 3-Hexenal ; (Z)-3-	M+H ; M+H ; M+H ; M+H ; M+H ; M+H ; M+H ; M+H ; M+H ; M+2Na ; M+2Na ; M+2Na ; M+2Na ; M+2Na ; M+2Na ; M+2Na ; M+2Na ; M+2Na ; M+2Na ; M+2Na ; M+2Na ; M+2Na ; 3M+K ; 3M+K ; M+2Na ; M+2Na ; M+2Na ; M+2Na ; M+2Na ; 2M+Na ; M+3H ; 2M+Na ; 2M+H+Na ; M+Na ; M+Na ; M+Na ; 2M+Na ; 2M+Na ; 2M+Na ; 2M+Na ; 2M+Na ; 2M+Na ; M+Na ; M+Na ; M+Na ; M+Na ; M+Na ; 2M+Na ; 2M+Na ; M+Na ; M+Na ; 2M+Na ; M+Na ; M+Na ; M+Na ; M+Na ; M+Na ; M+Na ; M+2H+Na ; M+Na ; M+Na ; 2M+Na ; 2M+Na ; M+Na ; M+Na ; M+2H+Na ; M+2H+Na ; M+Na ; M+Na	C9H18N2O4 ; C9H18N2O4 ; C9H18N2O4 ; C9H18N2O4 ; C9H18N2O4 ; C9H18N2O4 ; C9H18N2O4 ; C9H18N2O4 ; C9H18N2O4 ; C24H40O4 ; C24H40O4 ; C24H40O4 ; C24H40O4 ; C24H40O4 ; C24H40O4 ; C24H40O4 ; C24H40O4 ; C24H40O4 ; C24H40O4 ; C24H40O4 ; C24H40O4 ; C3H8O ; C3H8O ; C24H40O4 ; C24H40O4 ; C24H40O4 ; C24H40O4 ; C24H40O4 ; C6H10O ; C35H60O7P2 ; C6H10O ; C11H17N3O ; C12H20O2 ; C12H20O2 ; C12H20O2 ; C6H10O ; C6H10O ; C6H10O ; C6H10O ; C6H10O ; C6H10O ; C12H20O2 ; C12H20O2 ; C12H20O2 ; C12H20O2 ; C12H20O2 ; C6H10O ; C6H10O ; C12H20O2 ; C12H20O2 ; C6H10O ; C12H20O2 ; C12H20O2 ; C12H20O2 ; C12H20O2 ; C12H20O2 ; C12H20O2 ;
44989_287.	1.746303	0.154083	3.6405	0.004533	2.3436	0.055419	342.4945	HMDB31376 ; HMDB36656 ; HMDB38608 ; HMDB38886 ; HMDB39075 ; HMDB39076 ; HMDB39517 ; HMDB39946 ; HMDB40120 ; HMDB40942	Congmuyenoside A ; Asiaticoside ; Soyasaponin A3 ; Mabioside A ; Hoduloside I ; Hoduloside II ; Soyasaponin V ; Centellasaponin C ; Centellasaponin A ; Mabiogenin 3-rhamnosyl-(1-6)-glucosyl-(1-2)-glucoside	M+3Na ; M+3Na ; M+3Na ; M+3Na ; M+3Na ; M+3Na ; M+3Na ; M+3Na ; M+3Na ; M+3Na	C48H78O19 ; C48H78O19 ; C48H78O19 ; C48H78O19 ; C48H78O19 ; C48H78O19 ; C48H78O19 ; C48H78O19 ; C48H78O19 ; C48H78O19
73531_75.48	1.758591	0.282923	-3.6363	0.004565	2.3406	0.055531	398.0274	HMDB01155 ; HMDB15528 ; HMDB29177 ; HMDB30237 ; HMDB41831 ; HMDB60274 ; HMDB60414	Diadenosine triphosphate ; Quazepam ; 3-O-Methyl-(-)-epicatechin-7-O-sulphate ; Syzyginin B ; Azosemide ; Sedoheptulose 17-bisphosphate ; 6-Methylthiopurine 5-monophosphate ribonucleotide	M+H+K ; 2M+H+Na ; 2M+Na+K ; M+H+K ; 2M+3H2O+2H ; 2M+3H2O+2H ; 2M+H+K	C20H27N10O16P3 ; C17H11ClF4N2S ; C16H15O8S ; C33H24O21 ; C12H11ClN6O2S2 ; C7H16O13P2 ; C11H15N4O7PS
6067_179.8	1.732426	0.395429	-3.6365	0.004563	2.3407	0.055531	345.1056	HMDB01212 ; HMDB06061 ; HMDB34322	Hydantoin-5-propionic acid ; 4-Hydroxyphenylacetylglutamine ; L-alpha-Amino-5-oxo-2(5H)-isoxazolepropanoic acid	2M+H ; M+ACN+Na ; 2M+H	C6H8N2O4 ; C13H15NO6 ; C6H8N2O4

769392_25(	1.761841	0.91911	3.6324	0.004594	2.3378	0.055756	336.0769	HMDB01849 ; HMDB12226 ; HMDB13943 ; HMDB14835 ; HMDB15115 ; HMDB29829 ; HMDB30121 ; HMDB31148 ; HMDB39125 ; HMDB41954 ; HMDB60554 ; HMDB60964 ; HMDB41837	Propranolol ; Entacapone ; Alpha-hydroxyalprazolam ; Tizanidine ; Ramelteon ; Norchalciporyl propionate ; Kuwanon L ; Protochlorophyllide ; Coumaperine ; Norbutorphanol ; 4-hydroxyalprazolam ; 5-Chloro-4-(2-imidazolin-4-on-2-ylamino)-213-benzothiazdiazole ; Berberine chloride	M+2K-H ; 2M+Na+K ; 2M+H+Na ; M+2ACN+H ; M+2K-H ; M+2K-H ; M+2Na ; M+Na+K ; M+2K+H ; M+2K-H ; 2M+H+Na ; M+2ACN+H ; M+H-2H2O	C16H21NO2 ; C14H15N3O5 ; C17H13ClN4O ; C9H8ClN5S ; C16H21NO2 ; C16H21NO2 ; C35H30O11 ; C35H30MgN4O5 ; C16H19NO2 ; C16H21NO2 ; C17H13ClN4O ; C9H8ClN5S ; C20H18ClNO4
9501_54.7!	1.76282	0.13423	-3.619	0.004697	2.3282	0.056261	NA	NA	NA	NA	NA
'85532_128	1.752933	0.644327	3.6216	0.004677	2.33	0.056261	292.5786	HMDB33762 ; HMDB33937 ; HMDB36739 ; HMDB41884 ; HMDB60822 ; HMDB60823	Ginkgetin ; Isoginkgetin ; Yuccaol C ; Doxorubicinol ; Doxorubicin-semiquinone ; Doxorubicinol aglycone	M+H+NH4 ; M+H+NH4 ; M+ACN+2H ; M+H+K ; M+H+K ; M+H+K	C32H22O10 ; C32H22O10 ; C30H22O10 ; C27H31NO11 ; C27H31NO11 ; C27H31NO11
2656_223.3	1.75052	0.331807	3.6214	0.004679	2.3298	0.056261	173.0427	HMDB02666 ; HMDB00098 ; HMDB00283 ; HMDB00366 ; HMDB00621 ; HMDB00646 ; HMDB00751 ; HMDB01644 ; HMDB03371 ; HMDB11719 ; HMDB12194 ; HMDB12325 ; HMDB14564 ; HMDB15202 ; HMDB15471 ; HMDB29941 ; HMDB29942 ; HMDB30831 ; HMDB30871 ; HMDB31760 ; HMDB31898 ; HMDB33088 ; HMDB33564 ; HMDB33565 ; HMDB33911 ; HMDB38677 ; HMDB39281 ; HMDB39282 ; HMDB39283 ; HMDB39284 ; HMDB39285 ; HMDB39742 ; HMDB41648 ; HMDB41721 ; HMDB59753 ; HMDB59967 ; HMDB60254 ; HMDB61117	Thiamine monophosphate ; D-Xylose ; D-Ribose ; 2-Deoxyribonic acid ; D-Ribulose ; L-Arabinose ; L-Threo-2-pentulose ; D-Xylulose ; L-Ribulose ; Homovanillic acid sulfate ; Beta-D-ribopyranose ; Arabinofuranose ; Promazine ; Promethazine ; Salsalate ; D-Apiose ; Arabinose ; Alternariol ; Isogentisin ; Gentisin ; Porric acid C ; ()-2-(34-Dihydroxyphenyl)-13-benzodioxole-5-carboxaldehyde ; 23-Dihydro-2-methylthiophene ; 23-Dihydro-5-methylthiophene ; 568-Trihydroxy-2-methylbenzogchromen-4-one ; Alfafuran ; 2-O-Galloylsucrose ; 6-O-Galloylsucrose ; 1-O-Galloylsucrose ; 4-O-Galloylsucrose ; 6-O-Galloylsucrose ; 4-O-Methyl-a-D-glucosyl-(1-2)-b-D-xylosyl-(1-4)-D-xylose ; 2-Hydroxy-4-methoxyacetophenone 5-sulfate ; Dihydrocaffeic acid 3-sulfate ; 2-Deoxypentonic acid ; 3-hydroxy-3-(3-hydroxyphenyl)propanoic acid-O-sulphate ; Aldehydo-D-xylose ; 3-(35-dihydroxyphenyl)-1-propanoic acid sulphate	M+2H ; M+Na ; M+Na ; M+Na ; M+Na ; M+Na ; M+Na ; M+Na ; M+Na ; M+2ACN+2H ; M+Na ; M+Na ; M+Na+K ; M+Na+K ; 2M+3H ; M+Na ; M+Na ; 2M+3H ; 2M+3H ; 2M+3H ; 2M+3H ; 2M+3H ; 3M+2Na ; 3M+2Na ; 2M+3H ; 2M+3H ; M+2H+Na ; M+2H+Na ; M+2H+Na ; M+2H+Na ; M+2H+Na ; M+H+2Na ; M+2ACN+2H ; M+2ACN+2H ; M+Na ; M+2ACN+2H ; M+Na ; M+2ACN+2H	C12H17N4O4PS ; C5H10O5 ; C5H10O5 ; C5H10O5 ; C5H10O5 ; C5H10O5 ; C5H10O5 ; C5H10O5 ; C5H10O5 ; C9H10O7S ; C5H10O5 ; C5H10O5 ; C17H20N2S ; C17H20N2S ; C14H10O5 ; C5H10O5 ; C5H10O5 ; C14H10O5 ; C14H10O5 ; C14H10O5 ; C14H10O5 ; C14H10O5 ; C5H8S ; C5H8S ; C14H10O5 ; C14H10O5 ; C19H26O15 ; C19H26O15 ; C19H26O15 ; C19H26O15 ; C19H26O15 ; C17H28O15 ; C9H10O7S ; C9H10O7S ; C5H10O5 ; C9H10O7S ; C5H10O5 ; C9H10O7S
97326_168.	1.735544	0.615207	-3.6196	0.004693	2.3286	0.056261	201.5097	HMDB33193	Avenanthramide L	M+2K	C18H15NO5

7382_100.[illegible]	1.693753	1.008364	3.6182	0.004703	2.3276	0.056261	424.0647	HMDB14599 ; HMDB33442 ; HMDB34674 ; HMDB37423 ; HMDB38042 ; HMDB38252 ; HMDB38769 ; HMDB38960	Cefalotin ; Mytilin B ; (S)-23-Dihydro-35-dihydroxy-2-oxo-3-indoleacetic acid 5-glucoside ; Isoscoparin 2-(6-(E)-p-coumaroylglucoside) ; Isovitexin 2-O-(6-feruloyl)glucoside ; Isorhamnetin 3-O-4-Hydroxy-E-cinnamoyl-(-6)-b-D-glucopyranosyl-(1-2)-a-L-rhamnopyranoside ; Kaempferol 3-sophoroside 7-glucuronide ; Zeanoside C	2M+3H2O+2H ; M+2K-H ; M+K ; M+2K ; M+2K ; M+2K ; M+Na+K ; M+K	C16H16N2O6S2 ; C14H23N2O8 ; C16H19NO10 ; C37H38O18 ; C37H38O18 ; C37H38O18 ; C33H38O22 ; C16H19NO10
0761_124.[illegible]	1.689216	0.688662	-3.6236	0.004662	2.3315	0.056261	NA	NA	NA	NA	NA
9265_167.	1.702284	0.127541	-3.6165	0.004717	2.3264	0.056287	330.0609	HMDB02044 ; HMDB15413 ; HMDB15621 ; HMDB29191 ; HMDB32956 ; HMDB60156	8-Hydroxyguanosine ; Sulfadoxine ; Sulfadimethoxine ; 5-(34-Dihydroxyphenyl)-gamma-valerolactone sulfate ; 2-O-p-Coumaroyltartronic acid ; 3-oxo-(2S)-Methylisocapryloyl-CoA	2M+Na+K ; 2M+H+K ; 2M+H+K ; M+ACN+H ; M+ACN+Na ; M+3Na	C10H13N5O6 ; C12H14N4O4S ; C12H14N4O4S ; C11H12O7S ; C12H10O7 ; C30H50N7O18P3S
1093_168.[illegible]	1.720525	0.145589	-3.6092	0.004774	2.3211	0.056835	402.0121	HMDB52141	TG(24:1(15Z)24:1(15Z)24:1(15Z))	M+3Na	C75H140O6
1876_282.[illegible]	1.778899	0.427496	3.603	0.004824	2.3166	0.056881	649.1912	HMDB30613 ; HMDB31286 ; HMDB34290 ; HMDB35318 ; HMDB36523 ; HMDB41076 ; HMDB60116	19-Octacosanediol ; erythro-79-Octacosanediol ; 128-Octacosanediol ; Apiosylglucosyl 4-hydroxybenzoate ; Licoagroside B ; erythro-68-Octacosanediol ; Dibenzoalpyrene	3M+H+NH4 ; 3M+H+NH4 ; 3M+H+NH4 ; 3M+2H ; 3M+2H ; 3M+H+NH4 ; 2M+2Na-H	C28H58O2 ; C28H58O2 ; C28H58O2 ; C18H24O12 ; C18H24O12 ; C28H58O2 ; C24H14
2488_52.9[illegible]	1.760349	0.080142	-3.6035	0.00482	2.317	0.056881	NA	NA	NA	NA	NA
2879_75.3[illegible]	1.737695	0.37646	3.6056	0.004803	2.3185	0.056881	438.1373	HMDB02376 ; HMDB14902 ; HMDB14969 ; HMDB34942 ; HMDB40891 ; HMDB41582	Tricrocin ; Mometasone ; Trifluoperazine ; Geniposidic acid ; 345-Trimethoxycinnamyl alcohol acetate ; Sapidolide A	M+Na+K ; 2M+H+Na ; 2M+Na+K ; M+ACN+Na ; 3M+2K ; 3M+2K	C38H54O19 ; C22H28Cl2O4 ; C21H24F3N3S ; C16H22O10 ; C14H18O5 ; C14H18O5
5861_98.7[illegible]	1.700942	0.06342	-3.605	0.004808	2.318	0.056881	258.0496	HMDB00422 ; HMDB00448 ; HMDB00752 ; HMDB00858 ; HMDB02074 ; HMDB02173 ; HMDB02399 ; HMDB06900 ; HMDB28728 ; HMDB28740 ; HMDB28770 ; HMDB29034 ; HMDB29904 ; HMDB30620 ; HMDB30797 ; HMDB31816 ; HMDB32077 ; HMDB33756 ; HMDB33837 ; HMDB39913 ; HMDB41114	2-Methylglutaric acid ; Adipic acid ; Methylglutaric acid ; Monomethyl glutaric acid ; 22-Dimethylsuccinic acid ; Solerol ; Cystathionine sulfoxide ; (S)-2-Aceto-2-hydroxybutanoic acid ; Asparaginyl-Cysteine ; Asparaginyl-Serine ; Cysteinyl-Asparagine ; Serinyl-Asparagine ; Kanzonol T ; Morusinol ; Mulberranol ; 3-Hydroxyflavone ; Dibenzyl disulfide ; Albanin C ; Dimethyl succinate ; 16-Dihydroxy-37-dimethoxy-2-(3-methyl-2-butenyl)-8-(2-oxo-3-methyl-3-butenyl)-xanthone ; Artonin V	3M+2K ; 3M+2K ; 3M+2K ; 3M+2K ; 3M+2K ; 3M+2K ; 2M+H+K ; 3M+2K ; M+Na ; M+K ; M+Na ; M+K ; M+2K ; M+2K ; M+2K ; 2M+H+K ; 2M+H+Na ; M+2K ; 3M+2K ; M+2K ; M+2K	C6H10O4 ; C6H10O4 ; C6H10O4 ; C6H10O4 ; C6H10O4 ; C6H10O4 ; C7H14N2O5S ; C6H10O4 ; C7H13N3O4S ; C7H13N3O5 ; C7H13N3O4S ; C7H13N3O5 ; C25H26O7 ; C25H26O7 ; C25H26O7 ; C15H10O3 ; C14H14S2 ; C25H26O7 ; C6H10O4 ; C25H26O7 ; C25H26O7

[illegible]3206_21.5[illegible]	1.776551	0.47665	3.6009	0.004841	2.3151	0.056953	442.3523	HMDB13339 ; HMDB13340 ; HMDB08026 ; HMDB08053 ; HMDB08085 ; HMDB08118 ; HMDB08150 ; HMDB08191 ; HMDB08224 ; HMDB08256 ; HMDB08276 ; HMDB08285 ; HMDB08308 ; HMDB08340 ; HMDB08350 ; HMDB08382 ; HMDB08414 ; HMDB08446 ; HMDB08479 ; HMDB08533 ; HMDB08542 ; HMDB08543 ; HMDB08564 ; HMDB08572 ; HMDB08573 ; HMDB08595 ; HMDB08604 ; HMDB08635 ; HMDB08766 ; HMDB08792 ; HMDB08797 ; HMDB08798 ; HMDB09247 ; HMDB09510 ; HMDB11202 ; HMDB11355 ; HMDB11380 ; HMDB13466 ; HMDB43046 ; HMDB43802 ; HMDB43884 ; HMDB59929	3-Hydroxy-11Z-octadecenoylcarnitine ; 3-Hydroxy-9Z-octadecenoylcarnitine ; PC(16:1(9Z)24:1(15Z)) ; PC(18:022:2(13Z16Z)) ; PC(18:1(11Z)22:1(13Z)) ; PC(18:1(9Z)22:1(13Z)) ; PC(18:2(9Z12Z)22:0) ; PC(18:3(6Z9Z12Z)24:1(15Z)) ; PC(18:3(9Z12Z15Z)24:1(15Z)) ; PC(18:4(6Z9Z12Z15Z)24:0) ; PC(20:020:2(11Z14Z)) ; PC(20:022:4(7Z10Z13Z16Z)) ; PC(20:1(11Z)20:1(11Z)) ; PC(20:2(11Z14Z)20:0) ; PC(20:2(11Z14Z)22:2(13Z16Z)) ; PC(20:3(5Z8Z11Z)22:1(13Z)) ; PC(20:3(8Z11Z14Z)22:1(13Z)) ; PC(20:4(5Z8Z11Z14Z)22:0) ; PC(20:4(8Z11Z14Z17Z)22:0) ; PC(22:018:2(9Z12Z)) ; PC(22:020:4(5Z8Z11Z14Z)) ; PC(22:020:4(8Z11Z14Z17Z)) ; PC(22:1(13Z)18:1(9Z)) ; PC(22:1(13Z)20:3(5Z8Z11Z)) ; PC(22:1(13Z)20:3(8Z11Z14Z)) ; PC(22:2(13Z16Z)18:0) ; PC(22:2(13Z16Z)20:2(11Z14Z)) ; PC(22:4(7Z10Z13Z16Z)20:0) ;	M+H ; M+H ; M+ACN+2H ; M+ACN+2H ; M+ACN+2H ; M+ACN+2H ; M+ACN+2H ; M+H+NH4 ; M+H+NH4 ; M+H+NH4 ; M+ACN+2H ; M+H+NH4 ; M+ACN+2H ; M+ACN+2H ; M+H+NH4 ; M+H+NH4 ; M+H+NH4 ; M+H+NH4 ; M+H+NH4 ; M+ACN+2H ; M+H+NH4 ; M+H+NH4 ; M+ACN+2H ; M+H+NH4 ; M+H+NH4 ; M+ACN+2H ; M+H+NH4 ; M+H+NH4 ; M+H+NH4 ; M+ACN+2H ; M+H+NH4 ; M+H+NH4 ; M+3ACN+2H ; M+3ACN+2H ; M+ACN+2H ; M+3ACN+2H ; M+3ACN+2H ; M+2ACN+2H ; M+2K ; M+2K ; M+2K ; M+ACN+H	C25H47NO5 ; C25H47NO5 ; C48H92NO8P ; C48H92NO8P ; C48H92NO8P ; C48H92NO8P ; C48H92NO8P ; C50H92NO8P ; C50H92NO8P ; C50H92NO8P ; C48H92NO8P ; C50H92NO8P ; C48H92NO8P ; C48H92NO8P ; C50H92NO8P ; C50H92NO8P ; C50H92NO8P ; C50H92NO8P ; C50H92NO8P ; C48H92NO8P ; C50H92NO8P ; C50H92NO8P ; C48H92NO8P ; C50H92NO8P ; C50H92NO8P ; C48H92NO8P ; C50H92NO8P ; C50H92NO8P ; C50H92NO8P ; C48H92NO8P ; C50H92NO8P ; C50H92NO8P ; C43H86NO7P ; C43H86NO7P ; C48H92NO8P ; C43H86NO7P ; C43H86NO7P ; C45H89N2O7P ; C52H102O5 ; C52H102O5 ; C52H102O5 ; C23H44O5
[illegible]2293_281.[illegible]	1.77021	0.167173	3.5946	0.004891	2.3106	0.057141	NA	NA	NA	NA	NA

7604_103.(	1.764303	1.135722	-3.595	0.004889	2.3108	0.057141	166.0628	HMDB11672 ; HMDB32655 ; HMDB33299 ; HMDB33697 ; HMDB33817 ; HMDB37253 ; HMDB37315 ; HMDB37955 ; HMDB38494 ; HMDB39381 ; HMDB39740 ; HMDB00957 ; HMDB01555 ; HMDB01936 ; HMDB02434 ; HMDB15510 ; HMDB31180 ; HMDB31667 ; HMDB32037 ; HMDB33002 ; HMDB33115 ; HMDB33127 ; HMDB33662 ; HMDB37621 ; HMDB37772 ; HMDB39465 ; HMDB39468 ; HMDB40030 ; HMDB40186 ; HMDB41802 ; HMDB42004 ; HMDB59734	Aflatoxin B1 dialcohol ; (R)-2-Feruloyl-1-(4-Hydroxyphenyl)-12-ethanediol ; 7-Hydroxy-3-(3-hydroxy-4-methoxybenzyl)-5-methoxy-4-chromanone ; Melilotocarpan C ; 4-Hydroxy-239-trimethoxypterocarpan ; 5-Hydroxy-3-(4-hydroxybenzyl)-78-dimethoxy-4-chromanone ; Cerasinone ; ()-34-Methylenedioxy-57-dimethylepicatechin ; Americanol ; Americanol A ; Isoamericanol A ; Pyrocatechol ; Pyridoxamine 5-phosphate ; Doxylamine ; Hydroquinone ; Flunitrazepam ; (EE)-24-Hexadienedial ; Nor-psi-tropine ; 13-Benzenediol ; 5-Methyl-2-furancarboxaldehyde ; 6-Methylquinoline ; 2-Acetylfuran ; (S)-5-Deoxy-5-(methylsulfinyl)adenosine ; 4-Methyl-4-(methylthio)-2-pentanone ; 8-Acetylegelolide ; S-Methyl hexanethioate ; S-Methyl 4-methylpentanethioate ; 1-(1-Pyrrolidinyl)-2-propanone ; 3-(Methylthio)hexanal ; 2-Aminonaphthalene ; Quinaldine ; Pyrazin-2-carboxylic acid	M+2H ; M+2H ; M+2H ; M+2H ; M+2H ; M+2H ; M+2H ; M+2H ; M+2H ; M+2H ; M+2H ; 3M+2H ; M+2ACN+2H ; M+Na+K ; 3M+2H ; M+H+NH4 ; 3M+2H ; M+K ; 3M+2H ; 3M+2H ; M+Na ; 3M+2H ; M+H+NH4 ; 2M+H+K ; M+H+Na ; 2M+H+K ; 2M+H+K ; M+K ; 2M+H+K ; M+Na ; M+Na ; M+ACN+H	C18H18O6 ; C18H18O6 ; C18H18O6 ; C18H18O6 ; C18H18O6 ; C18H18O6 ; C18H18O6 ; C18H18O6 ; C18H18O6 ; C18H18O6 ; C18H18O6 ; C6H6O2 ; C8H13N2O5P ; C17H22N2O ; C6H6O2 ; C16H12FN3O3 ; C6H6O2 ; C7H13NO ; C6H6O2 ; C6H6O2 ; C10H9N ; C6H6O2 ; C11H15N5O4S ; C7H14OS ; C16H20O6 ; C7H14OS ; C7H14OS ; C7H13NO ; C7H14OS ; C10H9N ; C10H9N ; C5H4N2O2
6368_105.!	1.730176	0.241908	-3.5964	0.004877	2.3119	0.057141	111.5136	HMDB33843	Polyvidone	M+2Na	C6H13NOP2

4415_113.7	1.746764	0.543868	-3.5861	0.004961	2.3045	0.057818	185.0704	HMDB02269 ; HMDB29486 ; HMDB30467 ; HMDB30686 ; HMDB33086 ; HMDB33344 ; HMDB34112 ; HMDB35526 ; HMDB36324 ; HMDB38225 ; HMDB38488 ; HMDB38528 ; HMDB38873 ; HMDB00182 ; HMDB01106 ; HMDB01888 ; HMDB02134 ; HMDB03405 ; HMDB03559 ; HMDB03584 ; HMDB05028 ; HMDB05038 ; HMDB05796 ; HMDB11737 ; HMDB12114 ; HMDB12115 ; HMDB14338 ; HMDB15676 ; HMDB31365 ; HMDB31382 ; HMDB32715 ; HMDB32716 ; HMDB33094 ; HMDB33095 ; HMDB33111 ; HMDB33164 ; HMDB33224 ; HMDB33225 ; HMDB33226 ; HMDB33290 ; HMDB34558 ; HMDB35609 ; HMDB36423 ; HMDB36677 ; HMDB36779 ; HMDB36782 ; HMDB36891 ; HMDB36892 ; HMDB37002 ; HMDB37003 ; HMDB38314 ; HMDB38611 ; HMDB38979 ; HMDB39056 ; HMDB39910 ; HMDB40309 ;	Curcumin ; Integrin ; Artocarpetin A ; Cyclointegrin ; Cyclocurcumin ; Mollicellin H ; 9-O-Methylglyceofuran ; Glycyrrhizaisoflavone C ; Glicoricone ; Glycycoumarin ; Gancaonin B ; Gancaonin N ; Isoglycycoumarin ; L-Lysine ; 3-Aminopropionaldehyde ; NN-Dimethylformamide ; Aminoacetone ; D-Lysine ; Gibberellin A3 ; Taurocyamine ; Escitalopram ; Citalopram ; Picrocrocin ; Gamma Glutamylglutamic acid ; (3S)-36-Diaminohexanoate ; (3S5S)-35-Diaminohexanoate ; Indecainide ; Difluprednate ; Gibberellin A94 ; Portuloside A ; Lepidine B ; Lepidine D ; Lepidine F ; Lepidine E ; Epijasminoside A ; Acetyl-T2 Toxin ; (1R4R5S)-5-Hydroxyfenchone glucoside ; (1R4S6R)-6-Hydroxyfenchone glucoside ; (1S4R)-10-Hydroxyfenchone glucoside ; Junosmarin ; 6-Hydroxy-2-bornanone glucoside ; Gibberellin A68 ; Cynaropicrin ; Strigol ; Diosbulbin E ; Diosbulbin G ; Gibberellin A22 ; 11beta-Hydroxygibberellin A7 ; Mozambioside ; Cafamarine ;	M+2H ; M+2H ; M+2H ; M+2H ; M+2H ; M+2H ; M+2H ; M+2H ; M+2H ; M+2H ; M+2H ; M+2H ; M+2H ; M+K ; 2M+K ; 2M+K ; 2M+K ; M+K ; M+H+Na ; M+NH4 ; M+2Na ; M+2Na ; M+H+K ; 2M+3H ; M+K ; M+K ; M+Na+K ; M+H+2Na ; M+H+Na ; M+H+K ; M+H+Na ; M+H+Na ; M+H+Na ; M+H+Na ; M+H+K ; M+H+2Na ; M+H+K ; M+H+K ; M+H+K ; M+H+Na ; M+H+K ; M+H+Na ; M+H+Na ; M+H+Na ; M+H+Na ; M+H+Na ; M+H+Na ; M+H+Na ; M+H+2Na ; M+H+2Na ; M+H+Na ; M+H+2Na ; M+H+K ; M+H+K ; M+3H ; M+H+Na ; M+H+Na ; M+H+Na ; M+H+Na ; 2M+3H ; 2M+K ; M+3ACN+2H ; M+H+2Na	C21H20O6 ; C21H20O6 ; C21H20O6 ; C21H20O6 ; C21H20O6 ; C21H20O6 ; C21H20O6 ; C21H20O6 ; C21H20O6 ; C21H20O6 ; C21H20O6 ; C21H20O6 ; C21H20O6 ; C6H14N2O2 ; C3H7NO ; C3H7NO ; C3H7NO ; C6H14N2O2 ; C19H22O6 ; C3H9N3O3S ; C20H21FN2O ; C20H21FN2O ; C16H26O7 ; C10H16N2O7 ; C6H14N2O2 ; C6H14N2O2 ; C20H24N2O ; C27H34F2O7 ; C19H22O6 ; C16H26O7 ; C20H18N4O2 ; C20H18N4O2 ; C20H18N4O2 ; C20H18N4O2 ; C16H26O7 ; C26H36O10 ; C16H26O7 ; C16H26O7 ; C16H26O7 ; C19H22O6 ; C16H26O7 ; C19H22O6 ; C19H22O6 ; C19H22O6 ; C19H22O6 ; C19H22O6 ; C19H22O6 ; C19H22O6 ; C26H36O10 ; C26H36O10 ; C19H22O6 ; C26H36O10 ; C16H26O7 ; C16H26O7 ; C26H32O13 ; C19H22O6 ; C19H22O6 ;

2631_64.9	1.737527	0.370771	-3.579	0.00502	2.2993	0.058375	531.9663	HMDB05401 ; HMDB05470 ;<br>HMDB14394 ; HMDB35705 ;<br>HMDB36566 ; HMDB42232 ;<br>HMDB42260 ; HMDB42266 ;<br>HMDB42435 ; HMDB42493 ;<br>HMDB42499 ; HMDB42615 ;<br>HMDB42637 ; HMDB42681 ;<br>HMDB42696 ; HMDB42817 ;<br>HMDB43960 ; HMDB43986 ;<br>HMDB43992 ; HMDB44019 ;<br>HMDB44176 ; HMDB44203 ;<br>HMDB44209 ; HMDB44235 ;<br>HMDB44242 ; HMDB44289 ;<br>HMDB44344 ; HMDB44364 ;<br>HMDB44419 ; HMDB44485 ;<br>HMDB44505 ; HMDB44532 ;<br>HMDB44767 ; HMDB44798 ;<br>HMDB44824 ; HMDB44974 ;<br>HMDB44980 ; HMDB45000 ;<br>HMDB45032 ; HMDB45039 ;<br>HMDB45110 ; HMDB45162 ;<br>HMDB45182 ; HMDB45235 ;<br>HMDB45299 ; HMDB45318 ;<br>HMDB45344 ; HMDB45546 ;<br>HMDB45570 ; HMDB45576 ;<br>HMDB45601 ; HMDB45719 ;<br>HMDB45725 ; HMDB45745 ;<br>HMDB45751 ; HMDB45775 ;	TG(18:020:020:4(5Z8Z11Z14Z))iso6 ;<br>TG(18:2(9Z12Z)20:1(11Z)20:1(11Z))is<br>o3 ; Idoxuridine ; Zeaxanthin<br>dipalmitate ; Xantofyl palmitate ;<br>TG(14:022:022:4(7Z10Z13Z16Z)) ;<br>TG(14:024:020:4(5Z8Z11Z14Z)) ;<br>TG(14:024:020:4(8Z11Z14Z17Z)) ;<br>TG(14:020:3(5Z8Z11Z)24:1(15Z)) ;<br>TG(14:024:1(15Z)20:3(5Z8Z11Z)) ;<br>TG(14:024:1(15Z)20:3n6) ;<br>TG(14:020:3n624:1(15Z)) ;<br>TG(14:020:4(5Z8Z11Z14Z)24:0) ;<br>TG(14:022:2(13Z16Z)22:2(13Z16Z)) ;<br>TG(14:022:4(7Z10Z13Z16Z)22:0) ;<br>TG(14:020:4(8Z11Z14Z17Z)24:0) ;<br>TG(16:020:022:4(7Z10Z13Z16Z)) ;<br>TG(16:022:020:4(5Z8Z11Z14Z)) ;<br>TG(16:022:020:4(8Z11Z14Z17Z)) ;<br>TG(16:024:018:4(6Z9Z12Z15Z)) ;<br>TG(16:020:3(5Z8Z11Z)22:1(13Z)) ;<br>TG(16:022:1(13Z)20:3(5Z8Z11Z)) ;<br>TG(16:022:1(13Z)20:3n6) ;<br>TG(16:024:1(15Z)18:3(6Z9Z12Z)) ;<br>TG(16:024:1(15Z)18:3(9Z12Z15Z)) ;<br>TG(16:018:3(6Z9Z12Z)24:1(15Z)) ;<br>TG(16:020:3n622:1(13Z)) ;<br>TG(16:020:4(5Z8Z11Z14Z)22:0) ;<br>TG(16:022:4(7Z10Z13Z16Z)20:0) ;	M+3ACN+2H ; M+3ACN+2H ;<br>3M+2H ; M+H+NH4 ;<br>M+H+NH4 ; M+3ACN+2H ;<br>M+3ACN+2H ; M+3ACN+2H ;	C61H110O6 ; C61H110O6 ;<br>C9H11IN2O5 ; C72H116O4 ;<br>C72H116O4 ; C61H110O6 ;<br>C61H110O6 ; C61H110O6 ;
5492_101.7	1.772946	1.051509	3.5689	0.005105	2.292	0.059226	267.5685	HMDB60032	(beta-1-O-N-(2-formyl-3-chlorophenyl)anthraniloyl-D-glucupyranuronic acid)	M+2ACN+2H	C20H18ClNO9

[illegible]1073_150.[illegible]	1.768328	0.374062	-3.5658	0.005132	2.2897	0.059258	139.0831	HMDB00925 ; HMDB12136 ; HMDB12176 ; HMDB29350 ; HMDB30428 ; HMDB30895 ; HMDB31383 ; HMDB31384 ; HMDB32054 ; HMDB32209 ; HMDB32291 ; HMDB32343 ; HMDB33150 ; HMDB33228 ; HMDB34673 ; HMDB35287 ; HMDB35887 ; HMDB36032 ; HMDB36730 ; HMDB36880 ; HMDB37195 ; HMDB37279 ; HMDB37284 ; HMDB38136 ; HMDB38138 ; HMDB38196 ; HMDB38197 ; HMDB38247 ; HMDB38248 ; HMDB38250 ; HMDB39130 ; HMDB39745 ; HMDB40273 ; HMDB40281 ; HMDB41589 ; HMDB59959	Trimethylamine N-oxide ; 1-Amino-propan-2-ol ; 5-Aminopentanamide ; Geranyl 3-methylbutanoate ; Linalyl isovalerate ; (4R5S7R11x)-1112-Dihydroxy-1(10)-spirovetiven-2-one 12-glucoside ; (3S4S6R7S)-110-Bisaboladiene-34-diol ; beta-Kessyl alcohol ; alpha-Terpinyl pentanoate ; Citronellyl trans-2-methyl-2-butenoate ; Geranyl 2-methylbutyrate ; Isobornyl 2-methylbutyrate ; (4R5S7R11S)-1112-Dihydroxy-1(10)-spirovetiven-2-one 11-glucoside ; Guaidiol ; Calamendiol ; Daucol ; Germacrenone ; 3beta-7-Drimene-311-diol ; Tricyclohumuladiol ; 169-Farnesatriene-311-diol ; 2-Propenyl cyclohexanehexanoate ; 210-Bisaboladiene-14-diol ; 5(1-10)-Abeo-112-patchoulanediol ; Curcumadiol ; alpha-Bisabolol oxide C ; alpha-Bisabolol oxide A ; alpha-Bisabolol oxide B ; Bornyl valerate ; Bornyl isovalerate ; Isobornyl isovalerate ; Methyl (ZZ)-58-tetradecadienoate ; (2E6E9xi)-Farnesol ; Terpenyl isovalerate ; Geranyl valerate ; alpha-Kessyl alcohol ; 2-(2-Dimethyl(2-[illegible]	M+ACN+Na ; M+ACN+Na ; M+Na ; M+H+K ; M+H+K ; M+3H ; M+H+K ; M+H+K ; M+H+K ; M+H+K ; M+H+K ; M+H+K ; M+3H ; M+H+K ; M+H+Na	C3H9NO ; C3H9NO ; C5H12N2O ; C15H26O2 ; C15H26O2 ; C21H34O8 ; C15H26O2 ; C15H26O2 ; C15H26O2 ; C15H26O2 ; C15H26O2 ; C15H26O2 ; C21H34O8 ; C15H26O2 ; C10H28N2OP2
[illegible]5374_119.[illegible]	1.703014	0.449799	-3.5652	0.005136	2.2894	0.059258	243.0025	HMDB00174 ; HMDB00849 ; HMDB02712 ; HMDB03081 ; HMDB05876 ; HMDB10207 ; HMDB12327 ; HMDB15045 ; HMDB29196 ; HMDB33050 ; HMDB33051 ; HMDB33821 ; HMDB38183 ; HMDB39783 ; HMDB41500 ; HMDB59624 ; HMDB59625 ; HMDB60267	L-Fucose ; Rhamnose ; 15-Anhydrosorbitol ; Beta-D-Fucose ; 3-Deoxyfructose ; L-Rhamnulose ; 2-Deoxygalactopyranose ; Zonisamide ; D-Fucose ; Butyl isopropyl disulfide ; Butyl propyl disulfide ; 2-O-Methyl-D-xylose ; 3-(4-Methyl-3-pentenyl)thiophene ; 1-(Methylthio)-1-hexanethiol ; 1-Deoxy-D-glucitol ; Alpha-L-fucose ; beta-L-fucose ; L-Fuculose	M+2K+H ; M+2K+H ; M+2K+H ; M+2K+H ; M+2K+H ; M+2K+H ; M+2K+H ; 2M+Na+K ; M+2K+H ; M+2K+H ; M+2K+H ; M+2K+H ; M+2K-H ; M+2K+H ; M+2K-H ; M+2K+H ; M+2K+H ; M+2K+H	C6H12O5 ; C6H12O5 ; C6H12O5 ; C6H12O5 ; C6H12O5 ; C6H12O5 ; C6H12O5 ; C8H8N2O3S ; C6H12O5 ; C7H16S2 ; C7H16S2 ; C6H12O5 ; C10H14S ; C7H16S2 ; C6H14O5 ; C6H12O5 ; C6H12O5 ; C6H12O5

8488_99.87	1.702674	0.805233	-3.5644	0.005144	2.2887	0.059258	218.0338	HMDB03974 ; HMDB29613 ; HMDB34884 ; HMDB37818 ; HMDB37855 ; HMDB37868 ; HMDB37869 ; HMDB37870 ; HMDB37871 ; HMDB38792	Oxalosuccinic acid ; 9-Azabicyclo331nonan-3-one ; 1234-Tetrahydro-6-propanoylpyridine ; 2-Pentyloxazole ; 4-Butyl-2-methyloxazole ; 24-Dimethyl-5-propyloxazole ; 45-Dimethyl-2-propyloxazole ; 45-Diethyl-2-methyloxazole ; 45-Dimethyl-2-(1-methylethyl)oxazole ; 5-Pentyloxazole	2M+3H2O+2H ; M+2K+H ; M+2K+H ; M+2K+H ; M+2K+H ; M+2K+H ; M+2K+H ; M+2K+H ; M+2K+H ; M+2K+H	C6H6O7 ; C8H13NO ; C8H13NO ; C8H13NO ; C8H13NO ; C8H13NO ; C8H13NO ; C8H13NO ; C8H13NO ; C8H13NO
9352_54.55	1.746955	0.891864	3.5619	0.005165	2.287	0.059349	269.9209	HMDB05356 ; HMDB10413 ; HMDB42065 ; HMDB42124 ; HMDB42153 ; HMDB42181 ; HMDB42991 ; HMDB43047	TG(16:016:016:0)iso ; TG(16:014:018:0)iso6 ; TG(14:014:020:0) ; TG(14:016:018:0) ; TG(14:018:016:0) ; TG(14:020:014:0) ; TG(15:015:018:0) ; TG(15:018:015:0)	M+3H ; M+3H ; M+3H ; M+3H ; M+3H ; M+3H ; M+3H ; M+3H	C51H98O6 ; C51H98O6 ; C51H98O6 ; C51H98O6 ; C51H98O6 ; C51H98O6 ; C51H98O6 ; C51H98O6
1383_119.3	1.695373	0.887826	-3.5607	0.005175	2.2861	0.059349	120.5191	HMDB06409	Tyramine-O-sulfate	M+H+Na	C8H11NO4S
8943_276.7	1.773946	0.251774	3.5535	0.005237	2.2809	0.059922	NA	NA	NA	NA	NA
4603_88.49	1.775865	0.622506	-3.5492	0.005276	2.2777	0.05995	285.1195	HMDB13129 ; HMDB29868 ; HMDB30593 ; HMDB30845 ; HMDB33428 ; HMDB34679 ; HMDB35475 ; HMDB36982 ; HMDB39912 ; HMDB41048 ; HMDB60841	Glutaconylcarnitine ; Mangostanol ; Edulisin II ; Archangelicin ; ()-Setoclavine ; Cinncassiol D2 glucoside ; Simmondsin 2-ferulate ; Atrovirisidone ; Mangostenol ; Physagulin B ; N-Despropyl-rotigotine	2M+H+Na ; 2M+3H ; 2M+3H ; 2M+3H ; 2M+Na+K ; M+H+K ; M+H+NH4 ; 2M+3H ; 2M+3H ; M+H+Na ; 2M+H+Na	C12H19NO6 ; C24H26O7 ; C24H26O7 ; C24H26O7 ; C16H18N2O ; C26H42O11 ; C26H33NO12 ; C24H26O7 ; C24H26O7 ; C30H39ClO7 ; C16H19NOS
1179_264.1	1.715189	0.399574	-3.5502	0.005266	2.2785	0.05995	420.0412	HMDB60506 ; HMDB11747 ; HMDB30000 ; HMDB37553 ; HMDB39940 ; HMDB14636 ; HMDB37132	S-(22-Dichloro-1-hydroxy)ethyl glutathione ; Ciliatine ; Bissulfine ; SS-Ethylidene dithioacetate ; S-2-Propenyl 2-propene-1-sulfonothioate ; Cefotaxime ; (2R)-2-Hydroxy-2-(4-hydroxyphenyl)ethyl glucosinolate	M+H ; 3M+2Na-H ; 2M+ACN+Na ; 2M+ACN+Na ; 2M+ACN+Na ; M+H-2H2O ; M+H-2H2O	C12H19Cl2N3O7S ; C2H8NO3P ; C6H10O2S2 ; C6H10O2S2 ; C6H10O2S2 ; C16H17N5O7S2 ; C15H21NO11S2
3523_58.99	1.69552	0.334801	3.5507	0.005262	2.2789	0.05995	NA	NA	NA	NA	NA

[illegible]3009_278.[illegible]	1.770342	0.189215	3.5463	0.005301	2.2757	0.059963	757.2213	HMDB34396 ; HMDB35004 ; HMDB37353 ; HMDB37411 ; HMDB37427 ; HMDB37566 ; HMDB37959 ; HMDB38253 ; HMDB38470 ; HMDB38768 ; HMDB39951 ; HMDB40294 ; HMDB40543 ; HMDB41470 ; HMDB29459 ; HMDB30682 ; HMDB31996 ; HMDB33027 ; HMDB34213 ; HMDB35525 ; HMDB36157 ; HMDB36158 ; HMDB36160 ; HMDB38774 ; HMDB39005 ; HMDB41123 ; HMDB41290	Kaempferol 3-gentiobioside 7-rhamnoside ; Isovitexin 47-diglucoside ; Clovin ; Lucenin 27-O-rhamnoside ; Kaempferol 3-sophoroside 7-rhamnoside ; Wyomin ; Manghaslin ; Isorhamnetin 3-O-b-D-xylopyranosyl-(1-2)-a-L-rhamnopyranosyl-(1-6)-b-D-glucopyranoside ; Cynarotrioside ; Kaempferol 3-glucosyl-(1-3)-rhamnosyl-(1-6)-galactoside ; Kaempferol 3-(6-rhamnosylsophoroside) ; Quercetin 3-rhamnosyl-(1-2)-rhamnosyl-(1-6)-glucoside ; Kaempferol 7-galactoside 3-rutinoside ; Isorhamnetin 3-(2G-apiosylrutinoside) ; Xanthoxylol ; Cubebin ; Licorice glycoside E ; Licoagrodione ; Kievitone ; Licofuranone ; 15-Deacetylneosolaniol ; 4-Deacetylneosolaniol ; 8-Acetyl-T2 tetrol ; 5-Deoxykievitol ; Hv-NCC-1 ; Kinobeon A ; 18-Dihydroxy-35-dimethoxy-2-prenylxanthone	M+H ; M+H ; M+H ; M+H ; M+H ; M+H ; M+H ; M+H ; M+H ; M+H ; M+H ; M+H ; M+H ; M+H ; 2M+2Na-H ; 2M+2Na-H ; M+ACN+Na ; 2M+2Na-H ; 2M+2Na-H ; 2M+2Na-H ; 2M+2K-H ; 2M+2K-H ; 2M+2K-H ; 2M+2Na-H ; M+2K+H ; 2M+2Na-H ; 2M+2Na-H	C33H40O20 ; C33H40O20 ; C33H40O20 ; C33H40O20 ; C33H40O20 ; C33H40O20 ; C33H40O20 ; C33H40O20 ; C33H40O20 ; C33H40O20 ; C33H40O20 ; C33H40O20 ; C33H40O20 ; C33H40O20 ; C20H20O6 ; C20H20O6 ; C35H35NO14 ; C20H20O6 ; C20H20O6 ; C20H20O6 ; C17H24O7 ; C17H24O7 ; C17H24O7 ; C20H20O6 ; C35H42N4O10 ; C20H20O6 ; C20H20O6
[illegible]9543_100.[illegible]	1.739038	0.639755	-3.547	0.005295	2.2761	0.059963	553.172	HMDB30669 ; HMDB32401 ; HMDB39959 ; HMDB39960 ; HMDB14911	3-O-Feruloylquinic acid ; (-)-3-(2-methyl-3-furyl)thio-2-butanone ; 3-O-Caffeoyl-1-O-methylquinic acid ; 3-O-Caffeoyl-4-O-methylquinic acid ; Etoposide	3M+2H ; 3M+H ; 3M+2H ; 3M+2H ; M+H-2H2O	C17H20O9 ; C9H12O2S ; C17H20O9 ; C17H20O9 ; C29H32O13
[illegible]8146_100.[illegible]	1.756881	0.985398	-3.5384	0.005371	2.2699	0.060592	296.0658	HMDB01235 ; HMDB33261 ; HMDB00824 ; HMDB02338 ; HMDB05781 ; HMDB14407 ; HMDB14572 ; HMDB14659 ; HMDB29522 ; HMDB30768 ; HMDB31428 ; HMDB31720 ; HMDB34127 ; HMDB36629 ; HMDB39166 ; HMDB39578 ; HMDB41758 ; HMDB41776	5-Aminoimidazole ribonucleotide ; Mulberrofuran M ; Propionylcarnitine ; Biochanin A ; Glycitein ; Carmustine ; Streptozocin ; Albendazole ; 57-Dihydroxy-3-methoxyflavone ; 2-(6-Hydroxy-2-methoxy-34-methylenedioxyphenyl)benzofuran ; Farnisin ; Xenognosin B ; Prunetin ; (-)-Maackiain ; Cajaquinone ; 15-Dihydroxy-2-methoxy-6-methylanthraquinone ; Melanettin ; Stevenin	M+H ; M+2H ; M+2K+H ; 2M+H+Na ; 2M+H+Na ; M+2ACN+H ; 2M+Na+K ; 2M+Na+K ; 2M+H+Na ; 2M+H+Na ; 2M+H+Na ; 2M+H+Na ; 2M+H+Na ; 2M+H+Na ; 2M+H+Na ; 2M+H+Na ; 2M+H+Na ; 2M+H+Na	C8H14N3O7P ; C34H22O10 ; C10H19NO4 ; C16H12O5 ; C16H12O5 ; C5H9Cl2N3O2 ; C8H15N3O7 ; C12H15N3O2S ; C16H12O5 ; C16H12O5 ; C16H12O5 ; C16H12O5 ; C16H12O5 ; C16H12O5 ; C16H12O5 ; C16H12O5 ; C16H12O5 ; C16H12O5

1817_282.3	1.739367	0.059052	3.5373	0.005381	2.2692	0.060592	611.4618	HMDB07063 ; HMDB07280 ; HMDB07309 ; HMDB07336 ; HMDB07337 ; HMDB07763 ; HMDB56155 ; HMDB56304 ; HMDB56367 ; HMDB07014 ; HMDB07015 ; HMDB07025 ; HMDB07026 ; HMDB07042 ; HMDB07052 ; HMDB07053 ; HMDB07099 ; HMDB07106 ; HMDB07127 ; HMDB07133 ; HMDB07134 ; HMDB07154 ; HMDB07182 ; HMDB07211 ; HMDB07273 ; HMDB07302 ; HMDB07330 ; HMDB07444 ; HMDB07473 ; HMDB07501 ; HMDB07530 ; HMDB31042 ; HMDB41092 ; HMDB55961 ; HMDB55962 ; HMDB55971 ; HMDB55977 ; HMDB56015 ; HMDB56031 ; HMDB56040 ; HMDB56139 ; HMDB56145 ; HMDB56164 ; HMDB56171	DG(14:1(9Z)22:6(4Z7Z10Z13Z16Z19Z) 0:0) ; DG(18:3(6Z9Z12Z)18:4(6Z9Z12Z15Z)0 :0) ; DG(18:3(9Z12Z15Z)18:4(6Z9Z12Z15Z) 0:0) ; DG(18:4(6Z9Z12Z15Z)18:3(6Z9Z12Z)0 :0) ; DG(18:4(6Z9Z12Z15Z)18:3(9Z12Z15Z) 0:0) ; DG(22:6(4Z7Z10Z13Z16Z19Z)14:1(9Z) 0:0) ; DG(14:1n50:022:6n3) ; DG(18:3n60:018:4n3) ; DG(18:3n30:018:4n3) ; DG(14:018:1(11Z)0:0) ; DG(14:018:1(9Z)0:0) ; DG(14:020:4(5Z8Z11Z14Z)0:0) ; DG(14:020:4(8Z11Z14Z17Z)0:0) ; DG(14:1(9Z)18:00:0) ; DG(14:1(9Z)20:3(5Z8Z11Z)0:0) ; DG(14:1(9Z)20:3(8Z11Z14Z)0:0) ; DG(16:016:1(9Z)0:0) ; DG(16:018:4(6Z9Z12Z15Z)0:0) ; DG(16:1(9Z)16:00:0) ; DG(16:1(9Z)18:3(6Z9Z12Z)0:0) ; DG(16:1(9Z)18:3(9Z12Z15Z)0:0) ; DG(18:014:1(9Z)0:0) ; DG(18:1(11Z)14:00:0) ;	M+H ; M+H ; M+H ; M+H ; M+H ; M+H ; M+H ; M+H ; M+H ; M+2Na-H ; M+2Na-H ; M+Na ; M+Na ; M+2Na-H ; M+Na ; M+Na ; M+2Na-H ; M+Na ; M+2Na-H ; M+Na ; M+Na ; M+2Na-H ; M+2Na-H ; M+2Na-H ; M+Na ; M+Na ; M+Na ; M+Na ; M+Na ; M+Na ; M+Na ; 2M+K ; M+Na ; M+2Na-H ; M+2Na-H ; M+Na ; M+Na ; M+2Na-H ; M+Na ; M+2Na-H ; M+Na ; M+Na ; M+Na ; M+Na	C39H62O5 ; C39H62O5 ; C39H62O5 ; C39H62O5 ; C39H62O5 ; C39H62O5 ; C39H62O5 ; C39H62O5 ; C39H62O5 ; C35H66O5 ; C35H66O5 ; C37H64O5 ; C37H64O5 ; C35H66O5 ; C37H64O5 ; C37H64O5 ; C35H66O5 ; C37H64O5 ; C35H66O5 ; C37H64O5 ; C37H64O5 ; C35H66O5 ; C35H66O5 ; C35H66O5 ; C37H64O5 ; C37H64O5 ; C37H64O5 ; C37H64O5 ; C37H64O5 ; C37H64O5 ; C37H64O5 ; C17H34O3 ; C37H64O5 ; C35H66O5 ; C35H66O5 ; C37H64O5 ; C37H64O5 ; C35H66O5 ; C37H64O5 ; C35H66O5 ; C37H64O5 ; C37H64O5 ; C37H64O5 ; C37H64O5

3861_44.06	1.759336	0.328299	-3.5358	0.005394	2.2681	0.060611	219.1339	HMDB14515 ; HMDB28916 ; HMDB28938 ; HMDB29042 ; HMDB29043 ; HMDB29074 ; HMDB29137 ; HMDB33675 ; HMDB41313 ; HMDB00348 ; HMDB00361 ; HMDB00384 ; HMDB00411 ; HMDB00438 ; HMDB00478 ; HMDB00514 ; HMDB00518 ; HMDB00626 ; HMDB00664 ; HMDB00686 ; HMDB00733 ; HMDB00811 ; HMDB00820 ; HMDB00863 ; HMDB00946 ; HMDB02451 ; HMDB02488 ; HMDB02536 ; HMDB02585 ; HMDB03315 ; HMDB12187 ; HMDB30332 ; HMDB30354 ; HMDB30867 ; HMDB30945 ; HMDB31030 ; HMDB31196 ; HMDB31496 ; HMDB31497 ; HMDB31498 ; HMDB31563 ; HMDB31564 ; HMDB31692 ; HMDB32051 ; HMDB32193 ; HMDB32331 ; HMDB32901 ; HMDB33549 ; HMDB33714 ; HMDB34903 ; HMDB35157 ; HMDB35239 ; HMDB36048 ; HMDB36599 ; HMDB37301 ; HMDB38270 ;	Meprobamate ; Isoleucyl-Serine ; Leucyl-Serine ; Serinyl-Isoleucine ; Serinyl-Leucine ; Threoninyl-Valine ; Valyl-Threonine ; L-Lysopine ; Pantothenamide ; 3b12a-Dihydroxy-5a-cholanoic acid ; 3b7a-Dihydroxy-5b-cholanoic acid ; 3a7a-Dihydroxycholanoic acid ; 3a12b-Dihydroxy-5b-cholanoic acid ; 3b12a-Dihydroxy-5b-cholanoic acid ; Allodeoxycholic acid ; Allochenodeoxycholic acid ; Chenodeoxycholic acid ; Deoxycholic acid ; Isohyodeoxycholic acid ; Isoursodeoxycholic acid ; Hyodeoxycholic acid ; Murocholic acid ; Propyl alcohol ; Isopropyl alcohol ; Ursodeoxycholic acid ; 7b12a-Dihydroxycholanoic acid ; 7a12b-dihydroxy-5b-Cholan-24-oic acid ; Isodeoxycholic acid ; 3b12b-Dihydroxy-5b-cholanoic acid ; Cyclohexanone ; All-trans-heptaprenyl diphosphate ; 2-Methyl-4-pentenal ; Arenaine ; Dihydro-5-(2-octenyl)-2(3H)-furanone ; Methyl 2-undecynoate ; 6-Heptyl-56-dihydro-2H-pyran-2-one ; 4-Methyl-2-pentenal ; 2-Hexenal ; 3-Hexenal ; (Z)-3-	M+H ; M+H ; M+H ; M+H ; M+H ; M+H ; M+H ; M+H ; M+H ; M+2Na ; M+2Na ; M+2Na ; M+2Na ; M+2Na ; M+2Na ; M+2Na ; M+2Na ; M+2Na ; M+2Na ; M+2Na ; M+2Na ; M+2Na ; 3M+K ; 3M+K ; M+2Na ; M+2Na ; M+2Na ; M+2Na ; M+2Na ; 2M+Na ; M+3H ; 2M+Na ; 2M+H+Na ; M+Na ; M+Na ; M+Na ; 2M+Na ; 2M+Na ; 2M+Na ; 2M+Na ; 2M+Na ; 2M+Na ; M+Na ; M+Na ; M+Na ; M+Na ; M+Na ; 2M+Na ; 2M+Na ; M+Na ; M+Na ; 2M+Na ; M+Na ; M+Na ; M+Na ; M+Na ; M+Na ; M+Na ; M+2H+Na ; M+Na ; M+Na ; 2M+Na ; 2M+Na ; M+Na ; M+Na ; M+2H+Na ; M+2H+Na ; M+Na ; M+Na	C9H18N2O4 ; C9H18N2O4 ; C9H18N2O4 ; C9H18N2O4 ; C9H18N2O4 ; C9H18N2O4 ; C9H18N2O4 ; C9H18N2O4 ; C9H18N2O4 ; C24H40O4 ; C24H40O4 ; C24H40O4 ; C24H40O4 ; C24H40O4 ; C24H40O4 ; C24H40O4 ; C24H40O4 ; C24H40O4 ; C24H40O4 ; C24H40O4 ; C24H40O4 ; C24H40O4 ; C3H8O ; C3H8O ; C24H40O4 ; C24H40O4 ; C24H40O4 ; C24H40O4 ; C24H40O4 ; C6H10O ; C35H60O7P2 ; C6H10O ; C11H17N3O ; C12H20O2 ; C12H20O2 ; C12H20O2 ; C6H10O ; C6H10O ; C6H10O ; C6H10O ; C6H10O ; C6H10O ; C12H20O2 ; C12H20O2 ; C12H20O2 ; C12H20O2 ; C12H20O2 ; C6H10O ; C6H10O ; C12H20O2 ; C12H20O2 ; C6H10O ; C12H20O2 ; C12H20O2 ; C12H20O2 ; C12H20O2 ; C12H20O2 ; C12H20O2 ;
6278_59.47	1.739012	0.918617	3.5319	0.00543	2.2652	0.060786	214.0063	HMDB01439 ; HMDB15276	Phosphoribosyl formamidocarboxamide ; Sulfoxone	M+Na+K ; M+H+Na	C10H15N4O9P ; C14H16N2O6S3

7749_105.:	1.712837	0.639084	3.5308	0.00544	2.2644	0.060786	271.0678	HMDB00978 ; HMDB01128 ; HMDB06766 ; HMDB10333 ; HMDB10335 ; HMDB10353 ; HMDB10354 ; HMDB29262 ; HMDB30414 ; HMDB37220 ; HMDB37542 ; HMDB37937 ; HMDB40487 ; HMDB60328 ; HMDB60901	4-(2-Aminophenyl)-24-dioxobutanoic acid ; 5-Phosphoribosylamine ; Estriol-16-Glucuronide ; Estriol-17-glucuronide ; Estriol-3-glucuronide ; 15-Hydroxynorandrostene-317-dione glucuronide ; 16-alpha17-beta-estriol 17-beta-D-glucuronide ; Quercetin 374-O-triglucoside ; Quercetin 3-glucosyl-(1-2)-galactosyl-(1-2)-glucoside ; Quercetin 3-(2-glucosylgalactoside) 7-glucoside ; Patuletin 3-(2-apiosylgentiobioside) ; Quercetin 3-(2G-glucosylgentiobioside) ; Moracetin ; 1-Nitro-56-dihydroxy-dihydronaphthalene ; Clozapine glucuronide	M+ACN+Na ; M+ACN+H ; M+2K ; M+2K ; M+2K ; M+2K ; M+2K ; M+2H+Na ; M+2H+Na ; M+2H+Na ; M+2H+Na ; M+2H+Na ; M+2H+Na ; M+ACN+Na ; M+H+K	C10H9NO4 ; C5H12NO7P ; C24H32O9 ; C24H32O9 ; C24H32O9 ; C24H32O9 ; C24H32O9 ; C33H40O22 ; C33H40O22 ; C33H40O22 ; C33H40O22 ; C33H40O22 ; C33H40O22 ; C10H9NO4 ; C24H27ClN4O6
4588_22.4(	1.684486	0.706501	-3.53	0.005447	2.2639	0.060786	433.2845	HMDB01160 ; HMDB35638 ; HMDB35642 ; HMDB35649 ; HMDB35650 ; HMDB41050 ; HMDB41070	Tricosanoic acid ; 6-Hydroxy-8-tricosanone ; 8-Hydroxy-6-tricosanone ; 4-Hydroxy-6-tricosanone ; 6-Hydroxy-4-tricosanone ; (3beta22E24R)-Ergosta-468(14)22-tetraen-3-ol ; erythro-68-Tricosanediol	M+2K+H ; M+2K+H ; M+2K+H ; M+2K+H ; M+2K+H ; M+K ; M+2K-H	C23H46O2 ; C23H46O2 ; C23H46O2 ; C23H46O2 ; C23H46O2 ; C28H42O ; C23H48O2
39388_123.	1.730143	0.718667	-3.5278	0.005467	2.2623	0.060874	227.6939	HMDB15662 ; HMDB31274 ; HMDB31745 ; HMDB38525 ; HMDB39182 ; HMDB39185 ; HMDB39368 ; HMDB39369 ; HMDB39370	Fosaprepitant ; 134-Trigalloyl-beta-D-glucopyranose ; 124-Trigalloyl-beta-D-glucopyranose ; 3-(10-Heptadecenyl)phenol ; 126-Trigalloyl-beta-D-glucopyranose ; 146-Trigalloyl-beta-D-glucopyranose ; 125-Tri-O-galloylhamamelofuranose ; Kurigalin ; 3-O-Galloylhamamelitannin	M+3Na ; M+H+2Na ; M+H+2Na ; M+3ACN+2H ; M+H+2Na ; M+H+2Na ; M+H+2Na ; M+H+2Na ; M+H+2Na	C23H22F7N4O6P ; C27H24O18 ; C27H24O18 ; C23H38O ; C27H24O18 ; C27H24O18 ; C27H24O18 ; C27H24O18 ; C27H24O18

2278_31.9	1.754951	0.346331	-3.5145	0.00559	2.2526	0.06169	253.1182	HMDB00027 ; HMDB00318 ; HMDB00787 ; HMDB00969 ; HMDB01414 ; HMDB01434 ; HMDB11754 ; HMDB29668 ; HMDB30657 ; HMDB31260 ; HMDB31869 ; HMDB32776 ; HMDB32966 ; HMDB34474 ; HMDB34672 ; HMDB34984 ; HMDB35053 ; HMDB35228 ; HMDB36604 ; HMDB36686 ; HMDB36694 ; HMDB36821 ; HMDB36824 ; HMDB36897 ; HMDB36900 ; HMDB36945 ; HMDB37776 ; HMDB38047 ; HMDB38529 ; HMDB38760 ; HMDB38761 ; HMDB40233 ; HMDB41002 ; HMDB41231 ; HMDB41349 ; HMDB41636 ; HMDB59658 ; HMDB59756 ; HMDB60243 ; HMDB60747	Tetrahydrobiopterin ; 34-Dihydroxyphenylglycol ; Sapropterin ; 125-Dihydroxyvitamin D3-2623-lactone ; Putrescine ; 3-Methoxytyrosine ; Methyldopa ; Eremopetasinorol ; Carinol ; (all-E)-179-Heptadecatriene-111315-triyne ; (SE)-Lyratol propanoate ; 3-Oxo-carbofuran ; Furaneol acetate ; Niveusin C ; (5alpha8beta9beta)-59-Epoxy-36-megastigmadien-8-ol ; (5R6S)-56-Epoxy-7-megastigmen-9-one ; Gibberellin A52 ; Theaspirone A ; N-Methylcalystegine B2 ; 2267-Tetramethylbicyclo430nona-1(9)4-diene-78-diol ; 15-Hydroxyleptocarpin ; 3-Hydroxy-beta-ionone ; Acetal R ; Gibberellin A17 ; Gibberellin A66 ; Gibberellin A125 ; 59:69-Diepoxy-3-megastigmene ; Carvyl propionate ; 59:69-Diepoxy-4-megastigmene ; Herierin III ; Herierin IV ; 235688a-Hexahydro-2558a-tetramethyl-7H-1-benzopyran-7-one ; (1E4Z6a8b10a)-8-(2-Methylbutanoyloxy)-1015-dihydroxy-3-oxo-1411(13)-germacratrien-126-olide ; Gibberellin A99 ; Gibberellin A102 ; (3R8E)-3-	2M+H+Na ; M+2ACN+H ; 2M+H+Na ; M+Na+K ; 2M+2K-H ; M+ACN+H ; M+ACN+H ; M+2Na-H ; 2M+3H ; 2M+Na+K ; M+2Na-H ; M+NH4 ; M+2ACN+H ; 2M+3H ; M+2Na-H ; M+2Na-H ; 2M+3H ; M+2Na-H ; M+ACN+Na ; M+2Na-H ; 2M+3H ; M+2Na-H ; M+2Na-H ; 2M+3H ; 2M+3H ; 2M+3H ; M+2Na-H ; M+2Na-H ; M+2Na-H ; M+2ACN+H ; M+2ACN+H ; M+2Na-H ; 2M+3H ; 2M+3H ; 2M+3H ; M+2Na-H ; 2M+H+Na ; M+2ACN+H ; 2M+2K-H ; M+ACN+H	C9H15N5O3 ; C8H10O4 ; C9H15N5O3 ; C27H40O5 ; C4H12N2 ; C10H13NO4 ; C10H13NO4 ; C13H20O2 ; C20H26O7 ; C17H18 ; C13H20O2 ; C12H13NO4 ; C8H10O4 ; C20H26O7 ; C13H20O2 ; C13H20O2 ; C20H26O7 ; C13H20O2 ; C8H15NO4 ; C13H20O2 ; C20H26O7 ; C13H20O2 ; C13H20O2 ; C20H26O7 ; C20H26O7 ; C20H26O7 ; C13H20O2 ; C13H20O2 ; C13H20O2 ; C8H10O4 ; C8H10O4 ; C13H20O2 ; C20H26O7 ; C20H26O7 ; C20H26O7 ; C13H20O2 ; C9H15N5O3 ; C8H10O4 ; C4H12N2 ; C10H13NO4
97933_113.	1.743857	0.855152	-3.5094	0.005637	2.2489	0.06169	294.5798	HMDB15381	Gliquidone	M+Na+K	C27H33N3O6S
33105_93.2	1.716257	0.392698	-3.5155	0.005581	2.2533	0.06169	NA	NA	NA	NA	NA
3056_97.7	1.706364	1.126279	-3.5172	0.005564	2.2546	0.06169	284.0803	HMDB36254 ; HMDB36255 ; HMDB10356 ; HMDB13970 ; HMDB15702 ; HMDB30630 ; HMDB33935 ; HMDB40977 ; HMDB60861	Gladiatoside C2 ; Gladiatoside C1 ; Estriol 3-sulfate 16-glucuronide ; Dehydroxyzyleuton ; Ticagrelor ; Rubraflavone C ; Anigorufone ; Brassitin ; OR-1896	M+2H ; M+2H ; M+H+Na ; M+ACN+Na ; M+2Na ; M+2K ; 2M+H+Na ; M+ACN+Na ; M+K	C29H26O12 ; C29H26O12 ; C24H32O12S ; C11H12N2OS ; C23H28F2N6O4S ; C30H34O6 ; C19H12O2 ; C11H12N2OS ; C13H15N3O2
0391_129.5	1.698794	0.651784	3.5101	0.005631	2.2494	0.06169	235.004	HMDB00926 ; HMDB01544 ; HMDB06524 ; HMDB06802 ; HMDB31665 ; HMDB33044 ; HMDB33045	Pyridine ; m-Chlorobenzoic acid ; 3-Indoleacetonitrile ; O-Phospho-4-hydroxy-L-threonine ; Daucic acid ; 1-(Methylthio)propyl propyl disulfide ; Ethyl 1-(ethylthio)propyl disulfide	2M+2K-H ; 3M+2H ; M+2K+H ; 2M+H+K ; 2M+Na+K ; M+K ; M+K	C5H5N ; C7H5ClO2 ; C10H8N2 ; C4H10NO7P ; C7H8O7 ; C7H16S3 ; C7H16S3

1438_23.72	1.689741	1.22588	-3.5096	0.005636	2.249	0.06169	728.5241	HMDB10226 ; HMDB10227 ; HMDB56404 ; HMDB56405 ; HMDB56458 ; HMDB56468 ; HMDB56493 ; HMDB56494 ; HMDB56523 ; HMDB56527 ; HMDB56618 ; HMDB56674 ; HMDB56707 ; HMDB56711 ; HMDB56715 ; HMDB56716 ; HMDB56781 ; HMDB56833 ; HMDB56949 ; HMDB56953 ; HMDB56975 ; HMDB57011 ; HMDB57053 ; HMDB57077 ; HMDB57078 ; HMDB57204 ; HMDB57215 ; HMDB57241 ; HMDB57463 ; HMDB57464 ; HMDB57667 ; HMDB57871 ; HMDB58117 ; HMDB58334 ; HMDB58530 ; HMDB58697 ; HMDB58698 ; HMDB58719 ; HMDB02392 ; HMDB07266 ; HMDB07293 ; HMDB07294 ; HMDB07322 ; HMDB07323 ; HMDB07350 ; HMDB07462 ; HMDB07491 ; HMDB07518 ; HMDB07519 ; HMDB07547 ; HMDB07548 ; HMDB07574 ; HMDB07575 ; HMDB07686 ; HMDB07713 ; HMDB07714 ;	CL(18:1(11Z)18:1(11Z)18:1(11Z)18:2(9Z12Z)) ; CL(18:1(9Z)18:1(9Z)18:1(9Z)18:2(9Z12Z)) ; CL(16:016:018:022:5(4Z7Z10Z13Z16Z)) ; CL(16:016:018:022:5(7Z10Z13Z16Z19Z)) ; CL(16:016:022:5(4Z7Z10Z13Z16Z)18:0) ; CL(16:016:022:5(7Z10Z13Z16Z19Z)18:0) ; CL(16:018:016:022:5(4Z7Z10Z13Z16Z)) ; CL(16:018:016:022:5(7Z10Z13Z16Z19Z)) ; CL(16:018:022:5(4Z7Z10Z13Z16Z)16:0) ; CL(16:018:022:5(7Z10Z13Z16Z19Z)16:0) ; CL(16:018:1(11Z)18:2(9Z12Z)18:2(9Z12Z)) ; CL(16:018:1(9Z)18:2(9Z12Z)18:2(9Z12Z)) ; CL(16:018:2(9Z12Z)18:1(11Z)18:2(9Z12Z)) ; CL(16:018:2(9Z12Z)18:1(9Z)18:2(9Z12Z)) ;	M+2H ; 3M+H+K ; M+ACN+Na ; 2M+3H2O+2H ; 2M+3H2O+2H ; 2M+3H2O+2H ; 2M+3H2O+2H ; 2M+3H2O+2H ; 3M+H+NH4 ; 2M+3H2O+2H ; 3M+H+K ; 3M+H+K ; 3M+H+K ; 3M+H+K ; 3M+H+K ; 3M+H+K ; 3M+H+K ; 3M+H+K ; 3M+H+K ; 3M+H+K ; 3M+H+K ; 3M+H+K ;	C81H148O17P2 ; C81H148O17P2 ;
3489_72.55	1.681435	0.267281	-3.5093	0.005639	2.2488	0.06169	303.1663	HMDB13016 ; HMDB02171 ; HMDB02248 ; HMDB04985 ; HMDB04987 ; HMDB14884 ; HMDB15692 ; HMDB28758 ; HMDB28947 ; HMDB35340 ; HMDB35354 ; HMDB36485 ; HMDB39228 ; HMDB41579 ; HMDB60994	Neuromedin B (1-3) ; Glycylprolylhydroxyproline ; Gamma glutamyl ornithine ; Aspartylysine ; Alpha-Aspartyl-lysine ; Deferoxamine ; Isothipendyl ; Aspartyl-Lysine ; Lysyl-Aspartate ; Ganoderic acid Mi ; Ganoderic acid Mj ; Trigoneoside XIb ; (3-Aminopropoxy)guanidine ; 78-Dihydro-3b6a-dihydroxy-alpha-ionol 9-apiosyl-(1-6)-glucoside ; alpha-Hydroxymetoprolol	M+H ; M+NH4 ; M+ACN+H ; M+ACN+H ; M+ACN+H ; M+2Na ; M+NH4 ; M+ACN+H ; M+ACN+H ; M+Na+K ; M+Na+K ; M+3H ; 2M+K ; M+2ACN+2H ; 2M+H+K	C12H22N4O5 ; C12H19N3O5 ; C10H19N3O5 ; C10H19N3O5 ; C10H19N3O5 ; C25H48N6O8 ; C16H19N3S ; C10H19N3O5 ; C10H19N3O5 ; C33H52O6 ; C33H52O6 ; C44H74O19 ; C4H12N4O ; C24H42O12 ; C15H25NO4
6903_59.74	1.670916	1.045958	3.511	0.005622	2.2501	0.06169	274.0597	HMDB39751 ; HMDB14965 ; HMDB60396 ; HMDB60559	alpha-D-Galactopyranuronosyl-(1-4)-alpha-D-galactopyranuronosyl-(1-4)-D-galacturonic acid ; Cinoxacin ; 5-Fluorouridine ; Acetaminophen cystein	M+2H ; 2M+H+Na ; 2M+H+Na ; 2M+H+K	C18H26O19 ; C12H10N2O5 ; C9H11FN2O6 ; C11H14N2O3S

9997_65.28	1.740298	0.71854	3.5033	0.005695	2.2445	0.062035	311.09	HMDB38936 ; HMDB38937 ; HMDB01867 ; HMDB14383 ; HMDB29303 ; HMDB30579 ; HMDB30740 ; HMDB31986 ; HMDB33018 ; HMDB39523 ; HMDB40681 ; HMDB41484 ; HMDB41686 ; HMDB41696 ; HMDB42029 ; HMDB59723 ; HMDB37250	Garcilivin A ; Garcilivin B ; 4-Aminohippuric acid ; Nevirapine ; 1-(3-Methoxy-4-hydroxy)-phenyl-67-dihydroxy-isochroman ; CI Natural Red 20 ; 11-Methoxyyangonin ; CI Pigment Red 149 ; 10-Methoxyyangonin ; Isomelitric acid A ; Melitric acid A ; 5-Acetyl-24-dimethylthiazole ; 5-Methoxy-O-desmethylangolensin ; 6-Hydroxyangolensin ; Tetrazepam ; Pyridylacetylglycine ; Muscomin	M+2H ; M+2H ; 3M+H+K ; M+2Na-H ; M+Na ; M+Na ; M+Na ; M+H+Na ; M+Na ; M+2ACN+2H ; M+2ACN+2H ; 2M+H ; M+Na ; M+Na ; M+Na ; 3M+H+K ; M+H-2H2O	C36H28O10 ; C36H28O10 ; C9H10N2O3 ; C15H14N4O ; C16H16O5 ; C16H16O5 ; C16H16O5 ; C40H26N2O4 ; C16H16O5 ; C27H22O12 ; C27H22O12 ; C7H9NOS ; C16H16O5 ; C16H16O5 ; C16H17ClN2O ; C9H10N2O3 ; C18H18O7
5467_103.6	1.691683	0.216157	-3.5043	0.005686	2.2452	0.062035	252.5045	HMDB34994 ; HMDB41891	Chikusetsusaponin Ia ; Famprofazone	M+3H ; 2M+3H	C41H70O12 ; C24H31N3O
9783_51.82	1.767714	0.275211	3.4972	0.005754	2.2401	0.062539	202.937	HMDB15677	Dimercaprol	M+2K+H	C3H8OS2
7759_103.4	1.676836	1.202236	-3.4906	0.005817	2.2353	0.063092	344.5838	HMDB01373 ; HMDB06824 ; HMDB36319 ; HMDB55950 ; HMDB55951 ; HMDB59645	Dephospho-CoA ; Dihydroneopterin phosphate ; (R)-Skyrin 2-xyloside ; TG(22:6(4Z7Z10Z13Z16Z19Z)22:6(4Z7Z10Z13Z16Z19Z)o-18:0) ; TG(22:6(4Z7Z10Z13Z16Z19Z)o-18:022:6(4Z7Z10Z13Z16Z19Z)) ; beta-nicotinamide D-ribonucleotide	M+2H ; 2M+H+NH4 ; M+H+NH4 ; M+3Na ; M+3Na ; 2M+H+NH4	C21H35N7O13P2S ; C9H14N5O7P ; C35H26O14 ; C65H104O5 ; C65H104O5 ; C11H16N2O8P
9628_35.61	1.739686	0.076199	-3.4883	0.005839	2.2336	0.063195	203.139	HMDB28690 ; HMDB28691 ; HMDB28900 ; HMDB28922 ; HMDB06246 ; HMDB11143 ; HMDB11624 ; HMDB15596 ; HMDB30896 ; HMDB31181 ; HMDB31408 ; HMDB32531 ; HMDB35246 ; HMDB35411 ; HMDB35679 ; HMDB35680 ; HMDB38108 ; HMDB39339 ; HMDB39590	Alanyl-Isoleucine ; Alanyl-Leucine ; Isoleucyl-Alanine ; Leucyl-Alanine ; Tetracosatetraenoic acid (24:4n-6) ; MG(18:0e0:00:0) ; Decyl alcohol ; Vildagliptin ; Momordicilin ; Homodihydrojasmone ; ()-3-Decanol ; 2-trans-6-cis-Dodecadienal ; 37-Dimethyl-3-octanol ; (R)-Dihydrocitronellol ; 1-Hydroxy-1-phenyl-3-octadecanone ; 3-Hydroxy-1-phenyl-1-octadecanone ; cis-Quinceoxepane ; Tricycloekasantalol ; (2E4E)-24-Dodecadienal	M+H ; M+H ; M+H ; M+H ; M+2Na ; M+Na+K ; M+2Na-H ; 2M+3H ; M+3Na ; M+Na ; M+2Na-H ; M+Na ; M+2Na-H ; M+2Na-H ; M+2Na ; M+2Na ; M+Na ; M+Na ; M+Na	C9H18N2O3 ; C9H18N2O3 ; C9H18N2O3 ; C9H18N2O3 ; C24H40O2 ; C21H44O3 ; C10H22O ; C17H25N3O2 ; C36H60O3 ; C12H20O ; C10H22O ; C12H20O ; C10H22O ; C10H22O ; C24H40O2 ; C24H40O2 ; C12H20O ; C12H20O ; C12H20O

9118_72.62	1.760425	0.256605	-3.4826	0.005896	2.2295	0.063283	193.0819	HMDB29048 ; HMDB00812 ; HMDB03470 ; HMDB03828 ; HMDB14439 ; HMDB30960 ; HMDB31091 ; HMDB31208 ; HMDB31379 ; HMDB31577 ; HMDB31856 ; HMDB32025 ; HMDB32415 ; HMDB33562 ; HMDB36183 ; HMDB38934 ; HMDB39163 ; HMDB39803 ; HMDB40245 ; HMDB41493 ; HMDB60080 ; HMDB60273	Serinyl-Serine ; N-Acetyl-L-aspartic acid ; N-Formyl-L-glutamic acid ; D-Pantethine ; Etonogestrel ; Obtusilactone A ; 3-Methyl-5-pentyl-2-furannonanoic acid ; 1-Phenyl-1-pentanone ; Annosquamosin B ; Berteroin ; ()-2-Methyl-3-(4-methylphenyl)propanal ; 4-Isopropylacetophenone ; 2-Methyl-4-phenylbutyraldehyde ; 3-Methyl-2-phenylbutanal ; 4-(4-Methylphenyl)-2-butanone ; Ssioriside ; D-N-(Carboxyacetyl)alanine ; 44alpha56-Tetrahydro-7-methyl-2(3H)-naphthalenone ; ()-2-Methyl-3-(2-methylphenyl)propanal ; 4-Isopropylphenylacetaldehyde ; Thyrotropin releasing hormone ; 2-Amino-3-oxoadipate	M+H ; M+NH4 ; M+NH4 ; M+2H+Na ; M+Na+K ; M+2K ; M+2K ; 2M+Na+K ; M+2K ; M+NH4 ; 2M+Na+K ; 2M+Na+K ; 2M+Na+K ; 2M+Na+K ; 2M+Na+K ; M+2H+Na ; M+NH4 ; 2M+Na+K ; 2M+Na+K ; 2M+Na+K ; M+H+Na ; M+NH4	C6H12N2O5 ; C6H9NO5 ; C6H9NO5 ; C22H42N4O8S2 ; C22H28O2 ; C19H32O3 ; C19H32O3 ; C11H14O ; C19H32O3 ; C7H13NS2 ; C11H14O ; C11H14O ; C11H14O ; C11H14O ; C11H14O ; C27H38O12 ; C6H9NO5 ; C11H14O ; C11H14O ; C11H14O ; C16H22N6O4 ; C6H9NO5
4953_65.57	1.742481	0.734074	3.4847	0.005874	2.231	0.063283	291.0645	HMDB38641 ; HMDB41121 ; HMDB15016 ; HMDB29733 ; HMDB30109 ; HMDB30668 ; HMDB31536 ; HMDB32653 ; HMDB33160 ; HMDB33706 ; HMDB34733 ; HMDB38551 ; HMDB39235 ; HMDB40862	1-49-Dihydro-2-(methylthio)-13-thiazino65-bindol-4-yl-2-propanone ; 2-(4-Hydroxyphenyl)naphthalic anhydride ; Chlorhexidine ; 2-Ethyl-5-methylthiophene ; Albanol B ; 1-(246-Trimethoxyphenyl)-13-butanedione ; Myricatomentoside I ; (Z)-3-(Methylsulfinyl)-1-propenyl 2-propenyl disulfide ; 2-Propylthiophene ; Salviaflaside methyl ester ; Vaccinoside ; Methyl 345-trimethoxycinnamate ; Citrusin F ; Piceatannol 4-galloylglucoside	M+H ; M+H ; M+2K ; 2M+K ; M+H+Na ; M+K ; M+2K ; M+2ACN+H ; 2M+K ; M+2Na ; M+2Na ; M+K ; M+Na+K ; M+H+Na	C14H14N2OS2 ; C18H10O4 ; C22H30Cl2N10 ; C7H10S ; C34H22O8 ; C13H16O5 ; C26H32O10 ; C7H12OS3 ; C7H10S ; C25H28O13 ; C25H28O13 ; C13H16O5 ; C22H32O14 ; C27H26O13
9973_116.5	1.709402	1.323192	3.4824	0.005898	2.2293	0.063283	251.5175	HMDB00580 ; HMDB60358 ; HMDB60381	Chondroitin sulfate ; 23-Dihydro-2-S-glutathionyl-3-hydroxy bromobenzene ; 34-Dihydro-3-hydroxy-4-S-glutathionyl bromobenzene	M+H+K ; M+H+Na ; M+H+Na	C13H21NO15S ; C16H22BrN3O7S ; C16H22BrN3O7S

79291_27.46	1.690294	0.997327	3.4851	0.005871	2.2313	0.063283	220.1179	HMDB00210 ; HMDB12204 ; HMDB12518 ; HMDB14369 ; HMDB15217 ; HMDB15434 ; HMDB29569 ; HMDB29602 ; HMDB29640 ; HMDB30065 ; HMDB31566 ; HMDB31617 ; HMDB31619 ; HMDB32306 ; HMDB33092 ; HMDB33176 ; HMDB33789 ; HMDB35175 ; HMDB35925 ; HMDB40348 ; HMDB59897 ; HMDB60813	Pantothenic acid ; Cis-zeatin ; 11-Carboxy-gamma-tocotrienol ; Indinavir ; Pilocarpine ; Forasartan ; Dibutyl disulfide ; 4-Phenyl-2-butenal ; 3-(4-Methylphenyl)-2-propenal ; Colupulone ; 2-Methyl-3-phenyl-2-propenal ; Benzylideneacetone ; 2-Phenyl-2-butenal ; 4-Heptenal diethyl acetal ; 3-(1-Hydroxymethyl-1-propenyl)pentanedioic acid ; 23-Dimethylbenzofuran ; 2-O-Methyl-L-fucose ; 2-Amino-16-dimethylfuro32-eimidazo45-bpyridine ; 4-(1-Methylethenyl)benzaldehyde ; 18-Octanedithiol ; Benzofuran 47-dimethyl- ; Descarbonyl-lacosamide	M+H ; M+H ; M+H+K ; M+H+2Na ; 2M+H+Na ; M+H+Na ; M+ACN+H ; 3M+2H ; 3M+2H ; M+H+K ; 3M+2H ; 3M+2H ; 3M+2H ; M+NH4 ; M+NH4 ; 3M+2H ; M+ACN+H ; M+NH4 ; 3M+2H ; M+ACN+H ; 3M+2H ; 2M+H+Na	C9H17NO5 ; C10H13N5O ; C25H36O4 ; C36H47N5O4 ; C11H16N2O2 ; C23H28N8 ; C8H18S2 ; C10H10O ; C10H10O ; C25H36O4 ; C10H10O ; C10H10O ; C10H10O ; C9H14O5 ; C9H14O5 ; C10H10O ; C7H14O5 ; C10H10N4O ; C10H10O ; C8H18S2 ; C10H10O ; C11H16N2O2
04441_130.3	1.698309	0.574879	3.4743	0.005978	2.2235	0.063923	417.0294	HMDB03705 ; HMDB14098 ; HMDB41567	Phosphoguanidinoacetate ; 5-Hydroxytenoxicam ; Lithol Rubine	2M+Na ; M+ACN+Na ; 2M+Na+K	C3H8N3O5P ; C13H11N3O5S2 ; C18H14N2O6S
08193_58.67	1.675258	1.008623	3.4738	0.005983	2.2231	0.063923	172.0308	HMDB15034 ; HMDB01067 ; HMDB13911 ; HMDB13912 ; HMDB13913 ; HMDB13914 ; HMDB29512 ; HMDB30718 ; HMDB38091 ; HMDB38811	Triazolam ; N-Acetylaspartylglutamic acid ; 12-Hydroxynevirapine ; 2-Hydroxynevirapine ; 8-Hydroxynevirapine ; 3-Hydroxynevirapine ; 45-Dihydroxy-7-methoxy-6-methylflavone ; Sayanedin ; Peonidin 3-rhamnoside ; Alfalone	M+2H ; M+H+K ; M+Na+K ; M+Na+K ; M+Na+K ; M+Na+K ; M+2Na ; M+2Na ; M+3Na ; M+2Na	C17H12Cl2N4 ; C11H16N2O8 ; C15H14N4O2 ; C15H14N4O2 ; C15H14N4O2 ; C15H14N4O2 ; C17H14O5 ; C17H14O5 ; C22H23O10 ; C17H14O5

26579_113.(	1.712563	0.590416	-3.4721	0.006	2.2219	0.063962	178.0327	HMDB38591 ; HMDB13742 ; HMDB14831 ; HMDB14944 ; HMDB29609 ; HMDB30111 ; HMDB33298 ; HMDB33683 ; HMDB33692 ; HMDB33797 ; HMDB33924 ; HMDB37251 ; HMDB37477 ; HMDB37481 ; HMDB37749 ; HMDB39825 ; HMDB40145 ; HMDB40357 ; HMDB41234 ; HMDB41790	Brassicanal A ; 2-Furanmethanol ; Fluorescein ; Pentoxifylline ; 5-Methyl-2(3H)-furanone ; Homoferreirin ; 3-(34-Dihydroxybenzyl)-7-hydroxy-5-methoxy-4-chromanone ; Melilotocarpan E ; Melilotocarpan D ; Olivin ; Cajanol ; 58-Dihydroxy-3-(4-hydroxybenzyl)-7-methoxy-4-chromanone ; 57-Dihydroxy-3-(3-hydroxy-4-methoxybenzyl)-4-chromanone ; 35-Dihydroxy-47-dimethoxyflavanone ; 47-Dihydroxy-25-dimethoxyisoflavanone ; Cyclocalopin F ; 5-Hydroxy-4-pentenoic acid d-lactone ; 611-Dihydroxy-22-dimethylpyrano32-cxanthen-7(2H)-one ; Artocarpanone A ; Violanone	M+H ; 3M+Na+K ; M+H+Na ; M+2K ; 3M+Na+K ; M+H+K ; M+H+K ; M+H+K ; M+H+K ; M+H+K ; M+H+K ; M+H+K ; M+H+K ; M+H+K ; M+H+K ; M+Na+K ; 3M+Na+K ; M+2Na ; M+H+K ; M+H+K	C9H7NOS ; C5H6O2 ; C20H12O5 ; C13H18N4O3 ; C5H6O2 ; C17H16O6 ; C17H16O6 ; C17H16O6 ; C17H16O6 ; C17H16O6 ; C17H16O6 ; C17H16O6 ; C17H16O6 ; C17H16O6 ; C17H16O6 ; C15H18O6 ; C5H6O2 ; C18H14O5 ; C17H16O6 ; C17H16O6
3996_283.7	1.748335	0.716157	3.4693	0.006028	2.2198	0.064129	506.744	HMDB06902 ; HMDB39399 ; HMDB40688	Cobinamide ; Basellasaponin A ; (3x5x10x)-910-Didehydroisohumbertiol O-rhamnopyranosyl-(1-4)-rhamnopyranosyl-(1-2)-(E)-feruloyl-(-4)-rhamnosyl-(1-6)-glucoside	M+H+Na ; M+ACN+2H ; M+H+NH4	C48H72CoN11O8 ; C47H70O21 ; C49H70O21
2372_39.12	1.688244	0.410498	3.4656	0.006066	2.2171	0.064396	443.0824	HMDB01022 ; HMDB01269 ; HMDB01372 ; HMDB02135 ; HMDB02255 ; HMDB02310 ; HMDB33420 ; HMDB35038 ; HMDB35052 ; HMDB36890 ; HMDB36898 ; HMDB39623 ; HMDB40324 ; HMDB40651 ; HMDB60494	Succinyl-CoA ; Methylmalonyl-CoA ; Thiamine pyrophosphate ; S-(3-oxo-3-carboxy-n-propyl)cysteine ; R-Methylmalonyl-CoA ; S-Methylmalonyl-CoA ; Gibberellin A55 ; Gibberellin A50 ; Gibberellin A49 ; Gibberellin A72 ; Gibberellin A76 ; Gibberellin A79 ; (7R8R)-3-Methoxy-34799-pentahydroxy-84-oxyneolignan ; Gibberellin A91 ; N-Acetylmuramoyl-Ala	M+H+NH4 ; M+H+NH4 ; M+NH4 ; 2M+H ; M+H+NH4 ; M+H+NH4 ; M+2K+H ; M+2K+H ; M+2K+H ; M+2K+H ; M+2K+H ; M+2K+H ; M+2K+H ; M+2K+H ; M+2K+H	C25H40N7O19P3S ; C25H40N7O19P3S ; C12H19N4O7P2S ; C7H11NO5S ; C25H40N7O19P3S ; C25H40N7O19P3S ; C19H24O7 ; C19H24O7 ; C19H24O7 ; C19H24O7 ; C19H24O7 ; C19H24O7 ; C19H24O7 ; C19H24O7 ; C14H24N2O9
16003_91.78	1.709304	0.909339	-3.4627	0.006095	2.215	0.064569	382.9816	HMDB01570	Thymidine 35-cyclic monophosphate	M+2K+H	C10H13N2O7P
013218_154	1.702716	0.308416	-3.4559	0.006166	2.21	0.065174	140.9913	HMDB14841 ; HMDB33205	Methazolamide ; Roxarsone	M+2Na ; M+H+NH4	C5H8N4O3S2 ; C6H6AsNO6

6614_58.4[illegible]	1.692737	0.763571	3.4543	0.006181	2.2089	0.065202	293.1197	HMDB30187 ; HMDB33048 ; HMDB33049 ; HMDB36317 ; HMDB38834 ; HMDB59732 ; HMDB59772	(EE)-Piperlonguminine ; Ethyl 1-(propylthio)propyl disulfide ; Butyl 1-(methylthio)propyl disulfide ; Canavalioside ; (2E)-Piperamide-C5:1 ; 2-(2-Phenylacetoxy)propionylglycine ; N-Phenylacetylglutamic acid	2M+H+K ; M+2ACN+H ; M+2ACN+H ; M+H+K ; 2M+H+K ; 2M+3H2O+2H ; 2M+3H2O+2H	C16H19NO3 ; C8H18S3 ; C8H18S3 ; C26H42O12 ; C16H19NO3 ; C13H15NO5 ; C13H15NO5
6942_101.[illegible]	1.73751	0.693499	-3.4491	0.006236	2.2051	0.065642	290.1457	HMDB28705 ; HMDB28747 ; HMDB00145 ; HMDB12150 ; HMDB15219 ; HMDB32673 ; HMDB37778	Arginyl-Aspartate ; Aspartyl-Arginine ; Estrone ; 2-Keto-6-acetamidocaproate ; Primaquine ; 15-Octadecene-91113-triynoic acid ; Ganoderiol I	M+H ; M+H ; 2M+H+K ; 3M+H+NH4 ; 2M+Na+K ; 2M+H+K ; M+2K	C10H19N5O5 ; C10H19N5O5 ; C18H22O2 ; C8H13NO4 ; C15H21N3O ; C18H22O2 ; C31H50O5
7568_22.2[illegible]	1.744401	0.436846	3.4432	0.006298	2.2008	0.066015	444.3588	HMDB43451 ; HMDB43536 ; HMDB43621 ; HMDB43654 ; HMDB43677 ; HMDB43682 ; HMDB43710 ; HMDB43732 ; HMDB43738 ; HMDB43766 ; HMDB43787 ; HMDB43794 ; HMDB48090 ; HMDB48136 ; HMDB48376 ; HMDB48378 ; HMDB52514 ; HMDB52645 ; HMDB52750 ; HMDB52757 ; HMDB52764 ; HMDB52871 ; HMDB53063 ; HMDB53091 ; HMDB53182 ; HMDB53366 ; HMDB53479 ; HMDB53570 ; HMDB53582 ; HMDB54030 ; HMDB54919 ; HMDB55160 ; HMDB55254 ; HMDB55268 ; HMDB55366 ; HMDB55542 ; HMDB11701 ; HMDB11704 ; HMDB11708 ; HMDB13820 ; HMDB30507 ; HMDB32145 ; HMDB34919 ; HMDB35124 ; HMDB35265 ; HMDB35274 ; HMDB35817 ; HMDB36016 ; HMDB36659 ; HMDB39243 ; HMDB42346 ; HMDB42348 ; HMDB42376 ; HMDB42378 ; HMDB42520 ; HMDB42521 ;	TG(15:018:3(6Z9Z12Z)22:6(4Z7Z10Z13Z16Z19Z)) ; TG(15:020:4(5Z8Z11Z14Z)20:5(5Z8Z11Z14Z17Z)) ; TG(15:022:5(4Z7Z10Z13Z16Z)18:4(6Z9Z12Z15Z)) ; TG(15:018:3(9Z12Z15Z)22:6(4Z7Z10Z13Z16Z19Z)) ; TG(15:018:4(6Z9Z12Z15Z)22:5(4Z7Z10Z13Z16Z)) ; TG(15:018:4(6Z9Z12Z15Z)22:5(7Z10Z13Z16Z19Z)) ; TG(15:020:4(8Z11Z14Z17Z)20:5(5Z8Z11Z14Z17Z)) ; TG(15:020:5(5Z8Z11Z14Z17Z)20:4(5Z8Z11Z14Z)) ; TG(15:020:5(5Z8Z11Z14Z17Z)20:4(8Z11Z14Z17Z)) ; TG(15:022:5(7Z10Z13Z16Z19Z)18:4(6Z9Z12Z15Z)) ; TG(15:022:6(4Z7Z10Z13Z16Z19Z)18:3(6Z9Z12Z)) ; TG(15:022:6(4Z7Z10Z13Z16Z19Z)18:3(9Z12Z15Z)) ; TG(14:1(9Z)18:2(9Z12Z)22:6(4Z7Z10Z13Z16Z19Z)) ; TG(14:1(9Z)20:2n622:6(4Z7Z10Z13Z16Z19Z)) ;	M+2H ; M+2Na ; M+2Na ; M+2Na ; 2M+ACN+Na ; 2M+H+K ; 2M+H+K ; 2M+H+K ; 2M+H+K ; 2M+H+K ; 2M+H+K ; 2M+H+K ; 2M+H+K ; 2M+H+K ; 2M+H+K ; M+2Na ; M+2Na ; M+2Na ; M+2Na ; M+2Na ; M+2Na ; M+H+Na ; M+H+Na ; M+2Na ; M+2Na ; M+H+Na ; M+H+Na ; M+H+Na ; M+H+Na ; M+H+Na ; M+H+Na ; M+2Na ; M+2Na ; M+H+Na ; M+2Na ; M+2Na ; M+H+Na ; M+H+Na ; M+H+Na ; M+H+Na ; M+2Na ; M+H+Na ; M+H+Na ; M+2Na ; M+H+Na ; M+H+Na ; M+2Na ; M+H+Na ; M+H+Na ; M+2Na ; M+H+Na ; M+2Na ; M+H+Na ; M+H+Na ; M+H+Na ; M+H+Na ; M+H+Na	C58H94O6 ; C54H98O6 ; C54H98O6 ; C54H98O6 ; C14H22 ; C30H48O ; C30H48O ; C30H48O ; C30H48O ; C30H48O ; C30H48O ; C30H48O ; C30H48O ; C30H48O ; C30H48O ; C54H98O6 ; C54H98O6 ; C54H98O6 ; C54H98O6 ; C54H98O6 ; C54H98O6 ;

2899999_1(	1.664772	1.119571	-3.4439	0.00629	2.2013	0.066015	282.556	HMDB05432 ; HMDB10411 ; HMDB10422 ; HMDB12105 ; HMDB42064 ; HMDB42073 ; HMDB42079 ; HMDB42123 ; HMDB42137 ; HMDB42144 ; HMDB42151 ; HMDB42421 ; HMDB42543 ; HMDB42601 ; HMDB42753 ; HMDB42990 ; HMDB43004 ; HMDB43011 ; HMDB43018 ; HMDB43177 ; HMDB43179 ; HMDB43401 ; HMDB43424 ; HMDB43459 ; HMDB43627 ; HMDB43843 ; HMDB43850 ; HMDB47755 ; HMDB47757 ; HMDB47889 ; HMDB47910 ; HMDB47911 ; HMDB47932 ; HMDB47955 ; HMDB47977 ; HMDB48568 ; HMDB48569	TG(16:1(9Z)16:1(9Z)16:1(9Z))iso ; TG(16:014:016:0)iso3 ; TG(16:1(9Z)14:018:2(9Z12Z))iso6 ; SM(d18:123:0) ; TG(14:014:018:0) ; TG(14:014:020:3(5Z8Z11Z)) ; TG(14:014:020:3n6) ; TG(14:016:016:0) ; TG(14:016:018:3(6Z9Z12Z)) ; TG(14:016:018:3(9Z12Z15Z)) ; TG(14:018:014:0) ; TG(14:020:3(5Z8Z11Z)14:0) ; TG(14:018:3(6Z9Z12Z)16:0) ; TG(14:020:3n614:0) ; TG(14:018:3(9Z12Z15Z)16:0) ; TG(15:015:016:0) ; TG(15:015:018:3(6Z9Z12Z)) ; TG(15:015:018:3(9Z12Z15Z)) ; TG(15:016:015:0) ; TG(15:014:1(9Z)18:2(9Z12Z)) ; TG(15:014:1(9Z)20:2n6) ; TG(15:018:2(9Z12Z)14:1(9Z)) ; TG(15:018:3(6Z9Z12Z)15:0) ; TG(15:020:2n614:1(9Z)) ; TG(15:018:3(9Z12Z15Z)15:0) ; TG(16:014:018:3(6Z9Z12Z)) ; TG(16:014:018:3(9Z12Z15Z)) ; TG(14:1(9Z)15:018:2(9Z12Z)) ; TG(14:1(9Z)15:020:2n6) ;	M+H+2Na ; M+3Na ; M+H+2Na ; M+H+2Na ; M+3Na ; M+H+2Na ; M+H+2Na ; M+3Na ; M+H+2Na ; M+H+2Na ; M+3Na ; M+H+2Na ; M+H+2Na ; M+H+2Na ; M+H+2Na ; M+3Na ; M+H+2Na ; M+H+2Na ; M+3Na ; M+H+2Na ; M+H+2Na ; M+H+2Na ; M+H+2Na ; M+H+2Na ; M+H+2Na ; M+H+2Na ; M+H+2Na ; M+H+2Na ; M+H+2Na ; M+H+2Na ; M+H+2Na ; M+H+2Na ; M+H+2Na ; M+H+2Na ; M+H+2Na ; M+H+2Na ; M+H+2Na	C51H92O6 ; C49H94O6 ; C51H92O6 ; C46H93N2O6P ; C49H94O6 ; C51H92O6 ; C51H92O6 ; C49H94O6 ; C51H92O6 ; C51H92O6 ; C49H94O6 ; C51H92O6 ; C51H92O6 ; C51H92O6 ; C51H92O6 ; C49H94O6 ; C51H92O6 ; C51H92O6 ; C49H94O6 ; C51H92O6 ; C51H92O6 ; C51H92O6 ; C51H92O6 ; C51H92O6 ; C51H92O6 ; C51H92O6 ; C51H92O6 ; C51H92O6 ; C51H92O6 ; C51H92O6 ; C51H92O6 ; C51H92O6 ; C51H92O6 ; C51H92O6 ; C51H92O6 ; C51H92O6 ; C51H92O6
3433_251.(	1.725819	1.116092	3.4413	0.006318	2.1994	0.066084	NA	NA	NA	NA	NA
3166_127.1	1.692675	0.971375	3.4322	0.006415	2.1928	0.066958	128.0193	HMDB01240 ; HMDB01329 ; HMDB01497 ; HMDB03929 ; HMDB32739 ; HMDB38182 ; HMDB60679	Pseudooxynicotine ; 2-Hydroxynicotine ; Nicotine-1-N-oxide ; 5-Aminoimidazole ; S-Methyl methanesulfinothioate ; 47-Dihydro-5-(4-methyl-3-pentenyl)-123-trithiepin ; Glycinexylidide	M+2K ; M+2K ; M+2K ; M+2Na-H ; M+NH4 ; M+H+Na ; M+2K	C10H14N2O ; C10H14N2O ; C10H14N2O ; C3H5N3 ; C2H6OS2 ; C10H16S3 ; C10H14N2O
6731_101.6	1.733755	0.946956	-3.4303	0.006435	2.1915	0.067029	NA	NA	NA	NA	NA
7899_55.2	1.74283	0.384551	3.4285	0.006455	2.1901	0.067098	421.8698	HMDB31798 ; HMDB31959 ; HMDB56120 ; HMDB56122	()-Leptophos ; Lablaboside D ; DG(24:00:018:2n6) ; DG(24:00:020:2n6)	2M+H+Na ; M+3H ; M+3ACN+2H ; M+3ACN+2H	C13H10BrCl2O2PS ; C60H94O28 ; C46H86O5 ; C46H86O5
6112_119.3	1.694847	1.116867	3.4257	0.006486	2.188	0.067278	387.996	HMDB41087	Furoparadine	M+2K-H	C17H13NO5
6028_54.2	1.69372	0.285559	3.423	0.006515	2.1861	0.067439	112.0506	HMDB00630 ; HMDB00962 ; HMDB01932 ; HMDB06037 ; HMDB13960 ; HMDB14568 ; HMDB14712 ; HMDB38461 ; HMDB40700	Cytosine ; Lipoamide ; Metoprolol ; 8-Hydroxy-7-methylguanine ; Donepezil metabolite M4 ; Hyoscyamine ; Atropine ; 1-Isothiocyanato-6-(methylsulfinyl)hexane ; Dihydroferuperine	M+H ; M+H+NH4 ; M+3Na ; M+ACN+2H ; M+H+2Na ; M+H+2Na ; M+H+2Na ; M+H+NH4 ; M+H+2Na	C4H5N3O ; C8H15NOS2 ; C15H25NO3 ; C6H7N5O2 ; C17H23NO3 ; C17H23NO3 ; C17H23NO3 ; C8H15NOS2 ; C17H23NO3

3177_116.4	1.73388	0.162967	-3.4195	0.006553	2.1835	0.0677	253.0933	HMDB01262 ; HMDB03213 ; HMDB03539 ; HMDB06599 ; HMDB06857 ; HMDB11729 ; HMDB11730 ; HMDB29910 ; HMDB29921 ; HMDB29922 ; HMDB29929 ; HMDB29930 ; HMDB29937 ; HMDB33673 ; HMDB33748 ; HMDB34072 ; HMDB35322 ; HMDB38851 ; HMDB38852 ; HMDB38853 ; HMDB39703 ; HMDB39704 ; HMDB39706 ; HMDB39707 ; HMDB39708 ; HMDB40181 ; HMDB40984 ; HMDB41948 ; HMDB60468 ; HMDB02302 ; HMDB03581 ; HMDB14567 ; HMDB14970 ; HMDB29030 ; HMDB29135 ; HMDB29738 ; HMDB32867 ; HMDB34064 ; HMDB36398 ; HMDB36600 ; HMDB37892 ; HMDB37942 ; HMDB38558 ; HMDB60797 ; HMDB60811 ; HMDB61013 ; HMDB61170	Maltotriose ; Raffinose ; Levan ; 3-Galactosyllactose ; Dextrin ; 1-Kestose ; Melezitose ; Gentiotriose ; Neokestose ; Galactotriose ; 6-O-Glucosylmaltose ; beta-D-Fructofuranosyl alpha-D-glucopyranosyl-(1-4)-D-glucopyranoside ; Panose ; 6-Kestose ; Umbelliferose ; Gentianose ; Fagopyritol B2 ; beta-D-Galactopyranosyl-(1-2)-beta-D-galactopyranosyl-(1-4)-D-galactose ; beta-D-Galactopyranosyl-(1-4)-beta-D-galactopyranosyl-(1-4)-D-galactose ; beta-D-Galactopyranosyl-(1-3)-beta-D-galactopyranosyl-(1-6)-D-galactose ; 3-beta-Gentiobiosylglucose ; alpha-D-Glucopyranosyl-(1-6)-alpha-D-glucopyranosyl-(1-2)-D-glucose ; 4-beta-Laminaribiosylglucose ; alpha-D-Glucopyranosyl-(1-4)-alpha-D-glucopyranosyl-(1-6)-D-glucose ; 3-beta-Cellobiosylglucose ; Fagopyritol A2 ; Sophorotriose ; Nephritogenoside ; D-Gal alpha 1-6D-Gal alpha 1-6D-Glucose ; 3-Indolepropionic acid ; Dethiobiotin ; Methocarbamol ; Phensuximide ; Prolyl-Valine ; Valyl-	M+2H ; M+ACN+Na ; M+K ; 2M+H+Na ; M+ACN+Na ; M+K ; M+K ; M+ACN+Na ; 3M+2H ; M+2K ; M+2ACN+2H ; M+H+K ; M+H+2Na ; M+Na ; M+H+K ; M+H+K ; M+Na ; M+Na+K ; M+H+NH4	C18H32O16 ; C11H11NO2 ; C10H18N2O3 ; C11H15NO5 ; C11H11NO2 ; C10H18N2O3 ; C10H18N2O3 ; C11H11NO2 ; C12H8O ; C26H36O5 ; C20H22O10 ; C24H34O9 ; C34H48O16 ; C13H14N2O2 ; C24H34O9 ; C25H32F2O6 ; C13H14N2O2 ; C27H29FN4O ; C22H24F3NO8

'5329_67.0(	1.746423	0.008869	-3.4081	0.00668	2.1753	0.068213	207.0975	HMDB14725 ; HMDB01908 ; HMDB01979 ; HMDB02513 ; HMDB03235 ; HMDB03247 ; HMDB04241 ; HMDB04242 ; HMDB05099 ; HMDB06070 ; HMDB12110 ; HMDB12144 ; HMDB12633 ; HMDB12635 ; HMDB14615 ; HMDB14794 ; HMDB28809 ; HMDB29103 ; HMDB29119 ; HMDB29161 ; HMDB31005 ; HMDB31483 ; HMDB31486 ; HMDB31487 ; HMDB31488 ; HMDB31539 ; HMDB31540 ; HMDB31550 ; HMDB31551 ; HMDB31569 ; HMDB32301 ; HMDB33667 ; HMDB33827 ; HMDB34177 ; HMDB34372 ; HMDB34678 ; HMDB34873 ; HMDB36021 ; HMDB36171 ; HMDB36361 ; HMDB36854 ; HMDB38105 ; HMDB40339 ; HMDB40521 ; HMDB60661	Cinalukast ; 19-Hydroxy-PGE2 ; 615-Diketo1314-dihydro-PGF1a ; Lithocholate 3-O-glucuronide ; Prostaglandin G2 ; 20-Hydroxy-PGE2 ; 6-Ketoprostaglandin E1 ; 11-Dehydro-thromboxane B2 ; Thromboxane B3 ; Pregnanetriol ; 5(6)-Epoxy Prostaglandin E1 ; 2-Amino-4-oxo-6-(123-trihydroxypropyl)-diquinoid-78-dihydroxypterin ; 20-COOH-1011-dihydro-LTB4 ; 20-dihydroxyleukotriene B4 ; Fluoxetine ; Trazodone ; Glutaminyl-Tyrosine ; Tyrosyl-Glutamine ; Tyrosyl-Gamma-glutamate ; gln-tyr ; 3-Methyl-5-pentyl-2-furanundecanoic acid ; (Z)-4-Heptenal ; 3-Hepten-2-one ; (E)-4-Hepten-2-one ; 2-Hepten-4-one ; 2-Methylcyclohexanone ; 4-Methylcyclohexanone ; 5-Methyl-3-hexen-2-one ; 5-Methyl-5-hexen-2-one ; 4-Methyl-1-phenyl-2-pentanone ; trans-2-trans-4-Heptadien-1-ol ; (R)-3-Methylcyclohexanone ; 2-Heptenal ; (S)-4-(4-Methylphenyl)-2-pentanone ; xi-3-Methyl-3-cyclohexen-1-ol ; Cinncassiol D2 ; Ethyl 2-hydroxy-3-(3-indolyl)propanoate glucoside ;	M+2H ; M+2Na ; M+2Na ; M+3Na ; M+2Na ; M+2Na ; M+2Na ; M+2Na ; M+2Na ; M+2K ; M+2Na ; M+3ACN+2H ; M+2Na ; M+2Na ; 2M+3H ; M+ACN+2H ; 2M+3H ; 2M+3H ; 2M+3H ; 2M+3H ; M+2K ; 3M+2K ; 3M+2K ; 3M+2K ; 3M+2K ; 3M+2K ; 3M+2K ; 3M+2K ; 3M+2K ; 2M+Na+K ; 3M+2K ; 3M+2K ; 3M+2K ; 2M+Na+K ; 3M+2K ; M+2Na ; M+H+NH4 ; 2M+Na+K ; 2M+Na+K ; M+2H+Na ; M+2Na ; M+2K ; 3M+2K ; M+2H+Na ; M+3H	C23H28N2O3S ; C20H32O6 ; C20H32O6 ; C30H48O9 ; C20H32O6 ; C20H32O6 ; C20H32O6 ; C20H32O6 ; C20H32O6 ; C21H36O3 ; C20H32O6 ; C9H15N5O6 ; C20H32O6 ; C20H32O6 ; C17H18F3NO ; C19H22ClN5O ; C14H19N3O5 ; C14H19N3O5 ; C14H19N3O5 ; C14H19N3O5 ; C21H36O3 ; C7H12O ; C7H12O ; C7H12O ; C7H12O ; C7H12O ; C7H12O ; C7H12O ; C7H12O ; C12H16O ; C7H12O ; C7H12O ; C7H12O ; C12H16O ; C7H12O ; C20H32O6 ; C19H25NO8 ; C12H16O ; C12H16O ; C36H40N2O6 ; C20H32O6 ; C21H36O3 ; C7H12O ; C36H40N2O6 ; C33H38N4O8
06216_67.6	1.69086	1.194082	3.4126	0.006629	2.1785	0.068213	229.9806	HMDB32846	5-Chloro-6-methoxy-2(3H)-benzoxazolone	2M+Na+K	C8H6ClNO3
'0677_290.:	1.690175	0.888252	3.4114	0.006643	2.1776	0.068213	714.9771	HMDB12333 ; HMDB12343 ; HMDB12353 ; HMDB12363 ; HMDB38344	PS(14:016:1(9Z)) ; PS(14:1(9Z)16:0) ; PS(16:014:1(9Z)) ; PS(16:1(9Z)14:0) ; Spirolide C	2M+H+NH4 ; 2M+H+NH4 ; 2M+H+NH4 ; 2M+H+NH4 ; 2M+H+NH4	C36H68NO10P ; C36H68NO10P ; C36H68NO10P ; C36H68NO10P ; C43H63NO7
3715_129.4	1.685114	0.848813	3.4076	0.006685	2.1749	0.068213	412.0504	HMDB05014 ; HMDB37648 ; HMDB38359	Celecoxib ; 3-Galloylprodelphinidin B2 ; Theasinensin B	2M+Na+K ; M+Na+K ; M+Na+K	C17H14F3N3O2S ; C37H30O18 ; C37H30O18
1412_99.79	1.682407	0.001236	-3.4084	0.006676	2.1755	0.068213	160.0401	HMDB04068 ; HMDB15058 ; HMDB15081 ; HMDB28796 ; HMDB28817 ; HMDB28833 ; HMDB29148 ; HMDB30420 ; HMDB32173 ; HMDB32987 ; HMDB41304 ; HMDB42044	Melanin ; Levosimendan ; Phenprocoumon ; Glutaminyl-Glutamate ; Glutamyl-Glutamine ; Glutamyl-Gamma-glutamate ; Gamma-glutamyl-Glutamate ; Strobilurin A ; Bakers yeast extract ; Ipriflavone ; Asparenyol ; Tolmetin glucuronide	M+2H ; M+H+K ; M+H+K ; M+2Na ; M+2Na ; M+2Na ; M+2Na ; M+Na+K ; M+2Na ; M+H+K ; M+H+K ; M+H+2Na	C18H10N2O4 ; C14H12N6O ; C18H16O3 ; C10H16N3O6 ; C10H16N3O6 ; C10H16N3O6 ; C10H16N3O6 ; C16H18O3 ; C19H14O2 ; C18H16O3 ; C18H16O3 ; C21H23NO9

5402_53.74	1.674347	1.235623	3.41	0.006659	2.1766	0.068213	192.0145	HMDB60600 ; HMDB14404 ; HMDB29611 ; HMDB31868	Olsalazine-O-sulfate ; Sulfanilamide ; Asparagusic acid ; Monomethyl phenylphosphonate	M+2H ; 2M+H+K ; M+ACN+H ; 2M+H+K	C14H10N2O9S ; C6H8N2O2S ; C4H6O2S2 ; C7H9O3P
6248_28.51	1.729352	0.240075	3.4031	0.006736	2.1716	0.06859	298.0966	HMDB06408 ; HMDB14802 ; HMDB30188 ; HMDB30840 ; HMDB31935 ; HMDB32240 ; HMDB33758 ; HMDB38840 ; HMDB59806 ; HMDB60014 ; HMDB60438 ; HMDB60966 ; HMDB61009 ; HMDB61037	4-Hydroxy tolbutamide ; Sulfametopyrazine ; N-Isobutyl-248-decatrienamide ; 2-Methoxy-9-phenyl-1H-phenalen-1-one ; Blumenol C O-rhamnosyl-(1-6)-glucoside ; N-37-Dimethyl-26-octadienylcyclopropylcarboxamide ; L-Agaritine ; Sarmentine ; Phenylglucuronide ; Phenol glucuronide ; Benzoapyrene-78-diol ; norzolmitripan ; 4-amino-MX ; 44-methanol-bisbenzonitrile	2M+H+Na ; M+NH4 ; M+2K-H ; 2M+H+Na ; M+2K ; M+2K-H ; 2M+Na+K ; M+2K-H ; 2M+3H2O+2H ; 2M+3H2O+2H ; 2M+H+Na ; M+K ; 2M+Na+K ; M+ACN+Na	C12H18N2O4S ; C11H12N4O3S ; C14H23NO ; C20H14O2 ; C25H42O11 ; C14H23NO ; C12H17N3O4 ; C14H23NO ; C12H14O7 ; C12H14O7 ; C20H14O2 ; C14H17N3O2 ; C12H17N3O4 ; C15H10N2O
0154_267.6	1.714285	1.084528	-3.4007	0.006763	2.1699	0.068722	372.204	HMDB15049 ; HMDB15359 ; HMDB33241 ; HMDB35944 ; HMDB35946 ; HMDB36365 ; HMDB60644 ; HMDB60933 ; HMDB61151 ; HMDB61155 ; HMDB14490	Anileridine ; Encainide ; Glycylalanylprolylmethionylphenylalanylvalinamide ; Cyclopassifloside I ; Cyclopassifloside IV ; Cyclopassifloside X ; cis-Hydroxy Perhexiline ; Propofol glucuronide ; Hydroxylated N-acetyl desmethyl frovatriptan ; Monohydroxyperhexiline ; Alfuzosin	2M+H+K ; 2M+H+K ; M+3ACN+2H ; M+2Na ; M+2Na ; M+2Na ; M+2K+H ; M+NH4 ; M+2ACN+H ; M+2K+H ; M+H-H2O	C22H28N2O2 ; C22H28N2O2 ; C29H45N7O6S ; C37H62O12 ; C37H62O12 ; C37H62O12 ; C19H35NO ; C18H26O7 ; C15H19N3O3 ; C19H35NO ; C19H27N5O4

2273_224.5	1.669641	0.020134	-3.3996	0.006776	2.169	0.068722	186.0642	HMDB00788 ; HMDB05810 ; HMDB15055 ; HMDB15487 ; HMDB30847 ; HMDB31534 ; HMDB31911 ; HMDB32707 ; HMDB33710 ; HMDB34294 ; HMDB34720 ; HMDB36409 ; HMDB37314 ; HMDB37786 ; HMDB38575 ; HMDB39104 ; HMDB40314 ; HMDB40756 ; HMDB40762 ; HMDB40980 ; HMDB41651 ; HMDB28819 ; HMDB28840	Orotidine ; Eriodictyol ; Spectinomycin ; Acenocoumarol ; Aromadendrin ; ()-Galeon ; Porric acid B ; Dalbergioidin ; 1-(4-Hydroxy-3-methoxyphenyl)-7-phenyl-35-heptanedione ; 1311-Tridecatriene-579-triyne ; Gingerenone C ; 12-Dihydrodehydroguaiaretic acid ; Norartocarpanone ; S-(45-Dihydro-2-methyl-3-furanyl) ethanethioate ; (Z)-N-Feruloyl-5-hydroxyanthranilic acid ; (3E5Z)-135-Tridecatriene-7911-triyne ; 2457-Tetrahydroxyflavanone ; 3-Hydroxy-68-dimethoxy-7(11)-eremophilen-128-olide ; 55-Diisopropyl-22-dimethylbiphenyl-3344-tetrone ; Valdiate ; 3457-Tetrahydroxyisoflavanone ; Glutamyl-Glycine ; Glycyl-Glutamate	M+2ACN+2H ; M+2ACN+2H ; M+H+K ; M+H+NH4 ; M+2ACN+2H ; M+2Na ; M+2ACN+2H ; M+2ACN+2H ; M+2Na ; 2M+H+K ; M+2Na ; M+2Na ; M+2ACN+2H ; 2M+3H2O+2H ; M+ACN+2H ; 2M+H+K ; M+2ACN+2H ; M+Na+K ; M+2Na ; M+Na+K ; M+2ACN+2H ; M+H-H2O ; M+H-H2O	C10H12N2O8 ; C15H12O6 ; C14H24N2O7 ; C19H15NO6 ; C15H12O6 ; C20H22O4 ; C15H12O6 ; C15H12O6 ; C20H22O4 ; C13H10 ; C20H22O4 ; C20H22O4 ; C15H12O6 ; C7H10O2S ; C17H15NO6 ; C13H10 ; C15H12O6 ; C17H26O5 ; C20H22O4 ; C17H26O5 ; C15H12O6 ; C7H11N2O5 ; C7H11N2O5
2645_139.4	1.67681	0.745862	-3.3983	0.006791	2.1681	0.068732	185.1193	HMDB14708 ; HMDB14920 ; HMDB35150 ; HMDB35853 ; HMDB59773 ; HMDB60995 ; HMDB00792 ; HMDB31510 ; HMDB40196 ; HMDB59708 ; HMDB59719 ; HMDB59754	Cinnarizine ; Propantheline ; 2-Apo-beta-carotenal ; Fasciculol C ; S-3-oxodecanoyl cysteamine ; 10-alpha-methoxy-910-dihydrolysergol ; Sebacic acid ; R-2-Hydroxy-3-methylbutanoic acid 3-Methylbutanoyl ; Oxalic acid dibutyl ester ; 2-Ethylsuberic acid ; Heptylmalonic acid ; 3-Methylazelaic acid	M+2H ; M+2H ; M+H+2Na ; M+H+2Na ; M+3ACN+2H ; M+2ACN+2H ; M+H-H2O ; M+H-H2O ; M+H-H2O ; M+H-H2O ; M+H-H2O ; M+H-H2O	C26H28N2 ; C23H30NO3 ; C37H48O ; C30H52O6 ; C12H23NO2S ; C17H22N2O2 ; C10H18O4 ; C10H18O4 ; C10H18O4 ; C10H18O4 ; C10H18O4 ; C10H18O4
0621_101.1	1.715695	0.065999	-3.3957	0.006821	2.1662	0.068895	233.5311	HMDB00742 ; HMDB02108 ; HMDB14958	Homocysteine ; Methylcysteine ; Tadalafil	3M+Na+K ; 3M+Na+K ; M+2K	C4H9NO2S ; C4H9NO2S ; C22H19N3O4
5333_56.65	1.714017	0.73002	3.3858	0.006935	2.159	0.069628	462.8615	HMDB01999 ; HMDB03598 ; HMDB15655 ; HMDB35692 ; HMDB36703 ; HMDB36709 ; HMDB36775 ; HMDB36805 ; HMDB36811 ; HMDB36826 ; HMDB38172 ; HMDB38687 ; HMDB39486 ; HMDB41928	Eicosapentaenoic acid ; Retinyl ester ; Methyltestosterone ; 815-Isopimaradien-18-oic acid ; Siderone ; Yucalexin B14 ; Yucalexin A16 ; 8(17)12-Labdadiene-1516-dial ; Isopimaric acid ; Cascarillone ; 813-Abietadien-18-oic acid ; (ent-15beta16beta)-1516-Epoxy-3-kauranone ; ent-8(17)13(16)14-Labdatrien-18-oic acid ; Methenolone	3M+H+NH4 ; 3M+H+NH4 ; 3M+H+NH4 ; 3M+H+NH4 ; 3M+H+NH4 ; 3M+H+NH4 ; 3M+H+NH4 ; 3M+H+NH4 ; 3M+H+NH4 ; 3M+H+NH4 ; 3M+H+NH4 ; 3M+H+NH4 ; 3M+H+NH4 ; 3M+H+NH4	C20H30O2 ; C20H30O2 ; C20H30O2 ; C20H30O2 ; C20H30O2 ; C20H30O2 ; C20H30O2 ; C20H30O2 ; C20H30O2 ; C20H30O2 ; C20H30O2 ; C20H30O2 ; C20H30O2 ; C20H30O2
6741_100.2	1.687674	0.010907	3.3858	0.006935	2.159	0.069628	NA	NA	NA	NA	NA

4543_88.4	1.670494	0.088444	-3.3876	0.006914	2.1603	0.069628	348.0925	HMDB06587 ; HMDB14574 ; HMDB29726 ; HMDB33746 ; HMDB35453 ; HMDB41820 ; HMDB60776 ; HMDB00912 ; HMDB37475 ; HMDB60825	Lactodifucotetraose ; Cefpiramide ; 2-Vinylthiophene ; Benzenethiol ; Pelargonidin 3-O-b-D-Glucopyranosyl-(1-2)-4-hydroxy-3-methoxy-(E)-cinnamoyl-(-6)-b-D-glucopyranoside 5-O-(6-O-malonyl-b-D-glucopyranoside) ; 8-Oxoguanine ; 5-Hydroxydantrolene ; Succinyladenosine ; 4beta-(2-Aminoethylthio)catechin ; Hydromorphone-3-sulphate	M+Na+K ; M+2ACN+2H ; 3M+NH4 ; 3M+NH4 ; M+2H+Na ; 2M+NH4 ; M+NH4 ; M+H-2H2O ; M+H-H2O ; M+H-H2O	C24H42O19 ; C25H24N8O7S2 ; C6H6S ; C6H6S ; C46H51O26 ; C5H3N5O2 ; C14H10N4O6 ; C14H17N5O8 ; C17H19NO6S ; C17H19NO6S
9636_250.	1.713706	1.044977	3.3824	0.006974	2.1565	0.069743	187.014	HMDB00229 ; HMDB32886 ; HMDB32902 ; HMDB37836 ; HMDB59913	Nicotinamide ribotide ; Orange I ; Sodium 6-hydroxy-5-(phenylazo)-2-naphthalenesulfoniate ; 4-(2-Hydroxy-1-naphthalenyl)azobenzenesulfonic acid ; (5E)-6-Oxo-5-(phenylhydrazono)-56-dihydro-2-naphthalenesulfonic acid	M+H+K ; M+2Na ; M+H+Na ; M+2Na ; M+2Na	C11H15N2O8P ; C16H12N2O4S ; C16H11N2NaO4S ; C16H12N2O4S ; C16H12N2O4S
0121_277.	1.681572	0.760489	3.3833	0.006964	2.1571	0.069743	NA	NA	NA	NA	NA
5761_277.	1.673119	0.121313	3.3806	0.006996	2.1552	0.06982	596.6496	HMDB12055	Ganglioside GT3 (d18:018:0)	M+2H+Na	C81H144N4O37
3351_100.1	1.720571	1.075227	-3.3778	0.007029	2.1531	0.069868	210.5284	HMDB11610 ; HMDB29286 ; HMDB33211 ; HMDB37845 ; HMDB39157	5-Taurinomethyl-2-thiouridine ; Avenanthramide 2s ; Avenanthramide 2 ; N-(1-Deoxy-1-fructosyl)tyrosine ; Clovamide	M+H+Na ; M+Na+K ; M+Na+K ; M+2K ; M+Na+K	C12H19N3O8S2 ; C18H17NO7 ; C18H17NO7 ; C15H21NO8 ; C18H17NO7
0935_97.9	1.718933	0.656153	-3.3779	0.007028	2.1532	0.069868	537.1941	HMDB06602 ; HMDB06610 ; HMDB06628 ; HMDB38170	Lacto-N-hexaose ; Lacto-N-neohexaose ; p-Lacto-N-hexaose ; Isolimonic acid	M+2H ; M+2H ; M+2H ; 2M+Na+K	C40H68N2O31 ; C40H68N2O31 ; C40H68N2O31 ; C26H34O10
07002_65.3	1.718614	0.497154	3.3748	0.007064	2.1509	0.07008	290.0807	HMDB13690 ; HMDB29298 ; HMDB29754 ; HMDB32900 ; HMDB33973 ; HMDB33974 ; HMDB37347 ; HMDB37471 ; HMDB37968 ; HMDB38366 ; HMDB38367 ; HMDB38368 ; HMDB38369 ; HMDB38741 ; HMDB41239 ; HMDB15522 ; HMDB36348	Procyanidin ; 4-O-85-5-Dehydrotriferulic acid ; Procyanidin B1 ; Epicatechin-(6-8)-epicatechin ; Procyanidin B2 ; Procyanidin B3 ; Terniflorin ; 2-O-p-Coumaroylvitexin ; ent-Epicatechin(4alpha-8)catechin ; Procyanidin B5 ; Procyanidin B6 ; Procyanidin B7 ; Procyanidin B8 ; Genistein 7-O-(2-p-coumaroylglucoside) ; ent-Epicatechin-(4alpha-6)-ent-epicatechin ; Sulfamethazine ; Vitisin A	M+2H ; M+2H ; M+2H ; M+2H ; M+2H ; M+2H ; M+2H ; M+2H ; M+2H ; M+2H ; M+2H ; M+2H ; M+2H ; M+2H ; M+2H ; 2M+H+Na ; M+H+NH4	C30H26O12 ; C30H26O12 ; C30H26O12 ; C30H26O12 ; C30H26O12 ; C30H26O12 ; C30H26O12 ; C30H26O12 ; C30H26O12 ; C30H26O12 ; C30H26O12 ; C30H26O12 ; C30H26O12 ; C30H26O12 ; C30H26O12 ; C12H14N4O2S ; C26H25O14

72248_250.4	1.718639	1.061413	3.372	0.007097	2.1489	0.070131	207.5272	HMDB05008 ; HMDB14912 ; HMDB29329 ; HMDB29332 ; HMDB29827 ; HMDB31554 ; HMDB33524 ; HMDB36168 ; HMDB38418	Lansoprazole ; Hydroflumethiazide ; Gravacridonolchlorine ; 5-Methoxynoracronycine ; Junosidine ; Sucralose ; Lambertine ; Bis(2-methyl-3-furanyl)tetrasulfide ; Glucoconringiin	M+2Na ; M+2ACN+2H ; M+H+K ; M+2K ; M+2K ; M+H+NH4 ; M+2K ; M+3ACN+2H ; M+H+Na	C16H14F3N3O2S ; C8H8F3N3O4S2 ; C19H18ClNO5 ; C20H19NO4 ; C20H19NO4 ; C12H19Cl3O8 ; C20H19NO4 ; C10H10O2S4 ; C11H21NO10S2
0478_272.7	1.710316	0.28378	3.3728	0.007088	2.1495	0.070131	NA	NA	NA	NA	NA
21503_280.8	1.650059	0.337407	3.368	0.007146	2.1459	0.070473	386.0822	HMDB34756 ; HMDB41063 ; HMDB41207 ; HMDB29399	Methyl 345-trimethoxycinnamate arabinosyl-(1-3)-glucosyl-(1-6)-glucosyl ester ; 12-Dimethoxy-13-methyl-13benzodioxolo56-cphenanthridine ; Dide-O-methylsimmondsin ; Ochratoxin A	M+2K ; M+K ; M+K ; M+H-H2O	C29H42O19 ; C21H17NO4 ; C14H21NO9 ; C20H18ClNO6
45285_121.3	1.698845	0.66634	-3.3661	0.007168	2.1446	0.070552	212.0245	HMDB01095 ; HMDB01499 ; HMDB06369 ; HMDB06557 ; HMDB12128 ; HMDB14328 ; HMDB15516 ; HMDB31186 ; HMDB32742 ; HMDB32934 ; HMDB35057 ; HMDB36562 ; HMDB37287 ; HMDB37291 ; HMDB40601 ; HMDB40602 ; HMDB41189 ; HMDB59882 ; HMDB60779	GDP-L-fucose ; GDP-D-Rhamnose ; ADP-Mannose ; ADP-glucose ; (R)-Amphetamine ; Amphetamine ; Dextroamphetamine ; N-Nitrosothialdine ; Di-O-methylcrenatin ; 3-Butylpyridine ; 1-(3-Hydroxy-4-Methoxyphenyl)-12-ethanediol 3-O-b-D-glucoside ; Aucubin ; 3-(2-Methylpropyl)pyridine ; 2-(2-Methylpropyl)pyridine ; (1xi2xi)-1-(4-Hydroxyphenyl)-123-propanetriol 2-O-beta-D-glucopyranoside ; (1xi2xi)-1-(4-Hydroxyphenyl)-123-propanetriol 3-O-beta-D-Glucopyranoside ; 345-Trimethoxyphenyl glucoside ; Benzyl chloride ; 5-Carboxy meloxicam	M+H+2Na ; M+H+2Na ; M+H+2Na ; M+H+2Na ; M+2K-H ; M+2K-H ; M+2K-H ; 2M+H+K ; M+2K ; M+2K-H ; M+2K ; M+2K ; M+2K-H ; M+2K-H ; M+2K ; M+2K ; M+2K ; 3M+2Na ; M+ACN+2H	C16H25N5O15P2 ; C16H25N5O15P2 ; C16H25N5O15P2 ; C16H25N5O15P2 ; C9H13N ; C9H13N ; C9H13N ; C6H12N2OS2 ; C15H22O9 ; C9H13N ; C15H22O9 ; C15H22O9 ; C9H13N ; C9H13N ; C15H22O9 ; C15H22O9 ; C15H22O9 ; C7H7Cl ; C14H11N3O6S2
68838_101.4	1.727882	0.958015	3.361	0.007231	2.1408	0.070867	267.0669	HMDB01534 ; HMDB05973 ; HMDB15037 ; HMDB28727 ; HMDB28748 ; HMDB32926 ; HMDB33961 ; HMDB37463 ; HMDB39741	5-Formiminotetrahydrofolic acid ; Dimethyltryptamine ; Didanosine ; Asparaginyl-Aspartate ; Aspartyl-Asparagine ; Polycartine B ; 2-(3-Methylbutyl)-1H-pyrrolo23-bpyridine ; Spinosin C ; alpha-L-Rhamnopyranosyl-(1-3)-alpha-D-galactopyranosyl-(1-3)-L-fucose	M+Na+K ; M+2K+H ; 2M+Na+K ; 2M+H+K ; 2M+H+K ; M+2K+H ; M+2K+H ; M+H+2Na ; M+Na+K	C20H24N8O6 ; C12H16N2 ; C10H12N4O3 ; C8H13N3O6 ; C8H13N3O6 ; C12H16N2 ; C12H16N2 ; C37H38O17 ; C18H32O14

7911_42.6	1.686026	0.725485	-3.36	0.007243	2.1401	0.070867	164.1178	HMDB01043 ; HMDB02177 ; HMDB03450 ; HMDB03634 ; HMDB03667 ; HMDB03701 ; HMDB06036 ; HMDB13324 ; HMDB14573 ; HMDB14996 ; HMDB15334 ; HMDB15345 ; HMDB29740 ; HMDB30386 ; HMDB30892 ; HMDB30893 ; HMDB30906 ; HMDB30907 ; HMDB30908 ; HMDB31854 ; HMDB32120 ; HMDB32242 ; HMDB32536 ; HMDB33113 ; HMDB33114 ; HMDB34715 ; HMDB34916 ; HMDB34973 ; HMDB34974 ; HMDB34975 ; HMDB34985 ; HMDB35054 ; HMDB35077 ; HMDB35078 ; HMDB35092 ; HMDB35100 ; HMDB35120 ; HMDB35125 ; HMDB35158 ; HMDB35203 ; HMDB35241 ; HMDB35277 ; HMDB35280 ; HMDB35600 ; HMDB35604 ; HMDB35622 ; HMDB35656 ; HMDB35706 ; HMDB35737 ; HMDB35738 ; HMDB35741 ; HMDB35743 ; HMDB35744 ; HMDB36044 ; HMDB36079 ; HMDB36080 ;	Arachidonic acid ; Cis-8111417-Eicosatetraenoic acid ; (-)-trans-Carveol ; Perillyl alcohol ; Alpha-Pinene-oxide ; Dimethylbenzimidazole ; Mesterolone ; 2-Octenoylcarnitine ; Carboprost Tromethamine ; Drostanolone ; Nadolol ; Metipranolol ; 1H-Indole-3-methanamine ; Myosmine ; p-Menth-3-en-9-al ; Isocitral ; 14-Dimethylpyrrolo12-apyrazine ; 34-Dimethylpyrrolo12-apyrazine ; 13-Dimethylpyrrolo12-apyrazine ; 3-Methyl-5-propyl-2-cyclohexen-1-one ; 26-Dimethyl-17-octadien-3-one ; (E)-37-Dimethyl-157-octatrien-3-ol ; 2-(trans-2-Pentenyl)cyclopentanone ; 1-Methylpyrrole ; 2-Methylpyrrole ; p-Mentha-1(6)8-dien-3-ol ; cis-2-Thujen-4-ol ; (R)-Campholenic aldehyde ; (R)-Carvotanacetone ; Piperitone ; ()-Fenchone ; (1S4R)-p-Mentha-28-dien-1-ol ; (1R4R)-p-Mentha-28-dien-1-ol ; Geranial ; cis-Citral ; 2-Pinen-10-ol ; Dehydrolinalool ; Darwinol ; 12-Epoxy-p-menth-8-ene ; (S)-910-Cyclo-p-menth-1-en-4-ol ; (Z)-2-Methyl-6-methylene-27-octadien-1-ol ; (2S4R)-	M+H+Na ; M+H+Na ; 2M+H+Na ; 2M+H+Na ; 2M+H+Na ; M+NH4 ; M+H+Na ; M+ACN+2H ; M+3H ; M+H+Na ; M+H+NH4 ; M+H+NH4 ; M+NH4 ; M+NH4 ; 2M+H+Na ; 2M+H+Na ; M+NH4 ; M+NH4 ; M+NH4 ; 2M+H+Na ; 2M+H+Na ; 2M+H+Na ; 2M+H+Na ; M+2ACN+H ; M+2ACN+H ; 2M+H+Na ;	C20H32O2 ; C20H32O2 ; C10H16O ; C10H16O ; C10H16O ; C9H10N2 ; C20H32O2 ; C15H27NO4 ; C25H47NO8 ; C20H32O2 ; C17H27NO4 ; C17H27NO4 ; C9H10N2 ; C9H10N2 ; C10H16O ; C10H16O ; C9H10N2 ; C9H10N2 ; C9H10N2 ; C10H16O ; C10H16O ; C10H16O ; C10H16O ; C5H7N ; C5H7N ; C10H16O ;
508_100.2	1.643621	0.442384	3.3621	0.007217	2.1416	0.070867	170.0051	HMDB00538 ; HMDB01440 ; HMDB04078 ; HMDB30882 ; HMDB32838 ; HMDB39027 ; HMDB59593 ; HMDB59596	Adenosine triphosphate ; dGTP ; Cinnavalininate ; (S)-Rutaretin ; Dorsteniol ; Celereoin ; 2-hydroxy-dATP ; ETF	M+3H ; M+3H ; M+H+K ; M+2K ; M+2K ; M+2K ; M+3H ; 3M+H+K	C10H16N5O13P3 ; C10H16N5O13P3 ; C14H8N2O6 ; C14H14O5 ; C14H14O5 ; C14H14O5 ; C10H16N5O13P3 ; C2H3F3O
5391_100.	1.705191	0.438534	-3.3548	0.007306	2.1363	0.071305	170.0475	HMDB30589 ; HMDB30619 ; HMDB02349 ; HMDB02432 ; HMDB06331 ; HMDB32923 ; HMDB32988 ; HMDB59762 ; HMDB60941	O-Methylsterigmatocystin ; Ovalitenone ; trans-trans-Muconic acid ; Sumikis acid ; ciscis-Muconic acid ; Kojic acid ; 5-Hydroxymaltol ; 23-Methylenesuccinic acid ; Rosuvastatin 5 S-lactone	M+2H ; M+2H ; 2M+3H2O+2H ; 2M+3H2O+2H ; 2M+3H2O+2H ; 2M+3H2O+2H ; 2M+3H2O+2H ; 2M+3H2O+2H ; M+H+2Na	C19H14O6 ; C19H14O6 ; C6H6O4 ; C6H6O4 ; C6H6O4 ; C6H6O4 ; C6H6O4 ; C6H6O4 ; C22H26FN3O5S
3288_58.0	1.683558	0.905624	3.354	0.007316	2.1357	0.071305	297.9673	HMDB31154	Allitridin	3M+Na+K	C6H10S3
3931_297.	1.670095	0.848175	3.3507	0.007357	2.1333	0.071567	NA	NA	NA	NA	NA
32591_100.	1.728217	0.517165	-3.3474	0.007397	2.1309	0.071818	221.0183	HMDB00793 ; HMDB15024 ; HMDB29940	Monoiodothyronine ; Bumetanide ; Alginic acid	M+ACN+2H ; M+2K ; M+H+Na	C15H14INO4 ; C17H20N2O5S ; C12H20O12P2
215_56.22	1.656823	1.049588	3.3454	0.007423	2.1294	0.071926	152.0222	HMDB00273 ; HMDB14809 ; HMDB15394	Thymidine ; Cefixime ; Telbivudine	M+Na+K ; M+3H ; M+Na+K	C10H14N2O5 ; C16H15N5O7S2 ; C10H14N2O5

7687_101.(	1.720715	1.161182	-3.34	0.007491	2.1255	0.072307	NA	NA	NA	NA	NA
3894_72.44	1.69893	0.445748	3.3404	0.007486	2.1258	0.072307	89.10739	HMDB12189 ; HMDB31506 ; HMDB31641	Aminopropylcadaverine ; 44-Diaminodibutylamine ; Pyrrolidine	M+H+NH4 ; M+H+NH4 ; M+NH4	C8H21N3 ; C8H21N3 ; C4H9N
6945_45.7(	1.682776	0.036439	-3.3364	0.007536	2.1228	0.072325	163.1157	HMDB32380 ; HMDB00832 ; HMDB13116 ; HMDB30200 ; HMDB33526 ; HMDB41540 ; HMDB59745	(S1)-Methoxy-3-heptanethiol ; Capryloylglycine ; Valproylglycine ; Hovenine A ; Chalciporone ; N-(5-Methyl-3-oxohexyl)alanine ; N-Acetylaminooctanoic acid	M+H ; M+3ACN+2H ; M+3ACN+2H ; M+3H ; 2M+3H ; M+3ACN+2H ; M+3ACN+2H	C8H18OS ; C10H19NO3 ; C10H19NO3 ; C27H42N4O4 ; C16H21NO ; C10H19NO3 ; C10H19NO3
5935_38.9!	1.672025	0.061554	3.337	0.007529	2.1233	0.072325	427.1086	HMDB00890 ; HMDB00916 ; HMDB00936 ; HMDB14008 ; HMDB14009 ; HMDB29515 ; HMDB31769 ; HMDB33009 ; HMDB33011 ; HMDB33148 ; HMDB33219 ; HMDB33236 ; HMDB33645 ; HMDB33712 ; HMDB34786 ; HMDB34874 ; HMDB39014 ; HMDB39469 ; HMDB41102 ; HMDB41546 ; HMDB60971 ; HMDB61164	Uroporphyrin IV ; Uroporphyrin III ; Uroporphyrin I ; Hydroxylansoprazole ; Lansoprazole sulfone ; Licoricone ; Carbendazim ; Foeniculoside VIII ; Foeniculoside IX ; Artocarpetin B ; (1S2S4R5S7S)-257-Fenchanetriol 2-O-b-D-glucoside ; cis-10-Hydroxylinalyl oxide 7-glucoside ; (1S2S4S5S)-247-Thujanetriol 4-glucoside ; Glycyrin ; (1S2S4S)-18-Epoxy-p-menthane-27-diol 2-O-b-D-glucoside ; Foeniculoside V ; Nepetariaside ; (1S2S4R)-p-Menth-8-ene-1210-triol 2-glucoside ; Kanzonol O ; Foeniculoside VII ; 5-hydroxylansoprazole ; N-Trifluoroacetyladriamycin	M+H+Na ; M+H+Na ; M+H+Na ; M+ACN+H ; M+ACN+H ; M+2Na-H ; 2M+2Na-H ; M+2K+H ; M+2K+H ; M+2Na-H ; M+2K+H ; M+2K+H ; M+2K+H ; M+2Na-H ; M+2K+H ; M+2K+H ; M+2K+H ; M+2K+H ; M+2Na-H ; M+2K+H ; M+ACN+H ; 2M+3H	C40H38N4O16 ; C40H38N4O16 ; C40H38N4O16 ; C16H14F3N3O3S ; C16H14F3N3O3S ; C22H22O6 ; C9H9N3O2 ; C16H28O8 ; C16H28O8 ; C22H22O6 ; C16H28O8 ; C16H28O8 ; C16H28O8 ; C22H22O6 ; C16H28O8 ; C16H28O8 ; C16H28O8 ; C16H28O8 ; C22H22O6 ; C16H28O8 ; C16H14F3N3O3S ; C29H28F3NO12
9221_86.9	1.61217	0.517336	-3.3374	0.007523	2.1236	0.072325	238.0378	HMDB29418 ; HMDB15424 ; HMDB15563 ; HMDB29356 ; HMDB29708 ; HMDB31255 ; HMDB32686 ; HMDB38463 ; HMDB38789 ; HMDB41147 ; HMDB41258 ; HMDB41418	S-Cysteinosuccinic acid ; Cefoperazone ; Pargyline ; N-Feruloylglycyl-L-phenylalanine ; 5678-Tetrahydro-24-dimethylquinoline ; Fluorocitric acid ; beta-D-3-5-Deoxy-5-(dimethylarsinyl)ribofuranosyloxy-2-hydroxy-1-propanesulfonic acid ; 3-(Isothiocyanatomethyl)-1-methoxy-1H-indole ; Torvanol A ; Isochinomin ; Homomangiferin ; Taxifolin 3-arabinoside	M+H ; M+3Na ; M+2K+H ; M+2K ; M+2K-H ; 2M+3H2O+2H ; M+2ACN+2H ; 2M+H+K ; M+H+Na ; M+H+K ; M+H+K ; M+H+K	C7H11NO6S ; C25H27N9O8S2 ; C11H13N ; C21H22N2O6 ; C11H15N ; C6H7FO7 ; C10H21AsO9S ; C11H10N2OS ; C20H20O10S ; C20H20O11 ; C20H20O11 ; C20H20O11

[illegible]2837_221.[illegible]	1.649329	0.217541	-3.331	0.007605	2.1189	0.072842	225.1233	HMDB14386 ; HMDB29347 ; HMDB29348 ; HMDB30421 ; HMDB39573 ; HMDB39749 ; HMDB00068 ; HMDB00512 ; HMDB00819 ; HMDB00860 ; HMDB00947 ; HMDB01010 ; HMDB02042 ; HMDB02373 ; HMDB02832 ; HMDB15652 ; HMDB29455 ; HMDB29564 ; HMDB30572 ; HMDB30838 ; HMDB31313 ; HMDB32044 ; HMDB32325 ; HMDB32448 ; HMDB33551 ; HMDB33618 ; HMDB33736 ; HMDB33848 ; HMDB34134 ; HMDB34230 ; HMDB34363 ; HMDB34456 ; HMDB34458 ; HMDB34460 ; HMDB34635 ; HMDB36034 ; HMDB36229 ; HMDB38237 ; HMDB38267 ; HMDB38523 ; HMDB39215 ; HMDB39274 ; HMDB39356 ; HMDB40171 ; HMDB40193 ; HMDB40266 ; HMDB41426 ; HMDB41427 ; HMDB41559 ; HMDB59831 ; HMDB59860 ; HMDB60608 ; HMDB60818	Butalbital ; Acuminoside ; Neryl arabinofuranosyl-glucoside ; L-Linalool 3-xylosyl-(1-6)-glucoside ; Geranyl arabinopyranosyl-glucoside ; Kenposide B ; Epinephrine ; N-Acetyl-L-phenylalanine ; Normetanephrine ; Phenylpropionylglycine ; Undecanoic acid ; Nicotine imine ; 3-Phenylpropionylglycine ; 46-Dimethylnonanoic acid ; Methylnoradrenaline ; Levonordefrin ; Ginkgotoxin ; Bufotenine O-glucoside ; ()-Anisoxide ; Feniculin ; 2-Pentyl-3-phenyl-2-propenal ; Hexyl 3-methylbutanoate ; 2-Hexyl-45-dimethyl-13-dioxolane ; Octanal propyleneglycol acetal ; 5-Ethyl-3-hydroxy-4-methyl-2(5H)-furanone ; 3-Methylbutyl hexanoate ; Homofureanol ; Methyl decanoate ; Octyl propanoate ; Mesifurane ; Monocrotaline ; 9-Methyldecanoic acid ; Isobutyl enanthate ; Heptyl butanoate ; Esculentoside E ; 66-Dimethoxy-255-trimethyl-2-hexene ; Pentyl hexanoate ; xi-8-Methyldecanoic acid ; 77-Diethoxy-3-heptene ; Methyl 2-(10-heptadecenyl)-	M+H ; M+2H ; M+2H ; M+2H ; M+2H ; M+2H ; M+ACN+H ; M+NH4 ; M+ACN+H ; M+NH4 ; M+K ; M+ACN+Na ; M+NH4 ; M+K ; M+ACN+H ; M+ACN+H ; M+ACN+H ; M+2ACN+2H ; M+Na ; M+Na ; M+Na ; M+K ; M+K ; M+K ; M+2ACN+H ; M+K ; M+2ACN+H ; M+K ; M+K ; M+2ACN+H ; M+3ACN+2H ; M+K ; M+K ; M+K ; M+2H+Na ; M+K ; M+K ; M+K ; M+K ; M+Na+K ; M+K ; M+Na ; M+Na ; M+K ; M+K ; M+K ; M+2ACN+H ; M+2ACN+H ; M+K ; M+K ; M+K ; M+NH4 ; M+2H+Na	C11H16N2O3 ; C21H36O10 ; C21H36O10 ; C21H36O10 ; C21H36O10 ; C21H36O10 ; C9H13NO3 ; C11H13NO3 ; C9H13NO3 ; C11H13NO3 ; C11H22O2 ; C10H13N2 ; C11H13NO3 ; C11H22O2 ; C9H13NO3 ; C9H13NO3 ; C9H13NO3 ; C18H26N2O6 ; C14H18O ; C14H18O ; C14H18O ; C11H22O2 ; C11H22O2 ; C11H22O2 ; C7H10O3 ; C11H22O2 ; C7H10O3 ; C11H22O2 ; C11H22O2 ; C7H10O3 ; C16H23NO6 ; C11H22O2 ; C11H22O2 ; C11H22O2 ; C35H54O11 ; C11H22O2 ; C11H22O2 ; C11H22O2 ; C11H22O2 ; C25H40O3 ; C11H22O2 ; C14H18O ; C14H18O ; C11H22O2 ; C11H22O2 ; C11H22O2 ; C7H10O3 ; C7H10O3 ; C11H22O2 ; C11H22O2 ; C11H22O2 ; C11H13NO3 ; C35H54O11
[illegible]0876_58.2[illegible]	1.73105	0.451777	3.326	0.00767	2.1152	0.073183	223.9721	HMDB40147 ; HMDB05874	2-Bromo-1H-indole-3-carboxaldehyde ; 3-Bromotyrosine	M+H ; M+H-2H2O	C9H6BrNO ; C9H10BrNO3

[illegible]0592_69.0[illegible]	1.666655	0.452292	3.3268	0.007659	2.1158	0.073183	131.0469	HMDB32102 ; HMDB00108 ; HMDB01858 ; HMDB02048 ; HMDB02055 ; HMDB03119 ; HMDB15084 ; HMDB28853 ; HMDB29105 ; HMDB29451 ; HMDB29700 ; HMDB30819 ; HMDB32121 ; HMDB32585 ; HMDB32662 ; HMDB32950 ; HMDB32951 ; HMDB33895 ; HMDB34223 ; HMDB35608 ; HMDB36029 ; HMDB36819 ; HMDB37179 ; HMDB38894 ; HMDB39387 ; HMDB40286 ; HMDB40833 ; HMDB40988 ; HMDB60359 ; HMDB60426 ; HMDB60565 ; HMDB60920 ; HMDB60921	1234-Tetrahydro-b-carboline-13-dicarboxylic acid ; Ethanol ; p-Cresol ; m-Cresol ; o-Cresol ; Benzyl alcohol ; Felbamate ; Glycyl-Tyrosine ; Tyrosyl-Glycine ; gamma-Glutaminyl-4-hydroxybenzene ; Cinnamyl benzoate ; Aesculetin ; Annuionone B ; 4-Methoxychalcone ; (S)-9-Hydroxy-10-undecenoic acid ; 57-Dihydroxy-4H-1-benzopyran-4-one ; 47-Dihydroxy-2H-1-benzopyran-2-one ; Anisole ; 23456-Penta-O-acetyl-D-glucose ; Monotropein ; Clarycet ; Dehydrovomifoliol ; 3-Nonanon-1-yl acetate ; Isoamyl p-anisate ; Methyl 26-dihydroxy-4-quinolinecarboxylate ; Benzyl cinnamate ; 3-4-Hydroxy-3-(3-methyl-2-butenyl)phenyl-2-propenal ; Falimint ; 23-Epoxymenaquinone ; 8-Methoxykynurenate ; 1-Hydroxyibuprofen ; 2-Hydroxyibuprofen ; 3-Hydroxyibuprofen	M+2H ; 2M+K ; M+Na ; M+Na ; M+Na ; M+Na ; M+H+Na ; M+H+Na ; M+H+Na ; M+H+Na ; M+H+Na ; M+2ACN+2H ; M+H+K ; M+H+Na ; M+Na+K ; M+2ACN+2H ; M+2ACN+2H ; M+Na ; M+3H ; M+3H ; M+Na+K ; M+H+K ; M+Na+K ; M+H+K ; M+ACN+2H ; M+H+Na ; M+2Na ; M+H+Na ; M+3Na ; M+ACN+2H ; M+H+K ; M+H+K ; M+H+K	C13H12N2O4 ; C2H6O ; C7H8O ; C7H8O ; C7H8O ; C7H8O ; C11H14N2O4 ; C11H14N2O4 ; C11H14N2O4 ; C11H14N2O4 ; C16H14O2 ; C9H6O4 ; C13H18O3 ; C16H14O2 ; C11H20O3 ; C9H6O4 ; C9H6O4 ; C7H8O ; C16H22O11 ; C16H22O11 ; C11H20O3 ; C13H18O3 ; C11H20O3 ; C13H18O3 ; C11H9NO4 ; C16H14O2 ; C14H16O2 ; C11H14N2O4 ; C21H24O3 ; C11H9NO4 ; C13H18O3 ; C13H18O3 ; C13H18O3
[illegible]1131_100.[illegible]	1.691437	1.090417	-3.3228	0.007711	2.1129	0.073439	369.1091	HMDB14857 ; HMDB14980 ; HMDB29839 ; HMDB31076 ; HMDB31095 ; HMDB32959 ; HMDB33444 ; HMDB33538 ; HMDB34989 ; HMDB35879 ; HMDB36137 ; HMDB38015 ; HMDB39240 ; HMDB39824 ; HMDB41350 ; HMDB41867 ; HMDB60970 ; HMDB60975 ; HMDB61121 ; HMDB61175	Azatadine ; Oxazepam ; Ascorbigen ; Gibberellin A105 ; Gibberellin A108 ; 1-Octen-3-yl glucoside ; 15-Acetyl-4-deoxynivalenol ; Fragransol B ; Gibberellin A62 ; Hericenone A ; Acetyldeoxynivalenol ; Gibberellin A121 ; Gibberellin A88 ; O-Acetylcyclocalopin A ; Gibberellin A95 ; Demoxepam ; norclobazam ; nortemazepam ; 3-Hydroxynordiazepam ; p-Hydroxynordiazepam	M+2K+H ; M+2ACN+H ; M+ACN+Na ; M+K ; M+K ; M+2K+H ; 2M+Na+K ; M+K ; M+K ; M+K ; 2M+Na+K ; M+K ; M+K ; 2M+Na+K ; M+K ; M+2ACN+H ; M+2ACN+H ; M+2ACN+H ; M+2ACN+H ; M+2ACN+H	C20H22N2 ; C15H11ClN2O2 ; C15H15NO6 ; C19H22O5 ; C19H22O5 ; C14H26O6 ; C17H22O7 ; C19H22O5 ; C19H22O5 ; C19H22O5 ; C17H22O7 ; C19H22O5 ; C19H22O5 ; C17H22O7 ; C19H22O5 ; C15H11ClN2O2 ; C15H11ClN2O2 ; C15H11ClN2O2 ; C15H11ClN2O2 ; C15H11ClN2O2

3549_83.7	1.638369	0.08362	-3.3209	0.007736	2.1115	0.073532	176.1224	HMDB01126 ; HMDB03116 ; HMDB34892 ; HMDB36142 ; HMDB39831 ; HMDB39832 ; HMDB39983 ; HMDB39984 ; HMDB39985 ; HMDB39986 ; HMDB39987 ; HMDB39988	Nornicotine ; Stanozolol ; 3-Ethyl-5-methyl-2-vinylpyrazine ; 67-Dihydro-23-dimethyl-5H-cyclopentapyrazine ; 67-Dihydro-25-dimethyl-5H-cyclopentapyrazine ; 67-Dihydro-26-dimethyl-5H-cyclopentapyrazine ; 25-Dimethyl-3-(2-propenyl)pyrazine ; 35-Dimethyl-2-(2-propenyl)pyrazine ; 23-Dimethyl-5-(2-propenyl)pyrazine ; 25-Dimethyl-3-(1-propenyl)pyrazine ; 35-Dimethyl-2-(1-propenyl)pyrazine ; 23-Dimethyl-5-(1-propenyl)pyrazine	2M+3H2O+2H ; M+H+Na ; 2M+3H2O+2H ; 2M+3H2O+2H ; 2M+3H2O+2H ; 2M+3H2O+2H ; 2M+3H2O+2H ; 2M+3H2O+2H ; 2M+3H2O+2H ; 2M+3H2O+2H ; 2M+3H2O+2H ; 2M+3H2O+2H	C9H12N2 ; C21H32N2O ; C9H12N2 ; C9H12N2 ; C9H12N2 ; C9H12N2 ; C9H12N2 ; C9H12N2 ; C9H12N2 ; C9H12N2 ; C9H12N2 ; C9H12N2
7799_78.8	1.649775	0.416493	-3.319	0.007761	2.1101	0.073632	385.1288	HMDB06809 ; HMDB29833 ; HMDB37125 ; HMDB37263 ; HMDB41513	Nicotinate D-ribonucleoside ; Cyclodopa glucoside ; Pisumoside B ; HMBOA-Glc ; Isopropyl apiosylglucoside	3M+2H ; 2M+3H2O+2H ; M+2K ; 2M+3H2O+2H ; 2M+Na+K	C11H14NO6 ; C15H19NO9 ; C32H52O16 ; C15H19NO9 ; C14H26O10
31559_23.5	1.725703	0.679066	3.3133	0.007836	2.1059	0.074077	269.1282	HMDB30132 ; HMDB32708 ; HMDB33452 ; HMDB33803 ; HMDB34337 ; HMDB36435 ; HMDB37036 ; HMDB39003 ; HMDB40744 ; HMDB40949 ; HMDB41444 ; HMDB60974	Isonuatigenin 3-rhamnosyl-(1-2)-glucoside ; 1-Naphthaleneacetic acid ; Koenoline ; Nuatigenin 3-rhamnosyl-(1-2)-glucoside ; Polypodosaponin ; Lucidenic acid D2 ; Glycinoeclepin C ; Fistuloside A ; Polypodoside B ; Melongoside F ; Mukoline ; cycloguanil	M+3Na ; M+2ACN+H ; M+ACN+H ; M+3Na ; M+3Na ; M+H+Na ; M+H+Na ; M+3Na ; M+3Na ; M+3Na ; M+ACN+H ; M+NH4	C39H62O13 ; C12H10O2 ; C14H13NO2 ; C39H62O13 ; C39H62O13 ; C29H38O8 ; C29H38O8 ; C39H62O13 ; C39H62O13 ; C39H62O13 ; C14H13NO2 ; C11H14ClN5
3749_104.2	1.683296	0.417002	-3.3132	0.007837	2.1058	0.074077	156.5184	HMDB01410 ; HMDB14468 ; HMDB61025	2-Amino-4-oxo-6-(12-dioxoprolyl)-78-dihydroxypteridine ; Tolcapone ; 5-Chloro-13-dihydro-1-(4-piperidinyl)-2H-benzimidazol-2-one	M+2Na ; M+H+K ; M+Na+K	C9H9N5O5 ; C14H11NO5 ; C12H14ClN3O
0626_96.7	1.714828	0.640725	3.3109	0.007867	2.1042	0.074218	NA	NA	NA	NA	NA
9661_95.0	1.711183	0.173646	-3.3056	0.007938	2.1003	0.074321	235.0387	HMDB13696 ; HMDB32259 ; HMDB32877 ; HMDB32937 ; HMDB32997 ; HMDB32999 ; HMDB33342 ; HMDB41737 ; HMDB41738 ; HMDB41739 ; HMDB41832	Urolithin B ; Ethyl 4-(acetylthio)butyrate ; Glucorhein ; Methyl 4-chloro-1H-indole-3-acetate ; 2-Hydroxyxanthone ; 4-Hydroxyxanthone ; Mollicellin E ; Genistein 4-O-glucuronide ; Genistein 5-O-glucuronide ; Genistein 7-O-glucuronide ; Baicalin	M+Na ; M+2Na-H ; M+H+Na ; 2M+H+Na ; M+Na ; M+Na ; M+H+Na ; M+H+Na ; M+H+Na ; M+H+Na ; M+H+Na	C13H8O3 ; C8H14O3S ; C21H18O11 ; C11H10ClNO2 ; C13H8O3 ; C13H8O3 ; C22H19ClO8 ; C21H18O11 ; C21H18O11 ; C21H18O11 ; C21H18O11
7112_295.4	1.691904	0.097418	3.3062	0.00793	2.1007	0.074321	NA	NA	NA	NA	NA

:8308_114.:	1.66697	1.009298	3.3075	0.007912	2.1017	0.074321	153.0328	HMDB01343 ; HMDB02994 ; HMDB04136 ; HMDB14391 ; HMDB28730 ; HMDB28814 ; HMDB30564 ; HMDB30565 ; HMDB31422 ; HMDB33341 ; HMDB33980 ; HMDB36294 ; HMDB41447 ; HMDB60917	Mevalonic acid-5P ; Erythritol ; D-Threitol ; Ziprasidone ; Asparaginyl-Glutamate ; Glutamyl-Asparagine ; trans-Piceid ; (Z)-Resveratrol 4-glucoside ; cis-Piceid ; Mollicellin C ; 7-Methoxy-2-methylisoflavone ; (E)-2-Glucosyl-345-trihydroxystilbene ; (E)-4-5-(4-Hydroxyphenoxy)-3-penten-1-ynylphenol ; 4-Hydroxyflurbiprofen	2M+3H ; 2M+Na+K ; 2M+Na+K ; M+H+2Na ; M+2Na ; M+2Na ; M+3Na ; M+3Na ; M+3Na ; M+H+2Na ; M+H+K ; M+3Na ; M+H+K ; M+2Na	C6H13O7P ; C4H10O4 ; C4H10O4 ; C21H21ClN4OS ; C9H14N3O6 ; C9H14N3O6 ; C20H22O8 ; C20H22O8 ; C20H22O8 ; C22H20O8 ; C17H14O3 ; C20H22O8 ; C17H14O3 ; C15H13FO3
:7011_98.0:	1.649607	1.115151	3.3059	0.007934	2.1005	0.074321	360.0367	HMDB33954 ; HMDB37850 ; HMDB38286	Juzirine ; Myricetin 33-digalactoside ; Eujambin	M+2K+H ; M+2K ; M+Na+K	C17H15NO3 ; C27H30O18 ; C30H26O17
:2529_47.8:	1.68958	0.262196	3.3002	0.008011	2.0963	0.074871	274.8733	HMDB07954 ; HMDB08592 ; HMDB08981 ; HMDB09008 ; HMDB09040 ; HMDB09073 ; HMDB09105 ; HMDB09212 ; HMDB09231 ; HMDB09241 ; HMDB09242 ; HMDB09263 ; HMDB09273 ; HMDB09295 ; HMDB09338 ; HMDB09371 ; HMDB09403 ; HMDB09436 ; HMDB09468 ; HMDB09489 ; HMDB09500 ; HMDB09520 ; HMDB09521 ; HMDB09531 ; HMDB09532 ; HMDB09552 ; HMDB09562 ; HMDB09563 ; HMDB09593 ; HMDB09625 ; HMDB09658 ; HMDB09749 ; HMDB09756	PC(15:022:2(13Z16Z)) ; PC(22:2(13Z16Z)15:0) ; PE(16:1(9Z)24:1(15Z)) ; PE(18:022:2(13Z16Z)) ; PE(18:1(11Z)22:1(13Z)) ; PE(18:1(9Z)22:1(13Z)) ; PE(18:2(9Z12Z)22:0) ; PE(18:4(6Z9Z12Z15Z)24:1(15Z)) ; PE(20:020:2(11Z14Z)) ; PE(20:022:5(4Z7Z10Z13Z16Z)) ; PE(20:022:5(7Z10Z13Z16Z19Z)) ; PE(20:1(11Z)20:1(11Z)) ; PE(20:1(11Z)22:4(7Z10Z13Z16Z)) ; PE(20:2(11Z14Z)20:0) ; PE(20:3(5Z8Z11Z)22:2(13Z16Z)) ; PE(20:3(8Z11Z14Z)22:2(13Z16Z)) ; PE(20:4(5Z8Z11Z14Z)22:1(13Z)) ; PE(20:4(8Z11Z14Z17Z)22:1(13Z)) ; PE(20:5(5Z8Z11Z14Z17Z)22:0) ; PE(22:018:2(9Z12Z)) ; PE(22:020:5(5Z8Z11Z14Z17Z)) ; PE(22:1(13Z)18:1(11Z)) ; PE(22:1(13Z)18:1(9Z)) ; PE(22:1(13Z)20:4(5Z8Z11Z14Z)) ; PE(22:1(13Z)20:4(8Z11Z14Z17Z)) ; PE(22:2(13Z16Z)18:0) ; PE(22:2(13Z16Z)20:3(5Z8Z11Z)) ; PE(22:2(13Z16Z)20:3(8Z11Z14Z)) ;	M+2H+Na ; M+2H+Na ; M+2H+Na ; M+2H+Na ; M+2H+Na ; M+2H+Na ; M+2H+Na ; M+3H ; M+2H+Na ; M+3H ; M+3H ; M+2H+Na ; M+3H ; M+2H+Na ; M+3H ; M+3H ; M+3H ; M+3H ; M+3H ; M+2H+Na ; M+3H ; M+2H+Na ; M+2H+Na ; M+3H ; M+3H ; M+2H+Na ; M+3H ; M+3H ; M+3H ; M+3H ; M+3H ; M+2H+Na ; M+3H	C45H86NO8P ; C45H86NO8P ; C45H86NO8P ; C45H86NO8P ; C45H86NO8P ; C45H86NO8P ; C45H86NO8P ; C47H84NO8P ; C45H86NO8P ; C47H84NO8P ; C47H84NO8P ; C45H86NO8P ; C47H84NO8P ; C45H86NO8P ; C47H84NO8P ; C47H84NO8P ; C47H84NO8P ; C47H84NO8P ; C47H84NO8P ; C45H86NO8P ; C47H84NO8P ; C45H86NO8P ; C45H86NO8P ; C47H84NO8P ; C47H84NO8P ; C45H86NO8P ; C47H84NO8P ; C47H84NO8P ; C47H84NO8P ; C47H84NO8P ; C47H84NO8P ; C45H86NO8P ; C47H84NO8P

7728_268.(	1.641282	0.479002	-3.2972	0.008052	2.0941	0.075113	205.0858	HMDB33303 ; HMDB35095 ; HMDB31920 ; HMDB34998 ; HMDB36970 ; HMDB37241 ; HMDB37242 ; HMDB37299 ; HMDB60830 ; HMDB15145 ; HMDB15185 ; HMDB30810 ; HMDB32841 ; HMDB32842 ; HMDB35404 ; HMDB35405 ; HMDB36822	Anofinic acid ; 7-Ethoxy-4-methyl-2H-1-benzopyran-2-one ; 9-Hydroxycalabaxanthone ; Bakkenolide D ; Garcimangosone B ; 89-Dihydro-5-hydroxy-8-(1-hydroxy-1-methylethyl)-6-(2-methyl-1-oxopropyl)-4-phenyl-2H-furo23-h-1-benzopyran-2-one ; Mammea AAC cyclo F ; 68-Dihydroxy-17-diprenylxanthone-2-carboxylic acid ; Melatonin glucuronide ; Edrophonium ; Novobiocin ; Porson ; 5-Megastigmen-7-yne-39-diol 9-glucoside ; 5-Megastigmen-7-yne-39-diol 3-glucoside ; Isogingerenone B ; Gingerenone B ; (3S7E9S)-9-Hydroxy-47-megastigmadien-3-one 9-glucoside	M+H ; M+H ; M+2H ; M+2H ; M+2H ; M+2H ; M+2H ; M+2H ; M+2H ; M+K ; M+3H ; M+H+Na ; M+H+K ; M+H+K ; M+H+Na ; M+H+Na ; M+H+K	C12H12O3 ; C12H12O3 ; C24H24O6 ; C21H28O6S ; C24H24O6 ; C24H24O6 ; C24H24O6 ; C24H24O6 ; C19H24N2O8 ; C10H16NO ; C31H36N2O11 ; C22H26O6 ; C19H30O7 ; C19H30O7 ; C22H26O6 ; C22H26O6 ; C19H30O7
3493_37.4	1.702445	0.568402	3.2919	0.008124	2.0902	0.075556	592.2113	HMDB60986 ; HMDB00181 ; HMDB00609 ; HMDB05383 ; HMDB05388 ; HMDB05397 ; HMDB05403 ; HMDB05424 ; HMDB05437 ; HMDB10445 ; HMDB10446 ; HMDB15393 ; HMDB29626 ; HMDB31142 ; HMDB31144 ; HMDB33718 ; HMDB35757 ; HMDB35784 ; HMDB36544 ; HMDB38336 ; HMDB38750 ; HMDB42171 ; HMDB42315 ; HMDB42344 ; HMDB42374 ; HMDB42402 ; HMDB42460 ; HMDB42461 ; HMDB42489 ; HMDB42664 ; HMDB43090 ; HMDB43092 ; HMDB43398 ; HMDB43456 ; HMDB43903 ; HMDB44037 ; HMDB44064 ; HMDB44090 ; HMDB44144 ; HMDB44145 ; HMDB44199 ; HMDB44226 ; HMDB44389 ; HMDB44685 ; HMDB44867 ; HMDB44892 ; HMDB44917 ; HMDB44918 ; HMDB44944 ; HMDB44970 ; HMDB45023 ; HMDB45514 ; HMDB45516 ; HMDB45666 ; HMDB45690 ; HMDB45691 ;	Zafirlukast metabolite M5 ; L-Dopa ; DL-Dopa ; TG(16:018:1(9Z)20:1(11Z))iso6 ; TG(16:018:2(9Z12Z)20:0)iso6 ; TG(18:018:018:2(9Z12Z))iso3 ; TG(18:018:1(9Z)18:1(9Z))iso3 ; TG(16:1(9Z)18:020:1(11Z))iso6 ; TG(16:1(9Z)18:1(9Z)20:0)iso6 ; TG(18:1(11Z)18:018:1(11Z))iso3 ; TG(18:1(11Z)18:018:1(9Z))iso6 ; Darunavir ; 5alpha-Ethoxy-6beta-hydroxy-56-dihydrophysalin B ; Glycerol 13-di-(9Z-octadecenoate) 2-octadecanoate ; Glycerol 12-di-(9Z-octadecenoate) 3-octadecanoate ; Clitocine ; Cascaroside C ; Aloinoside B ; 1-Acetoxyeugenol acetate ; 2-Hydroxy-3-(34-dihydroxyphenyl)propanamide ; N-Hydroxy-L-tyrosine ; TG(14:018:022:2(13Z16Z)) ; TG(14:016:1(9Z)24:1(15Z)) ; TG(14:018:1(11Z)22:1(13Z)) ; TG(14:018:1(9Z)22:1(13Z)) ; TG(14:020:1(11Z)20:1(11Z)) ; TG(14:022:1(13Z)18:1(11Z)) ; TG(14:022:1(13Z)18:1(9Z)) ; TG(14:024:1(15Z)16:1(9Z)) ;	M+H ; 3M+H ; 3M+H ; 2M+3H ; 2M+3H ; 2M+3H ; 2M+3H ; 2M+3H ; 2M+3H ; 2M+3H ; 2M+3H ; M+2Na-H ; 2M+H+K ; 2M+3H ; 2M+3H ; 2M+NH4 ; 2M+3H2O+2H ; 2M+3H2O+2H ; 2M+ACN+Na ; 3M+H ; 3M+H ; 2M+3H	C31H33N3O7S ; C9H11NO4 ; C9H11NO4 ; C57H106O6 ; C57H106O6 ; C57H106O6 ; C57H106O6 ; C57H106O6 ; C57H106O6 ; C57H106O6 ; C57H106O6 ; C27H37N3O7S ; C30H36O11 ; C57H106O6 ; C57H106O6 ; C9H13N5O6 ; C27H32O13 ; C27H32O13 ; C14H16O5 ; C9H11NO4 ; C9H11NO4 ; C57H106O6 ;

[illegible]4903_62.4[illegible]	1.687015	0.748249	3.2915	0.00813	2.0899	0.075556	179.0485	HMDB00078 ; HMDB28775 ; HMDB28838 ; HMDB00227 ; HMDB04304 ; HMDB12140 ; HMDB15186 ; HMDB15484 ; HMDB29404 ; HMDB30148 ; HMDB30383 ; HMDB30402 ; HMDB30594 ; HMDB30599 ; HMDB31639 ; HMDB31724 ; HMDB32277 ; HMDB32374 ; HMDB32375 ; HMDB33950 ; HMDB34861 ; HMDB34878 ; HMDB35220 ; HMDB35732 ; HMDB36181 ; HMDB36194 ; HMDB36979 ; HMDB38158 ; HMDB38784 ; HMDB39071 ; HMDB40152 ; HMDB40755 ; HMDB41229 ; HMDB59629 ; HMDB59705 ; HMDB60325 ; HMDB60349 ; HMDB60892	Cysteinylglycine ; Cysteinyl-Glycine ; Glycyl-Cysteine ; Mevalonic acid ; Nivalenol ; (R) 23-Dihydroxy-3-methylvalerate ; Penicillin G ; Cefacetrile ; (S)-a-Amino-25-dihydro-5-oxo-4-isoxazolepropanoic acid N2-glucoside ; Prehumulinic acid ; Zapotidine ; L-Hexahydro-3-imino-124-oxadiazepine-3-carboxylic acid ; Edulisin I ; 5-(4-Hydroxypheny)ethenyl-2-(3-methyl-1-butenyl)-13-benzenediol ; Glycerol 1-propanoate ; Moringyne ; (-)-3-(Ethylthio)butanol ; 2-Mercapto-2-methyl-1-pentanol ; (-)-4-Mercapto-4-methyl-2-pentanol ; Parakmerin A ; 3-Hydroxychavicol 1-glucoside ; 3-Mercapto-2-methylpentanol ; Cryptotanshinone ; Dukunolide D ; 2-(Isopropylthio)methylfuran ; 4-Methoxy-2-methyl-2-butanethiol ; 6b-Hydroxy-8a-methoxy-7(11)-eremophilen-128-olide ; Jangomolide ; (E)-Arachidin II ; exo-Dehydrochalepin ; 3-Mercapto-1-hexanol ; 3b-Hydroxy-6b-methoxy-7(11)-eremophilen-128a-olide ; Methyl (3b11x)-3-Hydroxy-8-oxo-6-eremophilen-12-oate ; (R)-	M+H ; M+H ; M+H ; 2M+Na+K ; M+2Na ; 2M+Na+K ; M+H+Na ; M+H+NH4 ; M+H+Na ; M+2K ; 2M+H+Na ; 2M+H+K ; M+H+2Na ; M+Na+K ; 2M+Na+K ; M+2Na ; M+2Na-H ; M+2Na-H ; M+2Na-H ; M+Na+K ; M+2Na ; M+2Na-H ; M+Na+K ; M+3Na ; M+Na ; M+2Na-H ; M+2K ; M+3Na ; M+Na+K ; M+Na+K ; M+2Na-H ; M+2K ; M+2K ; 2M+Na+K ; 2M+Na+K ; M+Na ; M+Na ; M+2Na	C5H10N2O3S ; C5H10N2O3S ; C5H10N2O3S ; C6H12O4 ; C15H20O7 ; C6H12O4 ; C16H18N2O4S ; C13H13N3O6S ; C12H18N2O9 ; C16H24O4 ; C7H9N3S ; C5H9N3O3 ; C28H26O8 ; C19H20O3 ; C6H12O4 ; C15H20O7 ; C6H14OS ; C6H14OS ; C6H14OS ; C19H20O3 ; C15H20O7 ; C6H14OS ; C19H20O3 ; C26H28O8 ; C8H12OS ; C6H14OS ; C16H24O4 ; C26H28O8 ; C19H20O3 ; C19H20O3 ; C6H14OS ; C16H24O4 ; C16H24O4 ; C6H12O4 ; C6H12O4 ; C11H8O ; C11H8O ; C22H16O2

5761_112.2	1.670714	0.659911	-3.2866	0.008197	2.0864	0.076036	185.0766	HMDB00401 ; HMDB00545 ; HMDB00659 ; HMDB02032 ; HMDB02121 ; HMDB04225 ; HMDB14423 ; HMDB14552 ; HMDB29337 ; HMDB29527 ; HMDB29852 ; HMDB29870 ; HMDB30090 ; HMDB30622 ; HMDB30793 ; HMDB30807 ; HMDB30852 ; HMDB31302 ; HMDB31420 ; HMDB31471 ; HMDB31620 ; HMDB32314 ; HMDB32442 ; HMDB32664 ; HMDB33199 ; HMDB33243 ; HMDB33291 ; HMDB33309 ; HMDB34011 ; HMDB34012 ; HMDB34077 ; HMDB34114 ; HMDB34115 ; HMDB34193 ; HMDB34438 ; HMDB34781 ; HMDB35135 ; HMDB36177 ; HMDB36239 ; HMDB36749 ; HMDB36753 ; HMDB36755 ; HMDB37276 ; HMDB37320 ; HMDB37322 ; HMDB37489 ; HMDB38268 ; HMDB38448 ; HMDB38697 ; HMDB39502 ; HMDB39613 ; HMDB40285 ; HMDB40867 ; HMDB41110 ; HMDB41726 ; HMDB60285 ;	28-Dihydroxyadenine ; Chenodeoxycholic acid disulfate ; Deoxycholic acid disulfate ; 8-Hydroxyguanine ; Carnosol ; 2-Oxoarginine ; Argatroban ; Loxapine ; Oxoglaucine ; Melilotocarpan B ; Methylgingerol ; 37-Dihydroxy-4-methoxyisoflavanone ; Sakuranetin ; (R)-Oxypeucedanin ; Moracin B ; 34-Dihydro-8-hydroxy-3-(3-hydroxy-4-methoxyphenyl)-1H-2-benzopyran-1-one ; Asperxanthone ; Methyl 2-octynoate ; Heliannone C ; Diplodiatoxin ; Vestitone ; 24-Hexadienyl propionate ; 2-Nonenoic acid gamma-lactone ; Corchorifatty acid A ; (4beta5beta6beta14beta15alpha20S22R)-56-Epoxy-41415-trihydroxy-1-oxowitha-224-dienolide ; Corchorifatty acid D ; 38-Dihydroxy-9-methoxypterocarpan ; Moracin F ; 2-(24-Dihydroxyphenyl)-56-dimethoxybenzofuran ; 34-Dihydroxy-9-methoxypterocarpan ; Withaperuvin C ; 6alpha-Hydroxyisomedicarpin ; 6alpha-Hydroxymedicarpin ; 2425-Epoxywithanolide D ; (R)-Pabulenol ;	M+NH4 ; M+3H ; M+3H ; M+NH4 ; M+H+K ; 2M+H+Na ; M+H+2Na ; M+ACN+2H ; M+H+NH4 ; M+2ACN+2H ; M+Na+K ; M+2ACN+2H ; M+2ACN+2H ; M+2ACN+2H ; M+2ACN+2H ; M+2ACN+2H ; M+2ACN+2H ; 2M+Na+K ; M+2ACN+2H ; M+Na+K ; M+2ACN+2H ; 2M+Na+K ; 2M+Na+K ; M+Na+K ; M+3Na ; M+Na+K ; M+2ACN+2H ; M+2ACN+2H ; M+2ACN+2H ; M+2ACN+2H ; M+3Na ; M+2ACN+2H ; M+2ACN+2H ; M+3Na ; M+2ACN+2H ; M+Na+K ; 2M+Na+K ; 2M+Na+K ; 2M+Na+K ; M+H+K ; M+H+K ; M+H+K ; 2M+Na+K ; M+2ACN+2H ; M+2ACN+2H ; M+2ACN+2H ; 2M+Na+K ; M+2ACN+2H ; 2M+Na+K ; M+ACN+2H ; M+H+K ; 2M+Na+K ; M+Na+K ; M+3Na ; M+2ACN+2H ; 2M+Na+K ; M+ACN+2H	C5H5N5O2 ; C24H40O10S2 ; C24H40O10S2 ; C5H5N5O2 ; C20H26O4 ; C6H11N3O3 ; C23H36N6O5S ; C18H18ClN3O ; C20H17NO5 ; C16H14O5 ; C18H28O4 ; C16H14O5 ; C16H14O5 ; C16H14O5 ; C16H14O5 ; C16H14O5 ; C16H14O5 ; C9H14O2 ; C16H14O5 ; C18H28O4 ; C16H14O5 ; C9H14O2 ; C9H14O2 ; C18H28O4 ; C28H38O7 ; C18H28O4 ; C16H14O5 ; C16H14O5 ; C16H14O5 ; C16H14O5 ; C28H38O7 ; C16H14O5 ; C16H14O5 ; C28H38O7 ; C16H14O5 ; C18H28O4 ; C9H14O2 ; C9H14O2 ; C9H14O2 ; C20H26O4 ; C20H26O4 ; C20H26O4 ; C9H14O2 ; C16H14O5 ; C16H14O5 ; C16H14O5 ; C9H14O2 ; C16H14O5 ; C9H14O2 ; C18H17NO5 ; C20H26O4 ; C9H14O2 ; C18H28O4 ; C28H38O7 ; C16H14O5 ;

7936_87.4	1.703492	0.82406	3.2845	0.008226	2.0848	0.076125	171.0958	HMDB01091 ; HMDB40799 ; HMDB00673 ; HMDB03797 ; HMDB04995 ; HMDB05047 ; HMDB05048 ; HMDB06007 ; HMDB06227 ; HMDB06270 ; HMDB13036 ; HMDB13865 ; HMDB15091 ; HMDB29800 ; HMDB30208 ; HMDB30212 ; HMDB30248 ; HMDB30255 ; HMDB30430 ; HMDB30957 ; HMDB30958 ; HMDB31051 ; HMDB31097 ; HMDB31262 ; HMDB31269 ; HMDB31527 ; HMDB31599 ; HMDB31653 ; HMDB31834 ; HMDB32535 ; HMDB32667 ; HMDB32691 ; HMDB32962 ; HMDB33554 ; HMDB33647 ; HMDB33772 ; HMDB33777 ; HMDB33794 ; HMDB34758 ; HMDB34761 ; HMDB34764 ; HMDB34845 ; HMDB35916 ; HMDB35926 ; HMDB35927 ; HMDB37001 ; HMDB37150 ; HMDB37170 ; HMDB38067 ; HMDB38400 ; HMDB38447 ; HMDB38647 ; HMDB38725 ; HMDB38977 ; HMDB39461 ; HMDB39767 ;	3-Hydroxyquinine ; 11-Hydroxytubotaiwine ; Linoleic acid ; Bovinic acid ; Codeine ; 9E11E-Octadecadienoic acid ; 10E12Z-Octadecadienoic acid ; Isopentanol ; 1-a24R25-Trihydroxyvitamin D2 ; Linoelaidic acid ; 1-Pentanol ; CGP72383 ; Hydrocodone ; Mangiferic acid ; Koenigicine ; Mukonicine ; Neopine ; Erysodine ; Linalyl caprylate ; (3Z6Z)-36-Nonadien-1-ol ; (Z)-6-Nonenal ; Ethyl 2E4Z-hexadecadienoate ; 5-Octadecynoic acid ; 26-Nonadien-1-ol ; 2-Nonenal ; (S)-2-Methyl-1-butanol ; 2-Pentanol ; 3-(Methylthio)-1-propene ; ()-26-Dimethyl-5-heptenal ; trans-4-Nonenal ; 59-Epidioxy-3-hydroxyergost-7-en-6-one ; (3beta5alpha6alpha7beta14alpha22E24R)-56-Epoxyergosta-822-diene-3714-triol ; Secoclausenamide ; 2-Methyl-2-octenal ; 56:89-Diepoxyergost-22-ene-37beta-diol ; 2-Methyl-2-butanol ; 3-Methyl-2-butanol ; ()-226-Trimethylcyclohexanone ; 8-Nonen-3-one ; 5-Nonen-2-one ; 3-Nonen-2-one ; 24-Nonadien-1-ol ; (E)-	M+2H ; M+2H ; M+Na+K ; M+Na+K ; M+ACN+2H ; M+Na+K ; M+Na+K ; 3M+2K ; M+3Na ; M+Na+K ; 3M+2K ; M+3H ; M+ACN+2H ; M+Na+K ; M+H+NH4 ; M+H+NH4 ; M+ACN+2H ; M+ACN+2H ; M+Na+K ; 2M+Na+K ; 2M+Na+K ; M+Na+K ; M+Na+K ; 2M+Na+K ; 2M+Na+K ; 3M+2K ; 3M+2K ; M+2ACN+H ; 2M+Na+K ; 2M+Na+K ; M+3Na ; M+3Na ; M+ACN+2H ; 2M+Na+K ; M+3Na ; 3M+2K ; 3M+2K ; 2M+Na+K ; 2M+Na+K ; 2M+Na+K ; 2M+Na+K ; 2M+Na+K ; 2M+Na+K ; 2M+Na+K ; 2M+Na+K ; 2M+Na+K ; 2M+Na+K ; 2M+Na+K ; 2M+Na+K ; 2M+Na+K ; M+3ACN+2H ; M+ACN+2H ; M+ACN+2H ; M+3Na ; 2M+Na+K ; M+ACN+2H ; M+2ACN+H ; M+3H ; 2M+Na+K ; M+2ACN+H ; M+ACN+2H ; M+ACN+2H ; M+H+NH4 ; M+ACN+2H	C20H24N2O3 ; C20H24N2O3 ; C18H32O2 ; C18H32O2 ; C18H21NO3 ; C18H32O2 ; C18H32O2 ; C5H12O ; C28H44O4 ; C18H32O2 ; C5H12O ; C29H32N7O2 ; C18H21NO3 ; C18H32O2 ; C20H21NO3 ; C20H21NO3 ; C18H21NO3 ; C18H21NO3 ; C18H32O2 ; C9H16O ; C9H16O ; C18H32O2 ; C18H32O2 ; C9H16O ; C9H16O ; C5H12O ; C5H12O ; C4H8S ; C9H16O ; C9H16O ; C28H44O4 ; C28H44O4 ; C18H21NO3 ; C9H16O ; C28H44O4 ; C5H12O ; C5H12O ; C9H16O ; C9H16O ; C9H16O ; C9H16O ; C9H16O ; C9H16O ; C9H16O ; C9H16O ; C9H16O ; C9H16O ; C9H16O ; C9H16O ; C9H16O ; C10H19NS2 ; C18H21NO3 ; C18H21NO3 ; C28H44O4 ; C9H16O ; C18H21NO3 ; C4H8S ; C30H38O7 ; C9H16O ; C4H8S ; C18H21NO3 ; C18H21NO3 ; C20H21NO3 ;
1314_65.2	1.687949	1.016292	3.2837	0.008237	2.0842	0.076125	309.0941	HMDB40692 ; HMDB03178 ; HMDB03546 ; HMDB15188 ; HMDB29482 ; HMDB30766 ; HMDB32367 ; HMDB33262 ; HMDB34023 ; HMDB36049 ; HMDB36052 ; HMDB36662 ; HMDB37325 ; HMDB37487 ; HMDB38331 ; HMDB38964 ; HMDB41186 ; HMDB41187 ; HMDB60405	4R5R6S-Trihydroxy-2-hydroxymethyl-2-cyclohexen-1-one 6-(2-hydroxy-6-methylbenzoate) ; Heme ; Salicin ; Mimosine ; Didymin ; 11-Hydroxy-12-methoxydihydrokawain ; L-Menthone 12-glycerol ketal ; Solanolone ; Isosalicin ; Marasmone ; Isomarasmone ; 2beta9xi-Dihydroxy-8-oxo-1(10)411(13)-germacratrien-126alpha-olide ; Achimilic acid ; Poncirin ; 3-Hydroxybenzyl alcohol glucoside ; 3-Hydroxy-46-heptadiyne-1-yl 1-glucoside ; Ilicifolinoside A ; (Z)-2-Methyl-2-butene-14-diol 4-O-beta-D-Glucopyranoside ; 5-Deoxy-5-fluorocytidine	M+H ; M+2H ; M+Na ; 3M+H+Na ; M+H+Na ; 2M+Na+K ; 2M+H+Na ; 2M+Na+K ; M+Na ; 2M+Na+K ; 2M+Na+K ; 2M+Na+K ; 2M+Na+K ; M+H+Na ; M+Na ; M+Na ; M+2Na-H ; M+2Na-H ; M+ACN+Na	C15H16O7 ; C34H32FeN4O4 ; C13H18O7 ; C8H10N2O4 ; C28H34O14 ; C15H18O5 ; C16H15N3OS ; C15H18O5 ; C13H18O7 ; C15H18O5 ; C15H18O5 ; C15H18O5 ; C15H18O5 ; C28H34O14 ; C13H18O7 ; C13H18O7 ; C11H20O7 ; C11H20O7 ; C9H12FN3O4

4923_250.8	1.685108	1.062602	3.2815	0.008268	2.0826	0.076134	335.0735	HMDB29249 ; HMDB29737 ; HMDB33278 ; HMDB34759 ; HMDB37085 ; HMDB37361 ; HMDB37375 ; HMDB37409 ; HMDB37410 ; HMDB37422 ; HMDB37443 ; HMDB37500 ; HMDB37743 ; HMDB37744 ; HMDB37745 ; HMDB37746 ; HMDB38749 ; HMDB38813 ; HMDB39124 ; HMDB39313 ; HMDB29383 ; HMDB60420	Luteolinidin ; 1H-Indole-3-carboxaldehyde ; 8-Acetoxy-4-methoxypinoresinol 4-glucoside ; Methyl 5-(1-Propynyl)-2-thiophenepropanoate ; Isorhamnetin 3-O-b-D-glucopyranosyl-(1-2)-a-L-rhamnopyranoside ; Azaleatin 3-rutinoside ; Isorhamnetin 3-glucoside 4-rhamnoside ; 38-Diglucosyldiosmetin ; 68-Diglucosyldiosmetin ; Isoscoparin 2-O-glucoside ; Sexangularetin 3-glucoside 7-rhamnoside ; Subulin ; Pasternoside ; Isorhamnetin 3-O-b-L-rhamnofuranosyl-(1-6)-D-glucopyranoside ; Calendoflavoside ; Keioside ; Osmanthuside B ; Scoparin 2-glucoside ; Crosatoside A ; 4-(4-Hydroxyphenyl)-2-butanone O-2-galloyl-6-cinnamoylglucoside ; Chlorogenoquinone ; 7-Hydroxymethyl-12-methylbenzaanthracene sulfate	M+ACN+Na ; 2M+2Na-H ; M+2K ; 3M+2Na ; M+2Na ; M+2Na ; M+2Na ; M+2Na ; M+2Na ; M+2Na ; M+2Na ; M+2Na ; M+2Na ; M+2Na ; M+2Na ; M+2Na ; M+2K ; M+2Na ; M+2Na ; M+Na+K ; M+H-H2O ; M+H-H2O	C15H11O5 ; C9H7NO ; C29H36O13 ; C11H12O2S ; C28H32O16 ; C28H32O16 ; C28H32O16 ; C28H32O16 ; C28H32O16 ; C28H32O16 ; C28H32O16 ; C28H32O16 ; C28H32O16 ; C28H32O16 ; C28H32O16 ; C28H32O16 ; C29H36O13 ; C28H32O16 ; C28H32O16 ; C32H32O12 ; C16H16O9 ; C20H16O4S
8951_282.7	1.659039	0.305622	3.2826	0.008253	2.0834	0.076134	648.7639	HMDB59637	3-(35-Diiodo-4-hydroxyphenyl)pyruvate	3M+2H	C9H6I2O4
8327_66.20	1.686662	0.838166	3.2787	0.008308	2.0805	0.076218	310.0868	HMDB15461 ; HMDB02455 ; HMDB11602 ; HMDB14004 ; HMDB15573 ; HMDB29374 ; HMDB30639 ; HMDB31304 ; HMDB32414 ; HMDB32992 ; HMDB33103 ; HMDB35529 ; HMDB36187 ; HMDB38663 ; HMDB40794 ; HMDB41959 ; HMDB60868	Glycodiazine ; Pyruvatoxime ; 3-Oxoalanine ; Norhydromorphone ; Niflumic Acid ; (EE)-Trichostachine ; ()-Tembamide ; Osmundalin ; 2-Methyl-3 or 5 or 6-(furfurylthio)pyrazine (mixture of isomers) ; Furaneol 4-glucoside ; (R)-Higenamine ; Chlorosesamone ; 2-(2-Furanylmethyl)thio-6-methylpyrazine ; D-1-(3-Carboxypropyl)amino-1-deoxyfructose ; 5-Hydroxy-4-methoxy-6-canthinone 3-N-oxide ; Normorphine ; Phenytoin quinone	M+H ; 3M+H ; 3M+H ; M+K ; 2M+3H2O+2H ; M+K ; M+K ; 2M+H+K ; 3M+2H ; 2M+H+K ; M+K ; M+NH4 ; 3M+2H ; M+2Na-H ; 2M+3H2O+2H ; M+K ; 2M+3H2O+2H	C13H15N3O4S ; C3H5NO3 ; C3H5NO3 ; C16H17NO3 ; C13H9F3N2O2 ; C16H17NO3 ; C16H17NO3 ; C12H18O8 ; C10H10N2OS ; C12H18O8 ; C16H17NO3 ; C15H13ClO4 ; C10H10N2OS ; C10H19NO7 ; C15H10N2O4 ; C16H17NO3 ; C15H10N2O4
5393_250.2	1.683772	1.090656	3.2789	0.008305	2.0807	0.076218	357.0555	HMDB30590 ; HMDB31795 ; HMDB33100 ; HMDB36343 ; HMDB37570	Dihydrosterigmatocystin ; Iprodione ; Atroviridin ; O-Demethylforbexanthone ; Demethoxykanugin	2M+Na+K ; 2M+3H2O+2H ; 2M+Na+K ; 2M+Na+K ; 2M+Na+K	C18H14O6 ; C13H13Cl2N3O3 ; C18H14O6 ; C18H14O6 ; C18H14O6

5832_293.4	1.675911	0.150993	-3.2774	0.008325	2.0796	0.07624	NA	NA	NA	NA	NA
92118_250.	1.702374	0.815512	3.2715	0.008408	2.0753	0.076719	196.0192	HMDB00044 ; HMDB01412 ; HMDB03454 ; HMDB04089 ; HMDB04291 ; HMDB06355 ; HMDB13030 ; HMDB14544 ; HMDB30567 ; HMDB31193 ; HMDB32806 ; HMDB33548 ; HMDB33586 ; HMDB33787 ; HMDB36582 ; HMDB37086 ; HMDB38318 ; HMDB39720 ; HMDB40936 ; HMDB41645 ; HMDB41650 ; HMDB41672 ; HMDB41682 ; HMDB41694 ; HMDB41697 ; HMDB41700 ; HMDB41703	Ascorbic acid ; 78-Dihydropteroic acid ; 4-Pyridoxolactone ; Formylanthranilic acid ; 5-Pyridoxolactone ; D-Glucurono-63-lactone ; Noradrenochrome ; Griseofulvin ; (-)-349-Trimethoxypterocarpan ; 123-Propanetricarboxylic acid ; p-Hydroxyphenethyl trans-ferulate ; 4-Hydroxy-5-methyl-3(2H)-thiophenone ; 2-Hydroxy-446-trimethoxychalcone ; 239-Trimethoxypterocarpan ; 6-Methoxy-2(3H)-benzoxazolone ; 58-Dimethoxychalepensin ; (R)-2-Hydroxy-2H-14-benzoxazin-3(4H)-one ; a-L-threo-4-Hex-4-enopyranuronosyl-D-galacturonic acid ; beta2-Dihydroxy-46-dimethoxy-3-methylchalcone ; 2-Hydroxyenterolactone ; 2-Hydroxyenterolactone ; 4-Hydroxyenterolactone ; 4-Hydroxyenterolactone ; 5-Hydroxyenterolactone ; 6-Hydroxyenterolactone ; 6-Hydroxyenterolactone ; 7-Hydroxyenterolactone	2M+H+K ; M+2K ; 2M+Na+K ; 2M+Na+K ; 2M+Na+K ; 2M+H+K ; 2M+Na+K ; M+H+K ; M+2K ; 2M+H+K ; M+2K ; 3M+2H ; M+2K ; M+2K ; 2M+Na+K ; M+2K ; 2M+Na+K ; M+H+K ; M+2K ; M+2K ; M+2K ; M+2K ; M+2K ; M+2K ; M+2K ; M+2K ; M+2K	C6H8O6 ; C14H14N6O3 ; C8H7NO3 ; C8H7NO3 ; C8H7NO3 ; C6H8O6 ; C8H7NO3 ; C17H17ClO6 ; C18H18O5 ; C6H8O6 ; C18H18O5 ; C5H6O2S ; C18H18O5 ; C18H18O5 ; C8H7NO3 ; C18H18O5 ; C8H7NO3 ; C12H16O12 ; C18H18O5 ; C18H18O5 ; C18H18O5 ; C18H18O5 ; C18H18O5 ; C18H18O5 ; C18H18O5 ; C18H18O5 ; C18H18O5
8457_55.95	1.688343	0.906717	-3.2718	0.008404	2.0755	0.076719	463.9788	HMDB60660	6-Mercaptopurine ribonucleoside 5-diphosphate	2M+H+K	C10H14N4O10P2S

3052_95.3	1.671482	0.267436	-3.2633	0.008527	2.0692	0.077375	170.0593	HMDB60244 ; HMDB00172 ; HMDB00557 ; HMDB00687 ; HMDB01645 ; HMDB01901 ; HMDB03640 ; HMDB05785 ; HMDB13243 ; HMDB13773 ; HMDB28914 ; HMDB28998 ; HMDB29168 ; HMDB30204 ; HMDB31716 ; HMDB32501 ; HMDB33121 ; HMDB33923 ; HMDB33957 ; HMDB36382 ; HMDB37209 ; HMDB37832 ; HMDB38024 ; HMDB38544 ; HMDB38782 ; HMDB39134 ; HMDB39358 ; HMDB40009 ; HMDB40011 ; HMDB40185 ; HMDB40374 ; HMDB41061 ; HMDB41475 ; HMDB60561 ; HMDB60650 ; HMDB00734	Phosphodimethylethanolamine ; L-Isoleucine ; L-Alloisoleucine ; L-Leucine ; L-Norleucine ; Aminocaproic acid ; Beta-Leucine ; Indole-3-carbinol ; Leucyl-phenylalanine ; D-Leucine ; Isoleucyl-Phenylalanine ; Phenylalanyl-Isoleucine ; 3-Aminocaproic acid ; 1-(2-Furanylmethyl)-1H-pyrrole ; 3-(Methylthio)-1-propanol ; Santalyl acetate ; Paraquat dichloride ; ()-erythro-Isoleucine ; Crithmumdiol ; 6-Deoxyfagomine ; alpha-Santalyl acetate ; Vetiveryl acetate ; beta-Santalyl acetate ; Longistylin C ; 1-Heptadecene-46-diyne-39-diol ; Ginsenoyne B ; Ginsenoyne D ; 4-(2-Furanylmethylene)-34-dihydro-2H-pyrrole ; 3-(2-Furanylmethyl)-1H-pyrrole ; 3-Mercapto-2-butanol ; Ginsenoyne I ; Nigellidine ; 4-Mercapto-2-butanol ; Albendazole sulfone ; N-(2-Hydroxyethyl)-morpholine ; Indoleacrylic acid	M+H ; M+K ; M+K ; M+K ; M+K ; M+K ; M+K ; M+Na ; M+Na+K ; M+K ; M+Na+K ; M+Na+K ; M+K ; M+Na ; M+ACN+Na ; M+2K ; M+2ACN+2H ; M+K ; M+2K ; M+K ; M+2K ; M+2K ; M+2K ; M+Na+K ; M+2K ; M+2Na ; M+2K ; M+Na ; M+Na ; M+ACN+Na ; M+2K ; M+2Na ; M+ACN+Na ; M+ACN+2H ; M+K ; M+H-H2O	C4H12NO4P ; C6H13NO2 ; C6H13NO2 ; C6H13NO2 ; C6H13NO2 ; C6H13NO2 ; C6H13NO2 ; C9H9NO ; C15H22N2O3 ; C6H13NO2 ; C15H22N2O3 ; C15H22N2O3 ; C6H13NO2 ; C9H9NO ; C4H10OS ; C17H26O2 ; C12H14Cl2N2 ; C6H13NO2 ; C17H26O2 ; C6H13NO2 ; C17H26O2 ; C17H26O2 ; C17H26O2 ; C20H22O ; C17H26O2 ; C17H23ClO2 ; C17H26O2 ; C9H9NO ; C9H9NO ; C4H10OS ; C17H26O2 ; C18H18N2O2 ; C4H10OS ; C12H15N3O4S ; C6H13NO2 ; C11H9NO2
6456_80.2	1.671108	0.250786	3.2637	0.00852	2.0696	0.077375	254.0806	HMDB37352 ; HMDB02991 ; HMDB14420 ; HMDB15158 ; HMDB15325 ; HMDB15353 ; HMDB28750 ; HMDB28771 ; HMDB32991 ; HMDB33080 ; HMDB33081 ; HMDB34659 ; HMDB35690 ; HMDB39359 ; HMDB40326 ; HMDB40731 ; HMDB40938 ; HMDB40990 ; HMDB41122 ; HMDB60150 ; HMDB60453	Caryatin glucoside ; Cysteamine ; Olmesartan ; Felodipine ; Brinzolamide ; Budesonide ; Aspartyl-Cysteine ; Cysteinyl-Aspartate ; 7-Methoxy-6-methyl-2H-1-benzopyran-2-one ; Antibiotic CJ 15544 ; Antibiotic CP 412065 ; Homofukinolide ; 6-O-Methylarmillaridin ; Methyl (ZZ)-10-hydroxy-28-decadiene-46-diynoate ; 7-Hydroxy-25-dimethyl-4H-1-benzopyran-4-one ; Avenalumic acid ; Coixinden A ; 3b6b-Diangeloyloxy-7(11)-eremophilen-128b-olide ; (S)-23-Dihydro-5-hydroxy-2-methyl-14-naphthoquinone ; Cysteaminium ; Chondroitin 6-sulfate	M+2H ; 3M+Na ; M+Na+K ; M+3ACN+2H ; M+3ACN+2H ; M+2K ; M+NH4 ; M+NH4 ; M+ACN+Na ; M+2K ; M+2K ; M+2K ; M+2Na ; M+ACN+Na ; M+ACN+Na ; M+ACN+Na ; M+ACN+Na ; M+2K ; M+ACN+Na ; 3M+Na ; M+H+NH4	C24H26O12 ; C2H7NS ; C24H26N6O3 ; C18H19Cl2NO4 ; C12H21N3O5S3 ; C25H34O6 ; C7H12N2O5S ; C7H12N2O5S ; C11H10O3 ; C25H34O6 ; C25H34O6 ; C25H34O6 ; C25H31ClO6 ; C11H10O3 ; C11H10O3 ; C11H10O3 ; C11H10O3 ; C25H34O6 ; C11H10O3 ; C2H7NS ; C16H27NO14S

69261_70.5	1.622216	0.151706	3.2649	0.008503	2.0704	0.077375	131.0469	HMDB32102 ; HMDB00108 ; HMDB01858 ; HMDB02048 ; HMDB02055 ; HMDB03119 ; HMDB15084 ; HMDB28853 ; HMDB29105 ; HMDB29451 ; HMDB29700 ; HMDB30819 ; HMDB32121 ; HMDB32585 ; HMDB32662 ; HMDB32950 ; HMDB32951 ; HMDB33895 ; HMDB34223 ; HMDB35608 ; HMDB36029 ; HMDB36819 ; HMDB37179 ; HMDB38894 ; HMDB39387 ; HMDB40286 ; HMDB40833 ; HMDB40988 ; HMDB60359 ; HMDB60426 ; HMDB60565 ; HMDB60920 ; HMDB60921	1234-Tetrahydro-b-carboline-13-dicarboxylic acid ; Ethanol ; p-Cresol ; m-Cresol ; o-Cresol ; Benzyl alcohol ; Felbamate ; Glycyl-Tyrosine ; Tyrosyl-Glycine ; gamma-Glutaminyl-4-hydroxybenzene ; Cinnamyl benzoate ; Aesculetin ; Annuionone B ; 4-Methoxychalcone ; (S)-9-Hydroxy-10-undecenoic acid ; 57-Dihydroxy-4H-1-benzopyran-4-one ; 47-Dihydroxy-2H-1-benzopyran-2-one ; Anisole ; 23456-Penta-O-acetyl-D-glucose ; Monotropein ; Clarycet ; Dehydrovomifoliol ; 3-Nonanon-1-yl acetate ; Isoamyl p-anisate ; Methyl 26-dihydroxy-4-quinolinecarboxylate ; Benzyl cinnamate ; 3-4-Hydroxy-3-(3-methyl-2-butenyl)phenyl-2-propenal ; Falimint ; 23-Epoxymenaquinone ; 8-Methoxykynurenate ; 1-Hydroxyibuprofen ; 2-Hydroxyibuprofen ; 3-Hydroxyibuprofen	M+2H ; 2M+K ; M+Na ; M+Na ; M+Na ; M+Na ; M+H+Na ; M+H+Na ; M+H+Na ; M+H+Na ; M+H+Na ; M+2ACN+2H ; M+H+K ; M+H+Na ; M+Na+K ; M+2ACN+2H ; M+2ACN+2H ; M+Na ; M+3H ; M+3H ; M+Na+K ; M+H+K ; M+Na+K ; M+H+K ; M+ACN+2H ; M+H+Na ; M+2Na ; M+H+Na ; M+3Na ; M+ACN+2H ; M+H+K ; M+H+K ; M+H+K	C13H12N2O4 ; C2H6O ; C7H8O ; C7H8O ; C7H8O ; C7H8O ; C11H14N2O4 ; C11H14N2O4 ; C11H14N2O4 ; C11H14N2O4 ; C16H14O2 ; C9H6O4 ; C13H18O3 ; C16H14O2 ; C11H20O3 ; C9H6O4 ; C9H6O4 ; C7H8O ; C16H22O11 ; C16H22O11 ; C11H20O3 ; C13H18O3 ; C11H20O3 ; C13H18O3 ; C11H9NO4 ; C16H14O2 ; C14H16O2 ; C11H14N2O4 ; C21H24O3 ; C11H9NO4 ; C13H18O3 ; C13H18O3 ; C13H18O3
52539_110.2	1.715827	0.148774	3.2571	0.008616	2.0647	0.077739	164.5263	HMDB12266	N-Succinyl-2-amino-6-ketopimelate	M+H+K	C11H15NO8
90504_53.3	1.675219	0.125117	-3.2582	0.0086	2.0655	0.077739	NA	NA	NA	NA	NA
3482_217.8	1.642707	0.465962	3.2586	0.008595	2.0658	0.077739	466.0435	HMDB34623 ; HMDB39268 ; HMDB39896	N-(4E8Z)-13-dihydroxyoctadeca-48-dien-2-ylhexadecanamide 1-glucoside ; Heterophylliin E ; xi-8-Acetonyldihydrosanguinarine	2M+3H ; M+H+Na ; M+2K-H	C40H75NO8 ; C40H28O25 ; C23H19NO5
06335_98.05	1.609779	0.935184	-3.2562	0.008629	2.064	0.077739	197.5206	HMDB11761 ; HMDB15093	Cer(d18:018:0) ; Carboplatin	M+2H+Na ; M+H+Na	C36H73NO3 ; C6H12N2O4Pt
90442_290.	1.679448	1.247993	3.2464	0.008773	2.0568	0.078896	714.809	HMDB00265 ; HMDB60074	Liothyronine ; Reverse-triiodthyronine	M+ACN+Na ; M+ACN+Na	C15H12I3NO4 ; C15H12I3NO4
39332_168.	1.652692	0.563774	-3.2388	0.008887	2.0512	0.079775	383.0389	HMDB39287 ; HMDB34590	Theogallin ; Shoyuflavone C	M+K ; M+H-2H2O	C14H16O10 ; C19H14O11

0764_100.6	1.645684	0.763196	-3.2375	0.008907	2.0503	0.079812	206.5071	HMDB07021 ; HMDB07049 ; HMDB07101 ; HMDB07102 ; HMDB07129 ; HMDB07157 ; HMDB07185 ; HMDB07214 ; HMDB07357 ; HMDB07385 ; HMDB14971 ; HMDB55963 ; HMDB56016 ; HMDB56017 ; HMDB56041 ; HMDB56065	DG(14:020:1(11Z)0:0) ; DG(14:1(9Z)20:00:0) ; DG(16:018:1(11Z)0:0) ; DG(16:018:1(9Z)0:0) ; DG(16:1(9Z)18:00:0) ; DG(18:016:1(9Z)0:0) ; DG(18:1(11Z)16:00:0) ; DG(18:1(9Z)16:00:0) ; DG(20:014:1(9Z)0:0) ; DG(20:1(11Z)14:00:0) ; Cefaclor ; DG(14:00:020:1n9) ; DG(16:00:018:1n7) ; DG(16:00:018:1n9) ; DG(18:00:016:1n7) ; DG(20:00:014:1n5)	M+2H+Na ; M+2H+Na ; M+2H+Na ; M+2H+Na ; M+2H+Na ; M+2H+Na ; M+2H+Na ; M+2H+Na ; M+2H+Na ; M+2H+Na ; M+2Na ; M+2H+Na ; M+2H+Na ; M+2H+Na ; M+2H+Na ; M+2H+Na	C37H70O5 ; C37H70O5 ; C37H70O5 ; C37H70O5 ; C37H70O5 ; C37H70O5 ; C37H70O5 ; C37H70O5 ; C37H70O5 ; C37H70O5 ; C15H14ClN3O4S ; C37H70O5 ; C37H70O5 ; C37H70O5 ; C37H70O5 ; C37H70O5
2704_54.8	1.69142	0.794207	3.2356	0.008936	2.0489	0.079924	NA	NA	NA	NA	NA
3717_44.4	1.698115	0.536252	-3.2339	0.008961	2.0476	0.08001	189.1234	HMDB02226 ; HMDB14362 ; HMDB30002 ; HMDB30252 ; HMDB31153 ; HMDB31198 ; HMDB31265 ; HMDB31359 ; HMDB31565 ; HMDB31728 ; HMDB31732 ; HMDB31799 ; HMDB32112 ; HMDB32540 ; HMDB33546 ; HMDB33916 ; HMDB35676 ; HMDB35677 ; HMDB36190 ; HMDB36815 ; HMDB38910 ; HMDB60992	Adrenic acid ; Bethanidine ; (-)-Nopol ; Limonene aldehyde ; Diisobutylcarbinol ; ()-355-Trimethyl-1-hexanol ; 1-Nonanol ; 24-Undecadienal ; Dihydrojasmone ; 2-Hexylidenecyclopentanone ; 3-Nonanol ; Malonoben ; 124-Nonadecanetriol ; 266-Trimethyl-1-cyclohexen-1-acetaldehyde ; 25-Undecadienal ; 2-Nonanol ; 1-Hydroxy-1-phenyl-3-hexadecanone ; 3-Hydroxy-1-phenyl-1-hexadecanone ; 2-Heptylfuran ; Norecasantalol ; 2-Methyl-5-(8-pentadecenyl)-13-benzenediol ; 2-hydroxydesipramine	M+2Na ; 2M+H+Na ; M+Na ; M+Na ; M+2Na-H ; M+2Na-H ; M+2Na-H ; M+Na ; M+Na ; M+Na ; M+2Na-H ; 2M+3H ; M+Na+K ; M+Na ; M+Na ; M+2Na-H ; M+2Na ; M+2Na ; M+Na ; M+Na ; M+2Na ; 2M+3H	C22H36O2 ; C10H15N3 ; C11H18O ; C11H18O ; C9H20O ; C9H20O ; C9H20O ; C11H18O ; C11H18O ; C11H18O ; C9H20O ; C18H22N2O ; C19H40O3 ; C11H18O ; C11H18O ; C9H20O ; C22H36O2 ; C22H36O2 ; C11H18O ; C11H18O ; C22H36O2 ; C18H22N2O
0906_65.4	1.694703	0.490709	3.2276	0.009057	2.043	0.080723	245.0591	HMDB01413 ; HMDB02015 ; HMDB30106 ; HMDB31764 ; HMDB33324 ; HMDB33325 ; HMDB34447 ; HMDB35067 ; HMDB39337 ; HMDB31863 ; HMDB31969 ; HMDB33743 ; HMDB38635	Citicoline ; Cystathionine ketimine ; Neosilyhermin A ; Nifursol ; Silyhermin ; Silandrin ; Ptelatoside B ; Melleolide L ; Tetramethylquercetin 3-rutinoside ; gamma-Glutamyl-S-methylcysteine sulfoxide ; 3-Hydroxy-2-(4-methylbenzoyl)-4H-1-benzopyran-4-one ; Neodunol ; Methoxybrassenin A	M+2H ; M+ACN+H ; M+H+Na ; M+3ACN+2H ; M+H+Na ; M+H+Na ; M+Na+K ; M+H+K ; M+3Na ; M+H-2H2O ; M+H-2H2O ; M+H-2H2O ; M+H-2H2O	C14H26N4O11P2 ; C7H9NO4S ; C25H22O9 ; C12H7N5O9 ; C25H22O9 ; C25H22O9 ; C20H28O10 ; C23H27ClO7 ; C31H38O16 ; C9H16N2O6S ; C17H12O4 ; C17H12O4 ; C13H16N2OS2

[illegible]4434_48.9[illegible]	1.61085	1.060866	-3.224	0.009113	2.0403	0.081075	162.1124	HMDB00062 ; HMDB00596 ; HMDB00648 ; HMDB11531 ; HMDB11562 ; HMDB31841 ; HMDB32946 ; HMDB36132 ; HMDB36174 ; HMDB39995 ; HMDB39996 ; HMDB41345	L-Carnitine ; Glucosylsphingosine ; Galactosylsphingosine ; MG(0:014:1(9Z)0:0) ; MG(14:1(9Z)0:00:0) ; 25-Dimethyl-3-propylpyrazine ; 35-Diethyl-2-methylpyrazine ; 25-Diethyl-3-methylpyrazine ; 2-Methyl-3-(2-methylpropyl)pyrazine ; 35-Dimethyl-2-propylpyrazine ; 23-Dimethyl-5-propylpyrazine ; 23-Diethyl-5-methylpyrazine	M+H ; M+2H+Na ; M+2H+Na ; M+H+Na ; M+H+Na ; 2M+H+Na ; 2M+H+Na ; 2M+H+Na ; 2M+H+Na ; 2M+H+Na ; 2M+H+Na ; 2M+H+Na	C7H15NO3 ; C24H47NO7 ; C24H47NO7 ; C17H32O4 ; C17H32O4 ; C9H14N2 ; C9H14N2 ; C9H14N2 ; C9H14N2 ; C9H14N2 ; C9H14N2 ; C9H14N2
[illegible]5455_59.7[illegible]	1.654817	0.477946	-3.2115	0.009309	2.0311	0.082523	227.0485	HMDB00434 ; HMDB14499 ; HMDB29495 ; HMDB29645 ; HMDB29648 ; HMDB29788 ; HMDB30571 ; HMDB32028 ; HMDB32061 ; HMDB32365 ; HMDB35721 ; HMDB37177 ; HMDB39427 ; HMDB40256 ; HMDB40637 ; HMDB41219 ; HMDB60398 ; HMDB60733 ; HMDB60735	Homoveratric acid ; Aztreonam ; Trigoforin ; Xanthoxylin ; 245-Trimethoxybenzaldehyde ; Kanzonol V ; ()-3-(34-Methylenedioxyphenyl)-12-propanediol ; (E)-3-(2-Methylpropylidene)-1(3H)-isobenzofuranone ; 3-Butylidene-1(3H)-isobenzofuranone ; Maltyl isobutyrate ; (S)-Batatic acid ; 1-(245-Trihydroxyphenyl)-1-butanone ; 2-Hydroxy-3-(4-methoxyphenyl)propanoic acid ; (S)-2-(4-Methoxyphenoxy)propanoic acid ; 4-Hydroxy-6-methyl-3-(1-oxobutyl)-2H-pyran-2-one ; Allyl cinnamate ; 5-Hydroxyconiferyl alcohol ; 3-(3-Hydroxyphenyl)-2-methyllactic acid ; 3-(34-Dihydroxyphenyl)-2-methylpropionic acid	2M+Na+K ; M+H+NH4 ; M+K ; 2M+Na+K ; 2M+Na+K ; M+2K ; 2M+Na+K ; M+K ; M+K ; 2M+Na+K ; 2M+Na+K ; 2M+Na+K ; 2M+Na+K ; 2M+Na+K ; 2M+Na+K ; M+K ; 2M+Na+K ; 2M+Na+K ; 2M+Na+K	C10H12O4 ; C13H17N5O8S2 ; C12H12O2 ; C10H12O4 ; C10H12O4 ; C24H24O4 ; C10H12O4 ; C12H12O2 ; C12H12O2 ; C10H12O4 ; C10H12O4 ; C10H12O4 ; C10H12O4 ; C10H12O4 ; C10H12O4 ; C12H12O2 ; C10H12O4 ; C10H12O4 ; C10H12O4

[illegible]4145_40.0[illegible]	1.630486	0.578441	-3.2117	0.009305	2.0313	0.082523	262.1284	HMDB13133 ; HMDB33865 ; HMDB34777 ; HMDB39082 ; HMDB34346 ; HMDB03905 ; HMDB15443 ; HMDB15686 ; HMDB28778 ; HMDB28779 ; HMDB28904 ; HMDB28926 ; HMDB29876 ; HMDB32156 ; HMDB33156 ; HMDB33462 ; HMDB60724 ; HMDB60741 ; HMDB60742 ; HMDB37844	Methylmalonylcarnitine ; Lotaustralin ; Epidermin ; Camelinin ; 4-Deoxyphysalolactone ; Imidazole-4-acetaldehyde ; Heptabarbital ; Pipazethate ; Cysteinyl-Isoleucine ; Cysteinyl-Leucine ; Isoleucyl-Cysteine ; Leucyl-Cysteine ; Paucine ; alpha-Acetolactate decarboxylase (enzyme preparation from bacillus subtilis recombinant) ; Methoxypyrazine ; 4-Coumaroyl-2-hydroxyputrescine ; 24-Diamino-67-dimethoxyquinazoline ; 3-Hydroxy-4-aminopyridine ; 3-Hydroxymelatonin ; N-(1-Deoxy-1-fructosyl)valine	M+H ; M+H ; M+H ; M+H ; M+2H ; 2M+ACN+H ; 2M+H+Na ; M+3ACN+2H ; 2M+3H2O+2H ; 2M+3H2O+2H ; 2M+3H2O+2H ; 2M+3H2O+2H ; 2M+H+Na ; M+3ACN+2H ; 2M+ACN+H ; 2M+H+Na ; M+ACN+H ; 2M+ACN+H ; 2M+H+Na ; M+H-H2O	C11H19NO6 ; C11H19NO6 ; C11H19NO6 ; C12H23NOS2 ; C28H39ClO7 ; C5H6N2O ; C13H18N2O3 ; C21H25N3O3S ; C9H18N2O3S ; C9H18N2O3S ; C9H18N2O3S ; C9H18N2O3S ; C13H18N2O3 ; C21H25N3O3S ; C5H6N2O ; C13H18N2O3 ; C10H12N4O2 ; C5H6N2O ; C13H18N2O3 ; C11H21NO7
[illegible]5234_36.0[illegible]	1.680162	0.066408	3.2072	0.009376	2.028	0.082972	498.2885	HMDB00523 ; HMDB00540 ; HMDB00550 ; HMDB02984 ; HMDB03218 ; HMDB13156 ; HMDB13199 ; HMDB14759 ; HMDB34415 ; HMDB34650 ; HMDB36362 ; HMDB36437 ; HMDB39072 ; HMDB40640 ; HMDB42042	5-Androstene-3b16b17a-triol ; 5-Androstene-3b16a17a-triol ; 5-Androstenetriol ; 11-Hydroxyandrosterone ; Withanolide ; 16-alpha-Hydroxyandrosterone ; 5-Androstene-3alpha-16b17b-triol ; Oxandrolone ; Withanolide A ; Soyasaponin II ; 28-Glucosyl-(1-6)-glucosyloleanolic acid 3-arabinoside ; Methyl lucidenate F ; Hoduloside III ; 8-Paradol ; Tiropramide	3M+2K ; 3M+2K ; 3M+2K ; 3M+2K ; 2M+3H2O+2H ; 3M+2K ; 3M+2K ; 3M+2K ; 2M+3H2O+2H ; M+2ACN+2H ; M+2ACN+2H ; 2M+3H2O+2H ; M+2ACN+2H ; 3M+2K ; 2M+Na+K	C19H30O3 ; C19H30O3 ; C19H30O3 ; C19H30O3 ; C28H38O6 ; C19H30O3 ; C19H30O3 ; C19H30O3 ; C28H38O6 ; C47H76O17 ; C47H76O17 ; C28H38O6 ; C47H76O17 ; C19H30O3 ; C28H41N3O3
[illegible]5335_110.[illegible]	1.689645	0.011051	-3.2049	0.009413	2.0263	0.083112	284.1355	HMDB28799 ; HMDB28883 ; HMDB28899 ; HMDB29151 ; HMDB11657 ; HMDB15190 ; HMDB28964 ; HMDB29132 ; HMDB31873 ; HMDB39090 ; HMDB40893	Glutaminyl-Histidine ; Histidinyl-Glutamine ; Histidinyl-Gamma-glutamate ; Gamma-glutamyl-Histidine ; 26-Diamino-4-hydroxy-5-N-methylformamidopyrimidine ; Echothiophate ; Lysyl-Valine ; Valyl-Lysine ; 17-Diphenyl-4-hepten-3-one ; 78-Dihydro-8-hydroxyreticulataxanthin ; (EE)-17-Diphenyl-46-heptadien-3-ol	M+H ; M+H ; M+H ; M+H ; 3M+H+NH4 ; 2M+3H2O+2H ; M+K ; M+K ; 2M+H+K ; M+2K ; 2M+H+K	C11H17N5O4 ; C11H17N5O4 ; C11H17N5O4 ; C11H17N5O4 ; C6H9N5O2 ; C9H23NO3PS ; C11H23N3O3 ; C11H23N3O3 ; C19H20O ; C33H46O3 ; C19H20O
[illegible]2566_101.[illegible]	1.627774	0.558348	-3.2042	0.009425	2.0257	0.083112	NA	NA	NA	NA	NA

2644_62.0	1.654476	1.251788	3.2027	0.009448	2.0246	0.083172	207.9983	HMDB15242 ; HMDB00272 ; HMDB01721 ; HMDB11635 ; HMDB33384 ; HMDB34782 ; HMDB59996 ; HMDB60445 ; HMDB60447	Miconazole ; Phosphoserine ; DL-O-Phosphoserine ; p-Cresol sulfate ; CI Direct Red 45 ; Benzyl sulfate ; Dihydronaringenin-O-sulphate ; Bromobenzene-23-dihydrodiol ; Bromobenzene-34-dihydrodiol	M+2H ; M+Na ; M+Na ; 2M+H+K ; M+3Na ; 2M+H+K ; M+Na+K ; M+NH4 ; M+NH4	C18H14Cl4N2O ; C3H8NO6P ; C3H8NO6P ; C7H8O4S ; C24H17N3O7S3 ; C7H8O4S ; C15H14O8S ; C6H7BrO2 ; C6H7BrO2
9236_98.8	1.677679	0.615371	-3.2011	0.009475	2.0234	0.083201	177.0319	HMDB04284 ; HMDB14548 ; HMDB29563 ; HMDB29671 ; HMDB29672 ; HMDB30764 ; HMDB31519 ; HMDB32036 ; HMDB32136 ; HMDB32137 ; HMDB32139 ; HMDB32159 ; HMDB32237 ; HMDB32290 ; HMDB32430 ; HMDB32945 ; HMDB34241 ; HMDB35176 ; HMDB36649 ; HMDB39096 ; HMDB39802 ; HMDB39843 ; HMDB40174 ; HMDB40177 ; HMDB40533 ; HMDB40889 ; HMDB41607	Tyrosol ; Alprazolam ; 3-Acetyl-25-dimethylfuran ; 14-Dimethoxybenzene ; 13-Dimethoxybenzene ; Acetylpterosin C ; 4-Mercapto-4-methyl-2-pentanone ; 4-Ethoxyphenol ; 2-Methoxy-4-methylphenol ; 26-Dimethyl-14-benzenediol ; 12-Dimethoxybenzene ; 2-Acetyl-35-dimethylfuran ; 25-Dimethyl-3-mercaptotetrahydrofuran ; 1-(2-Furyl)butan-3-one ; 4-(Methylthio)-2-pentanone ; 1-Phenyl-24-pentadiyn-1-one ; 4-Methoxybenzyl alcohol ; 3-Mercapto-2-methylpentanal ; Sinalbin B ; N-gamma-Glutamyl-S-propylcysteine ; 1-(2-Furanyl)-1-butanone ; S-Methyl 3-methylthiobutyrate ; 4-Ethyl-12-benzenediol ; 3-Ethyl-12-benzenediol ; Artomunoxanthentrione epoxide ; (all-E)-17-bis(4-hydroxyphenyl)-146-heptatrien-3-one ; 2-Phenoxyethanol	M+K ; M+2Na ; M+K ; M+K ; M+K ; M+2K ; M+2Na-H ; M+K ; M+K ; M+K ; M+K ; M+K ; M+2Na-H ; M+K ; M+2Na-H ; M+Na ; M+K ; M+2Na-H ; 2M+3H ; M+Na+K ; M+K ; M+2Na-H ; M+K ; M+K ; M+3Na ; M+Na+K ; M+K	C8H10O2 ; C17H13ClN4 ; C8H10O2 ; C8H10O2 ; C8H10O2 ; C16H20O4 ; C6H12OS ; C8H10O2 ; C8H10O2 ; C8H10O2 ; C8H10O2 ; C8H10O2 ; C6H12OS ; C8H10O2 ; C6H12OS ; C11H6O ; C8H10O2 ; C6H12OS ; C12H12N2OS2 ; C11H20N2O5S ; C8H10O2 ; C6H12OS ; C8H10O2 ; C8H10O2 ; C26H22O8 ; C19H16O3 ; C8H10O2
4852_56.4	1.621822	0.869842	-3.1994	0.009502	2.0222	0.083201	452.9575	HMDB00269 ; HMDB36699	Sphinganine ; Chlorophenol red	3M+2H ; 2M+Na+K	C18H39NO2 ; C19H12Cl2O5S
4513_272.	1.598193	0.260884	3.2001	0.009491	2.0227	0.083201	251.5175	HMDB00580 ; HMDB60358 ; HMDB60381	Chondroitin sulfate ; 23-Dihydro-2-S-glutathionyl-3-hydroxy bromobenzene ; 34-Dihydro-3-hydroxy-4-S-glutathionyl bromobenzene	M+H+K ; M+H+Na ; M+H+Na	C13H21NO15S ; C16H22BrN3O7S ; C16H22BrN3O7S
4991_111.	1.673604	0.854463	-3.1972	0.009537	2.0206	0.083367	259.0385	HMDB15606 ; HMDB29467 ; HMDB29473 ; HMDB39860 ; HMDB40055 ; HMDB41442 ; HMDB60002	Lofexidine ; Eugenitin ; Isoeugenitin ; Propylene glycol alginate ; Ethyl 3-(2-furanylmethyl)thiopropanoate ; Hispolone ; Indole-3-carboxilic acid-O-sulphate	M+H ; M+K ; M+K ; M+2K ; M+2Na-H ; M+K ; M+NH4	C11H12Cl2N2O ; C12H12O4 ; C12H12O4 ; C17H28O13 ; C10H14O3S ; C12H12O4 ; C9H7NO5S

3436_282.[illegible]	1.648636	0.452091	3.1951	0.009571	2.019	0.083519	611.8883	HMDB04845 ; HMDB11917 ; HMDB11918	Ganglioside GM3 (d18:118:0) ; Ganglioside GM3 (d18:018:1(11Z)) ; Ganglioside GM3 (d18:018:1(9Z))	M+ACN+2H ; M+ACN+2H ; M+ACN+2H	C59H108N2O21 ; C59H108N2O21 ; C59H108N2O21
7356_97.3[illegible]	1.639425	0.102336	-3.1935	0.009597	2.0179	0.083597	452.5737	HMDB00781 ; HMDB00814 ; HMDB00841 ; HMDB01339 ; HMDB30436 ; HMDB59619 ; HMDB60310	N-Acetylgalactosamine 4-sulphate ; N-Acetylglucosamine 6-sulfate ; N-Acetylgalactosamine 6-sulfate ; Glutaryl-CoA ; Emblicanin B ; (S)-ethylmalonyl-CoA ; (2R)-Ethylmalonyl-CoA	3M+2H ; 3M+2H ; 3M+2H ; M+H+Na ; M+3ACN+2H ; M+H+Na ; M+H+Na	C8H15NO9S ; C8H15NO9S ; C8H15NO9S ; C26H42N7O19P3S ; C34H20O22 ; C26H42N7O19P3S ; C26H42N7O19P3S
5248_183.[illegible]	1.697238	0.01091	-3.1894	0.009665	2.0148	0.08385	365.0675	HMDB34913 ; HMDB37554 ; HMDB39864 ; HMDB33287	Imazamethabenz-methyl ; ()-Rollipyrrole ; 3-O-beta-D-Galactopyranosylcinnamtannin B1 ; Theaflagallin	M+2K-H ; M+2K-H ; M+3Na ; M+H-2H2O	C16H20N2O3 ; C16H20N2O3 ; C51H46O23 ; C20H16O9
9925_282.[illegible]	1.662064	0.162278	3.1887	0.009676	2.0143	0.08385	593.549	HMDB05368 ; HMDB05381 ; HMDB05395 ; HMDB05422 ; HMDB10433 ; HMDB13622 ; HMDB30970 ; HMDB34386 ; HMDB35575 ; HMDB41850 ; HMDB42135 ; HMDB42164 ; HMDB42192 ; HMDB42220 ; HMDB42221 ; HMDB42249 ; HMDB42307 ; HMDB42336 ; HMDB42366 ; HMDB42395 ; HMDB42454 ; HMDB42483 ; HMDB43002 ; HMDB43366 ; HMDB43841 ; HMDB43896 ; HMDB43948 ; HMDB43949 ; HMDB43975 ; HMDB44002 ; HMDB44029 ; HMDB44056 ; HMDB44083 ; HMDB44138 ; HMDB44193 ; HMDB44678 ; HMDB44730 ; HMDB44781 ; HMDB44807 ; HMDB44859 ; HMDB44885 ; HMDB44911 ; HMDB44938 ; HMDB45484 ; HMDB45534 ; HMDB45535 ; HMDB45559 ; HMDB45584 ; HMDB45659 ; HMDB46260 ; HMDB46261 ; HMDB46309 ; HMDB46333 ; HMDB47007 ; HMDB47054 ; HMDB07078 ;	TG(16:018:020:1(11Z))iso6 ; TG(16:018:1(9Z)20:0)iso6 ; TG(18:018:018:1(9Z))iso3 ; TG(16:1(9Z)18:020:0)iso6 ; TG(18:018:018:1(11Z))iso3 ; Nonadeca-10(Z)-enoic acid ; Glycerol 13-dioctadecanoate 2-(9Z-octadecenoate) ; Glycerol 12-dioctadecanoate 3-(9Z-octadecenoate) ; 46-Nonadecanedione ; Cefodizime ; TG(14:016:024:1(15Z)) ; TG(14:018:022:1(13Z)) ; TG(14:020:020:1(11Z)) ; TG(14:022:018:1(11Z)) ; TG(14:022:018:1(9Z)) ; TG(14:024:016:1(9Z)) ; TG(14:016:1(9Z)24:0) ; TG(14:018:1(11Z)22:0) ; TG(14:018:1(9Z)22:0) ; TG(14:020:1(11Z)20:0) ; TG(14:022:1(13Z)18:0) ; TG(14:024:1(15Z)16:0) ; TG(15:015:024:1(15Z)) ; TG(15:024:1(15Z)15:0) ; TG(16:014:024:1(15Z)) ; TG(16:016:022:1(13Z)) ; TG(16:020:018:1(11Z)) ;	2M+3H ; 2M+3H ; 2M+3H ; 2M+3H ; 2M+3H ; 2M+H ; 2M+3H ; 2M+3H ; 2M+H ; 2M+H+NH4 ; 2M+3H ; M+H-H2O ; M+H-H2O ; M+H-H2O	C57H108O6 ; C57H108O6 ; C57H108O6 ; C57H108O6 ; C57H108O6 ; C19H36O2 ; C57H108O6 ; C57H108O6 ; C19H36O2 ; C20H20N6O7S4 ; C57H108O6 ; C38H74O5 ;

0721_101.[illegible]	1.624506	0.58284	3.1902	0.009651	2.0154	0.08385	389.1041	HMDB01957 ; HMDB02928 ; HMDB06791 ; HMDB12779 ; HMDB15191 ; HMDB29360 ; HMDB29401 ; HMDB29651 ; HMDB29911 ; HMDB40937 ; HMDB59978 ; HMDB59979	Pentacarboxyl porphyrinogen III ; Maltitol ; Melibiitol ; 4-Hydroxyestrone sulfate ; Praziquantel ; Isorheagenine ; Ochratoxin B ; Phlorisobutyrophenone 2-glucoside ; 1-O-alpha-D-Glucopyranosyl-D-mannitol ; Lactitol ; 4-Hydroxy-5-(dihydroxyphenyl)-valeric acid-O-sulphate ; 4-Hydroxy-5-(dihydroxyphenyl)-valeric acid-O-sulphate III	M+2K ; M+2Na-H ; M+2Na-H ; M+Na ; M+2K-H ; 2M+H+K ; 2M+H+K ; 2M+Na+K ; M+2Na-H ; M+2Na-H ; M+2ACN+H ; M+2ACN+H	C37H40N4O10 ; C12H24O11 ; C12H24O11 ; C18H22O6S ; C19H24N2O2 ; C20H19NO6 ; C20H19NO6 ; C16H22O9 ; C12H24O11 ; C12H24O11 ; C11H14O8S ; C11H14O8S
[illegible]1952_56.6[illegible]	1.61197	0.407361	-3.1865	0.009713	2.0127	0.084018	520.9432	HMDB08782 ; HMDB51354 ; HMDB51405 ; HMDB53166 ; HMDB53236 ; HMDB53257 ; HMDB53262 ; HMDB53976 ; HMDB54009 ; HMDB54231 ; HMDB54297 ; HMDB54331 ; HMDB54337 ; HMDB54864 ; HMDB54904 ; HMDB55044 ; HMDB55100 ; HMDB55142 ; HMDB55308 ; HMDB55343 ; HMDB55355 ; HMDB55488 ; HMDB55635 ; HMDB55665 ; HMDB55681 ; HMDB55736 ; HMDB55760 ; HMDB55854 ; HMDB55875	PC(24:024:0) ; TG(20:3(5Z8Z11Z)20:5(5Z8Z11Z14Z17Z)o-18:0) ; TG(20:3(5Z8Z11Z)o-18:020:5(5Z8Z11Z14Z17Z)) ; TG(18:3(6Z9Z12Z)22:5(4Z7Z10Z13Z16Z)o-18:0) ; TG(18:3(6Z9Z12Z)22:5(7Z10Z13Z16Z19Z)o-18:0) ; TG(18:3(6Z9Z12Z)o-18:022:5(4Z7Z10Z13Z16Z)) ; TG(18:3(6Z9Z12Z)o-18:022:5(7Z10Z13Z16Z19Z)) ; TG(20:3n620:5(5Z8Z11Z14Z17Z)o-18:0) ; TG(20:3n6o-18:020:5(5Z8Z11Z14Z17Z)) ; TG(20:4(5Z8Z11Z14Z)20:4(5Z8Z11Z14Z)o-18:0) ; TG(20:4(5Z8Z11Z14Z)20:4(8Z11Z14Z17Z)o-18:0) ; TG(20:4(5Z8Z11Z14Z)o-18:020:4(5Z8Z11Z14Z)) ; TG(20:4(5Z8Z11Z14Z)o-18:020:4(8Z11Z14Z17Z)) ; TG(22:4(7Z10Z13Z16Z)18:4(6Z9Z12Z15Z)o-18:0) ; TG(22:4(7Z10Z13Z16Z)o-18:018:4(6Z9Z12Z15Z)) ; TG(22:5(4Z7Z10Z13Z16Z)18:3(6Z9Z12Z)o-18:0) ; TG(22:5(4Z7Z10Z13Z16Z)18:3(9Z12Z15Z)o-18:0) ;	M+2ACN+2H ; M+3ACN+2H	C56H112NO8P ; C61H104O5

5013_65.15	1.671901	0.706916	3.1771	0.009868	2.0058	0.085213	310.095	HMDB10319 ; HMDB40830 ; HMDB59804 ; HMDB06825 ; HMDB12267 ; HMDB29466 ; HMDB29472 ; HMDB29775 ; HMDB30818 ; HMDB31656 ; HMDB32952 ; HMDB33530 ; HMDB34310 ; HMDB35232 ; HMDB35271 ; HMDB35303 ; HMDB36627 ; HMDB38261 ; HMDB38264 ; HMDB41037 ; HMDB60849 ; HMDB61068	Inodxyl glucuronide ; N-Feruloylaspartic acid ; Indoxylglucuronide ; Tetrahydrofolyl-Glu(2) ; N-Succinyl-LL-26-diaminopimelate ; Eugenitol ; Isoeugenitol ; Vinyl caffeate ; Scoparone ; Jaceidin 4-glucuronide ; Citropten ; (2E4E6Z)-246-Decatrienoic acid dehydropiperidide ; Cordeauxione ; Rotundine B ; Rotundine A ; 3-Methoxy-45-methylenedioxycinnamaldehyde ; Eugenin ; ()-7-epi-Syringaresinol 4-glucoside ; 356-Trihydroxy-347-trimethoxyflavone 3-glucuronide ; 8-Deoxy-11-hydroxy-13-chlorogrosheimin ; NN-Didesmethyltramadol ; S-methylazathioprine	M+H ; M+H ; M+H ; M+2Na ; 2M+H+K ; 3M+2H ; 3M+2H ; 3M+2H ; 3M+2H ; M+2ACN+2H ; 3M+2H ; M+2K+H ; M+NH4 ; M+2K-H ; M+2K+H ; 3M+2H ; 3M+2H ; M+H+K ; M+2ACN+2H ; 2M+H+Na ; M+2K-H ; M+NH4	C14H15NO7 ; C14H15NO7 ; C14H15NO7 ; C24H30N8O9 ; C11H18N2O7 ; C11H10O4 ; C11H10O4 ; C11H10O4 ; C11H10O4 ; C24H24O14 ; C11H10O4 ; C15H21NO ; C14H12O7 ; C15H23NO ; C15H21NO ; C11H10O4 ; C11H10O4 ; C28H36O13 ; C24H24O14 ; C15H19ClO4 ; C15H23NO ; C10H10N7O2S
7656_118.9	1.608277	0.246414	-3.1717	0.009959	2.0018	0.085854	257.6808	HMDB35112 ; HMDB35624 ; HMDB35843	(3beta17alpha23R)-1723-Epoxy-329-dihydroxy-27-norlanost-8-ene-1524-dione ; (17alpha23S)-Epoxy-2829-dihydroxy-27-norlanost-8-ene-324-dione ; (23S24S)-1723-Epoxy-2429-dihydroxy-27-norlanost-8-ene-315-dione	M+ACN+2H ; M+ACN+2H ; M+ACN+2H	C29H44O5 ; C29H44O5 ; C29H44O5
9623_39.13	1.604343	1.025689	-3.1666	0.010046	1.998	0.086451	562.327	HMDB10384 ; HMDB11128 ; HMDB30361	LysoPC(18:0) ; LysoPC(0:018:0) ; (-)-Aspidospermine	M+K ; M+K ; 3M+Na+K	C26H54NO7P ; C26H54NO7P ; C22H30N2O2
9151_55.39	1.639766	0.690075	-3.1638	0.010093	1.996	0.08671	441.9969	HMDB29200 ; HMDB41748 ; HMDB59587 ; HMDB60874	Ferulic acid 4-sulfate ; Isoferulic acid 3-sulfate ; Perfluorooctanoic acid ; Thioguanosine 5-diphosphate	3M+Na+K ; 3M+Na+K ; 2M+3H2O+2H ; M+H-H2O	C10H10O7S ; C10H10O7S ; C8HF15O2 ; C10H15N5O10P2S

8021_109.8	1.667023	0.176803	-3.1624	0.010118	1.9949	0.086778	267.1088	HMDB02096 ; HMDB02421 ; HMDB11174 ; HMDB11175 ; HMDB12994 ; HMDB14437 ; HMDB14801 ; HMDB15262 ; HMDB15611 ; HMDB28915 ; HMDB28937 ; HMDB32412 ; HMDB32582 ; HMDB33655 ; HMDB34446 ; HMDB39656 ; HMDB41526	3-Indolebutyric acid ; 7-Sulfocholic acid ; L-isoleucyl-L-proline ; L-leucyl-L-proline ; Leukotriene D5 ; Etomidate ; Flumethasone Pivalate ; Prednicarbate ; Methsuximide ; Isoleucyl-Proline ; Leucyl-Proline ; Methyl beta-naphthyl ketone ; 2-Biphenylol ; 711-Bisdeacetylvaltrate 7-(3-methylpentanoate) 11-(3-hydroxy-3-methylbutanoate) ; Diphenyl ether ; 5-(2-Furanyl)-123456-hexahydro-7H-cyclopentabpyridin-7-one ; Isosalsolidine	M+ACN+Na ; M+2Na ; M+K ; M+K ; M+H+K ; M+Na ; M+H+K ; M+2Na ; M+ACN+Na ; M+K ; M+K ; 3M+H+Na ; 3M+H+Na ; M+H+K ; 3M+H+Na ; M+ACN+Na ; M+ACN+Na	C12H13NO2 ; C24H40O8S ; C11H20N2O3 ; C11H20N2O3 ; C25H38N2O6S ; C14H16N2O2 ; C27H36F2O6 ; C27H36O8 ; C12H13NO2 ; C11H20N2O3 ; C11H20N2O3 ; C12H10O ; C12H10O ; C26H38O9 ; C12H10O ; C12H13NO2 ; C12H13NO2
9899_261.4	1.654458	0.329468	3.1436	0.010446	1.9811	0.089176	380.835	HMDB07087 ; HMDB07619 ; HMDB15275 ; HMDB55993	DG(15:022:1(13Z)0:0) ; DG(22:1(13Z)15:00:0) ; Dichlorphenamide ; DG(15:00:022:1n9)	M+3ACN+2H ; M+3ACN+2H ; M+2K-H ; M+3ACN+2H	C40H76O5 ; C40H76O5 ; C6H6Cl2N2O4S2 ; C40H76O5
1638_58.95	1.638973	0.47504	3.1433	0.010452	1.9808	0.089176	90.05502	HMDB04362 ; HMDB31263 ; HMDB31271 ; HMDB31514 ; HMDB31531 ; HMDB32265 ; HMDB32307 ; HMDB32413 ; HMDB32491 ; HMDB33167 ; HMDB34716 ; HMDB35307 ; HMDB36208 ; HMDB36222 ; HMDB37023 ; HMDB37496 ; HMDB37618 ; HMDB37629 ; HMDB38272 ; HMDB38275 ; HMDB39736 ; HMDB39794 ; HMDB40210 ; HMDB59728 ; HMDB59817 ; HMDB59840 ; HMDB59894 ; HMDB60286	4-Hydroxynonenal ; 24-Nonanedione ; 2-Nonenoic acid ; 6-Butyltetrahydro-2H-pyran-2-one ; Dihydro-5-pentyl-2(3H)-furanone ; cis-Ethyl 4-heptenoate ; Hept-trans-2-en-1-yl acetate ; Methyl 2-octenoate ; Prenyl isobutyrate ; Ethyl cyclohexanecarboxylate ; 4-Oxononanal ; Quercuslactone a ; 2-Propenyl 2-ethylbutanoate ; Cyclohexyl propionate ; 2-Hydroxy-266-trimethylcyclohexanone ; Methyl (E)-2-octenoate ; (E)-3-Heptenyl acetate ; Isobutyl angelate ; 2-Hexenyl propionate ; cis-3-Hexenyl propanoate ; 6-Hydroxy-46-dimethyl-3-hepten-2-one ; Methyl 4Z-octenoate ; 2-Propenyl hexanoate ; Methyl 3-Octenoate ; (Z)-Oak lactone ; (E)-2-Ethyl heptenoate ; (E)-Oak lactone ; 34-Epoxynonanal	M+H+Na ; M+H+Na	C9H16O2 ; C9H16O2

[truncated: 15,032,300 more chars]
